
JOURNAL INFORMATION
==============================
NLM Title Abbreviation: Mol Oncol
Journal ID: Mol Oncol
NLM Journal ID: 101308230
Journal ID: MOL2

Molecular Oncology

ISSN: 1574-7891
EISSN: 1878-0261
Publisher: Wiley

ARTICLE INFORMATION
==============================
PMCID: PMC10250143
PMID: 37290960
DOI: 10.1002/1878-0261.13471
Article ID: MOL213471
Article version: 1
Subjects: Abstracts; EACR 2023: Innovative Cancer Science, 12‐15 June 2023, Torino, Italy; Abstracts

Abstracts

Electronic publication date: 2023 Jun 8
Publication date: 2023 Jun
Volume: 17
Issue: Suppl 1
Issue title: EACR 2023: Innovative Cancer Science, 12‐15 June 2023, Torino, Italy
First page: 1
Last page: 597
Copyright: © 2023 The Authors. Molecular Oncology is published by John Wiley & Sons Ltd on behalf of Federation of European Biochemical Societies
License: This is an open access article under the terms of the http://creativecommons.org/licenses/by/4.0/ License, which permits use, distribution and reproduction in any medium, provided the original work is properly cited.
License URL: https://creativecommons.org/licenses/by/4.0/

Figure count: 0
Table count: 0
Page count: 597

source-schema-version-number: 2.0
cover-date: June 2023
details-of-publishers-convertor: Converter:WILEY_ML3GV2_TO_JATSPMC version:6.2.9 mode:remove_FC converted:08.06.2023

==============================

Molecular Oncology

Volume 17, Supplement 1, June 2023

### EACR 2023 Congress Abstracts

### Contents

**PROFFERED PAPER PRESENTATIONS ....... 2**

Rising Star and Top Abstracts ..... 2
Symposium: Interface between the Microbiome and Immune System ..... 3
Symposium: Drug Resistance ..... 4
Symposium: Mechanism Based Clinical Trial ..... 5
Symposium: Challenging Drug Targets ..... 7
Joint EACR-SIC Symposium: Spatial Transcriptomics ..... 8
Symposium: Cell Adaptation and Competition ..... 9
Symposium: Tumour Metabolism and Epigenetic Control .. 10
Joint EACR-AACR Symposium: Computational Biology and AI in Research and Cancer Care ..... 11
Symposium: Normal Tissue and the Biology of Early Cancer ..... 12
Symposium: Drugging the RAS Pathway ..... 13
Symposium: Advanced T-Cell Therapy ..... 14
Symposium: Cancer Models ..... 15
Symposium: Tumour Ecology and Evolution at Single Cell Level ..... 16
Symposium: Tumour Dormancy / Persistence / Senescence 17
Symposium: Neoantigens & Vaccines ..... 19
Symposium: Liquid Biopsies ..... 20
Symposium: Chemical Biology ..... 21

**POSTER PRESENTATIONS (Tuesday/Wednesday) ..... 22**

Bioinformatics and Computational Biology ..... 22
Biomarkers in Tissue and Blood ..... 52
Cancer Cell Biology ..... 107
  Poster in the Spotlight ..... 220
Cancer Genomics ..... 224
  Poster in the Spotlight ..... 248
  Poster in the Spotlight ..... 249
Carcinogenesis ..... 252
Drug Resistance ..... 264
  Poster in the Spotlight ..... 268
Epigenetics ..... 314
  Poster in the Spotlight ..... 316
Experimental / Molecular Therapeutics, Pharmacogenomics ..... 328
Immunotherapy ..... 388
Molecular and Genetic Epidemiology ..... 413
Prevention and Early Detection ..... 416
Radiobiology / Radiation Oncology ..... 420
  Poster in the Spotlight ..... 431
Signalling Pathways ..... 432
Translational Research ..... 449
Tumour Biology ..... 480
  Poster in the Spotlight ..... 536
Tumour Evolution and Heterogeneity ..... 554
  Poster in the Spotlight ..... 556
  Poster in the Spotlight ..... 559
Tumour Immunology ..... 570
  Poster in the Spotlight ..... 580
  Poster in the Spotlight ..... 582
  Poster in the Spotlight ..... 593

### Proffered Papers

10-minute talks awarded for the highest scored abstracts, embedded in the scientific symposia sessions. These presentations are not accompanied by a poster.

### Posters in the Spotlight

Tuesday 13 June, 17:30 - 18:30, Poster and Exhibition Hall
Wednesday 14 June, 17:15 - 18:15, Poster and Exhibition Hall

Dedicated sessions taking place in the spotlight area within the Poster and Exhibition Hall. Poster presenters with high scoring abstracts will give short presentations of up to 10 minutes. Their posters will also be available to view during the Poster Discussion Sessions.

Molecular Oncology 17 (Suppl. 1) (2023) 1–597
© 2023 The Authors. Molecular Oncology is published by John Wiley & Sons Ltd on behalf of Federation of European Biochemical Societies
This is an open access article under the terms of the Creative Commons Attribution License, which permits use, distribution and reproduction in any medium, provided the original work is properly cited.

### PROFFERED PAPER PRESENTATIONS

### Rising Star and Top Abstracts

### EACR23-0090

### High risk of metastatic recurrence in colorectal cancer by residual EMP1+ tumor cells

A. Cañellas Socias[1], C. Cortina[1], X. Hernando-Momblona[1], S. Palomo-Ponce[1], C.S.O. Attolini[2], E. Batlle[1]
[1]IRB Barcelona, Cancer Science, Barcelona, Spain
[2]IRB Barcelona, Biostatistics, Barcelona, Spain

**Introduction**

30-40% of colorectal cancer (CRC) patients undergoing curative resection of the primary tumor will relapse in the following years. In these patients, disseminated tumor cells are undetectable until they regenerate the disease in foreign organs, such as the liver and lungs. The identity and features of the residual tumor cells responsible for CRC relapse remain elusive due to the impossibility of analyzing this clinically occult population in patients.

**Material and Methods**

By analyzing the transcriptomes of individual tumor cells in multiple primary CRC patient samples, we discovered that genes associated with an elevated risk of relapse are expressed by a defined subset of tumor cells that we named High Relapse Cells (HRCs). To investigate HRCs, we established a human-like CRC mouse model that, following surgical resection of the primary tumor, undergoes metastatic relapse. We also developed methodology to isolate residual disseminated tumor cells before metastases are detectable.

**Results and Discussions**

HRCs are abundant at invasion fronts, retain an epithelial program and express genes involved in cell adhesion, locomotion and extracellular matrix remodeling. In addition, single-cell profiling demonstrated that residual tumor cells occult in mouse livers after primary CRC surgery resembled the HRC population present in patients. Over time, HRCs in micrometastasis gave rise to multiple cell types, including Lgr5+ stem cell-like cells, and generated macrometastases that can kill the host. Using Emp1 (epithelial membrane protein 1) as a marker gene for HRCs, we tracked and selectively eliminated this cell population. Genetic ablation of HRCs prior to extirpation of the primary CRC prevented metastatic recurrence and mice remained disease-free after surgery. Furthermore, the analysis of the tumor microenvironment revealed that micrometastases generated by HRCs are initially T cell infiltrated but become excluded during metastatic outgrowth coinciding with fibroblast and macrophage recruitment. Treatment with neoadjuvant immunotherapy had limited impact on the primary CRC yet eliminated micrometastases and saved mice from relapsing after surgery.

**Conclusion**

Our findings reveal the features of the tumor cell population responsible for CRC recurrence and anticipate that therapies targeting HRCs, including neoadjuvant immunotherapy, may help prevent metastatic relapse.

### EACR23-0616

### Interferon-encompassing 9p21.3 deletions promote immune evasion and metastasis

F.M. Barriga[1,2], K.M. Tsanov[2], Y.J. Ho[2], N. Sohail[3], A. Zhang[4], T. Baslan[2], A.N. Wuest[2], I. Del Priore[2], B. Meskauskaite[3], G. Livshits[2], D. Alonso-Curbelo[2,5], J. Simon[2], A. Chaves-Perez[2], D. Bar-Sagi[6], C. Iacobuzio-Donahue[7], F. Notta[4], R. Chaligne[3], R. Sharma[3], D. Pe´er[3,8], S.W. Lowe[2,8]
[1]VHIO, Preclinical and Translational Research, Barcelona, Spain
[2]MSKCC, Cancer Biology and Genetics, New York, United States
[3]MSKCC, Computational and Systems Biology, New York, United States
[4]OICR, PanCuRx Translational Research Initiative, Toronto, Canada
[5]IRB Barcelona, Cancer Science, Barcelona, Spain
[6]NYU, Biochemistry, New York, United States
[7]MSKCC, Pathology, New York, United States
[8]HHMI, Howard Hughes Medical Institute, Chevy Chase, United States

**Introduction**

Somatic chromosomal deletions are prevalent in cancer, yet their functional contributions remain ill-defined. Among these alterations, loss of chromosome 9p21.3 is the most frequent homozygous deletions across cancers and portends the worse prognosis of all copy number alterations. The biology of 9p21.3 loss has been largely ascribed to the disruption of tumor suppressor genes CDKN2A/B, which activate the p53 and RB pathways. However, 9p21.3 loss frequently encompasses a cluster of 16 type I interferons (IFNs), whose role in cancer remains poorly understood.

**Material and Methods**

To dissect the biology of 9p21.3 and other large deletions, we developed Molecular Alteration of Chromosomes with Engineered Tandem Elements (MACHETE), which is a rapid and flexible approach based on CRISPR-Cas9 that enables the creation of megabase-sized deletions in cellular models. By applying MACHETE to mouse models of pancreatic cancer and melanoma, we engineered 9p21.3 syntenic deletions: loss of *CDKN2A/B* (Small, DS) or concomitant loss of the type I IFN cluster and *CDKN2A/B* (Large, DL). Using orthotopic transplantation of DS and DL cells in syngeneic hosts, we measured tumor initiation, progression, and response to immune checkpoint blockade. Tumors were then analyzed by immunophenotyping, bulk and single cell transcriptional profiling, and further functional studies.

**Results and Discussions**

We identified that IFN-deficient DL cells evaded immunoediting, had higher rates of metastasis, and showed blunted responses to immune checkpoint blockade. These phenotypes were dependent on an adaptive immune response. Characterization of immune infiltrating cells showed that tumor-derived IFNs dictated the activation of the IFN pathway in antigen presenting cells and to an anti-tumor state in CD8 T cells, which was conserved in human cancers. By using an extended allelic series of deletions, we identified that the tumor-specific Interferon epsilon (*Ifne*) was necessary and sufficient to blunt metastasis.

© 2023 The Authors. Molecular Oncology is published by John Wiley & Sons Ltd on behalf of Federation of European Biochemical Societies

**Conclusion**
Our study identified that 9p21.3 coordinates cell intrinsic and extrinsic tumor suppression by simultaneous disruption of *CDKN2A/B* and type I IFNs. We identify a novel tumor suppressive activity in this locus driven by the poorly characterized *Ifne*, which elicits an anti-metastatic response via adaptive immunity. 9p21.3 affects 15% of all cancers, making this one of the most frequent genetic mechanisms of immune evasion. Overall, the experimental framework we developed will enable the study of large genomic deletions across cancers.

### EACR23-1186
### Single-strand mismatch and damage patterns revealed by single-molecule DNA sequencing

*M.H. Liu[1], C. Benjamin[1], U. Choi[1], R. Bandler[1], E. Lassen[2], M. Grońska-Pęski[1], A. Schwing[1], Z. Murphy[1], D. Rosenkjær[2], S. Picciotto[3], V. Bianchi[4], L. Stengs[4], M. Edwards[4], C. Loh[1], T. Truong[1], R. Brand[5], A.B. Skytte[2], U. Tabori[4], J. Shoag[3], G. Evrony[1]*
*[1]NYU Grossman School of Medicine, Center for Human Genetics & Genomics, New York City, United States*
*[2]Cryos Sperm & Egg Bank, Cryos International, Aarhus, Denmark*
*[3]Case Western Reserve University School of Medicine, Department of Urology, Cleveland, United States*
*[4]Hospital for Sick Children, Division of Haematology/Oncology, Toronto, Canada*
*[5]University of Pittsburgh School of Medicine, Division of Gastroenterology, Pittsburgh, United States*

**Introduction**
Mutations accumulate in the genome of every cell of the body throughout life, causing cancer and other genetic diseases. Almost all of these mosaic mutations begin as nucleotide mismatches or damage in only one of the two strands of the DNA prior to becoming double-strand mutations if unrepaired or misrepaired. However, current DNA sequencing technologies cannot resolve these initial single-strand events from which mutations originate.
**Material and Methods**
We developed a single-molecule, long-read sequencing method that achieves single-molecule fidelity for single-base substitutions when present in either one or both strands of the DNA. It also detects single-strand cytosine deamination events, a common type of DNA damage. This was accomplished by: (1) a large number of independent sequencing passes for each molecule to create a high-quality consensus sequence for each strand; (2) eliminating *in vitro* artifacts; and (3) a computational pipeline distinguishing single- from double-strand changes.
**Results and Discussions**
We profiled 110 human samples from diverse tissues, including from individuals with cancer-predisposition syndromes and tumors. We define the first single-strand mismatch and damage signatures: SBS10ss, SBS14ss, and SBS30ss (ss, single-strand). We find correspondences between these single-strand signatures and known double-strand mutational signatures, which resolves the identity of the initiating lesions. Tumors deficient in both mismatch repair and replicative polymerase proofreading show distinct single-strand mismatch patterns compared to samples deficient in only polymerase proofreading. In the mitochondrial genome, our findings support a mutagenic mechanism occurring primarily during replication.
**Conclusion**
We demonstrate the highest fidelity for single base changes of any DNA sequencing method to date, and the first detection of single-strand changes to DNA. Since the double-strand DNA mutations interrogated by prior studies are only the endpoint of the mutation process, our approach to detect the initiating single-strand events at single-molecule resolution will enable new studies of how mutations arise in healthy tissues and in cancer.

### Symposium: Interface between the Microbiome and Immune System

### EACR23-0186
### INKT cells contribute to colorectal cancer progression inducing neutrophils pro-tumorigenic functions

*G. Lattanzi[1], F. Strati[2], F. Facciotti[2]*
*[1]University of Milan, Department of Oncology and Hemato-oncology, Milano, Italy*
*[2]University of Milano Bicocca, Department of Biotechnology and Biosciences, Milan, Italy*

**Introduction**
invariant Natural Killer T cells (iNKT) account for a relevant fraction of tissue resident, effector T cells in the intestine and are considered an attractive platform for cancer immunotherapies since they overcome major limitations related to the use of conventional T cells. Although iNKT cells are cytotoxic lymphocytes, their functional role in colorectal cancer (CRC) is still controversial, limiting their therapeutical use in CRC.
The tumor microenvironment (TME) is an important factor of CRC. Immune cells and the microbiota, which are elements of the TME, influence each other affecting CRC developmental trajectory.
**Material and Methods**
To gain insights into the functionality of iNKT cells in CRC and their crosstalk with the TME, we examined the immune cell composition and iNKT cell phenotype in CRC patients (n=118) and in three different murine models of CRC. We performed high dimensional single-cell flow cytometry and metagenomic analysis of paired tumor and non-tumor tissue sites. Functional hypothesis were validated *in vitro, in vivo* and with RNA-seq experiments. Survival analyses of CRC patients were performed to clinically translate our findings.
**Results and Discussions**
We observed that iNKT cells are enriched in CRC lesion and express a pro-tumor phenotype with the expression of IL17 and GM-CSF. The tumor-associated pathobiont *Fusobacterium nucleatum,* enriched in our CRC cohort, induced IL17 and GM-CSF expression in iNKT cells blunting their cytotoxic capability and promoting iNKT cell-mediated recruitment of neutrophils with myeloid derived suppressor cells like (MDSCs) phenotype and functions. Additionally, iNKT cell depletion decreased tumor burden by reducing the recruitment of immune suppressive neutrophils. *In*

© 2023 The Authors. Molecular Oncology is published by John Wiley & Sons Ltd on behalf of Federation of European Biochemical Societies

*vivo* activation of iNKT cells with αGalCer restored their anti-tumor functions suggesting that iNKT cells can be modulated to overcome CRC-associated pro-tumor phenotype. CRC patients' survival analyses demonstrated that tumor co-infiltration by iNKT cells and neutrophils correlates with negative clinical outcomes.

**Conclusion**

Our results reveal a functional plasticity of human intestinal iNKT cells in CRC, suggesting a pivotal role of iNKT cells in shaping the TME and cancer developmental trajectory with implications for treatment of CRC.

### EACR23-1485

### Stability of the Gut Microbiota during Anti-PD1 Immunotherapy Defines Complete Response in Melanoma Patients

*A.D. Macandog[1], C. Catozzi[1], A. Nabinejad[1], M. Capone[2], G. Madonna[2], P.F. Ferrucci[3], N. Segata[4], E. Cocorocchio[5], P. Ascierto[2], T. Manzo[1], L. Nezi[1]*

*[1]Istituto Europeo di Oncologia - IRCCS, Experimental Oncology, Milano, Italy*

*[2]Istituto Nazionale Tumori - IRCCS Fondazione "G. Pascal e", Melanoma Cancer Immunotherapy and Development Ther apeutics Unit, Napoli, Italy*

*[3]Istituto Europeo di Oncologia - IRCCS, Tumor Biotherapy Unit, Milano, Italy*

*[4]University of Trento, Department CIBIO, Trento, Italy*

*[5]Humanitas-Gavazzeni, Medical Oncology, Bergamo, Italy*

**Introduction**

The advent of immune checkpoint inhibition (ICI) therapy markedly improved the outcome for melanoma. However, response remains heterogeneous, with about half of the patients being refractory or developing relapse. Although a causal link between the gut microbiota and modulation of antitumor response has been established, current knowledge is limited to findings from cross-sectional analyses. Here, we follow the gut microbiota of melanoma patients over the course of anti-PD1 therapy, to delineate gut changes related to host response and identify gut and host factors involved.

**Material and Methods**

To study response-related gut microbiota changes during ICI, patients with unresectable melanoma from two Italian Hospitals (n=19) were followed at baseline and over the course of anti-PD1 immunotherapy (n=13) to collect fecal and blood samples. Patients were annotated following RECIST 1.1 classification and PFS. Additionally, baseline fecal samples from tumor-free subjects were used as reference to compare gut diversity trends between response groups at therapy. Finally, cross-study validation was carried on metagenomes (n=281) from Europe (n=4), UK (n=2) and USA (n=3) baseline cohorts.

**Results and Discussions**

Our results demonstrate that the gut microbiota is fairly variable during ICI therapy. However, changes are less pronounced among complete responders (CR), especially at later cycles. We identify and validate a core of longitudinally stable gut microbiota taxa in CR, which comprise mostly Clostridia taxa. These core CR taxa associate consistently with positive systemic markers, supporting their immune modulatory potential. At the functional level, our data demonstrate a key role for specific bacterial cell components in driving a productive immune response.

**Conclusion**

Overall, we propose microbiota stability during ICI therapy as a consequential feature of an immune-beneficial Clostridia-rich gut among complete responders.

### Symposium: Drug Resistance

### EACR23-0444

### Exportin 1 inhibition prevents neuroendocrine transformation on targeted therapy in the lung and prostate through SOX2 downregulation

*A. Quintanal-Villalonga[1], V. Durani[1], A. Sabet[1], E. Redin-Resano[1], K. Kawasaki[1], S. Zaidi[1], H. Sridhar[1], P. Manoj[1], W. Karthaus[2], M. Haffner[3], C.L. Sawyers[4], C.M. Rudin[1]*

*[1]Memorial Sloan Kettering Cancer Center, Medicine, New York, United States*

*[2]École polytechnique fédérale de Lausanne, Medicine, Lausanne, Switzerland*

*[3]Fred Hutchinson Cancer Center, Pathology, Seattle, United States*

*[4]Memorial Sloan Kettering Cancer Center, Human Oncology & Pathogenesis Program, New York, United States*

**Introduction**

In lung and prostate adenocarcinomas (ADs) treated with targeted inhibitors, lineage plasticity mediates neuroendocrine (NE) transformation in up to 14% and 30% of *EGFR*-mutant lung and AR-dependent prostate tumors respectively, and leads to extremely poor prognosis. Even if ADs at high risk of transformation (*TP53*/*RB1* loss) can be identified, no therapy to prevent transdifferentiation is available. Previously we have described a strong dependency of small cell lung carcinomas (SCLCs) on exportin 1 (XPO1), a nuclear protein exporter involved in cancer with potent and safe inhibitors available in the clinic, such as selinexor. The present work aims to study the role of XPO1 in NE transformation.

**Material and Methods**

Exportin 1 expression was assessed in transforming clinical specimens. Isogenic *TP53*/*RB1*-knock out AD cell lines were generated and sensitivity to the exportin 1 inhibitor selinexor was assessed in these. Patient-derived xenograft (PDX) models of NE transformation were treated with targeted therapy, selinexor or their combination, and transcriptome sequencing was performed in these, followed by functional validation.

**Results and Discussions**

XPO1 expression was upregulated in clinical specimens undergoing NE transformation. Interestingly, increased exportin 1 levels were observed already in pre-transformation ADs, suggesting that exportin 1 upregulation may occur at early stages of transformation. Consistently, we observed increased XPO1 mRNA expression on *TP53*/*RB1*-mutant lung and prostate AD clinical samples relative to wild type tumors, as well as increased exportin 1 expression and sensitivity to selinexor after *TP53*/*RB1*-knock out in isogenic AD cell lines, suggestive of an increased dependency on exportin 1 in tumors at high risk of NE transformation. In this line,

© 2023 The Authors. Molecular Oncology is published by John Wiley & Sons Ltd on behalf of Federation of European Biochemical Societies selinexor prevented NE transformation and dramatically delayed relapse in lung and prostate xenograft models treated with targeted therapy. In these, exportin 1 inhibition prevented upregulation of SOX2, a transcription factor necessary for NE-T. SOX2 ectopic overexpression rescued the NE transformation phenotype in selinexor-treated NE transformation models, suggesting that SOX2 downregulation by selinexor is the mechanism by which NE transformation is inhibited.

**Conclusion**

We nominate exportin 1 as a therapeutic target to constrain plasticity and prevent NE transformation. The clinical availability of selinexor will allow immediate clinical translation of these results in a disease setting with extremely limited therapeutic options.

### EACR23-0879
### Sequential redox vulnerabilities with therapeutic potential during the acquisition of drug resistance in BRAFV600E lung adenocarcinoma

*D. Santamaria[1], M.J. Nokin[2], E. Darbo[3], E. Richard[3], S. San José[1], S. de Hita[1], V. Prauzet-Mauleon[3], B. Turcq[3], B. Koopmansch[4], F. Lambert[4], J. Horne[5], E. Ziemons[5], A. Villanueva[6], A. Biomme[7], D. Cataldo[2], P. Porporato[8], E. Nadal[9], P. Lito[10], M. Awad[11], C. Ambrogio[8]*

*[1]CIC-IBMCC CSIC- Molecular Mechanisms of Cancer Program, CSIC-University of Salamanca, Salamanca, Spain*

*[2]Laboratory of Biology of Tumor and Development LBTD-GIGA-Cancer, University of Liege, Liege, Belgium*

*[3]Bordeaux Institute of Oncology BRIC, INSERM U1312- University of Bordeaux, Bordeaux, France*

*[4]University Hospital Center of Liege, Department of human genetics, Liege, Belgium*

*[5]Laboratory of Pharmaceutical Analytical Chemistry- CIRM, University of Liege, Liege, Belgium*

*[6]Catalan Institute of Oncology ICO, Oncobell Program- Bellvitge Biomedical Research Institute IDIBELL, Barcelona, Spain*

*[7]Laboratory of Cancer Signaling- GIGA-Stem Cells, University of Liege, Liege, Belgium*

*[8]Molecular Biotechnology Center- University of Torino, Department of Molecular Biotechnology and Health Sciences, Torino, Italy*

*[9]Catalan Institute of Oncology- Oncobell Program- IDIBELL, Department of Medical Oncology, Barcelona, Spain*

*[10]Memorial Sloan Kettering Cancer Center, Department of Medicine, New York, United States*

*[11]Dana–Farber Cancer Institute, Department of Medical Oncology, Boston, United States*

**Introduction**

The current therapy for *BRAF*$^{V600E}$-driven lung cancer consists of a dual blockade of RAF and MEK kinases often combining dabrafenib with trametinib (D/T). This regimen results in extended survival when compared to single agent treatments but, as with other targeted therapies, disease progression is unavoidable. There is limited information of how *BRAF*$^{V600E}$-driven lung adenocarcinomas adapt to this targeted treatment and persist before clinical relapse is detected. At this point, a significant fraction of resistant tumours display mutations in the RAS-ERK pathway and counteract the inhibitors effect by reactivating oncogenic signalling. While a number of molecular mechanisms mediating disease progression have been suggested, this has not yet resulted in novel second-line targeted treatments. As a consequence, patients displaying resistant disease are treated with rather inefficient palliative chemo-immunotherapy regimens.

**Material and Methods**

In this work, we have performed whole genome Crispr-screening, generated PDX-derived cell lines and implemented drug treatments *in vivo* to investigate the mechanistic basis and associated vulnerabilities of early stage D/T resistance in *BRAF*$^{V600E}$-driven lung cancer.

**Results and Discussions**

We demonstrate here that oxidative stress together with the concomitant induction of antioxidant responses is an early feature boosted by D/T treatment in *BRAF*$^{V600E}$-mutant lung cancer. Yet, the nature of the oxidative damage and the choice of redox detoxification systems display substantial differences during the process leading to drug resistance. While persister cells suffer from lipid peroxidation and rely on GPX4 to prevent ferroptosis-driven cell death, D/T resistant tumours harbouring *NRAS* secondary mutations enhance cystine transport to boost antioxidant responses. Accordingly, timely inhibition of these detox programs by GPX4 or HDAC inhibitors decrease resistant cell viability and extend therapeutic efficacy.

**Conclusion**

- Redox-dependent vulnerabilities are a common feature during the process leading to the acquisition of D/T resistance in *BRAF*$^{V600E}$-driven lung cancer.
- The nature of the oxidative detoxification programs elicited by early persister cells and fully resistant tumours are mechanistically different and identify stage-specific therapeutic vulnerabilities.
- Timely inhibition of these detox programs by GPX4 or HDAC inhibitors decrease resistant cell viability in a ROS dependent manner and extends both therapeutic efficacy and survival *in vivo* using PDX-derived models.

### Symposium: Mechanism Based Clinical Trial

### EACR23-0842
### Anaplastic Lymphoma Kinase receptor can be effectively targeted in Consensus Molecular Subtype 1 (CMS1) colorectal cancer

*M. Mazzeschi[1], M. Sgarzi[1], D. Romaniello[1], F. Ambrosi[2], A. Morselli[1], C. Girone[1], F. Pagano[1], K. Rihawi[3], G. D'Uva[1], M. Ferracin[1], M. Fiorentino[1], A. Ardizzoni[1], B. Győrffy[4], R. Palmer[5], N. Li[6], C.J. Lengner[7], Y.P. Mossé[8], M. Lauriola[1]*

*[1]University of Bologna, Department of Medical and Surgical Sciences, Bologna, Italy*

*[2]Maggiore Hospital- AUSL Bologna, Pathology Unit, Bologna, Italy*

*[3]IRCSS Azienda-Ospedaliero Universitaria di Bologna, Medical Oncology, Bologna, Italy*

*[4]Semmelweis University,*

© 2023 The Authors. Molecular Oncology is published by John Wiley & Sons Ltd on behalf of Federation of European Biochemical Societies

Department of Bioinformatics and 2nd Department of Pedi atrics, Budapest, Hungary
[5]Institute of Biomedicine- Sahlgrenska Academy- Universit y of Gothenburg,
Department of Medical Biochemistry and Cell Biology, Gothenburg, Sweden
[6]School of Veterinary Medicine- University of Pennsylvani a, Department of Biomedical Sciences, Philadelphia, United States
[7]Perelman School of Medicine and Institute for Regenerati ve Medicine- University of Pennsylvania,
Department of Cell & Developmental Biology, Philadelphia, United States
[8]Perelman School of Medicine at the University of Pennsyl vania- Division of Oncology and Center for Childhood Ca ncer Research- The Children's Hospital of Philadelphia, Department of Pediatrics, Philadelphia, United States

**Introduction**

Colorectal cancer (CRC) is characterized by specific molecular features that contribute to a significant degree of inter-individual heterogeneity. This heterogeneity underscores the need for personalized medicine to treat patients effectively. The Consensus Molecular Subtypes (CMSs) classification stratifies CRC into four well-defined molecular subgroups, providing support to the use of targeted therapies. Unfortunately, so far only a few targetable biomarkers are known in the CRC setting, leaving a big portion of patients not eligible for any individualized treatment regimen. Through a bioinformatic meta-analysis of a dataset of 1700 CMS-stratified CRC patients, we determined that high levels of anaplastic lymphoma kinase (ALK) expression were negatively correlated with relapse-free survival (RFS) exclusively in the CMS1 subtype[1]. Conversely, we did not observe such a correlation in the other 3 subgroups.

**Material and Methods**

Stemming from these observations, we generated the hypothesis that ALK pharmacological inhibition may elicit therapeutic potential in CMS1 patients. Thus, we tested both small ALK-TKIs and an ALK-directed antibody-drug conjugate (ADC) on many CRC models stratified according to the CMS classification, through several *in vitro* (2D-3D) and *in vivo* assays. To unveil the mechanism, we applied single-cell sequencing on CRC patients and tested for the abundance of ALK ligands.

**Results and Discussions**

Notably, ADC-based inhibition of ALK had a remarkable effect in mice, by blunting tumor growth. This may be due in part to the activity of the two ALKAL1 and ALKAL2 ligands, which activate ALK signaling and may contribute to the initiation of cell migration and invasion, thus facilitating metastatic spread. Interestingly, by single-cell RNA sequencing, we found that the above-mentioned ligands are expressed both in the epithelial tumor tissue and in the cancer-associated fibroblasts, suggesting a paracrine secretion sustaining cancer cells, which may explain the stronger efficacy of the ADC compared to the TKIs. Mechanistically, we found that CMS1 cells display several mRNA copies of both ALK and ALKAL2 ligand, along with a higher ALK protein amount compared to the other subtypes, suggesting a role for ALK abundance in the differential response to its inhibition.

**Conclusion**

Collectively, our data suggest that ALK inhibition by means of ALK-directed antibody-drug conjugate may represent an attractive target that may broaden the therapeutic opportunities for CMS1 colorectal cancer patients.

### EACR23-1011

### The diversity of T-cell repertoire in tumor micro-environment is correlated with the mutations induced by temozolomide treatment in the ARETHUSA clinical trial

G. Crisafulli[1], A. Sartore-Bianchi[2], L. Lazzari[3], F. Pietrantonio[4], P. Battuello[5], A. Bartolini[6], F. Di Nicolantonio[7], S. Marsoni[3], S. Siena[2], A. Bardelli[8]
[1]IFOM,
Bio Informatic Group BIG & Laboratory of Molecular Onc ology, Milano, Italy
[2]Niguarda Cancer Center and Department of Oncology an d Hemato-Oncology,
Grande Ospedale Metropolitano Niguarda and Università degli Studi di Milano, Milano, Italy
[3]IFOM, Precison Oncology Lab, Milano, Italy
[4]Fondazione IRCCS Istituto Nazionale dei Tumori, Department of Medical Oncology, Milano, Italy
[5]IFOM and University of Torino,
Bio Informatic Group BIG & Laboratory of Molecular Onc ology, Milano, Italy
[6]Candiolo Cancer Institute- FPO - IRCCS, Fondazione del Piemonte per Oncologia, Candiolo, Italy
[7]Candiolo Cancer Institute- FPO - IRCCS and University o f Torino, Department of Oncology, Candiolo, Italy
[8]University of Torino and Candiolo Cancer Institute, Present address: Department of Oncology- University of T orino- Italy and IFOM ETS – The AIRC Institute of Molecu lar Oncology, Milano, Italy

**Introduction**

Mismatch repair (MMR) proficient (MMRp) metastatic colorectal cancers (mCRCs) are usually unresponsive to immunotherapy, while MMR deficient (MMRd) tumors often respond to immune checkpoint blockade (ICB). In the ARETHUSA clinical trial (NCT03519412), we reported that the MMR machinery can be pharmacologically inactivated by temozolomide (TMZ) and that this process can be monitored through mutational signature analysis and MMR gene mutations. We demonstrated that a subset of initially MMRd CRC patients achieved long lasting disease stabilization from ICB after receiving temozolomide (TMZ) priming treatment which caused the emergence of an inactivating *MSH6* mutation and the TMZ mutational signature. We reported that TMZ treatment can induce heterogeneous genomic consequences, leading to three different patient subtypes, identifiable through blood and tissue samples analysis. In particular, we described a dose-dependent accumulation of mutations with a molecular signature visible at the clonal level (subtype B2) in 2/21 patients, at subclonal level (subtype B1) in 15/21 patients and not present in 4/21 patients (subtype A).

**Material and Methods**

The abundance and diversity of T-cell receptors (TCRs) in tumor micro-environment after TMZ treatment were analysed in patients enrolled in the ARETHUSA trial.

© 2023 The Authors. Molecular Oncology is published by John Wiley & Sons Ltd on behalf of Federation of European Biochemical Societies

**Results and Discussions**
We identified expanded clonotypes in patients that acquired a clonal increase of mutations due to the priming treatment. TCR diversity of immune infiltrate confirmed the three-class classification based on Tumor Mutational Burden (TMB) and mutational signatures analysis. We also found a linear correlation (Spearman rank corr, pvalue 0.0048) between diversity of T-cells (Simpson Index) in the micro-environment and the number of mutations induced by TMZ. According to this model, increasing the number of TMZ-induced mutations would increase the likelihood that TMZ-driven neoantigens will produce a clonal expansion of T-cells in the tumor. Indeed, the subset of TMZ-treated patients whose tumors displayed acquired *MSH6* mutation, TMZ mutational signature and increased TMB not only achieved temporary disease stabilization upon ICB, but also showed clonal expansion of T-cells in the tumour microenvironment post TMZ treatment.
**Conclusion**
Collectively, these findings indicate that the inactivation of MMR achieved through TMZ priming is able to reshape the immune microenvironment and could modulate the immune response in CRC.

### Symposium: Challenging Drug Targets

### EACR23-0016
### Integrative multi-omic analyses of lung cancer clinical samples proves AKT and MYC as regulators of lung adenocarcinoma to squamous cell lung cancer transdifferentiation

*T. Sen*[1]
[1]*Tisch Cancer Institute- Mount Sinai, Oncological Sciences, New York, United States*
**Introduction**
Lineage plasticity, the ability to transdifferentiate among distinct phenotypic identities, facilitates therapeutic resistance in multiple cancers. In lung adenocarcinomas (LUADs), this phenomenon includes small cell and squamous cell (LUSC) histologic transdifferentiation in the context of acquired resistance to targeted inhibition of driver mutations. The incidence of transdifferentiation into squamous carcinoma in *EGFR* mutant tumors, the setting where this histologic shift has been most extensively described, occurs in up to 9% of cases relapsed on osimertinib and has been associated with poor prognosis. The paucity of well-annotated pre- and post-transdifferentiation clinical samples has precluded the performance of informative molecular analyses: little is known about the molecular mechanisms leading to this histological transition.
**Material and Methods**
Multi-parameter profiling of mixed histology (LUAD/LUSC) tumors, together with pre- and post-transdifferentiation clinical samples, could provide insight into factors licensing lineage plasticity between these histologies and promoting squamous transdifferentiation of LUAD. We performed detailed genomic (whole-exome sequencing), epigenomic, transcriptomic (RNAseq), proteomic (antibody arrays), and single-cell RNAseq and ATACseq characterization. Clinical findings were validated in preclinical models including cell lines and patient-derived xenograft treatments.
**Results and Discussions**
Our results suggest that LUSC transdifferentiation is primarily driven by transcriptional reprogramming rather than mutational events, and indicate that the resulting squamous tumors retain transcriptomic and methylation profiles of their previous LUAD state. We observed coordinated upregulation of PI3K/AKT, MYC, and PRC2 pathway genes in the LUSC component of mixed histology tumors. Concurrent activation of PI3K/AKT and MYC induced squamous features in *EGFR*-mutant LUAD preclinical models, further augmented under the selective pressure of osimertinib. Pharmacologic inhibition of EZH1/2 in combination with osimertinib prevented relapse and squamous transdifferentiation in an EGFR-mutant patient-derived xenograft model, and inhibition of EZH1/2 or PI3K/AKT signaling re-sensitized resistant transdifferentiated LUSC tumors to osimertinib.
**Conclusion**
Our findings provide the first comprehensive molecular characterization of LUSC transdifferentiation, suggesting putative drivers and promising therapeutic targets to constrain or prevent lineage plasticity in this setting.

### EACR23-0212
### SET, Inhibitor 2 of phosphatase PP2A is a new molecular target in KMT2A (MLL) rearranged acute myeloid leukaemia

*A. Di Mambro*[1]*, Y. Arroyo*[1]*, F. Tiziana*[2]*, M. Randles*[3]*, L. Cozzuto*[4]*, V. Rajeeve*[5]*, A. Cevenini*[2]*, G. Esposito*[2]*, J. Ponomarenko*[4]*, C. Lucas*[3]*, P. Cutillas*[5]*, J. Gribben*[5]*, O. Williams*[6]*, Y. Calle*[1]*, B. Wrench*[5]*, M.T. Esposito*[1]
[1]*University of Roehampton, Life and Health Sciences, London, United Kingdom*
[2]*University of Napoli Federico II, Dipartimento di Medicina Molecolare e Biotecnologie Med iche, Napoli, Italy*
[3]*University of Chester, Medical School, Chester, United Kingdom*
[4]*Centre Genomic Regulation, Centre Genomic Regulation, Barcelona, Spain*
[5]*Queen Mary University of London, Barts Cancer Institute, London, United Kingdom*
[6]*University College London, Great Ormond Street Institute of Child Health, London, United Kingdom*
**Introduction**
Acute leukaemias associated with the chromosomal translocations involving the *Histone-Lysine-N-methyltransferase 2A (KMT2A)* gene are highly aggressive and chemorefractory. Transcriptomics-based characterization and chemical interrogation identified kinases as key drivers of *KMT2A*-rearranged (*KMT2A*-R) leukemic cells' survival and resistance. In contrast, the role of protein phosphatases is unexplored. In this study, we uncover the essential role of the oncogene *SET,* encoding the endogenous inhibitor of the tumor suppressor SER/THR phosphatase PP2A, in the *KMT2A*-R leukaemia pathogenesis.

© 2023 The Authors. Molecular Oncology is published by John Wiley & Sons Ltd on behalf of Federation of European Biochemical Societies

**Material and Methods**

The expression of *SET* was analysed in a large acute myeloid leukaemia (AML) RNA-seq dataset. The *in vitro* experiments were performed on *KMT2A*-R leukemic cell lines, primary samples and patient-derived xenotransplants (PDX). *SET* silencing was achieved by lentiviral vector-mediated delivery of specific shRNAs. The cellular effects of Fingolimod (FTY720) were evaluated by proliferation, cell cycle and apoptotic cell death assays; the effects on gene expression and protein-phosphorylation were analysed by RNA-seq and phospho-proteomics, respectively.

**Results and Discussions**

*SET* mRNA was found expressed in all the AML subtypes and SET expression positively correlated with poor overall survival in AML. Silencing of *SET* by shRNA completely abolished the clonogenic ability of *KMT2A*-R leukemic cells and transcription of the *KMT2A* target genes *HOXA9* and *HOXA10*. Immunoprecipitation and ChIP assays showed that SET interacted with both KMT2A wild-type and fusion proteins and was recruited to the *HOXA10* promoter. Consistent with these results, treatment with FTY720 disrupted SET-PP2A interaction, led to cell cycle arrest and increased the response of *KMT2A*-R PDX to standard chemotherapy *in vitro*. These effects were mirrored by the decreased phosphorylation of key PP2A targets, namely ERK1, ARKB and MYC and reduced the expression of key genes critical for sustaining this disease, as revealed by phospho-preoteomics and RNA-seq. Silencing *PPP2CA*, the gene encoding the catalytic subunit of PP2A, restored phospho-ERK1 levels and rescued the KMT2A-R cells from FTY720-induced apoptosis, indicating that the FTY720-effects observed in *KMT2A*-R cells specifically depended on PP2A activation.

**Conclusion**

Our results identify SET as a novel player in *KMT2A*-R leukaemia and provide evidence that SET antagonism could serve as a novel strategy to treat this aggressive and poorly treatable form of leukaemia.

### Joint EACR-SIC Symposium: Spatial Transcriptomics

### EACR23-0574
### Phenotypic and spatial ecological heterogeneity in breast cancer patient-derived tumour xenografts

*G. Lerda[1], Y. Eyal-Lubling[1], C. Mulvey[1], A. Fatemi[1], T. Whitmarsh[2], S.F. Chin[1], R. Manzano Garcia[1], C.R.U.K. IMAX-T Grand Challenge Team[1], C. Caldas[1]*
*[1]University of Cambridge, CRUK Cambridge Institute, Cambridge, United Kingdom*
*[2]University of Cambridge, Institute of Astronomy, Cambridge, United Kingdom*

**Introduction**

Tumours are complex ecosystems where several neoplastic and non-transformed populations (the tumour microenvironment – TME) interact in heterogeneous and dynamic ways. Descriptive studies in patients often lack the temporal and spatial resolution needed to fully recapitulate this variability. Mouse models allow longitudinal and spatially extensive studies, representing a necessary complement to descriptive human analyses. Among *in vivo* models, patient-derived xenografts (PDTXs) have been excluded from ecological studies due to the absence of a human TME and the immune-compromised status of the host. Evidence however suggests that PDTXs might be good candidates for ecosystem analyses because they contain composite tumour and TME compartments where cells form functional interactions. To demonstrate this, we perform the first in-depth description of PDTX ecosystems and confirm their potential as model systems for tumour ecology.

**Material and Methods**

We characterize the ecosystem of selected breast cancer xenografts using single cell RNA-sequencing (scRNA-seq), three-dimensional imaging with serial two-photon tomography (STPT), two-dimensional *in situ* transcriptomics and imaging mass cytometry (IMC).

**Results and Discussions**

High-resolution scRNA-seq reveals unprecedented complexity in the PDTX ecosystem. We describe multiple TME cell types that overlap with populations found in other pre-clinical and clinical studies. These cells have varying frequencies in different PDTXs and include immune populations with tumour-reactive phenotypes. Expanding previous descriptions, we show pervasive inter- and intra-model transcriptional variation in the PDTX tumour compartment. Using ligand-receptor analysis, we study how different populations interact with each other and generate complex ecological dynamics. Using STPT, IMC and *in situ* transcriptomics we show that tumour and TME cell distribution is largely model-specific, suggesting a concerted organisation of PDTX ecosystems by the cell-autonomous compartment. In agreement with this, we show that regional variations in tumour phenotypes result in heterogeneous TME features within specific samples.

**Conclusion**

Our work unveils the phenotypic and spatial heterogeneity of PDTX ecosystems, showing their variable organisation between and within models. By providing evidence of potential interactions between different populations, our study suggests the functional relevance of this variation. Overall, these findings support a wider application of PDTXs for the study of tumour ecology.

### EACR23-1499
### Transcriptome Profiling of Metastatic-Niche in a Murine Model of Triple-Negative Breast Cancer Lung Metastases

*S. Lin[1], T. Carroll[2], B. Elshenawy[1], V. Bridgeman[3], L. Ombrato[3], S. Smart[1], P. Kinchesh[1], D. Allen[1], V. Kersemmans[1], I. Malanchi[3], A. Banham[4], A. Harris[1]*
*[1]University of Oxford, Oncology, Oxford, United Kingdom*
*[2]University of Oxford, Ludwig Institute of Cancer Research, Oxford, United Kingdom*
*[3]Francis Crick Institute, Tumour-Host Interaction Laboratory, London, United Kingdom*
*[4]University of Oxford, Radcliffe Department of Medicine- Nuffield Division of Clinical Laboratory Sciences-, Oxford, United Kingdom*

© 2023 The Authors. Molecular Oncology is published by John Wiley & Sons Ltd on behalf of Federation of European Biochemical Societies

**Introduction**
20% of all breast cancers is attributed to triple-negative breast cancer (TNBC). TNBC metastasizes to visceral organs including the lungs. Secondary lung metastases have shown modest efficacy and/or resistance to traditional targeted therapies and chemotherapy. Poor response to therapies addresses the complexity of tumour and the surrounding microenvironment. Thus, interrogating tumour microenvironment (metastatic-niche) is needed to understand resistance mechanisms and find new therapeutic targets.
**Material and Methods**
An experimental lung metastasis *in vivo* model was generated via intravenous inoculation of murine syngeneic TNBC cell line (E0771). MRI imaging and histological staining of metastatic lungs were performed to characterize tumour growth and vascularization. For transcriptional analysis, spatial FAC-sorting of metastatic-niche cells was achieved using a new niche labelling technology. Single-cell RNA-seq droplet-based platform was used for gene expression profiling of isolated metastatic-niche cells and stromal cells from healthy lungs.
**Results and Discussions**
MRI *in vivo* imaging and histological analysis of metastatic lungs showed that E0771 TNBC adopt non-angiogenic and angiogenic growth patterns. Single-cell expression analysis of early- and late-metastatic niches reveals spatial and temporal remodelling of metastatic-niche stroma compared to the normal lung counterpart. At early stage of metastases, a subset of capillary type II ECs upregulates MHC class II antigen-processing machinery indicating its role in generating an immunosuppressive tumour microenvironment early on. In addition, inflammatory mesothelial cells increased with an enrichment of interferon and cytokine-mediated pathways. In advanced metastases, several stromal cells emerge including neophalanx/breach/tip ECs and cancer-associated mesothelial (CAM) cells. Neophalanx/breach/tip ECs and capillaries present in the late niche upregulate genes related to glycolysis and oxidative phosphorylation and downregulate sphingolipid metabolism demonstrating high production of energy to support angiogenesis. CAM cells detected at late stage of metastases exhibited heterogenous phenotype with an upregulation of epithelial–mesenchymal transition (EMT) pathway.
**Conclusion**
Targeting those identified immunosuppressive ECs and CAM cells could be an effective strategy for treatment of metastatic disease. However, further investigation of the mechanisms driven by CAM cells to support angiogenesis and metastatic progression is warranted.

### Symposium: Cell Adaptation and Competition

### EACR23-0105
### Early dissemination highly enhances liver metastasis development during intestinal tumorigenesis

*J. Pannequin[1], Z. Homayed[1], G. Belthier[1], T. Lahlou[1], L. Bassaganyas[1], C. Bouclier[1], L. Bansard[1], M. Newger[2], J.F. Bourgaux[3], A. Erturk[2]*
*[1]Institut de Génomique Fonctionnelle, Physiology and Cancer, Montpellier, France*
*[2]Institute for Tissue Engineering and Regenerative Medicin e, Helmholtz Center, Munich, Germany*
*[3]Carémeau Hospital, Service d'Hépato-Gastroentérologie, Nîmes, France*
**Introduction**
Although the vast majority of cancer deaths are due to metastasis, the mechanisms underlying this process remain poorly described. The literature on the subject mainly focuses on the late stages of tumorigenesis, ignoring early dissemination, especially in colorectal cancer CRC. Our first goal is to show that a process of early tumor dissemination occurs in CRC. Our second goal is to assess the role of early disseminated cells (eDTC) in distant organs.
**Material and Methods**
We have generated an inducible mouse model that enables us to lineage trace dissemination at very early stages of tumoral development thanks to the expression of RFP and deletion of the APC gene specifically in the intestinal epithelium. eDTCs were searched in the liver, the main organ that is prone to getting colonized by metastatic CRC cells, using tissue clearing followed by microscopy, intravital live imaging, and immunolabelling. The impact of eDTCs in the liver was assessed using different global approches: scRNAseq, spatial transcriptomics, CyTOF/hyperion and validated with immunostaining. Elisa assays were performed to quantified circulating cytokines in the plasma of mice.
**Results and Discussions**
In the liver of mice bearing intestinal adenomas, we first demonstrate the presence of eDTCs along with a microenvironmental remodeling characterized by a strong infiltration of myeloid cells and a change in the extracellular matrix. Concomitantly, in mouse plasma, we detect higher level of cytokines involved in the myeloid cell enrichment and this was validated in the blood of patients with intestinal adenomas. Finally, this liver remodeling has a strong impact on metastatic colonization where we have demonstrated that it enhances the welcoming of future waves of late metastatic cells.
**Conclusion**
This project functionally validates for the first time the existence of an early dissemination process in CRC in mice and proposes a causal role of these cells in an early pre-metastatic niche preparation. Further studies to deeply characterize the liver remodeling are ongoing in order to identify potential actionable targets to prevent this early pre-metastatic niche preparation.

### EACR23-0758
### Pathway-derived molecular subtyping unveils a subtle shift in proliferative-to-differentiated homeostasis associated with outcome in colorectal cancer

*S. Malla[1], R.M. Byrne[1], M.W. Lafarge[2], S.M. Corry[1], N.C. Fisher[1], P.K. Tsantoulis[3], S.J. Leedham[4], V.H. Koelzer[2], O.J. Sansom[5], P.D. Dunne[1]*
*[1]Queen's University Belfast, The Patrick G. Johnston Centre for Cancer Research, Belfast, United Kingdom*

© 2023 The Authors. Molecular Oncology is published by John Wiley & Sons Ltd on behalf of Federation of European Biochemical Societies

[2]University Hospital of Zürich, Department of Pathology and Molecular Pathology, Zürich, Switzerland
[3]Université de Genève, Faculty of Medicine, Geneva, Switzerland
[4]University of Oxford, Wellcome Trust Centre Human Genetics, Oxford, United Kingdom
[5]Cancer Research UK Beatson Institute, Cancer Research UK Beatson Institute, Glasgow, United Kingdom

**Introduction**

Individual gene expression-based subtyping, such as consensus molecular subtyping (CMS) of colorectal cancer (CRC), has been able to establish transcriptomic CRC tumour landscape relating to its molecular features and prognostic significance. Here, we propose, rather than individual gene-level classification, pathway-level subtyping may offer broader insights into biological behaviour of a tumour, which ultimately may be more informative for stratification and therapeutic decision-making.

**Material and Methods**

Single sample pathway scores were generated from CRC transcriptomic dataset (n=361 primary tumours diagnosed at all stages; GSE156915) and were utilised to perform unsupervised *k*-means clustering for class discovery and classifier development. Subsequently, the defined tumour clusters were comprehensively characterised with a series of *in silico* methods, immunohistochemistry (IHC) and computational image analysis. For validation, independent bulk (*n*=>1000 patients) and single cell (n=60000 cells) cohorts were used, including both human CRC tissue and mouse models.

**Results and Discussions**

Pathway-level CRC classification defined three "**Pathway-Derived Subtypes**" (PDS1-3), molecularly and biologically unique in their transcriptomic landscape. While PDS2 represent tumours with stroma/immune-dense, regenerative stem-like features with elevated pathways such as TGF-β and inflammatory response, PDS1 and PDS3 display contrasting epithelial biology. PDS1 tumours showed enrichment for MYC targets, highly proliferative with stem-like features and good prognosis, while PDS3 revealed a heterogeneous yet differentiated-like phenotype associated with reduced polycomb repressive complex 2 (PRC)-mediated gene repression and poor prognosis. Furthermore, the inverse correlation between MYC-PRC targets emerged in association with PDS1 and PDS3 across many bulk and single cell CRC cohorts, which was determined to represent a subtle shift in proliferative vs repressive biology using trajectory analysis in mouse and human CRC tissue.

**Conclusion**

This study proposes a novel PDS classification of CRC with biologically and clinically divergent tumours between more proliferative, better prognosis PDS1 and phenotypically 'lethargic', poor prognosis PDS3. This underlying tumour biology reflects the MYC-PRC targets association underpinning stem-to-differentiated-like shift that may expose therapeutic vulnerabilities and inform future treatment strategies.

### Symposium: Tumour Metabolism and Epigenetic Control

### EACR23-0270
### Non-liver cancers rewire liver metabolism in the host

O. Goldman[1], E. Hajaj[1], L. Adler[1], C. Tommaso[2], N. Darzi[1], H. Tishler[1], Y. Ariav[1], L. Fellus-Alyagor[3], R. Oren[3], E. David[4], R. Seger[5], N. Erze[6], I. Amit[4], A. Tanay[7], J.S. Lee[8], I. Wolf[9], A. Erez[1]
[1]Weizmann Institute of Science, Molecular Cell Biology., Rehovot, Israel
[2]Weizmann Institute of Science, Brain Science, Rehovot, Israel
[3]Weizmann Institute of Science, Veterinary Resources, Rehovot, Israel
[4]Weizmann Institute of Science, System Immunology, Rehovot, Israel
[5]Weizmann Institute of Science, Immunology and Regenerative Biology, Rehovot, Israel
[6]Tel Aviv University, Sackler Faculty of Medicine, Tel Aviv, Israel
[7]Weizmann Institute of Science, Computer Science and Applied Mathematics, Rehovot, Israel
[8]Sungkyunkwan University, Artificia Intelligence, Suwon, South Korea
[9]Tel Aviv University, Sourasky Medical Center, Tel Aviv, Israel

**Introduction**

During the late stages of tumor progression, 80% of cancer patients are prone to deteriorate to a metabolic phenomenon termed cancer-associated cachexia (CAC). CAC manifests as weight loss and muscle and adipose tissue atrophy and is responsible for at least 20% of cancer deaths. To date, no biomarkers or efficient therapy for counteracting CAC exists.

The liver is the main metabolic organ in mammals. One of the crucial metabolic activities in the liver is disposing of excess toxic ammonia by converting it to urea via the urea cycle (UC). We previously demonstrated a downregulation of the UC in the livers of Breast cancer bearing mice and children. Thus, we hypothesized that non-hepatic cancers could regulate liver metabolism and contribute to cancer growth and the systemic manifestation of CAC.

**Material and Methods**

Using cutting edge technologies such as scRNAseq, CyTOF, AAV vectors, and live infusions of labeled isotopes, we characterized change in liver metabolism throughout carcinogenesis.

**Results and Discussions**

We found in breast and pancreatic cancer bearing mice liver metabolic alterations at the transcriptional, translational, and functional levels starting early during carcinogenesis. Interestingly, we find that the liver secretes increasing levels of CCL2, resulting in recruitment of myeloid cells to the liver. Liver NPCs scRNAseq, together with the hepatocytes RNAseq, suggest inter-cellular crosstalks between immune cells and hepatocytes, resulting in activation of ERK-IL6-STAT3 pathway. This activation subsequentially causes for depletion of HNF4α via miRNA-24. Furthermore, in-vivo inhibition of this

© 2023 The Authors. Molecular Oncology is published by John Wiley & Sons Ltd on behalf of Federation of European Biochemical Societies pathway maintained the expression levels of HNF4α, restored liver metabolism, and reduced tumor size. Notably, the consequent changes in liver metabolism result in elevated metabolites that promote cancer proliferation and restrict T-cell survival and activation. Re-expression of the HNF4α in vivo by viral therapy preserves liver metabolism, decreases tumor growth, and alleviates CAC. Our findings further suggest that early changes detected in a routine liver biochemical profile before and at the time of diagnosis in patients without known liver metastasis can predict CAC and worse cancer outcomes in human patients.

**Conclusion**

We demonstrated that systemic metabolic events that lead to CAC occur at early carcinogenesis and can be identified by standard liver biochemical tests. Furthermore, our findings suggest that targeting the ERK-IL6-STAT3-HNF4α signaling pathway can restrict pre-CAC manifestations.

### EACR23-0805

### Linoleic acid potentiates CD8+ T cell metabolic fitness and anti-tumor immunity

*C. Nava Lauson[1], S. Tiberti[1], P. Corsetto[2], P. Tyagi[3], P.D. Greenberg[4], J.B. Huppa[5], I. Caruana[6], S. Rodighiero[1], L. Nezi[1], T. MANZO[1]*

*[1]European Institute of Oncology, Department of Experimental Oncology, Milan, Italy*

*[2]University of Milan, Department of Pharmacological and Biomolecular Science s, Milan, Italy*

*[3]European Institute of Oncology, Department of Experimental Oncology-, Milan, Italy*

*[4]Fred Hutchinson Cancer Research Center, Clinical Research Division and Program in Immunology, Seattle, United States*

*[5]Institute for Hygiene and Applied Immunology, Medical University of Vienna- Center for Pathophysiology- Infectiology and Immunology, Vienna, Austria*

*[6]University Hospital of Würzburg, Department of Paediatric Haematology- Oncology and Ste m Cell Transplantation, Würzburg, Germany*

**Introduction**

Adoptive T Cell Therapy (ACT) aims to re-educate the immune system against cancer, enabling unprecedented clinical responses. Yet, efficacy of ACT in solid tumors is still marginal. The metabolic state represents a major hurdle limiting ACT efficacy. Indeed, specific lipids can harm $CD8^+$ T cell (CTL) mitochondrial integrity, leading to defective anti-tumour responses. However, the extent to which lipids can affect CTL functions and fate remains unexplored.

**Material and Methods**

We screened different lipids and assess their ability to shape CTL differentiation and activity using multi-parametric flow cytometry, functional assays, together with a complete transcriptomic and epigenomic profiling. Metabolic profile of CTL cells was examined by bioenergetic flux measurements paired with metabolomic and lipidomic analysis. Finally, the anti-tumour responses of metabolically-edited CTL was evaluated in a pre-clinical mouse model, known to poorly respond to immunotherapy, as well as in the clinical setting of human CAR-redirected T cells.

**Results and Discussions**

We identified Linoleic Acid (LA) as a major positive regulator of CTL activity by improving metabolic fitness, preventing exhaustion and stimulating a memory-like phenotype with superior effector functions. Mechanistically, LA treatment enhances the formation of ER-mitochondria contacts (MERC), which in turn promotes calcium ($Ca^{2+}$) signaling, mitochondrial energetics, and CTL effector functions. As a direct consequence, the anti-tumour potency of LA-instructed CD8 T cells is superior towards different type tumors, both *in vitro* and *in vivo* following adoptive transfer into tumour bearing mice.

**Conclusion**

In conclusion, our study demonstrates that LA could be considered as a molecular switch to fine-tune memory T cell formation and metabolic fitness maintenance, linking lipid metabolism to anti-tumour surveillance. This pave the way for a new generation of adoptive T cell-based therapies, where LA can be used to during *ex vivo* CAR- and TCR- T cell manufacturing to achieve metabolic reprogramming and long-term functionality, broadening the therapeutic efficacy of ACT to a wide range of malignancies. Thereby, we propose here a novel strategy to boost ACT efficacy by implementing CTL long-term functionality, metabolic fitness and preventing exhaustion through lipid-induced mitochondrial rewiring (PCT/EP2022/081824).

### Joint EACR-AACR Symposium: Computational Biology and AI in Research and Cancer Care

### EACR23-0087

### Pan-cancer whole genome comparison of primary and metastatic solid tumors

*F. Martinez-Jimenez[1,2], A. Movasati[3], S.R. Brunner[3], L. Nguyen[3], E. Cuppen[3,4], A. Van Hoeck[3]*

*[1]Vall d'Hebron Institute of Oncology VHIO, Pre-clinical and translation department, Barcelona, Spain*

*[2]Hartwig Medical Foundation, Data mining, Barcelona, The Netherlands*

*[3]University Medical Center Utrecht- Utrecht, Human Genetics, Utrecht, The Netherlands*

*[4]Hartwig Medical Foundation, Clinical and pre-clinical, Barcelona, The Netherlands*

**Introduction**

Metastatic cancer remains almost inevitably a lethal disease. Despite the many efforts to understand these phenomena, we still have limited knowledge of the contribution of genomic changes that equip tumors with these extraordinary capacities. Thus, it is essential to characterize genomic differences between primary and metastatic cancers and quantify their impact on therapy resistance to be able to understand and harness therapeutic interventions that establish more effective and more personalized therapies.

**Material and Methods**

Here, we present a large-scale unified analysis of more than 7,000 whole-genome sequenced (WGS) tumor

© 2023 The Authors. Molecular Oncology is published by John Wiley & Sons Ltd on behalf of Federation of European Biochemical Societies samples (re-)analyzed by the same data-processing pipeline. Importantly, this dataset encompasses unpaired primary and metastatic tumor samples from 71 cancer types, including 23 cancer types with large representation from both clinical stages.

**Results and Discussions**

In general, our analysis shows that metastatic tumors have a low intra-tumor heterogeneity, high genomic instability and increased frequency of structural variants with comparatively a modest increase in the number of small genetic variants. However, these differences are cancer type specific and are heavily impacted by the exposure to cancer therapies. Five cancer types, namely breast, prostate, thyroid, kidney renal clear cell carcinoma and pancreatic neuroendocrine, are a clear exception to the rule, displaying an extensive transformation of their genomic landscape in advanced stages. These changes were supported by increased genomic instability and involved substantial differences in tumor mutation burden, clock-based molecular signatures and the landscape of driver alterations as well as a pervasive increase in structural variant burden. The majority of cancer types had either moderate genomic differences (e.g., lung adenocarcinoma and colorectal carcinomas) or highly consistent genomic portraits (e.g., ovarian serous carcinoma and skin melanoma) when comparing early- and late-stage disease. Exposure to treatment further scars the tumor genome and introduces an evolutionary bottleneck that selects for known therapy-resistant drivers in approximately half of treated patients.

**Conclusion**

Our data showcases the potential of whole-genome analysis to identify distinctive features of late-stage tumors and provides a valuable resource to further investigate the biological basis of cancer and resistance to cancer therapies.

### EACR23-1138
### Mutational processes and genomic aberrations driving cancer evolution in 1011 lung tumours

*K. Thol[1], M. Litovchenko[1], R. Bentham[1], M. Dietzen[1], C. Bailey[2], A. Frankell[2], O. Pich[2], T. Baker[3], K. Litchfield[1], C. Hiley[1], C. Swanton[2], N. McGranahan[1]*
*[1]University College London, Department of Oncology, London, United Kingdom*
*[2]The Francis Crick Institute, Cancer Evolution and Genome Instability Laboratory, London, United Kingdom*
*[3]The Francis Crick Institute, Cancer Genomics Laboratory, London, United Kingdom*

**Introduction**

Lung cancer is the leading cause of cancer-related deaths worldwide. Lung cancer genomes have been shown to be heterogeneous, highly mutated and subject to frequent copy number aberrations. However, less is known about the specific genomic aberrations driving lung tumorigenesis and the biological processes that induce them. Here, we aim to fully unravel the extent to which lung cancer genomes are disrupted, and distinguish which disruptions have a 'cancer driver' functionality. Additionally, we aim to identify the processes that induce genomic disruptions in the genome and their timing during the course of lung tumour evolution.

**Material and Methods**

1011 lung tumour samples with matched normal tissue, including 8 distinct histological subtypes, from the 100,000 genomes project were subject to whole genome sequencing (WGS), and mutation, copy number and structural rearrangement calling. Five bioinformatics tools were employed and combined for *de-novo* coding and non-coding cancer gene discovery. Mutational signatures were extracted *de novo* using hierarchical Dirichlet processes.

**Results and Discussions**

Within our cohort we find a subset of tumours that only have identified cancer driver mutations or genomic rearrangements in the non-coding parts of the genome. This subset would have been entirely missed without high quality WGS data. Using mutational signatures, we detected significant differences in mutational processes between histological subtypes of lung cancer, with different smoking-induced genomic aberrations found in lung adenocarcinomas and squamous cell carcinomas. Moreover, smoking-induced mutations enabled us to infer smoking history where this information is missing. The timing of occurrence of copy number aberrations varied between adenocarcinomas with and without mutations associated with tobacco smoking, emphasising that lung tumours with an aetiological link to smoking are genomically distinct from those without. We observe that constraints imposed by histology on single base substitutions are stronger at earlier evolutionary time points, however, subsequently, these constraints weaken and the evolutionary space expands.

**Conclusion**

This work presents the largest whole genome analysis of lung cancer to date and demonstrates the importance of WGS for clinical decision making. It also contributes to our insight into the evolutionary histories of lung tumours, which may ultimately underpin improvements in the discovery of clinical biomarkers and treatment strategies.

### Symposium: Normal Tissue and the Biology of Early Cancer

### EACR23-0211
### Epithelial Grem1 drives ectopic stem cell niche formation through stromal remodelling and tissue co-evolution in intestinal cancers

*E. Mulholland[1], H. Belnoue-Davis[1], G. Valbuena[1], E. Gil Vazquez[1], A. Ligeza[1], S. Leedham[1]*
*[1]University of Oxford, Wellcome Centre for Human Genetics, Oxford, United Kingdom*

**Introduction**

Polarised Wnt and BMP gradients restrict stem cells to the intestinal crypt base. Grem1, a BMP antagonist, has been identified as the cause of ectopic stem cell niches and crypt foci (ECF) (ECF) in Hereditary Mixed Polyposis syndrome (HMPS) and sporadic traditional serrated adenomas (TSA). Although Grem1 expression in epithelial cells has been linked to ECF development, its impact on intestinal homeostasis and stromal remodelling remains unclear. In

© 2023 The Authors. Molecular Oncology is published by John Wiley & Sons Ltd on behalf of Federation of European Biochemical Societies this study, we examined the effect of epithelial Grem1 on cell-fate determination and stromal remodelling. Additionally, we investigated the role of Wnt signalling in Grem1-driven ECF formation and tested a new anti-Grem1 antibody as a potential therapy.

**Material and Methods**

We generated Vill-Grem1 mice which is a model of HMPS with epithelial Grem1 over-expression, and analysed their intestinal morphology, cell-fate determination, and stromal remodelling. We inhibited Wnt signalling using a PORCN inhibitor (LGK974) and examined the impact on ECF formation. We also tested the efficacy and safety of a novel anti-Grem1 antibody in preventing and regressing ECF phenotype in Vill-Grem1 mice.

**Results and Discussions**

Epithelial Grem1 disrupted homeostatic intestinal morphogen gradients and altered cell-fate determination, promoting the persistence of Olfm4+, AXIN2+, Lgr5- progenitor cells that have exited the stem-cell niche. These cells formed ECF, proliferated, accumulated somatic mutations, and initiated intestinal neoplasia. Grem1 also promoted the expansion of Wnt2b+ CD55+ fibroblasts in the stromal compartment. Inhibition of Wnt signalling using a PORCN inhibitor significantly reduced polyp size and ECF number in Vill-Grem1 mice compared to vehicle controls. Treatment with the anti-Grem1 antibody prevented polyp initiation in Vill-Grem1 mice when given early and induced polyp regression in aged mice, significantly prolonging lifespan in both treatment arms. Prolonged treatment (>500 days) with the anti-Grem1 antibody was safe in these models, with no adverse effects identified.

**Conclusion**

Epithelial Grem1 disrupts intestinal homeostasis and promotes ECF formation by altering cell-fate determination, leading to ectopic stem cell persistence. Grem1 promotes stromal remodelling and Wnt2b+ CD55+ fibroblast expansion. Inhibiting Wnt signalling or targeting Grem1 with an anti-Grem1 antibody may prevent or regress ECF in TSA or HMPS patients, offering new targets for colorectal cancer treatment.

### EACR23-1245
### Lung adenocarcinoma promotion by air pollutants

*W. HILL*[1], *E. Lim*[1], *C. Weeden*[1], *C. Lee*[1], *M. Augustine*[2], *K. Chen*[2], *F.C. Kuan*[3], *F. Marongiu*[4], *E. Evans*[4], *D. Moore*[5], *E. Gronroos*[1], *J. Downward*[6], *C. Carlsten*[7], *T. Jacks*[8], *I. Malanchi*[9], *A. Hackshaw*[10], *K. Litchfield*[2], *J. DeGregori*[4], *M. Jamal-Hanjani*[2], *C. Swanton*[1]

[1]*The Francis Crick Institute, Cancer Evolution and Genomic Instability Laboratory, London, United Kingdom*
[2]*UCL Cancer Institute, CRUK Lung Cancer Centre of Excellence, London, United Kingdom*
[3]*Chang Gung Memorial Hospital, Department of Hematology and Oncology, Chiayi, Taiwan*
[4]*University of Colorado Anschutz Medical Campus, Department of Biochemistry and Molecular Genetics, Aurora, United States*
[5]*UCL Cancer Institute, Cancer Research UK Lung Cancer Centre of Excellence, London, United Kingdom*
[6]*The Francis Crick Institute, Oncogene Biology Laboratory, London, United Kingdom*
[7]*Vancouver Coastal Health Research Institute, Department of Medicine, Vancouver, Canada*
[8]*Koch Institute for Integrative Cancer Research at MIT, The Jacks Lab, Cambridge, United States*
[9]*The Francis Crick Institute, Tumour-Host Interaction Laboratory, London, United Kingdom*
[10]*UCL Cancer Institute, Cancer Research UK & University College London Cancer Trials Centre, London, United Kingdom*

**Introduction**

A complete understanding of how environmental exposures may promote cancer formation is lacking. Exposure to air pollution particulate matter < 2.5μm (PM2.5) is associated with lung cancer incidence but the underlying mechanism is unclear. Over 70 years ago, tumour formation was proposed to occur in a two-step process: an initiating step which induces mutations in normal cells, followed by a promoter step which triggers cancer development. We investigated *EGFR*-driven lung cancer, more common in never-smokers, and its association with air pollution.

**Material and Methods**

In this multidisciplinary study, we integrate epidemiological analysis of 32,957 *EGFR*-driven lung cancer cases in four within-country cohorts, mechanistic studies using genetically engineered mouse models, *in vitro* organoid culture and ultradeep mutational profiling of histologically normal lung tissue from 295 individuals from 3 clinical cohorts.

**Results and Discussions**

We find a positive association between *EGFR* mutant lung cancer incidence and PM2.5 levels and evidence that 3 years of exposure may be sufficient for this association. In mouse models, air pollutants induce macrophage influx to the lung and interleukin-1β (IL-1β) release which is sufficient to provoke a progenitor-like state within *EGFR* mutant epithelia fuelling tumorigenesis. Anti-IL-1β therapy inhibited pollutant-promoted tumour formation. Driver mutations in *EGFR* and *KRAS* were identified in 18% and 53%, respectively, of human normal lung samples, where environmental black carbon deposits were associated with *EGFR* mutant cell expansion. In normal human lung samples, driver mutations in *EGFR* and *KRAS* were identified in 18% and 53% of individuals respectively. In addition, environmental black carbon deposits in the lung (anthracosis) were associated with *EGFR* mutant cell expansion.

**Conclusion**

This work supports a model by which PM2.5 promotes lung adenocarcinoma formation from pre-existing *EGFR* mutant cells via an inflammatory axis, shedding light on the aetiology of lung cancer in never smokers. Our results reveal a mechanistic basis for PM2.5 driven lung cancer providing opportunities for molecular targeted cancer prevention in at-risk individuals.

### Symposium: Drugging the RAS Pathway

© 2023 The Authors. Molecular Oncology is published by John Wiley & Sons Ltd on behalf of Federation of European Biochemical Societies

### EACR23-0367
### Collective MAPK activity waves are emergent properties that control breast cancer cell response to therapy

P.A. Gagliardi[1], A. Frismantiene[1], M. Dobrzyński[1], O. Pertz[1]
[1]University of Bern, Institute of Cell Biology, Bern, Switzerland

**Introduction**
The development of fluorescent biosensors of ERK (MAPK) kinase activity has revealed the temporal dimension of single-cell signaling. More recently, we have shown that individual cell dynamics can self-organize in signaling waves, such as the apoptotic MAPK waves, that induce survival of the neighbors in normal epithelia.
**Material and Methods**
Here, by combining fluorescent biosensors, automated time-lapse microscopy, single-cell image and data analysis, and computational modeling, we explored the role of collective MAPK activity waves in cancerous mammary cell response to chemotherapy in vitro.
**Results and Discussions**
In isogenic variants of MCF10A cells harboring the oncogenic mutations PIK3CA E545K and H1047R, or in the breast cancer cell line MCF7, we observed apoptosis-induced collective MAPK-activity waves in response to doxorubicin treatment. In addition, we observed spontaneous, apoptosis-independent, collective MAPK-activity waves, making these cellular systems highly dependent on cell-cell communication of MAPK activity. Co-treatment of doxorubicin with inhibitors of the MAPK-activity waves, reduces MAPK activity and survival in the neighboring cells, amplifying the effect of doxorubicin and causing a faster collapse of the cancerous cellular community.
**Conclusion**
This shows the importance of dynamic communication among cancer cells. Inhibition of collective MAPK-activity waves might be a general strategy to improve the effect of chemo- or targeted therapies.

### EACR23-1198
### Optimizing RAS inhibition with dual blockade of oncogenic and wild-type RAS

M. Holderfield[1], S. Cai[1], J. Dinglasan[1], N. Montazer[1], L. Lai[1], B. Lee[1], K. Yang[1], J.A.M. Smith[1], M. Singh[1], D. Wildes[1]
[1]Revolution Medicines- Inc., Biology, Redwood City, United States

**Introduction**
Mutant-selective KRAS G12C(OFF) inhibitors have clinically validated the targeting of oncogenic KRAS. However, onset of resistance to these inhibitors is often coincident with RTK activation or the emergence of secondary RAS mutations, highlighting the need for therapeutic strategies that more broadly suppress RAS signaling. While the current approved KRAS G12C inhibitors target the inactive, GDP-bound state of KRAS (RAS(OFF)), at Revolution Medicines, we have pursued a novel drug mechanism, which is specifically designed to target RAS(ON). Our compounds enter a tumor cell, bind initially to a widely expressed second protein called cyclophilin A, and form a binary complex. This binary complex presents a new protein binding surface that is optimized for a selective high-affinity interaction with a particular RAS(ON) target. Formation of the tri-complex rapidly inactivates RAS signaling by sterically preventing binding and activation of downstream RAS pathway effectors.
**Material and Methods**
We have developed several in vitro models with acquired or engineered resistance to KRAS G12C(OFF) or tri-complex KRAS G12C(ON) inhibitors that recapitulate and validate many of the putative resistance mutations observed in patient tumors during escape from first generation KRAS G12C(OFF) inhibitors.
**Results and Discussions**
Tumors with secondary mutations in the KRAS Switch II domain demonstrated resistance only to KRAS G12C(OFF) inhibitors and remained sensitive to tri-complex KRAS G12C(ON) inhibitors. Other escape mechanisms such as activating RTK mutations, which caused strong resistance to KRAS G12C(OFF) inhibition, induced relatively modest potency shifts for a KRAS G12C(ON) inhibitor, which was reversed by addition of a SHP2 or RTK inhibitor. Additionally, the combination of a KRAS G12C(ON) inhibitor with a RAS MULTI inhibitor (a tri-complex RAS(ON) inhibitor that targets both wild-type and multiple oncogenic forms of K- H- and NRAS proteins) not only improved response to KRAS G12C inhibition and overcame many RTK-mediated resistance mechanisms, but also suppressed expansion of secondary non-G12C RAS mutations.
**Conclusion**
Collectively these data suggest that blockade of both oncogenic and wild-type RAS signaling may be necessary to maximize anti-tumor response and highlight the utility of combining a mutant selective RAS(ON) inhibitor with RAS MULTI to forestall resistance.

### Symposium: Advanced T-Cell Therapy

### EACR23-0044
### Transient cell-in-cell formation underlays tumor escape from immunotherapy

Y. Carmi[1], A. Gutwillig[2]
[1]Tel-Aviv University, Pathology, Ramat Aviv Tel Aviv, Israel
[2]Tel-Aviv university, Pathology, Tel Aviv, Israel

**Introduction**
Despite the remarkable successes of cancer immunotherapies, the majority of patients will experience only a partial response followed by a relapse of resistant tumors. While treatment resistance has frequently been attributed to clonal selection and immunoediting, comparisons of paired primary and relapsed tumors in melanoma and breast cancers indicate that they share the majority of clones.
**Material and Methods**
Whole genome and mRNA sequencing were used to compare the neoantigen burden on primary and immunotherapy-relapsed tumor cells. Super-resolution electron and confocal microscopy were used to identify tumor cell-in-cell structures.

© 2023 The Authors. Molecular Oncology is published by John Wiley & Sons Ltd on behalf of Federation of European Biochemical Societies

**Results and Discussions**
we demonstrate in both mouse models and clinical human samples that tumor cells evade immunotherapy by generating unique transient cell-in-cell structures, which are resistant to killing by T cells and chemotherapies. While the outer cells in this cell-in-cell formation are often killed by reactive T cells, the inner cells remain intact and disseminate into single tumor cells once T cells are no longer present. This formation is mediated predominantly by IFNγ-activated T cells, which subsequently induce phosphorylation of the transcription factors signal transducer and activator of transcription 3 (STAT3) and early growth response-1 (EGR-1) in tumor cells. Indeed, inhibiting these factors prior to immunotherapy significantly improves its therapeutic efficacy.
**Conclusion**
Overall, this work highlights a currently insurmountable limitation of immunotherapy and reveals a previously unknown resistance mechanism which enables tumor cells to survive immune-mediated killing without altering their immunogenicity

### EACR23-0980
### Single-Cell Signalling Analysis of Engineered γδ T cell Biotherapeutics for the Treatment of Colorectal Cancer

*C. Nattress[1], V. Petra[1], D. Fowler[2], J. Sufi[1], F. Cardoso Rodriguez[1], M. Ramos Zapatero[1], A. Campbell[1], X. Qin[1], J. Opzoomer[1], M. Barisa[2], J. Fisher[2], C. Tape[1]*
*[1]University College London Cancer Institute, Department of Oncology, London, United Kingdom*
*[2]University College London- Great Ormond Street Institute of Child Health, Developmental Biology and Cancer, London, United Kingdom*

**Introduction**
Colorectal cancer (CRC) is a devastating disease that kills ~700,000 people worldwide annually, with immunotherapies struggling against the immunosuppressive CRC tumour microenvironment (TME). Tumour infiltrating γδ T cells confer a prognostic benefit to CRC patients and can kill cancer via multiple innate and adaptive mechanisms, including antibody-dependent cellular cytotoxicity (ADCC) against tumour antigens (e.g., B7-H3). We hypothesise that γδ T cells can be exploited as an 'off-the-shelf' anti-CRC biotherapeutic but their complex interactions within the CRC TME need to be elucidated, as well as any heterogeneity that exists between γδ T cell donors.
**Material and Methods**
We performed 3D cultures of CRC patient-derived organoids (PDOs) and human Vγ9Vδ2 T cells, either unmodified or engineered to secrete a modified IL15 cytokine (stIL15-γδs). The addition of anti-B7-H3 IgG also allows anti-PDO ADCC, with antigen specificity assessed using CRISPR-Cas9 PDO models. Using 126-plex Thiol Organoid Barcoding in situ (TOB*is*) mass cytometry (MC), we measured over 60 parameters per cell across 510 PDO- and 780 γδ-cultures. Cell-type specific signalling analysis across multiple γδ donors and CRC PDOs including post-translational modifications (PTMs), cell-state and immunological phenotype were analysed computationally.
**Results and Discussions**
stIL15-γδs exhibit superior proliferation, purity and viability, with responses to cytokine engineering varying between different γδ donors (e.g., $^{127}$IdU and pSTAT5 [Y694] expression). Multiple stIL15-γδs demonstrated significant cytotoxicity against CRC PDOs (cCaspase3 [D175]+), commonly outperforming standard-of-care chemotherapies. ADCC varies between γδ donors and is associated with FcγR CD16 expression. Furthermore, the amount of PDO killing achieved via ADCC varies across different PDOs. Following co-culture with PDOs, stIL15-γδ signalling is regulated in a PDO-specific manner, with significant regulation of stIL15-γδ cell-state (e.g., pRB [S807/811] and $^{127}$IdU), PTMs (e.g., pSTAT3 [Y705] and pERK1/2 [T202/Y204]) and immunological phenotype (e.g., Granzyme B and CD69) observed. Moreover, we detect differential regulation of individual stIL15-γδ cell-cycle phases following co-culture with CRC PDOs.
**Conclusion**
Candidate stIL15-γδ donors demonstrate rapid and substantial cytotoxicity against a range of CRC PDOs when compared to standard-of-care chemotherapies. The proportion of ADCC killing, as well as the regulation of stIL15-γδs by PDOs, happens in a PDO-specific manner.

### Symposium: Cancer Models

### EACR23-0169
### A time-resolved single-cell analysis of melanoma dermal invasion

*N. Van Raemdonck[1], O. Marín-Béjar[2], J. Poźniak[1], M. Ciwinska[1], M. Planque[1], J. van den Oord[3], C. Scheele[1], S.M. Fendt[1], J.C. Marine[1]*
*[1]KU Leuven - VIB, Oncology, Leuven, Belgium*
*[2]Germans Trias i Pujol Research Institute, Cancer department, Barcelona, Spain*
*[3]KU Leuven, Imaging & Pathology, Leuven, Belgium*

**Introduction**
Cutaneous melanoma initiates with the abnormal proliferation of malignant epidermal melanocytes. A key step towards the development of an aggressive and metastatic disease is when some malignant cells cross the basement membrane and enter the dermis. The molecular and cellular mechanisms enabling dermal invasion of melanoma cells are largely unknown. This is due to a lack of accurate *in vivo* model systems that recapitulate this event.
**Material and Methods**
To fill out this gap, we have developed a novel melanoma mouse model, which allows activation of a Braf$^{V600E}$-driven melanomagenic program in tail epidermal melanocytes and *in vivo* fate mapping of the melanoma cells. The model takes advantage of an X-linked spontaneous *Eda$^{Ta-6j}$* mutation (Tabby) which abrogates the formation of hair follicles in the tail, thereby ensuring that melanoma early development remains strictly confined to the epidermis.
**Results and Discussions**
Using intravital microscopy, we observed that individual melanoma cells change their morphology before entering the dermis and cross the basement membrane through a limited number of gaps created by "leading" cells. We leveraged single-cell RNA sequencing (10X Genomics) to

© 2023 The Authors. Molecular Oncology is published by John Wiley & Sons Ltd on behalf of Federation of European Biochemical Societies portray the transcriptional heterogeneity of early melanoma lesions and to monitor the transcriptional changes that accompany dermal invasion. We discovered that this process associates with a partial de-differentiation of melanoma cells and the upregulation of a hypoxia-related gene signature.

**Conclusion**

All in all, we have established a suitable *in vivo* model to study the cellular and molecular mechanisms underlying melanoma dermal invasion. This model provides a unique platform for the discovery of novel biomarkers of dermal invasion and therapeutic interventions that intercept the disease before its lethal dissemination to vital organs.

### EACR23-0433

### An automation workflow for high-throughput manufacturing scaffold-supported 3D tissue arrays and single-cell-based analysis

*R. Cao[1], N. Li[2], S. Latour[2], J. Cadavid[3], C. Tan[2], A. Forman[4], H. Jackson[4], A. McGuigan[3]*
*[1]University of Toronto, Institute of Biomedical Engineering, Toronto, Canada*
*[2]University of Toronto, Department of Chemical Engineering and Applied Chemistry, Toronto, Canada*
*[3]University of Toronto, Department of Chemical Engineering and Applied Chemistry- Institute of Biomedical Engineering, Toronto, Canada*
*[4]Univeristy of Toronto, Department of Molecular Genetics, Toronto, Canada*

**Introduction**

Patient-derived tumour organoids have emerged as a useful tool to model tumour heterogeneity and 3D cancer biology. Currently, a major barrier to progress is the ability to scale these patient-specific culture models and enable detailed analysis of distinct cell populations that exist in the patient. To address this challenge, we developed an automated workflow to generate engineered microtissues and integrated with downstream single-cell-based and multiplexed analysis methods.

**Material and Methods**

One strategy to enable high-throughput organoid cultures that also enables easy image-based analysis is the Scaffold-supported Platform for Organoid-based Tissues (SPOT) platform developed by our research group. SPOT allows the generation of flat, thin and low-volume microtissues in 96- and 384-well plate footprints that are compatible with image-based readouts. To improve scalability, in this study, we optimize an automation approach to generate 3D-engineered microtissues in SPOT using a liquid handler. Furthermore, we develop an automated cell extraction protocol to support downstream single-cell-based analysis, and showing its potential for multiplexed, high-dimensional analysis.

**Results and Discussions**

Using the optimized automated seeding protocol, we have shown comparable intra- and inter-sample variation to standard manual manufacturing. As a proof-of-value demonstration, we generated 3D complex tissues containing distinct tumour-stromal cell compositions and performed single-cell-based end-point analysis. We probed the impact of co-culture on the tumour cell population and captured physiologically-relevant cell responses. Using three orthogonal methods: high-throughput flow cytometry, cytometry by time of flight (CyTOF), and high-content imaging analysis, we show a correlated increase of proliferation in cancer cells when co-cultured with stromal cells. We have also incorporated primary patient-derived organoids into the pipeline to capture patient-level tumour heterogeneity.

**Conclusion**

The SPOT platform coupled with the automation workflow, particularly multiplexed CyTOF, is a powerful and novel way for studying cellular responses in a tissue-mimicking 3D microenvironment at scale. We envision that our work will offer new avenues for discovering novel personalized therapeutics as it offers the potential for investigating patient-level heterogeneity, with the possibility of combining with big-data analysis in the future.

### Symposium: Tumour Ecology and Evolution at Single Cell Level

### EACR23-0359

### Tracing growth and metastasis dynamics in melanoma at single cell resolution

*P. Karras[1], I. Bordeu[2], J. Pozniak[1], A. Nowosad[1], C. Pazzi[1], N. Raemdonck[1], E. Landeloos[1], Y. Herck[3], D. Pedri[1], R. Browaeys[4], Y. Saeys[4], F. Bosisio[5], A. Rustgi[6], O. Bechter[3], C. Blanpain[7], B. Simons[2], F. Rambow[1], J.C. Marine[1]*
*[1]VIB-KU Leuven, Oncology, Leuven, Belgium*
*[2]University of Cambridge, Department of Applied Mathematics and Theoretical Physics- Centre for Mathematical Sciences, Cambridge, United Kingdom*
*[3]UZ Leuven, Department of General Medical Oncology, Leuven, Belgium*
*[4]VIB Center for Inflammation Research, Data Mining and Modeling for Biomedicine Group, Ghent, Belgium*
*[5]KU Leuven, Department of Imaging and Pathology, Leuven, Belgium*
*[6]Columbia university, Herbert Irving Comprehensive Center- Columbia University Irving Medical Center, New York, United States*
*[7]Free University of Brussels, Laboratory of Stem Cells and Cancer, Brussels, Belgium*

**Introduction**

**Cellular plasticity** endows cancer cells with capacity towards a phenotypic state. Except from the genetic origin of cellular plasticity, cancer cells can transit to reversible phenotypic states as a result of microenvironmental cues and are often driven by stochastic epigenetic and/or transcriptional fluctuations. Melanoma is a prime example of heterogeneous and plastic tumors where the **origin and magnitude** of cell state diversity and dynamics remain poorly understood. Equally is not known the origin and molecular features of **metastatic initating cells (MICs)**.

**Material and Methods**

Here we performed a comprehensive and exhaustive approach to address the mode of tumor growth and the

© 2023 The Authors. Molecular Oncology is published by John Wiley & Sons Ltd on behalf of Federation of European Biochemical Societies origin of metastasis in melanoma. We generated **new clinically-relevant lineage tracing reporter mouse models of melanoma** to trace growth dynamics of cancer cells and the plasticity of MICs. We combined them with advanced 3D imaging, quantitative mathematical modelling and single-cell and spatial transcriptomics.

**Results and Discussions**

We demonstrated that tumors follow a **hierarchical model of growth** supported by a population of **Melanoma Stem-like Cells** (MSCs) that exhibit a transcriptomic signature of pre-migratory neural crest cells established transiently during embryonic development. Multimodal analysis unraveled unique cell type and state interactions and importantly demonstrated that MSCs reside in **perivascular niches** favouring tumor growth. **Endothelial Cells** were found to have a key role in the acquisition of stemness properties. Co-culture assays led to melanoma dedifferentiation and proliferation advantage. Of note, supplementing melanoma cells with ECs accelerated the growth of tumors *in vivo*, where MSCs pool was increased. Consistent with a model in which only a fraction of cells are fated to fuel growth, temporal single-cell tracing of a population of melanoma cells harbouring a **mesenchymal-like state** driven by the Transcription Factor PRRX1 revealed that these cells do not contribute to primary tumour growth but, instead, constitutes **a pool of metastatic-initiating cells** that switch cell identity while disseminating to secondary organs.

**Conclusion**

Taken together, these results uncouple growth and metastatic dissemination and pave the way for the development of strategies that exploit the "chameleonic" nature of cancer cells to target MSC niche-dependent specification mechanisms and inhibit early dissemination of melanoma.

### EACR23-0480
### Spatial Architecture of Myeloid and T Cells Orchestrates Immune Evasion and Clinical Outcome in TRACERx

*M. Angelova[1], K.S.S. Enfield[1], A. Magness[1], E. Colliver[1], C. Lee[1], D.A. Moore[2], M. Sivakumar[2], K. Grigoriadis[1], O. Pich[1], T. Karasaki[1], P. Hobson[3], R. Salgado[4], E. Nye[5], N. McGranahan[6], S.A. Quezada[7], C.T. Hiley[1], J. Downward[8], E. Sahai[9], C. Swanton[1]*

[1]*The Francis Crick Institute, Cancer Evolution and Genome Instability, London, United Kingdom*

[2]*University College London Hospitals, Department of Cellular Pathology, London, United Kingdom*

[3]*The Francis Crick Institute, Flow Cytometry, London, United Kingdom*

[4]*GZA-ZNA Hospitals, Department of Pathology, Antwerp, Belgium*

[5]*The Francis Crick Institute, Experimental Histopathology, London, United Kingdom*

[6]*University College London Cancer Institute, Cancer Research UK Lung Cancer Centre of Excellence, London, United Kingdom*

[7]*University College London Cancer Institute, Department of Haematology-, London, United Kingdom*

[8]*The Francis Crick Institute, Oncogene Biology Laboratory, London, United Kingdom*

[9]*The Francis Crick Institute, Tumour Cell Biology Laboratory, London, United Kingdom*

**Introduction**

The role tumour microenvironment (TME) in cancer is tightly linked to the cell phenotypes and their spatial context, which can be modulated by cancer cells in favour of immune escape and tumour promotion. An improved understanding of the TME during tumour evolution is critical to defining optimal therapeutic strategies. Multiplexed imaging is a powerful tool for profiling cell phenotypes and spatial tissue organisation; however, data analysis often involves complex steps and substantial manual intervention.

**Material and Methods**

To characterise the co-evolution of the tumour and its TME, we performed imaging mass cytometry (IMC) of 151 tumour and 49 normal regions from 81 patients within TRACERx, a longitudinal, prospective study of early-stage non-small cell lung cancer (NSCLC). To address the need for reproducible, benchmarked, and interpretable workflows for multiplexed imaging analysis, we first developed TRACERx-PHLEX, a user-friendly computational pipeline for comprehensive analysis of the TME, validated and compared against standard tools. PHLEX encapsulates deep learning-enhanced cell segmentation, detection of spatially resolved protein expression, automated cell-type annotation and interpretable spatial analysis.

**Results and Discussions**

Fully automated, open-source and portable, TRACERx-PHLEX outputs spatial catalogues of cell-type specific co-expression of therapeutic targets and produces clinically relevant spatial information. We applied PHLEX on IMC and integrated it with paired genomics data in TRACERx. We showed that tumours with high clonal neoantigen burden had high intra-tumour CD8 T cell infiltration and spatial hubs of CD8 T cells and M2-like macrophages. Immune cold tumours were distinguished by dense fibroblast arrangements. Tumour-Associated Neutrophils (TANs) defined a distinct TME that supports subclonal expansion, glycolysis and hypoxia, characterised by sparse T cell infiltration and modulated by cancer-neutrophil crosstalk through PI3K and RAS signalling. Finally, we demonstrated that TANs predict clinical outcome in NSCLC. Histopathology-derived TAN scoring revealed a high risk of relapse and metastasis in TAN-high cancers in independent discovery (n=44) and validation (n=331) TRACERx cohorts.

**Conclusion**

Our findings support a central role of the neutrophil-rich TME in driving metastasis and delineate optimal therapeutic strategies based on spatial heterogeneity of immune-checkpoint expression.

### Symposium: Tumour Dormancy / Persistence / Senescence

### EACR23-0260
### Clearance of apoptotic cells by neutrophils triggers an anti-inflammatory and

© 2023 The Authors. Molecular Oncology is published by John Wiley & Sons Ltd on behalf of Federation of European Biochemical Societies

### regenerative response in colorectal cancer

R. Oehler[1], V. Schimek[1], V. Brandel[1], A. Beer[2], M. Sachet[1], C. Ramos[1], V. Gerakopoulos[1], M. Bergmann[1], A. Assinger[3], P. Starlinger[1]
[1]Department of General Surgery, Medical University of Vienna, Vienna, Austria
[2]Department of Pathology, Medical University of Vienna, Vienna, Austria
[3]Department of Vascular Biology and Thrombosis Researc h, Medical University of Vienna, Vienna, Austria

**Introduction**

Cancer therapies lead to apoptosis of cancerous and sometimes also of non-malign cells. Apoptotic cell debris is removed by various categories of phagocytes via efferocytosis. Here we show that neutrophils play a central role in the clearance of apoptotic cells in primary and metastatic colorectal cancer (CRC) and generate a tumor-supportive microenvironment.

**Material and Methods**

Blood and cancer tissue samples of patients with primary or metastatic CRC were analyzed by immunohistochemistry and flow cytometry to characterize immune cell infiltration or apoptotic cell death as defined by the presence of caspase-cleaved cytokeratin-18 (M30). Apoptosis was induced in both primary patient-derived and established CRC cell lines by combined TNFalpha-treatment and UV-C irradiation or chemotherapeutic agents. Neutrophils were prepared from blood of healthy volunteers and co-cultured with either autologous monocyte/macrophages, apoptotic tumor cells, or their extracellular vesicle (aEVs).

**Results and Discussions**

Apoptosis led to a substantial release of neutrophil-attracting chemokines, most importantly interleukin-8 (IL-8). Accordingly, tumor associated neutrophils accumulated at sites of apoptotic tumor cells in CRC tissue. The same areas were also highly infiltrated by macrophages, while T cells were virtually absent. Attracted neutrophils induced an M2-like CD86low CD163+ CD206+ phenotype in co-cultured monocyte-derived macrophages and suppressed the release of pro-inflammatory cytokine. Furthermore, we found apoptotic cell derived extracellular vesicles (aEVs) in the circulation of patients with CRC liver metastases. Efferocytosis of aEVs by neutrophils induced an activated phenotype (CD11bhigh CD16high CD66bhigh CD62Llow), however, classical inflammatory responses such as NETosis, respiratory burst, degranulation, or secretion of pro-inflammatory cytokines could not be observed. Instead, efferocytosing neutrophils released various growth factors including fibroblast growth factor-2 and hepatocyte growth factor (HGF). Accordingly, we observed a correlation of free HGF and of HGF+ neutrophils with apoptotic debris in the circulation.

**Conclusion**

These data suggest that removal of apoptotic cells results in a population of non-inflammatory but regenerative neutrophils that create an immunosuppressive and resolution-promoting tumor microenvironment, supporting the growth of tumor cells that survived anti-tumor treatment.

### EACR23-1430
### Clearance of senescent macrophages ameliorates tumorigenesis in KRAS-driven lung cancer

D. Muñoz-espin[1], S. Haston[2], E. Gonzalez-Gualda[3], S. Morsli[4], J. Ge[4], V. Reen[5], J. Gil[5], J.P. Martinez-Barbera[2]
[1]UNIVERSITY OF CAMBRIDGE, ONCOLOGY, CAMBRIDGE, United Kingdom
[2]Institute of Child Health UCL, Developmental Biology and Cancer Programme, London, United Kingdom
[3]Early Cancer Institute, Oncology University of Cambridge, Cambridge, United Kingdom
[4]Early Cancer Institute, Oncology Cambridge, Cambridge, United Kingdom
[5]Institute of Clinical Sciences, Faculty of Medicine Imperial College London, London, United Kingdom

**Introduction**

Cellular senescence was initially thought to function primarily as a potent tumour suppressive mechanism that limits the ability of oncogenically-stressed cells to proliferate, and this is often accompanied by a process of immunoclearance. However, upon persistent damage, accumulation of senescent cells in the tumour microenvironment can drive a variety of cancer-promoting activities through the proinflammatory senescence-associated secretory phenotype (SASP), but this remains poorly understood in the case of lung cancer.

**Material and Methods**

We have generated a new mouse model, termed *p16-FDR*, that can be used to identify, isolate, trace and ablate p16$^{INK4a}$-expressing cells in vivo. We use the p16-FDR model in combination with distinct oncogenic KRAS-driven non-small cell lung carcinoma (NSCLC) murine models and naturally-aged mice to investigate the role that p16INK4a-expressing cells play in influencing lung tumour development. Our experimental settings include molecular characterisation by single cell transcriptomic studies, a variety of functional and histopathology analyses, and validation/proof-of-concept with NSCLC clinical samples.

**Results and Discussions**

Using a new *p16-FDR* mouse line, we show that macrophages and endothelial cells are the predominant senescent cell types in murine KRAS-driven lung tumours. Single cell transcriptomics identify a population of tumour-associated macrophages (TAMs), expressing a unique array of pro-tumourigenic SASP factors and surface proteins. Interestingly, this population of TAMs with senescent features is also present in normal aged lungs. Genetic or senolytic ablation of senescent cells, or macrophage depletion following CSF1R blockade, result in a significant reduction in tumour burden and increased mouse survival of KRAS-driven lung cancer models. Mechanistically, senescent macrophages promote tumour immunosuppression by reducing CD8+ T cell accumulation and increasing numbers of regulatory T cells, while they stimulate the tumour vasculature maintenance. Of translational relevance, we reveal the presence of macrophages with senescent features in human lung premalignant lesions, including atypical adenomatous hyperplasia and adenocarcinoma in situ, but not in adenocarcinomas.

© 2023 The Authors. Molecular Oncology is published by John Wiley & Sons Ltd on behalf of Federation of European Biochemical Societies

**Conclusion**
Together, our results have uncovered a population of (targetable) senescent macrophages contributing to the initiation and progression of lung cancer, thus opening potential therapeutic avenues and cancer preventative strategies for patients bearing lung multifocal primary lesions.

### Symposium: Neoantigens & Vaccines

### EACR23-0072
### Unconventional effectors of immune checkpoint blockade in HLA class I-deficient cancers

*J. van de Haar*[1], *N.L. de Vries*[2], *V. Veninga*[1], *M. Chalabi*[3], *T.N. Schumacher*[1], *L.F.A. Wessels*[4], *F. Koning*[5], *N.F.C.C. de Miranda*[2], *E.E. Voest*[1]
[1]*The Netherlands Cancer Institute, Department of Molecular Oncology and Immunology, Amsterdam, The Netherlands*
[2]*Leiden University Medical Center, Department of Pathology, Leiden, The Netherlands*
[3]*The Netherlands Cancer Institute, Gastrointestinal Oncology, Amsterdam, The Netherlands*
[4]*The Netherlands Cancer Institute, Division of Molecular Carcinogenesis, Amsterdam, The Netherlands*
[5]*Leiden University Medical Center, Department of Immunology, Leiden, The Netherlands*

**Introduction**
Immune checkpoint blockade (ICB) is thought to primarily activate conventional $CD8^+$ αβ T cells, which recognize tumor antigens presented by HLA class I molecules. However, mismatch repair-deficient (MMR-d) cancers with HLA class I defects are resistant to these cytotoxic effectors and yet can show deep responses to ICB, offering a unique model to study powerful unconventional antitumor immunity.
**Material and Methods**
To identify unconventional effectors of ICB in HLA class I-negative cancers, we applied a suite of genomic, (single-cell) transcriptomic and imaging CyTOF-based techniques on MMR-d cancers with/without *B2M* mutations before and after ICB. This included patients from TCGA and our DRUP and NICHE clinical trials. The findings were further validated and studied mechanistically through *in vitro* experimentation with cell lines and patient-derived organoid (PDTO) – immune cell co-culture systems.
**Results and Discussions**
In the DRUP study, we paradoxically observed significantly enhanced responsiveness to ICB of *B2M*-mutant (vs *B2M*-wildtype) MMR-d cancers. Across multiple cohorts, *B2M*-mutant MMR-d cancers showed profound infiltration by γδ T cells, whereas the levels of other effectors (including $CD4^+$ and NK cells) were unaffected by *B2M* status. These γδ T cells were mainly composed of Vd1 and Vd3 subsets, and expressed high levels of PD-1, markers of activation and proliferation, a broad repertoire of cytotoxic molecules, and killer-cell immunoglobulin-like receptors (KIRs). *In vitro*, γδT cells isolated from MMR-d colon cancers exhibited a cytolytic response towards MMR-d colon cancer cell lines and PDTOs, which could be markedly enhanced by knocking out *B2M*. Paralleling observations in αβ T cells, *in vitro* tumor reactivity of γδ T cells was largely confined within PD-1 expressing subsets. Blocking experiments uncovered central determinants of tumor recognition by γδ T cells in this context. Finally, by comparing paired tumour samples of MMR-d colorectal cancer patients obtained before and after dual PD-1 and CTLA-4 blockade in the NICHE trial, we found that ICB specifically increased the intratumoral frequency of cytotoxic γδ T cells in HLA class I-negative cancers.
**Conclusion**
These data indicate that γδ T cells are unconventional effectors of ICB therapy in patients with HLA class I-negative, MMR-d colon cancers. This underlines the potential of γδ T cells in cancer immunotherapy, in particular for cancers with antigen presentation defects.

### EACR23-0350
### Endogenous retroelements as cancer-specific antigens

*T. Hofland*[1], *R. Thompson*[1], *J. Attig*[1], *G.R. Young*[1], *G. Kassiotis*[1]
[1]*Francis Crick Institute, Retroviral Immunology, London, United Kingdom*

**Introduction**
Immunotherapy relies on cancer-specific antigens to target T cell responses to tumour cells. Although neo-antigens have been the main focus as T cell targets, their individualistic nature makes it difficult to translate these therapies for multiple patients or tumour types. Our lab has recently described increased expression of endogenous retroelements (ERE) across cancer types. We identified ERE-transcripts with cancer-specific expression that could serve as tumour antigens, with broad expression across patients and multiple tumour types, highlighting their immunotherapeutic potential. However, it is unknown if effective anti-tumour T cell responses can be induced to ERE antigens.
**Material and Methods**
To determine the immunogenicity of ERE peptide epitopes, we co-cultured human peptide-pulsed dendritic cells (DC) with CD8 T cells *in vitro*. ERE-specific T cells were identified by tetramer staining, and sorted for 10X single cell TCR profiling. Additionally, we used HHD mice (which express HLA-A2) to expand ERE-specific CD8 T cells *in vivo*. HHD mice were immunized with peptide-pulsed DCs and ERE-specific CD8 T cells were sorted for TCR sequencing. Isolated human and murine TCRs were cloned into an expression vector and transduced into Jurkat cells to study their functionality.
**Results and Discussions**
We expanded human T cells recognizing peptide epitopes from several cancer-specific ERE-transcripts that associate with multiple cancer types (e.g. melanoma, oesophageal, colon, stomach and lung cancer), leading to the identification of multiple ERE-specific TCRs. This level of T cell expansion indicates that EREs can serve as immunogenic cancer targets. ERE-specific TCRs showed variable sensitivities to their cognate antigen, but it is unclear how sensitive a TCR needs to be in order to induce

© 2023 The Authors. Molecular Oncology is published by John Wiley & Sons Ltd on behalf of Federation of European Biochemical Societies anti-tumour responses. We therefore immunized HHD mice to the same ERE epitopes (which are not tolerized in mice) to compare the sensitivity of human TCRs (which may be partly tolerized) with murine TCRs, which would define the therapeutic potential of our human TCRs. Anti-tumour responses of ERE-specific TCRs are currently being examined in *in vitro* co-cultures with primary human cancer cells and in *in vivo* tumour challenge experiments in mice.

**Conclusion**

We show that EREs harbour immunogenic epitopes that can serve as cancer-specific antigens. T cell responses to EREs could be a novel addition for immunotherapeutic strategies that could potentially benefit a large number of patients across cancer types.

### Symposium: Liquid Biopsies

### EACR23-0017
### Minimally invasive proteome sampling with electroporation-based biopsy carries features discriminating between cutaneous squamous cell carcinoma and basal cell carcinoma

*A. Golberg[1], E. Vitkin[2], W. Julia[2], A. Berl[3], O. Shir-az[3], B. Gabay[1], Z. Yakhini[4], A. Shalom[3]*
*[1]Tel Aviv University, Faculty of Exact Sciences, Tel Aviv, Israel*
*[2]Tel Aviv University, Environmental Studies, Tel Aviv, Israel*
*[3]Meir Medical Center, Department of Plastic Surgery, Kfar Sava, Israel*
*[4]Reichman University, Reichman University, Herzliya, Israel*

**Introduction**

Although the clinical features of cutaneous squamous cell carcinoma (cSCC) and basal cell carcinoma (BCC) are well described, and in the absolute majority of cases the clinical diagnosis is done according to the subsequent histological verification, sometimes the clinical phenotypes of these cancers are ambiguous and discrepancies between the clinical presentations and histologic analyses rise. To address the need in improved tissue-spatial sampling of biomarkers, and to extend the technology state of the art in enabling precise personal medicine, we developed a novel tissue liquids sampling approach with molecular biopsy using electroporation. We show that electroporation-based molecular sampling, coined “e-biopsy”, selectively extracts liquids from solid tissues with informative proteomes.

**Material and Methods**

We report the development of a novel tissue sampling approach with molecular biopsy using electroporation. The method, coined e-biopsy, enables non-thermal permeabilization of cells in the skin for efficient vacuum-assistant extraction of informative biomolecules for rapid diagnosis. We used e-biopsy for *ex vivo* proteome extraction from 3 locations per patient in 21 cSCC and in 21 BCC pathologically validated human tissue samples. The total 126 extracted proteomes were digitalized with mass spectrometry. The obtained mass spectra presented significantly different proteome profiles for cSCC and BCC with several hundreds of proteins significantly differentially expressed in each tumor in comparison to another.

**Results and Discussions**

Notably, 17 proteins were uniquely expressed in BCC and 7 were uniquely expressed in cSCC patients. Statistical analysis of differentially expressed proteins found 31 cellular processes, 23 cellular functions and 10 cellular components significantly different between cSCC and BCC. Machine Learning classification models constructed on the sampled proteomes allowed separation of cSCC patients from BCC with 81% accuracy, 78.7% precision and 92.3% recall, which is comparable to manual initial diagnostics in clinical setup. Finally, the protein-protein interaction analysis of 11 most informative proteins, derived from Machine Learning framework, enabled detection of a novel protein-protein interaction network valuable for further understanding of skin tumors.

**Conclusion**

Our results provide evidence that the e-biopsy could potentially be used as a tool to support cutaneous tumors classification with rapid molecular profiling.

### EACR23-0102
### Individualised Predictions of Response to Treatment in Metastatic Breast Cancer using ctDNA

*S. Karapanagiotis[1], E.J. Beddowes[2], M. Ortega-Duran[2], O. Rueda[1], C. Caldas[2]*
*[1]University of Cambridge, MRC Biostatistics Unit, CAMBRIDGE, United Kingdom*
*[2]University of Cambridge, Cancer Research UK Cambridge Institute, Cambridge, United Kingdom*

**Introduction**

Evaluation of response to treatment is essential in the management of metastatic disease. Monitoring of circulating tumour DNA (ctDNA) has been proposed as an attractive (inexpensive and minimally invasive) method to track response to treatment. Here we describe a probabilistic framework to predict response to treatment in metastatic breast cancer (mBC) using longitudinal ctDNA data.

**Material and Methods**

A cohort of 188 mBC patients had DNA extracted from serial plasma samples (total 1098, median=4; median (range) follow-up 1.15 (0-12.4) years). Plasma DNA was assessed using sWGS and the ctDNA levels were estimated using ichorCNA. Response to treatment was determined through CT imaging. Both the evolution of the longitudinal ctDNA levels and the trajectories of the response to treatment were modelled.

**Results and Discussions**

We show that longitudinal ctDNA monitoring predicts response to treatment in mBC. We found that (1) the incorporation of ctDNA to monitor response to treatment offers clinical utility, outperforming CA 15-3, and (2) the model allows for individualised dynamically updated predictions as additional ctDNA measurements become available.

**Conclusion**

Our results demonstrate the promise of ctDNA monitoring in predicting treatment response and its potential for personalised clinical decision-making. We provide

© 2023 The Authors. Molecular Oncology is published by John Wiley & Sons Ltd on behalf of Federation of European Biochemical Societies quantitative estimates of response to treatment. Our proposal considers the probability of treatment response for each patient individually, by doing so, it can identify individual patients with extremely high-risk of non-response to treatment for which the treatment should be discontinued. We anticipate our modelling framework will be a starting point for more sophisticated models in additional cancer types.

### Symposium: Chemical Biology

### EACR23-0419
### Biochemical and biological characterization of novel, irreversible ALDH1A3 inhibitors for use in breast cancer therapy

*A. Constantinescu[1], R. Pequerul Pavon[2], R. Rebollido-Rios[3], X. Parés[2], D. Colignon[4], A. Berrou[5], G. Fournet[6], G. Martin[1], J. Farrés[2], M. Pérez-Alea[1]*
*[1]Advanced BioDesign, Research and Development, Saint Priest, France*
*[2]Faculty of Biosciences- Universitat Autonoma de Barcelo na, Biochemistry and Molecular Biology, Barcelona, Spain*
*[3]Faculty of Medicine and Univerity Hospital of Cologne, Internal Medicine, Cologne, Germany*
*[4]Currently- Admescope, Analytical Chemistry, Oulu, Finland*
*[5]Currently- CEISAM-UMR CNRS6230, Chimie Moléculaire, Nantes, France*
*[6]Université de Claude Bernard-Lyon1, Institut de Chimie et Biochimie Moléculaire et Supramoléc ulaire, Lyon, France*

**Introduction**
Aldehyde dehydrogenase 1 isoforms (ALDH1) are cancer stem cell (CSC) markers and, in breast cancer, they are associated with triple-negative/basal-like subtypes and aggressive disease. Gene silencing and functional validation studies on ALDH1A3 suggest that this isoform could be a target in breast cancers that are commonly dependent on its activity for tumour growth. Nonetheless, the translation of research into clinical medicine remains a challenge, mainly due to the dearth of selective inhibitors.
**Material and Methods**
To facilitate the identification of novel inhibitors of this enzyme, a compound library was generated in order to increase the chemical and biological relevance of the 4-dimethylamino-4-methylpent-2-ynthioic acid S-methyl ester (DIMATE), a small-molecule inhibitor of ALDH1, currently in clinical evaluation. The most promising compounds were subjected to in-depth biochemical and enzymatic characterization using human recombinant ALDH enzymes. Automated capillary-based immunoassay in combination with flow cytometry were implemented for the identification of proteins involved in the cell death response signalling. Cell migration studies and preclinical efficacy studies were conducted in various cell lines and in orthotopic breast cancer xenograft models.
**Results and Discussions**
Through a tiered screening approach, two structural analogues of DIMATE were identified as irreversible, label-selective inhibitors of ALDH1A3, with higher inhibitory potency than that of DIMATE. Both compounds induced apoptosis mediated by ROS-dependent, prolonged JNK activation. Evaluation of the antitumor activity showed a significant inhibition of primary breast tumour growth and metastasis to lymph node and lungs, enhancing the *in vivo* activity of DIMATE.
**Conclusion**
Our work confirms the role of ALDH1A3 as an important target in breast cancer cells and proposes new promising molecules for translational clinical research.

### EACR23-1129
### Discovery of I-0436650, a potent and selective SHP2 allosteric inhibitor for the treatment of RAS driven solid tumors

*F. Puca[1], D. Fabbrini[2], F. Scalabrì[1], C. Alli[3], V. Pucci[4], C. Montalbetti[2], R. Di Fabio[2], A. Petrocchi[2], A. Carugo[1], C. Toniatti[1]*
*[1]IRBM Spa, Oncology, Pomezia, Italy*
*[2]IRBM Spa, Small Molecules R&D department, Pomezia, Italy*
*[3]IRBM Spa, Translational Research department, Pomezia, Italy*
*[4]IRBM Spa, Experimental Pharmacology department, Pomezia, Italy*

**Introduction**
SHP2 (Src homology region 2-containing protein tyrosine phosphatase 2) is a target of interest for cancer therapy due to its key role in the regulation of RAS/MAPK signal transduction downstream of Receptor Tyrosine Kinases (RTKs). We report here the identification of I-0436650, a novel, highly potent, orally available SHP2 allosteric inhibitor, with potential for the treatment of tumors with dysregulated RTK/RAS/ERK signaling pathways.
**Material and Methods**
High-throughput biological, biochemical and pharmacodynamic (PD) assays were used to inform Structure Activity Relationship studies which led to the identification of a chemical series of potent SHP2 inhibitors. Iterative optimization of physicochemical and pharmacological properties resulted in the identification of I-0436650, an orally available SHP2 inhibitor with excellent drug-like properties. The therapeutic potential of I-0436650 was evaluated in a series of ex vivo and in vivo experiments.
**Results and Discussions**
I-0436650 is a potent inhibitor of the SHP2 enzyme in vitro (IC50 = 5 nM) with a very slow koff rate (2.42E-05 1/s). When tested in cell line, it strongly inhibits pERK, a downstream marker of MAPK pathway activity, in a dose dependent manner. I-0436650 exhibits significant anti-proliferation activity against RAS or EGFR mutant cancer cell models alone and in combination with different FDA approved drugs. In particular, the combination of I-0436650 with the KRAS G12C inhibitors Sotorasib and Adagrasib, the EGFR inhibitor Osimertinib and the CDK4/6 inhibitor Ribociclib results in synergistic growth inhibition of multiple RTK/KRAS-driven cancer cell lines. Further, I-0436650 effectively inhibits tumor growth in vivo in SHP2-dependent human xenograft models.
**Conclusion**
I-0436650 is a potent, selective and orally available SHP2 inhibitor with opportunity for once-a-day dosing in humans. Of note, its synergistic effect when combined with

© 2023 The Authors. Molecular Oncology is published by John Wiley & Sons Ltd on behalf of Federation of European Biochemical Societies selected pharmacological agents makes it a valuable therapeutic option against a multitude of RTK/KRAS-driven tumors and metastases.

### POSTER PRESENTATIONS (Tuesday/Wednesday)

### Bioinformatics and Computational Biology

### EACR23-0022
### Facilitating drug discovery and structural biology of large protein assemblies such as the Ras-Raf signalosome by simulations of biomolecular binding from the first principles

Y. Shan[1], Z. Zhou[2], C. Ambrogio[3], K. Westover[4], J. Pasi[5], V. Mysore[6], J. Kapp[7], A. Pluckthun[7]

*[1]Antidote Health Foundation / Broad Institute, Stanley Center, Cambridge, United States*

*[2]UT Southwest Medical Center, Dept of Biochemistry and Radiation Oncology, Dallas, United States*

*[3]University of Turin, Molecular Oncology, Turin, Italy*

*[4]UT Southwest Medical Center, Dept of Biochemistry and Radiation Oncology, Dallas- Texas, United States*

*[5]Dana Farber Institute, Thoracic Oncology, Boston- Massachusetts, United States*

*[6]Nvidia, Dept of Principal Solutions, Castro Valley- California, United States*

*[7]University of Zurich, Dept of Biochemistry, Zurich, Switzerland*

**Introduction**

It is increasingly feasible to simulate the physical process of protein-protein and protein-small molecule binding and to generate native structures of biomolecular complexes using molecular dynamics from first principle of physics. With experimental validation, such simulations can produce atomic-detailed structural models of protein-small molecule and protein-protein complexes, including structure of large functional protein assemblies.

**Material and Methods**

The work discussed here were based on the so-called "swimming" molecular dynamics simulations combined with a number of experimental validations

**Results and Discussions**

In this talk we will show that such swimming simulations correctly recapitulated the small-molecule binding of several drug target and provided critical insights to drug discovery at molecular level. We will also present our modeling the structure of megadalton membrane-anchored Ras-Raf signalosome underlying the activity of MAPK pathway using such simulations.

**Conclusion**

The structural model of the Ras-Raf signalosome provides explanations for a large body of divers experimental findings under one framework. It suggests that disrupting the assembly of the Ras-Raf signalosome is essential to success of drug discovery aiming to attenuate the activity of the MAPK pathway and minimize drug resistance.

### EACR23-0042
### The world's first digital cell twin in cancer electrophysiology: a new direction in cancer research (Part I)

C. Baumgartner[1], S. Langthaler[1]

*[1]Graz University of Technology, Institute of Health Care Engineering with European Testin g Center of Medical Devices, Graz, Austria*

**Introduction**

The introduction of functional in-silico models, in addition to in-vivo tumor models such as cell line-derived xenograft or patient-derived xenograft models opens up new possibilities in cancer research and drug development with the increasing availability of experimental data. The world's first digital twin of the A549 cell's electrophysiology in the human lung adenocarcinoma, unveiled in 2021, enables for the first time the investigation and evaluation of new research hypotheses about modulating the function of ion channels in the cell membrane, which are important for a better understanding cancer development and progression, as well as for the development of new drugs and predicting treatments.

**Material and Methods**

The developed mathematical model which is based on a hidden Markov (HMM) description of the underlying ion channel mechanisms of the electrophysiologcial system, allows virtual simulations of the cell's rhythmic oscillation of the membrane potential $V_m$ that can trigger the transition between cell cycle phases. The model consists of 11 known functionally expressed ion channels in the cell's plasma membrane with their specific voltage and calcium dependence.

**Results and Discussions**

The cell twin is able to predict the promotion or interruption of cell cycle progression through targeted activation and inactivation of ion channels, leading to abnormal hyper- or depolarization of the membrane potential, a potential key signal for the known cancer hallmarks. For example, model simulations of blockade of transient receptor potential cation channels (TRPC6), which are highly expressed during S-G2/M transition, results in a strong hyperpolarization of the cell's membrane potential that can suppress or bypass the depolarization required for the S-G2/M transition, allowing for possible cell cycle arrest and inhibition of mitosis. All simulated research hypotheses could be verified by experimental studies in the literature.

**Conclusion**

Functional, non-phenomenological digital twins, ranging from single cells to cell-cell interactions and 3D tissue models, representing a mirror image of the real biological system at different levels of abstraction, open new avenues for modern cancer research through "dry lab" approaches that optimally complement established in-vivo and in-vitro methods. In particular, the A549 cell model confirms that the changing membrane potential is a potential key signal for the known cancer hallmarks which should be paid more attention in research and development in cancer electrophysiology.

© 2023 The Authors. Molecular Oncology is published by John Wiley & Sons Ltd on behalf of Federation of European Biochemical Societies

### EACR23-0043
### The world's first digital cell twin in cancer electrophysiology: simulation and prediction of ion channel mediated cell proliferation (PART II)

S. Langthaler[1], C. Baumgartner[1]
[1]Graz University of Technology, Institute of Health Care Engineering with European Testin g Center of Medical Devices, Graz, Austria

**Introduction**
Advances in molecular targeting of ion channels involved in tumor development and progression may open up new avenues for therapeutic approaches in cancer based on the cells' bioelectric properties. However, the complexity of the underlying mechanisms requires a holistic view and strategy to effectively identify and explore potential key targets. In silico models can therefore provide deeper insight into the complex role of electrophysiological properties in cancer and reveal the impact of altered ion channel expression and aberrant changes of the membrane potential on malignant processes. With the vision of establishing such approaches in the context of cancer cells, we recently introduced a first model of the A549 lung cancer cell line, describing the electrophysiological processes underlying cell proliferation. The model focusses solely on channels of the plasma membrane, not considering the interplay of inner membrane channels. To further increase the accuracy, we now propose an extension of the model by taking into account the complex local intracellular calcium dynamics, which significantly affect the entire electrophysiological properties of the cell and control cell cycle progression.

**Material and Methods**
The initial model was extended by a spatio-temporal modeling approach, addressing the heterogenous calcium profile and dynamics in the ER-PM junction provoked by local calcium entry of store operated calcium channels (SOCs), and release from the ER by SERCA pumps, RyR and $IP_3$ receptors. Changes of cytosolic calcium levels due to diffusion from the ER-PM junction and responding plasma membrane channels were simulated and the dynamics evaluated based on calcium imaging data.

**Results and Discussions**
Calcium signaling and hyperpolarization of the cell membrane are considered important key signals for the transition from G1 to S phase. Corresponding simulations of a calcium depletion from intracellular stores in G1 phase using the model lead to a reliable hyperpolarization of the membrane potential, providing an electrophysiological explanation of the processes behind G1/S transition.

**Conclusion**
The local calcium distribution and time evolution in microdomains of the cell is highly significant for the activity of responding ion channels and, subsequently, the electric properties of the cell membrane. Thus, this further development represents an important step on the route towards a profound and valid model for oncological research and development of novel therapeutic strategies supported by model simulations.

### EACR23-0118
### Study on suppression of dual-phosphorylation to Ser218/Ser222 by allosteric inhibitors targeting MEK1/2 kinase

S.K. Mudedla[1], H.Y. Lee[2], J.J. Kim[2], S.H. Jang[1], J. Sridhara[3], J. Shin[1], H. Lee[1], G. Mukherjee[1], M.R. Doddared[3], S. Wu[1,4], J.J. Park[2]
[1]PharmCADD, R&D Center, Busan, South Korea
[2]Isu Abxis, New Drug Discovery Division, Seongnam-si- Gyeonggi-do, South Korea
[3]PharmCADD, R&D Center, Hyderabad- Telangana, India
[4]Pukyong National University, Department of Physics, Busan, South Korea

**Introduction**
The co-crystallized structures of MEK1/2 and their allosteric inhibitors reveal distinct interactions between the allosteric inhibitors and the MEK1/2 activation loop residues. The suppression mechanism of phosphorylation of MEK1/2 at Ser218/222 for each allosteric inhibitor is different. The structural analyses will determine whether, and how, distinct inhibitors suppress the phosphorylation of MEK1 and MEK2 and may guide future therapeutics development.

**Material and Methods**
*Computational Details*
In this study, we explored the suppression mechanism of the phosphorylation process in the presence of MEK allosteric inhibitors such as selumetinib, trametinib, cobimetinib, and CH5126766 by employing molecular dynamics simulations and quantum mechanics/molecular mechanical calculations.**Results and Discussions**
The simulations of wildtype MEK1/2 shows that Ser222 can come close to γ-phosphate but not Ser218. We have found the conformation where Ser222 is within 5 Å of distance which makes Ser222 accessible for γ-phosphate. The activation energy barriers from QM/MM study for the transfer of γ-phosphate to Ser222 are in close agreement with previous report. The conformation analysis from the simulations of MEK1/2 in the presence of allosteric inhibitors, reveals that the inhibitor restricts the flexibility of Ser222 through strong interactions with the activation loop, Lys97, and water mediates interactions with amino acids in the vicinity. The strong interaction of water molecules with halogen atoms (Br, Cl, and I) in the allosteric inhibitor further stabilizes the complex and may also contribute to water-mediated MEK/RAF interactions. The results reveal that all the inhibitors act as screeners between the activation loop and Mg-ATP and restricting the flexibility of the activation loop through strong interaction causes the suppression of the phosphorylation process of MEK1/2.

**Conclusion**
The results were compared using CHARMM General Force Field (CGenFF) and in-house built AI force fields for allosteric inhibitors. The results conclude that a strong interaction of allosteric inhibitors with the activation loop restricts the movement of Ser218/222 towards Mg-ATP, which could be the dominant factor for the suppression of phosphorylation in MEK1/2. This research will provide novel insights to design effective anti-cancer therapeutics for targeting MEK1/2 in the future.

© 2023 The Authors. Molecular Oncology is published by John Wiley & Sons Ltd on behalf of Federation of European Biochemical Societies

### EACR23-0215
### miRcuit: A tool for integrating analysis of mRNA and miRNA expression data to infer regulatory circuits

*B. Karaoglu[1], E. Dallı[2], V. Mazlum[3], H. Ogul[4], B. Gur Dedeoglu[1]*
*[1]Ankara University Biotechnology Institute, System Biotechnology, Ankara, Turkiye*
*[2]Ege University, Department of Biology, Izmir, Turkiye*
*[3]Cankaya University, Department of Computer Engineering, Ankara, Turkiye*
*[4]Østfold University College, Department of Computer Secience and Communication, Halden, Norway*

**Introduction**

Gene expression is controlled by many factors. While transcription factors (TFs) perform this control at the transcriptional level, microRNAs (miRNAs) are involved at the post-transcriptional level. Biological networks and circuits based on TFs, miRNAs and their targets are extremely important for a better understanding of cellular processes. For this purpose, we developed an algorithm and designed a web tool that creates biological circuits by using experiment-specific and literature-based data together.

**Material and Methods**

Circuit analysis tool was created using Python software language (v.3.7.4) to construct regulatory circuits from the high-throughput data containing expression values of genes and miRNAs. The datasets to be uploaded are mRNA (also including TFs) and miRNA files that show significant expression changes under two different conditions like tumor and normal tissues. Then, the absolute log(FC) values were calculated by performing the log2 transformation for each molecule in the sets (mRNA list and miRNA list) by Pandas and Math libraries offered by Python. Each molecule is labelled "up" or "down" and the direction of the intermolecular relationship is determined.

**Results and Discussions**

The mRNA (TF) and miRNA lists showing significant expression changes were combined and all possible mRNA (TF)-miRNA pairs were obtained. Significant relationships were extracted by comparing all possible pairs with known pairs in databases (Data bases and direction of the relationship: TargetScan: miRNA-mRNA relationship, miRNA ----| mRNA or miRNA----| TF. TransmiR: TF-miRNA relationship, TF ----> miRNA. hTFtarget: TF-mRNA relationship, TF ----> mRNA). All TF-mRNA, TF-miRNA, miRNA-mRNA and miRNA-TF pairs showing significant expression changes were obtained and intersected with each other. As a result, two different types of open circuit and one closed type circuit files were created.

**Conclusion**

The algorithm developed to create biological circuits extracting the TF – miRNA – mRNA relationships will soon be available as a web tool under the name "Integrative Regulatory Circuit Finder".

### EACR23-0221
### 3D-Quantitative Structure-Activity Relationship approach in the design of novel Rho-associated protein kinases (ROCK) inhibitors

*M. Beljkaš[1], S. Oljačić[1], M. Petković[2], K. Nikolić[1]*
*[1]Faculty of Pharmacy- University of Belgrade, Department of Pharmaceutical Chemistry, Belgrade, Serbia*
*[2]Faculty of Pharmacy- University of Belgrade, Department of Organic Chemistry, Belgrade, Serbia*

**Introduction**

Rho-associated protein kinases (ROCK1 and ROCK2) are involved in various cellular functions as the key regulators of numerous substrates. Alterations in ROCK activities are associated with many diseases, including cancer. ROCKs play a critical role in the initiation and growth of cancer through the activation and stabilization of some oncogenes and in cancer progression through influence on migration, invasion, and angiogenesis. Recent studies have shown an association between the overactivity of ROCKs and lower survival in patients with the most aggressive carcinomas, such as pancreatic ductal adenocarcinoma (PDAC). Moreover, the activity of ROCK is related to tumor cells and the tumor environment, which contributes to tumor progression. Therefore, ROCKs are very good drug targets in anticancer research. In this study 3D-quantitative structure-activity relationship (3D-QSAR) method was applied to examine the structures of ROCK1 and ROCK2 pharmacophores and design dual ROCK1 and ROCK2 inhibitors as novel anticancer agents.

**Material and Methods**

The dataset of structurally diverse ROCK1 and ROCK2 inhibitors with $IC_{50}$ values was downloaded from the ChEMBL database. Conformations of all compounds were optimized by use of Hartree-Fock with 3-21G basis set method. The bioactive conformations of the ligands for the 3D-QSAR study were generated by a molecular docking study that was performed by GOLD software 2022.1.0. Pentacle software 1.07 was used to generate 3D-QSAR models for ROCK1 and ROCK2 isoforms. All compounds were divided into a training set and a test set to prepare both 3D-QSAR models.

**Results and Discussions**

The molecular determinants responsible for ROCK1 and ROCK2 inhibiting activities have been identified by 3D-QSAR modeling and comparative analysis of the ROCK1 and ROCK2 pharmacophores. Both created 3D-QSAR models met all the internal and external validation criteria. Validated 3D-QSAR models were used to design new ROCK1 and ROCK2 inhibitors and predict their activities. The most active designed compounds were further evaluated using *in silico* ADMET (Absorption, Distribution, Metabolism, Excretion, Toxicity) profiling. Based on these findings, novel dual ROCK1/ROCK2 inhibitors were selected for further synthesis.

**Conclusion**

According to the obtained results, both 3D-QSAR models can be considered as a reliable prediction of the ROCK1 and ROCK2 inhibiting activity of novel compounds and thus significantly facilitate the selection of the best candidates for further synthesis and biological evaluation.

### EACR23-0251
### A transcriptome-wide gene expression

© 2023 The Authors. Molecular Oncology is published by John Wiley & Sons Ltd on behalf of Federation of European Biochemical Societies

### outlier analysis led to the identification of PRMT5 as synthetic lethal target in MTAP-deleted colorectal cancer cells

*E. Mariella[1], G. Grasso[1], M. Miotto[1], K. Buzo[1], N.M. Reilly[1], P. Andrei[1], P.P. Vitiello[1], G. Crisafulli[2], S. Arena[1], G. Rospo[1], G. Corti[1], A. Lorenzato[1], C. Cancelliere[3], L. Barault[1], G. Gionfriddo[1], M. Linnebacher[4], M. Russo[1], F. Di Nicolantonio[1], A. Bardelli[1]*
*[1]University of Torino, Department of Oncology, Candiolo, Italy*
*[2]IFOM- The AIRC Institute of Molecular Oncology, Genomics of Cancer and Targeted Therapies, Milano, Italy*
*[3]Candiolo Cancer Institute FPO-IRCCS, Molecular Oncology, Candiolo, Italy*
*[4]University Medicine Rostock, Department of General Surgery, Rostock, Germany*

**Introduction**

The number of effective therapies approved for metastatic colorectal cancer (mCRC) is still limited and multiple strategies are continuously explored to expand the drug target repertoire. Among these, the identification of overexpressed genes has prompted the discovery of actionable oncogenic dependencies in multiple tumour types.

**Material and Methods**

Starting from RNA sequencing data, we identified transcriptome-wide gene expression outliers, defined as samples showing abnormal expression for a particular gene, across 226 CRC cell lines, considering both overexpression and underexpression events as positive or negative outliers. Then, the distance of each outlier from gene-specific reference points, absolute expression values and differential expression values were considered in a multi-filter strategy to select extreme gene expression outliers, with the hypothesis that they are more likely to be functionally relevant in cancer cells. We also profiled genetic and epigenetic features of CRC cell lines based on whole exome sequencing and DNA methylation microarray data.

**Results and Discussions**

Extreme positive and negative gene expression outliers were found for 3,533 and 965 genes, respectively, and only some of them were associated with underlying genetic and epigenetic alterations. Gene expression alterations with known therapeutic or diagnostic value in CRC were pinpointed as extreme positive and negative outliers thus confirming the validity of the approach. Annotation of overexpressed enzyme genes according to the Target Development Level (TDL) classification revealed numerous enzymes for which inhibitors are already available. We next explored underexpression events to identify potential synthetic lethal targets. Intriguingly, we found that CRC models lacking expression of the *MTAP* gene were sensitive to treatment with an inhibitor of the PRMT5:MTA complex currently under clinical development.

**Conclusion**

We found that mapping extreme and transcriptome-wide positive and negative gene expression outliers in CRC cell lines is an effective strategy to identify putative drug targets and biomarkers, independently from the underlying genetic or epigenetic alterations. We indeed present a comprehensive atlas of CRC extreme gene expression outliers which includes events with diagnostic or therapeutic relevance. This resource could also serve as a reference for further discoveries in CRC and other tumour types.

### EACR23-0441
### Cancer signatures for reproducible gene expression analysis of single-cell and spatial transcriptomic data: the computational way to achieve precision medicine

*S. Pirrotta[1], L. Masatti[1], A. Corrà[1], F. Pedrini[1], P. Martini[2], D. Risso[3], C. Romualdi[1], E. Calura[1]*
*[1]University of Padova, Biology, Padova, Italy*
*[2]University of Brescia, Molecular and Translational Medicine, Brescia, Italy*
*[3]University of Padova, Statistical Sciences, Padova, Italy*

**Introduction**

Cancer is a complex disease, characterized by extensive genomic aberrations with an impact on gene expression and cell biological processes. Many studies and some clinical trials proposed gene expression signatures as a valuable tool for understanding cancer mechanisms, defining subtypes, monitoring patient prognosis and therapy efficacy. However, one of the major concerns is the lack of a computational implementation of the proposed signatures. That would provide detailed signature definition and would assure reproducibility, dissemination and usability of the classifier. An additional challenge is to define if these signatures, derived by bulk data analysis, are transferable to the new single-cell and spatial transcriptomic data, allowing the evaluation of intra-sample heterogeneity and the dissection of cell type contribution.

**Material and Methods**

A list of gene expression signatures covering numerous cancer hallmarks was collected from the literature. Then, they were implemented with R, the main programming language in computational biology.

**Results and Discussions**

With the aim of providing a tool able to improve reproducibility and usability of gene expression signature, we proposed signifinder, an R package that collects and implements more than 40 expression-based signatures from cancer literature. Collected signatures can attribute a score for each sample that summarizes many different tumor aspects, such as predict the response to therapy or the survival association, as well as quantify the environmental statuses, such as hypoxia status, or the activity of the immune response. signifinder can be applied to spatial and single-cell transcriptomic data with the attempt to study patient, sample and cell heterogeneity. For the first time, these new package features allow the analysis of the cancer signatures on these innovative types of transcriptomic data. Further, to help the researcher in dealing with the data interpretation signifinder is equipped with a workbox with plenty of graphical visualizations to allow an easy results exploration. signifinder has been included in the Bioconductor project since the 3.16 release.

**Conclusion**

© 2023 The Authors. Molecular Oncology is published by John Wiley & Sons Ltd on behalf of Federation of European Biochemical Societies

With the promise of tailored and optimized predictions for individual cancer patients, gene-expression signatures collected by signifinder can help in automatically investigating tumor samples, leading us a step closer to reproducible expression signature analysis for precision medicine.

### EACR23-0464
### Explainable Machine learning identifies survival predictors in metastatic colorectal cancer patients

*C. Martínez-Cortés[1], M.A. Beltrán Videla[2], E. Rodriguez-Braun[3], D. Torres-Moreno[4], A.J. Banegas-Luna[1], M.D. López-Abellán[5], G. Luengo-Gil[2], A.M. Hurtado[2], H.E. Perez-Sanchez[1], P. Conesa-Zamora[6]*
*[1]San Antonio Catholic University from Murcia, Structural Bioinformatics and High Performance Computi ng, Murcia, Spain*
*[2]Santa Lucia University Hospital, Molecular Pathology and Pharmacogenetics, Cartagena, Spain*
*[3]Santa Lucia University Hospital, Medical Oncology, Cartagena, Spain*
*[4]Santa Lucia University Hospital, Pathology, Cartagena, Spain*
*[5]Santa Lucia University Hospital, Laboratory Medicine, Cartagena, Spain*
*[6]Santa Lucia University Hospital, Molecular Diagnostics, Cartagena, Spain*

**Introduction**
Colorectal cancer (CRC) is the leading cause of cancer death in Western countries, after lung cancer in men and breast cancer in women. About 50% of patients with localized CRC will develop metastases, and 21% of patients already have metastases at diagnosis. The 5-year survival rate for metastatic CRC (mCRC) is 14%. Therefore, it is crucial to identify survival predictors at the time of metastasis diagnosis to provide these patients with proper and risk-adjusted treatment and follow-up. However, the enormous number of patient-associated information which could impact survival prediction is difficult to manage with conventional bioinformatics methods. The application of artificial intelligence (AI), and more specifically explainable machine learning (XML) is suitable to transform such data into real understandable and useful knowledge such as survival prediction.
**Material and Methods**
A total of 488 patients with mCRC from Murcia Region, Spain were enrolled in this study which is part of the REVERT project consortium (GA848098; www.revert-project.eu). Available information included demographic, lifestyle, comorbidities, clinical, histological, molecular, variables and blood tests at CRC diagnosis. Data were processed with the SIBILA XML tool (https://github.com/bio-hpc/sibila), where different machine learning models were geratedtrained (KNN, XGBOOST, RLF, RP, ANN,RF, SVM), and XML techniques were applied (LIME, SHAP, permutation importance and counterfactuals) to yield information about the most relevant factors that influence survival.
**Results and Discussions**
Binary classification ML models were obtained with XGBOOST, RLF and RF (AUC values of 0.915, 0.926 and 0.953, respectively). In general terms, older age and hypertension were predictors of worse survival although the best ones were related to metastasis localization; liver and bone metastasis being associated with worse and better survival, respectively. Synchronic metastasis, n-stage and tumor budding predicted bad survival as well as *BRAF* mutation, *PIK3CA* or *KRAS* native status. Some blood test results such as high lymphocyte, low neutrophil counts, and low gamma-glutamyl transferase were associated with better survival.
**Conclusion**
XML models based on clinical, histological and biochemical data could be useful to predict which patients will have worse outcome and thus, be candidates for a more aggressive or intense treatment or follow-up.

### EACR23-0468
### Quantitative Digital Image Analysis of Ultra-High Plex Immunofluorescence on Tissue Microarrays of Lung Cancer

*B. Ben Cheikh[1], R. Nandigama[2], N. Ma[3], R. Savai[2], O. Braubach[3]*
*[1]Akoya Biosciences- Inc, Marketing, LOS ANGELES, United States*
*[2]Max Planck Institute for Heart and Lung Research, Oncology Basic Research Unit, Munich, Germany*
*[3]Akoya Biosciences- Inc, Marketing, Menlo Park, United States*

**Introduction**
Ultra-high plex tissue imaging platforms, such as the PhenoCycler-Fusion, are transforming immuno-oncology research by enabling the simultaneous detection of more than 100 protein biomarkers at single-cell resolution. Quantitative image analysis of such large and complex datasets requires careful development of computational methods to accurately extract meaningful information. Tissue microarrays (TMA), in particular, can pose several challenges to analyses, owing primarily to batch effects and technical variability across large numbers of cores. We describe the development of an image analysis workflow for TMA datasets.
**Material and Methods**
200 lung cancer tissues were collected from patients with different EGFR and KRAS mutation status ($EGFR^+/KRAS^-$, $EGFR^-/KRAS^+$ and $EGFR^-/KRAS^-$) and placed on 4 TMA slides. The samples were then imaged using PhenoCycler-Fusion with a 42-antibody panel including, immune, tumor, proliferation, and apoptosis markers. A total of 382 TMA cores were analyzed using an advanced computational workflow. The first step of the workflow consists of nuclear and cell segmentation using a fine-tuned Deep Learning model, resulting in a total of 2.16 million cells across the whole dataset. This was followed by calculation of average protein expressions from the corresponding cell compartments, i.e. membrane vs. nucleus. Batch effect correction was then performed using a spectral graph method to minimize the technical variability between TMA cores. Unsupervised clustering using a GPU-accelerated Leiden algorithm was then performed on the batch-corrected feature matrix. A total of 36 clusters were identified and manually annotated into 15 cell phenotypes based on their protein expression patterns on a hierarchical clustering heatmap. Cell phenotype

© 2023 The Authors. Molecular Oncology is published by John Wiley & Sons Ltd on behalf of Federation of European Biochemical Societies percentages were calculated from each TMA core and compared between the 3 mutation groups, as well as spatial proximity and cellular neighborhood.

**Results and Discussions**

When compared to EGFR$^-$/KRAS$^-$ group, EGFR$^+$/KRAS$^-$ and EGFR$^-$/KRAS$^+$ tumors showed a significant increase in the percentages of tumor cell subtypes and a decrease in certain immune cell subtypes. Spatial analyses indicated statistically significant differences in the organization of these cell types between the 3 groups.

**Conclusion**

The results of our study show that ultra-high plex immunofluorescence on TMA slides combined with adequate computational tools provides a unique opportunity to better understand the spatiotemporal architecture of lung cancer and identify new therapeutic targets.

### EACR23-0503
### Deep Learning Based Algorithm For Postoperative Glioblastoma MRI Segmentation: A Promising New Tool For Tumor Burden Assessment

*M. Bonada[1], A. Bianconi[1], L.F. Rossi[2], P. Zeppa[1], R. De Marco[1], A. Melcarne[1], F. Cofano[1], L. Morra[2], L. Mainardi[3], D. Garbossa[1]*

*[1]University of Turin, Neurosurgery Department, Turin, Italy*

*[2]Politecnico di Torino, Dipartimento di Automatica e Informatica, Turin, Italy*

*[3]Politecnico di Milano, Dipartimento di elettronica- informazione e bioingegneria, Milan, Italy*

**Introduction**

Clinical and surgical decisions for glioblastoma patients depend on a tumor imaging-based evaluation. Artificial Intelligence (AI) can be applied to Magnetic Resonance Imaging (MRI) assessment to support clinical practice. In a real-world context, the current obstacles for AI are low-quality imaging and post-operative reliability. The aim of this study is to train an automatic algorithm for glioblastoma segmentation on a clinical MRI dataset and to obtain reliable results both pre- and post-operatively. To reproduce a real clinical scenario, the dataset includes cases with missing sequences and non-volumetric scans.

**Material and Methods**

The dataset used for this study comprises 237 (71 preoperative and 166 postoperative) MRIs from 71 patients affected by a histologically confirmed Grade IV Glioma. The implemented U-Net architecture was trained by transfer learning to segment the resection cavity (RC), the enhancing tumor volume (ET) and the whole tumor volume (WT) in post-operative images. The learning phase was carried out first on BraTS2021 dataset for preoperative segmentation. Performance is evaluated using DICE score (DS) and Hausdroff 95%.

**Results and Discussions**

The overall mean DS are 91.09 (± 0.60) and 72.31 (± 2.88), respectively obtained in pre-operative and post-operative scenarios. The overall mean Hausdroff 95% is 8.35 (± 1.12) for preoperative segmentation and 23.43 (± 7.24) for the postoperative assessment. These results are obtained using the two most informative MRI sequences (T1ce and T2-FLAIR). Remarkably, the RC segmentation obtained a mean DS of 63.52 (± 8.90) in postoperative MRIs.

Due to the benefits granted by informatic tools and strategies, our results are in line with the existing literature on this topic. Differently from previous studies, this work is not biased by restrictive inclusion/exclusion criteria for MRI scans. Therefore, we present this work as a starting point to apply AI to clinical practice for glioblastoma with remarkable reliability both in the preoperative and postoperative context.

**Conclusion**

The performances achieved by the algorithm are consistent with previous literature for both pre-operative and post-operative glioblastoma's MRI evaluation. Through the proposed algorithm, it is possible to reduce the impact of low-quality images and missing sequences. Some strategies have been proposed in this work to overcome current limitations, with promising results. Clinical applicability of this tool requires further confirmation from multi-institutional datasets.

### EACR23-0516
### Cancer cell states from high-throughput expression data: the definition of cancer heterogeneity through a collection of cancer signatures

*L. Masatti[1], S. Pirrotta[1], A. Corrà[1], F. Pedrini[1], P. Martini[2], D. Risso[3], C. Romualdi[1], E. Calura[1]*

*[1]University of Padova, Biology, Padova, Italy*

*[2]University of Brescia, Molecular and Translational Medicine, Brescia, Italy*

*[3]University of Padova, Statistical Sciences, Padova, Italy*

**Introduction**

Over the years, transcriptional signatures have been frequently studied because of their potential to show ongoing cancer activities. Gene expression signatures, defined as a specific collection of genes, can be summarized into a score that provides single-sample predictions about all complex traits of the disease such as response to therapy, hypoxia, immune response activity, epithelial-to-mesenchymal transition and others. Similarly, a new emerging trend in new high-resolution technologies, aims to identify cancer cell states, defined through the use of gene expression modules that have been summarized into cell-specific scores. All studies conducted so far, proposing gene expression signatures as tools to dissect and understand cancer mechanisms, lack of reproducibility and public open-source implementations. The R package *signifinder*, finally provides the infrastructure to collect, implement and compare expression-based signatures from cancer literature, improving their reproducibility and usability.

**Material and Methods**

Here, we present three applications of the Bioconductor *signifinder* R package on the analysis of bulk, single cell and spatial transcriptomic datasets. The three analyses characterized and discussed the bulk ovarian cancer (OVC) transcriptome dataset of The Cancer Genome Atlas (TCGA); the glioblastoma single-cell

© 2023 The Authors. Molecular Oncology is published by John Wiley & Sons Ltd on behalf of Federation of European Biochemical Societies dataset of Darmanis et al. and finally the spatial transcriptomic 10x Visium data called "Human Breast Cancer: Ductal Carcinoma In Situ, Invasive Carcinoma (FFPE).

**Results and Discussions**

Through the analysis of TCGA-OVC bulk data, we were able to identify areas of biological interest (i.e., signatures guided by the same transcriptional regulatory programs), and show the co-occurrence of different processes. This demonstrates that identifying the main biological characteristics of samples is made easier by using combinations of transcriptional signatures. In addition, the use of *signifinder* on new high-resolution technologies, such as single-cell RNA-seq and spatial transcriptomics, implement the definition of the intra-tumor cell heterogeneity and highlights spatial-specific patterns of expression signatures.

**Conclusion**

Cancer cells exploit existing gene expression modules, expressing them at different levels and with different levels of heterogeneity. With *signifinder*, heterogeneity in these systems can be finally evaluated, quantified, and weighted to evaluate the importance of the signature prediction at the patient level, having a role in prediction of treatment options.

### EACR23-0519

### CopyClust: A Reliable DNA Copy Number-Based Machine Learning Algorithm for Breast Cancer Integrative Cluster Classification

*C. Young[1], C. Caldas[1], O. Rueda[2]*

*[1]University of Cambridge, Cancer Research UK Cambridge Institute and Department of Oncology, Cambridge, United Kingdom*

*[2]University of Cambridge, MRC Biostatistics Unit, Cambridge, United Kingdom*

**Introduction**

Breast cancer is a major cause of cancer-related deaths among women worldwide. Integrative Cluster subtypes (IntClusts) classify breast cancer tumours into 10 distinct groups based on DNA copy number and gene expression, each with unique biologic drivers of disease and clinical prognoses. Gene expression data is often lacking, and accurate classification of samples into IntClusts with DNA copy number data only is essential. Current classification methods achieve low accuracy (~70%) when gene expression data is absent, warranting the development of new approaches.

**Material and Methods**

1,980 breast cancer samples from METABRIC (internal validation) and 1,075 samples from TCGA (external validation) with available DNA copy number and gene expression data were used to train and validate a multi-class XGBoost machine learning algorithm (*CopyClust*). IntClust label was assigned from the original dataset for METABRIC samples and determined via the *iC10* R package for TCGA samples using DNA copy number and gene expression data. Using METABRIC samples, a piecewise constant function was fitted for each IntClust and unique breakpoints across the 10 profiles were identified and converted into ~500 distinct genomic regions. The mean copy number in each region was calculated for each sample and used as features for *CopyClust*. Model classification performance was internally validated on 392 held-out METABRIC samples and rebuilt and externally validated on the TCGA dataset.

**Results and Discussions**

*CopyClust* achieved high recall (82%), top-2 recall (97%), and precision (82%) of IntClust subtype on internal validation and performed similarly during external validation. Class-specific recall remained high (>75%) among all groups despite the small sample size of some IntClusts. Misclassification was identified among IntClusts with similar copy number profiles, indicating that gene expression data may have driven the initial development of the IntClusts.

**Conclusion**

*CopyClust* achieves a significant improvement over current methods (>10%) in classification accuracy of IntClust subtypes for samples without available gene expression data and is an easily implementable algorithm for IntClust classification of breast cancer samples with DNA copy number data.

### EACR23-0593

### Integrative multiomics analysis of poor risk AML rationalizes differential drug responses in cases with mature and primitive molecular landscapes

*P. Casado Izquierdo[1], A. Rio-Machin[1], J.J. Miettinen[2], N. Khan[3], F. Bewicke-Copley[1], S. Krizsán[4], C. Bödör[4], C. Heckman[2], J. Fitzgibbon[1], P.R. Cutillas[1]*

*[1]Barts Cancer Institute / Queen Mary University of London , Centre for Genomics and Computational Biology, London, United Kingdom*

*[2]Institute for Molecular Medicine Finland / University of H elsinki, iCAN Digital Precision Cancer Medicine Flagship, Helsinki, Finland*

*[3]Institute of Immunology and Immunotherapy / University of Birmingham, Clinical Immunology Service, Birmingham, United Kingdom*

*[4]Semmelweis University Budapest, 1st Department of Pathology and Experimental Cancer Res earch, Budapest, Hungary*

**Introduction**

Acute myeloid leukemia (AML) is a disease in which myeloid precursor cells stop differentiating into functional blood cells and increase their proliferation leading to bone marrow failure. Low survival rates for poor-risk karyotype cases accentuates the need of new targeted therapies. Here, we used a multiomic approach to investigate relationships between immunophenotype, signaling pathway activation and ex-vivo drug responses in AML cases with poor-risk karyotypes.

**Material and Methods**

We reanalyzed data from a study of 52 AML poor-risk cases, consisting of genomic (NGS for 54 genes), transcriptomic (33,567 transcripts), proteomic (6,637 proteins, 26,710 phosphopeptides and 84 kinase activities), immunophenotypic (35 differentiation markers, DM) and *ex-vivo* drug response (357 compounds) datasets.

**Results and Discussions**

© 2023 The Authors. Molecular Oncology is published by John Wiley & Sons Ltd on behalf of Federation of European Biochemical Societies

Expression of each DM correlated with the expression and phosphorylation of hundreds of proteins and the sensitivity to multiple compounds. Classification of AML cases into primitive (n=12) and mature (n=10) revealed that primitive cells were more sensitive to 22 compounds including azacitidine and navitoclax. In contrast, mature cells were preferentially sensitive to 17 compounds including the MLC1 inhibitor A-1210477, the TLR8 agonist motolimod, the ROS inducer auranofin and 4 IAPs inhibitors. To rationalize specific drug sensitivities of each group we determined the molecular differences between mature and primitive cases across omics data. Mature and primitive samples, respectively, significantly increased the expression of 360 and 567 proteins, and the phosphorylation of 2767 and 2313 sites. Primitive cells increased the expression of SOD1 and VDAC, which are linked to sensitivity to BCL2 inhibitors. Mature cells increased the phosphorylation of stress response proteins like ASK1, P38A, JNK1 at and ATF7 at regulatory sites, but were more resistant to the P38 and JNK inhibitors ralimetinib and tanzisertib, respectively.

**Conclusion**

The higher activity of the stress response pathway in mature cells could be the reason for their higher sensitivity to compounds that either induce (Auranofin) or modulate (IAPs inhibitors) the stress response. Overall, our integrative analysis is a rich source of molecular information to rationalize specific drug sensitivities of poor risk AML cases with mature and primitive phenotypes. This knowledge could be used for the implementation of precision medicine in AML by selecting therapeutic approaches based on specific immunophenotypic and proteomic signatures.

### EACR23-0605
### Establishment and validation of prognostic risk model based on Neutrophil Extracellular Traps gene signature in urothelial carcinoma of bladder cancer patients

*S.Y. Huang*[1], *Y.L. Su*[1], *C.T. Lin*[1]
[1]*Kaohsiuang Chang Gung Memorial Hospital, Division of Hematology Oncology- Department of Internal Medicine, KAOHSIUANG, Taiwan*

**Introduction**

Urothelial carcinoma of bladder cancer (UBC) remains a global challenge with limited therapeutic options. Although immunotherapy made major breakthrough within years, the picture is vague in anticancer immunity among tumor microenvironment (TME). As we known, neutrophils are the main component of myeloid cells in the TME. However, the clinical significance and function of the neutrophils remain unclear in UBC. Neutrophil extracellular traps (NETs) are known to play an important role in chemotherapy resistance, tumor metastasis and immune escape of cancers. Our study aimed to investigate NET-related genes and their clinical prognostic value in patients with UBC.

**Material and Methods**

We identified NET-related genes expressed in multiple public gene sets (TCGA-BLCA, GSE series) simultaneously. In order to avoid overfitting and confirm the practicability, we set up a prognostic risk model through univariable, least absolute shrinkage and selection operator (LASSO) and multivariable Cox algorithms. A nomogram was further used to explore the clinical value of the model. The discriminatory value of the nomogram was evaluated using the receiver operating characteristic (ROC) and area under curve (AUC). Internal and external validation were conducted to test the clinical applicability and reproducibility.

**Results and Discussions**

Four NET-associated genes were selected to constructed the risk model. In the training cohort, the low-risk UC patients showed significantly better overall survival (OS) than that of in high-risk UC patients ($p<0.0001$). The nomogram of Kaplan–Meier (K-M) analysis also showed a significant prognostic value, and the area under the curve (AUC)  value at 1, 3 and 5 years are 0.66, 0.64, 0.63 respectively. Internal validation further strengthened the credibility of the clinical prognostic model with acceptable K-M analysis ($p<0.05$) and AUCs (0.82 at 1 years, 0.76 at 3 years and 0.77 at 5 years). Similar to internal validation, external validation also showed significant results($p<0.05$). In clinical feature with muti-variate cox regression, advanced stage (Stage III: HR 1.6, $p=0.029$; Stage IV: HR 2.5, $p<0.001$) showed comparative differences to the risk score (HR 2.1, $p<0.001$). Moreover, the difference in immune infiltration was significant in UC patients with different risks, especially in neutrophil and macrophage(M2).

**Conclusion**

NET-related gene signature is identified to constructed a novel prognostic risk model in UBC patients.

### EACR23-0614
### Deep Learning Methods for Predicting Homologous Recombination Deficiency from Histopathology Slides and Gene Expression Profile Data

*S. PAN*[1], *M. Secrier*[1]
[1]*University College London, Research Department of Genetics- Evolution and Environ ment GEE, London, United Kingdom*

**Introduction**

Homologous recombination deficiency (HRD) is often associated with sensitivity to DNA damage response inhibitors. Recent advancements in AI-based methods offer the capability to predict expression profiles for certain genes or even complex phenotypes like microsatellite instability directly from histopathology slides. Diagnosing HRD in H&E-stained slides would be a cost-effective way to inform treatment allocation in the clinic, but studies to date have only developed models based on BRCA1/2 mutation status and do not consider the current HRD state of the tumour. Here, we present a deep learning-based framework that employs a Graph Neural Network (GNN) and Multiple Instance Learning (MIL) to predict and interpret the HRD status in breast cancer directly from H&E-stained slides.

**Material and Methods**

We employ a transcriptomic signature of HRD developed in breast cancer to explore key genes involved in the process and the ability to detect this phenotype in H&E

© 2023 The Authors. Molecular Oncology is published by John Wiley & Sons Ltd on behalf of Federation of European Biochemical Societies slides. Our pipeline begins with a GNN module to rank genes involved in HRD based on their gradient importance, which helps prioritise master regulators of this process. This is followed by an MIL module that is trained on matched RNA-seq and H&E slides from the TCGA-BRCA cohort to predict HRD status only from diagnostic H&E slides.

**Results and Discussions**

Our importance score highlighted 22 significant genes from an expanded list of 130 genes in the original transcriptional signature, resulting in a reduced gene signature with excellent classification capability, i.e. AUC = 85% for distinguishing HRD from HR proficient tumours in the TCGA-BRCA dataset. The MIL module outperformed transfer learning, achieving an AUC of 79% in the diagnostic H&E slides from TCGA-BRCA.

**Conclusion**

The proposed deep learning methods, including the GNN-based and MIL-based modules, exhibit promising results in predicting HRD status from RNA-Seq and H&E slides. The proposed importance score can provide valuable insights for gene selection and prioritization for further validation or investigation. Further work will aim to improve the performance of the proposed pipeline and expand our initial experiments to other datasets.

### EACR23-0647

### Leveraging RNA-seq variant discovery for breast cancer intratumoral heterogeneity studies.

*R. Manzano Garcia[1], O.M. Rueda[2], J. Ezike[3], J. Hess[3], G. Getz[3], C. Caldas[4]*

*[1]University of Cambridge - Cancer Research UK Cambridge Institute, Caldas Lab, Cambridge, United Kingdom*

*[2]University of Cambridge, MRC Biostatistics Unit, Cambridge, United Kingdom*

*[3]Broad Institute of MIT and Harvard, Getz lab, Cambridge, United States*

*[4]Cancer Research UK Cambridge Insitute, Caldas Lab, Cambridge, United Kingdom*

**Introduction**

The genome and transcriptome of a tumour provide complementary information essential for establishing robust molecular profiles. Mutations in DNA are well-studied and used to direct targeted treatments in clinic. Approximately 30-50% of these mutations are detectable in matched RNA. However, identifying mutations directly from RNA is fraught with technical challenges. Moreover, there is a subset of somatic RNA-only (RO) variants that could have a regulatory role and are understudied. Here, we present a pipeline to accurately and efficiently detect variants in RNA bulk sequencing data. This work is an extension of the previously published RNA-MuTect pipeline in Yizhak *et al.,* 2019 (Science).

**Material and Methods**

**RNA Variant Calling**: the pipeline uses STAR-aligned RNA-seq BAMs files as input. We follow GATK best practices for the pre-processing of the reads followed by variant calling with three different mutation callers: mutect2, strelka2 and sage. A voting consensus approach is followed and filtering is applied following in Yizhak *et al.,* 2019 methods. The pipeline is written in Nextflow.

**Data**: subset of 241 samples were taken from The Cancer Genome Atlas (TCGA) as a training set. The panel of normals were generated with samples from The Genotype-Tissue Expression (GTEx) project and normal adjacent tumour for TCGA.

**Results and Discussions**

We have improved the previously published work in detecting variants from RNA, creating a robust pipeline to detect mutations in RNA. Using a subset of 241 tumours from The Cancer Genome Atlas (TCGA) we have compared our pipeline against the original version of RNA-MuTect and showed more than 80% detected variants overlap with the two methods. We demonstrate the robustness of our consensus approach, as artifacts (more common in RNA-seq data) are removed. Furthermore, the pipeline leverages Nextflow's parallelisation capabilities and robust error handling mechanisms to ensure efficient and reliable performance. We have started to apply the new pipeline to breast cancer datasets and perform computational analysis using both DNA and RNA sequencing data to characterize ITH profiles.

**Conclusion**

The pipeline that we present here is able to robustly detect somatic events in RNA-seq data and it also compares these RNA events with SNVs identified in DNA from matched samples. The introduction of the consensus approach removes background noise from the results, enabling more accurate downstream analysis. The pipeline is now used by us to study intratumoral heterogeneity.

### EACR23-0655

### Challenging the cancer genotype-to-phenotype dogma; KRAS mutation does not lead to a distinct transcriptional state in established colorectal tumours

*R. Byrne[1], S.B. Malla[1], S.M. Corry[1], E. Rogan[1], N.C. Fisher[1], R. Amirkhah[1], S. Sakhnevych[1], K.L. Redmond[1], P.D. Dunne[1]*

*[1]Queen's University Belfast, Patrick G Johnston Centre for Cancer Research, Belfast, United Kingdom*

**Introduction**

Oncogenic *KRAS* mutations are one of the most pervasive in cancer, found in approximately 40-50% of colorectal cancers (CRCs). Preclinical models, in combination with the ease and affordability of molecular profiling, have enabled the development of transcriptional signatures that can be used as surrogate biomarkers of *KRAS*-driven biologies. Although such tightly controlled pre-clinical systems are well suited to the development of transcriptional signatures to represent mechanistic cascades following genotype induction, it remains to be seen how representative the phenotypic trajectories of such models align to the observable complexity and heterogeneity across human tumours.

**Material and Methods**

Five RAS-related gene expression signatures, identified from the Molecular Signatures Database (MSigDB) and the literature, were applied to four independent CRC cohorts with bulk transcriptomic data (n=1,695 in total) and one large single-cell RNA-seq cohort (n=42,954 epithelial cells from n=62 patients) that all had accompanying mutational status data. The association between these signatures and

© 2023 The Authors. Molecular Oncology is published by John Wiley & Sons Ltd on behalf of Federation of European Biochemical Societies the *KRAS* mutational status of samples was assessed using Gene Set Enrichment Analysis (GSEA), single sample GSEA (ssGSEA) and Receiver Operating Characteristic (ROC) curves.

**Results and Discussions**

While GSEA assessment of a series of RAS-related signatures indicated some significant associations with mutational status, when tested across a series of independent cohorts these analyses revealed no consistent significant findings. There was no significant association between ssGSEA scores for any signature and *KRAS* mutational status. A lack of association remained even when *BRAF* mutant samples or samples with mutations in other key MAPK/PI3K pathway genes were excluded from these analyses. Moreover, when these analyses were broken down according to specific *KRAS* mutations, there remained no association between signature expression levels and specific *KRAS* mutation. Even, in the single-cell RNA-Seq cohort where only the epithelial cells were analysed, eliminating the potential confounding effect of the tumour microenvironment, RAS-related signatures were again, remarkably, unrelated to *KRAS* mutational status.

**Conclusion**

Data presented here indicates that some of the most widely used RAS-specific transcriptional signatures are independent of *KRAS* mutational status.

### EACR23-0678
### Identification of differentially A>I(G) RNA-edited sites by ADAR in genotoxic drugs sensitivity in breast cancer

*Y. Bernal*[1], *A. Blanco*[1], *R. Armisén*[1]

[1]*Instituto de Ciencias e Innovación en Medicina- Facultad de Medicina- Clínica Alemana- Universidad del Desarroll o,*
*Laboratory of Cancer Functional Genomics- Centro de Ge nética y Genómica., Santiago, Chile*

**Introduction**

The most abundant RNA editing in mammals is the conversion from adenosine to inosine, translated as guanosine (A>I(G)), and it is catalyzed by RNA-specific adenosine deaminase (ADAR). The A>I(G) RNA editing has been implicated in various diseases, including cancer. Breast cancer (BC) patients with poor overall survival have a high tumoral expression of ADAR. Our aim is to identify A>I(G)RNA-edited sites of high confidence that are associated with high- or low-sensitivity to genotoxic drugs (PARP inhibitors, anthracyclines, and alkylating agents) in BC cell lines. In addition, we modeled the genotoxic drug sensitivity with a machine-learning approach using the differentially RNA-edited sites identified.

**Material and Methods**

Drug sensitivity data (IC50) was obtained from *The Genomics of Drug Sensitivity in Cancer Project* (GDSC) to classify BC cell lines and RNA-seq data from the *Cancer Cell Line Encyclopedia*. Identification of A>I(G) RNA-edited sites is performed using REDITools and excluded common and somatic variants and includes strict filters. Differential RNA edited sites are assessed with REDITs based on a beta-binomial model and annotated using Ensembl Variant Effect Predictor. Random forest and logistic regression are used to model drug sensitivity.

**Results and Discussions**

From the GDSC database, we selected 25 BC cell lines with high- (<p25 of IC50) and low-sensitivity (>p75 of IC50) to genotoxic drugs. Of all identified RNA editing sites that passed our filters of coverage (>30x) and a minimum of 5 edited reads, on average 6,737 (±326) that represent 2.3% were differential RNA editing sites. PARP inhibitors, anthracyclines, and alkylating agents shared only 957 (6%) of all differential RNA-edited sites. In our analysis of functional consequences interpretation, we highlight that RNA-edited sites affect splicing and missense changes with probable and possible predicted damage. We found that the regulation of biological and RNA metabolic processes is enrichment in the Gene Ontology analysis of 957 RNA-edited sites. Finally, we prioritize only high-confidence and top 25 RNA-edited sites with higher edition levels to predict the response to drugs in BC.

**Conclusion**

Identifying differential A>I(G) RNA-edited sites between high- and low-sensitivity to genotoxic drugs in BC cell lines could be relevant to understanding the drug response from an epitranscriptome view.

### EACR23-0717
### Integrative omic analysis of faecal samples shows novel miRNA-mediated host-microbiota interactions in colorectal cancer

*G. Ferrero*[1], *B. Pardini*[2], *S. Tarallo*[2], *B. Zwinsová*[3], *L. Vodickova*[4], *P. Vodicka*[4], *N. Segata*[5], *F. Cordero*[6], *E. Budinska*[3], *A. Naccarati*[2]

[1]*University of Turin,*
*Department of Clinical and Biological Sciences, Orbassano, Italy*
[2]*Italian Institute of Genomic Medicine,*
*Molecular epidemiology and exposomics, Candiolo, Italy*
[3]*Masaryk University, RECETOX- Faculty of Science, Brno, Czech Republic*
[4]*Institute of Experimental Medicine of the Czech Academy of Sciences, Department of Molecular Biology of Cancer, Prague, Czech Republic*
[5]*University of Trento, Department CIBIO,*
*Department of Cellular- Computational and Integrative Bi ology, Italy*
[6]*University of Turin, Department of Computer Science, Turin, Italy*

**Introduction**

Dysbiosis of the gut microbiota has been linked to colorectal cancer (CRC). Alterations of human microRNAs (miRNAs) are also implicated in CRC and recent findings support a crosstalk between gut microbiota and miRNAs released in the gut lumen. miRNAs and microbial species can be profiled from fecal samples representing a reliable biospecimen to explore host-microbial interactions. In a pilot study, we demonstrated that combining fecal miRNA and microbial species levels can accurately distinguish CRC patients from healthy (Tarallo et al., 2019. *mSystems*. 4(5):e00289-19; doi:10.1128/mSystems.00289-19). Moreover, we observed that this combined information can also accurately classify subjects with specific dietary habit (Tarallo et al., 2022. *Gut*. 71(7):1302-1314. doi:

© 2023 The Authors. Molecular Oncology is published by John Wiley & Sons Ltd on behalf of Federation of European Biochemical Societies

10.1136/gutjnl-2021-325168; and Francavilla et al., 2023. *Gut Microbes* 15(1):2172955; doi: 10.1080/19490976.2023.2172955).

**Material and Methods**

To evaluate candidate host-microbial interactions mediated by miRNAs in CRC, we performed an integrative analysis of shotgun metagenomes and small RNA-Sequencing (sRNA-Seq) on stool samples from 442 healthy subjects, 162 patients with inflammatory or diverticular diseases, 64 with precancerous lesions, or 281 CRC from three independent European cohorts. Microbial profiling was performed using MetaPhlAn 4.0, while Docker4Seq was applied for miRNA quantification. Rank regression models were applied to compute the miRNA-microbial associations.

**Results and Discussions**

The levels of fecal miRNAs dysregulated in CRC patients were evaluated in relation to the microbial abundances observing significant associations (adj. $p<0.05$), including a correlation between miR-1276 and *Fusobacterium nucleatum* and *Parvimonas micra* levels, all increasing in CRC patients, and an anticorrelation between miR-6777-5p and *Escherichia coli* levels. The association significance progressively increased/decreased from healthy subjects to late-stage tumors. In addition, significant associations were observed also considering miRNA levels measured in tumor/adjacent tissue by sRNA-Seq.

**Conclusion**

Our data suggest that a specific microbial composition may regulates the expression and release of miRNAs in the gut lumen, with their subsequent detection in stool. Conversely, these miRNAs may regulate microbial gene expression defining network of cross-kingdom molecular interactions.

### EACR23-0734
### IDPredict: A machine learning framework for the prediction of disordered proteins as potential predictive biomarkers for targeted therapies based on network topological data

*K. Schulc*[1], *G. Kovács*[2], *B. Kovács*[1], *P. Csermely*[1], *D.V. Veres*[2]
[1]*Semmelweis University, Department of Molecular Biology, Budapest, Hungary*
[2]*Turbine Ltd., Turbine Ltd., Budapest, Hungary*

**Introduction**

The identification of predictive oncotherapeutic biomarkers is a promising area of precision medicine. In the *IDPredict* framework, we suggest a machine learning method based on network topological motif analysis to the prediction for intrinsically disordered proteins (IDPs) with predictive biomarker properties. With the analysis of motifs containing IDPs and oncotherapeutic targets, their complex regulation is well modelled. A Biomarker Probability Score was developed to assess the biomarker potential of each disordered protein-target pair.

**Material and Methods**

Motifs were identified on the directed edges of three networks using the FANMOD program. Cytoscape plugins were used for network analysis. IDPs and biomarkers were annotated based on the DisProt and CIViCmine databases, respectively. Machine learning models were developed with the Python scikit-learn, XGBoost and SHAP packages. A Biomarker Probability Score was developed based on the ranks of eight independent predictions.

**Results and Discussions**

We have shown that IDPs form common motifs with known oncotherapeutic targets, where many IDPs are predictive biomarkers for the given target *(Human Cancer Signaling Network: 23%)*. Thus, we developed machine learning models based on topological data and biological characteristics of 109 target-IDP pairs, whose predictive biomarker properties were previously known. After multiple cross-validations and testing, eight different models with >0.88 LOOCV accuracy were used to predict potential predictive biomarker properties for the other identified 695 pairs in our networks. The resulting Biomarker Probability Score was showed to perform well in the discrimination of potential predictive biomarkers. As one of the examples, a clinically relevant predictive biomarker was identified for ponatinib, a multikinase-inhibitor with BCR-ABL as its main target.

**Conclusion**

We show based on network topology analysis that intrinsically disordered proteins have great potential as predictive biomarkers. Our results imply that investigation of their motifs helps to identify novel predictive biomarkers, which can be both further studied individually and validated experimentally. As predictive biomarkers are vital for therapeutical decision making, developing a tool for predictive biomarker identification may have effect on the daily clinical practice.

### EACR23-0787
### Cell-of-origin identification based on gene mutational profiles and chromatin state in melanoma

*P. Štancl*[1], *R. Karlić*[1]
[1]*Faculty of science- University of Zagreb, Department of biology, Zagreb, Croatia*

**Introduction**

Cancers of unknown primary origin (CUP) pose a challenge for clinicians as they make up 3-5% of all cancers. Identification of the correct cell-of-origin (COO) of cancers can facilitate accurate diagnosis and administration of appropriate treatment. Various WGS-based methods for the prediction of COO have been developed. However, whole-exome (WXS) sequencing methods of similar accuracy are still lacking. The aim of our research was to develop a model based on mutational profiles in genes and epigenetic features of the COO to identify the COO.

**Material and Methods**

We analyzed a publicly available melanoma cohort from the International Cancer Genome Consortium. We downloaded ChIP-seq data for six histone modifications from 68 cell-types was downloaded from the Roadmap Epigenome project and calculated FPKM values over all genes on the hg19 human genome. We trained a multiple linear regression model with 10-fold cross-validation to compute the amount of variance of aggregated mutations across genes explained by the epigenome of each COO. The model with the highest variance explained indicates the predicted COO. We applied wavelet transformation to

© 2023 The Authors. Molecular Oncology is published by John Wiley & Sons Ltd on behalf of Federation of European Biochemical Societies normalize different datasets to a common scale and analysed the performance of models trained on distinct sets of genes, grouped according to biotype and function.

**Results and Discussions**

The model based on gene mutational profiles predicted the correct cell of origin of melanoma. Wavelet transformation showed a significant increase in prediction accuracy, with an explained variance of around ~60% for both all and only protein-coding genes. Residual and over-representation analyses detected a specific group of protein-coding genes involved in melanin metabolic processes and pigmentation as informative for predicting COO. Genes involved in tumorigenesis annotated by COSMIC showed the highest explained variance of ~65% as well as the largest percentage of correctly identified COO in individual melanoma patients.

**Conclusion**

The study demonstrates that the cell's epigenome and cancer's gene mutation profile can be used to predict the cell-of-origin. Developing a gene-based method that can utilize variants from WXS could be useful for diagnosing and treating cancer patients as it is more cost and time-efficient compared to WGS data.

### EACR23-0788

### Mechanistic insights into the interactions between cancer drivers and the tumour immune microenvironment

*H. Misetic*[1], *M.R. Keddar*[1], *J.P. Jeannon*[2], *F. Ciccarelli*[1]
[1]*King's College London, School of Cancer & Pharmaceutical Sciences, London, United Kingdom*
[2]*King's College London, Department of Head & Neck Surgery, London, United Kingdom*

**Introduction**

The crosstalk between cancer and the tumour immune microenvironment (TIME) has attracted significant interest in the latest years because of its impact on cancer evolution and response to treatment. Despite this, cancer-specific tumour-TIME interactions and their mechanisms of action are still poorly understood.

**Material and Methods**

Here, we compute significant interactions between cancer-specific genetic drivers and five anti- and pro-tumour TIME features in 32 cancer types using Lasso regularised ordinal regression. Focusing on head and neck squamous cancer (HNSC), we then rebuild the functional networks linking specific TIME driver alterations to the TIME state.

**Results and Discussions**

The 477 TIME drivers that we identify are multifunctional genes whose alterations are selected early in cancer evolution and recur across and within cancer types. Tumour suppressors and oncogenes have an opposite effect on the TIME and the overall anti-tumour TIME driver burden is predictive of response to immunotherapy. TIME driver alterations predict the immune profiles of HNSC molecular subtypes, and perturbations in keratinization, apoptosis and interferon signalling underpin specific driver-TIME interactions.

**Conclusion**

Overall, our study delivers a comprehensive resource of TIME drivers, gives mechanistic insights into their immune-regulatory role, and provides an additional framework for patient prioritisation to immunotherapy.

### EACR23-0810

### A novel approach for methylation data analysis leads to the discovery of a candidate subtype in LGG with the worst prognosis.

*A. Velle*[1], *C. Ambrosino*[2], *C. Romualdi*[1], *S.M. Pagnotta*[2]
[1]*University of Padova, Department of Biology, Padova, Italy*
[2]*University of Sannio, Dept. of Science and Technology, Benevento, Italy*

**Introduction**

DNA methylation is known to regulate gene expression by altering the accessibility of DNA to the transcription machinery. These epigenetic modifications have been shown to play a crucial role in cancer's pathological origin and development.

In this study, we characterize Low-Grade Glioma (LGG) by methylation profiles downloaded from TCGA. Most LGG have an excellent long-term prognosis, so being able to molecularly characterize bad prognosis groups is crucial to inform treatment.

A novel bioinformatics procedure has been designed for unsupervised methylation data clustering and a downstream analysis to identify the methylated regions associated with the clusters. The pipeline results confirm known Low-Grade Glioma subtypes and detect a new group with the worst prognosis in the cohort and evident proliferative functional traits.

**Material and Methods**

The cohort analyzed in this study comprises 516 LGG samples from TCGA. Bioconductor package TCGAbiolinks was exploited to download methylation beta values obtained from the Illumina Infinium HumanMethylation450 platform. After data pre-processing and a features reduction step according to the AWST protocol, an unsupervised hierarchical clustering (with euclidean distances and Ward's linkage) on methylation data has been run. yaConsensus CRAN package allowed to check the clustering stability. The Cox-Proportional Hazard models were adopted to study each cluster's survival profile (corrected by age). The new pipeline includes a novel way to think about enrichment analysis in the case of methylation data. massiveGST methodology paired with a new notion of a gene set allowed us to associate probes to genes and genomic regions.

**Results and Discussions**

The clustering is stable, as suggested by the analysis of the consensus similarity matrix with a sampling rate of 75%, and is not associated with a few probes. In fact, 15% of the features still reproduce the same partition.

The clusters obtained with our procedure partially overlap with subtypes from supervised procedures in the literature. In addition, a new subgroup in the molecular IDH-wildtype subtype arises, segregating samples associated with the worst prognosis. Consistently with the bad prognosis, we detected increased proliferation processes and decreased immune-related activities and cell adhesion.

**Conclusion**

© 2023 The Authors. Molecular Oncology is published by John Wiley & Sons Ltd on behalf of Federation of European Biochemical Societies

The new pipeline can reconstruct known results and suggest the existence of new groups. In this study, a new candidate subtype with the worst prognosis has been identified and functionally characterized.

### EACR23-0839
### In Silico Saturation Mutagenesis to Identify Clonal Hematopoiesis Driver Mutations

*S. Demajo[1,2], J.E. Ramis-Zaldivar[1], F. Muiños1[1,2], M. Grau[1], A. González-Pérez[1,2,3], N. López-Bigas[1,2,3,4]*
*[1]Institute for Research in Biomedicine IRB Barcelona, The Barcelona Institute of Science and Technology, Barcelona, Spain*
*[2]Centro de Investigación Biomédica en Red en Cáncer, Instituto de Salud Carlos III, Barcelona, Spain*
*[3]Research Program on Biomedical Informatics, Universitat Pompeu Fabra, Barcelona, Spain*
*[4]Institució Catalana de Recerca i Estudis Avançats ICREA, Catalunya, Barcelona, Spain*

**Introduction**
Clonal hematopoiesis (CH) is a common condition characterized by a clonal expansion in the blood caused by somatic mutations in hematopoietic stem cells that confer them a selective advantage. CH is usually linked to aging or to chemotherapy exposure, and is associated with increased risk of hematological cancer and other diseases such as certain solid tumors. Although in recent years the main CH driver genes have been characterized, identifying which specific mutations in those genes are capable of driving CH is still an unsolved problem. Here, we propose a machine learning-based approach for in silico saturation mutagenesis of CH genes to identify CH driver mutations.
**Material and Methods**
We repurposed a machine learning method originally devised to identify cancer driver mutations to apply it to blood somatic mutations from more than 36,000 individuals. With this repurposed method, BoostDM-CH, we built gene-specific models that identify CH driver mutations. We evaluated our method by identifying CH mutations in independent cohorts in comparison with the state-of-the-art rule-based approaches. We further validated our approach by studying the association of CH with several medical conditions in close to 470,000 individuals with whole-exome sequencing from the UK Biobank.
**Results and Discussions**
We obtained reliable BoostDM-CH models for twelve genes, including the most common CH drivers DNMT3A, TET2, and ASXL1. These models provide a thorough picture of all potential driver mutations in each gene, defining the specific features that characterize them and providing a better understanding of the mechanisms leading to clonal expansion in the blood. The evaluation of BoostDM-CH in independent cohorts evidenced that it has an accuracy comparable to state-of-the-art manually curated rules in identifying CH driver mutations, with the advantage of being automatic and unbiased. Using the large cohort from the UK Biobank, we showed that CH driver mutations identified by BoostDM-CH models highly correlate with age while non-drivers show no association. Similarly, only BoostDM-CH drivers are associated with an increased risk of hematological cancer (especially myeloid neoplasms), and some solid tumors such as lung and breast cancers, and sarcoma.
**Conclusion**
We developed and validated gene-specific machine learning models for in silico saturation mutagenesis to identify CH driver mutations. These comprehensive models may support the identification and clinical interpretation of CH mutations in newly sequenced individuals.

### EACR23-0840
### Characterisation of the Immune and Genomic Landscape of Ovarian Cancer Uncovers Key Drivers of Heterogeneity Across Subtypes

*J. Boyle[1], J. Zaucha[1], F. Ng[1], A. Spitzmüller[2], M. Vuko[2], F. Segerer[2], M. Azqueta Galvadon[2], M. Testori[2], A. Raymond[3], B. Nuttall[3], A.E. Storti[4], S. Gulati[5], S. Gill[6], M. Surace[7], P. Marco-Casanova[6], B. Sidders[1], J.C. Barrett[3], H. Angell[6]*
*[1]AstraZeneca, Oncology Data Science- Research and Early Development- Oncology R&D, Cambridge, United Kingdom*
*[2]AstraZeneca, Computational Pathology GmbH, Munich, Germany*
*[3]AstraZeneca, Translational Medicine- Oncology R&D, Boston, United States*
*[4]AstraZeneca, Oncology Data Science Platforms- Oncology R&D, Munich, Germany*
*[5]AstraZeneca, Oncology Data Science Platforms- Oncology R&D, Cambridge, United Kingdom*
*[6]AstraZeneca, Translational Medicine- Oncology R&D, Cambridge, United Kingdom*
*[7]AstraZeneca, Translational Medicine- Oncology R&D, Gaithersburg, United States*

**Introduction**
Ovarian cancers with similar histopathologic profiles but diverse immunogenomic profiles may respond very differently to the same treatment, leading to distinct clinical outcomes. There is thus considerable value in identifying the main immunogenomic drivers of inter-tumour heterogeneity within ovarian cancer. We analysed somatic mutation, mRNA expression, and multiplex immunofluorescence data, together with clinical metadata, from 197 patients with ovarian cancer, most of whom were sensitive to platinum-based chemotherapy.
**Material and Methods**
Analysis was done using Multi-Omics Factor Analysis, an extension of Principal Component Analysis to multi-modal settings. Joint modelling of the different types of data enabled the detection of multi-modal patterns that defined subtypes of ovarian cancer. The cohort enabled the comparison of high grade serous ovarian cancer (HGSOC) with other disease subtypes, in particular clear cell and endometrioid.
**Results and Discussions**
Gene expression and immune response were the predominant drivers of inter-tumour heterogeneity. We observed a pattern of mutually exclusive mutations in TP53 vs ARID1A, KRAS, PIK3CA and CTNNB1

© 2023 The Authors. Molecular Oncology is published by John Wiley & Sons Ltd on behalf of Federation of European Biochemical Societies

(p<0.01). HGSOC patients exhibited a high prevalence of TP53 mutation compared to other subtypes (p<0.01). Higher levels of immune infiltration of macrophages and CD8 T-Cells were associated with HGSOC (p<0.01). Two distinct subtypes of HGSOC were identified, one exhibiting immune exclusion and one with immune infiltration into the tumour core. High expression levels of MHC Class II genes was correlated with increased density of CD8 T-Cells and macrophages (Spearman's rho > 0.7 in the tumour core), and inversely correlated with the presence of proliferating tumour cells. This relationship was substantially stronger in the tumour core than in the invasive margin.

Several combinations of somatic mutation, gene expression and immune response patterns were identified as key drivers of inter-tumour heterogeneity, with many displaying significant relationships with histology and/or survival outcomes. Additionally, differences were identified in the relationship between gene expression and the immune response in the tumour core and in the invasive margin.

**Conclusion**

The histopathologically homogeneous HGSOC patient group is immunogenomically heterogeneous. These findings highlight opportunities for immuno-oncology-based therapies for early stage ovarian cancer patients beyond the existing standard chemotherapy regimens.

### EACR23-0890
### A PK/PD Simulation Model for Supporting Doxorubicin Therapy in Multiple Myeloma

*D. Andrean[1], R. Doshmanziari[2], F. Da Ros[3], M. Mazzucato[3], M.G. Pedersen[1], R. Visentin[1]*
*[1]University of Padova, Information Engineering, Padova, Italy*
*[2]Norwegian University of Science and Technology, Department of Engineering Cybernetics, Trondheim, Norway*
*[3]National Cancer Institute CRO Aviano, Dipartimento della ricerca traslazionale e della diagnostic a avanzata dei tumori, Aviano, Italy*

**Introduction**

Doxorubicin (DOXO) is a well-known chemotherapy drug for Multiple Myeloma(MM). However, maximizing DOXO treatment efficacy with low toxicity is challenging due to its possible side effects. Here we propose a pharmacokinetic/pharmaco-dynamic (PK/PD) simulation environment, developed based on an in vitro experimental and modeling approach to support DOXO treatment optimization.

**Material and Methods**

The PK/PD simulator allows defining a desired DOXO treatment regimen (i.e., dose amount and duration) and simulating the resulting DOXO PK and its effects on MM1R cells. It consists of two modules. The first one models DOXO PK by three compartments, describing DOXO administration in the extracellular space and its diffusion into MM cell cytoplasm and nucleus, where it binds to the DNA, causing its damage. This model was developed from DOXO concentration data collected from in vitro experiments with MM1R cells exposed for 3 hrs to two DOXO doses (200 and 450nM). The second module is a logistic model, linking the DOXO dose to the number of living cells in time, based on a concurrent action of cell proliferation and death rates. PK and PD models are interconnected by a Hill function, where DNA-bound DOXO (available from PK model) controls cell death rate. The PK/PD model was identified on MM1R counts measured for 20 days after 3-hr DOXO administration at different doses (0, 10, 20, 40, 50, 200, 450, 900nM),proving to well capture both cell growth and death dynamics.

**Results and Discussions**

In order to describe the simulator functioning, we run two 20-day simulations, each one with different DOXO regimens given after 50 hrs of cell proliferation: 1) a single3-hr administration of 200nM; 2) a repeated 3-hr administration of 50nM every three days. Setting the initial cell number at 5000 cells, the maximum cell count reached was similar in the single- vs. repeated-dose trial: 25418 and 25638. After20 days, MM1R proliferation was suppressed in both trials, resulting in 1642 vs.17 cells alive, with the single- vs. repeated-dose regimen, respectively. A lower peak of DNA-bound DOXO concentration was obtained with the repeated-low-dose regimen (71nM vs 132nM). Results suggest that multiple low dosing allows effective cell death with potentially lower toxicity than a single high DOXO dose.

**Conclusion**

The proposed PK/PD simulator represents a supportive solution to guide future DOXO testing in MM, allowing optimization of the DOXO regimen by limiting DOXO exposure and preventing potential side effects.

### EACR23-0895
### The association of FGFR2 expression and splice isoforms with breast cancer subtypes.

*R. Duarte[1], T. Dix-Peek[1], C. Dickens[1], J. Valcarcel[2]*
*[1]University of the Witwatersrand, Internal Medicine, Johannesburg, South Africa*
*[2]Centre for Genomic Regulation, Gene Regulation- Stem Cells and Cancer, Barcelona, Spain*

**Introduction**

Gene expression profiling of breast cancer tumours has identified 5 molecular subtypes, each with different growth rates, treatment responses and clinical outcomes.
Over the past 15 years, genome-wide association studies have identified loci associated with breast cancer. The Fibroblast Growth Factor Receptor 2 (*FGFR2*) gene was a top-scoring candidate and the association was verified in a series of replication studies.
FGFR2 belongs to a receptor tyrosine kinase family involved in many biological processes and undergoes mutually exclusive splicing giving rise to isoforms that are expressed either in epithelial (*FGFR2 IIIb*) or mesenchymal cells (*FGFR2 IIIc*).
Using data retrieved from The Cancer Genome Atlas and a series of publicly available databases we investigated the association between *FGFR2* expression, isoform expression, and breast cancer subtype.

**Material and Methods**

The cBioPortal for cancer genomics was used to interrogate *FGFR2* expression.  Plot data of log

© 2023 The Authors. Molecular Oncology is published by John Wiley & Sons Ltd on behalf of Federation of European Biochemical Societies transformed *FGFR2* gene expression were downloaded in .csv format for PAM50 subtypes. *FGFR2* splice variants were downloaded from Ensemble Genome Browser 108 together with reference genome information, and transcript variants aligned using MUSCLE. The TCGA Splice Seq database was used to interrogate *FGFR2* splice patterns. All data were analysed using STATA v14.2.

**Results and Discussions**

Elevated *FGFR2* expression was significantly associated with luminal, estrogen receptor (ER+) positive and invasive lobular carcinomas, whereas lower *FGFR2* expression was associated with basal, epidermal growth factor receptor 2 (HER2) positive, or triple-negative breast cancer and invasive ductal carcinomas. The *FGFR2 IIIb* isoform was significantly enriched in ER+ breast cancer, while the mesenchymal *FGFR2 IIIc* isoform was significantly prevalent in HER2+ cancer. Increased levels of *FGFR2* and *IIIb* splice isoform were associated with less aggressive breast cancer phenotypes, while decreased levels of *FGFR2* expression and increased *IIIc* splice isoform expression were associated with more aggressive phenotypes.

**Conclusion**

As FGFRs are increasingly being considered as therapeutic targets for a variety of cancers, including breast, there is a need to determine the mechanism by which they facilitate progression of cancer. The expression of *FGFR2*, and its isoforms, relate to breast cancer subtypes. *FGFR2* expression is related to maintaining epithelial characteristics of breast cancer whereas low *FGFR2* is associated with more aggressive, basal forms.

### EACR23-0911
### Mutation-Attention (MuAt): deep representation learning of somatic mutations for tumour typing and subtyping

*P. Sanjaya*[1], *K. Maljanen*[1], *R. Katainen*[2], *S. Waszak*[3], *L. Aaltonen*[2], *O. Stegle*[4], *J. Korbel*[5], *E. Pitkänen*[1]

[1]*Institute for Molecular Medicine Finland FIMM, Helsinki Institute of Life Science- University of Helsinki, Helsinki, Finland*

[2]*University of Helsinki, Department of Medical and Clinical Genetics- Faculty of Medicine, Helsinki, Finland*

[3]*Centre for Molecular Medicine Norway NCMM, University of Oslo and Oslo University Hospital, Oslo, Norway*

[4]*German Cancer Research Center DKFZ, Division of Computational Genomics and Systems Genetics , Heidelberg, Germany*

[5]*European Molecular Biology Laboratory EMBL, European Bioinformatics Institute- Wellcome Genome Ca mpus, Hinxton, United Kingdom*

**Introduction**

Cancer genome sequencing enables accurate classification of tumours and tumour subtypes. However, prediction performance is still limited using exome-only sequencing and for tumour types with low somatic mutation burden such as many pediatric tumours. Moreover, the ability to leverage deep representation learning in discovery of tumour entities remains unknown.

**Material and Methods**

We introduce here Mutation-Attention (MuAt), a deep neural network to learn representations of simple and complex somatic alterations for prediction of tumour types and subtypes. In contrast to many previous methods, MuAt utilises the attention mechanism on individual mutations instead of aggregated mutation counts. We trained MuAt models on 2,587 whole cancer genomes (24 tumour types) from the Pan-Cancer Analysis of Whole Genomes (PCAWG), and 7,352 cancer exomes (20 types) from the Cancer Genome Atlas (TCGA). For external validation, we tested MuAt on 9,796 whole-genome sequenced (WGS) tumours across 7 tumour types available in the 100,000 Genomes Project, Genomics England (GEL), and WGS of 256 colorectal cancer cases from Katainen et al.

**Results and Discussions**

MuAt achieved prediction accuracy of 89% for whole genomes (PCAWG) and 64% for whole exomes (TCGA), and a top-5 accuracy of 97% and 90%, respectively. MuAt models were found to be well-calibrated, and perform well in over 10,000 independent cancer genomes. We show MuAt to be able to learn clinically and biologically relevant tumour entities including acral melanoma, SHH-activated medulloblastoma, SPOP-associated prostate cancer, microsatellite instability, POLE proofreading deficiency, and MUTYH-associated pancreatic endocrine tumours without these tumour subtypes and subgroups being provided as training labels. Finally, scrutiny of MuAt attention matrices revealed both ubiquitous and tumour-type specific patterns of simple and complex somatic mutations.

**Conclusion**

Integrated representations of somatic alterations learnt by MuAt were able to accurately identify histological tumour types and identify tumour entities, with potential to impact precision cancer medicine.

Acknowledgement:

We thank CSC – IT Center for Science, Finland, for generous computational resources. This research was made possible through access to the data and findings generated by the 100,000 Genomes Project; http://www.genomicsengland.co.uk.

### EACR23-0924
### AI-based pathomics biomarkers predict outlier response to first line treatment in metastatic colorectal cancers

*V. Giannini*[1,2], *G. Nicoletti*[1,3], *G. Mauri*[4,5,6], *C. Marchiò*[7,8], *A. Sartore-Bianchi*[5,6], *C. Aquilano*[9], *A. Bardelli*[4,10], *L. Lazzari*[11], *S. Marsoni*[11], *D. Regge*[2]

[1]*University of Turin, Surgical Sciences, Torino, Italy*

[2]*Candiolo Cancer Institute- FPO - IRCCS, Radiology, Candiolo, Italy*

[3]*Polytechnic of Turin, Department of Electronics and Telecommunications, Torino, Italy*

[4]*IFOM-Istituto Fondazione di Oncologia Molecolare ETS, Genomics of cancers and targeted therapies, Milano, Italy*

[5]*Grande Ospedale Metropolitano Niguarda, Hematology- Oncology- and Molecular Medicine, Milano, Italy*

© 2023 The Authors. Molecular Oncology is published by John Wiley & Sons Ltd on behalf of Federation of European Biochemical Societies

[6]Università degli Studi di Milano, Oncology and Hemato-Oncology, Milano, Italy
[7]University of Turin, Medical Sciences, Torino, Italy
[8]Candiolo Cancer Institute- FPO - IRCCS, Pathology, Candiolo, Italy
[9]Grande Ospedale Metropolitano Niguarda, Surgical Pathology and Cytogenetics, Milano, Italy
[10]University of Turin, Oncology, Torino, Italy
[11]IFOM-Istituto Fondazione di Oncologia Molecolare ETS, Precision Oncology, Milano, Italy

**Introduction**

The standard of care(SOC) treatment for microsatellite stable metastatic colorectal cancer(mCRC) is a backbone of "one-fits-all" 5-Fluorouracil-based chemotherapy combined with oxaliplatin, and/or irinotecan. Complete or long-lasting responses(CR-LLR) occur in 20-30% of patients, while 15-20% are refractory. In mCRC first-line(1L) response is a proxy of survival, refractory patients are thus exposed to side effects lowering their quality of life. The development of biomarkers to predict 1L SOC response is a pivotal unmet clinical need in mCRC to optimize the cost-benefit balance in individual patients. Our study aims at developing an unsupervised bag-of-words artificial intelligence (AI)-based model based on pathological images to predict 1L outcomes in patients with outlier response (progression or CR/LLR).

**Material and Methods**

32 patients were retrospectively enrolled at Niguarda Cancer Center, and classified as "sensitive" if they achieved CR or >10 months LLR to any 1L(N=10), or "refractory" if progression occurred at first disease reassessment(N=22). H&E slides of the primary CRC resections were digitalized to obtain whole slide images (WSI), that were were first divided into tiles of 224x224 pixel(0.5μm/pixel), and classified either as tumor or not using a VGG19 convolutional neural network. First-order and texture features were extracted from all tumoral tiles, which were consequently grouped into homogenous clusters through a 3x3 self-organized map (SOM). Finally, for each patient, the percentage of tiles belonging to each tiles' cluster was computed and used by a dendrogram to create clusters of similar patients.

**Results and Discussions**

6 clusters of patients were identified: 3 were composed of all but one refractory patients, 1 of a majority of sensitive patients, and 2 contained both groups. Each of these patients' clusters was characterized by the presence of the majority of tiles belonging to only one cluster of tiles. When the three clusters of refractory patients were pooled together to classify patients either as refractory or sensitive, a negative predictive value (NPV) of 92% (12/13) was obtained. In this study, NPV is the most clinically relevant metric to ensure that sensitive patients are not wrongly prevented to receive treatment.

**Conclusion**

We demonstrated the potential of a pathomics signature to predict outcomes of 1L SOC in mCRC patients. Preliminary findings should be further validated on a larger cohort of patients that we are collecting through a multi-institutional study.

### EACR23-1001
### Activation of developmental branching morphogenesis marks aggressive lung adenocarcinomas

K. Bienkowska[1], G. Thomas[1], C. Hanley[1]
[1]University of Southampton, Cancer Sciences- Faculty of Medicine, Southampton, United Kingdom

**Introduction**

Non-small cell lung cancers (NSCLC) are a heterogenous group of tumours often heavily infiltrated by stromal and immune cells, which can influence tumour progression and response to therapy.

**Material and Methods**

**Single-sample Gene Set Enrichment Analysis (ssGSEA)** was used to calculate enrichment scores in bulk transcriptome data for previously described developmental lung programmes, and cell-specific gene expression modules - identified by **WGCNA (Weighted Gene Co-expression Network Analysis)** of single-cell RNA-seq samples (42 LUAD, 13 LUSC, 17 Normal). **Survival analysis** was performed using log-rank tests. In vitro analysis was carried out using **collagen-embedded 3D tri-cultures** (consisting of H441, MRC5 and THP1 cells) and **qPCR**.

**Results and Discussions**

As observed during development, branching morphogenesis (BM) and alveogenesis (ALV) programmes were inversely correlated in NSCLC (R=-0.83, p<2.2e-16). Cell-specific gene expression modules showed this progression involved the replacement of alveolar type II (AT2) cells with basal epithelial cells, in a stepwise manner from Normal, through adenocarcinomas (LUAD), to squamous cell carcinomas (LUSC). Increased expression of the BM programme was associated with poor prognosis in LUAD (p<0.0001, n=508) and changes in the tumour microenvironment, including macrophage and myofibroblast recruitment/activation. In vitro analysis showed that fibroblasts and macrophages induced downregulation of ALV markers and upregulation of BM markers in H441 (LUAD) spheroids.

**Conclusion**

Activation of BM in LUAD creates more aggressive tumours, likely due to a transdifferentiation of AT2 cells into basal-like cells, which may be regulated by interactions with fibroblasts and macrophages.

### EACR23-1015
### The impact of different methods for mutational signature analysis on colorectal cancer profiling

P. Battuello[1], G. Crisafulli[2], G. Corti[3], A. Lorenzato[4], A. Bartolini[5], M. Russo[6], F. Di Nicolantonio[6], A. Bardelli[7]
[1]University of Turin and IFOM ETS - The AIRC Institute of Molecular Oncology, BioInformatic Group BIG & Genomics of Cancer and Targeted Therapies Unit, Milan, Italy
[2]IFOM ETS - The AIRC Institute of Molecular Oncology, BioInformatic Group BIG & Genomics of Cancer and Targeted Therapies Unit, Milan, Italy
[3]University of Turin, BioInformatic Group BIG & Genomics of Cancer and Targeted Therapies Unit, Orbassano, Italy
[4]University of Turin, Oncology, Orbassano, Italy

© 2023 The Authors. Molecular Oncology is published by John Wiley & Sons Ltd on behalf of Federation of European Biochemical Societies

[5]Candiolo Cancer Institute- FPO - IRCCS,
FPO - Fondazione del Piemonte per l'Oncologia, Candiolo, Italy
[6]University of Turin and Candiolo Cancer Institute - FPO - IRCCS, Oncology, Orbassano, Italy
[7]University of Turin and Candiolo Cancer Institute - FPO - IRCCS,
Present address: Department of Oncology- University of Torino- Italy and IFOM ETS – The AIRC Institute of Molecular Oncology, Milan, Italy

**Introduction**

Mutational signature fitting analysis identifies specific patterns of alterations in order to estimate the prevalence of mutational processes that have been active over time in a genome. This analysis requires to arbitrarily set up several parameters and currently there is no standardized procedure. We wondered whether different approaches may lead to discrepant signatures due to technical aspects rather than biological differences. To address this issue, we performed a comparative study investigating the impact of specific arbitrary parameters on fitting mutational signatures in a sample.

**Material and Methods**

We exploited the highly heterogeneous genetic profile of colorectal cancer (CRC) to evaluate how arbitrary parameters impact on mutational signature fitting. We exploited a large preclinical dataset of 230 CRC cell lines encompassing the main molecular subtypes and a second clinical validation dataset of 152 CRC patients from TCGA. We compared the performance of five different tools, different sized reference datasets and data from multiple sequencing workflows (Whole-Genome Sequencing, Whole-Exome Sequencing, and Pan-Cancer Panel). For each feature, we performed a technical and biological evaluation assessing the ability of mutational signatures to stratify CRCs based on their genetic profile, considering microsatellite stable mismatch repair proficient, mismatch repair deficient and POLE mutated subtypes.

**Results and Discussions**

The use of multiple algorithms and reference datasets led to statistically different results, highlighting how arbitrary choice of different parameters can determine variability in calling the mutational signature contributions. Using independent cohorts of both germ-line matched and tumor samples, we identified the minimum number of mutations necessary for the analysis. In addition to the actual number of alterations, also tumor mutation quality showed a profound impact on the results. Therefore, we recommended the use of a metanormal when the germinal matched sample is not available. Finally, considering that tumor specimens are not generally profiled at whole-genome level, we determined the feasibility of mutational signature analysis using a Pan-Cancer Panel that is often used in the clinic.

**Conclusion**

Our work demonstrates that different arbitrary choices can impact on CRC mutational signatures profiling, highlighting the need of a standardized method for performing such analyses before implementing this biomarker in clinical laboratories.

### EACR23-1054
### PROTACs in oncology: learned lessons applied to the design of novel β-catenin degraders

M. Rossi Sebastiano[1], G. Apprato[1], D. García Jiménez[1], P. Rossetti[1], G. Ermondi[1], G. Caron[1]
[1]University of Turin,
Molecular Biotechnology and Health Sciences Department, Turin, Italy

**Introduction**

Proteolysis Targeting Chimeras (PROTACs) are a novel promising therapeutic strategy in oncology. They act as proximity-inducing agents, recruiting a ubiquitin ligase (E3) and a protein of interest (POI). The association of the ternary complex (TC) triggers the POI ubiquitination and degradation, disrupting its catalytic and scaffolding functions. This allows targeting previously undruggable pathways and minimizes resistance. Instances of POIs which are targeted by oncologic PROTACs are the Androgen Receptor (AR), and the Bromodomain-containing protein 4 (Brd4).

However, there are some limitations: first, a rational drug design is limited by difficulties in determining the 3D structure of TCs. Second, PROTACs are larger than classical small molecules, displaying limitations to oral dosing. Consequently, novel *ad hoc* strategies are needed for an all-round design optimization. To this aim, we applied a plethora of chromatographic and computational tools.

**Material and Methods**

Computational tools: Maestro (conformational sampling, CS), NAMD2 (molecular dynamics, MD); SwissDock, Chimera 1.16, MOE (TC modeling, virtual screening); R (Machine Learning, ML). Molecular properties were experimentally measured via HPLC as previously described.

**Results and Discussions**

We first employed machine learning models to rationalize the *in vitro* degradation activity of published AR-targeting PROTACs. Then, with the aid of Artificial Intelligence-predicted protein structures, we set up a method to model 3D TCs and explain their structure-activity relationship. Interestingly, we discovered that the common cell-based degradation assays are strongly influenced by poor cell permeability.

We then took a series of PROTAC candidates designed against Brd4 and, with a set of validated chromatographic indexes, we experimentally characterized molecular properties related to oral bioavailability (polarity, lipophilicity). Moreover, we applied advanced computational simulations to rationalize (and predict) their experimental behavior.

We utilized the learnt lessons to computationally design PROTAC candidates targeting β-catenin. First, we identified molecules able to bind the POI, then we generated a library of potential compounds. Finally, we modeled ternary complexes and run simulations to select the most promising ones.

**Conclusion**

We report two case studies providing design strategies to overcome major PROTAC limitations: we applied them to design novel potential β-catenin degraders.

© 2023 The Authors. Molecular Oncology is published by John Wiley & Sons Ltd on behalf of Federation of European Biochemical Societies

### EACR23-1067
### Integrative bioinformatical analysis of RNA-Seq for non-small cell lung cancer biomarkers identification

K. Widzisz[1], J. Zyla[1]
[1]Silesian University of Technology, Department of Data Science and Engineering, Gliwice, Poland

**Introduction**

Non-small cell lung cancer (NSCLC) is the most prevalent subtype of lung cancer. It is one of the main causes of cancer-related deaths globally. Due to the limited efficacy of current treatments for NSCLC, it remains necessary to identify new tumour-associated biomarkers to improve patients' diagnosis and prognosis.

**Material and Methods**

To conduct this study, two NCBI-GEO datasets containing RNA expression profiles of NSCLC and healthy tissues were collected. In total the dataset included 179 samples from NSCLC-affected lung tissues and 151 samples from healthy tissues. As the data comes from various experiments their integration through the removal of batch effect was performed. In further steps, the analysis was carried out using bioinformatics tools and techniques, including dimensionality reduction techniques (UMAP, PCA). Next, two GLMs to find differentially expressed genes (DEGs) by comparing a group of NSCLC and healthy tissues were constructed. Both models took into account the batch effect, but additionally, the second model incorporated also the tissue type which varies in healthy samples. The obtained model p-values were corrected for multiple testing and adjusted p-value of <0.05 with $|log_2FC|>0.6$ states DEG.

**Results and Discussions**

After normalization and filtration of the RNA-seq data, the 14873 genes common to NSCLC and healthy tissues were analysed. Among these genes, 75 DEGs (5 up-regulated, 70 down-regulated) for GLM adjusted for batch effect and 124 DEGs (11 up-regulated and 113 down-regulated) for GLM adjusted for batch and tissue type were extracted. It should be noted that all of the differentially expressed genes identified by the first GLM were also found by the second GLM. The most significantly up-regulated DEGs were HLA-G, PRR4, LGALS4, PTCH2, and CAPN6, all of which are involved in cell proliferation and differentiation.

**Conclusion**

In summary, we provided an integrative analysis of RNA-seq human samples from various sources. As a result of bioinformatical investigation 75 and 124 DEGs in NSCLC by two GLMs were obtained. Future research will focus on exploring the potential of the identified DEGs as biomarkers and on investigating their clinical significance in NSCLC diagnosis and treatment through pathway enrichment analysis. The findings could potentially contribute to the development of new biomarkers for NSCLC, improving its diagnosis and treatment. To reveal more complex relation between DEGs and investigated phenotype the pathway enrichment analysis needs to be performed.

### EACR23-1069
### Mitochondrial mutation and dysfunction in High Grade Serous Ovarian Cancer

R. Silk[1], A. Ewing[1], A. Meynert[1], B. Dougherty[2], P. Roxburgh[3], C. Gourley[4], C. Semple[1]
[1]Institute of Genetics and Cancer, MRC Human Genetics Unit, Edinburgh, United Kingdom
[2]AstraZeneca, Translational Medicine- Oncology R&D, Waltham- MA, United States
[3]Beatson West of Scotland Cancer Centre, Wolfson Wohl Cancer Research Centre, Glasgow, United Kingdom
[4]Institute of Genetics and Cancer, Cancer Research UK Edinburgh Centre, Edinburgh, United Kingdom

**Introduction**

In recent years, there has been a growing body of evidence linking somatically acquired mitochondrial dysfunction to cancer.  However, robust estimates of the prevalence, patterns and impact of these events remain limited due to small sample sizes and incomplete analyses. Therefore, it is not yet understood how these alterations could provide a mechanism for tumour initiation and growth, and also how they may affect a patient’s response to treatment. This lack of systematic analysis for mitochondrial dysfunction is most prevalent in ovarian cancer.

**Material and Methods**

Here, we have analysed 324 whole-genome sequenced High Grade Serous Ovarian Cancer (HGSOC) samples with blood matched normals using pipelines that navigate the bioinformatic complexities of the mitochondrial genome.

**Results and Discussions**

We find frequent somatic mutations in the tumours mitochondrial DNA, the most deleterious of which are associated with reduced overall survival.

**Conclusion**

This may constitute a novel biomarker for HGSOC patient prognosis and lead to greater stratification of patients.

### EACR23-1103
### Elucidating tumor plasticity and drug resistance mechanisms on 3D organoids of lethal prostate cancer using a network-based framework for single-cell multimodal data

A. Vasciaveo[1], J.J. Li[2], C. Abate-Shen[3], M.M. Shen[2], A. Califano[1]
[1]Columbia University, Department of Systems Biology, New York City, United States
[2]Columbia University, Department of Genetics and Development, New York City, United States
[3]Columbia University, Department of Molecular Pharmacology and Therapeutics, New York City, United States

**Introduction**

Although major mechanisms of resistance to targeted therapies include mutation of the drug target, recent evidence suggests cell-adaptive mechanisms as alternative avenues for escaping treatment. In several cancers, tumor

© 2023 The Authors. Molecular Oncology is published by John Wiley & Sons Ltd on behalf of Federation of European Biochemical Societies cells become plastic in response to target inhibition, and change their identity and molecular programs without acquiring further mutations. In the case of castration-resistant prostate cancer (CRPC) after standard-of-care androgen deprivation therapy (ADT), tumor cells often switch lineage to survive drug pressure, acquiring neuroendocrine (NE) features and transforming into neuroendocrine prostate cancer (NEPC). Existing targeted therapies for CRPC and NEPC provide limited benefit, representing an unmet clinical need**.** Hence, the elucidation of tumor plasticity and consequent heterogeneity that contribute to drug resistance is of paramount importance to identify novel therapeutic opportunities.

**Material and Methods**

The Califano, Shen, and Abate-Shen labs have recently developed a network-based, computational and experimental framework to characterize molecular and drug sensitivity profiles of ~100 Genetically-Engineered Mouse Models (GEMM)-derived tumors of prostate cancer. This study revealed that tumors derived from GEMMs with loss of function of both Pten and Trp53 (NPp53) display significant heterogeneity in regulatory programs and histopathologic features, including NE differentiation. In particular, using 3D organoid lines from the NPp53 model generated by the Shen lab, we have further characterized regulatory programs that promote tumor plasticity and heterogeneity. Specifically, we reverse-engineered genome-wide regulatory networks using multimodal single-cell RNA-Seq and ATAC-Seq data to identify Master Regulator (MR) proteins implementing tumor cell identities, and characterized their epigenetic landscape.

**Results and Discussions**

Our network-based regulatory analysis has identified several distinct cell populations in CRPC, including cells activating epithelial-to-mesenchymal (EMT) programs and NE differentiation, all resistant to ADT. Cross-species analysis has successfully aligned these cells to human CRPC and NEPC tumors.

**Conclusion**

We have leveraged single-cell, multimodal data from mouse CRPC and NEPC primary tumors and organoids to characterize their heterogeneity and elucidate regulatory programs of drug-resistant, lethal prostate cancer.

### EACR23-1123
### Homologous Recombination deficiency classification and PARP inhibitor response of reference-free colorectal cancer samples

*G. Corti[1], K. Buzo[1], E. Berrino[1], M. Miotto[1], M. Lentini[1], M. Russo[1], C. Marchiò[1], F. Di Nicolantonio[1], S. Arena[1], A. Bardelli[1]*

*[1]University of Torino, Department of Oncology, Candiolo, Italy*

**Introduction**

PARP inhibitors (PARPi) are known to promote a synthetic lethal interaction with tumors bearing the homologous recombination deficiency (HRD) phenotype (also defined as "BRCAness"), therefore accurate HRD prediction represents an urgent medical need to select patients candidate for these therapies.

Although BRCAness is well known to occur in a fraction of breast, ovarian, prostate and pancreatic tumors, recent evidence has shown that up to 15% of colorectal cancers (CRC) carry defects in the HR pathway, opening up new avenues for new therapeutic options in CRC patients. HRDetect is a recently developed tool that is able to predict HRD in tumors, by the concomitant analysis of somatic and matched germline DNA; however, the latter is not always available.

**Material and Methods**

We used WGS data from more than 70 breast samples from the original HRDetect work as a validation dataset to develop a new tool called HRDirect, based on the HRDetect pipeline.

We also exploited a collection of CRC patient-derived organoids with matched germline DNA to confirm that our tool could be applied to tumor types other than breast or ovarian origin.

Finally we validated the power of HRDirect in predicting response to olaparib by comparing the performance of our test and the commercial assay AmoyDx HRD by Amoy Diagnostics in a set of more than 30 CRC cell lines.

**Results and Discussions**

We proved that HRDirect is able to predict HRD from a reference-free tumor sample. HRDirect predictions on tumor-only samples reproduced the expected results with a very low discordance (Cohen's kappa: 0.97).

After having confirmed that our tool performs well also on matched CRC organoids, we obtained scores from HRDirect and AmoyDX HRD: both approaches resulted equivalent at predicting the sensitivity of CRC cell lines to PARPi.

Both tests were unable to identify HRD in samples characterized by ATM loss, a feature characterized by lack of molecular scars. For this reason, we propose to combine the HRDirect scoring with ATM immunohistochemistry (IHC) analysis to improve the overall HRD prediction.

**Conclusion**

Our results highlight that combining HRDirect and ATM IHC can potentiate the identification of HRD tumors, with an immediate translational and clinical impact.

This finding results of interest not only for the analysis of clinical samples missing the germline DNA, but also for preclinical models that have been demonstrated to be valuable platforms for olaparib or other PARP inhibitors.

### EACR23-1142
### Mathematical model for cancer clonal evolution using advanced branching process

*D. Volpatto[1], S. Pernice[1], E. Genuardi[2], E. Grassi[3], L. Trusolino[3], A. Bertotti[3], R. Sirovich[4], S. Ferrero[2], M. Ladetto[5], F. Cordero[1]*

*[1]University of Torino, Computer science, Turin, Italy*
*[2]University of Torino, Molecular Biotechnologies and Health Sciences, Turin, Italy*
*[3]University of Torino, Oncology, Turin, Italy*
*[4]University of Torino, Mathematics G. Peano, Turin, Italy*
*[5]Antonio e biagio e cesare Arrigo hospital, Division of Hematology, Alessandria, Italy*

**Introduction**

© 2023 The Authors. Molecular Oncology is published by John Wiley & Sons Ltd on behalf of Federation of European Biochemical Societies

The aim of this work is to refine stochastic processes used for cancer modelling taking into account passenger mutations and differentiating cells based on their genetic profile, providing a mathematical description of the tumor's clonal evolution. This is particularly useful since the subclone multiplicity and the degree of intratumor heterogeneity have been reported as prognostic biomarkers. Furthermore the simulation of the cancer evolution based on genetic profiles can be used to forecast the courses of the disease and to simulate the target therapy effects.
**Material and Methods**
Branching processes, widely used in population dynamics analysis, are the main mathematical tools used to study the cancer evolution. This type of process is capable of describing how probable it is for an individual to have a given progene, something that perfectly matches with cancer evolution. We implemented the model on R to simulate a tumor expansion and we used data of both solid and liquid tumors (such as colorectal carcinoma and mantle cell lymphoma) to calibrate the model and to verify the results obtained.
**Results and Discussions**
We built a branching process capable of describing mutations that change tumor fitness and ability to further mutate: our model requires a multidimensional approach and relies on two kinds of parameters (i) the growth rate of cells associated with a given genetic profile and (ii) the rate at which new mutations are acquired. The selective advantage that each driver mutation provides makes the growth rate increase accordingly, while accumulation of somatic (also known as, passenger) mutations increases the probability of driver genes to mutate. Whenever a driver mutation is supposed to occur, we let it be randomly chosen according to a probability distribution given by genes dependencies knowledge. Finally carrying capacity of the tissue is taken into account limiting the expansion otherwise of explosive kind and competitive behaviour between clones sharing space and resources are included.
**Conclusion**
Compared to pre-existing mathematical models, our approach takes into account both driver and passenger mutations, and it is able to distinguish individual driver mutations instead of just counting them. Moreover, our branching approach is able for each subclone to (i) profile it from a genomic point of view and (ii) to estimate the number of cells. To the best of our knowledge, this represents a step towards the realisation of a virtual tumor to encompass the challenge of personalized medicine.

### EACR23-1153
### Image integrative spatial transcriptomics analysis to uncover tumor heterogeneity

R. Gindra[1], S. Bärthel[2], D. Saur[2], T. Peng[1]
[1]*Helmholtz Munich - German Research Center for Environ ment and Health, Institute of AI for health, München, Germany*
[2]*Center for Translational Cancer Research - TranslaTUM, School of Medicine - Technical University of Munich, München, Germany*
**Introduction**
Spatial transcriptomics (ST) technologies aid in understanding cellular patterns in healthy and diseased tissues by mapping gene-expression across them. The popular sequencing based ST approaches currently are limited by sequencing resolution with spots containing multiple cells. To overcome this, several cell deconvolution methods exist to comprehensively map the cell types in-situ for diverse tissues thus allowing to understand the spatial context at a higher resolution. However, precise spatial localization of cell-types on a pixel level in tissues with high structural variability like cancer tissues remains challenging.
**Material and Methods**
Here, we developed the i-Stanly toolkit, a multi-modal representation learning model that uses single cell reference gene-expressions, spatial gene-expression, and corresponding histology images to perform tissue region prediction. We use the learning mechanism as a precursor to precisely localize/segment cell-types at a pixel level. In particular, two complementary modalities, cell-type gene expressions estimated from transcriptomic data using Cell2location, a bayesian deconvolution model and rich morphological features from histological images using CTransPath, a self-supervised image model are integrated in a co-attention mechanism to perform region prediction. The network is trained in a supervised setting, posing it as a classification problem while learning relevance of different cell-type expressions on the corresponding images.
**Results and Discussions**
We assess the i-Stanly toolkit in two different cancer tissue cohorts (Her2 positive breast cancer, Pancreatic ductal adenocarcinoma) and show higher resolution mapping/segmentation of cell-types across different technologies and spot resolutions. To compare the performance with single modality methods, we also train an image only model and a gene-expression only model to perform tissue region prediction. We show that i-Stanly exceeds classification performance compared to gene-expression model and performs equivalent to the image-only model.
**Conclusion**
Our results present i-Stanly as a highly adaptable pipeline for pixel-level mapping/segmenting heterogeneous cell-types, thereby introducing a multimodal computational toolkit for detailed exploration of the tumor microenvironment composition, spatial neighborhoods and complex cell-cell communication networks.

### EACR23-1178
### 'MedClass' application for normalisation and classification of thyroid cancer types based on gene signatures

A. Staśczak[1], H. Langer-Macioł[2], A. Morel[2], T. Tyszkiewicz[3], P. Tudrej[3], D. Rusinek[3], M. Oczko-Wojciechowska[3], S. Student[1]
[1]*Silesian University of Technology, Biotechnology Center and Department of Systems Biology and Engineering, Gliwice, Poland*
[2]*Silesian University of Technology, Faculty of Automatic Control Electronics and Computer Sc ience, Gliwice, Poland*
[3]*Maria Sklodowska-Curie National Research Institute of Oncology- Gliwice Br anch, Department of Clinical and Molecular Genetics,*

© 2023 The Authors. Molecular Oncology is published by John Wiley & Sons Ltd on behalf of Federation of European Biochemical Societies

*Gliwice, Poland*

**Introduction**

Significant progress has been made in recent years through targeted therapies and immunotherapy in the treatment and diagnosis of thyroid cancer. Using personalised therapies, the quality and level of treatment can change for the better, considering the need for surgery and other complementary therapies, while minimizing side effects and their excessive aggressiveness. Molecular classifiers may be used to help identify thyroid malignant nodules and choose the best option of treatment. An attempt was made to create an application that allows normalisation of the data entered, molecular classification of thyroid cancer subtypes based on selected gene signatures in the model entered by the user, and graphical interpretation to help with the analysis.

**Material and Methods**

The interactive application was created using Shiny package, the programming language R, free version of the integrated development environment RStudio. Part of the analysis was done through Bioconductor use. The application normalises data using the comparative normalisation algorithm. It is based on calibration samples and housekeeping genes, whose expression values remain constant regardless of the type and origin of the sample on a microarray set. The normalisation process acquired expression values manifested as multiplicities of the calibration sample. The classification process is based on the loaded or prepared model according to the specified guidelines.

**Results and Discussions**

The work results in an application that normalises data and classifies them according to gene signatures and the Bethesda System for Reporting Thyroid Cytopathology. The result consists of a class: malignant/benign, determined by probability, and a graphical analysis representation displayed as a table and visualised on interactive graphs (the user can analyse all results simultaneously or separately). At the end of the analysis, the user can also download the customised report. The application is structured and contains among others "Home", "Normalisation", "Classification", "Report" and "Instruction", which have possible sub-tabs.

**Conclusion**

The MedClass application was designed to be implemented in the patient's diagnostic and treatment system. The concept of the application assumed the creation of a user-friendly graphical interface and intuitive functions. Using it, staff can easily and quickly assess and analyse the results.

### EACR23-1229
### Profiling of the olfactory receptor family expression in colon carcinoma by using in silico approaches

*Y. Oztemur Islakoglu[1], P. Korhan[1,2], E. Bagirsakci[1,3], G. Solmaz[1,2], N. Atabey[1,2]*
*[1]Izmir Biomedicine and Genome Center, Basic and Translational Research Program, Izmir, Turkiye*
*[2]Izmir Tınaztepe University, Galen Research Center, Izmir, Turkiye*
*[3]Dokuz Eylül University, Izmir International Biomedicine and Genome Institute, Izmir, Turkiye*

**Introduction**

Colon adenocarcinomas (CC) are the most common type of colorectal cancer, with approximately 600.000 deaths/per year. Early diagnosis options are very limited for CC, and reliable biomarkers are crucial to its clinical practice. Olfactory receptors (ORs), the most prominent family of GPCRs, are previously thought to be associated only with a sense of smell, are expressed in many normal tissues besides olfactory epithelium and sensory neurons, and play a role in many physiological processes, including blood pressure regulation, triglyceride metabolism, sperm movement, and muscle regeneration. Recent studies showed the significance of ORs in the development and progression of different cancers and revealed that many ORs are essential markers for cancer. However, up to date there are limited studies are focusing on importance of ORs in CC. In this study, we aimed to investigate important ORs, that family has nearly 850 members in CC using bioinformatics approaches.

**Material and Methods**

This study aims to determine specific OR types expressed in the colon. For this purpose, we created OR-related meta-data obtained from different cohorts, such as TCGA, using bioinformatics tools and databases such as UALCAN, cBioPortal, and UCSC Xena Browser. We analyzed all OR family members and determined that (i) different ORs are expressed in colon tissue and (ii) expression levels of specific OR types differ in normal colon and CC samples.

**Results and Discussions**

In light of these bioinformatics data, we identified ORs in all primary CC tumors, and their adjacent normal, and OR51E1 is an important candidate for CC. Furthermore, it is shown that OR51E1 is expressed in colon tissue, and it is differentially expressed (DE) in colon cancer samples when compared to normal samples. In addition, increased OR51E1 levels were determined during tumor progression. Lastly, we grouped patients according to OR51E1 expression pattern and identified DE genes between high and low-expressed patient samples. Consequently, our data suggests that OR51E1 may be an important biomarker candidate for CC.

**Conclusion**

This study provides the opportunity to combine patient data from a large number of samples and presents new and comprehensive information to identify the roles of ORs in CC.

This study is supported by the TUBITAK project, #122S547.

### EACR23-1248
### Sequence homology of bacterial and mitochondrial genes implicated in cancer pathogenesis

*A. Basgaran[1], S. Paschke[1], E. Lymberopoulos[1], M. Hobbs[1], D. Delanoue[1], J. Arney[1], M. Alomari[1], N. Sharma[1]*
*[1]BioCorteX LtD, BioCorteX LtD, London, United Kingdom*

**Introduction**

Bacteria have been implicated in the pathogenesis of various cancers. While the precise mechanisms remain unclear, it has been suggested that epigenetic reprogramming, immune evasion, and dysregulated mitochondrial function could play a role. Given the

© 2023 The Authors. Molecular Oncology is published by John Wiley & Sons Ltd on behalf of Federation of European Biochemical Societies bacterial origins of mitochondria, we explore the overlap between mitochondrial genes implicated in cancer pathogenesis and genes from bacterial species.

**Material and Methods**

Using the BioCorteX CarbonMirrorTM platform, we examine the nucleotide sequences of key mitochondrial genes implicated in cancer pathogenesis (ATP6, ATP8, CO1, CYB, ND3, ND4, ND5, TL1). We examine for sequence homology against a subset of 8,570 bacterial species and 63,000 strains with thresholds of 70% in sequence homology and 80% in length similarity. The platform includes a deterministic engine based upon first principles - note it did not include any a priori bacteria-cancer connections.

**Results and Discussions**

Within the subset of 6026 strains processed, 5290 strains contain at least one of the eight mitochondrial genes at the required threshold. Four mitochondrial genes in total had bacterial overlap: ATP8, CO1, ND3 and TL1.

The mitochondrial gene ATP8 was present in 18 out of 6026 strains investigated. 10 of these were *Helicobacter pylori* strains, and both this species and the ATP8 gene have established causative links with gastric cancer through gene mutation.

Similarly, the mitochondrial gene ND3 was found in 2 bacterial strains of the species *Campylobacter jejuni*, a common cause of diarrhoea. Again, both this bacterial species and ND3 mutations have been implicated in colorectal tumorigenesis.**Conclusion**

The BioCorteX engines have rediscovered the mechanisms underlying two key bacteria and cancer pathogenesis from first principles: *Helicobacter pylori* and gastric cancer through the ATP8 mitochondrial pathway, and *Campylobacter jejuni* and colorectal cancer through the ND3 mitochondrial pathway. With the application of a more extensive list of oncogenes and bacterial strains, the BioCorteX CarbonMirrorTM platform can be further leveraged to discover thousands of mechanistic actionable insights into the microbiome's role in cancer pathogenesis, opening a novel field for cancer therapeutics.

### EACR23-1253
### Game-theoretic image segmentation in medical and synthetic images

T. Żytecki[1], J. Mielczarek[1], D. Borys[2]
[1]Silesian University of Technology, Faculty of Automatic Control- Electronics and Computer S cience, Gliwice, Poland
[2]Silesian University of Technology, Department of Systems Biology and Engineering, Gliwice, Poland

**Introduction**

There are many ways to segment images, they can be divided into two groups - finding the edges of the object and dividing the image into regions. Division of the image into regions uses the pixel values themselves, which means that local interference does not significantly affect the final result. Unfortunately, there is a tendency to oversegmentation in this group of methods. The methods for finding the edges of objects use the gradient of the image, which makes them much better at locating the right object. Goals of this thesis were to implement and study the algorithm described in A. Chakraborty's and J. S. Duncan's work "Game-Theoretic Integration for Image Segmentation''.

**Material and Methods**

In order to conduct the research, synthetic, natural and dermoscopic images from a publicly available PH2 database were used. Segmentation was carried out for 20 different photos of moles, and for each the Dice index was calculated. The openCV library was used to work with images in Python, and the scipy library for optimization. The openCV library for Python is based on the numpy library. In the edge-seeking module, the cost function is optimized using the Nelder-Mead minimalization function, while the edge-dividing module uses the ICM algorithm to segment the image.

**Results and Discussions**

Integration used does indeed improve the performance of the respective segmentation methods. The algorithm used is able to match the input contour of the image to the object to some extent. However, this approach requires initialization, which is not problematic in the region splitting module but requires a fairly accurate representation of the true edge of the object in the edge detection module. With a good mapping of the object's initial edge, this game-theory integration gives better segmentation results than using the involved methods separately.

**Conclusion**

The research shows that the selected approach to segmentation in its original form gives not good enough results to be practical. The most problematic was the module searching for edges, in which a function was used that was not suitable for effective optimization by known methods. However, game theory seems to be a potential and promising strategy in the segmentation of medical images.

### EACR23-1262
### Deep multiple instance learning of single-cell transcriptomics predicts the anti-cancer response to immune checkpoint therapy

Y. Chung[1], D. Kim[1], K. Im[2], J. Lee[2]
[1]SungKyunKwan University, Department of Artificial Intelligence, 2066- Seobu-ro- Jangan-gu- Suwon-si- Gyeonggi-do, South Korea
[2]SungKyunKwan University, School of Medicine, 2066- Seobu-ro- Jangan-gu- Suwon-si- Gyeonggi-do, South Korea

**Introduction**

Immune checkpoint inhibitors (ICIs) provide durable clinical responses, but only a subset of patients benefit from the treatment. The identification of predictive biomarkers for ICI response is a pressing question in the field; nonetheless, the complexity of the immune response and the limitation of data make it difficult to tackle. In recent years, artificial intelligence (AI) has emerged as a promising tool for predicting ICI response based on patient-specific characteristics. In this work, we present an AI model that leverages single-cell RNA-seq (scRNA-seq) data from peripheral blood mononuclear cells (PBMCs) to predict ICI responses in patients with solid tumors.

**Material and Methods**

© 2023 The Authors. Molecular Oncology is published by John Wiley & Sons Ltd on behalf of Federation of European Biochemical Societies

Multiple instance learning (MIL) is one of the forms of weakly supervised learning where the set of training data is called a bag, and a label is provided for the entire bag. The scRNA-seq data consists of transcriptomic profiles of a large number of cells, while the ICI response label is assigned to each patient, which naturally formulates a MIL problem. We develop TRIPS – a Transformer-based deep multiple instance learning framework leveraging scRNA-seq data from cancer patients to predict responses to ICI treatment.

**Results and Discussions**

TRIPS consists of two major components: i) Extracting the features using a pre-trained model, and ii) Aggregating the features to predict ICI response. Specifically, we make use of a pre-trained model trained on large-scale scRNA-seq data to learn a robust representation for scRNA-seq data. Then, we propose an inter/intra-cellular self-attention (IICSA), which aggregates the features by applying a self-attention framework both intracellularly and intercellularly. We apply TRIPS to a PBMC scRNA-seq dataset of 55 NSCLC patients treated with anti-PD1 to predict the response to the therapy. TRIPS exhibits highly predictive performance (AUC=0.790, ACC=0.764), that surpasses the existing PDL1 score-based prediction (AUC=0.730, ACC=0.759). In our study, we have unveiled the potential of natural killer cells, gamma delta T cells, and proliferating T cells as predictors of anti-PD1 responsiveness by utilizing attention scores for the class token in our model.

**Conclusion**

To our knowledge, this is a pioneering study that proposes a predictive deep learning model for ICI response based on scRNA-seq data for the first time. Once validated and tested in the clinic, the scRNA-seq based biomarkers of PBMC samples have a great potential to enable minimally invasive prediction of the response to ICI.

### EACR23-1296

### A better data integration for cancer prevention: proposal of a cancer research framework including the integration of environmental and internal exposome data in Cancer Registries.

*C. Ambrosino*[1], *F. Savoia*[2], *C. Calì*[2], *M.F. Vitale*[3], *V. Ciullo*[3], *A. Pizzolante*[4], *F. Mario*[3], *C. Enzo*[5]

[1]*CNR-IEOS, Molecular oncology, Napoli, Italy*

[2]*A.O.R.N. Santobono-Pausilipon - Ospedale Pausilipon, Childhood Cancer Registry Campania, Via Posillipo- 226- 80123 Napoli NA, Italy*

[3]*AslNA3Sud, Cancer Registry, Brusciano NA, Italy*

[4]*Istituto Zooprofilattico del Mezzogiorno, Ambiente e Salute, Portici NA, Italy*

[5]*ASL-BT, Cancer Registry, Andria, Italy*

**Introduction**

Several European initiatives suggest the need to reach a better use of data. The EOSC4Cancer project aims to prepare and enrich services, data, and tool from the cancer research community, extending the use and integration of data relevant to cancer research. Cancer Registries currently represent the most complete tool for collecting population cancer data and, therefore, the best tool for promoting research on environmental exposures as risk factors for the onset of cancer. Web-based interactive platforms of geo-referenced cancer incidence data are now available, which allow the analysis of data from small areas; this approach is currently considered the best tool for detecting environmental effects on health by also estimating the weight of social and behavioural determinants as risk factors. We propose to establish an open science workflow in which the cancer registries data are integrated to external and internal exposome data.

**Material and Methods**

The georeferenced incident cancer cases of the ASLNa3Sud Cancer Registry are integrated with the georeferenced environmental and socio-economic data of the same area with the aim of describing the geographical variability of the risk of lung cancer and investigating the association with environmental and socio-economic factors. A recently developed Municipal Environmental Pressure Index (MIEP) was used by weighted integration of multiple geospatial variables, as measured in the SPES study (doi: 10.2144/fsoa-2020-0164.) and the Deprivation Index (DI), as calculated by ISTAT. Spatial analysis was conducted through hierarchical Bayesian models that calculate relative risks for each geographic unit; crude models were compared with those that introduce environmental and socioeconomic risk factors.

**Results and Discussions**

The geographic pattern of lung cancer risk shows a marked component of heterogeneity between areas in both males and females. The association between lung cancer incidence and DI was found, while the relationship between MIEP and lung cancer incidence appears weak.

**Conclusion**

We propose that contextual assessment and correction for each of the individual risk factors, MIEP and DI, is important for predicting and weighing the impact that environmental factors and/or socioeconomic factors have on the incidence of different types of cancer.

### EACR23-1299

### Characterization of ovarian high-grade serous carcinoma phenotypes using inferred transcription factor activities

*K. Zhang*[1], *A. Häkkinen*[1], *K. Lavikka*[1], *K. Huhtinen*[2], *A. Virtanen*[3], *S. Hietanen*[4], *J. Hynninen*[4], *S. Hautaniemi*[1]

[1]*University of Helsinki, Research Program in Systems Oncology- Research Programs Unit- Faculty of Medicine, Helsinki, Finland*

[2]*University of Turku, Cancer Research Unit- Institute of Biomedicine and FICAN West Cancer Centre, Turku, Finland*

[3]*Helsinki University Hospital, Department of Pathology- University of Helsinki and HUS Diagnostic Center, Helsinki, Finland*

[4]*University of Turku and Turku University Hospital, Department of Obstetrics and Gynecology, Turku, Finland*

**Introduction**

Dysregulation of transcription factor (TF) can lead to cancer progression, metastasis and chemotherapy resistance. Ovarian high-grade serous carcinoma (HGSC) is the most common and aggressive subtype of ovarian cancer. We hypothesized that characterization of transcriptional phenotypes based on TF activities allows

© 2023 The Authors. Molecular Oncology is published by John Wiley & Sons Ltd on behalf of Federation of European Biochemical Societies biomarker-based prediction and targeting of chemoresistance in HGSC.

**Material and Methods**

We constructed TF-target regulatory networks based on gene coexpression and cis-regulatory motif enrichment analysis of 350 fresh HGSC tumor samples from 160 patients, resulting in 339 TF regulons. For each regulon, we modeled the TF and copy number regulations on gene expression using Gaussian Bayesian networks. We assumed the target gene expression levels depend linearly on their DNA copy number levels and on the latent TF activities. Given the observed gene expression and copy number profiles, we estimated the regulatory weights and the local distribution of the latent TF activities using an expectation-Maximisation (EM) procedure.

**Results and Discussions**

With estimated TF activities in each tumor sample, we identified seven TF modules: chromatin remodeling-associated TFs (e.g., EP300, KMT2A), mesenchymal-associated TFs (e.g., ZEB1, TWIST1), AP-1 TFs (e.g., FOS, JUN), two inflammation-associated TF modules (mesenchymal-dependent and -independent inflammatory modules, e.g. NFKB1/2, STAT3), proliferation-associated TFs (e.g., E2F1/2, MYBL1/2) and interferon-associated TFs (e.g., IRF1/7, STAT1). Our findings highlight that tumors with distinct TF activity profiles coexist at different anatomic sites within an individual patient before chemothreapy. Ascites samples are significantly different from solid tumors in the mesenchymal and inflammatory TF modules, and omentum and peritoneum metastases have higher activities of mesenchymal and proliferation modules than tubo-ovarian samples. Survival analysis demonstrated that mesenchymal, mesenchymal-dependent inflammatory and the AP-1 TF modules are associated with significantly shorter platinum-free interval and overall survival, suggesting their roles in promoting chemoresistance.

**Conclusion**

We characterized the transcriptional heterogeneity over different anatomic sites in HGSC based on the inferred TF activities. Our results show that the mesenchymal, mesenchymal-dependent inflammatory and the AP-1 TF modules are associated with poor patient prognosis.

### EACR23-1303

### Are prostate cancer cell lines clinically relevant? An integrated clinicomics approach to validating 42 cell lines against well characterised global patient cohorts.

*Z. Ahmed[1], S. Heavey[1]*

*[1]University College London, Division of Surgery and Interventional Sciences, London, United Kingdom*

**Introduction**

The majority of cancer drug discovery pipelines involve widespread in vitro screening at early stages, despite the well documented imperfections of cell line models. A range of in silico, in vivo and ex vivo approaches are gaining in popularity, with the aim of partially or fully replacing the use of cell lines, however we cannot ignore the plethora of historical data from cell lines, nor write off any use for them in the future – especially within advanced models such as 3D bioengineered models, organoids and tumour-on-a-chip. Therefore, we must also ask ourselves if and how cell lines hold clinical relevance, and which ones are relevant in which ways.

**Material and Methods**

Here, we dissect the clinical and biological characteristics of 42 prostate cancer cell lines, comparing them to over 10,000 well characterised human cases from 23 studies in 8 countries, and where possible, against broader global epidemiological information. We firstly present baseline comparisons between cell lines and human samples including age, race, cancer stage, cancer subtype, site of sample and other clinical data, as well as biological comparisons including somatic, germline and driver mutations, gene expression, deletions, and copy number alterations.

**Results and Discussions**

We find that the most commonly used cell lines are accurately representative of only a very small proportion of actual prostate cancer cases, with poor validity relating to sample site and patient race, among others. We also suggest potential sub-cohorts of publicly available datasets for validation of specific cell line findings, as well as inclusion and exclusion criteria for prospective ex vivo work, and hypothesize which patients could ultimately benefit from specific cell line findings. Finally we contextualise the utility of each of the most used cell lines, highlighting their relevance to each stage of the prostate cancer clinical pathway, for use in discovery research, and the most relevant phases for them to be used within drug development pipelines.

**Conclusion**

With cancer drug discovery success rates remaining poor, we must work to improve the clinical validity of pre-clinical models. Here, we identify specific sub-cohorts of publicly available human prostate cancer samples that are recapitulated well by specific cell lines, allowing researchers to validate and further investigate their cell line findings in relevant subsets of publicly available datasets, and offer clinical sub-cohorts for consideration where existing findings may be most relevant.

### EACR23-1305

### Metabolic enzymes essential for GB cell aggressive behaviors identified from single cell transcriptome analyses.

*M.S. Saurty-Seerunghen[1], L. Bellenger[2], T. Daubon[3], H. Chneiweiss[1], C. Antoniewski[2], E.A. El-Habr[1], M.P. Junier[1]*

*[1]Institute Biology Paris Seine - IBPS, Neuroscience Paris Seine-NPS- CNRS UMR8246- Inserm U1130- UPMC- Sorbonne Universités, Paris, France*

*[2]Institute Biology Paris Seine - IBPS, ARTbio Bioinformatics Analysis Facility, Paris, France*

*[3]Institut de Biochimie et Génétique Cellulaires, CNRS UMR5095- Inserm U1029- Université de Bordeaux, Bordeaux, France*

**Introduction**

Glioblastoma is a devastating brain tumor. It is characterized by a diversity of genomic anomalies, and the ability of its cancer cell populations to adapt their behavior to the constantly changing environment of a growing tumor

© 2023 The Authors. Molecular Oncology is published by John Wiley & Sons Ltd on behalf of Federation of European Biochemical Societies and to therapies. Because metabolism is an obligatory step in shaping cell behavior, we looked for metabolic activities required for sustaining two glioblastoma cell properties critical for tumor aggressiveness, motility and tumorigenicity, regardless of the diverse genetic contexts encountered in patients' glioblastoma.

**Material and Methods**

To obtain a systemic view of the metabolic pathways at play in the complex setting of fully-grown tumors as observed at diagnosis, we combined analyses of publicly available single cell RNA-sequencing data obtained from glioblastoma resections with in vitro and in vivo experimental manipulations.

**Results and Discussions**

Data reduction based on experimentally-defined molecular signatures allowed to group cells according to their potential properties, and to identify their corresponding sets of overexpressed genes encoding metabolic enzymes. Computational analyses integrating expression network modeling or trajectory modeling disclosed metabolic enzymes essential for sustaining glioblastoma cell motile and tumor-initiating properties, respectively. The soundness of the prediction of the computational modeling was experimentally verified using a collection of human glioblastoma cells, tissue organoids and intracerebral xenografts combined with pharmacological and genetic manipulations and a variety of cell biological and biochemical assays. As a result, the cysteine metabolism enzyme 3-Mercaptopyruvate sulfurtransferase (MPST) and the most downstream component of the polyunsaturated fatty acid synthesis pathway ELOVL2, were demonstrated to be essential for GB cell motility and tumorigenicity, respectively.

**Conclusion**

Our result show that specific metabolic reprogramming are linked to specific cancer cell behaviors and maintained across heterogeneous genomic landscapes. They further show the relevance of analyses of single cell transcriptomes for unraveling metabolic dependencies of cell malignancy.

### EACR23-1324

### Pan-cancer evaluation of apoptosis, necroptosis, pyroptosis, ferroptosis and netosis in terms of prognosis and tumor microenvironment

*T. Sanavia*[1], *V. Alberini*[1], *L. Nicolè*[2], *P. Fariselli*[1]

[1]*University of Torino, Department of Medical Sciences, Torino, Italy*

[2]*Ospedale Dell'Angelo, Unit of Surgical Pathology & Cytopathology, Mestre, Italy*

**Introduction**

Apoptosis, necroptosis, pyrptosis, ferroptosis and netosis represent different pathways of programmed cell death that play important roles in the development and treatment of cancer. These types of cell death are often disrupted during neoplastic onset and progression, resulting in survival and proliferation of cancer cells. In this study we investigated their mechanisms and effects at a pan-cancer level, focusing on the expression of the related genes, their impact on the patients' prognosis and their correlation with the immune infiltrate characterizing the tumor microenvironment.

**Material and Methods**

Gene expression RNA-seq data were obtained from The Cancer Genome Atlas (TCGA), investigating 32 different tumor types and considering 10,788 samples. The signatures of genes characterizing five types of cell death pathways (apoptosis, necroptosis, pyroptosis, ferroptosis and netosis) were identified through literature review and biological annotation from databases (e.g. KEGG and Wikipathways). Differential gene expression analysis between tumor types was carried out using Kruskall-Wallis and Wilcoxon tests. Survival analysis was then applied to identify the most prognostic genes through Cox regression model, while Spearman's rank correlation test was used to assess, in each tumor type, the association between gene expression and the immune infiltrate, retrieved by deconvolution methods on RNA-seq data. Enrichment analysis using Fisher's exact test, PPI annotations from STRING database and hierarchical clustering helped in interpreting and visualizing the obtained results.

**Results and Discussions**

Overall, 383 genes were associated with the five cell death pathways. Higher differential gene expression distributions across the tumor types was mainly observed in a subset of tumors: CHOL, DLBC, UCS, ACC, MESO and ESCA. Genes significantly associated with the patients' prognosis were mainly identified in MESO, ESCA, PAAD and UVM. In particular, ferroptosis-related prognostic genes were found in ESCA and mainly associated with CD4 and T-helper cell populations, while pyroptosis-related prognostic genes in UVM were found linked with macrophages and B cell populations.

**Conclusion**

This preliminary analysis has allowed a first stratification in each tumor type of different types of cell death and immune cell populations. Interesting conclusions have been drawn for specific tumors which should be carried out in further studies.

### EACR23-1345

### The spatial transcriptomic landscape of prostate cancer within the tumour microenvironment: analysis of compartmentally and pathologically segmented radical prostatectomy specimens from SCREEN cohort

*V. Thavarajah*[1], *G. Akman*[1], *A. Freeman*[2], *H. Aiman*[1], *J. Crompton*[1], *A. Enica*[1], *W. Hayley*[1], *U. Okoli*[1], *U. Cheema*[1], *S. Heavey*[1]

[1]*University College London, Division of Surgery and Interventional Sciences, London, United Kingdom*

[2]*University College London Hospital, Department of Histopathology, London, United Kingdom*

**Introduction**

Within the prostate cancer field, the spatial transcriptomics era has begun in earnest, with a small number of studies showcasing the competing technologies and providing ample food for thought in terms of the biological complexity they can reveal. As hoped, we are now teasing apart the complex interplay between intratumoural

© 2023 The Authors. Molecular Oncology is published by John Wiley & Sons Ltd on behalf of Federation of European Biochemical Societies heterogeneity, microenvironment, immune infiltration and tissue architecture at the RNA level. Here, we set out to provide a novel dataset using the GeoMx Digital Spatial profiler (DSP) platform from NanoString, to thoroughly characterise a cohort of radical prostatectomy specimens from the SCREEN study.

**Material and Methods**

We present data from 3 Gleason 7 radical prostatectomy specimens, recruited using the PEOPLE method of MRI-guided biobanking, including 3 tumour biopsy samples containing 21 regions of interest (ROIs) of which 3 are adjacent benign. 3 distant benign samples were also scanned. Region selection was based on pathologist-contouring into 27 ROIs, further segmented into PanCK+ and - staining, providing a total of 54 areas of interest for sequencing using the Whole Transcriptome Atlas (WTA). We compared benign prostate epithelium versus its surrounding stromal microenvironment, in both tumour-adjacent regions and distant patient matched benign regions.

**Results and Discussions**

Notably we found that benign tissue in the immediate tumour environment appeared altered, broadly mirroring the gene expression of both the tumour itself, and the distant benign tissue. Additionally, within the adjacent benign tissue, epithelial and stromal tissues were difficult to distinguish from each other, whereas within tumour tissue and distant benign tissue, expression appeared to be broadly segregated into compartment specific genes as expected. We also present overviews of the spatial expression of commonly investigated prostate cancer genesets, expression of inflammatory markers and immune infiltration, and the morphology of the specimens using fluorescent markers, as well as offering comparisons to existing spatial and bulk transcriptomic data from the literature.

**Conclusion**

Overall, we conclude that spatial transcriptomic evaluation of radical prostatectomy specimens in a compartment specific design is feasible, confirms some theories suggested from previous bulk and spatial work, and offers food for thought for future work. Chiefly, if adjacent benign tissue behaves similarly to tumour tissue – what happens to it when treated with therapeutics, and can we profile this response spatially?

### EACR23-1366

### Can we recapitulate the true prostate tumour microenvironment via short term explant culture? A spatial transcriptomic interrogation of explants and matched primary specimens from the SCREEN cohort

*V. Thavarajah*[1], *G. Akman*[1], *L. Maria Carmona Echeverria*[1], *G. Shaw*[2], *A. Sridhar*[2], *J. Kelly*[1], *H. Whitaker*[1], *O. Uzoamaka*[1], *U. Cheema*[1], *S. Heavey*[1]
[1]*University College London, Division of Surgery and Interventional Sciences, London, United Kingdom*
[2]*University College London Hospitals, University College London Hospitals, London, United Kingdom*

**Introduction**

The success of drug development pipelines for cancer treatment remains both lower than desired, and hard to predict. We posit that enhancing the clinical relevance of models used at strategic points in pre- clinical and clinical development can improve our ability to predict which drugs will, and will not, offer clinical utility. The field of in vitro and ex vivo modelling of cancer has evolved rapidly in recent years, incorporating organoid, tumour on a chip and explant methods among others. Researchers typically reason that these models should offer improved clinical relevance over typical cell line or xenograft approaches. However, the vast majority of published models are not well validated against patient matched tissue, nor are they characterised in depth using the latest endpoint analyses, such as spatial transcriptomics. This leaves significant questions unanswered, that we must investigate if we can truly improve drug development success rates via use of such models.

**Material and Methods**

We studied a total of 145 areas of interest using the GeoMx Digital Spatial Profiler Whole Transcriptome Atlas. We compared the pathology and morphology of explants versus matched patient samples, as well as gene expression of a wide range of biologically and clinically relevant pathways, framed under the recently updated 14 hallmarks of cancer, with the addition of prostate-specific genes and pathways of interest.

**Results and Discussions**

Our findings suggest that explant morphology, tissue organisation and architecture is broadly reflective of matched prostate specimens, however key morphological differences emerge, including some epithelial deterioration, visible at both the pathological and transcriptomic levels. Encouragingly, the explants appear to be immunocompetent, suggesting a potential role for screening certain immunotherapies via explant culture in future. However, we also observed potential phenotypic plasticity, with the cultured explants exhibiting some alteration in key Androgen Receptor related genes and pathways, and perhaps taking on a more neuroendocrine phenotype post culture.

**Conclusion**

Here we showcase the clinically relevant aspects of explant culture over competing in vitro, in vivo and ex vivo models, and the hope it may provide in improving drug development, as well as identifying key hurdles to overcome to ensure clinical relevance.

### EACR23-1384

### Chemical imaging for segregation of colorectal cancer response in patient-derived xenograft models.

*M.R. Rafsanjani*[1], *A.C. O'Farrell*[2], *M. A. Jarzabek*[2], *L. Shiels*[2], *A. T. Byrne*[2], *J. H. M. Prehn*[2], *A. D. Meade*[1,3]
[1]*Technological University Dublin, Radiation and Environmental Science Centre- Focas Resea rch Institute, Dublin, Ireland*
[2]*Royal College of Surgeons in Ireland, Department of Physiology and Medical Physics-, Dublin, Ireland*
[3]*Technological University Dublin,*

© 2023 The Authors. Molecular Oncology is published by John Wiley & Sons Ltd on behalf of Federation of European Biochemical Societies

*School of Physics- Clinical & Optometric Sciences- Centra l Quad, Dublin, Ireland*

**Introduction**

Dysfunctional apoptosis is a key characteristic of the development of chemotherapy resistance in cancer. Small molecules sensitizers of apoptosis such as APT-199, that target anti-apoptotic BCL-2 family proteins can restore or induce cell death signalling in cancer cells.

**Material and Methods**

Here two patient-derived xenograft (PDX) mice models of metastatic colorectal cancer (mCRCs; CRC0076 and CRC0344) were interrogated for spectral imaging markers discriminating on response to 5-fluoro-uracil(FU)- based chemotherapy (FOLFOX), ABT-199, and a combination of both. Spectral imaging referenced previous findings *in vivo* which reported differential tumour growth responses towards ABT-199 [1].

**Results and Discussions**

Here, we used Fourier transform infrared (FTIR) spectroscopy approach to generate chemical images from the two PDX models providing detailed spatially resolved information about the biochemical composition and molecular structure of mCRC tissue. Traditional and advanced machine learning approaches were used to segregate tissues from each PDX on treatment pathway and response, identifying spectral biomarkers differentiating on response -ABT199, FOLFOX and the combined regimen.

**Conclusion**

We predict that using FTIR spectoscopy, we are able to deploy more accurate machine learning model that will uncover the biochemicall and spatial information of mCRCs due to the massive spectral data generated from chemical images.

**References**[1] A. C. O'Farrell *et al.*, "Implementing Systems Modelling and Molecular Imaging to Predict the Efficacy of BCL-2 Inhibition in Colorectal Cancer Patient-Derived Xenograft Models," *Cancers (Basel).*, vol. 12, no. 10, pp. 1–19, Oct. 2020, doi: 10.3390/CANCERS12102978.

### EACR23-1386
### Mechanistic disease models for drug repurposing in Soft-tissue Sarcomas (STSs)

*M. Peña-Chilet[1], M. Paya-Milans[2], M. Esteban-Medina[2], C. Loucera[2], J. Dopazo[2]*
*[1]Fundación Progreso y Salud - CIBERER, Clinical Bioinformatics Area, Sevilla, Spain*
*[2]Fundación Progreso y Salud, Platform for Computational Medicine, Seville, Spain*

**Introduction**

Soft-tissue Sarcomas (STS) account for less than 1% of adult and 15% of pediatric tumors, representing an heterogeneous group of rare cancers, with an average life expectancy remaining at 1.5 years. The search for systemic treatments that improve overall survival is an urgent need. Drug repurposing is an interesting approach in diseases with a lack of dedicated resources, such as sarcoma. Our group has developed a methodology that uses cell mechanisms modeling together with machine learning methodologies in order to evaluate the potential impact of certain drugs over a disease. Here we have obtained a list of candidate drugs for repurposing in STSs.

**Material and Methods**

To evaluate the deregulation of signaling pathways in STS, we used RNA-Seq data from TCGA and GTEx repositories, preprocessed with the same pipeline and available through Recount2. We then used Hipathia tool to conduct a mechanistic pathway analysis of STSs compared with paired normal tissue.
We constructed the map of action by extracting all the pathway's circuits (receptor-effector) that were deregulated in STS. We selected genes target of known drugs (KDTs) from the Drugbank DB and implemented Multi-Output Random Forest (with SHAP for per-circuit explainability) to use KDTs expression data to predict the circuits activity values, thus to infer the effect of the selected drug targets over the activity of the STS' map.
The circuits were annotated using uniprot and CHAT tool, and manually curated.

**Results and Discussions**

We obtained significantly deregulated mechanisms for the different STS' subtypes, with only a few mechanisms being shared by all subtypes, which supports the heterogeneity shown in STSs, most of them were related to tumorigenesis, angiogenesis and EMT. Using these deregulated circuits, we constructed the STS mechanistic map.
Moreover, the application of multi-output regression methodologies revealed relevant potential known drug targets for each STS subtype that are functionally related to hallmarks of cancer.

**Conclusion**

We have used a machine learning methodology for the prediction of potentially causal relationships between known drug targets and cell activities related with STS subtypes and hallmarks, using the constructed map of the disease as a functional frame. These findings would lead to a more efficient drug repurposing for STS' patients' treatment.

### EACR23-1388
### Development and validation of innovative methods to dissect longitudinal data improving outcome prediction.

*F. Cordero[1], S. Pernice[1], R. Sirovich[2], M. Beccuti[1], E. Grassi[3], A. Bertotti[3], L. Trusolino[3], S. Ferrero[4], E. Genuardi[4], M. Ladetto[5]*
*[1]University of Turin, Computer Science, Turin, Italy*
*[2]University of Turin, Mathematics, Turin, Italy*
*[3]University of Turin, Oncology, Turin, Italy*
*[4]University of Turin, Molecular Biotechnologies and Health Sciences, Turin, Italy*
*[5]Antonio e Biagio e Cesare Arrigo Hospital, Division of Hematology, Alessandria, Italy*

**Introduction**

Longitudinal data are useful to explore the evolution of a given event. This type of data consists of portions of functions or curves, with quantities observed as they evolve through time. The advances in technologies give us the possibility to study surrogate tissues to monitor the phenomena of interest over time. From the computational point of view, the analysis of longitudinal data is limited in fitting the data with a specific function (i.e. exponential)

© 2023 The Authors. Molecular Oncology is published by John Wiley & Sons Ltd on behalf of Federation of European Biochemical Societies and then comparing the parameter values of the function to aggregate similar evolution trends.

**Material and Methods**

We implemented CONNECTOR a computational methodology for unsupervised analysis of longitudinal data. It can analyze any sample consisting of measurements collected sequentially over time by a model-based approach for clustering functional data. CONNECTOR is based on a functional clustering algorithm based on a mixed-effect model that is particularly effective when observations are sparse and irregularly spaced. A new CONNECTOR' module for the prediction of the risk class label was implemented. This was performed using Bayes' optimal allocation rule, on the estimated probabilities of belonging to each class given the observed values.

**Results and Discussions**

Two cases of study are proposed. In the first, ASO RQ-PCR data to follow the minimal residual disease (MRD) was generated from bone marrow and peripheral blood samples of 117 patients in the FIL MCL0208 trial, offering first-line high-dose chemoimmunotherapy and autologous transplantation to younger mantle cell lymphoma (MCL) patients. CONNECTOR identified four patient risk clusters predicting patients' outcomes: median TTP was not reached for favorable MRD kinetics clusters while 36 and 27 months for unfavorable MRD kinetics, $p<0.0001$. 89 out of 95 patients (94%) were correctly reclassified. An independent validation on a European cohort, on behalf of the MCL network, is now running. In the second case study, more than 1500 growth curves of patient-derived xenograft derived from metastatic colorectal cancer, the clusters generated by CONNECTOR allow the identification of a subset of cetuximab-resistant tumors associated with novel unrecognized molecular and phenotypic features. Other cases are now evaluated for prediction purposes.

**Conclusion**

The CONNECTOR framework and its modules are a user-friendly tool to efficient comprehended longitudinal data, providing hints to increase interpretability and molecular accuracy and to use the new model found for improving the outcome prediction.

### EACR23-1391
### Evaluating Microsatellite Instability Tools and Benchmarks

*H. Anthony*[1], *C. Seoighe*[1]
[1]*University of Galway, School of Mathematical and Statistical Sciences, Galway, Ireland*

**Introduction**

Microsatellite instability (MSI) is a phenomenon seen in several cancer types which has been used as a biomarker for immune checkpoint inhibitor efficacy. The variation in microsatellite size that is the hallmark of MSI is largely attributed to a cell's inability to correct errors during DNA synthesis, leading to an increase in the number of insertions and deletions. Microsatellite instability has frequently been identified by PCR of five microsatellites and through IHC staining of the mismatch repair proteins. However, with the advent of next generation sequencing (NGS), researchers have created computational tools to categorize samples as MSI high (MSI-H) or low (MSI-L) or being microsatellite stable (MSS) using NGS data. Most of these tools have been solely trained and tested on whole exome sequencing data (WXS) from The Cancer Genome Atlas (TCGA). This can be problematic as the tools were published with claims that they can be used with a variety of sequencing types, tumor purities, coverage thresholds and, in some cases, without a matched normal sample. This raises the question of how accurate these tools are for the task of classifying tumor samples using real-world data from different platforms or scenarios where a large set of normal samples is not available.

**Material and Methods**

We benchmarked several leading MSI tools on datasets resembling different real-world sequencing scenarios. These included 10 random MSI-H, MSI-L, and MSS samples from the TCGA whole genome sequencing (WGS) datasets, 20 random MSI-H, MSI-L, and MSS samples from the TCGA WXS dataset, and 92 TSO-500 gene panel samples. We then used best practice settings for each tool to determine MSI status for all samples. Accuracy and precision were then compared for all tools on each dataset.

**Results and Discussions**

Early results suggest that the accuracy of MSI tools that do not require a matched normal sample is significantly impacted by reducing the number of normal samples used to create a baseline, and we found a lack of concordance between tools trained on WXS data and the results of the TSO-500 pipeline. Concerningly, the accuracy of most tools was diminished on the WGS data and there are potential issues with reproducing the original results of the WXS data.

**Conclusion**

There is a need to thoroughly test tools which have clinical and research applications. Although most MSI tools claim accuracies close to 100%, we found discrepancies when testing them outside their optimal conditions and on different sequencing types.

### EACR23-1393
### From small to Great: a new computational workflow for data analysis and modelization

*D. Tortarolo*[1], *S. Pernice*[1], *F. Clapero*[2], *M. Beccuti*[1], *G. Serini*[2], *F. Cordero*[1], *D. Valdembri*[2]
[1]*University of Turin, Computer Science, Turin, Italy*
[2]*IRCCS Candiolo Cancer Institute, Oncology, Turin, Italy*

**Introduction**

In recent years, there has been a collective effort to uncover how multiple levels of regulations and signaling pathways integrate during the onset and progression of complex diseases such as cancer. In this context, it has become evident that a shift has to be made toward a more holistic perspective, and computational modeling arises as instrumental in gaining a functional understanding of complex data. However, we are still missing a comprehensive workflow which can support scientists in all phases of scientific discovery, from raw data analysis to computational model creation and analysis.

**Material and Methods**

Herein, we propose a workflow for (i) the analysis of the large amounts of heterogeneous data produced in a lab, and (ii) the creation, (iii) parametrization and (iv) analysis of a computational model of the phenomenon of interest. We

© 2023 The Authors. Molecular Oncology is published by John Wiley & Sons Ltd on behalf of Federation of European Biochemical Societies developed a new Shiny application, based on R, that integrates tools specifically developed or adapted for the elaboration of raw Western Blot (WB), Reverse Transcription-quantitative PCR (RT-qPCR) and Enzyme-Linked ImmunoSorbent Assay (ELISA) experiments, together with well-known tools, such as ImageJ, for raw Immunofluorescence (IF) and live IF data processing. All lab data can be integrated into a computational model, that can be intuitively drawn, parametrised and analysed using GreatMod software, a quantitative modeling framework based on the graphical formalism Petri Net and its generalizations.

**Results and Discussions**

Our workflow provides biologists a comprehensive set of tools for the analysis of the most common lab experiments. It was developed with a keen attention to data analysis standardisation and reproducibility since it is able to provide all the preliminary and intermediate steps of data analysis. Our workflow is accessible to scientists without advanced R language knowledge, and can provide the right supporting tools for integration of lab data into a computational model, an often burdensome step that discourages cellular biologists from exploiting computational techniques. To showcase ourworkflow's functionalities and potential, we developed a toy model of integrin recycling in endothelial cells, which plays a role in the malignant potential of a tumour.

**Conclusion**

Our workflow provides an exhaustive platform where scientists can experience the discovery process from the analysis of a single datum to the creation of a complex computational model, all in one place and with an emphasis on reproducibility.

### EACR23-1398
### Interventional constraints for representation learning in cancer omics

*D. Kirkham[1], O.M. Rueda[1], S. Mukherjee[1]*
*[1]University of Cambridge, MRC Biostatistics Unit, Cambridge, United Kingdom*

**Introduction**

Many recent machine learning (ML) applications in cancer make use of protein-protein interaction (PPI) networks to constrain models. PPI networks are general and lack specialisation to a cell type or particular task. Through transfer learning, we propose the use of large-scale loss-of-function screens carried out on cancer cell lines (CCLs) to more appropriately guide model constraints, providing a domain-specific, experimentally derived approach to constraining ML models. Our method also provides a route to directly quantify the usefulness of loss-of-function screens in providing insight into outcomes such as drug response in CCLs.

**Material and Methods**

We evaluate the usefulness of providing constraints derived from the large-scale CRISPR knockout screen DepMap (n=973) to a semi-supervised machine learning model based on a variational autoencoder. The output of this model is a constrained low-dimensional representation of RNA-Seq gene expression for a CCL. Our method maps the information contained in the gene expression input which is associated with sensitivity of the CCL to gene knockouts into individual dimensions of the representation. These constraints allow the formation of direct associations between CRISPR screening outcomes (gene dependency) and other outcomes in cancer-related datasets. We use these constrained representations as inputs to models predicting drug response in selected cell lines from the GDSC2 dataset (n=684) and in further datasets covering patient-derived xenografts (PDXs) and patients.

**Results and Discussions**

We demonstrate the effect of constraining representations to encode sensitivity to gene loss-of-function by generating inputs for models of drug response. For the MAPK pathway, we directly encode information related to CCL dependency on genes in this pathway into representations of gene expression, then use these representations to predict response to MAPK inhibitors. This reproduces known relationships between gene dependency and drug response and suggests potential novel interactions. We also consider the effect of added constraints on model robustness in making predictions across cancer types and subtypes.

**Conclusion**

We show the transferability of large scale loss-of-function screens by using them to constrain representations of omics data used as inputs for tasks such as predicting drug response. This allows us to directly draw relationships between gene dependency and drug response, and also produce an interpretable and robust representation of RNA-Seq data.

### EACR23-1408
### Mathematical modelling of cell cycle shortening on the basis of an microfluidic experiment

*R. Cichorowski[1], S. Student[2]*
*[1]Silesian University of Technology, Department of Data Science and Engineering- Gliwice- Poland, Gliwice, Poland*
*[2]Silesian University of Technology, Department of Systems Biology and Engineering- Gliwice-Poland, Gliwice, Poland*

**Introduction**

The necessity of mathematical modelling is important in current times. The improvement of computational technology makes it possible to simulate very complex models. The creation of a model of examining phenomena shed new light on these phenomena. The creation of mathematical descriptions requires an appropriate approach to the problem. There are three main steps in the modelling process. First step is a good understanding of modelling phenomena to select experimental methods to gain valuable and important data. Second step is the selection of mathematical methods which describe phenomena in the best way - different phenomena have specific behavior, which determine the way of mathematical description. Third step is verification of the constructed model in the light of a considered phenomenon. Nowadays mathematical modelling has a crucial role in different branches of biology, especially in oncology, to understand a lot of various processes on different levels of biological matter organization. The aim of this work is to find a proposal of a mathematical model of cell cycle shortening on the basis of a microfluidic experiment with HeLa cell line.

© 2023 The Authors. Molecular Oncology is published by John Wiley & Sons Ltd on behalf of Federation of European Biochemical Societies

**Material and Methods**
The experiment was performed on a HeLa cell line in an appropriately designed microfluidic system. It examines a HeLa cells' development under the same microenvironment conditions during the same period of time for each run. The most important stage was to find and select the most influential features to the model with particular emphasis on the neighborhood of the single cell. The experimental data were collected as a series of images for each run. Then the features were found through the sets of numerical data extracted from images series and the most significant were selected. The results were verified in the current state of cell and molecular biology knowledge to correct the proposed models.
**Results and Discussions**
Proposed model was used for the numerical simulation of cell population development based on the computer implementation of the cellular automaton. The results of the many simulations showed how modelling are computational effective, and how the results are statistically significant and how accurate in the light of current biological knowledge is.
**Conclusion**
Results of this work can show the cell cycle shortening influence on cell population development especially on cancer cells population.

### EACR23-1433
### CrossWGCNA identifies mediators of trumor-stroma interactions from laser capture microdissection and spatial transcriptomics data

*A. Savino*[1], *F. Iorio*[1], *P. Provero*[2], *V. Poli*[3]
[1]*Human Technopole, Computational Biology, Milan, Italy*
[2]*University of Turin, Department of Neurosciences "Rita Levi Montalcini", Turin, Italy*
[3]*University of Turin, Molecular Biotechnology and Health Sciences, Turin, Italy*
**Introduction**
Tumors are surrounded by several cell types influencing their growth and migration. Despite their well-recognized role, experimentally studying these communications is laborious, and mostly limited to the analysis of one signal-receptor interaction at a time.
Gene co-expression networks have been widely employed and proven valuable in inferring genome-wide gene interactions but have been exclusively applied to individual tissues or samples comprising a non-separable admixture of cell types. Nevertheless, the principle of co-expression networks could be extended to the study of inter-cellular communication, to provide a simple way of selecting genes mediating inter-tissue interactions, which could be then assessed experimentally.
**Material and Methods**
We developed crossWGCNA, an adaptation of the WGCNA algorithm (Weighted Gene Co-expression Network Analysis) to study inter-tissue interactions. In our method, expression measures of the same gene in different tissues or cell types define different nodes. Edge weights are based on genes' correlations and self-loops are removed. Intra- and inter-tissue connectivities ($k_{int}$ and $k_{ext}$) are defined as the sum of nodes' edges with other nodes of the same or the alternate cell compartment, and $k_{ext}/k_{int}$ is used as the main metric for prioritizing highly interacting genes. We applied crossWGCNA to breast cancer data from laser capture microdissected tumors, analysed separately in their stromal and epithelial compartments, and implemented a set of functions for applying crossWGCNA to spatial transcriptomics data and identifying coordinated gene sets at the epithelium-stroma boundaries.
**Results and Discussions**
As expected, genes with functions in signaling display high communication scores. Moreover, we show the reliability of our approach with extensive computational validations, making use of epithelial cells – fibroblasts co-cultures and single-cell data from primary tumors. We explored genes expressed in the stroma that are coupled with specific pro-tumoral pathways , and highly correlated gene sets and gene pairs. For example, in a spatial transcriptomic dataset of breast cancer, EFNA1, known to regulate adhesion and migration, is coupled with high COL6A2 expression in the stroma, indicating an interaction between ephrins expression and extracellular matrix composition.
**Conclusion**
In conclusion, crossWGCNA allows quantifying the transcriptional coupling between tissues and to identify potential mediators of inter-tissue interactions.

### EACR23-1457
### CROCoDILE: A Robust Deconvolution Pipeline for Identifying Clinically Relevant Rare-Cell Populations from Bulk Transcriptomics Data

*K. Im*[1], *J. Lee*[1]
[1]*Sungkyunkwan university, Medicine, Suwon, South Korea*
**Introduction**
Identifying clinically relevant rare-cell populations is essential in cancer research. Tumor microenvironment heterogeneity is one of the major obstacles in accurately predicting treatment response. While single-cell sequencing technologies can identify rare cell populations that shed light on resistance mechanisms, such technologies are limited in availability and require robust cell-type markers. The deconvolution of bulk transcriptomics provides a complementary method for estimating cell-type specific expression, but it requires auxiliary labels.
**Material and Methods**
We develop a novel computational pipeline, termed Clinically relevant and Objective Cluster Detection In buLk sEquencing Data (CROCoDILE), that integrates single-cell and bulk transcriptomics to identify clinically relevant rare cell populations from bulk RNAseq data, where the phenotypic label is available. CROCoDILE uses a cell-type specific signature matrix from single-cell RNAseq (scRNA-seq) data and applies deconvolution methods to bulk RNA-seq data, identifying cell types including rare cell populations relevant to specific phenotypes. We evaluate the meaningful clusters based on the association with the given clinical phenotype, and iteratively refine the clustering until convergence We analyze two lung cancer immunotherapy cohorts with bulk

© 2023 The Authors. Molecular Oncology is published by John Wiley & Sons Ltd on behalf of Federation of European Biochemical Societies pretreatment bulk RNAseq data (n=228 and 43 patients, respectively) based on the scRNA-seq reference profiles from cancerous and healthy lung tissues. We compare the identified rare-cell populations between the two cohorts and investigate their associations with immunotherapy response.

**Results and Discussions**

Our analysis confirms CD8 T progenitors as positively associated with ICI response and identifies several cell clusters expressing high levels of NNMT, MDK, and IGHG1 as negatively associated with immunotherapy response. We also identify lncRNA RP11-16E12.2 as negatively associated and KB-1980E6.3 as positively associated with ICI response, potentially serving as ICI prediction markers. CROCoDILE balances cell-type information and phenotypic information in bulk transcriptomics, providing a novel approach to identify clinically relevant rare-cell populations.

**Conclusion**

CROCoDILE offers a novel approach to identifying rare-cell populations in bulk transcriptomics data, providing potential ICI response markers and highlighting the importance of considering rare-cell populations in cancer patients' treatment response and prognosis.

### EACR23-1523
### Developmental Deconvolution for classification of cancer origin

E. Moiso[1], S. Mildrum[2], A. Farahani[2], A. Hendricks[1], H. Marble[2], S. Levine[1], J. Lennerz[2], S. Garg[1]
[1]MIT, Koch Institute, Cambridge, United States
[2]MGH, Department of Pathology, Boston, United States

**Introduction**

Cancer is partly a developmental disease, wiith maliganncies named based on cell of tissue of origin. However, a sustematic atlas of tumor originis is lacking.

**Material and Methods**

We map the transcriptional profile of more than 1000000 cells, grouped in 56 developmental trajectories to the trascriptome of over 10000 tumors across 33 cancer types. We deconvolute tumor transcriptomes into signals for individual developmental trajectories. Using these signals as input we train a developmetal multilayer perceptron (D-MLP) that outputs cancer origin.

**Results and Discussions**

We analyze tumors from patients with cancer of inknown primary (CUP), selecting the most difficult cases in which extensive miltimodal workup yeld no definitive tumor type.

inyerstingly CUPs formfor groups distinguished by developmental trajectories, and classification reveals diagnosis for patient tumors.

**Conclusion**

Out framework represents the first quantitative and systematic measurement of the contribution of developmental programs to tumor formation.

Moreover it exploits this informatiin in conjunction with a newly developed AI model to assist pathologists in CUP diagnosis.

### EACR23-1525
### Multi-modal machine learning approach for cancer outcome prediction

D. Meerzaman[1], Q. Chen[2], G. Flanigan[3]
[1]NIH/NCI, CBIIT, Rockville, United States
[2]NIH/NCI, Ccomutational biology and bioinformatics branch, Rockville, United States
[3]NIH/NCI, Computational Biology and Bioinformatics Branch, Rockville, United States

**Introduction**

The question of how *O*$^6$-alkylguanine DNA alkyltransferase (MGMT) methylation status can be used as a marker for clinical subtyping or treatment prediction in glioblastoma multiforme (GBM) has been a topic of much recent discussion. From the 2021 move from the World Health Organization to look at MGMT expression through the lens of mutation as predictive of patient survival to the controversial Brain Tumor Segmentation (BraTS) challenge subtask to predict methylation status from MRI scans, the topic of MGMT in GBM has generated interest but still leaves many open questions about the exact relationship of methylation to how GBM manifests and how an individual's MGMT is determined as methylated or unmethylated.

**Material and Methods**

We use hematoxylin and eosin stained whole slide histopathology images from The Cancer Genome Atlas to predict methylation beta values for individual CpG islands within the MGMT promoter region. This was done with a 23 CpG positions from a set of 355 cases analyses with either Illumina 27k or Illumina 450k methylation arrays. We ran the same procedure looking at the 144 CpG positions only covered in the 225 cases analyzed using the Illumina 450k methylation array. We use a ResNet based feature extractor combined with a deep neural network classifier to predict the beta values.

**Results and Discussions**

While predictive accuracy varies with position, we have achieved an accuracy of 65% with a loss around 0.05 in 50 epochs for many promising positions. This should help to address some of the disparities in how MGMT is classified as methylated or not methylated, as there is currently no clear standard methodology.

**Conclusion**

The results provided can be used as a basis to move towards a by-position method of determining MGMT methylation instead of a number or proportion of positions methylated approach, improving accuracy and inter-institution comparability of future studies.

### Biomarkers in Tissue and Blood

### EACR23-0023
### A phosphorylation mapping tool for monitoring tyrosine kinase inhibition in lung cancer patients

A. Wuethrich[1], M. Ahmed[1], N. Constantin[1], K. Karthik[1], P. Mainwaring[1], A. Kulasinghe[2], L. Carrascosa[1], A.I. Sina[1], K. O'Byrne[3], M. Trau[1]
[1]The University of Queensland,

© 2023 The Authors. Molecular Oncology is published by John Wiley & Sons Ltd on behalf of Federation of European Biochemical Societies

Australian Institute for Bioengineering and Nanotechnolog y, Brisbane, Australia
[2]The University of Queensland Diamantina Institute, Institute for Molecular Bioscience, Brisbane, Australia
[3]Princess Alexandra Hospital, Medical Oncology, Brisbane, Australia

**Introduction**

Protein phosphorylation is one of the most common post-translational modifications that regulates key cellular functions. This modification is frequently impaired in kinase pathways in lung cancer resulting in a permanently active constitutive phosphorylation to initiate tumour growth. Although aberrations to phosphorylation pathways are targeted with tyrosine kinase inhibitors (TKI), the development of drug non-responsiveness limits the long-term clinical benefits of TKI. A liquid biopsy test to probe for changes in kinase phosphorylation has significant potential to aid TKI selection and for therapy monitoring. We herein describe a multiplexed phosphoprotein analyser chip (MPAC) that enables sensitive detection of kinase phosphorylation status derived from circulating extracellular vesicles (EVs). Using MPAC, we monitored kinases involved in MAPK and PI3K/AKT/mTOR pathways in non-small cell lung cancer (NSCLC) models and patient samples.

**Material and Methods**

MPAC involves the extraction of kinases from plasma extracellular vesicles (EVs) followed by electrochemical read-out of extracted kinase to infer phosphorylation status. MPAC is applied to profile the phosphorylation levels of EVs-derived EGFR, ALK, ROS1, BRAF, ERK, mTOR, and PD-L1 in TKI-treated cell lines and plasma of advanced NSCLC patients (n=33). In a second cohort of 65 early to advanced NSLC patients, MPAC derived EVs phospho-PD-L1 levels are corelated against tissue proportion score PD-L1.

**Results and Discussions**

MPAC enabled phosphoproteomic analysis of kinases requiring an input of 250 µl plasma or approx. 0.5 ng of extracted kinase. MPAC is sensitive to detect 2% phosphorylated kinase spiked into 98% non-phosphorylated kinase. Using EGFR and ALK inhibitors in cell line models, we monitor changes in phosphorylation of kinase pathways. To indicate aberrations in cellular pathways, we generated a phosphorylation heatmap by EVs phosphoproteomic profiling of seven kinases in NSCLC patients and non-cancer individuals. Interestingly, we find that EVs phospho-PD-L1 correlates with tissue proportion score PD-L1 (Pearson, r=0.9892), potentially enabling blood-based assessment of tumour PD-L1 expression.

**Conclusion**

MPAC enables streamlined assessment of phosphorylation status in kinases using a simple electrochemical read-out. The technology generated heatmaps of seven EVs-derived kinases in plasma of NSCLC patients that provide a dynamic snapshot of which pathways are over-activated and could be targeted by TKI.

### EACR23-0025
### Metabolomics approach in search for potential diagnostic biomarkers from Glioblastoma liquid biopsy.

*A.C. Ferrasi[1], R. Puttini[1], P.T. Hamamoto-Filho[2], G.A. Kurokawa[1], A.F. Galvani[1], J. Delafiori[3], R.R. Catharino[3], M.I.M.C. Pardini[1], M.A. Zanini[2], E. Lima[1]*
*[1]São Paulo State University, Department of Internal Medicine, Botucatu, Brazil*
*[2]São Paulo State University, Department of Neurology- Psychology and Psychiatry, Botucatu, Brazil*
*[3]University of Campinas, Innovare Biomarkers Laboratory, Campinas, Brazil*

**Introduction**

Among the Central Nervous System (CNS) tumors, glioblastoma (GBM) is the most common primary malignant one (49.1%), and only 6.8% of patients survive five years post-diagnosis. In the early stages, brain tumors may not show well-defined and characteristic clinical symptoms, delaying diagnosis. Although genetic alterations have been associated with CNS tumor prognosis, they are not completely clear, and depend on biopsy collection, what limits early diagnostic approaches. Searching for early GBM markers are of particular importance, especially in samples of easier access, such as peripheral blood. Then, the aim of this study was to explore molecular targets in blood samples through metabolomics approach, which might reflect GBM metabolic alterations and indicate differential targets as potential tools for early diagnosis.

**Material and Methods**

The work was approved by the Research Ethics Committee (Protocol 3.491.414), and the subjects freely consented to participate. Blood samples were collected from 50 healthy volunteers (Control group) and 24 patients with glioblastoma (GBM group). Plasma samples were prepared and submitted to mass spectrometry analysis (MS). Multivariate statistical analysis was applied through partial least-squares discriminant analysis (PLS-DA), followed by analysis of Variable Importance in Projection (VIP) score. With a VIP score > 2.5, the most important GBM markers were selected; a heatmap and Receiver Operating Characteristic - ROC curves were built, and the area under the curve (AUC) was evaluated. The selected biomarkers were identified through tandem MS, metabolomics databases analysis and literature search.

**Results and Discussions**

The PLS-DA showed good separation between CT and GBM groups and a promising biomarkers profile for GBM was achieved (VIP score > 2.5): *m/z* 143 (5-Hydroxymethyluracil), *m/z* 931 (3-O-Sulfogalactosylceramide), *m/z* 111 (Pyruvate), *m/z* 294 (Arginyl-Proline), and *m/z* 819 (phosphatidylserine). Pyruvate and 5-hydroxymethyluracil ROC curve presented the better AUC when analyzed together, with AUC = 0.986. Considering the biological importance, pyruvate is commonly found in tumors due to Warburg effect; 5-hydroxymetyluracil might represent a byproduct of DNA-demethylation process, involved in tumorigenesis.

**Conclusion**

Although the present results are still preliminary and must be validated in a larger casuistry, they highlight potential plasma metabolites for GBM early diagnosis from a liquid biopsy.

© 2023 The Authors. Molecular Oncology is published by John Wiley & Sons Ltd on behalf of Federation of European Biochemical Societies

### EACR23-0030
### Molecular biomarker analysis for high grade glioma recurrence risk

A.F. Galvani[1], A.C. Ferrasi[1], D.B. dos Santos[1], G.A. Kurokawa[1], E.O. Lima[1], M.A. Zanini[2], P.T. Hamamoto-Filho[2]
[1]São Paulo State University - Botucatu Medical School, Department of Internal Medicine, Botucatu, Brazil
[2]São Paulo State University - Botucatu Medical School, Department of Neurology- Psychology and Psychiatry, Botucatu, Brazil

**Introduction**
Glioblastoma (GBM) are responsible for the majority of deaths caused by primary brain tumors with a higher recurrence rate, greater resistance to treatments, and poor prognosis. A lot of efforts are not completely efficient in preventing relapses and increasing patient survival. Long non-coding RNAs (LncRNAs) have been coverd in several studies as promising biomarkers for use as potential therapeutic or diagnostic targets in many tumor types, including gliomas. The LncRNA RP11-731F5.2 (RP11) has been associated with evolution of gastric cancer, chronic obstructive pulmonary disease, and hepatocellular carcinoma. However, RP11 expression data associated with GBM genesis, tumor progression and risk of recurrence do not exist. Thus, the aim of this project was to analyze the differential expression of RP11 in healthy glial cells, primary GBM and recurrent GBM.

**Material and Methods**
All patients consented to participate in this study. Five patients accomited by recurrent GBM, operated by the Neurosurgery service - Botucatu Medical School - were included. The patients had: First surgery for GBM resection (CNS-G); Second surgery for recurrent GBM resection (CNS-RG). Control Group (CG) was composed by 5 healthy dead glia cell donors. Total RNA was isolated from each sample. Random primers were used to synthesize cDNA, which was analyzed as a template in the real-time polymerase chain reaction (qPCR) using Power SYBR Green PCR Master Mix at the StepOne Plus™ System®. The qPCR reaction was carried out as follows: 50°C for 2 min; 95°C for 2 min; 40 cycles: 95°C for 15 sec. and 62°C for 1 min. Gene expression was normalized with Beta-Actin to calculate relative expression using the $2^{-\Delta\Delta Ct}$ method.

**Results and Discussions**
The mean age was 62 years old (56-68) for both CNS-G and CNS-RG patients, and 58 years old (54-64) for CG. Biological groups (CG; CNS-G; CNS-RG) were created to analyze the differential expression of RP11, adopting CG as a reference group. The data showed up-regulation of RP11 in CNS-G group (RQ (log2) =7.60) and in CNS-RG group (RQ (log2) =5.50) when compared to CG. *In silico* analyses showed a strong chemical interaction between RP11 and LncRNA KCNQIOTI, which plays an important function associated with genesis, tumor progression and chemoresistance in gliomas through intracellular signaling cascade pathways.

**Conclusion**
The RP11 could be a biomarker involved in gliomagenesis and its relapses, but complementary studies and larger number of cases are necessary.

### EACR23-0040
### Plasma lncRNA RP11-731F5.2 level as a risk biomarker for Hepatocellular Carcinoma in chronic Hepatitis C

A.C. Ferrasi[1], G.J. Fernandez[2], A.F. Galvani[1], D.B. Santos[1], E.O. Lima[1], G.F. Silva[1], M.I.D.M.C. Pardini[1]
[1]Sao Paulo State University, Internal Medicine, Botucatu, Brazil
[2]Universidad de Antioquia, Grupo Biologia y Control de Enfermedade Infeciosas, Medelìn, Colombia

**Introduction**
Hepatocellular carcinoma (HCC) has a high mortality rate. Chronic infection with hepatitis B and C viruses is among the main causes of HCC. Although advances in the treatment of infection have contributed to reduce the risk of HCC, the diagnosis of infection and cancer has occurred late for most patients. Due to nonspecific symptoms, early detection requires monitoring of individuals high risk using imaging tests and alpha-fetoprotein dosage. Such tests are limited, making the search for early HCC markers essential. LncRNAs have regulatory roles in tumorigenesis and progression. LncRNAs are released by tumors into body fluids, suggesting their usefulness as non-invasive markers in liquid biopsies. Thus, the aim of this study was to verify the differential expression of lncRNA RP11-731F5.2 in the plasma of advanced chronic hepatitis C (CHC) patients, evaluating it as a risk marker for HCC.

**Material and Methods**
Approval for this study was granted by the Research Ethics Committee. Plasma was separated from the blood of 21 CHC patients with advanced fibrosis (METAVIR F3/F4), (male, mean age 57) and 12 healthy donors (male, mean age 57,6). Three biological groups were created: HCCpos and HCCneg; grouped according to the development or not of HCC in up to 5 years of follow-up) and, Control Group (healthy donors). RNA was extracted by Plasma Circulating RNA Purification kit. RNA was reverse transcribed to cDNA and qPCR was performed using the Power SYBR Green PCR Master Mix, according to the manufacturer's protocol. Gene expression was normalized with Beta-Actin to calculate relative expression using the $2^{-\Delta\Delta CT}$ method.

**Results and Discussions**
RP11-731F5.2 was upregulated in HCCneg and HCCpos groups when compared to the healthy donor group, (RQ (log2) = 3.108 and 4.108). Comparison between HCCneg and HCCpos showed that patients affected by HCC were upregulated (RQ (log2) = 0.827). Due to the lack of data about function and interactions of this LncRNA, we performed *in silico* analyzes looking for interactions with other targets. In an unprecedented way, it was found a strong interaction between this RP11 and another LncRNA, KCNQIOTI, which plays a key role in proliferation, apoptosis and chemoresistance in HCC.

**Conclusion**
RP11-731F5.2 is more expressed in plasma of patients with chronic hepatitis C than in healthy donors. It is also upregulated in the HCCpos when compared to HCCneg, which suggests that this LncRNA may be a good biomarker of HCC risk in chronic hepatitis C. Other studies are needed to validate these results.

© 2023 The Authors. Molecular Oncology is published by John Wiley & Sons Ltd on behalf of Federation of European Biochemical Societies

### EACR23-0049
### Integrating liquid biopsy with computational analysis of circulating microRNA for precision diagnostics of prostate cancer

A. MANOJ[1], M.K. Ahmad[1], A.A. Mahdi[1], M. Kumar[2]
[1]King George's Medical University, Biochemistry, Lucknow, India
[2]King George's Medical University, Urology, Lucknow, India

**Introduction**
Current biomarkers devise challenges for accurate and efficient Prostate Cancer (PCa) diagnosis, thus leading to invasive procedures, over-diagnosis, and over-treatment. This trend demands highlighting the need for novel biomarkers. The most widely used clinical methods for early detection to date are still digital rectal examination (DRE) and testing for prostate-specific antigen (PSA), although they were unable to predict clinical behavior. The rapid advances in molecular technologies have allowed the identification of various potential biomarkers for PCa. MicroRNA are highly conserved small non-coding RNA that affect the central dogma at the translational level. Thus, they modulate genes involved in numerous biological processes and form part of complex networks that play a significant role in prostate cancer initiation and progression. In keeping this view, we have designed our study to investigate the non-invasive biomarker potential of miRNA 125b.

**Material and Methods**
In the present study, there are two cohorts: the Discovery cohort (n=40) and the validation cohort (n=80). The discovery cohort consists of healthy and PCa tissues, while the validation cohort consists of healthy male, benign prostatic hyperplasia (BPH), PCa, and castration-resistant prostate cancer (CRPC) serum samples. The expression pattern and biomarker potential of miRNA 125b were determined by Real time-PCR and ROC curve analysis, in both discovery and validation cohorts. Further, miRNA 125b targeted significant genes and pathways were explored using In-Silico tools like TargetScan, miRDB, miRWalk3.0, and miRTargetLink 2.0 and performed gene and pathway enrichment analysis using DAVID 6.8, gProfiler, and STRING App.

**Results and Discussions**
Our observation shows that miRNA 125b is significantly downregulated in tissues (1.88-fold-change decrease) and serum (BPH-0.17; PCa-1.4; CRPC-0.3 fold-change decrease) with significantly ($p<0.001$) great AUC, for both the cohort (PCa tissue- 0.926; BPH-0.959, PCa-0.916, CRPC-0.938)as compared with their respective controls. The in-silico analysis reveals the involvement of miRNA 125b in PCa progression by directly targeting E2F3, PTEN, EGFR, TP53, MYC, E2F2, and SRF. These genes form a complex network that aids in PCa progression and development.

**Conclusion**
Consequently, our findings suggest the tumor suppressor role of miRNA 125b with significant non-invasive biomarker potential in better and more efficient PCa diagnosis and its target gene indicate involvement of miRN125b in pathogenesis of PCa.

### EACR23-0081
### Significance of the HER2 overexpression in urothelial carcinoma of the bladder

A. Bdioui[1], L. Zeineb[1], M. Sarra[1], B. Oussema[1], M. Wiem[1], S. Sihem[1], N. Missaoui[2]
[1]Medicine Faculty of Sousse, Pathology Department of Sahloul University Hospital, Sousse, Tunisia
[2]Medicine Faculty of Sousse, LR21ES03 Oncogenesis and Tumor Progression, Sousse, Tunisia

**Introduction**
Bladder cancer is the first urological cancer in Tunisia. It constitutes a public health problem, given the absence of effective adjuvant treatment, apart from surgery. The prognosis of invasive forms remains pejorative. Indeed, one of the main challenges in the fight against bladder cancer is the lack of tools specifically targeting the molecular alterations of this cancer, which can lead to better therapeutic management of patients and can improve overall survival. Although the utility of HER2 overexpression is well established in breast and stomach cancer, it remains contentious in bladder cancer, especially in Tunisia. In this study, we analysed the expression profile of HER2 proto-oncogene in urothelial carcinoma of the bladder and we investigated its clinicopathological significance in Tunisian patients.

**Material and Methods**
We conducted a cross-sectional and analytical study of 58 urothelial carcinomas diagnosed in the Pathology Department of the Sahloul University Hospital of Sousse, Tunisia, during 2020-2022. Clinicopathological particularities were recorded. HER2 expression was explored by immunohistochemistry technique on whole sections of archived material. Equivocal HER2 immunostaining was further tested by *in situ* chromogenic hybridization.

**Results and Discussions**
The median age was 67.5 years, and a male predominance was found (sex-ratio = 11). Urothelial carcinoma with the HER2 immunolabeling score 0 was the most frequent (80%), followed by the score 3 (12%) and score 1 (8%). HER2 immunostaining score 2 was observed only in one tumour and in situ chromogenic hybridization confirmed the absence of HER2 overexpression. Overall, HER2 overexpression was identified in 12% of analysed cancers. Furthermore, no significant association was identified with clinicopathological features, including tumour grade ($p=0.327$), vascular emboli ($p=1.00$), peri-nervous invasion ($p=0.392$), stroma type ($p=0.467$), local infiltration ($p=0.107$), and nodal metastases ($p=0.061$).

**Conclusion**
On the whole, HER2 overexpression was observed in only 12% of bladder cancers. No correlation was found with histoprognostic factors. Our results disagreed with most of previous studies, which confuse an association of HER2 overexpression with tumour grade and local infiltration. Larger multicentre studies with additional molecular investigation are required to more explore the overexpression of HER2 in urothelial carcinoma as well as in low-grade and non-invasive bladder tumours in the Tunisian population.

© 2023 The Authors. Molecular Oncology is published by John Wiley & Sons Ltd on behalf of Federation of European Biochemical Societies

### EACR23-0111
### Significance of Survivin expression combined with fever and LDH on the prognosis of patients with neuroblastoma

*F. Pinhatti[1], A. Castro[2], M. Olandoski[1], L. Noronha[1], S. Elifio-Esposito[1]*
*[1]Pontificia Universidade Católica do Paraná, Graduate Program in Health Sciences, Curitiba, Brazil*
*[2]Hospital Pequeno Principe, Oncology and Hematology, Curitiba- PR, Brazil*

**Introduction**
Survivin is a 16.5 kDa protein identified as a potential therapeutic target as it is highly expressed in many tumors. While the efficacy and safety of its therapeutic inhibition are still under investigation, its application as a prognostic marker has been little explored in neuroblastoma. This work aimed to investigate the associations of SVV immunoexpression with clinical variables concerning the outcomes of death and progression of neuroblastoma.
**Material and Methods**
Data from 130 patients treated for neuroblastoma at Pequeno Príncipe Pediatric Hospital and Erasto Gaertner Cancer Hospital, both in Curitiba-PR, Brazil, between 1990 and 2016, were collected from medical records. FFPE tumor samples in TMA were subjected to immunohistochemical staining and analyzed by color morphometry. The associations of SVV with the study variables were investigated concerning death and progression. Kaplan-Meier curves were built, and the Log-rank test was used to compare two or more groups. Cox regression models were adjusted to assess the association of quantitative variables with survival or time to progression.
**Results and Discussions**
Death and disease progression were significantly ($p<0.001$) correlated with each other, and with age, stage, risk, MYCN amplification, bone metastasis at diagnosis, Shimada ($p=0.003$), and disease extent at diagnosis ($p=0.003$). Death also showed a positive correlation with elevated LDH levels at diagnosis ($p=0.021$), fever at diagnosis ($p=0.001$), and medullary infiltration ($p=0.050$). Fever at diagnosis associated with low SVV showed a higher risk of death than fever alone. Conversely, high SVV expression reduced the risk of death. Serum LDH was transformed into a dichotomous categorical variable by fitting a ROC curve for death. The resulting cutoff point was 734 U/L, with a sensitivity of 62.9% and a specificity of 79.9%. A significant risk of death increases when a high LDH level is combined with low SVV. We also noted the more prolonged survival for elevated LDH and high SVV.
**Conclusion**
The immunohistochemical expression of SVV analyzed by morphometry showed potential as a good prognostic factor for patients diagnosed with malignant NB. The higher expression of SVV may define a better response subgroup in this population, as shown for fever and LDH.

### EACR23-0139
### Systemic Immune-Inflammation index is an indicator of patient relapses in breast cancer: a pilot study

*A.A. Zergoun[1,2], S. Braikia[3], M.W. Boubnider[4], K. Bouzid[3], C. Touil-Boukoffa[1]*
*[1]Faculty of Biological Sciences, Laboratory of Cellular and Molecular Biology, Algiers, Algeria*
*[2]Faculty of Sciences, Department of Biology, Boumerdes, Algeria*
*[3]Centre Pierre et Marie Curie, Medical oncology service, Algiers, Algeria*
*[4]Centre Pierre et Marie Curie, Surgery service "B"- Senology, Algiers, Algeria*

**Introduction**
NLR, PLR and SII index are widely used to assess patient's outcome in many types of neoplasms including breast cancer. The aim of this study was to select the best parameter with high prognostic value by defining the optimal cutoff of each parameter in patients undergoing mastectomy.
**Material and Methods**
This is a retrospective monocentric pilot study enrolling 59 patients. The ROC curve was used for the obtention of the optimal cutoff value of NLR, PLR and SII. The Kaplan Meier curve and log-rank test were used to assess RFS, DMFS and OS. The three indicators were compared to clinicopathological characteristics such as molecular subtypes, clinical stages and histological types.
**Results and Discussions**
Among the three parameters, we obtained the optimal cutoff for SII and NLR but not PLR (AUC $\leq 0.5$)**.** To assess the RFS, the optimal cutoff for SII and NLR were defined as 472 (AUC= 0.578) and 2.11 (AUC= 0.568), respectively. Only high SII was associated to shorter RFS (HR: 2.223; 95% CI: 1.066-4.573; p= 0.0368). For DMFS, the optimal cutoff for SII and NLR were 472 (AUC= 0.634) and 1.60 (AUC= 0.59), respectively. The Kaplan-Meier curve showed that both high SII and high NLR were associated to shorter DMFS with (HR: 3.094; 95% CI: 1.083-6.898; p=0.0345 and HR: 3.584; 95% CI: 0.9413-6.947; p=0.0672, respectively). No significant results were observed between levels of SII, NLR and PLR compared to clinical parameters.
**Conclusion**
The SII index is the best indicator that fit with patients' outcomes and showing a promising predictive tool of both locoregional and distant metastasis.

### EACR23-0154
### 2C as a possible treatment for ovarian cancers: a nanomechanical characterization

*S. Bonin[1], D. Tierno[1], E. Azzalini[1], R. Farra[2], S. Drioli[3], F. Felluga[3], G. Grassi[2], M. Lazzarino[4]*
*[1]University of Trieste, DSM-Dept. of Medical Sciences, Trieste, Italy*
*[2]University of Trieste, DSV-Department of Life Sciences, Trieste, Italy*
*[3]University of Trieste, DSCF- Department of Chemical and Pharmaceutical Scien ces, Trieste, Italy*
*[4]CNR, IOM- Istituto officina dei materiali, Trieste, Italy*

**Introduction**
Epithelial ovarian cancers (EOCs) are heterogeneous tumors at molecular and clinical level. During the past

© 2023 The Authors. Molecular Oncology is published by John Wiley & Sons Ltd on behalf of Federation of European Biochemical Societies decades, few improvements have been achieved in EOC management and treatment efficacy. 2C is a non-selective and irreversible isopeptidases inhibitor (Cersosimo et al., 2015) of possible use in tumor treatment (Tomasella et al., 2014). In addition of inhibiting DUBs, a class of isopeptidases, this drug shows parallel activity in triggering a cellular necrotic pathway with cytoskeletal reorganization. As ubiquitin specific peptidase 5 has been shown to promote ovarian cancer cell proliferation, 2C can represent a possible new treatment for EOC. Possible utility of 2C in ovarian cancer can also be related to its activity on AKT molecules (Ciotti et al., 2018), which have already been reported in EOCs (Azzalini et al., 2022).

**Material and Methods**

Eight EOC cell lines were analyzed in the present study, namely SKOV3, IGROV1, HEY, OVCAR8, OVCAR4, TYKNU, TYKNU CpR and OAW42. Each cell line was treated for 24 hours at increasing concentrations of 2C. Afterward, an assessment of cell viability for each concentration was performed to obtain the IC50. Invasion assay as well as nanomechanical characterization by Atomic force microscopy were carried out in untreated as well as cell lines treated with 20 μM of 2C.

**Results and Discussions**

Cell lines presented two patterns of the Young modulus distribution: Gaussian/unimodal and bimodal. 2C treatment led to an overall decrease of the average elastic modulus. An association between the coefficient of variation of cellular stiffness after 2C treatment (CV%), the IC50 and the stiffness distribution pattern was found. Cell lines with bimodal pattern had lower CV% ($p = 0.01$) and higher resistance to 2C ($p < 0.001$). A negative linear correlation between the CV% and the IC50 was observed only when the stiffer population in bimodal cell line distribution was considered ($p = 0.04$). Cell lines with fibroblastic-like morphology resulted more invasive than those with epithelial morphology ($p = 0.0002$). In addition, a negative linear correlation between the stiffness and the mean number of invasive cells was found only when the softer population in bimodal cell line distribution was considered ($p = 0.05$).

**Conclusion**

EOC cell lines are characterized by inter and intra-mechanical heterogeneity. 2C can represent a possible treatment of EOCs and due to the effect of 2C on the cytoskeleton the coefficient of variation of cell stiffness could represent a possible biomarker of 2C response.

### EACR23-0155
### Exosome micro RNA as liquid biopsy biomarkers for skin melanomas

*S. Bonin[1], E. De Martino[1], I. Gandin[1], E. Azzalini[1], C. Massone[2], M.A. Pizzichetta[1,3], E. Giulioni[4], S. Javor[5], C. Pinzani[6], C. Conforti[1], I. Zalaudek[1,7]*

*[1]University of Trieste, DSM-Dept. of Medical Sciences, Trieste, Italy*

*[2]Ospedali Galliera, Dermatology Unit & Scientific Directorate, Genova, Italy*

*[3]Centro di Riferimento Oncologico CRO- IRCCS, Oncologic Dermatology Prevention, Aviano, Italy*

*[4]AS FO Azienda sanitaria Friuli Occidentale, Unit of Dermatology, Pordenone, Italy*

*[5]Ospedali Galliera, Dermatology Unit, Genova, Italy*

*[6]Centro di Riferimento Oncologico CRO- IRCCS, Oncologic Dermatology Prevention Unit, Aviano, Italy*

*[7]ASU GI- Azienda Sanitaria Universitaria Giuliano Isontin a, Unit of Dermatology, Trieste, Italy*

**Introduction**

In last decade, cutaneous melanoma incidence has dramatically increased and early diagnosis is crucial for an appropriate tumor management. Liquid biopsy has attracted attention as a non-invasive method for early diagnosis, monitoring and treatment response evaluation. There is a urgent need of new biomarkers to follow-up patient with melanoma negative for BRAF and/or NRAS hot spot mutations.

**Material and Methods**

In this prospective multicenter study blood samples for miRNA profiling were obtained from consecutive patients with clinical and dermoscopic suspected diagnosis of melanoma and a control group without melanoma. Seven miRNAs, namely *hsa-miR-149-3p*, *hsa-miR-150-5p*, *hsa-miR-21-5p*, *hsa-miR-200c-3p*, *hsa-miR-134-5p*, *hsa-miR-144-3p* and *hsa-miR-221-3p* were profiled by quantitative PCR (qPCR) in plasma from patients with malignant melanoma and age and gender matched controls.

**Results and Discussions**

Our results showed that three out seven miRNAs, namely *hsa-miR-200c-3p*, *hsa-miR-144-3p* and *hsa-miR-221-3p* were differentially expressed in plasma-derived exosomes from melanoma patients and controls. Furthermore, *hsa-miR-200c-3p* expression was significantly higher in stage III and IV melanomas (Mann-Whitney test, p=2e-5). Contrarily, *hsa-miR-144-3p* and *hsa-miR-221-3p* were over-expressed in patients with stage I and II melanomas (Mann-Whitney test, p=9e-4 and p=9e-4, respectively).

**Conclusion**

The combined expression level *hsa-miR-200c-3p, hsa-miR-144-3p* and *hsa-miR-221-3p* resulted to be a strong candidate biomarker for discriminating between nevi and melanoma with high accuracy. If validated, this finding has possible implication both for early diagnosis and follow-up procedures.

### EACR23-0157
### BIOCHEMISTRY APPROACH TO INVESTIGATE LUMINAL B HER2-NEGATIVE PHENOTYPES

*A. Razzoli[1], D. Schiroli[2], L. Merolle[2], G. Gavioli[1], M. Ragazzi[2], E. Gasparini[2], R. Belli[3], D. Peroni[3], C. Marraccini[2]*

*[1]University of Modena and Reggio Emilia, PhD program in Clinical and Experimental Medicine- Dep artment of Biomedical- Metabolic and Neural Sciences, Reggio Emilia, Italy*

*[2]AUSL-IRCCS di Reggio Emilia, Department of Oncology and Advanced Technologies, Reggio Emilia, Italy*

*[3]University of Trento, Department of Cellular- Computational and Integrative Bi ology CIBIO, Trento, Italy*

**Introduction**

Showing high proliferative index, poor response to therapy and high incidence of distant metastases, luminal B HER2- represents one of the most aggressive breast cancer

© 2023 The Authors. Molecular Oncology is published by John Wiley & Sons Ltd on behalf of Federation of European Biochemical Societies subtypes. Clinical treatment consists of surgical resection, which can be followed by adjuvant hormonal therapy and chemotherapy, although the benefit of the latter in the early stages is still debated and clinical decisions often rely on expensive non-equivalent genomic tests. This project aims at identifying specific immunohistological markers to be used in the clinic to stratify the risk of recurrence in luminal B HER2- patients and help to discriminate those who would benefit from adjuvant chemotherapy. We also expect to discover novel pharmacological targets and to gain insight into the subtype molecular heterogeneity.

**Material and Methods**

25 luminal B HER2- patients with at least 10 years of follow-up were selected. Patients were homogeneous in terms of grade, stage, and positivity for the routine predictive markers, and selected to belong to 4 different groups with respect to the therapy received and outcome at 10 years from diagnosis: i) relapse-free without adjuvant chemotherapy, ii) relapse-free with adjuvant chemotherapy, iii) relapsed without adjuvant chemotherapy, iv) relapsed with adjuvant chemotherapy. Formalin-fixed paraffin-embedded tissues from biopsies were obtained from our institute's biobank and subjected to proteomic analysis and data were analysed using bioinformatics tools. Validation of the results will be performed by *in vitro* experiments and then by immunohistochemistry on a larger cohort of patients.

**Results and Discussions**

Analysis of the data allowed patients to be classified as having a high, intermediate or low risk of distant metastasis, and to identify differentially expressed proteins and thus a specific protein panel for each risk class. Up and downregulation of the selected proteins will enable *in vitro* characterization of the pathways involved and elucidation of the molecular mechanisms. The most promising proteins will then be validated in a larger cohort of patients.

**Conclusion**

The study will allow the identification of protein signatures correlating with the different luminal B HER2- phenotypes and, therefore, will be of clinical value in stratifying patients at diagnosis and identifying those who should receive adjuvant therapy, minimising overtreatment and identifying chemoresistant patients that may require a different therapeutic approach. The study will also identify targets for innovative therapies.

### EACR23-0170
### Circulating histone signature of pediatric Diffuse Intrinsic Pontine Glioma (DIPG)

*D. Buzova[1], L.L. Petrilli[2], S.D. Bianco[3], M.R. Braghini[4], A. Alisi[4], O. Lo Re[5], J. Cerveny[1], T. Mazza[3], M. Vinci[2], M. Vinciguerra[5]*

*[1]Czech Globe, Department of Adaptive Biotechnologies, Brno, Czech Republic*

*[2]Ospedale Pediatrico Bambino Gesu, Department of Onco-hematology, Roma, Italy*

*[3]IRCCS Casa Sollievo della Sofferenza, Laboratory of Bioinformatics, Rome, Italy*

*[4]Ospedale Pediatrico Bambino Gesu, Research Unit of Molecular Genetics of Complex Phenotyp es, Roma, Italy*

*[5]Medical University of Varna, Department of Stem Cell Biology and Transplantology, Varna, Bulgaria*

**Introduction**

Diffuse intrinsic pontine glioma (DIPG) is usually diagnosed when children are aged ten or below. It is a devastating and fatal disease with a median overall survival of less than 12 months after diagnosis. Radiological imaging is the gold standard for DIPG diagnosis while the use of invasive and risky biopsy focuses on the understanding its molecular biology, such as the histone H3K27M mutation, identified in ~30% of the cases. The urgent need to improve the survival encourages targeting biofluids such as cerebrospinal fluids (CSF) and blood plasma for optimizing molecular diagnoses in DIPG. Here, we propose a new, fast, imaging and epigenetics based approach to diagnose DIPG in the plasma of pediatric patients.

**Material and Methods**

A total of 20 healthy children (mean age 10.5] and 25 children diagnosed with DIPG (mean age 8.5) were recruited. 8/25 DIPG patients displayed histone H3K27M mutation. Individual histones, histone dimers and nucleosomes (histone tetramers) were assayed in serum samples by means of a new advanced flow cytometry ImageStream(X)-adapted method.

**Results and Discussions**

We implemented successfully a multi-channel flow methodology on ImageStream(X), to image single histone staining (H2A, H2B, H3, H4, macroH2A1.1 and macroH2A1.2). We report here a significant upregulation of histone dimers and tetramers (macroH2A1.1/H2B vs control: p-value<0.0001; macroH2A1.2/H2B vs control: p-value<0.0001; H2A/H2B vs control: p-value<0.0001; H3/H4 vs control: p-value =0.008; H2A/H2B/H3/H4 vs control: p-value<0.0001) and a significant downregulation of individual histones (H2B vs control: p-value<0.0001; H3 vs control: p-value<0.0001; H4 vs control: p-value<0.0001). Moreover, using a sample subset we show that individual histones and histone complexes are also detectable with a robust signal in the CSF of DIPG children.

**Conclusion**

In summary, we identified a new circulating histone signature able to discriminate the presence DIPG in children, using a rapid and non-invasive ImageStream(X)-based imaging technology. The patterns observed suggest the differential involvement of histone chaperone complexes in histone extracellular release in DIPG children plasma.

### EACR23-0171
### MGMT methylation in extracellular vesicle-based liquid biopsy as a tool for glioma patient management

*I. Ibanez de Caceres[1], R. Rosas-Alonso[1], O. Pernia[1], M. Burdiel[1], C. Rodriguez-Antolin[1], I. Losantos-Garcia[1], R. Moreno-Velasco[1], I. Esteban-Rodriguez[1], V. Martinez[1], J. de Castro[1]*

*[1]FIBHULP-idipaz, Genetics. Experimental therapies and novel biomarlkers.Id iPAZ, MADRID, Spain*

**Introduction**

© 2023 The Authors. Molecular Oncology is published by John Wiley & Sons Ltd on behalf of Federation of European Biochemical Societies

Glioblastoma (GB) is a devastating tumour of the central nervous system characterized by a poor prognosis. The only well-established predictive and prognostic biomarker in patients with GB is $O^6$-methylguanine-DNA methyltransferase (MGMT) methylation (*mMGMT*), which is associated with improved treatment response and survival. To date, all efforts to monitor patients with GB through *mMGMT* detection have failed, making small extracellular vesicles (sEVs) a key element that could reinvent the clinical diagnosis by opening new possibilities for liquid biopsy. The aim of this study was to determine whether sEV-based liquid biopsy is a useful tool for disease monitoring and management of patients with glioma.

**Material and Methods**

We performed a prospective exploratory study to analyse the sensitivity and specificity of sEV-based liquid biopsy to determine m*MGMT* status relative to the tumour sample. The association of sEV-DNA methylation with overall survival was also evaluated, as well as its usefulness in the follow-up of patients.

**Results and Discussions**

We evaluated for the first time in liquid biopsy the methylation of the *MGMT* (mMGMT) genepromoter in the DNA from extracellular vesicles (sEV-DNA), obtaining concordant results with respect to the analysis carried out in tissue. Our study reached a sensitivity of 85.7% in the detection of mMGMT in liquid biopsy, the best result achieved to date. Furthermore, we demonstrate that liquid biopsy assessment of sEV-DNA is a powerful tool for disease monitoring and for the detection of disease progression in glioblastoma patients, providing relevant prognostic information for the patient.

**Conclusion**

Molecular under-detection leads not only to missed treatment opportunities, but also to misdiagnoses, resulting in the use of ineffective therapies. Therefore, the results obtained in this study represent an important contribution to the field of sEV-DNA-based liquid biopsy, reflecting the tumour tissue heterogeneity and presenting itself as a promising tool for the detection of biomarkers of the nervous system, specifically for gliomas. Funding:Instituto de Salud Carlos III and the European Regional Development Fund/European Social Fund FIS [ERDF/ESF], Una Manera de Hacer Europa (PI18/050; PI21/0145; DTS20/029; JR21/03; FI19/061). MICINN PLEC2021-08034, and GHETTI2021.

### EACR23-0208

### Discrepancy in microsatellite instability status as determined by MSI-PCR versus MMRD-immunohistochemistry

*M.L. Nádorvári[1], T. József[1], R. Erzsébet[1], B. Tamás[1]*
*[1]Semmelweis University, Department of Pathology- Forensic and Insurance Medicine, Budapest, Hungary*

**Introduction**

Diagnostic testing for mismatch repair deficiency/microsatellite instability is necessary to screen patients for Lynch syndrome and/or for eligibility for immune checkpoint inhibitor therapy. Recent guidelines suggest a primary immunohistochemical (IHC) testing for MMR proteins of the tumor and in case of abnormal staining or clinical history positivity, a confirmation of the status by MSI-PCR.

**Material and Methods**

Throughout the years of 2021 and 2022, we have tested 660 tumor samples for MSI status using Ventana® antibodies for MLH1, MSH2, MSH6 and PMS2 in Benchmark® automatic stainer for immunohistochemistry or by Promega® Oncomate MSI-Dx PCR test.

**Results and Discussions**

Using immunohistochemistry, MMRD rate in colorectal cancer was 8.8% while PCR resulted in 8,9% of MSI rate. In case of gastric cancer, IHC resulted in 21% as compared to 15% by PCR. In endometrial cancer, IHC identified MMRD in 35% as compared to 30% MSI rate by PCR. The 436 cases where the two methods have been performed parallel provide a basis for the comparison of the diagnostic efficacies: the two diagnostic techniques gave different results in 11.7% of the cases. The MSI-high/MMR proficient discrepancies were rare (1.1%) compared to MSS/MMR deficiency(4.1%). In colorectal cancer, the same figures were 1.5% versus 4.2%. Furthermore, MSI-low/undetermined status was detected in 6.4% among which MMR deficiency was found in 0.7%, while the majority was MMR proficient (5,7%). Considering the low rate of MMRD/MSI in most cancers, these differences seem to be significant.

**Conclusion**

To screen colorectal patients for Lynch syndrome, IHC selection is very sensitive to detect MMR protein loss, which must be followed by mutation testing. However, the requirement to select patients for immunotherapy is the MSI-high genomic status, which seems to be more appropriate by PCR testing methodology. IHC testing in our study incorrectly defined MSI status in 5%, which can be considered as a high error rate. We suggest parallel testing as an alternative approach for MSI/MMRD detection in malignant tumors to achieve correct identification of patients with Lynch syndrome and to detect the ones who qualify for immunotherapy.

### EACR23-0217

### Circulating tumor cell-based PSMA mRNA in localized prostate cancer

*J.S. Chung[1], H. Cho[2], J. Cha[2], J.I. Chung[3], S.D. Kim[4], K.H. Han[2]*
*[1]INJE University Haeundae Paik Hospital, Urology, BUSAN, South Korea*
*[2]INJE University, Nanoscience and Engineering Center for Nano Manufacturing, Kimhae, South Korea*
*[3]INJE University Busan Paik Hospital, Urology, Busan, South Korea*
*[4]Jeju National University, Urology, Jeju, South Korea*

**Introduction**

Localized prostate cancer (LPCa) have a broad spectrum, ranging from indolent to cancer that requires aggressive treatment. Patients with LPCa can be treated by radical prostatectomy (RP) but 30-40% of them experienced tumor recurrence. Biomarker capable of finding micrometastasis, which is not easily detected by conventional imaging after RP in LPCa, can help guide clinical decision-making. Recently, PSMA-PET CT, which visually measures PSMA expression, are being performed to detect micrometastasis

© 2023 The Authors. Molecular Oncology is published by John Wiley & Sons Ltd on behalf of Federation of European Biochemical Societies or biochemical recurrence (BCR) after RP. The aim of this study was to determine whether circulating tumor cell (CTC)-based PSA mRNA or PSMA mRNA can be used as a marker to predict BCR after RP in LPCa.

**Material and Methods**

Peipheral blood samples were obtained from 92 patients who had LPCa. After isolating CTCs by microfluidic magnetophoresis, the expression of PSA mRNA and PSMA mRNA in CTCs were measured by droplet digital PCR. The outcomes of interest was biochemical recurrence-free survival (BCRFS) in LPCa, which was estimated using the Kaplan-Meier method and Cox proportional hazards models.

**Results and Discussions**

The detection rate of CTCs were 80.4% (74/92) in LPCa or LAPCa. The PSA mRNA and PSMA mRNA expression in CTCs were observed in 31.1% (23/74) and 48.6% (36/74). The CTC count and detection rate of PSA mRNA or PSMA mRNA expression increased for more advanced PCa. The 2-year BCRFS after surgery were 72.5% for PSA mRNA negative and 60.9% for positive (P=0.537). The corresponding rates were 55.6% for PSMA mRNA positive and 81.6% for negative (P=0.012). The BCRFS were also affected by preoperative CTC count. (82.8% vs. 50.2%, P =0.001). However, In Cox hazards models, only PSMA mRNA positive were significantly different from their PSMA mRNA negative (HR: 3.922 95% CI: 1.441-10.671, P = 0.007).

**Conclusion**

Our study showed that CTC-based PSA mRNA were not related to BCR, but PSMA mRNA had a significant association. The quantified PSMA gene expression in CTC can be used as a useful marker to predict BCR after RP in LPCa

### EACR23-0224

### Can saliva be considered a proxy of gastric tumors? A bioinformatic analysis towards biomarker discovery and early diagnosis

*C. Lopes[1,2,3], T. C. Almeida[1], A. Brandão[4], M. Dinis-Ribeiro[1,5], C. Pereira[1,3]*

*[1]Portuguese Institute of Oncology of Porto/Porto Comprehensive Cancer Center, Precancerous Lesions and Early Cancer Management Group- Research Center/Rise@CI-IPOP, Porto, Portugal*

*[2]ICBAS-UP – Institute of Biomedical Sciences Abel Salazar, International Doctoral Programme in Molecular and Cellular Biotechnology Applied to Health Sciences BiotechHealth, Porto, Portugal*

*[3]CINTESIS – Center for Health Technology and Services Research, Clinical & Translational Research, Porto, Portugal*

*[4]Portuguese Institute of Oncology of Porto/Porto Comprehensive Cancer Center, Cancer Genetics Group- Research Center/Rise@CI-IPOP Health Research Group, Porto, Portugal*

*[5]Portuguese Oncology Institute of Porto, Department of Gastroenterology, Porto, Portugal*

**Introduction**

In the last decade, the potential of saliva as a non-invasive, self-collected liquid biopsy has been explored in the diagnosis of several diseases, including gastric cancer (GC). It may represent an opportunity for screening and early diagnosis, improving patients' quality of life, since it enables the empowerment of each citizen in their own assessment of disease risk. Thus, the aim of this study was to evaluate saliva as a proxy of gastric tissue by comparing the transcriptomic profile between saliva and tissue in GC, thus understanding which pathways are commonly dysregulated.

**Material and Methods**

Two microarray datasets on gene expression in Asian individuals were downloaded from Gene Expression Omnibus (GEO), GSE54129 and GSE64951, including 111 GC patients and 21 healthy controls and 63 GC patients and 31 controls, respectively. Affy and limma package from R software (version 4.2.1) were used for normalization and base-2 logarithm conversion of the matrix data and for screening of the differentially expressed genes (DEGs), respectively. Functional enrichment analysis of those genes was performed using the goprofiler2 package. P-value < 0.05 and adjusted P-value < 0.05 were considered statistically significant.

**Results and Discussions**

Overall, 5875 DEGs were identified in tissue of GC patients, of which 3167 genes were down regulated and significantly enriched in several biological processes associated with metabolism, including carbohydrate and lipid metabolic processes, biosynthetic processes, and glycosylation. On the other hand, the 2708 up regulated genes were involved in processes such as cell migration and motility, response to stimulus, leukocyte migration and chemotaxis, and extracellular matrix-associated terms. Over 17% of DEGs found in tissue were also dysregulated in saliva from GC patients. The 1838 down regulated genes were significantly enriched in metabolism-associated processes, similarly to what was found in tissue, but they were mostly related to proteins rather than carbohydrates or lipids. Furthermore, and although not observed in tissue, protein modification processes, particularly ubiquitination, were evidenced in the top biological processes of DEGs found in saliva.

**Conclusion**

Although not all DEGs found in tissue are mirrored in saliva, nearly 700 genes shared the same expression profile across biopsies, supporting the potential of salivary transcriptomics as a source of GC biomarkers and proxy of gastric tissues as a strategy for GC early screening and diagnosis.

### EACR23-0246

### Proteomics of the nucleoli-enriched fractions reveals alterations in renal cell carcinoma

*A. Piekielko-Witkowska[1], B. Skupień-Rabian[2], U. Jankowska[2], M. Grzanka[1], B. Rybicka[1], J. Bogusławska[1], P. Popławski[1]*

*[1]Centre of Postgraduate Medical Education, Department of Biochemistry and Molecular Biology, Warsaw, Poland*

*[2]Jagiellonian University, Proteomics and Mass Spectrometry Core Facility- Malopolska Centre of Biotechnology, Cracow, Poland*

**Introduction**

© 2023 The Authors. Molecular Oncology is published by John Wiley & Sons Ltd on behalf of Federation of European Biochemical Societies

Clear cell renal cell carcinoma (ccRCC) is the most common subtype of kidney cancers. ccRCC tumor grading (Fuhrman, ISUP/WHO) is based on nucleolar morphology changes that correlate with ccRCC progression. We hypothesized that nucleolar pleomorphy may result from disturbed expression of nucleolar proteins. Here, we: i) analyzed nucleolar proteomes and transcriptomes of ccRCC cells and normal proximal tubules; ii) searched for microRNAs potentially affecting the expression of nucleolar proteins.

**Material and Methods**

Nucleoli were isolated from ccRCC cell lines (786-O, Caki-1) and control cell line (RPTEC/TERT1) using sucrose gradient centrifugation. Proteomic analysis was performed using label-free approach and data-dependent acquisition. The expression of ccRCC genes was retrieved from our recently published microarray analysis. Nucleolar miRNA expression was analyzed using RNAseq. The expression of microRNAs/proteins in >500 ccRCC tumors was evaluated using ENCORI and UALCAN/CPTAC.

**Results and Discussions**

The expression of 233 proteins was altered in ccRCC cell lines when compared with normal kidney proximal tubules. 37 proteins were detected exclusively in ccRCC cell lines and were not detectable in control cell lines. GO PANTHER Overrepresentation test showed that upregulated proteins were enriched in nucleolus and nucleolar rRNA processing. The expression changes of 72 proteins were reflected by similar alterations in gene expression. There were 161 proteins of which genes were not altered, suggesting that they were selectively retained/expelled from the nucleolus. The expression of 88 proteins correlated with ccRCC tumor grades. Top altered nucleolar proteins included upregulated: EEF1A2, GLTSCR2/NOP53 and MINA, as well as downregulated DSP, TERT, and MYOF. Expression of EEF1A2, GLTSCR2/NOP53, DSP and MYOF proteins correlated with nucleolar alterations/tumor grade. Targetscan predictions revealed miR-31-5p to target EEF1A2 and GLTSCR2, miR-582-5p and miR-335-5p to target GLTSCR2 and MINA, miR-425-5p to target DSP and MYOF, while miR-139-5p to target TERT and MYOF. miRNAs' expressions were altered in opposite directions to protein expression changes in ccRCC cell lines and tumors when compared to their non-cancerous counterparts.

**Conclusion**

The expression of nucleolar proteins is disturbed in ccRCC, possibly due to the altered levels of their targeting miRNAs.

Financed by National Science Center, Poland grant 2019/35/B/NZ5/00695.

### EACR23-0253
### Flowing through the immune system – deep single-cell immunophenotyping of immune cells in liquid biopsies

*J. Pedersen*[1], *K.R. Gammelgaard*[1], *M.R. Jakobsen*[1]
[1]*Aarhus University, Department of Biomedicine, Aarhus, Denmark*

**Introduction**

The immune system plays a pivotal role during cancer development as it can both fight and facilitate tumor growth. Consequently, understanding how the immune system functions during tumorigenesis is vital for developing and improving cancer treatments and has already resulted in several successful immunotherapies. Common to the immunotherapies is that a significant part of the patients does not respond well to treatment. The precise mechanism remains unknown and warrants further investigations into the interplay between therapy, cancer, and the immune system. Here, we present a 39-parameter spectral flow panel that enables deep single cell immunophenotyping of all major immune cell types.

**Material and Methods**

Spectral flow has revolutionized flow cytometry and enabled construction of +40-marker panels while maintaining the benefits of high-throughput analysis from conventional flow cytometry. Using spectral flow as the platform, we designed a 39-marker panel consisting of extra- and intracellular markers for analysis of a broad range of immune cells from liquid biopsies such as fresh and frozen whole blood and bronchoalveolar lavage samples.

**Results and Discussions**

Our spectral flow panel on whole blood samples was able to quantify the frequency of all main immune cell types including CD4 and CD8 T cells, B cells, NK cells, monocytes, dendritic cells, plasmacytoid dendritic cells, basophils, eosinophils, and neutrophils. Using additional lineage and differentiation markers T cells, NK cells, monocytes, and dendritic cells could be further divided into minor subtypes. Finally, with 15 different activation and checkpoint markers, we were able to perform a profound functional characterization of the individual immune cell subsets. Tracking the expression of checkpoint molecules will be particularly valuable in the setting of cancer immunotherapy as many approved and upcoming checkpoint inhibitors directly target these molecules.

**Conclusion**

Here, we present a validated 39-marker spectral flow panel for deep single-cell immunophenotyping of all major immune cell subsets found in different biospecimens. In the future this immune profiling method will not only expand our knowledge in the immune-oncology field leading to development of new therapies but can also become a novel tool for guiding personalized treatment plans of cancer patients in general.

### EACR23-0279
### Transcriptomic differences between localized and metastatic prostate cancer using circulating tumor cells isolated by lateral magnetophoretic microfluidic technology

*H. Cho*[1], *C. Jiwon*[1], *R. Jaewook*[1], *P. Suhee*[1], *K.H. Han*[1], *J.S. Chung*[2]
[1]*Inje University, Nanoscience & Engineering, Gimhae, South Korea*
[2]*Haeundae Paik Hospital, Urology, Busan, South Korea*

**Introduction**

We aimed to isolate circulating tumor cells (CTCs) from prostate cancer (PCa) patients using microfluidic technology based on lateral magnetophoresis and evaluate the transcriptomic difference between localized and metastatic PCa from purified CTCs.

© 2023 The Authors. Molecular Oncology is published by John Wiley & Sons Ltd on behalf of Federation of European Biochemical Societies

**Material and Methods**
A total of 189 samples of patients who underwent treatment for prostate cancer between 04/2020 and 05/2022 were enrolled for CTC collection. Approximately 10 ml of whole blood was drawn from prostate cancer patients. Patients were divided into two subgroups of localized PCa and metastatic PCa. The 5 mL of blood samples were used for CTCs enumeration, and remained 5 mL of samples were used for transcriptomic analysis after the CTC isolation process. The 48 gene expression levels in CTCs were measured by droplet digital PCR.
**Results and Discussions**
The detection rate of isolated CTCs were 81.6% and 100% in localized PCa and metastatic PCa, respectively ($P < 0.05$). The enumerated CTCs were increased depending on their clinical stages. Most of the androgen receptor-related genes(AR, AR-V7, NKX3-1,HGF) and cancer stem cell-related gene(PSCA) were highly expressed more than 10 times in metastatic PCa than in localized PCa ($p<0.001$). DNA repair related gene, such as ATM, CDK12, BRCA1, BRCA2 were also highly overexpressed more than 100 times in metastatic PCa ($P < 0.05$). However, cell signaling and sex reversal-related genes did not show distinctive differences except for the TSPAN8 gene. The DKK-1 gene showed a high expression rate (>100 times) at metastatic stages ($P < 0.05$). The cell cycle progression-related gene(CCND1) and tumor-associated inhibitor gene(SPINK1) were also overexpressed at metastatic stages more than 5 times. Especially, prostate-specific genes, PSA and PSMA, were highly detectable and expressed at metastatic PCa.
**Conclusion**
Our data showed that considerable selected genes were overexpressed at metastatic PCa. Transcriptomic analysis via CTC-based multigene profiling may guide the early diagnosis of advanced PCa and provide an additional means of risk stratifying PCa.

### EACR23-0297
### Epidermal growth factor receptor (EGFR) expression shows distinctive functional and spatial dynamics in high-risk and low-risk thyroid neoplasms partly regulated by miRNAs

A. Al-abdallah[1], I. Jahanbani[1], B. AlShammary[1], J. Prasanth[1]
[1]*Kuwait university, Pathology, kuwait, Kuwait*
**Introduction**
Identification of molecular alterations associated with tumor behavior is necessary to guide clinical management. The 2022 WHO classification has grouped the thyroid follicular cell-derived neoplasms into benign, low-risk and high-risk neoplasms, and emphasized the value of biomarkers that may provide differential diagnostic and prognostic information to avoid overtreatment of low-risk neoplasms. This work aims to study the epidermal growth factor receptor (EGFR) expression, functional and spatial dynamics in relation to specific miRNAs alterations in papillary thyroid cancer (PTC) and in non-invasive follicular thyroid neoplasm with papillary-like nuclear features (NIFTP) considered as models of high-risk and low-risk thyroid tumors respectively.
**Material and Methods**
Paraffin embedded tissues from 80 cases diagnosed as PTC, NIFTP and follicular nodular disease were used for real time PCR, immuno-fluorescence stain and confocal microscopy experiments. Primary thyroid cultured cells were used for miRNA gain/loss of function and luciferase reporter assays. Ethical approval to conduct this study was obtained from Kuwait Ministry of Health and Kuwait University Health Sciences Center ethics committee.
**Results and Discussions**
Our results showed that in PTC, EGFR mRNA is reduced as an effect of miR-146b-5p upregulation. The EGF expression is low and the ERK pathway is inhibited. The EGFR protein high cytoplasmic expression and colocalization with the endosomal/exosomal markers, ALIX and CD63, suggest the occurrence of stress-induced EGFR internalization, accumulation in endosomal vesicles and secretion via exosomes. In NIFTP, EGFR transcription is increased in association with downregulation of miR-7-5p. Protein expression is weak at the tumor core and the EGFR/ERK pathway is active.
**Conclusion**
High EGFR protein in PTC (high-risk tumor) is not due to gene overexpression, but rather caused by accumulation of non-degraded protein arrested in endosomal compartments and disseminated through exosomes to the extracellular milieu. In NIFTP (low-risk tumor) EGFR gene is overexpressed and follows the canonical pathway of signaling through ERK pathway followed by internalization and degradation. These patterns can be used as diagnostic features in addition to the histopathological criteria. This work also showed two microRNAs that have combined effect on EGFR expression which may have important use in EGFR related cancers' prognosis and therapy.

### EACR23-0298
### A rapid, selective, and ultrasensitive voltammetric and gravimetric protocol for MMP-1 active form detection

A. Kowalczyk[1], M.K. Nisiewicz[1], A. Kasprzak[2], M. Bamburowicz-Klimkowska[3], A.M. Nowicka[1]
[1]*University of Warsaw, Faculty of Chemistry, Warsaw, Poland*
[2]*Warsaw University of Technology, 2Faculty of Chemistry, Warsaw, Poland*
[3]*Medical University of Warsaw, Faculty of Pharmacy, Warsaw, Poland*
**Introduction**
Matrix metalloproteinases (MMPs) are key enzymes in the digestion of extracellular matrix components. MMPs are involved in healthy cells apoptosis. Whereas in cancer cells, they participate in chemokine cleavage, thus preventing inflammatory cell chemotaxis and their recruitment to cancerous tissues. Moreover, they significantly influence the growth of metastatic tumor cells and increase the mobility of epithelial cells by regulating dynamic endothelial permeability and transendothelial migration in metastasis. They also promote angiogenesis, the formation of capillary blood vessels from a preexisting vascular system after extravasating tumor cells to create a metastatic niche to allow the growth of tumor cells in an unfavorable environment. That is why MMPs are

© 2023 The Authors. Molecular Oncology is published by John Wiley & Sons Ltd on behalf of Federation of European Biochemical Societies considered to be predictive and prognostic biomarkers, which might strengthen the current predictive systems.

**Material and Methods**

The activity of MMP-1 is related with its proteolytic properties towards the peptide bond between glycine and isoleucine. The application of voltammetric techniques requires the presence of the redox probe in the receptor layer. In this purpose the conjugate of redox probe with tripeptide $(Cys\text{-}Gly\text{-}Ile)_2$ was synthesized in seven steps using the solid-phase peptide synthesis (SSPS) methodology.

**Results and Discussions**

Here we present a rapid, selective, and ultrasensitive voltammetric and gravimetric protocol for the detection of the active form of MMP-1. The detection was based on MMP-1 hydrolytic activity; as a consequence, the receptor fragment with redox probe was removed from the sensor surface. The biosensors were characterized by a wide dynamic concentration response range (from 1.0 $pg \cdot mL^{-1}$ to 1.0 $mg \cdot mL^{-1}$) and a low detection limit (33 $fg \cdot mL^{-1}$), without the amplification step. One of the important advantages of the proposed biosensors is that they can be directly used to analyze the content of the active form of MMP-1 in clinical samples without the dilution step and any other preparation step. Contrary to the commercial ELISA tests, which are typical for the analysis MMPs in clinical samples, the proposed biosensors can detect MMP-1 in plasma samples with sensitivity at the level of hundredths of $pg \times mL^{-1}$.

**Conclusion**

The proposed biosensor showed an extremely low detection limit, high sensitivity, and a wide range of linear responses without any amplifiers, e.g., metal nanoparticles. In addition, the functionality of the developed biosensors was confirmed by the analysis of plasma samples.

### EACR23-0299

### Effective voltammetric tool for simultaneous detection of MMP-1, MMP-2, and MMP-9; important non-small cell lung cancer biomarkers

*A.M. Nowicka[1], A. Kowalczyk[1], M. Nisiewicz[1], M. Bamburowicz-Klimkowska[2], A. Kasprzak[3]*

*[1]University of Warsaw, Faculty of Chemistry, Warsaw, Poland*

*[2]Medical University of Warsaw, Faculty of Pharmacy, Warsaw, Poland*

*[3]Warsaw University of Technology, Faculty of Chemistry, Warsaw, Poland*

**Introduction**

Among the various cancers in humans, lung cancer is one of the most common and least predictable. Research over the past decade shows that metalloproteinases play a key role in the growth and metastasis of cancer. High levels of matrix metalloproteinases (MMPs) have been shown to correlate with poor overall survival in virtually all solid malignancies. In particular, MMP-1, MMP-2, and MMP-9 are important indicators of non-small cell lung cancer development and angiogenesis. Simultaneous detection of these biomarkers can allow to reduce the costs of medical diagnostics, and thus improve the accuracy and effectiveness of disease diagnosis and prognosis.

**Material and Methods**

We aimed to develop a novel, low cost, single-electrode, antibody-free system for simultaneous detection of active forms of MMP-1, MMP-2 and MMP-9. The sensor matrix was constructed using a G2 polyamidoamine dendrimer containing amino, carboxyl, and sulfhydryl groups providing the independent introduction of three receptors. The recognition process was based on specific enzymatic cleavage of the Gly-Ile peptide bond by MMP-1, Gly-Leu bond by MMP-2, and Gly-Met bond by MMP-9, and monitoring was done by square wave voltammetry. The activity of metalloproteinases was detected based on the change of current signals of redox receptors (dipeptides labeled with redox probes) covalently anchored onto the electrode surface.

**Results and Discussions**

The analytical response of the proposed biosensor was in the range from $1.0 \times 10^{-8}$ to 1.0 $mg \times L^{-1}$, and the limit of detection for MMP-1, MMP-2, and MMP-9 was 0.35, 0.62, and 1.10 $fg \times mL^{-1}$, respectively. The proposed biosensor was tested in a mouse model of NSCLC. The levels of the active form of MMP-1, MMP-2, and MMP-9 were determined in tumor and lung tissue extracts as well as in plasma collected after 30 and 50 days of transplantation of A549 cells. The data obtained from the measurements of the concentration of MMPs using the biosensor and the ELISA test indicated that all mean concentrations determined in these measurements were equal.

**Conclusion**

We developed an ultrasensitive method for simultaneous and selective determination of lung cancer biomarkers. Compared with conventional assay methods, our proposed device is a sensitive and selective nanoplatform for simultaneous detection of MMP-1, MMP-2, and MMP-9 in plasma and tissue and tumor extracts. We believe that this novel device may allow early and fast-track diagnosis as well as treatment monitoring based on MMP profiling in cancer patients.

### EACR23-0301

### Novel electrogravimetric biosensors for the ultrasensitive detection of plasma matrix metalloproteinase-2 considered a potential tumor biomarker

*M. Nisiewicz[1], A. Kowalczyk[2], A. Kasprzak[3], M. Bamburowicz-Klimkowska[4], A.M. Nowicka[1]*

*[1]University of Warsaw, Faculty of Chemistry, Warsaw, Poland*

*[2]University of Warsaw, Faculty of Chemistry, Warszawa, Poland*

*[3]Warsaw University of Technology, Faculty of Chemistry, Warsaw, Poland*

*[4]Medical University of Warsaw, Faculty of Pharmacy, Warsaw, Poland*

**Introduction**

Metalloproteinases (MMP-2) found in the extracellular matrix are enzymes that degrade extracellular proteins. Many diseases, including cancers, are associated with the overexpression and activity of these enzymes. In the human body, MMPs are involved in various processes, such as embryogenesis or bone formation and play an important role in neoplastic processes. As the neoplastic

© 2023 The Authors. Molecular Oncology is published by John Wiley & Sons Ltd on behalf of Federation of European Biochemical Societies disease progresses, the level of metalloproteinases in body fluids, as well as their activity, increases in different fashions. Their increased expression in the neoplastic infiltration is considered a valuable prognostic factor and allows determining the effectiveness of treatment in the course of the neoplastic disease. Due to the increased activity of MMPs, adjuvant treatment can be initiated in the intermediate stages of the disease.

**Material and Methods**

The developed sensors are costeffective, require a very less amount of reagents, and are time-saving. The detection of MMP-2 with using gravimetric immunosensor was based on antigene-antibody recognition. Whereas, the basis of the voltammetric sensor operation was the ability of MMP-2 to cleave glycine-leucine peptide bond. The three dimensional bioplatform of the sensors consisted of a cationic polyethyleneimine (PEI) polymer facilitating robust immobilization of the dipeptide labeled with redox probe (anthraquinone), or antibody molecules in appropriate density, which is crucial for recognition process. The detection process was performed using quartz crystal microbalance with dissipation and voltammetry.

**Results and Discussions**

We developed novel, simple gravimetric immunosensor and voltammetric sensor for the ultrasensitive detection of active matrix metalloproteinase-2 in plasma. The results showed that the developed sensors were characterized by high stability, wide analytical range (from 2.0 pg·mL$^{-1}$ to 5.0 mg·mL$^{-1}$), and low detection limit (*ca.* 10 fg·mL$^{-1}$). They also exhibited excellent efficiency in the determination of active MMP-2 in real samples, such as plasma. As a reference method of the determination of MMP-2 in plasma ELISA test were used. Almost perfect agreement between results obtained with using proposed biosensor and applied ELISA test clearly indicated that the as-developed biosensor can be successfully applied in plasma samples.

**Conclusion**

The developed biosensors exhibited excellent efficiency in the determination of active MMP-2 in real samples, such as blood plasma. The developed sensors may hold great promise for the early diagnosis of cancers.

### EACR23-0311
### Immunoregulatory role of extracellular vesicles in advanced non small cell lung cancer

*P. Ghidotti[1], C. Proto[2], M. Brambilla[2], D. Signorelli[3], F. Pontis[1], I. Petraroia[1], G. Sozzi[1], E. Jachetti[4], O. Fortunato[1]*

*[1]Fondazione IRCCS Istituto Nazionale dei Tumori, Epigenomic and Biomarkers of solid tumors Unit, Milan, Italy*

*[2]Fondazione IRCCS Istituto Nazionale dei Tumori, Thoracic Oncology Unit, Milan, Italy*

*[3]Grande Ospedale Metropolitano Niguarda-Milano, Niguarda Cancer Center, Milan, Italy*

*[4]Fondazione IRCCS Istituto Nazionale dei Tumori, Molecular Immunology Unit, Milan, Italy*

**Introduction**

Programmed death ligand-1 (PD-L1) expression on tumor cells is the only predictive biomarker used in clinical practice for Immune Checkpoint Inhibitors (ICIs) alone or in combination with chemotherapy in NSCLC.

Extracellular vesicles (EVs) are lipid bilayered particles described as biomarkers for cancer progression and as modulators of anticancer immune response. We previously described that high percentage of CD81+ EVs in patients with low PD-L1 expression treated with ICIs therapy alone correlated with a better Overall Survival (OS) and outcome. Here we aimed to evaluate EVs of advanced NSCLC patients with low PD-L1 expression in order to find biomarkers for combinational therapy and to investigate their role as regulator of anti-tumor immune response.

**Material and Methods**

Plasma-EVs were isolated using ultracentrifuge from Responder (R) and Non Responder (NR) advanced NSCLC patients treated with ICI plus chemotherapy. EVs characterization was performed following MISEV guidelines. Tetraspanins expression was assessed via super resolution microscopy. MiRNA expression within EVs was evaluated by using miRCURY LNA miRNA Focus Panel. EVs subpopulations were sorted through FACS or immuno capture beads. T cells were isolated and stimulated with CD3/CD28 beads in presence of different EV subpopulations and analyzed by flow cytometry.

**Results and Discussions**

R- and NR-EVs did not differ in terms of size and concentration. Despite we did not observe differences in CD9 expression in WB analysis, super resolution microscopy revealed a slight increase in CD9$^{+}$ EVs in NR samples. Class comparison analysis found 9 miRNAs differentially expressed between R and NR patients. Among them, hsa-mir-142-3p was the most expressed. Starting from bulk plasma EVs samples, we successfully isolated different EVs subpopulations based on their expression of CD9 and CD45. In functional experiments, CD9$^{-}$CD45$^{-}$ EVs were able to affect both the percentage and Granzyme B production of CD4$^{+}$ and CD8$^{+}$ T cells. No differences were observed in terms of T cells proliferation and immune checkpoint inhibitor markers expression.

**Conclusion**

Our preliminary results showed that R and NR EVs differ in terms of 9 different miRNAs and that potentially could be used as signature to discriminate the two groups. Proteomic analysis will improve the cargo characterization of patient-EVs and potentially find other biomarkers. Further studies are needed to elucidate the role of EV subpopulations in immune response modulation in lung cancer.

### EACR23-0340
### Protein signature of extracellular vesicles derived from tumors for breast cancer diagnosis

*J.Y. Hyon[1], K.A. Hyun[2], M.W. Kim[3], Y. Yang[1], H. Lee[1], S.I. Kim[3], H.I. Jung[2], Y.H. Chung[1]*

*[1]Korea Basic Science Institute, Research Center for Bioconvergence Analysis, Ochang, South Korea*

*[2]Yonsei University, School of Mechanical Engineering, Seoul, South Korea*

*[3]Yonsei University, College of Medicine, Seoul, South Korea*

**Introduction**

© 2023 The Authors. Molecular Oncology is published by John Wiley & Sons Ltd on behalf of Federation of European Biochemical Societies

Tumor-derived extracellular vesicles (tdEVs) have been emerging as potential biomarkers for cancer diagnosis because the tdEVs precisely reflect tumor cell alterations with significantly increased production. The proteomic profiling study of tdEVs represents a promising approach in a non-invasive manner to novel biomarker discovery for early detection and targeted therapy of cancer.

**Material and Methods**

Previously, we have developed a novel microfluidic chip for rapid and selective isolation of tdEVs. This microfluidic chip enables the selection of two types of EVs by using breast tumor-derived proteins (EpCAM & CD49f) within two minutes. In this study, we compared and analyzed proteomics of EVs isolated from several breast cancer cell lines by using a commercialized EV isolation kit based on the CD63 marker and our microfluidic chip which can selectively isolate tdEVs with the cancer cell-specific markers EpCAM & CD49f.

**Results and Discussions**

There were 3,700 proteins found in total. A Gene Ontology (GO) analysis of 474 unique proteins isolated from EVs by CD63 revealed that these proteins were involved in EV production and transport, including exocytosis and endocytosis. GO analysis revealed that 389 proteins expressed only in tdEVs were associated with signal transduction and RNA metabolism. This may be closely related to the production of miRNA in EV, which exhibits a distinct expression difference in breast cancer patients. Based on this result, we investigated the proteome of tdEVs extracted from the plasma of breast cancer patients and healthy individuals. As a result of comparative analysis, 179 out of 389 proteins unique to tdEVs were identified, and 103 proteins (57.5%) were confirmed to exhibit significant differences. In particular, five potential EV markers associated with the progression and relapse of breast cancer were significantly elevated in TNBC patient-isolated EVs compared to normal control samples. The best EV protein marker distinguished patients with early and advanced breast cancer from normal controls with 83% sensitivity, 80% specificity, and over 0.8 area under the receiver operating characteristic curve. Moreover, several other EV protein markers had comparable diagnostic utility.

**Conclusion**

Proposed proteomic biomarkers from tdEVs are anticipated to serve as a novel diagnostic and management tool for breast cancer patients in the future.

### EACR23-0354

### Transcriptomic signatures of circulating tumour cells predict outcome in Oral Squamous Cell Carcinoma patients.

*<u>A. Chauhan</u>[1], G. S Boora[1], S. Ghosh[2], M. Parsana[3], S. K Patra[2], R. K Verma[3], J. Bakshi[3], D. Chatterjee[4], R. Srinivasan[5], S. Ghoshal[6], A. Maitra[2], A. Pal[1]*

*[1]Post Graduate Institute of Medical Education and Research, Biochemistry, Chandigarh UT, India*

*[2]National Institute of Biomedical Genomics, National Institute of Biomedical Genomics, Kalyani- West Bengal, India*

*[3]Post Graduate Institute of Medical Education and Research, Otolaryngology, Chandigarh UT, India*

*[4]Post Graduate Institute of Medical Education and Research, Histopathology, Chandigarh UT, India*

*[5]Post Graduate Institute of Medical Education and Research, Cytopathology & Gynecological pathology, Chandigarh UT, India*

*[6]Post Graduate Institute of Medical Education and Research, Radiotherapy, Chandigarh UT, India*

**Introduction**

Oral Squamous Cell Carcinoma (OSCC) patients often have poor prognoses due to locoregional metastasis/relapse. CTCs (Circulating tumour cells), an essential element of “Liquid Biopsy”, are key factors in metastasis/relapse. However, molecular analysis of CTCs in management of OSCC patients remains minimal.

**Material and Methods**

We developed an in-house FACS-based strategy for CTC isolation, which overcomes the disadvantages of currently available strategies, like using a single CTC marker or size-based selection. Our approach combines the enrichment of cancer cells from blood by depleting CD45+ cells and FACS-based positive selection of CTCs using 4 markers, namely EpCAM, EGFR, CK and vimentin, labelled with a single fluorophore, i.e., FITC. The whole transcriptome of CTCs and paired primary tumours were analysed by RNAseq, followed by validation by qRT-PCR. Transcriptomic signatures of CTCs were correlated with prognosis.

**Results and Discussions**

CTCs were detected in 72% (50/69) of our patients. The CTC-positivity rates were similar in early (25/35;71%) and late-stage (25/34;74%) patients. We observed that CTCs express EpCAM, EGFR, CK and vimentin markers in various combinations in different patients, suggesting extensive phenotypic heterogeneity in CTCs. Patients with EpCAM-negative CTCs had worse OS (p=0.044*) than those with EpCAM-positive counterparts, suggesting the limitation of the EpCAM-based approach for CTC detection. Unsupervised hierarchical clustering of RNAseq data suggested CTCs are transcriptionally different from primary tumours. There was extensive transcriptomic heterogeneity in CTCs, while primary tumours were less heterogeneous. Various pathways like EMT, IL-6 JAK STAT3 signalling, UV response, inflammatory response, complement, and ECM receptor interaction were positively enriched in CTCs. CTC transcriptome data also stratified the patients in two different cohorts having a significant difference in OS (p=0.0004***) and DFS (p=0.0097***), indicating transcriptomic signatures in CTCs between patients is an important marker for prognostication. TNF-alpha signalling and KRAS-signalling pathways were enriched in CTCs with bad prognostic traits. Expression of CTSS, NAMPT and BCL2A1 in CTCs were validated to be associated with poor OS and DFS.

**Conclusion**

Here, we report that transcriptomic signatures of CTCs identified by a novel, cost-effective CTC isolation technique suitable for developing countries are associated with disease outcomes in OSCC patients.

### EACR23-0361

### Improved enrichment of circulating tumor cells from diagnostic leukapheresis product

*<u>M. Stevens</u>[1], A. Mentink[1], A. Nanou[1], F. Coumans[1],*

© 2023 The Authors. Molecular Oncology is published by John Wiley & Sons Ltd on behalf of Federation of European Biochemical Societies

K. Isebia[2], J. Kraan[2], P. Hamberg[3], J. Martens[2], L. Terstappen[1]
[1]University of Twente, Medical cell biophysics, Enschede, The Netherlands
[2]Erasmus MC, Medical Oncology, Rotterdam, The Netherlands
[3]Franciscus Hospital, Oncology, Rotterdam, The Netherlands

**Introduction**

The median number of circulating tumor cells (CTCs) detected in 7.5 mL of peripheral blood by CellSearch (PB-CS) in patients with metastatic prostate cancer is in the order of 1-10, which means many samples have insufficient tumor cells for comprehensive characterization. A significant increase in blood volume is obtained through Diagnostic LeukApheresis (DLA), however, only 2-3% of the DLA product can be processed per CellSearch test, limiting the gain in blood volume.

**Material and Methods**

Aliquots from DLA product consisting of $0.2 \cdot 10^9$ leukocytes were processed using CellSearch (DLA-CS). The Reduced Enrichment Reagent protocol (RER) was used to process $0.2 \cdot 10^9$ leukocyte aliquots with 10-fold less enrichment reagents than DLA-CS. For thirty samples from metastatic prostate cancer patients, the number of tumor cells and the total number of captured cells was determined using the CellTracks Analyzer. Additionally, for six DLA samples, a $1.0 \cdot 10^9$ leukocyte aliquot was processed (RER+), using 2-fold less enrichment reagents than DLA-CS.

**Results and Discussions**

No difference in tumor cell recovery was found between DLA-CS and RER methods (Wilcoxon Signed Ranks Test, p=0.953), with a median 2.7-fold reduction in leukocyte co-enrichment. Using $1.0 \cdot 10^9$ leukocyte aliquots a 4-fold increase in tumor cells compared to DLA-CS and a 24-fold increase compared to PB-CS was obtained.

**Conclusion**

Using 10-fold less CellSearch capture reagent, we processed standard leukapheresis aliquots with no loss in tumor cell recovery, while attaining a higher purity. Using this method, 26% of the total leukapheresis sample can be processed using the CellSearch reagents from a single test, enabling the obtainment of a sufficient number of CTC for tumor cell characterization in most metastatic prostate cancer patients.

### EACR23-0368

### Measuring antigen expression of cancer cell lines and circulating tumour cells

A. Mentink[1], K. Isebia[2], J. Kraan[2], L. Terstappen[1], M. Stevens[1]
[1]University of Twente, Medical cell biophysics, Enschede, The Netherlands
[2]Erasmus MC, Medical Oncology, Rotterdam, The Netherlands

**Introduction**

When evaluating EpCAM-based enrichment technologies for circulating tumour cells (CTCs), the cell lines used should closely resemble real CTCs, meaning the EpCAM expression of CTCs needs to be known, but also the EpCAM expression of cell lines at different institutions and times is important.

**Material and Methods**

As the number of CTCs in blood is low, we enriched CTCs through depletion of leukocytes from diagnostic leukapheresis products of 13 prostate cancer patients and measured EpCAM expression using quantitative flow cytometry. Antigen expression was compared between multiple institutions by measuring cultures from each institution. Capture efficiency was also measured for one of the used cell lines.

**Results and Discussions**

Results show CTCs derived from prostate cancer patients have a varying but relatively low EpCAM expression, with median expression per patient ranging from 35 to 89,534 (mean 24,993) molecules per cell. A large variation in the antigen expression of identical cell lines cultured at different institutions was found, resulting in recoveries when using the CellSearch system ranging from 12 up to 83% for the same cell line.

**Conclusion**

We conclude that large differences in capture efficiency can occur while using the same cell line. To closely resemble real CTCs, a cell line with a relatively low EpCAM expression should be used, and its expression should be monitored frequently.

### EACR23-0383

### Proof-of-mechanism study for a diagnostic probe compound generating D5-ethanol as an on-breath reporter molecule for lung cancer – Evolution phase 1

M. Ferreira Leal[1], C. Frederick Labuschagne[1], R. Smith[2], M. P van der Schee[1], B. Boyle[3], M. Allsworth[3], P. Crosbie[4], R. Rintoul[5]
[1]Owlstone Medical, Clinical and Translational Science, Cambridge, United Kingdom
[2]Owlstone Medical, Analytical Science, Cambridge, United Kingdom
[3]Owlstone Medical, Leadership Team, Cambridge, United Kingdom
[4]Hospital of South Manchester, Respiratory Medicine, Wythenshawe, United Kingdom
[5]Papworth Hospital, Thoracic Oncology, Cambridge, United Kingdom

**Introduction**

Analysis of volatile metabolites in breath represents an attractive potential diagnostic modality for lung cancer. One of the key challenges for the realisation of this potential is to optimise the signal to noise ratio in the fundamentally complex matrix that breath is. Owlstone Medical has funded this proof-of-mechanism study to evaluate whether administration of a probe compound, specific to tumour-associated extracellular β-glucuronidase, results in the production of a unique exogenous volatile organic compound (EVOC) on breath. Such an EVOC probe could prove of significant diagnostic value as a breath-based test for lung cancer.

**Material and Methods**

We developed D5-ethyl-βD-glucuronide (D5-EtGlu) as a hydrophilic substrate probe that upon hydrolysis by β-glucuronidase releases D5-ethanol as a unique volatile reporter. To establish proof of mechanism, two complementary approaches were used: Firstly, samples

© 2023 The Authors. Molecular Oncology is published by John Wiley & Sons Ltd on behalf of Federation of European Biochemical Societies from primary lung tumors, lymph node metastases and otherwise normal lung tissue were immunostained to evaluate the presence of β-glucuronidase. Secondly, as part of a phase 1 single ascending dose trial, D5-ethyl-βD-glucuronide was administered to 43 individuals to establish safety and evaluate EVOC levels on breath using various breath collection and analytical approaches.

**Results and Discussions**

Immunostaining of 140 lung resection specimens showed elevated levels of extracellular β-glucuronidase in 88% of stage 1 non-small cell lung cancers (48), 83% of stage 2 (18), 95% of stage 3 (20). For adjacent non-malignant tissue in lung cancer subjects (38) and non-malignant lung tissue resection in controls (16), staining was restricted to intracellular sources, particularly within macrophages. Intravenous administration of the probe to subjects with lung cancer and controls revealed an excellent safety profile. The cleavage product D5-ethanol could be detected on breath in a subset of participants, and this showed a relationship with the volume of breath collected.

**Conclusion**

This study demonstrates proof of mechanism for the in human cleavage of the volatile reporter molecule D5-ethanol from D5-ethyl-βD-glucuronide. Such an EVOC probe approach is attractive as it has the potential to optimise the signal to noise ratio of a breath test. These results provide a promising foundation for a phase 2 dose-finding study designed to explore diagnostic performance of this innovative breath test approach for lung cancer.

### EACR23-0399
### Characterization of the immune and genomic profile of a large cohort of advanced KRAS-driven non-small cell lung cancer

*P. Gausà-Busquets[1,2,3], E. Marin[1,2,3], S. Hijazo-Pechero[3], A. Arcocha[1,2], R. Reyes[1,2], C. Teixido[2,3,4], N. Reguart[1,2,3]*

*[1]Hospital Clínic de Barcelona, Department of Medical Oncology- Thoracic Oncology Unit , Barcelona, Spain*

*[2]Hospital Clínic de Barcelona, Unitat Funcional de Tumors Toràcics, Barcelona, Spain*

*[3]Institut d'Investigacions Biomèdiques August Pi i Sunyer I DIBAPS, Translational Genomics and Targeted Therapeutics in Solid Tumors, Barcelona, Spain*

*[4]Hospital Clínic de Barcelona, Department of Pathology, Barcelona, Spain*

**Introduction**

*KRAS* is the most frequent oncogene in non-squamous non-small cell lung cancer (NSCLC). The recent development of KRAS inhibitors has opened the possibility of a new targeted therapy for a large subset of patients (pts). Thus, the improvement of patient selection and the definition of rational combinations for these pts have become an important clinical need.

To gain insights into potential predictors of therapy response, we conducted a study examining the genomic co-alterations, PD-L1 expression, and prognosis in samples from *KRAS*-driven NSCLC pts.

**Material and Methods**

Formalin-fixed paraffin-embedded tissue and blood samples were collected from 173 pts with advanced *KRAS*-driven NSCLC. Different next-generation sequencing (NGS) techniques were used to detect genomic co-alterations in solid samples, including Oncomine (ThermoFisher) and nCounter (Nanostring). Guardant360 CDx (Guardant Health) and InVisionFirst®-Lung (Inivata) NGS tests were used for blood samples. PD-L1 expression was evaluated using a 22C3 pharmDx assay clone (Agilent Technologies) and results were reported as the tumor proportion score (TPS) categorized as negative (<1%), low (1-49%), or high (≥50%).

**Results and Discussions**

*KRAS* mutations were identified in codons G12 (88%), Q61 (6%), G13 (5%), and A146 (<1%), with G12C (49%) being the most common subtype followed by G12V (19%). Co-alterations were detected in 61% of samples, with the most frequent ones observed in tumor suppressor genes *TP53* (39%) and *STK11* (20%). PD-L1 expression was detected in 68% of samples; with 31% low, 37% high, and 32% negative TPS. PD-L1 expression did not differ significantly when stratified by *KRAS* mutation.

Overall survival (OS) was evaluated in 171/173 pts. Multivariate analysis revealed that pts with *KRAS* G13 or G12C mutations tended to have worse OS (HR2.4,p=0.053, and HR1.6,p=0.07, respectively). Interestingly, pts with *STK11* co-mutation were associated with a 90% decrease in OS (HR1.9,p=0.01). Furthermore, pts treated with immunotherapy (IO) had better OS than those receiving chemotherapy (HR0.4,p=0.002). PD-L1 expression did not appear to impact OS.

**Conclusion**

Our study provides valuable insights into the molecular and clinical characteristics of *KRAS*-driven NSCLC and highlights the potential benefits of IO in this population. *KRAS* is a complex genetic mutation associated with heterogeneity in PD-L1 expression and co-alterations, which could serve as biomarkers for differential outcomes. *STK11* co-mutation appears to be a poor prognostic biomarker in this context.

### EACR23-0414
### The miRvana of the pleural effusion. Can miRNAs provide information about the progression of a malignant pleural effusion and enable us to discriminate between benign and malignant conditions?

*M. Pont Gòdia[1], M. Marqués[1], I. Hidalgo[1], M.A. Sorolla[1], E. Parisi[1], P. Sancho[1,2], A. Salud[1,3], A. Sorolla[1], J.M. Porcel[1,2]*

*[1]Biomedical Research Institute of Lleida, Medicine, Lleida, Spain*

*[2]Arnau de Vilanova University Hospital, Department of Internal Medicine - Pleural Medicine Unit, Lleida, Spain*

*[3]Arnau de Vilanova University Hospital, Oncology Department, Lleida, Spain*

**Introduction**

Malignant pleural effusion (MPE) is a serious pathologic condition usually caused by the metastatic growth of an existing primary tumour. It is associated with poor prognosis; its treatment is currently palliative, and the

© 2023 The Authors. Molecular Oncology is published by John Wiley & Sons Ltd on behalf of Federation of European Biochemical Societies diagnostic sensitivity of pleural fluid (PF) analyses is low to moderate.
In this study, the crosstalk between stromal and tumour cells *in vitro* has been investigated to discover factors present in the PF which could mediate an MPE formation. Overall, this project aimed to decipher the biological effects of PFs and their miRNA content from patients with benign and MPEs on pleural mesothelial cells in vitro. In addition, we intended to identify a miRNA signature able to highly discriminate between benign and MPEs.
**Material and Methods**
The MeT-5A cells, a normal mesothelial cell line, was exposed to different pleural fluids to study their biological effects. For this, benign and malignant PFs samples were collected at the Pleural Unit of Hospital Arnau de Vilanova de Lleida (HUAV) and processed.
On the one hand, Met5A cells were treated with 10% of PF in volume and different functional assays such as cell viability, cell proliferation, cell migration assays, and Western Blot were performed.
On the other hand, to study the effect of the miRNA of these PFs, on the MeT-5A cells, cells were treated with the previously extracted miRNA fraction, equivalent to 10% of PF and subjected to the same functional assays.
Finally, exosome-miRNA Sequencing through next-generation sequencing technology coupled with bioinformatics analysis was performed to profile the miRNA content in the PF samples.
**Results and Discussions**
Treatment of Met-5A cells with malignant PFs increased cell viability, cell proliferation, cell migration, and activated different survival signalling pathways, e.g. AKT and mitogen-activated protein kinase pathways. In addition, we identified differentially expressed miRNAs between benign and malignant PFs that could be responsible for those changes. Consistently, the bioinformatic analysis revealed an enrichment of migration-related processes with the discovered miRNAs. Such a miRNA signature had a high discriminative potential between benign and malignant PFs, being able to reclassify all the MPEs with a false-negative cytological examination in our study.
**Conclusion**
Our work adds valuable knowledge about the molecular processes underlying an MPE and opens the possibility of using certain miRNA signatures for diagnostic purposes.

### EACR23-0415
### Association of circulating markers of Gut Barrier Integrity and Inflammation with Pancreatic Ductal Adenocarcinoma

*N. Daniel[1], P. Souček[2], S. Susova[3], D. Hughes[1]*
*[1]Conway Institute- School of Biomedical and Biomolecular Sciences- University College Dublin, Cancer Biology and Therapeutics Laboratory, Dublin, Ireland*
*[2]Biomedical Centre- Faculty of Medicine in Pilsen- Charles University-, Laboratory of Pharmacogenomics, Pilsen, Czech Republic*
*[3]National Institute of Public Health- Czech Republic, Toxicogenomics Unit, Prague, Czech Republic*
**Introduction**
Pancreatic ductal adenocarcinoma (PDAC) has a five-year survival rate of 10%. Disturbances to normal gut microbiota (dysbiosis) and resultant reductions in gut barrier integrity are increasingly implicated in the development of cancers of the digestive system. Intestinal fatty acid binding protein (iFABP) leaks from enterocytes and enters circulation following gut barrier damage. Increased levels of toll like receptor 4 (TLR4) have been associated with intestinal tight junction permeability. Zonula occludens 1 (ZO-1) links tight junction proteins with the cytoskeleton and constitutes a marker of tight junction integrity. Calprotectin is a marker of inflammation. Increased serum concentrations of inflammatory cytokines including IFNy, IL6, IL10, and TNFα have been associated with impaired gut integrity and functioning. We investigated the association between gut barrier damage and PDAC development.
**Material and Methods**
Concentrations of circulating inflammatory cytokines and gut barrier protein biomarkers were assessed in serum from a PDAC case (n=90) -control (n=60) cohort from the Czech Republic. TLR4, ZO1 and iFABP were assessed by ELISA. Calprotectin and IFNy, IL1β, IL2, IL4, IL6, IL8, IL10, IL-12p-70, IL13 and TNFα were assessed by mesoscale assay (Mesocale Discovery, Rockville, Md, United States). Protein and inflammatory biomarkers were also assessed by ELISA and mesoscale assay respectively in a second Czech PDAC case cohort (n=45).
**Results and Discussions**
Markers indicating impaired gut barrier function were elevated in PDAC cases of the first cohort relative to controls; iFABP (p=0.024), TLR4 (p=0.03) and ZO1 (p=0.008). Calprotectin was also elevated in PDAC cases relative to controls (p=0.014). Five of the ten assessed inflammatory cytokines were elevated in PDAC cases relative to controls; IFNy (p=0.025), IL10 (p=0.002), IL6 (p=0.001), IL8 (p=0.004) and TNFα (p=0.045). Calprotectin (p=0.019), iFABP (p=0.016), ZO1 (p=0.053), IL10 (p=0.004), IL6 (p=0.026), IL8 (p=0.008) were elevated in the second PDAC cohort relative to controls while no difference was detected for TLR4, IFNy or TNFα.
**Conclusion**
These findings suggest that a reduction in gut barrier integrity and attendant intestinal/systemic inflammation is associated with PDAC development. Next steps will be to assess correlations between these data, tumour microbiome profiles and survival outcomes.

### EACR23-0443
### Complementarity of miR-203a-3p and BANCR sequences may influence thyroid capsule invasion in papillary thyroid carcinoma

*T. Išić Denčić[1], S. Stojanović[1], I. Đorić[1], J. Janković Miljuš[1], V. Živaljević[2], M. Milinković[3], S. Šelemetjev[1]*
*[1]Institute for the Application of Nuclear Energy- INEP- University of Belgrade, Department for Endocrinology and Radioimmunology, Belgrade, Serbia*
*[2]Clinical Center of Serbia, Clinic for Endocrine Surgery, Belgrade, Serbia*

© 2023 The Authors. Molecular Oncology is published by John Wiley & Sons Ltd on behalf of Federation of European Biochemical Societies

[3]Clinical Center of Serbia, Department of Pathology, Belgrade, Serbia

**Introduction**

Thyroid neoplasms are characterized by a great diversity of histopathological features, which are the consequence of a great diversity of their genetic and epigenetic background. Long non-coding RNAs (lnRNAs) and microRNAs (miRs) are a class of molecules known to be important regulators of thyroid cancer progression and development. Furthermore, lnRNAs may compete with other transcripts for shared miRs or bind miRs in a complementary manner.

**Material and Methods**

MiR-203a-3p levels were determined in 10 cases of nodular goiter (NG), in 12 follicular thyroid adenomas (FTA), 76 papillary thyroid carcinomas (PTC) and matched nonmalignant thyroid epithelial tissues (NMT) by quantitative RT-PCR. BANCR levels were determined in the same group of PTC patients. The results were correlated with the clinicopathological characteristics of the patients. The fold change of the tested molecules was calculated as the ratio of relative expression in thyroid neoplasia versus relative expression in paired NMT.

**Results and Discussions**

The relative expression of miR-203a-3p did not vary between diverse thyroid tissue samples (total NMT 0.004 [0.002–0.007], NG 0.006 [0.001–0.023], FTA 0.001 [0.0003–0.002], PTC 0.002 [0.001–0.006]). However, the fold change of miR-203a-3p depended on the thyroid neoplasm type (NG 3.02 [0.074–7.85], PTC 0.658 [0.123–2.032], FTA 0.137 [0.110–0.810]), ($p<0.05$). The relative expression of BANCR in NMT was 0.167 [0.070–1.504] and in PTC was 0.045 [0.070–0.288]. BANCR was down-regulated in PTC, its fold change was 0.223 [0.041–1.053]. Bioinformatic analysis and model prediction revealed that miR-203a-3p might interact with BANCR in a complementary manner. In PTC, individually high relative expression of miR-203a-3p and BANCR correlated with the presence of extrathyroid invasion ($p<0.05$). However, there was no correlation between mutually high expression of miR-203a-3p and BANCR and the presence of invasion of the thyroid capsule ($p<0.05$).

**Conclusion**

Down-regulation of miR-203a-3p indicates the presence of thyroid tumor (PTC or FTA), whereas its up-regulation indicates NG. High individual expression of miR-203a-3p and BANCR correlates with extrathyroid invasion in PTC, while their co-occurrence does not. Therefore, BANCR might serve as a sponge for miR-203a-3p and influence the aggressiveness of PTC.

### EACR23-0461
### Prostate Specific Membrane Antigen (PSMA) expression on Circulating Tumor Cells (CTC) and tumor-derived Extracellular Vesicles (tdEV) from metastatic Castration Naïve Prostate Cancer (mCNPC) patients

*E. Dathathri[1], K. Isebia[2], A. Nanou[1], F. Abali[1], M. Stevens[1], J. Kraan[2], M. Lolkema[2], L. Terstappen[1], R. Bansal[1], J. Martens[2]*

*[1]University of Twente, Medical Cell Biophysics, Enschede, The Netherlands*

*[2]Erasmus MC Cancer Institute, Department of Medical Oncology, Rotterdam, The Netherlands*

**Introduction**

PSMA has a variety of clinical implications in the management of prostate cancer and has now emerged as a treatment target. PSMA expression on cancer cells is essential for PSMA-targeted applications to be effective. In this study, we investigated the PSMA expression on individual CTCs and tdEVs obtained from Diagnostic Leukapheresis (DLA) of mCNPC patients to determine inter- and intra-patient heterogeneity in PSMA expression and investigate whether tdEVs have potential to be used as a surrogate biomarker for CTCs.

**Material and Methods**

PSMA expression was determined in 25 mCNPC patients with ≥3 CTCs in 7.5mL of peripheral blood (PB) as per standard CellSearch assessment, and additionally DLA was performed. DLA aliquots of 2mL containing $2x10^8$ mononuclear cells were immunomagnetically enriched with the CellSearch system and stained with CK-FITC, PSMA-PE, CD45-APC, and DNA-DAPI. The samples were scanned using CellTracks Analyzer II and images were analyzed using the "Full Detection" processor in the open-source Automated CTC Classification Enumeration and PhenoTyping software. Gates were set to identify CTCs and tdEVs that are CK+/PSMA+, CK+/PSMA-, and CK-/PSMA+.

**Results and Discussions**

We have analyzed CTC and tdEV counts along with their CK and/or PSMA expression from the DLA of 25 mCNPC patients. The CTC counts ranged from 0-1034 (median 48) and the tdEV counts ranged from 17-6227 (median 333). The tdEV counts were 9 (± 7.93) fold higher than CTC counts in the DLA. PSMA expression was detected in >25%, >50%, and >75% of CTCs in 88%, 56%, and 24% of patients respectively. PSMA was detected in >25%, >50%, and >75% of tdEVs in 80%, 64%, and 36% of patients respectively. The positivity of CTCs and tdEVs for PSMA showed a positive correlation (Spearman's rho = 0.78, $p<0.001$). Heterogeneity in PSMA expression was observed in CTCs irrespective of CK positivity. The CK-,PSMA+ CTCs ranged from 0-100% (median 28) and molecular confirmation that these CTCs are indeed cancer cells is currently being investigated.

**Conclusion**

Heterogeneity in PSMA positivity between patients was observed for CTCs, and tdEVs, and results were concordant between CTCs and tdEVs. Considering this high concordance, both could help to stratify patients who might benefit from PSMA-targeted therapy. tdEVs might next to CTCs also serve for an accurate determination of PSMA expression in PB. Our current dataset will be extended with PB samples and the added value of analysis of PSMA heterogeneity in CTCs and tdEVs in PB versus DLA will be assessed.

### EACR23-0467
### Modelling drug response with multi-omics using machine learning in a PDTC high-throughput drug-screening.

*R. Masina[1], D. Guerrero Romero[1], E. Esmaeilishirazifard[1], G. Lerda[1], D. Georgopolou[1], A. Shea[1], W. Greenwood[1], R. Manzano[1], S.F. Chin[1], S. Cosulich[2], C. Crafter[2],*

© 2023 The Authors. Molecular Oncology is published by John Wiley & Sons Ltd on behalf of Federation of European Biochemical Societies

M. Lawson[2], A. Bruna[1], M. Callari[3], O. Rueda[4], C. Caldas[1]
[1]CRUK Cambridge Institute,
Caldas Laboratory - Breast Cancer Functional Genomics, Cambridge, United Kingdom
[2]AstraZeneca,
Bioscience- Oncology- Early Oncology R&D, Cambridge, United Kingdom
[3]Michelangelo Foundation, Department of Oncology, Milan, Italy
[4]MRC Biostatistics Unit, Statistical Omics, Cambridge, United Kingdom

**Introduction**

Intra-tumoral heterogeneity represents a major challenge for the effective implementation of precision oncology and underlies the modest predictive value of biomarker discovery based on cancer cell lines. Previous work by our group has shown that breast cancer patient-derived xenografts (PDTXs) and PDTX-derived tumour cells (PDTCs) robustly recapitulate intra-tumoral heterogeneity. It also developed a platform for high-throughput drug screening in PDTCs. This study aims to integrate the baseline molecular landscape of tumours with response data from breast cancer PDTC high-throughput drug screening using machine learning to characterize pharmaco-omic associations.

**Material and Methods**

PDTCs from 34 different models were treated ex vivo with 67 different compounds at 7 different concentrations. The compounds were either approved cancer treatments or drugs targeting key cancer pathways. Viability was measured by Cell-Titer-Glo (CTG) after 7 days and area under the dose-response curve (AUC) was calculated to determine therapy effectiveness. Shallow whole genome sequencing, whole exome sequencing, RNA sequencing, methylation-sequencing, protein mass cytometry and reverse phase protein array profiles were obtained from the same models. A defined set of metrics were computed and integrated to build a predictive model of drug-response using machine learning characterizing the landscape of pharmaco-omic associations.

**Results and Discussions**

Compounds were filtered based on the response pattern across the models. 67/67 drugs tested had at least 2 models showing response (AUC>0.2) and at least 2 models showing resistance (AUC<0.2). A wide range of low- and high-level features were computed. We show that several molecular features such as tumour mutational burden and chromosomal instability are associated with in vitro drug response in high-throughput drug screening, and that results recapitulated known mechanisms of sensitivity and resistance while identifying multiple new candidate biomarkers. In addition, we show that the integration of multi-omic features using machine learning achieves superior predictive performance after cross-validation and suggests novel biomarkers.

**Conclusion**

PDTXs and PDTCs are a powerful platform in pre-clinical cancer research. Here, we have demonstrated the potential of coupling high-throughput drug screening with multi-omic profiling for novel biomarker discovery and drug development.

### EACR23-0485
### Biomarker potential of the transcript PHF19-207 in colon cancer

S. Ignjatovic[1], D. Pavlovic[1], T. Babic[1], S. Dragicevic[1], A. Nikolic[1]
[1]Institute of Molecular Genetics and Genetic Engineering, Laboratory for Molecular Biology, Belgrade, Serbia

**Introduction**

Recent data from a comprehensive pan-cancer transcriptome analysis demonstrated differential activity of two alternative *PHF19* gene promoters in malignant vs. non-malignant gut mucosa. The promoter found to be up-regulated in colon and rectal cancer gives rise to the transcript PHF19-207. This finding has pointed to the biomarker potential and possible tumor-promoting role of this transcript. Our study aimed to evaluate the expression of PHF19-207 in colon cancer, as well as to investigate its potential function using *in silico* tools.

**Material and Methods**

Immortalized colonic epithelial cell line isolated from healthy tissue (HCEC-1CT) and a set of colon cancer cell lines (Caco-2, HCT116, HT29, DLD-1, SW480 and SW620) were used. The expression analysis of PHF19-207 transcript was performed using qPCR. For *in silico* analysis, Coding Potential Calculator tool and AnnoLnc tool were used.

**Results and Discussions**

The expression analysis demonstrated that the expression of PHF19-207 was increased in all malignant cell lines in comparison to the non-malignant cell line HCEC-1CT (2 to 5-fold). Also, the more prominent increase was observed in the cell lines originating from later stages of colon tumors. Based on Coding Potential Calculator tool, PHF19-207 was classified as non-coding, with coding probability of 0.2. The AnnoLnc tool indicates its downregulation of PHF19-207 in normal colon tissue and its upregulation in cancer tissue samples. The same tool indicates nuclear localization of the PHF19-207 transcripts.

**Conclusion**

The results of expression analysis confirm potential of PHF19-207 as diagnostic biomarker, while the results of *in silico* analysis suggest that this transcript may be a lncRNA with role in gene expression regulation. Further research on this RNA molecule should aim for functional studies to investigate its role in colon carcinogenesis.

### EACR23-0495
### An activity-based biomarker for identifying homologous recombination deficiencies across cancer types in real-time

H.Y. Chi[1], C.Y. Lee[1], W.F. Cheng[2], P.H. Lin[3], Y.L. Chen[2], S.H. Huang[1], K.H. Lei[1], K.Y. Chang[1], M.Y. Ko[1]
[1]National Taiwan University,
Institute of Biochemical Sciences, Taipei, Taiwan
[2]National Taiwan University Hospital,
Department of Obstetrics & Gynecology, Taipei, Taiwan
[3]National Taiwan University Hospital,
Department of Medical Genetics, Taipei, Taiwan

**Introduction**

Homologous recombination (HR)-mediated DNA repair is a prerequisite for maintaining genome stability. Cancer cells displaying HR deficiency (HRD) are selectively

© 2023 The Authors. Molecular Oncology is published by John Wiley & Sons Ltd on behalf of Federation of European Biochemical Societies eliminated by poly(ADP-ribose) polymerase inhibitors (PARPi), which benefit survival rates among breast, ovarian, pancreatic, and prostate cancer patients. To date, sequencing pathogenic mutations in HR-associated *BRCA1/2* genes and analyzing genome instability are used as clinical biomarkers for PARPi therapy. However, these genetic tests cannot truly reflect HR status in real-time.

**Material and Methods**

We have developed an activity-based functional assay that directly quantifies real-time cellular HR activity.

**Results and Discussions**

Our functional assay establishes a universal activity threshold for identifying HRD among colon, ovarian, and triple-negative breast cancers, validated by PARPi sensitivity and BRCA status. Notably, this fluorescence biomarker can be applied to primary ovarian cancer cells from patients to reflect their level of HRD. Thus, our work demonstrates a new predictive functional biomarker that can be deployed alongside PARPi.

**Conclusion**

Our work shows the clinical applicability of our activity-based biomarker in primary ovarian cancer cells. Apart from its correlation with *ex vivo* PARPi sensitivity, the HR status detected by our activity-based analysis is also associated with patients' clinical responses to platinum-based first-line treatment.

### EACR23-0514

### Preliminary results of the THRuST trial: early identification of recurrence in patients with resectable colon cancer by longitudinally following a personalized molecular signature from a blood test

*M. Macagno[1], A. Kudriavtsev[2], P. Fernandez Calotti[3], N. Saoudi-Gonzalez[4], A. Mellano[5], G. Crisafulli[6], V. Pessei[5], M. Ychou[7], A. Vivancos[4], A. Sapino[5], C. Marchiò[1], D. Azuara[3], R. Salazar[3], C. Santos[3], E. Elez[4], F. Di Nicolantonio[6], A. Thierry[8]*

*[1]Fondazione del Piemonte per l'Oncologia, Department of Oncology, Candiolo, Italy*

*[2]IRCM- Institute of Research in Oncology of Montpellier, Inserm U1194, Montpellier, France*

*[3]Institut Català d'Oncologia- IDIBELL, L'Hospitalet de Llobregat, Barcelona, Spain*

*[4]Vall d'Hebron Institute of Oncology VHIO, Gastrointestinal Cancer Unit, Barcelona, Spain*

*[5]Fondazione Piemontese per l'Oncologia, Department of Oncology, Candiolo, Italy*

*[6]University of Torino, Department of Oncology, Torino, Italy*

*[7]ICM- Regional Institute of Cancer of Montpellier, University of Montpellier, Montpellier, France*

*[8]IRCM- Institute of Research in Oncology of Montpellier, Department of Medical Oncology, Montpellier, France*

**Introduction**

More than 50% colon cancer (CC) patients are diagnosed when the disease is amenable to surgical resection leading potentially to curative treatment. Adjuvant treatment is currently administered to all stage III and high-risk stage II patients. Tumors shed cell free DNA (cfDNA) into the blood stream. The analysis of tumor informed DNA in plasma samples could enable precision oncology in CC and identify patients with micrometastatic disease who need more aggressive adjuvant therapies, as well as those who are cured by surgery alone and may be spared the toxicity of prolonged or combinatorial chemotherapy regimens. There is no biomarkers for revealing recurrence of the disease at an early stage.

**Material and Methods**

Thrust is a prospective, multicenter and blinded study on stage II and stage III CC patients (https://www.transcanfp7.eu/index.php/abstract/thrust.html ). A molecular signature of selected genetic alterations is defined for each CC patient based on next-generation sequencing of tumor exome at diagnosis. Individual genetic signatures are assessed by an advanced qPCR based method (IntPlex), including quantitative cfDNA parameters such as the total concentration of cfDNA, the mutant allele fractions of cfDNA, and the cfDNA fragmentation index.

**Results and Discussions**

Out of the 293 CC patients initially screened across four centers, 88 were stage III. Whole exome or target panel sequencing was performed on 60 primary tumor tissue samples. Using a cut off of 10% mutant reads (Variant allele frequencies) to discriminate the more likely subclonal from the clonal variants, a total of 6647 unique genetic variants were identified. We found 4063 genes affected. We identified recurrent alterations in the RAS-MAPK, PI3K, WNT, TGF-b and p53 pathways, well-known to be deregulated in CC. These analyses allowed the design of patient specific probes to track molecular signatures in the blood of CC patients.

**Conclusion**

We developed a wet and computational NGS workflow to identify relevant molecular alterations in CC tissues. We achieved a comprehensive view of tumor genomic landscape in a set of stage III tissue samples. We managed to select patient specific genomic variants that are prospectively used to track ctDNA in the corresponding plasma samples.

This ERA-NET TRANSCAN-2 project has received funding from the European Union's Horizon 2020 research and innovation programme under grant agreement No 643638.

### EACR23-0528

### Exploring Changes in Biochemical, Tumor and Cytokine Markers in Early versus Advanced Breast Cancer Stages: Towards Identifying the Most Probable Blood Based Biomarkers for Early Cancer Detection

*M. Masih[1], S. Agarwal[2], P.K. Gautam[2]*

*[1]All India Institute of Medical Sciences AIIMS, Biochemistry, New Delhi, India*

*[2]All India Institute of Medical Sciences, Biochemistry, New Delhi, India*

**Introduction**

Early detection of cancer is the need of the hour. Researchers are now starting to realize the importance of non-invasive detection of cancer in its earliest stages. Tumor cells are known to secrete certain chemicals into the blood stream which stimulate differentiation and self-

© 2023 The Authors. Molecular Oncology is published by John Wiley & Sons Ltd on behalf of Federation of European Biochemical Societies proliferation of various cells in the tumor microenvironment. These cancer associated chemicals are produced by different cells present in the tumor microenvironment at a particular stage of the disease progression which may be unique and specific. The aim of this study is to detect the serum levels of these different tumor antigens, cytokines, and biochemicals in the different stages of breast cancer and analyze their pattern/trend.

**Material and Methods**

Around 5 ml whole blood was collected from healthy controls, treatment naive early stage and advanced stage breast cancer patients and further serum and plasma were isolated and stored at -80°C. The study was divided into three parts. The first part included analysis of tumour markers and protiens like Cancer Antigen 27-29 (CA 27-29), Carcinoembryonic Antigen (CEA), Aldo-Keto Reductase1B10 protein (AKR1B10) by ELISA and glutamine by LCMS. The second part included estimation of serum cytokines by high sensitivity ELISA for CXCL8, CXCL9, CXCL 12, Interleukin-6, TNF-α and TGF-β. Thirdly biochemical parameters like, potassium, calcium, phosphate, uric acid was estimated.

**Results and Discussions**

Results obtained were analyzed and compared statistically which showed that AKR1B10, CEA and CA27-29 were significantly higher in metastatic group as compared to the control group. CA27-29 was also significantly higher in locally advanced group. Serum glutamine levels were higher in early group than the controls while no significant difference was observed between advanced groups. Higher cytokine levels of CXCL8, CXCL9, CXCL12, Interleukin-6 and TNF-α in early and advanced stage of breast cancer patient's serum as compared to control group was seen, while decreased level of TGF-β was seen in early and advanced stage as compared to control group.

**Conclusion**

Our results suggested increase in specific parameters during early stage of disease progression which may correlate with the inflammatory tumor microenvironment cells secreting them. We concluded that, knowing the trend of such specific cytokines and proteins in different stages of the disease could provide new insights into early-stage detection, thereby planning early intervention and therapeutic strategies for patients with breast cancer.

### EACR23-0548
### Elucidation of Blood-Based Biomarkers for Prostate Cancer Using Proteomic Approach

*S. Agarwal[1], M. Masih[1], P.K. Gautam[1]*
*[1]All India Institute of Medical Sciences, Biochemistry, Delhi, India*

**Introduction**

The detection of cancer in its early stage is considered one of the holy grails of patient care in the fight against the disease. Therefore, aim of this study was to identify blood-based biomarkers for prostate cancer that was specific for corresponding to stage progression of the disease.

**Material and Methods**

In this study, blood was collected from patient and healthy subjects. Serum samples were separated from blood and then stored at −80°C for further analysis. We performed serum protein profiling on patients diagnosed with prostate cancer and healthy subjects. A proteomic approach based on LC-MS was used to identify serum biomarker for prostate cancer detection. Subsequently, we validated the cancer related biomarkers using specific antibodies. We also analysed the level of prostate cancer associated genes such as Caveolin -1, CASP 3, PTN, PSMA and PSCA using RT-PCR. RNA extraction from PBMCs was performed by RNAiso plus reagent. The quantity of RNA was confirmed by Nano drop. For RT-PCR, 1µg RNA was reverse transcribed into complementary DNA by cDNA synthesis kit based on the manufacturer's protocols. Finally, the cDNA samples were kept at -80°C until used for PCR.

**Results and Discussions**

Most patients had adenocarcinoma. They were assessed and classified based on Gleason score and PSA level. We identified more than 200 proteins by MS based proteomic analysis. We have found the differential expression of protein using Western blot. The gene expression level of mRNA biomarkers in blood such as Caveolin -1, CASP 3, PTN, PSMA and PSCA were significantly elevated in prostate cancer patients as compared to control.

**Conclusion**

Our results suggested that prostate cancer associated genes could serve as a biomarker. Our finding indicates that the analyses of blood based biomarkers could improve the accuracy of non-invasive detection of early-stage prostate cancer. We will continue to explore more biomarkers in prostate cancer detection. The future goal is to develop portable, cost-efficient and user-friendly point-of-care diagnostic device for early-stage cancer biomarkers.

### EACR23-0554
### Afadin (AF6) and SIPA1 coexpression in breast cancer and the prognostic implication

*Y. Yu[1], T. Martin[1], W. Jiang[1], E. Davies[2]*
*[1]Cardiff University School of Medicine, Cardiff China Medical Research Collaborative Division of Cancer and Genetics, Cardiff, United Kingdom*
*[2]University Llandough Hospital, Wales Breast Centre, Cardiff, United Kingdom*

**Introduction**

AF6 (Afadin or MLL4, Myeloid/Lymphoid Or Mixed-Lineage Leukemia; Translocated To 4) is a protein suspected to regulate the formation of adherens junctions and possibly in the homotypic and heterotypic cell-cell adhesion. Although little is known as to how AF6 is regulated intracellularly, it has been shown that SIPA1 (Signal-Induced Proliferation-Associated 1) is a prospective regulator for the action of AF6 in the cells including cancer cells. Here, we explored the expression and co-expression pattern of AF6 and SIPA1 in human breast cancer and their potential clinical value.

**Material and Methods**

AF6 and SIPA transcripts were determined breast cancer tissues (n=127) together with normal mammary tissues. The expression patterns were examined against the clinical, pathological and clinical outcome of the patients, as well as the hormone receptor status of the patients

**Results and Discussions**

© 2023 The Authors. Molecular Oncology is published by John Wiley & Sons Ltd on behalf of Federation of European Biochemical Societies

In breast cancer tissues, AF6 significantly correlated with SIPA1 (r=0.32, p=0.002). In contrast, no correlation was found between the two in normal mammary tissues (p=0.97). Co-expression of AF6 and SIPA1 showed significant survival benefits for patients with non-triple negative breast cancers (p=0.034, Hazard Ratio (HR)=0.519), in EGFR low expression (p=0.016, HR=0.340) and in Her2 over-expressing cancers (p=0.048). AF6 and SIPA1 signficantly correlated with ERBB4 in tumours (r=0.244 and p=0.018 for AF6; r=0.396, p<0.001). However, no additional survival benefit was seen when ERBB4 and ER were considered.

**Conclusion**

AF6 and SIPA1, a complex of cell adhesion and adhesion regulators, are favourable prognostic indicators for patients with non-triple negative breast cancers, arguing the importance of the cell adhesion complex in clinical breast cancer.

### EACR23-0555
### WAVEs (Wiskott-Aldrich Syndrome Protein Family Members) expression in clinical pancreatic cancer and the clinical significance

*Y. Yu*[1], *T. Martin*[2], *W. Jiang*[2], *C. Hao*[3], *J. Ji*[3]
[1]*Cardiff University School of Medicine, Cardiff China Medical Research Collaborative Division of Cancer and Genetics, Cardiff, United Kingdom*
[2]*Cardiff University School of Medicine, Cardiff China Medical Research Collaborative Division of Cancer and Genetic, Cardiff, United Kingdom*
[3]*Peking University Cancer Hospital and Institute, Key Laboratory of Carcinogenesis, Beijing, China*

**Introduction**

WAVEs (Wiskott-Aldrich Syndrome Protein Family Members, or WASFs) are a small intracellular protein family, together with the rac/rho GTPases and actin regulated proteins (ARPs), regulating cellular migration and membrane events linked to the migrations. Targeting WAVE associated molecules can ameliorate cancer cell migration. In clinical cancers, it has been reported that levels of certain WAVE may have role in the clinical course of the patients. The present study investigated the clinical and prognostic value of WAVE expression in pancreatic cancer.

**Material and Methods**

Expression of WAVE-1, WAVE-2 and WAVE-3 was evaluated in a cohort of clinical pancreatic cancer (n=224). The expression levels of the WAVE transcripts were correlated with the clinical, survival and pathological parameters of the patients.

**Results and Discussions**

High levels of WAVE-1 and WAVE-2 were seen in pancreatic tumours compared with in normal pancreas tissues. High levels of WAVE-1 were seen in advanced tumour stages. High levels of all three WAVE-1 and WAVE-2 were seen in node positive tumours and in tumours from patients with pancreatic cancer incidence. Whilst WAVE-1 and WAVE-2 were seen in patients who died of pancreatic cancer than those who remained live, WAVE-3 was in a contrast pattern. In EGFR overexpressed patients, high levels of WAVE-1 and WAVE-2 were related a significantly shorter overall survival (p=0.017, Hazard Ratio 1.53) and disease free survival (p=0.009, Hazard Ratio 2.16).

**Conclusion**

Members of the WAVE family expressed aberrantly in pancreatic cancer and linked with survivals of the patients with EGFR over-expression. This may present an opportunity for targeting.

### EACR23-0603
### Depletion of bone marrow cells for optimized detection of disseminated cancer cells

*L.M. Rudhart*[1], *E. Raya*[1], *L.M. Köhler*[1], *A. Damböck*[1], *C. Irlbeck*[1], *H. Koerkel-Qu*[1], *C. Fisch*[1], *C. Botteron*[2], *M. Werner-Klein*[1], *M. Meyer*[3], *M. Weber*[4], *P. Ugocsai*[5], *S. Seitz*[5], *C.A. Klein*[1]
[1]*University of Regensburg, Chair of Experimental Medicine and Therapy Research, Regensburg, Germany*
[2]*Fraunhofer Institute for Toxicology and Experimental Me dicine, Division of Personalized Tumour Therapy, Regensburg, Germany*
[3]*University Hospital Regensburg, Department of Orthopaedic Surgery, Bad Abbach, Germany*
[4]*Hospital Barmherzige Brüder Regensburg, Department of Trauma Surgery, Regensburg, Germany*
[5]*University Hospital Regensburg- St. Josef, Department of Gynaecology and Obstetrics, Regensburg, Germany*

**Introduction**

Disseminated cancer cells (DCC) in bone marrow (BM) aspirates of pre-metastatic breast cancer (BC) patients are very rare, in the range of $10^{-6}$ to $10^{-5}$. So far detection has relied on the expression of histogenetic markers, such as cytokeratin or EpCAM, and enrichment strategies employed mostly CD45 depletion. Our standard protocol further comprises CD11b, CD33, and CD235a, however detection of EpCAM-positive DCC is still laborious, particularly since EpCAM is not specific for cancer cells. We therefore set out to identify markers that improve depletion strategies.

**Material and Methods**

We collected BM aspirates from BC patients and non-cancer patients for control. After depletion with our standard cocktail and EpCAM staining we picked EpCAM-positive cells, called non-cancer cells (NCC) when from controls and DCC when from cancer patients. After whole transcriptome amplification and comparative gene expression analysis, we identified CD27 and CD319 as characteristic markers for the confounding population of EpCAM-positive cells in controls. Additional markers were determined that may be used to deplete further physiological BM cells. With these markers we created a flow cytometry staining panel and checked the expression on protein levels before and after depletion.

**Results and Discussions**

CD319 and CD27 are expressed on EpCAM-positive NCC but not on EpCAM-positive DCC. Adding the two markers to the depletion protocol, we could successfully deplete those cells. We are currently evaluating further

© 2023 The Authors. Molecular Oncology is published by John Wiley & Sons Ltd on behalf of Federation of European Biochemical Societies improvements of our protocol by additional depletion markers.

**Conclusion**

We optimized our DCC enrichment method by depletion of CD27-positive and CD319-positive cells, the major confounders for the specific detection of EpCAM positive DCC in BM. The enrichment factor by adding additional depletion markers is currently assessed.

### EACR23-0683
### Usefulness of circular RNA hsa_circ_0116796 as a novel prognostic biomarker of human hepatocellular carcinoma

*K. Hur[1], H. Do[2], G. Kim[3], Y.R. Lee[4], Y.K. Jo[2], M.K. Kang[5], J.G. Park[5], S.Y. Park[4], W.Y. Tak[4]*
*[1]Kyungpook National University, Department of Biochemistry and Cell Biology- School of Medicine, Daegu, South Korea*
*[2]Kyungpook National University, Department of Biomedical Science and Technology- School of Convergence, Daegu, South Korea*
*[3]Kyungpook National University, Department of Biochemistry and Cell Biology- School of Medicine, Daegu, South Korea*
*[4]Kyungpook National University, Department of Internal Medicine, Daegu, South Korea*
*[5]Yeungnam University Medical Center, Department of Internal Medicine, Daegu, South Korea*

**Introduction**

Hepatocellular carcinoma (HCC) is the third leading cause of cancer-related deaths worldwide. It lacks sufficient diagnostic and prognostic markers, which leads to a poor prognosis. The survival rate of HCC patients is still low due to its highly metastatic nature. According to recent studies, non-coding RNAs play a vital role in cancer pathogenesis and may be a new diagnostic target for HCC. Circular RNA (circRNA) is a type of non-coding RNA with a closed loop structure formed by mRNA back-splicing. Its functions are miRNA sponging, translating to proteins and gene regulation. Recently, it has been found that circRNAs play a potential role at angiogenesis, metastasis, and drug resistance in many types of cancers. Thus, we aim to investigate the clinical relevance of hsa_circ_0116796 in HCC.

**Material and Methods**

Total RNAs from 100 pairs of HCC and corresponding normal liver (NL) tissues with different stages were extracted using QIAzol. CircRNA primers were coded including the gap junction for specificity. The expression levels were determined by quantitative real-time PCR (qRT-PCR), and its expression was normalized with GAPDH. In addition, we analyzed the correlation between hsa_circ_0116796 expression and various clinicopathological features of HCC patients. The target miRNAs were predicted in silico analyses.

**Results and Discussions**

We found that hsa_circ_0116796 was significantly decreased in HCC tumor tissues compared to corresponding NL tissues (P<0.001). Suppression of hsa_circ_0116796 expression was associated with HCC progression (P<0.001). Moreover, hsa_circ_0116796 expression was significantly associated with T-stage (P=0.009), TNM stage (P=0.005), BCLC stage (P=0.0132), and survival (P<0.001).

**Conclusion**

We provided the novel evidence for usefulness of hsa_circ_0116796 expressions as strong potential biomarkers for diagnosis and predicting prognosis of HCC patients.

### EACR23-0687
### Development of Early Cancer Detection System using Multi-miRNA Markers in Liquid Biopsy

*J.H. Bae[1], M.S. Kil[1], Y.H. Nam[2], S.H. Lee[1]*
*[1]Clinomics, Cancer Genomics Research Institute, Cheongju-si, South Korea*
*[2]NuriBio, R&D Center, Anyang-si, South Korea*

**Introduction**

There are 20 million new cancer cases worldwide, and cancer mortality is increasing due to low rates of early diagnosis, resulting in 10 million deaths each year. As an alternative to overcome this, early cancer screening through liquid biopsy has been proposed, and for this, a technique for accurately detecting a very small amount of miRNA isoform is required. However, it is difficult to satisfy the detection performance with conventional techniques so far, and in particular, a multi-marker technique for simultaneous detection/analysis of miRNA and ctDNA has not been developed.

**Material and Methods**

Plasmas were isolated from blood samples collected from 87 patients with lung cancer, 51 patients with pancreatic cancer, 51 patients with colorectal cancer, and 83 healthy people. The isolated RNAs from plasma were synthesized to cDNA using Poly A tailing methods. Total 35 significant target miRNAs and 2 internal controls (reference genes) were finally selected compare normal with patient blood.

**Results and Discussions**

Artificial Neural Network (ANN) training was conducted using the data of 150 cancer patients (50 lung cancer, 50 colorectal cancer, 50 pancreatic cancer) and 50 healthy people, a total of 200 cases. Training was performed with 100 randomly selected data out of 200, validation was performed with 50 data, and test was performed with 50 data. As a result, 90% accuracy was secured. Based on the established training data, it showed a final accuracy of 97%, 98% sensitivity and 95% specificity in 70 clinical samples. In particular, it showed a high accuracy of 95.7% in the carcinoma classification.

**Conclusion**

Through these results, the validity of 'cancer-related miRNA diagnosis' was confirmed, and cancer diagnosis with high accuracy, sensitivity, and specificity is expected to be possible. In the future, it is expected that a large-scale patient sample will be used to determine the possibility of diagnosis by stage and detailed site, and we believe that an automated system will be built to add miRNAs related to various cancer types. By learning the miRNA expression patterns of each of lung cancer, pancreatic cancer, and colorectal cancer used in this study with a neural network, it is expected that accurate diagnosis of more cancer types will be possible.

© 2023 The Authors. Molecular Oncology is published by John Wiley & Sons Ltd on behalf of Federation of European Biochemical Societies

### EACR23-0688
### Development of Automatic All-in-one System for Mutation Detection in Liquid Biopsy

*M.S. KIL*[1], *J.H. Bae*[1], *C.M. Choi*[2], *J.C. Lee*[3], *S.H. Lee*[1]
[1]*Clinomics, Cancer Genomics Research Institute, Cheongju-si, South Korea*
[2]*Asan Medical Center- University of Ulsan College of Med icine, Departments of Pulmonary and Critical Care Medicine- D epartments of Oncology, Seoul, South Korea*
[3]*Asan Medical Center- University of Ulsan College of Med icine, Departments of Oncology, Seoul, South Korea*

**Introduction**

Body fluid such as blood contain various substances that are released by cancerous tissue. The investigation and analysis of these substances in body fluids plays a pivotal role in the diagnosis of various diseases. Therefore, it is important to accurately isolate and detection, and many techniques are used for this. To this day, many studies have focused on accurately identifying fewer substances. In recently, liquid biopsy has still limitation for the cancer detection, however it is very simple and grown up to good method through by analysis advance.

**Material and Methods**

We made prototypes of device and disc. For primer accuracy and limit of detection (LOD) determination, 121 clinical blood samples of normal and EGFR mutant types were used.

**Results and Discussions**

EGFR mutation is well known as related with lung cancer. In a once operation, four mutation region such as E19del, T790M/C797S, L858R can be detected in real-time PCR step using two individual whole blood samples. When compared to other commercial detection kits, the concordance was 94% in 121 clinical samples. For the mutation detection sensitivity, LOD tests were performed using EGFR mutant cell lines. The LOD was 10 copies and the wild-type cell line was indicated completely negative.

**Conclusion**

Liquid biopsy is simple and free of time and place limitation. It has an advantage for both the patients and the hospital. To further enhance these strengths, we developed an all-in-one system that can automatically perform from sample separation after biopsy to real-time PCR for mutation detection. Moreover, it has the advantages of good acquisition yield, short operation time, and guarantees high reproducibility.

### EACR23-0714
### Multi-omics data integration shows the connection between metabolomic and circulating microRNA content in Lynch syndrome and reveals the top predictors of carrier status

*T. Jokela*[1], *T. Sievänen*[1], *T.M. Korhonen*[1], *J. Karppinen*[1], *J.P. Mecklin*[2], *T. Seppälä*[3], *E. Laakkonen*[1]
[1]*University of Jyväskylä, Gerontology Research Center and Faculty of Sport and He alth Sciences, Jyväskylä, Finland*
[2]*Hospital Nova of Central Finland, Hospital Nova, Jyväskylä, Finland*
[3]*University of Tampere, Department of Clinical Medicine- Faculty of Medicine and Health Technology, Tampere, Finland*

**Introduction**

Hereditary Lynch syndrome (LS), caused by mutations in DNA mismatch repair genes, causes up to 90% lifetime risk of developing cancer. However, cancer risk can be modified by lifestyle factors. The current challenge is that there are no valid biomarkers to predict when cancer emerges. Potential translational biomarkers would be easily accessible through liquid biopsy. Multi-omics approach has promoted biomarker discovery. From the blood-based omics integration studies, E.g. circulating microRNAs (cmiR) and metabolomics have shown potential for biomarker discovery. In addition to that, those studies have revealed new biological insights into different disease pathways.

**Material and Methods**

Here we are looking for biomarker candidates by utilizing omics data sets analyzed from LS carriers and control groups' serum samples; cancer-free non-carrier group (n=27), non-carrier rectal cancer patient group (n=24), and LS-carrier group (n=71). From the serum samples we analyzed cmiRs by using high-throughput sequencing and utilized Nightingale's NMR-based blood biomarker analysis service to measure >200 serum metabolite variables. To study and visualize omics data sets, we use unsupervised dimension reduction algorithms; principal component analysis and Principal Coordinates Analysis. To identify biomarkers, we use machine learning-based feature selection tools; information gain and Least Absolute Shrinkage and Selection Operation. We use Sparse Partial Least Squares analysis to study interactions between two omics data sets.

**Results and Discussions**

We have identified biomarkers that can classify our study groups. Our results show that currently, cancer-free LS carriers cmiRs and metabolomics have strong similarities with cancer patients' omics profiles. Also, we collect cancer incidence data from the LS group and the future goal is to test if our data can also reveal biomarkers that can predict future cancer. Also, we are looking for interactions between cmiR and metabolomics datasets to show biological pathways and connections. For example, lipid particles analyzed in metabolomics data can act as miR carriers in circulation and our preliminary results revealed interactions between a specific type of lipid particles, such as LDL and VLDL particles with different sizes and lipid composition features, and cmiRs. Overall, this study presents unique blood-based omics integration in LS.

**Conclusion**

This study has potential to identify novel cancer risk biomarkers and reveal biological interactions between cmiRs and metabolomics.

### EACR23-0725
### Development of SERS-Based Liquid Biopsy for Early Detection of Recurrence in Acute Leukemia: A Promising New Diagnostic

© 2023 The Authors. Molecular Oncology is published by John Wiley & Sons Ltd on behalf of Federation of European Biochemical Societies

### Approach

M. Akdeniz[1,2], F. Oktem[3], Z. Al-Shaebi[1,2], M. Keklik[3], O. Aydin[1,2,4,5]
[1]Erciyes University, Department of Biomedical Engineering, Kayseri, Turkiye
[2]Erciyes University, NanoThera Lab- ERFARMA- Drug Application and Research Center, Kayseri, Turkiye
[3]Erciyes University, Department of Hematology- Faculty of Medicine, Kayseri, Turkiye
[4]Erciyes University, ERNAM- Nanotechnology Research and Application Center, Kayseri, Turkiye
[5]Erciyes University, ERKAM- Clinical Engineering Research and Implementation Center , Kayseri, Turkiye

**Introduction**

Acute leukemia (AL) is a type of blood cancer that can be classified into two subtypes: acute myeloid leukemia (AML) and acute lymphocytic leukemia (ALL). Early diagnosis of AL is critical for successful treatment, but traditional methods such as bone marrow biopsy can be invasive and may have limited sensitivity and specificity. An alternative approach is liquid biopsy, which involves analyzing biomarkers like circulating tumor cells (CTCs), exosomes in blood. However, the deficiency of liquid biopsy can be improved by utilizing Surface-enhanced Raman Scattering (SERS), a highly sensitive and specific method that enhances Raman signals by interacting with plasmonic nanostructures. By analyzing various biomarkers with SERS, we can increase accuracy of liquid biopsy and improve our ability to diagnose AL. Machine learning algorithms can then be trained on these data to develop models that can predict the likelihood of AL recurrence. This study highlights the potential of SERS and machine learning in liquid biopsy-based diagnosis and prediction of AL and could lead to improved diagnostic and therapeutic strategies for AL.

**Material and Methods**

To investigate potential of using SERS and machine learning for diagnosis and prediction of AL, the study collected serum samples from patients with AML, ALL patients, and healthy individuals. The serum samples were dropped onto an AgNPs-based SERS substrate to collect SERS spectra. To discriminate between the three groups, unique spectral profiles of serum were defined, and machine learning algorithms, including support vector machine (SVM), random forest (RF), and k-nearest neighbors (kNN), were used to predict recurrence or diagnose AL.

**Results and Discussions**

SERS spectra of the serum samples revealed peaks assigned to proteins, lipids, and nucleic acids. Peak intensity ratios were compared using a heatmap graph. The results showed significant differences in the intensities of several peaks between the groups. Among the machine learning algorithms, RF showed the best classification performance, with an accuracy of 95.4%, and predicted recurrence of AML and ALL with accuracies of 95.8% and 99.2%, respectively. These findings demonstrate the potential of SERS and machine learning as a method for detecting AL and predicting its recurrence.

**Conclusion**

The utilization of SERS and machine learning holds promise as a sensitive and specific method for the detection and prediction of AL from blood serum.

### EACR23-0739
### Evaluation of prognostic biomarkers in First-Line Cetuximab Plus Platinum-Based Chemotherapy in Recurrent/Metastatic Head and Neck Cancer.

D. Lenoci[1], E. Rosso[2], F. Cordero[2], S. Cavalieri[3], L. Licitra[3], L. De Cecco[1]
[1]IRCSS Istituto Nazionale dei tumori, Department of Research-Integrated biology of rare tumors, Milan, Italy
[2]University of Torino, Department of Computer Science, Turin, Italy
[3]IRCSS Istituto Nazionale dei tumori, Head and Neck Medical Oncology Department, Milan, Italy

**Introduction**

B490 (EudraCT#2011-002564-24) was a multicenter study that included patients with recurrent/metastatic (R/M) head and neck squamous cell carcinoma treated with cetuximab plus platinum-based chemotherapy. The present study aimed to characterize biomarkers such as genes, small non-coding RNAs (sncRNAs), and their networks, to stratify patients based on progression-free survival (PFS).

**Material and Methods**

Patients were treated from September 2011 to 2017, follow-up data were updated in July 2020. Primary tumors were collected at primary diagnosis, and archived as FFPE blocks. Total RNA was extracted using the Qiagen RNeasy Mini Kit. Transcriptome and sncRNAs libraries were generated using the TruSeq Library Prep (Illumina) and QIAseq miRNA Library Kit respectively. Samples were sequenced on NextSeq500. Patients were divided in long- (PFS>12 months) and short- (PFS<6 months) groups. Differential gene expression (DGE) analysis of genes and sncRNAs associated to PFS was available for 123 cases. Enrichr and sncRNAs target prediction were performed.

**Results and Discussions**

Genes and sncRNAs DGE analysis stratified patients in short- and long- PFS with a significative p-value. In long-PFS: eight genes were up- (IF35, IFIT3, OAS3, PML, SP100, TAP1, TAPBP, TNFSF10) and two down- (MROH2A, RPL7L1P11) regulated; Six sncRNAs were up-(miR-449a, miR-4632-5p, miR-6125, miR-636, miR-6860, piR-019050) and three down- (miR-1246, miR-200c-3p and miR-335-5p) regulated. Enrichr showed that the deregulated genes were involved in viral response in head and neck mucosa, interferon and apoptotic signaling pathways. SncRNAs target prediction revealed that, miR-1246 targeted TAPBP, miR-335-5p PML and miR200c-3p TNFSF10 and IFIT3. The expression level of these genes regulated by miRNAs were confirmed by DGE. Mir-204c-3p presented the best p-value and resulted up-regulated in short-PFS. This result is concordant with the literature, in fact mir-204c-3p expression was associated with poor overall survival in metastatic breast cancer and in high-grade serous ovarian cancer.

**Conclusion**

© 2023 The Authors. Molecular Oncology is published by John Wiley & Sons Ltd on behalf of Federation of European Biochemical Societies

The present study evaluated biological biomarkers associated to PFS in B490. The down-regulation of miRNAs that targeted genes involved in interferon signaling seems to be protective in this setting. Validation of identified genes and sncRNAs in an external dataset is ongoing to: confirm our results; generate hypothesis for this standard of care treatment used for PDL1-negative R/M; select cases after immunotherapy failure.

### EACR23-0740
### Optimized identification of EpCAM positive DCC in the bone marrow of breast cancer patients

*E. Raya[1], L. Rudhart[1], L.M. Köhler[1], A. Damböck[1], C. Irlbeck[2], H. Koerkel-Qu[1], M. Werner-Klein[1], S. Seitz[3], C.A. Klein[1]*
*[1]University of Regensburg, Chair of Experimental Medicine and Therapy Research, Regensburg, Germany*
*[2]Fraunhofer Institute for Toxicology and Experimental Medicine, Personalized Tumour Therapy, Regensburg, Germany*
*[3]University Hospital Regensburg St. Josef, Department of Gynaecology and Obstetrics, Regensburg, Germany*

**Introduction**
It has become firmly established that dissemination of cancer cells from primary to distant sites often occurs very early, long before the diagnosis of the primary tumor, and that DCCs from earliest lesions are able to proliferate and kill their hosts. While it is important to reveal the transcriptional traits of these early DCCs, they are a very small and hard-to-find population of cells in the bone marrow (BM). Our current approach to detect DCCs is to pick EpCAM-positive cells and then confirm their malignant origin, for example by subjecting these cells to whole genome sequencing at low coverage (low pass sequencing) to reveal copy number alterations. Since this is a lengthy and expensive method when applied to a large number of cells, we sought to find markers that can differentiate these cells from their non-cancer cell (NCC) counterparts in the BM of breast cancer patients.
**Material and Methods**
Microarray data from EpCAM-positive cells collected from patients with breast cancer as opposed to non-cancer donors revealed candidate genes that were exclusively expressed in DCCs and in NCCs. Based on these profiles, a qPCR assay was designed to check for the expression of these NCC/DCC markers in EpCAM-positive cells picked from pre-metastatic breast cancer patients in order to classify the picked cells as DCCs or NCCs depending on their transcription profile. The results were then compared to RNA-seq as well as lowpass classification of the same picked cells.
**Results and Discussions**
The results of these experiments show a strong match between the RNA-seq classification, and the qPCR signature established for the EpCAM-positive cells. This will allow the reliable exclusion of picked NCCs from further analysis. The molecular characterization of the isolated DCC is currently underway.
**Conclusion**
The characterization of DCCs which would allow the analysis of the metastasis founder cells requires a more efficient method of profiling these cells in the bone marrow of pre-metastatic patients. The application of the NCC/DCC signature could potentially improve our selection criteria of what cells are to be considered DCCs. Eventually, the information can be used for reliable quantification of DCC-load in bone marrow for diagnostic purposes.

### EACR23-0745
### Low salivary Cornulin, a marker for tissue differentiation, predicts poor prognosis in Head Neck Squamous Cell Carcinoma patients.

*R. Kaur[1], A. Chauhan[1], D. Chatterjee[2], J. Bakshi[3], R. Srinivasan[4], S. Ghoshal[5], A. Pal[1]*
*[1]Post Graduate Institute of Medical Education and Research, Department of Biochemistry, Chandigarh, India*
*[2]Post Graduate Institute of Medical Education and Research, Department of Histopathology, Chandigarh, India*
*[3]Post Graduate Institute of Medical Education and Research, Department of ENT, Chandigarh, India*
*[4]Post Graduate Institute of Medical Education and Research, Department of Cytology and Gynaecological Pathology, Chandigarh, India*
*[5]Post Graduate Institute of Medical Education and Research, Department of Radiotherapy and Oncology, Chandigarh, India*

**Introduction**
Cornulin, a relatively unexplored protein, was reported to be downregulated in oesophageal carcinoma. In our preliminary study, based on TMT-based LC-MS/MS analysis, the salivary Cornulin was also found to be downregulated ~10-fold in Head Neck Squamous Cell Carcinoma (HNSCC) patients. Although a few studies reported the association of low tissue Cornulin level with advanced disease in squamous cell carcinomas of the oesophagus and oral cavity, the exact pathophysiological role of Cornulin in HNSCC is yet to be explored. Here we investigated the potential role of Cornulin as a biomarker as well as its role in the pathophysiology of HNSCC.
**Material and Methods**
Sandwich ELISA and immunohistochemistry were performed to estimate the salivary and tissue levels of Cornulin, respectively, in HNSCC patients. The mRNA levels of Cornulin from TCGA database were also analysed using GEPIA2. The role of Cornulin in the pathophysiology of HNSCC was checked by upregulation of Cornulin levels by lentiviral transduction in low Cornulin expressing Cal27, oral squamous cell carcinoma cell line. The molecular signalling pathway was elucidated by analysing transcriptome data generated by RNAseq using various tools.
**Results and Discussions**
The pre-treatment salivary Cornulin levels were significantly low **($p<0.0001$)** in HNSCC patients ($n=128, 146.4\pm5.589$pg/mL) with respect to healthy controls ($n=84, 185.2\pm7.170$pg/mL). Also, the tumour tissue expression of Cornulin ($n=113$, H-score$=12.70\pm2.396$) was significantly downregulated **($p<0.0001$)** in comparison to the tumour-

© 2023 The Authors. Molecular Oncology is published by John Wiley & Sons Ltd on behalf of Federation of European Biochemical Societies free margin (n=72, H-score=139.6±10.34). The patients showing complete clinical response regained normal salivary within 6 months of completion of treatment (**p<0.0001**). ROC analysis for the potential diagnostic biomarker reveals a significant score with area under the curve of 66% (**p<0.0001**), taking 189.6pg/mL as a cut-off. More importantly, ***low salivary Cornulin level at diagnosis was associated with poor overall survival*** **(p=0.0282),** indicating it to be ***a potential prognostic marker***. TCGA data also showed a significant association between low-tissue Cornulin mRNA in HNSCC patients with poor disease outcomes. Intriguingly, the cell viability, migration and invasion were decreased in Cornulin overexpressed cells, indicating the anti-tumour role of Cornulin. Further GSEA revealed Cornulin to be involved in PI3K/Akt/mTOR signalling pathway.

**Conclusion**

Here, we report for the first time Cornulin as a potential prognostic marker and its role in the pathophysiology of HNSCC.

### EACR23-0771
### Deep Amplicon Sequencing of POLE Gene Using fastGEN Technology

*J. Stranska[1,2,3], R. Slavkovsky[1,3], N. Ptakova[4], G. Korinkova[5], B. Blumova[1], J. Drabek[1,3], V. Seidlova[6], K. Pehlikova[7], P. Broz[8,9], M. Hajduch[1,3,10]*

*[1]Faculty of Medicine and Dentistry Palacky University Olo mouc, Institute of Molecular and Translational Medicine, Olomouc, Czech Republic*

*[2]Olomouc University Hospital, Department of Neurology, Olomouc, Czech Republic*

*[3]Olomouc University Hospital, Laboratory of Experimental Medicine, Olomouc, Czech Republic*

*[4]Bioptická laborator s.r.o., Molecular Genetics, Pilsen, Czech Republic*

*[5]Olomouc University Hospital and Palacky University Olo mouc, Department of Clinical and Molecular Pathology, Olomouc, Czech Republic*

*[6]BioVendor Group, BioVendor MDx, Brno, Czech Republic*

*[7]BioVendor Group, BioVendor LM, Brno, Czech Republic*

*[8]BIOXSYS s.r.o., BIOXSYS s.r.o., Usti nad Labem, Czech Republic*

*[9]Charles University Prague and Faculty Hospital Motol Pr ague, Department of Biology and Medical Genetics- 2nd Faculty of Medicine, Prague, Czech Republic*

*[10]Palacky University Olomouc, Czech Advanced Technology and Research Institute, Olomouc, Czech Republic*

**Introduction**

*POLE* gene encodes the central catalytic subunit of DNA polymerase epsilon. Its DNA testing is a prerequisite for a tumor risk-group assessment and personalized treatment in endometrial carcinoma. *POLE* mutated tumors lower the risk, thus, the intensity of the adjuvant chemotherapy should be lowered. 7 – 12 % of endometrial cancers harbor *POLE* mutations and have been associated with a high tumor mutation burden. It was shown that pathogenic *POLE* mutations were related to a clinical benefit to an immune checkpoint inhibitor therapy, thus, further thorough prospective studies are warranted to validate the *POLE* mutation as a predictive biomarker.

**Material and Methods**

A high potential to be a suitable method for the simultaneous detection of somatic mutations within hotspot regions with a defined detection limit down to 1 % minor allelic frequency (MAF) has a deep amplicon sequencing (DAS). We have developed and validated a unique fast method known as fastGEN using Illumina platforms. Formalin-fixed paraffin-embedded endometrial tumors were genotyped for hotspot mutations of the *POLE* exonuclease domain and sequenced on MiSeq (Illumina).

**Results and Discussions**

We have analyzed 74 endometrial tumors with a 100 % success rate. Pathogenic variants were found in 7 samples (2 with *POLE* p.P286R, 3 with p.V411L, 1 with p.M444K, and 1 with p.S459F). fastGEN results were validated using larger somatic NGS panels (Nonacus Pancancer TMB/MSI; Qiagen QIAseq TMB Panel; Archer VariantPlex GyNcore and Illumina TSO 500). Using samples (n = 10), where results of both methods were available, we observed a concordance with 100 % specificity and sensitivity. The variant detection using fastGEN *POLE* was highly reproducible (n = 4, *POLE* p.S459F, MAF = 29.8 % ± 1.7 %) and sensitive (n = 3, POLE p.P286R, MAF = 23,17 % ± 3.5 %, input = 1 ng DNA). A minimum turn-around time (sample to final report) was less than 24 hours. fastGEN technology is routinely performed for tumor testing of *POLE, RAS*, *BRAF*, *EGFR*, *IDH1/2*,and *PIK3CA* in our lab, other genes are under development. The technology was licensed by the partner BioVendor Group.

**Conclusion**

fastGEN technology used for the detection of *POLE* somatic mutations is really fast and easy to perform with a high success rate, including samples with low amount and low quality DNA. Together with other often requested predictive biomarkers, fastGEN kits can be easily implemented in laboratories with Illumina sequencers. A user-friendly and robust bioinformatics pipeline is based on Genovesa fastGEN platform.

### EACR23-0777
### The role of Extracellular Vesicles in Non-Small-Cell Lung Cancer, the unknowns, and how New Approach Methodologies can support new knowledge generation in the field

*S. Mullen[1], D. Movia[1]*

*[1]Trinity College Dublin, Radiation Therapy, Dublin, Ireland*

**Introduction**

Extracellular vesicles (EVs) are nanosized particles released from most human cell types and containing a variety of cargos responsible for mediating cell-to-cell and organ-to-organ communications. Current knowledge demonstrates that EVs are a driving factor in Non-Small-Cell Lung Cancer (NSCLC) progression. The aim of our work was to review knowledge to date in the field, analyse which models have been used in clinical and/or preclinical research to gather such information, and analyse whether

© 2023 The Authors. Molecular Oncology is published by John Wiley & Sons Ltd on behalf of Federation of European Biochemical Societies such models have hindered the full understanding of EV function in NSCLC determining knowledge gaps.

**Material and Methods**

A thorough literature search was carried out using PubMed, Embase, and Google Scholar, limiting the results to primary and secondary sources published between January 2017 and January 2023.

**Results and Discussions**

Current knowledge demonstrate that EVs play critical roles in many aspects of the progression of Non-Small-Cell Lung Cancer (NSCLC). Their roles range from increasing proliferative signalling to inhibiting apoptosis, promoting cancer metastasis, and modulating the tumour microenvironment to support cancer development. However, due to the limited availability of patient samples, intrinsic inter-species differences between human and animal EV biology, and the complex nature of EV interactions *in vivo*, where multiple cell types are present and several events occur simultaneously, the use of conventional preclinical and clinical models has significantly hindered reaching conclusive results. New Approach Methodologies (NAMs) such as microfluidic platforms, organoids, and spheroids have been shown to overcome these limitations and have been or could be used to gather further knowledge in the EV cancer field.

**Conclusion**

EVs play many biological roles in NSCLC, but gaps are still present in today's knowledge. Several NSCLC models have been used to define currently-known EV functions; however, limitations are associated with their use in this field. NAMs can be used to overcome these limitations, effectively supporting future exciting discoveries in the NSCLC field and the potential clinical exploitation of EVs.

### EACR23-0781

### Exploring the use of Genomic Instability Metric (GIM) to determine Homologous Recombination Deficiency (HRD) in cancer

*M. Vasileva*[1], *R. Dodova*[2], *M. Georgiev*[2], *V. Petkova*[2], *R. Dimitrov*[3], *S. Slavov*[3], *V. Ivanova*[4], *R. Kaneva*[2]

[1]*Molecular Medicine Center, Department of Medical Chemistry and Biochemistry- Medical Faculty- Medical University Sofia, Sofia, Bulgaria*

[2]*Molecular Medicine Center, Department of Medical Chemistry and Biochemistry- Medical Faculty- Medical University Sofia, Sofia, Bulgaria*

[3]*University Hospital of Obstetrics and gynecology, Department of Obstetrics and gynecology- Medical University Sofia, Sofia, Bulgaria*

[4]*Medical Faculty- Medical University Sofia, Department of Pathology, Sofia, Bulgaria*

**Introduction**

Homologous recombination deficiency (HRD) tests have emerged as promising biomarkers of genome instability, useful for the identification of cancer patients that would benefit from poly ADP ribose polymerase inhibitors (PARPi). In this study we aimed to evaluate the new tool called GIM (genome instability matrix) for HRD assessment, based on Oncomine Comprehensive Assay Plus panel (OCA Plus) (Thermo Fischer Scientific). The panel detects SNVs and CNVs across 500+ genes, as well as complex biomarkers, such as tumour-mutational burden (TMB), microsatellite instability (MSI), and loss of heterozygosity (LOH). GIM score is produced using different measures of instability and a cut off of 16 is recommended to define HRD+ and HRD- samples.

**Material and Methods**

DNA was isolated using 24 FFPE tissues from solid tumours, including 14 ovarian cancers - 7 with known *BRCA1/2* mutations, 7 without mutations (wt), of which one was HRD+ control, 3 pancreatic, 2 colorectal cancers, and 5 single rare cancer cases. Library preparation was done with OCA plus assay. Sequencing was completed on the IonGene Studio S5 and data analysis was carried out with Ion Reporter Software version 5.20 (Thermo Fischer Scientific).

**Results and Discussions**

Three samples (12,5%) failed due to low DNA quality. After running the OCA plus panel, all known *BRCA1/2* mutations were detected, 4 in *BRCA1*, and 1 in *BRCA2* gene. Interestingly, 3 of the samples with *BRCA1* mutation had a GIM ≥16 and defined as HRD+, while 2 of them had GIM of 15 (*BRCA2* - c.6402_6406delTAACT) and GIM of 8 (*BRCA1* - c.1016delA). Among the OC samples without *BRCA1/2* mutations, 43% (3/7) showed GIM ≥16, and were defined as HRD+. One OC sample had a GIM of 15. As expected, all OC samples were classified as MSS and had a low TMB (≤5 mutations/Mb). Only 1 CRC case was MSI-H, with high TMB and low GIM score, confirming that MSI and HRD are mutually exclusive mechanisms of genomic scaring. All the rest rare tumours were MSS and showed low GIM score, with variations in the TMB.

**Conclusion**

The calculated GIM score reliably defines HRD+ tumours and could be used to identify patients that might benefit from therapy with PARP inhibitors. Criteria and thresholds for the estimation of genomic instability vary among the existing tests and still need to be properly established to reflect the underlying repair deficits.

Acknowledgements: NSF, MES: D01-395/18.12.2020, D01-278-14.12. 2022, D01-302/17.12.2021, D01 165/28.07.2022; KP-06-OPR03/1719.12.2018/NSF

### EACR23-0792

### T-cell immunoglobulin and mucin-domain containing molecule-3 (Tim-3) may promote tight junction deterioration of blood brain barrier (BBB) of cerebral endothelial cells

*X. Cao*[1,2,3], *B. Cong*[1,3], *W. Jiang*[1], *T.A. Martin*[1]

[1]*Cardiff University School of Medicine, Cardiff China Medical Research Collaborative- Division of Cancer & Genetics, Wales Cardiff, United Kingdom*

[2]*Tianjin Medical University- National Clinical Research Center for Cancer- Tianjin's Clinical Research Center for Cancer- Key Laboratory of Cancer Prevention and Therapy- Key Laboratory of Breast cancer Prevention and Therapy, Tianjin Medical University cancer institute and Hospital, Tianjin, China*

[3]*Shandong Cancer Hospital and Institute- Shandong First Medical University and Shandong Academy of Medical Sciences, Breast Cancer Centre, Jinan, China*

**Introduction**

© 2023 The Authors. Molecular Oncology is published by John Wiley & Sons Ltd on behalf of Federation of European Biochemical Societies

T-cell immunoglobulin and mucin-domain containing molecule-3 (Tim-3)/Hepatitis A virus cellular receptor 2 (HAVCR2) decreases tight junction (TJ) formation in vascular endothelial cells. Its role in blood brain barrier (BBB) of cerebral endothelium remains unknown. This study aimed to investigate the association/functionality of Tim-3 in TJ of BBB.

**Material and Methods**

Human cerebral microvessel endothelial cells (hCMEC/D3) were used to model BBB in vitro. Tim-3 was knocked down by siRNA transfection in hCMEC/D3 cell lines. Changes were observed of TJ molecules using quantitative polymerase chain reaction (qPCR). Electric cell impedance sensing (ECIS) was used for assesssing cell function. Changes in TJ behaviour were further assessed using transendothelial resistance (TEER)/paracellular permeability (PCP). An in vitro transwell cell invasion assay was set up to measure the invasion of breast cancer cells lines.

**Results and Discussions**

Tim-3 highly expressed in hCMEC/D3 cells, was knocked down via siRNA. Knockdown Tim-3 decreased TJ formation of cerebral endothelial cells by decreasing the expression levels of claudins-1/-5/-10/, occludin, marvelD3, JAM-1/-2 and ZO1. Knockdown cells displayed lower electric resistance during initial attachment, spreading and migration, suggesting Tim-3 might inhibit TJ function. TEER in knockdown cells were also reduced. Higher PCP fluorescence signals were detected in knockdown cell lines. There are significantly more breast cancer cells invasion Tim-3 knockdown hCMEC/D3 cells which further proved that Tim-3 influence the integrity of cerebral endothelium TJ.

**Conclusion**

Tim-3 may have a function as part of the regulatory apparatus for TJ in cerebral endothelial cells by influencing TJ protein expression.

### EACR23-0804

### Deciphering the potential of CD44v6 as biomarker and therapeutic target to improve personalized therapy in bladder cancer disease management

*I. Lodewijk*[1,2,3], *C. Rubio*[1,2,3], *I. A. Reina*[3], *M. Alonso-Sánchez*[3], *O. Alberquilla*[4,5], *R. Sánchez-Domínguez*[4,5], *R. García-Martin*[6], *L. Parrilla*[6], *J.M. Paramio*[1,2,3], *M. Dueñas*[1,2,3]

[1]*Institute of Biomedical Research- Hospital Universitario 1 2 de Octubre Imas12, Cellular and Molecular Oncology Genitourinary Tumor G roup, Madrid, Spain*

[2]*Centro de Investigación Biomédica en Red Cáncer CIBER ONC, Mecanismos de Progresión Tumoral, Madrid, Spain*

[3]*Centro de Investigaciones Energéticas- Medioambientales y Tecnológicas CIEMAT, Molecular Oncology Unit, Madrid, Spain*

[4]*Centro de Investigaciones Energéticas- Medioambientales y Tecnológicas CIEMAT and Centro de Investigación Bio médica en Red de Enfermedades Raras CIBER-ER, Division of Hematopoietic Innovative Therapies, Madrid, Spain*

[5]*Instituto de Investigación Sanitaria Fundación Jiménez Díaz IIS-FJD/UAM, Advanced Therapy Unit, Madrid, Spain*

[6]*Hospital Universitario 12 de Octubre, Pathology Department, Madrid, Spain*

**Introduction**

Limited advances in bladder cancer (BC) management represent a major problem. Therefore, the improvement of diagnostic and predictive tools as well as the identification of new therapeutic targets for personalized medicine in the BC area represent an urgent need. The objective of this study is the characterization of CD44v6 (splice variant of CD44 glycoprotein) as potential biomarker and therapeutic target in BC.

**Material and Methods**

We evaluated CD44 and CD44v6 expression among non-muscle invasive BC (NMIBC) and muscle invasive BC (MIBC) patient samples by tissue microarrays. Specimens were annotated with clinical information, including tumor stage, grade and recurrence.

Among 11 human BC cell lines evaluated for CD44 and CD44v6 receptor expression by flow cytometry, RT112 and J82 cell lines were selected for further experimentation. Using fluorescent activated cell sorter, we isolated and generated stable cell lines CD44 Low, CD44 High and CD44v6 High from each parental cell line. These cell lines were used for molecular profiling (complete transcriptome by RNAseq and miRNA by Nanostring) as well as for *in vitro* and *in vivo* experimentation, evaluating cell proliferation, invasion and migration capacity (XTT, Wound Healing assay and Migration & Invasion Transwell analysis) as well as tumor growth and tumor phenotype (tumor disaggregation, flow cytometry and immunohistochemistry), among others.

**Results and Discussions**

We found a positive correlation of CD44 and CD44v6 expression in higher stage and grade NMIBC samples. Currently, MIBC samples are being analyzed as well as correlation to other clinical data.

Using Nanostring data and unsupervised hierarchical clustering, we observed a shared miRNA profile for the CD44v6 cell lines compared to their parental, showing 27 miRNAs being upregulated and 14 miRNAs downregulated in the CD44v6 cell lines. RNA sequencing preliminary results show similarities between the selected cell lines with different molecular subtypes, which reinforces correlations with clinical parameters observed in the CD44v6 positive tumors.

We found significant differences in proliferative, migration and invasion capacity between the newly established cell lines *in vitro*, and clear differences in tumor growth *in* vivo. Tumor phenotypes are being evaluated.

**Conclusion**

We show preliminary data indicating that CD44v6 might be a potential diagnostic, prognostic and/or predictive biomarker for specific BC subtypes. Additionally, CD44v6 might be a potential therapeutic target for personalized treatment.

### EACR23-0853

### A miRNA signature for COPD clinical management: diagnostic implications for lung cancer screening.

*O. Pernía Arias*[1], *A. Arauzo*[2], *J. Colmenarejo*[1], *A. Garcia-Guede*[1], *M. Burdiel*[1], *E. Martínez Cerón*[3], *R. Galera*[4],

© 2023 The Authors. Molecular Oncology is published by John Wiley & Sons Ltd on behalf of Federation of European Biochemical Societies

J. de Castro[1], F. Garcia-Rio[3], I. Ibanez de Caceres[1]
[1]IdiPAZ-HULP, Experimental Therapiues and novel biomarkers in cancer, Madrid, Spain
[2]IdiPAZ-HULP, Experimental Therapies and novel biomarkers in cancer, Madrid, Spain
[3]IdiPAZ-HULP, Experimental Respiratory disease group- respiratory servi ce-, Madrid, Spain
[4]IdiPAZ-HULP, Experimental Respiratory disease group- respiratory servi ce., Madrid, Spain

**Introduction**

Chronic obstructive pulmonary disease (COPD) is a common, preventable and treatable disease characterised by the concurrence of chronic respiratory symptoms and persistent airflow limitation. There is a strong relationship between COPD and the role of epigenetic alterations in the development of lung cancer. Specially, changes in the activity of different microRNAs.

**Material and Methods**

A cohort of 119 COPD patients from the high-risk lung cancer screening programme were collected in a prospective study at the Hospital La Paz. The following clinical parameters were obtained: FEV1, FVC, FEV1/FVC, FRC and DLCO (spirometry) and MLD, P15 and inspiration-expiration MLD. Total RNA and DNA was isolated from buccal swabs. miR-124 and -55745 levels were assessed by qRT-PCR. Relative quantification of each miRNA was performed by 2-ΔΔCt. DNA was modified with sodium bisulfite. Methylation levels of miR-7 were assessed by qMSP using the following equation Cmeth = 100/ [1+2(CTCG - CTTG)].

**Results and Discussions**

We observed an inversely proportional relationship between increased methylation levels of miR-7 and decreased FEV1 and DLCO parameters (p=0.036 and p=0.032 respectively). These results suggest that the study of miR-7 methylation could be used as a parameter for the detection of emphysema using functional techniques. This would indicate the severity of the disease in the upper airways. For miR-124, we observed that both, inspiratory and expiratory P15 are significant (p=0.027 and p=0.0049), and MLD is very close to significance (p=0.061). Regarding miR-55745, the decrease in its levels is accompanied by a significant decrease in the MLD insp-esp ratio, calculated as the difference between inspiration and expiration (p=0.035), as well as in the inspired and exhaled P15 values (p=0.047 and p=0.046). Therefore, miR-55745 and miR-124 levels could be used as potential biomarkers for early stage prediction in support of imaging techniques, as they are associated with earlier onset clinical parameters (MLD and P15) related to lower tract entrapment.

**Conclusion**

Our results suggest that the combination of the three miRNAs, and especially miR-55745, would help to profile high-risk patients for screening, improve early diagnosis and reduce lung cancer mortality. Funding:Instituto de Salud Carlos III and the European Regional Development Fund/European Social Fund FIS [ERDF/ESF], Una Manera de Hacer Europa (PI18/050; PI21/0145; PI22/01764).

### EACR23-0857
### Survival analysis based on Circulating Tumor Cells identified by Deep Learning

F. Coumans[1], A. Nanou[1], L.W.M.M. Terstappen[1], P. Ansaloni[2], N. Gentili[2], R. Miserendino[2], G. Signorini[2], L. Biasiolli[2], G. Medoro[2]
[1]University of Twente, Medical Cell BioPhysics, Enschede, The Netherlands
[2]Menarini Silicon Biosystems, Research & Development, Bologna, Italy

**Introduction**

The presence of Circulating Tumor Cells (CTC) assessed with the CELLSEARCH® system is strongly associated with poor overall survival in metastatic cancer. CELLSEARCH presents a gallery of images to an operator who classifies the objects as CTC when they display cell like features, express DAPI and Cytokeratin and lack CD45. This classification is time intensive and is prone to subjective interpretations. Recently a Deep Learning (DL) method showed promise to replace the operator and improve the relation with clinical outcome. Here, we present a cloud-based Artificial intelligence (AI) algorithm incorporating the DL method to process and analyze CELLSEARCH images.

**Material and Methods**

CELLSEARCH images archived from the original IMMC38 study of castration resistant prostate cancer patients were used to evaluate the prognostic performance of AI CTC counts. This includes 131 image sets from 131 patients before initiation of a new line of therapy and 127 image sets from 127 patients at several time points after initiation of treatment.

**Results and Discussions**

CTC counts of all 258 image sets scored by the operator ranged from 0 to 5925 (median 4, mean 89.4, SD 426.4) and AI counts of the same set ranged from 0 to 6559 (median 6, mean 105.1, SD 465.5). Correlation between operator CTC counts and AI CTC counts was $R^2 = 0.988$ with a slope of 1.09 and an intercept of 8.09. The concordance rate between the operator and AI assigning patients to favorable (<5) and unfavorable (≥5) CTC group was 93.4%. Hazard ratio (HR) of Overall survival (OS) of 131 patients using baseline log-transformed CTC as a continuous variable was 1.57 (95% CI: 1.35-1.84) for operator CTC count and 1.63 (95% CI: 1.36-1.95) for AI CTC count. Using Favorable (<5) and Unfavorable (≥5) CTC, the HR of the OS was 2.49 (95% CI: 1.64-3.78) for operator CTC count and 2.93 (95% CI: 1.90-4.53) for AI CTC count.

**Conclusion**

We introduced an AI algorithm (only for research) to eliminate operators' bias and review time in CTC assignment. Automated and manual CTC counts were strongly associated. The HR results on this dataset suggest that AI can perform similarly to or better than human reviewers in predicting OS. Therefore, our cloud-based AI algorithm allows for fast, reproducible and bias-free enumeration of CTCs that are clinically relevant.

### EACR23-0862
### PATZ1 expression and nuclear localization are regulated in NSCLC, negatively

© 2023The Authors. Molecular Oncology is published by John Wiley & Sons Ltd on behalf of Federation of European Biochemical Societies

### correlate with PD-L1, and suppress the malignant phenotype

*S. Lucà[1], R. Franco[1], A. Napolitano[2], V. Soria[2], A. Ronchi[1], F. Zito Marino[1], C. Arra[3], S. Battista[2], L. Cerchia[2], M. Fedele[2]*
*[1]University of Campania "Luigi Vanvitelli", Pathology Unit- Department of Mental and Physical Healt h and Preventive Medicine, Naples, Italy*
*[2]Institute of Experimental Endocrinology and Oncology IE OS - CNR, Biomedical Sciences, Naples, Italy*
*[3]Istituto Nazionale Tumori – IRCCS - Fondazione G. Pasc ale, S.S.D. Sperimentazione Animale, Naples, Italy*

**Introduction**

Non-small cell lung cancer (NSCLC), the leading cause of cancer death worldwide, is still an unmet medical problem due to the lack of both effective therapy against advanced stages and markers to allow a diagnosis of the disease before it progresses to advanced stages. Immunotherapy targeting the PD-1/PD-L1 checkpoint is promising for many cancers, including NSCLC, but its success depends on the tumor expression of PD-L1. PATZ1 is an emerging cancer-related transcriptional regulator and diagnostic/ prognostic biomarker in different malignant tumors, but its role in lung cancer is still obscure.

**Material and Methods**

A local cohort of 104 NSCLCs, including lung squamous cell carcinomas (LUSCs) and adenocarcinomas (LUADs), was retrospectively analyzed for the expression of PATZ1 and PD-L1 by immunohistochemistry. The results were correlated with each other and with the clinical characteristics. Different publicly available transcriptomic datasets were also analyzed. Then, two NSCLC cell lines, transiently transfected with a plasmid overexpressing PATZ1, were analyzed by western blot for the expression of PD-L1, and by different functional assays for their capacity to proliferate, migrate and invade. Finally, we histologically characterized the lung tumor phenotype occurred in heterozygous mice for the Patz1 knockout gene.

**Results and Discussions**

We showed on the one hand a positive correlation between the elevated expression of PATZ1 and the LUSC subtype and, on the other hand, a negative correlation between PATZ1 and PD-L1, which was validated at the mRNA level in independent NSCLC datasets. Consistently, the two NSCLC cell lines transfected with a PATZ1-overexpressing plasmid showed PD-L1 downregulation, suggesting a role for PATZ1 in the negative regulation of PD-L1. We also showed that PATZ1 overexpression inhibits NSCLC cell proliferation, migration, and invasion, and that Patz1-knockout mice develop LUAD, indicating that PATZ1 has a key role in suppressing malignant features of NSCLC cells, and suggesting a putative tumor suppressor role.

**Conclusion**

PATZ1 expression and subcellular localization are regulated in NSCLC, where high expression and nuclear localization are associated with the LUSC subtype and low tumor PD-L1 expression. High PATZ1 in NSCLC cells inhibits malignant features, suggesting a tumor suppressor role. This was supported by the development of NSCLC in mice knockout for the PATZ1 gene.

### EACR23-0869
### Novel diagnostic DNA methylation biomarkers for renal cell carcinoma

*K. Lommen[1], S.M.J. van Kuijk[2], A. Koch[1], N. Buekers[1], K.A.D. Wouters[1], J. van der Meer[1], T. Marcelissen[3], J.G. van Roermund[3], M.J.B. Aarts[4], T. Kerkhofs[4], F. Smit[5], J. Vandersmissen[5], E. Oosterwijk[6], P. Mulders[6], J. Schalken[6], V. Melotte[1], I.J. Samarska[1], L.J. Schouten[7], M. van Engeland[1], K. Smits[1]*
*[1]GROW – School for Oncology and Reproduction- Maastri cht University Medical Center, Pathology, Maastricht, The Netherlands*
*[2]GROW – School for Oncology and Reproduction- Maastri cht University Medical Center, Clinical Epidemiology and Medical Technology Assessmen t, Maastricht, The Netherlands*
*[3]GROW – School for Oncology and Reproduction- Maastri cht University Medical Center, Urology, Maastricht, The Netherlands*
*[4]GROW – School for Oncology and Reproduction- Maastri cht University Medical Center, Medical Oncology, Maastricht, The Netherlands*
*[5]MDxHealth, MDxHealth, Irvine, United States*
*[6]Radboudumc, Urology, Nijmegen, The Netherlands*
*[7]GROW – School for Oncology and Reproduction- Maastri cht University Medical Center, Epidemiology, Maastricht, The Netherlands*

**Introduction**

As the 5-year survival of renal cell carcinoma (RCC) decreases to 12% in advanced disease stages, early RCC diagnosis is crucial to increase survival rates. Urine is regarded as a non-invasive source of early detection biomarkers, such as DNA methylation biomarkers, that can potentially have a high clinical impact. Currently, published potential urinary DNA methylation biomarkers for RCC do not exceed 65% sensitivity and 89% specificity with Level of Evidence III, making them inapplicable for clinical use. We used a novel *in silico* approach to identify DNA methylation biomarkers for the early detection of RCC.

**Material and Methods**

Publicly available data from The Cancer Genome Atlas (TCGA) was used to identify potential RCC biomarkers. These markers were validated in 85 RCC tissue samples, and 63 normal, healthy kidney tissue samples with quantitative methylation specific PCR to select the most promising biomarkers for urinary validation. These were further validated in cfDNA from an independent population consisting of 92 RCC patients' urine samples, and 115 urine samples from individuals without cancer. ROC curves were created and analyzed to determine methylation cutoffs. Subsequently, a diagnostic model was created using stepwise backward logistic regression analysis. To internally validate the model and correct for optimism, bootstrapping was used.

**Results and Discussions**

Twelve potential diagnostic DNA methylation biomarkers were identified through TCGA analysis; nine biomarkers were suitable for subsequent validation in clinical samples. Individual sensitivities ranged from 13-84%, and individual specificities ranged from 44-98% in tissue samples. The six most promising biomarkers were selected for further evaluation in urine samples. In urine samples,

© 2023 The Authors. Molecular Oncology is published by John Wiley & Sons Ltd on behalf of Federation of European Biochemical Societies individual sensitivities ranged from 3-86% and individual specificities ranged from 7-99%. The final diagnostic model consisted of 4 biomarkers (marker names anonymized to enable patent applications), sex and age, with an optimism-corrected AUC of 0.87. Although not directly linked to RCC, previously these genes have been linked to cell adhesion, invasion, metastasis, apoptosis, oxidative stress and progression in cancer.

**Conclusion**

This DNA methylation biomarker panel for diagnosing RCC in urine showed to be a robust model in the sample set studied here. Therefore, it serves as a promising starting point for further validation, and extension by addition of other types of biomarkers to further improve this model.

### EACR23-0885
### Single molecule localization microscopy for nanoscale detection of extracellular vesicles in cancer

*M. Triki[1], M. Pagliuca[2], C. Schietroma[1], C. Butler[1], B. Verret[2], A. Italiano[3], F. André[2]*
*[1]Abbelight, 191 avenue Aristide Briand, Cachan, France*
*[2]Gustave Roussy Institute, U981 Molecular Predictors and New Targets in Oncology, Villejuif, France*
*[3]Gustave Roussy Institute, Drug Development Department, Villejuif, France*

**Introduction**

Extracellular vesicles (EVs) are nano-sized lipid bilayer vesicles constitutively released from cells known as mediators of cell-to-cell communication. EVs have diverse biological activities, ranging from roles in homeostasis to cancer progression. They have been shown to have great potential as liquid biopsies; unfortunately, the clinical applicability of these biological nanoparticles has yet to be proven. This is because EVs are extremely heterogeneous in size, and molecular composition, thus making them very difficult to characterize. Here, we developed an integrated workflow based on single molecule localization microscopy (SMLM) to characterize individual EVs from patients with advanced cancer. We demonstrate that SMLM can provide unique insights about EVs subpopulations based on EVs size and biomarker distribution.

**Material and Methods**

Plasma from healthy and advanced cancer patients were diluted in PBS (1:50) and loaded on slides with capture monoclonal antibodies (Ab) (CD9; CD63; CD81; etc..) for SMLM imaging. Captured EVs were stained using a mix of anti-tetraspanin Ab (CD9+CD63+CD81) labelled with AF647 fluorophores. Single molecule imaging (dSTORM) was performed using Abbelight SMART-kit buffer on a SAFe360 Abbelight super-resolution module mounted on an Nikon Ti2 inverted microscope. Fluorophore labelled EVs were excited with the 640nm laser at 50% of nominal power over a ROI of 80*80 micrometers, by Abbelight Aster technology for homogeneous laser illumination; for each dataset 10000 frames were collected at 40 FPS, with one-two technical replicates per sample (RAW data). Single molecule localization in 3D was performed on the RAW data using Abbelight Neo_Analysis Software, and localization clusters corresponding to labelled EVs were extracted using DBSCAN and K-Ripley clustering algorithms.

**Results and Discussions**

Single molecule localization microscopy was used to generate EVs high-resolution images. The quantification of biomarker distribution in each patient's sample revealed variations in the size and number of clusters. These findings support the great potential of SMLM technology to detect and characterize individual EVs in biological clinical samples.

**Conclusion**

The association of tetraspanin labeling with SMLM imaging enables the detection of demonstrable differences in plasma samples obtained from healthy individuals versus those with advanced cancer. Further studies are conducted to refine this technology and render it suitable for use for clinical implementation.

### EACR23-0906
### Identification of high risk patients in stage II pT3N0 microsatellite-stable colorectal cancer

*E. Budinska[1], T. Catela Ivkovic[2], M. Hrivnakova[1], O. Slaby[2], B. Bencsikova[3], V. Popovici[1]*
*[1]Masaryk University, RECETOX - Fac. of Science, Brno, Czech Republic*
*[2]Masaryk University, Central European Institute of Technology, Brno, Czech Republic*
*[3]Masaryk Institute of Oncology, Masaryk Institute of Oncology, Brno, Czech Republic*

**Introduction**

The risk of recurrence of patients with stage II colorectal cancer (CRC) without adjuvant chemotherapy is, in general, 15-20%. The benefit of adjuvant chemotherapy is unclear, with an estimated 2% reduction of 5-year relapse likelihood. In the absence of other risk factors, patients with stage II pT3N0 CRC are considered low risk and not given adjuvant chemotherapy. However, about 10% of these patients relapse within five years. Here, we identify the main markers for risk of relapse for untreated pT3N0 CRC patients, proposing an approach for predicting those that may benefit from adjuvant treatment.

**Material and Methods**

Two cohorts of patients with stage II pT3N0 microsatellite-stable CRC were used (one in-house cohort and a subset of publicly available dataset E-MTAB-863 (ArrayExpress)), with sample size of 39 and 150, respectively. “Early relapse” was defined as relapse within 5 years after tumor resection, while “late relapse” was defined as no relapse for at least 6 years. Differential gene expression followed by gene set enrichment analysis were used for identifying the main differences between the groups at gene and pathway levels. Elastic net regression was used for building predictive models using single sample pathway activation scores as features and performance was estimated using 5-fold cross-validation.

**Results and Discussions**

The strongest differences at pathway levels between the two groups were found in epithelial-to-mesenchymal transition, TNF-alpha signalling, interferon alpha and gamma response, and hypoxia response. In addition, early relapsing tumors were enriched in myofibroblastic cancer-

© 2023 The Authors. Molecular Oncology is published by John Wiley & Sons Ltd on behalf of Federation of European Biochemical Societies associated fibroblasts (ECM-myCAF and wound-myCAF). The predictive model achieved an estimated accuracy of 76.9% (95% CI: 60.3-88.3) and 71.33% (95% CI: 63.3-78.3) on the two datasets. The patients relapsing within 5 years were identified with a precision of 80% (95% CI: 55.7-93.4) and 73.2% (95% CI:59.5-83.8), respectively.

**Conclusion**

Several major pathways are differentially activated between patients relapsing within 5 years and those with no relapse for at least 6 years. Interestingly, the presence of specific fibroblasts seem also to be indicative of shorter time to relapse. A classifier built on pathway activation scores was able to predict relapse with good sensitivity in untreated pT3N0 patients, selecting those that may benefit from adjuvant therapy.

### EACR23-0916
### miR-124 as a liquid biopsy prognostic biomarker in small extracellular vesicles from NSCLC patients

*Á. García[1], D. Sanchez-Cabrero[1], O. Vera[1], L. Gutierrez[1], O. Higueras[1], I. Estaban[1], C. Rodriguez-Antolin[1], I. Losantos[2], J. De Castro[1], I. Ibanez de Caceres[1]*
*[1]IdiPAZ-FIBHULP, Experimental Therapies and Novel biomarkers in cancer, madrid, Spain*
*[2]IdiPAZ-FIBHULP, Biostatistcs Unit, madrid, Spain*

**Introduction**

Due to Lung cancer is the most common form of cancer, there is great scientific interest in finding new biomarkers to help predict the development of the disease and optimise clinical decisions. Thus, miRNAs emerge as a good oncological biomarker for both tissue and liquid biopsy samples. In this study we have evaluated the potential prognostic role of 7 candidate miRNAs selected in a previous microarray screening study performed in patients with early NSCLC.

**Material and Methods**

In this study, a total of 120 samples were collected from three cohorts of 88 NSCLC patients:

- 48 samples were paired non-tumour and tumour tissue, plus plasma samples from 16 Stg (I/II) patients
- 21 plasma samples were collected from an additional cohort of Stg (I/II) patients.
- 51 plasma samples were collected from a cohort of Stg (III/IV) patients.

The miRNA fraction was obtained by standard manual protocol (Trizol) for tissue samples and by specific isolation of and extraction kits for free circulating and exosomal miRNA samples. The miRNAs levels in the different sample types were analyzed by qRT-PCR using Taqman probes. Survival analyses were performed considering the clinicopathological data and KM. The study was completed with an *in silico* analysis for the identification of miR-124 target genes with clinical implication in 2952 NSCLC patients from two public database.

**Results and Discussions**

In this study we found we found that lower tissue levels of miR-10 and higher levels of miR-124 and miR-7 in plasma were significantly associated with both relapse and exitus in early-stage patients. We found both miR-124 and miR-132 circulating levels significantly increased in samples from advanced-stage patients. Our translational approach in terms of survival indicate that only exosomal levels of miR-124 significantly predict both PFS and OS in advanced stage NSCLC patients. *In silico* results show that miR-124 may be a regulator of specific genes with a potential oncogenic role in NSCLC.

**Conclusion**

Plasma levels of miR-124 could be used as a potential novel biomarkers in liquid biopsy to predict the evolution of NSCLC in terms of PFS and OS. Funding:Instituto de Salud Carlos III and the European Regional Development Fund/European Social Fund FIS [ERDF/ESF], Una Manera de Hacer Europa (PI18/050; PI21/0145; La Caixa impulse CI20-00182)

### EACR23-0920
### Adjuvant therapy in cutaneous melanoma: monitoring disease recurrence by circulating tumor DNA

*S. Marchisio[1], A.A. Ricci[1], A. Russo[1], E. Berrino[1], E. Ortolan[1], G. Matullo[1], C. Marchiò[1], R. Senetta[2], A. Funaro[1], P. Quaglino[1]*
*[1]University of Turin, Department of Medical Sciences, Turin, Italy*
*[2]University of Turin, Department of Oncology, Turin, Italy*

**Introduction**

Adjuvant treatment with targeted therapies (TT) or immune checkpoint inhibitors (ICI) has drastically reduced the risk of tumor relapse in patients with resected cutaneous melanoma (CM) at stage III/IV. The short-term benefit associated with adjuvant treatment highlighted the need for markers to predict and monitor the disease course. To this aim, we investigated the landscape of somatic mutations in CM sites and circulating tumor DNA (ctDNA).

**Material and Methods**

37 CM patients receiving adjuvant treatment (TT or ICI) were enrolled. DNA was extracted from primary tumor surgical specimens and 523 cancer genes were sequenced by next generation sequencing (NGS). In 23 patients of the cohort, plasma was collected monthly during therapy. We performed longitudinal analysis of plasma ctDNA using droplets digital PCR (ddPCR) in CM harboring $BRAF^{V600}$ (n=15) or $NRAS^{Q61}$ (n=5) mutations in their primary sites. In patients wild-type for both driver genes (WT; n=3) we monitored both $BRAF^{V600}$ and $NRAS^{Q61}$ ctDNA mutations.

**Results and Discussions**

By NGS, we identified 53 somatic non-synonymous pathogenic or likely pathogenic coding variants, including mutations never described in CM. Considering the driver genes, 26/37 patients carried $BRAF^{V600}$ mutations, which were mutually exclusive with $NRAS^{Q61}$ alterations (5/37). 6/37 were WT for BRAF and NRAS. At the beginning of adjuvant therapy, ddPCR confirmed mutant ctDNA detection in 1/23 individuals, supporting the disease-free status of the patients after surgical eradication of the tumor. In the positive case, who had no evidence of relapse, ctDNA became undetectable at subsequent time points. Mutant ctDNA was analysed with the disease status in relapsed (n=7; $BRAF^{V600}$=2, $NRAS^{Q61}$=2, WT=3) and disease-free (n=16; $BRAF^{V600}$=13, $NRAS^{Q61}$=3) patients. At the time of clinical relapse, mutant $BRAF^{V600}$ ctDNA

© 2023 The Authors. Molecular Oncology is published by John Wiley & Sons Ltd on behalf of Federation of European Biochemical Societies was detected in 2/2 patients. Notably, mutant $BRAF^{V600}$ ctDNA was detectable even 3 months before clinical relapse. Among the WT group, in 1/3 patients $BRAF^{V600}$ mutant ctDNA was detected at clinical relapse, and persisted until the end of therapy, suggesting acquisition of a new mutation potentially contributing to therapy resistance. $NRAS^{Q61}$ ctDNA was undetectable, probably due to low sensitivity of our detection approach. Specific mutant ctDNA was undetectable in all 16 disease-free patients.

**Conclusion**

Our preliminary results suggest that mutant $BRAF^{V600}$ ctDNA might be an easy-to-use tool for early detection of CM recurrence and better management of patients during adjuvant treatment.

### EACR23-0978
### Plasma EV-derived hsa-miR-4521 as a potential biomarker of clear cell Renal Cell Carcinoma aggressiveness

*F. Dias[1], A.L. Teixeira[1], M. Mastik[2], I. Vieira[3], J. Siva[3], J. Maurício[4], A. Morais[3], B. Costa-Silva[5], K. Kok[6], R. Medeiros[1]*
*[1]Portuguese Oncology Institute of Porto Research Center- Porto Comprehensive Cancer Center Porto.CCC & RISE @CI-IPOP Health Research Network, Molecular Oncology and Viral Pathology Group, Porto, Portugal*
*[2]University Medical Center Groningen, Department of Pathology and Medical Biology, Groningen, The Netherlands*
*[3]Portuguese Oncology Institute of Porto, Department of Urology, Porto, Portugal*
*[4]Portuguese Oncology Institute of Porto, Department of Medical Oncology, Porto, Portugal*
*[5]Champalimaud Centre for the Unknown, Systems Oncology Group, Lisbon, Portugal*
*[6]University Medical Center Groningen, Department of Genetics, Groningen, The Netherlands*

**Introduction**

The identification of clear cell Renal Cell Carcinoma (ccRCC) patients likely to develop metastatic disease, despite an apparent low risk at the time of surgery, represents a key clinical issue. This is particularly important for the identification of subgroups of patients' whose clinical outcome is not correctly predictable based on the conventional scoring systems. Due to the unique function of the kidneys, both blood and urine are valuable biofluids with abundant extracellular vesicles (EVs), which are readily accessible sources for biomarkers discovery through minimally invasive approaches. Thus, our aim was to study the plasma EVs microRNA abundance in ccRCC patients at different phases of the disease to explore new potential prognosis biomarkers.

**Material and Methods**

We recruited two groups of ccRCC patients: Group A (N=17) made by patients with low risk ccRCC that collected blood before and after surgery, and Group B (N=14) made by patients with metastatic disease, that were considered low risk at diagnosis but developed metastasis after surgery and collected blood once. EVs were isolated from the patients' plasma samples and characterized by Transmission Eletron Mycroscopy, Nanoparticle Tracking analysis and Flow cytometry. The microRNAs were analyzed using the NanoString Human v3 miRNA Panel, which is a technology capable of detecting 799 known microRNAs per sample at once.

**Results and Discussions**

When comparing the pre-surgery samples with the post-surgery samples (group A), we observed a decrease in the abundance of 4 EV-derived miRNAs (hsa-miR-1233-3p, has-miR-142-5p, hsa-miR-133b and hsa-miR-4521) after tumor removal and an increase in the abundance of 9 EV-derived miRNAs. Functional enrichment bioinformatic analysis showed that the 4 miRNAs that decreased after surgery were involved in regulation of intrinsic apoptotic signalling pathways, regulation of oxidative stress, cell aging, central carbon metabolism in cancer and adherens junction.On the other hand, when we compared the post-surgery samples (group A) with the metastatic group (group B), we observed a decrease of 11 EV-derived miRNAs and an increase of hsa-miR-4521.

**Conclusion**

EV-derived hsa-miR-4521 can be a potential prognostic biomarker for ccRCC aggressiveness since its EV abundance decreases after tumor removal and increases when metastatic disease is settled. These results need to be validated in a larger cohort of patients and more data is needed regarding the functional impact of has-miR-4521 in ccRCC disease progression.

### EACR23-0984
### Circulating microRNAs as cancer biomarkers in Lynch syndrome

*T. Sievänen[1], J. Karvanen[2], T. Jokela[1], T.M. Korhonen[1], K. Pylvänäinen[3], S. Sipilä[1], J.P. Mecklin[4], E. Sillanpää[1], T.T. Seppälä[5], E. Laakkonen[1]*
*[1]University of Jyväskylä, Faculty of Sport and Health Sciences, Jyväskylä, Finland*
*[2]University of Jyväskylä, Faculty of Mathematics and Statistics, Jyväskylä, Finland*
*[3]Central Finland Health Care District, Department of Education and Research, Jyväskylä, Finland*
*[4]Central Finland Health Care District, Department of Surgery, Jyväskylä, Finland*
*[5]University of Tampere, Faculty of Medicine and Health Technology, Tampere, Finland*

**Introduction**

Lynch syndrome (LS) is an inherited cancer predisposition syndrome that manifests in high cancer risk which is modified by sex and body weight. Circulating microRNAs (c-miRs) are lifestyle modifiable gene regulators that contribute to all carcinogenesis stages and display biomarker potential. We have shown that the c-miR profile of cancer-free LS carriers does not differ from the corresponding profile of sporadic cancer patients and deviates LS carriers from the healthy population. Here, we investigated if c-miR profile can predict cancer event in LS within 2-3 year follow-up.

**Material and Methods**

c-miR data was collected with high-throughput sequencing from cancer-free LS carriers (n=117, 52% males) included in Finnish LS Research Registry. Of them, 18 (72% males) developed cancer during the follow-up. Data was randomly

© 2023 The Authors. Molecular Oncology is published by John Wiley & Sons Ltd on behalf of Federation of European Biochemical Societies split to training (n=59) and validation group (n=58). With the training group, Lasso-regularized Cox model with age as time scale was used to screen the best predictor c-miRs from the previously characterized LS-specific signal of 40 c-miRs. Model performance was evaluated with Harrel's C-index using the validation group. To avoid overfitting, risk sum was calculated for the of Lasso-obtained c-miRs by multiplying each c-miRs' expression with the respective Cox regression coefficient. Body mass index (BMI) was calculated by dividing the subject's weight with squared height. The final multivariate Cox model included c-miR risk sum adjusted with sex and BMI.

**Results and Discussions**

Lasso identified miR-10b, miR-27a and miR-3613 as the best predictors of LS cancer event with C-index 0.83. Model performance in the validation group was moderate with C-index 0.66. Multivariate Cox model showed that the risk sum predicted LS cancer event (hazard ratio 2.72, 95% confidence interval 1.43-5.16). When this model was adjusted with sex and BMI, the risk sum remained as an independent and more robust predictor of LS cancer event (hazard ratio 3.70, 95% confidence interval 1.61-8.54). These preliminary results show that higher expression of miR-10b, miR-27a and miR-3613 predict LS cancer event when potential confounding by sex and BMI is adjusted. In support to our findings, all these miRs have been shown to have various roles in several sporadic cancer types.

**Conclusion**

Our results suggest that c-miRs can predict cancer event in LS and may be useful in identifying individuals at higher risk. However, larger sample is needed to confirm these findings.

### EACR23-1002
### Evaluation of the Tie-2-expressing monocytes in patients suffering from ovarian cancer as a diagnostics biomarker

*W. Skiba[1], A. Pawłowska[1], A. Kwiatkowska-Makuch[2], W. Kuryło[3], B. Barczyński[2], R. Tarkowski[2], J. Kotarski[1], I. Wertel[1]*

*[1]Medical University of Lublin, Independent Laboratory of Cancer Diagnostics and Immunology, Lublin, Poland*

*[2]Medical University of Lublin, I Chair and Department of Gynaecologic Oncology and Gynaecology, Lublin, Poland*

*[3]Medical University of Lublin, The Student Research Group- Independent Laboratory of Cancer Diagnostics and Immunology, Lublin, Poland*

**Introduction**

One of the most deadly female genital tract cancers is ovarian cancer (OC). The prognosis for OC is poor, and the mortality rate is devastating. There are no effective screening methods and biomarkers for OC. According to the literature, one subset of monocytes/macrophages, Tie-2-expressing monocytes/macrophages (TEMs), plays a key role in the tumor microenvironment, tumor progression, and tumor spread. The role of these cell subsets in the development of OC has not been fully explored. This study aimed to determine if Tie-2-expressing monocytes (MO) could be used as a biomarker of ovarian cancer progression.

**Material and Methods**

There were 30 OC patients in the study group, and 8 healthy blood donor individuals in the control group included in the study. The percentage of Tie-2 expressing MO was investigated in the peripheral blood by flow cytometry. The obtained results were compared to the control group and the clinical characteristics of the patients.

**Results and Discussions**

The percentage of TEMs ($CD14^{++}Tie\text{-}2^{+}$) was evaluated in monocytes, dividing them into two subpopulations ($CD14^{++}CD16^{-}Tie\text{-}2^{+}$ - classical and $CD14^{++}CD16^{+}Tie\text{-}2^{+}$ - intermediate). The frequency of classical $Tie\text{-}2^{+}$ monocytes in OC patients was significantly higher ($p<0.0001$) than the classical MO. TEMs percentage among classical MO (median 0.22; range 0.01 – 8.12) did not differ significantly ($p>0.05$) between OC patients and the healthy control group (median 0.10; range 0.02 – 0.24). The same tendency was observed in intermediate $Tie\text{-}2^{+}$ MO (median 2.25; range 0.06 – 37.58) in the study group in comparison to the control group (median 1.14; range 0.08 – 5.99) ($p>0.05$). The percentage of TEMs in classical and intermediate MO did not differ significantly ($p>0.05$) in patients with intermediate-grade tumors (G2) compared to high-grade tumors (G3) as well as in the I-II FIGO stage in comparison to III-IV stage ($p>0.05$). There was no correlation between the percentage of classical TEMs and CA125 level ($p>0.05$). Likewise, the correlation between intermediate TEMs and CA125 was not observed ($p>0.05$).

**Conclusion**

The percentage of Tie-2 expressing monocytes did not differ significantly between OC patients and the control group. The percentage of TEM did not differ significantly between patients with different clinical manifestations of ovarian cancer. This subset of monocytes has limited use as a biomarker of OC progression.

### EACR23-1006
### Molecular profiling of newly diagnosed gliomas by liquid biopsy of cerebrospinal fluid

*F. Orzan[1], F. De Bacco[1,2], E. Lazzarini[3], G. Crisafulli[4], A. Gasparini[3], A. Dipasquale[5], C. Giovenino[1], M. Macagno[6], P. Persico[5], F. Pessina[7], B. Bono[7], L. Giordano[5], P. Zeppa[8], P. Cassoni[9], D. Garbossa[8], A. Santoro[5], P.M. Comoglio[10], S. Indraccolo[3,11], M. Simonelli[5,12], C. Boccaccio[1,2]*

*[1]Candiolo Cancer Institute- FPO-IRCCS, Laboratory of Cancer Stem Cell Research, Candiolo, Italy*

*[2]University of Turin Medical School, Department of Oncology, Candiolo, Italy*

*[3]Veneto Institute of Oncology IOV-IRCCS, Basic and Translational Oncology Unit, Padua, Italy*

*[4]IFOM ETS – The AIRC Institute of Molecular Oncology, BioInformatic Group BIG & Genomics of Cancer and Targeted Therapies Unit, Milan, Italy*

*[5]IRCCS Humanitas Research Hospital, Medical Oncology and Hematology Unit, Rozzano, Italy*

*[6]Candiolo Cancer Institute- FPO-IRCCS, Laboratory of Cancer Epigenetics, Candiolo, Italy*

*[7]IRCCS Humanitas Research Hospital, Neurosurgery Department, Rozzano, Italy*

*[8]University of Turin Medical School,*

© 2023 The Authors. Molecular Oncology is published by John Wiley & Sons Ltd on behalf of Federation of European Biochemical Societies

Department of Neurosciences, Turin, Italy
[9]University of Turin Medical School,
Department of Medical Sciences, Turin, Italy
[10]IFOM ETS – The AIRC Institute of Molecular Oncology, Scholar, Milan, Italy
[11]University of Padova,
Department of Surgery- Oncology and Gastroenterology, Padua, Italy
[12]Humanitas University,
Department of Biomedical Sciences, Pieve Emanuele, Italy

**Introduction**

Molecular profiling is recommended by diagnostic guidelines as a means of classifying glioma patients into prognostic and treatment subgroups. If the tumor tissue is inaccessible, liquid biopsy may be the sole alternative for performing molecular profiling. The presence of the blood-brain-barrier hinders glioma DNA release into circulation, restricting the practicality of blood-based strategies. To overcome this limitation, the use of cerebrospinal fluid (CSF) as an alternative source of circulating tumor DNA is currently investigated. Here we present a prospective study exploring CSF-based liquid biopsy for genetic profiling and stratification of patients with newly diagnosed malignant glioma.

**Material and Methods**

CSF samples were retrieved from two cohorts of newly diagnosed glioma patients after obtaining informed written consent and based on institutionally approved studies conducted in accordance with the Declaration of Helsinki. In cohort 1 (n=45) CSF was collected in proximity of the tumor (peritumoral CSF), while in cohort 2 (n=39) CSF was collected by lumbar puncture (LP-CSF), immediately prior to surgery. Glioma tissues were also recovered. Comprehensive Next Generation Sequencing (NGS) or targeted methodologies (droplet digital or BEAMing PCR) were used to characterize DNA samples from CSF and matched gliomas. Correlations between tumor clinical features and CSF DNA characteristics were analyzed and molecular diagnosis based on liquid biopsy was compared with conventional histopathological diagnosis.

**Results and Discussions**

Peritumoral CSF samples contained abundant tumor DNA, whereas LP-CSF samples had lower levels, often below the sensitivity threshold of NGS. However, our study found that in over 60% of LP-CSF samples, there was still sufficient tumor DNA to analyze a selected panel of relevant genetic alterations (including IDH and TERT promoter mutations, EGFR amplification, CDKN2A/B deletion, and MGMT methylation: ITEC protocol) that allowed for tissue-agnostic identification of major diffuse glioma molecular subtypes. Notably, the absence of tumor DNA in LP-CSF samples was associated with a better prognosis.

**Conclusion**

This study reveals both the potential and limitations of using CSF liquid biopsy to achieve molecular diagnosis of gliomas at the initial clinical presentation. Additionally, the study proposes a flexible protocol able to maximize the diagnostic information that can be obtained from CSF DNA.

### EACR23-1020
### Analysis of PLA2G12A gene as a promising biomarker for colorectal cancer prognosis

I. Hidalgo Muñoz[1], R. Montal[2], A. Casali[3], A. Salud[2], M.A. Sorolla[1], E. Parisi[1]
[1]IRBlleida, Cancer, Lleida, Spain
[2]Arnau de Vilanova University Hospital,
Medical Oncology, Lleida, Spain
[3]Universitat de Lleida,
Department of Basic Medical Sciences, Lleida, Spain

**Introduction**

Colorectal cancer (CRC)is one of the most frequently diagnosed cancers worldwide. Although early detection and screening programs have reduced its incidence and associated mortality, it is estimated that between 40-50% of patients with CRC will have metastases, which is the most common cause of cancer-related death. For this reason, the identification of new biomarkers to better recognize patients with high risk of metastasis is still needed.

**Material and Methods**

Previous results from our group in a CRC Drosophila melanogaster in vivo model showed differences in circulating tumor cells (CTCs) and in tumor burden when certain genes were inhibited, among them PLA2G12A, a phospholipase which functions are still unknown. We checked the implication of that gene in cancer processes using in silico analysis from TCGA data and three human colon cancer cell lines (HCT116, HT29 and SW480). From TCGA analysis we have elucidated some of the pathways in which PLA2G12A can be involved. Later, we used in vitro models where we suppressed the expression of PLA2G12A with interference RNA and we analyzed the effect in cell migration, proliferation and invasion through the scratch wound healing, colony formation and transwell invasion assay, respectively.

**Results and Discussions**

Our results reveal a significant increase on migration, proliferation and invasion rates when PLA2G12A is downregulated.

**Conclusion**

These findings provide evidence that PLA2G12A deficiency promotes tumor growth and dissemination, and encourage further analysis in CRC human cohorts to validate PLA2G12A as a good predictive biomarker of metastasis.

### EACR23-1027
### Multi-cancer CpG methylation biomarkers accurately predict tumour status

T. Vanpoucke[1], J. Vandenhoeck[1], I. Neefs[1], J. Ibrahim[1], A. Suls[1], D. Peeters[2], A. Hoischen[3], M. Peeters[4], G. Van Camp[1], K. Op de Beeck[1]
[1]University of Antwerp and Antwerp University Hospital, Centre for Medical Genetics - Centre for Oncological Rese arch Antwerp, Edegem, Belgium
[2]Antwerp University Hospital, Department of Pathology, Edegem, Belgium
[3]Radboud University Medical Centre,
Department of Human Genetics and Radboud Institute for Molecular Life Sciences- Department of Internal Medicine and Radboud Centre for Infectious Diseases, Nijmegen, The Netherlands
[4]University of Antwerp and Antwerp University Hospital, Centre for Oncological Research Antwerp, Edegem,

© 2023 The Authors. Molecular Oncology is published by John Wiley & Sons Ltd on behalf of Federation of European Biochemical Societies

*Belgium*

**Introduction**

Cancer is characterized by a variety of genetic and epigenetic changes that include DNA methylation modifications. Methylation aberrations occur across the entire genome and develop early in disease, making the methylome a rich source of biomarkers. Previously, our lab discovered the *in-silico* possibility to discriminate 14 different cancer types from controls and from each other using methylation biomarkers. For enrichment of specific methylated sites, our lab developed MSRE-smMIP-seq, a new technique combining targeted capture via single-molecule molecular inversion probes with methylation sensitive restriction enzymes.

**Material and Methods**

1791 CpG sites with at least 25% higher methylation in tumour tissue in 8 of the most lethal cancers worldwide compared to adjacent normal tissue and normal blood cells were selected from TCGA array data for validation. These potential biomarker sites were then tested on 35 whole blood and 225 fresh frozen samples, of which 111 were tumour samples and 114 adjacent normal.

All locations were enriched by MSRE-smMIP-seq, amplified by PCR and sequenced. After quality control, sequence mapping and normalization, a dataset with the counts for all targets for each sample was obtained. Using this dataset, a linear discriminant analysis (LDA) model was built per CpG site, predicting tumour status for each sample using 5-fold cross validation. Finally, a global model was made, containing the 358 best CpG site LDA models.

**Results and Discussions**

The single CpG LDA models performed well, with 939 CpG models having a cross-validated area under the curve (cvAUC) over 0.8, with the best model even achieving 0.92 cvAUC. The global model has a sensitivity of 0.84, a specificity of 0.95 and an accuracy of 0.90. Accuracy per cancer type ranged from 0.71 to 1, with breast tumours being discerned with perfect accuracy, while colorectal and liver tumours performed worst. While the sensitivity of the final model is not perfect (0.84), for every sample there are single-smMIP models that make the correct prediction. Therefore, all tumour samples are distinguishable from normal tissue at least at one of the selected CpG sites.

**Conclusion**

We discovered 939 CpG sites that can be used for prediction of tumour status in tissue samples. Combining the 358 best performing markers, a model was constructed with a sensitivity of 0.84, a specificity of 0.95 and an accuracy of 0.90.

### EACR23-1030
### A serum-based multi-OMICS signature for early detection of pancreatic cancer

*A. Gavish[1], B. Sarvin[2], O. Kronfeld[3], E. Kepten[3], S. Shaham-Niv[4], T. Shlomi[4]*
*[1]MetaSight, Medical Affairs, Rehovot, Israel*
*[2]MetaSight, Chemistry, Rehovot, Israel*
*[3]MetaSight, Data Science, Rehovot, Israel*
*[4]MetaSight, Research and development, Rehovot, Israel*

**Introduction**

Pancreatic ductal adenocarcinoma (PDAC) is one of the deadliest cancers, accounting for approximately 50,000 deaths per year in the United States. Unfortunately, it is very often diagnosed at a late stage when unresectable. To date, there are no approved efficient and cost effective screening methods for PDAC, which would allow earlier diagnosis and potentially better outcome. Here, we developed a novel cost-effective multi-OMICS diagnostic signature for PDAC based on metabolomic, lipidomic, and proteomic markers, which is robust more than 6 months prior to diagnosis and amenable for general population screening.

**Material and Methods**

Serum samples from ~500,000 subjects were collected as part of standard clinical routine by a central Israeli HMO , within the ongoing Israeli multi-OMICS Serum Screening (IMOSS-500K) study. Electronic health records were used to identify 97 samples from patients with PDAC diagnosed after serum was collected and then manually validated by a health specialist. Age and gender matched controls were identified for comparison (n=117). All samples were analyzed with high-throughput liquid-chromatography mass-spectrometry (LC-MS) based metabolomics, lipidomics and proteomics. Machine learning was used to identify a molecular signature distinguishing cases from controls.

**Results and Discussions**

We identified a novel molecular signature for PDAC consisting of 2 metabolites, 1 lipid and 1 protein. In a standard cross-validation test, our signature demonstrated ROC AUC of 0.89. Tested in specific time windows of 0-3 months (n=46), 3-6 months (n=28) and 6-10 (n=18) months prior to diagnosis, we got ROC AUCs of 0.91, 0.87 and 0.81, respectively. Of note, CA19-9 decreases considerably when tested 6-12 months prior to diagnosis, reaching a ROC AUC of 0.7 (Fahrmann J. et al, Gastroenterology 2021). Importantly, the diagnostic performance of our signature remains high also when comparing PDAC cases with intra papillary mucinous neoplasm cases (IPMN, n=73) up to 12 months prior to IPMN diagnosis, with a ROC AUC of 0.83.

**Conclusion**

We propose a novel, non-invasive, serum-based method for early diagnosis of PDAC. Our results suggest that our signature can distinguish PDAC cases from controls several months before these patients would have otherwise been diagnosed. The cost-efficiency of metabolomics and proteomics methods would allow screening large at-risk populations, such as patients diagnosed with NOD, enabling earlier diagnosis and more treatment options for this deadly disease.

### EACR23-1032
### miRNA dysregulation as an emerging tool for oral cancer diagnosis

*N. Mehterov[1], A. Sacconi[2], C. Pulito[3], B. Vladimirov[4], G. Haralanov[4], D. Pazardjikliev[5], B. Nonchev[6], I. Berindan-Neagoe[7], G. Blandino[8], V. Sarafian[1]*
*[1]Medical University-*
*Plovdiv- Research Institute- Medical University-Plovdiv, Department of Medical Biology-, Plovdiv, Bulgaria*
*[2]IRCCS Regina Elena National Cancer Institute, UOSD Clinical Trial Center- Biostatistics and Bioinformat ics, Rome, Italy*
*[3]Regina Elena National Cancer Institute,*

© 2023 The Authors. Molecular Oncology is published by John Wiley & Sons Ltd on behalf of Federation of European Biochemical Societies

Translational Oncology Research Unit- Department of Research- Advanced Diagnostic- and Technological Innovation, Rome, Italy
[4]Medical University-Plovdiv,
Department of Maxillofacial Surgery, Plovdiv, Bulgaria
[5]Medical University-Plovdiv,
Department of Otorhinolaryngology, Plovdiv, Bulgaria
[6]Medical University-Plovdiv,
Department of Endocrinology, Plovdiv, Bulgaria
[7]"Iuliu Hatieganu" University of Medicine and Pharmacy,
Research Center for Functional Genomics- Biomedicine and Translational Medicine, Cluj-Napoca, Romania
[8]IRCCS- Regina Elena National Cancer Institute,
Translational Oncology Research Unit- Department of Research- Advanced Diagnostic- and Technological Innovation, Rome, Italy

**Introduction**

Oral squamous cell carcinoma (OSCC) survival has not been improved over the last decades and its development is accompanied by aberrant microRNAs (miRNAs) expression.

**Material and Methods**

We applied bioinformatical and molecular methods to identify miRNAs with possible clinical significance as biomarkers in OSCC. A set of 10 miRNAs were selected via an *in silico* approach by analysing the 3'untranslated regions (3'UTRs) of cancer-related genes. RT-qPCR was used to compare the expression of *in silico* identified miRNAs in OSCC and their normal counterparts (n=32).

**Results and Discussions**

Among the miRNAs analysed, miR-21-5p ($p < 0.0001$), miR-93-5p ($p < 0.0197$), miR-146b-5p ($p < 0.0012$), miR-155-5p ($p < 0.0001$), miR-182-5p ($p < 0.0001$) were significantly induced, whereas miR-133b ($p < 0.05$) was significantly repressed in OSCC tissues. This was further confirmed in two additional OSCC validation cohorts: Regina Elena National Cancer Institute (IRE cohort, N=74) and The Cancer Genome Atlas Data Portal (TCGA cohort, N=354). T1/T2 stages tumors expressed more miR-133b ($p < 0.0004$) compared to T3/T4 stages. miR-93-5p ($p < 0.0003$), miR-133b ($p < 0.0017$) and miR-155-5p ($p < 0.0004$) were identified as markers for HPV-positive tumors. The 6 miRNAs as a signature predicted shorter disease-free survival (DFS) and could efficiently distinguish OSCC cases from healthy controls with areas under the curve (AUC) of 0.91 with sensitivity and specificity of 0.98 and 0.6, respectively. Further target identification analysis showed enrichment of genes participating in FOXO, longevity, glycan biosynthesis and p53 cancer-related signaling pathways. Also, the selected targets were weekly expressed in OSCC tissues and showed clinical significance related to overall survival (OS) and DFS.

**Conclusion**

Our results demonstrate that miR-21-5p, miR-93-5p, miR-133b, miR-146b-5p, miR-155-5p and miR-182-5p panel could be used as OSCC-specific molecular signature with diagnostic and prognostic significance related to OS and DFS.

### EACR23-1037

### Assessing homologous recombination deficiency in a tumour molecular profiling program

H. Domenech[1], S. Simonetti[2], A. Llop-Guevara[1], C. Viaplana[3], C. García-Durán[4], M. Oliveira[5], I. Pimentel[5], P. Romero[6], E.A. Harrington[7], A. Oaknin[4], P. Nuciforo[2], S. Aguilar[8], V. Ana[6], R. Dienstmann[3], C. Saura[5], J. Balmaña[5], V. Serra[1]
[1]Vall d'Hebron Institute of Oncology,
Experimental Therapeutics Group, Barcelona, Spain
[2]Vall d'Hebron Institute of Oncology,
Molecular Oncology Group, Barcelona, Spain
[3]Vall d'Hebron Institute of Oncology,
Oncology Data Science Group, Barcelona, Spain
[4]Vall d'Hebron University Hospital- Vall d'Hebron Institute of Oncology, Gynaecologic Cancer Programme, Barcelona, Spain
[5]Vall d'Hebron University Hospital- Vall d'Hebron Institute of Oncology, Medical Oncology, Barcelona, Spain
[6]Vall d'Hebron Institute of Oncology,
Cancer Genomics Laboratory, Barcelona, Spain
[7]AstraZeneca, Translational Medicine, Cambridge, United Kingdom
[8]Vall d'Hebron Institute of Oncology,
Molecular Prescreening Program, Barcelona, Spain

**Introduction**

Homologous recombination deficiency (HRD) is associated with platinum and PARPi response in breast cancer (BC) and in high-grade ovarian cancer (HGOC). Genetic/genomic HRD tests have shown a limited predictive value as biomarkers, while functional tests such as detection of RAD51 foci might be more accurate. We implemented the RAD51 test in the molecular profiling program at VHIO with the aim to test its clinical feasibility, validity and utility.

**Material and Methods**

We prospectively analysed tumour samples from advanced BC (n=101) and newly diagnosed HGOC patients (n=59). A custom gene capture sequencing of 435 genes was performed on FFPE tumour samples to report pathogenic mutations in HRR genes (*BRCA1*, *BRCA2*, *PALB2*, *RAD51C* and *RAD51D*) and a genomic instability (GI) score based on genomic LoH, LST and TAI. The RAD51 test was performed on FFPE tumour sections by manual immunofluorescence (IF) staining and scoring. We also studied the impact of time-to-fixation (0-24h) on the evaluability of the RAD51 test in 4 HR proficient (HRP) patient-derived xenografts (PDX).

**Results and Discussions**

Tumour panel sequencing and the RAD51 test were informative in 152/160 (95%) and 125/186 (67%) of tumours, respectively. A higher proportion of surgical specimens delivered non-informative results for RAD51 than for tumour biopsies (26% vs 7%), an effect that was not observed for tumour sequencing (3% vs 2%). Tumours from 4 PDX were used to identify variables impacting the evaluability of the RAD51 test. We observed that fixation after 4h impacts cell/nucleus integrity, geminin signal/background, pan-nuclear RAD51 signal and yH2AX foci. These parameters were used to develop a tissue quality score, which was then applied to assess the quality of HGOC samples. Surgical specimens had a lower quality score than paired intraoperative biopsies, suggesting that delayed time-to-fixation impacts the evaluability of the RAD51 test. Regarding comparisons among HRD

© 2023 The Authors. Molecular Oncology is published by John Wiley & Sons Ltd on behalf of Federation of European Biochemical Societies biomarkers in the tumour molecular profiling program, we noted that 48% of tumours with HRR gene mutations showed functional HRD by RAD51. In addition, genomic and functional HRD tests were 70% concordant and identified potential HRD samples in tumours without HRR gene mutations (57%).

**Conclusion**

RAD51 testing is feasible in an "intent-to-test" population. Delayed sample fixation affects the viability and integrity of the tissue, thus impacting biomarker staining and scoring. RAD51 complements gene panel sequencing results and GI score by providing evidence of functional HRD.

### EACR23-1062
### THERAPY TAILORING IN GASTRIC CANCER: IDENTIFICATION OF BIOMARKERS OF RESPONSE TO ANTI-EGFR TREATMENTS

*D. Conticelli[1], C. Orrù[1], C. Simona[1], S. Giordano[1], C. Migliore[1]*
*[1]Candiolo Cancer Institute- FPO-IRCCS- Candiolo- Italy- University of Torino-, Department of Oncology, Candiolo, Italy*

**Introduction**

Gastroesophageal adenocarcinoma (GEA) represents the third cancer related cause of death worldwide. Despite intense preclinical work, target therapies failed to significantly change GEA clinical practice: compared to other malignancies, few target therapies have been approved so far. Thus, new effective therapeutic strategies are urgently needed for appropriate patients' selection and treatment. EGFR (Epidermal Growth Factor Receptor) targeting in GEA patients has been actively investigated, but the disappointing failure of some phase II/III clinical trials, very likely due to wrong or absent patient selection, determined its resting far from the approval.

**Material and Methods**

To analyze responsiveness to EGFR inhibitors we exploited a proprietary human Gastro-Esophageal Adenocarcinoma (GEA) annotated platform of patient-derived xenografts (PDXs) and PDX-derived primary cells on which we performed *in vivo* and *in vitro* experiments.

**Results and Discussions**

We explored the sensitivity of a subset of 27 primary GEA cell lines to EGFR targeting. 22% of the cell lines were sensitive to the monoclonal antibody cetuximab. These results have been validated *in vivo* by means of xenotrials: tumors were serially transplanted in mice since a cohort of 6 mice per experimental arm with the same tumor was generated. When the tumor of the cohort reached an average volume of 250mm$^3$, mice were treated or not with cetuximab. *In vitro* sensitive tumors (but not the resistant ones) displayed a strong pharmacological response *in vivo*, in some cases reaching a complete response. We reasoned that if EGFR targeting affects the viability of sensitive models, the deregulation of some molecules linked to EGFR pathway may be involved. Indeed sensitive cells displayed a marked overexpression of HER3 and of the AREG and EREG ligands. HER3 targeting (by means of the novel antibody drug conjugate patritumab deruxtecan) and AREG/EREG silencing strongly affected cell viability, suggesting that sensitive models rely on these pathways for their growth.

**Conclusion**

We identified a subset of GEA sensitive to EGFR targeting drugs and propose HER3 and AREG/EREG expression as markers for patients' selection. Further validation on GEA samples derived from clinical trials evaluating effectiveness of EGFR targeting is needed. We believe that, despite the negative results of clinical trials so far, EGFR can represent a suitable target in molecularly selected GEA patients.

### EACR23-1063
### MiRNA-182-5p targets Cyld and Foxo1 in diet-induced NAFLD/NASH/HCC C57BL/6J mouse models and is involved in HCC pathogenesis

*C. Compagnoni[1], V. Zelli[1], R. Capelli[1], A. Corrente[1], F. Colaianni[1], I. Flati[1], M. Di Vito Nolfi[1], E. Alesse[1], F. Zazzeroni[1], A. Tessitore[1]*
*[1]University of L'Aquila, Department of Biotechnological and Applied Clinical Scie nces, L'Aquila, Italy*

**Introduction**

The non-alcoholic fatty liver disease (NAFLD) is a relevant chronic illness worldwide. Variable percentages of NAFLD cases progress from steatosis to steato-hepatitis (NASH), cirrhosis and, eventually, hepatocellular carcinoma (HCC). MicroRNAs are short, non-coding RNAs able to fine-tune gene expression. In a previous work, we described miR-182-5p early dysregulation in a mouse model of diet-induced NAFLD/NASH/HCC progression. In this study, we aim at deepening the role of miR-182-5p in liver damage and HCC pathogenesis.

**Material and Methods**

MiR-182-5p expression was assessed by qRT-PCR in hepatic tissues/tumors from NAFLD/NASH/HCC C57BL/6J mouse models, high fat (HF)- or low fat-high carbohydrate (LF-HC)-diet fed. MiR-182-5p target genes were identified by bioinformatics analysis and validated by miRNA mimics/antagomiR transfection in HepG2 cells. Target genes' protein products were assessed in mouse hepatic tissues by immunoblotting. Publicly available datasets were used to analyse miR-182-5p/target genes expression in human HCC.

**Results and Discussions**

MiR-182-5p increase, more marked in HF-fed mice, was early detected in livers as NAFLD damage progressed, and in tumor compared to peritumor normal tissues. After bioinformatics analysis, Cyld and Foxo1, both with tumor suppressor activity, were identified as interesting putative target genes. *In vitro* assay on HepG2 cells confirmed the miR-182-5p-mediated regulation. According to miR-182-5p levels, decrease of Cyld and Foxo1 protein expression was observed in mouse tumors compared to peritumor tissues. Analyses of publicly available human HCC datasets revealed miR-182-5p, Foxo1 and Cyld expression levels consistent with those from our NAFLD/NASH/HCC mouse models. MiR-182-5p ROC curve showed very good diagnostic accuracy to distinguish normal from tumor tissues.

**Conclusion**

© 2023 The Authors. Molecular Oncology is published by John Wiley & Sons Ltd on behalf of Federation of European Biochemical Societies

In this study, we showed early miR-182-5p overexpression in hepatic tissues obtained from diet-induced NAFLD/NASH/HCC mouse models, and identified two target genes with tumor suppressor activity, Cyld and Foxo1, thus highlighting miR-182-5p involvement in NAFLD/NASH/HCC pathogenesis and progression. Analysis of human HCC publicly available datasets confirmed results obtained from our animal models, emphasizing in particular miR-182-5p diagnostic accuracy to distinguish normal from cancer tissues and opening to further studies to assess its potential role as biomarker of liver disease progression or therapeutic target.

### EACR23-1064
### Novel diagnostic/predictive biomarkers in familial-hereditary breast/ovarian cancer: focus on circulating miRNA-signatures

*V. Zelli[1], C. Compagnoni[1], E. Ricevuto[1], C. Ficorella[1], T. Sidoni[2], A. Di Marco[3], A. Bianchi[3], F. Zazzeroni[1], E. Alesse[1], A. Tessitore[1]*
*[1]University of L'Aquila, Department of Biotechnological and Applied Clinical Scie nces, L'Aquila, Italy*
*[2]St. Salvatore Hospital- L'Aquila, Medical Oncology, L'Aquila, Italy*
*[3]University of L'Aquila, Department of Information Engineering- Computer Scienc e- and Mathematics, L'Aquila, Italy*

**Introduction**
Circulating microRNAs (miRNAs) are emerging as very promising non-invasive biomarkers in cancer. Here, we performed a circulating miRNA profiling in hereditary-familial breast/ovarian cancer (BC/OC) cases, grouped based on the presence/absence of germline pathogenic variants in the high-penetrance susceptibility genes *BRCA1/2*, in order to identify differentially expressed miRNAs of potential clinical relevance as diagnostic/predictive biomarkers.

**Material and Methods**
Plasma miRNAs of a series of 32 familial BC/OC patients, including 18 *BRCA1/2* positive (BRCA) and 14 *BRCA1/2* negative (non-BRCA) cases, were analyzed by miRNA-sequencing using Illumina technology. Five age-matched healthy controls were also included in the study. *A bioinformatic pipeline comprising* Bowtie1 tool for alignment to miRBase v.22 and the reference genome GRCh38, as well as DESeq2 package for differential expression analysis was used. Differentially expressed miRNAs were filtered based on a log2 fold change <-1 or >1 (down-/up-regulated miRNAs respectively), and an FDR adjusted p-value ≤ 0.05. Receiver operating characteristic (ROC) curves were built to determine miRNAs' diagnostic potential by calculating the area under the curve (AUC) with 95% confidence intervals (CI).

**Results and Discussions**
miR-320e emerged as the most relevant miRNA able to distinguish with good accuracy (AUC 0.79; CI: 0.68-1; p-value=0.04) BC/OC cases and controls, showing down-regulation in BC/OCs compared to healthy controls. A total of 23 differentially expressed miRNAs, 12 up-regulated and 11 down-regulated, were found in non-BRCA compared to BRCA cases. Among them, 12 miRNAs were also differentially expressed between non-BRCA cases and control group, thus representing a specific miRNA expression pattern of non-BRCA patients; ROC curves showed very good diagnostic accuracy (mean AUC 0.80; p-value<0.05), suggesting that this miRNA-panel could potentially be used as diagnostic/predictive signature in hereditary-familial non-BRCA BC/OC.

**Conclusion**
Overall, these results suggest that the analysis of circulating miRNAs expression levels, based on the *BRCA1/2* germline mutational status, could provide important information for characterizing and putatively distinguishing familial-hereditary BC/OC cases, particularly non-BRCA group, and might be considered as potentially applicable in screening and prevention programs.

### EACR23-1070
### Fallopian tube lavage proteomics: towards new biomarkers for early ovarian cancer detection

*M. Seaton[1], R. O'Cualain[2], J. Selley[2], D. Knight[2], D. Brison[3], R. Edmondson[4], C. Schmidt[5]*
*[1]Manchester Cancer Research Centre, Genome Stability Laboratory, Manchester, United Kingdom*
*[2]The University of Manchester, The Biological Mass Spectrometry Core Research Facility, Manchester, United Kingdom*
*[3]Manchester Academic Health Sciences Centre, Department of Reproductive Medicine, Manchester, United Kingdom*
*[4]Manchester University NHS Foundation Trust, Division of Gynaecology, Manchester, United Kingdom*
*[5]The University of Manchester, Division of Cancer Sciences, Manchester, United Kingdom*

**Introduction**
High grade serous ovarian cancer (HGSOC) is the most common and aggressive type of ovarian cancer, responsible for 70-80% of the ~200,000 deaths caused by ovarian cancer annually. This is partly due to over 70% of cases not being diagnosed until an advanced stage. Overwhelming evidence now suggests that the vast majority of HGSOCs begin as pre-invasive lesions in the fallopian tube, known as serous tubal intraepithelial carcinoma lesions, which migrate to the ovary and beyond after approximately 6-7 years. There is currently no diagnostic test for these pre-invasive lesions, and existing ovarian cancer diagnostic tests fail to detect almost 40% of early-stage ovarian cancers. Biomarkers capable of detecting ovarian cancer at early and pre-invasive stages are therefore urgently needed to reduce mortality.

**Material and Methods**
To address this challenge, we collected fallopian tube lavages from patients diagnosed with, or having risk-reducing surgery to prevent, HGSOC, as well as controls with normal fallopian tubes and ovaries. Proteomic analysis of these samples was carried out using mass spectrometry.

**Results and Discussions**
Our analysis identified 51 proteins more abundant in the high-risk/cancer patients, which have the potential to be used as biomarkers. Several of the proteins we detected are already known to be involved in the development and

© 2023 The Authors. Molecular Oncology is published by John Wiley & Sons Ltd on behalf of Federation of European Biochemical Societies metastasis of ovarian cancer, highlighting the feasibility of our approach for detecting relevant proteins. Interestingly, we found changes in the MAPK/ERK pathway, calcium signalling, ciliary protein trafficking and cell adhesion to be potential early events in the establishment of pre-invasive lesions.

**Conclusion**

Future work will show whether the identified proteins can also be detected in other biological fluids, and whether they could be used in a diagnostic test for early-stage and pre-invasive cancer. The dysregulated pathways we observed form the perfect groundwork for further understanding the initiation of HGSOC, which could open new opportunities for advances in prevention, early detection and treatment of this debilitating disease.

### EACR23-1085

### Plasma extracellular vesicles-AGO2-related microRNAs as potential prognosis biomarkers in clear-cell renal cell carcinoma

*A.L. Teixeira[1], F. Dias[1], A.S. Patrão[2], M. Morais[1], A. Silva[3], M. Vilhena[3], A. Morais[3], J. Maurício[2], B. Costa-Silva[4], R. Medeiros[1]*

*[1]Portuguese Oncology Institute of Porto / Porto Comprehensive Cancer Center Porto.CCC & RISE@CI-IPOP Health Research Network, Molecular Oncology and Viral Pathology Group- Research Center of IPO Porto CI-IPOP, Porto, Portugal*

*[2]Portuguese Oncology Institute of Porto, Medical Oncology Department, Porto, Portugal*

*[3]Portuguese Oncology Institute of Porto, Urology Department, Porto, Portugal*

*[4]Champalimaud Centre for the Unknown, Systems Oncology Group-, Lisboa, Portugal*

**Introduction**

Renal cell carcinoma(RCC) is one of the leading urologic cancers worldwide, with incidence rate increasing in the last decades, being the clear-cell RCC(ccRCC) the most frequent subtype. About 33% of ccRCC patients are diagnosed with local or distant metastases, and ~40% of patients submitted to surgery will develop metastasis. Therefore, it is important to identify new biomarkers to improve the patients' prognosis. Extracellular vesicles(EVs) present a key role in the cell communication established in tumor microenvironment, being able to promote metastasis formation through cargo transfers among cells. This study aims to investigate the potential of EVs-derived miRNAs involved in AGO2 expression modulation as prognosis biomarkers.

**Material and Methods**

A bioinformatic analysis was performed to select miRNAs that target AGO2. The candidates were analyzed in the plasma EVs fraction from ccRCC patients(n=69), by real-time qPCR during the course of the disease in order to analyze their effect on overall survival(OS). Secretion of the EVs-derived miRNAs profile was also assessed by digital PCR in tumor ccRCC(FG-2, 786-O, Caki-1) and normal(HKC-8) kidney cell lines.

**Results and Discussions**

5 AGO2-related miRNAs were selected to be analyzed in plasma EVs from ccRCC patients. We found that ccRCC patients with localized disease present miR-15b-5p($P$=0.033) and miR-376a-3p($P$=0.046) higher plasma levels compared to metastatic disease patients. Moreover, we observed a trend of higher levels of miR-132-3p($P$=0.079) in patients with localized disease. Patients with low levels of miR-132 present a lower OS(220 *vs* 116 months, Log rank test, $P$=0.033). Cox regression model shows that these patients present a higher risk of death after adjustment for age and ISUP grade(HR=16.12, $P$=0.038), being also associated with an increase of concordance (c) index. Moreover, we observed that EVs from 786-O, Caki-1 and FG-2 present a lower quantity of miR-132 and miR-15 than HKC-8, but, we did not find the presence of miR-376 in EVs from these cell lines.

**Conclusion**

Results shows that EVs from tumor cells present miR-132 low levels compared to normal cells. Moreover, low plasma levels of this miRNA were associated with metastatic disease and poor OS. The low secretion of EVs-derived miR-132 could lead to a microenvironment where oncogenic functions associated with AGO2 overexpression is potentiated. Larger clinical setting validation studies are needed in order to validate the potential application of miR-132 as ccRCC biomarker.

### EACR23-1096

### The determination of Homologous Recombination Deficiency by comprehensive genomic profiling panel associates with clinical outcome in Ovarian Cancer patients.

*C. Roma[1], R. Esposito Abate[1], A. Sacco[1], F. Bergantino[1], D. Califano[2], L. Arenare[3], F. Perrone[3], S. Pignata[4], N. Normanno[1]*

*[1]Istituto Nazionale Tumori-IRCCS-Fondazione G. Pascale, Cell Biology and Biotherapy Unit, Naples, Italy*

*[2]Istituto Nazionale Tumori-IRCCS-Fondazione G. Pascale, Microenvironment Molecular Targets Unit, Naples, Italy*

*[3]Istituto Nazionale Tumori-IRCCS-Fondazione G. Pascale, Clinical Trial Unit, Naples, Italy*

*[4]Istituto Nazionale Tumori-IRCCS-Fondazione G. Pascale, Department of Urology and Gynecology, Naples, Italy*

**Introduction**

Homologous Recombination Deficiency (HRD) status predicts response to treatment with Poly(ADP-ribose) polymerase inhibitors (PARPi) in Ovarian Cancer (OC) patients. The only test approved for the assessment of HRD is the Myriad myChoice CDx assay. The Oncomine Comprehensive Assay Plus (OCA) is a targeted sequencing panel that covers over 500 unique genes, including 46 key genes in the homology recombination repair pathway. Here, we evaluated the concordance in determining HRD status as well as the prognostic ability of Myriad and OCA assays, in a cohort of OC patients enrolled in the MITO16A/MaNGO-OV2 trial.

**Material and Methods**

HRD analysis with OCA was performed on DNA from Formalin-Fixed and Paraffin-Embedded tumor samples of 97 untreated OC patients for which Myriad assay results were available. For OCA, the Genomic Instability Metric (GIM) was determined by measuring the genomic fraction

© 2023 The Authors. Molecular Oncology is published by John Wiley & Sons Ltd on behalf of Federation of European Biochemical Societies that carries unbalanced copy number. A HRD-positive status was assigned in case of positive test for deleterious or suspected deleterious mutations in BRCA genes, or in case of a GIM≥16 for OCA or a Genomic Instability Score (GIS) ≥42 for Myriad. HRD status determined with both assays was associated to Progression-Free Survival (PFS) after first line bevacizumab and platinum-based therapy and Overall Survival (OS) in univariate and multivariate analysis.

**Results and Discussions**

HRD status obtained by OCA was compared with the reference Myriad assay in 86/97 samples for which both test results were available. HRD-positive cases by Myriad were also positive by OCA in 51/52 cases (sensitivity 98.1%). HRD-negative status by Myriad was confirmed by OCA test in 27/34 samples (specificity 79.4%). OCA test showed a good overall concordance with the gold standard assay despite these panels use different approach to calculate HRD (90.7%; K Cohen= 0.80). In univariate analysis, both tests did not associate with improved PFS (Myriad HR=0.68 HRD positive vs negative, p=0.1; OCA HR=0.65, p=0.09). However, both tests resulted prognostic in multivariate analysis for PFS (Myriad HR=0.53, p=0.01; OCA HR=0.46, p=0.006). In univariate analysis for OS, OCA was prognostic (HR=0.44, p=0.016) but not Myriad (HR 0.71, P=0,3). Multivariate analysis for OS was not performed due to low number of events.

**Conclusion**

These data suggest the feasibility of OCA testing for assessing HRD status, with a good concordance with the gold standard assay and association with clinical outcome.

### EACR23-1097

### Impaired IgG antibody response to Merkel cell polyomavirus in patients affected by malignant pleural mesothelioma and workers ex-exposed to asbestos

*J.C. Rotondo[1], C. Mazziotta[1], C.F. Cervellera[1], G. Badiale[1], I. Vitali[1], I. Bononi[2], R. Libener[3], A. Maconi[3], F. Grosso[4], M. Tognon[1], F. Martini[1]*

*[1]University of Ferrara, Medical Sciences, Ferrara, Italy*

*[2]University of Ferrara, Translational Medicine, Ferrara, Italy*

*[3]Research Training and Innovation Infrastructure, Department of Integrated Research and Innovation Activiti es DAIRI- AO SS. Antonio e Biagio e Cesare Arrigo, Alessandria, Italy*

*[4]Mesothelioma Unit - SS. Antonio and Biagio and Cesare, Arrigo Hospital, Alessandria, Italy*

**Introduction**

Malignant pleural mesothelioma (MPM) is a rare but aggressive tumor of the serosal cavities. Previous data suggest that alongside the inhalation of carcinogenic asbestos, other factors are involved in the MPM onset, including oncogenic viruses. Merkel cell polyomavirus (MCPyV) is a ubiquitous small DNA tumor virus linked to Merkel cell carcinoma, a skin tumor that primarily arises in conditions of immune suppression. The MCPyV seroprevalence in MPM patients and in workers ex-exposed to asbestos (WEAs) is unknown.

**Material and Methods**

Herein, an indirect enzyme-linked immunosorbent assay (ELISA) method, with two peptides mimicking MCPyV antigens, was used to investigate immunoglobulins G (IgGs) to MCPyV in sera from MPM patients (n=108), WEA individuals (n=62) and healthy subjects (HS) (n=110). MCPyV seroprevalence and serological profiles were determined. To assess the humoral immune status of MPMs and WEAs, the total serum IgGs were evaluated.

**Results and Discussions**

A lower rate of serum anti-MCPyV IgGs was detected in MPM (29.6%) and WEA (29%) compared to HS (61%) (p<0.001). Serological profile indicated lower optical densities (ODs) in MPM and WEA compared to HS (p<0.05). The mean total IgG concentration resulted similar among MPM, WEA and HS groups (p>0.05). Receiver-operating characteristic (ROC) curves indicated that MCPyV serology proved high sensitivity (77.2-91.2%) and specificity (77.8-93.5%) in distinguishing both MPM and WEA from HS, with moderate J indexes (0.55-0.84); areas under the curves (AUCs) (0.85-0.98) were within the moderate/accurate reference range, and higher than that of a worthless test (0.5, p<0.001). WEAs with the highest asbestos exposure had the lower rate of serum anti-MCPyV IgGs (5.5%) compared to WEAs (45.5%) with the lower exposure. Serological profile confirmed these data.

**Conclusion**

We provided the first evidence of the MCPyV serology in MPM and WEA. Our data suggest that MPMs and WEAs are experiencing a specific impairment of their immune response to MCPyV, which might rely to the immunomodulatory effect of asbestos, a well-known immunosuppressive mineral. Both MPMs and WEAs may be predisposed to a reduced ability to present MCPyV antigens, thus resulting to a possible increase in viral activity. Verifying how and whether MCPyV plays a role in MPM is a future area of study. Evaluating MCPyV serology may complement current follow-up analyses, thus giving additional information on exposed subjects who are at risk of developing asbestos-related tumors, such as MPM.

### EACR23-1111

### Establishment and characterization of preclinical models derived from circulating tumor cells for breast cancer dissemination and drug screening.

*Z. Kahounová[1], M. Pícková[1,2,3], S. Drápela[4], R. Víchová[1], J. Procházková[1], J. Navrátil[5], P. Fabian[6], K. Souček[1,2,3]*

*[1]Institute of Biophysics of the Czech Academy of Sciences- v.v.i, Department of Cytokinetics, Brno, Czech Republic*

*[2]Masaryk University, Faculty of Science- Department of Experimental Biology, Brno, Czech Republic*

*[3]St. Anne's University Hospital, International Clinical Research Center, Brno, Czech Republic*

*[4]H. Lee Moffitt Cancer Center & Research Institute, Department of Molecular Oncology, Tampa- FL, United States*

*[5]Masaryk Memorial Cancer Institute, Department of Comprehensive Cancer Care, Brno, Czech Republic*

© 2023 The Authors. Molecular Oncology is published by John Wiley & Sons Ltd on behalf of Federation of European Biochemical Societies

[6]Masaryk Memorial Cancer Institute, Department of Oncological Pathology, Brno, Czech Republic

**Introduction**

Circulating tumor cells (CTCs) are mediators of tumor dissemination that enter the peripheral circulation from both the primary tumor and metastases and are further transported to distant sites in the body. Due to their easy accessibility from peripheral blood, CTCs have been extensively studied as a possible insight into the metastatic cascade. CTCs-derived preclinical models such as xenografts (CDXs) and *in vitro* cell cultures are urgently needed to understand the biology of CTCs, their role in dissemination and to find potential CTCs-targeting drugs. In this work, we present the establishment and characterization of CDX *in vivo* model and CDX-derived *in vitro* cell culture of a progressive breast cancer.

**Material and Methods**

CTCs-enriched fraction was obtained from 9 ml of patient whole blood using CD45 depletion (RosetteSep™ Human CD45 Depletion Cocktail) and implanted under the renal capsule of female NRG mouse. Single cell suspension from CDX1 tumor was stained for human CD298 marker and $CD298^+$ cells were sorted using cell sorter and expanded in 3D spheroids *in vitro*. Tumorigenic and metastatic potential of the established *in vitro* culture was evaluated *in vivo* in NRG mice. Spectral flow cytometry was used for evaluating the epithelial-mesenchymal phenotype of the models.

**Results and Discussions**

CTCs obtained from patient with advanced progressing breast cancer were able to form tumor under the renal capsule 6 months after implantation (CDX). We were able to propagate the formed CDX subcutaneously in several passages. Further, *in vitro* spheroid culture was established from CDX tumor-derived single cell suspension. Moreover, we evaluated the stem-like potential of established cell line in clonogenic assay. To examine the tumorigenic and metastatic potential of the established cell line, implantation subcutaneously or into the mammary fat pad was performed. Finally, several surface markers were evaluated by spectral flow cytometry to assess epithelial/mesenchymal phenotype of each model.

**Conclusion**

Characterization of the CTCs-derived model may help us understand the plasticity and behavior of these cells during tumor progression. Moreover, this model can be further exploited for drug screening.

This work was supported by a grant from the Czech Health Research Council (NU21-08-00023).

### EACR23-1122
### COLLECTION AND PRESERVATION OF FIRST-VOID URINARY ANALYTES FOR LIQUID BIOPSY APPLICATIONS

K. MacDonald[1], M. Richer[1], T. Dang[1], A. Rioscortes[2], C. Wood[1], N. Meers[2], K. Beyers[2], G. Morse[1], T. Crawford Parks[1]
[1]DNA Genotek, R and D, Ottawa, Canada
[2]Novosanis NV, R and D, Wijnegem, Belgium

**Introduction**

In recent years, liquid biopsy has become increasingly important for cancer screening, detection and monitoring, as cancer-specific biomarkers can be captured non-invasively, reducing patient risk, while also allowing serial and at-home sampling compared to tissue biopsy. In particular, first-void urine (FVU) offers distinct advantages, such as ease of collection and large available volumes. However, FVU requires important pre-analytical considerations, including standardized collection and prevention of biomarker degradation. Colli-Pee® UAS™ FV-5040 is a FVU self-collection device pre-filled with UAS™, a non-lytic liquid preservative. Here, we evaluated the performance of the device for FVU collection and analyte preservation.

**Material and Methods**

FVU samples were collected from healthy male and female donors with the Colli-Pee® UAS™ FV-5040 device to determine total sample volume (n=22) or evaluate analyte preservation (n=14). Samples were stored at room temperature (RT), with aliquots processed at baseline and 8 days, or after freeze/thaw cycling (-20°C to 40°C) used to simulate variability in transport conditions. Cell-free DNA (cfDNA, Qiagen Circulating Nucleic Acid Kit) and extracellular vesicle RNA (EV RNA, Qiagen exoRNeasy Maxi Kit) were extracted from sample supernatants and evaluated for analyte preservation via qPCR assays targeting β-globin (cfDNA) or GAPDH (EV RNA). Cellular pellets were obtained by centrifugation and DNA was extracted (Qiagen PowerFecal Pro DNA Kit). Host cell integrity was inferred using a qPCR assay specific for human DNA (TS143), and microbial growth determined via 16S qPCR. Data are presented as mean ± SD.

**Results and Discussions**

The total collected volume was 39.14 mL ± 1.92 mL. Urinary cfDNA was successfully preserved after RT storage (ΔCt: 0.01 ± 1.04), as was EV RNA (0.58 ± 1.15) and host cell integrity (0.00 ± 0.50). In addition, microbial growth was prevented (ΔCt: 0.06 ± 0.51). Under simulated ambient temperature transportation conditions, cfDNA (ΔCt: 0.57 ± 1.58), EV RNA (1.05 ± 0.94), and host cell integrity (0.63 ± 0.73) were successfully preserved.

**Conclusion**

Urinary cfDNA, EV RNA, and host cell integrity from FVU samples were effectively preserved up to 7 days post-collection at RT, and under simulated ambient temperature transportation. By enabling standardized volumetric FVU collection and analyte preservation, the Colli-Pee® UAS™ FV-5040 device emerges as a non-invasive FVU self-sampling device for oncology applications, simplifying serial and at-home collection.

### EACR23-1143
### Model to predict brain metastatic ability using tumor derived extracellular vesicles: Above seed soil hypothesis

V. Upadhyay[1], K. Bhadresha[2], A. Mandoli[1], R. Rawal[2]
[1]National Institute of Pharmaceutical Research NIPER Ah medabad, Department of Biotechnology, Ahmedabad, India
[2]Gujarat University, Department of Life Sciences, Ahmedabad, India

**Introduction**

Brain metastasis is a life-limiting complication in cancer patients with up to 30% of cancer patients develops it during their disease course, with lung cancer, breast cancer,

© 2023 The Authors. Molecular Oncology is published by John Wiley & Sons Ltd on behalf of Federation of European Biochemical Societies melanoma and renal cell cancer. Currently, no specific biomarkers are available in clinical settings for early detection. Liquid biopsy is one of widely accepted non-invasive method for diagnosing cancer and other diseases. Here, we demonstrate that extracellular vesicles (EVs) derived from breast cancer brain metastatic patients exhibited a great capacity to promote the progression of breast cancer cells.

**Material and Methods**

We analyzed the EMT markers FN1,Vimentin, HIF1, VEGFA, CD44, SNAIL1, TWIST 1 from the exosomes isolated from peripheral blood of breast brain metastasis patients (n=10) and primary breast cancer patients (n=10) and healthy individuals (n=10) by qRT-PCR. To study this *in vitro*, an uptake assay was designed to demonstrate how breast cancer cells uptake EVs from breast brain metastatic patients by co-culturing it with breast cells MDA-MB-231. However, assays post uptake like cell migration, invasion, proliferation, cell cycle analysis and expression analysis were analyzed.

**Results and Discussions**

These EMT markers expression initially when measured in the patient samples, showed a marked difference in the extracellular vesicles of brain metastasis patients from the primary breast cancer patient samples. However, the in-vitro model using these EVs was also developed as a proof of concept to support the hypothesis. EVs obtained from brain metastasis patients when incubated with MDA-MB-231 cells, were efficiently observed to be ingested by breast cancer cells. Even more, these extracellular vesicles when taken by breast cancer cells, it affected breast cancer cell proliferation, migration, invasion, and also could measure some cell cycle changes. This also increased the expression of FN1, VEGFA, CD44, HIF1, SNAIL, TWIST, and VIMENTIN genes in treated MDA-MB-231 cells.

**Conclusion**

The data obtained from this study, support to the possibility that circulating EVs play a significant role in the formation of the pre-metastatic niche, eventually leading to metastasis. These can also be the probable reason of breaking the blood brain barriers and reaching this isolated organ

### EACR23-1156

### Characterizing the neoantigen-specific CD4 T cell receptors in Lynch Syndrome carriers for cancer prevention strategies

*M.S. Benitez-Cantos[1,2,3], S. Garcia-Rodriguez[4,5], R. DIaz de la Guardia[1,6], M.A. Lopez-Nevot[1,7,8], A. Poyatos-Andujar[9], A.M. Serrano-Mira[10], A. Delgado-Maroto[11], P. Lizardi[3], M. Cuadros[1,2,3], C. Cano[5]*

*[1]University of Granada, Department of Biochemistry and Molecular Biology III and Immunology, Granada, Spain*

*[2]Health Research Institute of Granada Ibs.Granada, Cancer Genetics- Biomarkers and Experimental Therapies Group, Granada, Spain*

*[3]Centre for Genomics and Oncological Research- Pfizer/University of Granada/Andalusian Regional Government Genyo, Gene Expression Regulation and Cancer Group, Granada, Spain*

*[4]Centre for Genomics and Oncological Research- Pfizer/University of Granada/Andalusian Regional Government Genyo, Pharmacogenetics Group, Granada, Spain*

*[5]University of Granada, Department of Computer Science and Artificial Intelligence, Granada, Spain*

*[6]Centre for Genomics and Oncological Research- Pfizer/University of Granada/Andalusian Regional Government Genyo, Gene Regulation- Stem Cells and Development Group, Granada, Spain*

*[7]Hospital Universitario Virgen de las Nieves, Clinical Analysis and Immunology Service, Granada, Spain*

*[8]Health Research Institute of Granada Ibs.Granada, Immunogenetics- Systemic and Skin Diseases, Granada, Spain*

*[9]Hospital Universitario San Cecilio, UGC de Laboratorios, Granada, Spain*

*[10]Hospital Universitario Juan Ramón Jiménez, Unidad de Genética. Servicio de Análisis Clínicos, Huelva, Spain*

*[11]Hospital Universitario Torrecárdenas, Unidad de Gestión Clínica de Aparato Digestivo, Almería, Spain*

**Introduction**

Lynch syndrome (LS) confers an 80% lifetime risk of developing colorectal cancer (CRC). LS is caused by germline mutations in the DNA mismatch repair genes, leading to an accumulation of frameshift mutations in microsatellites even before the onset of cancer. CD4+ T cells can recognize neoantigens in premalignant lesions via their antigen-specific T cell receptors (TCRs), making LS an attractive model to study the feasibility of early cancer detection by identifying neoantigen-specific TCRs in peripheral blood. This study aims to identify neoantigen-specific TCRs in LS carriers before tumor development.

**Material and Methods**

We performed TCR-Seq of circulating CD4+ T cells from 20 healthy LS mutation carriers and 19 non-carriers that we complemented with TCRs extracted from public bulk RNA-Seq of 125 LS-CRC tumors and TCR-Seq data from a cohort of 666 healthy donors. To cluster sequences with sequence similarity across all datasets, we employed the GIANA algorithm. Additionally, we used the SelTarBase database to identify the most frequently mutated coding microsatellites in CRC and subsequently ran the neural-network-based tool NetMHCIIpan with a panel of representative MHC class II alleles to predict which LS neoantigens are most likely to be presented.

**Results and Discussions**

We gathered ~15M distinct TCR sequences from LS carriers, non-carriers, public CRC RNA-Seq samples (caTCRs) and an external healthy cohort. After sequence similarity clustering, we identified 7 potential TCR candidates, defined as 1) disease-specific, i.e. TCRs present in carrier samples that cluster only with caTCRs and not with TCRs from healthy individuals; 2) shared among different LS carriers and 3) detectable by current sequencing technologies due to their abundance. To assess the antigen specificity of the candidate TCRs, we predicted that only 240 out of ~8000 15-aa neoepitopes resulting from LS mutations were likely to be presented by MHC class II molecules and thus recognized by CD4+ T cells.

**Conclusion**

© 2023 The Authors. Molecular Oncology is published by John Wiley & Sons Ltd on behalf of Federation of European Biochemical Societies

We explored the utility of cutting-edge computational tools to address the needle-in-a-haystack problem of identifying tumor-associated TCRs in LS. Our candidates are undergoing experimental validation in a T cell antigen specificity assay using a panel of MHC class II neoantigens. This study lays the groundwork for the development of a non-invasive cell-free monitoring of cancer development in LS patients.

### EACR23-1157
### Protocol optimization for cfRNA-based liquid biopsies

*L. Cabús[1], S. Belmonte[1], C. Tuñi[1], J. Lagarde[1], J. Curado[1], E. Lizano[1], J. Perez-Boza[1]*
*[1]Flomics Biotech, Research and development, Barcelona, Spain*

**Introduction**
During the last decade, liquid biopsies have become increasingly important diagnostic tools in early-stage cancer due to their high sensitivity and minimal invasiveness. Cell-free RNA (cfRNA) is informative of both the circulating signature of the tumour and the systemic response of the host against it. Because of this, cfRNA is an invaluable tool for cancer early detection, when the number of tumour cells is limited. Unfortunately, since the field of cfRNA-based liquid biopsies is still young, several technical limitations remain, limiting the reproducibility and applicability of this approach.
**The goal of this project is to study all the steps involved in the processing of plasma samples to determine their cfRNA profiles and define a standard, reliable and robust protocol for cfRNA analysis.**
**Material and Methods**
We extracted blood from three donors, each in three different blood collection tubes. From each tube, we performed two different plasma isolation protocols. Then we tested two different commercial RNA isolation methods, three different DNAse treatment protocols and two different commercial library preparation kits.
After sequencing, we processed the data using an open-source bioinformatic pipeline and computed the correlation between the gene expression and performed multiple statistical tests between the samples to identify which of the processing steps affected the most the quality of the samples or changed the most their cfRNA profile.
**Results and Discussions**
Our results show very high variability between all the different methodologies. We observed that the blood collection tube and the RNA extraction and library preparation kits cause the highest variation in the cfRNA profiles, causing the greatest effect on the quality of the sample. However, we found that other steps, such as the blood plasma isolation and DNAse treatment, don’t affect the samples as drastically as we initially thought.
**Conclusion**
Our results show that possible variations of the protocol have a high effect on the final cfRNA profile, masking the biological variability and biassing the results, highlighting the need for a defined and thoroughly tested protocol. Here we propose the optimal combination of methods for robust and reproducible cfRNA studies. Although there is still work to be done in order to obtain an optimised standard protocol, we have identified the most critical steps in the procedure, focusing on those that are crucial to generate the most accurate results.

### EACR23-1167
### MicroRNA-based signature in liquid biopsy for early detection of lung cancer

*G.M. Stanfoca Casagrande[1], R. Sampaio Chiarantano[1], A. Polo Siqueira[1], A.C. Laus[1], E. Ramos Martins Cabral[1], A. Neves Oliveira Filatieri[1], R.E. Noleto da Nobrega Oliveira2[2], L. Sussuchi da Silva[1], R.M. Reis[3], L. Ferro Leal[1]*
*[1]Barretos Cancer Hospital, Molecular Oncology Research Center, Barretos, Brazil*
*[2]Barretos Cancer Hospital, Department of Medical Oncology, Barretos, Brazil*
*[3]University of Minho, Life and Health Sciences Research Institute ICVS- School of Medicine, Braga, Portugal*

**Introduction**
Lung cancer is the deadliest cancer worldwide. Programs screening has been applied in high-risk subjects (smokers; 50-75 yrs) and has proven to reduce lung cancer deaths in high-income countries. Biomarkers detection in body fluids may guide more precise selection of high-risk subjects but this approach has not been currently employed for lung cancer screening. **Aims:** To identify a miRNA-based signature for early detection of lung cancer in liquid biopsies.
**Material and Methods**
We analyzed plasma and sputum samples from high-risk subjects (n=54), and patients (n=60) diagnosed with non-small cell lung cancer (NSCLC), no metastatic. Samples were subjected to RNA isolation followed by miRNA expression using Human v3 miRNA panel (NanoString technologies). Counts were normalized by housekeeping candidates, and differentially expressed miRNAs were filtered out by Rosalind software according to fold-change (≥±1.3), p-value (≤0.01), and ROC Curve (AUC>0.70).
**Results and Discussions**
Overall, high-risk subjects were stratified in LungRADS1 (n=14) and LungRADS2 (n=40), and cancer patients were diagnosed with adenocarcinoma (n=28), squamous cell carcinoma (n=27) and others (n=5). No differences were observed related to age, sex, and tobacco exposure in high-risk and cancer groups (p>0.05). For plasma, we observed seven differentially expressed miRNAs able to distinguish cancer from non-cancer samples, and three of them presented with high accuracy (AUC = 0.70 – 0.74). We observed a significantly different miRNA expression profile in plasma samples between early stage and locally advanced disease (fold-change ≥±1.5; p-value ≤0.01). For sputum, we observed four differentially expressed miRNAs able to distinguish cancer from non-cancer samples, and one of them presented with high accuracy (AUC = 0.70).
**Conclusion**
We identified fluid-specific miRNA signatures in liquid biopsies potentially to be employed in lung cancer screening programs to better guide selection of high-risk subjects for early detection of lung cancer.

### EACR23-1195
### Correlation between genetic and

© 2023 The Authors. Molecular Oncology is published by John Wiley & Sons Ltd on behalf of Federation of European Biochemical Societies

### proteomic expression of predictive biomarkers of response to neoadjuvant chemoradiotherapy in locally advanced rectal cancer identified by DIA mass spectrometry

A. Stefanovic[1], V. Lygirou[2], S. Stojanović-Rundić[3,4], R. Janković[1], S. Castellvi-Bel[5], R. J.A. Fijneman[6], A. Vlahou[2], J. Zoidakis[2,7], M. Samiotaki[8], M. Čavić[1]
[1]Institute for Oncology and Radiology of Serbia, Experimental Oncology Department, Beograd, Serbia
[2]Biomedical Research Foundation- Academy of Athens, Department of Biotechnology, Athens, Greece
[3]Clinic for Radiation Oncology and Diagnostics- Departm ent of Radiation Oncology- Institute for Oncology and Rad iology of Serbia,
Clinic for Radiation Oncology and Diagnostics- Departme nt of Radiation Oncology, Belgrade, Serbia
[4]University of Belgrade, Faculty of Medicine, Belgrade, Serbia
[5]Fundació Clínic per la Recerca Biomèdica-
Institut d'Investigacions Biomèdiques August Pi i Sunyer F RCB-
IDIBAPS- Centro de Investigación Biomédica en Red de E nfermedades Hepáticas y Digestivas CIBERehd- Hospital Clínic- University of Barcelona,
Gastroenterology Department, Barcelona, Spain
[6]the Netherlands Cancer Institute,
Department of Pathology, Amsterdam,
Netherlands Antilles
[7]National and Kapodistrian University of Athens, Department of Biology, Athens, Greece
[8]Biomedical Sciences Research Center "Alexander Fleming ", Institute for Bioinnovation, Vari- Attica, Greece

**Introduction**

Neoadjuvant chemoradiotherapy (nCRT) has become common in treating locally advanced rectal cancer (LARC) to improve local control and survival rates, but the response to treatment varies. To identify who may benefit most from nCRT, previous research using DIA-MS identified 915 differentially expressed proteins between responder and non-responder groups. The current study aimed to evaluate the correlation between genetic and proteomic expression levels of candidate markers of response.

**Material and Methods**

We used the STRING analysis tool to understand better the interaction between differentially expressed proteins (DEPs). After the analysis was performed the transcription-level tool ROCplotter was used to shortlist potential biomarkers. The study included 42 patients with locally advanced rectal cancer (LARC), categorized as responders or non-responders, and selected promising biomarkers with an AUC> 0.7, ROC p-value <0.05, and Mann Whitney p-value <0.05. *In silico* analysis was used to determine candidates for validation in a cohort of 45 patients treated with neoadjuvant chemoradiotherapy. Gene expression analysis was performed on candidates with the best predictive potential using qRT-PCR and TaqMan Gene Expression Assay. Statistical analysis was performed using Mann Whitney t-test and ROC curve analysis (p-value <0.05).

**Results and Discussions**

After performing STRING analysis, the data showed that the responder group had several protein-rich groups with high levels of interactions, particularly in pre-mRNA processing. There was a strong correlation between proteins involved in information RNA processing and genes whose protein products participate in translation. The PPI enrichment p-value was less than 1.0e-16. Out of 915 differentially expressed proteins (DEPs), ROCplotter analysis identified 23 promising biomarkers that met all three criteria. Out of 23 DEPs, we selected the top four genes (CRKL, HAS1, COPB1, and MGLL) to validate in our cohort of samples. However, after statistical analysis, there was no correlation between gene expression and protein expression in the analyzed cohort of samples (p>0.05).

**Conclusion**

Changes in the expression profile of analyzed genes may be regulated on the post-transcriptional level. Translation control is essential in differentiating responses to neoadjuvant chemoradiotherapy in patients with locally advanced rectal cancer.

### EACR23-1196
### Metabolic Profiling of Extracellular Vesicles from Ascitic Fluids of Ovarian Cancer Patients

G. Trindade[1,2], R. Mendes[1,2], I. Muzaferovic[1,2], L. Rodrigues[1,2], F. Silva[3], A.C.L. Guerreiro[1,2], S.D. Silva[1,2], L. Gafeira[2], P.M. Alves[1,2], C. Brito[1,2], P. Gomes-Alves[1,2], A. Félix[3,4], I.A. Isidro[1,2]
[1]iBET, Instituto de Biologia Experimental e Tecnológica, Oeiras, Portugal
[2]Instituto de Tecnologia Química e Biológica António Xavi er, Universidade NOVA de Lisboa, Oeiras, Portugal
[3]CEDOC - Chronic Diseases Research Centre - NOVA Me dical School, Universidade NOVA de Lisboa, Lisboa, Portugal
[4]IPOLFG,
Instituto Português de Oncologia de Lisboa Francisco Gen til, Lisboa, Portugal

**Introduction**

The accumulation of ascitic fluid (AF) in the peritoneal cavity is a hallmark of Ovarian Cancer (OvC), the most lethal gynecological malignancy. AF is enriched in cells and their secreted products, which contain cues that promote tumor growth and dissemination. One of such products, extracellular vesicles (EVs), acts as versatile cell-cell communication mediators and have been described to be involved in the pathogenesis of cancer, namely promoting metastasis and chemoresistance. As such, EVs have emerged as potential non-invasivecirculating biomarkers in liquid biopsies for the early detection and prognosis of diseases. Yet EVs remain largely under-studied regarding their metabolic content. Here we present a platform for the isolation and metabolomic characterization of AF-derived EVs from OvC patients which can be applied to identify novel disease biomarkers and therapeutic targets.

**Material and Methods**

EVs were isolated from 14 OvC patient biofluids, either AF (11) or peritoneal washes (3), using an ultracentrifugation-based protocol, and further

© 2023 The Authors. Molecular Oncology is published by John Wiley & Sons Ltd on behalf of Federation of European Biochemical Societies characterized by western blot, nanoparticle tracking analysis, and transmission electron microscopy. A targeted LC-MS metabolomics and unsupervised analysis approach was employed to assess the feasibility of metabolic profiling of EVs as a source of early detection and prognostic biomarkers.

**Results and Discussions**

We successfully isolated EVs from OvC patient biofluids which showed enrichment of typical protein markerTSG101, the predicted size (74.2 % of the particles within 50 to 250 nm), and expected cup-shaped morphology. Metabolic profiling of AF and derived EVs identified up to 154 metabolites, of which 65% were lipids. Within EVs at least 77 metabolites were detected, of which 70 were lipids. EVs were enriched in metabolites related to the TCA cycle, regulation of DNA and histone methylation, and mediation of redox processes balance when compared with their original biofluid. Further clustering analysis of EVs revealed two major groups of samples, differentiated mainly by the profile of acylcarnitines and phosphatidylcholines.

**Conclusion**

The platform presented allows the metabolic characterization of EVs starting from patient biofluids such as AF, thus opening avenues for uncovering potential biomarkers which may be applied in early diagnosis and prediction of prognosis or therapy response, and supporting future applications in precision medicine.

### EACR23-1226

### Evaluation of circulating microRNAs as predictive and prognostic biomarkers for advanced melanoma patients

*G. Durante[1], F. Comito[1,2], F. Longo[1], G. Gallerani[1], M. Naddeo[1,2], A. Ardizzoni[1,2], M. Ferracin[1,2]*

*[1]University Of Bologna, Department of Medical and Surgical Sciences - DIMEC, Bologna, Italy*

*[2]IRCCS, Azienda Ospedaliero-Universitaria di Bologna, Bologna, Italy*

**Introduction**

Metastatic melanoma is the deadliest form of skin cancer whose incidence has been rising dramatically over the last few decades. The advent of Immune checkpoint inhibitors (ICIs) has strongly improved the survival of melanoma patients. However, a high fraction of patients shows primary resistance or will develop secondary resistance during therapy. For these reasons, it is of utmost importance to develop biomarkers that could predict response or resistance to therapies. Among the most promising biomarkers analyzed in peripheral blood there are circulating miRNAs, which are small non-coding RNA of 20-22 nucleotides in length. The aim is to identify a microRNAs signature able to predict prognosis and therapy response before clinical relapse.

**Material and Methods**

We collected longitudinal blood draws from metastatic melanoma patients treated with ICIs at IRCCS Azienda Ospedaliero-Universitaria di Bologna. We separated plasma from whole blood, extracted RNA from plasma samples and analysed the absolute levels of miR-155-5p, miR-424-5p, and miR-320a using the droplet digital PCR.

**Results and Discussions**

We found that patients with high expression of miR-155-5p, miR-320a, and mir-424-5p had longer progression-free survival (PFS), and overall-survival (OS). Moreover, when we considered a more homogeneous group of patients, we found that miR-155-5p could have a predictive value for response to first-line anti-PD-1 treatment.

**Conclusion**

These preliminary findings can provide potential biomarkers to predict response to immunotherapy and guide clinical decision-making in melanoma patients.

### EACR23-1228

### Exploring circular MET RNA as a potential biomarker in tumors exhibiting high MET activity

*F. Bersani[1], F. Picca[1], L. Righi[2], M. Russo[3], P. Bironzo[4], F. Pietrantonio[5], F. Di Nicolantonio[3], A. Bardelli[3], S. Novello[4], R. Taulli[1]*

*[1]University of Torino, Department of Oncology, Orbassano, Italy*

*[2]University of Torino, Pathology Unit- Department of Oncology at San Luigi Hospital, Orbassano, Italy*

*[3]University of Torino, Department of Oncology and Candiolo Cancer Institute FPO-IRCCS, Candiolo, Italy*

*[4]University of Torino, Thoracic Unit and Medical Oncology Division- Department of Oncology at San Luigi Hospital, Orbassano, Italy*

*[5]Fondazione IRCCS Istituto Nazionale dei Tumori, Medical Oncology Department, Milano, Italy*

**Introduction**

MET-driven acquired resistance is emerging with unanticipated frequency in patients relapsing upon molecular therapy treatments. However, the determination of *MET* amplification remains challenging using both standard and next-generation sequencing-based methodologies. Liquid biopsy is an effective, non-invasive approach to define cancer genomic profiles, track tumor evolution over time, monitor treatment response and detect molecular resistance in advance. Circular RNAs (circRNAs), a family of RNA molecules that originate from a process of back-splicing, are attracting growing interest as potential novel biomarkers for their stability in body fluids.

**Material and Methods**

We identified a circRNA encoded by the *MET* gene (circMET) and exploited blood-derived cell-free RNA (cfRNA) and matched tumor tissues to identify, stratify and monitor advanced cancer patients molecularly characterized by high MET activity, generally associated with genomic amplification.

**Results and Discussions**

Using publicly available bioinformatic tools, we discovered that the *MET* locus transcribes several circRNA molecules, but only one candidate, circMET, was particularly abundant. Deeper molecular analysis revealed that circMET levels positively correlated with MET expression and activity, especially in *MET*-amplified cells. We developed a circMET-detection strategy and, in parallel, we performed standard FISH and IHC analyses in the same specimens to assess whether circMET quantification could identify patients displaying high MET

© 2023 The Authors. Molecular Oncology is published by John Wiley & Sons Ltd on behalf of Federation of European Biochemical Societies activity. Longitudinal monitoring of circMET levels in the plasma of selected patients revealed the early emergence of *MET* amplification as a mechanism of acquired resistance to molecular therapies.

**Conclusion**

We found that measurement of circMET levels allows identification and tracking of patients characterized by high MET activity. Circulating circMET (ccMET) detection and analysis could be a simple, cost-effective, non-invasive approach to better implement patient stratification based on MET expression, as well as to dynamically monitor over time both therapy response and clonal evolution during treatment.

### EACR23-1242
### The novel urine biomarker, EnBi1, can differentiate deep endometriosis from superficial and no endometriosis groups, confirming laparoscopy results.

*B. Guinn[1], J. Allen[2], K. Cunningham[2], L. Cooksey[1], K. Phillips[2], H. Draper[1]*
*[1]University of Hull, Centre for Biomedicine- Hull York Medical School, Hull, United Kingdom*
*[2]Hull University Teaching Hospitals NHS Trust, Department of Obstetrics and Gynaecology, Hull, United Kingdom*

**Introduction**

Endometriosis is a benign condition which exhibits the features of malignancy including invasion, metastasis, and angiogenesis. There is an increasing body of evidence demonstrating an important role for endometriosis-associated metabolic changes. Despite causing debilitating pain and subfertility in 190 million women worldwide, there are no accurate non-invasive tests in clinical use resulting in significant diagnostic delay. EnBi1 has been identified as a candidate urine biomarker using two RNA-sequencing databases; this study aims to assess its diagnostic accuracy.

**Material and Methods**

Patients with symptoms of endometriosis undergoing elective laparoscopy were recruited into this prospective observational study. Patients were allocated into deep, superficial or no endometriosis groups by an BSGE-accredited endometriosis surgeon. Urinary EnBi1 expression was determined by ELISA (AFG Bioscience) and normalised to creatinine (InvitrogenTM assay). A further group of healthy volunteers are currently being recruited.

**Results and Discussions**

Preliminary results of 85 patient samples were analysed, 69 women with endometriosis and 16 symptomatic controls. Mean age of participants was 33 (range 19-49) and BMI 27 (range 16-42). There was no difference between groups for age (p=0.89) or BMI (p=0.74). EnBi was detectable in every sample with a mean level of 36pg/mg creatinine (range 6.16-138.09).
Urinary EnBi1 levels were significantly increased in women with deep endometriosis compared to both superficial disease (p=0.02) and symptomatic controls (p=0.04) regardless of hormonal medications or menstrual phase. There was no difference between superficial disease and control. EnBi1 levels were significantly reduced by hormonal medications in women with endometriosis (p=0.04), but not symptomatic controls (p=0.23). Conversely, EnBi1 levels were significantly increased during the proliferative menstrual phase compared to secretory (p=0.04).

**Conclusion**

EnBi1 is expressed in the urine of patients with endometriosis symptoms and appears to be a biomarker for deep endometriosis. Recruitment is ongoing to investigate its clinical utility together with its association with menstrual cycle, hormonal medications and asymptomatic controls.

### EACR23-1246
### Exploratory analysis of serum fascin concentration in patients with colorectal cancer

*E. Rodríguez-Braun[1], F. Postigo-Corrales[2], A. Albaladejo-González[3], A. Beltrán-Videla[3], M.D. López Abellán[4], D. Torres-Moreno[5], M. Soler Giménez[6], A.M. Hurtado[3], G. Luengo-Gil[7], P. Conesa-Zamora[4]*
*[1]Santa Lucia University Hospital, Medical Oncology, Cartagena, Spain*
*[2]Universidad Católica San Antonio de Murcia, Molecular Pathology, Cartagena, Spain*
*[3]Fundación para la Formación e Investigación Sanitarias, Molecular Pathology and Pharmacogenetics, Cartagena, Spain*
*[4]Santa Lucia University Hospital, Laboratory Medicine, Cartagena, Spain*
*[5]Santa Lucia University Hospital, Pathology, Cartagena, Spain*
*[6]Santa Lucia University Hospital, Surgery, Cartagena, Spain*
*[7]Santa Lucia University Hospital, Molecular Pathology, Cartagena, Spain*

**Introduction**

Fascin is an actin-bundling protein is essential for the formation of filopodia and other cellular structures of the cytoskeleton. Fascin is absent or expressed at low levels in most healthy adult epithelial cells. However, its overexpression in numerous tumors plays a causal role in the development of the invasive and metastatic phenotype, and its expression is associated with tumors with poor prognosis, including colorectal cancer (CRC). Circulating fascin has been described as a potential new minimally-invasive prognostic marker in advanced adrenocortical carcinoma or head and neck cancer. Our objective was to investigate whether serum fascin levels were associated with anatomopathological characteristics of the tumor, as well as a prognostic marker in CRC.

**Material and Methods**

A prospective study including 250 patients with non-metastatic CRC was performed on serum collected right before surgery. After obtaining informed consent, histological, biochemical and clinical variables were included in the study database. Fascin levels were measured in serum using FineTest Human FSCN1(Fascin) ELISA Kit (Cat. No.: EH1915) in automated plate reader. Non-parametric statistical analysis was performed in SPSS statistics. The time-to-recurrence (TTR) was defined as the time from date of curative surgery to the time of

© 2023 The Authors. Molecular Oncology is published by John Wiley & Sons Ltd on behalf of Federation of European Biochemical Societies recurrence. The study was previously approved by the Clinical Research Ethics Committee of the hospital.

**Results and Discussions**

Preliminary results indicate that fascin levels were not associated with clinical or histological features including age, sex, CEA levels, ECOG, chronic kidney disease, colon sideness, tumor growth pattern, grade, histological subtype (conventional, serrated, mucinous, primary signet-ring cell, cribriform carcinoma), KRAS mutation status, pT, pN and clinical stage. The survival analysis could be performed on 162 patients after a median follow-up of 18 months. Serum fascin levels below median expression were associated with shorter TTR (log-rank test, $p$=0.014). This result was also significant in the multivariate analysis including pT and pN (Cox regression, HR: 0.419; 95%CI: 0.193-0.910; $p$=0.028).

**Conclusion**

Low levels of circulating fascin, measured before surgery in CRC serum patients, were associated with worse prognosis. TTR, even when pT and pN were included in the analysis. These data suggest that circulating fascin levels measured from date of curative surgery could be a useful prognostic marker of recurrence in colorectal cancer.

### EACR23-1251

### Digital PCR based liquid biopsy test to understand dynamics of circulating tumor HPV DNA as a treatment monitoring tool in patients with cervical cancer

*P. Parida[1], K. Sharan[2], S. Salins[2], R.R. Damerla[1]*
*[1]Kasturba Medical College Manipal, Department of Medical Genetics, Manipal, India*
*[2]Kasturba Medical College Manipal, Department of Radiotherapy, Manipal, India*

**Introduction**

Detecting circulating tumor DNA (ctDNA) in the blood using minimally invasive techniques known as a "liquid biopsy", is gaining tremendous popularity in oncology research. Integration of human papillomavirus (HPV) DNA and the overexpression of the E6 and E7 oncogenes are crucial steps in the development of cervical cancer. We investigated the potential utility of HPV ctDNA as a biomarker for monitoring treatment outcomes in patients with cervical cancer.

**Material and Methods**

We plan to use droplet digital PCR (ddPCR) to measure the amount of HPV16/18 cfDNA in the plasma of cervical cancer patients. The HPV standard GP5+/6+ primers were used to determine HPV positivity in our atients. The primers were designed to amplify specifically the E6 oncogene of HPV16 and HPV18. Hydrolysis probes were designed for the L1 and E6 genes of HPV. Plasmid constructs were made using the PCR-purified products of HeLa and SiHa cell lines which would act as a positive control for the assay. HPV16 and 18 plasmids were serially diluted and then tested in qPCR as a template using different primer sets. Standard curves were made from the recombinant plasmids. This will serve as a positive control and will also provide a correlation between the sensitivity and specificity of qPCR vs ddPCR.

**Results and Discussions**

We hypothesize that our dPCR test will be able to detect minute quantities of HPV ctDNA in plasma of cervical cancer patients as ctDNA levels are expected to be decrease as treatment progresses. The assay sensitivity and specificity will be obtained by comparing results to QPCR. The study samples involve a tumor biopsy and a blood sample at the baseline prior to the therapy and serial blood sampling in different stages of treatment and follow-ups. These samples will also serve for treatment monitoring of these patients through ddPCR. Tumor samples are used to confirm the HPV status of patients using HPV-specific GP5+/GP6+ qPCR. We have recruited 75 cervical cancer patients with majority of them being squamous cell carcinoma and stage IIB disease. The qPCR results from tumor biopsy revealed HPV 16 was most prevalent (32/43), followed by HPV18 (6/43) and HPV 33(2/43).

**Conclusion**

we will now use Qiagen digital PCR system to understand HPV ctDNA levels in our patient cohort. we believe that our comprehensive sample collection after every treatment milestone and thorough follow-up for upto 2 years after diagnosis will allow us to study the utility of this sensitive test in monitoring disease and relapse.

### EACR23-1255

### Circulatory plasma proteomic biomarkers combined with high-throughput proteomic screen of patient-derived organotypic tumor spheroids predict responses to immunotherapy in melanoma patients

*M. Rucevic[1], M. Forster[2], J. Jenkins[3], A. Mehta[3], G. Boland[4], M. Sade-Feldman[5], N. Hacohen[6], A. Schneider[7]*
*[1]Olink Proteomics, Scientific Affairs, Boston, United States*
*[2]Olink Proteomics, Scientific Affairs, Montreal, Canada*
*[3]MGH, Medical Oncology, Boston, United States*
*[4]MGH, Surgical oncology, Boston, United States*
*[5]MGH, Medicine, Boston, United States*
*[6]Broad Institute, Medicine, Boston, United States*
*[7]Broad, Medical, Boston, United States*

**Introduction**

The majority of patients treated with immunotherapy do not have durable treatment responses. Therefore, there is an urgent need to identify early non-invasive biomarkers for treatment response.

**Material and Methods**

In this study, we performed plasma proteomic analysis of >700 proteins at three timepoints on 174 metastatic melanoma patients treated with immune checkpoint blockade (ICB). We then expanded our analyses to >3000 proteins performed on a larger cohort of 250 patients for deeper exploration of baseline and early on-treatment predictive biomarkers for response to ICB treatment.

**Results and Discussions**

As a result, we built a predictor of immunotherapy response that outperforms several tissue-based approaches. From the differentially expressed proteins between ICB responders (R) and non-responders (NR), we identified a co-regulated module of proteins associated with treatment resistance comprising IL-6, IL-8, MIA, LIF and GDF-15 enriched in certain NR patients. By analyzing single-cell RNA-sequencing data of tumor biopsies from 32 patients

© 2023 The Authors. Molecular Oncology is published by John Wiley & Sons Ltd on behalf of Federation of European Biochemical Societies and bulk RNA-sequencing data from 70 patients, we determined the relative contribution of cells in the tumor to proteins in circulation, and associated plasma protein levels with tumor immune microenvironment (TME) phenotypes. The major TME subsets driving the expression of the non-response module proteins were tumor and myeloid cells. Amongst myeloid cells, a subset of tumor-associated macrophages (TAMs) with a suppressive phenotype were identified as potential key drivers of non-response, having the highest expression of all the proteins in the co-regulated NR module. Lastly, we performed a deep proteomic screen of the secretome obtained from patient-derived organotypic tumor spheroids (PDOTS) treated with ICB to validate identified biomarkers and obtain deeper insight into actionable biology of melanoma resistance in a biomimetic tumor microenvironment.

**Conclusion**

In summary, an integrated longitudinal analyses of circulatory plasma proteins, combined with TME transcriptomics, provides deeper insight into the biology of immunotherapy resistance, and demonstrates prognostic significance and utility of plasma proteomics in biomarker discovery for cancer immunotherapy.

### EACR23-1273

### Early detection of colorectal cancer with RNA-Seq-based liquid biopsies

*C. Tuñí i Domínguez*[1], *L. Cabús*[1], *S. Belmonte*[2], *J. Curado*[1], *E. Lizano*[2], *J. Pérez*[2], *J. Lagarde*[1]

[1]*Flomics Biotech SL, Bioinformatics department, Barcelona, Spain*

[2]*Flomics Biotech SL, Genomics department, Barcelona, Spain*

**Introduction**

Colorectal cancer (CRC) is the third most prevalent type of cancer both in women and men worldwide. The mortality of CRC is still high, accounting for 14.6% of all cancer-related deaths. Half of the CRC cases diagnosed today are detected in late stages when the chances of recovery go from 90% (stage I and II) to 64% (stage III) and 8% (stage IV). Early diagnosis is key to improve these rates. A good option for population screening are liquid biopsies. Nucleic acids that are present in circulation are promising targets for this approach. RNAs can be extracted from blood and used as biomarkers to improve diagnostic options. **The objective of this project is to develop a high-throughput diagnostic method for colorectal cancer, using liquid biopsies and cell-free RNA (cfRNA) profiling.**

**Material and Methods**

Plasma samples were obtained from patients with CRC in the various stages of the disease, and from healthy donors. After RNA isolation and library preparation, samples were analyzed using NGS. The processing of the data was done following standard pipelines to obtain the relative abundance of all genes detected. Gene quantification profiles are fed to Least Absolute Shrinkage and Selection Operator algorithm, to create a classification method based on a patient score: genes are given a coefficient and a linear model is created. After the application of this model, patients get a score that classifies them as either "cancer" or "non-cancer". To assess the validity of the score, samples are separated into a training cohort, which is used to obtain a signature, and a validation cohort, to which the signature is applied and tested.

**Results and Discussions**

Over 15,000 genes were detected, but no significant differences were observed in expression. After testing the machine learning algorithms, a signature was defined using the combined relative abundance of 8 genes. This signature had a sensitivity of 85% and a specificity of 100% in the validation cohort, with all control samples correctly classified. The total accuracy of the test was 90,4%.

**Conclusion**

More extensive validation is needed, the signature proposed in this study suggests an alternative to the current methods of screening. The state-of-the-art for CRC population testing is based on the detection of blood in feces and the method has very low sensitivity in early-stage cancer. The results of this study show the first steps to develop a more accurate and sensitive method of screening, promoting early detection and improving patient diagnosis and survival rates.

### EACR23-1284

### Unveiling the diagnostic and therapeutic potential of the splicing factors SNRNP200, SRSF3, and SRRM1 in prostate cancer

*A.J. Montero Hidalgo*[1], *A.D.L.S. De la Rosa-Herencia*[1], *E. Gómez-Gómez*[2], *P. Sáez-Martínez*[1], *F. Porcel-Pastrana*[1], *A. Sarmento-Cabral*[1], *M.D. Gahete*[3], *J.M. Jiménez-Vacas*[1], *R.M. Luque*[1]

[1]*Maimonides Institute for Biomedical Research of Cordoba IMIBIC, OncObesity and Metabolism, Cordoba, Spain*

[2]*Maimonides Institute for Biomedical Research of Cordoba IMIBIC, Genitourinary diseases, Cordoba, Spain*

[3]*Maimonides Institute for Biomedical Research of Cordoba IMIBIC, Molecular Hepatology, Cordoba, Spain*

**Introduction**

Prostate cancer (PCa) is the second most common cancer type among men population worldwide. The serum prostatic specific antigen (PSA) levels are used as a screening method to detect PCa. Unfortunately, PSA has several significant limitations, including low sensitivity, and it may be elevated for reasons unrelated to cancer (e.g., inflammation). Therefore, the identification of more reliable non-invasive biomarkers for PCa is a critical unmet clinical need. In this context, the dysregulation of the splicing process has emerged as a main hallmark of PCa development/progression. Interestingly, several splicing factors (SFs) have been found to be secreted by cancer cells. Herein, we aimed to analyze the potential role as non-invasive biomarkers and therapeutic targets of SNRNP200, SRSF3, and/or SRRM1 in PCa since we had previously reported that these SFs could play a relevant pathophysiological role in this tumor pathology.

**Material and Methods**

Plasma and urine levels of SNRNP200, SRSF3, and SRRM1 were determined by ELISA in samples from a well-characterized cohort of control individuals ($n$=41) and PCa patients ($n$=175). Moreover, clinical and molecular associations with SNRNP200, SRSF3, and SRRM1 were interrogated. Based on the results generated, the therapeutic value of SRRM1 in PCa (i.e., silencing) was tested *in vivo*.

**Results and Discussions**

© 2023 The Authors. Molecular Oncology is published by John Wiley & Sons Ltd on behalf of Federation of European Biochemical Societies

SNRNP200 and SRRM1 plasma levels were higher, while urine levels were lower, in PCa patients, and were able to discriminate between control individuals vs. PCa patients (AUC>0.65; p-value<0.05), while plasma and urine SRSF3 levels did not differ across groups. Also, high plasma SNRNP200 and SRRM1 levels were associated with key clinical features of aggressiveness, including the development of biochemical recurrence and metastasis at diagnosis. Interestingly, plasma SNRNP200 and SRRM1 levels were higher in PCa patients treated with androgen-deprivation therapy vs. treatment-naïve patients. Notably, plasma SRRM1 levels directly correlated with *AR* expression levels and AR activity [mRNA expression of androgen response genes (e.g., *ACSL3*, *FKBP5, KLK3*)] in PCa tissues. Finally, the *in vivo* silencing of SRRM1 expression reduced aggressiveness features in PCa-derived xenografted tumors.

**Conclusion**

Altogether, our data revealed that circulating SNRNP200 and SRRM1 levels could represent useful diagnostic and prognostic non-invasive biomarkers in PCa, and that SRRM1 might also serve as a novel therapeutic target for this tumor pathology.

Fundings: ISCIII (DTS20/00050), FPU18/02485

### EACR23-1326

### Characterization of the immuno-oncological transcriptomic landscape of uterine smooth muscle tumors of uncertain malignancy potential (STUMP) according to clinical outcome

*L. Mangherini[1], A.A. Ricci[1], M. Goia[2], I. Castellano[1], S. Cosma[3], M. Preti[3], C. Benedetto[3], P. Cassoni[1], F. Borella[3], L. Bertero[1]*

*[1]Pathology Unit- University and City of Health and Scienc e- University of Turin, Department of Medical Sciences, Turin, Italy*

*[2]City of Health and Science University Hospital, Pathology Unit, Turin, Italy*

*[3]Division of Gynecology and Obstetrics 1- City of Health a nd Science University Hospital- University of Turin, Department of Surgical Sciences, Turin, Italy*

**Introduction**

Uterine smooth muscle tumors of uncertain malignancy potential (STUMP) are a diagnostic and clinical challenge, due to their rarity, borderline histopathological features and recurrence potential, even as malignant sarcomas with a dismal outcome. Novel diagnostic/prognostic markers are warranted to improve STUMP clinical management.

**Material and Methods**

Eleven uterine STUMP samples, diagnosed according to the 2020 WHO Classification of Female Genital Tumours (5$^{th}$ed.), were collected as well as representative series of uterine leiomyomas and leiomyosarcomas. Gene expression profiling was analyzed using the nCounter PanCancer IO 360 Panel (NanoString® Technologies) targeting 770 mRNAs related to tumor biology, immune microenvironment and immune response.

**Results and Discussions**

The analyzed series was composed of 11 STUMPs, including 5 relapsed (rSTUMP) and 6 non relapsed cases (nrSTUMP), 11 leiomyosarcomas (LMS) and 11 leiomyomas (LM).

The comparison between all STUMP and LM did not show any differentially expressed genes, but nrSTUMPs exhibited the downregulation of *CXCL8* (p=0.0033), *CCNO* (p=0.00519), *CCNB1* (p=0.0 0704), *S100A8* (p=0.00779), *BRIP1*(p=0.0286) and *S100A9* (p=0.0286) compared to LMS. Similarly, several pathways were downregulated in nrSTUMP compared to LMS: DNA damage repair, cell proliferation, angiogenesis, epigenetic regulation, and those related to immune modulation. Immune cell-related signatures showed reduced signatures of neutrophils and exhausted CD8 T cells suggesting a less immunosuppressive tumor microenvironment. This finding was similar to what observed between LM and LMS.

*EZH2* (p=0.0192), *BIRC5* (p=0.0192), *H2AFX* (p= 0.0213), *SLC7A5* (p=0.0213), *FANCA* (p=0.037), *BRIP1* (p=0.039) genes were upregulated in rSTUMP versus LM. Cell proliferation, DNA damage repair, hypoxia, metabolic stress, epigenetic regulation pathways, were upregulated both in rSTUMP and LMS compared to LM.

Interestingly, none significantly differentially expressed genes were observed when comparing rSTUMP with LMS and nrSTUMP with LM.

**Conclusion**

The here presented data demonstrates the heterogeneity of the immune-related transcriptomic landscape of uterine STUMP based on their clinical behavior. Specifically, nrSTUMP and rSTUMP have been shown to harbor molecular profiles closer to LM and LMS, respectively. These differences could be exploited to pinpoint novel diagnostic/prognostic markers and correctly stratify STUMP according to their malignant potential.

### EACR23-1339

### Gene signatures with clinical value for lung adenocarcinoma patients.

*I. Lemuqui Tegami[1], Stanfoca Casagrande G.M.[1], L. Sussuchi da Silva[2], R. Oliveira Cavagna[1], M.F. Gonçalves[1], F. Augusto Ferreira da Silva[3], E. Caetano Albino da Silva[4], V. Duval da Silva[5], P. De Marchi[6], R. Manuel Reis[1], L. Ferro Leal[1]*

*[1]Barretos Cancer Hospital, Molecular Oncology Research Center, Barretos, Brazil*

*[2]Diagnoses of the America S. A., Not applicable, São Paulo, Brazil*

*[3]Barretos Cancer Hospital, Departament of Clinical Oncology of Thorax, Barretos, Brazil*

*[4]Barretos Cancer Hospital, Departament of Pathology, Barretos, Brazil*

*[5]Bacchi Laboratory – Pathology Consultancy, Not applicable, Botucatu, Brazil*

*[6]Oncoclinics Rio de Janeiro, Not applicable, Rio de Janeiro, Brazil*

**Introduction**

The management of lung adenocarcinoma (LUAD) has improved significantly in the last two decades. Despite the availability of personalized therapies for actionable alterations, half of the patients do not harbor any of these

© 2023 The Authors. Molecular Oncology is published by John Wiley & Sons Ltd on behalf of Federation of European Biochemical Societies actionable variants. Differentially expressed genes (DEGs) offer a promising approach, serving as possible diagnostic, prognostic and predictive biomarkers. Thus, the aim of this study was to identify diagnostic, prognostic and predictive biomarkers for lung adenocarcinoma.

**Material and Methods**

We evaluated fresh-frozen tumoral tissues from LUAD patients (n=53). Gene expression analysis was performed using bulk RNA, and applying the nCounter® PanCancer Pathways panel (NanoString® Technologies). DEGs were selected according to fold-change (FC) and p-value using the ROSALIND® software, and hierarchical clustering was used to stratify patients for downstream analysis. *In silico* validation was conducted using The Cancer Genome Atlas Lung Adenocarcinoma dataset on The Lung Cancer Explorer platform. Enrichment analysis was performed using the STRING database. Median normalized counts were used as cut-off for stratification of genes for survival analysis by Cox regression.

**Results and Discussions**

We identified a 78-gene signature that stratified LUAD tissues from non-tumoral lung tissues ($FC \geq |2|$; $p \leq 0.01$), of which 77 genes were validated ($p<0.05$) and considered biologically connected (PPI p-value=1.0e-16). Patients were stratified into hierarchical clusters, as follows: cluster I, cluster II, and cluster III. Cluster I exhibited a favorable outcome, while cluster II showed an intermediate survival rate with a hazard ratio (HR) of 1.56 (95%CI:0.4-5.8; p=0.5), and cluster III had an unfavorable outcome with a HR of 4.2 (95%CI:1.1-16.1; p=0.037). The downregulation of a subset of the 78-gene signature was associated with a worse overall survival, in which the CD19 gene had a HR of 3.14 (95%CI:1.34-7.33; p=0.008), and the IL1R2 gene had a HR of 4 (95%CI:1.65-9.8; p=0.002). Furthermore, when comparing driver-mutated and wild-type samples, a 37-gene signature was identified ($FC \geq |2|$; $p \leq 0.01$) exhibiting biological interconnectivity (PPI p-value=2.63e-8). Among these DEGs, the expression of CHEK2 and BRCA1 genes might be promising predictive biomarkers.

**Conclusion**

We identified potential gene signatures with diagnostic, prognostic and predictive value for LUAD patients. These findings may contribute to a tailored management for lung cancer patients, although further studies are needed.

### EACR23-1378
### MASTL/GREATWALL KINASE CORRELATES WITH GOOD PROGNOSIS AND RADIOTHERAPY RESPONSE IN HEAD AND NECK SQUAMOUS CELL CARCINOMAS

*E. Pozo Agundo*[1,2,3], *M. Álvarez-González*[1,3], *V. García-de-la-Fuente*[1,3], *A. de-Martino*[4], *J.R. Tejedor*[3,5,6], *J.P. Rodrigo*[1,2,3,7], *J.M. García-Pedrero*[1,2,3], *M. Álvarez-Fernández*[1,3]

[1]*Health Research Institute of Asturias ISPA, Head and Neck Cancer Research, Oviedo, Spain*
[2]*Spanish Biomedical Research Network in Cancer CIBER ONC, Instituto de Salud Carlos III ISCIII, Madrid, Spain*
[3]*University Institute of Oncology of Asturias IUOPA, University of Oviedo, Oviedo, Spain*
[4]*Spanish National Cancer Research Centre CNIO, Histopathology Unit, Madrid, Spain*
[5]*Health Research Institute of Asturias ISPA, Cancer Epigenetics and Biomedicine, Oviedo, Spain*
[6]*Nanomaterials and Nanotechnology Research Centre, Consejo Superior de Investigaciones Científicas, Principality of Asturias, Spain*
[7]*Central University Hospital of Asturias HUCA, Department of Otolaryngology, Oviedo, Spain*

**Introduction**

Microtubule-associated serine/threonine kinase-like (MASTL/Greatwall) is an essential mitotic kinase controlling cell division that may also participate in DNA damage/repair pathways. This kinase is known to be overexpressed in several cancer types, such as breast or colon cancer, in which it correlates with tumor progression and bad prognosis. Head and neck squamous cell carcinoma (HNSCC) is a tumor type originating in the larynx, pharynx and oral cavity epithelium. Besides surgery, the standard of care for HNSCC patients is chemo- and/or radiotherapy, which involve high toxicity and resistance development, leading to a 5-year survival of around 50%. Moreover, there is a striking lack of early diagnosis and predictive biomarkers in HNSCC that would facilitate the management of these patients. Thus, the purpose of this work is to investigate the role of MASTL/Greatwall in HNSCC prognosis and treatment response.

**Material and Methods**

MASTL protein expression was analyzed by immunohistochemistry in two independent cohorts of patients treated at the Hospital Universitario Central de Asturias (HUCA). The first cohort was comprised of 355 HNSCC samples from patients that underwent surgery with no previous treatment; and the second cohort included 64 HNSCC samples from patients who received induction chemotherapy after sample collection. Additionally, we used MASTL mRNA expression data in HNSCC tumors from the TCGA database as an independent cohort for validation purposes.

**Results and Discussions**

In contrast to reported data from other tumor types, we found that MASTL expression correlates with good prognosis in HNSCC patients, specifically in pharyngeal and well-differentiated tumors. Furthermore, multivariate Cox analysis revealed that MASTL expression and lymph node infiltration (N status) were the only parameters independently associated with good prognosis. Surprisingly, this association is solely observed in those patients who received post-operative radiotherapy, but not in patients only surgically treated. Curiously, no association was found with chemotherapy, suggesting a specific association with radiation treatment. Importantly, the same correlation was found in the TCGA dataset, in which MASTL expression associated with good prognosis only in HNSCC patients treated with radiotherapy.

**Conclusion**

Given our findings in two independent cohorts of HNSCC patients, we conclude that MASTL/Greatwall might emerge as a new independent predictive biomarker of good response to radiotherapy in HNSCC.

### EACR23-1405
### Differentially expressed genes associated

© 2023 The Authors. Molecular Oncology is published by John Wiley & Sons Ltd on behalf of Federation of European Biochemical Societies

**with neoadjuvant chemotherapy non-response in breast cancer Latino patients, a molecular subtype approach.**

*H.M. Guevara-Nieto[1], R. Parra-Medina[2], J.C. Mejía-Henao[3], P. López-Correa[2], S. Diaz-Casas[4], J. Garai[5], J. Zabaleta[6], L. Lopez-Kleine[7], A.L. Cómbita[1]*
*[1]Instituto Nacional de Cancerologia, Cancer Biology Research Group, Bogotá, Colombia*
*[2]Instituto Nacional de Cancerologia, Pathology, Bogotá, Colombia*
*[3]Fundación Valle de Lili, Pathology, Cali, Colombia*
*[4]Instituto Nacional de Cancerologia, Breast and Soft Tissue Department, Bogotá, Colombia*
*[5]Louisiana State University Health Science Center, Stanley S. Scott Cancer Center, New Orleans- LA, United States*
*[6]Louisiana State University Health Science Center, Department of Interdisciplinary Oncology, New Orleans- LA, United States*
*[7]Universidad Nacional de Colombia, Bioinformatics and Systems Biology group GiBBS, Bogotá, Colombia*

**Introduction**

The most common invasive malignancy in women is breast cancer (BC). Given BC heterogeneity, hub genes may explain disparities in responsiveness to neoadjuvant chemotherapy (NAC). This study examines gene expression profiles connected with incomplete pathological response to NAC in Colombian women with invasive BC, focusing on intrinsic molecular BC subtypes.

**Material and Methods**

RNA sequencing was performed on 87 pre- and post-NAC paraffin-embedded samples from 64 female patients from National Cancer Institute Colombia with invasive BC (stage IIB to IIIC). Two comparison analyses were performed to discover differentially expressed genes (DEGs): one compared baseline and follow-up samples from nonresponders to NAC, and the other one compared baseline samples between responders and non-responders. Similar molecular intrinsic subtypes were divided into four groups and compared in both comparative analyses: All luminal, LuminalBHer2+/-, LuminalBHer2+ & HER2 enriched, and TNBC+Her2-enriched. Genes in a different patient cohort were verified by RT-PCR. To compare BC and normal tissue expression, protein expression, and prognosis, DEGs were externally validated using UALCAN and the Human Protein Atlas.

**Results and Discussions**

29 of 64 patients exhibited pathological complete response (pCR), whereas 35 did not. Non-responders' analysis had 1526 significant DEGs and baseline analysis 160. Responders and nonresponders had distinct gene signatures in all categories. From these analyses, 19 DEGs were found in common. Subtype grouping found 4 genes in common (across nonresponders and baseline analyses) for All luminal samples (CGA, NTNG1, OR9A2, ZNF385B), 7 genes for LuminalBHer2+/- (AGTR1, APOD, CGA, NPY1R, SLITRK1, WDR5B), 1 gene for LuminalBHer2+ & HER2 enriched (CCL19), and 2 genes for TNBC+Her2-enriched (ECEL1, PCSK1). Thirteen genes with the strongest correlation between RNAseq and RT-PCR ($p<0.001$) were selected for RT-PCR verification and were overexpressed in non-responders. DEGs differed between BC and normal tissues and predicted survival probability.

**Conclusion**

There is a significant DEG signature between responders and non-responders. Overexpression of CGA, ECEL1, NTNG1, TPRG1, WDR5B, and ZNF385B may become predictive biomarkers of therapy response and may prevent patients from responding to NAC. Their use in clinical practice may also improve patient risk stratification, long-term survival, and the ability to pinpoint Latino (Colombian) patients who will benefit from NAC.

### EACR23-1432
### Resistance to BRAF inhibition interrogated through single Circulating Tumor Cell molecular profiling in BRAF-mutant Non-Small-Cell Lung Cancer

*M. Oulhen[1], L. Mezquita[2], A. Aberlenc[1], M. Deloger[3], M. Aldea[4], K. Howarth[5], B. Besse[4], N. Manaresi[6], D. Planchard[4], F. Farace[1]*
*[1]Gustave Roussy, Gustave Roussy- Université Paris-Saclay- "Rare Circulating Cells" Translational Platform- CNRS UAR3655 – INSERM US23 AMMICA- F-94805- Villejuif- France- INSERM- U981 "Identification of Molecular Predictors and new Targets for Cancer Treatment"- F-94, Villejuif, France*
*[2]Hospital Clinic of Barcelona, Medical Oncology Department- Laboratory of Translational Genomics and Targeted Therapies in Solid Tumors- IDIBAPS, Barcelona, Spain*
*[3]Gustave Roussy, Gustave Roussy- Université Paris-Saclay- Bioinformatics Platform- CNRS UAR3655 – INSERM US23 AMMICA- F-94805- Villejuif- France, Villejuif, France*
*[4]Gustave Roussy, Gustave Roussy- Université Paris-Saclay- Department of Medicine- F-94805- Villejuif- France, Villejuif, France*
*[5]Inivata, Inivata- Global Locations- Babraham Research Park- Cambridge- UK Research Triangle Park- NC- USA, Cambridge, United Kingdom*
*[6]Menarini Silicon Biosystems, Menarini Silicon Biosystems S.p.A- I-40013- Bologna- Italy, Bologna, Italy*

**Introduction**

Resistance mechanisms to combination therapy with dabrafenib plus trametinib are poorly known in patients with *BRAF*$^{V600E}$-mutant advanced non-small cell lung cancer (NSCLC). Circulating tumor cells (CTCs) could provide a comprehensive genomic picture of tumor resistance. We examined resistance to BRAF inhibition by single CTC sequencing in *BRAF*$^{V600E}$-mutant NSCLC.

**Material and Methods**

Blood samples for CTCs and cfDNA sequencing were collected in seven *BRAF*$^{V600E}$-mutant NSCLC patients at failure to dabrafenib plus trametinib. Matched tumor tissue was available for four patients. Single CTC isolation was performed by fluorescence-activated cell sorting following enrichment and immunofluorescence staining (Hoechst33342/CD45/pan-cytokeratins). Hotspot regions over 48 cancer-related genes were examined for mutational

© 2023 The Authors. Molecular Oncology is published by John Wiley & Sons Ltd on behalf of Federation of European Biochemical Societies analysis. Low-pass whole genome sequencing was performed for copy-number alteration (CNA) profiling.

**Results and Discussions**

*BRAF*$^{V600E}$ was found in 4/4 tumor biopsies and 5/7 cfDNA samples. CTC mutations were mostly found in MAPK-independent pathways and only 1/26 CTCs was *BRAF*$^{V600E}$ mutated suggesting that resistance was not driven by *BRAF*$^{V600E}$-mutant CTCs. CTCs encompassed the majority of matched tumor biopsy CNAs but 72.5% to 84.5% of CTC CNAs were exclusive to CTCs. Extensive diversity involving RTK/RAS/PI3K, cell-cycle, DNA repair and immune response pathways was observed in CTCs and missed by analyses on tumor biopsies and cfDNA. Driver alterations in clinically relevant genes such as *ATM* or *HER* family were found recurrently altered in CTCs.

**Conclusion**

High intra- and inter-patient tumor genomic heterogeneity were found in CTCs compared to tumor and cfDNA at failure to BRAF inhibition, in *BRAF*$^{V600E}$-mutant NSCLC, including clinically relevant alterations that may represent future treatment opportunities and guide precision medicine.

### EACR23-1436

### Additional markers of response to anti-EGFR therapy in metastatic colon cancer.

*E. Budinska[1], V. Vasylieva[1], M. Hrivnakova[1], R. Nenutil[2], O. Slaby[3], B. Bencsikova[4], V. Popovici[1]*
*[1]Masarykova Univerzita, Faculty of Science- RECETOX, Brno, Czech Republic*
*[2]Masaryk Memorial Cancer Institute, Oncological Pathology Department, Brno, Czech Republic*
*[3]Masarykova Univerzita, Central European Institute of Technology, Brno, Czech Republic*
*[4]Masaryk Memorial Cancer Institute, Complex oncology clinic, Brno, Czech Republic*

**Introduction**

Our previous research shows that tumour morphology is associated with specific transcriptomic profiles. We were interested to see whether the same applies for mutational profiles in order to develop more sensitive patient stratification strategies. In metastatic setting, colon cancer patients with KRAS and NRAS wild type tumours receive anti-EGFR agents as first line treatment. The question is whether low incidence genomic aberrations that would become apparent under multiple-region tumour sampling could be used as predictors of shorter time-to-progression and, hypothetically, selection of additional (or subsequent) treatment targets. For this, we performed multiple-region deep targeted sequencing of a selected set of primary tumours and compared it to whole-tumour sections for the purpose of predicting patients with short progression-free survival.

**Material and Methods**

We extracted DNA of whole tumour and multiple morphologically homogenous regions from consecutive FFPE slides of primary tumours from 40 *RAS wt colon cancer patients with anti-EGFR+ct therapy in the 1st line treatment (20 early progressors <6months after treatment + 20 late progressors >12 month after treatment). Targeted sequencing was performed using QIAGEN Human Comprehensive Cancer QIASeq DNA Panel and150bp paired-end reads at Illumina NextSeq 500. Variants were called using Mutect2 and annotated to the ClinVar db (1.69). We used Fishers exact test to test the frequency of variants between early and late progressors as well as their association with morphologies.

**Results and Discussions**

We found specific variants associated with early progression in subgroups of patients, such as PFGFRA c.1507T>C, PTCH1 c4325G>A, or BRAF c.1919T>A (p.Val640Glu). Interestingly, the BRAF variant was more frequently present in mucinous and serrated regions – morphologies, that were also significantly associated with early progression. Regardless of the progression status we found several morphology specific variants, such as MLH1 gene c.1624C>T (p.Gln542Ter) in desmoplastic and solid/trabecular regions RAD50 c.2165del (p.Lys722fs) found in mucinous and complex tubular regions.

**Conclusion**

The presence of certain morphologies (mucinous, serrated) in colon tumours is indicative of specific mutations associated with the anti-EGFR treatment outcome. Assessment of tumour morphological heterogeneity would be a reasonable tumour sampling strategy that could maximise the utility-to-costs ratio of personalized treatment.

### EACR23-1458

### Integrating transcriptomics, imaging and genetic mutations reveals biomarkers potentially associated to immunotherapy response in lung cancer

*K. Leila[1], A. Essabbar[1], M. Hurtado[1], J. Mazieres[2], V. Pancaldi[1]*
*[1]CRCT- UMR1037 Inserm / Université Toulouse III Paul S abatier, Centre de Recherches en Cancérologie de Toulouse, Toulouse, France*
*[2]Centre Hospitalier Universitaire de Toulouse, Hopital Larrey, Toulouse, France*

**Introduction**

Lung cancer is the leading cause of cancer death worldwide. Despite great progress in therapies, many patients do not respond. Despite the incredible progress made with immunotherapies targeting immune checkpoints involved in interactions between T cells and cancer cells, for most patients these strategies are not successful. Patient stratification based on omics data for the personalization of these therapies is needed to optimize resources and improve patient outcomes.

**Material and Methods**

In this project, we aimed to characterize the relationships between immune cells infiltrating the tumour as detected by deconvolution of bulk RNAseq samples, expression of classic and novel immune checkpoints, including PD1 and VISTA (VSIR) amongst others and genetic mutations. We performed the analysis on a cohort of 80 samples from IUCT Oncopole in Toulouse and a validation cohort of 90 patients from Vanderbilt university. We developed a pipeline to perform cell type deconvolution using a variety of methods and signatures and designed an approach for feature selection and dimensionality reduction that

© 2023 The Authors. Molecular Oncology is published by John Wiley & Sons Ltd on behalf of Federation of European Biochemical Societies incorporates principal component analysis (PCA), linear discriminant analysis (LDA), and Weighted Correlation Network Analysis.

**Results and Discussions**

We report an incomplete association of stage with levels of immune infiltration, the existence of multiple tumour microenvironment profiles that are partially related to the activity of specific transcription factors, and interesting correlations between the presence of specific markers and spatial patterns of cells in the tissue detected by network analyses. Analysis of a reduced set of samples for which we have response to anti-PD1 and public datasets in other cancer types suggests that some of the features could be predictive of response.

**Conclusion**

Despite the number of available methods for cell types deconvolution using bulk RNAseq data, there is no clear way to perform a benchmark of these methods. Combining results based on an automated feature grouping algorithm is a promising approach to uncover clinically relevant biomarkers. Finally, we implemented the tool in a user-friendly graphical interface (R Shiny) for researchers and clinicians.

### EACR23-1469
### Exploring the Tumor Microenvironment in Pancreatic Ductal Adenocarcinoma (PDAC): Multiplexed Imaging of Immune Cell Lineages and Cell to Cell Interactions in the Hypoxic Tumor

*R. Prioli[1], L. Arvidson[1], M. Jacquet[2], S. Jensen[1], M. Smith[3], K. White[3], D. Pointu[4], H. Sasaki[5], T. Phan[3], C.C. Huang[3], M. Angus-Hill[3]*

*[1]Cell Signaling Technology, Cell, Boston, United States*
*[2]Cell Signaling Technology, Cell, Leiden, Netherlands Antilles*
*[3]Leica Microsystems, Cell, Bellevue, United States*
*[4]Leica Microsystems, Cell, Paris, France*
*[5]Leica Microsystems, Cell, Tokyo, Japan*

**Introduction**

Understanding the evolution of the tumor microenvironment (TME) during tumorigenesis and therapeutic response is critical for developing personalized treatments that can improve cancer therapy outcomes. A powerful approach for probing the TME is to use immune biomarker antibodies and multiplexed imaging technologies to interrogate immune cell lineages and structures in conjunction with specific oncology biomarker antibodies. In this way, the immune response within the TME can be captured in various neoplasms.

**Material and Methods**

The Cell DIVE$^{TM}$ Multiplex Imaging Solution allows for the probing and imaging of dozens of biomarkers on a single tissue section using an iterative staining and dye inactivation workflow. We selected antibodies from a broad portfolio of robust IHC-validated antibodies from Cell Signaling Technology (CST). Antibodies were directly conjugated to dyes, to enable the detection of key proteins in the TME. Validation of the AB dye conjugate required minimal time, and assembly of antibody panel was straightforward, since there are no abnormal antibody interactions or antibody exclusion with this methodology. The novel CST panel of immuno-oncology antibody biomarkers was used to probe pancreatic ductal adenocarcinoma (PDAC) to define the immune cell landscape in the hypoxic tumor, in the normoxic tumor and in normal pancreas.

**Results and Discussions**

The CST panel accurately defined the immune cell landscape in the hypoxic PDAC tumor, with minimal optimization required for development. Complex cell types were identified, and their cell-to-cell interactions within the TME were revealed. This approach provides novel insights and spatial resolution of myeloid and lymphoid immune cell populations and includes immune cell subtypes that are potential targets for immunotherapy. Cell DIVE, in combination with CST's broad portfolio of robust IHC-validated antibodies, enables the accurate detection and phenotyping of immune cells in tissue.

**Conclusion**

In conclusion, multiplexed imaging technologies and specific biomarker antibodies offer a powerful approach for probing the TME, and Cell DIVE, in conjunction with CST's broad portfolio of robust IHC-validated antibodies, is particularly effective for this purpose. The results of this study provide novel insights into the complex cellular interactions within the heterogenous TME and defines the tumor and immune cell interactions, which can ultimately be used to develop personalized cancer treatments that improve therapy outcomes.

### EACR23-1500
### A method for Early Diagnosis of Lung Cancer from Tumor Originated DNA Fragments using plasma cfDNA Methylome and Fragmentome Profiles

*S.H. Lee[1], Y.J. Kim[2], H. Jeon[2], S. Jeon[2], B.C. Kim[2], J.H. Kim[3], B.C. Kim[2], J. Bhak[4], I.J. Oh[5]*

*[1]Clinomics, Cancer Genomics Research Institute, Cheongju-si, South Korea*
*[2]Clinomics, R&D Center, Ulsan-si, South Korea*
*[3]Dongnam Institute of Radiological and Medical Sciences Cancer Center, Cardiothoracic surgery, Busan-si, South Korea*
*[4]Ulsan National Institute of Science and Technology UNIS T, Department of Biomedical Engineering- College of Inform ation and Biotechnology, Ulsan-si, South Korea*
*[5]Chonnam National University Medical School- and Hwas un Hospital, Department of Internal Medicine, Hwasun-si, South Korea*

**Introduction**

Early detection is critical for minimizing mortality from cancer. Plasma cell-free DNA (cfDNA) contains the signatures of tumor DNA, allowing us to quantify the signature and diagnose early-stage tumors.

**Material and Methods**

TOF utilizes the amount of ctDNA predicted from the methylation density information of each cfDNA read mapped on 6,243 lung-tumor-specific CpG markers. The 6,243 tumor-specific markers were derived from lung tumor tissues by comparing them with corresponding normal tissues and healthy blood from public methylation data. TOF also utilizes two cfDNA fragmentomic

© 2023 The Authors. Molecular Oncology is published by John Wiley & Sons Ltd on behalf of Federation of European Biochemical Societies signatures: 1) the short fragment ratio, and 2) the 5' end-motif profile. We used 298 plasma samples to analyze cfDNA signatures using enzymatic methyl-sequencing data from 201 lung cancer patients and 97 healthy controls.

**Results and Discussions**

The TOF score showed 0.98 of the area under the curve in correctly classifying lung cancer from normal samples. The TOF score resolution was high enough to clearly differentiate even the early-stage non-small cell lung cancer patients from the healthy controls. The same was true for small cell lung cancer patients. To be sure that our method is generally applicable to all cancer types, we should perform the deconvolution of other cancer types from heterogeneous specimens for more precise diagnosis, as DNA methylation patterns are tissue- and cell-type specific in general. An extended investigation on many cancer epigenomes is necessary to distinguish the tissue-specific markers and pan-cancer markers for various clinical applications.

**Conclusion**

We characterized and quantified tumor-originated fragments in plasma cfDNA by investigating the cfDNA fragment properties, such as DNA methylation, fragment size, and fragment end-motif of lung cancer patients compared to healthy individuals for early-stage lung cancer detection as a new liquid biopsy method.

### EACR23-1524
### Uncovering gene fusions with 3D genomics: from clinical validation to actionable insights for undiagnosable solid tumors

*Sikkink K.[1], Galbraith K.[1], Movahed-Ezazi M.[1], Jour G.[1], Schmitt A.[2], Snuderl M.[1]*

*[1]Departement of Pathology, NYU School of Medicine, New York, NY, 10016, USA*

*[2]Arima Genomics Inc, Carlsbad, CA, 92011, USA*

Identifying gene fusions in tumor biopsies is critical for understanding disease etiology, however, clinical NGS panels often fail to yield clear genetic drivers. A key challenge is that RNA-seq does not perform well in FFPE tissue blocks due to RNA degradation and/or RNA panel design, and cannot detect breakpoints outside of the gene body, which are established clinical biomarkers with mechanistic significance and clinical utility in solid hematological cancers. We developed a novel DNA-based partner-agnostic approach for identifying fusions from FFPE tumors using 3D genomics based on Arima-HiC technology and NGS. We profiled 184 FFPE tumors across various tumor types. This cohort includes 33 tumors with known gene fusions detected by RNA panels for clinical concordance analysis. It also includes 151 FFPE tumors, including 62 CNS tumors, 59 gynecological sarcomas, and 22 solid heme tumors, with no detectable genetic drivers from prior DNA and RNA panel CLIA assays. For clinical concordance, Arima-HiC technology detected 33/33 fusions previously detected by RNA panels. For clinical validation and utility studies in our driver-negative cohort, Arima-HiC detected 1 or more fusion in 72% (109/151) of tumors. To attribute clinical significance, we compared the genes implicated in our fusion calls with NCCN and WHO guidelines, and OncoKB, and assigned which tumors had an FDA-approved therapeutic level biomarker ("Tier 1"), a biomarker targeted in an ongoing clinical trial ("Tier 2"), or a diagnostic/prognostic biomarker ("Tier 3"). Arima-HiC analysis found 33.8% (51/151) of tumors had Tier 1 biomarkers, 4.0% (6/151) had Tier 2 biomarkers, and a further 14.6% (22/151) had Tier 3 biomarkers, indicating an overall yield of clinically actionable biomarkers at 52.3%. Several cases with Tier 1-3 fusions underwent confirmatory immunohistochemistry testing for oncoprotein expression, and 91.6% (11/12) showed diffuse or focally positive staining. To highlight examples from prospectively analyzed cases from this cohort, we identified a novel PD-L1 rearrangement in a pediatric glioma tumor that was not detected by DNA or RNA panels, which we confirmed by PD-L1 IHC, and the patient was administered pembrolizumab after tumor recurrence and has since exhibited stable disease. We also identified a MYBL1 fusion in a glioma that spared the patient unnecessary chemotherapy post-resection. Our findings provide evidence of clinical concordance, validation, and utility, and underscores the need for 3D genome profiling to increase diagnostic yield by finding clinically actionable fusions in FFPE solid tumors.

### Cancer Cell Biology

### EACR23-0012
### The changes of the plaque inflammation assessed by 18F-FDG PET after radioimmunotherapy with 131I-rituximab in patients with lymphoma

*B. Byeon[1]*

*[1]Korea Cancer Center Hospital- Korea Institute of Radiolo gical and Medical Sciences, Department of Nuclear Medicine, Seoul, South Korea*

**Introduction**

Atherosclerosis is a chronic inflammatory disease modulated by many immune cells, including B cells. Preclinical and several small-scale human studies have reported the reduction of plaque inflammation by using rituximab which depletes atherogenic B-2 cells. We assessed the influence of radioimmunotherapy with $^{131}$I-rituximab on the plaque inflammation assessed by $^{18}$F-FDG PET in patients with lymphoma.

**Material and Methods**

We retrospectively analyzed $^{18}$F-FDG PET scans of patients treated with $^{131}$I-rituximab for the first time for relapsed or refractory CD20(+) B cell lymphoma. $^{18}$F-FDG PET scans were performed before (PET1), 5 days (PET2), and 1 m (PET3) after the administration of $^{131}$I-rituximab. On each PET dataset, target-to-background ratio (TBR) was calculated from the ratio of maximum standardized uptake value of the right carotid artery compared with mean standardized uptake value of inferior vena cava. TBRs measured in PET1, PET2, and PET3 were designated as TBR1, TBR2, and TBR3, respectively. Statistical analysis was performed using the Wilcoxon-test for comparison between the groups. In addition, we divided patients into 2 groups (patients with higher TBR1

© 2023 The Authors. Molecular Oncology is published by John Wiley & Sons Ltd on behalf of Federation of European Biochemical Societies vs equal to or lower TBR1 than the median value of TBR1) and compared TBRs on each PET dataset.

**Results and Discussions**

A total of 32 patients were analyzed for 32 radioimmunotherapies. The mean and SD values of TBR1, TBR2, and TBR3 were 1.49 ± 0.28, 1.36 ± 0.21, and 1.35 ± 0.19, respectively. TBR2 (P = 0.008) or TBR3 (P = 0.002) was statistically significantly lower than TBR1, while there was no significant difference between TBR2 and TBR3. In patients with higher TBR1 (n =16), TBR significantly decreased in PET2 (mean TBR = 1.46, P = 0.001) or PET3 (mean TBR = 1.42, P < 0.001) compared with TBR in PET1 (mean TBR = 1.69). In patients with lower TBR1 (n =16), however, TBR in PET2 (mean TBR = 1.26) nor PET3 (mean TBR = 1.29) showed significant difference compared with TBR in PET1 (mean TBR = 1.29).

**Conclusion**

In lymphoma patients with higher plaque inflammation, the degree of plaque inflammation measured by $^{18}$F-FDG PET significantly decreased after radioimmunotherapy with $^{131}$I-rituximab. On the other hand, in patients with lymphoma with lower plaque inflammation, no significant decrease in plaque inflammation was observed after radioimmunotherapy. It is presumed that plaque inflammation was alleviated by B cell depletion by radioimmunotherapy with $^{131}$I-rituximab.

### EACR23-0026
### Erythrocyte-membrane vesicles as drug carriers

*N. kostevšek[1], I. Serša[2], P. Miklavc[1], G. Della Pelle[1]*
*[1]Jožef Stefan Institute, Department for nanostructured materials, Ljubljana, Slovenia*
*[2]Jožef Stefan Institute, Condensed Matter Physics Department, Ljubljana, Slovenia*

**Introduction**

Among different circulatory cells, erythrocytes are the most abundant and thus can be isolated in sufficiently large quantities to decrease complexity and cost of the treatment compared to other cell-based vaccines. Therefore, we propose to use erythrocyte membrane vesicles (EMVs) as a drug delivery system. We present the preparation of EMVs as magnetic resonance imaging (MRI) contrast agents and as carriers for cyanine dyes as photo-thermal and imaging agents.

**Material and Methods**

Magneto-EMVs and cyanine dye-containing EMvs were characterized using zeta potential and particle size measurements, TEM and cryo-TEM analysis, thermal- and photo-stability studies, MRI measurements and hemotoxicity tests.

**Results and Discussions**

Encapsulation of 5-nm magnetic nanoparticles in the liposomes and EMVs leads to a significant improvement in their relaxivity, where r2 values increased to r2 = 188 ± 2 mM-1s-1 for magneto-liposomes and r2 = 269 ± 3 mM-1s-1 for magneto-erythrocyte membranes, compared to a "free" IO NPs with an r2 value of 12 ± 1 mM-1 s-1, measured at 9.4 T MRI scanner. The superiority of magneto-erythrocyte membranes in terms of MRI contrast efficacy is clearly shown on T2-weighted MR images. A hemotoxicity study revealed the safety of developed contrast agents in the MRI-relevant concentration range.

Cyanine fluorescent dyes were encapsulated into liposomes and EMVs. The optical properties of both dyes were investigated in different biological relevant media. Then, temperature- and photo-stability of dyes in a free form and encapsulated into liposomes and EMVs were evaluated. Nano-carriers can efficiently protect dyes from thermal degradation. However, they cannot prevent photo-induced degradation. Finally, a hemotoxicity study revealed that EMVs seem to be less hemotoxic dye carriers than clinically approved liposomes.

**Conclusion**

The superiority of magneto-erythrocyte membranes in terms of MRI contrast efficacy is clearly shown on $T_2$-weighted MR images. We found that encapsulation of dyes into liposomes and EMVs can improve dyes' thermal stability. However, nano-carriers cannot prevent photo-induced degradation. Liposomes were found to be hemotoxic at as high concentrations as 5 mM, which shine the light on the superiority of EMVs regarding the hemocompatibility profile. In conclusion, the use of body’s own cells as nano-carriers for dyes and other active components is a highly attractive strategy that might help to overcome the challenges the current nanomedicine is facing.

### EACR23-0053
### KDM5B's role in creating radiation synthetic vulnerabilities and its effects on sequential therapy combinations in melanoma

*B. Shannan[1], J. Matschke[2], P. Braß[1], D. Schadendorf[1], V. Jendrossek[2], A. Roesch[1]*
*[1]University Hospital Essen, Dermatology, Essen, Germany*
*[2]University Hospital Essen, Cell Biology, Essen, Germany*

**Introduction**

Cutaneous melanoma is the most aggressive form of skin cancer, and survival rates dramatically drop following distant organ metastases. Moreover, it is not fully understood which molecular mechanisms of adaptation are responsible for the observed melanoma cells escape from initial therapeutic hits such as targeted (MAPKi) or radiation-induced killing, nor how to target these surviving cells. We have previously described the existence of a small subpopulation of slow-cycling cells that survives multiple available drugs and re-populates the tumor. This multi-drug-resistance and universal self-renewal capacity seems to be dependent on the upregulation of the histone H3K4 demethylase KDM5B. We have also recently shown an important role of KDM5B in melanoma repopulation after combined targeted plus radiation therapy (RT) and that the degree of therapy resistance may be even dependent on the temporal treatment sequence chosen. However, the molecular mechanisms and dynamics downstream of KDM5B that mediate cell survival and radio-tolerance and -resistance are not known.

**Material and Methods**

In this study, we attempted to define the role of KDM5B in mediating tolerance and acquired radio-resistance induced by chronic cycling hypoxia and to identify protein targets

© 2023 The Authors. Molecular Oncology is published by John Wiley & Sons Ltd on behalf of Federation of European Biochemical Societies that may drive radiation resistance through cell survival assays and an RPPA screen.

**Results and Discussions**

We show that cells undergoing initial repeated cycles of hypoxia and intermittent reoxygenation become more tolerant to RT and have an increased endogenous expression level of KDM5B. What is more, our RPPA analyses show that KDM5B has a role in regulating the cell cycle, metabolism and survival following radiation as well as being involved in phenotype switching. We also demonstrate that prolonged cycles of hypoxia have the opposite effects, where the cells have a decreased expression level of KDM5B and a proliferative phenotype.

**Conclusion**

These results highlight the importance of characterizing possible vulnerabilities in the context of KDM5B to unravel the involved cell survival pathways.

### EACR23-0064
### Multilayered N-glycosylation dependent control of FGF/FGFR signaling and cell fate by extracellular galectins

*D. Zukowska[1], A. Gedaj[1], P. Gregorczyk[1], M. Pozniak[1], N. Porebska[1], L. Opalinski[1]*
*[1]University of Wroclaw, Department of Protein Engineering, Wroclaw, Poland*

**Introduction**

FGF/FGFR signaling is critical for the development and homeostasis of the human body and imbalanced FGF/FGFR contributes to the progression of severe diseases, including cancers. FGFRs, as well as the vast majority of FGFs, are N-glycosylated, but the role of these modifications is largely unknown.

**Material and Methods**

We use a wide set of biophysical (DLS, BLI), protein engieering (recombinant proteins, galectin variants of tuned valency) and cell biology techniques (cell culture, scratch assay, cell proliferation measurements, fluorescence microscopy, western blotting, pull down, galectin arrays)

**Results and Discussions**

Here, we demonstrate that a precise set of extracellular multivalent lectins, galectins -1, -3, -7 and -8, differentially read the information stored in the N-glycans of FGFRs and FGFs to adjust FGF/FGFR signaling and cell physiology at multiple levels. At the stage of the receptor, galectins directly bind the N-glycans of the D3 domain of FGFR1 and trigger receptor activation. Using engineered galectins with controlled valency, we show that N-glycosylation-dependent clustering of FGFR1 constitutes a mechanism for FGFR1 stimulation by galectins. We also show that N-glycans of FGFR1 constitute intracellular targeting signal, determining FGFR1 transport to the plasma membrane or its accumulation in the nuclear envelope. At the ligand level, galectins directly interact with the N-glycans of the vast majority of FGFs. We show that galectins differentially attract N-glycosylated FGF4 to cells, forming a reservoir of growth factor at the cell surface. Furthermore, galectins moderate cellular trafficking and signaling of FGF4, and the multivalency of galectins is critical for these activities. Importantly, the consequences of galectin/FGFR and galectin/FGF4/FGFR signaling for the cell physiology are markedly different from the effects induced by FGF/FGFR and FGF4/FGFR signaling units.

**Conclusion**

Our data identify a novel regulatory module within FGF and FGFR signaling, in which the glyco-code in FGFs and FGFRs provides previously unanticipated information about their spatial distribution that is differentially deciphered by distinct multivalent galectins, affecting signal transmission and cell fate.

*This research was funded by SONATA BIS grant (2019/34/E/NZ3/00014) from the National Science Centre awarded to L.O.*

### EACR23-0066
### The path to CRAF degraders: learnings and pitfalls of high-throughput PROTAC screening.

*A. Frost[1], V. Vetma[1], N. Chessum[2], L. Casares Perez[1], N. Braun[2], T. Gerstberger[2], P. Stolt-Bergner[2], A. Ciulli[1], W. Farnaby[1], M. Koegl[2]*
*[1]University of Dundee, Centre for Targeted Protein Degradation, Dundee, United Kingdom*
*[2]Boehringer Ingelheim RCV GmbH & Co KG, Cancer Research, Vienna, Austria*

**Introduction**

The RAF family of proteins (ARAF, BRAF and CRAF) are serine/threonine kinases involved in the well-known RAS-RAF-MEK-ERK signalling pathway. This pathway is dysregulated in up to 30% of all cancers, leading to uncontrolled proliferation of malignant cells. In both mouse tumour models and human cancer cell lines, RAS mutated cancers have been shown to depend on the presence of CRAF, but not on its kinase catalytic activity. This suggests that targeted protein degradation (TPD) of CRAF may be of therapeutic value compared to CRAF inhibition in RAS mutated tumours. TPD is a novel strategy based on the use of heterobifunctional molecules, such as proteolysis-targeting chimeras (PROTACs), to recruit a cellular target to an E3 ligase for its ubiquitination and subsequent proteasomal degradation.

**Material and Methods**

A toolbox library of ~5800 bifunctional compounds was designed using 13 different RAF binders and a selection of available E3 ligands. This library was screened by both biophysical and cellular methods to interrogate their binding affinity for both CRAF and E3 ligase, and their ability to degrade HiBiT-tagged CRAF as well as endogenous CRAF. A novel high-throughput assay based on Promega's Lumit™ platform was optimised to enable large-scale screening for endogenous degradation of CRAF.

**Results and Discussions**

Unexpectedly, cellular reporter assays were proven to be unreliable in this case as the half-life of HiBiT-tagged CRAF was found to be much shorter than the endogenous protein. Furthermore, unstable CRAF made this project more vulnerable to false positives related to neosubstrates of E3 ligase CRBN as well as cytotoxic compounds. On the other hand, the Lumit™ platform was confirmed to be an accurate, affordable and high-throughput way to screen for degradation of endogenous CRAF as evidenced by comparison with automated Western Blot. To the best of

© 2023 The Authors. Molecular Oncology is published by John Wiley & Sons Ltd on behalf of Federation of European Biochemical Societies our knowledge, this is the first time Lumit™ has been used in the field of TPD for such a purpose.

**Conclusion**

Our findings highlight the importance of reliable screening assays in a high-throughput setting and how confounding factors such as off-target effects can lead to false positives in TPD projects.

### EACR23-0071
### CD36 is a novel biomarker for desaturase inhibitors in triple negative breast cancer

*F. Fai[1], B. Malacrida[2], J.L. Jones[1], L. Hazlehurst[3], J.A. Copland III[4], F. Balkwill[5], A. Finch[6], K. Hodivala-Dilke[5], B. Peck[1]*

*[1]Barts Cancer Institute- Cancer Research UK Centre of Ex cellence- Queen Mary University of London- Charterhouse Square- London- EC1 6BQ- UK, Centre for Tumour Biology, London, United Kingdom*

*[2]Barts Cancer Institute- Cancer Research UK Centre of Ex cellence- Queen Mary University of London- Charterhouse Square- London- EC1 6BQ- UK, Centre for Tumour Microenvironment-, London, United Kingdom*

*[3]School of Medicine- West Virginia University- Morgantow n WV 26501- USA, Cancer Center, Morgantown, United States*

*[4]Mayo Clinic- Jacksonville- Florida 32224- USA, Department of Cancer Biology, Florida, United States*

*[5]Barts Cancer Institute- Cancer Research UK Centre of Ex cellence- Queen Mary University of London- Charterhouse Square- London- EC1 6BQ- UK, Centre for Tumour Microenvironment, London, United Kingdom*

*[6]Barts Cancer Institute- Cancer Research UK Centre of Ex cellence- Queen Mary University of London- Charterhouse Square- London- EC1 6BQ- UK, Centre for Tumour Biology-, London, United Kingdom*

**Introduction**

Triple Negative Breast cancer (TNBC) is a tumour type of significant clinical unmet need due to the lack of actionable biomarkers and efficacious targeted therapies. Indeed, TNBCs make up 20% of all breast cancer cases and do not express the hormone receptors, meaning there are limited therapeutic options. TNBCs are aggressive and display high invasiveness and metastatic potential, which is associated with a poor prognosis. Indeed, the median overall survival for metastatic TNBCs is about 1 year versus approximately 5 years for the other subtypes. TNBCs are metabolically distinct and known to be dependent on multiple metabolic enzymes for their growth. We previously identified Stearoyl-CoA desaturase (SCD), which controls the first committed desaturation step in lipogenesis, to be significantly up-regulated and be a metabolic vulnerability in hormone-independent cancers, such as TNBCs. Currently, there are no known, robust biomarkers that impart sensitivity to SCD inhibition. Therefore, we sought to study the interplay of extracellular lipid transporter expression and lipid desaturation dependency in human breast cancer cells.

**Material and Methods**

HAP1 and TNBC lines grown under physiologically relevant conditions utilising both 2- and 3-dimensional models to recapitulate the unfavourable tumour microenvironment *in vitro*. Various viability and analytical biochemical assays were performed to assess SCD activity, expression and drug sensitivity.

**Results and Discussions**

We have found that the Monounsaturated Fatty Acid (MUFA) receptor CD36, also known as fatty acid translocator (FAT), is lost in a significant proportion of human breast cancers (7%) and that SCD is significantly upregulated in these tumours. Utilising our *in vitro* haplo-insufficient genetic CRISPR models and TNBC cell lines we have shown that CD36 loss imparts sensitivity to SCDi, even in lipid-replete conditions. Moreover, this sensitivity was rescued by reinstating intracellular MUFA levels via a CD36-independent mechanism.

**Conclusion**

In conclusion, we have found that CD36 loss results in increased SCD activity and response to SCDi in cancer cells, under various stress conditions. As SCD targeted therapies enter clinical testing, for example SSI-4 (Modulation Therapeutics), this study provides the necessary tools and patient stratification rationale for validating CD36 loss as a biomarker for SCDi response in human TNBCs. Moreover, it furthers our knowledge of the role of lipid desaturation in cancer aggressiveness and treatment response.

### EACR23-0083
### Leverage of Microenvironmental Ornithine in Triple Negative Breast Cancer Cells under Glutamine Starvation

*G. Verch[1], M. Berishaj[2], H.P. Schlemmer[1], K.R. Keshari[2]*

*[1]German Cancer Research Center, Radiology, Heidelberg, Germany*

*[2]Memorial Sloan Kettering Cancer Center, Radiology, New York, United States*

**Introduction**

As the most abundant amino acid in circulation, glutamine plays a pivotal role in the metabolism of proliferating breast cancer cells. Strategies to reduce tumor's ability in glutamine utilization, most notably targeted inhibition of the enzyme glutaminase, show little success in clinical trials. To address this question, we sought to identify pathways which could overcome resistance to glutamine deprivation. In this regard tumor associated macrophages (TAMs), when polarized to an M2-phenotype in the tumor microenvironment (TME) have been shown to upregulate cytosolic arginase (ARG1). Here we describe not only the role of arginine metabolism in rescuing triple negative breast cancer (TNBC) cells but also connect changes in arginine metabolism with the TME.

**Material and Methods**

Detection of metabolic changes in glycolysis and pentose phosphate pathway in TNBC cells in glutamine depleted conditions was performed by $^{13}$C-NMR tracing from 1,2-$^{13}$C-glucose. In addition we conducted a targeted metabolite analysis by LC-MS tracing from $^{13}$C-arginine. To further demonstrate the symbiosis between TNBC cells and TAMs, we compare inhibitory effects of TAM depletion by Clodronate® combined with glutaminase inhibition by CB-839® on tumor growth in vivo in mice xenografted with MDA-MB-468 and MDA-MB-231 tumors.

**Results and Discussions**

© 2023 The Authors. Molecular Oncology is published by John Wiley & Sons Ltd on behalf of Federation of European Biochemical Societies

We show that upon supplementation with arginine and ornithine tumor growth, in a panel of breast cancer cell lines that are resistant to glutaminase inhibition, can be rescued in glutamine depleted conditions. This is accompanied by an increase of NAD:NADH as well as NADP:NADPH levels and requires expression of mitochondrial enzymes ornithine-acetyltransferase (OAT) and pyrroline-5-carboxylate reductase 1 (PYCR1). The results of the in vivo study show a lower tumor burden in CB-839® resistant MDA-MB-468 Xenografts upon TAM depletion highlighting the importance of TAMs as a pathophysiological provider of ornithine in TNBC.

**Conclusion**

Taken together, this work expands the knowledge of a tumor-immunological symbiosis between tumor cells and TAMs, which should be considered a new target in TNBC.

### EACR23-0104
### Therapeutic effect of proteasome inhibitor Carfilzomib against HPV negative Cal-27 by PI3K/Akt/mTOR and p21 signaling pathways

*J. Kim*[1], *L. Hye-Yeon*[1], *J. Woo-Jin*[1]
[1]*Seoul National University Bundang Hospital, Otorhinolaryngology-Head & Neck Cancer Lab, bundang-gu, South Korea*

**Introduction**

Squamous cell carcinoma of the head and neck (HNSCC) presents at heterogeneous sites, a variety of etiologies, and risk factors, and has a diverse genetic background conferring different responses to treatment, including chemotherapy. Nevertheless, chemotherapy for head and neck cancer has been classically based on cisplatin, and its therapeutic results have been stagnant for decades. We sought to identify pathways that differentially affect HNSCC according to the genetic background, therefore identifying targets and suitable candidate for novel treatment. The purpose of this study was to lay the foundation for targeted treatment of HPV-negative head and neck cancer, which has relatively poor therapeutic results compared to HPV-positive head and neck cancer.

**Material and Methods**

We previously found through chemical genetic library screening that targeted therapies acting on the PSMD1 pathway performed significantly better in HPV-negative head and neck cancer cell lines. HNSCC cells were treated with Carfilzomib (CFZ) alone and was analyzed for cell viability, apoptosis, fluorescence- activated cell sorting (FACS) and western blot to evaluate anti-tumor effects *in vitro* and in xenograft *in vivo* models.

**Results and Discussions**

In vitro validation of CFZ showed an $IC_{50}$ that was more than 4-fold lower in HPV-negative Cal-27 than other HNSCC cells. Knockdown of PSMD1 transcripts by transfected siRNAs significantly reduced Cal-27 cells viability. In this study, we observed that low doses of the proteasome inhibitor CFZ caused apoptosis of Cal-27 cancer cells. In addition, CFZ inhibited p-Akt and p-S6 and activated p21 which increased growth inhibition and apoptosis in Cal-27 cells. In mice bearing xenografted HPV-negative Cal-27 cells, we confirmed that CFZ reduced tumor growth and weight. Collectively, treatment of CFZ is very effective for HPV-negative Cal-27. The cytotoxic effects induced by CFZ involve cell growth inhibition and apoptosis via the PI3K/Akt/mTOR and p21 signaling pathways.

**Conclusion**

This suggests that CFZ is a novel therapeutic agent that can overcome the existing cisplatin resistance used in the treatment of HPV-negative head and neck cancer.

### EACR23-0128
### Itaconate induces metabolic reprogramming in ER-positive breast cancer cells

*H.C. Wang*[1], *Y.C. Lai*[1], *D.Y. Lee*[2], *M.C. Hung*[1]
[1]*China Medical University- Taiwan, Graduate Institute of Biomedical Sciences, Taichung, Taiwan*
[2]*China Medical University, Graduate Institute of Integrated Medicine, Taichung, Taiwan*

**Introduction**

Changing intracellular metabolites to meet energy demand and function as non-metabolic signals is essential for tumor development and progression. Itaconate, synthesized from cis-aconitate in the TCA cycle by IRG1, is an anti-inflammatory metabolite via directly alkylating target proteins on cysteine residues. However, it has yet to be made clear about the role of itaconate in tumor cells per se and development. In this study, we hypothesized that itaconate is crucial in determining breast cancer cell fate and potentially therapeutically treating breast cancer.

**Material and Methods**

Exogenous itaconate and itaconate derivative, 4-octyl-itaconate, were evaluated for their effects on breast cancer cell growth detected with colony formation, EdU cell proliferation, and apoptosis analyses. Stable expressions of IRG1 cells were established to examine the impacts of endogenous itaconate on the cells. A proteomic approach was applied to systematically explore the subsequent events following IRG1 activation in the cells. Changes in selected intermediates of glycolysis, the TCA cycle, and the lipid metabolism profile were measured using mass spectrums.

**Results and Discussions**

ER-positive breast cancer cells were more responsive to itaconate-induced cell death than ER-negative cells. Notably, hormone therapy-resistant breast cancer cells were also sensitive to itaconate-induced cell death. Itaconate caused cell cycle arrest via DNA proliferation inhibition and apoptosis due to the resulting energy stress. Mechanically, itaconate induced the activation of the AMP-activated protein kinase (AMPK) pathway in response to metabolic stress, accompanied by the change in glycolysis, the TCA cycle, and the lipid metabolites. Consequently, itaconate triggered metabolic stress via metabolic reprogramming, leading to mitochondrial dysfunction and cell death in ER-positive breast cancer cells.

**Conclusion**

This study demonstrated that itaconate rewrote biochemical pathways, which increased metabolic stress from energy imbalance and inhibited ER-positive cancer cell growth. Our discoveries may show promise in

© 2023 The Authors. Molecular Oncology is published by John Wiley & Sons Ltd on behalf of Federation of European Biochemical Societies facilitating the design for developing more efficacious therapeutic and preventive strategies for breast cancer.

### EACR23-0129
### Alisol A induces heme oxygenase-1 transactivation-mediated apoptotic cell death of oral cancer cells by triggering p38 signaling pathway

S.F. Yang[1], M.Y. Tsai[1], C.W. Lin[2]
[1]*Chung Shan Medical University, Institute of Medicine, Taichung, Taiwan*
[2]*Chung Shan Medical University, Institute of Oral Sciences, Taichung, Taiwan*

**Introduction**
Oral cancer is a common malignancy globally. At present, comprehensive treatment strategies have been available for oral cancer treatment. However, oral cancer still has a poor prognosis and high recurrence. Alisol A, a protostane-type tetracyclic triterpenoid, has been documented to elicit various oncostatic properties alone or in combination with established therapeutics. Here, we further explored the anticancer effect and the underlying molecular mechanism of Alisol A on oral cancer cells.
**Material and Methods**
Cell viability and flow cytometry analysis for apoptosis profile were evaluated by MTT assay and flow cytometric analysis. Human apoptosis array and Western blotting were used to explore the apoptotic effect and its mechanisms of Alisol A in oral cancer cells.
**Results and Discussions**
Our results showed that Alisol A dose-dependently decreased the cell viability of on human SCC-9 and HSC-3 oral cancer cells. Moreover, the human apoptosis array showed that heme oxygenase (HO)-1 and cleaved caspase-3 expressions had significant increases after Alisol A treatment in SCC-9 cells. Mechanistically, Alisol A treatment induced caspase-mediated apoptosis via upregulating the initiators of both extrinsic caspase 8 and intrinsic caspase 9, and significantly increased cleaved PARP expression in SCC-9 and HSC-3 oral cancer cells. Moreover, Alisol A -mediated upregulation of HO-1 and cleavage of caspase-9, -8 and -3 were significantly inhibited by pharmacological blockage of p38 kinase.
**Conclusion**
Collectively, these data revealed that Alisol A arrested cell cycle progression and elicited cell apoptosis in oral cancer cell lines, reshaping a p38-dependent profile of apoptotic proteome. Our findings provided novel insights into the therapeutic implications of Alisol A on the management of oral cancer.

### EACR23-0130
### Curcumin analogue L48H37 induces apoptosis by activating caspase cascades and downregulating the inhibitor of apoptosis proteins through JNK/p38 signaling in human oral cancer cells

C.W. Lin[1], S.F. Yang[2]
[1]*Chung Shan Medical University, Institute of Oral Sciences, Taichung, Taiwan*
[2]*Chung Shan Medical University, Institute of Medicine, Taichung, Taiwan*

**Introduction**
Oral cancer is a life-threatening mouth malignancy in Asia and Taiwan. Surgical resection combined with radiotherapy or chemotherapy are the common treatments for oral cancer; however, the efficacy is still unsatisfactory. L48H37 is a synthetic curcumin analog and has anticancer potentials. Here, we further explored the anticancer effect of L48H37 on oral cancer cells and its mechanistic acts.
**Material and Methods**
Cell viability and cell cycle distribution were assessed by MTT assay and flow cytometric analysis. Apoptosis was elucidated by staining with PI / Annexin V and activation of the caspase cascade. Cellular signaling was explored using apoptotic protein profiling, Western blotting, and specific inhibitors.
**Results and Discussions**
Our findings showed that L48H37 significantly reduced the cell viability of SCC-9 and HSC-3 cells, resulting in sub-G1 phase accumulation and increased apoptotic cells. Apoptotic protein profiling revealed that L48H37 increased cleaved caspase-3, and downregulated cIAP1 and XIAP in SCC-9 cells, and the downregulated cIAP1 and XIAP in both oral cancer cells were also demonstrated by Western blotting. Meanwhile, L48H37 triggered the activation of caspases and mitogen-activated protein kinases (MAPKs). The involvement of c-Jun N-terminal kinase (JNK) and p38 MAPK (p38) in the L48H37-triggered apoptotic cascade in oral cancer cells were also elucidated by specific inhibitors.
**Conclusion**
Collectively, these findings indicate that L48H37 has potent anticancer activity against oral cancer cells, which may be attributed to JNK/p38-mediated caspase activation and the resulting apoptosis. This suggests a potential benefit for L48H37 for the treatment of oral cancer.

### EACR23-0135
### Lactate preserves genome integrity and extends proliferative potential

V. Rossi[1], M. Govoni[1], G. Di Stefano[1]
[1]*University of Bologna, Department of Medical and Surgical Science- DIMEC, Bologna, Italy*

**Introduction**
The metabolic reprogramming described in tumor cells is based on increased glucose uptake and glycolysis and reflects the metabolic features of embryonic and stem cells. This metabolic asset results in enhanced production of lactate, a metabolite found to be involved in the modulation of gene expression. By using colon and breast cancer cells, we investigated on a possible direct effect of lactate in preserving genome integrity and promoting proliferative potential.
**Material and Methods**
As a model of colon cancer, we used SW620 cells, which can be maintained in a glucose-free medium. To assess the effects of lactate on DNA damage repair, SW620 cells were exposed to 20mM lactate and their response to cisplatin was investigated. 20 mM lactate is in line with the metabolite concentration detected in neoplastic tissues. By using RT-PCR, we also evaluated the effect of lactate on

© 2023 The Authors. Molecular Oncology is published by John Wiley & Sons Ltd on behalf of Federation of European Biochemical Societies gene expression in SW620 cultures and in MCF7 breast cancer cells.

**Results and Discussions**

Lactate-exposed SW620 cells showed significantly reduced DNA damage signatures after cisplatin treatment, which correlated with an increased expression of genes involved in mismatch DNA repair. RT-PCR experiments also showed overexpression of PCNA, MKI67 and RFC4, which are indicative of enhanced proliferative potential. To confirm the link between lactate and proliferative potential, we also used MCF7 cells. We found that these cells are characterized by a moderate level glycolysis, with reduced lactate production. In this model, the effect caused by increased lactate exposure on gene expression was not superimposable to that observed in SW620 culture. Interestingly, among the lactate-upregulated genes we found TAZ, TERT and TERC (the RNA and catalytic component of telomerase complex, respectively). TAZ overexpression was found to promote EGFR signaling, leading to AKT/ERK activation and increased cell proliferation. By adopting a functional assay of telomerase activity, we verified that the overexpression of TERT and TERC genes led to a higher-level activation of the telomerase complex in lactate-exposed MCF7 cells.

**Conclusion**

Our results suggest that lactate helps cancer cells in maintaining DNA integrity and increasing cell proliferation, by triggering different molecular mechanisms according to the cell type. These effects are in line with the role of glycolytic metabolism in promoting embryonal development and maintenance of the stem compartment in normal tissues. However, in cancer cells they facilitate the onset of drug resistance.

### EACR23-0143
### Morin suppresses human osteosarcoma metastasis by inhibiting signal transducer and activator of transcription 3 to decrease urokinase plasminogen activator

*J.S. Yang*[1], *K.H. Lu*[2], *S.F. Yang*[3]
[1]*Chung Shan Medical University Hospital, Department of Medical Research, Taichung, Taiwan*
[2]*Chung Shan Medical University Hospital, Department of Orthopedics, Taichung, Taiwan*
[3]*Chung Shan Medical University, Institute of Medicine, Taichung, Taiwan*

**Introduction**

Osteosarcoma, the most common primary bone cancer that affects adolescents worldwide, has the early metastatic potential to be responsible for high mortality rates. Morin has a versatile role in myriad cancers, whereas little is known about its role in osteosarcoma metastasis. Therefore, we postulated that morin represses the biological behaviors of the migratory potential and the invasive activities of human osteosarcoma cells.

**Material and Methods**

First of all, human osteosarcoma cell lines U2OS and HOS were used to investigate the cell viabilities treated by morin through MTT assays. Furthermore, Boyden chamber assay was performed to test the effect of morin on migratory capacity and the invasive potential of U2OS and HOS cells *in vitro*. Western blot analysis was conducted to explore the protein levels of molecular mechanism and signaling pathways of morin in U2OS and HOS cells.

**Results and Discussions**

Up to 100 μM of morin, without cytotoxicity, reduced invasion and migration capabilities, and urokinase plasminogen activator (uPA) expression in human osteosarcoma U2OS and HOS cells. Although morin decreased phosphorylation of the signal transducer and activator of transcription (p-STAT)3, no evident influence on focal adhesion kinase (FAK), Src, extracellular signal-regulated protein kinase (ERK), c-Jun N-terminal kinase (JNK), p38, and their phosphorylation was observed. After STAT3 overexpression, the decrease of the migratory potential and uPA expression caused by 100 μM of morin in U2OS cells was countered, indicating that STAT3 contributes to the antimetastatic property of morin in human osteosarcoma cells by reducing uPA.

**Conclusion**

Collectively, these results suggested that morin contributes to the suppression of human osteosarcoma U2OS and HOS cellular invasion and migration by inhibiting uPA through the upstream STAT3 pathway, not through MAPKs or FAK-Src signaling. Thus, morin may be a potential candidate for the antimetastatic treatment of human osteosarcoma.

### EACR23-0159
### An androgen receptor antagonist improves conventional therapy for inflammatory breast cancer.

*B. Crespo Cortés*[1], *S. Cáceres*[1], *G. Silvan*[1], *M.J. Illera*[1], *J.C. Illera*[1]
[1]*Veterinary medicine School- University Complutense of M adrid, Animal Physiology, Madrid, Spain*

**Introduction**

Inflammatory breast cancer is an aggressive and highly metastasic form of breast cancer. Its is characterized by a poor survival and a high relapse rate. Many of inflammatory breast cancers have an absence of expression of the classical hormone receptors (ER, PR and HER-2) on which hormone therapy is usually focused. The conventional therapy applied in this cases is based on chemotherapy with anthracyclines and taxanes. However, the pathological response is limited. it has been demonstrated that inflammatory breast cancer present a strong hormonal influence. The androgen receptor presence, which is highly expressed, has been related to a better prognosis.

**Material and Methods**

Thus, this study is focus on the combination of an androgen receptor (AR) antagonist with conventional therapies, in order to improve treatment effectiveness. IPC-366, a triple negative breast cancer-AR+ and inflammatory mammary cancer cell line, has been used for this purpose, as it has been considered a good research model for inflammatory breast cancer. Bicalutamide was combined with the current treatment (Doxorubicin + Docetaxel). Besides, the two current drugs independently, were combined with Bicalutamide (Doxorubicin + Bicalutamide and Docetaxel + Bicalutamide). Sensitivity, proliferation and migration assays *in vitro* were performed. In addition, Balb/SCID mice were inoculated with IPC-366 and treated with those combined treatments.

© 2023 The Authors. Molecular Oncology is published by John Wiley & Sons Ltd on behalf of Federation of European Biochemical Societies

**Results and Discussions**

The sensitivity assay revealed a half-maximal effective concentration (EC-50) *in vitro* of 100nM for each compound, this dose was used on subsequent assays. Results revealed that the most effective combination *in vitro* was Docetaxel with Bicalutamide, which significantly reduced cell viability (75.01%), and cell migration (37.91%). The other combinations reduced cell migration but not cell proliferation. However, *in vivo* results showed significant reductions with all treatments, being the triple combination the most effective one. The use of taxanes (Docetaxel) in inflammatory breast cancer has been associated with better results than the anthracyclines (Doxorubicin) application *in vitro*, being able to avoid the irreversible side effects that produce the use of anthracyclines.

**Conclusion**

It can be concluded that the addition of Bicalutamide to the current treatment produce higher tumor reductions *in vivo*. Therefore, the AR antagonist addition to conventional treatment for inflammatory breast cancer could translate into an improvement in the effectiveness of the treatment.

### EACR23-0163

### SPINK1-induced tumor plasticity provides a therapeutic window for chemotherapy in hepatocellular carcinoma

*K.F. Man*[1], *L. Zhou*[2], *H. Yu*[1], *K.H. Lam*[1], *Y.T. Lam*[1], *J. Yu*[3], *J.P. Yun*[4], *X.Y. Guan*[5], *M. Liu*[6], *S. Ma*[1]

[1]*The University of Hong Kong, School of Biomedical Sciences, Hong Kong, Hong Kong- China*

[2]*Sun Yat-Sen University, Precision Medicine Institute, Guangzhou, China*

[3]*The Chinese University of Hong Kong, Department of Medicine and Therapeutics, Hong Kong, Hong Kong- China*

[4]*Sun Yat-Sen University Cancer Centre, Department of Pathology, Guangzhou, China*

[5]*The University of Hong Kong, Department of Clinical Oncology, Hong Kong, Hong Kong- China*

[6]*Guangzhou Medical University, School of Basic Medical Science, Guangzhou, China*

**Introduction**

Intratumor molecular heterogeneity of hepatocellular carcinoma (HCC) is partly attributed to the presence of cancer stem cells (CSCs) which represents a root of tumor recurrence and chemoresistance. CD133 is known to represent a critical marker of liver CSCs. We have demonstrated CD133 to enrich after chemotherapy while CD133/Prom1-depletion enhanced HCC tumors' chemosensitivity. Unfortunately, CD133 is not specific to HCC but is also expressed in normal regenerating liver. Identifying critical factors expressed specifically in liver CD133+ CSCs, but not in liver normal CD133+ stem/progenitor cells may offer new therapeutic opportunities overcoming chemoresistance in HCC.

**Material and Methods**

RNA-seq profiling compared sorted CD133+ and CD133- subsets of normal regenerating liver induced by DDC diet and HCC induced by N-nitrosodiethylamine/carbon tetrachloride or hydrodynamic tail vein injection of proto-oncogenes NRAS and AKT (NRAS/AKT), identifying candidate genes distinctly expressed in the CD133+ subset of HCC but not normal regenerating liver. The functional role of SPINK1 in tumorigenicity, dedifferentiation/stemness and chemoresistance was explored by gene manipulation, recombinant protein and neutralizing antibody approaches. The upstream mechanism enhancing SPINK1 transcriptional activity and the downstream signaling where SPINK1 regulates various oncogenic properties were explored. A proof-of-principle therapeutic experiment was performed by treating NRAS/AKT HCC mouse model with Spink1 knockdown and chemotherapy.

**Results and Discussions**

SPINK1 was distinctly expressed in the CD133+ subset of HCC but not normal regenerating liver. Its expression increased during early liver progenitor development, peaked in the premature hepatocyte stage, decreased in hepatocyte maturation and increased progressively from well to poorly differentiated HCCs. Enhanced SPINK1 transcriptional activity was mediated by promoter binding of ELF3, which like CD133, both increased after chemotherapy. SPINK1 inhibition reduced tumor initiation, self-renewal and chemoresistance. Mechanistically, secretory SPINK1 bound to EGFR activating ERK/cyclin D1/E2F2 signaling, overcoming G1/S checkpoint to promote cell cycle progression and transcribe stemness, oncogenic dedifferentiation and chemoresistance-related genes.

**Conclusion**

Our findings suggest SPINK1 to play a critical role in HCC and that SPINK1 monoclonal antibody may represent a novel therapeutic option for HCC by targeting CD133+ CSC tumor roots and overcoming chemoresistance.

### EACR23-0176

### Prevalence and fate of pro-oncogenic clones in the human colon

*N. Skoufou-Papoutsaki*[1], *S. Adler*[1], *R. Kemp*[1], *D. Winton*[1]

[1]*University of Cambridge, CRUK Cambridge Institute, Cambridge, United Kingdom*

**Introduction**

Normal aged tissues are known to exist as a patchwork of mutations. However, our appreciation of the prevalence of cancer-driver mutations in normal human colonic epithelium is limited.

**Material and Methods**

We used a targeted-amplicon multiplexing sequencing approach on a large patient cohort of normal colon FFPE samples focusing on genes frequently mutated in colorectal cancer (N=288 patients and around 5 million crypts in total). The method was applied on three sampling scales to capture different clone sizes, while retaining the spatial information. This allowed the inspection of the tissue areas with potential mutations and further characterisation of the clones in situ.

**Results and Discussions**

Cancer-driver mutations in important colorectal cancer driver genes APC, KRAS, TP53, FBXW7 and CTNNB1 were detected. This is the first time that APC mutations, which are the most frequent event in colorectal cancer, are detected in histologically normal tissue. Cancer driver mutations were associated with biases in the processes of

© 2023 The Authors. Molecular Oncology is published by John Wiley & Sons Ltd on behalf of Federation of European Biochemical Societies clone fixation and expansion, leading to a high mutational burden in the normal human colon. KRAS mutations were associated with the highest bias for clonal expansion. When comparing the frequency of such events in the normal tissue versus colorectal cancer, it became obvious that the potential of clones for neoplastic conversion does not scale with the mutational burden. In addition, while crypts with APC and TP53 mutations appeared histologically normal, there was a level of heterogeneity in the morphological manifestation of KRAS mutant clones, with some of them exhibiting an abnormal crypt morphology. The morphological manifestation seemed dependent on the specific KRAS amino acid change and clone size. Finally, a role of the immune cell compartment was revealed in relation to the expansion of mutant KRAS patches.

**Conclusion**

The normal human colon appears to contain more cancer-driver events than originally thought. However, not all mutant clones have an equal potential to drive conversion of the normal tissue to cancer. Understanding the processes by which some clones have a higher potential for transformation can help us understand how cancer starts.

### EACR23-0179
### PIH1D1 of PAQosome interacts with human papillomavirus oncoprotein E7 to disrupt the pRB-E2F1 complex

*M. Shadang[1], R. Yadav[1], S. Mathur[2], V. Iyer[2], S. Singhal[3], S.S. Chauhan[1], R.A. Mir[1]*
*[1]All India Institute of Medical Sciences- New Delhi, Biochemistry, Delhi, India*
*[2]All India Institute of Medical Sciences- New Delhi, Pathology, Delhi, India*
*[3]All India Institute of Medical Sciences- New Delhi, Obstetrics and Gynecology, Delhi, India*

**Introduction**

The PAQosome (particle for the arrangement of quaternary structure) is a heteromultimeric co-chaperone to the Hsp90 chaperone involved in the stabilization, assembly and maturation of multiprotein complexes. This intricate complex consists of four core subunits- RUVBL1, RUVBL2, PIH1D1 and RPAP3 (R2TP complex) along with eight other subunits. PIH1D1 recognises bridging proteins phosphorylated by casein kinase II (CK2). The human papillomavirus oncoprotein E7 has an SXXE/D motif recognised by CK2.

**Material and Methods**

The interaction of E7 and PIH1D1 was analyzed by performing a GST pull-down assay and co-immunoprecipitation. E7 mutants, defective of their CK2 phosphorylation site were generated by site-directed mutagenesis. In vitro competition assay was set up to study the binding of E7 and PIH1D1 to pRB.
Immunofluorescence staining was done to co-localize E7, PIH1D1 and RPAP3. siRNA knockdown of PIH1D1 was done to study the impact on cell cycle and proliferation. Cell lines used in this study were CasKi, HeLa, HEK293T, T98G and C33A. Immunohistochemistry was performed with archival formalin-fixed, paraffin-embedded tissue specimens of normal cervical epithelia and cervical carcinoma of 30 patients.

**Results and Discussions**

Here we show that PIH1D1 pulls down both E7 and RB proteins in a complex. In vitro CKII-phosphorylated E7 interacted with PIH1D1 whereas unphosphorylated E7 couldn't. Mutation of both the serine residues in the SXXE/D motif abolishes the phosphorylation of E7 and the interaction between E7 and PIH1D1. The difference in interaction with high-risk and low-risk E7 proteins was then analyzed. In cells, PIH1D1 and RPAP3 were co-localized to the nucleus and cytoplasm. In aiding E7 to disrupt the RB-E2F complex, we also analyzed E2F1-dependent transcription after knocking down PIH1D1. Silencing of PIH1D1 also led to cell cycle arrest and defects in the proliferation and migration of cell lines. Immunohistochemical analysis shows that the R2TP complex is overexpressed in cervical carcinoma but absent to minimal expression in controls. This upregulation could be a factor to drive malignant transformation.

**Conclusion**

Casein kinase II phosphorylated HPV oncoprotein E7 interacts with the PIH1D1 subunit of PAQosome. The interaction dissociates the pRb-E2F complex and likely promotes the various activities of E7 responsible for tumorigenesis. This investigation can yield a potential route for therapeutic intervention in HPV-induced malignancy.

### EACR23-0181
### Relevance of RAF kinases localization at the membrane as key feature to sustain oncogenic MAPK activity

*E. Berlinska[1], R. Scardaci[1], D. Santamaría[2], C. Ambrogio[1]*
*[1]Molecular Biotechnology Center- University of Torino, Department of Molecular Biotechnology and Health Scienc es, Turin, Italy*
*[2]Cancer Research Center, Centro de Investigación del Cáncer, Salamanca, Spain*

**Introduction**

RAF kinases are among the most relevant known RAS effectors and have an important role in the MAPK pathway. Their activation in the RAS signalosome requires direct contact with active RAS proteins localized at the membrane and leads to the initiation of a sequence of downstream phosphorylation events starting from MEK to different cellular targets, that will orchestrate a variety of biological responses. However, a detailed understanding of RAS-RAF membrane dynamics is still far from being complete. This study investigates whether forced localization of RAF kinases at the cell membrane can be *per se* an oncogenic trigger that leads to hyperactivation of the MAPK signaling even in the absence of oncogenic mutations of KRAS. We evaluated the oncogenic potential of constitutive or inducible localization of RAF kinases at the cell membrane *in vitro*.

**Material and Methods**

*Ras*-less or *Raf*-less MEFs devoid of all *Ras* or *Raf* isoforms respectively were transduced by lentiviral delivery with constitutive or doxy-inducible A-, B- or CRAF isoforms engineered with a consensus sequence known as CAAX motif, which allows their association with plasma membranes. With no interference from the endogenous wild-type Raf or Ras genes, our system enabled direct and robust comparison between

© 2023 The Authors. Molecular Oncology is published by John Wiley & Sons Ltd on behalf of Federation of European Biochemical Societies various Raf isoforms in terms of regulation of MAPK levels and cell fitness.

**Results and Discussions**

Our results showed that, by forcing RAF localization at the membranes, the MAPK pathway is activated by all individual isoforms, increasing pMEK and pERK activity. However, only in the case of ARAF, cells show high tolerance to its exogenous expression in both *Ras*-less or *Raf*-less MEFs without causing cellular toxicity. Forced membrane localization of BRAF and CRAF in *Ras*-less cells caused a dose-dependent toxicity due to excessive MAPK signaling, resulting in the selection of cells with lower lever of exogenous RAF-CAAX expression in the constitutive context.

Most intriguingly, none of the isoforms have a cytotoxic effect in *Raf*-less cells, implying that RAS proteins can buffer RAF activity and/or expression fluctuation to prevent MAPK-dependent toxicity.

**Conclusion**

- We demonstrate that in the absence of RAS proteins, increased expression of specific RAF isoforms at the cell membrane is sufficient to activate the MAPK pathway and promote cell growth.
- *Ras*-less cells are particularly sensitive to excessive membrane-localized BRAF and CRAF expression.
- *Raf*-less cells are insensitive to variation of expression of membrane-localized RAF isoforms.

### EACR23-0189
### Riproximin mediated cytostatic effects and expression modulations of cell cycle related genes in cancers

*A. Pervaiz[1], K. Umer[1], M.R. Berger[2]*
*[1]University of Health Sciences,*
*Institute of Biomedical and Allied Health Sciences, Lahore, Pakistan*
*[2]German Cancer Research Centre DKFZ- Heidelberg Univ ersity, Toxicology & Chemotherapy Unit, Heidelberg, Germany*

**Introduction**

Breast, liver, lungs and colorectal cancers are leading malignancies affecting the human population. Advanced stages of these cancers are challenging and with limited therapeutic options, 5-year survival rate is low. De-regulated cell cycle is a major functional property being investigated and targeted therapeutically in cancers. In this study, we explored the potential cytostatic effects of a purified/sequenced plant protein (riproximin) on cell cycle related properties and genes in representative cell lines of breast, liver, lungs and colorectal cancers.

**Material and Methods**

Cytostatic effects imposed by riproximin in the representative cell lines of breast (MDA-MB-231), liver (HepG2), lungs (H1299) and colorectal (SW620) cancers were evaluated via MTT dye reduction assay and flow cytometry (FACS). Expressional modifications in 84 cell cycle related genes in response to riproximin exposure were figured out by using real-time PCR panels in the four cell lines.

**Results and Discussions**

Riproximin inhibited the proliferation of cancer cell lines in a concentration format. The protein imposed a major halt in S phase of the cell cycle in the selected panel of cell lines as demonstrated by FACS analysis. Real-time PCR data showed the substantial potential (≥2fold) of riproximin to alter the expression levels of multiple cell cycle related genes in selected cancer cell lines. Precisely, these expressional modifications were more prominent in liver, breast, lungs and colorectal cancer cells, respectively.

**Conclusion**

Riproximin effectively induces cytostatic effects and can alter expression of multiple related genes in cancer cells representing various malignancies. Further investigations are deemed crucial to understand the cytostatic nature of this plant protein for therapeutic purposes.

### EACR23-0192
### Dual inhibition of CDK4/6 and XPO1 induced senescence with acquired vulnerability to CRBN-based PROTAC drugs

*H. Wang[1], C. Wang[1], W. Qin[1]*
*[1]Shanghai Cancer Institute,*
*State Key Laboratory of Oncogenes and Related Genes, Shanghai, China*

**Introduction**

Almost all currently approved systemic therapies for liver cancer failed to achieve satisfactory therapeutic effects, highlighting the need for more effective treatments. CDK4/6 inhibitors have demonstrated promising results in treating HR+/HER2− breast cancers by inducing cell cycle arrest or cellular senescence. Inducing senescence may be a promising strategy for treating liver cancer, especially when such pro-senescence therapy is combined with a drug that selectively eliminates senescent cancer cells.

**Material and Methods**

A compound screen was conducted to identify inhibitors that could synergistically induce senescence when combined with CDK4/6 inhibitor. The combination effects of CDK4/6 inhibitor and XPO1 inhibitor on cell proliferation and cellular senescence were investigated in a panel of human liver cancer cell lines, mouse model of liver cancer, patient derived organoid, and PDX. Proteomics analysis RNAseq, and IHC staining were used to identify the mechanism of the synergistic effects of CDK4/6 inhibitor and XPO1 inhibitor. A senolytic-drug screen was performed to identify the drug that selectively killed senescent liver cancer cells.

**Results and Discussions**

The combination of CDK4/6 inhibitor and XPO1 inhibitor synergistically induces senescence of liver cancer cells *in vitro* and *in vivo*. The XPO1 inhibitor works by blocking RB in the nucleus, leading to decreased E2F signaling and promoting senescence induction by the CDK4/6 inhibitor. Through a senolytic-drug screen, CRBN-based PROTAC ARV-825 was identified as an agent that can selectively kill senescent liver cancer cells. Upregulation of CRBN was a vulnerability of senescent liver cancer cells, making them highly sensitive to CRBN-based PROTAC drugs. Furthermore, the positive correlation between the expression of CRBN and USP2 was validated in both cell lines and clinical samples. Mechanistically, USP2 directly interacts with CRBN, leading to the deubiquitination and stabilization of CRBN in senescent liver cancer cells.

© 2023 The Authors. Molecular Oncology is published by John Wiley & Sons Ltd on behalf of Federation of European Biochemical Societies

**Conclusion**
Our study demonstrates the striking synergy of inducing senescence in liver cancer cells through the combination of CDK4/6 inhibitor and XPO1 inhibitor. These findings also shed light on the molecular processes underlying the vulnerability of senescent liver cancer cells to CRBN-based PROTAC therapy and suggest a potential therapeutic strategy for liver cancer treatment.

### EACR23-0194
### Lamin A phosphorylation by AKT2 leads to nuclear deformation and genome instability during epithelial-mesenchymal transition

*H.C. Chen*[1], *J.R. Fan*[1], *S.N. Chang*[1], *C.T. Chu*[1]
[1]*National Yang Ming Chiao Tung University, Biochemistry and Molecular Biology, Taipei, Taiwan*

**Introduction**
Nuclear deformation or dysmorphia has long been considered to associate with the malignancy of tumors. However, its underlying mechanism and biological significance in tumorigenesis remain unclear.
**Material and Methods**
To address these questions, we employed the human lung cancer A549 cell line as a model in context with transforming growth factor β (TGFβ)-induced epithelial-mesenchymal transition.
**Results and Discussions**
Upon TGFβ stimulation, AKT2 is activated in a PI3K- and Smad3-dependent manner, which then translocates into the nucleus where it directly phosphorylates lamin A mainly at Ser390. The increased phosphorylation of lamin A at Ser390 may result in relaxed nuclear lamina. Subsequently, the TGFβ-induced increases in actomyosin-mediated contractility and heterochromatin may respectively exert inward and outward force to cause nuclear deformation, accompanied by a loss of nuclear lamina integrity. The defects in the nuclear lamina then induce mis-localization and/or downregulation of DNA repair proteins, such as ATR and 53BP1, finally leading to genome instability.
**Conclusion**
The present study not only unravels a significant role of AKT2 in lamin A phosphorylation and nuclear deformation, but also demonstrates a tight association of nuclear deformation with genome instability during TGFβ-induced epithelial-mesenchymal transition.

### EACR23-0213
### Dietary intervention as novel cancer therapeutics: Targeting the methionine dependence of Hepatocellular Carcinoma

*Z.Y. Ooi*[1], *K.K.L. Kwan*[1], *J.W.S. Cheu*[1], *Y. Chen*[1], *H. Deng*[1], *C.C.L. Wong*[1,2]
[1]*Li Ka Shing Faculty of Medicine- The University of Hong Kong, Department of Pathology, Hong Kong, Hong Kong- China*
[2]*The University of Hong Kong, State Key Laboratory of Liver Research, Hong Kong, Hong Kong- China*

**Introduction**
Metabolic reprogramming is a hallmark of cancer. An emerging aspect of cancer metabolic rewiring is the dependence on methionine, an essential amino acid that is involved in a myriad of cellular functions such as methylation reactions, redox maintenance, and polyamine synthesis. Its biological impact has been explored in the context of ageing, metabolic diseases, and cancer development. Previous studies suggested methionine transporter expression as the rate-limiting factor for methionine cycle activity in immune cells, however, the dependence of cancer cells on methionine uptake for cancer progression remains unexplored.
**Material and Methods**
Here, we explored the transcriptomic expression of methionine transporters from The Cancer Genome Atlas public database and checked the mRNA levels from our in-house Hepatocellular Carcinoma (HCC) patient samples. Identified methionine transporter encoding genes were knocked down using short hairpin RNA and methionine levels in the culture medium and rodents' diet were customised for *in vitro* and *in vivo* experiments respectively. Tumors were dissociated into single-cell suspensions, and flow cytometry was used to analyze the tumour-infiltrating immune cells.
**Results and Discussions**
We identified two methionine transporters, SLC7A6 and SLC43A2 that were significantly upregulated in HCC tumors and were associated with poor prognosis. Knocking down either or both the methionine transporters impairs the proliferation of HCC cells *in vitro*. Similar results were observed in multiple HCC cell lines when we depleted methionine from the culture medium. As diet is the primary nutrient source for tumors, we aimed to target this vulnerability using dietary methionine restriction (MR). Indeed, *in vivo* HCC growth were hindered upon MR diet treatment. We demonstrated that short-term intermittent MR regimens effectively restricted HCC development with minimal toxicity. Interestingly, tumors from MR-treated mice are generally less immunosuppressive with decreased expression of exhaustion markers on NK cells as well as decreased levels of M2-like tumor-infiltrating macrophages, suggesting the potential of MR in activating antitumor immune response.
**Conclusion**
Our work highlighted dietary intervention as an attractive therapy and the potential of methionine transporter in predicting the sensitivity of cancer cells towards methionine restriction. Moving forward, we aim to explore the potential of dietary methionine restriction in enhancing the efficacies of conventional therapies.

### EACR23-0214
### Micro RNA miR-27a-3p negatively affects prostate cancer migration

*S. Conrad*[1,2], *K. Erdmann*[3], *S. Füssel*[3], *M. Rauner*[1,2], *L. Hofbauer*[1,2]
[1]*Uniklinikum Dresden, Department of Medicine III, Dresden, Germany*
[2]*Uniklinikum Dresden, Center for Healthy Aging, Dresden, Germany*
[3]*Uniklinikum Dresden, Department of Urology, Dresden, Germany*

**Introduction**

© 2023 The Authors. Molecular Oncology is published by John Wiley & Sons Ltd on behalf of Federation of European Biochemical Societies

Prostate cancer shows a high incidence of cancer-related death in men and preferably forms metastases in the bone. Micro RNAs (miRNAs) are known to either promote or suppress the tumorigenesis of different cancer types. However, how miRNAs orchestrate the bone prostate cancer cell crosstalk remains unclear. Here, we evaluated the effects of prostate cancer miRNAs miR-26a-5p, miR-27a-3p, and miR-30e-5p *in vitro* and in human serum samples.

**Material and Methods**

MiRNAs were downregulated in human prostate cancer cell lines (PC3, C42B, DU-145) and in human epithelial prostate cells (RWPE-1) using specific simiRNAs. To assess the effect of simiRNA-mediated knockdown, proliferation (BrdU), vitality (CellTiterGlo), apoptosis (Caspase 3/7) and wound healing assays were performed. Further, human serum samples from patients with primary prostate cancer (n=14) or prostate cancer metastases (n=10) were analyzed for miRNA expression.

**Results and Discussions**

Analysis of proliferation and apoptosis revealed no significant differences after knockdown of miR-26a-5p, miR-27a-3p, and miR-30e-5p in human prostate cancer cell lines. However, knockdown of miR-27a-3p showed an increased viability of PC3 cells after 24 h [+28,9%, P<0.05] compared to non-targeting controls. MiR-27a-3p knockdown also resulted in 4% less migration of PC3 cells after 24 h [P<0.05]. Furthermore, lower miR-26a-5p, miR-27a-3p, and miR-30e-5p levels were measured in the serum from patients with prostate cancer metastasis compared to patients with primary prostate cancer tumors without metastasis [P<0.01, P<0.001].

**Conclusion**

Our results highlight the importance of miR-27a-3p on the migration potential of prostate cancer cells *in vitro* suggesting its importance for intercellular crosstalk between prostate cancer cells and bone underlined by the human serum data.

### EACR23-0216

### The cytotoxic and apoptotic effect of Pleurotus florida against human breast and colon cancer cells

*A. Chinnaiyan*[1], *S. Kannaiyan*[2], *S. Govindarajan*[3], *S. Kathiresan*[4]

[1]*Thanthai Periyar Government Arts and Science CollegeAutonomous-Affiliated to Bharathidasan University, Post Graduate and Research Department of Biochemistry, Tiruchirappalli, India*

[2]*A.V.V.M Sri Pusphpam CollegeAutonomous-Affiliated to Bharathidasan University, Post Graduate and Research Department of Microbiology, Thanjavur, India*

[3]*A.V.V.M Sri Puspham CollegeAutonomous-Affiliated to Bharathidasan University, Post Graduate and Research Dept. of Microbiology, Thanjavur, India*

[4]*Annamalai University, Biotechnology and Biochemistry, Chidambaram, India*

**Introduction**

Cancer is the world second leading cause of death after the coronary heart diseases. Breast and colon cancers incidences and deaths are rising in both developing and developed nations. The cytotoxic effect of *Pleurotus florida* methanolic extract on HT-29 and MCF-7 cell lines was investigated in this study.

**Material and Methods**

*The P. florida* mushrooms were cultivated and the fruit body was collected, air dried, powdered and extracted in methanol and the resulting extract were used for the study. HT-29 and MCF-7 cell lines were obtained from National Centre for Cell Science (NCCS), Pune, India. The cell viability was analysed by MTT assay.

**Results and Discussions**

The effective doses of methanolic extract of *P. florida* were calculated by different concentrations (10-100 µg/ml) treated with the cell lines showed significant cytotoxic activity against HT-29 cell line with an $IC_{50}$ value of 10µg/ml and MCF-7 cells achieving an $IC_{50}$ value of 20 µg/ml. The significant anti cancer compounds of this extract showed antioxidants activities as confirmed by analysis of lipid peroxidation, MDA formation, superoxide dismutase, catalase, glutathione peroxidase, reduced glutathione and intracellular reactive oxygen species (ROS) levels. The activity of thiobarbituric acid reactive substance (TBARS) conjugated dienes and lipid hydroperoxide levels were increased in both the cancer cells compared to control cells. The results of antioxidant enzymes superoxide dismutase, catalase, glutathione peroxidase, and reduced glutathione in *P.florida* extract treated cells showed significant changes. Morphological changes examined using floid cell imaging station revealed control cells had a highly green fluorescence nucleus which indicates the presence of live cells. *P.florida* extract treated cells showed early apoptotic cells stained orange in color and red stained fragments nuclei indicating late apoptosis at different points. Apoptotic cells were significantly increased in extract treated HT-29 and MCF-7 cells.

**Conclusion**

The overall results confirmed that *P.florida* extract exhibit cytotoxic activity by inhibiting cell proliferation through ROS dependent mitochondrial mediated apoptosis as evidenced by elevated ROS generation resulting in loss of mitochondrial membrane potential and oxidative DNA damage. This necessitates further studies on the active components for proper assessment for a possible development as promising anticancer drug.

### EACR23-0222

### A P-cadherin-dependent actin signalling pathway can explain the progression of premalignant breast cells malignancy

*L. Faria*[1], *S. Canato*[2], *T. Teles Jesus*[1], *M. Gonçalves*[3], *P. S. Guerreiro*[4], *C. S. Lopes*[3], *I. Meireles*[1], *E. Morais de Sá*[3], *J. Paredes*[1], *F. Janody*[1]

[1]*i3S-Instituto de Investigação e Inovação em Saúde, Ipatimup, Porto, Portugal*

[2]*Champalimaud Foundation, Physiology and Cancer Program, Lisboa, Portugal*

[3]*i3S-Instituto de Investigação e Inovação em Saúde, IBMC-Instituto de Biologia Molecular e Celular, Porto, Portugal*

[4]*Vector B2B - Associação Para Investigação Em Biotecnologia, Drug Developing, Lisboa, Portugal*

**Introduction**

© 2023 The Authors. Molecular Oncology is published by John Wiley & Sons Ltd on behalf of Federation of European Biochemical Societies

Only 20-50% of premalignant breast lesions will develop into breast cancer if left untreated. However, the actual risk that a pre-cancerous condition will progress to an overt malignancy remains unknown. Aberrant expression of the cell-cell adhesion molecule P-cadherin (P-cad) is an early event in hyperplastic transformation of some tissues. In the breast, P-cad is enriched in Basal-like Triple-Negative Breast Cancer (TNBC), which remains by far the most lethal subtype of breast cancer, being significantly associated with a worse disease-free and overall patient survival. Therefore, P-cad and its downstream effectors could constitute a risk factor for malignant progression.

**Material and Methods**

We have established a clinically relevant *in vivo* model for functional exploration of P-cad effectors by expressing the human gene encoding for P-cad in *Drosophila*. P-cad effectors were then validated in a human mammary epithelial cell line with conditional activation of the Src oncogene, which recapitulates the multistep progression of basal-like breast cancer cells.

**Results and Discussions**

We show that the consequences of expressing human P-cad in the fly wing disc epithelium are reminiscent to those of expressing P-cad in breast cancer cells. We identified actin nucleators, MRTF and SRF as downstream P-cad effector in fly.

Validation of these findings in Src-induced MCF10A cells indicates that prior to acquiring malignant features, cells transiently upregulate P-cad and accumulate MRTF-A, which translocates to the nucleus where it upregulates the expression of SRF target genes. The transient upregulation of SRF target genes depends on P-cad and actin filaments, as knocking-down P-cad or preventing F-actin polymerization with Latrunculin A abrogates their transient upregulation. Blocking MRTF-A nuclear translocation with CCG-203971 prevents the acquisition of proliferative, invasive and stemness abilities.**Conclusion**

These findings suggest that by promoting a transient boost of MRTF-A-SRF signalling activity in premalignant basal-like TNBC, P-cad could trigger their malignant progression, opening the possibility of using P-cad as a prognosis factor predicting malignant progression.

### EACR23-0230

### Metabolism meets function: the mitochondrial chaperone TRAP1 in DLBCL

L. Ferrone[1], A. Tosato[2], E. Carraro[2], M. Pillon[2], M. Pizzi[3], A. Rasola[1], L. Mussolin[2]

[1]*University of Padua, Department of Biomedical Sciences, Padova, Italy*

[2]*University of Padua, Department of Women's and Children's Health, Padova, Italy*

[3]*University of Padua, Department of Medicine, Padova, Italy*

**Introduction**

Diffuse large B-cell lymphoma (DLBCL) arises from Germinal Center (GC), where neoplastic B cells undergo transcriptional changes caused by dysregulated chromatin remodeling factors. Repression of differentiation and cell cycle checkpoint genes favors lymphomagenesis. This process requires H3K27 trimethylation by methyltransferases. The demethylation mirror reactions are catalyzed by demethylases, allosterically regulated by TCA cycle intermediates, with succinate as an inhibitor. Our group found that the chaperone TRAP1 is a key component of the mitochondrial machinery, and its activity allows tumor cells progression. We have shown that TRAP1 prompts a pro-neoplastic succinate accumulation in diverse cancer cells by inhibiting succinate dehydrogenase (SDH), but no TRAP1-dependent epigenetic change has ever been reported.

**Material and Methods**

Bioinformatic analysis of RNAseq DLBCL patients' cohort (n=385); western blot; RT-qPCR; FACS cell cycle analysis with propidium iodide. Treatments with: TRAP1 inhibitor; succinate.

**Results and Discussions**

My results stem from the observation of a lower overall survival in DLBCL patients with higher TRAP1 RNA levels. TRAP1 was enriched in DLBCL subtypes that present the worst prognosis. These data were corroborated by immunohistochemical analyses, which documented a TRAP1 positivity in all tested DLBCL samples compared to non-neoplastic counterpart. After checking TRAP1 expression in DLBCL cells, I found that its genetic and pharmacological suppression, the latter achieved by using selective TRAP1 inhibitor, upregulates SDH activity. I have analyzed H3K27me3 levels, finding that the absence of TRAP1 dramatically decreases them. In addition, treatment with succinate avoid H3K27 demethylation in TRAP1 KO DLBCL cells. Strikingly, genetic TRAP1 ablation increases BLIMP1 expression, the master regulator of plasma-B cells differentiation. Hence, these observations are consistent with a TRAP1-dependent inhibition of BLIMP1 and of B cell differentiation *via* histone methylation. Moreover, absence of TRAP1 correlates with a downregulation of the cell cycle.

**Conclusion**

These findings support the involvement of TRAP1 in repression of B cells differentiation through metabolic rewiring and chromatin remodeling, and that its effect can be manipulated by treating cells with selective TRAP1 inhibitors. These data represent the starting point in the definition of a novel clinical and prognostic indicator, possibly improving the therapeutic armamentarium against DLBCL.

### EACR23-0232

### Inhibition of glutaminolysis augments the anti-leukemic effect of L-asparaginase treatment in B-cell childhood acute lymphoblastic leukaemia cells in vitro

B. Vergov[1], Y. Sbirkov[1,2], V. Sarafian[1,2]

[1]*Medical University of Plovdiv, Medical Biology, Plovdiv, Bulgaria*

[2]*Research Institute at Medical University of Plovdiv, Division of molecular and regenerative medicine, Plovdiv, Bulgaria*

**Introduction**

L-asparaginase is a key component of standard chemotherapy in childhood acute lymphoblastic leukaemia (cALL) due to the dependency of lymphoblast cells on exogenous asparagine. Nevertheless, prolonged exposure

© 2023 The Authors. Molecular Oncology is published by John Wiley & Sons Ltd on behalf of Federation of European Biochemical Societies to this enzyme often leads to resistance, which may be due to an immunological response to its bacterial origin, but also to altered gene expression and/or metabolic rewiring of the lymphoblasts. Therefore, elucidating the molecular mechanisms of L-asparaginase action and finding novel targets that may augment its effect on cell proliferation and viability before resistance develops could be of utmost clinical significance.

**Material and Methods**

REH, Sup-B15, Sup-PR, SEM, and SEM-K2 B-cell childhood ALL cell lines were examined after treatment with L-asparaginase and the inhibitor of glutamate dehydrogenase epigallocatechin gallate (EGCG). Metabolomics analysis of cells, MTT assays, glutamine/glutamate luminescent assay, Annexin V/PI apoptosis test were applied, as well as assessment of mitochondrial function and ATP production by Seahorse XFp Analyzer (Agilent).

**Results and Discussions**

In attempt to characterise the effect L-asparaginase may exert on cell metabolism *in vitro* we used metabolomics analysis in combination with functional mitochondrial activity assays. We found that L-Asparaginase alters a number of amino acid biosynthesis pathways as well as purine and pyrimidine synthesis. These metabolic changes are accompanied by a significant reduction of mitochondrial function and ATP production. Of note, further pharmacological inhibition of glutaminolysis, one of the top hits from our metabolite set enrichment and pathway analyses, showed significant additive anti-leukaemic effect when combined with L-Asparaginase.

**Conclusion**

Our data suggest previously undescribed mechanisms of action of L-asparaginase, which leads to considerable cell metabolic changes, besides deamination of asparagine and glutamine. Further inhibition of glutaminolysis has a pronounced additive effect. Thereby, this study presents the basis for further investigations in the context of augmenting the efficacy of this critical arm of chemotherapy in cALL.

### EACR23-0240

### Tumor Budding in colorectal cancer: association with clinopathological parameters and prognostic impact in stages II and III

*P.G. Giordano*[1], *A.G. Díaz Zelaya*[2], *Y.Y. Aguilera Molina*[1], *N.O. Taboada Mostajo*[1], *Y. Ajete Ramos*[1], *R. Ortega García*[1], *E. Peralta De Michelis*[1], *J.C. Meneu Díaz*[1]

[1]*Complejo Hospitalario Ruber Juan Bravo, General Surgery, Madrid, Spain*

[2]*Complejo Hospitalario Ruber Juan Bravo, Pathology Department, Madrid, Spain*

**Introduction**

Tumor Budding (TB) is considered as an independent adverse prognostic marker in colorectal cancer (CRC). The prognostic impact of TB at the tumor invasive front in CCR remains unclear, hence institutional practices on the description of TB and methods for its assessment widely vary. This study was undertaken to clarify the clinicopathologic and pronostic implications of TB in patients with stage I to III CRC.

**Material and Methods**

Between 01/2017 and 12/2022, patients undergoing colectomy or attempted rectal resection after neoadjuvant therapy for CCR were identified. Patients with diagnosis of colorrectal adenocarcinoma, stage M0 at the moment of surgery, and description of the TB status in pathological report were included in our study. The effect of TB on histological factors, clinical stage, local recurrance rate, disease-free (DFS) and overall survival (OS) was assessed.

**Results and Discussions**

Of 78 cases of CRC, TB at the definitive pathological description was present in 56 patients (71,8%), including low grade in 22 (39,3%), intermediate grade 17 (30,4%) and high grade 17 (30,4%) patients. The proportion of patients showing regional lymph node metastasis, lymfovascular and perineural invasión was significantly higher in patients with TB (26,8% vs 0 %, p=0,008; 41,1% vs 4,5%, p=0,002; 16,1% vs 0% p=0,054; respectively). Moreover, pathological T1 stage group showed a significantly higher proportion in TB negative group than the TB positive group (31,8% vs 8,9, p=0,031). DFS was 86,3% in TB low, 75,3% in TB intermediate, and 70,3% in TB high grade, respectively. Intermediate and high grade TB were associated with shorter OS compared to low TB (93,7% and 75,4% vs 100%, respectively p=0,0021).

**Conclusion**

These results suggest that the TB expression may be a useful risk factor for lymph node metastasis, local recurrence and distant mestastasis. TB at the tumor invasive front is associated with shorter overall survival after curative surgery for CRC.

### EACR23-0242

### Establishment of isoliquiritigenin as a novel autophagy-ferritinophagy regulator in pancreatic cancer adjuvant chemotherapy

*J. Ko*[1], *Z. Zhang*[2]

[1]*Hong Kong Baptist University, Centre for Cancer and Inflammation Research, Kowloon, Hong Kong- China*

[2]*Hong Kong Baptist University, Department of Biology, Hong Kong, China*

**Introduction**

We have determined that isoliquiritigenin (ISL), a chalcone derivative of licorice, is a natural antioxidant and redox regulator (Antioxidant 11:1349, 2022). In the present study, we attempted to investigate the chemotherapeutic mechanism of ISL through differential regulation of autophagy in the tumor microenvironment of pancreatic ductal adenocarcinoma (PDAC).

**Material and Methods**

The formation of autophagosomes was analyzed by immunofluorescence microscopy and transmission electron microscopy. Molecular docking assay was used to assess the structural interaction of ISL with p38 protein. The molecular mechanism was elucidated by using qRT-PCR and Western immunoblotting. Inhibition of ISL on tumor growth and tumor immunity was determined *in vivo* using mice xenograft model.

© 2023 The Authors. Molecular Oncology is published by John Wiley & Sons Ltd on behalf of Federation of European Biochemical Societies

**Results and Discussions**
ISL possessed synergistic anticancer effects with gemcitabine and inhibited pancreatic cancer progression by accumulation of autophagosome via blockade of late stage autophagic flux. Moreover, autophagy inducer rapamycin enhanced ISL-evoked cell growth inhibition and promoted apoptosis, while inhibition of autophagosome formation by *siAtg5* attenuated ISL-induced apoptosis. Molecular docking analysis has indicated that ISL acted by direct targeting of p38 MAPK, which was confirmed by ISL-induced phosphorylation of p38. Autophagy flux induced by p38 inhibitor SB203580 was blocked by ISL, with increase in cytotoxicity in pancreatic cancer cells. We have revealed that ISL reduced the protein expression of nuclear receptor coactivator 4 (NCOA4), a selective cargo receptor for the turnover of ferritin, and increased the gene expression of ferritin heavy polypeptide 1 (FTH1) in PANC-1 cells. ISL also upregulated the gene expression of GPX4 and SLC7A11, the negative regulator of ferroptosis. Despite the view that ferritinophagy-mediated ferroptosis may contribute to tumor suppression, alternative reports also indicated that elevated ferritinophagy expression signature predicts poor prognosis in PDAC patients while ferroptotic cell death could aggravate pancreatic cancer by reprogramming of tumor immunity. In our study, ISL exhibited reduction of myeloid-derived suppressor cell numbers in both PDAC tumor tissues and the peripheral blood, with concurrent increase in $CD4^+$ and $CD8^+$ T cell numbers, together with attenuation of M2-polarization in tumor-associated macrophages.
**Conclusion**
Our findings have suggested that ISL can be established as a novel autophagy-ferritinophagy regulator in adjuvant chemotherapy of pancreatic cancer.

### EACR23-0243
### The oncogenic role of Hedgehog pathway co-receptors in Rhabdomyosarcoma: expanding knowledge to discover new therapeutic targets

*J. Roma[1], P. Zarzosa[1], L. Garcia-Gilabert[1], J. Sansa-Girona[1], G. Pons[1], G. Gallo-Oller[1], M. Segura[1], S. Gallego[1], L. Moreno[1]*
*[1]Vall d'Hebron Research Institute, Childhood Cancer and Hematologic Diseases, Barcelona, Spain*

**Introduction**
Rhabdomyosarcoma (RMS) is the most common soft tissue sarcoma in children. The mortality rate for RMS still remains between 35-40%. A persistent activation of the Hedgehog (Hh) signaling pathway is well established and associated with worse prognosis. Our group has been the first in publishing the important role of Hh Ligands in RMS, proposing an autocrine Hh activation model. The standard pathway activation entails ligand binding to Patched receptor, which allows the activation of Smoothened and the subsequent activation of Gli proteins, the main effectors of the pathway. However, there are other co-receptors (Gas1, CDO and BOC) that are also able to bind with ligands and even seem to be necessary for complete Hh activation.
**Material and Methods**
Since the role of these co-receptors in RMS has not yet been characterized, we propose their study with the aim of finding new molecular targets and opening up new therapeutic possibilities. First of all, a consistent expression of these co-receptors in RMS tumors was verified, as a previous step to attribute them an oncogenic role. Moreover, we genetically downregulated BOC and CDO expression and studied the underlying molecular and functional consequences of their absence.
**Results and Discussions**
The results obtained permitted us to rule out an essential oncogenic role of BOC. Conversely, CDO genetic or pharmacologic inhibition caused strong anti-oncogenic effects in vitro as decreased cell proliferation, arrested cell cycle and, finally, the induction of both cell differentiation and apoptosis. A remarkable reduction of tumor growth was also observed in in vivo models in immunocompromised mice.
**Conclusion**
In conclusion, we propose the inhibition of CDO as a novel and potent therapeutic target against rhabdomyosarcoma, with potential for all Hedgehog-driven cancers.

### EACR23-0244
### Investigating the anti-cancer effects of cannabidiol on prostate cancer

*J. Cosgrave[1], E. O' Reilly[2], M. Prencipe[2], W. Gallagher[2], A.S. Perry[1]*
*[1]University College Dublin, School of Biology and Environmental Science, Dublin, Ireland*
*[2]University College Dublin, School of Biomolecular and Biomedical Science, Dublin, Ireland*

**Introduction**
Cannabidiol (CBD) has shown promising anticancer effects in multiple cancer types, including prostate cancer (PCa). Studies illustrate that CBD can reduce cell proliferation and increase programmed cell death pathways. Epigenetic alterations are widespread in PCa, with changes seen early in disease progression. Additionally, studies have suggested that cannabinoids can modulate the epigenomes of brain, skin, and other cell types. This study aims to investigate the anti-cancer effects of CBD in PCa and to explore any role that it has in modifying the PCa epigenome and transcriptome.
**Material and Methods**
Five cell lines (3 PCa lines: DU145, PC3, LNCaP and 2 non-cancerous lines: PWR1E and HFF2) were treated with CBD (0- 100 μM) for 72 hours to determine the half-maximal inhibitor concentration ($IC_{50}$). Effects on cell viability were measured via MTT, and on proliferation by clonogenic assays. The effect of CBD on total PCa DNA methylation levels was determined using an ELISA-based 5-methylcytosine (5mC) assay, and its effect on the PCa transcriptome was measured using RNAseq.
**Results and Discussions**
CBD reduced the viability of all PCa cells, with $IC_{50}$ concentrations ranging from 11.07 - 13.31 μM. These concentrations were significantly reduced in DU145 cells to 2.95 mM (p=0.02) and in PC3 cells to 3.11 mM (p=0.02) grown in serum-free conditions, showing that

© 2023 The Authors. Molecular Oncology is published by John Wiley & Sons Ltd on behalf of Federation of European Biochemical Societies serum affects CBD efficacy. The viability of the noncancerous cells was also affected by CBD (and the presence/absence of FBS), although to a lesser degree. Toxic concentrations of CBD decreased the proliferation of DU145 cells by 14% and PC3 cells by 46% (p=0.04) but not the non-cancerous cell lines. CBD treatment also resulted in a change in the total 5-methylcytosine levels in androgen-dependent LNCaP but not in the androgen-independent PC3 and DU145 cells. CBD also altered the expression of numerous genes involved in DNA replication, cell division and the cell cycle, although this analysis is still ongoing.

**Conclusion**

CBD reduces the viability and proliferation of PCa cells and is affected by the presence or absence of serum. CBD also altered total 5mC levels in addition to gene expression. Thus, showing the anti-cancer effect of CBD in PCa whilst also suggesting that the epigenome and transcriptome play a role. Overall, these results make CBD an attractive possible PCa therapeutic.

### EACR23-0248

### Human amniotic membrane extract as an approach for hepatocellular carcinoma: effects of protein fractionation, thermal denaturation and a 2D electrophoresis comparative study

*R. Teixo[1,2,3,4], M. Laranjo[1,2,3,4], M.J. Carvalho[1,2,3,4,5], B. Serambeque[1,2,3,4], A.M. Abrantes[1,2,3,4], P. Domingues[6], M.F. Botelho[1,2,3,4]*

*[1]Institute of Biophysics, Faculty of Medicine of University of Coimbra, Coimbra, Portugal*

*[2]Institute for Clinical and Biomedical Research iCBR area of Environmental Genetics and Oncobiology CIMAGO, Faculty of Medicine of University of Coimbra, Coimbra, Portugal*

*[3]Clinical Academic Center of Coimbra, University of Coimbra, Coimbra, Portugal*

*[4]Center for Innovative Biomedicine and Biotechnology CIB B, University of Coimbra, Coimbra, Portugal*

*[5]Gynecology Service, Coimbra Hospital and University Centre, Coimbra, Portugal*

*[6]Mass Spectrometry Center- LAQV-REQUIMTE- Department of Chemistry, University of Aveiro, Aveiro, Portugal*

**Introduction**

Hepatocellular carcinoma (HCC) is most common liver cancer and presents poor prognosis due to treatment failure and high recurrence rates. Human amniotic membrane (hAM) presents interesting anti-tumor properties, such as anti-angiogenic and pro-apoptotic activity. We previously investigated the effects of total human amniotic membrane extract (hAME) on HCC cell lines and showed decreased viability and increased cell death. Therefore, we aimed to assess effects of hAME fractions and thermal denaturation on HCC cells and studied possible key partners involved by a preliminary 2D electrophoresis (2D-E) comparative study.

**Material and Methods**

Fractionation was performed considering solubility, through ammonium sulphate (AS) precipitation. by sequentially adding 10, 25 and 50% AS to hAME, on ice, 15min, centrifuged at 14000G, 15min. Precipitated fractions (10P/25P/50P) were resuspended on PBS; soluble fractions (10S/25S/50S) were submitted to salting out with PBS by centrifugation (4000G, 60min), on VivaSpin® tubes (30kDa cutoff). hAME thermal denaturation was performed at 100ºC, 5min. HepG2, Hep3B and HuH7.sil cells were incubated with total hAME, fractions and thermal denaturated hAME (1µg/µL), for 72h. Metabolic activity was accessed by MTT assay. For 2D-E comparative study, cells were incubated with hAME and fractions as previously described. After, protein extracts were obtained with RIPA and a 2D-E were performed in a PROTEAN® i12™ IEF cell (BIORAD). Gels were stained with silver staining and spots were compared.

**Results and Discussions**

Total hAME induced a decrease on metabolic activity on HCC cells, compared to control (HepG2:57.07±9.26; Hep3B:50.66±9.78; HuH7.sil:58.16±6.05). Incubation with hAME fractions induced a lower metabolic activity compared to total hAME, in a cell-dependent manner (2-30%). Thermal denaturation induced a partial inhibition of total hAME effects. Preliminary results of 2D-E comparative study seem to indicate that protein spots profile is altered when HCC cells are incubated with hAME.

**Conclusion**

Our study indicates that fractions could improve hAME effects in HCC cells. Preliminary 2D-E comparative effects seem to demonstrate an altered protein profile induced by hAME incubation.

**Funding:** Foundation for Science and Technology (FCT), Portugal, through Strategic Projects UID/NEU/04539/2019, UIDB/04539/2020, UIDP/04539/2020, and by COMPETE-FEDER, reference number POCI-01-0145-FEDER-007440. Scholarship grants from FCT and European Social Funding (SFRH/BD/116794/2016, 2020.07672.BD).

### EACR23-0265

### Cancer stem-like cells: a new therapeutic target for the treatment of Uveal Melanoma

*A. Loda[1], S. Calza[1], A. Giacomini[1], C. Ravelli[1], A.M. Krishna Chandran[1], C. Tobia[1], G. Tabellini[1], S. Parolini[1], F. Semeraro[2], R. Ronca[1], S. Rezzola[1]*

*[1]University of Brescia, Department of Molecular and Translational Medicine, Brescia, Italy*

*[2]University of Brescia, Department of Medical and Surgical Specialties- Radiological Sciences and Public Health, Brescia, Italy*

**Introduction**

Cancer stem-like cells (CSCs) are a subpopulation of tumor cells responsible for tumor initiation, metastasis, chemoresistance, and relapse. Recently, CSCs have been identified in Uveal Melanoma (UM), which represents the most common primary tumor of the eye. UM is highly resistant to systemic chemotherapy and effective therapies aimed to improve overall survival of patients are eagerly required.

© 2023 The Authors. Molecular Oncology is published by John Wiley & Sons Ltd on behalf of Federation of European Biochemical Societies

**Material and Methods**
Herein, taking advantage from a pan Fibroblast Growth Factor (FGF)-trap molecule, we singled out and analyzed a UM-CSC subset with marked stem-like properties. A hierarchical clustering of gene expression data publicly available on The Cancer Genome Atlas (TCGA) was performed to identify patients' clusters.
**Results and Discussions**
By disrupting the FGF/FGF receptor (FGFR)-mediated signaling, we unmasked an FGF-sensitive UM population characterized by increased expression of numerous stemness-related transcription factors, enhanced aldehyde dehydrogenase (ALDH) activity, and tumor-sphere formation capacity. Moreover, FGF inhibition deeply affected UM-CSC survival *in vivo* in a chorioallantoic membrane (CAM) tumor graft assay, resulting in the reduction of tumor growth. At clinical level, hierarchical clustering of TCGA gene expression data revealed a strong correlation between FGFs/FGFRs and stemness-related genes, allowing the identification of three distinct clusters characterized by different clinical outcomes.
**Conclusion**
Our findings support the evidence that the FGF/FGFR axis represents a master regulator of cancer stemness in primary UM tumors and point to anti-FGF treatments as a novel therapeutic strategy to hit the CSC component in UM.

### EACR23-0277
### Metformin suppresses cancer cell invasion by inhibiting the secretion of amphiregulin from cancer-associated fibroblasts

H.Y. Lee[1], B.Y. Jeong[2], S.J. Cho[1], J.H. Hong[3], C.G. Park[1]
[1]*College of Medicine. Konyang University, Pharmacology, Daejeon, South Korea*
[2]*School of Medicine- Oregon Health Science University, Cell- Development and Cancer Biology, Portland, United States*
[3]*Chungnam National University College of Medicine, Pharmacology, Daejeon, South Korea*

**Introduction**
Metformin, a biguanide derivative, is a well-known first-line drug for type 2 diabetes and has been shown to attenuate various cancer cell growth and metastasis. Additionally, growing evidence shows that metformin suppresses cancer cell progression by blocking bidirectional interactions between cancer cells and the tumor microenvironment. In the present study, we aimed to determine the underlying mechanism by which metformin suppresses cancer cell invasion in the tumor microenvironment.
**Material and Methods**
Metformin was obtained from Sigma-Aldrich. Cancer-associated fibroblasts (CAFs) were isolated from breast cancer patients. The depletion of amphiregulin (AREG) in CAF cells was carried out using CRISPR/Cas-9. Immunoblotting was used to analyze the expression of proteins. Matrigel was used to analyze 3D cancer cell invasion. For tumor metastasis studies, cancer cells were mixed with an immortalized human mammary fibroblast cell line (NF), CAFs, and AREG knock-out CAFs, and injected into the peritoneum or tail vein of athymic-nu/nu mice.
**Results and Discussions**
Pretreatment of CAFs with metformin reversed the lysophosphatidic acid-induced downregulation of phospho-YAP. Additionally, metformin inhibited lysophosphatidic acid-induced AREG transcript expression and secretion into the media. Furthermore, metformin dramatically reduced the invasiveness of cancer cells in 3D Matrigel. Importantly, metformin significantly attenuated peritoneal growth and metastasis of cancer cells co-cultured with CAFs, suggesting that metformin inhibits AREG expression and secretion from CAFs into the tumor microenvironment.
**Conclusion**
Taken together, the present study provides evidence that metformin attenuates cancer cell invasion and metastasis by inhibiting YAP expression and subsequently reducing the secretion of AREG from CAFs, highlighting the therapeutic potential of metformin for cancer patients.

### EACR23-0288
### Induction of an aggressive breast cancer phenotype upon nuclear deformation during transmigration across tissue interfaces

C. Clemens[1], P. Riedl[1], J. Sapudom[2], L. Orgus[1], R. Gehring[1], J.C.M. Teo[2], T. Pompe[1]
[1]*University of Leipzig, Institute of Biochemistry, Leipzig, Germany*
[2]*New York University Abu Dhabi, Division of Engineering, Abu Dhabi, United Arab Emirates*

**Introduction**
During migration from the primary tumor to the adjacent healthy tissue invasive breast cancer cells are challenged by a rapid decrease in microenvironmental stiffness and extracellular matrix density. Previous studies showed that MDA-MB-231 breast cancer cells that migrated across clearly defined interfaces between two differently porous collagen-I matrices altered their migratory phenotype, suggesting a direct influence of such tissue interfaces on tumor cellular behavior.
**Material and Methods**
Based on such biomimetic matrix interfaces, we now revealed further insights into the phenotype switch, indicating a more invasive and aggressive phenotype, and involved mechanisms. Using the sequentially reconstituted three-dimensional collagen-I matrices we investigated the changes in gene expression, proliferation and chemoresistance of the transmigrating cells.
**Results and Discussions**
Besides the previously reported increased migration directionality, we observed a strongly elongated morphology as well as higher proliferation rates, and an increase of aggressive markers in the genetic profile. These phenotypic changes were persistent in prolonged cell cultures of days within homogeneous matrices. Mechanistically, we observed a peak incidence of DNA damage directly at the matrix interface, suggesting the involvement of the mechanosensitive nuclei and chromatin as modulators of the more aggressive phenotype. Ongoing studies using other invasive breast cancer cell lines verify the principle we postulate of instructive matrix interface-

© 2023 The Authors. Molecular Oncology is published by John Wiley & Sons Ltd on behalf of Federation of European Biochemical Societies induced phenotype changes during transmigration of tumor cells.

**Conclusion**

Our findings suggest that the rapid change in extracellular mechanical signaling during transmigration of tumor cells from the tumor to the surrounding tissue may induce a strong deformation of the nucleus, which in turn subsequently alters gene expression.

### EACR23-0289
### Antiproliferative effect of tubastatin A on vemurafenib sensitive and resistant A375 melanoma cells

*A. Vuletić[1], V. Đinović[1], N. Tišma Miletić[1], T. Srdić-Rajić[1], K. Mirjačić Martinović[1]*

*[1]Institute of oncology and radiology of Serbia, Department of experimental oncology, Belgrade, Serbia*

**Introduction**

Although treatment of metastatic melanoma harboring $BRAF^{V600}$ mutation with BRAF inhibitor vemurafenib showed clinical benefits, acquired resistance occurs in most patients. Histone deacetylase 6 (HDAC6) is often overexpressed in melanoma and has recently emerged as an attractive therapeutic target as its inhibition is not associated with severe toxicities. The aim of this study is to investigate the antitumor effect of HDAC6 inhibitor tubastatin A on A375 melanoma cells with $BRAF^{V600E}$ mutation and its potential to overcome resistance to BRAF inhibition.

**Material and Methods**

A375 human melanoma cells cultured in RPMI1640 cell culture medium (CM) were treated for 72h with vemurafenib, tubastatin A or their combination at 37°C and 5% $CO_2$ in humid atmosphere. Cell survival and IC50 values were estimated by MTT assay. Cell cycle analysis and PI/annexinV assay for apoptosis were performed by flow cytometry. Statistical differences between the control (CM) and treatment and parental and resistant cell line were estimated by one-way ANOVA and Student’s t-test.

**Results and Discussions**

MTT assay showed significant decrease in cell viability after 72 h of treatment with increasing concentrations of vemurafenib (0.1-5µM, IC50=2.60µM) and tubastatin A (12.5-50µM, IC50=26.61). Cytotoxic effect of combination treatment of tubastatin A and vemurafenib on A375 cells was evaluated by Chou-Talalay model that showed synergism for concentrations approximate to IC50. Furthermore, vemurafenib resistant A375 cells (A375R) were generated after 3-month cultivation with increasing concentrations of vemurafenib. A375R compared to parental cells had shorter doubling time (13h vs 24h) and five times higher IC50 value for vemurafenib. After 72h treatment, cell cycle analysis of parental cells showed G0/G1 phase arrest, decrease in the percentage of cells in S and G2M phases in response to vemurafenib, while A375R cell cycle distribution remained unaffected. However, in response to tubastatin A, A375R cells showed significantly lower IC50 (12.90µM) compared to the parental cells indicating increased sensitivity of A375R cells to tubastatin. Tubastatin A induced G2/M phase arrest and decrease in S phase in parental cells and increase in subG1 phase in both cell lines that was more profound in A375R and associated with increased fraction of cells in early and late apoptosis.

**Conclusion**

Our findings may provide the additional rationale for further development of HDAC6 inhibitor therapy for BRAF inhibitor resistant melanoma.

### EACR23-0291
### PJA2 ubiquitin ligase in glioblastoma: role in the preservation of the telomere integrity and cancer stem cell proliferation

*S.M. Pagnotta[1], V. Nittoli[2], F. De Vita[3], R. Flammia[1], F. Russo[2], C. Reale[2], N.A. Russo[2], L. Rinali[4], A. Feliciello[4], C. Ambrosiono[1]*

*[1]University of Sannio, Dept. of Science and Technology, Benevento, Italy*

*[2]BIOGEM, Genes and Environment Lab, Ariano Irpino, Italy*

*[3]University of Campania “Luigi Vanvitelli”, Department of Experimental Medicine, Napoli, Italy*

*[4]University of Naples “Federico II”, Department of Molecular Medicine and Medical Biotechno logy, Napoli, Italy*

**Introduction**

Telomeres regulate essential cellular processes, including chromosomal stability, also observed in glioblastoma multiforme (GBM). Protein ubiquitination has been involved in carcinogenesis and in maintaining the telomere length. Here, we report the result of a study to investigate the role of the E3-ubiquitin ligase Praja2 (PJA2) in molecular pathogenesis processes in GBM, including the telomere length.

**Material and Methods**

We conducted the bioinformatics analysis of RNA-Seq data from U87-MG cells transfected with PJA2 shRNA and of single cells RNA-Seq dataset from patients suffering glioblastoma, available at TCGA. Pja2KO U87-MG cells were generated (CRISPR/Cas9) to evaluate the levels of telomere length (qPCR), telomerase and other proteins (western blotting), and lncRNA TERRA (RTqPCR). ChIP-Seq experiments were conducted to reveal the presence of PJA2 at the telomeres.

A zebrafish line carrying the glial specific inactivation of the pja2 gene by CRISPR/Cas9 approach and overexpressing cMyc (Tol2 mediated transgenesis) was generated.

**Results and Discussions**

Bioinformatics analysis of RNA-seq data, as in material and methods, suggests the role of PJA2 in maintaining the telomere length. It was also confirmed experimentally by measuring telomere length, by qPCR, in Pja2KO U87-MG and control cells. Their shortening was evidenced in the former and the reduction of lncRNA TERRA, whose deletion was shown to promote telomere shortening in other cell lines. Concordantly with what was observed in GBM samples, we also detected an increase in TERT protein. In the Pja2KO U87-MG also cMyc protein increased, an event previously associated with glioma cancer cell proliferation and survival.

Further, we conducted ChIP experiments to evidence the interaction of PJA2 protein I U87-MG cells, evidencing its specific binding to telomeres. Finally, we developed a zebrafish line carrying the glial-specific inactivation of the

© 2023 The Authors. Molecular Oncology is published by John Wiley & Sons Ltd on behalf of Federation of European Biochemical Societies pja2 (Pja2 KO-zebrafish) gene and overexpressing or not cMyc. While the latter did not develop cancer up to 24 months, the former did it at different ages, starting from 12 months. Furthermore, we confirmed in zebrafish larvae that cMyc is a target of PJA2, being increased in Pja2 KO-zebrafish vs. wild-type fishes.

**Conclusion**

PJA2 activity could favor GBM development by two different mechanisms: promotion of the telomere shortening and increase of the cellular content of cMyc, an event promoting the proliferation of GBM cancer stem cells.

### EACR23-0294

### Mast cells promote breast cancer aggressiveness through the induction of stem-like traits

R. Bongiorno[1], M.T. Majorini[1], M.P. Colombo[1], D. Lecis[1]

[1]Fondazione IRCCS Istituto Nazionale dei Tumori, Department of Experimental Oncology, Milan, Italy

**Introduction**

Interactions that take place in the tumor microenvironment can have a strong impact on cancer cell behavior and aggressiveness. In this context, little is known about the role of mast cells (MCs), since they seem to play an opposite role in different cancer types. Indeed, MCs release several classes of mediators with a pro- or anti-tumor effect depending on cancer cell characteristics. In the case of breast cancer (BC), an increased density of MCs is associated with worse prognosis, even if the underlying mechanisms are largely unknown. We hypothesize that MCs promote major aggressiveness by inducing the acquisition of stem-like traits in tumor cells.

**Material and Methods**

Stem-like features were evaluated in vitro in human and mouse mammary cancer cells, testing the capacity of MCs to increase the expression of genes involved in stemness and to induce the formation of spheres, since only cells with stem-like characteristics are able to propagate as spheroid bodies. Moreover, in vivo experiments performed with mice that lack MCs, were instrumental to confirm the role of MCs in promoting breast cancer aggressiveness.

**Results and Discussions**

To test whether MCs are involved in stemness induction, we analyzed the effect of MC-conditioned medium (CM) on spheres formation. We found that this treatment increased the capability to form spheres. Moreover, this stem-like feature conferred by MCs was confirmed also by the increased expression of the stem-related gene Sox2 and by in vivo experiments, where the co-injection of BC cells with MCs increased the engraftment rate. One of the mechanisms that may induce stemness involves the estrogen receptor. Since we previously demonstrated that MCs activate estrogen receptor, we tested the effect of tamoxifen on spheres formation, finding that only cells treated with CM are able to form spheres upon tamoxifen treatment. Analyzing the mechanism through which MCs stimulate the expression of estrogen receptor we found that MC enzyme heparanase induced the expression of still to-be defined soluble factors that stimulated the expression of MUC1 in cancer cells. This mucin was in turn involved in the estrogen receptor upregulation, so favoring the stem related features.

**Conclusion**

Our findings highlight the possibility that MCs could promote the formation of cancer stem cells in an estrogen receptor-dependent manner. However, further work is necessary to understand if the targeting MCs may represent a novel therapeutic strategy for BC treatment.

### EACR23-0302

### Anticancer effects of Thymus vulgaris and Thymus serpyllum essential oils from Montenegro

I. Pasic[1], I.Z. Matić[1], N. Petrović[1,2], K. Preljević[3], T. Stanojković[1], S. Perović[3]

[1]Institute for Oncology and Radiology of Serbia, Department of Experimental Oncology, Belgrade, Serbia

[2]"VINČA" Institute of Nuclear Sciences- National Institute of the Republic of Serbia- University of Belgrade- Belgrade- Serbia, Laboratory for Radiobiology and Molecular Genetics, Belgrade, Serbia

[3]University of Montenegro- Faculty of Natural Sciences and Mathematics, Department of Biology, Podgorica, Montenegro

**Introduction**

The phytochemicals present in essential oils derived from aromatic plants of genus *Thymus* have been reported to exert antioxidant, anti-inflammatory, and anticancer effects. The aim of the research was to examine the cytotoxic activity and the mechanisms of anticancer action of the two essential oils obtained from *Thymus vulgaris* and *Thymus serpyllum* grown in Montenegro.

**Material and Methods**

The cytotoxic activity was determined against four human cancer cell lines: cervical adenocarcinoma HeLa, malignant melanoma A375, colorectal adenocarcinoma LS 174T, and lung carcinoma A549, as well as against normal lung fibroblasts MRC-5 by MTT assay. The cell cycle phase distribution of HeLa cells and the potential activation of caspase-3, caspase-8, and caspase-9 were investigated by flow cytometry. Gene and microRNA expression levels in HeLa cells were measured using RT-qPCR. The intracellular levels of reactive oxygen species (ROS) in MRC-5 cells were measured by flow cytometry.

**Results and Discussions**

Both essential oils exerted strong cytotoxicity against cancer cell lines with $IC_{50}$ concentrations in the range from 0,20 to 0,24 µL/mL for *T. vulgaris* and from 0.32 to 0.49 µL/mL for *T. serpyllum*. Strong cytotoxicity was observed against lung fibroblasts MRC-5. The remarkable increases in the percentage of HeLa cells in the subG1 phase of the cell cycle after 24 h treatment with *T. vulgaris* and *T. serpyllum* essential oils were observed in comparison to the control cells. Pretreatment of HeLa cells with caspase inhibitors showed that *T. vulgaris* oil induced apoptotic cell death through caspase-3 and caspase-8, while *T. serpyllum* oil induced apoptosis through caspase-3 activation. Both essential oils decreased intracellular ROS levels in MRC-5 cells and reduced levels of oxidative stress induced by hydrogen peroxide. The treatment of HeLa cells with *T. vulgaris* oil lowered the *MMP2* expression levels, increased *MMP9* and *VEGFA* levels when compared with control cells, while *T. serpyllum* oil decreased the levels

© 2023 The Authors. Molecular Oncology is published by John Wiley & Sons Ltd on behalf of Federation of European Biochemical Societies of *MMP2* and *MMP9*, and increased *VEGFA* levels. HeLa cells treated with *T. vulgaris* oil had increased levels of miR-21, miR-16, and miR-34a, while cells treated with *T. serpyllum* oil had lower miR-16 and miR-34a levels, but higher miR-21 levels.

**Conclusion**

These initial findings suggest that *Thymus* essential oils might have significant potential as cancer-chemopreventive and cancer-therapeutic agents, but further studies are necessary to evaluate their *in vivo* efficacy and safety.

### EACR23-0303
### Senescence induced by UVB in skin cells promotes melanoma progression

*C. Ge*[1], *E. Fitsiou*[1], *A. Altulea*[1], *M. Demaria*[1]
[1]*University Medical Center Groningen UMCG, European Research Institute for the Biology of Ageing ERI BA, Groningen, The Netherlands*

**Introduction**

Excessive exposure to ultraviolet radiation B (UVB) is associated with skin damage and dysfunction, eventually leading to an increased risk of developing melanoma. In culture, UVB light can promote cells to enter a state of premature senescence characterized by irreversible cell cycle arrest and a hypersecretory phenotype (SASP). However, the potential pathological function of UVB-induced senescence and whether UVB-induced senescent cells contribute to melanoma development remains poorly characterized.

**Material and Methods**

In the study presented here, we used transgenic senescent reporter mice to monitor the induction of senescent cells after UVB irradiation in vivo, and verify senescence induction by immunofluorescence staining. In addition, we used different types of UVB-induced senescent human skin cells ex vivo to perform unbiased transcriptomic studies and identify potential mechanisms associated with UVB-induced senescence.

**Results and Discussions**

Our data show that exposure to intermittent UVB radiation is sufficient to promote the premature induction of several senescence markers in mice. RNAseq data indicate that a conserved phenotype of UVB-induced senescence in human skin cells is the upregulation of the SASP marker Decoy receptor 1 (DcR1). UVB-induced senescent skin cells can secrete DcR1, thus potentially inhibiting TNF-related apoptosis-inducing ligand (TRAIL)-dependent apoptosis. In accordance, conditioned media collected from UVB-induced skin senescent cells can protect melanoma cells from TRAIL-mediated apoptosis. Mechanistically, overexpression of DcR1 in senescent cells depends on p53 activity, as using siRNA against p53 normalize DcR1 levels in senescent cells.

**Conclusion**

UVB radiation is sufficient to induce senescence in humans and mice. DcR1 secreted by UVB senescence leads to an anti-apoptotic mechanism that protects melanoma cells from immune-mediated apoptosis, potentially favoring tumor progression. Blocking DcR1 signaling in senescent cells has the potential to serve as a potent tumor-suppressive intervention.

### EACR23-0305
### Tomatine-mediated inhibition of Notch signaling induces apoptosis in human oral and laryngeal cancer cells

*S. kathiresan*[1], *T. Azamuthu*[1], *I.C. Senkuttvan*[2], *N. ahamed*[3], *P. Ravichandran*[1]
[1]*Annamalai university, biochemistry and biotechnology, Chidambaram, India*
[2]*annamalaiuniversity, biochemistry and biotechnology, chidambaram, India*
[3]*annamalai university, biochemstry and biotechnology, chidambaram, India*

**Introduction**

The Notch pathway is related with significant occasions supporting cancerogenic advancement: cell multiplication, self-renewal, angiogenesis, and safeguarding of a favorable to oncogenic microenvironment. Moreover, Notch is essential for cell cycle regulation and plays a dual role as both an oncogene and a tumour suppressor. The constitutive initiation of the Notch pathway has been demonstrated in different kinds of malignancies, yet this signaling pathway and its context of Head and neck squamous cell carcinoma (HNSCC) stay disputable.

**Material and Methods**

Tomatine is a glycoalkaloid derived from leaves and unripe fruit of tomato species, conventionally used as medicinal herb. It exhibits pharmacodynamic, anti- oedematous, anti-tumoranti-inflammatory, and Vaso protective properties.We determine the anticancer effects of tomatine on human oral carcinoma (KB) cells and human laryngeal carcinoma (HEp-2) cellsby targeting Notch signaling.

**Results and Discussions**

Treatment of KB cells with 25µM and HEp-2 cells with 22µM tomatinefor 24 hours suppressed proliferation in these cells and induced morphological changes consistent with apoptosis. Exposure of both cell lines to tomatine resulted in marked increase in reactive oxygen species, loss of cell viability, and reduction of cellular glutathione levels resulting in depolarization of mitochondrial membrane potential thereby increasing oxidative DNA damage in KB and HEp-2 cells. Furthermore, tomatine-mediated apoptosis in these cells progress via notch signaling as shown by downregulation of Notch1, Notch2, and Jagged-1, HES1, and HEY1 expression

**Conclusion**

Taken together, our results demonstrate that tomatine initiate apoptosis in KB and HEp-2 cells by increasing oxidative damage implying that tomatine might be developed as a potential chemotherapeutic agent for the treatment of human oral and laryngeal cancer.

### EACR23-0323
### POU1F1-mediated breast cancer cell plasticity

*L. Avila*[1], *S. Rodriguez*[1], *S. Seoane*[1], *M.E. Arias*[2], *M. Macia*[2], *R. Perez-Fernandez*[1]
[1]*University Santiago de Compostela, Department of Physiology, Santiago de Compostela, Spain*
[2]*University Santiago de Compostela, Department of Obstetrics and Gynecology, Santiago de Compostela, Spain*

**Introduction**

© 2023 The Authors. Molecular Oncology is published by John Wiley & Sons Ltd on behalf of Federation of European Biochemical Societies

POU1F1 is a transcription factor that plays a critical role in pituitary cell differentiation during organogenesis. POU1F1 has been also related to malignant neoplastic processes such as breast cancer. It has been previously reported that POU1F1 overexpression induces cellular proliferation, promotes metastasis, and increases tumoral cell resistance. In addition, POU1F1 induces key factors that take part in the epithelial–mesenchymal transition (EMT) generating plasticity changes in tumoral cells.

**Material and Methods**

In this study, we explore the possibility that breast cancer cells with deregulation of the POU1F1 undergo changes that modify their phenotype, leading them into cells that have mesenchymal and/or stem cell-like characteristics. After overexpression of POU1F1 in the low invasive human breast cancer MCF-7 and BT-474 cell lines, and knock-down in the highly invasive MDA-MB-231 cells, we evaluated for 21 days the mRNA expression of breast cancer stem cell (BCSCs) markers, such as CD24, CD44, CD133, ALDH1A1, and ALDH activity

**Results and Discussions**

Our results indicate that overexpression of POU1F1 increases CD24, CD44, CD133, and ALDH1A1 mRNA expression, as well as increases ALDH activity in both MCF-7 and BT-474 cells. POU1F1 knock-down in the triple-negative MDA-MB-231 cells reduces CD24 and increases CD44, CD133, and ALDH1A1 mRNA, and reduces ALDH activity. Thus, POU1F1 leads to phenotypic changes in breast cancer cells, suggesting that depending on its expression in a specific type of cell may induce cell sub-populations with similar phenotypic features to epithelial, mesenchymal, and/or BCSCs

**Conclusion**

Our data seems to indicate that POU1F1 modifies cell plasticity in breast tumor cells, and that phenotype switching could cause a change in the invasive capacity and resistance to drugs and chemotherapy treatments.

### EACR23-0341
### HuR regulates Triple Negative Breast Cancer (TNBC) progression via aerobic glycolysis

*A. Dev J R*[1], *A. Gogia*[1], *S.R. Mathur*[2], *S. Deo*[3], *C.P. Prasad*[1]
[1]*All India Institute Of Medical Sciences New Delhi, Medical Oncology, Ansari Nagar, India*
[2]*All India Institute Of Medical Sciences New Delhi, Pathology, Ansari Nagar, India*
[3]*All India Institute Of Medical Sciences New Delhi, Surgical Oncology, Ansari Nagar, India*

**Introduction**

Human Antigen R (HuR/ELAVL1) is a ubiquitously expressed RNA-binding protein that post-transcriptionally regulates mRNAs that contain U/AU-rich sequences in their 3' UTR. Increased cytoplasmic HuR expression is associated with aggressive and high-grade malignancies, poor prognosis, chemo- and radio-therapy resistance in various cancers. The present study investigates the oncogenic role of HuR in TNBCs, an aggressive subtype of Breast Cancer.

**Material and Methods**

Survival curves were plotted for HuR transcript and protein levels using meta-analysis tools. Human TNBC cell lines MDA-MB-231 and MDA-MB-468 were used for *in vitro* studies after substantiating cytoplasmic HuR accumulation in these cells via Immunofluorescence. The RNA binding ability of HuR was disrupted by treatment with CMLD2 (a specific HuR inhibitor), and subsequent physiological changes were studied using functional assays like MTT, wound healing, and colony formation assay. An exploratory investigation of HuR-modulated metabolic changes was performed by nCounter XT gene expression assay using the metabolic pathways panel. Investigation and/or validation of the downstream molecular changes were done via immunoblotting.

**Results and Discussions**

Meta-analysis using Liu-2014 TNBC dataset correlated high HuR protein expression with reduced patient survival ($p=0.028$). Inhibiting HuR with CMLD2 resulted in the downregulation of the protein and its downstream targets CDK2, MMP9, and β catenin. HuR inhibition compromised proliferation, migration, clonogenicity, and EMT (downregulated E-Cadherin, N-Cadherin, and Vimentin) in TNBC cell lines. Transcriptomics studies using nCounter XT showed that HuR inhibition in MDA-MB-231 altered the profile of glycolytic genes in addition to other pathways such as cell cycle, DNA damage repair, and amino acid synthesis. In accordance with this, the expression of glycolytic proteins LDHA, PFKP, and MCT1 was found to be reduced upon CMLD2 treatment. Consistent results were obtained on HuR knockdown by siRNA suggesting that HuR-mediated effects on TNBC progression might be in part through its modulation of aerobic glycolysis.

**Conclusion**

To our knowledge, this is the first report that demonstrates HuR as a positive regulator of aerobic glycolysis in TNBCs and the ability of CMLD2 to inhibit HuR provides a rationale to consider it as a potential anticancer agent for TNBCs.

### EACR23-0351
### METABOLIC CHANGES IN BREAST CANCER CELL LINES RESISTANT TO TAMOXIFEN

*K. Gotvaldová*[1], *J. Špačková*[1], *J. Truksa*[2], *K. Smolková*[1]
[1]*Institute of Physiology of the Czech Academy of Sciences, Laboratory of Mitochondrial Physiology, Prague, Czech Republic*
[2]*Institute of Biotechnology of the Czech Academy of Sciences, Laboratory of Tumour Resistance, Prague, Czech Republic*

**Introduction**

Breast cancer is the leading type of cancer among women and its incidence is increasing. Despite the targeting of estrogen receptor (ER)-positive cases by hormone therapy, primary and secondary resistance often lead to cancer progression and death. It is critical to identify predictive molecular biomarkers and understand the molecular mechanisms behind primary and secondary resistance. We aim to search for diagnostic or therapeutic markers within metabolic phenotype.

**Material and Methods**

In this project, we used a novel cell model of secondary resistance to tamoxifen (Tam5R) in breast carcinoma cell lines MCF7 and T47D. We use metabolomics and

© 2023 The Authors. Molecular Oncology is published by John Wiley & Sons Ltd on behalf of Federation of European Biochemical Societies lipidomics to characterize metabolic phenotype, with a focus on metabolic pathways commonly upregulated or downregulated in both cell lines.

**Results and Discussions**

Metabolomics and lipidomics profiling revealed several mitochondrial and extramitochondrial metabolic pathways, upregulated in the Tam5R phenotype. We have focused on the synthesis of glycerophospholipids and ceramides. The synthesis of phosphatidylinositol and cardiolipin was significantly increased in Tam5R cells compared to control cells. Silencing of several enzymes affected cell growth of Tam5R cells but not control cells.

**Conclusion**

The purpose of the study was to identify metabolic pathways associated with resistance to tamoxifen in breast cancer cells. We have identified several metabolic targets, which might contribute to the development or maintenance of resistance to hormone treatment. We aim to perform a functional analysis of these metabolic pathways to understand their contribution to cancer development and potential growth advantage. Our findings have important implications for the development of novel targeted therapies for tamoxifen hormone therapy in breast cancer and for improving patient outcomes. The study provides a valuable basis for further investigations of anabolic metabolism in cancer biology. *Supported by Czech Science Foundation grant 23-06208S to KS.*

### EACR23-0352

### The mitochondrial biogenesis on high-fat induced hepatic stellate cell activation in liver fibrosis progression and regression by anti-inflammation strategy

*S.C. Wang[1], J.F. Huang[2], C.Y. Dai[3], W.L. Chuang[4], M.L. Yu[5]*

*[1]Kaohsiung Medical University., Department of Medical Laboratory Science and Biotechnology., Kaohsiung, Taiwan*

*[2]Kaohsiung Medical University Hospital, Hepatobiliary Division- Department of Internal Medicine, Kaohsiung, Taiwan*

*[3]Kaohsiung Medical University, Faculty of Internal Medicine- School of Medicine- College of Medicine, Kaohsiung, Taiwan*

*[4]Kaohsiung Medical University Hospital-, Hepatobiliary Division- Department of Internal Medicine, Kaohsiung, Taiwan*

*[5]National Sun Yat-sen University, School of Medicine- College of Medicine, Kaohsiung, Taiwan*

**Introduction**

Overweight and obesity are rising worldwide due to the change in Western lifestyles with high-fat and high-sugar diets. Excessive accumulation of liver fat, associated with inflammation and metabolic disorders, paves the way for nonalcoholic fatty liver disease (NAFLD) and metabolic-associated fatty liver disease (MAFLD) with a high risk of developing hepatocarcinoma (HCC). The new MAFLD definition is based on a metabolic disorder and recognize as one of the most common liver diseases with a prevalence of about 25% and increasing worldwide. The concomitant presence of metabolic disorders promotes liver fibrosis and the progression of hepatocellular carcinoma. To better understand the molecular mechanism underlying the pathogenesis of MAFLD and the progression of the disease, animal models showing the progression of hepatic steatosis to MAFLD are needed.

**Material and Methods**

we developed a MAFLD animal model and a cell model with adipocytes in a lipid-rich environment. We established a reproducible MAFLD animal model of a western diet containing high-fat, high-sucrose, and high cholesterol ((42% Kcal/fat diet, 41% sucrose, and 1.25% cholesterol), with a high sugar solution (23.1 g/L d-fructose and 18.9 g/L d-glucose) given C57BL/6J mice. This mouse model recapitulates the progressive stages of human fatty liver disease, from simple steatosis to inflammation, fibrosis, and MAFLD with metabolic risk factors.

**Results and Discussions**

The early stage of the 3-month MAFLD animal model shows insulin resistance and fat accumulation in the liver; the late stage of the 7-month MAFLD animal model further shows extensive fibrosis and inflammation in the liver. We found that the endogenous anti-inflammatory 5-methoxytryptophan has a significant effect on the resolution of fibrosis in the MAFLD liver section by switching mitochondria of HSCs from oxidative metabolism to a more glycolytic metabolism to restore ATP synthesis, which contributes to the regression of fibrosis in a lipid-rich environment.

**Conclusion**

The anti-inflammatory 5-methoxytryptophan plays an important role in the resolution of fibrosis to highlight the regulation of mitochondrial function as a key mechanism for the progression and regression of metabolism-associated fatty liver disease. The research results can provide more evidence of the influence of mitochondrial biogenesis on liver fibrosis.

### EACR23-0357

### Role of Orphan Nuclear Receptors in breast cancer cell proliferation

*M.A. Caricasulo[1], G. Paroni[1], A. Zanetti[1], A.V. Cavallaro[1], M. Terao[1], E. Garattini[1]*

*[1]Mario Negri Institute for Pharmacological Research, Biochemistry and Molecular Pharmacology, Milan, Italy*

**Introduction**

Nuclear receptors (NR) are transcription factors playing pivotal roles in development and adult cell homeostasis. Their dysregulation has been associated to pathological states such as hypertension, diabetes, cardiovascular disease, metabolic syndrome and cancer. In breast cancer the role of the estrogen receptor and the progesterone receptor as predictive and prognostic factors as well as therapeutic targets has been largely studied. In particular, Orphan Nuclear Receptors (ONR) represent a class of poorly studied NR whose ligand has not been yet identified and that resulted differentially expressed between normal and tumor samples. In the present study we analyze the impact of ONR on breast cancer cell proliferation.

**Material and Methods**

The effect of ONR silencing on breast cancer cell proliferation was assessed by RNA interference. For each ONR analyzed (n=10), a panel of five breast cancer cell

© 2023 The Authors. Molecular Oncology is published by John Wiley & Sons Ltd on behalf of Federation of European Biochemical Societies lines was transiently silenced by the means of reverse transfection. Cell proliferation was evaluated at 2,4- and 6-days post transfection by sulforhodamine B assay. shRNA stable transfected cell lines were generated to validate screening results. Transcriptome analysis of NR2F6 wild type and NR2F6 silenced cells were performed to investigate the ONR mechanism of action.

**Results and Discussions**

We identified NR2F6 as an ONR involved in breast cancer cell proliferation. Indeed, NR2F6 silencing by transient siRNA transfection was found to inhibit cell proliferation. This effect occurs across all the breast cancer cell line analyzed, independently of the breast cancer subtype. MCF-7 that constitutively express a shRNA against NR2F6 have a low rate of proliferation compared to control cells. Furthermore, colony assay revealed that a down-regulation of NR2F6 induced a reduction of MCF-7 clonogenic properties. These data are supported by the transcriptomic analysis of the gene pathways primarily affected by NR2F6 silencing which demonstrated that the cell cycle is among the biological processes mainly involved.

**Conclusion**

NR2F6 silencing inhibits the proliferation of breast cancer cells and reduces their clonogenic ability. These findings are supported by transcriptomic analysis that underlines a down-regulation of genes associated to cell cycle progression in NR2F6 silenced cells. These data support an oncogenic role for NR2F6 in breast cancer that requires further investigation.

### EACR23-0363
### MNT EFFECTS ON CELL MIGRATION AND DNA REPAIR THROUGH CCDC6 INTERACTION

*V. Junco[1], J. Liaño Pons[1], M.D.C. Lafita-Navarro[1], S. Lastra[1], M.D. Delgado[1], J. León[1]*

*[1]Instituto de Biomedicina y Biotecnología de Cantabria, Departamento de Señalización Celular y Molecular, Santander, Spain*

**Introduction**

Proteins of MXD family, including MAX network transcriptional repressor (MNT), antagonize the activity of *MYC*, the oncogene most prevalently dysregulated in human cancer. To better understand the MYC-MAX-MXD network and its implications in cancer, our group focused on new biological functions of MNT by investigating the effect of MNT knockdown on transcriptome and proteome. We discovered a new interaction between MNT and coiled-coil domain-containing protein 6 (CCDC6), which is involved in DNA damage response and fuses to several genes in various cancers. Given their relevance in cancer, we aim to study the effect of MNT and CCDC6 knockdown, their physical interaction and its biological consequences.

**Material and Methods**

To analyse the biological effects of MNT knockdown, we used the HAP1 MNT-KO cell line, from human chronic myeloid leukemia. We performed RNA-seq using the Illumina platform and analyzed the data with Cufflinks, DESeq2 and RNA eXpress. We analyzed the effect of MNT knockdown on cell proliferation and migration. MNT interactome was analyzed using Affinity Purification-Mass Spectrometry in two rat pheochromocytoma cell lines, without and with inducible MAX. MNT-CCDC6 interaction was confirmed by co-immunoprecipitation (Co-IP) in different cell lines, and the domains involved were studied using deletion mutants. The localization of the complex was determined by subcellular fractionation followed by Co-IP.

**Results and Discussions**

Analysis of the RNA-seq data in wild-type and MNT-deleted cells showed that MNT may act as a transcriptional repressor and activator in HAP1 cells. MNT knockdown alters the expression of 460 genes, including upregulation of *THBS1* (Thrombospondin 1), which inhibits angiogenesis and promotes cell migration. Consistently, HAP1 MNT-KO also showed increased migration and proliferation. We identified an interaction between MNT and CCDC6, which mainly takes place in the cytoplasm, and found that CCDC6 knockdown increased the resistance to DNA damage. The interaction may need, at least, the 101-223 amino acids of CCDC6, together with the N-terminal and the leucine zipper domain of MNT. Together, these findings suggest that MNT may act as a tumor suppressor via its interaction with CCDC6, playing a role in apoptosis and DNA damage response. Experiments are underway to elucidate the mechanisms involved.

**Conclusion**

We have discovered MNT-CCDC6 interaction, which opens a new path to the understanding of MNT´s functions and its effects as a modulator of MYC-mediated oncogenic activity.

### EACR23-0377
### Using exogenous volatile organic compound (EVOC) probes to target tumour-associated aldo-keto reductase activity: a potential tool to detect lung cancer

*M. Ferreira Leal[1], A. Martin[2], B. Taylor[2], S. Rory[2], C. Clarke[2], C. Frederick Labuschagne[1], R. Smith[2], M. Hart[3], M. Allsworth[3], B. Boyle[3]*

*[1]Owlstone Medical, Clinical and Translational Science, Cambridge, United Kingdom*

*[2]Owlstone Medical, Analytical Science, Cambridge, United Kingdom*

*[3]Owlstone Medical, Leadership team, Cambridge, United Kingdom*

**Introduction**

Detection and treatment of early-stage lung cancer confer the best chance of long-term cure. Non-invasive breath tests targeting cancer metabolism could improve detection. Aldo-keto reductase (AKR) enzymes including AKR1B10 and AKR1B15 are upregulated in lung cancers to reduce aldehydes, produced by lipid peroxidation, into corresponding alcohols. These aldehydes and alcohols are volatile and could be detected in breath to detect cancer. Here, we measure AKR1B10/B15 activity in lung cancer cells by administering aldehydes as exogenous volatile organic compound (EVOC) probes and monitoring alcohol production through *in vitro* headspace analysis.

**Material and Methods**

We modulated AKR activity using small compounds (tolrestat and JF0064) in A549 and H460 lung cancer cell lines, as well as by developing A549 CRISPR-cas9

© 2023 The Authors. Molecular Oncology is published by John Wiley & Sons Ltd on behalf of Federation of European Biochemical Societies

AKR1B10/B15 knockouts. Vehicle groups and mock-Cas9 (wildtype) were used as controls. AKR activity was measured using a colorimetry assay and eVOC probe. For this last approach, we treated cells with aldehydes (multiple classes) and collected supernatants at multiple timepoints for the analysis of volatiles. Substrate and bioproduct levels were analyzed using headspace HiSorb extraction and GC-MS. To assess evaporation, a parallel plate was set up with the same aldehydes spiked in the cell culture medium (no cells).

**Results and Discussions**

Using a colorimetric assay, a dose-dependent effect of tolrestat in AKR activity in A549 and H460 was observed. However, JF0064 does not affect overall AKR activity in both cell lines. In addition, a reduction in AKR activity of over 50% was detected in AKR1B10 knockouts and variable levels were observed in AKR1B15 knockout, with one clone showing AKR activity more similar to wild-type cells. Then, using our headspace analysis platform, we detected lower aldehyde and higher alcohol levels in wildtype and vehicle control samples compared to evaporation controls, confirming that AKRs are active in these cells. Moreover, cells with a knockout or inhibited AKR activity, including using JF0064 AKR inhibitor compound, show reduced production of alcohol bioproducts.

**Conclusion**

Using *in vitro* study of lung cancer cells, we showed the potential to monitor the metabolic conversion of administered EVOC Probe aldehydes into alcohols by AKRs. Our data suggest the potential to use these aldehydes as EVOC Probes for cancer early detection on the breath by targeting upregulated AKR activity in lung cancer tissue.

### EACR23-0380
### Regulatory functions of Androgen Receptor splice variant V7 in prostate stemness and plasticity

*T. Furlan[1], F. Handle[2], J. Céraline[3], F. Santer[1]*
*[1]Medical University of Innsbruck, Division of Experimental Urology, Innsbruck, Austria*
*[2]Medical University of Innsbruck, Institute for pathology, Innsbruck, Austria*
*[3]University of Strasbourg, Institut de génétique- biologie moléculaire et cellulaire, Illkirch, France*

**Introduction**

Therapy options for advanced and castration-resistant prostate cancer (CRPC) include the use of drugs targeting the ligand binding domain (LBD) of androgen receptor (AR) such as enzalutamide (Enza) and abiraterone acetate. However, primary or acquired treatment failures are frequently observed and have been associated with the expression of truncated androgen receptor variants devoid of the LBD. In particular, the AR variant AR3, also known as ARV7, is detected in over 80% of CRPC specimen and circulating tumor cells. In PC models ARV7 expression causes treatment resistance to Enza. Moreover, ARV7 expression leads to increased expression of mesenchymal markers and stem cell signature genes. CDH2 (N-Cadherin) expression is increased by ARV7 binding to regulatory elements in intron 1 of CDH2, which is antagonized by full length (FL) AR. Based on these observations we hypothesize that ARV7 is a marker and regulator of PC stemness and/or plasticity.

**Material and Methods**

ARFL or ARV7 mRNA and protein was detected in cell lines and tissue samples by qRT-PCR, RNA in situ hybridization (RISH), immunofluorescence (IF) and western blotting using the monoclonal ARV7 antibody RM7. ARFL or ARV7 stably expressing LNCaP were obtained by lentiviral transduction. LNCaP cells resistant to Enza (LNCaP-Enza) were generated by exposure to increasing concentrations (up to 5 µM) of the drug. Transcriptome analysis in LNCaP-Enza vs. LNCaP was done by RNAseq and Gene Set Enrichment Analysis.

**Results and Discussions**

ARV7 mRNA was detectable in LNCaP, albeit gene expression was 2.3% - 2.6% of the known ARV7 positive cell lines 22Rv1 and VCaP, respectively. RISH and IF demonstrated that only a fraction (~10%) of LNCaP cells expressed ARV7 mRNA and nuclear localized ARV7 protein. ARV7 mRNA was also detected in isolated epithelial and stromal areas in treatment-naïve prostate cancer specimen by RISH. In contrast, all LNCaP-Enza cells were positive for nuclear ARV7 protein. Transcriptome analysis of LNCaP-Enza cells showed enrichments for published ARV7 gene expression signatures.

**Conclusion**

Insular spots of ARV7 mRNA were detected in treatment-naïve tissue and in isolated cells of the castration-sensitive cell line LNCaP suggesting that these cells could be intrinsically resistant against LBD-targeting drugs and responsible for the failure of various endocrine therapies. This project will further analyze whether this subpopulation possesses stem cell (-like)/plasticity characteristics and whether these are regulated by ARV7.

### EACR23-0382
### Detection of metabolic interactions in tumor cells by Surface-Enhanced Raman Scattering

*P.S. Valera Sapena[1], J. Plou[2], I. García[3], I. Astobiza[1], L.M. Liz-Marzán[2], A. Carracedo[1]*
*[1]CIC BioGUNE, Cancer Cell Signaling and Metabolism Lab, Derio, Spain*
*[2]CIC BiomaGUNE, Bionanoplasmonics Lab, Donostia-San Sebastián, Spain*
*[3]CIBER-BBN, Biomedical Research Networking Center in Bioengineering - Biomaterials- and Nanomedicine, Donostia-San Sebastián, Spain*

**Introduction**

Solid tumors are dynamic pseudo-organs governed by complex relationships between cancer cells and the stroma, which work in symbiotic relationships. As a result, the Tumor Microenvironment (TME) presents unique and continuous evolving properties based on a metabolite-based communication. Hence, tumor-secreted metabolites can either support the maintenance and growth of the tumor or act impairing antitumor immunity. Consequently, understanding the role of such interactions in tumor development could encourage new drug targets and cancer therapies. In this scenario, Surface-Enhanced Raman

© 2023 The Authors. Molecular Oncology is published by John Wiley & Sons Ltd on behalf of Federation of European Biochemical Societies

Scattering (SERS) stands as a promising new approach for the label-free detection of diverse metabolites of interest among extracellular components.

**Material and Methods**

Nanostructured AuNPs substrates and Paper-based AgNPs plasmonic substrates were used to monitor selected tumor metabolites by Surface-Enhanced Raman Scattering (SERS). Microfluidic devices were developed combining conventional PDMS soft lithography with SLA 3D printed molds.

**Results and Discussions**

Applied substrates showed a good performance for detecting the metabolic activity of Indoleamine 2,3-dioxygenase 1 (IDO-1) enzyme, overexpressed in many tumor cell types and responsible for the Tryptophan/Kynurenine metabolism. Additionally, we have also tested our system for the monitoring of MTAP deleted tumor dynamics. Finally, to upscale our metabolomics studies to high-throughput modalities, we devised ad hoc microfluidic chips, to readily conduct SERS measurements through a prototype relying on capillary pumps made of filter paper, which eventually would function as the SERS substrates.

**Conclusion**

The developed strategy may pave the way towards a faster implementation of SERS into cell secretome classification, which could be extended even to laboratories lacking highly specialized facilities.

### EACR23-0384

### Cannabigerol in vitro effect on Human Pancreatic Ductal Adenocarcinoma

*C. Aguzzi[1], M. Nabissi[1], L. Zeppa[1], O. Marinelli[1], M.B. Morelli[1], F. Maggi[1], C. Amantini[1], G. Santoni[1]*

*[1]University of Camerino, Experimental Medicine, Camerino, Italy*

**Introduction**

Pancreatic Ductal Adenocarcinoma (PDAC) is the most common type of pancreatic cancer. The five years survival is about 5% and the poor prognosis is correlated to the absence of specific symptoms, preventive diagnosis biomarkers, great invasiveness and treatment resistance. New strategies to treat this tumour are necessary. Cannabinoids, compounds derived from *Cannabis sativa*, showed promising results in several cancer models, but only few data are available regarding the anticancer effect of cannabigerol (CBG). In this work, it was investigated the *in vitro* effect of CBG on two human PDAC cell lines.

**Material and Methods**

CBG cytotoxicity on two PDAC cell lines was evaluated by MTT assay. The type of cell death induced by CBG was evaluated by Annexin V-FITC/PI staining and cytofluorimetric analysis and confirmed by the evaluation of Caspase-3 protein expression and activation by western blot. The modulation of genes involved in cancer cell aggressiveness was evaluated by western blot and by Milliplex assay. Modulation of autophagy was evaluated with western blot and acridine orange dye. The cytotoxicity of the combination of CBG and chemotherapeutic drugs was evaluated by MTT assay and the synergism was calculated with SynergyFinder.

**Results and Discussions**

CBG is cytotoxic on PDAC cell lines in a dose dependent manner and it induces apoptotic cell death. CBG modulates EGFR/Akt/mTOR and RAS pathways, involved in cancer cell aggressiveness, and induces autophagy in PDAC cell lines. Moreover, CBG potentiates the cytotoxic effect of gemcitabine and paclitaxel, the main chemotherapeutic drugs used in PDAC therapy, showing a synergistic effect with them.

**Conclusion**

Data show that CBG displays anticancer effects *in vitro* on PDAC cell lines, inducing cell death, interfering with many pathways involved in cancer progression and aggressiveness, and synergizing with chemotherapeutic drugs.

### EACR23-0386

### Adipocyte-derived FABP4 promotes non-alcoholic fatty liver disease (NAFLD)-induced hepatocellular carcinoma by driving Wnt/β-catenin signaling cascade

*C.N. Chu[1], C.O.N. Leung[1], S. Gurung[1], K.P.S. Chung[1], T.K.W. Lee[1]*

*[1]Hong Kong Polytechnic University, Department of Applied Biology and Chemical Technology, Hong Kong, Hong Kong- China*

**Introduction**

Nonalcoholic fatty liver disease (NAFLD)-induced hepatocellular carcinoma (HCC) is an emerging malignancy in the developed countries. However, the mechanisms that contribute to its formation are largely unknown. Given the role of cancer stem cells (CSCs) in tumor initiation and therapeutic resistance, we hypothesize that adipocytes, one of the key cellular factors within the tumor microenvironment of NAFLD-induced HCC, may play a critical role in HCC development and drug resistance via regulation of liver CSCs.

**Material and Methods**

The interaction between differentiated adipocytes and HCC cells was evaluated by co-culture system. The conditioned medium of adipocytes was profiled by Orbitrap Liquid Chromatography-Mass spectrometry. The functional role of FABP4 in regulation of liver CSCs was evaluated by various CSC functional assays. In vivo functional characterization of FABP4 was examined in FABP4 knockout mice. Molecular pathways mediating the phenotypic alterations was identified through RNA sequencing analysis coupled with pathway analysis.

**Results and Discussions**

Using a co-culture system in which differentiated adipocytes were grown with HCC cells, we found that adipocytes enhanced the self-renewal ability of HCC cells through indirect paracrine secretion. HCC cells pre-incubated with conditioned medium (CM) of adipocytes showed enhanced liver CSC properties including self-renewal, tumorgenicity, invasiveness and chemo-resistance to doxorubicin and sorafenib. Secretome profiles showed that FABP4 to be preferentially secreted by adipocytes and its level was further augmented when co-cultured with HCC cells. Concurrently, rrecombinant FABP4 enhanced CSC properties of HCC cells; while FABP4 inhibitor (BMS309403) abolished the CSC enhancing effect of adipocytes CM. Drastic delay in the onset of tumor

© 2023 The Authors. Molecular Oncology is published by John Wiley & Sons Ltd on behalf of Federation of European Biochemical Societies development in FABP4$^{-/-}$ mice upon DEN-injected and high fat diet–induced mouse models of NAFLD-HCC. KEGG enrichment analysis showed significant downregulation of Wnt/β-catenin pathway in tumors of FABP4$^{-/-}$ mice. Further functional analysis showed that adipocyte-derived FABP4 promotes liver CSC function via driving Akt mediated β-catenin activation.

**Conclusion**

Adipocyte-derived FABP4 may crucial role in development of NAFLD-induced HCC. Targeting the adipocyte-derived, FABP4-mediated PI3/Akt/ β-catenin cascade may be a therapeutic strategy for the treatment of this disease.

### EACR23-0387
### Galactoside 2-alpha-L-fucosyltransferase 2 (FUT2) as a novel stemness driver and therapeutic target in hepatocellular carcinoma driven by PTEN suppression

*T.M. Wong[1], T.L. Wong[1], L.S. Yeung[1], S. Ma[1]*
*[1]The University of Hong Kong, School of Biomedical Sciences, Hong Kong, Hong Kong- China*

**Introduction**

Current first-line treatment for hepatocellular carcinoma (HCC) involves mostly pan-tyrosine kinase inhibitors administered alone or in combination with immunotherapy. Yet, treatment efficacy is unpredictable due to the complex genetic landscape of HCC and the presence of cancer stem cells (CSC). In HCC, homozygous deletion of tumor suppressor PTEN directly affects 5-10% cases, with its downregulation observed in more than 50% of patients. Here, we attempted to delineate factors which promote HCC driven by PTEN loss, in hope to formulate novel targeted therapies.

**Material and Methods**

HCC organoids were derived from tumors developed via hydrodynamic tail-vein injection of oncogenic plasmids Pten-KO/c-Myc or c-Myc control into C57BL/6 mice. RNA-sequencing and pathway analyses were performed on these organoids. The clinical relevance of FUT2 was examined with publicly available datasets and by immunohistochemistry using tissue sections. The upstream regulation of FUT2 expression was investigated by manipulation of PTEN and ELF3 expression, and reporter assays in HCC cells. The role of FUT2 in cancer stemness and proliferation was explored by lentiviral-based overexpression and knockdown of the protein. As a prove of concept, AAV8-mediated liver-directed therapy of FUT2 suppression was introduced into mice with Pten-KO HCC as a therapeutic approach.

**Results and Discussions**

RNA-sequencing revealed glycosphingolipid biosynthesis to be activated in mice organoids derived from Pten-KO/c-Myc HCC, compared to control organoids. Fut2 was the top differentially upregulated gene in the discovered pathway. Elevated FUT2 expression portends aggressive stemness features and adverse prognosis specifically for patients with a background of PTEN loss. ELF3 was predicted to be an upstream regulator of FUT2 and the co-upregulation of the proteins was observed in PTEN-knockout HCC cells. Functionally, FUT2 level positively affected the ability of cancer cells to proliferate and form spheroids. Meanwhile, shRNA-mediated suppression of Fut2 was able to prolong survival of mice in HCC driven by Pten-KO.

**Conclusion**

By introducing clean driver mutation into mice and coupling that with organoid system, we were able to identify and study, in detail, the molecular features specific to HCC driven by PTEN suppression. As an essential player in glycoprotein fucosylation, FUT2 may shed light on unique post-translational modification promoting HCC, which can be exploited for novel therapy.

### EACR23-0388
### Effect of Cannabidiol, Melatonin and Oxygen/Ozone combination in preclinical models of human Pancreatic Ductal Adenocarcinoma

*L. Zeppa[1], M. Nabissi[1], C. Aguzzi[1], O. Marinelli[1], M. Luongo[2]*
*[1]University of Camerino - School of Pharmacy, Experimental Medicine Department, Camerino MC, Italy*
*[2]University of Campania Luigi Vanvitelli, Medicine, Napoli, Italy*

**Introduction**

Pancreatic Ductal Adenocarcinoma (PDAC) is one of the most aggressive types of solid malignancy and new therapeutical approaches are necessary. Nowadays the interest in using natural molecules as integrated therapy to support common chemotherapy is growing. Several *in vitro* and *in vivo* studies demonstrated the potential anticancer effect of cannabidiol (CBD), a non-psychotropic cannabinoid from *Cannabis sativa*, and melatonin (MLT), a hormone produced by pineal gland mainly involved in circadian rhythm regulation. Oxygen/Ozone therapy ($O_2/O_3$) was found to ameliorate chronic pain and inflammation. In addition, some *in vitro* evidences demonstrated that $O_2/O_3$ inhibited the growth of different human tumor cells. Herein, we evaluated the anticancer efficacy of a combination of CBD, MLT and $O_2/O_3$, alone and in combination with gemcitabine, the chemotherapeutic drug commonly used in PDAC therapy, in *in vitro* and *in vivo* models of human PDAC, studying cancer cell death and evaluating the potential modulation of protein involved in PDAC aggressiveness.

**Material and Methods**

Firstly, the potential cytotoxicity induced by the combination of CBD, MLT and $O_2/O_3$, alone and with gemcitabine, was evaluated by MTT assay and cytofluorimetric cell death analysis in PDAC cell lines. Then, the anticancer effect of the combination was evaluated in Athymic Nude-Foxn1nu mice injected with PANC-1 cells into the pancreas tail exploiting the echo-guided procedure. Ultrasound and photoacoustic imaging were used to evaluate the engraftment and the development of tumoral mass and in the end macroscopic necroscopy was performed. The modulation of proteins involved in pathways associated to PDAC aggressiveness was evaluated both in PDAC cell lines and tumoral tissues collected from mice, by Milliplex assay.

**Results and Discussions**

© 2023 The Authors. Molecular Oncology is published by John Wiley & Sons Ltd on behalf of Federation of European Biochemical Societies

Data showed that the combination of CBD, MLT and $O_2/O_3$ therapy was able to reduce PDAC cancer cells viability *in vitro* and to potentiate the efficacy of gemcitabine. In *in vivo* PDAC models, the combination induced a significant reduction of tumor volume and, combined with gemcitabine, increased its anticancer efficacy. In addition, the combination therapy leaded to a modulation of PDAC aggressiveness pathways, that was observed both *in vitro* and *in vivo*.

**Conclusion**

These preclinical data evidenced that the combination of CBD, MLT and $O_2/O_3$ was able to increase gemcitabine anticancer effect, thus could be used as adjuvant in PDAC chemotherapy.

### EACR23-0390
### Identification of the Transcriptional Drivers of Metastasis Newly Diagnosed Metastatic Prostate Cancer

*N. martin-martin[1], J. Corres[1], I. Mendeizabal[1], D. Gonzalo[2], A. Loizaga[2], M. Unda[2], A. Carracedo[1]*
*[1]cicbiogune, Cancer Cell Signaling And Metabolism Lab, Derio, Spain*
*[2]Basurto University Hospital, Service of Urology, Bilbao, Spain*

**Introduction**

Newly diagnosed metastatic (NDM) prostate cancer (PCa) refers to prostate cancer that has spread beyond the prostate gland to other parts of the body, such as the bones, liver, or lungs at time of diagnosis. Although the incidence of NDM PCa is relatively low (5-10%) it accounts for the 50% of the PCa related deaths. NDM patients have not yet been treated with any systemic therapy, such as chemotherapy or hormone therapy so they offer a unique opportunity to study aggressive PCa in a treatment-naïve context.

**Material and Methods**

To elucidate the molecular drivers and therapeutic vulnerabilities of this entity we compare formalin fixed paraffin embedded needle biopsies from primary tumors of patients with localized PCa (n=47) and NDM PCa (n=31). For functional validation of transcriptional drivers of the NDM disease, genetically modified PCa cell lines were interrogated in *in vitro* and *in vivo* assays to evaluate the aggressiveness and metastatic capacity of these modified cells.

**Results and Discussions**

RNAseq analysis reveal striking differences between the two entities, with almost 6000 differentially expressed genes. Differentially expressed genes found by RNAseq were then assessed by enrichment analysis revealing processes linked to neural signaling, developmental processes and extracellular matrix organization. Interestingly, 31% of the transcription factors found in the enrichment analysis belong to either POU or SOX families. Transcriptional programs controlled by these families are involved in developmental processes, such as cell commitment and differentiation. Their expression is extremely restricted both temporally and tissue specifically and is known to be dysregulated in cancer. Through the genetic manipulation of these transcriptional drivers, we have uncovered how members of SOX family enhance aggressiveness and metastatic capacity of PCa cell lines both *in vitro* and *in vivo* assays.

**Conclusion**

NDM prostate cancer is an entity strikingly different from localized prostate cancer. NDM PCa presents characteristic associated to neural signaling and cell commitment that can be partially driven by transcriptional programs of the SOX family of transcription factors.

### EACR23-0391
### IER5L at the core of cancer aggressiveness

*J. Crespo[1], N. Martin-Martin[1], O. Carlevaris[1], A. Zabala-Letona[1], I. Mendizabal[1], A. Ercilla[1], A. Carracedo[1]*
*[1]Cic Biogune, Cancer Cell Signaling and Metabolism, Derio, Spain*

**Introduction**

Prostate cancer is the second most common cancer in men and is responsible for more than 350,000 deaths every year, making the disease one of the leading causes of cancer-associated deaths worldwide. The morbidity is predominantly associated to the acquisition of aggressive and metastatic features. Our main aim is to discover new factors that sustain prostate cancer aggressiveness contributing to the development of metastasis.

**Material and Methods**

By means of gene expression and disease-free survival analyses from publicly available datasets, we performed our bioinformatics and enrichment analysis. For *in vitro* assays, we performed colony formation, migration, cell cycle analysis, spheroids, soft agar, in different prostate cancer cell lines. For *in vivo assays*, we used nude mice to develop xenografts and intracardiac injections to evaluate the metastatic effect though IVIS technology. Furthermore, mechanistic analyses were based on the application of transcriptomics, proteomics and phosphoproteomics strategies that were integrated in the search for robust IER5L effectors.

**Results and Discussions**

Our bioinformatics screening has revealed that the immediate Early Response gene 5L, IER5L, is consistently up regulated in colon, lung, breast and prostate cancer and its expression is strongly correlated with worse prognosis. We observe that invasion, migration and proliferation under stress conditions are compromised when IER5L levels are decreased in several prostate cancer cell lines. Moreover, since the role of IER5L in the cells is not extensively described, we have performed omics studies, such as RNA-seq, proteomics and phosphoproteomics analysis, to elucidate the molecular pathways through which IER5L is contributing to the acquisition of these metastatic features. We identify a PP2A activity-dependent genetic program downstream IER5L that might be responsible for its aggressive features.

**Conclusion**

Altogether, our data suggests that IER5L acts driving prostate cancer, although further studies are needed to fully understand the molecular mechanisms underlying this process.

### EACR23-0393
### Elevated D2HG does not cause features of tumorigenesis

*J. Adam[1], A. Finch[2], S. Kriaucions[1], K. Kranc[3], F. Szele[4], P. Ratcliffe[1], T. Soga[5], I. Tomlinson[1], C. Bardella[6]*

© 2023 The Authors. Molecular Oncology is published by John Wiley & Sons Ltd on behalf of Federation of European Biochemical Societies

[1]Ludwig Cancer Research,
Nuffield Department of Medicine, Oxford, United Kingdom
[2]ICGS,
College of Medical and Dental Sciences- University of Bir mingham, Birmingham, United Kingdom
[3]Barts Cancer Institute, Queen Mary University of London, London, United Kingdom
[4]DPAG, Department of physiology anatomy and genetics, Oxford, United Kingdom
[5]IAB, Keio University, Tokio, Japan
[6]ICGS, College of Medical and Dental Sciences, Birmingham, United Kingdom

**Introduction**

Mutation R132H in isocitrate dehydrogenase (IDH1$^{R132H}$) is a driver event in gliomas and other malignancies, which possibly causes tumorigenesis through D2-hydroxyglutarate accumulation (D2HG), although the downstream mechanisms remain unclear. In physiological conditions IDH1 converts isocitrate to α-ketoglutarate (αKG), but when mutated, IDH1 possesses a novel enzymatic function that reduces αKG to D2HG. D2HG is thought to act as an oncometabolite, mostly by inhibiting αKG-dependent enzymes, which include enzymes involved in DNA and histone demethylation among others. Studies are undergoing to clarify the causative role of D2HG in IDH-mutant tumours, but it is still not clear whether D2HG is the main driver/oncometabolite. Our aim is to understand the role of D2HG in developing and adult mouse tissues and whether its accumulation might cause features of tumorigenesis.

**Material and Methods**

A constitutive *D2hgdh* Knock-out mouse (*D2hgdh* KO) was generated and the relative molecular and cellular analysis were performed.

**Results and Discussions**

Brains dissected from *D2hgdh* KO mice appeared to be histologically normal. No differences were found in the proliferation and labelling retaining capacity of neural stem and progenitors cells (NSC/NPC) of the D2hgdh KO mice compared to controls. A comprehensive metabolites analysis showed that *D2hgdh* KO mouse accumulated D2HG in various organs and tissues, included total brains and in the NSC/NPC microdissected from the subventricular zone, the site of origin of many human gliomas. The DNA amount of 5mC and 5hmC extracted from brains of *D2hgdh* KO mice was similar to controls. A normal number of haematopoietic progenitors was also found.

**Conclusion**

Although *D2hgdh* KO mice accumulated D2HG in all tissues analysed, they did not develop any abnormalities and remained completely asymptomatic. This suggests that the only increment of D2HG in developing and adult tissues may be not sufficient to cause tumorigenesis (and gliomagenesis), leading some doubts on the oncogenic roles of the D2HG in IDH-mutant tumours.

### EACR23-0400
### Spatio-temporal fate mapping of a melanoma-stem cell population

<u>*C. Pazzi*</u>[1], *A. Nowosad*[1], *C.A. Pulido Vicuna*[1], *L. Vanwynsberghe*[1], *G. Bervoets*[1], *J. Pozniak*[1], *M. Dewaele*[1], *F. Rambow*[1], *P. Karras*[1], *J.C. Marine*[1]
[1]VIB-KU Leuven Center for Cancer Biology, Department of Oncology, Leuven, Belgium

**Introduction**

Cutaneous melanoma is a highly heterogeneous disease, characterized by phenotypically distinct cell states that communicate with each other and with the tumor microenvironment (TME). Although cellular heterogeneity can have a genetic origin, the interaction with the TME can lead melanoma cells to acquire diverse phenotypic traits, including diverging proliferative, invasive, and stemness features. Our lab previously identified a fully dedifferentiated population, the melanoma stem cell (MSC) state, which resides close to blood vessels and is endowed with stem-like transcriptional properties. In this study, we aim to test the hypothesis that the MSC state sits at the top of the tumor cellular hierarchy, thereby establishing the perivascular niche as a major driver of melanoma hierarchical structure.

**Material and Methods**

We leveraged an NRASQ61K-driven melanoma mouse model, which develops cutaneous monoclonal melanoma lesions. This model is implemented with color-based lineage tracing and co-injection xenografts to map the MSC population *in vivo*, and to address the tumor-stromal interplay. On top of that, we aim to integrate analysis of single-cell chromatin accessibility (scATACseq) and gene expression (scRNAseq) to unravel unique promoter and enhancer regions that regulate the MSC transcriptional activity. We will also deploy Hi-plex RNAscope and protein multiplex (CODEX) approaches to study the spatial positioning of the MSC population and the MSC-niche composition.

**Results and Discussions**

Our data revealed that MSC cells express specific stemness markers as NOTCH3. Mechanistically, we found that endothelial cells foster the expression of differentiation-inhibiting genes in melanoma, thereby promoting a stem-like transcriptional profile. Moreover, subcutaneous co-injection of melanoma and endothelial cells *in vivo* results in boosted tumor growth, increased metastatic burden, and reduced overall survival. In this regard, the combination of lineage tracing *in vivo* models and single-cell multi-omics approaches will unravel the functional role of MSC cells in melanoma progression and its interplay with other TME components.

**Conclusion**

Our findings will provide valuable insights into melanoma heterogeneity and determine the interplay between the MSCs and the TME in tumor growth. Ultimately, this knowledge will aid the development of innovative and effective therapies to tackle melanoma.

### EACR23-0402
### p140Cap modulates the mevalonate pathway decreasing cell migration and enhancing drug sensitivity in breast cancer cells

*G. Centonze*[1], *D. Natalini*[1], *V. Salemme*[1], *E. Turco*[1], *C. Riganti*[2], <u>*P. Defilippi*</u>[1]
[1]University of Torino, Molecular Biotechnology and Health Sciences, Torino, Italy

© 2023 The Authors. Molecular Oncology is published by John Wiley & Sons Ltd on behalf of Federation of European Biochemical Societies

[2]University of Torino, Oncology, Torino, Italy

**Introduction**

p140Cap is an adaptor protein acting as a tumor suppressor in Her2-amplified breast cancer (BC) and neuroblastoma patients, where its expression correlates with a better prognosis. Here we study the role of p140Cap in the modulation of the mevalonate (MVA) pathway, responsible for the biosynthesis of cholesterol and non-sterol isoprenoids in BC cells. The MVA pathway is often deregulated in cancer.

**Material and Methods**

MDA-MB-231, SKBR3, HEK293T, and 4T1 cell lines were obtained from ATCC; TuBo cells from F. Cavallo's lab; antibodies from different sources for WB analysis; simvastatin, doxuribicin, vinorelbine, paclitaxel from Sigma-Aldrich. Five-week-old female Balbc mice form Charles River.

Retroviral infection and transfections; Measurements of the MVA pathway metabolic flux; HMGCR immunoprecipitation and activity; tumor growth and tumor metabolic activity; luciferase activity; qRT–PCR; cholesterol depletion, loading, and intracellular cholesterol measurement; cholesterol efflux; ABC transporter activity; membrane cholesterol measurement; membrane fluidity measurement; wound healing migration assays; lipid rafts flow-cytometer analysis; lipid rafts isolation; cell viability.

**Results and Discussions**

We found that both *in vitro* and *in vivo*, p140Cap cells show an increased flux through the MVA pathway by positively regulating the pace-maker enzyme of the MVA pathway, the 3-hydroxy-3-methyl-glutaryl-coenzyme A reductase (HMGCR), via transcriptional and post-translational mechanisms. The higher cholesterol synthesis is paralleled with enhanced cholesterol efflux. Moreover, p140Cap promotes cholesterol localisation in the plasma membrane, and reduces lipid rafts-associated Rac1 signalling, impairing cell membrane fluidity and cell migration in a cholesterol-dependent manner. Finally, p140Cap BC cells exhibit decreased cell viability upon treatments with statins, alone or in combination with chemotherapeutic drugs.

**Conclusion**

Overall, our data highlight a new unexpected role for p140Cap in BC cells, namely its ability to regulate the MVA pathway, affecting tumor properties such as cell migration and drug sensitivity. In line with previous data showing the tumor-suppressor function of p140Cap, these results also highlight p140Cap as a key regulator of cell viability to conventional therapeutics and combined treatment with statins, thus paving the way to the use of p140Cap as a potent biomarker to stratify patients for possible new therapeutic options.

### EACR23-0413
### Dickkopf-1 (DKK1) mediates proteostatic stress-induced cytokine response in prostate and breast cancer

S. Pählig[1], N. Jaschke[1], A. Göbel[1], T. Rachner[1]
[1]Universitätsklinikum Dresden,
Division of Endocrinology- Diabetes- and Bone Diseases-Department of Medicine III, Dresden, Germany

**Introduction**

Dickkopf-1 (DKK1) is an inhibitor of canonical Wnt signaling. In homeostasis, DKK1 acts as a regulator of bone metabolism and embryonal development. Conversely, DKK1-overproduction is a hallmark of numerous pathologies including cancer. Tumor cells encounter a high proteostatic burden resulting from their secretory and proliferative activities, but also due to hypoxia or nutrient deprivation in their microenvironment. We hypothesized, that proteostatic stress underlies DKK1 overproduction in cancer, which triggers an adaptive response. Additionally, DKK1 may function as a mediator of the inflammatory response, further supporting tumor growth and metastasis.

**Material and Methods**

Human prostate cancer cells (PC3, DU145) and breast cancer cells (MDA-MB-231) were treated with the chemical endoplasmic reticulum stress inducers thapsigargin (Tg) or tunicamycin (Tm). Ralimetinib was used for p38-signaling inhibition. Gene silencing was performed using siRNA and stable shRNA clones. Gene expression was assessed by real-time PCR. Protein expression was analyzed by immunoblotting. Vitality measurements were performed using the Cell Titer Glo® assay.

**Results and Discussions**

We confirmed a strong DKK1 protein expression in all cell lines. To assess a potential regulation of DKK1 by proteostatic stress, cells were treated with Tg and Tm, respectively. DKK1 mRNA was significantly increased, which was paralleled by upregulated IL1b and IL6 transcript levels. Knockdown of DKK1 significantly prevented the cytokine response, pointing to a mechanistic link between DKK1 and cytokines upon ER stress induction. Suppression of the effector kinase of the unfolded protein response (UPR) revealed, that PERK but not ATF6 or IRE1 were required for stress-mediated DKK1 induction and the resulting inflammatory response. In addition, combined treatment with Tg and a p38 inhibitor showed, that cancer cells required p38 signaling to mount DKK1 response upon ER stress. Moreover, treatment of PC3-shDKK1 clones with both ER stressors showed an exaggerated loss in viability compared to the control cells. These results indicate that DKK1 deficiency sensitizes PC3 cells to proteostatic stress.

**Conclusion**

Our data indicate DKK1 as a potential target in sensitizing tumor cells to ER stress inducers. Additional experiments and *in vivo* models are needed to further dissect the role of DKK1 in tumor cell ER stress.

### EACR23-0418
### p140Cap inhibits β-Catenin in the breast cancer stem cell compartment instructing a protective anti-tumor immune response

V. Salemme[1], A. Piccolantonio[1], A. Morellato[1], L. Conti[1], S. Ugel[2], M. Iezzi[3], D. Tosoni[4], E. Turco[1], S. Pece[4], P. Defilippi[1]
[1]University of Turin,
Department of Molecular Biotechnology and Health Scienc es, Turin, Italy
[2]University of Verona,
University Hospital and Department of Medicine- Immunol ogy Section, Verona, Italy
[3]G. d'Annunzio University of Chieti-Pescara,

© 2023 The Authors. Molecular Oncology is published by John Wiley & Sons Ltd on behalf of Federation of European Biochemical Societies

Department of Neurosciences- Imaging and Clinical Sciences, Chieti, Italy
[4]University of Milan,
European Institute of Oncology IRCCS, Milan, Italy

**Introduction**

The existence of a subpopulation of tumor-initiating cells(TIC), able to resist therapies and drive disease progression and metastasis, is a major underlying cause of Breast Cancer(BC) heterogeneity. The aberrant Wnt/β-Catenin signaling pathway is involved in the acquisition of stemness traits. Recent evidence has highlighted a key role for the Wnt/β-Catenin pathway in the bidirectional cross talk between tumor cells and TME cells. p140Cap is an adaptor protein, encoded by the *SRCIN1* gene, whose expression is associated with a significantly reduced probability of developing distant recurrence and improved overall survival, in particular in HER2-positive BC patients, mainly due to its intrinsic ability to interact with proteins involved in different cancer-associated biological networks. Noteworthy, the dysfunction of tumor suppressor pathways relevant to BC can hijack the Wnt/β-Catenin signaling pathway to instruct a tumor-promoting immune response in the TME has been poorly investigated.

**Material and Methods**

TuBo and 4T1 BC cells were injected in Balb/c mice to asses the *in vivo* tumor growth, metastasis formation and tumor immune infiltrate. Mammosphere formation assay and confocal microscopy to investigate the TIC compartment and the Wnt/β-Catenin pathway. Clinical studies to evaluate the correlation between p140Cap expression and tumor-infiltrating lymphocytes(TILs).

**Results and Discussions**

We highlight an original function of p140Cap in orchestrating local and systemic tumor-extrinsic events that result in inhibition of the polymorphonuclear myeloid-derived suppressor cell(PMN-MDSCs) function in creating an immunosuppressive tumor-promoting environment in the primary tumor and premetastatic niche. Our data reveal that p140Cap controls an epistatic axis where, through the β-Catenin inhibition, restricts tumorigenicity and self-renewal of TIC limiting the release of the inflammatory cytokine G-CSF, required for PMN-MDSC to exert their local and systemic tumor conducive function.
Mechanistically, p140Cap inhibition of β-Catenin depends on its ability to localize in and stabilize the β-Catenin destruction complex, promoting β-Catenin inactivation. Clinical studies show that p140Cap expression correlates with high TILs stromal infiltration in a large cohort of real-life BC patients.

**Conclusion**

Collectively, our findings highlight p140Cap as a biomarker for therapeutic intervention targeting the β-Catenin/Tumor-initiating cells/G-CSF/PMN-MDSC cell axis to restore an efficient anti-tumor immune response.

### EACR23-0423
### Protein phosphatase PP2A-based triple-strike therapy for medulloblastoma

O. Denisova[1], R. Huhtaniemi[1], J. Merisaari[1], X. Qiao[1], M. Pääkkönen[1], M. Ohlmeyer[2], O. Kauko[1], J. Westermarck[1]
[1]University of Turku & Åbo Akademi University, Turku Bioscience Centre, Turku, Finland
[2]Atux Iskay LLC, Atux Iskay LLC, Plainsboro, United States

**Introduction**

Deregulation of protein phosphatase PP2A by overexpression of PP2A inhibitor proteins (PIPs) such as protein phosphatase methylesterase 1 (PME-1), Cancerous Inhibitor of PP2A (CIP2A), SET nuclear proto-oncogene (SET) have been proposed as a potential general cause for kinase inhibitor resistance in cancer. While overexpression of PIPs has been studied in various adult cancers, their relevance in pediatric brain cancer is poorly understood. Here, we highlight the implications of PME-1 in growth of pediatric brain tumor medulloblastoma (MB) and present a novel combination therapy approach based on PP2A activation.

**Material and Methods**

In vitro and in vivo studies were done on established MB cell lines, DAOY (SHH group) and D283-Med (Group 3). CRISPR/Cas9 approach was used for PME-1 inhibition. All animal experiments were authorized by the National Animal Experiment Board of Finland.

**Results and Discussions**

Overall survival analysis of MB patients revealed that mRNA expression of several PIPs is associated with poor patient prognosis. Interrogation of CRISPR/Cas9 loss-of-function data showed many PP2A and PIP genes among the 10 top preferentially essential genes, especially in DAOY cells. Intracranial in vivo experiments validated the importance of PME-1 for MB growth, with significantly prolonged survival in mice carrying intracranial PME-1 knockout DAOY and D283-Med cells.
To validate the role of PP2A inhibition in kinase inhibitor resistance in MB, we next tested the impact of recently developed triplet kinase/phosphatase combination therapy on DAOY cells. A triplet combination of AKT inhibitor, mitochondrial PDK1-4 inhibitor, and PP2A reactivating compounds (SMAPs) induced very robust cell killing in long-term colony growth assays. Moreover, orally dosed triplet therapy significantly increased the survival of mice carrying intracranial DAOY xenograft. Using mass spectrometry phosphoproteomics analysis, we molecularly profiled the triplet therapy effect in tumors validating its impact on both apoptosis and the cell cycle. Furthermore, triplet therapy inhibited phosphorylation of the activation loop of AKT1, 2, and 3, and impacted several phosphoproteins linked to mTOR signaling.

**Conclusion**

These results highlight PP2A inhibitor protein PME-1 as a novel potential MB oncoprotein and therapy resistance driver. Further, the triplet kinase/phosphatase therapy is a promising lead for the development of novel combination therapy approaches for MB.

### EACR23-0429
### Tracing main c-Myc isoforms endogenous expression for targeted anti-cancer therapies

A. Kubickova[1], M. Hajduch[1]
[1]Institute of Molecular and Translational Medicine- Palacky University Olomouc,
Czech Advanced Technology and Research Institute- Institute of Molecular and Translational Medicine- Palacky University in Olomouc, Olomouc, Czech Republic

**Introduction**

© 2023 The Authors. Molecular Oncology is published by John Wiley & Sons Ltd on behalf of Federation of European Biochemical Societies c-Myc is one of the most well-studied multifunctional transcription factors involved in several processes, including proliferation, differentiation, apoptosis, ribosome biogenesis, protein translation, angiogenesis, metabolism, DNA repair, immune surveillance and stem cell formation. In addition to selectively targeting more than 15% of the human genome, c-Myc acts indirectly as a transcriptional enhancer. Therefore, its deregulation promotes the malignant transformation that leads to the hallmark features of more than half of human cancers. This project aims to determine the effect of compounds from the LOPAC and Prestwick repurposing libraries on the physiological levels of the two major isoforms of the c-Myc transcription factor. Using two novel technologies (CRISPR/Cas9 and NanoLuc), reporter cell lines were established to monitor physiological levels of the c-Myc transcription factor. The uniqueness of these reporters is that they allow easy quantification of the two major isoforms of c-Myc, whose locus has a rather complex structure. The two isoforms are structurally very similar, although their properties differ. The p64 isoform is referred to as oncogenic, whereas the p67 isoform is associated with an anti-oncogenic activity.

**Material and Methods**

Validation of the obtained reporter was performed by verifying the molecular weight of the tagged proteins using HiBiT blot. We then proceeded to validate the specificity of the reporters using siRNA targeting c-Myc itself and pathways closely associated with it. The tested pathways are involved in c-Myc stabilization and degradation and transcription factors that directly affect c-Myc expression and c-Myc transcriptional targets.

**Results and Discussions**

Since we will use high throughput screening (HTS) to determine the activity of compounds in the LOPAC and Prestwick libraries, the NanoLuc assays were optimized to a 384-well plate format, and candidate reference antagonists were tested. The selection was made from compounds interfering with the binding of c-Myc to the promoters of its target genes. In addition, BRDi or MEKi were chosen from compounds reducing c-Myc expression. Potential reference antagonists were tested in a dose-dependent manner for 6 hours.

**Conclusion**

For HTS alone, 10074-G5 and OTX015 at a concentration of 50 µM were preferred according to their highest activity. HTS of LOPAC and Prestwick libraries will soon be performed using our facility's upgraded, fully automated robotic platform.

### EACR23-0445

### Targeting the Hippo pathway in NF2-null schwannoma and meningioma tumours.

*L. Laraba[1], L. Hillson[1], J. Grimm de Guibert[1], A. Hewitt[1], T. Tang[2], L. Post[2], D. Jagger[3], O. Hanemann[1], D. Parkinson[1]*
*[1]University of Plymouth, School of Medicine, Plymouth, United Kingdom*
*[2]Vivace Therapeutics Inc, 1500 Fashion Island Blvd.- Suite 102, San Mateo- CA 94404, United States*
*[3]University College London, UCL Ear Institute, London, United Kingdom*

**Introduction**

Loss of function of the Merlin (NF2) protein is associated with multiple tumour types, including schwannoma, meningioma and ependymoma. Our work has focussed upon meningioma and schwannoma using in vitro and in vivo models to understand both the genetics of these tumours and trial new therapies.

Here we use small-molecule TEAD auto-palmitoylation inhibitors in the two most clinically relevant models of schwannoma and meningioma, the Periostin;Cre-*NF2*$^{fl/fl}$ schwannoma model, in which schwannomas develop spontaneously in the vestibular ganglia (VGs) and dorsal root ganglia (DRGs), and an orthotopic xenograft mouse meningioma model.

**Material and Methods**

Experiments are performed in both primary human tumour cells in vitro and in pre-clinical mouse models of schwannoma and meningioma.

**Results and Discussions**

Genetic deletion of either YAP or TAZ on an *NF2*-null background significantly reduced proliferation and macrophage infiltration in Periostin;Cre-*NF2*$^{fl/fl}$ mice schwannoma tumours. Deletion of TAZ but not YAP also ameliorated the severe neuronal loss seen in DRGs, highlighting the overlapping but distinct functions of YAP and TAZ in schwannoma development. TEAD inhibitor drug treatment in Periostin;Cre-*NF2*$^{fl/fl}$ mice resulted in significant reductions in proliferation and an increase in apoptosis in both VGs and DRGs compared to vehicle-treated littermate controls. TEAD inhibition also significantly reduced DRG and VG schwannoma tumour volume following 21d treatment in 9-month old animals compared to vehicle controls. qPCR and western blot analysis showed target engagement by downregulation of multiple Hippo target genes such as CTGF and Cyr61. TEAD inhibitors were also seen to significantly reduce proliferation in primary human meningioma cells with nanomolar $IC_{50}$. Orthotopic injection of meningioma cell lines led to tumour establishment in NSG mice and treatment with TEAD inhibitors significantly reduced proliferation and tumour size in vivo.

**Conclusion**

For *NF2*-related schwannomatosis patients, drugs that effectively target schwannomas, meningiomas and ependymomas are the ultimate goal. Aberrant Hippo pathway activity is recognised to drive tumour phenotypes in *NF2*-null schwannoma and meningioma. Here we show that TEAD autopalmitoylation inhibitors can potently block and even reduce tumour growth in schwannoma *in vivo* and can inhibit meningioma tumour growth in vivo. This new class of inhibitors show great promise in the treatment of these two tumour types.

### EACR23-0456

### Participation of metabolic flexibility in adaptation of glioma cells to chemotherapeutic stress

*M. Pudełek[1], S. Kędracka-Krok[2], J. Czyż[1]*
*[1]Jagiellonian University, Department of Cell Biology, Kraków, Poland*
*[2]Jagiellonian University,*

© 2023 The Authors. Molecular Oncology is published by John Wiley & Sons Ltd on behalf of Federation of European Biochemical Societies

*Department of Physical Biochemistry, Kraków, Poland*

**Introduction**

Despite the recent development of glioma treatment strategies, commonly used cytostatic drugs can still promote the drug-resistance mechanisms in glioma neoplasms. Their efficiency may depend on the metabolic flexibility of glioma cells, which are able to adapt the profile and intensity of their mitochondrial metabolism to chemotherapeutic stress. Consequently, the tumors often rebuild their malignancy after the cessation of chemotherapy. Taking into account these issues, we focused on the short- and long-term shifts of metabolic phenotype induced in T98G glioma cells by the pulse doxorubicin treatment.

**Material and Methods**

Human T98G cells (ATCC; CRL-1690) were exposed to doxorubicin (DOX, pulse treatment; 1μM, 48h) followed by short and long-term (48h – 14 days after drug removal) phenotypic analysis. Measurements of cellular respiration were performed with Seahorse XFp analyzer. Experiments were supported by enzymatic determination of intracellular ATP, NAD(P)H, 2-oxoglutarate and glutamate content. Mass spectrometry (MS), assisted by Perseus MaxQuant and STRING database analyses, was used to follow the levels of proteins involved in mitochondrial metabolism.

**Results and Discussions**

DOX-resistant fraction of T98G cells displayed noticeable metabolic flexibility, which was illustrated by their ability to increase the mitochondrial metabolism efficiency in response to chemotherapeutic stress. This effect was associated with enhanced respiration and intracellular pool of ATP and NAD(P)H. Concomitantly, noticeably increased intracellular levels of 2-oxoglutarate and glutamate indicated the stimulation of the coupling between TCA cycle and glutamate metabolism. MS-assisted analysis showed the elevated levels of key mitochondrial enzymes involved in OXPHOS, β-oxidation, TCA cycle and glutamate utilization pathways. Moreover, the compensatory role of intracellular accumulation of energetic equivalents was confirmed by significant up-regulation of ATP-binding proteins that are involved in cellular anabolic and stress responses.

**Conclusion**

Collectively, our observations support the hypothesis claiming the role of mitochondrial metabolism in the maintenance of T98G homeostasis. In conjunction with the data on the kinetics of T98G recovery following the doxorubicin-induced stress, they show that metabolic plasticity is crucial for tumor survival in stress conditions, and for the renewal and spreading of gliomas after chemotherapeutic cycles.

### EACR23-0457
### ProNGF promotes brain metastasis through TrkA/EphA2 induced Src activation in triple negative breast cancer cells

*J. Cicero[1], S. Trouvilliez[1], C. Lagadec[1], E. Vandenhaute[2], P. Giacobini[3], F. Gosselet[4], N. Maubon[2], X. Le Bourhis[1], C. Mysiorek[4], R.A. Toillon[1]*

*[1]Univ. Lille- CNRS- Inserm- CHU Lille- UMR9020-U1277 - CANTHER - Cancer Heterogeneity Plasticity and Resistance to Therapies- F-59000 Lille- France, Biology, Lille, France*

*[2]HCS Pharma- F-59120 Loos- France, Biotechnology, Loos, France*

*[3]Univ. Lille- Inserm- CHU Lille- Équipe développement et plasticité du cerveau neuroendocrine- Lille Neuroscience et Cognition- UMR-S1172- F-59000 Lille Cedex- France., Neurosciences, Lille, France*

*[4]Univ. Artois- UR 2465- Laboratoire de la Barrière Hémato-Encéphalique LBHE- F-62300 Lens- France, Biology, Lens, France*

**Introduction**

The majority of breast cancer deaths are due to metastasis. Triple-Negative Breast Cancer (TNBC) is particularly aggressive, and its metastasis to the brain has a significant psychological impact on patients' quality of life, in addition to reducing survival. Therefore, understanding the mechanisms behind the development of brain metastases is essential to fight them. To date, the mechanisms that induce brain metastasis in TNBC are poorly understood.

**Material and Methods**

Using an *in vitro* human blood-brain barrier (BBB) model, an *in vitro* 3D organotypic extracellular matrix, an *ex vivo* mouse brain slices co-culture and in an *in vivo* xenograft experiment, key step of brain metastasis were recapitulated to study TNBC behaviors. These models were coupled with single cell imaging to fellow the signaling pathway involved in these biological processes.

**Results and Discussions**

In this study, we demonstrated for the first time the involvement of the precursor of Nerve Growth Factor (pro-NGF) in the development of brain metastasis. More importantly, our results showed that proNGF acts through TrkA independent of its phosphorylation to induce brain metastasis in TNBC. In addition, we found that proNGF induces BBB transmigration through the TrkA/EphA2 signaling complex. More importantly, our results showed that combinatorial inhibition of TrkA and EphA2 decreased TBNC brain metastasis in a preclinical model.

**Conclusion**

In this study, we demonstrate for the first time the prominent role of proNGF, a growth factor expressed in the central nervous system, in brain tropism and the different stages of brain metastasis, such as Blood Brain Barrier crossing and micrometastases. These disruptive findings provide new insights into the mechanisms underlying brain metastasis with proNGF as a driver of brain metastasis of TNBC and identify TrkA/EphA2 complex as a potential therapeutic target.

### EACR23-0466
### mTOR involvement in STAT3 S-P at the ER and regulation of calcium-mediated apoptosis

*D. Viavattene[1], A. Savino[1], L. Ponzone[1], A. Lobascio[1], A.N. Kouakou[1], A.R. Marchetti[1], L. Conti[1], F. Cavallo[1], E. Calautti[1], L. Avalle[1], V. Poli[1]*

*[1]Molecular Biotechnology Center "Guido Tarone", Department of Molecular Biotechnology and Health Sciences, Turin, Italy*

**Introduction**

Signal Transducer and Activator of Transcription 3 (STAT3) is a pleiotropic transcription factor often

© 2023 The Authors. Molecular Oncology is published by John Wiley & Sons Ltd on behalf of Federation of European Biochemical Societies constitutively activated in tumors including breast cancer where, among other effects, it triggers resistance to apoptosis and chemotherapy. We have recently described STAT3 ability to control ER $Ca^{2+}$ release and $Ca^{2+}$-mediated apoptosis in STAT3-dependent Triple Negative Breast Cancer (TNBC) cells, localizing both to the Endoplasmic Reticulum (ER) and Mitochondrial Associated Membranes (MAMs), where it interacts with the $Ca^{2+}$ channel inositol 1,4,5-risphosphate receptor type 3 (IP3R3). Mechanistically STAT3, when phosphorylated on Serine 727 (S-P), triggers IP3R3 degradation, and indeed its silencing correlates with increased IP3R3 levels. Accordingly, STAT3 and IP3R3 protein levels are inversely correlated in the highly aggressive human basal-like breast tumors, where STAT3 is often constitutively activated.

**Material and Methods**

To investigate the mechanisms regulating STAT3 S-P at the ER, we searched for STAT3 interactors known to localize to the ER, and selected the mammalian target of rapamycin (mTOR) as the most promising one.

**Results and Discussions**

STAT3-mTOR interaction was confirmed in several human TNBC cells, where both proteins co-localize to the Protein Disulfide Isomerase (PDI)-positive perinuclear region corresponding to the ER. Pan-mTOR inhibition by Torin-1 – but not mTOR Complex 1 inhibition via Rapamycin – could prevent both IP3R3 degradation and $Ca^{2+}$-mediated apoptosis, mimicking the effects obtained upon STAT3 silencing and correlating with reduced ER localization of serine-phosphorylated STAT3. These observations suggest that STAT3 S-P may indeed be regulated directly at the ER by mTOR Complex 2. We therefore generated mTORC1 and mTORC2 KO TNBC cells by CRISPR CAS9-mediated disruption of their respective scaffolding proteins Raptor and Rictor in MDA-MB-468 cells. Preliminary data indicate that STAT3-mTOR might directly interact, since they can still co-immunoprecipitate in the absence of either complex. The assessment of the relative role of mTORC1 and 2 in regulating STAT3 S-P at the ER, $Ca^{2+}$ release from the ER to the mitochondria, and $Ca^{2+}$-mediated apoptosis is in progress.

**Conclusion**

Dissecting these molecular details, in addition to contributing to characterize the multiple non-canonical mechanisms of STAT3 action, may reveal novel therapeutic targets to disrupt apoptotic resistance in STAT3-dependent TNBC cells.

### EACR23-0479
### Assessing the anti-cancer potential of ONC201 and ONC206 on human prostate cancer using two-dimensional (2D) and three-dimensional (3D) in vitro cell models

*S. Hachem*[1], *A. Yehya*[1], *F. Ghamlouche*[1], *T. Abou-Antoun*[2], *W. Abou-Kheir*[1]
[1]*American University of Beirut, Anatomy- Cell Biology and Physiological Sciences, Beirut, Lebanon*
[2]*Washington University in Saint Louis, Department of Pediatric Hematology-Oncology and Genetics, Washington, United States*

**Introduction**

Prostate cancer (PC) is an alarming health issue among men worldwide. Although many therapeutic approaches have been used to manage PC, it often develops resistance and progresses into a lethal state, known as metastatic castration-resistant prostate cancer (mCRPC). Therefore, defining new targets and elucidating novel therapeutics for managing PC are of utmost priority. Imipridones represent a novel class of anti-cancer compounds that selectively antagonize the G protein-coupled receptor (GPCR) dopamine receptor 2 (DRD2) and agonize the mitochondrial protease (ClpP). DRD2 and/or ClpP are up-regulated and differentially expressed in certain malignancies, including PC. ONC201, the first-in-class clinical imipridone, showed to have anticancer effects in PC. ONC206, an analog of ONC201, possesses enhanced nano-molar potency. Thus, the aim of this study is to investigate the anti-cancer potential of ONC206, in comparison to ONC201, on human PC using two-dimensional (2D) and three-dimensional (3D) in vitro cell models.

**Material and Methods**

ONC201 and ONC206 drugs were tested on two PC cell lines (PC-3 and DU-145) using several in vitro assays. MTT assay was performed to evaluate the cytotoxic effect of a wide range of concentrations of ONC201 and ONC206 on PC cells. Trypan blue exclusion assay was then used to assess the effect of both drugs on cellular viability. In addition, cell migration ability was investigated using the "wound-healing" scratch assay. Furthermore, the 3D sphere-forming assay was applied to examine the effect of both drugs in targeting the enriched population of PC stem/progenitor cells. Moreover, the effect of both drugs was tested in PC cell-derived organoids. Patient-derived organoids will be also used to validate the results.

**Results and Discussions**

Our MTT data showed that ONC206 exerts a more potent cytotoxic effect on the DU-145 and PC-3 cell lines compared to ONC201, in a time and dose-dependent manner. These results were confirmed through the trypan-blue viability assay. Similarly, ONC206 displayed a more significant attenuation in the migration ability of PC cells in comparison to ONC201. Importantly, these results were validated in a 3D culture system with the sphere-forming and PC cell-derived organoid assays.

**Conclusion**

Imipridones represent a novel approach to therapeutically target DRD2 and/or ClpP in PC. ONC206 shows more potent anti-cancer effects on PC cells than ONC201, paving the way for new effective therapeutics and better management of PC.

### EACR23-0492
### Gene fusions juxtapose 'B' (closed) with 'A' (open) chromatin domains leading to gene activation in cancer

*M.D. Bashyam*[1], *A. Gupta*[1]
[1]*Centre for DNA Fingerprinting and Diagnostics, Laboratory of Molecular Oncology, Hyderabad, India*

**Introduction**

Gene fusions (GFs) are of proven diagnostic/prognostic/therapeutic utility in several cancers.

© 2023 The Authors. Molecular Oncology is published by John Wiley & Sons Ltd on behalf of Federation of European Biochemical Societies

However, a possible connection between chromatin architecture/domains and GFs remains largely unexplored.
**Material and Methods**
We applied Arriba, FusionCatcher and SOAPfuse on RNA-seq data generated in-house followed by validation through RT-PCR and Sanger sequencing, to identify GFs in early onset sporadic rectal cancer (EOSRC). A parallel analysis was performed using TCGA-CRC and CRC cell line RNA-Seq data. GFs detected for 31 cancer types were accessed through https://portal.gdc.cancer.gov. We analysed FANCD2 ChIP-Seq and MiDAS-Seq data to correlate GF breakpoints with common fragile sites (CFSs). HiC data for two colorectal and one breast (BRCA) cancer cell line were assessed to determine association of chromosomal contacts, topologically associating domains (TADs) and chromatin ('A' (open) / 'B' (closed)) domains with GF breakpoints. Transcript levels of the GF 5' and 3' partners were assessed from RNA-Seq data and validated by quantitative RT-PCR.
**Results and Discussions**
A comparable average per sample GF frequency and presence of several common and recurrent GFs including *RNF43-SUPT4H1* and *DNM2-QTRT1* were identified in EOSRC and TCGA-CRC. Gene Ontology (GO) analysis revealed enrichment of distinct 'Biological Processes' with no overlap between 5' and 3' partners or between EOSRC and TCGA-CRC. In contrast, RNA binding, cadherin binding and ubiquitin ligase binding were frequent GO 'Molecular Function' terms associated with both 5' and 3' genes and enriched in EOSRC and TCGA-CRC. We generated a network of genes contributing to GFs; genes with high degree and centrality scores such as *WWOX*, *ACTB*, *KRT8* (in both data sets), *YHWAZ* (only in EOSRC), and *FHIT* (only in TCGA-CRC), overlapped with CFSs. We identified distinct frequency of intra and inter chromosomal involvement in GFs in different cancer types which correlated with the chromosomal contacts as well as TADs. As expected, majority of GFs in EOSRC, TCGA-CRC and BRCA arose from 'A' chromatin domain ('A-A'). Interestingly, we detected a significant induction of expression of the 3' partner in 'A-B' GFs, revealing a hitherto unrecognized mode of gene activation in cancer.
**Conclusion**
We present the first comprehensive assessment of GFs in EOSRC that reveal interesting correlation with CFSs, TADs and chromatin domains. This is the first report of gene activation in cancer due to altered chromatin domain arising from GF.

### EACR23-0496
### Relevance of cathepsins B and X for treatment of breast cancer stem cells

A. Mitrović[1,2], J. Kos[1,2]
[1]*Jožef Stefan Institute, Department of Biotehnology, Ljubljana, Slovenia*
[2]*University of Ljubljana, Faculty of Pharmacy, Ljubljana, Slovenia*
**Introduction**
The effectiveness of anti-tumor therapy is limited by development of resistance and cancer recurrence, the events, associated with the presence of small population of cancer stem cells (CSCs) that are resistant to the most conventional antitumor therapies. This calls for the identification of the therapeutic modalities that would be effective also towards CSCs. In this study we evaluated cathepsins B and X in CSCs and their relevance as possible new targets to direct antitumor therapy towards CSCs. Lysosomal cysteine cathepsins B and X are well-established targets in different types of cancer and participate in various stages of development and progression of cancer including invasive tumor growth and metastasis formation. Cathepsins have been recognized as an important part of dynamic response in anticancer therapy that can be selectively regulated at multiple levels, including specific small molecular inhibitors.
**Material and Methods**
CSCs were isolated from breast cell lines based on their ability to form tumorspheres. Western blot, ELISA, qPCR, and enzyme kinetics activity were used to evaluate expression and activity of cathepsins B and X in CSCs. Next, selective, reversible small molecular inhibitors of cathepsins B and X were used to test the relevance of cathepsins B and X in breast CSCs alone or in a combination with conventional chemotherapeutics. The effect of cathepsin inhibition on cell signaling was evaluated using western blot and enzyme kinetics.
**Results and Discussions**
We demonstrated that cathepsins B and X are present in higher levels in breast CSCs following tumorsphere formation compared to single adherent differentiated tumor cells. Next, we showed cathepsin B and X inhibitors effected CSC phenotype by decreasing the expression of stemness markers and markers of mesenchymal cell phenotype. Cathepsins' inhibition increased differentiation of CSCs, thus improving the effectiveness of the conventional chemotherapy. Additionally, our results show that cathepsin inhibition effects signaling pathways important for tumor progression.
**Conclusion**
In conclusion our results highlight the targeting of cathepsin B and X as new promising approach to improve existing antitumor therapy and address main challenges that currently limit the effectiveness of cancer treatment.

### EACR23-0499
### Antitumor activity of miR-99b-5p in TNBCs is mediated by the regulation of cell proliferation and apoptosis

S. Noyan[1], H. Gurdal[2], B. Gur Dedeoglu[1]
[1]*Ankara University, Biotechnology Institute, Ankara, Turkiye*
[2]*Ankara University, Faculty of Medicine- Department of Pharmacology, Ankara, Turkiye*
**Introduction**
Breast cancer is the most common cancer diagnosed worldwide, affecting approximately one in eight women. It is a highly heterogeneous disease and clinically categorized into four subtypes based on the presence of ER, PR, HER2 and ki67. TNBC, which is like basal tumors, represents tumors without the expression of ER, PR and HER2. Identification of these subtypes is particularly important for the decision of treatment approaches. Although drug applications for different subtypes have been determined, the benefit gained from these drugs loses their

© 2023 The Authors. Molecular Oncology is published by John Wiley & Sons Ltd on behalf of Federation of European Biochemical Societies effectiveness throughout the course of the disease due to genetic heterogeneity and acquired resistance. That's why the concept of combining different therapeutic agents for synergistic benefits in treatment has been utilized for decades. miRNAs comprise a class of 19–22 nucleotides long, non-coding, endogenous RNA molecules, which regulate a variety of cellular pathways.

**Material and Methods**

In this study, it is aimed to investigate the molecular function of miR-99b-5p in breast cancer, which we have determined in our preliminary experiments that its expression decreases cell proliferation in breast cancer cells and increases the activity of trastuzumab and tamoxifen. To find out the clinical significance of miR-99b-5p we analyzed its expression profile in the TCGA breast cancer cohort (BRCA). Then, we decreased the expression of miR-99b-5p through siRNA transfection in TNBC cells and the molecular mechanism of its anti-proliferative effect was investigated by apoptosis and cell cycle experiments in three different TNBC cell lines.

**Results and Discussions**

miR-99b-5p has been studied in many cancer types so far and its expression was found to be significantly lower in all cancer types in the literature compared to normal samples. The increase in the expression of miR-99b-5p in breast tumors contrary to all cancers in the literature according to our TCGA data analysis suggests that this miRNA may provide activity through a different mechanism in breast cancer. Additionally, among the subtypes of breast cancer the highest expression of this miRNA was in basal like tumors. The downregulation of miR-99b-5p in TNBCs effectively reduced cell proliferation and this reduction was related to G1 arrest of the cells.

**Conclusion**

It can be concluded that the regulation of miR-99b-5p may play a biological role in the signaling pathways related to proliferation in breast cancer cells and can be proposed as a potential therapeutic target in breast cancers particularly in TNBCs.

### EACR23-0506
### miR-214 induces cell metabolism rewiring, which leads to cancer progression

*S. cozzubbo[1], M. Coco[1], S. Cucinelli[1], A. Larcher[1], I.C. Salaroglio[2], J. Kopecka[2], G. Morciano[3], F. Orso[4], P. Pinton[3], C. Riganti[2], D. Taverna[1]*

*[1]Molecular Biotechnology Center "Guido Tarone", Department of Molecular Biotechnology and Health Scienc es, Torino, Italy*

*[2]University of Torino, Department of Oncology, Torino, Italy*

*[3]University of Ferrara, Department of Medical Science, Ferrara, Italy*

*[4]University of Piemonte Orientale, Department of Translational Medicine DIMET, Novara, Italy*

**Introduction**

microRNAs (miRNAs) are involved in the regulation of multiple cellular events in cancer from proliferation to cell metabolism. Reprogrammed energy metabolism, especially the Warburg effect (highly glycolytic), is one of the most common tumor metabolic phenotypes. We previously demonstrated that miR-214 is a pro-metastatic miRNA overexpressed in both melanoma and breast cancer by activating a dissemination program. Specifically, miR-214 silences Transcription Factors (TFs), such as TFAP2C, suppresses the anti-metastatic miR-148b and upregulates specific adhesion molecules, such as ALCAM and ITGA5, direct-miR-148b targets. Strong evidence indicates an intense crosstalk between tumor progression and metabolic rewiring, hence we aim at understanding the role of miR-214 on the metabolic switch.

**Material and Methods**

We evaluated the metabolic profiles of miR-214 overexpressing melanoma and breast cancer cells in culture and in xenotranplants in mice. We worked on the identification of the main metabolic players coordinated by miR-214 and on the impact of the metabolic rewiring on metastatic features.

**Results and Discussions**

We showed that miR-214 promotes a Warburg effect. In fact, an increase in glucose uptake, glycolysis, lactate production, glycolytic enzyme activity was observed in miR-214-overexpressing cells while miR-214-depleted cells revealed an opposite phenotype. Consistently, a decrease in Tricarboxylic acid (TCA), electron transport chain and fatty acid oxidation enzymes activity was evidenced in miR-214-high level cells. In line, a diffused mitochondrial damage (altered morphology and function) was found. We are currently investigating the molecular mechanisms underlying the observed metabolic rewiring and the mitochondrial altered phenotype. Mitofusin 2, a mitochondrial GTPase, and a proven miR-214 direct target, able to mediate the outer membrane fusion and to contribute to the mitochondrial network maintenance, is potentially responsible for the mitochondria damage. When we assessed the impact of miR-214-dependent metabolism rewiring on metastatic traits, we evidenced altered cell adhesion and cell motility which suggests a direct interplay between metabolic changes and tumor dissemination.

**Conclusion**

Our data support the key role played by miR-214 in promoting malignancy and tumor progression by driving metabolism reprogramming in melanoma and breast cancer cells.

### EACR23-0507
### Tocotrienol-Rich Fraction Regulates Proteomic Changes That Induce Cellular Response to Stress Associated Pathways in Resistant Glioblastoma Cell Line

*D. El-Rabie Osman[1], K. Bai Magalingam[1], M. Noor Alfarizal Kamarudin[1], S. Asnawi Zainal Abidin[1], A. Kutty Radhakrishnan[1], S. Bhuvanendran[1]*

*[1]Monash University Malaysia, Jeffrey Cheah School of Medicine and Health Sciences, Bandar Sunway, Malaysia*

**Introduction**

Glioblastoma (GBM), a grade IV intracranial tumour that is widely known for its highly aggressive and heterogeneous nature, contributing to treatment resistance. Tocotrienol-rich fraction (TRF), a natural extract of vitamin E derived from palm oil, has been reported to exhibit potent anti-cancer effects. However, scanty is known on the effect of TRF on GBM, while the underlying

© 2023 The Authors. Molecular Oncology is published by John Wiley & Sons Ltd on behalf of Federation of European Biochemical Societies molecular mechanism remains unclear. This study aimed to uncover the anti-cancer mechanism exerted by TRF on a resistant human cell model of GBM (LN-18 cell line) at the proteome level.

**Material and Methods**

The half maximal inhibitory concentration (IC50) of TRF on LN-18 cells was identified from 24 - 72 hrs using a range of concentrations (0-40 μg/mL) treated. Protein was extracted from LN-18 cells following 72 hrs exposure to TRF, then label-free quantitative proteomic profiling was utilized to analyse the proteomic changes and determine the differentially expressed proteins (DEPs) between the TRF-treated and untreated control. The identified DEPs were further analysed using bioinformatic databases to recognise functional classification of proteins and pathway enrichment analysis.

**Results and Discussions**

TRF treatment in GBM demonstrated a concentration and time-dependent reduction in cell viability with IC50 values 25, 18 and 15μg/mL for 24, 48 and 72 hrs, respectively. A total of 65 DEPs were identified from the proteome of TRF-treated LN-18 cells as compared to the untreated control. Bioinformatic analysis reveals that 65 DEPs modulated by TRF are involved in the cellular response to stress (CRS) pathways. PANTHER functional analysis demonstrated these DEPs are characterised into metabolic conversion enzymes (11 proteins, mainly in glycolysis process), cytoskeletal proteins (8 proteins), chaperones (4 proteins), chromatin-binding proteins (11 proteins), and translational proteins (7 proteins). Further analysis demonstrated that chaperone (HSPA5), chromatin-binding proteins (H1 and H2B histone proteins) and translational proteins (60S and 40S ribosomal proteins) are found to be associated with the induction of CRS by TRF in LN-18 cells.

**Conclusion**

The current findings suggest that TRF treatment induced anti-proliferative effect through the modulation of DEPs that are associated with CRS pathways, This is further supported by the alignment of the DEPs to functional classes as chaperones, chromatin-binding proteins and translational proteins which are incorporated to the CRS pathways.

### EACR23-0508
### Dysregulated lipid metabolism supports acquired resistance to anti-EGFR therapy in head and neck squamous cell carcinoma

*V. Van den bossche[1,2], J. Vignau[1], E. Vigneron[1], H. Zaryouh[3], J. Ambroise[4], A. Wouters[3], J.P. Machiels[2,5], O. Feron[1], S. Schmitz[5,6,7], C. Corbet[1]*

*[1]UCLouvain, Pole of Pharmacology and Therapeutics- Institut de Recherche Expérimentale et Clinique, Brussels, Belgium*

*[2]Cliniques Universitaires Saint Luc, Department of Medical Oncology, Brussels, Belgium*

*[3]UAntwerp, Center for Oncological Research- Integrated Personalized and Precision Oncology Network, Wilrijk, Belgium*

*[4]UCLouvain, Center for Applied Molecular Technologies- Institut de Recherche Expérimentale et Clinique, Brussels, Belgium*

*[5]UCLouvain, Pole of Molecular Imaging- Radiotherapy and Oncology- Institut de Recherche Expérimentale et Clinique, Brussels, Belgium*

*[6]Cliniques Universitaires Saint Luc, Department of Head and Neck Surgery, Brussels, Belgium*

*[7]Cliniques Universitaires Saint Luc, King Albert II Cancer Institute, Brussels, Belgium*

**Introduction**

Head and neck squamous cell carcinoma (HNSCC) is associated with dismal prognosis for patients with locally advanced or recurrent/metastatic disease. Clinical response to anti-EGFR therapy (cetuximab; CTX) in these patients is strongly limited by the occurrence of acquired resistance. While no genetic alteration is clearly associated with cetuximab resistance, tumor microenvironment is thought to actively contribute to disease progression and clinical relapse in HNSCC. We aim to characterize the metabolic preferences and molecular mechanisms supporting cetuximab resistance in HNSCC.

**Material and Methods**

2D and 3D cultures of cetuximab-sensitive (-S) and -resistant (-R) HNSCC cell lines, as well as patient-derived xenograft models of HNSCC, were combined with transcriptomic, proteomic, metabolomic and lipidomic studies. Seahorse-based metabolic profiling, flow cytometry, immunoblotting, real-time PCR, and functional phenotypic assays were also used. To investigate the communication between HNSCC cells and cancer-associated fibroblasts (CAFs), we used either direct co-culture assays or supplementation with CAF-conditioned media.

**Results and Discussions**

Untargeted proteomics unraveled enhanced fatty acid oxidation (FAO) in CTX-R cells, as confirmed by higher oxygen consumption rates upon FA treatment and increased levels of the mitochondrial fatty-acyl-CoA transporter CPT1A, while showing higher levels of the FA transporter CD36 and a higher uptake of fluorescently labelled palmitate. Lipidomic analysis and BODIPY 493/503 staining revealed lower neutral lipid accumulation in CTX-R cells, while the fraction of monounsaturated FA was higher. RNA sequencing data from HNSCC PDTX confirmed deregulated lipid metabolism and identified a PPARα-related gene signature to orchestrate the resistance phenotype. Importantly, we showed an increased Erk1/2 phosphorylation, at least partly controlled by the dysregulated lipid metabolism, in CTX-R cells. Pharmacological inhibition of CPT1 and PPARα could overcome CTX resistance *in vitro* and *in vivo*. Finally, we documented that CAFs, unlike patient-matched healthy fibroblasts, exerted a protective role towards CTX in HNSCC cells, through a lipid-based metabolic communication between the two cell types.

**Conclusion**

We report a role for enhanced FAO to support CTX resistance in HNSCC and we highlight a metabolic cooperation between CAF and cancer cells, providing new potential therapeutic opportunities.

### EACR23-0511
### Inhibition of TGFβ1-activated NDRG1 leds to a reduction of cancer stem cells in triple-negative breast cancer.

© 2023 The Authors. Molecular Oncology is published by John Wiley & Sons Ltd on behalf of Federation of European Biochemical Societies

A. López Tejada[1], C. Griñán-Lisón[1], A. González-González[2], F.E. Cara[2], J.L. Blaya-Cánovas[2], A. Navarro-Ocón[2], J. Calahorra[2], J.A. Marchal[3], S. Granados-Principal[1]
[1]University of Granada- GENYO- Centre for Genomics an d Oncological Research- Pfizer/University of Granada/And alusian Regional Government, Biochemistry and Molecular Biology 2, Granada, Spain
[2]GENYO- Centre for Genomics and Oncological Research- Pfizer/University of Granada/Andalusian Regional Gover nment, Genyo, Granada, Spain
[3]University of Granada, Department of Human Anatomy and Embryology- Biopath ology and Regenerative Medicine Institute IBIMER, Granada, Spain

**Introduction**

Triple-negative breast cancer (TNBC) presents the lowest patient survival, high chemoresistance, metastases, and heterogeneity, mainly due to cancer stem cells (CSCs). *NDRG1* (N-myc downstream regulated gene 1) is either a metastasis suppressor or promoter in various types of cancer, but the mechanisms of its pleiotropy are still unclear. We reported that TGFβ1 induces NDRG1 to promote metastasis depending on the progression stage of TNBC cell lines, as well as its phosphorylation regardless of the progression stage of tumor cells, suggesting a deeper interplay between both molecules. As TGFβ1 is a known inducer of CSCs, NDRG1's role is hypothesized.

**Material and Methods**

TNBC cell lines from pleural effusion (MDA-MB-231, MDA-MB-436) and primary tumor (SUM159, BT549) were used. Changes in phospho(p)-NDRG1(Thr346) and NDRG1 were assessed by immunofluorescence in MDA-MB-231 cells treated or not with TGFβ1 (10ng/ml) for 48h. *NDRG1* gene was inhibited with siRNA (50nM, 48h) after stimulation with TGFβ1 (10ng/ml) for 8h (MDA-MB-231, MDA-MB-436) or 14 days (SUM159, BT549). CSCs' self-renewal was studied with mammospheres (MS) and soft-agar colony assays and different CSCs populations (ALDH1$^{+}$, CD44$^{+}$/CD24$^{-/low}$, and side population) by flow cytometry.

**Results and Discussions**

Confocal imaging in MDA-MB-231 cells showed that TGFβ1 enhanced the number of positive cells expressing NDRG1 and p-NDRG1(Thr346), which confirms our previous results of western-blot. In presence of TGFβ1, the formation of secondary MS was significantly decreased by *NDRG1* inhibition in all four cell lines compared to scramble control. Moreover, in SUM159 and MDA-MB-231, a reduction of tertiary mammosphere-forming efficiency was also seen. Soft-agar colony formation provided similar findings as only knockdown of NDRG1 with TGFβ1 led to a reduced number of colonies in all cell lines. Regarding CSC populations, ALDH1$^{+}$ population was reduced in all cell lines after NDRG1 knockdown with TGFβ1 compared with their negative controls. However, an evident diminution of CD44$^{+}$/CD24$^{-/low}$ population was only found in SUM159, whereas very little or no change was seen in MDA-MB-231 and MDA-MB-436, respectively. Similarly, the side population was modified only in SUM159.

**Conclusion**

Overall, our findings suggest that NDRG1 can be involved in the maintenance of different CSCs subpopulations in TNBC, depending on the tissue of origin of the cell lines (primary tumor or pleural effusion), and their grade of differentiation into a more aggressive phenotype induced by TGFβ1.

### EACR23-0517
### Unravelling the molecular landscape of Desmoplastic Small Round Cell Tumor

L. Lobo Selma[1], J. Olmedo[1], C. Rodríguez Martín[2], J. Alonso[2], J. Díaz Martín[1], R. Rodríguez[3], A.T. Amaral[1], C. Salguero Aranda[1], E. de Álava[1]
[1]Institute of Biomedicine of Seville IBiS, Department of Normal and Pathological Cytology and Hist ology, Seville, Spain
[2]Institute for Rare Disease Research IIER- Carlos III Healt h Institute ISCIII, Department of Human genetics, Madrid, Spain
[3]Sarcomas and Experimental Therapeutics Laboratory- Ins titute for Health Research of the Principality of Asturias IS PA- Hospital Universitario Central de Asturias, Human genetics, Oviedo, Spain

**Introduction**

Desmoplastic small round cell tumor (DSRCT) is a rare and aggressive sarcoma whose molecular hallmark is a chromosomal translocation that generates a gene fusion (GF) between *EWSR1* and *WT1*, a chimeric transcription factor (TF) which is the main driver of the malignant process. Unravelling functional interactions with other cooperating TF is essential to gain deeper knowledge of the biology of this tumor.

**Material and Methods**

Transformed Human Mesenchymal Stem Cells (hMSC) were transduced with two lentiviral plasmids harbouring the most prevalent GF variants of *EWSR1::WT1* fused to the hemagglutinin epitope. GF expression was confirmed by western blot and q-RT-PCR. RNAs were used to perform a ClarionS expression array and analysed using TAC4.0 software.

JN-DSRCT-1 cells expressing the cas9-GFP genes were infected with a library of lentiviral guideRNA particles targeting 18479 TFs to perform a screening by CRISPR. Next-generation sequencing was done after antibiotic selection and analysed with Galaxy software.

Finally, data from the GF ectopic expression, TFs CRISPR screening and inducible short hairpin RNAs model targeting the GF (public database, GSE180031) were integrated to identify candidate TFs.

Cell proliferation (MTT assay), cell cycle (propidium iodide staining) and clonogenic assays are currently undergoing through small interference RNAs (siRNAs) targeting the selected genes.

**Results and Discussions**

Data from GF ectopic expression in hMSC showed 3370 upregulated and 261 downregulated genes. Genes such as *EWSR1* and *WT1*, and the well-known target *LRRC15*, were significantly overexpressed in the presence of the GF. TFs screening by CRISPR revealed 515 depleted genes and 1460 enriched genes.

Data integration among the different analyses identified potential gene candidates such as *SNAI2*, implicated in epithelial-mesenchymal transition, and *MYBL2*, involved in cell cycle regulation. siRNA preliminary results confirmed that SNAI2 and MYBL2 expression are GF-dependent, as their expression is meaningfully reduced after WT1 siRNA

© 2023 The Authors. Molecular Oncology is published by John Wiley & Sons Ltd on behalf of Federation of European Biochemical Societies treatment. Interestingly, cell proliferation and clonogenic capacity were significantly impaired when cells were transfected with SNAI2 and MYBL2 siRNAs at 40 nM, indicating the important role of these TFs in DSCRT cell biology.

**Conclusion**

Preliminary results show that *MYBL2* and *SNAI2* genes play a key role in DSRCT malignancy. Understanding of the regulation of these genes by the GF and how they relate to cell survival is instrumental in the design of new therapeutic approaches.

### EACR23-0523
### FADS1/2-mediated lipid metabolic reprogramming drives ferroptosis sensitivity in metastatic triple negative breast cancer

*N. Lorito[1], A. Subbiani[1], A. Smiriglia[1], M. Bacci[1], I. Meattini[1], P. Chiarugi[1], A. Bachi[2], D.L. Longo[3], A. Morandi[1]*

*[1]University of Florence, Department of Experimental and Clinical Biomedical Sciences, Florence, Italy*

*[2]IFOM Foundation, The FIRC Institute of Molecular Oncology, Milan, Italy*

*[3]Institute of Biostructures and Bioimaging IBB, Italian National Research Council CNR, Turin, Italy*

**Introduction**

Triple negative breast cancer (TNBC) is the most lethal subtype lacking the expression of hormone receptors and HER2. Consequently, TNBC patients have limited therapeutic options and show high grade of recurrence and distant metastasis. Metastatic TNBC cells exhibit a high metabolic flexibility depending on their metastatic potential and site of metastasis. Importantly, fatty acid (FA) metabolism is crucial in TNBC aggressiveness and therefore exploitable for therapeutic and/or diagnostic purposes.

**Material and Methods**

We investigated the lipid metabolic dependencies underlying the differential metastatic capacities of a panel of murine and human TNBC cell lines, ranging from non-invasive to highly metastatic. Genome-wide profiling and subsequent gene set enrichment analysis (GSEA) together with an array of complementary techniques including Seahorse analysis and confocal microscopy revealed enhanced *de novo* FA biosynthesis in the highly metastatic models when compared to the less aggressive cells.

**Results and Discussions**

Since no significant difference between poorly and metastatic cells was observed when we comparatively assessed cell survival changes induced by agents targeting lipid metabolism, we postulated that the increased FA synthesis could be relevant as it alters lipid composition and FA complexity. Indeed, metastatic cells showed increased expression of the key enzymes of FA desaturation (FADS1 and 2), that could be used as a metabolic vulnerability by impacting the intracellular availability of polyunsaturated FA (PUFA) that represent essential peroxidation substrates promoting ferroptosis, a form of iron-dependent cell death. Accordingly, we observed that the more aggressive models were sensitive to the ferroptosis induction mediated by both RSL3 and Erastin and displayed increased reactive oxygen species (ROS) levels and accumulation of toxic lipid peroxides. Crucially, targeting FADS1/2 prevented the drug-induced ferroptosis, whereas the inhibition of stearoyl-CoA desaturase 1 (SCD1) failed since this enzyme catalyzes the rate-limiting step in the production of monounsaturated FA (MUFA). To note, altering PUFA/MUFA ratio by exogenous MUFA administration prevented drug-induced ferroptosis in the metastatic models.

**Conclusion**

Collectively, our data suggest that FADS1/2 are associated with lipid peroxidation and iron-dependent cell death and will offer novel potential predictive biomarkers that can be also used for synthetic lethality therapeutic approaches in TNBC.

### EACR23-0524
### Trop-2 induces apoptosis via transcriptional activation of TRAIL, FAS/FASL, CD40/TNFRSF5

*S. Alberti[1], M. Trerotola[2], A. Moschella[3], E. Guerra[2]*

*[1]University of Messina, BIOMORF, Messina, Italy*

*[2]Center for Advanced Studies and Technology University of Chieti, Department of Medical- Oral and Biotechnological Sciences, Chieti Scalo, Italy*

*[3]University of Messina, Biomedical Sciences, Messina, Italy*

**Introduction**

Trop-2 is a transmembrane $Ca^{2+}$ signal transducer that is broadly upregulated in human cancers, where it drives tumor growth and metastasis. However, this does not occur in all tumor types, as Trop-2 is associated to better disease outcome of distinct subtypes, e.g. of breast and lung cancers. A prototypic pattern of Trop-2 expression is shown in normal epithelia by non-proliferating normal cells, from the supra-basal to the corneum layer of multistratified epithelia. The corresponding expression gradient parallels induction of programmed cell death in skin keratinocytes. This led us to think that Trop-2 could act as trigger of apoptosis.

**Material and Methods**

We assessed NS-0 myeloma Trop-2 transfectants for apoptosis induction by dynamic cell morphometry, membrane blebbing and cell fragmentation. Early time points were assessed for phosphatidyl-serine membrane exposure. Late time points were assessed for propidium iodide permeability, nuclear pyknosis / karyorrhexis and cell death fraction. DNA fragmentation was quantified by TUNEL and DNA laddering.

**Results and Discussions**

Trop-2 expression induced a dramatic increase of apoptotic cell death. Transcriptomic analysis at early time points of apoptosis induction indicated overexpression of TRAIL, FAS/FASL and CD40/TNFRSF5 transmembrane death receptors. This paralleled upregulation of downstream death-inducing signaling complex components and of executioner Caspases 8 and 3, suggesting Trop-2 as a trigger of extrinsic apoptosis pathways. Apoptosis induction translated into diminished tumor growth in vivo, which was shown to be proportional to Trop-2 expression levels.

© 2023 The Authors. Molecular Oncology is published by John Wiley & Sons Ltd on behalf of Federation of European Biochemical Societies

**Conclusion**
Trop-2 is an apoptotic inducer of expressing cells. Trop-2 apoptosis induction paralleled stimulation of NS-0 cell growth and of defence mechanisms from apoptosis, inhibitory transcription factors, Rb-binding protein, survivin, Myd-118. These findings suggests a previously unappreciated balance between stimulation of cell growth and of death-triggering mechanisms by Trop-2, and may pave the way for selective anticancer therapy approaches in Trop-2-expressing cancers.

### EACR23-0529
### Stresses induction of BCL2A1 promotes metastatic aggression in ovarian cancer

P. Jiao[1], M.M.H. Yung[1], M.K.Y. Siu[1], R. Liang[1], A.N.Y. Cheung[2], H.Y.S. Ngan[1], D.W. Chan[1,3], K.K.L. Chan[1]
[1]The University of HongKong, Department of Obstetrics & Gynaecology, HONGKONG, Hong Kong- China
[2]The University of HongKong, Department of Pathology, HongKong, Hong Kong- China
[3]The Chinese University of Hong Kong, School of Medicine-, ShenZhen, Hong Kong- China

**Introduction**
Ovarian cancer (OvCa) is one of the most lethal female malignancies. Recent evidence has suggested that adaptation of metastatic OvCa cells to tumor microenvironmental stresses such as hypoxia and nutrient deprivation is critical for promoting malignant metastatic progression. Furthermore, autophagy has been implicated in the survival mechanism for stressed cells, which are found to be correlated with early invasion of malignancies. Our previous cDNA microarray profiling analysis revealed that BCL2A1, a BCL2 family member, was upregulated in hypoxic OvCa cells compared with normoxic counterpart. Importantly, it has been reported that BCL2A1 was also involved in autophagy. Here, we sought to characterize the role of BCL2A1 in mediating the metastatic potential of OvCa cells and the autophagic flux in stressed conditions.
**Material and Methods**
Different physiological stresses were conducted on OvCa cells. RT qPCR, Western blot and IHC analyses were applied to investigate the activities of BCL2A1 and associated signalings. TEM (transmission electron microscope) and flow cytometry were performed to evaluate the process of autophagy.
**Results and Discussions**
Our findings showed that BCL2A1 was induced in OvCa cells at earlier phase of physiological stresses including hypoxia, glucose starvation, anoikis and ascitic fluid, followed by a gradual reduction, indicating an auto-regulation system in OvCa cells for BCL2A1 upon stresses. Functional studies indicated that augmented BCL2A1 in OvCa cells enhanced cell migration and invasion under hypoxic or ascitic fluid treatments. IHC analysis additionally proved the clinical relevance that BCL2A1 and p-IκB were frequently upregulated in metastatic OvCa, inferring NFκB/BCL2A1 axis is required for OvCa metastatic progression. Further investigations disclosed that overexpression of HIF-1α led to concomitant increase in p-IκB and BCL2A1. By contrast, BCL2A1 was not apparently activated when HIF-1α was stimulated under hypoxia condition along with knockdown of NFκB p65, alluding that HIF-1α is conceivably involved as the upstream regulator of BCL2A1 induction via NFκB signaling. Consistently, our data validated that stresses induced BCL2A1 was able to trigger autophagy in OvCa cells by interacting with Beclin1 and facilitating the turnover of LC3, leading to cell survival of OvCa cells in stressed conditions.
**Conclusion**
Taken together, our results provide a scientific basis for exploring NFκB/BCL2A1 signaling axis as a therapeutic target to impede metastatic dissemination of OvCa.

### EACR23-0530
### A low anticoagulant bovine heparin fraction prevents lung metastasis in mice by interfering with microemboli formation

J.M. Motta[1], C. Roberto-Fernandes[1], K.V.A. Micheli[1], P.A.S. Mourão[1], M.S. Pereira[1]
[1]Federal University of Rio de Janeiro, Medical Biochemistry Institute, Rio de Janeiro, Brazil

**Introduction**
Metastasis is the leading cause of death in cancer patients. During metastatic dissemination, circulating tumor cells need to survive in the bloodstream and their interaction with platelets through P-selectin-ligand seems to be crucial. It has been demonstrated that heparin (from porcine mucosa, porcine UFH), a glycosaminoglycan composed by repetitive disaccharides of uronic acid and glucosamine, may interfere with P-selectin interaction. Recently our research group purified by ion exchange chromatography a fraction of bovine heparin displaying very low anticoagulant potential, named LABH (~20 IU/mg LABH x ~200 IU/mg porcine UFH x ~100 IU/mg bovine UFH). The advantage of bovine UFH and, especially of LABH, is their reduced risk of bleeding side effect. Our aim in this work was to investigate the potential use of LABH as an antimetastatic agent *in vivo* and evaluate *in vitro* its ability to interfere with tumor cell-platelet interaction, tumor cell binding to P-selectin as well as determine coagulation pathway contribution.
**Material and Methods**
For this, we challenged 8-12 weeks C57Bl/6 mice with 4 mg/kg of porcine UFH, bovine UFH or LABH followed by intravenous injection of B16F10 cells (murine melanoma cell line). After 21 days, lungs were analyzed and metastatic foci were counted.
**Results and Discussions**
Metastatic foci were significantly reduced when animals were pretreated with heparins (50-70 foci in control mice and 10-15 in treated mice). In other attempt, isolated platelets from healthy volunteers were incubated for 1h with MV3 cells (human melanoma cell line) *in vitro* in the presence or absence of heparin. All three heparins tested were efficient in blocking MV3-platelet interaction in a dose-response manner, but LABH required higher doses than porcine and bovine UFH. When analyzing the direct binding of U937 cells (human lymphoma cell line) to immobilized P-selectin, we could observe that porcine and bovine UFH inhibited at the same level (~ 60% of inhibition with 100 μg/mL), while LABH was less effective (~ 45% of inhibition with 100 μg/mL). Tumor cells-induced platelet aggregation was also analyzed. Data

© 2023 The Authors. Molecular Oncology is published by John Wiley & Sons Ltd on behalf of Federation of European Biochemical Societies showed that porcine UFH, bovine UFH and LABH reduced the aggregation at 2, 4 and 100 μg/mL, respectively.
**Conclusion**
Collectively our results demonstrate that despite presenting distinct patterns of sulfation and anticoagulant potential, all heparins significantly reduced lung metastasis, platelet-tumor cell adhesion and P-selectin binding with minimal contribution of coagulation pathways.

### EACR23-0534
### Exploiting CDK4/6 inhibitors-induced senescence in a synthetic lethal approach to treat advanced prostate cancer

*J. Brandariz[1], D. Aguilar[1], S. Cordoba-Terreros[1], L. de Llobet Cucalon[1], A. Sole Casaramona[1], A. Salca[1], V. Esquefa Fauro[1], S. Arce-Gallego[1,2], J. Mateo[1,3], N. Herranz[1,2]*
*[1]Vall d'Hebron Institute of Oncology, Prostate Cancer Translational Research Group, Barcelona, Spain*
*[2]Vall d'Hebron Institute of Research, Vall d'Hebron Hospital Campus, Barcelona, Spain*
*[3]Vall d'Hebron University Hospital, Medical Oncology, Barcelona, Spain*

**Introduction**
Metastatic prostate cancer (PC) is a lethal disease and an unmet medical need. Cyclin-dependent kinases 4 and 6 (CDK4/6) inhibitors are approved in breast cancer and are currently being tested in clinical trials in PC. Since CDK4/6i potent cytostatic effects associate with senescence, their combination with senolytic drugs is being explored. We hypothesize that CDK4/6i-induced senescence could facilitate tumor cell survival towards later development of advanced PC and that selective killing of senescent cells is beneficial.
**Material and Methods**
We explored the emergence of a senescence phenotype after continuous/intermittent CDK4/6i in PC cell lines (LNCaP, 22Rv1, PC3 and DU145) by assessing cell proliferation (CCK8 and BrdU), cell viability (Annexin/PI), senescence-associated β-galactosidase (SAβG) activity and RNAseq. In-vivo, CDK4/6i-induced senescence was characterized by SAβG activity and protein expression of Ki67 (proliferation), CC3 (apoptosis) and p16. Killing capacity of senolytics was assessed by calculating the senolytic index (SI; IC50 of control cells/IC50 of senescent cells). Migratory capacity was assessed using Boyden chamber assay.
**Results and Discussions**
Both in-vitro and in in-vivo, CDK4/6i (7 days) induced a strong and stable proliferation arrest not compromising cell viability and correlated with a robust induction of senescence features (SAβG activity, p16 upregulation, without a pro-inflammatory SASP). CDK4/6i-induced senescent cells were highly sensitive to the senolytics ARV-825 (LNCaP SI=11.03; 22Rv1 SI=7.11) and Navitoclax (LNCaP SI=7.01). Results were validated in LNCaP xenografts. Intermittent CDK4/6i in breast cancer patients is common due to side effects. However, in our models, CDK4/6i withdrawal resulted in reversal of the senescent phenotype and a significant decreased sensitivity to senolytic drugs. Interestingly, we observed that, once cells reversed, re-induction of senescence was less efficient with each round of CDK4/6i re-exposure. In addition, reversed/re-induced cells showed a higher migratory capacity than control counterparts.
**Conclusion**
CDK4/6 inhibition results in senescence induction in prostate cancer models; when coupled with senolytic therapies, the result is tumor cell kill. However, the senescent phenotype is reversible upon drug withdrawal and re-induction of senescence upon drug re-exposure is poor. Hence, our results highlight the importance of eliminating PC senescent cells at first occasion and could lead to repurposing CDK4/6 inhibition in prostate cancer.

### EACR23-0539
### TRIM33 is a Novel Regulator of Estrogen Receptor Alpha in Breast Cancer

*B. Romo[1], A. Kettenbach[2], L. Cressey[3], B. Brauer[3], T. Miller[4]*
*[1]Geisel School of Medicine at Dartmouth, Molecular and Systems Biology, Lebanon, United States*
*[2]Geisel School of Medicine at Dartmouth, Department of Biochemistry and Cell Biology- Dartmouth Cancer Center, Lebanon, United States*
*[3]Geisel School of Medicine at Dartmouth, Department of Biochemistry and Cell Biology, Lebanon, United States*
*[4]Geisel School of Medicine at Dartmouth, Molecular and Systems Biology-Dartmouth Cancer Center, Lebanon, United States*

**Introduction**
Approximately 70% of breast cancers express estrogen receptor alpha (ER). Such patients are commonly treated with adjuvant endocrine therapies, but 1/3 eventually experience disease recurrence that, while initially managed with further endocrine and tumor-targeted agents, ultimately proves fatal. A potential explanation for endocrine resistance is alteration of coregulatory proteins that affect ER activity.

**Material and Methods**
Proteomic interactome studies used the TurboID biotin ligase system. ER+ breast cancer cells were stably transfected with fusion constructs encoding FLAG-TurboID-Control and FLAG-TurboID-ER constructs. Cells were supplemented ±100 uM biotin ± 1 nM 17b-estradiol for 1 h, and lysates were subjected to streptactin pulldown for mass spectrometry-based proteomics. For TRIM33 functional assays, stable cell lines were generated by either shRNA knockdown or constitutive overexpression of TRIM33, and cells were analyzed by cycloheximide pulse-chase assay, RT-qPCR, and growth assay.
**Results and Discussions**
Proteomic profiling revealed enrichment for 93 and 68 proteins that interacted with ER (FLAG-TurboID-ER) compared to control (FLAG-TurboID-Control) under hormone-depleted and E2-treated conditions, respectively; 23 proteins including TRIM33 were enriched in both conditions. In analyzing 3 ER interactome datasets, TRIM33, which has E3 ubiquitin ligase activity, was common to all datasets. TRIM33 knockdown decreased ER stability in both the presence and absence of the ER ligand 17b-estradiol, suggesting that TRIM33 prevents ER degradation. Gene expression analysis of ER-inducible

© 2023 The Authors. Molecular Oncology is published by John Wiley & Sons Ltd on behalf of Federation of European Biochemical Societies transcripts showed that TRIM33 knockdown reduced sensitivity to 17b-estradiol. Upon knockdown of TRIM33, cells showed reduced sensitivity to the growth-stimulating effects of 17b-estradiol. TRIM33 overexpression increased ER stability without altering transcriptional response to 17b-estradiol.

**Conclusion**

ER interactome profiling revealed TRIM33 as a novel 17b-estradiol-inducible interactor. TRIM33 regulates ER activity as evidenced by its ability to stabilize ER. TRIM33 loss reduced ER transcriptional activity and growth response to 17b-estradiol. These findings promote targeting of TRIM33 to inhibit ER-driven breast cancer cell growth. Further investigation is warranted on the role of TRIM33 in the development of endocrine resistance, specifically in the setting of hormone-independent growth due to the observed increase in ER protein levels.

### EACR23-0540

### The novel therapeutic Diiminoquinone exhibits anticancer effects on human prostate cancer cells in 2D and 3D in vitro models

*A. Yehya[1], A. A Monzer[2], O. Hadadeh[1], K. Wakimian[1], F. Ghamlouche[1], N. Saheb[3], A. Tawil[3], H. Gali-Muhtasib[4], D. Mukherji[5], A. El-Hajj[6], W. Abou-Kheir[1]*

*[1]American University of Beirut, Department of Anatomy- Cell Biology and Physiological S ciences, Beirut, Lebanon*

*[2]American University of Beirut, Department of Biology, Beirut, Lebanon*

*[3]American University of Beirut Medical Center, Department of Pathology and Laboratory Medicine, Beirut, Lebanon*

*[4]American University of Beirut, Department of Biology and Center for Drug Discovery, Beirut, Lebanon*

*[5]American University of Beirut Medical Center, Department of Internal Medicine- Division of Hematology/ Oncology, Beirut, Lebanon*

*[6]American University of Beirut Medical Center, Department of Surgery- Division of Urology, Beirut, Lebanon*

**Introduction**

Prostate cancer (PC) is a leading cause of cancer-related morbidity and mortality in men worldwide. Despite its curability with castration, radiation, and hormonal therapies, a significant proportion of patients with high-risk localized disease and all patients with advanced disease at diagnosis experience progression to metastatic castration-resistant prostate cancer (mCRPC). This has been mainly attributed to the presence of a subpopulation of cancer stem cells (CSCs) within the tumor bulk. So far, there have been no effective drugs to target CSCs. The identification of novel therapeutics that can simultaneously target both tumor bulk cells and CSCs remains a major hurdle for successful treatment in cancer patients. Diiminoquinone (DIQ), a novel quinone derivative, has shown promising effects in targeting CSCs in colon cancer. Yet, limited research is available on the effect of DIQ in eradicating CSCs in PC. Herein, we aim to investigate the anticancer potential of DIQ on PC in two-dimensional (2D) and three-dimensional (3D) *in vitro* culturing models.

**Material and Methods**

The cytotoxic effect of DIQ on PC3 and DU145 PC cell lines was determined using the 3-(4,5 dimethylthiazol-2-yl)-2,5-diphenyltetrazolium bromide (MTT) and trypan blue exclusion assays. In addition, the 3D sphere-forming assay was performed to examine the effect of DIQ in targeting the enriched population of PC stem/progenitor cells. Besides, 3D organoids were established from PC cell lines and from fresh tissue samples from different stages of PC. The samples were obtained from consented patients undergoing radical prostatectomy at the American University of Beirut Medical Center (AUBMC) according to the appropriate Institutional Review Board (IRB) approval guidelines. This model was used to assess the potency of DIQ on the organoids' growth, which was evaluated by quantifying the number of organoids formed (OFC) and calculating their average size (diameters).

**Results and Discussions**

Our results showed that DIQ significantly reduced cell proliferation and viability in PC3 and DU145 PC cell lines. DIQ also attenuated the sphere-forming unit (SFU) and the size of PC3 and DU145 prostatospheres at sub-toxic doses. Potently, DIQ displayed a significant reduction in both the number and the size of the PC cell line- and patient-derived organoids.

**Conclusion**

This study provides promising clues for the use of the novel anticancer therapeutic 'DIQ' in targeting CSCs, findings that may have promising therapeutic implications for PC patients.

### EACR23-0557

### EMP3 facilitates the EGFR/CDK2 signaling axis in glioblastoma

*A. Krauß[1,2,3], A.A. Martija[1,3,4], L. Doth[1,3,4], C. Herold-Mende[5], A. von Deimling[1,3], S. Pusch[1,3]*

*[1]German Cancer Research Center DKFZ, Clinical Cooperation Unit Neuropathology, Heidelberg, Germany*

*[2]Heidelberg University, Medical Faculty, Heidelberg, Germany*

*[3]Heidelberg University Hospital, Department of Neuropathology, Heidelberg, Germany*

*[4]Heidelberg University, Faculty of Biosciences, Heidelberg, Germany*

*[5]University Hospital Heidelberg, Division of Neurosurgical Research - Department of Neuro surgery, Heidelberg, Germany*

**Introduction**

Epithelial membrane protein 3 (EMP3) is a transmembrane protein that is highly expressed in IDH wild-type glioblastoma (IDH-wt GBM). EMP3 overexpression is associated with shorter patient survival, and it is therefore proposed to facilitate tumor progression. However, it remains unclear how EMP3 influences oncogenic programs responsible for cell survival. The aim of this study was to identify signaling pathways affected by EMP3 and how it contributes to oncogenic characteristics of IDH-wt GBM cells.

**Material and Methods**

© 2023 The Authors. Molecular Oncology is published by John Wiley & Sons Ltd on behalf of Federation of European Biochemical Societies

Transcriptomic, phosphoproteomic, and phenotypic analyses were performed on control and EMP3 CRISPR/Cas9-knockout (KO) DK-MG and U-118 cells to identify pathways affected by the loss of EMP3. The phenotypic impact of EMP3 was further evaluated in proliferation assays using patient-derived GBM spheroids transduced with non-targeting and EMP3-targeting short hairpin RNAs (shRNAs). Additionally, we assessed whether treatment with a CDK2 inhibitor synergizes with EMP3 knockdown in vitro.

**Results and Discussions**

Through transcriptomic analysis we discovered 125 cell cycle-related genes that are significantly downregulated in EMP3 KOs. Gene set enrichment analysis identified EGFR gene signatures indicating that EMP3 KO negatively affects EGFR signaling. EMP3 KO U-118 cells showed greater susceptibility to osimertinib, an EGFR inhibitor, further confirming EGFR inhibition upon EMP3 KO. Phosphoproteomics revealed an increase of dephosphorylated CDK2 targets in EMP3 KOs compared to controls, suggesting that CDK2 is likewise inhibited in EMP3 KOs. Given that CDK2 is a known downstream target of EGFR, we hypothesized that EMP3-dependent inhibition of EGFR culminates in CDK2 inactivation. To confirm whether EMP3 facilitates EGFR/CDK2 signaling, phenotypic effects of shRNA-mediated EMP3 knockdown in patient-derived EGFR-high versus EGFR-low GBM spheroids were compared. Inhibition of EMP3 in an EGFR-high GBM spheroid cell line led to reduced proliferation compared to the control. Additionally, treatment with K03861, a CDK2 inhibitor, showed a synergistic effect with EMP3 knockdown in another EGFR-high GBM spheroid line, but not in EGFR-low spheroids.

**Conclusion**

Our results demonstrate that EMP3 supports the EGFR/CDK2 signaling axis in GBM. Loss of EMP3 inhibits EGFR signaling and inactivates CDK2 and downstream targets. These molecular effects reduce proliferation and increase sensitivity to drugs targeting EGFR and CDK2. Our results suggest that EMP3 may be a potential target for GBM therapy.

### EACR23-0566

### Creatine kinase B depletion and cyclocreatine treatment affect proliferation, migration and metastasis of osteosarcoma cells

*J. Pokludová[1,2], K. Říhová[1,2], L. Knopfová[1,2], P. Beneš[1,2]*

*[1]Masaryk University- Faculty of Science, Department of Experimental Biology, Brno, Czech Republic*

*[2]St. Anne´s University Hospital, International Clinical Research Centre, Brno, Czech Republic*

**Introduction**

Creatine kinase B (CKB) is an important enzyme involved in the cellular energy metabolism. However, this protein also has other functions, including regulation of the cell cycle or immune system response. Altered expression of CKB has been observed in various cancer types such as breast cancer, colorectal cancer, or metastatic melanoma. Nevertheless, the function of CKB in regulation of proliferation, chemosensitivity or metastatic activity of osteosarcoma cells is still not fully understood. The aim of this study is to clarify the role of CKB in progression of osteosarcoma, the most common type of primary bone cancer.

**Material and Methods**

We derived highly metastatic SAOS-2 LM5 osteosarcoma cells with depleted expression of *CKB*. Later, we examined the effect of CKB deficiency on growth, colony-forming capacity, migration, metastasis, amount of ATP and energy metabolism of these osteosarcoma cells. Similar experiments were conducted with two other osteosarcoma cell lines (143B and Dunn LM8). In this case, however, cyclocreatine (CCr), the most effective creatine analogue, was used to inhibit CKB activity.

**Results and Discussions**

Depletion of CKB expression decreased cell migration as well as metastatic potential in immunodeficient mice. On the protein level we found altered expression of different genes related to cell migration and the epithelial-mesenchymal transition (fibronectin, Twist, p21 or p-Akt) between SAOS-LM5 *CKB* KO cells and control cells. CKB depletion did not affect proliferation rate of SAOS-2 LM5 cells, their chemosensitivity or cellular metabolism. Treatment with creatine kinase inhibitor CCr inhibits both cell proliferation, clonogenic capacity and migration suggesting that, besides CKB, other creatine kinases are important regulators of osteosarcoma growth and motility.

**Conclusion**

Our results showed that CKB modulate migration and metastasis of osteosarcoma cells and that other creatine kinases regulate growth and motility of osteosarcoma cells.

*This project was supported by European Regional Development Fund - Project ENOCH (No. CZ.02.1.01/0.0/0.0/16_019/0000868).*

### EACR23-0580

### MYCN mediates ferroptosis sensitivity in neuroblastoma

*H. Alborzinia[1], Z. Chen[2], U. Yildiz[3], F.C.E. Vogel[4], A. Schulze[4], A. Trumpp[1], J.P. Friedmann Angeli[2]*

*[1]Heidelberg Institute for Stem Cell Technology and Experi mental Medicine- Heidelberg- Germany, Stem Cells and Cancer, Heidelberg, Germany*

*[2]Rudolf Virchow Zentrum RVZ- University of Würzburg, Center for Integrative and Translational Bioimaging, Würzburg, Germany*

*[3]European Molecular Biology Laboratory, Genome Biology Unit, Heidelberg, Germany*

*[4]German Cancer Research Center DKFZ, Division of Tumor Metabolism and Microenvironment, Heidelberg, Germany*

**Introduction**

MYC is a family of oncogenes deregulated in approximately 50% of human tumors, including lymphomas, lung, breast, neuroblastomas, and melanomas. However, specific therapies that efficiently target and eliminate *MYC(N)* overexpressing cancer cells are unavailable. We have recently shown that the oncogene MYCN sensitizes neuroblastoma cells to ferroptosis. Our findings disclose a yet-unaccounted mechanism of

© 2023 The Authors. Molecular Oncology is published by John Wiley & Sons Ltd on behalf of Federation of European Biochemical Societies selective ferroptosis induction that might be explored as a therapeutic strategy for high-risk neuroblastoma and potentially other *MYCN*-amplified entities.

**Material and Methods**

Genome-wide and single-cell transcriptomics CRISPR-activation (CRISPRa) screens. Orthotopic *in-vivo* models of neuroblastoma.

**Results and Discussions**

We identify novel regulators of ferroptosis and detect shared transcriptional signatures and states regulating ferroptosis hypersensitivity. Specifically, we identify the low-density lipoprotein receptor (LDLR) related protein 8 (LRP8, also known as APOER2) as a critical bottleneck in selenium/selenocysteine metabolism in *MYCN*-amplified entities. We found that the MYCN-associated vulnerability is due to the incompatibility of *MYCN*-amplified cells to activate alternative selenium/selenocysteine pathways, such as SLC7A11, required to support selenoprotein translation.

**Conclusion**

Our work demonstrates that different selenium/selenocysteine acquisition pathways are associated with unique metabolic consequences that can be accompanied by severe metabolic disruption in specific oncogenic contexts. The identification of these metabolic vulnerabilities offers unanticipated opportunities to specifically, selectively, and safely induce ferroptosis for therapeutic benefit.

### EACR23-0585

### Establishment of murine hepatocellular carcinoma unravels key reprogramming of glutamine metabolism which correlates with tumor behavior

*V. Tambay*[1], *V.A. Raymond*[1], *M. Bilodeau*[1,2]

[1]*Centre de recherche du Centre Hospitalier de l'Université de Montréal, Laboratoire d'hépatologie cellulaire, Montréal, Canada*

[2]*Université de Montréal, Département de médecine, Montréal, Canada*

**Introduction**

Metabolic reprogramming has become a key area of interest in the pathophysiology of hepatocellular carcinoma (HCC). Glutamine (gln) is a key metabolite involved in hepatocellular metabolism and has been linked with cancer development. This study aimed to evaluate the reprogramming of gln metabolism during experimental hepatocarcinogenesis.

**Material and Methods**

Hepatocarcinogenesis was induced in C57BL/6 mice by intrasplenic injection of highly tumorigenic murine HCC Dt81Hepa1-6 cells. To study the progression of HCC over time, whole livers were collected 3.5, 7, 14, 21, or 28 days after injection. Then, whole-liver gln and glutamate (glu) were quantified using targeted LC/MS metabolomics. A screening of gln-associated gene expression was performed by qPCR.

**Results and Discussions**

During hepatocarcinogenesis, whole-liver gln decreased ($p<0.05$) whereas glu increased ($p<0.001$) over time. The resulting gln/glu ratio decreased from 2.12±0.12 (3.5 days) to 0.36±0.12 (28 days; $p<0.0001$), highlighting that metabolic reprogramming in expanding HCC tumors is characterized by increased gln breakdown and decreased gln synthesis. Key gln metabolism-related genes were altered over the course of HCC progression. Gln synthetase (GS) decreased over time, from 123.1±39.5 arbitrary units (AU) at 3.5 days to 70.9±14.4AU at 28 days ($p<0.05$). Kidney-type glutaminase (GLS1) increased from 3.2±0.7 to 29.1±8.5AU ($p=0.065$). Liver-type glutaminase (GLS2), inversely, decreased from 372.5±60.7 to 145.3±61.4AU ($p<0.05$). Given GLS1 is a higher affinity glutaminase than GLS2, the upregulation of GLS1 in tumor-bearing livers could represent the dependence of HCC on rapid gln metabolism. GS and GLS2 expression positively correlated with gln/glu ratio (respectively $R^2=0.43$, $p<0.001$ and 0.37, $p<0.001$). GLS1, however, negatively correlated with gln/glu ratio ($R^2=0.41$, $p<0.001$), suggesting that increased gln breakdown is linked to GLS1. In HCC livers, GLS1 and GLS2 were negatively correlated ($R^2=0.55$, $p<0.001$). GLS2 positively correlated with albumin ($R^2=0.66$, $p<0.001$), a marker of hepatocellular differentiation. GLS1 positively correlated with AFP ($R^2=0.86$, $p<0.001$), EpCAM ($R^2=0.91$, $p<0.001$), two biomarkers of HCC, as well as tumor burden ($R^2=0.78$, $p<0.001$).

**Conclusion**

In conclusion, metabolic reprogramming in HCC encompasses major alterations in gln metabolism. Gln may be a limiting nutrient in HCC tumors, which could be explained by its increased breakdown. Importantly, HCC tumors express a switch in glutaminase expression from GLS2 to GLS1.

### EACR23-0587

### Targeting cyclin-dependent kinase 9 to control lethal prostate cancer

*A. Gondane*[1], *S. Yalala*[1], *S. Pallasaho*[1], *J. Liang*[1], *H. Itkonen*[1]

[1]*University of Helsinki, Department of Biochemistry and Developmental Biology, Helsinki, Finland*

**Introduction**

Prostate cancer is the most common cancer in males and the disease is driven by the nuclear hormone transcription factor, androgen receptor. Anti-androgens initially halt the progression of the disease; however, many patients develop castration-resistant prostate cancer (CRPC), a disease with no curative treatment options. In the CRPC-setting, pro-proliferative transcriptional networks are sustained despite the anti-androgen therapy, and effective means to suppress these networks should block tumor growth. Cyclin-dependent kinase 9 (CDK9) phosphorylates RNA polymerase II to promote transcription of protein-encoding genes in all of our cells. Small molecule inhibitors targeting CDK9 halt the proliferation of cancer cells, and these compounds have gained significant success in clinical trials. However, all cells depend on transcription, and it is therefore not clear why cancer cells are so sensitive against compounds targeting CDK9: understanding this will enable identification of the patients that most likely benefit from CDK9 inhibitors and support the design of rational combinatoric treatment strategies.

**Material and Methods**

We have used multiomic-profiling to understand how both androgen-dependent and CRPC cells respond to depletion

© 2023 The Authors. Molecular Oncology is published by John Wiley & Sons Ltd on behalf of Federation of European Biochemical Societies of CDK9 activity using highly selective probe compounds and clinically-relevant drugs.

**Results and Discussions**

Prostate cancer cells respond to decreased CDK9 activity by rewiring metabolic, transcriptional-, splicing- and proteomic-networks. These changes reveal both adaptive and detrimental responses, and identify actionable combinatoric lethal targets. Integrating genetic information from the patient's tumor may enable discovery of actionable synthetic lethal interactions.

**Conclusion**

Compounds targeting CDK9 elicit comprehensive rewiring of prostate cancer cells and provide a candidate treatment strategy against CRPC.

### EACR23-0591
### Green Tea (Camellia sinensis) and Olive (Olea europaea L.) Leaf Extracts for Cancer Chemoprevention: The Tumor Suppressor p53 as a Potential Molecular Target

*D. Ferraz[1], R. Santos[1], E. Dutra[1], T.A. Santos[1], H. Pessoa[1], L. Zago[1], J. Silva[2], L. Rangel[3]*
*[1]Rio de Janeiro State University, Nutrition Institute, Rio de Janeiro, Brazil*
*[2]Federal University of Rio de Janeiro, National Institute of Science and Technology for Structural Biology and Bioimaging, Rio de Janeiro, Brazil*
*[3]Federal University of Rio de Janeiro, Pharmacy Faculty, Rio de Janeiro, Brazil*

**Introduction**

Increasing evidence indicates an inverse correlation between the consumption of plant-based foods and cancer incidence. Phytochemicals naturally found in such foods, particularly polyphenols, modulate multiple cancer-related biological pathways. Green tea (*Camellia sinensis*) and olive (*Olea europaea L.*) bioactive compounds have been shown to play an essential role in chemoprevention, but the related molecular mechanisms need to be further explored. We hypothesized that green tea and olive leaf extracts derived from their respective food matrices could promote p53-mediated anticancer effects on human cancer cells.

**Material and Methods**

Green tea extract (GTE) and olive leaf extract (OLE) were previously obtained by our group and tested on human breast cancer cell lines and hepatocellular carcinoma. Cell viability and cell migration were measured by Alamar Blue® and wound-healing assays, respectively. p53, p21, and related proteins were identified by western blotting and immunocytochemistry.

**Results and Discussions**

MCF-7 (WT-p53) and MDA-MB-231 (p53 mutant) were first exposed to GTE (31.2–1250 µg/mL) for 24-48 h and cell viability was assessed in the presence of the p53 inhibitor pifithrin-α. GTE selectively targeted breast cancer cells while having no effect on non-tumoral MCF-10A cells. p53 inhibition increased viable cells, particularly in MCF-7, indicating that p53 is involved in GTE-induced cytotoxicity. We also observed that GTE had no effect on peripheral blood mononuclear cells (PBMC) viability. Additionally, GTE was effective in reducing MCF-7 and MDA-MD-231 cell migration by 30 and 50%, respectively. An increment in p53 and p21 expression stimulated by GTE was observed in MCF-7, and the opposite phenomenon was found in MDA-MB-231. GTE and OLE are currently being studied by our group for their anticancer properties in hepatocellular carcinoma cells HepG2 (WT-p53) and Hep3B (p53 null). Preliminary data show that GTE reduced the viability of HepG2 and Hep3B in a dose-dependent manner. OLE had a less pronounced effect on HepG2 cell viability, in comparison to GTE, but when these cells were grown in a 3D model, we noticed significant changes in the morphology and size of the spheroids after OLE exposure. Future experiments are planned to investigate the role of p53 in GTE and OLE-induced effects in hepatocarcinoma.

**Conclusion**

Our findings provide insights into the actions of GTE and OLE from the food matrix and support their antitumor potential in breast cancer cells and hepatocellular carcinoma.

### EACR23-0607
### Elucidating the tumor suppressor role of RBM10 in lung adenocarcinoma

*A. Utrilla Maestre[1], P. Peinado Fernández[2], A.M. Matia Gonzalez[1], M.D.L.A. Becerra Rodriguez[3], P.P. Medina Vico[1]*
*[1]Pfizer-University of Granada- Junta de Andalucia Centre for Genomics and Oncological Research GENYO, Department of Biochemistry and Molecular Biology I- University of Granada, Granada, Spain*
*[2]The Francis Crick Institute, neurosciences, London, United Kingdom*
*[3]Bioscience Research Institute, School of Biochemistry and Cell Biology- University College Cork, Cork, Ireland*

**Introduction**

Recent studies have defined the RNA-binding motif 10 protein (RBM10) as a tumor suppressor in lung adenocarcinoma (LUAD), though its specific molecular mechanism remains elusive. Several RNA-binding proteins (RBPs) are known to be involved in the biogenesis of circular RNAs (circRNAs), defined as non-coding RNAs produced by backsplicing whose aberrant expression can be associated with cancer. Given the canonical function of RBM10 in the regulation of alternative splicing, we wonder whether this RBP could execute its tumor suppressor function regulating circRNAs biogenesis in LUAD.

**Material and Methods**

Viability and clonogenic assays were performed to confirm the tumor suppressor role of RBM10. To this extent, LUAD cell lines lacking RBM10 were selected and the expression of the protein was transiently restored.
To identify differentially expressed circRNAs an RNA-seq in the aforementioned LUAD cell lines was carried out and data was analysed using circEXPLORER2. circRNAs candidates were experimentally validated by RT-qPCR. RBM10 and circRNAs expression correlation in patients was measured by qPCR. Subcellular location of the potential candidate circRNAs was analysed by cellular fractionation assays. Direct interaction between RBM10 and candidate circRNAs was assessed by biotin pull-downs and monitored by immunoblot. The phenotypical effect of

© 2023 The Authors. Molecular Oncology is published by John Wiley & Sons Ltd on behalf of Federation of European Biochemical Societies the circRNAs candidates was studied performing knock-down experiments with silencing RNAs followed by cell viability and clonogenic assays.

**Results and Discussions**

The knockdown of RBM10 resulted in a higher proliferation rate and clonogenic capacity, confirming its tumor suppressor role.

In addition, we observed that several circRNAs were differentially expressed upon RBM10 restoration, among which four of them were experimentally validated. Indeed, deregulation of these circRNAs was further confirmed in LUAD patient samples where a strong correlation between RBM10 and circRNA expression was observed. All these circRNAs are mainly expressed in the cytoplasm whereas RBM10 is localised in the nucleus. Strikingly, we observed that RBM10 specifically binds to the flanking regions of the validated circRNAs. Importantly, knocking-down one of the validated circRNA phenocopied RBM10 restoration.

**Conclusion**

Overall, this work demonstrates the tumor suppressor role of RBM10 in LUAD and shows for the first time evidence of its relevant function in the biogenesis of oncogenic circRNAs.

### EACR23-0626
### Exploring anti-cancer effects of cannabinoids and medicinal mushrooms and their interactions with conventional treatments in breast and colon cancer

*E. Oron[1], I. Angel[2], B. Fares[1], V. Bar[3], A. Zundelevich[3], E. Eisenberg[4], T. Peretz-Yablonski[5]*
*[1]Cannabotech LTD, Integrative Oncology, Herzliya, Israel*
*[2]Angel Pharmaceutical Consulting & Technologies, A.P.C.T. Ltd., Nes-Ziona, Israel*
*[3]Curesponse, Curesponse, Tel Aviv, Israel*
*[4]Technion, Israel Institute of Technology, Haifa, Israel*
*[5]Hadassah Medical Center and Hebrew University, Faculty of medicine, Jerusalem, Israel*

**Introduction**

Growing evidence in a wide spectrum of tumor cells both *in vivo* and *in vitro* suggest that *Cannabis*-derived substances and medicinal mushrooms (used in traditional medicine practices) have anticancer activity brought about by a wide range of mechanisms that may be cancer type and even subtype specific. In addition, the fact that these botanical substances are commonly used by cancer patient's alongside standard medical care raises questions regarding possible interactions between them.

In this study we explore the anti-cancer effects of cannabinoids and medicinal mushrooms on various breast and colon cancer subtypes and test interactions with common conventional treatments.

**Material and Methods**

Anti-cancer efficacy of cannabinoid and mushroom in vitro was evaluated through cell viability and proliferation assays (MTT, BrdU). For each cancer i.e. Breast and Colon, 4 different cell lines, representing different defined subtypes, were tested. Ex Vivo analysis of cancerous tissue from patients was done using cResponse™ platform. This Ex Vivo Organ Culture System, uniquely maintains the tissue's 3D structure and the tumor microenvironment (TME), including stromal cells and the immune system, and thus allows evaluation of drug efficacy in living tumor tissue with an intact TME.

**Results and Discussions**

We show that anti-cancer efficacy of both cannabinoids and mushrooms varies among subtypes of a specific cancer. Furthermore, combination of cannabinoids has increased activity compared to individual cannabinoids (entourage effect). Intriguingly we find that both cannabinoids and mushroom combinations interact with some conventional treatments. For example, synergistic interaction between cannabinoids and Taxol, and interfering interaction between cannabinoids and Tamoxifen.

**Conclusion**

Our results strongly suggest that cannabinoids and medicinal mushrooms may have effects on the tumor itself in addition to alleviating treatment related side effects and improving the patient's quality of life. The results also warrant additional analysis in the future to further investigate the potential benefit of combining cannabinoids with specific conventional treatments.

### EACR23-0628
### Nanoparticles based treatment of glutamine addiction in pancreatic cancer: a double hit strategy

*M.C. De Santis[1], A. Amaolo[1], J.P. Margaria[1], E. Di Gregorio[1], J.M. Backer[2], G. Ferrauto[1], E. Hirsch[1], M. Martini[1]*
*[1]Molecular Biotechnology Center- University of Torino, Dept. of Molecular Biotechnology and Health Sciences, Torino, Italy*
*[2]Albert Einstein College of Medicine, Dept. of Molecular Pharmacology, Bronx- NY, United States*

**Introduction**

Pancreatic cancer (PC) is on path to become the second leading cause of cancer-related death by 2030. The late diagnosis and the resistance to chemotherapy are the main reasons for the poor clinical outcome, thus new therapeutic approaches are urgently required. mTOR-dependent metabolic adaptation to nutrient availability, such as glutamine, is a key feature of PC, that can be therapeutically exploited. To overcome the tumor microenvironment constraints, like the desmoplasia reaction, alternative strategies for drug delivery are required. We previously demonstrated that a class II PI3K, PI3K-C2g, is a negative regulator of mTOR and a predictive marker for mTOR and glutaminase inhibitors in PC patients. Understanding how glutamine impacts on PI3K-C2g activity would help to develop novel therapeutic strategies and drugs encapsulation in nanoparticles would represent a valid drug delivery option to increase tumor targeting.

**Material and Methods**

We will model PI3K-C2g loss in a panel of preclinical models of human PC (Panc1, MiaPaca2 cells) to validate the impact of glutamine availability on PI3K-C2g activity. mTOR (everolimus and AZD2014) and glutaminase (CB839, BPTES and DON) inhibitors will be encapsulated in nanoparticles in order to identify the best formulation for in vitro and ex vivo testing. To use a model that recapitulates as close as possible the complexity of PC

© 2023 The Authors. Molecular Oncology is published by John Wiley & Sons Ltd on behalf of Federation of European Biochemical Societies microenvironment, alternative 3D culture systems will be tested. Cells will be treated with drugs alone or in addiction to standard chemotherapy in order to tailor more effective strategies.

**Results and Discussions**

Upon glutamine deprivation, PI3K-C2g localization and targets were affected, suggesting a possible involvement in Rab-mediated vesicle trafficking toward lysosomes, where PI3K-C2g regulates mTOR. In 3D culture models, AZD2014 in combination with BPTES improved the cytotoxic effect of gemcitabine. BPTES and AZD2014 nanoparticles had an improved pharmacokinetics and efficacy compared to un-encapsulated drugs.

**Conclusion**

We demonstrated that the combinatorial targeting of mTOR and glutamine metabolism is an innovative therapeutic option in PC and we validated a novel strategy of drug delivery, based on NP encapsulation, to enhance tumor targeting and increase tumor drug exposition. The study of glutamine-mediated regulation of PI3K-C2g activity and the manipulation of glutamine addiction would help to identify the best combinatorial treatment that fits with patients and the most suitable drug delivery to improve the clinical outcome.

### EACR23-0630

### Voltage-gated-sodium channel and its role in human glioblastoma cancer stem cells

*F. Brandalise[1], C. Biella[1], E. Pastorelli[1], F. Cianci[1], F. Giammello[1], M. Amat Di San Filippo[1], M. Mazzanti[1]*

*[1]University of Milan, Department of Biosciences, Milan, Italy*

**Introduction**

Glioblastoma multiforme (GBM) is the most common and aggressive primary malignant brain tumor. Despite decades of research, we still lack an efficient treatment and the average survival rate is only about 2 years. The variability in cell type composition combined with the presence of glioblastoma stem cells (GSCs), which are known for their high tumorigenic capacity, make GBM an unsolved clinical issue. It is universally acknowledged that an aberrant functional expression of ion channels is required to sustain the growth of solid cancer cells. Sodium, potassium, calcium, and chloride channels have been correlated with carcinogenesis. However, the significance of membrane ionic permeability regulation in the GBM progression and relapse is not known yet.

**Material and Methods**

Experiments have been performed on human GSCs obtained from surgical specimen at the Neurosurgery Department of IRCCS-AOU San Marino IST (Genova, Italy), from patients who had not received therapies before intervention. The genetic profile and the transcript expression of stemness markers were evaluated both in control condition and in the presence of the $Na_v$ channel blocker Tetrodotoxin (TTX, 30mM). Stemness markers protein content was quantified using Western-Blot analysis. $Na_v$-mediated currents were recorded from single cells and measured in voltage clamp by applying consecutive voltage steps of +10 mV from a holding potential of -70 mV and up to +60 mV. Transient inward current was calculated on the peak subtracting the baseline leak currents. Current density (pA/pF) was calculated as the ratio between the peak current recorded at +20 mV and the capacitance of the cell.

**Results and Discussions**

We have identified a subpopulation of GBM cells expressing TTX-sensitive inward currents. We have shown that $Na_v$ density positively correlates with a depolarized resting membrane potential (RMP) in GBM cells. By adding TTX to our GBM primary cultures, we were able to shift the RMP to more hyperpolarized values. Pharmacological blockade of $Na_v$-mediated currents has shown a significant impact on stemness markers mRNA and protein expression. Moreover, the blockade of $Na_v$-mediated currents regulates cell cycle progression and proliferation.

**Conclusion**

Our preliminary evidence suggests that $Na_v$-mediated currents play a crucial role in a subpopulation of GBM cells. As a result, the present study intends to demonstrate how certain membrane channel conductance's overexpression is fundamental during GBM progression and in its strong resistance mechanisms.

### EACR23-0635

### The ying and yang of polyamines in prostate cancer

*A. Zabala[1,2,3], M. Pujana-Vaquerizo[1], O. Carlevaris[1], A. Ercilla[1], L. Valcarcel[4,5], C. Frezza[5], A. Carracedo[1,2,3,4,6]*

*[1]CICbioGUNE, Cancer cell Signaling and Metabolism, Derio, Spain*

*[2]CIC bioGUNE-Basurto- Biocruces Bizkaia Health Research Institute, Translational Prostate Cancer Research Lab, Bilbao, Spain*

*[3]CIBERONC, Centro de Investigación Biomédica En Red de Cáncer, Madrid, Spain*

*[4]University of Basque Country U.P.V.-E.H.U., Biochemistry and Molecular Biology Department, Leioa, Spain*

*[5]CECAD Research Centre, CECAD Research Centre, Cologne, Germany*

*[6]IKERBASQUE, Basque Foundation for Science, Bilbao, Spain*

**Introduction**

Prostate cancer (PCa) is among the most frequent cancers in men. Although it has been largely studied and many therapeutic approaches have been tested preclinical and clinically, it continues to cause a large number of deaths in western societies[1].

In the last years we have described how polyamine biosynthesis is elevated in PCa with profound implications in the progression of the disease[2]. However, polyamine deprivation therapy has failed in patients in the last years and currently, in spite of the conclusive data about the contribution of polyamines to cancer biology, their molecular effectors remain obscure, and this leads to failed therapeutic approaches in cancer.

In this study we aimed to understand the first molecular effectors and biological consequences derived from polyamine deprivation.

**Material and Methods**

We have undertaken a multiomics strategy in PCa cell lines and validate this data in mice *in vivo*.

© 2023 The Authors. Molecular Oncology is published by John Wiley & Sons Ltd on behalf of Federation of European Biochemical Societies

**Results and Discussions**
Here, we describe how prostate cancer cells acquire survival properties and resistance to therapies when targeting polyamine metabolism. Furthermore, we define a possible mechanism orchestrated by ATF4 signaling through which cells acquire this capacity to counteract these types of stresses.
**Conclusion**
Overall, we have uncovered unprecedented processes under the control of polyamines that lead us to propose unexpected new functions for these metabolites in health and disease.

### EACR23-0638
### Targeting mTOR kinases enhances GPX4-mediated ferroptosis and offers a potential therapeutic intervention in SCCHN

*Y. Huang[1], R. Carter[1], S. Varadarajan[1]*
*[1]University of Liverpool, Molecular and clinical cancer medicine, Liverpool, United Kingdom*

**Introduction**
Recently ferroptosis has earned a great amount of attention in cancer research. This iron- and ROS- dependent form of regulated cell death is mechanistically and morphologically distinct from apoptosis and offers a novel therapeutic strategy in cancer. In this study, we report that pharmaceutical targeting of mTOR kinases can induce ferroptosis, mediated by GPX4 inhibition, in Squamous cell carcinoma of the head and neck (SCCHN), thereby offering a novel and promising combination regimen in SCCHN.
**Material and Methods**
RNA interference and pharmacological inhibition were employed to specifically target components of mTORC1 (Rictor) and mTORC2 (Raptor and Sin1) to perform clonogenic survival and cell death assays in a panel of SCCHN cell lines. Expression analysis and pharmaceutical inhibition of ferroptosis-related proteins, GPX4, xCT and FSP1 in SCCHN cell lines were conducted to confirm the functional role of different ferroptosis inducers in SCCHN cell lines.
**Results and Discussions**
Our results indicate that specific inhibition of mTORC2 (instead of mTORC1) and GPX4 (instead of xCT and FSP1) by Torin-1 and RSL3, respectively could induce extensive ferroptosis in SCCHN cell lines. The further investigation revealed that mTORC2-Akt/PKB-SREBP1 signalling pathway suppresses ferroptosis *via* fatty acid synthase (FASN), and the small-molecular inhibitor targeting FASN, GSK2194069, extends therapeutic effect of ferroptosis induction in SCCHN cell lines.
**Conclusion**
The combination of FASN inhibition and ferroptosis induced shown promising therapeutic outcome in SCCHN cell lines. Current work was to extend this combination strategy to *ex vivo* SCCHN tumour tissue.

### EACR23-0643
### Effect of Plasma-Activated Media in MCF-7 and HCC1806 Breast Cancer Cell Lines

*C. Almeida-Ferreira[1], R. Silva-Teixeira[2], F. Caramelo[3], M.F. Botelho[1], M. Laranjo[1]*
*[1]Biophysics Institute - Institute for Clinical and Biomedical Research iCBR area of Environment Genetics and Oncobiology CIMAGO, Faculty of Medicine- University of Coimbra, Coimbra, Portugal*
*[2]Hospital Center of Vila Nova de Gaia/Espinho, Department of Cardiology, Vila Nova de Gaia, Portugal*
*[3]Biophysics Institute - Institute for Clinical and Biomedical Research iCBR, Faculty of Medicine- University of Coimbra, Coimbra, Portugal*

**Introduction**
Breast cancer (BC) disease has been growing worldwide, achieving the major incidence and mortality in women in 2020. Unfortunately, BC therapies continue to be incredibly challenging, and new approaches are still needed. Some patients present poor responses, and treatments are frequently associated with several side effects. In the last decade, cold atmospheric plasma (CAP), the fourth state of matter, a gas with an equal number of positive and negative particles, has been investigated as a potential anti-tumour therapy. Plasma-activated media (PAM) seem to allow delivering CAP anti-tumour properties conditioned solutions. Thus, the main goal of this study was to determine the cytotoxicity activity produced by PAM in breast cancer cell lines through metabolic activity and protein content evaluation after several time exposures.
**Material and Methods**
This study used two BC cell lines representative of a hormonal receptor positive (MCF7) and a triple negative (HCC1806) BC. The media of each cell line was submitted to plasma treatment using homemade atmospheric plasma equipment during several time exposures: 60, 120, 180, 195, 210, 225 and 240 seconds. Cells were cultured, plated, and PAM was transferred. Cells were evaluated through colourimetric MTT and SRB assays 24 hours later to assess metabolic activity and protein content.
**Results and Discussions**
Both cell lines showed a decreased metabolic activity and protein content as the exposure time increased. MCF-7 cell culture demonstrated a marked reduction of metabolic activity (37.22±9.51)% and protein content (35.94±15.67)% after 60 seconds, as verified in our previous studies with CAP direct. HCC1806 seemed less sensitive than the hormone-dependent cell line concerning cell proliferation. However, our results showed a decrease in the metabolic activity of these cells (53.50±6.19)% and protein content (29.08±4.87)% after 180 seconds of exposure.
**Conclusion**
The effects of PAM treatment suggest that atmospheric plasma is cytotoxic in both BC cell lines with different time exposure. These findings encourage further studies to examine relevant signalling pathways influenced by CAP.
**Funding:** PhD studentship grant by the Portuguese National Funding Agency for Science and Technology (FCT) and European Social Funding (2022.12228.BD). Institutional funding through FCT strategic projects (UIDB/04539/2020, UIDP/04539/2020) and by COMPETE-FEDER (POCI-01-0145-FEDER-007440).

© 2023 The Authors. Molecular Oncology is published by John Wiley & Sons Ltd on behalf of Federation of European Biochemical Societies

### EACR23-0651
### Loss of MKP-3 Upregulates PD-L1 on Extracellular Vesicles

H.S. Kim[1]
[1]Inha University, Molecular Medicine, Incheon, South Korea

**Introduction**

Tumor cells evade immune surveillance by upregulating the surface expression of programmed death-ligand 1(PD-L1), which interacts with programmed death-1 (PD-1) receptor on T cells to inhibits the anti-cancer immune response. The abundance of programmed death-ligand 1 on extracellular vesicles derived from cancer cells plays a role in immune escape by reducing T-cell activity and promoting tumor growth.

**Material and Methods**

We applied the CRISPR-Cas9 technology to target MKP-3 gene at the DNA level in mouse CT26 colon cancer cell lines. Extracellular vesicles from cell culture-conditioned medium were isolated by ultracentrifugation. The exosomal markers and PD-L1 in the extracellular vesicles were examined by western blotting. We also examined the tumorigenesis and growth of mouse CT26 colon cancer cell lines in syngeneic Balb/c mice.

**Results and Discussions**

We found that the PD-L1 on extracellular vesicles from MKP-3 knockout CT26 cells was higher than that from wildtype CT26 cells. In addition, MKP-3 knockout CT26 tumor grew markedly faster than wildtype CT26 cells.

**Conclusion**

Our data suggest that MKP-3 is involved in the up-regulation of PD-L1 on extracellular vesicles derived from mouse CT26 colon cancer cell lines, and thus may play a major role in tumor growth and tumor immune evasion.

### EACR23-0652
### Membrane-associated heat shock protein mHsp70 involved in cancer cell invasion and brain tumor recurrence as a novel target for theranostics

M. Shevtsov[1], N. Yudintceva[2], D. Bobkov[2], R. Tagaeva[2], K. Samochernych[3], A. Kim[4], A. Nechaeva[3], E. Fedorov[4], V. Fedorov[3], E. Nazaralieva[4], A. Ten[5], S. Combs[1]
[1]Technical University of Munich, Klinikum rechts der Isar, Munich, Germany
[2]Institute of Cytology of the Russian Academy of Sciences RAS, Laboratory of Biomedical Nanotechnologies, St.Petersburg, Russia
[3]Almazov National Medical Research Centre, Personalized Medicine Centre, St.Petersburg, Russia
[4]Almazov National Medical Research Centre, Polenov Neurosurgical Institute, St.Petersburg, Russia
[5]Far Eastern Federal University, Institute of Life Sciences and Biomedicine, Vladivostok, Russia

**Introduction**

Heat shock proteins (HSPs) constitute a large family of highly conserved proteins acting as molecular chaperones that play a key role in intracellular proteostasis. Apart from their intracellular localization, members of different HSPs families such as Hsp70 have been found to be localized on the plasma membrane of malignantly transformed cells, including multiforme glioblastoma (GBM). However, the role of the membrane-bound mHsp70 has not yet been elucidated in the pathophysiology of GBM and as a target in the tumor theranostics.

**Material and Methods**

mHsp70 expression as related to tumor cell invasive potential was assessed in biopsies obtained from neuro-oncological patients employing inverted confocal microscopy (Leica Microsystems) and CellVoyager CQ1 Benchtop High-Content Analysis System (Yokogawa). Application of membrane-bound mHsp70 for targeted therapies was evaluated in the intracranial human NCH421k, NCH644, and U87 glioblastoma models in immunodeficient mice employing various chaperone inhibitors (i.e., pifithrin-μ (PES), JG-98, MKT-077) as a monotherapy or in combination with a single dose (10Gy) of stereotactic irradiation (SARRP).

**Results and Discussions**

Live-cell imaging of the patient-derived biopsies revealed the increased cellular density of mHsp70-positive tumor cells particularly in the tissue along the tumor-brain margin (biopsy was derived 3 mm from contrast-enhancing margin and also within hyperintense FLAIR). Time-lapse analysis of the tumor sample further demonstrated the migration mHsp70-positive cells into the surrounding normal tissues. Subsequent high-powered multiplex immunofluorescence analysis (Akoya Biosystems Inc.) confirmed markedly increased single-cell infiltration of mHsp70+ tumor cells (that were also co-immunofluorescently stained for SOX2, Nestin, and Oct4) in the normal brain tissues. Application of the mHsp70 inhibitors as a monotherapy or combined with radiotherapy in GBM animal models inhibited tumor progression (as shown by MRI) and significantly increased the animal survival.

**Conclusion**

Expression of the mHsp70 on the plasma membrane of tumor cells corresponds to the highly invasive potential of the GBM cells and recurrence of these tumors in neuro-oncological patients. Application of the small molecular inhibitors of Hsp70 significantly decreased the GBM progression and invasion of tumor cells thus prolonging the overall survival of glioma-bearing animals. Development of mHsp70-targeting agents represents a promising approach in treatment of malignant brain tumors.

### EACR23-0658
### miR-148b-mediated regulation of cell metabolism affects tumour progression in melanoma and breast cancer

M. Coco[1], S. Cozzubbo[1], I.C. Salaroglio[2], J. Kopecka[2], G. Morciano[3], F. Orso[4], P. Pinton[3], C. Riganti[2], D. Taverna[1]
[1]Molecular Biotechnology Center "Guido Tarone", Department of Molecular Biotechnology and Health Sciences- University of Torino, Torino, Italy
[2]University of Torino, Department of Oncology, Torino, Italy
[3]Laboratory for Technologies of Advanced Therapies LTTA- Section of Experimental Medicine, Department of Medical Science- University of Ferrara, Ferrara, Italy
[4]Università del Piemonte Orientale, Department of Translational Medicine DIMET, Novara,

© 2023 The Authors. Molecular Oncology is published by John Wiley & Sons Ltd on behalf of Federation of European Biochemical Societies

*Italy*
**Introduction**
microRNAs (miRNAs) are often abnormally expressed in pathological conditions and affect cancer progression by coordinating a variety of cell processes, including metabolism. miR-148b is an antimetastatic small non-coding RNA often downregulated in cancer, including breast carcinoma and melanoma, where it inhibits malignancy by impairing tumour cell dissemination. Since a complex interplay occurs between tumour progression and metabolic rewiring, we aim at understanding the impact of miR-148b on cell metabolism.
**Material and Methods**
We characterized the metabolic profile of miR-148b-overexpressing or downregulated cells and investigated the intervention of this small non-coding RNA on the main players of glycolysis and mitochondrial functions in cell cultures and in xenotransplants in mice.
**Results and Discussions**
We evidenced decreased glucose uptake, glycolysis and lactate production in miR-148b-overexpressing cells, while the opposite conditions were found in miR-148b depleted cells. In presence of high levels of miR-148b reduced activity and expression of specific glycolytic players was found, among them, the glucose transporter 1 (GLUT1), a predicted direct target of miR-148b. In parallel, PI3K/AKT and WNT/β-catenin pathway downmodulation was observed. Importantly, miR-148b-dependent glycolysis modulations could affect breast cancer and melanoma cell metastatic traits suggesting a direct link with tumour dissemination. Decreased mitochondrial oxidative phosphorylation was also found upon miR-148b overexpression, as revealed by decreased electron transport chain activity and mitochondrial ATP production. In parallel, alterations in mitochondrial morphology, content and ROS production were detected. miR-148b overexpression also led to the downregulation of mitofusin 1 and OPA1 mitochondrial dynamin like GTPase, essential for mitochondrial fusion. Some preliminary data suggest that the impairment of miR-148b-dependent mitochondrial function could depend on the downregulation of peroxisome proliferator-activated receptor-gamma coactivator-1alpha (PGC1α), a master regulator of mitochondrial biogenesis and a predicted target of miR-148b, downregulated in miR-148b-overexpressing cells. The link between miR-148b dependent mitochondria alterations and inhibition of tumour cell dissemination is underway.
**Conclusion**
Altogether, these data suggest that miR-148b affects glycolysis and mitochondrial functions which can contribute to metastasis inhibition.

### EACR23-0661
### Exploiting MYB-dependent vulnerabilities for novel therapeutics in acute myeloid leukaemia.

N. Che[1], D. O'Connor[2], O. Williams[1]
*[1]UCL Great Ormond Street Institute of Child Health, Department of Developmental Biology & Cancer, London, United Kingdom*
*[2]UCL Cancer Institute, Research Department of Haematology, London, United Kingdom*
**Introduction**
Acute myeloid leukaemia (AML) is highly dependent on a master regulator, MYB, for cell survival and disease progression. However, MYB targeting has proven elusive, none of the current studies leading to successful development of a clinically approved MYB-directed treatment for AML. This is mainly due to MYB being a transcription factor, proteins which are inherently "undruggable" through traditional approaches, usually lacking any clear pocket or interface for the docking of small molecules.
**Material and Methods**
Here, we conducted a genome-wide CRISPR dropout screen on MYB-overexpressing THP-1 cells to reveal the integrated networks which are controlled by MYB and to exploit these MYB-dependent vulnerabilities to identify novel therapeutic targets in AML. Briefly, we established clones from THP-1 cells with stabilised MYB mutant (ΔMYB) overexpression, and transduced them with a lentiviral CRISPR-knockout library. Genomic DNA of ΔMYB-overexpressing clones and control clones was harvested after 14 cell doublings and barcoded sgRNAs were detected by Next-Generation Sequencing.
**Results and Discussions**
From the screen, we found that MYB was responsible for AML metabolic reprogramming. MYB-overexpressing cells showed a higher dependency on aerobic glycolysis instead of oxidative phosphorylation. Moreover, increased extracellular lactate was detected in MYB-overexpressing cells and vice versa in MYB-silenced cells. Interestingly, we discovered that MYB-overexpressing cells were more dependent on the first rate-limiting enzyme in glycolysis, HK2.
**Conclusion**
Taken together, our study suggests that targeting glycolysis may be a promising therapeutic strategy for AML, particularly in cases where MYB is overexpressed. The expression level of MYB could also be used as a prognostic marker for anti-glycolytic treatment. Overall, the study provides valuable insights into the integrated networks controlled by MYB in AML and identifies potential therapeutic targets for this disease.

### EACR23-0673
### Identification of the transcription factor ELF3 as a candidate MOC driver gene

S. Abdirahman[1], C. Salazar[1], B. Galea[1], K. Gorringe[1]
*[1]Peter MacCallum Cancer Centre, Cancer Evolution and Metastasis Program, Melbourne, Australia*
**Introduction**
Mucinous ovarian cancer (MOC) is a rare subtype of epithelial ovarian cancer with limited therapeutic options and poor prognosis when diagnosed at advanced stages. Recent genetic sequencing studies of all the histological grades of MOC revealed its molecular and mutational landscape. E74-like ETS transcription factor 3 (ELF3), a member of the E-twenty-six family of transcription factors whose role varies in different types of cancers was identified to be among several significantly mutated genes not previously linked to MOC. However, the role and

© 2023 The Authors. Molecular Oncology is published by John Wiley & Sons Ltd on behalf of Federation of European Biochemical Societies specific mechanisms of ELF3 in the development of MOC still remains understudied.

**Material and Methods**

We performed a comprehensive analysis of the gene expression data available for MOC according to the mutation status of ELF3 in 40 MOC patient samples, in addition to genetic studies on 184 MOC samples. Next, we performed immunohistochemical analysis of MOC tumour tissue to evaluate the expression pattern of ELF3 in patient tumours. Finally, we used CRISPR/Cas9-mediated ELF3 knockout in established MOC cell lines to identify the role of ELF3 in MOC.

**Results and Discussions**

We identified that ELF3 was mutated in 8.2% of our sample cohort and may possibly act as a tumour suppressor in MOC; however, its role might depend on the presence of coexisting mutations. Expression of ELF3 in MOC tissue was elevated and knocking out of ELF3 in MOC cells increased the proliferation, migration and invasion capability of the cells. Preliminary results indicate that the function of ELF3 may be mediated through epithelial-mesenchymal transition (EMT) pathway.

**Conclusion**

Our preliminary data indicate that ELF3 is a negative regulator of the EMT transcription factor ZEB1, however, a detailed understanding of the biological mechanisms is required. In summary, ELF3 presents a promising prognostic biomarker and a therapeutic target in MOC patients.

### EACR23-0675
### NKX2-1 Mediates Chemokine Release and Neutrophil Recruitment in Lung Adenocarcinoma

*M.L. Wang[1], A. Silas[2], Y.C. Chen[1], J.C.Y. Chen[2], M.L. Tsai[1], S.H. Chiou[1]*
*[1]Taipei Veterans General Hospital, Department of Medical Research, Taipei, Taiwan*
*[2]National Yang Ming Chiao Tung University and Academi a Sinica, Taiwan International Graduate Program in Molecular Me dicine, Taipei, Taiwan*

**Introduction**

Lung cancer has the highest burden of mortality and is the most diagnosed cancer worldwide. The survival rate of lung cancer is 19% with a life span of about five years. Considering the poor survival of lung cancer patients, several attempts to ameliorate the severity of lung cancer by using numerous therapeutic approaches have been made but patients are often faced with drug resistance. NK2 homeobox 1 (NKX2-1) is well-known as a lineage transcription factor that regulates pulmonary differentiation. In lung adenocarcinoma (LUAD), low expression of NKX2-1 results in an abnormal differentiation program, leading to the loss of pulmonary cell identity and high tumor metastasis. However, the NKX2-1 involving molecular mechanisms in lung cancer progression is still waiting for further illustration.

**Material and Methods**

We applied molecular biology methods, public domain database analysis, tissue array staining, syngeneic mouse models, and single-cell NGS to show that the downregulation of NKX2-1 in high-grade LUAD is mediated by TGF-β and leads to neutrophil infiltration through CXCLs signaling activation.

**Results and Discussions**

We demonstrated that the low expression of NKX2-1 in LUAD tissue is associated with EGFR-TKI resistance and tumor metastasis. Silencing NKX2-1 with shRNA leads to increased cell motility, tumor growth, and tumor metastasis of LUAD. We found that the cellular expression levels of NKX2-1 are modulated by TGF-β in LUAD cells; a high level of TGF-β inhibits NKX2-1 expression and induces epithelial-to-mesenchymal transition (EMT). We also found from public databases that LUAD patients who express a pattern of NKX2-1$^{high}$/TGF-β$^{low}$ had better survival outcomes than those with NKX2-1$^{low}$/TGF-β$^{high}$. NKX2-1 knockdown can increase the expression and secretion of several CXC chemokines in LUAD tumor cells. Even though TGF-β can promote the expression of these chemokines, ectopic expression of NKX2-1 hampered TGF-β-induced CXC chemokine expression. Furthermore, we found that NKX2-1 can regulate the expression of these CXC chemokines through histone methylation. The syngeneic mouse model showed increased neutrophil infiltration in the NKX2-1/KD tumor, which can be reversed upon inhibition of CXC signaling.

**Conclusion**

In summary, we characterize the role of NKX2-1 as a *bona fide* tumor suppressor in LUAD, whose loss leads to multiple oncogenic effects that include creating a pro-oncogenic tumor microenvironment due to infiltration with neutrophils.

### EACR23-0676
### Remodeling the brain tumor microenvironment by targeting MALT1 to overcome macrophage immunosuppression.

*J. Hofstatter Azambuja[1], G.N. Debom[2], S.S. Yerneni[3], L.M. Maurer[1], H.E. Butterfield[1], L.R. Klei[1], G. Kohanbash[2], P.C. Lucas[4], L.M. McAllister-Lucas[1]*
*[1]University of Pittsburgh, Pediatrics, Pittsburgh, United States*
*[2]University of Pittsburgh, Neurological Surgery, Pittsburgh, United States*
*[3]Carnegie Mellon University, Chemical Engineering, Pittsburgh, United States*
*[4]University of Pittsburgh, Pediatrics and Pathology, Pittsburgh, United States*

**Introduction**

Glioblastoma (GBM) is the most common and aggressive brain tumor. Despite advances in treatment modalities, GBM remains largely incurable. An innovative strategy for therapy is to combine the inhibition of cancer cell-intrinsic oncogenic signalling with the activation of the tumor microenvironment (TME). MALT1 represents a candidate to enable such a dual approach by engaging only a single target. Initially identified as a proto-oncoprotein in lymphoma, MALT1 has recently been implicated in multiple solid tumors, where its inhibition may abrogate tumor progression. Interestingly, blockade of MALT1 protease has also been shown to selectively reprogram tumor infiltrating Treg cells into antitumor effector cells. Based on these observations, we hypothesized that MALT1

© 2023 The Authors. Molecular Oncology is published by John Wiley & Sons Ltd on behalf of Federation of European Biochemical Societies acts both within tumor cells and within cells of the TME to promote GBM tumorigenesis and MALT1 inhibition could thus have dual benefit.

**Material and Methods**

*In vitro* and *in vivo* experiments were performed to evaluate the effect of MALT1 pharmacological or genetic inhibition on MALT1 oncogenic function in GBM and immunological activation of the brain TME.

**Results and Discussions**

MALT1 blockade in GBM cells, using siRNA or MALT1 protease inhibitors, reduces viability and clonogenic potential, suggesting that inhibition of MALT1 could impair tumor cell survival. In addition, GBM cells induce MALT1-dependent NF-kB activation within macrophages and this is associated with polarization towards an M2-like immunosuppressive phenotype. Treating macrophages with MALT1-protease inhibitor prevents GBM-induced M2-polarization. In syngeneic orthotopic mouse models, GBM tumor growth is impaired and survival is extended when GL261 or CT2a GBM tumor cells are implanted into MALT1-protease dead (PD) host mice (mice harbor a point mutation in the MALT1-protease catalytic cysteine) as compared to when implanted into wild-type mice. MALT1-PD mice demonstrate a less immunosuppressive TME with an increased M1/M2 macrophage ratio. Further, a similar macrophage phenotype switch in the TME and decrease in GBM tumor growth was observed in mice treated with a MALT1 protease inhibitor, thus confirming a critical role for MALT1 in tumor-induced macrophage reprogramming and GBM progression.

**Conclusion**

Our studies nominate MALT1 as a new therapeutic target in GBM whose inhibition could offer dual therapeutic benefit by simultaneously acting in tumor cells and in cells of the TME to impair tumor cell proliferation and survival and reverse tumor-induced immunosuppression.

### EACR23-0700
### RIPK4 inactivation as a driver mutation in skin carcinogenesis

*B. Stepic[1,2], L.A. Herron[1,2], G. Tanghe[1,2], C. Urwyler-Rösselet[1,2], M. Devos[1,2], B. Gilbert[1,2], J. Deckers[1,2], J. Maelfait[1,2], P. Vandenabeele[1,2], W. Declercq[1,2]*
*[1]VIB-UGent, Center for Inflammation Research, Ghent, Belgium*
*[2]Ghent University, Department of Biomedical Molecular Biology, Ghent, Belgium*

**Introduction**

Correct epidermal differentiation is essential for preventing development of several pathologic conditions, including cancer. Receptor-Interacting Protein Kinase 4 (RIPK4) is a serine-threonine kinase and a crucial regulator of epidermal differentiation. Unpublished data from our group show that epidermal-specific RIPK4 deficient mice develop spontaneous skin tumors. Somatic RIPK4 mutations occur regularly in human squamous cell carcinomas. However, the impact of these mutations on RIPK4 activity is unclear. Inducible skin-specific RIPK4 ablation ($RIPK4^{iEKO}$) is accompanied by IL23-IL17A pro-inflammatory signaling, a signaling cascade known to contribute to several skin inflammatory diseases and malignancies. Here we investigated the contribution of the IL23 response to skin inflammation in $RIPK4^{EKO}$ mice and we characterized the skin SCC-associated RIPK4 mutations.

**Material and Methods**

To address the role of IL23 as a driver of the pro-inflammatory response in RIPK4-deficient epidermis, we generated $RIPK4^{iEKO}$; $IL23^{-/-}$ mice. To study the impact of SCC-associated RIPK4 mutations we cloned all these mutants and analyzed their signaling capacity in reporter assays. Moreover, we created HaCaT $RIPK4^{-/-}$ cells and used primary keratinocytes to study the molecular impact of RIPK4-deficiency on keratinocyte differentiation.

**Results and Discussions**

Within 4 weeks of RIPK4 ablation $RIPK4^{iEKO}$ mice displayed epidermal hyperproliferation, which was characterized by increased levels of S100A9, TSLP, IL1a, IL23 and infiltrating CD8+ IL17A producing T cells. Within 8 months spontaneous well-differentiated tumors form around head and neck area. However, a full knockout of IL23 did not prevent the hyperproliferation or Tc17 response in RIPK4-deficient epidermis, indicating that IL23 does not contribute significantly to the inflammatory Tc17 response observed in RIPK4 deficient skin.
The analysis of the SSC-associated RIPK4 mutations revealed that about 40% of these mutations had an inactivating effect on autophosphorylation activity and on RIPK4-dependent NF-kB and IRF6 activation. Moreover, we show that RIPK4 regulates the expression of terminal keratinocyte differentiation markers, such as filaggrin and claudin-4.

**Conclusion**

Our data suggest that epidermal RIPK4 deficiency triggers an IL23-independent Tc17 response. In addition, our experiments suggest that RIPK4 deficiency in SCCs contributes to carcinogenesis by preventing proper keratinocyte differentiation.

### EACR23-0702
### eIF4F controls AMPK activity in malignant melanoma

*N. Vadovicova[1,2], B. Valcikova[1,2], K. Kozdonova[1,2], D. Potesil[3], Z. Zdrahal[3], S. Uldrijan[1,2]*
*[1]International Clinical Research Center- St.Anne's University Hospital Brno, Cancer Plasticity, Brno, Czech Republic*
*[2]Masaryk University, Department of Biology, Brno, Czech Republic*
*[3]Masaryk University, Central European Institute of Technology, Brno, Czech Republic*

**Introduction**

Oncogenic mutations in the RAS-RAF-MEK-ERK signaling pathway are drivers of metastatic melanoma. Patients usually respond to BRAF/MEK inhibitors, but resistance often rapidly emerges. **The eukaryotic translation initiation complex (eIF4F)** can promote resistance to small-molecule drugs targeting BRAF/MEK kinases. Furthermore, simultaneous inhibition of BRAF and eIF4F synergized in killing cancer cells. Therefore, we aimed to precisely characterize the **crosstalk between ERK and eIF4F** signaling pathways in melanoma in a proteomic screen.

**Material and Methods**

We performed a proteomic analysis of changes induced in NRAS- and BRAFV600E-mutant melanoma cell lines in

© 2023 The Authors. Molecular Oncology is published by John Wiley & Sons Ltd on behalf of Federation of European Biochemical Societies response to MEK and eIF4F inhibitors. The proteomic screen was followed by the validation of targets using RNAi-mediated **knockdown** and western blotting.

**Results and Discussions**

**The proteomic analysis** revealed several potential common targets of the ERK and eIF4F pathways in melanoma. Interestingly, among them were regulators of the **AMP-dependent protein kinase (AMPK)**, a primary cellular energy sensor, such as MO25, part of an AMPK-activating complex (LKB1-STRAD-MO25), and PP2Aα, an AMPK-inhibiting phosphatase. In addition, RNA interference data demonstrated **LKB1-independent AMPK activation** in melanoma cells upon eIF4F inhibition, also confirmed in LKB1-deficient BRAFV600E-mutant cells. Furthermore, PP2Aα seems to play an essential role in AMPK activity control in melanoma, as RNAi-mediated knockdown of PP2Aα and a small-molecule PP2A inhibitor both potently promoted AMPK activity.

**Conclusion**

Our results indicate cooperation between the ERK and eIF4F pathways in controlling the essential cell energy sensor AMPK in a non-canonical, LKB1-independent manner.

**Acknowledgments**

This work was supported by the European Regional Development Fund - Project ENOCH (No. CZ.02.1.01/0.0/0.0/16_019/0000868), the Czech Science Foundation (GA22-30397S), and Masaryk University (MUNI/A/1393/2022).

### EACR23-0706
### EFFECT OF TRANSMEMBRANE EMP24 DOMAIN PROTEIN TMED9 IN MULTIPLE MYELOMA PROLIFERATION

*Ş. Punar[1], B. Salman Yaylaz[1], S. Sırma Ekmekci[1], N. Abacı[1]*
*[1]Aziz Sancar Institute of Experimental Medicine- Institute o f Graduate Studies in Health Sciences- Istanbul University, Genetics, Istanbul, Turkiye*

**Introduction**

Multiple myeloma (MM) is a malignancy of terminally differentiated plasma cells. MM is one of the most common cancers among hematological malignancies. It is characterized by the overproduction and accumulation of non-functional immunoglobulins or immunoglobulin chains in the plasma cells. The product of the *TMED9* gene is the carrier protein expressed on the endoplasmic reticulum (ER) membrane. It plays a role in the transport of proteins. As we previously found the high RPKM value of *TMED9* gene in MM patients with the transcriptome study, we hypothesized *TMED9* may have a role in the pathogenesis of MM. Therefore, in this study, we aimed to investigate the function of the *TMED9* gene in MM cell lines.

**Material and Methods**

*TMED9* gene expression was suppressed by siRNA with the electroporation method in U266 and RPMI8226 cell lines. *TMED9* gene was overexpressed by using expression vector in both cell lines. After gene suppression and overexpression, the expression levels were determined by qPCR. The change in the protein level of *TMED9* was shown by western blot analysis. Cells were stained with three fluorescent dyes which are Apopxin green,7-AAD, and CytoCalcein Violet 450. Cells were visualized under confocal microscopy to determine apoptosis in the control and treated groups. The number of apoptotic and viable cells were counted with the ImageJ program. The cell proliferation was determined with the MTT assay.

**Results and Discussions**

In siRNA-treated groups, the suppression of TMED9 was calculated by ΔΔCT method at 24 and 48 hours compared to control groups. Likewise, the overexpression level in the vector-treated cells was calculated by ΔΔCT method compared to control groups. Suppression and overexpression of *TMED9* were demonstrated at the protein level by Western Blot. As a result of the suppression of siRNA-mediated gene expression, it was shown, the rate of apoptotic cells increased compared to the control group. MTT results revealed that cell proliferation decreased siRNA-treated group compared to the control group. On the other hand, *TMED9* overexpression showed increased cell viability and proliferation compared to the control group.

**Conclusion**

In this study, we found that suppression of *TMED9* gene expression decreased the viability of MM cells and triggered apoptosis. Our results are promising in MM, as *TMED9* has been shown to be associated with poor prognosis in a different types of cancers. It is also the first functional study with the *TMED9* gene in U266 and RPMI8266 cells.

### EACR23-0715
### MED12 promotes prostate cancer (PCa) cell proliferation through the androgen receptor (AR) and c-MYC signalling

*C. Andolfi[1], E. Morales[1], J. Guzman[2], S. Wach[2], H. Taubert[2], A. Aigner[3], F. Handle[4], Z. Culig[1]*
*[1]Medical University of Innsbruck, Dept. of Urology- Division of Experimental Urology, Innsbruck, Austria*
*[2]Universitätsklinikum Erlangen, Dept. of Urology and Pediatric Urology, Erlangen, Germany*
*[3]University of Leipzig, Dept. Clinical Pharmacology, Leipzig, Germany*
*[4]Medical University of Innsbruck, Institute of Pathology- Neuropathology & Molecular Patho logy, Innsbruck, Austria*

**Introduction**

The Mediator complex is a multi-subunit assembly that plays a genome-wide role in the regulation of gene expression. In the last decade, some of its subunits were observed to promote prostate cancer (PCa) by inducing androgen receptor (AR) activity. Among them, preliminary evidence showed that MED12 is upregulated in advanced PCa tissues, as well as MED12 knockdown inhibited PCa cell line proliferation. Therefore, we aim to explore MED12 role in the promotion of PCa, focusing on its possible relation to AR and the acquirement of AR-targeted therapy resistance.

**Material and Methods**

We analyzed RNA-seq, CRISPR- and RNAi-based databases (DepMap portal) to assess MED12 mRNA expression in PCa cell lines and the effects of its knockdown on their growth. We compared these results

© 2023 The Authors. Molecular Oncology is published by John Wiley & Sons Ltd on behalf of Federation of European Biochemical Societies between $AR^+$ (MDA-PCa2b, 22Rv1, LNCaP, and VCaP) and $AR^-$ cell lines (DU145, PC3). We downregulated MED12 expression in two PCa cell lines (22Rv1 and PC3) by siRNA pool transfection. Cell proliferation was quantified by fluorescent-based counting of nuclei. We performed RNA-sequencing and pathway analysis in MED12-depleted 22Rv1 cells and confirmed the results through qPCR and Western Blotting. AR activity was additionally quantified by measuring PSA protein levels in cell medium.

**Results and Discussions**

MED12 transcript is expressed at a higher level in the $AR^+$ cell lines compared to the $AR^-$ cells. Concordantly, $AR^+$ PCa cell lines are more dependent on MED12 for their growth in comparison to $AR^-$ cells (DepMap portal). However, in our confirmation experiments, we observed that siRNA-mediated MED12 knockdown significantly inhibited cell proliferation (~ -30%) in both 22Rv1 ($AR^+$) and PC3 ($AR^-$) cells. In 22Rv1, MED12 downregulation significantly inhibited c-MYC and AR pathways (RNA-seq data), both of them being major activators of PCa cell proliferation. Indeed, MED12 downregulation consistently decreased the mRNA and protein expression of c-MYC in both 22Rv1 and PC3 cells. In 22Rv1 cells, MED12 downregulation significantly inhibited PSA secretion (-66%) and AR-V7 protein expression (-60%), a ligand-independent variant of AR responsible for acquiring therapy resistance.

**Conclusion**

Our results suggest that MED12 drives PCa cell proliferation in both AR-dependent and independent ways. Due to its positive correlation with AR activity, we plan to study its involvement in the acquirement of AR-targeted therapy resistance.

### EACR23-0723
### Cancer Stem Cell glycosylation markers: A promising biomarker for prognosis and patients follow up

*B. Chandouri-Faize[1], T. Naves[1], S. Durand[1], M. Yassine[1], J. Pannequin[2], A. Chaunavel[3], N. Girard[4], V. Carré[5], F. Lalloué[1]*
*[1]Omega Health, INSERM U1308 CAPTuR- Université de Limoges- CHU de Limoges, Limoges, France*
*[2]Institut de Génomique Fonctionnelle- Centre National de la Recherche Scientifique UMR5203, Université de Montpellier, Montpellier, France*
*[3]Plateforme HISTOLOGIE BISCEm, CHU de Limoges, Limoges, France*
*[4]Curie-Montsouris Thorax, Thoracic Surgery, Paris, France*
*[5]Carcidiag Biotechnologies, Direction, Guéret, France*

**Introduction**

Lung cancer is one of the leading causes of cancer deaths worldwide. Despite scientific advances, its diagnosis and the patient management are still difficult due to the lack of biomarkers to predict tumor aggressiveness and the risk of recurrence. However, growing evidences support that cancer stem cells (CSCs) are responsible for cancer initiation, aggressiveness and therapeutic resistance. Thus, CSCs might afford useful prognosis biomarkers for monitoring lung cancer progression and preventing recurrences. In this context, this work focuses on specific and early CSCs detection using a new diagnostic and prognostic approach based on the recognition of specific CSCs glycosylation patterns. The LungSTEM kit based on a mix of biotinylated lectins was developed by Carcidiag Biotechnologies and provide a new tool for detecting and sorting CSCs from a heterogeneous tumor cell population.

**Material and Methods**

The efficiency of kit for detecting specific glycosylated patterns was assessed on two distinct tumor cell subpopulations expressing or not glycosylated markers by cell sorting (MACS and FACS). Functional tests were performed on both A549 sorted cell subpopulation to evaluate clonogenicity, drug resistance and tumorigenicity. To determine whether cell sorting based on this new tool was relevant, we compared it with conventional CSCs biomarker, CD133. In parallel, the correlation between the detection of CSCs with the MIX and patient's overall survival was analyzed by Immunohistochemistry from 235 patients recruited in Lyon civil hospices.

**Results and Discussions**

Our results demonstrated that MIX+ tumor cells are significantly enriched in CSCs compared to MIX- cells and that the CSC rate is significantly increased in Mix+ cells compared to CD133-sorted cells. In a similar way, tumorigenesis is significantly increased in MIX+ cells compared to MIX-. Kaplan-Meier analysis demonstrates that survival of MIX-positive patients was decreased in the early stages compared to MIX-negative patients. Furthermore, the ROC curve showed a specificity of 100% and a sensitivity of 72%.

**Conclusion**

These promising results suggest that this new tool is relevant and efficient for detecting lung CSCs. Clinical studies also showed its significance as a prognosis biomarker for predicting tumor aggressiveness at early stages. Altogether these results are of prime importance to assess the prognosis value on therapeutic response in lung cancer and to improve patient management.

### EACR23-0728
### LncRNA TAZ-AS202 promotes lung cancer progression through the regulation of the E2F1 transcription factor

*G. Gobbi[1], A. Grieco[1], F. Torricelli[1], E. Sauta[2], G. Santandrea[3], E. Zanetti[3], M. Vohra[4], S.V. Salad[4], A. Ciarrocchi[1], V. Sancisi[1]*
*[1]Azienda USL-IRCCS di Reggio Emilia, Laboratory of Translational Research, Reggio Emilia, Italy*
*[2]IRCCS Humanitas Clinical and Research Center, AI Center, Milano, Italy*
*[3]Pathology Unit, Azienda USL-IRCCS di Reggio Emilia, Reggio Emilia, Italy*
*[4]Harvard Medical School, Department of Otolaryngology- Head and Neck Surgery-Massachusetts Eye and Ear Infirmary, Boston, United States*

**Introduction**

Lung cancer represents the leading cause of cancer mortality worldwide, being responsible for more than 1.8 million deaths every year. Non-small cell lung cancer (NSCLC) is the most common subtype, representing 80% of cases. Long-non coding RNAs (lncRNAs) are increasing

© 2023 The Authors. Molecular Oncology is published by John Wiley & Sons Ltd on behalf of Federation of European Biochemical Societies reported to regulate several physiological and cancerous processes. TAZ-AS202 is a lncRNA transcribed from the TAZ genomic locus in antisense orientation. E2F1 is a key transcription factor in cell cycle progression and apoptosis regulation. Recently, E2F1 has been shown to play a key role also in the acquisition of malignant proprieties in different cancer types.

**Material and Methods**

We used a siRNA approach to downregulate TAZ-AS202, E2F1 or EPHB2 expression in NSCLC cell lines A549 and NCI-H23. On these cells, we performed functional assays, including proliferation, migration and invasion. To determine TAZ-AS202 expression in lung cancer patients, we used RNA-scope. To dissect the pathway downstream TAZ-AS202, we performed an RNAseq analysis, followed by validation experiments, including qRT-PCR, ChIP, Western blot and functional assays. E2F1 was identified by bioinformatic analysis and its stabilization was confirmed by Western blot.

**Results and Discussions**

In this work, we identify and characterize a novel lncRNA, TAZ-AS202, which is overexpressed in lung adenocarcinoma compared with healthy lung tissue. TAZ-AS202 enhances proliferation, migration and invasion of NSCLC cell lines by regulating a set of genes belonging to cancer- associated pathways, such as WNT, EPH-Ephrin signaling and vescicular traffic. TAZ-AS202 expression is under the control of YAP/TAZ-containing transcriptional complexes. Conversely,TAZ-AS202 does not affect TAZ or YAP expression or localization, but instead stabilizes the E2F1 protein. E2F1, in turn, transcriptionally regulates a large set of TAZ-AS202 target genes. The silencing of both E2F1 and EPHB2 recapitulates TAZ-AS202 silencing cellular phenotype, indicating that they are essential mediators of its activity.

**Conclusion**

Our study indicates that the lncRNA TAZ-AS202 supports lung cancer cells proliferation and acquisition of aggressive features through the stabilization of the transcriptional factor E2F1. The TAZ- AS202/E2F1 axis is a new pro-oncogenic pathway which supports NSCLC tumorigenesis and that could be further investigated in order to identify new biomarkers and new therapeutic targets.

### EACR23-0730
### Recapitulating thyroid cancer histotypes through engineering human embryonic stem cells

*F. Verona[1], V. Veschi[2], A. Turdo[2], C. Modica[2], S. Di Franco[2], M. Gaggianesi[2], S. Di Bella[1], M. Lo Iacono[1], V.D. Pantina[2], G. Porcelli[1], L.R. Mangiapane[1], P. Bianca[1], I. Pillitteri[1], M. Todaro[1], G. Stassi[2]*

*[1]University of Palermo, Promise, Palermo, Italy*

*[2]University of Palermo, Dichirons, Palermo, Italy*

**Introduction**

Thyroid carcinoma (TC) is the most common malignancy of endocrine organs. The preponderance of TCs arises from endodermal-derived follicular cells and can be distinguished into well differentiated and undifferentiated carcinomas. Although the majority of differentiated TCs can be cured by standard treatments, 5-20% of them recur and relapse. Anaplastic TCs (ATCs) are highly refractory to current therapies and behave very aggressively by invading adjacent tissues and metastasizing distant organs. The cell subpopulation in the lineage hierarchy that serves as cell of origin for the different TC histotypes remains unknown.

**Material and Methods**

Human embryonic stem cells (hESCs) with appropriate *in vitro* stimulation undergo sequential differentiation into thyroid progenitor cells (TPCs-day22), which maturate into thyrocytes (day 30). We generated follicular cell-derived TCs of all the different histotypes based on specific genomic alterations delivered by CRISPR-Cas9 in hESC-derived TPCs.

**Results and Discussions**

TPCs harboring $BRAF^{V600E}$ or $NRAS^{Q61R}$ mutations generate papillary or follicular TC, respectively, whereas addiction of $TP53^{R248Q}$ generate undifferentiated TCs. Of note, TCs arise by engineering TPCs, whereas mature thyrocytes have a very limited tumorigenic capacity. The same mutations result in teratocarcinomas when delivered in early differentiating hESCs.

The integration of transcriptomic analyses in primary and metastatic TC lesions of human patients and xenografts derived from TPCs harboring $BRAF^{V600E}$ or $NRAS^{Q61R}$ in combination with $TP53^{R248Q}$ mutations, emerged TIMP1/MMP9/CD44 complex as fundamental for TC promotion, while the activation of KISS1/KISS1R signaling crucial for the metastatic growth.

**Conclusion**

The identification of the molecular mechanisms driven by the different mutational profiles that characterize the histotypes of TCs, will allow to identify new target molecules for the diagnosis and therapy of advanced TCs. Particularly we found KISS1R and TIMP1 targeting increase NIS expression and restoring the functional radioiodine uptake by representing a therapeutic adjuvant strategy for undifferentiated TCs resistant to the standard radiometabolic therapy.

### EACR23-0737
### PhenDC3 kills canine lymphoid cells by stabilizing G-quadruplexes

*B. Hernández Suárez[1], A. Pawlak[1]*

*[1]Wroclaw University of Environmental and Life Science, Pharmacology and Toxicology, Wroclaw, Poland*

**Introduction**

Lymphoma and leukemia are among the most common spontaneous tumors occurring in dogs. Unfortunately, with the use of conventional chemotherapy, most of the patients' relapse [1]. Targeted therapy is a promising tool that needs to be explore in canine cancer. One possible therapeutic target unexplored in dogs are the G-quadruplexes (G4s). G4s are RNA or DNA structures of guanines joined together by Hoogsteen hydrogen bonds. G4s can regulate mRNA translation, specifically it has been shown to be related to control gene expression in cancer [2], and they can cause genomic instability [3]. The aim of the project is to explore the use of the G4 ligand PhenDC3 as a possible therapeutic tool.

**Material and Methods**

Canine lymphoma and leukemia cell lines were used in the study. Cells were treated with PhenDC3 for 24 and 48 hours. MTT assay was performed to identify the IC50 of

© 2023 The Authors. Molecular Oncology is published by John Wiley & Sons Ltd on behalf of Federation of European Biochemical Societies the drug. Western blot was used to check DNA damage. A portion of the cells were stained with Annexin V and propidium iodide to analyze by flow cytometry the portion of apoptotic cells. Cells were incubated for 24 hours with the biotracker BioCyTASQ to visualize under the microscope the G4 structures.

**Results and Discussions**

The presence of G4 structures was confirmed in the canine cells by immunofluorescence microscopy. A group of apoptotic cells have been observed by flow cytometry after treatment with 30 µM of PhenDC3 for 48 hours. An increase in DNA damage after treatment with PhenDC3 as well as an increase of Rad51 expression were observed in the cells by western blot, confirming the fact that the stabilization of G4 induced DNA damage, and caused cell dead.

**Conclusion**

Based on the obtained results, targeting the G4 with the use of PhenDC3 may be a promising direction in the treatment of lymphoid malignancies in dogs.

**References**:

[1] D. H. Thamm, "Novel Treatments for Lymphoma," *Veterinary Clinics of North America: Small Animal Practice*, vol. 49, no. 5, pp. 903–915, Sep. 2019, doi: 10.1016/j.cvsm.2019.04.004.

[2] P. Herviou *et al.*, "hnRNP H/F drive RNA G-quadruplex-mediated translation linked to genomic instability and therapy resistance in glioblastoma," *Nat Commun*, vol. 11, no. 1, p. 2661, Dec. 2020, doi: 10.1038/s41467-020-16168-x.

[3] Y. Wu, K. Shin-ya, and R. M. Brosh, "FANCJ Helicase Defective in Fanconia Anemia and Breast Cancer Unwinds G-Quadruplex DNA To Defend Genomic Stability," *Mol Cell Biol*, vol. 28, no. 12, pp. 4116–4128, Jun. 2008, doi: 10.1128/MCB.02210-07.

### EACR23-0742

### Investigating a potential key miRNA player within Rhabdomyosarcoma-derived exosomal cargo in cancer progression and metastasis

*F. Ramadan[1,2,3,4], N. Hussein[2], R. Saab[5,6,7], F. Ghamloush[5], B. Badran[2], R. Khoueiry[8], Z. Herceg[8], P. Clezardin[1,4], S.E. Ghayad[3,9], P.A. Cohen[1,4]*

*[1]Université Lyon 1, Faculty of Medicine, Lyon, France*

*[2]Laboratory of Cancer Biology and Molecular Immunolog y- Department of Chemistry and Biochemistry- Faculty of Science I, Lebanese University, Hadat, Lebanon*

*[3]Department of Biology- Faculty of Science II, Lebanese University, Beirut, Lebanon*

*[4]INSERM- Research Unit UMR_S1033, LyOS, Lyon, France*

*[5]Department of Pediatrics and Adolescent Medicine- Child ren's Cancer Institute, American University of Beirut, Beirut, Lebanon*

*[6]Department of Pediatrics, Stanford University School of Medicine, Palo Alto, United States*

*[7]Department of Anatomy- Cell Biology and Physiology- Fa culty of Medicine, American University of Beirut, Beirut, Lebanon*

*[8]International Agency for Research on Cancer, World Health Organization, Lyon, France*

*[9]Aix-Marseille University- INSERM 1263- INRAE 1260, Center for CardioVascular and Nutrition Research C2VN, Marseille, France*

**Introduction**

Embryonal rhabdomyosarcoma (ERMS) is a pediatric soft tissue sarcoma and an officially registered rare disease. It is characterized by the failure of skeletal myoblasts to complete myogenesis. Presence of metastasis remains a challenge that contributes to poor survival outcomes. Exosomes can deliver nucleic acid and protein cargo to recipient cells, altering the tumor microenvironment and contributing to cancer metastasis. Several exosomal proteins or nucleic acids show promise as biomarkers of diagnosis or as therapeutic targets. ERMS-derived exosomes are enriched in miRNA compared to the donor ERMS cells, and the profiling of this miRNA cargo revealed a common enrichment in 2 miRNAs across several cell lines. MiR-1246 is one of these miRNAs and an interesting candidate as a potential biomarker of metastasis or target of treatment due to its correlation with metastasis in different types of cancer. We therefore investigated the impact of miR-1246 on the tumor microenvironment and ERMS progression.

**Material and Methods**

We functionally inhibited miR-1246 in ERMS cells using miRZip-lentiviral technology and confirmed this inhibition by looking at the levels of expression of its validated mRNA targets using RTq-PCR. Exosomes were isolated from the modified ERMS cells using differential ultracentrifugation and recipient fibroblasts were treated with the different exosomes. Alternatively, we created DOTAP-mimic lipoplexes carrying miR-1246 mimics and delivered the liposomes to recipient fibroblasts. We then performed proliferation assays as well as trans-well migration and invasion assays on the recipient fibroblasts to determine functional behavioral changes.

**Results and Discussions**

Functional inhibition of miR-1246 inhibited both exosome-mediated proliferation and enhanced migration and invasive capacity of recipient fibroblasts. Interestingly, liposome-mediated delivery of the miRNA alone mimicked the observed effects of ERMS-derived exosomes indicating that the observed changes in recipient cells could be a direct effect of this miRNA and that it could be a key player in ERMS metastasis. Furthermore, our results reveal that miR-1246 targets epigenetic regulator *JARID2* as well as *GSK3β*, a major player in Wnt signaling pathway which have been linked to metastasis in different types of cancer.

**Conclusion**

Our results suggest that miR-1246 in ERMS-derived exosomes is a key player within the ERMS exosomal cargo that could be a potential new candidate biomarker of metastasis and/or potential therapeutic target in ERMS.

### EACR23-0744

### Exploring L-Asparaginase effect on solid tumors: differential response to treatment in adenocarcinoma cell lines

*G. Pessino[1], C. Scotti[1], O. Cazzalini[1], I. Guardamagna[2], O. Iaria[2], L. Lonati[2], G. Baiocco[2], P. Perucca[1], L.A. Stivala[1], M. Maggi[1]*

*[1]University of Pavia,*

© 2023 The Authors. Molecular Oncology is published by John Wiley & Sons Ltd on behalf of Federation of European Biochemical Societies

Molecular Medicine- Unity of Immunology and General Pa thology, Pavia, Italy
[2]University of Pavia,
Physics- Laboratory of Radiation Biophysics and Radiobio logy, Pavia, Italy

**Introduction**

The quest for effective therapies in solid tumors is a challenging field in cancer research. Tumor metabolic rewiring makes amino acids appealing targets, as they can become essential for growth. In Acute Lymphoblastic Leukemia (ALL), blasts survival depends on asparagine (Asn), since cells cannot synthetize it *ex novo*. Asparaginase (ASNase), leading to Asn and glutamine (Gln) depletion, is a key drug in ALL treatment. However, sensitivity to ASNase in solid tumor is poorly predictable. Here, we compared two epithelial solid tumor cell lines, 786-O (renal adenocarcinoma) and MCF7 (breast adenocarcinoma), which show an opposite behavior upon ASNase treatment, at the cell cycle level and for expression of ASNase sensitivity markers.

**Material and Methods**

786-O and MCF7 cells were cultured in complete RPMI 1640 and DMEM-HG, respectively. For cell cycle analysis, 72 h after ASNase treatment (3.0, 1.0, 0.50, 0.05, 0.01 U/ml), cells were incubated with EdU (5-ethynyl-2'-deoxyuridine) for 1 h and collected. Cell cycle S-phase duration was determined by Continuous Fluorescence Intensity (CFI) analysis: 48 h after ASNase treatment (1 U/ml), EdU was added and cells were collected at regular timepoints for 13 h total.

**Results and Discussions**

Inhibition of cell proliferation after ASNase treatment was only observed in 786-O cells. Levels of ASNase genetic sensitivity markers (ASNS, GLUL, SLC1A3, ATF4) highlighted the overexpression of ASNS gene in treated 786-O (fold change: 7.2), as well as a lower expression of GLUL when compared to treated MCF7 cells (fold change: 0.4). Cell cycle analysis showed a reduction of new DNA synthesis in treated 786-O cells with a steady decrease at 0.05, 1 and 3 U/ml of ASNase dosage, not seen in treated MCF-7 cells. This differs from what is described for leukemic blasts, where ASNase typically lead to a G1-phase block. The CFI assay, performed to monitor the S-phase progression in 786-O cells, showed a lower increase of EdU fluorescence intensity peak over time in treated cells, suggesting a progressively slower S-phase as a result of ASNase treatment.

**Conclusion**

These data suggest that ASNase effect on 786-O cells may differ from the canonical one observed in blasts. Our hypothesis is that the reason for new DNA synthesis impairment lies in the interference in the nucleotide synthesis pathways, and, in particular, in Gln or Asn/Gln depletion. Further knowledge on Gln role must be investigated to describe the basis of a possible differential mechanism of action of ASNase in solid tumors.

### EACR23-0746
### Bone Marrow Niche Dynamics and Metastatic Dormancy

A. Schäffer[1], I. Tsoukala[1], M. Mosa[2], D. Soetopo[1], W. Kisel[3], J. Rivière[4], R. Wehner[5], C. Brandts[6], M. Schmitz[5], B. Drotleff[7], T. Alexandrov[7], L. Sevenich[2], T. Broggini[6], M. Czabanka[6], H. Bönig[8], K. Götze[4], K. Pantel[9], L. Hofbauer[5], M. Rauner[5], H. Medyouf[1]
[1]Georg-Speyer-Haus – Institute for Tumor Biology and Experimental Ther apy, Medyouf Group, Frankfurt Main, Germany
[2]Georg-Speyer-Haus – Institute for Tumor Biology and Experimental Ther apy, Georg-Speyer-Haus, Frankfurt Main, Germany
[3]Uniklinikum Dresden, Uniklinikum Dresden, Dresden, Germany
[4]Technische Universität München, Technische Universität München, Munich, Germany
[5]Technische Universität Dresden, Technische Universität Dresden, Dresden, Germany
[6]Universitätsklinikum Frankfurt, Universitätsklinikum Frankfurt, Frankfurt Main, Germany
[7]Mcf EMBL, Mcf EMBL, Heidelberg, Germany
[8]Institute for Transfusion Medicine and Immunohematolog y, Institute for Transfusion Medicine and Immunohematology , Frankfurt Main, Germany
[9]UKE Hamburg, UKE Hamburg, Hamburg, Germany

**Introduction**

Despite substantial advances in the treatment of primary tumors, disseminated disease remains the major source of cancer-related deaths. In breast cancer, bone is one of the major sites of metastasis and the clinical relevance of disseminated tumor cells (DTCs) is highlighted by the fact that their detection at the time of diagnosis correlates with poor outcome. While cues leading to the activation of dormant DTCs remain largely elusive, advanced age is one of the most significant predictors of overt bone metastasis occurrence.

**Material and Methods**

Here, we used a newly generated immunocompromised model that recapitulates *bona-fide* features reminiscent of physiological aging, to demonstrate that age-related alterations in the bone marrow niche, dictate growth dynamics of human breast cancer DTCs in the bone.

**Results and Discussions**

These observations point to a prominent tumor-extrinsic control of dormancy that is linked to aging of structural niche cell types. To confirm this in a fully human setting and dissect the cellular origin of the observed phenotype, we used bone metastatic cell lines and patient-derived circulating tumor cells (CTCs) in a newly established 3D human organotypic marrow environment (3D-HOME) system to demonstrate that age-related alterations in the cell-fate decision of mesenchymal niche components drive the awakening of quiescent metastatic cancer cells. Metabolomic analysis revealed specific dependencies, targeting of which, effectively reverted the outgrowth phenotype.

**Conclusion**

Notably, although early DTC activation is boosted by an adipo-rich environment, a prominent feature of aged bone, late-stage metastatic lesions are devoid of adipocytes and characterized by the accumulation of cancer-associated fibroblasts, hinting towards a dynamic re-programing of the tumor microenvironment and potentially stage-dependent vulnerabilities. Improved understanding of these evolving niche dependencies may enhance our ability to propose disease-stage adapted therapeutic interventions

© 2023 The Authors. Molecular Oncology is published by John Wiley & Sons Ltd on behalf of Federation of European Biochemical Societies and inform the rational design of new treatment strategies for patients with bone metastasis.

### EACR23-0755
### Dehydrogenase/Reductase 2 (DHRS2) can be the novel cell cycle regulator in breast cancer cells

B. Salman-Yaylaz[1], Ş. Punar[1], S. Sırma Ekmekci[2], N. Abacı[2]
[1]İstanbul University Institute of Graduate Studies in Health Sciences & Aziz Sancar Institute of Experimental Medicine, Genetics, İstanbul, Turkiye
[2]İstanbul University Aziz Sancar Institute of Experimental Medicine, Genetics, İstanbul, Turkiye

**Introduction**

The cell cycle regulatory proteins are still crucial for cancer treatment approaches. The dysregulation of cyclins and cyclin-dependent kinases has vital roles in cancer progression at a cellular level. In addition to the core regulators, many oncogenes and tumor suppressor genes, such as p53 and Mdm2, join these regulation mechanisms. (Gordon *et al.,* 2018) The Dehydrogenase / Reductase 2 gene (DHRS2), also known as Hep27, has been explored as an accumulated protein in HepG2 cells under sodium butyrate treatment. Butyrate blocks the cells at G0/G1 phase. (Donadel G. *et al.* 1991; Gabrielli F. *et al.* 1995) c-MYB implicates many genes such as DHRS2, p53, and CCNB1 as a transcription factor, and DHRS2 binds the center domain of Mdm2 and regulates levels of p53, p21, and other cell cycle components in the cell. (Pellegrini *et al.,* 2002; Deisenroth C. *et al*., 2010) In breast cancer, there are many inducers of cell division. We decided to investigate cell cycle regulation based on the DHRS2 expression level.

**Material and Methods**

MCF10A, MCF7, T47D, and MDA MB 231 are four human breast cell lines used in this study. A fluorescence ubiquitination cell cycle indicator, FUCCI (Premo™ FUCCI Cell Cycle Sensor *BacMam 2.0*), which contains viral particles, was given to the cells following the kit protocol for labeling. After that, DHRS2 expressions were manipulated with gene-specific siRNA and gene-expression lentiviral vectors with liposome-dependent transfection. The highest DHRS2 silencing and overexpression were determined via the qRT-PCR method and used for analysis. Western blot was made using cyclins and cyclin-dependent kinase antibody cocktails to investigate protein levels of cell cycle components. The mentioned cell cycle gene expressions were also analyzed in RNA levels.

**Results and Discussions**

In all cells, DHRS2 protein levels affect cell cycle regulation. In confocal microscopy, we visualized the cells with the fluorescence indicator and then confirmed western blotting. Although gene expression vector transfected cells did not enter the G1 phase, siRNA knockdown cells were one step ahead compared to non-transfected control cells. There was a noticeable difference between cell lines. Furthermore, our previous network analysis showed that CCNE1, p21, and MDM2 were correlated in different ways according to breast cancer subtypes manipulated with DHRS2 gene expression.

**Conclusion**

DHRS2 is a novel gene for cell cycle regulation in breast cancer cell lines. This study may be a guide for further studies.

### EACR23-0761
### Dissecting the cellular and molecular interaction networks that promote melanoma stemness at the perivascular niche

A. Nowosad[1], C. Pazzi[1], J. Pozniak[1], J. Pauwels[2], K. Gevaert[2], A. Hendrix[3], S. Guelfi[1], G. Bergers[1], P. Karras[1], J.C. Marine[1]
[1]VIB-KULeuven, Center for Cancer Biology, Leuven, Belgium
[2]VIB uGent, Center for Medical Biotechnology, Gent, Belgium
[3]Ugent, Cancer Research Institute Ghent, Gent, Belgium

**Introduction**

Being the deadliest skin cancer with continuously increasing incidence rate, cutaneous melanoma represents a major clinical and societal challenge. Melanomas are made up of not only malignant cells but also the supporting once-normal stroma, consisting mainly of immune cells, fibroblasts and blood vessels. We have recently identified a malignant cell population with stemness properties which fuel tumour growth.

**Material and Methods**

Using multiplex spatial single cell technologies, in combination with state-of-the-art bioinformatics, we shown that that MSCs are predominantly located in the perivascular niche around blood vessels. By using 2D and 3D co-culture models, we aim to dissect the complexity of cell-cell interactions within a perivascular niche and to identify signals driving melanoma stemness.

**Results and Discussions**

Our results show that co-culture of melanoma cells with endothelial cells promotes MSC phenotype via extracellular vesicles-mediated paracrine signalling. Driven by transcriptomic and proteomic data, we identified a series of molecular targets to disrupt the perivascular niche and eradicate MSCs.

**Conclusion**

Together, our results show that extrinsic cues emanating from TME dictate the transcriptional program of melanoma cells, providing new therapeutic opportunities targeting MSC-promoting signalling.

### EACR23-0764
### The heme synthesis-export system is a druggable pan-cancer essentiality.

V. Fiorito[1], A.L. Allocco[1], C. Riganti[2], G. Ferrauto[3], S. Bellini[4], M. Forni[1], S. Petrillo[1], F. Bertino[1], F. De Giorgio[1], G. Ammirata[1], E. Quarta[1], D. Chiabrando[1], F. Altruda[1], C. Ambrogio[1], E. Tolosano[1]
[1]University of Torino_Molecular Biotechnology Center "Guido Tarone",
Department of Molecular Biotechnology and Health Sciences, Torino, Italy
[2]University of Torino, Department of Oncology, Torino, Italy
[3]University of Torino_Molecular Imaging Center, Department of Molecular Biotechnology and Health Scienc

© 2023 The Authors. Molecular Oncology is published by John Wiley & Sons Ltd on behalf of Federation of European Biochemical Societies es, Torino, Italy
[4]University of Torino_Laboratory of Diabetic Nephropathy, Department of Medical Sciences, Torino, Italy

**Introduction**

Heme, a porphyrin ring encaging the great majority of iron in humans, is one of the most utilized cofactors in biological redox reactions, as well as a molecule able to bind diatomic gases, to act as a nitrogen sensor and to modulate the function of transcription factors and ion channels. Heme is both essential and toxic for cells, therefore its production and trafficking are highly regulated processes. Accumulating evidence indicates that increased heme synthesis and export represent pan-cancer metabolic dependencies, proposing their targeting for therapeutic purposes.

**Material and Methods**

We perturbed heme synthesis and export in different kinds of tumors both *in vitro* (tumor cell lines) and *in vivo* (mouse) models and analyzed the consequences in terms of tumor growth, dissemination and metabolic adaptation.

**Results and Discussions**

Our data unveil the existence of a functional axis between heme synthesis and heme export, where heme export by the Feline Leukemia Virus Subgroup C Receptor 1 a (FLVCR1a) participates to the heme-mediated feedback control of the heme biosynthetic enzyme 5-aminolevulinate synthase 1 (ALAS1). We demonstrate that this system is promoted in cancer cells to restrict oxidative metabolism, and that its disruption results in a metabolic rewiring characterized by high oxidative metabolism and reduced glycolysis. This is associated to reduced tumor growth and dissemination, confirming the essentiality of the pathway for tumors, as well as to increased sensitivity to drugs targeting oxidative phosphorylation, as metformin.

In the attempt to implement a clinically applicable strategy to selectively downmodulate heme synthesis in tumors, we postulated to elicit the heme-mediated feedback inhibition of ALAS1 by using the safe and tumor-selective drug 5-aminolevulinic acid hydrochloride (ALA), a known imaging probe and photosensitizer used in tumor fluorescence-guided surgery and photodynamic therapy, respectively. The collected data prove that ALA administration results in heme accumulation and ALAS1 downmodulation, phenocopying the metabolic reprogramming, compromised cell proliferation and increased sensitivity to metformin observed upon *FLVCR1a* silencing.

**Conclusion**

Altogether, these results support the targeting of the FLVCR1a-ALAS1 axis as a promising strategy to counteract tumor growth and to increase the sensitivity to antitumor agents targeting oxidative metabolism. Finally, they sustain ALA repositioning as anticancer agent.

### EACR23-0767
### Exploring the mucus matrix potential for supporting growth of peritoneal metastases in PMP

*M. McAllister*[1], *R. Nagaraju*[1], *D. Thornton*[1], *J. Barriuso*[1], *O. Aziz*[1,2], *S. O'Dwyer*[2], *P. Caswell*[1]
[1]*University of Manchester, Faculty of Biology Medicine and Health, Manchester, United Kingdom*
[2]*The Christie Hospital NHSFT, The Colorectal and Peritoneal Oncology Centre, Manchester, United Kingdom*

**Introduction**

Pseudomyxoma Peritonei (PMP) is a rare malignancy caused by peritoneal metastases (PM) arising from appendiceal mucinous neoplasms (AMN), and is characterised by significant mucus accumulation within the peritoneum. To date, basic research and pre-clinical models are sparse, resulting in limited treatments and therapeutic advancements. Aberrant extracellular matrix (ECM) remodelling is key during metastasis, with heavily glycosylated mucins seen to promote ECM remodelling and chemoresistance in various cancers. Therefore, we aim to elucidate how the mucus matrices adapt at sites of PM to support cancer growth.

**Material and Methods**

We generated a patient-derived library of 12 primary appendix and 25 PM organoid models in laminin-rich basement membrane matrices from low and high grade AMN patients. Furthermore, we collected 20 intraperitoneal mucus samples and 15 whole tissue samples from AMN patients and performed cutting-edge proteomics and glycosylation profiling. Purified mucins were generated from patient mucus and used to treat organoids, assessing growth and proliferation.

**Results and Discussions**

Established organoid cultures expressed tumour associated markers that recapitulate AMNs including CDX2, CK20, Ki67, MUC2, and MUC5AC. In AMN mucus samples, we found that low-grade AMN mucus was heavily glycosylated with α2-3-linked sialic acid and α- or β-linked N-acetylgalactosamine versus high-grade AMN ($P<0.005$). Proteomic and imaging data identified matrix associated proteins within AMN mucus including COL11A1, FB1, CLCA1 and metalloproteases suggesting surprising structural and matrix remodelling components of intraperitoneal mucus. We identified elevated secreted mucin expression including MUC2, MUC5AC and MUC5B following the development of PMP and used this information to mimic the mucus ECM in organoids. Interestingly, when mucin rich fractions were isolated from low grade AMN mucus and supplemented into organoid cultures, we found significant stimulation of organoid growth when compared to untreated organoids over 7 days ($P<0.05$).

**Conclusion**

Our data demonstrate that mucins support growth of AMN and PMP organoids. By elucidating insoluble ECM and mucus composition we will further tune the matrix microenvironment to create improved organoid systems to study PMP. Assessing the role intraperitoneal mucus plays in AMN/PMP growth offers potential in the identification of new potential therapeutic targets.

### EACR23-0772
### Characterizing chemotherapy-surviving tumor cells: understanding cellular senescence in drug resistance

*E. Bajtai*[1,2,3], *G. Szakács*[1,4], *J. Tóvári*[2], *A. Füredi*[1,5]
[1]*Research Centre for Natural Sciences, Institute of Enzymology, Budapest, Hungary*

© 2023 The Authors. Molecular Oncology is published by John Wiley & Sons Ltd on behalf of Federation of European Biochemical Societies

[2]*National Institute of Oncology,
Department of Experimental Pharmacology, Budapest, Hungary*
[3]*Semmelweis University,
Doctoral School of Pathological Sciences, Budapest, Hungary*
[4]*Medical University of Vienna,
Institute of Cancer Research, Vienna, Austria*
[5]*Centre for Energy Research,
Institute of Technical Physics and Materials Science, Budapest, Hungary*

**Introduction**

The treatment of cancer remains a significant challenge in clinical practice. The emergence of drug resistance in tumor cells often limits treatment efficacy, resulting in only temporal response. It is known that residual cancer cells do not just survive chemotherapy, but they can lead to relapse months or even years after treatment. In this study, we established an *in vitro* experimental system to model the characteristics and behavior of tumor cells that survive high-dose chemotherapy treatment and test a promising new approach in treating chemotherapy-surviving tumors.

**Material and Methods**

Various breast cancer cell lines (MCF7, T47D, MDA-MB-231, Hs578T) were treated with high doses of doxorubicin in the range of 70nM to 200nM, which could effectively eliminate most of the cells, while also enable reappearance of proliferating cells after several weeks. We observed the cultures from the initial treatment until repopulation and monitored the morphological and molecular changes of the surviving cells.

**Results and Discussions**

Our observations revealed that all surviving cells in all cell lines, despite having different genetic background and breast cancer type origin, showed characteristics of cellular senescence. Surprisingly, despite cellular senescence is considered irreversible, in our experimental system chemotherapy-surviving tumor cells were able to overcome senescence and repopulate the cultures. Moreover, these survivor cells were proved to be resistant to a plethora of other compounds, suggesting that even sequential use of different chemotherapeutics would be ineffective against the senescent cancer cell phenotype. Our results indicate a promising new approach in treating chemotherapy-surviving tumors by eliminating senescent cells through the use of senolytic treatments. This approach has the potential to bypass drug resistance and potentially delay or prevent relapse when combined with chemotherapy. We tested this hypothesis using navitoclax, a Bcl-2 inhibitor senolytic drug and the results showed that targeting senescent cells after chemotherapy effectively prevented or delayed the repopulation of surviving tumor cells.

**Conclusion**

Our findings suggest that chemotherapy-surviving senescent cells represent a drug resistance mechanism which ensure the subsistence of the tumor, and the targeting of these cells could provide a new and promising strategy for the treatment of chemotherapy-resistant tumors. These results highlight the potential of senolytic treatments in cancer therapy.

### EACR23-0773
### Investigating key molecular players in putative stem cell subpopulations of prostate cancer

*W. Cheng[1], S. Karkampouna[1], M. De Menna[2], F. Bonollo[1], P. Chouvardas[1], G. N. Thalmann[3], M. Kruithof-de Julio[1]*
[1]*University of Bern,
Department for BioMedical Research- Urology Research Laboratory, Murtenstrasse 24- 3008 Bern- Switzerland, Switzerland*
[2]*University of Bern,
Translational Organoid Models- Department for BioMedical Research, Murtenstrasse 24- 3008 Bern- Switzerland, Switzerland*
[3]*Inselspital- Bern University Hospital,
Department of Urology, Bern, Switzerland*

**Introduction**

Androgen deprivation therapy is the standard treatment for prostate cancer (PCa). Nevertheless, despite initial effectiveness, pre-existing cancer stem cell (CSC) populations invariably lead to incurable castration-resistant prostate cancer (CRPC). CSCs are a subset of cancer cells possessing self-renewal properties, driving tumor progression and regrowth. CD44+ PCa cells exhibit more stemness features and are enriched in tumorigenic and metastatic progenitor cells. Here, we aim to explore different subpopulations in tumors based on levels of CD44 expression and investigate whether they have distinct molecular properties and functional characteristics.

**Material and Methods**

The number of CD44+ cells was evaluated in a tissue microarray from primary prostate cancer patient samples (EMPACT cohort) with clinical follow-up. Flow cytometry sorted the CRPC model LAPC9 tumor into CD44 high (CD44-H) and CD44 low (CD44-L) cells. RNA sequencing was performed on these subpopulations to explore their transcriptomic profiles. CD44 expression was validated at the cDNA and protein levels using qPCR and Western blot (WB) analysis. Sorted cells maintained as organoids in vitro.

**Results and Discussions**

Patients with elevated CD44 expression of CD44 (higher number of CD44+ cells) at the time of surgery exhibited a greater propensity for clinic progression (5-year progression-free survival: 75% vs 95%, P=0.03). Flow cytometry data revealed that 26% of CD44-H cells also displayed high ALDH activity, another CSC marker in PCa. Furthermore, CD44-H cell ratio increased after castration treatment for 14 days, indicating that CD44-H cells are able to survive after treatment. Subsequent WB analysis confirmed that CD44 was highly expressed in sorted CD44-H cells, validating the flow cytometry results. Analysis of the CD44 RNA-seq data showed differential expression of CD44 transcript variants between sorted CD44-H and CD44-L cells. Specifically, total mRNA levels were non-significant between the two sorted subpopulations, however, CD44v10 (CD44-201) and CD44v7-10 (CD44-209) were notably upregulated. In organoid culture, sorted CD44-H cells displayed enhanced formation, indicating they possessed higher proliferative capacity and clonogenicity than CD44-L cells.

**Conclusion**

Elevated CD44 expression is related to an increased risk in primary PCa. In addition, CD44 high cells exhibit high tumorigenicity in organoid cultures. Furthermore, the

© 2023 The Authors. Molecular Oncology is published by John Wiley & Sons Ltd on behalf of Federation of European Biochemical Societies upregulation of CD44v10 and v7-10 in the CD44-H subpopulation, may promote tumor formation and metastasis.

### EACR23-0790
### Exploring the role of RAC1 in melanoma brain metastasis

*I. Todoran- née Stejerean[1], P.A. Gimotty[2], P. Brafford[3], H. Li[3], Z. Bonilla Del Rio[1], G.B. Mills[4], M.A. Davies[4], M. Herlyn[3], A. Vultur[1], I. Bogeski[1]*
*[1]University Medical Center Göttingen- Georg August University of Göttingen, Molecular Physiology, Göttingen, Germany*
*[2]University of Pennsylvania School of Medicine, Department of Biostatistics- Informatics and Epidemiology, Philadelphia, United States*
*[3]The Wistar Institute, Program of Cellular and Molecular Oncogenesis- Melanoma Research Center, Philadelphia, United States*
*[4]MD Anderson Cancer Center- University of Texas, Department of Melanoma Medical Oncology, Houston, United States*

**Introduction**
With around 50% of patients with stage IV melanoma being diagnosed with brain lesions, melanoma, the deadliest of skin cancers, has the highest brain tropism of all cancer types. Despite recent therapeutic advances, melanoma brain metastases (MBMs) continue to pose clinical challenges. Additionally, biomarkers predicting brain dissemination are not clear, differences with other metastatic sites are poorly understood and few human cell-based models are available for drug studies.
**Material and Methods**
To identify brain-specific melanoma properties and molecular processes, we used a genetically heterogeneous panel of human-derived MBM cell lines and conducted functional and molecular studies.
**Results and Discussions**
We observed that MBM cells display a slower intrinsic growth rate *in vitro* compared to extracranial cells. Reverse phase protein array analyses highlighted MBM-specific protein expression in targets associated with proliferation, survival, adhesion and migration. These targets pointed towards RAC1, an essential protein that regulates critical cellular functions; RAC1 was later shown as being upregulated in the MBM cells. Knockdown of RAC1 using shRNA or its inhibition using small molecule inhibitors (alone or in combination with BRAF inhibitors) contributed to a less aggressive MBM phenotype in two- and three-dimensional cultures, while RAC1 knockdown *in vivo* resulted in reduced tumor volumes and delayed tumor appearance. Finally, cellular processes associated with MBM aggressiveness were altered in the presence of insulin and brain-derived soluble factors and were affected by RAC1 levels *in vitro*.
**Conclusion**
Our findings indicate that MBM can engage specific molecular processes such as RAC1 signaling to adapt to the brain microenvironment and this can be used for the development of novel and personalized therapeutic approaches against brain lesions.

### EACR23-0803
### Combined inhibition of Insulin Growth Factor-1 Receptors and Autophagy to hinder Colorectal Cancer Metastasis: A Novel Therapeutic Approach

*M. Saber-Ayad[1], E. Omar[2], K. Bajbouj[3], S. Ahmed[4], S. Hafezi[2], L. Aldohaji[2], M. Al Mashhadani[5], I. Talaat[1], W. Abdel-Rahman[6], R. Hamoudi[1]*
*[1]University of Sharjah- College of Medicine, Clinical Sciences, Sharjah, United Arab Emirates*
*[2]University of Sharjah, Sharjah Institute for Medical Research, Sharjah, United Arab Emirates*
*[3]University of Sharjah- College of Medicine, Basic Medical Sciences, Sharjah, United Arab Emirates*
*[4]Sheffield Hallam University, College of Health Wellbeing and Life Sciences, Sheffield, United Kingdom*
*[5]Mohammed Bin Rashid University of Medicine and Health Sciences, Mocular Biology, Dubai, United Arab Emirates*
*[6]University of Sharjah- College of Health Sciences, Medical Lab Sciences, Sharjah, United Arab Emirates*

**Introduction**
Metabolic syndrome generates a chronic inflammatory milieu that promotes colorectal cancer (CRC) metastasis. Insulin-like growth factor receptors (IGFRs) are physiologically expressed in the muscular and mucosal layers of the colon and highly expressed in CRC. Autophagy provides the CRC cells with substrates for energy support needed for further proliferation and migration. Combined suppression of the IGF-IGFR-axis and autophagy is hypothesized to inhibit cell migration.
**Material and Methods**
Based on previous transcriptomic analysis of 18 FFPE patients' samples from different CRC stages, autophagy pathways and *IGF-1R* gene were of the top differentially expressed in advanced stage (III) compared to early CRC stages (I and II). In this study, CRISPR-Cas9 using ribonucleoprotein was performed to knock out the HCT116 (CMS4) CRC cell line for autophagy related gene-5 (ATG5) and ATG7. We compared the autophagy genes expression, migration (wound healing assay) as well as the expression epithelial-mesenchymal transition markers; E-cadherin, N-cadherin, Snail1 and Zeb1 (Western Blotting) in different cell lines [isogenic cell lines, HCT116, LOVO, SKCO1, treated with IGF-1, knocked down for IGFR or using its inhibitor (picropodophyllin; PPP)].
**Results and Discussions**
There was an increase in basal autophagosome formation as well as autophagy flux subsequent to IGF-1 treatment; revealed by ATG5 and ATG7 increased levels (WB (, whereas suppressed by PPP. Combined IGF-1R and autophagy inhibition reduces CRC cells migration, as revealed from wound healing experiments at 0, 24, and 48 h of treatment. HCT116, ATG7-/- and NC cells treated with IGF-1 displayed higher migration rate compared to the untreated cells after 48 hrs. IGF-1R siRNA knock down upregulated E-cadherin and downregulated N-cadherin expression in HCT116, SKCO1 cell lines, same for ATG5-/- and ATG7-/- cells. Both ZEB1 and Snail1 were significantly downregulated in ATG5-/- and ATG7-/-. Collectively, we observed potent modulation of the EMT

© 2023 The Authors. Molecular Oncology is published by John Wiley & Sons Ltd on behalf of Federation of European Biochemical Societies markers in CRC cells when we concurrently inhibit IGF-1R and autophagy.

**Conclusion**

Concurrent Inhibition of the autophagy flux and IGF-1 genetically or pharmacologically suppresses CRC cell migration and EMT marker expression. Combined suppression of IGF-1 and autophagy represents a potential therapeutic modality for fighting metastasis in advanced colorectal cancer.

**The study is funded by MBRU-Al-Mahmeed Research Award (ALM#1914).*

### EACR23-0816
### The antiproliferative activity of metformin in glioblastoma stem cells is mediated by the direct binding and functional inhibition of the Chloride Intracellular Channel 1 protein

*F. Cianci[1], I. Verduci[1], R. Cazzoli[2], G. Cannavale[1], M. Ranucci[1], P. Luca[1], S. Minucci[2], T. Florio[3], M. Mazzanti[1]*

*[1]University of Milan, Department of Biosciences, Milano, Italy*

*[2]IEO- Istituto Europeo di Oncologia, Departmend of Experimental Oncology, Milano, Italy*

*[3]Università di Genova, Internal Medicine, Genova, Italy*

**Introduction**

Glioblastoma (GB) is the deadliest brain tumor. Its heterogeneity and invasiveness make GB difficult to be treated and recurrence is inevitable. There is scientific consensus that tumor relapse starts from progenitor/stem-like cells known as glioblastoma stem cells (GSCs). Thus, GSCs represent the target of newly designed therapies. In recent years, it has been highlighted that assumption of the antidiabetic drug metformin has been positively linked with a decrease in the risk of several cancer types and cancer-related mortality, including glioblastoma in patients. It is widely agreed that metformin must be internalized for its pharmacological activity, but there has been no direct evidence of metformin's specific membrane receptor in cancer cells. Here, we show that Chloride Intracellular Channel 1 transmembrane form(tmCLIC1) acts as a privileged metformin receptor in GB stem-like cells (GSCs). tmCLIC1 contributes to the progression of GB both *in vitro* and *in vivo,* promoting GSCs oxidative stress. In addition, its specific localization on GSC plasma membrane makes tmCLIC1 as a prime pharmacological target for GB.

**Material and Methods**

We evaluated the action of Metformin in GSCs in several genetic backgrounds: WT cells, knockout for CLIC1 and rescued for the WT protein and mutated for Arginine 29 (R29A), the putative binding site of metformin located inside the pore. We performed experiment on cancer cell proliferation in 2D and 3D model, metabolism assays, electrophysiology experiments, and NMR investigation to demonstrate the direct binding between tmCLIC1 and metformin. We also executed *in vivo* experiments in zebrafish embryos and mice orthotopically engrafted with GB cells and treated with metformin.

**Results and Discussions**

Our experiments have shown that metformin acts on GSCs only when tmCLIC1 is expressed in its native form. Metformin treatment on WT and rescued WT GSCs results in the impairment of mitochondrial respiration, with a consequent decrease of cell proliferation in vitro and in vivo. KO and R29A cells are completely insensitive to the biguanide. We demonstrated a direct binding between tmCLIC1 and metformin, not KO and R29A cells. There is a strong dependency between metformin and CLIC1 protein: when the interaction is disrupted cells not only become resistant to metformin but there are no obvious metabolic perturbations.

**Conclusion**

The ability of KO and the R29A mutants to revert effects of metformin on GBM strongly supports the idea of tmCLIC1 as primary metformin interactor.

### EACR23-0821
### LAMTOR1 is essential for the control of AMPK activity in melanoma cells

*K. Koždoňová[1,2], N. Vadovičová[1,2], V. Palušová[3], S. Uldrijan[1,2]*

*[1]International Clinical Research Center- St. Anne´s University Hospital, Cancer Plasticity, Brno, Czech Republic*

*[2]Faculty of Medicine- Masaryk University, Department of Biology, Brno, Czech Republic*

*[3]Central European Institute of Technology- Masaryk University, Centre for Molecular Medicine, Brno, Czech Republic*

**Introduction**

Targeted therapy of malignant melanoma often aims at the components of the hyperactivated ERK pathway, especially BRAF and MEK kinases. However, patients treated with ERK pathway inhibitors usually develop resistance within several months. We recently identified new molecular mechanisms involving the metabolic stress sensor AMPK that could affect ERK signaling in melanoma despite the presence of *NRAS* and *BRAF* oncogenic mutations, leading to the suppression of melanoma cell growth. In the current project, we focused on the LAMTOR1 subunit of the Ragulator complex, a known AMPK and ERK regulator, and its role in AMPK and ERK signaling activation in the cellular context of melanoma.

**Material and Methods**

We performed analyses of melanoma cells´ response to metabolic stress after disrupting the Ragulator complex using small molecule compounds or RNA interference. The activity of ERK and AMPK signaling pathways was analyzed by western blotting. Changes in protein interactions after metabolic stress were studied using proximity ligation assays and immunoprecipitations.

**Results and Discussions**

We identified a partial disruption of the Ragulator complex in response to compounds promoting the LAMTOR1 subunit accumulation on the interfaces of enlarged endolysosomes in melanoma. Crucially, the AMPK activation by metabolic stress was disrupted under these circumstances, indicating an essential function of LAMTOR1 in AMPK activation in melanoma. Furthermore, the indispensable role of this Ragulator subunit for AMPK activation after metabolic stress in

© 2023 The Authors. Molecular Oncology is published by John Wiley & Sons Ltd on behalf of Federation of European Biochemical Societies melanoma was also verified using RNA interference targeting *LAMTOR1* gene expression.

**Conclusion**

Our results indicate the importance of the lysosomal Ragulator complex for AMPK kinase activation in response to metabolic stress in melanoma.

**Acknowledgments**

This work was funded by the European Regional Development Fund - Project ENOCH (No. CZ.02.1.01/0.0/0.0/16_019/0000868), the European Union – Next Generation EU - the project National Institute for Cancer Research (Programme EXCELES, Project No. LX22NPO5102), and Masaryk University (MUNI/A/1393/2022).

### EACR23-0824

### Targeting the cell and non-cell autonomous regulation of 47S synthesis by GCN2

*M. Piecyk[1], M. Triki[1], P.A. Laval[1], C. Duret[1], J. Fauvre[1], L. Cussonneau[2], N. Rama[1], B. Gibert[1], G. Ichim[1], F. Catez[1], F. Bourdelais[1], S. Durand[1], J.J. Diaz[1], I. Coste[1], T. Renno[1], S. Manié[1], N. Aznar[1], S. Ansieau[1], C. Ferraro-Peyret[1], C. Chaveroux[1]*

*[1]Centre de Recherche en Cancérologie de Lyon CRCL, Rhône, Lyon, France*

*[2]INRAE Auvergne Rhône Alpes, Auvergne, Saint Genes Champanelle, France*

**Introduction**

Nutrient availability is a key determinant of tumor cell behavior. While nutrient-rich conditions favor proliferation and tumor growth, scarcity, and particularly glutamine starvation, promotes cell dedifferentiation and chemoresistance. Here, we link ribosome biogenesis adaptation to the nutritional environment and cell fate. Our data unveil that the stress kinase GCN2 maintains ribosome biogenesis plasticity according to the nutritional environment, ensuring cell survival and proliferation.

**Material and Methods**

2D and 3D models of colon adenocarcinomas (cancer cell lines and primary tumor cells) and tightly-defined experimental settings mimicking the intratumor heterogeneity of nutrients availability were used *in vitro.* A pharmacological inhibitor and RNA interference were used to study the impact of GCN2 inhibition. Ribosome biogenesis and nucleolar homeostasis were characterized by northern blotting and immunofluorescence staining. Cell viability assays and live-cell imaging were performed to investigate the impact on cell fate. Molecular mechanism was explored by RNA sequencing, western blotting of mTORC1 and autophagic markers (P-S6, P-4EBP1, P-Ulk1, LC3, p62) and monitoring of autophagic flux.

**Results and Discussions**

Under metabolic stress, we show that the amino acid sensor GCN2 represses the expression of the precursor of ribosomal RNA, 47S ribosomal RNA (rRNA). In this condition, the blockade of GCN2 triggers cell death by an irremediable nucleolar stress and subsequent TP53-mediated apoptosis in patient-derived models of colon adenocarcinoma. In nutrient-rich conditions, GCN2 activity supports cell proliferation, independently of the canonical ISR axis. Indeed, impairment of GCN2 activity prevents nuclear translocation of the methionyl tRNA synthetase (MetRS) participating in the transcription of 47S rRNA in the nucleolus. This event leads to a nucleolar stress, inhibition of mTORC1 pathway and the induction of a protective autophagic flux. Finally, inhibition of the GCN2-MetRS axis drastically improves the cytotoxicity of RNA polymerase I inhibitors, including the first-line chemotherapy oxaliplatin, on patient-derived colon adenocarcinoma tumoroids.

**Conclusion**

Our data thus reveal that GCN2 differentially controls the ribosome biogenesis according to the nutritional context. Furthermore, pharmacological co-inhibition of the two GCN2 branches and the RNA polymerase I activity may represent a valuable strategy for elimination of proliferative and metabolically-stressed colon adenocarcinoma cell.

### EACR23-0833

### Exploring Glycosphingolipid-Related Epitopes Together with Surface EMT Markers in Breast Tumor and Non-Tumor Tissue Using Single-Cell Profiling

*J. Prochazkova[1], B. Kvokačková[1,2,3], R. Fedr[1,3], B. Hradilová[1,2], J. Slavík[4], M. Machala[4], J. Navrátil[5], P. Fabián[6], K. Souček[1,2,3]*

*[1]Institute of Biophysics of the CAS, Cytokinetics, Brno, Czech Republic*

*[2]Faculty of Science- Masaryk University, Department of Experimental Biology, Brno, Czech Republic*

*[3]International Clinical Research Center of St. Anne's Unive rsity Hospital, Cancer Plasticity, Brno, Czech Republic*

*[4]Veterinary Research Institute, Pharmacology and Toxicology, Brno, Czech Republic*

*[5]Masaryk Memorial Cancer Institute, Department of Comprehensive Cancer Care, Brno, Czech Republic*

*[6]Masaryk Memorial Cancer Institute, Department of Oncological Pathology, Brno, Czech Republic*

**Introduction**

Breast cancer (BCa) tumor microenvironment is composed of many cellular types with each displaying unique surface fingerprint. On the other hand, microenvironment of healthy mammary tissue is in definition complex as well. Considering this, we asked a question if single-cell profiling of cellular surfaces present in non-tumor and breast tumor tissue may serve as a tool for reproducible description of BCa-related heterogeneity and phenotypic plasticity driven, among others, by epithelial-to-mesenchymal transition?

**Material and Methods**

Multicolor spectral flowcytometry and liquid chromatography combined with tandem mass spectrometry were used to describe protein and lipid-based markers of epithelial-to-mesenchymal transition in *in vitro* cell cultures of normal and breast cancer tissue. Panel of antibodies recognizing identified protein- or glycosphingolipid-related epitopes was optimized for the multicolor flow-cytometry analysis of native clinical samples of non-tumor and tumor breast tissue.

© 2023 The Authors. Molecular Oncology is published by John Wiley & Sons Ltd on behalf of Federation of European Biochemical Societies

**Results and Discussions**

Surface profiles of epithelial- (EPCAM, TROP2, CD9), mesenchymal- (CD29, CD49c) and glycosphingolipid-related (Gb3, SSEA1, SSEA3, SSEA4, GD2) epitopes were analyzed in a panel of *in vitro* breast tissue-related models (HMLE, HMLE-EMT, MCF10A LXSN, MCF10A LXSN V12, SKBR3, T47D, MCF7, BT549, MDA-MB-231) to reveal association of tested epitopes with phenotypic status of cells (epithelial vs. mesenchymal). These associations were then investigated in clinical samples of breast non-tumor and tumor tissue. Our data indicate, that in comparison with stromal-like/mesenchymal cells, breast epithelial cells appeared more positive for surface SSEA1 staining. Promising enhancement of Gb3 positivity was observed in stromal-like cells of tumor origin when compared with paired non-tumor counterparts. However, further analyses are essential to validate more precisely the unique associations between the surface presence of specific GSL-related epitopes (e.g. Gb3) and distinct cellular phenotypes (e.g. tumor stromal-like cells).

**Conclusion**

Single-cell profiling of surface GSL-related epitopes may help to describe more precisely heterogenic subpopulations of epithelial and stromal-like cells present in breast tissue microenvironment (supported by the Czech Science Foundation project GA CR 21-11585S).

### EACR23-0836
### Deciphering the role of endothelin-1/ZEB1/gasdermin E axis in the metastatic progression of high-grade serous ovarian cancer

*R. Sestito*[1], *V. Caprara*[1], *P. Tocci*[1], *C. Roman*[1], *V. Di Castro*[1], *A. Sacconi*[2], *G. Blandino*[2], *A. Bagnato*[1]
[1]*IRCCS-Regina Elena National Cancer Institute, Preclinical model and new therapeutic agents Unit, Rome, Italy*
[2]*IRCCS-Regina Elena National Cancer Institute, Traslational oncology research Unit, Rome, Italy*

**Introduction**

Gasdermin E (GSDME) is a key executor of pyroptotic cell death recently implicated in tumor growth and immune response. Novel evidence pinpoints a role of the core epithelial-to-mesenchymal (EMT) factor, ZEB1, in controlling GSDME abundance. However, knowledge about the molecular determinants underlying GSDME-mediated effects beyond pyroptosis remains elusive. Considering the ability of endothelin-1 (ET-1)/endothelin A receptor ($ET_AR$) axis to drive EMT and metastatic progression in high-grade serous ovarian cancer (HG-SOC), by promoting ZEB1 activity, in this study we evaluate whether GSDME signaling may be intertwined with the ET-1/ZEB1 axis to favor the acquisition of aggressive traits of HG-SOC.

**Material and Methods**

Mechanistic studies, including co-immunoprecipitation and chromatin immunoprecipitation assays, were performed in patient-derived (PD)-HG-SOC cells and cell lines. The therapeutic efficacy of ET-1 receptor (ET-1R) antagonist was analyzed *in vitro* and in PD-HG-SOC xenografts. Survival analyses were conducted in HG-SOC patients by using The Cancer Genome Atlas (TCGA) datasets.

**Results and Discussions**

Here we show that in PD-HG-SOC cells $ET_AR$ activation increased GSDME expression by tethering the ZEB1 recruitment on the GSDME promoter. Moreover, ET-1 promoted the nuclear accumulation of GSDME and its physical interaction with ZEB1. Of therapeutic interest, the treatment of PD-HG-SOC cells with the FDA approved ET-1R antagonist macitentan, curbing the ZEB1/GSDME interplay, inhibited ET-1-triggered EMT and cell invasion, as well as the metastatic dissemination *in vivo*. High expression of the $ET_AR$/ZEB1/GSDME signature marked mesenchymal HG-SOC patients and associated with poor prognosis.

**Conclusion**

This study contributes to unravel whether the poor prognosis of mesenchymal HG-SOC can be associated to a greater susceptibility to GSDME-mediated effects that lay in the intersection between pyroptotic cell death and rewiring of immune microenvironment, offering the opportunity to hinder its function by ET-1R blockade in metastatic HG-SOC.

### EACR23-0837
### PKP1 as a novel therapeutic target in squamous cell lung carcinoma

*F. Ritoré Salazar*[1], *A.M. Arenas*[1], *A.M. Matia-Gonzalez*[1], *A.B. Rovsing*[2], *E.A. Thomsen*[2], *M.I. Rodríguez*[3], *J.G. Mikkelsen*[2], *P.P. Medina*[1]
[1]*Pfizer-University of Granada-Junta de Andalucía Centre for Genomics and Oncological Research GENYO, Department of Biochemistry and Molecular Biology I- Uni versity of Granada, Granada, Spain*
[2]*Aarhus University, Department of Biomedicine, Aarhus, Denmark*
[3]*Pfizer-University of Granada-Junta de Andalucía Centre for Genomics and Oncological Research GENYO, Department of Biochemistry and Molecular Biology III- U niversity of Granada, Granada, Spain*

**Introduction**

Plakophilin-1 (PKP1) is a key protein in desmosomes with a tumor suppressor role, as opposed to what it has been observed in squamous lung cancer (LUSC), where it is one of the most overexpressed proteins. PKP1 can be associated with the desmosomes or the translation machinery. Desmosomal-PKP1, dephosphorylated, exhibits a tumor suppressor role, and has functions related to adhesion. Conversely, translator-PKP1, usually phosphorylated by AKT, promotes the translation of MYC, playing an oncogenic role in LUSC.

PKP1-targeting therapies are being designed for the treatment of LUSC. However, cells develop resistances to targeted therapies by rewiring their intracellular pathways and developing non-mutational bypass mechanisms, known as collateral dependencies (CDs). Therefore, a study of the vulnerabilities that appear upon PKP1 inhibition is essential to ensure that future targeted therapies against PKP1 in LUSC are successful.

**Material and Methods**

© 2023 The Authors. Molecular Oncology is published by John Wiley & Sons Ltd on behalf of Federation of European Biochemical Societies

We studied the potential of AKT inhibitors to redirect translator-PKP1 towards desmosomal-PKP1 by calculating $IC_{50}$ in PKP1-KO and WT LUSC cell lines at 48h and 72h and monitoring protein and mRNA levels after treatment by immunoblot and qPCR, respectively. Additionally, phenotypic assays as wound healing, immunofluorescence and clonogenic assays were performed. To assess potential CDs upon PKP1-targeted treatments, a CRISPR-screening was performed in PKP1-KO LUSC cell lines.

**Results and Discussions**

We observed a reduction of phosphorylated PKP1, p-AKT and C-MYC protein levels upon Perifosine treatment. Phenotypic assays showed that WT cells exhibit a lower migration rate and have higher levels of desmosomal PKP1 that colocalize with desmosomal markers after treatment. In addition, these cells revealed a tumor suppressor role when exposed to the drug.

The CRISPR-screening showed that mitochondrial gene expression and processing of mitochondrial mRNA are the most affected pathways, hinting at an increased dependence of LUSC cells on mitochondrial transcription-related genes when PKP1 is depleted.

**Conclusion**

These results allow us to establish a novel strategy for lung cancer therapy in which we can redistribute PKP1 to the desmosome with tumor suppressor function. Also, we have observed a mitochondrial function vulnerability that could be explored as a future co-therapy along with PKP1 inhibition in LUSC.

### EACR23-0844
### Enolase 2 is positively associated with prostate cancer progression

Y. Zhou[1], G. Richards[1], N. Wang[1]

[1]The Medical School, Department of Oncology and Metabolism, Sheffield, United Kingdom

**Introduction**

Prostate cancer (PCa) is the most frequently diagnosed cancer in men and preferentially metastasizes to bone. Enolase 2 (ENO2) is a widely-used biomarker for neuroendocrine PCa, but there are no studies to determine whether ENO2 is functionally involved in PCa progression and metastasis. Therefore, we hypothesize that ENO2 positively contributes to PCa bone metastasis.

**Material and Methods**

ENO2 expression was retrospectively examined using online datasets (GDS1439 and GDS3289). Kaplan-Meier curves were generated via GEPIA to reveal the association between ENO2 expression and PCa patient survival. ENO2 expression at transcriptional and translational levels were examined in a panel of human PCa cells (PC3, DU145, 22RV1, LNCaP, LNCaP-LN3, C4-2, C4-2B4) and benign prostatic cells (WPMY-1) using qRT-PCR and Western Blotting. After siRNA transient knockdown and CRISPR/cas9 knockout (KO) ENO2 in PC3 and 22Rv1 cell lines, cell viability, proliferation and apoptosis were examined using AlamarBlue™, CyQuant™ and Cell Meter™ Caspase 3/7 assays, respectively.

**Results and Discussions**

Retrospective studies suggested that higher ENO2 expression associates with advanced metastatic PCa ($p<0.05$). Kaplan-Meier curves shows a ~20% decreased survival at 100th month in patients with high ENO2 expression. PCa cell lines with higher metastasis potentials, e.g. 22Rv1, has higher ENO2 expression at both transcriptional ($p<0.001$) and translational ($p<0.0001$) levels. 24-hours post siRNA transfection, mRNA expression of ENO2 in PC3 and 22Rv1 cells decreased by 94.6% and 92.5%, respectively. Knockdown of ENO2 reduced viability (~4.9% in PC3, $p<0.01$, and ~23.6% in 22Rv1, $p<0.05$) and proliferation (~30.6% in PC3, $p<0.01$, and ~36.6% in 22Rv1, $p=0.0584$) but increased apoptosis (~61.7% in PC3, $p<0.05$, and ~25.9% in 22Rv1, $p=0.0773$), compared to mock transfected controls at 48-hour post-transfection. Successful KO of ENO2 in two PC3 clones were verified at both mRNA and protein levels. Genetic knockout of ENO2 significantly reduced viability (~39% in PC3 ENO2 KOs, $p<0.001$) and proliferation (~20% in PC3 ENO2 KOs, $p<0.05$), but increased apoptosis (~18.3% in PC3 ENO2 KO #1, $p=0.0814$ and ~20.1% in PC3 ENO2 KO #2, $p=0.0783$).

**Conclusion**

ENO2 is positively associated with PCa viability and proliferation. Genetic and pharmaceutical manipulation of ENO2 will be adopted to further explore its functional involvements in PCa progression and the potential as a pharmaceutical target.

### EACR23-0845
### GAK kinase activity is not required for synthetic lethality with FBXW7

M. Unbekandt[1], M. Rapti[2], S. Howard[3], P. Day[2], M. Ahn[1], M. Wade[1], C. Valenzano[2], C. Richardson[1], J. Lyons[1], N. Wallis[1]

[1]Astex Pharmaceuticals, Biology, Cambridge, United Kingdom

[2]Astex Pharmaceuticals, Molecular Sciences, Cambridge, United Kingdom

[3]Astex Pharmaceuticals, Chemistry, Cambridge, United Kingdom

**Introduction**

FBXW7 is an E3 ubiquitin ligase involved in the degradation of key oncogenic pathways (e.g., c-Myc, Cyclin E, MCL1). FBXW7 gene locus is deleted in a third of cancers and copy loss occurs in 15%, although no targeted therapy is available for the FBXW7-deficient patient population. A synthetic lethal relationship has been reported between FBXW7 and GAK (Dolly et al., British journal of cancer, 2017), a kinase involved in clathrin mediated endocytosis and a potential target for a small molecule kinase inhibitor. To assess the validity of GAK kinase as a drug discovery target in FBXW7-deficient cancers, we further tested the FBXW7/GAK synthetic lethality hypothesis and the significance of GAK kinase function in this context.

**Material and Methods**

*Cell models*: Cell lines used included an isogenic knockout pair of HCT116 wild type (wt) or FBXW7-/-, a HCT116 knock in GAK D191N/D191N and a panel of FBXW7 proficient or deficient breast cancer cell lines.

*GAK siRNA:* The effect of GAK siRNA knockdown on cell viability was investigated in colony formation assays.

*Compound treatment*: To assess the role of GAK kinase activity, the effect of published GAK kinase inhibitors on cell viability was tested using CellTiterGlo.

© 2023 The Authors. Molecular Oncology is published by John Wiley & Sons Ltd on behalf of Federation of European Biochemical Societies

*Rescue experiment:* HCT116 FBXW7-/- cell lines expressing siRNA resistant GAK wt or D191N were tested for their sensitivity to GAK siRNA knockdowns in colony formation assays.

**Results and Discussions**

GAK siRNA knockdowns did not significantly affect the viability of the FBXW7 proficient cell lines tested. GAK knockdown significantly reduced viability in the HCT116 FBXW7-/- cell line and FBXW7 deficient breast cancer cell lines, validating the synthetic lethality of GAK and FBXW7 as previously described.

HCT116 FBXW7-/- cells did not display an increased sensitivity to published GAK kinase inhibitors as compared to HCT116 wt cells. We introduced a mutation of GAK amino acid D191N in the ATP binding pocket which should prevent ATP binding and render the kinase inactive. In rescue experiments, both wt GAK and GAK D191N, were able to rescue the GAK/FBXW7 synthetic lethality. Furthermore, HCT116 wt or GAK D191N/D191N were not differentially sensitive to FBXW7 siRNA knockdowns.

**Conclusion**

We confirmed that GAK knockdown was synthetically lethal with FBXW7 loss in multiple cellular models, supporting GAK as a valid target in FBXW7-deficient cancers. However, this synthetic lethality was not dependent on GAK kinase activity, suggesting the need for targeting an alternative mechanism of action.

### EACR23-0851

### Anticancer potential of two Helichrysum italicum extracts obtained by supercritical CO2 extraction

*I. Matic*[1], *N. Petrovic*[1,2], *V. Tadic*[3], *S. Maksimovic*[4], *T. Stanojkovic*[1], *I. Zizovic*[5]

[1]*Institute for Oncology and Radiology of Serbia, Department of Experimental Oncology, Belgrade, Serbia*

[2]*"VINČA" Institute of Nuclear Sciences- National Institute of the Republic of Serbia- University of Belgrade- Belgrade- Serbia, Laboratory for Radiobiology and Molecular Genetics, Belgrade, Serbia*

[3]*Institute for Medicinal Plant Research "Dr Josif Pančić", Department of Pharmaceutical Research and Development, Belgrade, Serbia*

[4]*University of Belgrade- Faculty of Technology and Metallurgy, Department of Organic Chemical Technology, Belgrade, Serbia*

[5]*Wroclaw University of Science and Technology- Faculty of Chemistry, Group of Micro- Nano and Bioprocess Engineering, Wroclaw, Poland*

**Introduction**

The plant species belonging to the large genus *Helichrysum* are a valuable source of bioactive compounds. The aim of this study was to investigate the anticancer properties of the two *Helichrysum italicum* extracts obtained from plant material by supercritical $CO_2$ extraction.

**Material and Methods**

The cytotoxicity of the extracts was examined against six human cancer cell lines: cervical adenocarcinoma HeLa, lung carcinoma A549, prostate adenocarcinoma PC-3, breast adenocarcinoma MCF-7, melanoma A375, and chronic myelogenous leukemia K562, as well as against two human normal cell lines: lung fibroblasts MRC-5 and keratinocytes HaCaT. Cell cycle analysis was performed by flow cytometry. Gene and microRNA expression levels were measured by RT-qPCR.

**Results and Discussions**

The *H. italicum* extracts exerted concentration-dependent and selective cytotoxic effects on cancer cells. HeLa, A375, and K562 cells were the most sensitive to the cytotoxic activity of both extracts. The extracts showed lower intensities of cytotoxic activity against normal HaCaT cells when compared with intensities of activity against K562, A375, and HeLa cells. An increases in the percentages of cells within subG1 and S phases of the cell cycle were observed in HeLa cells incubated for 24 h with $IC_{50}$ and $2IC_{50}$ concentrations of the extracts in comparison with control cells. A similar effect on the cell cycle phase distribution was seen in A375 cells after 24 h incubation. The G2/M phase arrest was also detected in A375 cells exposed to $IC_{50}$ concentrations of the extracts. Both extracts induced apoptosis in HeLa cells through the activation of effector caspase-3. The extracts triggered apoptosis through the intrinsic pathway mediated by caspase-9 and the extrinsic pathway mediated by caspase-8. Each of the extracts decreased expression levels of *MMP2* in HeLa cells, slightly increased levels of *MMP9*, and increased levels of *VEGFA.* Up-regulations of genes involved in cancer invasion and progression, *MMP2, MMP9*, and *VEGFA,* were observed in A375 cells after 24 h treatment with extracts. The *H. italicum* extracts increased the expression levels of miR-16, miR-21, and miR-155 in HeLa cells, and decreased the expression levels of these miRNAs in A375 cells, which could be explained by their cancer-specific roles and signatures.

**Conclusion**

The results of this study point out promising cancer-suppressive properties of *Helichrysum italicum* extracts rich in bioactive phytochemicals.

### EACR23-0855

### Regenerative potential of mesenchymal stem cells can be modulated by metabolic reprogramming

*J. Špačková*[1], *K. Gotvaldová*[1], *Y. Petrenko*[2], *K. Smolková*[1]

[1]*Institute of Physiology of the Czech Academy of Sciences, Laboratory of Mitochondrial Physiology, Prague, Czech Republic*

[2]*Institute of Experimental Medicine of the Czech Academy of Sciences, Department of Biomaterials and Biophysical Methods, Prague, Czech Republic*

**Introduction**

Multipotent mesenchymal stromal cells (MSCs) are currently the most applied cell type in regenerative medicine (RM) applications, owing to their differentiation, immunomodulatory, and paracrine potential. MSCs secrete a variety of growth factors, cytokines, and immunomodulatory molecules, which support the recovery and function of cells within the damaged tissues and organs. The regenerative properties of multipotent mesenchymal stromal cells (MSC) are largely affected by energy metabolism. Our working hypothesis is that the

© 2023 The Authors. Molecular Oncology is published by John Wiley & Sons Ltd on behalf of Federation of European Biochemical Societies extensive cell expansion and related metabolic adaptations in MSCs affect their regenerative potential and subsequent fate, survival, and secretory activity. In this project, we aim to explore possibilities to suppress OXPHOS activity in MSCs to enhance their paracrine action and, thus, therapeutic properties towards neural regeneration.

**Material and Methods**

We use MSC isolated from Wharton´s jelly and adipose tissue from multiple donors. We prime MSCs with TNFα/IFNγ (10 ng/ml of each) for 24 hrs. Additionally, we challenge MSCs metabolically (substrate variations, inhibitors of metabolic enzymes). We assess bioenergetic features, growth factor production, and metabolomics/lipidomics changes. For experiments, we utilize physiological levels of glucose and glutamine concentrations, i.e. 5 mM glucose and 2 mM glutamine.

**Results and Discussions**

To summarize, the removal of glucose levels leads to significant increase in priming efficiency toward induction of ECAR (glycolytic rate) both basal and maximal. We focus mainly on ECAR because it was reported to be determinant in growth factor release by MSC in response to priming. However, glutamine removal, although oxidative substrate, significantly reduces both OCR and ECAR and makes cells irresponsive to cytokine priming (ECAR maximal and basal). Priming in the combination with glucose and glutamine removal affects production of growth factors, namely FGF10, LIF, and SCF in a positive manner in WJ-derived and AT-derived MSC cultures.

**Conclusion**

Our data indicate that metabolic signaling contributes to regenerative phenotype of MSC. Priming of MSC with inflammatory cytokines also affects bioenergetic features, expressed as respiration and glycolysis rate. A combination of energy substrate availability and cytokine priming can be exploited in modulating MSC growth and a regenerative phenotype.

Supported by Czech Science Foundation project 22-31457S to YP and KS.

### EACR23-0856

### Restoring p53 activity as an approach to target aggressive stem-like childhood sarcomas

*L. Curylova[1,2], I. Staniczkova Zambo[3], J. Neradil[1,2], N. Jurackova[3], P. Chlapek[1,2], J. Almeida[4], M. Kyr[1,5], R. Veselska[1,2], J. Sterba[5], L. Saraiva[4], J. Skoda[1,2]*

*[1]Masaryk University, Department of Experimental Biology. Faculty of Science, Brno, Czech Republic*

*[2]St. Anne's University Hospital Brno, International Clinical Research Center, Brno, Czech Republic*

*[3]St. Anne's University Hospital Brno and Faculty of Medici ne. Masaryk University, First Department of Pathology, Brno, Czech Republic*

*[4]Universidade do Porto, LAQV/REQUIMTE. Laboratório de Microbiologia. Depart amento de Ciências Biológicas. Faculdade de Farmácia, Porto, Portugal*

*[5]University Hospital Brno and Faculty of Medicine. Masar yk University, Department of Pediatric Oncology, Brno, Czech Republic*

**Introduction**

The tumor suppressor p53 is frequently inactivated in aggressive childhood sarcomas characterized by drug resistance and disease recurrence, both generally associated with cancer stemness. As p53 is a known regulator of stemness, we aimed to explore whether the dysregulated p53 pathway might represent a target that could be exploited in sarcoma stem-like cells to improve sarcoma therapy.

**Material and Methods**

Correlation between the deregulated p53 pathway and expression of stemness-associated transcription factors was assessed by immunoblotting in a panel of 36 patient-derived and established osteosarcoma, rhabdomyosarcoma and Ewing's sarcoma cell lines. Models with the highest and lowest calculated stemness potential were tested for their tumorigenic capacity in NSG mice. Obtained xenografts were processed to construct a tissue microarray and were analyzed for selected stemness-associated and p53 pathway proteins by immunohistochemistry (IHC). Sensitivity to drugs targeting the p53 pathway and their effects on stem-like traits were tested in paired tumorigenic and nontumorigenic cell lines derived from all three sarcoma subtypes.

**Results and Discussions**

The comprehensive screening using the panel of cell lines suggested the crucial roles of the transcription factors SOX2, OCT4 and NANOG (limited to Ewing's sarcoma) in sarcoma stemness and tumorigenicity. Importantly, the most aggressive phenotypes, reflected by rapid tumor initiation and growth in mice, showed cell lines with mutated or dysregulated p53. However, subsequent IHC analyses revealed that only SOX2 expression was maintained in a majority of xenograft tumors and this was concurrent with deregulated p53 expression. Drugs that target the p53 pathway (mutant p53 re-activator PRIMA-$1^{MET}$, dual MDM2/MDMX inhibitor RO-5963, and LEM3, a new molecule targeting interactions of MDM2/p53 family proteins) markedly inhibited viability of tumorigenic Ewing's sarcoma cells, demonstrating their increased vulnerability to restoring the p53 activity compared with nontumorigenic cells. Treatment with RO-5963 and LEM3 in tumorigenic cells stabilized p53 and induced apoptosis in a dose-dependent manner.

**Conclusion**

SOX2 expression and the p53 pathway deregulation are associated with sarcoma stemness. Our results indicate that enhanced p53 activity might effectively target aggressive stem-like childhood sarcomas, therefore showing promising clinical potential for p53 pathway re-activation in sarcoma therapy.

### EACR23-0860

### The effect of amino acid deprivation on the growth signaling in malignant melanoma

*A. Dorotíková[1,2], N. Vadovičová[1,2], S. Uldrijan[1,2]*

*[1]International Clinical Research Center- St. Anne's Univer sity Hospital, Cancer Plasticity, Brno, Czech Republic*

*[2]Masaryk University- Faculty of Medicine, Department of Biology, Brno, Czech Republic*

**Introduction**

© 2023 The Authors. Molecular Oncology is published by John Wiley & Sons Ltd on behalf of Federation of European Biochemical Societies

The mTORC1 signaling pathway is an essential regulator of cancer cell survival, growth, and metabolism. In addition to growth factors, energy status, and cellular stress, mTORC1 responds to the level of amino acids, sensed by specific sensors. Methionine, an essential amino acid, is sensed indirectly through its metabolite S-adenosylmethionine, which binds to SAMTOR and enables mTORC1 activation. Its presence is vital for the growth and proliferation of many cancer types. Here we analyzed the effect of methionine and glutamine deprivation on the growth signaling in malignant melanoma.

**Material and Methods**

We used a panel of *BRAF*- and *NRAS*-mutant melanoma cell lines for the mTOR signaling analysis. We used growth media without methionine to induce methionine deprivation. For the inhibition of glutaminolysis, we used the small molecule glutaminase inhibitor BPTES. Samples were analyzed by Western blotting.

**Results and Discussions**

We analyzed the impact of methionine deprivation on mTORC1 activity in *BRAF*- and *NRAS*-mutated melanoma cells. Based on the current knowledge, we expected that methionine restriction would cause mTORC1 inhibition due to a lack of S-adenosylmethionine. However, in *BRAF*-mutant cells, we observed an unexpected increase in mTORC1 activity. Notably, a similar increase was also observed after the inhibition of glutamine metabolism.

**Conclusion**

Our data indicate that another backup mechanism is involved in the mTORC1 regulation of *BRAF*-mutant melanoma cells under the methionine restriction. It could also respond to the shortage of other amino acids, including glutamine. We hypothesize that the mTORC2, AMPK, or ERK signaling pathways, all of which affect the mTORC1 activity, could participate in this mechanism.

**Acknowledgments**

This work was funded by the European Regional Development Fund - Project ENOCH (No. CZ.02.1.01/0.0/0.0/16_019/0000868), the European Union – Next Generation EU - the project National Institute for Cancer Research (Programme EXCELES, Project No. LX22NPO5102), Masaryk University (MUNI/A/1393/2022), and Brno city municipality (Brno Ph.D. Talent Scholarship).

### EACR23-0868

### M1 macrophages drive prostate cancer stem cell plasticity by stimulating the expression of NANOG, SOX2 and CD44

*K. Kainulainen[1], E. Niskanen[1], K. Hartikainen[1], S. Pasonen-Seppänen[1], K. Ketola[1]*

*[1]University of Eastern Finland, Institute of biomedicine, Kuopio, Finland*

**Introduction**

Inflammation is one hallmark of cancer – the reactive tumor microenvironment is maintained by pro- and anti-inflammatory cytokines secreted both by tumor and stromal cells. These inflammatory mediators drive oncogenic processes and eventually suppress immune responses against tumor. Macrophages are an abundant immune cell type in the inflammatory tumor microenvironment, where they have distinct anti- and protumor functions. Previous studies have shown that TAMs accumulate in the same areas with cancer stem cells, that have increased ability for drug resistance, and they are associated with immune evasion and progression of metastasis. In this study, we investigated the role of pro-inflammatory (M1) and immunosuppressive (M2) macrophages on prostate cancer cell plasticity.

**Material and Methods**

THP-1 monocytes were differentiated to macrophages (M0) using PMA. Thereafter, cells were polarized to M1 and M2 type macrophages with LPS and IFNγ, or alternatively with IL4 and IL13. The effects of secreted factors from macrophages on the expression of *SOX2, NANOG* and *CD44* in LNCaP prostate cancer cells were studied with RNAseq, western blot and immunofluorescence imaging.

**Results and Discussions**

Our results showed that M1 macrophages induce the expression of NANOG, *SOX2* and *CD44* in LNCaP cells. Moreover, secreted factors from M1 macrophages suppressed AR and *PSA* expression. Blocking of NFκB pathway with IKK inhibitor reversed the M1 macrophage-induced expression of *NANOG, SOX2* and *CD44,* and downregulation of *PSA* was suppressed. These results suggest that M1 macrophages promote the expression of NANOG, *SOX2* and *CD44* via the activation of NFκB signaling.

**Conclusion**

Our results indicate that secreted factors from M1 macrophages promote LNCaP prostate cancer cell plasticity by suppressing AR expression and activating NFκB signaling, which leads to upregulation of *SOX2, CD44* and NANOG. These results suggest that pro-inflammatory M1 macrophages promote cancer stem cell plasticity and thereby drive aggressive phenotype of prostate cancer cells.

### EACR23-0876

### A link between CIP2A and DNA damage response in basal like-triple negative breast cancer

*P. Doan[1,2], S. G. Nagelli[1,2], O. Kauko[1], J. Westermarck[1,2]*

*[1]Turku Bioscience Centre, University of Turku and Åbo Akademi University, Turku, Finland*

*[2]Institute of Biomedicine, University of Turku, Turku, Finland*

**Introduction**

Basal-like breast cancer (BLBC) is one of the most aggressive human cancer subtypes. About 75% of BLBCs belong to the triple-negative breast cancer subtype (BL-TNBCs). BL-TNBCs have the highest mutational burden due to acquisition of BRCA mutations, or other defects in the homologous recombination (HR) pathways which required for response to double-stranded DNA breaks. CIP2A (PP2A inhibitor) is associated with clinical aggressivity and promotes the malignant growth of BL-TNBC. Interestingly, recent phosphoproteome data indicate that CIP2A inhibits PP2A-mediated dephosphorylation of several proteins associated with DNA damage response (DDR). However, the role of these phosphorylation sites in BL-TNBC cells and in DNA damage response in general is not known. In this project, we aim to characterize a functional link between CIP2A

© 2023 The Authors. Molecular Oncology is published by John Wiley & Sons Ltd on behalf of Federation of European Biochemical Societies and DDR which could aid in understanding of signaling dysregulation in BL-TNBCs.

**Material and Methods**

Codependence analysis was performed using data from Depmap to figure out the functional association of CIP2A with critical DNA damage response proteins. In addition, a site-specific CRISPR/Cas9 mutagenesis screen was performed for CIP2A-regulated phosphosites in BL-TNBC cells. To further understand the link between CIP2A and DDR, the cytotoxicity and colony assay were conducted using PP2A binding defective CIP2A mutant TNBC cells, and a series of DNA damage inhibitors.

**Results and Discussions**

CIP2A was found to be functionally co-dependent in cancer with several critical DDR proteins. Additionally, based on our recent phosphoproteomics analysis of CIP2A-regulated phosphosites, CIP2A inhibits PP2A-mediated dephosphorylation of several proteins in the TopBP1 associated DDR complex. We observed that the PP2A binding defective CIP2A mutant TNBC cells are differentially sensitive to DNA damage inhibitors.

**Conclusion**

The data suggest the role of CIP2A in phosphoregulation of critical DDR proteins modulating BL-TNBC cell therapy responses. By using a site-specific CRISPR/Cas9 mutagenesis we expect to have an expanded DDR protein network regulated by CIP2A which explain the molecular mechanism essential for BL-TNBC cells survival. Finally, we expect the data to be beneficial in developing a novel targeted therapy for BL-TNBCs.

### EACR23-0882

### Defining the Chronology of Epithelial Transformation in Renal Cell Carcinoma Using a Novel Mouse Model

*S. Clerici*[1], *D. Spies*[1], *A. Boletta*[1]
[1]*San Raffaele Scientific Institute, Molecular Basis of Cystic Kidney Disorders Unit, Milan, Italy*

**Introduction**

Chronic mTORC1 activation drives several types of cancer, including renal cell carcinoma (RCC). Our lab previously generated a mouse model carrying kidney-specific inactivation of *Tsc1* (repressor of mTORC1 activity) (*Tsc1*$^{f/f}$*;KspCre*, *Tsc1*kKO) in distal tubules/collecting ducts of the nephron. Mice develop progressive cyst (at P20), papillae and cystadenomas (at P50) and carcinomas (at P80) with full penetrance (Drusian et al, Cell Reports, 2018). These features allow us to investigate the mechanisms underlying RCC initiation.

**Material and Methods**

Since mutant cells lose markers upon transformation, we crossed our *Tsc1*kKO model with a *mTmG* strain, carrying a membrane Tomato marker (mT) recombining to a membrane GFP (mG) upon Cre expression (*Tsc1*kKO;*mT/mG*) . Mutant kidneys were characterized by histological and immunofluorescence analysis. Single cell suspensions were obtained pairing mechanical and enzymatic dissociation and characterized by cytofluorimetric analysis. An initial 10x chromium scRNA sequencing was performed at P80, when cystadenomas and cancerous lesions are present.

**Results and Discussions**

*Tsc1*kKO;mT/mG mutant kidneys confirmed the progressive transformation of the epithelium lining cysts and papillae to cystadenomas and carcinomas with identical timing and frequency. Moreover, all the structures were mG$^+$, allowing to identify and track *Tsc1* KO cells over time. Flow cytometry revealed 20% of mG$^+$ cells in the mutants at P20 (comparable with controls) expanding to 65% of mG$^+$ cells at P80, in line with the sustained proliferation observed during progression.
scRNA sequencing identified clusters of cells deriving from all the segments of the nephron in both control and mutant kidney, with the opportunity to identify mG$^+$ cells on the base of *Egfp* transcripts. As expected, mutants displayed expansion of the epithelial cells derived from distal tubule and collecting duct, where the *Tsc1* is deleted. Interestingly, we identified sub-clusters of cells with a proliferative and de-differentiated profile. Moreover, we observed expansion of the immune compartment in the mutant kidney. Further analysis is underway.

**Conclusion**

We applied scRNA-seq to a mouse model of RCC characterized by progressive epithelial transformation to carcinoma. Preliminary data at late timepoints identify unique signatures present after transformation. We will next analyze early timepoints to identify initiating driving events of epithelial transformation.

### EACR23-0884

### TGFβ signaling mediates OXPHOS inhibition-based cell death through disruption of metabolism programming

*Y. Cao*[1], *W. Qin*[1], *B. Rene*[1], *C. Wang*[1]
[1]*Shanghai Cancer Institute, State Key Laboratory of Oncogenes and Related Genes, Shanghai, China*

**Introduction**

Mitochondria are not only essential for cellular energy metabolism, but they also play a critical role as modulators of cellular responses to microenvironmental stress. Numerous studies have demonstrated that interventions targeting mitochondria function may hold promise as potential approaches for the treatment of various types of cancer.

**Material and Methods**

A kinome CRISPR screen was conducted to identify the kinase whose knockout can synergies with IACS-01059, an oxidative phosphorylation inhibitor of complex I. The combination effects of Dual Specificity Tyrosine Phosphorylation Regulated Kinase 1A (DYRK1A) inhibitor and IACS-01059 on cell proliferation were investigated in a panel of human liver cancer cell lines, mouse models of liver cancer, and patient-derived xenografts. Metabolomics, metabolic flux and transcriptomics analysis were used to elucidate the mechanism of the synergistic effects of DYRK1A inhibitor and IACS-01059.

**Results and Discussions**

The suppression of DYRK1A had a synergistic effect with IACS-010759 both in vitro and in vivo, by inhibiting metabolic reprogramming. We also discovered that the TGFβ signaling pathway is required for the sensitivity of oxidative phosphorylation inhibitors. Mechanistically, SMAD3 can bind to the promoter of SLC1A5, a

© 2023 The Authors. Molecular Oncology is published by John Wiley & Sons Ltd on behalf of Federation of European Biochemical Societies transporter of glutamine, and inhibit its transcription. Additionally, DYRK1A phosphorylates SMAD3 at a non-classical threonine residue at position 132, thereby negatively regulating the suppressive action of SMAD3 on SLC1A5.

**Conclusion**

Our data has uncovered the crucial role of the TGFβ signaling pathway in determining the sensitivity of oxidative phosphorylation inhibitors. Furthermore, our findings suggest that the combination of a DYRK1A inhibitor and OXPHOS inhibitors could represent a promising therapeutic strategy for targeting mitochondria.

### EACR23-0886
### CCR3 knockdown promotes lung tumorsphere formation

T.J. Peng[1,2], K.H. Sun[1,2,3]
[1]National Yang Ming Chiao Tung University, Department of Biotechnology and Laboratory Science in M edicine, Taipei, Taiwan
[2]National Yang Ming Chiao Tung University, Cancer Progression Research Center, Taipei, Taiwan
[3]Taipei City Hospital, Department of Education and Research, Taipei, Taiwan

**Introduction**

Cancer stem cells harbored in the highly heterogenous tumor were identified as the culprit to the therapeutic resistance, organ metastasis, tumor initiation, and recurrence then consequently promoted malignant progression. Chemokine family within the tumor microenvironment intricately orchestrated the tumor progression, while the regulatory mechanisms are not fully defined. In this study, we aim to dissect the role of chemokine axis in lung cancer progression and cancer stemness properties.

**Material and Methods**

A549 and H1299 lung cancer cells were used to initiate the experimental study. Lentiviral-mediated CCL13 overexpression and CCR3 knockdown were transduced to establish stable cell lines. Cell proliferation assay, wound-healing assay, transwell migration/invasion assay, and soft-agar assay were performed with adherent cells. A549 and H1299 lung cancer stem cells were derived from serum-free suspension culture to generate lung tumorspheres and determined the sphere-forming capacity, chemo-drug toxicity resistance, and stemness gene expression. RNA-sequencing followed by RT-qPCR analysis was adopted to scrutinize the transcriptomic alteration.

**Results and Discussions**

Semi-quantitative cytokine antibody array showed a two-fold increase of CCR3 ligands including CCL11, CCL13, and CCL26 in the conditioned medium from H1299 lung tumorsphere, suggesting the potential role of CCLs/CCR3 axis in cancer stemness property. CCL13 overexpression promoted migration, invasiveness, and sphere-forming capacity. The exogenous treatment with CCL13 recombinant protein induced similar effects on cancer cells. Additionally, CCL13-induced chemotaxis was diminished by CCR3 knockdown. On the other hand, CCR3 knockdown impaired cell proliferation, clonogenicity, motility, and invasiveness, while promoting anchorage-independent growth, tumorsphere formation, and chemo-drug resistance. RNA-sequencing followed by RT-qPCR analysis showed that CCR3 knockdown in H1299 tumorspheres rewired the chemokine expression profile, upregulated a cluster of tumor-promoting genes, and sculpted a pro-tumoral transcriptomic.

**Conclusion**

We found that CCL13 induced chemotaxis, which was alleviated by CCR3 knockdown, and promoted sphere-forming capacity. Furthermore, CCR3 showed contrary roles in the malignancy of adherent cells and tumorspheres. RNA-sequencing suggested a cluster of tumor promoters were upregulated in CCR3-knockdown tumorspheres, of which regulatory mechanisms and contributions await further investigation.

### EACR23-0904
### Amino acid amides of cortienic acids as potential antitumour agents

Z. Zizak[1], V. Dobricic[2], O. Cudina[2], I. Besu[1]
[1]Institute of Oncology and Radiology of Serbia, Department of Experimental Oncology, Belgrade, Serbia
[2]Faculty of Pharmacy- University of Belgrade, Department of Pharmaceutical Chemistry, Belgrade, Serbia

**Introduction**

Glucocorticoids act through the glucocorticoid receptor (GR) and have numerous functions including growth inhibition and induction of apoptosis, which is in contrast to the action to receptors for other steroid hormones that are considered oncogenes and promote cell growth. The aim of this work was to investigate the antiproliferative activity and mode of cell death induced by three newly synthesized amino acid amides of betamethasone- and dexamethasone-derived cortienic acids against three human tumor and one control normal cell lines *in vitro*, and compare them with the cytotoxicity of the parent compounds.

**Material and Methods**

Stock solutions of phenylalanine methyl ester derivative of betametasone-derived cortienic acid (BF), histidine methyl ester derivative of dexamethasone-derived cortienic acid (DHIS) and tryptophan methyl ester derivative of dexamethasone-derived cortienic acid (DTRP) were dissolved in DMSO at concentrations of 10 mM, and afterwards diluted by nutrient medium to various final concentrations. Target cells used were malignant human cervix carcinoma HeLa, myelogenous leukemia K562, prostate cancer PC-3 and normal lung fibroblast MRC-5 cells. Antiproliferative activity of investigated compounds was assessed, measuring cell survival in standard, 72 h MTT test. In order to determine the mode of cell death, microscopic examination of morphological characteristics of acridine orange and ethidium bromide stained HeLa cells were performed.

**Results and Discussions**

Investigated compounds exerted a dose dependent antiproliferative action towards all cell lines with good selectivity in their action to tumor cells in comparison to normal control cells. Moreover, the newly synthesized compounds showed an order of magnitude higher activity than the parent betamethasone and dexamethasone and other frequently used glucocorticoids in clinical practice (hydrocortisone, prednisolone and fluocinolonacetonide). The most potent activity showed DHIS against K562 cells

© 2023 The Authors. Molecular Oncology is published by John Wiley & Sons Ltd on behalf of Federation of European Biochemical Societies

($IC_{50}$ = 17.4 µM vs 184 µM for dexamethasone), but best selectivity in activity toward tumor cells in comparison to normal control cells showed DTRP ($IC_{50}$ = 21.3 µM against PC-3 cells vs 114 µM against control MRC-5 cells). Microscopic examination of the mode of HeLa cell death induced by investigated compounds, showed morphological appearance of apoptosis.

**Conclusion**

Results obtained showed that investigated compounds, could be promising agents for the treatment of human tumors, and are candidates for further analyses on experimental animals *in vivo*.

### EACR23-0907
### Liver X Receptors acts as a promoter of epithelial-mesenchymal transition in advanced prostate cancer

*E. Bouchareb[1], J. Bunay[1], J.M. Lobaccaro[1], F. Degoul[1], C. De Joussineau[1], L. Morel[1], A. Kocer[1], S. Baron[1]*
*[1]Genetique Reproduction and Developpement, Endocrinology- Signaling- Cancer, Clermont-Ferrand, France*

**Introduction**

Prostate cancer (PCa) is the most frequent cancer in men. Most prostate cancer related deaths correlates with the metastatic potential of the tumor. One of the main processes involved in the metastatic spread is the "Epithelial-Mesenchymal Transition" (EMT), in which epithelial cells lose their adhesive properties and acquire a mesenchymal-like phenotype, allowing the cells to migrate and invade distant organs. Liver X Receptors (LXRs) are nuclear receptor that have been shown to downregulate EMT in many cancers. In this project, we are defining how LXRs pathway controls EMT in prostate cancer using *ex vivo* and *in vivo* models.

**Material and Methods**

To study the underlying molecular mechanisms by which LXRs signaling controls EMT, we characterized main EMT markers in several human metastatic prostate cancer cell lines: LNCaP (Lymph node metastasis), DU145 (brain metastasis) and PC3 (bone metastasis) using molecular (RT qPCR, *Western Blot*) and immunohistochemistry approaches in presence and absence of LXRs agonist (GW3965). We also assessed EMT function by studying migration (Scratch assay) and invasion (Boyden chamber). *In vivo*, we used Nod-Scidγ immunodeficient mice xenografted with PC3-GFP, allowing us to study metastatic spread and the effect of LXRs activation on this process (permission has been obtained from the ethic committee authority).

**Results and Discussions**

Our data show that PC3 displays the most mesenchymal phenotype regarding of EMT markers. Surprisingly, LXRs activation increases the accumulation of Vimentin, a protein involved in EMT. Moreover, migration and invasion of PC3 cells is significantly increased, demonstrating an upregulation of EMT by LXRs activation in this cell line. Interestingly, *in vivo*, LXRs activation significantly augments the number of metastasis in the lungs and lymph nodes in our mice model. Finally, public data (TCGA) shows a positive correlation between LXRs target genes and Vimentine in human metastasis from prostate cancer samples.

**Conclusion**

LXRs have been described to inhibit EMT in several cancers. However, in this project, we highlight a novel role of LXRs in advanced prostate cancer. Our results indicate that LXRs activation increases the accumulation of Vimentin, migration and invasion of a metastatic cell line, suggesting an activation of EMT by LXRs. *In vivo*, we show that treatment with LXRs agonist augments metastatic spread. Altogether, this work identifies a promoting effect of LXRs on epithelial-mesenchymal transition in advanced prostate cancer.

### EACR23-0909
### Effect of neoadjuvant FOLFOXIRI plus Bevacizumab treatment of colorectal peritoneal metastasis on local and systemic immune cells

*M. Sachet[1], C. Müller[1], A. Beer[2], H. Birnleitner[1], M. Rainer[1], T. Bachleitner-Hofmann[3], R. Oehler[1]*
*[1]Medical University of Vienna, Department of General Surgery – Research Laboratories, Vienna, Austria*
*[2]Medical University of Vienna, Department of Pathology, Vienna, Austria*
*[3]Medical University of Vienna, Department of General Surgery, Vienna, Austria*

**Introduction**

The immune system plays a crucial role in the outcome of colorectal cancer. Systemic chemotherapies modulate the immune cell composition and thus interfere with the sensitivity of colorectal cancer to immunotherapy. Little is known about these changes in peritoneal metastasized colorectal cancer. Thus, we aimed to characterize local and systemic immune cells in the course of systemic chemotherapy. We included 20 patients with peritoneal metastasized colorectal cancer in our exploratory study. All patients received systemic FOLFOXIRI plus bevacizumab.

**Material and Methods**

Tumour tissue and peritoneal fluid were collected before and after systemic chemotherapy. Peripheral blood was taken at different times. The main immune cell subtypes were characterized using flow cytometry and immunohistochemistry.

**Results and Discussions**

Neutrophils and the neutrophil-to-lymphocyte ratio significantly declined in response to systemic chemotherapy while circulating T cells increased (CD8+ p=0.015, CD4+ p=0.041). In peritoneal fluid we observed a decrease of CD25+/FOXP3+/CD4+ regulatory T cells (p=0.049). T cell infiltration in the tumour microenvironment showed a considerable variability between patients. However, the number of tumour infiltrating lymphocytes was not significantly changed by the application of chemotherapy. Our data show that immune cell distribution after systemic chemotherapy changes in peripheral blood. Interestingly, in peripheral fluid only the inhibitory Treg population decreased and local T cells within peritoneal metastases remain unaffected.

**Conclusion**

These data indicate little to no effect of neoadjuvant chemotherapy on the local immune system supporting the

© 2023 The Authors. Molecular Oncology is published by John Wiley & Sons Ltd on behalf of Federation of European Biochemical Societies need for new therapeutic options and perhaps opening new opportunities for immunotherapy.

### EACR23-0913
### PML hinders senescence in clear cell renal cell carcinoma by restraining p53 activity

*M. Simoni*[1], *C. Menegazzi*[2], *C.C. Biffi*[2], *F. Genova*[3], *J. Brugarolas*[4,5], *R. Bernardi*[1]
[1]*IRCCS San Raffaele Scientific Institute, Milan, Italy*
[2]*Vita-Salute San Raffaele University, Milan, Italy*
[3]*Centre for Omics Sciences, IRCCS San Raffaele Scientific Institute, Milan, Italy*
[4]*Hematology-Oncology Division, Department of Internal Medicine, University of Texas Southwestern Medical Center, Dallas, USA*
[5]*Kidney Cancer Program, Simmons Comprehensive Cancer Center, University of Texas Southwestern Medical Center, Dallas, USA*

Introduction: Clear cell renal cell carcinoma (ccRCC) is the most common type of renal cancer and accounts for 4% of all adult malignancies leading to ~134,000 deaths per year worldwide. Currently, ccRCC is amongst the most difficult-to-treat cancers. Five years survival rates drop from 70-90% for localized tumors, to 13% for metastatic disease, and patients often develop resistance to treatment. Therefore, finding new therapeutic strategies is an urgent clinical need. Here, we identified the promyelocytic leukemia protein (PML) as a novel druggable target in ccRCC. PML has long been described as a tumor-suppressor gene, but more recent evidence shows that it is endowed with oncogenic functions in specific tumor types. We found that PML is overexpressed in ccRCC, where it plays fundamental functions of tumor promotion, and its targeting halts tumor progression. Materials and methods: We performed in vivo and in vitro functional assays following PML knock-down with an inducible shRNA system or PML pharmacological targeting with arsenic trioxide. We systematically characterized the oncogenic functions of PML in ccRCC and defined the mechanistic basis of PML dependency. RNA sequencing was used to identify a PML-dependent transcriptional signature of cell proliferation. Results and discussion: We found that PML is overexpressed in ccRCC, where it correlates with increased mortality. By silencing PML in ccRCC cell lines and patient-derived xenograft (PDX), we found that PML inhibition halts proliferation in vitro and in vivo by unleashing cellular senescence. RNA sequencing confirmed the down-regulation of pro-proliferative oncogenic pathways and the up-regulation of senescence-related pathways. Mechanistically, we found that PML knock-down leads to dramatic p53 accumulation, a finding that has important therapeutic implications as p53 is rarely mutated in ccRCC. Finally, we found that pharmacological inhibition of PML with arsenic trioxide, an antineoplastic agent used as first-line treatment for acute promyelocytic leukemia patients, inhibits ccRCC cell proliferation in vitro and in vivo, promotes p53 accumulation, thereby recapitulating PML genetic manipulation. Conclusion: Here we report for the first time that ccRCC is addicted to PML expression for proliferation. On these bases, PML targeting with arsenic trioxide may represent a new therapeutic opportunity for ccRCC.

### EACR23-0915
### The Role of Transforming Growth Factor Beta Induced (TGFBI) in Cancer-Associated Fibroblasts

*S.C. Arandkar*[1], *S. Ramanathan*[1], *S. Mukherjee*[1], *G.D. Yengkhom*[1]
[1]*Advanced Centre for Treatment- Research and Education in Cancer- Tata Memorial Centre, Cancer Research Institute, Navi Mumbai, India*

**Introduction**

Cancer-Associated Fibroblasts (CAF), a predominant population in the tumor stroma is regulated by the TGF-β signaling cascade. Our work decodes the dynamics of stromal modulation, especially CAFs in NSCLC, by TGFBI, a 683 amino acid ECM binding protein downstream to the TGF-β pathway.

**Material and Methods**

Differential expression of TGFBI and CAF markers between NF and CAF cells upon conditioned media treatment and co-culture was estimated by qRT PCR, Immunoblotting and Immunofluorescence assays. RNAi was employed to knockdown TGFBI in CAFs to assess its functional importance through Collagen contraction, Trans-well migration and Gap closure assays.

**Results and Discussions**

TGFBI was highly expressed in CAFs at both mRNA and protein levels which was also reflected at a single-cell level through Immunofluorescence. While upon conditioned media treatment and co-culture with tumor cells, TGFBI and ACTA -2 were upregulated in both NFs and CAFs. The importance of TGFBI in CAFs was confirmed through knockdown resulting in the loss of collagen contraction property. We observed an overall decrease in the migration of TGFBI knockdown in CAFs and a decrease in tumor cell migration towards TGFBI knockdown CAFs compared to its control counterpart.

**Conclusion**

TGFBI is upregulated in CAFs, and its expression increases further upon tumor cell contact. While its knockdown reduced CAF contraction, migratory and chemo-attractant properties suggest that TGFBI is a key molecule in regulating CAF biology.

### EACR23-0925
### The MEK/MYC axis is required to support metastatic stem-like cells from Cancer of Unknown Primary

*F. Verginelli*[1], *G. Gambardella*[2], *E. Cascardi*[3], *E. Candiello*[1], *A. D'Ambrosio*[1], *A. Pisacane*[3], *A. Iuliano*[4], *E. Geuna*[5], *F. Montemurro*[5], *A. Sapino*[3], *P. Comoglio*[6], *C. Boccaccio*[1]
[1]*Candiolo Cancer Institute- FPO-IRCCS, Cancer Stem Cell Laboratory, Candiolo, Italy*
[2]*Telethon Institute of Genetics and Medicine TIGEM, Computational Biology for precision Medicine, Pozzuoli, Italy*
[3]*Candiolo Cancer Institute- FPO-IRCCS, Pathology Unit, Candiolo, Italy*
[4]*Telethon Institute of Genetics and Medicine TIGEM,*

© 2023 The Authors. Molecular Oncology is published by John Wiley & Sons Ltd on behalf of Federation of European Biochemical Societies

*Bioinformatics Core, Pozzuoli, Italy*
[5]*Candiolo Cancer Institute- FPO-IRCCS, Multidisciplinary Oncology Outpatient Clinic-, Candiolo, Italy*
[6]*IFOM- FIRC Institute of Molecular Oncology, Scholar, Milan, Italy*

**Introduction**

Cancer of unknown primary (CUP) is a rare but rapidly lethal malignancy, characterized by widespread metastases without an identifiable primary tumor or specific tissue markers for classification and treatment planning. Although presenting with different metastatic patterns and histopathological features, CUP patients invariably experience aggressive disease progression, resistance to conventional therapies and, often, a lack of biomarkers exploitable for a targeted approach. To shed light on the enigmatic pathogenesis of this malignancy and its therapeutic liabilities, we have recently isolated and characterized cancer stem-like cells, called 'agnospheres', from a cohort of CUPs patients. We reported that the majority of CUPs share upregulated MAPK-MYC signaling and that treatment with the MEK inhibitor trametinib halt cell proliferation and triggers apoptosis *in vitro,* as well as inhibits the growth experimental tumors at injection and metastatic sites. Here, we investigated the requirements of downstream MEK signaling targets for the CUP phenotype.

**Material and Methods**

Agnospheres were treated with trametinib or vehicle in time-course experiments. RNA and proteins were analyzed with 3'UTR RNAseq analysis and Mass-Spec, respectively. GSEA of Cancer Hallmarks analysis were performed and normalized enrichment score with FDR<10% were considered statistically significant. Data where validated with western blot and qPCR. Agnospheres were treated with the MYC inhibitor JQ1 and their viability and apoptotic response were measured. Tissue microarrays were generated with tramentinib vs vehicle-treated agnospheres and analyzed by immunohistochemistry.

**Results and Discussions**

Here, we show that in agnospheres: (i) sensitivity to MEK inhibition correlates with prominent downregulation of gene sets modulated by MYC; (ii) MYC down-regulation by JQ1 mimics trametinib-induced cell proliferative arrest and death; (iii) cell death induced by trametinib or JQ1 is accompanied by agnosphere pseudo-differentiation, characterized by acquisition of tissue markers likely reminiscent of a hypothetical tissue of origin; (iv) pseudo-differentiation tend to persist in cells surviving treatment, with implications for further tissue-specific approaches.

**Conclusion**

Altogether our results highlight the key role of the MEK/MYC axis in CUP pathogenesis, and suggest potential therapeutic strategies aimed at targeting these players and/or their downstream effectors.

### EACR23-0933
### Reduced Neurocan in pancreatic cancer is associated with poor prognosis.

*Y. Xu[1], W.G. Jiang[1], Y. Lin[1], H. Chunyi[2]*
[1]*Cardiff University- Medical School, Cardiff China Medical Research Collaborative, Cardiff, United Kingdom*
[2]*Key laboratory of Carcinogenesis and Translational Rese arch Ministry of Education- Beijing- Peking University Cancer Hospital & Institute, Department of Hepato-Pancreato-Biliary Surgery, Beijing, China*

**Introduction**

NCAN,a member of the lectican family, promotes cell division,spheroid formation,and enhances malignant traits of cancerous cells in brain. Pancreatic cancer is one of the most lethal malignant neoplasms with high mortality rate. Novel biomarkers are urgently needed to achieve accurate and early diagnosis.To date,involvement of NCAN in pancreatic cancer remains unknown,this study aims to evaluate the expression of NCAN in pancreatic cancer and corresponding clinical implication.

**Material and Methods**

NCAN transcripts were determined in a discovery cohort containing 137 pancreas ductal adenocarcinoma(PDAC) tissues and 133 adjacent normal tissues using real time PCR. Relationship between NCAN and pathology features were determined by non-parametric statistical methods: One-way ANOVA, Mann–Whitney test and Wilcoxon rank test.E-GEOD-71729, E-GEOD-21501 and E-MTAB-6134 were employed for further analyses. NCAN knockdown and overexpression were established using lentiviral vectors respectively followed by cellular functional assays.

**Results and Discussions**

Reduced NCAN transcript levels were exhibited in pancreas tumours tissues in comparison with paired adjacent non-tumours in the discovery cohort($p<0.0001$). Lower expression levels of NCAN were observed in adenocarcinoma compared with other cancer types($p=0.0149$) except for ductal carcinoma. Although lack of statistical significance, a trend of decreased NCAN levels was seen in advanced tumours according to the T staging. An increased expression of NCAN was also evident in highly differentiated tumours in comparison with moderate differentiated($p=0.0493$). Furthermore, reduced expression of NCAN is associated with shorter overall survival($p=0.016$) with a median of 19.7 months, compared with 23.0 months in patients with higher expression levels of NCAN. Reduced expression of NCAN is also associated with shorter disease-free survival with a median survival of 14.7 months, $p=0.009$ compared with patients with a higher NCAN expression tumour(median survival of 16.6 months). Furthermore, NCAN significantly enhanced adhesion and proliferation of pancreatic cancer cells.

**Conclusion**

NCAN was downregulated in pancreatic cancer and the reduced expression was associated with poor prognosis. Further investigation will shed light on the influence of NCAN on cellular functions of pancreatic cancer and underlying molecular mechanisms and will also provide proof for its potential as a biomarker or prognostic factor.

### EACR23-0936
### Cancer stem cell marker ALDH1A3 promotes breast cancer metastasis by regulating the plasminogen activation pathway

*M. McLean[1], A. Bharadwaj[1], M. Dahn[1], H. Cahill[1],*

© 2023 The Authors. Molecular Oncology is published by John Wiley & Sons Ltd on behalf of Federation of European Biochemical Societies

M.C. Wasson[1], R.P. Arun[1], O. Walker[1], P. Barnes[1], G. Bethune[1], G. Knapp[2], L. Helyer[2], C. Giacomantonio[3], B. Cruickshank[2], D. Waisman[4], P. Marcato[5]
[1]Dalhousie University, Pathology, Halifax, Canada
[2]Dalhousie University, Surgery, Halifax, Canada
[3]Dalhousie University, Pathology and Surgery, Halifax, Canada
[4]Dalhousie University, Pathology and Biochemistry and Molecular Biology, Halifax, Canada
[5]Dalhousie University, Pathology- Microbiology and Immunology and Nova Scoti a Health Authority, Halifax, Canada

**Introduction**

Breast cancers with high levels of cancer stem cell marker aldehyde dehydrogenase 1A3 (ALDH1A3) have poor patient outcomes and increased rates of metastasis. This study aims to investigate the molecular mechanism of ALDH1A3-mediated metastasis in breast cancer.

**Material and Methods**

To investigate the molecular mechanism of ALDH1A3-mediated metastasis in breast cancer, we assessed the effects of ALDH1A3 on the expression of protease families that regulate the degradation of the extracellular matrix, an essential early step for metastasis. These analyses implicated that ALDH1A3 regulates the plasminogen activation pathway and the serine protease, plasmin. RT-qPCR, western blots, tissue plasminogen activator (tPA) and urokinase plasminogen activator (uPA) activity assays were used to examine expression levels of plasminogen activators in breast cancer cells manipulated to have varying levels of ALDH1A3. Plasminogen activation assays were also used to assess the effect of ALDH1A3 expression in breast cancer cells on plasmin generation. Transwell invasion assays assessed for effects on invasion. Fixed sections of patient tumor tissues were stained by multiplex immunofluorescence for ALDH1A3, tPA, uPA, and plasminogen activator inhibitor 2 (PAI-2) levels and assessed for co-localization and clinical correlations. Immunodeficient mice were orthotopically implanted with breast cancer cells, with or without knockdown of tPA, and assessed for effects on tumor growth and metastasis.

**Results and Discussions**

ALDH1A3 increased tPA and uPA and decreased PAI-2 levels in breast cancer cells. ALDH1A3 increased plasmin generation by increasing tPA. Plasmin generation is required for ALDH1A3-mediated invasion. In fixed breast cancer patient tumor samples, expression of ALDH1A3 was highly correlated with tPA and high numbers of ALDH1A3+/tPA+ tumor cells correlated with increased tumor grade and the development of metastasis. Knockdown of tPA in tumor xenografts resulted in decreased metastasis to the lymph nodes of mice, consistent with the role of tPA in mediating metastasis.

**Conclusion**

These analyses provide new mechanistic information for the increased breast cancer metastasis associated with increased expression of cancer stem cell marker ALDH1A3. This study demonstrates that ALDH1A3 increases the expression of tPA leading to increased plasmin generation, invasion, and metastasis in breast cancer. Targeting the plasminogen activation pathway in ALDH1A3 high tumors could be a novel therapeutic strategy.

### EACR23-0952
### Role of DTX3L and PARP9 in breast cancer

G. Paroni[1], M.A. Caricasulo[1], A. Zanetti[1], A.V. Cavallaro[1], M.C. Barbera[2], E. Garattini[1], M. Terao[1]
[1]Mario Negri Institute for Pharmacological Research, Biochemistry and Molecular Pharmacology, Milan, Italy
[2]Mario Negri Institute for Pharmacological Research, Oncology, Milan, Italy

**Introduction**

DTX3L and PARP9 form a complex with both ubiquitin ligase and ADP-ribosyl transferase activity and endowed with anti-viral properties. The complex is well studied in the context of the host defense to viruses, as it is induced by these organisms and by interferons. Both DTX3L and PARP9 are required for a proper antiviral response. High levels of DTX3L/PARP9 are expressed in Diffuse Large B Cell Lymphoma characterized by a prominent ineffective immune infiltrate and in prostate cancer cell-lines showing increased IFN/STAT1 signaling.

**Material and Methods**

Transcriptomic data analysis of breast cancer patients and cell lines were done using on-line tools (cBioPortal for Cancer Genomics). DTX3L and PARP9 protein levels were assessed by WesternBlot analysis. DTX3L sh-RNA silenced models were generated by lentiviral infection of SKBR3 and HCC70 cell lines. Pattern Recognition Receptor (PRR) pathway activation was carried-out by poli(I:C) transfection and evaluated by WesternBlot analysis of its members. Drug activity on cell proliferation was measured by Sulforhodamine assay.

**Results and Discussions**

DTX3L and PARP9 expression is higher in the neoplastic tissue as compared to the normal counterpart and correlates with high interferon I signaling and levels of gene products involved in antiviral responses. In breast cancer cell lines, PARP9 and DTX3L are co-expressed and form a protein complex. Their expression pattern is heterogeneous with high expression levels associated with sustained expression of proteins involved in antiviral defense signaling. Mimicking viral infection and activation of the pattern recognition receptor pathway by poli(I:C) transcriptionally induced DTX3L and PARP9 expression. DTX3L/PARP9 increased expression levels occurs early and before the appearance of poli(I:C) triggered apoptosis. The inhibition of the PRR pathway by the use of a TBK-1 inhibitor, abrogates DTX3L/PARP9 up-regulation suggesting that the complex participates in the PRR signaling process. DTX3L upregulation occurs also after treatment of breast cancer cell lines with viral mimicry inducing agents such as retinoic acid and a KDM5 inhibitor.

**Conclusion**

DTX3L and PARP9 are up-regulated in breast cancer and their expression associates with tumors expressing viral defense signaling proteins at high levels. In breast cancer cells the PRR pathway activation governs transcriptionally their expression that occurs early before apoptosis induction. Further studies are required to decipher DTX3L/PARP9 role in breast cancer biology.

© 2023 The Authors. Molecular Oncology is published by John Wiley & Sons Ltd on behalf of Federation of European Biochemical Societies

### EACR23-0958

### Selected clones of MDA-MB-231 after chronic treatment with 2-deoxy-D-glucose or combined metformin + 2-deoxy-D-glucose treatment show increased population of cells in anchorage-independent conditions.

T. Snedec[1], J. Repas[1], M. Pavlin[1,2]
[1]Faculty of Medicine- University of Ljubljana, Institute of biophysics, Ljubljana, Slovenia
[2]Faculty of Electrical Engineering- University of Ljubljana , Group for Nano- and Biotechnological applications, Ljubljana, Slovenia

**Introduction**

Cancer cells exhibit altered metabolism and immune escape. Metformin is a metabolic drug with promising anti-cancer effects whose mechanisms of action on cancer are yet to be fully understood. It exhibits synergistic effects in combination with 2-deoxy-D-glucose (2DG). High concentrations of 2DG and the combination of metformin and 2DG cause reversible detachment of a population of viable triple-negative breast cancer cells (MDA-MB-231) retaining proliferation capacity after reseeding, resembling early steps of metastasis formation. Though detached and attached cells differ metabolically, the underlying mechanism remains unclear. Treated cells have increased mitochondrial mass and decreased PD-L1 expression. Our study aims to investigate how cancer cells adapt to chronic treatment with metformin and 2DG and to analyze differences between chronically treated cells - selected floating and unselected cells. This will help us understand the mechanisms of adaptations to treatments and mechanisms that support survival in an anchorage-independent state.

**Material and Methods**

MDA-MB-231, triple-negative breast cancer cells were treated for 72 h with 4.8 mM 2DG or 5 mM metformin + 0.6 mM 2DG, non-treated cells were also grown on poly-HEMA. Floating cells were harvested and plated to reattach. Selection lasted for four cycles.

**Results and Discussions**

We observed an increase in the floating fraction of cells after selection in both treatments by up to 30 % with over 90 % of viable cells. Treated selected cells proliferated slower than untreated cells. We observed no differences in mitochondrial mass between the detached and the attached cells, selected clones had increased mitochondrial mass compared to unselected cells. Treated selected clones had increased ROS production. Attached cells had higher ROS production than detached cells. Interestingly, treated selected clones had in general decreased PD-L1 expression.

**Conclusion**

With chronic treatment, we have selected clones that are more likely to detach and survive in a detached state. Selected clones express lower PD-L1, which makes them more prone to be eliminated by the immune system and have adapted to increased ROS production. Selected clones had increased mitochondrial mass, which has already been linked to anchorage independence.

### EACR23-0968

### Hippo pathway targeting in TP53-mutant Uterine Leiomyosarcoma

A. Costa[1], L. Gozzellino[1], T. Palumbo[2], M. Nannini[3], M. Urbini[4], G. Pasquinelli[5], M.A. Pantaleo[3], A. Astolfi[1]
[1]University of Bologna, Department of Medical and Surgical Medicine, Bologna, Italy
[2]University of Bologna, Alma Mater Institute on Healthy Planet, Bologna, Italy
[3]IRCCS Azienda Ospedaliero - Universitaria di Bologna, Division of Oncology, Bologna, Italy
[4]IRRCS Istituto Romagnolo per lo Studio dei Tumori IRST ' 'Dino Amadori", Biosciences Laboratory, Meldola, Italy
[5]IRCCS Azienda Ospedaliero - Universitaria di Bologna, Division of Pathology, Bologna, Italy

**Introduction**

Uterine Leiomyosarcoma (uLMS) is a rare malignant neoplasm accounting for ~1% of female genital tumors. uLMS is characterized by an aggressive clinical course and a scarce response to chemotherapy. The high frequency of *TP53* inactivation (>60% of patients) is linked to uLMS progression and therapy resistance. Recent evidence prove that the Hippo signaling cooperates with wild-type p53 as tumor suppressor to induce senescence and apoptosis in response to stress conditions, while mutant p53 forms a complex with YAP promoting the transcription of key cell cycle regulators. Thus, we explored Hippo pathway deregulation in *TP53*-mutated uLMS and in gene-edited induced Pluripotent Stem Cells (iPSC) differentiated towards the mesodermal layer.

**Material and Methods**

The effects of the YAP inhibitor Verteporfin (VP) on cell viability, proliferation and apoptosis were measured in two *TP53*-mutated uLMS cell lines (SK-UT-1 and SK-LMS-1) by MTT assay, Brdu Cell Profliferation assay and cytometry by Annexin-V kit, respectively. Deregulation of the Hippo signaling was analyzed by quantitative PCR on the main readouts of the pathway (CTGF and Cyr61). RNA sequencing was performed with the Illumina Truseq mRNA stranded kit. VP-treatment was performed on *TP53*$^{-/-}$ iPSC clones, previously obtained by *TP53* knockout genome editing and then differentiated towards the mesodermal layer.

**Results and Discussions**

VP treatment induced cell viability decrease on both SK-UT-1 ($IC_{50}$=3,4 uM) and SK-LMS-1 ($IC_{50}$=3,7 uM), and resulted in reduced cell proliferation, increased percentage of apoptotic cells and decreased expression of the YAP-target genes. Gene expression profiling of VP-treated cell lines showed an upregulation of the p53 pathway and a downregulation of genes involved in myogenesis and epithelial to mesenchymal transition. Further, pluripotent and differentiated *TP53*$^{-/-}$ iPSC clones showed upregulation of the Hippo pathway readouts with respect to their wild-type counterpart and displayed a viability decrease upon VP treatment with an $IC_{50}$ of 1,3 uM.

**Conclusion**

These results prove that interfering with Hippo pathway deregulation in *TP53*-mutated uLMS could represent a novel approach for an aggressive disease orphan of effective therapies. In this context, we demonstrate the possibility to study pathway activation and interference in

© 2023 The Authors. Molecular Oncology is published by John Wiley & Sons Ltd on behalf of Federation of European Biochemical Societies an iPSC model, that mimics the disease both with respect to the oncogenic signature and cell lineage commitment.

### EACR23-0970
### Unraveling the Trop-2 Interactome: Insights into the Role of Trop-2 and Desmoglein 2 in Breast Cancer Metastasis

*O. Vacek[1,2,3], K. Gömöryová[3], T.W. Radaszkiewicz[3], T. Bárta[4], P. Jirgalová[1,2,3], J. Remšík[5], P. Beneš[2,3], A. Hampl[2,4], V. Bryja[1,3], K. Souček[1,2,3]*
*[1]Institute of Biophysics of the CAS - v. v. i., Department of Cytokinetics, Brno, Czech Republic*
*[2]St. Anne's University Hospital, International Clinical Research Center, Brno, Czech Republic*
*[3]Masaryk University, Department of Experimental Biology - Faculty of Science, Brno, Czech Republic*
*[4]Masaryk University, Department of Histology and Embryology - Faculty of Med icine, Brno, Czech Republic*
*[5]Memorial Sloan Kettering Cancer Center, Human Oncology and Pathogenesis Program, New York, United States*

**Introduction**
The majority of cancer-related deaths result from metastasis, and epithelial-mesenchymal plasticity (EMP) is one of the crucial mechanisms during dissemination. Epithelial marker Trop-2 is considered to be linked with worse outcome of patients with breast and other carcinomas. Despite efforts to develop Trop-2 antibody-based cancer therapy, its role during metastatic cascade is still unclear. As Trop-2 is involved in organization of epithelium, we investigated the relationship between Trop-2 loss and mesenchymal phenotype of cancer cells. We therefore concentrated on identifying novel interactions of Trop-2 that influence the epithelial phenotype of tumor cells by strengthening cell-cell adhesion. For this purpose, we performed Trop-2 interactome screen using proximity-dependent biotin identification assay (BioID).
**Material and Methods**
BioID assay identified ineractome in breast epithelial HMLE and cancer MDA-MB-231 cells. Selected candidates were validated in HaCaT cells using immunocytochemistry and proximity ligation assay. Western blot analysis and fluorescence microscopy was used for quantification of desmoglein 2 (DSG2) level in HaCaT and T-47D Trop-2 KO cells. Integrity of the epithelial monolayer measured using transepithelial resistance assay, permeability assay and Dispase assay.
**Results and Discussions**
We detected interaction of Trop-2 and desmogleins in breast non-tumor and tumor epithelial cells and confirmed this interaction in epithelial cell in general. Interaction is affecting DSG2 function as the loss of Trop-2 is accompanied by DSG2 protein level downregulation and change in its localization on the membrane of breast cancer cell model. Trop-2 KO cells show decreased DSG2 level and altered subcellular localization of DSG2. Trop-2 loss is associated with decreased intercellular adhesion manifested as reduced epithelial monolayer integrity and this phenotype is reflected after DSG2 loss.
**Conclusion**
We first describe the Trop-2 interactome using the BioID assay. One of the identified interactions - DSG2 - plays a significant role alongside Trop-2 during EMP as both proteins enhance the integrity of epithelial cell-cell connections. Remodeling of surface molecules is a crucial step during metastasis, and impact of EMP on both Trop-2 and DSG2 level fits in the formulated hypothesis.
This work was supported by Czech Science Foundation, grant nr. 21-11585S.

### EACR23-0977
### Role of the mitochondrial fission protein DRP1 on tumorigenic potential and docetaxel resistance in prostate cancer

*F. Roa[1], I. González[1], D. Savic[2], J. Van Goubergen[3], F.R. Santer[3], M. Puhr[3], J.R. Toledo[1], I. Skvortsova[2], Z. Culig[3], H. Klocker[3]*
*[1]University of Concepcion, Pathophysiology, Concepcion, Chile*
*[2]Medical University of Innsbruck, Therapeutic Radiology and Oncology, Innsbruck, Austria*
*[3]Medical University of Innsbruck, Urology, Innsbruck, Austria*

**Introduction**
Mitochondrial dynamics refers to the process of continuous movement of mitochondria and changes in their morphology through mitochondrial fission (fragmentation) and fusion (elongation). This process is regulated by the fission protein DRP1 and the fusion proteins MFN1, MFN2 and OPA1. Mitochondrial dynamics has been described as unbalanced in cancer, with a fragmented mitochondrial network associated to tumorigenesis. However, if mitochondrial dynamics is involved in prostate cancer progression and chemotherapy resistance remains unclear. The aim of this work was to study the role of DRP1 and OPA1 on tumorigenic potential and docetaxel resistance in prostate cancer cells.
**Material and Methods**
Prostate cancer cell lines DU145 and docetaxel-resistant DU145 (DU145-DR) were used. DU145-DR were previously generated by exposure to increasing concentrations of docetaxel. DRP1 and OPA1 knockdown was performed by siRNA transfection and the silencing efficiency was analyzed by western blot. The effect of the knockdown in mitochondrial morphology, proliferation and cell motility was analyzed by immunofluorescence, colony formation assays, cell counting and migration assays. Energy metabolism analysis was performed in a Seahorse XF Analyzer. Transcriptome analysis was performed by RNAseq and Gene Set Enrichment Analysis.
**Results and Discussions**
Microscopy analysis of mitochondrial morphology in both cell lines showed that DRP1 and OPA1 silencing produced more elongated or fragmented mitochondria, respectively. DRP1 silencing decreased proliferation, colony formation capacity and migration, while OPA1 silencing produced no effect. Seahorse experiments showed that DRP1 silencing decreased mitochondrial respiration and ATP production, while OPA1 silencing increased them. RNAseq analysis showed that DRP1 and OPA1 silencing produced changes in gene sets related to mitochondrial function and cancer-associated pathways. Interestingly, silencing DRP1 produced a stronger effect in DU145-DR than DU145

© 2023 The Authors. Molecular Oncology is published by John Wiley & Sons Ltd on behalf of Federation of European Biochemical Societies when analyzing proliferation, colony formation capacity, migration and mitochondrial respiration.

**Conclusion**

Changes in mitochondrial dynamics mediated by DRP1 silencing decrease tumorigenic potential in prostate cancer cells, with a stronger effect in docetaxel-resistant cells. Our results suggest that DRP1 induced-mitochondrial fission promotes prostate cancer malignant potential and resistance to docetaxel. This might be mediated by an increased mitochondrial respiration, which confers a metabolic advantage to cancer cells.

### EACR23-0985

### The endothelin 1 receptors shape extracellular matrix remodeling by cancer associated-fibroblasts through β-arrestin-1.

*D. Del Rio[1], V. Caprara[2], I. Masi[1], F. Ottavi[1], V. Licursi[1], M. Kunkl[3], L. Tuosto[3], A. Bagnato[2], F. Spadaro[4], L. Rosanò[1]*
*[1]Institute of Molecular Biology and Pathology- CNR, Dipartimento di Scienze Biomediche, Rome, Italy*
*[2]Regina Elena National Cancer Institute, Modelli Preclinici e Nuovi Agenti Terapeutici, Rome, Italy*
*[3]Università La Sapienza, Biologia e Biotecnologie Charles Darwi, Rome, Italy*
*[4]Istituto Superiore di Sanità, Confocal Microscopy Unit-Core Facilities, Rome, Italy*

**Introduction**

Cancer-associated fibroblasts (CAFs) are the major effectors of extracellular matrix (ECM) remodeling through secretion of collagens, cross-linking enzymes and proteases, and engagement of integrins especially integrin β1 (Intβ1). Tumor-related endothelin-1 (ET-1) controls the crosstalk between cancer and stromal cells supporting serous ovarian cancer (SOC) progression, acting through ETA and ETB receptors and the recruitment of β-arrestin-1 (β-arr1) which regulate active signaling complexes. However, how the ET-1/βarr-1 axis might "educate" stroma to become permissive to SOC invasion needs to be investigated.

**Material and Methods**

Human primary ovarian fibroblasts (HOF) cells were cultured with ET-1 and/or Ambrisentan or BQ123 (ETAR antagonists), and/or BQ788 (ETBR antagonist), and/or Bosentan (ETA/B R antagonist), or ATN-161 (Intβ1 antagonist). sh-β-arr1 HOFs were obtained with lentiviruses infection. Protein expression/activation was evaluated by Western Blotting, immunofluorescence (IF) and pulldown (PD) assays. Gene expression patterns were evaluated by qRT-PCR analysis and RNA-seq. Promoter activity was evaluated by Luciferase Reporter Assay. Invadosome activity was analyzed by IF and gelatin degradation assays. In vivo assays were performed using HOF-Luc+ SKOV3-Luc (SOC cells) treated or not with Ambrisentan (5mg/kg) or Bosentan (10mg/kg) or sh-β-arr1-HOF Luc + sh-β-arr1 -SKOV3 Luc i.p injected in mice (5 weeks).

**Results and Discussions**

HOFs secrete ET-1 and express both ET1 receptors along with β-arr1. ET-1 increases CAF markers expression, cell proliferation, secretion of proinflammatory cytokines, collagen contraction, and cell motility, while these effects were lost in β-arr1-silenced cells, or upon ET1 receptor antagonist treatment. Of note, ET-1 increases Col1 expression through nuclear β-arr1, while sh-β-arr1 HOFs exhibit an altered ECM proteins expression, in particular collagen-containing ECM. Moreover, ET-1 promotes co-localization of Intβ1 with the invadosome marker cortactin, while sh-β-arr1 or ET1-R antagonists induce a loss of ET-1-dependent ECM degradation. In vivo, Bosentan or βarr1-depleted cells significantly inhibit SOC cell growth and spreading.

**Conclusion**

These data indicate that ET-1/βarr-1 axis educates the stromal HOF to become permissive for SOC invasion by promoting ECM remodeling and desmoplasia.

### EACR23-0986

### Beyond the MAPK Pathway: Unraveling the Importance of RKIP in Lung Adenocarcinoma

*A. Raquel-Cunha[1], J. Pinheiro[1], D. Cardoso-Carneiro[1], P. Fontão[1], A. Mendes[1], M. Gabriela-Freitas[1], R.M. Reis[1,2], O. Martinho[1,2]*
*[1]ICVS- Life and Health Sciences Research Institute, BiTCancer Team, Braga, Portugal*
*[2]Hospital de Amor, Molecular Oncology Research Center, Barretos, Brazil*

**Introduction**

Lung cancer continues to be the most fatal cause of cancer-related deaths worldwide, being late diagnosis a major contributing factor, strongly influencing patients' response to therapy. The most frequent subtype of non-small cell cancer, lung adenocarcinomas, often shows activating changes in the MAPK pathway, which are strongly linked to resistance mechanisms and tumor aggressiveness. In light of this, Raf kinase inhibitor protein (RKIP) is known to be a regulator of such pathway and other cancer-relevant ones, mostly exerting an inhibitory effect. Importantly, this tumor suppressor protein is downregulated in several types of tumors and its loss is associated with metastasis development and increased tumor aggressiveness. However, the role of RKIP in lung adenocarcinomas is not fully explored. Hence, this study aims to explore RKIP's role in lung tumorigenesis and its clinical significance.

**Material and Methods**

Thus, two lung adenocarcinoma cell lines, PC9 and HCC827, were genetically altered to knockout (KO) and overexpress (OE) RKIP. The impact of RKIP dysregulation was evaluated in vitro (migration, viability, clonogenicity, 3D assay) and in vivo (subcutaneous, tail vein xenograft mouse model). Nanostring analysis (PanCancer Pathways Panel) was performed to identify genes whose expression could be altered by RKIP OE. Western blot and qPCR were done to assess the expression and/or activation of RKIP, its targets, and relevant signaling pathways. I*n silico* analysis was attained using TCGA data, to infer RKIP alterations and their clinical significance.

**Results and Discussions**

Interestingly, the dysregulation of RKIP had a more significant impact in vivo than on in vitro experiments, according to our results. Upon KO of RKIP tumor growth in mice happened faster compared with its control and, on

© 2023 The Authors. Molecular Oncology is published by John Wiley & Sons Ltd on behalf of Federation of European Biochemical Societies the contrary, overexpressing RKIP impaired tumor growth. Nanostring data showed that, upon RKIP OE, genes related to focal adhesion, TNF, IL18 pathway, and apoptosis were overexpressed and, genes behind MAPK and PI3K/AKT were downregulated. Further, in silico data revealed that loss of RKIP was associated with worse overall survival in adenocarcinoma patients.

**Conclusion**

Altogether, these data show that RKIP's loss of expression induces lung adenocarcinoma aggressiveness. Importantly, this work pointed to the importance of exploring the role of RKIP, together with the tumor microenvironment and the immune system, to fully understand the consequences of RKIP's loss in tumorigenesis.

### EACR23-0990
### A role for PROM2 in cancer: Implications for metastasis and resistance to cell death in cancers

*J. PARIS[1], C. Wilhelm[2], C. Lebbé[3], G. Falgarone[1], A.T. Phan[4], G. Bousquet[1]*
*[1]Université Sorbonne Paris Nord, Inserm UMR_S942- MASCOT, Bobigny, France*
*[2]Institut Curie, Laboratoire Physico Chimie Curie, Paris, France*
*[3]Université Paris Cité, Human Immunology- Pathophysiology- Immunotherapy, Paris, France*
*[4]Nanyang Technological University, Institute of Structural Biology, Singapore, Singapore*

**Introduction**

Metastasis is still a leading cause of cancer-related death. The identification of new targets that can prevent or slow down the progression of metastasis is essential. Our team previously identified PROM2 as a biomarker predictive of distant metastases in human melanoma. Here, we investigated the significance of PROM2 overexpression in cancer cell using a new pathophysiological *in vivo* model.

**Material and Methods**

We injected intravenously 6-weeks old nude mice with human melanoma A375 cell lines overexpressing PROM2 or not. After six weeks, mice were euthanized and metastatic cells were harvested after macrodissection and reinjected to adult mice, repeating this loop for 3 times. In parallel to PROM2 quantification, we assessed epithelial-to-mesenchymal transition markers and ferroptosis markers, as well as migration and invasion using Boyden chamber. Patient-derived xenografts and exceptional sequential human metastasis biopsies (melanoma, breast or renal cell carcinoma) were used to assess PROM2 implication in different types of cancers.

**Results and Discussions**

We showed that PROM2 overexpression gradually increased during the amplification loop in metastatic cells. This was associated with the increase of ZEB1, ZEB2, SNAI1, SNAI2, TWIST1, TWIST2, VIM expression, displaying an epithelial-to-mesenchymal transition (EMT) phenotype. This was concomitantly associated to ferroptosis resistance. Using an antisense oligonucleotide (ASO) to downregulate PROM2 expression, we observed that it decreased the EMT phenotype, invasion and ferroptosis resistance, restoring the native melanoma cell line phenotype. PROM2 was found to be involved in a positive feedback loop, contributing to aggravating the metastatic process. Preliminary results seemed to confirm the same role of PROM2 in patients derived xenografts from renal cell carcinoma and triple negative breast cancer.

**Conclusion**

Our study demonstrates the role of PROM2 overexpression in cancer metastasis and cell death resistance. PROM2 is strongly associated with poor patient outcomes in various types of cancer and may serve as serum prognosis biomarker. Moreover, inhibiting PROM2 expression through ASOs may offer a promising therapeutic approach. Targeting PROM2 could be promising for various types of cancer therapy.

### EACR23-0991
### Caspase-9 as a regulator of cell migration: Proteomic and functional analysis

*K. Rihova[1,2], L. Knopfova[1,2], J. Pokludova[1,2], P. Lapcik[3], B. Vesela[4], P. Bouchal[3], E. Matalova[4,5], P. Benes[1,2]*
*[1]Masaryk University, Department of Experimental Biology, Brno, Czech Republic*
*[2]St. Anne's University Hospital, International Clinical Research Centre, Brno, Czech Republic*
*[3]Masaryk University, Department of Biochemistry, Brno, Czech Republic*
*[4]Czech Academy of Sciences, Institute of Animal Physiology and Genetics, Brno, Czech Republic*
*[5]Veterinary University, Department of Physiology, Brno, Czech Republic*

**Introduction**

Caspase-9 (casp-9) is the key initiator component of the intrinsic apoptotic pathway. However, recent studies describe its involvement in various non-apoptotic processes related to development and tumorigenesis in many tissues including bone. Ontogenesis and tumorigenesis often share signalling/regulatory molecules as they control the same major cellular processes.

**Material and Methods**

To analyze the non-apoptotic functions of casp-9 we derived mouse osteoblastic MC3T3-E1 cell line deficient in casp-9 using CRISPR/Cas9 approach. In the next step, we analysed the proteome of control and casp-9-deficient cells using liquid chromatography-mass spectrometry (LC-MS) analysis in data-independent (DIA) mode. GSEA analysis was implemented to identify enriched pathways. Cell migration was evaluated using scratch assay and Xcelligence transwell real-time cell monitoring. Deregulation of candidate proteins involved in regulation of cell migration was confirmed using immunoblotting. Phenotypic analysis of cells treated with casp-9 inhibitor was performed to confirm the results obtained with casp-9 KO cells.

**Results and Discussions**

We found upregulation of 283 and downregulation of 141 protein groups from the total 7669 protein groups identified by the proteomic screen. Subsequent GSEA analysis of differentially expressed protein groups identified cell migration as the most significantly enriched pathway. Casp-9 KO cells showed reduced migratory capacity in both scratch assay and transwell system. Similarly, pharmacological inhibition of casp-9 resulted in

© 2023 The Authors. Molecular Oncology is published by John Wiley & Sons Ltd on behalf of Federation of European Biochemical Societies reduced migration thus confirming the functional role of casp-9 in regulation of MC3T3-E1 cell motility. Subsequently, we found upregulated level of casp-9, both on mRNA and protein level, in aggressive metastatic LM8 osteosarcoma mouse cell line compared to parental non-metastatic Dunn cells suggesting the possible role of casp-9 in regulation of metastasis in osteosarcoma.

**Conclusion**

Our data provide evidence that genetic or pharmacological inhibition of casp-9 inhibits migration of osteoblastic and osteosarcoma cell lines. New possible substrates of casp-9 were identified using proteomic screen that are currently evaluated as metastatic/prognostic markers in osteosarcoma.

*This research was supported by the Czech Science Foundation (project 19-14727S) and by National Institute for Cancer Research (Programme EXCELES, ID Project No. LX22NPO5102) - Funded by the European Union - Next Generation EU.*

### EACR23-0996
### Proline dehydrogenase affects lung cancer cell proliferation by inducing ROS and cellular senescence

*E. Berno[1], P. Chiofalo[1], R. Cinquetti[1], M. Gallazzi[2], A. Bruno[1], M. Zana[1], E. Zini[1], P. Campomenosi[1]*

*[1]University of Insubria, Department of Biotechnology and Life Sciences, Varese, Italy*

*[2]IRCCS MultiMedica, Unit of Molecular Pathology- Biochemistry and Immunolo gy, Milano, Italy*

**Introduction**

Proline dehydrogenase (PRODH) is a mitochondrial flavoenzyme that catalyzes proline oxidation. Its catabolism is linked to basic cellular metabolism. Moreover, the electrons generated from oxidation can be used to generate ATP or ROS, potentially affecting several cellular processes, such as survival, apoptosis, and senescence. PRODH is frequently expressed in lung adenocarcinoma (LUAD), yet little is known about the correlation between the expression of this enzyme and its role in lung cancer development. Lung cancer is one of the most frequent types of tumor and the leading cause of cancer-related deaths. Availability of biomarkers for early diagnosis, prognosis, and prediction of response to therapy would improve patients survival. Thus, we aim to investigate if PRODH could represent a diagnostic and prognostic marker. To do so, we are elucidating its role in LUAD cell lines.

**Material and Methods**

To investigate how PRODH affects growth and proliferation of lung cancer cells, we stably transfected the NCI-H1299 LUAD cell line, not expressing PRODH, with a PRODH expression construct or the empty vector (control). To compare the ability of PRODH expressing cells to survive and proliferate compared to control cells, we performed clonogenic assays and MTT assays. ROS production as well as apoptosis and cellular senescence were also investigated.

**Results and Discussions**

We observed a decrease in survival and proliferation in PRODH clones compared to control clones. PRODH clones were found to produce higher levels of ROS using the 2′,7′-dichlorofluorescin diacetate assay. In order to identify the biological process(es) involved in the observed phenotype, we tested induction of apoptosis. We found no difference in the number of apoptotic cells between PRODH and control clones. Instead, a greater number of senescent cells was observed in PRODH clones with the senescence-associated β-galactosidase assay. Induction of cell senescence was confirmed by the increase in senescence markers. We conclude that PRODH promotes cellular senescence in LUAD cells via ROS production and that senescence is the mechanism underlying the decrease in proliferation observed in PRODH expressing cells.

**Conclusion**

The results obtained from our study allow to better understand the role of PRODH in lung cancer development. Induction of cellular senescence by ROS production was shown to affect LUAD cell proliferation. We envisage that the senescent phenotype induced by PRODH may also affect the response to therapy of cancer cells.

### EACR23-0998
### Microtubule-associated protein MAP2 promotes drug resistance and cancer stemness in hepatocellular carcinoma through integrin dysregulation

*U.K. LOU[1], T.L. Wong[1], K.H. Lam[1], H. Yu[1], J.J. Loh[1], K.F. Man[1], L. Zhou[2], C.H. Yu[1], S.K.Y. Ma[1]*

*[1]The University of Hong Kong, School of Biomedical Sciences, Hong Kong, Hong Kong- China*

*[2]Precision Medicine Institute, The First Affiliated Hospital- Sun Yat-Sen University, Guangzhou, China*

**Introduction**

Integrins mediate cell adhesion and transmit mechanical and chemical signals to the cell interior. Various mechanisms deregulate integrin signaling in cancer, empowering tumor cells with the ability to drive multiple cancer stem cell (CSC) functions, including tumor initiation, epithelial plasticity, and resistance to therapies. However, the mechanisms underlying these alterations remain poorly understand. This study aims to decipher the interplay of cancer stemness and integrin signaling in driving hepatocellular carcinoma (HCC).

**Material and Methods**

Transcriptome profiling was performed to compare sorted $CD133^{+/-}$ cells isolated from mouse models representing liver regeneration or proto-oncogene (NRAS+AKT) driven HCC. Targeted depletion of Prom1/CD133 in HCC using $Prom1^{C-L/+}$;$Rosa26^{DTA/+}$ mice were also used. HCC cell lines, patient-derived organoids, and tissues were used to assess the role of MAP2 in driving stemness and resistance to standard therapy. Pathway analysis of HCC datasets and transcriptome profiling of $CD133^{+/-}$ or HCC cells with or without MAP2 expression manipulated were also carried out by GO and GSEA.

**Results and Discussions**

HCC cells marked by CD133 represents an important functional subset in HCC tumors, displaying a dedifferentiated status with stem cell traits. Transcriptome

© 2023 The Authors. Molecular Oncology is published by John Wiley & Sons Ltd on behalf of Federation of European Biochemical Societies profiling of epithelial-specific 'normal' $CD133^+$ cells isolated from regenerating liver against 'HCC' $CD133^+$ cells isolated from NRAS+AKT-driven HCC revealed a specific downregulation of integrin α signaling in HCC but not regenerating liver. Of note, one of the most differentially upregulated genes identified in $CD133^+$ HCC cell profiling, MAP2, demonstrated the ability to suppress integrin expression. MAP2 is frequently overexpressed in HCC and correlated with aggressive clinical and stemness features. Epigenetic modification by H3K27Ac contributed to MAP2 upregulation. MAP2 promoted cancer stemness and proliferation, and conferred resistance to targeted therapy sorafenib. Estramustine Phosphate (EMP), reported to inhibit the interaction of MAP2 with actin filaments, attained a synergistic effect in suppressing tumor initiation cell frequency and growth of HCC cell lines and patient-derived organoids when used in combination with sorafenib.

**Conclusion**

MAP2 inhibition may represent a potential novel therapy for HCC by targeting its CSC roots and altering integrin signaling. Future research will focus on the study of MAP2 regulation on integrin signaling and cell behaviors in the maintenance of a more stemness state in HCC.

### EACR23-1000

### Acute promyelocytic cure is linked to a PML-dependent metabolic rewiring

*G. Fortin*[1,2]*, F. Qiu*[2]*, M. Kawakita*[2]*, H. De Thé*[1,2]

[1]*Collège de France, Oncologie Cellulaire et Moléculaire, Paris, France*

[2]*Université Paris Cité, Institut de Recherche Saint-Louis, Paris, France*

**Introduction**

PML/RARA fusion protein expression leads to acute promyelocytic leukemia through the disruption of nuclear structures called PML nuclear bodies. These domains modulate stress response, particularly senescence, through the recruitment and post-translational modifications of a variety of partner proteins. All-Trans Retinoic Acid (ATRA) and Arsenic TriOxide (ATO), the current standard of care, cure the vast majority of patients, and both PML and PML/RARA have been identified as the direct targets of the treatment. However, the precise role of PML in APL cure still remains unclear. Previous work has failed to identify the actual functions of PML required for treatment response, including in metabolism. Moreover, multiple studies have revealed the role of the metabolism for therapy response and cure in cancer. Yet, any role of metabolism in APL cure remains largely unexplored.

**Material and Methods**

We generated a variety of PML/RARA expressing mice in backgrounds where mutations in Pml abolish specific functions of PML (nuclear localization, partner recruitment or nuclear bodies formation). Then, APL blasts were transplanted in syngenic immunocompetent receivers to assess APL response to ATRA+ATO treatment. Single-cell RNA-seq was performed at different time points in these PML-mutant APLs. Finally, APL cells were treated for short times and their metabolites were analyzed by mass spectrometry.

**Results and Discussions**

We found that PML delays APL onset and aggressiveness in untreated and treated acute transplant recipients. Critically, APLs defective for PML nuclear bodies formation or partner recruitment initially responded to ATRA+ATO treatment, but quickly relapsed, while mice transplanted with $Pml^{+/+}$ APL could be cured. $Pml^{-/-}$ APL cells exhibit a treatment-resistance signature in the form of a highly upregulated expression of mitochondrial metabolism. This signature reveals the existence of a wide metabolic stress in $Pml^{-/-}$APL cells. This stress, highlighted by reduced amounts of numerous metabolites linked to energy production, could drive treatment resistance.

**Conclusion**

Our work demonstrates that PML nuclear body formation and partner recruitment are required for APL cure and that the later is linked to metabolic rewiring. These findings emphasize that metabolism plays an essential role in response and resistance to anti-cancer drugs, including targeted therapies.

### EACR23-1009

### Long pentraxin-3 as a tumor promoter in medulloblastoma.

*S. Filiberti*[1]*, C. Tavani*[1]*, D. Capoferri*[1]*, S. Mussi*[1]*, R. Ronca*[1]*, R. Bortolozzi*[2]*, E. Rampazzo*[2]*, G. Viola*[2]

[1]*University of Brescia, Department of Molecular and Translational Medicine, Brescia, Italy*

[2]*University of Padova, Department of Woman's and Child's Health, Padova, Italy*

**Introduction**

Medulloblastoma (MB) is an aggressive solid tumor of the central nervous system and represents the most common malignant brain tumor of the childhood. MB comprises four distinct subgroups: WNT, SHH, group 3, and group 4. It has an annual incidence of about 5 cases per 1 million individuals and it is estimated that 30%-40% of MB patients experience tumor recurrence.

The Long Pentraxin 3 (PTX3) is a component of the innate immunity that is also involved in several aspects of tumorigenesis. However, it remains unclear whether PTX3 acts as a good or bad player in cancer. In fact, it has been reported that PTX3 acts as an extrinsic oncosuppressor by modulating FGF/FGFR signalling and inflammation in several tumors, while in other cancers it seems to have a pro-tumoral role. Nowadays, no data is available regarding the impact of PTX3 in MB.

**Material and Methods**

PTX3 expression was analysed in MB cell lines and subgroups, by Western blot (WB) and R2 database analysis. Then, we focused on DAOY cells (representing SHH subgroup) and generated both PTX3 silenced MB cells, using short hairpin RNA (shRNA) and PTX3 knock-out (KO) clones, using CRISP/Cas9 technology.

The silenced cells and KO clones were used to elucidate the role of PTX3 in MB performing in vitro assays of proliferation, colony formation and migration. Furthermore, the angiogenic capacity of these cells was characterized in vivo using the chick embryo chorioallantoic membrane (CAM) assay.

**Results and Discussions**

Western blot revealed that PTX3 is expressed, at protein level, in various human MB cell lines (DAOY, HD-MB03,

© 2023 The Authors. Molecular Oncology is published by John Wiley & Sons Ltd on behalf of Federation of European Biochemical Societies

CHLA-01-MED and D283-Med), as well as in primary patient's derived cell lines, and that DAOY cells express the higher levels. Accordingly, R2 Analysis in Cavalli's dataset, confirmed that PTX3 expression is upregulated in SHH subgroup, in line with the higher levels of PTX3 expression in DAOY cells, which belong to SHH-MB subgroup.

In addition, both the knock down (shRNA) an the knock out (CRISPR/Cas9) of PTX3 in DAOY cells resulted in a significant impairment of relevant tumor features, such as proliferation, migration and clonogenic potential, in vitro. Moreover, when tested for their angiogenic potential in the CAM assay, PTX3 downregulation resulted in a significant reduction of the angiogenic activity of DAOY cells.

**Conclusion**

Our preliminary findings support the hypothesis that PTX3 might exert a pro-tumor effect in the SHH subgroup of MB and provide the basis to better study the molecular pathways driven by PTX3 in this tumor type.

### EACR23-1021

### Pancreatic cancer treatment strategy using a subtypeconversion

H.Y. YUN[1], H. Jang[2], C.W. Yun[2], S. Han[2], H. Jang[1]

*[1]National Cancer Center Graduate School of Cancer Scien ce and Policy- Goyang 10408- Republic of Korea, Department of Cancer Biomedical Science, Goyang, South Korea*

*[2]National cancer center, Department of Research in Cancer Biology, Goyang, South Korea*

**Introduction**

Pancreatic ductal adenocarcinoma (PDAC) is an aggressive cancer with a 5-year survival rate of less than 8%. PDAC can be classified into two molecular subtypes based on whole-genome expression profiling, and the basal-like subtype is generally more undifferentiated and has a lower survival rate than the classical subtype. Clinical research has shown that the classical subtype is more responsive to chemotherapy than the basal-like subtype, making the conversion of PDAC into the classical subtype a promising therapeutic strategy. In this study, we aimed to identify targets or combinations of targets that can effectively convert PDAC into the classical subtype.

**Material and Methods**

mRNA sequencing is used by public datas. Histological feature is analyzed by mouse xenograft tumor model. Stable cell line is generated using lenti-viral system.

**Results and Discussions**

First, to establish an in vitro model for subtype conversion, we classified PDAC cell lines based on their molecular subtypes. We compared the relative expression of classical and basal-like subtype gene sets by mRNA sequencing. We also assessed the expression level of GATA6, a representative classical subtype marker. Furthermore, we confirmed the degree of differentiation by histological analysis. As a results, MIA PaCa-2 and PANC-1 were classified as undifferentiated basal-like subtypes with low expression of GATA6, while PATU-8988S was classified as a well-differentiated classical subtype with high expression of GATA6. BxPC-3 and HPAC exhibited moderate expression of GATA6, and a mixture of well-differentiated and undifferentiated tissues.

Next, we conducted Ingenuity pathway analysis to identify genes and pathways that exhibit the greatest differences between basal-like and classical subtype cell lines. To validate the predicted result, we generated MIA PaCa-2 cell lines with specific genes regulated alone or in combination. We observed the differences in organoid formation and the degree of histological differentiation between the generated cell lines and the control group.

**Conclusion**

In this study, we established criteria for distinguishing subtypes based on the level of GATA6 expression and degree of differentiation, and used it to classify cell lines representing subtypes of pancreatic cancer patients. Furthermore, we confirmed that regulating specific genes can affect the ability to form organoids and the degree of histological differentiation. These results suggest that subtype conversion could be a therapeutic strategy for pancreatic cancer.

### EACR23-1042

### FOXP3 highly expressed population harbors a stemness feature and promotes chemoresistance in pancreatic cancer

R. Gong[1], H. Ren[2]

*[1]The Affiliated Hospital of Qingdao University, Department of Gastroenterology, Qingdao, China*

*[2]The Affiliated Hospital of Qingdao University, Center for GI Cancer Diagnosis and Treatment- Tumor Im munology and Cytotherapy- Medical Research Center- The Affiliated Hospital of Qingdao University- Qingdao- Chin a, Qingdao, China*

**Introduction**

Pancreatic ductal adenocarcinoma (PDAC) is a highly aggressive disease for which no effective treatment is available. Tumor cell plasticity is the root of tumor heterogeneity, and cellular trans-differentiation is not only an important aspect of tumor plasticity, but also throughout the process of pancreatic carcinogenesis and evolution. From the perspective of epithelial cell transdifferentiation to lymphocytes, we found that there are subpopulations of pancreatic cancer epithelial cells that mimic the phenotype and function of regulatory T cells.

**Material and Methods**

We first labeled FOXP3+E-Cadherin- cells in pancreatic cancer tissue samples by immunohistochemical staining and performed clinicopathological and survival prognostic analysis of the staining results. We isolated and cultured the subgroup of cells by limiting dilution in vitro, and conducted experiments on cell invasion, migration, proliferation, drug resistance and immunology.

**Results and Discussions**

Our results revealed a subpopulation of pancreatic ductal epithelial cells that express the transcription factor FOXP3 in the nucleus but are deficient in E-Cadherin expression. This cell subpopulation was significantly associated with poor prognosis in PDAC patients. Therefore, we isolated this subpopulation of cells in vitro and studied their biological functions, and found that these cells are morphologically distinct from epithelial and mesenchymal cells, are round and spherical in shape, and have a reduced skeletal structure with the soft characteristics of stem cells.

© 2023 The Authors. Molecular Oncology is published by John Wiley & Sons Ltd on behalf of Federation of European Biochemical Societies

Although their expression of molecules related to epithelial-mesenchymal transition was elevated, their invasive and migratory abilities were weak. Therefore, we further explored additional biological features of this subpopulation of cells and found that this subpopulation was able to significantly promote angiogenesis in vivo and in vitro, and was resistant to gemcitabine. Notably, when co-cultured with cytotoxic T lymphocytes, this subpopulation of cells was found to be less capable of recruiting CD8+ T cells compared to FOXP3-E-Cadherin- cells, favoring the promotion of an immunosuppressive microenvironment. In addition, PD-L1 was highly expressed in the FOXP3+E-Cadherin- cell population, providing an alternative strategy for these cells to evade immune surveillance.

**Conclusion**

These findings provide insight into the biological significance of FOXP3 expression in PDAC cells and reveal a novel mechanism of how epithelial cell subpopulations evade immunosurveillance.

### EACR23-1045
### Dedifferentiated esophageal cells constitute a reservoir for pre-cancerous metaplasia

<u>A. Vercauteren Drubbel</u>[1], B. Beck[2]

[1]*Université libre de Bruxelles - ULB, IRIBHM, Brussels, Belgium*

[2]*Université libre de Bruxelles - ULB, Institut de Recherche Interdisciplinaire en Biologie Humai ne et moléculaire, Brussels, Belgium*

**Introduction**

Esophageal adenocarcinoma (eAC) incidence has been multiplied by 4 in USA over the past 4 decades. Esophageal adenocarcinoma is the consequence of a multistep process in which chronic gastroesophageal reflux disease progressively induces esophagitis, Barrett esophagus (BE), dysplasia and finally eAC. BE is defined by the replacement of the squamous epithelium by an intestinal-like epithelium. Several studies have shown that BE-like metaplasia and eAC arise from the squamo-columnar junction (SCJ) *in vivo* but not from esophageal cells. Still, it has been demonstrated that rare squamous progenitors can give rise to BE-like metaplasia *in vivo* but the mechanisms involved in their transdifferentiation are still partially unknown.

Recently, we showed that the hedgehog (HH) pathway is activated in the esophageal cells upon chronic acid reflux. Although this pathway is not sufficient to trigger the development of specialized metaplasia, it induces the dedifferentiation of esophageal cells into embryonic-like progenitors *in vivo*. Interestingly, recent multi-omics data suggest that human metaplasia and eAC may originate from a pool of undifferentiated cells. We thus hypothesized that HH-dedifferentiated cells could be a reservoir for columnar metaplasia and/or eAC initiation.

**Material and Methods**

In this project, we analyzed publicly available copy number variation data in human eAC and developed new transgenic mouse models to mimic eAC-associated gene amplification in esophagus epithelium. We followed mutated esophageal cells by lineage tracing and characterized them by histology and RNA sequencing.

**Results and Discussions**

We found that *GATA4* is one of the most frequently amplified genes in eAC. Moreover, GATA4 is absent from normal esophagus and overexpressed in human BE samples. Since GATA4 is a transcription factor necessary and sufficient for columnar epithelial morphogenesis in the stomach, it is a good candidate to drive columnar metaplasia and/or eAC.

GATA4 ectopic expression in esophageal cells has virtually no phenotype. On the opposite, when expressed in HH-dedifferentiated esophageal cells, GATA4 induces a transcriptomic profile that resemble gastric-like metaplasia and a columnar phenotype. We repeated the same experiments with the intestinal transcription factor CDX2 and found analogous results.

**Conclusion**

In conclusion, our data suggest that embryonic-like esophageal progenitors constitute a cellular reservoir that can accumulate mutations and initiate metaplasia.

### EACR23-1071
### SHIP2 regulates esophageal cancer cell growth

<u>B. Beck</u>[1], A.R. Ramos[1], G. Bregni[1], C. Bodart[1]

[1]*Université Libre de Bruxelles, IRIBHM - faculty of medicine, Brussels, Belgium*

**Introduction**

Esophageal squamous cell carcinoma (eSCC) is among the deadliest cancers worldwide. Current management, consisting of surgery preceded by neo-adjuvant radiochemotherapy, is poorly efficient. Its relapse carries a short-term grim prognosis because of rapid resistance to therapy and the limited number of efficient drugs available. In this context, it is crucial to develop novel targeted therapies against eSCC.

The phosphoinositide (PI) 3-kinase (PI3K) pathway is one of the most frequently mutated signaling pathways in cancer and is being actively pursued today as a therapeutic target. The role of PIP3, the product of PI3K is often considered “oncogenic”, but other PIs such as PI(3,4)P2 are also critical signal molecules. SHIP2 (SH2 domain-containing PI-3,4,5-trisphosphate 5-phosphatase 2) is an enzyme able to catalyze the formation of PI(3,4)P2 and plays a crucial role in AKT activation. Phosphorylation of the AKT protein, one of the mechanisms leading to the activation of the pathway, shows higher expression in eSCC than in corresponding normal tissue, supporting the relevance of the PI3K-AKT path in eSCC development. However, virtually nothing is known about the role of SHIP2 in eSCC.

**Material and Methods**

In this study, we used publicly available transcriptomics and genomics dataset, a panel of 22 human eSCC cell lines, siRNA and pharmacological inhibitors (K149 and AS194940), xenografts, histology and RNA sequencing.

**Results and Discussions**

By analyzing data from the Cancer Genome Atlas, we found that eSCC is the most frequent cancer type with amplification of *INPPL1* coding SHIP2 (20%) and SHIP2 is the PI 5-phosphatase with the highest expression in human eSCC cells. We used siRNA-mediated SHIP2 knockdown and found that SHIP2 modulates cell growth in several eSCC lines. We then tested the impact of

© 2023 The Authors. Molecular Oncology is published by John Wiley & Sons Ltd on behalf of Federation of European Biochemical Societies pharmacological inhibitors and found that SHIP2 inhibition decreases eSCC cell survival *in vitro* and *in vivo*. To determine how SHIP2 regulates cell survival, we profiled eSCC cells treated with inhibitor or siRNA and found that this SHIP2 regulates cell cycle progression. SHIP2 inhibition is associated to a decrease in the level of AKT phosphorylation, thus suggesting that SHIP2 modulates eSCC cell growth through the regulation of AKT.

**Conclusion**

In conclusion, SHIP2 may constitute a good target to inhibit AKT-dependent eSCC cell growth in the future.

### EACR23-1072
### Associations between transforming growth factor beta type II receptor (TβR2) and estrogen receptor (ER) subtypes expression in prostate cancer (PCa)

M. Puchinskaya[1]
[1]*Minsk City Clinical Oncologic Dispensary, Out-patient, Minsk, Belarus*

**Introduction**

Epithelial-mesenchymal transition (EMT) is important for cancer metastasis and treatment resistance. A number of molecules regulate EMT process, transforming growth factor beta being one of the most important of them. It regulates intracellular processes through direct binding to TβR2, which causes receptor heterodimerization with other family members and further downstream signaling. In PCa ER alpha and beta play an important role in tumor development and progression. However, their role in EMT regulation in PCa is not fully understood. The aim of the present study was to assess the correlations between TβR2 as an EMT regulator and ER subtypes expression in PCa.

**Material and Methods**

56 cases of PCa were studied. Tissue was obtained during radical prostatectomy, 1 representative slide per case was analyzed. ER subtypes were stained on the same slide using double immunofluorescence technique, TβR2 was stained on a consecutive slide. Primary mouse antibodies to ERα (ThermoFischer, 1:30) and rabbit to ERβ (BioGenex, 1:500) and TβR2 (ThermoFischer, 1:100) were used, as well as secondary goat antibodies labeled with AlexaFluor 488 (anti-mouse) and 555 (anti-rabbit) (ThermoFischer, 1:200). Staining was assessed *ad oculus* semiquantitatively in 3-15 random non-crossing high power fields (HPFs) with calculating total staining scores (TSS), multiplying proportion of stained cells (0-3) and staining intensities (1-3). A mean TSS per case (TSSc) was used to compare levels of markers stained on different slides. Nuclear ER staining was assessed separately in cancer epithelium and stroma, and for TbR2 only membranous staining in cancer cells was assessed.

**Results and Discussions**

As no direct matching of HPFs for slides with TβR2 and ER staining was possible, only TSSc were compared. No statistically significant correlations ($p_{Spearman}$, p>0,05) were found between membranous TβR2 expression and ER subtypes in stroma and epithelium, with the only exception of direct moderate correlation between membranous TβR2 and stromal ERβ (r=0.31). This may be due to a possible paracrine signaling from ERβ positive stromal cells to PCa cells. However, it is not clear whether receptor up-regulation in these circumstances will lead to enhanced downstream signaling and EMT promotion.

**Conclusion**

ERβ expression in PCa stroma correlates with TβR2 expression, while Era and epithelial ERβ do not. That may point to a role of stromal ERβ in PCa EMT regulation, though further studies are needed.

### EACR23-1074
### Vemurafenib and diclofenac counteract the Hif-1α-mediated glycolytic phenotype and synergistically restrain cell viability of BRAF-mutated thyroid carcinomas

S. Cataldi[1], M. Aprile[1], C. Perfetto[1], A. Ciccodicola[1], V. Costa[1]
[1]*National Research Council, Institute of Genetics and Biophysics "A. Buzzati Traverso", Napoli, Italy*

**Introduction**

B-raf inhibitors (BRAFi) - e.g. vemurafenib, VMR and dabrafenib, DBR - are effective for *BRAF*-mutated papillary (PTC) and anaplastic (ATC) thyroid carcinomas, but acquired resistance frequently occurs, limiting drug efficacy. The mechanisms underlying the adaptation to anti-tumor therapies include the metabolic reprogramming. Here, we investigated the expression patterns - and the metabolic vulnerabilities - of *BRAF*-mutated thyroid tumors, analyzing the effects of BRAFi on the glycolytic phenotype of these tumors, and proposing new combinatorial approaches to maximize BRAFi efficacy.

**Material and Methods**

Tumor subtype-specific expression and methylation patterns were identified in *BRAF*-mutated tumors combining multiple omic data and key transcription factors were selected by in silico screening metabolic genes' promoters. *HIF1A* silencing and its chemical stabilization (by siRNAs and $CoCl_2$, respectively) were performed to assess its role in this process. Gene expression, glucose uptake, lactate efflux and cell viability were analyzed in PTC, ATC and normaloid cell lines (BCPAP, 8505c and Nthy-3-ori, respectively) treated (or not) with BRAFi and diclofenac to evaluate the anti-metabolic properties.

**Results and Discussions**

A metabolic gene signature - characterized by increased glucose uptake and lactate efflux - was identified as hallmark of *BRAF*-mutated TCs. The alteration of metabolic genes - not fully recapitulated by changes in DNA methylation – is mediated by Hif1α, identified as a key regulator of energy metabolism-related genes. Our data show that MAPK activation and Hif1α stabilization induce metabolic genes whereas BRAFi and *HIF1A* knockdown repress them. Moreover, Hif1α stabilization counteracts VMR effects on energy metabolism-related genes and tumor cell viability. Notably, both VMR and diclofenac restrain the glycolytic phenotype of *BRAF*-mutated PTC and ATC cells and diclofenac maximizes BRAFi efficacy even at low doses, by synergistically reducing tumor cells' viability, even compared to the combination of BRAFi and MEKi ( trametinib).

**Conclusion**

We define a specific metabolic vulnerability of *BRAF*-mutated thyroid carcinomas, identifying HIF1α as crucial

© 2023 The Authors. Molecular Oncology is published by John Wiley & Sons Ltd on behalf of Federation of European Biochemical Societies mediator of the glycolytic phenotype. We also report the synergistic ability of BRAFi and diclofenac to restrain cell viability, opening new interesting therapeutic perspectives for maximizing drug efficacy and counteract the acquired resistance.

### EACR23-1075
### Modeling the development of Clear Cell Ovarian Carcinoma (CCOC) using organoid culture and single cell RNA sequencing

*F. Kalantari[1], D. Cochrane[1], Y. Wang[1], W. Yang[1], T.G. Trigo-gonzalez[1], M. Douglas[1], J. Mcalpine[2], D. Huntsman[1]*
*[1]University of British Columbia, Molecular Oncology, Vancouver, Canada*
*[2]University of British Columbia, Department of Obstetrics & Gynecology, Vancouver, Canada*

**Introduction**

Clear Cell Ovarian Carcinoma (CCOC) is an uncommon epithelial ovarian cancer which is inherently resistant to chemotherapy. *ARID1A*, a subunit of SWI/SNF chromatin remodeling complex, is frequently mutated in CCOC. Our lab has discovered inactivating mutations of *ARID1A* in about 50% of CCOC, together with a *PIK3CA* activating mutation. CCOC is strongly associated with endometriosis (functional endometrial tissue outside of the uterine cavity). *ARID1A* and *PIK3CA* mutations occur with high prevalence in precursor lesions adjacent to CCOC suggesting this mutation is an early event in tumor development. In this project, I will use organoid culture of normal human endometrium to model early initiation events of cancer progression. We hypothesized that ARID1A deficiency in CCOC results in epigenetic and transcriptomic alterations which may provide insights on the biology of CCOC and suggest potential therapeutic options.

**Material and Methods**

Organoid culture was derived from primary normal human endometrial cells in which ARID1A and PIK3CA mutations achieved by lentiviral transduction. The organoids were maintained over many passages and morphology was assessed at each passage. Single cell RNA and ATAC sequencing was performed to compare gene expression profiles between the non infected and mutant organoids.

**Results and Discussions**

The mutant organoids demonstrate phenotypic differences with 3 times larger than uninfected organoids and, they manifest CCOC histopathology in H&E staining. Single cell gene expression profiles from passage1(LogFC0.84 P=1.08e-58) and passage6 (logFC2.23 P=7.2E-33) experiments showed upregulation of S100A4, a known metastasis gene, in the ARID1A knockout cells. These data correlated with the increased accessibility of the chromatin of the S100A4 gene in ATAC-seq analysis. The inverse correlation between ARID1A and S100A4 was validated in ARID1A wild type and mutant CCOC and endometrial cancer cell lines using western blot analysis. Additionally, the IHC results from CCOC (P=0.022) and endometrial cancer (P=1.8e-06) from tissue microarrays (TMAs) showed significant association between S100A4 and ARID1A, with higher A100A4 expression observed in ARID1A loss in patient tumors

**Conclusion**

In this project,S100A4, a known cancer metastasis gene was one of the genes to be upregulated in our data. Further functional analysis of S100A4 in CCOC is ongoing with the hope that understanding the biological function of S100A4 protein may lay the groundwork for the development of new therapeutics for CCOC.

### EACR23-1091
### FGFR2 gene amplification is delivered in extracellular vesicles in two models of cancer of unknown primary

*I. Salamon[1], G. Storci[2], B. Fontana[1], N. Bortoletto[1], S. Seravalle[3], S. Santi[4], M. Guescini[5], M. Bonafè[1], M. Ferracin[1]*
*[1]University of Bologna, Department of Medical and Surgical Sciences – DIMEC, Bologna, Italy*
*[2]IRCCS Azienda Ospedaliero-Universitaria di Bologna, Immunobiologia dei trapianti, Bologna, Italy*
*[3]IRCCS Azienda Ospedaliero-Universitaria di Bologna, Department of Pediatrics, Bologna, Italy*
*[4]National Research Council of Italy CNR, Institute of Molecular Genetics, Bologna, Italy*
*[5]University of Urbino Carlo Bo, Dipartimento di Scienze Biomolecolari, Urbino, Italy*

**Introduction**

The extracellular vesicle (EV) route is essential for cell-to-cell communication. Cells release EVs in the extracellular space, which can interact with recipient cells inducing regulation of gene expression, activation of specific pathways in pathological settings and stimulation of cancer progression. We investigated the EVs released by two cell line models of cancer of unknown primary site (CUP) we recently developed. This tumor type comprises 3-5% of new cancer cases and presents with metastases of unknown or uncertain origin and no apparent primary tumor. It has been recognized that tumors use circular extrachromosomal DNA (ecDNA) as a way to increase oncogenic amplification. Therefore, we hypothesized that FGFR2 amplification in our CUP cell lines could be linked to ecDNA generation and that this ecDNA could be loaded as cargo inside EVs.

**Material and Methods**

We developed two CUP cell line models that are both characterized by FGFR2 gene amplification. FGFR2 copy number was quantified using a probe-based droplet digital PCR assay and FISH assay. EVs were isolated through ultracentrifugation and the circular nature of the FGFR2 ecDNA was assessed using the Plasmid-Safe ATP-dependent DNAse to digest linear DNA. The delivery capabilities of the oncogene through the EVs was tested administering isolated CUP#96 exosomes to a recipient cell lines (NCI-N87).

**Results and Discussions**

First, we identified the different nature of FGFR2 amplification in the two models: CUP#55 presented a homogeneus staining region instead CUP#96 displayed the double minuts. The FGFR2 DNA was detected also in the cytoplasm of cells, therefore we isolated the extracellular vesicles from culture medium of the two models and the

© 2023 The Authors. Molecular Oncology is published by John Wiley & Sons Ltd on behalf of Federation of European Biochemical Societies

FGFR2 DNA inside the vesicles was confirmed. Since ecDNA contributes to cancer genome remodeling also forming chimeric circles, we assessed the circular nature of the FGFR2 loaded in EVs. Finally, after the administration of CUP#96 exosomes in NCI-N87, we observed an increase of FGFR2 DNA in NCI-N87, then we detected an increase of FGFR2 transcript and finally we evaluated the effect on cells proliferation.

**Conclusion**

In conclusion, we identified a model of oncogene amplification and delivery in our two cell lines of cancers of unknown primary that could explain the high metastatic potential of this cancer type.

### EACR23-1092
### SH3BP5L reroutes Integrin beta-1 (ITGB1) recycling in aggressive breast cancer

*J.P. Margaria[1], H. Li[1], L. Gozzelino[1], M. Jenkins[2], F. Tucci[3], M.C. De Santis[1], F. Gulluni[1], L. Prever[1], C. Zanini[1], M. Forni[1], C.C. Campa[4], I. Franco[5], M. Martini[1], J. Burke[2], S. Pece[3], G. Serini[4], E. Hirsch[1]*

*[1]Molecular Biotechnology Center- UNITO, Molecular Biotechnology and Health Science, Turin, Italy*

*[2]University of Victoria, Department of Biochemistry and Microbiology, Victoria, Canada*

*[3]Università degli Studi di Milano, Department of Oncology and Hemato-Oncology, Milan, Italy*

*[4]Candiolo Cancer Institute - FPO- IRCCS, Istituti di Ricovero e Cura a Carattere Scientifico, Candiolo TO, Italy*

*[5]Università Vita-Salute San Raffaele, Division of Genetics and Cellular Biology- Molecular Basi s of Cystic Kidney Disorders Unit, Milan, Italy*

**Introduction**

Metastatic breast cancer lacks valid therapeutic targets making its prognosis generally poor. Circulating cancer cells expose and recycle a large variety of plasma membrane proteins including integrins to extravasate and eventually colonize the metastatic niche. Here we explored how SH3BP5L, a protein involved in RAB11A mediated recycling, controls metastatization through integrin beta 1 (ITGB1) trafficking in aggressive breast cancer cells.

**Material and Methods**

To study metastatization we used a model of triple negative breast cancers (TNBC) cells, the MDAMB231. We engineered this cell line with Cas9 knock-out technology and analysed its protein expression and RAB11A activity. To better investigate the cellular role of SH3BP5L, a newly designed bioinformatic pipeline named APSQ is used to automatically determine single cell subcellular distribution of the fluorescent tagged gene through four sequential steps: acquisition, prediction, segmentation, and quantification. We eventually use tail vein injection of MDAMB231 cells in mice and intracardiac injection in zebrafish to study their metastatic ability.

**Results and Discussions**

SH3BP5L, one of the two known guanine nucleotide exchange factors (GEF) activating Rab11A, is amplified and overexpressed in triple negative breast cancers (TNBC) and contribute to the metastatic ability of this tumor subtype. Using APSQ, a novel high content analysis (HCA) microscopy pipeline to detect single cell subcellular localization signatures, we found an overlap between SH3BP5L distribution and active integrin beta 1 (ITGB1) localization patterns. Interfering with SH3BP5L GEF activity not only redirects active ITGB1 trafficking from the plasma membrane toward lysosomes but also reduces the ability of TNBC cells to metastatize in fishes and mice. By immunoprecipitating SH3BP5L we find by mass spectrometry two interactors of interest: RAB11A and the anterograde motor protein KIF5B. Next, we modulate the GEF activity of SH3BP5L to find that it stabilizes the interaction with KIF5B and active RAB11A, eventually pushing the trafficking of active ITGB1 towards the plasma membrane and promoting metastatization. Ultimately, we demonstrate in vivo that a weakening of SH3BP5L protein expression or its GEF function impairs the metastatic potential of MDAMB231 in fishes and mice and can be rescued by a constitutively active Rab11A.

**Conclusion**

Our data reveals a key role of SH3BP5L in the control of KIF5B/RAB11A-mediated recycling of active ITGB1 in TNBC metastatization.

### EACR23-1093
### Prognostic value of LAMP proteins and tumor budding in colorectal cancer

*D. Molander[1,2], T. ivanova[1,2], Y. Sbirkov[1,2], D. Dikov[1], M. Kazakova[1,2], V. Sarafian[1,2]*

*[1]Medical University Plovdiv, Medical Biology, Plovdiv, Bulgaria*

*[2]Research Institute at Medical University of Plovdiv, Division of Molecular and Regenerative Medicine, Plovdiv, Bulgaria*

**Introduction**

Long-term management of colorectal cancer (CRC) relies on effective chemotherapy and the prevention of metastatic disease, which ultimately is the main cause of mortality. However reliable prognostic biomarkers for better stratification and treatment of CRC patients are currently missing. At present the fundamental role of autophagy in maintaining cell homeostasis is well defined with lysosomes playing a major role in it. Nevertheless, in oncogenesis a dual role of autophagy is also indicated, either in promoting or inhibiting tumor growth. Here we show the concurrence of the spatial immunochemical expression of the lysosome associated membrane proteins LAMP1 and LAMP2, and tumor budding in CRC patient samples, as well as their prognostic significance.

**Material and Methods**

CRC patient tissue sections (n=31) were analyzed by immunohistochemistry and LAMP1 and LAMP2 expression levels were evaluated in normal and tumor colon samples. The clinicopathological characteristics as tumor differentiation, lymphatic and vascular invasion, budding were also considered.

**Results and Discussions**

To predict the risk of lymph node metastases and cancer infiltration, tumor bud counts were evaluated and a significative association between the tumor budding and tumor emboli in the lymphatic vessels was found. However no correlation with the tumor grade or patient mutation status was detected. Interestingly, LAMP protein levels in the tumor stroma, parenchyma, and tumor front were

© 2023 The Authors. Molecular Oncology is published by John Wiley & Sons Ltd on behalf of Federation of European Biochemical Societies significantly increased in contrast to the normal colon. The consistent accumulation of LAMP2 in the tumor front with the tumor budding, indicated a possible role of LAMP2 in cancer progression.

**Conclusion**

The current study proposes a novel strategy to predict the invasive potential of cancer cells and may reveal new opportunities to optimize standard chemotherapy regimens in CRC.

### EACR23-1095

### Awaken the tumor suppressive mechanism of senescence in patient-derived model of KRAS-mutated colorectal cancer

*L. di Blasio*[1], *V. Monica*[2], *V. Cepas Lopez*[1], *I. Cantarosso*[1], *A. Puliafito*[1], *L. Primo*[1]
[1]*University of Torino- Candiolo Cancer Institute FPO-IRCCS, Department of Oncology, Candiolo, Italy*
[2]*University of Torino, Department of Oncology, Candiolo, Italy*

**Introduction**

Among the RAS family of oncogenes, KRAS is mutated in 40% of colorectal tumors. Currently used therapies are completely ineffective in patients with KRAS mutation, resulting almost untreatable. Oncogene-induced senescence (OIS) represents the first barrier against cancer development after oncogene activation. Thus, we propose to re-activate the primary effect of KRAS as inducer of senescence in colorectal cancer (CRC).

**Material and Methods**

We exploited the XENTURION collection of PDX-derived organoids (PDO), recently established in our Institute from metastatic CRC. Since OIS requires the blocking of Cyclin D/CDK4/6 complex, we essayed Palbociclib, a CDK4/6 inhibitor, as senescence inducer in PDO expressing either wild-type or mutated KRAS. In these models, we evaluated cell viability and senescence induction after CDK4/6 inhibition. Moreover, we tested the sensitivity to Palbociclib of colorectal cancer cells lines expressing different p21 protein levels (HCT116, SW837).

**Results and Discussions**

Our results show that treatment with Palbociclib decreased the growth of several KRAS-mutated colorectal cancer organoids. In contrast, KRAS-wild type PDO were generally less sensitive to this treatment. Notably, all Palbociclib-responder PDO kept a reduced proliferation rate after drug withdrawal.

The treatment with Palbociclib increased the amount of senescent bGAL-positive cells in most KRAS-mutated CRC PDO, as well as the expression of p16 and p21, both cyclin-dependent kinase inhibitors recognized as senescence markers. Moreover, we observed the loss of nuclear envelope protein laminB1 in the majority of KRAS-mutated PDO after Palbociclib treatment.

In addition, several cytokines, such as IL-6, IL-1A and B, IL-8, were upregulated, indicating the activation of the so-called senescence-activated secretory phenotype (SASP). We noticed that the majority of PDO less responsive to Palbociclib were those either expressing low level of p21 or not able to upregulate the protein in the nucleus.

**Conclusion**

Our results indicate that CDK4/6 inhibition activates a senescence program in KRAS-mutated PDO, reducing their ability to grow. This senescent phenotype appears to be in most cases irreversible, opening new possibilities for the treatment of metastatic colorectal cancers harboring KRAS mutations.

### EACR23-1098

### Development of novel nanoparticles to treat breast cancer by targeting autophagy

*Y. Akkoc*[1], *Ö. Unal*[2], *M. Kocak*[3], *E. Nalbat*[3], *A. Dogan-Ekici*[4], *H. Yagci-Acar*[5,6], *D. Gozuacik*[1,7]
[1]*Koç University,*
*Koç University Research Center for Translational Medicin e KUTTAM, İstanbul, Turkiye*
[2]*Koç University,*
*Graduate School of Material Science and Engineering, İstanbul, Turkiye*
[3]*Sabancı University,*
*Nanotechnology Research and Application Center SUNUM , İstanbul, Turkiye*
[4]*Acıbadem Mehmet Ali Aydınlar University School of Medi cine, Department of Pathology, İstanbul, Turkiye*
[5]*Koç University, Department of Chemistry, İstanbul, Turkiye*
[6]*Koç University,*
*Surface Science and Technology Center KUYTAM, İstanbul, Turkiye*
[7]*Koç University, School of Medicine, İstanbul, Turkiye*

**Introduction**

MicroRNAs (miRNAs) are small, evolutionary conserved and 19–25 nucleotides in length that are involved in diverse biological functions through the repression of target genes. As important gene regulatory elements, miRNAs have expanded therapeutic opportunities as oligonucleotides, however, efficient miRNA delivery strategies need to be developed. SPIONs are FDA-approved superparamagnetic iron oxide (Fe3O4) nanoparticles that can be detected and manipulated using magnetic fields, and their usage as gene therapy agents is under investigation. In order to achieve an effective miRNA delivery vehicle, we designed and modified SPION (Super Paramagnetic Iron Oxide Nanoparticle)-based functionalized, theranostic, innovative RNA-loading nanoparticles to carry miRNAs into specific breast cancer cells.

**Material and Methods**

We propose for the first-time, the usage of the microRNA machinery RISC complex component AGO2 (Argonaute 2) protein as a microRNA stabilizing agent and a delivery vehicle. In this study, AGO2 protein-coated, anti-HER2 antibody-linked and fluorophore-tagged SPION nanoparticles were developed (SP-AH nanoparticles) and used as a carrier for an autophagy inhibitory miRNA, *MIR376B*.

**Results and Discussions**

These functionalized nanoparticles could selectively deliver an effective amount of the miRNA into HER2-positive breast cancer cell lines *in vitro* and in a xenograft nude mice model of breast cancer *in vivo*, and post-translationally orchestrate autophagic activity in cancer successfully. Furthermore, combination of the chemotherapy agent cisplatin with *MIR376B*-loaded SP-

© 2023 The Authors. Molecular Oncology is published by John Wiley & Sons Ltd on behalf of Federation of European Biochemical Societies

AH nanoparticles increased the efficacy of the anti-cancer treatment both *in vitro* in cells and *in vivo* in the nude mice.

**Conclusion**

Here, we target not only the tumor-specific pathways but also cell metabolism through targeting autophagy. We developed non-toxic, bio-compatible SPION-based AGO2 protein containing theranostic nanoparticles that are efficiently used as innovative gene therapy tools for targeted cancer therapy.

### EACR23-1099

### Targeting the Sigma-1 receptor increases the anti-proliferative effects of chemotherapeutic drugs and reduces motility in a triple-negative breast cancer cell line

*P. Borde*[1], *E. Rajab*[1], *S. Safrany*[1]
[1]*RCSI Bahrain,*
*School of Postgraduate Studies and Research, Busaiteen, Bahrain*

**Introduction**

Triple-negative breast cancer (TNBC) is an aggressive disease with a high risk of relapse and metastasis. TNBC is characterized by the lack of hormone receptors and HER2 amplification which makes it unamenable to standard targeted therapies. Conventional chemotherapy, which is limited by off-target toxicity, remains the main systemic treatment strategy. The sigma-1 receptor (Sig1R), a stress-activated chaperone, is frequently dysregulated in breast cancer but its capacity as a druggable target in TNBC has not been explored. We have previously shown Sig1R is highly expressed in TNBC tumours and its presence correlates with poor survival in patients with oestrogen receptor- and Her2 receptor- negative tumours [1].

**Material and Methods**

MDA-MB-231 cells were treated with the Sig1R antagonists *N*-[2-(3,4-dichlorophenyl)ethyl]-*N*-methyl-2-(dimethylamino)ethylamine dihydrobromide (BD1047) or 1-(4-iodophenyl)-3-(2-adamantyl)guanidine (IPAG) either 24 hours before or simultaneously with paclitaxel or doxorubicin for 72 hours. Cell viabilities were determined by MTS assays. MDA-MB-231 cells were incubated with BD1047 or IPAG and the motility of treated cells evaluated by scratch (wound healing) assays. The impact of Sig1R activity on stemness was evaluated by culturing MDA-MB-231 mammospheres in the presence of BD1047 or IPAG.

**Results and Discussions**

Compared to vehicle, 24 hours of IPAG pre-treatment lowered the half maximal inhibitory concentrations of both doxorubicin and paclitaxel in MDA-MB-231 cells. Simultaneous treatment with BD1047 and paclitaxel reduced viability more than either agent administered singly. These results suggest a combination effect between Sig1R antagonism and chemotherapeutic agents which will be further investigated. The migratory capacity of treated cells, as estimated by wound closure over 48 hours, was significantly reduced by both Sig1R antagonists in a dose dependent manner. Untreated mammospheres appeared as compact clusters of tightly bonded cells with clearly visible borders. In contrast, mammospheres grown in the presence of BD1047 were diffuse looking aggregates with indistinct borders.

**Conclusion**

Our data indicate Sig1R has a role in TNBC stemness and metastatic potential, and that Sig1R antagonists might reduce the dosage requirement of chemotherapeutic drugs. Our ongoing work in other TNBC models will expand the understanding of cellular processes in this disease and accelerate Sig1R's development as a therapeutic target.

1. Borde, P., et al., Cancer Gene Ther. 2023 Feb;30(2):368-374.

### EACR23-1100

### The p53 short isoforms are activated by the integrated stress response to enhance survival in wild-type p53 cancer cell lines

*A.C. Ramalho*[1], *M.J. López-Iniesta*[2], *R. Lacerda*[1], *S.N. Parkar*[2], *L. Romão*[1], *M.M. Candeias*[2]
[1]*National Institute of Health Dr Ricardo Jorge,*
*Department of Human Genetics, Lisboa, Portugal*
[2]*Kyoto University,*
*MaRCU – Molecular and RNA Cancer Unit- Graduate School of Medicine, Kyoto, Japan*

**Introduction**

The full-length p53 (FLp53) protein is a transcriptional factor that mediates cellular stress responses. The appropriate response is modulated according to the nature and extent of the damage, which means that p53 may promote cell cycle arrest and DNA repair or apoptosis in different situations. In all cases, p53 manages the response by modulating the expression of a wide range of target genes, as it contains two N-terminal transactivation domains. However, this gene also encodes for shorter isoforms that lack the N-terminal region and display oncogenic functions: Δ133p53 and Δ160p53. In contrast to the protective nature of the full-length protein, these two isoforms promote cell survival, proliferation and invasion. Both are typically overexpressed in tumours, while in normal tissues their levels are low or undetectable.
Data from this study indicate that the elevated levels of these isoforms may be owed to the Integrated Stress Response (ISR), which is typically a pro-survival programme, and it is commonly activated in cancer cells. The ISR is initiated by a group of kinases in response to different stress stimuli, converging in the phosphorylation of the alpha subunit of eukaryotic translation initiation factor 2 (eIF2α). This blocks global translation, while promoting the expression of activating transcription factor 4 (ATF4), the main effector of ISR.

**Material and Methods**

In this work, cancer cell lines with endogenous p53 were used to verify the expression of its isoforms during ISR, which was induced by treatment with thapsigargin and tunicamycin. To investigate if they were translated internally, bicistronic construct systems were employed. The interaction of the isoforms with FLp53 was verified by co-immunoprecipitation and their effect on the mRNA levels of p53 target genes was evaluated.

**Results and Discussions**

The translation of Δ160p53 and Δ133p53 was promoted by ISR induction in the cell lines tested via internal translation from FLp53 mRNA. Interaction of both isoforms with FLp53 was also confirmed, and it was demonstrated that

© 2023 The Authors. Molecular Oncology is published by John Wiley & Sons Ltd on behalf of Federation of European Biochemical Societies

Δ160p53 selectively acts as a regulator of p53 transcriptional activity on some genes.

**Conclusion**

These results hint at a physiological role for p53 short isoforms in the modulation of p53 target gene expression during ISR. On the other hand, their activation by abnormal ISR induction in cancer cells supports oncogenicity. The uncovering of an intersection between p53 isoforms and ISR could open a new path for future cancer therapies.

### EACR23-1105

### The impact of TP53 on calcium channel expression and regulation in triple-negative breast cancer (TNBC)

*K. E Rabab[1], A. Murphy[1], A. White[1], P. Buchanan[2], A.J. Eustace[1]*

*[1]Dublin City University, National Institute of Cellular Biotechnology, Dublin, Ireland*

*[2]Dublin City University, School of Nursing- Psychotherapy and Community Health, Dublin, Ireland*

**Introduction**

Triple Negative Breast Cancer (TNBC) is an aggressive form of breast cancer (BC) with poor patient outcomes. Despite resistance, chemotherapy remains a treatment option for TNBC, highlighting the need for personalized medicine. 80% of TNBC are TP53 mutant, which greatly reduces the effectiveness of chemotherapy. A non-transcriptional function of TP53 has been demonstrated in which TP53 is recruited to the ER and mitochondria. The mitochondrial $Ca^{2+}$ overload mediated from ER by TP53 promotes apoptosis. Mutations in TP53 disrupt these processes, resulting in apoptotic resistance. This study investigated how TP53 mutations affect calcium channels and calcium levels in TNBC.

**Material and Methods**

To investigate calcium channels linked to TP53, we cultured TNBC cell lines with both wildtype (CAL51) and mutant TP53 profiles (HDQ-P1 and MDA-MB-157). qRT-PCR was used to analyze the expression of store-operated calcium channels linked to TP53. Changes in intracellular calcium were determined using the ratiometric fluorescent calcium dye, Fura-2AM. Measuring store release with 4 uM thapsigargin (TG) in 0 mM physiological saline solution, and resulting store-operated calcium entry (SOCE) by addition of external $CaCl_2$ (2 mM). COTI-2 at 100 nM was used to reactivate TP53 in mutant cell lines. Additionally, we investigated the functional impact of calcium channel targeting on TNBC cancer cell biology using cell proliferation via the acid phosphatase method.

**Results and Discussions**

TP53 mutant cell lines exhibited significant downregulation of TRPC6 and $Ca_V1.3$ compared to WT TP53 TNBC cells. HDQ-P1 and MDA-MB-157 showed 3- and 11-fold decreases in $Ca_V1.3$ gene expression, respectively. Measurements of cytosolic calcium levels showed that a decrease in gene expression correlated with significantly reduced SOCE in MDA-MB-157 and HDQ-P1. Treatment of HDQ-P1 and MDA-MB-157 with COTI-2 significantly increased $Ca_V1.3$ expression by 10-fold and restored SOCE to levels observed in CAL-51. Cell proliferation data indicated that CAL51 was more sensitive to ER stress induced by TG treatment than HDQ-P1 and MDA-MB-157, correlating with less SOCE in the latter two cell lines which is typically associated with apoptotic resistance

**Conclusion**

Mutations in TP53 reduce SOCE and sensitivity to TG, highlighting associated apoptosis resistance. In TP53 mutant cells, calcium entry was increased by COTI-2, indicating that TP53 reactivation enhances SOCE. This suggests calcium channels may be targeted to improve treatment sensitivity in TP53 mutant TNBC

### EACR23-1115

### Scheduling of ATM and ATR inhibitors with Cisplatin revealed potent anti-cancer effects in models of TP53 mutant cancer

*A. White[1], L. Ivers[1], M. Obinu[1], N.T. Conlon[1], F. Matra[2], A. Bracken[2], M. Lowery[3], J. Crown[4], A.J. Eustace[1,5]*

*[1]Dublin City University, National Institute for Cellular Biotechnology, Dublin, Ireland*

*[2]Trinity College Dublin, The Smurfit Institute of Genetics, Dublin, Ireland*

*[3]Trinity College Dublin, School of Medicine, Dublin, Ireland*

*[4]Saint Vincent's University Hospital, Department of Medical Oncology, Dublin, Ireland*

*[5]Dublin City University, School of Biotechnology, Dublin, Ireland*

**Introduction**

The DNA damage response (DDR) pathway is activated in response to DNA damage and functions to arrest the cell cycle enabling DNA repair. However >50% of cancers harbor a TP53 mutation, which forces the cancer cells to rely on alternative DDR pathways to maintain genomic stability. ATM and ATR are kinases that are regulators of DDR and represent novel therapeutic options for cancer. Our hypothesis is that dual inhibition of ATM and ATR in combination with Cisplatin can induce synthetic lethality in TP53 mutant cells due to existing defects in DDR. However prior data confirms this treatment strategy is associated with significant patient toxicity. Therefore we want to design a scheduling strategy which aims to reduce toxicity of drug combination.

**Material and Methods**

A panel of breast, ovarian, lung, and pancreatic cancer TP53 mutant cell lines (n=10), as well as MCF10A normal breast epithelial cells were treated with AZD1390(ATMi), AZD6738(ATRi) and Cisplatin alone and in combination for 120h using the acid phosphatase method. Combination Index values were calculated using Calcusyn to determine synergy. Apoptotic induction was measured over 120h using the Caspase-3/7 dye on the Incucyte live cell imaging system in both lung and pancreatic cancer TP53 mutant cells. Pharmaco-dynamic analysis of both DDR and cell cycle regulators were assessed at 6, 24, 48, 72h using WB. In a pancreatic cancer TP53 mutant cell model we dosed cells using both synchronous and sequential delivery strategies to identify optimal dosing strategies.

**Results and Discussions**

In the cell line panel, the antitumor efficacy of the triple combination showed the most synergy, whilst in TP53 WT

© 2023 The Authors. Molecular Oncology is published by John Wiley & Sons Ltd on behalf of Federation of European Biochemical Societies

MCF10A cells IC50 values were higher and exhibited antagonism at ED50 and below. WB analysis of DDR proteins revealed that treatments with ATMi had significantly lower levels of CHK-2 phosphorylation. The triple combination was significantly more effective at inducing apoptosis after 60h. However, it was proven that treatment of cancer cells every 24h with ATRi, then Cisplatin, then ATMi induced a level of apoptosis that was comparable to when drugs were delivered concurrently.

**Conclusion**

Delivering ATMi, ATRi and Cisplatin using a scheduling approach exhibits a similar antitumor/apoptotic effect as when given concurrently in an in vitro model. These results confirm that giving the treatments over time does not reduce the potency of the treatment.This has the potential to reduce the side effect burden on patients. Further study is warranted in in vivo models.

### EACR23-1124

### Mutant p53 sustains serine-glycine synthesis and essential amino acids intake promoting breast cancer growth

*C. tombari*[1,2], *A. Zannini*[1,2], *R. Bertolio*[1,2], *L. Triboli*[1,2], *A. Rustighi*[1,2], *F. Di Camillo*[1,2], *F. Mantovani*[1], *G. Blandino*[3], *B. Belletti*[4], *G. Baldassarre*[4], *S. Bicciato*[5], *C. Tripodo*[6], *N. Mitro*[7,8], *G. Del Sal*[1,2,9]

[1]*University of Trieste, Life Sciences, Trieste, Italy*

[2]*ICGEB, International Centre for Genetic Engineering and Biotechnology, Trieste, Italy*

[3]*IRCCS Regina Elena National Cancer Institute, Translational Oncology Research Unit, Rome, Italy*

[4]*Centro di Riferimento Oncologico di Aviano CRO- IRCCS - National Cancer Institute, Unit of Molecular Oncology, Aviano, Italy*

[5]*University of Modena and Reggio Emilia, Center for Genome Research, Modena, Italy*

[6]*University of Palermo, Tumor Immunology Unit- Department of Health Science-Human Pathology Section- School of Medicine, Palermo, Italy*

[7]*University of Milan, Department of Pharmacological and Biomolecular Sciences, Milano, Italy*

[8]*IEO- European Institute of Oncology IRCCS, Department of Experimental Oncology, Milano, Italy*

[9]*IFOM, the AIRC Institute of Molecular Oncology, Milano, Italy*

**Introduction**

Amino acids are crucial nutrients for cancer cells since they provide metabolic and energetic intermediates and promote their survival in challenging environments. Tumors are indeed avid for amino acids and, consequently aberrantly increase amino acids intake, biosynthesis and catabolism. This metabolic reprogramming represents an Achille's heel of tumors. Thus, unveiling the oncogenic drivers that reprogram amino acid metabolism in cancer is fundamental to understand disease progression and to find therapeutic opportunities.

**Material and Methods**

We combined metabolomic and transcriptomic analyses on breast cancer (BC) cells expressing missense mutant p53 and grown under different nutritional conditions. We utilized various strategies, including knockdown, overexpression, or the use of inhibitors, to modulate mutant p53 signaling in BC cells grown in 2D and 3D as spheroids. We then performed quantitative mRNA and protein expression analyses as well as proliferation/survival assays. Our results were supported by immunohistochemical and bioinformatic analyses of invasive BCs and were validated using *in vivo* mouse models and patient-derived tumor organoids (PDOs).

**Results and Discussions**

We discovered that in BC missense mutant p53 oncoproteins stimulate *de novo* serine/glycine synthesis and essential amino acids intake, thereby promoting cancer growth. Mechanistically, mutant p53 controls the expression of serine-synthesis-pathway enzymes and L-type amino acid transporter 1 (LAT1)/CD98 heavy chain heterodimer. Both amino acid scarcity and increased mechanical stress exacerbated this effect, suggesting a link between amino acid availability and mechanosensing in activating a mutant p53-dependent metabolic adaptive response to stressful conditions. Indeed, when cells encounter amino acid scarcity, mutant p53 protein is stabilized and induces an amino acid transcriptional program, sustaining cancer cell proliferation. Pharmacological targeting of either serine-synthesis-pathway and LAT1-mediated transport or mechanosignaling, synergizes with amino acid shortage in blunting mutant p53-dependent growth.

**Conclusion**

In sum, we disclosed a novel metabolic activity of missense mutant p53 in BC cells, i.e. promoting synthesis of serine/glycine and intake of essential amino acids. Our findings indicate that mutant p53, unleashing this metabolic program, supports metabolic adaptation to environmental stresses, thus revealing vulnerabilities potentially exploitable for tackling breast tumors bearing missense *TP53* mutations.

### EACR23-1127

### Associations between epithelial-mesenchymal transition (EMT) markers and estrogen receptor (ER) expression in prostate cancer (PCa)

*M. Puchinskaya*[1]

[1]*Minsk City Clinical Oncologic Dispensary, Out-patient, Minsk, Belarus*

**Introduction**

PCa is an androgen dependent disease, however, estrogens acting through binding to ER alpha and beta also play an important role in PCa development and progression. EMT is crucial for cancer metastases and treatment resistance, and its regulatory mechanisms may be a target for novel drugs development. Data on the role of ER subtypes in EMT in PCa are scarce. The aim of the present work was to assess correlations between EMT markers (E-cadherin (E-cad), N-cadherin (N-cad)) and ER expression in clinical PCa samples.

**Material and Methods**

56 radical prostatectomy specimens were studied. 1 representative slide per case was analyzed. Double immunofluorescence technique was used to stain two markers on the same slide: ERα and ERβ, as well as E- and

© 2023 The Authors. Molecular Oncology is published by John Wiley & Sons Ltd on behalf of Federation of European Biochemical Societies

N-cad on a consecutive slide. Primary mouse antibodies to E-cad (1:150), ERα (ThermoFischer, 1:30) and rabbit to N-cad (ThermoFischer, 1:1500), ERβ (BioGenex, 1:500) were used, as well as secondary goat antibodies labeled with AlexaFluor 488 (anti-mouse) and 555 (anti-rabbit) (ThermoFischer, 1:200). Staining was assessed semiquantitatively with calculating weighed staining index (WSI – percentage of stained cells with discrimination of proportion of cells with weak, moderate or strong staining among all stained cels) for N-cad, total staining scores per case (TSSc, mean of multiplying scores for proportion of stained cells and staining intensities in 3-15 x40 high power fields) for ERα, ERβ separately in tumor stroma and epithelium.

**Results and Discussions**

Median WSI for N-cad was 8 (0-70). As E-cad was expressed in all cancer cells, only staining intensity was scored, which was weak in 14, moderate in 34 and strong in 7 cases. Mean TSSc for ERα were 0.96 (0-6) and 1.55 (0-6.9) in epithelium and stroma, and for ERβ 4.73 (0.14-9) and 3.85 (0-8.75), correspondingly. No significant differences in N-cad WSI were found between groups with different E-cad staining intensities ($p_{Kruskal\text{-}Wallis}=0.46$), as well as no differences in ER subtypes TSSc in both stroma and epithelium. The only possible signal (p=0.09) was seen for stromal ERβ that tended to be higher in high E-cad cases. Also N-cad WSI did not correlate significantly with all ER TSSc ($p_{Spearman}>0.05$).

**Conclusion**

In the present cohort no significant associations were found between EMT markers and ER subtypes expression. This may be due to absence of direct influence of ER signaling on EMT features in PCa, as well as a result of more complex interactions not based solely on ER expression levels.

### EACR23-1128

### The therapeutic potential of MYC inhibition in aggressive prostate cancer with low PGC1α expression.

*I. HERMANOVA*[1], *A. Pintor-Rial*[1], *A. Schaub-Clerigue*[2], *A. Macchia*[2], *J.E. Martin Rodriguez*[3], *A.M. Aransay*[3], *A. Carracedo*[2], *V. Torrano*[1]

[1]*University of the Basque Country - UPV/EHU, Biochemistry and Molecular Biology, Leioa, Spain*

[2]*CIC bioGUNE- Bizkaia Technology Park- 801 building, Cancer Cell Signaling and Metabolism, Derio, Spain*

[3]*CIC bioGUNE- Bizkaia Technology Park- 801 building, Genome Analysis Platform, Derio, Spain*

**Introduction**

Despite the positive clinical outcomes of prostate cancer (PCa) therapy, some individuals will still develop metastatic disease that cannot be predicted or effectively treated. In this context, we identified the transcriptional co-regulator PGC1α as a prognostic factor in PCa with tumor and metastasis suppressive activity (PMID: 27214280, 31594836), however the molecular mechanism that can point us in the direction of efficient treatments is yet unknown.

**Material and Methods**

Our project integrates GSEA (PMID: 16199517) of publicly available PCa datasets (PMID: 20579941, 24071849, 22722839, 28068672, 15067324) and *in vitro* functional tests using PC3 cells with doxycycline inducible expression of PGC1α (PMID: 27214280) and MYC dominant mutant omomyc (PMID: 9824157, 12067996).

**Results and Discussions**

To uncover the biological pathways linked with differential PGC1α mRNA level, we performed GSEA on five PCa patient cohorts. We identified MYC targets among the top ranked gene sets enriched in PCa patients with low PGC1α expression. In line with the result, PGC1α and MYC mRNA expression was inversely correlated independently of the presence of MYC amplification. The relation between PGC1α tumor suppressive effect and MYC inhibition was confirmed *in vitro*. RNAseq followed by GSEA of PC3 cells with inducible expression of the tumor suppressor showed that PGC1α re-expression is associated with a profound repression of MYC, interestingly along with activation of IFN pathway. A time course experiment revealed that MYC repression precedes increased expression of IFN pathway-related genes. We next attempted to phenocopy PGC1α tumor suppressive effect by inhibiting MYC using omomyc in aggressive PCa cells (PC3-PGC1α negative cells). Our data show that omomyc induces a significant reduction in 2D proliferation and 3D invasive growth, coupled with a cell cycle arrest and activation of the IFNα pathway. In order to find potential combinatory therapy, we next analyzed PGC1α-driven transcriptome using iLINCS connectivity map (PMID: 35945222). HDAC inhibitors were among the top chemical perturbagen signatures that mimic PGC1α. Indeed, the antiproliferative effect caused by MYC inhibition was intensified when combined with HDACi vorinostat.

**Conclusion**

Overall, our results suggest that monitoring PGC1α transcriptional landscape opens new avenues for the treatment of lethal aggressive PCa with MYC inhibition as a therapeutic strategy.

Grant IJC2019-040709-I funded by MCIN and, as appropriate, by the European Union.

### EACR23-1154

### YAP MEDIATES THE SENSITIVITY TO FK866 IN TRIPLE NEGATIVE BREAST CANCER

*Á. Carreira*[1], *A. Provenzani*[1]

[1]*University of Trento, CIBIO - Laboratory of Genomic Screening, Trento, Italy*

**Introduction**

Disruption of the balance between cell proliferation and cell death disturbs cell and tissue homeostasis and often drives pathological conditions. YAP/TAZ are the downstream effectors of the Hippo pathway and function as transcriptional co-activators of context-specific pro or anti-survival target genes. YAP and TAZ coordinate several $NAD^+$-dependent processes like glycolysis, fatty acid oxidation, and glutaminolysis in nutrient-deprivation conditions. However, correlations between NAD(H) levels and YAP/TAZ metabolic function are poorly understood.

**Material and Methods**

YAP stable silenced cells were obtained by infecting the triple-negative breast cancer (TNBC) cell line MDAMB231 (MDA) with lentiviral particles containing

© 2023 The Authors. Molecular Oncology is published by John Wiley & Sons Ltd on behalf of Federation of European Biochemical Societies pLKO-shYAP. Metabolic and non-metabolic viability assays were used to assess the sensitivity to the $NAD^+$-depleting agent FK866 in attachment conditions, while the soft agar assay was performed to evaluate the ability of cells to grow in anchorage-independent conditions. Mitochondrial respiration was assessed through the Seahorse MitoStress test. Real-Time PCR and Western Blot were performed to determine the expression levels of genes/proteins of interest.

**Results and Discussions**

We showed that FK866 treatment decreases YAP phosphorylation status at serine 127, thus increasing its nuclear translocation in a cancer cell model of TNBC, which is sensitive to FK866. The stable silencing of YAP in MDA rescued FK866 toxicity both in 2D and 3D culturing, promoting a dose-dependent FK866-resistant like phenotype. At the metabolic level, the silencing of YAP on MDA p. induces the FK866-dependent expression of the mitochondrial biogenesis gene PGC1-α and increases the mitochondrial respiratory capacity. These features were previously associated with TNBC FK866-resistant phenotype by our group.

**Conclusion**

We identified a correlation between YAP activation and sensitivity to FK866, which can partially sustain the acquirement of resistance to FK866 in TNBC, likely through the modulation of mitochondrial metabolic traits.

### EACR23-1161
### Genetically and phenotypically heterogeneous cancer stem cells can be isolated from single glioblastomas by alternative selective pressures

*F. De Bacco[1,2], F. Orzan[1], G. Crisafulli[3], M. Prelli[1,2], C. Isella[2,4], E. Casanova[1], R. Albano[5], G. Reato[1,2], A. Bartolini[5], M. Panero[6], L. Casorzo[6], F. Pagani[7], A. Melcarne[8], P. Zeppa[9], D. Garbossa[9], S. Pellegatta[10], P.M. Comoglio[11], G. Finocchiaro[12], P.L. Poliani[7], C. Boccaccio[1,2]*

*[1]Candiolo Cancer Institute- FPO-IRCCS, Laboratory of Cancer Stem Cell Research, Candiolo, Italy*
*[2]University of Turin Medical School, Department of Oncology, Turin, Italy*
*[3]IFOM ETS - The AIRC Institute of Molecular Oncology, BioInformatic Group BIG & Genomics of Cancer and Targeted Therapies Unit, Milan, Italy*
*[4]Candiolo Cancer Institute- FPO-IRCCS, Oncogenomics, Candiolo, Italy*
*[5]Candiolo Cancer Institute- FPO-IRCCS, Core Facilities, Candiolo, Italy*
*[6]Candiolo Cancer Institute- FPO-IRCCS, Unit of Pathology, Candiolo, Italy*
*[7]University of Brescia, Department of Molecular and Translational Medicine, Brescia, Italy*
*[8]Città della Salute e della Scienza, Neurosurgery Unit, Turin, Italy*
*[9]University of Turin Medical School, Department of Neurosciences, Turin, Italy*
*[10]Fondazione IRCCS Istituto Neurologico C. Besta, Brain tumor immunotherapy, Milan, Italy*
*[11]IFOM ETS - The AIRC Institute of Molecular Oncology, Scholar, Milan, Italy*
*[12]IRCCS Ospedale San Raffaele, Department of Neurology, Milan, Italy*

**Introduction**

Glioblastoma (GBM) is an invariably lethal brain tumor, known for its genetic and transcriptional heterogeneity, which limit the effectiveness of current treatments. GBM contains a population of stem-like cells (GSCs), essential for tumor onset, progression, and therapeutic resistance. GSCs are typically isolated from patient biopsies using a culture technique producing "neurospheres" (NS) in a serum-free medium supplemented with EGF and FGF2. The current methodology mostly relies on tissue samples of limited size, which may not fully capture the entire range of GBM heterogeneity. Moreover, standard culture conditions may select for a limited number of subclones that have the highest fitness in those conditions, potentially leading to an incomplete picture of the genetic diversity of the tumor. Hence, there is a need for alternative methods of GSC isolation that can better capture GBM heterogeneity and facilitate the study of subclonal evolution within the tumor.

**Material and Methods**

We collected a cohort of human GBMs surgically removed as ultrasonic aspirates (UA; n=31), virtually allowing to recover the entire tumor mass. From each single UA we established multiple NS parallel cultures ('NS families') by applying different positive selective pressures, represented by different cocktails of growth factors (EFG, FGF2, PDGFBB, and HGF). Four representative NS families underwent full genomic, transcriptomic and phenotypic characterization.

**Results and Discussions**

Within each NS family, NS members displayed homogeneous driver gene mutations but, when present, heterogeneous EGFR or MYC gene amplifications, in terms of amplification levels and modalities (clusters vs. double minutes). However, the tumorigenic potential of each NS wasfound to better correlate with its transcriptional profile rather than its genetic landscape. Across multiple GBMs, classical NS were invariably more malignant than mesenchymal GSCs *in vitro* and *in vivo*. Interestingly, from a tumor including two histopathologically distinct areas, a conventional GBM and a more aggressive primitive neuronal component, we established a NS family whose members displayed homogeneous genetic features but distinct transcriptional and biological features, and were able to reproduce one or the other tumor's component.

**Conclusion**

Our methodology allows to propagate distinct GSCs from individual tumors, which, collectively, better recapitulate the original GBM and represent a resource to study GBM heterogeneity.

### EACR23-1162
### Expression profiling of ANKRD1 in rhabdomyosarcoma cell lines

*E. Milosevic[1], J. Jasnic[1], M. Novkovic[1], V. Cenni[2,3], A. Bavelloni[4], S. Kojic[1]*

*[1]Institute of Molecular Genetics and Genetic Engineering, Laboratory for Molecular Biology, Belgrade, Serbia*
*[2]Institute of Molecular Genetics "Luigi Luca Cavalli-*

© 2023 The Authors. Molecular Oncology is published by John Wiley & Sons Ltd on behalf of Federation of European Biochemical Societies

*Sforza"- National Research Council of Italy IGM-CNR, Unit of Bologna, Bologna, Italy*
*[3]IRCCS, Istituto Ortopedico Rizzoli, Bologna, Italy*
*[4]IRCCS, Istituto Ortopedico Rizzoli- Laboratory for Experimental O ncology, Bologna, Italy*

**Introduction**

Rhabdomyosarcoma (RMS) is the most common soft tissue malignancy in children and adolescents. Respecting the age of the patients and the tumor aggressiveness, investigation of the molecular mechanisms of RMS tumorigenesis is essential, most notably due to the possible identification of novel therapeutic targets. To contribute to a better understanding of the molecular pathology of RMS, we investigated ANKRD1 (ankyrin repeat domain 1) gene, considered a potential RMS diagnostic marker. The changes in its expression are related to carcinogenesis and resistance to chemotherapy in several types of tumors.

**Material and Methods**

In this study, we used three RMS cell lines, SJRH30, RD and HS-729. The expression pattern of endogenous ANKRD1 was determined by RT-PCR, qPCR, Western blot and immunoprecipitation, and the intracellular localization of the protein was examined by immunocytochemistry. Sanger sequencing was used to check for possible alterations in the ANKRD1 open reading frame. Transient transfections of cells were performed with the eukaryotic expression vector encoding ANKRD1 open reading frame. Proteasomal degradation was inhibited by the incubation of cells with MG132.

**Results and Discussions**

RMS cell lines expressed similar amounts of wild-type ANKRD1 transcript, but the protein level was different. ANKRD1 protein was expressed at detectable levels in the SJRH30 and RD cells (SJRH30>RD), but not in the HS-729. Immunocytochemistry revealed the predominant nuclear localization of ANKRD1 and its presence in unidentified nuclear bodies. Overexpression of ANKRD1 was not achieved in RMS cell lines, although it was successful in other cell types. This was potentially due to the proteasomal degradation of ANKRD1, as inhibition of proteasomes with MG132 led to an increase of ANKRD1 protein level in the RMS cells.

**Conclusion**

ANKRD1 protein is expressed at different levels in RMS cell lines and its regulatory role is supported by nuclear localization. ANKRD1 propensity for proteasomal degradation indicates that ANKRD1 overexpression may be lethal for RMS cells. These observations suggest that ANKRD1 warrants further consideration as RMS therapeutic target.

### EACR23-1165
### Deregulation of BID in malignant thyroid neoplasm

F.M. Orlandella[1], N. Luciano[2], M. Braile[3], K. Pane[3], M. Franzese[3], G. Salvatore[1]
*[1]Università degli studi di Napoli "Parthenope", Dipartimento di Scienze Motorie e del Benessere, Naples, Italy*
*[2]Università degli Studi di Napoli "Federico II", Dipartimento di Scienze Biomediche avanzate, Naples, Italy*
*[3]IRCCS Synlab SDN S.p.A., IRCCS Synlab SDN, Naples, Italy*

**Introduction**

BID (BH3 Interacting Domain Death Agonist) is a proapoptotic protein belonging to the BCL-2 family that plays a central role in apoptotic signaling pathway. Several papers reported that deregulated expression of BID is associated with poor outcome in cancer patients.
Although the majority of papillary thyroid cancer (PTC) patients respond well to therapy, no effective therapeutic strategies are available for the treatment of the aggressive forms of this neoplasm. Given the importance of the apoptotic process during the human carcinogenesis, we purpose to assess the expression of BID in thyroid cancer (TC) tissues.
microRNAs (miRNAs), regulating gene expression, have an important role in the molecular pathogenesis of TC. In this field, our preliminary data showed that BID is one of downregulated proteins in TC cells overexpressing the miR-331-5p, suggesting that this miRNA could regulate the expression of BID in this malignancy.

**Material and Methods**

Expression studies in thyroid cancer tissues were investigated by *in silico* analysis. Bioinformatic program, western blot and luciferase assays were applied to unveil that BID is a direct target of miR-331-5p.

**Results and Discussions**

By interrogating The Cancer Genome Atlas (TCGA-THCA), we found that BID is overexpressed in thyroid cancer compared to normal thyroid tissues. The ectopic expression of miR-331-5p in thyroid cancer cells leads to the downregulation of BID protein. The luciferase assay confirmed the direct targeting. Co-expression analysis across 509 thyroid cancer tissues unveils an inverse correlation between miR-331-5p and BID. Interestingly, the proliferation marker KI67 is inversely correlated with miR-331-5p and positively correlated with the mRNA of BID.

**Conclusion**

These altogether findings suggest that the altered expression of BID/miR-331-5p could be a novel potential candidate biomarker for the malignant thyroid neoplasm.

### EACR23-1180
### Engineering Target Tissue in Lab-on-a-chip Devices for Predicting Homing Choices of Metastatic Cancer

G. BATI AYAZ[1,2], D. Pesen Okvur[3]
*[1]Izmir Institute of Technology, Bioengineering, Izmir, Turkiye*
*[2]Izmir Biomedicine and Genome Center, Basic and Translational Research, Izmir, Turkiye*
*[3]Izmir Institute of Technology, Molecular Biology and Genetics, Izmir, Turkiye*

**Introduction**

Breast cancer is the most common cancer in women, and lung is one of the primary sites of metastasis. Metastasis is the leading cause of cancer related deaths. One of the most important steps of the metastatic cascade is the extravasation of cancer cells into distant organs. Vascularization is a critical component of engineering the tumor microenvironment. Recently developed lab-on-a-chip platforms strive to mimic the in vivo

© 2023 The Authors. Molecular Oncology is published by John Wiley & Sons Ltd on behalf of Federation of European Biochemical Societies microenvironment that support complex cell-cell interactions comprising tri-culture of endothelial, epithelial and stromal cells.

**Material and Methods**

3D printing was used for the mold fabrication of LOC devices. The design consists of a homing channel at the middle of LOC and two lateral side channels for medium reservoirs. Different hydrogel combinations were tested to form target microenvironments with vasculature using fibrin, matrigel, and collagen with various concentrations. Hydrogel mixture comprising of growth factor reduced matrigel (3mg/ml): fibrin (3mg/ml): fibroblasts (3T3-L1/WI38: 1×106): epithelial cells (MCF10A/BEAS2B: 1×106): endothelial cells (b.End3: 6×106) was loaded into matrix channel. After the polymerization step, medium was added into lateral side channels. LOCs were incubated overnight in 37˚C incubator 5% CO2 and humidified incubator for the vasculature of breast and lung target tissues. 40 kDa fluorescent dextran was introduced into the lateral medium channels to determine permeability of target tissue. Fluorescently labeled breast cancer cells were introduced into the homing channel. 3D images of target tissues were acquired with a confocal microscope and analysis was performed using Fiji depending on day1 images.

**Results and Discussions**

Hydrogel mixture comprising growth factor reduced matrigel : fibrin was found to be the best combination because the tumor microenvironment with vasculature formed faster compared to the other combination of hydrogels. Permeability and formation of vascularization was confirmed by the diffusion of 40 kDa fluorescent dextran and phalloidin staining. The analysis of extravasation and intravasation showed that more cells extravasated into the lung tissue microenvironment, while more cells intravasated in the breast tissue microenvironment.

**Conclusion**

We have optimized tri-culture of lung and breast tissue microenvironments including a vascular network in a LOC device. Our assay can be used for basic and applied research for breast cancer including but not limited to mechanistic studies and drug development.

### EACR23-1184
### Targeting CDK4/6 in Head and Neck Squamous Cell Carcinoma: A new therapeutic strategy

*M. Álvarez González[1], P.A. Esperanza[1], V.G. Alfonso[2], L.C. Daniela[3], L. Fernando[4], A. Aurora[3], G.P. Juana M.[1], R.T. Juan P.[4], Á.F. Mónica[1]*

*[1]Investigación Sanitaria del Principado de Asturias ISPA-Oviedo- Spain, Department of Otolaryngology, Oviedo, Spain*

*[2]Radiophysics Unit- Hospital Universitario Central de Asturias HUCA-, Radiophysics Unit, Oviedo, Spain*

*[3]Biobanco del Principado de Asturias BBPA- ISPA-IUOPA, Biobanco, Oviedo, Spain*

*[4]Hospital Universitario Central de Asturias HUCA, Department of Otolaryngology, Oviedo, Spain*

**Introduction**

Head and neck squamous cell carcinoma (HNSCC) is a tumor type arising from epithelial cells in the oral cavity, pharynx[MÁF1] or larynx. The major risk factors for HNSCC are tobacco and alcohol consumption, and human papilloma virus (HPV) infections., HNSCC remains a tumor type with low survival rate. Surgery and/or radiotherapy often in combination with cisplatin-based chemotherapy remains the standard of care for advanced HNSCC tumors. However, only a limited number of patients respond to these treatments. CDK4/6 are serine/threonine kinases that modulate cell cycle entry by phosphorylation of retinoblastoma protein (RB). Inhibition of CDK4/6 is a promising therapeutic target in HNSCC, in which this pathway if often deregulated.

Interestingly it has recently been shown that CDK4/6 inhibitors prevent the recovery from the DNA damage induced by genotoxic agents, suggesting its potential use as chemo-sensitizing drugs.

We therefore hypothesize that CDK4/6 inhibition in combination with chemo- and/or radiotherapy might be a new therapeutic strategy for treating HNSCC patients.

**Material and Methods**

We have performed colony formation assays in several HPV-negative HNSCC cell lines and in one HPV-positive cell line in the presence of cisplatin or radiotherapy combined with CDK4/6 inhibitors following a sequential schedule: cisplatin/radiotherapy on day 1, followed by 5 days with CDK4/6 inhibitors and 1 day of drug-holiday. HNSCC patient-derived organoid models (PDOs) generated in our group have also been used for validation studies.

**Results and Discussions**

Treatment of HNSCC cells with cisplatin followed by CDK4/6 inhibition improve the therapeutic effect of cisplatin/radiotherapy alone in HPV-negative HNSCC tumor cells but not in HPV-positive ones, likely due to RB inactivation by the HPV viral oncogene E7. Importantly, our preliminary data in PDOs also show a significant reduction in cell viability in the combined treatment (cisplatin-CDK4/6 inhibitor). We are now exploring the molecular basis for both chemo- and radiosensitizing effects of CDK4/6 inhibition in HPV-negative HNSCC tumor cells.

**Conclusion**

The sequential combination of CDK4/6 inhibitors with the standard of care for HNSCC might be a new therapeutic strategy to improve the clinical management of HPV-negative HNSCC patients. Importantly, CDK4/6 inhibitors might represent a less toxic alternative to cisplatin to potentiate the effect of radiotherapy.

### EACR23-1187
### SOS1 deficiency protects from KRASG12D-driven lung adenocarcinoma

*F. CALVO BALTANAS[1,2], R. García-Navas[1], P. Rodríguez-Ramos[1], N. Calzada[1], C. Cuesta[3], J. Borrajo[4], R. Fuentes-Mateos[1], A. Olarte-San Juan[1], N. Vidaña[1], E. Castellano[3], E. Santos[1]*

*[1]CIC-IBMCC Universidad de Salamanca-CSIC and CIBERONC, Lab 1, Salamanca, Spain*

*[2]Instituto de Biomedicina de Sevilla IBiS and Hospital Universitario Virgen del Rocío, Fisiología Médica y Biofísica, Sevilla, Spain*

*[3]CIC-IBMCC Universidad de Salamanca-CSIC, Lab 5, Salamanca, Spain*

© 2023 The Authors. Molecular Oncology is published by John Wiley & Sons Ltd on behalf of Federation of European Biochemical Societies

[4]Universidad de Salamanca,
Ciencias Biomédicas y del Diagnóstico, Salamanca, Spain

**Introduction**

Lung adenocarcinoma (LUAD) is the most common subtype of pulmonary cancers. About one third of LA are driven by KRAS mutations. Small-molecule inhibitors of $KRAS^{G12C}$ and $KRAS^{G12D}$ have been recently tested in clinical trials but drug resistances frequently arise and few other therapeutic options are yet available for RAS-driven LUAD. The SOS1 and SOS2 Ras-GEFs are recognized as the foremost RAS activators in mammalian cells and specific inhibitors of SOS1-RAS functional interactions have been recently developed.

**Material and Methods**

Using our tamoxifen-inducible SOS1/2 KO animal model, here we evaluated the *in vivo* functional impact of genetic (Tamoxifen-induced SOS1 gene disruption) SOS1/2 ablation on development, progression, regression and tumor microenvironment of a murine model of $KRAS^{G12D}$-driven LUAD.

**Results and Discussions**

SOS1 single ablation in $KRAS^{G12D}$ mice significantly increased animal survival and, strikingly, SOS1 or SOS2 single depletion delayed LUAD tumor initiation/progression, although the effect of SOS2 ablation was significant only during the initial steps of tumor progression. Tumor size, number of surface tumors and pathological grade of tumoral lesions were significantly reduced in SOS1/2-ablated as compared to $SOS^{WT}$ animals, although with a more significant effect in $SOS1^{KO}$ than in $SOS2^{KO}$, showing SOS1 mutant mice a huge reduction in tumor cell proliferation. Notably, SOS1 ablation also reduced tumor-induced macrophage responses and activation of cancer-associated fibroblasts in the tumor microenvironment, potentially attenuating the deleterious effects of tumor microenvironment.

**Conclusion**

Our data indicate that SOS1 is critically required for the development of $KRAS^{G12D}$-driven LUAD tumors through its action over tumor cells and tumor microenvironment-related cells populations, and support the consideration of the SOS GEFs as potential, *bona fide* therapy targets for $KRAS^{mut}$ LUAD and other RAS-dependent diseases. Finally, it will be highly interesting to use the same in vivo mouse models employed here to compare in future our current genetic ablation data with the pharmacological ablation of SOS1 mediated by recently developed inhibitors against this particular RAS-GEF.

### EACR23-1189
### SOS RAS-GEFs in Mitochondrial Metabolism of Immortalized MEFs: Implications for Cellular Homeostasis and Metabolic Adaptation

*P. Rodríguez Ramos*[1], *R. García Navas*[1], *A. Fernández Medarde*[1], *F. C. Baltanás*[2], *E. Santos*[1]
[1]*CIBERONC-IBMCC-USAL- Centro de Investigación del Cáncer, Laboratory 1, Salamanca, Spain*
[2]*Instituto de Biomedicina de Sevilla IBiS- Hospital Universitario Virgen del Rocío- CSIC- Universidad de Sevilla, Departamento de Fisiología Médica y Biofísica, Sevilla, Spain*

**Introduction**

RAS GTPases were first related to cancer in 1982. Since then, they have been recognized as oncogenes, being mutated in about 30% of cancers. The family members of RAS GTPases behave as signal transductors and molecular switches that cycle between their active (GTP-bound) and inactive (GDP-bound) states, a process regulated by GTPase activating proteins and guanine nucleotide exchange factors (GEFs). SOS proteins (SOS1 and SOS2) are the most important RAS-GEFs in mammals regarding their ubiquitous expression and functional relevance. Our laboratory developed a mouse model based on a conditional SOS1 *knockout* (KO) and constitutive SOS2 KO to study their functional specificity/redundancy, finding different physiological defects related to SOS1 ablation, including mitochondria and redox balance alterations, in primary mouse embryonic fibroblasts (MEFs) derived from this model. This led us to hypothesize that the observed defects are caused by a lack of RAS GTPases activation by SOS and that, therefore, rescuing RAS signaling would reverse observed defects.

**Material and Methods**

We immortalized WT, SOS1-KO, SOS2-KO and SOS1/2-DKO MEFs derived from our mouse model, obtaining a genetically manipulable *in vitro* model to test our hypothesis. We characterized the respiratory metabolism phenotype of our immortalized MEFs (iMEFs) to confirm their genotype and to study different activation pathways by Western Blot (WB), the production of Reactive Oxygen Species (ROS) by flow cytometry and mitochondrial morphology and metabolism by confocal microscopy and real-time metabolic assays respectively. In addition, we infected iMEFs with different active RAS isoforms to assess its effect on mitochondrial metabolism through the same set of experiments.

**Results and Discussions**

Successful immortalization and genotype induction was confirmed by WB. Respiratory metabolism characterization showed an increase in ROS levels in SOS1/2-DKO iMEFs. Moreover, SOS2-KO and SOS1/2-DKO genotypes displayed alterations in mitochondrial morphology, whereas respiratory defects were observed in SOS1-devoid genotypes. Interestingly, different active RAS isoforms were able to partially rescue mitochondrial respiratory phenotype of different genotypes on iMEFs.

**Conclusion**

Our results confirm SOS importance in mitochondrial metabolism, unveiling the fundamental role of SOS/RAS signaling in mitochondrial metabolism and cellular homeostasis, and suggest a possible functional specificity of RAS isoforms regarding mitochondrial respiratory metabolism.

### EACR23-1200
### Targeting the mitochondrial protease ClpP unveils novel vulnerabilities in Multiple Myeloma

*R. Del Pizzo*[1], *T. Perini*[1], *P. Zordan*[1], *L. Oliva*[1], *M. Materozzi*[1], *L. Cassina*[2], *U. Orfanelli*[1], *A. Boletta*[2], *S. Cenci*[1]
[1]*Ospedale San Raffaele, Age Related Diseases Unit, Milano, Italy*

© 2023 The Authors. Molecular Oncology is published by John Wiley & Sons Ltd on behalf of Federation of European Biochemical Societies

[2]Ospedale San Raffaele,
Molecular Basis of Polycystic Kidney Disease Unit,
Milano, Italy

**Introduction**

Despite the efficacy of targeted therapies, multiple myeloma (MM) is still incurable. Mitochondria are emerging as promising targets for cancer therapy, and recent evidence suggests that MM increasingly depends on mitochondria and oxidative phosphorylation (OXPHOS) as disease progresses. ClpP is an ATP-dependent protease in charge of mitochondrial protein quality control, and one of the main gatekeepers of mitochondrial homeostasis. Of note, targeting ClpP has recently emerged as an anti-cancer strategy against OXPHOS dependent malignancies. Prompted by its distinctive expression in malignant plasma cells, we manipulated ClpP to investigate its function and challenge it as a potential anti-myeloma target.

**Material and Methods**

We analyzed the effects of ClpP manipulation in MM cells both *in vitro* and *in vivo* by combining viability and growth assays together with Seahorse, proteomics, and metabolomics.

**Results and Discussions**

Acute ClpP KD induced remarkable toxicity in MM cells, due to apoptosis or cell cycle arrest, in both partially and in predominantly glycolytic lines. Surprisingly, ClpP inhibition failed to affect mitochondrial oxygen consumption, suggesting an energy-independent mechanism of toxicity. Indeed, untargeted metabolomics unveiled an unexpected deregulation of polyamines metabolism upon ClpP KD, with depletion of spermidine. Inducible ClpP KD confirmed reduced MM growth *in vitro*, and *in vivo* upon xenotransplant into immunocompromised recipient mice, and impacted polyamine biosynthesis possibly through a deregulation of urea and TCA cycles. Moreover, ClpP KD also boosted the expression of interferon-stimulated genes (ISG) in MM cells, likely due to release of mitochondrial DNA. Attesting to a possible immunomodulatory effect, human dendritic cells (DCs) cultured in the presence of medium conditioned by ClpP KD MM cells showed increased markers of activation, production of IL-1b and IL-12, and higher capacity to induce $CD8^+$ T cells proliferation and production of IFN-g.

**Conclusion**

Overall, our data suggest that ClpP is crucial to MM cells due to novel non-bioenergetic functions. Its manipulation unveils an unprecedented role of mitochondrial homeostasis in regulating polyamine biosynthesis, and causes intracellular activation of inflammatory pathways, suggesting the possibility to exploit ClpP as a novel target against MM to exert direct cytotoxicity and increase anti-tumor immunity.

### EACR23-1203
### Unclustering the Centrosomes by Nek2: A matter of life for cancer cells with extra centrosomes

B.M. Kalkan[1], C. Özcan[2], E. Çiçek[1], C. Açılan Ayhan[3]
[1]Koç University, Graduate School of Health Sciences, Istanbul, Turkiye
[2]Koç University,
Research Center for Translational Medicine, Istanbul, Turkiye
[3]Koç University, School of Medicine, Istanbul, Turkiye

**Introduction**

Modern chemotherapeutics frequently suffer from non-selective targeting of cancer cells.Contrast to normal cells, cancer cells frequently exhibit extra centrosomes (ECs), which tend to form multipolar spindles (MPS), triggering cell death. Nevertheless, cancer cells can divide successfully by coalescing their ECs into two poles. Nek2 is a mitotic kinase regulating several mitotic processes. We showed that while reduction of Nek2 activity favors centrosome clustering, its overexpression unclusters ECs. Thus, revealing the molecular mechanism for centrosomal unclustering via Nek2 in cells with ECs may be an alternative strategy for selective targeting for cancer cells.

**Material and Methods**

To investigate the mechanism of Nek2 overexpression for the induction of MPS, IF staining, cell viability, apoptosis and competition assays were performed. Initially, the known targets of Nek2 with relevant function (C-NAP1, Rootlein, Trf1, Hec1, Gas2L1) were assessed by knock out or siRNA mediated silencing. New candidates were determined via investigation of Nek2 interactome using TurboID proximity labelling followed by Mass-Spec. The potential Nek2 substrates were further analyzed via co-IP.

**Results and Discussions**

We showed that cancer cells with ECs were selectively depleted from the population upon Nek2 overexpression *in vitro* and *in vivo.* We observed no significant change on centrosome clustering when known Nek2 substrates were targeted. The effect of Nek2 overexpression on MPS was additive with other known unclustering factors, suggesting an independent mechanism. TurboID proximity labelling revealed potential targets of Nek2 that were either localized to the centrosome or microtubules. One of these proteins, NuMA, a previously known unclustering factor, was significantly enriched. NuMA did not co-IP with Nek2 but its knockdown reverted the unclustering. Significantly enriched motor protein, Kif2C not only reverted Nek2 activity, but also co-IP'ed with Nek2. Moreover, we identified MAPRE3, a regulator of microtubule dynamics, as interaction partners of both Nek2 and Kif2C. Our data suggests that Nek2 partners with Kif2C and MAPRE3, regulating centrosome clustering.

**Conclusion**

We assigned a novel function for Nek2 in centrosome clustering. We are currently elucidating the detailed mechanism of action for how target proteins interact with Nek2 regulating its centrosome unclustering activity. Understanding the molecular players in this process will provide new translational approaches for targeted cancer therapies.

### EACR23-1210
### Targeting USP7 in Colorectal Cancer

E. Agnew[1], P. Sung[2], M. Wappett[2], T. Harrison[2], D. Longley[1], S. McDade[1]
[1]Queen's University Belfast,
Patrick G Johnston Centre for Cancer Research, Belfast, United Kingdom
[2]Almac Discovery, Health Sciences Building, Belfast, United Kingdom

**Introduction**

© 2023 The Authors. Molecular Oncology is published by John Wiley & Sons Ltd on behalf of Federation of European Biochemical Societies

Ubiquitination is a post-translational modification important for cellular homeostasis and its dysregulation has been linked to various cancers including colorectal cancer (CRC). Ubiquitination involves E3 ubiquitin ligases adding ubiquitin to target proteins and deubiquitinase enzymes (DUBs) reversing this process. Ubiquitin-specific protease 7 (USP7) is one of the most well studied DUBs, with target proteins including MDM2, EZH2, β-catenin, FOXO4 and UHRF1. These proteins are involved cancer-related processes; DNA damage, immune signalling, epigenetic regulation and apoptosis. Much research has focused on the role of USP7 in regulation of the p53-MDM2 axis, and inhibiting USP7 has been explored as a way of activating p53 in cancer.

**Material and Methods**

Using a panel of novel, CRISPR generated, p53-knockout isogenic CRC cell line models, we determined the p53-dependent and -independent effects of a novel USP7 inhibitor, AD04, using protein analysis, apoptotic assays and RNA-seq. We also investigate how AD04 impacts response to the backbone of standard-of-care 5-Fluorouracil (5FU) treatment and how this is affected by interactions within the tumour microenvironment. We used imaging-based analysis for this and more complex 2D and 3D co-culture models, to mimic more clinically relevant models.

**Results and Discussions**

We identified the expected p53-dependent effect of AD04 on cell viability, that correlated with an increase in p53 stabilisation and its transcriptional targets p21 and MDM2. Co-treatment with AD04 and 5FU induced a significant reduction in cell viability compared to treatment with AD04 or 5FU alone. Notably, this synergy was independent of p53 status, albeit a greater effect was observed in p53-WT cells. Again, in co-cultures we found that AD04 enhances the effects of 5FU independent of p53 status. Moreover, co-cultures with activated T-cells further enhanced the effects of AD04/5FU in both 2D and 3D co-cultures. Although this immune cell-mediated enhancement was a caspase-dependent process, indicative of apoptosis, no enhanced expression of pro-apoptotic p53-target genes such as PUMA was observed in the co-treatment group compared to 5FU alone.

**Conclusion**

Altogether, these results reveal a novel synergism between USP7 inhibition and 5FU that is independent of p53 *in vitro*.

### EACR23-1214
### Extracellular HSP90 co-chaperone Morgana drives extracellular matrix remodelling

<u>*F. Zuppini*</u>*[1], P. Poggio[1], M. Brancaccio[1]*

*[1]University of Turin, Molecular Biotechnology Center, Turin, Italy*

**Introduction**

Heat shock proteins are a class of molecules upregulated by cells in response to stressful stimuli. Since cancer cells are stressed, they are “chaperone-addicted”. It has been described that many chaperones are secreted by cancer cells in the extracellular milieu where they can exert different functions.

Hsp90 is the most abundant and important chaperone and it is crucial in sustaining survival and growth in cancer cells. Morgana is a highly conserved HSP90 co-chaperone; we found that Morgana is secreted by cancer cells in the extracellular microenvironment where, in association with Hsp90 and other receptors, it stimulates cell migration.

**Material and Methods**

Cell treatment was performed using a recombinant Morgana fused to maltose binding protein (mbp) whereas mbp was used as control.

Extracellular matrix protein and integrin levels in total cell extract and conditioned medium were evaluated through Western Blot; the levels of the same molecules on cell surface were evaluated through Flow Cytometry. The RNA levels were evaluated through Real Time PCR.

**Results and Discussions**

**Secreted Morgana induces a remodelling of the extracellular matrix**: Cancer cell migration is strongly supported by the reshaping of the extracellular matrix (ECM). We found that cell treatment with the recombinant Morgana induces an increase of some ECM protein levels such as Fibronectin and Laminin in cell conditioned medium. However, we did not observe an increase in the RNA levels of such proteins. These results suggest that extracellular Morgana induces cancer cell migration through the remodelling of the ECM composition.

**Extracellular Morgana induces the recycling of the integrins**: Integrins constitute the principal adhesion receptors for the extracellular matrix and their recycling, together with other components of the ECM, is crucial for cell migration. We found that cells treated with the recombinant Morgana showed an increase in the exposition of integrins on cell surface, but we didn’t observe an increase in integrin RNA levels. These results suggest that extracellular Morgana could be involved in integrin based recycling of some ECM components.

**Conclusion**

In conclusion, we demonstrated that the chaperone Morgana has a role in the remodelling of the extracellular matrix composition and in the integrin-based recycling of its components. Since we couldn’t see a modulation in the RNA levels we speculate that Morgana preserves integrin integrity on cell membrane avoiding their degradation.

### EACR23-1218
### Adaptor protein Ruk/CIN85 contributes to drug resistance and metastasis of osteosarcoma cells

<u>*I. Horak*</u>*[1,2], L. Drobot[1], L. Knopfová[2]*

*[1]Palladin Institute of Biochemistry- National Academy of S ciences of Ukraine, Department of Cell Signaling, Kyiv, Ukraine*

*[2]Masaryk University, Department of Experimental Biology, Brno, Czech Republic*

**Introduction**

Previous studies demonstrated that a high expression level of adaptor protein Ruk/CIN85 is associated with increased resistance to anticancer therapeutics, elevated motility, and metastatic potential of many types of tumor cells, including breast, cervix, colon, and melanoma. The aim of the present study was to investigate the role of adaptor protein Ruk/CIN85 in the acquisition of malignant properties of osteosarcoma (OS) cells.

**Material and Methods**

© 2023 The Authors. Molecular Oncology is published by John Wiley & Sons Ltd on behalf of Federation of European Biochemical Societies

Human HOS and SAOS-2 lines and mouse Dunn osteosarcoma cells, corresponding drug-resistant and highly metastatic derivatives, as well as HOS sublines with Ruk/CIN85 overexpression (or corresponding mock-control cells) were used in the experiments. mRNA expression was assessed by RNAseq and supported by datamining of expression datasets available at GEO or TNMplot databases. Protein content was studied using Western-blotting.

**Results and Discussions**

According to TNMplot database https://tnmplot.com, Ruk/CIN85-encoding gene *SH3KBP1* is significantly overexpressed in human OS samples. Analysis of RNA sequencing data of HOS cells revealed elevated levels of *SH3KBP1* transcript levels in cells resistant to doxorubicin and methotrexate compared to parental cells. By immunoblotting we confirmed higher amount of Ruk/CIN85 in the doxorubicin- and methotrexate-resistant SAOS-2 cells and doxorubicin-resistant HOS cells compared to wt cells. Analysis of parental OS cells and their metastatic derivatives showed that Ruk/CIN85 expression was higher in metastatic 143B cells compared to parental HOS cells, and in LM8 cells compared to parental Dunn cells. These finding were confirmed by *SH3KBP1* expression data. Therefore, HOS subclones with Ruk/CIN85 up-regulation were generated, and it was found that these cells are characterized by elevated expression levels of epithelial-mesenchymal transition (EMT) markers vimentin, SNAIL, and SLUG, and decreased E-cadherin content. Also, Ruk/CIN85 overexpression resulted in increased content of PARP and Bcl-XL, indicating resistance to apoptosis.

**Conclusion**

Thus, elevated expression of adaptor protein Ruk/CIN85 in osteosarcoma cells is associated with resistance to chemotherapeutics and may also influence metastasis propensity.

This study was supported by Masaryk University programme “Masaryk helps Ukraine”.

### EACR23-1220

### Simultaneous apoptosis and autophagy occurrence in colorectal cancer cells by the synergism of doxorubicin and tocotrienol

*S.H. Loh*[1], *S.Y. Tham*[1], *Z.H. Yap*[1], *J.Y. Fu*[2], *C.W. Mai*[3], *S.M. Then*[4]

[1]*University of Nottingham Malaysia, School of Biosciences, Semenyih- Selangor, Malaysia*

[2]*Malaysian Palm Oil Board, Nutrition Unit- Product Development and Advisory Service s Division, Kajang, Malaysia*

[3]*UCSI University, Faculty of Pharmaceutical Sciences, Kuala Lumpur, Malaysia*

[4]*University of Nottingham Malaysia, Division of Biomedical Sciences, Semenyih- Selangor, Malaysia*

**Introduction**

Colorectal cancer (CRC) is the third most prevalent cancer and second most lethal cancer worldwide. Doxorubicin (Dox) has been widely used in chemotherapy for treating various cancers, including CRC although it is not the first-line CRC chemo-drug. The effectiveness of Dox is often limited by high-dose toxicity and tendency of resistance development. As an approach to chemo-sensitize Dox, tocotrienol (T3) was applied in combination on CRC cells.

**Material and Methods**

This study covered the *in vitro* evaluations on Caco-2 and SW48 cell lines with the synergistic combination of tocotrienol and doxorubicin (T3+Dox) for the antiproliferative effects, morphological changes, DNA damages, cell cycles and protein expression profiles in relation to apoptosis and autophagy.

**Results and Discussions**

The combined treatment (T3+Dox) resulted pharmacological synergisms and exerted significant anti-clonogenic survivals on Caco-2 and SW48 cells even at low concentrations. Morphologically, the Caco-2 and SW48 cells treated with low-concentration T3+Dox exhibited apoptotic features and great cellular stress. This combined treatment increased rate of apoptosis, DNA breaks and fragmentation. Mitochondrial membrane permeabilization (MMP) was observed in treated Caco-2 and SW48 cells. The inhibition of MMP using cyclosporine A successfully suppressed cell death suggesting that the combined treatment targets at mitochondria. Despite activations of caspase-8 and -3 were detected, the application of caspase inhibitors was unable to improve cell viability signifying the possibility of caspase-independent cell death occurrence. Besides, the combined treatment induced an enhanced autophagy in the treated cancer cells as evidenced by the formations of acidic vacuolar organelles, phagophores and autophagosomes, as well as increments of monodansylcadaverine intensity and microtubule-associated protein 1A/1B light chain 3 (LC3)-II expression. The involvement of autophagic cell death is further confirmed when the application of autophagy inhibitors, namely 3-methyladenine and bafilomycin A1 successfully reverted cell death.

**Conclusion**

Overall, current findings supported that T3+Dox combined treatment exerted both apoptosis and autophagy in Caco-2 and SW48 cells, where these multitargeted actions are highly imperative for treating the invasive type of CRC in the future.

### EACR23-1224

### Metabolic profiling of luminal and basal breast tumours reveals fatty acid synthesis and choline pathway as potential therapeutic targets.

*M. Golinska*[1], *E. Brown*[2], *L. Hacker*[1], *I. Quiros Gonzalez*[3], *M. Basetti*[1], *A. Shaw*[1], *C. Sekhar Reddy Chilamakuri*[1], *S. Bohndiek*[1]

[1]*Cancer Research UK Cambridge Institute, Department of Oncology, Cambridge, United Kingdom*

[2]*Washington University USA, School of Medicine, St Louis, United States*

[3]*FINBA-IUOPA- University of Oviedo, Cell Biology Department, Oviedo, Spain*

**Introduction**

Breast cancer subtypes are classified by their hormone receptor status and show differences in treatment response and clinical outcomes. Here, we explore the relationship between hormone receptor expression and metabolism to

© 2023 The Authors. Molecular Oncology is published by John Wiley & Sons Ltd on behalf of Federation of European Biochemical Societies determine the potential for using metabolic targeted therapies in breast cancer.

**Material and Methods**

Frozen tissues, collected from breast cancer patient-derived xenograft (PDX) models AB580 (estrogen and progesterone receptors, ER+/PR-, n=13) and STG-139 (ER-/PR-, n=19) as well as MCF7 (ER+/PR+, n=8) and MDA-MB-231 (ER-/PR-, n=8) xenograft models, were subjected *to ex* vivo HR MAS metabolic and RNAseq analyses. IHC staining on ER and PR and fatty acid synthase (FASN) was performed on paraffin-embedded samples. The findings in mouse models were verified using human tissue microarrays (TMAs).

**Results and Discussions**

HR MAS metabolic profiling revealed that choline, glycerophosphocholine (GPC) and total choline were significantly higher in ER- PDX tumours as was the GPC/phosphocholine ratio ($p<0.01$), in line with prior work. Our data suggests that differences in ER expression alone may contribute to this shift in choline metabolism. Differential gene expression analysis on RNA-seq data from both PDXs and xenografts showed higher expression of PLD1 in ER- tumours, suggesting increased breakdown of membranes containing choline phospholipids. This analysis also revealed activation of AMPK pathway in ER-/PR- tumours.

FASN staining was lower in ER-/PR- MDA-MB-231 xenografts as compared to ER+/PR+ MCF7 xenografts ($p<0.01$), in which FASN expression correlated positively with both ER and PR staining ($r=0.75$ and 0.9 respectively). Similarly, in the breast cancer PDX models, which lacked PR and HER2, we observed a higher FASN staining in ER+ compared to ER- models ($p<0.001$). Supporting these observations in mouse models, analysis of two independent breast cancer TMAs showed that FASN staining correlates positively with the expression of both ER and PR and that it is lowest in triple negative breast cancer.

**Conclusion**

Our data shows that the loss of ER expression influences choline and fatty acid metabolism and AMPK signalling. A strong correlation between FAS and PR/ER can be observed in patient samples as well as in breast cancer PDX and xenograft models. Development of metabolic blockers against those pathways holds promise for improved and personalised therapy.

### EACR23-1232

### Investigating novel vulnerabilities of drug-tolerant persisters

*M. Chen[1], L. Wang[1], S. Mainardi[1], J. Kuiken[1], C. Lieftink[1], I.D. Rink[1], H. Jin[1], R. Jansen[1], K.V.D. Mijn[1], N. Proost[1], M. Boeije[1], F. Jochems[1], R. Beijersbergen[1], R. Bernards[1]*

*[1]The Netherlands Cancer Institute, Molecular Carcinogenesis, Amsterdam, The Netherlands*

**Introduction**

Drug tolerant persisters (DTPs) are rare subpopulation of cancer cells which survive targeted or chemo therapy through non-genetic mechanism. Although mounting literature suggests that different mechanisms are involved in DTP formation, very few studies have reported on the vulnerabilities of DTPs.

**Material and Methods**

CRISPR genetic screen; Compound screen; scRNA seq; Xenograft animal models.

**Results and Discussions**

Using both kinome based genetic screen and compound screen, we identified BET inhibition as a vulnerability of DTPs. Subsequent functional validation studies showed that BET inhibitors suppressed DTEPs in a broad spectrum of cancer types. Although DTPs shared some similarities with senescent cells, cFLIP overexpression failed to rescue BET inhibition mediated killing, indicating they have different vulnerabilities. Mechanistically, scRNA seq unveiled tens of DTP marker genes involved in regulation of the NF-kB pathway, apoptotic pathway, anti-oxidative pathway, EMT and stemness features. Consistent with previous findings that DTPs have an elevated ROS level, we found surprisingly that BET inhibition induced lethal level of ROS in DTEPs through a novel mechanism of suppression of redox regulating DTP markers including GPX2, ALDH3A1 and MGST1. In vivostudies showed that BET inhibitor delayed tumor relapse in both melanoma and lung cancer xenograft models. Compared to the pre-clinical GPX4 inhibitor RSL3 which can't be applied in vivo, BET inhibitors have shown to be a well tolerated .

**Conclusion**

Our study suggest that apart from using the synergy testing as a gold stand, combining standard of care therapy (that yield DTPs) with another drug targeting the residual persisting tumor cells could be a potential theraputic strategy in the future.

### EACR23-1243

### In vitro functional analysis of HERC2 gene and evaluation of its variants' pathogenicity in non-small cell lung cancer

*B. Souza[1], G. Casagrande[1], R. Cavagna[1], A. Evangelista[1], F. Da Silva[1], P. De Marchi[2], W. Dos Santos[1], R. Reis[3], R. Oliveira[1], L. Leal[1]*

*[1]Barretos Cancer Hospital, Molecular Oncology Research Center, Barretos, Brazil*

*[2]Oncoclínicas, Cabeça & Pescoço e Tórax, Rio de Janeiro, Brazil*

*[3]Minho University, Medicine Course, Braga, Portugal*

**Introduction**

Previous data from our group revealed the *HERC2* gene as a potential new driver for Non-Small Cell Lung Cancer (NSCLC). This gene is involved in several biological pathways of tumoral development, but we know little about its relevance to lung cancer. Our aim was to evaluate the pathogenicity of *HERC2* variants found in a Brazilian series of NSCLC and describe the functional role of this gene *in vitro*.

**Material and Methods**

We evaluated *HERC2* mutational frequency in an NSCLC cohort (N=123) previously sequenced through WES. We predicted the impact of these variants using the Cancer Genome Interpreter (CGI) and associated the *HERC2* status with clinical features and genetic ancestry. We queried databases - cBioPortal, IntOGen, GDC-NCI, *KM Plotter* - for *HERC2* mutational frequency, gene expression, and clinical or survival associations in NSCLC. Statistical associations were estimated using the chi-square and Fisher's exact test, Kaplan-Meier method,

© 2023 The Authors. Molecular Oncology is published by John Wiley & Sons Ltd on behalf of Federation of European Biochemical Societies

Log-rank test, and Cox regression. The *in vitro* evaluation was performed with SK-MES-1(*HERC2*$^{WT}$) cell line, in which we transfected esiRNA, directed against *HERC2*, to achieve gene silencing. Subsequently, we analyzed cell proliferation (xCELLigence), cell migration (trans-well) and invasion (matrigel), tumorigenesis (colony forming assay), and cell cycle (flow cytometry).

**Results and Discussions**

*HERC2* mutational frequency in NSCLC was 10,5% (adenocarcinoma: 9,4%; squamous cell carcinoma: 17,9%). The variants were classified as polymorphism (N=1), passengers (N=7), and drivers (Tier1= 9; Tier2= 6). All of the *HERC2*$^{MUT}$ cases were ever-smokers (p=0,12). The median overall survival was 22.6 months in *HERC2*$^{WT}$ and 6 months in *HERC2*$^{MUT}$ (HR= 1.2; p=0.58). In the public databases, adenocarcinomas were mutated in 1.69-13% of cases, while squamous cell carcinoma (SqCC) showed a mutational frequency of 8.2-15%. Higher expression of *HERC2* in adenocarcinomas was associated with better overall survival (HR=0.73; p=0.02). *HERC2* knockdown successfully abolished *HERC2* protein expression. In functional assays, *HERC2*-depleted cells showed a decreased proliferation (90% less than non-targeting control), an arrest of the cell cycle in the S phase, and a diminished capacity to form colonies.

**Conclusion**

*HERC2* gene harbors potential driver variants associated with clinical features in a Brazilian series of NSCLC with a higher frequency in SqCC. Loss of *HERC2 in vitro* showed a less tumorigenic phenotype.

### EACR23-1247
### Carboxylesterase 1 (CES1) supports autophagy-mediated lipid catabolism and metabolic adaptation in ovarian cancer cells

*I. FLATI*[1], *D. Verzella*[1], *M. Di Vito Nolfi*[1], *C. Compagnoni*[1], *D. Vecchiotti*[1], *F. Dall'Aglio*[1], *E. Alesse*[1], *G. Franzoso*[2], *F. Zazzeroni*[1], *D. Capece*[1]
[1]*University of L'Aquila, Department of Biotechnological and Applied Clinical Scie nces DISCAB, L'Aquila, Italy*
[2]*Imperial College London, Department of Immunology and Inflammation, London, United Kingdom*

**Introduction**

During nutrient deprivation, the catabolic pathway of autophagy is upregulated to sustain aggressive malignant phenotypes, making this metabolic process a cancer vulnerability. However, how to effectively target autophagy in cancer is still under investigation. Recently, Carboxylesterase 1 (CES1) has been depicted in colorectal carcinoma (CRC) as a NF-κB-regulated lipase associated with advanced disease progression. CES1 promotes the metabolic adaptation of CRC cells by increasing free fatty acids availability to fatty acid oxidation (FAO). Like CRC, ovarian carcinoma (OC) preferentially metastasizes to peritoneal cavity and infiltrates the omentum, a fat rich organ. An increasing number of efforts are focused on how deregulated lipid metabolism impacts on ovarian cancer aggressiveness and drug resistance. Therefore, we investigated whether CES1 could play a role in the autophagy-mediated metabolic adaptation of OC cells.

**Material and Methods**

Public datasets of OC patients were analysed. A panel of OC cell lines were tested for *CES1* expression, metabolic phenotype, autophagy flux and survival under energy stress conditions with or without specific CES1 inhibitor were evaluated by qRT-PCR, seahorse, Western Blot and viability assay. *In vitro* enzymatic assay was used to measure CES1 activity in presence of CES1 inhibitors.

**Results and Discussions**

We found that elevated *CES1* expression correlates with worse prognosis in OC patients. Accordingly, we showed that CES1 was expressed in all OC lines when cultured under energy stress (ES), suggesting that CES1 could be relevant for their adaptation to harsh metabolic environment. Indeed, pharmacological CES1 blockade by commercially available GR-148672X inhibitor impaired bioenergetic parameters, blocked autophagy flux and caused cell death in Carboplatin-resistant OC cell lines under ES. Notably, oleate supplementation (circumventing the requirement for lipid catabolism) rescued OC cells from ES-induced toxicity. However, GR-148672X shows poor drug-like properties which preclude its clinical development in OC. Accordingly, we tested derivates of GR-148672X demonstrating that they retain specific activity and are effective in blocking autophagic flux and inducing cell death in OC cells under ES.

**Conclusion**

These data underscore the actionability of CES1 inhibition to block autophagy-mediated lipid catabolism and counter OC progression, especially in chemotherapy-refractory settings.

### EACR23-1252
### Cancer cells depend on wobble tRNA modification to sustain translation under mTORC1 inhibition

*J. Hermann*[1], *R. Kalis*[2], *A. Kowar*[3], *T. Bortecen*[4], *M. Schneider*[5], *C. Pechincha*[1], *D. Helm*[5], *J. Krijgsveld*[4], *F. Loayza Puch*[3], *J. Zuber*[2], *W. Palm*[1]
[1]*German Cancer Research Center DKFZ, Cell Signaling and Metabolism, Heidelberg, Germany*
[2]*Research Institute of Molecular Pathology IMP, Finding and understanding cancer dependencies, Vienna, Austria*
[3]*German Cancer Research Center DKFZ, Translational Control and Metabolism, Heidelberg, Germany*
[4]*German Cancer Research Center DKFZ, Proteomics of Stem Cells and Cancer, Heidelberg, Germany*
[5]*German Cancer Research Center DKFZ, Proteomics Core Facility, Heidelberg, Germany*

**Introduction**

The kinase mechanistic target of rapamycin complex 1 (mTORC1) is a central regulator of protein translation and commonly dysregulated in cancer. Because mTOR inhibitors strongly suppress cell proliferation in cell culture, they were developed as cancer therapeutics, but failed in many clinical studies. To systematically identify genes that are required for cancer cell proliferation under mTORC1 inhibition, we performed a proliferation-based

© 2023 The Authors. Molecular Oncology is published by John Wiley & Sons Ltd on behalf of Federation of European Biochemical Societies

CRISPR screen under mTOR inhibition in a pancreatic cancer cell line.

**Material and Methods**

Proliferation-based CRISPR Screen, ribosome profiling, (nascent) proteome analysis, cell proliferation assays under different metabolic conditions

**Results and Discussions**

Particularly striking hits in our screen were genes that modify tRNA wobble bases of NAA codons to form 5-methoxycarbonylmethyl-2-thiouridine ($mcm^5s^2U$): Elp2, Elp3 and Elp6, which are components of the elongator complex catalyzing the $mcm^5$ modification, and Ctu1/ Ctu2, which form a heterodimer that catalyzes the thiolation of uridine. Loss of any of these genes substantially decreased cell proliferation only when mTORC1 was inhibited. Pharmacologically blocking the upstream PI3K-Akt signaling pathway similarly suppressed proliferation of Ctu1 knock-out cells. To understand the relative contribution of wobble tRNA modification and mTORC1 inhibition on protein translation, we performed ribosome profiling. Deletion of Ctu1 or Elp3 led to ribosome accumulation specifically at NAA codons, which encode the amino acids lysine, glutamine and glutamate. Analyzing the nascent proteome revealed that deletion of Ctu1 decreased the synthesis in particular of ribosomal proteins, which are enriched in lysine AAA codons. At the proteome level, deletion of Ctu1 / Elp3 or mTORC1 inhibition only slightly decreased levels of ribosomal proteins, whereas deletion of Ctu1/ Elp3 in combination with mTORC1 inhibition dramatically reduced levels of ribosomal proteins. Consequently, a combination of Ctu1 / Elp3 deletion and mTORC1 inhibition resulted in suppression of global protein synthesis.

**Conclusion**

Our data shows that wobble tRNA modifications synergize with mTORC1 to mediate translation of ribosomal proteins. Thus, cancer cells depend on wobble tRNA modifications to sustain ribosomal biogenesis and thus global protein translation under mTORC1 inhibition. This novel mechanistic link between a class of tRNA modifications and mTORC1 in translation regulation may open therapeutic avenues in cancer treatment.

### EACR23-1254

### Evaluation of the role of Syncytin-1 and its receptor ASCT2 in cancer cell fusion

*M. Sieler[1], T. Dittmar[1]*

*[1]Witten/Herdecke University- Institute of Immunology, Department of Medicine- Center for Biomedical Education and Research ZBAF, Witten, Germany*

**Introduction**

Cell fusion is a biological process crucial in the development and homeostasis of mammals, as it is required in fertilization or wound healing, for example. Additionally, cell fusion was observed in a pathological context when cancer cells fuse with other cells. The resulting cancer hybrid cells can show more malignant properties, like a higher metastatic potential. To investigate how cancer cells can fuse with other cells, the human endogenous retroviral envelope protein Syncytin-1 and its receptor ASCT2, known to be fusogenically active in the placenta, were examined.

**Material and Methods**

To study the impact of Syncytin-1 and ASCT2 on the fusion of breast cancer cells with breast epithelial cells, either Syncytin-1 was stably overexpressed or its receptor ASCT2 was knocked out (KO) by CRISPR/Cas9 in the human breast epithelial cell line M13SV1. To quantify the cell fusion events between M13SV1 cells and three different human breast cancer cell lines (MDA-MB-231, MDA-MB-435, HS578T), two fluorescence-based assays were used: the fluorescence double reporter assay and the dual split protein assay. In addition, the cytokine TNFα and a Syncytin-1 inhibitory peptide were used to either mimic the inflammatory environment of cancers or examine whether fusion events could be reduced by blocking Syncytin-1.

**Results and Discussions**

Homogenic fusion of differently altered M13SV1 cells as well as heterogenic fusion with the different breast cancer cell lines showed that Syncytin-1 expression is sufficient to induce cell fusion with cells expressing its receptor ASCT2. Vice versa, cell fusion was prevented by ASCT2 KO or reduced by usage of the Syncytin-1 inhibitory peptide. TNFα was capable to further increase the cell fusion rate of M13SV1 cells overexpressing Syncytin-1 with two out of the three used breast cancer cell lines. The results suggest that the expression of Syncytin-1, which is normally restricted to the placenta, can lead to fusion of cancer cells and thus to the formation of cancer hybrid cells.

**Conclusion**

Cell fusion of cancer cells can lead to more malignant cancer hybrid cells, making it imperative to understand and possibly prevent the mechanism of cell fusion. Since Syncytin-1 and its receptor ASCT2 have been detected in various cancers, they represent a potential therapeutic target to prevent cell fusion and thus the formation of cancer hybrid cells.

### EACR23-1256

### Elucidating the tumor suppressor role of STAG2 in bladder cancer

*E. Lapi[1], M. Ramal[1], J. Martínez de Villareal[1], M. Marques[1], A. Losada[2], F.X. Real[1]*

*[1]Spanish National Cancer Research Centre - CNIO, Epithelial Carcinogenesis Group, Madrid, Spain*

*[2]Spanish National Cancer Research Centre - CNIO, Chromosome Dynamics Group, Madrid, Spain*

**Introduction**

Bladder cancer is a highly prevalent, under-researched, tumor with a high cost to the health systems.

STAG2, a cohesin component involved in chromosome organization and transcriptional regulation, was identified by our lab, and others, as one of the most important tumor suppressor genes involved in bladder cancer. STAG2 mutations are inactivating, associate with FGFR3 genetic alterations and with luminal tumours of low stage and grade, but there is a lack of understanding of how they contribute to tumor development. Accumulating evidence indicates that STAG2 loss is an early genetic event that participates in bladder carcinogenesis through mechanisms distinct from chromatin segregation.

**Material and Methods**

To assess how STAG2 loss contributes to BC development, we generated a conditional knockout (KO)

© 2023 The Authors. Molecular Oncology is published by John Wiley & Sons Ltd on behalf of Federation of European Biochemical Societies mouse model and established mouse bladder organoids and immortalized cultures of primary murine normal urothelial cells (NU1), both of which recapitulate urothelial differentiation. These tools allowed us to conduct valuable genomic studies.

**Results and Discussions**

Stag2 depletion in proliferating NU1 cells has minor transcriptomic effects but it has higher impact in differentiated cells, consistent with an enrichment in STAG2 localization at promoters and enhancers using ChIP-Seq. Although cell cycle genes are significantly upregulated in differentiated NU1 cells upon STAG2 loss, Stag2-knockdown cells do not show increased proliferation. Stag2 inactivation in the urothelium (Upk3a-CreERT) results in the upregulation of cell cycle genes and KI67 expression. Preliminary data suggest that changes in transcription factor positioning, rather than altered chromatin accessibility, account for cell cycle gene upregulation. While STAG2 loss in the murine urothelium does not have major histological effects in homeostatic conditions, concomitant STAG2 deletion and cyclophosphamide induced urothelial damage lead to hyperplasia and Stag2 KO urothelial cells display higher organoid-forming capacity.

**Conclusion**

In summary, Stag2 inactivation in the urothelium is not enough to trigger neoplastic transformation but results in cell cycle gene upregulation and primes cells for proliferation upon damage. Identifying the underlying mechanism will allow us to optimize therapies, prevent tumor progression, or overcome resistance.

### EACR23-1257
### Understanding the role of SS18L2, a novel cell cycle regulator, with proximity labelling approach in triple negative breast cancer

*B. Esin[1], Ö. Yedier- Bayram[1], K. Alişan[1], A.C. Aksu[1], N. Pınarbaşı- Değirmenci[1], A. Cingöz[1], H. Syed[2], M. Philpott[3], N.E. Özkan- Küçük[4], N. Özlü[5], T. Önder[6], T. Bağcı- Önder[6]*

*[1]Koç University Research Center for Translational Medicine, Graduate School of Health Sciences, Istanbul, Turkiye*
*[2]Koç University Research Center for Translational Medicine, Biostatistics- Bioinformatics and Data Management Core, Istanbul, Turkiye*
*[3]University of Oxford, Botnar Research Centre, Oxford, United Kingdom*
*[4]Koç University Research Center for Translational Medicine, Graduate School of Sciences and Engineering, Istanbul, Turkiye*
*[5]Koç University Research Center for Translational Medicine, Koç University College of Sciences, Istanbul, Turkiye*
*[6]Koç University Research Center for Translational Medicine, Koç University School of Medicine, Istanbul, Turkiye*

**Introduction**

Triple Negative Breast Cancer (TNBC) is a highly invasive breast cancer subtype which conventional targeted therapies fail. The lack of such therapeutic options and the complexity of the genetic pathways underlying this disease prompted us to investigate epigenetic modifiers as potential targets regulating TNBC cell cycle and survival. To identify these targets, we designed a custom curated CRISPR-Cas9 Library (Epigenome Wide CRISPR-Cas9 Knock- Out Library, EPIKOL), which identified SS18L2 as a novel epigenetic regulator through pooled screening in various TNBC cell lines. SS18L2 is a Synovial Sarcoma Translocation Gene, which is shown to be related to BAF chromatin remodeling complex. However, the effects of SS18l2 on a specific cancer type is not known or studied yet.

**Material and Methods**

Pooled CRISPR/Cas9 based library screens were conducted in multiple TNBC lines and non-malignant breast epithelial cells. Colony formation and cell viability assays were utilized to validate the function of screen hits. RNA sequencing and qPCR experiments were used to identify differentially regulated genes upon SS18L2 knock-out. Molecular engineering approach was applied to generate BirA* tagged expression constructs; and Immunofluorescence was utilized to address the cellular localization of SS18L2 and its homologs. Proximity labelling approach was conducted to biotin tag the interactomes of the fusion proteins, pulled down by streptavidin pull down experiments. All experiments were confirmed by western blotting; moreover, the interactomes are currently being analyzed by mass spectrometry.

**Results and Discussions**

Our CRIPSR-Cas9 based library screen identified multiple epigenetic modifiers, specifically, SS18L2, as a TNBC-specific cell cycle regulator, through RNA sequencing experiments and functional assays. Its suppression led to arrest of cells in G2/M phase and halted cell survival. Our results also indicate that SS18L2 has different effects of TNBC cell viability when compared to its homologous genes SS18 and SS18L1. The specific protein-protein interactions of SS18L2 in MDA-MB-231 cells are being studied by BioID experiments, to discover its functions and mechanisms further, which might lead to novel interactions related to TNBC cell fitness and survival.

**Conclusion**

SS18L2 was identified as one of the highest hits from EPIKOL screens, which arrests TNBC cells in G2/M phase. Interactomes that will be analyzed from mass spectrometry will enlighten the roles of SS18L2 further in TNBC cell viability.

### EACR23-1271
### Ras promotes lysosomal albumin catabolism by suppressing the albumin recycling receptor FcRn

*R. Paschoal de Campos[1], A. Inal[1], W. Palm[1]*

*[1]German Cancer Research Center, Cell Signaling and Metabolism, Heidelberg, Germany*

**Introduction**

Cancer cells commonly reside in poorly vascularized, nutrient-deprived tumor microenvironments. As a metabolic adaptation, oncogenic Ras signaling promotes macropinocytosis, a non-selective endocytic pathway that allows cancer cells to utilize albumin as a nutrient source. However, internalized albumin normally escapes lysosomal degradation through endosomal recycling to the extracellular space mediated by the neonatal Fc receptor (FcRn). Here, we investigate how oncogenic Ras

© 2023 The Authors. Molecular Oncology is published by John Wiley & Sons Ltd on behalf of Federation of European Biochemical Societies circumvents FcRn-mediated albumin recycling to promote lysosomal albumin catabolism.

**Material and Methods**

The effects of oncogenic Ras signaling on FcRn were evaluated in two cellular models: pancreatic cancer cells harboring oncogenic Ras mutations and Ras-transformed fibroblasts. FcRn expression was analyzed through qPCR and Western blot under Ras signaling modulation by growth factor deprivation and treatment with inhibitors of the downstream kinases of the MAPK pathway. Proliferation assays were conducted to evaluate the ability of cancer cells to grow using albumin as an amino acid source. Albumin uptake and catabolism were assessed with microscopy using fluorescent-labeled albumin.

**Results and Discussions**

We found that oncogenic Ras signaling potently represses the expression of FcRn at the transcriptional level. Ras-dependent FcRn suppression was mediated through the activation of the downstream MAPK signaling pathway. FcRn levels could be restored by pharmacological inhibition of Mek or Erk. Transcription factors of the Ets family and Myc, which act downstream of the Ras/MAPK pathway, repressed FcRn expression. Ectopic expression of FcRn in Ras-mutant cancer cells decreased lysosomal albumin catabolism and suppressed the ability of Ras-transformed cells to grow under amino acid deprivation using albumin as a nutrient.

**Conclusion**

Our findings reveal a novel mechanism through which Ras controls the fate of internalized albumin by repressing the expression of the albumin recycling receptor FcRn. This suggests that oncogenic Ras signaling promotes the lysosomal catabolism of albumin by activation of macropinocytosis and concomitant inhibition of endosomal recycling. Our results indicate that FcRn repression is a mechanism through which oncogenic Ras supports the proliferation of cancer cells that consume extracellular albumin as a nutrient.

### EACR23-1289
### MNT’s Role in Proliferation and Viability of Cutaneous T-Cell Lymphoma Cells

*L. Garcia-Gutierrez[1], T. Soto[1], J. Liaño-Pons[2], M. Merino[3], J.P. Vaqué[3], J. León[1]*
*[1]IBBTEC-UC, Molecular Biology, Santander, Spain*
*[2]Karolinska Institutet, Microbiology- Tumor and Cell Biology, Stockholm, Sweden*
*[3]IDIVAL-UC, Molecular Biology, Santander, Spain*

**Introduction**

The MYC-MAX-MXD network of transcription factors regulates the expression of many genes involved in essential cellular processes. While MYC is one of the most deregulated oncogenes in cancer, MXD proteins are considered MYC antagonists, both competing for their partner MAX. MNT is the most relevant MXD protein due to its implication in tumorigenesis. However, there is discrepancy regarding its role in cancer since some data suggest a tumour suppressive role of MNT, while others report an oncogenic role of MNT cooperating with MYC (i.e., MNT loss reducing MYC-inducing lymphomagenesis). Cutaneous T-Cell Lymphoma (CTCL) represents ~4% of non-Hodgkin lymphomas and shows the highest MNT mutational rate. Around 50% of the cases of Sezary Syndrome, the most aggressive form of CTCL, shows loss of one MNT alleles. Since MYC plays a pivotal role in CTCL development, we aim to decipher whether MNT loss cooperates with MYC in CTCL.

**Material and Methods**

Cell lines Myla, Seax, Hut78 (CTCL) and Jurkat (T cells). Gene expression knock down achieved by lentiviral transduction of specific shRNA. Compounds 10058-F4, 10074-G5 and KJ-Pyr-9 used to disrupt MYC-MAX interaction. RNA and protein levels analysed by RT-qPCR and western blot respectively. Trypan blue and intracellular ATP levels used to assess cell proliferation and viability.

**Results and Discussions**

Knocking down MNT expression in Jurkat and CTCL cell lines led to a drastic decrease in cell proliferation and viability, along with a reduction in MYC protein levels and cell cycle markers. MNT downregulation also impaired NF-kB signalling, in agreement with MNT’s role in the regulation of this pathway that we previously reported in other models. In fact, CTCL shows a strong activation of the NF-kB pathway. Consistently, NF-kB inhibitors also impaired proliferation of CTCL cell lines. On the other hand, treatment of Jurkat and CTCL cell lines with MYC-MAX (and likely MNT-MAX) disruptors resulted in a decrease in cell viability, accompanied by a decrease in MYC target genes and the destabilization of MYC protein.

**Conclusion**

In line with MNT’s suggested role as a MYC-cooperating factor, downregulation of MNT in CTCL cell lines results lethal, suggesting an important role for MNT in this type of cancer. While LOH for *MNT* locus has been recurrently reported in CTCL, there is no evidence of complete loss of MNT expression. This suggest a fine tuned balance of MNT levels in which reduced MNT dosage may be beneficial for MYC oncogenic activity while MNT complete loss is lethal.

### EACR23-1290
### Mutant p53 promotes tumor progression through its transmission between tumor cells in a prion-like manner

*L. Pereira Rangel[1], M. M. Paz[1], G. D. S. Ferretti[2], N. Oliveira da Silva[1], A.L. Barbosa e Vasconcelos[1], L. H. Amorim[3], J. Artunduaga Bonilla[3], L. Nimrichter[3], Y. Cordeiro[1], J. L. Silva[2]*
*[1]Universidade Federal do Rio de Janeiro, Faculdade de Farmácia, Ilha do Fundão- Rio de Janeiro, Brazil*
*[2]Universidade Federal do Rio de Janeiro, Instituto de Bioquímica Leopoldo de Meis, Ilha do Fundão- Rio de Janeiro, Brazil*
*[3]Universidade Federal do Rio de Janeiro, Instituto de Microbiologia Paulo de Góes, Ilha do Fundão- Rio de Janeiro, Brazil*

**Introduction**

p53 is a tumor suppressor. Missense mutations of the TP53 gene are the most common and promote the expression of misfolded proteins that lead to aggregates with amyloid characteristics. These aggregates create loss of function of the wild-type protein (WTp53) or gain of oncogenic

© 2023 The Authors. Molecular Oncology is published by John Wiley & Sons Ltd on behalf of Federation of European Biochemical Societies functions (GoFs) of the mutant protein (mutp53), which lead to characteristics that favor tumor progression. Also, a dominant-negative effect promoted by mutp53 on WTp53 suppresses its function and is related to a prion-like behavior that favors tumor progression. In this work, we sought to study the influences of mutp53 aggregates on tumor cells with WTp53.

**Material and Methods**

We used the conditioned media (CM) or cell lysate (CL) of a mutp53 cell line (MDA-MB-231, mutant R280K) to treat a cell line expressing WTp53, MCF-7. We used western blotting (WB) and immunoprecipitation to evaluate protein levels, fluorescence confocal microscopy to detect aggregates in the recipient cells, optical microscopy to evaluate morphological alterations, clonogenic assay to evaluate the formation of colonies and wound healing assay to evaluate the migration of cells. A 3D cell culture model using agar-coated plates was used. Thioflavin T tracked amyloid aggregation of p53 in vitro. Extracellular vesicles (EVs) were isolated by differential centrifugation and characterized through WB, transmission electron microscopy and dynamic light scattering.

**Results and Discussions**

Our data show colocalization between p53 and amyloid oligomers and the increase in p53 levels in cells treated with CL and CM, which indicates the transmission and amplification of these aggregates in WTp53 cells. CL of different cell lines were used to observe the prion-like potential of mutant p53 in vitro. Morphology and colony formation of cells treated with CM were altered, as well as their migration potential. We also observed the inhibition of MCF-7 spheroids formation by MDA-MB-231. Finally, EVs isolated from the CM of MDA-MB-231 demonstrated the presence of amyloid-state p53, which confirms mutp53 transmission.

**Conclusion**

The GoFs observed and the detection of amyloid p53 indicate the cell-to-cell transmission that define prion-like effects of mutp53 that promote tumor progression, leading a WTp53 cell to acquire a more aggressive pattern. We now intend to understand the genes activated by them and protein interactions engaged in the recipient cells, in order to improve our understanding of this new pharmacological target for antitumor therapy.

### EACR23-1292
### Cancer metabolic rewiring favors PEPCK-M against PEPCK-C to fuel tumor progression in CRC

*M. Tormo-Fabios[1], M. Aragó[1], P. Hyroššová[1], A. Méndez-Lucas[1], J.C. Perales Losa[1]*

*[1]University of Barcelona-IDIBELL, Department of Physiological Sciences- School of Medicine, L'Hospitalet del Llobregat, Spain*

**Introduction**

Metabolic adaptations in tumors frequently portray enzyme isoform switches that select oncofetal forms. For example, in tumors of the bowel or the liver, the expression of the PEPCK pair, PEPCK-M (PCK2) and PEPCK-C (PCK1), are asymmetrically regulated upon malignant transformation. PCK2 overexpression upon amino acid limitation and ER-stress in an ATF4-dependent manner, argues for a functional relationship with metabolic adaptations for cell survival. In this study, we aim to evaluate the contribution of the pair to CRC, specifically the role of PEPCK-M, beyond its already reported activity supplying biosynthetic intermediaries in the absence of glucose.

**Material and Methods**

We analyzed public tumor datasets from the TGCA using cBioportal and the KM plotter, for PCK1 and PCK2 gene effects on specific phenotypes in CRC, and scRNAseq to query spatial-temporal expression in fetal and adult intestinal epithelium. Wild-type HCT-116 cells were compared to PCK2 gain-of-function (PCK2-overexpressed) or loss-of-function (iPEPCK-2, treated cells) models, as previously described in our laboratory. GSEA analysis from RNAseq data, together with thorough phenotyping, metabolomics, and cell proliferation analyses, were performed to ascertain the participation of PCK2 in CRC.

**Results and Discussions**

PEPCK-M expression is maintained in epithelia of the bowel from the fetal to the adult stages, and is selectively retained upon dedifferentiation and tumor progression. In contrast, PEPCK-C is confined to adult, healthy tissue. Significant silencing of the PEPCK-C isoform in CRC correlated with promoter methylation, suggesting an oncofetal transition for this gluconeogenic isozyme pair that might carry functional and biological consequences to tumor progression. The PCK2/PCK1 gene expression ratio had a negative impact in the survival of colon cancer patients. Interestingly, PCK2 expression alone also had a negative impact in survival in patients with no lymph node infiltration. Changes in PEPCK-M activity in colorectal carcinoma cells had significant effects on cell proliferation and the regulation of key cancer-related signaling axes.

**Conclusion**

We conclude that in tumors originating from tissues where both the mitochondrial and cytosolic PEPCK enzymes have a role in healthy tissue, the former is specifically selected in malignant cells. Our data suggest that PEPCK-M might participate in the mechanisms to regulate proteostasis in well fed tumor cells. We provide molecular clues into the clinical relevance of PEPCK-M in CRC.

### EACR23-1300
### CRISPR/Cas9-based identification of melanoma brain metastatic drivers

*A. Vidal[1], F. Grebien[1], T. Redmer[2]*

*[1]University of Veterinary Medicine Vienna VetMed, Medical Biochemistry, Vienna, Austria*

*[2]University of Veterinary Medicine Vienna VetMed, Institute of Pathology, Vienna, Austria*

**Introduction**

Brain tumors are still among the deadliest forms of cancer. Unlike primary brain tumors, which develop from the brain's own cells, secondary brain tumors emerge by metastatic dissemination of solid extracranial tumors, mainly melanoma, lung cancer and breast cancer. Brain metastases develop frequently in 20-40% of melanoma patients during the course of disease, despite therapeutic progress. However, more than 80% of autopsied melanoma patients show brain metastatic lesions, suggesting that only a subset of dormant micro-metastases form growing tumors controlled by yet not defined and understood mechanisms.

© 2023 The Authors. Molecular Oncology is published by John Wiley & Sons Ltd on behalf of Federation of European Biochemical Societies

Therefore, understanding the mechanisms that facilitate invasion and tumor growth of brain metastatic tumor cells is critical for developing new strategies for the successful treatment of melanoma brain metastases (MBM) and improving the quality of life of patients.

**Material and Methods**

We established stably expressing Cas9-GFP cell clones from brain metastatic-derived cell (BMC) lines. BMCs were established from brain metastases of melanoma patients and serve as *in vitro* system for the screen of candidate genes. Candidate genes were selected regarding their potential role for MBM progression, maintenance and druggability by comparative analysis of MBM and normal brain tissue. We combined CRISPR/Cas9-based screening with live cell imaging for directly tracking the effectivity and consequence of knockouts for proliferation and migration or invasion in 3D collagen matrices.

**Results and Discussions**

The comparative analysis revealed 24 druggable candidate drivers that potentially control molecular programs driving cellular dependencies in MBM. CRISPR-screens for proliferation identified growth dependencies on 4 of those genes: two leucine zippers, a membrane receptor and a heat-shock stress response chaperone. CRISPR-screens for migration identified additional candidates**.** To further delimit the proliferation and migration dependencies and get our model system closer to the physiological conditions, we have established 3D wound-closure assays based on collagen hydrogels. This system gives us further details on matrix degradation properties during the process of MBM cell invasion.

**Conclusion**

Our study sheds light on factors that control molecular processes such as proliferation and invasion in MBM. However, further transcriptomic studies are needed to better understand the functional role of these factors and how they can be used for targeted therapy.

### EACR23-1304

### Lactate sustains prostate cancer aggressiveness through the activation of collagen-DDR1 axis

*L. Ippolito[1], A. Duatti[1], M. Iozzo[1], G. Comito[1], E. Pardella[1], G. Sandrini[2], C.V. Catapano[2], A. Morandi[1], E. Giannoni[1], P. Chiarugi[1]*

*[1]University of Florence, Department of Biomedical Experimental and Clinical Scie nces 'Mario Serio', Firenze, Italy*

*[2]Università della Svizzera italiana, Institute of Oncology Research IOR, Bellinzona, Switzerland*

**Introduction**

The major source of extracellular matrix (ECM) within a tumor is the stroma compartment, but increasing evidence have highlighted cancer cell-derived ECM production and its involvement in the acquisition of aggressive features. Recently, non-cell autonomous metabolic reprogramming in tumors can provoke striking transcriptional and metabolic changes within tumor cells allowing them to enhance their malignancy. Lactate sustains a prostate (PCa) cancer cells-cancer-associated fibroblasts (CAF) crosstalk. However, how stromal lactate can impact on the function of tumor-derived ECM remodeling has been poorly explored.

**Material and Methods**

We used PCa cell lines (DU145,PC3) conditioned by *ex vivo* isolated CAF (CAF-CM) or lactate-poor HPF (healthy fibroblasts) and lactate (i.e. lactic acid, 20mM) to mimic CAF metabolic supply. Expression of key molecules were assessed by WB, RT-PCR or immunofluorescence analysis. SCID mice were used for metastasis assay.

**Results and Discussions**

Gene Set Enrichment Analysis in DU145 cells exposed to exogenous lactate demonstrated that genes involved in collagen synthesis and remodeling pathways were significantly enriched in lactate-treated cells. To corroborate the importance of CAF-derived lactate, we observed that the collagen prolyl hydroxylase P4HA1 is overexpressed upon CAF-CM and lactate exposure in PCa cells and MCT1 lactate transporter inhibition impairs this. In keeping, we assessed a higher proline hydroxylation (i.e.P4HA1 activity) as well as higher levels of collagen type I in PCa cells when exposed to CAF-CM and lactate, highlighting the role of this metabolite in sustaining cancer cell-derived collagen synthesis. We found that P4HA1 targeting is detrimental for lactate-induced invasiveness *in vitro* and *in vivo* as well as for collagen I deposition. Interestingly, we identified a non-integrinic collagen I receptor – discoidin-domain collagen receptor 1 (DDR1) – as responsible for triggering tumour collagen-dependent signalling. Silencing of DDR1 impairs lactate-induced invasion and prostaspheres formation. We highlighted that DDR1 acts via STAT3 activation and its inhibition results in a decreased collagen I expression and inefficient prostaspheres formation in lactate-treated cells. Finally, *in vivo* silencing of DDR1 impairs lung metastatization of lactate-treated tumor-xenografts.

**Conclusion**

Overall these findings uncover a novel role of CAF-derived lactate in the activation of P4HA1-collagen-DDR1 axis, thereby sustaining prostate cancer cell dissemination.

### EACR23-1309

### In vivo Isotope tracing reveals how acetate fuels the mitochondrial metabolism of tumours.

*L. de Benito Gómez[1], J. Concetti[2], P.M. Nunes[2], P. Kreuzaler[3], P.C. Driscoll[2], J.C. Perales[1], X. Chen[4], J.I. MacRae[2], M. Yuneva[2], A. Méndez-Lucas[1]*

*[1]Facultad de Medicina y Ciencias de la salud de Bellvitge UB - IDIBELL, Ciencias Fisiológicas, L`Hospitalet de Llobregat, Spain*

*[2]The Francis Crick Institute, Oncogenes and Tumour Metabolism Laboratory, London, United Kingdom*

*[3]CECAD Research Center, Zentrum für Biochemie, Köln, Germany*

*[4]University of California, Department of Bioengineering and Therapeutic Sciences a nd Liver Center, San Francisco, United States*

**Introduction**

Energy-producing metabolic pathways change in the process of neoplastic transformation. Glucose and glutamine derived carbons constitute the main fuel that supports proliferation of cancer cells. However, alternative

© 2023 The Authors. Molecular Oncology is published by John Wiley & Sons Ltd on behalf of Federation of European Biochemical Societies carbon sources have been shown to contribute to the metabolism of cancerous cells, including lactate, various amino acids and lipids as well as acetate. Indeed, acetate has emerged as a critically important nutrient under different metabolic situations, and previous studies have found that the cyto/nuclear-localized form of the acetyl-CoA synthetase enzyme (ACSS2) plays an important role in cancer cells. Nonetheless, the fate of acetate in tumours developing in their natural microenvironment has not been yet fully traced, in particular with regard to the effect of the subcellular localization of the enzymes involved in acetate utilization, including the mitochondrial forms, ACSS1 and ACSS3.

**Material and Methods**

We performed in vivo infusions with [13C2]acetate, in mouse models of liver, breast and lung cancer, including patient-derived xenografts, coupled with exhaustive metabolic analysis by GC/MS, LC/MS and NMR. These results were combined with gene expression (transcriptomics) profiling and analysis of protein expression. Human cell lines were used to perform gain-of-function and loss-of-function studies to assess the partial contribution of the three isoforms. Small molecule inhibitors against metabolic pathways were used to investigate the potential of acetate to compensate for a shortage of other carbon sources.

**Results and Discussions**

We provide proof for extensive metabolic plasticity in cancers, which can rewire their metabolism and overexpress a mitochondrial isoform, ACSS1, to drive acetate metabolism primarily as a mitochondrial fuel. In vivo metabolic labelling demonstrated that [13C]acetate feeds the mitochondrial metabolism in patient-derived xenografts, and mouse models of liver, and breast cancer. Our results demonstrate that the subcellular distribution of the ACSS enzymes determines the fate of acetate, and that some tumours use this nutrient preferentially to replenish the Krebs cycle, and all adjunct pathways.

**Conclusion**

Mitochondrial ACSS1-driven acetate metabolism provides an additional level of flexibility to the metabolism of tumours, affecting both proliferation, and survival under shortage of glucose carbons.

### EACR23-1319

### The lysosomal enzyme trafficking factor LYSET enables cancer cells to generate nutrients from extracellular proteins

*C. Pechincha*[1], *S. Groessl*[1], *R. Kalis*[2], *A. Zanotti*[3], *A. Schleiffer*[4], *S. Jabs*[5], *D. Haselbach*[2], *M. Lemberg*[6], *J. Zuber*[2], *W. Palm*[1]

[1]*German Cancer Research Center DKFZ, Cell Signalling and Metabolism, Heidelberg, Germany*

[2]*Research Institute of Molecular Pathology IMP, Cancer drug targets, Vienna, Austria*

[3]*Center for Molecular Biology of Heidelberg University Z MBH, Mechanism and function of intramembrane proteases, Heidelberg, Germany*

[4]*Research Institute of Molecular Pathology IMP, Department of Bioinformatics, Vienna, Austria*

[5]*Institute of Clinical Molecular Biology- Christian-Albrechts-University and University Medical Center Schleswig-Holstein, Epitranscriptome Regulation in Host-Microbe-Interactions, Kiel, Germany*

[6]*Center for Biochemistry and Cologne Excellence Cluster on Cellular Stress Responses in Aging- Associated Disease s CECAD, Mechanism and function of intramembrane proteases, Cologne, Germany*

**Introduction**

Cancer cells commonly reside in poorly vascularized, nutrient-deprived tumours. To adapt cancer cells can exploit macropinocytosis and lysosomal catabolism of extracellular proteins as an alternative nutrient source. However, the molecular pathways that enable cells to feed on extracellular proteins remain incompletely understood.

**Material and Methods**

We set out to identify genes essential for proliferation through albumin degradation in pancreatic cancer, a nutrient-poor, highly macropinocytic tumor. To this end, we conducted a genome-wide CRISPR screen where cancer cells grow either by import of free amino acids or by the uptake and lysosomal degradation of extracellular proteins. We identified an unknown protein (TMEM251, hereafter referred to as LYSET) and characterised its function in lysosomal metabolism, cancer cell proliferation, and tumour growth.

**Results and Discussions**

Genetic ablation of LYSET potently supressed proliferation of pancreatic cancer cells as well as cells derived from colorectal, bladder and lung carcinoma that depend on albumin as an amino acid source in vitro. Mechanistically, we characterized LYSET as novel component of the Golgi-resident GlcNAc-1-phosphotransferase complex, which is responsible for tagging catabolic enzymes with the lysosomal trafficking signal, mannose-6-phosphate (M6P). In the absence of LYSET, GlcNAc-1-phosphotransferase becomes destabilized leading to its degradation. Consequently, LYSET-deficient cells display a loss of M6P modification, catabolically inactive lysosomes, and thus impaired capacity to degrade macropinocytic and autophagic cargoes. Although LYSET-deficient cancer cells grew normally under nutrient-rich conditions, they displayed a severely reduced ability to form subcutaneous and orthotopic pancreatic tumours in mice.

**Conclusion**

Our CRISPR screens in pathologically adequate nutrient conditions comprehensively identified genetic dependencies of cancer cells that use extracellular proteins as nutrients. In particular, we identified LYSET, which was widely characterized as non-essential under standard cell culture conditions, but is required for tumour growth in vivo. As a selective cancer dependency, LYSET and the lysosomal enzyme trafficking pathway might be a promising target to suppress a key metabolic adaptation in cancer.

### EACR23-1321

### TRPML1 expression and function in myeloid leukemia: in search of an innovative target

*F. Maggi*[1], *M.B. Morelli*[2], *G. Santoni*[2], *C. Amantini*[1]

[1]*University of Camerino, School of Bioscience, Camerino,*

© 2023 The Authors. Molecular Oncology is published by John Wiley & Sons Ltd on behalf of Federation of European Biochemical Societies

*Italy*
[2]*University of Camerino, School of Pharmacy, Camerino, Italy*

**Introduction**

Although successful results were obtained with therapies, chemotherapy resistance, associate with relapse, is still challenges for curing acute and chronic myeloid leukemia. For this reason, the development of new therapeutic strategies and the identification of new targets represent an attractive goal. In this regard, several researches have demonstrated the role of Transient Receptor Potential (TRP) channels as possible targets, being implicated in the modulation of cancer growth, metastasis and chemoresistance. We previously demonstrated that the activation of TRP vanilloid 1 or 2 stimulates cell growth inhibition and shows synergistic effects with imatinib treatment in chronic myeloid leukemia. Given that mitochondria and lysosomes strongly contribute to progression and relapse of myeloid leukemia and the mucolipin TRP channel subfamily 1 (TRPML1) plays a pivotal role in regulating calcium dynamics at the mitochondria-lysosome contacts, we investigated its role and functions in myeloid leukemia.

**Material and Methods**

We firstly evaluated, by *in silico* analysis, the expression levels of TRPML1 in acute or chronic myeloid leukemia affected patients. Then, we analyzed the expression and the subcellular localization of TRPML1 in myeloid leukemia cells by using western blot and confocal microscopy. Moreover, calcium flux was investigated by flow cytometry and cell growth was evaluated by MTT assay and Tripan blu cell count. Signaling pathways associated with cell growth were investigated by western blot.

**Results and Discussions**

TRPML1 is expressed in both acute and chronic myeloid leukemia affected patients and its levels are modulated with respect to the cancer aggressiveness and progression. Although the expression was evidenced in all different myeloid leukemia cell lines analyzed, the highest level of TRPML1 was found in KU812 and THP-1 cells. The activation of TRPML1 by using specific agonists activates pathways involved in cell growth inhibition and modulation of calcium influx.

**Conclusion**

Our results, showing the expression and function of TRPML1 in myeloid leukemia cells, prompt the potential role as a pharmacological target of this channel receptor.

### EACR23-1327
### The paracrine effects of fibroblasts on Doxorubicin-treated breast cancer cells

A.M. Engelbrecht[1], C. Fourie[1]
[1]*Stellenbosch University, Physiological Sciences, Stellenbosch, South Africa*

**Introduction**

The most common cancer among women in both developed and developing countries is breast cancer and it is a major health problem throughout the world. The current standard treatment for breast cancer patients is radiation, surgery and chemotherapy or a combination of surgery with chemotherapy. The unresponsiveness of cancer cells to chemotherapeutics, however, is still a main concern. During chemotherapeutic treatment with doxorubicin, normal and healthy neighboring cells are also targeted. Apoptotic or senescent fibroblasts in the tumor microenvironment can then secrete a variety of bioactive molecules which promotes tumor growth, metastasis and drug resistance.

**Material and Methods**

MEFs were cultured and treated with doxorubicin to induce apoptosis and senescence respectively. An SA-ß-gal stain was used to determine the number of senescent cells in the cell population and Western blots were used to determine the expression of apoptotic and senescent markers. Conditioned media was collected from the MEFs after apoptosis and senescence induction and used to determine the paracrine effects between fibroblasts and E0771 cells.

**Results and Discussions**

Doxorubicin (1 µM) was able to significantly induce apoptosis in MEFs after 24 hours. During senescence induction, 2 µM of Doxorubicin treatment for 4 hours was able to induce senescence in the MEF population. The Western blot analyses showed that the expression of many apoptosis and senescence markers significantly changed after doxorubicin treatment. Furthermore, the results indicate that senescent fibroblasts are able to significantly increase cell viability in E0771 cells following treatment with doxorubicin.

**Conclusion**

Healthy, neighboring stromal cells such as fibroblasts are affected by chemotherapeutic agents, such as doxorubicin, to secrete paracrine factors that enhance breast cancer growth and therapeutic resistance by evading cell death.

### EACR23-1329
### Cathepsin S activity regulates antigen presentation and shapes the tumor microenvironment in B-cell lymphoma

M. Bruand[1], A. Petruzzella[1], D. Srivastava[1,2], N. Katanayeva[1], J. Thévenet[3], S. Tissot[3], G. Ciriello[4], E. Oricchio[1]
[1]*Ecole Polytechnique de Lausanne EPFL, Swiss Institute for Experimental Cancer Research, Lausanne, Switzerland*
[2]*University of Lausanne, Department of Computational Biology and Swiss Institute of Bioinformatics, Lausanne, Switzerland*
[3]*CHUV, Centre for Experimental Therapies- Department of Oncolo gy, Lausanne, Switzerland*
[4]*Univeristy of Lausanne, Department of Computational Biology and Swiss Institute of Bioinformatics, Lausanne, Switzerland*

**Introduction**

Cathepsin S (CTSS) is a key regulator of antigen processing and MHC-class II maturation in antigen-presenting cells including B cells, macrophages, and dendritic cells. Recently, we reported that cathepsin S is overactivated by a hot-spot gain of function mutation (Y132D) or over-expressed in patients with indolent B-cells lymphoma (Dheilly et al., Cancer Cell, 2020), indicating that in these patients antigen processing and tumor microenvironment composition could be altered.

**Material and Methods**

© 2023 The Authors. Molecular Oncology is published by John Wiley & Sons Ltd on behalf of Federation of European Biochemical Societies

To decipher the contribution of cathepsin S activity in normal B cell development and lymphomagenesis, we have developed a new transgenic mouse model expressing the mutated and overactivated murine form of Ctss$^{Y141D}$, which mimics the alteration observed in lymphoma patients. Using histopathological and spatial transcriptomics analyses, flow cytometry, sc-RNAseq analyses and co-culture assays, we studied the role of CTSS in the crosstalk with the tumor microenvironment *in vivo* and *in vitro.*

**Results and Discussions**

Histopathological analyses of the secondary lymphoid organs revealed that Ctss$^{Y141D}$ increases the expression of germinal center (GC) B cells-related markers and leads to an accumulation of proliferating cells in the spleen follicles, accelerating tumor development and shortening survival in animals that develop FL. Interestingly, targeting CTSS activity by using genetic knock-out animals induces changes in GC architecture reducing the number of malignant B-cells and CD4$^{+}$ T follicular helper cells. The remaining B cells show increased PD-L1 and MHC II protein promoting inflammation in the tumor microenvironment and changing the interactions between B and T cells. Clearly, loss of cathepsin S activity delayed lymphoma development and significantly prolonged survival of the mice, indicating that cathepsin S represents an ideal therapeutic target in B cell malignancies.

**Conclusion**

Overall, CTSS alterations are key drivers in lymphoma development since it regulates B-cell proliferation and modulation of the interactions with the TME. Further ongoing studies will help us to understand better the mechanistic contribution of CTSS in these processes.

### EACR23-1334
### Stromal lipid species dictate melanoma metastasis and tropism

S. Gurung[1], T. Budden[1], K. Mallela[1], A. Viros[1]
[1]Cancer Research UK Manchester Institute, Skin Cancer and Ageing, manchester, United Kingdom

**Introduction**

Cancer cells utilize different molecules and lipids to support uncontrolled cellular division and dissemination from the primary site; and lipid metabolism is often altered in cells acquiring a malignant phenotype. Adipocytes in the skin are the main provider of lipids to the cutaneous environment in homeostasis. In the skin, ageing profoundly transforms the hypodermis, and there are no studies comparing the impact of cutaneous adipocytes on cancer by age. Here we describe how age modifies the contribution adipocytes to melanoma progression and metastasis.

**Material and Methods**

We used preadipocytes from donors of different age groups and successfully differentiated them into functional adipocytes. We collected the secretomes and exposed them to melanoma cells to study the effect. We injected murine melanoma cells *in vivo* to study the metastatic burden and tropism imposed by either young or old adipocytes.

**Results and Discussions**

We found that age reduces the total amount of soluble lipids and modifies the lipid species adipocytes provide to the cutaneous environment. Melanoma cells exposed to aged adipocytes uptake fewer lipids for lipid oxidation, OXPHOS and energy production, and *in vivo*, lower oxidative stress promotes visceral metastasis. In contrast, melanoma cells exposed to lipid-rich, young adipocytes oxidize lipids for energy production, generating high ROS and oxidative stress. This limits their visceral metastatic capacity.

**Conclusion**

Our study shows that cutaneous adipocytes, lipid availability and lipid metabolism are key determinants of melanoma metastatic behaviour.

### EACR23-1348
### Single-Cell Lipidomics Reveals Phenotypic Differences in Pancreatic Ductal Adenocarcinoma (PDAC)

J. Delafiori[1], M. Shahraz[1], M. Trajkovic-Arsic[2,3], C. Münch[2,3], A.M. Winkelkotte[2,3], S.S. Lueong[2,3], S. Liffers[2,3], J. Siveke[2,3], T. Alexandrov[1,4,5,6]
[1]European Molecular Biology Laboratory - EMBL, Structural and Computational Biology Unit, Heidelberg, Germany
[2]University Hospital Essen, Bridge Institute of Experimental Tumor Therapy- West German Cancer Center, Essen, Germany
[3]Deutsches Krebsforschungszentrum - DKFZ, Division of Solid Tumor Translational Oncology- German Cancer Consortium DKTK- partner site Essen, Heidelberg, Germany
[4]BioInnovation Institute, Bio Studio, Copenhagen, Denmark
[5]Heidelberg University, Molecular Medicine Partnership Unit- EMBL and Heidelberg University, Heidelberg, Germany
[6]European Molecular Biology Laboratory - EMBL, Metabolomics Core Facility, Heidelberg, Germany

**Introduction**

Pancreatic ductal adenocarcinoma (PDAC) is associated with a low survival rate resulting from late diagnosis and aggressive tumor progression. During cancer cell epithelial-to-mesenchymal transition (EMT), distinct lipid signatures influence cell mobility characteristics leading to PDAC subtypes: epithelial/classical and quasi-mesenchymal (QM) phenotypes. These lipid alterations translate PDAC intratumoral heterogeneity and phenotypic profiles at the single-cell level. Aiming to differentiate PDAC classical from QM phenotypes, we applied the SpaceM method for single-cell lipidomics while integrating data from standardized cell lines and patient-derived xenografts (PDXs).

**Material and Methods**

Lipid differences between epithelial and QM phenotypes were assessed using four established cell lines (HPAC, HPAFII, MiaPaca2, and PSN1) and four PDXs with status confirmed by E-cadherin/Vimentin western blots. Cells were grown on glass slides reaching adequate confluency, fixed with paraformaldehyde, and stained with 4′,6-diamidino-2-phenylindole (DAPI). Brightfield and fluorescence microscopy images were acquired on desiccated cells before spraying the cells with 2,5-Dihydroxybenzoic acid (DHB) and after mass spectrometry (MS) analysis. Matrix-Assisted Laser Desorption/Ionization (MALDI)-imaging MS was used for data acquisition on positive ion mode at 25 μm step size

© 2023 The Authors. Molecular Oncology is published by John Wiley & Sons Ltd on behalf of Federation of European Biochemical Societies and m/z range of 600-1000. METASPACE software assisted with lipid annotation, the SpaceM method on single-cell data assignment, and Scanpy on data analysis.

**Results and Discussions**

Single-cell lipidomics profiles of epithelial and QM phenotypes were distinct, with the enrichment of phosphatidylcholines (PC) in the epithelial phenotype. In addition, PCs' lipid composition had a clear enrichment of unsaturated fatty acids with longer carbon chains. As previously reported for the QM phenotype, sphingolipids play a crucial role in lipid membrane fluidity and cell migration: QM cells presented a unique distribution of hexosylceramides, sphingomyelins, and ceramide 1-phosphates, as well as phosphatidic acid species among the top-ranked markers. Remarkably, the highlighted lipid markers associated with integration methods contributed to grouping cells by phenotypes (epithelial or QM), regardless of sample source (established cell lines or PDX cells). The expansion of the sample cohort is in progress.

**Conclusion**

Therefore, those findings reinforce the potential of the SpaceM method, and the single-cell lipidomics toward translational application.

### EACR23-1354
### Breakage and loss of distal 11 q in breast cancer: impact on RNA expression and response to ionising radiation

*K. Salciute[1], J. Passos[1], B. Arleri[1], D. Moralli[2], C. Green[2], A. Flaus[3], H. Dodson[1]*
*[1]University of Galway, Anatomy, Galway, Ireland*
*[2]University of Oxford, Chromatin Dynamics Group- Wellcome Trust Centre for Human Genetics, Oxford, United Kingdom*
*[3]University of Galway, Biochemistry, Galway, Ireland*

**Introduction**

The long arm of chromosome 11 (11q) is commonly altered in hormone positive, luminal breast cancer (BC). Genomic data reveals that the 11q13 locus, where the well-known oncogene *CCND1* maps to, is often amplified (amp). Additionally, one copy of the distal 11q arm where several DNA damage response (DDR) genes and potential tumour suppressors (including *MRE11, ATM, H2AX, CHEK1)* are located is often broken and lost. This breakage event may lower expression of these DDR genes and drive the formation of tumours with defective DDR which are more aggressive and resistant to endocrine and radiation therapy.

**Material and Methods**

The effects of 11q loss in luminal BC was modelled in cell lines with normal and altered 11q. These include MCF10A (normal breast), BT474 (11q13 amp), MCF7 (11q re-arrangement), MDA-MB-134-VI and CAMA-1 (11q13 amp/11q loss) and BT483 (11q loss). Cytogenetic analysis and FISH were used to determine the ploidy, 11q structure and copy number of housekeeping gene *MRPL19* at early and late passages. Probes targeting the *CCND1* gene, proximal to the break site, and probes to the *H2AX* and *CHEK1* genes, distal to the break site were optimized. RNA expression of genes in the amp and lost segments of 11q was determined by qPCR. Radiation sensitivity was assessed by clonogenic survival assay.

**Results and Discussions**

Most cell lines had a stable triploid karyotype which matched the copy number of *MRPL19.* 11q loss was associated with *CCND1* amp which is characteristic of the breakage-fusion-bridge mechanism of chromosomal instability. Contrary to published genomic data, the BT483 cell line showed no evidence of 11q loss.
Oncogene *CCND1* was overexpressed and *ATM* was underexpressed in all cell lines compared to MCF10A. Following 11q loss, MDA-MB-134-VI appears to compensate for the loss by upregulating expression of some DDR genes on the arm, this does not happen in CAMA-1. Despite this increase in expression, MDA-MB-134-VI was the most radiosensitive cell line and had a radiation response similar to MCF10A. The BT474 cell line was the most radioresistant which could be due to this cell line being derived from a more aggressive luminal HER2+ tumour.

**Conclusion**

Distal 11q loss is a common event in BC and care should be taken when selecting and verifying models of the disease using published genomics data. 11q loss does not always result in lower gene expression, however may result in increased radiosensitivity. Future studies should investigate which genes drive the radiosensitive phenotype as potential targets.

### EACR23-1357
### Inhibition of SOX18 homodimerization properties of by Small-Molecule 4 induces S-phase cell cycle arrest triggering p21 upregulation

*O. Rodak[1], M. Olbromski[1], M. Mrozowska[1], A. Rusak[1], A. Gomułkiewicz[1], P. Dziegiel[1]*
*[1]Wroclaw Medical University, Department of Human Morphology and Embryology, Wroclaw, Poland*

**Introduction**

SOX18 is an attractive target for cancer research due to its role in the regulation of vascular system development, endothelial barrier, wound healing, and potential involvement in cancer cell proliferation and metastasis. Previous studies have provided limited insight into its specific activity pathways. Therefore, we aimed to explore the effects of small-molecule 4 (Sm-4) on SOX18 activity in lung cancer.

**Material and Methods**

LXF289 and SK-MES-1 lung cancer cell lines were used in the study. Sm-4 cytotoxicity was evaluated with MTT tests and cells were subsequently treated with 10 and 20 μM concentrations of Sm-4. Cell cycle progression was evaluated with flow cytometry, and expression of transcription factors SOX18, SOX7, and SOX17, cyclins A1, E, and D1, and p21 were measured by PCR and western blotting.

**Results and Discussions**

Sm-4 showed concentration-dependent cytotoxicity in LXF289 and SK-MES-1 cell lines. Cells in S-phase were significantly accumulated after treatment with 20 μM Sm-4. Despite increased cyclin gene expression, no increase was observed at the protein level, indicating cell cycle arrest. In SK-MES-1 and LXF289 cell lines, Sm-4 treatment decreased cyclin D and cyclin E expressions,

© 2023 The Authors. Molecular Oncology is published by John Wiley & Sons Ltd on behalf of Federation of European Biochemical Societies respectively. Contrary to our primary hypothesis, the upregulation of SOX17 and SOX7 triggered by SOX18 activity inhibition was not confirmed. This is because the protein levels of neither SOX7 nor SOX17 responded to treatment. Following the findings of other researchers, we measured the expression of p21, a cyclin-dependent kinase inhibitor that controls the transition from the S to the G2/M phase. Our results confirmed a significant increase in p21 levels in both cell lines.

**Conclusion**

Presented results suggest that SOX18 plays a role in regulating cell cycle progression in lung cancer cells. Observed S-phase arrest is due to p21 upregulation triggered by SOX18 inhibition, indicating a link between SOX18 activity and p21 expression. Overall, our study highlights the importance of SOX18 in regulating cell cycle progression in lung cancer cells and suggests that targeting SOX18 with Sm-4 may represent a novel therapeutic strategy for lung cancer treatment.

The work was supported by the National Science Centre, Poland, under research project OPUS, agreement number 2018/31/NZ5/02238.

### EACR23-1362
### miR-214 mediated stroma-tumor cell crosstalk during tumor progression

*F. Orso[1], S. Cucinelli[2], M. Coco[2], S. Cozzubbo[2], I.C. Salaroglio[3], J. Kopecka[3], S. Zanivan[4], C. Riganti[3], D. Taverna[2]*

*[1]University of Eastern Piedmont, Dept. Translational Medicine, Novara, Italy*

*[2]Molecular Biotechnology Center "Guido Tarone", Dept. Molecular Biotechnology and Health Sciences- University of Torino, Torino, Italy*

*[3]University of Torino, Dept. Oncology, Torino, Italy*

*[4]Cancer Research UK Beatson Institute, Cancer Research UK Beatson Institute, Glasgow, United Kingdom*

**Introduction**

Cancer and stroma cells continuously interact during tumor progression and influence each other. Secreted microRNAs (miRNAs) have recently been implicated in metabolic tumor-stroma crosstalk. We previously observed high expression of miR-214 in human breast cancers and melanomas and evidenced its pro-metastatic role. We also detected elevated levels of miR-214 in stroma cells and unraveled a positive correlation between miR-214 and stroma signatures in human tumors. Relevantly, we uncovered that upon IL-6 secretion and STAT-3 signaling activation, stroma cells increase their miR-214 content and release it via Extracellular Vesicles (EVs), which are then taken by cancer cells. Here, miR-214 is able to activate a pro-metastatic program which leads to tumor dissemination. Due to the relevance of stromal miR-214 in tumor progression, we aim at understanding the impact of stromal-derived miR-214 on tumor cell metabolism.

**Material and Methods**

We are characterizing the metabolic and proteome profiles of melanoma and breast cancer cells upon stimulation with Conditioned Medium (CM) derived from intact or miR-214-depleted stromal cells and aim at identifying the main miR-214-dependent metabolic players. More importantly, we are investigating the intervention of stromal miR-214 on tumor metabolism and dissemination in miR-214 overexpressing and null mouse models.

**Results and Discussions**

We provided evidence that stromal miR-214 has an impact on tumor cell metabolism. In fact, following miR-214 depletion in stromal cells, we observed decreased glycolysis, glycolytic enzyme activity and expression accompanied by increased Tricarboxylic acid (TCA), electron transport chain, fatty acid oxidation and glutaminolysis enzyme activity in CM-treated tumor cells, compared to controls. In line, mitochondrial damage (function) rescuing was observed. The opposite results have been observed in xenotransplants grown in miR-214 expressing mice. Analysis in miR-214 overexpressing and miR-214 null mice is ongoing. In order to highlight the molecular mechanisms underlying stroma-miR-214-dependent metabolic alterations in tumor cells, we are currently performing proteomic analysis of tumor cells treated with stroma CM and data will be presented.

**Conclusion**

Our results underline the relevance of "stromal miR-214" for metabolic rewiring and metastasis formation and suggest the possibility of a double-edge therapeutic approach based on miR-214 targeting and metabolic players targeting in tumor and/or stroma cells.

### EACR23-1364
### Lysosomal lipid switch sensitises to nutrient deprivation and mTOR targeting in pancreatic cancer

*M.C. De Santis[1], C. Andrea[1], E. Di gregorio[1], C. Paola[1], G. Elisa[2], C. Vincenzo[3], P. Porporato[1], A. Morandi[4], E. Hirsch[1], M. Martini[1]*

*[1]University of Torino, Molecular Biotechnology and Health Sciences, Turin, Italy*

*[2]Cancer Center Amsterdam, Department of Medical Oncology, Amsterdam, Netherlands Antilles*

*[3]University of Verona, Department of Diagnostics and Public Health, Verona, Italy*

*[4]University of Firenze, Department of Biomedical- Experimental and Clinical Sciences, Firenze, Italy*

**Introduction**

Pancreatic ductal adenocarcinoma (PDAC) is an aggressive disease with limited therapeutic options. However, metabolic adaptation to the harsh PDAC environment can expose liabilities useful for therapy. Targeting the key metabolic regulator mechanistic target of rapamycin complex 1 (mTORC1) and its downstream pathway shows efficacy only in subsets of patients but gene modifiers maximising response remain to be identified.

**Material and Methods**

Three independent cohorts of PDAC patients were studied to correlate PI3K-C2γ protein abundance with disease outcome. Mechanisms were then studied in mouse (KPC mice) and cellular models of PDAC, in presence or absence of PI3K-C2γ (WT or KO). PI3K-C2γ-dependent metabolic rewiring and its impact on mTORC1 regulation were assessed in conditions of limiting glutamine availability. Finally, effects of a combination therapy

© 2023 The Authors. Molecular Oncology is published by John Wiley & Sons Ltd on behalf of Federation of European Biochemical Societies targeting mTORC1 and glutamine metabolism were studied in WT and KO PDAC cells and preclinical models.

**Results and Discussions**

PI3K-C2γ expression was reduced in about 30% of PDAC cases and was associated with an aggressive phenotype. Similarly, loss of PI3K-C2γ in KPC mice enhanced tumour development and progression. The increased aggressiveness of tumours lacking PI3K-C2γ correlated with hyperactivation of mTORC1 pathway and glutamine metabolism rewiring to support lipid synthesis. PI3K-C2γ-KO tumours failed to adapt to metabolic stress induced by glutamine depletion, resulting in cell death.

**Conclusion**

Loss of PI3K-C2γ prevents mTOR inactivation and triggers tumour vulnerability to RAD001 (mTOR inhibitor) and BPTES/CB-839 (glutaminase inhibitors). Therefore, these results might open the way to personalised treatments in PDAC with PI3K-C2γ loss.

### EACR23-1383
### MLK4 depletion induces mitochondrial metabolic alterations and enhances metastasis in pancreatic cancer

*N. Ghiglione[1], L. Gozzelino[1], C. Andrea[1], A. Morandi[2], E. Giovannetti[3], E. Hirsch[1], M. Martini[1]*
*[1]Molecular Biotechnology Center, Department of Molecular Biotechnology and Health Scienc es, Turin, Italy*
*[2]University of Firenze, Dipartimento di Scienze Biomediche- Sperimentali e Clinic he 'Mario Serio', Florence, Italy*
*[3]Cancer Center Amsterdam, Department of Medical Oncology, Amsterdam, The Netherlands*

**Introduction**

Pancreatic cancer (PC) is an extremely lethal form of cancer. The majority of PC cases involve the oncogenic KRAS mutation, which activates intracellular signaling pathways to promote cell proliferation and metabolic changes required to meet the increased energy and biosynthetic demands of the cancer. In order to develop alternative therapeutic approaches based on the specific metabolic features of PC, it is crucial to identify critical molecular mechanisms underlying the pathogenesis of this cancer. Mixed lineage kinase 4 (MLK4) is a serine/threonine kinase downstream of KRAS with both oncogenic and tumor suppressing functions. While downregulated in approximately 20% of PC patients, the role of MLK4 in PC progression remains unclear.

**Material and Methods**

To evaluate the impact of MLK4 on PC development, we knocked out MLK4 in pancreatic cancer cell lines with wild-type or mutated KRAS. We then performed metabolic assays and Seahorse analysis to assess changes in cell metabolism and bioenergetics upon modulation of MLK4 expression. We evaluated glycolytic rates, oxygen consumption rates, and glutamine metabolism. In vivo metastatic assays were conducted to determine the metastatic potential of WT and KO tumor cells.

**Results and Discussions**

Immunohistochemical analysis showed that MLK4 expression is reduced in around 20% of PC patients and is associated with reduced survival. Our results demonstrated that MLK4 plays a crucial role in regulating glucose and glutamine metabolism for energy generation and macromolecular synthesis. MLK4 loss increased glucose consumption and lactate production in PC cells, suggesting a role for MLK4 in modulating glycolytic rates. Additionally, loss of MLK4 increased oxidative stress and led to mitochondria fragmentation. In vitro and in vivo metastatic assays confirmed an increase in the metastatic potential upon MLK4 loss.

**Conclusion**

Our findings highlight the central role of MLK4 in sustaining tumor metabolic rewiring and suggest that this pathway could be exploited to develop new therapeutic strategies for PC. Given the metabolic addictions of PC cells, a better understanding of these metabolic alterations could provide new insights into the molecular mechanisms underlying the metabolic adaptation of PC cells.

### EACR23-1390
### Phosphoinositides control over cytokinesis: PI4KA-loss in tetraploidization, CIN and breast cancer progression.

*L. Prever[1], A. Amadori[1], H. Li[1], M.C. De Santis[1], E. Scarpa[2], F. Gulluni[1], E. Hirsch[1]*
*[1]University of Turin - Molecular Biotechnology Center, Dept. of Molecular Biotechnology and Health Sciences, Torino, Italy*
*[2]University of Cambridge, Dept. of Physiology- Development and Neuroscience, Cambridge, United Kingdom*

**Introduction**

Cytokinesis is a spatially and temporally regulated process involving sequential changes in membrane lipid composition. We recently identified a Phosphatidylinositol-4-phosphate (PI(4)P) pool at the midbody membranes taking part of the lipid changes during cytokinesis completion. What is the source of PI(4)P at the midbody membranes and whether its loss affects cytokinesis are open questions with relevant implications. Cytokinesis failure causes cell refusion and tetraploidization, an aneuploid condition generating chromosomal instability (CIN), that drives breast cancer (BC) evolution. Thus, we hypothesized that perturbations of PI(4)P production during cytokinesis enhances BC cells tetraploidization and CIN, fueling tumor progression and defining vulnerabilities to aneuploidy-enhancing drugs.

**Material and Methods**

Using selective inhibitors (GSK-A1, PIK93, In-10) on HeLa Kyoto cells we inhibited lipid kinases to monitor cytokinesis completion (by confocal live imaging) and ploidy profiling (Flow Cytometry). Transgenic zebrafish morphants were lived-imaged using confocal spinning disk microscopy. For BC progression analysis, we generated human TNBC (MDA-MB-231, MDA-MB-453) or murine BC (4T-1) lines with doxycycline-inducible sh-RNAs targeting PI4KA. NSG or BALB mice were used to orthotopically inject BC cells and monitor tumor growth. MPI-0479605 was used as Mps1 inhibitor.

**Results and Discussions**

We demonstrated that PI4KA functions during cytokinesis ensuring midbody attachment at plasma membrane and proper cell division. Loss of PI4KA leads to cell refusion and binucleation. Publicly available databases showed that

© 2023 The Authors. Molecular Oncology is published by John Wiley & Sons Ltd on behalf of Federation of European Biochemical Societies

39.6% of BC patients display reduced *PI4KA* mRNA levels with an increased aneuploidy score (TCGA, PanCancer). In accordance, modulation of PI4KA in BC cell lines generated CIN-prone tetraploid cells marked by DNA damage, micronuclei and chronic cGAS-STING pathway activation, together tuning tumor microenvironment and BC progression. Furthermore, we showed that modulation of PI4KA activity in BC cell lines decreased viability *in vitro* and tumor growth *in vivo* upon Mps1 inhibition.

**Conclusion**

Our results showed that PI4KA catalytic activity regulates cytokinesis and that its loss enhances tetraploidization and CIN in BC cells, fueling tumor progression. However, Mps1 inhibition forced low-PI4KA BC cells beyond a critical ploidy threshold, leading to mitotic catastrophe and cell death, thus suggesting a therapeutic approach for low-*PI4KA* tumors.

### EACR23-1395
### PML, a cellular hub required for response to chemotherapy in Acute Myeloid Leukemia

*D. REROLLE*[1,2], *G. Fortin*[1,2], *C. Berthier*[1,2], *K. Lanchais*[2], *H. De Thé*[1,2]
[1]*Collège de France, Cirb, Paris, France*
[2]*Umr7212 - U944, GenCellDis- IRSL, Paris, France*

**Introduction**

Acute myeloid leukemia (AML) is characterized by uncontrolled proliferation of myeloid progenitors in the bone marrow. Only few AML patients are cured, due to relapse occurring in more than half of them. Our laboratory focuses on Promyelocytic Leukemia protein (PML). PML polymerizes to form nuclear bodies (NBs), particularly upon oxidative stress. Biochemically, PML NBs recruit many partner proteins within their core, and these will undergo post-translational modifications, notably SUMOylation. Functionally, PML NBs are involved in fundamental pathways such as metabolism, senescence, oxidative stress response or apoptosis. PML was discovered in the context of acute promyelocytic leukemia (PML/RARA translocation), in which arsenic and retinoic acid cure patients in a PML-dependent manner. As recent studies showed that PML participates to specific AML responses, we wondered whether it is generally involved in AML response to conventional chemotherapy.

**Material and Methods**

*Mll/Enl* oncogene was retrovirally transduced into *Pml*$^{+/+}$, *Pml*$^{-/-}$ or PML-mutant mouse hematopoietic progenitors. These cells were engrafted in syngenic mice that rapidly developed leukemia. We treated leukemic mice with doxorubicin and cytarabine, the classic 7+3 regimen used in AML patients.

**Results and Discussions**

We observed that mice bearing *Pml*$^{-/-}$ leukemia exhibited a very poor response to the treatment compared to *Pml*$^{+/+}$ ones. Accordingly, mice bearing *Pml*$^{+/+}$ *Mll/Enl* leukemia survived significantly longer after treatment than those bearing *Pml*$^{-/-}$ *Mll/Enl* AMLs. Studies of different PML-mutant Mll/Enl leukemia showed that a sufficient quantity of PML and its partners recruitment at PML NBs are necessary for efficient response to chemotherapy.
We then explored the downstream mechanisms through which PML might be involved in chemotherapy response. Importantly, P53 and its targets appear to be quite similarly activated in both genotypes. We report here that PML plays a role in modulating metabolism of AML cells at the basal state and during the chemotherapeutic course, in particular mitochondrial function. We found that global SUMO conjugaison depends in part on PML status and could explain basal differences as well as differential response to treatment.

**Conclusion**

Our results demonstrate the key role of PML in chemotherapy response in AML, probably though control of protein SUMOylation and mitochondrial fitness. Taken the frequent downregulation of PML expression in advanced solid tumors, PML targeting could be of broader significance in cancer.

### EACR23-1396
### Oncogenicity of recurrent ERBB4 mutations and potential as predictive markers

*V.K. Ojala*[1], *S. Ahonen*[1], *A. Jokilammi*[1], *O. Esparta*[1], *P. Suominen*[1], *T. Airenne*[2], *M.S. Johnson*[2], *L.D. Eli*[3], *K. Elenius*[1], *K.J. Kurppa*[1]
[1]*University of Turku, MediCity Research Laboratory, Turku, Finland*
[2]*Åbo Akademi University, Faculty of Science and Engineering, Turku, Finland*
[3]*Puma Biotechnology Inc., Translational Medicine and Diagnostics, Los Angeles, United States*

**Introduction**

Hundreds of somatic *ERBB4* (HER4) mutations have been described in various cancer tissues with very limited information available about their functional significance. Understanding the activity of *ERBB4* mutations is needed to assess the relevance of targeting ERBB4 in human cancers, such as clinically used pan-ERBB inhibitors neratinib. Neratinib is an irreversible pan-ERBB tyrosine kinase inhibitor that potently inhibits ERBB4. It is currently approved for early stage and metastatic HER2+ breast cancers, and is under clinical evaluation for EGFR-, ERBB2-, and ERBB4-mutant cancers, including in the SUMMIT clinical trial (NCT01953926).

**Material and Methods**

We assessed the oncogenicity and predictive value of 18 *ERBB4* mutations, selected from cBioPortal data based on their 1) high recurrence, 2) analogy to oncogenic mutations in other ERBB family members and/or 3) structural location suggesting functional relevance. We utilized mouse lymphoid Ba/F3 cells and human mammary epithelial MCF10A cells in *in vitro* studies and analyzed clinical data obtained from the SUMMIT study, cBioPortal and AACR Genie.

**Results and Discussions**

10/18 of the *ERBB4* mutations demonstrated transforming potential in lymphoid or epithelial cell models. Structural analysis suggested that the three most potent mutations (S303F, E452K, L798R) enhance ERBB4 activity by stabilizing receptor dimers. Moreover, the mutants appear to utilize heterodimerization with ERBB3 in their oncogenic functions. Interestingly, the most potent mutation was S303F, which is analogous to the well-

© 2023 The Authors. Molecular Oncology is published by John Wiley & Sons Ltd on behalf of Federation of European Biochemical Societies known oncogenic *ERBB2* mutation S310F that strongly stabilizes heterodimers with ERBB3. All the oncogenic *ERBB4* mutations were found to render cells sensitive to clinically approved pan-ERBB inhibitors. The SUMMIT trial enrolled six patients with tumors harboring *ERBB4* mutations of which four were analyzed also in our study (R544W, R711C, L798R, V840I). Patients with oncogenic *ERBB4* mutations (n=3) harbored also TP53 alterations, which may contribute to the lack of response to neratinib, as has been suggested for ERBB2-mutant patients.

**Conclusion**

Many of the recurrent *ERBB4* mutations are oncogenic. Further investigation into potential utility of clinically used pan-ERBB inhibitors, such as neratinib, for patients whose tumors harbor these *ERBB4* mutations is warranted.

### EACR23-1399

### The therapeutic efficacy of Talazoparib as PARP inhibitor in metastatic castration-resistant prostate cancer

G. Guney Eskiler[1]

[1]*Sakarya University- Faculty of Medicine, Medical Biology, Sakarya, Turkiye*

**Introduction**

Metastatic castration-resistant prostate cancer (mCRPC) is an aggressive form of prostate cancer with a worse prognosis and approximately 20% of PC patients carry germline mutations in homolog recombination-related genes. In this context, Poly-ADP ribose polymerase inhibitors (PARPi) are promising therapeutic strategies for the treatment of mCRPC due to leading to synthetic lethality in HR-deficient cancer types. Olaparib and rucaparib PARPi have been approved for the treatment of mCRPC. However, despite ongoing clinical trials, the potential therapeutic effects of Talazoparib (TAL) as PARPi remain unclear on mCRPC at the molecular level. In this study, we, for the first time, assessed the underlying molecular mechanisms of TAL-induced cell death in two different mCRPC cells *in vitro*.

**Material and Methods**

PC-3 and DU-145 mCRPC cells were treated with 0.5 and 1 nM TAL for six days. Following treatment, TAL-induced cell death was assessed by Annexin V, cell cycle, AO/PI staining, western blot analysis and immunofluorescence staining.

**Results and Discussions**

TAL significantly reduced the viability of PC-3 and DU-145 cells in a dose and time-dependent manner ($p<0.05$). The percentage of the total apoptotic cell was 62.9% and 53.5% in PC-3 and DU-145 cells, respectively, through the increased p-γH2AX, cleaved PARP and caspase-3 protein levels at 1 nM TAL. Additionally, nuclear blebbing, chromatin condensation and increased nuclear γH2AX and PARP levels were observed in mCRPC cells. On the other hand, TAL treatment resulted in G2/M arrest in PC-3 cells, whereas the accumulation of the cells in the G0/G1 phase significantly increased in DU-145 cells following 1 nM TAL treatment. Therefore, the underlying molecular mechanisms of TAL-induced cell death and its association with DNA damage response should be further analyzed.

**Conclusion**

Our findings suggest that TAL induces apoptosis in mCRPC cells, and thus TAL treatment as PARPi could be a promising therapeutic modality for treating mCRPC. However, the response of mCRPC cells to TAL was different due to probably different genetic profiles.

### EACR23-1400

### Mesenchymal stem cell induced migratory and invasive activities of glioblastoma cells

M. Shevtsov[1], D. Goncharova[2], R. Tagaeva[3], N. Yudintceva[3], D. Bobkov[3]

[1]*Technical University of Munich, Klinikum rechts der Isar, Munich, Germany*

[2]*Almazov National Medical Research Centre, Personalized Medicine Centre, St.Petersburg, Russia*

[3]*Institute of Cytology of the Russian Academy of Sciences RAS, Laboratory of Biomedical Nanotechnologies, St.Petersburg, Russia*

**Introduction**

The development of novel strategies for the inhibition of glioblastoma cells invasion depends crucially on our understanding of the characteristics of the interaction between tumor cells and cells of the tumor microenvironment. In the current study, the system of co-cultivation of human mesenchymal stem cells (MSCs) and glioblastoma cells was employed to explore the properties of the motility of cancer cells interacting with normal cells.

**Material and Methods**

U251 human glioblastoma cells and FetMSC mesenchymal stem cells derived from bone marrow of 5–6 week human embryos were used in the studies. Extracellular vesicles (EVs) and conditioned medium (CM) obtained from FetMSCs were added to glioblastoma cells, and vice versa, FetMSCs were treated with EVs and CM obtained from U251 cells. Additionally, U251 and FetMSC were directly co-cultivated with each other. These three types of experimental setups were carried out for 24 hours, while the cell motility was recorded using the CellVoyager CQ1 Benchtop High-Content Analysis System (Yokogawa).

**Results and Discussions**

The results obtained indicate that EVs from FetMSC caused an increase in the speed of U251 cells migration. The speed increased even more under the influence of CM derived from FetMSC, and was further increased in U251 cells when the latter were co-cultured with FetMSC. For FetMSC cells, the opposite effect was observed - under the influence of EVs derived from U251, stem cells reduced the speed of movement, that was further reduced when FetMSC were co-cultured with U251 cells. In addition to speed, we also studied the sinuosity of cell movement tracks. The tortuosity of U251 did not change, while sinuosity of FetMSC significantly increased both under the influence of EVs obtained from U251 and during co-cultivation with tumor cells.

**Conclusion**

Human MSCs are suppressed in motility while U251 glioblastoma cells' motility is increased when the two cells are co-cultured. The acquired results imply that the secretome produced by MSCs has the ability to accelerate the invasion of cancer cells, while intercellular contacts also play an important role and can be used in conjunction with the secretome's action. Cancer cells actively

© 2023 The Authors. Molecular Oncology is published by John Wiley & Sons Ltd on behalf of Federation of European Biochemical Societies reorganizing the tumor microenvironment can reduce MSC motility.

### EACR23-1407
### Ferroptotic regulated cell death in lipid peroxidation resistant cell lines

*M. Adamiec-Organiściok[1,2], A. Dawicka[1,2], M. Węgrzyn[3], D. Gendosz de Carrillo[4,5], M. Skonieczna[1,2]*
*[1]Silesian University of Technology, Department of Systems Biology and Engineering, Gliwice, Poland*
*[2]Silesian University of Technology, Biotechnology Centre, Gliwice, Poland*
*[3]Silesian University of Technology, Students' Scientific Society of Biotechnology at the Biotechnology Centre, Gliwice, Poland*
*[4]Medical University of Silesia, Department of Physiology- Faculty of Medical Science Medicine, Katowice, Poland*
*[5]Medical University of Silesia, Department of Histology and Cell Pathology- Faculty of Medical Science, Zabrze, Poland*

**Introduction**
Regulated cell death, ferroptosis, occurred in cells with sensitiveness to lipid peroxidation, such as melanoma 1205-Lu cell line.

**Material and Methods**
We have found oxidized lipids accumulation after 24-houres cells incubation with erastin (5 and 10 μM). Surprisingly, p53 k.o. mutants, HCT116 p53-/- and K562 cells, survived such treatments and showed resistance to ferroptosis – reduction of oxidized lipids was sufficient to activate survival pathways, p53 not-dependent. That phenomenon was presented in our previous studies, where ferroptosis-resistant healthy keratinocytes, HaCaT showed a lipids-recovery strategy.

**Results and Discussions**
Here, for the first time, we present such results in colorectal cancer HCT116 p53-/- and myelogenous leukemia K562 cell lines. Reduction of oxidized lipids was visible in resistant HaCaT cells, also in cancer HCT116 p53-/- and K562, whereas not in ferroptosis-sensitive 1205-Lu cells. Although, the Fenton reaction inducer, erastin improved reactive oxygen species (ROS) formation, what correlated with elevated receptor TFRC (Transferrin Receptor) and enzyme ACLS4 (Acyl-CoA Synthetase Long Chain Family Member 4) genes expression, in resistant and p53-mutants the cellular death not occurred.

**Conclusion**
Ferroptosis suppressor protein 1 (FSP1) was found as a cellular guard, with p53 regulators cancer cells develop defence and survive death induction. Novel findings, about intracellular crosstalk, within p53 dependent pathways and regulated cell death are now discussed.

Funding: SUT in Gliwice, Poland, grant No 02/040/BK_23/1035.

### EACR23-1416
### Identification of a novel p53-induced, NFkB-regulatory signalling complex

*A. Lees[1], J. Holloway[1], E. Rogan[1], D. Longley[1], S. McDade[1]*
*[1]Queen's University Belfast, Patrick G Johnston Centre for Cancer Research, Belfast, United Kingdom*

**Introduction**
Appropriate regulation of cell fate in response to stress or damage is critical for the survival of all multi-cellular organisms. Maintaining such cellular homeostasis is a key function of the tumour suppressive transcription factor, p53, which has been shown to modulate cell fate by controlling an extensive gene regulatory network. However, exactly how p53 signals to enact distinct cellular outcomes (*i.e.* cell survival vs cell death) remains poorly understood. In recently published work we demonstrated that p53 directly activates the expression of FLIP(L) in colorectal cancer cells to inhibit cell death and also modulate p53 target gene expression in favour of cell survival. Importantly our results suggest that survival is dependent on the formation of a novel ligand-independent, FLIP(L) containing complex.

**Material and Methods**
To investigate the composition and downstream signalling consequences of this p53-induced complex, here we utilise a combination of siRNA-mediated protein knockdown, transcriptomics, immunoprecipitation and western blot analyses. These analyses were carried out in a panel of CRISPR-cas9 cell-death-associated-protein knockout models of colorectal cancer, alongside FLIP(L) overexpressing models, to dissect the molecular and phenotypic consequences of perturbing complex formation.

**Results and Discussions**
We have now identified a p53-induced signalling complex that is regulated by FLIP(L) and that, interestingly, modulates the activity of the NFkB pathway. This complex is comprised of the putative p53 target TRAIL-R2/DR5, as well as caspase-8, FADD, and indeed, FLIP(L). Activation of this complex does not require canonical TRAIL-ligand binding, however, upon siRNA-mediated FLIP(L) depletion and p53 activation it effectively induces colorectal cancer cell death. Mechanistically, this results in upregulation and nuclear translocation of NFkB family members, enhanced expression of canonical NFkB targets (*e.g.* cIAP2), and may also regulate the expression of inflammatory-associated proteins.

**Conclusion**
This work identifies p53-induced FLIP(L) as an important regulatory node which modulates the activity of key cell signalling pathways. Uncovering this novel cellular biology may help to further elucidate the molecular mechanisms which govern the p53-dependent switch from cell cycle arrest and survival to cell death. Moreover, a better understanding of the ways in which tumour cells exploit this signalling network in favour of cell survival may illuminate novel therapeutic opportunities.

### EACR23-1434
### Glutamine regulates BCR/Abl expression in hypoxic chronic myeloid leukemia cells via fatty acids metabolism

*A. Biagioni[1], C. Mancini[1], G. Menegazzi[1], S. Peppicelli[1], D. Guasti[2], D. Bani[2], G. Pieraccini[3], P. Dello Sbarba[1]*
*[1]University of Florence, Department of Experimental and Clinical Biomedical Scie*

© 2023 The Authors. Molecular Oncology is published by John Wiley & Sons Ltd on behalf of Federation of European Biochemical Societies nces "Mario Serio", Florence, Italy
[2]University of Florence, Department of Experimental and Clinical Medicine, Florence, Italy
[3]University of Florence, Mass Spectrometry Center CISM, Florence, Italy

**Introduction**

Under very low oxygen tension, Chronic Myeloid Leukemia (CML) cells undergo the suppression of the BCR/Abl oncoprotein, whereas a BCR/Abl-independent subset of cells, commonly referred to as leukemia stem cells, is maintained. Such cells retain the capacity, when transferred to normoxic conditions, to generate a BCR/Abl-expressing progeny which is, in vivo, responsible for the relapse of the disease, demonstrating to be also resistant to the tyrosine kinase inhibitors (TKi) by lacking their molecular target. Moreover, under oxygen restriction, glutamine plays a major role, stabilizing c-Myc expression and inducing cancer cells to diverge towards a more pronounced fatty acids (FA) metabolism.

**Material and Methods**

K562 and KCL22 cell lines were subjected to glucose and/or glutamine deprivation in hypoxic conditions (96hrs at 0.1% $O_2$). Cells metabolic profile was generated through the Seahorse XFe96 Analyzer while L-Glutamine-$^{13}C_5$ was exploited via LC/MS to determine its contribution in FA de novo synthesis. BODIPY 493/503 was used to measure the intracellular neutral lipid droplets in confocal microscopy and flow cytometry whose presence and morphology were also determined via transmission electron microscopy. BCR/Abl was evaluated via Western Blotting whilst CD36 was determined through flow cytometry.

**Results and Discussions**

We observed that glutamine is capable to boost glycolysis, leading to a faster BCR/Abl downregulation in hypoxic conditions, and decrease the basal and maximal cell respiration capacity. We also identified that under oxygen and glucose shortage, CML cells were characterized by numerous lipid droplets. Such an augmented neutral lipid content was due to a glutamine-dependent CD36 upregulation, which is capable to uptake FA from the extracellular milieu. In these conditions, CML cells rapidly lose BCR/Abl expression, a phenomenon which was validated by the treatment with exogenous BSA-Palmitate, capable to reduce BCR/Abl expression, while the use of the sulfosuccinimidyl oleate, a specific CD36 inhibitor, sustained the oncoprotein maintenance instead.

**Conclusion**

Our results suggest that FA may play a fundamental role in hypoxic-induced BCR/Abl suppression and that such FA degradation might be needed for the oncoprotein re-expression once in normoxic conditions. This phenomenon might be therefore exploited to sustain BCR/Abl expression in hypoxic cells to be more susceptible to TKi.

### EACR23-1449
### Establishment and characterization of human organoids derived from medullary cancer and serrated adencocarcinoma; rare histological variants of colorectal cancer

*R. Figueiredo[1], A. Hurtado[2], G. Luengo Gil[2], L. Feist[1], A Albaladejo-González[2], F. Postigo Corrales[2], A. Beltrán-Videla[2], D. Torres-Moreno[2], B. Rotter[1], P. Conesa Zamora[2]*
*[1]GenXPro, Biomarkers & Translatoinal Medicine, Frankfurt am Main, Germany*
*[2]Institute for Biomedical Research from Murcia, Santa Lucia University Hospital, Cartagena, Spain*

**Introduction**

Medullary cancer (MC) and serrated adenocarcinoma (SAC) are rare subtypes of colorectal cancer (CRC) representing <1% and <9% of all CRC diagnoses, respectively. Both have poor prognoses compared to conventional CRC and are end-points of different pathological routes. In this study, we generated Patient-derived organoids (PDOs) representing MC and SAC that were analysed by 3' mRNA profiling to determine the Consensus Molecular Subtype (CMS) as well as to collect characteristic clinical and biochemical features for *in sillico* drug sensitivity analyses.

**Material and Methods**

PDOs established from surgical specimens comprising tumoral and/or healthy cells were cultured in a 3D matrix. Sequential passages, cryopreservation, DNA and RNA extraction and paraffin-embedded organoid pellets were obtained. *KRAS* and *NRAS* mutations were determined by ddPCR, *BRAF* status by pyrosequencing. MACE-seq data (3'mRNA sequencing) was used for consensus molecular subtype (CMS) scoring and for *in silico* drug sensitivity prediction. For the latter, a database consisting of characteristic gene expression profiles for clinically relevant features was set up from public RNA-Seq data. Clinically relevant information for the organoids was compiled in a report for therapeutic decision support ("ClinXPro-Report").

**Results and Discussions**

Five tumoral PDOs were successfully established. One PDO was derived from a high-grade MC infiltrating subtype with MSI and *BRAF* mutation, the other from a low-grade SAC with MSS and KRAS mutation. Three PODs were derived from low grade (CC); one *BRAF*-mutated and two natives for *KRAS/NRAS/BRAF*. Non-tumoral PDO were also established from CC and SAC patients as controls. Principal component analysis showed that two CC are closer to the controls than the rest of CRC-PDOs. The MACE-seq-based CMS prediction indicate a CMS4 for SAC and CC; CMS3 for CC and CMS1 for MS and CC. The ClinXPro-Report revealed a potential sensitivity of the different organoids to drugs such as the ERK1/ERK2 inhibitor Ulixertinib, the PI3K inhibitor Pilaralisib, the MEK inhibitor Refametinib or the S6K1 inhibitor PF-4708671.

**Conclusion**

CRC-PDOs can be used as tools to model different histological subtypes of CRC. Notably, the less frequent subtypes SAC and MC exhibited, in our analysis, significantly greater differences in gene expression compared to CCs. Our results suggest that further characterization of less frequent subtypes is necessary and that PDOs can have potential implications in the development of personalized therapies.

### EACR23-1452
### Evaluation of therapy-induced senescence and senolytic efficacy in non-small cell

© 2023 The Authors. Molecular Oncology is published by John Wiley & Sons Ltd on behalf of Federation of European Biochemical Societies

**lung cancer using a high-throughput labeling strategy.**

J. Verswyvel[1], A. Domen[1,2], C. Deben[1], C. Hermans[3], H. Lambrechts[1], E. Cardenas De La Hoz[4], M. Lecompte[1], W. De Vos[5], F. Lardon[1], A. Wouters[1]
[1]University of Antwerp, Center for Oncological Research CORE and Integrated Personalized and Precision Oncology Network IPPON, Wilrijk, Belgium
[2]Antwerp University Hospital, Department of Oncology, Edegem, Belgium
[3]Antwerp University Hospital, Department of Pathology, Edegem, Belgium
[4]University of Antwerp, Industrial Vision Lab InViLab, Wilrijk, Belgium
[5]University of Antwerp, Laboratory of cell biology and histology, Wilrijk, Belgium

**Introduction**

Non-small cell lung cancer (NSCLC) remains a leading cause of cancer-related deaths worldwide with long-term survival still hard to achieve. Cellular senescence, an emerging hallmark of cancer and found to be induced by several anticancer therapies (i.e., therapy-induced senescence (TIS)), is often considered as an endogenous tumor suppressor mechanism. However, mainly through their distinctive senescence-associated secretory phenotype (SASP), these senescent cells can paradoxically affect the surrounding tumor microenvironment, ultimately leading to cancer relapse and metastasis. To better understand the role of cellular senescence in cancer and to evaluate potential senolytic therapies, adequate and high-throughput detection of senescence in preclinical and clinical cancer research is becoming increasingly important.

**Material and Methods**

A high-throughput analysis method was developed that labels cells based on the presence or absence of senescent phenotypic features: (i) an enlarged nucleus (blue nuclear dye Hoechst); (ii) cytoplasmic senescence-associated β-galactosidase (SA-β-Gal, CellEvent™ Senescence Green probe); and (iii) an enlarged area of SA-β-Gal as proxy for cell size. The accuracy of this labeling strategy was validated by immunohistochemistry staining using four conventional markers for senescence: SA-β-Gal, p53, $p21^{WAF/Cip1}$ and Ki67. Our labeling strategy was then used to evaluate the percentage TIS after a 5-day treatment with a concentration range of five SOC chemotherapeutic agents in eight different NSCLC cell lines. Finally, the potency of three senolytics to selectively eliminate senescent NSCLC cells 96 hours post treatment was evaluated with our labeling strategy.

**Results and Discussions**

Our analysis is able to accurately label senescent cells, as similar percentages of TIS were obtained using IHC (e.g., using 7500nM cisplatin, 61% TIS using the high-throughput strategy, 64% TIS using IHC). TIS induction was mainly observed in A549 cells, using all SOC compounds, with the highest levels of TIS using cisplatin (64%), carboplatin (61%) and pemetrexed (54%). Finally, the Bcl-2 inhibitor Venetoclax was identified as the most effective and selective senolytic compound.

**Conclusion**

We have successfully developed an accurate and high-throughput strategy to label senescent NSCLC cells in preclinical cancer research. We further demonstrated that SOC NSCLC therapies induce TIS in *in vitro* conditions and that senolytics are capable of selectively killing senescent NSCLC cells.

### EACR23-1454
### POSTER IN THE SPOTLIGHT
### Paradoxical activation of oncogenic signaling pathways as a cancer treatment strategy

M. dos Santos Dias[1], A. Friskes[2], S. Wang[3], J.M. Fernandes Neto[1], F. van Gemert[4], S. Mourragui[5], H. Jin[1], A. Villanueva[6], R. Medema[2], R. Bernards[1]
[1]Netherlands Cancer Institute, Molecular Carcinogenesis, Amsterdam, The Netherlands
[2]Netherlands Cancer Institute, Cell Biology, Amsterdam, The Netherlands
[3]Shanghai Cancer Institute, State Key Laboratory of Oncogenes and Related Genes, Shanghai, China
[4]Netherlands Cancer Institute, Tumor Biology & Immunology, Amsterdam, The Netherlands
[5]Hubrecht Institute-KNAW, Hubrecht Institute-KNAW, Utrecht, The Netherlands
[6]Bellvitge Biomedical Research Institute IDIBELL, Medical Oncology, Barcelona, Spain

**Introduction**

The homeostasis of cancer cells relies on a fine-tuned balance between the activated oncogenic pathways driving tumorigenesis and the engagement of stress-response programs that counteract the inherent toxicity of such aberrant signaling. Most of the current clinical and experimental cancer therapy is based on targeted inhibition of mitogenic signaling pathways. Unfortunately, long-lasting control of advanced cancers with such approach remains virtually elusive due to the rewiring of signaling pathways and the emergence of resistance. It is becoming increasingly clear that further activation of the same pathways driving tumorigenesis can also disrupt cancer cells' homeostasis and cause lethality. We hypothesized that a "paradoxical" deliberate overactivation of oncogenic signaling pathways could be combined with inhibition of the stress response pathways that help cancer cells survive in the presence of hyperactive oncogenic signaling.

**Material and Methods**

Using colorectal cancer cells as primary models, we performed stress-focused drug screens and genome-wide CRISPR screens to identify synthetic lethalities with the Protein Phosphatase 2A (PP2A) inhibitor LB-100. The findings were validated across colorectal, pancreatic, and cholangiocarcinoma cancer cell panels. Live-cell microscopy, DNA combing, and flow cytometry were used to address the DNA replication and mitotic dynamics. Single-cell RNAseq was used to investigate the phenotype of resistant cells

**Results and Discussions**

We show here that inhibition of Protein Phosphatase 2A (PP2A) hyperactivates multiple oncogenic pathways and engages stress responses while restraining the viability of colon cancer cells. Genetic and compound screens identified combined inhibition of PP2A and Wee1

© 2023 The Authors. Molecular Oncology is published by John Wiley & Sons Ltd on behalf of Federation of European Biochemical Societies synergistic in multiple cancer models. Mechanistically, sublethal doses of LB-100 and the Wee1 inhibitor adavosertib combine to collapse DNA replication and trigger premature mitosis followed by cell death. This combination suppressed the growth of patient-derived tumors and was tolerated *in vivo*. Remarkably, acquired resistance to this drug combination led to the down-modulation of oncogenic signaling, suppressing the malignant phenotype of colon cancer cells and their ability to form tumors *in vivo*

**Conclusion**

These data show preclinical efficacy and mechanistic basis for the combination of LB-100 and Wee1 inhibition in colorectal cancer cells. Moreover, our data suggest that paradoxical activation of oncogenic signaling can result in tumor suppressive acquired resistance

### EACR23-1461
### Mechanisms of PML nuclear body formation and therapeutic targeting by arsenic

*<u>P. BERCIER</u>[1], Q.Q. Wang[2], O. Ferhi[1], C. Berthier[1], M.C. Geoffroy[1], M. Abou-Ghali[1], H. Naranmandura[2], C. Zhou[2], H. De Thé[1], V. Lallemand-Breitenbach[1]*
*[1]Collège de France, Nuclear Organization and Post-Translational Control in Physiopathology, Paris, France*
*[2]Zhejiang University, Department of Toxicology, Hangzhou, China*

**Introduction**

PML (ProMyelocytic Leukemia) is the sole organizer of membrane-less organelles: the PML nuclear bodies (NBs). NBs recruit a wide variety of partner proteins and control their post-translational modifications, in particular their sumoylation, regulating their activity, playing versatile roles in biological processes. PML NBs are disorganized in acute promyelocytic leukemia (APL) where a chromosomal translocation between PML and RARα leads to the production of the PML-RARα oncoprotein. A combination treatment of arsenic and retinoic acid induces APL cure in 95% of the cases without additional chemotherapy. Critically, arsenic can directly bind to PML and induce NB reformation and subsequent senescence activation, leading to the loss of leukemic stem cell self-renewing activity, responsible for APL cure. However, the mechanism by which arsenic binding onto PML induces NB assembly remains unknown. NBs are lost in other kinds of cancers making it an interesting therapeutic target. Here, we aimed at identifying the mechanisms underlying PML NB assembly driving APL cure upon arsenic treatment.

**Material and Methods**

In this study, we determined the tridimensional crystallographic structure of purified PML peptides. We also led a comparative analysis of PML dynamics at NBs using FRAP in *Pml KO* Mefs stably expressing a GFP-PML protein harboring discrete substitution mutations on key amino acids.

**Results and Discussions**

We focused on the B2 domain of PML, which is found mutated in arsenic-resistant APL patients and revealed its central role in basal PML NB assembly dynamics. We determined that this domain controls PML dynamics at NBs through the formation of a homo-trimer coordinated by weak and reversible hydrophobic interactions between α-helixes. Moreover, these interactions regroup three free cysteine residues at the core of the trimer which play a central role in controlling the assembly dynamics of NBs. Arsenic targets this cysteine triad, transforming weak transient interactions into covalent ones and induces a liquid-like to gel-like transition of NBs. Our results suggest that arsenic-induced loss of self-renewing activity in cells transformed by PML-RARα may also depend on this cysteine triad.

**Conclusion**

Thereby, linking stereo-selectivity and intracellular dynamic, we solved the first step of the mechanism by which arsenic induces NB formation. These results pave the way for the development of new structure-based activators, which mimic arsenic and activate NB assembly to improve the therapeutic outcome.

### EACR23-1463
### Mechanisms of PML nuclear body formation and therapeutic targeting by arsenic

*<u>P. BERCIER</u>[1], Q.Q. Wang[2], H. Naranmandura[2], C. Zhou[2], H. De Thé[1], V. Lallemand-Breitenbach[1]*
*[1]Collège de France, Nuclear Organization and Post-Translational Control in Physiopathology, Paris, France*
*[2]Zhejiang University, Department of Toxicology, Hangzhou, China*

**Introduction**

PML (ProMyelocytic Leukemia) is the sole organizer of membrane-less organelles: the PML nuclear bodies (NBs). NBs recruit a wide variety of partner proteins and control their post-translational modifications, in particular their sumoylation, regulating their activity, playing versatile roles in biological processes. PML NBs are disorganized in acute promyelocytic leukemia (APL) where a chromosomal translocation between PML and RARα leads to the production of the PML-RARα oncoprotein. A combination treatment of arsenic and retinoic acid induces APL cure in 95% of the cases without additional chemotherapy. Critically, arsenic can directly bind to PML and induce NB reformation and subsequent senescence activation, leading to the loss of leukemic stem cell self-renewing activity, responsible for APL cure. However, the mechanism by which arsenic binding onto PML induces NB assembly remains unknown. NBs are lost in other kinds of cancers making it an interesting therapeutic target. Here, we aimed at identifying the mechanisms underlying PML NB assembly driving APL cure upon arsenic treatment.

**Material and Methods**

In this study, we determined the tridimensional crystallographic structure of purified PML peptides. We also led a comparative analysis of PML dynamics at NBs using FRAP in *Pml KO* Mefs stably expressing a GFP-PML protein harboring discrete substitution mutations on key amino acids.

**Results and Discussions**

We focused on the B2 domain of PML, which is found mutated in arsenic-resistant APL patients and revealed its central role in basal PML NB assembly dynamics. We determined that this domain controls PML dynamics at NBs through the formation of a homo-trimer coordinated

© 2023 The Authors. Molecular Oncology is published by John Wiley & Sons Ltd on behalf of Federation of European Biochemical Societies by weak and reversible hydrophobic interactions between α-helixes. Moreover, these interactions regroup three free cysteine residues at the core of the trimer which play a central role in controlling the assembly dynamics of NBs. Arsenic targets this cysteine triad, transforming weak transient interactions into covalent ones and induces a liquid-like to gel-like transition of NBs. Our results suggest that arsenic-induced loss of self-renewing activity in cells transformed by PML-RARα may also depend on this cysteine triad.

**Conclusion**

Thereby, linking stereo-selectivity and intracellular dynamic, we solved the first step of the mechanism by which arsenic induces NB formation. These results pave the way for the development of new structure-based activators, which mimic arsenic and activate NB assembly to improve the therapeutic outcome.

### EACR23-1504

### Characterisation of SUMOylation in human cancer cells and Naked mole-rat tissues.

M. Salih[1]

[1]Univesity of Bradford, School of Pharmacy and Medical Sciences- Institute of Ca ncer Therapeutics, Bradford, United Kingdom

**Introduction**

SUMOylation is a post-translational modification where small ubiquitin-like modifier (SUMO) proteins are conjugated to lysine residues of target proteins. SUMO proteins exist in all eukaryotes and participate in a catalytic cycle. SUMOylation is known to regulate numerous molecular regulatory mechanisms. In addition, SUMOylation dysregulation is known to promote several diseases, including cancer cell survival. The unique traits of phenotypic and molecular adaptations found in naked mole rats (NMR) suggest high stability and effective functioning of the molecular machinery that counteract damage accumulation in its genome. NMR lifespan can reach up to 32 years and acquires a very efficient mechanism of resistance to cancer. This study aims to investigate the regulation of SUMO machinery between human cancer cells and NMR tissues, in order to find novel targets for cancer treatment.

**Material and Methods**

Basic Local Alignment Search Tool (BLAST) (https://blast.ncbi.nlm.nih.gov/Blast.cgi) was used to identify similar regions of human and naked mole-rat SUMO nucleotides and proteins. The qPCR and western blotting analysis were used to investigate the expression of SUMO components at the mRNA and protein levels in NMR tissues and cancer cells lines.

**Results and Discussions**

The investigation of pairwise alignment identified the similarity of both SUMO genes (95% - 87%) and proteins (100% - 66.9%) between NMR and human. Low expression of SUMO components at the mRNA level in NMR brain and intestinal tissues compared to MCF-7, DLD-1, and SH-SY5Y cell lines. On the contrary, PIAS4 expression is significantly higher in the NMR tissues compared to cancer cell lines. Protein analysis performed on transfected MCF-7, DLD-1, and SH-SY5Y was also investigated compared to control cell.

**Conclusion**

Expression of SUMOylation in human cancer cells and NMR tissues is being established. Interestingly, lower expression of mRNA level in SUMO components was observed in NMR tissues compared to all human cancer cells. However, PIAS4 expression was significantly higher in the NMR tissues compared to cancer cell lines. This data revealed important differences in NMR SUMOylation machinery which provides a future therapeutic avenue to treat cancer. This data revealed important differences in NMR SUMOylation machinery which provides a future therapeutic avenue to treat cancer.

### EACR23-1506

### Fumarate hydratase functions as a tumor suppressor in endometrial cancer by inactivating EGFR signaling

H.H. Yuan[1]

[1]Kaohsiung Medical Univ, Graduate Institute of Medicine, Kaohsiung, Taiwan

**Introduction**

Fumarase hydratase is an enzyme that catalyzes the reversible hydration and dehydration of fumarate to malate in the tricarboxylic acid cycle. In this study, we addressed the role of fumarate hydratase in endometrial cancer.

**Material and Methods**

In clinical study, we observed that the expression of fumarate hydratase was significantly lower in endometrial cancer tissues compared to normal endometrial tissues. Furthermore, the decreased fumarate hydratase expression in endometrial cancer tissues was significantly associated with increased tumor size and lymph node metastasis. Further in vitro study showed that the cell proliferation, migration and invasion abilities were increased when we knockdowned the expression of fumarate hydratase in the endometrial cancer cells. In contrast, overexpression of fumarate hydratase in endometrial cancer cells decreased their cells proliferation and migration and invasion abilities.

**Results and Discussions**

Mechanistic studies showed that the expression of vimentin and twist, two well-studied mesenchymal markers, in endometrial cancer cells were upregulated in fumarate hydratase-knockdowned cells. In addition, phosphokinase array analysis demonstrated that the expression of phospho-EGFR (Y1086), which promoted carcinogenesis in cancers, was increased in endometrial cancer cells when fumarate hydratase was knockdowned.

**Conclusion**

In conclusion, our studies suggest that fumarate hydratase is a tumor suppressor and inhibits endometrial cancer cell proliferation and metastasis by inactivation of EGFR. Further studies are required to potentiate its role as a prognostic biomarker and therapeutic target for endometrial cancer.

### EACR23-1507

### The interaction of cellular senescence and compromised DNA repair in oral carcinogenesis and the development of novel therapeutics

Y.Y. Wang[1]

© 2023 The Authors. Molecular Oncology is published by John Wiley & Sons Ltd on behalf of Federation of European Biochemical Societies

[1]Kaohsiung Medical University, School of Dentistry, Kaohsiung, Taiwan

**Introduction**

Oral cancer is the fourth most common cancer among men in Taiwan, and the fourth leading cause of death from cancer among men in Taiwan. According to epidemiological reports, its morbidity and mortality also show a global increasing trend.

**Material and Methods**

In this study, we addressed how betel nut and its components play a role in oral carcinogenesis by inducing cellular senescence and declining DNA repair function by in vitro, in vivo and clinical studies to elucidate the interaction, role and molecular mechanism of cellular senescence and decreased DSB repair function during oral carcinogenesis. We will study how areca nut extract induces oral carcinogenesis by affecting cellular senescence and DSB repair-related markers in the oral microenvironment in a hamster model. Finally, we will develop a topical therapy for oral precancerous lesions (OPMDs) targeting cellular senescence and DSB repair-related biomarkers in a hamster model.

**Results and Discussions**

Normal cells showed cellular senescence and decreased protein expression of DNA double-strand break (DSB) repair proteins MRE11 and RAD51 after areca nut extract (ANE) treatment. Since cellular senescence and declined DNA repair function are the two major characteristics of cancer-prone elderly people, the role of these two in betel nut-induced oral carcinogenesis deserves further study.

**Conclusion**

This study will provide new molecular evidence for the influence of ANE on the carcinogenesis of the oral microenvironment. Furthermore, novel topically administered targeted therapy for treatment of oral precancerous lesions and prevention of oral cancer will be developed accordingly.

### EACR23-1511
### Differential responses to senescent cell removal between aged male and female livers

M. Klaver[1], B. Wang[1], Y. Lin[1], M. Demaria[1]
[1]UMCG, ERIBA, Groningen, The Netherlands

**Introduction**

Cellular senescence is characterized by a state of irreversible cell cycle arrest with upregulation of p16 and p21 expression, which is often accompanied by the secretion of a senescence-associated secretory phenotype (SASP). In human patients, the incidence of liver cancer is higher in males compared to females. In our cohort of aged mice, we observed similar results that a part of the males developed spontaneous liver cancer, whereas females did not. Senescent cells and the associated SASP factors are shown to promote tumorigenesis. Interestingly, our preliminary data also indicated that aged male livers burden higher senescent cells and SASP. Moreover, depletion of persistent senescent cells is shown to improve age-related functional decline in many aspects, however the sex disparity is never investigated. The aim of our study is to investigate whether there is a differential response to senescent cell removal in aged livers of males and females.

**Material and Methods**

Our p16-3MR mouse model allows p16-positive senescent cells to be tracked by Renilla luciferase, sorted by RFP, and eliminated by GCV. We also used transcriptomic, proteomic and lipidomic approaches to analyse the livers upon senescent cell depletion.

**Results and Discussions**

Here we show that senescent cells gradually accumulate with age systemically and in the livers by imaging and FACS. Constant treatments of GCV can remove senescent cells in both aged males and females in a comparable level. However, females seem to benefit more from senescent cell removal in the context of grip strength decline and tissue degeneration. Notably, our multi-omics data also showed clear sex disparity in the aged livers upon senescent cell removal.

**Conclusion**

Ageing leads to increased senescence burden in the liver, which results in different responses in males and females. The differential senescence responses in the liver might explain the difference in liver cancer incidence.

### EACR23-1522
### Species-specific differences in Malignant Transformation: Role of PP2A and its implications for cancer therapy

N. Gupta[1,2], O. Kauko[1], Y. Zhao[3], V. Gorbunova[4], N. Wang[1,2], T. Aittkallio[2,5], J. Westermarck[1,2]
[1]University of Turku, Turku Bioscience Centre, Turku, Finland
[2]University of Turku, InFLAMES Research Flagship Center, Turku, Finland
[3]Zhejiang University, School of Medicine, Hangzhou, China
[4]University of Rochester, Rochester Aging Research Center, Rochester, United States
[5]University of Helsinki, Institute of Molecular Medicine, Helsinki, Finland

**Introduction**

Malignant cellular transformation is a multi-stage process where oncogene activation and tumor suppressor inhibition co-operate. Previous studies have firmly established that in mouse cells, RAS can transform the cells in combination with inhibition of only one tumor suppressor, whereas RAS-mediated transformation of human cells requires inhibition of Protein Phosphatase 2A (PP2A). However, even after 20 years, the species-specific requirement of PP2A inhibition for cellular transformation remains as an unsolved major question in human cancer biology. Medically solving this question is very relevant as PP2A inhibition has a major role in protecting cancer cells from cell death and in driving cancer drug resistance.

**Material and Methods**

To address these fundamental research questions serial transformation models from mouse and human has been generated using piggyback systems. Cell line models were validated, surveyed, and subjected to comprehensive multi-omics analysis using in-house state of art of facilities including (i) RNA- sequencing with Illumina workflow (ii) proteomics/Phosphoproteomics using highly sensitive and robust Orbitrap Fusion Lumos Tribrid Mass spectrometer,

© 2023 The Authors. Molecular Oncology is published by John Wiley & Sons Ltd on behalf of Federation of European Biochemical Societies with a specific goal to identify targets that are dependent on PP2A inhibition in human cells only.

**Results and Discussions**

Current data suggest that RAS overexpression is sufficient for anoikis resistance in both mouse and human cells but not for the transformation of human cells, whereas PP2A inhibition is required for their transformation as measured by anchorage-independent growth assay. Moreover, RAS overexpression in mouse cells has an overall smaller impact on transcriptome and proteome regulation as compared to human cells, and PP2A inhibition in human cells further increased the gap between the species in the multi-OMICS level. Further analysis will include validation of the observed OMICS changes and their relevance to transformation using functional experiments. Very importantly, we are also in the process of assessing how signal rewiring changes in humans as compared to mouse cells upon transformation, and how these differential transformation requirements contribute to cancer drug sensitivity.

**Conclusion**

Collective this project may revolutionize our understanding of the species-specific differences in cellular transformation mechanisms and the results also have direct medical implications for understanding cancer therapy responses between species.

### Cancer Genomics

### EACR23-0018
### Integrative multi-omic analysis of malignant pleural mesothelioma PDX library reveals unique pathway alterations and novel therapeutic targets

T. Sen[1]

[1]*Tisch Cancer Institute- Mount Sinai, Oncological Sciences, New York, United States*

**Introduction**

Despite recent treatment advances, malignant pleural mesothelioma (MPM) is an aggressive, recalcitrant malignancy. The histologic subtype (epithelioid/non-epithelioid/biphasic) is the primary prognostic factor; other potential biomarkers to guide therapeutic strategies remain elusive. Even with multimodality therapies, recurrence is high in early-stage diseases. In the unresectable/metastatic setting, there are only two FDA-approved regimens, both in the first-line setting: cisplatin/pemetrexed and ipilimumab/nivolumab. Unfortunately, most who respond to first-line treatment experience disease progression within a year. Therapeutic and diagnostic advances in DPM are hindered by a scarcity of well-annotated preclinical models which can faithfully recapitulate the complex genomic interplay of the disease.

**Material and Methods**

We established a library of patient-derived xenografts (PDX) from patients with DPM. We performed multi-omic analyses on available PDX and patient samples to deconvolute the mutational landscapes, global expression profiles, and molecular subtypes. Targeted next-generation sequencing, immunohistochemistry, and histologic subtyping were performed. RNA sequencing was performed on all available PDX samples. Clinical outcomes and treatment history were annotated for all patients. Platinum-doublet progression-free survival (PFS) was determined from the start of chemotherapy until radiographic/clinical progression and grouped into $<$ or $\geq 6$ months.

**Results and Discussions**

The mutational landscapes of PDX models strongly correlated with paired tumor samples. There were some differences in *CDKN2A/B* mutations and relative enrichment of *NF2* with fewer *BAP1* alterations, the significance of which is being investigated. When compared by histological subtype, we observed an upregulation of genes involved in NOTCH and EMT signaling in the epithelioid models. Models derived from patients with shorter overall survival or poor response to platinum doublet had higher expression of WNT/β-catenin signaling, hedgehog pathway, and epithelial-mesenchymal transition signaling as well as downregulation of immune-activation pathways, including type I and II interferon signaling and inflammatory response pathways.

**Conclusion**

This library of MPM PDXs, the largest to date, effectively mimics human disease and provides unprecedented insight into the genomic, transcriptomic, and protein landscape of MPM. These PDX models will inform future clinical investigations and provide an important new preclinical resource.

### EACR23-0144
### Role of Lead and cadmium exposure risk, as well as CYP1A1 gene mutation, in benign prostatic hyperplasia and prostate cancer: A case-control study

M.K. Singh[1], V. Singh[1], A. Kumar[1]

[1]*King George Medical University, Urology, Lucknow, India*

**Introduction**

The prostatic condition is linked to benign prostatic hyperplasia (BPH) and prostate cancer (CaP). Evidently, dominant transcription factors and signalling pathways characterise their interaction. The etiology of a prostatic disease is multifaceted, including heavy metal toxicity such as lead (Pb), Cadmium (Cd), and hereditary factors. This study investigates the relationship between heavy metal toxicity (Pb, Cd, and CYP1A1 gene polymorphism) in BPH and CaP.

**Material and Methods**

a case-control study with (BPH, n=149), (CaP, n=78) and (controls, n=137) patients. Heavy metal Pb and Cd estimation by atomic absorption spectrophotometer. The polymorphism of the CYP1A1 gene was analyzed by polymerase chain reaction (PCR) followed by restriction fragment length polymorphism method

**Results and Discussions**

Higher levels of Pb and Cd were found in BPH and CaP followed by the control group (p-value: $< 0.05$). Pb and Cd show a significant correlation between prostate volume in CaP and BPH. Additionally, PSA, IPSS score, and pre-void volume were positively co-related with Pb in BPH patients. The posthoc test defines the level of Pb and Cd as

© 2023 The Authors. Molecular Oncology is published by John Wiley & Sons Ltd on behalf of Federation of European Biochemical Societies significantly elevated in the mutant genotype, highest among homozygous mutant genotypes of the CYP1A1 gene among BPH. In CaP, Pb is significantly higher among the homozygous mutant type of the CYP1A1 gene. The risk is also influenced by smoking, tobacco, and alcohol.

**Conclusion**

The heavy metal toxicity Pb and Cd were reported to raise the risk of BPH and CaP. However, a person with heavy metal toxicity especially in BPH has a high-risk genetic susceptibility to the CYP1A1 gene in the north Indian population

### EACR23-0158
### A new scalable tool to enable Full-Length V(D)J Immune Repertoire sequencing for cancer research

S. CHATTERJEE[1], R. Bombardi[2], M. Morikado[2], Q. Han[2], E. Jaeger[2], F. Chen[2], G. Schroth[2], D. Lok[2], S. Wehrkamp-Richter[2]
[1]Illumina, Cancer Research, San Diego, United States
[2]Illumina, Research & Development, San Diego, United States

**Introduction**

The ability of the immune system to protect us from countless pathogens and diseases comes through an evolutionarily refined process that produces a massively diverse repertoire of antigen receptors. The fields of tumor immunology and cancer immunotherapies have recently attracted increased attention. Applications of cancer vaccines, immune checkpoint inhibitors and cellular therapies are getting clinically recognized. Unfortunately, immunotherapy only reaches a minority of patients with specific tumor types. To drive wider adoption of personalized cancer therapies, and monitor genetic responses to immunotherapies, cancer researchers are looking to scale up methods for immune repertoire sequencing (IR-Seq).

**Material and Methods**

The routine use of immune repertoire sequencing in clinical discovery and diagnostic applications has remained elusive due to several challenging biological and technological factors. Our team used multiple commercial kits to generate IR-Seq libraries from human healthy donor samples and sequenced them on the latest 600-cycle flow cells on the Illumina NextSeq 1000/2000 systems.

**Results and Discussions**

Compared to currently available methods, we observed significantly improved base-calling quality of each V(D)J molecule (due to $\geq$ 80% of bases higher than Q30 at 2 $\times$ 300 bp) which resulted in overall higher sample-quality following fastq generation and secondary analysis. Importantly, we observed higher unique molecular identifier (UMI) recovery that ultimately lead to improved clone and clonotype detection.

**Conclusion**

Our research team wanted to challenge current limitations in IR-Seq methods, and in this poster, we describe enhancements to IR-Seq metrics and scalability upon use of the latest P1 and P2 600-cycle flow cells on the NextSeq 1000/2000 systems. Combination of advanced IR-seq with relevant Multi-omic approaches using Illumina as an Universal readout technology may support significant improvements for the Cancer Research and Immunotherapy fields.

### EACR23-0166
### A Robust and Scalable Workflow for Extraction of DNA, RNA, and Protein from FFPE Tissues

A. Garding[1], A. Dhingra[1]
[1]Covaris, Applications, Brighton, United Kingdom

**Introduction**

Confident characterization and monitoring of alterations occurring to DNA, RNA, and proteins during to onset and progression of any disease type is critical for optimizing therapeutic regime. However, inefficient sample preparation methodologies often impede the process of developing a robust protocol that can be applied by researchers regardless of the matrix or analyte complexity. In this study, we report development and optimization of a robust and comprehensive solution that allows reliable and reproducible isolation of DNA, RNA, and proteins from Formalin Fixed Paraffin Embedded (FFPE) tissues, scalable to 96-well format.

**Material and Methods**

This report will showcase the use of Adaptive Focused Acoustic® (AFA®) technology for deparaffinization and rehydration of FFPE tissue without the need of any hazardous, organic solvents enabling multiOMIC analysis from archived samples. We have developed workflows to isolate RNA and DNA from the same sample scalable to 96-well format and integration into lab-automation as well as workflows to characterize the proteome.

**Results and Discussions**

For nucleic acids, a workflow demonstrating isolation of DNA and RNA from the same sample is demonstrated, which is scalable to 96-well format and can be fully automated on liquid handlers starting from the deparaffinization step. Isolation of high-quality RNA and DNA from the same sample enables direct comparison of gene expression to DNA mutations and methylation profiles even from scarce material.

For protein analysis, an efficient and fast protein extraction from FFPE tissues suitable for downstream analyses (such as LC-MS) was developed, which in comparison to fresh frozen input samples demonstrates improved protein solubilization and high recovery for low abundance or difficult to isolate proteins without any technical bias.

**Conclusion**

In summary our workflows for FFPE tissues allow for comprehensive, scalable and reliable analysis of biomolecules from solid tumor material enabling integral understanding of underlying molecular characteristics of diseases.

### EACR23-0237
### Associations between whole genome duplication, MHC-II depletion and survival in high grade serous ovarian cancer

N. Burdett[1,2], M. Willis[1], A. Pandey[1], D. Bowtell[1,2], E. Christie[1,2]
[1]Peter MacCallum Cancer Centre, Cancer Research, Parkville, Australia
[2]The University of Melbourne,

© 2023 The Authors. Molecular Oncology is published by John Wiley & Sons Ltd on behalf of Federation of European Biochemical Societies

Sir Peter MacCallum Department of Oncology, Parkville, Australia

**Introduction**

Whole genome duplication (WGD) is frequently observed in cancer, and its prevalence indicates that it is advantageous to cancer cells. A high frequency of WGD (up to 80% of tumours) in our prior analysis of end-stage, homologous recombination (HR) deficient high grade serous ovarian cancer (HGSC) indicates that WGD provides a fitness advantage under the selection pressure of therapy. Therefore we aimed to identify potential therapeutic vulnerabilities in HGSC with WGD.

**Material and Methods**

Whole genome sequencing and bulk RNA sequencing data for 79 primary HGSC samples from the ICGC study were analysed to evaluate WGD status and to determine differentially expressed genes between WGD and non-WGD samples. Analyses were replicated on 166 HGSC samples from TCGA.

**Results and Discussions**

Across the two cohorts of HGSC samples, less than 100 genes were differentially associated with WGD status. Strikingly, HLA class II genes were significantly downregulated in WGD tumours, as was the Class II Major Histocompatibility Complex Transactivator gene (*CIITA*), which is known as the master-regulator of MHC-II activity. Pathway analysis showed transcriptional downregulation of immune response in samples with WGD, primarily in those with early WGD. IHC confirmed that MHC-II was expressed in tumour as well as adjacent cells of the tumour microenvironment, with the lowest tumour-specific expression occurring in early WGD cancers. Both progression free survival and overall survival were significantly worse in patients whose tumors had early WGD compared to cases with late or no WGD.

**Conclusion**

We demonstrate here novel findings that early WGD is associated with downregulation of *CIITA,* the driver of MHC-II expression, and, crucially, that it is associated with shorter survival in patients with HGSC. Our findings suggest that early WGD is associated with immune evasion, enabling tolerance of the genomic instability associated with WGD. This has not previously been recognised and has implications for therapeutic intervention.

### EACR23-0317

### Predictive modelling of response to neoadjuvant therapy in HER2+ breast cancer

*N. Cosgrove[1], A.J. Eustace[2], P. O'Donovan[1], S.F. Madden[3], B. Moran[4], P.G. Morris[5], L. Grogan[5], O.S. Breathnach[5], C. Power[6], M.J. Allen[6], J.M. Walshe[7], J. Crown[7], A.D. Hill[6], S. Das[8], M. Milewska[9], J. Fay[10], E. Kay[11], S. Toomey[12], B.T. Hennessy[5], S.J. Furney[1]*

*[1]RCSI University of Medicine and Health Sciences, Department of Physiology and Medical Physics, Dublin, Ireland*

*[2]National Institute for Cellular Biotechnology, School of Biotechnology, Dublin, Ireland*

*[3]RCSI University of Medicine and Health Sciences, Data Science Centre, Dublin, Ireland*

*[4]University College Dublin, Conway Institute, Dublin, Ireland*

*[5]Beaumont Hospital, Department of Medical Oncology, Dublin, Ireland*

*[6]RCSI University of Medicine and Health Sciences, Department of Surgery, Dublin, Ireland*

*[7]St Vincent's University Hospital, Department of Medical Oncology, Dublin, Ireland*

*[8]RCSI University of Medicine and Health Sciences, School of Pharmacy and Biomolecular Sciences, Dublin, Ireland*

*[9]Royal College of Surgeons in Ireland, Medical Oncology Group- Department of Molecular Medic ine, Dublin 9, Ireland*

*[10]RCSI University of Medicine and Health Sciences, RCSI Biobank Service- Beaumont Hospital, Dublin, Ireland*

*[11]RCSI University of Medicine and Health Sciences, Department of Pathology- Beaumont Hospital, Dublin, Ireland*

*[12]Royal College of Surgeons in Ireland, Medical Oncology Group- Department of Molecular Medic ine, Dublin, Ireland*

**Introduction**

HER2-positive (HER2+) breast cancer accounts for 20-25% of all breast cancers. Predictive biomarkers of neoadjuvant therapy response are needed to better identify patients with early stage disease who may benefit from alternate treatments in the adjuvant setting.

**Material and Methods**

As part of the TCHL phase-II clinical trial (ICORG10-05/NCT01485926) whole exome DNA sequencing (WXS) was carried out on normal-tumour pairs collected from 22 patients. Tumour samples included 22 pre-treatment, 4 post-treatment cycle one (Day-20) biopsies, 1 surgical resection specimen and 3 metastatic tumours. Recurrent somatic copy number alterations (SCNA), single nucleotide variants (SNVs), InDels, mutational signatures and estimated T cell fraction was identified from analysis of WXS data. Furthermore, tumour evolutionary analysis was carried out for 5 / 22 cases for which high depth WXS was available from samples taken at multiple timepoints during the course of treatment.

**Results and Discussions**

Here, predictive modelling of neoadjuvant therapy response using clinicopathological and genomic features of pre-treatment tumour biopsies identified age, estrogen receptor (ER) status and level of immune cell infiltration are together important for predicting response. Clonal evolution analysis of longitudinally collected tumour samples showed subclonal diversity and dynamics were evident with potential therapy resistant subclones detected.

**Conclusion**

The sources of greater pre-treatment immunogenicity associated with a pathological complete response is largely unexplored in HER2+ tumours. However, here we point to the possibility of APOBEC associated mutagenesis, specifically in the ER-neg/HER2+ subtype as a potential mediator of this immunogenic phenotype.

### EACR23-0332

### Profiling of tamoxifen response modulator genes using genome-wide CRISPR screening

Molecular Oncology 17 (Suppl. 1) (2023) 1–597

© 2023 The Authors. Molecular Oncology is published by John Wiley & Sons Ltd on behalf of Federation of European Biochemical Societies

A.J.W. Lui[1], D. Georgopoulou[1], A. Shah[2], L.V. Nguyen[1], D. Walter[3], S.F. Chin[1], C. Caldas[1]
[1]University of Cambridge, Cancer Research UK Cambridge Institute, Cambridge, United Kingdom
[2]AstraZeneca, AstraZeneca & Cancer Research Horizons Functional Genomics Centre, Cambridge, United Kingdom
[3]Cancer Research Horizons, AstraZeneca & Cancer Research Horizons Functional Genomics Centre, Cambridge, United Kingdom

**Introduction**

Tamoxifen is often used as first-line endocrine therapy for estrogen receptor-positive (ER+) breast cancer. Recognising genetic alterations that confer tamoxifen resistance or sensitivity is of prognostic significance and may provide therapeutic opportunities. Here we sought to identify tamoxifen response modulator genes in MCF7, an ER+ breast cancer cell line, with a genome-wide CRISPR screen.

**Material and Methods**

MCF7 cells stably expressing Cas9 (MCF7 Cas9) were generated by lentiviral transduction of MCF7 cells with lentiCas9-Blast and continuous blasticidin selection. Subsequently, MCF7 Cas9 cells were transduced with a lentiviral genome-wide single-guide RNA (sgRNA) library based on the auto-pick top 3 guides for each gene in the VBC-score database (19,078 genes, 57,521 sgRNAs). Transduced cells were selected by puromycin treatment 3 days after transduction. After 3 days of puromycin selection, cells were treated in duplicate with 4-hydroxytamoxifen (4OHT) or DMSO control and maintained for >12 population doublings. DNA was extracted from cells, and amplicon sequencing of integrated sgRNA sequences performed. MAGeCK and Chronos was utilized to identify hits, genes that exhibited negative or positive selection in response to 4OHT relative to the DMSO control.

**Results and Discussions**

Titration experiments determined the optimal dose of library lentivirus, minimum maintenance culture duration for 12 population doublings, and 4OHT concentration yielding ~30% doubling inhibition. Parameters during screening were within limits; transduction efficiency was 48% while viral library coverage at transduction and maintenance culture was >600, and >1100 cells/sgRNA, respectively. After 18 days maintenance culture, 13 population doublings occurred in the DMSO control arm, with 37% inhibition after 4OHT treatment. Upon sequencing, >250 reads/sgRNA on average per sample was obtained. Hits identified by CRISPR screening included both known and previously unknown genes that modulated response to tamoxifen. Enrichment analysis revealed pathways potentially amenable to therapeutic intervention that can enhance response to endocrine therapy.

**Conclusion**

CRISPR screening represents a valuable tool for identifying novel genes and pathways involved in tamoxifen response, which may have implications for the management of breast cancer patients.

### EACR23-0335
### Mechanisms underlying genomic instability at CTCF/Cohesin Binding Sites in cancers

S. Radhakrishnan[1], E.E. Faseela[1], D. Notani[1]
[1]National Centre for Biological Sciences, Bioinformatics, Bengaluru- Karnataka, India

**Introduction**

CCCTC-binding factor (CTCF) and Cohesin play a major role in the formation of chromatin loops and topologically associating domains (TADs) that controls gene regulation and DNA replication. CTCF/Cohesin binding sites (CBS), which are present at the loop anchors and TAD boundaries, are frequently mutated in cancers. However, the molecular mechanisms underlying this remain unclear. In this study, we propose that the increased somatic mutations observed at CBS could result from replication constraints imposed by the CTCF/Cohesin complex on the DNA, and the consequent activation of error-prone repair pathways.

**Material and Methods**

To study if the CTCF and Cohesin proteins are bound to DNA during replication phase, we used cancer cell line (HeLa) as a model and performed ChIP-seq of CTCF and RAD21 in S-phase. Further, to study the replication stress at these CTCF/Cohesin bound sites in S-phase we performed ChIP-seq of STN1, MRE11, gH2AX and RAD51 proteins, which are involved in sensing of DNA replication stress and repair, under normal and stress conditions (cells treated with HU and etoposide). Finally, to study the impact of genetic perturbations in STN1/MRE11 genes on genomic instability at CTCF/Cohesin bound sites, we used somatic mutations from whole-genome sequencing of cancer samples (from PCAWG study).

**Results and Discussions**

By using chromatin fractionation and ChIP-sequencing, we find that CTCF and Cohesin are bound to the DNA during the S phase in HeLa cells. Examining replication stress by immunostaining proteins associated with replication stress (STN1 and MRE11) and DNA double-strand breaks (gH2AX) revealed that they are colocalized with CTCF/Cohesin in the S phase compared to the asynchronous population of cells. Further, with ChIP-sequencing we assessed the DNA occupancy of the above proteins in the S phase and found that they are highly enriched at CBS as compared to the flanks and unbound sites. Moreover, analysis of somatic mutations from cancer genomes supports that the enrichment of mutations at CBS sites is higher in samples having somatic alterations in STN1 and MRE11 as compared to wild-type samples.

**Conclusion**

Our study reveals that the binding of CTCF/Cohesin on the DNA during the S phase causes replication stress and genome instability. This could explain the increased rate of somatic mutations observed at the CBS sites in multiple cancer types. This result will help to construct the background (null) mutation rate models to predict recurrent driver mutations in regulatory regions.

### EACR23-0348
### Genetic Polymorphism of Pro-Inflammatory Cytokines Interleukin-6, Interleukin-1-β In Non-Muscle Invasive Bladder Cancer

© 2023 The Authors. Molecular Oncology is published by John Wiley & Sons Ltd on behalf of Federation of European Biochemical Societies

A. Kumar[1], M.K. Singh[1], V. Singh[1]
[1]King George's Medical University, Urology, Lucknow, India

**Introduction**

The recurrence and progression of tumour is regulated by the inflammation and potentially oppose the therapeutic response. Thus, this study aimed to target the role of pro-inflammatory cytokines in tumour progression of BCG failure non muscle invasive bladder cancer (NMIBC) patient.

**Material and Methods**

In total, 220 bladder tumour patients were included from August 2020 to August 2022 (BCG failure n= 120 and BCG responsive n= 100) with mean age (years) ± SD 58.11 ±9.47. The patients were histopathological confirmed NMIBC. The BCG treatment consists 6 weekly instillation (induction + maintenance). The cystoscopic examination after 3 and 6 month to assess progression of tumor for the follow -up of BCG failure. Simultaneously blood samples were taken from each patients for genotyping. IL-1β-511 G/A (rs16944), and IL-6-572 C/G (rs1800796), using Taq-Man Probe based real-time polymerase chain reactions.

**Results and Discussions**

Interleukin-1 β -511's A, allele frequency, and Interleukin-6-genotype, 572's G allele frequency, were all substantially greater in BCG failure than in BCG responsive tumours (P <.05). IL-1β-511 AA/GG+AG and IL-6-572 GG/CC+CG genotype expression were shown to be substantially linked with BCG failure and tumour progression (AA/GG+AG; P=.015, GG/CC+CG; P=.048).

**Conclusion**

Identification of these genetic signatures in NMIBC paves the way to monitor disease progression due to BCG failure. Patients with the interleukin-1β-511 AA (rs16944) and IL-6-572 GC (rs1800796) genotype showed a high risk of bladder tumour progression.

### EACR23-0420

### Renal cancer genomes of patients with documented dietary acrylamide intake history harbour the mutational signature of acrylamide/glycidamide

K. Smits[1], F. Virard[2], B. Chavanel[3], M. Zhivagui[4], M.P. Cros[3], M. Churchwell[5], F. Beland[5], M. Korenjak[3], L. Schouten[6], J. Zavadil[3]
[1]Maastricht University, Department of Pathology, Maastricht, The Netherlands
[2]University Claude Bernard Lyon 1 INSERM U1052–CNRS UMR5286,
Cancer Research Center- Centre Léon Bérard, Lyon, France
[3]International Agency for Research on Cancer WHO, Epigenomics and Mechanisms Branch, Lyon, France
[4]University of California San Diego,
Moore's Cancer Center, La Jolla- CA, United States
[5]US Food and Drug Administration National Center for To xicological Research, Division of Biochemical Toxicology, Jefferson- AR, United States
[6]Maastricht University, Department of Epidemiology, Maastricht, The Netherlands

**Introduction**

Acrylamide (ACR) is a probable human carcinogen (IARC Group 2A) present in heated starchy foods and tobacco smoke. However, epidemiological studies proved inconclusive about the association between ACR exposure and cancer formation. We previously identified the experimentally derived mutational signature of glycidamide (GA), the reactive metabolite of ACR, in 30% of ~1,600 ICGC PCAWG tumour genomes from 19 human tumour types from 14 organs (PMID 30846532). Among these, clear-cell renal cell carcinomas (ccRCCs) stood out as substantially enriched for the GA signature (70% of 111 total), consistent with epidemiological studies reporting elevated non-significant risk associated with dietary ACR. Here we aimed to establish a molecular link between ACR intake and ccRCC using whole-genome sequencing (WGS) and mutational signature analysis conducted in a unique human sample set with available dietary ACR exposure information.

**Material and Methods**

Within the prospective Netherlands Cohort Study on Diet and Cancer (NLCS) involving 120,852 subjects of whom 480 developed RCC, we selected 20 never-smokers with ccRCC and a history of high *versus* low (10 cases per group) dietary ACR intake assessed by a food frequency questionnaire reflecting chemical analysis of relevant Dutch foods (PMID 18469268). DNA was isolated from the tumour and adjacent non-tumour FFPE tissue pairs by microdissection. Illumina NovaSeq 6000 was used for WGS analysis of somatic mutations. SigProfilerExtractor tool was used to extract *de novo* mutational signatures and for their decomposition into known COSMIC signatures. Mutational Signature Analysis (MSA) tool was used for optimized computational per-sample assignment of the GA signature alongside the extracted COSMIC signatures.

**Results and Discussions**

The NLCS ccRCC genome analysis identified endogenous COSMIC signatures SBS1, SBS5, SBS40 in all samples, not associating with either of the dietary ACR exposure groups. In contrast, MSA signature assignment showed a relative enrichment of the GA signature in the high ACR exposure (6 of 10 cases), compared to the low exposure patient group (3 of 10 cases).

**Conclusion**

Our results reveal for the first time GA mutagenesis in human renal cancer with documented dietary ACR exposure, indicating potential contributing effects of ACR. Upon confirmation in a larger sample set, these findings may have important implications for cancer prevention aimed at the reduction of human exposure to acrylamide.

### EACR23-0436

### Generation of a robust experimental model for CRISPR screening in muscle invasive bladder cancer

D. Smith[1], S. Lunj[1], A. Adamson[2], S. Nagarajan[3], T. Smith[1], A. Hudson[4], P. Hoskin[1], A. Choudhury[1]
[1]University of Manchester, Division of cancer science, Manchester, United Kingdom
[2]University of Manchester,
Faculty of biology- medicine and health, Manchester, United Kingdom
[3]University of Manchester,
Division of molecular and cellular function, Manchester,

© 2023 The Authors. Molecular Oncology is published by John Wiley & Sons Ltd on behalf of Federation of European Biochemical Societies

*United Kingdom*
*[4]The Christie NHS foundation trust, Urology, Manchester, United Kingdom*

**Introduction**

With 500K cases a year, bladder cancer is the 10th most common cancer worldwide. Chemotherapy resistance is a problem in muscle invasive bladder cancer (MIBC) due to intratumoural hypoxia. Whole genome CRISPR screening (WGCS) is a powerful tool for discovering novel genes involved in therapy resistance but has not yet been applied to MIBC. This research aims to characterise and generate a cell line model for future WGCS experiments studying hypoxia upregulated genes driving chemotherapy resistance in MIBC.

**Material and Methods**

*Cell death assay*: MIBC cell lines (T24, J82 & UMUC3) were seeded in 6-well plates ($2\times10^5$ cells/well) and cell death determined using Annexin V/7AAD flow cytometric staining in at 24, 48 and 72h (n=3).

*Cisplatin dose curves:* T24(n=4), J82 and UMUC3 (n=2) cells were seeded in 6-well plates ($1\times10^5$ cells/well) and treated with cisplatin (0.001µM–100µM). Cell viability was determined at 24, 48 and 72h under normoxic (21% $O_2$) and hypoxic conditions (0.1%, 1% $O_2$).

*Growth curves*: T24 wild type (WT) and T24 cells transduced with a lentiviral Cas9 (T24 Cas9) expression vector were seeded in 6cm plates ($0.6\times10^5$ cells) and incubated under normoxic (21% $O_2$) and hypoxic conditions (0.1% and 1% $O_2$) for 5 days (n=3). Cell counts were taken daily using a countess cell counter and cell viability determined by trypan blue exclusion.

**Results and Discussions**

Rapidly dividing cancer cells can have high cell death rates. Establishing accurate seeding densities accounting for this is essential prior to WGCS. Cell viability of T24 (66.2±2.2) cells was significantly ($p<0.001$) lower than in J82 (90±0.3) and UMUC3 (94.9±0.8) cells at 48h timepoint (unpaired t-test).

T24 cells showed significant ($p<0.05$) cisplatin resistance at 1µM cisplatin (peak plasma concentration in patients) at both 1% (61.5±8) and 0.1% $O_2$ (53.7±3.6) in comparison to normoxia (31.8±5.7). No cisplatin resistance was found in J82 or UMUC3 cells.

Expression of Cas9 had no significant effect on T24 cell growth in both normoxic and hypoxic conditions at all timepoints as determined by growth curves.

**Conclusion**

Three widely used MIBC cell lines were screened, and a robust workflow was developed to characterise the suitability of these cell models for use in future WGCS experiments to study the genetic drivers of cisplatin resistance in MIBC. T24 cells were the most suitable for our study. Ongoing studies include generating a monoclonal T24 Cas9 population and assessment of the cutting efficiency of Cas9.

### EACR23-0438
### High resolution mapping of the breast cancer tumor microenvironment using integrated single cell, spatial and in situ analysis

*K. Halldórsdóttir[1], A. Janesick[2], R. Shelansky[2], F. Wagner[2], M. Rouault[1], G. Beliakoff[2], C.A. Morrison[2], T.Y. Drennon[2], S.E.B. Taylor[2]*
*[1]10x Genomics, Research and development, Stockholm, Sweden*
*[2]10x Genomics, Research and development, Pleasanton, United States*

**Introduction**

The 10x Genomics Chromium platform enables single cell isolation and whole transcriptome profiling while the 10x Visium platform allows spatial whole transcriptome profiling of tissue sections at 55 µm resolution. The novel Xenium In Situ analysis platform enables targeted gene expression analysis at subcellular resolution with high sensitivity and high specificity. One key benefit of Xenium compared to other in situ platforms is that the expression data can be integrated with classical histological stainings, such as H&E and standard immunofluorescence (IF) on the same tissue section. This is particularly useful in dense heterogeneous tissue compartments and tumor microenvironments, maximizing data that can be obtained from the same tissue section.

**Material and Methods**

We profiled serial sections of a FFPE human breast cancer tissue using these three technologies - Xenium In Situ (using a 313-plex probe panel), Chromium (single cell fixed RNA profiling; scFFPE), and Visium (spatial transcriptomics). Chromium and Visium enabled annotation of the cell types, which was further refined by Xenium, by assigning transcripts to segmented cells and spatial cell type cluster analysis. After the Xenium workflow, we performed H&E staining and immunofluorescence on the same section.

**Results and Discussions**

Integration of data sets identified three molecularly distinct tumor subtypes and unique cellular compositions with differentially expressed genes. Because of the high resolution and sensitivity, Xenium identified a small ESR1+, PGR1+, and ERBB2+ region that did not stand out in the Chromium and Visium cell clusters. However, analyzing the corresponding tissue region in the Visium data showed array spots containing those triple positive cells. Analyzing the whole transcriptome data for this group of cells revealed novel differentially expressed genes, illustrating the power of combining all three platforms to retrieve maximum density of information from a tumor sample.

**Conclusion**

In summary, we demonstrate an integrated approach using Xenium In Situ in conjunction with whole transcriptome technologies to derive highly complementary and additive biological information.

### EACR23-0453
### Understanding the causes of head and neck cancer using mutational signature analysis

*L. Torrens[1], S. Moody[2], S. Senkin[1], C. De Carvalho[1], V. Gaborieau[1], B. Abedi-Ardekani[1], J. Atkins[1], P. Chopard[1], J. Wang[2], S. Fitzgerald[2], S. Cheema[2], T. Cattiaux[1], M. Diaz-Gay[3], E.N. Bergstrom[3], A. Khandekar[3], L. Humphreys[2], L.B. Alexandrov[3], M.R. Stratton[2], S. Perdomo[1], P. Brennan[1]*
*[1]International Agency for Research on Cancer IARC/WHO*

© 2023 The Authors. Molecular Oncology is published by John Wiley & Sons Ltd on behalf of Federation of European Biochemical Societies

, Genomic Epidemiology Branch, Lyon, France
[2]Wellcome Sanger Institute - Wellcome Genome Campus, Cancer Ageing and Somatic Mutation, Cambridge, United Kingdom
[3]UC San Diego Health, Moores Cancer Centre, La Jolla, United States

**Introduction**

The known causes of head and neck cancer (HNC) include smoking, alcohol, and human papillomavirus (HPV). Tobacco use, alone or combined with alcohol, accounts for most of the disease burden, while alcohol alone is responsible for only 4% of the cases, suggesting a limited impact of alcohol consumption among never smokers. This raises the question of whether alcohol is a carcinogen in the absence of tobacco. In addition, a subset of HNC cases has no history of exposure to risk factors. In this study, we used a mutational signature approach to 1) track the exposure to known and putative risk factors for HNC across multiple geographical regions and subsites, 2) evaluate whether an alcohol signature is present in HNC, and 3) assess its interaction with smoking or HPV infection.

**Material and Methods**

Whole-genome sequence data was generated from 265 HNC cases from retrospective HNC studies in Europe and South America with available lifestyle and environmental exposure data. Single-base substitution (SBS), double-base substitution (DBS), and insertion-deletion (ID) signatures were extracted with SigProfilerExtractor and decomposed into COSMIC reference signatures. Associations between the mutational signatures, epidemiological data, and germline variants were assessed using multivariate regression analysis.

**Results and Discussions**

We extracted 18 SBS, 4 DBS, and 12 ID COSMIC signatures, as well as 3 novel non-decomposable signatures (SBS1536I, SBS1536L, and DBS78D). APOBEC-driven signatures (SBS2, SBS13) were identified in 97% (258/265) of cases. We observed differences in the tobacco-related signatures across anatomical subsites. While known tobacco signatures (SBS4, SBS92, DBS2, and ID3) were found only in larynx cases, the novel SBS1536I signature was enriched in samples from smoker patients in all subsites (all $p<0.05$). SBS16, DBS4, and ID11 signatures were associated with alcohol consumption, with higher burdens among ever drinkers-ever smokers compared to ever drinkers-never smokers (all $p<1e-5$). UV-related signatures (SBS7a-c, DBS1, and ID13) were detected mostly in oral cavity cases from anatomical sites other than the external lip, suggesting a potential role of UV exposure in HNC carcinogenesis.

**Conclusion**

Mutational signature analysis points towards APOBEC signaling, tobacco, and alcohol as strong mutagenic agents in HNC. Our study provides molecular evidence suggesting that tobacco could enhance the oncogenic effects of alcohol and that UV exposure may act as a putative risk factor for oral cavity cancers.

### EACR23-0458
### miRNA-mRNA integrative analysis of carboplatin-resistant ovarian cancer cells with acquired mesenchymal-like phenotype reveals miR-103a-3p and miR-107 with a role in migration and invasion

M. Pernar Kovač[1], J. Kralj[1], V. Tadić[1], S. Orešković[2], M. Milković Periša[3], V. Banović[2], I. Babić[2], W. Zhang[4], Z. Culig[5], A. Brozovic[1]
[1]Ruđer Bošković Institute, Molecular biology, Zagreb, Croatia
[2]University Hospital Center Zagreb, Department of Obstetrics and Gynecology, Zagreb, Croatia
[3]University Hospital Center Zagreb, Department of Pathology and Cytology, Zagreb, Croatia
[4]Dalian University of Technology, Department of Engineering Mechanics, Dalian, China
[5]Medical University of Innsbruck, Department of Urology, Innsbruck, Austria

**Introduction**

Ovarian cancer (OC) has the highest death rate among malignancies that affect the female reproductive tract. Epithelial-mesenchymal transition (EMT), a process involved in metastasis, is described as an important factor in tumor spreading and failure of the therapy. Epigenetic signatures, such as DNA methylation, histone modifications, and microRNAs (miRNAs) affect OC progression and response to therapy.

**Material and Methods**

Epithelial ovarian cancer cell lines MES-OV, OVCAR-3, SK-OV-3 and their previously established and characterized carboplatin-resistant variants MES-OV CBP, OVCAR-3 CBP and SK-OV-3 CBP, with acquired mesenchymal-like phenotype (AMP), were used. MiRNA profiling was performed on MES-OV and MES-OV CBP cells. Epigenetic modulation, RT-qPCR validation and additional two cell pairs were used for the selection of miRNAs of interest. The integration of miRNA-predicted target genes and differentially expressed genes, pathway analysis using DAVID, and functional analysis (scratch assay for migration and Matrigel-coated trans-well inserts for invasion analysis) were used for forecasting their biological role. Finally, data mining was performed to determine their possible prognostic and predictive values.**Results and Discussions**

MiRNA profiling resulted in 29 up- and 48 downregulated miRNAs in MES-OV CBP cells with CBP-triggered AMP. Epigenetic modulations impacted AMP cells' drug sensitivity and decreased their migratory and invasive capacity. Of fourteen selected miRNAs, nine were validated as changed, and seven of these restored their expression upon treatment with epigenetic inhibitors. Only three miRNAs had similar expression patterns in additional OC cell lines. MiRNA-mRNA integrative analysis resulted in 56 upregulated target genes. Pathway analysis revealed that deregulated target genes are involved in Ras and ErbB signaling pathways, connected to cell adhesion, migration, and invasion. The functional analysis confirmed the role of miRNA-103a-3p and miRNA-107 in cell invasion, while data mining showed their prognostic and predictive values.

**Conclusion**

In summary, high throughput miRNA and cDNA profiling coupled with pathway analysis and data mining delivered evidence for miRNAs which can be epigenetically regulated in AMP OC cells as possible markers to combat

© 2023 The Authors. Molecular Oncology is published by John Wiley & Sons Ltd on behalf of Federation of European Biochemical Societies therapy-induced short overall survival, CBP-resistance and tumor metastatic potential.

### EACR23-0476
### Single cell genome and targeted proteome profiling

A. Alyasin[1], C. Botteron[2], C.A. Klein[1], Z.T. Czyż[1]
[1]Experimental Medicine and Therapy Research, University of Regensburg, Regensburg, Germany
[2]Division of Personalized Tumor Therapy, Fraunhofer ITEM-R, Regensburg, Germany

**Introduction**

The simultaneous genome and proteome analysis of very rare single cells would enable addressing important biological and diagnostic questions particularly in cancer. Here we introduce a novel genome and proteome (G&P) analytic workflow, a method allowing comprehensive analysis of single-cell DNA and targeted profiling of protein expression in single cells. As a proof of concept, we demonstrate the capability of the method by quantifying copy number of the ERBB2 gene alongside with protein expression levels of HER2 and phospho-HER2 in single cells.

**Material and Methods**

The G&P workflow utilizes antibodies conjugated with DNA oligonucleotides designed to enable their amplification alongside the single-cell genome subsequent quantification by either quantitative PCR (qPCR) or sequencing. We utilized antibodies targeting cytokeratin 8/18/19, HER2 and phospho-HER (Tyr1248). Performance of each DNA-conjugated antibody was assessed by immunofluorescence staining in a variety of breast cancer cell lines (MDA-MB231, MDA-MB453, BT474 and MCF7) and sample types. Stained cells were isolated by manual micromanipulation and subjected to whole genome amplification (WGA). Protein expression levels, derived from quantification of the amplified DNA tags, were measured by qPCR. Shallow whole genome sequencing was employed to facilitate genome-wide profiling of copy number variations (CNVs) and assess copy number of the ERBB2 locus.

**Results and Discussions**

We successfully developed and tested a G&P workflow allowing parallel genome-wide CNV profiling and quantification of three target proteins. The G&P protocols were optimized to decrease the non-specific background signal as well as to normalize the quantification of protein expression levels. We validated our workflow by analyzing cells derived from cell lines exhibiting distinct levels of HER2 and phospho-HER2 expression as well as different DNA copy numbers of the ERBB2 gene.

**Conclusion**

Our novel, multiplex G&P technique is capable of generating multiparametric measurement linking DNA copy numbers and expression levels of selected genes of interest. The method is optimized for analysis of single cells from rare cell populations and is readily translatable to analysis of circulating tumor cells (CTCs) and disseminated cancer cells (DCCs).

### EACR23-0489
### Rapid whole-slide spatial analysis of FFPE tissues with true multiomic panels enables the discovery of key cellular niches

L. Hernandez[1], J. Kennedy-Darling[2], W. Agnew-Svoboda[2], S. Pathare[2], S. Trivedi[2], A. Gamboa-Aldeco[2], C. Reid[2], C. Davies[2], S. Bommakanti[2], N. Le[2], J. Eng[2]
[1]Akoya Biosciences, R&D, Menlo Park, United States
[2]Akoya Biosciences, Research and development, Menlo Park, United States

**Introduction**

Spatial biology analyses are critical to understanding key cellular interactions. While there have been tremendous advancements in spatial technologies, there was always a trade-off between plex, resolution, and speed. Akoya's PhenoCycler®-Fusion spatial biology platform solves this tradeoff by enabling rapid spatial phenotyping of whole FFPE slides using protein targets, RNA targets, or a combination of both. The PhenoCycler-Fusion utilizes high-speed whole-slide imaging at single-cell resolution, proprietary data compression algorithms, and an ecosystem of software partners to streamline various analysis needs.

**Material and Methods**

Akoya's PhenoCycler®-Fusion was used to detect RNA and protein targets in whole FFPE slides of human cancer cell pellet arrays and tissue multi-arrays. RNA and protein markers were detected both individually on serial tissue sections and simultaneously in single tissue sections.

**Results and Discussions**

This workflow is inherently unbiased, and simultaneous detection of RNA and protein provides a comprehensive view of tumor biology. Here, we demonstrate the robustness and scalability of our true multiomics chemistry by spatial phenotyping 46 samples, 23 tissue types, and more than a million cells. Protein and RNA biomarkers play complementary roles in defining cell phenotypes and cell states, respectively, in a tissue sample. Proteins are the key effectors of cell function and detecting cell surface protein markers can serve as a ground truth for cell identity. RNA markers such as cytokines and chemokines can provide an insight into signaling pathways that result in changes in cell states, arising from interactions within the tumor microenvironment. Measuring both analytes within the same tissue can give researchers an accurate picture of tumor progression and response to therapy. The high-throughput capability of the platform, coupled with data compression makes analyzing whole slide sections routine and opens the door to cohort analysis for spatial phenotyping. By detecting 100+ targets and mapping every cell, new tissue structures and functions were discovered previously undetected by sequencing methods and traditional IHC readouts.

**Conclusion**

The uniqueness of the PhenoCycler chemistry to detect both RNA and protein targets simultaneously on a tissue section means critical questions related to signaling pathways, immune infiltration, and tumor environment can be answered directly using the optimal target panel.

### EACR23-0535
### Single-cell transcriptomic analysis of gingivo-buccal oral cancer exhibits heterogeneous cell states and gene expression programs in tumor ecosystem

© 2023 The Authors. Molecular Oncology is published by John Wiley & Sons Ltd on behalf of Federation of European Biochemical Societies

S. Roy[1], S. Kurkalang[1], A. Acharya[1], S. Mazumder[1], P. Mazumder[2], S. Patra[1], S. Ghosh[1], S. Sarkar[1], N.K. Biswas[1], S. Ghose[2], P.P. Majumder[1], A. Maitra[1]
[1]National Institute of Biomedical Genomics, nibmg, Kalyani, India
[2]Dr. R. Ahmed Dental College and Hospital, Oral Pathology, Kolkata, India

**Introduction**

Globally, oral squamous cell carcinoma (OSCC) is one of the most common cancers, with an annual incidence of > 3M. In India, OSCC of the gingivo-buccal region (OSCC-GB) is the most prevalent form of cancer among men and sixth most prevalent among females. It is largely associated with tobacco chewing, presentation at advanced stages, and a high rate of loco-regional metastasis and treatment failure. Heterogeneity of OSCC-GB tumor cells is a key determinant for the progression, recurrence, and overall survival. We undertook this study to profile the landscape of cellular diversity in OSCC-GB tumors using single-cell RNA sequencing (scRNA-seq), and obtained novel information on the heterogeneity of cell types and cell states in the tumor ecosystem.

**Material and Methods**

Twelve freshly resected, treatment-naïve OSCC-GB tumor biopsies were minced and dissociated by enzymatic digestion. Cell suspension with ≥75% viable cells were then processed for single cell 3' gene expression analysis. Clustering of cells, malignant cell identification, and gene set enrichment analysis were undertaken after processing the raw data.

**Results and Discussions**

Non-malignant cells were found clustered by cell type whereas patient-specific clustering of malignant cells was observed. Malignant cells exhibited two dominant cellular programs – fetal cellular recapitulation (enrichment with fetal cell-type signatures) and partial epithelial-mesenchymal transition (pEMT). Cells belonging to fetal cellular and pEMT programs were also enriched with expression of immune-related pathway genes known to be involved in the anti-tumor immune response. Cancer testis antigens and cancer cell-derived immunoglobulin genes were significantly enriched in tumors with OSMF background. We observed a higher infiltration of immune cells than stromal cells in contrast to other head and neck cancers. We detected double negative T cells, TCR positive macrophages and cells with intermediate M1-M2 macrophage polarization. We also observed a phenotypic shift of fibroblast population, in oral tumors associated with OSMF.

**Conclusion**

In summary, OSMF-associated OSCC-GB tumors exhibit an expression profile distinct from tumors not associated with OSMF. Fetal cellular reprogramming and pEMT appear to play major roles in OSCC-GB tumorigenesis. Thus, our study provides the first set of observations on the nature and extent of the diversity of cell types and gene expression states in the tumor ecosystem of OSCC-GB.

### EACR23-0544
### Multiomic Spatial Phenotyping of Head and Neck Cancers Reveals Distinct Immune and Cytokine Signatures in the Tumor Immune Microenvironment

H. Ji[1], N. Jhaveri[1], J. Yuan[2], A. Dikshit[3], E. Doolittle[4], S. Zhou[4], M. Srinivasan[4], D. Mason[5], F. Schneider[6], J. Mansfield[5], A. Kulasinghe[7], O. Braubach[1]
[1]Akoya Biosciences, Discovery Applications, Menlo Park, United States
[2]Akoya Biosciences, Research and Development, Menlo Park, United States
[3]Advanced Cell Diagnostics, Spatial Biology Applications, Newark, United States
[4]Advanced Cell Diagnostics, Research and Development, Newark, United States
[5]Visiopharm, Research Business Development, Horsholm, Denmark
[6]Visiopharm, Product Management, Horsholm, Denmark
[7]The University of Queensland, Frazer Institute- Faculty of Medicine, Brisbane, Australia

**Introduction**

The identification and evaluation of predictive markers for cancer immunotherapies requires a deep understanding of the factors that shape the tumor immune microenvironment (TiME). Cytokines, in particular, play an important role in modulating the proliferation and differentiation of immune cells to elicit immune responses. Here, we used a multiomic spatial biology approach that utilizes whole-slide spatial phenotyping using the PhenoCycler®-Fusion paired with RNAscope® ISH technology to study cellular and cytokine spatial signatures within the TiME. This two-step approach is compatible with human FFPE tissues and enables researchers to characterize the spatial biology of the TiME more accurately by simultaneously assessing protein and RNA markers on the same tissue.

**Material and Methods**

We performed ultrahigh-plex spatial phenotyping on the PhenoCycler®-Fusion on FFPE head and neck cancer tissue sections, using a >60-plex PhenoCode™ Discovery Panel designed for deep immune cell phenotyping. Using serial sections from the same tissue blocks, we then ran the automated RNAscope® HiPlex v2 assay on the PhenoCycler®-Fusion system. The latter consisted of 12-plex immuno-oncology panel of RNA target probes, which were selected to detect chemokines, cytokines, and key immune cell lineages. We used Phenoplex™ software to analyze the protein and RNA datasets and to compute cell phenotypes and spatial associations.

**Results and Discussions**

Ultrahigh-plex spatial phenotyping revealed unique cell phenotype compositions within the TiME of head and neck cancer tissues. Subsequently, the addition of RNA detection on serial sections identified spatial signatures that implicate cytokine expressions and immune phenotypes involved in both tumor progression and regression. We have identified areas of high and low CXCL9 and CXCL10 expression in several tumor regions that reflect immune-cell infiltration landscapes associated with resistance and sensitivity to immunotherapy.

**Conclusion**

The multiomic spatial phenotyping demonstrated in this study provides a more complete landscape of immune cells and their interaction with cytokines within the TiME, thereby enriching our understanding of tumor biology underpinning immunotherapy responsiveness and resistance.

© 2023 The Authors. Molecular Oncology is published by John Wiley & Sons Ltd on behalf of Federation of European Biochemical Societies

### EACR23-0581
### Spatial characterization of the epithelial-to-mesenchymal transition within the breast tumour microenvironment

E. Withnell[1], M. Secrier[1]
[1]UCL Genetics Institute,
Department of Genetics- Evolution and Environment,
London, United Kingdom

**Introduction**

The epithelial to mesenchymal transition (EMT) is a cellular process in which polarized epithelial cells undergo multiple molecular and biochemical changes, and lose their identity in order to acquire a mesenchymal phenotype. The EMT is not a binary process; instead, multiple hybrid EMT states underlie and drive the transition from epithelial to mesenchymal states. How EMT arises spatially within the tumour and how this, in turn, shapes the tumour microenvironment (TME) architecture is poorly understood, but could have great relevance to cancer progression and treatment.

**Material and Methods**

Using NMF and transfer learning approaches, we mapped 32,845 breast cancer 10X Genomic Visium spatial transcriptomic spots onto an EMT "timeline" quantified from single cell RNA-seq data across multiple stimuli. This allowed us to estimate the EMT transformation level within a spatial transcriptomic slide. We created networks from the spatial transcriptomic spots, with nodes labeled as the cell types present (as estimated using cellular deconvolution techniques). Using these networks, we analysed cell types and spatial structures associated with different states. We developed a graph neural network classifier to determine the extent to which EMT states are a function of their microenvironment.

**Results and Discussions**

We captured six discrete EMT states in multiple scRNA-seq datasets measuring EMT response, including an epithelial, four intermediate and a mesenchymal state. We projected these states onto the spatial transcriptomic datasets and observed specific enrichment of surrounding cells in the TME depending on the EMT state of cancer cell spots. We found that tumour cells occupying a quasi-mesenchymal intermediate state form the highest number of interactions with other cells in their microenvironment, most notably with CAFs ($p<0.001$) and CD4+ and CD8+ T-cells ($p<0.001$). We further employ graph neural networks to show that we can predict EMT states based on the spatial structure surrounding the cell, and can use this method to understand the importance of different microenvironment components on the EMT.

**Conclusion**

We uncover TME components that are dictated by the EMT state of surrounding cancer cells, with a quasi-mesenchymal intermediate state appearing most immunogenic and potentially most easily targetable via checkpoint inhibition approaches. We further show we can quantify the extent to which different EMT states are a function of their microenvironment.

### EACR23-0671
### Spatial single cell mapping of human solid tumors using high-plex in situ gene expression.

M. Faria de Oliveira[1], A. Kim[2], F. Wagner[3], S. Mohabbat[2], R. Shelansky[3], V. Gonzalez Munoz[2], S. Taylor[1]
[1]10x Genomics, Applications, Pleasanton, United States
[2]10x Genomics, Cell Biology, Pleasanton, United States
[3]10x Genomics, Computational Biology, Pleasanton, United States

**Introduction**

Cancer is a complex biological system of individually distinct pathologies with extensive intratumor and cross-patient heterogeneity. High-plex technologies that profile single cell gene expression within a whole tissue can revolutionize the study of tumor biology and pathology. Here, we used the Xenium Analyzer ® (10x Genomics) to map in situ gene expression of large formalin-fixed, paraffin-embedded (FFPE) solid cancer samples.

**Material and Methods**

Xenium human tissue-specific gene panels were designed (breast: 280; brain: 260; lung: 290 target genes). Coronal FFPE serial sections (5 µm in duplicates) of breast, glioblastoma, and lung cancer samples were placed on 12 x 24 mm Xenium slides for deparaffinization and de-crosslinking. Tissue-specific DNA probes were hybridized to mRNA. Each probe has two target binding regions and a gene-specific barcode that identifies the target transcript. The target binding regions bind and ligate to form a circular DNA probe, which is then amplified. On the Xenium instrument, fluorescently labeled detection probes were hybridized to the amplification products to generate a unique optical signature for each target gene. An on-instrument analysis pipeline decoded the fluorescent signals, segmented cell boundaries and assigned transcripts to cells for downstream visualization in Xenium Explorer ®.

**Results and Discussions**

We obtained three tissue-specific Xenium datasets for breast, glioblastoma, and lung cancer samples (average of 130 transcripts/cell and 300,000+ cells per sample), with transcript counts that were highly correlated between duplicates (R value < 0.95). We annotated and mapped cell composition in each tissue type at sub-cellular resolution, including immune cells' distribution in the TME of tumor lesions. Due to the non-destructive nature of the Xenium assay, H&E staining was performed on the same sections post-Xenium workflow, allowing us to register RNA-based information to the conventional histology.

**Conclusion**

The Xenium in situ assay is high-plex, robust and pinpoints single cell and sub-cellular spatial information from large FFPE tissues. The availability of tissue-specific gene panels allowed us to accurately resolve and map cell composition, which is a powerful tool to study TME in different tissue types. As Xenium enables the addition of up to 100 genes to the tissue-specific gene panels, future studies can interrogate specific markers and pathways to enable researchers to gain a deeper understanding of tumor biology.

### EACR23-0703
### Molecular Genetic Aspects of Gliomas

G. Abildinova[1], B.B. Zhetpisbaev[2], A. Borovikova[1], M. Solomadin[3], J. Zhabakova[1], A. Esentayeva[1],

© 2023 The Authors. Molecular Oncology is published by John Wiley & Sons Ltd on behalf of Federation of European Biochemical Societies

A. Rakhimova [1]
[1]Hospital of the Medical Centre of the Presidential Admini stration, Genetic Laboratory, Astana, Kazakhstan
[2]JSC National Centre for Neurosurgery Republic of Kazak hstan- Astana, Surgery, Astana, Kazakhstan
[3]Karaganda Medical University Karaganda- Republic of K azakhstan, School of Pharmacy, Karaganda, Kazakhstan

**Introduction**

Gliomas are the most common invasive primary brain tumours and represent a heterogeneous group of tumours of neuroepithelial origin. According to WHO guidelines (2021), the classification of gliomas should consider not only the histological features of the tumor cells, but also reflect the molecular features of glia cells for diagnosis of tumor subtypes, prescription of chemotherapy and radiotherapy.

**Purpose of study**: To study the mutational status of neuroglia tumour cells to clarify tumour type.

**Material and Methods**

Tumour tissue samples fixed in formalin and embedded in paraffin blocks were used for molecular genetic study. A commercial FFPE DNA PowerPlex Fusion System kit (USA) was used for DNA isolation according to the manufacturer's instructions. A mixture of P088, P105, ME012 probes (MRC Holland, Amsterdam, The Netherlands) was used for MLPA method according to the manufacturer's protocol.

**Results and Discussions**

Seventy patients who underwent surgery at the National Centre for Neurosurgery were included in the study. There were 34 males and 35 females. In 4% of cases the study was not carried out due to low DNA concentration.
The IDH1/IDH2 mutation was detected in 40 cases, of which 58% had IDH1/IDH2 mutations with a 1p/19q co-deletion, and 1 case had an IDH2 mutation identified. IDH1/IDH2 mutation was identified in 42% of neuroglial tumours of different localisation.
Pilocytic astrocytoma was confirmed in 12 cases, of which a BRAV600E point mutation was found in 25% of cases and a KIAA1549 BRAF mutation in 75%.
Homozygous deletion of the CDKN2A/B gene in astrocytomas is important for the prognosis of the disease; the mutation has been diagnosed in 10 cases, and in one case the deletion was combined with a 1p/19q co-deletion. EGFR mutation was identified, with 2 cases of deletion and 3 cases of duplication. The EGFR gene deletion was combined with both a homozygous CDKN2A/B deletion and a CDKN2A/B duplication.
Hypermethylation in the MGMT promoter region was studied in 22 patients, with rates ranging from 8.6% to 85.6%.

**Conclusion**

The results show that molecular profiling of tumour cells is essential for proper diagnosis of tumour subtype, mutations are prognostic markers and predictors of response when prescribing targeted therapy.

### EACR23-0704
### A comprehensive single-cell map of Inflammatory Breast Cancer

P. Schwerd-Kleine[1], T. Cheytan[1], R. Würth[1], L. Michel[2], V. Thewes[3], P. Lichter[3], A. Schneeweiss[2], A. Trumpp[1], M. Sprick[1]
[1]German Cancer Research Center DKFZ Heidelberg Instit ute for Stem Cell Technology and Experimental Medicine HI-STEM gGmbH, Division of Stem Cells and Cancer, Heidelberg, Germany
[2]National Center for Tumor Diseases NCT, Division of Gynecological Oncology, Heidelberg, Germany
[3]German Cancer Research Center DKFZ- National Center for Tumor Diseases NCT, Division of Molecular Genetics-, Heidelberg, Germany

**Introduction**

Inflammatory breast cancer (IBC) has been emerging as a clinically distinct breast cancer entity. Affected patients face worse prognosis, with fast progression and frequent therapy resistance. Even though IBC is rare (2-4% of breast cancer cases), it accounts for a disproportionately high number of deaths (7-10% of breast cancer mortality). Despite the apparent clinical need, molecular understanding of the disease is still missing to date. Previous attempts of identifying an IBC signature through transcriptomic and genetic studies have not been successful. Though some evidence has emerged indicating a key role for the microenvironment, and in particular lymphatic vessel colonisation and reciprocal growth stimulation, in promoting the aggressiveness and invasive potential of the disease.
We thus analysed a unique collection of IBC samples at single-cell level, to further elucidate the biology with focus on the microenvironmental factors driving this severe condition.

**Material and Methods**

We collected tissue samples from 10 patients with IBC, and subtype-matched non-IBC breast cancer samples. Upon receipt, we digested the samples, to obtain single-cell suspensions using a protocol optimised for hard-to-digest cell types. Subsequently we ran single-cell transcriptomic profiling (Chromium 3' v3.1, 10X) and analysed the resulting data using various computational methods, including Seurat, differential expression analysis, gene set enrichment, Liana interaction meta-analysis, cell2cell, pseudotime, RNA velocity and others.

**Results and Discussions**

We created a comprehensive single-cell map of inflammatory breast cancer and its microenvironment. Overall, we find a similar distribution of major cell lineages between the IBC and non-IBC samples. However, there are unique gene programmes active in IBC. Interestingly, we find significantly different cellular crosstalk, involving the myeloid compartment and the endothelium of IBC, including heightened TGF-β signalling and angiogenic cues.

**Conclusion**

Our dataset is the first of its kind, and allows us to analyse the microenvironment of inflammatory breast cancer at a previously unattained depth. It is a valuable resource and adds another piece to the unsolved puzzle of IBC biology.

### EACR23-0711
### Multigenic testing of somatic mutations in solid tumor cells

G. Abildinova[1], A. Borovikova[1], A. Rakhimova [1], M. Solomadin[2], A. Yessentayeva[1], J. Zhabakova[1]
[1]Hospital of the Medical Centre of the Presidential Admini stration, Genetic Laboratory, Astana, Kazakhstan

© 2023 The Authors. Molecular Oncology is published by John Wiley & Sons Ltd on behalf of Federation of European Biochemical Societies

[2]Me,
Karaganda Medical University Karaganda- Republic of Kazakhstan, Karaganda, Kazakhstan

**Introduction**

Currently, the treatment of cancer patients is based on personalized selection of drugs depending on the molecular profiling of tumor cells. The study of the mutational status of a tumor is aimed at establishing the risk of developing a tumor, clarifying the prognosis of the disease, and predicting the response to treatment.
The aim of the study was to search for mutations in the tumor tissues fixed with paraffin in order to optimize the prescription of targeted treatment.

**Material and Methods**

Material and methods. The material of the study was tumor cells fixed with formalin and embedded in paraffin. DNA and RNA were extracted from tumor cells using the RecoverAll™ Multi-Sample RNA/DNA kit (Termofisher Scientific, USA) according to the manufacturer's instructions. The method: Targeted semiconductor sequencing (Ion Gene Studio S5 Plus (Termofisher Scientific, USA). Preparation of the DNA and RNA library followed by sequencing of the amplified fragments was carried out in the Ion PGM™ system according to the manufacturer's protocol. Bioinformatic analysis of the results was carried out using the Ion Reporter™ software Software, Oncomine™ Focus Assay Module.

**Results and Discussions**

Research results. The study group consisted of 147 patients, in 33% of cases with solid tumors of various localization, genome changes were detected and 114 mutations were identified. In 75% of cases, missense mutations [BRAF (Val600Glu); KRAS (Gly12Val); IDH1(R132H); JAK3 (S493C); PIK3CA (E545Q; E542K; p.(H1047L)] - point mutations in the DNA nucleotide sequence. In 6 patients, a combination of 2 or more mutations [PIK3CA (E547K), FGFR3(G697S); PIK3CA (E542K), KRAS (Gly12Val) ; EGFR(A289T), JAK3(S493C); BRAF (G469V), KRAS (Gly12Val); TP53, FGFR4, CREBBP, SMARCA4]. In 10% of cases, an increase in the number of copies of the EGFR and KRAS genes was found, and in 10% of cases, a fusion gene (RNA) MET- MET.M13M15, TMPRSS2-ERG.
The identified genome changes were processed by the server through the FDA, NCCN, EMA, ESMO registries to search for the most appropriate therapy options that exist in world practice for a specific type and localization of the established mutation.

**Conclusion**

Conclusion. For personalized prescription of targeted drugs, it is more efficient to use multigene diagnostics for a comprehensive study of the mutational status of a tumor.

### EACR23-0718
### RNA-seq for the optimization of pediatric medulloblastoma classification

*I. Martinez de Estibariz[1], M. García-Ariza[2], L. Zaldumbide[3], L. Mosteiro[3], L. Alaña[4], I. Astigarraga[4], L.F. Callado[5], Á. Gutiérrez-Camino[4], D. Sinnett[6], I. Martin-Guerrero[1], N. Bilbao-Aldaiturriaga[4]*
*[1]University of the Basque Country, Department of Genetics- Physical Anthropology and Animal Physiology, Leioa, Spain*
*[2]Biocruces Bizkaia Institute/University of the Basque Country, Pediatric Oncology Group, Barakaldo, Spain*
*[3]Hospital Universitario de Cruces, Pathology, Barakaldo, Spain*
*[4]Biocruces Bizkaia Institute/University of the Basque Country, Pediatric Oncology Group, Leioa, Spain*
*[5]University of the Basque Country, Pharmacology, Leioa, Spain*
*[6]CHU Sainte-Justine Research Center, Hematology-Oncology, Montreal, Canada*

**Introduction**

Medulloblastoma (MB) is an aggressive brain tumor accounting for 30% of all malignant central nervous system (CNS) cancers in childhood. A multiomic point of view has allowed the classification of MB into four different molecular subtypes: WNT-MB, Sonic hedgehog (SHH)-MB (*TP53* mutated or wild-type) and non-WNT/SHH Group 3 (G3) and Group 4 (G4) MB. Nevertheless, some studies suggest that this classification does not account for existing heterogeneity. In this context, RNA-seq technology is gaining relevance as a powerful tool to unravel this complexity. This work aimed to characterize a retrospective series of Spanish MB samples to assess the capacity of RNA-seq to classify MB patients compared with *immunohistochemistry (IHC)* and methylation profile-based classifications.

**Material and Methods**

The study population included 55 pediatric patients (age<16 years at diagnosis) and 5 pediatric cerebellum donnors recruited from different national hospitals. Patients were classified based on their IHC features, methylation status (MS-MIMIC profiling), and transcriptomic profiling (total RNA, paired-end sequencing on a NovaSeq 6000 System, Illumina) at the Integrated Center for Pediatric Clinical Genomics (Montreal, Canada). After alignment (STAR) and quantification (featureCounts), bioinformatic study included differential expression (DESEeq2) and variant calling (VarScan and HaplotypeCaller) analyses.

**Results and Discussions**

RNA-seq analysis showed that 69% of the patients matched the classification provided by IHC and/or methylation status, while one patient previously classified as G4 was re-assigned to WNT subtype. Moreover, 84,6% of the unclassified or uncertainly classified patients were accurately grouped into the G3 or G4 subtypes. Only 9% of the patients presented uncertain classification using RNA-seq expression profiling. Variant calling analysis revealed 52 relevant pathogenic variants from which 10 had been already associated with MB. Indeed, all the variants detected in clinic previously were validated in RNAseq analysis. Interestingly, variants in *KBTBD4*, *KDM6A* and *KMT2C* were specific to G3 and G4 while variants in *HYDIN*, *RECQL4* and *SDHA* genes were present exclusively in G4 patients.

**Conclusion**

These results highlight that RNA-seq technology may be useful for adding resolution to the MB classification at diagnosis.

Grant references: This work was funded by Asociación Pablo Ugarte APU (BC/A/14/015), Pequerropa (BC/A/15/010), and the projects from EITB Media (BIO20/CI/013BCB and BIO20/CI/15BCB)

© 2023 The Authors. Molecular Oncology is published by John Wiley & Sons Ltd on behalf of Federation of European Biochemical Societies

### EACR23-0722
### Phosphodiesterase 4D inhibition as a mechanism to suppress GNAS-mutated colorectal cancers

S. Zafar[1], P. Nummela[2], E. Veikkolainen[1], V. Cinella[1], A. Ayo[3], M.Y. Asghar[4], I. Ukkola[5], N. Välimäki[2], E. Naakka[6], K. Törnquist[7], A. Karhu[8], P. Laakkonen[3], T. Salo[6], L.A. Aaltonen[1], A. Ristimäki[1]
*[1]University of Helsinki, Applied Tumor Genomics Research Program- Research Pr ograms Unit, Helsinki, Finland*
*[2]University of Helsinki, Applied Tumor Genomics Research Program- Research Pr ograms Unit-, Helsinki, Finland*
*[3]University of helsinki, Translational Cancer Medicine Research Program- Resear ch Programs Unit, Helsinki, Finland*
*[4]University of Helsinki, Cell and Tissue Dynamics Research Program- Institute of Biotechnology- HiLife, Helsinki, Finland*
*[5]University of Helsinki and Helsinki University Hospital, 2Department of Pathology- HUSLAB- HUS Diagnostic Ce nter, Helsinki, Finland*
*[6]University of Helsinki, Department of Oral and Maxillofacial Diseases, Helsinki, Finland*
*[7]Minerva Foundation Institute, Minerva Foundation Institute for Medical Research, Helsinki, Finland*
*[8]University of Helsinki, Department of Medical and Clinical Genetics, Helsinki, Finland*

**Introduction**
Colorectal cancer is the third most prevalent malignancy and the second leading cause of cancer death. Activating mutations of the *GNAS* codon 201 have been shown to activate the downstream adenylate cyclase and lead to constitutive cAMP signaling. According to several reports, GNAS mutation has been implicated with colorectal carcinogenesis, notably in mucinous carcinomas. In rare intestinal mucinous carcinomas, activating GNAS mutation is present in over 60% of the cases.

**Material and Methods**
The aim of this study was to clarify the mechanism of action of GNAS mutation in carcinogenesis, which remains largely unknown. We employed colorectal cancer cell lines edited with an activating GNAS mutation by CRISPR/Cas9 technology, RNA sequencing, and multiple functional *in vitro* and *in vivo* assays.

**Results and Discussions**
The most prominent upregulated gene in GNAS-mutated cells was the cAMP hydrolyzing phosphodiesterase 4D (PDE4D). This induction could further be suppressed by knocking-down GNAS expression through siRNAs, which also decreased the cAMP levels. GNAS-mutated cells showed increased migration and invasion as compared to the parental cells. Interestingly, inhibition of PDE4D activity using either pan-PDE4 inhibitor or PDE4D selective inhibitor increased the cAMP levels and resulted in a marked decrease in the oncogenic properties of GNAS-mutated cells, including cell proliferation, migration, and invasion, without affecting the parental cells.

**Conclusion**
In conclusion, oncogenic GNAS mutation causes a constitutive cAMP induction leading to upregulated PDE4D expression. Inhibition of PDE4D activity in GNAS-mutated cells leads to suppression of their oncogenic properties, suggesting that PDE4D inhibition could offer a potent means to control GNAS mutated tumors.

### EACR23-0752
### PHD2 (EGLN1) is a novel therapeutic target in KRAS-mutated lung carcinoma

S. Strocchi[1], F. Reggiani[1], E. Sauta[2], F. Torricelli[1], E. Zanetti[3], G. Santandrea[3], G. Damia[4], M. Paci[3], A. Ciarrocchi[1], V. Sancisi[1]
*[1]Azienda USL Reggio Emilia IRCCS, Scientific Direction, Reggio Emilia, Italy*
*[2]Humanitas Clinical and Research Center- IRCCS, AI Center, Rozzano- Milan, Italy*
*[3]Azienda USL Reggio Emilia IRCCS, Oncology Department, Reggio Emilia, Italy*
*[4]Istituto di Ricerche Farmacologiche Mario Negri IRCCS, Department of Oncology, Milan, Italy*

**Introduction**
Despite the introduction of innovative therapeutics, lung cancer is still the leading cause of cancer-related death world-wide. Patients harboring a KRAS mutation, comprising about 30% of lung adenocarcinoma cases, are particularly difficult to treat and often show a worse prognosis. For this reason, KRAS-driven lung cancer still requires deep molecular characterization to identify targets that can be used to develop novel drugs.

**Material and Methods**
To identify dependency genes that can be used as new therapeutic targets, we undertook an integrative approach, combining functional genomics, bioinformatics and cell biology. First, we combined the data from a CRISPR/Cas9 dependency screening performed in our laboratory with the dependency data from the DepMap Project, comprising 73 lung cancer cell lines. Next, to detect relevant therapeutic targets, we integrated dependency data with the Drug-Gene Interaction database (DGIdb) pharmacological data, The Cancer Genome Atlas (TCGA) gene expression and mutational profiles.

**Results and Discussions**
Through this analysis, we identified and validated EGLN1 as a novel druggable dependency gene, associated with KRAS-mutated lung cancer. The EGLN1 gene encodes the PHD2 prolyl-hydroxylase, the oxygen sensor, regulating the HIF transcription factor activity. PHD2 is overexpressed in tumor tissue compared to healthy surrounding tissue and its high expression correlates with worse prognosis in lung cancer patients. In lung cancer cell lines PHD2 supports proliferation, migration, colony formation and 3D growth. Pharmacological inhibition of PHD2 exerts anti-proliferative effects both in NSCLC cell lines and patient-derived organoids. We further characterized the processes underlying dependency on PHD2, showing that it promotes cell proliferation through at least two different molecular mechanisms, one HIF1a dependent and one HIF1a independent.

**Conclusion**

© 2023 The Authors. Molecular Oncology is published by John Wiley & Sons Ltd on behalf of Federation of European Biochemical Societies

Overall, we employed a functional genomics approach to identify and validate PHD2 as a novel therapeutic target in KRAS-mutated lung cancer. Our results uncover a pro-oncogenic role of PHD2 in lung cancer, partially relying on a HIF-independent mechanism. Moreover, our work suggests that PHD2 inhibitors, already in clinical trials for anemia, could be effective in KRAS-driven lung cancer treatment. Overall, our emerging data lay the basis for the development of new therapeutic strategies in KRAS-mutated adenocarcinoma patients.

### EACR23-0793
### WHOLE EXOME SEQUENCING TECHNOLOGIES COMPARISON

*Z. Rozankova*[1], *P. Zizkovicova*[1], *H. Jurtikova*[1], *H. Jurtikova*[2], *B. Blumova*[1], *V. Holinkova*[2], *J. Drabek*[1], *J. Drabek*[2], *M. Hajduch*[1], *M. Hajduch*[3]
[1]*Faculty of Medicine and Dentistry- Palacky University Olomouc, Institute of Molecular and Translational Medicine, Olomouc, Czech Republic*
[2]*University Hospital Olomouc, Laboratory of Experimental Medicine, Olomouc, Czech Republic*
[3]*Czech Advanced Technology and Research Institute, Institute of Molecular and Translational Medicine, Olomouc, Czech Republic*

**Introduction**
Massively parallel sequencing generates large amounts of sequencing data faster and cheaper than before. Like the different data sizes obtained in whole genome sequencing (WGS), protein-coding sequences (WES), or region of interest (panel sequencing), different approaches also can cause diversity in the sequencing data obtained. The aim of this study is to compare 5 library preparation and 2 sequencing methods in an attempt to determine whether these parameters cause differences in the quantity and quality of sequencing data.
**Material and Methods**
Five gDNA samples were involved in this study, input amount was 50-400 ng based on specific LibPrep protocol. Sequencing libraries were prepared as follows: Agilent SureSelect QXT Target Enrichment + V6 All exons Kit Probes, Twist Human Core Exome EF Multiplex Complete Kit + Twist Exome Probes, KAPA HyperPlus + KAPA HyperExome Probes, MGIEasy Exome Universal Lib PrepSet + Agilent V6 All exons Kit Probes, MGIEasy Exome Universal Lib PrepSet + MGI Exome Capture V4 ProbeSet. After quality control of all libraries, sequencing was performed using two platforms, Illumina and MGI. Bioinformatic analysis was performed using an in-house pipeline, and additional quality parameters control steps by FastQC, Qualimap, SAMtools, and BEDtools.
**Results and Discussions**
In order to compare sequencing technologies, the quality parameters of raw .fastq files were assessed using the FastQC. Parameters (Mean Quality Scores, Per Sequence GC content, and General Error Rate) show that the quality of data is satisfactory and comparable for both technologies.WES panel design was compared with three approaches. The coverage analysis was done on 3 levels for all 5 methods – with 3 different .bed files (universal uscs-exome+10.bed file; vendor‘s .bed file; intersect .bed file – the intersection of identical regions from all vendor‘s designs). Roche Kit had the highest on-target percentage of mapped reads and the highest mean coverage. The lowest duplicate rate was shown in Twist Kit. Next approach was Per base coverage analysis, which was calculated using the vendor‘s .bed file at particular thresholds. Besides Agilent probes, all 3 methods got above 90% of bases covered at 30x. Variant calling analysis of SNVs and INDELs was performed for all methods.
**Conclusion**
Both sequencing technologies have comparable outputs without significant differences. In term of the LibPrep method, Roche and Twist Kits reports better parameters than competitive kits for our purposes.

### EACR23-0801
### Mutational processes creating passenger hotspots across the genome

*C. Arnedo-Pac*[1], *F. Muiños*[2], *A. Gonzalez-Perez*[2], *N. Lopez-Bigas*[2]
[1]*MRC Toxicology Unit- University of Cambridge, Aitken Lab, Cambridge, United Kingdom*
[2]*IRB Barcelona, Biomedical Genomics Lab, Barcelona, Spain*

**Introduction**
Understanding how mutations accumulate in the human genome under neutrality is key to generating accurate models of mutagenesis. The analysis of whole genome sequences from cancer genomes has revealed that recurrent passenger somatic mutations at the exact same position, or hotspots, can be formed across cancer types. Yet, the mechanisms underlying passenger hotspots formation across tumours have not been systematically explored. Here, we study hotspots along tumor genomes as a means to understand the determinants of mutation rate variability at single nucleotide resolution.
**Material and Methods**
We built a new computational tool, HotspotFinder, to identify hotspots of somatic mutations across cancers and applied it to more than 7,500 whole genome sequences from 49 different cancer types. We next extracted mutational signatures and computed the propensity of 14 common mutational processes to create passenger hotspots across the genome. We developed a mathematical model for the expected number of hotspots that accounts for trinucleotide composition, regional mutation rate and covariates known to modulate mutation rate. We explored the underlying biological mechanisms of the differential hotspot propensity across mutational signatures and their implications beyond cancer genomes.
**Results and Discussions**
Mutational signatures 1 (SBS1) and 17a and b (SBS17a and SBS17b) showed the highest propensity to form passenger hotspots across cancers, showing from 5 to 78 times larger hotspot rates than other common mutational processes. Trinucleotide mutational probabilities, genome sequence composition and mutation density measured at the 10 Kbp genomic segments only accounted for 5-11% of the increased SBS17a/b hotspot propensity. This pointed to the role of other local genomic features in SBS17a/b hotspots, among which we found CTCF binding sites as a minor contributor. Conversely, most (80-100%) SBS1 hotspots were explained by the differential genome-wide methylation of CpG sites. We also uncovered the increased

© 2023 The Authors. Molecular Oncology is published by John Wiley & Sons Ltd on behalf of Federation of European Biochemical Societies hotspot propensity of SBS1 across normal tissues and human *de novo* germline variants.

**Conclusion**

Altogether, our findings provide new insights into the understanding of mutation rates under neutrality, which is key to modelling background mutagenesis to study tumour drivers, evolutionary trajectories and genome sequence evolution.

### EACR23-0802
### A subset of malignant pleural mesothelioma is sensitive to drugs targeting DNA damage response proteins

*N. Congiusta*[1], *C. Gigliotti*[1], *G. Corti*[1], *R. Libener*[2], *V. Pessei*[1], *M. Macagno*[1], *F. Maione*[1], *G. Gionfriddo*[1], *S. Lamba*[1], *C. Riganti*[3], *R. Taulli*[3], *L. Righi*[3], *P. Bironzo*[3], *G. Scagliotti*[3], *S. Novello*[3], *A. Bardelli*[1], *F. Bussolino*[1], *F. Di Nicolantonio*[1]

[1]*Candiolo Cancer Institute- FPO-IRCCS, Department of Oncology, Candiolo, Italy*

[2]*Azienda Ospedaliera SS. Antonio e Biagio Cesare Arrigo, Department of Integrated Activities Research and Innovati on, Alessandria, Italy*

[3]*University of Torino, Department of Oncology, Torino, Italy*

**Introduction**

Malignant pleural mesothelioma (MPM) is a rare and mostly lethal thoracic malignancy. Unmet medical needs in MPM are the lack of early detection strategies/biomarkers, the intrinsic chemoresistance and the presence of an immunosuppressive tumor microenvironment. The paucity of preclinical models has hampered research progress in this tumor type. In this work, we aimed to perform a multi-layer functional characterization of MPM patient derived models to fill the gap between descriptive molecular studies of MPM and patient management.

**Material and Methods**

Primary human cancer cells from MPM patients were obtained from the Biological Bank of Malignant Mesothelioma in Alessandria. Whole genome sequencing of 22 MPM cell lines was performed at 60X coverage. Matched PBMCs from individual patients were also sequenced to confirm the somatic status of genomic variants. Long-term cell proliferation assays were performed with DNA damage response (DDR) inhibitors targeting ATR, ATM, WEE1 or CHEK1.

**Results and Discussions**

Genomic and protein analyses revealed molecular alterations leading to disruption of known oncosuppressor proteins. A total of 74% cell lines had loss of CDKN2A and CDKN2B expression. Loss of BAP1 protein occurred in 52% of cases. Hotspot mutations of the LATS2 serine/threonine kinase protein in the Hippo signaling pathway were detected in 40% models. Cell lines recapitulated the molecular landscape of MPM clinical samples. Drug profiling assays revealed that a small subset of MPM lines displayed sensitivity to all DDR inhibitors. No recurrent alterations were found in genes encoding for proteins of the homologous recombination or DNA damage response signaling pathways. The pattern of sensitivity to DDR inhibitors did not correlate with BAP1 status or the mutational landscape of the cell lines. Drug resistant cell models showed either sarcomatoid or biphasic histology, while drug sensitive lines exhibited epithelioid histology.

**Conclusion**

The limited availability of preclinical MPM models has slowed progress to find targets and effective therapeutic options for this rare tumor type. We provide an extensive molecular characterization of novel MPM lines which can be exploited in future functional studies. In an initial drug screening effort, we report that a subset of MPMs is sensitive to DDR inhibitors. Histological subtypes rather than molecular alterations are correlated with increased sensitivity to these agents.

### EACR23-0846
### Integrated genomic mutational landscape of vulvar squamous cell carcinoma

*F. Lombardo*[1], *T. Zwimpfer*[2], *N. Rimmer*[1], *S. Goetze*[3], *F. Singer*[4], *A. Bertolini*[4], *C. Kurzeder*[5], *F. Jacob*[1], *V. Heinzelmann-Schwarz*[5]

[1]*University Hospital and University Basel- Biomedicine- B asel- Switzerland, Department of biomedicine DBM- Basel, Basel, Switzerland*

[2]*University Hospital Basel- Gynecological Oncology- Base l- Switzerland- Peter MacCallum Cancer Centre- Bowtell Laboratory- Melbourne- Australia, University Hospital Basel- Gynecological Oncology- Basel - Switzerland, Basel, Switzerland*

[3]*ETH zürich, Proteomics Plattform D-HEST, Zürich, Switzerland*

[4]*NEXUS Personalized Health Technologies, NEXUS Personalized Health Technologies, Zürich, Switzerland*

[5]*University Hospital Basel- Gynecological Oncology- Base l- Switzerland, University Hospital Basel- Gynecological Oncology- Basel - Switzerland, Basel, Switzerland*

**Introduction**

Vulvar cancer responds poorly to systemic treatment and in contrast to other gynecological cancers, targeted therapies remain at an early stage for this rare cancer type. Recent advances in molecular oncology may lead to personalized therapy with integration of targeted, immuno-, and traditional therapy. Here, we characterized the proteogenome of vulvar squamous cell carcinoma (vSCC) and provide a computational workflow to integrate publicly available data to study this rare and aggressive cancer type.

**Material and Methods**

Our cohort incorporates clinicopathological, whole exome sequencing, bulk RNA-sequencing, and proteomics data derived from 23 patients with vSCC. Raw reads underwent standard quality control based on read trimming, adapter removal, and fastQC. In addition, a total of 5543 women-derived TCGA genomic and transcriptomics sequencing data were obtained for this analysis conducted using R/Bioconductor.

**Results and Discussions**

Our cohort consisted of 34.8% human papillomavirus (HPV)+ and 65.2% HPV- vSCC patients. Early FIGO stage was significantly associated with HPV- status (12% (1/8) vs. 86% (12/15), $p$<0.001). The most prevalent pathological mutated genes were *FAT1* 52%, *KMT2D* 48%, *KMT2C* 39%, *TP53* 35%,

© 2023 The Authors. Molecular Oncology is published by John Wiley & Sons Ltd on behalf of Federation of European Biochemical Societies

*FBXW7* 26%*, NOTCH2* 17%*, PIK3CA* 17%, and *ATM* 17%. *TP53* and *CDKN2* were mutually exclusive to HPV+ with a frequency of 53% and 13.3% in HPV- patients, respectively. In contrast, HPV+ patients revealed higher frequency in *PIK3CA* mutations with 37.5% vs. 6.7% in HPV-. We identified the following mutational signatures in vSCC with *SBS1*, *SBS39*, *SBS15*, *SBS5*, *SBS6*, *DBS4*, and *DBS7* as the most prevalent signatures indicating defective DNA mismatch-repair and correlating with age at time of the cancer diagnosis. Tongue SCC (tSCC) was identified as a comparison cohort supported by different clustering methods of conjoint RNA-seq and clinicopathological data. vSCC was associated with disadvantageous immune cell types such as elevated activated mast cells ($p$=0.0031), monocytes ($p$=0.0027), and M2 macrophages ($p$<0.001) compared to tSCC independent of the HPV-status.

**Conclusion**

Our ongoing integrated proteogenomic characterisation of vSCC has revealed novel insights with 1) distinction of vSCC by HPV status, 2) main characteristics on DNA level, 3) TME finding, and 4) identification of tSCC as a similar cancer tissue. Further analysis envisions finding molecular signatures serving as biomarkers in vSCC and suggesting alternative treatment options of drugs already in use in tSCC or other cancer types.

### EACR23-0893
### Novel MET tyrosine kinase receptor variants found in South American lung cancer patients evidence actionable traits

*S. Rivas[1], C. Estay[1], V. Soto[1], J.I. Tapia[1], E. González-Feliú[1], R. Armisén[1]*

*[1]Institute of Science and Innovation in Medicine- Universid ad del Desarrollo, Genetics and Genomics, Santiago, Chile*

**Introduction**

In cancer, gene driver variants provide a selective growth advantage for carcinogenesis but also enable the development of targeted drugs to revert these cell effects imprinted by the driver alteration, decreasing the tumor clone expansion. This cell response is known as the actionability of the gene/variant, which has a powerful impact on patients’ prognosis and in the development of personalized medicine. Lung cancer is the principal cause of cancer death in 89/185 countries, and the IARC estimated that by 2040 will have an increase of 80% in lung cancer death in South America, evidencing, among other factors, the poor implementation of personalized medicine and the deficient progress in the study of the local actionable variants. Hence, we have searched and validated the most frequent novel predicted actionable variants.

**Material and Methods**

We sequenced 1732 DNA/RNA tumors of patients diagnosed with lung cancer from Chile, Brazil, and Peru using a next-generation sequencing panel that interrogates 52 cancer genes. This allowed us to identify the most frequent novel variants predicted as actionable. Later, we designed and expressed the novels *MET* variants in non-tumor BEAS-2B, HEK-293 cells, and lung cancer cells H1993 cultured 3-Dimensionally to evaluate if they might activate the Met tyrosine kinase receptor, proliferation, cell migration, and response to MET inhibitors.

**Results and Discussions**

Here we discovered that the actionable *MET* gene was mutated in 7% of the cohort. Moreover, we discovered that 36.5% of all variants were predicted as novels, which means they have not been registered in public cancer databases. The most frequent variants were found in the MET gene in the juxtamembrane and tyrosine kinase domains in positions T992 and H1094, respectively. Both novel variants induced robust MET activation, focal adhesions formation, and cell migration but not cell proliferation. Finally, spheroid lung cancer cells expressing *MET* variants showed high sensitivity to *savolitinib*. Meanwhile, we are testing the cell response to two additional MET target drugs. Notably, cells expressing novel variants showed a more sensitive response to the drug, even more than those cells expressing the actionable and well-known *MET* exon 14 skipping variant, for which drugs had been indicated.

**Conclusion**

As South American patients experience 7% of *MET* variants compared to the 3% of MET alterations in the US, Europe, and Asia, broadening the prescription of MET inhibitors could open more personalized therapy options.

### EACR23-0928
### Noncoding mutations as biomarkers of sensitivity and response to treatment in breast cancer

*A. Cuy Saqués[1], C. Monger[2], A. Bracken[2], F. Matra[2], A. Armesto Jimenez[2], S. Kennedy[3], D. Kaukonen[4], S. Madden[5], J. Crown[6], A. Eustace[1]*

*[1]Dublin City University, National Institute for Cellular Biotechnology, Dublin, Ireland*

*[2]Trinity College Dublin, Smurfit Institute of Genetics, Dublin, Ireland*

*[3]Technological University Dublin, Grangegorman Campus, Dublin, Ireland*

*[4]Karolinska Intitutet, Department of Medical Epidemiology and Biostatistics, Solna, Sweden*

*[5]Royal College of Surgeons in Ireland, Data Science Centre, Dublin, Ireland*

*[6]St. Vincent’s University Hospital, Medical Oncology Department, Dublin, Ireland*

**Introduction**

The PI3K/AKT pathway is the most mutated signalling pathway in breast cancer (BC). Coding mutations in this pathway are used as biomarkers of response to PI3K inhibitors (PI3Ki), but not all PIK3CA mutated patients respond to PI3Ki. Non-coding mutations, which are common in BC, can affect kinome gene expression and may play a role in controlling response or resistance to PI3Ki.

**Material and Methods**

Noncoding mutations surrounding kinome related genes were obtained from 3 BC Whole Genome Sequencing (WGS) datasets (n=915 patients) and classified based on frequency in specific BC subtypes. We used the Activity by Contact (ABC) model(Fulco *et al.*, 2019) to

© 2023 The Authors. Molecular Oncology is published by John Wiley & Sons Ltd on behalf of Federation of European Biochemical Societies bioinformatically predict the target gene of non-coding genomic regions significantly mutated in our BC cohort. Mutations predicted to interact with genes were cloned into lentiviral vectors to assess via reporter assays if they changed the expression of the luciferase gene in target cells. We used BC cell CCLE data to correlate whether those mutations impacted on protein/gene expression levels and drug sensitivity in-silico (MUT n=5, WT n=10). Drugs with increased proliferative inhibition in MUT cell lines were tested alone and in combination with the PI3Ki alpelisib to assess their potential as novel treatments in BC.

**Results and Discussions**

An A:TGC insertion in chromosome 17:39959177, inside the HER2 amplicon, was found in 15% of the HER2+ BC patients analysed(n=11/73). The ABC model predicted its interaction with GSDMA and LRRC3C, two inflammation and apoptosis related genes. In-silico data from 9177 mutated BC cells (MUT) demonstrated increased expression of genes up to 300kb from the mutation. In-vitro luciferase assays confirmed that presence of the mutation caused a 2.5±0.2-fold increase in luciferase gene expression. Reverse phase protein data analysis identified decreased expression of apoptosis-regulating proteins SMAD4 and Mre11 and increased JNK expression in MUT cells(p=0.034/0.047/0.171). These results correlated with increased response to the proapoptotic drugs talazoparib, teniposide and the IRAK4 inhibitor emavusertib(p=0.043/0.037/0.027). The combination of emavusertib and alpelisib had the greatest synergy in MUT and WT HER2 BC cells.

**Conclusion**

The 9177 mutation, present in 15% of HER2+ patients, impacts expression of HER2 amplicon genes and altered expression of apoptosis regulating proteins. The dual inhibition of IRAK and PI3K pathways has synergistic anti-proliferative effects and represents a potential novel treatment for HER2 BC patients.

### EACR23-0939

### Increased activities of endogenous mutational signatures characterize oral cancer in non-smokers non-drinkers with no other identified risk factors

*S. Deneuve[1], B. Fervers[2], S. Senkin[3], L. Bouaoun[4], O. Perol[2], B. Chavanel[5], I. Coste[6], T. Renno[6], J. Zavadil[5], F. Virard[6]*

*[1]CHU de Rouen, ENT Department, Rouen, France*

*[2]Centre De recherche En Cancerologie De Lyon, INSERM UMR 1296- "Radiations: Defense- Sante- Environnement", Lyon, France*

*[3]International Agency for Research on Cancer WHO, Genomic Epidemiology Branch, Lyon, France*

*[4]International Agency for Research on Cancer WHO, Environment and Lifestyle Epidemiology Branch, Lyon, France*

*[5]International Agency for Research on Cancer WHO, Epigenomics and Mechanisms Branch, Lyon, France*

*[6]Centre De recherche En Cancerologie De Lyon, Tumor Escape- Resistance and Immunity, Lyon, France*

**Introduction**

During the last 20 years, increased incidence has been observed for oral cavity squamous cell carcinomas (OCSCC), mainly occurring in the mobile tongue of younger patients with no identified risk factors (NIRF). This emerging subgroup of OCSCC of unknown etiology has not yet been recognized as a distinct clinical entity with characteristic molecular profiles or biomarkers.
To address this gap, we comprehensively analyzed the public head-and-neck cancer genomics data for mutational signatures and distribution of mutation classes affecting cancer driver genes in patients with oral (OC) or laryngeal (LX) tumors, specifically considering their NIRF status compared to known risk factors (HPV status, tobacco smoking and alcohol consumption).

**Material and Methods**

We devised a highly sensitive analysis approach of *de novo* mutational signature extraction using the non-negative matrix factorization module of the SigProfilerExtractor tool, innovatively coupled with per-sample fitting of the COSMIC signature database contents by optimized simulation- and bootstrap-based non-negative least squares algorithm using the MSA (Mutational Signature Analysis) tool. This approach comprehensively identified mutational signatures in 307 OC and 109 LX cancer cases from the TCGA-HNC collection. Recurrently mutated genes under selective pressure were identified by computing the non-synonymous to synonymous mutation ratios (dNdScv tool).

**Results and Discussions**

We observed that the NIRF OSCCs exhibited increased activities of signatures SBS2/13 (APOBEC enzyme mutagenesis) and/or SBS1 (endogenous clock-like mutagenesis). In tobacco smokers, we identified distinct anatomical site-specific mutagenic outcomes involving signatures SBS4 and SBS92 in cases with LX cancer, and SBS16 in cases with OC cancer. We propose that in OSCCs the signature SBS16, previously linked to alcohol consumption, rather reflects the combined effects of drinking and smoking. Lastly, we observed that the top identified driver genes, including NIRF-specific recurrently mutated genes, were selected based on a given mutational process, thus reflecting distinct natural disease histories.

**Conclusion**

Our results show that the NIRF OSCCs are likely driven by endogenous mutagenesis, with no apparent direct link to known exogenous exposures, and that these cancers have distinguishable disease histories. Our study provides a basis for more comprehensive future investigations of this emerging cancer subtype of unclear etiology and increasing incidence.

### EACR23-0944

### Evaluation of a KRAS gene expression signature in lung cancer

*A. Lovše[1], J. Otoničar[1], J. Kokošar[1], L. Ausec[1]*

*[1]Genialis Inc., Bioinformatics, Boston MA, United States*

**Introduction**

Lung adenocarcinoma is a complex disease driven by multiple oncogenic drivers including KRAS mutations which occur in approximately 30% of tumours. Gene expression signatures have been reported in literature that stratify KRAS-mutated lung cancers into phenotypic subgroups that are in part defined by a particular combination of genomic variants. Validation of these

© 2023 The Authors. Molecular Oncology is published by John Wiley & Sons Ltd on behalf of Federation of European Biochemical Societies subgroups in independent datasets is lacking. This study aimed to validate a previously identified lung adenocarcinoma gene expression classifier and investigate the association of the three subtypes with known genomic alterations.

**Material and Methods**

We reimplemented an 18-gene expression signature classifier (Skoulidis et al., 2015) and evaluated its performance using cross-validation on the original data set. Next, we applied the classifier to a previously described RNA-seq dataset from an independent cohort of Korean individuals with lung adenocarcinoma (Seo et al., 2012). To complement existing genetic characterisation, we developed a pipeline for RNA-seq-based variant calling to identify additional common oncogenic mutations. Finally, we explored and interpreted the association between expression-based subgroups, KRAS co-mutations, and other biological characteristics using enrichment analysis, descriptive statistics, and literature curation.

**Results and Discussions**

Our classifier based on linear regression demonstrated similar performance on the original training dataset to that reported in the original publication. Unsupervised clustering using gene expression of signature genes identified distinct subgroups of samples in the independent cohort. We found that the KRAS co-mutational landscape varied significantly between the predicted subtypes in the independent data set. Further biological characterisation of subgroups revealed differences in the expression of immune genes, metabolism, and signalling pathways. Finally, we discuss the signature's potential for patient stratification and ways of improving it.

**Conclusion**

In summary, our study provides independent validation of the 18-gene expression signature for lung adenocarcinoma subtypes and discusses the utility of the signature.

### EACR23-0950
### A liquid biopsy approach for the genetic characterization of cancers of unknown primary

*R. Roncarati*[1,2], *I. Pace*[2], *B. Fontana*[2], *G. Gallerani*[2], *N. Laprovitera*[2], *M. Riefolo*[3], *A. D'Errico*[3], *K. Rihawi*[3], *A. Ardizzoni*[3], *M. Ferracin*[4]

[1]*Istituto di Genetica Molecolare IGM CNR, Dip. Scienze Mediche e Chirurgiche - DIMEC, Bologna, Italy*

[2]*Università di Bologna, Dip. Scienze Mediche e Chirurgiche - DIMEC, Bologna, Italy*

[3]*IRCCS Azienda Ospedaliero-Universitaria di Bologna, Dip. Scienze Mediche e Chirurgiche - DIMEC, Bologna, Italy*

[4]*Università di Bologna, Dip. Scienze Mediche e Chirurgiche - DIMEC, _Bologna, Italy*

**Introduction**

Cancer of unknown primary (CUP) patients present with a heterogeneous metastatic disease without apparent primary tumor. The tumor undifferentiated morphology and undetermined histology prevent the tumor tissue-of-origin identification. CUP genetic testing is often limited by the reduced availability of tumor biopsy material, poor DNA quality or insufficient DNA amount, and the presence of unusual genetic alterations, which could not be included in common diagnostic gene panels. Liquid biopsy with a CUP-dedicated gene panel could represent the most suitable approach to overcome these limitations and guide therapeutic decisions.

**Material and Methods**

In this study, we profiled the mutational landscape of 36 circulating cell free DNA (ccfDNA) samples from 34 consecutive CUP patients using next generation sequencing (NGS). A custom panel including 92 genes frequently mutated in CUPs was generated using Agilent SureSelect XT Low Input kit with the intent to identify variants responsible of the metastatic process, actionable mutations/genes and gene pathways that can explain this peculiar cancer condition.

**Results and Discussions**

All CUP samples had circulating tumor DNA (ctDNA) in their ccfDNA fraction. More than 50% of the tested genes carried functional variants, and some of them with recognized clinical significance. Some genetic alterations are directly actionable, including EGFR, FGFR, MET, ALK mutations. Some of the remaining mutated genes are associated with evidence of synthetic lethality. ARID1A mutations are, for instance, indicators of response to PARP and BET inhibitors, and constitute one of the most frequently mutated gene in our cohort of CUP patients. Some mutations provide indications of a possible tissue of origin.

**Conclusion**

This study demonstrates the feasibility of liquid biopsy genetic testing of CUP patients and support the relevance of using a CUP dedicated cancer panel to identify clinically relevant mutated genes.

### EACR23-0965
### Implementation of BRCA1/2 mutation testing in patients with sporadic ovarian cancer in Serbia

*K. Zivic*[1], *I. Boljevic*[1], *M. Nedeljkovic*[1], *M. Tanic*[1], *R. Jankovic*[1]

[1]*Institute of Oncology and Radiology of Serbia, Department for Experimental Oncology, Belgrade, Serbia*

**Introduction**

Ovarian cancer is the ninth most lethal gynecological cancer globally, and Serbia is one of the countries with a high age-standardized incidence. Family history of disease is the most significant risk factor, accounting for 10-15% of all cases of disease. Other group of ovarian cancer cases is sporadic with high grade serous subtype being the most common, which has been observed to reach 20–25% mutations in *BRCA1* and *BRCA2* genes. The classification of patients for therapy with PARP inhibitors depends on the evaluation of *BRCA* gene mutational status in sporadic epithelial ovarian cancer. In Serbia PARP inhibitors have been approved in treatment of ovarian cancer sence 2016.

**Material and Methods**

FFPE samples for this study are collected at IORS from all health facilities in Serbia, and genetic material is extracted using comrcial kits for isolation (QIAGENE, Hilden, Germany). *BRCA1* and *BRCA2* mutation screening was carried out using the AmpliSeq for Illumina BRCA Panel (San Diego, CA, USA) and the MiSeq Reagent Kit 300v.

© 2023 The Authors. Molecular Oncology is published by John Wiley & Sons Ltd on behalf of Federation of European Biochemical Societies

Pair-end reading was used and cut-off of 10% for the Variant Allele Frequency was applied. The Sophia DDM program (Sophia Genetics, Saint-Sulp, Switzerland) and manual database searches were used for data analysis and to interpret pathogenicity.

**Results and Discussions**

Every woman with newly diagnosed or recurrent serous ovarian cancer, was eligible for testing at IORS to determine their *BRCA* status. In the period from 2016. until 2022. 701 patients required *BRCA* testing. Testing was successful in the group of 557 patients. The average patient age at diagnosis was 60,65 years. Pathogenic variants of *BRCA1/2* genes were found in 119 samples (21.36%) and variants of uncertain significance were found in 32 samples (5.75%). *BRCA1* mutations were discovered in 92 samples, with the most frequent one being c.5266dupC (rs80357906), which is consistent with results from other analyzed cohorts. *BRCA2* mutations were found in 58 samples, with c.5576_5579del (rs80359520) and c.5645C>A (rs80358785) mutations being more common than others. Provided results shows slightly higher proportion of *BRCA1/2* mutations then those from other study cohorts in the literature, also higher proportion of VUS variants.

**Conclusion**

The utilization of a tumor sample for molecular analysis of the complete coding sequence of both *BRCA1* and *BRCA2* has considerably increased the total number of patients who may benefit from targeted therapy with PARP inhibitors.

### EACR23-0976

### Comprehensive variant analysis of tumours in patients with non-small cell lung cancer treated with immunotherapy

*J. Svensson[1,2], E. A. Eklund[3,4,5], M. Yhr[1,2], N. Dutta[6], L. Akyürek[7], P. Torstensson[8], V. I. Sayin[3,4], A. Hallqvist[5,9], S. Raghavan[6,10], A. Rohlin[1,2]*

*[1]Sahlgrenska University Hospital, Department of Clinical Genetics and Genomics, Gothenburg, Sweden*

*[2]Institute for Biomedicine, Department of Laboratory Medicine- Sahlgrenska Academ y- University of Gothenburg, Gothenburg, Sweden*

*[3]Institute for Clinical Sciences, Department of Surgery- Sahlgrenska Center for Cancer Re search- University of Gothenburg, Gothenburg, Sweden*

*[4]Wallenberg Centre, Molecular and Translational Medicine- University of Goth enburg, Gothenburg, Sweden*

*[5]Sahlgrenska University Hospital, Department of Oncology, Gothenburg, Sweden*

*[6]Institute for Biomedicine, Department of Microbiology and Immunology- University of Gothenburg, Gothenburg, Sweden*

*[7]Institute for Biomedicine, Department of Clinical Pathology- Sahlgrenska University Hospital, Gothenburg, Sweden*

*[8]Skaraborg Hospital, Department of Pulmonary Medicine, Skövde, Sweden*

*[9]Institute for Clinical Sciences, Department of Oncology- Sahlgrenska Academy- Universit y of Gothenburg, Gothenburg, Sweden*

*[10]Sahlgrenska Univerity Hospital, Departement of Clinical Immunology and Transfusion Med icine, Gothenburg, Sweden*

**Introduction**

Treatment of non-small cell lung cancer (NSCLC) was revolutionised with immunotherapy. Particularly important is immune checkpoint blockade (ICB) targeting PD-1/PD-L1; nevertheless, two thirds are unresponsive to ICB. Better genetic biomarkers are warranted besides the FDA approved tumour mutational burden (TMB). Genetic variants in a few selected genes have been suggested to predict response to ICB alone or in combinations as co-mutations. This study aims to interpret variants in both oncogenes and tumour suppressor genes, and in addition analyse mutational signatures of the tumours, that might be used as predictive biomarkers.

**Material and Methods**

The prospective study cohort includes n=50 stage III-IV NSCLC patients that received ICB as first- or second line of treatment. Blood and tumour tissue was sequenced with next-generation sequencing (NGS) with a panel of 591 cancer-associated genes. A comprehensive variant classification approach was used to subclass somatic variants into 6 different categories based on standard workflows, in combination with several databases and prediction tools. In addition, mutational signatures were extracted using SigProfiler tools and analysed.

**Results and Discussions**

In total 977 variants were identified. These included 41 pathogenic, 101 likely pathogenic and 793 variants of unknown significance (VUS). The VUS:es were further subclassed into different categories (VUS-, VUS, VUS+ and VUS++) to identify those with higher or lower driver properties and probability of pathogenicity. By using this approach 35 VUS++ and 83 VUS+ were identified. Frequently mutated genes, number of variants in different classes and their pathogenicity were related to ICB response, as was mutational signatures.

**Conclusion**

Understanding the genetic landscape and identifying biomarkers of ICB are two key considerations for development of personalised treatment for patients. The approach of a thorough classification including subclassification of the VUS:es led to identification of variants that can potentially function as biomarkers, in combination with pathogenic and likely pathogenic variants. The analysis of the combination of mutational signatures and genetic variants further enhanced refinement of biomarkers of response to ICB.

### EACR23-1140

### COMETT lncRNA: a new potential target in BRAF-mutated thyroid carcinomas?

*C. Perfetto[1], S. Cataldi[1], M. Aprile[1], A. Ciccodicola[1], V. Costa[1]*

*[1]National Research Council, Institute of Genetics and Biophysics "A.Buzzati-Traverso", Naples, Italy*

**Introduction**

The deregulation of long non-coding RNAs (lncRNAs) is reported in several tumor types and their targeting can affect cancer progression, representing a potential therapeutic strategy. We previously identified *COMETT* as

© 2023 The Authors. Molecular Oncology is published by John Wiley & Sons Ltd on behalf of Federation of European Biochemical Societies a new lncRNA highly expressed in papillary thyroid carcinomas (PTCs), demonstrating that its siRNA-mediated silencing impairs the oncogenic properties of *RET*-rearranged PTC cells. Here, we investigated the molecular and functional effects of *COMETT* knockdown in *BRAF*-mutated PTC and anaplastic thyroid (ATC) cells by analyzing the changes in tumor cells' transcriptome in the oncogenic properties as well as in the sensitivity to vemurafenib (i.e. a FDA-approved B-raf inhibitor).

**Material and Methods**

LNA GapmeRs technology was used to knockdown (KD) *COMETT* lncRNA in B-CPAP (PTC) and in 8505c (ATC) cells, where RNA-Seq, cell viability, colony forming capacity and migration were assayed upon *COMETT* silencing. *COMETT* pull-down, followed by mass spectrometry (MS), and SUnSET assays were carried out in B-CPAP cells to identify *COMETT* protein interactors.

**Results and Discussions**

*COMETT* KD significantly reduces cell viability and colony forming capacity of *BRAF*-mutated ATC and PTC cells. Transcriptome analysis on *COMETT*-KD B-CPAP cells indicated a marked repression of EGFR, WNT and JAK-STAT signaling and of DNA replication and cell cycle processes, paralleled by the increase of pro-inflammatory and apoptotic genes. Preliminary data from MS analysis reveal the binding of *COMETT* to splicing factors (SR and hnRNP) as well as to multiple ribosomal proteins. Accordingly, we verified that *COMETT* silencing affects SR expression and phosphorylation, and impairs global translation rates, supporting a key role in mRNA splicing and cytosolic translation. Moreover, *COMETT*-KD ATC and PTC tumor cells also display an enhanced responsiveness to vemurafenib treatment (even at low doses).

**Conclusion**

Overall, we assessed that the targeting of *COMETT* lncRNA in *BRAF*-mutated PTC and ATC cells restrains their oncogenic properties, also increasing the responsiveness to vemurafenib. Moreover, our analysis suggests that *COMETT* silencing affects multiple cancer-related signaling pathways, mRNA splicing and cytosolic translation. Therefore, targeting this lncRNA may help the current therapeutic approach – based on B-raf inhibition – and counteract cancer progression and drug resistance in *BRAF*-mutated thyroid tumors.

### EACR23-1147

### Identification of direct transcriptional target genes of NUP98::KDM5A reveals regulatory networks in Acute Myeloid Leukemia

*S. Troester[1], J. Schmoellerl[2], T. Eder[1], N. Wukowits[1], G. Manhart[1], R. Meisel[3], G. Winter[4], J. Zuber[2], F. Grebien[1,5]*

*[1]University of Veterinary Medicine Vienna, Institute for Medical Biochemistry, Vienna, Austria*

*[2]Research Institute of Molecular Pathology - IMP, Research Institute of Molecular Pathology, Vienna, Austria*

*[3]Heinrich Heine University, Department of Pediatric Oncology, Duesseldorf, Germany*

*[4]Research Center for Molecular Medicine of the Austrian A cademy of Sciences, CeMM, Vienna, Austria*

*[5]St. Anna Children's Cancer Research Institute - CCRI, St. Anna Children's Cancer Research Institute, Vienna, Austria*

**Introduction**

Oncogenic fusion proteins involving the Nucleoporin 98 (*NUP98*) gene are recurrently found in acute myeloid leukemia (AML) and are associated with particularly poor prognosis. A better understanding of how NUP98-fusions induce deregulation of gene expression programs is required for the development of tailored treatments. In this study, we aimed to generate new models to decipher the epigenetic and transcriptional landscape of NUP98::KDM5A-driven AML to enable the identification of immediate critical effectors of the NUP98::KDM5A fusion protein.

**Material and Methods**

We developed a new model for degradation tag (dTAG)-mediated ligand-induced degradation of the NUP98::KDM5A protein. We used H3K4me3 and H3K27ac-directed CUT&Tag to understand how NUP98::KDM5A alters global epigenetic patterns. Direct transcriptional effects of NUP98-fusion-dependent gene regulation were studied by nascent RNA-seq. In parallel, we conducted a genome-scale CRISPR/Cas9 loss-of-function screen in a NUP98::KDM5A-driven AML cell line to unravel functional genetic dependencies associated with this fusion protein. Cells from a NUP98::KDM5A PDX model were used to validate potential therapeutic targets.

**Results and Discussions**

Complete loss of the dTAG-NUP98::KDM5A fusion protein was achieved within one hour after ligand addition, resulting in cell cycle arrest, terminal differentiation and apoptosis of leukemia cells. CUT&Tag revealed that fusion protein degradation caused global changes in the epigenetic patterns of the activating histone marks H3K27ac and H3K4me3. Global analysis of nascent mRNA expression by SLAM-seq identified 45 immediate NUP98::KDM5A target genes. Among these, 12 were classified as essential factors for NUP98::KDM5A cell growth from a genome wide CRISPR/Cas9 screen. This subset of genes was characterized by highly enriched NUP98::KDM5A binding as well as high levels of H3K27ac and H3K4me3 at their promoters. Among essential NUP98::KDM5A targets, CDK12 was a promising candidate that has previously been implicated in a wide range of solid tumors. NUP98::KDM5A AML cell lines as well as primary cells from a NUP98::KDM5A-expressing AML patient were highly sensitive to a CDK12 degrader.

**Conclusion**

Using a new model for ligand-induced degradation of NUP98::KDM5A, we identified epigenetic patterns and direct transcriptional target genes of NUP98::KDM5A that are functionally essential in AML. Among these, we could pharmacologically validate CDK12 as a promising therapeutic target in cells of a NUP98::KDM5A PDX model.

### EACR23-1150

### A HERV-H mediated transcript can silence TLR7 & TLR8, and is correlated with better survival in lung cancer patients.

© 2023 The Authors. Molecular Oncology is published by John Wiley & Sons Ltd on behalf of Federation of European Biochemical Societies

C. Hall[1], G. Kassiotis[1]
[1]Francis Crick, Retroviral Immunology, London, United Kingdom

**Introduction**

Endogenous Retroviral Elements (EREs) are ubiquitous in the Human genome. These elements have largely been silenced, though many have evolved roles in healthy and diseased tissues. The HERV (Human Endogenous Retrovirus) family of EREs is a relatively recent inclusion in the genome. One HERV element is situated approximately 16kb proximally of the genes TLR7 and TLR8 which are part of the innate immune system and detect single stranded RNA in endosomes. Our analysis of novel transcripts initiating in the HERV suggest a novel method of TLR7 and TLR8 control, which is differentially regulated in patients with lung adenocarcinoma or ovarian serous carcinoma.

**Material and Methods**

In previously published work, a *de novo* pan-cancer transcriptome was assembled, with LTR reads including HERVH|X|12939865|12945052 annotated. Correlative analysis provided evidence that these transcripts were highly expressed in a subset of lung adeno- and ovarian serous carcinomas. TCGA and CCLE expression data analysed for transcription relating to the HERV using Salmon and BLAST. A cell line lacking the HERVH cassette was manufactured using CRISPR and measured for response to TLR7 and TLR8 agonists.

**Results and Discussions**

Novel analysis performed by our lab has found a number of EREs which are differentially regulated in Cancer. The HERV-H element HERVH|X|12939865|12945052 on Chromosome X has been shown to be differentially regulated in Lung Adenocarcinomas and Ovarian Serous Carcinomas. Our analysis reveals antisense transcripts originating in this HERV which extend across the TLR7 and TLR8 cassette, and correlate with better survival in lung cancer patients. We hypothesise that this transcription can not only cause silencing of TLR7&8, but also that the silencing can be specific to either gene. Analysing of cell lines, we found that expression of TLR7, TLR8 and the antisense transcription were mutually exclusive, suggesting a mechanism of controlling TLR7 and TLR8 expression and downstream function with HERVH mediated transcription.

**Conclusion**

Transcription initiated in HERVH|X|12939865|12945052 provides a further example of ERE dysregulation in cancer, with a functional effect on genes involved in immune regulation. Expression of this transcript also linked to better survival in lung cancer patients, and TLR7 and TLR8 have also been suggested as candidate targets for cancer therapy. An understanding of this transcription and how it silences these genes could provide insights into how this treatment can best be used in the clinic.

### EACR23-1155
### p53 Functional Activity Prediction Improves Upon Mutation Status Alone In Colorectal Cancer.

E. Rogan[1], D. Longley[1], P. Dunne[1], S. McDade[1]
[1]Queen's University Belfast, Patrick G Johnston Centre for Cancer Research, Belfast, United Kingdom

**Introduction**

The tumour suppressor TP53 is mutated in approximately half of colorectal cancers (CRC), however the impact of mutation (MT) on p53 functional activity is not yet fully understood. This is at least in part due to the complex mutational spectrum, which can potentially have differential effects on protein function.

Moreover, tumours harbouring wild type (WT) p53 may repress canonical p53 function through a range of non-mutational mechanisms. As a result, TP53 mutation status does not currently inform prognosis or treatment selection in this highly heterogenous disease.

To better understand deregulation of p53 activity and determine if functional activity estimation provides further information regarding p53 function, prognosis, and treatment response in CRC, we assessed transcriptomic estimation of p53 activity using p53 Pathway-related Gene Sets (p53PGS).

**Material and Methods**

Leveraging CCLE data, single sample activity estimations were benchmarked against indicators of TP53 functional activity including sensitivity to Nutlin-3A, siRNA and CRISPR knock out of TP53 and negative regulator MDM2, to assess the utility of p53PGS in cell lines.

The Target Gene Regulation Database was used to characterise p53 target genes to investigate the influence of Mode of Regulation (MOR) and refine gene sets for directional p53 functional activity estimations which were applied to clinical CRC datasets to assess patient prognosis and treatment response.

In these datasets, estimation of repression of proliferation via p53-mediated activation of cell cycle regulator p21 was highly confounded by the presence of tumor stroma. We therefore limited this analysis to epithelial-rich patient subgroups.

**Results and Discussions**

A refined method of p53 functional activity estimation is proposed here. When applied to CCLE data, this method classified Nutlin-3A sensitivity in cancer cell lines with high accuracy, identifying non-functional p53 WT samples. This activity score significantly predicts response to chemotherapy in CRC, while mutation status does not.

**Conclusion**

This analysis highlights heterogeneity across published p53PGS and the need to account for p53 target gene MOR when estimating p53 functional activity. This most benefits stratification of epithelial-rich CRCs, improving upon mutation status alone and previously published transcriptomic methods of estimating p53 functional activity to identify tumors with true baseline p53 activity, associated with differential prognosis and response to standard of care chemotherapy.

### EACR23-1174
### Detection of isoforms and genomic alterations by high-throughput full-length single-cell RNA sequencing for personalized oncology

U. Lischetti[1], A. Dondi[2], F. Jacob[1], F. Singer[3], N. Borgsmüller[2], T.P. Consortium[4], V. Heinzelmann-Schwarz[1], C. Beisel[2], N. Beerenwinkel[2]

© 2023 The Authors. Molecular Oncology is published by John Wiley & Sons Ltd on behalf of Federation of European Biochemical Societies

[1]University Hospital Basel and University of Basel, Department of Biomedicine, Basel, Switzerland
[2]ETH Zürich, Department of Biosystems and Engineering, Basel, Switzerland
[3]ETH Zürich, NEXUS Personalized Health Technologies, Basel, Switzerland
[4]ETH Zürich- University Hospital Zürich- University of Zü rich- University Hospital Basel- Roche, Swiss Tumor Profiler, Basel- Zürich, Switzerland

**Introduction**

Cancer research and precision cancer diagnostics would greatly benefit from genotype-phenotype information at single-cell resolution. Unlike standard short-read single-cell RNA sequencing (scRNA-seq) that only sequences transcript ends for gene expression detection, long-read scRNA-seq capturing full-length transcripts has the inherent capability to profile isoform-level gene expression as well as genomic alterations such as mutations or gene fusions. The aim of this study was to (1) improve and overcome previous limitations in long-read sequencing output and (2) apply the technology to a clinical oncological cohort.

**Material and Methods**

We applied long-read scRNA-seq to the field of oncology. We freshly processed five samples from three patients with metastatic high-grade serous ovarian cancer and compared scRNA short-read Illumina and long-read PacBio sequencing. Leveraging multiple experimental strategies including artefact removal and transcript concatenation, we were able to increase long-read sequencing output by 50-fold corresponding to a sequencing depth of 12k reads per cell.

**Results and Discussions**

The increase in sequencing output allowed for short read-independent cell type annotation and gene expression measurement. Our approach captured 152,000 isoforms, of which over a third has never been reported. We detected cell type- and cell-specific isoform usage, and revealed differential isoform expression in tumor and mesothelial cells. Differential insulin-like growth factor pathway usage was detected in ovarian cancer cells and cells undergoing mesothelial-to-mesenchymal transition in the tumor stroma. Furthermore, we identified gene fusions, including a novel scDNA sequencing-validated IGF2BP2::TESPA1 fusion. This was misclassified as high TESPA1 expression in matched short-read data thus highlighting that care should be taken when interpreting short-read scRNA-seq gene expression data from tumor types that display high amounts of genome instability. Last, we successfully called somatic and germline mutations (e.g. transcribed TP53 mutations in cancer cells), confirming targeted NGS cancer gene panel results.

**Conclusion**

We demonstrate the potential of long-read scRNA-seq to provide combined genotype-phenotype information for clinical cancer samples. With multiple new opportunities we envision long-read scRNA-seq to become increasingly relevant in oncology and personalized medicine.

### EACR23-1237
### Functional Characterization of BRCA1 and BRCA2 Variants of Uncertain Significance (VUS) in Patients with Hereditary Cancer Syndrome

S. Ustun Yilmaz[1], K. Manto[2], N.B. Agaoglu[3], C. Uras[4,5], O. Uysal Sonmez[6], M. Muftuoglu[1,7], U. Özbek[2,8]
[1]Department of Medical Biotechnology, Institute of Health Sciences, Acibadem Mehmet Ali Aydinlar University, Istanbul, Turkey
[2]Department of Genome Studies, Institute of Health Sciences, Acibadem Mehmet Ali Aydinlar University, Istanbul, Turkey
[3]Department of Medical Genetics, Umraniye Training and Research Hospital, University of Health Sciences, Istanbul, Turkey
[4]Department of General Surgery, School of Medicine, Acibadem Mehmet Ali Aydinlar University, Istanbul,Turkey
[5]Research Institute of Senology, Acibadem Mehmet Ali Aydinlar University, Istanbul, Turkey
[6]Department of Medical Oncology, School of Medicine, Acıbadem Mehmet Ali Aydınlar University, Istanbul, Turkey
[7]Molecular Biology and Genetics, Faculty of Arts and Sciences, Acibadem Mehmet Ali Aydinlar University, Istanbul, Turkey
[8]Department of Medical Genetics, School of Medicine, Acibadem Mehmet Ali Aydinlar University, Istanbul, Turkey

**Introduction**

The development of genetic sequencing has dramatically increased the detection of variants with next-generation sequencing (NGS) based gene panel testing in hereditary cancers. Identifying variants in actionable genes may lead to risk reduction strategies such as enhanced surveillance, preventative surgery, and lifestyle changes to lessen morbidity and death. In addition to pathogenic/likely pathogenic variants, NGS also identifies variants of uncertain significance (VUS), and the role that plays in cancer development is obscure. The variant interpretations should be accurate for clinical usage, and the combination of disease and population databases, *in-silico* pathogenicity prediction algorithms, segregation data, and functional assay results should be evaluated. However, these evidence codes cause a variant interpretation between laboratories. From this point of view, we aim to construct a new fast, highly efficient, easily applicable, and aid in diagnosis functional analysis workflow to determine the clinical effects of missense variants classified as VUS in the *BRCA1* and *BRCA2* genes, which are involved in the homologous recombination (HR) DNA repair pathway and play a role in the repair of DNA double-strand breaks (DSB).

**Material and Methods**

We used population datasets and variant pathogenicity predictors to classify the variants for in-silico analysis. A consensus score was tallied for BRCA1/2 gene variants prone to be pathogenic. We also employed protein 3D structure-based techniques to predict the protein-level detrimental effect of these missense variant alterations. The blood samples of individuals will be collected to investigate the effects of candidate VUSs on the DSB repair function of BRCA1/2 proteins. PBMC will be isolated, and the accumulation of DSB in the cell by endogenous (non-induced) and induced by the DSB agent will be measured by Comet assay and γ-H2AX analyses.

**Results and Discussions**

© 2023 The Authors. Molecular Oncology is published by John Wiley & Sons Ltd on behalf of Federation of European Biochemical Societies

We have retrospectively screened 1119 variations in 913 HBOC patients, who were analyzed by hereditary cancer panels, and determined 549 VUS (57 of them BRCA1/2) according to the ClinVar database. Blood samples from 10 individuals will be collected and isolated PBMC examined by Comet Assay after being induced by a DNA-damaging agent. Additionally, γ-H2AX protein phosphorylation levels will be measured.

**Conclusion**

We will combine all outputs to (re)classify the variants and construct a new and effective VUS analysis pipeline for missense VUSs to be interpreted easily for clinical diagnostics.

### EACR23-1301
### Deciphering FGFR3-TACC3 oncogenic fusions

*J. Yemelyanenko[1], D. Zingg[1], J. Bhin[1,2,3], S. Klarenbeek[4], J.Y. Song[4], C. Lutz[1], M. van de Ven[5], S. Ganesan[6], L.F.A. Wessels[2], J. Jonkers[1]*

*[1]Netherlands Cancer Institute, Department of Molecular Pathology, Amsterdam, The Netherlands*
*[2]Netherlands Cancer Institute, Department of Molecular Carcinogenesis, Amsterdam, The Netherlands*
*[3]Yonsei University College of Medicine, Biomedical System Informatics, Seoul, South Korea*
*[4]Netherlands Cancer Institute, Experimental Animal Pathology, Amsterdam, The Netherlands*
*[5]Netherlands Cancer Institute, Mouse Clinic for Cancer and Aging, Amsterdam, The Netherlands*
*[6]Rutgers Cancer Institute of New Jersey, Department of Medicine- Division of Medical Oncology, New Brunswick- NJ, United States*

**Introduction**

Chromosomal rearrangements of the fibroblast growth factor receptor (*FGFR*) genes that give rise to fusions are one of several mechanisms by which the FGF/FGFR signaling axis can become deregulated and result in enhanced signaling in cancer. We have previously identified truncation of exon (E) 18 of *FGFR2* as a potent single-driver alteration in cancer, independently of the rearrangement (RE) partner. In contrast, the same does not appear hold true for its E18-truncated ortholog *FGFR3*.

**Material and Methods**

We mined human oncogenomic datasets from Hartwig Medical Foundation (>2,500 WGS profiles) and Foundation Medicine (>200,000 hybrid-capture panel-seq profiles) for alterations affecting *FGFR3*. Our findings led to the generation of a compendium of *FGFR3* structural variants which we then functionally tested *in vitro* and *in vivo*.

**Results and Discussions**

Examination of structural variants affecting *FGFR3* across multiple oncogenomic datasets found that 85% of all E18-truncating *FGFR3* REs involved transforming acidic coiled-coil-containing protein 3 (*TACC3*) as the downstream fusion partner gene. Additionally, there was a clear predominance of self-interacting domains (95%) among all *FGFR3* RE partners, suggesting enhanced receptor dimerization and downstream signaling capacity for the majority of the *FGFR3* fusions. *In vitro* testing of mouse mammary epithelial cells expressing *Fgfr3*$^{\Delta E18}$-*Tacc3* fusion variants showed that both E18-truncation and a fusion partner were required for 3D outgrowth and signaling induction. *In vivo* evaluation of the oncogenic capacity of *Fgfr3* variants using somatic delivery of lentiviruses to the mouse mammary gland and lung found that *Fgfr3*$^{\Delta E18}$-*Tacc3* fusion variants rapidly induced mammary and lung tumor formation in wild-type and *Trp53*$^{F/F}$ mice, respectively. In contrast, *Fgfr3*$^{full\text{-}length(FL)}$, *Fgfr3*$^{\Delta E18}$ *and Fgfr3*$^{FL}$*–Tacc3* were non-tumorigenic. Noteworthy, mammary tumors driven by *Fgfr3*$^{\Delta E18}$-*Tacc3* fusions were sensitive to the FGFR inhibitor AZD4547 during a drug intervention study.

**Conclusion**

In summary, our findings show that E18-truncating *FGFR3* alterations are recurrent across human cancers and, as opposed to *FGFR2* E18-truncating alterations, *FGFR3* appears to depend both on E18-truncation and an additional fusion partner with dimerizing capacity. Notably, *TACC3* is the most recurrent 3' fusion partner across all *FGFR3* E18-truncating alterations, and somatic tumor modeling has shown that *Fgfr3*$^{\Delta E18}$-*Tacc3* fusions drive mouse mammary and lung tumorigenesis, and are sensitive to FGFR-targeted therapy

### EACR23-1302
### Gaining a clue from 1q: A high-risk chromosomal gain in pediatric posterior fossa ependymoma

*C. Richman[1,2], A. Rasnitsyn[1,2], D. Przelicki[2,3], G. Macleod[4], K. Kharas[2,3], J. Lee[2,3], W. Ong[2,3], R. Suarez[2], S. Angers[4], A. Michealraj[5], M.D. Taylor[2,6]*

*[1]University of Toronto, Medical Biophysics, Toronto, Canada*
*[2]The Hospital for Sick Children, Developmental & Stem Cell Biology, Toronto, Canada*
*[3]University of Toronto, Laboratory Medicine and Pathobiology, Toronto, Canada*
*[4]Donnelly Centre for Cellular & Biomolecular Research, Cell & Systems Biology, Toronto, Canada*
*[5]University of Pittsburgh, Neurological Surgery, Pittsburgh, United States*
*[6]Texas Children's Hospital, Pediatric Neuro-Oncology Research Program, Houston, United States*

**Introduction**

Malignancies of the central nervous system are the leading cause of cancer related death in children and adolescents. Ependymomas are neuroepithelial tumours that can be classified into distinct molecular subgroups based on their anatomical location, genetic alterations, and expression profile – each exhibiting a unique clinical outcome. The most aggressive and abundant subgroup of ependymoma occurs within the cerebellum and is known as posterior fossa group A (PFA). Efforts to identify actionable targets have traditionally been hampered by the fact that PFAs have no highly recurrent driver mutations. However, approximately 25% of PFA tumours exhibit gains of the chromosome arm 1q which is associated with extremely poor prognosis despite aggressive treatment. By interrogating the multi-omic landscape of PFAs harbouring this copy number variation, we aimed to find genetic

© 2023 The Authors. Molecular Oncology is published by John Wiley & Sons Ltd on behalf of Federation of European Biochemical Societies dependencies from which an actionable therapeutic can be derived.
**Material and Methods**
Proteomic (LC-MS/MS) and transcriptional analyses were conducted on patient tissues and primary patient-derived cell lines to gain insight into the pathways that drive 1q gain pathogenesis. Further, the contribution of essential genes was investigated using genome wide CRISPR knockout screens. These methodologies were complemented by chromatin immunoprecipitation sequencing of H3K27me3 and H3K27Ac marked histones for global epigenetic analyses.
**Results and Discussions**
Through the comparison of 1q gain and balanced PFA samples, a characteristic gene signature was derived using both bulk and single cell sequencing approaches. Our multi-omic analyses identified the upregulation and essentiality of known oncogenic drivers, now implicated in the pathobiology of these tumours. Investigation of these candidate genes have shown a convergence on pathways related to ciliogenesis with therapeutic potential.
**Conclusion**
This combined approach has allowed for the derivation of a functional cancer signature underlying 1q gains in PFA ependymoma, and genetic vulnerabilities that may be leveraged for therapy. These targets will inform drug screening using our unique cell lines *in vitro*, to be further validated using our established xenograft mouse models for pre-clinical testing. PFA ependymoma is a dreadful disease whose complex biology has so far made it immune to therapy, and new insights obtained from this project will allow us to test and identify the first effective therapies for this vulnerable patient population.

### EACR23-1325
### SCREEN: Spatial transcriptomic effects of a panel of pre-clinical and clinical targeted therapeutic combinations in a clinically relevant prostate explant model
*U. Okoli[1,2], G. Akman[1], T. Poolman[3], J. Griffin[4], S.R. Ohayi[5], B. Simpson[1], H. Pye[1], H. Whitaker[1], U. Cheema[1], S. Heavey[1]*
*[1]University College London, Department of Targeted Intervention-Division of Surgery and Interventional Science, London, United Kingdom*
*[2]University of Nigeria Nsukka, Department of Pharmacology and Therapeutics, Enugu, Nigeria*
*[3]University College London, Department of Structural & Molecular Biology, London, United Kingdom*
*[4]Sheffield Teaching Hospital NHS Foundation Trust, Histopathology Department, Sheffield, United Kingdom*
*[5]Enugu State Teaching Hospital and College of Medicine, Department of Histopatology, Enugu State, Nigeria*
**Introduction**
Prostate Cancer remains a global threat, with nearly 400,000 deaths per year despite the wide-ranging breakthroughs in its detection and treatment. One issue contributing to this is poor success rates of drug development, which we posit is intrinsically linked with over-reliance on cell lines and murine models at the pre-clinical stage. SCREEN was established to help overcome this. We aimed to take one of the most clinically relevant prostate cancer models available for drug screening – the gelatin sponge explant method – and scrutinize its true clinical relevance via spatial transcriptomics, compared head-to-head with patient-matched specimens. With these aims now complete (and presented separately at this conference), we can now offer a detailed spatial transcriptomic analysis of the effects of treatment of these explants with a panel of pre-clinical and clinical drug combinations drugs.
**Material and Methods**
The drugs chosen we previously studied and published in standard cell lines and a pilot explant study, showing promising results. These drugs were AZD-1208, a pan-PIM kinase inhibitor; BEZ235/Dactolisib, a pan-PI3K-mTOR dual inhibitor, a combination of both AZD-1208 and BEZ235, and AUM-302 – a preclinical PIM, PI3K, mTOR triple inhibitor. We compared these treatments in tumour versus benign tissue, epithelial versus stromal tissue, across distinct pathological regions and in comparison with matched untreated cultured samples and matched uncultured samples.
**Results and Discussions**
AZD-1208 treated samples expressed genes that are regulated by genes associated with senescence within stromal regions. BEZ235 treated samples expressed genes that coregulate AR expression. AUM302 induced more spatially resolved transcriptomic variation when compared to the other drug treatments. Downstream analysis revealed that most drug treatments induced apoptosis. However, AZD-1208 activated PI3K cascade. MKi67 and PIM genes activity switched between different cell types in response to the different treatments, which may be compensatory.
**Conclusion**
We conclude that pre-clinical drug development can and should be carried out not just on cancer cells but on complex models, including epithelium, stroma, and benign areas, and that when such drug screening is carried out, advanced endpoint analyses such as spatial transcriptomics are warranted, in order to properly assess both the promise and the pitfalls of drug candidates in clinically relevant settings.

### EACR23-1342
### Identifying metastasis-associated genes in bone sarcoma using circulating tumour cells
*S. Llaneza-Lago[1], E. Bull[1], W.D. Fraser[1], D. Green[1]*
*[1]University of East Anglia, Medical School, Norwich, United Kingdom*
**Introduction**
Primary bone cancer (PBC) is a predominantly childhood cancer with poor survival rates, particularly when metastasis is present. Understanding the molecular and cellular mechanisms of PBC metastasis is critical for developing effective therapeutic strategies. Circulating tumour cells (CTCs) offer a minimally invasive approach to investigate mechanisms of cancer spread. Identifying the master regulators that drive pathological gene expression in CTCs is a challenge, especially in childhood cancer.
Here, we present an approach using CTC capture and single-cell RNA-sequencing to compare the expression profiles of tumour cells versus prometastatic CTCs. Our

© 2023 The Authors. Molecular Oncology is published by John Wiley & Sons Ltd on behalf of Federation of European Biochemical Societies objective is to identify metastasis-associated genes and determine their role in PBC spread.

**Material and Methods**

Live and viable CTCs were isolated from 7.5 mL of whole blood from 10 patients with high-grade osteosarcoma or Ewing sarcoma using the ClearCell FX system. Single CTCs were manually picked for RNA-seq and total RNA was extracted and sequenced using the Illumina NovaSeq 6000. Reads were aligned to the human genome, and transcript count matrices were generated. Cells were clustered and analysed using Monocle3, and differentially expressed (DE) transcripts were identified using the DESeq2 package in R ($log_2$ fold change ≥3, p-adjusted ≤ 0.001). KEGG pathway and GO enrichment analyses were performed on the selected transcripts using the R package ClusterProfiler.

**Results and Discussions**

Cluster analysis revealed a clear separation between CTCs and tumour cell controls. This demonstrates the significant transcriptomic differences between tumour-based cells and CTCs, reinforcing the value of CTCs as a valuable tool for investigating metastasis.

We identified 161 DE transcripts, which were enriched for immune response regulation. Of these, 20 were pseudogenes with no current known function. Our data suggest the value of these genes as both markers and therapeutic targets for metastasis.

**Conclusion**

This first stage of a larger planned study provides comprehensive insights into the transcriptomic changes underlying bone sarcoma metastasis and highlights the potential importance of pseudogenes in disease progression. These data emphasize the urgent need for novel therapeutic strategies to target the biological pathways leading to metastasis in childhood cancer. Our findings also suggest that the use of CTCs as a diagnostic tool holds promise for improved cancer detection and monitoring, allowing real-time analysis of tumour progression and response to therapy.

### EACR23-1392

### POSTER IN THE SPOTLIGHT

### The Curated Cancer Cell Atlas: single-cell cancer biology at an unprecedented scale

*M. Tyler[1], A. Gavish[1], R. Tschernichovsky[1], C. Barbolin[1], I. Tirosh[1]*

*[1]Weizmann Institute of Science, Molecular Cell Biology, Rehovot, Israel*

**Introduction**

Over the last decade, single cell RNA-seq (scRNA-seq) has emerged as a powerful method to study cancer biology, with a rapidly growing list of studies that characterise tumour samples from patients at single cell resolution. However, most studies investigate a relatively small number of tumours, severely limiting their statistical power. Moreover, the ability to compare data across different studies is hindered by considerable technical limitations, including batch effects and inconsistency in format, quality and cell and sample annotations.

**Material and Methods**

To overcome these challenges, we curated a large repository of over 130 published cancer scRNA-seq datasets, together comprising over 2000 tumours. We assigned cell types within each dataset by combining the original studies' annotations with further validation and refinements, resulting in consistent annotations that enable combined analysis.

**Results and Discussions**

We exploited the size of this data resource to pursue multiple pan-cancer analyses, including (i) defining the hallmarks of transcriptional intra-tumour heterogeneity; (ii) quantifying proliferation rates and cell cycle phase bias; (iii) characterising EMT programs and their context-specificity; and (iv) identifying expression patterns associated with clinical features. Finally, we created a website that makes all data, annotations and analyses freely available for exploration and download. Collectively, this effort establishes the Curated Cancer Cell Atlas (3CA).

**Conclusion**

We present the Curated Cancer Cell Atlas (3CA) and describe multiple examples of its application. 3CA serves as a central source of data and analyses for all cancer researchers, and its magnitude opens new avenues in cancer research.

### EACR23-1394

### Spatially resolved gene and protein analysis in multiple cancer tissues

*A. Patel[1], S. Williams[2], L. Gutgesell[1], B. Veire[1], A. Santiago[1], H. Singh[1], R. Chan[1], G. Kamath[2], S. Russell[3], D.J. Sukovich[1]*

*[1]10X Genomics, Molecular Biology, Pleasanton- CA, United States*

*[2]10X Genomics, Computational Biology, Pleasanton- CA, United States*

*[3]10X Genomics, Product Marketing, Pleasanton- CA, United States*

**Introduction**

The tumor microenvironment (TME) is composed of highly heterogeneous cell types that dynamically interact with each other. Traditional tissue-based studies of the TME can be limited to a small number of target analytes, which can limit biological insights. Here we use the Visium CytAssist instrument from 10x Genomics to profile whole transcriptome gene and protein expression from multiple formalin-fixed paraffin-embedded (FFPE) cancer tissues. The Visium CytAssist Spatial Gene and Protein Expression Assay enables multiomic analysis of tissues using a whole transcriptome probe-based approach to detect and quantify mRNA expression with spatial context, in combination with antibody-conjugated probes for spatially accurate detection of immune populations and oncology markers.

**Material and Methods**

Tumor FFPE tissues (including breast, colon, lung, and ovarian cancer) were spatially profiled using the Visium CytAssist instrument and Visium Spatial Solutions. Tissues were mounted on glass slides, H&E or IF stained, and imaged to select the target region for whole transcriptome analysis. Following incubation with gene and antibody-conjugated probes, the samples were prepared for transfer to spatially barcoded Visium slides with 6.5 x 6.5 or 11 x 11 mm capture areas. The captured probes were used in a downstream workflow to generate sequencing-ready libraries.

© 2023 The Authors. Molecular Oncology is published by John Wiley & Sons Ltd on behalf of Federation of European Biochemical Societies

**Results and Discussions**
Using the CytAssist workflow, we showcase the ability to spatially resolve oncogenes and immune cells associated with multiple tumor tissues, including an array of human breast cancer punches. Expression of these markers map back to distinct morphological features within the samples, allowing identification of differentially expressed genes and proteins associated with those areas.
**Conclusion**
Overall, these data highlight the value of Visium CytAssist Spatial Gene and Protein Expression Assay in immuno-oncology studies through the integration of spatially resolved transcriptomic and immune cell marker data. The spatial distribution of immune cells with respect to malignant cells can directly impact patient prognosis and overall survival outcomes. Our data provide a comprehensive understanding of cellular behavior in and around tumors yielding new insights into disease progression and therapeutic response.

### EACR23-1411
### POSTER IN THE SPOTLIGHT
### The Cancer Proteome Atlas, a large pan-cancer landscape based on mass spectrometry

*C. Jimenez*[1], *F. Böttger*[1], *M. Lyu*[2], *T. Pham*[1], *J. Knol*[1], *F. Rolfs*[1], *M. Nunes Monteiro*[1], *A. Vallés-Martí*[1], *R. de Goeij de Haas*[1], *I. Bijnsdorp*[1], *F. Zhang*[2], *J. Aa*[2], *B. Sitek*[3], *J. Lehtiö*[4], *J.P. Medema*[5], *A. Henneman*[1], *J. Koster*[5], *S. Piersma*[1], *T. Guo*[2]
[1]*Amsterdam University Medical Center, Medical Oncology- OncoProteomics Laboratory, Amsterdam, The Netherlands*
[2]*Westlake University, Westlake Laboratory of Life Sciences and Biomedicine, Hangzhou, China*
[3]*Medizinisches Proteom-Center, Clinical Proteomics, Bochum, Germany*
[4]*Karolinska Institutet, Science for Life Laboratory, Stockholm, Sweden*
[5]*Amsterdam University Medical Center, Center for Experimental Molecular Medicine, Amsterdam, The Netherlands*

**Introduction**
Next generation quantitative proteomics based on data-independent acquisition mass spectrometry (DIA-MS) enables large scale clinical proteomics. Here we report a multi-laboratory collaborative cancer proteome profiling effort, **T**he DIA-MS-based **C**ancer **P**roteome **A**tlas (**TCPA**) project. We generated a pan-cancer proteome landscape consisting of 1236 tissue samples including 999 primary cancers to better understand cancer biology and to identify core and cancer type enriched molecular therapeutic targets and biomarkers.
**Material and Methods**
Fresh-frozen, FFPE and RNAbee left-over tissue samples were included. Samples were processed via in-gel and in-solution digest workflows. HeLa tryptic digest injections were included in each batch. Mass spectrometry data were acquired in DIA mode and searched using DiaNN and the DPHL pan cancer spectral library. Label-free quantitation was performed using the IQ package which uses the MaxLFQ algorithm.
**Results and Discussions**
The total dataset filtered for 30% data presence per cancer type includes 9663 proteins. Unsupervised analyses as based on UMAP, hierarchical clustering and ssGSEA of cancer hallmarks show remarkable cancer type clustering despite heterogeneous sample types. Known cancer biology is rediscovered by cancer hall mark analysis and Weigthed Gene Coexpression Analysis, along with novel cancer type associated proteins. On-going analyses focus on the development of a cancer type classifier and on the 195 colorectal cancers in the landscape, comparing RNA-based CMS subtypes, proteomic consensus subtypes in relation to immune subsets and survival.
**Conclusion**
Our pan-cancer proteomics landscape comprises a unique data resource for the cancer research community that will be expanded in the coming years.

### EACR23-1417
### Profiling extrachromosomal circular DNA heterogeneity at the single cell level with scEC&T-seq

*M.D.R. Chamorro Gonzalez*[1], *T. Conrad*[2], *M. Stöber*[3], *R. Xu*[1], *M. Giurgiu*[1], *E. Rodriguez-Fos*[1], *K. Haase*[1], *R. Koche*[4], *A. Henssen*[1]
[1]*Charité, Department of Pediatric Oncology and Hematology, Berlin, Germany*
[2]*Max Delbrück Center for Molecular Medicine MDC, Genomics Technology Platform, Berlin, Germany*
[3]*Max Delbrück Center for Molecular Medicine MDC, Berlin Institute for Medical Systems Biology BIMSB, Berlin, Germany*
[4]*Memorial Sloan Kettering Cancer Center, Center for Epigenetic Research, New York, United States*

**Introduction**
Extrachromosomal DNA (ecDNA) amplification is a common event in cancer associated with worse patient outcome. One key feature of ecDNA is its ability to randomly segregate during mitosis, which promotes rapid intercellular heterogeneity allowing tumors to rapidly evolve and scape therapy. Smaller, non-amplified extrachromosomal circular DNAs are also abundantly identified in both healthy and malignant tissues, but their function in cancer is still unknown. Understanding how extrachromosomal circular DNA contributes to intercellular heterogeneity in cancer remains crucial, however methods for an unbiased characterization of ecDNA in single cells are lacking. We introduce *scEC&T-seq* (single cell extrachromosomal circular DNA and transcriptomic sequencing), a method for parallel detection of ecDNA and full-length mRNA in single cells.
**Material and Methods**
In scEC&T-seq, single cells are isolated and their transcriptomic content is captured and processed separately from their DNA using full-length transcript sequencing. As with most state-of-the-art circular DNA isolation protocols, single cell's DNA is subjected to exonuclease digestion to deplete linear DNA, followed by rolling circle amplification to further enrich for circular DNA elements. The method was first tested in neuroblastoma cell lines and

© 2023 The Authors. Molecular Oncology is published by John Wiley & Sons Ltd on behalf of Federation of European Biochemical Societies further validated in primary tumors and non-malignant T-cells.

**Results and Discussions**

scEC&T-seq was able to capture and recapitulate the structural complexity of ecDNAs in single neuroblastoma cells, and the matching transcriptomic data allowed the identification of fusion transcripts resulting from the rearranged extrachromosomal structures. More interestingly, scEC&T-seq was able to identify intercellular differences in ecDNA structure allowing the inference of ecDNA structural dynamics in primary tumors. Additionally, our method revealed how intercellular differences in ecDNAs' content and copy number can drive differences in oncogene levels in single neuroblastoma cells. Besides oncogene-containing ecDNA, scEC&T-seq also characterized hundreds of smaller, non-amplifed circular DNAs per single cell. Our data revealed that whereas ecDNAs were clonally present in most cancer cells, only a very small fraction of small circular DNAs was recurrently identified in single cells indicating yet unknown prerequisites for their maintenance and propagation.

**Conclusion**

We envision that scEC&T-seq will help unravel yet unanswered questions about the biology of small and large circular DNA in cancer and beyond.

### EACR23-1420

### HER2 overexpression induces breast tumorigenesis in a non-cell autonomous manner by inducing oxidative stress in the tissue microenvironment

*S.B. Gurler[1], F. Chen[1], C. Lawless[2], R. Pedley[1], D. Lili[1], W. Oliver[1], P. Kyriaki[1], A. Gilmore[1], K. Brennan[1], A. Ucar[1]*

*[1]The University of Manchester, Division of Cancer Sciences- School of Medical Sciences- Faculty of Biology- Medicine and Health, Manchester, United Kingdom*

*[2]The University of Manchester, Wellcome Trust Centre for Cell Matrix Research- Division of Cell Matrix Biology and Regenerative Medicine- School of Biological Sciences Faculty of Biology- Medical and He alth Sciences, Manchester, United Kingdom*

**Introduction**

Breast cancer (BC) is the most common cancer type in the world. 20% of invasive BC cases are associated with HER2 overexpression/amplification, although almost 70% of the pre-malignant stage BCs -ductal carcinoma in situ (DCIS)- overexpress HER2. High levels of therapy resistance and tumour relapse are related to breast cancer stem cells (BCSCs) in patients with HER2-positive invasive BC, even though HER2-targeted therapies can significantly improve the prognosis. Biological mechanisms of HER2 function in breast tumour cells and BCSCs have been extensively studied, but the role of HER2 during pre-malignant stages of tumorigenesis is still not well understood.

**Material and Methods**

We investigate the *HER2* overexpression driven cellular events at the premalignant stage of breast tumorigenesis using *in vitro*, *in vivo and ex vivo* models. We have performed mammosphere assays using primary cells obtained from a HER2+ BC mouse model and clinical samples of patients with DCIS to investigate BCSCs during tumour initiation. Proteomics and metabolomics analyses of mammary ducts from MMTV-Neu mice before development of histologically obvious tumours allowed us to identify pre-malignant stage alterations in the mammary epithelial cells *in vivo*. Immunostaining on histological sections of the mammary glands of MMTV-Neu mice were used to detect oxidative stress markers.

**Results and Discussions**

Our results have shown that BCSCs reside in HER2-negative populations both in patient samples of DCIS and murine tumours. Moreover, BCSCs arise from Neu-negative lineage in MMTV-Neu tumours. At a molecular level, *HER2/Neu* overexpression leads to an upregulation of glucose and lipid metabolism resulting in elevated reactive oxygen species levels in mammary epithelia that consequently cause induction of oxidative stress and accumulation of DNA damage not only in Her2-positive cells but also in neighbouring Her2-negative cells at the premalignant stage.

**Conclusion**

HER2 overexpression in normal mammary epithelia may lead to a genotoxic tissue microenvironment that may consequently result in the accumulation of further mutations in HER2-negative cell populations, which has been shown here to contain BCSCs. The accumulation of a specific set of mutations may underlie the observed tumour evolution during the earliest stages of breast tumorigenesis. Further understanding of the role of HER2 overexpression at early pre-malignant stage breast tumorigenesis will allow us to develop more efficient therapeutic and novel preventive strategies against BC.

### EACR23-1438

### Whole Genome versus 50-gene panel sequencing: diagnostic yields on real-world data.

*J. van Putten[1], M. Baksi[1], E. Cuppen[1,2], P. Roepman[1,3]*

*[1]Hartwig Medical Foundation, Medical, Amsterdam, The Netherlands*

*[2]University Medical Centre Utrecht, Centre for Molecular Medicine and Oncode Institute, Utrecht, The Netherlands*

*[3]Netherlands Cancer Institute, Department of Pathology, Amsterdam, The Netherlands*

**Introduction**

Clinical-grade Whole Genome Sequencing (WGS) has been shown to have equal performance and turn-around-time as panel-based Next-Generation Sequencing (pNGS), and is increasingly used for routine diagnostics in certain countries and settings. However, pNGS aimed at multi-gene analysis is still the most commonly used diagnostic approach, although a limited number of genes or exons are covered. An important question is to what extent diagnostically relevant events are missed by that approach, and what the added value of WGS is. In this study, we assessed the diagnostic yield of a widely used NGS panel by an *in silico* analysis of a large 'real-world' WGS database of patients with metastatic cancer.

**Material and Methods**

*In silico* analysis of Single Nucleotide Variant (SNV) coverage was performed on data of 6,068 metastatic

© 2023 The Authors. Molecular Oncology is published by John Wiley & Sons Ltd on behalf of Federation of European Biochemical Societies tumour samples in the Hartwig Medical Database. The common gene panel that was considered was the 50-gene AmpliSeq® for Cancer Hotspot Panel v2. The pNGS design was integrated and queried across the SQL database. To represent realistic diagnostic yields, WGS findings were restricted to aberrations in genes associated with high-level clinical evidence (level A/B from JAX CKB) and included multiple filtering steps, such as driver likelihood and bi-allelic status for tumour suppressors.

**Results and Discussions**

WGS identified a total of 25,564 tumour-specific SNVs (14,901 distinct) in 106 clinically relevant genes. Of these, 7,485 SNVs (158 distinct) in 39 genes were covered by pNGS, indicating a calculated pNGS yield of only 29%. Calculated yields for SNVs per gene range from 3% for MLH1 to nearly 100% for KRAS. Calculated pNGS yields of other genes include BRAF 84%, EGFR 71%, FGFR3 35%, KIT 57%, and TP53 87%, because only part of the genes is covered in the target design. This initial analysis will be expanded to include copy-number alterations, gene fusions and mutational signatures, for which the added value of WGS is expected to be higher. Future analyses will also include filtering based on driver likelihood and ESCAT level of evidence, and will also be repeated for larger comprehensive gene panels (e.g. TSO500®).

**Conclusion**

In this study, we investigated the *in silico* yield of 50-gene panel sequencing compared to WGS on real-world data for genes associated with clinical relevance. Overall diagnostic yield of pNGS was calculated to be 29% but varied strongly between genes (range: 3% to 100%). In future analyses, driver likelihood, ESCAT levels, and other (larger) panels will also be considered.

### EACR23-1446
### An excellent target for therapy of metastatic Group 3/ 4 Medulloblastoma

*V. Ferrucci[1], F. Bibbò[1], F. Asadzadeh[2], P. De Antonellis[2], C. Sorice[3], P. Penfornis[4], A. Mouton[5], M. Ibrahim[6], S. Amente[2], F. Gorini[2], E. Pedone[7], D. Diana[7], R. Fattorusso[7], M.E. Errico[8], V. Donofrio[8], L. Quaglietta[9], L. De Martino[9], G. Cinalli[10], P.P. Claudio[4], M. Zollo[2]*
*[1]Federico II University of Naples, Department of Molecular Medicine and Medical Biotechnology, Naples, Italy*
*[2]Federico II- University of Naples, Department of Molecular Medicine and Medical Biotechnology, Naples, Italy*
*[3]CEINGE Franco Salvatore- Biotecnologie Avanzate, CEINGE Franco Salvatore- Biotecnologie Avanzate, Naples, Italy*
*[4]University of Mississippi Medical Center, Department of Pharmacology & Toxicology, Jackson, United States*
*[5]University of Mississippi Medical Center, Department of Physiology and Biophysics, Jackson, United States*
*[6]University of Mississippi, National Center for Natural Product Research, Oxford, United States*
*[7]Federico II- University of Naples, Dipartimento di Farmacia, Naples, Italy*
*[8]Santobono-Pausilipon Children's Hospital, Surgical Pathology, Naples, Italy*
*[9]Santobono-Pausilipon Children's Hospital, Department of Onco-Hematology- Unit of Neuro-oncology, Naples, Italy*
*[10]Santobono-Pausilipon Children's Hospital, Department of Neurosciences- Unit of Pediatric Neurosurgery, Naples, Italy*

**Introduction**

Medulloblastoma (MB) is an embryonal tumor of the cerebellum constituting ~ 20% of pediatric brain tumors. To date, four MB molecular groups (further stratified in twelve subtypes) have been described. Among them, Groups 3 and Group 4 MB have the poorest prognosis due to their high metastatic potential. Recently, we have reported a novel metastatic axis driven by Prune1 overexpression in MB Group3 characterized by canonical TGF-β signaling enhancement, upregulation of OTX2 and inhibition of PTEN, thus affecting the epithelial-mesenchymal transition.

**Material and Methods**

We have generated a novel murine genetically engineered mouse model (GEMM) of metastatic MB driven by PRUNE1. NGS approaches, RNAseq analyses and cytokine arrays were used in preclinical in vivo studies to decipher those inflammatory pathways of immune cells within the brain tumor microenvironment. Real-time cell proliferation assays and Seahorse analyses were performed to study the anti-proliferative and anti-Warburg effects, respectively, of a novel anti-PRUNE1 inhibitor.

**Results and Discussions**

Here, we have developed a not toxic pyrido-pyrimidine derivative with the ability to impair Prune-1-driven-axis, thus ameliorating the survival rate of a new GEMM of metastatic MB Group3 characterized by overexpression of human PRUNE1 gene in the cerebellum. This small molecule is showing immunomodulatory functions by inhibiting the conversion of tumor-infiltrating T lymphocytes (TILs) to immunosuppressive regulatory T cells (Tregs) in vivo via impairing the secretion of inflammatory cytokines from MB cells. This molecule also exerts an anti-Warburg action, thus affecting tumor cell metabolism via lowering the Oxygen Consumption rate (OCR) by targeting those mitochondria-related functions. This molecule can also act synergistically with the currently used chemotherapy (e.g., Vincristine), with epigenetics drugs (e.g., LSD1/KDM1A inhibitors) or anti-glycolytic drugs (Gnetin-H).

**Conclusion**

Altogether these results are of importance for future targeted therapies of high-risk metastatic MB.

### EACR23-1464
### Stratifying tumour heterogeneity through imaging and transcriptomic profiling of colorectal organoids.

*P.L. White[1], Y. Hadzhiev[1], J. Wragg[1], L. Tee[1], A. Beggs[1], F. Müller[1]*
*[1]University of Birmingham- College of Medical and Dental Sciences, Institute of Cancer and Genomic Sciences, Birmingham, United Kingdom*

**Introduction**

Tumour heterogeneity is one of the biggest clinical challenges in cancer research with non-genetic diversity playing a key role in tumour aetiology. Recent findings from our lab show that transcription start site (TSS) usage within and among transcriptional promoters correlates with

© 2023 The Authors. Molecular Oncology is published by John Wiley & Sons Ltd on behalf of Federation of European Biochemical Societies global tumour responsiveness to irradiation and may act as a potential marker of tumour heterogeneity. However, extensive phenotypic variability exists within a single tumour, which prompted us to explore promoter usage in cancer cell states at the single-cell level.
**Material and Methods**
In this study, we carried out single-cell 5' captured RNA sequencing of colorectal cancer organoids with high precision of TSS detection. To determine cellular states, unsupervised clustering and gene ontology analysis of variable markers was performed. Promoter usage was determined through CAGEr analysis to identify transcription start sites, and to determine promoter utilisation patterns. In parallel, spatiotemporal mapping of cellular states was achieved through high-resolution 3D imaging using light sheet microscopy.
**Results and Discussions**
Our results show that not only is promoter usage attributable to whole tumour phenotype but also shows distinct variability at a subpopulation level. In vivo imaging analysis highlighted the continuum of cancer cell states and their transitions during organoid growth and have been correlated with transcriptomic profiles to identify the underlying molecular signatures of observed cell behaviour heterogeneity.
**Conclusion**
Our data highlights the importance of further investigation of an often-overlooked part of transcription regulation, the promoter, and the penetrance of its role present even at a single cell level.

### EACR23-1479
### The Role of Nucleotide Excision Repair (NER) in the mutagenicity of tobacco and alcohol

*F. Rajabi*[1], *A. Yurchenko*[1], *S. Nikolaev*[1]
[1]*Institut Gustave Roussy, Molecular Medecine, Villejuif, France*

**Introduction**
Xeroderma Pigmentosum (XP) is a genetic disorder caused by a constitutive deficiency of nucleotide excision repair (NER). XP patients are characterized by a 10'000-fold increased risk of skin cancer due to their inability to repair UV-induced DNA lesions. It is *shown that XP patients have increased risks of internal cancer. In XP-C patients, we have recently shown an increased intensive damage-induced mutagenesis associated with the purine residues.* The etiology of this mutational process is not well understood. However, it may be associated with genotoxins such as acetaldehyde (AC) or formaldehyde (FA), which are produced in the cell and can have endogenous but also exogenous origins such as benzo[a]pyrene. This project aims to identify the mutational consequences of chronic exposures to broadly distributed genotoxic substances.
**Material and Methods**
in vitro: We generated XPC-KO from the RPE-1 cell line. XPC-KO and NER proficient cell lines are treated with Benzo(a)pyrene Diol Epoxide, BPDE, AC, and FA chronically for 2 months. WGS has been done on the cells. In vivo: WGS of hematopoietic stem cells and liver tissue of XPC-/- and WT mice that were treated with genotoxins. Bioinformatic analysis: WGS data from over 6000 sporadic tumors (PCAWG and HARTWIG consortiums) are retrieved from the repositories. Identified signatures from in vivo and in vitro studies have been used to deconvolute mutational signatures in the cancer cohorts.
**Results and Discussions**
*The WGS results showed 5 times more mutational burden caused by BPDE in XPC-KO cells than XPC expressing cells. However, mutagenesis of AC and FA in XPC-KO cells showed a non-significant difference with XPC-expressing cells.*
Comparative analysis of the mutational profiles from in-vitro and in-vivo experiments may reveal the nature of the mutational process-induced above-listed genotoxins. *We further assess the impact of attenuation of NER (predominant pathway repairing DNA lesions that are induced by these genotoxins) on the mutagenesis. This project will provide clearer information concerning the increased risk of developing internal cancers in individuals with NER mutations.*
**Conclusion**
The results of the WGS displayed different mutational signatures with internal cancers in XP patients. We conclude that tested endogenous or exogenous chemicals are not underlying the unique mutational signature associated with XP patient-internal cancers. The etiology of the revealed signature can be due to the different roles of XPC in DNA repair, which investigation is ongoing.

### Carcinogenesis

### EACR23-0177
### Periprostatic adipose tissue is a target of the obesogen tributyltin empowering its pro-tumorigenic actions

*M. Feijó*[1], *C. R. Casanova*[1], *L. R. S. Fonseca*[1], *L. A. Brás*[1], *E. Kiss-Toth*[2], *S. Correia*[1], *S. Socorro*[1]
[1]*CICS-UBI - Health Sciences Research Centre, University of Beira Interior, Covilhã, Portugal*
[2]*Department of Infection- Immunity and Cardiovascular Di sease, University of Sheffield, Sheffield, United Kingdom*

**Introduction**
The "obese" periprostatic adipose tissue (PPAT) has been correlated with the aggressiveness of prostate cancer (PCa). Also, substantial evidence has linked the emergence of PCa and obesity with environmental influences. Several endocrine-disrupting chemicals capable of deregulating adipose tissue have been identified. So-called obesogens, these compounds alter adipocytes' phenotype and change metabolism favouring fat storage. This study hypothesizes that obesogens deregulate PPAT, being a driving force in PCa.
**Material and Methods**
PPAT isolated from 4-month-old rats was exposed *ex vivo* to the first described obesogen tributyltin (TBT, 0, 1, 10 and 100 nM) for 48 hours. Phenotypical features of PPAT and its secretome were evaluated by histological analysis, MTT assays, and colorimetric assays. Conditioned media assays were performed by exposing neoplastic (PC3) and non-neoplastic (PNT1A) human

© 2023 The Authors. Molecular Oncology is published by John Wiley & Sons Ltd on behalf of Federation of European Biochemical Societies prostate cells to the secretome of TBT-treated PPAT (and control) for 24 hours. Prostate cell viability and proliferation were analysed by MTT assays and Ki67 immunocytochemistry, respectively.

**Results and Discussions**

TBT exposure promoted adipocyte enlargement in the PPAT cultured *ex vivo*, whereas it did not affect cell viability. Phenotypic changes were accompanied by alterations in the oxidative and inflammatory status as indicated by the decreased superoxide dismutase activity and nitrite levels in the TBT-treated PPAT secretome, respectively. Moreover, TBT highly increased the free-fatty acids (FFAs) content in the PPAT secretome, accompanied by higher levels of lipid peroxidation. The deregulation of the oxidative, inflammatory and metabolic status of PPAT in response to TBT with a higher content of FFAs that seems to be made available to cancer cells, augmented lipid peroxidation and altered nitrite levels are supportive of a pro-tumorigenic environment sustaining PCa growth. Indeed, the impact on prostate cell fate of the PPAT alterations driven by TBT was demonstrated. TBT-treated PPAT secretome augmented the viability and proliferation of both PNT1A and PC3 prostate cells compared to the control.

**Conclusion**

Leaning over the PCa-obesity-environment triad, this study brings functional evidence about the capability of TBT exposure being a driving force in prostate carcinogenesis. It also presents the foundation for exploring the mechanistic underlying obesogens-induced PPAT dysfunction enhancing prostate cells' survival and proliferation.

### EACR23-0182

### Tumor-derived FVII is associated with poor prognosis and has a pro-oncogenic role in breast cancer

*B. Kocaturk[1,2], C. Kroone[2], C. Tieken[2], M. Paauwe[2], B. Ünlü[2], J.T. Buijs[2], H.H. Versteeg[2]*

*[1]Hacettepe University Cancer Institute, Basic Oncology Department, Ankara, Turkiye*

*[2]Leiden University Medical Center, Department of Internal Medicine, Leiden, The Netherlands*

**Introduction**

Coagulation activation and tumor progression are interlinked processes. Cancer is associated with a hypercoagulable state and conversely coagulation factors promote cancer progression. Interestingly, the use of prophylactic anticoagulation fails to inhibit tumor stemming thus studies aiming to better understand the role of coagulation factors in tumor progression are needed. Among these factors, FVII is produced both by liver and cancer cells whereas any relations of of tumor-derived and liver-derived FVII to breast cancer progression have not been examined.

**Material and Methods**

The association between FVII expression levels and clinopathological features were determined in a cohort of 574 breast cancer patients. FVII was stably overexpressed in MDA-MB-231 cells. The level of angiogenic and invasive markers were detected by means of qPCR and invasion was analyzed using matrigel coated chambers. Tumor growth was assessed via the orthotopic injection of cells to NOD-Scid-gamma mice and liver-specific FVII knockdown was achieved through tail vein injection of FVII siRNA.

**Results and Discussions**

Tumor FVII protein expression was associated with tumor grade, T-status increased distal metastasis and lower overall survival. Breast cancer cells overexpressing FVII had higher expression of angiogenic and epithelial-mesenchymal transition(EMT) markers accompanied with enhanced invasion *in vitro* and augmented metastasis and tumor growth *in vivo*. In contrast, recombinant FVII displayed an inhibitory role in EMT-related gene expression. In line with this finding liver-specific knockdown of FVII resulted in higher metastasis to liver. A possible explanation for the opposing roles of tumor- and liver-derived FVII was found to be their binding partners, Endothelial Protein C Receptor (EPCR) and Tissue Factor (TF) respectively. Our results indicate that targeting the tumor-derived FVII/EPCR driven signaling cascade might be a useful target for breast cancer tratment.

**Conclusion**

Tumor-derived and liver-derived FVII have opposing effects in breast cancer progression thus targeting tumor-derived FVII driven signaling may arise as a new therapy option for breast cancer patients.

### EACR23-0193

### Therapeutic inhibition of the β-catenin-aerobic glycolysis axis impairs the Triple Negative Breast Cancer (TNBC) progression

*S.M. Umar[1], S.R. Mathur[2], A. Gogia[3], S.V.S. Deo[4], C.P. Prasad[1]*

*[1]All India Institute of Medical Sciences, Medical Oncology Lab, Delhi, India*

*[2]All India Institute of Medical Sciences, Pathology, Delhi, India*

*[3]All India Institute of Medical Sciences, Medical Oncology, Delhi, India*

*[4]All India Institute of Medical Sciences, Surgical Oncology, Delhi, India*

**Introduction**

The aggressive breast cancer subtype known as triple-negative breast cancer (TNBC) is frequently linked to a poor prognosis and an early relapse. Conventional chemotherapy is the mainstay for treating TNBCs because these tumors are clinically negative for the hormones estrogen, progesterone, and HER2/neu receptor. Hence, finding new therapeutic targets in TNBCs is an unmet need. We have previously demonstrated that TNBCs are highly glycolytic, and β-catenin oncoprotein might be one of its positive regulators.

**Material and Methods**

In the present study, Immunohistochemical analysis for β-catenin, PFKP, and MCT1 was performed in 100 TNBC patients. Furthermore, for evaluating the effect of β-catenin inhibition on aerobic glycolysis on TNBC cell lines i.e., MDA-MB-231, MDA-MB-468, and 4T1 cells were treated with inhibitors of β-catenin i.e., XAV939 and Axitinib. Changes in physiological characteristics like proliferation, migration, and invasion were evaluated in the treated cells,

© 2023 The Authors. Molecular Oncology is published by John Wiley & Sons Ltd on behalf of Federation of European Biochemical Societies compared to controls. For *in vivo* evaluation, the 4T1-orthotopic balb/c mice model was used.

**Results and Discussions**

Analysis of TNBCs tumor specimens by immunohistochemistry showed that protein expression of β-catenin was elevated in around 55% of cases, and its expression was positively associated with aerobic glycolysis proteins, suggesting the elevated β-catenin-PFKP-MCT1 signaling axis in TNBC patients. On the therapeutic side, we found that axitinib (a small molecule tyrosine kinase inhibitor) happens to be a better inhibitor of β-catenin than XAV939, as it significantly inhibits β-catenin-PFKP-MCT1 signaling axis in TNBC cell lines. Furthermore, compared with XAV939, axitinib significantly impaired the lactate production, cell migration, and clonogenic potential of TNBCs cells. Using the 4T1-orthotopic balb/c mice model, we further demonstrated that axitinib significantly inhibited the tumor size, when compared with the controls.

**Conclusion**

Overall, our present study advocates the use of axitinib for the treatment of β-catenin positive TNBC patients.

### EACR23-0261
### Synthetic lethality among DNA polymerase theta inhibitors and DDR genes in cancer cells

*O. Manners[1], A. Cerutti[1], V. Grinkevich[1], L. Geo[1], R. Heald[1], K. Katjusa[1], S. Menon[1], J. Neves[1], G. Smith[1], H. Robinson[1]*

*[1]ArtiosPharma, Biology team, Cambridge, United Kingdom*

**Introduction**

DNA polymerase theta (Polθ) is essential for DNA double strand break repair through microhomology-mediated end-joining (MMEJ)[1]. Polθ has very restricted expression in normal tissues but is frequently overexpressed in cancer cells[2]. Its loss sensitises cancer cells, but not normal cells, to DNA damaging agents, such as irradiation[3] as well as targeted agents such as PARP inhibitors[4]. This highlights Polθ as a promising drug target for the selective killing of tumour cells.

**Material and Methods**

We recently developed potent and highly selective Polθ inhibitors showing *in vitro* and *in vivo* efficacy in cancer models[4-6]. To explore the possible therapeutic applications of these inhibitors, we performed CRISPR KO screens in various cancer cell lines with Polθ inhibitors.

**Results and Discussions**

In agreement with published data[4,7], key DDR regulators were identified as the strongest hits as sensitivity biomarkers for Polθ inhibitor treatment. These factors were validated by various approaches, including a multicolor competition assay.

**Conclusion**

Collectively, our results show that DDR deficiencies enhance the sensitivity to Artios Polθ inhibitors, facilitating patient stratification in clinical trials for Polθ inhibitors.

1. D.A. Ramsden et al., Nat Rev Mol Cell Biol 2021.
2. Higgins G et al., OncoTarget, 2010.
3. Higgins G et al., Cancer Research, 2010.
4. Zatreanu et al., Nature communications 2021.
5. Rodriguez-Berriguete G et al., Clin Cancer Res, 2023.
6. Martin L. Stockley et al., J Med Chem, 2022.
7. Dev H et al., Nat Cell Biol 2018.

### EACR23-0434
### Investigating the role of HIF2A in ccRCC using acute protein degradation and targeted in vivo CRISPR screening

*A. Dyas[1], J. Ge[1], L. Castillon[2], L. Wesolowski[3], D. Muñoz-Espin[1], S. Vanharanta[2]*

*[1]University of Cambridge, Department of Oncology, Cambridge, United Kingdom*

*[2]University of Helsinki, Faculty of Medicine, Helsinki, Finland*

*[3]University of Cambridge, MRC Cancer Unit, Cambridge, United Kingdom*

**Introduction**

VHL loss is a key early event in clear cell Renal Cell Carcinoma (ccRCC) development, leading to constitutive HIF2A activation. HIF2A is a genetically and clinically validated target in ccRCC, but there is significant *de novo* and acquired resistance. It is therefore critical to understand the mediators of HIF2A-dependent tumorigenesis.

**Material and Methods**

To unbiasedly characterise functionally relevant HIF2A targets *in vivo*, we first generated a system for acute HIF2A degradation using AID2 technology in a human metastatic ccRCC cell line. We validated this *in vivo* using xenograft tumours and subcutaneous or intravenous injection of 5-Ph-IAA. Tumour regression was monitored by IVIS imaging and protein level changes were characterised with immunohistochemistry. Acute expression changes following HIF2A loss were determined by RNA-seq and further validated by GSEA and intersection with publicly available ChIP-seq and TCGA datasets. Genes significantly regulated in the RNA-seq data were functionally characterised using *in vivo* CRISPR screening.

**Results and Discussions**

Applying the AID2 system to study HIF2A in ccRCC *in vivo*, we demonstrate loss of HIF2A upon 5-Ph-IAA treatment and tumour regression. Studying acute kinetics of this system, we find a dramatic loss of HIF2A protein levels following intravenous 5-Ph-IAA injection from as early as 15 minutes. By combining the AID2 system with RNA-seq, we observed downregulation of known HIF2A targets, prior to signatures for broad, downstream cell cycle inhibition. These RNA-seq results were consistent with HIF2A ChIP-seq data. Acute and sustained downregulated genes were significantly enriched in clear cell vs. papillary RCC in TCGA data. The resulting list of genes significantly downregulated upon acute HIF2A degradation was subsequently targeted in an *in vivo* CRISPR screen to identify HIF2A targets essential for tumour growth.

**Conclusion**

The AID2 system achieves rapid, functional degradation of HIF2A *in vivo*, demonstrating the power of AID2 for studying transcription factors in cancer biology. Acute protein degradation combined with RNA-seq identifies HIF2A target genes prior to broad downstream transcriptomic changes. Combining these studies with *in vivo* CRISPR screening provides an unbiased method for

© 2023 The Authors. Molecular Oncology is published by John Wiley & Sons Ltd on behalf of Federation of European Biochemical Societies characterizing functionally relevant HIF2A target genes in ccRCC.

### EACR23-0552
### HELICOBACTER PYLORI GENOTYPES IN GASTRIC PRE LESIONS RELATED TO INTESTINAL SUBTYPE IN BRAZIL

*M. Macambira Noronha[1], L. Magalhães Albuquerque[1], G. De Sousa Velozo[1], S.H. Barem Rabenhorst[1]*
*[1]Federal University of Ceara, Department of Pathology and Forensic Medicine, Fortaleza, Brazil*

**Introduction**
Gastric cancer develops through pre-injuries in steps of increasing severity. The first step is chronic gastritis (CG). The presence of neutrophils, associated with DNA damage, determines active gastritis (AG). *Helicobacter pylori* (HP) infection is the leading cause of CG which can evolve into intestinal metaplasia (IM) and cancer. These changes are attributed to HP infection and may depend on the strain genome.

**Material and Methods**
The patients (211 cases) were from the Cancer Institute of Ceara, Brazil. Among them, the cases were classified (Sidney's System) as IG(63); AG(148), and IM(37). The presence and genotype of *H. pylori, (vacA* alleles, *cagPai* genes: *cagA*, *cagE, cagM, cagG*) were identified using PCR. Chi-square ($\chi2$) and Fisher's exact tests were done for group comparations using EPINFO 16.0 software, with $p \leq 0.05$ as significant.

**Results and Discussions**
*vacAs*1 strain was present in: IG 55(30,2%); AG 127(69,8%) and IM 34(91,2%). Among them, all *cag*Pai genes studied had similar frequency, however when grouped considering 3 genes, in the analysis of IG vs AG, for all combinations for 3 genes, positivity showed GA risk outcome. It's interesting that the higher risk was observed for *cagA*(+)*cagG*(–)*cagE*(+) combination with 8.2 (2.7-25.0) which decrease with *cagG* (+) OR 7.3 (2,5-21) and drop down with *cagE*(-) OR 2.9 (1,1-7,8). Also, a high risk is observed for *cagA(+)cagE(+)cagM(+). A* variation of risk is also observed when *cagE or cagA are* combined with *cagG* and cagM, where *cagE(+)cagM(+)cagG(+)* was the highs risk, decreasing with the *cagG (-);* conversely, *cagA(+)cagM(+) cagG(–)*was the highs risk but decreased with *cagG*(+). In all combinations only *cagM*(+) or *cagG*(+) were not significant; while only *cagA(+)* or *cagE(+)* was found significant. Comparing GA with IM, protection was observed with the presence of *cagG,* in all combinations. These data are consistent with *Do Santos* study in which strain *cagE(+)* was associated with intestinal and *cagG* (+)*cagE(-*) with the diffuse outcome. A protection was associated with *cagG(+) cagE(*+) *cagM(+)* OR: 0.1 (p=0.0080). However, when we analyzed 2 genes, a risk was observed with *cagG(-) and cagM (+)* OR=5.7 (p= 0.0062), showing an ambivalence of *cagM.* As IM had a low number of cases further analyses should be done.

**Conclusion**
Positivity for all studied genes increases the risk for GA, lightning the *cagA,* and *cagE.* Furthermore, cagG(-) appears to be associated with GA risk and *cagG(–)cagM(*+) to IM risk, Thus, it seems that the risk is dependent on the strain genes composition.

### EACR23-0553
### Microbial Composition of Gastric Lesions: Differences Based on Helicobacter pylori Virulence Profile.

*S.H. RABENHORST[1], M. Barboza[1], V. Mello[2], A. Ferrasi[3]*
*[1]Medical School, Pathology and Forensic Medicine, Fortaleza, Brazil*
*[2]Federal University of Ceará, Science Center., Fortaleza, Brazil*
*[3]Medical School, Medical Clinic, Botucatu, Brazil*

**Introduction**
Globally Gastric Cancer (GC) ranks fourth for mortality. *Helicobacter pylori* (HP) infection is a major risk, with precursor lesions for the intestinal subtype in which severe gastric disease is dependent on virulence factors. However, colonization by other microorganisms may act as cofactors. In fact, RNA16S analysis revealed that the stomach harbors a distinct and complex ecosystem. However, in current metagenome studies, there isn't association with HP virulence. Thus, we aimed to investigate the difference in microbiota composition among gastric lesions, including GC, according to HP virulence (*cagA, cagE*).

**Material and Methods**
Fifty-four patients were included: intestinal GC (n=15), intestinal metaplasia (IM n=12), active chronic gastritis (ACG n=12), inactive chronic gastritis (ICG n=15), sub-grouped according to HP virulence profile, and normal gastric tissues (n=4). 16S rRNA sequencing was performed using the Illumina platform.

**Results and Discussions**
The phylum composition varied among the gastric lesions (p = 0.0292). Draws attention, to the large abundance of the phylum *Firmicutes* in the control group which decrease in the gastric lesions, and the presence of *Actinobacteria* in all gastric lesions but not in normal tissue. The microbial composition differed among the groups according to inflammation status (p=1.26e-11), HP presence (p=0.000133), and virulence (p=0.044126). The IM group differed from the control group (p=6.73e-09) and GC group (p=0.049779) and, a negative association between *Firmicutes* and *Helicobacte*r spp was observed (p=1.45e-07). *Helicobacter* spp. was more abundant in the IM than in the other lesions. The analysis of indicator genus revealed 13/270 genus as primarily responsible for the differences between groups highlighting *Raoultella* and *Leuconostoc* associated with the Cancer/HP non-virulent group (found in digestive tracts, patients with reduced immunity, treated with broad-spectrum antibiotics; some species are the cause of human disease while *Sarcina* and *Centipeda* in the Cancer/HP virulent group (associated with patients with gastric perforation; Immunosuppressive effects). *Lactobacillus* was the genus linked to the global Cancer group. *Salinicola* was an indicator of the ACG (isolated from a saltwater sample).

**Conclusion**
The phylum *Firmicutes* was an indicator of normal tissue. The variation in microbial composition was dependent on the HP strain development. The genus's indicator found in

© 2023 The Authors. Molecular Oncology is published by John Wiley & Sons Ltd on behalf of Federation of European Biochemical Societies

GC according to the HP virulence and gastritis may be a co-factor of HP for GC development.

### EACR23-0747
### Targeting ERK5 nuclear localization restores the antiproliferative effect of the ERK5 kinase inhibitor AX15836

*Z. Lombardi*[1], *A. Kashchuk*[2], *A. Menconi*[1], *L. Gardini*[2], *M. Lulli*[1], *I. Tusa*[1], *M. Capitanio*[2], *E. Rovida*[1]
[1]*University of Florence, SBSC Mario Serio, Florence, Italy*
[2]*University of Florence, LENS - European Laboratory for Non-Linear Spectroscopy, Sesto Fiorentino, Italy*

**Introduction**
The extracellular signal-regulated kinase 5 (ERK5) is emerging as a possible target for melanoma treatment. ERK5 pro-proliferative activities are linked to its presence in the nucleus, but the mechanisms involved in ERK5 nuclear translocation are poorly characterized. We focused on the elucidation of this process using single molecule tracking and searching compounds able to prevent ERK5 nuclear shuttling, in order to design new strategies for cancer treatment.
**Material and Methods**
HeLa cells were transfected with a vector for ERK5, linked to HaloTag, alone or with a vector for a constitutively active form of the ERK5 activator MEK5 (MEK5DD). The cell-permeable chromophore JaneliaFluor646 was used for the detection in super resolution imaging. In addition, HEK293T cells, transfected with ERK5 and MEK5DD, and A375 melanoma cells were treated with the α/β1 importin-inhibitor ivermectin (IVM) or with two siRNA targeting importin-β1. MTT, 2D-colony forming assays and apoptosis evaluation were performed in A375 or HeLa cells treated with IVM in combination with the ERK5-i AX-15836. A375 and HeLa spheroids have been used to evaluate the effect of IVM and AX-15836 on a 3D model of in vitro tumour growth.
**Results and Discussions**
The HaloTag technology provided the selective binding of JaneliaFluor646 to ERK5, and Highly Inclined and Laminated Optical sheet (HILO) microscopy allowed to collect the signal of single ERK5 instances. Data showed that in ERK5-transfected cells the protein is mainly localized in the cytoplasm, whereas it moves to the nucleus with the activator MEK5DD and this effect is partially reverted in cells treated with IVM. Moreover, ERK5 amount in the nuclear fraction of lysates from IVM treated-cells and from KPNB1 KD cells is reduced, confirming a role of importin β1 in ERK5 nuclear transport. Finally, we found that ERK5i AX-15836, which has been reported to induce ERK5 paradoxical activation by inducing its nuclear translocation, reduced melanoma cell proliferation only in combination with IVM.
**Conclusion**
The present study demonstrated the involvement of importin α/β1 in ERK5 nuclear translocation. Our data showed that impairment of ERK5 nuclear localization restores sensitivity to AX15836, suggesting that the actors involved in ERK5 nuclear shuttling could be exploited as novel targets for ERK5 inhibition, and therefore for additional anticancer therapies. The described super-resolution technique will also help future studies to investigate the mechanism of action of ERK5 in the nucleus.

### EACR23-0748
### Identification of an interplay between MEK5-ERK5 pathway and hypoxia inducible Factor 1 α

*A. Menconi*[1], *A. Gentilini*[2], *I. Tusa*[1], *G. Lori*[2], *F. Marra*[2], *E. Rovida*[1]
[1]*Università degli Studi di Firenze, Department of Experimental and Clinical Biomedical Scie nces "Mario Serio", Firenze, Italy*
[2]*Università degli Studi di Firenze, Department of Experimental and Clinical Medicine, Firenze, Italy*

**Introduction**
Cholangiocarcinoma (CCA) is the second most common liver cancer after hepatocellular carcinoma, and constitutes a heterogeneous group of malignancies that arise from the epithelium of the biliary tree. In particular, intrahepatic cholangiocarcinoma (iCCA) is an aggressive liver malignancy with limited therapeutic options, and its incidence is increasing in the Western countries. Recently, our group reported the importance of the mitogen-activated protein kinase extracellular signal regulated kinase 5 (ERK5) in supporting the survival and proliferation of CCA cells both in vitro and in vivo. In order to identify additional molecular targets in CCA, we investigated on the possible functional relationship between ERK5 and hypoxia inducible factors α, the main regulators of the response to hypoxia, a condition that is typical of the tumour microenvironment.
**Material and Methods**
Two CCA cell lines (CCLP-1 and HUCCT-1) were grown at different time points under normoxia and hypoxia conditions. Gene silencing was performed with short harpin RNA for ERK5 gene (MAPK7). Protein expression analysis was investigated by Western Blot. For the pharmacological treatments, HIF and ERK5 inhibitors effects were evaluated in term of cell viability using 3-(4,5-dimethylthiazol-2-yl)-2,5-diphenyl-2H-tetrazolium bromide (MTT) assay.
**Results and Discussions**
We found that ERK5 phosphorylation and HIF1α expression are increased in hypoxia. The increased activity of the latter in hypoxia was confirmed by the consistent increase of its target genes, Carbonic Anhydrase 9 (CAIX) and Glucose transporter 1 (GLUT1). We also found a functional relationship between ERK5 and HIF-1α; indeed, following ERK5 knockdown the increase of CAIX and GLUT1 was reduced in hypoxia compared to that observed in control cells (shNT). Combined treatment of ERK5i and HIFi in vitro and found a greater effect than the single treatments in hypoxic conditions. All together, these results lead to the idea to deepen this co-therapy to treat CCA, given the low oxygen concentration in tumor environment.
**Conclusion**
These findings led to the identification of a functional relationship between ERK5 and HIF-1α in the regulation of CCA homeostasis, and put light on a new possible therapeutic option for CCA.

© 2023 The Authors. Molecular Oncology is published by John Wiley & Sons Ltd on behalf of Federation of European Biochemical Societies

### EACR23-0769
### Mutation spectrum in Bulgarian patients with thyroid cancer and struma nodosa

R. Dodova[1], M. Vasileva[1], V. Petkova[1], K. Vidinov[2], R. Kaneva[1]
[1]Medical University of Sofia, Molecular Medicine Center- Department of Medical Chemistry and Biochemistry, Sofia, Bulgaria
[2]Medical University of Sofia, Department of Endocrinology, Sofia, Bulgaria

**Introduction**

About a quarter of Bulgarians between the ages of 20 and 80 have nodules in the thyroid gland. Thyroid cancer is the most common malignant tumor of the endocrine system. Environmental factors, lifestyle, and family history play a significant role in their development, but the mutation spectrum among Bulgarian patients was been investigated in details.

**Material and Methods**

Fresh frozen tissues from 27 patients with thyroid cancer and 27 with struma nodosa were collected. DNA was isolated from tumor tissue and NGS was performed with Ion AmpliSeqCancer HotSpot Panel v2 on the Ion PGM System platform. The results were analyzed by Torrents Suite (ThermoFisher) and VarSeq (Golden Helix) software.

**Results and Discussions**

In the studied papillary carcinoma patients, 111 different variants in 30 genes were found. After the analytical processing, depending on their clinical effect, they were divided into: pathogenic (28.83%), probably pathogenic (39.64%), with unclear clinical effect (17.12%), probably non-pathogenic (1.80%), and non-pathogenic (12.61%). The most frequently observed pathogenic and likely pathogenic variants were in TP53, BRAF, KIT, STK11, ERBB4, and APC. Accordingly, in patients with multinodular goiter, comparable number of variations were found - 94 variants in 29 genes distributed as follows: pathogenic (23.40%), probably pathogenic (40.42%), with unclear clinical effect (53 %), probably non-pathogenic (2.13%) and non-pathogenic (8.51%). In patients with struma nodosa, the pathogenic variants were observed mainly in TP53, NRAS, PTEN, and likely pathogenic in TP53, ABL1, APC, and STK11. Pathogenic and likely pathogenic variants but at lower frequencies were also found in CDKN2A, CTNNB1, EGFR, ERBB4, FBXW7, FGFR2, FGFR3, GNAS, HNF1A, KRAS, MET, PIK3CA, PTPN11, RB1, RET, SMAD4, STK11, and VHL.

**Conclusion**

Genetic factors play an important role in the etiology of many thyroid diseases. Surprisingly, pathogenic and likely pathogenic variations in similar number of genes were found in carcinoma and benign multiple nodules. The distinction between somatic and germline variants will assist in determining the correct diagnosis, but also to identify other family members at risk of disease and provide informed genetic counseling. Understanding the molecular genetic mechanisms of the development of the analysed thyroid disorders will allow better schemes for treatment and follow-up.

Acknowledgment. NSF,MES:D01-395/18.12.2020, D01-278-14.12. 2022, D01-302/17.12.2021, D01 165/28.07.2022; NSF,MES: KP06-H23/9.18.12.2018

### EACR23-0919
### Leveraging R-loop induced DNA damage in ER-overexpressing breast cancer

A. Roberts[1], N. Traphagen[1], E.C. Duffy[1], E. Demidenko[2], T. Miller[1]
[1]Dartmouth, Molecular and Systems Biology, Lebanon, United States
[2]Dartmouth, Biomedical Data Science, Lebanon, United States

**Introduction**

A newly identified but mechanistically undefined risk to genome stability in ER+ breast cancer is the R-loop. R-loops are three-stranded DNA:RNA hybrids formed during fundamental cellular processes such as transcription. R-loop formation can elicit DNA damage across a spectrum of diseases including cancer. Overexpression or genetic amplification of estrogen receptor alpha (ER) can occur naturally in endocrine-resistant metastatic breast cancers. Estrogens including 17b-estradiol have been used for decades to treat metastatic breast cancer, but their mechanism of anti-cancer action remains unclear, and strategies to enhance response remain to be developed.

**Material and Methods**

Using ER-overexpressing and endocrine-resistant breast cancer cell lines and patient-derived xenograft models, we analyzed the effects of 17b-estradiol on growth, R-loop formation, DNA damage, and DNA damage response through immunofluorescence, flow cytometry, and western blotting. The efficacy of combination therapy with the PARP inhibitor olaparib and 17b-estradiol was analyzed *in vitro* and *in vivo*.

**Results and Discussions**

High ER levels converted the estrogen 17b-estradiol from a growth promoter to a growth suppressor, increasing R-loop formation, DNA damage, and apoptosis. Cells incurring such DNA damage were in S-phase, implicating DNA replication in 17b-estradiol/ER/R-loop-induced DNA damage. Resolving R-loops through RNase H1 expression prevented such DNA damage. Inhibition of the DNA damage response through combination therapy with the PARP inhibitor olaparib synergized with 17b-estradiol to suppress cell growth *in vitro*, and to induce tumor regression and prevent recurrence of xenografts. Characterization of the DNA damage response showed activation of CHK1 and CHK2, offering these checkpoint kinases as other potential targets for combination with 17b-estradiol.

**Conclusion**

R-loop-induced DNA damage has the potential to be therapeutically leveraged in breast tumors expressing high ER levels through combination therapy with 17b-estradiol and inhibitors of the DNA damage response. Clinical testing of the combination of 17b-estradiol and a PARP inhibitor is warranted.

### EACR23-0972
### Neuroligin-2 regulates Hippo dependent contact inhibition trough cell polarity maintenance in PanIN progression.

E. Middonti[1], E. Astanina[1], J. Metovic[2], M. Papotti[2], P. Allavena[3], F. Novelli[4], P. Cappello[4], A. Scarpa[5], M. Di Maio[6], F. Bussolino[1]

© 2023 The Authors. Molecular Oncology is published by John Wiley & Sons Ltd on behalf of Federation of European Biochemical Societies

[1]Università degli studi di Torino, Oncology, Candiolo, Italy
[2]Università degli studi di Torino, Oncology, Torino, Italy
[3]IRCCS Humanitas, Clinical and Research Center, Rozzano, Italy
[4]Città della Salute e della Scienza di Torino, Center for Experimental Research and Medical Studies, Torino, Italy
[5]Università di Verona, Applied Research Center ARC-NET, Verona, Italy
[6]Università degli studi di Torino, Medical Oncology- Ordine Mauriziano Hospital, Torino, Italy

**Introduction**

Pancreatic ductal adenocarcinoma (PDAC) is a tumor with dismal prognosis that arises from precursor lesions called Pancreatic intraepithelial neoplasias (PanINs). PanINs are classified as low- and high-grade (LGP and HGP) and progress to PDAC by accumulating mutations in driver genes. While LGP have no clinical significance, their switch to HGP is characterized by loss of polarity and increased proliferation, it is considered the tumor initiation and a deeper understanding of this switch is needed. We identified Neuroligin-2 (NLGN2) as a candidate regulator of key mechanisms that drive the transition from LGP to HGP by modeling cell polarity and proliferation.

**Material and Methods**

We used transgenic mice models which express mutated K-Ras in pancreas, leading to PanINs onset and progression. Cells from HGP were extracted and screened for modulated genes from normal pancreas and LGP, resulting in NLGN2 as a candidate. NLGN2 expression was validated in mice models and in 204 human samples. NLGN2 was modulated in vitro in Human pancreatic ductal epithelial cells (HPDE), which were used to characterize cell polarity and proliferation in response to NLGN2 silencing. Cell polarity was evaluated by establishing 3D cultures and characterization of cyst morphology.

**Results and Discussions**

NLGN2 emerged as a candidate, which expression is lost in HGP in mice and humans. Human PDAC exhibited heterogeneous NLGN2 expression and Kaplan–Meier survival analysis indicated that loss of NLGN2 expression was associated with worse survival, but despite a clear separation of the curves this trend was not significant due to limited statistical power. While NLGN2 silencing had no effect in 2D subconfluent HPDE proliferation, it blocked contact inhibition in confluent cultures, allowing overcrowded cell growth. NLGN2 silencing in HPDE also lead to aberrant cyst morphology and proliferation. We examined NLGN2 effect on Hippo pathway, due to its role in integrating polarity and contact inhibition. NLGN2 silencing reduced the maturation of Crumbs (CRB3/PALS1/PATJ) polarity complex, reducing YAP inhibition in confluent cultures.

**Conclusion**

Trough NLGN2 silencing we modeled LGP to HGP transition, which features loss of polarity and contact inhibition as a consequence of Crumbs complex instability and YAP overactivation. With this work we want to characterize and highlight the relevance of extending pancreatic cancer research in early PanIN detection and intervention.

### EACR23-0981
### Adaptation to hypoxia influences somatic mutagenesis in normal cells of the kidney tubule

*G.B. Pipitone[1], A. Larcher[2], I. Rowe[2], G. Distefano[3], F. Corea[4], J.P. Margaria[5], G. Mancassola[1], U. Capitanio[2], P. Carrera[4], A. Salonia[2], A. Boletta[6], I. Franco[5]*
[1]San Raffaele Hospital, Laboratory of Clinical Molecular Genetics, Milano, Italy
[2]San Raffaele Hospital, Urological Research Institute URI, Milano, Italy
[3]San Raffaele Hospital, Division of Genetics and Cellular Biology, Milano, Italy
[4]San Raffaele Hospital, Center for OMICS Sciences, Milano, Italy
[5]Università Vita-Salute San Raffaele, Division of Genetics and Cell Biology, Milano, Italy
[6]San Raffaele Hospital, Division of Genetics and Cell Biology, Milano, Italy

**Introduction**

Somatic genetic changes accumulate during a lifetime and promote tumor initiation. However, very little is known about cellular processes that affect mutagenesis in aging tissues and how this is connected to cancer risk. In the kidney, 90% of tumors harbor inactivating mutations in the tumor suppressor *VHL*. Individuals that carry germline mutations in one copy of this gene develop kidney cancer at young age with high penetrance, following a second-hit inactivation model. The anti-oncogenic role of pVHL is mainly related to its activity of restraining intracellular signaling that mediates adaptation to low oxygen availability (hypoxia). Hypoxia is commonly observed in kidney tubules in association with exaggerated kidney workload. Here, we have explored how adaptation to hypoxia affects genome integrity in normal cells of the kidney tubule during adult life.

**Material and Methods**

To study somatic mutations in single genomes of normal kidney cells, we established a method based on isolation of tubule cells from the urine and *in vitro* clonal expansion. Each clone was subjected to whole genome sequencing and gene expression analyses. The landscape of somatic variants was analyzed in multiple cells from 5 control healthy individuals and 4 VHL-disease patients in the age range 24-56 and compared to a published dataset of normal genomes from clonally expanded tubule cells from kidney biopsies.

**Results and Discussions**

In control individuals, mutations accumulated steadily with age. However, clones with exceptionally high mutation burdens and a peculiar mutational profile appeared in 60+ individuals. Characteristic mutational features included enhanced mutation rates in highly transcribed DNA and a unique pattern of transcriptional strand bias. These mutation-prone cells expressed VCAM1, an inflammatory marker known to be turned on in scattered tubular cells exposed to ischemic damage and hypoxic environment *in vivo*. VCAM1+ clones showing high mutation burden and the characteristic mutation landscape were absent in control individuals younger than 60, while they were observed in 2 out of 4 VHL patients, aged 42 and 56. The characteristic mutational landscape observed in sparse

© 2023 The Authors. Molecular Oncology is published by John Wiley & Sons Ltd on behalf of Federation of European Biochemical Societies normal genomes was also noticed in 6/6 tumor genomes analyzed from the 56-years-old VHL patient, indicating that the related mutational process was enriched in kidney tumors.

**Conclusion**

Our study suggests that adaptation to hypoxia enhances mutagenesis, thus uncovering a link between tissue stress and tumorigenesis in aging human kidneys.

### EACR23-1019
### SOS-RAS GEFs as potential therapeutic targets in cancer and metabolism

*A. Olarte*[1], *R. García*[1], *N. Calzada*[1], *C. Gomez*[1], *R. Fuentes-Mateo*[2], *J. Vaquero*[3], *E. Santos*[4]

[1]*Centro de Investigación del Cáncer, Laboratory 1, Salamanca, Spain*

[2]*Groningen Research Institute for Asthma and COPC- Uni vestisity of Groningen, Deparment of molecular pharmacology, Groningen, The Netherlands*

[3]*HGU Gregorio Marañón-IiSGM-CIBEREHD., Servicio de Aparato Digestivo, Madrid, Spain*

[4]*Centro de Investigación del Cáncer, Microbiologia, Salamanca, Spain*

**Introduction**

RAS mutations are frequent (about 30%) in various human cancers. The activity of RAS proteins is positively regulated by guanine nucleotide exchangers (GEFs) of the SOS1/2 family that is homogeneously expressed in different tissues and stages of development. Recent work in our laboratory has shown that SOS1-mediated signalling is involved in controlling mitochondrial metabolism, skin homeostasis, organismal viability, and carcinogenesis. In addition, SOS-RAS-MAPK signalling plays an essential role in metabolism. Since obesity and fat intake are known risk factors for cancer, understanding the functional relevance of SOS1/2 GEFs in lipid metabolism under physiological and pathological contexts is crucial.

**Material and Methods**

To evaluate the relevance of SOS proteins in adipogenesis, mouse embryonic fibroblasts (MEFs) and genetically modified 3T3-L1 cells were used. We induced adipogenic differentiation both without and with several adipogenic-pathway inhibitors, to establish the mechanism by which SOS mediates differentiation. To analyze the importance of RAS-GEFs in obesity, a conditional murine model of TAM-inducible Cre-ER treated with a high-fat diet (HFD) was used. We followed two experimental approaches; 1) SOS1 deletion was induced before starting the HFD; 2) SOS1 deletion was induced 60 days after starting the HFD. To establish the role of SOS1 and SOS2 in the development of hepatocarcinoma, we will use diethylnitrosamine (DEN) as a pro-carcinogenic agent. Subsequently, we will separate those animals into two groups, one with chow-diet and the other with HFD.

**Results and Discussions**

Deletion of both SOS1/2 isoforms in MEFs or 3T3-L1 cells caused a significant loss of their ability to differentiate into adipocytes, whereas single SOS2KO cells kept a normal adipocytic differentiation capacity. Interestingly, SOS2KO mice showed a substantial increase in body weight compared to WT or SOS1KO mice after prolonged feeding with HFD. The HFD also caused significant fat accumulation in the livers of SOS2KO and WT mice, in sharp contrast to the livers of single SOS1KO mice fed with the same diet. SOS1KO mice showed a decrease in tumor number compared to WT and SOS2KO mice after DEN treatment and chow diet.

**Conclusion**

Our data suggest that the SOS1 and SOS2 GEFs play differential functional roles regarding the homeostasis of the adipose tissue and the liver of adult mice, which may lead to identifying relevant, novel therapeutic targets to treat metabolic diseases and/or cancer.

### EACR23-1043
### The role of Notch1 mutations in cancer: Modulation of 4-nitroquinoline 1-oxide (4NQO)-induced mice tumours in the oral cavity

*I. Tomas*[1], *K. Sipila*[1], *I. Sequeira*[1,2], *M. Battilocchi*[1], *F. Watt*[1]

[1]*King's College London, Centre for Gene Therapy and Regenerative Medicine, London, United Kingdom*

[2]*Queen Mary University of London, Centre for Oral Immunobiology and Regenerative Medicin e, London, United Kingdom*

**Introduction**

HNSCC (Head and Neck Squamous Cell Carcinoma) is causing close to 450 000 deaths yearly, with the 5-year survival rate still standing at 66%. HPV-negative patients have worst prognosis and are associated with smoking. The aim of the project is to understand which populations in the tumour microenvironment of 4NQO (4-nitroquinoline 1-oxide)-induced tongue tumours are affected differently upon exacerbated inflammation and how is this interaction altered, mimicking tobacco use.

**Material and Methods**

We administrated 4NQO in the drinking water of mice for 16 weeks and followed end-point experiment with normal water until 32 weeks. We analysed tumour ecology and mutational landscape of 4-NQO-induced tongue tumours, in C57/Bl6 mice. Subsequently, we induced Notch1 conditional knockout in squamous epithelium and combined it with the same 4NQO treatment resulting in tongue tumours.

**Results and Discussions**

In wildtype animals, we determined the mutational landscape of tumour progression in HNSCC and the correlation with smoking patterns. Interestingly, the tumours with Notch1 mutations had a higher infiltration of immune cells (CD45+), without correlation to subgroups like T-lymphocytes and macrophages.

In Notch1 knockout animals, we observed more frequent and quicker development of lesions, with no differential grading of diagnosis. The epithelium of these animals shows an increase in the density of proliferative cells although being significantly thinner. We thoroughly analysed immune cell infiltrate, T cells and regulatory T cells, proliferation and morphology of the lesions, in normal and tumour areas, within control mice and Notch1 knockout mice. Histology characterization revealed preliminary evidence of cell-to-cell adhesion impairment, like Pemphigus vulgaris. These areas affected both

© 2023 The Authors. Molecular Oncology is published by John Wiley & Sons Ltd on behalf of Federation of European Biochemical Societies phenotypes equally, not being mediated by complement C3, or differentially affecting desmoglein 1 or 3.

**Conclusion**

Overall, this suggests that the tumour suppressive role of Notch1 in the tongue might be related to inflammation and cell adhesion leading to a bigger susceptibility to cancer and higher incidence.

### EACR23-1066
### The insulin receptor isoform A stimulates liver carcinogenesis through non-cell autonomous mechanisms

*F. LEANDRE[1], C. Godard[1], A. Caldiero[1], A. Iddir[1], S. Pinto[1], I. Lagoutte[2], A. Alves[1], S. Colnot[1], A. Gougelet[1], C. Desbois-Mouthon[1]*

*[1]Centre de Recherche des Cordeliers, Inserm U1138, Paris, France*

*[2]Institut Cochin, Inserm U1016-CNRS UMR8104, Paris, France*

**Introduction**

The insulin receptor (IR) plays a crucial role in the liver by regulating metabolism and proliferation. In the adult liver, these functions are relayed by IRB. IRA, the fetal form, has been reported to be expressed during the progression of hepatocellular carcinoma (HCC), the most common liver cancer. IR isoforms result from the alternative splicing of exon 11 in *Insr* pre-mRNA. Recently, studies detected IRA in murine and human livers with hepatitis or cirrhosis which are risk factors for HCC. These data raise the question of the role of IRA in the pathogenesis of HCC. Therefore, the goal of our study is to investigate for the first time the impact of IRA on liver carcinogenesis *in vivo*.

**Material and Methods**

An *in vivo* CRISPR strategy using AAV8 expressing saCas9 nuclease and small guide RNAs was developed to delete exon 11 (IRB specific) in *Insr* gene and substitute IRB for IRA in adult mouse hepatocytes. This strategy was conducted in wild-type (WT) mice and in mice with beta-catenin activation in the liver due to hepato-specific APC loss ($APC^{KO}$) or beta-catenin mutation ($BCAT^{Dex3}$). $APC^{KO}$ and $BCAT^{Dex3}$ mouse models develop differentiated and undifferentiated tumors. *Insr* gene editing was evaluated by PCR. Glucose homeostasis was assessed with glucose and insulin tolerance tests. Tumor development was followed by ultrasonography.

**Results and Discussions**

Short-term expression of IRA (2 mo) in a WT liver did not induce disturbances in liver histology and homeostasis while long-term expression of IRA (12 mo) induced tumors in 3 mice out of 10. Expression of IRA in a liver parenchyma with constitutive activation of beta-catenin (IRA/$APC^{KO}$ and IRA/$BCAT^{Dex3}$) led to significantly earlier tumor initiation and increased tumor multiplicity. A higher proportion of differentiated tumors was observed in IRA/$APC^{KO}$ mice compared to $APC^{KO}$ mice (85% vs 44%, $p<0.0001$). The vast majority of spontaneous and beta-catenin activated tumors were not edited at the *Insr* gene, indicating that CRISPR/Cas9-targeted hepatocytes did not engage in transformation but that IRA may promote liver carcinogenesis by non-cell autonomous mechanisms. This may route through the alteration of the environment since a significant increase in myeloid markers and p21 expression was observed in the adjacent non tumoral liver of IRA/$APC^{KO}$ mice.

**Conclusion**

IRA promotes HCC development and could be considered as a risk marker. Further studies are needed to characterize the underlying mechanisms, focusing in particular on inflammation and cellular senescence.

### EACR23-1078
### The influence of tobacco smoking on the somatic mutational landscape of alveolar stem cells

*M. Przybilla[1], A. Ammar[2], R.C. Khaw[2], A.R.J. Lawson[1], N. Pantelis[1], H. Jung[1], Z. Frazer[2], I. Martincorena[1], P. Campbell[1], S.M. Janes[2]*

*[1]Wellcome Sanger Institute, Cancer- Ageing and Somatic Mutation Programme, Hinxton, United Kingdom*

*[2]University College London, UCL Respiratory, London, United Kingdom*

**Introduction**

Despite recent recognition of the diverse somatic variation in human tissues, the initial stages of the carcinogenic process are poorly understood. We previously found that normal bronchial epithelium in the proximal airway, where squamous cell carcinoma (SCC) is formed, accumulates DNA damage in proportion to an individual's smoking habits1. In contrast, the genomics of the distal airway, the site of lung adenocarcinoma (LUAD) formation, have not yet been studied.

**Material and Methods**

We devised a methodology to generate single-cell derived alveolar type II (AT2) organoids, a hypothesised cell of origin of LUAD2, from human tissue of 9 patients (3 never-, ex-, and current-smokers). We performed WGS on >800 organoids to analyse the landscape of somatic variation, including single nucleotide (SNVs), copy number (CNVs) and structural variants (SVs). Moreover, we used single-molecule sequencing of known cancer genes to analyse driver mutations in 30 individuals with heterogeneous smoking history3.

**Results and Discussions**

Our analysis of SNVs indicated that almost all organoids (785/803) were single cell derived, as estimated using previously demonstrated methods for clonality estimation4. Immunostaining for SFTPC and SFTPB further confirmed that our organoids expressed AT2-specific marker genes. In equivalence to the proximal airway, tobacco smoking was the major driver of elevated mutation burden in ex- and current- compared to never-smokers. Remarkably, however, we did not find a significant difference in the mutation burden of ex- and current-smokers (mean 49.6 and 51 SNVs per year), suggesting that smoking damage in the distal airway is more long-term, thus potentially explaining the constant risk for LUAD after smoking cessation5–7.

When investigating the selective landscape, we detected significant positive selection on SFTPB and SFTPC, mirroring findings in the liver, where albumin as a housekeeping gene of hepatocytes is frequently mutated8,9. In addition, we observed positive selection on TP53, as well as SLC34A2, a gene implicated in pulmonary dysfunction10. Single-molecule sequencing

© 2023 The Authors. Molecular Oncology is published by John Wiley & Sons Ltd on behalf of Federation of European Biochemical Societies demonstrated an unprecedented diversity in driver mutations in AT2 cells, including mutations at hotspots of BRAF, EGFR, KRAS.

**Conclusion**

Here, we provide a potential explanation for long-term risk for LUAD development in ex-smokers. We describe the diversity of cancer-related mutations, including BRAF or KRAS. Overall, we expect this work to provide insights into the contribution of somatic variation to lung carcinogenesis.

### EACR23-1090

### Establishing the priming potential of oncogenic and regenerative mutations in chronic inflammation

*E. Moutin[1], G. Giavara[1], F. Lourenco[1], R. Kemp[1], D. Winton[1]*

*[1]CRUK Cambridge Institute, Oncology, Cambridge, United Kingdom*

**Introduction**

Increased expansion of anti-inflammatory mutations has been described in the epithelium of ulcerative colitis patients, suggesting a role in mediating tissue regenerative responses. As chronic inflammation in a known risk factor for cancer, we asked whether pro-oncogenic mutations could also be favoured for expansion in this context.

**Material and Methods**

The *Muc2*$^{KO}$ murine model of colitis was used in which impairment of the mucus barrier leads to colitis. Clonal expansion was followed using the multicolour reporter confetti in *Muc2*$^{KO}$ mice that were further treated with the chemical mutagen ENU. At endpoint, the colon tissue was collected and optically cleared before confocal imaging to obtain a 3D image. Targeted multiplex amplicon sequencing was then used to identify mutations with anti-inflammatory or pro-oncogenic functions.

**Results and Discussions**

Inflammation, characterised by stromal and immune infiltrates, abscesses and high-grade dysplasia, is found at specific sites along the colon of *Muc2*$^{KO}$ mice. Quantification of clone sizes in these regions reveals significantly increased clonal expansion. On one hand, molecular characterisation using bulk RNA sequencing shows upregulation of a foetal signature previously linked with regeneration after epithelial damage. On the other hand, using chemical mutagenesis in the *Muc2*$^{KO}$ model, we see tumours developing in inflamed regions of the colon which underlines the role of inflammation in promoting tumorigenesis. Tumours, which can be detected through their expression of nuclear beta-catenin, arise in flat dysplasia, reminiscent of human colitis-associated-cancer. Taken together, this data suggests the co-existence of beneficial clonal expansions necessary for tissue repair and detrimental ones stemming from cancer-driver-mutated cells in chronically inflamed tissue. Sequencing in inflamed regions of the *Muc2*$^{KO}$ colon tissue confirms presence of both anti-inflammatory and pro-oncogenic mutations. Variant Allele Frequencies can be mapped back to 3D images to quantify clonal expansion of either function in chronically inflamed settings.

**Conclusion**

We developed a murine model of colitis-associated-cancer, in which we showed that anti-inflammatory and oncogenic mutations co-exist. Quantification of their expansion to establish priming potential can inform targeted action to limit oncogenic expansion without affecting tissue regeneration.

### EACR23-1149

### Deciphering the crosstalk between the intercellular communication via tunneling nanotubes, the BMP pathway and mammary cell transformation.

*C. CUELLA MARTIN[1,2], B. Guyot[1], C. Marquette[2,3], E. Petiot[2,3], V. Maguer-Satta[1], E.I. Pécheur[1]*

*[1]Centre de Recherche en Cancérologie de Lyon- UMR Inserm U1052/CNRS 5286, Auvergne Rhône-Alpes, Lyon, France*

*[2]Université Claude Bernard Lyon 1, Auvergne Rhône-Alpes, Lyon, France*

*[3]3d.Fab - CNRS UMR5246, Auvergne Rhône-Alpes, Villeurbanne, France*

**Introduction**

Breast cancer is the first cause of death by cancer in women. Unraveling the underlying mechanisms of mammary cell transformation is crucial. Mammary cells interact with their neighbours via actin-based tunneling nanotubes (TNTs), among others. TNTs are thin and long cell-cell connections, enabling cytoplasmic continuity and the transport of mitochondria among others. During breast carcinogenesis, several signalling pathways are altered, including the Bone Morphogenetic Proteins (BMP) pathway. The presence of TNTs in mammary cells and their impact on cancer metastases and chemoresistance have already been described. However, their impact on early stages of mammary cell transformation and their crosstalk with the BMP pathway remain unexplored.

**Material and Methods**

Using mammary cell lines of various degrees of transformation, we structurally characterised TNTs by scanning and transmission electron microscopy. Their dynamics and the intercellular exchanges were studied by live imaging and flow cytometry, using cocultures of fluorescent transformed and non transformed cells. BMP receptor genes expression was silenced by siRNA.

**Results and Discussions**

Scanning and transmission electron microscopy revealed that most mammary TNTs are branched and open. We observed a progressive increase of TNTs number and length with transformation, allowing long-range intercellular communication between transformed cells. Live imaging revealed that TNTs were forming and disassembling within minutes. Our fluorescent cocultures revealed that TNTs preferentially connected transformed to non transformed cells, with an intercellular exchange of information in the same direction. We quantified by flow cytometry the fluorescent material received by non transformed cells from transformed ones. We also observed a differential localisation of BMP Ia/Ib receptors at TNTs, BMPRIa being at their membrane potentially harboring a structural function and BMPRIb transiting in their lumen. Silencing BMPRIa or Ib expression resulted in a drastic reduction of the overall number of TNTs, and long TNTs (> 50 µm). In addition, the development of a mammary 3D-bioprinted model will allow us to study this

© 2023 The Authors. Molecular Oncology is published by John Wiley & Sons Ltd on behalf of Federation of European Biochemical Societies potential crosstalk in a more physio-pathologically relevant environment.

**Conclusion**

Our studies highlight a possible crosstalk between TNTs and BMP pathway, which could have an impact on initiation, propagation and/or maintenance of transformation. Our ongoing experiments currently aim to dissect the molecular mechanisms underlying mammary tumour initiation.

### EACR23-1181
### Silencing SEMA5A gene expression impairs the invasion capacity of chordoma cells in vitro

*N. Ekimci Gurcan[1,2], M. Bayindir Bilgic[1,2], A. Kuskucu[1], O.F. Bayrak[1]*

*[1]Yeditepe University School of Medicine, Department of Medical Genetics, Istanbul, Turkiye*

*[2]Yeditepe University- Institute of Natural and Applied Sciences, Biotechnology, Istanbul, Turkiye*

**Introduction**

Chordoma is a rare, slow-growing bone cancer that arises from remnants of the embryonic notochord. Chordomas are locally invasive and difficult to treat due to their resistant characteristics to traditional therapies and tumor locations in the spine and skull base. Despite recent advances in chordoma treatment, there is still a need for novel therapeutic targets to improve patient outcomes. A recent transcriptomic study we established showed increased expression of SEMA5A in chordoma tumorspheres. Several studies have shown that Sema5A, a member of the semaphorin family of signaling proteins, has been implicated in promoting tumor growth and metastasis in several types of cancer. However, the role of SEMA5A in migration and invasion capacity of chordoma cells has not been investigated.

**Material and Methods**

RNA samples extracted from UM-Chor1 and MUG-Chor1 chordoma cell line-derived tumorspheres and parental cell lines were used for transcriptomic profiling via Clariom D. Differential gene expression in tumorspheres compared to parental cell lines was analyzed via TAC software. Expression of SEMA5A gene was altered using siRNA in chordoma cell lines. Differential expression in tumorspheres and subsequent inhibition of gene expression in UM-Chor1 and MUG-Chor1 cells were validated via qRT-PCR. Downstream functional effects on migration and invasion capacity were assessed via Boyden chamber assay.

**Results and Discussions**

Differential transcriptomic profiling showed that chordoma tumorspheres exhibited dysregulation in the focal adhesion: PI3K-AKT-mTOR signaling pathway and upregulation in SEMA5A expression, compared to parental cell lines. Consistently several studies have shown that PI3K-Akt-mTOR pathway is upregulated in chordoma, and may play a role in the development and progression of the disease. Furthermore, SEMA5A has been shown to promote cancer cell migration and invasion through the activation of this pathway. Further analysis showed that siRNA-aided silencing of SEMA5A significantly impaired the invasion and migration capacities of UM-Chor1 cells, as well as the invasion capacity of MUG-Chor1 cells.

**Conclusion**

Our findings suggest that dysregulation of Sema5A expression may contribute to the aggressiveness of chordoma tumors, potentially through its effects on cell invasion and migration. Further studies are needed to fully elucidate the mechanisms underlying these effects and to explore the potential of SEMA5A as a therapeutic target for chordoma.

This project was funded by TUBITAK (118S691).

### EACR23-1215
### Gender-dependent HCC development in the X/MYC bi-transgenic mouse model

*A. Paturel[1], V. Iebba[2], G. Canarruto[3], L. Alice[1], C.D.T. Francesca[1], P. Silvano[3], L. Massimo[1], C.D.F. Claude[1], F. GUERRIERI[1]*

*[1]CRCL, UMR Inserm 1052 - CNRS 5286, Lyon, France*

*[2]university of trieste, Department of Medical- Surgical and Health Sciences, trieste, Italy*

*[3]ICGEB, International Centre for Genetic Engineering and Biotechnology, Trieste, Italy*

**Introduction**

Hepatocellular carcinoma (HCC), the most frequent primary tumor of the liver, is among the leading causes of cancer-related death worldwide. Most of HCCs is associated with chronic liver disease due to a known underlying etiology, including chronic viral hepatitis, alcohol intake and non-alcoholic steatohepatitis (NASH). It has been reported that epigenetics plays an important role in liver oncogenesis and that gut microbiota changes in HCC patients, but the relationship between them during carcinogenesis is not clear. Finally, epidemiological reports indicate that the incidence of HBV-related HCC is higher in males athan in premenopausal females, while the cause underlying this observation is largely unknown. **Aim:** To investigate the interplay between the transcriptomic profile and the microbiome during hepatocarcinogenesis by using the bi-transgenic X/MYC mouse model.

**Material and Methods**

X/Myc mice express both HBV HBx under control of viral regulatory elements and c-myc in the liver. They develop HCC with 100% penetrance, but later in females than in males. The mice were used, first to perform a RNA-seq analysis on liver tumor (T) and peri-tumor (PT) and second, to sequence 16s rRNA of stools during initiation and progression of liver tumors.

**Results and Discussions**

Transcriptome analysis of T and PT indicated that HCC developed by X/MYC mice correlated with the HCC subclass S2 described in human. This subclass is characterized by proliferation, stemness features and a poor prognosis. WT, PT and T tissues showed a different transcriptomic profile depending on the gender. This difference could explain the delayed onset of HCC in female mice. rRNAs 16S analysis confirmed this sex disparity both at early and late phases of HCC development. Males and females X/MYC mice had a significant different microbiota composition. Female mice, while presenting a dysbiosis, were enriched in protectives bacterial species, that have been previously reported to have a role in clinical response to anti-PD-1

© 2023 The Authors. Molecular Oncology is published by John Wiley & Sons Ltd on behalf of Federation of European Biochemical Societies immunotherapy. Finally, we defined a specific network of relevant RNA and bacterial species that characterized females and males hepatocarcinogenesis

**Conclusion**

Our results validate the X/Myc bi-transgenic mice as a relevant model for studying the human HCC subclass S2. Moreover, they highlight the existence of a sex-dependent RNA-gut microbiota cross talk during liver tumor development. Altogether, they provide molecular data that support the different incidence of HCC observed between males and females.

### EACR23-1263
### Targeting extracellular HSP90 chaperone complexes inhibits breast cancer progression

P. Poggio[1], L. Seclì[1], F. Fusella[1], F. Zuppini[1], L. Renzullo[1], E. Turco[1], M. Brancaccio[1]

[1]*Università degli studi di Torino, Dipartimento di Biotecnologie Molecolari e Scienze per la Salute, Turin, Italy*

**Introduction**

In order to survive and proliferate in a highly stressful environment cancer cells upregulate the expression of chaperone proteins. A wide range of cancer cells also secrete chaperones in the extracellular milieu to sustain their aberrant evolution toward malignancy. Heat Shock Protein 90 (HSP90) is one of the most abundant secreted chaperones and its role in promoting cancer cell survival, migration and invasion through autocrine mechanisms is well established. However, little is known on how extracellular HSP90 (eHSP90) cooperates with other secreted chaperones to activate cell surface receptors and extracellular clients.

**Material and Methods**

Protein–protein interactions were investigated performing immunoprecipitations followed by mass spectrometry, pull-down assays and gel filtration chromatography analysis. Different breast cancer cell lines were silenced for HSP90 (both α and β isoforms). The ability of cancer cells to respond to extracellular chaperone complexes was evaluated through wound healing, proliferation and invasion assays in presence or not of different inhibitors (small molecules or antibodies) and/or recombinant proteins. Syngeneic breast cancer mouse models were used to evaluate tumor growth in response to eHSP90 complexes inhibition.

**Results and Discussions**

Our data indicates that eHSP90 is capable of forming high molecular weight complexes in the extracellular milieu of breast cancer cells but not in the one of non-tumorigenic cell lines. We found that these aberrant eHSP90 complexes promote cancer cell growth and migration and consist of previously unidentified eHSP90 interactors, including its co-chaperone Morgana and Nucleophosmin. By using monoclonal antibodies against the newly identified eHSP90 interactors, we were able to interfere with eHSP90 extracellular complexes and block their functions. This approach impairs cancer progression both *in vitro* and *in vivo*.

**Conclusion**

Our research reveals that in the extracellular space of breast cancer cells eHSP90 forms complex chaperone networks that actively supports cancer progression. A deep understanding of the eHSP90 interactome represents a starting point to provide novel potential targets for drug intervention in cancer, preserving intracellular chaperones activity and avoiding toxicity associated with general HSP90 inhibition.

### EACR23-1308
### KRAS dosage variation drives dosage dependent EMT programs

M. Kessler[1], S. Mueller[1], R. Maresch[1], M. Najajreh[2], A. Papargyriou[2], N. de Andrade Krätzig[1], T. Engleitner[1], M. Reichert[2], D. Saur[3], R. Rad[1]

[1]*Klinikum rechts der Isar - Hospital of the Technical Unive rsity of Munich, Institute of Molecular Oncology and Functional Genomics, Munich, Germany*

[2]*Klinikum rechts der Isar - Hospital of the Technical Unive rsity of Munich, Klinik und Poliklinik für Innere Medizin II, Munich, Germany*

[3]*Klinikum rechts der Isar - Hospital of the Technical Unive rsity of Munich, Institute of Translational Cancer Research and Experiment al Cancer Therapy, Munich, Germany*

**Introduction**

Oncogenic dosage variation plays an important role in pancreatic adenocarcinoma (PC) biology and phenotypic diversification. Dosage variation of *KRAS* evolves along distinct evolutionary routes, where different *KRAS* dosages are licensed by defined inactivation stages of hallmark tumour suppressors such as *CDKN2A* or *TP53*. Using novel cellular systems, we model *KRAS* dosage dependent evolutionary trajectories *in vitro* and investigate the molecular and phenotypic effects of $KRAS^{G12D}$ dosage gain during initiation and progression in PC and lung adenocarcinoma (LC).

**Material and Methods**

To analyse the role of *KRAS* dosage variation during tumour evolution, different cell lines were transduced with doxycycline inducible $KRAS^{G12D}$ constructs: i) To model tumour initiation, non-transformed human pancreatic and bronchial epithelial cells were used. ii) To model tumour progression, murine PC cells with $Kras^{G12D}$ mutation ($Kras^{mut}$) and heterozygous *Cdkn2a* deletion ($Cdkn2a^{HETdel}$), as well as isogenic cells with CRISPR/Cas9-engineered *Cdkn2a* ($Cdkn2a^{HOMdel}$), were generated. $KRAS^{G12D}$ dosage dependent effects on cellular phenotypes were investigated using 2D and 3D assays. In addition, accompanying transcriptomic alterations were interrogated by RNA-Seq. *KRAS* dosage variation in PC and LC were analysed using corresponding cohorts of The Cancer Genome Atlas.

**Results and Discussions**

Titration of $KRAS^{G12D}$ dosage in non-transformed epithelial cells showed a strong positive correlation with induction of membrane protrusions, indicating epithelial-mesenchymal transition (EMT). This observation was further supported by dosage dependent *VIM* expression levels and transcriptomic enrichment of EMT gene sets. In fully transformed mouse PC cells increased $KRAS^{G12D}$ dosage caused further increase in mesenchymal phenotypes. These effects were dependent on engineered $Cdkn2a^{HOMdel}$, while parental cells with $Cdkn2a^{HETdel}$ showed signs of oncogene-

© 2023 The Authors. Molecular Oncology is published by John Wiley & Sons Ltd on behalf of Federation of European Biochemical Societies induced senescence. Furthermore, we show that increase of oncogenic *KRAS* dosage is frequent in human PC and LC cohorts.

**Conclusion**

Here we provide functional evidence that *KRAS* dosage variation drives dosage dependent EMT programs during tumorigenesis. Through modelling non-transformed (early) and transformed (late) stages of tumour evolution, we show that this dosage dependency is highly relevant during tumour initiation and progression. Taken together, the high frequency of *KRAS* gene dosage variation in human pancreas and lung cancer cohorts suggests broad relevance of our findings across different cancers.

### EACR23-1332
### The role of SSX in the early development of ovarian cancer

*A. Fearn[1], K. Grayson[1], H. Wilkinson[1], B. Guinn[1]*
*[1]University of Hull, Centre for Biomedicine, Hull, United Kingdom*

**Introduction**

Ovarian Cancer (OC) is the 6th most common cancer in women with around 7500 cases diagnosed each year in the UK. It is often diagnosed late due to its nebulous symptoms that often correspond with the menopause. The 5-year survival rates are as high as 95% when diagnosed at stage I but drop to as low as 15% at stage IV, meaning an earlier diagnosis could significantly improve survival rates. Previous research in our group has shown the presence of SSX in early stage OC, with SSX2 specifically presenting more commonly in early stage disease than either CA125 or HE4. Recent evidence has indicated that the most common and aggressive forms of epithelial OC derive from the cells of the fallopian tubes. Here we aim to determine whether SSX proteins are an early transformation event in OC by transfecting immortalised fallopian tube cells with SSX genes.

**Material and Methods**

Immunocytochemistry was used to confirm presence of the SSX proteins of interest in OC cell lines (OVCAR-3, A2780, SKOV-3). Immortalised fallopian tube cells were transfected with either SSX2A, SSX2B, SSX3, SSX4, or a vector control and grown into stable cell lines. Stable transfections were confirmed using qPCR and immunocytochemistry. The biological effects of the transfections were investigated by examining cell proliferation using trypan blue exclusion assays, foci formation using crystal violet staining, cell adhesion on collagen plates and cell migration in scratch assays.

**Results and Discussions**

After transfection with SSX, increases in proliferation and adhesion were seen when compared to the vector control and the parental cell line. SSX4 appears to have a greater effect on the cell proliferation and adhesion ($p<0.05$) than other SSX family members. It is unclear as to whether the differences between the biological effects of SSX family members are due to the SSX proteins or the different levels of plasmids taken up during transfection. Cells have been cloned so that the impacts of different levels of expression can be investigated further. What is clear however, is that the addition of the SSX plasmids does induce transformative effects on the fallopian tube cells.

**Conclusion**

SSX has transformative effects on immortalised fallopian tube cells however further research needs to be conducted on the effects of the levels of expression on biological change. Further screening of OC patient samples could lead to SSX being a potential biomarker and/or target for immunotherapy in early stage disease.

### EACR23-1520
### Wound memory establishes an epigenetic field cancerization

*C. Levran Levron[1], M. Watanbe[1], V. Proserpio[1], G. Piacenti[1], A. Lauria[1], C. Duval[1], K. Natsuga[2], T. Hagai[3], S. Oliviero[1], G. Donati[1]*
*[1]University of Torino, Molecular Biotechnology Center and Dep. of Life Sciences and Systems Biology, Torino, Italy*
*[2]Hokkaido University, Department of Dermatology, Sapporo, Japan*
*[3]Tel Aviv University, Shmunis School of Biomedicine and Cancer Research, Tel Aviv, Israel*

**Introduction**

Field cancerization (FC), which is the development of large areas with cancer-primed cells, often in the absence of an abnormal phenotype, is now recognized to guide the early steps of carcinogenesis in many organs such as skin, oral cavity, lung, esophagus, breast, colon and prostate. This phenomenon has been observed in patients, but functionally characterized only in genetic mouse models.

**Material and Methods**

Here, through lineage tracing and omics approaches, we provide evidence that a spatially defined FC is initiated in skin by a physiological alteration of the epithelial epigenetic landscape, rather than DNA mutations.

**Results and Discussions**

We found that, as a consequence of wound healing, specific epidermal stem cells expressing Lrig1, originate long-lasting wound memory progenitors distant from the original injury. This *distal epidermal memory* is established at the chromatin level in cells residing in a wide area surrounding the wound. They become primed (pre-activated) to repair more efficiently in case of a second injury, thanks to enhanced metabolism, faster migration and better survival with respect to untrained cells. Despite being advantageous from a tissue repair perspective in a large area surrounding the healed injury, the *distal memory* unfortunately represents an epigenetic FC component which favors the onset of pre-cancerous lesions as actinic keratosis, and squamous cell carcinoma, upon UVB treatment.

**Conclusion**

Overall, we show that sub-organ scale adaptation to injury relies on spatially organised memory-dedicated cells, characterised by an actionable cell state that establishes an epigenetic field cancerization and predisposes to tumour onset.

### Drug Resistance

© 2023 The Authors. Molecular Oncology is published by John Wiley & Sons Ltd on behalf of Federation of European Biochemical Societies

### EACR23-0055
### Cell-in-cell structures during the development of chemoresistance of colorectal cancer

I. Druzhkova[1], N. Ignatova[1], M. Bugrova[1], M. Lukina[1], L. Shimolina[1], E. Zagaynova[2], M. Shirmanova[1]
[1]Privolzhsky research medical institute, Research institute of experimental oncology and biomedica l technologies, Nizhny Novgorod, Russia
[2]Nizhny Novgorod State University, Rectoraty, Nizhny Novgorod, Russia

**Introduction**

Cell-in-cell (CIC) structure formation, or entosis, is a phenomenon in which one cell engulfs another whole cell without its obligatory death. The majority of inner cells undergo degradation inside outer cells by an autophagy–like cell death mechanism. But in some cases inner cells undergo cell division inside the outer cell or can be escaped. It is suggested that entotic cell death confers a survival advantage to the outer cells under stress conditions. Most studies demonstrate a pro-tumorigenic role of CIC structures in different cancer types. In general, the functions and outcomes of such structures in cancer remain poorly understood. The goal of this study was to elucidate the role of entosis in chemoresistance of colorectal cancer.

**Material and Methods**

- Colorectal cancer cell lines (CaCo-2, HCT116, HT29) and their chemoresistant analogs
- Chemotherapeutic drugs (oxaliplatin, irinotecan, 5-fluorouracil)
- Laser scanning fluorescent microscopy
  Transmission electron microscopy (TEM)
  Standard viability assays

**Results and Discussions**

First, the ability of three colorectal cell lines to form CICs was investigated using time-lapse imaging on a laser scanning fluorescent microscope. The CICs structures were additionally verified by TEM. It was found that the CaCo-2 cells formed the largest number of CICs and the HT29 cells were the least active. At the same time CaCo-2 cell line was intrinsically less chemosensitive than others. During the development of chemoresistance in vitro through the prolonged incubation with the increasing drugs concentration the ability to form CICs increased. CICs structures were also identified in tumor xenografts in mice obtained from the corresponding cell lines. The number of CICs was higher in CaCo-2 tumors and chemoresistant HCT116 tumors. Also, we were able to identify CICs in the histopathological slides from patients' colorectal tumors of severe stages.

**Conclusion**

Our results clearly demonstrate the intrinsic differences in the ability to form CICs between colorectal cancer cell lines and its correlation with chemosensitivity. The increased CICs formation by chemoresistant cells indicates the potential role of entosis in the acquired drug resistance.
Funding: The study was supported by the Ministry of Health of the Russian Federation (Government Assignment «Morphological structures cell-in-cell as a prognostic factor in oncological disease»).

### EACR23-0060
### Enhancing melanoma sensitivity to Interferon-gamma by c-MYC inhibition

O. Harush[1,2], E. Markovits[1,2], H. Moalem[1,2], M. Besser[1,3], G. Markel[1,3]
[1]Tel-Aviv University, Clinical Microbiology and Immunology, Tel-Aviv, Israel
[2]Sheba Medical Center, Ella Lemelbaum Institute for Immuno-oncology, Ramat-Gan, Israel
[3]Rabin Medical Center, Davidoff Cancer Center, Petah-Tikva, Israel

**Introduction**

Immunotherapy has revolutionized the treatment of patients with melanoma, however a large portion of patients still do not respond. Transcriptome analysis revealed that melanoma patients with primary resistance to immunotherapy demonstrated decreased expression of genes related to interferon-gamma (IFNg) signaling. Furthermore, non-responsiveness to IFNg was correlated with higher expression of c-MYC . Here, we aim to define the mechanism of IFNγ resistance mediated by MYC, as well as evaluate the potential of targeting MYC or MYC related proteins to reverse immune resistance.

**Material and Methods**

Melanoma cell lines derived from patients were retrovirally transduced to overexpress c-MYC. Mock control and MYC overexpression cells were either transfected with siRNA targeting MAX or treated with small molecule inhibitors along with IFNγ treatment. Melanoma cells were harvested for protein and mRNA analysis.

**Results and Discussions**

To examine the underlying mechanism of c-MYC expression on IFNg signaling and consequently on tumor resistance, we cultured melanoma cell lines with IFNg ; mRNA and protein level analysis indicate impaired IFNg signaling in melanoma cells overexpressing c-MYC, which reflected in the reduction of pSTAT1 and JAK2 expression, along with a reduction in interferon response genes. Moreover, antigen specific T cells following co-incubation with c-MYC transfected melanoma cells exhibited reduced T cell effector activity. Importantly, the inhibitory effect could be reversed by silencing c-MYC and its co-activator MAX. To further examine the effect of MYC inhibition on IFNg sensitivity, we cultured melanoma cells with small-molecule inhibitors of either c-MYC or its targets which resulted in sensitization of melanoma cells to IFNg. c-MYC inhibition promoted the expression of JAK2 and pSTAT1, as well as MHC class I and PD-L1, which indicate the suppressive effect of c-MYC on immunogenicity.

**Conclusion**

Altogether, our findings suggest an association between c-MYC expression levels and clinical resistance to immunotherapy, which is explained mechanistically by its ability to impair interferon signaling and T cell effector functions. These results suggest the potential of c-MYC targeted therapy in combination with immunotherapy approaches to improve clinical response.

### EACR23-0068
### Dissecting the cell state transition from

© 2023 The Authors. Molecular Oncology is published by John Wiley & Sons Ltd on behalf of Federation of European Biochemical Societies

### persistence to resistance associated with KRAS G12C-specific inhibitor response in lung cancer

A. MIRA[1], I. Hernandez[2], E. Mariella[3], E. Patrucco[1], A. Bardelli[4], M. Russo[3], C. Ambrogio[5]
[1]Molecular Biotechnology Center, Department of Molecular Biotechnology and Health Scienc es- University of Turin, Turin, Italy
[2]Spanish National Cancer Research Centre CNIO, H12O-CNIO Lung Cancer Clinical Research Unit, Madrid, Spain
[3]Candiolo Cancer Institute- FPO-IRCCS, Department of Oncology- University of Turin, Turin, Italy
[4]IFOM-FIRC Institute of Molecular Oncology, Department of Oncology- University of Turin, Milan, Italy
[5]Molecular Biotechnology Center, Department of Molecular Biotechnology and Health Scienc es- University of Turin, Turin, Italy

**Introduction**

Drug resistance is a bottleneck in the clinical treatment of cancer, limiting durable therapeutic benefits. Cancer cells, rather than being either sensitive or resistant, can be dynamic and transient, highlighting a generalized adaptive mechanism of survival due to non-genetic variations and resumption of drug sensitivity upon drug removal. This particular state lying between sensitivity and resistance- the “drug-tolerant persister” (DTP) state, limits therapeutic efficacy and explains the generation of resistant clones. The development of direct inhibitors of $KRAS^{G12C}$ (G12Ci) has improved the clinical management of KRAS-mutant lung adenocarcinoma. Despite high initial efficacy, tumors develop secondary resistance in almost all patients. Several genomic alterations have been identified to explain acquired resistance to G12Ci, but adaptive mechanisms of resistance arising from persister cells have not been described in lung cancer yet. Exploring the signaling dynamics of DTPs in this setting could highlight new vulnerabilities targetable for delaying resistance.

**Material and Methods**

We used human and murine cell lines harbouring a $KRAS^{G12C}$ mutation to study *in vitro* the formation of persister/resistant cells over time upon treatment with G12Ci, The number of residual cells was monitored over time and RNA and proteins were collected at different time points for molecular and gene expression analysis.

**Results and Discussions**

Our preliminary results suggest that lung cancer cells treated with G12Ci for one week undergo a G1-arrest and downregulation of cyclin D1 followed by apoptosis of sensitive cells, while two weeks treatment allows the emergence of drug-tolerant subclones with a slow-cycling phenotype that could be reverted upon drug removal. A one-week drug holiday period is sufficient to restore proliferation of persister cells consistent with reactivation of KRAS signaling pathway and upregulation of cyclin D1, as well as sensitivity to G12Ci treatment. Long-term schedules give rise to resistant clones with upregulation of different RAS isoforms.

**Conclusion**

Given the limited duration of the clinical response to the current G12Ci inhibitors, finding alternative therapies to delay or exclude the emergence of resistance is a pressing medical need. The molecular characterization of these drug-refractory cells before acquisition of permanent resistance is crucial to explain the onset of clinical relapse in those patients where acquired alterations are not detected and to identify new therapeutic vulnerabilities.

### EACR23-0109
### Sequential treatment with direct KRAS inhibitors: how to improve the management of KRAS G12C drugs

S. Vietti Michelina[1], P. Scaparone[1], E. Patrucco[1], S. Peirone[2,3], M. Cereda[2,3], A. Mira[1], C. Ambrogio[1]
[1]University of Torino, Molecular Biotechnology and Health Sciences, Torino, Italy
[2]Università degli Studi di Milano, Department of Biosciences, Milano, Italy
[3]Italian Institute for Genomic Medicine, c/o IRCCS, Candiolo, Italy

**Introduction**

lung cancer is responsible for 12% of cancer-related deaths worldwide (https://www.wcrf.org). Mutant KRAS, particularly $KRAS^{G12C}$, is the driver mutation in more than 25% of cases. Recently, two KRAS direct inhibitors, sotorasib (AMG510) and adagrasib (MTRX849), have been FDA approved for $KRAS^{G12C}$ LUAD treatment. Even if they are effective, patients develop resistance to sotorasib or adagrasib monotherapy, leaving limited possibility for further clinical treatment with targeted therapies. Our research aims to determine whether treating in a sequential mode $KRAS^{G12C}$ cells who are resistant to sotorasib with adagrasib –and viceversa– can be successful in prolonging the response *in vitro* and *in vivo*.

**Material and Methods**

we generated *Ras*-less cells expressing $KRAS^{G12C}$ alone (LOH) or in presence of the WT endogenous *Kras* allele. We obtained adaptive-resistant cells to sotorasib (AMG-R) and adagrasib (MRTX-R) by treating them with increasing concentrations of drugs and we sequenced them in order to identify potential selection of acquired alterations. In parallel, we also generated *bona fide* resistant *Ras*-less cells with mutations *in cis* with G12C (Y96D/R68S/H95R/H95Q). We investigated the sequential treatment efficacy *in vitro* and *in vivo.*

**Results and Discussions**

Proliferation assays on adaptive-resistant cells revealed that, while MRTX-R cells were resistant to AMG, AMG-R cells were still sensitive to MRTX treatment. Afterwards, we studied the effects on MAPK pathway, which showed that both models were characterized by lower pERK levels upon sequential treatment with the reciprocal drug. Constitutively resistant models showed different drug-response profile dependent on the mutation: KRAS G12C/Y96D and G12C/R68S were resistant to both drugs, whereas KRAS G12C/H95R and G12C/H95Q were resistant to MRTX849 but still slightly sensitive to AMG510. In this situation, combination therapy appeared to be more effective.
Adaptive resistant *in vivo* models confirmed that AMG-R tumors were partially responsive to MRTX treatment whereas MRTX-R tumors were resistant to AMG treatment.

**Conclusion**

© 2023 The Authors. Molecular Oncology is published by John Wiley & Sons Ltd on behalf of Federation of European Biochemical Societies

- MRTX-R models are resistant to sotorasib treatment whereas AMG-R cells are still partially sensitive to adagrasib
- G12C/Y96D and G12C/R68S cells are resistant to both drugs
- G12C/H95Q and G12C/H95R are resistant to adagrasib, but slightly sensitive to sotorasib
- *in vivo,* MRTX-R tumors are resistant to sotorasib treatment whereas AMG-R tumors are still partially sensitive to adagrasib

### EACR23-0126
### Epidermal growth factor receptor inhibitor and Doxorubicin synergistically inhibits proliferation and drug efflux in MDA-MB 231 and MCF-7 cells in vitro.

B. Abrahams[1], D. Hiss[2]
[1]*University of the Free State, Basic Medical Sciences, Bloemfontein, South Africa*
[2]*University of the Western Cape, Medical Biosciences, Cape Town, South Africa*

**Introduction**
The lack of effective treatments has contributed to breast cancer being the leading cause of mortality in females worldwide. Endocrine therapy for hormone receptor positive cancers including anthracycline treatment using Doxorubicin (Dox) against Triple negative breast cancer (TNBC), has become inefficient due to unfavorable pathological complete response in patients and resistance becoming a common occurrence. In this study we examined the *in vitro* synergistic therapeutic potential of Dox and Epidermal growth factor receptor inhibitor (EGFRi), including its ability to impair P-glycoprotein (P-gp) function in MDA-MB 231TNBC and MCF-7 estrogen receptor positive cells.

**Material and Methods**
Growth inhibition of single and combination of Dox and EGFRi on MDA-MB 231 and MCF-7 was assessed using a MTT assay. Drug-interaction effects for synergy or antagonism were assessed using the Combenefit software tool based on Loewe additivity and Bliss independence models. P-gp's function as an efflux pump was assessed using a Calcein-AM drug resistant assay. Caspase 3/7 activity was assessed ($p<0.05$) whilst *EGFR gene* expression was evaluated ($p<0.001$) using RT-PCR. Statistical analysis entailed non-linear regression analysis for the $IC_{50}$ and 95% confidence intervals for all treatments.

**Results and Discussions**
EGFRi exhibited time dependent inhibition following 48h to 72h single drug administration on MDA-MB 231($IC_{50}$=7.05µM & $IC_{50}$=6.03µM) and MCF-7 cells($IC_{50}$=5.57µM & $IC_{50}$=3.96µM) respectively, whilst a Dox-EGFRi combination potentiated inhibition in MDA-MB 231($IC_{50}$=0.46µM) and MCF-7 cells($IC_{50}$ =0.01µM) at 72h. Drug synergism was observed at 72h with Bliss synergy scores of 44 and 67 recorded in MDA-MB 231 and MCF-7 cells respectively, for drug combination. Calcein-AM retention, a substrate of P-gp, increased in MCF-7 following EGFRi exposure (100µM; p=0.004) however Calcein-AM was effluxed after Dox treatment. Drug combination (Dox 10µM:EGFR 10µM) increased substrate retention in MDA-MB 231 and MCF-7 (Dox 1µM:EGFR 1µM & Dox 10µM:EGFR 10µM) respectively. Caspases was induced in MDA-MB 231 cells 1µM(p=0.007) for EGFRi, and Dox at 0.1µM (p=0.008). *EGFR gene* expression in MCF-7 and MDA-MB 231 cells were downregulated after single EGFRi and Dox exposure of 1µM, with MDA-MB 231 cells more sensitive to both drugs at 0.1µM($p<0.001$).

**Conclusion**
Our study highlight the *in vitro* synergistic activity of Dox-EGFRi combination in both MDA-MD 231 and MCF-7 cell lines, including the ability of EGFRi to attenuate P-gp function in MCF-7 cells.

### EACR23-0142
### Macrophages-cancer cell interaction affects ferroptosis induction capability in triple-negative breast cancer cells

Y. Haga[1], H. Konishi[1], H. Tsujino[1,2], K. Higashisaka[1,3], Y. Tsutsumi[1,4,5]
[1]*Osaka University, Graduate School of Pharmaceutical Sciences, Osaka, Japan*
[2]*Osaka University, The Museum of Osaka University, Osaka, Japan*
[3]*Osaka University, Institute for Advanced Co-Creation Studies, Osaka, Japan*
[4]*Osaka University, Institute for Open and Transdisciplinary Research Initiatives, Osaka, Japan*
[5]*Osaka University, Global Center for Medical Engineering and Informatics, Osaka, Japan*

**Introduction**
To overcome refractory cancers, there is increasing attention to ferroptosis as a novel mechanism to eradicate cancer. Ferroptosis is a new type of cell death discovered in 2012 and caused by the accumulation of ferrous ion-mediated lipid peroxidase. However, there is little knowledge of how cell-cell interaction could affect ferroptosis sensitivity. Here, we focused on macrophages, a main cell type in tumor microenvironment (TME), and tried to identify the ferroptosis sensitivity in triple-negative breast cancer cells by several types of co-culture systems.

**Material and Methods**
4T1-luc, murine triple-negative breast cancer cells, and RAW 264.7, murine macrophage cells, were used. RSL3 was used to induce ferroptosis. In indirect co-culture system, transwell system was used. To evaluate the direct cell-cell interaction, 4T1-luc cells were labeled with GFP. Ferrous ion and lipid peroxidase were detected in confocal microscopy and flow cytometry. Proteomics analysis was performed by LC-MS.

**Results and Discussions**
First, we tested whether macrophage conditioned medium (CM) could affect the ferroptosis sensitivity. As a result, cell viability of 4T1-luc cells on RSL3 treatment did not change with or without CM. Next, we tested the effect of in-direct interaction by using transwell co-culture system. As a result, cell viability of 4T1-luc cells on RSL3 treatment was significantly decreased in co-culture group. We also evaluate whether direct co-culture affects the ferroptosis sensitivity. After co-culture of GFP-labeled 4T1-luc cells with RAW264.7 cells in the same dish, cell viability 4T1-luc cells on RSL3 treatment was significantly decreased. These results indicate that cell-cell interaction

© 2023 The Authors. Molecular Oncology is published by John Wiley & Sons Ltd on behalf of Federation of European Biochemical Societies alters ferroptosis susceptibility. In order to explore the detailed mechanisms, we evaluated the intercellular ferrous ion and lipid peroxidase contributing to ferroptosis. Flow cytometric and imaging analysis revealed that ferrous ion and lipid peroxidase were increased in co-culture condition. Proteomics analysis of mono- and co-culture condition was performed to further explore the molecular mechanisms. As a results, several ferroptosis-related pathways (EGFR signaling, mitochondrial pathway) were increased in differentially upregulated proteins in co-culture condition.

**Conclusion**

We demonstrated that indirect and direct co-culture of cancer cells with macrophages enhanced the ferroptosis induction capacity. These findings suggest the importance to consider TME to apply ferroptosis inducers as a therapeutic option for cancer.

### EACR23-0197
### Regulation of Acquired Drug Resistance by miRNAs in High Grade Serous Ovarian Cancer

*E.M. Aydın*[1], *İ. DURMAZ ŞAHİN*[2]
[1]*Koc University, Research Center for Translational Medicine KUTTAM, Istanbul, Turkiye*
[2]*Koc University, School of Medicine, Istanbul, Turkiye*

**Introduction**

Epithelial Ovarian Cancer (EOC) is the 8th most common cause of cancer-related deaths in women worldwide. High-grade serous ovarian cancer (HGSOC) is the predominant subtype. Cytoreductive surgery and platinum-based chemotherapy are the main treatment modalities for HGSOC. However, 25% of patient develop resistance after first-line chemotherapy within 6 months. Poly (ADP-ribose) polymerase (PARP) inhibitors are used for the maintenance treatment of recurrent HGSOC patients. Due to the poor prognosis associated with a high recurrence rate in 3 years (>75%) after second-line chemotherapy, it is vital to investigate new treatment strategies to overcome drug resistance. miRNAs are small, non-coding RNA molecules playing significant roles in tumor progression. Aim of this study is to determine the effects of miRNAs on drug resistance and examine the possible combinational drug therapy strategies.

**Material and Methods**

Total 40 FFPE sample of patients diagnosed with HGSOC were included in this study in collaboration with Koç University Hospital, Gynecology and Oncology Department. (Koç University Ethics Committee approved the use of human material (2019.257.IRB2.079)). Differential miRNA gene expression levels between 20 good prognosis (sensitive) and 20 bad prognosis (resistant) FFPE samples were determined by microarray and qRT-PCR assays. For *in vitro* studies, resistant HGSOC cell lines were generated by stepwise dose-escalation method. miRNA mimics were generated and transfected into resistant cells. Downstream molecular investigations with mimics only or in combination with conventional therapy, were performed by western blot and flow cytometry experiments.

**Results and Discussions**

Resistant index values for olaparib-resistant OVCAR-3, OVSAHO, and CAOV-3 cells were determined as; 5, 4.5, and 2.5, and for carboplatin-resistant OVCAR-3, OVSAHO, and CAOV-3 cells; 2, 5.7, and 1.5 respectively. Depending on differential expression analysis of miRNAs, *let7b-5p* and *188-5p* were found as significantly downregulated in resistant FFPE samples and *in vitro* resistance models. In consideration of combinational drug and miRNA-mimic treatment results, most synergistic miRNA and drug combination was selected for further downstream molecular assays to re-sensitize resistance cells to therapy.

**Conclusion**

Within the scope of this study, the role of miRNAs in the context of resistance in HGSOC was explored and synergistic effects of miRNA with drugs were tested. This information will shed light on the alternative treatment of HGSOC.

### EACR23-0257
### POSTER IN THE SPOTLIGHT
### EML4-ALK mediates resistance to KRAS G12C inhibition

*P. Scaparone*[1], *A. Mira*[1], *T.C. Cheong*[2], *E. Patrucco*[1], *B. Ricciuti*[3], *I. Savinelli*[1], *C. Voena*[1], *M. Awad*[3], *R. Chiarle*[1,2], *C. Ambrogio*[1]
[1]*University of Turin, Department of Molecular Biotechnology and Health Sciences, Turin, Italy*
[2]*Boston Children's Hospital and Harvard Medical School, Department of Pathology, Boston, United States*
[3]*Dana-Farber Cancer Institute, Lowe Center for Thoracic Oncology, Boston, United States*

**Introduction**

KRAS is one of the most frequently mutated oncogenes in cancer and has been considered undruggable for decades, till the development of two $KRAS^{G12C}$ inhibitors, sotorasib and adagrasib, that were approved by FDA between 2021 and 2022. The response rate in $KRAS^{G12C}$-tumors represented a major clinical breakthrough, nevertheless therapy resistance invariably occurs. Secondary on-target mutations have been found in patients, as well as potential by-pass mechanisms that, however, remain to be validated. Among patients enrolled in the KRYSTAL-1 clinical trial, two relapsed patients showed acquired EML4-ALK fusion. This work investigates whether EML4-ALK drives the resistance to adagrasib via the reactivation of the MAPK pathway and we validated EML4-ALK as a therapeutic target in adagrasib-resistant tumors.

**Material and Methods**

By CRISPR-Cas9 approach, we engineered the EML4-ALK fusion in $KRAS^{G12C}$ NSCLC cell lines that were tested *in vitro* and *in vivo*. We kept these cells in constant presence of adagrasib to mimic the clinical scenario where acquired alterations are selected upon treatment.

**Results and Discussions**

*In vitro*, the acquisition of EML4-ALK in $KRAS^{G12C}$ NSCLC cell lines conferred resistance to adagrasib compared to parental cell lines. The reactivation of the MAPK pathway, which was triggered by an increase of GTP-KRAS, was blocked upon concomitant treatment with adagrasib and the ALKi alectinib. Consistently,

© 2023 The Authors. Molecular Oncology is published by John Wiley & Sons Ltd on behalf of Federation of European Biochemical Societies double mutant KRAS$^{G12C}$/EML4-ALK xenografts were resistant to adagrasib as single agent *in vivo*, but sensitive to adagrasib in combination with ALKi. When cells were subjected to constant treatment with adagrasib, their oncogenic signaling rewired towards dependency on EML4-ALK signaling and became sensitive to ALKi alone while maintaining the resistance to adagrasib. Mechanistically, the MAPK pathway remained active with high levels of GTP-KRAS in the presence of adagrasib, while it was inhibited by ALKi treatment, alone or in combination with adagrasib. Likewise, ALKi both as single agent and in combination with adagrasib showed significant therapeutic activity *in vivo*.

**Conclusion**

- EML4-ALK causes resistance to adagrasib in KRAS$^{G12C}$ lung cancer cell lines and patients.
- Resistance is caused by MAPK pathway reactivation through RAS-GTP increase.
- *In vitro*, constant drug pressure results in a switch from KRAS to EML4-ALK oncogene dependency.
- The signaling switch of KRAS to EML4-ALK-driven oncogene dependency can be targeted by an ALKi alone *in vitro* and *in vivo*.

### EACR23-0282
### Akkermansia muciniphila: A key gut microbiota in development and immune resistance of non-alcoholic fatty liver disease (NAFLD)-induced hepatocellular carcinoma

*F. Ying[1], K.P.S. Chung[1], X.Q. Wu[1], T.K.W. Lee[1]*
*[1]Hong Kong Polytechnic University, Department of Applied Biology and Chemical Technology, Hong Kong, Hong Kong- China*

**Introduction**

Nonalcoholic fatty liver disease (NAFLD)-induced hepatocellular carcinoma (HCC) is an emerging malignancy in the developed world; however, the mechanisms contributing to its formation are largely unknown, and immune checkpoint therapy is ineffective. Recently, several studies have reported that the intestinal microbiota plays an important role in the pathogenesis of NAFLD. However, the exact role of gut microbiota in NAFLD-induced HCC remains exclusive.

**Material and Methods**

16S rRNA sequencing was employed to identify the changes of gut microbiota composition during the course of DEN/HFD-induced mouse HCC model. MCD-diet induced HCC mouse model was employed for functional characterization of Akkermansia muciniphila (AKK). The role of AKK in membrane integrity was evaluated by immunofluorescence staining, qPCR, mass spectrometry analysis, and ELISA assay. The effect of AKK in immune microenvironment was evaluated by single cell RNA sequencing (scRNA-seq), coupled with immune profiling analysis. The therapeutic value of AKK alone in combination with PD1 treatment was investigated *in vivo.*

**Results and Discussions**

*AKK was* decreased by ~40-folds from healthy to HCC tissues during the course of HCC tumor development; and daily administration of AKK could effectively attenuate the development of NAFLD-induced HCC. Given the physiological function of AKK in the maintenance of intestinal integrity, AKK *was* shown to repair the intestinal lining as evidenced by increase in tight-junction proteins, with concurrent decrease in the serum concentration of LPS and bile acid metabolites. Since LPS/ bile acid metabolites is regarded as an important regulator of innate immune cells, we examined the effect of AKK on the regulation of innate immunity in the tumor microenvironment by scRNA-seq and immunoprofiling analyses. We found that AKK decreased the populations of immunosuppressive cells, including monocytic myeloid-derived suppressor cells (mMDSCs) and M2 macrophages. By Trajectory analysis, AKK suppressed differentiation of M2 macrophages/mMDSCs from monocytes, which may lead to T cell proliferation and activation. AKK administration in combination with PD1 treatment exerts the maximal growth suppressive effect, which is accompanied with increased infiltration of CD4/8 T cells.

**Conclusion**

AKK in critically involved in development and immune resistance of NAFLD-induced HCC. Interestingly, AKK may serve as a biomarker for prediction of PD1 response in these HCC patients.

### EACR23-0304
### TFEB DRIVES CHEMO-IMMUNO-RESISTANCE IN LUNG CANCER

*M. Akman[1], C. Ariano[1], G. Doronzo[1], T. Mohr[2], F. Bussolino[1], C. Riganti[1]*
*[1]University of Torino, Department of Oncology, Torino, Italy*
*[2]Medical University of Vienna, Comprehensive Cancer Center, Vienna, Austria*

**Introduction**

Transcription factor EB (TFEB) is a leucine zipper protein and a major regulator of lysosomal biogenesis and autophagy. These two events confer chemoresistance in solid tumors, by sequestrating chemotherapeutic drugs, and modulating the immune-recognition. In this study, we investigated if TFEB affects the response to chemotherapy and to Vγ9δ2 T- lymphocytes in non-small cell lung cancer (NSCLC).

**Material and Methods**

Changes in the expression of TFEB and ABC transporters (ABCA1, ABCB1, ABCC1), and their networks, as well as their effect on survival in NSCLC were analyzed by using the TCGA-LUAD dataset. We silenced TFEB in NCI-H441 and NCI-H2228 cells. Changes in ABC transporters, in upstream signaling pathways, and metabolic pathways involved in chemo-immunoresistance were measured by RT-PCR, immunoblotting, radiolabeling, and ChIP. Co-cultures between NSCLC cells and Vγ9δ2 T-lymphocytes were set up to measure their expansion and cell killing. Sensitivity to cisplatin was evaluated by the WST-1 assay. Wild-type (WT) and shTFEB NSCLC xenografts implanted in Hu-CD34$^{+}$ NSG mice were used for in vivo validation.

**Results and Discussions**

Bioinformatic analysis showed that the TFEB$^{high}$ABCA1$^{high}$ABCC1$^{low}$ phenotype is associated with overall survival. ChIP assay indicated that ABCA1 is a direct target of TFEB. By reducing the pERK1/2, shTFEB cells had reduced ERK-1/2-mediated activation of

© 2023 The Authors. Molecular Oncology is published by John Wiley & Sons Ltd on behalf of Federation of European Biochemical Societies

SREBP2, which modulates genes of cholesterol homeostasis. As such, TFEB silencing down-regulated genes of cholesterol synthesis, decreased expression and activity of the cholesterol/IPP transporter ABCA1, the efflux of IPP, and the NSCLC killing by Vγ9δ2 T-lymphocytes. In parallel, shTFEB cells had increased expression of ABCB1 and ABCC1 and significantly higher IC50 to cisplatin. This effect is likely mediated by the de-inhibition of transcriptional activity of HIF-1 upon TFEB silencing, resulting in ABCB1/ABCC1 up-regulation, and/or by an increased production of mitochondrial ATP that fuels these transporters. The results of immune xenografts confirmed that shTFEB tumors were more resistant to cisplatin than wild-type counterparts. The combination of cisplatin and NZ was effective in reversing the chemo-immuno-resistance of shTFEB tumors.

**Conclusion**

We propose TFEB as a driver of chemo-immuno-resistance in NSCLC. Future experiments including a tumor single-cell transcriptomic profile are clarifying which cell populations and pathways make TFEB a controller of chemo-immuno-resistance in NSCLC.

Supported by the AIRC (Grant No. IG21408).

### EACR23-0338

### RNA editing by ADAR1 modulates lipid metabolism to drive resistance to chemotherapy in gastric cancer

*T.L. Wong[1,2], J.J. Loh[1], S. Lu[3], H.H.N. Yan[4], H.C. Siu[4], R. Xi[5], L. Chen[5,6], J.P. Yun[3], S.Y. Leung[4,7], S. Ma[1,2]*

*[1]The University of Hong Kong, School of Biomedical Sciences, Hong Kong, Hong Kong- China*

*[2]The University of Hong Kong – Shenzhen Hospital, Clinical Oncology Center, Shenzhen, China*

*[3]Sun Yat-Sen University Cancer Centre, Department of Pathology, Guangzhou, China*

*[4]The University of Hong Kong, School of Clinical Medicine, Hong Kong, Hong Kong- China*

*[5]National University of Singapore, Cancer Science Institute of Singapore, Singapore, Singapore*

*[6]National University of Singapore, NUS Centre for Cancer Research, Singapore, Singapore*

*[7]The University of Hong Kong, The Jockey Club Centre for Clinical Innovation and Disco very, Hong Kong, Hong Kong- China*

**Introduction**

Combination of fluoropyrimidine and platinum-based drugs (FP) is one of the most widely used chemotherapy for various cancer types. In gastric cancer (GC), FP plays a central role as peri-operative chemotherapy and palliative treatment for patients at advanced stage. However, treatment response is dismal, and resistance develops during treatment. Organoid model has been proven to recapitulate response to therapeutics in patients and serves as an excellent model for studying mechanisms of drug resistance. Here, we used GC patient-derived organoids to study the mechanism of development of resistant to FP treatment.

**Material and Methods**

5-fluorourcail (5FU) and cisplatin (CDDP) were used as example for FP treatment. GC organoids resistant to 5FU/CDDP (GC-Org-Res) were developed by escalating the dose progressively up to $IC_{50}$. The mutation and expression profile were analysed with whole exome sequencing (WES) and RNA sequencing (RNASeq). Lipidomics analysis was carried out with LC-MS/MS. Response to 5FU/CDDP was tested *in vivo* by organoid xenograft model in NSG mice.

**Results and Discussions**

GC organoids were developed with resistance to 5FU/CDDP treatment. Gene set enrichment analysis found interferon α and γ response, and JAK-STAT signaling being the top enriched pathways in GC-Org-Res. Expression of adenosine deaminase RNA specific 1 (ADAR1), a downstream effector of JAK-STAT signaling, was also concomitantly increased in GC-Org-Res. ADAR1 converts adenosine (A) to Inosine (I) (A-to-I) in RNA, leading to change in coding sequence or stability of RNA. By comparing the sequencing reads from WES and RNASeq, we identified 713 potential A-to-I editing sites that are commonly hyperedited in GC-Org-Res. Combined with RNASeq analysis, genes involved in lipid metabolism were found to be hyperedited and deregulated in GC-Org-Res. Lipidomics analysis confirmed altered fatty acid compositions in GC-Org-Res. As a proof-of-concept, stearoyl-CoA desaturase-1 (SCD1), a key lipid metabolism enzyme, was selected for follow-up. Knockdown of ADAR1 suppressed while overexpression of ADAR1 enhanced A-to-I editing of SCD1 mRNA and protein expression of SCD1. Suppression of SCD1 by shRNA or inhibitor, SSI4, re-sensitise GC-Org-Res to 5FU/CDDP. Clinically, a ADAR1/SCD1 signature predicted response to FP treatment.

**Conclusion**

Development of resistance towards 5FU/CDDP treatment in gastric cancer requires increased RNA editing of genes involved in lipid metabolism (e.g. SCD1). Targeting SCD1 reverses resistance towards 5FU/CDDP.

### EACR23-0366

### Resistance of melanoma to combined BRAF/MEK-targeted therapy is mediated by drug tolerant persister cells that possess multiple vulnerabilities

*R. Saup[1], K. Held[1], N. Nair[1], J. Shen[1], C. Hill[2], B.K. Garvalov[1], J.P. Sleeman[1]*

*[1]European Center for Angioscience ECAS, Medical Faculty Mannheim of the University of Heidelberg , Mannheim, Germany*

*[2]The Francis Crick Institute, Developmental Signalling Laboratory, London, United Kingdom*

**Introduction**

Melanoma, the most lethal type of skin cancer, is driven by activating BRAF mutations in about 50% of cases. Combined BRAF and MEK inhibition (BRAFi/MEKi) has significantly increased the survival time of BRAF-mutant melanoma patients. Unfortunately, the majority of patients quickly develop resistance to BRAFi/MEKi therapy. An emerging paradigm is that this resistance often emerges

© 2023 The Authors. Molecular Oncology is published by John Wiley & Sons Ltd on behalf of Federation of European Biochemical Societies through the reversible epigenetic reprogramming of slow cycling drug tolerant persister cells (DTPs).

**Material and Methods**

BRAF-mutant Yale University Mouse Melanoma (YUMM) 1.7 and 5.2 cells were treated for 12 months with increasing concentrations of the BRAFi/MEKi combinations vemurafenib + cobimetinib (VC) or dabrafenib + trametinib (DT). Resistant cells were functionally characterized using proliferation, adhesion, migration and invasion assays. Transcriptomic changes were identified by RNA-sequencing and functionally validated using pharmacological inhibition.

**Results and Discussions**

We have developed four mouse melanoma models for DTP-based BRAFi/MEKi resistance, using either VC or DT, drug combinations that are approved for clinical use. Three of these models exhibit a robust but reversible resistance to BRAFi/MEKi, which is rapidly lost after drug withdrawal, but quickly regained upon drug re-treatment, consistent with DTP-mediated resistance. The fourth model exhibits permanent acquired resistance. All four resistance models have common phenotypic alterations, including slower proliferation but increased invasiveness. Transcriptomic analysis indicated profound reprogramming in the resistant cells, and identified many key pathways that are commonly regulated in all four models, the most prominent being extracellular remodelling. These changes are reversible upon withdrawal of targeted therapy, even in the model that exhibits permanent acquired resistance, suggesting that DTP phenotypic reprogramming is still induced even after acquisition of irreversible resistance. Importantly, pharmaceutical intervention in a number of these resistance-associated functional pathways, including extracellular matrix-related ones, re-sensitized the resistant cells to the combined BRAF/MEK-targeted therapy.

**Conclusion**

Our findings have afforded new insights into the molecular basis of reversible adaptive resistance, and have allowed us to demonstrate that resistant DTPs have multiple vulnerabilities that can be targeted to overcome combined resistance to BRAF/MEK-targeted therapy.

### EACR23-0372

### New clues into the metabolic reprogramming of sunitinib- and pazopanib-resistant renal cell carcinoma cells

*F. Amaro[1,2], M. Carvalho[1,2,3,4], M.D.L. Bastos[1,2], P. Guedes de Pinho[1,2], J. Pinto[1,2]*

*[1]UCIBIO-REQUIMTE, Department of Biological Sciences- Laboratory of Toxicology- Faculty of Pharmacy- University of Porto, Porto, Portugal*

*[2]Associate Laboratory i4HB, Department of Biological Sciences- Laboratory of Toxicology- Faculty of Pharmacy- University of Porto, Porto, Portugal*

*[3]Faculty of Health Sciences, University Fernando Pessoa, Porto, Portugal*

*[4]Institute of Research- Innovation and Development, University Fernando Pessoa, Porto, Portugal*

**Introduction**

Tyrosine kinase inhibitors (TKIs), such as sunitinib and pazopanib, changed the therapeutic landscape of metastatic renal cell carcinoma (RCC). However, TKIs resistance and disease progression within one year have been observed even in patients who initially respond to treatment. Hence, understanding the metabolic mechanisms associated with TKIs resistance is of utmost importance to reverse this issue and improve RCC treatment guidelines. This work applied a metabolomics approach to investigate the metabolic dysregulations underlying sunitinib and pazopanib resistance in a metastatic RCC cell line (Caki-1).

**Material and Methods**

Caki-1 cell line was continuously (6 months) exposed to increasing concentrations of sunitinib and pazopanib to induce resistance. Resistance was confirmed through the MTT assay by a 4.9- and 2.8-fold increase in the $IC_{50}$ values of sunitinib and pazopanib-resistant cells compared with the parental cells, respectively. In the metabolomics assay, eight independent passages were considered for TKI-resistant and parental cells. Intracellular and extracellular metabolites were analyzed by proton nuclear magnetic resonance ($^1$H NMR) spectroscopy. Statistical analysis comprised multivariate and univariate methods, and biological interpretation was performed through pathway analysis.

**Results and Discussions**

Partial least squares discriminant analysis (PLS-DA) showed clear intracellular and extracellular metabolic dysregulations for both TKI-resistant Caki-1 cells compared with the parental cell line. Sunitinib- and pazopanib-resistant cells revealed a common reprogramming in the amino acid, glycerophospholipid, and nicotinate and nicotinamide metabolisms. Sunitinib-resistant cells were also characterized by an enhanced cellular antioxidant capacity supported by a significant increase in the intracellular levels of glutathione and *myo*-inositol, and a significantly higher uptake of glutamine. On the other hand, pazopanib-resistant cells exhibited marked changes in several metabolites (e.g., glucose, lactate, pyruvate, acetate, succinate, fumarate) participating in energy metabolism.

**Conclusion**

Our findings demonstrate for the first time a distinct pattern of metabolic alterations associated with sunitinib and pazopanib resistance in metastatic RCC cells. Targeting these dysregulations may constitute a promising strategy to restore cell sensitivity to treatment with these TKIs.

### EACR23-0374

### Identification of PARP inhibitor resistance in High-Grade Serous Ovarian Cancer patient-derived serum-free cell lines

*J. Schwickert[1], V. Thiel[2], F. Zickgraf[2], M. Sprick[2], A. Trumpp[2]*

*[1]German Cancer Research Center DKFZ, Stem Cells and Cancer, Heidelberg, Germany*

*[2]HI-STEM gGmbH @ German Cancer Research Center DKFZ,*

© 2023 The Authors. Molecular Oncology is published by John Wiley & Sons Ltd on behalf of Federation of European Biochemical Societies

*Stem Cells and Cancer, Heidelberg, Germany*

**Introduction**

Inhibitors of poly(ADP-ribose) polymerase (PARPi) are established in the treatment of homologous recombination deficient cancers such as High Grade Serous Ovarian cancer (HGSOC) in the clinic. There PARPi have demonstrated promising response rates among patients, however extended treatment usually leads to acquired PARPi resistance. At the moment multiple resistance mechanisms could be identified in preclinical studies. However, only a minority of those mechanisms seem to account for the observed resistances in the clinic (mainly by a re-activation of the Homologous directed repair pathway). Here we showed that PARPi resistant cells and can develop a potential DNA-repair independent resistance mechanism to different PARPi, which are commonly used in the clinic.

**Material and Methods**

Here, we use primary patient-derived cell lines cultured in a serum-free medium. To obtain PARPi resistant cell lines we treated ovarian cell lines over several passages with increasing doses of olaparib resulting at the end in PARPi-resistant cell lines. In addition we performed whole Exome sequencing, RNA sequencing as well as metabolomic analysis to investigate the underlying resistance mechanism.

**Results and Discussions**

The PARPi resistant cells derived from the treatment regime with olaparib are also cross-resistant to other approved PARPis such as niraparib, talazoparib and rucparib. In contrast, treating the cells with DNA-damage inducing agents (oxaliplatin & methyl methane sulfonate) resulted in the same drug efficiency seen in the sensitive cells. Furthermore, the DNA-repair capacity between the PARPi resistant and sensitive cells remained unchanged under treatment with DNA-damaging agents. These results suggest a so far unknown, PARPi resistance mechanism characterized by a metabolic switch and affecting the cell cycle of PARPi-resistant cells but is independent of the DNA-repair capacity of the cells.

**Conclusion**

In this study, we could identify, a so far, unknown PARPi-specific resistance mechanism, which seems to be independent of DNA-repair capacity of the cells.

### EACR23-0442
### PATIENT-DERIVED TUMOR XENOGRAFT IN EXPLORING THE FORMATION OF VEMURAFENIB-RESISTANCE IN BRAF-MUTANT MELANOMA

*M. Cserepes[1], D. Vári-Mező[1], A. Kigyós[2], J. Tóvári[1]*
*[1]National Institute of Oncology, Department of Experimental Pharmacology and the National Tumour Biology Laboratory, Budapest, Hungary*
*[2]KINETO Lab Ltd., Budapest, Hungary*

**Introduction**

Malignant melanoma is not the most prevalent among skin cancers, yet, due to high metastatic potential and often occurring therapy resistance, it is causing the most deaths. about half of melanomas are positive for BRAF V600E single nucleotide polymorphism, offering a good opportunity for targeted therapeutics binding to and inhibiting BRAF with the mutation. Multiple inhibitors have been introduced and used in clinics, such as vemurafenib or dabrafenib. A classical mechanism can reinforce the Growth Factor Receptor (GFR) signaling pathway independently from BRAF activity. This lead to successful combination of MAPK inhibitors (trametinib, cobimetinib) with BRAF inhibitors. Although, as cancer cells can develop different mechanisms of resistance, novel approaches are needed, too. The use of Patient-derived tumor xenograft (PDTX) models offer rapid and repeatable experimental model for tumor evolution upon treatment.

**Material and Methods**

We used a PDTX model of a melanoma patient heterozygous for BRAF V600E to model treatment. We used NOD-SCID male mice, and from tumor size of 100mm^3 we applied 100mg/kg vemurafenib per os every weekdays. On excessive tumor growth or animal age beyond 6 months, we transplanted tumor tissues to new animals, and collected tissue for analysis at each time point. 3-3 parallel samples from initial tumors, ones following 6 or 12 months of treatment were prepared for mRNA sequencing.

**Results and Discussions**

NGS screen contained 434 differentially expressed genes after both treatment times. We examined 12 genes with known role in cancer patient survival and consistent expression change over three-fold. Eight of these gene expression patterns were further confirmed by qPCR on 42 samples over 7 generations of mice. Expression increased in case of AGAP9, CD27, ABCB1, GNRHR, PDGFA, GPR39, SLC15A3 and decreased expression of IFI27.

**Conclusion**

Our data suggests that these changes, especially the intensive expression of multidrug-resistance related ABCB1 transporter, can set up a previously unreported model of vemurafenib resistance of melanomas, urging alternative therapeutic approaches for subgroups of patients with different genetic characteristics.

*This work is supported by the National Laboratory Programme (2022-2.1.1-NL-2022-00010) and the Thematic Excellence Programme (TKP2021-EGA-44) in the National Institute of Oncology.*

### EACR23-0448
### Elucidating 14-3-3σ's role in head and neck squamous cell carcinoma chemoresistance

*M. Bataller[1], A. Sánchez-García[1], C. Mir[1], Y. García-Mayea[1], L. Solé-Ingla[1], M. Miera-Maluenda[1], M.E. Lleonart Pajarín[1]*
*[1]Vall d'Hebron Research Institute VHIR, Pathology, Barcelona, Spain*

**Introduction**

Head and neck squamous cell carcinoma (HNSCC) is the sixth most commonly diagnosed cancer worldwide. However, HNSCC treatment has hardly evolved in the last decades, being cisplatin (CDDP) the main chemotherapeutic. Unfortunately, resistance to CDDP and subsequent relapse of HNSCC patients is common and explains HNSCC high mortality. We aim to identify target proteins involved in the acquisition of CDDP resistance to improve HNSCC treatment. By a comparative proteomics study 14-3-3σ was revealed as a protein potentially involved in CDDP-resistance in HNSCC. 14-3-3σ

© 2023 The Authors. Molecular Oncology is published by John Wiley & Sons Ltd on behalf of Federation of European Biochemical Societies

(*SFN* gene) is a Ser/Thr binding protein involved in many cellular processes such as cell cycle, cell proliferation, growth, motility and apoptosis.

**Material and Methods**

A comparative proteomics study analyzing HNSCC cells CCL-138 (metastatic pharynx), CCL-138-R (CDDP-resistant) and CCL-138-derived cancer stem cells (CSCs) was performed and 14-3-3σ was found to be overexpressed in CCL-138-R and CSCs in comparison to CCL-138. The results in this cell line were validated by western blot in JHU029 cell line (larynx). Depletion of 14-3-3σ by shRNA technology was used to elucidate 14-3-3σ's role in proliferation, apoptosis and CDDP-resistance. Moreover, CRISPR/Cas9 technology was used to obtain 14-3-3σ-knockout (*SFN*-KO) cells to validate shRNA results of CDDP-resistance and to perform an *in vivo* experiment in NMRI-Foxn1nu/Foxn1nu mice.

**Results and Discussions**

We found that 14-3-3σ depletion decreased cell proliferation, induced apoptosis and sensitized HNSCC cells to CDDP treatment. In addition, in 14-3-3σ-depleted cells p53 protein levels decreased. Furthermore, we demonstrated that JHU029 *SFN*-KO cells develop smaller tumors *in vivo* than control cells. On the other hand, in 71% of HNSCC patients 14-3-3σ was overexpressed in tumor tissue compared to normal one. TMAs staining analysis from a cohort of ~400 HNSCC patients' biopsies showed that 14-3-3σ expression was positively associated with p21, active Src (p-Src) and E-cadherin proteins.

**Conclusion**

Overall, this study suggests that 14-3-3σ is important for cell response to DNA damage, proliferation, cell survival and CDDP-resistance in HNSCC.

*Supported by grants from ISCIII (PI20/00556), co-financed by ERDF (CP03/00101), AECC (GC16173720CARR) and La Marató TV3 (202116-30).*

### EACR23-0449

### Genetic and pharmacological inhibition of SDCBP modulates stemness and chemoresistance in Head and Neck Squamous Cell Carcinoma

*C. Mir Pérez[1], M. Bataller[1], A. Sánchez-García[1], Y. García-Mayea[1], M. Miera-Maluenda[1], L. Solé-Ingla[1], M.E. Lleonart Pajarín[1]*

*[1]Vall d'Hebron Research Institute, Pathology, Barcelona, Spain*

**Introduction**

Head and Neck Squamous Cell Carcinoma (HNSCC) is the sixth most common cancer in the world. The 5-year survival rates for HNSCC patients have remained at ~50% over the last 40 years, largely due to frequent tumor recurrence and/or metastatic dissemination. Being drug resistance the principal limiting factor to achieving good survival rates in patients, the identification of potential biomarkers to design new molecular-targeted treatments, will be essential to improving HNSCC patient outcomes. So, the sensitization of resistant cells and cancer stem cells (CSCs) by inhibiting crucial proteins involved in cancer resistance could be a more effective therapeutic strategy.

**Material and Methods**

To unravel the mechanisms that govern chemoresistance, we performed a comparative proteomic study analyzing HNSCC cells: CCL-138 (parental), CCL-138-R (cisplatin-resistant), and CSCs. **Syntenin-1** (SDCBP) was upregulated in CCL-138-R cells and CSCs over parental cells. Then, SDCBP depletion and overexpression in HNSCC cell lines were characterized *in vitro* and/or *in vivo*.

**Results and Discussions**

On the one hand, SDCBP depletion sensitized biopsy-derived and established HNSCC cell lines to cisplatin (CDDP) and reduced CSC markers, being Src activation the main SDCBP downstream target. In mice, SDCBP-depleted cells formed tumors with decreased mitosis, Ki-67 positivity, and metastasis over controls. Moreover, the fusocellular pattern of JHU029-R cell-derived tumors reverted to a more epithelial morphology upon SDCBP silencing. Importantly, SDCBP expression was associated with Src activation, poor differentiated tumor grade, advanced tumor stage, and shorter survival rates in a series of 382 HNSCC patients.

On the other hand, through a virtual screening, sixteen new SDCBP ligands have been identified as candidates for HNSCC therapy. Biophysical interaction between the hits and the PDZ1 domain of SDCBP has been demonstrated. Two chemical compounds were selected form the preliminary hits and, were tested *in vitro*, analyzing anti-proliferative efficacy and biological implications, and *in vivo*, including toxicological and efficacy assays.

**Conclusion**

Our results reveal that SDCBP has clinical relevance and oncogenic function in HNSCC model and that genetic and pharmacological targeting of SDCBP could be a potential therapeutic strategy to effectively eliminate CSCs and CDDP resistant tumors.

*Supported by grants from ISCIII (PI20/00556), co-financed by ERDF (CP03/00101), AECC (GC16173720CARR) and La Marató TV3 (202116-30).*

### EACR23-0450

### Crucial proteins involved in Cisplatin Resistance in Head-and-Neck Squamous Cell Carcinoma

*Y. GARCÍA MAYEA[1,2], L. Benítez-Álvarez[2], A. Sánchez-García[1], M. Bataller[1], C. Mir[1], O. Companioni[3], S. Benavente[1], M. Miera-Maluenda[1], L. Solé-Ingla[1], M.E. LLeonart[1]*

*[1]Vall d'Hebron Research Institute, Pathology, Barcelona, Spain*

*[2]Universitat de Barcelona, Departament de Genètica- Microbiologia i Estadística, Barcelona, Spain*

*[3]Massey Cancer Center- Virginia Commonwealth Universi ty, Department of Biochemistry, Richmond- VA, United States*

**Introduction**

Acquisition of chemoresistance is the leading cause of recurrences, metastases and death in most cancers, including Head and neck squamous cell carcinoma (HNSCC).

**Material and Methods**

To identify novel genes involved in HNSCC chemoresistance, we explored the expression profiles of

© 2023 The Authors. Molecular Oncology is published by John Wiley & Sons Ltd on behalf of Federation of European Biochemical Societies the following cisplatin (CDDP) resistant (R) versus parental (sensitive) cell lines by RNA-sequencing (RNA-seq): JHU029 (RRID:CVCL_5993), HTB-43 (FaDu) and CCL-138 (Detroit 562). Moreover, we crossed-checked the RNA-seq results with the proteomic profiles of HTB-43-R (versus HTB-43) and CCL-138-R (versus CCL-138) cell lines. The results were validated by RT-PCR and the relevance of target gene inhibition was assessed by proliferation assays and *in silico* studies.

**Results and Discussions**

Using the parental condition as a control, 30 upregulated and 85 downregulated genes were identified for JHU029-R cells; 263 upregulated and 392 downregulated genes for HTB-43-R cells, and 154 upregulated and 68 downregulated genes for CCL-138-R cells. For the HTB-43-R cells, 21 upregulated and 72 downregulated targets overlapped between the proteomic and transcriptomic data; whereas in CCL-138-R cells, four upregulated and three downregulated targets matched. Following an extensive literature search, six genes from the RNA-seq (CLDN1, MAGEB2, CD24, CEACAM6, IL1B and ISG15) and six genes from the RNA-seq and proteomics crossover (AKR1C3, TNFAIP2, RAB7A, LGALS3BP, PSCA and SSRP1) were selected to be studied by qRT-PCR in 11 HNSCC patients: six resistant and five sensitive to conventional therapy. Interestingly, the high MAGEB2 expression was associated with resistant tumors and is revealed as a novel target to sensitize resistant cells to therapy in HNSCC patients.

**Conclusion**

Our findings demonstrated that omics technologies have a great potential to identify future therapeutic targets. In our case, we have applied these studies in the HNSCC model with the idea of suggesting alternative options for HNSCC patients who do not respond to conventional treatment. RNA-seq and proteomics studies have allowed us to identify 12 genes as potential targets. In particular, the most promising gene resulting from this study is MAGEB2, whose downregulation *in vitro* induces the sensitization of cells to CDDP. Importantly, the MAGEB2 expression can distinguish between the CDDP-responsive and CDDP-unresponsive patients.

*Supported by ISCIII: PI20/00556, and co-financed by the ERDF (CP03/00101) and AECC (GC16173720CARR)*

### EACR23-0451

### Pharmacological inhibition of TSPAN1-signaling decreases HNSCC tumorigenesis

*A. Sánchez García[1], Y. García-Mayea[1], M. Bataller[1], C. Mir[1], M. Miera Maluenda[1], L. Solé-Ingla[1], J. Castellvi[1], M. Lleonart-Pajarín[1]*

*[1]Vall d'Hebron Research Institute VHIR, Pathology, Barcelona, Spain*

**Introduction**

Head and Neck Squamous Cell Carcinoma (HNSCC) is a particularly aggressive cancer type since half the patients do not survive more than five years after diagnosis. Several studies have described a marked involvement of tetraspanin 1 (TSPAN1) in models of prostate, colon, pancreatic and gastric cancer. Our group has also confirmed its oncogenic role *in vitro* and *in vivo* models of HNSCC and its involvement in the acquisition of resistance to chemotherapy. TSPAN1 increased in cisplatin (CDDP) resistant cells and its depletion in parental and CDDP resistant HNSCC cells reduced cell proliferation, induced apoptosis, decreased autophagy and sensitized to chemotherapeutic agents. In addition, this inhibited several signaling cascades, where the phospho-SRC (p-Src) inhibition was the major common target. *In vivo*, TSPAN1 downregulation decreased size and proliferation rates of parental and CDDP resistant tumors and also reduced metastatic spreading.

**Material and Methods**

1- Through a virtual screening, possible drugs capable of blocking TSPAN1 and its possible molecular interactions were determined.

2- The possible *in vitro* biological activity of these compounds was characterized in HNSCC cell lines (parental and CDDP resistant): a) The treatment doses were selected by determining the corresponding IC50 values. b) Subsequently, the possible biological implications of TSPAN1 inhibition were evaluated by analyzing several molecular pathways.

3- Four drugs where selected to performed toxicological assays in mice, evaluating weight, biomarkers in serums and histopathology. Further it was performed *in vivo* tumorigenic and metastasis assays in mice.

**Results and Discussions**

Twenty completely new compounds were selected from the pharmacological point of view. *In vitro,* four drugs inhibited correctly TSPAN1 at the IC50 value and affected signaling cascades, where p-SRC was severe inhibited. In addition, apoptosis, autophagy, proliferation and morphology of the cell lines were affected. At the moment, *in vivo* toxicological assays demonstrated no significant negative effect in mice. Two drugs reduced significantly size and weight rates of HNSCC CDDP resistant tumors, control vs drug since week 1 of treatment till the end of the study.

**Conclusion**

Pharmacological targeting of TSPAN1 could be a possible cancer treatment (maybe in combination with the actual chemotherapeutics) for HNSCC tumors. *Supported by ISCII:P20/00556 and co-financed by thge ERDF (CP03/00101) and AECC (GC16173720CARR)*

### EACR23-0454

### Combined targeting the Integrated Stress Response, RAS-MAPK and STAT5 signaling to eradicate leukemic cells resistant to tyrosine kinase inhibitors

*K. Piwocka[1], W. Dudka[1], G. Hoser[2], L. Turos-Korgul[1], S.S. Mondal[1], A. Kominek[1], M. Wiech[1], P. Firmanty[1], M. Machnicki[3], T. Stoklosa[3]*

*[1]Nencki Institute of Experimental Biology, Laboratory of Cytometry, Warsaw, Poland*

*[2]Medical Center for Postgraduate Education, Laboratory of Cytometry, Warsaw, Poland*

*[3]Medical University of Warsaw, Department of Immunology, Warsaw, Poland*

**Introduction**

Integrated Stress Response (ISR) enables cellular adaptation and in cancer it is a driver of survival, progression and resistance. In leukemia ISR is activated by oncogenic signaling, bone marrow microenvironment

© 2023 The Authors. Molecular Oncology is published by John Wiley & Sons Ltd on behalf of Federation of European Biochemical Societies conditions including hypoxia or therapy as a protective signaling. Therefore, it is proposed as a therapeutic target, nevertheless, no specific strategy is proposed till now, especially for hematological malignancies. Here we validated the potential of the combined strategy based on pharmacological targeting of ISR together with TKI to eradicate resistant cells.

**Material and Methods**

We used CML cells lines, NSG mice bearing BCR-ABL1 cells and PDX mouse model with primary CD34+ CML-BC imatinib-resistant cells. PDX with *PTPN11*-mutated primary cells, resistant to TKIs, were used to verify effect on RAS overactivation and therapeutic potential. ISR was inhibited by small molecule ISRIB to target effector part of ISR. To assess clinical relevance and mechanism, cells viability, clonogenic potential, spleen and bone marrow engraftment and mice survival as well as signaling pathways and RNA-seq were checked. *PTPN11* mutations were identified by NGS.

**Results and Discussions**

ISR inhibition blocked the ATF4-dependent response, but also a regulatory network with BCR-ABL1-STAT5 signaling, leading to increased sensitivity to imatinib and eradication of CML resistant cells. Combined ISRIB+imatinib treatment inhibited STAT5 and RAS/RAF/MEK/ERK pathways, recognized as drivers of resistance. Moreover, ISRIB protected from ISR activation in response to low-dose imatinib, increasing sensitivity. To verify the clinical potential we used CD34+ TKI-resistant CML blasts with *PTPN11* gain-of-function mutation, that in CML/AML leukemias is responsible for uncontrolled hyperactivation of RAS/RAF/MAPK and JAK/STAT5 pathways, therapy resistance and bad prognosis. In PDX mice bearing CD34+ TKI-resistant *PTPN11* mutated cells, ISRIB+imatinib decreased leukemia engraftment and decreased expression of genes related to RAS/RAF/MAPK, JAK2/STAT5 and IFNγ as well as attenuated expression of STAT5-target genes responsible for proliferation, viability and stress response.

**Conclusion**

Our findings show potential clinical benefits of ISRIB plus imatinib combination and indicate that such drug treatment might improve therapeutic outcome of TKI-resistant leukemias with RAS/RAF/MAPK and STAT5 hyperactivation-mediated resistance in myeloid leukemias.

*National Science Center, Poland: 2015/19/N/NZ3/02267, 2021/41/B/NZ5/04077, 2014/14/M/NZ5/00441*

### EACR23-0483
### HDAC6 inhibition and HER2 blockade combination overcome Trastuzumab resistance in preclinical models of HER2+ positive breast cancer

*M. Cortesi[1], S. Ravaioli[1], M. Zanoni[1], M.M. Tumedei[1], F. Pirini[1], R. Maltoni[1], A. Rocca[2], E. Petracci[1], P. Ulivi[1], S. Bravaccini[1]*
*[1]Istituto Romagnolo per lo Studio dei Tumori "Dino Amadori" - IRST S.r.l., Biosciences Laboratory, Meldola, Italy*
*[2]University of Trieste, Department of Medical-Surgical and Health Sciences, Trieste, Italy*

**Introduction**

Adjuvant trastuzumab is the standard of care for HER2+breast cancer(BC). However, >40% of patients become resistant. Histone deacetylase 6 (HDAC6) is a promising therapeutic target and its inhibitors have been evaluated in preclinical models of several tumors, including BC. Recently, it has been reported that HDAC6 could suppress HER2-overexpressing BC paving the way to novel mechanisms of HER2-driven carcinogenesis and suggesting the possibility of combined HER2/HDAC6 targeting. In the present work, we identified in a HER2+ BC case series HDAC6 as unfavorable prognostic factor in patients treated with adjuvant trastuzumab. Then, we established two HER2+ trastuzumab-resistant cell lines (HR) to investigate HDAC6 inhibition and its relationship with trastuzumab effectiveness.

**Material and Methods**

26 relapsed and 26 not relapsed patients were selected among HER2+BC cases treated with adjuvant trastuzumab. Total RNA was isolated from FFPE primary tumor. The expression of 770 genes was analyzed using NanoString platform. Trastuzumab resistance was induced exposing SKBR3 and BT474 (ATCC) trastuzumab-sensitive cells to increasing concentrations of trastuzumab for 12 months. Cytotoxicity was evaluated using CellTiter 96®AQueous One Solution Cell Proliferation Assay. Molecular analyses were performed by realtime qRT-PCR and western blot. Immunohistochemistry was performed using the Ventana Benchmark Ultra staining system and HDAC6 activity was assessed using the HDAC-Glo™ I/II Assays and Screening System.

**Results and Discussions**

Univariate and multivariate analyses indicated HDAC6 as unfavorable prognostic factor, associated with increased risk of relapse ($p<0.05$). We established two trastuzumab-resistant subclones of HER2+ cell lines (SKBR3 and BT474) expressing different HDAC6 levels. In BT474HR we observed an overexpression trend of HDAC6 with respect to the wild-type cells(WT). NexturastatA antitumor activity was assayed in both WT and HR cells and was effective in reducing cell viability. Interestingly, increasing NextA concentrations, a reduction of HDAC6 selective inhibition was observed. Furthermore, HDAC6 activity was 15% to 40 % lower in HR subclones as compared to the WT. We finally exposed cells to a combination of low NextA concentrations and Trastuzumab, observing a synergistic effect of the drugs and a reversal of drug resistance in BT474HR subclone.

**Conclusion**

Our findings encourage the exploration of the mechanism underlying the effects of HDAC6 inhibition and HER-2 signaling blockade combination.

### EACR23-0518
### Investigating the Molecular Mechanisms of Combination Therapy on Drug-resistant Chronic Myeloid Leukaemia

*A. Polidano Vella[1], A. Cassar[1]*
*[1]University of Malta, Anatomy, Msida, Malta*

**Introduction**

The project aims to bring chronic myeloid leukaemia (CML) closer to a cure through the use of combination therapy using promising drugs from successful preliminary *in vitro* experiments. CML is characterised by

© 2023 The Authors. Molecular Oncology is published by John Wiley & Sons Ltd on behalf of Federation of European Biochemical Societies the uncontrolled proliferation of myeloid cells in the bone marrow and may be controlled using mono-therapeutic drugs such as the tyrosine kinase inhibitor (TKI) imatinib, however, it is increasingly common that patients develop resistances to said drugs.
87% of CML patients in the advanced phases of this leukaemia with resistances to imatinib had detectable genetic mutations within the oncogenic fusion gene. This project aims using combination therapy to target the mechanisms that induce said resistances based on the different genetic mutations.

**Material and Methods**

Imatinib resistant K562 cells have been grown into their clonal populations using methylcellulose-based medium, with each colony harbouring different and potentially unknown resistance mutations.
Cell colonies have been treated with different drug combinations, particularly; epigenetic modifiers, retinoic acids (RA) and imatinib. Cells from each colony had gene portions sequenced to identify the mutation and shed light on potentially unknown drug resistance mutations.
This is being followed by studies in molecular mechanisms, namely, cell surface marker detection using CD marker quantification and gene expression assays using qPCR, to identify differentiation and anti-proliferative pathways respectively.

**Results and Discussions**

Results show that combination treatments not only increased the leukaemia's susceptibility to imatinib, but also re-sensitized imatinib-resistant cells to the drug. Furthermore, these combinations showed no toxicity to healthy white blood cells (WBCs).
Cell colonies are also responding differently to different combination treatments, suggesting a difference in drug resistance mechanisms. Flow Cytometry analysis on CD cell surface markers show an increase in CD markers that are indicative of differentiation and a reduction in CD markers that suggest a stem-like nature.

**Conclusion**

Combination therapy outperformed the mono-therapeutic options that are currently being administered clinically. They have shown to re-sensitize drug resistant cells and pose no threat to healthy WBCs. Treatments also indicate differentiation of leukemic cells into less proliferative counterparts. The combination treatments can also be tailored to treat a specific resistance mutation hence leading the way to more personalised medicine.

### EACR23-0525

### Acetyl-CoA carboxylase 1 controls lipid droplets-peroxisomes metabolic axis and is a vulnerability of ER+ breast cancer resistant to estrogen deprivation.

*N. Lorito[1], M. Bacci[1], A. Smiriglia[1], A. Subbiani[1], F. Bonechi[1], A. Bachi[2], K. Havas[2], M. Benelli[3], I. Meattini[1], P. Chiarugi[1], E. Marangoni[4], A. Morandi[1]*
*[1]University of Florence, Department of Experimental and Clinical Biomedical Sciences, Florence, Italy*
*[2]IFOM Foundation, The FIRC Institute of Molecular Oncology, Milan, Italy*
*[3]Azienda USL Toscana Centro, Bioinformatics Unit, Prato, Italy*
*[4]Institut Curie, PSL Research University- Translational Research Department, Paris, Italy*

**Introduction**

Aromatase inhibitors (AI) deprive $ER^+$ breast cancer of estrogens and are an effective therapeutic approach for this tumor. However, AI resistance limits their clinical efficacy. We have previously demonstrated that AI resistant cells show enhanced metabolic plasticity supported by a deregulation of central carbon and amino acid metabolism. Now we have evidence that also lipid metabolic reprogramming and fat storage are involved in the response and adaptation to long-term estrogen deprivation (LTED), a condition that mimics AI resistance.

**Material and Methods**

LTED and parental ER+ breast cancer cells were subjected to Gene Expression Microarray analysis and subsequent Gene Set Enrichment Analysis (GSEA). Lipid metabolic phenotype and lipid storage were characterized by using an array of complementary techniques including radiolabeled nutrients assay, Seahorse analysis, and confocal microscopy.

**Results and Discussions**

We found that *de novo* lipogenesis, lipid uptake, and accumulation of fatty acids (FA) into lipid droplets (LD) are increased in LTED cancer cells, and this aberrant lipid metabolic reprogramming enhances their metabolic plasticity. Indeed, we observed that AI resistant cells mobilize lipids from LD to sustain their metabolic demands when challenged by nutritional stress conditions. Crucially, the inhibition of LD mobilization and usage impairs LTED respiratory capacity and ultimately their survival. Importantly, we showed that this reprogramming is controlled by Acetyl-CoA Carboxylase 1 (ACC1), whose targeting unbalances redox homeostasis selectively in the LTED cells thereby compromising their cell survival. Since the ACC1-derived malonyl-CoA is required for FA elongation, we hypothesized that the catabolism of very long chain FA (VLCFA) and branched chain FA (BCFA) mediated by peroxisomes could have a crucial role in the regulation of redox homeostasis and metabolic adaptability of the resistant tumors. Accordingly, an exogenous supplementation of these complex FA reverts ACC1 targeting while failing when peroxisomal activity is impaired.

**Conclusion**

Collectively, our data suggest that a high lipid metabolic plasticity is involved in the acquisition of adaptive features that allow the survival of $ER^+$ breast cancer cells under estrogen deprivation and will offer a series of novel potential predictive biomarkers and/or therapeutic targets implicated in AI response and resistance.

### EACR23-0536

### Mitochondrial fission mediator DRP1 is modulated by chemotherapy but does not mediate drug resistance in pediatric sarcomas.

*K. Borankova[1,2], M. Krchniakova[1], M. Solny[1], J. Skoda[1,2]*
*[1]Masaryk University, Faculty of Science, Brno, Czech Republic*

© 2023 The Authors. Molecular Oncology is published by John Wiley & Sons Ltd on behalf of Federation of European Biochemical Societies

[2]St. Anne's University Hospital, International Clinical Research Center, Brno, Czech Republic

**Introduction**

Altered mitochondrial dynamics has been implicated in cancer initiation and progression. Enhanced expression and activity of a key mitochondrial fission mediator, dynamin-related protein 1 (DRP1), have been suggested to promote chemoresistance and cancer stemness and associates with poor outcomes in various tumors. However, mitochondrial fission and fusion in pathogenesis of pediatric sarcomas remains underexplored. Here, we investigated the role of mitochondrial dynamics in chemoresistance of highly malignant sarcomas, rhabdomyosarcoma and osteosarcoma, to identify potential druggable targets.

**Material and Methods**

Changes in mitochondrial fission- and fusion-related proteins were analyzed by immunoblotting in embryonal rhabdomyosarcoma and osteosarcoma cells (in-house derived, NSTS-11, NSTS-46 & OSA-13, and commercially available, RD & SAOS-2, respectively) after 72-h treatment with $IC_{50}$ of chemotherapeutics (cisplatin, doxorubicin, vincristine). Stable DRP1 knockdown clones, and respective controls, were prepared using lentiviral delivery of constructs encoding target-specific shRNA under a constitutive promoter. Growth rates and sensitivity to chemotherapeutics were assessed by MTT assay and $IC_{50}$ values were determined by non-linear regression.

**Results and Discussions**

Chemotherapeutic treatments in sarcoma cells did not have any impact on the levels of mitochondrial fusion-related proteins, MFN1, MFN2, and OPA1. However, a cell line-specific modulation of protein levels and activating phosphorylation of DRP1 at S616 was observed in rhabdomyosarcoma cells. This suggested that the fine-tuning of mitochondrial dynamics might play a role in drug-induced resistance. Surprisingly, the stable downregulation of DRP1 had no marked effects on the cell growth and did not sensitize SAOS-2 and RD cell clones to various treatments, such as conventional chemotherapy (doxorubicin, vincristine, topotecan), molecularly targeted inhibitors (sunitinib, trametinib), or an autophagy inhibitor (bafilomycin). In contrast with reports in carcinomas, reduced levels of DRP1 resulted in a 2.3-fold increased resistance to cisplatin in rhabdomyosarcoma RD cells.

**Conclusion**

Overall, our results revealed that mitochondrial fission mediator DRP1 is modulated by exposure to chemotherapy but does not mediate drug resistance in osteosarcoma and rhabdomyosarcoma. Our findings point to a potential compensatory DRP1-independent fission mechanisms which require further investigation in pediatric sarcomas.

### EACR23-0559
### Combined downregulation of MNK1/2 signaling and BET bromodomain inhibition reveal novel vulnerability in melanoma.

*A. Méant[1], O. Moussa[1], B. Lebeau[1], C. Gonçalves[1], V.R. Richard[2], R.P. Zahedi[2], M. Tyers[3], W.H. Miller Jr.[1], M. Witcher[1], S.V. del Rincon[1]*
*[1]Lady Davis Institute - McGill University, Department of Oncology, Montréal, Canada*
*[2]Segal Cancer Proteomics Centre - Lady Davis Institute - McGill University, Department of Oncology, Montréal, Canada*
*[3]Institute for Research in Immunology and Cancer - University of Montréal, Department of Medicine, Montréal, Canada*

**Introduction**

Melanoma is the deadliest form of skin cancer. Despite clinical benefits of targeted therapy against BRAF-mutated melanoma and immunotherapy with immune checkpoint inhibitors, these therapies are limited by rapidly emerging resistance mechanisms, highlighting the urgent medical need to develop new treatment strategies. MNK1/2 kinases are activated downstream of upregulated NRAS and BRAF signaling pathways in melanoma. Our group has previously shown that MNK1/2 inhibitors (MNKi) serve a dual purpose as therapies that inhibit tumor progression and immunomodulatory agents that can be used in combination with immunotherapies in metastatic melanoma. Therefore, we hypothesized that MNKi represent a promising therapeutic option when combined with other agents against melanoma progression.

**Material and Methods**

We carried out a CRISPR/Cas9 screen using MNKi to identify synthetic lethality. We pharmacologically validated candidate hits using cell-based assays. We performed quantitative LC-MS/MS analysis and bulk RNA-sequencing to determine the molecular underpinnings of response to combined MNKi and specific candidate hit inhibition. We used melanoma cells resistant to clinically available therapies to test the efficiency of our dual targeting as a novel therapeutic approach.

**Results and Discussions**

Genome-wide CRISPR screen shows that MNKi displays synthetic lethality with bromo- and extra-terminal domain (BET) protein BRD2 deficiency. We confirmed that clinically tested BET inhibitors (BETi) in combination with MNKi significantly impairs the growth of NRAS-mutated and BRAF-mutated melanoma cells *in vitro*, compared to either monotherapy. Transcriptomic and proteomic analysis reveal that MNKi and BETi combination downregulate molecular effectors involved in extracellular matrix organization including the tissue transglutaminase TGM2 protein. We confirmed that MNKi and BETi combination downregulate TGM2 expression and showed that TGM2 protein is overexpressed in therapy-resistant melanoma cells. Finally, we demonstrated that silencing of TGM2 expression but also MNKi and BETi association significantly reduces therapy-resistant melanoma progression.

**Conclusion**

Overall, our data demonstrate that the combination of MNKi and BETi decrease melanoma growth and are promising drugs to overcome therapy-resistant disease.

### EACR23-0563
### Inhibition of the Cystine-Glutamate Transporter SLC7A11/xCT system increases sensitivity to platinum-based drugs in human lung cancer cells

*A. Jensen[1], G. Sharbeen[2], Z. Ma[1], R. Ignacio[1], S.W. Wong[1], J. Youkhana[2], J. Kokkinos[2], M. Kavallaris[1], P. Phillips[2], J. McCarroll[1]*

© 2023 The Authors. Molecular Oncology is published by John Wiley & Sons Ltd on behalf of Federation of European Biochemical Societies

[1]The Children's Cancer Institute, The Lowy Cancer Research Centre- University of New South Wales, Sydney, Australia
[2]Pancreatic Cancer Translational Research Group- Adult Cancer Program,
The Lowy Cancer Research Centre- University of New South Wales, Sydney, Australia

**Introduction**

Lung cancer is the leading cause of cancer-related deaths worldwide, and chemoresistance is a major clinical challenge. Platinum-based chemotherapy drugs are used as first-line treatments in lung cancer. These drugs cross-link DNA to inhibit cell proliferation, but they also produce intracellular reactive oxygen species (ROS). SLC7A11 is a key regulator of amino acid metabolism and is highly expressed in cancer cells. SLC7A11 protects cells from chemotherapy-induced ROS by promoting glutathione synthesis to stabilize ROS. We hypothesize that inhibiting SLC7A11 function using a pharmacological inhibitor Sulfasalazine (SSZ) which is used to treat arthritis or gene silencing using siRNA will increase platinum-based chemotherapy efficiency in lung cancer cells. The aim of this study is to determine the effectiveness of SSZ or SLC7A11 siRNA in combination with platinum chemotherapy drugs to inhibit lung cancer cell growth.

**Material and Methods**

SLC7A11 protein expression was measured using western blotting in human lung cancer cells (A549, H1299, H460, H441, H1975). Lung cancer cell growth in the absence or presence of SSZ (0.5-2mM), cisplatin (1.5-6uM), carboplatin (10-20uM) or oxaliplatin (1-4uM) was examined using trypan blue staining, IncuCyte® proliferation analysis, and clonogenic assays. Apoptosis and ROS accumulation were measured using Annexin V and C11-BODIPY (581/591) and flow cytometry. Glutathione levels were measured using an enzyme assay. SLC7A11 siRNA was delivered to lung cancer cells using Lipofectamine 2000®, and cell viability and drug sensitivity assessed by trypan blue staining and Alamar blue drug assays.

**Results and Discussions**

Lung cancer cells express SLC7A11. In A549 and H1299 cells, SSZ significantly decreased cell viability and proliferation when used in combination with cisplatin, carboplatin and oxaliplatin (*$p \leq 0.05$) compared to drugs alone. Mechanistically, we confirmed a significant depletion of glutathione and its active enzyme facilitator, glutathione peroxidase 4 (GPX4) after SSZ treatment within 24h (*$p<0.05$) and an increase in apoptosis when used in combination with platinum chemotherapy (**$p \leq 0.01$). SLC7A11-siRNA also decreased cell viability (*$p<0.05$) and sensitised A549 cells to cisplatin treatment (*$p \leq 0.05$).

**Conclusion**

These findings suggest that SLC7A11 is a promising therapeutic gene target to increase the effectiveness of platinum-based chemotherapy drugs in lung cancer cells.

### EACR23-0565
### ITGB1 and DDR activation are involved in acquired resistance to osimertinib in EGFR-mutant NSCLC

C. DE ROSA[1], V. De Rosa[2], C. Tuccillo[1], F. Ciardiello[1], F. Morgillo[1], F. Iommelli[2], C.M. Della Corte[1]
[1]University of Campania Luigi Vanvitelli,
Department of Precision Medicine, Naples, Italy
[2]National Research Council,
Institute of Biostructures and Bioimaging, Naples, Italy

**Introduction**

Among the broad mechanisms associated with resistance to osimertinib (OR), recent studies support the contribution of epithelial-to-mesenchymal transition (EMT) and the association with impaired DNA damage response (DDR). During EMT, integrin β1 (ITGB1) downstream pathways (NF-kB, PI3K, Src, Ras-MAPK) activation facilitate migration and metastasis. ITGB1 is also associated with chemo- and radio-resistance by modulating DDR. However, the link between EMT and DDR is unknown in this setting. We hypothesized that the interplay between ITGB1 and DDR contribute to progression in OR-NSCLC tumours, providing a basis for their use as diagnostic biomarkers or therapeutic targets.

**Material and Methods**

Four NSCLC cell lines, parental (PC9, H1975) and previously established OR cells (PC9OR, H1975OR) were utilized. Acquired resistance of PC9OR and H1975OR cell lines was preliminarily tested by MTS assay after 72h exposure to osimertinib. Flow Cytometry (FC), Western Blot (WB), and Transwell Migration Assay were performed following previously published protocols.

**Results and Discussions**

Both OR cells displayed increase in migration ability, EMT markers (SNAIL, MMP9, TACE, Vimentin) and DDR (γH2AX, PARP) compared to parental cells. EGFR was strongly downregulated, whereas the MAPK pathway (p-ERK1/2, p-MEK1/2, p-P38) remained fully active in PC9OR and H1975OR. The overexpression of ITGB1, together with activation of intracellular signalling (p-FAK, p-STAT3), contributed to persistence of MAPK pathway beyond OR. MAPK inhibition with selumetinib (AZD6244) reduced migration, EMT markers and P38 levels in both OR cell lines. In addition, the ability to repair DNA damage was still impaired in OR cells following exposure to selumetinib. ITGB1 total expression (by WB) was significantly reduced in selumetinib-treated PC9OR, but not in H1975OR cells. The membrane expression of ITGB1 (by FC) is increased in PC9OR compared to parental PC9, whereas it is similar between H1975 and H1975OR, implying that intracellular ITGB1 levels confer resistance by interacting with DDR proteins.

**Conclusion**

Our preliminary data suggest that overexpressed ITGB1 may be a biomarker for progression after prolonged therapy with osimertinib. The susceptibility of OR cells to DNA damage repair may provide potential options for using appropriate DDR inhibitors in combination with selumetinib in OR patients. Moreover, since DDR modulation is highly correlated with immune-responsiveness features in NSCLC, future studies may investigate novel immunotherapy combinations.

### EACR23-0567
### A promising therapeutic target for cancer treatment: AKR1B10-mediated anthracycline metabolism could be

© 2023 The Authors. Molecular Oncology is published by John Wiley & Sons Ltd on behalf of Federation of European Biochemical Societies

### inhibited by Bruton's tyrosine kinase inhibitors

*L. Čermáková[1], J. Želazková[1], E. Novotná[1], L. Laštovičková[1], V. Wsól[1]*
*[1]Faculty of Pharmacy in Hradec Králové Charles Universi ty, Department of Biochemical Sciences, Hradec Králové, Czech Republic*

**Introduction**

Aldo-keto reductase 1B10 (AKR1B10) has been suggested as a therapeutic target for the treatment of several types of cancer (e.g., breast, hepatocellular), in which it is frequently overexpressed. To physiological functions of AKR1B10 belonging, among others, the biotransformation of carbonyl-containing drugs. In the case of anthracyclines, AKR1B10 is responsible for their reduction to alcohol metabolites that show less antineoplastic properties[1]. Bruton's tyrosine kinase inhibitors ibrutinib (IBR) or zanubrutinib (ZAN) may be promising therapeutic agents. Tyrosine kinase inhibitors (TKI) have effective antitumor activity and have been proven active agents that suppress tyrosine kinase signaling. They are also targeting proteins involved in cancer multidrug resistance, such as drug transporters and biotransformation enzymes. Recent studies described that TKIs are able to enhance the cytotoxicity of anthracyclines[2].

[1]Zhong, L., et al.: Toxicol. Appl. Pharmacol. 2011, 255 (1), 40−47.
[2]Magni M., et al.: Br J Cancer; 2019; 121(7):567-577.

**Material and Methods**

Using the inhibition assays, we described the interactions of IBR and ZAN with recombinant AKR1B10 and determined the $IC_{50}$, inhibition constant, and type of inhibition. Also using transiently transfected HCT116 cells, we investigated the ability of IBR and ZAN to block the AKR1B10-mediated metabolism of anthracycline daunorubicin.

**Results and Discussions**

The achieved results exhibited a potent inhibitory effect of IBR and ZAN on recombinant AKR1B10. The interactions were confirmed at the level of intact cells as well.

**Conclusion**

In summary, our data introduce Bruton's tyrosine kinase inhibitors IBR and ZAN as effective modulators of AKR1B10 suggesting that their combination with daunorubicin might become an effective approach for the prevention of anthracycline resistance in clinical practice.

**Acknowledgements:**
The study was supported by the Grant Agency of Charles University No. 315221.

### EACR23-0569
### Targeting Sp1 to inhibit EMT-driven resistance to EGFR TKIs in mutant NSCLC

*V. De Rosa[1], C. De Rosa[2], C.M. Della Corte[2], S. Del Vecchio[3], F. Iommelli[1]*
*[1]National Research Council, Institute of Biostructures and Bioimaging, Naples, Italy*
*[2]University of Campania "Luigi Vanvitelli", Department of Precision Medicine, Naples, Italy*
*[3]University of Naples "Federico II", Department of Advanced Biomedical Sciences, Naples, Italy*

**Introduction**

A great clinical benefit is reported in NSCLC patients treated with EGFR TKIs. However, the majority of tumors relapse due to the occurrence of resistance that may be caused by multiple mechanisms. In particular, the activation of epithelial mesenchymal transition (EMT) has been detected in most aggressive tumors and has been associated with treatment failure and metastases. Here we hypothesized that Sp1 may have a central role in the activation of EMT-mediated resistance to osimertinib and in the concurrent upregulation of CSC (cancer stem cell) pathways, c-Myc and β1 signaling. We planned to test our hypothesis in mutant EGFR NSCLC cells and demonstrate that Sp1 may be a good therapeutic target to avoid tumor spreading and resistance.

**Material and Methods**

NSCLC cell lines bearing EGFR with activating and/or T790M mutations were treated with osimertinib to test drug sensitivity and to assess its effect on EGFR, CSC, β1 and EMT signaling after 72h of treatment. Western blot analysis of whole cell lysates and nuclear extracts was been also performed to detect Sp1 levels in untreated and treated cells. Furthermore, selective inhibition of Sp1 transcription or DNA binding in osimertinib treated cells was performed using selective siRNA or mithramycin A, respectively. The effects on cell migration, proliferation and apoptosis were also evaluated.

**Results and Discussions**

Western blotting of whole cell lysates from untreated and treated NSCLC cells showed that treatment with osimertinib 1 μM for 72h was able to reduce cell viability and increase cell death. However, despite such favourable drug response, we observed a concomitant activation of EMT, c-Myc, β1 and CSC signaling. These effects resulted in an increased cell migration as assessed by analysis of MMP9 levels and migration assay. Parallel experiments showed an increase of Sp1 levels in both whole cell lysates and nuclear extracts of treated cells. In these cells, downregulation of Sp1 by selective siRNA and treatment with mithramycin A were able to inhibit the activation of EMT, CSC and β1 signaling and reduce MMP9 and Slug levels.

**Conclusion**

EMT-mediated resistance may occur very early in response to osimertinib and it is characterized by Sp1 dependent upregulation of EMT, CSC and β1 signaling. Combined treatment with osimertinib and mithramycin A may be helpful to prevent treatment failure and reduce cancer cell migration in NSCLC patients.

### EACR23-0571
### Identifying Clonal Evolution of Drug Resistance in 3D Cell Culture System Using Cellular Barcoding technology

*G.D. Yalcin[1], K. Yilmaz[1], T. Dilber[1], A. Acar[1]*
*[1]Middle East Technical University, Biological Sciences, Ankara, Turkiye*

**Introduction**

Colorectal cancer (CRC) is one of the deadliest types of cancer. In the treatment of CRC, treatment options inadvertently become ineffective due to the development of drug resistance. Intratumor heterogeneity (ITH) provides

© 2023 The Authors. Molecular Oncology is published by John Wiley & Sons Ltd on behalf of Federation of European Biochemical Societies distinct molecular and phenotypic profiles, enabling the development of diverse resistance mechanisms and hence the tumour progression. ITH-induced drug resistance can be tracked using next-generation sequencing-based cellular barcoding technology, allowing us to identify functional subclones associated with the resistance phenotype. Numerous studies have demonstrated that the use of 3D cell culture is a suitable experimental model system to better mimic and study tumour physiology and drug resistance [1].

**Material and Methods**

In this study, for the first time, 3D cell culture models of HCT-116 and HT-29 cell lines harbouring unique cellular barcodes were established and irinotecan and dabrafenib resistant derivatives of these cell lines were generated, respectively. The frequencies of barcodes from resistant cell lines were determined. Whole exome sequencing (WES) analysis were carried out to identify copy-number changes and somatic mutations associated with the resistance phenotype.

**Results and Discussions**

Barcode frequency measurements revealed clonal dynamics of both resistant pre-existing and de novo barcodes. Furthermore, Whole-Exome Sequencing (WES) performed on drug resistant cell lines demonstrated Copy Number Variation (CNV) in several cancer-related genes, which were then validated by the qRT-PCR analysis. Moreover, somatic mutations that might lead to the resistance phenotype were found to be enriched in 3D drug-resistant cell lines.

**Conclusion**

Overall, in this study, for the first time, we show the generation of barcoded 3D drug-resistant cancer cell lines to track drug-induced clonal evolution and resistance-associated genomic alterations.

1 Yalcin GD, Danisik N, Baygin RC, Acar A. Systems biology and experimental model systems of cancer. *J Pers Med*. 2020;**10**(4).

**Funding:**

METU BAP Career Support Projects (AGEP).

### EACR23-0611

### Deciphering the impact of glycosylation on resistance to HER2-targeting antibodies in breast cancer

*R. Vincken[1], D. Wolf[2], A. Yoo[3], V. Steri[4], L. van 't Veer[2], M. Bordoy[1], J. Martens[5], D. Huylebroeck[1], M. Moasser[3], A. Ruiz-Saenz[1]*

*[1]Erasmus MC, Cell Biology, Rotterdam, The Netherlands*
*[2]University of California, Laboratory Medicine, San Francisco, United States*
*[3]University of California, Helen Diller Family Comprehensive Cancer Center, San Francisco, United States*
*[4]University of California, Preclinical Therapeutics Core, San Francisco, United States*
*[5]Erasmus MC, Medical Oncology, Rotterdam, The Netherlands*

**Introduction**

Worldwide, 25% of breast cancers are driven by overexpression of HER2. This dependence has been exploited therapeutically with HER2-targeting therapies that have revolutionized the treatment for HER2-positive cancer patients. Unfortunately, many patients develop resistance to the treatment. An extensive body of work has revealed a plethora of mechanisms of resistance. However, a key aspect of cancer cells has been largely overlooked: their aberrant glycosylation profile. Aberrant glycosylation is a hallmark of cancer and can affect receptor activation and immune invasion. Yet its impact on response to HER2-targeting therapies remains unknown.

**Material and Methods**

A set of 126 glycosylation-related genes was interrogated for association with trastuzumab response in patients. Selected glycogenes were analysed in trastuzumab-sensitive and trastuzumab-resistant cells generated *in vitro* and *in vivo* for their impact on viability upon trastuzumab treatment. Candidate genes were validated with the development and application of a co-culture system consisting of engineered natural killer cells and breast cancer cells. We are currently using 3D co-culture spheroids and advanced live microscopy to validate our findings in 3D and real-time.

**Results and Discussions**

Bioinformatics analysis of clinical data revealed the association of specific glycogenes with response to the HER2-targeting agent trastuzumab in HER2-positive breast cancer patients. RNA sequencing of resistant versus sensitive tumors from cell line xenografts revealed upregulation of glycogenes that were also associated with resistance in patients. We validated these findings in cell models using gain and loss of function assays. Furthermore, given that trastuzumab acts by recruiting natural killer cells inducing antibody dependent cellular cytotoxicity, we developed a relevant co-culture system to mimic *in vitro* the cytotoxicity induced by trastuzumab *in vivo*. Our results suggest that reducing the expression of specific glycogenes sensitizes HER2-positive breast cancer cells to trastuzumab-mediated cytotoxicity. In addition, we are currently addressing the impact of aberrant glycosylation on the efficacy of trastuzumab using live imaging of 3D cancer spheroids.

**Conclusion**

Using a valuable co-culture system, we have shown that specific glycogenes confer resistance to trastuzumab. Our work reveals new glycomarkers of response to HER2-targeting therapies that could be further explored as a biomarkers or therapeutic targets.

### EACR23-0613

### Darifenacin as novel chemosensitizer in pancreatic cancer

*H. Branco[1,2], S. Sousa[1,2,3], A. Avan[4,5], A. Palmeira[3,6], E. Giovannetti[7,8], M.H. Vasconcelos[1,2,9], C.P.R. Xavier[1,2,9]*

*[1]i3S - Instituto de Investigação e Inovação em Saúde- Univ ersidade do Porto, Cancer Drug Resistance, Porto, Portugal*
*[2]IPATIMUP - Institute of Molecular Pathology and Immun ology- University of Porto, Cancer Drug Resistance, Porto, Portugal*
*[3]Faculty of Pharmacy- University of Porto, LQOF – Laboratory of Organic and Pharmaceutical Chem istry- Department of Chemical Sciences, Porto, Portugal*
*[4]Mashhad University of Medical Sciences, Metabolic Syndrome Research Center, Mashhad, Iran*
*[5]Mashhad University of Medical Sciences,*

© 2023 The Authors. Molecular Oncology is published by John Wiley & Sons Ltd on behalf of Federation of European Biochemical Societies

Medical Genetics Research Center- Faculty of Medicine, Mashhad, Iran
[6]CIIMAR – Interdisciplinary Centre of Marine and Environ mental Research,
Medicinal chemistry: drug discovery and drug design, Matosinhos, Portugal
[7]Amsterdam UMC- VU University- Cancer Center Amsterd am, Department of Medical Oncology, Amsterdam, The Netherlands
[8]Fondazione Pisana per La Scienza, Cancer Pharmacology Lab, San Giuliano, Italy
[9]Faculty of Pharmacy- University of Porto, Department of Biological Sciences, Porto, Portugal

**Introduction**

Pancreatic ductal adenocarcinoma (PDAC) is one of the most aggressive malignancies worldwide. Indeed, PDAC remains associated with an extremely poor prognosis owing to drug resistance and to the lack of alternative therapeutic strategies. Our previous work demonstrated the involvement of Chitinase 3-like 1 (CHI3L1) in PDAC cellular resistance to gemcitabine (GEM), identifying CHI3L1 as a promising therapeutic target for PDAC. Hence, this work aimed to identify *in silico* putative CHI3L1 inhibitors, and to assess the chemosensitizing effect of darifenacin (DF), one of the identified CHI3L1 inhibitors, on PDAC cells.

**Material and Methods**

*In silico* analysis (AutoDock Vina) using 11,741 molecules from the DrugBank database was performed on the CHI3L1 protein. PDAC cell lines, BxPC-3 and PANC-1, and primary PDAC cells obtained from a resected patient, were used to evaluate the effects of DF on the % of cell growth, by the sulforhodamine B assay (SRB), used alone or in combination with GEM or with GEM plus paclitaxel (PAC). The cytotoxic effect of DF against normal immortalized pancreatic ductal cells (HPNE) was also assessed. Recombinant protein was used to confirm the impact of DF on CHI3L1-induced PDAC cellular resistance to therapy (SRB assay). The effect of DF on Akt activation was analysed by ELISA. The association between cholinergic receptor muscarinic 3 (CHRM3) expression and therapeutic response was evaluated by immunohistochemistry of paraffin-embedded tissues from surgical resections of a 68 patients' cohort.

**Results and Discussions**

*In silico* screening revealed that 568 molecules presented higher affinity towards CHI3L1 than a well-known ligand, chitotetraose. A score value of -11.8 kcal/mol was obtained for the muscarinic receptor antagonist DF, revealing the ability of this drug to target CHI3L1 with high efficiency. DF inhibited the growth of BxPC-3 and PANC-1 cell lines, with $GI_{50}$ concentrations of 26 and 13.6 µM, respectively. A similar effect was observed in primary cells ($GI_{50}$, 30 µM), while concentrations above 50 µM were not affecting the growth of the normal HPNE cells. Additionally, DF sensitized human PDAC cell lines to GEM or to GEM plus PAC treatments, and reverted CHI3L1-induced therapeutic resistance. Mechanistically, DF decreased Akt phosphorylation. Importantly, high expression of CRHM3 in paraffin-embedded tissues was found to be associated with poor therapeutic response to GEM.

**Conclusion**

This work shows evidence that DF is a chemosensitizer of PDAC to GEM or to GEM plus PAC treatments.

### EACR23-0641
### Integrin-linked kinase promotes epithelial-mesenchymal transition and osimertinib persistence/resistance in EGFR driven lung adenocarcinoma

R. Shi[1,2], D. Farnsworth[1,2], T. Atwal[1], J. Chow[1], Y. Inoue[1], S. Awrey[1], P. McDonald[1], S. Dedhar[1,2], K. Bennewith[1,2], W. Lockwood[1,2]
[1]BC Cancer Research Institute, Integrative Oncology, Vancouver, Canada
[2]University of British Columbia, Interdisciplinary Oncology, Vancouver, Canada

**Introduction**

Lung cancer is the leading cause of cancer-related deaths, with ~20% of lung adenocarcinoma (LUAD) cases driven by activating mutations in EGFR. The 3rd-generation EGFR tyrosine kinase inhibitor (TKI), osimertinib (Osi), greatly improved patient outcomes, but tumours inevitably develop resistance. Epithelial-mesenchymal transition (EMT) is an established resistance mechanism that is also linked with poor outcome. One known factor in promoting drug persistence/resistance is the interactions between tumour cells and the extra-cellular matrix (ECM) in the tumour microenvironment. Integrin-linked kinase (ILK) is a protein involved in signal transduction following the binding of integrins to ECM, and ILK has been shown to induce EMT in other cancers. Recently, high ILK expression was correlated to worse prognosis in patients treated with EGFR-TKIs. Taken together, we hypothesize that ILK promotes EMT and Osi resistance in EGFR-driven LUAD.

**Material and Methods**

Gene Set Enrichment Analysis was performed on LUAD patient databases. ILK was knocked down using inducible shRNA constructs. Cell viability and clonogenic assays were used to evaluate Osi sensitivity. Osi-resistant (OsiR) cells were made by dose-escalation. RNAseq and western blots were performed to assess expression levels.

**Results and Discussions**

EMT gene signature was found to be significantly enriched in EGFR-mutant LUAD patient tumours with high ILK expression. HCC4006 OsiR cells were enriched for an EMT gene signature in the drug-resistant state compared to its parental counterpart. Interestingly, HCC4006 also had the highest ILK expression in the parental state relative to other EGFR-mutant LUAD cells. Gene ontology analysis identified an upregulation in biological processes that ILK is known to regulate, namely genes involved with cell-to-ECM adhesion, in HCC4006 OsiR cells. Knocking down ILK in HCC4006 sensitized the parental, but not OsiR cells, to Osi and lowered the expression of EMT markers in response to Osi treatment, suggesting that ILK may mediate the transition towards resistance by EMT. Indeed, we found that knocking down ILK in HCC4006 cells reduced the viability of drug-tolerant-persister cells and knocking down ILK during the resistance-transformation process reduced the expression of mesenchymal markers.

**Conclusion**

Our results show that ILK is important in promoting EMT and drug tolerance/resistance in EGFR-driven LUAD and

© 2023 The Authors. Molecular Oncology is published by John Wiley & Sons Ltd on behalf of Federation of European Biochemical Societies support targeting ILK as a viable strategy to combat Osi resistance.

### EACR23-0645
### Identification of new drug resistance mechanisms in pancreatic ductal adenocarcinoma

T. Vorberg[1], M. Reitberger[2], B. Rodriguez Martin[3], M. Starostecka[3], O. Kossi[4], V. Vogel[1], J. Korbel[3], A. Trumpp[1], M. Sprick[1]
[1]German Cancer Research Center & Heidelberg Institute f or Stem Cell Technology and Experimental Medicine, Stem Cells and Metastases, Heidelberg, Germany
[2]Freudenberg & Co. KG, Home and Cleaning Solutions GmbH, Weinheim, Germany
[3]European Molecular Biology Lab, Genome Biology Unit, Heidelberg, Germany
[4]BioLabs, BioLabs Heidelberg, Heidelberg, Germany

**Introduction**

Pancreatic ductal adenocarcinoma (PDAC) is the fourth leading cause of cancer-related deaths in Western Countries and has been predicted to become the second leading cause of cancer-related deaths by 2030. Difficulties in early detection as well as strong chemoresistance of the disease prevent the successful treatment of PDAC patients. Although we have previously identified CYP3A5 as a mediator of paclitaxel resistance, further targets remain elusive. Thus, we set out to identify these CYP3A5-independent mechanisms which could lead to the identification of novel biomarkers and drug targets to improve PDAC treatment.

**Material and Methods**

Pancreatic cancer cell lines were obtained via orthotopic transplantation of PDAC samples into the pancreas of NSG mice and subsequent culturing *in vitro*. To induce paclitaxel resistance, we exposed the cells to increasing drug concentrations using a pulsed treatment regimen. Next, we characterized the cells using whole genome as well as RNA sequencing and validated potential candidate genes *in vitro*.

**Results and Discussions**

Multi-drug resistance protein 1 (ABCB1) was highly upregulated in our differential gene expression analysis and confirmed as a CYP3A5-independent mediator of acquired paclitaxel resistance using cell viability assays. On the one hand, we identified genomic rearrangements leading to the expression of a functional ABCB1-SEMA3C fusion transcript. On the other hand, ABCB1 expression and copy number were significantly enhanced by the generation of extrachromosomal DNA (ecDNA) upon drug treatment. As a next step, we combined targeted enrichment of the ecDNA with short-read sequencing as well as long-read optical mapping approaches for a deeper characterization of these molecules. This strategy revealed a precise structure of the most abundant ecDNAs and further supported their strong intra-tumoral heterogeneity.

**Conclusion**

Our results suggest the clinical relevance of ABCB1 as a mediator of paclitaxel resistance in PDAC and we plan to validate our findings in patient samples. However, no clinically applicable drug is available due to severe side effects. Therefore, we want to further analyze the regulatory pathways associated with enhanced ABCB1 expression which might offer unknown therapeutic potential.

### EACR23-0646
### Primaquine derivatives reverse multidrug resistance of tumor cells by modulation of ABCG2 transporter activity

M. Mioč[1], Á. Telbisz[2], M. Beus[3], Z. Rajić[4], B. Sarkadi[2], M. Kralj[1]
[1]Ruđer Bošković Institute, Department of Molecular Medicine, Zagreb, Croatia
[2]ELKH Research Centre for Natural Sciences, Membrane research group, Budapest, Hungary
[3]Institute for Medical Research and Occupational Health, Analytical Toxicology and Mineral Metabolism Unit, Zagreb, Croatia
[4]Faculty of pharmacy and biochemistry- University of Zagr eb, Department of pharmaceutical chemistry, Zagreb, Croatia

**Introduction**

Multidrug resistance (MDR) is a well-known cause of chemotherapy treatment failure. Although several cellular mechanisms are responsible for this phenomenon, the overexpression of ATP-binding cassette (ABC) transporters named ABCB1 (P-glycoprotein) and ABCG2 (BCRP) by cancer cells seems to be the most important. The ability to modulate their expression or block their function is of great clinical importance to achieve more effective antitumor therapy.
It was shown that the antimalarial drug primaquine has an adjuvant or direct antitumor effect and can inhibit the activity of the ABCB1 transporter and reverse tumor cell resistance to chemotherapeutic agents that are substrates of this transporter. Modifications of primaquine structure are often made to develop primaquine derivatives with improved antimalarial activity and lower toxicity and as new drugs with different biological activities. In this study, we investigated the effect of two groups of primaquine derivatives - primaquine and halogenaniline fumardiamide and *bis*-urea on ABCG2 transporter activity and expression.

**Material and Methods**

We performed various cellular and biochemical assays: a fluorescent substrate accumulation assay to investigate the effect of primaquine derivatives on ABCG2 activity, a conformation-sensitive 5D3 antibody shift assay, and an ATPase activity assay to investigate the nature of the compound-transporter interaction, as well as Western blot analysis and immunocytochemistry to see if the tested compounds impact ABCG2 expression. Finally, to see if tested compounds sensitize ABCG2-overexpressing cells to chemotherapeutic, an MTT test with a combination of a chemotherapeutic and tested compound was done. Experiments were done in ABCG2-overexpressing model cell lines and isolated membrane vesicles bearing ABCB1 or ABCG2.

**Results and Discussions**

We demonstrated that a few compounds act as strong ABCG2 inhibitors, and their inhibitory effect is comparable to potent ABCG2 inhibitor Ko143. These compounds efficiently sensitize cancer cells to the conventional chemotherapeutic mitoxantrone, the substrate of the ABCG2. We have also shown that these compounds

© 2023 The Authors. Molecular Oncology is published by John Wiley & Sons Ltd on behalf of Federation of European Biochemical Societies are substrates of the ABCB1, making them selective inhibitors of ABCG2.

**Conclusion**

These data are promising and indicate that primaquine derivatives could be used in cancer therapy due to their ability to reverse MDR. This work also contributes to the development of new compounds with even better inhibitory properties.

### EACR23-0648

### Pin1-Notch3 axis as a novel "druggable" biomarker to address the challenge of Platinum resistance in Ovarian Cancer

*M.V. Giuli[1], A. Mancusi[1], B. Natiello[1], S. Checquolo[2]*

*[1]Sapienza University of Rome, Molecular Medicine, Rome, Italy*

*[2]Sapienza University of Rome, Department of Medico-Surgical Sciences and Biotechnology, Latina, Italy*

**Introduction**

Ovarian Cancer (OC) represents one of the most lethal female-related malignancies. Primary cytoreductive surgery and platinum (PT)-based chemotherapy are the standard treatments for OC-bearing patients. Nonetheless, the insurgence of drug resistance challenged the efficacy of the therapies. Therefore, restoring PT-sensitivity is an urgent need in OC. Notably, Notch receptors are involved in PT-resistance. Notch3 (N3) is altered in a wide panel of OC. Moreover, it has been demonstrated that N3 sustains PT-resistance in OC cell lines, thereby supporting that N3 targeting may overcome PT-resistance. In this scenario, we previously demonstrated that Pin1 positively regulates N3 in T-cell acute leukemia (T-ALL) and we wondered whether this relationship occurs also in OC context. Collectively, the main aim of the present work is to investigate whether and how Pin1-Notch3 axis is involved in PT-resistance in OC.

**Material and Methods**

We used a wide panel of established OC cell lines. We performed: 1. *in silico* analysis on protein data collected by OC-bearing patients; 2. immunohistochemistry (IHC) on OC tissue samples; 3. mass spectrometry(MS)-based proteomics; 4. lentiviral transductions for the generation of N3-overexpressing cell lines; 5. long-term PT treatments for the generation of isogenic PT-resistant OC cell lines; 6 *in vitro* studies such as pharmacological treatments, cell viability and colony formation assays; and 7. *in vivo* experiments: xenografts in NOD/SCID immuno-deficient mice.

**Results and Discussions**

In keeping with existing literature, we first documented that N3 is involved in PT-resistance *in vitro* and *in vivo* by performing experiments on stable N3-overexpressing clones. Moreover, we performed MS-based proteomics on the above-mentioned clones to identify a potential N3-dependent protein signature which could be involved in PT-resistance. We next demonstrated that PT treatment induced an increased Pin1-Notch3 endogenous interaction, finally resulting in N3 stabilization. Consistently, Pin1 inhibition restored PT-sensitivity *in vitro*. Moreover, we performed IHC on primary lesions from OC-bearing patients and we documented that a consistent proportion of patients displays high expression of both proteins, which is also consistent with a poor prognosis.

**Conclusion**

These observations strongly suggest that Pin1-Notch3 axis might represent a novel "tunable" molecular pathway in Ovarian Cancer to maximize PT efficacy.

### EACR23-0679

### RING E3 Ubiquitin ligase RNF128 in response to irinotecan and 5-fluorouracil treatment of KRAS and BRAF mutated CRC

*O. Tubau[1], A. Rodriguez[1,2], A. Castells[1,2,3], E. Sánchez Tilló[1,2]*

*[1]IDIBAPS, Gastrointestinal and Pancreatic Oncology Group, Barcelona, Spain*

*[2]Biomedical Research Network in Gastrointestinal and Liver Diseases CIBEREHD, Carlos III National Health Institute ISCIII, Barcelona, Spain*

*[3]Hospital Clinic and University of Barcelona School of Medicine, Dept. of Gastroenterology, Barcelona, Spain*

**Introduction**

Colorectal cancer (CRC) displays a 5-year survival rate of 93 to 6% according to the diagnosed stage. KRAS and BRAF testing define effective treatments to reduce mortality. Irinotecan (IRI) or 5-fluorouracil (5-FU) improve response rates and survival. Eventually, tumor recurrence emerges due to drug resistance mechanisms, including the expression of RING E3 ubiquitin ligases. We have determined the role of RNF128 E3, expressed in primary tumors of colon adenocarcinomas, in response to chemotherapeutic treatment of KRAS or BRAF-mutated CRC.

**Material and Methods**

In vitro experiments were done with SW480 (KRAS mut) and HT29 (BRAF mut) CRC cell lines lentivirally infected to overexpress RNF128. IRI, 5-FU, and oxaliplatin (OXA) drug toxicity were analyzed by ELISA acid phosphatase and Annexin V apoptosis assays. *X*enografts were done in Nude mice, and drugs were i.p. administered. Tumors were immunohistochemistry stained. Tissue expression and drug response were determined with UALCAN and ROCplot.

**Results and Discussions**

RNF128 correlates with worse CRC disease-specific survival (DSS). However, in the presence of KRAS mutations in stem-like "serrated" C4 and "conventional adenoma" C5 subtypes, RNF128 is a good prognosis factor. In vivo tumorigenesis in SW480 and HT29 xenografts overexpressing RNF128 showed that tumor growth was reduced the half in presence of RNF128 in KRAS mutant background while it was promoted in oncogenic BRAF. Drug response and apoptosis were increased in presence of RNF128 in KRAS-mutated CRC, while RNF128 confers resistance in BRAF-mutated CRC. Xenografts inoculated with SW480 showed that RNF128 significantly improves response to IRI in both sexes and to 5-FU in males. Otherwise, in HT29 xenografts, RNF128 significantly enhanced IRI resistance in females. No response differences were seen to OXA. The suitability of RNF128 as a predictive biomarker of response to chemotherapy in CRC receiver-operating characteristic (ROC) and area under the curve (AUC) analysis was demonstrated for IRI response in colon cancer patients

© 2023 The Authors. Molecular Oncology is published by John Wiley & Sons Ltd on behalf of Federation of European Biochemical Societies

(AUC 0.748) and for fluoropyrimidines monotherapy (AUC 0.857) in young CRC patients.

**Conclusion**

RNF128 expression is a prognostic factor in KRAS-mutated colon cancer. Based on KRAS or BRAF oncogenic background, RNF128 inhibits or promotes CRC tumorigenesis, and sensitizes or enhances IRI and 5-FU resistance, respectively. RNF128 is a predictive biomarker of IRI resistance in colon cancer and fluoropyrimidines in young CRC patients with potential clinical utility.

### EACR23-0707
### HDAC7 expression improves Diffuse Large B Cell Lymphoma response to rituximab

*M. Gusi Vives[1], A. Meler[1], B. Győrffy[2], J.T. Navarro[3], J.I. Martín-Subero[4], O. Collazo[1], O. de Barrios[1], M. Parra[1]*
*[1]Josep Carreras Leukaemia Research Institute, Lymphocyte Development and Disease Group, Badalona, Spain*
*[2]Semmelweis University, Department of Bioinformatics, Budapest, Hungary*
*[3]ICO-Hospital Germans Trias i Pujol, Servicio de Hematología, Badalona, Spain*
*[4]Institut d'Investigacions Biomèdiques August Pi i Sunyer I DIBAPS, Biomedical Epigenomics, Barcelona, Spain*

**Introduction**

The generation of B lymphocytes is a highly complex process that consists of successive transitions from one to another cellular stage. The action of specific transcription factors drives lineage commitment and cell differentiation. Throughout this process, histone deacetylase HDAC7 acts as a crucial transcriptional repressor that silences non-lymphoid genes and other epigenetic regulators.Therefore, its deregulation can lead to B-cell malignancies.
Diffuse Large B Cell Lymphoma (DLBCL) is the most common and aggressive type of non-Hodgkin lymphoma. Cyclophosphamide, doxorubicin, vincristine and prednisone (CHOP) treatment regimen has been used for the last decades. The addition of rituximab, a monoclonal antibody against CD20 antigen, to this therapy (R-CHOP) is the gold standard treatment combining both immunotherapy and chemotherapy. However, patients with low CD20 expression are highly resistant to R-CHOP.

**Material and Methods**

Human DLBCL cells, healthy human germinal center (GC) B cells and DLBCL cell lines were used for RNA extraction and quantitative-PCR (qPCR).
Inducible HDAC7 expression systems were generated in DLBCL cell lines (MD-901, HBL-1, KARPAS-231) and protein and mRNA levels were analyzed by Western Blotting and Flow Cytometry, and by qPCR, respectively.

**Results and Discussions**

Outcome data associated to DLBCL patients treated with R-CHOP demonstrated that high expression of HDAC7 correlates with an improved overall survival, and focusing on R-CHOP non-responsive patients (i.e. with low CD20 levels), a stronger effect was found.
Primary samples from DLBCL patients, as well as from DLBCL cell lines, displayed low expression of HDAC7 when compared to tonsil-derived GC B cells, both at protein and mRNA levels. Importantly, low levels of HDAC7 correlated to low CD20 expression, suggesting a potential association of the expression of the two proteins. In this sense, induction of HDAC7 in DLBCL cell lines triggered an increased activation of CD20 . As a consequence, the expression of HDAC7 enhanced response to R-CHOP therapy in these cell lines compared to standard CHOP treatment, measured by an induction of apoptosis in DLBCL cells. DLBCL cell lines overexpressing HDAC7 or with high levels of HDAC7 showed an increase in apoptosis when treated with R-CHOP instead of CHOP alone.

**Conclusion**

Our data suggest that precise induction of HDAC7 in DLBCL induces CD20 expression, increasing sensitivity of resistant cells to R-CHOP therapy and potentially improving final outcome of DLBCL-diagnosed patients.

### EACR23-0753
### Phosphorylation of intestine-specific homeobox by ERK1 modulates oncogenic activity and sorafenib resistance

*S.N. Wang[1]*
*[1]Kaohsiung Medical University Hospital, Division of General and Digestive Surgery- Department of Surgery, Kaohsiung, Taiwan*

**Introduction**

Nuclear translocation regulated by phosphorylation is a key step in providing activated mitogen-activated protein kinases (MAPKs) access to their nuclear targets; however, the mechanisms linking MAPK-induced nuclear translocation and target gene expression mediating oncologic activity remain obscure.

**Material and Methods**

In-vitro and in-vivo assay were used in this study.

**Results and Discussions**

Here, we show that the MAPK extracellular signal-regulated kinase (ERK) 1, but not ERK2, phosphorylated intestine-specific homeobox (ISX), leading to its nuclear translocation and downstream oncogenic signaling. Mechanistically, ERK1 phosphorylated serine 183 of ISX, facilitating its nuclear translocation and downstream target gene expression. In contrast, dominant-negative ERK1 expression in hepatoma cells inhibited the nuclear translocation of ISX and the expression of downstream genes involved in cell proliferation, malignant transformation, and epithelialmesenchymal transition in vitro and in vivo. An activating mutation in ISX (S183D) exhibited a constitutive nuclear localization and resistance to sorafenib. Additionally, in 576 paired clinical hepatocellular carcinoma (HCC) samples and adjacent normal tissues, ERK1 and ISX were co-expressed in a tumor-specific manner at mRNA and protein levels, while their mRNA levels showed significant correlation with survival duration, tumor size, number, and stage.

**Conclusion**

These results highlight the significance of ERK1/ISX signaling in HCC progression and its potential as a prognostic and therapeutic target in HCC.

### EACR23-0776
### The NEDD8 pathway as a therapeutic target in HER2 amplified colorectal cancer

*F. INVREA[1], C. Petti[2], A. D'Andrea[2], M. Cortese[1],*

© 2023 The Authors. Molecular Oncology is published by John Wiley & Sons Ltd on behalf of Federation of European Biochemical Societies

E. Torchiaro[1], A. Carugo[3], S. Arena[1], A. Bardelli[4], G.F. Draetta[5], E. Medico[1]
[1]Candiolo Cancer Institute FPO-IRCCS, Oncology, Candiolo, Italy
[2]University of Turin, Oncology, Candiolo, Italy
[3]IRBM, Oncology, Pomezia, Italy
[4]University of Turin and IFOM ETS, Oncology, Milano, Italy
[5]UT MD Anderson Cancer Center, University of Texas, Houston, United States

**Introduction**

Colorectal cancer (CRC) is a heterogeneous disease with a wide spectrum of clinical outcomes, from indolent resectable disease to aggressive-metastatic cases. Primary and acquired resistance limits the efficacy of available treatments, and the identification of effective drug combinations is needed to further improve patients' outcomes. We previously found that the NEDD8-activating enzyme inhibitor pevonedistat induces tumor stabilization in preclinical models of poorly differentiated, clinically aggressive CRC resistant to available therapies.

**Material and Methods**

To identify drugs that can be effectively combined with pevonedistat, we performed a drop-out loss-of-function synthetic lethality screening with a shRNA library covering 200 drug-target genes in four different CRC cell lines.

**Results and Discussions**

Multiple screening hits were found to be involved in the EGFR signaling pathway, suggesting that, rather than inhibition of a specific gene, interference with the EGFR pathway at various levels could be effectively leveraged for combination therapies based on pevonedistat. Exploiting both BRAF-mutant and RAS/RAF wild-type CRC models, we validated the therapeutic relevance of our findings by showing that combined blockade of NEDD8 and EGFR pathways led to increased growth arrest and apoptosis both *in vitro* and *in vivo*. Our results suggested possible therapeutic opportunities in specific CRC clinical settings. We further focused on HER2 amplified CRC and found marked sensitivity to pevonedistat in a CRC cell line carrying HER2 amplification. In these cells, long-term *in vitro* treatment with a combination of lapatinib and trastuzumab induces the emergence of colonies of long-term persisters, i.e., cells surviving to several weeks of HER2/EGFR blockade. These persisters display extremely slow growth and senescence features, however they fully recover upon removal of the blockade, mimicking disease relapse in patients after treatment suspension. Interestingly, persister colonies maintained sensitivity to pevonedistat, displaying a marked decrease when pevonedistat was added after six weeks of HER2/EGFR blockade, independently of the continuation of the blockade. Furthermore, persister cells resulted responsive to the senolytic agent navitoclax, suggesting senescence as a potential drug-tolerance mechanism towards acquired resistance.

**Conclusion**

These results unveil the possibility of employing pevonedistat in HER2-amplified CRC patients subsequently to tumor stabilization or regression by HER2/EGFR blockade.

### EACR23-0779
### NAMPT in drug-resistant melanoma: linking NAMPT-dependent metabolic reprogramming and immune regulation.

I. Fiorilla[1], A.M. Todesco[1], L. Ponzone[2], E. Moiso[3], A. Ponzano[1], R. Piraino[1], S. Sdelci[4], M. Manfredi[5], V. Calautti[2], V. Audrito[1]
[1]University of Eastern Piedmont, Department of Science and Technological Innovation, Alessandria, Italy
[2]University of Turin, Department of Molecular Biotechnology and Health Scienc es- Molecular Biotechnology Center "Guido Tarone", Turin, Italy
[3]Broad Institute of Harvard and MIT, Broad Institute of Harvard and MIT, Cambridge- MA, United States
[4]CRG-Centre for Genomic Regulation, CRG-Centre for Genomic Regulation, Barcelona, Spain
[5]University of Eastern Piedmont, Department of of Translational Medicine, Novara, Italy

**Introduction**

Targeted-therapy and immune checkpoint inhibitors (ICIs) have notably improved the treatment of *BRAF*-mutated metastatic melanoma (MM) patients; however resistance to treatment dramatically impacts on the survival of patients. BRAF(i)nhibitors-resistant MM cells showed increased amounts of NAD, an essential redox cofactor, supporting their metabolic adaptations underlying the acquisition of drug resistance. This was obtained selectively overexpressing the rate-limiting NAD-biosynthetic enzyme nicotinamide phosphoribosyltransferase (NAMPT). NAMPT-NAD axis becomes a driver of melanoma progression and resistance to BRAFi, that it may be therapeutically targeted.

**Material and Methods**

NAMPT-NAD axis was studied using biochemical, enzymatic, immunohistochemical assays. Immunoprecipitation of NAMPT following mass spectrometry (MS) analysis was used to define NAMPT interactome. In silico analysis using TCGA database, western blot analysis and RT-PCR were used to detect and confirm protein expression and signaling pathways activation.

**Results and Discussions**

The BRAF oncogenic signaling and NAMPT expression are molecularly linked. We found that mutations in the *BRAF* oncogene positively correlate with NAMPT expression in TCGA melanoma cohort and in tissue from MM patients. The over-expression of NAMPT correlates with its gene amplification. Analyzing the nature of this genetic amplification we found a co-amplification of others 40 genes including *PIK3CG* that we started to evaluate functionally.
A second set of preliminary data revealed a significant increase in the nuclear localization of NAMPT in resistant cells, also as NAMPT nuclear fraction chromatin-bound. We performed NAMPT immunoprecipitation following MS in cellular extract to identify NAMPT-interacting proteins. Data showed enrichment of NAMPT-interacting nuclear proteins and proteins involved in RNA processing, translation, metabolic processes, cellular response to stress and immune response among others. Starting to analyze

© 2023 The Authors. Molecular Oncology is published by John Wiley & Sons Ltd on behalf of Federation of European Biochemical Societies

NAMPT-immune gene signature relationship we found in TCGA melanoma cohort a positive correlation between *NAMPT* expression and interferon signaling, including *CD274, IRF1, STAT1* genes that will be further investigated.

**Conclusion**

The multiple roles of NAMPT as intracellular and soluble protein are known; here we speculate that NAMPT could have an essential and unknown function in the nucleus and in regulating immune responses, with a possible impact on ICIs activities.

### EACR23-0789
### Exploring IRAK1: a new mediator of chemo-immuno-resistance in Non-Small Cell Lung Cancer?

*S. Digiovanni[1], P. Bironzo[2], F. Napoli[3], V. Ramundo[1], J. Kopecka[1], L. Righi[3], S. Novello[2], G.V. Scagliotti[2], C. Riganti[1], I.C. Salaroglio[1]*
*[1]University of Torino, Department of Oncology, Torino, Italy*
*[2]AOU San Luigi Hospital, Thoracic Oncology Unit- Department of Oncology, Orbassano, Italy*
*[3]AOU San Luigi Hospital, Pathology Unit- Department of Oncology, Orbassano, Italy*

**Introduction**

Among the potential mechanisms shaping chemo-immunoresistance in non-small lung cancer (NSCLC) there is the high expression of ABCB1 (P-glycoprotein) and ABCC1 (Multidrug Resistance-related Protein 1), both involved in the efflux of chemotherapeutic drugs, and the low expression of ABCA1, a transporter promoting the immuno-recognition of the tumour. ABCB1/ABCC1$^{high}$ABCA1$^{low}$ cells are resistant to chemotherapy and immune killing, but the underlying molecular pathways are unknown.

**Material and Methods**

We screened 28 cell lines of NSCLC with a chemo-immunoresistant (ABCB1/ABCC1$^{high}$, ABCA1$^{low}$) and a chemo-immunosensitive (ABCB1/ABCC1$^{low}$, ABCA1$^{high}$) phenotype. We built a CRISPR KO kinome library on NCI-H2228 chemo-immunoresistant cell line to decipher the kinases supporting the chemo-immuno-resistant phenotype.

**Results and Discussions**

Among the top 10 kinases that produced the shift to “chemo-immunoresistance-> chemo-immunosensitivity”, we identified interleukin 1 receptor associated kinase 1 (IRAK1), a kinase activated by chronic inflammation causing chemoresistance and reducing ABCA1 in cardiovascular system. IRAK1-silencing in chemo-immune-resistant NSCLC NCI-H2228 cells transcriptionally decreased ABCC1 and ABCB1, and increased ABCA1, while IRAK1-overexpression in chemo-immune-sensitive NCI-H1975 cells produced the opposite effects. IRAK1-silenced NCI-H2228 tumors implanted in NSG Hu-CD34$^{+}$ mice were re-sensitized to cisplatin compared to wild-type tumors. Moreover, the transcriptomic profile of 110 NSCLC patients treated with cisplatin-based chemotherapy, shown that IRAK1 was significantly more expressed in tumours than in non-tumoral lung tissues. According to the median value of IRAK1 in tumours, patients categorized as IRAK1$^{high}$ had worse progression free survival and overall survival.

**Conclusion**

Our preliminary results indicate IRAK1 as a new factor inducing chemo-immunoresistance in NSCLC and as a potentially additional predictive factor of chemoresistance and poor outcome in NSCLC patients.

### EACR23-0791
### Evaluation of the second generation histone deacetylase inhibitor (HDACi) quisinostat as therapeutic option to overcome cisplatin resistance

*S. Meneceur[1], M.J. Hoffmann[1], G. Niegisch[1]*
*[1]Medical Faculty and University Hospital- Heinrich-Heine-University Düsseldorf, Department of Urology - Research Laboratory, Düsseldorf, Germany*

**Introduction**

Cisplatin-based chemotherapy is the standard of care for urothelial cancer (UC). Resistance however hinders tumour control, underlying the need for new therapies. Epigenetic priming to restore chemosensitivity represents an interesting approach to target resistance. Clinical studies currently evaluate the potential of HDAC inhibitors (HDACi) to re-sensitise resistant tumours to chemotherapy. We previously showed that quisinostat, a second generation HDACi, had moderate antineoplastic effect as single agent in UC *in vitro* and *in vivo*. Combining quisinostat with either cisplatin or the PARP inhibitor (PARPi) talazoparib improved its efficiency in UC cell lines. In this study, we aimed at evaluating the sensitising effect of quisinostat to cisplatin and talazoparib in cisplatin resistant UC sublines.

**Material and Methods**

Parental lines ($IC_{50}$<10 µM) and previously generated cisplatin resistant sublines (LTT: long-term treated) with different degrees of resistance to cisplatin were used: J82LTT ($IC_{50}$=26.4 µM, moderately resistant), T24LTT ($IC_{50}$=72.3 µM, resistant) and RT112LTT ($IC_{50}$=200 µM, highly resistant). Dose-response curves were done for quisinostat, cisplatin, talazoparib and the combinations at fixed dose ratio 72h post-treatment. Effects of the combinations at low dose ratios (0.5x$IC_{50}$ and 0.75x$IC_{50}$) on cell cycle, apoptosis induction, DNA damage and long term proliferation were analysed by FACS, caspase 3/7 activity, western blots and clonogenicity assay, respectively. The Chou-Talalay method was used to assess synergism.

**Results and Discussions**

Quisinostat synergised with cisplatin and talazoparib in the parental lines. Addition of quisinostat to cisplatin led to a decrease in cisplatin $IC_{50}$ in J82LTT (3.2 x) and T24LTT (2x), indicating re-sensitisation; not in RT112LTT. In J82LTT and T24LTT, both cisplatin and talazoparib synergised with quisinostat at low dose ratio (CI<1) and a marked decrease in cell viability was observed. Further, both combinations increased caspase 3/7 activity (+122% for quisinostat+cisplatin; +69% for quisinostat+talazoparib in T24LTT), PARP cleavage and γH2AX levels. Long

© 2023 The Authors. Molecular Oncology is published by John Wiley & Sons Ltd on behalf of Federation of European Biochemical Societies term proliferation ability was markedly decreased, and cell cycle was disrupted.

**Conclusion**

Quisinostat re-sensitises moderately resistant and resistant LTT to cisplatin and the PARPi talazoparib. Synergy mechanisms involve caspase-dependent apoptosis and cell cycle disruption. RNA-Seq analysis and *in vivo* experiments are ongoing to better characterise these interesting therapy options.

### EACR23-0795
### MLK4 drives chemoresistance in triple-negative breast cancer and regulates the signalling network between breast cancer cells and the tumour microenvironment

*A. Marusiak*[1], *A. Mazan*[1], *D. Mehlich*[1]
[1]*IMol Polish Academy of Sciences, Laboratory of Molecular OncoSignalling, Warsaw, Poland*

**Introduction**

Breast cancer is one of the most common malignancies in women, and the development of better therapies for breast cancer patients remains a major unmet need in breast oncology. In the past few years, multi-omics profiling studies identified kinase-driven molecular alterations across molecular TNBC subtypes, which opened new possibilities for targeted therapeutic strategies. Recent cancer genomics data indicated that MLK4 (Mixed-Lineage Kinase 4) is amplified and overexpressed in over 50% of triple-negative tumours, facilitating the aggressive growth and migratory potential of breast cancer cells.

**Material and Methods**

We use various *in vitro* phenotypic assays, RNA sequencing, phosphoproteomics and *in vivo* models to investigate the role of MLK4 in breast cancer.

**Results and Discussions**

MLK4 is a member of the MLK family of serine/threonine kinases. We recently demonstrated that MLK4 promotes TNBC chemoresistance by regulating the pro-survival response to DNA-damaging therapies. Our new data suggest that MLK4 might also control autocrine/paracrine signalling loops in cancer cells and the tumour microenvironment. Using co-culture models, we also found that MLK4 regulates the phenotypic changes of breast cancer cells induced by the components of the tumour microenvironment. Furthermore, our research demonstrates mechanistic details of how MLK4 contributes to breast cancer development, drug resistance and progression to metastasis.

**Conclusion**

We identified an oncogenic function of MLK4 in breast cancer, highlighting that the inhibition of this kinase could be an effective strategy for breast cancer treatment.

### EACR23-0798
### New markers of cisplatin resistance of ovarian tumors

*P. Harvanik*[1]
[1]*University of Pavol Jozef šafárik in Košice, Medical biology, Košice, Slovakia*

**Introduction**

Cisplatin (CDDP) is a chemotherapeutic agent used for a wide range of solid tumours. Sometimes, and especially in the case of recurrent conditions, so-called cisplatin resistance occurs. This mechanism is characterized by a change in gene expression, which ensures the inhibition of cisplatin. Most often, these mechanisms are divided into four groups: proteins of efflux activity, antioxidant apparatus, proteins involved in DNA repair and others.

**Material and Methods**

In our work, we chose cell models A2780 (ovarian adenocarcinoma line) and its cisplatin-resistant derivative A2780cis as models for understanding new mechanisms of cisplatin resistance. We used western blotting analysis, quantitative real time PCR, wound healing essay, MTS essay and comparative proteomic analysis.

**Results and Discussions**

By comparing the metabolic activity of the parietal and resistant lines after exposure to CDDP for 72 hours using the MTS assay, the half maximal inhibitory concentration (IC50) for A2780cis was determined to be 27.5 μM, which is 3.8 times more than in the case of A2780 (7.2 μM). Comparative proteomic analysis revealed more than 20 significantly upregulated and downregulated genes. Among these genes, we found that have already been mentioned in the literature, such as CD70, SNCG, MEST, PHGDH, but also some that have not been mentioned yet. Wound healing essay showed that the migration ability of A2780 cells is higher compared to the cisplatin resistant A2780cis line. Also, western blotting analysis and quantitative real time PCR showed changes in protein composition and gene expression between the lines.

**Conclusion**

Our work suggest that there are a lot of unknown in field of cisplatin resistance. We have found a new proteins that are worthy of depper studing.

### EACR23-0819
### Inhibition of N Terminus MLL Complexes Menin and LEDGF Reverts Taxane Resistance in Castration-Resistant Prostate Cancer

*I. bulut*[1], *B. Cevatemre*[2], *H. Syed*[1], *S.C. Ozcan*[2], *B.M. Kalkan*[1], *C. Acilan Ayhan*[3]
[1]*Koc University, Graduate School of Health Sciences, Istanbul, Turkiye*
[2]*Koc University, Research Center for Translational Medicine, Istanbul, Turkiye*
[3]*Koc University, Medicine Faculty, Istanbul, Turkiye*

**Introduction**

Prostate cancer (PC) is usually hormone-dependent, and androgen deprivation therapy (ADT) is the standard of care for advanced tumors [1]. Patients eventually progress to a castration-resistant (CR) stage, where tumor growth becomes independent of the male hormone androgen [2]. Conventional chemotherapeutics such as Docetaxel (DTX) and Cabazitaxel (CBZ) are used frequently either in combination or as a follow-up of hormone therapies, yet some tumors still relapse [3].

**Material and Methods**

We generated resistant CRPC cell lines, and global proteomics and RNA sequencing approaches were used to compare the resistant and sensitive cell populations. Library drug screens revealed epigenetic targets and they

© 2023 The Authors. Molecular Oncology is published by John Wiley & Sons Ltd on behalf of Federation of European Biochemical Societies were knocked out via the CRISPR cas9 system, furthermore, RNA-sequencing and ChIP experiments were performed to uncover the essential effects of these factors in the resistant cell genome.

**Results and Discussions**

Proteomics and RNA sequencing approaches revealed that, the previously described ABCB1, was the top hit, between DTX and CBZ-resistant cells. Our epigenetic screen showed, two MLL complex inhibitors MI-2 (iMLL-Menin) and WDR5-0103 (iMLL-WDR5) synergized with both taxanes and significantly induced apoptosis when combined with the taxols. To determine the roles of individual proteins in the MLL complex, we knocked out MLL, and its known partners using CRISPR-Cas9 technology. The absence of the N-terminus MLL partners Menin and LEDGF had no effect on the parental cells, they significantly halted the growth of the resistant cells. Parental cells require Menin expression for the acquisition of drug resistance. Menin and LEDGF knockout cells were examined through RNA-sequencing and ChIP experiments. Menin targets were identified and verified through qPCR. Our chromatin pulldown showed that Menin is highly enriched in Myc promoter in the resistant cells.

**Conclusion**

MLL-Menin and MLL-WDR5 inhibition, reverts taxane resistance in CRPC. Menin knockout showed vulnerability in resistant cells and Menin expression is crucial for the acquisition of DTX resistance. Our future studies will elucidate the molecular role of Menin and LEDGF in the acquisition and reversion of taxol resistance.

### EACR23-0848
### TIMP-1 overexpression mediates chemoresistance in triple negative breast cancer cells

*L. Agnello[1], A. d'Argenio[1], A. Caliendo[1], R. Nilo[1], A. Zannetti[2], M. Fedele[1], S. Camorani[1], L. Cerchia[1]*
*[1]Institute of Experimental Endocrinology and Oncology "G . Salvatore", National Research Council of Italy, Naples, Italy*
*[2]Institute of Biostructures and Bioimaging, National Research Council of Italy, Naples, Italy*

**Introduction**

Triple-negative breast cancer (TNBC) is among the most aggressive subtypes of breast cancer (BC). Despite an initial response to neoadjuvant chemotherapy, a large proportion of tumors commonly recurs, which are highly metastatic and chemoresistant. Therefore, the identification of novel drug targets that might impact chemotherapy resistance, is an important medical need. Tissue inhibitor of metalloproteinases-1 (TIMP-1) is a secreted protein with an established tumor suppressor function. Increasing evidence indicate that TIMP-1, by binding to CD63 on cancer cells surface, may activate oncogenic signaling pathways. Moreover, primary tumor levels of TIMP-1 have been reported to correlate with resistance to chemotherapy in patients with metastatic BC.

**Material and Methods**

TNBC cell lines resistant to cisplatin (MDA-MB-231/Cis-Pt) and doxorubicin (MDA-MB-231/Dox) were generated by a chronic treatment of MDA-MB-231 cells with the two chemotherapeutics. The response to Cis-Pt and Dox of chemoresistant cells silenced for TIMP-1 expression, and parental cells treated with exogenously added TIMP-1 (recombined protein or TIMP-1-enriched conditioned media), was assessed by MTT and EC50 calculation. Cell death was assessed by flow cytometry analysis following Annexin-V and propidium iodide staining and caspase activation assays. The interaction of TIMP-1 with CD63 on TNBC cells was assessed by confocal microscopy and immunoprecipitation analyses.

**Results and Discussions**

We show that TIMP-1 is overexpressed in MDA-MB-231/Cis-Pt and MDA-MB-231/Dox and its knock-down hampers chemoresistance, causing a strong reduction of AKT activation. Additionally, TIMP-1 is released in the conditioned medium obtained from TNBC MDA-MB-231 and BT-549 cells, and chemoresistant TNBC cell lines. In contrast, triple-positive BC BT-474 (positive for estrogen and progesterone receptors, and epidermal growth factor receptor 2) and normal breast MCF10A cell lines, showed no detectable TIMP-1 levels in either cell extracts or in the media. Noteworthy, parental MDA-MB-231 exposed to exogenous TIMP-1 acquired resistance to both Cis-Pt and Dox. We proved that released TIMP-1 reassociates with plasma membrane of chemoresistant cells by binding to CD63 receptor.

**Conclusion**

Taken together, these results identify TIMP-1 as a new biomarker involved in chemoresistance of TNBC cells and, therefore, lay the groundwork for in vivo investigations to evaluate whether TIMP-1 blockade is a viable strategy for the treatment of TNBC.

### EACR23-0854
### Improving cancer patient outcomes through molecular and genetic profiling based on a personalized medicine approach

*C. Fourie[1], M. Du Plessis[1], A.M. Engelbrecht[1]*
*[1]Stellenbosch University, Physiological Sciences, Stellenbosch, South Africa*

**Introduction**

Gynaecological cancers represent a serious public health problem, especially in low-and middle income countries. Patients are often receiving suboptimal treatment which can result in relapse and cancer recurrence. Furthermore, current cytotoxic chemotherapeutic treatment regimens are often ineffective and present with high levels of treatment resistance. Treatment resistance may often be patient specific and is determined by a wide range of factors, including genome alterations. Therefore, the development of new treatment protocols personalized for an individual patient are required to overcome these challenges.

**Material and Methods**

Ethical clearance was obtained from the Stellenbosch University Research Ethics committee. With their consent, cervical tumour biopsies and liquid biopsies were collected at Tygerberg hospital from cervical cancer patients, with stage II or stage III diagnosis. Subsequently, tumour biopsies were washed and mechanically dissected and allowed to grow for approximately 2-3 weeks, until 70-80% confluency was reached. These cells (a mixed population of cancer cells and fibroblasts) were then treated with various cytotoxic chemotherapeutic agents,

© 2023 The Authors. Molecular Oncology is published by John Wiley & Sons Ltd on behalf of Federation of European Biochemical Societies followed by various cell viability, immunocytochemistry and qPCR experiments. Furthermore, circulating tumour cells (CTCs) were isolated from the liquid biopsies by using MetaCellÒ sized based separation tubes and were then subjected to qPCR and immunocytochemistry. Lastly, differential blood tests were also performed for each patient.

**Results and Discussions**

This study shows that after treatment with various cytotoxic chemotherapeutic agents, patients showed different treatment responses and often present with chemotherapy treatment resistance. These effects were associated with the under or over expression of genes commonly associated with treatment resistance such as *TP53, KRAS1/2, BRCA1/2 and PIK3CA* in both primary cultured cells and CTCs. Furthermore, these patients also often presented with an altered blood clotting profile.

**Conclusion**

We highlight that understanding the resistance/sensitivity profile of each patient's case prior to the initiation of treatment offers the ability to optimize treatments and time-dependent clinical outcomes. Therefore, the timely identification of chemotherapy drug resistance based on a personalized approach is critical for optimal therapy management.

### EACR23-0870

### Ribosomal protein uL3 as regulator of ferroptosis in 5-FU resistance

*C. Brignola*[1], *A. Pecoraro*[1], *P. Carotenuto*[2], *B. Franco*[3], *G. Russo*[1], *A. Russo*[1]

[1]*University of Naples "Federico II", Department of Pharmacy, Naples, Italy*

[2]*The Institute of Cancer Research, Cancer Therapeutics Unit, London, United Kingdom*

[3]*University of Naples "Federico II", Medical Genetics - Department of Translational Medical Sciences, Naples, Italy*

**Introduction**

The human ribosomal protein L3 (uL3) has been identified as a stress sensing molecule essential for cell response to 5-Fluorouracil (5-FU)-induced nucleolar stress in colorectal cancer (CRC) cells lacking active p53. Specifically, our previously data demonstrated that uL3 status is strictly associated to 5-FU resistance; downregulation of uL3 positively correlates with alteration of epithelial-mesenchymal transition program, increasing in cell migration and proliferation, inhibition of apoptosis, enhancement of autophagy, overexpression of drug transporters.

Ferroptosis, a novel form of programmed cell death, involves iron-dependent lipid reactive oxygen species (ROS) accumulation, making it morphologically and molecularly distinct from other types of regulated cell death. Emerging evidence indicates that cancer cells are characterized by higher basal iron and ROS levels than normal cells. Thus, induction of ferroptosis may become a new therapeutic approach for CRC.

**Material and Methods**

We utilized metabolomic and transcriptomic data to explore ferroptosis-related signalling pathways in p53 deleted colorectal cancer cells (HCT $116^{p53-/-}$) and in a derivative cell subline stably silenced for uL3 (uL3Δ HCT $116^{p53-/-}$). Real-Time PCR and immunoblotting analysis were performed to examine the expression profile of key ferroptosis biomarkers upon 5-FU treatment of both cell lines.

**Results and Discussions**

Here, we demonstrated that 5-FU treatment significantly induced ferroptosis in CRC cells expressing uL3. Specifically, 5-FU reduced glutathione and cysteine contents through the downregulation of cystine/glutamate antiporter system $xc^{-}$ expression, resulting in ROS accumulation and ferroptosis. In addition, HCT $116^{p53-/-}$ cells exposure to 5-FU modulated the expression levels of specific ferroptosis biomarkers. In contrast, in condition of uL3 silencing this process was partially inhibited, suggesting that the activation of the antioxidant defence response depends on uL3 status.

**Conclusion**

In conclusion, our results indicate that uL3 plays a critical role in promoting cancer cell response to 5-FU treatment via ferroptosis process. The restoration of uL3 could enhance the activity of many chemotherapeutic drugs by promoting apoptosis and ferroptosis.

### EACR23-0874

### Primary-derived colorectal cancer cell line resist to oxaliplatin treatment after triggering epithelial-mesenchymal transition

*T. Martinez-Bernabe*[1], *D.G. Pons*[1], *J. Sastre-Serra*[2], *J. Oliver*[2]

[1]*University of Balearic Islands UIB - Balearic Islands Health Research Institute IdISBa, Department of Fundamental Biology and Health Sciences, Palma de Mallorca, Spain*

[2]*University of Balearic Islands UIB - Balearic Islands Health Research Institute IdISBa - Ciber Fisiopatología Obesidad y Nutrición CB06/03, Department of Fundamental Biology and Health Sciences, Palma de Mallorca, Spain*

**Introduction**

Oxaliplatin is successfully used to eradicate micro-metastasis and improve survival, whereas benefit of adjuvant chemotherapy in early stages of colorectal cancer (CRC) remains controversial. In fact, near 40% of patients of CRC presents a relapse after treatment. There are different biological process managing the relapse, such as epithelial-mesenchymal transition (EMT) or treatment resistance by drug transporters. This study focuses on analyze the effect of oxaliplatin on EMT and drug transporters in a primary- and metastatic-derived CRC human cell lines.

**Material and Methods**

Primary- (SW480) and metastatic-derived (SW620) CRC cell lines, isolated from the same patient 1 year apart, were treated with oxaliplatin 5 μM for 48 hours to analyze cell viability, EMT markers and drug transporters mRNA expression by RT-qPCR and western blot and migration by wound healing. Also, cell viability was determined in SW480 cell line after cotreatment with oxaliplatin 5 μM for 48 hours and elacridar, as an inhibitor of ABCG2 drug transporter, at different concentrations (1.25, 2.5, 5 and, 10 μM).

© 2023 The Authors. Molecular Oncology is published by John Wiley & Sons Ltd on behalf of Federation of European Biochemical Societies

**Results and Discussions**
After oxaliplatin treatment for 48 hours, EMT markers mRNA expression was increased in both CRC cell lines. However, despite that N-cadherin protein expression increased in both CRC cell lines, Vimentin protein expression only increased in SW480 cell line after oxaliplatin treatment. This data correlates with findings related to migratory capacity after oxaliplatin treatment. On the other hand, the decrease of cell viability after oxaliplatin treatment was more prominent in SW620 cell line. Also, SW480 presents an increase of ABCG2 drug transporter after oxaliplatin treatment, so that increase may be a possible reason for the resistance to oxaliplatin.
**Conclusion**
Oxaliplatin is effective as an antitumoral treatment in metastatic-derived CRC cell line, not only decreasing cell viability but also not triggering EMT process. On the other hand, primary-derived CRC cell line showed a drug resistance against oxaliplatin and the capacity of trigger EMT. Thus, as we observed in this study, oxaliplatin treatment is not useful for early stages of CRC.

### EACR23-0883
### Metabolic reprogramming towards fatty acid oxidation contributes to BH3 mimetic resistance in haematological malignancies

_R. Carter_[1], _S. Varadarajan_[1]
[1]*University of Liverpool, Molecular and Clinical Cancer Medicine, Liverpool, United Kingdom*

**Introduction**
BH3 mimetics, and particularly the BCL-2 inhibitor, venetoclax, have demonstrated success in the treatment of haematological malignancies. However, initial clinical response is often followed by relapse and concomitant chemoresistance. Although resistance has often been attributed to upregulation of alternate anti-apoptotic BCL-2 family proteins, we and others have shown that the pro-apoptotic BH3 only proteins can be dispensable in BH3 mimetic-mediated apoptosis, suggesting other mechanisms may be involved.
**Material and Methods**
Electron microscopy was utilised to analyse changes in mitochondrial structure upon chemoresistance, both in primary Chronic Lymphocytic Leukaemia (CLL) treated using a navitoclax regimen and in haematological cell lines developed to exhibit resistance to venetoclax. The cell lines were further interrogated via Seahorse respiratory assays and Western blot analysis of BCL-2 family and metabolic protein levels, to identify druggable targets whose activities may be implicated in chemoresistance. Immunoprecipitations were employed in parallel to investigate interactions between key resistance proteins and whether their disruption could overcome resistance.
**Results and Discussions**
Two independent BH3 mimetic resistance models revealed a loss of mitochondrial cristae upon chemoresistance, which was coupled with reductions in specific electron transport chain proteins and impaired oxidative phosphorylation. Although a reduction in BCL-2 and increase in BCL-$X_L$ levels were also observed, immunoprecipitations indicated that BCL-$X_L$'s role in venetoclax resistance was not due to its ability to bind and sequester pro-apoptotic BH3 family members. In fact, metabolic reprogramming in resistant cells resulted in an increased reliance upon lipogenesis - specifically fatty acid oxidation (FAO) - and BCL-$X_L$ was discovered to exist in a complex with the palmitoyl carnitine transferase CPT1B. Further, inhibition of CPT1 (or upstream FASN) was able to resensitise cells to venetoclax.
**Conclusion**
Mitochondria undergo dramatic structural changes during BH3 mimetic chemoresistance, that not only perturb mitochondrial function but also result in metabolic reprogramming; this can be modulated to revert chemoresistance. It appears that BCL-$X_L$ and BAD exist in a complex with the FAO-specific protein CPT1B, and this can be disrupted by specific inhibitors to restore sensitivity to venetoclax.

### EACR23-0887
### A CHEMOKINE RECEPTOR ANTAGONIST PRODUCES RESISTANCE CHANGES IN THE GASTRIC CANCER CELL WITH CISPLATIN-RESISTANCE PHENOTYPE

_P. Brebi_[1], _M. Reyes_[1,2], _B. Mora_[2], _C. Ili_[1], _Y. Mora_[1], _K. Buchegger_[3], _I. Riquelme_[2], _L. Zanella_[1]
[1]*Universidad de La Frontera, Millennium Institute on Immunology and Immunotherapy. Laboratory of Integrative Biology LIBi- Centro de Excelencia en Medicina Traslacional CEMT- Scientific and Technological Bioresource Nucleus BIOREN, Temuco, Chile*
[2]*Universidad Autonoma de Chile, Instituto de Ciencias Biomédicas- Facultad de Ciencias de la Salud- Universidad Autónoma de Chile- Temuco- Chile., Temuco, Chile*
[3]*Universidad de La Frontera, Centro de Ciencias Biomédicas y Morfofuncionales CEBIM- Department of Basic Sciences- School of Medicine- Universidad de La Frontera- Temuco- Chile, Temuco, Chile*

**Introduction**
Introduction: Gastric cancer (GC) is one of the deadliest and significant public health problem worldwide, emphasizing Chile. GC's most widely used chemotherapy regimen is based on platinum drugs such as cisplatin (CDDP). Unfortunately, the high recurrence rate of GC is predominantly attributable to chemoresistance. The CCR5/CCL5 axis, that participates in inflammatory process, has been associated with the development and progression of cancer. However, its role in GC chemoresistance has not been fully elucidated. This study aimed to determine the effects of the blockade of the CCR5/CCL5 axis by a chemokine receptor inhibitor (CRI), on AGS cells (a human gastric adenocarcinoma cell-line) resistant to CDDP (AGS R-CDDP).
**Material and Methods**
Methodology: AGS R-CDDP cells were obtained based on a stepwise dosing drug protocol. CCL5 candidate was selected through transcriptomic analysis, and the CCL5 expression level was validated by qRT-PCR. Flow cytometry techniques were applied to evaluate the induction of apoptosis and cell cycle. The cytotoxicity was determined by MTT assays. CRI was used alone and in combination with CDDP in all assays.
**Results and Discussions**

© 2023 The Authors. Molecular Oncology is published by John Wiley & Sons Ltd on behalf of Federation of European Biochemical Societies

The cytotoxicity assays showed that CRI/CDDP combination re-sensitized AGS R-CDDP cells, decreasing cell viability, on the other hand, not increasing apoptosis. After 48 hours of treatment, the cell cycle assay revealed cells mainly arrested in S Phase. CCL5 showed a decrease in mRNA levels after the CRI/CDDP combination, possibly correlating with allosteric inhibition of the CCR5 receptor.

**Conclusion**

Our results indicate that CRI/CDDP combination sensitized AGS R-CDDP cells to CDDP treatment revealing it is potential coadjuvant in GC therapy.

### EACR23-0900
### Role of CDK8/19 inhibition in sensitization of chronic myelogenous leukemia cells to Bcr-Abl antagonists

*A. Khamidullina[1], M. Yastrebova[1], J. Nuzhina[1], A. Bruter[2], A. Khrustaleva[3], C.U. Lim[4], I. Roninson[4], A. Shtil[1], V. Tatarskii[1]*
*[1]Institute of Gene Biology Russian Academy of Sciences, Laboratory of Molecular Oncobiology, Moscow, Russia*
*[2]Institute of Gene Biology Russian Academy of Sciences, Center for Precision Genome Editing and Genetic Technol ogies for Biomedicine, Moscow, Russia*
*[3]Institute of Gene Biology Russian Academy of Sciences, Department of the Bioinformatics, Moscow, Russia*
*[4]University of South Carolina, Drug Discovery & Biomedical Sciences, Columbia- SC, United States*

**Introduction**

The pathogenesis of chronic myelogenous leukemia (CML) is due to the activity of chimeric tyrosine kinase Bcr-Abl. Selective inhibitors of Bcr-Abl (prototype – imatinib mesylate, IM, Gleevec®) cause a therapeutic effect in the treatment of the primary process. However, subsequently, resistance sometimes develops due, not only mutations, but also adaptive changes in gene transcription and activation of survival mechanisms in tumor cells. Transcription reprogramming is mediated by cyclin-dependent protein kinase CDK8 (or its paralog CDK19) in complexes with Cyclin C and the Mediator components. Inhibition of CDK8/19-mediated transcription reprogramming is aimed at preventing the protection of CML cells from the effect of Bcr-Abl inhibitors.

**Material and Methods**

The work was carried out on Bcr-Abl positive CML lines K562 and KU812. The K562 with inducible p27Kip1 expression was created by using the lentiviral transduction. Cell cycle distribution and cell death induction were studied by flow cytometry. Quantitative real-time PCR and RNA sequencing were performed to study gene expression. Changes in protein (total and phosphorylated) levels were studied by Western blotting. Senexin B (SenB) and SNX631 were used for selective inhibition of CDK8/19, to suppress Bcr-Abl – IM, dasatinib, nilotinib, PF-114.

**Results and Discussions**

It was found that CDK8/19 inhibition by SenB alone does not have an antiproliferative effect on CML cells. In K562 cell line, SenB prevents the G1 phase arrest causing by IM and other Bcr-Abl inhibitors and increase cell death rate and level significantly. Transcriptome analysis revealed changes signaling pathways related to cell cycle and metabolism differentially regulated by SenB, IM and their combination. The mechanism of sensitization includes a decrease in the levels of p27Kip1 and p18INK4c (CDK inhibitors, CKIs), an increase in PARP cleavage and activation of caspases 3 and 9, as well as a decrease in STAT3 S727 phosphorylation and an increase in c-Myc transcription factor. These changes were not demonstrated in KU812, where neither SenB sensitization, nor changes in expression of CKIs and c-Myc level were detected.

**Conclusion**

Inhibition of CDK8/19 helps to overcome the delay of the cell cycle caused by the Bcr-Abl antagonist in CML cells and increase the death of tumor cells. The absence of general toxicity of CDK8/19 inhibitors during prolonged treatment under experimental conditions allows us to recommend CDK8/19 inhibition with targeted therapy of Bcr-Abl-positive tumors in prospect.

### EACR23-0903
### NRAS Mutants Contribute to a Drug-Resistant Phenotype in Acute Myeloid Leukaemia

*F. Healy[1], V. Marensi[2], J. Woolley[1], D. MacEwan[1]*
*[1]Pharmacology and Therapeutics, University of Liverpool, Liverpool, United Kingdom*
*[2]Chester Medical School, University of Chester, Chester, United Kingdom*

**Introduction**

Acute Myeloid Leukaemia (AML) has poor prognosis, high relapse rate and extensive heterogeneity. Patients are first treated with chemotherapy comprising cytarabine and an anthracycline. Best outcomes are typically seen with a haematopoietic stem cell transplant (HSCT) after chemotherapy. However, HSCT is often unsuitable for older patients, where diagnoses are most common. Targeted therapy is thus being increasingly trialled and used, including FLT3-targeting agents midostaurin, gilteritinib and quizartinib. FLT3 mutations are the most common AML driver mutation. However, whilst these drugs are initially beneficial in FLT3-ITD-mutated AML, resistance remains problematic, and is partly attributed to NRAS mutations. Here, we discuss our use of NRAS over-expressing AML cell line models to assess the contribution of individual NRAS mutants to drug resistance.

**Material and Methods**

MOLM-13 (FLT3-ITD+/NRAS-WT) cells were transduced with lentivirus encoding NRAS WT, G12C, G12D or Q61K, to constitutively over-express the mutants, with success confirmed by Western blotting. Annexin V/Propidium Iodide staining determined sensitivity of these cells, parental and previously-generated drug-resistant cell lines with NRAS mutants, to various AML-associated drugs. Proliferative capacity was quantified by trypan blue and CFSE staining, over 96h. Cell cycle status was measured by 7AAD staining. Colony forming capacity was counted after 10 days culture in methylcellulose. Fluorescent staining was quantified by flow cytometry. RNA was extracted from over-expressing cells and the sequence analysed.

**Results and Discussions**

G12C over-expression increased sensitivity to cytarabine, gilteritinib and quizartinib compared to parental cells, whereas Q61K decreased cytarabine sensitivity. Over-

© 2023 The Authors. Molecular Oncology is published by John Wiley & Sons Ltd on behalf of Federation of European Biochemical Societies expression of the three NRAS mutants increased proliferative capacity, and colony forming capacity significantly increased in MOLM-13-NRAS-G12C and MOLM-13-NRAS-G12D (*P*<0.05), compared to parental, NRAS WT over-expressing and drug-resistant controls. There was a level of cell cycle arrest in G1 phase, compared to the NRAS WT over-expressing cells.

**Conclusion**

Some NRAS mutations decrease sensitivity to AML therapies versus the parental FLT3-ITD+ cell line. This resistance is greatest with NRAS Q61K, though G12 mutants are perhaps more leukaemogenic. RNASeq data will be analysed to further deduce mechanisms contributing to resistance. Overall, data thus far suggests potential for treatment stratification using the NRAS mutational signature.

### EACR23-0905
### Urushiol V suppresses cell proliferation and enhances antitumor activity of 5-FU in human colon cancer cells by inhibiting FoxM1

*J.H. Jeong[1], J.H. Ryu[1]*
*[1]Sookmyung Women's University, Research Institute of Pharmaceutical Sciences and College of Pharmacy, Seoul, South Korea*

**Introduction**

5-Fluorouracil (5-FU) is a commonly used chemotherapeutic drug for colorectal cancer (CRC) patients. However, the development of drug resistance in tumor chemotherapy can seriously reduce therapeutic efficacy of 5-FU. Recent data show that FoxM1 is associated with 5-FU resistance in CRC. FoxM1 plays a critical role in the carcinogenesis and drug resistance of several malignancies. In this study, we investigated the anticancer activity of urushiol V in CRC cells and explored its mechanism. In addition, the efficacy of urushiol V on restoring the antitumor activity of 5-FU in a 5-FU resistant SW480 colon cancer (SW480/5-FUR) cells was determined.

**Material and Methods**

Urushiol V was isolated from the cortex of *Rhus verniciflua* Stokes. A human CRC cell line, SW480, was used. We generated a 5-FU-resistant cell line (SW480/5-FUR). Inhibitory effect of urushiol V on CRC cell proliferation was evaluated by MTT and colony formation assays. Flow cytometry assays were employed to analyze the distribution of cell cycle phases. RT-PCR and Western blot assays were used to detect alterations in mRNA and protein expression, respectively.

**Results and Discussions**

Urushiol V reduced cell proliferation and induced S-phase arrest of SW480 colon cancer cells. It inhibited protein expression level of FoxM1 through activation of AMPK. Moreover, urushiol V and 5-FU combined significantly reduced FoxM1 expression and consequently reduced cell growth and colony formation in 5-FU resistant colon cancer cells (SW480/5-FUR).

**Conclusion**

Overall, these findings suggest that urushiol V can suppress cell proliferation by inhibiting FoxM1 and enhance the antitumor capacity of 5-FU. Therefore, urushiol V may act as a promising candidate for drug resistance in human CRC chemotherapy.

### EACR23-0938
### Phosphoproteomics for target discovery and response prediction in colorectal cancer

*A. Henneman[1], R. Beekhof[1], A. Bertotti[2], F. Boettger[1], T. Pham[1], J. Knol[1], R. de Goeij-de Haas[1], S. Piersma[1], M. Labots[1], H. Verheul[3], L. Trusolino[2], C. Jimenez[1]*
*[1]Amsterdam UMC, Medical Oncology, Amsterdam, The Netherlands*
*[2]University of Turin, Department of Oncology, Turin, Italy*
*[3]Erasmus MC, Medical Oncology, Rotterdam, The Netherlands*

**Introduction**

Aberrant kinase activity is one of the drivers of tumorigenesis and cancer progression and results in altered phosphorylation abundance of downstream substrates. In the oncology context, where personalized treatment requires analysis of single samples, phosphoproteomics coupled to Integrative Inferred Kinase Activity (INKA) analysis has emerged as tool that can prioritize actionable kinases for targeted inhibition (1-3).

In this project we performed phosphoproteomics of genomically and pharmacologically characterized mCRC patient-derived xenografts to investigate the molecular basis of response to EGFR blockade and identify alternative drug targets to overcome resistance.

**Material and Methods**

Mass-spectrometry-based proteomics, global (TiO2) and pTyr-based phospho-proteomics were perfomed of 30 mCRC-PDX tumors (Bertotti et al., Nature 2015)

**Results and Discussions**

Both tyrosine and global phosphoproteome as well as proteome harbored discriminatory response signatures. We found increased pathway activity related to MAPK inhibition and abundant tyrosine phosphorylation of cell junction proteins in sensitive tumors, while epithelial-mesenchymal transition and increased MAPK and AKT signaling were more prevalent in resistant tumors. Ranking of kinase activities in single samples confirmed driver activity of ERBB2, EGFR, and MET in cetuximab-resistant tumors. Moreover, high kinase activity was found of SRC and Ephrin kinase family members in 2 models with genomically unexplained resistance. Inhibition of these hyperactive kinases, alone or in combination with cetuximab, resulted in growth inhibition of in PDX-derived organoids and in vivo.

**Conclusion**

Together, these findings highlight the potential value of phosphoproteomics to improve our understanding of anti-EGFR treatment and response prediction in mCRC and bring to the fore alternative drug targets in cetuximab-resistant tumors.

**References**

1. Beekhof R, Henneman AA, Jimenez CR et al,. INKA, an integrative data analysis pipeline for phosphoproteomic inference of active kinases. Mol Syst Biol. 2019 May 24;15(5):e8981.
2. Cordo' V, Jimenez CR, Meijerink JPP et al., Phosphoproteomic profiling of T cell acute lymphoblastic leukemia reveals targetable kinases and combination

© 2023 The Authors. Molecular Oncology is published by John Wiley & Sons Ltd on behalf of Federation of European Biochemical Societies treatment strategies. Nat Commun. 2022 Feb 25;13(1):1048.
3.Piersma SR, Henneman AA, Jiménez CR et al.,. Inferring kinase activity from phosphoproteomic data: Tool comparison and recent applications. Mass Spectrom Rev. 2022 Sep 26:e21808.

### EACR23-0943
### PRC2 and MYC pathways regulate fetal conversion in drug-tolerant persister cells

L. Solé[1], T. Lobo-Jarne[1], Á. Montoto[1], J. Cabré[1], A. Bigas[1], L. Espinosa[1]
[1]IMIM, Cancer, Barcelona, Spain

**Introduction**

Emergence of drug tolerant-persister cells (DTP cells) has recently been described as an important mechanism of drug resistance in cancer. Upon treatment with DNA-damaging agents, cells can acquire a slow-proliferating state linked to a YAP1-dependent fetal-like conversion, as described by our group (Solé&Lobo-Jarne et al., 2022). DTP cells are capable to resume cell cycle and contribute to tumor relapse after a variable period of latency. Importantly, we have also defined a fetal signature that can predict poor patient's outcome in colorectal cancer (CRC), which is already present in untreated patients, implying that other factors apart from treatment are inducing fetal conversion. Thus, understanding which mechanisms regulate fetal conversion is essential to identify possible therapy targets.

**Material and Methods**

We have used three CRC patient datasets (Marissa, Jorissen and TCGA), cell lines and a biobank of patient-derived organoids (PDOs) developed by our group to analyze the molecular pathways regulating fetal conversion at transcriptional and protein levels.

**Results and Discussions**

By using the fetal signature that we have previously determined, we obtained the differentially expressed genes between fetal and non-fetal like tumors of three published CRC patient cohorts. ChEA analysis of deregulated genes showed that SUZ12 (member of PRC2) and MYC transcription factors could be regulating expression of up- and downregulated genes, respectively. We also observed that fetal-like tumors present a down-regulation of important elements of these two pathways. Importantly, we have demonstrated that common SUZ12 and MYC targets in the three cohorts have potential to predict patient's outcome. Downregulation of some PRC2 elements were also observed in treated-CRC cell lines and PDOs, showing that this mechanism is shared in both treated and untreated tumors. Finally, by using inhibitors of both PRC2 and MYC pathways we have been able to validate their role in regulating fetal genes, being both necessary to obtain the complete phenotype.

**Conclusion**

Our study indicates that not only DTP cells but also cells present in untreated tumors can display fetal-like conversion, responsible of poor prognosis of CRC patients. Fetal conversion is mainly regulated by PRC2 and MYC pathways, which could represent possible therapeutic targets to treat fetal-like tumors to prevent relapse and drug resistance.

### EACR23-0948
### Identifying targetable metabolic adaptations in drug tolerant BRAFV600E mutant lung adenocarcinoma

R. Crake[1], M. Thiry[1], O. Peulen[1], N. Kiweler[2], J. Meiser[2], A. Blomme[1], D. Cataldo[1], M.J. Nokin[1]
[1]University of Liege, GIGA-Cancer, Liège, Belgium
[2]Luxembourg Institute of Health, Department of Cancer Research, Luxembourg City, Luxembourg

**Introduction**

Acquisition of resistance to targeted therapies is one of the greatest challenges faced by precision oncology. Drug tolerant expanded persister (DTEP) cancer cells are considered as the reservoir from which genetically resistant cells subsequently emerge. Thus, targeting DTEP cells represents an arguably more efficient therapeutic strategy than targeting genetically resistant cells. $BRAF^{V600E}$ mutant lung adenocarcinoma (LUAD) patients often develop acquired/genetic resistance to BRAF (dabrafenib) and MEK (trametinib) inhibitor combination, however, little is known about the preceding drug tolerance mechanisms and associated vulnerabilities. We aimed to identify targetable metabolic adaptations in drug tolerant $BRAF^{V600E}$ mutant LUAD.

**Material and Methods**

DTEP cells were generated by treating HCC364 $BRAF^{V600E}$ mutated LUAD cells with increasing concentrations of dabrafenib/trametinib for several months. Mitochondrial structure, localisation, number and function, of parental compared to DTEP cells, was assessed by MitoTracker™ staining (and confocal microscopy), transmission electron microscopy (TEM), mtDNA content analysis (real-time PCR), oxygen consumption rate (XF-Flux analysis), and effect of glutamine starvation on cell viability. Glycolytic flux potential was assessed by measuring extracellular acidification rate (XF-Flux analysis) and glucose uptake (flow cytometry of fluorescent 2-NBDG; glucose analogue).

**Results and Discussions**

Compared to parental cells, HCC364-DTEP cell mitochondria showed a noticeable shift in network distribution, with hyperfusion, less-functional configurations, and more and larger mitochondria per cell. In concordance, mitochondrial oxygen consumption was significantly decreased in HCC364-DTEP cells. In addition, glutamine starvation decreased viability of parental cells more than DTEP cells, highlighting the reliance of parental HCC364 cells on mitochondrial function and HCC364-DTEP cells on alternative metabolic pathways for energy and survival. Interestingly, in response to glucose injection, HCC364-DTEP cells show a greater increase in rates of extracellular acidification, yet basal glucose uptake rates were similar between parental and DTEP cells, suggesting a higher glycolytic flux potential in DTEP cells.

**Conclusion**

Our preliminary results demonstrate that drug tolerant expanded persister cells undergo metabolic rewiring in $BRAF^{V600E}$ mutant LUAD. With further investigation, we expect to better understand the metabolic adaptations and target the associated vulnerabilities.

© 2023 The Authors. Molecular Oncology is published by John Wiley & Sons Ltd on behalf of Federation of European Biochemical Societies

### EACR23-0967
### Caveolin-1 as a modulator of sorafenib resistance in Hepatocellular Carcinoma

P. Korhan[1,2], E. Bagirsakci[1,3], Y. Islakoglu Oztemur[1], A. Bursali[1], G. Karakülah[4,5], N. Atabey[1,2]
[1]Izmir Biomedicine and Genome Center, Cancer Biology and Signaling Lab, Izmir, Turkiye
[2]Izmir Tınaztepe University, Galen Research Center, Izmir, Turkiye
[3]Izmir International Biomedicine and Genome Institute, Department of Molecular Biology and Genetics, Izmir, Turkiye
[4]Izmir Biomedicine and Genome Center, BIP Bioinformatics Platform, Izmir, Turkiye
[5]Izmir International Biomedicine and Genome Institute, Dokuz Eylul University, Izmir, Turkiye

**Introduction**

Sorafenib (SOR), a multikinase inhibitor, has been the effective first-line therapy for advanced hepatocellular carcinoma (HCC) patients. However, its efficacy is limited by drug resistance in some patients. Based on our previous studies describing the role of Cav-1 in acquiring aggressive phenotype, we tested whether Cav-1 regulates SOR resistance by bringing signaling molecules together in caveolar structures.

**Material and Methods**

First, we developed SOR-resistant HCC cell lines and examined their Cav-1 expression levels using qRT-PCR, Western Blot, and Confocal Microscopy. Then we overexpressed and silenced CAV-1 to understand whether Cav-1 modulates SOR resistance. We investigated the expression level of Cav-1 and downstream signaling pathways and performed electron microscopy (EM) to evaluate the caveolar structures. Additionally, we investigated Cav-1 expression in publicly available transcriptome data of patients who received SOR (GEO accession ID: GSE109211). We identify differentially expressed genes based on Cav-1 level between SOR-resistant and sensitive patients. We determined molecular signatures that can discriminate resistant patients from sensitive patients. Lastly, we performed biological assays including motility, invasion, proliferation, apoptosis, spheroid formation, and branching-tubulogenesis.

**Results and Discussions**

Both in vitro and bioinformatics analysis revealed that overexpression of Cav-1 and its downstream targets such as beta-catenin, vimentin, and E-cadherin, is related with SOR resistance in HCC cells and patients, respectively. EM analysis showed that the number of potential caveolar structures increased in SOR-resistant clones. Bioinformatics analysis revealed that caveolar structure molecules such as cavin-1, dynamin, pascin2, and EHD2 expressions are higher in SOR-resistant patients than in sensitives. As expected, the IC50 value of SOR was higher in SOR resistance and CAV-1 overexpressed clones compared to wild-type counterparts. Biological assays demonstrated that SOR resistance accelerated cellular mobility, however, it inhibited proliferation and did not affect apoptosis. Furthermore, Cav-1 silencing reduced motility and invasion of SOR-resistant cells.

**Conclusion**

Here we describe for the first time the relationship between Cav-1, caveolar structure, and sorafenib resistance in HCC and highlight the impact of Cav-1 in modulating signaling molecules in gaining more aggressive phenotype during SOR resistance. This project is funded by TUBITAK (project #118Z186).

### EACR23-1003
### Fatty Acid Oxidation and enhanced OXPHOS capacity sustain chemotherapy resistance in medulloblastoma

L. Manfreda[1], E. Mariotto[1], E. Rampazzo[1], C. Cerqua[1], C. Marchioro[1], M. Simonato[1], M. Canton[1], L. Persano[1], G. Viola[1], R. Bortolozzi[1]
[1]University of Padova, Department of Women's and Children's Health, Padova, Italy

**Introduction**

Medulloblastoma (MB) is an aggressive CNS tumor that most commonly affects children under the age of 10. MB patients frequently experience tumor recurrence (30%) and, despite the application of multimodal therapy including surgery, chemotherapy, and radiotherapy, the most aggressive MB have an extremely poor outcome in terms of overall survival (40-50%). The rarity of this tumor and the lack of matched samples of diagnosis and relapse make the study of recurrence a difficult challenge to overcome. In this context, we established an in vitro model of MB resistance to chemotherapy by weekly exposing MB cell lines to a cocktail of chemotherapeutics used in MB treatment. (Vincristine, Etoposide, Cisplatin, Cyclophosphamide – VECC).

**Material and Methods**

To evaluate mitochondrial morphology, lipid content, and FAO rate, both flow cytometry analyses and confocal microscope analyzes were carried out using MitotrackerGreen/DeepRed and the Bodipy493-503 and BodipyFL-C16. The enzymatic activity of ETC complexes and citrate synthase was measured spectrophotometrically using a Cary UV 100 spectrophotometer. All these analyzes were integrated by the use of Western Blot and Seahorse XFe96 Analyzer. Untargeted Metabolomics was performed using UHPLC-Q Exactive™ mass spectrometer with ESI source(Thermo Scientific).

**Results and Discussions**

Preliminary multi-omics data analysis highlight metabolism as one of the most deregulated hallmarks in MB-resistant cells, with enrichment in fatty acid metabolism, pyruvate metabolism, TCA cycle, mitochondrial biogenesis, and oxidative phosphorylation. These changes functionally reflect a different mitochondrial morphology and function, with an increase in the activity of some electron transport chain enzymes coupled with a decreased glucose uptake. Moreover, chemoresistant cells display an enhanced FAO rate and heterogeneous lipid accumulation. In this context, the transient silencing of CPT1a demonstrates the fundamental importance of this protein for cell proliferation and the response to VECC treatment in resistant MB.

**Conclusion**

Recent studies highlight the relevance of the metabolic plasticity of cancer cells in chemotherapy adaptation and in the maintenance of high antioxidant power and macromolecule biosynthesis, to counteract the damage caused by chemotherapy. Our data suggest that MB-

© 2023 The Authors. Molecular Oncology is published by John Wiley & Sons Ltd on behalf of Federation of European Biochemical Societies resistant cells change their metabolic behaviour and enhance their dependency on FAO required to sustain the increased OXPHOS, revealing a novel vulnerability to selectively target chemotolerant MB cells.

### EACR23-1008
### Patient-derived glioblastoma organoids reveal glioblastoma resistance to standard therapies

*B. Majc[1], A. Habič[1,2], M. Marta[3], S. Aleksander[3], A. Porčnik[4], J. Mlakar[5], M. Mlinar[1], T. Lah Turnšek[1], B. Breznik[1], M. Novak[1]*
*[1]National Institute of Biology, Department of Genetic Toxicology and Cancer Biology, Ljubljana, Slovenia*
*[2]Jožef Stefan International Postgraduate School, Nanosciences and Nanotechnologies, Ljubljana, Slovenia*
*[3]Faculty of Computer and Information Science University of Ljubljana, Artificial Intelligence Laboratory, Ljubljana, Slovenia*
*[4]University Medical Centre, Department of Neurosurgery, Ljubljana, Slovenia*
*[5]Institute of Pathology, Medical Faculty, Ljubljana, Slovenia*

**Introduction**
Glioblastoma (GB) remains a lethal disease despite current standard treatment with maximal surgical resection, radiation, and temozolomide (TMZ) therapy. One aspect that hinders drug development and study of GB therapeutic response is the lack of an appropriate model that represents the complexity of patients' tumours. To address these limitations, we investigated the effects of irradiation and TMZ using novel GB organoids (GBOs) that reflect the tumour heterogeneity and resistance to clinical therapies.
**Material and Methods**
We established 3D *in vitro* model of GBOs prepared from patient-derived tumours and stored in the Slovenian GlioBank, which contains glioma tissue samples, cell models, and associated clinical data. We compared patient-derived GBOs with the original tumour tissue by immunofluorescence staining of cell type markers and qPCR analysis of the expression of selected target genes. The therapeutic response of GB was analysed in GBOs from 11 patients, exposed to a single dose of irradiation (IR) (10 Gy), a one-week treatment with TMZ (50 μM), or a combination of both. The effects of therapy were assessed by viability and invasion assays. Expression levels of a number of genes related to GB subtypes, epithelial-mesenchymal transition, stem cells, DNA damage responses, cell cycle, cytokines, and immune cell markers were compared between treated and untreated GBOs.
**Results and Discussions**
Organoids recapitulated the cellular composition and gene expression patterns of the original tumour tissue. We confirmed the presence of GB stem and differentiated GB cells along with other cells of the tumour microenvironment (TME), e.g., macrophages and microglia, lymphocytes, and endothelial cells. We demonstrated that GBOs from most patients were resistant to IR and TMZ, as no significant effects on GBO viability or invasion were observed. Further analysis revealed that certain target genes were differentially expressed in the treated GBOs, such as E3 ubiquitin-protein ligase (MDM2), cyclin-dependent kinase inhibitor 1A (CDKN1A), and serine/threonine kinases ATM and ATR. Our results suggest activation of p53-related signalling pathways in the TME of GB tumours and shed light on possible mechanisms underlying GB therapy resistance.
**Conclusion**
Patient-derived GB organoids recapitulate the key features and complex composition of patient tumour tissues and provide a clinically relevant *in vitro* cell culture system for assessing the specific responses of GB patients to therapy.

### EACR23-1018
### CCR7-PI3Kgamma signaling supports persistence and resistance to tyrosine kinase inhibitors in ALK-rearranged lymphoma

*C. Voena[1], C. Mastini[1], G. Mura[1], M. Campisi[2], C. Ambrogio[1], E. Patrucco[1], S. D. Turner[3], C. Gambacorti-Passerini[4], E. Hirsch[1], R. Chiarle[1]*
*[1]University of Torino, Department of Molecular Biotechnology and Health Scienc es, TORINO, Italy*
*[2]Dana Farber Cancer Institute, Dana Farber Cancer Institute, Boston, United States*
*[3]University of Cambridge, Department of Pathology, Cambridge, United Kingdom*
*[4]University of Milano-Bicocca, Department of Medicine and Surgery, Monza, Italy*

**Introduction**
The ALK TKI crizotinib has shown therapeutic efficacy for chemotherapy-refractory/ relapsed ALK+ Anaplastic Large Cell Lymphoma (R/R ALK+ ALCL) and has been approved by FDA in 2021. However, in patients that achieve complete remission, crizotinib discontinuation causes rapid disease relapses, likely due to a rapid expansion of drug-resistant persister lymphoma cells. In the early phase of the disease, ALK+ ALCL cells colonize the intravascular and perivascular spaces in lymph nodes, supporting the idea that pro-survival signals originate from the microenvironment. We investigated the contribution of the perivascular niche in ALK+ ALCL persistence.
**Material and Methods**
We performed RNA-seq analysis on ALK+ALCL patient samples who developed resistance to ALK TKIs and crizotinib-resistant ALK+ ALCL cells generated *in vitro*. We performed scRNA-seq analysis in ALK+ ALCL primary samples and patient-derived xenografts (PDX). We developed a 3D microfluidic chip of the perivascular niche and evaluated cell viability in the presence of ALK TKIs.
**Results and Discussions**
We found that the perivascular niche induced a pro-survival pathway mediated by PI3Kγ-dependent CCR7 signaling that is derepressed during ALK TKI treatment. Resistant ALCL cells showed a consistent upregulation of PI3Kγ. We demonstrated that PI3Kg over-expression reduced TKI sensitivity in ALCL cell lines and in NPM-ALK transgenic mice the expression of a constitutively active PI3Kγ accelerated ALK-mediated lymphomagenesis. The expression of PI3Kγ, PI3Kδ and CCR7 were suppressed by ALK activity via STAT3 and

© 2023 The Authors. Molecular Oncology is published by John Wiley & Sons Ltd on behalf of Federation of European Biochemical Societies restored by ALK inhibition or degradation that caused a CCR7/ PI3Kγ-dependent activation of the MAPK pathway for cell survival. In a 3D microfluidic chip, endothelial cells that secrete the CCR7 ligands CCL19/21 protected $CCR7^{WT}$ ALCL cells from crizotinib-induced apoptosis. The protective effect was lost in $CCR7^{KO}$ ALCL cells. In the 3D model, both the PI3Kγ/δ inhibitor duvelisib and the PI3Kγ inhibitor eganelisib increased the efficacy of crizotinib and in ALCL patient-derived xenografts (PDX), combining duvelisib and crizotinib conferred a prolonged disease-free survival than crizotinib alone.

**Conclusion**

ALK+ ALCL cells exploit the upregulation of CCR7/ PI3Kγ to engage survival signals from the microenvironment during ALK-signaling blockade. The disruption of this survival axis by PI3Kγ inhibition or CCR7 knockout potentiates the efficacy of ALK TKIs supporting the use of combination therapies to eradicate persistent ALCL.

### EACR23-1025
### PROLINE DEHYDROGENASE AS A PLAYER IN ACQUIRED RESISTANCE TO TYROSINE KINASE INHIBITORS

*P. CHIOFALO[1], E. Berno[1], R. Cinquetti[1], V. Ciaramella[2], C.M. Della Corte[2], F. Morgillo[2], A. Bruno[1], M. Gallazzi[3], M.T. Palano[3], P. Campomenosi[1]*

*[1]University of Insubria, Biotechnology and Life Sciences, VARESE, Italy*

*[2]University of Naples "L. Vanvitelli", Department of Precision Medicine, Naples, Italy*

*[3]IRCCS MultiMedica, Unit of Molecular Pathology- Biochemistry and Immunology, Milan, Italy*

**Introduction**

Lung cancer is one of the biggest killers among tumors. Lung adenocarcinoma (LUAD) is the prevalent subtype and is characterized by recurrent molecular alterations, including EGFR mutations. This makes possible targeted therapy with Tyrosine-Kinases Inhibitors (TKIs). Invariably, the onset of resistance leads to cancer progression. Prognostic and predictive biomarkers would be beneficial to identify in advance patients outcome and response to therapy.

Proline dehydrogenase (PRODH) is a mitochondrial flavoenzyme, catalyzing proline oxidation. Its activity influences biological processes like senescence, apoptosis, and cell survival via ROS or ATP production.

Elevated PRODH expression was observed in LUAD clinical samples, but not in LUAD cell lines. However, we found that PRODH expression was increased in EGFR-mutant lung cell lines with *in vitro* acquired resistance to TKIs. We hypothesized that PRODH may be involved in this process, influencing metastasizing ability and survival of lung cancer cells, and the tumor microenvironment. Moreover, we aimed to test if mutant EGFR or activation of its downstream signaling pathways could modulate PRODH expression.

**Material and Methods**

PC9 and HCC827 cell lines, both harboring EGFR exon 19 deletion, and their derivatives PC9-OR and HCC827-GR5, with *in vitro* acquired resistance to Osimertinib or Gefitinib, respectively, were used to investigate the role of PRODH in the onset of TKIs resistance.

PRODH was silenced or inhibited to study its role in cell survival and migration ability in PC9-OR and HCC827-GR5. Transfection experiments with EGFR or STAT3 expression constructs were used to investigate direct regulation of PRODH expression. Secretome analyses were performed.

**Results and Discussions**

PRODH transcript and protein expression was increased in EGFR-mutant LUAD cell lines with *in vitro* acquired resistance to TKIs. The increase in transcript was not observed upon ectopic expression of EGFR activating mutations or constitutively active STAT3C. Secretome analyses showed that PRODH ectopic expression increased the levels of several cytokines involved in macrophage recruitment and polarization, and in angiogenesis. Ongoing PRODH inhibition or silencing experiments will clarify its contribution to the different phenotypes of TKI-resistant cells.

**Conclusion**

Although preliminary, our results suggest that PRODH may play a role in acquired resistance to TKI and that it may be used as a prognostic and/or predictive biomarker during therapy in TKI-resistant LUAD.

### EACR23-1031
### Exploring LSD1 inhibition as a novel therapeutic strategy to overcome proteasome inhibitors resistance in multiple myeloma and other B-cell malignancies

*C. Bandini[1], E. Mereu[1], T. Paradzik[2], M. Labrador Granados[1], M. Maccagno[1], M. Cumerlato[1], L. Prever[1], V. Manicardi[3], F. Gay[4], G. Merlo[1], E. Hirsch[1], A. Ciarrocchi[3], A. Neri[5], R. Piva[1]*

*[1]University of Torino, Department of Molecular Biotechnology and Health Sciences, Torino, Italy*

*[2]Rudjer Boskovic Institute, Department of Physical Chemistry, Zagreb, Croatia*

*[3]Azienda Unità Sanitaria Locale-IRCCS, Laboratory of Translational Research, Reggio Emilia, Italy*

*[4]Città Della Salute e della Scienza Hospital, Department of Molecular Biotechnology and Health Sciences, Torino, Italy*

*[5]University of Milano, Department of Oncology and Hemato-oncology, Milano, Italy*

**Introduction**

Despite the success of proteasome inhibitors (PIs), like carfilzomib (CFZ) and bortezomib (BTZ) in treating multiple myeloma (MM) and mantle cell lymphoma (MCL), innate and/or acquired resistance and toxic side effects remain major challenges for therapy menagment. Finding new compounds that can work synergistically with PIs to overcome resistance and broaden their applicability to other B-cell malignancies or solid tumors is crucial.

**Material and Methods**

We conducted genetic and drug screens to identify new synthetic lethal (SL) partners to PIs, and validated candidates in PI-sensitive and PI-resistant MM cells. We also tested promising candidate in other B-cell

© 2023 The Authors. Molecular Oncology is published by John Wiley & Sons Ltd on behalf of Federation of European Biochemical Societies malignancies, such as MCL, Burkitt's and diffuse large B-cell lymphomas (BL and DLBCLs). We evaluated the toxicity of combination treatments in normal peripheral blood mononuclear cells (PBMCs) and bone marrow stromal cells (BMSCs). We further confirmed the synergistic effects of the combination treatment ex vivo in primary CD138+ cells from MM patients, and in vivo in different MM xenograft models. We used RNA-Seq and Reverse-Phase Protein Arrays (RPPA) to investigate the molecular mechanisms of the synergy.

**Results and Discussions**

Our analysis identified lysine-specific demethylase 1 (LSD1) as a top candidate whose inhibition can synergize with CFZ treatment. LSD1 silencing enhanced CFZ sensitivity in both PI-resistant and -sensitive MM cells, resulting in increased tumor cell death. Small-molecule LSD1 inhibitors, such as SP2509, SP2577, and CC-90011, triggered synergistic cytotoxicity in combination with different PIs in MM and other B-cell tumors. CFZ/SP2509 exhibited no side effects on PBMCs and BMSCs. The synergistic/additive effects were also confirmed in primary $CD138^+$ cells, mice, and zebrafish MM xenograft models, where combo treatment impaired tumor growth. We found that DNA damage and cell cycle checkpoint signaling were the most affected pathways by CFZ/SP2509 combined treatment.

**Conclusion**

The present study preclinically demonstrated that LSD1 inhibition could provide a valuable strategy to enhance PI sensitivity and overcome drug resistance in MM patients and that this combination might also be exploited for the treatment of other B-cell malignancies, thus extending the therapeutic impact of the project. Further studies are needed to explore the clinical potential of this combination therapy.

### EACR23-1060
### Caveolae-dependent endocytosis is responsible for the selective effect of Silver Nanoparticles in Prostate Cancer Sensitive and Resistant to Androgen Deprivation Therapy

*M. Morais*[1,2], *P. Figueiredo*[3], *F. Dias*[1], *I. Tavares*[1,2], *K. S. Mikkonen*[3,4], *A.L. Teixeira*[1], *R. Medeiros*[1,2,5,6,7]
[1]*Research Center of IPO Porto CI-IPOP- Portuguese Oncology Institute of Porto IPO Porto, Molecular Oncology and Viral Pathology, Porto, Portugal*
[2]*University of Porto, ICBAS-Abel Salazar Institute for the Biomedical Sciences, Porto, Portugal*
[3]*Faculty of Agriculture and Forestry- FI-00014-University of Helsinki, Department of Food and Nutrition, Helsinki, Finland*
[4]*University of Helsinki, Helsinki Institute of Sustainability Science HELSUS FI-00014-, Helsinki, Finland*
[5]*Faculty of Health Sciences- Fernando Pessoa University UFP, Biomedical Reasearch Center CEBIMED, Porto, Portugal*
[6]*LPCC- Portuguese League Against Cancer NRNorte, Research Department, Porto, Portugal*
[7]*University of Porto, FMUP- Faculty of Medicine, Porto, Portugal*

**Introduction**

Castration-resistant prostate cancer(CRPC) is the most aggressive and poorer prognosis PC form, characterized by resistance to androgen deprivation therapy(ADT) and few therapeutic options. Silver nanoparticles(AgNPs) were shown cytotoxic against 2 CRPC cell lines(PC-3 and Du-145), but not against an ADT sensitive one(LNCaP). Uptake is a key influencer in drug's efficacy. Among endocytosis pathways, the caveolae-dependent raises interest since caveolins(CAVs) levels are higher in CRPC patients' plasma. This study aims to understand the caveolae-dependent endocytosis' role in the differences observed in AgNPs cytotoxicity.

**Material and Methods**

AgNPs were added to PC-3, Du-145 and LNCaP and their location was assessed by TEM. CAV1 and CAV2 mRNA and protein levels were assessed using q-PCR and western-blot. Caveolae-mediated endocytosis was inhibited using Genistein. After, 1500 μg/mL (PC-3) or 2500 μg/mL (Du-145) AgNPs were added for 24h and cells' viability was assessed by resazurin assay. Similarly, CAV1 and CAV2 were inhibited by siRNA transfection and, 24h after AgNPs addition, the viability was assessed. LNCaP cells were transfected with CAV1 and CAV2 plasmid, and 24h after 2500 μg/mL AgNPs addition, the viability was assessed.

**Results and Discussions**

TEM showed that AgNPs were located in different organelles of CRPC cells but outside of LNCaP. CAVs were upregulated in CRPC cells compared with LNCaP. In PC-3, AgNPs addition after: genistein treatment led to 70.61% viability($p<0.001$); CAV1 inhibition led to 61.25% viability($p=0.008$); CAV2 inhibition led to 59.32% viability($p<0.001$); CAV1+2 inhibition led to 54.76% viability($p=0.004$); compared with 29.78% viability in cells not inhibited. In Du-145, AgNPs addition after: genistein treatment led to 83.78% viability($p<0.001$); CAV1 inhibition led to 93.79% viability($p<0.001$); CAV2 inhibition led to 100% viability($p<0.001$); CAV1+2 inhibition led to 61.82%($p<0.001$) viability; compared with 26.99% viability in cells not inhibited. In LNCaP, AgNPs addition after induction of CAV1+2 led to 57.02%($p<0.001$) viability compared with 100% viability in cells not inducted.

**Conclusion**

We have demonstrated that AgNPs enter PC cells through caveolae mediated endocytosis, showing that this pathway is vital to AgNPs uptake and selective target of CRPC cells. This highlights the key role of uptake pathways in drug's efficacy. Validation studies are needed, but this preliminary research shows AgNPs could be potential future therapeutic agents to be considered in CRPC.

### EACR23-1106
### BRCA2 Germline Mutations Identify Gastric Cancers Responsive to PARP Inhibitors

*S. Giordano*[1,2], *A. Petrelli*[1], *S. Rizzolio*[2], *C. Orru'*[1,2], *S. Corso*[1,2]
[1]*UNITO, oncology, candiolo Torino, Italy*
[2]*Candiolo Cancer Institute- FPO-IRCCS, Molecular biology of cancer lab, Candiolo torino, Italy*

**Introduction**

© 2023 The Authors. Molecular Oncology is published by John Wiley & Sons Ltd on behalf of Federation of European Biochemical Societies

Despite negative results of clinical trials conducted on the overall population of gastric cancer (GC) patients, PARP inhibitor (PARPi) therapeutic strategy still might represent a window of opportunity for a subpopulation of GC patients. An estimated 7-12% of gastric cancers exhibit a mutational signature associated with homologous recombination (HR) failure, suggesting that these patients could potentially benefit from PARPi.

**Material and Methods**

To analyze responsiveness of GC to PARPi, we exploited a gastro-esophageal adenocarcinoma (GEA) platform of patient-derived xenografts (PDXs) and PDX-derived primary cells and selected 10 PDXs with loss-of-function mutations in HR pathway genes.

**Results and Discussions**

Cell viability assays and preclinical trials showed that olaparib treatment was effective in PDXs harboring *BRCA2* germline mutations and somatic inactivation of the second allele. Olaparib responsive tumors were sensitive to oxaliplatin as well. Evaluation of HR deficiency (HRD) and mutational signatures efficiently stratified responder and non-responder PDXs. A retrospective analysis on 57 GEA patients showed that *BRCA2* inactivating variants were associated with longer PFS upon platinum-based regimens. Trying to explain primary resistance in non-responsive cases, we hypothesized that it could be associated with the MSI status, since resistant tumors were genetically similar to responsive ones but had MSI-high status. In order to verify the possible association between a MSI status and insensitivity to PARP inhibition, we inactivated the *MLH1* gene in PARPi responsive cells, by means of CRISPR–Cas9 mediated genome editing. *MLH1* inactivation led to loss of sensitivity to PARPi but not to oxaliplatin. This was not a gastric cancer specific effect, as we observed it also pancreatic carcinoma cell lines.

Five out of 7 patients with *BRCA2* germline mutations carried the p.K3326* variant, classified as “benign”. However, familial history of cancer, the absence of RAD51 foci in tumor cells and a high HRD score suggest a deleterious effect of this mutation in gastric cancer.

**Conclusion**

PARP inhibition is a potential strategy for treating gastric cancer patients with mutated *BRCA2* or homologous repair deficiency, including familial intestinal gastric cancer patients, for whom *BRCA2* germline testing should be recommended.

### EACR23-1107

### In Pursuit of Novel Cancer Therapies: Identification of the Transcriptional Regulators of ATP7B gene by Genomic Locus Proteomics and Their Effect on Cisplatin Resistance

*A. Açar[1], B. Altay[1], B. Sergi[1], S.C. Ozcan[2], K. Kiselyov[3], C. Açılan Ayhan[4]*

*[1]Koç University, Graduate School of Health Sciences, İstanbul, Turkiye*

*[2]Koç University, Research Center for Translational Medicine, İstanbul, Turkiye*

*[3]University of Pittsburgh, Department of Biological Sciences, Pittsburgh, United States*

*[4]Koç University, Research Center for Translational Medicine & School of Medicine, İstanbul, Turkiye*

**Introduction**

ATP7B is a $Cu^+$ transporting ATPase, which gets shuttled from Golgi to lysosomes when $Cu^+$ levels are increased in the cell and eventually remove $Cu^+$ via exocytosis. Not surprisingly, this important function requires tight regulation, and changes in ATP7B activity is linked to several diseases. Lack of its activity disrupts the copper metabolism and leads to Wilson’s disease. On the other hand, elevated ATP7B levels leads to expelling platinum-based drugs along with $Cu^+$, such as cisplatin and result in chemotherapeutic drug resistance. Therefore, understanding how ATP7B is regulated is crucial to shed light on the physiology of these conditions.

**Material and Methods**

To investigate the transcription factors regulating ATP7B gene expression, we initially performed an in-silico analysis using TRANSFAC/PROMO software and have found that several transcription factors may bind to ATP7B promoter, which were focused around -3000 to +1 region. To investigate which of the predicted factors bind to the ATP7B promoter, the Genomic Locus Proteomics methodology was used. In this technique, deadCas9 (dCas9) protein is fused to the APEX2 enzyme and guided to the ATP7B promoter via specific gRNAs. The efficient gRNAs spanning -3000 to +1 region were determined by T7E assay and chromatin immunoprecipitation (ChIP). Then, using the gRNAs with the best targeting efficiency, we identified the biotinylated the proteins adjacent to the target region with dCas9APEX2 system followed by mass spectrometry analysis.

**Results and Discussions**

Depending on their enrichment scores and whether they have a previously described function associated with cancer progression, we chose several transcription factors and investigated their role in the regulation of ATP7B gene expression. To test this, the candidate proteins (ATF3, LEF1, PINX1, POU3F2, PRDM16, TOX4, TP53) were either overexpressed or knocked out via CRISPR-Cas9 technology. We successfully showed that these transcription factors could alter ATP7B expression and tested their ability to revert cisplatin resistance or sensitize cells to cisplatin. We are currently at the stage of confirming the binding of these factors on ATP7B promoter region via ChIP assay.

**Conclusion**

In summary, the results from our study will illuminate not only the mechanism of ATP7B gene regulation but also how the dysregulation of ATP7B result in physiological disorders. Targeting the regulators of ATP7B gene would have translational effects in overcoming chemotherapeutic drug resistance in cancers.

### EACR23-1108

### Identification of resistance mechanisms for the combination of SHP2 and ERK inhibitors in KRAS-mutant Pancreatic Cancer

*A. Mulero-Sánchez[1], A. Bosma[1], C. Lieftink[1], R. Beijersbergen[1], R. Bernards[1], S. Mainardi[1]*

© 2023 The Authors. Molecular Oncology is published by John Wiley & Sons Ltd on behalf of Federation of European Biochemical Societies

[1]*Netherlands Cancer Institute, Molecular Carcinogenesis, Amsterdam, The Netherlands*

**Introduction**

Over 90% of pancreatic tumors carry a driving mutation in the KRAS oncogene, which is also altered in 30-40% of lung and colorectal cancers, thus representing an ideal target for precision oncology. Nevertheless, despite the recent emergence of isoform-specific KRASG12C-inhibitors, most mutant KRAS isoforms, including the ones frequently associated with pancreatic ductal adenocarcinoma (PDAC), cannot be targeted directly in the clinical setting. We previously discovered that tumors carrying activating KRAS mutations are sensitive to the inhibition of SHP2, a phosphatase helping the transmission of the growth-promoting signals from the cell surface receptors to RAS. Moreover, SHP2 inhibitors cooperate with inhibitors of the RAS downstream effectors MEK and ERK to achieve superior disruption of the MAPK pathway, increased apoptosis and better tumor growth control, in the absence of *in vivo* toxicity. Based on those compelling results, we begun to investigate the combination of the SHP2 inhibitor RMC4360 with the ERK inhibitor LY3214996, in a Phase I/Ib clinical trial (SHERPA, **SH**P2 and **ER**K inhibition in **pa**ncreatic cancer, NCT04916236).

**Material and Methods**

In order to identify potential resistance mechanisms, which could lead to useful dynamic biomarkers of resistance, we performed a series of unbiased CRISPR-based enrichment screenings. In parallel, we derived spontaneously resistant cell lines.

**Results and Discussions**

Sequential genome-wide and focused resistance screenings led to the identification of two major resistance mechanisms: PTEN loss, leading to the activation of the PI3K/AKT/mTOR pathway, and suppression of the DET1/COP1 complex, leading to stabilization and hyperactivation of c-JUN.

In parallel, spontaneous resister cell lines also demonstrated increased PI3K/AKT/mTOR pathway and c-JUN activation, which interestingly co-existed in single cell-derived monoclones, suggesting a convergence or intersection between the two pathways.

Additionally, we found that the increased dependency of the resistant cells to the mTOR pathway represents a new vulnerability, that could potentially be exploited therapeutically with a sequential mTOR inhibitor intervention.

**Conclusion**

In conclusion, alteration of nodes in the PI3K/AKT/mTOR and in the c-JUN pathway may serve as markers for sensitivity/resistance to the drug combination investigated in the SHERPA trial. Ongoing *in vivo* studies are exploring the feasibility of a sequential treatment using mTOR inhibitors after tumors have relapsed upon SHP2 + ERK inhibition.

### EACR23-1120

### Gene expression signature as a predictive tool to select between target therapy or immunotherapy in BRAF-mutated cutaneous melanoma patients

*C. Solé-Blanch*[1], *S. España*[1,2], *A. Bernat-Peguera*[1], *A. Boada*[3], *A. Quer*[4], *L. Perez-Roca*[5], *M. Gonzalez-Cao*[6], *J.L. Manzano*[1,2], *A. Martínez-Cardús*[1]

[1]*Germans Trias i Pujol Research Insitute IGTP, Badalona Applied Research Group in Oncology B·ARGO-CARE Program, Badalona, Spain*

[2]*Catalan Institute of Oncology ICO Badalona, Medical Oncology Service, Badalona, Spain*

[3]*Hospital Universitari Germans Trias i Pujol, Dermatology Department, Badalona, Spain*

[4]*Hospital Universitari Germans Trias i Pujol, Pathology Department, Badalona, Spain*

[5]*Hospital Universitari Germans Trias i Pujol, Tumor Bank, Badalona, Spain*

[6]*Translational Cancer Research Unit, Instituto Oncológico Dr Rosell- Dexeus University Hospita l, Barcelona, Spain*

**Introduction**

In the current clinical practice, BRAF-mutated metastatic melanoma patients have two therapeutic options available at first-line of treatment: target therapy (BRAFi+MEKi) or immunotherapy (ITH). However, there is no molecular or translational evidence to choose between these two therapies and only clinical parameters are taken into account. In this context, we identified a gene expression signature (GHR, CCL21 and CXCL8) associated with clinical benefit, oppositely in both treatment cohorts. Hence, we have analyzed the impact of these biomarkers on survival, individually and in combination, to elucidate their robustness for predicting the best therapeutic option.

**Material and Methods**

Candidate genes were identified using Nanostring PanCancer IO 360 panel and nsolver software comparing high versus low clinical benefit (CB) groups, in 19 paraffin-embedded tumor tissues from BRAF-mutated melanoma patients treated with target therapy and 8 treated with immunotherapy. These genes have been validated by qPCR in the same cohort. Then, their expression has been correlated with progression-free survival (PFS) and overall survival (OS) using Kaplan-Meier plots and log-rank function. Cox Regression Univariate and Multivariate analysis have also been performed. P-value under 0.05 was considered statistically significant.

**Results and Discussions**

By using survival analysis, GHR downregulation is associated with a better outcome in BRAFi+MEKi cohort (p-value=0.01), also showing an opposite trend in the ITH cohort. Low levels of CXCL8 correlate with better outcome in the latest (p-value=0.07). CCL21 downregulation is related to good response in the BRAFi+MEKi cohort (p=0.01) and show an opposite trend in the ITH cohort. Interestingly, when all the genes of the signature are analyzed together, they show a higher capacity to predict survival in both cohorts (p-value=0.0003).

**Conclusion**

In conclusion, expression of GHR, CXCL8 and CCL21 predict treatment outcome in an opposite manner in both cohorts. Moreover, their predictive power increase when they are analysed as a signature, being postulated as biomarkers for selecting the best therapeutic choice. However, further validations in a prospective cohort are warranted to elucidate the clinical potential of this signature.

© 2023 The Authors. Molecular Oncology is published by John Wiley & Sons Ltd on behalf of Federation of European Biochemical Societies

### EACR23-1160
### Resistance Mechanisms to c-MET inhibition in Hepatocellular Carcinoma

R. Qian[1], W. Cun[1]
[1]State Key Laboratory of Oncogenes and Related Genes, Shanghai Cancer Institute, Shanghai, China

**Introduction**

c-MET has emerged as a promising therapeutic target for hepatocellular carcinoma (HCC), and several clinical trials of c-MET inhibitors for HCC are currently ongoing globally. Capmatinib and tepotinib are two recently approved c-MET selective inhibitors by the FDA in 2020. However, the development of resistance in single-agent therapies is an inevitable challenge in clinical applications, which highlights the need to understand the mechanism of c-MET inhibitors resistance.

**Material and Methods**

A panel of HCC cell lines was treated with increasing concentrations of c-MET inhibitors. To systematically identify the kinase whose inhibition confers resistance to capmatinib in primary sensitive HCC cells, we conducted a CRISPR-Cas9 functional genetic screen. Multiple long-term colony formation assays and western blot were used to study the synergistic effects of the combination of c-MET inhibitors and other potential therapeutic inhibitors.

**Results and Discussions**

We identified that *PTEN* knockout leads to robust resistance to c-MET inhibitors in MHCC97H and HCCLM3 cells. Molecular mechanism studies showed that the level of AKT phosphorylation can serve as a biomarker for the response to c-MET inhibitors. Furthermore, suppression of c-MET in HCC leads to feedback activation of upstream receptor tyrosine kinases ERBB2 and ERBB3, which in turn upregulate the AKT signaling pathway and confer resistance to c-MET inhibitors. Therefore, drugs that inhibit both ERBB2 and ERBB3 can reverse unresponsiveness to c-MET inhibitors in c-MET-resistant HCC cells.

**Conclusion**

Our findings demonstrated the resistance mechanism for c-MET inhibitors in HCC. More importantly, we propose following solutions for HCC cell lines which are resistant to c-MET inihibitors: (i) combination of c-MET inhibitors and AKT inhibitor MK2206; (ii) combination of c-MET inhibitors and ERBB2/ERBB3 inhibitors Afatinib or Dacomitinib.

### EACR23-1164
### Nrf2 role in the BRAFi/MEKi acquired resistance in melanoma

M.A. Cucci[1], M. Grattarola[1], C. Monge[2], A. Roetto[1], C. Dianzani[2], S. Pizzimenti[1]
[1]University of Turin, Clinical and Biological Science, Torino, Italy
[2]University of Turin, Scienza e Tecnlogia del Farmaco, Torino, Italy

**Introduction**

Melanoma is one of the most aggressive cancers with the poorest prognosis. However, the use of specific inhibitors towards mutant BRAF (BRAFi) and MEK (MEKi) in BRAF-mutated patients has significantly improved progression-free and overall survival. Nevertheless, half of the patients still develop resistance within the first year of therapy. Therefore, understanding the mechanisms of BRAFi/MEKi acquired resistance has become a priority for researchers. In these last few years, scientists have focused on the role of NF-E2-related factor 2 (Nrf2), the master regulator of the cytoprotective and antioxidant response, in acquired chemoresistance. Indeed, its expression and activity are upregulated in various cancer types resistant to several chemotherapic drugs. The aim of this study was to evaluate the contribution of Nrf2 in the BRAFi/MEKi acquired resistance in melanoma, as well as the mechanisms of its activity regulation.

**Material and Methods**

Starting from BRAF-mutated murine melanoma cell line D4M, we generated three subclones resistant to BRAFi (dabrafenib, D), MEKi (trametinib, T), and BRAFi/MEKi (double resistance, D+T). Then, we evaluated cell viability (MTT test), anchorage-independent cell growth (Sphere Formation and Soft Agar Assays), apoptosis (Annexin V/IP), Cell invasion (Transwell Boyden chamber), angiogenesis (Tube-Formation assay), intracellular oxidative stress (2′-7′-dichlorodihydrofluorescein diacetate, DCF-DA, assay), glutathione (GSH) levels (Ellman's method), gene expression (western blot, real-time PCR), and gene expression inhibition with specific siRNA.

**Results and Discussions**

After nine months of continuous treatments with D, T or D+T, we obtained the three resistant subclones. Compared with the sensitive clone, the resistant sublines showed higher resistance to D, T, or D+T treatments and an enhanced ability to anchorage-independent cell growth with increased migration and angiogenic capacity. These results allowed us to consider these cell lines good models of resistant melanoma cells toward targeted therapies. These cells showed increased oxidative stress and GSH levels. Nrf2 was upregulated at post-translational levels, with the involvement of deubiquitinase 3 (DUB3). Interestingly, Nrf2 or DUB3 inhibition sensitised cells to targeted therapies.

**Conclusion**

Nrf2 can contribute to the mechanism of targeted resistance in melanoma. A complete understanding of its role can contribute to developing increasingly effective therapies in advanced melanoma.

### EACR23-1171
### Cross-talk between TNBC cells and adipose mesenchymal stem cells contributes to maintaining a hostile acidic microenvironment and increases PDL-1 expression

A. Sarnella[1], Y. Ferrara[2], M.G. Caprio[2], L. Cerchia[3], G. De Simone[2], C.T. Supuran[4], A. Zannetti[2]
[1]Istituto di Biostrutture e Bioimmagini - Consiglio Nazionale delle Ricerche, National Research Council, Napoli, Italy
[2]Institute of Biostructures and Bioimaging-CNR, National Research Council, Napoli, Italy
[3]Institute of Experimental Endocrinology and Oncology "Gaetano Salvatore", National Research Council, Napoli, Italy
[4]Università di Firenze, Dipartimento NEUROFARBA- Sezione di Scienze Farmace

© 2023 The Authors. Molecular Oncology is published by John Wiley & Sons Ltd on behalf of Federation of European Biochemical Societies

*utiche, Firenze, Italy*

**Introduction**

Treatment strategies involving immune-checkpoint inhibitors (ICIs) have significantly improved survival for a subset of patients across a broad spectrum of advanced solid cancers including triple negative breast cancer (TNBC). Despite this, many patients fail to respond or develop resistance, suggesting that a significant population of patients stand to benefit from enhancing response rates to ICIs**.** The tumor microenvironment (TME) is a barrier to immune function, as the altered metabolism-related acidic microenvironment of solid tumors reduces immune activity. Crosstalk between tumor cells and stromal cells contributes to maintaining a hostile acidic TME. Therefore, in this study we investigated the role played by "tumor educated" adipose mesenchymal stem cells (TE-ADSCs) in supporting immune surveillance in TNBC through upregulation of hypoxia-induced carbonic anhydrase IX (CA IX), a well-known cell-surface enzyme involved in pH regulation of aggressive solid tumors. In addition, we evaluated the possibility of sensitizing TNBC to immunotherapy using the CA IX inhibitor SLC-0111 (in Phase Ib/II clinical trials).

**Material and Methods**

ADSCs were co-cultured with TNBC cell lines, MDA-MB-231 and BT-549, using a Boyden chamber with a porous membrane of 3 mm and vice versa. A conditioned medium (CM) obtained from the co-cultured cells was collected. The expression levels of CA IX, PDL-1, p-STAT3/STAT3, CD44, and Glut-1 were analyzed by western blotting in tumor and stromal cells. The expression levels of CA IX were silenced by using a specific siRNA. The induction of TNBC extracellular acidosis by TE-ADSCs and the ability of CA IX inhibitor, SLC-0111, to reverse it were analyzed by fluorometric pH assay.

**Results and Discussions**

When ADSCs were co-cultured with TNBC cell lines, MDA-MB-231 and BT-549, increased expression levels of CA IX were observed. CM obtained from ADSCs co-cultured with TNBC cell lines increased the expression levels of PDL-1, CA IX, and CD44 in MDA-MB-231 and BT-549, compared with CM obtained from naïve ADSCs. Inhibition of CA IX by SLC-0111 reduced the expression levels of PDL-1, p-STAT3, and Glut-1, and reversed TE-ADSC-induced acidic TME in TNBC cells.

**Conclusion**

Taken together our results demonstrate the crucial role played by TE-ADSCs in promoting immune surveillance by inducing an acidic TME in TNBC. Furthermore, we highlight the possibility to sensitize tumor cells to ICIs by targeting CAIX using SLC-0111.

### EACR23-1173
### The HMGA1/phospho-NUMB signaling pathway mediates the response of glioblastoma stem cells to hyperglycemia and caloric restriction mimetics.

*Y. Kuzay[1,2], S. Morlando[1], F. D'Alessio[1], T. Savarese[1], M. Fedele[2], A. Fusco[1], S. Battista[2]*

*[1]Facoltà di Medicina e Chirurgia- Università degli Studi " Federico II", Dipartimento di Biologia e Patologia Cellulare e Molecolare-, Naples, Italy*

*[2]CNR, Istituto di Endocrinologia ed Oncologia Sperimentale IEOS, Naples, Italy*

**Introduction**

Hyperglycemia (HG) and hypercaloric diet predispose to cancer and enhance drug resistance and mortality in many types of cancers, including glioblastoma (GBM), through not completely known mechanisms.

Brain tumor stem cells (BTSCs), which are responsible for GBM onset and resistance to therapy, show increased symmetric divisions (SDs) at the expenses of asymmetric divisions (ADs), leading to neoplastic transformation. NUMB phosphorylation regulates stem cell (SC) division mode. Recently, HG has been shown to downregulate NUMB in cancer cells, leading to radioresistance; conversely, NUMB upregulation inhibits hyperglycemic memory and radioresistance.

HMGA1 is an architectural transcription factor with a central role in stemness, cancerogenesis and drug resistance. High glucose levels favor HMGA1 binding to responsive promoters, stimulating transcriptional activation.

The pathways converting nutritional inputs into SC division mode decisions are not completely known: a better comprehension of these mechanisms would clarify the role of diet on cancer risk and may help to design dietetic strategies for cancer prevention and treatment.

**Material and Methods**

Two BTSC lines were silenced for HMGA1 expression using shRNAs and grown as tumor-spheres.

Paired-cell assays were performed to quantify SD/ADs, by pulse-chasing cells with BrdU, synchronizing them with nocodazole/thymidine/blebbistatin and performing immunofluorescences with anti-BrdU and anti-NUMB antibodies.

Cells were cultured in standard high-glucose medium conditions or in media reduced in insulin, glucose or growth factors, and protein expression was determined by Western blot.

**Results and Discussions**

We demonstrate that silencing of HMGA1 in BTSCs upregulates phospho-NUMB expression and recovers the ability to perform ADs.

In these cells, caloric restriction mimicking conditions also increase pNUMB expression, which appears to be a faithful sensor of nutrient conditions. Similarly, glucose reduction induces modifications in cell morphology and increases p-NUMB levels, which resulted to be inversely related to HMGA1 expression, suggesting a possible role of HMGA1 in hyperglycemic memory.

**Conclusion**

Collectively, these preliminary data provide a mechanistic explanation of the effect of glucose and nutrients on BTSC function and drug resistance and support a role for HMGA1 in converting dietetic inputs into division mode- and cell fate-determining signals, potentially linking HG to ADs impairment through NUMB regulation.

### EACR23-1182
### Characterization of New HDAC6 Inhibitors for the Treatment of Castration Resistant Prostate Cancer and Reversal of Taxane Resistance

© 2023 The Authors. Molecular Oncology is published by John Wiley & Sons Ltd on behalf of Federation of European Biochemical Societies

B. Yıldırım[1], B. Cevatemre[2], İ. Bulut[1], B.M. Kalkan[1], A. Lee[3], A. Ganesan[3], C. Acilan Ayhan[2]
[1]Koc University, Graduate School of Health Sciences, İstanbul, Turkiye
[2]Koc University, Research Center for Translational Medicine, İstanbul, Turkiye
[3]University of East Anglia, School of Pharmacy, East Anglia, United Kingdom

**Introduction**

Primary prostate cancers (PCa) have the potential to be treated with surgery and radiotherapy, but when cancer enters aggressive and metastatic "castration resistant" (CR) phase, treatment options are limited. Some patients benefit from taxane treatment at the beginning; but patients may develop drug resistance soon after. Epigenetic changes in tumors are known to cause development of resistant phenotypes and they are the potential drivers of drug resistance. There are important data showing that histone deacetylase inhibitors (HDACi's) may be useful in the treatment of CR-PCa. Among these, HDAC6 is the only HDAC, whose knockout is not lethal in mice, and its absence leads to reduction of tumor size, cellular migration, and proliferation, therefore can be used as a therapeutic target. The aim of this project is to find and characterize new HDAC6 inhibitors (HDAC6i's) that potentially synergizes with taxanes or reverses the taxane resistance in CR-PCa cells.

**Material and Methods**

We synthesized 38 HDAC6 inhibitor and tested their efficacy on CR-PCa and taxane resistant CR-PCa cells. We performed a viability screen in the presence of HDAC6i's via SRB and CTG assays. 9 of the inhibitors were picked for further testing, their efficacy was tested in vitro using HDAC6 enzyme activity assays, and their specificity was determined via their inhibition of HDAC1 and HDAC8 activity. The molecules, which failed to successfully inhibit HDAC6 or was not selective, were eliminated. Target engagement of HDAC6i in cells was determined via CETSA (Cellular Thermal Shift Assay). Their effects on invasion and migration were tested with wound healing and Matrigel invasion assays. We performed western blotting and qPCR to investigate the effect of HDAC6i on nuclear and cytosolic targets of HDAC6.

**Results and Discussions**

Three of the HDAC6 inhibitors, AF-4, AF-11 and AD-165, were more selective towards HDAC6, synergized with taxanes and partially reverted resistance in taxane resistant CR-PCa cells. Upon further analysis, we showed that while AF-4 and AF-11 did not affect the cell cycle, AD-165 effectively arrested cells in G2/M phase and exhibited the lowest $IC_{50}$ value, therefore was selected for additional studies.

**Conclusion**

Among the 38 HDAC6 inhibitors, AD-165 was the most potent HDAC6i, based on its $IC_{50}$, specificity, selectivity, and exhibition of the expected phenotypes. Currently, we are investigating the mechanism of action of AD-165 in KD-PCa through RNA-seq analysis and testing its efficacy in reduction of tumor size in mice.

### EACR23-1204
### Nanoparticles containing chemotherapeutic drugs and TLR2 inhibitors improve drug efficiency and reduce side effects in preclinical models of mammary cancer

C. Cossu[1], E. Di Gregorio[1], C. Romiti[1], A. Di Lorenzo[1], A. Amaolo[1], F. Cavallo[1], G. Ferrauto[1], L. Conti[1]
[1]Molecular Biotechnology Center "Guido Tarone", Department of Molecular Biotechnology and Health Sciences, Turin, Italy

**Introduction**

Toll-Like Receptor (TLR)2 acts as a double-edge sword in cancer. Besides its role in immune responses, TLR2 is expressed on breast cancer (BC) cells and is associated with poor prognosis in patients. We have already demonstrated that TLR2 promotes BC progression and represents a mechanism of chemoresistance, since chemotherapy induces the release of its activatory ligands by damaged cells. Indeed, TLR2 silencing or inhibition impair BC progression and restore sensitivity to chemotherapy. However, systemic chemotherapy causes side effects and requires high doses to reach an effective concentration in the tumor region. For these reasons, in this work nanosystems for combined therapy have been developed, involving both chemotherapeutic agents and TLR2 inhibitors to enhance drug delivery in the tumor region and to overcome side effects.

**Material and Methods**

Poly (lactic-co-glycolic acid) nanoparticles (PLGA-NPs) or liposomes containing chemotherapeutic agents (Docetaxel and Doxorubicin) or TLR2 inhibitor (CU-CPT22) were developed and functionalized or not with a cyclic Arg-Gly-Asp tripeptide (RGD) to target *αvβ3* integrins, overexpressed in breast cancer. Their therapeutic effect was assessed *in vitro* in breast cancer cell lines and *in vivo* in murine models.

**Results and Discussions**

Nanoparticles (NPs; both PLGA and liposomes) containing a FITC probe showed an increased internalization in breast cancer cell lines (4T1 and TUBO) *in vitro*, as shown by FACS and fluorescence microscopy experiments. Moreover, RGD-conjugated NPs enhance the chemotherapeutic effects of docetaxel and doxorubicin *in vitro*. In BALB/c mice injected subcutaneously with 4T1 cells, RGD-conjugated NPs containing docetaxel, doxorubicin or CU-CPT22 showed increased biodistribution in the tumor region and anti-tumor activity compared to non-conjugated NPs. Moreover, tumor growth in mice treated with non-conjugated NPs containing CUCPT22 was comparable to the control. The combination of NPs containing TLR2 inhibitor and chemotherapeutic drugs potentiate their anti-tumor effect *in vivo* and *in vitro*.

**Conclusion**

We demonstrated that TLR2 represent a mechanism of chemoresistance and that TLR2 inhibition can restore the sensitivity to chemotherapy. We also showed that the presence of the RGD mojety enhance tumor accumulation of the NPs helping the drugs to localize at the tumor site and to enhance their anti-tumor effects.

### EACR23-1207
### Aldo-keto reductase 1C3 as a novel off-target for the Bruton´s tyrosine kinase

© 2023 The Authors. Molecular Oncology is published by John Wiley & Sons Ltd on behalf of Federation of European Biochemical Societies

### inhibitor tirabrutinib

E. Novotná[1], L. Čermáková[1], L. Laštovičková[1], V. Wsól[1]
[1]Charles University- Faculty of Pharmacy in Hradec Kralove, Department of Biochemical Sciences, Hradec Kralove, Czech Republic

**Introduction**
Tirabrutinib is a small-molecule inhibitor of Bruton´s tyrosine kinase (BTKi) currently investigated in clinical trials for the treatment of different B-cell malignancies. In Japan, tirabrutinib has been approved to treat recurrent or refractory primary central nervous system lymphoma (PCNSL). Together with ibrutinib, tirabrutinib belongs to the effective drugs in the management of this diffuse large B-cell lymphoma subtype. Recent studies show that BTKis can enhance the cytotoxicity of anthracyclines and may improve the outcome of anthracycline-based chemotherapy, including that used to treat PCNSL[1,2,3]. However, the exact mechanism of this interaction remains to be elucidated.

**Material and Methods**
To determine whether tirabrutinib can increase the efficacy of anthracyclines by inhibiting their reductive metabolism, we screened its activity against recombinant anthracycline reductases AKR1A1, 1B1, 1B10, 1C3, and CBR1. The enzymes were expressed in *E. coli*. His-tagged proteins were purified using immobilized metal affinity chromatography. The results obtained with recombinant proteins were verified in experiments with transiently transfected cancer cells. Ultra-high performance liquid chromatography was used to detect the anthracycline metabolite daunorubicinol.

**Results and Discussions**
Tirabrutinib inhibits AKR1C3, the enzyme that converts doxorubicin and daunorubicin to their less active alcohol metabolites. In our experiments, tirabrutinib decreased the activity of recombinant AKR1C3 and was also effective at the level of intact cells. As investigated in the phase I study, the maximum serum concentration for tirabrutinib is 4.3 µM (1940 ng/ml, day 28, dose 480 mg once daily)[4]. This concentration is comparable to our experiments' inhibition constant (Ki). Therefore, it can be assumed that the effects observed in our *in vitro* experiments could also be seen in cancer patients.

**Conclusion**
The inhibition of AKR1C3 contributes to tirabrutinib mechanisms that lie beyond the targeting BTK and may affect the clinical outcome of the tirabrutinib-anthracycline combination, particularly in cancers with high expression of AKR1C3.

**Funding:** This work was supported by the InoMed project (project no. CZ.02.1.01/0.0/0.0/18_069/0010046) and by Charles University (SVV 260 550).

**References:** [1]Davies A. et al., Blood 2020, 136(Supplement 1): 38–39; [2] Lionakis M.S. et al., Cancer Cell 2017, 31(6): 833–843.e5; [3]Rotin L.E. et al., Leuk Lymphoma 2016, 57(10): 2432–2436; [4]Munakata W. et al., Cancer Sci 2019, 110(5): 1686–1964.

### EACR23-1217
### Tracing back primed resistance in ovarian cancer via sister cells

J. Dai[1], S. Zheng[1], M. Falco[1], J. Bao[1], J. Eriksson[1], S. Pikkusaari[1], S. Forstén[1], J. Jiang[1], W. Wang[1], L. Gao[1], F. Perez-Villatoro[1], O. Dufva[2], K. Saeed[2], Y. Wang[1], A. Amiryousefi[1], A. Färkkilä[1], S. Mustjoki[2], L. Kauppi[1], J. Tang[1], A. Vähärautio[1]
[1]University of Helsinki, Research Program in Systems Oncology, Helsinki, Finland
[2]University of Helsinki, Research Program in Translational Immunology, Helsinki, Finland

**Introduction**
Exploring the non-genetic evolution of cell states during cancer treatments has become attainable through recent advances in lineage-tracing methods. However, transcriptional changes that associate with resistant fates may be subtle, necessitating high-resolution analysis.

**Material and Methods**
We developed ReSisTrace which uses shared transcriptomic features of sister cells to predict the states that prime treatment resistance. We first applied ReSisTrace in a high-grade serous ovarian cancer (HGSOC) cell line to dissect primed resistance against olaparib, carboplatin or natural killer (NK) cells, and further explored in a more heterogeneous HGSOC patient-derived organoid context to understand intrinsic resistance to carboplatin.

**Results and Discussions**
We first confirmed that sister cells have significantly more similar transcriptomes than random pairs of cells, thus providing the biological basis to study primed resistance via sister cell inference. To address the interplay between genetic and non-genetic traits to primed resistance, we performed subclone analysis via inferred copy number variations (CNVs) and found inferred CNVs subclones closely matched the transcriptome-based clusters, and further analysis revealed that the subclonal enrichment contributes to pre-resistance signatures in both cell line and organoid contexts. Homologous recombination deficient (HRD) cancer cells are sensitive to olaparib because of synthetic lethality. Interestingly, we found that HRD-like cancer populations were also more susceptible to NK cells. Furthermore, by utilizing the pre-resistance signatures found from the cell line, we successfully identified small molecules that drive cells to sensitive states prior to each treatment.
In the more heterogeneous organoid context, we found stronger contrasts between carboplatin pre-resistant and pre-sensitive cells, whereas the control groups showed only mild differences. In line with our previous scRNA-seq analysis of paired HGSOC patient samples, stress-associated transcription factors (JUNB, FOS) were enriched in the carboplatin pre-resistant cells also in the organoid context.

**Conclusion**
In summary, ReSisTrace resolves pre-existing transcriptional features of treatment vulnerability, facilitating both molecular patient stratification for personalized treatments and discovery of synergistic pre-sensitizing therapies.

### EACR23-1259
### Resistance to PD-L1 inhibitor therapy in muscle-invasive urothelial carcinoma is associated with the EPHA1 receptor and

© 2023 The Authors. Molecular Oncology is published by John Wiley & Sons Ltd on behalf of Federation of European Biochemical Societies

### its ligands EFNA1, EFNA3 and EFNA4

Y.H. Yang[1], C. Cubuk[2], D. Castellano[3], A. Rodriguez-Vida[4], I. Durán[5], S. Crabb[6], F. Jackson-Spence[1], C. Toms[1], B. Szabados[1], T. Powles[1]
[1]Barts Cancer Institute, Centre for Experimental Cancer Medicine, London, United Kingdom
[2]William Harvey Research Institute, Centre for Experimental Medicine and Rheumatology, London, United Kingdom
[3]Hospital 12 de Octubre, Department of Medical Oncology, Madrid, Spain
[4]Hospital del Mar, Department of Medical Oncology, Barcelona, Spain
[5]Hospital Universitario Virgen del Rocio, Instituto de Biomedicina de Sevilla, Seville, Spain
[6]University of Southampton, Southampton Experimental Cancer Medicine Centre, Southampton, United Kingdom

**Introduction**

Erythropoietin-producing human hepatocellular (EPH) receptors and their ligands (EFN) are increasingly implicated in tumorigenesis. In urothelial carcinoma, EFN inhibition has demonstrated synergy with PD-L1 blockade in the metastatic setting. Using data from the ABACUS trial (NCT02662309), we present an exploratory analysis of the biological roles and clinical significance of EFN/EPH in the context of PD-L1 inhibitor therapy for muscle-invasive urothelial carcinoma.

**Material and Methods**

The ABACUS trial investigated the use of neoadjuvant atezolizumab before cystectomy for muscle-invasive urothelial carcinoma, and collected baseline and post-treatment samples for comparison. Patient demographics are as outlined in the original manuscript. 80 patients were assessable for biomarker analysis. All bioinformatic analysis was conducted in R. The DESeq2 package was used to process raw RNA-seq counts for baseline and post-treatment samples, and quantify changes in EFN/EPH expression following treatment. EFN/EPH expression was correlated against transcriptional signatures for four immune (T, B, NK and Myeloid dendritic cells) and two stromal (endothelial cells and fibroblasts) populations, and signatures for VEGF-dependent angiogenesis and hypoxia at baseline and post-treatment. Patients were grouped into high and low EFN/EPH expression using the median expression level for each gene. Survival analysis was performed using the Kaplan-Meier method and log-rank test. P-values were adjusted for multiple correction testing.

**Results and Discussions**

Expression of the EPHA1 receptor and its ligands EFNA1, EFNA3 and EFNA4 were associated with reduced immune, stromal and angiogenesis signatures and increased hypoxia post-treatment, but not at baseline. A reduction in the expression of these genes was seen in patients who achieved pathological complete response and/or major pathological response (N=27) (EFNA1: log2FC -0.92, $p=0.01$; EFNA3: log2FC -1.53, $p=0.0005$; EFNA4: log2FC -0.75, $p=0.01$; EPHA1: log2FC -1.77, $p<0.00001$) but not amongst patients who relapsed following cystectomy (N=17). Patients with high expression levels of EFNA1 ($p = 0.03$) and EFNA4 ($p = 0.03$) post-atezolizumab had a shorter relapse-free survival than those with low expression levels.

**Conclusion**

These findings suggest that EFNA-EPHA signalling is associated with treatment resistance to PD-L1 inhibitor therapy in muscle-invasive urothelial carcinoma, therefore representing a potential biomarker and treatment target.

### EACR23-1267
### Epigenetic Drug Library Screening Identifies Key Modulators To Overcome Lysosome-Mediated Tolerance of Tumor Cells To Cisplatin

B. Sergi[1], S.C. Ozcan[2], K. Kiselyov[3], C. Acilan Ayhan[2,4]
[1]Koc University, Graduate School of Health Sciences, Istanbul, Turkiye
[2]Koc University, Research Center for Translational Medicine KUTTAM, Istanbul, Turkiye
[3]University of Pittsburgh, Department of Biological Sciences, Pittsburgh, United States
[4]Koc University, School of Medicine, Istanbul, Turkiye

**Introduction**

One of the key mechanisms driving differential gene expression leading to oncogenic transformation and drug resistance is epigenetic changes. Lysosomes are critical components in signaling, expulsion, and sequestration of a wide range of components, including toxic metals and chemotherapeutic agents. Drug sequestration in the lysosomes, and expulsion through exocytosis, is a recently recognized strategy that cancer cells use to escape cell death. Our research focuses on the epigenetic regulation of these processes with the hypothesis that epigenetic modifier drugs (epidrugs), which are capable of inhibiting lysosomal activities could also serve as potential therapeutics and synergize with cisplatin.

**Material and Methods**

To test this idea, epidrugs were screened for their effect on lysosomal exocytosis (β-hex assay), lysosomal biogenesis (LysoTracker staining), and combinatorial cytotoxic activity with cisplatin (SRB viability assay).

**Results and Discussions**

Our results showed that among the few drugs that decreased exocytosis, MS023 (an arginine methyl transferase (PRMT) inhibitor) singled out as exhibiting promising activity in reduction of exocytosis and increasing cisplatin efficacy. Furthermore, we individually knocked down the known targets of MS023, PRMT1, -6, and -8, and evaluated how each target contributed to the phenotype. Interestingly, silencing of neither of the PRMTs effectively reduced exocytosis, suggesting the need for inhibition of multiple PRMTs at once or the presence of another unknown target. To assess the molecular changes in response to MS023, RNA-seq analysis, gene ontology, and GSEA analyses identified several biological processes and functions, which could be responsible for changes in exocytosis and drug response. We also handpicked three genes out of differentially expressed genes (DEGs), ABCA1, ABCA3 and SerpinE1, which have been previously associated with drug resistance. We further focused on ABCA3, as it exhibited the highest fold change,

© 2023 The Authors. Molecular Oncology is published by John Wiley & Sons Ltd on behalf of Federation of European Biochemical Societies and is known to be localized to the lysosomes. Similar to MS023, knockdown of all MS023 target PRMTs reduced ABCA3 expression.

**Conclusion**

We propose that lysosomal drug efflux is a significant burden on drug discovery and clinical use; therefore, suppressing it will be impactful. Overall, MS023 appears as a promising epidrug, which affects processes related to both secretory pathways and drug efflux. By establishing the action mechanism of candidate epidrugs, we wish to set future targets for cancer intervention and enhancing the drug efficacy.

### EACR23-1268
### Rictor/mTORC2 downregulation promotes metabolic rewiring and therapeutic resistance in BRAFV600E melanoma cells

*L. Ponzone[1], C. Levra Levron[1], V. Audrito[2], C. Landi[3], M. Gasparrini[4], N. Raffaelli[4], L. Bini[3], E. Calautti[1]*

*[1]Molecular Biotechnology Center, Department of Molecular Biotechnology and Health Sciences- University of Turin, Turin, Italy*

*[2]University of Eastern Piedmont, Department of Sciences and Technological Innovation, Alessandria, Italy*

*[3]University of Siena, Department of Life Sciences, Siena, Italy*

*[4]Polytechnic University of Marche, Department of Agricultural-Food and Environmental Sciences, Ancona, Italy*

**Introduction**

mTORC2 is a key regulator of cell survival and metabolism. Melanoma cells rely heavily on metabolic rewiring to acquire resistance to targeted therapies but the specific role of mTORC2 in this context still needs to be defined. This issue is especially relevant in the field of cancer therapy considering that mTOR inhibition, alone or in combination with other drugs, has shown so far a limited success in this context.

Rictor is an essential component of mTORC2 that is required for its signaling activity and integrity. To study how changes in mTORC2 activity affect melanoma cells responses to targeted therapy (BRAF/MEK inhibitors, BRAFi/MEKi) we analyzed therapeutic responses and metabolic profiles of Rictor-deficient and –proficient $BRAF^{V600E}$ melanoma cell lines**Material and Methods**

Stable downregulation of Rictor was achieved by shRNA lentiviral transduction in different $BRAF^{V600E}$ human melanoma cell lines. Metabolic profiles were analyzed by Seahorse assay.

Sensitivity of melanoma cells to BRAF inhibition was evaluated by clonogenic survival assay *in vitro* and by analysis of tumor xenograft growth *in vivo;* proteomic analyses were carried out by 2D-GE/Mass Spectrometry; interactome studies were carried out by Immunoprecipitation followed by Mass Spectrometry

**Results and Discussions**

Rictor downregulation induces a metabolic reprograming in $BRAF^{V600E}$ melanoma cells which affects their sensitivity to BRAFi/MEKi. Most prominent changes observed include alteration of enzymes involved in mitochondrial functions and NAD metabolism, and pharmacological inhibition of some of these pathways increased their sensitivity to BRAFi both in cultured cells *in vitro* and in tumor xenografts *in vivo*.

Proteomic analysis of Rictor-deficient and -proficient cells highlighted specific changes in expression and post-translational modifications of several proteins, most of which are involved in cellular metabolic functions. Subsequent interactome analyses of most interesting candidates showed distinct changes in protein-protein interactions, which may affect their function and activity. Pharmacological inhibition of these metabolic processes further sensitized melanoma cells to BRAFi, suggesting a potential benefit for their clinical application**Conclusion**

This work identifies several novel interconnections between Rictor/mTORC2 and the metabolic reprograming of $BRAF^{V600E}$ melanoma cells, which may represent new therapeutic vulnerabilities of BRAFi-resistant melanomas

### EACR23-1279
### Drug resistance mechanisms in KRAS mutant colorectal cancers

*T. Thakur[1], B. Cong[2], A. Campbell[1], O. Sansom[1], R. Cagan[2]*

*[1]CRUK Beatson Institute, CRUK Beatson Institute, Glasgow, United Kingdom*

*[2]University of Glasgow, School of Cancer Sciences, Glasgow, United Kingdom*

**Introduction**

Metastatic colorectal cancer (CRC) is the second leading cause of cancer-related mortality in the world, accounting for more than 900,000 deaths in 2020. A disproportionate number of these deaths are due to KRAS-mutant CRCs, which account for ~40% of all CRC cases and are notoriously resistant to most therapies. Despite showing great promise in preclinical studies, targeted therapies have performed sub-optimally in clinical trials for KRAS mutant cancers. The mechanisms by which RAS pathway inhibitors have failed to reduce tumor progression remains poorly understood and presents a huge clinically unmet need. We aim to explore this question using fruit-fly, mouse, and multi-omics approach. Our hypothesis is that drug resistance is an emergent feature of genetically complex tumors.

**Material and Methods**

To capture tumor genome complexity, we use a diverse panel of whole animal CRC models reflecting multigenic and heterogeneous nature of tumours. Our patient specific fruit-fly *avatars* and transgenic mouse models are designed to explore how genome complexity impacts drug response. Our models comprise alterations in atleast three primary pathways implicated in CRCs– APC, KRAS and TP53. Initially we screened our models against trametinib apart from other RAS-pathway inhibitors and multi-kinase inhibitors. We then used immunoprecipitation and immuno-histochemical analysis to explore RAS pathway and apoptosis upon trametinib treatment. We also studied the dynamics of cell-cycle progression using flow cytometry.

**Results and Discussions**

Corroborating earlier studies, we found that one-hit [KRAS] fly model demonstrated strong sensitivity while three-hit [APC-KRAS-P53] fly model was resistant to single agent trametinib. Similarly, APC-KRAS mouse intestinal organoids were more sensitive compared to APC-

© 2023 The Authors. Molecular Oncology is published by John Wiley & Sons Ltd on behalf of Federation of European Biochemical Societies

KRAS-P53 organoids. Further analysis showed short-term suppression of phospho-ERK in both APC-KRAS and APC-KRAS-P53 mouse small intestine and organoids. Moreover, we observed that APC-KRAS-P53 intestinal organoids predominantly arrest at G0/G1 stage whereas majority of APC-KRAS organoids underwent apoptosis upon trametinib treatment.

**Conclusion**

Our findings thus far show that functional p53 might be essential to therapy-induced apoptosis. As organoids that have lost p53, arrest at G0/G1 stage, we believe this pause from cell cycle progression contributes to the development of drug resistance mechanisms. We are currently exploring the dynamics of cell cycle progression and cell fate upon trametinib treatment in our models.

### EACR23-1280
### CRISPR activation screen identifies MDM2 as a modulator of proteasome inhibitor resistance in multiple myeloma

*M. Labrador[1], S. Cozzubo[1], E. Mereu[1], T. Paradzik[1], C. Bandini[1], M. Cumerlato[1], B. Donati[2], V. Manicardi[2], D. Ronchetti[3], A. Ciarrocchi[2], A. Neri[3], R. Piva[1]*
*[1]Molecular Biotechnology Center- Università di Torino, Molecular Biotechnology and Health Sciences, Torino, Italy*
*[2]Azienda USL-IRCCS di Reggio Emilia, Laboratory of Translational Research, Reggio Emilia, Italy*
*[3]University of Milano, Department of Oncology and Hemato-oncology, Milano, Italy*

**Introduction**

Multiple Myeloma (MM) is a neoplasm characterized by the uncontrolled proliferation of monoclonal plasma cells in the bone marrow. Despite the availability of different therapeutic regimens, the acquisition of drug resistance widely affects patients' response and prognosis. Investigating the basis of drug resistance, particularly with regard to proteasome inhibitors (PIs), is crucial.

**Material and Methods**

In this study, we performed a gain of function screen using the CRISPR-based SAM system and SAM sgRNA library targeting 23,430 genes, as well as a loss of function screen with a library of 320 small-molecule inhibitors under the selective pressure of carfilzomib (CFZ).

**Results and Discussions**

The Mouse Double Minute 2 homolog (MDM2) gene emerged from the genome-wide CRISPR activation screen as a potential modulator of carfilzomib (CFZ) resistance. MDM2 transactivation enhanced MM cell recovery after cytotoxic CFZ treatment and conferred selective advantage in cell competition assays. Complementary pharmacological screening revealed that the MDM2 selective inhibitor NVP-CGM097 significantly synergized with CFZ, increasing cell death in a panel of MM cell lines independently of their p53 status. Importantly, NVP-CGM097 was also effective against primary CD138+ cells from MM patients and was able to resensitize CFZ/BTZ-resistant cell lines to PI.

**Conclusion**

Our results suggest that MDM2 may be instrumental in driving PI resistance, and thus reinforce the interest in exploring the molecular mechanisms and therapeutic potentials of NVP-CGM097/CFZ combination to overcome PI resistance in MM and other hematological malignancies.

### EACR23-1294
### Role of fascin in docetaxel resistance acquisition in breast cancer cell models

*F. Postigo-Corrales[1], A.M. Hurtado-López[2], A. Albaladejo-González[2], P. Vera-Perez[3], P. Aneiros-Garcia[3], D. Pérez-Parra[4], D. Torres-Moreno[4], F. Ayala de la Peña[5], G. Luengo-Gil[2], P. Conesa-Zamora[6]*
*[1]Universidad Católica San Antonio de Murcia, Molecular Pathology, Cartagena, Spain*
*[2]Fundación para la Formación e Investigación Sanitarias, Molecular Pathology, Cartagena, Spain*
*[3]Santa Lucia University Hospital, Surgery, Cartagena, Spain*
*[4]Santa Lucia University Hospital, Pathology, Cartagena, Spain*
*[5]Morales Meseguer University Hospital, Medical Oncology, Murcia, Spain*
*[6]Santa Lucia University Hospital, Laboratory Medicine, Cartagena, Spain*

**Introduction**

Fascin is an actin-bundling protein whose expression is normal epithelial is virtually absent and plays a causal role in the development of the invasive and metastatic phenotype typical from several poor prognosis tumors, especially triple negative breast cancer (TNBC) where fascin is expressed in more than 85% of cases. Fascin has been involved in the chemotherapeutic resistance of breast cancer (BC) cells so our objective was to study the role of fascin in the progressive acquisition of resistance to docetaxel and to test its reversion by using the antidepressant imipramine which has been identified as an antifascin agent.

**Material and Methods**

MDA-MB-231 (TNBC) and MCF7 (luminal B BC) cell models were cultured under increasing concentrations of docetaxel for over 3 years. Fascin levels were evaluated by IHC and imipramine was tested against wild type and resistant cell lines. *In vitro* evaluation of drug effects included proliferation and migration.

**Results and Discussions**

From MDA-MB-231 wild type we were able to obtain cell lines resistant from 1 to 1000 nM docetaxel. However, from MCF7 only resistance to 100nM was achievable in the same period. In the TNBC cell line, fascin expression increased in parallel with acquisition of resistance.

In proliferation assays, imipramine reduced the viability of resistant MDA-MB-231 cell line in comparison to MDA-MB-231 wild type (IC50 of imipramine: 9.2±4.9 vs 21±1.2μM, $p<0.05$; IC50 of NP-G2-044: 5.2±1 vs 14.2±2.1μM, $p<0.05$). In migration assays with docetaxel resistant MDA-MB-231 and wild type cell line, imipramine has shown differences between control and 100μM of treatment (% of confluence of resistant MDA-MB-231: 33.9±8.2 vs 10.6±1.9, $p<0.04$; % of confluence in wild type: 86.8±4.6 vs 36.5±8.8, $p<0.0002$).

**Conclusion**

TNBC cell model MDA-MB-231 develop resistance much faster than the luminal model MCF7. During acquisition of resistance to docetaxel, fascin expression increases in a

© 2023 The Authors. Molecular Oncology is published by John Wiley & Sons Ltd on behalf of Federation of European Biochemical Societies dose-dependent manner. Imipramine reduced the viability and migration abilities of the docetaxel-resistant versus wild-type MDA-MB-231 cell line. This study could be a foundation for a better understanding of docetaxel resistance in TNBC and suggests that the repurposed drug imipramine could be contributing to the reversion of this phenotype.

### EACR23-1298
### Contribution of adipocytes in the tumor microenvironment as a novel strategy in breast cancer therapy

W. Brankiewicz[1]
[1]*Institute of Clinical Medicine, Department of Medical Genetics, Oslo, Norway*

**Introduction**

The tumor microenvironment (TME) is a heterogeneous ecosystem composed of infiltrating immune cells, mesenchymal support cells, and matrix components contributing to tumor progression. Adipocytes are the primary cellular component comprising the breast cancer (BC) microenvironment. Cancer-associated adipocytes (CAAs) are not only found adjacent to cancer cells but can also influence cancer cells by releasing various factors that result in enhanced tumor progression and resistance to treatment. Therefore the adipocyte-cancer cell crosstalk leads to phenotypical and functional changes in both cell types, which can further enhance tumor progression.

**Material and Methods**

A model of 3D adipose tissue culture and different co-culture assays of breast cancer cells with primary adipocytes from breast cancer patients was established to retrieve the comprehensive multi-omics characterization of each cell type.

**Results and Discussions**

This project is a comprehensive analysis of multi-omics data, focused on lipidomics, metabolomics and expression data of CAAs and tumor cells in breast cancer. It builds upon novel models, techniques and therapeutic approaches targeted at breast adipose tissue. Our approach will generate new knowledge needed for giving women personalized diagnoses and treatment options.

**Conclusion**

Our approach generates new knowledge needed for giving women personalized diagnoses and treatment options. This may prevent overtreatment and unnecessary side effects associated with less effective treatment regimens. As such, it could not only provide physical health benefits but also reduce the psychological and socioeconomic impacts of the disease, as well as the effects of modification on appearance and sexuality.

### EACR23-1317
### A GENOME-WIDE CRISPR/CAS9 LOSS-OF-FUNCTION SCREEN TO IDENTIFY MECHANISMS OF CHEMO- AND RADIOTHERAPY RESISTANCE IN HEAD AND NECK SQUAMOUS CELL CARCINOMAS

E. Pozo Agundo[1,2,3], *M. Álvarez-González*[1,3], *F. Hermida-Prado*[1,2,3], *J. Bestilleiro-Márquez*[1,3], *A. Villacé-Gallego*[4], *I. Varela*[5], *J.P. Rodrigo*[1,2,3,6], *J.M. García-Pedrero*[1,2,3], *M. Álvarez-Fernández*[1,3]
[1]*Health Research Institute of Asturias ISPA, Head and Neck Cancer Research, Oviedo, Spain*
[2]*Spanish Biomedical Research Network in Cancer CIBER ONC, Instituto de Salud Carlos III, Madrid, Spain*
[3]*University Institute of Oncology of Asturias IUOPA, University of Oviedo, Oviedo, Spain*
[4]*Central University Hospital of Asturias HUCA, Radiophysics Unit, Oviedo, Spain*
[5]*Institute of Biomedicine and Biotechnology of Cantabria, University of Cantabria-CSIC, Santander, Spain*
[6]*Central University Hospital of Asturias HUCA, Department of Otolaryngology, Oviedo, Spain*

**Introduction**

Head and neck squamous cell carcinomas (HNSSC) are tumors arising in the mucosal epithelium of the upper respiratory tract, i.e. larynx, pharynx and oral cavity. Despite advances in cancer therapy, survival rates in HNSCC have increased only marginally, with about 50% of patients dying of the disease. Treatment for HNSCC is mostly limited to surgery together with chemo- and/or radiotherapy. Yet, the downsides of these treatments are the high toxicity and frequent development of resistance, which, together with late diagnosis, lead to such high mortality rate in HNSCC patients. Importantly, no predictive markers are available for clinical use. We, therefore, decided to perform a genome-wide screen to identify mechanisms underlying cisplatin and radiation resistance, as a source of new targets and/or biomarkers that could improve patient management and survival

**Material and Methods**

We have used a lentiviral-pooled library of 90709 sgRNAs directed at 18010 genes across the human genome to perform a genome-wide loss-of-function CRISPR-Cas9 screen in HNSCC cell lines. Cells with constitutive expression of the endonuclease Cas9 were infected with the above mentioned sgRNA library, and treated with different doses of cisplatin and ionizing radiation. Genomic DNA was extracted from surviving cells, and integrated sgRNAs identified by PCR amplification followed by Illumina next generation sequencing. MAGecK software has been used to determine the differential abundance of sgRNAs in treated versus control cells

**Results and Discussions**

Our preliminary bioinformatic analysis has revealed interesting pathways enriched and depleted in cisplatin-resistant cells compared to control cells, with some candidates selected for validation studies. We are currently analyzing the data on radiated cells. Upon completion of this analysis we expect to provide the first comparative study of determinants of sensitivity to radiation versus chemotherapy in HNSCC tumor cells. This is of significant relevance since those treaments are frequently combined, and cisplatin is also used as a surrogate indicator for radiation response

**Conclusion**

Our data show the strong potential of CRISPR/Cas9-based screens to get a comprehensive view of the common and specific pathways determining the response to different treatments, which might bring new opportunities to identify biomarkers and therapeutic strategies to overcome treatment resistance

© 2023 The Authors. Molecular Oncology is published by John Wiley & Sons Ltd on behalf of Federation of European Biochemical Societies

### EACR23-1331

### Phosphoinositide signaling mediated by INPP4B controls tumour progression and chemotherapy resistance through the regulation of lysosomal functions

*L. SALMENA[1], J. Woolley[1], K. Chen[1], G. Saffi[1]*
*[1]University of Toronto, Pharmacology and Toxicology, Toronto, Canada*

**Introduction**

Latest efforts focused on Inositol polyphosphate 4-phosphate Type II (INPP4B), a 4-phosphate that hydrolyses phosphatidylinositol-3,4-bisphosphate (PI(3,4)P2) to form PI(3)P, have revealed novel signaling paradigms in cancer. Recent work has uncovered roles for INPP4B in leukemic stem cell maintenance and leukemia progression and a pan-cancer analysis revealed that pancreatic cancer patients expressing high levels of INPP4B had poor prognosis. We and others have previously reported that INPP4B may be a biomarker of aggressiveness and therapy resistance in some cancers. This work has prompted us to investigate cellular and molecular consequences of INPP4B overexpression in leukemia and pancreatic ductal adenocarcinoma (PDAC).

**Material and Methods**

Transcriptional analyses of patient samples have identified associations of *INPP4B* with the integrated stress response and lysosome functions. In AML, INPP4B and lysosomal signatures were associated with stemness and differentiation. In pancreatic cancer INPP4B and lysosomal transcripts demonstrated a strong correlation to cellular migration and other invasive properties of pancreatic cancer cells. Cellular and biochemical studies support a role for INPP4B in regulating lysosomal properties including lysosomal content, size, proteolytic capacity, and notably localization.

**Results and Discussions**

Overall, we propose that INPP4B drives cancer by promoting lysosomal functions, in turn we are exploring whether cancer patients with high INPP4B expression will benefit from therapeutic strategies targeting lysosomes and identify therapeutic strategies to exploit this novel signaling axis to mitigate stemness and metastases in AML and pancreatic cancer, respectively.

**Conclusion**

We conclude that phosphoinositide signalling mediated by INPP4B controls tumour progression and chemotherapy resistance by mediating the integrated stress response through the regulation of lysosomal functions

### EACR23-1335

### SMAC-mimetic enhances the effect of standard chemotherapy in ovarian cancer via MSLN-TNF alpha axis

*R. Coelho[1], B. Seashore-Ludlow[2], S. Schütz[3], L. Lombardo[3], A. Fedier[3], O. Kallioniemi[2], L. David[4], F. Jacob[3], V. Heinzelmann-Schwarz[5]*
*[1]University Hospital Basel, Biomedicine, Basel, Switzerland*
*[2]Karolinska Institutet, Oncology-Pathology, Stockholm, Sweden*
*[3]University of Basel, Biomedicine, Basel, Switzerland*
*[4]IPATIMUP, Differentiation and cancer, Porto, Portugal*
*[5]University of Basel and University Hospital of Basel, Biomedicine, Basel, Switzerland*

**Introduction**

In the era of targeted therapy and personalized medicine, women diagnosed with epithelial ovarian cancer (EOC) are still treated with classical chemotherapeutic agents. Despite recent advances in maintenance therapy, a valuable therapeutic option that can increase sensitivity to standard first-line chemotherapy is still missing. Considering its central role in EOC progression, the glycoprotein Mesothelin (MSLN) is an emerging candidate for targeted therapy. Here, we explore the relevance of MSLN in ovarian cancer treatment to identify a new and valuable therapeutic option.

**Material and Methods**

Drug-screening test was performed using a library of 528 oncology compounds, each in 5 concentrations in MSLN-engineered EOC cell lines (n=4) with either loss or gain of function. Drug-sensitive scores and drug synergies were determined using the BREEZE pipeline (https://breeze.fimm.fi/50166) and SynergyFinder+ software (https://synergyfinder.fimm.fi), respectively. Validation of hit compounds and their combinations was performed using co-culture experiments with normal mesothelial cells and ex vivo culture of patient-derived cells.

**Results and Discussions**

We identified the Second Mitochondria-derived Activator of Caspases (SMAC) mimetics as a family of compounds that preferentially target MSLN-low EOC cells with a relatively specific effect on cancer cells as shown by co-culture experiments with normal immortalized mesothelial cells. Subsequent combinatorial drug screenings in cell lines and ex vivo patient-derived cultures revealed a synergistic effect of chemotherapeutic agents carboplatin and paclitaxel with SMAC mimetics. Mechanistically, we show that the drug synergism is mediated through an MSLN-TNF-α dependent axis, leading to activation of the apoptotic pathway.

**Conclusion**

We provide mechanistic and applicable data proposing the use of SMAC mimetics in combination with chemotherapy in ovarian cancer. Data generated so far are the basis of phase I clinical trial to evaluate the use of SMAC mimetics to overcome intrinsic and acquired chemoresistance in ovarian cancer patients.

### EACR23-1337

### Regulation of YAP activity upon EGFR-targeted therapy in EGFR-mutant lung cancer

*M. Tienhaara[1], K. Kurppa[1], J. Westermarck[2]*
*[1]University of Turku, Institute of Biomedicine- MediCity Research Laboratories, Turku, Finland*
*[2]University of Turku- Åbo Akademi University, Turku Bioscience Centre- Institute of Biomedicine, Turku, Finland*

**Introduction**

Activation of YES-associated protein (YAP) is a widely observed mechanism of resistance to targeted therapies

© 2023 The Authors. Molecular Oncology is published by John Wiley & Sons Ltd on behalf of Federation of European Biochemical Societies in manycancer types. Specifically, recent studies have implicated activated YAP as a crucial factor facilitating the establishment of residual disease following EGFR-targeted therapy in EGFR-mutant lung cancer. The activity of YAP is regulated through phosphorylation by both an upstream signaling network, the Hippo pathway, as well by Hippo-independent mechanisms. Our goal is to identify upstream regulators of YAP activity in response to EGFR-targeted therapy in order to develop combination strategies to prevent YAP activation and thus limit residual disease and drug resistance.

**Material and Methods**

Our phosphoproteomics data revealed three Hippo-independent phosphorylation sites in YAP. To determine the functional role of these phosphosites for YAP activity, the sites were individually mutated into alanines in EGFR-mutant lung cancercells using CRISPR/CAS9 technology. YAP activation, apoptosis, and short- and long-term response to EGFR-targeted therapy were assessed using the established cells harboring the endogenous YAP phosphosite mutations.

**Results and Discussions**

One of the three phosphorylation sites, Serine 367, was identified as necessary for YAP activity following EGFR-targeted therapy. Mutation of this site to alanine resulted in increased apoptosis, enhanced sensitivity to EGFR-targeted therapy, and significant decrease of surviving residual cells following long-term treatment. More in-depth investigation of the upstream regulator(s) of YAP Serine 367 phosphorylation upon EGFR-targeted therapy could offer novel therapeutic approaches to prevent YAP activation, potentially limiting residual disease and drug resistance.

**Conclusion**

These results demonstrate that phosphorylation of Serine 367 is required for YAP function and modulates YAP-mediated cell survival upon EGFR-targeted therapy in EGFR-mutant NSCLC.

### EACR23-1344
### IDENTIFICATION OF MECHANISMS OF PRIMARY AND SECONDARY RESISTANCE TO ANTI-EGFR AND BRAF INHIBITION IN BRAFV600E CRC PDOs AND PDX

*A. Kotarac[1], H. Osumi[2], M. Benary[3], A. Malt[4], M. Alp[5], S. Hahn[6], C. Sers[7], S. Liebs[1], A. Dielmann[1], U. Krüger[8], C. Rose[9], F. Reichenbach[9], T. Zemojtel[8], P. Mertins[5], R. Yao[10], N. Ishaque[11], U. Keilholz[1], S. Stintzing[12], L. Vecchione[12]*

*[1]Charité Universitätsmedizin, Charité Comprehensive Cancer Center, Berlin, Germany*
*[2]Cancer Institute Hospital- Japanese Foundation for Cancer Research, Department of Gastroenterology, Tokyo, Japan*
*[3]Charité Universitätsmedizin Berlin, BIH-Core Unit Bioinformatics, Berlin, Germany*
*[4]Berlin Institute of Health and Charité Universitätsmedizin Berlin, Center for Digital Health, Berlin, Germany*
*[5]Max-Delbrück-Centrum für Molekulare Medizin, Proteomics Platform, Berlin, Germany*
*[6]Ruhr-Universität Bochum, Zentrum für Klinische Forschung, Bochum, Germany*
*[7]Charité Universitätsmedizin, Department of Pathology, Berlin, Germany*
*[8]Charité Universitätsmedizin Berlin, BIH-Core Unit Genomics, Berlin, Germany*
*[9]Pierre Fabre Pharma GmbH, Pierre Fabre Pharma GmbH, Freiburg, Germany*
*[10]Cancer Institute- Japanese Foundation for Cancer Research, Department of Cell Biology, Tokyo, Japan*
*[11]Berlin Institute of Health and Charite Universitätsmedizin Berlin, Center for Digital Health, Berlin, Germany*
*[12]Charité Universitätsmedizin, Department of Hematology- Oncology and Cancer Immunology, Berlin, Germany*

**Introduction**

The administration of the dual combination of BRAF inhibitor encorafenib (LGX818) and anti-EGFR inhibitor cetuximab (C225) is the first chemo-free regimen that was approved as treatment for *BRAF*$^{V600E}$ metastatic colorectal cancer (mCRC) patients after systematic therapy [1]. Despite improving the overall and progression-free survival, primary and secondary resistance occur and remain an area for active investigation [2,3]. Therefore, the aim of our current work was to identify mechanisms of both primary and secondary resistance by using patient-derived organoid (PDOs) and xenograft (PDX) models and eventually identify new combinational treatments.

**Material and Methods**

Eleven *BRAF*$^{V600E}$ CRC PDOs were treated with erlotinib (OSI-774), LGX818 and SN-38 as single agents as well as in combination (OSI-774+ LGX818 and OSI-744+SN-38). Sensitivity was defined based on IC50, AUC and Synergy score. All PDOs underwent whole exome sequencing (WES), RNAseq, mass spectrometry and human phospho-kinase array analysis. Additionally, three sensitive and three resistant models underwent RNAseq analysis upon 24 hrs treatment with OSI-774+LGX818. Finally, we induced secondary resistance in 3 sensitive PDO models and an independent cohort of ten *BRAF*$^{V600E}$ CRC PDX underwent single and combinational treatments as well as molecular analysis.

**Results and Discussions**

All PDOs were intrinsically resistant to single agents OSI-744, LGX818, while 6 models were intrinsically sensitive to SN-38 alone. Four out of eleven models were classified as sensitive to the combination of OSI-744+ LGX818. WES analysis confirmed the *BRAF*$^{V600E}$ mutation in all but one tested PDO. Full molecular analysis is currently ongoing. To the best of our knowledge, this is the first preclinical study using the largest cohort of *BRAF*$^{V600E}$ CRC PDOs and PDX aiming to identify mechanisms of primary and secondary resistance to the combination of anti-EGFR and BRAF inhibitors.

**Conclusion**

36% of our *BRAF*$^{V600E}$ CRC PDOs show sensitivity towards the combination of anti-EGFR and BRAF inhibitors. On the contrary, the vast majority of our models are intrinsically sensitive to SN-38 and the addition of an anti-EGFR inhibitor does not improve the response rates. The identification of biomarkers of response/resistance to the combination of anti-EGFR and BRAF inhibitors through molecular analysis as well as the integration of the PDX data will be presented at the conference.

**References**

[1] J. Tabernero *et al.*,PMID:33503393

© 2023 The Authors. Molecular Oncology is published by John Wiley & Sons Ltd on behalf of Federation of European Biochemical Societies

[2] D. Oddo *et al.*,PMID:27312529
[3] G. Middleton *et al.*,PMID:32047001

### EACR23-1355
### Inhibition of BRPF proteins reverses taxane resistance in castration resistant prostate cancers

*B. Dedeoğlu*[1], *İ. Bulut*[2], *B. Cevatemre*[3], *H. Syed*[4], *T. Bagcı Onder*[5], *C. Acılan*[4]
[1]*Koç University, Graduate School of Science and Engineering, Istanbul, Turkiye*
[2]*Koç University, Graduate School of Health Sciences, Istanbul, Turkiye*
[3]*Koç University, Translational Medicine Research Center KUTTAM, Istanbul, Turkiye*
[4]*Koç University, School of Medicine, Istanbul, Turkiye*
[5]*Koc University, School of Medicine, Istanbul, Turkiye*

**Introduction**
While localized prostate cancer can be treated with androgen suppression and surgery, many patients develop castration-resistant prostate cancer (CRPCa), which is more aggressive and/or metastatic. The standard of care for CRPCa patients involves taxanes (namely Docetaxel and Cabazitaxel), yet resistance against these drugs develops over time. Epigenetic regulation of cancer not only affects tumor development and progression but also resistance for chemotherapy. Hence, targeting these mechanisms appear as an alternative strategy to overcome drug resistance. Our previous studies using an epigenetic drug library and epi-targeted CRISPR screens using taxane resistant CRPCa cells (Du145 and 22Rv1) has shown that inhibition of BRPF proteins (epigenetic reader proteins containing bromodomain group) efficiently reverses taxane resistance. Currently, we aim to elucidate the molecular pathways involved in the reversion of taxane resistance through BRPFs in PCa cells.

**Material and Methods**
BRPF inhibition with small molecules resensitized taxane-resistant CRPCa cells to both taxanes under study. Efficient silencing of both BRPF1 and -2 using siRNAs were able to revert taxane resistance, albeit only mildly. On the other hand, knock-out of BRPF2 effectively reversed taxane-resistance and induced cell death. BRPF1 was essential for the viability of resistant cells, hence could not be generated despite several attempts. In order to investigate the transcriptomic differences between siBRPF and siControl treated cells, we performed RNA sequencing. Currently, we are investigating which other molecular players are involved in BRPF mediated drug resistance through ChIP sequencing.

**Results and Discussions**
Our RNA sequencing analysis showed that five genes were consistently differentially regulated in both Docetaxel and Cabazitaxel resistant cells and that inhibition of BRPFs can change the expression towards the parental-like sensitive cells. Furthermore, silencing or knock out of BRPFs led to downregulation of ABCB1, which encodes a permeability glycoprotein (Pgp), and suppressed its function, potentially explaining how cells may be resensitized to taxane treatment. This suppression seems to involve direct binding of BRPF1 to the ABCB1 promoter, as determined by ChIP-qPCR analysis.

**Conclusion**
BRPF inhibition appears as a promising anticancer strategy in taxane resistant CRPCa and the mechanism seems to involve inhibition of drug efflux. BRPF is shown to exert its effects through the regulation of ABCB1 expression.

### EACR23-1370
### miR-181a-5p/GLS axis: a potential biomarker for sorafenib response in Hepatocellular Carcinoma

*E. Bagirsakci*[1,2], *P. Korhan*[1,3], *Y. Oztemur Islakoglu*[1], *N. Atabey*[1,3]
[1]*Izmir Biomedicine and Genome Center, Cancer Biology and Signaling, Izmir, Turkiye*
[2]*Dokuz Eylul University- Izmir International Biomedicine a nd Genome Institute, Molecular Biology and Genetics, Izmir, Turkiye*
[3]*Izmir Tınaztepe University, Galen Research Center, Izmir, Turkiye*

**Introduction**
Hepatocellular carcinoma (HCC) is a leading cause of deaths globally due to a steady rise in the incidence of obesity and metabolic diseases and the limited efficacy offered by the existing therapy. Although, novel therapies have entered clinical practice in recent years, the prognosis of HCC remains poor. There are no biomarkers to predict sorafenib response or prognosis. Recent studies have highlighted the influence of miRNAs in development, progression, metabolic reprogramming and therapy responses in HCC. Our previous studies showed that decreased expression miR-181a-5p causes more aggressive phenotype in HCC. In this study, we aimed to identify a metabolism-related molecular signature regulated by miR-181a-5p that can predict sorafenib therapy responses in HCC.

**Material and Methods**
We investigated publicly available transcriptome data of patients who received SOR/placebo (GEO accession ID: GSE109211) and TACE (GEO accession ID: GSE104580). We identify genes in common that are expressed more in non-responders groups than responders. We selected miR-181a-5p specific metabolic targets and performed validation experiments by using qRT-PCR in wild-type and SOR-resistant cell lines we had developed previously. Then, we mimic and inhibit miR-181a-5p in SOR-resistant and wild-type cells, respectively. Then we performed Western Blotting to analyse the expression pattern of miRNA-181a-5p potential target molecules. We also examined expression patterns of miR-181a-5p and its target in HCC tumours and tumour-adjacent normal tissues obtained from patients who had sorafenib treatment.

**Results and Discussions**
We identified 297 genes in common that expressed more in non-responder groups. Our bioinformatic analysis revealed that the precursor of miR-181a-5p is downregulated in non-responders and specifically targets glutaminase (GLS). We determined that miR-181a-5p expression was lower in SOR-resistant cells compared to wild types, while GLS expression was higher. When we mimicked and inhibited miR-181a-5p in SOR-resistant and wild-type cells, respectively, we observed that miR-181a-5p and GLS expression patterns were opposite to each other. We observed that the GLS/miR-181a-5p axis is differentially regulated in wild type and SOR-resistant clones.

© 2023 The Authors. Molecular Oncology is published by John Wiley & Sons Ltd on behalf of Federation of European Biochemical Societies

**Conclusion**
Regarding the fact that miR-181a-5p regulates GLS in HCC, it would be fascinating to reprogram metabolism and target GLS at the same time using miR-181a-5p. The relation between GLS/miR-181a-5p and metabolism genes has been described for the first time in this project.

### EACR23-1374
### Dinaciclib as an effective treatment for Small Cell Lung Cancer

*L. Valdez Capuccino[1], F. Bonechi[1], T. Kleitke[1], Y. Wang[1], H. Walkzac[2], J. Brägelmann[1], M. Sos[1], N. Peltzer[1]*
*[1]Uniklinik Koeln, Department of Translational Genomics, Cologne, Germany*
*[2]Center for Biochemistry, Medical Faculty- University of Cologne, Cologne, Germany*

**Introduction**
Treatment-naïve Small Cell Lung Cancer (SCLC) is typically highly sensitive to standard-of-care chemotherapy consisting of a combination of Cisplatin or Carboplatin, and Etoposide. Yet, in the vast majority of cases, SCLC patients develop resistance to standard-of-care therapy and alternative therapies are urgently required to overcome this resistance. In this study, we tested the efficacy of Dinaciclib, an inhibitor of the kinase CDK9, among CDKs 1, 2 and 5, in SCLC. CDK9 inhibition has been shown to inhibit activation of RNA pol II and induce apoptosis by decreasing anti-apoptotic proteins in several cancer entities. We, therefore, hypothesize that Dinaciclib can overcome resistance to standard-of-care chemotherapy by downregulating anti-apoptotic proteins and engaging apoptosis in SCLC cells.

**Material and Methods**
To test the effect of Dinaciclib on lung cancer we used a panel of mouse cells derived from autochthonous mouse models of SCLC and NSCLC, as well as established human SCLC and NSCLC cell lines. We used SCLC cell lines intrinsically resistant to chemotherapy, as well as cell lines rendered resistant by chronic exposure to the chemotherapeutic agents. *In vitro* experiments measuring the effects of Dinaciclib, Cisplatin and Etoposide, and a specific CDK9 inhibitor, NVP-2, on tumour cells include viability by Cell Titer Glo and changes in key proteins by Western Blot. We further tested the efficacy of Dinaciclib *in vivo* using a syngeneic subcutaneous mouse model of SCLC.

**Results and Discussions**
We show that Dinaciclib displayed high killing potential in a panel of mouse and human SCLC cell lines when compared to NSCLC. Mechanistically, CDK9 inhibition by Dinaciclib led to a marked reduction in phosphorylated and total RNA poll II, with a subsequent reduction in the short-lived antiapoptotic proteins MCL-1 and cFLIP. This CDK9 dependency was corroborated with the use of a specific CDK9 inhibitor, NVP-2, yielding similar results as with Dinaciclib. Whereas CDK9 inhibition did not synergise with chemotherapy, it displayed high efficacy on chemotherapy-resistant cells. *In vivo*, Dinaciclib treatment effectively reduced tumour growth and improved survival in a syngeneic model of SCLC.

**Conclusion**
Together, this study shows that Dinaciclib, and CDK9 inhibition, is a promising therapeutic agent against SCLC, and sets the basis for the use of Dinaciclib in chemotherapy-resistant SCLC.

### EACR23-1377
### EWS::FLI1-DHX9 interaction alters R-loop metabolism promoting Ewing sarcoma sensitivity to genotoxic agents

*J. OLMEDO-PELAYO[1], D. Delgado-Bellido[1], E. Granado-Calle[2], L. Lobo-Selma[1], C. Jordan-Perez[1], A. Ehlers[3], S. Ohmura[3], I. Machado[4], A. Llombart-Bosch[5], J. Alonso[6], A. Montero-Carcaboso[7], T. Monteiro-Amaral[1], T. G.P. Grünewald[3], F. Gomez-Herreros[2], E. de Alava-Casado[1]*
*[1]Institute of Biomedicine of Seville/Hospital Virgen del Roc io/University of Seville/CIBERONC/FISEVI, Normal and Pathological Cytology and Histology, Seville, Spain*
*[2]Institute of Biomedicine of Seville/Hospital Virgen del Roc io/University of Seville/FISEVI, Genetics, Seville, Spain*
*[3]Division of Translational Pediatric Sarcoma Research- G erman Cancer Research Center DKFZ/Hopp-Children's Cancer CenterKiTZ/Heidelberg University Hos pital, Pathology, Heidelberg, Germany*
*[4]Instituto Valenciano de Oncología, Pathology, Valencia, Spain*
*[5]Valencia University, Pathology, Valencia, Spain*
*[6]Instituto de Investigación de Enfermedades Raras/CIBER ER/Instituto de Salud Carlos III, Unidad de Tumores sólidos infantiles, Madrid, Spain*
*[7]Institute de Recerca Sant Joan de Deu/Sant Joan de Deu h ospital, Pediatric Hematology and Oncology, Barcelona, Spain*

**Introduction**
Ewing sarcoma (EwS) is an aggressive neoplasm mainly affecting children, adolescents and young adults. Molecularly, EwS is characterized by pathognomonic gene fusions, most often involving the *EWSR1* and *FLI1* genes. Although long-term survival rates for patients with localized disease are of 70-80%, patients with metastatic disease still present a dismal prognosis with long-term survival rates of around 30%. In fact, >25% of patients present metastasis at diagnosis. Currently, EwS treatment remains based on conventional regimens, including genotoxic agents (GA) combined with radiotherapy and/or surgery. Therapeutic failure, lack of response and/or resistance occur in 25-50% of cases, which stresses the need for an in-depth understanding of the mechanisms underlying GA-sensitivity.

**Material and Methods**
EwS and non-EwS models were treated with Irinotecan/SN-38. A673/TR/shEF cell line (carrying an inducible shRNA against EWS::FLI1), was used to evaluate the role of EWS::FLI1 in drug sensitivity. The correlation between DHX9 levels and patient outcome was evaluated in a cohort of clinically annotated EwS patients (n=200).

**Results and Discussions**
We observed that EwS presents a higher sensitivity to GA *in vitro* and *in vivo* than other sarcomas. This is dependent on EWS::FLI1 fusion, and results from a strong induction of drug-induced DNA damage. Strikingly, our results indicate that the interaction between EWS::FLI1

© 2023 The Authors. Molecular Oncology is published by John Wiley & Sons Ltd on behalf of Federation of European Biochemical Societies and DHX9, an helicase implicated in R-loop metabolism, reduces the resolution of drug-induced R-loops. This leads to genome instability and GA cytotoxicity through the induction of replicative stress. Accordingly, DHX9 overexpression or inhibition of EWS::FLI1-DHX9 interaction by YK-4-279 inhibitor reduces drug-induced replicative stress and DNA damage, promoting drug resistance. Notably, we observed that the inhibition of replicative stress associated kinase ATR enhances GA cytotoxicity in EwS cells. Finally, we found a significant correlation between poor clinical outcome and higher expression levels of DHX9, further supporting the role of DHX9 in drug sensitivity in EwS.

**Conclusion**

Herein, we describe that EWS::FLI1-DHX9 interaction leads to a defect in the resolution of R-loops, suggesting a novel mechanism of drug sensitivity in EwS and a potential role of DHX9 as a predictive biomarker.

### EACR23-1380
### BIM Serine 87 phosphorylation mediates resistance to EGFR-targeted therapy in EGFR-mutant lung cancer

*Z. Song[1,2], S.B. Ficarro[3], J. Marto[3], K.J. Kurppa[1,2]*
*[1]University of Turku, Institute of Biomedicine, Turku, Finland*
*[2]University of Turku, Medicity Research Laboratory, Turku, Finland*
*[3]Dana-Farber Cancer Institute- Harvard Medical School, Department of Cancer Biology, Boston- MA, United States*

**Introduction**

Epidermal growth factor receptor (EGFR) tyrosine kinase inhibitors (TKIs) are the standard-of-care for the treatment of EGFR-mutant non-small cell lung cancer (NSCLC) with typically excellent response rates. However, no patient can be cured due to the development of acquired drug resistance. A major contributor to drug resistance are drug tolerant cancer cells that persist under targeted therapy (drug tolerant persisters, DTPs). As evading apoptosis is absolutely necessary for the establishment and survival of DTPs, understanding the mechanisms enabling apoptosis evasion may provide vulnerabilities that can be leveraged to limit drug tolerance, and thus, acquired drug resistance.

**Material and Methods**

To gain unbiased insight to the non-genetic signaling events associated with drug tolerance, we performed phosphoproteomics analysis in PC-9 cells under prolonged EGFR TKI treatment and identified elevated BIM Serine (S) 87 phosphorylation in the drug tolerant cells. We constructed doxycycline-induced overexpression of BIM S87A mutant in PC-9 cells and utilized CRISPR/Cas9 to create BIM S87A mutation in the endogenous locus in PC-9 cells. We conducted apoptosis assays and performed long-term treatment assays using Incucyte live-cell imaging to assess the effect of BIM Serine 87 phosphorylation on EGFR TKI response.

**Results and Discussions**

Overexpression of BIM S87A mutation in PC-9 cells resulted in significantly more enhanced apoptosis than overexpression of wild-type BIM, demonstrating that the pro-apoptotic function of BIM is dampened by BIM S87 phosphorylation. Correspondingly, PC-9 cells harboring endogenous BIM S87A mutation demonstrated increased apoptosis and more durable response under prolonged EGFR TKI treatment. Long-term treatment assays using live-cell imaging indicated that the more durable response is associated with cell cycle defects in the drug tolerant cells, suggesting that BIM S87 phosphorylation is required for escape from the DTP state. Further research on the underlying mechanisms of BIM S87 phosphorylation may provide rationale for combination strategies that improve the efficacy of EGFR-targeted therapy in EGFR-mutant NSCLC.

**Conclusion**

These results suggest that BIM S87 phosphorylation contributes to resistance to EGFR TKIs in EGFR-mutant NSCLC by dampening the apoptotic functions of BIM, resulting in evasion of the initial drug-induced apoptosis and escape from the drug tolerant state.

### EACR23-1387
### EHMT2 inhibition enhances carfilzomib sensitivity and overcomes drug resistance in multiple myeloma cell lines

*E. Mereu[1], D. Abbo[1], T. Paradzik[2], M. Cumerlato[1], C. Bandini[1], M. Labrador Granados[1], M. Maccagno[1], D. Ronchetti[3], V. Manicardi[4], A. Neri[5], R. Piva[1]*
*[1]Molecular Biotechnology Center, Department of Molecular Biotechnology and Health Sciences, Turin, Italy*
*[2]Rudjer Boskovic Insitute, Department of Physical Chemistry, 10000 Zagreb Croatia, Croatia*
*[3]University of Milan, Department of Oncology and Hemato-oncology, Milan, Italy*
*[4]Laboratory of Translational Research, Azienda USL-IRCCS di Reggio Emilia, Reggio Emilia, Italy*
*[5]Scientific Directorate- Azienda USL-IRCCS di Reggio Emilia, Azienda USL-IRCCS di Reggio Emilia, 42123 Reggio Emilia, Italy*

**Introduction**

Proteasome inhibitors (PIs) are extensively used for the therapy of multiple myeloma. However, patients continuously relapse or are intrinsically resistant to this class of drugs. In addition, adverse toxic effects such as peripheral neuropathy and cardiotoxicity could arise.

**Material and Methods**

To identify compounds that can increase the efficacy of PIs, we performed a functional screening using a library of small-molecule inhibitors covering key signaling pathways. Among the best synthetic lethal interactions, the Euchromatic Histone-lysine N-Methyltransferase 2 (EHMT2) inhibitor UNC0642 displayed a cooperative effect with carfilzomib (CFZ) in numerous MM cell lines, including drug-resistant models.

**Results and Discussions**

In MM patients, EHMT2 expression correlated to worse overall and progression-free survival. Moreover, EHMT2 levels were significantly increased in bortezomib-resistant patients. We demonstrated that CFZ/UNC0642 combination exhibited a favorable cytotoxicity profile toward peripheral blood mononuclear cells and bone marrow-derived stromal cells. To exclude off-target effects, we proved that UNC0642 treatment reduces EHMT2-related molecular markers and that an alternative EHMT2 inhibitor

© 2023 The Authors. Molecular Oncology is published by John Wiley & Sons Ltd on behalf of Federation of European Biochemical Societies recapitulated the synergistic activity with CFZ. Finally, we showed that the combinatorial treatment significantly perturbs autophagy and the DNA damage repair pathways, suggesting a multi-layered mechanism of action.

**Conclusion**

Overall, the present study demonstrates that EHMT2 inhibition could provide a valuable strategy to enhance PI sensitivity and overcome drug resistance in MM patients.

### EACR23-1442
### CARBONIC ANHYDRASE IX AS A TARGET TO OVERCOME DRUG RESISTANCE IN GASTRIC CANCER

*E. Andreucci[1], A. Biagioni[1], S. Peri[2], G. Versienti[1], F. Cianchi[2], L. Antonuzzo[2], D. Massi[3], L. Calorini[1], L. Magnelli[1], L. Papucci[1]*

*[1]University of Florence, Department of Experimental and Clinical Biomedical Sciences "Mario Serio", Florence, Italy*

*[2]University of Florence, Department of Experimental and Clinical Medicine, Florence, Italy*

*[3]University of Florence, Department of Health Sciences, Florence, Italy*

**Introduction**

Gastric cancer (GC) represents the fifth most frequently diagnosed malignancy and the fourth leading cause of cancer-related death worldwide. When upfront surgery is not pursuable, multimodal perioperative chemotherapy (pCT) is used to improve patients' overall survival. However, GC progressively gains chemoresistance, limiting therapies, thus the identification of suitable targets to overcome drug resistance is of fundamental interest. Amongst the potential biomarkers, the carbonic anhydrase IX (CAIX) has gained the most attention, since its overexpression is associated with a poor prognosis of several solid cancers.

**Material and Methods**

GC patients who underwent pCT FLOT (*i.e.*, Leucovorin, 5-Fluouracil, Docetaxel, and Oxaliplatin) followed by gastrectomy at the Azienda Ospedaliero Universitaria Careggi (AOUC) in Florence were classified as responder and non-responder, depending on the tumor regression grade (TRG 2 for responder, N=7 versus TRG 3-5 for non-responder, N=16). Formalin-fixed Paraffin-embedded sections of human samples were analyzed by immunohistochemistry to detect the CAIX levels. GC cell lines were analyzed via flow cytometry and sorted for CAIX expression. pRP-EGFP/Neo-CMV>hCA9 plasmid was used to generate CAIX-overexpressing GC cells. Drug-resistant GC cells were generated according to the “high-level laboratory models”. The ureido-benzene-sulphonamide SLC-0111 was used to inhibit the CAIX activity in GC cells. Colony formation, MTT, and caspase 3/7 flow cytometry assay were performed following GC cell treatment with single or combined SLC-0111/pCT. GC spheroids were treated as well and evaluated with the ReViSP software.

**Results and Discussions**

CAIX expression in GC patients correlates with TRG, being significantly higher in the non-responder group than in the responder one. In the experimental setting, the CAIX-high-expressing AGS GC cells were more resistant to pCT than the CAIX-low-expressing counterpart. Accordingly, the forced overexpression of CAIX in AGS cells significantly impaired CT response compared to control cells. AGS and ACC-201 GC cell lines that were experimentally induced to be resistant to 5-Fluouracil, Paclitaxel, Cisplatin, or FLOT overexpressed CAIX compared to the control. The SLC-0111 significantly improved the therapy response of both wild-type and resistant GC cells in 2D and 3D settings.

**Conclusion**

Overall, these data suggest a correlation between CAIX and GC drug resistance highlighting the potential effect of SLC-0111 in re-sensitizing resistant GC cells to pCT.

### EACR23-1460
### The expression of MCOLN2 in glioma stem-like cells regulates Temozolomide response.

*M.B. Morelli[1], M. Nabissi[1], C. Amantini[2], F. Maggi[2], L. Ricci-Vitiani[3], R. Pallini[4], C. Aguzzi[1], L. Zeppa[1], G. Santoni[1]*

*[1]University of Camerino, School of Pharmacy, Camerino, Italy*

*[2]University of Camerino, School of Biosciences and Veterinary Medicine, Camerino, Italy*

*[3]Istituto Superiore di Sanità, Department of Hematology- Oncology and Molecular Medicine, Rome, Italy*

*[4]Gemelli University Polyclinic Foundation- Scientific Hospitalization and Care Institute IRCCS, Institute of Neurosurgery, Rome, Italy*

**Introduction**

Glioblastoma (GBM) is the most aggressive brain tumor. The inter- and intra-heterogeneity of GBM, which inhibits proper treatment, is indicated to be related to poor patient outcomes. Moreover, a subpopulation of tumorigenic cells exhibiting stem-like characteristics, the glioblastoma stem-like cells (GSCs), was shown to be responsible for relapse, resistance to therapy, and tumor maintenance. As Temozolomide (TMZ) is the first drug of choice for chemotherapy, and TMZ is known to modulate the formation of lysosomes, our aim was to assess whether the endolysosomal MCOLN2 channel plays a role in responding to this drug.

**Material and Methods**

T98 and U251 glioma cell lines were purchased from DSMZ (Germany). GSC cell lines were isolated from surgical samples of sixteen adult patients with primitive brain tumors, that had undergone complete or partial surgical resection from 2006 to 2010 at the Institute of Neurosurgery, Catholic University School of Medicine in Rome (Italy). MCOLN2 mRNA expression was evaluated in GSCs by qRT-PCR. The response to TMZ was evaluated in MCOLN2-induced or silenced GSCs by MTT assay, qRT-PCR and western blot analysis. Using the STRING online database, we created a Protein–Protein Interaction (PPI) network to investigate the main pathways related to TMZ resistance. Through a drug transporters expression profiler, we evaluated the expression and modulation of the main ABC and SLC drug transporters in GSC cells transfected with MCOLN2. T98 and U251 lines

© 2023 The Authors. Molecular Oncology is published by John Wiley & Sons Ltd on behalf of Federation of European Biochemical Societies were used to investigate the same pathways in non-stem glioma cells.

**Results and Discussions**

Despite the identification of new therapeutic targets for GBM, the overall survival of GBM patients remains dismal due to tumor recurrence followed by chemoresistance. We demonstrated PI3K/AKT and JAK/STAT pathways, as well as ABC and SLC drugs transporters, are involved in resistance to TMZ induced by increased MCOLN2 mRNA levels. Furthermore, the expression of ADAR1, a dsRNA-editing enzyme, seems to be related to TMZ sensitivity and regulated by MCOLN2. Also T98 and U251 cells modulated ADAR1 expression according to MCOLN2 levels.

**Conclusion**

A strategy designed specifically to target GSCs or the pathways involved in stemness characteristics might prove useful in the clinic due to the resistance of GSCs to traditional therapies. Our results suggest that MCOLN2 could be a promising biomarker for GBM, even though more in-depth studies on resistance mechanisms are needed.

### EACR23-1468

### Characterization of the mechanisms of resistance to NAD-targeting anti-cancer agent in human acute myeloid leukemia

*S. Matsumoto[1], P. Biniecka[1], A. Bellotti[1], A. Nahimana[1], M. Duchosal[1]*

*[1]University Hospital of Lausanne CHUV, Department of Medical Laboratories, Lausanne, Switzerland*

**Introduction**

Nicotinamide adenine dinucleotide (NAD) is an essential metabolite in cell life. As cancer cells are particularly highly dependent on a sustained level of intracellular NAD due to their altered metabolism and aberrant proliferation, targeting NAD depletion in cancers has emerged as a promising therapeutic approach. APO866, a potent inhibitor of nicotinamide phosphoribosyltransferanse (NAMPT) demonstrates anti-cancer cytotoxic effects by blocking the major NAD biosynthetic pathway and inducing apoptotic cell death. However, APO866 failed to show objective tumor responses in clinical trials and tumor relapses are often observed in preclinical models. Here, we aimed to characterize the mechanism of resistance to APO866.

**Material and Methods**

To investigate the molecular mechanisms contributing to the development of resistance to APO866, we generated *in vivo*-acquired APO866-resistant human acute myeloid leukemia (AML) ML2 cells in tumor xenograft mouse models. We analyzed the whole transcriptomes of the resistant ML2 by RNA sequencing in comparison with sensitive ML2, as well as in drug-treated or untreated conditions.

**Results and Discussions**

We successfully generated models of *in vivo*-acquired resistance to APO866. Transcriptomic analyses revealed profound and constitutive modifications in their transcriptomic programs compared to the sensitive parental ML2, including an up-regulation of interleukin signalings and anti-apoptotic signaling pathways. Notably, we demonstrated that several kinase pathways including PI3K-Akt and ERK pathways were activated, leading to increased activity of AP-1 transcription factor. These alterations collectively conferred the resistance to APO866-induced apoptosis, which was reversed by the use of specific inhibitors.

**Conclusion**

We identified the mechanism of acquired resistance to NAMPT inhibitor APO866 in AML, which relied on the activation of PI3K/Akt, MAPK and AP-1 dependent pro-survival cellular signalings. Our findings suggest that combinatory approaches targeting these pathways are promising strategies to enhance the therapeutic efficacy of APO866.

### Epigenetics

### EACR23-0085

### A study of the role of glutamate transporter, SLC25A22, in the epigenetic machinery and treatment of gastric cancer

*S.H. Lin[1,2,3], K.S. Yu[4], Y.J. Tsai[2], K.T. Yeh[1], M.W.Y. Chan[5,6,7]*

*[1]Changhua Christian Hospital, Department of Pathology, Changhua, Taiwan*

*[2]Changhua Christian Hospital, Translational pathology core laboratory, Changhua, Taiwan*

*[3]National Chung Hsing University, Department of Post-Baccalaureate Medicine- College of Medicine, Taichung, Taiwan*

*[4]National Chung Cheng University, Department of Biomedical Sciences, Chiyi, Taiwan*

*[5]National Chung Cheng University, Department of Biomedical Sciences, Chiayi, Taiwan*

*[6]National Chung Cheng University, Center for Nano Bio-Detection, Chiayi, Taiwan*

*[7]National Chung Cheng University, Epigenomics and Human Disease Research Center, Chiayi, Taiwan*

**Introduction**

Gastric cancer remains the world most common and highly fatal malignancy. Epigenetic modifications are important mechanisms in regulating gene expression. Numerous cancers shut down various Tumor suppressor genes (TSG) through epigenetic modifications. Evidence showed that PCDHB15 (Protocadherin Beta 15) is TSG which is epigenetically silenced by SLC25A22, a mitochondria glutamate transporter that plays an important role in supplying energies to cancer cells and inhibiting epigenetic-related enzymes. We aimed to study the role of SLC25A22 in the epigenetic silencing of PCDHB15 in gastric cancer.

**Material and Methods**

Firstly, we used real-time polymerase chain reaction (qPCR) to determine mRNA expression on gastric cancer cells (AGS, MKN45) and GES (Human Gastric Epithelial Cells). Western blot was performed to determine protein amount in AGS (with or without PCDHB15 overexpression, SLC25A22 knock out) MKN45(with or without SLC25A22 overexpression), GES cells. Pyro-

© 2023 The Authors. Molecular Oncology is published by John Wiley & Sons Ltd on behalf of Federation of European Biochemical Societies sequencing uses to determine promoter methylation rate on PCDHB15 promoter region in GES, AGS, MKN45. Colony formation assay was performed in AGS, MKN45 with or without PCDHB15overexpression. Immunohistochemically (IHC) was performed to analyze gastric tumor samples from 130 patients in Changhua Christian Hospital and Kaplan-Meierplot to observe the survival rate. Based on different markers to classify 4 subtypes of gastric cancer (CIN/GS/MSI/EBV).

**Results and Discussions**

Gastric cancer cells showed higher promoter hypermethylation at PCDHB15 than normal cells, showing a negative correlation with SLC25A22 expression based on Pyro-sequencing and qPCR on AGS, MKN45, and GES. Overexpression of PCDHB15 in AGS and MKN45 decreased cell proliferation. Additionally, Kaplan Meier plots showed that a higher level of PCDHB15 increases the survival rate in several types of cancers. We expect that SLC25A22 plays an important role in regulating the epigenetic machinery. Targeting STAT3 and glutaminolysis may be a novel strategy for treating gastric cancer.

**Conclusion**

PCDHB15 may be a tumor suppressor which is suppressed by SLC25A22 in gastric cancer. The role of SLC25A22 in the epigenetic silencing of PCDHB15 deserves further investigation.

### EACR23-0147
### Association of TERT gene promoter methylation with the aggressiveness of differentiated thyroid cancer

*J. Periša[1], I. Šamija[1], I. Blažeković[1], M. Romić[1], D. Raos[2], N. Sinčić[2], T. Jukić[1], A. Fröbe[1], Z. Kusić[3]*

*[1]University Hospital Center Sestre milosrdnice, Department of Oncology and Nuclear Medicine, Zagreb, Croatia*

*[2]University of Zagreb School of Medicine, Department of Medical Biology, Zagreb, Croatia*

*[3]Croatian Academy of Sciences and Arts, Croatian Academy of Sciences and Arts, Zagreb, Croatia*

**Introduction**

Differentiated thyroid cancer (DTC) incidence has been increasing over decades and accounts for 1-2% of all cancers diagnosed. Overdiagnosis of relatively indolent DTC represents a financial burden to healthcare systems. New biomarkers of aggressiveness are being examined, amongst which molecular markers show promising potential. BRAF V600E mutations, RAS, and TERT promoter mutations (TPMs) are the most common genetic alterations in DTC. However, not all thyroid carcinomas with increased TERT expression harbored TPMs. The methylation of TERT promoter mutation is one of the less studied mechanisms that regulates TERT expression on an epigenetic level. This study aimed to examine the association of TERT gene promoter methylation in differentiated thyroid cancer tissue with metastatic status as a direct measure of the aggressiveness of the disease.

**Material and Methods**

This study included 166 patients (78 presented without metastatic disease, 64 with locoregional, and 24 with distant metastases). The region of interest upstream of the transcription start site was selected according to the literature and span over 5 CpGs representative for promoter methylation. DNA was isolated from formalin-fixed paraffin-embedded tissue of primary thyroid cancer. Bisulfite conversion, purification, and amplification preceded the pyrosequencing of the selected region. Average methylation of all 5 CpG sites was noted. Association of methylation and metastatic status was examined using the Kruskal-Wallis test (p-value $< 0.05$ was considered statistically significant). Research was funded by the Croatian Science Foundation project IP-2019-04-1130.

**Results and Discussions**

TERT promoter methylation medians and interquartile ranges (IQR) were 17 % (IQR 6% - 34%) for patients with non-metastatic disease, 23 % (IQR 7,5% - 33,5%) and for patients with locoregional metastases and 33% (IQR 24,5% - 43%) for patients with distant metastatic disease. Methylation of TERT promoter in distant metastatic group was higher and differed significantly from other two groups ($P = 0.045$, Kruskal-Wallis test, post-hoc analysis Conover).

**Conclusion**

In this study we confirmed association of TERT promoter methylation in primary cancer tissue with metastatic status of the disease indicating association of promoter methylation with aggressiveness of differentiated thyroid cancer.

### EACR23-0330
### Novel functional screening approach with CRISPR/Cas9 and dCas9-based paired gRNA library targeting differential CTCF binding motifs in IDH-mutant gliomas

*E. Yilmaz[1], A. Kayabolen[1], D. Ozturan[2], S. Sarac[1], N. Lack[1,2], T. Bagci-Onder[1]*

*[1]Koc University, School of Medicine, Istanbul, Turkiye*

*[2]University of British Columbia, Department of Urologic Sciences, Vancouver, Canada*

**Introduction**

Isocitrate dehydrogenase (IDH) mutations are frequently observed in gliomas, and their mutation status has been accepted as a CNS tumor classification marker. IDHs are enzymes that catalyze the conversion of isocitrate to alpha-ketoglutarate (α-KG). The mutations result in neomorphic enzymatic activity, by which D-2-hydroxyglutarate is produced from α-KG. This leads to the inhibition of α-KG-dependent enzymes, such as DNA and histone demethylases, thereby altering chromatin structure and gene expression profile. Therefore, further understanding the functions of hypermethylated DNA and histone profile in IDH-mutant gliomas are of utmost priority.

**Material and Methods**

Recent studies showed that IDH-mutant gliomas have hypermethylation at the binding sites of cohesin and CCCTC binding factor (CTCF), the methylation-sensitive insulator protein, leading to reduced CTCF binding, the loss of insulation between topological domains, and ultimately aberrant oncogene activation. We reanalyzed available CTCF ChIP-seq data of three IDH-wild type (wt) and three IDH-mutant glioma cell lines and determined 602 lost and 344 gained CTCF ChIP-seq regions. We then chose the intersection of differential methylated regions

© 2023 The Authors. Molecular Oncology is published by John Wiley & Sons Ltd on behalf of Federation of European Biochemical Societies and differential CTCF ChIP-seq peaks and aimed to explore their roles in cell survival. To investigate the effect of deletion, methylation, and demethylation of a specific CTCF binding motif, we designed a novel paired gRNA (pgRNA) library targeting the left and right sides of these differential regions.

**Results and Discussions**

This pgRNA library offers advantages over available libraries as it can be utilized with different CRISPR systems. Given the limitations of designing specific and efficient gRNAs directly targeting CTCF binding motifs because of motif sequence similarities, our pgRNA library would offer a powerful strategy for functional screens. In addition to Cas9-based screening, we will use this library with dCas9-TETv4 in IDH-mutant glioma cells and with dCas9-DNMT3A-DNMT3L in astrocytes and IDH-wt glioma cells.

**Conclusion**

We aim to find out important CTCF binding motifs and significant chromatin interactions regulating glioma cell survival by (epi)genetically targeting specific regions that alter the binding of CTCF and consequently regulate the boundaries between topological domains. This high-throughput (epi)genome editing approach will allow for identifying regulatory interactions leading to survival and drug response in IDH-wt and IDH-mutant gliomas and can be further applied to other cancer types.

### EACR23-0362
### A natural variant of the ZNF217 oncogene retains positive transcriptional activity and cooperates with a key epigenetic regulator

<u>P. Fahme</u>[1,2], P. Clézardin[1,2], P.A. Cohen[1,2]
*[1]Université Lyon 1, Faculté de Médecine, Lyon, France*
*[2]INSERM, Research Unit UMR_S1033- LyOS, Lyon, France*

**Introduction**

ZNF217 is an oncogenic transcription factor that promotes cell proliferation, invasion, and chemotherapy resistance. It is a component of chromatin remodeling complexes and, while primarily acting as a transcriptional repressor, ZNF217 also acts as a double-faceted transcription factor by positively regulating the expression of several genes, of which, the *ErbB3* gene. The expression of a candidate splice variant of *ZNF217* (*ZNF217-ΔE4*) has been recently observed in primary breast tumor samples, but its transcriptional activity has barely been investigated.
The *ZNF217-ΔE4* mRNA encodes for a ZNF217 protein isoform with a distinct C-terminus sequence. We aim to: (i) investigate whether the ZNF217-WT and ZNF217-ΔE4 isoforms have similar or distinct transcriptional activity and recruitment at the promoter of the *ErbB3* gene; (ii) decipher the role of the ZNF217 C-terminus region; and (iii) explore the impact of the interaction between the two ZNF217 isoforms and an epigenetic partner (EP) on their respective genomic activities.

**Material and Methods**

Ectopic overexpression of ZNF217-WT, ZNF217-ΔE4, truncated variants of ZNF217's C-terminus, or EP was performed in HEK293T cells. We conducted a chromatin immunoprecipitation assay to examine histone marks and the recruitment of ZNF217-WT, ZNF217-ΔE4, and EP at the *ErbB3* gene promoter. Transcriptional activity at the *ErbB3* gene promoter was explored using a reporter gene assay.

**Results and Discussions**

Our study highlighted that ZNF217-ΔE4 retains recruitment capacity at the *ErbB3* gene promoter and exhibits stronger positive transcriptional activity than that of ZNF217-WT (determined by gene reporter assay and enrichment of histone activation marks). Experiments conducted in the presence of ZNF217 truncated variants revealed that the ZNF217-ΔE4's C-terminus region is crucial for mediating these effects. Our data found that EP is recruited to the *ErbB3* promoter when ZNF217-WT or ZNF217-ΔE4 is present. Strikingly, EP can positively regulate the transcription at the *ErbB3* gene promoter, alone but also through cooperation with ZNF217-WT and ZNF217-ΔE4.

**Conclusion**

These findings reveal that the ZNF217-ΔE4 isoform possesses functional transcriptional activity, and there is a positive collaboration between ZNF217 isoforms and EP in controlling oncogenic transcriptional programs at the genomic level. These findings could lead to new therapeutic strategies for cancers driven by dysregulated transcriptional programs.

### EACR23-0416
### POSTER IN THE SPOTLIGHT
### Mechanisms of Fusion-driven sarcomas involving variant PRC1

<u>A. Pejkovska</u>[1], N. Benabdallah[1], S. Placke[1], D. Helm[2], M. Schneider[2], L. Wagner[1], F. Huth[1], H. Peterziel[3], I. Oehme[3], A. Banito[1]
*[1]German Cancer Research Center DKFZ, B380 Soft tissue Sarcoma, Heidelberg, Germany*
*[2]German Cancer Research Center DKFZ, W120 Protein analysis, Heidelberg, Germany*
*[3]German Cancer Research Center DKFZ, B310 Paediatric oncology, Heidelberg, Germany*

**Introduction**

BCL-6 co-repressor (BCOR) is part of the non-canonical Polycomb repressive complex 1.1 (PRC1.1) that promotes gene silencing by depositing ubiquitin onto lysine 119 of histone H2A (H2Aub). PRC1.1 is instrumental in development and differentiation. Recent data has uncovered new BCOR fusion oncogenes as recurrent events in several sarcomas. However, it remains unclear how they lead to sarcoma development. In this project, we investigated two oncogenic fusions involving BCOR: BCOR-CCNB3 and ZC3H7B-BCOR.

**Material and Methods**

We developed a research model based on immortalized human mesenchymal stem cells (hMSCs) which combines simultaneous induction of BCOR oncogenic fusions and silencing of the endogenous *BCOR* gene. Through RNA sequencing and comparison to patient-derived expression data, we verified whether the oncogenic fusions lead to transcriptional reprograming that mimics expression signatures of human tumours. We further investigated the impact of BCOR-CCNB3 and ZC3H7B-BCOR on the integrity and activity of the PRC1.1 complex by co-IP and an imaging-based *in vitro* chromatin recruitment assay. Using co-IP-MS, we identified novel interactors that are

© 2023 The Authors. Molecular Oncology is published by John Wiley & Sons Ltd on behalf of Federation of European Biochemical Societies recruited to PRC1.1 target genes by the oncogenic fusions. Finally, we developed and utilized a patient-derived cell line harbouring a BCOR-CCNB3 mutation to confirm the findings generated in the models where the BCOR fusions were exogenously expressed.

**Results and Discussions**

We discovered that expression of the BCOR fusions led to transcriptional reprogramming of hMSCs in a manner that recapitulated patient-derived transcriptional signatures. This included activation of developmental genes and differed from BCOR silencing. By a combination of methods that enabled the study of protein interactions, we showed that BCOR-CCNB3 and ZC3H7B-BCOR do not disrupt the PRC1.1 complex, or its ability to deposit H2Aub. Instead, the BCOR fusions recruit novel transcriptional activators and cofactors that lead to aberrant upregulation of target genes.

**Conclusion**

In summary, our data indicates a novel gain-of-function mechanism that leads to sarcomagenesis. Here we propose that BCOR-CCNB3 and ZC3H7B-BCOR are able to recruit transcriptional activators to PRC1.1 sites, resulting in their aberrant activation which in turn may lead to deregulation of normal differentiation programs.

### EACR23-0422
### Genome-wide DNA methylation sequencing methods for cancer biomarker discovery: potential and limitations

*A.R. de Abreu*[1], *J. Ibrahim*[1], *L. Mateiu*[2], *M. Peeters*[3], *G. Van Camp*[1], *K. Op de Beeck*[1]
[1]*University of Antwerp and Antwerp University Hospital, Center of Medical Genetics CMG / Center for Oncological Research CORE, Edegem, Belgium*
[2]*University of Antwerp, Center of Medical Genetics, Edegem, Belgium*
[3]*Antwerp University Hospital UZA, Department of Oncology, Edegem, Belgium*

**Introduction**

DNA methylation is an important gene expression marker in human cancer. It is extensively explored as a source of biomarkers for diagnosis, prognosis, prediction and follow-up. Numerous methods exist for profiling, but many do not provide a comprehensive understanding of the disease. Despite its shortcomings, bisulfite sequencing remains the reference method for investigating methylation. Although several methods for circumventing associated limitations have been proposed, there has yet to be a clear consensus on which is most appropriate for genome-wide methylation profiling.

**Material and Methods**

We extensively examinated different DNA methylation analysis methods to make recommendations on which method is most suited for various applications. We assessed DNA methylation profiles from a genome derived from blood and four human cancer genomes derived from fresh tissue (n=2) and cell lines (n=2). In addition, we sought to adapt to a clinical setting where formalin-fixed paraffin-embedded (FFPE) samples are routinely used and checked the ability to sequence FFPE-DNA. Genomic DNA was subjected to short-read Whole-Genome Bisulfite Sequencing (WGBS), MethylationEPIC BeadChip, Enzymatic Methyl-Sequencing (EMSeq) and long-read sequencing by Oxford Nanopore Technologies (ONT). We compared the methods based on feasibility, coverage, accuracy, cost, and applicability for clinical diagnostics.

**Results and Discussions**

After rigorous quality assessment, we found overall high concordance between the methods, but differences in the efficiency of read mapping, CpG calling, and coverage. ONT detected sites inaccessible to short-read assays, many of which may have biological significance. The methylation signals obtained by ONT are highly comparable to WGBS, EMSeq and ONT with average Pearson correlation values of $r = 0.925$. Furthermore, due to structural interference with the nanopores, the current ONT chemistry does not permit native sequencing of FFPE-DNA. Moreover, despite using restoration methods, EPIC only occasionally produces satisfactory data due to the fragmented state and poor quality of the DNA.

**Conclusion**

Our findings provide an exposition of the advantages, shortcomings, and overall performance of several methylation profiling methods. Notably, when using FFPE samples, EPIC and native ONT sequencing fail to generate (high quality) data. Thus, WGBS or EMSeq are great alternatives. Overall, we provide a decision-making resource based on budget, DNA input and -source for genome-wide DNA methylation profiling studies.

### EACR23-0424
### DNA damage and methylation loss triggers cfDNA release in colorectal cancer cells

*V. Pessei*[1], *M. Macagno*[1], *E. Mariella*[2], *G. Gionfriddo*[2], *S. Lamba*[1], *G. Crisafulli*[3], *P. Battuello*[2], *A. Lorenzato*[2], *D. Oddo*[2], *A. Bardelli*[2], *F. Di Nicolantonio*[2], *L. Barault*[2]
[1]*Candiolo Cancer Institute- FPO-IRCCS, Department of Oncology, Candiolo, Italy*
[2]*University of Torino, Department of Oncology, Torino, Italy*
[3]*IFOM- The AIRC Institute of Molecular Oncology, Department of Oncology, Milano, Italy*

**Introduction**

Liquid biopsy based on cell free DNA (cfDNA) analysis could assist the diagnosis, genotyping and monitoring of solid tumors. Human cancers release cfDNA in the bloodstream due to a combination of events, including cell death, active and passive release. However, the precise mechanisms leading to cfDNA shedding remain to be fully characterized. Addressing this question in patients is complicated by several confounding factors, including highly variable levels of cfDNA and release of nucleic acids from normal cells. In this work we exploited cancer models to dissect basic mechanisms of DNA release.

**Material and Methods**

We measured cell loss ratio, doubling time and cfDNA release in the supernatant of a colorectal cancer (CRC) cell line collection (N=76) representative of the clinical subtypes. Quantitative parameters of cfDNA release and cell growth were correlated to omics features. Functional assays were performed to test how modulation of DNA methylation and/or DNA damage repair impact on cfDNA release.

**Results and Discussions**

© 2023 The Authors. Molecular Oncology is published by John Wiley & Sons Ltd on behalf of Federation of European Biochemical Societies

High levels of cfDNA release were associated with several variables: slow cell cycle, cell crashes, lack of CpG Island Methylator Phenotype (CIMP), impaired DNA integrity and MSI phenotype. We exploited methylation microarrays to identify probes significantly associated with cfDNA shedding and derive a signature capable of discriminating high from low cfDNA releasers. We applied this methylation signature to an independent set of 165 cell lines to select models predicted to be low or high releasers. The methylation profile of these cells successfully correlated with the amounts of cfDNA released in the supernatant. Genetic ablation of DNA methyl-transferases stimulated cfDNA release in CRC lines. Pharmacological induction of demethylation by decitabine was able to increase cfDNA shedding in low releaser models. Increased loss of DNA integrity was confirmed in high cfDNA releaser cells by comet assay. Considering that loss of DNA integrity could be a consequence of DNA repair deficiency, we assessed cell models in which DNA damage repair genes had been inactivated using the CRISPR CAS approach. Genetic deletion of ATM and T53BP1 were associated with a significant increase in cfDNA release.

**Conclusion**

Methylation status and DNA integrity contribute to the variability of cfDNA amounts. These findings could inform strategies to artificially modulate cfDNA release and increase sensitivity of liquid biopsy assays.

### EACR23-0500

### Regulative role of miR-770-5p on PRMT5-EGFR axis in TNBC: focus on miR-770-5p / PRMT5 interaction and EGFR signal regulation

*S. Noyan*[1], *B. Gur Dedeoglu*[1], *M. Alikasifoglu*[2]

[1]*Ankara University, Biotechnology Institute, Ankara, Turkiye*

[2]*Hacettepe University, Faculty of Medicine- Department of Medical Genetics, Ankara, Turkiye*

**Introduction**

Breast cancer is a sporadic disease with genetic and epigenetic components. The concept of combining different therapeutic agents for synergistic benefits in treatment has been utilized for years. TNBC overexpresses the epidermal growth factor receptor, and this makes EGFR an attractive therapeutic target in TNBC. PRMT5, which is a histone methyl transferase is shown to be upregulated in breast cancers. Epigenetic dysregulation of a significant number of oncogenes and tumor suppressor genes in cancer have been reported over the last few decades. Targeting epigenetic processes to alter their role in tumorigenesis and/or malignancy could be a promising therapeutic strategy to treat or prevent breast cancer. In recent years, the emergence of miRNAs as cancer biomarkers has added an extra dimension to the molecular signatures of breast cancer. Although the use of miRNA candidates for the development of therapeutics are growing rapidly, little is known about all possible biological interactions of a candidate miRNA. The aim of this study is to claify how miR-770-5p regulates EGFR signaling through PRMT5 in TNBC cells.

**Material and Methods**

At first, to determine the relationship between miR-770-5p and putatative target, PRMT5, we explored the expression profiles of these genes in TCGA breast cancer cohort. The protein expression levels of PRMT5, EGFR and downstream elements were investigated by western blot analysis. The affect of PRMT5 inhibitor EPZ015666 on proliferation was analyzed by IncuCyte realtime imaging system.

**Results and Discussions**

According to TCGA data analysis, the expression of PRMT5 was significantly high in tumor samples and negatively correlated with miR-770-5p expression in tumor samples compared to normal tissues. Western blot experiments demonstrated that upon overexpressing miR-770-5p in TNBC cells PRMT5 expression was decreased. Concordant with our PRMT5 down-regulation data, EGFR and p-EGFR expression levels were decreased together with p-AKT and p-ERK in miR-770-5p transfected cells. Also, when miR-770-5p was overexpressed in MDA-MB-468 cells, the sensitivity was increased compared to only EPZ015666 treated cells.

**Conclusion**

Our previous findings and this study will enable us to explore the role of miR-770-5p in breast cancer in broader aspect. It is thought that reducing the expression of PRMT5, which is highly expressed in breast cancer, by miR-770-5p may provide inhibition of EGFR signal, and elucidation of this mechanism will add a new dimension to TNBC potential treatment approaches.

### EACR23-0621

### Convergence of RAS and PP2A activity on epigenetic gene regulation

*M. Sharma*[1], *A. Aakula*[1], *F. Tabaro*[2], *R. Nätkin*[2], *J. Kamila*[1], *H. Honkanen*[1], *M. Schapira*[3], *C.H. Arrowsmith*[4], *M. Nykter*[2], *J. Westermarck*[1]

[1]*Turku Bioscience Centre- University of Turku and Åbo Ak ademi University- Turku- Finland, Pathology, Turku, Finland*

[2]*Laboratory of Computational Biology- Faculty of Medicin e and Health Technology- Tampere University and Tays C ancer Centre- Tampere- Finland, Bioinformatics, Tampere, Finland*

[3]*Structural Genomics Consortium- University of Toronto-Toronto- Canada, Department of Pharmacology and Toxicology- University of Toronto- Toronto- Canada, Toronto, Canada*

[4]*Princess Margaret Cancer Centre- University Health Net work- Toronto- Canada, Department of Medical Biophysics- University of Toronto-Toronto- Canada, Toronto, Canada*

**Introduction**

Protein phosphatase 2A (PP2A) is a tumor suppressor while RAS proteins are potent oncogenes in different cancer types. It is well established that RAS-mediated oncogenic transformation requires simultaneous inhibition of PP2A, but the molecular basis of their interaction is poorly understood. To address the target mechanisms co-regulated by RAS and PP2A we performed a phosphoproteomics screen upon RAS or PP2A manipulation. Overall the results indicated that RAS and PP2A regulate overlapping cellular processes and that their

© 2023 The Authors. Molecular Oncology is published by John Wiley & Sons Ltd on behalf of Federation of European Biochemical Societies activities might in particular converge at epigenetic machineries.

**Material and Methods**

To study the RAS and PP2A synergy on transcriptional and epigenome regulation we performed multi-omics analysis of cancer cells in which RAS and PP2A activities were modulated. To investigate the DNA methylation changes and chromatin remodeling effects upon PP2A modulation we used RRBS (Reduced representation bisulfite sequencing) & ATAC-seq (Assay for Transposase-Accessible Chromatin using sequencing) respectively.

**Results and Discussions**

The study reveals that RAS and PP2A regulate common phosphosites on epigenetic proteins such as HDAC1/2, KDM1A, MTA1/2, RNF168, and TP53BP. Either pharmacological or siRNA mediated RAS or PP2A modulation significantly affected HDAC1/2 recruitment to the chromatin while mutagenesis of the co-regulated phosphosite on RNF168 affected its interaction with TP53BP1. Further RAS activation and PP2A inhibition resulted in derepression of a GFP reporter indicative of epigenetic silencing and global gene expression leading to oncogenic transcription. PP2A inhibition further resulted in global DNA hypomethylation and an open state of chromatin confirming its role as a global repressor of epigenetic processes.

**Conclusion**

Collectively these results identify a novel phosphorylation switch on chromatin repressor complex as a converge point of KRAS and PP2A activities in cancer. Generally the results provide first indications to global importance of PP2A-mediated phosphorylation regulation in epigenetic gene regulation in cancer.

### EACR23-0632
### KDM6A is a Druggable Regulator of AR-V7 in Castrate Resistant Prostate Cancer

*R. Nelson[1], N. Brittian[1], L. Walker[1], L. Gaughan[1]*
*[1]Newcastle University Centre for Cancer, Translational and Clinical Research Institute, Newcastle Upon Tyne, United Kingdom*

**Introduction**

Prostate Cancer (PC) progresses to a metastatic form of cancer called Castration-Resistant Prostate Cancer (CRPC) after treatment with androgen deprivation therapies (ADTs). Epigenetic reprogramming through altered expression and activity of histone modifier proteins is one major mechanism of tumour resistance. Histone lysine methyltransferases (KMTs) and demethylases (KDMs) are becoming important epigenetic targets in CRPC. Here, we have focused on investigating the function of lysine demethylase 6A (KDM6A), a H3K27 demethylase, in CRPC. KDM6A is mutated in ~13% of CRPC and its expression is positively correlated with that of androgen receptor variant 7 (AR-V7) suggesting its role in the generation and regulation of the clinically significant AR-Vs.

**Material and Methods**

An siRNA screen targeting KDMs in CW22RV1-AR-EK cells was conducted to identify which KDMs regulated expression of AR-V7. Next, we interrogated the TCGA-PRAD dataset to identify which KDMs were significantly up-regulated in AR-V7+ tumours. VCAP cells were grown in castrate conditions, to induce AR-V7 expression, prior to Chromatin immuo-percipitation (ChIP) experiments to assess KDM6A abundance on the AR locus. Bulk RNA-Seq analysis following KMD6A silencing, or inhibition revealed the transcriptional programmes driven by KDM6A in PC cells. *In vivo* studies revealed the effect of KDM6A inhibition on tumour growth.

**Results and Discussions**

KDM6A is highly mutated in CRPC and significantly higher expressed in AR-V7+ patient tumours compared to AR-V7-. Over-expression of KMD6A lead to increased expression of AR-V7 in multiple PC cell lines. Interestingly our ChIP data reveals a significantly higher abundance of KDM6A at Cryptic Exon 3 (CE3) in conditions that drive AR-V7 generation, suggesting a role in regulating AR-V7. Knockdown (KD) or therapeutic inhibition of KDM6A significantly reduced PC cell line proliferation and reduces expression of AR-V7 and AR-FL. Notably, we have shown that therapeutic inhibition of KDM6A resensitised CW22RV1 cells to Enzalutamide which will be further validated *in vivo.*

**Conclusion**

To conclude we have demonstrated a relationship between KDM6A and AR-V7. Our data indicates that during PC progression, deregulation of epigenetic enzymes, such as KDM6A, may be crucial for the generation and regulation of AR-Vs. Taken together, our data suggests targeting KDM6A in CRPC may be a novel strategy to inhibit tumour growth.

### EACR23-0674
### KDM6A/UTX Loss Promotes Plasticity Between Small Cell Lung Cancer Subtypes

*L. Duplaquet[1], Y. Li[1], M.A. Booker[2], Y. Xie[3], D. Hong[1], P. Cejas[3], M.Y. Tolstorukov[2], H.W. Long[3], M.C. Haffner[4], M.G. Oser[1]*
*[1]Dana Farber Cancer Institute- Harvard Medical School, Medical Oncology, Boston, United States*
*[2]Dana Farber Cancer Institute- Harvard Medical School, Department of Informatics and Analytics, Boston, United States*
*[3]Dana Farber Cancer Institute- Harvard Medical School, Center for Functional Cancer Epigenetics, Boston, United States*
*[4]Fred Hutchinson Cancer Research Center, Division of Human Biology, Seattle, United States*

**Introduction**

Small cell lung cancer (SCLC) exists broadly in four molecular subtypes: ASCL1, NEUROD1, POU2F3, and Inflammatory. Initially, SCLC subtypes were thought to be mutually exclusive, but recent evidence shows intra-tumoral subtype heterogeneity and plasticity between subtypes. KDM6A (also known as UTX), a H3K27me3 histone demethylase that also has scaffolding functions in the COMPASS complex that catalyze mono-methylation of H3K4, is involved in development and differentiation in various tissues. We therefore hypothesized that KDM6A genetic loss, which occurs in SCLC, could have consequences on neuroendocrine differentiation that impacts determination of SCLC subtype.

**Material and Methods**

To study the consequences of KDM6A loss in SCLC, we used our CRISPR-based genetically-engineered mouse

© 2023 The Authors. Molecular Oncology is published by John Wiley & Sons Ltd on behalf of Federation of European Biochemical Societies model to generate autochthonous SCLCs that were *Kdm6a*-Mutant or *Kdm6a*-WT. In this model, autochthonous SCLC lung tumors are generated by intratracheally injecting adenoviruses encoding Cre recombinase and sgRNAs targeting *Rb1*, *Trp53*, and *Rbl2* (to induce SCLC initiation) and *Kdm6a* or control sgRNAs into lox-stop-lox Cas9 mice. The tumors formed were harvested and used to perform both bulk and single-cell RNA- and ATAC-sequencing analysis. Cell lines were also established from these tumors to perform more functional mechanistic studies including ChIP-seq.

**Results and Discussions**

We found that while *Kdm6a*-WT tumors express only ASCL1, KDM6A inactivation induced plasticity from ASCL1 to NEUROD1 resulting in SCLC tumors that heterogeneously express ASCL1 and NEUROD1. Mechanistically, KDM6A normally maintains an active chromatin state that favors the ASCL1 subtype with its loss decreasing H3K4me1 and increasing H3K27me3 at enhancers of neuroendocrine genes leading to a cell state that is primed for ASCL1 to NEUROD1 subtype switching. KDM6A does not directly bind NEUROD1 and therefore based on this and KDM6A's canonical activity of maintaining chromatin in a state permissible for gene activation, our data suggests that NEUROD1 induction upon KDM6A loss is indirect. We found that the H3K4me3 methyltransferase MLL/MENIN complex, which is upregulated upon KDM6A loss, is necessary for NEUROD1 induction.

**Conclusion**

This work identifies KDM6A as an epigenetic regulator that controls ASCL1 to NEUROD1 subtype plasticity and provides a novel autochthonous SCLC GEMM to model ASCL1 and NEUROD1 subtype heterogeneity and plasticity, which is found in 35-40% of human SCLCs.

### EACR23-0709

### Crosstalk between alterations in SWI/SNF complex and canonical tumour pathway in lung cancer

*R. Marqueño[1], B. Monterde[1], R. Blanco[1], D. Martín[1], I. Varela[1]*

*[1]Instituto de Biomedicina y Biotecnologia de Cantabria IB BTEC, Señalización Molecular y Celular, Santander, Spain*

**Introduction**

SWI/SNF complex is an ATP-dependent chromatin remodeller involved in the regulation of chromatin structure through disruption of nucleosomes. Consequently, it is involved in transcriptional regulation. Over the last ten years, multiple evidence for their role in tumour suppression has emerged. Indeed, SWI/SNF is displayed as the second most frequent alteration in cancer as nearly 20% of all cancers present inactivating mutation in genes encoding SWI/SNF subunits. Nevertheless, little is known about their target molecular pathways.

**Material and Methods**

In this project, non-small cell lung cancer cell lines deficient for the SWI/SNF complex were generated. For that purpose, we produced inducible knockdown cell lines against *ARID1A*, *ARID2* and *SMARCA4*, the SWI/SNF genes with the highest rate of mutation. Once confirmed the reduction of the expression of the target genes by qPCR, we performed RNA-seq, ATAC-seq and Cut&Run experiments to identify the molecular pathways altered by SWI/SNF deficiency. Additionally, the cells were treated with TGF-β together with EGF to induce the epithelial to mesenchymal transition (EMT). We assessed differences in cell morphology and gene expression pattern between the wildtype and the deficient SWI/SNF cell line.

**Results and Discussions**

Disruption of the SWI/SNF complex can affect different cellular processes such as DNA repair, gene expression or cellular differentiation. Our results show that depletion of one subunit of SWI/SNF complex triggers changes in the gene expression pattern of multiple molecular pathways. Interestingly, we have identified a transcriptional program compatible with an easier epithelial to mesenchymal transition (EMT) in SWI/SNF deficient cells which is accompanied by a higher resistance to EGFR inhibitors. All this data suggests a possible interplay between SWI/SNF complex and EMT molecular pathway.

**Conclusion**

Our results suggest that SWI/SNF deficiency can promote tumour progression, in part through epigenetic rewiring promoting an easier epithelial to mesenchymal transition which affects the sensitivity to some current antitumoral treatments for lung cancer.

### EACR23-0727

### A novel bisulfite free, highly multiplexable assay to detect DNA methylation levels

*I. Neefs[1,2], J. Vandenhoeck[1,2], T. Vanpoucke[1,2], J. Ibrahim[1,2], A. Suls[1], A. Hoischen[3], M. Peeters[2], K. Op de Beeck[1,2], G. Van Camp[1]*

*[1]Centre for Medical Genetics, University of Antwerp, Edegem, Belgium*

*[2]Centre for Oncological Research, Antwerp University Hospital and University of Antwerp, Edegem, Belgium*

*[3]Radboud University Medical Centre, Department of Human Genetics and Radboud Institute for Molecular Life Sciences, Nijmegen, The Netherlands*

**Introduction**

DNA methylation is an ideal candidate for several types of cancer biomarkers. Despite recent advancements, a low-cost, bisulfite-free detection method for multiplex analysis is still needed. To address this, we designed and optimized a next-generation methylation detection assay (patent-pending) by combining methylation-sensitive restriction enzymes (MSREs) and single-molecule Molecular Inversion Probes (smMIPs).

**Material and Methods**

DNA is first digested using a combination of four MSREs (HpaII, HpyCH4IV, AciI and HinP1I). As such, methylated CpGs remain intact, while unmethylated CpGs are digested. Then, smMIPs that are specifically designed for CpGs with high methylation levels in cancer and low methylation levels in normal tissues and blood, capture the methylated targets. Circular formed DNA fragment are amplified using multiplex PCR. Lastly, purified sample pools are sequenced using NGS.

**Results and Discussions**

We present the successful creation of our technology. To demonstrate the efficacy of the MSREs, qPCR was performed using λ phage DNA and primers for the restriction sites. Results show that methylated DNA is not

© 2023 The Authors. Molecular Oncology is published by John Wiley & Sons Ltd on behalf of Federation of European Biochemical Societies digested by the MSREs (Ct=5.7), while unmethylated DNA is (Ct=22). Furthermore, the internal control (unmethylated λ phage DNA) showed that only 1% of samples is not properly digested.
Notably, DNA input can be as low as 5 ng. It shows comparable results as inputs of 1µg DNA. This low amount of DNA input makes our technique well suited for use in liquid biopsies. To mimic ctDNA, cell line DNA was sheared and spiked-in in normal cfDNA samples. Results show that 20% ctDNA (lowest spiked-in tested) has 56% more normalized sequencing counts than and just cfDNA.
Importantly, the technology is highly robust and reproducible. Technical validation showed a Pearson's correlation of r= 0.99. Within the bland-Altman analysis only 5/75 of samples lay outside the limits of agreement, demonstrating the technology's repeatability.
Lastly, a biomarker panel was tested with our technology in 225 fresh frozen samples from the 8 most mortal cancer types. A sensitivity of 84% and a specificity of 93,5% was reached combining almost 1000 smMIPs.

**Conclusion**

In conclusion, we successfully created an innovative DNA methylation detection technology. The technique is fully optimized for low inputs of DNA from tissue samples. Further optimisations for use in liquid biopsies are currently ongoing.

### EACR23-0754
### GENTETIC AND EPIGENETIC CHARACTERIZATION OF HUMAN COLORECTAL CANCER: FROM TISSUES TO ORGANOIDS

*T. Gagliano[1], E. Kerschbamer[2], E. Malavasi[2], C. Weichenberger[3], U. Baccarani[2], G. Terrosu[2], C. Brancolini[2]*
*[1]University of Udine, DAME, Udine, Italy*
*[2]University of Udine, Department of Medicine, Udine, Italy*
*[3]EURAC, Bioinformatics, Bolzano, Italy*

**Introduction**

Colorectal carcinoma (CRC) is among the most commonly diagnosed cancers worldwide. In the past decades the genetic mutations and intra-tumour heterogeneity of CRC have been largely studied. Despite heterogeneity is widespread, epigenetic changes are also responsible for phenotypic variation between cancer cells. Particularly in CRC, epigenetic deregulation can be responsible of cancer onset and progression, understanding how DNA mutations and epigenetic events are related can shed light on CRC carcinogenesis opening new path for diagnosis and treatment.

**Material and Methods**

Human CRC tissues and patients derived organoids (PDOs) were obtained from surgery specimens. Tumor tissue and PDOs were compared with matching healthy tissues  by whole exome sequencing to identify DNA mutations thus classifying tumors and the different organoids.. PDOs with mutations in TP53 were selected for treatments  with HDACs inhibitors (HDACIs). Response to treatment was investigated by HDAC activity inhibition, Caspase activityand confocal microscopy. Changes in gene expression and chromatin accessibility were evaluated by RNA-seq and ATAC-seq.

**Results and Discussions**

As expected, the analysis of tumor tissues and PDOs revealed a wide heterogeneity in DNA mutations among patients.On the basis of their mutations specific PDOs were selected and treated with SAHA (a non-selective-HDACI) and NKL54 (a Class I HDACi). While SAHA reduced cell viability and activated apoptosis in PDOs regardless of their mutational status, NKL54, did not exerted the same effects. SAHA was able to strongly and dose dependently reduced the enzymatic activity of class I/II HDACs, while NKL54 was much less efficient similarly to entinostat. . Gene expression analysis and ATAC-seqrevealed that SAHA promotes the modulation of a higher number of genes compared to NKL54 and, for those in common, with a stronger effect.**Conclusion**

Our experiments confirm that CRC is indeed a malignancy with wide intra-patient heterogeneity,. The non-selective HDACI SAHA is able to significantly reduce cell viability and to activate apoptosis, while affecting several signalling pathways in PDOs. By contrast, class I selective HDACIs are much less effective as single agents against PDOs growth. The differential regulation of chromatin adaptations, genes and pathways by the two HDACI will be discussed.

### EACR23-0817
### Identification of Novel Epigenetic Modulators Regulating Glioblastoma Survival and Temozolomide Resistance.

*E.Y. Kala[1,2], A.C. Aksu[1,2], F. Senbabaoglu[1,2], O. Yedier-Bayram[1,2], G. Karabiyik[1,2], D. Yigci[1], H. Syed[2,3], T. Bagci-Onder[1,2]*
*[1]Koç University, School of Medicine, Istanbul, Turkiye*
*[2]Koç University, Research Center for Translational Medicine-KUTTAM, Istanbul, Turkiye*
*[3]Koç University, Biostatistics- Bioinformatics and Data Management Core- KUTTAM, Istanbul, Turkiye*

**Introduction**

Glioblastoma (GBM) is the most aggressive primary brain tumor with poor survival. The therapeutic approach includes surgery, irradiation, and chemotherapy with Temozolomide (TMZ), an alkylating agent applied as standard of care. TMZ is highly effective, however, the efficacy of TMZ can be reversed by several epigenetic mechanisms, including the regulation of the expression of 0-6-methylguanine methyltransferase (MGMT) enzyme. Promoter methylation status of MGMT is an important prognostic factor as its epigenetic silencing leads to improved TMZ response. New epigenetic factors that regulate GBM survival and therapy resistance, like MGMT, are yet to be discovered.

**Material and Methods**

To study resistance mechanisms in GBM cells, we generated TMZ-resistant cell lines by applying dose-escalation regimen and further investigated resistant mechanisms with different cell-based assays. Transcriptome analysis was conducted to determine genes and pathways that are differentially regulated in TMZ-resistant cells compared to parental cells. TMZ-resistant cell lines show increased IC50 values along with higher colony forming abilities compared to naïve parental cell

© 2023 The Authors. Molecular Oncology is published by John Wiley & Sons Ltd on behalf of Federation of European Biochemical Societies lines, attesting to their resistant phenotype. MGMT was significantly upregulated at gene and protein expression level. Transcriptome analysis of TMZ-resistant cell lines uncovered key pathways for resistance. As resistance can be associated with adaptive epigenetic changes, we interrogated chromatin regulators' roles in TMZ-resistant cells. To this end, we conducted a focused CRISPR/Cas9-based screen with our Epigenetic Knock-Out sgRNA Library (EPIKOL) that targets various chromatin modifiers and epigenetic enzymes. We later validated the results of the screens using viability and competition assays.

**Results and Discussions**

Results of EPIKOL screens with TMZ-resistant cell lines demonstrated *MGMT* as a top scoring hit, attesting to the successful ability of EPIKOL to identify major regulators of TMZ response. We found novel epigenetic regulators modulating TMZ response, and their further investigation will allow us to identify novel mechanisms that can breach acquired TMZ resistance.

**Conclusion**

Together our results have the potential to develop new epigenetic-based therapeutic targeting strategies for GBM in the future.

### EACR23-0898

### Multiomic analysis of CDX2 loss in colorectal tumors unveils the clinical value of a refined CDX2-based risk scoring and provides new insights on the therapeutic benefit of epigenetic switching.

*F. Gimeno-Valiente[1], E. Duréndez-Sáez[2], B.R. Badia[3], C. Martínez-Ciarpaglini[4], M. Iglesias[5], J. Linares[3], C. Montagut[6], A. Cervantes[7], A. Calon[3], N. Tarazona[7]*

*[1]University College of London, UCL - Cancer Institute, London, United Kingdom*

*[2]INCLIVA Biomedical Research Institute, Medical Oncology, Valencia, Spain*

*[3]IMIM Hospital del Mar Medical Research Institute, Medical Oncology, Barcelona, Spain*

*[4]Hospital Clinico Universitario of Valencia. INCLIVA Bio medical Research Institute. University of Valencia. Institut o de Salud Carlos III- CIBERONC, Department of Pathology, Valencia, Spain*

*[5]IMIM Hospital del Mar Medical Research Institute- Unive rsitat Pompeu Fabra- CIBERONC, Department of Pathology, Barcelona, Spain*

*[6]IMIM Hospital del Mar Medical Research Institute- Unive rsitat Pompeu Fabra- CIBERONC, Medical Oncology, Barcelona, Spain*

*[7]Hospital Clinico Universitario of Valencia. INCLIVA Bio medical Research Institute. University of Valencia. Institut o de Salud Carlos III- CIBERONC, Medical Oncology, Valencia, Spain*

**Introduction**

In colorectal cancer (CRC), the lack of caudal homeobox 2 protein (CDX2) expression is related to a poor prognosis. The mechanisms causing CDX2 loss and their relationship to other genetic and transcriptomic abnormalities are unknown. Furthermore, there is an unmet need for clear guidelines to determine its expression. A better understanding of CDX2 function will improve patient classification and response to standard therapy.

**Material and Methods**

50 CRC patients were recruited. We used RNAseq to perform an analysis of tumour gene expression profiles and whole exome sequencing to determine mutational (single nucleotide variants, SNV) and copy number (CN) status. Furthermore, tumour methylation profiles were measured by an EPIC panel with 800k positions. Cancer cell aggressiveness was studied in CDX2 negative patient-derived organoids (PDO) genetically engineered with CRISPR-dCAS9 fused to catalytic TET and DNMT to restore CDX2 expression.

**Results and Discussions**

Based on Dalerba et al. histopathological score, we recognized 14/50 patients showing an unclassifiable (UC) pattern of CDX2 expression. We improved already known classification analyzing 5000 more variable CpG and 4/14 had a profile similar to CDX2 negative (UC-), whereas 9/14 were similar to CDX2+ (UC+). Using specific promoter CpG, we determined that methylation and gene expression levels of CDX2 were negatively correlated in CRC ($p=0.0076$). At the epi and transcriptomic level, UC- and CDX2- showed EMT, INFa and INFg response pathway activation versus UC+ and CDX2+ ($FDR<0.05$), with high correlations with EPHB2, -B6 and -A6. In addition, UC+ and CDX2+ had exclusive SNVs in *KRAS*, *APC* and *NRAS*, while UC- and CDX2- showed specific SNVs in *ERBB2*, *ARID3A* and *NSD1*. *KRAS* activation hallmark genes were overall under CN amplifications events in UC+ and CDX2+. The SNV, CN, transcriptomic and epigenomic patterns were maintained in UC- and CDX2- PDOs versus CDX2+ PDOs. UC- and CDX2- showed higher resistance to FOLFOX chemotherapy versus CDX2+ PDOs. Notably, restoring CDX2 expression in CDX2- PDOs by targeting methylation increased sensitivity to FOLFOX in resistant cancer cells.

**Conclusion**

Multiomic analysis of CDX2 expression loss in CRC provides relevant insight into cancer progression pathways triggered by CDX2, resulting in the development of a refined risk score based on CDX2 methylation and the discovery of novel therapeutic opportunities via epigenetic switching.

### EACR23-0899

### Global Gene Expression Regulation by TGFβ in Prostate Cancer through H3K4me3 and H3K9me3 Mark-A Complex Interplay between TGFβ and Histone Methylation.

*A. Naik[1], N. Dalpatraj[1], N. Thakur[1]*

*[1]Ahmedabad University, Biological and Life Sciences, Ahmedabad, India*

**Introduction**

Epigenetic alterations play an important part in carcinogenesis and affect different biological responses, including cell proliferation, migration, apoptosis, invasion, and senescence. In addition, growth factors, such as transforming growth factor-beta (TGFβ), are essential regulators of tumorigenesis. Our understanding of the mechanisms that establish transient TGFβ stimulation into stable gene expression patterns remains incomplete. Epigenetic marks like Histone H3 modifications are

© 2023 The Authors. Molecular Oncology is published by John Wiley & Sons Ltd on behalf of Federation of European Biochemical Societies directly linked with gene expression and play an essential role in tumorigenesis. However, the effects of TGFβ signaling on the genome-wide H3K4me3 and H3K9me3 landscape remain unknown.

**Material and Methods**

In this study, we performed chromatin immunoprecipitation-sequencing (ChIP-Seq) to identify the genome-wide regions that undergo changes in H3K4me3 and H3K9me3 occupancy in response to TGFβ stimulation in cancer.

**Results and Discussions**

We show that TGFβ induces the H3K4me3 mark on its ligands like TGFβ, GDF1, INHBB, GDF3, GDF6, and BMP5, suggesting a positive feedback loop. Most genes were involved in the positive regulation of transcription from the RNA polymerase II promoter in response to TGFβ. Other functional categories were intracellular protein transport, EMT, angiogenesis, antigen processing, histone H4 acetylation, positive regulation of cell cycle arrest, and genes involved in mitotic G2 DNA damage checkpoints. Conversely, H3K9me3 occupancy increases in intronic regions after short-term (6h) TGFβ stimulation and distal intergenic areas during long-term stimulation (24h). The epigenetic mechanisms-mediated regulation of gene expression by TGFβ was concentrated at promoters rich in SRY and FOXJ3 binding sites. Our results point toward a positive association between the oncogenic function of TGFβ and the H3K9me3 mark and provide a context for the role of H3K9me3 in TGFβ-induced cell migration and cell adhesion. Interestingly, these functions of TGFβ through H3K9me3 mark regulation seem to depend on transcriptional activation in contrast to the conventionally known repressive nature of H3K9me3. Interestingly, these functions of TGFβ through H3K9me3 mark regulation seem to depend on transcriptional activation compared to the conventionally known repressive nature of H3K9me3.

**Conclusion**

Our results link TGFβ stimulation to acute changes in gene expression through an epigenetic mechanism. These findings have broader implications on epigenetic bases of acute gene expression changes caused by growth factor stimulation.

### EACR23-0971

### Genome-wide DNA methylation changes and somatic mutational patterns of Lynch syndrome and Familial adenomatous polyposis-associated colorectal tumors

*S. Mäki-Nevala[1], A. Kauppinen[1], T. Nieminen[1], T. Seppälä[2], L. Renkonen-Sinisalo[2], A. Lepistö[2], J.P. Mecklin[3], P. Peltomäki[1]*

*[1]University of Helsinki, Department of Medical and Clinical Genetics, Helsinki, Finland*

*[2]Helsinki University Hospital, Department of Gastrointestinal Surgery, Helsinki, Finland*

*[3]Nova Hospital- Central Finland Health Care District, Department of Education and Science, Jyväskylä, Finland*

**Introduction**

Lynch syndrome (LS) and Familial adenomatous polyposis (FAP) are hereditary cancer predisposition syndromes characterized with early-onset colorectal cancer. Germline mutations in DNA mismatch repair genes (*MLH1*, *MSH2*, *MSH6*, and *PMS2*) cause predisposition to LS, and inherited mutations in the *APC* gene to FAP. We aim to characterize the methylomes and somatic mutations associated with MMR haploinsufficiency (LS) vs. constitutionally inactive APC (FAP) -driven tumorigenesis by studying normal mucosa, adenomas, and carcinomas from genetically predisposed individuals.

**Material and Methods**

Our study cohort is consisted of 100 LS patients and 30 FAP patients who went through colonic mucosa sampling during colonoscopy screenings. Sampling was done in three rounds, i.e. three sample sets for each patient were collected at different time points, whenever possible. Normal mucosa was sampled from at least one location within the colorectum (mostly three locations from LS patients), and polyps and carcinoma samples were collected whenever there was enough material after diagnosis purposes. All tissue samples were flash frozen in liquid nitrogen at the time of the collection.

For methylation study of LS patients, we analyzed 28 adenomas and five carcinomas paired with normal mucosa from the same colonic location. Normal mucosa samples from 16 LS carriers that had never been diagnosed with colon polyps or cancer were also available. Of FAP patients, all 30 paired polyp and normal colonic mucosa samples from the first sampling round were selected, and all carcinoma samples (at any round) were included (n=2). For somatic mutation study, all aforementioned tumorous samples with paired normal colon mucosa samples were selected together with patients' whole blood DNA. Genome-wide methylation was studied with Illumina Infinium MethylationEPIC array, and data was analyzed with R methylation analysis packages. Somatic mutations were studied by parallel sequencing targeting around 1000 cancer-associated genes.

The study is approved by the institutional ethical committees, and all patients have given informed consent.

**Results and Discussions**

Compared to paired normal mucosa, methylation changes are abundant in LS-associated adenomas, whereas FAP-associated polyps show less methylation changes. The investigation is ongoing, and initial results will be available for presentation and discussion.

**Conclusion**

Genome-wide methylation alterations are an integral part of the multistep colorectal tumorigenesis of LS and FAP.

### EACR23-1077

### A guide to successful CUT&RUN for analyses of epigenetic marks and mechanisms in disease

*M. Solman[1], T. Oliveira[1], A.H. Guo[2], C.R. Comeau[2], F. Chen[2], C.J. Fry[2]*

*[1]Cell Signaling Technology, Technical Support, Leiden, The Netherlands*

*[2]Cell Signaling Technology, Epigenetics, Danvers, United States*

**Introduction**

CUT&RUN (Cleavage Under Targets & Release Using Nuclease) is a powerful and versatile technique used for profiling protein-DNA interactions in a native chromatin context. CUT&RUN is a rapid, robust, and true low cell

© 2023 The Authors. Molecular Oncology is published by John Wiley & Sons Ltd on behalf of Federation of European Biochemical Societies number assay. Downstream, CUT&RUN can be combined with qPCR or NG-seq (Next-generation sequencing) to analyze histone modifications and binding of transcription factors, DNA replication factors, or DNA repair proteins at specific target loci or across the entire genome.

**Material and Methods**

CUT&RUN and NGS library preparation was performed using CST #86652, CST #56795, and CST#47538 according to manufacturer's instructions. Antibodies used in the CUT&RUN experiments are CTCF (CST #3418), SUZ12 (CST #3737), H3K4me3 (CST #9751), TCF4 (CST#2569), JARID2 (CST #13594), Glucocorticoid Receptor (CST #12041), and RING1B (CST #5694).

**Results and Discussions**

Unlike ChIP (chromatin immunoprecipitation), CUT&RUN is free from formaldehyde cross-linking, chromatin fragmentation, and immunoprecipitation. Compared to ChIP, CUT&RUN requires fewer starting cells (100K), is a much faster protocol (one day from cells to DNA), generates lower background signal (requires less sequencing depth), and offers spike-in control DNA for effective normalization of signal between samples and between experiments. The CST CUT&RUN Assay Kit is validated for use with fewer starting cells as input (5,000-20,000 cells) and fixed cells and tissues.

**Conclusion**

This poster discusses the basics of CUT&RUN and important factors to consider when setting up your experiment. In addition, I will provide data showing the versatility of this assay for mapping various histone modifications, transcription factor, and transcription cofactor binding sites in different cell and tissue types. Finally, I will discuss how the general protocol is optimized for greater signal to noise ratio, reduced number of starting cells, and provide an alternative digestion method for preparing input DNA as a critical control of CUT&RUN.

### EACR23-1135

### Deciphering epigenetic vulnerabilities of IDH mutant glioblastoma cells through genetic screens using custom-made CRISPR sgRNA library

*F.M. kucuktas*[1], *F. Seker-Polat*[1], *A. Kayabolen*[1], *T. Bagci-Onder*[1]

[1]*Koc University, Graduate School of Health Sciences, istanbul, Turkiye*

**Introduction**

Isocitrate dehydrogenase (IDH) has a critical role in citric acid cycle to convert isocitrate to a-ketoglutarate (a-KG). In low-grade glioma and secondary glioblastoma, IDH mutation which is thought to be an early-stage driver mutation is very common. Mutant IDH leads to 2-hydroxyglutarate (2-HG) accumulation, a potential oncometabolite. The oncometabolite causes DNA and histone hypermethylation in cells. IDH1/2 mutant gliomas lack curative treatments. We aimed to investigate the IDH wild-type and mutant phenotype specific epigenetic dependencies in glioblastoma via the CRSIPR/Cas9 based loss of function screens using Epigenome Wide sgRNA Library, EPIKOL, which targets all chromatin-related proteins and is designed by our lab.

**Material and Methods**

The EPIKOL Screen was performed on IDH wild type and mutant pair A172 cells. After the screen, we listed the hit genes specific for IDH wild-type and IDH-mutant cells according to p-values and log-fold changes. To validate the shortlisted hit genes specific to the wild-type and mutant cells, we produced Cas9-stable A172 IDH wild-type and -mutant cell lines and knocked out the hit genes on those cell lines. After confirmation of knock-out and gene expression levels, we applied colony formation and cell viability assays to observe phenotype.

**Results and Discussions**

We observed an IDH-mutant specific gene, RBBP7, which is essential to IDH-mutant glioblastoma. When knockout of the wild-type and mutant-specific genes does not show essentiality in IDH wild-type cells, interestingly, it was an essential gene for IDH-mutant cells according to the results of colony formation assays and cell viability assays.

**Conclusion**

RBBP7 shows IDH-mutant specific phenotype. In further studies, we plan to further investigate the relations between the hit genes with the IDH mutation. At the same time, which pathways the perturbation of these genes affect in IDH mutant cells and the mechanisms of action will be investigated.

### EACR23-1152

### Interferon Regulatory Factor 4 controls Epigenetic Silencing in Melanoma Cells

*U. Sobhiafshar*[1], *T. Emre*[1]

[1]*Bogazici University, Department of Molecular Biology & Genetics, Istanbul, Turkiye*

**Introduction**

Altered activities of transcription factors and epigenetic modifiers play central roles in defining the gene expression programs of cancer cells. Identification of the targets of these factors is a critical step in the development of new anti-cancer strategies, including epigenetic therapies. Interferon regulatory factor 4 (IRF4) is a transcription factor with important roles in the development and functioning of immune cells. We and others have previously shown dependence to IRF4 expression in a variety of immune cancers, and revealed its mechanisms of action. Several studies also implicate IRF4 in non-immune cells such as in melanocytes, and associate IRF4 genetic variation and expression to melanoma skin cancers. However, the role of IRF4 in melanoma remains poorly understood.

**Material and Methods**

We embarked on an investigation to identify the functions of IRF4 in melanoma using genome-wide, cellular and molecular biological approaches with melanoma cell line models. We performed transcriptomic and localization assays to identify IRF4 target genes, and integrative analysis of these and public datasets to uncover relevant pathways. We experimentally manipulated IRF4 levels by RNAi, CRISPR-Cas9 editing and overexpression via lentiviral systems. We employed expression, methylation and cell-based assays to evaluate the downstream consequences of IRF4 manipulation at gene, pathway and process levels

**Results and Discussions**

© 2023 The Authors. Molecular Oncology is published by John Wiley & Sons Ltd on behalf of Federation of European Biochemical Societies

In addition to other cancer-related processes, our investigation uncovered epigenetic silencing pathways, such as DNA and histone H3 lysine 27 methylation, as novel IRF4 targets. Expression of several tumor suppressive genes known to be controlled by these epigenetic modifications are regulated by IRF4. These include cyclin-dependent kinase inhibitors, PI3K pathway and primary cilium regulators. Accordingly, IRF4-dependent epigenetic regulation in melanoma cells modulates key oncogenic pathways. Consequently, experimental manipulation of IRF4 expression manifests as altered the cell cycle, proliferation and survival profiles, in line with the observation by us and others that IRF4 acts as a dependency factor also in melanoma cells. Moreover, IRF4 modulates the activity of the relevant epigenetic drugs in melanoma cells.

**Conclusion**

Taken together, we provide evidence for the critical role of IRF4 in melanoma cells, and novel insight into its mechanisms via epigenetic silencing, encouraging further studies on therapeutic targeting of IRF4 in melanoma.

### EACR23-1163
### Genome-wide DNA methylation in relation to ARID1A deficiency in ovarian clear cell carcinoma

*S. de Jong*[1], *S. Li*[1], *G.J. Meersma*[1], *G.B.A. Wisman*[2]
[1]*University Medical Center Groningen, Medical Oncology, Groningen, The Netherlands*
[2]*University Medical Center Groningen, Gynecologic Oncology, Groningen, The Netherlands*

**Introduction**

The poor chemo-response and high DNA methylation of ovarian clear cell carcinoma (OCCC) has attracted extensive attention. Around 50% of patients with OCCC harbour deleterious mutations in ARID1A, a component of the SWI/SNF chromatin-remodelling complex. This study aims to comprehensively investigate whether ARID1A loss and genome-wide DNA methylation are co-regulated in OCCC and to identify putative therapeutic targets that are epigenetically regulated in the context of mutant ARID1A.

**Material and Methods**

Whole genome DNA methylation of ARID1A mutant/knock-out (mt/KO) and ARID1A wildtype (wt) OCCC patient tumors and cell lines were analysed using the Infinium MethylationEPIC BeadChips. Expression of differentially methylated genes in ARID1Amt/ko vs ARID1Awt OCCCs was determined using publicly available mRNA expression profiles of OCCC cell lines. Three pre-ranked GSEAs were used to identify gene-sets and leading-edge genes that are differentially regulated in ARID1A mutant vs wild type OCCC. The leading-edge genes showing consistent ARID1A-related methylation change in patient tumors and cell lines, were verified by bisulfite sequencing and rt-qPCR.

**Results and Discussions**

The overall genome-wide methylation was not different between ARID1Amt/KO and ARID1Awt tumors or cell lines. However, ARID1A mutations in OCCC were associated with differentially methylated promoter or gene-body CpG islands of 2004 genes. These differentially methylated genes were enriched in EZH2- and H3K27me3-related gene-sets. From the 238 differentially methylated leading-edge genes, 13 genes passed the expression-based in-silico validation and exhibited potential clinical relevance. Validation of six differentially methylated genes showed that DNA methylation and mRNA expression data were consistent with Infinium MethylationEPIC BeadChip arrays and publicly available RNA expression data, respectively. The mRNA expression after demethylation treatment was upregulated in cells that had high DNA methylation and low/silenced mRNA expression, showing that these genes were indeed epigenetically regulated.

**Conclusion**

By comparing methylation of ARID1Amt/KO to ARID1Awt OCCC tumor and cell lines, genes regulated by ARID1A status dependent methylation were identified. Especially, enrichment of EZH2-related gene-sets was observed. These genes might be novel ARID1A-related druggable targets in OCCC and their potential therapeutic value is currently being investigated.

### EACR23-1223
### MTHFD2: a novel mediator of DNA methylation and decitabine response in breast cancer

*O. Walker*[1], *M. Dahn*[1], *M.C. Wasson*[1], *W. Fernando*[1], *J. Salsman*[1], *G. Dellaire*[1], *M. Nightingale*[2], *D. Gaston*[1], *P. Marcato*[1,3]
[1]*Dalhousie University, Department of Pathology, Halifax, Canada*
[2]*Dalhousie University, Genomics Core, Halifax, Canada*
[3]*Dalhousie University, Department of Microbiology and Immunology, Halifax, Canada*

**Introduction**

The DNA hypomethylating drug decitabine is used in the treatment of blood cancers and is being tested on solid tumours including breast cancer. Decitabine has multiple modes of action, including global hypomethylation and DNA damage leading to cell death. It is unclear which mode of action is most important for efficacy in breast cancer nor which genetic factors make breast tumours respond to decitabine treatment.

**Material and Methods**

To better understand the mechanism of decitabine and identify mediators of decitabine response in breast cancer, we performed an *in vivo* knockdown screen in NOD-SCID mice bearing MDA-MB-231 tumours containing an shRNA library. From the screen, we identified methylenetetrahydrofolate dehydrogenase 2 (MTHFD2) as the key mediator of decitabine response. We performed 'omics' analyses of the decitabine-treated MDA-MB-231 tumours with or without MTHFD2 knockdown to determine the mechanism.

**Results and Discussions**

Whole-exome sequencing revealed no difference in mutation count or penetrance between control, knockdown, or treatment groups, suggesting that *in vivo* decitabine does not induce mutation-causing DNA damage. In contrast, methylome profiling of the treated tumours revealed significant effects. In untreated tumours, MTHFD2 knockdown resulted in lower methylation of multiple genic regions, yet increased methylation compared to control in decitabine-treated tumours. In the context of decitabine

© 2023 The Authors. Molecular Oncology is published by John Wiley & Sons Ltd on behalf of Federation of European Biochemical Societies treatment, global demethylation occurred in all treated tumours; however, MTHFD2 knockdown reduced the number of differentially methylated regions (DMRs) induced by decitabine. Gene ontology enrichment of the DMRs demonstrated differences in the expression of several pathways between control and knockdown tumours, including histone modifications and DNA repair by homologous recombination. Metabolomics analysis of the tumours revealed decreased nucleotide pools and an increased abundance of folate-cycle precursors in the MTHFD2 knockdown tumours.

**Conclusion**

This study reveals that MTHFD2 is required for decitabine sensitivity. MTHFD2 knockdown likely imparts resistance to decitabine by decreased one-carbon metabolism resulting in decreased cell proliferation and DNA synthesis leading to decreased decitabine incorporation, rendering the MTHFD2-low breast tumours resistant to decitabine.

### EACR23-1322
### Investigating epigenomic profile changes during Multiple Myeloma progression and the key regulatory role of NRF1

*C. Cortile[1], T. Bruno[1], M.C. Cappelletto[1], F. De Nicola[1], S. Di Giovenale[1], S. Gumenyuk[2], A. Mengarelli[2], G. Corleone[1], M. Fanciulli[1]*

*[1]IRCCS Regina Elena National Cancer Institute, Department of Research- Advanced Diagnostic- and Technological Innovation, Rome, Italy*

*[2]IRCCS Regina Elena National Cancer Institute, Hematology Unit, Rome, Italy*

**Introduction**

Multiple myeloma(MM) remains a challenging hematological malignancy. While genetic and transcriptomic changes have been identified as contributing factors, recent evidence suggests that epigenetic alterations, specifically chromatin accessibility changes, also play a critical role in therapy response. In this study, we aimed to describe the regulatory profile at the epigenomic level that drives cancer progression from healthy to monoclonal gammopathy of undetermined significance(MGUS) to MM and to identify the main transcription factor(TF) players in this process.

**Material and Methods**

In this study, we conducted an epigenomic profiling of a cohort comprising 60 MM patients, 10 MGUS patients, and 5 healthy individuals to investigate the changes occurring during cancer progression. We employed computational analysis to identify the TFs that bind open chromatin in the three conditions and compared the regulatory profiles. The shared TFs in the tumour population were selected and histone modification profiles were investigated to support the activation state of those TFs binding sites. To identify possible genetic alteration sustaining chromatin shifts, we profiled somatic mutations in our tumour samples and filtered them based on their presence at selected TFs binding sites, and compared them with somatic panels mutations of an external cohort of ~1000 MM patients.

**Results and Discussions**

We showed that the regulatory profiles of healthy, MGUS and tumour conditions consist in a progressive increase in the chromatin accessibility. We analyzed the distribution of TFs in the MM population and identified a group of ~30 TFs that were detected in the majority of the population, suggesting a potentially relevant role in MM. Among them, NRF1 was found to have a significant impact on MM cell fitness when knocked out. We observed a dramatic shift of NRF1 biding positioning between the different cancer stages surveyed, suggesting variations in pathway regulation.

**Conclusion**

In conclusion, our study provides a comprehensive analysis of epigenetic changes that occur during MM progression, from healthy to MGUS to tumour stages. We identified specific TFs that are crucial in each stage, with NRF1 being particularly active in carcinogenesis. Our findings provide evidence on the fundamental role of epigenetic regulation in driving MM pathogenesis and highlight potential targets for future therapeutic investigation. Collectively, our results have important implications for developing precision medicine strategies for MM treatments.

### EACR23-1413
### Small non-coding RNA profiling in human tissue and stool samples for the identification of potential biomarkers for CRC early diagnosis

*C. Di Battista[1], S. Tarallo[1,2], G. Amedeo[1,3], G. Francescato[1,4], G. Ferrero[3,4], E. Budinska[5,6], V. Vymetalkova[7,8,9], P. Vodicka[7,8,9], B. Pardini[1,2], A. Naccarati[1,2]*

*[1]Italian Institute for Genomic Medicine, Genetic and Molecular Epidemiology, Candiolo TO, Italy*

*[2]Candiolo Cancer Institute, FPO IRCCS Candiolo, Candiolo TO, Italy*

*[3]University of Turin, Department of Computer Science, Turin, Italy*

*[4]University of Turin, Department of Clinical and Biological Sciences, Turin, Italy*

*[5]Masaryk University, RECETOX- Faculty of Science, Brno, Czech Republic*

*[6]Masaryk Memorial Cancer Institute, Research Centre for Applied Molecular Oncology RECAMO, Brno, Czech Republic*

*[7]Institute of Biology and Medical Genetics, First Faculty of Medicine- Charles University, Prague, Czech Republic*

*[8]Institute of Experimental Medicine of the Czech Academy of Sciences, Department of Molecular Biology of Cancer, Prague, Czech Republic*

*[9]Biomedical Centre, Faculty of Medicine in Pilsen- Charles University, Prague, Czech Republic*

**Introduction**

Despite great achievements in understanding the pathogenesis of colorectal cancer (CRC), its underlying mechanisms are still largely unknown. The identification of clinically relevant molecular biomarkers is crucial for early diagnosis of this cancer. Epigenetic alterations implicated in CRC tumorigenesis include changes in small non-coding RNAs (sncRNAs). After exploring the most studied microRNAs (miRNAs) (Tarallo et al, mSystems 2019, Pardini et al, in revision), we focused our attention on the extent of alterations of other sncRNAs of similar size analyzed in tumor tissues and stool samples (mainly P-

© 2023 The Authors. Molecular Oncology is published by John Wiley & Sons Ltd on behalf of Federation of European Biochemical Societies element-induced wimpy testis (PIWI)-interacting RNAs (piRNAs), transfer RNA (tRNA) fragments and small nucleolar RNAs (snoRNAs). Alterations in stool sncRNA profiles can reflect changes occurring in the intestinal tissue of CRC or adenoma patients.

**Material and Methods**

A total of 1,147 small RNA-sequencing experiments were performed in multiple biospecimens from a multicentric international study. 36,328 sncRNA profiles other than miRNAs were investigated in 103 tumor and paired adjacent mucosa tissue samples from an Italian cohort. Tissue sncRNA profiles were compared with those of fecal samples from an Italian and Czech cohort of (n=465, including 158 CRC, 80 polyps, 98 other intestinal diseases, 129 colonoscopy-negative controls), and validated in additional fecal samples from 141 CRC and 80 healthy volunteers contributing to science, and 102 fecal immunochemical tests (FIT) leftover samples from the screening. Differential expression analyses were performed between different groups with DESeq2 package.

**Results and Discussions**

In tissues, 1,069 sncRNAs resulted differentially expressed (adj. p<0.05) in tumor vs. non-malignant adjacent tissue samples. In particular, piRNAs and tRNAs were the most represented sncRNA biotypes identified as differentially expressed. In stool, preliminary results highlighted a set of sncRNAs differentially expressed in patients with cancer and precancerous lesions in comparison with controls. Interestingly, some DE sncRNAs were shared between stool and tissues.

**Conclusion**

Small RNA-sequencing identified several sncRNAs altered in tissue and stool samples from CRC patients and other gastrointestinal diseases providing evidence for the use of these molecules as biomarkers for a non-invasive accurate CRC diagnosis.

### EACR23-1503
### CircCDYL, a circular noncoding RNA, in the regulation of alternative splicing and chromatin remodeling in breast cancer cell lines

*Y. Pignochino[1], J.M. Robbin[1], I. Tarulli[2], L. Franchitti[1], F. Dal Bello[3], B. Pergolizzi[1], G. Ferrero[1], M. De Bortoli[1], S. CUTRUPI[1]*

*[1]University of Turin, Clinical and Biological Sciences, Torino, Italy*

*[2]Ecole Polytechnique Fédérale de Lausanne EPFL, Neuroscience, Lausanne, Switzerland*

*[3]University of Turin, Molecular Biotechnology and Health Sciences, Torino, Italy*

**Introduction**

Circular RNAs (circRNAs) are a new class of RNA molecules characterized by a covalently closed structure. The circularization process is mediated by a specific splicing process, called back-splicing. The dysregulation of splicing is a hallmark in cancer and is associated with an alteration of circRNAs expression. Their closed ends provide them a great stability in extracellular liquids, therefore they are applied as biomarkers. Since the functions and molecular mechanisms of circRNA are not yet fully understood, their study can unveil new candidate RNA-based cancer drugs. Here, we explored the molecular mechanism of circCDYL, hsa_circ_0008285, one of the most abundant circRNAs in breast cancer.

**Material and Methods**

We studied circCDYL in breast cancer cell lines, MCF-7 and MDA-MB-231. The effect of circCDYL downregulation was studied using RNA-sequencing. Chromatin immunoprecipitation followed by PCR was performed to determine the epigenetic profile of the *CDYL* isoform-specific promoters. We identified RNA binding proteins (RBPs) complex using bioinformatic tools validated through RNA pull-down followed by high-resolution mass spectrometry.

**Results and Discussions**

CircCDYL biological function is completely unknown. Silencing circCDYL altered the splicing pattern of several genes including its host gene, *CDYL*, with higher expression of the CDYL-204 isoform. H3K27Ac enrichment was present at the CDYL-204 promoter after circCDYL downregulation, while H3K27me3 decreased. On the contrary, the promoter of the main expressed isoform, CDYL-203 has H3K27Ac reduction upon circCDYL silencing suggesting a role of circCDYL on the chromatin remodeling. Considering the CDYL-204 protein structure, it still preserves the interaction site with EZH2, a PRC2 catalytic subunit altering gene expression by H3K27me3 deposition. Since the mediators of this chromatin remodeling are unknown, we hypothesized a circRNA-RBP complex formation regulating the transcription. We identified two heterogeneous nuclear ribonucleoproteins (hnRNPs), hnRNP-H and hnRNP-L as circCDYL interactors.

**Conclusion**

These RBPs can regulate CDYL isoform expression changes by acting directly on the transcriptional machinery or indirectly on the splicing of factors involved in the transcriptional regulation. circCDYL may work as a central hub in the cell to control cell growth by orchestrating RBPs function in the regulation of chromatin remodeling and splicing.

### EACR23-1526
### Identification of novel epigenetic regulators of ferroptosis by chemical screen in triple negative breast cancer cells

*Doğan-Yılmaz G.[1], Dalgıç D.[1], Pınarbaşı-Değirmenci N.[1], Bağcı-Önder T.[1]*

*[1]Research Center for Translational Medicine, Koç University School of Medicine, Istanbul, Turkey*

Introduction: Triple-negative breast cancer (TNBC) accounts for 15-20% of all breast cancers, which are among the most frequently observed cancers in women worldwide. Cancer cells develop resistance to the treatments by altering cell death mechanisms. Therefore, it is crucial to understand the molecular mechanisms behind cancer cell therapy resistance. Ferroptosis is an iron-dependent and excessive reactive oxygen species (ROS)-induced non-apoptotic cell death mechanism. It is by blocking cystine uptake through SLC7A11, or by directly inhibiting the glutathione-dependent antioxidant enzyme glutathione peroxidase 4 (GPX4) activity. On the other hand, ACSL4 initiates the pathways from lipid production to lipid hydroperoxide generation and activates ferroptosis.

© 2023 The Authors. Molecular Oncology is published by John Wiley & Sons Ltd on behalf of Federation of European Biochemical Societies

As a cell death mechanism, activating ferroptosis offers promise for cancer treatment, therefore it is crucial to identify genetic and epigenetic mechanisms underlying ferroptotic cell death. As many other cellular processes, ferroptosis machinery can be regulated at the expression level by chromatin based epigenetic mechanisms. In this study, we aim to identify novel epigenetic regulators of ferroptosis in TNBC. Materials and method: We generated MDAMB231 TNBC cell line models with varying degrees of ferroptotic ability by using CRISPR/Cas9 based GPX4, SLC7A11, or ACSL4 genetic ablation. We showed the differences in the response of engineered MDAMB231 TNBC lines to ferroptosis-inducers (FINs), Erastin, RSL3, ML162 and FIN56. Using these paired cell lines with varying ferroptosis capacities, we aim to identify epigenetic regulators of ferroptosis in TNBC by an unbiased chemical screening using an epigenetic probe library. The library is composed of 146 probes targeting chromatin regulators, such as histone methyltransferases, histone demethylases, histone acetyltransferases, histone deacetylases, arginine methyltransferases, bromodomain proteins, among others. Results and Discussion: qPCR and phenotype results indicated the successful knockout of ferroptosis regulators. GPX4 and SLC7A11 knockout MDAMB231 cell lines showed decreased viability when treated with FINs compared to ACSL4 knockout MDAMB231 cell lines. Ferrostatin, an inhibitor of Ferroptosis, reversed the FIN-induced cell death as well as the effects of GPX4 and SLC7A11 knockout. As proof-of-principle, epigenetic drugs such as Belinostat or GSK-J4 have been utilized along with FINs in combination. MDAMB231 cells were significantly affected by Erastin and Belinostat combination, compared to individual treatments suggesting a functional interaction between Histone deacetylases and ferroptosis. Conclusion: Our results will lead to the development of novel therapy approaches in which epigenetic drugs can be exploited to modulate ferroptotic cell death.

### Experimental / Molecular Therapeutics, Pharmacogenomics

### EACR23-0063
### DEAD-BOX RNA HELICASE DP103 PREDICTS STATIN SENSITIVITY IN TRIPLE NEGATIVE BREAST CANCER

A. Kumar[1]
[1]*National University of Singapore, Department of Pharmacology, Singapore, Singapore*

**Introduction**

Simvastatin, a lipophilic statin used for lowering cholesterol, inhibits 3-hydroxy-3-methylglutaryl-CoA reductase (HMGCR), the key enzyme of the mevalonate pathway. Studies have shown that cancer cells express deregulated level of HMGCR and statins exert anti-tumoral activities.

**Material and Methods**

We first assessed correlation between mevalonate pathway genes and DDX20 (DP103, Gemin-3) in 1325 breast cancer patients and observed a positive correlation between DDX20 and the mevalonate pathway genes. Having this data, we then proceeded to explore the effect of statins on DDX20 expression. We used various in vitro cell lines and in vivo statin clinical trial patients specimens, mouse xenograft, mouse intravenous tail injection and Drosophila (wild-type vs Gemin-3 knockdown vs Gemin-3 overexpression flies) models.

**Results and Discussions**

We show exposure to statin decreases the expression of DDX20. Through a series of add-back experiments, we show that the decrease in DDX20 expression by statins is via the mevalonate pathway and downstream of RhoA. In clinical specimens, we observed breast cancer patients with high baseline DDX20 positively correlates with high baseline YAPTEAD expression. Having known that SUMOylation of YAP maintains its activity and that DDX20 is a critical enhancer of the SUMOlyation machinery, we showed through a series of experiments that a physical interaction between DDX20 and YAP is crucial for maintaining SUMOylation of YAP; thereby decreasing its ubiquitination and degradation.

**Conclusion**

Interestingly, we also identified for the first time that DDX20 is a direct target of YAP-TEAD complex and that maintenance of DDX20 expression is needed as a positive feedback forming an Achilles heel for sustained YAP-TEAD activity.

### EACR23-0069
### Mannose inhibits the pentose phosphate pathway in colorectal cancer and enhances sensitivity to 5-fluorouracil therapy

N. Darwiche[1], *S. Al Hadeethi[1], C. El Baba[1], K. Araji[1], B. Hayar[1], I.A. Cheikh[1], R. El-Khoury[2], J. Usta[1]*
[1]*American University of Beirut, Biochemistry & Molecular Genetics, Beirut, Lebanon*
[2]*American University of Beirut, Pathology & Laboratory Medicine, Beirut, Lebanon*

**Introduction**

Colorectal cancer (CRC) is the third most diagnosed cancer type and the second cause of cancer deaths. 5-fluorouracil (5-FU) is the therapy of choice for CRC but results in toxicity and drug resistance. Tumorigenesis is characterized by deregulated metabolism, particularly that of glucose, which promotes growth and survival of tumor cells. The pentose phosphate pathway (PPP), a major glucose metabolic pathway in cancer cells providing riboses needed for the synthesis of nucleotides and NADPH for the regulation of reactive oxygen species (ROS), is upregulated in CRC. Recently mannose was shown to inhibit tumor growth and to enhance chemotherapy by impairing the PPP. Mannose inhibitory effect on tumor growth is inversely related to the levels of phosphomannose isomerase (PMI). We studied the anti-tumor effects, metabolic changes, and mechanism of action of mannose, alone or in combination with 5-FU, in human CRC cell and animal models.

**Material and Methods**

*In silico* analysis was performed to investigate the levels of PMI expression in human normal and CRC tissues. The effect of mannose and 5-FU alone or in combination on HCT116, HCT116 p53$^{-/-}$, and HCT116 5-FU resistant human CRC cell lines on cell growth (MTT, SRB) was

© 2023 The Authors. Molecular Oncology is published by John Wiley & Sons Ltd on behalf of Federation of European Biochemical Societies assessed. Synergistic effects were estimated using the Compusyn software. The PPP enzymes were measured, and ROS levels were determined by nitro-blue tetrazolium-based assay. TUNEL, flow cytometry, and immunoblotting assays were used to study the mode of action of the different treatments on cell cycle and cell death mechanism. The xenograft mouse model was selected to explore the anti-tumor potential of mannose alone or in combination with 5-FU treatment.

**Results and Discussions**

Low PMI levels were detected in human CRC tissues, suggesting high mannose sensitivity. Mannose resulted in a dose- and -time dependent inhibition of cell growth in CRC cells with different p53 and 5-FU resistance status. Mannose synergized with 5-FU treatment in all tested CRC cell lines. Mannose alone or in combination with 5-FU reduced the glucose-6-phosphate dehydrogenase activity of the key PPP enzyme and enhanced oxidative stress. Mannose/5-FU combination treatment induced S phase cell cycle arrest and DNA damage in CRC cells but not apoptosis. Importantly, mannose single or combination treatments with 5-FU were well tolerated and reduced tumor volumes in a mouse xenograft model.

**Conclusion**

Mannose alone or in combination with 5-FU may represent a promising novel combination treatment in CRC.

### EACR23-0073

### Effects of Removing Heat and Nourshing Yin Prescription on Malignant Phenotype and PI3K/AKT Signaling Pathway of Human Esophageal Carcinoma Cells

*F. Si*[1], *G. Si*[2]

[1]*Henan University of Chinese Medicine, Academy of Chinese Medicine Sciences, Zhengzhou, China*

[2]*Peking University Third Hospital, Orthopedics Department, Beijing, China*

**Introduction**

Esophageal carcinoma (EC) ranks eighth in the incidence of malignant tumors and the sixth mortality worldwide. This study aims to screen out the Chinese herbs with strong inhibitory effects on the proliferation of four kinds of EC cells EC9706, EC-1, TE-1 and Eca109 from 366 Chinese herbs produced in Henan Province, and study the effect of composed Removing Heat and Nourshing Yin Prescription(RHNYP) on the proliferation, migration, invasion and PI3K/AKT signaling pathway of four EC cells.

**Material and Methods**

MTT and orthogonal design was applied to screen out the best effective Chinese herbs and determine the optimal dosage ratio of prescription on EC9706, EC-1, TE-1, Eca109 cells from 366 Chinese herbs alcohol extracts, the effects of this prescription on the proliferation, migration, invasion, clone formation, cell cycle and apoptosis of four EC cells were investigated by MTT, RTCA, soft agarose colony forming test, flow cytometry and western blot analysis. Then exploring the effects of RHNYP on EC related signaling pathways and key genes expression by western blot and RT-PCR assay.

**Results and Discussions**

*Rhizoma Fagopyri Dibotryis*(Jinqiaomai), *Rhizoma Anema rrhenae*(Zhimu), *Asparagus Cochinchinensis*(Tiandong), a nd *Carpesium Abrolanoides Linne*(Heshi) were screened out and composed Removing Heat and Nourshing Yin Prescription(RHNYP) according to their efficacy and orthogonal design. RHNYP could obviously inhibit the proliferation of EC9706, TE-1, EC-1, ECa109 cells with dose-effect and time-dependent manner. IC50 values of Jinqiaomai, Zhimu, Tiandong, Heshi in RHNYP for EC9706 cell were 27.639, 40.015, 9.044, 11.208μg/ml respectively; for EC-1 cell were 24.575, 56.991, 9.361, 13.584μg/ml respectively; for TE-1 cell were 23.534, 25.862, 6.788, 8.911μg/ml respectively; for ECa109 cell were 33.990, 33.604, 19.873,15.227μg/ml respectively. RHNYP could effectively inhibit the proliferation, migration, invasion, cloning ability, cell cycle and reduce apoptosis of four EC cells. PI3K/AKT/mTOR pathway had closely relationship with the occurrence and development of EC and RHNYP could effectively regulate the proteins expression on the pathway. CDCK3, KIF4A and RAD51AP1 genes were negatively correlated with the overall survival of EC patients and RHNYP could significantly inhibit their expression in four EC cells.

**Conclusion**

RHNYP can effectively inhibit EC cell proliferation, migration, invasion and cell cycle, induce apoptosis, which may be related to the regulation of PI3K/AKT/mTOR pathway.

### EACR23-0074

### Effects of Four Prescriptions in Treatise on Febrile Diseases on the Malignant Phenotype of Esophageal Cancer Cells

*F. Si*[1], *S. Gao*[2]

[1]*Henan University of Chinese Medicine, Department of Basic Medicine, Zhengzhou, China*

[2]*Peking University Third Hospital, Orthopedics Department, Beijing, China*

**Introduction**

Esophageal cancer(EC) is the eighth most common malignancy and the sixth most common cause of cancer death. Finding new effective treatment methods and drugs of EC has always been a key problem in the study of esophageal cancer. *Treatise on Febrile Diseases* is the typhoid diseases part of *Treatise on Typhoid and Miscellaneous Diseases*, one of the classics of traditional Chinese medicine with recording 113 prescriptions. This study aims to screen out formula with strong inhibitory effects on human esophageal cancer cells of TE-1, EC-1, ECa109 and EC9706 from 113 prescriptions of Tre*atise on Febrile Diseases,* and investigate the effects of screened prescriptions in Treatise on Febrile Diseases on 4 kinds of EC cells malignant phenotype.

**Material and Methods**

Preparing water decoction of each prescription According to the compatibility ratio of the original prescription in *Treatise on Febrile Diseases*. MTT method was applied to screen out the best effective prescription and determine the IC50 value of prescription on esophageal cancer TE-1, EC-1, EC109 and EC9706 cells. The effects of screened prescriptions on the proliferation, migration, invasion, cell cycle of TE-1, EC-1, ECa109 and

© 2023 The Authors. Molecular Oncology is published by John Wiley & Sons Ltd on behalf of Federation of European Biochemical Societies

EC9706 cells were investigated by MTT assay, RTCA assay and flow cytometry.

**Results and Discussions**

Baitouweng Decoction(BD), Dahuang Huanglian Xiexin Decoction(DHXD), Xiaoxianxiong Decoction(XD) and Guadi Powder(GP) were screened out from 113 prescriptions, which all could obviously inhibit the proliferation of TE-1, EC-1, ECa109 and EC9706 cells with dose-effect and time-dependent manner. The IC50 values of BD, DHCD, XD and GP for TE-1 cell were 120.164, 284.95, 842.682, 11.692μg/ml respectively; for EC-1 were 148.403, 287.6, 712.873, 31.612μg/ml respectively; for ECa109 were 44.03, 126.417, 367.59, 16.773μg/ml respectively; for EC9706 were 364.848, 369.259, 695.873, 70.608μg/ml respectively. The four prescriptions all significantly affected the cell morphology, migration, invasion, cell cycle of TE-1, EC-1, ECa109 and EC9706 cells. BD induced G1 phase arrest in TE-1, ECa109, induced S phase arrest in EC-1, induced G2 phase arrest in EC9706; DHXD and XD induced G1 phase arrest in TE-1, ECa109, while induced S phase arrest in EC-1 and EC9706; GP induced G2 phase arrest in all four EC cells.

**Conclusion**

The four prescriptions Baitouweng Decoction, Dahuang Huanglian Xiexin Decoction, Xiaoxianxiong Decoction and Guadi Powder screened from *Treatise on Febrile Diseases* can inhibit the malignant phenotype of esophageal cancer.

### EACR23-0091
### PARP and ATR/CHK1 inhibitors employment call out ovarian cancer cell death through premature mitotic entry and genomic instability.

*A. Rogalska[1], A. Gajek[1], P. Gralewska[1], A. Marczak[1]*
*[1]University of Lodz, Department of Medical Biophysics, Lodz, Poland*

**Introduction**

Olaparib (PARP inhibitor) monotherapy, as primary treatment of BRCA-mutated ($BRCA^{MUT}$) ovarian cancer, can delay tumor progression. However, it does not improve significantly overall survival either in patients with $BRCA^{MUT}$ and BRCA wild-type ($BRCA^{WT}$) ovarian carcinomas. Combining olaparib with ATR/CHK1 inhibitors increases its effectiveness and may be a new opportunity for more effective ovarian cancer therapy.

**Material and Methods**

We examined efficacy of PARPi in combination with DNA damage response pathway proteins inhibitors - ATR (AZD6738, ATRi) and CHK1(MK8776, CHK1i) kinases in $BRCA^{MUT}$ (PEO-1) and $BRCA^{WT}$ (SKOV-3 and OV-90) cells. Cellular viability was assessed by MTT and colony formation assays. Cell cycle distribution connected with BrdU incorporation, micronucleus assay, metaphase spread, western blot and immunofluorescence staining were also performed.

**Results and Discussions**

PARPi in shorter incubation time (48 h) shows cytostatic effect. After prolonged incubation time (5 days) simultaneous use of olaparib with inhibitors of replication stress response proteins lead to cell death based on synthetic lethality. Combined treatment of PARPi+ATRi/CHK1i suppressed cancer cell growth in a time-dependent manner but independent of homologous recombination effectiveness. Monotherapy with ATR/CHK1 inhibitors reduced BrdU incorporation due to a slower rate of DNA synthesis, resulting from raised replication stress level. We confirmed that ATRi and CHK1i cytotoxicity was due to mitotic dysfunction. The combination of olaparib with inhibitors of the ATR/CHK1 pathway elevated chromosomal abnormalities, micronuclei (MN) formation and γH2AX expression at collapsed replication forks.

**Conclusion**

The final outcome of cell death depends on their molecular profile. The combination of PARPi with ATR inhibition suppressed ovarian cancer cell growth independent on BRCA status. There is undoubtedly a connection between ATR/CHK1 inhibition and DNA damage-induced cell-cycle disruption. ATR and CHK1 inhibitors provoke premature mitotic entry, leading to genomic instability and ultimately cell death. This research was funded by the National Science Centre, Poland, grant number: Sonata Bis 2019/34/E/NZ7/00056.

### EACR23-0114
### Development of CRISPR Gene Delivery "Smart" Nanocarriers (sNCs) against Rare Cancer Chordoma

*O. Aydin[1], A.U. Kutlu[1], G. Kursunluoglu[2], M. Ercan[3], C.U. Tunc[4], E. Aydemir[5], Y. Yuksel-Durmaz[3]*
*[1]Erciyes University, Biomedical Engineering, Kayseri, Turkiye*
*[2]Erciyes University, Drug Application and Research Center ERFARMA, Kayseri, Turkiye*
*[3]Istanbul Medipol University, Biomedical Engineering, Istanbul, Turkiye*
*[4]University of Utah, Department of Molecular Pharmaceutics, Salt Lake City, United States*
*[5]Biruni University, Biomedical Engineering, Istanbul, Turkiye*

**Introduction**

Chordoma is a rare malignant tumor that poses a significant challenge for effective treatment. The current therapeutic strategy for chordoma combines surgery and radiotherapy, but with limited success. Imatinib, a tyrosine kinase inhibitor, has shown promise in halting the progression of chordoma but it is not effective in achieving complete regression or eradication of the tumor. MUC1 gene is highly expressed in chordoma, correlating with the aggressive nature of the disease and reducing the effectiveness of the available treatments. Thus, it is critical need to develop a more efficient therapeutic approach for chordoma. The aim of this study is to develop a novel treatment strategy using a cocktail therapy of Imatinib and CRISPR MUC1 gene delivery with "smart" nanoparticles. The nanoparticles, which consist of hydrophobic HMA monomers that can rupture the endosome membrane and pH-sensitive DMAEMA monomers that can create a proton-sponge effect generating a synergistic effect leading to a higher endosomal escape mechanism, efficiently transport the pDNA to the cytoplasm. Further, positively

© 2023 The Authors. Molecular Oncology is published by John Wiley & Sons Ltd on behalf of Federation of European Biochemical Societies charged nuclear localization signal peptides complexed with negatively charged pDNA makes "smart" nanoparticles nucleus-targetable. This provides a potential therapeutic approach for the treatment of chordoma.

**Material and Methods**

Biocompatible nanocarrier for pDNA delivery was developed using FDA-approved β-cyclodextrin as a seed, cationic copolymers (TMAEMA) and alkyne-functional PEG with alkyne-functional copolymers. They characterized the particles and tested their biocompatibility in CH22 chordoma cells. The nucleolus-targeted/untargeted sNCs with MUC1 pDNA were analyzed in terms of the cellular internalization rates, efficacy of MUC1 protein expression level, and gene knock-down using fluorescence microscopy, a flow cytometer, WB and RT-PCR.

**Results and Discussions**

NMR and GPC results confirmed the proper synthesis of carrier systems. The pDNA complexed sNCs had a size of roughly 200nm. The targeted sNCs exhibited 5-fold higher cellular/nucleus uptake rates of pDNA compared to commercial agents. The MUC1 expression level was significantly reduced with sNCs treatment, and combination therapy with Imatinib/MUC1 complexed sNCs showed synergistic therapeutic effects

**Conclusion**

All in all, we increased the therapeutic effect of Imatinib with MUC1 CRISPR gene therapy and currently working on the combination therapy of CRISPR gene therapy with Imatinib in vivo chordoma model.

This study is supported by TUBITAK (120S938)

### EACR23-0119
### An actin-based motor molecule myosin-X regulates the oncogenic activity of KITENIN by stabilizing its dimerization

*S.J. Kim[1], I.J. Chung[2], H. Kim[3], K.K. Kim[1]*

*[1]Chonnam National University Medical School, Pharmacology, Gwangju, South Korea*

*[2]Chonnam National University Medical School, Hematology-Oncology, Gwangju, South Korea*

*[3]Sunchon National University, College of Pharmacy, Sunchon, South Korea*

**Introduction**

The functional KITENIN (KAI1 C-terminal interacting tetraspanin) complex controls colorectal cancer (CRC) cell invasion, which contributes to promoting metastasis and also plays an important role in colorectal carcinogenesis, suggesting that the KITENIN axis is a molecular target for developing therapeutics to CRC. However, it was unknown how KITENIN stability is regulated and which molecules are closely involved in this regulation.

**Material and Methods**

We here investigated to identify the biochemical features maintaining the structural integrity of KITENIN, which could be used to target KITENIN to develop new therapeutics for CRC patients.

**Results and Discussions**

We report that the homodimerization of KITENIN through the intracellular C-terminal domain (CTD) is one of the biochemical features contributing to maintaining the stability of KITENIN. Expression of the KITENIN-CTD alone interfered with the formation of the KITENIN homodimer, and the amino acid sequence from 463 to 471 within the KITENIN-CTD was the most effective. This sequence coupled with a cell-penetrating peptide was named a KITENIN dimerization-interfering peptide (KDIP). As KITENIN achieves stability by forming dimers in the cell membrane, we tried to find molecules that interact with KITENIN and mediate the effects of KDIP by performing immunoprecipitation analysis. We detected the myosin-X (Myo10), one of several actin-based motor molecules in the myosin superfamily, as the KITENIN-interacting protein. Myo10 stabilized the *cis* form of the KITENIN homodimer by tying up the two intracellular cytoplasmic loops of the transmembrane portion of KITENIN. After treatment with KDIP, the downregulation of Myo10 was induced via proteasomal degradation, which led to the further loosening and disintegration of the KITENIN dimer, thus to expose the RACK1-interacting motif (RIM), which caused increased interaction of RACK1 with the exposed RIM and thereby triggered degradation of KITENIN in an autophagy-dependent manner. In *in vivo* mouse tumor model with the higher KITENIN level, KDIP significantly suppressed colorectal liver metastasis. A positive correlation was found between the expressions of *KITENIN* and *Myo10* in colorectal adenocarcinoma of The Cancer Genome Atlas.

**Conclusion**

The present results therefore provide a tool for specifically blocking the oncogenic actions of KITENIN in CRC patients with higher KITENIN expression.

### EACR23-0120
### CRISPRi Screen for Modulators of Obinutuzumab-induced Lysosomal Membrane Permeabilization in B Cell Lymphoma

*Y. Kim[1], J.R. Kim[1], J.Y. Kim[1]*

*[1]Yonsei University College of Medicine, Department of Pharmacology- Graduate School of Medica l Science- Brain Korea 21 Project, Seoul, South Korea*

**Introduction**

Non-Hodgkin lymphoma (NHL) is a cancer of the lymphatic system that is treated by chemotherapy and CD20-based immunotherapy such as obinutuzumab (OBZ). OBZ is a second-generation Fc-engineered type II antibody with increased interaction with FcγRIIIa expressed on immune effector cells, and it differs from type I anti-CD20 antibodies in that it promotes greater antigen-dependent cell cytotoxicity and direct cell death (DCD). The major phenotypes of OBZ-induced DCD include homotypic adhesion and lysosomal membrane permeabilization (LMP). However, the mechanism of action of OBZ-induced LMP still remains unclear. Gaining further insight into OBZ's mode of action can aid in developing improved therapeutics to treat refractory and relapsed NHL. Here, we used a CRISPRi-based screening approach to identify negative and positive modulators of OBZ-induced LMP to improve NHL treatment.

**Material and Methods**

CRISPR interference (CRISPRi)-based screening platform is used for large-scale genetic perturbations to probe gene functions. CRISPRi uses a catalytically inactive dead Cas9

© 2023 The Authors. Molecular Oncology is published by John Wiley & Sons Ltd on behalf of Federation of European Biochemical Societies

(dCas9) fused to a KRAB repressor domain, and it uses a single guide RNA to tune down gene expression. We used a CRISPRi-based screening approach to identify modulators of OBZ-induced LMP. We generated a dCas9-expressing Raji cell line and lentivirally transduced a sgRNA sub-library targeting kinases, phosphatases and drug targets. sgRNA-dCas9-Raji cells were treated with OBZ and stained with Lysotracker deep red, an intracellular dye accumulates in acidic compartments, namely the lysosome. OBZ-treated Lysotracker-stained cells were subjected to fluorescence-activated cell sorting (FACS) and then processed for next generation sequencing (NGS).

**Results and Discussions**

We identified 199 negative and 2 positive effector sgRNAs (FDR <0.25), which indicate genes required for LMP and those conferring resistance to OBZ, respectively. We further selected candidate hits based on essentiality, cellular expression, cellular localization and inhibitor availability. We validated CRISPRi screen hits by introducing target-specific sgRNA, treating those cells with OBZ and Lysotracker deep red and analyzing LMP by flow cytometry.

**Conclusion**

Taken together, this study will extend our knowledge of OBZ-induced DCD to better understand B cell biology and develop more potent therapeutics for NHL.

### EACR23-0121
### APEX-Based Proximity Labeling to Uncover Cell Death Mechanisms by Anti-CD20 Antibodies

A. Choi[1], D.H. Lee[1], J.Y. Kim[1]
*[1]Yonsei University College of Medicine, Department of Pharmacology- Graduate School of Medical Science- Brain Korea 21 Project, Seoul, South Korea*

**Introduction**

Obinutuzumab (GAZYVA®, OBZ), a treatment for B cell non-Hodgkin Lymphoma (BNHL), is an anti-CD20 monoclonal antibody (mAb) that exhibits binding-induced direct cell death (DCD) via lysosome membrane permeabilization (LMP), which is not seen with rituximab (Rituxan®, RTX). An engineered form of ascorbate peroxidase (APEX2) enables proximity labeling of proteins located within approximately 20 nm using biotin phenoxyl radicals in living cells. Proximity labeling with APEX2 fused OBZ will reveal proteins related to OBZ-bound CD20 that process DCD. Here, we propose a novel approach to elucidate DCD mechanisms by combining APEX2 proximity labeling technology with mass spectrometry (MS) and provide new therapeutic strategies to improve BNHL treatment.

**Material and Methods**

APEX2 fused anti-CD20 mAbs were produced and confirmed similar potency of DCD related parameters, such as DCD and LMP along with the same binding affinity. Biotin phenol and H2O2 were treated to Raji cells for proximity labeling after each APEX2 fused antibody treatment. Biotinylated proteins were purified through streptavidin agarose resins and analyzed by high performance liquid chromatography (HPLC) and MS (timsTOF Pro 2 Bruker). The protein enriched in RTX-APEX2 or OBZ-APEX2 labeled samples were analyzed to elucidate the different binding positions in the cell membrane and the internalization pathway of each antibody, which can suggest a new cooperative target protein to enhance DCD. The proteins enriched in OBZ-APEX2 labeled sample were knocked down by doxycycline induced shRNA or overexpressed in Raji cells to verify contribution in LMP and DCD.

**Results and Discussions**

APEX2 fused anti-CD20 mAbs showed similar potency of DCD, LMP, and affinity to CD20. Total 483 proteins were detected, and more than 50 plasma membrane proteins with intensity scores higher than 18000 were selected as closely related proteins to antibody bound CD20s. Compare to RTX-APEX2, OBZ-APEX2 showed higher cytosol and lysosome protein abundancy than plasma membrane proteins which implies short residence of OBZ bound CD20s in the plasma membrane. In addition, the knockdown of 3 genes showed reduced LMP and DCD by OBZ binding.

**Conclusion**

We suggest that the presence of different proteins near CD20 bound to OBZ or RTX may guide explicating the mechanism of OBZ-specific DCD. Proteins that impact LMP and DCD level by OBZ upon knock-down or overexpression can improve the DCD of OBZ and may ultimately contribute to treatment development for BNHL.

### EACR23-0122
### A novel and small protein binders for human IL-4 receptor discovered through synthetic nanobody screening

T. Ahn[1], D.H. Lee[1], J.Y. Kim[1]
*[1]Yonsei university college of medicine, Department of pharmacology and Brain Korea 21 Four Project for Medical Science, Seoul, South Korea*

**Introduction**

Dupilumab (Dupixent®) is a monoclonal antibody targeting human IL-4 receptor (IL-4R). Its blocking mechanism enables dupilumab to be approved by FDA for treating patients with severe allergic diseases such as rhinitis, and nasal polyps for systemic therapy. However, it is currently treated by the injection due to the big molecular size (~150KD), which causes some systemic side effects such as injection site reaction and serum sickness-like reaction. Hereto, we screened out a novel hIL-4R targeting-molecule with smaller molecular weight (~15KD), which is called synthetic nanobody, for paracellular nasal application.

**Material and Methods**

Convex scaffold was used for library construction and CDR3 region was chosen to be randomized. Ribosome display and two rounds of phage display were carried out for the screening. A binding affinity of the selected nanobody against IL-4R expressing cell was analyzed by flow cytometry, and its IL-4/IL-13 cytokine signal blocking effect was measured by the reporter cell assay. The nanobody was administered to the human nasal epithelial (HNE) sample of rhinitis patient, cytokine blockade ability in the epithelia was assessed. For optimizing an affinity of the nanobody, prediction of its structure, multiple docking strategies were used for sb-target complex model generation, molecular dynamics (MD) simulation was performed for the complex model

© 2023 The Authors. Molecular Oncology is published by John Wiley & Sons Ltd on behalf of Federation of European Biochemical Societies evaluation, and then affinity maturation was performed. In addition, two identical nanobodies were connected with a simple linker to enhance its binding affinity.

**Results and Discussions**

After the screening process, four different nanobody candidates were drawn, and finally "H5" clone was selected as the best binder against the IL-4R through ELISA. The H5 showed significant binding affinity to the IL-4 overexpressing cell, and IL-4/IL-13 dual signal blocking ability which is comparable to the dupilumab. A topical treatment of H5 on the HNE sample exhibited remarkable IL-4/IL-13 signal blocking function compare to that of the dupilumab, due to higher paracellular permeability of the H5. Affinity maturation process through the *in-silico* method and dimerization of nanobody allowed to enhance binding affinity of the H5, which led reinforced signal blocking function of the nanobody.

**Conclusion**

A novel IL-4 receptor-targeting nanobody was discovered, its functional and physiological effect were assessed with a topical treatment. *In-silico* affinity maturation and structural engineering enabled overcome a low binding affinity of the synthetic nanobody.

### EACR23-0123

### Identification of modulators enhancing HER2 endocytosis using CRISPRi-dCas9 screening in Gastric Cancer cells

*D.H. Kim*[1], *J.Y. Kim*[1]

[1]*Yonsei University College of Medicine, Department of Pharmacology and Brain Korea 21 Four Pr oject for Medical Science, Seoul, South Korea*

**Introduction**

Gastric Cancer (GC) is a disease that places a great burden on national health due to its high incidence rate, but there is no specialized treatment other than resection. Trastuzumab is approved for treating HER2-overexpressing breast cancer (BC) as well as metastatic gastric cancer in the 10-20% of patients with poor outcome HER2 amplification. In addition, T-DM1 is also used to kill cancer by accumulating toxins inside of the cells using a trastuzumab mediated endocytosis mechanism. However, most of the GC patient had generally low HER2 expression, a method that can enhance the endocytosis of trastuzumab is highly demanded even in low HER2 expression cancer.

**Material and Methods**

ERBB2 expression data of BC cells & GC cells were collected from The Cancer Dependency Map. HER2 expression in surface of NCI-N87, MKN-7, SNU-1 cells were analyzed by FACS, using trastuzumab. Endocytosis rate of each cell was measured by pH-sensing dye, pH-Ab, conjugated trastuzumab and observed by confocal microscope. Viability of each cell treating T-DM1 was measured by ATP measurement assay which is compared to HER2 overexpressed cell and HER2 knock down cell. To identify an endocytosis modulator of trastuzumab, we used CRISPRi library screening system, a process treating 2650 of sgRNAs of membrane proteins to the SNU-1 cells which is stably expressing dCas9.

**Results and Discussions**

The experiments using 3 grades of HER2 expressing GC cell lines along with genetically engineered HER2 over-expressed- or knock-downed- cells showed that the endocytosis rate and T-DM1 resistance were dependent on HER2 expression. The successful establishment of dCas9 system was verified by the knock down of HER2 surface expression level by transduction of HER2 sgRNA in the SNU-1 cells stably expression dCas9. After the pH-Ab treatment, the cells that showed low fluorescence of pH-Ab and similar expression of HER2 were selected. Successively, sgRNAs from the selected cells were undergone to sequencing.

**Conclusion**

Our research is on the process of discovering a modulator of trastuzumab endocytosis which is able to be a secondary target for enhancing T-DM1 toxicity via enhancing accumulation of T-DM1 in spite of the low HER-2 condition.

### EACR23-0134

### A RAD51/BRCA2 small molecule inhibitor enhances the antineoplastic effect of the PARPi talazoparib in pancreatic cancer

*L. Poppi*[1], *S.H. Myers*[2], *V. Previtali*[2], *F. Rinaldi*[2], *F. Farabegoli*[1], *G. Di Stefano*[3], *M. Roberti*[1], *A. Cavalli*[2]

[1]*University of Bologna, Pharmacy and Biotechnology department FaBiT, Bologna, Italy*

[2]*Italian Institute of Technology, Computational and Chemical Biology, Genoa, Italy*

[3]*University of Bologna, Medical and Surgical Sciences department DIMEC, Bologna, Italy*

**Introduction**

PARP inhibitors (PARPi) are drugs showing efficacy in HR-defective cancers. The RAD51/BRCA2 interaction is a key step in HR, which is the main DNA double strand break (DSB) repair pathway. As this mechanism is activated following single strand break (SSB) repair inhibition by PARPi, the simultaneous impairment of HR and PARP should lead to increased antineoplastic power of PARPi. We characterized the mechanism of action of ARN24922 (**1**), which is a new promising RAD51/BRCA2 inhibitor, developed in house. Furthermore, we evaluated its potential to increase the effect of talazoparib (TLZ, a potent PARPi) on 2D and 3D models of pancreatic cancer (PAC).

**Material and Methods**

We evaluated the potential of **1** in inhibiting HR activity through the mClover HR assay, which is based on the insertion of a mClover-containing sequence into a Cas9-generated DSB in the LaminA gene; the subsequent DSB repair leads to cell fluorescence. To confirm HR inhibition, we examined RAD51 nuclear foci after the induction of DNA damage by cisplatin (CPL) and RAD51/BRCA2 inhibition by **1**. Finally, we studied the antineoplastic effect of the **1**-TLZ combination in different PAC cell lines (BxPC-3, HPAC, AsPC-1) and in 3D models of PAC (PT291 and PDM41 organoids), with each of the models being characterized by functional BRCA genes and HR.

**Results and Discussions**

By applying the mClover HR assay, we found that 50 μM **1** produced an 80% HR inhibition. Therefore, this dose was used for further studies. Preliminarily, we observed that the selected dose of **1** caused a significant reduction of

© 2023 The Authors. Molecular Oncology is published by John Wiley & Sons Ltd on behalf of Federation of European Biochemical Societies

RAD51 nuclear foci in CPL exposed cells. This finding is in agreement with the results obtained in the HR assay, supporting the expected mechanism of action of **1**. In 2D PAC cultures, we found that 50 μM **1** significantly increased the anti-proliferative effect of 2 μM TLZ. The cell death mechanism involved in this effect was studied in BxPC-3 cells and was characterized as apoptosis.
When **1** was co-administered with TLZ, in 3D PAC models, a significant increase in the antineoplastic activity of TLZ was also observed. In addition, in PT291 organoid, **1** was found to increase the DNA damage signatures caused by TLZ, as shown by immunofluorescence detection of γ-H2AX in cell nuclei.

**Conclusion**

The obtained data support the idea that the inhibition of RAD51/BRCA2 interaction can extend the use TLZ to HR proficient tumor forms, highlighting a promising anticancer strategy for PAC.

### EACR23-0137
### NOX1/ADAM17 enzymatic complex regulates soluble MCAM-dependent pro-tumorigenic activity in colorectal cancer

*J. stalin[1], O. Coquoz[1], R. Jeitziner[2], S. Jemelin[3], N. Desboeufs[1], M. Delorenzi[2], M. Blot-Chabaud[4], B.A. Imhof[3], C. Ruegg[1]*
*[1]University of Fribourg- Section of Medicine, Oncology- Microbiology- and Immunology- Chair of Patho logy, Fribourg, Switzerland*
*[2]SIB Swiss Institute of Bioinformatics, Bioinformatics Core Facility, Lausanne, Switzerland*
*[3]University of Geneva Medical School, Department of Pathology and Immunology, Geneva, Switzerland*
*[4]Aix-Marseille University, C2VN- Inserm 1263- Inra 1260, Marseille, France*

**Introduction**

The cellular and molecular mechanism behind blood vessel development in a tumor context is still under extensive investigation. This is due to the failure of anti-angiogenic therapies. Among the new pro-angiogenic growth factors, soluble Melanoma Cell Adhesion Molecule (sMCAM) offered new perspectives due to the ability to block blood vessel development without impairing blood vessel integrity mediated by the membrane form of the molecule. The growth factor sMCAM, released from endothelial and cancer cells, induces tumor angiogenesis and growth. However, the molecular mechanisms responsible for its generation in a tumor context are still unclear.

**Material and Methods**

To elucidate this mechanism, we performed in vitro experiments with endothelial/cancer cells and applied pharmacological methods in vivo with mouse and human colorectal cancer cells. We performed gene expression analyses on data sets from human colorectal tumor samples.

**Results and Discussions**

We found that soluble MCAM generation is governed by the proteolytic activity of ADAM17 and NOX1-regulating ADAM17 expression. Treatment of colorectal tumor-bearing mice with pharmacologic NOX1 inhibitors or tumor growth in NOX1 deficient mice reduced blood concentration of soluble MCAM and abrogates the anti-tumor effects of anti-soluble MCAM antibodies. Pharmacologic inhibition of ADAM17 in vivo reduces tumor growth and angiogenesis. Importantly, NOX1, ADAM17, and MCAM expression were more prominent in the angiogenic, consensus molecular subtype 4 of human colorectal cancer and high MCAM expression correlated with angiogenic and lymphangiogenic markers. Furthermore, we demonstrated that soluble MCAM also acts as a lymphangiogenic factor in vitro.

**Conclusion**

These results identify a role for NOX1/ADAM17 in soluble MCAM generation, with potential clinical therapeutic relevance to the aggressive, angiogenic CMS4 colorectal cancer subtype.

### EACR23-0149
### PDGF receptor targeting prevents breast cancer metastasis development

*J. stalin[1], M. Nepomuceno Tavarez[1], O. Coquoz[1], C. Ruegg[1]*
*[1]University of Fribourg- Section of Medicine, Oncology- Microbiology- and Immunology- Chair of Patho logy, Fribourg, Switzerland*

**Introduction**

Breast cancer is the first cause of cancer-related death in women and besides the improvements in detection and treatments, the incidence is still increasing. Among the different subtypes, triple-negative breast cancer (TNBC) is the most aggressive one, associated with a worse prognosis. Around 20 to 30% of breast cancer patients will develop metastasis, months or years after the primary diagnosis and treatment. Breast cancer metastasis is the fatal consequence of the development of the disease and leads to death. This is mainly due to poor therapeutic availability able to prevent either metastatic spreading from the primary tumor or the growth of disseminated cancer cells into distant organs. Improving the cellular and molecular characterization of primary tumors and metastasis in distant tissue is crucial for the development of optimized new therapeutic options.

**Material and Methods**

Single-cell sequencing RNA studies were performed on human and mouse TNBC primary tumors as well as in isolated mouse lung metastasis derived from the primary tumor by microsurgery. Bioinformatic studies were conducted to establish the cellular and molecular characteristics of the tumor microenvironment (TME) and cancer cells. Confirmation studies were performed on immunohistochemistry (IHC) on tissue sections. The cellular or molecular targets were validated in mouse models using pharmacological inhibitors.

**Results and Discussions**

Bioinformatic analysis of RNA single-cell sequencing from the primary tumors revealed that cancer-associated fibroblasts (CAFs) are abundantly represented in the TME. Interestingly, CAFs are equivalently present for all breast cancer subtypes. Molecular characterization showed several CAFs subtypes with specific mRNA profiling. We found that Platelet-Derived Growth Factor (PDGF) receptors are highly expressed by breast cancer-associated CAFs. Targeting PDGF receptors using pharmacological inhibitors impaired orthotopic 4T1 mouse breast tumor

© 2023 The Authors. Molecular Oncology is published by John Wiley & Sons Ltd on behalf of Federation of European Biochemical Societies growth and metastatic dissemination in monotherapy and adjuvant therapy settings.

**Conclusion**

These results highlight CAFs are a major component of breast tumor TME. These CAFs subtypes can express common markers such as PDGF receptors. Finally, targeting PDGF receptors in vivo prevents primary tumor development and associated metastasis.

### EACR23-0156
### Napabucasin, a novel inhibitor of STAT3, inhibits growth and synergises with the MDM2-p53 antagonist idasanutlin in acute lymphoblastic leukaemia

E. Aptullahoğlu[1], Ş. Güngördü[1]
[1]Bilecik SE University,
Biotechnology Application and Research Center, Bilecik, Turkiye

**Introduction**

Chemotherapy is the major form of treatment for acute lymphoblastic leukaemia (ALL). In order to improve the poor outcome, especially in patients with high-risk features, novel and more effective therapies need to be introduced. In this research, the combined use of a new generation MDM2 inhibitor idasanutlin (RG7388) and a STAT3 inhibitor napabucasin (BBI608) was investigated with pre-clinical methods in ALL cells as a novel treatment strategy. Both of these inhibitors are under investigation in different ongoing clinical trials.

**Material and Methods**

Five ALL cell lines (MOLT-4, REH, RS4;11, Nalm-6 and HAL-01) were exposed to BBI608 and/or RG7388 for 72 h. Cell viability was assessed by XTT assay. For concentration-response matrix designs, all the cell lines were seeded and exposed to a range of drug combinations; the effect on cell viability after 72 h was determined as mentioned above, then analysed using the SynergyFinder web-application to evaluate the response to drug combinations. The combined effect was quantified using the zero interaction potency (ZIP) model.

**Results and Discussions**

RG7388 decreased cell viability at low nanomolar concentrations in all ALL cell lines (mean $IC_{50}$ values range from 47 to 346 nM). The REH cell line carrying the heterozygous c.541C>T mutation in the *TP53* gene has a slightly higher $IC_{50}$ value for RG7388 compared to other cell lines (Nalm-6 $IC_{50}$=82±6 *vs.* REH $IC_{50}$=346±38; unpaired *t*-test, $p$=0.0022). BBI608 induced growth inhibition in all the cell lines at mean $IC_{50}$s between 562 and 1114 nM. No statistically significant $IC_{50}$ difference was observed against BBI608 in the MOLT-4 cells carrying heterozygous c.2186G>A and c.1217C>T mutations in the *STAT3* gene. Combined drug concentration-response matrix experiments demonstrated synergy in 4 different cell lines REH, RS4;11, Nalm-6 and HAL-01 that were wild-type for the *STAT3* gene. The delta synergy scores for these cell lines were calculated as 11.58, 13.58, 13.89 and 8.53, respectively, while the highest synergy scores seen in the combination synergy score heat map ranged between 12.99-25.68. For MOLT-4 cells carrying 2 different mutations in the *STAT3* gene, the synergy score was 1.23 and remained in the additive effect range, suggesting that the *STAT3* status was determinant in the combined treatment response.

**Conclusion**

This research presents the possibility of a combination treatment with MDM2 and STAT3 inhibitors as a potential treatment option for *STAT3*$^{WT}$ ALL and suggests further functional studies and *in vivo* evaluation.

### EACR23-0180
### Cytotoxic effect and modulation of cytochromes P450 expression by morin in a rhadbomyosarcoma cell line

E. López-Rodríguez[1], C. Torres-Zárate[1], D. Molina-Ortiz[1], A. Vences-Mejía[1], R. Santes-Palacios[1]
[1]Instituto Nacional de Pediatria,
Laboratorio de Toxicologia Genetica, Mexico City, Mexico

**Introduction**

In Mexico, pediatric rhabdomyosarcoma (RMS) is the fourth leading cause of cancer-associated death with a range of response to antineoplastic drugs of 20-30%, which decreases the efficacy of chemotherapy. In addition, conventional antineoplastic drugs generate toxicity in most patients. Morin hydrate is a naturally occurring polyphenolic compound that possesses anticancer activity through various mechanisms such as antioxidant activity, modulation of liver metabolism enzymes (cytochromes P450) or inhibition of cell proliferation, so it could be a novel candidate to treat RMS.

**Material and Methods**

Cell cultures of an RMS cell line (ATCC CRL-2061) were exposed to different concentrations (0-600 µM) of morin. Cell viability (MTT assay) was determined to calculate the Mean Cytotoxic Concentration (CC50) of morin. The cytotoxic effect was compared with that produced by the reference antineoplastics cyclophosphamide, ifosfamide, vincristine and doxorubicin.

To determine the possible antimetastatic effect of morin, wound healing assay was performed and wound repopulation was monitored in the untreated and morin-treated cells. Gene expression of CYP3A4, CYP3A5, CYP2E1 and CYP1B1 was assessed by real-time PCR in cell cultures treated with morin (150 µM) or vehicle (DMSO) for 48 h.

The *in vitro* experiments were complemented by *in silico* assays to determine the molecular interactions of morin with CYP1B1, CYP2E1, CYP3A4, and CYP3A5.

**Results and Discussions**

Morin is cytotoxic (CC50=281 ± 14 µM), even more so than the reference antineoplastics cyclophosphamide, ifosfamide and vincristine. It also decreases the migration of RMS cells by 69% at 6 h and modulates the expression of some cytochromes P450 involved in RMS pathogenesis and the response to chemotherapy. CYP3A4, CYP1B1 and CYP2E1 increased 4-fold, 34-fold, and 16-fold, respectively, compared to control cells after 48 h of morin exposure.

Molecular docking suggests that π-π stackings between morin and phenylalanine of the cytochrome P450 catalytic site are important for protein-ligand interaction.

**Conclusion**

The molecular pathways involved in the cytotoxicity of morin and its potential adverse effects in the pediatric population should be described in further studies. Morin

© 2023 The Authors. Molecular Oncology is published by John Wiley & Sons Ltd on behalf of Federation of European Biochemical Societies modulates the expression of enzymes involved in the activation of prodrugs such as CYP3A4 and CYP1B1, so it could be considered for the development of a new chemotherapeutic regimen by increasing cytotoxicity through of the coadministration of morin with a CYP-activated prodrug in RMS cells.

### EACR23-0187
### Progress in the development of a clinically viable MYC inhibitor

*M.E. Beaulieu[1], S. Casacuberta-Serra[1], S. Martínez-Martín[1], M.F. Zacarías-Fluck[2], F. Giuntini[2], S. López-Estévez[1], L. Foradada[1], I. González-Larreategui[2], J.R. Whitfield[2], L. Soucek[2]*
*[1]Peptomyc S.L., Not Applicable, Barcelona, Spain*
*[2]Vall d'Hebron Institute of Oncology, Preclinical and Translational Research Program, Barcelona, Spain*

**Introduction**
MYC is a most wanted target in cancer therapy. However, it has long been considered an "undruggable" target, and there is no clinically approved MYC inhibitor yet. We designed and validated Omomyc, the most characterised direct MYC inhibitor to date. An Omomyc-based mini-protein therapeutic developed by Peptomyc S.L. – OMO-103 – has recently successfully completed a Phase 1 clinical study, demonstrating safety and clear signs of target engagement. Here, we present the main findings associated with biomarker discovery, both at the preclinical and clinical level, and show data on promising drug combinations to be tested in future clinical studies.
**Material and Methods**
Different biomarkers of drug activity have been studied preclinically, in vitro and in vivo, and in tumour and liquid biopsies from clinical trial patients. Transcriptional changes and immune-modulating soluble biomarkers have been analysed by Digital Spatial Profiling (DSP), RNAseq and Luminex technology.
In addition, different preclinical models (cancer cell lines, cell-derived and patient-derived xenografts) of Triple-Negative breast cancer (TNBC), melanoma and Non-Small-Cell Lung Cancer (NSCLC) have been tested for combination therapies, including chemo and targeted therapies.
**Results and Discussions**
RNAseq and Digital Spatial Profiling analysis show shut down of MYC transcriptional signature as a consequence of Omomyc treatment in both preclinical models and patient tumour biopsies. Among the most commonly regulated gene sets, we detected clear modulation of cholesterol metabolism and cross-talk with the tumour microenvironment, with several anti-tumour immune-related markers being affected by Omomyc. In fact, a distinctive pharmacodynamic signature of soluble immune modulators correlating with drug activity was identified in patients. Most interestingly, another immune-modulating signature was found to be predictive of disease stabilisation and could help stratify patients in upcoming additional clinical studies.
On the other hand, we have identified interesting molecular mechanisms that indicate potential synergy between Omomyc and MEKi/RAFi in melanoma, PARPi in TNBC, and KRASi in NSCLC.
**Conclusion**
MYC inhibition is finally progressing through clinical trials and it is revealing new aspects of MYC biology, including in the context of immune modulation. The pleiotropic role of MYC in drug resistance and survival suggests that MYC inhibition could be useful to increase the efficacy of – and prevent resistance to – standard-of-care therapies.

### EACR23-0210
### RADIOPROTECTIVE EFFECTS OF MOLECULAR HYDROGEN (H2).

*R. Amir[1]*
*[1]Centre de Santé des Fagnes, Nuclear Medicine, Chimay, Belgium*

**Introduction**
During these last years, many studies have shown the potential usefulness of $H_2$ in the protection against radiations and some specific pathologies.In the cases of patients treated by radiotherapy, many side effects are believed to be associated with increased oxidative stress and inflammation. Most of the ionizing radiation induced damage is caused by hydroxyl radical (•OH) from radiolysis of $H_2O$. Damaging effects of radiation are divided into direct and indirect effect, which accounts for about most of induced lesions. Since the indirect effect mainly due to the abundant free radicals caused from radiation, so blocking and scavenging of free radicals become our most important protecting strategy. Some studies demonstrated that $H_2$ might have great radioprotective effects in 2010. Since then, application of $H_2$ on radioprotection was well investigated.
**Material and Methods**
The exposure to ionizing radiation can lead to many hazards including genetic mutations, induction of cellular death, and carcinogenesis. Among the most radiation sensitive organs are the hematopoietic system, the gastrointestinal (GI) system, the reproductive system and the skin .Radiation energy can cause direct damage effect when it is absorbed by critical target molecules, which include DNA, proteins, and others in the pathway of radiation. This report demonstrate the benefits of drinking hydrogen water in patients receiving radiation therapy for malignant tumors.Drinking hydrogen-rich water improved the quality of life of the patients and reduce the side effects of radiotherapy.
**Results and Discussions**
Recent basic and clinical research revealed that $H_2$ is an important physiological regulatory factor with antioxidant, anti-inflammatory and anti-apoptotic protective effects on cells and organs. $H_2$ was also demonstrated has radioprotective effects on cultured cells and mice.$H_2$ has several special traits that would make it an efficient radioprotective agent in medical use. These traits include the capacity to neutralize hydroxyl radicals directly in living cells, also including penetrating organelles like mitochondria, the ability to cross the blood-brain barrier, and its stability at room temperature, and low solubility in water as a very favorable tolerability profile.
**Conclusion**
$H_2$ can be easily applied with little adverse effects and great efficacy as a potential radioprotective agent.

© 2023 The Authors. Molecular Oncology is published by John Wiley & Sons Ltd on behalf of Federation of European Biochemical Societies

Nevertheless, the molecular mechanisms for $H_2$ and its primary molecular targets are still unclear.

### EACR23-0219
### Novel brain penetrant PI3K/WNT pathway inhibitor that reduces proliferation and cancer stemness in glioblastoma

V. Tandon[1], A. Fistrovich[2], J. Nogales[1], N. McArthur[1], L. Nicely[1], C. Cabel[2], C. Thorne[2], C. Hulme[2], S. Banerjee[1]
[1]University of Dundee, School of Medicine, Dundee, United Kingdom
[2]The University of Arizona, Department of Pharmacology and Toxicology, Tuscon, United States

**Introduction**

Glioblastoma is the most common primary brain cancer in adults, however patient survival of about 15 months and treatment strategies have remained stagnant over the past 40 years. Due to its heterogeneity and high percentage of cancer stem cells, recurrence is almost always inevitable in patients. Therefore, establishing an inhibitor that targets oncogenic proliferative signaling and reduces cancer stemness is imperative for treatment of GBM.

**Material and Methods**

Primary patient derived cell lines, neurospheres, glioma stem cells (GSCs), and rat neurons were treated with inhibitor DYR726 to evaluate cytotoxicity. Kinomescan was conducted to evaluate specificity of inhibitor. Western blot and proliferation assays were conducted to assess changes in proliferation. Western blot and RT-qPCR were conducted to determine the changes in Wnt signaling and stemness markers. Matrigel invasion assays were conducted to assess tumour cell invasion in vitro.

**Results and Discussions**

DYR726 is a soluble, brain-penetrant inhibitor that was rationally-designed to simultaneously target multiple pro-oncogenic signaling pathways in glioblastoma. The molecule can target diverse isoforms of kinases at nanomolar range yet retain remarkable specificity surpassing those exhibited by multiple other FDA-approved kinase inhibitors. DYR726 treatment completely ablates the PI3K-mTOR signaling pathway which leads to reduced proliferation and marked cytotoxicity in various patient derived primary cell lines. DYR726 also inhibits the common PI3K E545K and H1047R drug resistant mutants. DYR726 treatment also targets the Wnt signaling pathway which results in significant reduction in beta catenin, BTRC, DVL2, AXIN2, and c-myc levels in GSCs. This targeting of wnt signaling also leads to an overall reduction in stemness by reducing levels of GSC markers such as CD133, Oct4, SOX2, and Nanog. Therefore, treatment of stem-like neurospheres with DYR726 prevents the formation of neurospheres and significantly reduces diameter size of formed neurospheres. Intriguingly, treatment of DYR726 to primary rat neurons depicts a significant therapeutic window suggesting a potential to be cancer-cell specific.

**Conclusion**

These findings demonstrate a novel inhibitor for treatment of glioblastoma that simultaneously targets oncogenic proliferation and cancer stemness which could potentially change the prognosis of GBM patients worldwide.

### EACR23-0227
### Identification of natural products with immune checkpoint inhibitory activity in breast cancer

A.L. Capatina[1], T. Czechowski[1], B.R. Lichman[1], T. Tonon[1], I. Kourtzelis[2], I.A. Graham[1], W.J. Brackenbury[1], D. Lagos[2]
[1]University of York, Biology, York, United Kingdom
[2]University of York, Hull York Medical School, York, United Kingdom

**Introduction**

Immune checkpoint proteins are common therapeutic targets for breast cancer. Indoleamine pyrrole 2', 3' – dioxygenase 1 (IDO1) is a tryptophan catabolic enzyme expressed by cancer cells in response to IFN-γ stimulation. IDO1 induces T cell tryptophan starvation and Treg differentiation, through the tryptophan metabolite, kynurenine. Here, we describe adapting a low-throughput kynurenine assay for medium/ high-throughput screens and employing it to screen a library of 630 natural compounds in MDA-MB-231 breast cancer cells. We reveal a series of compounds with potential IDO1 inhibitory activity and their chemical features and propose several mechanisms of action.

**Material and Methods**

A library of 620 natural compounds, predominantly terpenoids, was curated. IDO1 function was assessed using a spectrophotometric assay. Gene and protein expression were quantified via qPCR and western blot. An in vitro enzyme activity assay was used to verify the mechanism of action of the selected compounds of interest.

**Results and Discussions**

Our screen identified a set of 60 compounds of interest. 23 IDO1 inhibitory and one enhancer compound were validated by a second round of screening. We identify 2 groups of compounds: artemether-derivatives and euphorbia factors, that share similarities between chemical structures and their effect on kynurenine, without impacting on IDO1 mRNA or protein levels. We propose that artemether derivatives might physically interact with IDO1 via an endoperoxide pharmacophore structure, also believed to be the active site for the anti-malaria effects of these drugs. For euphorbia factors we show that addition of functional groups to carbon 7 might be essential for IDO1 inhibition. These results were validated in breast cancer and primary epithelial cells*.
*Work with primary cells was approved by the Department of Biology Research Ethics Committee at the University of York and the primary cells were supplied by Lonza in accordance with all regulatory requirements.

**Conclusion**

This study provides structural information about the type of chemical interactions required for IDO1 inhibition and highlights the potential of using naturally derived compounds for breast cancer immune checkpoint-based therapies.

### EACR23-0235
### MI-2 : a potential inducer of ferroptosis for the treatment of liver cancer

J. Wang[1], C. Wang[1]
[1]Shanghai Cancer Institute, State Key Laboratory of

© 2023 The Authors. Molecular Oncology is published by John Wiley & Sons Ltd on behalf of Federation of European Biochemical Societies

*Oncogenes and Related Genes, Shanghai, China*

**Introduction**

Ferroptosis is a new form of regulated cell death characterized by the iron-dependent accumulation of lipid hydroperoxides. Inducing ferroptosis may be a promising therapeutic strategy for the treatment of liver cancer. However, the currently available ferroptosis inducers, such as RSL3 and ML210, have limited efficacy in vivo, likely due to their poor pharmacokinetic properties, low bioavailability, and off-target effects.

**Material and Methods**

A compound screen was performed to identify an inducer that can specifically trigger ferroptosis in HCC cell lines. To confirm the induction of ferroptosis, we assessed the upregulation of lipid ROS, alteration of mitochondrial morphology, and the rescue effects of Ferrostatin-1 following treatment with MI-2. Additionally, a non-biased CRISPR loss of function genetic screen, activity-based protein profiling and proteomics analysis were performed to investigate the mechanism of MI-2 on ferroptosis induction. Multiple *in vitro* and *in vivo* assays were employed to study the synergistic effects of the combining of MI-2 and sorafenib.

**Results and Discussions**

Through integrated bioinformatics analyses, we have identified GPX4 as a potential therapeutic target for liver cancer. Based on compound screen, we discovered MI-2, a ferroptosis inducer that upregulates lipid ROS and alters mitochondrial morphology, which confirming the induction of ferroptosis. MI-2 directly binds to and inhibits the activity of GPX4, which further leads to ubiquitous degradation of GPX4. For cancer cell lines are resistant to MI-2, we demonstrated that combining MI-2 with sorafenib results in strong synergy in inducing ferroptosis. These effects were further confirmed in multiple *in vivo* cancer models.

**Conclusion**

Our data suggests that MI-2 could be a novel inducer of ferroptosis for liver cancer. Furthermore, the findings underscore the potential of combining MI-2 with sorafenib to treat these sorafenib-approved cancer types.

### EACR23-0269

### Pharmacological evaluation of the thiosemicarbazones derivatives in pancreatic neoplastic cells

*V. Costa[1], D. Filho[2], M.D.C. Lima[2], M. Rêgo[2]*
[1]*Federal University of Pernambuco, Therapeutic Innovation Research Center - Suely Galdino, Recife, Brazil*
[2]*Federal University of Pernambuco, Laboratory of Chemistry and Therapeutic Innovation, Recife, Brazil*

**Introduction**

Pancreatic ductal adenocarcinoma (PDAC) is one of the most aggressive and lethal cancers. Gemcitabine or FOLFIRINOX have been chosen as first line chemotherapy in the majority of patients. However, there are few therapeutic options that can contribute to patient overall survival improvement. Thus, the aim of this study was to evaluate the anti neoplastic activity of thiophene-thiosemicarbazone derivatives in PDAC lineages.

**Material and Methods**

The cytotoxicity activity from the six thiophene-thiosemicarbazone derivatives (PR-12, PR-13, PR-16, PR-17, PR-19, PR-20) were investigated against BxPC-3, MIA PaCa-2 and PANC- cell lines by MTT. Also, after ethical board approval, PBMCs form health donors were used to obtain compound´s selectivity index. Cell cycle, death and reactive oxygen species were evaluated by flow cytometer using specific probes from each. Colonies formation inhibition was carried out using crystal violet method. Lastly, an *in silico* analysis was performed in order to determine ADME parameters using SwissADME plataform.

**Results and Discussions**

PR-16, PR-19 and PR-20 were toxic in PBMCs showing presenting an $IC_{50}$ between 24.60μM and 62.75μM. Because of that they were excluded form the next analyses. PR-12, PR-13 and PR-17 have presented a $IC_{50}$ between 3.15μM and 29.72μM, considering the 3 pancreatic cell lines. PR-17 activity stood out front of MIA PaCa-2 ($IC_{50}$ 8.9μM) promoting an increase in cell death and reactive oxygen species generation as well as cycle cells arrest in S/G2/M phases ($p < 0.05$). PR-17 *in silico* analyses has shown a low gastrointestinal absorption as well as limited water solubility. However the compound does not violate Lipinski rules and it is not a substrate for p-glycoprotein.

**Conclusion**

PR-17 derivative can be considered as a possible therapeutic strategy in PDAC, however it´s mechanism of action needs to be elucidate.

### EACR23-0271

### Human mature omental adipocytes as paclitaxel reservoir for drug delivery in ovarian cancer

*P. Andele[1], S. Palazzolo[2], I. Caligiuri[2], V. Canzonieri[2], F. Rizzolio[1]*
[1]*University of Ca' Foscari, Departement of Molecular Sciences and Nanosystems, Venezia, Italy*
[2]*IRCCS CRO Aviano National Cancer Institute, Department of Pathology, Aviano, Italy*

**Introduction**

The bidirectional communication between ovarian cancer (OC) cells and adipocytes in the complex tumor microenvironment give rise to lipolysis inside the mature adipocytes that consequently fuel cancer cells with fatty acids. This crosstalk is already explored through preadipocytes for drug delivery purposes, but their premature state and the variation between differentiation cocktails may hampered the translation of the biomaterial. Conversely, we newly introduce the use of human mature omental adipocytes extracted directly from patient's sample undergoing surgery. We cocultured Patient Derived Mature Omental Adipocytes and Patient Derived Tumor Organoids (PDTO) deriving from the same patient to mimic the proximity observed between cancer cells and adipocytes in ovarian cancer *in vivo*.

**Material and Methods**

We use IHC to show the expression of alanine serine cysteine transporter 1 (ASC-1) on extracted white adipocytes. We then loaded 2μg/ml of paclitaxel inside 500 μl of package volume (PCV) of adipocytes and named

© 2023 The Authors. Molecular Oncology is published by John Wiley & Sons Ltd on behalf of Federation of European Biochemical Societies them Living paclitaxel bullets (LPB). The absolute amount of paclitaxel in the uptake and release experiments was determined by reverse phase liquid chromatography coupled with high-resolution mass spectrometry (LC-HRMS). In addition, we established an *in vitro* culture of mouse derived liver organoids to test the toxicity of our formulation.

**Results and Discussions**

LPB exhibit a time dependent drug uptake close to 100% within one hour. In addition, LPB can transfer fluorescently labeled paclitaxel to PDTO. Thereafter, a live/dead assay on ovarian tumor organoids cocultured with LPB, both deriving from the same patient shows a significant increase of the propidium iodide positive cells in the treated tumor organoids respected to the untreated one. A method to keep adipocytes viable for long term culture could enlighten us on the exact mechanisms behind this drug delivery system. In this view, our future research aims to establish a more advanced method such as microfluidic system containing 3D cultured adipocytes and 3D PDTO to create a model that mimic better the *in vivo* drug delivery.

**Conclusion**

Omental adipocytes (OA), due to their fragility are complicated to handle when culture *in vitro*. However, it is still preferable to use OA for drug delivery purpose respect to preadipocytes cell lines because this latter do not recapitulate all the physiological conditions of the OA. However, standardization is still required for LPB before crossing the gap of its clinical application in personalized medicine.

### EACR23-0285
### Romo1 is the new target for development of chemotherapy sensitizer

Y.D. Yoo[1]
[1]*Korea University College of Medicine, Graduate School of Medicine, Seoul, South Korea*

**Introduction**

Chemotherapy in conjunction with surgical operations have been commonly used for the treatment of many tumors. However, a significant number of tumors fail respond to radiation therapy and/or chemotherapy because many forms of tumors appear to become less sensitive or resistant to radiation and anticancer drugs after consecutive treatments. Although extensive studies on the molecular mechanisms of resistance to chemo- and/or radiation therapy have been carried out, problems related to overcoming this resistance remain to be solved. Romo1 is a nuclear-encoded small transmembrane protein located in mitochondrial inner membrane. It is known to induce mitochondrial reactive oxygen species (ROS) production in response to various cellular stresses. For a decade, Romo1 has been studied in the context of mitochondrial ROS production, cancer cell invasion, inflammation, replicative senescence, and mitochondrial dynamics.

**Material and Methods**

We identified a Romo1 antagonist and tried to its efficacy as chemotherapy sensitizer using cancer cells and animal models.

**Results and Discussions**

A Romo1 antagonist can enhance the cellular levels of ROS, leading to tumor cell death. Its treatment induced the elevation of chemotherapy-induced oxidative damage of cancer cells. We also treated the Romo1 antagonist in combination with various chemotherapeutic agents.

**Conclusion**

We suggest that Romo1 antagonist can enhance the cellular levels of ROS, leading to elevation of chemotherapy-induced oxidative damage of cancer cells. We also suggest that Romo1 is the new target to identify effective substances for development of chemotherapy sensitizer.

### EACR23-0296
### 1,2-Steroidal epoxides as a promising therapeutic approach for cancer

*A.R. Gomes[1,2], S. Costa[3], A.M. Abrantes[1,4,5], C. Varela[4,5,6], M.F. Botelho[1,4,5], E.J. Tavares-da-Silva[2], F.M.F. Roleira[2], A.S. Pires[1,4,5]*
[1]*Coimbra Institute for Clinical and Biomedical Research i CBR area of Environment Genetics and Oncobiology CIM AGO- Biophysics Institute of Faculty of Medicine, University of Coimbra, Coimbra, Portugal*
[2]*CIEPQPF- Faculty of Pharmacy- Laboratory of Pharmac eutical Chemistry, University of Coimbra, Coimbra, Portugal*
[3]*Faculty of Pharmacy- Laboratory of Pharmaceutical Che mistry, University of Coimbra, Coimbra, Portugal*
[4]*Clinical Academic Center of Coimbra CACC, University of Coimbra, Coimbra, Portugal*
[5]*Center for Innovative Biomedicine and Biotechnology CIB B, University of Coimbra, Coimbra, Portugal*
[6]*CIEPQPF- Coimbra Institute for Clinical and Biomedical Research iCBR- Faculty of Medicine, University of Coimbra, Coimbra, Portugal*

**Introduction**

Cancer incidence and mortality rates have been increasing worldwide, which reinforces the need for new emerging therapies. Steroidal compounds are one of the most diversified therapeutic class of compounds and they were proven to be efficient against several types of cancer. Previous studies by our group demonstrated that 1,2-epoxides are also very potent against several types of cancer cell lines. Thus, we combine this chemical function with the steroidal backbone, by synthesizing steroidal epoxides and evaluating their potential antitumor activity against lung, prostate and triple negative breast cancer cells, ultimately to find new antitumor agents with fewer side effects.

**Material and Methods**

Compounds 1α,2α,4β,5β-diepoxyandrostane-3,17-dione (**EP2**) and 1α,2α-epoxyandrosta-4,6-diene-3,17-dione (**EP3**) and their parent compounds androsta-1,4-diene-3,17-dione (**OL2**) and androsta-1,4,6-triene-3,17-dione (**OL3**) were synthesized, and their cytotoxicity evaluated in cancer cell lines H1299, HCC1806 and PC3, through SRB assay after treating cancer cells with the compounds (1-75 μM). Cell viability, cell death profile and alterations on cell cycle were assessed by flow cytometry.

**Results and Discussions**

Compounds **EP2** and **EP3** decreased all cancer cell lines proliferation in a dose-dependent manner, with $IC_{50}$ values ranging from 1.95 to 3.67 μM for **EP2** and 12.64 to 15.10 μM for **EP3**. On the contrary, the parent compounds failed to decrease cancer cell proliferation, proving that the introduction of an epoxide function was, in fact, beneficial

© 2023 The Authors. Molecular Oncology is published by John Wiley & Sons Ltd on behalf of Federation of European Biochemical Societies for the antitumor activity displayed. Flow cytometry studies revealed that both **EP2** and **EP3** caused a decrease in cell viability in all cell lines, which was accompanied by the induction of apoptosis or necrosis depending on the cell line. This goes accordingly with the cell cycle studies that show a blockage at phases G2/M and S. These alterations are dose-dependent for both compounds in all cell lines.

**Conclusion**

Our results with **EP2** and **EP3** compounds showed a beneficial antitumor effect from the introduction of the epoxide function, which is mediated by apoptosis and/or necrosis. This effect encourages further studies on their mechanism of action and selectivity in order to discover new molecules for cancer treatment.

**Funding:** Foundation for Science and Technology (FCT), Portugal (Strategic Projects UID/NEU/04539/2019, UIDB/04539/2020, UIDP/04539/2020); COMPETE-FEDER (POCI-01-0145-FEDER-007440); PhD Fellowship from FCT and European Social Funding of Ana R. Gomes (UI/BD/150865/2021).

### EACR23-0306

### Discovery of phalbinib, a novel anticancer compound targeting copper homeostasis and inducing cell cycle arrest and autophagy

*P. LELIEVRE[1], C. Nogier[1], N. Renier[2], J.L. Coll[1], I. Jabin[3], L. Sancey[1], V. Hennie[2], D. Aurélien[4], B. Busser[1]*
*[1]University Grenoble Alpes- Inserm U 1209- CNRS UMR 5 309, Institute for Advanced Biosciences, Grenoble, France*
*[2]Université libre de Bruxelles ULB- Ecole polytechnique d e Bruxelles, Engineering Molecular NanoSystems, Brussels, Belgium*
*[3]Université libre de Bruxelles ULB- Faculty of Sciences, Laboratoire de Chimie Organique, Brussels, Belgium*
*[4]Univ. Grenoble Alpes-CNRS-CEA-IRIG, Laboratoire de Chimie et Biologie des Métaux, Grenoble, France*

**Introduction**

Copper (Cu) is an essential co-factor for many enzymes, and therefore a key player for many biological processes. Free Cu toxic for the cell that has developed very efficient mechanisms to control its homeostasis. In addition, dyseregulation of Cu metabolism also leads to structural abnormalities or loss of essential physiological functions. These disruptions in Cu homeostasis are found in numerous pathologies, including cancers where Cu promotes cell proliferation and angiogenesis. Thus, targeting Cu metabolism is an innovative therapeutic strategy in oncology. In this context, we identified a new chemical structure capable of disrupting Cu homeostasis in various cancer cell lines, including lung cancer cells.

**Material and Methods**

We investigated the anticancer effect of this new compound (phalbinib) in different cell lines. Viability experiments ($IC_{50}$) of phalbinib were performed on various lung cancer cell lines (PC9, H322, H358, and A549) and in a commercial panel of 60-tumor cell lines. We studied the cellular effects of phalbinib on several proteins involved in Cu metabolism (Ctr1, ATP7a, ATP7b, Atox1). We further evaluated the effects of phalbinib on the cell cycle via flow cytometry experiments, and its ability to induce reactive oxygen species (ROS), apoptotic cell death or autophagy. We combined phalbinib with different conventional chemotherapies, looking for synergistic effects.

**Results and Discussions**

The investigation of the antiproliferative effects of this compound appears to be extremely promising as we observe $IC_{50}$s in the range of ~1 μM in 60 cancer cell lines. Phalbinib did not induce apoptosis nor ROS production in lung adenocarcinoma cell lines. Importantly, phalbinib induced an irreversible cell cycle arrest. The combination of phalbinib with Cu ions supplementation resulted in synergistic antiproliferative effects. This combination may induce an autophagic response since the expression level of LC3B II protein was increased.

**Conclusion**

Considering the anticancer effects observed in vitro, phalbinib is a novel anticancer drug with an innovative mechanism of action, and its applications for cancer treatment were patented. Because of its hydrophobic properties, we are currently working on a formulation to allow the following preclinical studies to be performed in mice bearing lung tumours.

### EACR23-0308

### Development and Validation of the Chick Embryo Tumour Model for Assessment of Cancer Combination Therapies.

*M. Boyd[1], H. Gardiner[2], C. Mullen[2], S. McCabe[2], L. Horan[2], A. Mullen[2], R. Akram[2], Y. Farhan[2], A. Sorensen[2], A. Herman[3]*
*[1]University of Strathclyde, SIPBS, Glasgow, United Kingdom*
*[2]University of Strathclyde, Strathclyde Institute for Pharmacy and Biomedical Science s, Glasgow, United Kingdom*
*[3]University of Liverpool, Chick Embryo Facility, Liverpool, United Kingdom*

**Introduction**

The **Chorioallantoic Membrane (CAM)**, also known as the chorioallantois, is a highly vascularized membrane found in the eggs of certain amniotes like birds and reptiles. It is formed by the fusion of the mesodermal layers of two extra-embryonic membranes – the chorion and the allantois and it is the avian homologue of the mammalian placenta. It is the outermost extra-embryonic membrane which lines the non-vascular egg shell membrane. Given the characteristics of this membrane, researchers have been investigating the use of the CAM for as a cheaper in vivo model replacement for Murine models which can reduce and replace procedures in rodents.

**Material and Methods**

Procedures were undertaken using white leg horn chicken eggs which were grown in humidified rotating incubators for 3 days before undergo windowing, tumour cell implantation and treatment with drugs or irradiation or nanoparticles on specific days of gestation. A variety of cells were used to grow tumours on the CAM including; Pancreatic, Triple Negative Breast Cancer and Glioblastoma cells. The development of these tumours are monitored from implantation until the end point by visual inspection and imaging under microscope.

**Results and Discussions**

© 2023 The Authors. Molecular Oncology is published by John Wiley & Sons Ltd on behalf of Federation of European Biochemical Societies

The CAM model has successfully produced tumours from a variety of different cancers that have been transfected with GFP, to allow clear distinction between cancer cells and cells belonging to the embryo. We were able to track metastasis through the membrane and measure the volume of tumour growth on the CAM. It was established that the dose of chemotherapeutic drugs and irradiation that are used to treat developing tumours do not kill the embryos.

**Conclusion**

We have successfully developed the methodology from the CAM tumour models, and established the replicability of the model. Including which cell type established the more desirable tumours and allows for investigation of metastasis. our next stage of the investigation will be to develop and adapt irradiation methodologies in the developed tumours of the chick embryo model for single or combination treatment and to validate the model by comparison to our existing mouse xenograft data. Finally, we will screen for the efficacy of drugs or nanomaterials alone or in combination.

### EACR23-0316
### Inhibition of oncogenic mutant KRAS via oxidation at Cysteine 118

*M. Kramer-Drauberg*[1], *E. Petrini*[1], *E. Patrucco*[1], *R. Scardaci*[1], *A. Mira*[1], *M.J. Nokin*[2], *D. Santamaria*[3], *P. Porporato*[1], *C. Ambrogio*[1]

[1]*University of Torino, Department of Molecular Biotechnology and Health Sciences, Torino, Italy*

[2]*University of Liege, GIGA-Cancer- Laboratory of tumor and development biology, Liege, Belgium*

[3]*CSIC-University of Salamanca, Molecular Mechanisms of Cancer Program- Centro de Investigación del Cáncer, Salamanca, Spain*

**Introduction**

Oncogenic mutant RAS is one of the main drivers of cancer. The redox-biology of RAS involves a redox-sensitive Cysteine, C118. By reacting with C118, free-radical oxidants, predominantly nitric oxide, activate RAS by promoting a nucleotide exchange. Recently we discovered in *C. elegans* that the ortholog of mutant KRAS is inhibited via oxidation at C118 by the non-radical oxidant hydrogen peroxide. Based on this finding, we investigated whether the mechanism of mutant KRAS inhibition through oxidation at C118 is maintained in mammalian cells *in vitro* and *in vivo*, and we explored the therapeutic value of KRAS oxidation

**Material and Methods**

To investigate the effects of oxidation on KRAS activity, we generated a new model by transducing different oxidation-mimetic KRAS constructs into Ras-less cells (Drosten et al, EMBO J, 2010). To mimic a permanent oxidation of KRAS at C118 by hydrogen peroxide, we replaced Cysteine 118 with Aspartic acid in KRAS (C118D); to inhibit oxidation at C118, we replaced Cysteine 118 with Serine (C118S). These mutations were introduced either alone or *in cis* with the most common oncogenic mutations in KRAS (G12C/G12D/G12V)

**Results and Discussions**

We found that the C118D substitution inhibited the growth rate of KRAS-driven cells *in vitro*. Given the C118D substitution mimics the Cysteine Sulfinic acid oxidative modification (an intermediate state of Cysteine oxidation by hydrogen peroxide) without affecting the KRAS protein level, the GTP level, the effector binding and localization, and with minimal impairment to the MAPK pathway, our data indicates that human mutant KRAS is inhibited via oxidation at C118 by hydrogen peroxide. Moreover, treatment with pro-oxidants in combination with nitric oxide production inhibitor L-NAME inhibited mutant $KRAS^{G12V}$ activity. Further proving that C118 is the target of this oxidation, we found that the C118S substitution rendered mutant KRAS insensitive to the inhibitory effect of the aforementioned combination treatment. *In vivo*, we confirmed that C118D *in cis* with oncogenic G12V mutation significantly impaired tumor growth and prolonged overall survival

**Conclusion**

Mimicking oxidation by hydrogen peroxide at C118 inhibits mutant KRAS *in vitro* and *in vivo*.
A combination of nitric oxide production inhibition and reactive oxygen species (ROS) production inhibits mutant KRAS by targeting C118.
We conclude that oxidation at C118 presents a novel way to inhibit mutant KRAS, thereby paving the way to explore oxidation based anti-KRAS treatments in humans.

### EACR23-0326
### A robust pipeline for high-throughput drug screening in PDTX models

*D. Guerrero-Romero*[1], *E. Esmaeilishirazifard*[1], *A. Lui*[1], *A. Shea*[2], *W. Greenwood*[1], *A. Bruna*[2], *O.M. Ruedda*[3], *C. Caldas*[1]

[1]*University of Cambridge, Cancer Research UK Cambridge Institute, Cambridge, United Kingdom*

[2]*Institute of Cancer Research, Preclinical Modelling of Paediatric Cancer Evolution Team, London, United Kingdom*

[3]*MRC Biostatistics Unit, SOMX: Statistical Omics, Cambridge, United Kingdom*

**Introduction**

Patient-Derived Tumour Xenografts (PDTXs) have been beneficial for pre-clinical cancer drug development. However, conducting drug tests on these models can be costly and time-consuming. To address this, we established a method that involves growing the tumour in mice and then testing drugs on short-term *in vitro* cultures of tumour cells called PDTX-derived tumour cells (PDTCs). We have created an experimental pipeline for high-throughput drug screening that is customised, dynamic and backed by incorporating a new metric for drug response in such models.

**Material and Methods**

The frozen tissues of 40 PDTXs were dissociated into single-cell suspensions with physical and enzymatic methods and cultured in 384-well plates. Two different bioassays were used to profile cell growth and to quantify drug responses on days 0, 1, 2, 3, 6 and 7 of culture: the CellTiter-Glo assay (CTG), used for measuring cell viability at specific endpoints and the Real-TimeGlo MT Cell assay (RTG), which allows real-time monitoring of cell viability.

**Results and Discussions**

© 2023 The Authors. Molecular Oncology is published by John Wiley & Sons Ltd on behalf of Federation of European Biochemical Societies

We established a pipeline that ensures PDTCs are a reliable drug-testing tool. We calculated the growth dynamics for each model *in vitro* in the analysis to obtain an overall more robust interpretation of the drug responses. The robustness of this improved system is a result of consolidating the individual dose responses that occur over time by real-time monitoring with RTG of the growth rate inhibition.
We compare growth rates using both CTG and RTG to demonstrate the advantages of using the RTG protocol, allowing us to track the same group of cells over time. We compare biological and technical reproducibility and show RTG allows reducing the inherent noise in high-throughput drug testing. Finally, we calculate drug response metrics RTG experiments metrics such as AUC/IC50 and provide thorough comparisons at various time intervals, models and cell lines, which reveal variations in the impact of drugs over time and across models. Showing that PDTCs models are useful.

**Conclusion**

We tested and improved a bioassay using RTG to screen drug responses in PDTCs in large-scale. CTG has more technically inherent inter-well variability. RTG is technically more accurate and sensitive to detect significant changes during drug treatment because it allows continuous tracking of the growth rate inhibition on the same group of cells. By consolidating the effect of drug doses on PDTC growth throughout the experiment, this metric provides a more reliable assessment of drug response in PDTCs.

### EACR23-0327

### Unlocking the Potential: PARP Inhibitors and DNA-DA combined to create an effective treatment for Pancreatic Cancer

*E. Pereira[1,2,3], P. S. Vaz[1,2,3,4],*
*A. S. Pires[1,2,3], M.F. Botelho[1,2,3]*
*[1]Coimbra Institute for Clinical and Biomedical Research i CBR Area of Environment- Genetics and Oncobiology CIM AGO- Institute of Biophysics- Faculty of Medicine, University of Coimbra, Coimbra, Portugal*
*[2]Center for Innovative Biomedicine and Biotechnology CIB B, University of Coimbra, Coimbra, Portugal*
*[3]Clinical Academic Center of Coimbra CACC, University of Coimbra, Coimbra, Portugal*
*[4]Centro Hospitalar e Universitário de Coimbra CHUC, General Surgery Department, Coimbra, Portugal*

**Introduction**

Pancreatic Cancer (PanC), one of the deadliest cancers worldwide with a 5-year survival rate of ~5%, needs new and more effective treatment approaches. It is known that Poly(ADP-ribose) Polymerase (PARP) blocking prevents cancer cells from repairing their damaged DNA. Thus, the combination of PARP inhibitors (PARPi) with DNA-damaging agents (DNA-DA), can increase the amount of DNA damage overwhelming the repair mechanisms, improving treatment effectiveness, and decreasing adverse effects. This study aims to investigate the potential of combining different therapeutic approaches, namely PARPi with DNA-DA, to improve treatment effectiveness and reduce adverse effects in PanC.

**Material and Methods**

Olaparib (OLA) was used as PARPi and irinotecan (IRI) or oxaliplatin (OXA) as DNA-DA. The combinations effect was assessed in MIA PaCa-2 cell line. Firstly dose-response curves were plotted to determine the mean inhibitory concentration ($IC_{50}$) of each drug 24, 48, and 72 hours after treatment, using the SRB assay. Then, based on each $IC_{50}$, drugs were simultaneously combined at a constant ratio for 24, 48, and 72 hours. Synergistic effect was assessed using the Chou-Talalay method to calculate the Combination Index (CI) and the Dose-Reduction Index (DRI).

**Results and Discussions**

OLA and IRI combination at 24, 48, and 72 hours, demonstrated a moderate synergistic effect with a CI of 0.71, 0.77, and 0.72, respectively. The DRI was calculated to determine the amount of dose reduction achieved with the combination therapy. We observed a 2.8-, 2.6-, and 2.8-fold reduction in the concentration of OLA and IRI 24, 48, and 72 hours after treatment, respectively. OLA and OXA combination demonstrated an antagonistic effect with a CI of 1.51, and 1.64, 24 and 48 hours after treatment, and a moderate antagonistic effect with a CI of 1.37, 72 hours after treatment. In the DRI was observed a 1.33-, 1.22-, and 1.46-fold reduction in the concentration of OLA and OXA 24, 48, and 72 hours after treatment.

**Conclusion**

The results demonstrate the applicability of the simultaneous combination of OLA and IRI against PanC. Despite the antagonistic effect observed with the simultaneous combination of OLA and OXA, further investigation is warranted, such as evaluating its sequential combination.

**Funding:** Foundation for Science and Technology (FCT), Portugal (Strategic Projects UID/NEU/04539/2019, UIDB/04539/2020, UIDP/04539/2020); COMPETE-FEDER (POCI-01-0145-FEDER-007440); PhD Fellowship from FCT and European Social Funding (2021.05543.BD).

### EACR23-0329

### Characterisation of novel inhibitory antibodies targeting growth hormone signalling in breast cancer cell lines

*C. Buckley[1], M. Lu[2], Y. Wang[1], R.J. Langley[3], J.K. Perry[1]*
*[1]University of Auckland, Liggins Institute, Auckland, New Zealand*
*[2]University of Auckland, Auckland Cancer Society Research Centre, Auckland, New Zealand*
*[3]University of Auckland, Department of Molecular Medicine and Pathology, Auckland, New Zealand*

**Introduction**

Growth hormone (GH) contributes to the development and progression of multiple cancer types, including breast cancer. GH signalling is mediated by two receptors: the GH receptor (GHR) and the prolactin receptor (PRLR). In breast cancer, tumour expression of both GH and prolactin (PRL) has been shown to be associated with a poor outcome for patients and most breast cancer cell lines have high expression of both GHR and PRLR. The only clinically approved GH signalling inhibitor is pegvisomant, a GHR specific antagonist. One proposed method for complete inhibition of GH signalling is the use of

© 2023 The Authors. Molecular Oncology is published by John Wiley & Sons Ltd on behalf of Federation of European Biochemical Societies monoclonal antibodies (mAbs). The aim of this study was to characterise three neutralising anti-GH mAbs and determine their utility as anticancer agents in breast cancer cell lines.

**Material and Methods**

Inhibitory activity was initially assessed using a Ba/F3-hGHR cell viability assay. Cross reactivity to proteins closely related to GH was determined by ELISA. Binding affinity ($K_D$) was determined by bio-layer interferometry. Three breast cancer cell lines with high GHR and PRLR expression were chosen for *in vitro* assays. Inhibition of GH signalling was determined by measuring STAT5 phosphorylation by western blotting and by cell viability assays. The protein component of pegvisomant (B2036) was used for comparison.

**Results and Discussions**

Three mAbs with inhibitory activity against GH were identified and characterised (8-2, 32-1 & 46-3). mAb 8-2 and 46-3 exhibited strong inhibitory activity against GH-induced cell growth with $EC_{50}$ values of 1.00 ± 0.27 and 0.5 ± 0.06 µg/ml, respectively. All antibodies cross-reacted with placental lactogen and placental GH but not with mouse GH or human prolactin. mAb 8-2 had a $K_D$ for GH of 0.62 ± 0.05 nM, while mAb 46-3 had a $K_D$ of 2.68 ± 0.53 nM. mAbs 46-3, 8-2 and 32-1 inhibited GH-dependent signal transduction in T-47D, ZR-75-1 & MCF-7 breast cancer cell lines, and reduced GH-dependent cell growth in the T-47D cell line. B2036 did not inhibit GH-dependent signal transduction or GH-dependent cell growth in these cell lines. Ongoing studies will look at the utility of combined treatment with GHR & PRLR antagonists and PRLR knockdown by siRNA.

**Conclusion**

Targeting GH signalling in breast cancer requires inhibition of both the GHR and PRLR. Using monoclonal antibodies or combined treatment with GHR and PRLR antagonists may be more effective in breast cancer cells lines with high GHR and PRLR expression.

### EACR23-0331

### Unraveling the chemotherapeutic potential of Royleanone, a natural drug lead, against glioblastoma.

M. Magalhães[1], E.M. Domínguez-Martín[2], J. Jorge[3], A.C. Gonçalves[3], A.I. Duarte[4], P.J. Oliveira[4], T. Efferth[5], P. Rijo[6], C. Cabral[7]

*[1]Institute of Interdisciplinary Research- University of Coimbra, Coimbra Institute for Clinical and Biomedical Research iCBR- Clinic Academic Center of Coimbra CACC- Faculty of Medicine / CNC - Center for Neuroscience and Cell Biology / Center for Innovative Biomedicine and Biotechnology CIBB-, Coimbra, Portugal*

*[2]Faculty of Pharmacy- University of Alcalá de Henares, Department of Biomedical Sciences- Pharmacology Area Pharmacognosy Laboratory- University of Alcalá de Henares / CBIOS – Universidade Lusófona's Research Center for Biosciences & Health Technologies- Lisbon- Portugal, Madrid, Spain*

*[3]Faculty of Medicine- University of Coimbra, Laboratory of Oncobiology and Hematology- University Clinic of Hematology and Applied Molecular Biology / iCBR- Group of Environment Genetics and Oncobiology CIMAGO, Coimbra, Portugal*

*[4]University of Coimbra, CNC - Center for Neuroscience and Cell Biology / Center for Innovative Biomedicine and Biotechnology CIBB, Coimbra, Portugal*

*[5]Institute of Pharmacy and Biochemistry- University of Mainz, Department of Pharmaceutical Biology, Mainz, Germany*

*[6]Universidade Lusófona, CBIOS – Universidade Lusófona's Research Center for Biosciences & Health Technologies / Instituto de Investigação do Medicamento iMed.ULisboa- Faculty of Pharmacy- University of Lisbon, Lisbon, Portugal*

*[7]Faculty of Medicine- University of Coimbra, Coimbra Institute for Clinical and Biomedical Research iCBR- Clinic Academic Center of Coimbra CACC- Faculty of Medicine / Center for Innovative Biomedicine and Biotechnology CIBB / Centre for Functional Ecology- Department of Life Sciences, Coimbra, Portugal*

**Introduction**

Glioblastoma (GB), an adult-type diffuse glioma with high heterogeneity, is the most aggressive and frequent glioma of the Central Nervous System. Despite the efforts so far, GB remains with a low 5-year survival rate (~6%), mainly due to late diagnosis, poor prognosis and lack of effective therapeutic options. Thus, design of new therapies based on plant-based drug leads is a compelling starting point to further improve patients' survival and well-being.

**Material and Methods**

Royleanone (Roy), a natural drug lead, was isolated from the acetonic extract of *Plectranthus hadiensis* (Forssk.) Schweinf. *ex* Sprenger var. *hadiensis*. The antitumor mechanism of action of Roy was studied in a panel of 5-glioma cell lines (A172, U87, H4, U118, U373), under normal (cells maintained with new medium) and conditioned (cells maintained with medium containing secretome from co-cultures of GB and microglia cells) states. Briefly, the impact of Roy treatment on cells' metabolic activity was assessed by Rezasurin assay, while cell death, cell cycle regulation and mitochondrial membrane potential were evaluated by flow cytometry. Measurement of mRNA levels of genes associated with pro-apoptotic mechanisms, as well as, autophagy events was performed by qPCR and validation of signaling pathways was assessed by Western blot.

**Results and Discussions**

Evidence in this work revealed that Roy presents a chemotherapeutic profile against GB cells, being this activity substantially improved when cells are maintained with conditioned medium. Moreover, this natural drug lead needs a concentration ~9 times lower than the necessary concentration of temozolomide to inhibit cell proliferation by 50%. Data analysis showed that treatment of GB cells with Roy leads to activation of caspase-mediated cell death, although, the mechanism of action seems to be distinct for the different cell lines (different stages of the disease). Hence, treatment with Roy induces apoptosis via activation of the intrinsic mitochondria-dependent pathway, as well as, induces autophagic cell death through disruption of Beclin1/Bcl2 complex. Also, Roy showed strong antiproliferative and antitumor activity by impairing DNA repair and promoting the upregulation of *TP53* and *PTEN* genes with subsequent inhibition of TP53/MDM2 and PI3K/Akt pathways, respectively.

© 2023 The Authors. Molecular Oncology is published by John Wiley & Sons Ltd on behalf of Federation of European Biochemical Societies

**Conclusion**
The outcome of this work has the potential to be perceived as an initial step in the design of more effective therapies based on a plant-based compound as a drug lead in future chemotherapeutic approaches for GB.

### EACR23-0336
### Morusin Inhibits the Growth and Migration of Prostate Cancer through Inactivation of Akt/mTOR Signaling Pathway

*C.Y. Li[1], W. Hsin-En[1], S. Chia-Cheng[2], W. Shu-Chi[3], C. Wei-Chung[4], Y. Hsin-Chih[5], C. Yuan-Ru[1], H. Shu-Pin[6]*
*[1]Kaohsiung Medical University, Graduate Institute of Medicine, Kaohsiung, Taiwan*
*[2]Chi-Mei Medical Center, Division of Urology- Department of Surgery, Tainan, Taiwan*
*[3]Kaohsiung Medical University, Department of Medical Laboratory Science and Biotechnol ogy, Kaohsiung, Taiwan*
*[4]China Medical University, Graduate Institute of Biomedical Sciences- and Research C enter for Tumor Medical Science, Taichung, Taiwan*
*[5]Kaohsiung Medical University, Department of Urology- School of Medicine, Kaohsiung, Taiwan*
*[6]Kaohsiung Medical University, Graduate Institute of Clinical Medicine, Kaohsiung, Taiwan*

**Introduction**
Prostate cancer (PCa) is a prevalent cancer in men worldwide, with a high risk of progression to castration-resistant PCa (CRPC) and a need for new therapeutic strategies. This study investigated the effects of morusin, a flavonoid from Morus alba L., on PCa progression and the underlying mechanism.
**Material and Methods**
Cell growth were examined by MTT assay and colony formation assay. Cell migration and invasion were analyzed using wound-healing assay, cell motility assay and transwell invasion assay. Expression of EMT markers were detected by western blot. Cycle progression and cell apoptosis were examined using flow cytometry and TUNEL assay, while transcriptome analysis was performed by RNA-seq with results being further validated using real-time PCR and Western blotting. A xenograft PCa model was used to examine tumor growth.
**Results and Discussions**
Morusin inhibited the growth, migration, and invasion of human PCa cells and suppressed epithelial-mesenchymal transition. Morusin also induced cell cycle arrest and apoptosis in PCa cells and reduced tumor growth in a mouse model. RNA-seq analysis and Western blotting revealed that morusin regulated PCa cells via the Akt/mTOR signaling pathway.
**Conclusion**
These findings suggest that morusin has potential as a treatment for CRPC by targeting multiple aspects of PCa progression.

### EACR23-0337
### KRAS mutant lung cancer specific methuosis-like cell death induced by uncontrolled macropinocytosis with the downregulation of KRAS/PI3K/Akt/mTOR pathway

*D. Kim[1], D. Min[1], J. Kim[2], M.J. Kim[2], J.Y. Lee[1]*
*[1]korea university medical center, Pathology, Seoul, South Korea*
*[2]Sookmyung Women's University, Biological Science, Seoul, South Korea*

**Introduction**
Oncogenic KRAS mutation, the most frequent gene mutation in numerous cancers, including non-small cell lung cancer (NSCLC), serves aggressiveness risk in the tumor thus it has been considered ‘undruggable’ due to the unusual structural characteristics. Lately, the FDA-approved sotorasib in 2021 is the first in class KRAS-G12C inhibitor, but it still has a derivative barrier, which has no effect on other types of KRAS mutation except G12C, and will develop resistance, implying the need for other therapeutic strategies.
**Material and Methods**
KRAS mutant, and wildtype NLSLC cells were used in vitro cell analyses. Cell viability, proliferation, and death were measured by MTT, cell counting, colony analyses, and annexin V staining for FACS. Cell tracker dyes were used to investigate cell morphology, and tomohologr apy, and confocal microscope were used to examine cell images. Zebrafish and mouse xenograft models were used for *in vivo* analysis.
**Results and Discussions**
In this study, we investigated and discovered that the MDM2 antagonist (MDM2-A) selectively induced KRAS mutant cell death, but not in KRAS wildtype cells via inhibition of KRAS/PI3K/Akt/mTOR pathway. This cell death was associated with disruption of the fusion of both autophagosome, and macropinosome with the lysosome, resulting in autophagic flux inhibition, and methuosis-like cell death, which is the non-apoptotic cell death derived from abnormal macropinocytosis ultimately rupture of cells. Further, effect of MDM2-A in KRAS mutant cells was verified in both zebrafish, and mouse as *in vivo* models.
**Conclusion**
MDM2-A could be a considerable potential therapeutic strategy in KRAS-MT NSCLC cells.

### EACR23-0349
### Evaluation of proteasome inhibitor resistance in long-term ixazomib exposed CCRF-CEM acute lymphoblastic leukemia cells harboring PSMB5 mutations.

*M. Roeten[1], J. van Meerloo[1], D. Geerke[2], S. van Dijk[1], Q. Hansen[1], Z. Kwidama[1], G. Kaspers[3,4], S. Zweegman[1], G. Jansen[5], J. Cloos[1]*
*[1]Amsterdam UMC- location Vrije Universiteit, Department of Hematology, Amsterdam, The Netherlands*
*[2]Vrije Universiteit Amsterdam, Department of Chemistry and Pharmaceutical Sciences, Amsterdam, The Netherlands*
*[3]Princess Máxima Center of Pediatric Oncology, Pediatric Oncology, Utrecht, The Netherlands*

© 2023 The Authors. Molecular Oncology is published by John Wiley & Sons Ltd on behalf of Federation of European Biochemical Societies

[4]Emma Children's Hospital- Amsterdam UMC- location Vr ije Universiteit, Pediatric Oncology, Amsterdam, The Netherlands
[5]Amsterdam UMC- location Vrije Universiteit, Amsterdam Rheumatology and Immunology Center, Amsterdam, The Netherlands

**Introduction**

Bortezomib (BTZ) was the first proteasome inhibitor (PI) that showed clinical activity in hematological malignancies. However, limiting factors such as off-target effects and resistance have emerged. Ixazomib/Ninlanro (IXA/NIN) is a next-generation, reversible and orally available PI. We studied potential acquired resistance to IXA and its underlying mechanism(s) in acute lymphoblastic leukemia (ALL) cells.

**Material and Methods**

CCRF-CEM T-ALL parental cells (CEM WT) and four IXA/NIN-resistant cell lines (Table) adapted to 350 and 2000 nM IXA were sequenced for mutations in the *PSMB5* gene. Of the different mutations, *in silico* molecular docking analysis was performed to assess whether the PIs would still be able to bind the β5-subunit of the proteasome. Proteasome subunit inhibition and growth inhibitory potential (MTT-assay) of IXA, BTZ and the irreversible PI marizomib (MRZ) were tested.

**Results and Discussions**

The Ala20Val mutation was previously described in a relapsed multiple myeloma patient treated with BTZ (Barrio *et al*, Leukemia 2019), while the Ala49Val was found in BTZ-resistant leukemia cell lines (Franke et al, Leukemia 2012). BTZ displayed the highest cross-resistance against the Ala49Val mutation, which impairs β5-subunit binding for all PIs, while MRZ may still bind to the β5-subunit with an Ala50Val mutation in the NIN2000 cells. Indeed, MRZ was still able to inhibit 60% of β5-subunit activity at 50nM in NIN2000 cells, compared to 13% decrease at 100 nM MRZ in X2000 cells. However, MTT-assay showed similar and relatively low RF for MRZ in NIN2000 and X2000 cells, probably explained by other factors besides the mutation, such as irreversible binding.

| | | | 50% growth inhibition compared to control $IC_{50}$ (nM) (resistance factor, RF) | | |
|---|---|---|---|---|---|
| **Cell line** | **Mutation** | **Possible binding** | **Ixazomib** | **Bortezomib** | **Marizomib** |
| **CEM-WT** | | | 16.0 | 3.4 | 8.2 |
| **CEM-NIN350** | A50V | MRZ | 613 (38) | 68.9 (20) | 45.4 (6) |
| **CEM-X350** | A20V, S18S | MRZ (BTZ) | 812 (50) | 86.7 (26) | 59.0 (7) |
| **CEM-NIN2000** | A50V | MRZ | 3149 (197) | 301 (89) | 77.5 (9) |
| **CEM-X2000** | A20V, S18S, A49V | Clash w/all | 6138 (384) | 930 (275) | 70.9 (9) |

**Conclusion**

These results indicate that IXA-resistant T-ALL cells acquired multiple *PSMB5* mutations conferring high cross-resistance to BTZ and to a lesser extend to MRZ. This cross-resistance profile is partly corroborated by computational docking analysis, but conceivably the difference in reversible (BTZ and IXA) versus irreversible (MRZ) binding may also be a critical contributing factor.

### EACR23-0401
### Genetic editing of the transcription factor FOXC1 with CRISPR/Cas9 ribonucleoproteins reprograms triple negative breast cancer cells to a less malignant state

M. Marqués[1], I. Urdanibia[1], M. Pont[1], I. Hidalgo[1], A. Oronich[1], M.A. Sorolla[1], E. Parisi[1], R. Montal[1], A. Salud[1], A. Sorolla[1]
[1]Biomedical Research Institute of Lleida IRBLleida, Medicine, Lleida, Spain

**Introduction**

Breast cancer (BC) is the most common diagnosed cancer worldwide. Among all BC subtypes, triple negative breast cancer (TNBC) is one of the most aggressive subtypes, possessing the highest ratios of mortality and recurrence. TNBC do not express oestrogen receptor, progesterone receptor and HER2. Thus, conventional highly toxic treatments with secondary effects such as chemotherapy remains the mainstay option for the patients. Therefore, a more selective therapeutic approach for TNBC is highly needed. Oncogenic transcription factors (TF) are frequently overexpressed in TNBC. In particular, FORKHEAD BOX C1 (FOXC1) was found to be the most highly overexpressed TF in basal-like breast cancer, having a role in TNBC progression. However, due to their structure and intracellular localization, TFs have been largely been considered "undruggable", which we plan to address using CRISPR/Cas9 technology.

**Material and Methods**

The TNBC cell lines MDA-MB-231 and MDA-MB-468 were transfected during 48 hours with three different single guide RNAs (sgRNAs) complexed with CRISPR/Cas9 ribonucleoproteins (RNPs). A special lipofectamine was used for improving the transfection efficiency of the complexes. sgRNAs were delivered both separately and mixed all together. After that, stable FOXC1-inhibited cell lines were stablished. Assays assessing malignant capabilities such as cell viability (sulforhodamide b assay), cell proliferation (Ki-67 assay) cell migration (would healing assay) cell invasion (transwell assay), anchorage-independent cell growth (colony formation assay in soft-agar) were performed in TNBC cells.

**Results and Discussions**

The internalisation efficiency of CRISPR/Cas9 RNPs into MDA-MB-231 and MDA-MB-468 cells was around 20% and 85%, respectively. We achieved a strong downregulation of the FOXC1 protein levels according to Western Blot. Preliminary results regarding cell viability, proliferation, migration, and invasiveness suggest a decrease in all of these malignant capabilities on the TNBC FOXC1 knock-out cells compared to the control cells.

**Conclusion**

This revolutionary targeted therapeutic approach based on the inhibition of the FOXC1 gene using CRISPR/Cas9 RNPs appears to be very promising in vitro. It could have the potential in the future to be highly effective while

© 2023 The Authors. Molecular Oncology is published by John Wiley & Sons Ltd on behalf of Federation of European Biochemical Societies avoiding the secondary effects of TNBC treatment, leading to an increase in life expectancy of TNBC patients.

### EACR23-0428
### Biological characterization of new Antibody Drug Conjugates armed with Smoothened inhibitors

E. Crivaro[1], E. Petricci[2], F. Manetti[2], L. Maresca[1], B. Stecca[3], M. Taddei[2]
[1]*Istituto per lo Studio- la Prevenzione e la Rete Oncologica ISPRO, Tumor Cell Biology Unit – Core Research Laboratory, Firenze, Italy*
[2]*University of Siena, Department of Biotechnology- Chemistry and Pharmacy DBCF – University of Siena, Siena, Italy*
[3]*Istituto per lo Studio- la Prevenzione e la Rete Oncologica ISPRO, Tumor Cell Biology Unit –Core Research Laboratory- ISPRO, Firenze, Italy*

**Introduction**
Aberrant activation of the Hedgehog (HH) pathway has been reported in several cancer types, including melanoma. The transmembrane G-protein coupled receptor Smoothened (SMO) is the main transducer of the HH pathway, and several SMO inhibitors have been approved as anti-cancer therapeutics. Here we propose to test a new class of biopharmaceuticals called Antibody-drug Conjugates (ADCs) for the treatment of melanoma. ADCs are composed by three major components: a monoclonal antibody (mAb) against tumor cell surface antigens, a cytotoxic payload and a linker that connects these two portions, allowing efficient release after internalization. Our goal was to combine the high selectivity of mAb Cetuximab directed against EGFR with the anti-proliferative activity of a SMO inhibitor (SMOi).
**Material and Methods**
In order to choose the best melanoma cell lines to characterize the ADCs, we evaluated the protein expression of EGFR, GLI1 and GLI2 by Western blot. Antiproliferative activity of ADCs was tested by crystal violet staining. In order to confirm the higher potency of the conjugate, each ADC was compared with SMOi alone, Cetuximab alone, and the complex liker-payload. To evaluate the effect of ADCs on HH and EGFR pathways the expression of their downstream targets were assessed by Western blot and quantitative real-time PCR (qPCR).
**Results and Discussions**
A375 and SK-MEL-5 melanoma cell lines were selected for ADC characterization. A375 cells showed high levels of EGFR and GLI2, while SK-MEL-5 intermediate levels of EGFR and GLI1. Cell viability assay in A375 and SK-MEL-5 showed that ADCs at 0.1 ug/ul and 0.2 ug/ul yielded a better antiproliferative activity compared to Cetuximab alone, SMOi alone and the complex linker-payload at the same concentrations. Furthermore ADCs induced a reduction of HH and EGFR targets protein levels, such as p-EGFR, p-ERK1/2, p-P38, GLI2 and to a lesser extent GLI1. In addition ADCs treatment for 48 h downregulated the mRNA levels of *PTCH1*, *GLI2* and *EGFR*.
**Conclusion**
We demonstrated the antiproliferative effect of ADCs in melanoma cells and confirmed their ability to inhibit EGFR and HH signaling pathways. We will evaluate the effect of ADCs on cell cycle and apoptosis and we will investigate their internalization. Finally, we will evaluate ADCs conjugated with other SMO inhibitors and the best ADC will be tested in vivo in a xenograft model.

### EACR23-0459
### SPECIFIC DIRECT-BINDING SURVIVIN INHIBITOR THAT INDUCES SENSITIZATION TO CONVENTIONAL CHEMOTHERAPY IN LUNG CANCER

C. Benítez-García[1], M. Pérez-Hernández[1], D. Martínez-García[1], R. Pérez-Tomás[2], L. Díaz[3], R. Quesada[4], V. Soto-Cerrato[1]
[1]*Faculty of Medicine and Health Sciences- Universitat de Barcelona/Institut d'Investigació Biomèdica de Bellvitge I DIBELL,*
*Department of Pathology and Experimental Therapeutics/ Molecular Signaling- Oncobell Program,*
*L'Hospitalet de Llobregat, Spain*
[2]*Faculty of Medicine and Health Sciences- Universitat de Barcelona,*
*Department of Pathology and Experimental Therapeutics,*
*L'Hospitalet de Llobregat, Spain*
[3]*Nostrum Biodiscovery, Drug Discovery, Barcelona, Spain*
[4]*Universidad de Burgos, Department of Chemistry, Burgos, Spain*

**Introduction**
Overexpression of anti-apoptotic proteins, such as survivin, represents one relevant mechanism of treatment resistance. We propose to evaluate the approved drug Asenapine Maleate (AM), a novel specific direct-binding survivin inhibitor we have recently identified, for tumor sensitization to conventional chemotherapy.
**Material and Methods**
We used surface plasmon resonance (SPR) assays and non-denaturing gel electrophoresis of survivin dimers/monomers to confirm direct interaction between AM and survivin. We tested the specificity of AM for survivin compared to XIAP, both members of the inhibitor of apoptosis protein family, by western blot in human lung cancer cells (A549).
We evaluated the potential synergistic/additive effect of AM in combination with chemotherapeutics (cisplatin, carboplatin and gemcitabine) on A549 by cell viability assays. Effects on cell cycle and apoptosis of AM and cisplatin combination were evaluated by flow cytometry and western blot.
In the in vivo therapeutic efficacy assay of AM and cisplatin combination, we used an ectopic model of lung cancer: $5 \cdot 10^4$ LLC1 cells were inoculated to induce subcutaneous tumors. We treated C57BL6 mice with AM (5 mg/kg, for 5 days and 2 days to rest until the end of the experiment), cisplatin (3 mg/kg days 0, 3 and 6) and AM and cisplatin in the same schedule as the monotherapies. At the end, we compared tumor volume and weight among groups.
**Results and Discussions**
SPR showed high binding affinity of AM to survivin, while increasing AM concentrations showed the decrease of survivin homodimer, suggesting their interaction. AM decreased survivin but not XIAP levels, revealing its specificity for survivin.

© 2023 The Authors. Molecular Oncology is published by John Wiley & Sons Ltd on behalf of Federation of European Biochemical Societies

Cell viability assays showed that AM combination with conventional chemotherapy significantly increases their cytotoxic effect. The analysis of this data with CompuSyn Software revealed synergy of the drugs, especially in the combination of AM with cisplatin. According to flow cytometry and western blot results, AM is able to enhance apoptosis in cisplatin-treated cells.
Therapeutic in vivo analyses showed significant impairment in tumor growth in mice treated with AM and cisplatin combination.**Conclusion**
Our results demonstrate there is a sensitizing effect when combining AM, a novel specific direct-binding survivin inhibitor, with conventional chemotherapy, especially cisplatin.
*Funding:Instituto Salud Carlos III (PI18/00441;PI22/00256),Junta de Castilla y León (BU067P20) and ERDF. We thank CERCA Programme/Generalitat de Catalunya for institutional support.*

### EACR23-0460
### Overexpression of CDK2 and CCNE1 associate with sensitivity to the Wee1 inhibitor adavosertib in primary cultures

*C. Aguado[1], A. Martinez-Bueno[2], C. Mayo de las Casas[1], B. Garcia-Pelaez[1], A. Aguilar[2], M. Garzón[1], M. Gonzalez Cao[2], R. Rosell[4], M.A. Molina Vila[1]*
*[1]Pangaea Oncology., Laboratory of Oncology, Barcelona, Spain*
*[2]Instituto Oncológico Dr. Rosell IOR, Hospital Universitari Dexeus- Grupo Quirón Salud, Barcelona, Spain*
*[3]Department of Cancer Biology and Precision Medicine, Germans Trias I Pujol Research Institute IGTP, Badalona, Spain*

**Introduction**
Cyclin E1 (*CCNE1*) amplification has been associated with response to WEE1 G2 checkpoint kinase inhibition (Wee1) but the role of tumor protein p53 (*TP53*) mutations and cyclin dependent kinase 2 (*CDK2*) upregulation has not been clarified. Here, we analyzed *CCNE1* and *CDK2* levels, *TP53* mutations and sensitivity to adavosertib in primary cultures derived from lung and ovarian cancer patients.
**Material and Methods**
The levels of *CDK2* and *CCNE1* mRNA in lung (n = 14) and ovary (n = 3) primary cultures derived from pleural effusions and ascites were measured using a massive hybridization 770 mRNA probe commercial panel (IO360, NanoString® Technologies), while *TP53* mutation status was determined by NGS. The cut-off for high expression was established as the geomean plus the standard deviation of all samples. Five primary cultures with different *CDK2* and *CCNE1* mRNA levels and *TP53* status were treated with adavosertib.
**Results and Discussions**
Among the 17 samples analyzed by massive hybridization, an ovarian primary culture showed high mRNA levels of *CCNE1* while another ovarian and a lung primary presented high *CDK2* expression. The three primaries were treated with adavosertib, together with two additional lung primary cultures with low *CDK2* and *CCNE1*. The three cultures overexpressing *CDK2* or *CCNE1* were moderately sensitive to the Wee1 inhibitor, with half-maximal inhibitory concentrations (IC50s) 200-500 nM. In contrast, the two low expressing primaries were completely resistant to the drug, with IC50s > 10 μM. Regarding *TP53* status, one of the primaries was wild-type while the other four harbored loss-of-function mutations.
**Conclusion**
Elevated levels of *CDK2* and *CCNE1* mRNA expression associate with sensitivity to Wee1 inhibition in lung and ovarian primary cultures.

### EACR23-0465
### Preclinical validation of rilmenidine for repurposing in pancreatic ductal adenocarcinoma

*J. Grahovac[1], K. Živić[1], M. Pavlović[1], M. Ostojić[1], A. Đurić[1], T. Srdić Rajić[1], A. Pavić[2], D. Galun[3]*
*[1]Institute for Oncology and Radiology of Serbia, Experimental Oncology, Belgrade, Serbia*
*[2]Institute for Molecular Genetics and Genetic Engineering, Molecular Genetics, Belgarde, Serbia*
*[3]University of Belgrade Medical School, Surgery, Belgrade, Serbia*

**Introduction**
Pancreatic ductal adenocarcinoma (PDAC) has dismal prognosis, as there are no screening tests available, most often is diagnosed in the metastatic phase of the disease and is refractory to conventional, targeted and immunotherapy. We have examined the expression and role of the novel tumor suppressor nischarin (NISCH) in PDAC and the effects of treatment with the agonist rilmenidine (approved for treatment of hypertension) in order to determine the potential of nischarin agonists for repurposing in this deadly disease.
**Material and Methods**
Nischarin expression was examined by immunohistochemistry in PDAC tissue array. NISCH was knocked-down (KD) in two PDAC cancer cell lines and the effects were examined by transcriptome sequencing and gene set enrichment analysis. The effects of rilmenidine treatment on pancreatic cancer cells were examined in assays for proliferation, migration and invasion *in vitro* and in the Tg(fli1:EGFP) zebrafish model. Effect of rilmenidine treatment on cancer cell-cancer associated fibroblast (CAF) co-cultures was analyzed with dot blot cytokine array and growth factor qRT-PCR array. Ultimately, the effect of rilmenidine on the ex vivo PDAC tumor cultures was examined by western blot.
**Results and Discussions**
Nischarin was expressed in both the tumor and the stromal compartment of PDAC. In cancer cells NISCH KD induced changes associated with cell adhesion and vesicular transport. Treatment with rilmenidine *in vitro* did not decrease cancer cell viability at concentrations achievable in patients, but significantly decreased cancer cell adhesion, migration and cell invasion through matrices. In the zebrafish model, rilmenidine drastically reduced tumor invasion. In cancer cell-CAF co-cultures rilmenidine reduced production of pro-inflammatory cytokines. Ultimately, in the *ex vivo*patient tissues treatment with rilmenidine remodeled extracellular matrix.
**Conclusion**

© 2023 The Authors. Molecular Oncology is published by John Wiley & Sons Ltd on behalf of Federation of European Biochemical Societies

Taken together, nischarin agonist rilmenidine reduces PDAC cancer cell invasion *in vitro* and *in vivo* and has an impact on cancer-stroma interactions. Our study lays a ground for potential repurposing of antihypertensive drug rilmenidine as antimetastatic therapeutic in PDAC.

### EACR23-0478
### Lunatin-1: A scorpion venom peptide that induces instant cell death by necrosis in the breast cancer cell line MDA-231

*B. Raissa de Oliveira[1], M. José González[2], A. Monteiro de Castro Pimenta[3], D. Assis Gomes[2], M. Nunes de Melo-Braga[2], T. Verano-Braga[1]*
*[1]Federal University of Minas Gerais, INCT-Nanobiofar- Department and Physiology and Biophysics- I nstitute of Biological Science, Belo Horizonte, Brazil*
*[2]Federal University of Minas Gerais, Department of Biochemistry and Immunology. Institute of Biological Science., Belo Horizonte, Brazil*
*[3]McGill University, Segal Cancer Proteomics Centre at Jewish General Hospit al - Lady Davis Institute, Montreal, Canada*

**Introduction**
Previous studies from our group have shown that Lunatin-1, a peptide isolated from the *Hadruroides lunatus* scorpion venom, induce apoptosis in human promyelocytic leukemia HL-60 cell line and causes death in MCF-7 and MDA-231 human metastatic cancer cells lines by unknown mechanisms.

**Material and Methods**
Synthetic Lunatin-1 was purified by high-performance liquid chromatography (HPLC) and analyzed by mass spectrometry (MALDI-TOF/TOF). We conducted a similarity search of Lunatin-1 against human proteins using Blast tool and performed gene ontology analysis with David tool. To determine the IC50 of Lunatina-1 on MDA-231 cell line, we treated the cells with different concentrations of Lunatin-1 and evaluated cell viability using resazurin. We also treated the cells with 25 µM of Lunatin-1 to evaluate cell viability by measuring propidium iodide (PI) stain, and its morphology during 1h using a Cell Imaging Multimode Reader (Cytation).

**Results and Discussions**
Lunatin-1 induced cytotoxicity in MDA-MB-231 cell line with an experimental IC50 of 31.25 µM. Treatment with 25 µM of Lunatin-1 induced necrosis, as evidenced by the presence of IP positive-stained cells after 20 minutes ($p< 0.05$) compared to vehicle (DMSO 0.5%). Moreover, cell swelling was observed after 5 minutes of treatment ($p<0.05$). Instant cell death was also observed ($p<0.05$) when compared with untreated cells. Bioinformatics showed that Lunatin-1 has a high degree of similarity with transport membrane proteins. This, together with the observed rapid effects suggested us that Lunatina-1 may impair electrolytic cell homeostasis by binding to transport membrane proteins.

**Conclusion**
Lunatin-1 induces death of breast cancer cell line and may be used as an antitumoral drug lead.

### EACR23-0487
### CBFβ inhibitors suppress myeloma cell growth and invasion by targeting of both Runx1 and Runx2 in myeloma cells

*P.S. Gowda[1], T.N. Trotter[1], Y. Yang[1]*
*[1]University of Alabama at Birmingham, Department of Pathology, Birmingham, United States*

**Introduction**
Multiple myeloma (MM) is a plasma-cell malignancy. The aggressiveness of MM cells is governed by a complicated network of molecular signals. The coordination of multiple genes involved in the network of these molecular signals in MM cells may be under the control of a few transcription factors. Using raw data extracted from GEO datasets (accession number GSE6477), we found a significant increase in the expression of Runt-related transcription factor factor (Runx) 1 (Runx1) and 2 (Runx2) in MM cells of MM patients, compared with healthy donors. Both of Runx1 and Runx2 forms a heterodimeric complex with core-binding factor β subunit (CBFβ). CBFβ enhances the affinity of Runx proteins for DNA binding and protects them from proteasome-mediated degradation. In current study, we investigated (1) whether knockdown of Runx1 or Runx2 in MM cells inhibits MM progression, and (2) whether CBFβ inhibitor(s) suppress MM growth and invasion by targeting of both Runx1 and Runx2 in MM cells.

**Material and Methods**
Runx1 or Runx2 expression was knocked down in murine 5TGM1 MM cells by transduction with specific Runx1 or Runx2 shRNA lentiviruses or non-targeted (NT) shRNA control (Sigma). NT control or Runx1 knockdown (k/d) or Runx2- k/d 5TGM1 cells were injected into 6 week old C57BL/KaLwRij mice via the tail vein ($10^6$ cells/injection). Serum IgG2bκ levels (a soluble marker of 5TGM1 cells) were measured bi-weekly by ELISA. Small molecule inhibitors of CBFβ, AI-10-104 and AI-14-91, and a control compound, Al-4-88, were obtained from Dr. Bushweller's lab (University of Virginia, USA).

**Results and Discussions**
Our in vivo studies demonstrated that knockdown of either Runx1 or Runx2 in 5TGM1 MM cells significantly inhibited tumor growth in syngenic C57BL/KaLwRij mice, compared to NT-control 5TGM1 cells. Next, 5TGM1 murine MM cells and CAG human MM cells were treated with CBFβ inhibitors AI-10-104 or AI-14-91 or control compound Al-4-88 (40 µm) for 24, 48, 72h respectively. Real-time PCR and western blot showed significantly reduced levels of both Runx2 and Runx1 in the nucleus of AI-10-104 or AI-14-91 treated MM cells. MTT and invasion assays showed that the proliferation and invasion of both 5TGM1 and CAG MM cells were significantly inhibited by AI-10-104 and AI-14-91 (40 µM), as compared with Al-4-88 control.

**Conclusion**
our study uncovered novel roles of MM cell-expressed Runx1 and Runx2 in MM progression, and identified novel targets (MM cell-expressed Runx1 and Runx2) and new drugs (CBFβ inhibitors) for MM treatment.

### EACR23-0522
### Benefits of cannabidiol as an anti-breast cancer agent when combined with Exemestane

© 2023 The Authors. Molecular Oncology is published by John Wiley & Sons Ltd on behalf of Federation of European Biochemical Societies

C. Almeida[1], N. Teixeira[1], G. Correia-da-Silva[1], C. Amaral[1]
[1]UCIBIO.REQUIMTE- Laboratory of Biochemistry- Departtment of Biological Sciences- Faculty of Pharmacy- University of Porto, Biological Scieces, Porto, Portugal

**Introduction**

Estrogen receptor-positive ($ER^+$) breast cancer accounts for 70-85% of all breast cancer cases, being aromatase inhibitors (AIs), like the steroidal Exemestane (Exe), one of the first-line treatments. Nevertheless, prolonged use may lead to endocrine resistance, reinforcing the need for novel therapeutic approaches. Different therapies have been suggested, including the combination of AIs with CDK4/6, mTOR and PI3K inhibitors or androgen receptor (AR) antagonists. Our group has already demonstrated that cannabidiol (CBD) displays important anti-tumor effects on $ER^+$ breast cancer cells, through the inhibition of aromatase, the modulation of ERs and the promotion of cell death. Considering this, we investigated whether CBD was able to improve the anti-cancer effects of Exe.

**Material and Methods**

An $ER^+$ breast cancer cell line, MCF-7aro, was used. Cell viability was accessed by MTT assay and apoptosis was detected through the analysis of caspases-7/-8/-9 activities. The involvement of ERα, AR and $ERK_{1/2}$ pathway was studied by Western Blot, qPCR and siRNA.

**Results and Discussions**

When combined with Exe, CBD potentiated its pro-cell death effects, decreasing cell viability, increasing caspase's activity, and inhibiting $ERK_{1/2}$ activation. Moreover, a crosstalk between ERα and AR, responsible for the complete abolishment of the estrogen-like effect of Exe, was found and demonstrated through the impairment of ERα activation and prevention of the oncogenic role of AR.

**Conclusion**

This study reveals the benefits of CBD as an adjuvant therapy for $ER^+$ breast cancer, since it was verified that this phytocannabinoid potentiates the pro-cell death effects of Exe, improving its efficacy. In fact, this combination may potentially be more attractive than other approaches currently under clinical application which combine endocrine therapy with other agents that present several side effects and limited efficacy. Thus, this study reinforces the power of cannabinoids as anti-cancer agents in breast cancer and opens up a new potential and promising line of research for the improvement of $ER^+$ breast cancer therapy.

### EACR23-0542
### Enabling mRNA Medicine for Brain Tumors

S. Yerneni[1], J. Azambuja[2], P. Lucas[2], L. McAllister-Lucas[2], K. Whitehead[1]
[1]Carnegie Mellon University, Department of Chemical Engineering, Pittsburgh, United States
[2]UPMC Children's Hospital of Pittsburgh, Department of Pediatrics, Pittsburgh, United States

**Introduction**

mRNA is a new class of drugs that has the potential to revolutionize the treatment of brain tumors. Thanks to the COVID-19 mRNA vaccines and numerous therapy-based clinical trials, it is now clear that lipid nanoparticles (LNPs) are a clinically viable means to deliver RNA therapeutics. However, LNP-mediated mRNA delivery to brain tumors remains elusive. Over the past decade, numerous studies have shown that tumor cells communicate with each other via small extracellular vesicles, which are around 100 nm in size and consist of lipid bilayer membrane similar to synthetic lipid-based nanocarriers. We hypothesized that rationally designed non-toxic LNPs based on extracellular vesicle mimicry would enable efficient delivery of RNA therapeutics to brain tumors.

**Material and Methods**

We synthesized LNPs using four components similar to the formulation used in the mRNA COVID-19 vaccines (Moderna and Pfizer-BioNTech): ionizable lipid, cholesterol, helper lipid and polyethylene glycol (PEG)-lipid. For the in vitro screen, we tested ten classes of helper lipids based on their abundance in extracellular vesicle membranes, commercial availability, and large-scale production feasibility while keeping rest of the LNP components unchanged. The transfection kinetics of GFP mRNA encapsulated in LNPs was tested using GL261, U87 and SIM-A9 cell lines. The best LNP formulations were then tested in vivo to deliver reporter mRNA via intrathecal administration in a syngeneic glioblastoma (GBM) mouse model. LNP formulations were assessed for delivering Cas9 mRNA and CD81 sgRNA (model protein) in murine syngeneic GBM model to enable gene editing in brain tumor cells.

**Results and Discussions**

Several formations resulted in stable transfection (5 days) of GFP mRNA in all the cell lines tested in vitro. In a syngeneic mouse GBM model, mRNA was successfully delivered to tumor cells (80% transfected) and a range of different cells in the tumor microenvironment, including tumor-associated macrophages (85% transfected), neurons (36% transfected), neural stem cells (51%transfected), oligodendrocytes (75% transfected) and astrocytes (59% transfected). Sanger sequencing showed that CRISPR-Cas9 editing was successful in ~94% of brain tumor cells in vivo.

**Conclusion**

In conclusion, we have developed a library of LNPs that can transfect GBM cells in vivo with high efficacy. This technology can potentially be used to develop novel mRNA therapies for GBM by delivering single or multiple mRNAs and holds great potential for studying brain tumor biology.

### EACR23-0583
### Targeting Cancer Cell Migration with Pentamethinium Salts

K. Petrlakova[1], J. Navratil[1], J. Leischner Fialova[1], M. Masarik[1]
[1]Faculty of Medicine- Masaryk University in Brno- Czech Republic, Department of Pathological Physiology, Brno, Czech Republic

**Introduction**

In solid tumors, invasiveness and metastasis are responsible for more than 90 % of deaths. Even though cancer treatment is at the top level today, the category of drugs against invasiveness and metastasis is still missing. The main benefit of migrastatic treatment will be

© 2023 The Authors. Molecular Oncology is published by John Wiley & Sons Ltd on behalf of Federation of European Biochemical Societies to reduce the most dangerous ability of tumor cells - their invasion into the environment. The qualitative benefits of migrastatic treatment over standard therapies are related to the fact that cells during migrastatic treatment do not have to be exposed to cytotoxic stress. Pentamethinium salts are a group of compounds characterized by fluorescence and variable cytotoxicity depending on the structure of the molecule and can play an important role in influencing the migration potential of tumor cells. Specific fluorescent dyes from pentamethin family exhibit exceptional affinity and selectivity for cardiolipin in the inner mitochondrial membrane and have excellent photostability, fluorescence properties and low phototoxicity.

**Material and Methods**

Cell cultures

MTT assay

Confocal microscopy

Wound healing assay

ATP production rate assay by Seahorse XF24 analyzer

Confocal microscopy

**Results and Discussions**

The results will be discussed in detail in the poster.

**MTT assay**

Using the MTT test, we obtained the inhibitory concentrations IC10, IC25, and IC50, which were later used for further experiments.

**Confocal microscopy**

The results from confocal microscopy prove that PMS1 reliably binds to mitochondria.

**Wound healing assay**

The inhibitory concentration IC10 of the PMS1 substance significantly reduced the migration of selected cancer cell lines.

**ATP rate assay**

The results show that even the inhibitory concentration IC10 of the PMS1 substance is sufficient for complete inhibition of OXPHOS from the original 60 % total ATP production in the control group.

**3D Spheroid viability assay**

With increasing inhibition concentration, the growth rate of spheroids were decreasing over time.

**Conclusion**

Pentamethinium salts seem to be a promising migrastatics based on the results obtained. The values of inhibition concentrations are significantly higher than those of commonly used cytostatics, potentially resulting in lower risk of cumulative toxicity in cancer patients. Even low inhibition concentrations are sufficient to significantly suppress the migration of tumor cells, inhibit oxidative phosphorylation, reduce overall ATP production, and simultaneously inhibit the growth of spheroids at the 3D level.

The poster will provide a detailed discussion of the results.

### EACR23-0590

### A new target pathway to treat Chronic Lymphocytic Leukemia

*L. Ikhlef[1], M. Yassine[1], L. Rivière[2], M.O. Jauberteau[3], P.F. Gallet[1]*

*[1]OmegaHealth, INSERM U1308 CAPTuR- Faculty of Medicine, Limoges, France*

*[2]University Hospital, Faculty of Medicine, Limoges, France*

*[3]University Hospital, Immunology and Immunogenetics, Limoges, France*

**Introduction**

Chronic Lymphocytic Leukemia (CLL) is a malignant lymphoproliferative disease characterized by an accumulation of mature B lymphocytes (B CLL) in the blood, bone marrow and lymphoid organs, due to their resistance to programmed cell death or apoptosis. Current therapies that target the B-cell receptor (BCR) pathway or the inhibition of anti-apoptotic proteins do not prevent the progressive forms of the disease, have low long-term efficacy and are subject to therapeutic resistance. Deciphering the mechanisms of leukemic cell survival and searching for new specific targets therefore remain major challenges to improve the management of this disease. It was evidenced that NTSR2 (neurotensin receptor 2), through the recruitment of TrkB (tyrosine kinase B), induces survival pathways in leukemic B-cells. We are currently investigating the therapeutic potential of this protein complex as a new target.

**Material and Methods**

HEK293T cells were used as a model to express NTSR2 and TrkB, their interaction was studied using techniques such as site-directed mutagenesis, co-precipitation followed by western blotting, proximity ligation assays and mass spectrometry. A peptide targeting the domain of interaction was then designed, its internalization was evaluated by confocal microscopy, its ability to interact with its target was evaluated by co-immunoprecipitation and its functional properties were characterized using live-cell imaging *in vitro* on the CLL cell line MEC-1 and, *ex vivo,* on B-cells from CLL patients.

**Results and Discussions**

The binding domain of NTSR2 and TrkB was identified. The peptide designed to target it has the ability to bind TrkB and efficiently decreases the interaction of the complex. It is also effectively internalized by B CLL. It demonstrated a cytotoxic effect both *in vitro* and *ex vivo*.

**Conclusion**

Altogether, these results underline the therapeutic potential of the NTSR2 / TrkB protein complex as a target in CLL and open new perspectives for the development of targeted therapies.

### EACR23-0600

### Targeting ionic homeostasis through lactate anion transportation as a novel approach for cancer therapy

*A. Arias-Betancur[1,2], P. Fontova[3], J. Duis[2], D. Alonso-Carrillo[3], I. Carreira-Barral[3], V. Soto-Cerrato[2,4], R. Quesada[3], R. Pérez-Tomás[2]*

*[1]Universidad de La Frontera, Department of Integral Adults Dentistry, Temuco, Chile*

*[2]Universidad de Barcelona, Department of Pathology and Experimental Therapeutics, L'Hospitalet de Llobregat, Spain*

*[3]Universidad de Burgos, Department of Chemistry, Burgos, Spain*

*[4]Institut d'Investigació Biomédica de Bellvitge IDIBELL, Molecular Signaling- Oncobell Program, L'Hospitalet de Llobregat, Spain*

**Introduction**

© 2023 The Authors. Molecular Oncology is published by John Wiley & Sons Ltd on behalf of Federation of European Biochemical Societies

Targeting ionic homeostasis in cancer could be an interesting therapeutic approach due to the related role of dysregulated pH with cell proliferation, evasion of apoptosis, metabolic rewiring and metastasis. In this context, the use of small molecules with anionophoric activity against specific anions, such as lactate, represents a novel way to selectively eradicate cancer cells. Our goal was to assess the mechanism of action and anticancer potential of a novel click-tambjamine derivative named AS37, specially designed to transport lactate anions in cancer cells.

**Material and Methods**

Transmembrane anion transport studies in liposome models and MTT cell viability assays in a panel of different cell lines were performed. In addition, cell death and stress-related signalling pathways were evaluated through western blot and flow cytometry. Subcellular compound localization and organelle affectation were assessed through confocal microscopy. Moreover, lactate anion transportation across cell membranes and changes in ionic homeostasis (cellular pH) were determined through luminescent- or fluorescent-based methods. Lastly, combination therapy assays of AS37 with standard chemotherapeutics were also performed through the MTT cell viability assay.

**Results and Discussions**

AS37 showed a potent anionophoric activity in liposome models and was effective to reduce cell viability in a wide panel of cancer cell lines. Interestingly, flow cytometry and western blot analysis showed that AS37 triggered both apoptosis and necrosis. It was also confirmed the ability of AS37 to export lactate anions to the extracellular space in a cellular in vitro model. Localization analysis revealed its rapid accumulation in lysosomes which induced their basification coupled with a significant dose-dependent drop in cytosolic pH after 1 h of treatment. AS37 also induced rapid mitochondrial acidification and vacuolization (swelling) with an increase in ROS production mediated by activation of p38 MAPK and JNK signalling pathways. Finally, AS37 was able to sensitize A549 cells against cisplatin as a pre-treatment of 4 h being synergic even at lower doses.

**Conclusion**

These results suggest that AS37 could be a promising anticancer agent that take advantage of upregulated lactate production of cancer cells to induce cytotoxicity. In addition, the synergism between AS37 and standard chemotherapy (cisplatin) supports its use as a sensitizing agent for further in vivo studies.

### EACR23-0604

### Omomyc downregulates MYC transcriptional signature in preclinical models of solid tumours and shows long half-life in patient-derived tumour tissues

*S. Martínez-Martín[1], S. López-Estévez[1], L. Foradada[1], J. Kaur[2], S. Casacuberta-Serra[1], H. Thabussot[1], V. Castillo Cano[1], L. Nonell[3], M.E. Beaulieu[1], L. Soucek[1,2,4,5]*

*[1]Peptomyc S.L., Biomarkers, Barcelona, Spain*
*[2]Vall d'Hebron Institute of Oncology VHIO, Models of Cancer Therapies, Barcelona, Spain*
*[3]Vall d'Hebron Institute of Oncology VHIO, Bioinformatics, Barcelona, Spain*
*[4]Universitat Autònoma de Barcelona, Department of Biochemistry and Molecular Biology, Bellaterra, Spain*
*[5]ICREA, Institució Catalana de Recerca i Estudis Avançats ICREA, Barcelona, Spain*

**Introduction**

The MYC family of oncoproteins is deregulated in up to 70% of human cancers through various mechanisms, functioning as master modulators of the cancer transcriptome. Despite the broad therapeutic utility anticipated for a clinical MYC inhibitor, MYC remains considered undruggable, and no direct MYC inhibitor has been approved for clinical use.

Omomyc is a first-in-modality recombinant mini-protein that completed a Phase I clinical trial in October 2022, demonstrating excellent safety and clear target engagement. Omomyc acts as a MYC dominant-negative, sequestering MYC away from its target genes on DNA. Here, we describe the transcriptional reprogramming exerted by Omomyc supporting target engagement in preclinical models of various solid tumours, suggesting its applicability in multiple oncological indications. We also show the presence of Omomyc in the biopsies from the first-in-human, MYCure trial.

**Material and Methods**

We performed *in vitro* experiments, treating with Omomyc for 24 and 120h and analysed gene expression by RNAseq. We also assessed its efficacy after 3 weeks of intravenous (iv), weekly treatments in subcutaneous xenograft mouse models. Finally, we analysed the transcriptome of *in vivo* tumours by RNAseq after 24h or 22 days of treatment. Importantly, to characterise the PK/PD relationship, we quantified the amount of functional Omomyc present in tumour tissue and serum of the treated mice using a targeted proteomic approach. We extended such quantification to patients' biopsies.

**Results and Discussions**

All 6 cell lines treated with Omomyc showed the shutdown of MYC transcriptional signature, both at 24 and 120h, confirming the on-target activity *in vitro*. Moreover, all the Omomyc-treated xenografts showed reduced tumour growth and MYC transcriptional shutdown *in vivo*. Importantly, quantification of functional Omomyc revealed that 2h after iv administration, the drug reached higher concentrations in the tumours compared to serum, and persisted there at higher levels at least for 72h after dosing. In line with the preclinical observations, we also confirmed the presence of Omomyc in patients' biopsies.

**Conclusion**

Our results show evidence of the long-lasting half-life of functional Omomyc in patient-derived tumour tissues, suggestive of a different PK profile between serum and tissue. We also demonstrate *in vivo* target engagement and the therapeutic utility of this pan-MYC inhibitor in lung, colon and breast cancer models.

### EACR23-0620

### Telmisartan derivatives induce mitochondrial fission and modulate autophagy in melanoma cell lines

© 2023 The Authors. Molecular Oncology is published by John Wiley & Sons Ltd on behalf of Federation of European Biochemical Societies

A. Damjanovic[1], M. Vukovic[1], D. Vukadinovic[2], V. Dobricic[2], J. Grahovac[1]
[1]Institute for Oncology and Radiology of Serbia, Department for Experimental Oncology, Belgrade, Serbia
[2]Faculty of Pharmacy, Department of Pharmaceutical Chemistry, Belgrade, Serbia

**Introduction**

We have previously shown that the drug telmisartan, an angiotensin 1 receptor (AT1R) inhibitor, has anticancer effects by altering melanoma cell bioenergetics. To circumvent the potentially unwanted hypotensive effect on cancer patients, we synthetized derivatives without AT1R binding ability. New derivatives were designed by modification of the carboxylic group by adding amino acids to prevent its binding for the AT1R. We selected 3 new derivatives with the best cytotoxic effects against A375 and vemurafenib-resistant A375 (A375R) human melanoma cells to further investigate the mechanisms by which derivatives lead to cell death.

**Material and Methods**

The cytotoxicity of 3 new derivatives and telmisartan were examined using an MTT test after 72 h of treatment. The effects on apoptosis were investigated by flow cytometry using Annexin V-FITC/propidium iodide staining after 24 h of treatment. Morphological changes of mitochondria upon 4 or 24 h treatment were examined by fluorescence microscopy using MitoTracker Red staining. Mitochondrial potential upon treatment and release of the reactive oxygen species (ROS) were measured by flow cytometry. The autophagic flux was examined with fluorescent microscopy of LC3 upon treatment with telmisartan derivatives alone and in combination with known autophagy inducer and inhibitor – Everolimus or chloroquine – respectively.

**Results and Discussions**

In cytotoxicity and apoptosis assays telmisartan derivatives were twice more potent than telmisartan itself in both lines, with A375R being more sensitive. Amino acids used for synthesis of derivatives (n-methyl tryptophan, tyrosine, phenylalanine) alone did not cause cytotoxicity, nor was the effect of telmisartan potentiated when they were added in mixture, implying that the compounds have novel effects. Treatment induced generation of mitochondrial ROS and a decrease of mitochondrial membrane potential. All 3 derivatives and telmisartan after 24h of treatment caused mitochondrial fission, but we did not detect their subsequent degradation by mitophagy. Combining telmisartan and its derivatives with chloroquine, an autophagy inhibitor, further decreased cell numbers and prevented the accumulation of LC3-positive autophagosomes.

**Conclusion**

Our study shows that telmisartan derivatives induce melanoma cell death through induction of mitochondrial fission and possibly inhibition of the autophagic flux; and lay a ground for further preclinical research of their potential as anti-melanoma agents.

### EACR23-0623
### Enhancing the efficacy of estrogen therapy with chromatin remodeling agents for endocrine-resistant ER+ breast cancer

A. Johnson[1], A. Warren[1], N. Traphagen[1], E. Demidenko[2], O. Wilkins[3], T.W. Miller[1]
[1]Geisel School of Medicine at Dartmouth College, Molecular and Systems Biology, Lebanon- NH, United States
[2]Geisel School of Medicine at Dartmouth College, Biomedical Data Science, Lebanon- NH, United States
[3]Geisel School of Medicine at Dartmouth College, Dartmouth Cancer Center, Lebanon- NH, United States

**Introduction**

Estrogen therapy elicits clinical benefit in ~30% of patients with endocrine-resistant ER+ breast cancer, but its mechanism of anti-cancer action and strategies to increase efficacy remain undefined.

**Material and Methods**

Endocrine-resistant long-term estrogen-deprived (LTED) and engineered ER-overexpressing ER+ breast cancer cells were treated ± 17b-estradiol and the histone deacetylase inhibitors (HDACi) entinostat or panobinostat. Endpoints included growth, apoptosis, DNA damage, and levels of transcripts, proteins, and histone post-translational modifications. NSG mice bearing endocrine-resistant patient-derived xenografts were treated with 17b-estradiol and HDACi, and tumor volumes were serially measured. Multiplexed single-cell ATAC-seq/RNA-seq was performed to measure changes in chromatin accessibility and gene expression.

**Results and Discussions**

Treatment with entinostat or panobinostat induced histone acetylation (histone 3 K27). Single-agent HDACi or 17b-estradiol inhibited cell growth, while entinostat synergized with 17b-estradiol. Combination drug treatments provided higher levels of apoptosis than single agents *in vitro*. In mice, entinostat slowed tumor growth rate, while single-agent 17b-estradiol and the combination of entinostat/17b-estradiol induced complete regression. This drug combination synergized to induce more rapid tumor regression than 17b-estradiol alone, and the combination delayed time to tumor recurrence compared to single-agent 17b-estradiol.

**Conclusion**

ER overexpression drives endocrine resistance and sensitizes cells to estrogen therapy via ER transcriptional hyperactivation that causes DNA damage. Opening chromatin with remodeling agents may yield a transcriptionally vulnerable state to enhance the toxicity of estrogen-induced ER activity. 17b-estradiol in combination with an HDACi is more effective that either drug alone in endocrine-resistant models. The novel combination of an HDACi and 17b-estradiol has the potential to be an effective therapy for patients with endocrine-resistant ER+ breast cancer.

### EACR23-0625
### COMBINED TARGETING OF MEK5/ERK5 AND HEDGEHOG/GLI PATHWAYS IN HUMAN MELANOMA

I. Tusa[1], A. Tubita[1], A. Menconi[1], S. Gagliardi[2], Z. Lombardi[1], M. Lulli[1], B. Stecca[2], E. Rovida[1]
[1]University of Florence, Department of Experimental and Clinical Biomedical Scie nces Experimental Oncology and Pathology Unit, Florence, Italy

© 2023 The Authors. Molecular Oncology is published by John Wiley & Sons Ltd on behalf of Federation of European Biochemical Societies

[2]*Core Research Laboratory - Institute for Cancer Research and Prevention ISPRO,*
*Institute for Cancer Research and Prevention ISPRO, Florence, Italy*

**Introduction**

Malignant melanoma is among the most aggressive cancers and its incidence is increasing worldwide. We have reported that the mitogen-activated protein kinase ERK5 promotes melanoma growth in vitro and in vivo. Additionally, we have recently reported that ERK5 is required for the Hedgehog/GLI (HH/GLI)-dependent melanoma cell proliferation and that GLI1 positively regulates the expression of ERK5. Since the HH/GLI pathway may be activated in a non-canonical way by the MAPK ERK1/2, we explored whether ERK5 positively regulates the HH/GLI signaling.

**Material and Methods**

BRAFV600E-mutated (A375 and Sk-Mel-5) and wild-type BRAF (SSM2c) melanoma cell lines and murine NIH/3T3 fibroblasts were silenced for ERK5 using ERK5-targeting shRNAs or treated with a non-targeting shRNA (shNT) as a negative control. Luciferase assay using the GLI-binding site luciferase reporter was performed to evaluate GLI transcriptional activity. A constitutively active form of MEK5 (MEK5DD) was used to induce activation of endogenous ERK5 or overexpressed ERK5. Chemicals (small molecule inhibitors) used were: the ERK5 inhibitors JWG-071 and AX-15836; MEK5 inhibitors GW284543 and BIX02189; GLI1/2 inhibitor GANT61; SAG, an HH/GLI pathway activator. Activation of HH/GLI pathway was obtained by PATCH1 silencing. 3D speroid assays were performed in SSM2c and A375 cells treated with GANT61 in combination with the MEK5 inhibitors.

**Results and Discussions**

Treatment with ERK5 inhibitors reduced transcriptional activity of endogenous HH/GLI pathway in a dose dependent manner in NIH/3T3 cells. This effect was recapitulated upon ERK5 genetic inhibition, which determined a reduction of GLI1 and GLI2 proteins. MEK5DD overexpression, which determined ERK5 activation, further increased transcriptional activity of SAG-induced HH/GLI, while silencing of endogenous ERK5 reverted this effect. These results confirmed that ERK5 positively regulates the HH/GLI signaling. Consistently, MEK5DD overexpression increased GLI1 and GLI2 protein levels. In melanoma cells, genetic and pharmacological ERK5 inhibition similarly inhibited the expression and activity of GLI proteins. Interesting, the combination of GANT61 with MEK5 inhibitors was more effective than single treatments in reducing the volume of melanoma spheroids.

**Conclusion**

Combined targeting of the MEK5/ERK5 and HH/GLI pathways may be a useful approach to prevent resistance mechanisms frequently observed upon monotherapy in melanoma.

### EACR23-0637
### ANTI-CANCER PROPERTIES AND POTENTIAL MOLECULAR MECHANISMS OF COMBINATIONS OF HERBAL EXTRACTS AND CHEMOTHERAPEUTIC AGENTS

*N. Avtandilyan*[1], *H. Javrushyan*[1], *H. Karapetyan*[1], *A. Grigoryan*[1], *E. Nadiryan*[1], *G. Sevoyan*[2], *M. Ginovyan*[1], *A. Maloyan*[3]
[1]*Yerevan State University,*
*Department of Biochemistry- Microbiology and Biotechnology, Yerevan, Armenia*
[2]*L.A.Orbeli Institute of Physiology NAS RA,*
*Laboratory of Immunology and Tissue Engineering, Yerevan, Armenia*
[3]*Oregon Health & Science University,*
*Center for Developmental Health- Knight Cardiovascular Institute, Portland- Oregon, United States*

**Introduction**

Modulation of biochemical and immune functions using medicinal plants and their products in combination with chemotherapeutic agents has recently become an accepted therapeutic approach. Despite the current advances and achievements in systems biology and translational research, the current strategies for cancer therapy, such as targeted therapy, immunotherapy, and chemotherapy remain unsatisfactory. We hypothesized that phytoextracts in anti-cancer treatment, either single or in combination with chemotherapy compounds, may effectively modulate the immune system, can lead to inhibition of tumor proliferation and growth, cause tumor apoptosis, and increase the production of antioxidant proteins.

**Material and Methods**

The plant materials (12 herbs) were harvested from the Tavush region of Armenia. Plants were deposited to the Herbarium of Yerevan State University Voucher specimen serial numbers were given. The MTT test was performed to assess the inhibition of growth of HeLa, MCF7, and A549 cells exposed for 6, 24, or 72 h to different concentrations of the plant extracts and chemotherapeutic drugs. Mammary gland tumors were induced by a single dose of 25 mg of 7,12-dimethylbenz(a)anthracene diluted in soy oil (1 mL) given injected subcutaneously, the second and third pair of breasts. Nor-NOHA, L-NAME, and 5-FU were used as chemotherapeutic drugs. All test components were determined using ELISA and colorimetric assay kits.

**Results and Discussions**

We have detected the induction of immunostimulatory effect by up-regulation of IL-2, induce of tumor apoptosis (caspase-3↑), antioxidant (SOD↑, MDA↓) and anti-inflammatory (Arginase↓, NOS↓, polyamines↓) properties, and inhibition of cancer cell proliferation after treatment by several Armenian herbal extracts separately and in combination with chemotherapeutic drugs in cancerous cell lines *in vitro* and *in vivo* rat mammary cancer model. Our results showed the treatment model can affect the regulation of cancer cell metabolism, which has been reflected in changes in tumor size, numbers, histopathological alterations, and mortality rate during *in vivo* rat mammary cancer model. It is noteworthy that the combination of some tested herbal extracts and chemotherapeutic agents has a synergistic effect.

**Conclusion**

We selected a promising combination of plant and drug (The combo of *Rumex obtusifolius L.* and nor-NOHA) that exhibited a significant anti-cancer activity, which may potentially have a practical use in cancer treatment.

© 2023 The Authors. Molecular Oncology is published by John Wiley & Sons Ltd on behalf of Federation of European Biochemical Societies

### EACR23-0663
### Development of a new class of targeted cancer therapy for activation of the tumour suppressor p53

K. Kawahara[1], T. Furukawa[2]
[1]Kagoshima University- Graduate School of Medical and Dental Science, Molecular Oncology, Kagoshima, Japan
[2]Kagoshima University- Graduate School of Medical and Dental Science, Pathology, Kagoshima, Japan

**Introduction**

Clinical trials of MDM2 inhibitors have begun, raising hopes for targeted molecular therapies that activate the tumour suppressor p53. Nucleolar/ribosomal stress response has recently been identified as a new mechanism for activating the p53 pathway without DNA damage. We have previously shown that the inhibition of PICT1/RPL11 binding by PICT1 depletion induces a nucleolar stress response, in which free RPL11 inhibits MDM2 activity, resulting in 1) a marked increase in p53 and suppression of tumour cell growth, 2) tumour suppression in mice without developmental abnormalities, and 3) better prognosis in tumour patients. The high expression of PICT1 and RPL11 in hematopoietic tumour suggests that PICT1/RPL11 binding may be an attractive target to activate the p53 pathway preferentially in tumours. The aim of the present study was to develop novel p53-activating tumour therapeutics.

**Material and Methods**

We have constructed a unique cellular fluorescent reporter system that detects nucleolar stress responses. We screened a 200,000-scale compound library by reporter activity. The results obtained were validated through biochemical and cell survival assay.

**Results and Discussions**

Drug screening/validation identified seed compounds that increase p53 via nucleolar stress response and induce p53-dependent apoptosis in juvenile leukaemia cells without DNA damage. We then synthesised an optimised compound, based on the seed compound, that can be administered to animals. Notably, the IC50 value of this compound in human peripheral blood mononuclear cells was 100-fold of that in tumour cells. Further mechanistic examination revealed that the compound induces a nucleolar stress response by inhibiting PICT1/RPL11 binding. In addition, onco-panel assays identified a few solid tumours sensitive to this drug, and sensitivity marker molecules were identified. The MDM2 inhibitors also activate the p53 pathway in normal cells, causing severe myelosuppression. Reducing secondary carcinogenesis by DNA-damaging anticancer agents for paediatric patients with leukaemia is also an urgent issue. Our compound may be a DNA damage-free, molecularly targeted cancer drug that activates the p53 pathway via a novel mechanism with reduced side effects.

**Conclusion**

We identified a compound that suppressed juvenile leukaemia via a nucleolar stress response with reduced blood toxicity. Development of this compound may contribute to a new class of p53-activating leukaemia therapy with reduced side effects and no incidence of secondary carcinogenesis.

### EACR23-0666
### Discovery of novel DNAJB1-PRKACA inhibitors for fibrolamellar hepatocellular carcinoma(FL-HCC)

E. Ko[1], L. Myeonghwi[2], Y. Jiseong[3], K. Sunyoung[2], K. Seungchan[3], J. Hyungjin[1]
[1]Standigm Inc., Bio-pipeline team, Seoul, South Korea
[2]Standigm Inc., Chem-pipeline team, Seoul, South Korea
[3]Standigm Inc., Medicinal chemistry team, Seoul, South Korea

**Introduction**

Fibrolamellar hepatocellular carcinoma (FL-HCC) is a rare form of liver cancer that affects adolescents and young adults without any prior history of liver disease such as cirrhosis. Surgical resection is currently the only known cure for FL-HCC, as no standard of care or targeted therapy exists for systemic treatment. Recent research has identified the DNAJB1-PRKACA fusion protein as a crucial oncogenic driver in FL-HCC. In this study, we aimed to identify inhibitors that specifically target the kinase activity of DNAJB1-PRKACA and evaluate their efficacy against FL-HCC.

**Material and Methods**

We developed an artificial intelligence algorithm trained on the molecular structures of known kinase inhibitors to predict compounds with high binding affinity to the kinase domain of DNAJB1-PRKACA. Using this algorithm, we virtually screened a large compound library and identified several potential inhibitors. We then designed novel inhibitors based on the primary hit compounds and experimentally validated the predicted inhibitors by testing their ability to inhibit DNAJB1-PRKACA kinase activity and reduce intracellular signaling in liver cell lines.

**Results and Discussions**

Our study has successfully identified several novel inhibitors with different scaffold structures from competitive inhibitors, which are patentable. These inhibitors have effectively blocked the kinase activity of DNAJB1-PRKACA, demonstrating excellent kinase selectivity and good druggability and mouse PK profiles. Moreover, these inhibitors have significantly reduced intracellular downstream signaling in human liver cancer cell lines, showing promising potential for anti-cancer efficacy in FL-HCC. Currently, we are validating their anti-cancer activity in 3D ex vivo models derived from FL-HCC patients and in vivo PDX models.

**Conclusion**

Our findings suggest that the inhibitors we identified, which selectively block the kinase activity of DNAJB1-PRKACA, could provide a promising new treatment option for patients with FL-HCC, a rare and aggressive form of liver cancer.

### EACR23-0708
### Combined use of Olaparib and Ivabradine as a novel treatment strategy for triple negative breast cancer.

U.S. Khoo[1], M.H. Leung[1], H. Tsoi[1], E.P. Man[1], C. Gong[2]
[1]The University of Hong Kong, Pathology, Hong Kong, China
[2]University of Cambridge, Hematology, Cambridge,

© 2023 The Authors. Molecular Oncology is published by John Wiley & Sons Ltd on behalf of Federation of European Biochemical Societies

*United Kingdom*
**Introduction**
Triple-negative breast cancer (TNBC) lacks targetable proteins for treatment. The PARP inhibitor, Olaparib, interferes with DNA-repair to trigger apoptosis in breast cancer cells with mutated BRCA1/2. Ivabradine is an FDA-approved Hyperpolarization-activated cyclic nucleotide-gated channel (HCN 1-4) inhibitor, used to treat chronic angina. We previously reported that HCN2 and HCN3 are overexpressed in breast cancer, with high expression associated with poorer survival. Targeting HCN with Ivabradine can suppress tumor growth in vitro and in vivo through induction of ER-stress [1]. Since ER-stress induced by Ivabradine will disrupt mitochondrial function, leading to DNA damage, we hypothesized that co-administration of Ivabradine with Olaparib will further accumulate damaged DNA, enhancing the efficacy of Olaparib in non-BRCA mutated TNBC.
**Material and Methods**
Cell viability, apoptotic, DNA damage and mitochondrial functional assays were examined in MDA-MB-231 and MDA-MB-453 cell-lines. Both chemical and clinical grade Ivabradine and Olaparib drugs were used. Combination index (CI<1) was employed to assess for possible synergistic effect. The effect of combined Ivabradine and Olaparib treatment was also examined in xenograft models.
**Results and Discussions**
Whilst Ivabradine can disrupt mitochondrial function and lead to DNA damage, combined Olaparib and Ivabradine accumulated additional DNA damage and induced significantly more DNA fragmentation. The combined treatment also enhanced cell apoptosis via both caspase-dependent and -independent pathways. *In vitro* functional assays showed Ivabradine and Olaparib could synergistically induce apoptosis. *In vivo* study using xenograft models confirmed that clinical grade Ivabradine and Olaparib effectively suppressed tumour growth by both subcutaneous injection and oral administration.
**Conclusion**
Our findings support the potential use of Olaparib and Ivabradine in combination to treat non-BRCA mutated TNBC, offering a new and promising treatment strategy.
*Reference: [1] Mok KC, Tsoi H, Man EPS, Leung MH, Chau KM, Wong LS, Chan WL, Chan SY, Luk MY, Chan JYW, Leung JKM, Chan YHY, Batalha S, Lau V, Siu DCW, Lee TKW, Gong C, Khoo US. Repurposing hyperpolarization-activated cyclic nucleotide-gated channels as a novel therapy for breast cancer. Clin Transl. Med 2021 Nov;11(11): e578. doi: 10.1002/ctm2.578.*

### EACR23-0713
### Assessing the Feasibility of Implementing the Salt Bridge Approach for eEF2K Suppression in Breast Cancer Treatment using AgNPs-Quercetin Conjugates

*O.B. EKŞİ[1], O. Aydin[1,2,3,4]*
*[1]Erciyes University, Nanotechnology & Nanoscience, Kayseri, Turkiye*
*[2]Erciyes University, NanoThera Lab- Drug Application and Research Center, Kayseri, Turkiye*
*[3]Erciyes University, Clinical Engineering Research and Implementation Center , Kayseri, Turkiye*
*[4]Erciyes University, Biomedical Engineering, Kayseri, Turkiye*
**Introduction**
Breast cancer is a major health concern globally, with several treatment modalities available for management. However, triple-negative breast cancer (TNBC) remains a significant challenge due to its aggressive nature and lack of targeted therapies. This study aimed to develop a novel approach for the treatment of TNBC by combining chemotherapy with siRNA-based gene therapy.
**Material and Methods**
We utilized quercetin, a flavonoid known for its chemotherapeutic properties, to synthesize silver nanoparticles (AgNPs) as a nanocarrier. To stabilize the AgNPs, we used sodium borohydride ($NaBH_4$) as a reducing agent and collected them in the presence of salty water. By modifying the surface of the AgNPs with sodium/potassium cations, we facilitated the electrostatic interaction of eEF2K siRNA with the particles. The characterization of AgNPs was performed using dynamic light scattering (DLS) and zeta potential analysis.
**Results and Discussions**
Our characterization process confirmed the size of the AgNPs to be 30.8 nm, with a zeta potential of approximately -16 mV. The siRNA-based gene therapy in combination with chemotherapy was highly effective in reducing cancer cell viability. Our findings demonstrate the potential of this dual-functionality approach in vitro for the treatment of TNBC. Quercetin, as a chemotherapeutic agent, not only acts as a reducing agent to synthesize AgNPs but also enhances the therapeutic efficacy of eEF2K siRNA by facilitating its electrostatic interaction with AgNPs.
**Conclusion**
In summary, our study demonstrates the feasibility of utilizing quercetin-synthesized AgNPs as a nanocarrier for the delivery of eEF2K siRNA and its potential as a dual-functionality approach for the treatment of TNBC. This novel approach offers a promising avenue for the development of targeted therapies for TNBC, which has limited treatment options. Further studies are warranted to evaluate the efficacy and safety of this approach in vivo.

### EACR23-0732
### Development of New Oncolytic Virotherapy Targeting Breast Cancer Using the Low Pathogenic Enteroviruses

*O. Bezborodova[1], E. Plotnikova[1], A. Pankratov[1], E. Nemtsova[1], N. Morozova[1], Y. Venediktova[1], G. Trunova[1], A. Kaprin[1], P. Chumakov[2]*
*[1]P. Hertsen Moscow Oncology Research Institute – Branch of the National Medical Research Radiological Centre- Ministry of Health of the Russian Federation, Department of Experimental Pharmacology and Toxicology, Moscow, Russia*
*[2]Engelhardt Institute of Molecular Biology- Russian Academy of Sciences, Cell Proliferation Laboratory, Moscow, Russia*
**Introduction**
Breast cancer is the most common cancer in women worldwide, and triple-negative breast cancer (TNBC) is

© 2023 The Authors. Molecular Oncology is published by John Wiley & Sons Ltd on behalf of Federation of European Biochemical Societies highly refractory to current standard therapies. Due to the absence of estrogen and progesterone receptors, as well as the absence of overexpression or gene amplification of human epidermal growth factor receptor 2 (HER2), endocrine therapy and anti-HER-2 targeted therapy have been proven ineffective. Although chemotherapy has shown substantial efficacy in some TNBC patients, the occurrence of drug resistance and poor prognosis have prompted the exploration of new and effective treatment methods. The emerging concept of oncolytic viruses (OVs) provides a new platform to treat TNBC. In this study, the potential of oncolytic human enteroviruses (NPE930, NPE902, NPE961, RNPP-S3) and a set of four (EnteroMix) was investigated as a novel biotherapeutic agent against breast cancer.

**Material and Methods**

Human breast cancer cell lines BT-20, -483, -549, MCF7, DU4475 (ATCC®) were infected with human enteroviruses and EnteroMix, and viral replication and cytotoxic effects were evaluated in vitro. Enteroviruses-induced oncolysis was also investigated in nude mice bearing breast cancer xenografts in vivo. All animal procedures were carried out under specific-pathogen-free (SPF) conditions and in accordance with the approved animal use protocols of P. Hertsen Moscow Oncology Research Institute Laboratory Animal Center.

**Results and Discussions**

Human enteroviruses and EnteroMix cocktail infection killed breast cancer cells in a time- and titer-dependent manner. Nude mice transplanted with human breast cancer cells were successfully treated with both human enteroviruses and the set of four (both intratumoral and intravenous administrations). Antitumor efficacy increases in the following order: NPE930 ≈ NPE902 < NPE961 < RNPP-S3 ≈ EnteroMix. Importantly, mice treated with intravenous administrations enteroviruses showed very few adverse events.

**Conclusion**

RNPP-S3 and EnteroMix are strong oncolytic viruses' candidates for breast cancer due to its oncolytic efficacy and improved safety profile.

### EACR23-0735
### Combined inhibition of MDM2 and PARP lead to a synergistic anti-tumoral response in p53 wild-type rhabdomyosarcoma

*G. Pons Barcons[1], G. Gallo-Oller[1], L. García-Gilabert[1], P. Zarzosa[1], J. Sansa-Girona[1], N. Navarro[1], M.F. Segura[1], J. Sánchez de Toledo[2], S. Gallego[2], L. Moreno[2], J. Roma[1]*

*[1]Vall d'Hebron Institute of Research- VHIR, Laboratory of Childhood Cancer and Blood Disorders, Barcelona, Spain*

*[2]Hospital Universitari Vall d'Hebron, Pediatric Oncology and Hematology Department, Barcelona, Spain*

**Introduction**

Rhabdomyosarcoma (RMS) is a highly aggressive cancer that arises from errors during the myogenic differentiation process and predominantly affects children and adolescents. In contrast to most adult tumors, *TP53* loss-of-function mutations are relatively rare in RMS, being detected in about 5% of tumors. However, the tumor cell can resort to other mechanisms, such as *CDKN2A* deletions or *MDM2* overexpression/overactivation, to keep p53 under control, survive and proliferate. Thus, the disruption of the MDM2-p53 interaction is a promising strategy to release p53 from MDM2 control and impede RMS $p53^{WT}$ progression. In this study, we provide results demonstrating the therapeutic potential of combining Siremadlin (MDM2 inhibitor) and Olaparib (PARP inhibitor) for treating $p53^{WT}$ RMS.

**Material and Methods**

Cell proliferation, cell death and apoptosis assays were used to determine the combination effects of Siremadlin and Olaparib in comparison to individual treatments. Gene and protein expression of p53 targets was assessed by RT-qPCR and WB. Finally, the effects on tumor growth and survival of the combination between Siremadlin and Olaparib was tested in xenograft murine models.

**Results and Discussions**

Proliferation assays using distinct $p53^{WT}$ RMS revealed synergistic effects of combining Siremadlin and Olaparib *in vitro*. Cell death assays and apoptosis assays showed an increased induction of apoptosis and cell death in the combination group compared to individual treatments. Additionally, an increased gene and protein expression of p53 targets (CDKN1A, MDM2, BAX, FBXW7) was observed in combination group. Finally, combination of both drugs resulted in significant reduction of tumor growth and increased survival *in vivo* when compared to individual treatments and control groups. Combination of both drugs allowed to reduce the toxicity associated with Siremadlin while increasing the individual drug effect in tumor growth inhibition.

**Conclusion**

Overall, our study demonstrates the synergistic effect of the combination between Siremadlin and Olaparib in the inhibition of $p53^{WT}$ RMS tumor growth in vitro and in vivo. Our findings support the potential to study the combination of both drugs in clinical trials and warrants further investigation in other tumors with similar molecular features.

### EACR23-0738
### Combined chemotherapy with hyperthermia and calcitriol in pancreatic tumor models

*O. Wiecheć-Cudak[1], A. Murzyn[1], D. Szczygieł[1], I. Łakoma[1], M. Oliinyk[1], A. Słomiński[2], M. Elas[1], M. Krzykawska-Serda[1]*

*[1]Jagiellonian University in Kraków, Faculty of Biochemistry Biophysics and Biotechnology, Kraków, Poland*

*[2]University of Alabama at Birmingham, Department of Dermatology, Birmingham, United States*

**Introduction**

Combination therapies are a fully established trend in clinical cancer treatment. However, more information is needed on why some modalities work effectively in a certain order. Synergistic multimodal therapy results in remarkable effects, more powerful than any single therapy or their theoretical combination. The study focused on pancreatic ductal adenocarcinoma (PDAC) as a highly lethal and aggressive malignancy. The aim was to determine the effectiveness of combined chemotherapy with gemcitabine, calcitriol, and hyperthermia in mouse

© 2023 The Authors. Molecular Oncology is published by John Wiley & Sons Ltd on behalf of Federation of European Biochemical Societies and human pancreatic cancer cells *in vitro* and orthotopic PDAC model *in vivo*.

**Material and Methods**

*In vitro* experiments were performed on human (PANC-1) and murine (Panc02) pancreatic cancer cells. Cells were heated to 41˚C and treated with gemcitabine (0.001 - 1000 µM) and calcitriol at a dose of 100 nM. Cell proliferation and metabolic activity were determined.

Immunocompetent C57BL/6J were inoculated with Pan_O2 spheroids to generate an orthotopic murine model of pancreatic cancer. Gemcitabine (IV; 45 mg/kg BW), calcitriol (IP; 100 nM/mice) and local hyperthermia (infrared light, <42 °C, 30 min) were administered in 6 doses, 72 hours apart. Tumor growth was examined by ultrasonography (Vevo 2100, Fuji VisualSonic). Blood and tissue samples were analyzed by e.g. histochemistry and Western Blot. All experiments were performed with Local Ethics Committee permission no.353/2022.

**Results and Discussions**

Combining gemcitabine with calcitriol and hyperthermia efficiently reduced cancer cell number and their metabolic activity, significantly slowing down PDAC tumor growth. Combinatory therapy is well tolerated by animals and can lead to better anti-cancer effects.

**Conclusion**

Combination therapy can lead to better anti-cancer effects than chemotherapeutic, calcitriol, or hyperthermia alone. We hypothesize that hyperthermia increases drug perfusion during chemotherapy, whereas calcitriol prevents the development of resistance in cancer.

### EACR23-0750
### Smart Nanoparticles Targeting Chordoma Cancer with CRISPR-CAS9

*A.U. KUTLU*[1], *G. Kursunluoglu*[2], *C.U. Tunc*[3], *M. Ercan Ayra*[4], *E. Aydemir*[5], *Y. Yüksel Durmaz*[4], *O. Aydin*[1]

[1]*Erciyes University, Biomedical Engineering, Kayseri, Turkiye*

[2]*Erciyes University, Drug Application and Research Center ERFARMA, Kayseri, Turkiye*

[3]*University of Utah, Utah Center for Nanomedicine, Salt Lake City, United States*

[4]*Istanbul Medipol University, Biomedical Engineering, Istanbul, Turkiye*

[5]*Biruni University, Biomedical Engineering, Istanbul, Turkiye*

**Introduction**

Chordoma is a rare malignant bone tumor with a very high recurrence rate, and the success of treatment is low despite the general treatment approach of high-dose radiotherapy in conjunction with surgical intervention. In recent years, various nanoparticles designed in the field of nanomedicine have been used to effectively transport therapeutic agents such as genes and drugs to diseased cells. Over the past 6 years, CRISPR/Cas9 technology has become one of the powerful gene transfer methods in biomedical research and therapeutic applications due to its effective and easy gene transfer. Nanoparticle-based/polymeric carriers are promising gene delivery platforms. In this study, an innovative treatment method was aimed to be developed by using smart nanoparticles for gene delivery in chordoma treatment.

**Material and Methods**

For this purpose, FDA-approved β-cyclodextrin (β-CD) was used as the main core; a complex was formed with plasmid DNA (pDNA) and a positively charged peptide sequence with nuclear localization signal (NLS) feature. Thus, a pH-sensitive, hydrophobic, and cationic nanoparticle was obtained from monomers and grafted with PEG to make it biocompatible.

**Results and Discussions**

As a result of the toxicity experiments, it was shown that the designed smart gene carrier nanoparticle had no toxic effects on the CH22, chordoma cell line. The gel electrophoresis experiment proved that NLS+β-CD structure can form a complex with CRISPR/Cas9 pDNA. In addition, fluorescence imaging showed nuclear entry in the NLS peptide-containing pDNA+β-CD complex group at 6 hours, while no nuclear entry was observed in the group without NLS peptide. Moreover, GFP transfection was observed 48 hours after the application of NLS+β-CD+pDNA complex to the chordoma cell line, showing that the sent plasmid reached the nucleus and became active.

**Conclusion**

Our study highlights the potential of electrostatic interaction through forming a complex of high base pair CRISPR pDNA with smart nanoparticles for gene therapy against rare chordoma cancer.

### EACR23-0757
### Isoxanthohumol is a beneficial treatment for chemotherapy-induced sepsis

*T.H. Kao*[1], *C.Y. Li*[1], *Y.R. Chen*[1], *H.E. Wu*[1]

[1]*Graduate Institute of Medicine, College of Medicine, Kaohsiung, Taiwan*

**Introduction**

Cancer patients have higher risk of developing an infection and sepsis under chemotherapy.

Infection with pathogens, such as bacteria, viruses and fungi, frequently results in septicemia due to a severe inflammatory reaction that has a very high mortality rate. Macrophages are an important type of antigen- presenting cells that control both innate and adaptive immune responses.

During pathogen infection, activated macrophages secrete many proinflammatory mediators and cytokines such as nitric oxide (NO), TNF-α and IL-6 against the pathogens; however, an overwhelming inflammatory response causes many cells to become apoptotic and sepsis patients eventually die of multiple organ failure.

Therefore, we found an anti-inflammatory compound, isoxanthohumol, which inhibits the excessive inflammation triggered by pathogen infection and may be an effective strategy to reduce the mortality of sepsis patients.

**Material and Methods**

We used lipopolysaccharide (LPS) as a pathogenic stimulus to induce inflammatory responses in J774A.1 macrophages.

Cell viability was detected by MTT assay. Secretion of NO was analyzed by Griess reagent assay, while the production of TNF-α, IL-6, IL-1β was measured by enzyme-linked immunosorbent assay (ELISA).

© 2023 The Authors. Molecular Oncology is published by John Wiley & Sons Ltd on behalf of Federation of European Biochemical Societies

Expression of iNOS, COX-2 and MAPKs was examined by Western blotting.
NF-κB activity was analyzed by promotor reporter assay.
Activation of NLRP3 inflammasome was examined by ELISA and immunofluorescence staining.
In addition, we also examined the effect of isoxanthohumol on the secretion of pro-inflammatory cytokines by THP-1 cell.
**Results and Discussions**
We found that isoxanthohumol significantly suppressed the secretion of NO and attenuated the production of TNF-α and IL-6 by LPS-induced macrophages.
In addition, isoxanthohumol also inhibited the expression of iNOS and COX-2, suppressed the phosphorylation of JNK, and attenuated the activity of NF-κB by LPS-induced macrophages.
Moreover, isoxanthohumol decreased the activation of NLRP3 inflammasome by reducing the production of IL-1β and repressing the ASC speck formation of NLRP3 inflammasome in LPS/ATP-induced macrophages.
**Conclusion**
Our experimental results demonstrated that isoxanthohumol effectively inhibited LPS induced inflammatory responses in macrophages, suggesting that isoxanthohumol might have benefit for treating chemotherapy-induced sepsis, resulting in decreasing the mortality of cancer patients under chemotherapy-induced sepsis.

### EACR23-0759
### The antifibrotic agent Pirfenidone sensitizes non-small cell lung cancer to Vinorelbine and to a combination of Vinorelbine with Carboplatin

*H. Branco[1], C. Rodrigues[1,2], N. Mendes[3], C. Antunes[1], I. Amorin[4,5], J. Oliveira[2], L. Lara Santos[2,6], M.H. Vasconcelos[1,7], C. P.R. Xavier[1,7]*
*[1]IPATIMUP/i3S, Cancer Drug Resistance Group, Porto, Portugal*
*[2]IPO – Instituto Português de Oncologia, Experimental Pathology and Therapeutics Group, Porto, Portugal*
*[3]IPATIMUP/i3S, Histology and Electron Microscopy, Porto, Portugal*
*[4]IPATIMUP/i3S, Glycobiology in Cancer Group, Porto, Portugal*
*[5]ICBAS-UP – School of Medicine and Biomedical Sciences, Patologia e Imunologia Molecular, Porto, Portugal*
*[6]ICBAS-UP – School of Medicine and Biomedical Sciences, Anatomia, Porto, Portugal*
*[7]FFUP – Faculty of Pharmacy of the University of Porto, Department of Biological Sciences, Porto, Portugal*
**Introduction**
The main therapeutic option for patients with advanced non-small cell lung cancer (NSCLC) without targetable alterations and/or low PD-L1 expression continues to be platinum-based chemotherapy. Thus, new therapeutic strategies are required. Pirfenidone (PF), an antifibrotic drug, was previously shown to have antitumor potential. Our work aimed to study the sensitizing effect of PF to treatments with Vinorelbine (VR) or with VR plus carboplatin (CBP), in NSCLC pre-clinical models.
**Material and Methods**
Three NSCLC cell lines (A549, NCI-H322 and NCI-H460), were selected to assess the effect of the isolated drugs and drug combinations consisting of: i) PF with VR; and ii) PF with VR and CBP (named as “triplet”). The following was studied: 1) cell growth (sulforhodamine B assay), 2) cell cycle profile (flow cytometry following PI staining), 3) cell proliferation (BrdU incorporation assay), and 4) cell death (flow cytometry following Annexin V FITC/PI staining). Effects on growth of two non-tumorigenic cell lines (MCF-10A and MCF-12A) were evaluated. *In vivo* studies were performed, in nude mice xenografted with A549 cells. Animals were intraperitoneally injected with the vehicle, PF, VR with CBP (named as “duplet”) or the triplet, once or twice a week during five weeks. Organs and tumor tissues were collected for immunohistochemical and histopathological analyses, and blood plasma for biochemical analysis.
**Results and Discussions**
PF sensitized the three NSCLC cell lines to VR treatment, triggering a significant reduction in cell growth, major alterations in the cell cycle profile, a significant reduction in the % of proliferating cells and an increase in the % of cell death. A significant reduction in the % of cell growth was observed with the triplet, when compared to the duplet currently applied in the clinical practice. Furthermore, the triplet drug combination did not induce cytotoxicity towards non-tumorigenic cell lines, when compared to the duplet. Importantly, the triplet treatment reduced xenograft tumor growth, even though without a statistically significant effect when compared with the duplet. Most notably, the triplet caused less side effects to the mice than the duplet. Biochemical analysis also supported the observation of less toxicity with the triplet drug combination.
**Conclusion**
Our study revealed the benefit of using PF in combination with VR or with VR plus CBP, paving the way towards the possibility of repurposing PF as a perioperative measure for NSCLC treatment.

### EACR23-0782
### FGF signaling blockade inhibits c-Myc-driven B-cell non-Hodgkin lymphoma growth

*J. Faletti[1], G. Gazzaroli[1], R. Ronca[1], L. Lorenzi[1], F. Facchetti[1], A. Tucci[2], M. Presta[1], A. Giacomini[1]*
*[1]University of Brescia, Department of Molecular and Translational Medicine, Brescia, Italy*
*[2]ASST Spedali Civili of Brescia, Department of Hematology, Brescia, Italy*
**Introduction**
B-cell non-Hodgkin lymphomas (B-NHL) are a heterogeneous group of malignancies that arise from peripheral B-lymphocytes. Among them, a subset of diffuse large B-cell lymphomas (DLBCL) and all Burkitt’s lymphomas (BL) are c-MYC-driven aggressive B-NHL. Approaches targeting c-MYC may represent novel attractive therapeutic strategies for aggressive B-NHL. Recently we have demonstrated that activation of the

© 2023 The Authors. Molecular Oncology is published by John Wiley & Sons Ltd on behalf of Federation of European Biochemical Societies fibroblast growth factor (FGF) signaling is involved in c-MYC protein stabilization, FGF ligand or FGF receptor (FGFR) inhibition leading to the proteasomal degradation of c-MYC protein. Thus, FGF/FGFR blockade may represent a promising anti-c-MYC strategy in c-MYC-driven B-NHL.

**Material and Methods**

FGFs and FGFRs expression were assessed by qPCR. FGFR activation and c-MYC protein levels were assessed by western blot and immunohistochemical analyses. The effects of FGF/FGFR blockade were investigated by viable cell counting, TMRE, Mitosox, Annexin-V/PI stainings and cytofluorimetric analyses. In vivo experiments were performed in NOD/SCID mice.

**Results and Discussions**

DLBCL and BL samples from patients showed high levels of phosphorylated (p)FGFR. Interestingly, pFGFR was observed in c-MYC positive but not in c-MYC negative samples, suggesting a strong correlation between FGFR activation and c-MYC protein expression. To assess a possible role for the FGF/FGFR system in c-MYC-driven B-NHL, we tested the anti-lymphoma activity of the FGF trap molecule NSC12 and the FDA-approved FGFR TK inhibitor Erdafitinib in DLBCL (RI-1) and BL (RAJI) cell lines. Both cell lines express FGFRs and several FGF ligands and show high levels of pFGFR in the absence of exogenous stimuli, indicating the presence of an autocrine FGF stimulation. Interestingly, both NSC12 and Erdafitinib rapidly induced the degradation of c-MYC protein as early as 3 hours after treatment and co-treatment with the proteosome inhibitor MG132 was able to prevent c-MYC degradation. Importantly, the reduction of c-MYC protein levels was paralleled by a significant increase in ROS-mediated apoptosis. In vivo FGF/FGFR inhibition significantly reduced the growth of both RI-1 and RAJI tumor xenografts. Analyses of tumor samples from treated mice showed a strong reduction of pFGFR and c-MYC protein levels compared to controls. Interestingly, NSC12 or Erdafitinib in combination with the standard therapy R-CHOP exerted synergistic anti-lymphoma effects.

**Conclusion**

These findings open new therapeutic hints for the treatment of c-MYC-driven aggressive B-NHL.

### EACR23-0785

### Targeting GLI1 and GLI2 with small molecule inhibitors to suppress GLI-dependent transcription and tumor growth

*L. Maresca[1], E. Crivaro[1,2], A. Giammona[1], E. Dreassi[2], M. Taddei[2], F. Manetti[2], E. Petricci[2], B. Stecca[1]*

*[1]Istituto per lo Studio la Prevenzione e la Rete oncologica, Core research laboratory - Tumor Cell Biology Unit, Firenze, Italy*

*[2]University of Siena, Department of Biotechnology- Chemistry and Pharmacy, Siena, Italy*

**Introduction**

Aberrant activation of Hedgehog (HH) signaling in cancer is the result of genetic alterations of upstream pathway components (canonical) or other oncogenic mechanisms (noncanonical), that ultimately concur to activate the zinc-finger transcription factors GLI1 and GLI2. Therefore, inhibition of GLI activity is a good therapeutic option to suppress both canonical and noncanonical activation of the HH pathway. However, only a few GLI inhibitors are available, and none of them have the profile required for clinical development due to poor metabolic stability and aqueous solubility, and high hydrophobicity.

**Material and Methods**

A virtual screening was used to identify novel GLI inhibitors, whose mechanisms of action were investigated using cell-based HH-specific assays, chromatin immunoprecipitation and site-directed mutagenesis. Therapeutic efficacy was assessed in multiple human cancer cell lines in vitro and using human melanoma xenografts. Drug specificity was assessed by genetic silencing and CRISPR/Cas9. ADME profile and PK parameters were determined.

**Results and Discussions**

We present the development and preclinical characterization of three novel drug-like GLI inhibitors that show a strong antitumor efficacy in multiple human cancer cell lines harboring canonical and non-canonical HH pathway activation. Mechanistically, they impair GLI1 and GLI2 activities by interfering with their binding to DNA. Specificity towards GLI1 and GLI2 was demonstrated by lower activity of the inhibitors in GLI1- or GLI2-depleted cancer cells. Among these new small molecules, JC19 showed good pharmacokinetics parameters. This molecule inhibits GLI-dependent human melanoma xenograft growth in vivo, without signs of toxic effects in mice.

**Conclusion**

Our results highlight the potential of JC19 to inhibit GLI1 and GLI2 hyperactivation induced not only by upstream PTCH/SMO-dependent signals, but also by downstream bypass mechanisms that are responsible for the resistance to anti-SMO drugs.

### EACR23-0794

### Assessing the Feasibility of Implementing the Salt Bridge Approach for siRNA Suppression in Breast Cancer Treatment using Silver Nanoparticles with Quercetin Conjugates

*O.B. EKŞİ[1], O. Aydin[1,2,3,4]*

*[1]Erciyes University, Nanotechnology & Nanoscience, Kayseri, Turkiye*

*[2]Erciyes University, NanoThera Lab- Drug Application and Research Center, Kayseri, Turkiye*

*[3]Erciyes University, Clinical Engineering Research and Implementation Center , Kayseri, Turkiye*

*[4]Erciyes University, Biomedical Engineering, Kayseri, Turkiye*

**Introduction**

Breast cancer is a major health concern globally, with several treatment modalities available for management. However, triple-negative breast cancer (TNBC) remains a significant challenge due to its aggressive nature and lack of targeted therapies. This study aimed to develop a novel approach for the treatment of TNBC by combining chemotherapy with siRNA-based gene therapy.

© 2023 The Authors. Molecular Oncology is published by John Wiley & Sons Ltd on behalf of Federation of European Biochemical Societies

**Material and Methods**
We utilized quercetin, a flavonoid known for its chemotherapeutic properties, to synthesize silver nanoparticles (AgNPs) as a nanocarrier. To stabilize the AgNPs, we used another reducing agent and collected them in the presence of salty water. By modifying the surface of the AgNPs with sodium/potassium cations, we facilitated the electrostatic interaction of siRNA with the particles. The characterization of AgNPs was performed using dynamic light scattering (DLS) and zeta potential analysis.
**Results and Discussions**
Our characterization process confirmed the size of the AgNPs to be 30.8 nm, with a zeta potential of approximately -16 mV. The siRNA-based gene therapy in combination with chemotherapy was highly effective in reducing cancer cell viability. Our findings demonstrate the potential of this dual-functionality approach in vitro for the treatment of TNBC. Quercetin, as a chemotherapeutic agent, not only acts as a reducing agent to synthesize AgNPs but also enhances the therapeutic efficacy of siRNA by facilitating its electrostatic interaction with AgNPs.
**Conclusion**
In summary, our study demonstrates the feasibility of utilizing quercetin-synthesized AgNPs as a nanocarrier for the delivery of siRNA and its potential as a dual-functionality approach for the treatment of TNBC. This novel approach offers a promising avenue for the development of targeted therapies for TNBC, which has limited treatment options. Further studies are warranted to evaluate the efficacy and safety of this approach in vivo.

### EACR23-0811
### KRAS mutant gene editing prevents tumor growth in vivo and overcomes acquired resistance to KRASG12C inhibitor.

*G. Divita[1], V. Guzman-Gonzalez[2], A. Grunenberger[1], N. Durany[3], E. Czuba[1], W. Ngyien[3], V. Faure[2], N. Desai[3]*
*[1]DIVINCELL, Molecular Therapy, Nimes, France*
*[2]IAB, Institut for advanced biosciences, Grenoble, France*
*[3]AADIGEN, Molecular Therapy, Pacific Palisades, United States*

**Introduction**
KRAS mutations at codon-12 are among the most commonly observed mutations in cancers. To date, treatment targeting $KRAS^{G12C}$ mutation have been successful in the clinic. However, it is hampered by adaptive resistance, associated with the emergence of secondary KRAS mutations, feedback pathway activation and high amplification of $KRAS^{G12C}$ allele. We have developed a potent strategy combining gene editing with a tumor selective peptides, to impair cancer cell proliferation by directly targeting mutations at codon-12 of *KRAS* oncogene.
**Material and Methods**
ADGN-121, ADGN-122, ADGN-123 are gene-editing complexes containing sgRNA targeting specifically $KRAS^{G12D}$, $KRAS^{G12V}$ or $KRAS^{G12C}$ complexed with proprietary peptides. ADGNs were evaluated on pancreatic, colorectal, and lung cancer cells harboring $KRAS^{G12D,12V,12C}$ mutations. *In-vivo* efficacy of IV-administered ADGN-121 and ADGN-122 (0.25-1.0 mg/kg, day1-7) were evaluated in Panc1 ($KRAS^{G12D}$) and SW403 ($KRAS^{G12V}$) mouse xenografts, respectively. ADGN-123 was evaluated on H358 generated clones resistant to sotorasib and harboring R68M and Y96D secondary KRAS mutations.
**Results and Discussions**
ADGN-121,122 and 123 selectively silenced $KRAS^{G12D}$, $KRAS^{G12V}$ $KRAS^{G12C}$, respectively, in colorectal, pancreatic and lung cancer cells resulting in reduction of cell proliferation ($IC_{50}$:10-30 nM) and inhibition of ERK and AKT phosphorylation. Only two IV-administrations of ADGN-121 containing $gRNA^{G12D}$ are required to abolish Panc1 tumor growth in a dose dependent manner, resulting in tumor regression of 65% at 1.0mg/kg. ADGN-122 containing $gRNA^{G12V}$ abolished SW403 tumor growth with a tumor regression of 70% at 1.0 mg/kg. No effect on tumor growth was observed with nonspecific gRNA. We demonstrated a synergistic combination between ADGN-121/Abraxane in pancreatic carcinoma and between ADGN-122/Capecitabine in colon Adenocarcinoma. We showed, that ADGN-123 containing $gRNA^{G12C}$ can effectively reduce the proliferation and inhibit ERK phosphorylation of sotorasib/adagrasib acquired resistance cells harboring $KRAS^{G12C/R68M}$ and $KRAS^{G12C/Y96D}$ mutations. ADGN treatments are well tolerated, no sign of clinical toxicity or inflammatory response was detected after repeated administrations.
**Conclusion**
Our study provides a proof-of-concept that ADGN can be applied to target driver mutations of cancers *in vivo* and permanently disrupt the oncogenic alleles, leading to major tumor regression. ADGN-123 constitutes an alternative strategy to overcome resistance associated to inhibitors of $KRAS^{G12C}$.

### EACR23-0815
### Effects of Fasting Mimicking Diets in Acute and Chronic mice models of Cancer Cachexia

*L. Garcia Castillo[1], A. Thaseen Eliass[1], N. Cortez Penso[1], O. Blazevits[2], P. Costelli[1], V. Longo[2], F. Penna[1]*
*[1]University of Turin, Department of Clinical and Biological Sciences, Turin, Italy*
*[2]The AIRC Institute of Molecular Oncology, Longevity and cancer Lab, Milan, Italy*

**Introduction**
Cancer cachexia is a complex multifactorial syndrome associated with involuntary body weight loss, muscle wasting, and anorexia, which leads to an altered metabolic state. Nutritional strategies have been employed to try to ameliorate the disease and to improve the survival of cancer patients. Fasting-Mimicking Diets (FMDs), which are calorie restricted plant-based diets, have shown to impact tumor growth and have synergistic effects with chemotherapy, including diminished side effects. Nevertheless, the effect of FMDs on cancer cachexia is yet to be explored. Hence, the aim of this project is to investigate the preventive and therapeutic impact of FMDs on intestinal cancer-related cachexia.
**Material and Methods**
Two distinct *in vivo* experiments have been performed. For the first experiment, female C26 tumor-bearing mice were treated weekly with chemotherapy and with three 4-day

© 2023 The Authors. Molecular Oncology is published by John Wiley & Sons Ltd on behalf of Federation of European Biochemical Societies cycles of FMD, then compared to C26 Folfox-treated mice fed standard diet (SD) *ad libitum*. Forelimb grip strength test was measured the day after each chemotherapeutic treatment. The organs, including skeletal muscles, of animals that reached the endpoint of thirty-three days after tumor inoculation were excised, weighed, and fast frozen for further processing. For the second experiment, the more chronic and clinically relevant Msh2$^{loxP/loxP}$ Villin-Cre (VCM) mice model, which develops spontaneous intestinal tumors, is being used. Age- and sex-matched VCM mice are treated with two 4-day FMD cycles every month until endpoint. Body weight, food intake and muscle strength are recorded regularly.

**Results and Discussions**

In the C26 tumor-bearing mice experiment, the mean survival did not present significant differences among groups. Overall, both groups showed similar body weight loss, muscle strength impairment, and gastrocnemius muscle wasting. Preliminary results from the ongoing experiment with VCM mice show that although body weight significantly decreased during FMD periods in both males and females it was recovered during the refeeding periods. Noteworthy, during the FMD periods, there was a significant increase in blood ketones levels and a decrease in glucose levels.

**Conclusion**

Based on preliminary results, this study shows that FMD has no impact in cancer cachexia neither in an acute cancer chemotherapeutically induced cachectic mice model nor in a chronic- autochthonous one. Nevertheless, further statistical and molecular analysis are needed upon completion of the study.

### EACR23-0822
### Inhibition of Carbonic Anhydrase IX induces mitochondrial oxidative stress and apoptosis in multiple myeloma

*G. Gazzaroli[1], R. Ronca[1], F. Carta[2], J. Faletti[1], M. Presta[1], C.T. Supuran[2], A. Giacomini[1]*
*[1]University of Brescia, Department of Molecular and Translational Medicine, Brescia, Italy*
*[2]University of Florence, Department of NEUROFARBAUniversity of Florence, Florence, Italy*

**Introduction**

Multiple myeloma (MM) arises from the clonal proliferation of malignant plasma cells in the bone marrow (BM). Since BM niches are characterized by low oxygen levels, MM cells have to adapt their metabolism to the hypoxic BM milieu. This generates high quantities of toxic metabolic acids that induce the expression of various proteins involved in the prevention of $H^+$ ions accumulation and maintenance of intracellular pH. In this context, carbonic anhydrase IX (CA IX), a hypoxia-induced cell-surface enzyme, sustains cancer cell survival under hypoxic and acidosis conditions by regulating intracellular pH. So far, no data are available about the impact of CA IX in MM. Our preliminary data demonstrated that MM cells express high levels of CA IX under both normoxic and hypoxic conditions, suggesting that inhibition of CA IX activity may affect MM survival. Here we investigated the anti-myeloma activity of two CA IX inhibitors, the clinical grade sulphonamide SLC-0111 (Phase Ib/II clinical trials) and its analog FC-531.

**Material and Methods**

CA IX blockade effects were investigated by western blot analysis, viable cell counting, and TMRE, Mitosox, Annexin-V/PI stainings and cytofluorimetric analyses. In vivo experiments were performed in NOD/SCID mice.

**Results and Discussions**

In vitro, both CA IX inhibitors significantly reduced the growth and survival of human KMS-11 MM cells, FC-531 being more potent compared to SLC-0111. Interestingly, CA IX inhibition strongly induced mitochondrial ROS production, mitochondrial membrane depolarization and apoptotic cell death. Accordingly, Western blot analysis revealed a significant induction of ROS-mediated DNA damage and caspase activation, as demonstrated by increased levels of γH2AX and cleaved PARP and caspase 3. Interestingly, apoptotic cell death was rescued by treatment with the potent anti-oxidant vitamin E, demonstrating that mitochondrial oxidative stress is the main cause for MM cell death induced by CA IX inhibition. Importantly, CA IX inhibitors strongly reduced the growth and survival also of Bortezomib-resistant MM cells, and CA IX inhibitors combined with Bortezomib exerted an increased anti-myeloma activity compared to single treatments. Finally, both SLC-0111 and FC-531 significantly reduced the growth of MM tumor xenografts in NOD/SCID mice.

**Conclusion**

Our data suggest that CA IX inhibitors as single agents or in combination with proteasome inhibitors may represent a valid therapeutic approach for both naïve and proteosome inhibitor-refractory MM patients.

### EACR23-0823
### N-Myristolytransferase (NMT) inhibitors as novel potent payloads for Antibody Drug Conjugates (ADC)

*J. Walton[1], R. Solari[2], F. Orafidiya[1], N. D'arcy[3], F. Falciani[2], E. Tate[1], R. Carr[2]*
*[1]Imperial College London, Department of Chemistry, London, United Kingdom*
*[2]Myricx Pharma, Biology, London, United Kingdom*
*[3]Myricx Pharma, Operations, London, United Kingdom*

**Introduction**

There is a clinical need for ADC payloads with a high therapeutic index. Consequently, we have coupled potent and selective inhibitors of NMT to Trastuzumab and Sacituzumab, to test their potential in both *in vitro* and *in vivo* cancer models.

**Material and Methods**

A highly potent NMT inhibitor was conjugated to Trastuzumab, Sacituzumab and an isotype control (IgG1 clone B12) to produce ADCs with a DAR of 5. ADCs were tested *in vitro* in cytotoxicity studies using Her2+ and Her2- cell lines and subsequently tested *in vivo* in breast and gastric cancer xenografts.

**Results and Discussions**

Treatment with our first Trastuzumab-NMT inhibitor ADC (MYX-2449) resulted in cytotoxicity in Her2+ BT474 cells with an $EC_{50}$ of 0.2nM, with no cell death observed in the B12 isotype control at concentrations tested (50nM and

© 2023 The Authors. Molecular Oncology is published by John Wiley & Sons Ltd on behalf of Federation of European Biochemical Societies below). Furthermore MYX-2449 was inactive against Her2- cells, MCF7 at concentrations up to 200nM. MYX-2449 exhibited bystander effects *in vitro.* Plating MCF7 (Her2-) cells with BT474 (Her2+) at a 1:1 ratio, followed by MYX-2449 treatment resulted in cytotoxicity of the MCF7 cells at 0.2nM MYX-2449. Conversely no cytotoxicity was observed in the monoculture control wells.

5mg/kg MYX-2449 (IV once a week for four weeks) was tested in the BT474 xenograft model and compared to Trastuzumab and vehicle controls. Treatment with MYX-2449 resulted in TGI of 108% on D21, with 7/10 mice having undetectable tumour by D33. Trastuzumab resulted in only a partial response (TGI of 44%). 5mg/kg MYX-2449 (IV on D1 and D8) was subsequently tested in a gastric cancer xenograft, NCI-N87, and compared to the isotype control and Trastuzumab. Treatment with MYX-2449 resulted in a TGI of 280%, with 6/10 mice having undetectable tumours by D21. Trastuzumab treatment resulted in TGI of 193% at D21. No significant body weight loss was observed during either study.

Treatment of a panel of solid cancer cell lines with our Sacituzumab- NMT inhibitor ADC (MYX-2468) resulted in cytotoxicity in NCI-N87, IM95m, JIMT-1 and NCI-H292 lines, with all $IC_{50}$s below 4nM. *In vivo* experiments are ongoing.

**Conclusion**

Potent small molecule inhibitors of NMT can be readily conjugated to therapeutic monoclonal antibodies to generate novel highly effective and well tolerated ADC payloads. NMT inhibitors represent a totally novel class of ADC payloads that exploit cancer cell dependency on N-myristoylated proteins. The potential of this novel mechanism to deliver high efficacy with better TI is currently being investigated in preclinical models.

### EACR23-0827

### Novel Combination treatment of Hesperetin and the histone demethylase inhibitor GSK-J4 and their effect on TGFβ induced EMT, Invasion, and Migration in Prostate Cancer Cells.

*N. D*[1], *A. Naik*[1], *N. Thakur*[1]

[1]*Ahmedabad University, Cancer Biology, Ahmedabad, India*

**Introduction**

Minimizing side effects, overcoming cancer drug resistance, and preventing metastasis of cancer cells are of growing interest in current cancer therapeutics. Phytochemicals are being deeply researched as they are protective to normal cells and have lesser side effects. Hesperetin is a citrus bioflavonoid known to inhibit TGFβ induced Epithelial to Mesenchymal Transition (EMT), migration, and invasion of prostate cancer cells. Targeting epigenetic modifications that cause cancer is another class of upcoming therapeutics, as these changes are reversible. Global H3K27me3 levels have been found to be reduced in invasive prostate adenocarcinomas. A combination of a demethylase inhibitor and a known anti-cancer phytochemical is a unique approach to target cancer to attain the aforementioned objectives.

**Material and Methods**

We used an H3K27 demethylase (JMJD3/KDM6B**)** inhibitor to study its effects on TGFβ-induced EMT in prostate cancer cells. We then gave a combined Hesperetin and GSK-J4 treatment to the PC-3 and LNCaP cell lines. The outcomes were studied using techniques like western blot analysis, RT-qPCR, wound healing assay, and invasion assay, and the Compusyn software was used to study drug synergism.

**Results and Discussions**

There was a dose-dependent increase in cytotoxicity and inhibition of TGFβ-induced migration and invasion of prostate cancer cells after GSK-J4 treatment. GSK-J4 not only induced trimethylation of H3K27 but also induced trimethylation of H3K4 levels. Surprisingly there was a reduction in the H3K9me3 levels. We found that the combination of hesperetin and GSK-J4 was synergistic as there was also a drastic decrease in the individual doses of both compounds, along with inhibition of migration and invasion of PC-3 cells.The effects are more pronounced in cancer lines with low tumorigenicity (LNCaP) as compared to a highly tumorigenic cell line (PC-3). Our study also provides a new insight to combine an epigenetic inhibitor (GSKJ4) as well as a phytochemical (hesperetin) to prevent the TGFβ-induced EMT, migration and invasion of prostate cancer cells. The epigenetic modifications are reversible and phytochemicals are well known to be less toxic. This combination might be associated with lesser side effects as compared to conventional chemotherapeutic drugs.

**Conclusion**

GSK-J4 alone and a combination of Hesperetin and GSK-J4 treatment effectively inhibit the important hallmarks of cancer, like cell proliferation, migration, and invasion, by altering the epigenetic landscape of cancer cells.

### EACR23-0828

### Investigating the Role of an Immune-Related Kinase as a Potential Therapeutic Target in High Grade Serous Ovarian Cancer.

*J. Casey*[1], *D. Wall-Coughlan*[1], *P. Dowling*[1], *M. Butler*[1]

[1]*Maynooth University, Biology Department, Maynooth, Ireland*

**Introduction**

Ovarian Cancer (OvCa) represents the sixth most common cancer in Irish women. The High Grade Serous Ovarian Cancer (HGSOC) subtype accounts for approximately 70% of all Epithelial Ovarian Cancer (EOC) diagnosis and 70-80% of all EOC deaths. Due to the high frequency of advanced stage detection (<70%) and relatively high incidence of patients developing chemo-resistant disease (<80% of initial responders), HGSOC is currently the most lethal gynaecological malignancy worldwide and the most clinically relevant subtype of EOC. In order to tackle this relatively poor prognosis cancer novel therapeutic targets are required.

**Material and Methods**

We have investigated the role of a specific immune related kinase at the protein level through the use of immunohistochemistry (IHC) and commercially available tissue microarrays. Through the use of three HGSOC cell line models we have investigated the role of this immune

© 2023 The Authors. Molecular Oncology is published by John Wiley & Sons Ltd on behalf of Federation of European Biochemical Societies related kinase through stable knockdown and the use of targeted inhibitor approaches. We have used 2D and 3D assays that include methods to assess proliferation, colony formation, 3D Matrigel growth and anchorage independent growth. We used Western blot analysis to unravel a mechanism of action of this kinase in HGSOC.

**Results and Discussions**

By IHC, we found significantly higher protein levels of this kinase in tumour biopsies of HGSOC compared to normal adjacent tissue. Additionally, through knockdown and inhibitor approaches, we uncovered a role for this kinase in the proliferation, colony formation, and anchorage independent growth of HGSOC cell. At the mechanistic level, we have discovered a key target of this kinase which is known to be deregulated in Breast and Ovarian cancer.

**Conclusion**

HGSOC is a poor-outcome cancer and patients need new treatments to improve overall survival which is currently <40%. Indeed, the 10-year overall survival has remained unchanged for the past 30 years. We highlight a new target of interest to pursue in pre-clinical animal models of Ovarian cancer, alone and in combination drug strategies, for the treatment of HGSOC.

### EACR23-0859
### Towards Discovery of Novel Targets and Medicines by Antibody Phenotypic Screening on Glioblastoma Stem Cells

*L. Slater[1], A. Mohamed[1], M. Barnard[1], S. Ryman[1], L. Jenkinson[1], S. Haynes[1], M. Groves[2], H. Bulstrode[3], D. Rowitch[3], S. Pollard[4]*

*[1]Cancer Research Horizons, Therapeutic Innovation - Biosciences, Cambridge, United Kingdom*

*[2]AstraZeneca, Biological Engineering, Cambridge, United Kingdom*

*[3]University of Cambridge, Wellcome-MRC Cambridge Stem Cell Institute, Cambridge, United Kingdom*

*[4]University of Edinburgh, Centre for Regenerative Medicine, Edinburgh, United Kingdom*

**Introduction**

Glioblastoma multiforme (GBM), the most common and fatal glioma in adults, accounts for about 50% of gliomas with a dismal patient survival of ~15 months. Genomics and transcriptomic studies have identified four GBM subtypes with variable prognosis and therapy response (classical, mesenchymal, proneural and neural).

The SOX2 transcription factor is involved in the maintenance of the undifferentiated state of cancer stem cells in several tissues and marks rapidly dividing progenitors in both GBM and the foetal human brain. In GBM, a significant fraction of $SOX2^+$ malignant cells is seen across genetically diverse subtypes of GBM and expands to dominate the tumour mass at recurrence.

**Material and Methods**

To identify novel therapeutic targets, we isolated antibodies which bind to $SOX2^+$ primary patient derived GSCs by phage display phenotypic selections using a restricted framework scFv library from AstraZeneca. The phage display selection outputs were screened in a high throughput cell binding assay identifying IgGs with specific binding to GSCs versus normal cell types. We have also established a high-throughput high-content imaging assay for SOX2 expression in GSCs, and used this in a phenotypic screen with a curated antibody array from AstraZeneca consisting of 124 therapeutic grade antibodies currently in the clinic or clinical development.

**Results and Discussions**

We identified 29 IgGs with binding to four primary GSC cultures, 8 of which were specific for GSCs. Furthermore, we identified 12 IgGs currently in the clinic or clinical development which inhibited proliferation/induced cell kill and 4 IgGs which downregulated SOX2 expression.

**Conclusion**

In summary, we present a platform that has been used to identify novel antibodies that bind specifically to GSC cell surface targets, and novel therapeutic targets in GBM which control SOX2 expression and could be targeted by antibodies that are already in clinical development. This is a promising therapeutic avenue that could provide First in Class (FIC) medicines to improve current GBM treatment, overcome resistance and improve clinical outcome.

### EACR23-0875
### Studies on the in vitro and ex vivo metabolism of tumour protease activated taxane prodrugs

*A. Polykandritou[1], H. Spencer[1], A. Serre[1], F. Barnieh[1], G. Ribeiro Morais[1], A. Race[1], S. Shnyder[1], P. Loadman[1], R. Falconer[1]*

*[1]University of Bradford, Institute of Cancer Therapeutics, Bradford, United Kingdom*

**Introduction**

Membrane-type metalloproteinases (MT-MMPs) are endopeptidases involved both in physiological and pathological processes. Their overexpression in various cancer types is linked to the promotion of angiogenesis, invasion and metastasis. Their absence in normal tissues makes the enzyme an ideal target for the selective activation of a prodrug. ICT3205, a peptide prodrug of paclitaxel, and analogues, were designed to release selectively the cytotoxic warhead in the tumour environment upon MT1-MMP (MMP-14) recognition and activation, thereby preventing systemic toxicity.

**Material and Methods**

ICT3205 and the analogues were synthesised and after determination of their cytotoxicity by MTT assay, the *in vitro* metabolism assay was carried out with cells being incubated with the prodrug (1 μM, $1 \times 10^6$ cells/time point), and the release of taxane was monitored over time (0-60 min). For the metabolism *ex vivo*, ICT3205 was added to tumour xenograft (HT1080, PC3) or normal tissue homogenates, and for every time point samples were removed, in order to determine the metabolism of the prodrugs and release of taxanes. Both experiments were repeated with incubation with ilomastat, a broad-spectrum MMP inhibitor. The samples for all the above experiments were analysed by LC/MS. For the detection of tubulin destruction, cells were treated overnight with paclitaxel (10 nM) and ICT3205 (100 nM), followed by tubulin staining with rabbit (R) anti-tubulin mAb, anti-R-488 and DAPI.

**Results and Discussions**

© 2023 The Authors. Molecular Oncology is published by John Wiley & Sons Ltd on behalf of Federation of European Biochemical Societies

The *in vitro* metabolism experiments demonstrated that the release of taxanes from the prodrugs could be correlated to the level of MT1-MMP expression in each cell line, as measured by Western blot. Evidence for selectivity was provided through incubation with ilomastat, which successfully inhibited the metabolism of the prodrugs, while leupeptin (a serine protease inhibitor) did not. *Ex vivo*, the prodrug showed selective metabolism in tumour over normal tissues (liver, kidney), where the release of taxanes was barely detectable. Immunofluorescence revealed similar changes in tubulin filament structure upon treatment with both ICT3205 and paclitaxel, confirming effective taxane release from the prodrug.

**Conclusion**

These metabolic studies successfully demonstrated selective metabolism and release of taxanes from the prodrugs by MT1-MMP, present in tumours and absent in healthy tissues. This approach has significant potential for the selective delivery of highly cytotoxic chemotherapies minimising systemic toxicity.

### EACR23-0897

### Brusatol treatment causes cell death in aggressive lymphomas in vitro and synergizes with Bcl-2 inhibitor

*M. Szmyra*[1], *S. Haingartner*[1], *S. Jantscher*[1], *K. Pansy*[1], *B. Uhl*[1], *H.T. Greinix*[1], *P. Neumeister*[1], *E. Bernhart*[2], *M. Dengler*[3], *B. Klösch*[4], *A.J.A. Deutsch*[1]

[1]*Medical University of Graz, Division of Hematology- Department of Internal Medicine, Graz, Austria*

[2]*Medical University of Graz, Division of Molecular Biology and Biochemistry- Gottfried Schatz Research Center for Cell Signaling- Metabolism and Aging, Graz, Austria*

[3]*Medical University of Graz, Division of Oncology- Department of Internal Medicine, Graz, Austria*

[4]*Ludwig Boltzmann Institute for Arthritis and Rehabilitation, Institute for Pharmaceutical Sciences, Graz, Austria*

**Introduction**

Aggressive lymphomas represent the most common lymphoid malignancies in adults with an increasing incidence. Despite available therapy, one-third of patients experience treatment failure. Therefore, there is a great need to develop new therapeutic strategies. The aim of this study was to investigate the potential of Brusatol, which possesses anti-cancer activity (as previously shown) in the treatment of aggressive lymphomas.

**Material and Methods**

Ten lymphoma cell lines were treated with increasing concentrations of Brusatol (up to 10µM) to determine the IC50 values. Apoptosis induction (Annexin V staining, Caspase-3- and PARP-cleavage) and changes in cell cycle (PI staining) were assessed in cells over 48h of Brusatol treatment. Samples from untreated and Brusatol-treated cells were collected for Western blot analysis. Nascent protein synthesis was evaluated by click chemistry after 4h of Brusatol treatment. Further, co-treatment of Brusatol with inhibitors of Bcl-2, Bcl-XL and Mcl-1, respectively, was performed and Annexin V levels were measured after 24h. Finally, the effect of combining Brusatol with Bcl-2 inhibitor was determined using the Bliss independence model after 24h via TMRE staining.

**Results and Discussions**

Brusatol induced cell growth inhibition in a concentration-dependent manner in all tested cell lines. Based on the results of apoptosis assays, they can be grouped into more and less sensitive cell lines to Brusatol. In more sensitive cell lines, a higher percentage of cells in the subG1 phase was observed, as well as reduced levels of Bcl-2, Bcl-XL, Mcl-1, p53 and Myc upon treatment. mRNA expression analysis showed the reduction of affected proteins occurred mainly at the protein level, that was confirmed by click chemistry assay. Interestingly, cell lines with higher Myc levels were more sensitive to Brusatol treatment. Furthermore, co-treatment of Brusatol with Bcl-2, Bcl-XL and Mcl-1 inhibitors, respectively, revealed a higher apoptotic effect compared to these substances alone. Finally, the combination of Brusatol and Bcl-2 inhibitor synergistically increased lymphoma cell killing.

**Conclusion**

Our data indicate that Brusatol induces cell death in aggressive lymphoma cells, especially in those with higher Myc content. Additionally, the combination of Brusatol with Bcl-2 inhibitor results in enhanced induction of apoptosis. Thus, our study suggests that Brusatol, alone or in combination with Bcl-2 inhibitor, represents a very interesting agent for the development of novel anti-lymphoma therapies.

### EACR23-0901

### CITK catalytic activity inhibition through Lestaurtinb leads to DNA damage, cytokinesis failure and cell death in brain tumors.

*A. Ferraro*[1], *G. Pallavicini*[1], *G. Iegiani*[1], *R. Paolisi*[1], *F. Garello*[2], *V. Bitonto*[2], *E. Terreno*[2], *M. Gai*[2], *C. Mercurio*[3], *M. Varasi*[3], *F. Di Cunto*[1]

[1]*Neuroscience Institute Cavalieri Ottolenghi, Department of Neurosciences "Rita Levi Montalcini" - University of Turin, Turin, Italy*

[2]*University of Turin, Department of Molecular Biotechnology and Health Sciences- University of Turin- Italy, Turin, Italy*

[3]*Experimental Therapeutics Program, FIRC Institute of Molecular Oncology Foundation IFOM, Milan, Italy*

**Introduction**

Medulloblastoma (MB) and gliomas are the most frequent high-grade brain tumors (HGBT) in children and adulthood, respectively. The general treatment for these tumors consists in surgery, followed by radiotherapy and chemotherapy. Despite the improvement in patient survival, these therapies are only partially effective, and many patients still die, making these diseases an unmet medical challenge. Citron kinase (CITK), product of the primary microcephaly gene MCPH17, is required in neural progenitor cells for cytokinesis, mitotic spindle positioning and chromosomal stability. In vivo studies in xenograft models and in SHH MB arising in transgenic mice have shown that CITK deletion inhibits tumor progression. On this basis, we are working on the development of CITK inhibitors as a possible strategy for HGBT treatment.

© 2023 The Authors. Molecular Oncology is published by John Wiley & Sons Ltd on behalf of Federation of European Biochemical Societies

**Material and Methods**
Stemming from published binding data between kinase inhibitors and the kinome, we discovered Lestaurtinib as CITK inhibitor. We therefore tested the biological effects of this inhibitor on different MB and GBM patient derived cell lines and in vivo injecting the drug in MBs arising in SmoA1 transgenic mice. In parallel, we developed a screening aimed at obtaining CITK specific inhibitors.
**Results and Discussions**
Similar to CITK knockdown, treatment of MB cells with 100 nM Lestaurtinib reduces phospho-INCENP levels at the midbody and leads to cytokinesis failure. Moreover, Lestaurtinib impairs cell proliferation, leads to accumulation of DNA double strand breaks and cell death in MB and GBM cells. Finally Lestaurtinib treatment reduces tumor growth and increases mice survival. Moreover, our screening campaign produced several interesting hits that we are functionally validating.
**Conclusion**
Our data indicate that Lestaurtinib produces in MB cells and in GBM cells phenotypes that recapitulate CITK knockdown effects. Reduced cell proliferation and increased mice survival indicate that Lestaurtinib and more specific CITK inhibitors are promising candidates for HGBT treatment, deserving deeper investigation.

### EACR23-0902
### Targeting mitochondrial translation reveals a synthetic lethality strategy to overcome multidrug resistance in MYC-driven neuroblastoma

*K. Borankova[1,2], M. Krchniakova[1,2], L.Y.W. Leck[3,4], J. Neradil[1,2], A. Kubistova[1], P.J. Jansson[3,4], M.D. Hogarty[5,6], J. Skoda[1,2]*
*[1]Masaryk University, Department of Experimental Biology - Faculty of Science, Brno, Czech Republic*
*[2]St. Anne's University Hospital, International Clinical Research Center, Brno, Czech Republic*
*[3]The University of Sydney, Cancer Drug Resistance & Stem Cell Program - School of Medical Science, Sydney, Australia*
*[4]The University of Sydney, Bill Walsh Translational Cancer Research Laboratory - Ko lling Institute, Sydney, Australia*
*[5]The Children's Hospital of Philadelphia, Division of Oncology and Center for Childhood Cancer Re search, Philadelphia, United States*
*[6]University of Pennsylvania, Department of Pediatrics - Perelman School of Medicine, Philadelphia, United States*

**Introduction**
Multidrug resistance (MDR) remains a major challenge in cancer treatment. Mitochondria are central for cell fate decisions and regulation of cell death. We have recently identified a novel mitochondria-mediated mechanism of MDR in a lethal childhood tumor, neuroblastoma (NBL). Mitochondria in relapsed, therapy-resistant NBL have reduced contacts with endoplasmic reticulum, leading to apoptotic insensitivity. Here, we investigated mitochondria as direct targets to overcome this clinically relevant MDR.
**Material and Methods**
We screened potential mitochondrial vulnerabilities using specific inhibitors in paired NBL models (therapy-naïve vs. MDR) derived from tumors of the same children at diagnosis and at relapse. Mitochondrial ribosome (mitoribosome)-specific effects of the leading drug, doxycycline (DOXY), were validated by other ribosomal antibiotics and tested in 12 NBL cell lines, including MYCN-/MYC-amplified and non-amplified clones. We applied live-cell/confocal microscopy, flow cytometry, functional assays, and time-course expression analyses in multiple nervous system tumors and normal fibroblasts to pinpoint mechanisms of the DOXY anticancer activity.
**Results and Discussions**
Targeting mitochondrial translation markedly reduced viability of NBL cells regardless of their MDR but it showed no cytotoxicity in fibroblast controls. Blocking mitoribosomes in NBL cells by DOXY disrupts mitochondrial morphology, impairs cancer stem-like traits, and induces dose-dependent mitochondrial depolarization and apoptosis. Time-course analyses identified the mitochondrial protease OMA1-mediated integrated stress response (ISR) as a key event induced by inhibited mitochondrial translation. The ISR blocks global protein synthesis. We found this favored degradation of MYC proteins in MYC-driven NBL models that overexpressed c-MYC/N-MYC phosphorylated at T58, tagging MYC proteins for rapid degradation which could be fully rescued by proteasome inhibition prior DOXY treatment. Importantly, levels of T58-phosphorylated forms correlated with cell death that was rapidly induced upon inhibition of mitoribosomes in MYC-driven NBL but not in other (MYC-low) models.
**Conclusion**
We identify mitochondrial translation as a novel synthetic lethal target to overcome MDR in MYC-driven NBL. Our study reveals the phospho-dependent turnover of MYC proteins as a major determinant of the mitochondrial ISR-induced cell death, suggesting a mechanism that may underlie vulnerability of MYC-addicted cancers to mitochondria- and ISR-targeted drugs.

### EACR23-0910
### Improvement of Drug Delivery Systems for Hydrophobic Drugs on Ovarian Cancer Treatment

*C.U. Tunc[1,2], G. Kursunluoglu[2], M. Akdeniz[2,3], A.U. Kutlu[2,3], M.I. Han[4,5], M.B. Yerer[2,6], O. Aydin[2,3]*
*[1]University of Utah, Utah Center for Nanomedicine, Salt Lake City, United States*
*[2]Erciyes University, Drug Application and Research Center ERFARMA, Kayseri, Turkiye*
*[3]Erciyes University, Biomedical Engineering, Kayseri, Turkiye*
*[4]University of Auckland, Auckland Cancer Society Research Centre, Auckland, New Zealand*
*[5]Erciyes University, Department of Pharmaceutical Chemistry, Kayseri, Turkiye*
*[6]Erciyes University, Department of Pharmacology, Kayseri, Turkiye*

**Introduction**

© 2023 The Authors. Molecular Oncology is published by John Wiley & Sons Ltd on behalf of Federation of European Biochemical Societies

Ovarian cancer is recognized as a prevalent cancer type among women worldwide and is considered the deadliest among cancers that affect women. Chemotherapy is among the first-choice treatments in cancer treatment, but chemotherapy agents have limited water solubility, and resistance to these agents develops over time, and they are not target-specific. Drug delivery systems are the golden key to overcoming problems that limit the bioavailability of drugs, such as the solubility of drugs in water. In this study, different hydrophobic drugs with chemotherapeutic activity were synthesized and used, then the anti-cancer activity were evaluated of these drugs on ovarian cancer cell after drug delivery with gold nanoparticles (GNP). The low solubility of the synthesized hydrophobic drug candidates in water makes it difficult to evaluate their efficacy and causes them to be used in high doses. A drug delivery system has been developed to increase their bioavailability.

**Material and Methods**

The characterizations of the synthesized GNPs were carried out by SEM, UV-Vis and DLS. Hydrophobic drugs are complexed with GNP by covalent bonding, at room temperature with in shaking condition. The characterization and stabilization of the synthesized hydrophobic drug complexes-GNP were evaluated by comparing wavelengths, size, and charges. Cytotoxicity of these hydrophobic drugs form before and after complex with GNP were evaluated on ovarian cancer cells. In addition, the anti-cancer effect of hydrophobic drug complexes-GNP was performed by cell cycle and apoptosis analysis by flow cytometry.

**Results and Discussions**

Water solubility and distribution of hydrophobic drug complexes-GNP have increased compared to free drugs. Anti-cancer activity of hydrophobic drug complexes-GNPs enhanced on ovarian cancer cells and these complexes showed excellent promising stability for 3 months. With the formation of complexes of hydrophobic drugs, their solubility in water increased, so it was determined that the cytotoxic effect of the drug in ovarian cancer increased. Our results are also very promising for preventing the use of drugs in high doses. A significant increase in apoptosis in ovarian cancer cells was observed with the development of hydrophobic drug complexes-GNP.

**Conclusion**

It has been observed that the effectiveness of hydrophobic drug complexes-GNP is increased compared to free drugs. Complexing hydrophobic drugs gold nanoparticles by covalent bonding has been remarkable in increasing the bioavailability of drugs.

### EACR23-0914
### Sulphamoylated estrone analogues which selectively target the tumour micromilieu radiosensitize cancer cells in vitro

*A. Mercier*[1], *A. Joubert*[1]
[1]*University of Pretoria, Physiology, Pretoria, South Africa*

**Introduction**

Radiation therapy remains a mainstay in the treatment of both primary and metastatic neoplastic lesions. However, this modality is associated with adverse effects and radioresistance. Design of third-generation estrone analogues aimed at preferential tumour localization by increasing their binding affinity to carbonic anhydrase IX which is over-expressed in the acidic neoplastic micro-milieu. Additionally, these analogues are not substrates of the PgP glycoprotein efflux pumps. As microtubule disrupting agents with prominent signalling of the retinoblastoma tumour suppressor protein-, p27Kip1-, and the phosphoinositide 3-kinase pathways, we investigated their potential as radiosensitizing agents.

**Material and Methods**

Murine pre-osteoblastic (MC3T3-E1)- and pre-osteoclastic (RAW 264.7) bone cells, metastatic prostate (DU 145)- and breast (MDA-MB-231) cancer cells, human umbilical vein endothelial cells (HUVECs), as well as haematopoietic stem- and progenitor cells (HSPCs) were used. Cells were exposed to low-dose 2-ethyl-3-*O*-sulphamoyl-estra-1,3,5(10)16-tetraene (ESE-16) for 24 hours followed by a single dose of radiation (4 – 8 Gy) with termination 2-, 24- and 48 hours thereafter. Investigation of the consequent cellular responses was done via spectrophotometry, various microscopy techniques, Western blotting and flow cytometry.

**Results and Discussions**

Compound pre-exposure enhanced the cytotoxic response of the cancer cells to radiation, whereas the non-malignant cells appeared somewhat spared. The combination treatment increased the amount of DNA damage, as seen with an increased number of micronuclei formation and γ-H2A.X foci, whereas the DNA damage repair response was delayed and retarded as represented by Ku70 and ATM signalling. The treatment enhanced reactive oxygen formation, and abrogated cell cycle progression, augmenting the induction of cell death, markedly diminishing long-term survival. The pre-osteoclastic and HSP cells were relatively unaffected by the drugs and retained their ability to differentiate into the haematopoietic lineages, although their differentiated counterparts displayed more cytotoxicity. Cancer cells and HUVECs showed reduced metastatic- and angiogenic signalling.

**Conclusion**

The analogue appeared to have interesting radiosensitizing properties at low doses, decreasing metastatic signalling and tumour cell survival whilst preferentially non-neoplastic cells. Future studies will include 3-dimensional *in vitro*- and murine *in vivo* models.

### EACR23-0918
### Axl-miR-214sponge chimeric aptamer reduces breast cancer and melanoma dissemination

*L. Quirico*[1], *F. Orso*[1], *N. Vitale*[1], *S. Bertone*[1], *P. Cirilo*[1], *C.L. Esposito*[2], *M.B. Stadler*[3], *V. de Franciscis*[2], *D. Taverna*[1]
[1]*Molecular Biotechnology Center MBC "Guido Tarone", Dept. Molecular Biotechnology and Health Sciences- Univ ersity of Torino, Torino, Italy*
[2]*Institute of Endocrinology and Experimental Oncology- C NR-,*
*Department of Biomedical Sciences- National Research Co uncil CNR-, Napoli, Italy*
[3]*Friederich Miescher Institute and Swiss Institute of Bioinf ormatics, Department of Biomedicine- University of Basel.,*

© 2023 The Authors. Molecular Oncology is published by John Wiley & Sons Ltd on behalf of Federation of European Biochemical Societies

*Basel, Switzerland*

**Introduction**

microRNAs (miRNAs) are important regulators of gene expression and are frequently deregulated in tumor progression. We previously showed that the inhibition of the pro-metastatic miR-214 is able to inhibit metastasis formation.

**Material and Methods**

In order to selectively hit tumor cells and reduce miR-214 levels, we generated a chimeric aptamer (conjugate) called axl-miR-214sponge. In detail, we linked GL21.T (axl), an aptamer that specifically binds to *axl*, an oncogenic tyrosine kinase receptor abundantly expressed on cancer cells, but poorly present on normal ones, to miR-214sponge, an oligonucleotide sequence able to block miR-214 functions. qRT-PCR and Western Blot analyses as well as proliferation, motility and mammosphere assays were performed to evaluate the impact of axl-miR-214sponge conjugates or of their negative controls on gene expression and biological functions of treated neoplastic cells. Tumorigenesis and metastatization were also investigated in mice carrying xenotranplants following tail vein treatments with the conjugates.

**Results and Discussions**

Cells treated with axl-miR-214sponge conjugates showed reduced migration, invasion and mammosphere formation of *axl*-expressing breast cancer and melanoma cells, compared to controls. In parallel, expression of miR-214 direct and indirect targets resulted affected. Notably, no effect was detected in cells that did not express *axl*, demonstrating the chimeric aptamer selectivity. Importantly, axl-miR-214sponge conjugates induced necrosis and apoptosis in primary tumor masses and reduced breast cancer metastatization in mice carrying xenotransplants, following systemic treatments.

**Conclusion**

Our data evidence that axl-miR-214sponge chimeric aptamers specifically hit *axl*-expressing cells and are able to reduce metastatic traits and cancer spreading, thus representing new potential therapeutic tools for the treatment of malignant breast cancers and melanomas that could be transferred to the clinics in the near future.

### EACR23-0945

### The combination of the PPARγ agonist pioglitazone and the RXR agonist IRX4204 induces adipocytic differentiation in myxoid liposarcoma

*M. Meroni[1], F. Mirimao[1], I. Craparotta[1], N. Panini[1], P. Ubezio[1], E. Bello[1], M. D'Incalci[2], R. Frapolli[1]*
*[1]Istituto di Ricerche Farmacologiche Mario Negri IRCCS, Department of Oncology, Milan, Italy*
*[2]Humanitas University-, Department of Biomedical Sciences, Milan, Italy*

**Introduction**

Myxoid liposarcoma (MLS) is characterized by a chromosomal translocation leading to FUS-DDTI3 fusion protein expression, that prevents the terminal differentiation from adipoblasts into adipocytes. Trabectedin is able to displace FUS-DDTI3 from the promoters of its target genes restoring adipogenesis, but unfortunately some tumors become resistant. Recent data show that the PPARγ agonist pioglitazone reactivates the adipogenic pathway in MLS preclinical models resistant to trabectedin, improving trabectedin efficacy. However, this approach requires a long time to observe a tumor response, limiting its effectiveness in the presence of rapidly progressing tumors. Since PPARγ heterodimerizes with the retinoid X receptor (RXR), one possible strategy to enhance adipocyte differentiation is to combine trabectedin and pioglitazone with the RXR agonist IRX4204.

**Material and Methods**

DL221 human MLS cell line was treated with pioglitazone (1 μM) and/or IRX4204 (0.06 μM). To evaluate the adipocytic differentiation cells were stained with LD540 and DAPI for fluorescence microscopy and the expression of FABP4 and ADIPOQ was evaluated by RT-PCR. Drug efficacy and tolerability were assessed in MLS patient-derived xenografts ML017 (sensitive), ML017/ET and ML006 (resistant to trabectedin). Mice received pioglitazone (150 mg/kg p.o. qdx28), trabectedin (0.15 mg/kg i.v. q14dx2), IRX4204 (10 mg/kg i.p. qdx28) or their combinations.

**Results and Discussions**

In the MLS DL221 cell line pioglitazone and IRX4204 alone caused lipid droplet accumulation and morphological changes characteristic of mature adipocytes. These effects were more evident when pioglitazone and IRX4204 were combined together. In this group, FABP4 and ADIPOQ expression increased. In ML017 wt and in ML017/ET models, the addition of IRX4204 to trabectedin and pioglitazone further improved the efficacy of treatments with greater and faster tumor shrinkage. Interestingly, despite both pioglitazone and IRX4204 were inactive alone, their combination caused a significant inhibition of tumor growth and complete responses in ML017 wt, even without trabectedin. Also in the ML006 model, the addition of IRX4204 improved tumor growth inhibition during and after treatments.

**Conclusion**

IRX4204 combination with trabectedin and pioglitazone is able to improve their antitumor activity enhancing their differentiating capabilities, causing more rapid and long-lasting tumor responses, potentially allowing the application of this combination even in case of rapid tumor progression.

### EACR23-0989

### Inhibition of FGF-FGFR system as a therapeutic approach in medulloblastoma

*C. Tavani[1], S. Filiberti[1], R. Bortolozzi[2], E. Rampazzo[2], G. Viola[2], R. Ronca[1]*
*[1]Università degli Studi di Brescia, Immunologia e Oncologia Sperimentale, Brescia, Italy*
*[2]University of Padova, Department of Woman's and Child's Health, Padova, Italy*

**Introduction**

Medulloblastoma (MB) is one of the most common malignant brain tumors of pediatric patients, it involves the central nervous system and seems to originate from different populations of neuronal stem cells or progenitor cells. MB is a heterogeneous tumor comprising four distinct molecular subgroups named WNT, SHH, group 3, and group 4. After diagnosis, the treatment involves surgical approach, radiotherapy and adjuvant

© 2023 The Authors. Molecular Oncology is published by John Wiley & Sons Ltd on behalf of Federation of European Biochemical Societies chemotherapy.The Fibroblast Growth Factor (FGF)/FGF Receptor (FGFR) system is a pleiotropic ligand/receptor pathway composed by 23 FGF ligands and four main tyrosine kinase FGFRs that mediate a plethora of physiological and pathological processes. In cancer FGF/FGFR are responsible for key processes like tumor cell proliferation, metastasis, angiogenesis, drug resistance and others.In literature, an aberrant activation of this system has been reported and associated with different MB cases, but pivotal studies on the role of FGFR blockade in MB treatment are still missing.

**Material and Methods**

In this study we used human MB cell lines (DAOY, HD-MB03 and D283) and MB cells resistant to chemotherapy (DAOY-R and HD-MB03-R). The presence of the FGF/FGFR system was assessed by qPCR and its activation by Western blot for phospho-FGFR. The impact of FGFR inhibition was studied using a clinical grade selective pan-FGFR inhibitor (erdafitinib). Western blot was used to verify the inhibition of FGFRs, while the effect on proliferation was analysed using CCK-8 after 48 hours of treatment.

**Results and Discussions**

Western blot and qPCR revealed that all the MB cells analysed express FGFRs and FGFs and that the FGFRs are activated. Interestingly, D283 cells turned out to have the highest activation of FGFR, and both DAOY-R and HD-MB03-R cells resulted to have a higher activation of the receptor in comparison with the respective wild type cells. For all the cell lines we confirmed that increasing concentrations of erdafitinib were able to impair the activation of FGFR, and this resulted in a significant reduction of cell proliferation when MB cells were treated with the FGFR inhibitor for 48 hours. Starting from these data further analyses will be performed to identify the intracellular pathways and mediators affected by FGFR inhibition.

**Conclusion**

In conclusion, our preliminary results confirm that FGF/FGFR system is present in the different subgroups of MB and suggest that the inhibition of FGFR system can have a promising anti-tumoral effect.

### EACR23-0992

### Dopamine receptor D2 (DRD2) antagonists have anti-cancer effects in solid tumors and upregulate the expression of Heme Oxgenase-1

*M. Mei[1,2], B. Miao[1], J. Hoheisel[1], N. Giese[3], T. Hackert[4]*

*[1]German Cancer Research Center, Division of Functional Genome Analysis, Heidelberg, Germany*

*[2]Heidelberg University, Medical Faculty Heidelberg, Heidelberg, Germany*

*[3]Heidelberg University Hospital, Department of General Visceral and Transplantation Surgery, Heidelberg, Germany*

*[4]Medical Center Hamburg-Eppendorf UKE, Department of General- Visceral and Thoracic Surgery, Hamburg, Germany*

**Introduction**

Dopamine receptor D2 (DRD2) is a member of the G protein-coupled receptors and is highly expressed in various cancers. In a previous study, DRD2 antagonists were shown to have a strong positive effect on pancreatic cancer (PDAC), reducing tumor volume and basically stopping metastasis altogether (Gastroenterology 151, 1218-1231; doi: 10.1053/j.gastro.2016.08.040). We aimed at investigating the response of also other solid tumors to DRD2 antagonists and to explore, if the same or other mechanisms as in PDAC are responsible for a therapeutic effect.

**Material and Methods**

We used pan-cancer analysis to analyze DRD2 expression in various cancer types. We detected cytotoxity of DRD2 antagonists in eight different cell lines. Reversal experiments of pimozide-induced cytotoxicity were conducted using four different cell death inhibitors (apoptosis inhibitor; autophagy inhibitor; ferroptosis inhibitor; necroptosis inhibitor). Transcriptome analysis was performed comparing pimozide-treated and control cells. Potential gene candidates of interest were validated by qPCR and Western blot. Knockdown was performed using small interfering RNAs. Cell cycle and apoptosis assays were performed by flow cytometry.

**Results and Discussions**

DRD2 antagonists induced cytotoxicies in eight cell lines of four tumor types (pancreatic cancer, colorectal cancer, ovarian cancer and gastric cancer). Pimozide-induced cytotoxicities were partially reversal by apoptosis and autophagy inhibitors, indicating that pimozide induced apoptosis and autophagy in cancer cells. Transcriptome analysis revealed that the gene of Heme Oxgenase-1 (*HMOX1*) is upregulated in treated cells. Knockdown of *HMOX1* exacerbated pimozide-induced cell death and cell cycle arrest, suggesting that reduction of *HMOX1* sensitized tumor cells to pimozide.

**Conclusion**

We demonstrated that DRD2 antagonists induced different types of cell death in cell lines of four solid tumor types. They upregulated the expression of *HMOX1*.
Reduced *HMOX1* expression achieved better anticancer effects concomitantly with pimozide treatment.

### EACR23-1040

### Selective inhibition of the nuclear export is a therapeutic strategy to overcome resistance to tyrosine kinase inhibitors in ALK-rearranged lung cancer

*M.V. Di Marco[1], M. Costanza[2], F. Picca[3], R. Taulli[3], E. Patrucco[1], L. Bonello[1], S. Novello[3], S. Mathas[2], R. Chiarle[1], C. Voena[1]*

*[1]University of Torino, Department of Molecular Biotechnology and Health Sciences, Torino, Italy*

*[2]Charité-Universitätsmedizin Berlin and Max-Delbrück-Center MDC for Molecular Medicine, Department of Hematology- Oncology and Cancer Immunology, Berlin, Germany*

*[3]University of Torino, Department of Oncology, Orbassano, Italy*

**Introduction**

© 2023 The Authors. Molecular Oncology is published by John Wiley & Sons Ltd on behalf of Federation of European Biochemical Societies

EML4-ALK fusion has been established as a major oncogenic-driver in Non-Small Cell Lung Cancer (NSCLC), due to the constitutive activation of ALK-kinase activity leading to aberrant cell proliferation and survival. ALK tyrosine kinase inhibitors (TKIs) show potent efficacy in ALK-driven NSCLC, but the development of resistance limits their long-term clinical impact. Of note, there is an unmet medical need for ALK+NSCLC patients with co-occurring TP53 mutations, as they suffer from unfavorable outcomes after treatment. The nuclear export protein exportin 1 (XPO1) is necessary for tumor progression and survival and selective inhibitors of nuclear export (SINEs) represent a promising therapy in single or in combination with standard therapies. We investigated *in vitro* and *in vivo* the efficacy of XPO1 inhibitors of first (selinexor) and second (eltanexor) generation in combination with ALK TKIs in ALK TKI-sensitive and resistant ALK+NSCLC cell lines and in ALK TKI-resistant patient-derived organoids (PDOs).

**Material and Methods**

ALK+NSCLC cell lines, ALK TKI-resistant patient-derived ALK+NSCLC cell lines and PDOs were treated with SINEs alone and in combination with ALK TKIs. Cell viability and cell death were evaluated, and cell cycle distribution was determined by propidium iodide staining. Treatment efficacy was also evaluated *in vivo.*

**Results and Discussions**

SINE compounds significantly affected cell viability of ALK TKI-sensitive and -resistant ALK+ NSCLC cell lines and PDOs and potentiated the efficacy of ALK TKIs. XPO1 inhibition induced cell cycle arrest at the G1 phase followed by cell death and promoted the accumulation in the nucleus of p53 ALK+ NSCLC cell lines expressing wild-type p53. However, the p53 mutational status did not affect SINE compounds' efficacy since they induced apoptosis in both p53 wild-type and mutated ALK+ NSCLC cells. The expression levels of p21 increased upon XPO1 inhibition, whereas the expression levels of anti-apoptotic proteins proportionally decreased over time. Eltanexor showed a stronger effect than Selinexor potently inducing PARP cleavage. Consistently with *in vitro* data, we observed that selinexor impaired tumor growth *in vivo*, both in EML4-ALK transgenic mice and in ALK TKI-resistant xenograft tumors in mice.

**Conclusion**

These data support the therapeutic value of SINEs alone or in combination with ALK-TKIs for the treatment of ALK+NSCLCs, even after their loss of responsiveness to targeted therapy and regardless of their p53 mutational status.

### EACR23-1046

### Novel alginate-based and silica-based therapeutic nanoparticles functionalized with aptamers for specific triple-negative breast cancer cells treatment

*S. Camorani[1], L. Agnello[1], A. d'Argenio[1], R. Nilo[1], M. Fedele[1], A. Zannetti[2], M. Comes Franchini[3], L. Sambri[3], L. Ricciardi[4], L. Cerchia[1]*

*[1]Institute of Experimental Endocrinology and Oncology "G . Salvatore", National Research Council, Naples, Italy*

*[2]Institute of Biostructures and Bioimaging, National Research Council, Naples, Italy*

*[3]University of Bologna, Department of Industrial Chemistry "Toso Montanari", Bologna, Italy*

*[4]NANOTEC Institute of Nanotechnology, National Research Council, Rende CS, Italy*

**Introduction**

Triple-negative breast cancer (TNBC) is an aggressive tumor with poor prognosis. We selected a panel of nuclease-resistant 2'Fluoro-pyrimidine RNA aptamers able to specifically bind to surface proteins expressed on TNBC cells without recognizing other breast cancer subtypes and non-tumor cells. These aptamers have been extensively used to deliver drug-loaded PLGA-based polymeric nanoparticles to TNBC cells and tumors implanted in mice. Herein, we report the synthesis and characterization of two other types of aptamer-functionalized nanosystems that we are evaluating as platforms for combined chemotherapy, photodynamic therapy (PDT) and photothermal therapy (PTT) in TNBC.

**Material and Methods**

The first nanosystem consists of an alginate-derived core linked to red-emissive carbon dots and doxorubicin, and functionalized with NH2-terminated TNBC sTN58 aptamer (RCDs@Alg_2S-Doxorubicin-TN58). The second nanosystem is composed of a gold core and a silica shell, with photosensitive ability, thanks to the water-soluble iridium (III) complex (Ir) embedded into the nanoparticle (Ir-Au$SiO_2$). Ir-Au$SiO_2$ surface was conjugated with either the $NH_2$-terminated anti-EGFR CL4 (Ir-AuSiO2_CL4) or anti-PDGFRβ Gint4.T (Ir-Au$SiO_2$_Gint4.T), or both the aptamers for dual targeting. Cell targeting/uptake of nanosystems was evaluated by confocal microscopy. Cytotoxicity and photodynamic evaluation of the nanovectors were tested in TNBC cells and their chemoresistant derivatives. Nanoparticles unconjugated or conjugated with a scrambled aptamer were used as negative controls.

**Results and Discussions**

Our results show rapid and aptamer-guided internalization of both alginate- and silica-based nanosystems in TNBC MDA-MB-231 and BT-549 cells. RCDs@Alg_2S-Doxorubicin-TN58 resulted significantly more cytotoxic than free drug and untargeted nanovectors, both unconjugated and functionalized with the scrambled aptamer. Moreover, a significant improvement in PDT efficacy was obtained in the presence of Ir-AuSiO2_CL4 and Ir-AuSiO2_Gint4.T compared to untargeted nanovectors. Studies in TNBC animal models are on-going to confirm the tumor targeting and therapeutic utility of our nanovectors.

**Conclusion**

Our study proposes novel and safe cancer-targeted multifunctional nanosystems with excellent potential for combined PDT and PTT approaches in TNBC. Furthermore, the availability of a panel of aptamers targeting TNBC offers the possibility of using them to decorate different drug-loaded nanovectors for combination treatment of distinct TNBC phenotypes.

### EACR23-1057

### Communication is key: dental stem cell-mediated suicide gene therapy for oral

© 2023 The Authors. Molecular Oncology is published by John Wiley & Sons Ltd on behalf of Federation of European Biochemical Societies

### cancer

J. Van den Bosch[1], S. Fieten[1], N. Caz[1], I. Lambrichts[2], E. Wolfs[1]
[1]Biomedical research institute BIOMED- Hasselt University,
Lab for Functional Imaging and Research on Stem Cells FIERCE Lab, Diepenbeek, Belgium
[2]Biomedical research institute BIOMED- Hasselt University, Laboratory for histology and regeneration, Diepenbeek, Belgium

**Introduction**
Oral squamous cell carcinomas (OSCC) are malignancies in the oropharynx and the oral cavity. These tumors are often associated with alcohol and tobacco use as well as with HPV infection. To reduce (systemic) side effects and save healthy tissue, it is essential to establish targeted and specific therapeutic strategies. We aim to develop a new therapy for OSCC based on dental pulp stem cell (DPSC)-mediated suicide gene therapy. Hence, we use herpes simplex virus type 1-thymidine kinase (HSV1-TK)-overexpressing DPSC (HSV1-TK$^+$ DPSC) in combination with the non-toxic prodrug ganciclovir (GCV). Upon phosphorylation by HSV1-TK, GCV turns into its toxic metabolite and induces cell death. Gap junction intercellular communication between DPSC and OSCC cells is essential to pass the cytotoxic GCV to the OSCC cells, leading to a successful bystander killing effect of tumor cells.

**Material and Methods**
We optimized a DPSC/OSCC co-culture model in 2D and in 3D. Both cell types are seeded in a 1:1 ratio after cytoplasmic staining to enable later distinguishment. Gap junction formation between DPSC and OSCC cells in co-cultures is evaluated via immunocytochemistry for connexin-43. Moreover, HSV1-TK$^+$ DPSC-mediated OSCC cell killing is assessed in 2D and 3D hydrogels by Incucyte confluency analysis and alamarBlue viability assays.

**Results and Discussions**
The presence of connexin-43 in the 2D and 3D DPSC/OSCC co-cultures suggests gap junction formation. Moreover, we successfully demonstrated HSV1-TK$^+$ DPSC-mediated OSCC cell killing *in vitro* in our DPSC/OSCC co-culture system.

**Conclusion**
In conclusion, our results suggest that gap junctional intercellular communication is responsible for DPSC-mediated OSCC cell killing in 2D and 3D co-cultures *in vitro*. These data indicate that DPSC are promising carriers to be used in a novel targeted therapy for OSCC. Future research is required to gain more in-depth knowledge in their *in vivo* use.

### EACR23-1087
### NEW EXPERIMENTAL APPROACHES TO DEEPEN INTO THE ROLE OF TGF-β IN LIVER CANCER.

A. Cantos Cortés[1], I. Peñuelas-Haro[1], M. Ruiz de Galarreta[2], P. Molina-Sanchez[2], A. Lujambio[2], E. Ramos[3], T. Serrano[3], A. Alay[4], E. González-Sánchez[5], I. Fabregat[5]
[1]Bellvitge Biomedical Research Institute IDIBELL- CIBERehd ISCIII, TGF-beta and Cancer Group - Oncobell Program, Barcelona, Spain
[2]Icahn School of Medicine at Mount Sinai, Department of Oncological Sciences, New York, United States
[3]University Hospital of Bellvitge HUB - IDIBELL- Faculty of Medicine and Health Sciences- University of Barcelona UB- CIBERehd ISCIII, TGF-beta and Cancer Group. Oncobell Program, Barcelona, Spain
[4]Catalan Institute of Oncology ICO – IDIBELL, Unit of Bioinformatics for Precision Oncology, Barcelona, Spain
[5]Bellvitge Biomedical Research Institute IDIBELL- CIBERehd ISCIII, TGF-beta and Cancer Group- Oncobell Program, Barcelona, Spain

**Introduction**
Liver cancer has increased in incidence in recent years, shows a high mortality rate and moderate response to treatment. Transforming Growth Factor-beta (TGF-β) is emerging as a potential therapeutic target in cancer, particularly combined with immune checkpoint inhibitor therapies. However, it is a pleiotropic cytokine that could be exerting different functions. Molecular gene signatures reflecting the TGF-β oncogenic arm are being identified in hepatocellular carcinoma (HCC), but no efficient biomarkers of potential utility in the clinics have been proposed, except the circulating level of TGF-β1. Moreover, there are no evidence reflecting the dependency of TGF-β in stroma cells during cancer associated fibroblasts activation or immunosuppression in liver cancers.

**Material and Methods**
We present two new mouse model cell lines obtained from tumors induced by hydrodynamic tail vein injection of two different transposon vector construction. These cells represent a valuable model to analyze whether TGF-β inhibitors could be a therapeutic option in HCC.

**Results and Discussions**
The characterization of these cell lines show that they have a different phenotype and response to TGF-β. AL1099 cell line shows an epithelial phenotype and responds to TGF-β in terms of growth inhibition and apoptosis. However, they also undergo EMT in response to TGF-β. AL1184 cell line shows a mesenchymal phenotype and lack of response to TGF-β in these terms. These results indicate that these new cell models may be helpful to better analyze the molecular mechanism that produces this differential response to TGF-β. Additionally, we present new *in vitro* 3D co-culture models, where we study the effect of TGF-β activation or inhibition on the different cell types and the co-interactions among them. AL1099 cells cultured in 3D maintain the response to TGF-β in terms of tumor suppression both in absence and in presence of mouse macrophages. Finally, data about the expression of TGF-β or its targets in HCC patients show that those patients with high TGFB1 levels present higher microvascular invasion and poorer patient overall survival.

**Conclusion**
Overall, these new experimental models will help to advance in the knowledge about the functions of TGF-β in HCC tumor cells and stroma. The final aim is the

© 2023 The Authors. Molecular Oncology is published by John Wiley & Sons Ltd on behalf of Federation of European Biochemical Societies identification of biomarkers reflecting activation of the TGF-β oncogenic arms, related to its pro-tumorigenic/metastatic and immunosuppressive actions.

*This study is funded by Spanish Association for Cancer Research(AECC) #PRYGN211279FABR, Spain.*

### EACR23-1109
### Driving discovery of covalent inhibitors by measuring in cellulo kinetics of on-target engagement

*V. Kantae[1], R. Polanski[2], H.J. Lewis[3], A. Haider[1], D. Barratt[1], B. Srinivasan[1]*
*[1]AstraZeneca, Mechanistic and Structural Biology-Discovery Sciences, Cambridge, United Kingdom*
*[2]AstraZeneca, Global Portfolio and Project Management Organisation, Cambridge, United Kingdom*
*[3]AstraZeneca, Oncology R&D, Cambridge, United Kingdom*

**Introduction**
Covalent inhibition of kirsten rat sarcoma (KRAS) mutants is an emergent anti-cancer therapeutic strategy, especially due to prolonged target occupancy. However, primary engagement by such probes is generally monitored using biochemical assays and a gap exists in translating this to cellular target engagement (TE). We addressed this by developing a cellular mass spectrometry (MS) proteomics workflow to measure on-target engagement of covalent molecules.

**Material and Methods**
A $KRAS^{G12C}$ specific covalent probe, compound 25, was used to establish the proteomic workflow. Biochemical TE of intact protein or $KRAS^{G12C}$ peptide by compound 25 was confirmed using RapidFire-QTOF MS. TE of $KRAS^{G12C}$ peptide in a dose-dependent manner was then used to standardize an ultra-performance liquid chromatography (UPLC) coupled triple quadrupole (QQQ) multiple reaction monitoring (MRM) MS workflow. *In cellulo* studies were performed in the $KRAS^{G12C}$ heterozygous cell line NCI-H358 to quantify TE and allele selectivity of compound 25 using the UPLC-QQQ MRM MS platform. Cells were dosed with compounds 25 in 6-well plates at 10 μM for 4h for TE or at different doses (0.1, 0.3, and 1 μM) across several time-points (0-4 h) for TE kinetics. For generating dose response curves, cells were treated with 10-point logarithmic titration (0.3 nM to 30 μM) of compound 25 or close analogues in 96-well plates. In each case, following compound exposure, KRAS was immunoprecipitated in cell lysates with anti-RAS antibody and analysed using the UPLC-QQQ MRM MS workflow.

**Results and Discussions**
A biochemical second-order rate constant kmod/KIapp of 6232 M–1 s–1, indicative of high potency, was reported for compound 25 using the UPLC-QQQ MRM MS platform. In cellulo results using UPLC-QQQ MRM MS showed 96% TE with $KRAS^{G12C}$ peptide and demonstrated allele selectivity as dose-dependent TE was observed with $KRAS^{G12C}$ peptide, but not with WT peptide. *In cellulo* kinetics measurement estimated a value of 248 ± 73 M–1 s–1 for kmod/Kiapp, which may be limited by factors affecting actual concentration of inhibitor available in cells for TE. Finally, the cellular TE potencies for compound 25 and analogues estimated using this method correlated well with potency estimates from a target-distal cellular imaging assay.

**Conclusion**
The sensitive UPLC-QQQ MRM MS workflow established allows evaluation of biochemical and cellular target engagement kinetics of covalent inhibitors at a high-throughput scale, opening avenues for application in early drug discovery.

### EACR23-1119
### Using arrayed CRISPR screening approaches and CRISPR engineered cell lines in the drug discovery process

*M. Maerken[1], P. Deng[2], K. Holden[3]*
*[1]Synthego, Technical Specialist, Redwood City, United States*
*[2]Synthego, Product Marketing Manager, Redwood City, United States*
*[3]Synthego, Head of Science and Applied Research, Redwood City, United States*

**Introduction**
Over the past decade, the genome editing technology CRISPR-Cas has revolutionized translational medicine and biomedical research. One of the many applications of CRISPR/Cas is to create relevant cellular models, allowing researchers to better understand the impact of genetics on disease development across therapeutic areas. Being able to create these models at scale is a game changer for drug discovery and therapies alike.

**Material and Methods**
Synthego has developed automated platforms for synthesizing CRISPR sgRNAs, at both RUO and GMP scale and for engineering cell lines at scale using the CRISPR-Cas technology.In our gene knockout approach, guide RNAs are multiplexed to produce a high likelihood of gene disruption.

**Results and Discussions**
Here we show how libraries of gene knockout guide RNAs can be used in an arrayed CRISPR screening approach to identify genes associated with cell survival and death, which could be candidates for molecularly targeted drugs. In addition, we show that cells pre-engineered with gene knockouts are valuable for validation studies when identifying genes associated with cancer drug resistance.

**Conclusion**
Synthego's automated CRISPR platform offers great precision and increased throughput. It greatly impacts the drug discovery landscape by accelerating the progress of many research projects and potentially lowering attrition rates.

### EACR23-1136
### TYROSINE KINASE INHIBITORS AFFECT THE INTERNALISATION RATES OF ANTIBODY-DRUG CONJUGATES IN HER2-POSITIVE BREAST CANCER

*N. Ashfield[1], A.F. Mahdi[1], N.T. Conlon[1], J. Crown[1,2], D.M. Collins[1]*
*[1]Dublin City University, Cancer Biotherapeutics- National Institute for Cellular Bio*

© 2023 The Authors. Molecular Oncology is published by John Wiley & Sons Ltd on behalf of Federation of European Biochemical Societies technology, Dublin, Ireland
[2]Saint Vincent's University Hospital,
Department of Medical Oncology, Dublin, Ireland
**Introduction**
Targeted treatments for HER2-positive (HER2+) breast cancer (BC) include tyrosine kinase inhibitors (TKIs) lapatinib, neratinib and tucatinib, and antibody-drug conjugates (ADCs) TDM1 and TDXd. Research suggests that combining TKIs with ADCs may enhance cytotoxicity, and the internalisation rate of ADCs is a factor in their efficacy. This study investigates the impact of lapatinib, neratinib, tucatinib, and afatinib (approved in EGFR-mutant lung cancer) on internalisation rates and anti-proliferative effects of TDM1 and TDXd in two HER2+ BC cell lines: HCC1569 (oestrogen receptor (ER)-negative) and BT474 (ER-positive).
**Material and Methods**
Anti-proliferative effects of TKI/ADC combinations were assessed via 5-day acid phosphatase assay. Calcusyn software generated combination index (CI) values. A CI > 1 represents antagonism; CI < 1 is synergistic. Two-way ANOVA confirmed synergy or antagonism. ADC internalisation rates (with/without TKI co-treatment) were assessed over 24 hours using a pH-sensitive fluorescent antibody-label on an Incucyte live-cell imager.
**Results and Discussions**
In both cell lines, TDM1 showed greater internalisation than TDXd. For the HCC1569 cell line, TKIs enhanced internalisation rates of both ADCs. In this cell line, both ADCs showed synergy with afatinib and neratinib (CI: 0.87±0.10 and 0.85±0.07 for afatinib plus TDM1 and TDXd respectively; 0.77±0.07 and 0.85±0.06 for neratinib plus TDM1 and TDXd respectively). Lapatinib and tucatinib displayed antagonism (CI: 1.21±0.14 and 1.38±0.22 for lapatinib plus TDM1 and TDXd respectively; 1.37±0.16 and 1.28±0.11 for tucatinib plus TDM1 and TDXd respectively). However, by ANOVA, lapatinib+TDM1 showed no significant improvement over single-agent; all other combinations were significantly better than single-agent. In the BT474 cell line, lapatinib and tucatinib significantly reduced ADC internalisation. All TKIs showed antagonism with TDM1 (CI: 1.41±0.21; 1.39±0.10; 1.12±0.16; 2.07±0.45 for afatinib, lapatinib, neratinib and tucatinib respectively). CI values are not used for BT474 TDXd combinations as single-agent TDXd did not reach IC50. ANOVA showed that afatinib+TDM1, lapatinib+TDM1, lapatinib+TDXd and tucatinib+TDXd were significantly more cytotoxic than single-agent.
**Conclusion**
TDM1 shows greater internalisation than TDXd. TKI co-treatment results in similar or greater cytotoxicity of ADCs in both cell lines, but only enhances internalisation in HCC1569. Further work will identify optimal TKI/ADC combinations for clinical investigation.

### EACR23-1137
### Targeting non-oncogene addiction as new combined therapeutic strategy to overcome TKI-induced resistance in NSCLC

*C. Terlizzi[1], C. De Rosa[2], C.C. Tufano[3], A. Ariano[3], C.M. Della Corte[2], F. Iommelli[3], S. Del Vecchio[1], V. De Rosa[3]*
[1]University of Naples "Federico II", Department of Advanced Biomedical Sciences, Naples, Italy
[2]University of Campania Luigi Vanvitelli- Italy, Department of Precision Medicine, Naples, Italy
[3]National Research Council, Institute of Biostructures and Bioimaging, Naples, Italy
**Introduction**
In oncogene-driven NSCLC we previously demonstrated that oncogene inhibition by tyrosine kinase inhibitors (TKIs) causes a reversal of Warburg effect associated with increase of OCR and apoptosis and decrease of proliferation. Unfortunately, these targeted therapies led to the development of drug-resistant clones and metastases. Here, we investigated an innovative therapeutic strategy, called non-oncogene addiction (NOA), based on targeting essential genes that support tumor phenotype. In particular, we focused on ATM/ATR involved in DNA damage response (DDR); PKM2, PDK1, LDH-A and complex I of OXPHOS involved in energy metabolism and Bcl-2/Bcl-xL involved in antiapoptotic processes.
**Material and Methods**
H1993, H1975 and A549 oncogene-driven NSCLC cells were treated with TKIs (crizotinib, osimertinib or erlotinib) and in parallel with a combination of two of selected NOA inhibitors (DCA, benserazide, oxamate, IACS-10759, KU55933, M4344 and ABT-263). Firstly, we scheduled a different treatment doses and times to identify the best drug combination using the CompuSyn software. To evaluate the synergistic effects of drug combinations compared to TKIs, we investigated the expression levels of phosphorylated forms of oncogene signaling pathways (WB), metabolic phenotype switch (glucose, ATP, OCR and ECAR) and modulation of apoptotic (fluorescence microscopy) and proliferation markers.
**Results and Discussions**
We firstly demonstrated a non-canonical role of PDK1 as a negative regulator of apoptosis, its silencing is associated with an increase of BAD and a concomitant decrease of Bcl-2/Bcl-xL at mitochondria interface in NSCLC. This pro-apoptotic status was confirmed in ex vivo shPDK1 tumors associated with an increase of BIM and cleaved lamin A/C and a decrease of HIF-1α, Bcl-2/Bcl-xL and mutant p53. In addition, we observed that PKM2 and LDH-A silencing reduced c-Myc and cyclin D1 expression respectively, while ATM downregulation caused a slight increase of BIM levels and a decrease of key glycolytic proteins and OXPHOS complex subunit proteins. Finally, PDKs inhibition with DCA caused a significant dose-dependent decrease of glucose consumption and increase of OXPHOS subunits.
**Conclusion**
Our preliminary data suggest that targeting these proteins may destabilize tumor environment thus coupling metabolic phenotype and DDR to drug resistance. The major translational relevance of this study is to exploit these new targets for innovative and improved therapeutic strategies in comparison to TKI therapies in NSCLC patients.

### EACR23-1146
### All-trans retinoic acid modulates the retinoic acid receptor signaling pathway and exhibits antineoplastic activity in

© 2023 The Authors. Molecular Oncology is published by John Wiley & Sons Ltd on behalf of Federation of European Biochemical Societies

### Merkel cell carcinoma cells

C. Mazziotta[1], G. Morciano[1], C.F. Cervellera[1], G. Badiale[1], G. Tonnini[1], G. Di Mauro[1], P. Pinton[1], A. Touzé[2], P. Gaboriaud[2], M. Tognon[1], F. Martini[1], J.C. Rotondo[1]
[1]Universtià di Ferrara, Medical Sciences, Ferrara, Italy
[2]University of Tours, ISP "Biologie des infections à polyomavirus" Team- UMR INRAE 1282, Tours, France

**Introduction**

Merkel cell carcinoma (MCC) is a rare but aggressive skin cancer. About 80% of MCCs, are linked to oncogenic Merkel cell polyomavirus (MCPyV). Since the currently available MCC therapeutic options are remarkably limited, novel therapeutic approaches are required. The biological activity of all-trans retinoic acid (ATRA) is mediated by RAR/RXR receptors, which are ligand-dependent transcription factors that activate genes crucial for cell differentiation. Dysregulations of RAR/RXR receptors lead to carcinogenesis. Although a strong *in vitro/in vivo* antitumor activity of ATRA has been proved in different carcinoma types, the effect of this drug in MCC is still unknown.

**Material and Methods**

Herein, we investigated the antineoplastic effect of ATRA *in vitro* in MCPyV-positive (MCCP), i.e., PeTa and WaGa, and -negative (MCCN), i.e., MCC13 and MCC26, MCC cell lines and control, normal human lung fibroblasts MRC5. The antineoplastic effect of ATRA was evaluated by testing MCC cell proliferation, migration and colony formation abilities. Apoptosis/cell death were evaluated *via* Annexin-V and propidium iodide (P.I.) assays. Apoptosis was molecularly evaluated by RT$^2$ Profiler PCR mRNA array and by western blot (WB) analysis. Retinoic pathway was evaluated by RT$^2$ Profiler PCR mRNA array.

**Results and Discussions**

ATRA treatment led to a significant reduction in MCC cell proliferation, migration and clonogenicity, while increasing apoptosis/cell death in MCC cell lines compared to untreated cells. MCCP cells were slightly more ATRA-sensitive compared to MCCN cells. No significant effects have been found in the ATRA-treated control cell line. Gene expression array indicated a significant overexpression of several pro-apoptotic genes in MCC cells. High levels of pro-apoptotic proteins have been found following ATRA treatments in MCC cells, while being almost undetectable in untreated cells. Pro-apoptotic markers were almost undetectable in ATRA-treated MRC5. Numerous retinoic signaling genes, including BMP2, FOXA1 and MAFB, were differentially expressed in ATRA-treated MCC cells compared to untreated cells.

**Conclusion**

Overall, our *in vitro* data indicate that ATRA is effective in reducing MCC cell growth, while presenting strong pro-apoptotic effects and favoring cell death, by modulating the retinoic receptor pathway. These results, for the first time, point to ATRA as a potential novel effective antineoplastic drug for the MCC therapy.

### EACR23-1190
### Epigenetic targeting of MECOM dependency in ovarian cancers

I. Singh[1], M. Kumar[2], P. Agarwal[1]
[1]Amity University- Noida- Uttar Pradesh- India, Amity Institute of molecular medicine and stem cell researc h, Noida, India
[2]Amity University- Noida- Uttar Pradesh- India, Amity Institute of Genome Engineering, Noida, India

**Introduction**

Ovarian cancer is one of the most lethal gynecological cancers exhibiting poor survival rates and high incidences of cancer relapse. Patients develop resistance to standard chemotherapy while the use of targeted therapy is limited due to the non-availability of specific molecular targets. Hence, identifying targetable oncogenes that the tumor cells are addicted to, is required for better patient treatment and management. The Cancer Genome Atlas (TCGA) revealed 22% copy number amplification of MDS1 and EVI1 Complex Locus (MECOM) in tumor samples of ovarian cancer patients. The molecular function of MECOM is largely unknown. In this study, we unraveled MECOM as an oncogene regulating tumor cell proliferation that can be targeted directly or indirectly by epigenetic mechanism.

**Material and Methods**

Ovarian cancer OVSAHO and SKOV3 cells demonstrating copy-number amplification of MECOM were selected and overexpression was confirmed at RNA/protein levels. MECOM loss of function studies was conducted and tested for proliferation, cell cycle, and migration defects. Pan histone demethylase (HDM) inhibitor was used to understand the epigenetic regulation of MECOM.

**Results and Discussions**

MECOM silencing in OVSAHO/SKOV3 cells led to significant proliferation defects and G2/M arrest. MECOM deficiency also inhibited SKOV3 migratory potential by downregulating EMT genes. This is caused by a prominent reduction in ERK1/2 phosphorylation mediated by MECOM depletion. We investigated potential epigenetic ways to inhibit MECOM using available epigenetic inhibitors. We identified ZJIB-04, a pan-histone demethylase inhibitor, as an epigenetic regulator of MECOM expression. Interestingly, ZJIB-04 treatment led to proliferation defect, G2/M arrest, and diminished ERK1/2 signaling phenocopying MECOM depletion. We find that ERK1/2 signaling affected EGR1 levels which in turn reduced ZEB1 expression and reversed EMT. ERK1/2 signaling also mediated G2/M arrest by inducing GADD45A and GADD45B. ZJIB-04 most likely transcriptionally regulates MECOM expression via histone H3K9 methylation, which is under detailed investigation.

**Conclusion**

Collectively, our study demonstrates cancer cell dependency on MECOM for cellular proliferation and migration. Further, targeting MECOM directly or epigenetically by altering H3K9me3 might be a potential therapeutic strategy for MECOM-dependent ovarian malignancies.

### EACR23-1193
### The therapeutic potential of AZD-7648, a DNA-PK inhibitor, on acute myeloblastic leukemia

B. Lapa[1,2,3], M.I. Costa[1,2,3], D. Figueiredo[1], J. Jorge[1,2,3,4], R. Alves[1,2,3,4], A.R. Monteiro[1], M.R. Silva[5], L. Carvalho[5],

© 2023 The Authors. Molecular Oncology is published by John Wiley & Sons Ltd on behalf of Federation of European Biochemical Societies

A.B. Sarmento-Ribeiro[1,2,3,6], A.C. Gonçalves[1,2,3,4]
[1]Laboratory of Oncobiology and Hematology and Universi ty Clinic of Hematology, Faculty of Medicine- University of Coimbra, Coimbra, Portugal
[2]Center for Innovative Biomedicine and Biotechnology, University of Coimbra, Coimbra, Portugal
[3]Coimbra Institute for Clinical and Biomedical Research, Group of Environmental Genetics of Oncobiology- Faculty of Medicine- University of Coimbra, Coimbra, Portugal
[4]Clinical Academic Center of Coimbra, University of Coimbra, Coimbra, Portugal
[5]IAP-PM- Institute of Anatomical and Molecular Pathology, Faculty of Medicine- University of Coimbra, Coimbra, Portugal
[6]Hematology Service, Centro Hospitalar e Universitário de Coimbra, Coimbra, Portugal

**Introduction**

Cells have developed a complex network called DNA Damage Response (DDR) to protect the integrity of the genome and repair damaged DNA. The DNA-dependent protein kinase (DNA-PK) is a nuclear serine/threonine protein kinase whose main function is to repair DNA double-strand breaks by the non-homologous end joining repair pathway. Dysfunctional DDR alters DNA repair mechanisms and has been implicated in the pathogenesis of various hematological malignancies, such as acute myeloblastic leukemia (AML). We used *in vitro* models of AML to assess the therapeutic potential of the DNA-PK inhibitor AZD-7648 in monotherapy.

**Material and Methods**

HEL, K-562, and LAMA-84 cell lines were used in this study. DDR gene mutations were obtained from the COSMIC database. Chromosomal damage and DNA repair kinetics were measured by cytokinesis-block micronucleus assay. DDR genes expression levels and telomere length were evaluated by qPCR. Cells were incubated in the absence or presence of increasing concentrations of AZD-7648. Cell density and viability were analyzed, for 72 hours, by trypan blue assay. Cell death (Annexin V/7-AAD double staining, cleaved PARP and activated caspase-3 levels), cell cycle distribution (propidium iodide/RNAse), cell proliferation rate (BrdU incorporations levels), and γH2AX levels were assessed by flow cytometry. The results were statistically analyzed, considering a significance level of 95%.

**Results and Discussions**

The cell lines presented distinct mutations and damage levels. HEL cells had the highest levels of γH2AX and LAMA-84 cells of chromosomal damage. The shortest telomere length was found on K-562 cells and LAMA-84 cells showed the highest DDR genes expression levels. All cell lines demonstrated an efficient repair. AZD-7648 reduced cell proliferation and viability in a dose-, time- and cell line-dependent manner. HEL was the most sensitive cell line, with an $IC_{50}$ of approximately 150μM, at 24 hours, and K-562 was the most resistant with an $IC_{50}$>200μM, at any time point. This inhibitor increased the percentage of apoptotic cells and the levels of activated caspase-3 and cleaved PARP, induced cell cycle arrest in $G_0/G_1$ phase, and reduced cell proliferation. DNA and chromosomal damage levels increased after incubation with AZD-7648.

**Conclusion**

In conclusion, our results demonstrated the efficacy of AZD-7648 on AML models, thus supporting further investigation of the potential of this inhibitor as a new therapy in AML that may ultimately improve the outcome of AML patients.

### EACR23-1199

### Low-molecular weight inhibitors targeting RTKs represent a strategy for the treatment of Giant Cell Tumor of Bone

V. Knechtová[1,2], M. Mahdal[3], R. Veselska[1,2], J. Neradil[1,2]
[1]Masaryk University, Department of Experimental Biology. Faculty of Science, Brno, Czech Republic
[2]St. Anne's University Hospital Brno, International Clinical Research Center, Brno, Czech Republic
[3]St. Anne's University Hospital Brno and Faculty of Medici ne. Masaryk University, First Department of Orthopedic Surgery, Brno, Czech Republic

**Introduction**

Giant cell tumor of bone (GCTB) is a benign but locally aggressive osteolytic tumor with high recurrence rate. GCTB contains multinucleated osteoclast-like giant cells and neoplastic mononuclear stromal cells. The stromal cells overexpress RANKL and other growth factors, which causes the fusion of monocytes into the giant cells responsible for the resorption of the bone. Various adjuvant therapies are being used to lower the risk of recurrence. Denosumab is a human monoclonal antibody that binds RANKL and inhibits osteoclast-like giant cells. However, it does not affect neoplastic mononuclear cells and can be associated with increased local recurrence after discontinuation of treatment. Therefore, new therapy targeting neoplastic stromal cells is needed.
Using phospho-protein arrays we have previously shown that RTKs signaling is activated in GCTB stromal cells and has an important role in the regulation of cell proliferation. The phosphorylation spectrum of RTKs and downstream proteins differed based on denosumab treatment and RTKs with high levels of phosphorylation could be targeted by low-molecular weight inhibitors.

**Material and Methods**

Using immunoblotting, we have detected phosphorylated and total levels of selected RTKs and downstream proteins in patients' tissue, who were either treated or untreated with denosumab. Based on those and the previous phospho-protein results, we have selected druggable molecular targets, including EGFR, c-Met, ALK, MEK1/2, and ERK1/2. Next, we analyzed the sensitivity of GCTB cell lines to inhibitors of selected targets *in vitro* with MTT assay, specifically inhibitors lapatinib, gefitinib, crizotinib, and trametinib.

**Results and Discussions**

Regarding the EGFR inhibitors, lapatinib didn´t have any observable effect on the viability of selected cell lines. Gefitinib was able to decrease the viability to approximately 60%, however only at the highest concentration, 10μM. MEK1/2 inhibitor trametinib significantly decreased the viability of tested GCTB cell lines at 0,1; 1 and 10μM concentrations. We also observed an intensive decrease in viability after treatment with

© 2023 The Authors. Molecular Oncology is published by John Wiley & Sons Ltd on behalf of Federation of European Biochemical Societies crizotinib, an inhibitor of ALK and c-Met, with the highest sensitivity observed at concentration 10µM.

**Conclusion**

Taken together, our data show that highly phosphorylated RTKs and their downstream pathways are promising therapeutic targets and some low-molecular weight inhibitors, such as trametinib and crizotinib, are able to massively decrease the viability of GCTB stromal cells *in vitro*.

### EACR23-1205
### Exploration of therapeutic vulnerabilities exposed by 9p21 loss in bladder cancer cell lines.

*R. Bevilacqua[1], P. Gasperini[1], T. Cantore[1], T. Fedrizzi[1], V. Adami[1], M. Pancher[1], B.M. Faltas[2,3,4,5], F. Demichelis[1,2]*
*[1]Università degli Studi di Trento, Department of Cellular- Computational and Integrative Bi ology - CIBIO, Povo Trento, Italy*
*[2]Weill Cornell Medicine, Englander Institute for Precision Medicine- New York-Presbyterian Hospital, New York, United States*
*[3]Weill Cornell Medicine, Department of Cell and Developmental Biology, New York, United States*
*[4]Weill Cornell Medicine, Sandra and Edward Meyer Cancer Center, New York, United States*
*[5]Weill Cornell Medicine, Department of Medicine, New York, United States*

**Introduction**

The deletion of chromosome 9p21 locus is the most frequent homozygous deletion in bladder cancer. It causes loss of the tumor suppressors *CDKN2A/2B* and of the metabolic gene *MTAP*, involved in the methionine and adenine salvage pathway. Large-scale shRNA screens have shown enhanced dependency of *MTAP*-deleted cells on PRMT5 and MAT2A, which led to the development of highly specific inhibitors. Here, we performed a multi-parametric drug screening to uncover new pharmacological vulnerabilities of 9p21-deleted bladder cancer cells, a disease with limited treatment options in the advanced stages.

**Material and Methods**

We employed CRISPR/Cas9 technology to generate 9p21 locus isogenic clones (WT and *CDKN2A/2B/MTAP*-null, or 3KO) from three bladder cancer cell lines with different genomic backgrounds (HT1197, T24, and TCCSUP). The HT1197 isogenic pair was used to perform a multi-parametric drug screening testing 2,351 compounds from Anticancer compound (Selleck) and MicroSource Spectrum collections. Cells were fixed and stained with Hoechst and Alexa 594-conjugated phalloidin. Fluorescence images were acquired with Operetta® and analyzed for differential compound effects using 13 informative features.

**Results and Discussions**

HT1197 and TCCSUP 3KO clones have higher proliferation rates compared to WT clones. On the other hand, T24 clones proliferation is not altered by 9p21 loss. Our drug screening nominated 18 drugs selectively effective in 3KO cells. Three were confirmed in dose-response viability assays performed at different time points and with several WT and 3KO clones. We hypothesized that these agents could synergize with MAT2A and PRMT5 inhibition and with two drugs that, according to literature data, are more effective in *CDKN2A*-deleted cells. Therefore, we tested numerous drug combinations and observed that when administered together, MAT2A inhibition combines with three of these agents, increasing cytotoxicity in 9p21-deleted bladder cancer cells. We also show that replication stress is induced upon MAT2A inhibition in 3KO cells, and it is exacerbated when the treatments are combined.

**Conclusion**

Our data suggest promising therapeutic strategies for the management of bladder cancer patients with 9p21 loss. Specifically, the 3KO cells are selectively sensitive to multiple agents that could be combined to maximally exploit the vulnerabilities exposed by 9p21 loss.

### EACR23-1208
### Combination of novel antibody targeting the hERG1/β1 integrin complex with different drugs: a new strategy in the treatment of pancreatic adenocarcinoma

*R. COLASURDO[1,2], C. Duranti[1], J. Iorio[1], T. Lottini[1], C. Capitani[1,2], M. Martinelli[1,2], F.N. D'Alessandro[1,2], E. Lastraioli[1], A. Arcangeli[1]*
*[1]Università degli studi Firenze, Dipartimento di medicina sperimentale e clinica, Florence, Italy*
*[2]Università degli studi di Siena, Genomec, Siena, Italy*

**Introduction**

The development of new approaches in the diagnosis and treatment of cancer, has seen a growing interest in the development of antibodies directed against specific targets. An example are the bispecific antibodies (bsAbs), which can be directed against two different target. Ion channels are emerging as potential biomarkers in cancer.  It is known that hERG1, a voltage gated potassium channel is aberrantly expressed in several types of primary tumor samples and tumor cell lines and it is involved in tumor progression. The channel can form a macromolecular complex with the β1 integrin adhesion molecule, which is selectively expressed in cancer cells, it can modulate different cancer related signaling pathways. In this scenario, it was developed a bispecific antibody which have specifically target the complex hERG1/β1 integrin complex. Another interesting field is the combination of this new tools with drug, which are already used in therapy, to improve the efficacy of both the compound.

**Material and Methods**

Human pancreatic adenocarcinoma (PDAC) cells were used to perform in vitro (viability, calculation of $IC_{50}$, combination index) analysis using novel antibodies scDb hERG1/β1, directed against hERG1/β1 integrin complex, combined with Gemcitabine which has been long used as a standard of care in PDAC. The combination was tested on different PDAC cell lines (Panc-1, MiaPaca 2, BxPC3) and on PCS-RLT used as control.

**Results and Discussions**

Gemcitabine shows a very high efficacy in BxPC3, a good efficacy in PANC-1 and MiaPaca 2 cells, and a very low efficacy in PSC-RLT. On the other hand, scDb-hERG1-β1

© 2023 The Authors. Molecular Oncology is published by John Wiley & Sons Ltd on behalf of Federation of European Biochemical Societies showed a very good efficacy in PANC-1 and MiaPaca 2 cells, low efficacy in BxPC3 and null efficacy in PSC-RLT. Overall, $IC_{50}$ values, relative to the scDb-hERG1-β1, completely fits in well with the expression of the hERG1/β1 integrin complex in pancreatic cell lines. The calculation of the combination index (CI) clearly shows a synergistic effect of the combination of scDb-hERG1-β1 and Gemcitabine only in PDAC cells which express the hERG1/β1 integrin complex.

**Conclusion**

These data shown very promising way to novel therapeutic regimens in PDAC, based on the targeting ion channels with the scDb hERG1/β1, in combination with low dose of chemotherapeutic drugs. To: address the action directly to the tumor, reduce the cytotoxicity of the drugs, minimize the onset of resistance. Given these promising results, we are proceeding to test the combination of the antibody with oxaliplatin, cisplatin, irinotecan and 5-flouuracile.

### EACR23-1221

### A novel class of small molecules for oral application to enhance tumor-reactive T cell cytotoxicity against melanoma

*B. Gapp[1], A. Dohnal[1], A. Stütz[2], G. Zischinsky[3], P. Nussbaumer[3], M. Urban[1], N. Birnbacher[1], D. Schögl[4], A. Birbach[5], R. Gugenberger[1]*

*[1]invIOs, Research and development, Vienna, Austria*

*[2]Pharma R&D consulting Stuetz, not applicable, Vienna, Austria*

*[3]Lead Discovery Center, Medicinal chemistry, Dortmund, Germany*

*[4]invIOs, Intellectual Property, Vienna, Austria*

*[5]invIOs, CMC and Quality Assurance, Vienna, Austria*

**Introduction**

The introduction of targeted immunomodulating agents has transformed cancer treatment over the last decade by demonstrating unprecedented efficacy in patients who respond. However, limited clinical response rates as well as adverse events underline the need to identify additional novel modalities in cancer immunotherapy.

Here we report for the first time on the discovery of a novel class of low molecular weight compounds for oral application that selectively enhance tumor-reactive T cell cytotoxicity.

**Material and Methods**

Hit-to-lead development of compound hit series A2 is being performed based on medicinal chemistry to investigate structure-activity-relations. Newly synthesized compounds are tested for EC50 potency by CD69 expression on anti-CD3/CD28 stimulated T cells. Furthermore absorption, distribution, metabolism and excretion (ADME) profiling and pharmacokinetics (PK) behavior is investigated to select candidate compounds for proof-of-concept studies in a B16-SIY melanoma mouse model.

**Results and Discussions**

A2-306, a representative of the A2 compound class, induces increased T cell proliferation, elevated levels of the activation markers CD25 and CD69 as well as enhanced secretion of IFN-g, IL-2 and TNF-a upon anti-CD3/CD28 stimulation. A2-306 treatment without T cell receptor ligation has no effect on T cell activation, suggesting selective activation potential only in the context of an antigen encounter. Structurally related compounds show a similar profile with potencies down to nanomolar level. Furthermore, A2-306 strengthens T cell mediated killing of M21 melanoma cells and enhances T cell function in response to viral antigens by showing a Th1 signature in a dose-dependent manner. In a murine B16-SIY melanoma model, oral single-agent administration is well tolerated, shows good bioavailability in lymphoid organs and plasma, and results in significant tumor growth inhibition of more than 80% at a dose level of 3 mg/kg body weight (QD). These mice show a distinct cytokine signature in plasma specimens, indicative of Th1 anti-tumor immunity.

**Conclusion**

Taken together, we report on the identification of a novel class of small molecules possessing high potential for selective anti-tumor activation of the immune system upon oral administration.

### EACR23-1244

### Liposomal Encapsulation of Daunorubicin and an Emetine Prodrug for Improved Efficacy Against Acute Myeloid Leukaemia

*I.N. Reiten[1], P. Moreau[2], F. Giraud[2], E.T. Gundersen[1], T.T. Augedal[1], F. Anizon[2], L. Herfindal[1]*

*[1]University of Bergen, Department of Clinical Science, Bergen, Norway*

*[2]Université Clermont Auvergne, Institut de chimie de clermont-ferrand, Clermont-Ferrand, France*

**Introduction**

Acute myeloid leukaemia (AML) is a cancer of the blood and bone marrow. Most AML-patients undergo an intensive therapy of an anthracycline and cytarabine, which has been the standard for decades. Unfortunately, AML is still associated with high relapse rate and mortality, thus new options are needed.

Protein synthesis inhibitors, such as emetine, can increase anthracycline-induced AML cell death, especially if administered 0.5-1 hours after anthracyclines. Hence, we wanted to produce an prodrug with delayed intracellular conversion to emetine and incorporate this with daunorubicin (DNR) in liposomes. Co-encapsulation of DNR and an emetine prodrug would ensure simultaneous delivery of the drugs, while retarding the protein synthesis inhibition to enhance the effect on anthracycline induced cell death.

**Material and Methods**

The emetine prodrug was synthesized by coupling an acyloxy methyl ester to an amine on emetine shown to be essential for close interaction at the binding site of ribosomes.

Pegylated liposomes were produced in a pH 6.4 buffer. A transmembrane pH gradient was created by exchanging the suspension buffer to pH 8.0 by gel filtration, and the drugs were incorporated through the acid precipitation principle. Liposome size and drug loading were quantified by DLS and HPLC, respectively. Cytotoxicity of the liposomal formulations towards AML-cells was performed by assessing metabolic activity.

The liposomal formulation was also produced together with a fluorescent lipid to study the distribution in zebrafish larvae after intravenous injection.

**Results and Discussions**

© 2023 The Authors. Molecular Oncology is published by John Wiley & Sons Ltd on behalf of Federation of European Biochemical Societies

We developed a liposomal carrier with an average drug loading ratio of DNR and the prodrug of 1:0.7. The diameter of the liposomes was around 130 nm, and addition of drugs did not alter the size.
Liposomes loaded with both DNR and the prodrug were more potent than both liposomal DNR alone and a combination of DNR and the prodrug administered as separate liposomal formulations against the MOLM-13 AML cell line, demonstrating the advantage of simultaneous delivery of both drugs in the cancer cells. The liposomal formulation could be observed in the circulatory system of zebrafish larvae for at least 48 hours.
**Conclusion**
A long circulating, dual-effect liposome with DNR and an emetine prodrug can be produced which appears to be more potent against the MOLM-13 AML cell line than DNR liposomes alone and than administering the drugs in separate liposomes. However, further preclinical tests are needed to fully reveal the potential of our formulation.

### EACR23-1249
### Identification of Genes that Regulate POU2F3 in Small Cell Lung Cancer

L. Duplaquet[1], M.G. Oser[1]
[1]*Dana Farber Cancer Institute- Harvard Medical School, Medical Oncology, Boston, United States*

**Introduction**
Small cell lung cancer (SCLC) are driven by near universal loss of function mutations in tumor suppressor genes RB1 and TP53 and do not harbor recurrent mutations in a druggable oncogenic driver, which makes finding therapeutic targets especially challenging. Recent findings have shown that most SCLCs can be characterized by the expression of the transcription factors ASCL1, NEUROD1 or POU2F3 which function as oncoproteins and therefore could be good therapeutic targets. Unfortunately, these transcription factors are "undruggable". Inactivating genes that promote their mRNA transcription or protein stability are alternative targeting strategies.
**Material and Methods**
To do this, we've developed a novel approach using CRISPR/Cas9-mediated homologous recombination to knock-in the suicide gene DCK* and GFP (linked by P2A) into the endogenous POU2F3 locus in the H1048 human SCLC cell line which highly expresses POU2F3. In these "knock-in" (KI) cells, endogenous POU2F3 expression is completely replaced by an POU2F3-DCK*-P2A-GFP chimeric fusion protein. Addition of a synthetic nucleoside analog (BVdU) selectively kill cells that express the fusion. We used this approach to perform positive selection CRISPR/Cas9 screens to identify genes that, when inactivated, destabilize the POU2F3-DCK* fusion and allow the cells to survive in the presence of BVdU.
**Results and Discussions**
Previously, we've successfully used this approach by exogenously expressing an ASCL1-DCK* fusion in Jurkat cells and performed a screen using a small CRISPR/Cas9 sgRNA library of druggable enzymes and have identified that CDK2 inactivation destabilizes ASCL1 expression. We are now wondering whether this approach could be used to study both the transcriptional and post-translational regulation of the different transcription factors in the endogenous SCLC context. To do this, we've first validated the KI H1048 model that endogenously expresses POU2F3-DCK* showing BVdU sensitivity compared to parental cells. We've recently completed genome-wide CRISPR/Cas9 screens on the KI H1048 cells in replicates and found that *POU2F3* and *DCK* were the top 2 hits demonstrating the robustness of these screens results. With this approach, we have identified novel transcriptional regulators of POU2F3 and some of these regulators are druggable with small molecules.
**Conclusion**
This approach could uncover candidate druggable SCLC targets that destabilize these transcription factors and could identify novel targeted therapies for SCLC.

### EACR23-1264
### Machine Learning for Multi-Omics Data Identifies Vulnerabilities in a Subset of Squamous Cell Lung Cancers

D. Kottmann[1], W. Hwang[2], W. Guo[1], N. Han[2], F. McCaughan[1]
[1]*The Heart and Lung Research Institute, Department of Medicine- University of Cambridge, Cambridge, United Kingdom*
[2]*Milner Therapeutics Institute, Jeffrey Cheah Biomedical Centre- University of Cambridge, Cambridge, United Kingdom*

**Introduction**
Lung squamous cell carcinoma (LUSC) is a subtype of non-small cell lung cancer and accounts for about 25% of all lung cancer cases worldwide. Despite recent advancements in cancer therapeutics, there are no targeted therapies approved specifically for LUSC. Druggable alterations frequently found in lung adenocarcinoma are not implicated in LUSC.
The lineage-survival oncogene SOX2 is dysregulated by gene amplification in a large proportion of clinical LUSC specimens and is mechanistically implicated as a key driver in multiple preclinical functional studies. SOX2 is a transcription factor and makes a challenging drug target. There is a clear unmet need to identify new targeted therapies in LUSC. In this work we focus on defining potential therapeutic targets for SOX2-dependent LUSC.
**Material and Methods**
We developed a machine-learning-based computational pipeline to integrate and analyse large-scale multi-omics datasets from public databases and a novel customised SOX2-dependent model of early LUSC.
We conducted a network analysis to identify drug target candidates. Targets were then evaluated in cell-based models using small molecule inhibitors and CRISPR-Cas9. The effects of target inhibition/knockout were evaluated by cell viability assays, cellular competition assays, and the analysis of mRNA and protein.
**Results and Discussions**
We used the pipeline to define the SOX2 interactome in LUSC and identify candidate drug targets. We then scored and ranked the targets based on the SOX2 network and their druggability. The top 10 candidates were considered for experimental *in vitro* validation.
In ongoing work we have validated a subset of identified targets. Existing small molecule inhibitors identified the PI3K/AKT/mTOR pathway with valid drug targets. Targeting AKT and mTOR was validated in an

© 2023 The Authors. Molecular Oncology is published by John Wiley & Sons Ltd on behalf of Federation of European Biochemical Societies organotypic SOX2-dependent model of early LUSC. Two further transcription factors (KLF5, FOXM1) were validated (knockout and small molecule inhibition) as targets in a subset of LUSC. Certain identified targets have been implicated by other groups recently and further validate our machine learning pipeline independently.

**Conclusion**

We have developed a machine-learning-based pipeline to integrate multi-omics datasets and to identify druggable pathways with defined vulnerabilities. The bioinformatic pipeline identified new candidate targets which we have confirmed have therapeutic potential *in vitro*. We anticipate this approach may help meet unmet needs in LUSC and a broad range of cancer types.

### EACR23-1287
### SIGMA RECEPTORS: ATTRACTIVE TARGETS IN THE FIGHT AGAINST GLIOBLASTOMA

*M. Bedeschi[1], N. Marino[1], S. Collina[2], G. Rossino[2], R. Listro[2], A. Tesei[1]*

*[1]Istituto Romagnolo per lo Studio dei Tumori IRST "Dino Amadori", BioScience laboratory, Meldola, Italy*

*[2]University of Pavia, Department of Drug Sciences, Viale Taramelli 12- 27100- Pavia- Italy, Italy*

**Introduction**

Human glioblastoma (GBM) is one of the most feared primary malignant brain tumors. The lack of successful therapeutic treatments has boosted the research world to find new effective drugs. The sigma-1 receptor (S1R) is a transmembrane protein and is highly expressed in neurons and glia of multiple regions within the central nervous system (CNS) where it is involved in neuroprotection, neuroinflammation, neurotransmission, and neuroplasticity. Furthermore, it is over-expressed in different tumors, such as lung, prostate and breast cancer and is studied for neuropathic pain as well. Therapies targeting this receptor are growing interest in the scientific community in order to develop new anti-cancer drugs able to improve both overall survival and quality of life. For these reasons, we investigated the effect of pan-sigma modulator and S1R agonists on GBM patient-derived cells.

**Material and Methods**

The effect of a pan-sigma modulator and two S1R agonists was evaluated *in vitro* on three different GBM patient-derived cells and *in vivo* on zebrafish model to attest their safety on embryos development.

**Results and Discussions**

Al the compounds resulted efficient at the higher concentrations tested, causing cells' growth inhibition. Moreover, a slight dose-dependent toxicity on zebrafish embryos was observed for the compounds at the higher concentrations.

**Conclusion**

Despite sigma receptors (SRs) emerged as interesting targets in the area of oncology, their role in tumor biology remains enigmatic. Some anticancer drugs targeting SRs are studied in the preclinical-stage, yet no clinically available drugs still available. In this light, modulators of SRs and S1R agonists can play an important role. The data obtained on GBM patient-derived cells encourage further preclinical investigation on *in vitro* and *in vivo* oncological models.

### EACR23-1314
### The oncometabolite 2-hydroxyglutarate - approaches to exploit its cellular toxicity

*A. Tietzel[1,2,3], J. Zaman[1], C. Herold-Mende[4], S. Pusch[1,2], A. von Deimling[1,2,3]*

*[1]German Cancer Research Center, Clinical Cooperation Unit Neuropathology, Heidelberg, Germany*

*[2]University Hospital Heidelberg- Institute of Pathology, Department of Neuropathology, Heidelberg, Germany*

*[3]Heidelberg University, Faculty of Medicine, Heidelberg, Germany*

*[4]University Hospital Heidelberg, Department of Neurosurgery, Heidelberg, Germany*

**Introduction**

Mutations in isocitrate dehydrogenase (IDH) are frequently found in lower-grade glioma. Mutant IDH converts α-ketoglutarate to the oncometabolite D-2-hydroxyglutarate (2-HG). 2-HG acts as a competitive inhibitor of α-ketoglutarate dependent dioxygenases, interfering with several pathways, especially epigenetics and metabolism, and thus promotes tumorigenesis. However, 2-HG also has multiple negative effects on the cell.

The aim of the study was to test methods for inducing intracellular accumulation of the oncometabolite and exploiting this for further therapy. The study is focusing on the inhibition of 2-HG transport as one way to accumulate 2-HG in tumor cells.

**Material and Methods**

The effects of pharmacological inhibition of 2-HG transporters SLC13A3 and the SLC22A family on intra- and extracellular 2-HG levels were analyzed in human patient-derived IDH mutant glioma cells and a glioma cell line ectopically overexpressing $IDH1^{R132H}$.

Assuming that elevated 2-HG levels, due to the acidic properties of 2-HG, lead to lower intracellular pH (pHi), a flow cytometry-based method for measuring pHi was established to validate the presence of 2-HG accumulation in the cell.

**Results and Discussions**

Inhibition of the transporters with available drugs did not lead to an accumulation of 2-HG in the cells, most probably due to a lack of drug specificity.

Consequentially, the next step is a more specific inhibition. Therefore, a sequence specific downregulation and knockout of different SLC transporters is currently ongoing.

Comparative analysis of various IDH-mutant cells with and without 2-HG production did not confirm our hypothesis of a correlation between 2-HG concentration and pHi. Consequentially, pHi cannot be used as a reliable predictor of intracellular 2-HG accumulation.

**Conclusion**

In conclusion, we are still convinced that inhibition of 2-HG transport could be a promising approach to accumulate 2-HG in tumor cells thereby exploiting its cellular toxicity. However, ways of intracellular 2-HG enrichment need further investigation.

Direct visualization of intracellular 2-HG levels is of special interest for analysis of cellular biology under 2-HG pressure. Further research on suitable readout methods for cellular 2-HG detection is ongoing.

© 2023 The Authors. Molecular Oncology is published by John Wiley & Sons Ltd on behalf of Federation of European Biochemical Societies

### EACR23-1340
### Identifying modulators of YAP/TAZ activity in Glioblastoma Stem Cells

M. Harvey[1], J. Dawson[1], M. Frame[1], N. Carragher[1]
[1]University of Edinburgh,
Cancer Research UK Edinburgh Centre, Edinburgh, United Kingdom

**Introduction**

Glioblastoma (GBM) accounts for just under half of all primary malignant brain tumours, is classified as the most advanced (Grade IV) and has a very poor prognosis, median survival is between 14-16 months. There are few therapeutic options other than the current standard of care, which consists of surgery followed by concomitant radiotherapy and temozolomide. GBM often shows rapid tumour recurrence due to several factors including high heterogeneity, phenotypic plasticity, invasion and the pro-tumour microenvironment, allowing cells to survive treatment. These processes can be driven by a subset of cells capable of self-renewal known as glioblastoma stem cells (GSCs). YAP and TAZ are the main effectors of the Hippo pathway and have been identified as capable of regulating up to 70% of the GSC transcriptomic signature but there are few specific inhibitors available.

**Material and Methods**

A high-content phenotypic screen was used to quantify YAP/TAZ sub-cellular localisation and morphological changes following a screen of 3080 compounds on two patient-derived GSC lines. Imaging utilised Hoeschst, Phalloidin and antibodies against YAP and TAZ, images were acquired using the ImageXpress-Confocal microscope. Validation studies monitored live cell responses to perturbations using the IncuCyte S3, including proliferation, apoptosis and cell cycle with FUCCI reporter cell lines. Analysis was performed using a combination of CellProfiler, StratoMiner and RStudio.

**Results and Discussions**

Screening identified 32 compounds of interest, 12 of which continued to show activity against YAP/TAZ in secondary dose response analysis; these included Dasatinib, eCF506, LY2090314 and Ouabain. Dasatinib and eCF506 elicited a reduction of nuclear YAP/TAZ. Whereas, Ouabain decreased nuclear TAZ where LY2090314 caused an increase, independent of YAP localisation. Functional assays showed the aforementioned compounds capable of reducing proliferation, inducing cell cycle arrest and apoptosis . Ongoing work seeks to understand pathway level effects of drug treatments on GSCs using NanoString and reverse phase protein array platforms

**Conclusion**

Dasatinib and eCF506 showed activity against YAP and TAZ as well as slowing proliferation through cell cycle arrest. Ouabain and LY2090314 could selectively modulate TAZ, but not YAP, localisation and induce apoptosis. This demonstrates high-content screening as a effective tool discover selective upstream modulators of YAP and TAZ cellular localisation in GSCs.

### EACR23-1346
### AKR1B10 is an actionable molecular target for apigenin in colon cancer

V. Russo[1], G. Gambera[1,2], C.G. Leotta[1], A. Rescifina[3], C. Drago[2], G.M. Pitari[1]
[1]Vera Salus Ricerca S.r.l., Dream Factory Lab, Siracusa, Italy
[2]Consiglio Nazionale delle Ricerche CNR, Institute of Biomolecular Chemistry, Catania, Italy
[3]Università di Catania, Dipartimento di Scienze del Farmaco e della Salute, Catania, Italy

**Introduction**

Apigenin, a plant flavonoid present in fruits and vegetables, exerts broad anticancer actions. However, its underlying mechanisms remain unclear. In the era of personalized medicine, this lack of knowledge represents a major obstacle for successful incorporation of phytochemicals into effective treatment strategies for cancer patients. This study aimed to elucidate the antitumor pharmacology and main target of apigenin in responsive human cancers.

**Material and Methods**

Cell proliferative kinetics were assessed in several human cancer cell lines, including colon, lung and skin ones, by quantitative cell counting and real-time analyses. Apoptosis was determined by acridine orange and caspase activity assays. Generation of reactive oxygen species (ROS) was quantified with a redox-sensitive fluorescent probe. Apigenin molecular targets were selected with Swiss Target Prediction, DiGeNET and STRING. Molecular docking with AutoDock Vina was, in turn, employed for target identification. Finally, gene expression and cancer patient survival were assessed employing data from open repositories GEPIA and UALCAN.

**Results and Discussions**

Apigenin disproportionally inhibited proliferation in solid cancer cell lines, with HCT116 human colon adenocarcinomas as the most responsive. In colorectal cancer (CRC) cells, apigenin reduced tumour propagation *in vitro* by promoting caspase-3 activation and apoptosis, reflecting intracellular ROS elevations and metabolic regulation. Computational prediction analyses suggested the aldo/keto reductase AKR1B10 as the molecular target mediating specific apigenin effects in CRC. In agreement, molecular modelling and docking analyses confirmed AKR1B10 as the apigenin target, reflected by meaningful energy binding interactions within the enzyme active pocket. Target validation *in vitro* and *in silico* was achieved by selective pharmacological and genetic manipulation of AKR1B10, respectively. Finally, AKR1B10 expression levels negatively correlated with overall survival in CRC patients. These observations indicate AKR1B10 is a pharmacological target for apigenin in CRC, which could be exploited to improve patient responses to therapy.

**Conclusion**

Dietary constituents have emerged as key chemomodulatory agents in cancer. Here, the oxidoreductase AKR1B10 was identified as a pharmacologically actionable molecular target for the flavonoid apigenin in CRC. Present findings support the exploitation of apigenin as a phytochemical intervention in novel individualized therapies for CRC patients.

© 2023 The Authors. Molecular Oncology is published by John Wiley & Sons Ltd on behalf of Federation of European Biochemical Societies

### EACR23-1350
### Therapeutic modulation of RNA splicing to combat malignant rhabdoid tumors

P. Sobczuk[1,2,3], I. Odintsov[1,2], Z. Liu[1,2], D. Kubota[1,2], R. Somwar[1,2], N. Shukla[4], M. Ladanyi[1,2]
[1]Memorial Sloan Kettering Cancer Center, Department of Pathology, New York, United States
[2]Memorial Sloan Kettering Cancer Center, Human Oncology & Pathogenesis Program, New York, United States
[3]Maria Sklodowska-Curie National Research Institute of Oncology, Department of Soft Tissue/Bone Sarcoma and Melanoma, Warsaw, Poland
[4]Memorial Sloan Kettering Cancer Center, Department of Pediatrics, New York, United States

**Introduction**

Malignant rhabdoid tumor (MRT) is a rare pediatric mesenchymal tumor characterized by loss of *SMARCB1*. MRT prognosis is very poor, with only around 10% of patients surviving more than 5 years, due to its very aggressive behavior and the limited efficacy of systemic therapy. Thus, there is a high unmet need for novel therapies for MRT patients.

**Material and Methods**

We performed screening of focused chemical libraries consisting of 5,998 compounds, including a panel of 880 kinase inhibitors in MRT cell lines (A204 and G401). Compounds of interest were validated in a larger panel of MRT cell lines (A204, G401, TTC549, TM87-16, KYM-1, JMURTK2) for viability, cell cycle progression and apoptosis. Transcriptome profiling was conducted to shed light on mechanisms of action.

**Results and Discussions**

We began to characterized agents from the kinase library where 62/880 compounds were classified as “hits” (B-score <-3 at 4 µM) in A204 and G401 and found an over-representation of inhibitors of kinases involved in RNA splicing. We examined the efficacy of independent inhibitors of the same pathways (DYRK/CLK) and found several that inhibited growth at low nanomolar concentrations. One compound (SM09419) that is already clinically available was selected for further studies. SM09419 inhibited growth of 6 MRT cells lines with $IC_{50}$ values of 30-170 nM, median value of 44.5 nM (less active in a non-tumor murine cell line) and inhibited colony formation in A204 and G401 cells. Treatment of A204 and TTC549 cells with SM09419 caused increase expression of the cycle inhibitors p21 and p27 concomitantly with G0/G1 cell-cycle arrest, increased expression of the pro-apoptosis proteins cleaved-PARP, PUMA and BIM, and activation of caspase 3/7. Transcriptomic profile of SM09419-treated cells showed significant negative enrichment for *WNT* pathway genes and qPCR analysis confirmed presence of altered splicing of selected WNT pathway genes (increased intron retention in *DVL2* and *TCF7* mRNA).

**Conclusion**

Our results suggest that targeting RNA splicing may be a promising approach for treatment of therapy-refractory MRT. Further pre-clinical *in vivo* studies are warranted to fully establish a rationale for clinical evaluation of SM09419 in MRT patients.

### EACR23-1356
### Exploring ring-fused chlorins based photodynamic therapy as a conservative approach for endometrial cancer

B. Serambeque[1], M.J. Carvalho[2], G. Correia-Barros[1], N. AM Pereira[3], A.C. Gonçalves[4], M. Piñeiro[3], M.F. Botelho[5], T. MVD Pinho e Melo[3], M. Laranjo[5]
[1]Coimbra Institute for Clinical and Biomedical Research i CBR Area of Environment- Genetics and Oncobiology CIM AGO- Institute of Biophysics- Faculty of Medicine- University of Coimbra- Center for Innovative Biomedicine and Biotechnology CIBB, University of Coimbra, Coimbra, Portugal
[2]Coimbra Institute for Clinical and Biomedical Research i CBR Area of Environment- Genetics and Oncobiology CIM AGO- Institute of Biophysics- Faculty of Medicine- University of Coimbra- Center for Innovative Biomedicine and Biotechnology CIBB- Universitary Clinic of Gynecology- Faculty of Medicine- University of Coimbra- Clinical Academic Center of Coimbra CACC- Gynecology Service- Coimbra Hospital and Universitary Center, University of Coimbra- Coimbra Hospital and Universitary Center, Coimbra, Portugal
[3]University of Coimbra- Coimbra Chemistry Centre-Institute of Molecular Sciences and Department of Chemistry, University of Coimbra, Coimbra, Portugal
[4]Coimbra Institute for Clinical and Biomedical Research i CBR Area of Environment- Genetics and Oncobiology CIM AGO- Laboratory of Oncobiology and Hematology- Faculty of Medicine- University of Coimbra- Center for Innovative Biomedicine and Biotechnology CIBB- Clinical Academic Center of Coimbra CACC, University of Coimbra, Coimbra, Portugal
[5]Coimbra Institute for Clinical and Biomedical Research i CBR Area of Environment- Genetics and Oncobiology CIM AGO- Institute of Biophysics- Faculty of Medicine- University of Coimbra- Center for Innovative Biomedicine and Biotechnology CIBB- Clinical Academic Center of Coimbra CACC, University of Coimbra, Coimbra, Portugal

**Introduction**

Endometrial cancer (EC) mostly affects postmenopausal women. However, about 4% are young women with reproductive desire, facing limitations regarding currently available conservative therapies. Photodynamic therapy (PDT), as an anticancer approach, induces damage through production of reactive oxygen species (ROS), by activating a photosensitizer (PS) with visible light. PDT based on the 4,5,6,7-tetrahydropyrazolo[1,5-*a*] pyridine-fused chlorins (Px-PDT) developed by us, has demonstrated a potent activity against cancer cells [1]. To provide a new conservative and minimally invasive approach for EC, the efficacy of Px-PDT was explored.

**Material and Methods**

ECC-1 and RL95-2 were incubated with 0.1-10µM of chlorins, Px1 (dihydroxymethyl derivative), Px3 (dicarboxylic acid derivative), and Px4 (dimethylesther derivative) for 24 hours, and then light activated (7.5mW/cm$^2$, 10J). The MTT assay was performed. To assess cell cycle, cell viability, and Px-induced cell death, EC cells were evaluated through flow cytometry. ROS influence was assessed by an indirect strategy using ROS scavengers followed by the MTT.

© 2023 The Authors. Molecular Oncology is published by John Wiley & Sons Ltd on behalf of Federation of European Biochemical Societies

**Results and Discussions**
In the absence of irradiation, chlorins were not cytotoxic. Px1 and Px3-PDT decreased ECC-1 viability. An increase in the subG0/G1 phase of ECC-1, with an increase of G0/G1 and a decrease in S phase were seen when Px1 ($p$=0.003; $p$=0.009) and Px3 ($p$=0.013; $p$=0.025) were used. Additionally, Px1 and Px3 induced late apoptosis and necrosis ($p$<0.001). In RL95-2, a similar cell cycle response was seen while all Px reduced cell viability, showing apoptosis and necrosis at 0.1μM, and late apoptosis and necrosis at 1μM. ROS analysis revealed an imbalance of peroxides and superoxide anion levels in both cell lines. Moreover, RL95-2 results suggest a relevant role of singlet oxygen.
**Conclusion**
Px-PDT showed to be effective against EC cells, evidencing the involvement of superoxide anion and singlet oxygen in the photodynamic reaction. These results encourage the continued investigation of Px-PDT as a potential conservative strategy for EC.
[1] Pereira, N.A.M. et al. Novel 4,5,6,7-tetrahydropyrazolo[1,5-a]pyridine fused chlorins as very active photodynamic agents for melanoma cells. Eur J Med Chem, 2015, 103: 374-80.
Funding: CIBB (UIDB/04539/2020, UIDP/04539/2020) and CQC (UIDB/00313/2020, UIDP/00313/2020) are supported by FCT, co-funded by COMPETE. Project Prova de Conceito (CENTRO-45-2021-30; 01/SAICT/2021, nº 180078) funded by Portugal2020. PhD Scholarship from FCT/ESF to BS (2020.07672.BD).

### EACR23-1359
### Validation of a novel reporter system for the identification of eIF4F inhibitors using high-throughput screening

*K. Smolkova[1,2], B. Valcikova[1,2], M. Lisova[3], P. Bartunek[3], S. Uldrijan[1,2]*
*[1]Masaryk University- Faculty of Medicine, Department of Biology, Brno, Czech Republic*
*[2]St. Anne's University Hospital, International Clinical Research Center, Brno, Czech Republic*
*[3]Institute of Molecular Genetics of the Czech Academy of S ciences, CZ-OPENSCREEN research infrastructure, Prague, Czech Republic*
**Introduction**
The eIF4F translation initiation complex is a promising therapeutic target in cancer cells. However, the spectrum of available eIF4F inhibitors is limited, and none is in clinical use. One of the reasons could lie in the relative complexity of techniques used to identify new inhibitors of eIF4F-mediated translation. Here we report a unique cell-based reporter system suitable for the high-throughput identification of novel eIF4F inhibitors in small molecule compound libraries. The system was validated in 384- and 1536-well format, confirming its applicability for high-throughput screening (HTS).
**Material and Methods**
We identified several eIF4F-regulated pathways controlling cancer cell proliferation in a proteomic screen. Then we used a promoter of one of the eIF4F-controlled genes to build a reporter system, responding to eIF4F inhibition by changes in luciferase expression in a dose-dependent manner. Subsequently, we validated the system in a panel of cell lines, determining the impact of eIF4F inhibition on luciferase activity.
**Results and Discussions**
We assessed the performance of the luciferase-based reporter system and confirmed its high sensitivity to eIF4F inhibition. The system validation in 384- and 1536-well format brought excellent results, with a stable dose-dependent response to Rocaglamide A, a flavagline known to block eIF4F by trapping its eIF4A helicase subunit. These analyses verified the system's applicability for HTS of bioactive compounds.
**Conclusion**
The current state-of-the-art eIF4F inhibitor screening assays, such as the proximity ligation assay (PLA), have several limitations in the high-throughput mode. Our novel eIF4F activity reporter system is not only specific and suitable for HTS; it is also less cost-intensive, significantly faster, and does not require expensive fluorescent microscopy/image analysis equipment. Therefore, our approach could speed up the development of eIF4F-targeting compounds for cancer patient treatment.
**Acknowledgments**
This work was funded by the European Regional Development Fund - Project ENOCH (No. CZ.02.1.01/0.0/0.0/16_019/0000868), the European Union – Next Generation EU - the project National Institute for Cancer Research (Programme EXCELES, Project No. LX22NPO5102), Masaryk University (MUNI/11/ACC/2/2022), Brno city municipality (Brno Ph.D. Talent Scholarship), RVO: 68378050-KAV-NPUI and by the Ministry of Education, Youth and Sports of the Czech Republic (project number LM2023052).

### EACR23-1367
### Ring-fused chlorins are effective photosensitizers for oral squamous cell carcinoma treatment

*A.C. Gonçalves[1,2], I. Ribeiro[1,2], C.M. Marto[1,2,3,4,5,6], B. Serambeque[2,4,5], M. Piñeiro[7], M.F. Botelho[2,4,5,6], T.M.V.D. Pinho e Melo[7], M. Laranjo[2,4,5,6]*
*[1]University of Coimbra- Faculty of Medicine, Institute of Integrated Clinical Practice, Coimbra, Portugal*
*[2]University of Coimbra- Faculty of Medicine, Biophysics Institute, Coimbra, Portugal*
*[3]University of Coimbra- Faculty of Medicine, Institute of Experimental Pathology, Coimbra, Portugal*
*[4]University of Coimbra- Faculty of Medicine, Coimbra Institute for Clinical and Biomedical Research iC BR- area of Environment Genetics and Oncobiology CIMA GO, Coimbra, Portugal*
*[5]University of Coimbra, Center for Innovative Biomedicine and Biotechnology CIB B, Coimbra, Portugal*
*[6]Clinical Academic Center of Coimbra CACC, Na, Coimbra, Portugal*
*[7]University of Coimbra- Department of Chemistry, Coimbra Chemistry Centre- Institute of Molecular Sciences, Coimbra, Portugal*
**Introduction**

© 2023 The Authors. Molecular Oncology is published by John Wiley & Sons Ltd on behalf of Federation of European Biochemical Societies

Oral squamous cell carcinoma (OSCC) is the most common oral cavity malignancy and the seventh most frequent cancer. Treatment includes surgery, chemo, and radiotherapy, which present side effects and high recurrence. Thus, new and effective therapies are necessary. Photodynamic therapy (PDT) is conservative and promising for cancer treatment. Our group has previously developed new photosensitizers (PSs) with improved properties, namely the dihydroxymethyl derivative of 4,5,6,7-tetrahydropyrazolo[1,5-*a*]pyridine-fused tetraphenylchlorin [1]. Thus, this study aimed to evaluate this chlorin photocytotoxicity in OSCC cells.

**Material and Methods**

Two OSCC cell lines, BICR10 and FaDU, were used. 24h after PS administration (1 to 10,000 nM), irradiation was performed. Metabolic activity was determined using the MTT assay, a dose-response curve was traced, and the $IC_{50}$ value was calculated. Then, the 100, 500, and 1000 nM were selected for SRB and Crystal Violet assays and May-Grünwald-Giemsa staining, to access viability, cell morphology and types of cell death.

**Results and Discussions**

PDT was effective in both cell lines. The $IC_{50}$ was 100.5 nM for BICR10 cells, while FaDu were more susceptible, with $IC_{50}$ of 32.95 nM. In BICR10 cells, protein content decreased about 70% and DNA content about 30%-40%. Regarding FaDu, preliminary results showed an 80% decrease of protein content when 500 nM were applied, as well as, 75% in DNA content. Cell morphology of BICR10 evidences both apoptosis and necrosis.

Overall, the disclosed results reinforce the potential of the studied chlorin as PDT agent , being an effective PS for OSCC treatment at low concentrations. Further studies should explore the differences between the two cell lines sensitivity to PDT.

**Conclusion**

The dihydroxymethyl derivative of 4,5,6,7-tetrahydropyrazolo[1,5-a]pyridine-fused tetraphenylchlorin reveals high phototoxicity against OSCC cells. The efficacy of PDT, combined with its few side effects and a more localized action, supports its future potential use for OSCC treatment.

[1] Pereira, N.A.M. et al. Novel 4,5,6,7-tetrahydropyrazolo[1,5-a]pyridine fused chlorins as very active photodynamic agents for melanoma cells. Eur J Med Chem, 2015, 103: 374-80.

Funding: CIBB (UIDB/04539/2020, UIDP/04539/2020) and CQC (UIDB/00313/2020 and UIDP/00313/2020) are supported by the Foundation for Science and Technology, co-funded by COMPETE. Project Prova de Conceito (CENTRO-45-2021-30; 01/SAICT/2021, nº 180078) funded by Portugal2020.

### EACR23-1368
### Exploration of duocarmycin prodrugs for selective Rhabdomyosarcoma treatment

E. Arasanz Picher[1], S. Sneha[1], S. Shnyder[1], P. Loadman[1], K. Pors[1]

[1]*University of Bradford, Institute of Cancer Therapeutics, Bradford, United Kingdom*

**Introduction**

Rhabdomyosarcoma (RMS) is a rare Soft Tissue Sarcoma that mostly affects children and teenagers. Mortality rates remain exceptionally high in high-risk RMS patients. Duocarmycins (DMs) are natural, ultrapotent compounds as anticancer agents, although have they failed in the clinic due to intrinsic broad toxicity. These minor groove DNA alkylating agents have been reengineered to generate prodrugs that are selectively activated by certain cytochrome P450 (CYP) isoforms via hydroxylation, which show a cancer-associated overexpression (e.g. CYP1A1, CYP1B1, CYP2W1).

**Material and Methods**

CYP1A1, CYP1B1 and CYP2W1 expression in RMS cell lines were assessed using a combination of molecular biology techniques in 2D and 3D spheroidal models. CYP1-related enzymatic activity was measured by the fluorescence-based, EROD assay. We tested a library of DM drugs and CYP450-bioactivated prodrugs (e.g. ICT2700 and ICT2706, which are CYP1A1 and CYP2W1-bioactivated respectively) in both preclinical models using antiproliferative tests. DM prodrug efficacy was tested in normoxic vs hypoxic ($O_2\% = 0.1$) conditions, and the AhR-HIF-1a pathways crosstalk was analysed in qPCR experiments. We examined the potential synergism between DM prodrugs and the HDAC inhibitor Vorinostat.

**Results and Discussions**

Except for RH30 and RMSYM cells ($IC_{50}$=0.2-0.5 µM for ICT2700), the $IC_{50}$ for CYP1A1- and CYP2W1-bioactivated DMs (ICT2700 and ICT2706, respectively) were in the low micromolar range. Consistently, only RH30 and RMSYM cell lines displayed a high CYP1A1 expression and a CYP1-related enzymatic activity, indicating CYP1 involvement in ICT2700 antiproliferative activity. Our data suggested that CYP1A1 and CYP2W1 enzymes might be upregulated RMS spheroids. Hypoxic exposure did not hamper chemosensitivity of RMS cells to DM prodrugs; CYP1A1 and CYP1B1 gene expression remained unaltered, indicating that prodrug activation is still possible under low oxygen tension. An enhanced antiproliferative effect of ICT2700 when used in combination with Vorinostat via upregulation of CYP1A1 expression levels.

**Conclusion**

Evaluation of CYP target expression in RMS was carried out to assess if DM prodrugs can be progressed as a novel therapy. CYP expression in RMS cell lines seems to vary depending on the complexity of the *in vitro* model used. Hypoxic exposure did not reduce antiproliferative activity. Further penetration and drug distribution studies will provide valuable evidence on how efficient DMs might be at destroying the hypoxic fractions in solid tumours.

### EACR23-1372
### SELECTIVE CRISPR-BASED TARGETING OF KRAS DRIVER MUTATIONS IN LUNG CANCER

J. Sanjuán Hidalgo[1], J.C. Álvarez Pérez[1], A.M. Arenas Molina[1], Á. Andrades Delgado[1], P.P. Medina Vico[1]

[1]*University of Granada - Centre for Genomics and Oncolo gical Research GENyO, Biochemistry and Molecular Biology I, Granada, Spain*

**Introduction**

Lung cancer is the leading cause of cancer death worldwide, being adenocarcinoma (LUAD) the most

© 2023 The Authors. Molecular Oncology is published by John Wiley & Sons Ltd on behalf of Federation of European Biochemical Societies prevalent subtype. It is estimated that 30% of these tumours harbour mutations in *KRAS*, which are mainly located in the 12th codon of exon 2. These mutations are termed *driver*, as they turn *KRAS* into a hyperactive protein, promoting cell division that eventually leads to tumour development. Given the high frequency of these mutations in lung cancer and the reported acquired resistance to recently approved compounds, we have developed a CRISPR/Cas 9 targeting strategy against two of the most frequent mutations (G12C and G12D), which aims to be an alternative therapeutic approach for future lung cancer treatment.

**Material and Methods**

To precisely modify mutant alleles, we built ribonucleoprotein particles (RNPs) using a High Fidelity (HiFi) version of Cas9 coupled to a series of sgRNAs designs. We validated their specificity and efficiency through both T7 endonuclease assay and next-generation sequencing (NGS). The therapeutic potential of the system was assessed using adenoviral vectors (AdVs) in a panel of $KRAS^{Mut}$ and $KRAS^{WT}$ lung cancer cell lines using cell- and patient-derived xenografts (CDX and PDX, respectively).

**Results and Discussions**

Candidate gRNAs against G12C and G12D mutations yielded editing efficiencies of 82.7% and 74.4 %, respectively. Importantly, the presence of edition in $KRAS^{WT}$ cells was almost undetectable. Moreover, KRAS-dependent signalling was significantly inhibited and correlated with abrogation of cell viability in 2D and 3D *in vitro* culture. The use of AdVs in preclinical models of CDX and PDX led to significant tumour

**Conclusion**

We demonstrated that CRISPR/Cas9 can be implemented as an alternative therapeutic tool to treat lung cancer by selectively editing the most frequent *KRAS* mutations without affecting its wildtype version.

### EACR23-1389

### NOX4 is a better candidate than TGF-beta receptor I to target the TGF-beta signalling in the tumour microenvironment and halt cholangiocarcinoma progression

*J. Amengual[1], E. Gonzalez-Sanchez[2], C. Guiton[1], M. Vallete[3], L. Aoudjehane[4], L. Fouassier[3], D. Calvisi[5], A. Méndez-Lucas[6], I. Fabregat[2], J. Vaquero[7]*

*[1]Bellvitge Biomedical Research Institute IDIBELL, TGF-beta and Cancer Group- Oncobell Program, Barcelona, Spain*

*[2]Bellvitge Biomedical Research Institute IDIBELL- CIBER ehd ISCIII, TGF-beta and Cancer Group- Oncobell Program, Barcelona, Spain*

*[3]Sorbonne Université- INSERM, Centre de Recherche Saint-Antoine CRSA, Paris, France*

*[4]Sorbonne Université- INSERM, IHU-Innovation of Cardiometabolism and Nutrition IHU-ICAN, Paris, France*

*[5]Institute of Pathology, University of Regensburg, Regensburg, Germany*

*[6]Bellvitge Biomedical Research Institute IDIBELL- Nutrition Metabolism and Gene Therapy Group, Department of Physiological Sciences- Faculty of Medicine and Health Sciences- University of Barcelona, Barcelona, Spain*

*[7]Bellvitge Biomedical Research Institute IDIBELL- CIBER ehd ISCIII - Centro de Investigación del Cáncer and Instituto de Biología Molecular y Celular del Cáncer- CSIC- Universidad de Salamanca, TGF-beta and Cancer Group- Oncobell Program IDIBELL, Barcelona, Spain*

**Introduction**

TGF-beta plays a dual role on the malignant cell, acting as a suppressor at early stages, but contributing to tumour progression once cells escape from its cytostatic effects. Moreover, TGF-beta and its downstream mediator NOX4 modulate the response of stromal cells contributing to tumour progression and immune evasion. In cholangiocarcinoma (CCA), a bile duct malignancy, clinical trials with TGF-beta inhibitors, alone or in combination with chemotherapy or immune checkpoint inhibitors did not reach the expected results. Here, we aim to determine the effects of the TGF-beta-NOX4 axis in CCA.

**Material and Methods**

TGFB1-3, TGFBR1-3 and NOX4 were analysed by bioinformatic analyses in human CCA and by RT-QPCR in murine CCA models (syngeneic orthotopic and oncogene-induced). A panel of 8 murine and human CCA cell lines and human hepatic stellate cells (HSC) and CAF were cultured in 2D or 3D to analyse response to TGF-β, TGF-beta receptor I inhibitor galunisertib and dual NOX1/NOX4 inhibitor GKT137831. Cell viability was determined by cell counting and sphere size. Gene and protein expression was evaluated by RT-QPCR and Western blot.

**Results and Discussions**

TGFB1, 2 and 3, and receptors TGFBR1 and 2 were strongly upregulated in human CCA and murine CCA models, indicating that TGF-beta signalling is highly active in CCA. In vitro, 6 of 8 CCA cell lines showed response to TGF-beta in terms of a very strong reduction of cell viability accompanied by a potent arrest of cell growth and the induction of apoptosis. TGF-beta unresponsive cell lines showed alterations in members of the TGF-beta signalling pathway (SMAD3 and SMAD7) leading to impaired SMAD signalling. Treatment of CCA cell lines with galunisertib increased the number of CCA colonies in 2D assays and the size of CCA spheroids and CCA-fibroblast mixed spheroids in 3D assays. NOX4 expression was upregulated in human CCA, it was undetectable in CCA cell lines, and was upregulated in HSC and CAF by TGF-β1, concomitant to fibroblast activation. GKT137831 inhibited fibroblast activation and reduced the size of CCA-fibroblast mixed spheroids.

**Conclusion**

Due to the strong suppressor effect of TGF-beta on the CCA tumour cell, inhibitors of the TGF-beta pathway as anti-cancer therapies may lead to counterproductive effects. Targeting the tumour microenvironment by inhibiting downstream mediators of the TGF-beta pathway, such as NOX4, may represent a therapeutic opportunity for CCA.

*This study is funded by Spanish Association for Cancer Research (AECC) #PRYGN211279FABR.*

© 2023 The Authors. Molecular Oncology is published by John Wiley & Sons Ltd on behalf of Federation of European Biochemical Societies

### EACR23-1410
### Synthetic lethal drug combination of a novel MAP2K4 inhibitor with MAPK inhibitors in KRAS mutant cancers

*R. Jansen[1], M. dos Santos Dias[1], M. Chen[1], S. Mainardi[1], R. Bernards[1]*
*[1]Netherlands Cancer Institute, Molecular Carcinogenesis, Amsterdam, The Netherlands*

**Introduction**
The reactivation of KRAS and its downstream effectors upon drug treatment has shown to be one of the mechanisms of drug resistance. MEK and ERK inhibition results in a feedback activation of the parallel MAP2K4-JNK-JUN pathway, leading to transcriptional upregulation of HER Receptor Tyrosine Kinases (RTKs) and thereby reactivating the MAPK pathway upon its initial inhibition. Therefore, combining MAPK inhibitors with the inhibition of this feedback activation may show to be effective in overcoming the adaptive reactivation of KRAS signaling. **These results predict a therapeutic strategy in which MAP2K4 inhibitors would show strong synergy with MAPK inhibitors in *KRAS* mutant tumors.**
**Material and Methods**
To investigate the synthetic lethal interaction between MAPK inhibitors and the novel MAP2K4 inhibitor HRX-b, we tested the combination in a panel of non-small-cell lung cancer (NSCLC) and colorectal cancer (CRC) cell lines harboring a *KRAS* mutation.
**Results and Discussions**
We show that combination of the MAP2K4 inhibitor HRX-b and MEK, ERK, KRASG12C or SHP2 inhibitors show a strong synergy in multiple KRAS mutant cancer models. HRX-233 disables the feedback activation upon MAPK inhibition and thereby strongly enhances the sensitivity in KRAS-driven tumors.
After decades of drug-targeting efforts, the first KRAS G12C inhibitor AMG510 was approved for clinical use in metastatic NSCLC harboring this specific mutation. While most *KRAS G12C* mutant NSCLC patients experience clinical benefit from these selective inhibitors, patients with colorectal cancer harboring the same mutation hardly respond with response rates of only 7%. Like resistance to MEK inhibitors, adaptive resistance to KRAS G12C inhibition has also been described to occur via reactivation of RTKs. Remarkably, combining AMG510 with MAP2K4 inhibition also showed a strong synergy in these cancer cells. Combining KRAS G12C inhibition with MAP2K4 inhibition strongly increased apoptosis in already relatively sensitive cells as well as more resistant cancer cell lines.
**Conclusion**
**Taken together, our data demonstrate a strong synthetic lethal drug combination of a the novel MAP2K4 inhibitor HRX-b with several different MAPK pathway inhibitors.** Given the lack of clinical response to MAPK inhibition due to adaptive resistance and the unmet need for treatment options in KRAS mutant cancers, this combination shows potential as a novel combinational drug therapy.

### EACR23-1451
### Identification of a gene signature for cancers sensitive to N-Myristoylation inhibition

*E. Beauchamp[1], C. Cromwell[2], E. Moussa[3], A. Iyer[4], R. Pain[4], J. Gamma[5], M. Kostiuk[4], O. Julien[3], B. Hubbard[2], L. Berthiaume[1,4]*
*[1]Pacylex Pharmaceuticals inc., Discovery Biology, Edmonton, Canada*
*[2]University of Alberta, Pharmacology, Edmonton, Canada*
*[3]University of Alberta, Biochemistry, Edmonton, Canada*
*[4]University of Alberta, Cell Biology, Edmonton, Canada*
*[5]University of Alberta, Medicine, Edmonton, Canada*

**Introduction**
In humans, over 600 proteoforms are modified with the fatty acid myristate by two N-myristoyltransferases: NMT1 and NMT2. These include many proto-oncogenic proteins, metabolic regulators, and mitochondrial proteins. By inhibiting this essential protein modification process, PCLX-001 shows high therapeutic potential in multiple cancer cell lines and animal models. PCLX-001 is a new, orally bioavailable, N-myristoylation inhibitor, which has been under clinical evaluation for the treatment of lymphoma and solid tumors for over a year (NCT04836195).
**Material and Methods**
RNAseq datasets corresponding to 1200 cell lines treated with PCLX-001 or PCLX-002 were used to evaluate *NMT* expression profiles and NMT1 dependency. Since many hematologic cancer cell lines showed reduced*NMT2* expression, we performed bisulfite sequencing to evaluate *NMT2* locus methylation. We used mass spectrometry and RNAseq to characterize the proteomes and transcriptomes of individual or dual NMT1 and/or NMT2 CRISPR/Cas9 KO Hap 1 cells. Affected pathways were identified by Gene Set Enrichment Analysis.
**Results and Discussions**
*NMT2* expression is epigenetically repressed in multiple cancers. Since most hematologic cancer cells are NMT2-deficient, we hypothesise that by targeting the remaining NMT1, PCLX-001 selectively kills these cells in a manner reminiscent of synthetic lethality, thereby sparing normal human cells with two NMTs.
Surprisingly, PCLX-001 and NMT1 KO had a more drastic effect on mitochondrial respiratory complex I proteins than on myristoylated signaling proteins, leading to complex I misassembly and activity abrogation. Since complex I is essential for optimal oxidative phosphorylation, this seemingly ordinary observation could have transformational implications for cancer treatment since oxidative phosphorylation is critical for both cancer stem cell survival and metastasis.
Sensitivity towards PCLX-001 or -002 is not solely dependent on *NMT2* loss. Rather, we define a 91 gene set enriched in sensitive cells. The PCLX sensitivity score–91 (PSS-91) is higher in sensitive tumors compared to their associated normal tissue, in hematological cancers and in highly metabolically active tumors.
**Conclusion**
PCLX-001 is a drug that preferentially targets select tumor types in a manner reminiscent of synthetic lethality. In-depth analysis of sensitive cells allowed us to develop a PSS-91 sensitivity signature that will help identify future

© 2023 The Authors. Molecular Oncology is published by John Wiley & Sons Ltd on behalf of Federation of European Biochemical Societies cancer indications and patients that would benefit from N-myristoyltransferase inhibitor therapy.

### EACR23-1465

### A high grade serous ovarian cancer patient derived organoids model for personalized therapy

*E.A. Cavarzerani*[1], *I. Caligiuri*[2], *V. Canzonieri*[2], *F. Rizzolio*[3]
[1]*Ca' Foscari University of Venice, Department of Molecular Sciences and Nanosystems, Venezia, Italy*
[2]*Centro di Riferimento Oncologico di Aviano C.R.O. IRCC S, Pathology Unit, Aviano, Italy*
[3]*Ca' Foscari University of Venice, Department of Molecular Sciences and Nanosystems, Venice, Italy*

**Introduction**

Ovarian cancer (OC) is a silent fast deadly disease with the highest mortality rate among the gynecological cancer. Early diagnose is hard to achieve due to the lack of specific markers and symptoms. In fact, approximately 70% of women with OC are diagnosed at an advanced stage, leading to a 5-year survival rate lower than 45%. In this scenario, there is a need for the development of more appropriate biological models to enhance current therapeutic approaches. To date, it has been demonstrated that patient derived organoids (PDOs) are able to replicate the histopathological features and mutational landscape of the patient's primary tumor, being able to predict the patient's response to therapeutic drugs. Hence, the choice of the most effective drug in the shortest possible time could improve the overall survival of the patients.

**Material and Methods**

PDOs cultures were derived from primary tumors or ascites of HGSOC patients and were characterized for PAX8, CA125, WT1 and p53 by IHC. Long-term organoid cultures were compared with short-term cultures by IHC and proteomic analysis. PDOs were treated with 21 drugs for 96 hours and IC50s were calculated with an MMT-like assay. Finally, the data generated by the PDOs were compared with the patients' outcome.

**Results and Discussions**

A proteomic profile of the PDOs was established and more than 90% of the proteins analyzed undergo no significant changes in low- and high-passage organoids. IC50s of 21 standard and FDA approved drugs were calculated reporting no significative differences between short- and long-term organoids cultures. Organoids were able to replicate patients' response to the drugs. Among targeted therapies, CDK4/6, MEKi and AKTi were effective on more than 50% of the PDOs. Interestingly, a PDO resistant to Carboplatin and PARPi, derived from a BRCA1 mutated patient showed no benefit from Olaparib. Among emerging candidate targets, a Pin1 inhibitor developed by our group showed a good activity even on some platinum resistant patients. This highlight the potential utility of PDO even in the presence of clinically validated predictive biomarkers as BRCA mutations.

**Conclusion**

PDOs, recapturing the histological features of the parental tumor, could be a useful platform for drug screening in a short time, being able to predict drug responses before the administration to the patients. Moreover, it has been shown how this model is stable over time validating the advantages of having a biobank in which, inevitably, long-term cultures are generated.

AIRC IG23566

### EACR23-1466

### Plant-mediated green synthesis of gold-based nanoparticles for colon cancer applications and mechanisms

*K. Soto*[1], *A. Manzano Ramírez*[1], *I. Luzardo-Ocampo*[2]
[1]*CINVESTAV, MATERIALS, Queretaro, Mexico*
[2]*uaq, chesmestry, Queretaro, Mexico*

**Introduction**

Using plant extracts for the green synthesis of gold nanoparticles represents a viable alternative to traditional methods, which involve using chemical reagents and producing toxic by-products for humans and the environment. Plant-mediated synthesis is relatively mild, eco-friendly, cost and time effective; their phytochemicals and bioactive contents act as reducing, capping, and stabilizing agents. One of the most essential applications of gold nanoparticles is cancer treatment, which has become one of the leading causes of death in Mexico and seeks forms of treatment that replace conventional ones, whose side effects decrease patients' emotional and physical health

**Material and Methods**

The present work presents the synthesis of AuNPs with Roselle, and marigold ethanolic flower extracts, which will act as reducing and stabilizing agents; the extracts were evaluated by ABTS and DPPH methods for antioxidant activity and obtained a phenolic profile by HPLC. The synthesis of gold nanoparticles was carried out by monitoring the pH and temperature of the reaction, and the generated AuNPs were characterized by Transmission electron microscopy (TEM), Scanning electron microscope (SEM), X-ray diffraction (XRD), UV-visible spectroscopy, DLS analyses, thermogravimetric analyses (TGA), and the cytotoxic effect was evaluated in colon cancer cell lines (HT29 and SW480), the production of ROS and caspase-7 activation. The green nanoparticles were compared to AuNPs obtained with traditional methods.

**Results and Discussions**

The prepared nanoparticles have triangular, spherical, and hexagonal morphologies with diameters between 10 and 50 nm, and the time and quantity of reducing agent depend on the plant used. Marigold extracts require a lower quantity and reaction time to synthesize AuNPs; this can be related to the antioxidant activity and the type of compounds present in the extract. TGA analysis revealed that biomolecules present in the plant extract capped the nanoparticles and acted as stabilizing agents. The anticancer effect depends on the size and concentration of AuNPs; better results are obtained with Rosselle extracts (IC50:52.88 ug/mL in SW480) and high production of ROS species and caspasa7 activation.

**Conclusion**

Plant extracts synthesized AuNPs successfully acting as reducing and capping agents, AuNPs present cytotoxic activities against cancer cell lines. The activity presents a dose-response effect due to the production of ROS.

© 2023 The Authors. Molecular Oncology is published by John Wiley & Sons Ltd on behalf of Federation of European Biochemical Societies

### EACR23-1486
### PATIENT-DERIVED ORGANOIDS AS A PRECLINICAL PLATFORM TO IMPROVE THE TREATMENT FOR HEAD AND NECK SQUAMOUS CELL CARCINOMAS

*M. Álvarez-Fernández[1,2], M. Álvarez-González[1,3], E. Pozo-Agundo[1,2,3], B. de Luxán-Delgado[1,2], D. Corte[4], A. Villacé-Gallego[5], F. López[1,2,3,6], A. Astudillo[4], I. Fernández-Vega[4], J.P. Rodrigo Tapia[1,2,3,6]*
*[1]Instituto de Investigación Sanitaria del Principado de Ast urias ISPA, Head and Neck Cancer, Oviedo, Spain*
*[2]Instituto Universitario de Oncología del Principado de As turias IUOPA, University of Oviedo, Oviedo, Spain*
*[3]Centro de Investigación Biomédica en Red de Cáncer CIB ERONC, Instituto de Salud Carlos III, Madrid, Spain*
*[4]Biobanco del Principado de Asturias, Hospital Universitario Central de Asturias HUCA, Oviedo, Spain*
*[5]Hospital Universitario Central de Asturias HUCA, Radiophysics Unit, Oviedo, Spain*
*[6]Department of Otolaryngology, Hospital Central Universitario de Asturias HUCA, Oviedo, Spain*

**Introduction**

The expansion of the 3D organoid technology makes possible to develop patient-derived organoids (PDOs) that recapitulate histological features and physiological phenotypes of the tissue of origin. Head and neck squamous cell carcinoma (HNSCC) represent a tumor type arising from the epithelial cells of the larynx, pharynx, and nasal and oral cavities. Despite advances in cancer therapy, HNSCC remains with low survival rates (around 50%) mostly due to late diagnosis and limited treatment options. Surgery and/or concurrent chemo-radiotherapy remains the standard of care for advanced HNSCC tumors, but many patients do not response or develop resistance to treatment. And there are no predictive biomarkers available in the clinic.

Therefore, we aim to generate a biobank of HNSCC-derived PDOs as a preclinical model to evaluate the response to available treatments and new therapeutic strategies; and to evaluate its predictive value to guide therapy in functional precision medicine programs in the future.

**Material and Methods**

Fresh tissue samples have been obtained from HNSCC patients surgically treated or biopsied at Hospital Universitario Central de Asturias (HUCA). We have established organoid cultures following the protocol described by the laboratory of Hans Clevers. After expansion, PDOs have been cryopreserved, and also fixed and paraffin-embedded for histological evaluation.

**Results and Discussions**

More than 40 tumor organoids lines have been established and cryopreserved derived from HNSCC biopsies of different anatomic locations, mostly laryngeal. Organoids from oropharyngeal tumors have also been established, representing both Human papilloma virus (HPV) related and unrelated tumors. Interestingly, samples from recurrent or persistent tumors after chemo and/or radiotherapy treatment show lower outgrowth efficiency compared to biopsies from untreated tumors.

PDOs recapitulate the histological characteristics of the tumor of origin in terms of morphology and squamous differentiation. Moreover, these PDOs show differential response to radiation and cisplatin treatment, the standard of care for HNSCC.

**Conclusion**

To our knowledge, this is the largest collection of HNSCC-derived organoids, specially from laryngeal tumors. HNSCC PDOs constitute a powerful platform to study the biology of these tumors, but also to evaluate and predict treatment response in HNSCC patients.

### EACR23-1491
### Neuromodulation interventions for treating cancer-related pain: a systematic review and meta-analysis.

*D. Martinez-Magallanes[1], P.B. Kevin[1,2]*
*[1]Spaulding Rehabilitation Hospital and Massachusetts Gen eral Hospital- Harvard Medical School, Neuromodulation Center and Center for Clinical Research Learning, Boston- MA, United States*
*[2]Universidad San Ignacio de Loyola- Vicerrectorado de In vestigación, Unidad de Investigación para la Generación y Síntesis de Evidencias en Salud, Lima, Peru*

**Introduction**

Cancer-related pain is one of the most common complications whether related to metastasis, chemotherapy or radiation therapy. Either neuropathic or nociceptive, chronic cancer pain is a health priority given that it has a significant neuropathic pain component in about 40% of patients and its poor response to conventional pharmacological therapies. Therefore, innovative treatments such as neuromodulation techniques could be an option to manage chronic pain and it needs to be explored.

**Material and Methods**

We conducted a literature search in databases such as Pubmed and Embase to assess the implementation of neuromodulation techniques for treating cancer-related chronic pain. Interventions consisted in repetitive transcranial magnetic stimulation (rTMS), spinal cord stimulation (SCS), transcranial direct current stimulation (tDCS), dorsal root ganglion stimulation (DRGS). The primary outcomes were different pain scores such as Visual Analog Scale (VAS), Numeric Rating Scale (NRS) and Oral Morphine Equivalent (OME) levels. In total 15 (n=206) studies were included. We performed a random effect size meta-analysis.

**Results and Discussions**

We included 15 studies; 88% (13 studies, n= 117) were case reports or case series, and 12% were RCTs (2 studies, n=89). The most common neuromodulation techniques were SCS (67%), excitatory motor cortex rTMS (12%), and anodal motor cortex tDCS (5%). Only rTMS and tDCS have randomized evidence; the remaining techniques were tested in case reports and case series. The included populations were heterogeneous regarding cancer type and stage; however, most studies tested advanced cancer cases (palliative care setting) or cases with chemotherapy-related pain. The most common cancer types were hepatocellular carcinoma, non-small cell lung cancer, and breast cancer. Regarding efficacy data from RCTs, both studies have a low risk of bias, and the pooled effect size (VAS pain reduction) is SMD -1.12 (95% CI -1.57 to -0.67),

© 2023 The Authors. Molecular Oncology is published by John Wiley & Sons Ltd on behalf of Federation of European Biochemical Societies

I2=69.4%. None of the studies analyzed (RCT or case report) reported severe adverse effects, only minor temporary sensory side effects (e.g., skin redness, tingling and itching).

**Conclusion**

Neuromodulation techniques for cancer-related pain are a promising approach for pain management and reducing opioids usage, further studies for testing efficacy either as a treatment for cancer-related chronic pain or palliative care should be conducted.

### EACR23-1496

### Targeting cytochrome P450-expressing breast cancer cells for selective activation of duocarmycin bioprecursors

*S. Smarakan[1], E. Denasio[1], S. Shnyder[1], P. Loadman[1], H. Sheldrake[1], K. Pors[1]*

*[1]Institute of Cancer Therapeutics, Faculty of Life Sciences- University of Bradford- UK, Bradford, United Kingdom*

**Introduction**

Cytochrome P450 (CYP) enzymes play a critical role in the oxidation of both endogenous and xenobiotic compounds. The upregulation of CYP1A1, 1B1, and 2W1 in tumor tissue and surrounding stroma compared to adjacent normal tissue provides a unique opportunity for the development of targeted cancer therapeutics. The ultrapotent duocarmycins are ideal candidates for bioprecursor development and we have demonstrated that these can be re-engineered into derivatives selectively activated by CYP1A1, 1B1, and 2W1 in vitro and in vivo[1-3].

**Material and Methods**

Sensitive and drug-resistant breast cancer cells (MCF-7, MDA-MB-468, and MDA-MB-231) were used in the study to evaluate the effect of active duocarmycins and potential bio precursors. Synthesis and characterization of the compounds were performed as mentioned in the references below. Briefly, these include synthetic chemistry, the use of recombinant CYP bactosomes for metabolite identification using LC/MS, and chemosensitivity analysis using the MTT assay. Evaluation of CYP1-related functional enzymatic activity in breast cancer cells was measured by the fluorescence-based, 7-ethoxyresorufin-O-deethylase (EROD) assay. Phosphorylation of H2AX was used as a marker of DNA damage and the mechanism of cell death was studied by analyzing apoptosis markers using flow cytometry and western blotting.

**Results and Discussions**

A library of novel compounds was investigated *in vitro* and it was shown that the 3-modified CPI bio precursors were bioactivated to potent (10-100 nM) metabolites which were capable of damaging DNA as observed with a time-dependant increase in H2AX phosphorylation. The apoptosis assays showed an increased expression of cleaved PARP and cleaved caspase with increased drug incubation. LCMS confirmed the presence of hydroxylated seco-duocarmycins, and structure-activity relationship studies revealed that subtle changes to the substitution pattern of the DNA recognition motif resulted in changes to the CYP bioactivation and chemosensitivity. The duocarmycin bio precursors were intolerant by functionalization at selected positions on the DNA binding motif while position 5 was critical for potent bioactivation and anticancer activity.

**Conclusion**

Modifications to the structure of the seco-duocarmycins influence the extent of their CYP-mediated activation and indicate their potential for breast cancer chemotherapy.

[1] Sheldrake et al. J Med Chem. 2013, 56, 6273-7.[2] Travica et al. Clin Cancer Res. 2013, 19, 2952-61.[3] Pors et al. Chem Commun. 2011, 47, 12062-4.

### EACR23-1517

### Patient derived tumor cells identify mechanistically rational combinations for the PI3Kdelta inhibitor roginolisib in solid and haematologic malignancies.

*G. Di Conza[1], C. Riganti[2], C. Tarantelli[3], B. Kandathilparambil Sasi[4], M. Lahn[5], F. Bertoni[3], J.R. Brown[4]*

*[1]iOnctura, Research and Development, Geneva, Switzerland*

*[2]University of Turin, Dpt of Oncology, Turin, Italy*

*[3]Institute of Oncology Research- Universita della Svizzera Italiana USI, Faculty of biomedical sciences, Bellinzona, Switzerland*

*[4]Dana-Farber Cancer Institute- Boston- MA- USA, Dpt of Medical oncology, Boston, United States*

*[5]iOnctura SA, Research and Development, Geneva, Switzerland*

**Introduction**

Roginolisib (IOA-244) is a first in class allosteric modulator and non-ATP competitive PI3Kd inhibitor currently in a Phase 1 clinical study. In previous preclinical studies, roginolisib inhibits suppressive immune cells, such as Tregs and myeloid-derived suppressive cells (MDSC), while preserving proliferation and function of CD8 T cells. Consistent with prior PI3Kd inhibitors, roginolisib inhibits the *in vitro* growth of lymphoma cells. In contrast to other PI3Kd inhibitors, roginolisib activity is correlated with the expression levels of PIK3CD, suggesting cancer cell-intrinsic effects of IOA-244.

**Material and Methods**

We have used patient derived tumor cells to evaluate roginolisib in combination with immune-targeted or molecularly-targeted therapies to identify synergies that could be translated to future clinical studies.

**Results and Discussions**

Here, we show that in two ex vivo co-culture models of patient-derived mesothelioma cells with matched PBMC, the addition of roginolisib to cisplatin plus nivolumab specifically increased activated Ki67+/IFNg+ CD8 T cells and M1-like macrophages, and concomitantly decreased Tregs, exhausted TIM3+ CD8 T cells and MDSCs with an overall effect to increase the antitumoral immune response. A screen of 474 compounds in combination with roginolisib in one T cell and one B cell lymphoma cell lines prioritised several clinical compounds to test in patient derived tumor cells. In particular, Roginolisib synergised with BCL2 inhibitors in the cell lines, with validation in four patient-derived CLL cells from patients who had developed resistance to prior BTK inhibitor treatment.

**Conclusion**

© 2023 The Authors. Molecular Oncology is published by John Wiley & Sons Ltd on behalf of Federation of European Biochemical Societies

Fresh patient derived tumor cells are a powerful in vitro approach to identify potential combination to evaluate in cancer patients. Our data supports combining roginolisib with checkpoint inhibitors, for example in lung cancers, and targeted molecular therapies such as BCL2 inhibitors in CLL. The mechanistic synergy of these combinations has potential to provide greater patient benefit compared to the use of these medicines as single agents.

### Immunotherapy

### EACR23-0099
### Approach using « second generation » immune checkpoint inhibitors for the treatment of triple-negative breast cancer

M. Ancion[1], T. Lerho[1], C. Reynders[1], P. Roncarati[1], M. Luyckx[1], M. Renard[1], M. Herfs[1], P. Hubert[1]
[1]Liège University, GIGA Cancer- Laboratory of Experimental Pathology, Liège, Belgium

**Introduction**

Immunotherapy is revolutionizing cancer treatment. However, only a subset of patients benefit from it, the majority of them showing limited or no response. Triple-negative breast cancers (TNBC), highly heterogeneous and aggressive, represent 10-20% of invasive breast cancers and have no specific treatment yet. The blockade of novel "second generation" immune checkpoints could be promising to enhance the number of responders.

**Material and Methods**

This project aims at highlighting new immune checkpoints and studying the impact of their inhibition on TNBC progression. To this end, we selected potential immune checkpoints that showed high mRNA expression in TNBC using bioinformatic analyses. Next, we chose the targets displaying a higher protein expression in TNBC compared to the 3 other categories of breast cancer (LumA, LumB and HER2+) by using immunohistochemistry.
The proteins VISTA, sirp-α, CD47 and PVR were selected. Several murine syngeneic tumor models were used. Checkpoint expression was thoroughly investigated in 12 specific immune populations using flow cytometry. Monoclonal antibodies against said immune checkpoints were selected.
Using syngeneic mouse models, the effect of the monoclonal antibodies on tumor growth as well as on composition of the immune tumor microenvironment (TME) was assessed.

**Results and Discussions**

Tumor growth was significantly slowed down in Balb/C mice bearing 4T1 tumors treated with the anti-VISTA and anti-TIGIT (PVR ligand) antibodies, while no effect was shown in a comparable NOD-Scid model, hence confirming the effect is due to the reactivation of the immune system. In Balb/C mice, the anti-VISTA seems to elicit a drastic drop in the CD4+ and CD8+ regulatory T cells percentage in the treated group as well as an increase in the proportion of M1-like macrophages in the TME.
In C57Bl/6 mice bearing E0771 tumors, treatment with anti-VISTA, anti-sirp-alpha, anti-CD47 and anti-TIGIT antibodies significantly slowed down tumor growth. Here, similar effects on regulatory T cells were shown and the anti-VISTA seemed to increase the M1-like/M2-like macrophages ratio as well.**Conclusion**

We showed that blocking immune checkpoints such as VISTA remodelled the tumor immune microenvironment efficiently enough to slow down tumor growth in two syngeneic breast cancer models.
Further investigation will be carried out in order to highlight the activation status of the tumor resident immune cells and exact mechanisms at play.

### EACR23-0202
### "Second- generation" immune checkpoint inhibitors for the treatment of malignant pleural mesothelioma

M. Renard[1], M. Luyckx[1], P. Roncarati[1], M. Ancion[1], R. Célia[1], T. Lerho[1], P. Hubert[1], M. Herfs[1]
[1]University of Liège, Laboratory of Experimental Pathology, Liège, Belgium

**Introduction**

Malignant pleural mesothelioma (MPM) is a rare but aggressive form of cancer that is often diagnosed 30 to 50 years after exposure to asbestos. After diagnosis, patients with MPM have a life expectancy between 9 and 15 months, depending on treatment, histological subtype (epithelioid, biphasic or sarcomatoid), and stage of the disease. Recently, anti-PD-L1 and CTLA-4 combination immunotherapy has been shown to improve patient's outcome by 6 months compared to chemotherapy (cisplatin + pemetrexed). However, the overall response rate to these monoclonal antibodies remains relatively modest in most cases. Could second-generation immune checkpoint inhibitors represent a solution for treating MPM?

**Material and Methods**

In order to characterise the immune checkpoint expression profile of all MPM subtypes and their immune microenvironment, TCGA data as well as patient's samples included in our local Biobank will be used. Bioinformatics analyses and immunohistochemical experiments will be performed. Following this characterization, several syngeneic mouse models will be used and treated with the most promising immune checkpoint inhibitors.

**Results and Discussions**

Strikingly, we found that B7-H3, CD112 and the CD200/CD200R axis are much more highly expressed than PD-1, PD-L1 and CTLA-4 and, therefore, could represent relevant targets in the specific context of MPM. These results have already been confirmed at both mRNA and protein levels. Based on these results, we can now move on to the *in vivo* characterisation using syngeneic mouse models.

**Conclusion**

The immune checkpoints B7-H3, CD112, CD200 and CD200R seem promising for MPM treatment with increased expression compared to current immunotherapy. Of course, monoclonal antibodies targeting these so-called "second generation" immune checkpoints need to be tested *in vivo* to validate their therapeutic efficiency and decipher their mechanism of action.

© 2023 The Authors. Molecular Oncology is published by John Wiley & Sons Ltd on behalf of Federation of European Biochemical Societies

### EACR23-0204
### "Second-generation" Immune Checkpoint Inhibitors for the Treatment of Malignant Pleural Mesothelioma

*M. Luyckx[1], M. Renard[1], M. Ancion[1], C. Reynders[1], T. Lerho[1], P. Roncarati[1], P. Hubert[1], M. Herfs[1]*
*[1]University of Liège, Laboratory of Experimental Pathology, Liège, Belgium*

**Introduction**

Malignant pleural mesothelioma (MPM) is characterized by a poor prognosis with an overall survival ranging from 9 to 18 months depending on the histological subtypes, the stage of the disease and the treatment.

The combination of pemetrexed and cisplatin was the gold standard for the treatment of malignant pleural mesothelioma (MPM) and achieves a survival of 12 months. However, in 2021, the combination of two immune checkpoint inhibitors has been shown to significantly increase the survival compared to the standard-of-care chemotherapy (OS~18 months). This observation led to the direct FDA approval of this combination.

While the approved inhibitors are targeting three well-known immune checkpoints (PD-1, PD-L1 and CTLA-4), there is a growing interest in revealing the potential therapeutic efficiency of drugs targeting "second-generation" immune checkpoints.

This project aims at selecting promising immune checkpoints in the context of human pleural mesothelioma and studying the potential therapeutic effects of their inhibition.

**Material and Methods**

First, bioinformatic analyses were performed to select the most interesting/promising targets based in their mRNa levels. Then, the collected data were confirmed by immunohistochemistry (IHC) on 66 human specimens of pleural mesothelioma. Later, *in vitro* and *in vivo* models will be used to precisely decipher the immune microenvironment of MPM, as well as the immune populations involved in the potential therapeutic effect of immune checkpoint inhibitors of interest.

**Results and Discussions**

B7-H3, CD112 and the CD200/CD200R axis were selected as promising targets. We showed that all four were more highly expressed compared to PD-L1 both at the mRNA and protein levels. Although these immune checkpoints seem to be promising targets for future patient treatment due to their high expression profile in MPM, a high expression profile does not automatically mean that they are better targets. *In vitro* and *in vivo* models will be essential to clearly identify the impact of the blockade of these latter immune checkpoints.

**Conclusion**

MPM patients highly need better therapeutic strategies, which will significantly increase their survival. The approval of the combination of ipilimumab and nivolumab has highlighted the benefit of immunotherapy for the treatment of MPM. Our results suggest that other immune checkpoints could be more beneficial to patients, as they are more expressed by tumor cells and/or immune cells.

### EACR23-0207
### Multiplexed proximity ligation-based method uncovers immune checkpoint activation in the context of the tumor microenvironment

*S. Bodbin[1], J. Vennberg[1], D. Raykova[1], K.I. Au Ieong[1]*
*[1]Navinci Diagnostics, Research and Development, Uppsala, Sweden*

**Introduction**

The interaction of immune checkpoint (IC) proteins PD1 and PD-L1 plays a pivotal role in initiating T cell inhibition and preventing autoimmune responses in healthy cells. In cancer, it facilitates tumor evasion from the immune system, which makes ICs promising immunotherapy targets. However, the success of IC inhibition (ICI) has been limited to a poorly defined subset of patients. ICI therapy may fail because of insufficient antigen presentation/colocalization of T cells and antigen-presenting cells, CD8 T-cell exhaustion, etc. Further, identifying responders may better correlate with high levels of PD1/PD-L1 interaction than with the overexpression of either protein alone, which makes *in situ* proximity ligation technology a valuable tool in patient stratification. Here, we combined the power of interaction detection to identify PD1/PD-L1 axis activation with the concomitant visualization of relevant biomarkers to create an immune profile in aid of immunotherapeutic strategies.

**Material and Methods**

Deparaffinization and antigen retrieval on human FFPE healthy and tumor tissues was performed according to standard protocols. We then used proprietary Naveni *in situ*-plex technology based on proximity ligation and flexible choice of biomarkers to stain the tissues. The PD1/PD-L1 interaction and a combination of two single proteins e.g., cytokeratin and CD8, were visualized. A multiplex fluorescent readout was acquired via standard epifluorescent microscopy without a need for rehybridization or multiple imaging cycles.

**Results and Discussions**

Tissues were simultaneously stained for one IC interaction and two biomarkers of choice (for mature/cytotoxic/helper T cells, or tumor markers e.g., Ki67, cytokeratin). The detection of the PD1/ PD-L1 interaction has the potential to make a difference in immunotherapy as its presence indicates activation of the IC axis in the tumor, not merely IC protein expression. Since the communication between PD1 and PD-L1 is not strictly limited to immune cells but can also be used by cancer cells, the addition of relevant biomarkers was useful in demonstrating whether T cell inhibition had occurred as part of the normal immune process (when interaction-positive cells were two immune cells) or was a sign of tumor evasion (when communicating cells were positive for tumor markers too).

**Conclusion**

Our novel *in situ*-plex technology is a multiplexed solution which creates tissue profiles with application in immunooncology and can aid patient stratification for immunotherapy.

### EACR23-0218
### Targeting βig-h3-a key stromal protein in cancer

© 2023 The Authors. Molecular Oncology is published by John Wiley & Sons Ltd on behalf of Federation of European Biochemical Societies

A. Hennino[1], S. Bachy[2], K. Aguerra[1], C. de la Fouchardière[3]
[1]StromaCare, Research and Development, Lyon, France
[2]StromaCare, Research and Developement, Lyon, France
[3]Centre Léon Bérard, Oncology, Lyon, France

**Introduction**

Solid cancer is associated with a stromal reaction leading to immune escape and tumor growth. We aimed to study the impact of stromal components on the modulation of the antitumoral immune response and therapeutics in solid cancer. We identified βig-h3 stromal protein as a key actor of the immune paracrine interactions mechanism that drives solid cancer ie pancreatic, ovarian cancer and melanoma. This protein acts directly on tumor-specific CD61 expressing $CD8^+$ T cells in pancreatic cancer (PC) and melanoma and on unconventional T cells in ovarian cancer as well as F4/80 macrophages.

**Material and Methods**

We performed studies with mouse models of PC, ovarian cancer and melanoma. Some mice were given injections of anti-βig-h3 and anti-PD1 depleting antibodies. Tumor growth as well as modifications in the activation of local immune cells were analysed by flow cytometry, immunohistochemistry and immunofluorescence. Tissue stiffness was measured by atomic force microscopy.

**Results and Discussions**

We identified βig-h3 stromal-derived protein as a key actor of the immune paracrine interaction mechanism that drives several solid cancers. We found that βig-h3 is highly produced by cancer-associated fibroblasts and macrophages in the stroma of human and mouse. This protein acts directly on tumor-specific $CD8^+$ T cells and F4/80 macrophages. Depleting βig-h3 *in vivo* reduced tumor growth by enhancing the number of activated $CD8^+$ T cell within the tumor and subsequent apoptotic tumor cells. Furthermore, βig-h3 has also been shown to bind to several extracellular matrix molecules, such as collagens I. By using atomic force microscopy, we show that βig-h3 binds to type I collagen and establishes thicker fibers. The use of our mAb reduces the stiffness of the stroma leading increased cytotoxic $CD8^+$ T cell infiltration. We found that targeting βig-h3 in PD-1 resistant melanoma was able to unleash the efficacy of immune therapy when used in combination with PD-L1/PD-1 inhibition treatment.

**Conclusion**

Our data indicate that targeting stromal extracellular matrix protein βig-h3 improves the antitumoral response, consequently reduces tumor weight and reverse resistance to PD-1 therapy. Our findings present βig-h3 as an ubiquitary target in solid stromal cancer. We developed and humanized a specific mAb to deplete βig-h3 protein. The project is entering its toxicity phase in cynomolgus.

### EACR23-0236
### Antitumor dynamics of Natural Killer cells in 3D tumor organoid models

J. Shi[1]
[1]Hong Kong Baptist University, Department of Physics, Kowloon, Hong Kong- China

**Introduction**

Natural Killer (NK) cell plays a central role in cancer immunosurveillance and is considered a promising therapeutic candidate for cancer treatment.

**Material and Methods**

To acquire new dynamic insight into how NK cells move and interact with tumor cells in a 3D microenvironment, we develop novel 3D live-cell imaging assays and machine learning-assisted single cell analysis to comprehensively profile and quantify the interaction dynamics between primary NK cells from human blood and patient-derived gastric tumor organoids. Results from the 3D microscopy analysis allow us to investigate the dynamic control by which NK cells detect, migrate to and kill tumor cells in a complex 3D microenvironment.

**Results and Discussions**

Preliminary data of a selected panel of gastric tumor organoids of five distinctive molecular subtypes (MSI, Intestinal, Diffuse, Mixed and EBV) already revealed intriguing tumor variability in their NK cell responses, in particular the extent of directed NK cell motion towards the distinct tumor organoids and NK-tumor cell interaction after NK cell infiltration into the 3D tumor.

**Conclusion**

Further analysis of the underlying molecular regulators will not only identify potential targets to improve NK cell-based cancer immunotherapy but also serve as a paradigm to employ 3D imaging analysis of immune-tumor co-culture model to elucidate other important immuno-oncology dynamics, e.g., involved in T functions.

### EACR23-0238
### Exploring the anti-tumor activity of plasmacytoid dendritic cells

T.W. Bjerg[1], M.R. Jakobsen[1], R.O. Bak[1]
[1]Aarhus University, Department of Biomedicine, Aarhus C, Denmark

**Introduction**

Plasmacytoid dendritic cells (pDCs) are rare, unique immune cells that only make up 0.1% of human peripheral blood mononuclear cells (PBMCs). Recently, they were grouped as a new type of innate lymphocytes due to their broad immunological functions in modulating both the innate and adaptive immune system. In contrast to their well-described role in antiviral defense, little is known about pDC biology in cancer. This can be linked to the low numbers of pDCs in blood and tissue, combined with a low proliferation potential and resistance toward genetic manipulations. Some reports indicate that pDCs are present in tumor tissue and can be correlated with both a positive and a negative outcome for cancer patients. Cancer therapies targeting pDCs have shown clinical effects, but whether this is direct or indirect effects of pDCs remains unresolved

**Material and Methods**

Here we use a hematopoietic stem and progenitor cell (HSPC) model for generating human pDCs to gain a better biological understanding of different antitumoral effector mechanisms of pDCs. Our main focus is to explore whether and how pDCs mechanistically induce killing of cancer cells. A thorough immunophenotypic characterization and analysis of the transcriptomic landscape of activated pDCs were used to screen for potential genes and proteins with importance for the

© 2023 The Authors. Molecular Oncology is published by John Wiley & Sons Ltd on behalf of Federation of European Biochemical Societies induction of cell death. These targets were used to investigate the interaction between tumor cells and pDCs in co-culture studies *in vitro*.

**Results and Discussions**

In co-cultures of pDCs with different human cancer cell lines we demonstrate pronounced killing within 24 to 48 hours. Mechanistically, we demonstrate that killing is dependent on two different signals: an initial cell-to-cell contact had to be established, and in parallel a selective activation of pDCs was needed to elicit killing. The cytotoxic capabilities of pDCs could be correlated to an upregulation of proapoptotic genes including granzyme B.

**Conclusion**

Gaining more knowledge of pDCs and what drives the anti-tumor activity can help the field further understand the role of tumor infiltrating pDCs, and more importantly, it can lead the way for novel cancer therapies based on pDCs.

### EACR23-0249

### An unsurmountable immunologic barrier? A detailed view on the interaction of hypermutated colon cancer with autologous T cells

*S. Schwarz*[1], *M. Krohn*[1], *M. Loeffler*[2], *C.S. Linnebacher*[1], *A. Schlosser*[3], *M. Linnebacher*[1]

[1]*University Medical Center Rostock, General Surgery- Molecular Oncology and Immunotherap y, Rostock, Germany*

[2]*University Hospital Tübingen, Department of General- Visceral and Transplant Surgery, Tübingen, Germany*

[3]*University of Würzburg, Rudolf-Virchow Center- Center for Integrative and Translational Bioimaging, Würzburg, Germany*

**Introduction**

Recently, immunotherapies revolutionized cancer treatment, but the proportion of patients actually benefitting is still limited. Reasons for therapy failure are ambiguous, thus underlining the need for an improved understanding of the mutual relationship between autologous T and tumor cells.

**Material and Methods**

Two hypermutated colon cancer cases with retained HLA expression were selected. Patient T cells were from peripheral blood (pTc) or tumor infiltrate (TIL) and the proportion of exhausted as well as regulatory T cells was determined. In co-cultures, the interaction of respective cell populations was examined. The degranulation capacity of T cells was tested as: 1) pTc vs. TIL; 2) untreated coculture vs. treated coculture (immune checkpoint inhibitors (ICI), inhibitor of proteinase inhibitor 9 (PI-9i)) and 3) untreated vs. T cells stimulated with tumor-specific peptides.

**Results and Discussions**

TIL were dominated by $CD4^+$ T cells as well as exhausted T cells, explaining the less effective tumor recognition compared to pTc. With the amount of regulatory T cells not exceeding 8%, immune suppression by inhibitory T cells seemed, however, to be negligible. Highest relative degranulation was by pTc, not in contact with tumor cells before the assay. This highlights the immunosuppressive effects induced by the autologous tumor cells. This was not ascribable to IL-10 secretion, but decreased levels of pro-inflammatory cytokines were observed in co-cultures. Unexpectedly, treatment with ICI did not restore anti-tumor activity. Stimulation with tumor-specific peptides heightened degranulating cell amounts, but even then, ICI could not further improve tumor cell recognition.

In final kill assays, unstimulated T cells did not eliminate tumor cells (3%) but ICI and PI-9i improved killing (32 and 33%). Peptide-stimulated T cells were effective (77%), with weak effects of ICI and PI-9i addition (both 83%). Most surprising, combining both immune modifiers canceled out the anti-tumor effects in unstimulated (0%) and peptide-stimulated T cells (66%).

**Conclusion**

These results strengthen the supremacy of peptide-stimulated compared to unstimulated T cells. They further imply that for personalized immunotherapies, pTcs could not only substitute for but even be superior to tumor-infiltrating ones. The unexpectedly low effects of the applied inhibitors (ICI and PI-9i) underline the importance of further research on tumor immune evasion strategies to overcome immunologic barriers erected by a given tumor.

### EACR23-0281

### Combating anti-PD-L1 resistance in hepatocellular carcinoma

*G.F.W. Sit*[1,2], *D.K.C. Chiu*[1], *J.W.S. Cheu*[1,2], *V.W.H. Yuen*[1,2], *H. Deng*[1], *B.P.Y. Wong*[1,2], *K.K.L. Kwan*[1,2], *H.Y.H. Lau*[1], *Y. Zhang*[1,2], *C.C.L. Wong*[1,2,3]

[1]*The University of Hong Kong, Department of Pathology, Hong Kong, Hong Kong- China*

[2]*Centre for Oncology and Immunology, Hong Kong Science Park, Hong Kong, Hong Kong- China*

[3]*State Key Laboratory of Liver Research, The University of Hong Kong, Hong Kong, Hong Kong- China*

**Introduction**

Hepatocellular carcinoma (HCC) is characterized by an immunosuppressive tumor microenvironment.

Programmed death 1 (PD-1), one of the most upregulated immune checkpoints on T cells, binds with programmed death-ligand 1 (PD-L1) and fosters T cell exhaustion. Nivolumab, an anti-PD-1, was not superior to sorafenib, indicating resistance against immune checkpoint inhibitors (ICIs). Recently, FDA approved the combination of atezolizumab, an anti-PD-L1, plus bevacizumab, an antibody targeting vascular endothelial growth factor (VEGF), as a new 1$^{st}$ line treatment for advanced HCC due to its superiority to sorafenib, showing that ICI combination treatment is promising.

However, little is known about anti-PD-L1 resistance in HCC. Exploring how it modulates the tumor microenvironment might help unlock its potential. Therefore, we study its resistance mechanism by using cytometry by time of flight (CyTOF) and flow cytometry.

**Material and Methods**

Using hydrodynamic tail vein injection (HDTV), we established an anti-PD-L1 resistant HCC C57BL/6 model. Anti-PD-L1 was given, and tumors were analyzed by CyTOF and flow cytometry and processed using FlowJo. Data were analyzed by Student's t-test and one-way ANOVA.

**Results and Discussions**

© 2023 The Authors. Molecular Oncology is published by John Wiley & Sons Ltd on behalf of Federation of European Biochemical Societies

To mimic the tumor microenvironment of human HCC, we used HDTV for the spontaneous development of HCC. Anti-PD-L1 did not inhibit tumor growth and thus is ideal for studying its resistance.
To understand how anti-PD-L1 modulates the tumor infiltrating lymphocytes (TILs), we employed CyTOF to analyze over 30 surface markers simultaneously. Distribution of TILs was presented in 2D via UMAP, showing that anti-PD-L1 led to an enrichment of $CD8^+$ T cells. We applied Phenograph to classify the TIL subpopulations and identified 20 unsupervised TIL clusters. Clusters expanded by anti-PD-L1 were effector memory $CD8^+$ T cells demonstrating high expression of immune checkpoints, especially PD-1, TIGIT, CD96, LAG3 and CD39.
To verify CyTOF data, we repeated this experiment using flow cytometry. Similarly, there was an increase in tumor size and a significant increase in tumour-infiltrating $CD8^+$ T cells. PD-1, TIGIT, LAG3 and CD39 were also being upregulated significantly, except for CD96.

**Conclusion**

Through CyTOF and flow cytometry, we identified that immune checkpoints PD-1, TIGIT, LAG3 and CD39 were upregulated after anti-PD-L1 treatment. We thereby proposed that pairing anti-PD-L1 with inhibitors neutralizing either one of them is a novel combination treatment for advanced HCC.

### EACR23-0343
### Evaluating the safety and effect of chemotherapy and localised immunotherapy for the treatment of inoperable pancreatic cancer using an implantable device

*E. Minaei[1,2], R. Turner[1,2], S.J. Wade[1,2], C. Penney[1,2], M. Ranson[1,2], M. Aghmesheh[1,3]*
*[1]Illawarra Health and Medical Research Institute, University of Wollongong- Wollongong, Wollongong- NSW, Australia*
*[2]School of Chemistry and Molecular Bioscience, Molecular Horizons- University of Wollongong, Wollongong- NSW, Australia*
*[3]Nelune Comprehensive Cancer Centre, Bright Building- Prince of Wales Hospital, Sydney- NSW, Australia*

**Introduction**

Pancreatic cancer (PC) remains insensitive to immune checkpoint inhibitors (ICIs) due to its immunosuppressive microenvironment. While evidence suggests chemotherapy causes immunogenic cell death and promotes T cell activation, it has not enhanced the efficacy of ICIs in PC. Even the combination of chemotherapy and ICIs with other immune priming agents such as anti-CD40 antibodies (αCD40) are yet to improve treatment outcomes as the effective dose does not reach the tumour site and often results in immune-related adverse events. For this reason, we developed a biodegradable polymeric implant and assessed its safety and efficacy in delivering αPD1+αCD40 in a pancreatic mouse model compared to systemic administration of those agents with chemotherapy.

**Material and Methods**

Implants containing αCD40+αPD1 or empty implants (control) were prepared as described by our group previously and inserted into C57BL/6 mice bearing Luc-tagged KPC subcutaneous tumours. Anti-tumour efficacy against systemic treatment was determined by measuring tumour growth using Bioluminescence imaging (N=10 mice per cohort). Mice also received gemcitabine+nab-paclitaxel chemotherapy. Immune system activation was analysed using flow cytometry and LEGENDplex assay on the tumour-draining lymph node and plasma, respectively.

**Results and Discussions**

The radiance intensity of tumours was significantly lower in mice treated with immunotherapy implants compared to other groups indicating local treatment's efficiency in restraining tumour growth. Further, mice treated with the immunotherapy implants showed no evidence of metastasis. Inflammatory cytokines including GM-CSF were elevated in the systemic compared to the local treatment arm, leading to the activation of T cells marked by a higher percentage of CD8+PD1+ T cells compared to the local arm. In contrast, the proportion of M1 anti-tumour macrophages was significantly higher in the localised treated arm. At the later time point, there was an elevated amount of activating cytokines belonging to the IL1 family in the local compared to the systemic treatment arm, suggestive of a prolonged effect of the local treatment due to a gradual release of the drugs from the implantable device. This finding was confirmed by analyzing the implant's release profile. We found 8% of the antibodies were still present at the end of the study.

**Conclusion**

Local administration of αCD40+αPD1 immunotherapy with chemotherapy was safe and more efficient in restraining tumour growth and metastasis than systemic treatment.

### EACR23-0353
### Vinorelbine and intermittent cyclophosphamide sensitize an aggressive Myc-driven B-cell lymphoma to anti-PD-1 by an immunological memory effective against tumor re-challenge.

*S. Orecchioni[1], P. Falvo[1], G. Talarico[1], G. Bravetti[1], G. Mitola[1], P. Mancuso[1], P. Nicoli[2], F. Bertolini[1]*
*[1]European Institute of Oncology, Department of Hematology, Milan, Italy*
*[2]European Institute of Oncology, Department of Experimental Oncology, Milan, Italy*

**Introduction**

Immune checkpoint inhibitors (ICIs) have been successfully introduced in the therapy of a variety of cancer types. However, only a fraction of cancer patients benefits from ICIs, and the clinical benefit is limited in time. We described that a triple therapy (TT, Falvo et al, Cancer Res 2021) involving antigen-presenting cell activation by vinorelbine (V) and the generation of new TCF1+ stem cell-like T cell (scT) clones by an intermittent dosage of cyclophosphamide (C140) can significantly improve the efficacy of anti-PD-1 in two triple negative breast cancer (TNBC) models otherwise poorly sensitive to ICIs. TT effect was due to T cells, as it was abrogated by the in vivo depletion of CD4+ and/or CD8+ T cells.

© 2023 The Authors. Molecular Oncology is published by John Wiley & Sons Ltd on behalf of Federation of European Biochemical Societies

**Material and Methods**
In the present study we describe a model of a very aggressive hematological malignancy resistant to anti-PD-1, ie a disseminated, orthotopic, Myc-driven B-cell lymphoma, and compared TT with other therapies currently used in the clinic for this disease.
**Results and Discussions**
TT, but not other therapies including doxorubicin and platinum salts, sensitized lymphoma cells to anti-PD-1, controlled lymphoma progression and generated an immunological memory that in the large majority of mice avoided lymphoma generation even when lymphoma cells were re-injected in doses 2.5 times greater than the previous inoculum. Terminally exhausted T cells were abundant in the control, and decreased in TT-treated mice. TT was associated with a significant decrease in exhausted $CD8^+$ T cells compared to controls and C140-treated mice. A similar trend was also observed for exhausted $CD4^+$ T cells. These results suggest that TT reshapes T cell landscape and selects for anti-tumor effective T cell clones. We also analyzed exhausted $CD4^+$ and $CD8^+$ T cells along with their PD-$1^+$TIM$3^+$ subpopulations in the spleen. Exhausted T cells dramatically increased in the $CD4^+$ and $CD8^+$ subpopulations in lymphoma-bearing mice. At variance, in TT-treated mice were lymphoma did not grow after re-challenge we observed very few exhausted T cells. This evidence suggests a splenic selection for anti-tumor T cells. In TCR clonal analyses of splenic $CD8^+$ T cells, after tumor re-challenge we detected two specific peaks in mice resistant to lymphoma.
**Conclusion**
TT generates an anti-cancer immunological memory including scT cells with enhanced proliferative and anti-tumor potential, selected for a restricted T cell repertoire. When phenotypically defined, these cells might be considered for possible auto/allo cellular therapies.

### EACR23-0439
### Immunogenic cell death is required for the anticancer effect of high-dose vitamin C

*A. Cavaliere[1], M. Macagno[1], F. Maione[1], V. Amodio[2], R. Chilà[2], G. Germano[2], S. Lamba[1], P.P. Vitiello[2], A. Lorenzato[1], I. Cavaglià[1], N. Congiusta[1], V. Pessei[1], A. Bardelli[2], F. Di Nicolantonio[1]*
*[1]Candiolo Cancer Institute- FPO-IRCCS, Department of Oncology- University of Torino, Candiolo, Italy*
*[2]IFOM- The FIRC Institute of Molecular Oncology, Department of Oncology- University of Torino, Milan, Italy*
**Introduction**
Despite the success of immunotherapy in promoting long-term disease control in sone cancer patients, most cases are primarily resistant or become refractory to immunotherapy. There is a need to identify additional strategies that could stimulate anticancer immune response. Several anticancer agents are known to promote immunogenic cell death (ICD) by killing cancer cells, inducing antigen release and immune priming, which all trigger an anti-cancer immune response. We have previously shown that VitC can promote cytotoxic T cell activity and cooperate with anticancer immune checkpoint therapy in immunocompetent mice, but the molecular mechanisms underlying these observations remain to be clarified. We hypothesized that VitC could act as an ICD inducer.
**Material and Methods**
We employed BRAF mutant colorectal tumors (VBC9 and VPFB6) and orthotopic breast cancers (TS/A MMRd). We assessed ICD markers including high-mobility group box 1 protein (HMGB1), calreticulin (CALR). To functionally characterize the role of ICD in mediating the anticancer effects of VitC in vivo, we employed a CALR blocking antibody and immune profile analysis.
**Results and Discussions**
We found that administration of high dose VitC delayed tumor growth in syngeneic immunocompetent mice, but not in immunocompromised hosts. Analyses performed on tumors explanted from vitamin C treated mice revealed strong 8-hydroxyguanosine staining as a marker of oxidative stress. This was accompanied by induction of ICD markers CALR and HMGB1 in tumors from both immunocompetent and immunocompromised animals treated with VitC. Reactive oxygen species abrogation by concomitant administration of N-acetylcysteine and high-dose VitC largely prevented induction of ICD markers in vivo. The administration of a CALR blocking antibody was able to prevent CALR translocation and HMGB1 release in murine tumors. Notably, preventing ICD by anti-CALR antibody was able to impair the anticancer effect of VitC in breast and colon mouse tumors. The immune profiling performed on cancer bearing mice revealed that VitC enhanced the infiltration and activation of the NK and T lymphocytes and decreased the TREGs. CALR blockade was sufficient to abolish the reshape of immune tumor microenvironment in VitC treated mice.
**Conclusion**
Our data indicate that high dose VitC as monotherapy could foster ICD in several murine cancer models and that this effect is required for unleashing an anticancer immune response.

### EACR23-0455
### Adenovirus encoded anti-CTLA-4 is a potent genetic adjuvant of Adenovirus based neoantigen vaccine for more robust and effective anti-tumor response

*L. Nocchi[1], I. Garzia[1], L. Seclì[1], G. Cotugno[2], F. Troise[2], S. Allocca[2], G. Romano[2], A. Nicosia[3], E. Scarselli[1], A.M. D'Alise[1]*
*[1]Nouscom Srl, Immunology, Rome, Italy*
*[2]Nouscom Srl, Vector Construction, Rome, Italy*
*[3]University of Naples Federico II, Department of Molecular Medicine and Medical Biotechno logy, Naples, Italy*
**Introduction**
Genetic vaccines based on viral vectors represent a promising approach with the potential to become an important modality for cancer treatment. The discovery of tumor neoantigens has renewed the interest in the field of cancer vaccination, given their potential of inducing strong immunogenicity and effective anti-tumor activity compared with classical tumor-associated antigens. However, cancer vaccines still face the complexity of inducing a T cell response in the hostile tumor microenvironment and new vaccine adjuvant approaches

© 2023 The Authors. Molecular Oncology is published by John Wiley & Sons Ltd on behalf of Federation of European Biochemical Societies are needed to achieve more effective vaccines. We have previously shown that neoantigens-based Adenoviral (Ad) vaccines synergize with anti-PD-1 leading to cancer cure in 50% of treated animals in different tumour models.

**Material and Methods**

Here, we generated an Ad encoding an anti-CTLA-4 antibody (Ad-α-CTLA-4) to enhance immunogenicity of Ad-based cancer vaccine and to improve its anti-tumor efficacy.

**Results and Discussions**

The delivery of Ad-α-CTLA-4 co-administered with a neoantigen-based Ad vaccine results in significantly higher neoantigen specific T cell responses as compared to the delivery of the antibody in its proteinaceous form. Interestingly, the adjuvant activity requires the co-administration with Ad vaccine. In a setting of therapeutic vaccination in tumor bearing mice, the Ad-α-CTLA-4 adjuvanted vaccine combined to anti-PD-1 led to stronger anti-tumor activity, with tumor eradication observed in nearby 100% of treated animals, compared to the not adjuvanted vaccine and anti-PD-1. The higher efficacy correlated with the reduction of intratumoral Treg and increase of T effector cells. The enhancement of vaccine-induced immune response was confirmed also in the context of viral antigens.

**Conclusion**

These findings support the exciting potential of this approach to develop more effective genetic vaccines.

### EACR23-0602

### Development of an absolute quantitation method for genetically modified cell therapies

_E. Bugallo Blanco_[1], *J. Cleaver*[2], *J. Foster*[2], *R. Kasahni*[2], *S. Papa*[1], *J. Sosabowski*[2]

[1]*King's College London, Research Oncology, London, United Kingdom*

[2]*Barts Cancer Institute, Molecular Imaging, London, United Kingdom*

**Introduction**

Chimeric Antigen Receptor (CAR) T-cell immunotherapy has shown promising results in the treatment of haematological tumours; however, the success rate drastically decreases for solid tumours. In this case, CAR T cells face several challenges they need to overcome, such as, the need to migrate to the tumour site, extravasate, proliferate and persist to elicit a response, but little is known about these dynamic processes. We propose the use of non-invasive serial *in vivo* imaging of CAR T-cells to shed light on these challenges and improve our understanding of CAR T-cell behaviour, dosing, and reasons behind treatment outcomes; ultimately paving the way to safer and more efficacious immunotherapeutic drugs for solid tumours.

**Material and Methods**

In the present study, we co-expressed a prostate-specific membrane antigen (PSMA) targeting CAR with the human sodium iodide symporter (hNIS), to establish a mathematical model to quantify CAR T-cells *in vivo* using single-photon emission computed tomography-computerized tomography (SPECT/CT).

**Results and Discussions**

The CAR T cells were extensively characterized, showing strong antigen-specific lysis and proliferation. CAR T-cells phenotype consisted of a mixture of effector and memory cells, with further enrichment of effector memory T-cells after antigen exposure. Radionuclide influx and efflux by the CAR T cells was rapid and it did not alter their proliferative capacity or anti-tumoral response. Significantly, only high doses of tracer induced DNA damage, which was repaired within 24 hours. High sensitivity was observed with our SPECT system using the extra-extra ultra-high sensitivity collimator; the limit of detection being just under 4,000 CAR T cells.

*In vivo*, we demonstrate that the use of the clinically relevant tracer technetium pertechnetate can be used for the spatial and temporal monitoring of infiltrating CAR T cells in cancer-bearing mice. CAR T cell infiltration was confirmed *ex vivo* by immunohistochemistry and the number of infiltrating CAR T cells was quantified by flow cytometry.

**Conclusion**

These data demonstrate the feasibility of using the hNIS reporter gene as a universal tool for CAR T cell monitoring. We can study tracer kinetics and accumulation at the tumour site through dynamic SPECT imaging. We propose using a two-compartment model to quantify CAR T-cells homing to the tumour non-invasively. This model could provide much-needed information regarding dosing and quantity of CAR T-cells required to induce tumour regression.

### EACR23-0627

### Antibody blockade of PSGL-1, a novel immune checkpoint protein, enhances human T cell activation against lymphoma cells

_J.L. Pereira_[1], *F. Ferreira*[1], *A. Matos*[2], *D. Pereira*[3], *R.F. Santos*[4], *A.M. Carmo*[4], *M.J. Oliveira*[2], *J.C. Machado*[1], *N.R. dos Santos*[1]

[1]*i3S - Instituto de Investigação e Inovação em Saúde-Universidade do Porto, Intercellular Communication and Cancer, Porto, Portugal*

[2]*i3S - Instituto de Investigação e Inovação em Saúde-Universidade do Porto, Tumour and Microenvironment Interactions, Porto, Portugal*

[3]*IPO-Porto-Portuguese Oncology Institute of Porto, Service of Onco-Hematology, Porto, Portugal*

[4]*i3S - Instituto de Investigação e Inovação em Saúde-Universidade do Porto, Cell Activation & Gene Expression, Porto, Portugal*

**Introduction**

Lymphomas represent a diverse collection of malignancies, most of which were shown to be refractory to immune checkpoint therapies (e.g. anti-PD-1). Although P-selectin glycoprotein ligand-1 (PSGL-1) was found to be an immune checkpoint protein promoting mouse T cell exhaustion in the context of cancer and infection, an immune regulatory function in human T cells has so far remained to be demonstrated. In this study, we aimed to evaluate PSGL-1 expression dynamics on resting vs activated human T cells and test the potential of PSGL-1

© 2023 The Authors. Molecular Oncology is published by John Wiley & Sons Ltd on behalf of Federation of European Biochemical Societies antibody targeting to stimulate T cell responses against lymphoma.

**Material and Methods**

Human healthy donor T cells were cultured with irradiated Raji lymphoma B-cell line. T cell immunological synapses were analyzed by CD3 and PSGL-1 immunofluorescence staining. T-cell activation was assessed by flow cytometry detection of CD25, CD69, IL-2 and IFNγ. *In vitro* exhausted T cells were generated by repeated CD3/CD28 stimulation and flow cytometry detection of PD-1, TIM-3 and LAG-3. Patient lymphoma cells were obtained from excised lymph nodes. The PSGL-1 PL-1 mAb was used in cocultures.

**Results and Discussions**

We found that PSGL-1 expression decreased in healthy donor $CD4^+$ and $CD8^+$ T cells upon CD3/CD28 stimulation. While resting T cells showed PSGL-1 distributed across the cell membrane, PSGL-1 polarized to the opposite pole of the immunological synapse established between T and Raji antigen-presenting cells. This suggests that, not only PSGL-1 expression decreases upon activation, but relocalizes upon T cell encounter with a foreign antigen. When Raji-primed T cells were cocultured again with Raji cells, the percentage of $CD69^+$ and $CD25^+$ activated cells increased but to a larger extent with PL-1 treatment. Furthermore, pre-activated T cells upregulated CD69 and CD25 and increased IL-2 production upon coculture with Raji cells, but in a more sustained manner upon PSGL-1 antibody blockade. The PL1 mAb increased the percentage of $CD4^+CD69^+$ T cells and IFN-γ and IL-2 after coculture of *in vitro* exhausted-like T cells with Raji cells. Finally, we cultured an unsorted patient mantle cell lymphoma cell suspension, and found that the PSGL-1 antibody enhanced CD69 expression in T cells.

**Conclusion**

With our *in vitro* approach, we demonstrate for the first time that PSGL-1 antibody blockade enhances human T cell activation against lymphoma cells. Therefore, these findings support the notion that PSGL-1 can be a target for future immunotherapeutic options.

### EACR23-0629

### Antibody targeting of surface PSGL-1 leads to lymphoma cell apoptosis and inhibits tumorigenesis

*J.L. Pereira*[1], *N.R. dos Santos*[1]

[1]*i3S - Instituto de Investigação e Inovação em Saúde-Universidade do Porto, Intercellular Communication and Cancer, Porto, Portugal*

**Introduction**

Lymphomas are a heterogenous set of B and T cell malignancies, which treatment consists on chemotherapy cycles and, in some cases, radiotherapy and immunotherapy. However, for relapse patients there is a lack of therapeutic options that can improve outcome, especially in non-Hodgkin lymphomas. Our group has previously shown that P-selectin glycoprotein ligand-1 (PSGL-1) was associated with lymphoma development in a mouse model of T cell lymphoma. Considering that PSGL-1 is expressed in both T and B cells, our work aims to study the therapeutic potential of PSGL-1 targeting in lymphoma.

**Material and Methods**

Human lymphoma cell lines were purchased from the DSMZ repository. PSGL-1 surface expression levels were assessed by flow cytometry. Lymphoma cell lines were treated *in vitro* with the PL-1 mAb, which recognizes the PSGL-1 extracellular domain. Cell viability and apoptosis was determined by trypan blue and 7-AAD plus Annexin V staining, respectively. HUT-78 and Raji cell lines were injected s.c. in $Rag2^{-/-}$ $\gamma c^{-/-}$ immunodeficient mice. Mice were treated i.p. with PL-1 mAb or mouse IgG (5 injections at days 1, 4, 7, 11 and 14 post-injection) and tumor growth measured with caliper.

**Results and Discussions**

We found that PSGL-1 was expressed at high levels on the surface of cutaneous T cell lymphoma (HUT-78 and HH), Burkitt's lymphoma (Raji and Daudi), anaplastic large cell lymphoma (L-82 and SU-DHL-1) and Hodgkin's lymphoma (L-428, KM-H2 and HDLM-2) cell lines. PSGL-1 surface levels were lower in diffuse large B cell lymphoma cell lines (U-2932, DOHH-2, OCI-LY3 and SU-DHL-4). PL-1 antibody treatment for 24-48 h led to strong reduction of cutaneous T cell lymphoma cell lines (HUT-78, HH) viability, which was due to apoptosis. Other cell lines (RAJI, OCI-LY3 and L-428) also showed reduced cell viability after PL-1 treatment, but to a lower degree. While PL-1 mAb *in vivo* administration did not prevent Raji s.c. tumorigenesis, it significantly reduced HUT-78 tumor growth.

**Conclusion**

Our work shows that PSGL-1 antibody ligation induces apoptosis *in vitro* and tumorigenesis *in vivo* of lymphoma cell lines expressing high surface PSGL-1 levels. These findings suggest that PSGL-1 can be a potential therapeutic target against lymphoma, especially in cutaneous T cell lymphomas.

### EACR23-0633

### The impact of genetic disorders on the effectiveness of immunotherapy in patients with advanced NSCLC

*A. Rekowska*[1], *P. Rola*[1], *A. Bełżek*[1], *N. Krzyżanowska*[2], *I. Chmielewska*[2], *M. Wójcik-Superczyńska*[2], *T. Jankowski*[2], *A. Grenda*[2], *M. Gil*[2], *R. Kieszko*[2], *M. Szczyrek*[2], *K. Reszka*[3], *P. Krawczyk*[2], *J. Milanowski*[2]

[1]*Medical University of Lublin, Student Scientific Society at the Department of Pneumonol ogy- Oncology and Allergology, Lublin, Poland*

[2]*Medical University of Lublin, Department of Pneumonology- Oncology and Allergology, Lublin, Poland*

[3]*Institute of Genetics and Immunology, Genim LCC, Lublin, Poland*

**Introduction**

The effectiveness of immune checkpoint inhibitors (ICIs) in non-small cell lung cancer (NSCLC) patients with oncogenic driver alterations has not been sufficiently studied. Abnormalities in the *EGFR, ALK* and *ROS1* genes limit the effectiveness of ICIs, while mutations in the *KRAS* gene increase their effectiveness. However, the next-generation sequencing (NGS) technique allows the detection of many other genetic abnormalities with an unknown impact on the effectiveness of immunotherapy.

**Material and Methods**

© 2023 The Authors. Molecular Oncology is published by John Wiley & Sons Ltd on behalf of Federation of European Biochemical Societies

NGS was performed on 114 NSCLC patients using the Oncomine® Focus Assay on the S5 Ion Torrent® platform (Thermo Fisher, USA). DNA and RNA were isolated from formalin-fixed paraffin-embedded primary tumors or metastatic lesions. In all patients the most common abnormalities of the *EGFR, ALK* and *ROS1* genes had been previously excluded. 68 patients (median age 63 years, 37 women, 31 men, 15 non-smokers) received immunotherapy: 1st line (pembrolizumab alone or in combination with chemotherapy, n=44) or 2nd line (atezolizumab or nivolumab, n=24). The objective response rate (ORR), progression free survival (PFS) and overall survival (OS) in the groups of patients considering the molecular background of NSCLC.

**Results and Discussions**

112 genetic disorders were detected in 92 patients (in 22 patients no mutations were detected). The most common were: G12C mutation in the *KRAS* gene (28% of patients), other mutations in the *KRAS* gene (20.2% of patients), rare mutations in the *EGFR* gene (6.1% of patients) mutations in the *PIK3CA* gene (5.3% of patients), and mutations in the *BRAF* gene (5.3% of patients). Compared to other genetic abnormalities, mutations in the *KRAS* gene were significantly more common in smokers than in non-smokers (p=0.032, =4.63). Response to immunotherapy was found in 24.6% of patients, and disease stabilization in 47.7% of patients. ORR did not depend on the presence of mutations in the *KRAS* gene and other genetic disorders. The median PFS was only numerically higher in patients with *KRAS* mutations compared to patients without these mutations (6.3 vs 5.2 months, HR=0.882, p=0.7). *KRAS* mutations had no effect on OS. The combined analysis of all genetic abnormalities showed no impact on PFS and OS.

**Conclusion**

It appears that the effectiveness of immunotherapy is not greatly affected by *KRAS* mutations and other genetic disorders in patients with NSCLC who do not show abnormalities in the *EGFR, ALK,* and *ROS1* genes.

### EACR23-0657

### ALK chimeric antigen receptor T cells cooperate with ALK inhibitors to target neuroblastoma cells with low target density

E. Bergaggio[1], W.T. Tai[1], A. Aroldi[2], E. Landoni[3], M. Nuesch[1], I. Mota[1], J. Metovic[4], L. Ma[5], D. Alvarado[6], C. Ambrogio[7], C. Voena[7], R.B. Blasco[1], T. Li[8], D. Klein[8], D.J. Irvine[5], M. Papotti[4], B. Savoldo[3], G. Dotti[3], R. Chiarle[1]

[1]Boston Children's Hospital and Harvard Medical School, Department of Pathology, Boston - MA, United States

[2]University of Milano-Bicocca, Department of Medicine and Surgery, Monza, Italy

[3]University of North Carolina at Chapel Hill, Lineberger Comprehensive Cancer Center, Chapel Hill - NC, United States

[4]University of Torino, Department of Oncology, Torino, Italy

[5]MIT, Koch Institute, Cambridge - MA, United States

[6]Celldex, Therapeutics, New Haven - CT, United States

[7]University of Torino, Department of Molecular Biotechnology and Health Scienc es, Torino, Italy

[8]Yale University School of Medicine, Department of Pharmacology, New Haven - CT, United States

**Introduction**

Neuroblastoma is the most common extracranial solid tumor of childhood. It accounts for 12-15% of cancer-related deaths in children, with limited success in treating refractory or relapsed cases using current therapies. Chimeric antigen receptor (CAR) T cell therapy targeting GD2 has shown promise in neuroblastoma treatment, but relapses are associated with loss of antigen expression. The selection of the best target is critical for the therapeutic success of CAR-T cells in hematologic malignancies and solid tumors. The Anaplastic Lymphoma Kinase (ALK) receptor is expressed in most neuroblastoma cases, while virtually absent in the majority of normal tissues. ALK is an oncogenic driver in neuroblastoma and ALK inhibitors have also shown efficacy in clinical trials. All these features make ALK an attractive target for CAR-T cell therapy.

**Material and Methods**

We generated seven ALK.CAR constructs using single-chain variable fragments from different anti-ALK monoclonal antibodies and tested their ability to target and kill neuroblastoma cells expressing varying levels of ALK. We compared the activity of ALK.CAR-T cells to GD2.CAR-T cells and evaluated the combination of ALK inhibitors with CAR-T cell therapy *in vitro* and *in vivo*.

**Results and Discussions**

ALK.CAR-T cells demonstrated potent activity against neuroblastoma cells with high ALK expression, without on-target or off-target toxicity. Additionally, the combination with ALK inhibitors specifically enhanced the activity of ALK.CAR-T cells, but not GD2.CAR-T cells, against neuroblastoma with low ALK expression. In neuroblastoma cell lines and in a patient-derived xenograft (PDX), the combination of ALK inhibitors and ALK.CAR-T cells significantly reduced tumor growth and extended mice survival. Mechanistically, ALK inhibitors upregulated ALK expression and impaired tumor growth, thereby improving the targeting of neuroblastoma tumors by ALK.CAR-T cells.

**Conclusion**

These results indicate that ALK.CAR-T cells are a safe and effective monotherapy for neuroblastoma with high ALK expression. Additionally, the combination of ALK inhibitors with CAR-T cell therapy increases the efficacy of ALK.CAR-T cells by enhancing ALK targeting. These findings provide insights into the potential of ALK.CAR-T cells as a novel therapeutic strategy for neuroblastoma. A Phase I clinical trial to test ALK.CAR-T cells in combination with ALK TKIs in children with refractory/relapsed neuroblastoma is being implemented based on these results.

### EACR23-0664

### Immune predictors of response to immunotherapy in metastatic cSCC

B. Genenger[1,2], A. Thind[1,2], S. Napaki[3,4,5], J.R. Perry[1,2], B. Ashford[2,3], M. Ranson[1,2], D. Brungs[1,2,3,6]

[1]University of Wollongong,

© 2023 The Authors. Molecular Oncology is published by John Wiley & Sons Ltd on behalf of Federation of European Biochemical Societies

Molecular Horizons- School of Chemistry and Molecular B ioscience, Wollongong, Australia
[2]University of Wollongong, Illawarra Health and Medical Research Institute, Wollongong, Australia
[3]University of Wollongong, Graduate School of Medicine, Wollongong, Australia
[4]Southern IML Pathology, Sonic Healthcare, Wollongong, Australia
[5]Wollongong Hospital, Anatomical Pathology, Wollongong, Australia
[6]Wollongong Hospital, Illawarra Cancer Center, Wollongong, Australia

**Introduction**

Cutaneous squamous cell carcinoma (cSCC) is a very common skin malignancy that develops metastases in up to 5% of cases. While early stage cSCC can often be cured by local surgical resection, locally advanced and metastatic cSCC requires aggressive multimodal therapy. Prior to the introduction of immune checkpoint inhibitors, locally advanced disease required debilitating surgery and radiotherapy, and oligometastatic disease was universally fatal. Cemiplimab, an immune checkpoint inhibitor (ICI), demonstrated 47% overall response rate and received FDA approval for locally advanced or metastatic cSCC in 2018. To date, the ICI cemiplimab and pembrolizumab remain the only systemic therapies approved for cSCC not amenable for curative surgery or radiotherapy. However, there are currently no prognostic biomarkers available to predict ICI treatment outcome and the biology dictating therapy outcome remains poorly understood. In this study, we explore the expression of immune-related markers in one of the first cohort of patients with metastatic and advanced cSCC treated with immunotherapy.

**Material and Methods**

RNA was extracted from representative areas of cSCC tumors in FFPE samples of patients with advanced or metastatic cSCC who had undergone treatment with ICI targeting PD-L1/PD-1 and had responded or not responded. The gene expression of 770 immune-related genes was determined using the nCounter PanCancer IO 360 panel ™ (Nanostring, USA). Differential gene expression analysis was performed using DEseq2. CIBERSORTx digital cytometry was used to estimate the immune cell fractions.

**Results and Discussions**

Firstly, we explored the differential gene expression of responders *versus* non-responders to immune checkpoint inhibitors. Preliminary results suggest the increased expression of L-Selectin, a cell adhesion molecule with a role in tumor immunity, in non-responding patients. Digital spatial profiling of responders highlighted the relevance of the tumour immune microenvironment and its utility in prognosis.

**Conclusion**

In conclusion, this work provides a first indication for potential biomarkers and factors that govern the response to immune checkpoint inhibition in cSCC.

### EACR23-0686
### Development of Melanoma Treatment by Synthetic Stem-Loop RNA (sl-RNA) Derived from Sendai Virus Genome Combined with a Pyro-Drive Jet Injector

T. Nishikawa[1], C.Y. Chang[2], J.A. Tai[3], K. Yamashita[3]
[1]Osaka University- Graduate School of Medicine, Department of Device Application for Molecular Therapeu tics, Osaka, Japan
[2]Osaka University Graduate School of Medicine, Department of Gene and Stem Cell Regenerative Therapy, Osaka, Japan
[3]Osaka University Graduate School of Medicine, Department of Device Application for Molecular Therapeu tics, Osaka, Japan

**Introduction**

In this study, we developed synthetic stem-loop (sl-) RNA fragment which induces strong antitumor immunities. The sl-RNA sequence is derived from Sendai virus DI (defective interfering) particle genome. Previous studies showed that inactivated Sendai virus (hemagglutinating virus of Japan; HVJ) particles called HVJ-envelope (HVJ-E) have multiple-anti-cancer activities. One of the activities is activation of anticancer immunities through inactivation of Treg (regulatory T cell), promotion of NK cells acti vation and generation of CTL against cancers. Another is the cancer selective apoptosis by induction of proapoptotic genes such as TRAIL and Noxa in various human cancer cells but not in normal cells. Most of these anti-cancer activities are conducted by RNA genome fragments of HVJ-E through RIG-I/MAVS (retinoic-acid inducible gene-I, mitochondrial antiviral signaling protein) signal pathway.

**Material and Methods**

Sin-sl-RNA (114nt) and sl-RNA-57 (57nt) have been developed as a third generation of synthetic RNA fragment which originated in Sendai virus DI particle genome. Sin - sl-RNA is a single fragment which has complemental base sequence (25nt) at both ends of fragment. The complemental base sequence helps to forms double strand stem and single strand loop structure. In other words, sin-sl-RNA can form stem-loop structure by a single RNA fragment. On the other hand, sl-RNA-57 (57nt) is a half size of the sin-sl-RNA. Two sl-RNA-57 fragments are needed to be annealed to form the stem-loop structure. The sin-sl-RNA and sl-RNA-57 were injected to B16F10 (mouse melanoma) tumor by pyro-drive jet injector (PJI) three times. The PJI is a novel injector system capable of injection depth adjustment without a needle.

**Results and Discussions**

The melanoma (B16F10) tumor growth was strongly suppressed in sin-sl-RNA and sl-RNA-57 injected group. Chemokine and cytokine array revealed that MCP-2, IP-10, RANTES and MIP-2 secretions were increased in the B16F10 tumor tissues after sl-RNA-57 injections. Additionally, macrophage infiltrations and macrophage polarization to M1 (anti-tumorigenic) were observed in the sl-RNA-57 injected B16F10 tumor sections by F4/80 and NOS-2 immunostaining.

**Conclusion**

Originally HVJ-E was recognized as the main antitumor immunity inducing factor. Further investigations revealed that the core of antitumor immunity induction part might be the sl-RNA fragment derived from Sendai virus genome. These findings provide a novel nucleic acid medicine for the cancer treatment.

© 2023 The Authors. Molecular Oncology is published by John Wiley & Sons Ltd on behalf of Federation of European Biochemical Societies

### EACR23-0695

### T-cell derived extracellular vesicles prime macrophages for improved STING based cancer immunotherapy

A. Hansen[1], L. Jensen[1], K. Ryttersgaard[1], C. Krapp[1], J. Just[2], K. Gammelgaard[1], K. Jonsson[1], A. Etzerodt[1], B. Deleuran[1], M. Jakobsen[1]
[1]Aarhus University, Biomedicine, Aarhus, Denmark
[2]Aarhus University, Clinical Medicine, Aarhus, Denmark

**Introduction**

Despite recent years advancement reported for cancer immunotherapies within multiple cancer types, we still see a large group of patients failing to respond to treatment. Activation of the innate immune pathway controlled by STimulator of INterferon Genes (STING) has been considered as an attractive target for boosting cancer immunotherapy. Extracellular vesicles (EVs) are key players in shaping immune responses and are proposed to provide a danger signal for antigen-presenting cells. Here, we hypothesize that $CD4^+$ T-cell derived EVs (T-EVs) enhance innate immune cells function, such as macrophages, by priming the STING signaling pathway.

**Material and Methods**

We purified T-EVs from aCD3/aCD28 stimulated $CD4^+$ T cells by differential ultracentrifugation, and primed macrophages with these prior to stimulation with STING agonists. Subsequent activation was measured by induction of type I Interferons and phosphorylation of signaling molecules within the STING pathway. We further explored the in vivo efficacy of combining T-EV priming and STING agonists in mice with a syngeneic subcutaneous MC38 tumor.

**Results and Discussions**

We found that T-EVs sensitizes macrophages to respond more potently to STING activation. Importantly, this was independent on both surface-associated and intravesicular DNA. The priming of STING signaling was largely induced by IFNγ and TNFα carried by the T-EVs. We further showed that the T-EVs enhances the efficacy of STING agonist stimulation, by controlling tumor growth in syngeneic MC38 tumor models.

**Conclusion**

Our work support that T-EVs can disrupt the immune suppressive tumor microenvironment by reprogramming macrophages to a pro-inflammatory phenotype, and priming them for a robust immune response towards STING activation.

### EACR23-0721

### Carfilzomib-mediated immunogenic cell death in BRAF mutant preclinical models of colorectal cancer.

F. Maione[1], D. Oddo[1], F. Galvagno[2], C. Falcomatà[3], M. Pandini[2], M. Macagno[2], V. Pessei[2], L. Barault[2], C. Gigliotti[2], G. Corti[2], S. Lamba[4], C. Riganti[5], B. Castella[6], R. Rad[7], D. Saur[8], A. Bardelli[9], F. Di Nicolantonio[2]
[1]Candiolo Cancer Institute IRCCS FPO and University of Torino, Oncology, Candiolo, Italy
[2]Candiolo Cancer Institute IRCCS FPO and University of Torino, Oncology, Candiolo Torino, Italy
[3]ISMMS, Oncology, New York City, United States
[4]Candiolo Cancer Institute IRCCS FPO, Oncology, Candiolo Torino, Italy
[5]Interdepartmental Center of Research in Molecular Biotec hnology- University of Torino, Oncology, Orbassano- Torino, Italy
[6]Laboratorio di Immunologia dei Tumori del Sangue LITS- Centro Interdipartimentale di Ricerca in Biologia Molecol are CIRBM, Oncology, Torino, Iran
[7]Center for Translational Cancer Research TranslaTUM- School of Medicine- Technical University of Munich- Muni ch- Germany., Internal Medicine, München, Germany
[8]Division of Translational Cancer Research- German Canc er Research Center and German Cancer Consortium- Heid elberg- Germany, Internal medicine, München, Germany
[9]University of Torino and IFOM- The FIRC Institute of Mo lecular Oncology, Oncology, Torino, Italy

**Introduction**

Somatic *BRAF* mutations are found in 10-15% of colorectal cancer (CRC) patients, and identify a distinct subset of tumors with aggressive phenotype and poor outcome in the metastatic setting. Novel therapeutic strategies are needed to improve the therapeutic treatment of CRC patients carrying *BRAF* mutant tumors. We have previously reported that BRAF mutant CRC cells are characterized by a high rate of protein production causing a proteotoxic stress and are selectively sensitive to proteasome inhibitors bortezomib and carfilzomib. In this work we tested whether carfilzomib could restrain the growth of BRAF mutant CRC mouse models not only in a cancer cell autonomous manner, but also indirectly by eliciting immunogenic cell death (ICD), a mechanism by which dying cancer cells can induce an effective anti-tumor response by the immune system.

**Material and Methods**

We measured the release of ATP, Annexin A1 (ANXA A1) and High Mobility Group Protein 1 (HMGB1) and the exposure of calreticulin in *BRAF* mutant CRC cells upon carfilzomib administration in vitro. We employed syngeneic mouse models of *BRAF* mutant CRC to test the anticancer activity of carfilzomib. To investigate the ability of carfilzomib to promote ICD and modulate the tumor immune microenvironment, we performed confocal and cytofluorimetric analysis on carfilzomib –treated tumor tissues and controls.

**Results and Discussions**

We found that carfilzomib is able to trigger a robust endoplasmatic reticulum stress and autophagy, followed by the extracellular release of ATP, ANXA A1 and HMGB1 and calreticulin translocation in *BRAF* mutant CRC cells in vitro. We also observed that carfilzomib promotes ICD markers in BRAF mutant CRC murine tumors. Treatment with carfilzomib delayed the growth of BRAF mutant CRCs and favored the intratumor recruitment of activated cytotoxic T cells and Natural killers concomitant with the downregulation of Foxp3+ T cells.

**Conclusion**

Our findings confirms that the proteasome inhibitor carfilzomib is an ICD inducer, which promotes a tumor immune microenvironment reshape. These results will inform the design of clinical trials *BRAF* mutant metastatic CRC patients.

© 2023 The Authors. Molecular Oncology is published by John Wiley & Sons Ltd on behalf of Federation of European Biochemical Societies

### EACR23-0743
### PD-L1 network genes are potential glioblastoma biomarkers

A. Zottel[1], N. Šamec[1], I. Jovčevska[1]
[1]Faculty of Medicine- UL,
Institute of Biochemistry and Molecular Genetics- Center f or Functional Genomics and Biochips, Ljubljana, Slovenia

**Introduction**

Glioblastoma is the most common primary brain tumour with extremely low survival rate, as most patients die within 2 years of diagnosis. The main factors contributing to treatment failure are lack of reliable biomarkers and efficient therapy. In our research, we analysed gene expression of the PD-L1 network, one of the most de-regulated immune pathways in cancer, as potential glioblastoma biomarkers.

**Material and Methods**

PD-L1 network was constructed using Cytoscape, with STRING PubMed query and STRING protein query set to 0.7 confidence. Gene expression was analyzed using CGGA (mRNAseq_693 dataset), TCGA (TCGA GBMLGG dataset retrieved from GlioVis), and Rembrandt (retrieved from GlioVis) datasets. Survival analysis of GBM patients was determined based on optimal cutoff, calculated by surv_cutpoint function in R (version 4.1.1), using package survminer. For overexpression analysis, glioblastoma, glioma WHO grade 3, lower-grade glioma and normal brain tissue were included. Recurrent tumours were excluded from the study.

**Results and Discussions**

Enrichment data analysis shows that the genes are mainly involved in immune system processes. Next, we selected genes that were 1) overexpressed in glioblastoma compared with tumors of WHO grade 3 glioma and lower grade glioma, 2) overexpressed in glioblastoma compared with normal brain tissue and 3) their higher expression in glioblastoma was associated with poorer overall survival. The genes that met these criteria in at least two databases were *CASP4*, *CD276*, *CD44*, *RAB42*, *TNFRSF14*, *CD163*, *FKBP5*, *TNFSF14*, *CD40*, *ITGAM*, *PVR*, *TMEM205*, *CMT M6*, *ERBB2*, and *HOXD13*.

**Conclusion**

Our results suggest that the discovered genes may be novel biomarkers and therapeutic targets for glioblastoma that need to be further evaluated with in vitro and in vivo studies.

### EACR23-0762
### Design, characterization and preclinical validation of a combinatorial CAR-based immunotherapy against colorectal cancer with HER2 amplification

M. Cortese[1], E. Torchiaro[1], A. D'Andrea[2], S. Leto[1], F. Cottino[1], V. Vurchio[1], C. Petti[2], F. Invrea[1], S. Arena[2], E. Medico[2]
[1]Candiolo Cancer Institute FPO IRCCS, Oncology, Turin, Italy
[2]University of Turin, Oncology, Turin, Italy

**Introduction**

ACT based on CAR-T cells has led to successful treatment of some hematological malignancies, but it remains extremely challenging for solid tumors, mostly because of "on-target off-tumour" toxicity, as observed in the case of anti-HER2 CAR-T treatment of CRC with HER2 amplification. To enable ACT against $HER2_{amp}$ CRC, we therefore considered a combinatorial strategy based on the synNotch-based artificial regulatory network. A synthetic Notch receptor was employed in which the extracellular domain is an anti-HER2 scFv and the intracellular domain contains the GAL4VP64 artificial transcription factor. Engagement of the anti-HER2 domain by target cells drives GAL4VP64 cleavage and translocation to the nucleus, where it drives expression of a CAR under a GAL4UAS. In this way, only cells co-expressing both HER2 and the CAR target are killed. As a CRC-specific CAR target we selected CEA. CEA expression is restricted to the digestive tract and is increased in cancer.

**Material and Methods**

As effector cells, we chose the natural killer cell line NK-92. NK-92 cells transduced with the two lentiviral vectors encoding HER2-synNotch and CEA-CAR were repeatedly sorted in the OFF and ON state to select those with the best CAR induction after synNotch engagement. Subsequently, cloning of sorted cells led to identification of an optimally responsive clone (5F).

**Results and Discussions**

*In vitro*, the 5F clone displayed selective cytotoxicity against $HER2_{amp}$/CEA+ CRC cells, with minimal killing activity against $HER2_{amp}$/CEA- cells, or against HER2-/CEA+ cells. Additional assays on 3D organoids highlighted better recruitment and infiltration by clone 5F respect to NK-92 WT cells, only in $HER2_{amp}$ models. *In vivo*, the clone 5F significantly impaired tumor growth in two different $HER2_{amp}$ CRC models. To further improve survival, tumor penetration and *in vivo* efficacy of the NK-92-5F clone, we built a system in which HER2-synNotch engagement drives not only expression of the CEA-CAR but also of IL-2. 5F-IL-2 cells displayed a further increase of cytotoxicity *in vitro*, also at a particularly low effector:target ratio (1:50). *In vivo*, 5F-IL-2 cells drastically increased survival of mice carrying $HER2_{amp}$ CRC xenografts respect to the parental 5F clone.

**Conclusion**

The observed selective efficacy both *in vitro* and *in vivo* of the HER2-synNotch/CEA-CAR system, and its possible future evolutions, opens a perspective for possible clinical applications in cases of $HER2_{amp}$ CRC displaying primary or secondary resistance to HER2/EGFR blockade.

### EACR23-0775
### Nanoparticle-enabled CAR T cell therapy: Unlocking new frontiers in cancer immunotherapy

I. Pinto[1], R. Cordeiro[1,2], H. Faneca[1,2]
[1]Center for Neuroscience and Cell Biology, University of Coimbra, Coimbra, Portugal
[2]Institute of Interdisciplinary Research III, University of Coimbra, Coimbra, Portugal

**Introduction**

Some T-cell cancer immunotherapies, like chimeric antigen receptor (CAR) T-cell therapy, require gene-modified T cells to redirect their antigen specificity and overcome tumor escape mechanisms. The T-cells genetic modification has been done through viral vectors, which is associated with safety concerns, high cost and production

© 2023 The Authors. Molecular Oncology is published by John Wiley & Sons Ltd on behalf of Federation of European Biochemical Societies challenges, and more recently also through electroporation, which can be extremely cytotoxic. In this context, nanosystems constitute an alternative to overcome the challenges associated with current methods, resulting in a safe and cost-effective platform. In this work, we proposed to develop polymer-based nanosystems to efficiently deliver genetic material into T cells, with reduced cytotoxicity.

**Material and Methods**

A panel of polymers were screened as potential nanoplatforms for T cell engineering, using Jurkat cell line and primary T cells as models. These nanosystems were prepared in different polymer/genetic material ratios, submitted to physicochemical characterization, and evaluated in terms of transfection activity and toxicity. Their cellular internalization was also analyzed.

**Results and Discussions**

T cells exhibit different properties, including in terms of their uptake and intracellular trafficking mechanisms, when compared with other cell types. In this context, a strategy of polyplexes optimization in terms of composition and polymer/genetic material ratio was adopted. The best-performing formulation achieved high biological activity and reduced toxicity when compared to the gold standard bPEI-based polyplexes, even in primary T cells. The developed nanosystems resulted in high cellular uptake, being observed inside almost 80% of T cells, which can be correlated with the biological activity obtained. Moreover, they were able to deliver different types of genetic material to T cells and presented a size of 150 nm, high capacity to complex the genetic material and zeta potential of +30-35 mV. Thus, the positive charge of the polymeric mixtures led to strong electrostatic interactions with the nucleic acids, promoting the formation of stable and small polyplexes that resulted in high internalization in T cells.

**Conclusion**

The developed nanosystems not only exhibited suitable physicochemical properties for gene delivery, but also proved to be an effective strategy for T-cell engineering. With their continuous optimization, they may have a strong potential for future clinical applications, improving the current T-cell-based cancer treatments.

### EACR23-0786
### LOCAL INFLAMMATION WITH TOLL-LIKE RECEPTOR AGONISTS AS AN ALTERNATIVE THERAPY FOR ANTI-PD-1 RESISTANT NSCLC TUMORS DUE TO PTEN LOSS

*M. Houry[1], E. Francisco[1], R. Miriam[1], L. Teresa[2], A. Ramón[3], L.V. Sergio[1], S. Diego[1], L. Juan José[2], P. Katerina[4], C. Alfonso[1]*

*[1]CIMA, Solid Tumors, Pamplona, Spain*

*[2]CIMA, Immunology and Immunotherapy Program, Pamplona, Spain*

*[3]Vall d'Hebron Institute of Oncology VHIO, Thoracic Cancers translational Genomics Unit, Barcelona, Spain*

*[4]Yale Cancer Center, Department of Medicine, New Haven, United States*

**Introduction**

Targeted therapy and immunotherapy (IT) have revolutionized the way patients with non-small cell lung cancer (NSCLC) are treated. However, a large percentage of patients is unresponsive to IT or will acquire treatment resistance. *PTEN* is a tumor suppressor gene (TSG) whose mutation leads to hyperactivation of the PI3K/mTOR/AKT pathway and enhancement of proliferation, metabolism, and survival. This study shows that PTEN loss leads to IT refractoriness in NSCLC. Moreover, we demonstrate the efficacy of a novel therapy based on toll-like receptors.

**Material and Methods**

IHC was used to quantify PTEN in tumors from NSCLC patients treated with IT. We developed a *Pten*-null model using the UNSCC680 lung squamous carcinoma cell line in immunocompetent AJ mice. We then characterized immune-related cell populations in these tumors by performing multiplex immunofluorescence (mIF), and analyzed transcriptomic changes by RNAseq in these cells to understand α-PD-1 resistance. Treatment with poly(I:C)+resiquimod (P/R) alone or in combination with α-PD-1 or α-TGFβ was performed to assess whether resistance could be overcome.

**Results and Discussions**

We found that the *Pten*-null model was resistant to α-PD-1 therapy. Similarly, NSCLC patients with low PTEN levels were less responsive to IT. A significant increase in the number of CD4+/FOXP3+ cells (Tregs) in *Pten*-null cells compared to *Pten* wild type (WT) ones was found. Co-culture of *Pten*-null cells with CD4+ cells resulted in a higher conversion of CD4+ cells into Tregs compared to WT cells, also in agreement with higher TGFβ and CXCL10 levels. Moreover, *Pten*-null cells presented higher nuclear NFkB levels upon activation with TNFα, suggesting higher susceptibility to inflammatory responses through type I IFNs. Treatment with P/R, α-PD-1+P/R or α-TGFβ+P/R caused regressions in *Pten*-null tumors, thus showing this strategy's efficacy in overcoming resistance to α-PD-1. The most successful treatment was the administration of α-TGFβ+P/R, which cured 8/8 (100%), with all animals showing immunological memory.

**Conclusion**

Our results demonstrate that lack of PTEN participates in IT resistance in NSCLC. Besides, we have shown that reducing Tregs in combination with an induction of a local inflammatory response is an alternative strategy to overcome α-PD-1 resistance in this context.

### EACR23-0796
### COMBINATION OF DASATINIB AND IMMUNOTHERAPY TO ENHANCE THE THERAPEUTIC RESPONSE OF SMALL CELL LUNG CANCER

*N.B. Otegui García[1], E. Redín[1], M. Santos[2], M. Redrado[1], S. León[1], K. Politi[3], S. Vicent[1], L. Montuenga[1], A. Calvo[1]*

*[1]Center for Applied Medical Research CIMA, Program in Solid Tumors, Pamplona, Spain*

*[2]CIEMAT, Molecular Oncology Unit, Madrid, Spain*

*[3]Yale Cancer Center, Department of Pathology- Yale School of Medicine, New Haven, United States*

**Introduction**

© 2023 The Authors. Molecular Oncology is published by John Wiley & Sons Ltd on behalf of Federation of European Biochemical Societies

Small cell lung cancer (SCLC) is a highly aggressive and deadly tumor type with very poor survival (5-year survival rate lower than 6%), with no effective therapeutic treatments. Current regimes include chemotherapy in combination with immune checkpoint inhibitors (ICIs), but most patients rapidly relapse. SCLC is characterized by lack of MHC-I and PD-L1 expression in cancer cells, as well as an immunosuppressive tumor microenvironment (TME). We have previously shown that targeting YES1/SRC axis with dasatinib reactivates the immune system and enhance ICIs responses in non-small lung cancer models. Here we aimed to test dasatinib and ICIs (a-PD-1+ a-CTLA4) in murine SCLC models.

**Material and Methods**

Transplantable models of *Rb1/Tp53/p130* KO (RPP) (high YES1) and *Tp53/Rbl1/p130/Pten* KO (SCLC57) (medium YES1) cells were used in syngeneic immunocompetent mice. Flow cytometry, PCR and western blotting were used to characterize the cell lines. *In vivo* experiments, including depletion assays and multiplex immunofluorescence (mIF) were used to characterize the efficacy of dasatinib, ICI and dasatinib+ICI.

**Results and Discussions**

Both RPP and SCLC57 cells lacked MHC-I and PD-L1 expression even when stimulated with IFNg. Single treatments with a-PD-1 or a-CTLA4 had no effect on tumor volume whereas dasatinib (Das) alone reduced tumor growth by ~30% in the RPP model and had little effect on SCLC57 model. In contrast, a-PD-1+ a-CTLA4 (IT) had a very potent antitumor effect in both models, with >80% growth inhibition. However, addition of dasatinib to dual ICIs further increased overall survival in RPP model. Other dual combinations (Das+a-PD-1 or Das+a-CTLA4) had lower effect. A significant increase in CD11c+, CD4+ and CD8+ cells and a decrease in Tregs, together with high CCL5 and CXCL1 plasma levels were found for Das+ICIs. Depletion experiments showed that CD4+ T lymphocytes and MHC-II+ (likely dendritic cells) cells were responsible for the antitumor response. mIF also evidenced significant interaction between CD4+ and CD11c+ cells.

**Conclusion**

This study unveils the mechanisms underlying the antitumor response of the combination of dasatinib with immunotherapy in high YES1 SCLC models, highlighting the role of CD4 T lymphocytes and MHC-II+ cells in the therapeutic response.

### EACR23-0808
### Validation of anti-linker rabbit monoclonal antibodies for flow cytometric detection of scFv-based CARs

*M. Jacquet[1], G. Lima[2], J. Fisher[2], R. Sinapius[2], E. Wade[2], S. L'Heureux[2], J. Lohmueller[3], M. Kvorjak[3], R. Prioli[2], A. Singh[4]*

*[1]Cell Signaling Technology, Immunology, Leiden, The Netherlands*

*[2]Cell Signaling Technology, Flow Cytometry and Conjugation, Danvers, United States*

*[3]UPMC Hillman Cancer Center - University of Pittsburgh, Department of Surgery, Pittsburgh, United States*

*[4]Cell Signaling Technology, Immunology, Danvers, United States*

**Introduction**

Chimeric Antigen Receptor (CAR)-T cell therapy is a revolutionary form of adoptive cell immunotherapy that has proven to be successful in the treatment of various forms of hematologic malignancies. As this treatment modality continues to evolve, with a focus placed on engineering cells with greater persistence, there is a need in multiple phases of the CAR-T development pipeline for highly specific protein detection reagents. Many commercially available CAR detection reagents, however, either lack specificity or are not versatile in their ability to detect surface expressed CARs of differing antigen specificity. Here, we report on the validation of recombinant rabbit monoclonal antibodies raised against two linker sequences that are commonly employed in single-chain variable fragment (scFv)-based CARs. These antibodies can be used to monitor the surface expression of CARs directed toward distinct antigens.

**Material and Methods**

The recombinant rabbit monoclonal antibodies, E7O2V and E3U7Q, were generated by rabbits immunized with peptide sequences that are commonly used in the linker region of scFv based CARs, Gly4Ser and Whitlow/218, respectively. E7O2V and E3U7Q were directly conjugated to fluorophores and validated for specificity using flow cytometric analysis of CAR-transduced cell lines and primary human T cells.

**Results and Discussions**

Flow cytometric analysis of live Jurkat cells and primary human T cells transduced with CAR constructs revealed that E7O2V and E3U7Q could detect surface expressed CARs containing the appropriate linker sequence, independently of scFv specificity. No specific staining was observed on non-transduced cells. Furthermore, neither E7O2V nor E3U7Q cross-reacted with either a Whitlow/218 linker containing CAR or a Gly4Ser linker containing CAR, respectively.

**Conclusion**

In a live cell flow cytometry assay, E7O2V and E3U7Q specifically detect surface expressed scFv-based CARs containing either a Gly4Ser linker or a Whitlow linker, respectively. Furthermore, these recombinant monoclonal antibodies are versatile and can detect their intended linker sequence independent of scFv specificity. The potential exists to leverage these antibodies for CAR-T cell enrichment and for incorporation into multiparametric flow cytometry panels used to phenotype CAR-T cells during the discovery, manufacturing, and clinical phases of the development pipeline.

### EACR23-0896
### Taming the metabolism of tumor associated macrophages to fight NF1-related tumors

*F. scantamburlo[1]*

*[1]Università degli Studi di Padova, Biomedical Sciences, Padova, Italy*

**Introduction**

Neurofibromatosis type 1 (NF1) is a genetic mendelian disease due to germline inactivating mutations in the *NF1* gene that encodes for neurofibromin, a negative RAS regulator. A severe aspect of this disease is the

© 2023 The Authors. Molecular Oncology is published by John Wiley & Sons Ltd on behalf of Federation of European Biochemical Societies potential development of malignant peripheral nerve sheath tumors (MPNSTs), aggressive cancers of Schwann cell (SC) origin that are unresponsive to conventional treatments. MPNSTs display significant immune cell infiltrates, including tumor associated macrophages (TAMs). These immune cells are emerging as important players in the growth of many cancers, especially in advanced stages. Yet, *the molecular crosstalk between MPNSTs and TAMs is lacking*.
The aim of the project is to investigate the potential cross-talk between MPNSTs and macrophages infiltrating the peripheral nerve lesions with the final goal of identifying macrophage-specific metabolic actors that sustain neoplastic SC growth.

**Material and Methods**

We use in vitro models of cell crosstalk mechanisms (co-cultures, conditioned media) employing MPNSTs and bone marrow derived macrophages (BMDM). Through WB, ELISA and qPCR assays we study the ability of MPNST cells to drive a metabolic-based TAM phenotype acquisition. Using Boyden chamber and Matrigel assays of tumor cells co-cultured with M2-TAM like cells we investigate the effect of macrophage metabolism on invasion/migration and 3D growth of MPNSTs.

**Results and Discussions**

Our results indicate that MPNST cells induce a significant transition of BMDM toward a metabolic state characterized by a robust upregulation of ARG1 (Arginase 1), ARG2 (Arginase 2), CD206 (Mannose Receptor) and GLUL (glutamine synthetase) and a profile of induced VEGF-A (Vascular Endothelial Growth Factor), MGL1 (Macrophage Galactose-C type Lectin 1) and HIF1α (Hypoxia inducible factor 1α) expression. Such anti-inflammatory, M2-like, TAMs sustain *in vitro* MPNST 3D growth and migration, and endothelial cell angiogenesis. By ablating the mitochondrial chaperone TRAP1, a negative regulator of respiratory chain complex II, we find that the pro-tumoral functions of TAMs are impaired.

**Conclusion**

Our findings suggest a new crosstalk between MPNSTs and TAMs, in which macrophages exposed to conditioned media of tumor SCs acquire a M2-like metabolic state. This inter-cellular signaling could be crucial in facilitating tumor maintenance and invasion. Inhibition of TRAP1, a metabolic regulator previously identified as a target in MPNSTs, could be used as a therapeutic strategy to reverse this macrophage mis-education.

### EACR23-0934

### Improvement of cell fusion efficiency by novel cell fusion method and its application in dendritic cell-tumour cell fusion vaccines

*J. Tai[1], C.Y. Chang[2], T. Nishikawa[1], K. Yamashita[1,3]*
*[1]Osaka University Graduate School of Medicine, Device Application for Molecular Therapeutics, Suita-shi, Japan*
*[2]Osaka University Graduate School of Medicine, Gene and Stem Cell Regenerative Therapy, Suita-shi, Japan*
*[3]Daicel Corporation, Medical Device Research- Industry Business Unit- Safety Strategic Business Unit, Osaka-shi, Japan*

**Introduction**

Cell-to-cell fusion involves fusion of somatic cells into single hybrid cells. The process can potentially be applied to a wide range of cell engineering technologies, such as regenerative medicine, antibody engineering and cancer therapy. Polyethylene glycol (PEG) cell fusion is a chemical-based method of cell fusion that is simple and low-cost, but produces a low overall fusion efficiency and high cell cytotoxicity. In dendritic cell (DC)-tumour cell vaccines, DCs are fused with tumour cells to promote tumour antigen presentation and activate anti-tumour immune responses. In this study, we developed a new method to improve conventional PEG cell fusion and demonstrate its potential application in generating DC-tumour vaccines.

**Material and Methods**

To improve on the conventional PEG fusion method a mixture of NS-1 myeloma cells and C57BL/6N mouse splenocytes were subjected to standard PEG addition, followed by transient pressurisation using a modified closed volume vessel attached to the pyro-drive jet injector (PJI). Cell fusion efficiency and viability were analysed by flow cytometry and trypan blue staining respectively. To generate DC-tumour cell fusion vaccine, activated mouse DCs were subjected to fusion with inactivated B16-F10 mouse melanoma cells using PJI method. DC vaccines were analysed for anti-tumour functionality after tumour challenge by tumour growth observation and tumour-specific IFN-γ ELISpot assay.

**Results and Discussions**

PJI-PEG fusion method improved cell fusion efficiency, and decreased cell cytotoxicity during cell fusion process, compared to conventional PEG method. This was likely due to PJI-generated instantaneous transient pressure exerted onto the mixture of cells within a closed environment, increasing cell-to-cell contact, resulting in increased probability of cell fusion. Functionally, PJI-PEG-generated DC vaccine suppressed B16-F10 tumour growth, on par with PEG method, but elicited significantly higher tumour-specific IFN-γ immune activation. These results suggest PJI-PEG fusion method can produce viable DC vaccine with antigen presentation function capable of anti-tumour immune response activation.

**Conclusion**

Our novel cell fusion method using instantaneous transient pressure is an effective and reliable cell fusion method and can potentially improve currently existing methods in applications such as DC fusion vaccines or hybridoma production.

### EACR23-0955

### Tumor metabolism modulation increases DAMPs expression during treatment with plant extracts in a murine triple negative breast cancer model.

*C. Carlosama Puerta[1], M.C. Jimenez[1], P. Lasso[1], A. Barreto[1], S. Fiorentino[1]*
*[1]Pontificia Universidad Javeriana, Microbiology, Bogota, Colombia*

**Introduction**

© 2023 The Authors. Molecular Oncology is published by John Wiley & Sons Ltd on behalf of Federation of European Biochemical Societies

Plant extracts present different anticancer activities such as the inhibition on cell proliferation, the metabolism modulation and the increasing of immune response among others. We have obtained two different plant extracts: P2Et from *Caesalpnia spinosa* and anamu SC from *Petiveria alliacea*. Both have shown an anticancer potential in a breast mouse model increasing immune response and impairing the tumor metabolism. Here, we evaluated the relationship between metabolism and immunogenicity in tumor cells treated with plant extracts.

**Material and Methods**

4T1 murine triple negative breast cancer cell line was knocked down with *sh*RNA targeting *Hexokinase 2* (*Hk-2*) and *C1qbp* proteins associated with glycolytic and OXPHOS metabolism, respectively. Then, clones were treated with P2Et and Anamu SC and metabolism and DAMPs (Damage-associated molecular pattern) expression were evaluated.

**Results and Discussions**

4T1 *shHk2* clone was more sensitive to anamu SC treatment. Both clones reduced glucose uptake, ATP production and cell migration when they were treated with anamu SC meanwhile P2Et did not have any effect. On the other hand, both plant extracts increased DAMPs expression as Calreticulin, HSP70 and HSP90; however, in *shHk2* clone treated with anamu SC the DAMPs expression were considerably higher compared to the *sh-C1qbp* clone and the wild-type cell line.

**Conclusion**

These results show that glycolysis modulation increases DAMPs expression in response to treatment with plant extract anamu SC and this could increase the immune response and tumor elimination.

### EACR23-0961

### Differential cell cycle dependent nuclear PD-L1 variants of head and neck cancer cells

*D. Schulz*[1], *L. Feulner*[1], *M. Wetzel*[1], *D. Rubenich*[1], *S. Heimer*[1], *N. Schäfer*[2], *Y. Reiners*[3], *A. Wege*[4], *G. Brockhoff*[4], *T.E. Reichert*[1], *T. Ettl*[1], *R. Bauer*[1]

[1]*University Hospital Regensburg, Oral and Maxillofacial Surgery, Regensburg, Germany*

[2]*University Hospital Regensburg, Orthopaedic Surgery, Regensburg, Germany*

[3]*Leibniz-Institut für Analytische Wissenschaften- ISAS e.V., Analytische Wissenschaften, Dortmund, Germany*

[4]*University Hospital Regensburg, Gynecology and Obstetrics, Regensburg, Germany*

**Introduction**

The PD-L1/PD1 axis is mainly associated with immunoregulatory effects. Recently, there is emerging evidence for cell-intrinsic functions of PD-L1 in tumor cells. Recently, it has been demonstrated, that PD-L1 is not exclusively localized to the cell membrane.

**Material and Methods**

Experiments were performed with six head and neck cancer cell lines: A-253, D-562, FaDu, SCC-9, SCC-15 and PCI-52 as well as primary tumors from head and neck cancer patients. Subcellular fractions were maintained with the subcellular protein fractionation kit (Thermo Scientific). Cellular PD-L1 localisation was confirmed via immunofluorescence staining. For cell cycle inhibition the inhibitors palbiciclib (G1), aphidicolin (S) and nocodazole (G2/M) were used. Inhibitory concentrations were adjusted using cell cycle analysis and apoptosis experiments via flow cytometry. Protein expression was analyzed via western blotting. Specificity of PD-L1 expression was approved by PD-L1 siRNA knockdown (KD) experiments and LC-MS analysis. Co-Immunoprecipitation (Co-IP) and a proximity ligation assay (PLA) were used to detect interaction partners of PD-L1.

**Results and Discussions**

By separating the cell into distinct cellular fractions, PD-L1 could be detected in all cellular compartments including the nucleus of six well-characterized HNSCC cell lines. By means of immunodetection, we detected, the standard PD-L1 in its glycosylated and deglycosylated state with a molecular weight (MW) of approximately 42-50 kDa, also PD-L1 with a MW of approximately 70 kDa and >150 kDa in nuclear fractions. These findings were confirmed in tumor samples from HNSCC patients. The specificity of the immunodetection was confirmed by PD-L1 siRNA KD experiments and LC-MS protein analyses after PD-L1 co-immunoprecipitation. Furthermore, we demonstrated that nuclear variant expression is cell cycle dependent. Immunofluorescence staining against PD-L1 confirmed the observations in different cell cycle phases of synchronized HNSCC cells. In addition, using PLA and Co-IP, we detected a cell cycle-dependent interaction of vimentin with PD-L1.

**Conclusion**

Our data may provide an insight as to why differential response to PD-L1 antibody therapy occurs during HNSCC treatment. Our results suggest that there is a highly complex regulation of PD-L1 and that there are several tumor cell-intrinsic, rather complex functions of PD-L1 which are independent of immune regulation. These observations may eventually have a major impact on the therapeutic success of approved antibody therapies.

### EACR23-1026

### Anti-sarcoma activity of CAR-redirected Cytokine-Induced Killer lymphocytes is indirectly enhanced by PD-1 blockade

*C. Donini*[1], *F. Galvagno*[1], *L. Vitali*[1], *A. Massa*[1], *A. Proment*[1], *A. Merlini*[1], *E. Vigna*[1], *G. Grignani*[2], *G. Dotti*[3], *V. Leuci*[4], *D. Sangiolo*[4]

[1]*University of Turin, Department of Oncology, Orbassano, Italy*

[2]*Candiolo Cancer Institute FPO-IRCCS, Medical Oncology, Candiolo, Italy*

[3]*University of North Carolina, Department of Microbiology and Immunology, Chapel Hill - North Carolina, United States*

[4]*University of Turin - Candiolo Cancer Institute FPO-IRCCS, Department of Oncology, Orbassano, Italy*

**Introduction**

Cytokine-Induced Killer lymphocytes (CIK) are ex vivo expanded T-NK lymphocytes. Given their intrinsic HLA-independent antitumor activity, CIK are a promising platform for CAR-therapy against solid tumors. We recently reported initial data supporting the promising activity of CAR-CIK, redirected against the tumorigenic target CSPG4, against soft tissue sarcomas (STS) (Leuci V. Clin Cancer Res 2020). Emerging data support a potential

© 2023 The Authors. Molecular Oncology is published by John Wiley & Sons Ltd on behalf of Federation of European Biochemical Societies synergism of conventional CAR-T with PD-1 blockade, capable of enhancing their activity especially against challenging solid tumors. The impact of PD-1 blockade on CAR-CIK activity is currently not defined.
Purpose of this study is to investigate the modulation by PD-1 blockade on CAR-CIK activity, dissecting a direct effect from possible contributions deriving by immune-infiltrating components.

**Material and Methods**

Our experimental platform is based on patient-derived CAR.CIK and STS cell lines. CAR.CIK were generated by retroviral engineering with a 2nd generation anti-CSPG4 CAR, including the 4-1BB costimulatory domain. Three-dimensional (3D) patient-derived organotypic STS spheroids (PDO-STS), were generated from fresh surgical samples and the endogenous immune infiltrate characterized by flow cytometry.

**Results and Discussions**

Anti-CSPG4-CAR.CIK efficiently killed *in vitro* STS (n=12 different histotypes), with significant superiority as compared with unmodified controls ($p<0.0001$). Mature CAR.CIK presented a low membrane expression of PD-1 (14%±7) and the combination with anti-PD1 Ab did not enhance their anti-sarcoma activity *in vitro* ($p>0.05$). Consistently, anti-CSPG4 CAR-CIK effectively delayed *in vivo* growth of STS (Fibrosarcoma, GIST, UPS cell lines, $p<0.001$) xenografts and even in this model the combination with anti PD-1 Ab did not enhance their anti-sarcoma effects.
Next, we tested CAR.CIK against PDO-STS that retained the endogenous immune and stromal components, including variable rates of PD1+ T infiltrating lymphocytes (TILs; 40%). CAR.CIK were effective against PDO-STS and in this case the combination with anti-PD1 Ab enhanced their anti-sarcoma killing activity (56% vs 35%), supporting an indirect contribution by the activation of TILs.

**Conclusion**

PD-1 blockade enhances the anti-sarcoma activity of CAR.CIK by promoting a synergism with endogenous TILs. The integration of CAR.CIK cellular immunotherapy with PD-1 blockade may provide a powerful tool deserving exploration within the challenging and immunosuppressive microenvironment of advanced sarcomas.

### EACR23-1044

### A three-dimensional glioblastoma model for NK cell immunotherapy research

*A. Habič[1,2], B. Majc[1], E. Senjor[3], M. Perišić Nanut[3], M. Novak[1], B. Breznik[1]*
*[1]National Institute of Biology, Department of Genetic Toxicology and Cancer Biology, Ljubljana, Slovenia*
*[2]Jožef Stefan Institute, Jožef Stefan International Postgraduate School, Ljubljana, Slovenia*
*[3]Jožef Stefan Institute, Department of Biotechnology, Ljubljana, Slovenia*

**Introduction**

Glioblastoma (GB) remains one of the most aggressive malignancies of the brain with limited treatment options. Several novel therapies are currently under investigation, of which immunotherapy with natural killer (NK) cells holds great potential. The aim of the study was to establish 3D GB models for NK cell immunotherapy research and investigate the interactions between GB and NK cells.

**Material and Methods**

We set up and characterised a 3D GB spheroid model established from either GB stem cells (GSCs) or differentiated GB cells using the ClinoStar system. The expression levels of various ligands for activating and inhibitory NK cell receptors were compared between flask- and spheroid-cultured GB cells. Calcein-release assay and flow cytometry were used to compare the cytotoxicity of NK92 cells against GB cells in flask cultures and in spheroids. Infiltration of NK92 cells into spheroids and specific receptor-ligand interactions were additionally analysed by immunofluorescence and proximity ligation assay on formalin-fixed, paraffin-embedded sections of the co-cultures.

**Results and Discussions**

We optimised a method to produce uniformly sized GB spheroids from either suspension-cultured GSCs or adherent differentiated GB cells. These spheroids differ in their growth kinetics, but both can be cultured for several weeks, allowing for various applications. Several ligands for inhibitory and activating receptors on the surface of NK cells are expressed in GSCs and differentiated GB cells, including MHC class I, CD155, ULBPs, B7-H6, CD112, and CD54. The type of cell culture (flask culture vs. spheroids) affects the proportion of cells positive for specific NK cell ligands, most markedly for CD155 and ULBPs, which are significantly downregulated in spheroids of both cell lines. The differences in ratios of NK cell activating and NK cell inhibitory ligands likely result in lower cytotoxic activity of NK92 cells against GB cells in spheroids compared with their cytotoxicity against cells grown in flask culture. The contribution of the ligand CD155 and its interactions with activating (DNAM1, CD96) and inhibitory (TIGIT) receptors on NK92 cells were studied in more detail using the proximity-ligation assay.

**Conclusion**

We have established a 3D GB model that can be used for high-throughput evaluation of NK cell therapy effect and to study the patient-specific interactions between GB cells and NK cells. Our results highlight the importance of 3D *in vitro* tumour models for more precise immunotherapy research.

### EACR23-1053

### FS222, A Tetravalent Bispecific CD137 and PD-L1 Targeting Antibody, Modulates Anti-Tumor Immunity Preclinically and Demonstrates Pharmacology in Patients in an Ongoing Phase I Trial

*R. Russell[1], M. Lakins[2], D. Jones[1], L. Kayitalire[3]*
*[1]F-star Therapeutics Ltd, Translational Sciences, Cambridge, United Kingdom*
*[2]F-star Therapeutics Ltd, Biology, Cambridge, United Kingdom*
*[3]F-star Therapeutics Ltd, Clinical Development, Cambridge, United Kingdom*

**Introduction**

© 2023 The Authors. Molecular Oncology is published by John Wiley & Sons Ltd on behalf of Federation of European Biochemical Societies

FS222 is a novel tetravalent bispecific antibody targeting PD-L1, the immune checkpoint protein that is overexpressed on many solid tumors, and CD137 (4-1BB), a co-stimulatory molecule known to be upregulated on tumor-reactive CD8+ T cells. FS222 has been engineered to have reduced FcgR binding.

**Material and Methods**

FS222 and a surrogate mAb2 antibody have been characterised using *in vitro* T cell activation assays and *in vivo* mouse syngeneic efficacy studies respectively. Non-clinical safety assessment of FS222 was performed in non-human primates as per industry standards. Study endpoints included toxicology assessment as well as monitoring of pharmacokinetic (PK) and pharmacodynamic (PD) endpoints such as whole blood immunophenotyping.

A First-in-Human (FIH) Phase I clinical trial in patients with advanced malignancies was initiated in December 2020 (NCT04740424) with the aim of evaluating safety, PK/PD and biomarkers, and identifying a dose for exploration in Part B. A relational database and R Shiny app were implemented for data storage, standardisation and interactive data analysis.

**Results and Discussions**

In preclinical studies, FS222 demonstrated potent T cell activation *in vitro* with no evidence of a bell-shaped dose response. A mouse surrogate mAb2 antibody of FS222 showed enhanced T cell proliferation and significant in vivo survival benefit in two syngeneic tumor models. FS222 has also shown a good PK profile and favourable safety profile to date in non-human primates (NHP). Based on preclinical PK/PD, selected PD and biomarker endpoints have been incorporated into the FIH study design.

**Conclusion**

FS222 mechanism of action, as described in preclinical studies, appeared to translate to the emerging clinical pharmacology profile observed in patients in the Phase I study. Use of bioinformatics analysis pipelines enabled efficient handling of clinical pharmacology data.

### EACR23-1073

### A subset of colorectal cancers overexpressing mesothelin can be effectively targeted by natural killer cells expressing mesothelin-CAR

*E. Torchiaro[1], C. Petti[2], M. Cortese[1], F. Invrea[1], A. D'Andrea[2], D. Sangiolo[2], E. Medico[2]*

*[1]Candiolo Cancer Institute, Oncogenomics Lab, Candiolo, Italy*

*[2]University of Torino, Department of Oncology, Candiolo, Italy*

**Introduction**

Colorectal cancer (CRC) is the third most common cancer worldwide, with highly variable prognosis and response to treatment. A large subset of patients does not respond to standard treatments or develops resistance. As an alternative, CAR-T cells therapy has been proposed, however with significant on-target, off-tissue adverse events. We therefore evaluated different CAR targets and alternative effector cells.

**Material and Methods**

We analyzed RNA expression profiles of CRC datasets (500 patient-derived xenografts, 150 cell lines, 640 tumors) to identify suitable targets, i.e. genes expressed at high levels in a fractions of samples and known CAR targets in other tumor types. The natural killer cell line NK-92 was selected to generate CAR-killers due to more favorable toxic profile, and do not cause graft-versus-host disease. NK-92 cells were transduced with a lentiviral vector encoding the second-generation CAR, and next sorted and cloned. Activity of CAR-NK-92 cells against target-expressing CRC cells was assessed *in vitro* and *in vivo.*

**Results and Discussions**

The expression analyses highlighted Mesothelin (MSLN) as an interesting CAR target. MSLN is a GPI-anchored cell-surface protein poorly expressed in normal tissues and overexpressed in mesothelioma and ovarian cancer, for which CAR-T therapy has already been validated at the preclinical level. We found that about 15% of CRCs overexpress MSLN at levels comparable to those of the above cancers. Intriguingly, MSLN-overexpressing CRCs preferentially belong to a transcriptional subtype endowed with poor prognosis. Lentiviral transduction with MSLN-CAR of NK-92 was poorly efficient, and required multiple rounds of sorting and, finally, cloning. After clone prioritization and selection, one clone (NK-92cl45) expressing good and stable levels of MSLN-CAR was selected. Flow cytometry analysis confirmed that NK-92cl45 retained the NK phenotype and NK antigen markers. The clone showed high and specific *in vitro* lytic activity towards CRC cells overexpressing MSLN. When tested *in vivo* in mouse CRC xenografts, NK-92cl45 consistently impaired tumor growth only in MSLN-overexpressing xenografts, while wild-type NK-92 cells displayed negligible activity.

**Conclusion**

MSLN is a good target for CAR-based adoptive immunotherapy, being poorly expressed in normal tissues. Our results showed both *in vitro* and *in vivo* that MSLN CAR-NK-92 can be a valid alternative treatment in MSLN-overexpressing CRCs that belong to a poor prognosis subtype frequently resistant to standard care.

### EACR23-1089

### Fourth generation CAR-T cells secreting a TLR modified ligand exhibit an antitumor profile in in vitro experiments

*J. Magri[1,2,3], M. Menotti[1,2], R. Giampà[1,2], R. De Maria[4,5], T.L. Haas[1,2,6]*

*[1]Italian Institute for Genomic Medicine, Immunotherapy laboratory, Candiolo TO, Italy*

*[2]Candiolo Cancer Institute, FPO irccs, Candiolo TO, Italy*

*[3]UCSC - Università Cattolica del Sacro Cuore, Faculty of Medicine, Rome, Italy*

*[4]UCSC - Università Cattolica del Sacro Cuore, Department of Translational Medicine and Surgery, Rome, Italy*

*[5]Fondazione Policlinico Universitario "A. Gemelli", irccs, Rome, Italy*

*[6]UCSC - Università Cattolica del Sacro Cuore, Istituto di Patologia Generale, Rome, Italy*

**Introduction**

Chimeric Antigen Receptor (CAR)-T cell therapy still require efforts in overcoming main limitations in solid

© 2023 The Authors. Molecular Oncology is published by John Wiley & Sons Ltd on behalf of Federation of European Biochemical Societies cancer treatment. Immunosuppression in tumor microenvironment (TME), antigen heterogeneity and immune escape weaken its efficacy in many tumor types, mostly in those defined as “cold”. We propose to foster anticancer immunity by combining CAR-dependent tumor cell killing and adjuvanticity in one tool. We aim to produce 4th generation CAR-T cells secreting a modified TLR ligand directly into the TME. In our hypothesis, the ligand will trigger an innate immune response, induction of inflammation and immune cell infiltration, establishing an immune-stimulating TME.

**Material and Methods**

MC38 colon adenocarcinoma cells expressing a truncated human Epidermal Growth Factor Receptor were produced as tumor model. TLR ligand production and secretion, as well as its activity, were tested by western blot and colorimetric assays. Its ability to cross biological membranes was analyzed in a permeability assay. Primary murine T cells, isolated from spleens of C57BL/6 mice, were transduced with retroviral vectors carrying a cetuximab-based 3rd generation murine CAR and/or the TLR ligand with an IRES GFP as a reporter. Expression of the CAR and GFP were assessed by flow cytometry. Killing ability, activation status of CAR-T cells, cytokine production and TLR ligand secretion and activity were assessed upon co-culture with MC38 cells by luminescence or colorimetric assays, flow cytometry and ELISA.

**Results and Discussions**

The secreted TLR ligand activated TLR at levels comparable to its natural ligand when coupled with the IgK signal peptide alone or in combination with a repetition of eight arginines (polyR). Furthermore, the addition of the polyR helped the protein to overcome an impermeable cell monolayer, suggesting a transit through cell membranes and a better diffusion within the tumor mass. After 48 hours of co-culture, the viability of target cells was significantly reduced compared to control cells, indicating CAR-dependent target recognition and killing. Moreover, CAR-T cells expressed higher levels of CD25 and CD69 activation markers, which was accompanied by secretion of activation-induced cytokines such as IL-2 and IFN-γ. Finally, the TLR ligand secreted by CAR-T cells showed TLR activation.

**Conclusion**

Our results lay the foundation for a new potential weapon against cancer. Further characterization of our 4th generation CAR-T cells *in vitro* and *in vivo* will unravel their features in anticancer immunotherapy.

### EACR23-1116
### The Development of CAR-T Cell Therapeutics Targeting tumour-specific CD146

E. Uzuner[1], H. Mole[1], C.W. Wong[1], M.L. Fernandez Carro[1], K. Sefton[1], R. Salmi-Leshem[1], A.F.L. Hurlstone[1]
[1]University of Manchester, Faculty of Biology- Medicine and Health, Manchester, United Kingdom

**Introduction**

Chimeric antigen receptor (CAR) T cells are a promising class of “living drug” for treating cancer wherein primary T cells are engineered to express a synthetic antigen receptor that combines antigen recognition with (co-)stimulation. Despite success with B-cell malignancies, CAR T cells are far less effective with other cancer types, reflecting a lack of specific target proteins causing significant “on-target/off-tumour” toxicities. Herein, we attempt to improve the tumour specificity of CAR T cells by targeting a tumour-specific (ts) epitope of CD146. CD146 is a glycoprotein upregulated in various solid tumours including melanoma and ovarian cancer, but that is also expressed on benign cells, including endothelial cells, smooth muscle cells, and pericytes. Nonetheless, an antibody has been developed that binds selectively to tsCD146.

**Material and Methods**

Through rounds of protein engineering, we have progressed CAR constructs incorporating the antigen-binding moieties of this anti-tsCD146 antibody. The modifications include substituting or truncating the hinge domain, replacing the transmembrane domain, and adding an additional co-stimulatory domain and a chimeric co-stimulatory CAR (CCR) domain. Activity has been assessed using either flow cytometry to measure surface CD69 expression, an early activation marker, on CAR-expressing Jurkat cells co-cultured with several MCAM +ve and MCAM -ve human melanoma and ovarian cancer cell lines. The killing ability of CAR-primary T cells has been evaluated by the luciferase activity of target cells.

**Results and Discussions**

Our anti-tsCD146 CAR directs selective activation of Jurkat cells when co-cultured with MCAM +ve human melanoma and ovarian cancer cell lines cells as effectively as CAR targeting pan-CD146. Anti-tsCD146 CAR primary T cells have proportional killing activity for these target cells comparing pan-CD146 targeting primary T cells. Moreover, we have created MCAM knock-out human cancer cell lines to validate the effects of our CAR T cells. However, our CARs do not target MCAM +ve mouse cell lines.

**Conclusion**

We have successfully integrated the antigen-binding domain of an anti-tsCD146 antibody into a CAR scaffold. Our future work will be evaluating the tumour selectivity of our CARs as well as the anti-tumour capabilities of primary CAR T cells *in vitro* and *in vivo*.

### EACR23-1130
### Characterising the response of tumours to fractionated radiotherapy and immune checkpoint inhibitors

C.R. Bell[1], T. Katopodi[1], Y. Xin Ho[1], E. Wright[1], A. Cantrell[1], N. Moore[1], S. Brown[1], L. Mooney[1], J. Kendrew[1]
[1]Sygnature Discovery, Translational Oncology, Manchester, United Kingdom

**Introduction**

Radiotherapy is a main treatment modality for multiple cancer types, with approximately half of all cancer patients receiving radiotherapy as part of their treatment regimen. In addition to direct cytotoxic effects on cancer cells, it is now appreciated that irradiation (IR) of tumours can modulate the tumour immune microenvironment (TIME). Therapies which augment anti-cancer immune responses,

© 2023 The Authors. Molecular Oncology is published by John Wiley & Sons Ltd on behalf of Federation of European Biochemical Societies such as immune checkpoint inhibitors (ICIs), have revolutionised cancer treatment in recent years, and IR therapy in combination with ICIs is being investigated across multiple clinical trials. Characterising the effects of radiotherapy on the TIME and responses to IR and ICI in preclinical tumour models is therefore valuable to inform drug discovery research.

**Material and Methods**

The colorectal carcinoma cell line MC38 was implanted subcutaneously into C57BL/6 mice and tumour growth was monitored three times per week using callipers. Animals were randomised into treatment groups when average tumour volume reached approximately 0.1cm$^3$. Mice receiving IR therapy were administered targeted X-rays using a Xstrahl CIX3 cabinet irradiator, and animals were shielded using lead panels with only the tumour exposed for localised IR treatment. The effect of single high dose IR and different fractionated IR dosing regimens and ICIs on tumour growth and tolerability were evaluated. The effect of fractionated IR and ICIs on the TIME was evaluated using multi-colour flow cytometry.

**Results and Discussions**

Administering IR in a fractionated dosing regimen was efficacious and well tolerated in C57BL/6 MC38 tumour-bearing mice, inducing tumour growth delay and improving survival. Treatment with ICIs led to a mixed response, with tumour eradication obtained in a fraction of the animals treated. Characterising the TIME following fractionated IR and ICIs revealed that these therapies modulate the intratumoural immune infiltrate.

**Conclusion**

We have characterised and established dosing regimens for fractionated IR and ICIs and evaluated changes to the TIME. These data demonstrate the value of these models for immuno-oncology drug discovery research.

### EACR23-1192

### Nanoparticles loaded with Poly(I:C) and Resiquimod triggers tumor-associated macrophages for effective anti-tumoral immunity to treat primary solid tumors and their distant metastasis.

*F. Torres Andón[1], C. Anfray[2], A. Ummarino[2], I. Fernández Mariño[3], C. Fernández Varela[3], J. Crecente Campo[3], E. Fernández Megía[4], A. Mantovani[2], M.J. Alonso[3], P. Allavena[2]*

*[1]INIBIC/CHUAC, Oncology Unit- Complexo Hospitalario Universitario de A Coruña CHUAC, A Coruña, Spain*

*[2]Clinical and Research Hospital Humanitas, Laboratory of Cellular Immunology, Rozzano-Milano, Italy*

*[3]Universidade de Santiago de Compostela, CiMUS, Santiago de Compostela, Spain*

*[4]Universidade de Santiago de Compostela, CiQUS, Santiago de Compostela, Spain*

**Introduction**

In the tumor microenvironment, tumor-associated macrophages (TAMs) play a key immunosuppressive role that limits the ability of the immune system to fight cancer[1]. Toll-like receptors (TLRs) ligands, such as Poly(I:C) or Resiquimod (R848) are able to reprogram TAMs towards M1-like antitumor effector cells[2]. Here, we describe our work using nanoparticles loaded with Poly(I:C)+R848 to improve their stability, pharmacokinetic profile and systemic toxicity, thus allowing their application in the clinic.

**Material and Methods**

Polymeric nanocapsules (NCs) were developed by the solvent displacement and layer-by-layer methodologies; and characterized by DLS and TEM. Hyaluronic acid was chemically functionalized with mannose for the coating of the NCs to target TAMs. TLR-loaded-NCs were evaluated *in vitro* for toxicity and immunostimulatory activity by Alamar Blue, ELISA and flow cytometry (FACS) using primary human monocyte derived macrophages (HMDMs). Cytotoxic activity of macrophages towards cancer cells was evaluated with an *in vitro* functional assay by FACS. Biodistribution of mannose-HA-NCs was evaluated by IVIS. For *in vivo* experiments, the CMT167 lung cancer, 4T1 breast cancer, K8484 pancreatic cancer and the MN/MCA1 fibrosarcoma model metastasizing to lungs were used; tumor-infiltrating leukocytes were evaluated by FACS, PCR and multispectral immunophenotyping.

**Results and Discussions**

We have developed polymeric nanocapsules loaded with Poly(I:C)+R848. Among a series of 5 lead prototypes, protamine-NCs were selected based on their pharmaceutical properties (size, charge, stability) and *in vitro* analysis, showing no toxicity and ability to polarize macrophages towards M1-like antitumor effectors. *In vivo*, the intratumoral injection of Poly(I:C)+R848-protamine-NCs significantly prevented tumor growth and metastasis. For the intravenous administration, an additional polymeric layer of hyaluronic acid functionalized with mannose was implemented to target the CD206 receptors overexpressed on the surface of TAMs. These NCs presented higher accumulation in TAMs versus non-functionalized NCs[3]. The intravenous administration of mannose-HA-protamine-NCs loaded with Poly(I:C)+R848 showed antitumoral efficacy in preclinical murine tumor models.

**Conclusion**

Mannose-HA-protamine-NCs loaded with Poly(I:C)+R848 showed the capacity to repolarize TAMs, to reduce tumor progression and prevent metastasis in murine tumors.

### EACR23-1222

### Exploring the potential of a chimeric DNA vaccine targeting CSPG4 in comparative models of osteosarcoma

*L. Tarone[1], D. Giacobino[2], S. Iussich[2], L. Maniscalco[2], K. Mareschi[3], E. Tirtei[3], E. Morello[2], P. Buracco[2], F. Cavallo[1], F. Riccardo[1]*

*[1]University of Turin, Department of Molecular Biotechnology and Health Sciences, Torino, Italy*

*[2]University of Turin, Department of Veterinary Sciences, Grugliasco, Italy*

*[3]University of Turin, Department of Sciences of Public Health and Pediatrics, Torino, Italy*

**Introduction**

Osteosarcoma (OSA) is a rare pediatric tumor, characterized by high invasiveness and resistance to therapies. Despite current treatments, the outcome of metastatic OSA patients is poor and the finding of alternative therapies is needed. In the search of novel

© 2023 The Authors. Molecular Oncology is published by John Wiley & Sons Ltd on behalf of Federation of European Biochemical Societies tumor antigens to target, the chondroitin sulfate proteoglycan (CSPG)4 is gaining attention. CSPG4 is overexpressed in several solid tumors, where it sustains multiple pro-tumoral functions, while it is absent in normal tissues. Hence, CSPG4 is an attractive immunotherapeutic target. We previously investigated CSPG4 expression in a relevant translational pre-clinical model, such as canine OSA, to lay the basis for further investigations on the impact of anti-CSPG4 immunotherapy.

**Material and Methods**

We evaluated CSPG4 expression in a cohort of pediatric OSA patients through immunohistochemistry. We downmodulated CSPG4 through siRNAs on human OSA cells, and performed functional assays. We immunized mice with a chimeric human/dog (HuDo)-CSPG4 DNA vaccine, and adoptively transferred vaccine-induced CD8+ T cells and antibodies. Client-owned OSA-bearing dogs were enrolled in a clinical veterinary trial and adjuvantly treated with HuDo-CSPG4 vaccine combined with electroporation, and we evaluated the vaccine-induced immune response. We exploited human-surrogate cytotoxic assays using healthy donor-derived dendritic cells to stimulate autologous lymphocytes *in vitro*.

**Results and Discussions**

We found CSPG4 overexpression in most human OSA biopsies analysed. CSPG4 downmodulation significantly impaired human OSA cell malignant behavior *in vitro*, suggesting that anti-CSPG4 immunotherapy could have relevance for OSA management. We investigated the potential of HuDo-CSPG4 DNA vaccine in human OSA xenograft mouse models and dogs with spontaneous $CSPG4^+$ OSA. Vaccine-induced $CD8^+$ T cells and antibodies delayed OSA tumor growth and metastatization in human-OSA-bearing mice. HuDo-CSPG4 vaccination was safe in dogs. We observed the induction of both anti-CSPG4 humoral and cellular immunity. The vaccination resulted clinically effective; vaccinated dogs had a prolonged overall survival as compared to conventionally treated controls. Finally, HuDo-CSPG4 induced a cytotoxic response in a human surrogate setting.

**Conclusion**

Overall, based on these findings and considering the high predictive value of spontaneous canine tumors, these results might route the potential translation of this novel immunotherapeutic approach into a human setting.

### EACR23-1239

### Genetically engineered Salmonella Typhi as novel microbial immunotherapy for bladder cancer

*C. Prevosto[1], N. Glanville[2], M. Chol[3], O. Oke[3], A. Scott[3], S. Domingos-Pereira[4], L. Polak[4], N.H. Nardelli-Haefliger[4], A. Sevko[1], L. Deban[5]*

[1]*Prokarium Ltd, Translational Research, London, United Kingdom*

[2]*Prokarium Ltd, VP Research, London, United Kingdom*

[3]*Prokarium Ltd, Preclinical Research, London, United Kingdom*

[4]*Centre Hospitalier Universitaire Vaudois and University of Lausanne, Urology, Lausanne, Switzerland*

[5]*Prokarium Ltd, Chief Scientific Officer, London, United Kingdom*

**Introduction**

Intravesical Bacillus Calmette-Guérin (BCG) is the standard-of-care (SOC) for non-muscle invasive bladder cancer (NMIBC). Despite high initial efficacy, BGC treatment is associated with high rates of recurrence, significant side effects, intense treatment burden, product supply shortages. There is a significant unmet need for novel therapeutic approaches in NMIBC management. We established the preclinical safety and efficacy of live-attenuated *Salmonella enterica* Typhi strain ZH9 as a novel immunotherapy for NMIBC.

**Material and Methods**

ZH9 efficacy and mechanism of action (MOA) were tested in the murine orthotopic, syngeneic MB49 tumor model, compared to OncoTICE®BCG. Survival was analysed with log-rank test (Mantel-Cox). MOA was assessed by flow cytometry and Nanostring assays.

**Results and Discussions**

In contrast to SOC BCG, ZH9 demonstrated a significant survival benefit after a single intravesical (IVES) dose in both permissive (treatment 2 days post-tumour inoculation) and stringent (treatment 4-5 days post-tumour) models. Surviving ZH9-treated animals were protected from tumor rechallenge in the bladder or at a distant site, indicating induction of systemic anti-tumour immunity. ZH9 treatment induced strong immune activation in the bladder, resulting in accumulation of monocytes, NK, T and dendritic cells. The duration and magnitude of these immune responses were greater after single ZH9 dose than after an equivalent dose of BCG. Recent data suggest that intradermal BCG immunisation can improve clinical outcome of BCG SOC in NMIBC. Thus, we investigated the effect of ZH9 systemic priming prior to IVES ZH9. Immune cell recruitment and expression of genes associated with antigen processing, interferon response and the function of various immune cells were enhanced by priming. Now we are proceeding to phase1b ZH9 safety and tolerability trial in NMIBC patients. We will also include an extensive translational and biomarker investigational package to explore the mechanism of action of ZH9 and the local and systemic immune responses by analysing urine, blood, as well as tumor and healthy bladder layer tissue.

**Conclusion**

A single dose of ZH9 provides significant therapeutic benefit over BCG and induces robust local cellular immune responses. Systemic priming further enhances immune responses and may therefore result in greater efficacy. This suggests that ZH9 immunotherapy may improve outcomes for patients with NMIBC, allowing less aggressive treatment and more reliable manufacturing method than the current SOC.

### EACR23-1276

### Targeting Cancer-Associated Fibroblasts in Head and Neck Cancer through MAGED4B Vaccination

*J. Buckingham[1], C. Hanley[1], N. Savelyeva[2], G. Thomas[1]*

[1]*University of Southampton, Cancer Sciences- Faculty of Medicine, Southampton, United Kingdom*

[2]*University of Liverpool, Molecular & Clinical Cancer Medicine, Liverpool, United Kingdom*

**Introduction**

© 2023 The Authors. Molecular Oncology is published by John Wiley & Sons Ltd on behalf of Federation of European Biochemical Societies

A myofibroblastic CAF (myoCAF)-rich stroma is associated with poor prognosis in multiple cancers, including head and neck cancer (HNSCC). In part, this is due to myoCAF suppressing anti-tumour immunity and contributing to immunotherapy resistance. Depleting myoCAF from tumours could potentiate immunotherapy response. However, a lack of specific myoCAF markers has limited this therapeutic strategy.

**Material and Methods**

**scRNA-seq** and **immunohistochemistry** (IHC) were used to analyse cancer/testis antigen (CTA) expression in human HNSCC.

**MOC1 and MOC2 murine oral cancer models** were developed to test the effect of MAGED4B-directed vaccination in HNSCC. **myoCAF-rich tumour models** were generated through co-injection of MOC1 and MOC2 with TGF-β-treated murine tongue fibroblasts (myoMTF; 1:5 ratio). Resected tumours were studied through H&E and IHC analysis. Two pDOM DNA fusion vaccines against MAGED4B were constructed, and then tested *in vivo* on tumour-naïve mice. **Immunogenicity** was confirmed by *ex vivo* IFNγ ELISpot. To generate tumours that express MAGED4B in tumour cells and/or myoCAF, MOC1/MOC2 cells and MTF were **retrovirally transduced** with MAGED4B.

**Results and Discussions**

We identified CTA *MAGED4B* in the scRNA-seq HNSCC dataset and found its expression limited to myoCAFs and tumour cells. IHC analysis of HNSCC (n=53) confirmed high MAGED4B expression in tumour cells (43/53) and myoCAF (25/53).

*In vivo,* MOC1 cells produced tumours that were non-metastatic, moderately differentiated and composed of islands of tumour cells often showing central keratinisation. Comparatively, MOC2 tumours were poorly differentiated and commonly metastasised to regional and sometimes distant lymph nodes. Co-injection of MOC1/MOC2 cells with myoMTF significantly increased tumour growth (p=0.0015) and also increased αSMA expression, as detected by IHC (p=0.0041).

To generate tumours that express MAGED4B in tumour cells and/or myoCAF we retrovirally transduced MOC1/MOC2 cells and MTF with MAGED4B and confirmed expression by IHC. Vaccine efficacy experiments on MAGED4B-expressing tumours are ongoing.

**Conclusion**

Significant levels of myoCAF are present in around 50% of HNSCC, which have one of the lowest response rates to anti-PD1 immunotherapy (~15%). MAGED4B-directed vaccination has the potential to target both HNSCC cells and myoCAF simultaneously and has significant potential for combination immunotherapy in highly aggressive myoCAF-rich HNSCC.

### EACR23-1297
### Unleashing the Potential of CD47-Mediated Therapy: New Agonist Triggers Programmed Cancer Cell Death in Hematological Malignancies

*S. Skah*[1], *S. Matar*[1,2], *A. Yadav*[3], *H. Kiil Blomhoff*[3], *R.D. Pettersen*[1], *K. Hestdal*[1], *N. Richartz*[1,3]

[1]*Oslo Science Park- Oslo ShareLab, Caedo Oncology AS, Oslo, Norway*
[2]*University of Stavanger- Faculty of Science and Technology, Department of Chemistry-Bioscience and Environmental Engineering, Stavanger, Norway*
[3]*University of Oslo, Department of Molecular Medicine-Institute of Basic Medical Sciences, Oslo, Norway*

**Introduction**

The transmembrane glycoprotein CD47 plays a crucial role in regulating the immune response to cancer cells. When CD47 on target cells interacts with SIRPα on macrophages, it induces a "don't eat me" signal that prevents phagocytosis. Overexpression of CD47 in cancer cells is frequently associated with an unfavorable outcome, making CD47 an attractive target for cancer therapy. However, several studies have suggested that blocking the CD47-SIRPα interaction is not sufficient to achieve clinically meaningful efficacy in cancer. Furthermore, antibody binding to red blood cells (RBCs) is a frequent unwanted side effect. Here, we characterize CO-1, a novel IgG4 CD47 monoclonal antibody (mAb) that acts as a CD47 agonist. CO-1 induces potent and rapid programmed cancer cell death (PCCD) in addition to inducing phagocytosis of cancer cells by blocking the CD47-SIRPα interaction. Our data provide a novel treatment strategy for hematological malignancies.

**Material and Methods**

To study the therapeutic potential of CO-1 in hematological malignancies, we used cell lines derived from hematological cancers, cancer cells isolated from patients diagnosed with B cell precursor acute lymphoblastic leukemia (BCP-ALL), and murine xenograft models of BCP-ALL. PCCD was analyzed by Annexin V and TMRM staining. Hemagglutination assays were performed by incrementally adding CO-1 to a suspension of RBCs from healthy donors.

**Results and Discussions**

CO-1 selectively targets and eliminates cancer cells while sparing normal cells. CO-1 induces tumor cell phagocytosis by blocking the CD47-SIRPα interaction, as well as induces rapid (within 30 min) and potent PCCD at low concentrations (≤100 ng/ml) of the antibody. Importantly, CO-1 exhibits low binding affinity to RBCs and does not cause hemagglutination within this same concentration range. Compared to the previously published anti-CD47 antibodies Magrolimab and AO-176, CO-1 is superior both in terms of PCCD of hematological malignant cells and had higher treatment efficacy in the BCP-ALL xenograft model at low doses. Furthermore, CO-1 displayed a high safety profile in B cells derived from healthy human donors.

**Conclusion**

Compared with other anti-CD47 antibodies, our results demonstrate that CO-1 is superior both *in vitro* and *in vivo* in terms of its unique mechanisms of action and its enhanced efficacy. By inducing PCCD combined with selective phagocytosis of cancer cells, CO-1 may provide a new therapeutic strategy for improving the outcome of patients with hematological malignancies.

### EACR23-1310
### TET2-mutations reshape tumour infiltrating leukocytes to promote

© 2023 The Authors. Molecular Oncology is published by John Wiley & Sons Ltd on behalf of Federation of European Biochemical Societies

### immunotherapy response

R. Vanner[1], A. Zeng[2], M. Chan-Seng-Yue[2], S. Bansal[2], S. Genta[3], E. Sanz Garcia[3], L. Siu[3], M. Butler[4], A. Trumpp[5], J. Dick[2]
[1]DKFZ, Stem Cells and Cancer, Heidelberg, Germany
[2]Princess Margaret Cancer Centre, Stem Cells, Toronto, Canada
[3]Princess Margaret Cancer Centre, Drug Development Program, Toronto, Canada
[4]Princess Margaret Cancer Centre, Tumor Immunotherapy Program, Toronto, Canada
[5]DFKZ, Stem Cells and Cancer, Heidelberg, Germany

**Introduction**

Clonal Hematopoiesis (CH) occurs when a somatic mutation causes a hematopoietic stem cell's disproportionate contribution to blood. CH is present in up to 20% of solid cancer patients and has been associated with poor prognosis. The epigenetic modifier TET2 encodes one of the most frequently mutated CH driver genes, and in animal models its loss of function was linked to enhanced adaptive immunity in chronic infection and adoptive cell therapy. Here we investigate how TET2-mutant CH (TET2-CH) influences response to immune checkpoint blockade (ICB).

**Material and Methods**

658 ICB-treated patients were screened for CH with targeted sequencing (n=89) or publicly-available exome sequencing (n=569) and assessed for durable clinical benefit at 6 months. To model TET2-CH C57/Bl6 mice were rescued from lethal irradiation with bone marrow from Tet2-heterozygous null (-het), or wild type (wt) mice. MC-38 colon adenocarcinoma cells were subcutaneously implanted and mice were treated with anti-PD-1 or control antibodies +/- cell depleting antibodies or clodronate. Mouse tumour-infiltrating-leukocytes (TILs) were profiled with 10X 5' scRNA-seq and Illumina Infinium DNA methylation arrays.

**Results and Discussions**

CH was present in 88/658 of ICB treated patients (13%). While CH with any driver was not associated with likelihood of clinical benefit, in n=205 melanoma patients, the presence of TET2-CH was associated with significantly higher odds of clinical benefit (OR 6.0, 1.01-66.6). In an animal model of TET2-CH, anti-PD-1 treated tumours in Tet2-het mice were significantly smaller, and Tet2-het mice were significantly more likely to have a response, recapitulating the clinical effect. Anti-PD-1 therapy with targeted cell depletion revealed the TET2-CH effect depends on CD4 T Cells, CD8 T Cells and macrophages, but not NK cells. scRNAseq showed that in response to PD-1 blockade, Tet2-het TILs were enriched for effector and memory CD8 T cells plus memory and interferon-producing CD4 cells, while wt TILs were enriched for exhausted CD8 T cells and regulatory CD4 cells. Tet2-het TILs exhibited hypermethylation of genetic pathways regulating leukocyte differentiation.

**Conclusion**

TET2-CH is associated with increased odds of 6 month clinical benefit in immunotherapy-treated melanoma patients. In a mouse TET2-CH model, Tet2-loss of function reshapes the TIL epigenome and enhances effector/memory fate while relieving exhaustion and inhibiting regulatory T cell fate, to drive immunotherapy response.

### EACR23-1450
### Universal Cancer Vaccine derived engineered TCR: Genetic engineering and functional analysis of novel hTERT-specific HLA Class II-restricted TCR for Adoptive Cell Therapy

A. Kumar[1], J. Galaine[1], M. Fredon[1], A. Marguier[1], R. Loyon[1], F. Garnache-Ottou[1], O. Adotevi[1,2], Y. Godet[1]
[1]INSERM UMR1098 - Unit RIGHT, Team TICI, Besançon, France
[2]University Hospital of Besançon, Department of Medical Oncology, Besançon, France

**Introduction**

Adotevi *et al.* (J. Clin. Oncol., 2023) recently published the safety, immunogenicity, and efficacy of a novel cancer vaccine (UCPVax) through a phase Ib/phase IIa de-escalation study. They showed that UCPVax induced polyfunctional and long-lasting CD4+ T helper-1 response directed against human Telomerase (hTERT). Riding on this success, we isolated HLA-DR-restricted TCRs specific to UCPVax from the peripheral blood of cancer patients in order to develop T-cells based cell therapy and investigate the mechanism of these riveting clinical responses to UCPVax.

**Material and Methods**

TCR sequences were obtained from CD4+ T-cell clones specific for UCPVax, demonstrated previously (Galaine *et al.,* J. Immunol, 2016). The TCRs were chimerized using murine constant chain, and thereafter developed for lentiviral vector to produce lentiviral supernatant. T-cells from the peripheral blood of healthy donors (n=5) were isolated and thereafter stimulated by CD3/CD28 beads with IL-2 for two days, followed by lentiviral transduction at MOI 10. Positive cells were isolated at day 5 post-transduction, using the CD19 selection gene, and thereafter amplified on feeder cells. At last, the transgenic T cells were co-cultured with HLA-matched cancer cells loaded with UCPVax, in order to study T-cell activation through IFNγ-based flow cytometry.

**Results and Discussions**

We observed the transgenic TCR redirected primary T-cells exhibited TCR surface expression across CD4+ and CD8+ T-cells. Most interestingly, when co-cultured with peptide-loaded HLA-matched cancer cells, we obtained *in-vitro* antigen-specific activation for both CD4+ as well as CD8+ T-cells, suggesting co-receptor independent TCR downstream signaling.

**Conclusion**

We now aim to investigate the *in-vivo* efficacy and safety of the transgenic TCR, alongside their T-cell polarization, differentiation profile, and cytotoxic capacity. At last, we will employ non-viral genome editing to broaden hTERT-specific T-cell therapy for cancer treatment.

### EACR23-1456
### Developing enhanced TCR-based T-cell therapy using in silico devised receptor mutations

© 2023 The Authors. Molecular Oncology is published by John Wiley & Sons Ltd on behalf of Federation of European Biochemical Societies

C. Cohen[1], M. Radman[1]
[1]Laboratory of Tumor Immunology and Immunotherapy- B ar-Ilan University, Life Sciences, Ramat Gan, Israel

**Introduction**

T-cells can be genetically modified with tumor specific receptors such as CARs or TCRs, to provide them with the ability to recognize cancer cells. However, several factors may limit the effectiveness of this approach. The solutions for overcoming the technical obstacles of introducing exogenous TCR chains to cells that already express an endogenous TCR are either aimed at removing the endogenous TCRs, modifying the exogenous TCRs, or a combination of both. Some of these techniques, however, are not currently feasible in clinical settings, produce only a minor benefit, or are restricted to a single TCR.

**Material and Methods**

In this project, our goal was to increase the anti-cancer function of T-cells by increasing the stability of the constant region of the TCR and generating improved versions of several widely used TCRs. This can be achieved by modifying selected amino acids in the constant region. Using our modified TCRs in therapeutic settings will not require changing the well-established protocols currently in use.

Our method combines previously described modifications with an evolution-guided atomistic design to improve protein stability.

**Results and Discussions**

The combination of several approaches resulted in an increased exogenous TCR expression, which directly correlated in co-cultures with tumor cells with an enhanced cytokine secretion that ranged from a three-fold increase to a sixteen-fold increase, depending on the TCR and the targeted cell line. Moreover, improved mutant TCRs mediated an increased in expression of activation-associated differentiation markers (e.g., 41BB, CD25 and CD69) and these optimized designs could be applied to multiple TCRs targeting different antigens. Finally, T-cells expressing the mutated TCRs demonstrated an improved ability in targeting tumors in killing assays.

**Conclusion**

In conclusion, we demonstrate herein that it is feasible to generically devise in silico specific structural mutations applicable to multiple TCRs and can mediate superior anti-tumor activity.

### EACR23-1459
### Photodynamic treatment of cancer cell spheroids co-cultured with preprogrammed macrophages

K. Korotkaja[1], Ž. Rudevica[1], J. Jansons[1], D. Lapiņa[1], A. Zajakina[1]
[1]Latvian Biomedical Research and Study Centre, Cancer Gene Therapy, Riga, Latvia

**Introduction**

Photodynamic therapy (PDT) is a promising cancer therapy approach based on the activation of the photosensitive drug, e.g. chlorin e6 (Ce6), by near-infrared light after its introduction into the body (Kwiatkowski, *Biomed. Pharmacother.* **2018**). The effect of the therapy can thus be localized, and side effects reduced. It is known that macrophages can both promote and inhibit the progression of tumour growth (Zhou, *Front. Oncol.* **2020**). Previous studies have shown that PDT can program macrophages into anti-cancer phenotypes (Yu, *Front. Pharmacol.* **2022**). The main purpose of our study was to investigate the effect of PDT on cancer cell spheroids co-cultured with macrophages.

**Material and Methods**

4T1 mouse breast cancer cell spheroids were generated as previously described (Trofimova, *Vaccines* **2021**). Macrophages were derived from bone marrow monocytes of BALB/c mice and polarized using cytokines and TLR ligands: IFNγ and Pam3SCK4 to generate M1-like phenotype, and IL-4 to generate M2-like phenotype. Ce6 activated with a 650 nm LED light source was used for PDT. Flow cytometry was used to examine the profile of surface markers of 2D- and 3D-cultured macrophages. Co-culture models of macrophages and cancer cells were used for phagocytosis investigation.

**Results and Discussions**

4T1 treatment with Ce6 and subsequent exposure to 650 nm LED 3-5 J/cm2 induced dramatic cell death through ROS production in a Ce6/LED dose-dependent manner. Apoptotic cells showed calreticulin – the marker of the immunogenic cell death. Cancer cell culturing under 3D conditions revealed a lower degree of Ce6 penetration into cells and slow inhibitory effects under repeated light exposure compared to 2D cultures. Co-culturing of cancer cells with macrophages pre-differentiated into M1 and M2 phenotypes led to efficient phagocytosis of Ce6/LED treated 4T1 cells by both phenotypes. Ce6/LED significantly affected the viability of macrophages and led to a decrease of CD11b and MHC II markers. Furthermore, it was found that the treatment of macrophages did not affect the polarization process if the macrophages were cultured under 3D conditions.

**Conclusion**

PDT is less effective in 3D models and cannot reprogram macrophages towards the M1 phenotype. On the other hand, it is a promising approach for cancer therapy due to its ability to deplete cancer-associated macrophages and promote cancer cell phagocytosis. As PDT does not interfere with monocyte polarization, it has a high potency to be combined with immunomodulatory treatment.

### EACR23-1462
### Efficient tumor suppression of neoantigenic peptides identified by using a neoantigen prediction platform VACINUS based on tumor-reactive TILs TCR-pMHC ternary complex

S.M. Kim[1], S.H. Kim[1], B. Lee[1], K. Song[1], W.Y. Park[1]
[1]Geninus Inc., Drug Discovery Department, Seoul, South Korea

**Introduction**

Neoantigens are important target for tumor immunotherapy due to their strong immunogenicity and lack of expression in normal tissues. We have developed *in silico* neoantigen prediction platform 'VACINUS' by structure-based pairing with T cell receptors (TCRs) on tumor-reactive TILs from hepatocellular carcinoma (HCC) and showed *in vitro* mmunogenicity and tumor reactivity. In this study, we tried to validate our neoantigen prediction platform *in vivo* mouse model.

© 2023 The Authors. Molecular Oncology is published by John Wiley & Sons Ltd on behalf of Federation of European Biochemical Societies

**Material and Methods**
To evaluate the VACINUS platform, 7-week-old male mice were implanted subcutaneously on the flank with either MC38 or B16F10 cells (C57BL/6), respectively. Tumor tissues were obtained from mice implanted with MC38 or B16F10 cells. To select target TILs, tumor tissues were subjected to scRNA/TCR sequencing. Tier1 epitopes were derived from VACINUS platform which predicts neoantigen using ternary complex of target TILs TCR-peptide-MHC. After vaccination of mice with tier1 epitopes, immunogenicity was confirmed in splenocytes. Therapeutic model was tested in 5 groups after implanting MC38 or B16F10 cells: G1) control, G2) adjuvants (Poly-IC), G3) adjuvants/tier1 epitope, G4) adjuvants/anti-PD1 antibody, G5) adjuvants/anti-PD1 antibody/tier1 epitope.
**Results and Discussions**
Using VACINUS platform, we identified 3 of Tier1 peptides each in both MC38 and B16F10 mouse models, respectively. Immunogenicity of these neoantigenic peptides were evaluated using peptide-specific IFN-γ secretion by splenocytes. In MC38 mouse tumor model, combination of our Tier1 vaccines with an immune checkpoint anti-PD-1 antibody showed synergistic anti-tumor efficacy showing tumor regression, In B16F10 mouse tumor model, comparing to control group, our Tier1 vaccines showed not only anti-tumor efficacy in Tier1 single administration, but also synergistic effect in vaccines with anti-PD-1 combination. We also monitored survival of tumor bearing mice following vaccination. Compared to the control group, our Tier1 vaccine with anti-PD-1 combination saved mice more than 90%.
**Conclusion**
The VACINUS platform predicts high-quality neoantigens with higher immunogenicity and antitumor efficacy. Neoantigen prediction by structure-based pairing of neoantigens and phenotype-selected TILs showed promising potential to better select therapeutically-relevant cancer vaccines.

### EACR23-1477
### Armoring p95HER2 CAR Ts as an effective immunotherapy against a subset of HER2-positive tumors

*A. Grinyo i Escuer[1], M. Román Alonso[1], S. Duro-Sánchez[1], I. Rius Ruiz[1], M. Escorihuela[1], M. Bort Brusca[2], J. Arribas[2]*
*[1]Vall d'Hebron Institute of Oncology VHIO, Preclinical Research, Barcelona, Spain*
*[2]Hospital del Mar Medical Research Institute IMIM, Preclinical Cancer Research Programme, Barcelona, Spain*

**Introduction**
T lymphocytes can be effectively redirected against tumor-associated or tumor-specific antigens using bispecific antibodies, commonly known as bispecific T cell engagers (BiTEs), or chimeric antigen receptors (CARs). So far, T cell redirection has been a successful immunotherapy approach on the treatment of certain hematologic malignancies, leading to complete and durable responses and has had a more modest response against solid tumors. While most HER2-positive breast tumors diagnosed at early stages have already effective and available treatment, refractory and metastatic tumors still pose an unmet medical need, with some patients having to discontinue targeted therapies due to HER2-targeting associated toxicities. P95HER2 is a truncated form of the tyrosine kinase receptor HER2 which is expressed in 30% of HER2-amplified tumors, including breast, gastric and lung. Recently, we have identified it as a tumor-specific antigen, it is not expressed in normal tissue, which makes redirection of T cells to p95HER2 a promising safe option with no “on-target off-tumor” toxicity.
**Material and Methods**
Using buffy coats from healthy donors we have produced p95HER2 CAR Ts that can also secrete bispecific antibodies.
We have tested their efficacy in immunodeficient mice with Patient-Derived Xenografts (PDXs) and metastatic models.
To test their safety, we checked their effect in tumors derived from cells expressing basal levels of HER2.
**Results and Discussions**
We have developed p95CAR T cells that are effective in eliminating p95HER2-expressing cells in vitro and in vivo, and have a partial response in patient-derived xenografts (PDXs). To improve the antitumor activity, we armored the p95HER2 CAR T to secrete HER2 BITE that has shown to be highly effective against PDXs, as it has a dual targeting of antigens and can recruit bystander CD3 positive T cells. Which can help overcome antigen heterogeneity and the immunosuppressive environment found in solid tumors. To prevent HER2-targeting associated toxicities we have tuned the affinity of the HER2-targeting scFv, remarkably eradicating tumors in breast cancer PDX models, and which has no effect on tumors expressing normal levels of HER2. Indicating our strategy is a successful and safe treatment.
**Conclusion**
Our p95HER2 CAR T secreting HER2 targeting bispecific T cell engager represents a promising immunotherapy approach to treat HER2-positive tumors.

### EACR23-1482
### Role of the Galectin-9 in the Induction of Immunosuppressive Mature Regulatory Dendritic Cells by Nasopharyngeal Carcinoma Exosomes

*C. trioen[1], A. Lefebvre[1], B. Leroux[1], N. Delhem[1], O. Morales[1]*
*[1]INSERM, U1189 Oncothai, Lille, France*

**Introduction**
Nasopharyngeal Carcinoma (NPC) is characterized by an immunosuppressive microenvironment dominated by regulatory T lymphocytes and tumor-derived exosomes (Exo-NPC), both having immunosuppressive properties. Indeed, Exo-NPCs carry Galectin-9 (Gal-9), a lectin inducing T lymphocytes cells death by binding to the TIM3. Results of the laboratory have shown that Exo-NPC induced mature dendritic cells with tolerogenic properties (mregDC). ***In this context, our main objective is to investigate the implication of Gal9 in this induction to propose an immunotherapy able to target Gal-9 and so inhibit Exo-NPC.***
**Material and Methods**
We first isolated and characterized NPC-Exo, derived from a human xenotransplanted NPC in SCID mice. We

© 2023 The Authors. Molecular Oncology is published by John Wiley & Sons Ltd on behalf of Federation of European Biochemical Societies generated dendritic cells (DC) from human monocytes in the presence of recombinant Gal-9S (DC-Gal9) or with Exo-NPC-Gal9$^{+}$ (DC-Exo-NPC). The state of maturation of the DCs was then validated at the phenotypic (flow cytometry) and functional (ELISA, MLR) level. Finally, we evaluated the effect of Gal-9 blocking with a neutralizing antibody patented by the laboratory (1g3 clone) on Exo-NPC and DC suppressive function.

**Results and Discussions**

We noticed that DC-Gal9 and DC-Exo-NPC express the different maturation markers similarly to mature control (mDC). Unlike DC-Exo-NPC which secretes few pro-inflammatory cytokines, DC-Gal9 has a cytokine secretion profile identical to mDC. Nevertheless, our results suggest that both DC-Gal9 and DC-Exo-NPC possess immunosuppressive properties by inducing a decrease in CD3$^{+}$TL proliferation testifying to a tolerogenic function. Interestingly, the use of an antibody targeting Gal9 appears to be able to **(i)** inhibit the suppressive functions of Exo-NPC by restoring the PBMC proliferation and **(ii)** inhibit the suppressive function of DC-Exo-NPC and DC-Gal9 by restoring the proliferation of CD3$^{+}$TL.

**Conclusion**

Our work suggests for the first time that Gal9$^{+}$-NPC-Exo, induced mregDCs. In addition, the use of an antibody neutralizing Gal9 induces a reversion of the suppressive properties of Exo-NPC confirming the involvement of Gal9 in this mechanism. Despite good response to classical treatment, resistance and recurence are still fatal in NPC cases. These results open up very promising immunotherapeutic prospects in the management of patients with NPC.

### EACR23-1513
### Microbiological profile of Pediatric Febrile Neutropenia patients during COVID Pandemic

*V. Sharma[1], A. Dabas[2], A. Singh[3], K. Kriplani[1], R. Gaind[4], R. Gera[5]*

*[1]Vardhman Mahavir Medical College and Safdarjung Hos pital, Student, New Delhi, India*

*[2]Vardhman Mahavir Medical College and Safdarjung Hos pital, Resident Physician, New Delhi, India*

*[3]Vardhman Mahavir Medical College and Safdarjung Hos pital, Associate Professor, New Delhi, India*

*[4]Vardhman Mahavir Medical College and Safdarjung Hos pital, Head of Department Microbiology, New Delhi, India*

*[5]Vardhman Mahavir Medical College and Safdarjung Hos pital, Head of Department Pediatrics, New Delhi, India*

**Introduction**

Febrile neutropenia (FN) is the most common admitting diagnosis for paediatric oncology patients on chemotherapy. Over the past few decades, changes in hospital flora and antibiotics overuse and resistant patterns have dictated and altered the epidemiology of predominant causative pathogens in neutropenic patients. Previously this population was found to be more prone to bacterial and fungal infections; and less commonly mycobacterial, viral, and protozoal organisms. The most common aetiological agents included gram-negative bacteria E. coli, Klebsiella and Pseudomonas. However, the advent of COVID-19 infection, the imposition of a nationwide lockdown, the minimisation of healthcare visits, and strict interpersonal preventive measures are expected to change the prior trends.

**Material and Methods**

A prospective observational study of all eligible paediatric cancer patients(n=131) aged 1–12 years admitted with febrile neutropenia from December 2019 to May 2021(16 months) was done. The patients were assessed for causative pathogens and relevant lab investigations were sent on admission, day 3 and day 7.

**Results and Discussions**

Of the 131 children enrolled in the study, an aetiological agent could be identified in 72. SARS-CoV-2, Acinetobacter and Klebsiella accounted for over 50% of the infections. SARS-CoV-19 was found to be the most common pathogen. The trends were very different from those in the pre-pandemic era, non-SARS-CoV-19 coronaviruses were also detected, even more than E coli. This shift in the spectrum of infectious agents can be explained by the fact that the imposition of strict social distancing, travel restrictions and personal protective measures have resulted in decreased hospital-acquired and contact-transmitted infections but the risk of infection from the overgrowth of endogenous gram-negative flora remains unchanged. However, COVID-19 surpassed all etiological agents in causing fever in immunosuppressed children despite interpersonal preventive measures, possibly due to a peaked environmental prevalence and infection from subclinical carriers and close contacts.

**Conclusion**

Routine hygiene measures normally used by patients and their caregivers are effective in preventing contact-transmitted and hospital-acquired infections, but despite the strict implementation of interpersonal precautions, air-borne infections are still a major cause of concern in febrile neutropenia paediatric cancer patients.

### Molecular and Genetic Epidemiology

### EACR23-0065
### Investigation of DNA mismatch-repair protein immunoexpression in glioma

*N. Missaoui[1], S. Mestiri[2], A. Bdioui[2], O. Belkacem[2], S. Limam[3], M. Mokni[3], S. Hmissa[2]*

*[1]Medicine Faculty of Sousse, LR21ES03 Oncogenesis and Tumor Progression, Sousse, Tunisia*

*[2]Medicine Faculty of Sousse, Pathology Department of Sahloul University Hospital, Sousse, Tunisia*

*[3]Medicine Faculty of Sousse, Pathology Department of Farhet Hached University Hospit al, Sousse, Tunisia*

**Introduction**

Gliomas constitute a diversified group of malignant primary brain tumors with different biological and clinical characteristics. The gliomagenesis remains not fully established. Although the significance of the immunoexpression of the mismatch repair proteins of DNA (MMR) in colorectal cancer is well-recognized, it remains debated in extra-colorectal tumors, such as glial tumors.

© 2023 The Authors. Molecular Oncology is published by John Wiley & Sons Ltd on behalf of Federation of European Biochemical Societies

Herein, we investigated the MMR expression in glioma from Tunisian patients and then, we evaluated its clinicopathological and prognostic signification.

**Material and Methods**

We conducted a retrospective study of 95 glial tumors diagnosed in the Pathology Departments of Farhet Hached University Hospital and Sahloul University Hospital, Sousse (Tunisia), during 2008-2022. MMR expression was analyzed by immunohistochemistry technique on whole sections of archived material. Clinicopathological features and patient outcomes were collected. Survival analysis was explored using the Kaplan-Meier estimates and Log-Rank test

**Results and Discussions**

Overall, 59 glioma samples (62.1%) displayed an altered pattern of MMR protein expression. We detected a loss of MLH1 expression in 10.5% of cases, a loss of PMS2 in 43% of cases, a loss of MSH2 in 12.6% of cases and a loss of MSH6 in 25% cases. The expression deficiency of at least one MMR protein was mainly observed in patients diagnosed with IDH1 wild-type GBM (n=29 cases) and pilocytic astrocytoma (n=14 cases). Based on these results, microsatellite instability (MSI) phenotype was identified in 18 patients diagnosed with pilocytic astrocytomas (n=6), oligoastrocytoma anaplastic NOS (n=1), 1p19q IDH1 mutant oligodendroglioma (n=2) and IDH1 wild-type GBM (n=9). However, the remaining glioma samples (37.9%) exhibited a proficient MMR expression and, therefore, are considered of microsatellite stability (MSS) phenotype. Survival analysis showed a statistically significant difference between patients with IDH1 wild-type GBM of MSS and MSI phenotype (Log-Rrank test, $p<0.0001$).

**Conclusion**

Our study revealed the profile of deficient MMR glioma among Tunisian subjects. The loss of expression of MMR proteins was observed in glial tumors diagnosed in adults and children. This study contributes to the development of a standardized protocol for evaluating MSI in glioma. Nevertheless, a multicenter study of a larger series with additional molecular study will be necessary to more explore these tumors.

### EACR23-0881

### Germline tumor suppressor gene duplication of the SDHB locus as a novel mutational mechanism in hereditary pheochromocytoma/paraganglioma syndrome

*A. Bozsik[1,2], J. Papp[1,2], V.K. Grolmusz[1,2], A. Patócs[1,2,3], P. Mezei[4], H. Butz[1,2,3]*
*[1]National Institute of Oncology, Department of Molecular Genetics, Budapest, Hungary*
*[2]Hungarian Academy of Sciences - Semmelweis University, Hereditary Tumours Research Group, Budapest, Hungary*
*[3]Semmelweis University, Department of Laboratory Medicine, Budapest, Hungary*
*[4]Fejér Vármegyei Szent György Egyetemi Oktató Kórház, II. sz. Belgyógyászat, Székesfehérvár, Hungary*

**Introduction**

Loss-of-function (LoF) alterations are classical genetic mechanisms for the inactivation of tumor suppressor genes (TSGs). Duplications within TSGs leading to coding sequence changes are admitted genetic events that can result in LoF. However, duplications of whole TSGs are rarely recognized as a pathogenic mechanism in cancer development. Accordingly, a duplication affecting a whole tumor suppressor gene is usually classified as a variant of uncertain significance (VUS) according to the American College of Medical Genetics and Genomics (ACMG) guidelines. In this study, we characterized a duplication of a large genomic region including the whole tumor suppressor gene succinate dehydrogenase B (*SDHB*), a typical pheochromocytoma gene, in a patient diagnosed with adrenal pheochromocytoma.

**Material and Methods**

A clinical genetic examination was conducted, which included genetic counseling and pedigree analysis. Molecular genetic analyses of germline and tumor DNA were then performed using multigene panel testing, multiple ligation-dependent probe amplification (MLPA) and whole genome sequencing (WGS) for the characterization of structural rearrangements and identification of exact breakpoints. Quantitative polymerase chain reaction (RT-qPCR) validation was used to assess RNA levels, and immunostaining was employed to investigate the protein level.

**Results and Discussions**

In the germline DNA of the patient, multigene panel testing revealed a whole gene duplication on one allele of *SDHB*, which was validated by MLPA. The heterozygous duplication of the gene was also confirmed in the tumor tissue. RT-qPCR showed significant reduction of *SDHB* transcript in the germline. Loss of heterozygosity in the tumor was proved by immunohistochemistry, which showed the total loss of the SDHB protein. WGS of the germline and somatic DNA samples revealed that the duplicated genomic region extended further to the *SDHB* locus in both directions, affecting altogether a 162 kb genomic stretch on chromosome 1, spanning four additional coding genes, of which only *SDHB* had reported concern with tumor development.

**Conclusion**

DNA, RNA, and protein level analyses proved the pathogenicity of a complete, intact *SDHB* gene duplication, which can be considered a novel pathomechanism for hereditary tumor development. These data resulted in the reclassification of this variant from VUS to the likely pathogenic category, which has direct relevance for the patient and his family.

**Financial funding:** TKP2021-EGA-44 and EMMI IV/366-1/2022/EKF

### EACR23-1194

### Exploring the role of genetically determined BMI in infancy, childhood and adulthood on colorectal cancer development in later life

*D. Hughes[1], N. Papadimitriou[2], C. Bull[3], Z. Chen[4], W. Zheng[4], T. Richardson[3], N. Murphy[2], M. Jenab[2]*
*[1]UCD Conway Institute, Cancer Biology and Therapeutics Group, Dublin, Ireland*
*[2]International Agency for Research on Cancer, Nutrition and Metabolism Branch, Lyon, France*

© 2023 The Authors. Molecular Oncology is published by John Wiley & Sons Ltd on behalf of Federation of European Biochemical Societies

[3]Bristol Medical School, MRC Integrative Epidemiology Unit, Bristol, United Kingdom
[4]Vanderbilt University Medical Center, Division of Epidemiology- Vanderbilt Epidemiology Center , Nashville- TN, United States

**Introduction**

Compelling evidence implicates obesity as a major factor in colorectal cancer (CRC) development. We hypothesize that varying obesogenic inherited predisposition in early- and later-life stages may differentially impact the onset of adulthood CRC.

**Material and Methods**

Using GWAS-associated SNPs for body-size, we have conducted Mendelian randomization (MR) analyses with genetic instruments for 'recalled' early life body size at age 10 and measured adult BMI. These were then associated with CRC risk via a meta-analysis of three large, international genetic consortia (CORECT, CCFR, & GECCO). These analyses were also subsequently conducted in an Asia CRC consortium (ACCC). We thereafter constructed specific genomic risk scores (GRS) for obesity in infancy, childhood, and adulthood and assessed their association with CRC risk (Cox regression models, also stratified by sex and cancer subsite) utilizing the UK Biobank, EPIC, and GECCO.

**Results and Discussions**

In MR analyses, genetically predicted adult body size was estimated to increase risks of colorectal, colon, and proximal colon cancer (but not distal or rectal cancer) (Papadimitriou et al 2022, *BMC Med* doi: 10.1186/s12916-022-02702-9). After accounting for adult body size, genetically-predicted early life body size had no significant association with colorectal (OR: 0.97, 95% CI: 0.77-1.22) colon (OR: 0.97, 95% CI: 0.76-1.25) or distal colon cancer (OR: 1.27, 95% CI: 0.90-1.77), although the latter finding had a similar magnitude but more imprecise estimate to the significant result in the univariable analysis (OR: 1.25, 95% CI: 1.04-1.51). We have subsequently also observed an increased CRC risk with genetically predicted BMI for Asian adults only (OR: 1.12, 95% CI: 1.03-1.21). In the GRS analyses for birth and early life ($2 \leq age \leq 8$), there were generally non-statistically significant positive associations (with small point estimates) observed between higher scores and increased CRC risks. For adults, higher GRS scores for BMI & WHR were associated with CRC & colon cancer risks (e.g., HR for CRC per 1 SD unit increment of adult WHR in EPIC and UK Biobank, respectively = 1.11, 95% CI: 1.04-1.19 & 1.03, 95% CI: 1.00-1.06). The GRS analyses for infancy and childhood analyses were limited by the modest numbers of SNPs in the GRSs.

**Conclusion**

Childhood adiposity putatively influences CRC risk due to a long-term effect of remaining overweight throughout life. Further research is required to examine the role of early life body size at different colorectal subsites.

### EACR23-1320
### Prevalence of BRCA1 and BRAC2 Mutations among Breast and Ovarian Cancer Patients in Northern Emirates, UAE.

Y. Al Dhaheri[1]
[1]UAEU, Biology, Abu Dhabi- Al Ain, United Arab Emirates

**Introduction**

Breast cancer (BC) is the most common cancer and the second most cause of death among women. Mutations in *BRCA1* and *BRCA2* genes confer high susceptibility to both breast and ovarian cancer. However, data on the prevalence of the *BRCA1/2* mutations among breast and ovarian cancer patients is limited. The genetic component of breast cancer in UAE is largely unknown and no study has evaluated the *BRCA* mutations status in breast and ovarian cancer patients in UAE population. This retrospective study aimed to establish mutation frequencies of the *BRCA* genes in breast and ovarian cancer patients from Northern Emirates and sought to examine potential association of *BRCA* carriers and Triple- Negative Breast Cancer (TNBC).

**Material and Methods**

The study population included patients who underwent *BRCA* genetic testing at Sheikh Khalifa Specialty Hospital (SKSH) to determine hereditary breast/ovarian cancer. Mutations in *BRCA1* and *BRCA2* were analyzed by Sanger sequencing or next generation sequencing (NGS) along with multiple ligation probe amplification (MLPA).

**Results and Discussions**

Among 262 patients, 224 (85.5%) had no mutation. *BRCA* mutations were identified in 38 patients (14.5%). *BRCA1* and *BRCA2* mutations were detected in 6.9% and 7.6% of the patients, respectively. Variant of unknown significance in *BRCA1* was found in 0.4% of patients (one patient). TNBC accounted for 22% of all patients with breast cancer who underwent immunohistochemistry (28/127). Importantly, one novel *BRCA1* mutation: c. (80+1_81-1) _ (441+1_442-1) dup in exons 3,5,6,7 was observed in one patient with ovarian cancer who showed positive family history and age ≤ 45. Moreover, two novel deletion mutations were identified in the *BRCA2* gene. One deletion in exon 5 in ovarian cancer patient who showed positive family history and age ≤ 45. The second was a deletion in exons 10-13 observed in male patient with breast cancer with age ≤ 45.

**Conclusion**

In conclusion, this study provides a baseline information on the prevalence of BRCA mutation in hospital based population in northern emirates. The current findings indicate that 14.5% of unselected patients enrolled in this study carried BRCA mutations. The present data provide an evident contribution of BRCA1 and BRCA2 mutation in patients of breast and ovarian cancer. One limitation of this study is the low number of patients who underwent TNBC testing.

### EACR23-1495
### Integrated genomic analysis refines molecular classifications of hepatocellular carcinoma based on a large-scale cohort of 529 samples

L. PAN[1], S. Caruso[1], T. Hirsch[1], B. Gupta[1], S. Imbeaud[1], E. Letouze[1], J. Zucman-Rossi[1]
[1]Centre de Recherche des Cordeliers- Sorbonne Université

© 2023 The Authors. Molecular Oncology is published by John Wiley & Sons Ltd on behalf of Federation of European Biochemical Societies

- Inserm- Université Paris Cité,
team «Functional Genomics of Solid Tumors», Paris, France

**Introduction**

Liver cancer is a common malignancy and is the third cause of cancer-related death in the world and hepatocellular carcinoma (HCC) is the major type of liver cancer, accounting for ~90% of cases. Only 20 ~ 30% of patients can benefit from combined immunotherapy and targeted therapy due to the high inter-tumor heterogeneity of HCCs. This work aims to refine the molecular subtypes in HCCs based on a larger cohort, and further identify its potential utilization in clinical practice.

**Material and Methods**

We performed whole genome/exome sequence and RNA sequence on 529 HCCs from 461 patients collected mainly from France, which was used as discovery cohort. A combined cohort from TCGA, Japan and China with available genomic and transcriptomic data was used as validation cohort. Comprehensive genomic, transcriptomic and clinicopathological data were used to charactirize each HCC subgroup. The study was approved by institutional review board committees and written informed consents for all patients were acquired in accordance with legislation.

**Results and Discussions**

Based on integrated genomic and transcriptomic data, we classified HCCs into 3 commonly exclusive subgroups (*CTNNB1*$^{ex3}$ HCC 25%, *TP53* HCC 22%, *AXIN1* HCC 10%), 6 rare subgroups (*cyclin* HCC 5%, *BAP1* HCC 6%, *CTNNB1*$^{ex3}$;*TP53* HCC 6%, *IRF2* HCC 1%, *HNF1A* HCC 1%, *APC* HCC 1%), 1 unclassified-inflamed HCC (8%) and 1 unclassified-noninflamed HCC (14%). There are two new subgroups which have not been described before. One novel subgroup was identified by *HNF1A* biallelic inactivation, showing a homogenous molecular and clinical HCC subgroup with an enrichment of females, low fibrosis and an absence of classical HCC etiologies, which is associated with good prognosis. Besides, all of these HCCs belongs to clinically AFP-negative HCCs. Another subgroup was defined by both *CTNNB1*$^{ex3}$ and *TP53* mutations, which presented mixed features of *CTNNB1*$^{ex3}$ HCC and *TP53*-mut HCC. In clinicopathological and genomic level, this subgroup presented the characteristics of *TP53*-mut HCCs with an enrichment of HBV infection, higher Edmonson III-IV grade and CCND1-FGF19 amplification, more copy-number events and high proportion of whole-genome duplication and was associated with poor prognosis, while in the transcriptomic level, *CTNNB1*$^{ex3}$;*TP53* HCC clustered with *CTNNB1*$^{ex3}$ HCCs.

**Conclusion**

We refined the molecular classifications of HCC, which may be useful for therapeutic strategy.

### Prevention and Early Detection

### EACR23-0024
### A digital nanotechnology for phenotyping of extracellular vesicles in lung cancer screening

A. Wuethrich[1], J. Li[1], A.I. Sina[1], F. Antaw[1], D. Fielding[2], A. Möller[3], R. Lobb[1], M. Trau[1]
[1]The University of Queensland, Australian Institute for Bioengineering and Nanotechnolog y, Brisbane, Australia
[2]Royal Brisbane and Women's Hospital Herston, Department of Thoracic Medicine, Brisbane, Australia
[3]QIMR Berghofer Medical Research Institute, Tumour Microenvironment Laboratory, Brisbane, Australia

**Introduction**

Early and accurate stratification of lung lesions into benign and malignant is critical for efficient lung cancer screening. Yet, this is challenging as images obtained by low-dose computer tomography (LDCT) can show similar features between benign and malignant lesions, necessitating bronchoscopies to confirm diagnosis. Here, we assess the potential of profiling extracellular vesicles (EVs) as a non-invasive approach for lung cancer screening. To achieve multiplex profiling of EVs, we develop the single-molecule-resolution-digital-EVs-counting-detection (DECODE) chip that detects three lung cancer-associated and one generic EVs biomarker. We generate EVs profiles in patients with early malignant (stages I and II) and benign lung lesions having similar features in clinical imaging.

**Material and Methods**

The DECODE chip is based on an immunoassay that spatially confines individual EVs on a nanopillar array and applies nanoparticle barcodes to phenotypically characterise single EVs using surface-enhanced Raman spectroscopy. The DECODE chip targets the expression of lung cancer associated EVs surface proteins (THBS2, VCAN, TNC) and a generic EVs marker (CD63). After assessing the analytical performance of the DECODE chip on cell lines (i.e., HCC827, H1975, HCC78, HBEC), plasma derived EVs of 33 individuals with early malignant lung lesion (i.e., stages I and II), benign lung lesions, and healthy participants were profiled using DECODE. The utility of EVs profile to classify benign and malignant lung nodules was compared against clinical assessment by LDCT and PET scans.

**Results and Discussions**

The DECODE chip enabled multiplexing of EVs with single EVs resolution in cell line EVs and patient derived EVs, providing a detection limit of 12 EVs/µL. Multiplex analysis of EVs identified patients with early stage malignant (n=11) and benign (n=11) lung lesions by receiver operating characteristic (ROC) curve with an area under the curve (AUC) of 0.85. The acquired EVs profiles of the malignant group were phenotypically diverse, indicating a high level of EVs heterogeneity and requirement for multiplex analysis for accurate diagnosis.

**Conclusion**

We developed a digital, single EVs sensitive nanotechnology (DECODE chip) to profile EVs in plasma of patients with benign and malignant lung lesions. We discovered a multiplex EVs signature to stratify lung lesions into benign and malignant groups, showing the potential of EVs profiling for non-invasive lung cancer screening.

© 2023 The Authors. Molecular Oncology is published by John Wiley & Sons Ltd on behalf of Federation of European Biochemical Societies

### EACR23-0092

### Parental occupational exposure to combustion products, metals, silica and asbestos and risk of childhood leukaemia in their offspring

F. Onyije[1], A. Olsson[1], F. Erdmann[1,2], S. Schüz[1]
[1]International Agency for Research on Cancer IARC/WHO , Environment and Lifestyle Epidemiology Branch, Lyon, France
[2]Institute of Medical Biostatistics- Epidemiology and Informatics IMBEI- University Medical Center, Division of Childhood Cancer Epidemiology Institute of Medical Biostatistics- Epidemiology and Informatics IMBEI, Mainz, Germany

**Introduction**

Leukaemia is the most common malignancy diagnosis among children aged 0-14 years. The main subtypes of leukaemia are acute lymphoblastic leukaemia (ALL) which accounts for ~80% of leukaemia and acute myeloid leukaemia (AML) ~17%. Parental occupational exposures around conception (father) or during pregnancy (mother) have been hypothesized as potential predisposing factors for childhood leukaemia. We investigated parental exposure to several known occupational carcinogens and childhood leukaemia risk.

**Material and Methods**

We conducted a pooled analysis using case-control data included in the Childhood Cancer and Leukemia International Consortium (CLIC) from France, Germany, Greece and Italy (3362 childhood leukemia cases and 6268 controls). Parental occupational exposures to polycyclic aromatic hydrocarbons (PAH), diesel engine exhaust (DEE), chromium, nickel, crystalline silica, and asbestos were assessed by a general population job-exposure matrix (DOM-JEM). We estimated odd ratios (ORs) and 95% confidence intervals (CIs) using unconditional logistic regression models for all childhood leukaemia combined, by leukaemia type and by ALL subtype (B-lineage and T-lineage).

**Results and Discussions**

We found an association between high paternal occupational exposure to crystalline silica and childhood ALL (OR 2.20, CI 1.60-3.01) with increasing trend from no exposure to high exposure ($P$ = <0.001), and also for AML (OR 2.03, CI 1.04-3.97; *P for trend* = 0.008). ORs were similar for B- and T-lineage ALL. For ALL, ORs were also slightly elevated with wide confidence intervals for high paternal occupational exposure to chromium (OR 1.23, CI 0.77-1.96), and DEE (OR 1.21, CI 0.82-1.77). No associations were observed for paternal exposures to nickel, PAH and asbestos. For maternal occupational exposure we found several slightly elevated odds ratios but mostly with very wide confidence intervals due to low numbers of exposed mothers.

**Conclusion**

This is a first study suggesting an association between fathers' occupational exposure to crystalline silica and an increased risk of childhood leukaemia in their offspring. As this association was driven by certain occupations (field crop farmers and miners) where other potentially relevant exposures like pesticides and radon may also occur, more research is needed to confirm our findings of an association with silica, and if so, mechanistic studies to understand the pathways.

### EACR23-0328

### The Awareness of the Risk Factors, Signs, and Symptoms of Oral Cancers in younger adults in Scotland

A. sorensen[1], R. Taylor[1], C. Robertson[1], Z. Mahmood[1], S. Lyttle[2]
[1]University of Strathclyde, Strathclyde Institute of Pharmacy and Biomedical Science, Glasgow, United Kingdom
[2]NHS Lanarkshire, Department of Health Improvement, Glasgow, United Kingdom

**Introduction**

Cancers of the oral cavity are one of the top 10 occurring cancers worldwide with approximately 377,713 cases and 177,757 deaths in 2020. The main risk factors for oral cancer are largely preventable including tobacco, alcohol and UV exposure. Early detection of oral cancers increases the 5 year survival rate from 50% to 90% however the awareness of the signs and symptoms of oral cancer are poor. This study aimed to evaluate the awareness amongst younger adults in Scotland of the risk factors, signs, and symptoms of oral cancers which has an incidence rate of oral cancers double that of England and Wales.

**Material and Methods**

A self-directed questionnaire was developed in Qualtrics to address the aim. Participants were recruited through social media. A total of 425 17-24-year-olds (90 males, 330 females) completed the questionnaire.

**Results and Discussions**

Overall, 90.2% of participants had heard of oral cancer. However, in an open ended question only 54.8% were able to list any signs and symptoms of oral cancers. The most common response was ulcers (34.5%) followed by lumps (18.3%). Only 3.1% and 3.5% respectively listed white and red patches in the oral cavity, one of the most common symptoms of oral cancer. In a follow up question where participants were given a list of signs/symptoms 61% and 59% identified white and red patches and 86.2% identified long lasting ulcers. The risk factors which were most frequently listed by participants were tobacco smoking (45.2%) and alcohol consumption (16.4%) but less than 2.6% listed HPV as a risk factor. Overall, only 52.2% of participants were aware of any risk factors associated with oral cancers. When given a list of risk factors that may or may not be associated with oral cancers the majority of participants associated tobacco smoking (87.6%) and alcohol (61.9%) but less identified HPV (38.1%). Overall, 57.1% stated they rarely or never checked their mouths for abnormal changes.

**Conclusion**

This study highlights that younger adults have heard of oral cancers. Most participant were able to identify some of the common risk factors and signs and symptoms of oral cancer when presented with a list but less than 55% were able to do so in the absence of a list. Despite some awareness of signs associated with oral cancer less than 50% regularly checked their mouth for symptoms. This suggests that targeted public health campaigns could be of benefit to increase awareness of the signs/symptoms and

© 2023 The Authors. Molecular Oncology is published by John Wiley & Sons Ltd on behalf of Federation of European Biochemical Societies regular self check of the oral cavity in younger adults in Scotland.

### EACR23-0342
### Does scar remodeling improve outcome in mastectomy patients compared with sham laser therapy? - CARE-TOP - A comparative study in 48 patients

*S. Otto[1], R. Schleip[2], V. Fink[3], A. Bayat[4]*
*[1]Comprehensive Cancer Center Ulm CCCU, University Hospital Ulm, Ulm, Germany*
*[2]Chair of Conservative and Rehabilitative Orthopaedics- Faculty of Sport and Health Sciences, Technical University of Munich TUM, Munich, Germany*
*[3]Department of Gynaecology and Obstetrics, University Hospital Ulm, Ulm, Germany*
*[4]Plastic & Reconstructive Surgery Research, University of Manchester, Manchester, United Kingdom*

**Introduction**
After mastectomy, an estimated 25-60% of patients suffer from chronic pain, associated with physical, psychological and functional morbidity [1]. Protective attitudes due to scarring, as well as fear of scar complications, often go untreated, leading to a deterioration in quality of life. Further, scarring of tissue through surgery affects the fascia, and fascia mobility has an effect on wounds. Myofascial release stretches the elastin and changes the viscosity of the gel substance to increase gliding of the tissue. In light of this, a new scar treatment will be evaluated for its effect on improving scar tissue quality with a targeted focus on scar flexibility after bilateral mastectomy.

**Material and Methods**
This is a phase II, interventional, prospective, cluster randomized-controlled mixed model study (AB/BA design) to be conducted over a 12-week period in 48 patients undergoing bilateral mastectomy. The study treatment (A) consists in multimodal manual movement therapy (MMMT) including stretching and myofascial release. The control treatment (B) is a “high-tech” laser therapy (sham) with green light and gentle touch. Important endpoints such as improvement in scar tissue quality, stiffness and mechanical tissue mobility/elasticity, laboratory validation of skin scarring and wound healing, as well as ROM (Range Of Motion) and quality of life are evaluated in pre/post comparison.

**Results and Discussions**
We expect that the impact of a MMMT on the development of scar tissue stiffness in women after bilateral mastectomy will be of significantly higher quality than sham laser therapy.

**Conclusion**
Currently, there is no adequate treatment method that can prevent or improve the distressing physical and psychological symptoms after mastectomy. This approach can provide an improvement in scar management over sham laser therapy by leading to the highest level of functional movement, tissue repair, pain-free range of motion, and improvement in quality of life.

[1]Elzohry AAM, Abd Elhamed MF, Mahran MH. Post Mastectomy Pain Is No Longer Nightmare. *Journal of Family Medicine.* 2018;1:1.

### EACR23-0360
### REVISITING THE ARACHIDONIC ACID PATHWAY IN GASTRIC CANCER: A TRANCRIPTOMICS APPROACH TOWARDS THE DISCOVERY OF NOVEL BIOMARKERS

*T. Almeida[1], C. Lopes[1,2,3], A. Brandão[4], M. Dinis-Ribeiro[1,5], C. Pereira[1,3]*
*[1]Portuguese Institute of Oncology of Porto /Porto Comprehensive Cancer Center, Precancerous Lesions and Early Cancer Management Group- Research Center of IPO Porto/Rise@CI-IPOP, Porto, Portugal*
*[2]ICBAS-UP – Institute of Biomedical Sciences Abel Salazar, International Doctoral Programme in Molecular and Cellular Biotechnology Applied to Health Sciences BiotechHealth, Porto, Portugal*
*[3]CINTESIS – Center for Health Technology and Services Research, Clinical & Translational Research, Porto, Portugal*
*[4]Portuguese Institute of Oncology of Porto /Porto Comprehensive Cancer Center, Cancer Genetics Group - Research Center of IPO Porto /Rise@CI-IPOP Health Research Group, Porto, Portugal*
*[5]Portuguese Institute of Oncology of Porto /Porto Comprehensive Cancer Center, Department of Gastroenterology, Porto, Portugal*

**Introduction**
Inflammation is one of the key hallmarks of gastric cancer (GC) development, and the arachidonic acid (AA) pathway with more than 90 members has been shown to play a significant role in this process. Recent advances in omics technology have shed light on the molecular changes in cancer, providing a foundation for the identification of candidate biomarkers for use in diagnosis, prognosis, and screening. Thus, the purpose of this study was to revisit the AA pathway in GC using a transcriptomics approach, in order to identify which genes are particularly dysregulated and thus potentially representing targetable biomarkers.

**Material and Methods**
RNA-Seq gene expression data (Level 3) and clinical data of the stomach adenocarcinoma TCGA cohort (STAD), containing 414 tumor samples, was obtained using cBioportal (http://cbioportal.org) and UCSC Xena (http://xena.ucsc.edu/). Shapiro–Wilk normality test was used to determine variable normality, and statistically significant differences of gene expression levels between tumor tissue and adjacent normal tissue were evaluated using the nonparametric Wilcoxon test. Hazard ratios for prognosis assessment were calculated using a univariate Cox regression model. Differential gene expression (DGE) analysis was performed using limma and edgeR R-packages. All statistical analyses were performed using R software (version 4.2.1), and p-values $< 0.05$ were considered statistically significant.

**Results and Discussions**
A total of 53 genes were found to be differentially expressed in GC tumors compared to normal adjacent tissues. Overall, 29 genes were downregulated and 24 were upregulated, with the majority being involved in prostaglandin regulation and synthesis. The top

© 2023 The Authors. Molecular Oncology is published by John Wiley & Sons Ltd on behalf of Federation of European Biochemical Societies downregulated genes include *CYP4B1, AKR1C2*, and *HPGD*, which encode proteins involved in AA and prostaglandin metabolism.
*SOX9*, *GPX2,* and *PON1* account for the top 3 upregulared genes. In the univariate analysis, *ANXA5*, an upregulated gene, was associated with worse overall survival (HR = 1.452, P = 0.0005).
**Conclusion**
Overall, using a transcriptomic approach, the AA pathway was shown to be dysregulated in GC, with over half of the genes exhibiting altered expression, and going beyond inflammatory biomarkers, highlighting the pivotal role this pathway has in tumor development and progression and the potential for targetable actions.

### EACR23-0866
### hsa-miR-215-5p, hsa-miR-10b-5p, and hsa-miR-7a-5p expression are associated with persistent infection of hepatitis C virus-induced hepatocellular carcinoma

*S. Wen hsiu[1], C.S. Shabangu[2], S.C. Wang[3]*
*[1]Kaohsiung Medical University, Medical Laboratory Science and Biotechnology, Kaohsiung, Taiwan*
*[2]Kaohsiung Medical University, Medicine, Kaohsiung, Taiwan*
*[3]Kaohsiung Medical University, Department of Medical Laboratory Science and Biotechnology, Kaohsiung, Taiwan*

**Introduction**
MicroRNAs (miRNAs) are short non-coding RNAs of 22 nucleotides that mediate gene expression through various functions. Dysregulation of miRNA function has been associated with numerous diseases, including hepatocellular carcinoma (HCC). During the progression of HCV-HCC, the function of miRNAs may be altered thereby altering gene expression to favor HCC. Therefore, the involved miRNA-mRNA interaction needs to be further studied. In the current study, we aim to investigate the function and effects of persistent HCV-induced miRNAs expression in HCC progression and gene expression.
**Material and Methods**
We used HCV miRNA arrays to identify the differentially expressed in short-term(S-HCV) and long-term HCV(L-HCV) JFH1 replicon infected Huh751 cell, and uninfected Huh7.5.1 cells were used as the control group. Next, we identified miRNA-mRNA targets in 3 databases (miRDB; miRPathDB; TargetScan) and analyzed the targets for KEGG pathways using ShinyGO. The target miRNA-mRNA candidate genes were then compared with L-HCV NGS data to integrate miRNA-mRNA. The identified genes were analyzed in The Cancer Genome Atlas for survival, biological process, and expression in LIHC Big Data.
**Results and Discussions**
miRNAs in L-HCV and S-HCV were significantly differentially expressed compared with the control group.The results showed 5 of L-HCV miRNAs group was significantly associated with survival in LIHC. Hsa-miR-215, hsa-mir-10b, and hsa-let-7a of L-HCV miRNAs showed high significance for LIHC overall survival with a hazard ratio >1.0. Moreover, miRNAs associated with L-HCV survival were LIHC-specific but not in other cancers. We generated a Venn diagram using miRNA-mRNA targets from 3 specific miRNAs and compared them to L-HCV NGS mRNA data, resulting in 11,008 overlapping genes. Further analysis revealed that 102 of these genes were specifically affected by the regulation of these miRNAs. Through systematic integration of these findings, we identified HMOX1 and BMF genes as being specifically associated with HCC and regulated by hsa-miR-215-5p, hsa-miR-10b-5p, and hsa-miR-7a-5p.
**Conclusion**
The main cause of HCC is that HCV inhibits the expression of hsa-miR-215-5p, hsa-miR-10b-5p, and hsa-miR-7a-5p, both have a significant effect on survival, suggesting that these miRNAs may play an essential role in virus-related liver cancer. These findings provide a precision strategy to monitor HCC caused by chronic hepatitis C, reduce the incidence of HCC in chronic HCV-infected patients, and achieve the goal of precision medicine.

### EACR23-0947
### Exosomes as Imaging Biomarkers for Early-Stage Detection of Lung Metastases from Osteosarcoma: Preclinical Studies

*S. Almeida[1], A. Fonseca[2], J. Sereno[2], H. Ferreira[1], M. Lapo-Pais[2], T. Martins-Marques[1], T. Rodrigues[1], H. Girão[1], A. Abrunhosa[2], C. Gomes[1]*
*[1]Faculty of Medicine - University of Coimbra, iCBR - Coimbra Institute for Clinical and Biomedical Research, Coimbra, Portugal*
*[2]CIBIT/ICNAS - Institute for Nuclear Sciences Applied to Health- University of Coimbra, ICNAS Pharma, Coimbra, Portugal*

**Introduction**
Lung metastases represent the most adverse clinical factor and are the leading cause of osteosarcoma (OS)-related death. However, current imaging tools have limited ability to detect pulmonary metastases in the early stages. Exosomes (EXs) released by tumor cells are key determinants of metastatic organotropism, and have an intrinsic homing ability for donor cells, making them potential diagnostic tools and therapeutic vehicles for metastatic lesions. Herein, we developed an imaging tool based on exosomes derived from OS cells as targeted diagnostic imaging agents for the early and non-invasive detection of lung metastasis using positron emission tomography (PET).
**Material and Methods**
Swiss Fox$^{1nu}$ nude mice were *i.v.* injected with OS cells (143B-Luc+) into the tail vein. Lung metastasis formation was monitored by bioluminescence imaging (BLI) and magnetic resonance imaging (MRI). Exosomes were isolated from the supernatant of 143B cells and functionalized with a bifunctional chelator for complexation with the positron-emitter radionuclide copper-64 ($t_{1/2}$ = 12.7 h, 17.9% $\beta^+$, E = 0.653 MeV) via maleimide-thiol conjugation. Labeling efficiency and stability of the radiolabelled exosomes in biological fluids were assessed. Control and lung metastasis-bearing mice were *i.v.* injected with the radiolabelled EXs and imaged in a microPET/MRI at 24 h post-injection. OS-derived EXs were also loaded with DMNPE-caged luciferin or labeled

© 2023 The Authors. Molecular Oncology is published by John Wiley & Sons Ltd on behalf of Federation of European Biochemical Societies with a near-infrared (NIR) fluorescent dye for optical imaging.

**Results and Discussions**

The surface functionalization via maleimide-thiol conjugation does not affect the physicochemical properties of EXs or their targeting ability for OS cells and endows them with favorable pharmacokinetics for *in vivo* studies. Whole-body PET/MRI images obtained 24h after injection showed a clear uptake of EXs in the lung metastatic lesions, which was also observed by NIR fluorescence and confirmed by *ex vivo* biodistribution studies by gamma counting. No detectable PET or fluorescent signals was observed in healthy lungs devoid of metastasis, confirming the homing and selective affinity of EXs for homotypic donor cells.

**Conclusion**

Exosomes derived from OS cells possess an intrinsic homing ability for metastatic lesions and hold great potential as targeted imaging agents for the non-invasive early detection of lung metastasis by nuclear imaging. The clinical translation of this imaging agent has the potential to greatly improve the prognosis of OS patients.

### EACR23-1049
### Olive mill wastewaters: from byproducts to potential applications as nutraceuticals and cancer preventive agents

*A. Albini[1], L. Calabrone[2], V. Carlini[2], D. Morelli[2], M.M. Festa[3], S. Cristoni[4], C. Sansone[5], D.M. Noonan[6]*
*[1]IRCCS IEO European Institute of Oncology, Scientific Directorate, Milan, Italy*
*[2]IRCCS MultiMedica, Unit of Molecular Pathology- Biochemistry and Immunolo gy, Milano, Italy*
*[3]IRCCS MultiMedica, Laboratory of Cardiovascular Research, Milano, Italy*
*[4]I.S.B., Ion Source & Biotechnologies, Bresso- Milano, Italy*
*[5]Stazione Zoologica Anton Dohrn, sede Molosiglio Marina Acton, Napoli, Italy*
*[6]Università dell'Insubria- Varese - IRCCS MultiMedica, Unit of Molecular Pathology- Biochemistry and Immunolo gy, Milano, Italy*

**Introduction**

Several diet-derived compounds have been reported to exert anti-oxidant, anti-proliferative, anti-angiogenic and pro-apoptotic effects. Furthermore, they can target cancer cells and the tumor microenvironment though their metabolic derangements as well.

Extra virgin olive oil represents an important constituent of the Mediterranean diet. Compared to other vegetable oils, the presence of several phenolic antioxidants, including hydroxytyrosol, has been reported to prevent the occurrence of a variety of pathological processes. Selected diet-derived cancer preventive agents are involved in the inhibition of angiogenesis and inflammation.

We have assessed the anti-angiogenic and anti-tumor properties exerted by a purified extract (A009) from olive mill wastewaters (OMWWs), which represent a waste product from olive oil industry, compared to hydroxytyrosol alone, in *in vitro* and *in vivo* models.

**Material and Methods**

OMWWs ability to affect cell proliferation and survival on colorectal, lung, prostate and breast carcinoma cells was assessed by MTT. OMWWs interference with endothelial cell tube formation, migration and invasive capacities was studied by endothelial cell morphogenesis and migration assays. The inhibition of angiogenesis and tumor cell growth was evaluated *in vivo*.

**Results and Discussions**

OMWWs were able to hinder colorectal, lung, prostate and breast carcinoma cell growth in a dose dependent manner, exerting a stronger inhibitory effect than pure hydroxytyrosol alone. Molecular targets, among others, were CXCR4 and CXCL8. Moreover, OMWWs were able to inhibit HUVECs migration and capillary morphogenesis. OMWWs inhibited tumor angiogenesis and tumor cell growth *in vivo*. Furthermore the extract was able to protect from cardiac toxicity exerted by anticancer drgus such as 5-FU, cis-platin and doxorubicin.

**Conclusion**

Our results suggest that a polyphenol enriched extract from olive oil processing (OMWWs) has anti-angiogenic and anti-tumor potential. A pool of specific polyphenols (A009, Oliphenolia) were characterized by stronger anti-angiogenic/anti-tumor properties compared to hydroxytyrosol alone. A nutraceutical preparation with A009 can be considered a valid source of polyphenols suitable for angiopreventive approaches with natural compounds.

**Aknowledgements**

We thank Fattoria “La Vialla” (Castiglion Fibocchi-Arezzo) for collaboration and for the purified olive mill wastewaters.

### Radiobiology / Radiation Oncology

### EACR23-0019
### Hemostatic palliative radiotherapy for gastric cancer: A literature review

*O. Tanaka[1], T. Taniguchi[1], K. Adachi[1], S. Nakaya[1], T. Kiryu[2], C. Makita[3], M. Matsuo[3]*
*[1]Asahi University Hospital, Department of Radiation Oncology, Gifu, Japan*
*[2]Asahi University Hospital, Department of Radiology, Gifu, Japan*
*[3]Gifu University Hospital, Department of Radiology, Gifu, Japan*

**Introduction**

Gastric cancer has a high prevalence in Asia and may be detected only in the advanced stage. Therefore, patients with gastric cancer may experience fatal symptoms caused by bleeding or stenosis at the time of consultation. In this review, we describe the hemostatic radiotherapy (RT)

**Material and Methods**

Reports of 17 retrospective studies and 3 prospective studies are analyzed. Prescription dose, BED(10) and ECD2(10), response rate, survival prognosis and toxicities. Besides, possibility of non invasive examination using MRI follow up and treatment of rebleeding.

**Results and Discussions**

© 2023 The Authors. Molecular Oncology is published by John Wiley & Sons Ltd on behalf of Federation of European Biochemical Societies

Based on the 20 studies, the following observations were made: the hemostatic effect was approximately 80%, the survival time after irradiation was about 3 months on average, and a prescribed dose of 30 Gy/10 f and 20 Gy/5 fx was considered suitable. MRI based follow up is useful and re-irradiation were acceptable (in small number).

**Conclusion**

In this review, the studies on hemostatic irradiation presented in the literature have been summarized and the most optimal treatment method has been proposed. Like RT for bone metastasis, 30Gy/10 fractions and 20Gy/5 fractions were ideal. In addition, we have described the possibility of reirradiation, which has been reported in some case reports. Palliative care providers should refrain from invasive procedures, and this review suggests that noninvasive therapy and therapeutic effect measurement using magnetic resonance imaging can be used as an alternative to endoscopy.

### EACR23-0138
### Identification of molecular targets for increased uptake and therapeutic efficacy of radiolabeled minigastrin [177Lu]Lu-PP-F11N in cholecystokinin B receptor (CCKBR)-positive tumors

*M. Grzmil[1], Y. Qin[1,2], A. Blanc[1], S. Frank[3], R. Schibli[1,2], M. Behe[1]*
*[1]Center for Radiopharmaceutical Sciences, Paul Scherrer Institute, Villigen PSI, Switzerland*
*[2]Department of Chemistry and Applied Biosciences, ETH Zürich, Zürich, Switzerland*
*[3]Institute of Pathology, University of Basel, Basel, Switzerland*

**Introduction**

Radioligand therapy (RLT) selectively delivers a cytotoxic dose of radiation to cancer lesions. In 2018, the FDA approved Lutathera for the first-in-class peptide receptor radionuclide therapy of gastroenteropancreatic and neuroendocrine tumors. More recently, Pluvicto was approved for the treatment of metastatic prostate cancers. These therapeutic successes open a new era for RLT and bring many challenges related to insufficient delivery of the radioligands and cytotoxicity to the healthy tissues. The development of strategies for enhanced tumor uptake of radiopharmaceuticals, while sparing healthy organs, has a great potential to substantially improve the efficacy and safety of RLT. Here, we identified molecular targets and validated therapeutic strategies for tumor uptake enhancement of radiolabeled minigastrin, which binds to overexpressed cholecystokinin B receptor (CCKBR) and shows a favorable pharmacokinetics profile.

**Material and Methods**

Kinase and FDA-approved drug library screens were employed to identify compounds for increased uptake of lutetium-177-labeled minigastrin analog [$^{177}$Lu]Lu-PP-F11N in CCKBR-transfected human epidermoid carcinoma A431 cells. Internalization and viability assays verified cellular uptake and cytotoxicity *in vitro*. A431/CCKBR xenografted nude mice were used for biodistribution, single-photon emission computed tomography (SPECT) imaging as well as efficacy and safety study followed by the histopathological assessments of healthy organs.

**Results and Discussions**

The study identified and validated compounds, which increased cellular uptake of [$^{177}$Lu]Lu-PP-F11N including inhibitors of mammalian target of rapamycin complex 1 (mTORC1) or serotonin 5-HT-3 receptor (5HT3R). *In vivo*, pharmacological inhibition of mTORC1 by RAD001 or 5HT3R by ondansetron significantly enhanced uptake of [$^{177}$Lu]Lu-PP-F11N selectively in tumors without altering uptake in healthy organs such as the kidney or stomach. Furthermore, RAD001 enhanced the therapeutic efficacy of [$^{177}$Lu]Lu-PP-F11N in CCKBR-tumor-bearing mice without adverse effects suggesting further development of the combinatory treatment for clinical applications.

**Conclusion**

Our study established a screen-based workflow suitable for identifying molecular targets for enhanced uptake or radiolabeled ligands. The follow-up study demonstrates the potential of mTORC1 inhibitor RAD001 to substantially increase tumor uptake and therapeutic efficacy of radiolabeled minigastrin in CCKBR-positive cancers, feasible for clinical development.

### EACR23-0255
### Higher VEGFA expression levels are associated with earlier onset of acute breast skin reactions of BC patients treated with radiotherapy

*N. Petrović[1,2], I. Srbljak Ćuk[2], D. Marjanović Đorić[3], I. Petrović[4], P. Paunović[4], N. Medić Milijić[4]*
*[1] "VINČA" Institute of Nuclear Sciences- National Institute of the Republic of Serbia- University of B elgrade, Laboratory for Radiobiology and Molecular Genetics, Belgrade, Serbia*
*[2]Institute for Oncology and Radiology of Serbia, Experimental Oncology, Belgrade, Serbia*
*[3]Institute for Oncology and Radiology of Serbia, Department of Radiotherapy, Belgrade, Serbia*
*[4]Institute for Oncology and Radiology of Serbia, Department of Pathology, Belgrade, Serbia*

**Introduction**

Nearly 60% of patients with breast cancer (BC) undergo radiotherapy (RT). A significant proportion of patients experience side effects that can influence their quality of life and treatment effectiveness. Gene and microRNA expression patterns may be associated with individual patients' sensitivity to RT.

**Material and Methods**

Expression levels of miR-133b, miR-206, and their target genes matrix metalloproteinase 9 (*MMP9*) and vascular endothelial growth factor *(VEGFA)* were investigated in normal tissue adjacent to tumors of 27 BC patients. Sixteen out of 27 (59.3%) patients underwent RT, while 13 of them had breast skin reactions (acute dermatitis). Patients with acute dermatitis were divided into two groups, H-hypofractionated and F-standardly fractionated group. The H group underwent 16 fractions of RT (2.65 Gy), while the second group-F had 25 fractions (2 Gy) or 25 fractions plus boost. All patients from the F group had acute dermatitis before the 25$^{th}$ fraction, so the boost did not influence the time of toxicity occurrence. After the RNA extraction, followed by reverse transcription, expression levels of the

© 2023 The Authors. Molecular Oncology is published by John Wiley & Sons Ltd on behalf of Federation of European Biochemical Societies four molecules were measured by RT-qPCR. Relative quantity (RQ) units were calculated by a comparative $2^{-ddCt}$ method.

**Results and Discussions**

MiR-133b/206 and *MMP9*/*VEGFA* gene levels did not differ between patients from H and F groups (p = 0.573; p = 0.143; p = 0.727; p = 0.582, respectively). The F group was divided into two subgroups-earlier (E) subgroup with patients with skin reactions before or at 20th fraction, and the later (L) subgroup with patients with skin reactions after 20th fraction. *VEGFA* gene expression was significantly higher in the E group compared with the L group, indicating that it may be a specific predictor of earlier onset of acute skin reaction in the normal breast tissue of patients undergoing RT (p = 0.036, *Mann–Whitney U test*). MicroRNAs miR-133b, miR-206, and *MMP9* were not associated with earlier or later onset of skin reactions in this cohort (p = 0.343; p = 0.800; p = 0.786). It has been also shown that some *VEGFA* polymorphic regions can predict normal tissue reaction to RT in BC patients.

**Conclusion**

Higher levels of *VEGFA* are associated with earlier onset of skin reactions, but in patients undergoing conventionally fractionated RT. *VEGFA* expression difference indicates that neoangiogenesis capacity might be associated with individual risk of earlier occurrence of acute side effects following RT.

### EACR23-0263
### REPURPOSING DRUGS FOR THE DEVELOPMENT OF NOVEL COMBINATION THERAPIES FOR THE TREATMENT OF CANCERS

*H. Gardiner[1], S. McCabe[1], C. Mullen[1], R. Akram[1], Y. Farhan[1], A. Mullen[1], M. Boyd[1]*
*[1]University of Strathclyde, Strathclyde Institute of Pharmaceutical and Biomedical Sciences, Glasgow, United Kingdom*

**Introduction**

Despite recent advancements, certain hard to treat cancers such as brain, pancreatic and triple negative breast cancer (TNBC) still have a poor prognosis. Therapy resistance is one of the major contributing factors to the failure of cancer therapy, leading to relapse, metastasis and mortality. Radio- and chemoresistance can occur for a variety of reasons. One of the major contributing factors to therapy resistance is tumour heterogeneity. Thus, during treatment cancer cells continue to evolve and mutate often to therapy resistant phenotypes. Over the course of the disease and its treatment, cancers become more heterogeneous and are composed of pockets of tumour cells which are molecularly distinct from others and possess differential levels of sensitivity to therapies. As a result of this, many current cancer therapies have limited success and more optimal combination chemoradiotherapies are needed.

**Material and Methods**

TNBC cells were cultured in 2D and 3D models to investigate the efficacy of combination therapies designed from chemotherapy or irradiation and a pre-approved Drug X. Drug X has been shown to inhibit the NRF2 pathway and is currently used as a treatment for several autoimmune diseases. TNBC cells resistant to radiation and chemotherapy, developed in house by serial culture were used to investigate novel combination therapies to overcome resistance. Clonogenic assays and 3D spheroids were used to investigate toxicity and mechanistic studies such as comet assay & cell cycle analysis to understand the mechanisms underpinning observed effects.

**Results and Discussions**

Specifically scheduled combination therapies using chemotherapy and radiation with Drug X in toxicity studies, showed a statistically significantly reduction in survival when compared with the control and the individual treatments alone. Mechanistic studies suggest an increase in DNA damage, depletion in glutathione levels and cell cycle arrest between combination therapy, control and single treatments. These findings in TNBC cells were mirrored in the resistant cells, providing a possible combination therapy that can be used to overcome resistance in TNBC patients.

**Conclusion**

Our research focuses on utilising medicines already approved for other conditions and using them in combination with current therapies to improve the efficacy of the treatment and potentially reduce the amount of chemotherapy/radiotherapy required. The results found provide a promising potential treatment option for TNBC patients with recurring cancer.

### EACR23-0264
### Developing Novel Combination Therapies for Pancreatic Cancer

*C. Mullen[1], H. Gardiner[1], R. Akram[1], S. McCabe[1], A. Mullen[1], M. Boyd[1]*
*[1]University of Strathclyde, Strathclyde Institute of Pharmacy and Biomedical Sciences, Glasgow, United Kingdom*

**Introduction**

Pancreatic cancer is an aggressive malignancy, with an global 5-year survival rate of 9%. As the disease is often diagnosed in the later stages, the main treatment option available is chemotherapy. Despite recent advancements, gemcitabine is still regarded as the chemotherapy of choice for pancreatic cancer, however one of the major problems is that pancreatic tumours often develop resistance to this therapy. Therefore, in an attempt to combat this resistance we are investigating a repurposed drug (drug X) to develop novel combination therapies for the treatment of pancreatic cancers. Drug X was originally developed for the treatment of autoimmune disease and is already approved for use in humans, which would greatly reduce the time and cost required to bring any combinations developed to clinical trial.

**Material and Methods**

The pancreatic cancer cell lines Panc-1 and Mia PaCa-2 were utilised to test drug X in both 2D and 3D cell culture models. The clonogenic assay was used to assess the cytotoxic effect of drug X as both a monotherapy and in various scheduled combinations. Multicellular tumour spheroids (MTS) were also used to assess cytotoxicity of drug X and combinations in a 3D cell model. Finally, combination index analysis was used to assess the

© 2023 The Authors. Molecular Oncology is published by John Wiley & Sons Ltd on behalf of Federation of European Biochemical Societies synergism of drug X in combination, to determine the feasibility of the combinations.

**Results and Discussions**

Preliminary results show that drug X is cytotoxic in both pancreatic cancer cell lines tested in a dose-response manner when given as a monotherapy in 2D cell culture, and following combination index analysis drug X showed synergism in combination with gemcitabine and radiation. However in MTS, it did not reduce Mia PaCa-2 spheroid growth as a monotherapy, suggesting that there is alternative mechanisms of action in the 3D model when compare with 2D cell culture. The combinations developed in 2D culture showed little effect on MTS growth, suggesting alternative dosing or composition of combinations may be required.

**Conclusion**

Drug X shows potential as a treatment for pancreatic cancer and shows synergism in combination with gemcitabine and radiation. Further studies are required to fully elucidate the mechanism of action (e.g. apoptosis and DNA damage analysis). Further investigation into the differential activity of the combination in MTS will be required to determine if drug X is an appropriate potential treatment. Additionally the combination therapies will be assessed *in vivo* using the chick embryo model.

### EACR23-0283
### Potential Role of Exosomal circRNAs in Radiotherapy of Lung Cancer

*S. Rahimi[1], M. Asadi[1], C. Muftuoglu[1], U. Mert[2], A. Caner[3]*
*[1]Ege University, Basic Oncology, Izmir, Turkiye*
*[2]Ege University,*
*Atatürk Health Care Vocational School- Basic Oncology- Translational Pulmonary Research Center, Izmir, Turkiye*
*[3]Ege University,*
*Basic Oncology- Translational Pulmonary Research Cente r, Izmir, Turkiye*

**Introduction**

It has been shown that circRNAs play an important role in the diagnosis, treatment, and progression of cancer due to their stable structures. Also, circRNAs are more abundant in exosomes and contain a more stable structure. Exosomal circRNAs (ecircRNAs), which have the characteristics of the cell of origin and are found in various body fluids, have the capacity to be a suitable biomarker for the diagnosis and treatment evaluation of cancer. We aimed to investigate the effect of radiotherapy on circRNAs and differentially expressed circRNAs as treatment markers in lung cancer.

**Material and Methods**

Lung cancer epithelial (A549) and lung fibroblast (WI38) cell lines were used. To evaluate the effect of radiotherapy, both cell lines were irradiated with 4Gy after colony test, and exosomes were isolated by ultracentrifugation method. Exosomes was characterized by flow cytometry and dynamic light scattering particle size analysis. After total RNA isolation from exosomes, expression levels of ecircRNAs were examined by RT-qPCR. By bioinformatics methods, possible miRNAs interacting with ecircRNAs expressed in the groups were determined. Expressed ecircRNAs and their parental genes were evaluated by comparing data from non-small cell lung cancer patients undergoing radiotherapy in TCGA

**Results and Discussions**

Exosomes released from irradiated cancer cells were higher in density and size than those in cancer cells. Of the ecircRNAs, 4 ecircRNAs were significantly found in the cells groups. Notably, hsacirc0001900 and hsacirc0014235 are more regulated in A549 than in WI38 cells. After irradiation, the expression level of the former was significantly increased in A549 but decreased in WI38 cells, while the other was not expressed in both cell line. hsacirc0000190 was expressed only in cancer cells, while hsacirc0000003 gene was expressed only after irradiation of both cells, and this expression was significantly higher in irradiated healthy cells. Most of the miRNAs that interact with these circRNAs were found to be associated with lung cancer or other cancers. Expression of the parenteral S100A2 gene of hsacirc0014235 was found to be significantly higher in tumor tissue

**Conclusion**

The stable and resistant structures of exosomal-derived circRNAs found in body tissues and fluids are promising molecules in diagnosis and monitoring of cancer. hsacirc0001900 and hsacirc0014235 that are specific to lung cancer and differentiate with radiotherapy may be potential biomarker candidates in diagnosis and treatment follow-up

### EACR23-0312
### The use of gedatolisib for combination therapy of triple negative breast cancer

*Y. Farhan[1], H. Gardiner[1], C. Mullen[1], R. Akram[1], S. McCabe[1], S. Shamsaldeen[1], O. Starostenko[1], A. Mullen[1], M. Boyd[1]*
*[1]University of Strathclyde,*
*Strathclyde institute of pharmacy and biomedical sciences, Glasgow, United Kingdom*

**Introduction**

Triple negative breast cancer (TNBC) is an aggressive and invasive subtype that accounts for20% of all breast cancer cases. This subtype is heterogeneous and highly metastatic with low survival rates compared with other breast cancers. Due to the lack of hormonal receptors, the treatment of TNBC is challenging. Several types of chemotherapy and radiotherapy have been utilized clinically, however, high rates of tumour reoccurrence and therapeutic resistance have been demonstrated which result in low quality of life and poor prognosis. Phosphoinositide-3-kinase (PI3K)/mTOR pathway alterations are common in cancers including TNBC and this pathway could be a target for cancer treatments. Due to the frequency of such mutations in TNBC, Gedatolisib, a dual inhibitor of PI3K/mTOR pathways has potential as part of combination therapy regimens TNBC. The aim of this project is therefore to assess Gedatolisib in combination with gold standard TNBC therapies as a novel targeted combination therapy

**Material and Methods**

*In vitro* models including 2D clonogenic assay and 3D tumour spheroids models were utilised to assess the effectiveness of Gedatolisib as a single therapy and in combination with radiotherapy. As a single therapy, a range of concentrations 0.1-1 μM of Gedatolisib, and radiation were administered to TNBC lines and cytotoxicity assessed. After determination of the $IC_{25}$ and $IC_{10}$ the efficacy of combination were determined by

© 2023 The Authors. Molecular Oncology is published by John Wiley & Sons Ltd on behalf of Federation of European Biochemical Societies clonogenic assay and Combination Index analysis in 2D models and spheroid regrowth delay in spheroid models
**Results and Discussions**
The cytotoxicity of Gedatolisib in the MDA-MB-231 cell line was demonstrated via reduced clonogenic survival following drug administration in. Furthermore, this drug has been shown to delay the growth of MDA-MB-231 tumour spheroids in a concentration dependent manner. Additionally, a combination of 0.1 μM (IC25) Gedatolisib with a clinical radiation dose (2 Gray) resulted in a significant increase in cell killing and spheroidal growth delay in comparison with the single drug therapy.
**Conclusion**
The current findings have shown a promising combinational therapy for triple negative breast cancer. Future work will assess Gedatolisib in combination with chemotherapy drugs in double and triple combinations *in vitro* and *in vivo* utilising both the chick embryo and Murine xenograft models as well as investigation of mechanisms underpinning toxicity

### EACR23-0313
### Therapeutic targeting of neuropilin-1 mitigates the contribution of M2-polarized macrophages to radiation-induced lung fibrosis

*L. Gockeln[1], A. Haak[1], I. Bocci[1], S. Curras Alonso[2], J. Soulier[2], C. Fouillade[2], A. Londoño-Vallejo[2], M. Stuschke[3], V. Jendrossek[1], F. Wirsdörfer[1]*
*[1]University Hospital Essen, Institute of Cell Biology Tumor Research, Essen, Germany*
*[2]Institut Curie, Signaling- Radiobiology and Cancer Unit, Paris, France*
*[3]University Hospital Essen, Department of Radiotherapy, Essen, Germany*

**Introduction**
Radiation-induced lung fibrosis (RILF) constitutes a dose-limiting side effect of thoracic radiotherapies. The underlying pathogenesis is incompletely understood, but it is assumed that a sophisticated network of resident cells, immune cells (e.g., M2 macrophages) and soluble mediators fosters the inflammatory and fibrotic lung tissue alterations. In this context, TGFβ is considered as a pro-fibrotic key mediator. Here, we aimed to study the relevance of the TGFβ co-receptor neuropilin-1 (NRP1) in radiation-induced lung fibrosis, with focus on the macrophage compartment.
**Material and Methods**
To unravel the relevance of NRP1 for RILF pathogenesis, C57BL/6 mice received a single dose (12.5 Gy) whole thorax irradiation (WTI) and were additionally treated in the fibrotic phase (from week 16 post WTI onwards) with a small peptide inhibitor (A7R) for pharmacologic targeting of NRP1. At 25-30 weeks post irradiation, the fibrosis degree and TGFβ expression were assessed. scRNA seq was performed, to identify potential target cells of NRP1 inhibition, which were further analyzed by histological and RT-qPCR analyses in A7R-treated mice. The effect of A7R on the M2 polarization of macrophages was investigated in detail in a surrogate *in vitro* model with bone marrow-derived macrophages (BMDMs).
**Results and Discussions**
Irradiated C57BL/6 mice showed an increased expression of TGFb and NRP-1 in fibrotic foci. Treatment with the NRP1 inhibitor A7R ameliorated the degree of fibrosis, the expression of pro-fibrotic markers and TGFβ protein levels in irradiated lungs. Based on scRNA seq data and histological analyses, we identified pulmonary macrophages as potential target cells of NRP1 inhibition. *In vivo*, NRP1 inhibition reduced the organization of pro-fibrotic M2 macrophages in clusters and the mRNA expression of the M2 polarization marker arginase-1. *In vitro*, NRP1 was highly expressed on M2-like BMDMs and NRP1 inhibition mitigated the expression of M2 markers upon IL-4 stimulation. These results imply that NRP1 promotes the M2-polarization and organization of macrophages in RILF pathogenesis and that pharmacologic inhibition of NRP1 counteracts fibrosis development.
**Conclusion**
Based on our current investigations, we conclude that NRP1 has diverse therapeutically targetable functions in RILF pathogenesis, which include, but are not limited to the M2 polarization of pulmonary macrophages. Patients receiving thoracic radiotherapies might thus benefit from therapeutic targeting of NRP1, to attenuate normal tissue toxicities.

### EACR23-0314
### Optimization of soft tissue sarcoma especially HT1080 cell lines

*S. SHAMSALDEEN[1], C. Mullen[1], R. Akram[2], O. Starostenko[1], M. Almuqati[1], H. Gardiner[1], Y. Farhan[1], S. McCabe[1], A. Mullen[1], M. Boyd[1]*
*[1]University of Strathclyde, Strathclyde Institute of Pharmacy and Biomedical Sciences , Glasgow, United Kingdom*
*[2]University of Strathclyde, Strathclyde Institute of Pharmacy and Biomedical Science, Glasgow, United Kingdom*

**Introduction**
Sarcomas are rare tumours of the bones and soft tissues that affects all ages. Sarcomas contain special biological characteristics, that include a propensity for metastasis and a high incidence of aggressive local behaviour. Novel more efficacious therapies are slow to come to clinic in part because of the rarity of sarcoma that delays the progress of medical research. One type of sarcomas are soft tissue sarcomas (STS) that begins in the tissues that connect, support, and surround muscle, fat, blood vessels, nerves, tendons, and the lining of the patients' joints. The most common occurs in the arms and legs, and in the abdomen. Some STS types are more likely to affect children, while others affect mostly adults. Diagnosis includes advanced imaging techniques, biopsy and surgical removal is the most common treatment, radiation and chemotherapy may be recommended-depending on the class of tumour. New novel therapies that are more tumour specific and less toxic are therefore required particularly for the treatment of paediatric sarcomas
**Material and Methods**
A panel of STSs were purchased from ATCC and optimised in clonogenic assay, spheroid regrowth delay and Chick embryo tumour models. Once optimised this

© 2023 The Authors. Molecular Oncology is published by John Wiley & Sons Ltd on behalf of Federation of European Biochemical Societies assay cascade is now being utilised to assess the efficacy of novel combination therapies

**Results and Discussions**

Parameters were optimised for utilisation of STS cell lines in clonogenic assays. Due to the poor plating efficiency of STS cell lines large cell numbers had to be seeded to enable statistically relevant colony numbers to enable assessment of single therapies in clonogenic survival assays. Furthermore, several methodologies were assessed for growth of these cell lines as multicellular tumour spheroids. STS cell lines did not form spheroids using the spinner flask method and optimal spheroid growth was achieved by utilisation of low attachment plates. The cells are currently being assessed for their ability to form tumours in Chick embryo tumour models to assess whether this model can be utilised for novel combination therapy development

**Conclusion**

Development of an assay cascade of 2D, 3D and non-murine in vivo models will allow the assessment of novel combination therapies for STC. Once optimised the models will be utilised to assess the efficacy of combinations for STS, such as drugX and radiation.

### EACR23-0385
### Radium-223 induces a systemic immune response in men with metastatic prostate cancer

*S. Lunj[1], Y.P. Song[2], M. Reardon[1], H. Nightingale[2], K. Patel[2], H. Mistry[1], K. Reeves[1], T. Smith[1], F. Currell[3], C. West[1], J. Honeychurch[1], P. Hoskin[2], A. Choudhury[2]*

*[1]University of Manchester, Division of Cancer Sciences, Manchester, United Kingdom*

*[2]The Christie NHS Foundation Trust, Urology Department, Manchester, United Kingdom*

*[3]University of Manchester, The Dalton Cumbria Facility and the Department of Chemi stry, Manchester, United Kingdom*

**Introduction**

Radium-223 (Ra-223) is a form of targeted radiotherapy approved for treating metastatic castrate-resistant prostate cancer (mCRPC) that improves symptom control and survival in some but not all men. There is a need to increase understanding of the mechanism of action (MoA) of Ra-223 and identify how more men might benefit from the treatment. The primary MoA of ionising radiation is DNA damage leading to tumour cell death, but ongoing research suggests the immune system affects response to external beam radiotherapy (EBRT). EBRT has immunomodulatory effects that can be immune-stimulatory or -suppressive. The Ra-223 induced immune response has yet to be explored. We aimed to investigate whether Ra-223 induces systemic immuno-modulatory changes in men with mCRPC and whether these changes affect Ra-223 response.

**Material and Methods**

Plasma was collected from 43 men with mCRPC prior to each injection of Ra-223 (up to 6 injections). Using Luminex® technology we measured 61 immune-related molecules (cytokines, damage associated molecular patterns, checkpoint molecules and apoptotic clearance molecules). Response was defined as a ≥30% decrease in prostate specific antigen (PSA) levels at the end of treatment compared with baseline.

**Results and Discussions**

In men who demonstrated PSA response, Ra-223 induced a significant decrease in the levels (pg/ml) of 5 immune-related molecules after 4 injections of Ra-223 compared with baseline levels: AXL (11810.4±1505.8 vs. 8381.3±1172.5, *p=0.0039*), Mer (817.5±89.2 vs. 638.8±36.6, *p=0.0137*), interleukin 1 receptor antagonist (IL1RA - 336.8±118.6 vs. 182.6±58.4, *p=0.0195*), hepatocyte growth factor (HGF - 659.9±60.8 vs. 504.8±50.4, *p=0.0195*) and CD137 (52.2±17.1 vs. 26.7±4.5, *p=0.0371*). No significant changes in these 5 molecules were seen in men with no Ra-223 response.

**Conclusion**

Our data demonstrates that treatment with Ra-223 results in systemic immuno-modulatory changes which is different in responders and non-responders. The on-treatment decreases observed in responders were in molecules associated with immune suppression (CD137, Mer, HGF and IL1RA) and radio-resistance (AXL). Thus, Ra-223 may radio-sensitise tumour cells and enhance anti-cancer immune response in responding patients. Mechanistic experiments are ongoing to validate the contribution of the immune system to control disease progression following Ra-223 treatment.

### EACR23-0432
### Evaluating mechanisms of neuroprotection by MSC therapy against radiation induced brain damage

*C. Stockwell[1], N. Adán Castro[1], J.M. Sierra Párraga[1], P. Juárez Blázquez[1], C. Panadero Morón[1], L. Olmedo Moreno[1], C. Pinto Perea[1], V. Capilla González[1]*

*[1]Centro Andaluz de Biología Molecular y Medicina Regene rativa CABIMER, Integrative Pathophysiology and Therapies, Sevilla, Spain*

**Introduction**

Brain tumors are the most common solid tumors in pediatric patients and are often treated with radiation therapy. Due to the radiosensitivity of the developing brain, pediatric patients are more susceptible to neurological impairments post treatment. There are currently no effective strategies for preventing radiation induced brain damage available for these patients. Our group has previously shown that mesenchymal stem cells (MSCs) have neuroprotective effects when delivered through the intranasal route without compromising survival in tumor bearing mice that received whole brain irradiation. In this study, we aim to elucidate the mechanisms of neuroprotection of MSCs using in vitro models.

**Material and Methods**

Here we use two cellular systems to study the mechanisms of neuroprotection by mouse MSC conditioned media ($CM^{MSC}$) in vitro. First, cellular and molecular changes of irradiated murine primary glial cells (25 Gy) treated with $CM^{MSC}$ or control media were quantified using RT-qPCR, ELISA, MTT and immunofluorescence staining. Microglia and astrocyte crosstalk in the context of irradiation was also analyzed. Second, proliferation and migration of brain tumor cell lines Daoy and U87 were analyzed in the presence of $CM^{MSC}$ using Scratch assay and MTT assays.

© 2023 The Authors. Molecular Oncology is published by John Wiley & Sons Ltd on behalf of Federation of European Biochemical Societies

**Results and Discussions**
MSC treatment of irradiated primary glial cultures decreased markers of inflammation compared to control. Irradiated microglia exhibited ameboid morphology as previously reported, but when microglia were treated with $CM^{MSC}$ after irradiation, the microglia exhibited more ramified or rod like morphologies, consistent with neuroprotective or resting microglia. MSC treatment also modulates astrocyte reactivity in vitro when exposed to a radiation-like environment. Importantly, treatment of brain tumor cell lines with $CM^{MSC}$ did not result in accelerated tumorigenesis in vitro.
**Conclusion**
MSC secretome decreases glial cell inflammation triggered by radiation in vitro without promoting tumor progression. These results provide further evidence that MSC therapy could be a possible treatment for radiation induced brain damage in patients with brain tumors.

### EACR23-0527
### Developing an Ex Vivo Radiosensitivity Assay for Breast Cancer

*M. Bavelaar[1], D. van Gent[1], R. Kanaar[1], A. Jager[2], F. Froklage[3], M. Capala[3], M. Rovituso[4], I. Lauwers[3]*
*[1]Erasmus Medical Center, Molecular Genetics, Rotterdam, The Netherlands*
*[2]Erasmus Medical Center, Medical Oncology, Rotterdam, The Netherlands*
*[3]Erasmus Medical Center, Radiotherapy, Rotterdam, The Netherlands*
*[4]Holland Proton Therapy Center, Research and Development, Delft, The Netherlands*
**Introduction**
Proton therapy is currently applied for breast cancer (BC) when it has the potential of reducing complications for the tissue surrounding the tumor, based on the normal tissue complication probability (NTCP). Current NTCP models consider the physics, but not the biology of the tumor tissue. Taking into account the biological variation in tumor sensitivity might lead to possible dose reductions or better tumor control. We therefore aimed to develop a functional *ex vivo* sensitivity assay to determine tumor sensitivity to radiotherapy.
**Material and Methods**
Tumor material from BC patient-derived xenograft (PDX) models and primary BC samples was sliced into 300 µm slices and cultured in customized breast medium under constant rotation. Slices were irradiated with either photons (X-ray at Erasmus MC, Rotterdam) or protons (R&D proton beamline at HollandPTC, Delft). Applied doses ranged from 0 to 5 Gy and culture periods from 2 hours to 5 days, to determine optimal parameters for the sensitivity assessment. Tissue slices were fixed and stained to visualize tissue integrity, proliferation and apoptosis.
**Results and Discussions**
Experimental setups have been developed for tissue slice irradiation and subsequent processing. Tissue integrity was maintained throughout the *ex vivo* culture period, whereas an increase in apoptosis and decrease in proliferation was observed after irradiation. Automated and manual quantification of results is being investigated to optimize analysis. The automated analysis workflow is mainly important for eventual integration into clinical practice.
**Conclusion**
We have developed a method to assess radiosensitivity *ex vivo*. We compare irradiation with photons or protons, which will subsequently be developed into an assay to determine differential sensitivity to both regimens. Ultimately, this can be validated in a clinical study and be used to select the best treatment option for patients.

### EACR23-0533
### Reproductive outcomes of rectal cancer irradiation

*R. simaan[1], M. Zohar-Fux[1], T. Goshen-Lago[1], I. ben-aharon[1]*
*[1]Technion - Israel Institute of Technology, Medicine, haifa, Israel*
**Introduction**
Radiation is a key modality in the treatment paradigm of rectal cancer. Yet, there is lack of data regarding radiation-induced uterine toxicity, while former clinical data relies on whole pelvic radiation which significantly differs from field and constraints of current rectal radiation. Due to increasing incidence of rectal cancer in young population, revealing treatment outcomes on future fertility and pregnancy competence is crucial. We aimed to longitudinally study the impact of rectal radiation on the uterus and pregnancy outcomes in a mouse model.
**Material and Methods**
Pubertal ICR female mice at age 7 weeks were irradiated twice in a 1-week interval. Radiation plan consisted rectal field, 600cGy (equivalent to standard dose to rectum in human subjects; 5000cGy). Control mice were anesthetized only. Prior to radiation, female mice were hormonally synchronized. blood was drawn for Anti-Mulerian Hormone (AMH). Irradiated and control mice were mated at 1-week and 1-month post irradiation with control male mice and followed for pregnancies and litter size and individual pup weight. Mice were sacrificed after giving birth and uterus were removed and processed for immunohistochemistry of multiple fibrosis markers and evaluation of the thickness of all three uterus layers.
**Results and Discussions**
During mating, female from both 1-week and 1-month post irradiation could conceive, similarly to control mice, yet there were differences in pregnancy outcomes. 1-week post irradiation, we observed no significance change in the litter size, however, the control pup weight was significantly higher than the irradiated pup weight ($P<0.0001$). 1-month post irradiation, litter size of irradiated mice, was smaller compered to control ($P<0.05$), as well as pup weight that was significantly higher in the irradiated pup ($P<0.0001$). 1-month post irradiation, differences were observed in the uterine tissue of the irradiated mice, compared to control: we observed tissue fibrosis 1-month post radiation, mostly in the endometrium in dorsal sections, which was closer to the radiation filed (SMA, Vimentin and CD31 staining's; $P<0.05$). Moreover, MMP9 staining indicate on a tissue fibrosis in the middle section ($P<0.05$), and trichrome staining indicate on a tissue fibrosis in the serosa ($P<0.05$).
**Conclusion**
Our results indicate that rectal irradiation partially affect the uterine competency reflected by reduced litter size and pup weight. Histological analysis of irradiated uterus

© 2023 The Authors. Molecular Oncology is published by John Wiley & Sons Ltd on behalf of Federation of European Biochemical Societies revealed that the effect is more prominent in the endometrium.

### EACR23-0538
### Development and characterization of a radioresistant lung cancer model. Role of the immune system in the acquisition of resistance to irradiation

*S. León Valencia[1], D. Serrano[1], E. Redín[1], J. Barranco[1], M.C. Houry[1], F. Expósito[1], I. Tamayo[2], L. Montuenga[1], A. Calvo[1], J.J. Aristu[3]*
*[1]Centro de investigación Medica Aplicada CIMA, Solid Tumors, Pamplona, Spain*
*[2]Centro de investigación Medica Aplicada CIMA, Bioinformatic Platform, Pamplona, Spain*
*[3]Clinica Universidad de Navara, Radiation Oncology and Protontherapy Unit, Madrid, Spain*

**Introduction**
Treatment of stage III NSCLC patients includes radiotherapy (RT), but locoregional recurrence occurs in 10%-30% of the patients. In cases of exclusive local recurrence re-irradiation is the main curative-intent option available, but adverse effects and radioresistance are challenges in this setting. Current radioresistant (RR) models have been developed in immunodeficient mice, where the effect of the immune system cannot be evaluated. We have developed and characterized an immunocompetent RR locally invasive lung cancer model that can be used to study the role of the immune system and the effect of immunotherapy in a RR scenario.

**Material and Methods**
Lacun3RR cells were developed by several rounds of irradiation of Lacun3 parental cells. *In vitro* functional characterization, RNAseq, *in vivo* experiments in immunosuppressed (athymic nude) and immunocompetent (*Balb/C*) mice were used. The tumor microenvironment (TME) was analyzed by IHC and multiplex immunofluorescence (mIF).

**Results and Discussions**
Unlike Lacun3, Lacun3RR were resistant (up to 6 Gy irradiation), shown by clonogenic and apoptotic assays. Lacun3RR were more invasive, exhibited enrichment in cancer stem cell properties and alteration in DNA-repair, cell cycle and metabolic pathways compared to parental cells, but they did not form larger tumors. RR was demonstrated *in vivo* in *Balb/C* mice, using either single (10 Gy) or fractionated (3x8 Gy) RT, whereas the Lacun3 model was sensitive (~60% reduction in tumor volume). Surprisingly, tumor volume in the Lacun3RR model was also ~60% decreased when implanted in athymic nude mice, suggesting that RR in immunocompetent mice was mediated by a suppressive immune population. mIF analysis revealed decrease in CD8+ cells and increase in Tregs and PD-L1+ macrophages in Lacun3RR irradiated tumors, compared to the Lacun3 model.

**Conclusion**
Our immunocompetent RR model shows the possible effect of immunosuppressive cells on RT efficacy. Future studies will assess the functional role of Tregs, macrophages and myeloid-derived suppressive cells (MDSC) in the RR tumors and the effect of immunotherapy in this setting.

### EACR23-0543
### Unravelling the STING-dependent immune-activating effects of radiotherapy

*K. Lanng[1], A. Etzerodt[1], J.M. Gudbergsson[1], M.R. Horsman[2], M. Høyer[3], B.S. Sørensen[3], M.R. Jakobsen[1]*
*[1]Aarhus University, Department of Biomedicine, Aarhus C, Denmark*
*[2]Aarhus University Hospital, Department of Experimental Clinical Oncology, Aarhus, Denmark*
*[3]Aarhus University Hospital, DCPT – Danish Center for Particle Therapy, Aarhus, Denmark*

**Introduction**
Radiotherapy (RT) is known to induce a local inflammatory response, that triggers multiple parts of the immune system. However, it is not yet established how to fully exploit the immunostimulatory effect of RT in cancer treatment. Stimulator of Interferon Genes (STING) is an endogenously expressed protein involved in innate immune activation through sensing cytosolic DNA by the so-called cGAS-STING pathway. It is well-established that RT of tumor tissue can result in accumulated cytosolic DNA in cancer cells due to a dysfunctional DNA damage repair system. How and to what extent the cGAS-STING pathway and subsequent immune activation is essential for a proper anti-tumoral response has not been fully elucidated.

**Material and Methods**
Using a murine model of head-and-neck cancer (MOC1 and MOC2) we initiated a study to investigate the effect of STING pathway activation in response to photon irradiation. MOC1 and MOC2 are syngeneic cell lines, characterized by two remarkably different growth phenotypes and immunogenic profiles *in vivo.* Whilst MOC1 demonstrate an indolent growth phenotype and high immunogenicity, MOC2 demonstrates an aggressive growth phenotype, prone to metastasize and poor immunogenicity.

**Results and Discussions**
Wildtype (WT) and CRISPR-Cas9 generated STING knock-out (KO) murine cancer cell lines were challenged with 0 to 10 Gy photon irradiation *in vitro.* Preliminary experiments have shown that in both WT and KO cells, RT induces DNA damage and micronuclei formation. We found a RT dose-dependent induction of inflammatory cytokines which was dependent on cGAS and STING protein levels. To explore the immunological and antitumoral responses we next established the MOC1 and MOC2 tumors *in vivo*. Immunophenotypic characterization demonstrated that MOC1 tumors had a significantly higher frequency of infiltrating immune cells than MOC2 tumors (41.82 ±6.83% vs. 5.72±1.61% CD45+ cells, P = 0.0079). Currently, we are evaluating the effect of radiation *in vitro* and *in vivo*.

**Conclusion**
The immunological potential of RT is a double-edged sword, due to the ability of RT to either induce beneficial immune activation or detrimental immune inhibition. We hypothesize that STING is important for regulating the balance between immune activating and immune inhibitory

© 2023 The Authors. Molecular Oncology is published by John Wiley & Sons Ltd on behalf of Federation of European Biochemical Societies properties of RT. Here we show that modulation of STING affects the cancer intrinsic response to RT, both in relation to DNA damage and induction of an inflammatory response.

### EACR23-0576
### Investigating the Effect of Hypofractionated Radiotherapy in vitro and in vivo in a Murine Cerebral Glioblastoma Model

B. Karacam[1], I. Khan[1], S. Mahfooz[1], K. Akdur[2], E.B. Elbasan[1], G. Coban[3], M.A. Hatiboglu[2]
[1]Bezmialem Vakif University- Beykoz Institute of Life Sciences and Biotechnology, Molecular Biology, Istanbul, Turkiye
[2]Bezmialem Vakif University, Neurosurgery, Istanbul, Turkiye
[3]Bezmialem Vakif University, Pathology, Istanbul, Turkiye

**Introduction**
Glioblastoma (GBM) is one of the most aggressive brain tumors with a poor survival time. Radioresistance is an important factor hindering effective treatment. Hypofractionated radiotherapy (HFRT) has been thought to overcome radioresistance mechanisms in GBM. However, the role of HFRT has not been well established. Therefore, we hypothesized that HFRT would be more effective compared to single fraction radiotherapy (SFRT) via overcoming the radioresistance mechanisms in GBM.

**Material and Methods**
U87 MG cells were irradiated with 5 Gy, 10 Gy, 15 Gy, and 20 Gy for SFRT, and 1 Gy/fr, 2.5 Gy/fr, 5 Gy/fr, and 7.5 Gy/fr (three fractions for each dose) for HFRT using Gamma Knife 4C model. Cell viability was quantified with MTT assay. DAPI, MitoTracker Red CMX, and DCFH-DA stainings were done to investigate apoptotic nuclear condensation, mitochondrial membrane potential, and reactive oxygen species (ROS) production, respectively. The expressions of YAP1 and HSP90 were assessed using Western blot. An intracranial GBM murine model was established in athymic BALB/C mice using U87 MG cells. Animals were irradiated with 15 Gy for SFRT, and 5 Gy (three fractions) for HFRT. Overall survival time was recorded. The expressions of YAP1 and HSP90 were measured using IHC from brain tumor tissues.

**Results and Discussions**
The cytotoxic effect of HSRT was greater than SFRT. HSRT induced a higher accumulation of ROS compared to SFRT. DAPI and Mitotracker Red staining showed that both treatments induced apoptosis in U87 MG cells. However, the stimulation of apoptosis was higher with HSRT compared to SFRT. Western blotting indicated that inhibition of radioresistance related proteins YAP1 and HSP90 was more prominent with HSRT as compared to SFRT. Contrary to *in vitro* results, the survival time of the mice treated with SFRT was found to be longer than those treated with HFRT (49 days vs. 40 days). There may be some other contributing factors for higher survival in the animal model with SFRT. This requires further evaluation. Also, there was no significant difference between the levels of YAP1 and that of HSP90 with IHC from brain tumor tissues in SFRT and HFRT groups.

**Conclusion**
Our results suggested that the HSRT treatment regimen had a better effect on U87 MG cells *in vitro* compared to SFRT. However, a similar effect of HSRT was not observed in *in vivo* experiments. To better understand the optimal radiation treatment regimen in GBM, further investigations are warranted.

### EACR23-0940
### Radium-223 induces morphological changes associated with cell death in 3D spheroids of metastatic prostate cancer alterations

I. Marques[1,2,3], D. Cancelinha[1,4], A.S. Pires[1,2,5], G. Costa[6], A.M. Abrantes[1,2,5,7], M.F. Botelho[1,2,5]
[1]Institute of Biophysics- Coimbra Institute for Clinical and Biomedical Research iCBR area of CIMAGO- Faculty of Medicine, University of Coimbra, Coimbra, Portugal
[2]Center for Innovative Biomedicine and Biotechnology CIBB, University of Coimbra, Coimbra, Portugal
[3]Faculty of Pharmacy, University of Coimbra, Coimbra, Portugal
[4]Faculty of Sciences and Technology, University of Coimbra, Coimbra, Portugal
[5]Clinical Academic Center of Coimbra, cacc, Coimbra, Portugal
[6]Centro Hospitalar e Universitário de Coimbra CHUC, Department of Nuclear Medicine, Coimbra, Portugal
[7]Imaging and Radiotherapy Department, Polytechnic Institute of Coimbra- ESTESC-Coimbra Health School, Coimbra, Portugal

**Introduction**
The clinical outcomes of using Radium-223 dichloride (Ra-223) do not meet the clinical outcomes expected. Experts believe this is mostly justified by the doubts about Ra-223 accumulation n the metastatic site and how it interacts within the metastatic niche. Thus, our main goal is to analyze the radiobiological effects of Ra-223 in 3D models of metastatic prostate cancer (mPCa) that better mimic the tumor microenvironment, to deeper study its mode of action and radiobiological response.

**Material and Methods**
Spheroids of PC3 and LNCaP cells (5000 or 1000 cells/spheroid) were created and irradiated with increasing volumic activities of Ra-223 (55-7040 Bq/mL) for 24 h. Optical microscopic images were captured for 7d post-irradiation to analyze the morphological response of spheroids. Automated image analysis was performed by AnaSP freeware to measure spheroids' area, volume, solidity, and compactness. After 7d of irradiation, spheroids' viability was evaluated by CellTiter-Glo® 3D. Cell viability and cell death profile were also studied by double staining with AnnV/PI (confocal microscopy) followed by Giemsa staining.

**Results and Discussions**
It was observed a decrease in spheroid size, integrity, and viability with increasing activities of Ra-223, mainly for exposures to 5280 and 7040 Bq/mL. The viability of PC3 spheroids 7d post irradiation with 5280 and 7040 Bq/mL, for 24h, decreases to 32.2±1.7% ($p<0,0001$) and 23.1±1.8% ($p<0.0001$), respectively. Also, cell death increases with the increase of Ra-223 activities, mostly by apoptosis. Morphological changes are in accordance with

© 2023 The Authors. Molecular Oncology is published by John Wiley & Sons Ltd on behalf of Federation of European Biochemical Societies biological assays, with a decrease in spheroid area, volume, and compactness dependent on the increase of Ra-223 initial activity. This spheroids' disintegration was observed mainly in the peripheral region, which could be justified by the small range of alpha particles.

**Conclusion**

The results showed that Ra-223 induces a cytotoxic effect in mPCa spheroids. As expected, fewer radiobiological effects were observed in 3D cell models compared to our previous studies in 2D cell cultures, evidencing that 3D spheroids are a better tool to reproduce *in vivo* microenvironment. Effects on cell migration and cell-cell interactions are also under development.

Funding: PhD Scholarship grant from FCT and ESF to I.A.M. (SFRH/BD/136973/2018). Institutional funding from FCT strategic projects (UIDB/04539/2020, UIDP/04539/2020) and by COMPETE-FEDER (POCI-01-0145-FEDER-007440).

### EACR23-0956
### L-Asparaginase alters ionizing radiation response, migration and adhesion in Caco-2 cells

*I. Guardamagna[1], O. Iaria[1], L. Lonati[1], A. Mentana[1], A. Previtali[1], V. Uggè[1], C. Scotti[2], G. Pessino[2], M. Maggi[2], G. Baiocco[1]*

*[1]University of Pavia, Department of Physics, Pavia, Italy*

*[2]University of Pavia, Department of Molecular Medicine, Pavia, Italy*

**Introduction**

Colorectal cancer (CRC) is the 4$^{th}$ cancer for incidence and the 3$^{rd}$ for mortality worldwide (iarc, 2023). CRC's poor response to standard therapies is attributed to its high heterogeneity and complex genetic background. Dysregulation or depletion of the tumor suppressor p53 is involved in CRC transformation and its capability to escape therapy, with p53$^{null}$ cancer subtypes known to have a poor prognosis. In such context, new therapeutic approaches aimed at reducing CRC proliferation must be investigated. In the clinical practice, CRC chemotherapy is often combined with radiation therapy with the aim of blocking the expansion of the tumor mass or removing residual cancer cells. Contemporary targeting of amino acid metabolism has not yet been explored.

**Material and Methods**

We here propose an exploratory *in vitro* study, using Caco-2 cells (p53$^{null}$) as a model of radioresistant CRC, to analyze the combined effects of ionising radiation (IR) and L-Asparaginase (L-ASNase), a protein drug that blocks cancer proliferation by impairing asparagine and glutamine extracellular supply. Caco-2 cells were exposed to X-rays (0, 1, 3, 5, 10Gy) at a radiotherapy facility and treated with 1U/ml L-ASNase following different treatment schemes. We integrated results obtained from clonogenic survival, distribution in cell cycle phases and regulation of G2/M transition, DNA damage induction, regulation of Erk and Akt pathways and activation of matrix metalloproteases (MMPs) as an index of invasion potential. Finally, results on the combined effects have been complemented with the measurement of EcAII-induced changes in cell proliferation/migration and adhesion.

**Results and Discussions**

Caco-2 cells show a reduced proliferative index if exposed to X-rays in an amino acid deprivation state mediated by L-ASNase. In particular, the observed reduced clonogenic activity in the presence of L-ASNase can be linked to a less efficient DNA damage response, to the activation of the Erk-mediated autophagic process, to the reduction of Akt-mediated proliferation signalling, and to a reduced capability of the cells to adhere and migrate.

**Conclusion**

Taken together these observations pave the way for further studies on the combined effects of amino acid deprivation and X-rays in other CRC cell lines, also with different genetic background. In perspective, such studies could lead to advances in the treatment of highly aggressive and poorly treatable CRC, such as p53$^{null}$ subtypes.

### EACR23-1151
### Characterisation of HIF-mediated genoprotection in clear cell renal cell carcinoma

*S. ElBadry[1], S. El-Khamisy[1], F. van Eeden[1]*

*[1]University of Sheffield, School of Biosciences, Sheffield, United Kingdom*

**Introduction**

Clear cell renal cell carcinoma (ccRCC) is the most common form of renal cell carcinoma(RCC). Patients with ccRCC often have a remarkably high resistance to traditional radiotherapy and chemotherapy. Hypoxic cores are a common marker of various tumours, which trigger a genetic cascade causing the upregulation of genes crucial to cell survival, such as angiogenesis, cellular proliferation, and altered metabolism. Previous work suggests that increased resistance to radiotherapy/chemotherapy may be due to high levels of hypoxia-inducible factors (HIFs), which under normoxic conditions are targeted for degradation by the von-Hippel Lindau protein (VHL)/E3 ligase complex. Under hypoxia, HIF levels accumulate, allowing them to activate hypoxic response elements downstream. Mutations in VHL, which are commonly seen in ccRCC patients, cause constant upregulation of HIFs. Therefore, pathways promoting cell survival are constitutively upregulated, initiating tumour formation.This project seeks to understand what pathways are involved in HIF-mediated genoprotection and whether or not high levels of HIF provide a genuine genoprotective effect in humans.

**Material and Methods**

DNA damage assays, such as comet assays, clonogenics and cell titre blue assays were used to compare the DNA damage response between RCC4-VHL -/- and RCC4-VHL WT cell lines. Western blot and qPCR was used to compare protein expression between cell lines, focusing on VHL, HIFs, and DNA repair proteins. CRISPR/Cas9 was used to generate a RCC4-VHL -/- ; HIF2a -/- line to address the contribution of HIF2a to genoprotection.

**Results and Discussions**

The expression levels and activity of a key regulator of DNA damage response was highly upregulated in RCC4 VHL -/- cells, which correlates with high HIF expression. By knocking down HIF1a or HIF2a separately, I demonstrate that HIF2a is key for this upregulation. RCC4 VHL -/- cells show greater resistance to two DNA

© 2023 The Authors. Molecular Oncology is published by John Wiley & Sons Ltd on behalf of Federation of European Biochemical Societies damaging agents: camptothecin and olaparib, both of which demonstrate anti-cancer properties.

**Conclusion**

Initial results indicate that HIF knockdown is insufficient to resensitise RCC4 VHL -/- cells to camptothecin treatment, although both cell lines show clear differences in DNA damage pathways. Further work will be done utilising CRISPR/Cas9 cell lines to address whether long-term downregulation of HIF can provide a protective effect. RCC4 cell lines can be used to address key questions in hypoxia-related diseases.

### EACR23-1168

### Atomic Quantum Clusters of five atoms as Radiosensitizers in Cancer Therapy

*V. Porto González[1], N. Davila-Ferreira[1], B. Dominguez[1], A. Bertho[2], L. Salgueiro[3], B. Jakob[4], D. González-Castaño[5], Y. Prezado[2], M. Treder[6], F. Dominguez[1]*

*[1]Center for Research in Molecular Medicine and Chronic Diseases CIMUS, Physiology, Santiago de Compostela, Spain*

*[2]Intitute Curie, Translational research deparment, Orsay, France*

*[3]German Cancer Research Center, Cell Biology and Tumor Biology, Heidelberg, Germany*

*[4]GSI Helmholtzzentrum für Schwerionenforschung GmbH, Biophysics. Molecular Radiobiology & Imaging, Darmstadt, Germany*

*[5]University of Santiago de Compostela, Radiation physics, Santiago de Compostela, Spain*

*[6]Arjuna Therapeutics, Arjuna Therapeutics, Ames, Spain*

**Introduction**

Radiotherapy (RT) remains a cornerstone of cancer treatment. However, there are still a wide range of obstacles such as the resistance of the tumors and the adverse effects to normal tissues that compromise its efficacy. The introduction of new RT modalities such as protons and heavy ions improved RT efficacy by increasing damage to the tumor tissue and reducing the side effects. But, despite this, radioresistance and tumor recurrence are still observed. Radiosensitizers are agents with the ability to enhance the sensitivity to radiation specificDespite of this, ally in tumor cells.

Atomic Quantum Clusters of five atoms (Ag5-AQCs) are characterized by their size (<1nm), which is responsible for the loos of its typical metallic character and the acquisition of a molecular-like behavior, leading to the emergence of new and exclusive physico-chemical properties. Ag5-AQCs catalyze the selective oxidation of the thiol groups of glutathione (GSH) and the thioredoxin (Trx) family of proteins mediated by reactive oxygen species (ROS). Since redox homeostasis is fundamental to maintain cellular functions and ensuring cell survival, Ag5-AQCs cause rapid cell death by induction of apoptosis in cells with high levels of ROS such as cancer cells (Porto, V., et al, 2022). We hypothesize that Ag5-AQCs could benefit from the increase in ROS levels mediated by RT to oxidize GSH and Trx and therefore increase the RT efficacy both, obtaining the maximal biological effect using lower radiation dose -reducing side effects- and sensitizing resistant cells to radiation.

**Material and Methods**

Lung adenocarcinoma (A549), glioblastoma (U251) and hepatocellular carcinoma (HepG2) cell lines were treated with sub-lethal doses of Ag5-AQCs for 10 min and then irradiated using photons, protons and carbon ions (2, 4 and 6 Gy). Surviving fraction was determined via clonogenic assay. Radiation-induced DNA-damage was analysed by flow cytometry using the p-H2AX antibody.

**Results and Discussions**

Ag5-AQCs treatment synergistically decreased colony formation when combined with photons, protons and CIRT in all the cell lines tested. Moreover, pretreatment with Ag5-AQCs do not increase DNA damage suggesting that Ag5-AQCs acts through a different cell death mechanism, using ROS generated by radiation to accelerate irreversible protein oxidation.

**Conclusion**

The reported evidences suggest that the use of Ag5-AQCs in combination with RT provide a breakthrough for high ROS cancers and may improve RT efficacy without the need to increase the radiation doses.

### EACR23-1202

### CRAIN: A phase 1b clinical trial with dose escalation and dose expansion phases of Tolinapant in combination with standard chemoradiation in cervical cancer

*K. Bigos[1], L. Marina[2], S. Geoff[2], R. Kimberley[1], R. Rachel[1], W. Kaye[3], P. Hoskin[1], A. Choudhury[1]*

*[1]University of Manchester, Division of Cancer Sciences, Manchester, United Kingdom*

*[2]University of Southamptom, Clinical trials unit, Southamptom, United Kingdom*

*[3]University of Manchester, Division of Pharmacy and Optometry, Manchester, United Kingdom*

**Introduction**

Cervical cancer is the fourth most common cancer in women. Current standard of care for locally advanced cervical cancer (LACC) in the UK is chemoradiation with weekly cisplatin (CRT). Five-year overall survival rate is 65% with a distant relapse rate of 50%. Hypoxia, a common feature of LACC, up-regulates expression of apoptotic pathway inhibition mediated via the inhibitors of apoptosis (IAP) and induces cisplatin resistance. Tolinapant is an antagonist to IAPs.

CRAIN is a clinical study which aims to characterise the toxicity, response and efficacy of combining tolinapant with CRT in patients with hypoxic tumours for phase 2 trials. The aims of a parallel mechanistic study are to identify the effect of tolinapant in combination with cisplatin-based CRT in hypoxic cervical cell lines on apoptosis, proliferation and protein expression of IAPs.

**Material and Methods**

*Annexin V apoptosis assay:* HeLa and SiHa cervical cancer cell lines were seeded in a 96 well white/clear bottom plate (10,000 cells/well). Cells were incubated in normoxia or hypoxia (0.1% or 1% $O_2$) for 24h then treated with 1μM cisplatin, 1μM tolinapant, 1μM cisplatin and 1μM tolinapant or untreated (n=2). Following 2h treatment, cells were untreated (0 Gy) or irradiated (2 Gy), and Promega

© 2023 The Authors. Molecular Oncology is published by John Wiley & Sons Ltd on behalf of Federation of European Biochemical Societies

RealTime-Glo™ Annexin V reagent was added. Luminescence was measured at 24h.

**Results and Discussions**

Apoptosis (annexin V exposure) in HeLa cells was significantly ($p<0.001$) increased in cells treated with cisplatin and 2Gy irradiation compared with HeLa cells treated with 2Gy irradiation alone. Cisplatin treated cells showed a 0.3 fold increase in apoptosis compared to untreated cells. SiHa cells treated with 2Gy irradiation alone at 0.1% oxygen showed a significant fold change ($p< 0.01$) vs cisplatin and tolinapant combination at 2Gy and cisplatin alone at 2Gy. The treated cells had a fold change decrease of 0.5 and 0.6 respectively (two-way ANOVA).

**Conclusion**

The combination of cisplatin and tolinapant with irradiation in hypoxic conditions does not increase apoptosis. However, literature has shown that tolinapant induces necroptosis and later stage apoptosis. Therefore, future work will explore these pathways under hypoxic conditions. Additionally changes in cell proliferation and protein expression of IAPs will be measured following exposure of cervical cancer cell lines to different hypoxic conditions in combination with irradiation, cisplatin and tolinapant.

### EACR23-1265

### Radiomodulating potential of Zoledronic acid and Pavastatin (ZOPRA): effect on normal and cancerous prostate cell lines

*J. Fawaz[1], H. Fakhereddine[1], A. Yehya[1], F. Ghamlouche[1], R. Challita[2], C. Feghaly[2], L. Bodgi[2], W. Abou Kheir[1]*

*[1]American University of Beirut, Medicine, Hamra, Lebanon*

*[2]American University of Beirut, Radiation Oncology, Hamra, Lebanon*

**Introduction**

Prostate cancer (PCa) is one of the most common types of cancer among men. Radiotherapy is one of the main treatments for PCa. However, its efficacy depends on the radiosensitivity of both the tumor and the surrounding normal cells. Many studies have focused on developing drugs that can radiosensitize tumors while protecting normal tissues. Recently, a combination of bi-phosphonates (zoledronic acid) and statins (pravastatin), ZOPRA, was shown to radio-protect normal tissues by enhancing DNA double-strand breaks (DSB) repair mechanism. However no studies were yet conducted on cancer tissues. The purpose of this study is to access the potential use of ZoPra as a radio-sensitizing agent for radio-resistant prostate cancer cells.

**Material and Methods**

We are interested in assessing the radio-protecting and radio-sensitizing properties of these drugs, alone or in combination, on DU-145 and PC-3 (prostate cancer cell lines) and RWPE1 (prostate epithelial cell line) cells. The cells were cultured with a medium containing Zoledronic acid (Zo) and Pravastatin (Pra), individually and combined (ZoPra), and subjected to X-ray radiation.

MTT assay was performed to study the cytotoxic effect of different concentrations of Zoledronic acid and Pravastatin. Based on these results, a concentration of 1 μM was chosen to analyze the radiomodulating effect of ZO, PRA and their ZOPRA combination. Cells were treated with 1 μM Zo and Pra, alone and in combination, prior to a 2 Gy irradiation. Clonogenic assay was performed to assess cell survival and cellular radiosensitivity. Immunofluorescence analysis of pATM and γH2AX kinetics was performed to study DNA DSB repair kinetics.

**Results and Discussions**

A ZOPRA treatment before a 2Gy irradiation was shown to increase the residual number of γH2AX foci in DU-145 and PC3 cell lines while decreasing the residual number of γH2AX foci in RWPE-1 cells, when compared with a 2Gy irradiation alone. A significant decrease in cell survival in DU-145 and PC3 was observed. However, no significant change was observed in cell survival of RWPE-1 cells.

**Conclusion**

Our results show that ZOPRA has the potential to be a cost-effective radio-sensitizing agent for PCa, by decreasing the efficiency of DNA DSB repair mechanisms. ZOPRA also has the potential to improve the kinetics of DNA damage repair in normal tissues. The repurposing of a commercial FDA-approved drugs to widen the therapeutic ratio between local control and normal tissue complications is highly encouraging and requires further experimentation.

### EACR23-1423

### POSTER IN THE SPOTLIGHT

### Modeling post-radiotherapy recurrence in Medulloblastoma for innovative therapeutic strategies

*P. de Antonellis[1], C. Richman[2], A. Rasnitsyn[2], B. Holgado[2], M.C. Vladoiu[2], O. Saulnier[2], N. Abeysundara[2], K. Kharas[2], L. Garzia[2], A.S. Guerreiro Stucklin[2], M.D. Taylor MD[2]*

*[1]University of Naples, DIPARTIMENTO DI MEDICINA MOLECOLARE E BIOT ECNOLOGIE MEDICHE, NAPLES, Italy*

*[2]The Hospital for Sick Children, Developmental & Stem Cell Biology, Toronto, Canada*

**Introduction**

Medulloblastoma (MB) is among the most common paediatric malignant brain tumours. Advances in molecular profiling have established the existence of four distinct subgroups of MB including WNT, SHH, Group 3, and Group 4, each with their own distinct molecular and clinical characteristics. Treatment for MB is multimodal consisting of maximal surgical resection followed by radiotherapy of the whole cranio-spinal axis and adjuvant chemotherapy designed according to patient age and risk stratification. When the disease recurs (in 40% of cases), no therapies have been shown to confer a survival benefit. Group 3 (G3) MB carries the worst prognosis amongst all four subgroups with an urgent need for the development of novel therapeutics.

**Material and Methods**

We have established clinically relevant mouse models of recurrent Group 3 MB based on the administration of high-dose fractionated and CT-guided radiation to the brain of mice bearing patient-derived xenografts in the cerebellum. Radiation treatment has been shown to increase overall survival and induce the clonal selection of more resistant subpopulations of tumour cells. In order to compare recurrences to treatment naïve primary tumours, single cell

© 2023 The Authors. Molecular Oncology is published by John Wiley & Sons Ltd on behalf of Federation of European Biochemical Societies

RNA-sequencing was used to define a recurrent gene signature predictive of poor prognosis and radio-resistance.

**Results and Discussions**

By investigating the mechanism of acquired resistance to radiation in recurrent Group 3 MB, we discovered the overexpression of a distinct set of genes. Using a large patient cohort, this gene signature was clinically prognostic in human Group 3 MBs. Integrative pathway enrichment analysis of scRNA-seq differential expression data using three PDX cell lines (MB114, MB211, MB411) showed pathways converging on oxidative phosphorylation and nucleoside metabolism. Furthermore, correlation analysis revealed CA4, an actionable target, as a top gene correlated with the recurrent signature. We showed that CA4 inhibition via an FDA-approved drug in preclinical mouse models improves treatment efficacy without substantial systemic toxicity.

**Conclusion**

The discovery that recurrent G3 MBs are transcriptionally divergent from primary tumours and harbour a radiation-resistance gene expression signature reveals vulnerabilities that can be leveraged for therapy. Insights from this project present a candidate drug with the potential to be repurposed as an adjuvant to radiotherapy for the treatment or as a basis for evaluating radiotherapy dose reduction of G3 MB.

### EACR23-1515
### Inhibitors of the enzyme Arginine Methyltransferase (PRMT) are identified as radiosensitizers in glioblastoma cells through a chemical screen using an epigenetic probe library

*B.N. Köseoğlu*[1,2], *N. Pınarbaşı-Değirmenci*[1,2], *I. Sur-Erdem*[1,2], *U. Selek*[3], *T. Bağcı-Önder*[1,2]

[1]*Koç University School of Medicine, Brain Cancer Research and Therapy Laboratory, İstanbul, Turkiye*

[2]*Koç University, Research Center for Translational Medicine KUTTAM, İstanbul, Turkiye*

[3]*Koç University School of Medicine, Department of Radiation Oncology, İstanbul, Turkiye*

**Introduction**

Radiotherapy (RT) is a cornerstone of survival-prolonging treatment for glioblastoma (GBM), a largely incurable primary malignant brain tumor. The treatment failure relies on the adaptation of tumor cells to treatment and resistance, which is a major reason for inevitable recurrence. Understanding the molecular mechanisms behind this adaptive resistance and designing effective therapeutic strategies are of utmost priority. Therefore, finding epigenetic modulators of radiotherapy response, in other words, "radiosensitizers", will be crucial for glioblastoma patients.

**Material and Methods**

To investigate the epigenetic factors regulating glioblastoma cell survival, a chemical screen using 146 epigenetic drugs against chromatin modifiers, that involves inhibitors of the epigenetic writer and eraser enzymes, was conducted. From this screen, inhibitors of arginine methyltransferase (PRMT) family were identified as radiosensitizers. To examine the combined effect various PRMT inhibitors and RT, cell viability was measured with CellTiterGlo™ and colony formation assays in U373 GBM cells treated with 4 Gy radiation dose and different PRMT inhibitors (MS023, MS049, SGC707, and LLY283). As a complement to the chemical approach, the expression of genes encoding different PRMT family members was reduced by CRISPR/Cas9-based genetic ablation and the combined effect with RT was examined.

**Results and Discussions**

Results of the chemical screen indicated that inhibition of the PRMT family significantly increased the response to RT in GBM cells. A combination of low doses of PRMTi with RT was applied and cell viability and colony-forming capacity were observed to be significantly reduced. It has also been noticed that PRMT inhibitors alone did not affect cell survival. Arginine methylation is a common post-translational modification functioning as an epigenetic and cellular regulator of transcription and playing key roles in DNA damage signaling and cell fate decision. Its aberrant expression has been linked to tumor progression. However, the relationship between PRMT inhibition and RT response remains to be characterized.

**Conclusion**

Together, our study ascribes a role for PRMTs in regulating the RT response of glioblastoma cells, adding to the recently emerging functions of these enzymes in cancer. The effects of PRMTi on the transcriptome of the glioblastoma cells as well as the DNA damage response will be investigated to decipher the specific roles PRMTs play in the context of RT in glioblastoma.

### Signalling Pathways

### EACR23-0014
### Understanding and targeting mitogenic addiction as a therapeutic vulnerability in a neuroendocrine-driven subtype of small cell lung cancer

*T. Sen*[1]

[1]*Tisch Cancer Institute- Mount Sinai, Oncological Sciences, New York, United States*

**Introduction**

Small cell lung cancer (SCLC) is a high grade neuroendocrine tumor accounting for ~15% of all lung cancers. Whilst MAPK mutations can be found in roughly 30% of human cancers including non-small cell lung cancer (NSCLC), genomic and proteomic analyses have indicated suppression of MAPK pathway activity in SCLC. This striking difference is not well understood and previous attempts to determine whether this might be therapeutically important have had conflicting conclusions. SCLC has recently been defined by the relative expression of four major transcriptional regulators (*ASCL1, NeuroD1, POU2F3, YAP1*). In this study, we aimed to elucidate the effect of MAPK activation in these different SCLC subtypes and explore its therapeutic vulnerability.

**Material and Methods**

We used a doxycycline-inducible vector for expression of MEKDD$^{S217D/S221D}$ (MEK1) in a cohort of *ASCL1-*,

© 2023 The Authors. Molecular Oncology is published by John Wiley & Sons Ltd on behalf of Federation of European Biochemical Societies

*NEUROD1, POU2F3-* and *YAP1*-driven cell lines and mouse models.

**Results and Discussions**

Activation through MEK1 in *ASCL1*-driven SCLC cell lines resulted in a significant decrease in cell growth over 9 days. This was associated with a decrease in neuroendocrine markers ASCL1 and INSM1, a G2 cell cycle arrest and no significant increase in apoptotic cells. Remarkably, athymic mice injected with a MEK1-expressing ASCL1-driven cell line showed significantly slower tumor formation and longer survival than the ASCL1-driven cell line not expressing MEK1.

We observed strong upregulation of DUSP6, SPRY2, but not ETV5 upon MAPK activation. This was especially prominent in ASCL1-driven cell lines that changed from the normal phenotype of being in suspension to a more adherent morphology as a result of MAPK activation. Phosphokinase array in all four subtype cell lines after MEK1 activation demonstrated that, almost exclusively, the STAT pathways, in particular, STAT3 through phosphorylation at S727 were strongly upregulated in the ASCL1-driven subtype. Upon treatment with a STAT3 inhibitor, Stattic (1µM), ASCL1-driven SCLC cells reached their IC50 after 3-5 days in comparison to 9 days for other SCLC subtypes. NSCLC cell line was resistant to STAT3 inhibition.

**Conclusion**

These findings suggest that ASCL1-driven SCLC *in vitro* and *in vivo* is sensitive to activation of MAPK signaling in comparison to other SCLC subtypes. Whilst activation of the MAPK pathway might seem counterintuitive to current treatment strategies that aim to inhibit oncogenic signaling, we propose the use of a STAT3 inhibitor that has shown to be effective *in vitro.*

### EACR23-0141
### Ultra High-Plex Spatial Proteogenomic Investigation of Giant Cell Glioblastoma Multiforme Immune Infiltrates Reveals Distinct Protein and RNA Expression Profiles

*S. Bonnett[1], A. Rosenbloom[1], G. Ong[1], M. Conner[1], D. Newhouse[1], F. New[1], H. Sato[1], A. Zucca[1], G. Geiss[1], J. Beechem[1]*

*[1]Nanostring Technologies, Research and Development, Seattle, United States*

**Introduction**

The advancement of spatially resolved, multiplex proteomic and transcriptomic technologies has revolutionized approaches to complex biological questions pertaining to tumor heterogeneity, cellular interactions, and therapeutic response.  Most spatial technologies yield single analyte proteomic or transcriptomic datasets from separate FFPE tissues sections. Multiple studies have demonstrated poor correlation between RNA expression and protein abundance owing to transcriptional and translational regulation, target turnover, and post-translational protein modifications.  Therefore, a workflow that accurately measures RNA and protein simultaneously within a single tissue section with distinct spatial context is critical to a more complete biological understanding of cellular interactions and activities. Such multimodal omic datasets of protein and RNA have been termed "spatial proteogenomics".

**Material and Methods**

Here we present a novel spatial proteogenomic (SPG) assay on the GeoMx® Digital Spatial Profiler platform with NGS readout that enables ultra high-plex digital quantitation of proteins (147-plex) and RNA (whole transcriptome,>18,000-plex) from a single FFPE sample. We demonstrated high concordance, $R>0.85$, between the SPG assay and the single analyte GeoMx Whole Transcriptome Atlas and GeoMx NGS Protein assays. We used the SPG assay to interrogate 23 different glioblastoma multiforme samples across 4 pathologies.

**Results and Discussions**

We observed clustering of both RNA and protein based on cancer pathology and anatomic location. The in-depth investigation of giant cell glioblastoma multiforme (gcGBM) revealed distinct protein and RNA expression profiles compared to glioblastoma multiforme (GBM).  Spatial proteogenomics allowed simultaneous interrogation of critical protein post-translational modifications alongside whole transcriptomic profiles within the same distinct cellular neighborhoods.

**Conclusion**

Within our dataset, we observed >2-fold higher protein expression of phospho-GSK3β (Ser9) in gcGBM compared to GBM.  Inactivation of GSK3β through phosphorylation has been shown to enhance proliferation of GBM cells. We also observed differential protein expression of phosphorylated Tau variants. Phospho-Thr231 Tau was >2-fold higher in GBM compared to gcGBM.  Associated with neurodegenerative Alzheimer's disease, changes in Tau phosphorylation have also been observed in glioblastoma.  Our study exemplifies the utility of the SPG assay in expanding our understanding of glioblastoma molecular pathology.

### EACR23-0148
### Decoy extracellular vesicles protect against IFNγ antitumour effects

*B. Costa-Silva[1], J. Elzanowska[1], J. Maia[1]*

*[1]Fundacao Champalimaud PT507131827, Champalimaud Physiology and Cancer Programme, Lisbon, Portugal*

**Introduction**

IFNγ has been linked to decreased tumour cell proliferation and tumour apoptosis. Extracellular Vesicles (EVs), key players in the intercellular transport of molecules, play an important role in cellular homeostasis and in diseases. This includes the regulation of tumour immunity and the induction of niches supportive of metastasis. By transferring full-length membrane proteins from cells to biofluids, EVs also serve as scaffolds for protein interactions between soluble cytokines and their receptors at the surface of EVs. As such, in a still poorly studied mechanism, EV surface proteins can work as molecular decoys by regulating the activity of cytokines. Although EVs are key to cancer progression, it is still unclear whether they regulate the activity of tumour-suppressive cytokines, such as IFNγ.

**Material and Methods**

We developed the "EV-Precipitation" method to test whether IFNγR$^{+}$ EVs could interact with IFNγ. Three

© 2023 The Authors. Molecular Oncology is published by John Wiley & Sons Ltd on behalf of Federation of European Biochemical Societies different mixtures were ultracentrifuged separately: IFNγ alone, EVs alone, and a mix of EVs and IFNγ. To test the role of IFNγR in the binding of EVs to IFNγ, IFNγR1 expression was knocked-down (KD) in B16F10 cells, and as consequence, in B16F10 EVs. We hypothesised that IFNγR$^+$ EVs would act as a decoy for IFNγ, preventing it from exerting anti-tumour effects in tumour cells. To test that, B16F10 cells were treated with IFNγ alone or in combination with EVs from sh-Control (IFNγR1+ EVs) or from sh-IFNγR1 B16F10 cells (IFNγR1KD EVs) and analysed by MTS.

**Results and Discussions**

We found that while IFNγ alone or EVs alone did not produce a detectable IFNγ signal in the pellet, the presence of EVs caused IFNγ precipitation. This finding shows that IFNγR$^+$ EVs bind to IFNγ. Reducing IFNγR1 levels in EVs by 80% resulted in a 60% reduction in IFNγ binding to EVs, which demonstrates that IFNγ binding to tumour EVs is mediated by IFNγR1. We found that neither IFNγR1$^+$ EVs nor IFNγR1KD EVs alone altered B16F10 cell viability. IFNγ treatment alone reduced cell viability, while co-incubating IFNγ with IFNγR1$^+$ EVs prevented this effect, and co-incubating IFNγ with IFNγR1KD EVs did not.

**Conclusion**

Together, these results show that IFNγR1$^+$ tumour EVs can "shield" tumour cells from the tumoricidal effect of IFNy, playing a potential role in tumour immune evasion.

### EACR23-0252

### The HMGB1/RAGE interaction as a possible therapeutic target for the prevention of EMT and breast cancer cell migration.

*S.Y. Myashkova[1], D. Vladimirova[1], I. Ugrinova[1], J. Todorova[1]*

*[1]Bulgarian Academy of sciences, Institute of Molecular Biology, Sofia, Bulgaria*

**Introduction**

The main process that leads to metastasis is the epithelial-mesenchymal transition /EMT/. EMT refers to the transformation of differentiated epithelial cells into mesenchymal stem cells with enhanced cell movement. High Mobility Group box 1 (HMGB1) and Receptor for advanced glycated products (RAGE) are identified as a ligand-receptor pair, that plays an important role in tumorigenesis. HMGB1 induces epithelial-to-mesenchymal transition in various cancer cells and promotes cell migration and invasion. The role of HMGB1 and its receptor RAGE on the process of metastasis of breast cancer cells is still not well studied. Recently it was demonstrated that the antidiabetic drug metformin directly binds to HMGB1 and inhibits its inflammatory effect. Numerous studies suggest that metformin also reduces the risk of cancer and cancer progression, but the molecular mechanism of this effect remains unknown. **The aim** of this study is to investigate the role of HMGB1/RAGE pathway in epithelial-mesenchymal transition process and the migration of breast cancer cells.

**Material and Methods**

Breast cancer cell lines grown in 10% FBS at 37°C and 5% $CO_2$ were used. Cell movement was detected by wound-healing assay. EMT-markers were detected by Western blot analysis, and the cellular localization of some proteins was carried out by immunocytochemical staining of the cells.

**Results and Discussions**

Extracellular recombinant HMGB1 promotes dose-dependent cell migration and EMT-related change in gene expression of breast cancer cells: downregulation of E-cadherin, upregulation of N-cadherin and vimentin. To test the hypothesis that HMGB1 exerts this effect through RAGE signaling we silenced RAGE by esiRNA. Cells with downregulated RAGE, showed cell motility similar to the control. We found that HMGB1 induced epithelial-mesenchymal transition through RAGE signaling pathway. Moreover, treatment with metformin effectively blocked epithelial to mesenchymal transition and cell migration induced by HMGB1 probably by interrupting HMGB1/RAGE interaction.

**Conclusion**

Our results indicate that HMGB1 activates RAGE signaling pathways and induces EMT and cell migration in breast cancer cells. Impeding this interaction results in a reduction of cell movement. We hypothesize that HMGB1/RAGE pathway can be used as a potential target in cancer therapy strategy.

Acknowledgements: This work was supported by Bulgarian National Scientific Fund, project № КП-06-Н51/13, 17.11.2021.

### EACR23-0318

### Targeting aberrant Wnt and Notch signalling in meningioma progression

*A. Bukovac[1], B. Blažević[2], M. Katić[2], N. Njirić[3], D. Müller[4], M. Raguž[5], A. Jakovčević[6], A. Kafka[1], N. Pećina-Šlaus[1]*

*[1]Croatian Institute for Brain Research- School of Medicine - University of Zagreb, Laboratory of neurooncology, Zagreb, Croatia*

*[2]Faculty of Science- University of Zagreb, Department of Biology, Zagreb, Croatia*

*[3]University Hospital Center Zagreb, Department of Neurosurgery, Zagreb, Croatia*

*[4]University Hospital Dubrava, Clinical Department of Pathology and Cytology, Zagreb, Croatia*

*[5]University Hospital Dubrava, Department of Neurosurgery, Zagreb, Croatia*

*[6]University Hospital Center Zagreb, Department of Pathology, Zagreb, Croatia*

**Introduction**

Underlying mechanisms of progression of mostly benign meningiomas to very aggressive malignant forms with poor outcome for patients are still underexplored. Pinpointing aberrant signalling responsible for progression can lead to development of successful targeted treatments. Our study is focused on detecting the role of Wnt and Notch signalling in meningioma development. Nuclear translocation of β-catenin, central molecule of Wnt, as well as NOTCH1 and NOTCH2, central molecules of Notch signalling, can activate transcription of oncogenic genes, supporting malignant transformation.

**Material and Methods**

We performed methylation specific PCR on 46 samples of meningioma with different grades. Methylation of

© 2023 The Authors. Molecular Oncology is published by John Wiley & Sons Ltd on behalf of Federation of European Biochemical Societies promoter site was tested for *NOTCH2* and tumour suppressor gene *APC* responsible for β-catenin destruction. Localization and expression of main actors of Wnt and Notch signalling were analysed on formalin-fixed paraffin-embedded meningioma sections using DAB-labelled immunohistochemical reactions with antibodies that detect activated β-catenin, intracellular NOTCH1 domain and NOTCH2. Healthy brain tissue from cortex was also analysed, and results were compared to the ones of tumour samples.

**Results and Discussions**

Methylation of *APC* promoter was distinctive to anaplastic (p=0,000), while methylation of *NOTCH2* promoter was distinctive to atypical meningioma (p=0,000). Methylation of *APC* promoter was not correlated to the expression of β-catenin. Nuclear translocation was event more common for Notch signalling since around 90% of samples harboured NOTCH1 and NOTCH2 nuclear expression, while only 26% of samples harboured nuclear expression of β-catenin. However, stronger nuclear translocation of β-catenin was correlated to atypical meningioma (p=0,002). Nuclear translocation of NOTCH1, NOTCH2 and β-catenin was absent in healthy brain tissue.

**Conclusion**

Our studies have shown that both signalling pathways are activated in meningioma. Results on nuclear translocation suggest Notch as early leading process, while results on *APC* promoter silencing and β-catenin translocation suggest aberrant Wnt signalling as a later event in in meningioma progression.

### EACR23-0381
### SAMM50 affects mitochondrial dynamics in high-fat/high-ROS related non-alcoholic steatohepatitis

*Y.T. Liu*[1], *S.C. Wang*[1]
[1]*Kaohsiung Medical University, Medical Laboratory Science and Biotechnology, Kaohsiung, Taiwan*

**Introduction**

Nowadays,western cuisine have caused the prevalence of nonalcoholic steatohepatitis (NASH) and nonalcoholic fatty liver disease (NAFLD).NAFLD-related diseases have relatively higher potential to develop into complications such as fibrosis,cirrhosis,and even hepatocellular carcinoma (HCC).Mitochondrial dynamics are important in regulating energy balance,which regulates mitochondrial function.The Sorting and Assembly Machinery Component 50 homolog (SAMM50) is a mitochondrial outer membrane protein and plays an important role in the regulation of mitochondrial dynamics.The aim of the study was to investigate the effect of SAMM50 on the components of mitochondrial dynamics in a cellular and animal model with high-lipid content and high-ROS.

**Material and Methods**

In the study,we first explore gene variant research through genome-wide association studies (GWAS) with various databases such as SNiPA,PolyPhen2,UniprotKB and GEPIA.Indeed,we established an animal model with rapid progression of NAFLD by the high-fat western diet and a high sugar solution (HFSD) given for 7 months,containing 21.1% fat,41% Sucrose,and 1.25% Cholesterol and a high sugar solution (23.1g/L d-fructose and 18.9 g/L d-glucose).*In vitro* study,hepatocytes AML12 were cocultured with 3T3-L1 adipocytes conditioned medium (Adipo-CM) for 2 days and treated with $H_2O_2$ for 6 hours.

**Results and Discussions**

The database results show that PNPLA3,SAMM50 and EHBP1L1 are associated with NAFLD-related diseases.Moreover,only SAMM50 has a remarkable correlation with overall survival (OS) in HCC by GEPIA system. The HFSD animal model showed significant ballooning and steatosis as liver damage index by H&E and Masson staining.Protein immunoblotting of perfused hepatocytes showed significant upregulation of TGF-β1 and α-SMA,which was accompanied by significant downregulation of SAMM50 in the HFSD group compared with the ND group.*In vitro* study results found that the synergistic effect of Adipo-CM and $H_2O_2$ decreased ATP levels in AML12 and shRNA vector control.The SAMM50 shRNA further suppressed ATP production compared with transfected vectors under the synergistic effect of Adipo-CM and $H_2O_2$.In addition, we observed that the expression of mitochondria fusion, fission, and biogenesis proteins were all significantly reduced by knockdown of SAMM50 shRNA compared with transfected vectors under conditions of high lipid content and high ROS.

**Conclusion**

These results suggest that down-regulated of SAMM50 is associated with hepatocyte damage and affects mitochondrial dynamics.

### EACR23-0392
### Pro-invasive role of NMDAR/MET complex in breast cancer

*S. Gallo*[1], *A. Vitacolonna*[1], *T. Crepaldi*[1]
[1]*University of Turin, Department of Oncology - Candiolo Cancer Institute, Candiolo TO, Italy*

**Introduction**

High secretion of glutamate correlates with tumor aggressiveness. Some subsets of breast cancer, especially the triple negative breast carcinomas (TNBC), show “glutamine addiction”. Recent studies have shown that activation of the N-methyl-D-aspartate receptor (NMDAR), a glutamate-gated ion channel involved in excitatory synaptic transmission, stimulates a pro-invasive growth in different types of cancers, including breast cancer. MET and its natural cytokine ligand hepatocyte growth factor (HGF) are known to drive the invasive and metastatic phenotype and are highly expressed in TNBC. Our hypothesis is that in breast cancers a link exists between glutamate, NMDAR, and MET. However, functional and/or structural interactions between these mediators remain undetermined.

**Material and Methods**

NMDAR and MET expression were evaluated in TNBC by bioinformatic analysis, western blot, and immunohistochemistry staining. Western blot and immunofluorescence analysis were used to evaluate the phosphorylated level of NMDAR2B subunit. To evaluate the physical interaction between MET and NMDAR co-immunoprecipitation, confocal immunofluorescence, and proximity ligation assays were exploited. Specific inhibitors, JNJ-38877606 for MET and MK801 and ifenprodil for NMDAR, were used to decouple the receptor

© 2023 The Authors. Molecular Oncology is published by John Wiley & Sons Ltd on behalf of Federation of European Biochemical Societies crosstalk in the MET/NMDAR complex. Finally, wound healing and matrigel invasion assays were exploited to evaluate migration and invasion, respectively.

**Results and Discussions**

*GRIN2B* gene, encoding the NMDAR2B subunit, is the member of *GRIN* gene family most highly expressed in cancer samples and its high expression is a negative prognostic factor in human invasive breast carcinomas. TNBC cells express both glutamate (NMDAR) and MET receptors. HGF stimulation activates the MET kinase, which phosphorylates the NMDAR2B subunit of the glutamate receptor at the tyrosine 1252. Importantly, activated MET and phosphorylated NMDAR2B are physically associated. Specific inhibition of NMDAR by MK801 or ifenprodil completely blunt migration and invasion elicited by HGF treatment.

**Conclusion**

Overall, these results demonstrate that HGF-MET axis co-opts the glutamate neuronal signaling pathway in its invasive program and pave the way for a new combinatorial anti-cancer therapy for aggressive breast cancer.

### EACR23-0396

### Regulation of estrogen receptor α by PIM kinases in luminal A breast cancer

*W. Eccleshall[1], K.L. Mung[1], I. Laiho[2], C. Sahlgren[2], P. Koskinen[1]*

*[1]University of Turku, Department of Biology, Turku, Finland*

*[2]Åbo Akademi University, Department of Biosciences, Turku, Finland*

**Introduction**

The PIM family kinases are constitutively active serine/threonine kinases that phosphorylate multiple substrates to support signaling pathways important to cancer cell survival, proliferation, metabolism and motility (Santio NM & Koskinen PJ, *Int. J. Biochem. Cell. Biol.* 93:74, 2017). Here we provide evidence that estrogen receptor α (ERα) is a novel PIM substrate, which is phosphorylated in its N-terminal transactivation domain (AF1). This AF1 domain is important for recruiting transcriptional coregulators of estrogen-inducible gene expression.

**Material and Methods**

We have performed i*n vitro* kinase assays with recombinant PIM and ERα proteins to identify the PIM target site(s) in ERα. We have used MCF7 luminal A breast cancer cells and their CRISPR/Cas9-edited triple knock-out (TKO) derivatives lacking all three PIM family members to analyse the phosphorylation status of ERα and the expression of ERα target genes by luciferase reporter assays, qPCR and western blotting. We have also used pharmacological inhibitors targeting PIM or ERα.

**Results and Discussions**

We have identified serine 167 (S167) as the major PIM target site in ERα. We have observed that inhibition of PIM expression or activity reduces S167 phosphorylation in MCF7 cells. Data from luciferase reporter assays suggest that PIM TKO cells have a diminished ability to upregulate expression of ERα target genes in response to stimulation with estradiol. Furthermore, qPCR and western blotting data indicate that the estrogen-responsive genes TFF1, XBP1 and RET are downregulated in these cells. Based on these data, we hypothesize that phosphorylation at S167 regulates protein-protein interactions of ERα via AF1.

**Conclusion**

Post-translational modifications on ERα have been posited as a potential route by which breast cancer patients develop resistance to estrogen receptor antagonists such as 4-hydroxytamoxifen, leading us to speculate that by phosphorylating ERα at S167, PIM kinases may not only modulate ERα signaling, but also facilitate the development of treatment resistance in patients with luminal A breast cancer. To further investigate the physiological consequences of phosphorylation, we are in the process of mutating the PIM target site from the endogenously expressed ERα by CRISPR-based prime editing. We will also analyse the therapeutic opportunities provided by combinatory treatments with PIM and ERα inhibitors.

### EACR23-0409

### MET exerts pro-migratory and anti-apoptotic functions by counteracting NMDAR cleavage in breast cancer

*A. Vitacolonna[1], S. Gallo[1], T. Crepaldi[1]*

*[1]University of Turin, Department of Oncology, Candiolo Cancer Institute, Candiolo, Italy*

**Introduction**

MET is a master regulator gene for invasive growth of cancer cells and encodes the tyrosine kinase receptor for hepatocyte growth factor (HGF). The glutamate-gated ion channel N-methyl-D-aspartate receptor (NMDAR) has been associated to the pro-invasive growth of cancer cells. Recently, we demonstrated that MET physically interacts with NMDAR and that this complex is involved in the HGF-dependent migratory and invasive responses in triple negative breast cancer (TNBC) cells. In this work we elucidate the molecular mechanism(s) underlying the role of MET in its physical interaction with the glutamate receptor.

**Material and Methods**

To study the MET/NMDAR complex, transient transfections of TNBC and Hek293T cells with MET and NMDAR2B expressing constructs were exploited. Protein expression and MET/NMDAR physical interaction were evaluated through western blot and co-immunoprecipitation assays, respectively. Cytokines array was used to identify the signal(s) involved in the MET-dependent regulation of NMDAR cleavage. Specific inhibitors for MET (JNJ, JNJ-38877606), lysosomes (Bafilomycin), and metalloproteases (Batimastat) were used. Finally, migration and apoptosis were evaluated through wound healing and cleaved-caspase 3 assays, respectively.

**Results and Discussions**

Cells transfected with NMDAR2B construct alone show the low molecular weight form of 110 kDa corresponding to the cleaved product. In contrast, cells co-transfected with MET and NMDAR2B constructs show the high molecular weight form of NMDAR2B protein (200 kDa) corresponding to the full-size NMDAR2B. Moreover, MET preferentially interacts with the uncleaved NMDAR2B form. The MET-mediated protection from

© 2023 The Authors. Molecular Oncology is published by John Wiley & Sons Ltd on behalf of Federation of European Biochemical Societies

NMDAR2B cleavage was lost when MET and NMDAR2B co-transfected cells were treated with MET inhibitor (JNJ) or when the MET kinase dead mutant was used. These results suggest that activated MET physically interacts with NMDAR2B and prevents its cleavage. Superimposable results were obtained with the specific inhibitors of lysosomes and metalloproteases, suggesting that these two processes are involved in NMDAR cleavage. Finally, TNBC cells transfected with NMDAR plus MET constructs acquire pro-migratory phenotype and protection from apoptotic death.

**Conclusion**

Overall, these results demonstrate that activated MET counteracts NMDAR cleavage by physical interaction and allows cancer cells to acquire pro-migratory and anti-apoptotic functions.

### EACR23-0412
### AIM2 influences lung aenocarcinoma patients' prognosis.

*C. Colarusso[1], M. Terlizzi[2], R. Sorrentino[3]*

*[1]UNISA, Department of Pharamcy, Fisciano, Italy*

*[2]UNISA, Deaprtment of Pharmacy, Fisciano, Italy*

*[3]University of SAlerno, Department of Pharmacy DIFARMA, Fisciano, Italy*

**Introduction**

Cigarette smoking is a high-risk factor of lung cancer (LC), specifically non-small-cell lung cancer (NSCLC). Nevertheless, epidemiological studies have revealed that 40% of people who stop smoking are still at high-risk to develop LC, and in particular lung adenocarcinoma (LUAD). In our previous studies, we found that the inflammasome complex, herein AIM2 complex, was at the crossroad between the inflammatory pattern of both COPD and LUAD patients. Therefore, the aim of this study was to understand any differential molecular and cellular mechanism/s underlying LUAD in smokers and former smokers.

**Material and Methods**

To pursue this goal, we used human lung samples obtained by LUAD patients undergoing thoracic surgery and analyzed the expression of AIM2, correlated to the transcriptomic profile by using a public dataset (TCGA-LUAD). The underlying mechanism of the inflammatory role of AIM2 in the lung of both COPD and LUAD, was corroborated taking advantage of cigarette-smoke exposed mouse model.

**Results and Discussions**

AIM2 expression was higher in the lung of LUAD patients either in terms of protein or of transcript. The increased levels of AIM2 in the tumor mass was associated to lower survival rate of both smoker and former smoker LUAD patients, most likely due to higher immunosuppressive signature. This scenario was also observed in mice, whose lung was populated by AIM2 positive myeloid and regulatory T cells, which activity was fostered by IL-1-like cytokines and TGFβ. In addition to mice, human samples also showed higher presence of resting T memory cells, implying a reduced activity of the adaptive immune anti-tumor arm.

Our data demonstrate that AIM2 overexpression is associated to an immunosuppressive environment which facilitates a dismal prognosis of LUAD.

**Conclusion**

The involvement of AIM2 in LUAD establishment and progression highlights a novel molecular signature to pursue in order to discover new drugs.

### EACR23-0498
### Mass Spectrometry analysis of the Androgen Receptor and Serum Response Factor reveals common interactors and signalling pathways in castrate resistant prostate cancer

*H. Azam[1], Y. Basler[1], S. Marcone[2], W.M. Gallagher[1], M. Prencipe[1]*

*[1]University College Dublin, Cancer Biology & Therapeutics Lab, Dublin, Ireland*

*[2]Trinity Translational Medicine Institute- Trinity College Dublin, Department of Surgery, Dublin, Ireland*

**Introduction**

Advanced prostate cancer (PC) is managed using androgen ablation therapy (ADT), including anti-androgens such as enzalutamide which target the androgen receptor (AR). Although initially very effective, most patients develop ADT resistance. One approach to overcome this resistance is to target AR’s cofactors, indirectly abrogating AR function. One such cofactor is the Serum Response Factor (SRF), which is involved in cellular pathways relevant to cancer and was shown to cross-talk with AR. Furthermore, SRF is associated with PC progression, poor prognosis and resistance to AR inhibitors. The aim of this study was to investigate the molecular determinants of the SRF/AR relationship.

**Material and Methods**

To identify key molecular determinants of the SRF/AR axis, co-immunoprecipitation (Co-IP) of SRF and AR were performed in the castrate resistant LNCaP Abl cells. Conditions included transient knockdown of either SRF and AR, which served as a negative control for the analysis, or overexpression of SRF prior to Co-IP, and pulldown of endogenous AR. Samples were run on a timsTOF mass spectrometer. The interactome of the MS hits was investigated using STRING, reactome and ingenuity pathway analysis

**Results and Discussions**

These experiments identified 9 proteins which were co-precipitated with both AR and SRF, including heatshock proteins HSP70 and HSP90 alpha subunit, both involved in subcellular trafficking of AR. Mass Spec hits were associated with other cancer-related pathways including PI3k/Akt signalling and the mTOR pathway. Furthermore, Ingenuity Pathway Analysis revealed Twist1, previously implicated in CRPC progression, and an SRF transcriptional target, as an upstream regulator of the proteins co-precipitated with AR and SRF.

**Conclusion**

These experiments will help elucidating the key points in AR/SRF pathways, which can be targeted to disrupt the crosstalk. The relationship between SRF and AR is driven by common interactors which provide a pipeline for future therapeutic targets for CRPC.

### EACR23-0510
### Glucocorticoid receptor involved in triple-

© 2023 The Authors. Molecular Oncology is published by John Wiley & Sons Ltd on behalf of Federation of European Biochemical Societies

### negative breast cancer progression

H. Butz[1], É. Saskői[2], V. Vereczki[1], I. Likó[3], E. Tóth[4], K. Nagy[5], I. Kacskovics[5], A. Patócs[1]
[1]National Institute of Oncology, Department of Molecular Genetics and the National Tumor Biology Laboratory, Budapest, Hungary
[2]National Insitiute of Oncology, Department of Oncology Biobank, Budapest, Hungary
[3]Eötvös Loránd Research Network, Hereditary Tumours Research Group, Budapest, Hungary
[4]National Institute of Oncology, Department of Surgical and Molecular Pathology, Budapest, Hungary
[5]ImmunoGenes-ABS Ltd, ImmunoGenes-ABS Ltd, Budakeszi, Hungary

**Introduction**

Glucocorticoids are known to have a dual role in the pathophysiology of tumorigenesis. Glucocorticoid receptor (GR), like estrogen (ER) and progesterone receptor (PR), is a nuclear receptor that can influence breast cancer tumorigenesis via nuclear receptor cross-talk.

**Material and Methods**

First, we used an *in silico* investigation to 1) characterize the expression of GR in multiple cohorts including 24,256 breast cancer specimens on the RNA level and 220 samples on the protein level and to correlate these data with clinicopathological parameters, and 2) assess the potential effect on breast cancer survival in 1053 ER– and 2912 ER+ breast cancer samples. In the second, experimental part, we executed *in vitro* functional assays to evaluate the role of GR on breast cancer cell viability, proliferation, and migration. In addition, we investigated the GR activation state by identifying GR cellular localization in ER+ and triple-negative human breast cancer specimens using immunohistochemistry. Finally, we used RNA sequencing data from 15,780 breast cancer samples to determine the molecular basis of the GR effect on cancer cell migration.

**Results and Discussions**

While GR was found to be more abundant in males than females in normal breast tissue, we discovered the opposite in cancerous specimens. There was no difference in GR expression in primary vs. metastatic breast cancer cells, however, we found GR at a higher level in triple-negative cancer cells compared to ER+ ones. Higher GR expression was associated with better overall survival (OS) in ER+ and with a worse OS in ER– breast cancer types. Immunohistochemistry showed mostly cytoplasmic localization, but in some cells, both cytoplasmic and nuclear positivity were also seen, irrespective of ER status. GR transfection increased cell viability and proliferation while decreased cell migration in ER– cells but not in ER+ cells. Transcriptome sequencing indicated that GR transactivated genes were implicated mainly in cell migration.

**Conclusion**

We found that the presence of the GR in the context of the ER had a different effect on breast cancer cell behaviour supported by both survival and *in vitro* functional data. GR-transactivated genes were mostly involved in cell migration, emphasizing the importance of GR in disease progression.

### EACR23-0558
### Therapeutic insights into the homologous recombination-deficient tumour microenvironment in breast cancer

D. Jacobson[1], M.A. Clarke[1], S. Bazan[1], S. Pan[2], J. Fisher[1], M. Secrier[2]
[1]University College London, Cancer Institute, London, United Kingdom
[2]University College London, UCL Genetics Institute, London, United Kingdom

**Introduction**

Homologous recombination is a largely error-free method of DNA repair that relies on genes such as BRCA1 and BRCA2. Defects in these genes can lead to homologous recombination deficiency (HRD) which confers sensitivity to PARP inhibitors and drives widespread chromosomal instability, triggering cGAS/STING-mediated inflammation. This inflammation is often treated with immunosuppressors such as infliximab and tocilizumab, which raises questions about their combined effect with DNA damaging agents. The crosstalk between these processes complicates the search for safe and effective combination therapies, which calls for innovative computational approaches to predict optimised combination treatments and their mechanism of action.

**Material and Methods**

We used multinomial elastic net regression to develop and validate a 130-gene transcriptional HRD signature rom bulk RNA-seq data, and employed it to elucidate tumour-wide HRD prevalence and heterogeneity across >44,000 cells from 14 breast cancers. We applied CellphoneDB to interrogate whether HRD and HR-proficient cells interact distinctly with immune and stromal cells in the tumour microenvironment (TME). Finally, we developed an executable model describing the crosstalk between the DNA damage and cGAS/STING-mediated inflammatory responses, which we used to screen for effective and safe combination treatments.

**Results and Discussions**

We demonstrate that our signature predicts both HRD and BRCA status, and is associated with PARP inhibitor sensitivity in breast cancer cell lines and patients from the I-SPY2 trial. We show that HRD cells display a global reduction of interactions with the TME, especially with T-cells, typified by loss of TNF-alpha-mediated communication. Following this, we present an executable model that recapitulates experimentally validated behaviours of the DNA damage and inflammatory signalling responses and predicts the effects of DNA damaging agents in the context of immunosuppression.

**Conclusion**

We provide insights into the complex relationship between DNA repair and inflammatory responses at single cell resolution and demonstrate that HRD and HR-proficient tumour cells interact differentially with their TME, indicating variable patterns and mechanisms of immune evasion. Additionally, in modelling the crosstalk between these processes, we provide a resource for which novel therapeutic combinations can be screened and demonstrate its clinical significance in combining cancer treatment with immunosuppression and inflammatory targeting.

© 2023 The Authors. Molecular Oncology is published by John Wiley & Sons Ltd on behalf of Federation of European Biochemical Societies

### EACR23-0561
### IL-13-mediated PTP1B activation leads to glioblastoma growth and invasion through Schnurri-3 phosphorylation

*R.Á. Bartolomé[1], Á. Martín-Regalado[1], L. Pintado-Berninches[1], M.Á. Ramírez-González[2], I. Boukich[1], J. Robles[1], P. Sánchez-Gómez[2], I.V. Balyasnikova[3], J.I. Casal[1]*
*[1]Center for Biological Research Margarita Salas CSIC, Molecular Biomedicine, Madrid, Spain*
*[2]Instituto de Salud Carlos III, Functional Unit for Research in Chronic Diseases, Majadahonda, Spain*
*[3]Feinberg School of Medicine- Northwestern University, Department of Neurological Surgery, Chicago, United States*

**Introduction**
Phosphatase PTP1B is a key mediator of the interleukin 13 (IL-13)/IL13Rα2 signaling pathway that promotes glioblastoma (GBM) invasion. After IL-13 addition, PTP1B associates with IL13Rα2 $Tyr_{369}$ to promote Src activation, which in turn activates PI3K through FAM120A leading to cell adhesion, migration, invasion, proliferation and survival in tumor cells. However, downstream mechanisms remain unclear. This analysis clarify novel signaling pathways ultimately regulated by PTP1B after IL-13 addition to gain further insight into the role of PTP1B in cancer invasion and inflammation.
**Material and Methods**
We analyzed the PTP1B interactome using two complementary assays: immunoprecipitation (IP) and Bio-ID in U251 and Flp-In 293 cells, respectively. Flp-In 293 cells are tailored for BioID analyses. BioID identifies proteins in close proximity to PTP1B in a dynamic way during 24 h, whereas IP identifies proteins in the same protein complex of PTP1B at a determined moment. Furthermore, we carried out the analysis of the PTP1B-related phosphoproteome after IL-13 treatment.
**Results and Discussions**
PTP1B was associated with proteins involved in signal transduction, vesicle transport, cell cycle, and, in particular, with proteins from the NF-κB signaling pathway, including tenascin-C (TNC). PTP1B participates with NF-κB in TNC-mediated proliferation and invasion. Alterations in the phosphorylation pattern induced by PTP1B activation after treatment with IL-13 affected phosphoproteins involved in gene expression, cell cycle regulation, and cell signaling. Among others, we observed increased phosphorylation of the transcription factor Schnurri-3 (SHN3), a reported competitor of NF-κB. SHN3 silencing in GBM caused a strong inhibition in cell invasion and proliferation associated with the down-regulation of the Wnt/β-catenin pathway. In addition, knocking-down SHN3 caused an extensive inhibition of MMP9 expression and the subsequent inhibition of tumor growth in mice xenografts. Regarding clinical value, high expression of SHN3 was associated with poor survival in GBM in different datasets. Moreover, SHN3 showed a strong correlation with IL13Rα2 and MMP9-associated poor prognosis in different cancers.
**Conclusion**
In conclusion, we have uncovered the capacity of the IL-13/IL13Rα2/PTP1B pathway to induce SHN3 activation for promoting GBM progression by modulating the Wnt/β-catenin pathway and MMP9 expression. All these findings suggest a high therapeutic value for SHN3 in GBM and, likely, other tumors.

### EACR23-0564
### The suppressive role of protein AMPylation mediated signaling pathways in tumorigenesis

*W. li[1], W. ting[1]*
*[1]tianjin medical university, Department of Pharmacology- School of Basic Medicine, Tianjin province, China*

**Introduction**
Signaling pathways determine cell fate, and their abnormalities may cause tumor development. Protein modification is the biochemical basis of signal transduction. Therefore, protein-modifying enzymes are the main focus of signal transduction and important targets for cancer research and therapy. Although AMPylation, which transfers AMP to protein Ser/Thr/Tyr residues, has mainly been studied in prokaryotes, it has also been detected in mammalian cells. However, it is fully unknown whether AMPylation is involved in mammalian signaling pathways and tumorigenesis. AMPylating enzyme SELENOO(SELO) is conserved from prokaryotes to human cells and is clinically relevant to cancer. In this study, we identified, for the first time, the AMPylation-mediated signaling pathways in mammalian cells and their suppressive role in tumorigenesis by studying the function of SELO at the organismal, cellular, and molecular levels.
**Material and Methods**
**The functional investigation:** Firstly, we explored the correlation between SELO expression level and cancer prognosis through TCGA and our own cancer samples. We then established SELO knockout mice and induced primary liver cancer model using den/ccl4 to analyze the progression of liver cancer and its lung metastasis. The effect of SELO-catalyzed AMPylation on cell proliferation was explored through cultured cells and xenograft mouse model. The effect of SELO-catalyzed AMPylation on tumor metastasis was investigated through cell scratch and transwell experiments as well as small animal image in vivo.
**The indentification of signaling pathways:** AMPylation-specific antibodies were generated and used to enrich SELO-catalyzed substrates, and LC-MS/MS was used to explore the substrates and their AMPylation sites. The AMPylation sites were confirmed in cell lysates and purified proteins through experiments such as CO-IP and WB. Stable cell lines with mutations on the AMPylation site of SELO substrate were constructed to explore the functional role of SELO-catalyzed AMPylation. The downstream signaling molecules were explored through experiments such as CO-IP and WB by identifying changed molecular interactions by AMPylation.
**Results and Discussions**
This study uncovers the negative regulatory role of the SELO-(AMP)CDK5RAP3-ARF/PPM1D pathway in tumor cell proliferation and migration as well as their abnormality in related to tumor development.
**Conclusion**

© 2023 The Authors. Molecular Oncology is published by John Wiley & Sons Ltd on behalf of Federation of European Biochemical Societies

This is the first work to reveal the involvement of protein AMPylation in mammalian signaling pathway and its implications for cancer development.

### EACR23-0640
### Analysis of the prognostic interest of sortilin_dependent molecular profile in lung cancer

*M. Yassine*[1], *B. Chandouri*[1], *L. Ikhlef*[1], *S. Durand*[1], *J. Tricard*[2], *F. Lalloue*[1], *T. Naves*[1]
[1]*OmegaHealth, INSERM U1308 CAPTuR- Faculty of Medicine, Limoges CEDEX, France*
[2]*University Hospital, Thoracic surgery, Limoges CEDEX, France*

**Introduction**

Lung cancer is the leading cause of cancer-related deaths worldwide. Activating mutations in the epidermal growth factor receptor (EGFR) have made it an important target for therapeutic intervention. While tyrosine kinase inhibitors (TKIs) have shown promise in inhibiting EGFR and generating tumor responses, resistance can emerge due to EGFR internalization and degradation deregulation. Our previous studies have shown that sortilin is associated to a better prognosis and plays a crucial role in limiting EGFR signaling by promoting its internalization and degradation. Our recent observations suggest that sortilin may counteract EGFR's transcriptional program by interacting with chromatin, which has led us to focus on the transcriptional activity of sortilin in lung cancer cells in order to identify sortilin regulated-genes.

**Material and Methods**

To investigate the impact of sortilin expression on gene activity, we mimicked the EGFR-mutant in H1975 and H3255 lung cancer cells harboring EGFR mutations. We used these models to engineer a Tet-On system to restore sortilin expression. We created a HEK293T cell line with *SORT1* null background through CRISPR/Cas9 editing. This model was used to express both sortilin (knock-in, KI) and different EGFR mutants. A transcriptomic analysis was performed to reveal a specific molecular signature related to sortilin expression. In addition, live cell imaging was performed to analyze cell behavior upon sortilin expression.

**Results and Discussions**

Transcriptomic data revealed that *SORT1* expression would result in control of the expression of genes involved in cell cycle and DNA repair despite the oncogenic driving force of EGFR. While EGFR mutants drive higher proliferation, their dominance was limited following sortilin restoration. Indeed, cell proliferation decreased significantly in H1975 and H3255 Tet-On models, as well as in KI *SORT1* HEK293T models expressing different EGFR mutants. Likewise, size and number of spheroids derived from both Tet-On cell lines decreased significantly.

**Conclusion**

Our observations provide further evidence that sortilin may act as a tumor suppressor in lung cancer by likely inhibiting oncogenic signaling pathways and reducing the proliferation of cells with mutant EGFR. The molecular signature associated with sortilin expression could be a valuable tool for predicting tumor progression and resistance. These results highlight the potential of sortilin as a promising target for a new therapeutic strategy to improve the outcomes of lung cancer patients.

### EACR23-0672
### Hepatocyte Growth Factor and Epidermal Growth Factor signalling crosstalk is involved in tunneling nanotube formation in A549 human lung adenocarcinoma cells

*S. Banerjee*[1], *G. Awanis*[1], *R. Johnson*[1], *D. Warren*[1], *A. Sobolewski*[1]
[1]*University of East Anglia, School of Pharmacy, Norwich, United Kingdom*

**Introduction**

Non-small cell lung cancer accounts for 85% of all lung cancer cases and is often associated with overexpression of hepatocyte growth factor (HGF) and epidermal growth factor (EGF). Tunneling nanotubes (TNTs) are thin cytoplasmic protrusions involved in long-distance intercellular communication via cargo transfer and may encourage cancer development and metastasis. This study investigates the role of EGF/HGF crosstalk in TNT formation and defines markers and signalling pathways involved in TNT formation in A549 cells.

**Material and Methods**

The A549 cell line was cultured and treated with 100ng/mL EGF and HGF for 24h. White light microscopy was used to capture cell images and TNT analysis was undertaken using Fiji ImageJ. Pharmacological inhibitors of c-Met, EGFR, MEK, PI3K, Rac1, Cdc42, and the Arp2/3 complex and an siRNA against Paxillin were used to assess signalling pathways. Immunofluorescent labelling was performed to visualise TNT markers.

**Results and Discussions**

EGF, HGF and EGF+HGF induced TNTs in 42%, 39% and 46% of cells respectively, with TNT lengths ranging up to 300μm. Combined treatment of EGF and HGF yielded effects consistent with individual EGF and HGF effects, suggesting convergence of EGF and HGF signalling pathways. We also found the Ras/MAPK/MEK and PI3K/Akt pathways and the Arp2/3 complex regulated EGF and HGF-induced TNTs. While singular inhibition of MEK or PI3K diminished TNTs induced by HGF/EGF individually, this was not sufficient to inhibit EGF+HGF-induced TNTs, and simultaneous inhibition of both pathways was required for suppression of TNTs to basal levels. This is indicative of compensatory signalling between the pathways, wherein one pathway may be switching to the other uninhibited pathway, thus evading TNT inhibition. Furthermore, knockdown of Paxillin inhibited EGF/HGF-induced TNTs, thus emerging as an important scaffolding protein in TNT formation. Finally, the observed TNTs showed co-localisation of the novel markers c-Met and β1 integrin along the TNT length, in addition to expressing the classical TNT markers F-actin, α-tubulin and M-sec.

**Conclusion**

This study reports EGF/HGF crosstalk in TNT formation and provides insight into how the MAPK/MEK and PI3K/Akt pathways, traditionally thought to be independent, may participate in compensatory signalling to induce TNTs in A549 cells. Hence, their combined inhibition may be a more efficient therapeutic strategy.

© 2023 The Authors. Molecular Oncology is published by John Wiley & Sons Ltd on behalf of Federation of European Biochemical Societies

Future work will investigate the functional consequences of mitochondria/organelle transfer and chemoresistance via TNTs.

### EACR23-0831
### Lymphocyte Receptor Expression as a Therapeutic Target In Acute Myeloid Leukaemia

*J. Griffin[1], F. Healy[1], J. Slupsky[2], J. Woolley[1]*
*[1]University of Liverpool, Department of Pharmacology and Therapeutics, Liverpool, United Kingdom*
*[2]University of Liverpool, Department of Molecular and Clinical Cancer Medicine, Liverpool, United Kingdom*

**Introduction**

The lymphoid receptor CD7 is aberrantly expressed in AML in up to a third of AML patients. It has been reported that $CD7^+$ AML patients perform worse in key clinical outcomes, including rates of relapse, remission, and overall survival. Therefore, CD7 expression represents a biologically and clinically relevant target in AML. Dual expression of CD7 and CD33 is unique to AML blasts, presenting an opportunity to target these cells with a bispecific antibody drug conjugate (bi-ADC) against both antigens. However, little is known about the role of CD7 in AML. In this study, we aimed to investigate internalisation of the bi-ADC by CD7/CD33 dual expressing AML cells, to gain better mechanistic insights into ADC processing by AML. We also investigate the molecular function of CD7 in AML to better understand how expression contributes to poorer outcomes.

**Material and Methods**

$CD7^+$ AML cell lines were stimulated with anti-CD7 antibody or the CD7 ligand SECTM1, before lysates were collected and immunoblotted. A CD33-ADC and bispecific CD7-CD33-ADC were conjugated to Alexa-fluor-647 for immunofluorescence. $CD7^+$/$CD33^+$ AML cells were incubated with indicated compound for 1 hour at 4°C, then for 1 hour at 37°C. Cells were co-stained with LAMP-1-Alexa-fluor-488 and DAPI, and examined using the Zeiss Elyra 7 super-resolution microscope. HS5 stromal cell models of SECTM1 were developed to investigate the ligands role in CD7-AML.

**Results and Discussions**

Rapid internalisation and co-localisation of the bi-ADC with lysosomal marker LAMP-1 was observed. Incubation of $CD7^+$ AML cells with SECTM1 reduces binding of the bi-ADC, but importantly does not affect internalisation rate. Stimulation of CD7 using anti-CD7 antibody or SECTM1 activated downstream signalling through AKT and ERK, and increased protein levels of SECTM1. Both the soluble and integral membrane retained isoforms of SECTM1 are present in AML cells. Co-culture of SECTM1-expressing HS5 bone marrow stromal cells, with $CD7^+$ AML cell lines has been used to examine paracrine signalling-mediated phenotypic changes of AML cells induced by CD7 activation or attenuation.

**Conclusion**

Stimulation of $CD7^+$ AML cells with SECTM1 showed, for the first time, that CD7 is functional in AML and activates downstream signalling. CD7 antibody increasing SECTM1 levels suggests autocrine signalling through the CD7 receptor mediated by AML cells. Rapid internalisation of bi-ADC was observed, even in the presence of SECTM1, validating the feasibility of targeting the combination of CD7 and CD33.

### EACR23-0930
### α-phellandrene reduce 5-Fluorouracil-Induced inflammation in intestinal epithelial cells.

*C.C. Wu[1], S. Anggraeni[2], L. Hartajanie[2], S.L. Hsieh[3]*
*[1]Providence University, Department of Food and Nutrition, Taichung, Taiwan*
*[2]Soegijapranata Catholic University, Faculty of Agricultural Technology- Food Technology Mas ter Program, Semarang, Indonesia*
*[3]National Kaohsiung University of Science and Technology , Department of Seafood Science, Kaohsiung, Taiwan*

**Introduction**

5-Fluorouracil (5-FU) is commonly used in colorectal and breast cancer therapy. However, the side effect of 5-FU on intestinal mucositis is a leading case of poor prognosis. α-phellandrene (α-PA), one of the significant components of dill, has an anti-inflammation property. This study aims to investigate the reducing mucositis effect of α-PA in rat intestinal epithelium IEC-6 cells induced by 5-FU.

**Material and Methods**

In this study, α-PA was an experimental material, a normal rat epithelium IEC-6 cells cultured as an experimental model. After IEC-6 cells were treated with α-PA for 2 days, then α-PA was combined with 5-FU for another 2 days. The oxidative stress and inflammation response biomarkers were analyzed, and Nrf-2 and NF-κB signaling activation were also analyzed in IEC-6 cells.

**Results and Discussions**

The IL-6, TNF-α, and NO levels were reduced, and ROS and TBARS were also reduced after IEC-cells were treated with α-PA combined 5-FU. The results of Nrf-2 signaling activation analysis showed Nrf-2/β-catenin signaling was activated, including increased cytosol Nrf-2 and Keap-1protein levels, and the levels of transcript product, HO-1, GCLc and GCLm mRNA were increased after IEC-cells were treated with α-PA combined 5-FU. The results of NF-κB signaling activation analysis showed the levels of p-IκB and NF-κB translocate into nuclear were decreased after IEC cells were treated with α-PA combined 5-FU.

**Conclusion**

These results showed α-PA could regulate Nrf-2 and NF-κB signaling pathways to reduce inflammation in IEC cells induced by 5-FU

### EACR23-0937
### Biliary epithelial cell-specific RAGE mediates fibrosis upon chronic liver injury

*W.L.M. Lam[1], A. Damle-Vartak[1], G. Gabernet[2], D. Schneller[1], T. Poth[3], S. Jörs[4], N. Vartak[5], J. Hengstler[5], F. Geisler[4], P. Angel[1]*
*[1]German Cancer Research Center, Signal Transduction and Growth Control, Heidelberg, Germany*
*[2]Eberhard Karls University of Tübingen, Quantitative Biology Center QBiC, Tübingen, Germany*
*[3]University Hospital Heidelberg,*

© 2023 The Authors. Molecular Oncology is published by John Wiley & Sons Ltd on behalf of Federation of European Biochemical Societies

Center for Model System and Comparative Pathology CM CP, Heidelberg, Germany
[4]Technical University of Munich,
Clinic and Polyclinic for Internal Medicine II, Munich, Germany
[5]Leibnitz Research Centre for Working Environment and H uman Factors, System Toxicology, Dortmund, Germany

**Introduction**

The proliferation of biliary epithelial cells (BECs), also known as ductular reaction (DR), is a common characteristic of various liver diseases and is associated with increased risk of fibrosis. Using genetically modified mice harbouring germline deletion of the Receptor for Advanced Glycation End Products (RAGE), this molecule was identified as a critical mediator of DR and fibrosis in a liver cancer model. Yet, the specific cell type that contributes to RAGE-dependent DR-associated fibrosis remains unclear. Herein, we hypothesized that RAGE plays a distinctive role in regulating the activation of BECs, thus mediating the initiation of fibrosis.

**Material and Methods**

We utilized a biliary tracing reporter murine model (*$R26^{Tom}$Hnf1bCreER*) and deleted *Rage* conditionally in BECs. Choline-deficient ethionine-supplemented (CDE) diet was fed to the mice for three weeks to induce chronic liver injury. Utilizing the state-of-the-art intravital imaging, 3D immunofluorescence (IF) staining and RNA-seq, we interrogated the physiological functions of RAGE on BECs and the respective underlying genetic program in DR and fibrosis. *In vitro* co-culture assays of BECs and hepatic stellate cells (HSCs) combined with flow cytometry and mass spectrometry analysis were utilized to delineate the newly identified RAGE-dependent paracrine crosstalk between BECs and HSCs.

**Results and Discussions**

Although BEC-specific deletion of *Rage* abolishes DR, real-time intravital imaging of bile uptake kinetics in mouse liver demonstrated that *Rage* deletion recues the mice from obstructed bile flow during liver injury *in vivo*. RNA-seq data of primary isolated BECs from CDE-challenged mice revealed RAGE-dependent genetic programs in extracellular matrix organization and HSC activation. The HSC network is substantially diminished with lessened bridging fibrosis in BEC-specific *Rage* knockout mice. *In vitro* studies of directly and indirectly co-cultured BECs and HSCs, together with subsequent mass spectrometry analysis, showed that BEC-derived secretory JAG1 activates Notch signaling in HSCs in a RAGE-dependent manner *in trans*, and promote a myofibroblastic phenotype in HSCs.

**Conclusion**

The present study provides a novel insight into the adverse consequence of DR in fibrosis. We report that BECs activate Notch signaling in HSCs via a trans-regulatory mechanism in a RAGE-dependent manner, thereby establishing a pro-fibrotic milieu upon chronic liver injury. Therapeutically, RAGE could be a promising target in precluding cholestasis-associated fibrosis.

### EACR23-1023
### Deciphering the Mechanisms Contributing to the Role of Neddylated c-Met on the Response of Neddylation Inhibitor MLN4924 Treatment in Liver Cancer

Y. Yilmaz[1], S. İcier[2], G. Solmaz[3], B. Kaplan-Turkoz[4], N. Atabey[1,3]
[1]Basic and Translational Research Program,
Izmir Biomedicine and Genome Center, Izmir, Turkiye
[2]Institute of Health Sciences,
Department of Health Bioinformatics- Ege University, Izmir, Turkiye
[3]Galen Research Center, Izmir Tinaztepe University, Izmir, Turkiye
[4]Food Engineering Department, Ege University, Izmir, Turkiye

**Introduction**

As a post-translational modification, neddylation has recently gained attention in cancer studies and neddylation inhibitor Pevonedistat (MLN4924) has been in the spotlight for cancer clinical trials. Sustained activation of c-Met receptor tyrosine kinase has an essential promoting role in liver cancer (LC) progression. Recently we identified c-Met as a target of neddylation in LC, and here we aimed to focus on elucidating the role of c-Met neddylation on MLN4924 response in LC.

**Material and Methods**

To study our aim, we first created c-Met mutations on K1232 and K1360 which were revealed as potential neddylation sites depending on our previous data derived from computational analysis. Then, we analyzed cell cycle progression with cells transfected with GFP conjugated mutant c-Met plasmids (K1232A/K1360A), treated with HGF (10 ng/ml) and/or MLN4924 (4 uM) and stained with PI for subsequent flow cytometry analysis. The effect of mutations and MLN4924 on downstream signalling controlling survival and proliferation, Erk and Akt phosphorylations, was determined by western blotting. The role of MLN4924 on c-Met neddylation mutants on cell proliferation was determined by ki67 immunofluorescence staining. For SEC-SAXS experiments His-NEDD8 and c-Met kinase domain (KD) were expressed and purified using Ni-NTA and SEC columns, and SAXS experiments were carried at the EMBL-P12 beamline at Petra III DESY.

**Results and Discussions**

For deciphering the specific effect of c-Met neddylation, mutants and MLN4924 were tested for determining survival and proliferation capacities of LCs. Cell cycle analysis showed that MLN4924 treatment results in G2-M arrest and expressing WT/neddylation mutants of c-Met in cells reverses this effect. Supportingly, ki67 immunostaining also revealed diminished cell proliferation with MLN4924 treatment, whereas no difference was observed between WT or mutants. pErk and pAkt evaluations indicated that Erk activations were blocked upon MLN4924 treatment as expected and interestingly MLN4924 results an increase in Akt activation in WT-Met transfected cells, whereas a mild increase in 1232 mutant and no change in 1360. SAXS data analysis showed that NEDD8 was found as a monomer (10 kDa) in solution as expected whereas c-Met KD was tetrameric (146 kDa). The solution structures of each protein were modeled from the SAXS data.

**Conclusion**

Pevonedistat (MLN4924) could be a valuable drug in liver cancer treatment, and c-Met activation and neddylation

© 2023 The Authors. Molecular Oncology is published by John Wiley & Sons Ltd on behalf of Federation of European Biochemical Societies have profound roles in determining its effect in liver cancer.

### EACR23-1081
### Hypoxia and metabolic stress rewire the ERK MAPK optimum in malignant melanoma

S. Uldrijan[1], K. Smolkova[1], N. Vadovicova[1], B. Valcikova[2]
[1]*St. Anne's University Hospital Brno, International Clinical Research Center, Brno, Czech Republic*
[2]*Masaryk University, Faculty of Medicine, Brno, Czech Republic*

**Introduction**

Oncogenic *BRAF* and *NRAS* mutations activating the ERK mitogen-activated protein kinase (MAPK) pathway are drivers of metastatic melanoma. However, due to the high activating potential of the mutations, the intensity of ERK MAPK signaling must be strongly limited by various negative feedbacks to allow for optimal tumor growth. We studied the control of the MAPK optimum in melanoma cells responding to a lack of oxygen or vital nutrients.

**Material and Methods**

We used Western blotting and ERK activity reporter constructs to analyze the response of a panel of NRAS- and $BRAF^{V600E}$-mutant human melanoma cell lines to hypoxia and compounds targeting cell energy metabolism.

**Results and Discussions**

Our results suggest that the ERK MAPK optimum established in malignant melanoma cells bearing oncogenic *BRAF* and *NRAS* mutations is lower than the total signaling capacity of the ERK pathway in these cells. Metabolic stressors and compounds disrupting phosphatase-mediated negative feedbacks can rapidly disrupt the control of the MAPK optimum in melanoma. Furthermore, we show that hypoxia rewires the ERK signaling in melanoma cells. The MAPK optimum becomes even more dependent on the negative feedback control by phosphatases, indicating a potential for disrupting the MAPK signaling in hypoxic cancer cells.

**Conclusion**

We analyzed molecular mechanisms controlling the MAPK optimum in melanoma cells under normal and low oxygen conditions. We found that disruption of the negative feedback control potently dysregulated ERK signaling in hypoxic melanoma cells.

***Acknowledgments:*** *This work was funded by the European Regional Development Fund - Project ENOCH (No. CZ.02.1.01/0.0/0.0/16_019/0000868), the Czech Science Foundation (GA22-30397S), the European Union – Next Generation EU - the project National Institute for Cancer Research (Programme EXCELES, Project No. LX22NPO5102), and Masaryk University (MUNI/A/1393/2022).*

### EACR23-1082
### Polo-like kinase 1 (PLK1) and AURORA KINASE B modulate Histone Deacetylase 7 (HDAC7) activity in human colorectal cancer cells.

E. Malavasi[1], E. Zamprogno[1], C. Brancolini[1], T. Gagliano[1]
[1]*University of Udine, Department of Medicine, Udine, Italy*

**Introduction**

Colorectal carcinoma (CRC) is among the most diagnosed cancers worldwide. We recently demonstrate that the histone deacetylase 7 (HDAC7) is overexpressed in CRC tumor samples as compared to their "healthy "counterpart. In addition, we found that overexpression of HDAC7 in CRC cell lines increase cell viability and promotes cell invasion, while its silencing exerts opposite effects. We have performed RNAseq and ATACseq experiments to explore the downstream pathway of HDAC7. Currently, there are no specific HDAC7 inhibitors, thus understand how HDAC7 is regulated is crucial to modulates is activity. To this aim we employed a library of target specifics inhibitors to identify upstream regulators of HDAC7.

**Material and Methods**

We used Human CRC cell lines SW620_GFP-**WT,** and SW620-**HDAC7**-GFP.
opnME compound library was provided by Boehringer Ingelheim containing 129 compounds targeting different class of protein. Cell proliferation was evaluated by crystal violet, protein expression and acetylation was detected by western blot. Analysis of phosphorylation site/motif awere performed using phosphosite.org online tool.**Results and Discussions**

Screening with opnME library performed on WT cells, identified 9 compounds able to significantly reduce cell viability, among those PLK1 and AURORA B inhibitors were further evaluated. HDAC7 can be phosphorylated at SER155, once phosphorylated it can translocate from the cytoplasm to the nucleus. Intriguingly both PLK1 and AURORA B phosphorylation motive overlap with the sequence of HDAC7 serine 155.When tested on HDAC7 overexpressing cells, PLK1 and AURORA B inhibitors partially lost their ability to reduce cell viability. Furthermore, while both compounds were increasing y-H2AX levels in WT cells, in HDAC7 overexpressing cells this effect is partially lost. In addition, phosphorylation levels of HDAC7 are decreased by treatment with AURORA B and PLKL inhibitors, on the other hand treatment with the two compounds increased acetylated levels of histone H2B.

**Conclusion**

We characterised HDAC7 for its ability to promotes proliferation and invasion in CRC
cells, howeve, understand the pathways that modulated the activity of HDAC7 can be challenging. We have employed an original approach, a compound screening, to identify potential modulators of HDAC7. Our data suggest that PLK1 and AURORA B can play a pivotal role in modulate HDAC7 activity and localization, further experiments are needed to better characterized this exciting hypothesis

### EACR23-1088
### Role of universally conserved transcription factor SUPT5H in Lung Cancer progression.

V. PANDEY[1], Y. Pokharel[1]
[1]*South Asian University, Faculty of Life Sciences and Biotechnology, New Delhi, India*

**Introduction**

© 2023 The Authors. Molecular Oncology is published by John Wiley & Sons Ltd on behalf of Federation of European Biochemical Societies

SUPT5H is a universally conserved transcription factor, conserved across all three domains of life. The protein is known to perform essential function of regulating the promoter proximal pausing of the transcription apparatus which serves as additional check point in progression of cell cycle. it is known to be performing oncogenic function in several cancers by upregulating the expression various proteins and downregulating others.

**Material and Methods**

cBioPortal and UALCAN were used to retrieve protein level expression data of SUPT5H across various cancer. CRISPR/Cas9 system was used to knockdown the cells. Western blot analysis was performed to assess the expression level of the protein involved in different cancer proliferation pathway, cell cycle pathways and pathways involved in the angiogenesis and migration. Flow cytometry analysis was performed to determine cell death and cell cycle arrest.

**Results and Discussions**

SUPT5H is significantly upregulated across different cancers, both at the protein and the mRNA level suggestive of its importance in regulating cellular progression. Knocking down of SUPT5H results in the upregulation of the expression of TP53, P-TP53(Ser15/Ser20) as these proteins are involved in maintaining the genome stability there upregulation post SUPT5H knockdown is suggestive of restoration of protective regulatory function a property typically lost in cancer cells. p21, p27, Rb proteins show an upregulation upon the knockdown of the SUPT5H, suggestive of its role in positive regulation of cell cycle and cell cycle protein. This was also confirmed by a decline in the expression level of cyclins A2, B1 and several CDKs. Additionally, we also found an increase in the expression level of the several proapoptotic protein such as the Bax, cleaved caspase 3/9 . As these proteins are involved in the intrinsic pathways, we suggest that SUPT5H in tumor cells must be suppressing these proteins to maintain the tumor. Further, we assessed the expression level of various survival proteins in the knockdown cells and found a reduced expression of various MAPK pathway protein such as p38, P-p38, and p-ERK. Also, a reduced expression of c-Jun was also reported in the knockdown cells.

**Conclusion**

SUPT5H is a universally conserved gene and hence has potential to upregulate or suppress the expression level of various genes involved in cell survival and homeostasis. A proper understanding of the associated biology will be beneficial in designing therapies targeting cancer progression.

### EACR23-1185

### Intrinsic and extrinsic activation of Interferon pathway driven by the prostate tumor suppressor PGC1α

*A. Pintor-Rial*[1], *A. Schaub-Clerigué*[1,2], *L. Valcarcel-Jimenez*[3], *A. Carracedo*[1,2,4,5], *I. Hermanova*[1], *V. Torrano*[1,2,4]

[1]*University of The Basque Country, Biochemistry and Molecular Biology Department, Leioa, Spain*

[2]*CIC bioGUNE- Bizkaia technology park, 801 building, Derio, Spain*

[3]*University of The Basque Country, Biochemistry and Molecular biology, Leioa, Spain*

[4]*Centro de Investigación Biomédica, en Red de Cáncer CIBERONC, 28029 Madrid, Spain*

[5]*IKERBASQUE, Basque Foundation for Science, 48009 Bilbao, Spain*

**Introduction**

In the era of precision medicine, effective treatments emerge from the challenge to identify patients who will relapse and who could benefit from new therapeutic approaches. Despite favourable clinical responses, some prostate cancer (PCa) patients relapse and develop lethal metastasis. The transcriptional co-regulator PGC1α has been identified as a tumor and metastasis suppressor, whose expression is decreased as PCa progresses, and it has prognostic value (PMID: 27214280 & 31594836). Still, the molecular mechanism which drives PGC1α suppressive phenotype is unknown.

**Material and Methods**

To infer on the molecular pathways, RNAseq and subsequently GSEA analysis of the differential expressed genes were performed on PC3 cells with doxycycline-inducible PGC1α expression. The results were validated by RT-qPCR and WB and we used a JAK1/2 inhibitor to impair Interferon (IFN) pathway.To study the extrinsic effect, IFN-β levels of the secretomes produced by PGC1α negative versus positive cells were measured (IFN-β ELISA Assay). Moreover, we treated PCa cells with conditioned media from PGC1α expressing and PGC1α non-expressing cells and we co-cultivated PGC1α positive with PGC1α negative cells.

**Results and Discussions**

GSEA analysis showed that PGC1α induces the expression of IFN α and γ response genes. PGC1α re-expression initiates STAT1 phosphorylation at 16hr and precedes both STAT2 phosphorylation and IFN genes transcriptional activation at 72hr. In addition, JAK1/2 inhibition data suggested that activation of IFN pathway, both at mRNA and protein level, were dependent on JAK1/2 activity. However, induction of IFN pathway does not sustain the PGC1α suppressive activity as treatment with JAK1/2 inhibitor does not rescue cell autonomous proliferation. Extrinsically, PGC1α re-expression has non-cell autonomous anti-proliferative effect on aggressive PCa cells as this IFN-β enriched-secretomes activate IFN Type I signalling pathway and its transcriptional program in recipient and co-cultured cells.

**Conclusion**

Altogether, autocrine communication may be the trigger of the intrinsic signalling cascade and IFN pathway induction driven by PGC1α goes beyond cell intrinsic transcriptional changes. Deciphering how the IFN pathway is stimulated by PGC1α in a tumor suppressive context may help to design new therapeutic strategies for aggressive PCa and the implementation of therapeutic strategies based on PGC1α.

Grant PID2021-1233720B-100 funded by MCIN/AEI/ 10.13039/501100011033 and, as appropriate, by "ERDF A way of making Europe".

### EACR23-1191

### Regulation of the glycerophosphodiesterase EDI3 via HER2 signaling and its role in HER2 positive

© 2023 The Authors. Molecular Oncology is published by John Wiley & Sons Ltd on behalf of Federation of European Biochemical Societies

### breast cancer

K. Rohlf[1], A. Glotzbach[1], D. Göçener[1], S. Lüke[1], Ö. Demirci[1], M. Schmidt[2], W. Brenner[2], J.G. Hengstler[1], K. Edlund[1], R. Marchan[1]
[1]Leibniz Research Centre for Working Environment and H uman Factors, Toxicology, Dortmund, Germany
[2]University Medical Center Mainz, Department of Obstetrics and Gynecology, Mainz, Germany

**Introduction**

Alterations in choline metabolism have been reported in several malignancies, including breast cancer, and is considered a metabolic hallmark of cancer. The choline-releasing glycerophosphodiesterase EDI3 (Endometrial carcinoma differential 3, *GPCPD1*) is a key enzyme in choline metabolism, and has been shown to be associated with tumor cell migration, adhesion, and metastasis. Studying EDI3 in breast cancer revealed that EDI3 mRNA and protein expression are highest in ER-HER2+ breast cancer tissue and cells compared to other subtypes, indicating an important role of EDI3 in this specific subtype. In the present work, the regulation of EDI3 via HER2 signaling was explored as well as whether high EDI3 expression can be exploited therapeutically using both in vitro and in vivo models.

**Material and Methods**

To understand how EDI3 is regulated, HER2, as well as key enzymes downstream of HER2 major signaling pathways were inhibited. In addition, candidate transcription factors downstream of HER2 signaling with potential binding sites on EDI3's promoter region were examined. To study if high EDI3 expression provides the cells with a survival advantage, EDI3 was silenced in ER-HER2+ cells in vitro and in vivo and the effect on viability and tumor growth was studied in cells and in mice, respectively.

**Results and Discussions**

Silencing and pharmacological inhibition of HER2 and key enzymes in HER2 regulated pathways identified the pathway downstream of HER2/PI3K/Akt/mTOR and GSK3β as the most relevant in the regulation of EDI3. Furthermore, the transcription factors HIF1α, CREB, and STAT3 may be directly involved in regulating EDI3 expression. Silencing EDI3 significantly reduced viability in ER-HER2+ cells, in particular those more resistant to HER2-targeted therapy. Finally, pharmacological inhibition and silencing of EDI3 led to a reduction in tumor growth in HER2-targeted therapy-resistant breast tumors in vivo.

**Conclusion**

Our results indicate that targeting EDI3 may be an alternative in HER2-targeted therapy-resistant breast tumors which justifies further investigations of EDI3's potential as a therapeutic target in ER-HER2+ breast cancer.

This project was supported by the German Cancer Aid (Stiftung Deutsche Krebshilfe— grant number 70114035).

### EACR23-1285
### Inactive mutated VEGFR2 promotes melanoma growth via heterodimerization with wild-type receptor

E. Grillo[1], C. Ravelli[1], M. Corsini[1], R. Bresciani[1], S. Mitola[1]
[1]University of Brescia, Department of Molecular and Translational Medicine, Brescia, Italy

**Introduction**

In cancer, the activation of the VEGF/VEGFR2 pathway regulates both stromal and parenchymal cell biology and metabolism, supporting tumor progression. For this reason, VEGFR2-targeted tyrosine kinase inhibitors (TKi) are widely used in the clinic to treat different cancer types. However, primary or acquired resistance often occurs, likely due to the acquisition of novel mutations. Here we studied the mechanism of action of the most frequent non-synonymous mutation R1032Q of VEGFR2. Although this substitution entails a loss of function of the receptor, the expression of VEGFR2$^{R1032Q}$ in cancer cells promotes tumor growth.

**Material and Methods**

By using protein-protein interaction assays, molecular imaging (FRAP, FLIM/FRET) and enzymatic assays we studied the dimerization, membrane dynamics and activation of mutated receptor. Next, we set up a melanoma model with heterozygous R1032Q mutation of VEGFR2 which was exploited to characterize the pro-tumorigenic effects and drug response of VEGFR2$^{R1032Q}$.

**Results and Discussions**

VEGFR2$^{R1032Q}$ forms functional heterodimers with wild-type VEGFR2, altering the membrane dynamics of the wild-type receptor and increasing the VEGFR2-associated intracellular signaling in the absence of exogenous VEGF-A stimulation. In a melanoma model, heterozygous VEGFR2$^{R1032Q}$ triggers pro-oncogenic events modifying gene expression, cell metabolism, and increasing cell growth and metastasis *in vitro* and *in vivo*. Also, the expression of VEGFR2$^{R1032Q}$ increases melanoma cell resistance to the VEGFR2-targeted TKi linifanib and vatalanib.

Remarkably the R1032Q substitution of VEGFR2 occurs in a hot-spot residue of the kinase domain which is recurrently mutated in many other receptor tyrosine kinases (RTKs), including EGFR, KIT, FLT3, FLT4 and PDGFRA, among others. This underscores the importance of mutations found at this position. Moreover, mutations of corresponding residues across different proteins elicit similar effects and may be similarly targeted. Therefore, our results anticipate the effects and druggability of all other uncharacterized mutations corresponding to the substitution R1032Q of VEGFR2.

**Conclusion**

Our data reveal a possible ligand-independent inter-receptor kinase activation of VEGFR2/VEGFR2$^{R1032Q}$ heterodimers which drives tumor progression. This novel mechanism of activation of VEGFR2, which may be shared by other RTKs, could be exploited to develop new therapeutic approaches to treat tumors harboring the VEGFR2$^{R1032Q}$ mutation and possibly all other corresponding ones.

### EACR23-1306
### Gene co-expression networks in Basal-Like Breast Cancer allowed to identify

© 2023 The Authors. Molecular Oncology is published by John Wiley & Sons Ltd on behalf of Federation of European Biochemical Societies

### E2F3,TFDP1, TEAD1, PTTG1 and C/EBPg transcription factors as central hubs regulating aggressiveness features.

L. Avalle[1], A. Savino[1], M. Cugusi[1], S. Neralagundi[1], G. Di Blasio[1], G. Accetta[1], I. Miglio[1], A. Griffa[1], F. Anselmi[2], I. Molineris[2], P. Provero[1], V. Poli[1]
[1]University of Turin, Dept. Molecular Biotechnology and Health Sciences, Turin, Italy
[2]University of Turin, Department of Life Sciences and Systems Biology- Molecular Biotechnology Center- University of Turin-, Turin, Italy

**Introduction**

Breast cancer (BC) is a highly heterogeneous disease for which no general treatment is available, in particular, the aggressive basal-like BC (BLBC) subtype currently represents an unmet medical need. Intrinsic features of tumor cells determining their aggressiveness, such as survival, proliferation and invasion, are the result of the orchestrated activity of many components interacting with each other, determining specific gene regulation events. Gene coexpression networks are considered useful tools to define prognostic gene signatures, and identify centrally connected genes as potential therapeutic targets.

**Material and Methods**

Weighted Gene Coexpression Network Analysis was applied to METABRIC, a large primary BC dataset comprising 1981 samples belonging to all molecular subtypes. This led to the identification of 21 modules, representing groups of genes topologically close in the whole gene expression network. We next searched for modules tightly interconnected in the BLBC subtype and we posited network centrality (kWithin) as a reliable parameter to identify modules' regulators, selecting as potential activators of the module the most central Transcriptional Factors (TF). We thus selected 5 not yet studied TFs as candidates for functional validation and inactivated them in multiple BLBC cells, by means of both RNA silencing and CRISPR-mediated KO.

**Results and Discussions**

Reconstructing BC gene coexpression networks, we obtained groups of genes significantly correlated with survival and tumor grade. We next generated subtype-specific gene co-expression networks and identified a module, named bE2F-targets, where genes are more tightly connected in the highly aggressive BLBC subtype. The expression levels of the module strongly correlate with clinical features and poor prognosis, supporting its relevant biological functions. The most central TFs of bE2F-target module, namely PTTG1, TEAD4, E2F3, TFDP1 and C/EBPγ, were chosen as potential regulators of the entire module. *In vitro* validations showed that targeting these TFs reduced aggressiveness features in BLBC cells. Moreover, RNA sequencing showed that E2F3 silencing significantly disrupted the expression of the whole module.

**Conclusion**

Our work allowed to identify gene expression modules highly relevant in BC as well as their central TF hubs. Upon experimental validation, this approach could be successfully extended to other BC subtypes, and to all tumors for which gene expression data are increasingly available, allowing to identify druggable targets.

### EACR23-1343
### Validation of the Less Frequent RICTOR Amplification and Its Potential Role for Targeted Therapy Across Multiple Malignancies

D. Sztankovics[1], I. Krencz[1], G. Bedics[1], G. Papp[1], D. Moldvai[1], T. Dankó[1], G. Petővári[1], J. Pápay[1], C. Bödör[1], A. Sebestyén[1]
[1]Semmelweis University, Department of Pathology and Experimental Cancer Research, Budapest, Hungary

**Introduction**

mTOR signalling failures including *RICTOR* amplification support the growth, progression and metastasis of several cancers. The oncogenic role of mTORC2 (mechanistic target of rapamycin complex 2) hyperactivity and the overexpression of its main compartment, Rictor (rapamycin-insensitive companion of mTOR), has been documented in several cases. Rictor overexpression could contribute to uncontrolled tumour growth, influencing cellular survival, cytoskeletal reorganisation and migration. The amplification of *RICTOR* has been described in several tumours including lung cancer, breast cancer and melanoma, etc. Regarding these, *RICTOR* amplification highlights the importance of targeting mTOR signalling and mTORC2 complex hyperactivity in the personalised therapy of solid tumours.

**Material and Methods**

Between 2018 and 2022, more than 400 patients were sequenced with next-generation sequencing (NGS) for diagnostic purposes at our institute.

Detected *RICTOR* copy number alterations (described copy number variation–CNV), as a sign of potential *RICTOR* amplification in different malignant tumour tissues, were further studied using the newly established Droplet Digital PCR (ddPCR) and the "gold-standard" fluorescence *in situ* hybridisation (FISH). Additionally, the detected *RICTOR* amplification was compared to the *in situ* Rictor and phospho-Akt (Ser473) protein expressions assessed by immunohistochemistry (IHC).

**Results and Discussions**

Of the 420 malignant tumour samples, *RICTOR* gene amplification was suspected in 37 (8.8%) cases by NGS. *RICTOR* amplification was observed in 11 and 16 of the 37 cases (29.7%, 43,2%) by ddPCR and FISH, respectively. Elevated Rictor expression was detected in 12 and 14 (37.8, 32.4%) cases using two different Rictor antibody clones. Additionally, overexpression of phospho-Akt (Ser 473) was detected in 7 cases (18.9%). Higher copy numbers (CNV results higher than 3) detected by further NGS analyses could highlight the real *RICTOR* amplification, and in correlation with these the targetable high mTORC2 complex and mTOR signalling activity.

**Conclusion**

Based on our results, higher than 3 CNV of the *RICTOR* gene recorded by NGS can be a good indication of *RICTOR* amplification and initiates further validation by FISH or ddPCR. Additionally, *in situ* Rictor, phospho-Akt (Ser 473) protein expression studies may also

Molecular Oncology 17 (Suppl. 1) (2023) 1–597
© 2023 The Authors. Molecular Oncology is published by John Wiley & Sons Ltd on behalf of Federation of European Biochemical Societies be useful to validate Rictor or mTORC2 complex activation at the protein level in tumour tissue for administrating targeted therapies.

### EACR23-1347
### Characterization of the molecular mechanism of autophagy inhibition by HER2

S. Vega-Rubin-de-Celis[1]
[1]*Institute for Cell Biology Tumor Research, Molecular Cell Biology, Essen, Germany*

**Introduction**

Approximately 20% of breast cancer cases have amplifications in HER2. We previously showed that BECN1 (a protein essential for autophagy and codified by a haploinsufficient tumor suppressor) and HER2 interact, leading to autophagy inhibition and tumour development. Thus, we aim to understand the mechanism by which HER2 regulates autophagy and tumorigenesis. In addition, HER2 mutants inhibit autophagy through BECN1 phosphorylation, but wild-type HER2 does not appear to phosphorylate BECN1, and inhibits autophagy independent of BECN1 phosphorylation in an mTORC1-dependent manner. Therefore, we hypothesized that other proteins might mediate the effect of HER2 on BECN1, and on autophagy.

**Material and Methods**

We identified a new BECN1 interacting partner, based on data derived from a large-scale screen of the autophagy proteins network. *In vitro* approaches such as GFP-LC3 puncta formation assay, western blotting and HiBiT-LC3 reporter system were used to measure the autophagic flux. Besides, docking studies led to the identification of key residues in both BECN1 and its interactor implicated in regulating autophagy. The role of these proteins in tumorigenesis was determined *in ovo*, through the Chick Chorioallantoic Membrane (CAM) assay. Clinical data from the BRCA-TCGA and the METABRIC datasets gene expression was analyzed, as well as reverse phase protein arrays (RPPA) analysis.

**Results and Discussions**

Our data demonstrate that the BECN1/HER2 complex includes a novel BECN1 interacting protein. Knockdown of this protein induces autophagy and upregulates the catalytic activity of Vps34 in a manner that is independent of mTORC1. Mutation analysis lead to the identification of key regions for such binding including the ECD domain of BECN1. I*n ovo* CAM studies indicate that knockdown of this new interactor inhibits tumour growth. In addition, gene expression data analysis indicates a positive correlation of our new identified protein with markers of autophagy in breast cancer patient samples. Such correlation was even more prominent when protein levels were analyzed by RPPA, suggesting that patients with decreased protein X levels may have an induced autophagy. Furthermore, these patients with low protein X and p62 presented a better overall and progression-free survival than those with high levels of both proteins.

**Conclusion**

Taken together, our data suggest that a new BECN1-interacting protein mediates the binding of HER2 to BECN1and represents a potential new regulator of autophagy activity and tumour growth.

### EACR23-1409
### Targeting the RAF Kinase Independent Functions in NRAS and BRAF Mutant Melanoma

N. Aboud[1], L. García-Gutiérrez[2], W. Kolch[1], D. Matallanas[1]
[1]*Systems Biology Ireland, School of Medicine, Dublin, Ireland*
[2]*Instituto de Biomedicina y Biotecnología de Cantabria, Universidad de Cantabria, Santander, Spain*

**Introduction**

RAF-1 kinase independent functions were shown to inhibit apoptosis by binding to the Hippo core kinase MST2. We previously identified small compounds that disrupt the RAF-1/MST2 interaction to promote apoptosis. Our current work is aimed at exploring the therapeutic potential of targeting the RAF-1/MST2 interaction in NRAS and BRAF mutant melanoma.

**Material and Methods**

We downregulated RAF-1 using siRNAs in various melanoma cell lines, such as the NRAS mutant (SKMEL2), the BRAF mutant (A375) and the BRAF inhibitor resistant (A375R) and levels of apoptosis were measured using flow cytometry. We determined the effect of disrupting the RAF-1/MST2 interaction on apoptosis by treating the cell lines with the previously identified disruptors, VCC923573:16 and VCC199189:07. Protein-protein interactions were assessed by co-immunoprecipitation experiments and western blots. Reactivation of the Hippo pathway with treatment was assessed by measuring changes in protein phosphorylation levels in a time course experiment. Additionally, cells were treated with the disruptors in combination with an MST1/2 or LATS1/2 inhibitor to determine the role of the Hippo pathway in mediating treatment outcomes. Finally, we generated a zebrafish melanoma model with induced skin tumours driven by NRASQ61L or BRAFV600E, and are proficient or deficient in LATS1 expression. Histological analysis of the zebrafish sections was performed after H&E staining, and the proteome of the tumours was analysed by mass spectrometry.

**Results and Discussions**

We found that RAF-1 downregulation is lethal downstream NRASQ61 signalling, while mutations in BRAFV600 overcome this effect. Interestingly, the secondary mutations acquired during BRAF inhibitor resistance seem to resensitize the cells towards RAF-1 downregulation. Consistently, disruptor treatment increased apoptosis in the NRAS mutant SKMEL2 dependent on Hippo pathway reactivation, and in the BRAFi resistant A375R. Furthermore, our in vivo results have revealed differential tumour onset and progression in zebrafish dependent on the driver mutation and the status of LATS1 expression.

**Conclusion**

Our work highlights the importance of RAF-1 in NRAS mutant and BRAFi resistant melanoma. We have also shown that reactivating the Hippo pathway by disrupting the inhibitory RAF-1 kinase independent interaction is a potential therapeutic target in melanoma. Future work will focus on deciphering the disruptors' mechanisms of action and testing the effect of disrupting the RAF-1/MST2 interaction in our in vivo model.

© 2023 The Authors. Molecular Oncology is published by John Wiley & Sons Ltd on behalf of Federation of European Biochemical Societies

### EACR23-1414
### Analysis of cyclin docking specificity using a novel intracellular binding assay

M. Örd[1,2], M. Winters[3], P. Pryciak[3], N. Davey[2]
[1]University of Cambridge,
Cancer Research UK Cambridge Institute, Cambridge, United Kingdom
[2]The Institute of Cancer Research,
Division of Cancer Biology, London, United Kingdom
[3]University of Massachusetts Chan Medical School,
Department of Biochemistry and Molecular Biotechnology, Worcester, United States

**Introduction**
Short linear motifs (SLiMs) in intrinsically disordered regions of proteins play a key role in dynamic intracellular signalling. Missense mutations in SLiMs that mediate protein localization, degradation or protein-protein interactions have been identified as cancer drivers. However, only a small fraction of SLiMs have been experimentally studied due to a lack of robust high-throughput methods for characterisation of dynamic low affinity interactions. SLiMs play a crucial role in cell cycle regulation by cyclin-dependent kinases (CDKs), where cyclin-binding SLiMs in various proteins function as substrate recruitment modules directing CDK phosphorylation. Yet, the cyclin specificity and the specificity determinants of these SLiMs have remained unclear.

**Material and Methods**
We use systematic intracellular motif binding analysis (SIMBA), an *S. cerevisiae* competitive growth assay combined with deep sequencing, for deep mutational scanning of known cyclin binding motifs and for discovery of novel binding peptides. The competitive growth assay results are validated using peptide pulldowns and fluorescence polarisation. The impact of cyclin docking motif specificity and affinity on substrate phosphorylation in cell cycle is studied in human cell lines.

**Results and Discussions**
To characterise the full repertoire of cyclin docking SLiMs, we built a library of tiled peptides from 802 cell cycle proteins – the potential cyclin interactome – and then scored their binding to cyclins with SIMBA. We find that cyclins bind a greater variety of SLiMs than previously known and that peptide tiling enables precise identification of binding motifs. For a comprehensive understanding of SLiM specificity determinants, we performed deep mutational scanning of these motifs, which revealed sequence features that impart a broad range of binding strengths and diverse patterns of specificity for distinct cyclin types. Measuring the binding of over 100k peptides with cyclins provided the basis for a comprehensive map of cyclin docking specificity that regulates CDK substrate protein phosphorylation.

**Conclusion**
SIMBA enables discovery and detailed characterisation of SLiM-mediated interactions. Identifying novel motif-mediated interactions provides insight into signalling networks and could provide additional targets for cancer drug design. Deep mutational scanning allows characterisation of mutation effects in SLiMs on a large scale.

### EACR23-1415
### Validation of a novel inhibitor of TRAF6/NFκB axis in models of breast cancer metastasis

F. Zeng[1], S. Marino[1,2], I. Bassanini[3], S. Conrad[4], G. Carrasco[1], B. Li[1], P. Mollat[5], A. Sophocleous[6], M. Meli[3], E. Ferrandi[3], G. Morra[3], M. Rauner[4], A. Sparatore[7], A. Idris[1,2]
[1]University of Sheffiled,
Department of Oncology and Metabolism, Sheffield, United Kingdom
[2]University of Edinburgh,
Bone and Cancer Group- Edinburgh Cancer Research Cen tre, Edinburgh, United Kingdom
[3]Italian National Research Council,
Institute of Chemical Sciences and Technologies, Milan, Italy
[4]Technische Universität Dresden,
University Medical Center, Dresden, Germany
[5]Galapagos SASU, 102 Avenue Gaston Roussel, Romainville, France
[6]European University Cyprus, Department of Life Sciences, Nicosia, Cyprus
[7]University of Milan,
Department of Pharmaceutical Science, Milan, Italy

**Introduction**
The pro-inflammatory TRAF/NFκB signalling pathway plays a key role in triple-negative breast cancer (TNBC). The TRAF family constitutes of 7 adaptor proteins that exhibit distinct and overlapping functions. Thus, there is a need to explore and validate which TRAF represents a potential druggable target for difficult-to-treat breast cancer subtypes, such as TNBC.

**Material and Methods**
TRAF expression and activity were evaluated by DARTS, Western Blot, *in silico* and meta- and bioinformatics analyses. Cell viability, migration and invasion were assessed using AlarmarBlue, wound healing and transwell assays, respectively. Osteoclasts were visualized by TRAcP staining and osteolysis was measured by microCT.

**Results and Discussions**
Meta-analysis of relevant studies identified 14 *in vitro*, 11 *in vivo* and 4 human articles. Analysis of pooled studies showed that genetic and pharmacological inhibition of TRAF2/4/6 is associated with reduced breast cancer (BCa) cell behaviour *in vitro* and tumorigenesis and metastasis in mice. Bioinformatics validation confirmed that only TRAF6 expression is associated with bone metastasis and survival in BCa patients. Follow up functional and mechanistic studies in TNBC and bone models confirmed that TRAF6 is highly expressed in osteotropic (BT) clones of the TNBC human MDA-MB-231-BT and mouse 4T1-BT cells. Knockdown and pharmacological (using a panel of novel small-molecules called FSAS1-5) inhibition of TRAF6 suppressed cell growth in a dose and time dependant manner. The potent FSAS3 reduced the ability of MDA-MB-231-BT to migrate, invade and support the lineage commitment of macrophages (M0) into tumour-associated macrophages (M2) and 'bone-resorbing' multi-nucleated osteoclasts. FSAS3 also enhanced the *in vitro* cytotoxic efficacy of a panel of chemotherapeutic agents, particularly Docetaxel. *Ex vivo*, FSAS3 inhibited

© 2023 The Authors. Molecular Oncology is published by John Wiley & Sons Ltd on behalf of Federation of European Biochemical Societies

MDA-MB-231-induced osteolysis in mouse calvarial bone as assessed by microCT. Mechanistically, TRAF6 was identified as a target protein of FSAS3 using DARTS, and predicted pose from *in silico* docking analysis showed that it binds to CD40 and a novel pocket at the c-terminus of TRAF6. Consistently, FSAS3 inhibited TRAF6-IKK binding, IκB activation and NFκB-DNA binding, indicative of canonical NFκB inhibition, which was significantly blunted in TRAF6 deficient MDA-MB-231 cells.

**Conclusion**

TRAF6 inhibitors are of value in the treatment of advanced breast cancer. *In vivo* studies that test the anti-metastatic effect of FSAS3, alone and in combination with Docetaxel, are ongoing.

### EACR23-1431
### miR-29a as a factor potentially involved in the pathogenesis of diseases associated with endocrine disruptions

W. Losa[1]
[1]*Silesian University of Technology, Faculty of Automatic Control- Electronics and Computer Science, Gliwice, Poland*

**Introduction**

MicroRNAs are an evolutionarily conserved class of small non-coding RNAs that influence a broad spectrum of physiological processes in cells. One of the microRNAs, miR-29a, is well-connected with the development of different kinds of cancer. The expression of miR-29a can be regulated by hormones such as insulin, progesterone, or estradiol. The aim of the study was to check if miR-29a can be involved in the pathogenesis of diseases associated with endocrine disruptions.

**Material and Methods**

Based on the NCBI database, genes whose expression is regulated by miR-29a were selected. The Pathway Commons database was used to determine the signaling pathways associated with the retrieved genes. Based on the literature, diseases associated with disruption of signaling pathways regulated by miR-29a were selected. We then examined which of the selected diseases involve estradiol-, progesterone- or insulin-sensitive cells.

**Results and Discussions**

The NCBI database found 86 genes regulated by miR-29a. The proteins encoded by the genes found are mainly involved in the maturation and modification of mRNA. Diseases in which RNA processing has been found to be impaired include, in addition to cancer, neurodegenerative diseases, metabolic diseases, muscular dystrophy, cardiovascular disease, or diabetes. The cells in which the above pathological processes take place have estradiol, progesterone or insulin receptors. It suggests that endocrine disruption can affect miR-29a expression in these cells and consequently lead to serious diseases.

**Conclusion**

The results show that perturbation of miR-29-a expression caused by endocrine disruption can affect numerous genes and signaling pathways that lead to diseases. Thus, it seems that miR-29a may be a potential molecular target in the therapy of heart, lung and neurodegenerative diseases.

### EACR23-1445
### WNT5A activates MMP2 through WNT5A/PKC pathway in OSCC cells

Z. Prgomet[1], T. Andersson[2]
[1]*Malmö University, Oral Pathology- Faculty of Odontology- Biofilms - Research Center for Biointerfaces-, Malmö, Sweden*
[2]*Lund University, Cell and Experimental Pathology- Department of Translational Medicine, Malmö, Sweden*

**Introduction**

WNT5A, the non-canonical WNT signaling protein, activates WNT/$Ca^{2+}$/PKC pathway and increases the migration and invasion of oral squamous cell carcinoma (OSCC) cells. The invasion of OSCC starts with the degradation of the *basal lamina* and the extracellular matrix, which is mainly executed by matrix metalloproteinases (MMPs). WNT5A, MMP2, and MMP9 are all overexpressed in OSCC however, the association of these proteins with each other has not yet been examined. The objective of this study was to examine whether WNT5A regulates the activation of MMP2 and MMP9 in OSCC cells and through which pathway is this conducted.

**Material and Methods**

The expression of WNT5A protein in human OSCC tissue was evaluated by immunohistochemistry. The effect of rWNT5A on the secretion and activation of MMP2 and MMP9 in two OSCC cell lines, SCC9 and SCC25, was assessed by ELISA, zymography and western blot.

**Results and Discussions**

Cytoplasmic WNT5A protein expression was found in 84% of OSCCs. Stimulation of OSCC cells with rWNT5A led to increased secretion of both pro-MMP9 and pro-MMP2 in both SCC9 and SCC25 however, rWNT5A induced activation of only MMP2 in the highly invasive cell line i.e. SCC25. Furthermore, stimulation of SCC25 with rWNT5A in presence of two PKC inhibitors showed decreased MMP2 activity and thereby indicated that WNT5A activates MMP2 through PKC in SCC25.

**Conclusion**

Based on the present findings, we suggest that WNT5A activates MMP2 through PKC and thereby contributes to the invasiveness of OSCC.

### Translational Research

### EACR23-0050
### Spatial-Omics pipeline to aid personalization of precision medicine in metastatic recurrent Head & Neck Cancers.

J.L. Gonzalez Cruz[1], A. Causer[2], X. Tan[2], X. Lu[1], P. Moseley[1], C. Perry[3], I. Frazer[1], B. Panizza[3], R. Ladwa[4], Q. Nguyen[2]
[1]*The University of Queensland, Frazer Institute, Brisbane, Australia*
[2]*The University of Queensland, Institute of Molecular Biology, Brisbane, Australia*
[3]*Princess Alexandra Hospital, Department of Otolaryngology, Brisbane, Australia*

© 2023 The Authors. Molecular Oncology is published by John Wiley & Sons Ltd on behalf of Federation of European Biochemical Societies

[4]Princess Alexandra Hospital,
Department of Medical Oncology, Brisbane, Australia

**Introduction**

Immune checkpoint inhibitors are used to treat recurrent metastatic oropharyngeal squamous cell carcinomas (OPSCC). Unfortunately, <30% of patients benefit from this therapy. We postulate that spatial determinants in the tumor play a critical role in cancer therapy outcome.

**Material and Methods**

Thus, we performed spatial transcriptomics (ST) and *in-situ* multiprotein detection (CODEX) on tissue isolated from a patient diagnosed with metastatic OPSCC. Patient's primary oral tumor responded to chemo-radio therapy, followed by nivolumab. However, new soft pallet OPSCCs resurged (MAR21). Although, subsequent pembrolizumab + lenvatinib treatment had an initial effect, new oral tumors re-emerged (SEP21) suggesting drug resistance.

**Results and Discussions**

Unbiased clustering, based on differentially expressed genes, recapitulated tissue annotations and aid pathologists to resolve conflicted areas. From 11 tumor clusters (CL), CL4 & CL5 were the real carcinogenic tissue. Interestingly, these 2 CLs represented metabolically distinct tumor regions: CL4, highly proliferative cells *vs* CL5 enriched in genes participating in cell-cell interaction/migration and innate immune response. We confirmed these finding with CODEX and use the proteomic data to generate a new method of cell deconvolution (Spatial Proteomics-informed deconvolution method or SPiD) which outperformed the currently 4 most used packages. Focusing on the real tumor, we observed high expression of drug resistance genes including *SNAI2* and *SOX4* consistent with the disease aggressive behavior. Although, *PD-1/PD-L1* expression was absence, we identified 9 over-expressed druggable targets (*i.e.*, *EGFR, TF, VEGF*) and 9 pre-clinical patient-specific targets in CL4-5. To rank drugs' potential success, we measured the co-expression of each target ligand-receptor pair (L/R), reducing the candidates to two main pathways (TF/TFRC & VEGFA/NRP1). Lastly, the compassion between pembrolizumab + lenvatinib responsive (MAR22) and non-responsive (SEP22) OPSCCs showed shared CL4-5 phenotypes with PD-1/PD-L1$^{low}$ and VEGFA$^{high}$ expression, suggesting that SEP22 treatment failure could be linked to the reduction of Lenvatinib dose instead of a drug resistance mechanism.

**Conclusion**

We demonstrated the appropriateness of Spatial Omics as a medical tool for recapitulating patient disease and aiding in the personalized drug selection processes. The use of L/R interactions and pathway analysis provides a scientific-based rationale for drug selection, allowing the prioritization of targets.

### EACR23-0094

### Overcoming resistance to KRASG12C inhibitors with the novel RAF/MEK clamp avutometinib (VS-6766)

*C. caffarra malvezzi*[1], *A. Mira*[1], *S. Coma*[2], *J. Pachter*[2], *C. Ambrogio*[1]
[1]University of Torino,
Molecular Biotechnology and Health Sciences Molecular Biotechnology Center MBC, Torino, Italy
[2]Verastem, Verastem Oncology, Needham, United States

**Introduction**

*KRAS* is the most frequently mutated oncogene in solid tumors, including lung adenocarcinomas (LUADs), and it has been considered undruggable until a few years ago. Recently, sotorasib and adagrasib, two KRAS G12C inhibitors (G12Ci), have shown efficacy in patients with KRAS$^{G12C}$ cancers, receiving FDA approval in 2021 and 2022, respectively. Despite G12Ci showing remarkable clinical responses, treatment with G12Ci monotherapy inevitably leads to the development of resistance. Thus, the necessity to find new therapeutic approaches and combination therapies is urgent. Targeting the MAPK pathway at different levels may aid in deeper and longer-lasting responses. Avutometinib (VS-6766; avuto) is a novel RAF/MEK clamp that potently inhibits MEK kinase activity and induces dominant negative complexes of ARAF, BRAF and CRAF with MEK, preventing the typical compensatory re-activation of MEK induced by MEK-only inhibitors. Furthermore, MAPK pathway inhibition has been shown to induce focal adhesion kinase (FAK) as an adaptive resistance mechanism.

**Material and Methods**

Here, we tested the antitumor activity of avuto alone or in combination with sotorasib ± FAK inhibitor in RAS-less mouse embryonic cells (MEFs) engineered to express KRAS$^{G12C}$ with *in cis* Y96D mutation which has been reported to confer acquired resistance to G12Ci.

**Results and Discussions**

Avuto alone, both *in vitro* or *in vivo*, strongly inhibited proliferation and tumor growth in the G12Ci-resistant KRAS$^{G12C/Y96D}$ model through the inhibition of the MAPK pathway, while sotorasib was ineffective. Moreover, tumor growth inhibition was enhanced when avuto was administered in combination with FAK inhibition, and stronger tumor growth inhibition was observed with the combination of avuto, sotorasib and FAK inhibitor.

**Conclusion**

Our results support the ongoing clinical trial assessing avutometinib in combination with sotorasib (NCT05074810) for treatment of KRAS$^{G12C}$ NSCLC in both G12Ci naïve patients and patients progressing on G12Ci treatment. Moreover, these data suggest that adding the FAK inhibitor defactinib to the combination of avuto and sotorasib may further increase depth and duration of responses.

### EACR23-0100

### Effect of modulated electro-hyperthermia (mEHT) on angiogenesis in mouse triple negative breast cancer (TNBC) model

*S.M.Z. Bokhari*[1], *K. Aloss*[1], *P.H.L. Viana*[1], *C.A. Schvarcz*[1], *L. Danics*[1], *B. Besztercei*[1], *G. Nino*[1], *Z. Benyó*[1], *P. Hamar*[1]
[1]Semmelweis Univerisity, Translational Medicine Institute, Budapest, Hungary

**Introduction**

Triple negative breast cancer (TNBC) is a highly aggressive breast cancer type with no targeted therapy. Modulated electro-hyperthermia (mEHT) is a novel complementary therapy where a 13.56 MHz radiofrequency current targets cancer cells selectively, inducing tumor damage by thermal and electromagnetic effects. Complementary mEHT treatment can therefore

© 2023 The Authors. Molecular Oncology is published by John Wiley & Sons Ltd on behalf of Federation of European Biochemical Societies improve the efficacy of available TNBC treatments. However, the astounding ability of tumor cells to adapt and evade therapy is a constant challenge, thus a better understanding of the therapeutic effects of mEHT has great translational potential. We observed angiogenic alterations in mEHT-treated tumors and aimed to investigate the effects of mEHT on angiogenesis in our TNBC mouse model.

**Material and Methods**

TNBC murine isografts were treated three or five times with LabEHY 200 at 0.7 ± 0.3 W for 30 min every 48 hours. Tumor growth was monitored with ultrasound and digital calipers. Tumor destruction histology, blood capillary damage, and molecular changes were detected by immunohistochemistry, next generation sequencing, and Nanostring technology.

**Results and Discussions**

Capillary damage was detected as free red blood cells (RBCs) in the interstitium of TNBC isografts on HE-stained slides, 24h after 3 mEHT treatments in a time-dependent manner. The bleeding peaked at 12h and interstitial RBC count was significantly reduced by 24h. Similar bleeding was observed after five mEHT treatments and the number of viable blood vessels was significantly reduced.

Significant upregulation of stress-related genes in response to mEHT treatment was observed in NGS corroborated by nanostring and MS, which may be partially due to capillary damage-mediated hypoxia. Also, angiogenic repair in response to mEHT was detected by a reduction at 12h and subsequent continuous upregulation of tumor vasculature markers CD105 and CD-31 after 24 hours to sham level. Reduction of hypoxic stress by treatment with digoxin, a cardiac glycoside also known to reduce expression of Hypoxia Inducible Factor (HIF-1a), in combination with mEHT demonstrated a reduction in tumor hypoxia. We further aim to identify, if this reduction in tumor hypoxia is translated to reduced tumor angiogenesis and enhanced tumor damage.

**Conclusion**

mEHT induces blood capillary damage and triggers a stress response that repairs the tumor vasculature. Combining mEHT treatment with digoxin can potentially reduce this angiogenic repair in tumor cells and hence can enhance the effectivity of mEHT & other forms of cancer treatment.

### EACR23-0145

### A novel organ-on-chip-based in vitro approach for co-culturing 3D human cancer tissues and circulating capillary flow-driven immune cells for more predictive drug testing and human disease modeling

S. scaglione[1], E. Palama[1], C. Vitale[2], M. Aiello[1]
[1]*React4life, R&D, Genoa, Italy*
[2]*University of Genoa, dimes, Genoa, Italy*

**Introduction**

The human disease modeling for basic research and drug testing purposes is currently carried out through 2D static cell culture and animal models, but predictability, reliability, and complete immune compatibility remain important challenges. For this aim, novel 3D, fully humanized in vitro cancer tissue models have been recently optimized by adopting emerging technologies such as microphysiological systems (MPS) and 3D cell laden hydrogels. In particular, a novel Multi-In Vitro Organ (MIVO) MPS platform has been recently adopted to culture 3D clinically relevant size cancer tissues under proper physiological culture conditions to investigate the efficacy of anticancer treatments.

**Material and Methods**

Biologically relevant cancer samples have been developed by using cell laden alginate based hydrogels with tunable stiffness supporting the proper tumor cells viability, cluster formation and migration. Ovarian and and ovarian cell laden hydrogels have been cultured within the MIVO chamber, while either testing molecules (cisplatin) or human immune cells (Natural Killer cells, NK) respectively circulate in the MPS mimicking the blood capillary flow. The tumor cell proliferation and viability were investigated in such dynamic cell culture conditions to assess the cytotoxic efficacy of the treatment. When the systemic administration of cisplatin was simulated within the MPS, the anticancer drug efficacy was also tested and compared to the animal model. When NK cells were placed in circulation, their extravasation through a permeable barrier resembling the vascular barrier, and infiltration within the neuroblastoma cancer tissue were analyzed.

**Results and Discussions**

A human 3D ovarian model was developed and treated with Cisplatin in static conditions, within MIVO, and in the xenograft model. Similar tumor regression was observed in MIVO and in mice, while the static culture displayed an unpredictive chemoresistance, due to unreliable drug diffusion within the 3D matrix.

A human 3D neuroblastoma cancer model with proper immunophenotype was optimized to develop a complex tumor/immune cell coculture. Importantly, a tumor-specific NK cell extravasation was observed under dynamic culture, with NK cells able to infiltration within the tumor where they induced cancer cells apoptosis.

**Conclusion**

We generated a relevant human disease model, through the adoption of a MPS system, that can be efficiently employed as a drug screening platform but also for better investigating crosstalk among immune /tumor cells.

### EACR23-0150

### Increasing Plexity of Imaging Mass Cytometry for Tumor Tissue Analysis

R. Spada[1], E. Lopez[1], S. Ouladan[2], T. Pfister[2], D. Majonis[2], C. Loh[2]
[1]*Standard BioTools, Commercial Operations EMEA, Les Ulis, France*
[2]*Standard BioTools, Research and Development, Markham, Canada*

**Introduction**

Imaging Mass Cytometry™ (IMC™) is the leading platform for high-plex tissue imaging. IMC allows for detailed assessment of cell phenotype and function using 40-plus markers simultaneously at subcellular resolution on a single slide. A comprehensive IMC panel containing structural, functional, and immune markers enables us to reveal the complex heterogeneity of tumor tissues as well as the tumor microenvironment (TME). Driven by an

© 2023 The Authors. Molecular Oncology is published by John Wiley & Sons Ltd on behalf of Federation of European Biochemical Societies increase in the number of antibody markers and the addition of mRNA markers, there is an increasing demand for larger panels. In addition, increasing the number of investigated target markers on a single tissue enriches spatial characterization that may facilitate a more accurate prediction of disease progression and preclinical outcome measures in clinical research projects using tumor biopsies or tissue microarrays (TMAs). Therefore, to increase the plexity of IMC panels, it is essential to expand the number of available metal channels. Here, we demonstrate the incorporation of conjugated antibodies with yttrium ($^{89}Y$) and indium ($^{115}In$), two low-mass metals, for IMC application. These metal tags have been previously tested as putative channels for IMC application.

**Material and Methods**

We performed IMC analysis of various tissue types stained with panels of conjugated antibodies including the novel $^{89}Y$- and $^{115}In$-conjugated antibodies. At least 3 different regions of interest (ROIs) were assessed for each of the investigated tissue types.

**Results and Discussions**

We compared images for the $^{89}Y$- and $^{115}In$-conjugated antibodies with the images generated using Maxpar® catalog antibodies of the same clones, with a focus on marker specificity and background signal. Compared with the lanthanide-conjugated catalog antibodies, the $^{89}Y$- and $^{115}In$-conjugated antibodies showed equivalent specificity and staining quality.

**Conclusion**

Our results open a new avenue to assign markers to $^{89}Y$ and $^{115}In$, which enables a larger list of potential targets to be investigated in any IMC study. Expanding the number of markers to 40-plus in Imaging Mass Cytometry will improve the imaging results necessary to identify novel cell signatures (phenotype and interactions) in the TME.

### EACR23-0151
### Identifying Pathophysiological Features of Mouse Tumors Using Imaging Mass Cytometry

*R. Spada*[1], *E. Lopez*[1], *Q. Raza*[2], *L. Lim*[2], *T. Pfister*[2], *C. Loh*[2]
[1]*Standard BioTools, Commercial Operations EMEA, Les Ulis, France*
[2]*Standard BioTools, Research and Development, Markham, Canada*

**Introduction**

An obstacle in predicting therapeutic drug efficacy is the ability to quantitatively evaluate the multi-parametric post-treatment response in the tumor microenvironment (TME). Identification of immuno-oncological processes that dictate tumor growth, metastasis, and immune response is essential for selecting promising drug candidates for further clinical evaluation. Imaging Mass Cytometry™ (IMC™) is a vital and proven high-plex imaging technology that enables deep characterization of the complexity and diversity of tumor tissue without disrupting spatial context. The Hyperion™ Imaging System utilizes IMC technology to simultaneously assess 40-plus individual structural and functional markers in tissues, providing unprecedented insight into the organization and function of the TME. Here, we showcase the Maxpar® OnDemand™ Mouse Immuno-Oncology IMC Panel Kit for application on a variety of mouse tumor tissues.

**Material and Methods**

We compiled the antibody panel to quantitatively assess IO-related processes and applied it to a tissue microarray (TMA) containing a large variety of mouse tumors. We digitized high-plex data from mouse tissues using IMC and generated images demonstrating the detailed layout of the TME. We further conducted single-cell analysis to identify specific populations of tumor and immune cells in the TME.

**Results and Discussions**

This approach successfully identified pathophysiological processes such as immune cell infiltration and activation, signaling pathway activation, biomarkers of epithelial-to-mesenchymal transition (EMT), metabolic activity, growth, and the tissue architecture of the TME. Single-cell analysis of several highly relevant tumor types separated distinct cellular clusters representing tumor, immune, stromal, and vascular cells. Activation of cellular processes associated with signaling, growth, and metastasis were identified in tumor cells. In addition, cytotoxic and inflammatory activation in lymphoid and myeloid immune cell subtypes were detected.

**Conclusion**

Application of IMC based multiparametric analysis successfully identified the spatial landscape of the TME at single-cell resolution. Quantitative analysis of tumor composition revealed critical insights regarding prognostic parameters such as metastatic and growth potential of tumors, and identification and activation of immune cell infiltrates. Overall, we demonstrate the power of IMC and provide evidence of its successful application in mouse tumor models.

### EACR23-0184
### Comparison of clinical outcomes among cancer patients treated in and out of clinical trials

*J. Carreno*[1], *B. Carlos*[2], *N. Sanchez*[3]
[1]*Instituto Nacional de Cancerologia, Cancer Clinical Trials, Bogota, Colombia*
[2]*Cancer Treatment and Research Center CTIC, Oncology, Bogota, Colombia*
[3]*Cancer Treatment and Research Center CTIC, Clinical Research, Bogota, Colombia*

**Introduction**

It is unknown if participation in a cancer clinical trial confers clinical benefits to patients. There is not enough scientific evidence in this regard and the available publications are scarce and provide ambiguous and limited information. we compared overall and progression-free survival and response to treatment among those who met the eligibility criteria and accepted to participate and those who refused to participate in cancer clinical trials.

**Material and Methods**

An observational cross-sectional study with an analytical component was carried out, which included patients diagnosed with cancer who participated in phase III clinical trials and patients who, being eligible, refused to participate. The patients were cared for at the National Institute of Cancerology in Colombia between 2019 and 2022. Analysis of differences in proportions and means of sociodemographic and clinical variables was included;

© 2023 The Authors. Molecular Oncology is published by John Wiley & Sons Ltd on behalf of Federation of European Biochemical Societies overall survival and progression-free survival time were described and the survival curves between groups were compared. Variables related to survival were determined using a Cox regression model and Hazard Ratios were calculated.

**Results and Discussions**

112 patients were included (51 participants and 61 refusers). Median progression-free survival for participants was 18.1 months (95%CI 14.0-22.2) vs 24.0 months (95%CI 16.4-31.5) in refusers p=0.23 and median overall survival for participants was 21.9 months (95CI % 17.6-26.1) vs refusers 33.2 months (CI95% 24.0-42.3), for the participating women it was 21.6 months (14.7-28.5 IC95%) vs refusers 22.7 months (9.3-36.0 IC95%) and for the group of participating men was 16.7 months (14.3-19.1 IC95%) and for refusers 32.4 months (25.5-39.3 IC95%). In the Cox proportional hazards model, only ECOG 1 was a better predictor of survival HR 0.48 (95%CI 0.23-0.97) p=0.042.

**Conclusion**

In this research, we observed that the functional status determined by the ECOG was a significant predictor of survival HR 0.48 (CI95% 0.23-0.97) p=0.042.In summary, the results of this research should be taken with caution, considering the limitations of the design, the size of the sample, and the types of tumors that were included; however, these results may contribute to partially reducing the existing uncertainty about the effect of participation in clinical trials on some of the outcomes of cancer patients. Complementary studies are also required to help reduce the gap between the expectations of patients and the effect of participation on health status.

### EACR23-0188
### Clinical profiling of anticancer genes and targeting by a ribosomal inactivating plant protein (riproximin) in breast and colorectal cancers

*A. Pervaiz[1], K. Umer[1], M.R. Berger[2]*
*[1]University of Health Sciences, Institute of Biomedical and Allied Health Sciences, Lahore, Pakistan*
*[2]German Cancer Research Centre DKFZ- Heidelberg University, Toxicology & Chemotherapy Unit, Heidelberg, Germany*

**Introduction**

Anticancer genes are endogenous enemies of cancer cells and impose antineoplastic effects upon ectopic expression. Identifying the expression patterns of these genes is inevitable to explore their prognostic and therapeutic relevance in cancer cells. In this study, we identified the expression levels of three anticancer genes (NOXA, PAR-4, TRAIL) in breast and colorectal cancers. Additionally, potential of a purified/sequenced plant protein (riproximin) was evaluated for targeting the anticancer genes in breast and colorectal cancer cells.

**Material and Methods**

Expression profiles of anticancer genes were identified by immunohistochemistry and real-time PCRs methodologies in breast and colorectal cancer clinical isolates after due ethical approvals. Effects of the plant protein (riproximin) exposure on expression of the anticancer genes were identified via real-time PCRs in breast (MDA-MB-231, MCF-7) and colorectal cancer (SW480, SW620, HCT116) cell lines.

**Results and Discussions**

NOXA expression was evenly de-regulated, while PAR-4 was substantially down-regulated in majority of the breast and colorectal clinical isolates. TRAIL expression was not de-regulated uniformly in the two malignancies as shown by a dominant inhibition and up-regulation in colorectal and breast cancer tissues, respectively. Riproximin showed a substantial potential of inducing the anticancer genes in all selected breast and colorectal cancer cell lines at the transcriptomic levels.

**Conclusion**

Anticancer genes are de-regulated in cancer tissues and shows distinct expression patterns in breast and colorectal cancer patients. Riproximin can effectively induce the expression of selected anticancer genes in cancer cells and need due attention for therapeutic purposes.

### EACR23-0195
### Immune cell infiltration and sidedness in colon cancer.

*Y. Iliev[1]*
*[1]UMBAL Pulmed, Medical oncology, Ploviv, Bulgaria*

**Introduction**

Colorectal cancer (CRC) is a leading cause of cancer mortality worldwide. For 2020 it is third in terms of new cases per year- 1.9 million, but second in terms of mortality- 930 000 deaths per year.The World Health Organization (WHO) introduced the immune response at the tumor site, as a new essential and desirable diagnostic criterion for localised CRC alongside standard histological parameters, in the 5$^{th}$ edition of digestive tumors classification reference book for pathologists.Immunoscore®(IS) is standardized immune-based consensus assay, available for routine clinical management. Its prognostic value has been confirmed in International validation study in stage I-III colon cancer. Sidedness is an independent prognostic factor. Many studies have confirmed the prognostic and predictive power of tumor sidedness.

The purpose of this study is to elucidate the correlation between immune cell infiltration,quantitatively measured by Immunoscore® and sidedness.

**Material and Methods**

Single center retrospective study of patients ≥18 years old with stage II or stage III non-metastatic colon cancer who underwent a curative-intent surgery and who are potentially eligible to adjuvant chemotherapy. FFPE(formalin-fixed paraffin-embedded) tumor samples from each patient were retrospectively collected. Densities of CD3+ and CD8+ T cells in the tumor and invasive margin were determined by immunohistochemistry . Results are converted with predefined thresholds to Immunoscore®. Quantitatevely are divided in 5 groups: (IS-0,IS-1,IS-2,IS-3,IS-4) from low Immunoscore (I0) to high Immunoscore (I4).The Immunoscore® testing was done in laboratory HalioDx,Marseille,France,blinded for clinical data.

The patient's clinical data were analyzed for stage,

© 2023 The Authors. Molecular Oncology is published by John Wiley & Sons Ltd on behalf of Federation of European Biochemical Societies sidedness, DFS(disease free survival),postoperative treatment,levels of tumor markers,age and gender.

**Results and Discussions**

36 patients samples were tested. Total 29 patients had validated results from Immunoscore® and were included in this analysis.

There is difference in Immunoscore® results in left- versus right-sided tumors.

Total there are 16 patients with left colon cancer with median Immunoscore® -1.94(1,44-2,43).

There are 13 patients with right colon cancer with median Immunoscore® -2.23(1,79-2,67).

**Conclusion**

There is association of Immunoscore® and primary tumor sidedness, even in small patients number. We can hypothesize that this could be one of reasons for different prognosis and survival of left and right sided tumors.

### EACR23-0226

### Co-clinical studies reveal an association between NKX3.1 expression, oxidative stress, and metformin response in prostate cancer

*A. Papachristodoulou[1], I. Heidegger[2], R. Virk[3], M. Di Bernardo[1], C. Laplaca[1], M. Loda[4], J. McKiernan[5], A. Dutta[1], C. Abate-Shen[1]*

*[1]Columbia University Irving Medical Center, Department of Molecular Pharmacology and Therapeutics, New York, United States*

*[2]Medical University Innsbruck, Department of Urology, Innsbruck, Austria*

*[3]Columbia University Irving Medical Center, Department of Pathology and Cell Biology, New York, United States*

*[4]Weill Medical College of Cornell University, Department of Pathology and Laboratory Medicine, New York, United States*

*[5]Columbia University Irving Medical Center, Department of Urology, New York, United States*

**Introduction**

Prostate cancer is the most prevalent and second leading cause of cancer death in men worldwide. Our previous work has shown that the prostate-specific homeobox gene, *NKX3.1*, protects the prostatic epithelium from cancer-related mitochondrial oxidative stress, while its reduced expression, prevalent in early-stage prostate cancer, is associated with increased risk of progression to lethal disease and impaired response to preventative interventions. In the current study, we asked whether NKX3.1 expression and function associate with response to metformin, a widely used anti-diabetic drug with known anti-oxidative activities in other cancers and, whether intervention with metformin could provide an early treatment to suppress prostate cancer.

**Material and Methods**

We performed co-clinical studies using genetically engineered mouse models, human prostate cancer cells and tissues with retrospective clinical information. In particular, we analyzed *Nkx3.1* mutant mice, a model of early-stage prostate cancer, the LNCaP prostate cancer cells as well as, human prostate tissue organotypic slices to determine the effect of metformin for suppression of oxidative stress and prostate tumorigenesis. Tissue microarrays (TMA) from radical prostatectomy specimens of prostate cancer patients who received metformin due to diabetes mellitus type II were examined to determine the association of NKX3.1 expression and metformin response for clinical outcome.

**Results and Discussions**

Our co-clinical analyses demonstrate a functional and clinical association between NKX3.1 expression, mitochondrial oxidative stress, and metformin intervention in prostate cancer. Specifically, we found that metformin reduces oxidative stress and suppresses cancer progression in the prostates of *Nkx3.1* mutant mice. Additional studies in human prostate cancer cells and organotypic assays showed that metformin can restore mitochondrial function and inhibit tumorigenicity in the context of low or absent NKX3.1 expression. Finally, TMA analyses revealed that metformin treatment improved biochemical recurrence-free estimated survival only in patients with low NKX3.1 expression, suggesting that NKX3.1 expression status may act as a predictor of metformin response in prostate cancer.

**Conclusion**

Our data identifies metformin as a promising treatment to abrogate tumor-promoting mitochondrial oxidative stress in the absence of NKX3.1 and suggests that prostate cancer patients with low NKX3.1 expression status are more likely to benefit from metformin intervention.

### EACR23-0267

### Development of a Glioblastoma in the Chick Embryo Tumour Model with the Application of Raman for Gold Nanoparticle Detection

*S. McCabe[1], H. Gardiner[1], C. Mullen[1], L. Horan[1], A. Mullen[1], K. Faulds[2], M. Boyd[1]*

*[1]University of Strathclyde, Strathclyde Institute of Pharmacy and Biomedical Sciences , Glasgow, United Kingdom*

*[2]University of Strathclyde, Pure and Applied Chemistry, Glasgow, United Kingdom*

**Introduction**

Glioblastoma is an aggressive, high grade brain cancer in need of novel therapies. Patients who are usually treated with radio-chemotherapy suffer side effects caused by the inability to specifically identify tumour margins due difficulty in distinguishing between cancerous and non-cancerous tissues using conventional imaging. Researchers have been investigating the use of the chorioallantoic membrane (CAM) for a variety of *in vivo* studies to reduce and replace rodent studies. The chick embryo has a lower sentience than rodents, which is a step towards reducing suffering, is cheaper and potentially has higher throughput than murine models. The CAM is a highly vascularised membrane that forms around the chick embryo and develops from day 3 to day 14 of embryonic development. Gold nanoparticles (AuNPs) are advantageous in cancer therapy due to their optical and physical properties. In this study, AuNPs were designed to target Tenascin-C overexpressed in U87-MG glioblastoma by addition of a Tenascin-C antibody. Surface enhanced spatially offset Raman spectroscopy (SESORS) is an

© 2023 The Authors. Molecular Oncology is published by John Wiley & Sons Ltd on behalf of Federation of European Biochemical Societies analytical technique used to detect Raman scattered photons at depth with the aim to introduce non-invasive detection of analytes, such as AuNPs, below a surface in cancer nanomedicine

**Material and Methods**

U87-MG cells were resuspended in a mixture of PBS and matrigel (50:50) following windowing to create an accessible area on the CAM. Trauma was induced to a blood vessel and trypsin was added to aid in cell invasion. The cells were then added to the CAM and were incubated for 6 days. AuNPs were synthesised using a Raman reporter and detected using handheld SORS instrument.

**Results and Discussions**

We report for the first time the successful tumour growth of U87-MG cells on the CAM with tumours derived on day 13 after 7 days growth. Following termination of live embryos using a schedule one technique the tumour was excised, fixed in paraformaldehyde, and embedded in wax. Sections were collected to use immunohistochemistry and determine the Tenascin-C expression throughout the tumour. SESORS detection was applied to chick embryo tumour bearing AuNPs in situ with subsequent layers of tissue added to achieve optimal depth detection through tissue

**Conclusion**

We have developed a method for the implantation of U87-MG cells to the CAM of the chick embryo for tumour development. We have use the model to assess distribution of AuNPs and for the first time applied SESORS to successfully detect AuNPs conjugated with a Tenascin-C antibody through tissue

### EACR23-0280

### Dissecting the 3D invasive properties of cancer cell lines and patient primary tumour cells and the impact of stromal cell co-culture

*D. Roy Chowdhury[1], C. Amarkanth[1], M. Mitsi[2], R. Urbanet[2], B. Simona[2], A. Roy Chowdhury[1]*
*[1]Mestastop Solutions Pvt. Ltd., Core R&D, Bangalore, India*
*[2]Ectica Technologies AG, Research and development, Zurich, Switzerland*

**Introduction**

The node status of a primary tumour patient having no clinical metastasis is currently used to predict metastasis probability. However, it is not always accurate, with node-negative patients developing metastasis and node-positive patients not reporting metastasis. Therefore, it is imperative to create better predictive metastasis diagnostics to help identify high-risk patients.

**Material and Methods**

Our previous work has helped identify the critical rate-limiting steps of metastasis. Two essential steps are the initial dissemination ability from the primary tumour and the final colonization ability to form the secondary tumour. In this study, we created a baseline of dissemination behaviour of multiple epithelial carcinoma cell lines across different tumour tissue types. We used a proprietary synthetic hydrogel precast in imaging plates, 3DProSeed®, made of PEG bioconjugates and free of animal proteins. The hydrogel offers significant control over the culture conditions and underlying biology. After creating baseline data with cell lines, we tested multiple primary patient tumour samples on this in vitro 3D model, identifying two variables that drive successful dissemination.

**Results and Discussions**

2D migration and invasion data helped us identify the most disseminating lines amongst twelve CRC, TNBC and H&N cell lines. Interestingly, this data could not explain their metastatic behaviour as some non-metastatic cell lines showed high migration and invasion, whereas a few metastatic samples showed low migration and invasion. 3DProSeed® analysis of the same cell lines suggested that it was essential to understand the microspheroid formation ability of the cell lines along with their dissemination. A cell line's ability to disseminate (D) was directly proportional to its invasiveness (I) and inversely proportional to its spheroid formation ability (SFc). Analysis of patient samples on the said criteria and subsequent follow-up survival data suggests the appropriateness of this evaluation. Ongoing work aims to improve our understanding by integrating colonization information in the secondary microenvironment. This is achieved by using a stromal microenvironment in 3DProseed® that is formed prior to the addition of cancer cells.

**Conclusion**

We now present a method that could help evaluate the dissemination ability of primary tumour cells and might eventually help understand their summative metastatic ability, once integrated with the colonization data.

### EACR23-0290

### Imaging efficacy of a novel integrin-targeting fluorescent contrast agent during fluorescence-guided surgery in dogs with spontaneous tumors

*A. Cordaro[1], C. Brioschi[2], G. Valbusa[3], F. Blasi[2]*
*[1]Bracco Imaging SpA, BioImaging department, Colleretto Giacosa TO, Italy*
*[2]Bracco Imaging SpA, Early Stage Innovation, Colleretto Giacosa TO, Italy*
*[3]Bracco Imaging SpA, Digital Innovation, Colleretto Giacosa TO, Italy*

**Introduction**

Despite many recent improvements in the treatment of cancer, surgery remains the most effective therapeutic strategy for the majority of patients with solid tumors. Tissue palpation, visual inspection, and real-time frozen section analyses are commonly used by surgeons to identify the tumor during resection. Recurrence due to incomplete tumor resection is one of the major causes of death in human and canine cancer patients. Fluorescence-guided surgery is a novel technology that may facilitate surgical resection of solid tumors through real-time visualization of contrast agents that accumulate in tumor tissue.Integrins are an attractive cancer biomarker for targeted imaging-guided surgery. In particular, $\alpha_v\beta_3$ integrin is usually expressed at low or undetectable levels in most adult epithelia, but it is highly up-regulated in tumors and correlates positively with disease progression. This study employed a novel $\alpha_v\beta_3$-integrin

© 2023 The Authors. Molecular Oncology is published by John Wiley & Sons Ltd on behalf of Federation of European Biochemical Societies targeting near-infrared fluorescent (NIRF) contrast agent, intended for intraoperative use during tumor resection.

**Material and Methods**

The novel contrast agent was administered intravenously to dogs with superficial (cutaneous and subcutaneous) solid tumors. NIRF imaging was performed during surgery and on excised specimens to evaluate fluorescence intensities of tumor and adjacent (healthy) tissues. During surgery, additional wound bed tissue was excised if residual fluorescence was present after standard-of-care resection. Routine histopathology, immunohistochemistry and proteomic analyses were performed on further dissected specimens.

**Results and Discussions**

Thirty-two superficial solid tumors were removed in 24 client-owned dogs after administration of the probe. No side effects were observed. The mean intra-operative tumor-to-background ratio for mammary tumors, mast cell tumors and sarcomas was 2.2 ± 1.0, 2.5 ± 1.7, and 3.5 ± 1.3, respectively. After primary tumor resection, fluorescence signal was detected in 4 wound beds that contained residual disease (true positive) confirmed by histopathology. *Ex vivo*, the mean tumor-to-margin ratio for mammary tumors, mast cell tumors and sarcomas was 3.3 ± 1.6, 4.4 ± 3.7, and 3.3 ± 3.0, respectively. A greater contrast was detected in resected metastatic lymph nodes than in non-metastatic ones.

**Conclusion**

The novel contrast agent demonstrated safe and positive imaging performance during fluorescence-guided surgery in dogs with superficial tumors. Larger clinical trials are warranted for further evaluation of the contrast agent.

### EACR23-0293
### Identification of cellular targets and physiological modifications in the post-surgical microenvironment of glioblastoma toward the development of tailored treatments to avoid recurrences

*C. Bastiancich[1], E. Snacel-Fazy[2], S. Fernandez[3], S. Robert[4], B. Guillet[3], F. Debarbieux[5], R. Cavalli[1], E. Tabouret[2], M.A. Estève[2], A. Tchoghandjian[2]*

*[1]Università degli Studi di Torino, Department of Drug Science and Technology, Torino, Italy*

*[2]Aix Marseille Université, Institut de Neurophysiopathologie, Marseille, France*

*[3]Aix Marseille Université, Centre Européen de Recherche en Imagerie Médicale, Marseille, France*

*[4]Aix Marseille Université, Centre de Recherche Vasculaire de Marseille, Marseille, France*

*[5]Aix Marseille Université, Institut des Neurosciences de la Timone, Marseille, France*

**Introduction**

Glioblastoma (GBM) is an incurable primary brain tumour. Most GBM patients undergo surgery prior to chemoradiation, but recurrences inevitably lead to patient death. The brain physiological regenerative responses following tumor debulking have a beneficial role in the healing process. However, they also evoke characteristic time-dependent peritumoral immune responses which can promote the formation of recurrences. The crosstalk between glial, immune and GBM cells at the surgical borders and their impact on recurrences still lacks appropriate characterization. This project aims at dissecting the GBM post-surgical microenvironment (SMe) over time and space to identify therapeutic targets and develop tailored GBM therapies to avoid the onset of recurrences.

**Material and Methods**

We developed a tumor resection model in transgenic mice bearing GL261-DsRed tumors and established a chronic intracranial window post-surgery for biphotonic imaging. Nuclear imaging was performed at defined time points using $^{99m}$Tc-DTPA, $^{68}$Ga-RGD and $^{18}$F-FDG to evaluate neuroinflammation, neoangiogenesis, infiltrating tumor cells metabolism and BBB permeability. The dynamics of the inflammatory landscape following surgery was characterized by blood sampling and post-mortem analysis on brain samples (peripheral and local immunophenotyping by multiparametric flow cytometry; brain clearing and ultramicroscopy).

**Results and Discussions**

We analyzed the dynamics of recruitment and localization of immune cells coming to the surgical site from the brain parenchyma or from the periphery, as well as blood vessels, cell morphologies and motilities from surgery to recurrence. BBB disruption was observed post-surgery followed by a recovery within three days. Based on the results obtained we finally tested a therapeutic approach combining the local administration of lauroyl-gemcitabine lipid nanocapsules hydrogel with the repeated systemic administration of a SMAC-mimetic drug showing an increase in survival of GBM-bearing mice.

**Conclusion**

This comprehensive study expands the knowledge on the SMe by analyzing the impact of BBB disruption on immune cells recruitment and the role of lymphoid and myeloid populations - as well as macrophages and microglia - on the onset of tumor recurrences. Moreover, it proposes a rationally designed combinatory treatment able to target the identified cellular targets to delay or inhibit the onset of recurrences.

### EACR23-0310
### Galectins 1,4 and 9 Circulating levels in Gastric Adenorcinoma Patients: Determination of accuracy parameters and clinical association

*M. Rêgo[1], M.D.F. Souza[1], M. Pitta[1], S.F. Antonio[2], L. Lima[2], P. Michelly[3]*

*[1]Federal University of Pernambuco, Biochemistry, Recife, Brazil*

*[2]UPE, Garanhuns Campus, Garanhuns, Brazil*

*[3]Federal University of Pernambuco, Pharmacology and Physiology, Recife, Brazil*

**Introduction**

Gastric Adenocarcinoma (GA) is an aggressive malignancy showing a poor survival rate in advanced stages patients. GA serum markers have been shown poor accuracy parameters which limits their clinical application. Galectins are β-galactosides biding proteins with key roles in tumorigenesis and immunosurveillance. In this sense, the present study aimed to evaluate galectins-1, 4 and 9 circulating level and their association with

© 2023 The Authors. Molecular Oncology is published by John Wiley & Sons Ltd on behalf of Federation of European Biochemical Societies clinicopathological characteristics and accuracy parameters.

**Material and Methods**

Sixty-nine GA patients from the oncology service of Hospital das clinicas from Federal University of Pernambuco (UFPE) and 67 healthy subjects were included in the study. Circulating levels of those galectins were determined by ELISA. Receptor operating characteristics (ROC), sensitivity, specificity, and likelihood ratio (LR) were plotted using GraphPad Prisma software.

**Results and Discussions**

Galectins-1, -4 and -9 circulating levels were higher in patients with gastric adenocarcinoma compared to healthy subjects. Galectin-1 cut-off was 22935 pg/ml presenting 96.08% of specificity, LR:15.79, AUC: 0, 9153, (p< 0.0001). Circulating levels of galectin-1 were also associated with angiolymphatic invasion (p = 0.0496). Galectin-4 cut-off was 572.3 pg/ml showing a sensitivity of 98.55%, specificity of 84.62%, LR: 6.406 and AUC of 0.9632, (p<0 .0001). On the other hand galectin-9 cut-off point to distinguish the groups evaluated was 5517pg/ml (sensitivity 80.60%, specificity 97.01%, LR: 27.00) (AUC: 0.9414, p<0.0001).Circulating levels of galectin-9 were associated with sex (p = 0.0033), age (p = 0.0158) and histological grade (p = 0.0013). In addition, Gal-9 levels showed a moderate correlation with the absolute number of neutrophils in GA patients (r =0.4228; p = 0.0053)

**Conclusion**

Our findings showed that all evaluated Galectins have presented good accuracy parameters capable of distinguishing patients with gastric adenocarcinoma from healthy individuals. Also Galectin-1 and 9 showed association with angiolymphatic invasion and histological grade, respectively. Thus, these molecules emerge as potential GA markers.

### EACR23-0322

### A Suppressed Immune Activation Denotes an Aggressive Subset of High Budding Tumours in Early-stage Colorectal Cancer

*N. Fisher[1], K. Redmond[1], L. van der Schee[2], M. Loughrey[3], S. Richman[4], S. Leedham[5], D. Adams[6], L. Moons[7], M. Laclé[2], P. Dunne[1]*

*[1]Queen's University Belfast, Patrick G. Johnston Centre for Cancer Research, Belfast, United Kingdom*

*[2]University Medical Centre, Department of Pathology, Utrecht, Netherlands Antilles*

*[3]Royal Victoria Hospital- Belfast Health and Social Care T rust-, Department of Cellular Pathology, Belfast, United Kingdom*

*[4]St. James' University of Leeds, Leeds Institute of Medical Research, Leeds, United Kingdom*

*[5]University of Oxford, Wellcome Trust Centre for Human Genetics, Oxford, United Kingdom*

*[6]Wellcome Genome Campus, Wellcome Sanger Institute, Cambridge, United Kingdom*

*[7]University Medical Centre, Department of Gastroenterology and Hepatology, Utrecht, Netherlands Antilles*

**Introduction**

Accurate understanding of prognostic features, both histologically and biologically, is essential to enable identification, stratification and ultimately treatment of aggressive tumours. Tumour budding (TB) is the histological manifestation of local tumour cell dissemination and a poor prognostic feature in early-stage colorectal cancer (CRC). This is particularly timely, given the increased number of early-stage patients diagnosed since the introduction of the bowel cancer screening (BCS) programme, with stage I CRC accounting for 42.1% of BCS-detected tumours, compared to approximately 20% outside screening. Despite the prognostic value of TB, not all high TB tumours develop lymph node metastatic disease (LNMD), therefore further characterisation of high TB tumours with LNMD compared to those without LNMD is needed. The aim of this study was to understand the biology driving dissemination, ultimately leading to LNMD.

**Material and Methods**

A subset of CRC T1 tumours (n=249) from within the STONE (Studies on T1 CRC) cohort of the Dutch T1 CRC Working Group, were selected for extensive molecular and histological characterisation, to identify the biology driving early-dissemination. The cohort is comprised of surgical resections with definitive nodal status, enriched for aggressive tumours (n=64 with LNMD; n=185 without LNMD). Profiling includes 3' RNA sequencing, whole exome sequencing, low pass whole genome sequencing for copy number alterations, alongside representative histology with digital pathology assessment.

**Results and Discussions**

TB has been assessed manually (Bd1:117; Bd2:31; Bd3:21) and using a semi-automated approach. Alignment of the transcriptional data to molecular subtypes revealed a high TB severity was associated with Consensus Molecular Subtypes (CMS) ($p=1.2x10^{-6}$) with the stroma-rich CMS4 associated with Bd3 (5.6; $p=4.7x10^{-7}$), and wider biology indicative of a higher tumour stroma ratio. Preliminary assessments of high TB tumours with LNMD compared to those without LNMD revealed an association between the presence and extent of innate immune activation. Furthermore, stratification of a stage II CRC cohort based on innate immune activation status was sufficient to identify stroma-rich CRCs most likely to relapse following surgery.

**Conclusion**

Differentiating lethal early-stage cancer that require further clinical intervention from those non-lethal cancers that do not is a key priority, with the T1 cohort described here enabling further characterisation of the biology driving tumour dissemination.

### EACR23-0356

### Metastasis-On-A-Chip: A Microfluidic Device for Efficient Capture and Drug Screening of Cancer Cells

*A. Wong[1], M. Tang[2]*

*[1]University of Hong Kong, School of Biological Sciences, Hong Kong, Hong Kong- China*

*[2]MetTactics Limited, Research and Development, Hong Kong, Hong Kong- China*

**Introduction**

Metastatic cancer cells are responsible for the majority of cancer-related deaths. However, conventional methods for studying these cells, such as transwell migration or invasion assays and static adhesion assays, do not always

© 2023 The Authors. Molecular Oncology is published by John Wiley & Sons Ltd on behalf of Federation of European Biochemical Societies reflect the *in vivo* conditions due to tumor heterogeneity and the lack of physiologically relevant conditions. Capturing of the truly metastatic cancer cells therefore represents a major challenge for research, drug screening and precision medicine.

**Material and Methods**

To address these, we aim to develop a microfluidic device to resemble the fluidic tumor microenvironment of the peritoneal cavity *in vitro*. With precise fluid flow control, this PDMS-based device allows for real-time monitoring of cancer cell adhesion as well as capturing of those adhered cells.

**Results and Discussions**

Cells captured by this device showed increased adhesion potential of the captured cells onto the omentum, a common metastatic site for peritoneal cancers, in an *in vivo* adhesion assay in mice, when compared to the non-captured counterparts. Additionally, in a pilot drug screening, we found that two compounds showing adhesion inhibition in static adhesion assay were non-inhibitory in the dynamic adhesion assay using our device. More importantly, these two compounds were also non-inhibitory in the *in vivo* adhesion assay, suggesting that microfluidic device could better predict *in vivo* drug response than the traditional static adhesion assay by more closely mimicking the *in vivo* conditions.

**Conclusion**

Overall, the ability of our device to capture the truly metastatic cancer cells in a more clinically relevant manner make it an ideal tool for drug screening and precision medicine in cancer research.

### EACR23-0365
### Upconversion nanoparticles as labels for histopathological tissue evaluation

*K. Krawczyk[1], M. Mickert[1], K. Christiansson[2], S. Andersson-Engels[3], O. Andersson[1]*

*[1]Lumito AB, Research and Development, Lund, Sweden*

*[2]Lumito AB, Marketing, Lund, Sweden*

*[3]Tyndall National Institute, Biophotonics@ Tyndalli, Cork, Ireland*

**Introduction**

For decades, haematoxylin and eosin (H&E) stains together with a horseradish peroxidase (HRP) label and diamino benzidine (DAB) as a chromogenic substrate, have been the gold standard to visualise tissue morphology and to detect markers of interest. However, these methods suffer from a narrow dynamic range, difficulties in quantification and limited possibilities regarding multiplexing. Fluorescent IHC techniques open the possibility for a quantitative readout but suffer from photobleaching and spectral overlapping emission bands in multiplexed applications.

Here we present an upconversion nanoparticle (UCNP) based technique to visualise the breast cancer marker HER2 in tissue sections, that allows to overcome problems associated with commonly used labelling techniques.

**Material and Methods**

Formalin-fixed paraffin-embedded breast cancer cell line and human breast cancer tissue were sectioned and labelled. Upconversion imaging of the human tissue sections was conducted in our prototype device and compared with a standard DAB-based IHC. The combination of UCNP and haematoxylin counterstaining on the same slide was investigated.

**Results and Discussions**

Images obtained with our novel device demonstrate that our UCNP bioconjugates are excellent labels for the detection of cancer markers in tissue sections. Brightfield images prove that UCNPs do not interfere with the standard tissue evaluation by a pathologist. Additionally, brightfield and luminescent images can be merged to provide a better understanding of tissue morphology.

**Conclusion**

The emerging field of UCNP-based labeling techniques provides new possibilities for more accurate diagnosis. Staining solutions and a novel device developed by us keep the advantage of haematoxylin staining and combine it, in one image, with the UCNP luminescent data. The high-contrast images of the UCNP labeling – generated by our scanning device – set the foundation for generating ground truth for machine learning algorithms.

### EACR23-0369
### Neoantigen Based Therapeutic Cancer Vaccines: Rationale, Practice and Perspectives in Endometrial Cancer

*A. piermattei[1], E. De Paolis[2], A. Minucci[2], C. Nero[1], E. Micarelli[3], G. Leoni[3], G.F. Zannoni[1], G. Scambia[1], A. Urbani[2], F. Fanfani[1]*

*[1]Fondazione Policlinico Universitario Agostino Gemelli, Scienze della salute della donna- del bambino e di sanità p ubblica, Rome, Italy*

*[2]Fondazione Policlinico Universitario Agostino Gemelli, Dipartimento di scienze di laboratorio e infettivologiche, Rome, Italy*

*[3]Nouscom S.R.L, Via di Castel Romano-100, Rome, Italy*

**Introduction**

Endometrial cancer (EC) is one of the most prevalent gynecological tumors affecting women. The prognosis of recurrent or metastatic EC is poor. Molecular classification system identified four distinct EC subtypes: mismatch repair-deficient (dMMR), polymerase ε–hypermutated, p53-abnormal and p53-wild-type. EC with dMMR typically exhibit the Microsatellite Instability High (MSI-H) phenotype which results in high somatic mutation rates. Insertions/deletions (*indels*) dMMR-related mutational events in microsatellites of coding genes can result in the synthesis of "shared frameshift peptides" (FSPs) that are expected to be very strong and safe neoantigens (nAgs).

**Material and Methods**

Samples from 35 patients with a histological diagnosis of primary EC with MSI-H from 'Fondazione Policlinico Universitario A. Gemelli-IRCCS' were enrolled. The MSI status was defined according to immunohistochemistry results. DNA was extracted from tissue slides using MagCore Genomic DNA FFPE One-Step kit (RBC Bioscience®). Whole Exome Sequencing was carried out using the Illumina DNA Prep with Enrichment (S) Tagmentation kit on the Next Generation Sequencing NovaSeq6000® platform (Illumina®). FSPs were compared to the the previously validated pool of nAgs included in the Nous-209 vaccine at present under evaluation in metastatic MSI solid tumours by Nouscom® (NCT04041310).

**Results and Discussions**

© 2023 The Authors. Molecular Oncology is published by John Wiley & Sons Ltd on behalf of Federation of European Biochemical Societies

Of the 35 patients with dMMR, 4 showed an absence of protein expression for hMLH1, 20 for hMLH1/PMS2, 1 for hMSH2/hMSH6, 2 for hMSH6, 7 for PMS2, and 1 for hMSH2 alone. The comparative analysis between the EC FSPs and the validated Nous-209 genetic polyvalent vaccine helped to identify a relevant number of nAgs as optimal candidates for cancer vaccination. The sequencing data analysis revealed the presence of a median 16 [range 1-45] FSPs out of the 204 encoded by the Nous-209 vaccine. A total of 48 FSPs were shared in more than 10% of patients. Moreover, samples of our series that showed loss of expression of hMLH1 or hMLH1/PMS2 showed a higher number of FSPs (21 FSPs). Several potential *indels* leading to FSPs have been reported as well as the identification of a molecular-defect-based behaviour in nAgs enrichment and demonstration of the feasibility of the proteogenomic approach in nAgs prediction and targeting for immunotherapy.

**Conclusion**

nAgs have a special value combined as biomarker-driven therapy to novel drug combinations resulting in a novel therapeutic approach that could remodel the management of malignant gynecological cancers.

### EACR23-0370

### A multicellular organoid approach to model the tumour microenvironment of pancreatic cancer

*V. Kast*[1], *A. Nadernezhad*[1], *D. Pette*[1], *A. Gabrielyan*[1], *M. Fusenig*[1], *K.C. Honselmann*[2], *D.E. Stange*[3], *C. Werner*[1], *D. Loessner*[4]

[1]*Leibniz Institute of Polymer Research Dresden e.V., Max Bergmann Centre of Biomaterials, Dresden, Germany*

[2]*University Medical Center Schleswig-Holstein- Campus Lübeck, Department of Surgery, Lübeck, Germany*

[3]*University Hospital Carl Gustav Carus- Medical Faculty- Technical University Dresden, Department of Visceral- Thoracic and Vascular Surgery, Dresden, Germany*

[4]*Monash University, Department of Chemical and Biological Engineering and Department of Materials Science and Engineering-, Melbourne, Australia*

**Introduction**

Pancreatic cancer is a devastating disease with limited therapeutic options. The success of standard-of-care therapy is unsatisfactory, and immunotherapy has been thus far unsuccessful in clinical trials. Response to therapy is dictated by the tumour microenvironment (TME). Excessive amounts of extracellular matrix (ECM) molecules and immune cells accumulate and form a stiff and immunosuppressive TME that acts as a physical barrier for drug delivery. There is a lack of pre-clinical 3D models for pancreatic cancer that integrate different elements of the TME to study response to therapy. To address this limitation and explore the effects of immunotherapy in combination with chemotherapy, we developed a fully defined, biomimetic 3D cancer model using a protease-sensitive star-shaped poly(ethylene glycol) (star-PEG)-heparin hydrogel matrix.

**Material and Methods**

ECM mimicking starPEG-heparin hydrogels were formed, and human pancreatic cancer cells were grown encapsulated with patient-derived cancer-associated fibroblasts and myeloid cells, mimicking the cellular TME. Hydrogels were characterised by rheology and various high-resolution microscopy techniques. By applying our TME model, we assessed cell responses toward the novel CD11b agonist ADH-503 in combination with anti-PD-1 immunotherapy and chemotherapy. Cell viability was analysed by live/dead staining, flow cytometry, and metabolic activity and cell proliferation assays. Changes in biomechanics and cytokine secretion were determined.

**Results and Discussions**

The versatility of the starPEG-heparin hydrogels allowed us to precisely tune the mechanical properties of the matrix, resulting in covering the entire tissue stiffness range of 4.9-17.2 kPa, which is in line with reported data for patient-derived tissues. Over the analysed time frame, cells populated the hydrogel matrix and proliferated, forming multicellular cancer spheroids. Myeloid cells differentiated into macrophages without external stimuli, highlighting the presence of cancer-immune cell interactions. Using our TME model as a pre-clinical drug testing platform, 3D cultures were challenged with the CD11b agonist ADH-503 in combination with anti-PD-1 and chemotherapy. This treatment regimen significantly reduced cancer cell viability and cancer-promoting cytokine secretion and modulated the tumour-immune landscape.

**Conclusion**

We provide a novel, rationally designed, multicellular 3D cancer model based on starPEG–heparin, which allows the screening of immunomodulatory therapeutic strategies.

### EACR23-0395

### Synergism between methotrexate and modulated electro-hyperthermia is mediated by H19 lnc-RNA

*C. Schvarcz*[1,2], *L. Danics*[1], *G. Forika*[3], *P. Viana*[1], *T. Krenács*[3], *Z. Benyó*[1], *T. Vancsik*[3], *P. Hamar*[1]

[1]*Semmelweis University, Institute of Translational Medicine, Budapest, Hungary*

[2]*Eötvös Loránd Research Network and Semmelweis Univer sity (ELKH-SE) Cerebrovascular and Neurocognitive Disorders Resea rch Group, Budapest, Hungary*

[3]*Semmelweis University, Department of Pathology and Experimental Cancer Resear ch, Budapest, Hungary*

**Introduction**

Modulated electro-hyperthermia (mEHT) is a novel antitumor therapy, with strong tumor-progression inhibiting potential according to in vitro, in vivo experiments and clinical studies in monotherapy and as complementary therapy as well. Implantation of 4T1 cancer cells into immunocompetent mice serves as a reliable model for triple negative breast cancer (TNBC). H19 long non-coding RNA promotes breast cancer cell invasion, migration and metastasis.

**Material and Methods**

In-vitro 4T1 spheroids were treated with mEHT for 30 minutes. In-vivo TNBC cells (4T1, 4T07) were inoculated orthotopically in female BALB/c mice. Tumor growth was

© 2023 The Authors. Molecular Oncology is published by John Wiley & Sons Ltd on behalf of Federation of European Biochemical Societies monitored in vivo by digital caliper and ultrasound. Mice were randomized into groups treated with mEHT monotherapy or in combination with methotrexate (MTX) 2 or 3 times for 30 minutes. H19 expression was measured with RT-PCR from dissected tumors. Histological evaluation of tumor sections were performed by hematoxylin-eosin (H&E) and cleaved caspase-3 (cC3) immunohistochemistry stainings.

**Results and Discussions**

H19 expression correlated with tumor aggressiveness (4T07:0.006±0.004 vs 4T1:0.4±0.07, p<0.0001). Single mEHT treatment of 4T1 spheroids reduced H19 expression vs normothermic control (Ctrl: 0.004±0.0004, mEHT: 0.0006±0.0002, p<0.0001). In vivo, MTX diminished metastatic burden (meta/lung area: Ctrl: 5.942±4.500 %, MTX: 1.723±1.138 %). mEHT and MTX significantly reduced tumor size and weight in monotherapy and combination of mEHT with MTX demonstrated synergistic effectiveness. mEHT but not MTX induced apoptosis (cC3+ area: sham: 18.8±11.4, mEHT: 50.9±26.2, MTX: 15.4±9.5, mEHT+MTX: 57.6±13.5%, p<0.0001). Tumor size reduction was accompanied by reduction of H19 expression (sham: 0.068±0.044, mEHT: 0.033±0.024, MTX: 0.104±0,038, mEHT+MTX: 0.056±0.025, p<0.01). H19 reduction peaked 12 hours after mEHT, recovering slowly by 48-72 hours. Furthermore, MTX induced H19 overexpression was reversed by cotreatment with mEHT (sham: 0.16±0.07, mEHT: 0.11±0.06, MTX: 0.21±0.08, mEHT+MTX: 0.11±0.05, p<0.05).

**Conclusion**

MTX and mEHT synergize each other's tumor-killing effect, making their combination a potential therapeutic option. The observed synergism may be based on 1) different mechanisms of action: mEHT killing viable cells through apoptosis induction and MTX killing proliferating cells, and 2) inhibition of H19 lncRNA involved in both apoptosis and cancer cell proliferation.

*Funding: NVKP_16-1-2016-0042; ÚNKP-22-4-I-SE-15; STIA-OTKA to PH; 2021-1.2.4-TÉT-2021-00060*

### EACR23-0397

### Coupling an in vivo D.melanogaster tumour model with mass spectrometry imaging to study the efficacy of anticancer drug agents.

*K.F.C. Wong[1], Z. Markus[2], R.L. Griffiths[1], M. Georgiou[2], Z. Zhu[1]*

*[1]University of Nottingham, School of Pharmacy, Nottingham, United Kingdom*

*[2]University of Nottingham, School of Life Sciences, Nottingham, United Kingdom*

**Introduction**

*Drosophila melanogaster* is a useful tool to study complex cancer biology, due to the conservation of tumour-related pathways along with the genetic amenability of fruit flies. An *in vivo D.melanogaster* tumour model established by Dr Georgiou's group generates mutant clones expressing specific tumour phenotypes, enabling tumour clone behaviour to be observed with confocal imaging. Here we explore the applicability of the fly tumour model for cancer drug research and its compatibility with analytical atmospheric pressure matrix-assisted laser desorption/ionization (AP-MALDI) mass spectrometry (MS), which allows us to locate the drug and probe metabolomic changes to infer its mechanism of action. As such, we have developed a pre-clinical drug evaluation platform for anticancer agents.

**Material and Methods**

A proliferative cancer model (*lgl$^{4}$; TSC1KD*) was genetically crossed for this study and model flies were grown on untreated or drug-treated food from birth.
Live imaging of GFP-labelled tumour clones in sample pupae was performed using a Zeiss LSM880 inverted confocal microscope, and drug response was measured via quantitative analysis of tumour-related phenotypes.
AP-MALDI MS was conducted in positive ion mode with a MassTech ion source coupled to a ThermoFischer Q-Exactive, and samples were sprayed with 10mg/ml CHCA. MS-images were visualized using Spectral Analysis at 15 µm spatial resolution. 3D OrbiSIMS spectra were obtained in both ion modes using an Orbitrap.

**Results and Discussions**

Confocal experiments demonstrated the capability of the proliferative cancer model to investigate drug action. Rapamycin-treatment displayed anticancer activity by reversing TSC1-knockdown-induced upregulated cell proliferation. Drug-dose dependency of rapamycin was established using the model and determined an $IC_{50}$ of 570.6 nM. Optimal detection of purified drug compounds was conducted prior to tissue analysis. Native tissue metabolites and rapamycin drug metabolites in fly pupae samples could be distinguished via AP-MALDI MS imaging. Rapamycin metabolites were only detected in treated pupae samples, which validates the use of AP-MALDI MS for drug localization in tumour model tissue.

**Conclusion**

The fly tumour model can investigate anticancer drug action and coupling this with AP-MALDI MS for drug localization may prove to be an effective means for pre-clinical drug research. Future work will explore a drug's mechanism of action via proteomic and metabolomic MS analysis, and validate findings using 3D OrbiSIMS or immunostaining.

### EACR23-0403

### Inhibition of Triple-Negative Breast Cancer Growth in a Mouse Model – Enhancement of Modulated Electro-Hyperthermia (mEHT) Effects by Heat Shock Factor 1 Inhibition

*P.H. Leroy Viana[1], C. Schvarcz[1], L. Danics[1], B. Besztercei[1], K. Aloss[1], Z. Bokhari[1], N. Giunashvili[1], Z. Benyó[1], P. Hamar[1]*

*[1]Semmelweis University, Department of Translational Medicine, Budapest, Hungary*

**Introduction**

Female breast cancer is the most diagnosed cancer worldwide. Triple negative breast cancer (TNBC) is the most aggressive type and is not sensitive to endocrine therapy. Modulated electro-hyperthermia (mEHT) is a non-invasive complementary tumor therapy using an electromagnetic field generated by amplitude modulated 13.56 MHz frequency that induces tumor cell destruction. However, we have demonstrated a strong induction of the

© 2023 The Authors. Molecular Oncology is published by John Wiley & Sons Ltd on behalf of Federation of European Biochemical Societies heat shock response (HSR) by mEHT, which can result in thermotolerance. We hypothesized that downregulation of HSF1 gene sensitizes the transfected tumor cells to mEHT and reduce tumor growth.

**Material and Methods**

A Balb/C isogenic murine TNBC cell line (4T1) was used. HSF1 CRISPR/Cas9 lentiviral knockdown or wild type 4T1 cells were inoculated into mammary gland's fat pad. Further, wild type tumors were treated with the HSF-1 inhibitor KRIBB11, for 8 days. Four mEHT treatments were performed every two days and the tumor growth was followed by ultrasound and caliper. Tumor destruction histology and molecular expression changes were assessed.

**Results and Discussions**

Reduction of tumor size and weight, and enlargement of tumor destruction area were observed in HSF1-KO mEHT-treated mice vs HSF1-KO Sham (HSF1-KO: 43.66 mg ± 20.09 mg; 84.45% ± 15.66% vs Sham: 110.0 mg ± 44.54 mg; 32.1% ± 14.52%, respectively) and Empty Vector mEHT-treated (89.49 mg ± 24.8 mg; 77.24% ± 7.34%, respectively). HSF1 mRNA level was significantly reduced in the KO group (Sham: 0.006180 ± 0.0006644; mEHT-treated: 0.005832 ± 0.001073) when compared to Empty Vector group (Sham: 0.01330 ± 0.002487; mEHT-treated: 0.01732 ± 0.004167). CRISPR/Cas9 lentiviral construct was able to diminish the induction of HSP70 mRNA expression (mEHT-treated: 0.01046 ± 0.005662) when compared to Empty Vector mEHT-treated group (0.02273 ± 0.01385). Immunohistochemistry confirmed the molecular data. Combined therapy of mEHT and KRIBB11 significantly reduced tumor weight (160.3 mg ± 33.26 mg) further compared to monotherapy (mEHT: 236.8 mg ± 46.42 mg; KRIBB11: 312.3 mg ± 41.45 mg).

**Conclusion**

Combined mEHT-therapy with HSF1 inhibition can be a possible new strategy of treating TNBC with a great translational potential.

*Funding: SE250+ EFOP-3.6.3-VEKOP-16-2017-00009 / SH scholarship - Tempus Public Foundation / ÚNKP-22-4-I-SE-15 / TÉT Brazil: 2021-1.2.4-TÉT-2021-00060*

### EACR23-0430

### Non-steroid anti-inflammatory treatment enhances the efficacy of modulated electro hyperthermia on triple negative breast cancer and melanoma cancer models in vivo

*N. Giunashvili[1], J. Thomas[1], P. Leroy[1], K. Aloss[1], Z. Bokhari[1], Z. Koós[1], B. Dániel[1], Z. Benyó[1], C. Schvarcz[1], P. Hamar[1]*

*[1]Semmelweis University, Institute of Translational Medicine, Budapest, Hungary*

**Introduction**

Modulated electro-hyperthermia (mEHT) is an advanced option in the hyperthermia field, applying a 13.56 MHz radiofrequency electromagnetic current to induce tumor-specific damage. This study investigates the mEHT-induced molecular effect and the potential of combination non-steroid anti-inflammatory drugs (NSAIDs) to enhance its anti-tumor effects in 4T1 triple-negative breast cancer (TNBC) and B16F10 melanoma mouse models.

**Material and Methods**

4T1 TNBC and B16F10 melanoma cell lines were injected into Balb/C and C57BL/6 mice, respectively. They have been treated according to the protocol with only mEHT or mEHT combined with non-selective COX-inhibitors (Aspirin) or selective COX2 inhibitors (SC236). Tumor volume was monitored by ultrasound and a digital caliper. At the end of the experiments, mice were euthanized and tumors excised for molecular studies.

**Results and Discussions**

Our previous multiplex studies, demonstrated that mEHT induced a local acute phase response (APR) in TNBC. Here we report that mEHT monotherapy stimulates local IL1-beta and IL6, and consequently cyclooxygenase 2 (COX 2) production. These effects could be considered as part of a self-defensive, wound-healing reaction of the tumor to protect itself from the mEHT-induced stress. In the present study, we combined mEHT with non-steroid anti-inflammatory drugs (NSAIDs), the non-selective (Aspirin), or the selective COX2 inhibitor (SC236) in vivo. All of these therapies have already demonstrated antitumor effects in various cancer models as monotherapies. Here we demonstrate that NSAID treatment synergistically increased the effect of mEHT in 4T1 TNBC. Tumor weight and tumor volume (measured by ultrasound and a digital caliper) were lowest, and the tumor destruction ratio (TDR) was the highest in the combination treated (NSAID + mEHT) groups. Tumor damage was accompanied by a significant increase in cleaved caspase-3 (cC3), suggesting an important role for apoptosis. Similarly, in the B16F10 melanoma model, lungs nodules were significantly less in mice treated with mEHT + Aspirin.

**Conclusion**

NSAIDs effectively enhance the mEHT anti-tumor effect in TNBC and melanoma cancer models; they increase tumor destruction, where apoptosis may play a role. Disecting the exact molecular mechanisms further is under our current investigation.

*Funding: NVKP_16-1-2016-0042; ÚNKP-22-4-I-SE-15; STIA-OTKA to Péter Hamar; 2021-1.2.4-TÉT-2021-00060*

### EACR23-0484

### Novel functional immunoassay for identification of multidrug resistance markers in non-small cell lung carcinoma patient-derived cells

*A. Stepanović[1], J. Dinić[1], A. Podolski-Renić[1], S. Jovanović Stojanov[1], M. Dragoj[1], M. Jovanović[2], E. Lupšić[1], A. Milićević[3], S. Glumac[4], D. Marić[5], M. Ercegovac[6], M. Pešić[1]*

*[1]Institute for Biological Research "Siniša Stanković"- National Institute of the Republic of Serbia- University of Belgrade, Department of Neurobiology, Belgrade, Serbia*

*[2]Institute for Biological Research "Siniša Stanković"- National Institute of the Republic of Serbia- University of Belgrade, Department of Biochemistry, Belgrade, Serbia*

*[3]University Clinical Center Niš, Clinic for Pathology, Belgrade, Serbia*

*[4]University of Belgrade-Faculty of Medicine, Clinic for Pathology, Belgrade, Serbia*

*[5]Univerisity of Belgrade-Faculty of Medicine, Clinic for Pulmonology, Belgrade, Serbia*

*[6]University of Belgrade-Faculty of Medicine,*

© 2023 The Authors. Molecular Oncology is published by John Wiley & Sons Ltd on behalf of Federation of European Biochemical Societies

*Clinic for Thoracic Surgery, Belgrade, Serbia*
**Introduction**
Multidrug resistance (MDR) significantly hampers non-small cell lung carcinoma (NSCLC) drugs' efficacy. To evaluate the contribution of MDR markers to anticancer drugs' sensitivity, we performed pharmacological screening on patient-derived NSCLC cells *ex vivo* and assessed the expression of MDR markers in cancer and stromal (non-cancer) cells.
**Material and Methods**
Primary patient-derived cultures were established from the NSCLC resections. After short-term culturing (2-3 weeks), a mixed population of cancer and non-cancer cells were treated with 8 chemotherapeutics (cisplatin, carboplatin, paclitaxel, docetaxel, etoposide, vinorelbine, gemcitabine, and pemetrexed). The maximum concentration reached in human plasma to which the patient is exposed during therapy (Cmax) was set as an upper limit and four lower concentrations were also applied during the study. Immunofluorescence assay enabling discrimination of epithelial cancer cells positive to a cocktail of antibodies against cytokeratin 8/18 vs. negative mesenchymal non-cancer cells was conducted using high-content imager ImageXpress Pico (Molecular Devices) with CellReporterXpress 2.9 software. Within the same immunoassay, MDR markers (ABCB1, ABCC1, and ABCG2) were analyzed by corresponding antibodies.
**Results and Discussions**
Among all tested compounds, only gemcitabine increased the number of positive cancer cells to all MDR markers in all investigated primary cell cultures. Pemetrexed did not change the number of MDR-positive cancer cells. In a patient sample IIIA stage bearing EGFR mutation (L858R), the number of positive cancer cells to all MDR markers increased upon treatment with cisplatin, carboplatin, paclitaxel, docetaxel, etoposide, vinorelbine, and gemcitabine. Stromal (non-cancer) cells mainly followed the pattern of MDR observed in cancer cells.
**Conclusion**
Novel functional immunoassay can provide valuable information about the sensitivity of NSCLC to different drugs and possible treatment outcomes based on the expression of MDR markers.

### EACR23-0531
### Long-term gonadal effects of in-utero chemotherapy exposure

*R. simaan[1], H. Bar-Joseph[2], A. Eldar-Boock[2], T. goshen-lago[1], M. Zohar-Fux[1], R. shalgi[2], I. ben-aharon[1]*
*[1]Technion - Israel Institute of Technology, Medicine, haifa, Israel*
*[2]Tel Aviv University, medicine, tel aviv, Israel*
**Introduction**
Pregnancy-associated cancer represents a unique clinical scenario, which requires a delicate balance between risks and benefits for the integrity of both mother and fetus. In case of cancer diagnosis during in the 1'st trimester, termination of the pregnancy is advised. When cancer is diagnosed during 2'nd and 3'd trimester, several classes of chemotherapy are allowed while doxorubicin (DXR) is the main chemotherapy used during pregnancy. Current clinical evidence regarding safety of chemotherapy administration during pregnancy refer mainly to cardiac and neurocognitive effects, there is lack of data regarding the impact on gonadal reserve. Moreover, since gonadogenesis occurs during pregnancy the impact of chemotherapy may be critical. Here we aimed to further evaluate the long-term male reproductive effects of in utero exposure to chemotherapy in a mouse model.
**Material and Methods**
Pregnant ICR mice were treated once with DXR (10mg/kg) or saline on day E12.5, litter was followed longitudinally for several systematic outcomes. Gonadal outcomes were evaluated in male offspring at three time-points: Day0 (birth), 10weeks (pubertal) and 6 months (full puberty). Mice were sacrificed and testes were processes, sperm extracted and blood drawn for Anti-Mullerian Hormone (AMH). Immunohistochemistry was employed to evaluate morphological changes, gonadal markers and apoptosis. Sperm was processed for RNASeq.
**Results and Discussions**
At birth in–utero DXR-exposed mice resembled the control mice in terms of histological appearance (condensed, organized spermatogonial filled seminiferous tubules). Nevertheless, at 10 weeks seminiferous tubules architecture appeared mildly disrupted in the exposed mice that dramatically worsened at 6-month age. Moreover, sperm count was reduced in the exposed mice compared to controls. DXR-exposed mice displayed an increased AMH levels, indicating an abnormal AMH regulation that may be derived from a toxic effect on supporting Sertoli cells. Sperm RNAseq of full pubertal exposed mice revealed significant profile of genes associated with spermatogenesis, sperms mobility, maturation and differentiation of spermatozoa compared with control mice.
**Conclusion**
Our study is the first to evaluate male reproductive outcomes of in-utero exposure to chemotherapy. Our results indicate that following exposure to chemotherapy in 2'nd trimester there is a latent effect manifested by a significant disruption of seminiferous tubules architecture, inferior sperm counts and differential gene expression pattern.

### EACR23-0575
### Patient-derived gastric tumour organoids recapitulate pivotal tissue molecular features making them useful for functional precision medicine.

*M. Cabeza-Segura[1], A. Marín-Alonso[1], B. García-Micó[1], A. González-Vilanova[2], V. Gambardella[1], R. Villagrasa[3], C. Martínez-Ciarpaglini[4], A. Cervantes[1], T. Fleitas[1], J. Castillo[5]*
*[1]INCLIVA Biomedical Research Institute, Medical Oncology, Valencia, Spain*
*[2]INCLIVA Biomedical Research Institute, Bioinformatics Unit, Valencia, Spain*
*[3]Hospital Clínico Universitario de Valencia, Endoscopy Unit, Valencia, Spain*
*[4]INCLIVA Biomedical Research Institute, Pathology, Valencia, Spain*
*[5]University of Valencia, Biochemistry and Molecular Biology, Valencia, Spain*
**Introduction**

© 2023 The Authors. Molecular Oncology is published by John Wiley & Sons Ltd on behalf of Federation of European Biochemical Societies

Gastric cancer (GC) has poor prognosis and presents high heterogeneity with low treatment response rate and unknown mechanisms of drug resistance. Tumour organoids are *in vitro* derived 3D cell culture from self-organizing tumour stem cells. The establishment of gastric tumour organoids (GTO) represents a good strategy to evaluate tumour molecular characteristics. GTOs are also a new potential tool for screening drug resistance and for searching novel drugs by performing functional analyses, representing a relevant arm for precision medicine.

**Material and Methods**

A prospective biobank of GTOs derived from advanced GC patients, mostly from biopsies and palliative gastrectomy, was generated according to an in-house protocol from January 2019. NGS analyses were used to characterize the mutational profile and copy number variation (CNV) was studied by CytoScan-HD. Immunohistochemistry (IHQ) was used to evaluate ERBB2 expression. Gene expression of specific cell population markers was studied by quantitative reverse transcription PCR (RT-qPCR) to determine the presence of cancer stem cells (CD44), gastric goblet cells (MUC5AC), proliferative cells (MKI67) and gastric differentiated cells (KRT20) in each GTO line. Cell cycle analyses by flow cytometry were also performed. To assess drug sensitivity, cell viability was analyzed through luminescent Cell Viability Assay.

**Results and Discussions**

From February 2019, 29 GTO were established with a success rate of 65% (22 from gastroscopy, 5 from gastrectomy, 1 from ascites and 1 from pleural effusion). 65% of these tumours were intestinal, 23% were diffuse and 12% were mixed subtypes, according to Lauren classification. Despite tumour heterogeneity, NGS analyses showed a relevant molecular concordance between each GTO line and the tissue which they derived from. CNV results showed *ERBB2* amplifications in several organoids, highlighting an important correlation with the tissue IHQ evaluation. GTOs are composed by different epithelial cell types. Among them, those organoids with high presence of cancer stem cells are more proliferative, they express the most *MKI67* and have more cells in S phase and mitosis, accordingly to RT-qPCR and flow cytometry results, respectively. Interestingly, GTOs showed heterogeneous drug sensitivity to chemotherapy drugs and targeted agents (trastuzumab, lapatinib…), allowing us to detect drug-resistant organoids.

**Conclusion**

Patient-derived GTOs represent a potential preclinical tool to model tumour heterogeneity and to assess drug responses.

### EACR23-0599
### NON-INVASIVE OXYGEN MEASUREMENT OF PANCREATIC TUMORS AFTER MULTI-MODALITY-TREATMENT AS A TOOL TO MONITOR TUMOR RESPONSE

*A. Murzyn[1], G. Dziurman[1], O. Wiecheć-Cudak[1], A. Drzał[1], M. Serda[2], M. Elas[1], M. Krzykawska-Serda[1]*
*[1]The Faculty of Biochemistry Biophysics and Biotechnolog y Jagiellonian University,*
*Department of Biophysics and Cancer Biology, Kraków, Poland*
*[2]University of Silesia, Institute of Chemistry, Katowice, Poland*

**Introduction**

Pancreatic ductal adenocarcinoma (PDAC) is one of the most lethal solid tumors. Many studies indicate that low oxygen concentration is crucial in treating adverse treatment responses. Reducing the state of hypoxia in cancer could make the treatment more effective by making the tumor more sensitive to treatment. Electron Paramagnetic Resonance (EPR) spectroscopy and imaging with oximetric probes provide a sensitive and non-invasive way to study these phenomena. The aim of this study was to determine how tumor oxygenation correlates with therapy success during multi-modality treatment (a combination of chemotherapy and hyperthermia based on gold nanorods).

**Material and Methods**

Oximetry was carried out by inserting the OxyChip (lithium octa-butoxynaphthalocyanine - LiNc-BuO) oxygen probe into the tumor tissue in the C57BL/6J mouse ectopic PDAC model (Pan_O2). Oxygen concentration measurements were performed before, during, and after therapy using a surface coil with a continuous wave (CW) Bruker EPR spectrometer, and JIVA-25 Pulse EPR with a loop-gap 19 mm resonator. The therapy cycle included the administration of therapeutics: AuNRs-GEM (gold nanorods with gemcitabine) and hyperthermia with light in the near-infrared range of about 808 nm. The therapeutic sequence included 5 doses AuNRs (approx. 1ug/ml) together with GEM (approx. 45mg/kg BW) and near-infrared heating (approx. 30 min) administered every 72h. Tumors during therapy were under ultrasound control (Vevo 2100, Fuji VisualSonics). All experiments were performed with Local Ethics Committee permission no.151/2022.

**Results and Discussions**

Both CW and Pulse EPR spectroscopy allows for very fast *in vivo* measurements (<5 min) to obtain high-quality data during the course of the therapy. Average tumor oxygenation was around 10 mmHg with an increase up to 20 mmHg after the last dose of chemotherapy. Changes in the relaxation times (in relation to the level before treatment) can possibly predict promising and unsuccessful responses to therapy, e.g. the small increase in oxygenation during gemcitabine treatment correlated with better mouse survival.

**Conclusion**

Oxygenation of the tumor is an effective biomarker of therapeutic outcome.

### EACR23-0656
### Bridging the Healthcare literacy gap for people with a KRAS oncogene

*C. Conneran[1]*
*[1]KRAS Kickers, Advocate, Charlotte, United States*

**Introduction**

The KRAS oncogene is the most common biomarker associated with some of the deadliest types of cancer including lung, colorectal and pancreatic. People with this diagnosis are searching to find options, gain knowledge of their cancer, and join in community with others that are facing the same issues. In this fight for lives, there is an

© 2023 The Authors. Molecular Oncology is published by John Wiley & Sons Ltd on behalf of Federation of European Biochemical Societies ache to find current research and treatment options that can extend living with cancer.
January 2020, KRAS Kickers was formed by Terri Conneran, a KRAS Lung cancer patient frustrated by the limited amount of "patient friendly" information available. Her goal is to ensure that all KRAS cancer patients have access to resources and tools to better understand the science and medical options available for all KRAS cancer types.

**Material and Methods**

Terri's approach is to breakdown the medical and scientific jargon for KRAS cancer patients so they can make informed decisions working side by side with their medical team to advocate for their own survivorship. By breaking down the medical and scientific jargon for people, cancer patients are empowered to make informed decisions along side their medical team, The KRAS Kickers mission is to connect people to current research, resources, and community to kick cancer's KRAS! KRAS Kickers members engage with leading doctors, researchers, and advocates to learn about new developments and clinical trials, connect to resources, build community, and share their stories to give hope to all with a KRAS biomarker. Believing that KRAS Knowledge + Research + Advocacy = Survivorship

**Results and Discussions**

KRAS Kickers in was formed just over 3 years ago and has experienced substantial growth year over year not only in its membership numbers but in the programs they provide. The KRAS Kickers currently have close to 2,000 survivors in its Facebook groups, and members on its website, 3000 plus twitter followers and 6,400 LinkedIn connections with an international presence in over 65 countries.
The regular educational series with leading experts continues to provide unparalleled knowledge to the community by bridging the healthcare literacy gap. Surveys and participation establish it.

**Conclusion**

Formed three years ago, KRAS Kickers are worldwide tumor as a agnostic RAS oncogene advocacy group. Known across the research and clinical areas as an empowerment of patients and caregivers. Being included as an informed decision partner makes the difference one person, at a time with worldwide impact.

### EACR23-0669

### A silver jubilee for synthetic lethality in cancer treatment: Where do we stand?

*J.S. Lee[1], Y. Chung[2], A. Kammula[3], A. Schaffer[3], E. Ruppin[3]*
*[1]Sungkyunkwan University, School of Medicine, Suwon, South Korea*
*[2]Sungkyunkwan University, Department of Artificial Intelligence, Suwon, South Korea*
*[3]National Cancer Institute, Cancer Data Science Lab, Bethesda, United States*

**Introduction**

Synthetic lethality (SL) denotes a genetic interaction between two genes whose co-inactivation is detrimental to cells. Since the seminal work of Hartwell and colleagues has raised the possibility that SL can be used to devise highly selective cancer treatments, it has been one of the promising approaches for precision oncology and drug discovery. Many different avenues have so far been explored to bring this idea to the clinic. As 25 years have passed by now, we take stock and systematically and comprehensively chart the landscape of SL-based preclinical research and clinical trials.

**Material and Methods**

We have systematically mined both public and commercial databases to curate the preclinical and clinical landscape of the SL-based oncology studies. We have used PubMed to find preclinical synthetic lethality studies. We focused on those studies where synthetic interactions in cancer were investigated and validated for preclinical evidence in animal models. We have analyzed a comprehensive proprietary database, Trialtrove, to survey synthetic lethality-based oncology clinical trials.

**Results and Discussions**

Our analysis shows that the number of SL oncology studies is rapidly growing. Importantly, we find that the success rate of SL oncology trials is significantly higher than non-SL-based trials. While more than 70% of SL-oncology trials involve genes in the most-studied DNA damage response (DDR) pathways, the fraction of SL trials involving non-DDR pathways keeps growing since 2009. We further charted the landscape of SL triplets, which is a promising future higher-order extension of the conventional pairwise SL interactions. We find that only about 8% of preclinically validated SL triplets were clinically tested in trials, providing new opportunities for more refined clinical trial design. Our analysis further points that emerging opportunities in SL oncology arise from metabolic and paralogous interactions, disease-agnostic biomarkers, context-specific combinations against treatment resistance, artificial intelligence and data science approaches, and multi-omics patient stratification signatures.

**Conclusion**

We performed a large-scale systematic survey of the current landscape of the efforts to bring synthetic lethality to the clinic, from preclinical in vivo studies to clinical trials. Our study reinforces the hope that the synthetic lethality approach may serve as a key driver of precision oncology going forward.

### EACR23-0685

### The Road from Human Sample Collections to the First Rectal Cancer Biobank at the Institute for Oncology and Radiology of Serbia

*A. Djuric[1], M. Tanic[1], S. Bjelogrlic[1], S. Stojanovic-Rundic[2,3], M. Marinkovic[2], A. Stanojevic[1], R. Fijenman[4], S. Castellví-Bel[5], J. Zoidakis[6,7], M. Cavic[1]*
*[1]Institute for Oncology and Radiology of Serbia, Department for Experimental Oncology, Belgrade, Serbia*
*[2]Institute for Oncology and Radiology of Serbia, Clinic for Radiation Oncology and Diagnostics- Department of Radiation Oncology, Belgrade, Serbia*
*[3]University of Belgrade, Faculty of Medicine, Belgrade, Serbia*
*[4]The Netherlands Cancer Institute, Department of Pathology, Amsterdam, The Netherlands*
*[5]Fundació de Recerca Clínic Barcelona-*

© 2023 The Authors. Molecular Oncology is published by John Wiley & Sons Ltd on behalf of Federation of European Biochemical Societies

Institut d'Investigacions Biomèdiques August Pi i Sunyer- Centro de Investigación Biomédica en Red de Enfermedad es Hepáticas y Digestivas- Hospital Clínic- University of B arcelona, Gastroenterology Department, Barcelona, Spain
[6]Biomedical Research Foundation- Academy of Athens, Department of Biotechnology, Athens, Greece
[7]National and Kapodistrian University of Athens, Department of Biology, Athens, Greece

**Introduction**

Cancer patient samples have been collected and stored at the Institute for Oncology and Radiology of Serbia (IORS) for over 50 years for the purpose of specific studies. As the number of patients grew, it became necessary to collect, store and disseminate samples and related data in accordance with good biobanking practices. The aim of this study was to establish a proper procedural workflow for setting up a first Rectal Cancer Biobank (RCB) at IORS within the framework of the Horizon Europe project STEPUPIORS (101079217).

**Material and Methods**

Procedures were developed according to recommendations of the International Society of Biological and Environmental Repositories (ISBER), the Biobanking and BioMolecular resource Research Infrastructure (BBMRI), European Research Infrastructure Consortium (ERIC), and EU regulations followed by partner institutions. Ethical and legal regulations, respecting national and European legislation were followed. Biobank equipment and software were procured to ensure maximum accordance with infrastructural, storage and data protection requirements. Human capacities were developed through intensive online and in person trainings and expert visits to partner institutions' biobanks. Consensus consortium decisions were reached on all aspects during regular meetings.

**Results and Discussions**

A procedural basis for the establishment of the RCB with a planned cohort of around 100 locally advanced rectal cancer (LARC) patients was successfully introduced. Fifteen starting RCB standard operating procedures (SOPs) were developed to comply with good biobanking practices in terms of processing, storage, and sample dissemination. Scientific and management oversight committees comprised of members of all participating institutions were formed to assure high-quality biobank-related research and innovation that will advance the treatment of LARC patients. Fourteen IORS researchers (5 physicians, 3 biochemists, 4 molecular biologists, 2 pharmacists) were trained for various roles in the new biobank. Although primarily established for the purposes of the STEPUPIORS project, the LARC cohort might be further used for a large spectrum of future research approaches. The developed procedures and IT tools are expected to be reusable for future biobanks at IORS and potentially for other tumor biobanks in Serbia.

**Conclusion**

The establishment of the first RCB at the IORS was successful, although accompanied by various scientific, legal, infrastructural, procedural and human resource-related challenges.

### EACR23-0733
### FUT5 overexpression as a possible prognostic marker in ovarian cancer patients

A. Wong[1], A. Hassan[1], P. Ip[2]
[1]University of Hong Kong, School of Biological Sciences, Hong Kong, Hong Kong- China
[2]University of Hong Kong, Department of Pathology, Hong Kong, Hong Kong- China

**Introduction**

Ovarian cancer is the most lethal gynecological malignancy in the world, with 80% of the cases being detected at an advanced stage. The lack of an accurate and predictable prognostic factor has been a major hindrance towards improving this statistic. We recently demonstrated that fucosyltrasferase 5 (FUT5), a rate-limiting enzyme that catalyzes addition of fucose in the synthesis of sialyl Lewis$^x$ is critical for ovarian cancer metastasis. Despite the well-established role of fucosylation in cancer progression, the role of FUT5 in ovarian cancer prognosis remains elusive.

**Material and Methods**

We investigated the correlation between FUT5 expression and several clinicopathological features in 94 ovarian cancer patient samples of high-grade serous carcinoma (HGSC) (N=45), endometrioid carcinoma (N=3), and clear cell carcinoma (N=49) subtype by qPCR. Median expression of FUT5 was used as cut-off value to divide samples into high and low expression groups. Clinicopathological features of FUT5 high vs low groups were analyzed in all samples.

**Results and Discussions**

While there was no difference in FUT5 expression between the different histological subtypes, FUT5 expression was significantly higher among HSGC patients with advanced stage (Stage IIB-IV) tumor. Moreover, patients with higher FUT5 expression appeared to correlate with decreased overall and progression-free survival.

**Conclusion**

The data suggests that FUT5 may serve as a predictive marker for ovarian cancer progression, and highlights the potential utility of its expression level in clinical diagnosis (This research is supported by HMRF 08192286).

### EACR23-0736
### Evaluation of exhausted T-cell biomimetic nanoparticles for cancer therapy

J.L. Blaya-Cánovas[1], C. Griñán-Lisón[2], A. López-Tejada[2], I. Blancas[3], A. Navarro-Ocón[4], J. Calahorra[4], V. Delgado-Almenta[4], J.A. Marchal[5], R.M. Sánchez-Martin[5], S. Granados-Principal[2]
[1]Hospital Universitario de Jaén, UGC de Oncología Médica, Jaén, Spain
[2]Faculty of Pharmacy- University of Granada, Department of Biochemistry and Molecular Biology 2, Granada, Spain
[3]Hospital Universitario San Cecilio, UGC de Oncología, Granada, Spain
[4]GENYO- Centre for Genomics and Oncological Research- Pfizer/University of Granada/Andalusian Regional Gover nment, Genomic Oncology, Granada, Spain
[5]Instituto de Investigación Biosanitaria ibs.GRANADA, Instituto de Investigación Biosanitaria ibs.GRANADA,

© 2023 The Authors. Molecular Oncology is published by John Wiley & Sons Ltd on behalf of Federation of European Biochemical Societies

*Granada, Spain*
**Introduction**
Biomimetic nanoparticles (NPs) are coated with membranes that maintain the properties of the cell of origin. Tumor cells, such as metastatic triple-negative breast cancer (TNBC), can evade immunity by the expression of inhibitory checkpoint molecules that induce immune exhaustion. We hypothesized that biomimetic NPs coated with checkpoint-molecules-enriched membranes can efficiently target immune-evading tumor cells.
**Material and Methods**
PLGA cores (uNP) were synthesized and coated with exhausted T-cell-derived membranes (NExT) from TNBC patients (n=13), who signed the informed consent (Ethical Committee approval ref. PI19/01533/1626-N-19). Size, ζ-potential, and PDI of the NP were evaluated with Dynamic Light Scattering. Size, shape, and coating were confirmed by Transmission Electron Microscopy (TEM), purification of membranes by Western blot, and toxicity with WST1. The expression, preservation, and correct orientation of PD1, LAG3, and TIM3 both in T cells and NExT, as well as the cell uptake of coumarin-loaded uNP and NExT, were determined by flow cytometry. *In vivo* biodistribution of IR780-loaded uNP and NExT was investigated in SUM159 xenografts (n=6 mice) with an IVIS Imaging System.
**Results and Discussions**
NExT exhibited favorable size, ζ-potential, PDI, an appropriate coating, and a spherical shape. The high transmembrane ATPase alpha-1 subunit levels in both membrane and NExT confirmed the correct membrane purification and coating. uNP and NExT were stable during a two-week experiment and were non-toxic *in vitro*. T-cell activation resulted in elevated exhaustion markers, especially PD1, which remained at high levels in NExT. NExT was more rapidly internalized in SUM159PT cells (PD-L1$^{high}$), and it was modulated by the PD-L1/PD1 interaction as found in MDA-MB-468 cells (PD-L1$^{low}$) treated or not with IFNγ, which showed a higher uptake when IFNγ was added (PD-L1$^{high}$). *In vivo* biodistribution confirmed that NExT had tropism for PD-L1$^{high}$ tumors, as evidenced by a higher accumulation in SUM159PT tumors compared with uNP.
**Conclusion**
The development of our nanosystem, which efficiently targets and accumulates in TNBC cells by taking advance of their ability to evade the immune system, provides promising prospects for enhancing treatment outcomes in TNBC and other tumors expressing inhibitory immune checkpoint molecules. Importantly, the success of NExT derived from patient T-cells highlights the potential of personalized medicine in cancer treatment.

### EACR23-0741
### Tumor-targeted Bola4A-nanopaticle for delivery of imatinib chemotherapeutics

A. Wong[1], Z. Shi[1], M. Zhang[1], M. Artemenko[1], K. Chan[2], X. Liu[3], L. Peng[4]
[1]*University of Hong Kong, School of Biological Sciences, Hong Kong, Hong Kong- China*
[2]*University of Hong Kong, Department of Obstetrics and Gynecology, Hong Kong, Hong Kong- China*
[3]*China Pharmaceutical University, Institute of Pharmaceutical Sciences, Nanjing, China*
[4]*Aix-Marseille Université CNRS-, Centre Interdisciplinaire de Nanoscience de Marseille, Marseille, France*
**Introduction**
Chemoresistance and recurrence remain major obstacles in effective anticancer therapies. Overexpression of CD117 in the cancer stemness subpopulation makes it an attractive candidate to overcome drug resistance and relapse. However, there are limitations of imatinib mesylate that targets CD117 in ovarian cancer treatment.
**Material and Methods**
In this study, we encapsulated imatinib in different nanoformulations using film dispersion. MTT analysis was used for in vitro anticancer activity, and in vivo anticancer and biodistribution activity were studied in mice. LC-MS/MS was used to explore novel interacting partners.
**Results and Discussions**
We showed that Bola4A-amphiphilic dendrimer (Bola4A)-based nanoformulation outperformed others and imatinib alone *in vitro* with higher drug loading and enhanced anticancer activity. Bola4A-imatinib could also effectively mediate drug efflux. *In vivo* studies further showed targeted drug delivery and prolonged drug retention of the Bola4A-imatinib nanoformulation. Bola4A/imatinib and cisplatin or paclitaxel combined treatment showed an effective sensitization to chemotherapy, reducing tumor dissemination. Moreover, the nanoformulation, both alone and as a part of the combined therapy, was well tolerated to the tested animals. Mechanically, we further identified a novel β-catenin/HRP2 signaling which was potently inhibited by Bola4A-encapsulated imatinib. Furthermore, we observed a positive association between b-catenin and HRP2 in clinical samples. High expression of HRP2 tended to correlate with poor clinical outcomes.
**Conclusion**
Together, these studies illustrate an example of implementing dendrimer nanotechnology to advance anticancer drug potency and combat drug resistance (This work is supported by HMRF 06173496).

### EACR23-0749
### Tumour and microenvironment remodelling during chemo-free neoadjuvant treatment of HER2+/ER+ breast cancer patients with residual disease at surgery

M. Callari[1], M. Dugo[2], B. Gyorffy[3], M. Barreca[4], B. Galbardi[2], L. Viganò[2], A. Locatelli[2], G. Viale[5], L. Gianni[1], G. Bianchini[2]
[1]*Fondazione Michelangelo, Fondazione Michelangelo, Milan, Italy*
[2]*San Raffaele Hospital, Oncology, Milan, Italy*
[3]*Semmelweis University, Bioinformatics, Milan, Italy*
[4]*University of Milano Bicocca, Biotechnology and Biosciences, Milan, Italy*
[5]*European Institute of Oncology, Pathology, Milan, Italy*
**Introduction**
Serial molecular characterization of clinical tumours during neoadjuvant treatment could unveil mechanisms of resistance and inform on candidate alternative treatments.

© 2023 The Authors. Molecular Oncology is published by John Wiley & Sons Ltd on behalf of Federation of European Biochemical Societies

We investigated the potential of longitudinal molecular profiling in HER2+/ER+ breast cancer (BC) patients.
**Material and Methods**
HER2+/ER+ BC patients enrolled in the NA-PHER2 trial received neoadjuvant trastuzumab, pertuzumab, palbociclib with (n=30) or without (n=28) fulvestrant in two non-randomized cohorts. RNA-seq profiles, Ki67 and tumour infiltrating lymphocytes (TILs) centralised quantification were obtained pre-treatment, at day 14 and at surgery from 43 patients with residual disease. Ki67 was dichotomised in High or Low using a 10% threshold. Ki67 class at day 14 and at surgery identified the High-High, Low-High and Low-Low groups. Gene expression was adjusted for changes in tumour purity. Genes were tested for differential expression over time in the overall population, depending on the treatment arm or according to Ki67 groups. Differentially expressed (DE) genes (FDR<10%) were clustered and pathway analysis performed for each cluster. DE genes were also evaluated in single-cell data from HER2+ clinical breast cancers to identify the cell type predominantly expressing them.
**Results and Discussions**
A total of 655 genes were DE in the time-course analysis. Inflammation, interleukin signalling and activation of the leptin/complement cascade were upregulated at surgery, mostly expressed by the tumour microenvironment. CAF-expressed members of the WNT pathway were downregulated over treatment.
No major expression dynamics emerged between the two treatment arms, except for ER-regulated genes specifically downregulated at surgery in the arm receiving fulvestrant. Immune-related genes (e.g. B2M, STAT1), mostly expressed by myeloid cells, were highly expressed in the Ki67 High-High group, but downregulated over treatment in the Low-Low group. On the contrary, the natural killer marker CD56 was specifically upregulated in the Low-Low group at surgery. Global stromal and intra-tumoral lymphocytic infiltration was the highest in the High-High group and the lowest in the Low-Low group, both before and after treatment.
**Conclusion**
HER2+/ER+ BC receiving chemo-free neoadjuvant treatment underwent major transcriptional reprogramming both in tumour cells and in the tumour microenvironment. Intriguingly, the Ki67 High-High group, arguably the most resistant to treatment, has the highest immune infiltration, although ineffective in eradicating the tumour.

### EACR23-0756
### Evaluation of dual targeting of high-grade serous ovarian cancer via PI3K inhibition and lysosomes using patient derived tumor organoids

*A. SKORDA[1], A. Røssberg Lauridsen[1], A. Lahtinen[2], M. Lund Bay[1], K. Huhtinen[3], J. Hynninen[4], S. Hautaniemi[2], T. Kallunki[1]*
*[1]Danish Cancer Society Research Center, Cancer invasion and resistance, Copenhagen, Denmark*
*[2]University of Helsinki, Research Program in Systems Oncology- Research Programs Unit- Faculty of Medicine, Helsinki, Finland*
*[3]University of Turku, Cancer Research Unit- Institute of Biomedicine and FICAN West Cancer Centre, Turku, Finland*
*[4]University of Turku, Department of Obstetrics and Gynecology, Turku, Finland*
**Introduction**
High-grade serous ovarian carcinoma (HGSC) represents the most common type of ovarian cancers. Having median latency of more than 10 years, HGSC is diagnosed at an advanced disease stage. Tumor evolution associates with high disease heterogeneity and distinct-enriched pathways mediate progression and resistance towards the current standard-of-care treatments. The curative care becomes challenging unless we aim for combinatory treatment options. Lysosomes are emerging as targets to bypass cancer survival and resistance and thus, could be targeted in the refractory HGSC cases.
**Material and Methods**
Tissues collected from HGSC patients were grown as stable long-term 3-dimensional (3D) tumor organoid cultures (PDOs) which were DNA sequenced. Stemness and proliferation of PDOs was assessed via high-throughput immunofluorescent detection of marker proteins PAX8, Ki67, p53 with ImageXpress Confocal HT.ai. Alterations of distinct cell proliferative pathways in four PDOs were identified via RNA sequencing and bioinformatics analysis. PDOs' response to drug combinatory interventions were evaluated via cell death assays. All assays were performed under a 3D setup and image-based quantification was possible via MetaXpress analysis software. PDX models were set up for in vivo drug testing.
**Results and Discussions**
DNA sequencing verified that the established PDOs retain the molecular characteristics of the parental tumors. PDOs that resist chemotherapy become dependent on specific proliferative pathways that are drivers for relapses. Inhibition of the PI3K pathway sensitized PDOs towards cell death. Interestingly, combinatory targeting of multiple pathways may overcome the refractory disease. Among the lysosomal death inducing drugs are well tolerated antihistamines that were studied in the context of PI3K inhibition. In vivo testing will be the step further.
**Conclusion**
HGSC PDOs were established and used as rational and effective models to study drug interventions. Tumor evolutionary analysis based on data of longitudinal, prospective, and multiregional collected samples suggest pathways that mediate chemotherapy resistance but also, pinpoint targets for drug intervention for the relapse cases. Inhibition of these pathways with simultaneous targeting of other cellular functions, such as lysosomal-mediate apoptosis, may be highly effective alternative treatment options.

### EACR23-0766
### Modulated Electro Hyperthermia Enhances the Delivery and Antitumor Effects of Doxorubicin Encapsulated in Lyso-thermosensitive Liposome in 4T1 Mouse Model

*K. Aloss[1], C. Schvarcz[1], P. Leroy[1], M.Z. Bokhari[1], N. Guinasvilli[1], D. Bocsi[1], Z. Koós[1], P. Hamar[1]*

© 2023 The Authors. Molecular Oncology is published by John Wiley & Sons Ltd on behalf of Federation of European Biochemical Societies

[1]Semmelweis University, Translational Medicine Institute, Budapest, Hungary

**Introduction**

Modulated electro hyperthermia (mEHT) is an adjuvant cancer therapy which selectively targets the tumor. By the autofocusing of mEHT the selective and significant (+2.5°C) heating of even deeply situated tumors is possible. In this study, we investigated the possibility of using mEHT as a source of heat for thermosensitive liposomes (TSLs) to effectively enhance the delivery and in vivo efficacy of doxorubicin (DOX).

**Material and Methods**

a triple-negative breast cancer cell line (4T1), was orthotopically injected into Balb/C mice and treated with a combination of mEHT and lyso- thermosensitive liposomal doxorubicin (LTLD). Tumor growth inhibition was followed and DOX accumulation in the tumor was monitored by in vivo optical imaging

**Results and Discussions**

LTLD+ mEHT combination was more effective in tumor growth inhibition compared to free DOX and pegylated liposomal DOX (PLD- Caelyx) as demonstrated by both tumor volume and tumor weight reduction. Moreover, the highest tumor destruction ratio (TDR) was observed in (LTLD+mEHT) group on HE stained samples. Furthermore, LTLD combined with mEHT resulted in the highest DOX accumulation in the tumor at 1 h and 24 h after treatment. On the other hand, LTLD exhibited similar toxicity to free DOX as demonstrated by body weight loss

**Conclusion**

mEHT can be an effective tool to improve tumor delivery and anti-tumor activity of DOX-encapsulated in TSL.

*Funding: ID: ÚNKP-22-4-I-SE-15, 2021-1.2.4-TÉT-2021-00060, STIA-OTKA to PH*

### EACR23-0832
### Clinical and molecular characterization of extracranial metastases in glioblastomas

*J. Jacobsen[1,2], C. O'Rourke[3], A. Locallo[3,4], J.F. Carlsen[5,6], J.D. Ewald[7,8], D. Scheie[6,9], K. Grunnet[10], A.Y. Schmidt[11], L.C. Melchior[9], J. Weischenfeldt[3,4], V.A. Larsen[5], J.B. Andersen[3], H.S. Poulsen[10], H. Broholm[9], S.R. Michaelsen[1,2], B.W. Kristensen[1,2]*

*[1]Rigshospitalet- Bartholin Institute- Copenhagen Universit y Hospital, Department of Pathology, Copenhagen, Denmark*

*[2]University of Copenhagen, Department of Clinical Medicine and Biotech Research an d Innovation Center BRIC, Copenhagen, Denmark*

*[3]University of Copenhagen, Department of Health and Medical Sciences- Biotech Rese arch and Innovation Center BRIC, Copenhagen, Denmark*

*[4]Rigshospitalet, The Finsen Laboratory, Copenhagen, Denmark*

*[5]Rigshospitalet, Department of Radiology, Copenhagen, Denmark*

*[6]University of Copenhagen, Department of Clinical Medicine, Copenhagen, Denmark*

*[7]Odense University Hospital, Department of Pathology, Odense, Denmark*

*[8]University of Southern Denmark, Department of Clinical Research, Odense, Denmark*

*[9]Rigshospitalet- Copenhagen University Hospital, Department of Pathology, Copenhagen, Denmark*

*[10]Copenhagen University Hospital, The DCCC Brain Tumor Center- Department of Cancer Tr eatment, Copenhagen, Denmark*

*[11]Rigshospitalet, Center for Genomic Medicine, Copenhagen, Denmark*

**Introduction**

Glioblastoma (GBM) is the most common and malignant primary brain tumor in adults, but in contrast to many other malignancies they rarely metastasize. Metastasizing GBMs cause both diagnostic and therapeutic challenges and are generally poorly investigated. Therefore, our aim was to characterize these tumors clinically and molecularly.

**Material and Methods**

We collected and examined the largest cohort to date of tissue from 16 glioma patients (14 GBMs and 2 lower-grade gliomas) with extracranial metastases, including 10 distant metastases to lymph node, bone, liver, and scalp, and 6 extracranial extensions. After histopathological re-evaluation of the tumors, we assessed the associated MRI scans, investigated the tumor microenvironment by immunohistochemistry (IHC) followed by artificial intelligence-based quantification and lastly performed molecular analyses by genome-wide 850 K methylation profiling and NGS analysis with the TSO500 panel.

**Results and Discussions**

Clinically (gender, age, treatment, and survival), the patients were comparable to other GBM patients. MRI scans revealed proximity of the brain tumors to dura, large vessels, and ventricles in a high proportion of the patients. Paired samples from primary tumors, recurrences and metastases analyzed by genome-wide methylation profiling and copy number analysis overall confirmed the histological diagnoses and revealed a patient-specific methylation pattern, although differentially methylated regions in extracranial extensions and distant metastases vs. primary tumors, respectively, were also identified. A higher methylation-based stem-cell division rate in distant metastases vs. primary tumors was revealed, while cell-type deconvolution showed no changes in immune cell composition. Similarly, IHC of immune- and stem cell markers did not reveal major differences in the extracranial metastases vs. primary tumors. NGS analyses revealed that distant metastases clonally derive from primary tumors and not from recurrences.

**Conclusion**

In conclusion, patients with GBMs that metastasize were clinically comparable to other GBM patients. The molecular analyses confirmed the histological diagnoses and showed in the longitudinal sampling a patient-specific pattern. Surprisingly, the amount of immune cells appeared to be similar in primary tumors and extracranial metastases. Distant metastases were clonally derived from primary tumors and not recurrences.

### EACR23-0858
### Mutiplex Analysis of Modulated Electro-Hyperthermia-Induced Local Acute Phase Protein Production in Mouse TNBC Model

*P. Hamar[1], C. Schvarcz[1], L. Danics[1], T. Krenács[2], P. Viana[1], Á. Nagy[2], A. Szász[3], Z. Benyó[1]*

*[1]Semmelweis University, Institute of Translational Medicine, Budapest, Hungary*

© 2023 The Authors. Molecular Oncology is published by John Wiley & Sons Ltd on behalf of Federation of European Biochemical Societies

[2]Semmelweis University,
Department of Pathology and Experimental Cancer Resear ch, Budapest, Hungary
[3]Szent Istvan University- Oncotherm Kft.,
GINI - Kihelyezett Biotechnológiai Tanszék, Gödöllő, Hungary

**Introduction**

Triple-negative breast cancer (TNBC) is a highly aggressive breast cancer type with no anti-hormone and targeted therapy. Modulated electro-hyperthermia (mEHT) is able to selectively destroy solid tumors without harming healthy tissues, based on bioelectric properties of tumors. Our aim was to investigate tumor growth inhibiting effects of mEHT and reveal most significant molecular pathways, induced by mEHT treatment in a TNBC mouse model.

**Material and Methods**

410.4-derived mammary carcinoma TNBC cells were inoculated orthotopically into female BALB/c mice and randomized into sham and mEHT groups by tumor size. 30 minute long treatments were performed with Labehy 200 (Oncotherm Ltd.). Tumors were removed 4/12/24/48/72 hour after last treatment and processed for immunohistochemical (IHC) and molecular analysis. Tumor Destruction Ratio (TDR%) was evaluated on H&E slides. Cleaved caspase 3 (cC3) and heat shock protein 70 (HSP70) stainings were performed. RNA and protein isolated from tumors were investigated with NGS, Nanostring and mass spectrometry. mEHT in combination with heat shock inhibition (KRIBB11) was studied in vitro.

**Results and Discussions**

mEHT treatment effectively reduced tumor growth and resulted in significantly smaller tumor size (224±38 $mm^3$ vs. mEHT: 106±25 $mm^{3,}$ ) and weight (sham: 288±6 mg vs mEHT: 98±2 mg, $p<0.05$) in mEHT treated mice, compared to sham without toxicity. HSP70 expression was 6.1x increased and cC3+ tumor area was also significantly higher in treated tumors ($p<0.001$ and $p<0.01$). NGS revealed that upregulation of Acute Phase Protein expression was the most remarkable change in mEHT-treated tumors. Upregulated expression of protease inhibitors, fibrinogens, complement components and haptoglobin was validated on both RNA and protein level. KRIBB11 effectively decreased viability of 4T1 cells in vitro, accompanied with inhibition of HSP70 and C4b expression.

**Conclusion**

Repeated mEHT treatment efficiently inhibited tumor progression in our TNBC mouse model. The most prominent response is a local Acute Phase Protein production, which may be interpreted as a protective mechanism of cancer cells. APPs can serve as molecular targets for inhibitory therapy to potentiate the efficacy of mEHT and other cellular stress-inducing therapy modalities.

*Funding: NVKP_16-1-2016-0042; ÚNKP-22-4-I-SE-15; STIA-OTKA to PH; 2021-1.2.4-TÉT-2021-00060*

### EACR23-0894
### Identification of the Genomic Landscape and Drug Sensitivity of the Patient-Derived Organoids Established from Metastatic Colorectal Cancer Patients

G.D. Yalcin[1], K. Yilmaz[1], R. Klima[2], O. Kurtulan[3], D. Ozer Turkay[4], O.H. Aktepe[5], C. Sokmensuer[3], A.B. Dogrul[6], V. Oter[7], E.B. Bostanci[7], S. Yalcin[5], J. Fernandez-Mateos[8], L. Braga[2], A. Acar[1]
[1]Middle East Technical University, Biological Sciences, Ankara, Turkiye
[2]International Centre for Genetic Engineering and Biotech nology ICGEB, Functional Cell Biology Group, Trieste, Italy
[3]Hacettepe University Faculty of Medicine, Pathology, Ankara, Turkiye
[4]Ankara City Hospital, Pathology, Ankara, Turkey
[5]Hacettepe University Cancer Institute, Medical Oncology, Ankara, Turkiye
[6]Hacettepe University Faculty of Medicine, Department of General Surgery, Ankara, Turkiye
[7]Ankara City Hospital, Gastrointestinal Surgery, Ankara, Turkiye
[8]University Institute of Oncology of Asturias- University of Oviedo, Biochemistry and Molecular Biology, Oviedo, Spain

**Introduction**

Several studies have shown that three-dimensional organoid culture systems, specifically patient-derived organoids (PDOs), can be used to model various aspects of cancer biology [1,2]. We aimed to establish a living PDO biobank from metastatic colorectal cancer (mCRC) patients for the first time in Türkiye including their genomic characterization and sensitivity by a drug panel.

**Material and Methods**

To characterize the genomic profile of the PDOs, the next generation sequencing (NGS) approach based on Whole-Exome Sequencing and RNA sequencing was performed. Moreover, hematoxylin-eosin (H&E) staining was utilized to find the phenotypic resemblance of the PDOs with their corresponding parental tumors. The anti-cancer drug sensitivity of these organoids, which previously been treated chemotherapy and/or targeted therapy, was performed using a high-throughput drug screening approach.

**Results and Discussions**

We used Whole-Exome Sequencing (WES) to analyze matched tumor and PDO samples from 20 different mCRC patients and discovered a high degree of concordance in mutational and copy-number variation. Furthermore, bulk RNA sequencing performed on 20 different matched tumor and PDO samples exhibited the maintenance of transcriptomic signature. The independent pathologist observations confirmed that histologic features were retained in PDOs. Finally, high-content drug screening using a compound library demonstrated rapid and functional testing of effective hits in PDOs, indicating the platform's potential use and expansion into clinical decision making.

**Conclusion**

In summary, for the first time in Türkiye, we established a living biobank of PDOs from 20 mCRC patients, including histological, genomic, and drug sensitivity analyses, while preserving intratumor heterogeneity and recapitulating key molecular features in these PDOs with respect to their parental tumors. These findings indicate that our living PDO biobank could be useful for basic and translational research as well as testing therapeutic modalities for personalized medicine approaches.

© 2023 The Authors. Molecular Oncology is published by John Wiley & Sons Ltd on behalf of Federation of European Biochemical Societies

1. Yalcin GD, Danisik N, Baygin RC, Acar A. Systems biology and experimental model systems of cancer. *J Pers Med*. 2020;**10**(4).
2 .Vlachogiannis G, Hedayat S, Vatsiou A, Jamin Y, Fernández-Mateos J, Khan K, et al. Patient-derived organoids model treatment response of metastatic gastrointestinal cancers. *Science (1979)*. 2018;**359**(6378).
*Funding: The Scientific and Technological Research Council of Turkey (TUBITAK) grant number: 118C197. Turkish Academy of Sciences.*

### EACR23-0921
### MEK inhibitor encapsulation into innovative BBB-targeting liposomes exerts specific GBM targeting

*M. Mattioli[1], E. Stanzani[1], M. Pizzocri[1], V. Ribecco[1], E. Lauranzano[1], M.M. Ravanelli[1], F. Pessina[2], F. Re[3], M. Matteoli[1], L. Passoni[1]*
*[1]IRCCS Humanitas Research Hospital, Laboratory of Pharmacology and Brain Pathology, Milano, Italy*
*[2]IRCCS Humanitas Research Hospital, Neurosurgery Unit, Milano, Italy*
*[3]Universita degli Studi di Milano Bicocca, Nanomedicine Center- NANOMIB, Monza, Italy*

**Introduction**
Among the main aberrations occurring in GBM, those in MEK/ERK pathways predominate and confer GBM Stem Cells (GSCs) sustained proliferation and resistance to conventional therapy (radiotherapy plus adjuvant and concomitant Temozolomide). Recently our laboratory has provided proof-of-concept for a combination strategy based on radiation and adjuvant doxorubicin-loaded liposomes (LPs) conjugated with a modified Apolipoprotein E-derived peptide (mApoE), known to facilitate BBB crossing.
**Material and Methods**
A panel of eight patient-derived primary GSCs lines (GBM WHO IV grade; IDH-wt) displaying a basal activation of the MEK/ERK pathway were surveyed *in-vitro* and *in-vivo* for sensitivity to two small kinase MEK inhibitors, namely MEKi-1 and MEKi-2. The ability of MEKi-1 and -2 to cross the Blood Brain Barrier (BBB) and target GBM cells was investigated using a transwell-based human BBB in vitro model. As GSC-PDX pre-clinical model CD1-*Foxn1*$^{nu}$ mice were orthotopically injected with GSC trasduced with GFP-luciferase construct.
**Results and Discussions**
MEKi-1 and -2 caused a prompt phospho-ERK reduction in all the GSC lines and impaired gliomasphere formation already at very low concentrations (30 nM). However, cell death assessed at flow-cytometry by AnnexinV/PI staining demonstrated an overall higher capacity of MEKi-1 to induce apoptosis compared to MEKi-2 upon 72 hours incubation.The results on BBB model showed inefficient MEKi-1 and -2 BBB crossing suggesting a limited utility for GBM therapy. We, thus, assayed whether the association of MEKi to a brain specific drug delivery strategy might achieve a therapeutic effect. Given its higher in vitro performance, MEKi-1 anti-tumor activity was evaluated both as free drug or encapsulated into mApoE-LPs in GSC-PDX mice. Results demonstrate a striking reduction of systemic toxicity in mice treated with mApoE-MEKi1-LP compared to MEKi1 delivered as free drug (body weight loss below 20% in 71.43% of mice treated with MEKi-1 vs 0% mApoE-MEKi1-LP). Moreover, an improved efficacy of mApoE-MEKi1-LP in decreasing tumor growth compared to free drug MEKi-1 (two-way Anova p = 0.0007).
**Conclusion**
In conclusion, MEKi-1 represents a suitable molecule to target GSC sustained proliferation and our encapsulation strategy represents a valuable solution to overcame deliverable issues in Glioblastoma.

### EACR23-0951
### Solid and liquid biopsy approach to better detect aberrant TP53 in gastric adenocarcinoma

*E. Boldrin[1], M.A. Piano[1], R. Alfieri[2], F. Bernaudo[1], M.R. Biasin[3], I.M. Montagner[3], A. Rosato[1,4], A. Scapinello[3], P. Pilati[2], M. Curtarello[1]*
*[1]Veneto Institute of Oncology IOV-IRCCS, Immunology and Molecular Oncology, Padova, Italy*
*[2]Veneto Institute of Oncology IOV-IRCCS, Surgical Oncology of the Esophagus and Digestive Tract U nit, Padova, Italy*
*[3]Veneto Institute of Oncology IOV-IRCCS, Pathology Unit, Padova, Italy*
*[4]University of Padova, Oncology and Immunology Section- Department of Surger y Oncology and Gastroenterology, Padova, Italy*

**Introduction**
Chromosomal instability (CIN) is frequent in gastric adenocarcinoma (GAC) and it is characterized by *TP53* deletions/mutations, resulting in p53 nuclear accumulation, revealed by immunohistochemistry (IHC). Aiming to improve *TP53* aberrant detection, droplet digital PCR (ddPCR) was used to evaluate *TP53* deletion in solid and liquid biopsy.
**Material and Methods**
83 retrospective and 60 prospective GACs were IHC typed for p53, considering ≥70% stained nuclei as positive for aberration (Gonzalez IHC model). ddPCR was performed in FFPE-DNA and cell-free DNA (cfDNA). Different models' discriminatory power for *TP53* status was tested by receiver operating characteristic (ROC) curves. A custom NGS panel was used to analyze *TP53* mutations.
**Results and Discussions**
Based on ddPCR data, *TP53* was deleted at high frequency in both ≥70% and ≤2% positive nuclei retrospective FFPEs (71% and 77%). Thus, we hypothesized that low p53 staining percentage should be considered "aberrant" as high percentage. Then, we redefined the "Gonzalez IHC model" by incorporating as IHC positive also cases with ≤2% positive nuclei ("Modified IHC model"). A significant AUC of 0.6414±0.06334 (95% CI, 0.5172-0.7657; sensitivity 51.43%; specificity 76%; *p*-value=0.0272) was obtained, in contrast with non-significance of "Gonzalez IHC model" (AUC: 0.5263±0.0665; 95% CI, 0.3959-0.6567; sensitivity 40%; specificity 71.43%; *p*-value=0.697). NGS data on a subset of cases revealed that several samples with ≤2% IHC positive nuclei have premature stop codons (11/16=68.7%), while all ≥70% have missense mutations

© 2023 The Authors. Molecular Oncology is published by John Wiley & Sons Ltd on behalf of Federation of European Biochemical Societies

(21/21=100%).In prospective cohort, *TP53* deletion was analyzed in DNA-FFPE and in time-matched cfDNA. Based on their concordance, samples were classified in: i) both deleted (del/del); ii) both not deleted (not-del/not-del); iii) deleted in only one sample type (del/not-del). According to "Modified IHC model", del/del samples were found with a higher frequency in the positive IHC group compared to the IHC negative (40% *vs* 8%, *p*-value=0.007), while not-del/not-del were more represented in the negative group (36% *vs* 8.6%, *p*-value=0.019). Frequency of del/not-del was similar (51.4% *vs* 56%, *p*-value=0.79).

**Conclusion**

ddPCR and NGS in solid biopsy suggest that IHC, by considering p53 low level staining as negative, underestimates CIN frequency. In addition, ddPCR data, by combining analysis of solid and liquid biopsy, confirms a high frequency of aberrant *TP53* in ≤2% and ≥70% positive nuclei samples, and not aberrant *TP53* in intermediate (3-69%) p53 staining group.

### EACR23-0995
### MOLECULAR CHARACTERIZATION OF UNIFOCAL AND MULTIFOCAL PROSTATE CANCER

*L. Segalés Tañà[1], N. Juanpere[2], M. Lorenzo[3], D. López[3], A. Rodriguez-Vida[4], J. Bellmunt[4], L. Fumadó[5], L. Cecchini[5], J. Lloreta[2], S. Hernández-Llodrà[1]*

*[1]Universitat Pompeu Fabra, Department of Medicine and Life Sciences, Barcelona, Spain*

*[2]Hospital del Mar Medical Research Institute IMIM, Department of Pathology, Barcelona, Spain*

*[3]Hospital del Mar-Parc de Salut Mar, Department of Pathology, Barcelona, Spain*

*[4]Hospital del Mar Medical Research Institute IMIM, Department of Medical Oncology, Barcelona, Spain*

*[5]Hospital del Mar Medical Research Institute IMIM, Department of Urology, Barcelona, Spain*

**Introduction**

Role of tumor focality in prostate cancer (PCa) prognosis has been studied with conflicting results. Some authors reported that unifocal (UF) and multifocal (MF) PCa may be biologically different, with distinct clinical-pathological features and progression risk. Our group has stated that alterations in PTEN, SPOP, SLC45A3, ETV1 and ERG may be used as promising prognostic factors.

Our aim is to analyze the expression of these proteins in UF and MF PCa, to evaluate their worth as prognostic markers, and to study the role of tumor heterogeneity in MF disease.

**Material and Methods**

PTEN, SPOP, SLC45A3, ETV1 and ERG immunostaining was evaluated in 185 PCa, 51 UF and 134 MF, from 9 TMAs. In addition, in a subset of 69 MF cases, the more- and less-aggressive foci were compared. Heterogeneity was considered when both foci presented different expression patterns. Relationship with clinical-pathological features and PSA recurrence was analyzed.

**Results and Discussions**

Expression patterns in UF *vs* MF cases were compared and ETV1 overexpression was associated with UF disease (P=0.04). SLC45A3 loss in UF (P=0.05) and SPOP loss in MF PCa (P=0.04) were related to PSA recurrence. Immunostaining status in the more- and less-aggressive foci from 69 MF PCa was analyzed. PTEN loss (P=0.03), SLC45A3 loss (P<0.001), PTEN plus SPOP loss (P=0.02) and Triple Hit (P=0.003) were associated with the more-aggressive foci.

Data from expression patterns in both foci showed that homogeneous ETV1 overexpression was associated with extraprostatic extension (P=0.05), whereas homogeneous ERG overexpression showed a trend to be related to a younger age at diagnosis (P=0.08). Perineurial infiltration showed a trend to be associated with PTEN homogeneous and SPOP heterogeneous loss (P=0.08; P=0.08).

**Conclusion**

Our results indicated that UF and MF PCa might be different molecular entities, and that immunostaining analysis of these proteins could have prognostic role. In UF disease, ETV1 overexpression was more frequent but SLC45A3 loss was associated with PSA recurrence. On the contrary, in MF, SPOP loss was associated with PSA recurrence.

In addition, data from both foci in MF PCa showed that alterations frequently display a heterogeneous pattern, mostly associated with the more-aggressive foci. Interestingly, homogeneous alteration of PTEN, ETV1 and ERG, but heterogeneous alteration of SPOP, showed a trend or were associated with adverse clinical-pathological features, suggesting that their analysis may be used to predict PCa outcome.

### EACR23-1050
### Preclinical validation of biomimetic regenerative devices to treat bone metastases

*C. Cocchi[1], M. Tavoni[2], E. Lucarelli[3], A.N. Guerrieri[3], C. Liverani[1], M. Dapporto[2], S. Sprio[2], T. Ibrahim[3], A. De Vita[1], L. Mercatali[1]*

*[1]Istituto Romagnolo per lo studio dei Tumori "Dino Amadori" IRST IRCCS, Preclinic and Osteoncology Unit, Meldola, Italy*

*[2]Institute of Science- Technology and Sustainability for Ceramics ISSMC-CNR- former ISTEC-CNR, Institute of Science- Technology and Sustainability for Ceramics ISSMC-CNR- former ISTEC-CNR, Faenza, Italy*

*[3]Istituto Ortopedico Rizzoli IOR IRCCS, Osteoncology- Bone and Soft Tissue Sarcomas and Innovative Therapies Unit OSOTT, Bologna, Italy*

**Introduction**

In cancer patients, the development of bone metastases (BM) is very common. Osteolytic BM represent a prominent source of morbidity being associated with clinical complications, negatively affecting quality of life, functional autonomy and survival rate. Materials that can control tumor growth and bone resorption at the same time could be used for the treatment of BM. We functionalized self-hardening calcium phosphates cements (CPCs) with chemotherapeutic doxorubicin (Doxo) and an anti-RANKL antibody (an inhibitor of osteoclastic-induced bone resorption) and tested the efficacy in preclinical models of BM.

**Material and Methods**

Bone cements were synthesized, functionalized with Doxo and anti-RANKL antibody, and then characterized. Drug

© 2023 The Authors. Molecular Oncology is published by John Wiley & Sons Ltd on behalf of Federation of European Biochemical Societies release was checked to satisfy specific selected concentrations. MDA-MB-231 breast cancer (BC) cells, peripheral blood mononuclear cells (PBMCs) differentiated into Osteoclasts (OCs), and Mesenchymal Stem Cells (MSCs) differentiated into osteoblasts (OBs), were seeded on bone cements in mono- and co-cultures to test drug efficacy. Bone cell differentiation was checked by analysing markers of osteoclastogenesis and osteoblastogenesis with Real Time PCR and immunofluorescence. Patient-derived tumor fragments (PDTFs) of bone metastasis were used to validate the efficacy ex vivo. Viability assays, immunofluorescence, and confocal microscopy were performed as downstream analyses.

**Results and Discussions**

Bone cements were observed to have a good safety profile for all types of cells used. Devices functionalized with Doxo resulted in about an 80% inhibition of cancer cell growth with respect to non-medicated devices; the anti-RANKL antibody decreased cancer cell growth by about 20%. However, the effect of combining the two drugs was not additive. The co-coculture of cancer cells with either OCs or OBs did not affect cancer cell viability. Both drugs decreased the number of OBs/OCs and inhibited their differentiation**.** Results on PDTFs show a decreased viability in samples exposed to the combination of medicated CPCs.

**Conclusion**

Medicated bone cements provided a relevant impact on cancer cell death and on bone cell differentiation, particularly of osteoclasts. These observations are important as these two events are integral to the process of blocking the vicious cycle of bone metastases. These results provide a proof of concept of the clinical use of these medicated materials on patients with bone metastases.

### EACR23-1051
### Cyclodextrin-based nanomedicine as tailored platform for chemotherapy and immunotherapy for glioblastoma

*A. Scomparin[1], S.A. Rizzo[1], S. Pozzi[2], M. Argenziano[1], C. Dianzani[1], F. Caldera[3], F. Trotta[3], R. Satchi-Fainaro[2], U. Dianzani[4], R. Cavalli[1]*
*[1]University of Turin, Drug Science and Technology, Torino, Italy*
*[2]Tel Aviv University, Physiology and Pharmacology, Tel Aviv, Israel*
*[3]University of Turin, Chemistry, Torino, Italy*
*[4]University of Eastern Piedmont, Department of Health Sciences, Novara, Italy*

**Introduction**

Glioblastoma is the most aggressive of brain cancers and its treatment includes surgery, radiation, anti-angiogenic therapy, and chemotherapy. Temozolomide is the gold standard drug but a number of patients do not respond due to chemoresistance issues. In the last few years, with the general recognition of the efficacy of immunotherapy in cancer treatment, and the concomitant identification of lymphatic vessels in the brain, immune-checkpoint modulators, CAR T cells, and vaccines have been exploited for glioblastoma therapy. Recently, the tyrosine kinase inhibitor ibrutinib has been shown to be active in inhibiting glioblastoma, targeting tumorigenic glioma stem cells. The synergistic effect of two drugs with different mechanisms of action represents great therapeutic potential.

**Material and Methods**

In order to fully exploit this strategy, we developed a nano-sized polymeric system, based on β-cyclodextrin (β-CD), a natural cyclic oligosaccharide. The repeating units of β-CD were reacted with different cross-linking agents, to obtain linear, branched, or hyper-crosslinked polymers. The latter, defined as nanosponges (NS), are exploited to load both low molecular weight drugs and antibodies. The β-CD-based branched polymers were further modified to introduce positively charged amino groups, and are able to form electrostatic complexes with oligonucleotides.

**Results and Discussions**

NS are nano-sized (~250 nm) polymers with multiple domains, i.e. hydrophobic CD cavities and hydrophilic nanochannels, that can be exploited to load active molecules. They possess high encapsulation efficacy for temozolomide (92.08%, with a loading capacity of 7.56%) and ibrutinib (62.60%, with a loading capacity of 5.35%). The formulation retains the in vitro cytotoxicity of the drugs on a rat undifferentiated malignant glioma cell line (F98 cells). Furthermore, the NS can be conjugated to the inducible T cell co-stimulator (ICOS) protein, which has been demonstrated to have several implications in cancer progression. ICOS-decorated NS loaded with ibrutinib were able to inhibit F98 cells migration.

**Conclusion**

The newly developed β-CD-based polymers can be easily tailored to develop several types of nanomedicines able to improve the efficacy of anticancer drugs, oligonucleotides, and immunotherapeutic agents.

### EACR23-1058
### Generation of first PDX model of ALK+ histiocytosis demonstrates high sensitivity to ALK inhibition.

*E. Patrucco[1], L. Mologni[2], G. Mura[1], C. Mastini[2], C. Ambrogio[1], F. Pagni[2], R. Piazza[2], C. Voena[2], R. Chiarle[1,3], C. Gambacorti-Passerini[2,4]*
*[1]University of Torino, Molecular Biotechnology and Health Science, Torino, Italy*
*[2]University of Milano-Bicocca, Department of Medicine and Surgery, Monza, Italy*
*[3]Children's Hospital and Harvard Medical School, Department of Pathology, Boston, United States*
*[4]IRCCS San Gerardo, Hematology, Monza, Italy*

**Introduction**

Histiocytic disorders are rare diseases characterized by proliferation of differentiated macrophages, dendritic cells, or monocytes in various tissues and organs. Genetic analysis changed the understanding of these diseases from a primary inflammatory condition to a clonal neoplastic disease. Molecular alterations that drive neoplastic growth involve the MAP kinase pathway, the mTOR/PI3K/AKT pathway as well as the recently characterized ALK+ histiocytosis. We describe here the establishment of the first patient-derived xenotransplant (PDX) of an ALK+ histiocytosis and demonstrated its value to determine therapeutic approaches.

**Material and Methods**

© 2023 The Authors. Molecular Oncology is published by John Wiley & Sons Ltd on behalf of Federation of European Biochemical Societies

A 22-year-old patient with history of Hodgkin lymphoma in 2019 developed a paravertebral mass that was diagnosed as ALK+ histiocytic tumor (ALK+, CD45+, CD163+, CD68PGM1+, CD30-, Cytokeratin-, CD3-, CD20- by IHC). The patient was treated with the ALK TKI crizotinib in 2020, relapsed in 2021 and was shifted to lorlatinib obtaining a new remission. In November 2021, a residual 7.5 cm node was resected from the left lung composed of abundant necrotic and fibrotic tissue with few residual ALK+/CD163+ cells. We established a PDX by implanting fragments of the resected tumor in NSG mice. The PDX was studied by phenotyping, RNA-seq, scRNA-seq, Western Blot, and in a mouse clinical trial (MCT).

**Results and Discussions**

Immunohistochemical analysis of the PDX confirmed the ALK+, CD163+, phenotype identical to the patient's tumor. RNA-seq and scRNA-seq demonstrated a fusion between EML4(exon 2) and ALK(exon 20) and confirmed high expression of CD68, CD163, S100, with low expression of TTF1 and all cytokeratins. WB confirmed the presence of a phosphorylated ALK fusion protein. The MCT was implemented with cohorts of NSG mice weighing 25 grams. Mice bearing sub-cutaneous PDX were treated with the ALK inhibitors Crizotinib (100mg/kg/day) and Lorlatinib (10mg/kg/day). Both ALK TKIs showed potent anti-tumor activity by inducing tumor regression as single agent therapy. WB analysis showed a marked reduction in the phosphorylation of ALK and key downstream effectors ERK1/2 and STAT3 only in the treated groups.

**Conclusion**

We generated the first model of an ALK+ histiocytosis that closely recapitulates the phenotypic and molecular features of the patient's tumor. Like in patients, ALK+ PDX histiocytic tumors showed sensitivity to ALK TKIs and will offer a valid experimental model to study potential resistance mechanisms and additional therapies.

### EACR23-1059

### An in vitro and ex vivo validation of medicated devices to treat soft tissue sarcomas.

*A. De Vita[1], C. Cocchi[1], E. Lucarelli[2], C. Bellotti[2], A.N. Guerrieri[2], C. Liverani[1], G. Sbanchi[1], M.L. Focarete[3], T. Ibrahim[2], L. Mercatali[1]*

*[1]Istituto Scientifico Romagnolo per lo studio dei Tumori "D ino Amadori", Preclinic and Osteoncology Unit, Meldola, Italy*

*[2]Istituto Ortopedico Rizzoli IOR IRCCS, SC Osteoncology- Bone and Soft Tissue Sarcomas and Inn ovative Therapies Unit OSOTT, Bologna, Italy*

*[3]Bologna University, Department of Chemistry "Giacomo Ciamician", Bologna, Italy*

**Introduction**

Soft Tissue sarcomas (STS) are a heterogeneous group of rare tumors with a set of peculiar clinical challenges, such as tumor recurrence and wound complications. To improve patient outcomes, innovative therapeutic approaches must be developed. Materials with both antitumor and anti-inflammatory agents could be used after surgery to eliminate residual tumor cells and also to promote tissue repair. For this purpose, we developed a device that could be implemented in patients with STS and tested its efficacy in an in-vitro model.

**Material and Methods**

The device was made in the form of a patch to be flexible and suturable. The bioresorbable patch was composed of two outer layers made of polymeric nanofibrous multilayer (PLLA+ Pluronic acid) and a GelMA hydrogel inner layer. Epirubicin (an anticancer drug), was added to both outer layers and diclofenac (an anti-inflammatory drug) was added to the inner layer to obtain a controlled drug release over 7 days. The release of the drugs was quantified through HPLC-MS.

The effect of single or combined drugs was evaluated in-vitro using a commercial and a patient-derived STS cell line. Normal fibroblasts were used as a healthy component to mimic a soft tissue microenvironment. Viability assays, immunofluorescence, and confocal microscopy were performed as downstream analyses.

**Results and Discussions**

Epirubicin was released in a sustained manner over a period of about 3 days, whereas diclofenac was released in a sustained manner over a period of about 7 days. Epirubicin had a cytotoxic effect with inhibition of cancer cell growth. Diclofenac decreased fibroblast survival without any further effects on cancer cells. In addition, the results obtained on the patient-derived myxofibrosarcoma cell line (MF-R3) demonstrated a decrease in cell viability, with an increase in necrosis.**Conclusion**

Our results provide proof of concept of the efficacy of medicated patches for the treatment of STS in-vitro. These results establish the basis for the application of medicated materials to control tumor growth and induce soft tissue regeneration.

### EACR23-1061

### XENTURION, a multidimensional resource of xenografts and tumoroids from metastatic colorectal cancer patients for population-level translational oncology

*S.M. Leto[1], E. Grassi[1], M. Avolio[1], V. Vurchio[1], F. Cottino[1], M. Ferri[1], E.R. Zanella[1], L. Di Blasio[2], F. Galimi[1], M. Viviani[1], A. Puliafito[2], L. Primo[2], A. Bertotti[1], L. Trusolino[1]*

*[1]Candiolo Cancer Institute – FPO IRCCS, Laboratory of Translational Cancer Medicine, Candiolo Torino, Italy*

*[2]Candiolo Cancer Institute – FPO IRCCS, Laboratory of Cell Migration, Candiolo Torino, Italy*

**Introduction**

The breadth and depth at which cancer models are interrogated contribute to successful translation of drug discovery efforts to the clinic. In metastatic colorectal cancer (mCRC), model availability is limited by a dearth of large-scale collections of patient-derived xenografts (PDXs) and matched organoids (tumoroids). Here we describe XENTURION, a unique open-science resource of XENografts and Tumoroids for Research In ONcology that encompasses 129 sibling pairs of mCRC PDXs and PDX-derived tumoroids (PDXTs) with accompanying molecular and therapeutic characterization.

**Material and Methods**

© 2023 The Authors. Molecular Oncology is published by John Wiley & Sons Ltd on behalf of Federation of European Biochemical Societies

The vast majority of XENTURION models were analyzed for mutations (targeted next-generation sequencing of 116 relevant CRC genes), gene copy number architecture (DNA shallow sequencing) and global transcriptomics (RNAseq) and benchmarked against large patient datasets (TCGA and MSK-IMPACT). A PDXT-based population trial with the clinically approved anti-EGFR antibody cetuximab was performed in 116 PDXTs and 79 matched PDXs. Cetuximab response profiles were compared to the outcome of EGFR knock-out by CRISPR-Casp9 technology in 13 representative PDXTs. Adaptive signals upregulated by EGFR blockade were computationally and functionally prioritized; top candidates were screened in PDXTs; and surviving compounds were finally validated in PDXs.

**Results and Discussions**

XENTURION models were representative of the genetic heterogeneity of mCRCs and showed high genetic and transcriptional similarity between matched pairs. Cetuximab responses in PDXTs revealed variable sensitivities that were consistent with clinical response biomarkers, mirrored tumor growth changes in matched PDXs, and recapitulated the outcome of EGFR genetic deletion. A stepwise drug screen identified actionable co-extinction targets, whose inhibition increased the magnitude of response to cetuximab.

**Conclusion**

This platform addresses a long-standing quest for large-scale collections of extensively annotated CRC preclinical models for integrative *ex vivo* and *in vivo* translational applications. To our knowledge, this is the first large-scale study in which a systematic comparison of molecular and therapeutic profiles between PDXT-PDX pairs was attempted. As a publicly available resource, XENTURION will offer a knowledge base of disseminatable methods, resources and information to streamline preclinical studies and accelerate new treatments for patients with mCRC.

### EACR23-1083

### Peritoneal metastasis of colorectal cancer (pmCRC): Identification of predictive molecular signatures by a novel preclinical platform of pmCRC PDX models

M. Dahlmann[1,1,2,3], B. Brzezicha[2], J. Hoffmann[2], U. Keilholz[4], W. Walther[1], B. Rau[5], U. Stein[1,3]

*[1]Experimental and Clinical Research Center- Charité - University Medicine Berlin- and Max-Delbrück-Center for Molecular Medicine in the Helmholtz Association, Translational Oncology of Solid Tumors, Berlin, Germany*

*[2]Experimental Pharmacology & Oncology GmbH, Berlin-Buch, Berlin, Germany*

*[3]DKFZ, German Cancer Consortium, Heidelberg, Germany*

*[4]Charité – Universitätsmedizin Berlin, Charité Comprehensive Cancer Center, Berlin, Germany*

*[5]Charité – Universitätsmedizin Berlin, Department of Surgery, Berlin, Germany*

**Introduction**

Peritoneal metastasis of colorectal carcinoma (pmCRC) occurs in approximately 15% of CRC patients, behaves clinically distinct from hematogenous spread. Predictive molecular signatures to medical treatments have not yet been defined. The aim of the study was the establishment and characterization of patient-derived xenograft (PDX) models from surgical samples of pmCRC patients for individualized therapy concept to improve outcome of pmCRC patients and to define signatures to better guide therapy selection.

**Material and Methods**

pmCRC metastases were obtained during routine surgery and used to generate PDX models, which were treated with Standard-of-Care (SoC) regimen and targeted drugs, while growth response was monitored. All sample types were molecularly characterized by multi-omics technologies to identify putative predictive biomarkers.

**Results and Discussions**

14 pmCRC PDX models were established. Preclinical treatment response to 17 SoC and targeted therapies were integrated with multi-omics data and resulted in predictive biomarkers for 10 treatment options. Of note, enrichment of BRCA2 mutations in the pmCRC samples pointed to the use of PARP inhibitors as novel two-hit combination therapy, in which combination of olaparib with 5-FU or trametinib strongly improved the response of our PDX models resistant to respective monotherapy. Patterns of gene/protein expression, coding mutations, and protein phosphorylation of pmCRC metastases and their derived PDX models correlated well. Data integration revealed altered cellular processes and signaling pathways impacting therapy response as basis to molecularly tailor new therapies.

**Conclusion**

The novel preclinical drug testing platform of matched pmCRC models, characterized by multi-omics, facilitated the identification of predictive biomarkers and cancer relevant signatures for efficient and novel drug combinations to improve the outcome in pmCRC therapy, ready for validation in clinical cohorts.

### EACR23-1086

### Synergic activity of FGFR2 and MEK inhibitors in the treatment of FGFR2-amplified cancers of unknown primary

G. Gallerani[1], I. Salamon[1], A. Cavazzoni[2], C. Fumarola[2], M. Riefolo[3], K. Rihawi[4], B. Fontana[1], S. Cairo[5], A. Ardizzoni[4], M. Ferracin[1]

*[1]University of Bologna, Department of Medical and Surgical Sciences DIMEC, Bologna, Italy*

*[2]University of Parma, Department of Medicine and Surgery, Parma, Italy*

*[3]IRCCS Azienda Ospedaliero-Universitaria di Bologna, Pathology Unit, Bologna, Italy*

*[4]IRCCS Azienda Ospedaliero-Universitaria di Bologna, Oncology Unit, Bologna, Italy*

*[5]XenTech, XenTech, Evry, France*

**Introduction**

Patients with cancer of unknown primary (CUP) carry the double burden of an aggressive disease and reduced access to therapies, which are typically tumor-type oriented. Experimental *in vitro* and *in vivo* models summing up CUP features and heterogeneity are pivotal for CUP biology investigation and drug testing.

**Material and Methods**

© 2023 The Authors. Molecular Oncology is published by John Wiley & Sons Ltd on behalf of Federation of European Biochemical Societies

We established four CUP cell lines derived from different biological sources: CUP#96, CUP#55, CUP#157 from ascites and CUP#138 from metastases biopsy. We derived corresponding patient-derived xenografts (PDXs) from CUP#55 and CUP#96 cell lines. CUP cell lines and PDXs were subjected to histological, immune-phenotypical, molecular and genomic characterization to show their ability to recapitulate the original tumor. MicroRNA profile, target sequencing and low-pass Whole Genome Sequencing were applied to identify the possible sites-of-origin and therapeutic targets. FGFR2 gene amplification in both cell lines provided the rationale for evaluating the potential of targeting this receptor for therapeutic intervention. To target FGFR2 we first tested a panel of FGFR2 inhibitors, then we selected the most effective and combined this drug with a MEK inhibitor and compared with the standard therapy received by the patients.

**Results and Discussions**

Tumor primary sites predicted thought MicroRNA profiles were gastrointestinal for CUP#96 and cholangiocarcinoma for CUP#55 model. Genetic testing and FISH analysis identified FGFR2 amplification as druggable genetic alteration in both patients, which was also validated in cellular and animal models. Drug-screening assays were performed *in vitro* to test the activity of FGFR2 targeting drugs on cancer cell proliferation and viability. The permanence of MAPK signaling activity upon FGFR2 targeting prompted the combination treatment with MEK inhibitor, trametinib, which proved to be synergic and exceptionally active.

**Conclusion**

This study brings personalized therapy closer to CUP patients and paves the way to future applications of personalized medicine for metastatic patients with adverse prognosis.

### EACR23-1134
### Molecular imaging based evaluation of mRNA vaccine candidates

H. Youn[1], H.R. Oh[2], I.W. Kim[3], K.J. Hong[4]
*[1]Seoul National University Hospital, Cancer Imaging Center, Seoul, South Korea*
*[2]Seoul National University, Biomedical Sciences, Seoul, South Korea*
*[3]Seoul National University, Cancer Research Institute, Seoul, South Korea*
*[4]Gachon University, Microbiology, Incheon, South Korea*

**Introduction**

Reporter-based molecular imaging has provided a lot of important information for research in life science. Among various imaging techniques, bioluminescence imaging is widely used as *in vivo* preclinical imaging because of its advantages such as high sensitivity, high resolution, and high selectivity without external light excitation.The COVID-19 pandemic has accelerated the development of mRNA vaccines, and non-invasive tracking of mRNA vaccines is essential to evaluate vaccine efficacy and safety. In this study, we established a standard protocol for visualizing mRNA vaccine candidates containing luciferase-encoded mRNA and lipid nanoparticles (LNPs) at the preclinical level using bioluminescence imaging. This protocol was used to visualize and evaluate the in vivo luciferase expression of mRNA vaccine candidates with various mRNA platforms and LNPs under different injection routes.

**Material and Methods**

In vitro transcriptions of different mRNA platforms were performed to generate luciferase-coding mRNAs. Different mRNAs were encapsulated with LNPs by and EnParticles's microfluidics device (enCell Master, 10 ml/min). To visualize mRNA vaccine candidates by bioluminescence imaging, 3 mg of D-luciferin as a luciferase substrate, was intraperitoneally administered to BALB/c nude mice. Bioluminescence signals were acquired with IVIS spectrum and analyzed with Living Imaging software (ver.2.50.2).

**Results and Discussions**

Luminescence signals over >2,000 p/s/cm$^2$/sr were observed from 20 minutes after vaccination and peaked at 6 h. When the luciferase signal was tracked for several days, the peak of the luminescent signal was gradually decreased after 6 h, but signal remained even after 1 week. When different amounts of luciferase mRNA were injected, the correlation between the amount of mRNA and the luminescence signal was investigated, and a sufficient luminescence signal was observed even with 1 ug of mRNA. In addition, sufficient signals were observed in the injection site, nearby lymph nodes, spleen, and liver for all of the intramuscular, subcutaneous, and intravenous injections. When the mRNA platform and the composition of the LNP were different, the in vivo expression including the injection site and nearby lymph nodes showed a temporal and spatial difference.

**Conclusion**

Taken together, monitoring the *in vivo* expression of mRNA vaccines under various conditions using bioluminescence imaging can be effectively used to evaluate the efficacy and safety of vaccine candidates.

### EACR23-1176
### Patient-Derived Organoids to Identify Novel Therapeutic Targets in EGFR-Driven Lung Adenocarcinoma

V. Guzzeloni[1,2], F. Pedica[1,3], P. Muriana[4], P. Novellis[4], A. Nuccio[1,2], F. Ogliari[1,2], A. Bartolucci[1,3], M.G. Cangi[3], G. Arrigoni[3], G. Veronesi[1,4], A. Bulotta[2], G. Foggetti[1,2]
*[1]Vita-Salute San Raffaele University, Milan, Italy*
*[2]Department of Medical Oncology, IRCCS Ospedale San Raffaele, Milan, Italy*
*[3]Pathology Unit, IRCCS Ospedale San Raffaele, Milan, Italy*
*[4]Department of Thoracic Surgery, IRCCS Ospedale San Raffaele, Milan, Italy*

**Introduction**

Lung cancer is the leading cause of cancer deaths worldwide and lung adenocarcinoma (LUAD) is the most frequent histological subtype of this disease. Oncogenic alterations in *EGFR* are found in ~15% of LUADs and predict sensitivity to specific tyrosine kinase inhibitors (TKIs), which represent the current frontline therapy. Despite high tumor responses to TKIs, there is heterogeneity in the clinical outcomes and in the drug resistance mechanisms that inevitably emerge. Preclinical models of *EGFR* mutant tumors such as patient-derived organoids (PDOs) can recapitulate the complexity of human disease representing a valuable tool to investigate

© 2023 The Authors. Molecular Oncology is published by John Wiley & Sons Ltd on behalf of Federation of European Biochemical Societies determinants of reduced drug response and define novel therapeutic targets.

**Material and Methods**

We assessed the 3D culture conditions in human *EGFR* mutant cell lines and began to generate PDOs from tumor samples collected from patients with *EGFR* mutant LUAD who are enrolled in the '*Lung Cancer Tissue Collection*' protocol. After enzymatic and mechanic tissue dissociation, single cells were embedded in a basement membrane matrix and cultured using different culture media. We evaluated organoid formation and growth, as well as histological and molecular features. We paired our *in vitro* data with genomic and clinical data analysis.

**Results and Discussions**

We propagated organoids up to 28 days of culture. Individual cells proliferated and formed spherical organoid morphology that were maintained during the time of culture. We monitored organoid growth and viability by measuring the diameter of individual organoids and their metabolic activity. As expected, larger organoid diameters correlated with higher ATP levels. Organoids recapitulated LUAD histological features by staining for H&E and LUAD markers. Moreover, since we previously demonstrated that the KEAP1 pathway plays a role in mediating TKI sensitivity, we are evaluating the status of the KEAP1 pathway in our models to investigate the extent to which this pathway is dysregulated in *EGFR* mutant tumors.

**Conclusion**

We optimized the conditions to generate *EGFR* mutant PDOs mirroring the features of human specimens. Our current work focuses on establishing a cohort of PDOs that we will leverage to evaluate TKI sensitivity and investigate the mechanisms that could mediate a differential response to TKIs. Future studies with these unique models will help identify genotype-specific vulnerabilities and test potential therapeutic strategies for subsets of lung tumors to improve drug response and delay the occurrence of resistance.

### EACR23-1233

### ROS-responsive dextran nanocarriers as a new drug delivery system for head and neck cancer.

*N. Caz*[1], *S. Nayak*[2], *J. Van den Bosch*[1], *A. Ethirajan*[2], *E. Wolfs*[1]

[1]*Hasselt University- Biomedical Research Institute BIOME D, Laboratory for Functional Imaging & Research on Stem Cells FIERCE, Diepenbeek, Belgium*

[2]*Hasselt University- IMO-IMOMEC- IMEC, Nano-biophysics and soft matter interfaces NSI, Diepenbeek, Belgium*

**Introduction**

Chemoradiotherapy is the main treatment strategy for many cancers, including head and neck cancer. Despite successful tumour-killing, healthy tissue is also affected leading to adverse side-effects and a decreased patients' quality of life. In order to circumvent severe (systemic) side-effects, we aim to deliver chemotherapeutic agents using a dextran nanocarrier (NC)-based system that respond to reactive oxygen species (ROS). ROS are known to be increased in the tumour micro-environment, and will be used as a stimulus for the NCs to release their cargo.

**Material and Methods**

NCs consist of dextran polymers and are incorporated with thioketal moieties to enable ROS responsiveness. The NCs are loaded with a fluorescent dye, allowing visualization. ROS responsiveness is confirmed through release studies using a dialysis bag membrane. Cytotoxicity of these NCs is examined using a head and neck squamous cell carcinoma (HNSCC) cell line (UM-SCC-14C) via the alamarBlue cell viability assay. Moreover, cellular uptake of the NCs is assessed via confocal microscopy imaging and flow cytometry. Alterations in cellular redox state are evaluated based on microplate assays for general ROS levels, mitochondrial superoxide, and reduced GSH levels.

**Results and Discussions**

ROS release studies using the NCs display solid responsiveness to the cellular environment, outperforming the release induced by $H_2O_2$. Furthermore, NCs show excellent biocompatibility as indicated by a maintained cell viability after incubation with HNSCC cells. Cells also successfully take up ROS-responsive NCs. In addition, the endogenous intracellular ROS and reduced GSH levels are not changed upon incubation with NCs.

**Conclusion**

Our data confirm that the designed NCs are ROS responsive, biocompatible, and taken up by HNSCC cells. For future experiments, NCs can be loaded with a chemotherapeutic agent and tested for selective tumour-killing. However, our results indicate that these stimuli-induced NCs offer a promising drug delivery strategy to treat head and neck cancer.

### EACR23-1261

### Mesenchymal stem cell membrane-coated polymeric nanoparticles for TPCS2a delivery

*G. Avancini*[1], *L. Menilli*[1], *F. Moret*[1], *F. Mastrotto*[2]

[1]*University of Padova, Department of Biology, Padova, Italy*

[2]*University of Padova, Department of Pharmaceutical and Pharmacological Sciences, Padova, Italy*

**Introduction**

Breast cancer (BC) still represents a challenge in tumour treatment, especially for patients with advanced-stage disease. Photodynamic therapy (PDT) is gaining a lot of attention as therapeutic option for BC because of its selectivity and low off-target effects. However, the high hydrophobicity of photosensitizers (PSs) is a major obstacle to PDT application. PSs loading into a polymeric nanoparticle (NP) may represents a successful strategy to improve tumour accumulation and efficacy. In addition, the coating of NPs with mesenchymal stem cell-derived plasma membranes (mMSCs) may prolong NPs circulation time and confers tumor-homing ability to the NPs, due to the presence of cytokines/chemokines receptors associated to the membranes.

**Material and Methods**

The photosensitizer $TPCS_{2a}$ was loaded into PLGA NPs via microfluidic technique. $TPCS_{2a}$-PLGA NPs were coated with isolated mMSC by ultrasonication to obtain the biomimetic nanosystem (mMSC-$TPCS_{2a}$-NPs). Size distribution, zeta potential and morphology of NPs were assessed by DLS and TEM. $TPCS_{2a}$ loading efficiency was determined by RP-HPLC, while singlet oxygen production

© 2023 The Authors. Molecular Oncology is published by John Wiley & Sons Ltd on behalf of Federation of European Biochemical Societies by NPs was measured by fluorescence analysis using Sensor Green reagent. *In vitro* phototoxicity and cellular uptake of $TPCS_{2a}$-PLGA NPs and mMSC-$TPCS_{2a}$-NPs was measured on MDA-MB-231,MCF-7, and MCF10A breast cancer cell lines in 2D and 3D models. NPs uptake by human macrophages was also evaluated.

**Results and Discussions**

$TPCS_{2a}$-loaded NPs of 100 nm with an encapsulation efficiency of 88% were obtained. TEM images showed a core-shell structure for mMSC-$TPCS_{2a}$-NPs with a size of 115 nm. NPs release studies indicated a sustained $TPCS_{2a}$ release over 18 days. *In vitro* cellular uptake studies suggested that $TPCS_{2a}$-loaded PLGA NPs and mMSC-$TPCS_{2a}$-NPs were similarly internalized by cancer cells, with a slightly lower uptake of membrane coated NPs. Both formulations had comparable cell cytotoxic effects after red-light irradiation, in either 2D or 3D cell models, while no toxicity was observed in absence of irradiation. Lower uptake by macrophages was observed for mMSC-$TPCS_{2a}$-NPs as compared to $TPCS_{2a}$-loaded PLGA NPs.

**Conclusion**

Our results demonstrate the efficient loading of TPCS2a in PLGA-NPs and the successful coating of the nanosystem with mMSCs, thus avoiding PS aggregation, limiting the clearance by macrophages, and ensuring the production of singlet oxygen upon light irradiation.The *in vitro* uptake and cancer cell killing efficiency of mMSC-$TPCS_{2a}$-NPs was comparable to that of $TPCS_{2a}$-loaded NPs.

### EACR23-1282
### Microsurgical resection on glioblastoma causes ischemic microenvironment and proneural to mesenchymal tumor cells transition

*A. Ballestín[1], C. Pichol-Thievend[1], G. Bourmeau[1], B. Blanchard[1], A. Pettiwala[1], J. Reveilles[1], S. Leboucher[2], O. Anezo[1], G. Seano[1]*
*[1]Institut Curie, Tumor Microenviroment Laboratory UMR3347 CNRS / U1 021 INSERM, Orsay, France*
*[2]Institut Curie, Histology Facility, Orsay, France*

**Introduction**

Glioblastoma (GB) is the most common and lethal primary brain tumor. The standard of care is based on maximal safe surgical resection followed by fractionated radiotherapy with concomitant and adjuvant systemic chemotherapy with temozolomide. Despite optimal treatment, recurrence occurs in more than 90% of patients and usually begins with regrowth of highly invasive cells that spread from the peritumoral brain resection zone. Four GB cellular states have been identified, driving the intratumor heterogeneity of malignant cells influenced by genetics and the tumor microenvironment (TME). The impact of surgical resection on TME or plasticity of GB cells is unknown. Thus, the objective of this study was to perform morphological and transcriptomic phenotyping of residual tumor cells and TME after microsurgical resection.

**Material and Methods**

C57Bl6j mice and syngeneic GB IDH-wildtype cell lines CT2A and GL261 were used. GFP-transduced GB cells were orthotopically implanted. We developed a refined microsurgical resection protocol for GB in size-matched mice and carefully followed the same surgical steps performed in patients. The mice were randomly divided into four groups: control (prior to surgery) and 1, 3, and 7 days post-surgery. Tumor tissues were harvested for bulk and single-cell RNA sequencing and immunohistochemistry (IHC) studies.

**Results and Discussions**

Time-resolved transcriptomic and IHC analyses showed that surgical damage induced pronounced proneural-to-mesenchymal transition (PMT), stimulation of hypoxia-driven pathways, and induction of chromatin remodeling in residual post-surgery GB cells, independent of the transcriptional landscape of GB cell lines. Moreover, single-cell RNA-seq of post-resection tumors allowed us to observe significant changes in the TME that are in line with exposure to ischemia, specifically in microglia, T-cells, Tregs and endothelial cells.

**Conclusion**

Our results clearly demonstrated that surgical insult has important consequences on tumor fate. The early postoperative phenotypic landscape of GB tumor cells and TME is characterized by a transient ischemic TME that induces PMT in GB cells. In addition, we established a well-characterized and refined microsurgical resection model that may serve to evaluate therapeutic agents under reproducible and controlled conditions.

### EACR23-1421
### Consensus identification of future cancer research priorities in Europe: the UNCAN initiative

*J. Carmona[1], A. Piris[1], L. Villanova[2], I. Miguel[3], N. Bayó[1], E. Solary[4], R. de Maria[5], F. Locatelli[6], A. Bigas[3], J. Tabernero[7]*
*[1]Vall d'Hebron Institute of Oncology, Research Management Area, Barcelona, Spain*
*[2]Alliance Against Cancer, Project Management, Rome, Italy*
*[3]CIBERONC, Scientific Direction, Barcelona, Spain*
*[4]Institut National de la Santé et de la Recherche Médicale, Clinical Hematology, Villejuif, France*
*[5]Alliance Against Cancer, Translational Medicine and Surgery, Rome, Italy*
*[6]Alliance Against Cancer, Oncology- Hematology and Cell and Gene Therapy, Rome, Italy*
*[7]Vall d'Hebron Institute of Oncology, Medical Oncology, Barcelona, Spain*

**Introduction**

The launch of Europe's Beating Cancer Plan and the Horizon Europe's Mission on Cancer reflect the institutional commitment of the European Union (EU) to boost the strategy against cancer to improve the lives of citizens through better prevention, treatments, and quality of life. Consistent with this goal, the Mission's Board spurred the creation of a Europe-wide platform to **UN**derstand **CAN**cer (UNCAN.eu) to address the need for more investment on innovative research. Seeking to accelerate its implementation, one of the goals of the coordination and supportive action (CSA) "4.UNCAN.eu" is to define the research priorities for UNCAN.eu in six pre-defined research areas.

© 2023 The Authors. Molecular Oncology is published by John Wiley & Sons Ltd on behalf of Federation of European Biochemical Societies

**Material and Methods**
Following a European-wide open call, independent cancer researchers and patient representatives were invited to apply to join the six Expert Working Groups (EWG) of UNCAN. Through a systematic selection process, 300 candidates (50 per area) were selected to form the EWG in the areas of cancer prevention, early diagnosis, sensitivity and resistance to therapy, pediatric cancer, cancer and ageing and survivorship. Participants within each EWG provided input through a two-wave, online Delphi consensus process, and were given the chance to propose new items. After an initial online phase, experts were invited to attend two face-to-face workshops to discuss top-ranked research items, brainstorm about new funding mechanisms to accelerate scientific breakthroughs, and debate transversal aspects related to data, innovation, and patient engagement.
**Results and Discussions**
The consultation process with European experts identified challenges in cancer research to be addressed at the supranational level and generated recommendations on funding mechanisms and data resources needed to accelerate research and innovation in the six research areas. Moreover, the consensus exercise provided a framework to align the priorities of cancer researchers with the patients' needs and produced specific guidelines to enhance communication between researchers and patients and increase their involvement in research activities.
**Conclusion**
As one of the main objectives of the CSA "4.UNCAN.eu", this consensus-based prioritization exercise joins the efforts from other partners to set a strategic agenda of research priorities in cancer that seeks to guide the European Commission in the design of funding programmes and policies that enable the next breakthroughs in cancer research needed to reduce the disease burden for citizens.

### EACR23-1424
### A Mesoporous silica-based Nanodevice as a vehicle for Specific Drug Targeting and Improved Efficacy in the treatment of Multiple Myeloma

*C. Morelli*[1], *C. Longobucco*[1], *M. Fava*[1], *M. De Santo*[1], *P.A. Cavallaro*[1], *D. Sisci*[1], *A. Leggio*[1], *L. Pasqua*[2]
[1]*University of Calabria, Department of Pharmacy- Health and Nutritional Sciences, Rende, Italy*
[2]*University of Calabria, Department of Environmental Engineering, Rende, Italy*
**Introduction**
Multiple myeloma (MM) is a B-cell neoplasm characterized by uncontrolled growth of malignant plasma cells and monoclonal immunoglobulins secretion within the bone marrow. One of the main challenges in tumor treatment is to develop a therapeutic strategy able to selectively target cancer cells preserving normal tissues from undesired side effects. Localized drug delivery should cope this aim. A mesoporous silica-based nanodevice, bearing the antineoplastic drug bortezomib (BTZ), whose release is triggered by the acidic tumor environment, and grafted with the targeting function folic acid (FOL) on the external surface, was developed (FOL-MSN-BTZ) and tested *in vitro* and *in vivo* against multiple myeloma (MM) cells and in xenograft models, respectively.
**Material and Methods**
Efficacy studies were performed by growth experiments, TEM, TUNEL assay, and Western Blotting (WB). In vivo studies were conducted on SCID mouse models bearing RPMI 8226 (RPMI) cells derived from MM tumors and subsequently, the biodistribution of MSN was determined by (plasma-mass spectrometry (ICP-MS) analysis).
**Results and Discussions**
The FOL-MSN-BTZ nanosystem demonstrated selectivity in killing cancer cells that overexpress the folate receptor (FR+), while not affecting normal cells that do not overexpress FR (FR-). On the other hand, free BTZ showed toxicity for all tested cell lines, regardless of their FR expression. The *in vitro* and *in vivo* studies reveal significantly increased antitumor efficacy compared to conventional formulations of Bortezomib. The developed injectable formulation showed higher antitumor efficacy and a reduced toxicity trend in an MM mouse model. Based on the biodistribution results, a higher accumulation of FOL-MSN-BTZ nanodevice was found in the tumor tissue compared to free BTZ, which is likely due to its highly selective targeting ability.
**Conclusion**
Due to its specific targeting towards FR-expressing MM cells, the FOL-MSN-BTZ nanosystem demonstrates improved safety and significantly increased antitumor efficacy compared to conventional formulations of Bortezomib. As a result, it represents a promising strategy for the targeted and safe delivery of bortezomib in MM treatment.

### EACR23-1480
### Optimized immunohistochemistry and NGS panels for diagnostic biopsies to improve the molecular characterisation of gastric cancer.

*M.M. Ginestà*[1], *O. Serra*[2], *M.J. Paules*[3], *N. Garcia*[4], *M.C. Galan*[5], *R. Salazar*[6], *N. Sala*[7]
[1]*ICO-IDIBELL, Hereditary Cancer Program, L'Hospitalet de Llobregat, Spain*
[2]*Althaia Foundation, Oncology service and Gastrointestinal Oncology- Endosco py and Surgery research group GOES, Manresa, Spain*
[3]*Hospital de Bellvitge, Pathology Department, L'Hospitalet de Llobregat, Spain*
[4]*ICO-IDIBELL, PROCure Program -ONCOBELL Program, L'Hospitalet de Llobregat, Spain*
[5]*ICO-IDIBELL, Medical Oncology-ONCOBELL Program, L'Hospitalet de Llobregat, Spain*
[6]*ICO-IDIBELL, Medical Oncology- ONCOBELL Program, L'Hospitalet de Llobregat, Spain*
[7]*ICO-IDIBELL, Nutrition and Cancer Unit-Cancer Epidemiology Research Program - ONCOBELL Program, L'Hospitalet de Llobregat, Spain*
**Introduction**
Gastric Cancer (GC) is a deathly and heterogeneous disease. It is notoriously resistant to chemotherapy and newer biologic-based therapies have not showed promising results. Despite multidisciplinary approaches the prognosis

© 2023 The Authors. Molecular Oncology is published by John Wiley & Sons Ltd on behalf of Federation of European Biochemical Societies of GC patients remains overall poor. During the last years some well-recognized groups proposed a molecular classification for GC, although its implementation into the clinics is difficult. In the present study we present a novel approach based on the combination of IHC/ISH and NGS panels which can be used with FFPE endoscopic biopsies embedded in paraffin with a reduced hands-on time.

**Material and Methods**

A total of 162 consecutive patients visited at the Oncology Service of the ICO were included (February 2017-September 2020). Patient inclusion criteria were age > 18, confirmed gastric or gastro-oesophageal junction carcinoma by endoscopic biopsy analysis, and > 2 months of clinical follow-up. Patients who did not receive any cancer treatment were excluded for the response to treatment evaluation.

From each FFPE block obtained from endoscopic biopsy at diagnosis, we obtained 3-5μm sections for H&E staining and for the IHC/ISH study. In the last slide the pathologist selected and marked the best tumour areas to obtain 1 mm diameter punches (1-3) in order to perform DNA and RNA extraction. The IHC/ISH panel include: HER-2, EBV, MMR, CDH1, TP53 and MUC6. NGS panel is an amplicon custom panel from Paragon Genomics designed to assess the most frequent GC mutations (mutations in more than 0.46% of samples) according to cBioportal located in 75 different genes and the coding region of the most frequently mutated genes in GC (APC, ARID1A, CDH1, KRAS, PIK3CA, PTEN, RHOA, RNF43, SMAD4, TP53).

**Results and Discussions**

Applying the hierarchical clustering with the IHC/ISH panel we classified the tumours in 5 clusters. One of them showed better response to fluoropirimidine treatment, although there were not statistically significant differences in overall survival. Although biological material was scarce, informative results were obtained in most of the samples (66,9%) by using our developed NGS panel.

**Conclusion**

We propose here a novel GC diagnostic strategy that can be feasible in clinical practice. The identification of the different IHC/ISH clusters may be useful for future selection of therapeutic strategies. The development of a GC specific panel optimized for the analysis of paraffin-embedded endoscopic biopsies can increase the number of patients whose tumours can be molecularly characterised.

### EACR23-1484
### N-Cadherin Expression and KRAS mutation status could predicts response to Anti-FGFR Therapy

*S. García Borrego[1], C. Cirauqui[1], H. Wang[2], A. Santos[1], D. Parra[1], J. Gracia[3], C. Ambrogio[4], L. Paz-Ares[5], I. Ferrer[5]*

*[1]Hospital 12 de Octubre Research Institute, Thoracic Oncology, Madrid, Spain*
*[2]Tongji University, Tongji University, Shanghai, China*
*[3]Hospital 12 de Octurbe Research Institute, Thoracic Oncology, Madrid, Spain*
*[4]Molecular Biotechnology Center MBC-, Department of Molecular Biotechnology and Health Scienc es-, Torino, Italy*
*[5]Hospital 12 de Octubre, Thoracic Oncology, madrid, Spain*

**Introduction**

INTRODUCTION: Lung cancer is a leading cause of cancer-related deaths globally, with FGFR1 implicated in various cancers including lung cancer. FGFR1-targeted therapy has been explored as a treatment approach, but has limited success. Previous results from our group have identified N-Cadherin as a potential predictor of FGFR-targeted therapy efficacy in Non Small Cell Lung Cancer (NSCLC). Our prior research using Patient-Derived Xenografts (PDX) suggests that KRAS mutational status may also play a potential role in determining the efficacy of anti-FGFR therapy. This study aimed to analyze N-Cadherin expression and KRAS mutational status as potential predictive biomarkers for FGFR-targeted therapies..

**Material and Methods**

MATERIAL AND METHOD: To examine the response of lung adenocarcinoma (LUAD) cell lines to FGFR inhibition, we conducted a bioinformatic study using the GDSC repository. To elucidate potential KRAS-dependent mechanisms underlying resistance to FGFR1 inhibition, we used an isogenic cell-based platform containing the major KRAS mutant isoforms. This platform was subjected to combinatorial treatments with the FGFRi AZD4547, plus various KRAS or KRAS-signaling pathway inhibitors.

**Results and Discussions**

RESULTS AND DISCUSSION: Our study identified an high number of KRAS mutant LUAD cell lines resistant to RTKi, including FGFR1 signaling inhibitors. These results validate our prior studies on the potential involvement of KRAS in FGFR1i resistance in PDX. Furthermore, our analysis revealed that high N-Cadherin expression was potentially associated with increased sensitivity to FGFR1i, as expected, only in KRAS wild-type contexts. Our platform corroborated our results indicating the involvement of KRAS in FGFR1i resistance, particularly in the KRAS G12V mutant. Moreover, its potential utility as a tool for exploring combination strategies to overcome resistance to FGFRi mediated by KRAS signaling was shown.

**Conclusion**

CONCLUSION: Our study extends understanding of FGFRi resistance mechanisms by identifying the potential utility of N-Cadherin expression as a biomarker to predict response to FGFR inhibitors in KRAS wild-type lung adenocarcinomas. Our results provide further support for the involvement of KRAS signaling in resistance to FGFR1 inhibition, demonstrated in both LUAD cell lines and isogenic models of KRAS mutations. The validation of our findings provides a basis for future researchs identifying combination therapies that could overcome resistance to FGFR-targeted therapy in KRAS mutant tumors.

### EACR23-1493
### STING activating nanoparticles light the fire in poor outcome breast cancer

*E. Gilmore[1], E. Parkes[2], S. McQuaid[3], H. McCarthy[1], N. Buckley[1]*

*[1]Queen's University Belfast, School of Pharmacy, Belfast, Ireland*
*[2]Oxford University, Department of Oncology, Oxford,*

© 2023 The Authors. Molecular Oncology is published by John Wiley & Sons Ltd on behalf of Federation of European Biochemical Societies

*United Kingdom*
*[3]Queen's University Belfast, Patrick G Johnson Centre for Cancer Research, Belfast, Ireland*

**Introduction**

STING signalling in cancer is an important indicator of immune system activation and may determine response to immunotherapy. Previously we have developed a novel approach to quantify active STING signalling in breast cancer. We have shown low levels of STING activity predict poor outcome in breast cancer and that STING activity may be supressed due to MYC amplification. Therefore, to improve breast cancer outcome, we are developing and characterising a novel nanoparticle-based delivery system (RALA) to facilitate the delivery of cGAMP, the natural ligand for STING, and MYC siRNA, overcoming the commonly associated limitations such as intracellular delivery, stability and bioavailability. RALA has been previously shown to allow effective delivery of nucleotide-based therapeutics *in vivo* with no observed toxicity. Furthermore, RALA has been shown to accumulate in tumour tissues therefore facilitating minimally invasive direct on-target delivery.

**Material and Methods**

RALA/cGAMP and RALA/MYCsi nanoparticles (NPs) have been formulated and characterised according to appropriate DLS characteristics. Treatments have been performed to compare the ability to activate STING signalling and/or kill breast cancer cells.

**Results and Discussions**

Treatment with RALA/cGAMP NPs shows induction of pTBK1 and IFN luciferase activity in cell line models. We observe elevated expression of STING target genes such as CXCL10, PD-L1 and IFN at mRNA level.Treatment with RALA/MYCsi NPs shows decreased viability in a range of breast cancer cell lines, comparable with the level achieved using commercially available reagents that are limited to *in vitro* delivery only.

**Conclusion**

Using the RALA delivery system, cGAMP and MYC siRNA have shown promising functionality *in vitro*. Future work will demonstrate improved bioavailability and efficacy *in vivo* using this novel delivery system.

### EACR23-1527

### RGD-targeted nanoparticles as innovative drug carriers for improving drug efficiency in breast cancer combined therapy

*Romiti C.[1], Amolo A.[1], Di Gregorio E.[1], Di Lorenzo A.[1], Cavallo F.[1], Ferrauto G.[1], Conti L.[1]*
*[1]University of Turin, Department of Molecular Biotechnology and Health Sciences, Via Nizza 52, 10126 Turin, Italy*

Introduction Systemic chemotherapy remains the primary choice for treating breast cancer (BC), the leading cause of cancer-related death in women. However, systemic chemotherapy causes serious side effects to distal organs and requires high doses to achieve an effective concentration in the tumor, hence boosting chemoresistance. The use of targeted nanosystems for drug delivery together with novel adjuvant therapies holds the promise of overcoming these limitations. Herein, we formulated Doxorubicin-loaded liposomes and Poly (lactic-co-glycolic acid) nanoparticles (PLGA-NPs) containing CU-CPT22, an inhibitor of Toll-like receptor 2 (TLR2). The cyclic RGD tripeptide was linked to both the liposome and PLGA-NP surfaces to actively target αvβ3 integrins, which are overexpressed in BC. TLR2 activation was previously shown to be associated with cancer stem cells (CSCs) survival and tumor progression; accordingly, CU-CPT22 and Doxorubicin co-administration has a higher efficacy with respect to chemotherapy alone. Here, the two drugs were encapsulated inside the RGD-targeted nanoparticles and tested both in vitro and in vivo to achieve a controlled and specific combination treatment protocol. Material and Methods Poly (lactic-co-glycolic acid) (PLGA) Nanoparticles (NPs) and liposomes were prepared with the emulsification solvent evaporation or with the hydration of thin lipidic film method, respectively. The nanoparticles were loaded with the proper drug (CU-CPT22 in PLGA and Doxorubicin in liposomes). They were bound to cyclic RGD through a ligation reaction to target αvβ3 integrins. Physicochemical properties (size, ζ potential) were determined by Dynamic Light Scattering (DLS). The amount of encapsulated drug was measured through UV/vis and NMR spectroscopy. In vitro cytotoxicity was analyzed by MTT assay. In vivo, the therapeutic effect of the two nanoparticles, both alone and in combination, was assessed on transplantable triple-negative breast cancer (TNBC) murine models. The outcome of therapy was followed by multiparametric Magnetic Resonance Imaging (MRI). Results and Discussions The efficiency of targeted NPs in the treatment of BC was assessed by comparing, both in cellular systems and in vivo, RGD-targeted nanoparticles – which we already demonstrated to perform better than untargeted counterparts - with the free drugs. At first, we were able to set up the most suitable CU-CPT22-PLGA dose both for cellular and animal models. Successively, we reported the outcome of single vs. combined treatments in TNBC models in terms of mouse weight, tumor growth, and necrosis spread in the lesion. Conclusion The combined active delivery of the two nanoparticles loaded with different drugs significantly improved the efficiency of the treatment. The implied nanosystems were suitable for the chosen drugs and assured higher retention at the tumor site with respect to that achievable with the free drugs. Besides, the drug release was this way gradual and maintained over time. The developed platforms thus represent a promising strategy for the delivery of combined therapy in a more efficient and safer fashion.

### Tumour Biology

### EACR23-0005

### A cell circuit approach to dissect fibroblast-macrophage interactions in the tumor microenvironment

*S. Mayer[1], T. Milo[2], A. Isaacson[1], C. Halperin[1], S. Miyara[2], Y. Stein[1], M. Pevsner-Fischer[1], E. Tzahor[2], U. Alon[2], R. Scherz-Shouva[1]*
*[1]The Weizmann Institute of Science, Biomolecular Sciences, Rehovot, Israel*
*[2]The Weizmann Institute of Science,*

© 2023 The Authors. Molecular Oncology is published by John Wiley & Sons Ltd on behalf of Federation of European Biochemical Societies

*Molecular Cell Biology, Rehovot, Israel*
**Introduction**
The tumor microenvironment (TME) is composed of various nonmalignant cell types that interact with each other and with cancer cells, impacting all aspects of cancer biology. The TME is complex and heterogeneous, and thus simplifying systems and concepts are needed. Here we provide a tractable experimental system and powerful mathematical circuit concepts to identify the main molecular interactions that govern the composition of the TME. We focus on two major components of the TME - cancer associated fibroblasts (CAFs) and tumor associated macrophages (TAMs), define their interactions and verify our predictions in mouse and human breast cancer.
**Material and Methods**
We measure the population dynamics starting from many initial conditions of co-cultures of macrophages and organ-derived fibroblasts from mammary, lung, and fat, and explore the effects of cancer-conditioned medium on the circuits. We define the circuits and their inferred parameters from the data using a mathematical approach, and quantitatively compare the cell circuits in each condition.
**Results and Discussions**
We find that while the homeostatic steady-states are similar between the organs, the cancer-conditioned medium profoundly changes the circuit. Fibroblasts in all contexts depend on autocrine secretion of growth factors whereas macrophages are more dependent on external cues, including paracrine growth factors secreted from fibroblasts and cancer cells. Transcriptional profiling reveals the molecular underpinnings of the cell circuit interactions and the primacy of the fibroblast autocrine loop. The same fibroblast growth factors are shared by the co-cultures and mouse and human breast cancer.
**Conclusion**
The cell circuit approach thus provides a quantitative account of cell interactions in the cancer microenvironment.

### EACR23-0052
### Uncovering the interactions between fibroblasts and macrophages that facilitate melanoma lung metastasis

T. Shami[1], L. Monteran[1], N. Cohen[2], N. Erez[1]
[1]*Tel aviv university, Pathology, Tel aviv, Israel*
[2]*Tel aviv university, Pahtology, Tel aviv, Israel*
**Introduction**
Malignant melanoma is the deadliest of all skin cancers. The major cause of melanoma mortality is metastasis to distant organs, frequently to the lungs. Dissemination of cancer cells to distant organs is a multistage process, affected by the microenvironment. While the importance of the microenvironment at the primary tumor is established, the interactions of stromal and immune cells at the metastatic microenvironment are still largely unresolved. Cancer-associated fibroblasts (CAFs) are a key component in the crosstalk between tumor cells and their microenvironment that facilitate tumor growth. We previously demonstrated that CAFs mediate macrophage recruitment and modify the functional activation status of recruited macrophages in breast cancer. However, the reciprocal interactions between tumor-associated macrophages (TAMs) and CAFs at the metastatic microenvironment in melanoma are largely unknown. In this study, we characterized the crosstalk between fibroblasts and macrophages that facilitates melanoma lung metastasis.
**Material and Methods**
To address this question, we investigated CAF-TAM interactions *in vivo* in a model of spontaneous melanoma lung metastasis combined with Col1a1-YFP mice, that enable unbiased tracking and isolation of fibroblasts. We further characterized CAF-TAM interactions by functional assays (migration, collagen centration and wound repair), gene expression, and flow cytometry analyses.
**Results and Discussions**
We found that melanoma-activated lung fibroblasts modulated the functional reprogramming of macrophages. Reciprocally, secreted factors from melanoma-activated macrophages activated primary lung fibroblasts, and enhanced CAF-like functions. Moreover, we found that macrophages instigated pro-inflammatory signaling in lung fibroblasts which was mediated via CAF enhanced expression and signaling of TLR4, a pattern-recognition receptor known to be important in innate immunity.
**Conclusion**
Since there are currently no efficient therapies for lung metastasis, it is essential to identify molecular factors that play a role during the early, rate-limiting steps of metastatic colonization in lungs. Achieving control on CAF responses might prove as a novel therapeutic approach to inhibit lung metastatic relapse.

### EACR23-0054
### Fisetin additively affects the Temozolomide response of Glioblastoma cells under normoxia and hypoxia.

S. Ferah[1], M. Camlibel[1], M. Ercelik[1], C. Tekin[1], G. Tezcan[2], B. Tunca[1]
[1]*Bursa Uludag University, Health Science Institute- Faculty of Medicine Faculty- Medical Biology Department, Bursa, Turkiye*
[2]*Bursa Uludag University, Faculty of Dentistry- Fundamental Sciences Department, Bursa, Turkiye*
**Introduction**
Temozolomide (TMZ) resistance is a major challenge for Glioblastoma (GB). Hypoxia leads to aggressive GB tumor phenotype and TMZ therapy failure. Diet-derived flavonoids show chemopreventive effects on cancer. Among these flavonoids, fisetin (FIS) promises anticancer effects through its antiproliferative and apoptotic properties. Therefore, this study investigated the impact of FIS on GB and its potential as a complementary therapy to prevent TMZ resistance in hypoxic conditions in-vitro GB models.
**Material and Methods**
The effect of FIS and TMZ-FIS was analyzed in GB cell lines, TMZ-resistant T98G, and TMZ-sensitive A172. The murine fibroblast cell, L929, was used as a non-cancerous cell line. The type of combined effect of FIS and TMZ was detected by SynergyFinder (version 3.0). Cell proliferation was assessed by real-time cell monitoring and colony formation. An annexin V assay analyzed the cell viability in TMZ-resistant T98G cells. A scratch wound-healing

© 2023 The Authors. Molecular Oncology is published by John Wiley & Sons Ltd on behalf of Federation of European Biochemical Societies assay showed the difference in the migration rate of T98G and A172 cells in normoxia and hypoxia (1% O2, 5% CO2, 94% N2). Data were confirmed by a hypoxic GB tumor model using 3D tumor spheres of TMZ-resistant T98G cells.

**Results and Discussions**

The 13.78uM and 16.40uM of FIS were detected as IC50 values over 24 hours in A172 and T98G cells, respectively (p<0,05). In contrast, these concentrations were ineffective in the fibroblast cell, L929. In normoxia, FIS induced apoptosis (p<0.0001) and additively affected the apoptosis-promoting capacity of TMZ in TMZ-resistant T98G cells (p<0,0001). In addition, the number of colonies formed by T98G and A172 cells decreased, and their migration rates slowed upon FIS and FIS-TMZ treatments compared to untreated and TMZ-only treated cells (p<0.0001). In hypoxia, where GB cells became more resistant to chemotherapy, FIS reduced the excessive migration rate of TMZ-resistant T98G cells compared to untreated cells. Moreover, FIS-TMZ treatments increased the anti-invasive effect of TMZ in T98G cells (p<0.0001). In support of this, FIS and TMZ equally decreased the size of 3D tumor spheres formed by T98G cells, and FIS-TMZ decreased the size of tumor spheres more strongly compared to TMZ-only (p<0.0001).

**Conclusion**

Our findings showed that FIS reduces hypoxia-induced TMZ resistance and contributes to the tumor-healing effect of TMZ, suggesting that FIS could be a promising additive therapy to TMZ for GB patients. The underlying mechanism of this effect of FIS is an open area to clarify in future drug development research.

### EACR23-0056
### B-NDG B2m KO plus mouse model is a powerful tool for immunotherapy

T. Rothrock[1], H. Jiang[2], J. xiao[2]
[1]*Inotiv, RMS marketing, Indianapolis, United States*
[2]*Inotiv, Research models and services R&D, St. Louis, United States*

**Introduction**

The B-NDG (***B***iocyotgen; ***N***OD; ***D***NAPK null; IL2r***g*** knockout) mouse model was developed to be an ideal testing platform for anti-tumor therapies in Cell-Line and Patient Derived Xenograft Models (CDX and PDX respectively). B-NDG mice exhibit several notable advantages over classical NOD-*scid* mice, including increased life span, NK cell deletion, and significantly improved xenograft efficiency. However, like the NOD-*scid* mice, the utility of B-NDG mice in long-term studies is severely limited by a high incidence of severe and early-onset GvHD (***G***raft ***v***ersus ***H***ost ***D***isease) – limiting experimental durations. To reduce the incidence and severity of GvHD in B-NDG mice, Biocytogen developed the B-NDG *B2m* KO plus mice.

**Material and Methods**

B-NDG B2m KO plus mice was generated using Cas9 approach. Specifically, endogenous mouse *B2m* was deleted, while *B2m* cDNA was fused with *Fcgrt* . MHC Class I and Class II expressions, and immune markers were verified by flow cytometry. Concentration of IV administered human IgG was quantified by ELISA. CDX models were generated, and antitumor therapies were administered using established protocols described below.

**Results and Discussions**

Compared with NOD-*scid* and B-NDG mice, B-NDG B2m KO plus mice showed undetectable MHC Class I expression in all tested tissues, without compromising MHC Class II expression. Moreover, no significant change in immune profiles or IgG turnover was detected, demonstrating highly targeted disruption of MHC Class I expression in B-NDG B2m KO plus mice. In the human PBMC-induced GvHD model, B-NDG B2m KO plus mice showed significantly improved survival, delayed onset and reduced clinical severity throughout the course of the model. Compared with B-NDG mice, B-NDG B2m KO plus mice exhibits significantly prolonged usefulness, demonstrating utility for up to 50 days after engraftment. B-NDG B2m KO plus mice were engrafted with human PBMCs and tumorigenic RKO cells on Day 0 and 14 respectively, treated with either vehicle or pembrolizumab and ipilimumab on Day 20, and observed until Day 48. Pembro/ipi treatment significantly inhibited tumor growth. B-NDG B2m KO plus mice were engrafted with tumorigenic human NCI-H226 cells on Day 0, treated with either vehicle or CAR-T cells (5E5) once tumor size reached ~150 $mm^3$ (around Day 20), and observed until Day 56. CAR-T treatment significantly suppressed tumor growth.

**Conclusion**

B-NDG B2m KO plus mouse model is a powerful tool for extended term anti-tumor studies, especially for studies involving immuno- and CAR-T therapies.

### EACR23-0067
### MicroRNA-34 is a promising marker for the glioma-targeted therapy.

N. Missaoui[1], S. Limam[2], S. Susan[3], M. Mokni[4], S. Hmissa[5]
[1]*Medicine Faculty of Sousse, LR21ES03 Oncogenesis and Tumor Progression, Sousse, Tunisia*
[2]*Farhet Hached University Hospital- Sousse, Pathology Department, Sousse, Tunisia*
[3]*CHU St James, Department of Neuro-oncology, Leeds, United Kingdom*
[4]*Medicine Faculty of Sousse, Pathology Department of Farhet Hached University Hospital, Sousse, Tunisia*
[5]*Medicine Faculty of Sousse, Pathology Department of Sahloul University Hospital, Sousse, Tunisia*

**Introduction**

DNA methylation plays a crucial role in the initiation and progression of several tumours. However, the study of microRNAs (miRNAs) hypermethylation in glial tumours is slightly explored yet. In this work, we investigated the pattern of miRNA methylation in gliomas, and further, we investigated the cytotoxic effect of miRNAs on two glioblastoma multiform (GBM) cell lines in order to test their potential utility for the targeted therapy of glioma.

**Material and Methods**

In this study, 186 glial tumours were included. The methylation profile of miRNA promoters (miR-34, miR-9, miR-137, miR-127, and miR-148) was investigated by specific PCR-methylation technique after DNA bisulphite

© 2023 The Authors. Molecular Oncology is published by John Wiley & Sons Ltd on behalf of Federation of European Biochemical Societies modification. Next, the cytotoxic effect of miRNAs found hypermethylated in gliomas was examined by reverse transfection using mimic miRNAs on two GBM cell lines (U251 and KNS42).

**Results and Discussions**

The analysis of miRNA methylation profile showed the hypermethylation of miR-34 (42.9%), miR-9 (41.1%), miR-137 (34.8%), miR-127 (33.9%), and miR-148 (33.9%) among adult patients. However, in children, the hypermethylation of miR-34, miR-9, miR-137, miR-127, and miR-148 was 31.1%), 17.6%, 9.5%, 27%, and 0.0%, respectively. Based on the analysis of the cytotoxicity of these hypermethylated miRNAs in gliomas, only the mimic miR-34 displayed the highest Z scores in both U251 (-3.10) and KNS42 (-3.89) cell lines inducing the arrest of the proliferative activity as well as the cell death in GMB cell lines.

**Conclusion**

Altogether, these findings **s**uggest that miR-34 could be a useful and promising marker for the targeted therapy of gliomas in adult and paediatric patients.

### EACR23-0075
### Cancer Blockers and Facilitators through the prism of the E3 ubiquitin ligase SMURF2

M. Blank[1]

[1]*Bar Ilan University, The Azrieli Faculty of Medicine, Safed, Israel*

**Introduction**

SMURF2 recently emerged as an essential regulator and a coordinator of diverse molecular and cellular processes central to cancer initiation, disease progression and therapeutic response. These processes are in the focus of our investigation.

**Material and Methods**

To elucidate the role of SMURF2 in cancer, we use a variety of approaches ranging from genetics, molecular biology and biochemistry to animal pathophysiology and human sample analyses.

**Results and Discussions**

We discovered that SMURF2 acts as a potent tumor suppressor and epigenetic regulator. SMURF2 ablation exerted a profound impact on the ability of the depleted cells and tissues to maintain the epigenetic structure landscape and chromosomal architecture, adequately respond to cellular stress and repair DNA damage, protect the genome integrity and cell identity. Mechanistically, we found that SMURF2 regulates the stability and/or activity of several core epigenetic regulators, including histone protein ligase RNF20 and methyltransferase EZH2, DNA topology regulator Topo IIα, poly(ADP-ribose) polymerase PARP1, transcriptional co-repressor and chromatin modifier TRIM28/KAP1, as well as the epi-transcriptome regulator and RNA editase ADAR1p110. We also found that SMURF2 regulates the autophagic-lysosomal turnover of the nuclear structure protein lamin-A and its mutant form progerin, whose expression underlies the development of premature ageing syndrome HGPS and is associated with physiological ageing and cancer. Our investigations also revealed that in malignant tissues SMURF2 expression is significantly altered, and suggested a link between aberrant localization of SMURF2 and tumor aggressiveness.

**Conclusion**

Altogether, these findings point to SMURF2 as a critical factor regulating key molecular and cellular processes pertinent to malignant transformation and carcinogenesis.

### EACR23-0084
### Non-alcoholic fatty Liver Disease Promotes Pancreatic Cancer Liver Metastasis and Resistance to Chemotherapy

Q. Yu[1]

[1]*The Affiliated Hospital of Qingdao University, Tumor Immunology and Cytotherapy, Qingdao, China*

Introduction Pancreatic ductal adenocarcinoma (PDAC) has the highest mortality among all types of cancer and a striking tendency to metastasize. The globally rising rates of non-alcoholic fatty liver disease (NAFLD) is likely to contribute to an increase in the incidence of liver metastasis. The mechanisms by which a fatty liver mediates and enhances PDAC liver metastasis are poorly known. Material and Methods Non-contrast and contrast enhanced computed tomography (CT) were used to assess fatty liver and gemcitabine response of PDAC patients of The Affiliated Hospital of Qingdao University, respectively. Diet-induced NAFLD model was generated by feeding C57/B6 mice with a choline-deficient L-amino acid-defined, high-fat diet (CDAA-HFD) for 4 weeks, followed by implantation of LSL-KrasG12D/+; LSL-Trp53R172H/+; Pdx-1-Cre (KPC) cells into the spleen to construct a pre-clinical PDAC metastatic model. Integrative analyses of the transcriptomic and proteomic data were applied using liver metastases samples. Published exemplar genes for the defined PDAC subtypes previously described by Moffitt et al., Collisson et al., Bailey et al., and Henry et al. were used to cluster PDAC tumors from our experiments. Single cell sequencing (GSE192740 and GSE1556698) and CellChat were applied to identify the key molecules mediating the NAFLD-induced liver metastasis. Results and Discussions Tumor response to gemcitabine within the liver in patients with NAFLD was disappointing, with no patients achieving a confirmed partial response (PR) and 25% (5 out of 20) achieving stable disease (SD), whilst in patients without NAFLD, a PR in the liver was achieved in 13 patients (25%) and SD in 34 patients (65.4%), giving a 2-month liver disease control rate of 90.4%. Diet-induced NAFLD promoted PDAC extravasation and enhanced the growth of PDAC liver metastasis and chemoresistance partially by inducing a more fibrotic and immune-suppressive microenvironment. The proteomic analysis of PDAC liver metastases in NAFLD settings partially matched with the clinically relevant PDAC molecular subtypes identified by previous groups. Macrophage migration inhibitor factor (MIF) was identified by CellChat that might mediate fatty liver-induced liver metastasis. Conclusion Targeting MIF might be a promising treatment strategy for liver metastasis in patients with NAFLD.

### EACR23-0125
### Intracellular osteopontin controls the release of cytokines by mast cells to

© 2023 The Authors. Molecular Oncology is published by John Wiley & Sons Ltd on behalf of Federation of European Biochemical Societies

### restrain neuroendocrine prostate cancer

R. Sulsenti[1], R. Ferri[1], S. Sangaletti[1], V. Cancila[2], G. Renne[3], C. Tripodo[2], C. Pucillo[4], M.P. Colombo[1], B. Frossi[4], E. Jachetti[1]
[1]Fondazione IRCCS Istituto Nazionale dei Tumori- Milan, Department of Research- Molecular Immunology Unit, Milan, Italy
[2]University of Palermo, Department of Health Sciences- Tumor Immunology Unit, Palermo, Italy
[3]IEO- European Institute of Oncology IRCCS, Division of Uropathology and Intraoperative Consultation, Milan, Italy
[4]University of Udine, Department of Medicine-Immunology Section, Udine, Italy

**Introduction**

Fatal neuroendocrine prostate cancer (NEPC) often emerges in patients relapsing after hormone therapies. Besides, *de novo* NEPC can rarely occur in treatment-naïve patients. Treatment-related and *de-novo* NEPC have different genomic alterations but share a common transcriptional profile.

**Material and Methods**

Investigating the tumor microenvironment, we recently found that mast cells (MCs) accumulate within hormone-sensitive prostate cancer favoring its growth, whereas are excluded by *de-novo* NEPC both in patients and in the transgenic TRAMP spontaneous mouse model. TRAMP mice backcrossed with MCs-deficient $Kit^{Wsh}$ mice showed an increased frequency of *de-novo* NEPC. The frequency of *de-novo* NEPC similarly raised also in TRAMP mice deficient for the matricellular protein osteopontin (OPN). Reconstituting $Kit^{Wsh}$-TRAMP mice with wild type, but not with OPN-deficient, MCs lowered the frequency of NEPC to that of untreated TRAMP mice.

**Results and Discussions**

We found that MCs stain positive for OPN in tumor sections and in vitro cultures, but release a tiny amount of OPN in supernatants if compared to NEPC cells. Notably, OPN has both secreted (sOPN) and intracellular (iOPN) forms; the latter can bind to MyD88 and regulate the signaling downstream toll-like receptors (TLRs). *In vitro*, wild type, but not OPN-/- or MyD88-/-, MCs inhibited the proliferation of NEPC cells. Also, *in silico* analyses showed that genes related to inflammatory response and TLRs signaling are down regulated in human and murine NEPC.

**Conclusion**

Our data suggest that TLRs/MyD88/iOPN-mediated pathways induce MCs to release factor(s) able to restrain NEPC. Further studies are required to molecularly dissect this novel function of MCs, to identify actionable targets against NEPC.

### EACR23-0132
### CAFs from a panel of tumors influence the proliferation and treatment response of head and neck cancer cells differently when co-cultured in spheroids

K. Roberg[1]
[1]Division of Cell Biology- The Department of Biomedical and Clinical Sciences- Linköping University- Linköping- Sweden,
Department of Otorhinolaryngology in Linköping- Anaesthetics- Operations and Specialty Surgery Center- Region Östergötland- Sweden, Linköping, Sweden

**Introduction**

Cancer-associated fibroblasts (CAFs) are the major cellular component of the tumor microenvironment and are known to effect tumor growth and response to various treatments. We have recently showed that tumor matched CAFs co-cultured with head and neck squamous cell carcinomas (HNSCC) cells in spheroids increase the tumor cell proliferation and have impact on the cetuximab and cisplatin treatment response.
This study was undertaken to investigate if CAFs from different tumors effect proliferation and treatment response differently compared to tumor matched CAFs.

**Material and Methods**

The HNSCC cell lines LK0902, LK0923 and LK0412, CAFs from eight different tumors and normal oral fibroblasts (NOF) were used in this study. Tumor cells were mixed with CAFs at a ratio of 2:1 and seeded into 96-well ultra-low attachment round-bottom plates (10,000 tumor cells and 5,000 CAFs/well). After seven days the spheroids were fixed, embedded in paraffin and sectioned, and thereafter stained with antibodies for Ki-67. Proliferation was measured by calculation of the amount of Ki-67 positive cells/ spheroid area.
For treatment, spheroids were cultured for 48 hours before addition of cisplatin (1.5 or 3 µg/ml) and untreated spheroids were used as controls. After seven days, viability was measured via CellTiter-Glo 3d Viability Assay. Real-time quantitative reverse transcription PCR analysis was performed on the panel of CAFs and ACTA2, PDGFRB, FAP and S100A4 were analyzed.

**Results and Discussions**

In the two cell lines (LK0902 and LK0412) we found that the amount of Ki-67 positive cells/area varies between co-cultures with different CAFs and NOF (Figure 1).
Then we investigate if CAFs from different tumors have different effect on treatment response (Figure 2). In the combinations of LK0902/0861Fib and LK0412/1002Fib, a significant decrease in treatment response was found compared to co-cultures with their tumor-matched CAFs or NOFs. Next we analyzed four CAFs markers (ACTA2, PDGFRB, FAP and S100A4) in the panel of CAFs and found these markers differently expressed among the CAFs (Figure 3). The results indicate the CAFs are tumor specific and can affect tumor growth differently.

**Conclusion**

CAFs from different tumors can affect proliferation and to some extent, the treatment response of tumor cells differently compared to tumor matched CAFs.

### EACR23-0167
### Tumor budding and YKL-40 protein expression in colorectal cancer

M. Kazakova[1], T. Ivanova[1], D. Dikov[2], D. Molander[1], K. Simitchiev[3], Y. Sbirkov[1], V. Sarafian[1]
[1]Medical University – Plovdiv- Bulgaria, Department of Medical Biology, Plovdiv, Bulgaria
[2]Medical University – Plovdiv- Bulgaria, Department of General and Clinical Pathology, Plovdiv, Bulgaria
[3]University of Plovdiv,

© 2023 The Authors. Molecular Oncology is published by John Wiley & Sons Ltd on behalf of Federation of European Biochemical Societies

Department of Analytical Chemistry and Computer Chemis try, Plovdiv, Bulgaria

**Introduction**

Colorectal cancer (CRC) is the third most common neoplasm worldwide and is one of the leading causes of death among oncological patients as approximately 25% of patients are initially diagnosed at an advanced stage with metastases. Heterogeneity within the same tumor stages defines the need for additional prognostic biomarkers. Tumor buds were proposed as a poor prognostic factor for CRC but they are still not implemented into routine pathology reporting. Chitinase-3-like protein 1 (CHI3L1) also known as YKL-40, is regarded as a candidate circulating biomarker and a therapeutic target in CRC.

**Aim:** The aim of our study is to investigate tissue YKL-40 localization and tumor budding in CRC by searching a relationship between protein expression, tumor budding and clinico-pathological parameters.

**Material and Methods**

A retrospective record review was performed on thirty one patients who had undergone surgical resection for CRC. Normal samples from distal non tumoral colonic tissue from the main group of patients and 5 non-neoplastic colon tissue samples were used as, respectively, internal and external normal controls. Standard haematoxylin-eosin (HE), haematoxylin-eosin-safran (HES) stainings and immunohistochemical analyses were accomplished. The quantitative morphologic and statistical assessment of tumor budding was performed as recommended from the International Tumor Budding Consensus Conference.

**Results and Discussions**

Intensive immunostaining of YKL-40 in the front of tumor invasion was found in comparison with tumor parenchyma and normal controls. Lack of YKL-40 reactivity in normal colon and weak expression in macrophages within the tumor stroma was detected. The immunohistochemical expression of YKL-40 was higher in CRC compared to the normal mucosa. Low to moderate YKL-40 expression was noted in the glandular tumor parenchyma of CRC tissues. High YKL-40 presence in the tumor buds in the front of tumor invasion and as well as in peritumoral venous tumor emboli indicate its contribution to cancer malignancy.

**Conclusion**

We present novel data on YKL-40 expression and tumor budding in CRC suggesting that this multifunctional glycoprotein might be implicated in tumor invasiveness.

**Acknowledgments:** This study is financed by the European Union-NextGenerationEU, through the National Recovery and Resilience Plan of the Republic of Bulgaria, project № BG-RRP-2.004-0007-C01

### EACR23-0174
### Impact of microbiota in anticancer therapies: a study on Lacticaseibacillus rhamnosus GG in KRAS G12C cellular models

R. Scardaci[1], J. Keijer[1], P. Scaparone[1], E. Patrucco[1], C. Ambrogio[1]
[1]*Molecular Biotechnology Center- University of Torino, Department of Molecular Biotechnology and Health Scienc es, Torino, Italy*

**Introduction**

KRAS mutations are common in lung adenocarcinomas (LUADs) and associated with poor prognosis in patients. Although new targeted therapies for G12C mutations have been approved in clinics, resistance(R) has already emerged as a major challenge. The role of microbiota in cancer research has developed in this context, as the formation of a tumoral microenvironment (TME) leads to microbial dysbiosis, which may worsen the pathological setting and alter therapeutic responses. KRAS-driven LUAD in mice generated a peculiar TME that disrupted the microbiota by favoring certain species and exacerbating tumor severity. Also, the aerosolization of *Lacticaseibacillus rhamnosus* GG (GG) in the lungs prevented metastasis and improved response to standard chemotherapy. Our overall aim is decoding both the reciprocal relationship between microbial-dependent patterns and tumor cells and the influence of certain bacterial species on response and R to therapy. We describe here a preliminary study on the effect of GG on KRAS G12C cellular models.

**Material and Methods**

We generated Ras-less cells rescued with KRAS WT or KRAS G12C, both in naïve conditions and resistant to the targeted inhibitor sotorasib (AMG). In parallel, we selected R to AMG in human and murine KRAS G12C LUAD cell lines by exposing cells to increasing doses of AMG up to 5µM (AMGR cells). Monolayers of these cellular models were treated with GG (multiplicity of infection of 1:100) with or w/o AMG for up to 72 h. KRAS downstream signal modulation was assessed by WB, while cellular proliferation was analyzed by visual cell count.

**Results and Discussions**

GG activated the MAPK pathway to different extents in the different cell lines suggesting that a heterogeneous background can impact the responses to an external stimulus. In addition, when MEFs AMGR cells were treated with AMG + GG, pERK and pMEK levels decreased while the apoptotic marker BIM increased. The results were validated with counting. We have demonstrated *in vitro* that in KRAS-driven tumor cells a diverse biological setting results in distinct responses of lung cancer cells to probiotics, which had also a promising impact on R to therapies.

**Conclusion**

These preliminary data pose the basis for unraveling novel strategies for reversing therapy R and harnessing the microbiota-KRAS driven tumor cells relationship in LUADs. The ultimate objective of this study is not only to develop more effective treatments for KRAS-driven lung cancer but also to comprehend the complex scenario of microbiota-cancer interactions.

### EACR23-0185
### p53 co-factor JMY is required for the formation of paraspeckles during DNA damage.

I. Rodriguez Pastrana[1], E. Birli[1], A. Coutts[1]
[1]*Nottingham Trent University, School of Science and Technology- Bioscience, Nottingham- NG11 8NS, United Kingdom*

**Introduction**

The tumour suppressor p53 is a nuclear transcription factor that initiates a checkpoint cascade during DNA damage,

© 2023 The Authors. Molecular Oncology is published by John Wiley & Sons Ltd on behalf of Federation of European Biochemical Societies ending in a myriad of cellular responses, including cell cycle arrest, DNA repair and apoptosis. Approximately 50% of human tumours present mutations in the p53 gene, highlighting its critical role as a tumour suppressor. Our work involves JMY, an actin nucleator and DNA damage-responsive p53 transcriptional cofactor. JMY predominantly localises in the cytosol, where it promotes actin nucleation, essential for cell migration and invasion. In addition, JMY impacts cell survival by inducing actin nucleation at the autophagosomes necessary during their formation and maturation. Importantly, during genotoxic stress JMY undergoes nuclear accumulation, which can enhance the p53-dependent expression of targets involved in DNA repair and apoptosis.

**Material and Methods**

To investigate the wider role of JMY in transcriptional regulation during genotoxic stress, we performed a transcriptomic RNA-sequencing analysis to identify novel targets of JMY. Mechanistic investigation on how these JMY-mediated targets can impact cell fate during the DNA damage response included fluorescence in situ hybridisation, comet assays and cell proliferation and survival analyses.

**Results and Discussions**

Our transcriptomic data suggests that JMY can impact paraspeckles, which are stress-responsive membranelles subnuclear bodies enriched in transcription factors and splicing-related proteins. We show that the lack of JMY reduces the expression of essential paraspeckle components, including the lncRNA NEAT1_2 and paraspeckle-associated proteins, like NONO and SFPQ, during etoposide-induced DNA damage. Our results also show that JMY deficiency impairs the p53-dependent expression of NEAT1_2, resulting in decreased paraspeckle formation. Furthermore, we demonstrate that decreased levels of NEAT1_2 enhance the accumulation of DNA damage, reduce cell proliferation, and increase cell sensitivity to chemotherapeutic agents.

**Conclusion**

Together, our results suggest that JMY plays a role in the p53-mediated paraspeckle formation, which impacts DNA damage and cell survival during genotoxic stress.

### EACR23-0191
### Hypoxia affects ECM composition, structure and morphology through HIF dependent and independent mechanisms, impairing cell migration in bladder cancer

*C. Guerrero Quiles[1], J. Gonzalez Abalos[1], V. Smith[1], M. Reardon[1], J. Humphries[2], M. Humphries[3], A. Choudhury[1], C. West[1]*
*[1]Oglesby Cancer Research Centre, Cancer Sciences, Manchester, United Kingdom*
*[2]Manchester Metropolitan University, Life Sciences, Manchester, United Kingdom*
*[3]University of Manchester, Cell Matrix Biology & Regenerative Medicine, Manchester, United Kingdom*

**Introduction**

Bladder cancer is a prevalent disease (>500,000 cases annually), with 70% of patients expressing hypoxia markers. Hypoxia (low oxygen levels) promotes extracellular matrix (ECM) remodelling, affecting cell behaviour and migration. Radiotherapy, used in bladder cancer, has synergies with the ECM as it influences cell/ECM interactions. Here, we performed the first omics comprehensive study of hypoxia effects in cancer ECM remodelling, its regulatory mechanisms, effects in cell adhesion and migration, and its synergies with irradiation.

**Material and Methods**

UMUC3, J82, RT4 and T24 cell lines were cultured for 7 days in 21% (normoxia) or 0.1-0.2% (hypoxia) $O_2$. ECM was collected and analysed through mass spectrometry (MS). Spatial transcriptomics (n=47 patients) addressed changes in gene expression for hypoxic CA9 stained vs unstained areas for core and stromal tumour regions. ChIP-Seq determined gene promotor regions bound to HIF1A/HIF1B. Immunofluorescence (IF) reported changes in collagen (COL) 1, COL5 and fibronectin (FN) fibrogenesis and morphology. Cell adhesion, migration and cadherin co-localisation were studied with attachment, scratch and IF assays for unirradiated and irradiated (2-8 Gy) cells seeded onto hypoxic and normoxic ECM.

**Results and Discussions**

MS identified 66 ECM proteins significantly affected by hypoxia (p. adj.<0.05, fold change>±2). *In silico* analyses showed hypoxia influences ECM structure through HIF1/2 signalling, being collagen (COL) and fibronectin (FN) central players. Interestingly, ECM protease activity was regulated by non-HIF1/2 mechanisms. Spatial transcriptomics showed significant changes (p. adj.<0.05, fold change>±2) in core (n=252 genes) and stromal (n=28 genes) hypoxic tumour areas, confirming changes ECM structure, and highlighting ECM proteolysis is mediated by tumour cells. Hypoxia also changed COL and FN morphology, increasing FN but decreasing COL fibres, both colocalising with cadherin independently of irradiation. Hypoxic ECM increased cell adhesion but impaired cell migration dependently (T24, J82), or independently (UMUC3) of irradiation.

**Conclusion**

Hypoxia induces ECM remodelling with FN and COL as central players. While HIF1/2 signalling affects structural ECM proteins, tumour cells drive COL proteolysis through non-HIF1/2 mechanisms. Hypoxia impairs cell migration in ECM with absence of FN, an effect enhanced by irradiation, suggesting FN is key in cancer cell migration in core hypoxic tumour areas and should be consider when targeting cancer metastasis.

### EACR23-0203
### Glioblastoma chemoresistance is influenced by STAT3 enhancer RNA

*E. Stasevich[1], A. Simonova[1], A. Uvarova[1], K. Korneev[1], E. Zheremyan[1], A. Schwartz[2], D. Demin[1]*
*[1]Engelhardt Institute of Molecular Biology, Molecular Biology, Moscow, Russia*
*[2]University of Haifa, Department of Human Biology, Haifa, Israel*

**Introduction**

STAT3 gene is a key player in the development of glioblastoma. Recently, non-coding RNAs transcribed from enhancer regions were established as tissue-specific gene regulators affecting growth and drug resistance (Stasevich, 2022). In this study, we focused on an enhancer

© 2023 The Authors. Molecular Oncology is published by John Wiley & Sons Ltd on behalf of Federation of European Biochemical Societies

RNA (eRNA) capable of regulating the oncogene STAT3 in glioblastoma.

**Material and Methods**

SEARCH OF ERNA THAT POTENTIALLY REGULATE VIABILITY OF GLIOBLASTOMA CELLS
The eRIC database was used to search for potential regions of eRNAs that may contain eRNAs associated with glioblastoma. We used the Ensembl database release 109 to identify annotated non-coding RNAs in potential eRNA regions.
ERNA KNOCKDOWN AND GENE EXPRESSION ANALYSIS: The human glioblastoma cell line DBTRG-O5MG was used. The suppression of eRNA expression was performed using small interfering RNAs delivered with Lipofectamine RNAiMAX. The expression of STAT3 eRNA and STAT3 was measured by RT-PCR.
CELL PROLIFERATION AND CHEMORESISTANCE ASSAYS: The rate of proliferation was estimated by xCELLigence Real-Time Cell Analysis. Measurements were made continuously during 96 hours. We applied Temozolomide at concentration 20ug/ml and DMSO as a control.

**Results and Discussions**

Based on the articles, we selected 33 key genes for glioblastoma development and progression. 3 annotated non-coding RNAs located in the enhancer region and related to the key genes were selected. Among the 3 eRNAs, only one RNA affected the expression of a related gene (STAT3).
Knockdown of STAT3 eRNA decreased STAT3 mRNA expression 1.5 times in glioblastoma cells. Moreover, STAT3 eRNA suppression led to the increased sensitivity of cells to temozolomide.
The most common therapy for glioblastoma involves the drug temozolomide, which has considerable side-effects. Five-year survival rate remains around 10-20% (Sang Y. Lee, 2016). Thus the search for a new treatment approach is an urgent problem. STAT3 is a promising target for cancer therapy. Suppression of STAT3 reduces tumor cell proliferation, invasiveness and enhances the anti-tumour effects of immune cells (Haeri Lee, 2019). The control of eRNAs expression may allow for the cell specific management of the oncogene expression therefore limiting the impact of the therapy on the normal cells.

**Conclusion**

We showed that STAT3 eRNA suppression in glioblastoma cells resulted in decreased STAT3 expression and increased sensitivity of cells to temozolomide.We suggest STAT3 eRNA has potential as a target for glioblastoma therapy.

### EACR23-0228
### Elucidating the mechanisms via which the proto-oncogene PBF stimulates cell motility in tumour progression

*M. Kocbiyik[1], S. Manivannan[1], M. Alshahrani[1], L. Zha[1], K. Brookes[1], H.R. Nieto[1], M. Elasrag[2], M.L. Read[1], A. Beggs[2], C.J. McCabe[1], V.E. Smith[1]*
*[1]University of Birmingham, Institute of Metabolism & Systems Research IMSR, Birmingham, United Kingdom*
*[2]University of Birmingham, Cancer and Genomic Sciences, Birmingham, United Kingdom*

**Introduction**

The proto-oncogene pituitary tumor transforming gene binding factor (PBF/PTTG1IP) is overexpressed in multiple tumours and associated with tumour progression. In vitro, PBF potently induces cancer cell migration and invasion. Src kinase phosphorylates PBF at Y174 and mutation of this residue renders PBF unable to stimulate cell invasion. This study aimed to further elucidate the mechanisms by which PBF induces cancer cell motility.

**Material and Methods**

PBF-Y174 is a key residue in PBF's YXXΦ endocytosis motif. Several PBF mutants with disrupted PBF phosphorylation and/or endocytosis were employed. Cell motility was determined using scratch wound and Transwell assays. To elucidate molecular events downstream of PBF overexpression, phosphoproteomic and RNA-Seq analyses of a thyroid cell line with stable PBF overexpression were performed. We then utilised a novel Pbf knockout ($Pbf^{-/-}$) mouse model with CRISPR/Cas9-mediated deletion of Pbf exon 4 in C57BL/6N mice. Mouse embryonic fibroblasts (MEFs) were isolated at embryonic day 13.5 and used as primary cultures.

**Results and Discussions**

Both PBF phosphorylation and endocytosis mutants were unable to stimulate thyroid and breast cancer cell migration and invasion suggesting that each of these processes is essential for PBF-induced cell motility. Phosphoproteomic and RNA-Seq analyses revealed enrichment for molecules involved in cell adhesion and cytoskeleton organisation in response to PBF overexpression, prompting further investigation into a physiological role for PBF in cell motility. $Pbf^{-/-}$ MEFs showed a significant reduction in migration and invasion compared with wild-type ($Pbf^{+/+}$) MEFs. Interestingly, the loss of one functional copy of Pbf in heterozygote MEFs ($Pbf^{+/-}$) resulted in an intermediate decrease in motility suggesting a gene-dosage effect. Initial immunofluorescent studies of $Pbf^{-/-}$ MEFs suggest alterations in the structure and distribution of focal adhesions (FAs). Importantly, $Pbf^{-/-}$ MEFs demonstrated a significant reduction in focal adhesion kinase (FAK) and paxillin staining with smaller, punctate and more radially distributed FAs compared to $Pbf^{+/+}$ MEFs.

**Conclusion**

These findings highlight a requirement for both PBF phosphorylation and endocytosis in stimulating cell migration and invasion, and suggest a significant role for PBF in focal adhesion regulation. Overall, these studies demonstrate a physiological role for PBF in cell motility and further elucidate the mechanisms by which PBF induces cell motility in tumour progression.

### EACR23-0229
### PKA: a key player for the colonization of the bone niche by prostate cancer cells.

*P. Sanchis[1], N. Anselmino[2], S. Lage-Vickers[1], A. Sabater[1], E. Labanca[2], P. Valacco[1], N. Navone[2], E. Vazquez[1], J. Cotignola[1], G. Gueron[1]*
*[1]University of Buenos Aires, Department of Biological Chemistry, Ciudad Autónoma Buenos Aires, Argentina*
*[2]MD Anderson Cancer Center,*

© 2023 The Authors. Molecular Oncology is published by John Wiley & Sons Ltd on behalf of Federation of European Biochemical Societies

*Department of Genitourinary Medical Oncology, Houston- Texas, United States*

**Introduction**

The metastatic cascade is a dynamic scenario where metabolic plasticity is critical for the adaptation of tumor cells to the new organ. Understanding the metabolic rewiring occurring in prostate cancer (PCa) cells in the bone niche is an unmet need underlying metastases' formation. In this work, we aimed at identifying critical regulatory axes of this process.

**Material and Methods**

We assessed the transcriptome of PCa cells (PC3) modulated by soluble factors released by bone precursors (MC3T3 and Raw264.7) in an indirect co-culture system. We validated the transcriptional profile of metabolic genes using open-access datasets. Ingenuity Pathway Analysis (IPA) was used to delineate the regulators of these metabolic genes. Bone secretome was profiled in the conditioned media (CM) by ESI-MS/MS. We validated our results using a PDX pre-clinical model comparing gene expression levels in MDA-PCa-183 growing intrafemorally (*i.f.*) vs. subcutaneously (*s.c.*).

**Results and Discussions**

PC3 cells co-cultured with bone progenitors displayed an activation of lipidic categories, including PPAR-signaling and fat absorption/digestion. Accordingly, PC3 cells treated with the CM of the co-culture had an increased accumulation of neutral lipids as shown by Bodipy 493/503 staining. Unsupervised Clustering analysis using transcriptomic data from human PCa and bone metastatic samples (GSE74685) showed that the metabolic genes deregulated in PC3 by the co-culture accurately clustered samples in primary tumor vs. bone metastasis. Moreover, a signature of 5 lipid-associated genes, *PPARA*, *VDR*, *SLC16A1, PAPSS2* and *GPX1*, was associated with a 23-fold higher risk of death (SU2C-PCF dataset). Interestingly, this signature was also upregulated in the MDA-PCa-183 PDX growing *i.f. vs. s.c.*, suggesting that there are intrinsic bone factors altering PCa metabolism. IPA revealed that these genes are regulated by the Protein Kinase A (PKA). Accordingly, PKA inhibition led to a downregulation of these genes. Moreover, PC3 cells treated with the CM of the co-culture presented differential ATP levels, that were restored by PKA inhibition. Finally, secretome and protein-protein interaction analyses revealed bone-secreted type I collagen and Fn1 as factors regulating tumoral PKA activity.

**Conclusion**

We propose a novel lipidic gene signature driving PCa aggressive metastatic disease, triggered by the dialogue with bone cells. This signature is regulated by PKA, which responds to bone-secreted factors, and emerges as a potential hub to halt disease progression.

### EACR23-0231

### Pro-carcinogenic effects of colibactin-producing E. coli in the colon : Involvement of L-serine

*A. devaux*[1], *G. roche*[1], *B. diémé*[2], *M. lagrée*[2], *M. rodrigues*[1], *C. jousse*[2], *N. barnich*[1], *R. villéger*[1], *M. bonnet*[1]
[1]*University of Clermont Auvergne, Microbe- Intestine- Inflammation and Host Susceptibility, Clermont-Ferrand, France*
[2]*University of Clermont Auvergne, MetaboHUB, Clermont-Ferrand, France*

**Introduction**

The colonic tissues are abnormally colonized by colibactin-producing *Escherichia coli* (CoPEC) in colorectal cancer (CRC) patients. Colibactin is a genotoxin produced by enzymes encoded by the pks genomic island. Metabolomic studies have shown that CoPEC infection results in reprogramming of intestinal epithelial cell metabolism, leading to a decrease of L-serine concentration. The aim is to investigate whether serine allows CoPEC to better persist in the gastrointestinal tract and/or exert their pro-carcinogenic functions.

**Material and Methods**

Expression of genes coding for the serine utilization and biosynthesis operon was evaluated by RT-qPCR following infection with a reference CoPEC strain (11G5), its colibactin-negative mutant (11G5Δ-*pks*) or a commensal *Escherichia coli* (K12C600) in human colorectal carcinoma T84 cells. The impact of a serine- and glycine- depleted diet (SD) on the persistence and genotoxic effects of CoPEC (ɣH2Ax immunolabeling) and tumor development was studied on two mouse models of CRC: one spontaneous model ($APC^{min/+}$) and one subcutaneous transplant (MC38). The serine utilization on CoPEC virulence was verified both *in vitro* and *in vivo* with a 11G5-Δ*tdcA* mutant, unable to metabolize serine.

**Results and Discussions**

*In vitro*, we have shown that CoPEC uses serine from the enterocyte via the activation of serine-utilization-operon, which provides to CoPEC a competitive growth advantage over a commensal strain or its mutant (11G5-Δ*pks*). To highlight the specific role of L-serine, $APC^{min/+}$ mice were fed with a SD diet. SD diet induced an early and transient decrease in CoPEC 11G5 bacterial colonization associated with a decrease of DNA damages. In the same way, the pro-carcinogenic potential of CoPEC on MC38 tumors is significantly lower with the SD diet than with the Control diet. A lower persistence of the 11G5-Δ*tdcA* mutant was observed in T84 cells and in $APC^{min/+}$ mice. The pro-carcinogenic potential of CoPEC on MC38 tumors was strongly decreased with 11G5Δ-*tdcA* mutant in comparison to 11G5 strain suggesting that host L-serine utilization by bacteria is a key factor for CoPEC pro-tumoral effects.

**Conclusion**

Altogether, these data support that CoPEC would use the host L-serine, by activation of its serine operon utilization, to persist and promote its genotoxic and pro-carcinogenic effects. This work allows us to elucidate the mechanisms of action of CoPEC in colonic carcinogenesis and identify potential novel therapeutic targets.

### EACR23-0254

### miR-662 is associated with metastatic relapse in early-stage breast cancer and promotes metastasis by stimulating cancer cell stemness

*M. Puppo*[1], *M. Valluru*[2], *M. Croset*[3], *D. Ceresa*[4], *M. Iuliani*[5], *A. Khan*[6], *C. Ginestier*[7], *F. Pantano*[5], *P. Ottewell*[6], *P. Clezardin*[8]

© 2023 The Authors. Molecular Oncology is published by John Wiley & Sons Ltd on behalf of Federation of European Biochemical Societies

[1]Universite' Lyon 1- INSERM, UMR-S 1033, Lyon cedex 8, France
[2]University of Sheffield, Department of Infection-Immunity and Cardiovascular, Sheffield, United Kingdom
[3]Centre de Recherche en Cancérologie de Lyon, iNSERM U1052, Lyon, France
[4]Università degli studi di Genova, IRCCS AOU San Martino, Genova, Italy
[5]Campus Bio-Medico University of Rome, Medical Oncology Department, Rome, Italy
[6]University of Sheffield, Department of Oncology and Metabolism, Sheffield, United Kingdom
[7]Institut Paoli-Calmettes, Aix-Marseille Univ, Marseille, France
[8]INSERM, Research Unit UMR S1033, Lyon, France

**Introduction**

Breast cancer (BC) metastasis, which often occurs in bone, contributes substantially to mortality. MicroRNAs play a fundamental role in BC metastasis, although microRNA-regulated mechanisms driving metastasis progression remain poorly understood.

**Material and Methods**

MiRome analysis in serum from BC patients was performed by TaqMan™ low-density array (TLDA). MiR-662 was overexpressed following MIMIC-transfection or lentivirus transduction. Animal models were used to investigate the role of miR-662 in BC (bone) metastasis. The effect of miR-662-overexpressing BC cell conditioned media on osteoclastogenesis was investigated. ALDEFLUOR assays were performed to study BC stemness. RNA-sequencing transcriptomic analysis on miR-662-overexpressing cells was performed to evaluate gene expression changes.

**Results and Discussions**

High levels of hsa-miR-662 (miR-662) in serum from early-stage BC patients, at baseline (time of surgery), were associated with future recurrence in bone. Experimentally, we showed that at an early-stage of the metastatic disease, miR-662 could mask the presence of BC metastases in bone by inhibiting the maturation of bone-resorbing osteoclasts. Nonetheless, metastatic miR-662-overexpressing BC cells then progressed as overt osteolytic metastases thanks to increased stem cell-like traits.

**Conclusion**

1) MiR-662 may be further developed as a prognostic marker to identify early-stage BC patients at high risk of (bone) metastasis; 2) MiR-662 is involved in BC metastasis progression by enhancing cancer cell stemness.

### EACR23-0256
### Protective effects of miR-24 in breast-to-bone metastasis through tumour cell cycle modulation

*M. Puppo*[1], *D. Ceresa*[2], *M. Croset*[3], *M.K. Valluru*[4], *M. Hernandez-Guadarrama*[5], *M. Iuliani*[6], *F. Pantano*[6], *P.D. Ottewell*[7], *P. Clezardin*[8]
[1]Universite' Lyon 1- INSERM, UMR-S 1033, Lyon cedex 8, France
[2]Università degli studi di Genova, IRCCS AOU San Martino, Genova, Italy
[3]Centre de Recherche en Cancérologie de Lyon, Inserm U1052, Lyon, France
[4]University of Sheffield, Department of Infection- Immunity and Cardiovascular, Sheffield, United Kingdom
[5]Faculty of Medicine Lyon-Est, Inserm umr 1033, Lyon, France
[6]Campus Bio-Medico- University of Rome, Medical Oncology Department, Rome, Italy
[7]University of Sheffield, Department of Oncology and Metabolism, Sheffield, United Kingdom
[8]INSERM, Lyos- UMR 1033, Lyon, France

**Introduction**

Breast cancer (BC) is the most common cancer in women worldwide, with ~30% of cases evolving into metastatic disease, mainly in bone. MicroRNAs play a fundamental role in BC metastasis, although our understanding of how microRNA-regulated mechanisms drive BC metastasis remains unclear. MiR-24 has been largely investigated in cancer and other diseases, however its possible role in BC progression to bone is unknown.

**Material and Methods**

MiRome analysis in serum from BC patients was performed by TaqMan™ low-density array (TLDA). MiR-24 was overexpressed following MIMIC-transfection or lentivirus transduction in human MDA-MB-231 BC cell line. Animal models of bone metastasis were used to investigate the role of miR-24 in the metastatic progression. The effect of miR-24-overexpressing BC cell conditioned media on osteoclastogenesis was investigated. RNA-sequencing transcriptomic analysis on miR-24-overexpressing MDA-MB-231 and MCF7 cell lines was performed to evaluate gene expression changes. The effect of miR-24-overexpression in MDA-MB-231 on cell proliferation and cell cycle was investigated.

**Results and Discussions**

Low miR-24 levels in serum from early-stage BC patients, at baseline (time of surgery), were associated with high risk of bone relapse. MiR-24 overexpression in MDA-MB-231 cells reduced their proliferative and migratory properties. *In vivo*, miR-24-overexpressing BC cells reduced metastatic tumour burden, particularly in bone. Moreover, miR-24 overexpressing MDA-MB231 and MCF-7 conditioned media inhibited osteoclastogenesis *in vitro,* thus suggesting an effect also on bone resident cells. GSEA analysis conducted on RNA-sequencing data on two different miR-24-overexpressing BC cell lines showed that several gene networks involving proliferation and cell cycle were modulated compared to control cells. Further validation *in vitro* confirmed that miR-24 had an impact on cell cycle of BC cells, explaining at least in part its protective effect on metastasis progression.

**Conclusion**

The restoration of miR-24 expression levels in metastatic BC cells resulted in a reduction of the metastatic properties suggesting miR-24 could be a good candidate to be developed as therapeutic agent for metastatic BC.

### EACR23-0258
### Modelling CMS4 colorectal cancer using a 3D model to investigate interactions in the tumour microenvironment

*E. Reidy*[1,2,3,4], *N. Leonard*[1,2,3], *A. Ryan*[1,2,3,4]
[1]University of Galway, Discipline of Pharmacology and Therapeutics- School of

© 2023 The Authors. Molecular Oncology is published by John Wiley & Sons Ltd on behalf of Federation of European Biochemical Societies

*Medicine- College of Medicine- Nursing and Health Sciences, Galway, Ireland*
[2]*University of Galway, Regenerative Medicine Institute REMEDI- School of Medicine- College of Medicine- Nursing and Health Sciences, Galway, Ireland*
[3]*University of Galway, Lambe Institute for Translational Research- School of Medicine- College of Medicine- Nursing and Health Sciences, Galway, Ireland*
[4]*University of Galway, CÚRAM-SFI Research Centre for Medical Devices, Galway, Ireland*

**Introduction**

Colorectal cancer(CRC) is the 3rd most common cause of cancer-related deaths worldwide. It has been classified into four Consensus Molecular Subtypes(CMS) with CMS1 tumours having microsatellite instability, CMS2 having mutations in cellular metabolism pathways, CMS3 having KRAS mutations and CMS4 being rich in mesenchymal stromal cells(MSCs) with increased angiogenesis and an inflamed immune phenotype. CRC variable response to treatment could be due to genetic and molecular heterogeneity, with CMS1 often responding to immunotherapies, CMS2 and CMS3 respond better to chemotherapy but CMS4 has been associated with the worst disease-free progression survival. Understanding cell-cell and cell-ECM interactions in CMS4 is necessary for developing a treatment for patients with this poor prognosis. We aim to develop a 3D model which mimics aspects of CMS4 CRC tumour microenvironment(TME).

**Material and Methods**

Spheroids were established from HCT116 and HT29 human CRC cell lines, and embedded into collagen hydrogels. To recapitulate stromal dense tumour microenvironments, hMSCs were incorporated in the spheroids. Metabolic activity and proliferation were assessed using Alamar blue and Cyquant. As a measure of metastatic ability, outgrowth from spheroids was measured using ImageJ. Confocal microscopy was used to analyse viability staining and ECM components of the TME. Finally, to mimic the inflamed immunophenotype of CMS4 CRC patients, spheroids were co-cultured with Jurkat cells(T cell line) or macrophage isolated from healthy patients. Using flow cytometry immune cell activation and polarisation in the 3D model was assessed.

**Results and Discussions**

Collagen provided a suitable ECM-like material allowing cells to proliferate and be metabolically active over a period of 10 days. Incorporating MSCs leads to reduced cell death and increased outgrowth from spheroids, mimicking the increased metastatic ability of CRC in a stromal dense environment. Gels with hMSCs had significantly higher levels of fibronectin than gels with CRC cells alone mimicking fibronectin in CRC TME in-vivo. Co-culturing Jurkat cells with spheroids +hMSCs lead to an increase in exhaustion markers compared to with cancer cells alone.

**Conclusion**

Our 3D model provides a suitable tool for analysing interactions between various cell types and cell-ECM interactions in CMS4 CRC. As well as this, these models have the potential to develop personalised patient 3D models which could ideally be used for analysing therapeutics for these patients in the future.

### EACR23-0276
### Estimation the invasive potential of glioma to peripheral normal tissue by using functional MRI

[1]*National Tsing Hua University, Institute of Nuclear Engineering and Science, Hsinchu, Taiwan*
[2]*Chang Gung University, Institute for Radiological Research, Taoyuan, Taiwan*

**Introduction**

The abnormal blood vessels in tumors create a hypoxic environment, reducing the effectiveness of radiation therapy and chemotherapy for brain tumors.Along with the anomalous blood vessels within the tumor, an invasive front structure emerges at the interface of malignant brain tumors with elevated malignancy and normal tissues. This invasive front is presumed to be the primary cause of tumor recurrence and invasion after treatment. To study this area, DCE-MRI was utilized to analyze blood flow and its relation to vessel structure and tumor invasion. The objective is to employ imaging to assess the blood flow status in the tumor edge region of glioma before treatment and the likelihood of tumor progression and invasion after treatment.

**Material and Methods**

ALTS1C1 mouse glioma cells were implanted into the mouse brain to simulate glioma growth. After two weeks, MRI with dynamic contrast enhancement was performed to calculate the initial velocity (V0) of blood flow leaving the tumor surface. Tumor tissue was obtained for immunostaining analysis to distinguish the invasion front region from the non-invasion region. Tumor blood vessel structures, vascular function, fibroblast activation protein (FAP) expression, and aquaporin expression were observed separately using immunostaining methods.

**Results and Discussions**

Our staining results revealed disparities between the invasive and non-invasive areas. Specifically, CD31 demonstrated vascular expansion towards the exterior of the tumor in the invasive region and higher vascular permeability. NG2+ expression indicated a more developed vascular system in the invasive region. Moreover, FAP+ expression levels, which reflect invasiveness, were greater in the invasive area than in the non-invasive area, while AQP-4+ expression decreased, indicating that the tumor's invasion reduced astrocyte activity in normal tissue and subsequently decreased water channel expression. Additionally, comparing the results of tissue staining with DCE-MRI imaging analysis revealed that the maximum distance of blood flow conversion to initial blood flow velocity (V0) was greater in the invasive area at the tumor margin.

**Conclusion**

This study found a link between the initial velocity of blood flow and the invasion ability of glioma. DCE-MRI can be used to detect changes in tumor blood flow, predict glioma invasion, and evaluate the tumor edge's invasion area after treatment.

### EACR23-0284
### AGPAT4 as a Novel Metabolic Driver of Oncogenic Stemness and Dedifferentiation

© 2023 The Authors. Molecular Oncology is published by John Wiley & Sons Ltd on behalf of Federation of European Biochemical Societies

### in Hepatocellular Carcinoma

*K.Y. Ng[1], T.Y. Koo[1], T.L. Wong[1], Y. Gao[1], J.P. Yun[2], X.Y. Guan[3], M. Liu[4], C.Y. Chung[1], S. Ma[1]*
*[1]The University of Hong Kong, School of Biomedical Sciences, Hong Kong, Hong Kong- China*
*[2]Sun Yat-Sen University Cancer Centre, Department of Pathology, Guangzhou, China*
*[3]The University of Hong Kong, Department of Clinical Oncology- School of Clinical Medi cine, Hong Kong, Hong Kong- China*
*[4]Guangzhou Medical University, School of Basic Medical Science, Guangzhou, China*

**Introduction**

The liver is an unique organ in that it is responsible for many metabolic functions. During HCC development, these metabolic machineries are extensively reprogrammed to support the insatiable nutrient requirement of HCC. Cancer stem cells (CSCs) are a small and elusive subpopulation of self-renewing cancer cells with remarkable ability to initiate, propagate, and spread the malignant disease. Liver CSCs reprogram their metabolic pathways to match with the increased metabolic needs for cancer cell survival under adverse conditions. Identifying novel metabolic targets that are related to stemness can offer promising strategies for targeting CSCs and hence to kill and to control their growth.

**Material and Methods**

Human HCC cell lines, preclinical sporadic HCC tumor models, and tissues of patients with HCC were used to assess the role of AGPAT4 in driving HCC stemness and dedifferentiation. Activity-based compound screening was utilized for discovery of new therapeutic compounds against AGPAT4.

**Results and Discussions**

Pathway enrichment analysis of genes that linked metabolism and stemness identified aberrant glycerophospholipid metabolism of which AGPAT4 ranked a top-hit. AGPAT4 upregulation in HCC is tightly correlated with aggressive clinical features. Expression of AGPAT4 peaked during early liver progenitor development, decreased during hepatocyte maturation, and increased progressively from well-differentiated to poorly differentiated HCCs. Enrichment of AGPAT4 in HCC was mediated by promoter binding of SOX9 to drive AGPAT4 transcriptional activity. AGPAT4 inhibition could mitigate tumor initiation, self-renewal, metastasis, and sorafenib resistance. Mechanistic studies revealed an AGPAT4-mediated phosphatidic acid production axis to promote HCC through regulating mTOR signaling. Inhibition of Agpat4 by AAV8 shRNA reduced tumorigenicity and stemness and sensitized HCC tumors to sorafenib. AGPAT4 overexpression was able to predict sorafenib response in the clinic. Through high-throughput screening coupled with activity-based protein profiling, a cysteine-reacting compound with high binding affinity and selectivity towards AGPAT4 was identified and found to work synergistically with sorafenib to suppress HCC.

**Conclusion**

AGPAT4 is a novel metabolic driver of oncogenic stemness, dedifferentiation and metastasis in HCC. AGPAT4-induced tumor lineage plasticity may represent an Achilles heel for HCC. Inhibition of AGPAT4 may widen the therapeutic window for sorafenib treatment in the clinic.

### EACR23-0287
### Fibroblast subpopulations differentially influence tumorigenesis in colorectal cancer

*A.Á. Soós[1], A. Kelemen[1], Z. Szvicsek[1], A. Orosz[1], Z. Wiener[1]*
*[1]Semmelweis University, Department of Genetics- Cell- and Immunobiology, Budapest, Hungary*

**Introduction**

The enrichment of cancer-associated fibroblasts (CAF) results in a worse patient survival in colorectal cancer (CRC). However, classification of the CAFs is still not resolved. In pancreatic ductal adenocarcinoma, the myofibrotic CAFs produce mainly extracellular matrix components, and inflammatory CAFs secrete inflammatory cytokines. A study proved that $TF^{low}$ fibroblasts of the colon were associated with inflammatory bowel disease. Moreover, CAFs support a niche for CRC stem cells by secreting growth factors. Characterizing stromal fibroblast heterogeneity and therapeutic targeting of both cancer and stromal cells in CRC are thus major challenges.

**Material and Methods**

In this work, we aimed at characterizing fibroblast subpopulations that differentially support CRC tumorigenesis. We also tested novel compound combinations on CRC cells and fibroblasts, and we compared their effects on the tumor supporting fibroblast subpopulation. To accomplish our aims, we applied patient-derived organoids that maintain the cellular heterogeneity of the original tissue.

**Results and Discussions**

TGFβ (transforming growth factor β) is a main inducer of fibroblast activation in CRC. TGFβ induced the accumulation of $TF^{high}$ cells in CRC patient-derived fibroblast cultures. Moreover, fluorescently sorted $TF^{high}$ fibroblasts increased the organoid forming efficiency and the percentage of proliferating cells of organoids compared to $TF^{low}$ fibroblasts in cocultures. When testing novel compound combinations, we observed that CRC fibroblasts showed a higher resistance for MEKi (MEK inhibitor) and the combined MEKi+Hsp90i (heat shock protein-90 inhibitor), but they were more sensitive to Bcli (Bcl-xL inhibitor), JQ1 (bromodomain epigenetic inhibitor) and the combination of these two drugs compared to CRC organoids. Importantly, all the combinations of these drugs acted synergistically on CRC organoids. Interestingly, when applying the IC50 concentrations of the compounds, we observed a decreased percentage of TF+ fibroblasts only for Hsp90i.

**Conclusion**

These data suggest that fibroblasts with different TF levels have different organoid initializing effects in CRC. Since fibroblasts are sensitive to Bcli, and Hsp90i results in a negative selection of the tumor promoting $TF^{high}$ fibroblasts, this combination may target both tumor cells and CAFs efficiently in CRC.

*Funding: OTKA137554, TKP2021-EGA-24, ÚNKP-22-5-SE-10, János Bolyai Research Fellowship (BO/00131/20/8).*

© 2023 The Authors. Molecular Oncology is published by John Wiley & Sons Ltd on behalf of Federation of European Biochemical Societies

### EACR23-0292
### The identification of functional germline variants in 3´UTR of androgen receptor variant 7

J. Van Goubergen[1], F. Handle[2], M.V. Cronauer[3], F.R. Santer[1]
[1]Medical University of Innsbruck, Division of Experimental Urology- Department of Urology, Innsbruck, Austria
[2]Medical University of Innsbruck, Institute of Pathology- Neuropathology & Molecular Patho logy, Innsbruck, Austria
[3]Universitätsklinikum Bonn, Institute of Pathology, Bonn, Germany

**Introduction**

The Androgen Receptor (AR) is an oncogenic driver in prostate cancer (PCa). As a result, therapies targeting the AR signaling axis, i.e. inhibitors (ARSI), are currently the gold standard for treating advanced PCa. However, patients eventually progress on these therapies. One of the main mechanisms behind ARSI resistance is the upregulation of constitutively active AR-variants (AR-Vs). Clinically, the most relevant variant is AR-V7. Resulting from cryptic exon 3 inclusion, AR-V7 mRNA possesses a distinct 3´untranslated region (UTR) compared to the full-length AR (AR-FL). This observation suggests a differential 3´UTR mediated post-transcriptional regulation. Here, we set out to better characterize the regulatory role of AR-V7´s 3´UTR by analyzing the contribution of a single nucleotide polymorphisms (SNPs) in alternative splicing.

**Material and Methods**

SpliceAidv2 was used to evaluate the capacity of AR-V7 3´UTR SNPs to alter RNA binding protein motifs. SU2C data, representing metastatic PCa, was used to assess differentially expressed genes based on AR-V7 expression cohorts. CRISPR/CAS9 mediated non-homologous end joining (NHEJ) was used to experimentally assess these predictions using qPCR and Western blot. Furthermore, predicted RNA-protein interactions were validated via CLIP-qPCR. Finally, an AR-V7 minigene was used to assess allelic effects on AR-V7 splicing.

**Results and Discussions**

AR-V7 3´UTR SNP rs5918762 (minor allele frequency=0.32) was selected as it is in linkage disequilibrium with several PCa susceptibility tagSNPs (rs5919393 and rs5919732). Spliceosome protein SRSF9 was identified as a protein of interest, since it is predicted to bind AR-V7 3´UTR in an rs5918762 allele-specific manner. Moreover, SRSF9 expression correlates with AR-V7 expression. SRSF9´s interaction with AR-V7 mRNA was confirmed by CLIP-qPCR. Detailed mapping suggests that multiple binding sites of SRSF9 exist across AR-V7 3´UTR. The AR-V7 minigene assay showed an allele-specific involvement of rs5918762. Moreover, the addition of the pan-CLK/DYRK inhibitor SM08502, inhibiting the phosphorylation of SR-proteins, had a similar outcome.

**Conclusion**

This work suggests that rs5918762 affects splicing of AR-V7 by modulation of the binding capacity of the spliceosome component SRSF9. RNA-pull downs have to confirm this suggestion. Taken together, these results suggest an involvement of the common SNP rs5918762 in AR-V7 splicing mediated by SRSF9 and highlight SRSF9 as a potential target to overcome AR-V7 mediated resistance.

### EACR23-0300
### Targeting stromal Nox4 abrogates onco-supportive cancer-associated fibroblast-driven cancer cell interactions in the prostate tumour microenvironment

E. Brunner[1], L. Neumann[1], E. Damisch[1], S.T. Deichsler[1], L. Nommensen[1], S. Karkampouna[2], F. Bonollo[2], M. Kruithof-de Julio[2], G. Schäfer[3], Z. Culig[1], N. Sampson[1]
[1]Innsbruck Medical University, Department of Urology- Division of Experimental Urology, Innsbruck, Austria
[2]University of Bern, Department for BioMedical Research DBMR- Urology Research Laboratory, Bern, Switzerland
[3]Innsbruck Medical University, Division of Pathology, Innsbruck, Austria

**Introduction**

Prostate tissue homeostasis is maintained by tightly regulated stromal-epithelial cell interactions. In prostate cancer (PCa), stromal-epithelial crosstalk is perturbed due to extensive stromal tissue remodelling, wherein fibroblasts adjacent to the tumour become activated. Notably, studies in other tissues have described both tumour-promoting but also tumour-restricting cancer-associated fibroblast (CAF) phenotypes. In PCa, CAF subtypes remain poorly described. A better understanding of tumour-promoting CAF subtypes and their interactions with epithelial cells, is crucial to develop novel stroma-targeting therapeutic strategies.

In this study, we describe a population of CAFs in PCa that express elevated levels of NADPH oxidase 4 (Nox4) and localize immediately adjacent to tumour foci. To evaluate the potential clinical utility of targeting Nox4-expressing CAFs in prostate cancer, we characterised the molecular driver pathways regulated by Nox4 in CAFs and determined their influence on stromal-cancer cell interactions in the tumour microenvironment.

**Material and Methods**

We used pharmacological Nox4 inhibition using the small molecule inhibitor Setanaxib in diverse *in vitro* and *ex vivo* models. These include primary prostate CAFs isolated from patient biopsy cores, *ex vivo* tissue cultures as well as CAF-PCa cell coculture models which recapitulate the reciprocal interactions between stromal and cancer cells more closely. We are currently investigating the therapeutic potential of Nox4 inhibition in an orthotopic CAF-PCa xenograft model.

**Results and Discussions**

We demonstrate that Nox4-expressing CAFs are associated with numerous clinical hallmarks of poor prognosis and display a transcriptional program related to a myofibroblastic phenotype. Functional studies revealed that Nox4 plays a central role in coordinating tyrosine phosphorylation of the focal adhesion and mechano-transduction machinery in CAFs and that Nox4 inhibition attenuates the growth-promoting effects of CAFs on PCa cells. We are currently awaiting RNA sequencing results from cocultured primary Nox4+ CAFs and PCa cells treated with Setanaxib. These results will provide further

© 2023 The Authors. Molecular Oncology is published by John Wiley & Sons Ltd on behalf of Federation of European Biochemical Societies mechanistic insights into the effects of Nox4+ CAFs on tumour cells and the effects of Setanaxib treatment on CAF-tumour cell interactions.

**Conclusion**

Our data indicate that inhibition of stromal Nox4 abrogates CAF-driven onco-promoting interactions with prostate cancer cells and thus represents a promising tumour microenvironment-targeted therapeutic strategy.

### EACR23-0307

### Analysis of murine bladder tumours to better classify their relationship with human bladder cancer

*R. Reed[1], S. Lunj[1], K. Reeves[1], M. Reardon[1], E. Romano[1], D. Mukherjee[1], R. Walshaw[2], J. Honeychurch[1], P. Hoskin[3], A. Choudhury[3]*

*[1]University of Manchester, Division of Cancer Sciences, Manchester, United Kingdom*

*[2]The Clatterbridge Cancer Centre, NHS Foundation Trust, Manchester, United Kingdom*

*[3]The Christie, NHS Foundation Trust, Manchester, United Kingdom*

**Introduction**

Bladder cancer accounts for nearly 170,000 deaths annually worldwide[1]. Muscle invasive bladder cancer (MIBC) can either be confined to the bladder or metastasise distally, for patients that are organ-confined the standard of treatment is radical cystectomy (RC); however this procedure has post-operative concerns in terms of quality of life[2].

The aim of the study presented here is to provide a detailed analysis of two MIBC cell lines (MB49 and MBT2) to provide a better understanding of the relationship between these two murine models and the known classification of primary human MIBC. Comparing the tumour landscape of the two murine MIBC models to see how well they correlate with human disease will establish whether these are good tumour models for studying MIBC and immune responses in human disease.

**Material and Methods**

Mice were inoculated with MB49 (C57Bl/6, n=5) and MBT2 (C3H/Hen, n=7) cell lines, tumours excised at ~200mm$^3$ volume and provided as dissociated tumour cells. The data presented here involves assessing the micro environmental cytokine and chemokines using a 36-plex murine array (ThermoFisher) in order to assess if the tumour chemokine and cytokine profile in murine MIBC models is similar to human MIBC.

**Results and Discussions**

Data from dissociated tumours shows all measured chemokines/cytokines are detectable with the exception of IFNα. Of the 9 chemokines measured, 5 have significant differential expression using the Mann-Whitney test; Eotaxin $p$=0.0303, MCP-1 $p$=0.0101, MIP-1 alpha $p$=0.0480, MIP-2 $p$=0.0051 and RANTES $p$=0.0303 when comparing the MB49 with MBT2. Of the 27 cytokines measured, 11 have significant differential expression G-CSF $p$=0.0242, GM-CSF $p$=0.0043, IL-1 beta $p$=0.0051, IL-3 $p$=0.0286, IL-5 $p$=0.0126, IL-12p70 $p$=0.0051, IL-17A $p$=0.0076, IL-27 $p$=0.0303, IL-28 $p$=0.0429, IL-31 $p$=0.0028, LIF $p$=0.0051, when comparing the MB49 with MBT2.

**Conclusion**

Analysis is ongoing to further profile the landscape of the murine tumours. The second part of the validation study to be performed is immunohistochemistry (IHC). We propose to use 5 multiplex panels to evaluate basal/luminal markers, and immune profiling of T cells, macrophages, granulocytes, immunosuppressive monocytes and immune checkpoints. This will help assess whether the infiltrating immune contexture in murine MIBC models is similar to human MIBC.

The clinical impact of this work will enable more rationalised approaches to pre-clinical research aimed at developing new treatments for bladder cancer.

### EACR23-0320

### Tumor metabolic acidosis: a key piece in the puzzle of colorectal cancer heterogeneity

*E. Richiardone[1], J. Ambroise[2], B. Drotleff[3], S. Arena[4], A. Bardelli[5], C. Corbet[1]*

*[1]UCLouvain, Pole of Pharmacology and Therapeutics FATH- Institut de Recherche Expérimentale et Clinique IREC, Sint-Lambrechts-Woluwe, Belgium*

*[2]UCLouvain, Centre des Technologies Moléculaires Appliquées CTMA- Institut de Recherche Expérimentale et Clinique IREC, Sint-Lambrechts-Woluwe, Belgium*

*[3]EMBL Heidelberg, Metabolomics Core Facility, Heidelberg, Germany*

*[4]University of Torino, Dept of Oncology- Candiolo Cancer Institute- IRCCS-FPO, Torino- Candiolo, Italy*

*[5]University of Torino, Department of Oncology- Candiolo Cancer Institute- IRCC s-FPO, Torino- Candiolo, Italy*

**Introduction**

Colorectal cancer (CRC) is one of the most frequent and aggressive cancers worldwide. Inter- and intra-tumor heterogeneities at both genetic and transcriptomic levels make the clinical management of CRC patients very challenging. Besides genetic instability, tumor microenvironment (TME) is increasingly recognized to support intratumoral heterogeneity and disease progression. Acidosis is a common feature of TME in solid tumors, including CRC, and it has been shown to shape more aggressive cancer cell phenotypes. Here, we hypothesize that targeting acidosis-induced phenotypic traits, in particular metabolic preferences, may serve as a "one-size-fits-all" therapeutic strategy to thwart tumor aggressiveness in CRC, regardless of their initial genotype.

**Material and Methods**

Four CRC cell lines (DiFi, LIM1215, HCT-116 and HT-29), with distinct genotypes and microsatellite instability status, have been adapted to chronic acidosis (pH 6.5) and then characterized by genomic, transcriptomic, metabolomic and phenotypic analyses.

**Results and Discussions**

A common signature of 10 up- and down-regulated genes has been identified in the 4 acidosis-adapted CRC cells, as well as increased migratory capacity and anoikis resistance. Moreover, acidosis-adapted CRC cells display reduced glycolytic rate while mitochondrial respiration is increased. Another common metabolic feature displayed by

© 2023 The Authors. Molecular Oncology is published by John Wiley & Sons Ltd on behalf of Federation of European Biochemical Societies acidosis-adapted CRC cells is increased intracellular levels of proline and serine amino acids. Higher abundance of proline might be related to collagen synthesis and extracellular matrix organization so that favoring a pro-migratory phenotype. The relevance of our findings is strengthened by the observation that inhibiting serine biosynthesis pathway, with a PHGDH inhibitor, drastically reduces the viability of acidosis-adapted CRC cells. Interestingly, alpha-keto glutarate, but not serine supplementation, restores the viability of acidosis-adapted cells under PHGDH inhibition. Moreover, impairment of mitochondrial respiration reduces the viability of acidosis-adapted CRC cells as well as resistance to anoikis.

**Conclusion**

While data are now being validated in relevant pre-clinical CRC models, including 3D patient-derived tumor organoids, these preliminary results open new perspectives in the identification and further therapeutic targeting of metabolic preferences driven by tumor acidosis in CRC.

### EACR23-0321

### Identification and characterisation of the stromal heterogeneity in prostate cancer

*E. Damisch[1], E. Brunner[1], G. Fotakis[2], Z. Trajanoski[2], A. Pircher[3], I. Heidegger-Pircher[1], S. Sopper[3], L. Nommensen[1], G. Schäfer[4], N. Sampson[1]*

*[1]Medical University of Innsbruck, Department of Urology, Innsbruck, Austria*

*[2]Medical University of Innsbruck, Institute of Bioinformatics, Innsbruck, Austria*

*[3]Medical University of Innsbruck, Department of Haematology and Oncology- Internal Medicine V, Innsbruck, Austria*

*[4]Medical University of Innsbruck, Department of Pathology- Neuropathology and Molecular Pathology, Innsbruck, Austria*

**Introduction**

Whilst localised prostate cancer (PCa), the second most common cancer in men worldwide, is often curable, a subset of patients relapse for whom androgen deprivation remains the first line therapeutic strategy. However, most patients eventually progress to lethal castration-resistant disease. Consequently, there is a necessity for new therapeutic strategies that either prevent or target this final stage of PCa.

Stromal fibroblasts and mural cells are key contributors to the development, progression and therapy resistance of PCa. Thus, there is increasing interest in targeting the stromal component of disease. However, the tumour microenvironment displays functional heterogeneity with some types of cancer-associated stromal cells (CASCs) exhibiting either tumour-supportive or –restrictive properties. Consequently, a better characterisation of CASC subtypes is required in order to specifically target cancer-promoting CASCs.

This study aims to identify, isolate and characterise the CASC phenotypes present in the PCa microenvironment.

**Material and Methods**

We used scRNAseq of digested PCa biopsy cores to identify stromal clusters of interest. Further bioinformatic analyses resulted in prognostic gene signatures for individual scRNAseq clusters and identified distinctive markers for labelling the respective CASC phenotypes in patient tissues via IHC and ISH. Based on the preceding results, a surface marker panel was developed that distinguishes different CASC populations in freshly digested PCa biopsies and can be adapted for isolation of those using FACS.

**Results and Discussions**

We will present scRNASeq data that illustrate the stromal heterogeneity found in the TME of PCa as well as gene signatures of individual clusters with a prognostic value on the TCGA PCa dataset. Analysis of the flow cytometry dataset of freshly digested PCa biopsy cores derived from multiple patients indicates that specific combinations of surface markers enable the identification and isolation of discrete CASC phenotypes. Using distinctive markers for the CASC phenotypes in IHC and ISH, we could spatially describe them in tissue sections of benign versus high grade PCa patient samples.

**Conclusion**

It is hoped that these new findings will advance the understanding of the TME in PCa and provide a better characterisation of the different CASC phenotypes on a cellular level, so that their contribution to PCa pathophysiology and therapy resistance can be better evaluated.

### EACR23-0324

### Inflammatory tumour microenvironment in colorectal cancer alters macrophage function through mesenchymal stromal cell PD-1/PD-L1 signalling

*N. Leonard[1], S. Corry[2], E. Reidy[1], O. Treacy[1], T. Ritter[3], L.J. Egan[1], D. Loessner[4], P. Dunne[2], A. Ryan[1]*

*[1]University of Galway, Pharmacology and Theraputics, Galway, Ireland*

*[2]Queens University Belfast, School of Medicine- Dentistry and Biomedical Sciences, Belfast, United Kingdom*

*[3]University of Galway, School of Medicine, Galway, Ireland*

*[4]Monash University, Chemical & Biological Engineering, Melbourne, Australia*

**Introduction**

Colorectal cancer (CRC) is the 3$^{rd}$ leading cause of cancer-related deaths worldwide despite recent improvements in treatment options. CRC patients with mesenchymal stromal cell (MSC) rich tumours (CMS4 subtype) have the lowest survival rates and show high levels of immunosuppression and treatment resistance. However, the role MSCs play in tumour promotion, in particular, their interaction with macrophages, which are the most abundant immune cell in CRC, is not fully understood.

**Material and Methods**

Bioinformatics analysis of 433 primary CRC tumours assessed by microarray was used to determine transcriptional changes in CMS4 changes. These transcriptional changes were then further investigated using complex 2D conditioning and co-culture systems in both mouse and human. Primary MSC was isolated from CRC patient tumours and expanded ex vivo. A 3D Gelatin methacrylate hydrogel model of CMS4-like tumours was developed. This 3D model combines HCT116 cancer cells, THP1 monocyte cell line and bone marrow-derived MSC.

© 2023 The Authors. Molecular Oncology is published by John Wiley & Sons Ltd on behalf of Federation of European Biochemical Societies

**Results and Discussions**

In MSC-rich CMS4 tumours, we observed an enhanced TNF-α signalling signature. MSCs were conditioned with inflammatory tumour cells secretome (iTCS), and we observed an increased expression of PD-L1 and CD47 by both RNA-seq and flow cytometry. Analysis of primary CRC patient tumours revealed higher numbers of macrophages in the stromal compartment CRC. Conditioned MSCs were co-cultured with macrophages and reduced MHC-II and TNF-α expression, increased CD206 expression, and suppressed phagocytic function in macrophages was observed. Blocking PD-1, the receptors for PD-L1, on macrophages, restored macrophage phagocytosis levels in these cultures. Using the 3D CMS4 like model, we could show that addition of MSCs to the culture system increased the secretion of MIF, SerpinE1, IL-8, CXCL12 and CXCL1, increased the transcription of extracellular matrix remodelling genes while also increasing cancer cell expression of EGFR, supporting tumour growth and proliferation.

**Conclusion**

These results show that inflammatory tumour-associated MSCs alter macrophage function and promote tumour immune evasion in 2D and 3D models. Using physiologically relevant models we demonstrate that stromal cells enhance cell surface and secreted immunomodulatory molecule expression in the tumour microenvironment. These findings and experimental models may suggest novel therapeutic targets for patients with MSC-rich tumours and improve treatment stratification of patients for immunotherapy treatment.

### EACR23-0325
### The interaction of ovarian cancer stem cells with the tumor microenvironment revealed by a platform of patient-derived organotypic models

*C. Battistini[1], H. Kenny[2], V. Nieddu[1], V. Melocchi[3], A. Decio[4], B. Malacrida[5], A. Gatto[1], F. Bianchi[3], R. Giavazzi[4], N. Colombo[6], F. Balkwill[5], E. Lengyel[2], U. Cavallaro[1]*

*[1]Istituto Europeo di Oncologia, Unit of Gynecological Oncology Research, Milano, Italy*

*[2]University of Chicago, Department of Obstetrics and Gynecology, Chicago, United States*

*[3]Fondazione IRCCS Casa Sollievo della Sofferenza, Cancer Biomarkers Unit, San Giovanni Rotondo, Italy*

*[4]Mario Negri Institute for Pharmacological Research - IR CCS, Laboratory of Cancer Metastasis Therapeutics, Milano, Italy*

*[5]Queen Mary University of London, Barts Cancer Institute, London, United Kingdom*

*[6]Istituto Europeo di Oncologia, Division of Gynecologic Oncology, Milano, Italy*

**Introduction**

High-grade serous ovarian cancer (HGSOC) remains a difficult-to-treat and lethal disease, mainly due to the lack of successful strategies for preventing tumor relapse, which is often chemoresistant. This unfavorable prognosis is driven by a small subset of ovarian cancer stem cells (OCSC), which are endowed with tumor-initiating ability and are intrinsically resistant to cytotoxic treatments. These characteristics are sustained by signals coming from the tumor microenvironment (TME), which in the case of HGSOC is best exemplified by the omentum. Thus, defining the biological mechanisms of this OCSC/TME cross-talk may lead to innovative therapeutic strategies.

**Material and Methods**

We reconstructed the architecture of human omentum *in vitro* in 3D co-cultures of primary human omental fibroblasts and mesothelial cells. We then integrated in these organotypic cultures primary, patient-derived HGSOC cells, either as bulk cancer cells or as patient-matched OCSC. After co-culture with TME, tumor cells were re-isolated by FACS sorting and subjected to whole-transcriptome RNA sequencing, comparing them to control cells that have not been in contact with TME.

**Results and Discussions**

The TME induced a massive transcriptional reprogramming in HGSOC cells, and we found a number of pathways deregulated in bulk vs OCSC, shedding light on the impact of TME on OC stemness. Moreover, we identified a panel of transcription factors (TFs) specifically altered by the TME in OCSC. These TF-regulated gene networks represent novel OCSC vulnerabilities and, hence, potential targets in relapsing tumors. As a proof-of-concept, we focused on FOXM1, a TF with pleiotropic functions in cancer cells. FOXM1 pathway was strongly induced by the TME in OCSC, and we could elucidate the underlying molecular mechanism. Moreover, the pharmacological inhibition of FOXM1 significantly impaired OCSC fitness, supporting, on one hand, the key role of TME in orchestrating OC stemness and suggesting, on the other, a new approach for the eradication of OCSC from the omental niche.

**Conclusion**

Overall, our patient-derived platform offers the opportunity to unravel the impact of the TME on OCSC at the molecular level in experimental models with high clinical relevance, and can be exploited to design innovative HGSOC-eradicating treatments.

### EACR23-0339
### Intravital Imaging Method for Visualization of Cellular Mechanism of Melanoma Metastasis

*H. Kwon[1], H.S. Kim[1], K. Akyldiz[1]*

*[1]IVIM Technology, Aplication Specialist, Songpa-gu- Seoul, South Korea*

**Introduction**

*Melanoma* is a cancer that occurs on the skin or mucous membranes and is known to be caused by UV exposure and genetic factors. So many studies are being conducted on the treatment prognosis of melanoma because it is inadequate even if detected early. Lymph node (LN) metastasis is an important factor in determining the direction of a treatment since melanoma metastases through lymphatic vessels and is closely related to survival rates. For this reason, research for discovering biomarkers such as *Spag9*, sperm-associated antigen 9, has been actively conducted to diagnose early-stage LN metastasis. To determine whether *Spag9* expression directly impacts melanoma metastasis, we aimed to visualize melanoma metastasis in several steps *in vivo*.

© 2023 The Authors. Molecular Oncology is published by John Wiley & Sons Ltd on behalf of Federation of European Biochemical Societies

**Material and Methods**
We injected anti-CD31 and anti-LYVE1 antibodies conjugated with fluorescent dyes to label blood vessels and LN. After exposing melanoma metastasized to the inguinal lymph node (ILN) with skin-flap opening surgery, the LN capsule was specified with second harmonic generation and then visualized with an intravital microscope. After observing metastasis, *Spag9*-knockin or -knockout B16F10-EGFP melanoma cell lines were subcutaneously inoculated into footpads, and popliteal lymph node (PLN) was imaged *ex vivo* to investigate the effect of *Spag9* on metastasis and malignancy.
**Results and Discussions**
As a result of inoculating melanoma cells into footpads and imaging the ILN, it was possible to visualize the early stage of cancer metastasis. In the same way, after inoculating the *Spag9*-knockin or -knockout melanoma cell lines, individuals with melanoma metastasis to the PLN were selectively maintained. Moreover, 32 days after inoculation, melanoma was extracted and imaged *ex vivo* under the same fluorescence condition. We observed *Spag9*-Knockin melanoma was in contact with the capsule of PLN, and *Spag9*-Knockout melanoma cells were dispersed. We could visualize the early stages of melanoma metastasis *in vivo* with an intravital microscope and discovered that *Spag9* overexpression could affect both metastasis and malignancy.
**Conclusion**
Previously, only a single time point of the final stage could be studied, so it was impossible to determine the onset or early stage of metastasis. Also, it took work to reflect on individual differences. Intravital microscopy will be a powerful tool to overcome these difficulties, which can make repetitive imaging within a single animal model possible to reduce artifacts due to individual differences and minimize animal use.

### EACR23-0345
### TMEM119+ microglia with cathepsin expression orchestrate astrocytoma invasion in 2D, 3D, in vivo models

*S. Wu*[1], *C. Chiang*[1]
[1]*National Tsing-Hua University, Department of Biomedical Engineering and Environmental sciences, Hsinchu, Taiwan*

**Introduction**
Tumor-associated macrophages/microglia (TAMs) constitute the majority of the brain tumor microenvironment (TME). Multiple studies suggest that these immune cells could promote brain tumor progression. However, distinguishing microglia from macrophages in brain tumors remains challenging for researchers. Thus, how microglia affect tumors, especially in the invasion area, is still unclear. In this study, we explore the landscape of infiltrating TAMs in different regions of the brain TME via specific microglia markers and found microglia dominated in the invasion regions, not macrophages. We further test the migration and invasion ability of glioma cells communicating with either macrophages or microglia via 2D/3D cell-cell interactions models.
**Material and Methods**
An orthotopic astrocytoma model, ALTS1C1, was used. The brain tumor tissue was dissected into the regions of interest including, normal, invasion, edge, and core area. The microglia, macrophage marker TMEM119, and CD68 were stained. The immune fluorescence staining data was confirmed via flow cytometry. To establish cell-cell interaction models, a microglia cell line, BV2, and the macrophage cell line, RAW264.7 were co-cultured with ALTS1C1 in the 2D/3D migration/invasion models. RNA sequence was used to compare the difference between BV2 and RAW264.7.
**Results and Discussions**
The results showed that $TMEM119^{+}$ cells were 8 times more than the $CD68^{+}$ cells in the invasion area, indicating microglia upturn in the invasion area. The flow cytometry data also showed more $CD11b^{+}CD45^{low}$ cells in the tumor surrounding tissue, evidencing microglia more in the invasion area. The 2D/3D invasion models showed glioma cells invade and migrate more in the presence of BV2 than RAW264.7 cells. To compare the difference between the two cells, RNA sequencing is applied, revealing a significant upregulation of the cathepsin protease family by microglia. In addition, we also found that the expression of the cathepsin family is more intensively in the invasion area, not only in our murine model but also in human glioma patients, and mainly shown by microglia.
**Conclusion**
In summary, we found microglia are the main TAMs distributed in the brain tumor invasion area and microglia could increase the tumor cells' invasion and migration ability, also, this study provides models for cell-cell interaction research and found special cathepsin expressing microglia, which promotes tumor migration, thus constituting a new therapeutic target for prevention and treatment for glioma patients.

### EACR23-0346
### Effect of Approved Non-Oncology Drugs on the Metastatic Process: Risks & Benefits

*D. Roy Chowdhury*[1], *C. Amarkanth*[1], *S. Kushary*[1], *J. Tayal*[2], *A. Khemani*[3], *T. Singh*[4], *S. Prasad Rao*[3], *S. Kannan*[1], *A. Mehta*[2], *D. Doval*[5], *J. Ellingboe*[6], *A. Roy Chowdhury*[1]
[1]*Mestastop Solutions Pvt. Ltd., Core R&D, Bengaluru, India*
[2]*Rajiv Gandhi Cancer Institute and Research Centre, Biorepository, New Delhi, India*
[3]*Mestastop Solutions Pvt. Ltd., Consultant- Data Strategy and AI, Bengaluru, India*
[4]*Mestastop Solutions Inc., Consultant- Data Strategy and AI, New Jersey, United States*
[5]*Rajiv Gandhi Cancer Institute and Research Centre, Solid Tumours, New Delhi, India*
[6]*Mestastop Solutions Inc., Core R&D, New York, United States*

**Introduction**
Over the last decade, the average overall survival of patients treated with all FDA-approved drugs for solid tumours was 2.8 months. The progression-free survival of patients treated with these drugs was 3.3 months, mainly due to the absence of drugs that would delay the process of metastasis biology. A review of FDA approvals between

© 2023 The Authors. Molecular Oncology is published by John Wiley & Sons Ltd on behalf of Federation of European Biochemical Societies

2017–2021 for adult solid tumours indicated a low clinical benefit for a substantial population.

**Material and Methods**

We wanted to understand the gaps in metastasis biology and subsequently addressed them by creating platforms to facilitate novel drug discovery targeting the metastatic process. We developed an *in vitro* platform METAssay®, which dissected metastasis biology into multiple steps and differentiated between phenotypic properties of proliferating and metastasizing cells. The platform was validated and normalized with *ex-vivo* patient tumour samples, which helped identify the critical rate-limiting steps. A novel animal model, METVivo®, allowed us to correlate the hits from the *in vitro* platform with *in vivo* efficacy in mouse models. Next, as a proof of concept of drug repurposing, we tested ten approved non-oncology drugs in METAssay®. We showed the specificity of our platform, with an *in vitro* hit molecule delaying metastasis in the METVivo® model.

**Results and Discussions**

To further understand the clinical significance of our findings, we initiated a retrospective study. One hundred treatment-naive colorectal cancer primary tumour patients diagnosed between 2011 and 2016 with no clinical metastasis were selected. Survival status, medication history and patient follow-up data were collected in September 2022. Machine learning algorithms were used to identify weighted steps, including the effect of chronic medications (approved for non-oncology indications), that impacted survival or death. We show that, like our *in vitro* and *in vivo* findings, certain drugs positively impacted survival, whereas some negatively affected survival. We next selected four of these drugs and tested them on our *in-vitro* platform to evaluate their effect on the metastatic process. The *in vitro* rank ordering of anti-metastasis potency matched the retrospective clinical study survival effect for all these four compounds.

**Conclusion**

Our work suggests a preliminary concept that approved drugs can have both a positive and negative effect on the survival of primary tumour patients by affecting metastasis biology. We are currently working on retrospective studies to evaluate the hypothesis further.

### EACR23-0373
### Optimized workflow for the in-depth characterization of human colon cancer-associated fibroblasts

D. Agorku[1], A. Bosio[1], P. Ströbel[2], O. Hardt[1]
[1]*Miltenyi Biotec B.V. & Co. KG, R&D Reagents, Bergisch Gladbach, Germany*
[2]*Institute of Pathology, University Medical Center Göttingen UMG, Göttingen, Germany*

**Introduction**

Fibroblasts are one of the most prominent cell types within the tumor microenvironment and have been shown to play a key role in cancer progression and response to treatment in different cancers, including colorectal cancer (CRC). Besides affecting tumor cells e.g. by promoting stemness, they can additionally act on endothelial cells to generate novel blood vessels or manipulate different immune cell subtypes. Accordingly, attempts have been made to eradicate cancer-associated fibroblasts (CAFs) from the tumor microenvironment. However, such studies revealed that targeting all fibroblasts can lead to increased aggressiveness of tumors and to adverse effects. This highlights the need for means to specifically target fibroblast subtypes within the tumor microenvironment. In order to address these limitations, we have established a workflow to deeply characterize cancer-associated fibroblasts in CRC and decipher their heterogeneity.

**Material and Methods**

We have developed an optimized workflow for the isolation of fibroblasts from CRC, allowing for their in-depth characterization regarding molecular and phenotypic classification. The transcriptome of 22,700 isolated CAFs from 7 different patients was analyzed by single-cell RNA sequencing. We have confirmed the presence of novel subpopulations by spatial and single cell protein analysis using cyclic immunofluorescence and flow cytometry.

**Results and Discussions**

Transcriptomic analysis of isolated fibroblasts suggested phenotypic and functional heterogeneity of fibroblasts in the colon, distributed among 11 distinct clusters. To validate our findings, we performed proteomic analysis of different sub-clusters on sections of both normal adjacent colon tissue and colon tumor tissue. Next to known subtypes such as myCAFs and iCAFs, clusters indicating new subtypes of fibroblasts could be identified. These were characterized by ECM-remodeling and wound-healing features pre-dominantly among CAFs and potentially immune interacting fibroblasts found in both normal and tumor tissue.

**Conclusion**

Our workflow for characterization of fibroblasts derived from primary tissue and the integration of data from different approaches revealed novel subpopulations of fibroblasts in colorectal cancer. The identified profiles in CAFs suggest a functional heterogeneity towards immunomodulation. These findings might facilitate the identification and future targeting of unfavorable CAF subpopulations.

### EACR23-0378
### Unveiling the Protective Role of Platelets in Cancer Cell: a shield against chemotherapy

J. Cerón Hernández[1], A. García[2], G. Martínez-Navajas[1], I. Simón-Sáez[3], A. Rodríguez-Martínez[4], I. Aznar[2], M.P. Molina[2], P.J. Real[1], M.J. Serrano[5]
[1]*GENYO - Centro Pfizer- Universidad de Granada- Junta de Andalucía de Genómica e Investigación Oncológica, Biochemistry and Molecular Biology I, Granada, Spain*
[2]*GENYO - Centro Pfizer- Universidad de Granada- Junta de Andalucía de Genómica e Investigación Oncológica, Liquid Biopsy and Cancer Interception Lab, Granada, Spain*
[3]*Mayo Clinic, Mayo Clinic, Rochester- Minnesota, United States*
[4]*Hospital Costa del Sol, Hospital Costa del Sol, Málaga, Spain*
[5]*GENYO - Centro Pfizer- Universidad de Granada- Junta de Andalucía de Genómica e Investigación Oncológica, Hospital Universitario Virgen de las Nieves, Granada, Spain*

**Introduction**

© 2023 The Authors. Molecular Oncology is published by John Wiley & Sons Ltd on behalf of Federation of European Biochemical Societies

Platelets are active players in tumorogenesis, although their direct impact on tumor cells remain largely unknown. Preliminary studies in our laboratory showed that coculturing lung and other cancer cell lines with platelets enhances proliferation and induces phenotype changes. One of the many functions proposed of this interaction between platelets and tumor cells is that platelets protect tumor cells against cell death and apoptosis and they could be shielding against treatments. In order to demonstrate that, *in vitro* tests were carried out with three different lung cancer cell lines (A549, H1975 and H1299) and platelets from a healthy donor in coculture treated with different drugs.

**Material and Methods**

Platelets were obtained from peripheral blood samples from a healthy donor and were added at concentration of 100,000 plt/µL for performing different experiments. The tests were carried out with treatments CIS-platin, Docetaxel or Erlotinib at previously calculated IC50 concentration with or without platelets.

The effect on cell death and apoptosis was evaluated at 24 h after treatment by flow cytometry using annexin V / 7AAD kit.

We also carried out xCelligence assays analyzing cell proliferation and cytotoxicity. After that we evaluated the capability of migration by wound healing assays.

**Results and Discussions**

The IC50 concentration of all drugs increased in all tumour lines when cells had 24 h of contact with platelets. Apoptosis in the A549 cell line co-cultured with platelets and treated with CIS-platin showed a statistically significant downward at 24h versus cells without platelets treated with the same drug ($P<0.05$). However, cell death was decreased but not significant. In the coculture of H1975 cell line with platelets treated with CIS-platin apoptosis and cell death were decreased but not significant. Regarding the xCelligence proliferation assays, significant differences were found between the condition of treated co-culture and treated cells without platelets for both cell lines at 24 and 48 h after treatment ($P<0.05$).

No significant differences were found in the migration assay, but there was a strong trend that at 24 h the cells in contact with the platelets were able to move and they survived more.

**Conclusion**

These results suggest that tumor cells might be protected by platelets. Platelets are able to interact with tumor cells and prevent apoptosis. Furthermore, platelets promote growth of tumor cells even these cells are treated with different drugs.

### EACR23-0398

### Poly(I:C): a therapy to reduce relapse rates in stroma-rich colon cancer via activation of an anti-tumoral immune response.

*S. Corry[1], S. Sakhnevych1[1], N.E. Mohamed[2], K. Redmond[1], N. Fisher[1], S. Malla[1], O. Sansom[2], M. Lawler[1], P. Dunne[1]*

*[1]Queens University Belfast, Patrick G. Johnston Centre for Cancer Research, Belfast, United Kingdom*

*[2]Cancer Research UK Beatson Institute, Beatson Institute, Glasgow, United Kingdom*

**Introduction**

Stroma-rich tumours are the worst prognostic subtype in stage II/III colon cancer (CC), with high relapse rates and limited response to current standard therapies. Targeting features enriched in stroma-rich tumours relative to more epithelial subtypes e.g. Transforming Growth Factor Beta (TGF-b) have thus far been unsuccessful. Therefore, our aim was to identify biology that underpinned relapse specifically within stroma-rich CC tumours.

**Material and Methods**

Using *in silico* analysis the signalling underpinning relapse in stroma-rich CC was identified and characterised. The potential of the good prognostic biology to be induced was further investigated using *in vitro* data, followed by therapeutic efficacy being explored in a stroma-rich *in vivo* model.

**Results and Discussions**

Our analyses outlined that good-prognostic biology within stroma-rich tumours was characterised by an elevated viral response, interferon signalling and increased antigen processing and presentation. This cascade of activated innate-adaptive immune biology was inducible using the viral mimetic, poly(I:C). Using an *in vivo* metastasis model of stroma-rich CC, poly(I:C) monotherapy was sufficient to induce a strong anti-tumoral viral response and interferon signalling cascade, which in turn resulted in a significant increase of cytotoxic T cells ($p<0.05$) in the liver and significantly reduced tumour burden ($p<0.0002$).To identify early-stage CC primary tumours that would be most likely to benefit from poly(I:C) treatment, a transcriptional signature was developed based on the biology induced by poly(I:C) in different lineages, termed the poly(I:C) response score (PRS). When applied to data from stage II/III CC, the PRS stratified patients based on the enrichment of this anti-tumoral immune biology and other clinically relevant features.

**Conclusion**

Poly(I:C) has therapeutic potential for reducing relapse rates in the worst prognostic subtype of CC. The PRS provides a tool to characterise the activation state of the immune landscape across the different molecular subtypes. This signature highlights a unique window of opportunity for therapeutic deployment of poly(I:C) in the stroma-rich subgroup a group currently unresponsive to current standard therapies.

### EACR23-0406

### Peritoneal mesothelial cells promote ovarian cancer cell migration via soluble factors

*V. Shinkevich[1], W. Szymanski[2], J. Graumann[2], T. Worzfeld[1]*

*[1]Institute of Pharmacology, Biochemical Pharmacological Center- Philipps University, Marburg, Germany*

*[2]Institute of Translational Proteomics, Biochemical Pharmacological Center- Philipps University, Marburg, Germany*

**Introduction**

Peritoneal carcinomatosis in advanced stages of high-grade serous ovarian carcinoma (HGSOC) results from transcoelomic dissemination of ovarian cancer cells (OCC) through the malignant ascites. Human peritoneal mesothelial cells (HPMCs) play a key role in the tumor microenvironment to establish a metastatic niche.

© 2023 The Authors. Molecular Oncology is published by John Wiley & Sons Ltd on behalf of Federation of European Biochemical Societies

However, the functional role and the molecular mechanisms of the interaction between HPMC and OCC remain largely unclear.

**Material and Methods**

For our study, we used primary OCC, primary HPMC, and ascites derived from patients with HGSOC. Conditioned medium (CM) was obtained from HPMC to treat OCC in order to examine the effect on the migratory capacity of tumor cells. We performed secretome and transcriptome analyses of HPMC samples to identify molecular programs involved in cell-cell communication.

**Results and Discussions**

Our findings show that, unexpectedly, HPMC from HGSOC patients could promote migration of OCC. Our data indicate that this pro-migratory effect is mediated by an indirect communication mechanism, i.e. via the secretion of soluble factors. Moreover, we found that this secretion from HPMC is promoted by malignant ascites. To identify the pro-migratory factors released from HPMC in response to ascites, we performed transcriptomic and proteomic analyses. Based on these systematic unbiased analyses, we tested candidate genes and proteins in functional assays.

**Conclusion**

Overall our data strongly suggest that HPMC have an important function in the metastasis of ovarian cancer cells into peritoneal organs, and that molecules in the tumor microenvironment lead to the reprogramming of HPMCs, which in turn contribute to tumor motility by secreting soluble factors.

### EACR23-0410

### Oral cancer cells induce a cancer-associated fibroblast-like phenotype by releasing transforming growth factor beta 1 presenting extracellular vesicles

*C. Park*[1], *S. Hunt*[2], *H. Colley*[2]

[1]*University of Sheffield, Clinical Dentistry, Sheffield, United Kingdom*

[2]*The University of Sheffield, Clinical Dentistry, Sheffield, United Kingdom*

**Introduction**

Head and neck cancer is the 6th most common cancer worldwide with 90% being classified as oral squamous cell carcinoma (OSCC). Recent findings have implicated the tumour microenvironment (TME), which includes cells such as cancer-associated fibroblasts (CAFs), in cancer progression. Tumour cells are known to communicate with stromal cells via a network of cytokines, growth factors and extracellular vesicles (EVs). There is evidence that some cytokines and growth factors are presented on the surface of EVs. We therefore sought to determine if the potent stromal cell activator transforming growth factor beta 1 (TGF-β1) is associated with oral cancer EVs.

**Material and Methods**

EVs were enriched by size exclusion chromatography (SEC) and characterised by nanoparticle tracking analysis and western blotting. TGF-β1 was quantified by ELISA. Alpha smooth muscle actin (α-SMA) expression was assessed by quantitative polymerase chain reaction (qPCR), western blotting and immunofluorescence microscopy (IF).

**Results and Discussions**

There were significantly higher levels of TGF-β1 associated with EVs derived from oral cancer cells (H357) compared to normal cells (FNB6). TGF-β1 levels were significantly greater in EV preparations compared to conditioned media (CM). Treatment of NOFs with soluble recombinant TGF-β1 or H357 derived EVs led to an increase in α-SMA protein expression, indicating their activation to a CAF-like phenotype. EV knockout H357 cell line in a transwell co-culture showed a reduced ability to increase α-SMA protein in NOFs, indicating that CAF transition is EV dependent.

**Conclusion**

Our data suggests that the majority of TGF-β1 in oral cancer cell CM is associated with EVs and OSCC-derived EVs. Blocking of EV release might be a novel strategy to reduce stromal cell activation in the TME.

### EACR23-0421

### A novel microfluidic platform for PDAC organoid culture and drug screening

*S. Paternoster*[1], *M. Geyer*[2], *D. Schreyer*[3], *S. Pfeffer*[4], *L.M. Gaul*[2], *C. Pilarsky*[4], *K. Queiroz*[2]

[1]*Mimetas, Sales and Marketing, Oegstgeest, The Netherlands*

[2]*Mimetas, Model Development, Oegstgeest, The Netherlands*

[3]*University of Glasgow, School of Cancer Sciences, Bearsden, United Kingdom*

[4]*Universitätsklinikum Erlangen, Translational Research Center TRC, Erlangen, Germany*

**Introduction**

Pancreatic Ductal Adenocarcinoma (PDAC), the most common pancreatic cancer type, will become the second leading cause of cancer-related deaths by 2030 with mortality rates of up to 93%. Current standard-of-care for patients with PDAC includes chemotherapeutic regimens and pancreatic cancer surgery. However, only 20% of the patients are eligible for surgery due to late diagnosis. Although chemotherapeutic regimens are the leading treatment to PDAC patients, these are still very limited and the development of resistance to treatment is often observed. PDAC tumors are characterized by high-density stroma, therefore these present high interstitial pressure and hypoxia. These features potentially interfere with the efficiency of chemotherapeutic drugs and highlight the urgent need for novel PDAC screening platforms.

**Material and Methods**

Here, we describe the establishment of PDAC organoid cultures in the MIMETAS OrganoPlate®. The aim of this project was to determine the treatment response of PDAC organoids embedded within an extracellular matrix in the Organoplate® 2-lane in mono-, and co-culture with pancreatic stellate cells (PSCs) under hypoxic and normoxic conditions. To recapitulate *in vivo* like conditions, the 2-lane OrganoPlate® from MIMETAS was used to study organoid growth and sensitivity to treatment. Several compounds were tested with standard of care chemotherapeutic Gemcitabine for 72h.

**Results and Discussions**

PDAC organoids showed an overall better survival after drug treatments when grown in hypoxia compared to normoxia. Interestingly, organoids grown in monoculture

© 2023 The Authors. Molecular Oncology is published by John Wiley & Sons Ltd on behalf of Federation of European Biochemical Societies and co-culture showed either a higher or a lower survival under the different treatments. The effect of reduced or increased cancer cell survival came from an altered gene expression of cells under different conditions. This highlights the incorporation of PDAC specific tumor microenvironment conditions, such as hypoxia and co-culture with stromal cells to better understand drug responses.

**Conclusion**

The OrganoPlate® 2-lane provides an excellent platform for (co-) cultivation and high-throughput phenotypic drug screening of PDAC organoids, thereby potentially enabling the development of novel *in-vivo* like model systems for efficient patient stratification and drug discovery.

### EACR23-0426
### Stroma metabolic reprogramming as a regulator of prostate cancer aggressiveness

*I. Astobiza[1], C. Viera-Bardón[1], A. Martínez-González[1], A.R. Cortázar[1], S. García-Longarte[1], A. Loizaga-Iriarte[2], A. Ugalde-Olano[2], J.I. López[3], M. Unda[2], F. Calvo[4], S. Cascais[5], M. Castillo[5], V. Torrano[6], A. Carracedo[1]*
*[1]CICbioGUNE, Cancer Cell Signaling And Metabolism Lab, Derio, Spain*
*[2]Basurto University Hospital, Urology, Bilbao, Spain*
*[3]Cruces University Hospital, Anatomic pathology, Cruces- Barakaldo, Spain*
*[4]IBBTEC, Molecular and cell signaling, Santander, Spain*
*[5]Fundação Champalimaud, Molecular and Experimental Pathology Laboratory, Lisboa, Spain*
*[6]University of Basque Country, Biochemistry and Molecular Biology, Leioa, Spain*

**Introduction**

The study of molecular cues regulating cancer progression is a potential source of stratification biomarkers and therapeutic targets. We developed a transcriptomics-based strategy in order to identify prostate cancer progression-regulatory metabolic processes, since metabolic changes are central to cellular transformation and cancer progression. Following this strategy, we demonstrated the tumor suppressive role of the PGC1α-ERRα axis in this tumor type, and the prognostic potential of a signature of the activity of this complex. Emanating from these results, we hypothesized that other yet unidentified metabolic processes could be regulated at the level of gene expression PCa progression.

**Material and Methods**

We devised a manual curated strategy for the stratification of those metabolic genes with high prognostic potential in the available datasets of PCa. Individual analysis of genes with the highest prognostic capacity revealed a significant metabolic role of tumor-associated stroma in the acquisition of aggressive features in PCa. We deconstructed the mechanism of regulation of metabolic rewiring in tumor-associated fibroblasts and studied the effector metabolites contributing to the phenotype by using "in silico", "in vitro" and "in vivo" approaches.

**Results and Discussions**

Our results underscored ASPA as the metabolic gene with the most consistent and potent prognostic capacity in PCa and that it´s repression was taken place in cancer associated fibroblasts. Besides, ASPA expression could be a checkpoint for N-Acetyl-Aspartate availability, the metabolite that ASPA catabolizes, that epithelial cells are producing and secreting. This metabolite accumulation in the tumour microenvironment could be repolarizing macrophages towards a more protumoral phenotype that contributes to tumour progression.

**Conclusion**

Overall, our results identified an unprecedented tumor-regulated mechanism instructing the metabolic program of the fibroblast compartment to engage in a tumor-promoting process. Moreover, targeting NAA metabolism could also be employed to improve clinical outcomes.

### EACR23-0427
### Sorafenib inhibits tumor-driven CAF invasion in head and neck squamous cell carcinoma

*L. Prieto-Fernández[1,2,3], M.D.L.A. Villaronga[1,2,3], F. Hermida-Prado[1,2,3], I. Montoro-Jiménez[1,2,3], M. Otero-Rosales[1,2], S. T. Menéndez[1,2,3], J.P. Rodrigo Tapia[1,2,3], F. Calvo[4], J.M. García-Pedrero[1,2,3], S. Alvarez-Teijeiro[1,2,3]*
*[1]Instituto de Investigacion Sanitaria del Principado de Asturias ISPA, Department of Otolaryngology- Hospital Universitario Central de Asturias, Oviedo, Spain*
*[2]Instituto Universitario de Oncología del Principado de Asturias IUOPA, University of Oviedo, Oviedo, Spain*
*[3]CIBERONC, Instituto de Salud Carlos III ISCIII, Madrid, Spain*
*[4]Instituto de Biomedicina y Biotecnología de Cantabria, Consejo Superior de Investigaciones Científicas- Universidad de Cantabria, Santander, Spain*

**Introduction**

Cancer associated fibroblasts (CAFs) participate in several cancer hallmarks and are known to be leading cells driving cancer cell invasion. This work was aimed to study the effects of tumor-secreted factors on CAF biology, ultimately identifying putative therapeutic targets for co-adjuvant treatments in head and neck squamous cell carcinoma (HNSCC).

**Material and Methods**

Primary populations of CAFs and normal fibroblasts (NF) were treated with conditioned media (CM) from HNSCC cells and normal keratinocytes (Kc) to functionally investigate their effects on fibroblast cell viability and invasive abilities (3D collagen matrices). MMPs expresion was assessed by RT-qPCR and kinase-substrate enrichment analysis (KSEA) was performed by MS-phosphoproteomics. Pharmacological Inhibition of the identified kinases were preformed using PD153035 (EGFR inhibitor), Sorafenib Tosylate (RAF1/BRAF inhibitor), SP600125 (MEKK4/MEKK7 inhibitor), GM6001 (MMP inhibitor) and GSK690693 (Akt inhibitor).

**Results and Discussions**

Functionally, HNSCC-secreted factors specifically and robustly promoted fibroblast invasion, as well as MMP expression. Mechanistically, changes in phosphorylation patterns were identified upon treatment with HNSCC-derived CMs, and several kinases were identified to be hyperactive: MKK7, MKK4, ASK1, RAF1, BRAF, ARAF, COT, PDK1, RSK2 and AKT1. Among the pharmacological kinase inhibitors tested, RAF1/BRAF

Molecular Oncology 17 (Suppl. 1) (2023) 1–597
© 2023 The Authors. Molecular Oncology is published by John Wiley & Sons Ltd on behalf of Federation of European Biochemical Societies inhibitor Sorafenib was the most effective to block tumor-promoted fibroblast invasion.

**Conclusion**

HNSCC-secreted factors promoted invasion of stromal fibroblasts, which was robustly abolished by treatment with the RAF1/BRAF inhibitor Sorafenib, emerging as a rational approach to target the pro-invasive fibroblasts in the head and neck cancer microenvironment.

### EACR23-0437
### MiRNAs as potential biomarkers of hepatocellular carcinoma of non-viral origin

*V.R. Mallela[1], M. Rajtmajerova[1], A. Trailin[1], P. Hosek[1], L. Cervenkova[2,3], R. Palek[2,4], V. Liska[2,4], K. Hemminki[1,5], F. Ambrozkiewicz[1]*

*[1]Biomedical center- Faculty of Medicine in Pilsen- Charles University, Laboratory of Translational Cancer Genomics, Plzeň, Czech Republic*

*[2]Biomedical center- Faculty of Medicine in Pilsen- Charles University, Laboratory of Cancer Treatment and Tissue Regeneration, Plzeň, Czech Republic*

*[3]Third Faculty of Medicine- Charles University, Department of Pathology, Prague, Czech Republic*

*[4]Faculty of Medicine in Pilsen- Charles University, Department of Surgery, Plzeň, Czech Republic*

*[5]German Cancer Research Center, Department of Cancer Epidemiology, Heidelberg, Germany*

**Introduction**

In spite of all scientific effort to decipher the tumour biology, hepatocellular carcinoma (HCC) remains the third leading cause of cancer-related deaths. MicroRNAs (miRNAs), which are small, non-coding, single-stranded RNAs, regulate the expression of many target genes, including driver genes and their associated signalling axes in a number of human malignancies, including HCC. We aimed to assess miRNA profiles in non-viral HCC and their influence on patient outcome such as time to recurrence (TTR), disease-free survival (DFS) and overall survival (OS).

**Material and Methods**

We have collected FFPE samples of 51 HCC patients who underwent initial tumor resection and no neo-adjuvant treatment. Total RNA from paired tumour and non-tumour adjacent tissue was extracted. miRNA profilling was performed using Agilent microarray's. Diferrential expression analysis was performed in GeneSpring and survial analysis in MatLab.

**Results and Discussions**

We have found 28 significantly dysregulated miRNAs (P-value <0.05 and FC >1.5); 13 were upregulated and 15 were downregulated. Seven miRNAs showed significant impact on patient survival according to the Kaplan-Meier plot. In case of hsa-miR-200a-3p (DFS p=0.01, TTR p=0.02, OS p=0.02), hsa-miR-221-3p (DFS p=0.02, TTR p=0.04, OS p=0.01), and hsa-miR-222-3p (DFS p= 0.04, TTR p= 0.02, OS p=0.006) high expression was linked to decreased TTR, DFS, and OS, while their low expression was associated with prolonged TTR, DFS, and OS. Contrary, higher expression was associated with longer TTR and DFS in case of hsa-miR-4270 (DFS p=0.01, TTR p=0.02, OS p=0.02), hsa-miR-654-3p (DFS p=0.03, TTR p=0.01, OS p=0.01), hsa-miR-200b-3p (DFS p=0.04, TTR p=0.04, OS p=0.02) and hsa-miR-1972 (DFS p=0.04, TTR p=0.05, OS p=0.002) and low expression was associated with worse OS.

**Conclusion**

Our findings confirm that miRNAs have a considerable impact on patient survival and may be employed as a diagnostic or target for new therapeutics in prospective investigations of non-viral – associated liver cancer in the European population. Moreover, we have found some novel significantly dysregulated miRNAs which were not reported in any previous HCC research so far. This may be due to the fact that the vast majority of research on this topic is performed on HCC of viral origin, which further emphasizes the importance of our findings.

### EACR23-0446
### Deciphering SWI/SNF complexes role in lung cancer using the Tuba-seq tool in a mouse model of lung adenocarcinoma

*D. Martín Gutiérrez[1], B. Monterde[1], R. Marqueño[1], R. Blanco[1], I. Varela[1]*

*[1]Instituto de Biomedicina y Biotecnología de Cantabria IB BTEC, Departamento de Señalización Celular y Molecular, Santander, Spain*

**Introduction**

Lung cancer is the most common cause of cancer-related mortality worldwide. Although new targeted therapies have been developed in the last decade, survival rates have only slightly improved. Further efforts need to be made in the characterization of the molecular mechanisms involved in this disease. SWI/SNF chromatin remodeling complexes have emerged as a major focus of attention in cancer research. In lung cancer, several SWI/SNF subunits are often mutated but their precise role, if any, in lung cancer progression is still uncharacterized. We propose to study at the molecular level the impact of SWI/SNF deficiency in a mouse model of lung adenocarcinoma (LUAD) through tumor barcoding and high-throughput sequencing (Tuba-seq).

**Material and Methods**

We have generated different genetic constructs to induce the specific recombination of the inducible *Kras* and *Tp53* mutant alleles *in vivo* specifically in lung epithelia and to lead to the expression of guide RNAs. To validate the lentiviral constructs, *in vitro* experiments were performed, including fluorescence imaging and luciferase assays. Two cell lines were generated for use in lentiviral titration by flow cytometry quantification of $GFP^{+}$ cells. *Kras* $^{FRT\text{-}G12D\text{-}FRT/+}$; *Cas9* $^{tg/+}$ and *Kras* $^{FRT\text{-}G12D\text{-}FRT/+}$; *Tp53* $^{FRT/FRT}$; *Cas9* $^{tg/+}$ mice were infected intranasally. Tumor growth can be monitored by *in vivo* imaging.

**Results and Discussions**

A lentiviral vector containing a sgRNA scaffold and flippase and RedF-luc genes was designed. We probed that *in vitro* the vector effectively produced the expected proteins as well as their correct functionality. Additionally, we have generated a GFP reporting lung cancer cell line for virus titration. Kras-Cas9 and Kras-p53-Cas9 mice were

© 2023 The Authors. Molecular Oncology is published by John Wiley & Sons Ltd on behalf of Federation of European Biochemical Societies infected intranasally to validate the tumor-induction ability of our lentiviral particles. Once we could generate tumors in our mice and validate that they recapitulate LUAD anatomic characteristics, we will infect our two mice groups with a pool of barcoded lentiviral particles targeting the SWI/SNF subunits ARID1A, ARID1B, ARID2, SMARCA2 and SMARCA4. Barcoding sequencing will allow the determination of tumor number and size and, therefore, the assessment of this SWI/SNF subunits oncogenic potential. Further transcriptional experiments will allow us to identify the molecular pathways altered by SWI/SNF deficiency in our model.

**Conclusion**

We have successfully generated all necessary tools to perform Tuba-seq experiments to study at the molecular level the role of SWI/SNF alteration in a mouse model of LUAD.

### EACR23-0447
### Sorafenib and Lenvatinib induce vascular responses in patient derived HCC on-Chip models

*O. Mocellin[1], A. Robinson[1], A. Olcyk[1], S. Treillard[1], T. Olivier[1], C. Ng[1], D. Goubert[1], M. Vermeer[1], J. Heijmans[1], A. Stok[1], G. van Tienderen[2], M. Verstegen[2], S. Trietsch[1], H. Lanz[1], P. Vulto[1], J. Joore[1], K. Queiroz[1]*
*[1]Mimetas, Model Development, Oegstgeest, The Netherlands*
*[2]Erasmus MC-University Medical Center Rotterdam, Department of Surgery, Rotterdam, The Netherlands*

**Introduction**

Hepatocellular carcinoma (HCC) is the most common type of liver cancer. Its incidence is increasing, and is closely related to advanced liver disease. Interactions in the HCC microenvironment between tumor cells and the associated stroma actively regulate tumor initiation, progression, metastasis, and therapy response.

**Material and Methods**

In the present study, we used the OrganoPlate graft to establish a co-culture system consisting of dissociated HCC tumors (HCC 1-8) and cell lines, HCC derived fibroblasts and vasculature. Cultures were prepared and validated by assessing their response to Sorafenib and Lenvatinib (72 hours). Cultures had their viability (alamar blue assay), and chemokine/cytokine levels in the supernatant (Luminex) determined. In addition, the organization of the vasculature in the tumor compartment was studied through immunostainings, confocal imaging, and subsequent morphological analyses.

**Results and Discussions**

HCC models were characterized by a range of specific markers, tumor (albumin), endothelial (CD31 and VE-Cadherin) and stromal (aSMA) cells. CD31 immunostained cultures were imaged, and morphology changes quantified. Sorafenib and Lenvatinib induced changes in the tumor vasculature area and organization.

**Conclusion**

Hereby, we present vascularized patient-derived HCC models that include relevant cellular players of the HCC microenvironment. These co-cultures are highly suitable for studying specific cell types as well as patient-specific responses. We envision that this patient derived model will evolve to become a platform for understanding the interplay between angiogenesis, stroma and immune infiltrate in HCC.

### EACR23-0472
### Spatial transcriptomics identify local tumor microenvironment structures in glioblastoma

*F. Petitprez[1], S. Webb[1], G. Morrison[1], S. Pollard[1], J.W. Pollard[1]*
*[1]University of Edinburgh, Institute for Regeneration and Repair, Edinburgh, United Kingdom*

**Introduction**

Glioblastoma (GBM) is an aggressive form of primary brain tumors. Recent efforts to characterize GBM using single-cell or spatially-resolved transcriptomics have revealed a tremendous intra-tumoral heterogeneity between malignant cells and between different tumor areas. However, most of these efforts focused on malignant cells and the tumor microenvironment heterogeneity remains poorly understood, particularly with regards to immunosuppression.

**Material and Methods**

We generated spatially-resolved, full transcriptome, full slide, spatial transcriptomics data on 15 tumor sections from 8 primary GBM tumors using the 10x Genomics Visium technology, covering over 15,000 55-µm wide spots. Through multimodal data integration, we coupled this dataset with large public single-cell RNA-seq datasets to estimate the fine-grain cellular composition of each spot. We integrated all these estimates to establish a classification of local microenvironment structures.

**Results and Discussions**

Clustering of over 15,000 spot transcriptomic profiles enabled us to identify 11 classes of local tumor microenvironment with extremely variable cellular composition, highlighting the strong intra-tumoral heterogeneity of GBM. Some classes were restricted to one tumor only, but most classes spanned more than 50% of all tumors included in the study. We assessed differentially-expressed gene signatures from each of these classes. Application of these classes-specific gene signatures to the TCGA bulk transcriptomic database allowed to show that a certain class of local microenvironment, characterized by a moderate normal brain cell content, proliferative monocyte-derived macrophages, and fibrosis, was associated with a poorer overall survival for GBM patients.

**Conclusion**

The intra-tumoral heterogeneity of GBM is not only visible on malignant cells but GBM harbor variable local microenvironments in different tumor compartments. These local microenvironment patterns influence clinical outcome and could exhibit potential biomarkers of response to therapy or novel biomarkers.

### EACR23-0474
### Investigating the effects of neo-adjuvant chemotherapy on the immune tumour microenvironment of bladder cancer

*M. Reardon[1], J. Honeychurch[1,2], E. Cheadle[2], P. Oliveira[3], K. Reeves[1], P. Hoskin[1], A. Choudhury[1,4], R. Walshaw[5]*
*[1]University of Manchester,*

© 2023 The Authors. Molecular Oncology is published by John Wiley & Sons Ltd on behalf of Federation of European Biochemical Societies

Translational Radiobiology Group - Division of Cancer Sci ences, Manchester, United Kingdom
[2]University of Manchester, Targeted Therapy Group - Division of Cancer Sciences, Manchester, United Kingdom
[3]The Christie NHS Foundation Trust, Department of Pathology, Manchester, United Kingdom
[4]The Christie NHS Foundation Trust, Department of Clinical Oncology, Manchester, United Kingdom
[5]The Clatterbridge Cancer Centre NHS Foundation Trust, Clatterbridge Cancer Centre, Liverpool, United Kingdom

**Introduction**

Patients with bladder cancer (BCa) are offered the choice of radical cystectomy or chemoradiotherapy (CRT), with similar survival outcomes. Cisplatin-based neo-adjuvant chemotherapy (NAC) is standard-of-care in the UK prior to these definitive treatments. As cancers grow, a tumour microenvironment (TME) develops that contains anti-tumour immune cells such as cytotoxic CD8+ T cells, but which also contains pro-tumour immune cells such as cancer-associated fibroblasts (CAFs) and regulatory T cells (T-regs). BCa patients whose tumours have high levels of immune infiltration have better survival outcomes after CRT but, while NAC can affect immune infiltration in some cancers, the effects of NAC on the TME in BCa have not been fully explored. We hypothesise that targeting specific pro-tumour immune mechanisms improves patient responses to NAC in BCa.

**Material and Methods**

An RNA-Seq differential gene expression analysis was performed on matched pre- and post-NAC tumour samples from a cohort of 32 BCa patients treated with 3 cycles of cisplatin-based NAC followed by radical cystectomy. Gene expression immune signatures curated from the literature were calculated and analysed in conjunction with clinical outcomes. Correlation analyses of pro- and anti-tumour signatures were performed to investigate BCa tumour resistance mechanisms to NAC.

**Results and Discussions**

Significantly over-expressed genes were enriched *inter alia* for immune signalling, cell cycle, proliferation and response to hypoxia, demonstrating heterogeneous BCa responses to NAC. Seven tumours had low immune infiltration after NAC while 25 had high post-NAC infiltration of both pro- and anti-tumour immune cells. Changes in the CD8+ T cell signature in muscle-invasive tumours were mirrored closely by the exhausted CD8 signature (Pearson's $r = 0.80$, $p = 1.7 \times 10^{-6}$), suggesting that the CD8+ T cells recruited into the TME were inactive in these tumours. Significant proportional changes for some pro-tumour signatures, such as T-regs ($r = 0.56$, $p = 3.5 \times 10^{-3}$) and immune checkpoints ($r = 0.71$, $p = 6.7 \times 10^{-5}$) were seen, but not for others such as CAFs or M2 macrophages.

**Conclusion**

Our data suggest that the presence of T-regs or expression of immune checkpoints could be associated with the response of BCa to NAC. Validation analyses are ongoing in a number of external datasets.

### EACR23-0481
### Development of a High-Plex Antibody Panel for Spatial Phenotyping of FFPE Murine Tissues

N. Nikulina[1], J. Niyati[1], O. Braubach[1], T. Tam[1], J. Fisher[2], S. Jensen[2], P. Leung-Lee[2], J. Loebelenz[2]
[1]Akoya Biosciences- Inc., Applications/Marketing Department, Menlo Park, United States
[2]Cell Signaling Technology- Inc., Marketing, Danvers, United States

**Introduction**

Single cell spatial phenotyping with protein biomarkers is a well-established tool for scientists involved in cancer research with the primary goal of understanding the biology of the tumor microenvironment. This technology has been developed primarily for use on human tissues and there is an appreciable lack of available applications to study murine cancer models. Yet, mouse models are widely used for immuno-oncology research, it is thus essential to develop spatial phenotyping applications for use in mice.

**Material and Methods**

A 30+ plex mouse antibody panel was developed by selecting biomarkers for immune cell lineages, immune activation, checkpoints, proliferation and structural components. Purified antibodies for these targets, were IHC validated in mouse models by using standard protocols developed by Cell Signaling Technology, Inc. Antibodies were then conjugated to PhenoCycler® oligo barcodes using a standard conjugation protocol; conjugated antibodies were then validated to confirm specificity and sensitivity. Once assembled, the 30+ plex antibody panel was deployed on murine spleen and syngeneic tumor tissues, and ultrahigh-plex whole-slide imaging was performed on the PhenoCycler®-Fusion Platform. Data were analyzed via unsupervised clustering and phenotyping approaches.

**Results and Discussions**

In depth characterization was performed for normal mouse spleen and syngeneic tumor which revealed different cellular phenotypes as well as the presence of cellular neighborhoods. This study demonstrates the feasibility of conducting ultrahigh-plex spatial phenotyping in FFPE mouse tissues and developed panel can be implemented for cancer biology research.

**Conclusion**

The tools that were designed as part of this study can easily be deployed for discovery and translational research in mouse tissues and will thereby pave the way for studies aimed at improving our understanding of cancer biology.

### EACR23-0482
### Spatial Metabolic Phenotyping: Looking Beyond the Tumor-Immune Microenvironment of Bladder Cancer

N. Jhaveri[1], D. Klymyshyn[1], N. Nikulina[1], B. Ben Cheikh[1], B. Anand[2], N. Nelson[2], M. Prater[2], S. Basu[2], A. Horowitz[3], O. Braubach[1]
[1]Akoya Biosciences, Applications, Menlo Park, United States
[2]Abcam, Immuno-Oncology, Cambridge, United Kingdom
[3]Icahn School of Medicine at Mount Sinai, Precision Immunology Institute/ Tisch Cancer Institute,

© 2023 The Authors. Molecular Oncology is published by John Wiley & Sons Ltd on behalf of Federation of European Biochemical Societies

New York, United States
**Introduction**
Single-cell spatial phenotyping of human cancers has led to critical insights into the mechanisms that govern immune surveillance and suppression. With PhenoCode™ Discovery Panels (PDP), for example, researchers can elucidate the tumor-immune contexture of human FFPE tissues in an unprecedented detail. However, the complex dynamics of cancer evolution and resistance to immuno-oncology therapies warrant a more comprehensive and multi-factorial approach to studying tumor biology. To that end, we have developed a 20+ plex panel of markers encompassing cellular metabolism, apoptosis, and stress pathways. Complementing the 60-plex PDP deep immune-phenotyping panel, the addition of these metabolism and stress-centric modules further enhances our understanding of the key molecular determinants involved in bladder cancer pathogenesis and progression.
**Material and Methods**
Metabolic and Stress Modules encompassing key markers involved in glycolysis, TCA cycle, fatty acid metabolism, apoptosis and DNA damage response (DDR) were combined with a core PDP panel and deployed on human FFPE bladder cancer tissues. In sum, 80+ antibodies were imaged in iterative cycles of hybridization and removal with complementary oligo-labeled fluorescent dyes on the PhenoCycler®-Fusion platform. Data were acquired in whole-slide format with single-cell resolution and analyzed via unsupervised clustering, cellular neighborhood analysis and functional spatial phenotyping.
**Results and Discussions**
Immune suppression and metabolic dysregulation are key hallmarks of cancer progression. Leveraging a novel multiplex panel comprising markers for cell lineage, immune activation, apoptosis, DDR, and metabolism, we interrogated the spatial phenotype of each cell within the tumor to create a comprehensive map of the cellular and functional organization of bladder cancers. These data provide quantitative and spatial information to further our understanding of tumor heterogeneity at multiple levels – metabolic, immune and stress – and to uncover the mechanisms underlying differences in clinical responses to immunotherapies.
**Conclusion**
Our study demonstrates the power of high-resolution single cell spatial phenotyping combined with deep bioinformatic analyses to reveal critical biomarkers and spatial signatures that may pave the way for a comprehensive understanding of bladder cancer and provide better treatment options.

### EACR23-0486
### Identifying Spatial Signatures of Response to Immunotherapy by Deep Spatial Phenotyping in Non–Small Cell Lung Cancer
*N. Ma*[1], *A. Pratapa*[1], *J. Monkman*[2], *K. O'Byrne*[3], *A. Kulasinghe*[2], *O. Braubach*[1]
*[1]Akoya Biosciences, Applications, Menlo Park, United States*
*[2]University of Queensland, Frazer Institute- Faculty of Medicine, Brisbane, Australia*
*[3]Princess Alexandra Hospital, Princess Alexandra Hospital, Brisbane, Australia*
**Introduction**
Lung cancers are the leading cause of cancer-related deaths, with a 5-year survival of ~20%. Whilst immunotherapies have led to durable and prolonged survival, only a subset of patients remain responsive. New biomarkers are needed to better predict if patients will respond or develop resistance against immune checkpoint inhibitor (ICI) therapies. Spatial phenotyping of the tumor immune microenvironment (TiME) is now recognized as a proxy for ICI therapy outcomes. Our study employs an end-to-end spatial biology strategy to survey non-small-cell lung cancer (NSCLC) tissues for new biomarkers that could guide immunotherapy treatments.
**Material and Methods**
We phenotyped pre-treatment biopsies from non-small-cell lung cancer (NSCLC) patients treated with single-agent Nivolumab. We developed a spatial analysis of an NSCLC cohort using customizable PhenoCode Signature Panels (PSP), which combines the barcode-based antibody chemistry from the PhenoCycler® platform with the signal amplification of Opal chemistry from the PhenoImager® platform. The panels we applied are used for high-throughput immune profile (CD3/CD8/CD20/CD68/PanCK + CD4), immuno-contexture (CD8/CD68/PD-L1/FoxP3/PanCK + PD-1), T Cell Status (CD8, CD4, CD20, FOXP3, PD-1 + CD45RO) and M1/M2 Polarization (CD8, CD68, CD163, PD-1, PD-L1 + FoxP3 add-in) imaging.
**Results and Discussions**
Our whole-slide single-cell spatial phenotyping analyses revealed high phenotypic diversity in the TiME of patients responsive and resistant to ICI therapy. In addition, higher-throughput analyses of the same tissues using multiple targeted PSP revealed quantification of density and spatial distribution of immune cell lineages, which confirms the potential biomarker value of spatial associations in patient tissues for stratification and treatment. We discovered multiple quantifiable and statistically substantial spatial signatures and spatial scores that appear to predict treatment benefit, confirming the potential biomarker value of spatial associations in patient tissues.
**Conclusion**
This study amounts to a uniquely comprehensive Single Cell Spatial Phenotyping analysis of pre-treatment NSCLC biopsies from a single-agent Nivolumab study. Our data catalogue the diversity in the immune microenvironment of NSCLC but highlighted that immune cell quantification is insufficient to stratify patient cohorts. On the other hand, single-cell spatial phenotyping promises to reveal new biomarkers that may aid in better stratification of patients in pre-treatment evaluations.

### EACR23-0497
### miR-567 regulates the progression of melanoma cells and functions of macrophages
*N. Ma*[1], *M.H.T. Nguyen*[2]
*[1]National Central University, Department of Biomedical Sciences and Engineering, Taoyuan City, Taiwan*
*[2]National Central University, Department of Biomedical Sciences and Engineering,*

© 2023 The Authors. Molecular Oncology is published by John Wiley & Sons Ltd on behalf of Federation of European Biochemical Societies

Taoyuan, Taiwan
**Introduction**
Target therapy and immunotherapy are advancements in the treatment of melanoma; unfortunately, a subset of patients does not benefit from or produce drug resistance. Therefore, an alternative strategy should be investigated to further improve clinical benefits. Macrophages are the abundant immune cells related to the progression of melanoma and resistance to drugs. In this study, we explored the novel roles of miR-567 in the progression of melanoma and the effects of macrophages.
**Material and Methods**
miR-567 expression in nevus and melanoma tissues was analyzed by in situ hybridization. miR-567 was transfected into melanoma cells to investigate its biological effects through functional assays such as proliferation, colony formation, soft agar, and migration assays. In addition, miR-567-related signal transduction pathways in the melanoma cells were studied by western blot and next-generation sequencing analyses. Different phenotypes of macrophages polarized from THP-1 monocyte cells were used as *in vitro* model to investigate the effects of miR-567 on macrophages.
**Results and Discussions**
Our results showed that miR-567 expression levels were decreased in melanoma cells compared to melanocyte cells. Consistently, expression of miR-567 was 0.403-fold lower in melanoma tissues (n=118) as compared to nevus tissues (n=40). In addition, the receiver operating characteristic presented that the area under the curve value of miR-567 is 0.9495. Importantly, higher expression of miR-567 enhanced the overall survival of melanoma patients. Overexpression of miR-567 significantly reduced proliferation, survival, anchorage-independent growth, migratory, and invasive abilities of melanoma cells and BRAF-inhibitor-resistant cells. Furthermore, our results demonstrated that IGF1R, E2F1, and Cyclin B2 are direct targets of miR-567 and the knockdown of these genes attenuated the proliferation and survival of melanoma cells. Interestingly, the introduction of miR-567 downregulated MAPK/ERK and PI3K/AKT pathways in melanoma cells and regulated multiple pathways related to immune response. Of note, overexpression of miR-567 reduced the promoting melanoma growth induced by M2 or melanoma-associated macrophages, indicating the involvement of miR-567 in the regulation of macrophages.
**Conclusion**
miR-567 could be served not only as a biomarker but also as a potential molecular target for the prevention of melanoma progression.

### EACR23-0502
### Elucidating the role of Cancer-associated Fibroblasts in Prostate Cancer progression through the establishment of in vitro 3D co-cultures

*<u>F. Bonollo</u>[1], W. Cheng[1], N. Sampson[2], G. Thalmann[3], M. Kruithof-de Julio[1], S. Karkampouna[1]*
*[1]University of Bern, Department for BioMedical Research DBMR, Bern, Switzerland*
*[2]Medical University of Innsbruck, Department of Urology, Innsbruck, Austria*
*[3]University Hospital Inselspital, Department of Urology, Bern, Switzerland*
**Introduction**
Cancer-associated Fibroblasts (CAFs) represent the most abundant component in the Prostate Cancer (PCa) Tumor Microenvironment (TME), promoting metastatic progression. In this study, we aim to characterize the mechanisms of CAF-induced resistance to androgen-deprivation therapies using *in vitro* 3D co-culture systems.
**Material and Methods**
The PCa Patient-derived Xenograft (PDX) models PNPCa (androgen-dependent early metastatic model), and LAPC9 (androgen-independent bone metastatic model) are used. PCa cells and CAFs (human and mouse component from PDX tissue, respectively) are separated through Magnetic-associated Cell Sorting. Obtained CAFs are characterized by RT-qPCR, Western Blot, and Immunofluorescence. Tumor-CAF 3D co-cultures are obtained by combining the two components in direct (ultra-low attachment condition) or indirect setting (3D organoid culture with CAFs cultured in 2D in transwell inserts). 3D Cell Viability Assay is performed on organoids at transwell co-culture Day 9.
**Results and Discussions**
Typical prostate CAF markers (e.g. α-Smooth Muscle Actin, Tenascin C), as well as Androgen Receptor (AR) are expressed *in vitro* by PDX-derived CAFs at RNA and protein level. Expression of AR (protein level), and that of its target gene *Fkbp5* (RNA level) is upregulated after 24h of treatment with dihydrotestosterone (DHT) 10 nM, and this effect is abrogated upon co-treatment with the AR inhibitor enzalutamide (10μM), indicating functional AR signaling in CAFs. Indirect (transwell) co-culture with CAFs supports the viability of PNPCa tumor organoids, but not that of LAPC9 organoids, suggesting that PCa cells might have differential sensitivity to CAF-secreted factors at different tumor stages. This CAF growth-promoting effect is also observed on PNPCa organoids cultured in non-optimal DHT concentrations (DHT 0.5nM and 0.25nM versus the standard DHT 1nM). Moreover, in our direct co-culture method, CAFs self-assemble with PCa cells forming unified 3D organoid structures, allowing functional characterization of cell-cell interactions among tumor-stroma.
**Conclusion**
Our data suggest that PCa cells from advanced PCa modify the surrounding microenvironment to promote tumorigenesis, as PDX-derived fibroblasts, initially part of the mouse stroma, express prostate CAF markers, and show active AR signaling. Our transwell experiments suggest that CAFs secrete molecular factors which support tumorigenesis at least in the initial hormone naïve stage, but not necessarily required during the hormone independent tumor stage.

### EACR23-0509
### Bone marrow stroma inhibits differentiation of acute myeloid leukemia cells induced by low dose cytarabine

*<u>T. Smoljo</u>[1], B. Tomic[1], H. Lalic[1], V. Dembitz[1], D. Visnjic[1]*
*[1]University of Zagreb School of Medicine, Department of physiology and Croatian Institute for Brain*

© 2023 The Authors. Molecular Oncology is published by John Wiley & Sons Ltd on behalf of Federation of European Biochemical Societies

Research, Zagreb, Croatia

**Introduction**

Cytarabine (AraC) is the backbone of the standard induction therapy for acute myeloid leukemia (AML). However, majority of older patients are unfit for intensive chemotherapy so that an acceptable strategy includes hypomethylating agents or low-dose cytarabine (LDAC). Cytotoxic effect of high doses of AraC on AML cells is ascribed to apoptosis, but LDAC triggered AML remissions without toxicity and induced differentiation of leukemic cells *in vitro*. Bone marrow (BM) stroma is known to protect AML cells exposed to toxic doses of cytarabine, but the effect on differentiation induced by LDAC has not been investigated. The aim of this study is to test the effect of the presence of BM stroma on AML differentiation induced by LDAC.

**Material and Methods**

Mouse stromal MS-5 cells and their conditioned media were used to mimic BM environment. Human AML cell lines U937 or MOLM-13 were seeded on stromal layers with or without Transwell inserts. The effects of increasing doses of AraC on viability, cell cycle, apoptosis, reactive oxygen species (ROS) and differentiation were determined by multiparametric flow cytometry analysis. Morphology was analyzed by microscopy, the cytokines levels by LEGENDplex™ and the phosphorylation of Cdk1 by western blot.

**Results and Discussions**

The presence of MS-5 cells inhibited differentiation, cell cycle arrest and phosphorylation of Cdk1 in AML cells treated with LDAC. An increase in apoptosis was detected only in cells treated with a high dose of AraC and MS-5 cells prevented cytotoxicity, as previously described. However, a high dose of AraC significantly decreased viability and induced morphological changes of stromal cells alone, and these effects were partially rescued by the presence of AML cells. Transwell experiments suggested that the effects of stroma on AML differentiation were not dependent on cell-to-cell contact. The analysis of MS-5 conditioned media revealed high levels of CXCL12 and TGFb. However, preliminary data suggest that the effects on differentiation cannot be rescued by addition of plerixafor.

**Conclusion**

Although differentiation in response to cytarabine has been confirmed in several AML cell lines, the effect is rarely seen in patients treated with LDAC. Results of our study show that the presence of stroma inhibits LDAC-induced differentiation, which suggest that the impact of BM microenvironment on AML may be one of the reasons for the modest and/or rare differentiation effects observed in patients.

### EACR23-0512

### Preclinical models of adenoid cystic carcinoma predict cell-type-specific drug sensitivity.

*M. Fleming[1], A. Rose[1], J. Francis[1], J. Ning[2], R. Chauhan[3], A. Patrikeev[3], K. Harrington[1], A. Swain[1]*

*[1]Institute of Cancer Research, Cancer Biology, London, United Kingdom*

*[2]Institute of Cancer Research, Tumour Modelling Facility, London, United Kingdom*

*[3]Institute of Cancer Research, Genomics Facility, London, United Kingdom*

**Introduction**

Adenoid cystic carcinoma (ACC) is a rare cancer, mainly affecting the salivary glands and is slow growing but persistent. Surgical resection in early-stage disease is possible, but recurrence occurs due to perineurial space invasion or distant metastasis. Chemotherapy and targeted treatments show little benefit. For this reason, ACC represents an area of high unmet clinical, with a lack of accurate preclinical models hindering therapeutic development.

**Material and Methods**

6 previously unpublished patient-derived xenograft lines were established and from these, 2D and 3D models generated. All were optimised to recapitulate the phenotype of the original tumours. 2D and 3D in vitro drug studies determined the efficacy and mechanism of dBET6 with PDX lines used for in vivo studies. RNA-seq gene expression analysis of in vivo samples following treatment was carried out.

**Results and Discussions**

Using our in vitro models, we have identified dBET6, a BRD4 targeting PROTAC, as being efficacious in reducing ACC cell viability. Treatment with dBET6 led to degradation of BRD4 protein in vitro, resulting in reduced expression of MYB and putative MYB targets. We also demonstrate a long-term impairment of tumour growth in vivo following administration of dBET6. Differential gene expression of putative MYB target genes was observed however the most striking changes were to cell-type-specific marker expression. ACC is a biphasic tumour composed of myoepithelial and ductal cells and changes to the relative cellular composition of tumours following treatment were observed. Using our 3D organoid model, we demonstrate that different cell types have varying sensitivity to dBET6 treatment, with myoepithelial-like progenitor cells showing increased sensitivity compared to ductal-like cells upon treatment.

**Conclusion**

These promising results suggest that targeting BRD4 using the novel PROTAC class of drugs could be a useful strategy for ACC treatment. We also demonstrate the need to target both cell types within a tumour to induce tumour regression, suggesting a new strategy in ACC drug development.

### EACR23-0515

### Kras mutant cells avoid cell competition-driven elimination from adult pancreas via active Wnt signalling.

*B. Salvador Barbero[1], M. Alatsatianos[1], L. Hill[1], J. D'Ambrogio[1], T. Phesse[1], J. Morton[2], O. Sansom[2], C. Hogan[1]*

*[1]Cardiff University ECSCRI, Biosi, Cardiff, United Kingdom*

*[2]Beatson Institute, School of Cancer Sciences, Glasgow, United Kingdom*

**Introduction**

Pancreatic ductal adenocarcinoma (PDAC) is among the deadliest human cancers (12% 5-year survival). The main factor is the late diagnosis of the disease; therefore, understanding the early stages driving to pancreatic cancer

© 2023 The Authors. Molecular Oncology is published by John Wiley & Sons Ltd on behalf of Federation of European Biochemical Societies is crucial to develop new tools for cancer detection and prevention. Activation of oncogenic KRAS is the first genetic event occurring in PDAC (>90% patients) development, and it is associated with the appearance of pancreatic intraepithelial neoplasia (PanIN). Cell competition is the process by which genetically different cells compare 'relative cell fitness' establishing a loser-winner relationship in which 'loser' cells are eliminated. We previously demonstrated that when present in adult tissues in low numbers, cells expressing oncogenic KrasG12D are outcompeted and actively eliminated from pancreas tissue in vivo. We also find that someKrasG12D-expressing cells are never eliminated and remain in the pancreatic epithelium, eventually giving rise to PanINs. Here, we set out to understand what identifies these 'never-eliminated' KrasG12D cells and determine the molecular mechanisms underlying how this population of cells avoid cell elimination signals to remain in tissues.

**Material and Methods**

We used the Pdx-1Cre$^{ER}$; KRas$^{G12D/+}$; Rosa26$^{LSL-RFP}$ mouse model. Adult mice were treated with low dose tamoxifen to stochastically induce KrasG12D and RFP reporter expression in low numbers of pancreatic epithelial cells. Tissue was collected 5 weeks post induction, by which time cell competition has occurred. We studied cell dynamics of 'never eliminated' RFP-labelled KrasG12D cells through confocal imaging and RNA sequencing.

**Results and Discussions**

Bulk RNAseq revealed that 'never-eliminated' KrasG12D cells deregulate canonical cell competition pathways and enrich for gene signatures of EMT and Wnt signalling, while downregulating cell differentiation signatures. Using both in vivo and established cell competition assays in vitro, we demonstrate a functional requirement for Wnt signalling in normal-mutant cell competition, whereby active Wnt signalling promotes mutant cell retention.

**Conclusion**

KrasG12D-expressing cells are eliminated from adult pancreas tissues via cell competition with normal neighbours; however, some KrasG12D cells avoid cell elimination and progress towards neoplastic state. Our data show an important role of Wnt signalling in this process in vivo.

### EACR23-0532
### Small vesicles with vital functions: Exosomes in cellular communications between pancreatic cancer cells and neural cells

D. Karakas Zeybek[1,2], N. Aztopal[1], M. Erkisa[3], B. Ozpolat[4], G. Ceyhan[5], E. Ulukaya[6]
*[1]Istinye University, Faculty of Engineering and Natural Sciences- Molecular B iology and Genetics, Istanbul, Turkiye*
*[2]Acibadem University, Pancreatic Cancer Research Center, Istanbul, Turkiye*
*[3]Istinye University, Istinye University Molecular Cancer Research Center ISU MKAM, Istanbul, Turkiye*
*[4]Houston Methodist Research Institute, Marry and Ron Roy Neal Cancer Center, Houston, United States*
*[5]Acibadem University, General Surgery- HPB-Unit, Istanbul, Turkiye*
*[6]Istinye University, Faculty of Medicine-Clinical Biochemistry, Istanbul, Turkiye*

**Introduction**

Pancreatic cancer (PCa) is characterized by prominent intrapancreatic neuropathic alterations such as increased neural density and hypertrophy. Besides, PCa cells closely interact with nerves, and they can invade the perineurium and become associated with Schwann cells and the axons of neurons. This vicious interaction between tumor cells and nerves is closely associated with increased pain and poor prognosis in patients. Although some of the mechanisms were identified, cancer-nerve interaction mechanisms remain still unclear. Therefore, we investigated exosome-mediated communication between the PCa cells and neurons/Schwann cells.

**Material and Methods**

SH-SY5Y neuroblastoma cells were differentiated into neurons by all-trans-retinoic acid treatment. The efficiency of differentiation was confirmed by morphological analysis, immunofluorescent staining, and western blot. The exosomes were obtained from three cell sources; neurons, Schwann cells, and PCa cell lines (PANC-1 and BxPC-3). The number of exosomes was quantified by CD63 ELISA assay, and exosome uptake was confirmed by PKH67 staining. Schwann cells and neurons were treated with either PANC-1 or BxPC-3 cell-derived exosomes. Then, the changes in migration, invasion, and proliferation abilities of the cells were evaluated. Similarly, PCa cells were exposed to exosomes derived from Schwann cells or neurons. Afterward, the changes in cell migration, invasion, colony formation, proliferation, and the expression of metastasis-related proteins were investigated. In addition, PCa patient tissues were stained with S100A and GFAP antibodies for the nerves and CD9 or CD81 antibodies for the exosomes. Besides, relative expression levels of 84 different exosomal miRNAs were assessed by miRNA array in the exosomes. One of the overexpressed miRNAs was selected as a potential player in the aggressiveness of PCa, and their effects on cancer cells were evaluated via functional analyses (cell migration, invasion, etc.).

**Results and Discussions**

We showed that PCa cell-derived exosomes increase migration, invasion, and proliferation of Schwann cells. Similarly, the exosomes derived from Schwann cells induced aggressive behaviors of PCa cells. Besides, the selected miRNA, which is overexpressed in Schwann exosomes, induced migration of PCa cells.

**Conclusion**

Briefly, these results suggest that exosomes may serve as a messenger between cancer cells-Schwann cells/neurons. Further research is needed to enlighten this hostile communication, which contributes aggressiveness of PCa.

### EACR23-0537
### Tumor resident microbiota in breast cancer: a multiomic approach to reveal the functional role

F. Pirini[1], N. Hajjaji[2], S. Aboulouard[3], B. Fosso[4], M. Puccetti[5], M.M. Tumedei[1], M. Zanoni[1], L. Magnani[6], S. Bravaccini[1], M. Salzet[3]

© 2023 The Authors. Molecular Oncology is published by John Wiley & Sons Ltd on behalf of Federation of European Biochemical Societies

[1]IRCCS Istituto Romagnolo per lo Studio dei Tumori IRST "Dino Amadori", Laboratorio di bioscienze, Meldola, Italy
[2]Oscar Lambret Center, Breast Cancer Unit, Lille, France
[3]University of Lille, Laboratoire Protéomique-Réponse Inflammatoire et Spectrométrie de Masse PRISM, Villeneuve d'Ascq Cedex, France
[4]Institute of Biomembrane- Bioenergetics and Molecular Bi otechnologies - IBIOM-CNR, Laboratorio Genetica Molecolare e Microbiologia, Bari, Italy
[5]Azienda Unità Sanitaria Locale AUSL di Imola, Pathology Unit, Imola, Italy
[6]The Imperial Centre for Translational and Experimental Medicine- Imperial College, Department of Surgery and Cancer, London, United Kingdom

**Introduction**

The microbiota is a component of the tumor microenvironment (TME), however its functional role is still poorly understood.

**Material and Methods**

By integration of meta-proteomic, meta-transcriptomic and meta-genomics we investigated the microbial diversity and its contribution to the reservoir of the microbiota-derived proteins in an exploratory cohort of 12 formalin-fixed, paraffin-embedded (FFPE) breast cancer (BC) samples: 6 TNBC, 3 LumA and 3 LumB. DNA/RNA extraction and proteomics were performed on 10µm tissue slides. Three tumoral regions (peripheral tumor, tumor core, *in situ* carcinoma) and 2 healthy regions (intra-tumoral fibrosis, healthy tissue) were selected for the proteomic analysis, performed by nanoLC-MS/MS analysis and data analysis by Perseus and Maxquant. Meta-transcriptomics was performed on tumor tissues by NEBNext Ultra II Library Prep kit (Illumina) and analyzed by Kraken2; the 16S rRNA-seq (Illumina) was performed in tumor *vs.* healthy regions. To confirm the proteomic results we reanalyzed proteomic data of 76 BC patients (LumA/B) (validation set) with available stromal and tumor regions analysis.

**Results and Discussions**

Despite the low number of samples, we identified a significant α and β diversity of the microbiota in tumor *vs.* healthy samples in the exploratory cohort (P=0.042; P=0.005). Moreover, we detected 29 bacterial proteins: 13 chaperonins, 7 ATP synthase subunits, 7 metabolic enzymes and 2 elongation factors. Most of the proteins (16) were located in the intra-tumoral fibrosis. A Glyceraldehyde-3-phosphate dehydrogenase (gapA, P0A9B5) and three DnaKs (A6LM32, A0M353, B8IHL3) were overexpressed in the tumor *vs.* healthy tissue. Only B8IHL3 was associated with a single species: *Methylobacterium nodulans*. To define the potential species of origin of the remaining proteins, we analyzed the proteomic data in light of the taxonomic results obtained by meta-transcriptomics. The two DnaKs possibly belong to *Methylobacterium sp4-46 (*A0M353) and *Clavibacter michiganensis (A6LM32)*, while gapA to *Escherichia coli*. The meta-proteomic analysis of the validation set confirmed a significant overexpression in tumors *vs.* stroma of 2 DnaKs (Q3B577, A0M353) and one of them was in common with the exploratory cohort.

**Conclusion**

We here report the overexpression in BC tissues of DnaKs, bacterial chaperones able to interfere with important pathways as DNA damage response, cell-cycle and apoptosis and supposed to promote cancer, and of gapA, a known regulator of metabolism and gene expression.

### EACR23-0541
### The chorioallantoic membrane model in melanoma progression and vascularization studies

J. Sopel[1], A. Bienia[1], A. Kozińska[1], M. Szczygieł[1], A. Markiewicz[2], B. Romanowska-Dixon[2], M. Elas[1]
[1]Jagiellonian University, Faculty of Biochemistry Biophysics and Biotechnology- De partment of Biophysics and Cancer Biology, Cracow, Poland
[2]Jagiellonian University, Chair of Ophthalmology- Faculty of Medicine- Jagiellonia n University Medical College and Ophthalmology and Ocu lar Oncology Clinic, Cracow, Poland

**Introduction**

The chorioallantoic membrane (CAM) is a model enabling studying various biological processes, including normal and tumor angiogenesis. It also found application in research on cancer development and biology, tumor metastasis and cancer therapies, with particular emphasis on anti-vascular therapies.
Melanoma originates from melanocytes from skin or choroid, and aggressively metastasizes to various organs, including the liver and lungs. Our aim was to compare tumor growth and vascular structure development in several cutaneous and uveal melanoma lines, obtained both from primary and metastatic tumors. For this purpose, we used the *in vivo* CAM model.

**Material and Methods**

In our studies, we used commercial lines of cutaneous melanoma and uveal melanoma isolated from patient tumors. We used the chicken CAM as *in vivo*. Melanoma cells were implanted on the chorioallantoic membrane in 7 embryo development day (EDD). Tumor growth was observed over the next 6 days. In 13 EDD embryos were sacrificed and tumors isolated. The weight and volume of the tumors were analyzed. Histological (H&E) analysis of the tumor structure was performed. The preparations were analyzed for the presence of blood vessels.

**Results and Discussions**

In all melanoma lines used, we observe growth in the CAM model. Our results show that tumors from different types of melanoma cells can be obtained in the CAM model. Visible vascularization is observed in the formed tumors, a few days after implantation. In addition, melanoma cells do not lose their ability to produce pigment. Tumors located 'below' the membrane was heavily pigmented, and had faster growth, than tumors growing 'above' the membrane (amelanotic). Histological analysis showed a variety in the structure and location of the vascularity.

**Conclusion**

Our research has shown that the CAM model is a good model in the study of melanoma progression. We observed tumor growth, formation of blood vessels, as well as preservation of features of primary tumors (pigmentation).

### EACR23-0546
### CITK loss leads to DNA damage accumulation impairing homologous

© 2023 The Authors. Molecular Oncology is published by John Wiley & Sons Ltd on behalf of Federation of European Biochemical Societies

### recombination dynamics and BRCA1 recruitment in medulloblastoma

G. Iegiani[1], G. Pallavicini[1], M. Gai[2], S. Bielas[3], F. Di Cunto[1]
[1]Neuroscience Institute Cavalieri Ottolenghi, Department of Neuroscience 'Rita Levi Montalcini'- University of Turin, Turin, Italy
[2]Molecular Biotechnology Center, Department of Molecular Biotechnology and Health Sciences- University of Turin, Turin, Italy
[3]University of Michigan, Department of Human Genetics, Ann Arbor, United States

**Introduction**

Medulloblastoma (MB) is the most common malignant pediatric brain tumor. The current therapy consists in surgery, followed by radiation of the entire neuroaxis and high dose multi-agent chemotherapy. Despite the improvement in patient survival, many patients still die and those who survive suffer from neurological and endocrine disorders. Therefore, more effective therapies are needed. Citron Kinase (CITK) is validated as target for MB treatment as its depletion induces apoptosis and reduces tumor growth *in vivo*. Moreover, loss of CITK leads to DNA double strand breaks (DSBs) accumulation and it impairs homologous recombination (HR), one of the major pathways used to repair DNA damage.

**Material and Methods**

We resorted to two human medulloblastoma cells lines, ONS-76 and DAOY, that were silenced with established siRNA sequences against CITK, to assess CITK molecular mechanism in HR pathway. Moreover, we confirmed *in vitro* findings in organoids and *in vivo* MBs.

**Results and Discussions**

We found that CITK depleted MB cells show a reduced mobility of the DSBs foci. In this context, CITK knockdown reduced BRCA1 nuclear levels and BRCA1 loading at DNA DSBs sites without altering phospho-RPA recruitment; these results were confirmed also in human organoids and *in vivo*. Consequentially, CITK knockdown reduces the HR downstream effectors BRCA2 and RAD51 protein levels without altering their transcriptional levels.

**Conclusion**

These data indicate that HR impairment after CITK loss is due to an altered foci dynamics and reduced recruitment of BRCA1 protein at DSBs. Our future effort will be on uncover how CITK regulates DSBs dynamics and BRCA1 recruitment. This will be important to propose CITK inhibition combined with radiation or cisplatin, treatments used in clinical practice that increase DSBs load in tumor cells.

### EACR23-0573

### Long pentraxin 3 (PTX3) as a regulator of lymphangiogenesis and lymphogenous dissemination in melanoma

M. Turati[1], S. Rezzola[1], F. Maccarinelli[1], S. Mussi[1], A. Giacomini[1], T. Annese[2], D. Ribatti[2], R. Ronca[1]
[1]University of Brescia, Department of Molecular and Translational Medicine, Brescia, Italy
[2]University of Bari Medical School, Department of Basic Medical Sciences- Neurosciences and Sensory Organs, Bari, Italy

**Introduction**

Melanoma is one of the most aggressive forms of cutaneous tumor, being responsible for 90% of skin cancer-related deaths each year. Melanoma-associated lymphangiogenesis plays a pivotal role in tumor dissemination to the draining lymph nodes (LNs) and then to distant organs, turning melanoma into a life threatening cancer. The soluble pattern recognition receptor long pentraxin 3 (PTX3) exerts pleiotropic functions in physiopathological conditions, including cancer, where it acts as an extrinsic oncosuppressor by modulating FGF/FGFR signaling and inflammation. We have previously observed that PTX3 impairs melanoma growth and its metastatic features by affecting several aspects of cancer progression, but to date little is known about its role in tumor-associated lymphangiogenesis.

**Material and Methods**

Here, we treated lymphatic endothelial cells (LECs) with lymphangiogenic stimuli in the absence or in the presence of recombinant PTX3, the effect of which on LEC activation was verified in terms of proliferation, migration, sprouting and tube formation. The activation of FGF/FGFR system was investigated through western blot analysis. Moreover, Matrigel plug assay and lymphatic dissemination of melanoma cells were performed in a mouse model characterized by lymphatic expression of PTX3. The effect of lymphangiogenic stimuli and melanoma-derived factor on PTX3 expression in LECs was assessed *in vitro* through both real time PCR and western blot. RNAscope analysis was performed on human melanoma samples to investigate PTX3 lymphatic expression in the primary tumors.

**Results and Discussions**

This preliminary work sheds light on the regulatory role of PTX3 in lymphangiogenesis and in melanoma lymphogenous dissemination. Indeed, treatment with recombinant PTX3 reduces LEC activation by inhibiting FGF/FGFR signaling. Furthermore, we observed that lymphatic expression of PTX3 hampers lymphangiogenesis *in vivo* and significantly reduces the metastatic spreading of melanoma cells to the draining LN in mice. Moreover, we observed that lymphangiogenic and melanoma-derived factors can downregulate PTX3 expression in LECs. Accordingly, PTX3 downregulation occurs in lymphatic vessels of primary human melanoma specimens when compared to normal skin.

**Conclusion**

We hypothesize an inhibitory role of PTX3 in melanoma-associated lymphangiogenesis, its downregulation in LECs representing a pivotal step in melanoma lymphogenous metastatization. Thus, lymphatic PTX3 may have mechanistic, prognostic and therapeutic implications in melanoma.

### EACR23-0578

### Impact of Age and Strain on Inflammation-Associated Colon Cancer Development in Mice

K. Piatek[1], N. Kupper[1], G. Frisch[1], V. Gushchina[1], T. Manhardt[1], M. Salzmann[1], S. Derdak[2], I. Mesteri[3], E. Kallay[1], M. Schepelmann[1]
[1]Medical University of Vienna,

© 2023 The Authors. Molecular Oncology is published by John Wiley & Sons Ltd on behalf of Federation of European Biochemical Societies

Institute of Pathophysiology and Allergy Research, Vienna, Austria
[2]Medical University of Vienna, Core Facilities, Vienna, Austria
[3]Pathologie Überlingen, Institute of Pathology, Überlingen, Germany

**Introduction**

Colon cancer is a serious problem in the western world and causes many deaths every year. Two of the most important risk factors for development of colorectal cancer are age and inflammation. However, most of the current preclinical *in vivo* studies are conducted in young mice, putting their relevance for modeling an aged population in question. Therefore, our aim was to determine the impact of age on the development of inflammation-associated colorectal cancer (CAC).

**Material and Methods**

In this study, we used the Azoxymethane (AOM) /Dextrane-Sulphate-Sodium (DSS) model of inflammation-associated colorectal cancer in young (3 months old) and old mice (18 months old) in two different mouse strains BALB/c and C57BL/6. After the end of the treatment, we performed histological analysis of the colon sections to determine mucosal remodeling, dysplasia and inflammation in the colons. We also assessed immune cell infiltration (CD3 and CD20) and cell proliferation (KI67) by immunostaining. We performed RNAseq analysis and validated some of the results of inflammation-related genes using RT-qPCR.

**Results and Discussions**

The histological analysis showed that the C57BL/6 strain is more sensitive to the AOM/DSS treatment, their clinical symptoms being more severe. C57BL/6 mice also developed more tumors per mouse and their cumulative tumor score, which includes size, number and degree of severity of the tumor, was much higher. In C57BL/6 animals, we observed also differences between young and old mice. Young mice had more tumors, twice as many as the old ones. Gene expression analysis showed higher expression of IL6, TNFα, COX2 and Lgr-5 in old C57BL/6 mice compared with old BALB/c mice. IL6 gene expression was higher also in young C57BL/6 mice than in the young BALB/c mice. Data obtained in immunostaining is currently being analyzed.

**Conclusion**

Our current results suggest that both the strain and the age of the mice affect the severity of inflammation-associated colorectal cancer in this mouse model.

### EACR23-0579
### TGFβ1/LOX CROSS-TALK MODULATES INVADOPODIA- AND MATRIX STIFFNESS-DEPENDENT INVASION OF CLEAR CELL RENAL CELL CARCINOMA CELLS

B. Torsello[1], S. De Marco[1], I. Morabito[1], V. Cassina[1], N. Zucchini[2], G. Strada[3], C. Bianchi[1]
[1]Milano-Bicocca University, School of Medicine and Surgery, Monza, Italy
[2]ASST Monza San Gerardo Hospital, Anatomo-Pathology Unit, Monza, Italy
[3]ASST North Milan Bassini Hospital, Urology Unit, Cinisello, Italy

**Introduction**

Clear cell Renal Cell Carcinoma (ccRCC) is frequently diagnosed in the metastatic phase. The invasion and metastasis of solid tumors exploit F-actin rich cell protrusions with matrix-degradative activity known as invadopodia. TGFβ induces invadopodia maturation in cancer cells and specifically enhances ccRCC invasion. Furthermore, the extracellular matrix modifying enzyme Lysyl oxidase (Lox), which is overexpressed in ccRCC and has a role in the modulation of matrix stiffness, induces cell migration and invasion.
Here, we studied the role of cross-talk between TGFβ and Lox in the matrix stiffness modulation and the effects of increased stiffness in the modulation of the invadopodia-dependent matrix degradation induced by TGFβ in an *in vitro* model of primary cell cultures of ccRCC with different Furhman grade.

**Material and Methods**

We performed immunofluorescence analysis of invadopodia F-actin spots and degradation assay of matrixes with different stiffness using primary cell cultures from human ccRCC tissues of different Fuhrman grade, treated or not with TGFβ1 and TGFβ-receptor inhibitor (SB431542). Real-Time PCR and ELISA evaluated Lox expression. We quantified the stiffness of matrixes treated with ccRCC culture conditioned media by Atomic Force Microscopy.

**Results and Discussions**

Lox transcript and active protein secreted in culture media were more abundant in low grade ccRCC than in high grade cultures. Furthermore, TGFβ1 treatment of ccRCC cells increased Lox secretion and SB431542 prevented this increment. Conditioned media of TGFβ1-treated ccRCC cultures increased the matrix stiffness and Lox inhibition with bAPN reverted this increment. Invadopodia F-actin spots and matrix degradation activity of ccRCC cells increased with TGFβ1 treatment. Inhibition of TGFβ1 signaling with SB431542 reduced this effect. High grade (G3-G4) ccRCC cultures showed more invadopodia than low grade (G1-G2) cultures in all analyzed experimental conditions. Moreover, ccRCC cells showed more invadopodia when cultured on a stiffer matrix, regardless of grade.

**Conclusion**

Our studies show that: -) TGFβ1 induces in ccRCC cells invadopodia maturation and Lox secretion that is able to increase matrix stiffness: -) invadopodia-dependent matrix degradation by ccRCC cells is increased in stiffer matrixes. Thus, the cross-talk between TGFβ1 and Lox could increase ccRCC invasion and may be a therapeutic target in ccRCC metastatic disease.

### EACR23-0594
### Inflammatory-associated genetic and epigenetic alterations during the stepwise development of Colitis-Associated Cancer

S.M. El-Daly[1], S. Abd El-Rahman[2], S. Gouhar[1], E. Omara[3]
[1]National Research Centre, Medical Biochemistry Department, Cairo, Egypt
[2]Faculty of Veterinary Medicine- Cairo University, Department of Pathology, Cairo, Egypt
[3]National Research Centre, Pathology Department, Cairo,

© 2023 The Authors. Molecular Oncology is published by John Wiley & Sons Ltd on behalf of Federation of European Biochemical Societies

*Egypt*
**Introduction**
Chronic intestinal inflammation is strongly linked to colitis-associated cancer (CAC) development. CAC development is a stepwise process with an accumulation of genetic and epigenetic changes. The inflammatory alterations linked to CAC pathogenesis influence the inner mucosal layer structure, which impacts the expression of drug transporters. Altered levels of drug transporters may affect local drug actions in the gastrointestinal tract and impact drug bioavailability. Our study aimed to evaluate the dynamic expression of a panel of inflammatory mediators and drug transporters in addition to profiling the differential expression of inflammatory-associated miRNAs during the stepwise stages of CAC development.
**Material and Methods**
We used the stepwise colorectal tumorigenesis murine model, Azoxymethane /Dextran sodium sulfate model, to recapitulate the different stages of tumorigenesis and the stepwise histopathological and genetic progression similar to humans. Through this model, each group represented an induction phase during the CAC development process. We evaluated the histopathological alterations and the manifestation in crypt morphology during the induction stages. Immunohistochemistry, ELISA, and Real-time PCR were applied to evaluate the expression patterns of the inflammatory-associated panel of genetic and epigenetic targets.
**Results and Discussions**
The growth and morphological features of colonic crypts support the sequential tumorigenesis process that originates from normal epithelial cells to aberrant crypts, preneoplastic lesions, and eventually CAC development. This sequential transformation was accompanied by a significant elevation in the level of several cytokines and gradual consecutive activation of NF-κB and STAT-3, as can be detected from colonic tissue immunostaining. The observed modulation in the expression and localization of drug transporters across the stages, as represented in the stepwise increase in MRP2 or gradual decrease in MDR1, was associated with the intestinal inflammation stage. The expression profile of tissue miRNAs was either dynamically changing in correlation with the stepwise developmental stages of CAC, like miR-141, miR-15b, and miR-181b, or only elevated significantly in the last two stages of induction as in miR-21, miR-31, miR-146b, miR-221.
**Conclusion**
Our study provides a molecular mechanistic approach to evaluate the dynamic alteration in some inflammatory-associated genetic and epigenetic markers involved in the stepwise development of colitis-associated cancer

### EACR23-0615
### Ectopic ACTH-Secreting Metastatic Pancreatic Neuroendocrine Tumor Initially Presenting as Gastrinoma: A Case Report

*H. Dudipala*[1], *A. Hussein*[2], *M. Kulke*[2]
[1]*Boston University School of Medicine and Boston Medical Center, Department of Medicine, Boston, United States*
[2]*Boston University School of Medicine and Boston Medical Center, Department of Hematology and Medical Oncology, Boston, United States*
**Introduction**
Pancreatic neuroendocrine tumors (pNETs) account for only 1-2% of all pancreatic malignancies. Functional pNETs comprise about 10% of pNETs. Insulinomas and gastrinomas are the most common, and there are a few cases of ectopic adrenocorticotropic hormone (ACTH) secretion. However, it is rare for pNETs to transform from secreting one type of peptide hormone to another. Herein, we present a case of metastatic pNET that originally presented as a gastrinoma, but later transformed to an ectopic ACTH-secreting pNET.
**Material and Methods**
A 54 year old male presented to the emergency room with symptoms of nausea, vomiting, and weight loss. CT imaging showed a 3.8cm pancreatic mass without evidence of metastatic disease. Biopsy revealed a well-differentiated, grade 2 neuroendocrine tumor, with Ki67 index 2-20%. He had elevated gastrin levels consistent with gastrinoma, and was initiated on somatostatin analogue (SSA) therapy octreotide monthly. One year later repeat imaging revealed multiple liver lesions, increased pancreatic mass size, and peritoneal involvement, consistent with metastatic disease. His gastrin level was elevated at 1910pg/mL, so he was continued on monthly SSA. The patient was subsequently admitted 10 months later with nausea, vomiting, hyperglycemia, and hypokalemia. Further workup showed elevated ACTH to 370pg/mL, elevated 24-hour urine cortisol, and abnormal dexamethasone suppression test. MRI brain showed no pituitary mass. He was diagnosed with Cushing's syndrome secondary to ectopic ACTH secretion from his pNET. He underwent bilateral adrenalectomy and was continued on monthly SSA injections. His course was complicated by multiple admissions for encephalopathy and catatonic depression, and was not a candidate for further therapy, therefore eventually succumbing to his disease.
**Results and Discussions**
Ectopic ACTH syndrome (EAS) is rare and accounts for 5-10% of Cushing's syndrome. There are only a few reported cases of gastrinomas converting to ectopic ACTH production. We report an unusual case of a patient with gastrinoma who developed EAS 2 years after initial diagnosis. This highlights the importance of comprehensive hormonal testing in patients with pNETs. Moreover, the established diagnosis of a certain hormone-secreting tumor does not exclude the occurrence of a new hormonal activity.
**Conclusion**
Transformation of pNETs to secrete different peptide hormones is rare, but clinicians should remain vigilant for the appearance of new symptoms, which may indicate new functional tumor activity.

### EACR23-0622
### Neuroblastoma-on-a-chip to study the alternative vasculature in neuroblastoma

*A. Villasante*[1], *M.J. Lopez Martinez*[1], *A. Garcia-Lizarribar*[1], *J. Samitier*[1]
[1]*Institute for Bioengineering of Catalonia, Nanobioengineering Laboratory, Barcelona, Spain*
**Introduction**

© 2023 The Authors. Molecular Oncology is published by John Wiley & Sons Ltd on behalf of Federation of European Biochemical Societies

Neuroblastoma (NB) is a highly vascularized pediatric tumor originating from undifferentiated neural crest cells in the first years of life. NB displays an alternative vasculature constituted by cancer cells that can transdifferentiate into tumor-derived endothelial cells (TECs), a phenotype associated with drug resistance and tumor relapse. We currently lack treatments against TECs, mainly because of the challenge of developing predictive models to mimic this phenomenon in vitro.
In the last years, engineered human tumors are starting to be fabricated in a miniaturized fashion known as tumors-on-a-chip to resemble the cellular composition and to mimic selected functions of the native tumor. The main advantage of this novel technology is the highest predictive potential.
Due to the TECs subpopulation is not present in traditional plastic dishes, we aimed to develop an NB-on-a-chip including the "traditional" vascularization and alternative vasculature observed in patients.

**Material and Methods**

Neuroblastoma-on-a-chip set-up. "butterfly"-like microfluidic chips were fabricated, and LUC-1 Luer and male Luer integral 1/16 connectors were used to connect Tygon tubes to chip inlets and outlets. The culture medium was pumped using a high-precision IPC-N 8 peristaltic pump.Hydrogels preparation and characterization. hydrogels were fabricated using bovine collagen I, fibrinogen from bovine blood plasma, and cross-linked with thrombin. FX3 and FX5 formulations were prepared.

**Results and Discussions**

We proposed recapitulating the native NB stiffness using a collagen I/fibrin-based hydrogel to obtain vascularization and TECs in vitro. By modifying the concentration of the thrombin enzyme, we obtained two stable formulations in the range of native stiffness named FX5 (Emod = 0.87 ± 0.28 kPa) and FX3 (Emod = 0.56 ± 0.30 kPa).Then, A co-culture of NB cells and HUVEC were seeded within the hydrogel FX5 and cultured for 3 and 7 days under flow perfusion conditions. At day 3, cells formed visible aggregates within the 3D matrix, are alive and starting to sprout. A tissue-engineered was observed inside the main chamber on day 7 as a confluent network of cell aggregates connected by tubular structures. Co-stains of MYCN (NB marker) and CD31 (endothelial marker) of the tissues demonstrated the presence of cells expressing both markers, confirming the conversion of NB cells into TECs.

**Conclusion**

The NB-on-a-chip demonstrated a high level of mimicry and recapitulated the presence of TECs observed in patients.

### EACR23-0624
### Colonising the neuronal microenvironment: an in vitro murine organotypic model of brain metastases.

*L. Napier*[1], *F. Tamagnini*[2], *P. Dash*[1]
[1]*University of Reading, School of Biological Sciences, Reading, United Kingdom*
[2]*University of Reading, School of Pharmacy, Reading, United Kingdom*

**Introduction**

Brain metastases (BM) are the most common intracranial tumour type and are associated with poor patient outcomes. The emerging field of cancer neuroscience has begun to explore the unexpected ways BMs colonise the neuronal microenvironment. We have developed an *in vitro* murine organotypic slice model of BMs to examine early BM microenvironment interactions with a particular focus on intracellular $Ca^{2+}$ oscillations.

**Material and Methods**

Co-culture of five cancer cell lines representing common primary sites of brain metastases (U-87 MG, MDA-MB-231, SK-MEL-28, A549 and DU-145) in organotypic brain slices from 12-day old C57BL/6J mice. Confocal intracellular $Ca^{2+}$ imaging was performed on GCaMP6s positive cells before and during co-culture. The organotypic brain slice was tested for tissue viability and 25 murine cytokine expressions were measured.

**Results and Discussions**

The majority of murine cytokine analyte expressions were significantly lower after 14 days in brain slices with cancer cells transplanted compared to slices with no cancer cells. Lactate dehydrogenase secretions were lower in slices with cancer cells transplanted and trypan blue staining indicated that the cancer cells may have preserved the slice in culture overall despite localised cell death around cancer cell aggregates. Fast Fourier Transform (FFT) analysis indicated a variety of changes in intracellular $Ca^{2+}$ oscillations after 1 day and 14 days in co-culture in the organotypic slice. A consistent finding in each cell line was an increase in GCaMP6s fluorescence amplitude after 14 days.

**Conclusion**

This *in vitro* murine organotypic BM model has provided insights into the early behaviours exhibited by cancer cells when they first encounter the brain microenvironment. Further study will include RT-qPCR to investigate expression changes in cancer cell $Ca^{2+}$ homeostatic machinery and validate GCaMP6s findings.

### EACR23-0634
### Investigating the Role of Connexin Proteins in Migratory/Invasive Mechanisms of Highly Metastatic Cancers

*S. Paunikar*[1], *L. Tamagnone*[1], *E. Testa*[1]
[1]*Universita Cattolica Del Sacro Cuore, Histology and Embryology, Rome, Italy*

**Introduction**

Decisive communication between cells in the microenvironment is vital to maintain the tissue homeostasis and overall function of multicellular organisms. This cellular crosstalk occurs via direct contact between neighboring cells or the extracellular matrix using various types of junctions. Gap junctional intercellular communication (GJIC), realized through channels formed by Connexins (Cx) proteins, is important in mediating normal cell growth, differentiation and development; and mutation or loss or alteration of these channels are associated with many diseases and disorders, including cancer.

**Material and Methods**

In our study, to investigate the involvement of connexin proteins in metastatic landscape, we employed gap junctional blocking using carbenoxolone disodium (CBX)

© 2023 The Authors. Molecular Oncology is published by John Wiley & Sons Ltd on behalf of Federation of European Biochemical Societies on a panel of immortalized cancer cell lines to understand alterations in cellular characteristics or basal connexin protein levels. Subsequently, the cells were stimulated with Transforming Growth Factor Beta (TGF-β) at different time-points to test for intracellular connexin-43 (Cx43).

**Results and Discussions**

We aim at studying Cx43 to elucidate its role in intracellular signaling, particularly in context of TGF-β signaling pathway, which is a pivotal regulator of cancer cell behavior, promoting loss of cell-cell junctions, invasiveness and metastasis. Intriguingly, we found that Cx43 protein levels are significantly upregulated in cancer cells in response to TGF-β stimulation, independent of gap junction formation.

**Conclusion**

Connexin proteins are increasingly implicated in several non-canonical functions unrelated to GJIC, particularly Cx43, which has been implicated in inter-cellular as well as intra-cellular signaling, with multifaceted functions in cancers. Our observation underscores a potential functional relevance of this connexin in intracellular TGF-β signaling network, deserving experimental investigation.

### EACR23-0667
### The Role of S100A11-Enriched Small Extracellular Vesicles in Breast Cancer Metastasis

S.K. Tey[1], V.Y. Shin[1], A. Kwong[1]
[1]*The University of Hong Kong, Surgery, Hong Kong, Hong Kong- China*

**Introduction**

Small extracellular vesicles (sEV) is a subpopulation of extracellular vesicles, ranging from 30-150 nm in size, that secreted by any type of cells. Cancer cell derived sEV has been shown to mediate intercellular communication in tumor microenvironment by transferring cargos into the recipient cells. S100A11 is frequently dysregulated in human carcinomas including breast cancer. However, there is no study report on the role of S100A11-enriched sEV in breast cancer.

**Material and Methods**

sEV derived from MDA-MB-231 cells and AK571 cells, cells that established from lung metastasis lesion of MDA-MB-231 cells implanted in NSG mice, was isolated by serial ultracentrifugation. The identity of sEV was validated by immunoblotting of sEV markers, transmission electron microscopy and nanoparticle tracking analysis. Proteomic profiling of sEV was performed by mass-spectrometry analysis. Level of dysregulated proteins in sEV derived from serum of breast cancer patients was measured by ELISA. The protein expression of dysregulated proteins was examined by immunohistochemistry of tissue microarray of paired breast cancer tissues and adjacent non-cancerous tissues.

**Results and Discussions**

sEV was isolated and validated. Proteomic profiling of sEV derived from MDA-MB-231 and AK571 cells revealed distinct profile of proteins expression. Among the dysregulated proteins, S100A11 showed the largest fold differences and significantly increased in AK571 cells derived sEV. The level of S100A11 was found to increase in sEV derived from serum of breast cancer patients as compared to healthy controls. The protein expression of S100A11 was upregulated in breast cancer tissues compared to adjacent non-cancerous tissues, which corroborate previous studies reported on increased S100A11 expression in breast cancer. Importantly, further examination of sEV-S100A11 level in serum of metastatic and non-metastatic breast cancer patient revealed a significant increased expression of sEV-S100A11 in metastatic patients.

**Conclusion**

In conclusion, S100A11 might play important roles in modulating breast cancer metastasis. Further study is warranted to investigate the molecular mechanism of actions and signalling pathway involved.

### EACR23-0670
### Development and characterisation of immunocompetent models for metastatic oestrogen receptor positive breast cancer

D. Bull[1], J. Hazlett[1], A. McLellan[2], A. Dunbier[1]
[1]*University of Otago, Centre for Translational Cancer Research and Department of Biochemistry, Dunedin, New Zealand*
[2]*University of Otago, Department of Microbiology and Immunology, Dunedin, New Zealand*

**Introduction**

Approximately 80% of breast cancer diagnoses present as oestrogen receptor-positive (ER+), with ~10% of these patients developing metastatic ER+ breast cancer (mBC), which is still incurable. The distinct lack of models that fully recapitulate the biological processes involved in ER+ mBC has significantly slowed progress towards developing effective treatment options. This research aims to develop the first biologically relevant preclinical model of ER+ mBC in mice with fully intact immune systems.

**Material and Methods**

Trackable, ER+ breast cancer-like cells were developed for visualisation of tumour growth with bioluminescence in the 129S6/SvEvTac mouse background. Fluorescently labelled sleeping beauty vectors encoding Antares2 or Firefly luciferase were used to transfect SSM3 cells via electroporation. These tagged SSM3 cells were introduced to mice to model spontaneous and experimental metastasis by mammary fat pad or tail vein injections. IVIS Lumina X5 imaging captured luciferase activity at multiple time points over six months following primary tumour removal (spontaneous model) or cell line injection (experimental model). SSM3 metastasis was determine through ex vivo imaging and histological analysis. Primary tumours were processed for FACs, to investigate a potential increase in immunogenicity of the tagged SSM3 cells.

**Results and Discussions**

Mammary fat pad injections of tagged SSM3 cells produce primary tumours with strong luciferase activity. Tumour establishment occurs at a rate of ~50%. This take rate and the morphology of the primary tumours indicate that the tagged SSM3 cells may promote more immune cell recruitment than the parental SSM3 cell line. This is potentially due to the immunogenicity of fluorescent proteins expressed by the tagged cells. *Ex vivo* imaging has shown evidence of low level metastasis to the uterus and intestine. Tagged SSM3 cells were detected in the chest of

© 2023 The Authors. Molecular Oncology is published by John Wiley & Sons Ltd on behalf of Federation of European Biochemical Societies mice following tail vein delivery. The spontaneous ER+ mBC model is being further developed by establishing the mammary intraductal injection (MIND) method in this background. The experimental metastasis model is being optimised by increasing the number of injected cells and by the addition of exogenous oestradiol.

**Conclusion**

This research has further defined the advantages and limitations of the syngeneic 129S6/SvEvTac model for ER+ mBC. Continued development of this model will allow investigation of new therapeutic options, specifically therapies that harness the immune system for metastatic ER+ breast cancer.

### EACR23-0677
### Pre-clinical models from rare aggressive tumours: Establishment and characterisation of PDX models for drug development

*S. Abdirahman[1], O. Craig[1], C. Salazar[1], M. Pechlivanis[1], N. Rajadevan[1], C. Scott[2], O. McNally[3], K. Gorringe[1]*
*[1]Peter MacCallum Cancer Centre, Cancer Evolution and Metastasis Program, Melbourne, Australia*
*[2]The Walter and Eliza Hall Institute of Medical Research, Clinical Translation, Melbourne, Australia*
*[3]The Royal Women's Hospital, Gynaecological Oncology, Melbourne, Australia*

**Introduction**

Mucinous ovarian cancer (MOC) is a rare subtype of epithelial ovarian cancer with limited therapeutic options and poor prognosis when diagnosed at advanced stages. Chemotherapy response rates for MOC remain low, and the increased molecular heterogeneity of this subtype highlights the need for novel, effective and personalised treatment strategies. Our aim is to establish and characterise a cohort of MOC patient-derived tumour (or organoid) xenografts (PDXs) as a renewable resource to evaluate their use in pre-clinical therapeutic studies.

**Material and Methods**

To establish a novel xenograft model of MOC to enable pre-clinical molecular and drug response assessment of individual MOC patients, we utilised molecularly annotated organoid lines or cryopreserved patient tissue with confirmed MOC diagnosis for implantation into NOD-SCID-IL2rg mice. We compared the engraftment efficiency of standard transplantation approaches including subcutaneous, intraperitoneal in addition to the ovarian intrabursa which is the orthotopic site. To optimise the model, we generated a panel of MOC organoids expressing luciferase and green fluorescent protein (GFP) to allow for *in vivo* monitoring of orthotopic tumours. Tumours from successful xenografts have been serially transplanted, with each passage biobanked and available for pre-clinical studies. Haematoxylin and Eosin staining, immunohistochemical staining, western blotting and genomic profiling will be used to compare the expression signatures of the transplanted tumours with the original patient tumour (or organoids).

**Results and Discussions**

To date, we have transplanted four cryopreserved tissues and six luciferase-expressing organoid lines for the establishment of PDXs. Mice are monitored for tumour development by bioluminescence imaging and/or calliper measurement, and preliminary phenotyping results from three successful lines indicate that parental tumour fidelity is retained.

**Conclusion**

In summary, our established PDXs will serve as pre-clinical tools to investigate cellular and molecular mechanisms of drug sensitivity and resistance thus providing a unique model system to identify and validate biomarkers and to develop new therapeutic approaches.

### EACR23-0690
### Combination of immune check points inhibitors with conventional chemotherapeutic agents in metastatic tumor models

*S. Joshi[1], B. Ramachandran[1], S. Kumar[1], R. Venkidusamy[1], R. Patil[1], A. Meru[1], K. Haladasappa[1]*
*[1]Adgyl Lifesciences Private Limited- Formerly Invivo Business Unit of Eurofins Advinus Ltd, Pharmacology, Bangalore, India*

**Introduction**

Immunotherapy has revolutionized cancer treatment paradigm with promising approach and strategy for modulating tumor growth and metastatic spread with substantial improvement in patient outcomes. In less than a decade, anti-PD1 therapy has progressed practical therapeutic approach for primary and metastatic tumours. There are research gaps in preclinical evaluation of anti-PD1 antibodies in metastasis and advantage of combining immune check point inhibitors with chemo agents is an upcoming area of preclinical research.

**Material and Methods**

In this study we evaluated the preclinical efficacy of mouse PD1 antibody in three experimental lung metastasis models Lewis lung carcinoma (LLC), MCA205 and B16F10 in C57BL/6 mice. The early-stage disease was modelled by intravenous injection of LLC, MCA205, B16F10 cells via tail vein in mice. Three days later disease induction mimics the clinical presence of micrometastases and treatment was initiated with anti-PD1 10mg/kg,ip;Q4Dx4 doses and standard of care (Cisplatin 5mg/kg,i.v;Q5Dx4 doses or Doxorubicin 4mg/kg,i.v;Q4Dx4 doses). The body weight, clinicals signs, mortality were monitored upto 21 days. At the end of the study, lungs were harvested, weighed, perfused with Indian ink, and fixed in formalin for LLC & MCA205 models. For B16F10 model the lungs were perfused and fixed in Bouin's solution for histopathology evaluation.

**Results and Discussions**

In MCA205 model, anti-PD1 therapy efficiently abolished and significantly reduced the incidence of no. of metastatic lungs nodules and lung weight compared to vehicle control. In LLC model, anti-PD1 treatment resulted in moderate reduction of no. of metastatic lung nodules. In B16F10 model, the anti-PD1 treatment resulted in marginal metastasis inhibition when compared to standard chemotherapy treatment regimen. Further, histopathological examination of the lung tissues of LLC or MCA205 or B16F10 cells revealed significant number of metastatic pulmonary nodules with clear progressive

© 2023 The Authors. Molecular Oncology is published by John Wiley & Sons Ltd on behalf of Federation of European Biochemical Societies pattern in vehicle control group. Based on the data, the degree of efficacy ranking for anti-PD1 is MCA205>LLC>B16F10.The exact mechanism of inhibiting pulmonary metastasis in the LCC and MCA205 models remains to be investigated.

**Conclusion**

The current data demonstrated the anti-metastatic activity of anti-PD-1 antibody in combination with chemo agents. Further mechanistic studies are required to understand the anti-metastatic efficacy in preclinical models and might pave road for potential clinical evaluation of combination treatment in patients.

### EACR23-0691
### Investigating the composition and the spatial relationship of the metastatic brain tumour immune and extracellular matrix microenvironment

*S. Widodo[1], M. Dinevska[1], L. Cuzcano[2], M. Papanicolaou[3,4], T. Cox[3,5], S. Stylli[1,6], T. Mantamadiotis[1,2,7]*
*[1]University of Melbourne, Department of Surgery Royal Melbourne Hospital, Melbourne, Australia*
*[2]University of Melbourne, Department of Microbiology and Immunology, Melbourne, Australia*
*[3]The Garvan Institute of Medical Research and The Kinghorn Cancer Centre, Cancer Ecosystems Program, Sydney, Australia*
*[4]University of Technology Sydney, School of Life Sciences, Sydney, Australia*
*[5]University of New South Wales St Vincent's Healthcare Clinical Campus, School of Clinical Medicine, Sydney, Australia*
*[6]Royal Melbourne Hospital, Department of Neurosurgery, Parkville, Australia*
*[7]University of Melbourne, The Centre for Stem Cell Systems, Melbourne, Australia*

**Introduction**

Metastatic cancer is accountable for most cancer-related deaths and is the most common neoplasms affecting the central nervous system. The metastatic tumour biology is regulated by multiple cellular and non-cellular components in the tumour microenvironment (TME). However, the composition of these components, as well as how tumour cell origin influences this composition is not deeply understood.

**Material and Methods**

By combining multiplex immunohistochemistry and histopathological staining, and performing spatial analysis on metastatic brain tumour tissue, we investigated the composition and the spatial relationship between neoplastic cells, immune cells, and the extracellular matrix (ECM).

**Results and Discussions**

Our results showed that metastatic brain tumours display a unique ECM composition compared to glioblastoma, the most common primary brain cancer, including differences in collagen fibre density. Infiltrating macrophages and T-cells are the most common immune cells in metastatic brain tumors, with an enrichment of these cells within ECM-rich regions. The dominant macrophage subtype in metastatic brain tumors are immunosuppressive/anti-inflammatory macrophages, which preferentially localize to these ECM-rich regions.

**Conclusion**

Overall, our data shows that macrophages and T-cells are restricted within ECM-dense regions, which may prevent these cells from interacting with, and effectively killing neoplastic cells. We also identified tumor infiltrating macrophages as the likely key immune cells involved in establishing an immunosuppressive TME. The findings support the view that optimal therapy for patients with metastatic brain cancer would include drugs which modify the ECM, and drugs which target immunosuppressive macrophages, in addition to cytotoxic therapies.

### EACR23-0697
### Platelet-derived extracellular vesicles alter metastatic melanoma cell behavior in tumor microenvironment

*Z. Tavukcuoglu[1], A. de Sousa Faria[1], M. Palviainen[1], P.R.M. Siljander[1]*
*[1]University of Helsinki, Faculty of Biological and Environmental Sciences, Helsinki, Finland*

**Introduction**

Skin melanoma is one of the most aggressive and deadliest of cancers. In addition to hemostasis, platelets play a role in cancer progression. Upon activation platelets generate platelet-derived extracellular vesicles (PEVs). PEVs are thought to educate cancer cells to favor cell proliferation and enhance metastasis. Here, we investigated how the treatment of melanoma cells with different PEVs affected the 3D melanoma spheroid growth and transcriptional landscape.

**Material and Methods**

A set of agonists were used to generate PEVs, which were isolated by size exclusion chromatography and analyzed according to the MISEV2018 guidelines. Melanoma cells (MV3) were cultured as 3D spheroids by using GrowDex hydrogel. Spheroids were then treated with or without PEVs every 48 hours for 5 days. Spheroids were collected at 6h and 24h time-points after the last PEV treatment. RNA was isolated from spheroids by Trizol reagent and whole mRNA sequencing was performed with Illumina NovaSeq platform.

**Results and Discussions**

The growth of the MV3 spheroids was altered depending on the type of PEVs used to educate the spheroids. CLEC-2- and GPVI- induced PEVs increased significantly the spheroid size of the MV3 cells compared to non-treated cells. The gene expression of PEV-educated MV3 cells were altered showing differences e.g. in the interferon pathway.

**Conclusion**

Platelets and their EVs are novel immunomodulatory agents in the tumor microenvironment. PEVs modify the functions of 3D melanoma spheroids, e.g. proliferation and survival agonist-dependently. The study illuminates some of the possible molecular mechanisms associated with the crosstalk between PEVs and cancer cells, which may provide novel targets for therapeutics.

© 2023 The Authors. Molecular Oncology is published by John Wiley & Sons Ltd on behalf of Federation of European Biochemical Societies

### EACR23-0699
### Cancer-Associated Fibroblasts Demonstrate Significant Impact in Ovarian Cancer Cell Proliferation and Drug Response

G. Gudoityte[1], O. Sharma[1], L. Leuenberger[1], E. Wallin[2], J. Fernebro[2], U. Joneborg[2], O. Kallioniemi[1], B. Seashore-Ludlow[1]
[1]Karolinska Institutet, Oncology-Pathology, Solna, Sweden
[2]Karolinska Institutet, Women's and Children's Health, Solna, Sweden

**Introduction**

Ovarian cancer (OC) is a leading cause of gynecological cancers in women. Despite treatment advances, most patients succumb to disease. Little is known of how different microenvironmental features contribute to OC progression and response to treatment. Cancer-associated fibroblasts (CAFs) play a crucial role in tumor microenvironment, where they establish a complex network of cellular and molecular interactions. Studies suggest that CAFs can both suppress and support disease progression. Hence, here we sought to systematically investigate the impact of CAFs on cell proliferation and drug response in high-grade OC.

**Material and Methods**

To capture the impact of CAFs and CAFs conditioned media on OC cell proliferation we developed co-culture system where pre-stained cells were continuously imaged for 72 h. Additionally, cytokine profiling was conducted using Olink proteomics[a] to evaluate changes in signaling molecule secretion. To characterize alterations in drug response induced by co-culture, we employed a library of 528 different drugs and imaged after 72 h of treatment.

**Results and Discussions**

Our data shows significant impact of CAFs on OC cells proliferation, both in a contact-dependent and independent manner. Of the five cancer cell lines tested, one (TykNu) demonstrated unaltered proliferation rate regardless of environmental changes. Cytokine profiling analysis indicated altered secretion of molecules between cancer and fibroblast, with distinct profiles depending on each pair. HGF, IL-6, VEGFA, TFPI-2 were among the most altered cytokines under different experimental conditions. Interestingly, patient-derived fibroblasts had higher cytokine secretion compared to normal fibroblasts. In our drug screen, ~70% of the drugs had a similar effect on cancer cells regardless of culturing conditions. However, ~25% of the drugs were more effective in cells grown in co-culture, with only few showing higher effect in monoculture (~1%). Interestingly, drugs commonly used in ovarian cancer treatment (Carboplatin, Paclitaxel, Gemcitabine) exhibited stronger effect on co-culture. Hence, these results highlight the crucial need for further research to uncover the mechanisms causing the impact of stroma on OC response to the drugs and disease development.

**Conclusion**

These findings demonstrate impact of fibroblasts in cancer cell proliferation, cytokine production and drug response. This study highlights the importance of taking the microenvironment into account in drug discovery and functional precision medicine applications.

### EACR23-0705
### IL-9 driven pseudohypoxia promotes metastasis in Cutaneous T-cell lymphoma (CTCL) through the HIF-1α/Cofilin-1 axis

D. Mukherjee[1], S. Marathe[1], D. Attrish[1], V. Sawant[1], S. Wad[1], B. Dhamija[1], N. Sharma[2], H. Jain[2], R. Purwar[1]
[1]Indian Institute of Technology Bombay, Biosciences and Bioengineering, Mumbai, India
[2]Tata Memorial Hospital, Medical oncology, Mumbai, India

**Introduction**

In advanced-stage Cutaneous T-cell lymphoma (CTCL), malignant cells metastasize to internal organs. While IL-9 is known to confer severe tumor progression via increased malignant T-cell survival, the role of IL-9 in malignant T-cell metastasis remains poorly examined. In this study, we inspected the roles of IL-9 in T-cell migration and delineated the underlying mechanism of metastasis in CTCL.

**Material and Methods**

CTCL patients (n=5) and healthy individuals (n=5) were recruited and their skin biopsies were used to make formalin-fixed paraffin-embedded (FFPE) sections. Using 3D-migration assays and live-cell imaging, the effect of IL-9 in cell migration was quantified in healthy and malignant T-cells.  High-throughput label-free quantitative proteomic analysis was performed to identify the mechanism of pseudohypoxia-mediated migration. IL-9-mediated protein dysregulations and pathways were validated using western blotting, immunofluorescence (IF), and other functional assays.

**Results and Discussions**

IL-9 significantly increased the migration potential of Jurkat cells as well as healthy activated T cells by 2-fold, in an ECM-like environment. Western blotting revealed increased levels of HIF-1α in IL-9-stimulated malignant and activated T-cells, indicating the roles of IL-9 in pseudohypoxia. Moreover, upon chemical induction of pseudohypoxia, malignant T cells showed augmented migration potential. To further elucidate the pseudohypoxia-induced protein networks, label-free proteomics profiling was performed in Jurkat cells. The data revealed protein clusters with enhanced expression of Cofilin-1 which is known to enhance metastasis in several cancers through actin cytoskeleton remodeling. To functionally validate these findings, pharmacological inhibition of HIF-1α function by echinomycin attenuated the migration of malignant T cells with concomitant inhibition of Cofilin-1 expression, thus highlighting the IL-9/HIF-1α/Cofilin-1 axis in malignant T-cell metastasis. Finally, there was overexpression of HIF-1α and Cofilin-1 in CTCL skin biopsies, underscoring the importance of this axis as a clinically relevant therapeutic target in CTCL.

**Conclusion**

Through this study, we identified and validated the critical association of the IL-9/HIF-1α/Cofilin-1 axis in CTCL tumor metastasis. Our data thus supports echinomycin-based targeting of HIF-1α as a potential anti-metastatic therapy in T-cell malignancies.

© 2023 The Authors. Molecular Oncology is published by John Wiley & Sons Ltd on behalf of Federation of European Biochemical Societies

### EACR23-0726
### Protective effect of primary- or peritoneal metastatic- derived colorectal cancer fibroblasts on tumor spheroids upon chemotherapeutic treatment

*V. Gerakopoulos[1], S. Vintila[1], V. Nori[1], M. Sachet[1], C. Müller[1], C. Ramos[1], R. Oehler[1]*
*[1]Medical University of Vienna, General Surgery, Wien, Austria*

**Introduction**
Colorectal cancer (CRC) is one of the most common malignancies. Different chemotherapeutic regimes are applied to primary CRC patients, compared to peritoneal metastatic CRC (pmCRC). We have previously shown that fibroblasts from pCRC (CAFs) are different from pmCRC ones (MAFs), in terms of their effect on the immune system and the secretion of different factors of the IGF pathway, including IGF1 and IGFBP2. Here, we would like to test if CAFs and MAFs offer to tumor cells different levels of protection against chemotherapy.
**Material and Methods**
Previously, CAFs and MAFs were cultured in 2D and conditioned medium (CM) was collected. In this study, spheroid formation was induced in CG08 cells, a pmCRC cell line. 3D cultures were grown in the presence of basal growth medium supplemented or not with MAF or CAF CM, or in co-culture experiments in the presence or not of CAFs or MAFs. Spheroids were treated with doxorubicin, a drug used during HIPEC (Hyperthermic intraperitoneal chemotherapy) in peritoneal metastatic CRC and viability was measured using the CellTiter Glo 3D assay.
**Results and Discussions**
Addition of CAF or MAF CM in the basal medium, or co-culture of tumor spheroids with CAFs or MAFs, significantly increased chemoresistance, compared to spheroids cultured alone. On average, CAFs showed a trend to induce higher chemoresistance, in comparison to MAFs. Notably, individual CAFs and MAFs showed different levels of protection. Currently, more experiments are in progress.
**Conclusion**
Our assay recapitulates the in vivo crosstalk between tumor cells and fibroblasts in a more reliable manner compared to single cultivation of tumor cells and it is a promising surrogate assay for drug testing in vitro. CAFs and MAFs protect tumor cells against doxorubicin. This protective effect varies based on tissue location (pCRC vs pmCRC) and from patient to patient, providing an aspect of personalized medicine to our assay. Our future approach could focus on evaluating additional agents used in the clinical routine of pCRC and pmCRC, either as single drugs or as combinations. In addition, further characterization of specific CAF/MAF-derived growth factors that promote spheroid drug resistance could be important for the development of therapeutic strategies in CRC.

### EACR23-0770
### Identifying drugs to simultaneously target cancer cells and microenvironmental mesenchymal stem cells

*F. Vajda[1,2], Á. Szepesi[3], E. Zsuzsa[3], K. Szebényi[1], K. Német[3], G. Szakács[1], F. András[1,4]*
*[1]Research Centre for Natural Sciences, Institute of Enzymology, Budapest, Hungary*
*[2]Semmelweis University, Doctoral School of Molecular Medicine, Budapest, Hungary*
*[3]Creative Cell Kft., Creative Cell Kft., Budapest, Hungary*
*[4]Centre for Energy Research, Institute of Technical Physics and Materials Science, Budapest, Hungary*

**Introduction**
Tumor cells are surrounded by non-cancerous cells recruited from different parts of the body. Blood vessel cells, immune cells, fibroblasts/mesenchymal stem cells (MSCs) construct a complex cellular network called tumor microenvironment (TME). Under certain circumstances MSCs can have antitumor properties, however now it's commonly accepted that these cells are mostly supporting the growth and survival of the tumor. To identify compounds capable to kill both cancer cells and MSCs we compared the response of these cells to different treatments.
**Material and Methods**
Nine different widely used chemotherapeutical agents were tested on six human MSC and three cancer cell lines. Presto Blue based viability assay was used to determine $IC_{50}$ values at day5 in each cell line. Flow cytometry was used to analyze co-cultured MSC-gfp cells with A431-mCh cells.
**Results and Discussions**
As expected, MSCs showed lower sensitivity to cisplatin, irinotecan, vinblastine and mitoxantron due to their significantly lower proliferation rate. Surprisingly, four drugs (bendamustine, doxorubicin, methotrexate and TPEN) proved to be similarly toxic to both cancer and mesenchymal stem cells, while the wt p53 inhibitor nutlin-3a was only effective against p53 wt cells. Strikingly, despite four drugs killed both cancer cells and MSCs, neither apoptotic nor senescence pathways are activated in MSCs after 5 days of treatment, suggesting fundamentally different responses to the same therapy. Furthermore, 2D and 3D co-culturing showed that MSCs function as a 'feeder' core for cancer cells in spheroids and because of the intensive cell-cell interaction, a new double fluorescent (gfp+, mCherry+), hybrid cell population has been identified.
**Conclusion**
Our work highlights how MSCs and cancer cells respond differently to chemotherapy. These results suggest that a well-chosen combinational therapy (e.g. doxorubicin with nutlin-3a, bendamustine with mitoxantron) can be significantly more efficient therapeutic strategy, as killing the supporting cells adjacent to cancer cells deprive the tumor from important surviving factors.

### EACR23-0774
### Individualizing chemotherapy treatment protocols with algorithm-assisted therapy design: An in vivo study

*B. Gombos[1,2], A. Lovrics[1], B.G. Czakó[3], M. Siket[3], D.A. Drexler[3], L. Kovács[3], G. Szakács[1,4], A. Füredi[1,5]*
*[1]Research Centre for Natural Sciences,*

© 2023 The Authors. Molecular Oncology is published by John Wiley & Sons Ltd on behalf of Federation of European Biochemical Societies

Institute of Enzymology, Budapest, Hungary
[2]Semmelweis University, Doctoral School of Molecular Medicine, Budapest, Hungary
[3]Innovation and Service Center of Óbuda University, Physiological Controls Research Center within the Resear ch, Budapest, Hungary
[4]Medical University of Vienna, Institute of Cancer Research, Vienna, Austria
[5]Centre for Energy Research, Institute of Technical Physics and Materials Science, Budapest, Hungary

**Introduction**

Chemotherapy is one of the most effective and commonly used tools in the treatment of malignant tumors, but its use is hampered by several difficulties in the clinical setting. Most importantly, the currently used "one size fits all" strategy of therapeutic protocols, which neglects inter-patient differences in personal pharmacokinetics and response to treatment, is one of the main reasons why chemotherapy frequently fails and considered non-personlizeable. In this project, we investigate the efficacy of individualized chemotherapy protocols using a mouse model of tractable breast cancer and a novel, algorithm-assisted therapy design (AATD) approach. We present our workflow and show that chemotherapy can be substantially improved by AATD.

**Material and Methods**

The genetically engineered Brca1$^{-/-}$;p53$^{-/-}$ FVB mice were used to test AATD. By monitoring treatment response, tumor growth, drug concentrations in the blood, and several other tumor-specific parameters, we optimized therapeutic parameters such as administration time, dose, and sequencing. Pegylated liposomal doxorubicin (PLD, Caelyx®) was selected for the AATD experiments due to its superior efficacy over conventional doxorubicin.

**Results and Discussions**

Compared to conventional treatment regimens that apply the maximum tolerable doses, personalized treatment plans resulted in a significant increase in the overall survival of the mice. According to our results, AATD can enhance the therapeutic index of chemotherapy. We fine-tuned the experimental workflow to test AATD on a genetically engineered mouse model of triple negative breast cancer and to identify relevant biological parameters that can impact the effectiveness of therapy planning and treatment outcomes. Our analysis revealed that tumor size, response to treatment, and plasma drug concentration are crucial parameters that need to be accurately measured to achieve successful AATD.

**Conclusion**

AATD is a new approach for developing personalized treatment plans that can be easily implemented into the clinical practice. It uses readily available biological parameters to improve the effectiveness of conventional chemotherapy.

### EACR23-0783
### Adipose tissue prompts a permissive microenvironment for breast cancer progression.

G. Porcelli[1], M. Gaggianesi[2], A. Turdo[1], C. D'Accardo[1], F. Verona[1], V.D. Pantina[2], O.R. Brancato[2], P. Bianca[1], G. Stassi[2], M. Todaro[1]
[1]University of Palermo, ProMISE- Department of Health Promotion- Mother and C hild Care- Internal Medicine and Medical Specialties, Palermo, Italy
[2]University of Palermo, Department of Surgical-Oncological and Stomatological Sciences, Palermo, Italy

**Introduction**

Obesity (Ob) is a complex and chronic disease, recognized as a global pandemic, which correlates with the increased risk of development and progression of tumors, including breast cancer (BC). Cancer stem cells (CSCs) are regulated by paracrine interactions with the tumor microenvironment (TME), mainly influenced by adipose stem cells (ADSCs) in mammary tissue (AT). Tumor-infiltrating ADSCs release cytokines recruiting and boosting into the TME macrophage characterized by an immunosuppressive M2-like phenotype. BC patients undergo tumor recurrence and relapse, even years after diagnosis, due to the refractoriness of breast CSCs (BCSCs) to therapy. The interaction between cancer cells and M2 macrophages prompts early dissemination of cancer cells in distant organs. Disseminated cancer cells remain in a dormant state and reawake in response to different TME cues. Several studies showed that AT can influence BC dormancy, metastatic outgrowth, and immune escape. However, the precise mechanisms regarding early cancer cell dissemination remain to be clarified.

**Material and Methods**

BCSCs as well as ADSCs have been purified from BC patient tissues and characterized by specific expression markers. Cytokines released in the culture media of ADSCs were analyzed using custom Luminex assays. Moreover, BCSCs were co-cultured with Ob ADSCs, alone or in presence of monocytes for 48 hours. To evaluate changes induced by ADSCs, the presence of lipid droplets and the expression of specific markers were assessed by immunofluorescence and cytofluorimetric assays.

**Results and Discussions**

Interestingly, our results showed that our collection of BCSCs displays high expression levels of CD36, a fatty acid transporter. In fact, our BCSCs accumulate lipid droplets after co-culture with ADSCs isolated from Ob BC patients, which are also able to induce Thp1 polarization in M2 macrophages (CD163, CD206, TNFRII). Accordingly, we found in Ob BC patient tissues an elevated presence of M2 macrophages, which induce NR2F1 expression in neighboring BC cells, indicating induction of dormancy.

**Conclusion**

The obtained data pointed out that pro-inflammatory TME induces metabolic changes in BCSC and favors the recruitment of M2 macrophages. We further envision performing transcriptomic and epigenetic analysis to deeply investigate the role of ADSCs in reprogramming BCSCs and TME components. We expect that the results of our research might corroborate the development of novel effective BC anti-cancer therapeutic approaches.

### EACR23-0806
### Using Mouse 3D Decellularized Liver Extracellular Matrix Scaffolds to Model Liver Metastases in Healthy and Fibrotic Livers.

K. Perry[1], F. Tamagnini[2], P. Dash[1]

© 2023 The Authors. Molecular Oncology is published by John Wiley & Sons Ltd on behalf of Federation of European Biochemical Societies

[1]University of Reading, School of Biological Sciences, Reading, United Kingdom
[2]University of Reading, School of Pharmacy, Reading, United Kingdom

**Introduction**

Liver Metastases (LM) are the most common type of liver cancer and have a high patient mortality due to diagnostic difficulties. The most common primary sites of LMs are colon, breast, kidney and stomach. One major factor in the successful invasion and colonisation of the liver is the tumour interactions with liver microenvironment, of which the extracellular matrix (ECM) is the main component. Therefore, we are developing a 3D decellularized liver (dLiver) ECM mouse model to investigate the interactions of LMs and the liver microenvironment. Major study focuses will include cell migration speed, proliferation rates and intracellular $Ca^{2+}$ oscillations.

**Material and Methods**

We have developed an adult mouse liver decellularization system via perfusion to produce a dLiver ECM scaffold for 3D cell culture. 3D cell culture will be performed with multiple mCherry positive cancer cell lines representing common prior sites of LMs in healthy and obese mouse models (MDA-MB-231, BT-549, NCI-N87, AGS, 786-O, Caki-1 and SNU-475). In addition, DIC Timelapse microscopy will be used to track cell migration speed and proliferation on different surface coatings, and Confocal intracellular $Ca^{2+}$ imaging of cancer cell lines before, during and after 3D culture.

**Results and Discussions**

Results from Timelapse microscopy showed significant increase in cell migration speed of the MDA-MB-231 cell line on NativeCoat™ Liver ECM Surface Coating, but not on cancer cell proliferation. Results from Confocal $Ca^{2+}$ imaging showed that the MDA-MB-231 cell line had significantly higher frequency of $Ca^{2+}$ oscillations compared to 786-O cancer cell line. H&E staining of dLiver ECM scaffold showed maintained liver structure and absence of cells, showing successful decellularization.

**Conclusion**

Preliminary results indicate that cancer cell migration speed will be significantly affected during 3D culture in dLiver ECM scaffold. H&E staining showed the successful development of a dLiver ECM mouse model, but it is still yet to be tested for 3D cell culture. Developing a 3D cell culture dLiver ECM mouse model will allow for a deeper understanding of LMs and how they interact with the liver microenvironment. Future work includes 3D cell culture in dLiver ECM scaffold of mCherry positive cancer cell lines and complete cell characterization (migration speed, proliferation rate, morphology, and intracellular $Ca^{2+}$ oscillations) to be done before, during and after 3D cell culture in dLiver ECM scaffold.

### EACR23-0812

### P-cadherin integrates growth factor and mechanical cues during ovarian cancer metastasis

S.K.Y. To[1], K. Wang[1], V.C.L. Ng[1], A.S.T. Wong[1]
[1]The University of Hong Kong, School of Biological Sciences, Hong Kong, China

**Introduction**

Ovarian cancer is the most lethal among all gynecological cancers since patients are usually diagnosed at an advanced stage with widespread peritoneal metastasis. Overexpression of P-cadherin, a cell adhesion molecule essential for embryonic development and adult tissue maintenance, has been associated with distant metastasis and poor patient survival in ovarian cancer. Cadherins are known to integrate both biochemical and mechanical cues to mediate various biological processes, however, the microenvironmental factors that regulate P-cadherin remain largely unknown.

**Material and Methods**

Cells were treated with actinomycin D or cycloheximide at various time points, followed by Western blot and RT-PCR, to access the mRNA and protein stabilities of P-cadherin respectively. To measure rupture forces during cell-cell adhesion, atomic force microscopy-based single-cell force spectroscopy was used.

**Results and Discussions**

We showed that hepatocyte growth factor, which is abundant in the malignant ascites, could upregulate P-cadherin levels at both transcriptional and post-transcriptional levels by enhancing its mRNA and protein stability respectively. Interestingly, other soluble factors such as epidermal growth factor and tumour necrosis factor-α, did not induce any statistically significant change in P-cadherin expression. Tumor-mesothelial adhesion is the first key step for metastatic colonization. Using force spectroscopy, we further looked at the biophysical cues that could activate P-cadherin signaling. We found that metastatic cells in physical contact with mesothelial cells possessed significantly larger rupture forces as compared to non-metastatic cells. P-cadherin-specific siRNA or neutralizing antibody could reduce the rupture forces significantly by 50%, hence confirming a mechanical role of P-cadherin at cell-cell junctions.

**Conclusion**

These results suggest important roles of P-cadherin in microenvironmental sensing during ovarian cancer metastasis. (This work is supported by RGC GRF grant 17105919)

### EACR23-0826

### Novel somatic sarcoma mouse models reveal gene-specific oncogenic programs

R. Imle[1], D. Blösel[1], E. Stutheit-Zaoh[2], C. Blume[3], R. Autry[2], D. Lupar[1], P. Günther[4], S. Pfister[5], A. Banito[1]
[1]KiTZ/DKFZ Heidelberg, Soft-tissue sarcoma, Heidelberg, Germany
[2]KiTZ/DKFZ Heidelberg, Clinical bioinformatics, Heidelberg, Germany
[3]DKFZ Heidelberg, Computational Genomics and Systems Genetics, Heidelberg, Germany
[4]University Hospital Heidelberg, Pediatric Surgery, Heidelberg, Germany
[5]KiTZ/DKFZ Heidelberg, Pediatric Neurooncology, Heidelberg, Germany

**Introduction**

While sarcomas are overall rare, accounting for only about 2% of all human cancers, they are significantly overrepresented in young patients and children, where they account for about 20% of all cancers. Characterized by typically poor prognosis, a particular challenge hampering

© 2023 The Authors. Molecular Oncology is published by John Wiley & Sons Ltd on behalf of Federation of European Biochemical Societies therapeutic advancements for sarcoma patients is the incredibly broad spectrum of molecularly distinct sarcoma subtypes and the corresponding lack of suitable model systems to recapitulate such rare diseases. To overcome this predicament, we developed a fast and versatile *in vivo* modeling system to introduce different putative oncogenic genetic alterations directly into muscle tissue of adolescent (P30) and neonatal (P0) mice via *in situ* electroporation (EPO) of CRISPR- and transposon vectors.

**Material and Methods**

Muscle electroporation, optimized by *in vivo* bioluminescence imaging. Established genetically engineered mouse model (GEMM) tumors were analyzed towards histomorphology, transcriptome (RNA seq) and DNA methylation (285k array). Methods for snygeneic allograft models (SAMs) and cell line derivation were established and optimized based on preservation of histomorphology and DNA methylation profiles.

**Results and Discussions**

Somatic genetically-engineered electroporation-based mouse modeling (EPO-GEMM) allowed us to generate tumors with a total of 18 genetically distinct combinations of sarcoma-typical genes, falling into seperate groups in unsupervised clustering based on RNA-seq and DNA-methylation, including embryonal rhabdomyosracoma (eRMS), alveolar RMS, Synovial Sarcoma, Alveolar Soft Part Sarcoma (ASPS), Infantile fibrosarcoma (IFS) and undifferentiated peomorphic sarcoma (UPS). Integrated analysis of histomorphology, transcriptome and methylome revealed fusion gene expression and *Trp53* mutation status as major determinants of sarcoma biology, recapitulating human sarcoma phenotypes. Additionally, BCOR could be confirmed as a tumor suppressor in RMS and ETV-NTRK as a bona-fide sarcoma driver.

**Conclusion**

The novel EPO-GEMM method developed here, led to an unprecedented array of 10+ genetically distinct sarcoma models, recapitulating the biologically diverse spectrum of human sarcomas, including the first of a kind NTRK-driven sarcoma model. Established SAMs and cell lines showed a remarkably high correlation to parental GEMMs and provide a highly valuable resource for future preclinical treatment studies. All in all, this work marks a milestone towards a deeper biological understanding and improved therapy for sarcoma patients.

### EACR23-0861

### Claudin-10 in Blood Brain Barrier of brain endothelial cells and transendothelial invasion of breast cancer cells

<u>X. Zhuang</u>[1,2], *W. Jiang*[1], *F. Ruge*[1], *T. Martin*[1]

[1]*Cardiff University, School of Medicine- CCMRC, Cardiff, United Kingdom*

[2]*Xiamen University, School of Medicine - The First Affiliated Hospital of Xiamen University -, Xiamen, China*

**Introduction**

Claudin-10 (CLDN10) is a member of the tight junctional (TJ) Claudin family with an unubiquitous distribution in the body and with largely unknown functions. We investigated the expression profile of CLDN10 and its role in TJ, brain blood barrier of cerebral endothelium and brain metastasis of breast cancer cells.

**Material and Methods**

We tested transcript and protein expression of CLDN10 in a range of endothelial cells, breast cancer, mesothelial cells and fibroblasts. Brain endothelial cell hCMEC/D3, with or without CLDN10 knockdown, was used to create BBB models. The impact of CLDN10 expression in the hCMEC/D3 cells on the barrier and permeability properties was analysed via Transendothelial Resistance (TEER), Paracellular Permeability (PCP) and electric cell-substrate sensing (ECIS). Trans-endothelial drug delivery and breast cancer cell invasion were also evaluated.

**Results and Discussions**

Brain endothelial cells expressed high levels of CLDN10, compared with peripheral, endothelial, mesothelial and breast cancer cells which had very low CLDN10. CLDN10 knockdown rendered hCMEC/D3 with reduced electric resistance and increased paracellular permeability. Knockdown of CLDN10 in hCMEC/D3 also facilitated transendothelial invasion by breast cancer cells. Transendothelial chemodrug delivery models showed an increase in drug penetration through the CLDN10 knockdown hCMEC/D3 cell layer. Finally, inhibiting SRC pathway by SRC kinase inhibitor (SRCi) caused a decrease in impedance and an increase in permeability in BBB.

**Conclusion**

CLDN10, expressed at relative high levels in brain endothelial cells, is involved in barrier function, drug permeability and cancer invasiveness. CLDN10 may have an important influence on brain metastasis and drug therapies in brain metastasis.

### EACR23-0871

### Humanized mouse models for preclinical evaluation of novel immune cell therapies, check point inhibitors, and immune cell engagers

<u>M. Stecklum</u>[1], *A. Wulf-Goldenberg*[1], *B. Brzezicha*[2], *W. Walther*[1], *J. Hoffmann*[1]

[1]*EPO GmbH, Preclinical Immunoncology, Berlin, Germany*

[2]*EPO GmbH, PDX Models, Berlin, Germany*

**Introduction**

The preclinical evaluation of novel immune therapies demands humanized mouse models with functional human immune cells. In previous studies we have demonstrated, that either peripheral blood mononuclear cells (PBMC), subsets of PBMCs like T- and NK-cells or hematopoietic stem cells (HSC) can be used to establish a humanized immune system with functional T-, B-, and NK cells in immunodeficient mice. By transplantation of cell-line-derived (CDX) or patient-derived (PDX) tumor xenografts on humanized mice, we successfully generated a full human tumor-immune-cell model for different tumor entities. Finally, we validated the functionality of these models using checkpoint inhibitors, cell therapies and immune cell engagers.

**Material and Methods**

HSC-humanized mice were generated by i.v. transplantation of CD34+ stem cells to immunodeficient NOG mice. Engraftment of immune cells was monitored

© 2023 The Authors. Molecular Oncology is published by John Wiley & Sons Ltd on behalf of Federation of European Biochemical Societies by FACS analysis of blood samples. PBMC or isolated T- or NK-cell preparations were used to humanize mice by single or multiple i.v. injections. CDX and PDX from different entities were s.c. (i.e. lymphoma) and/or i.v. (leukemia) transplanted on those humanized mice. These models were used to evaluate novel immune therapies. Blood and tumor samples were analysed by FACS for immune cell infiltration and activation.

**Results and Discussions**

The transplanted HSCs engrafted in mice and established a functional human immune system with proliferation and differentiation. 14 weeks after HSC inoculation up to 20% of the human immune cells in the blood were functional T-cells, characterized by a high PD-1 expression. The selected CDX and PDX tumors successfully engrafted on humanized mice without significant differences in tumor growth compared to non-humanized mice. Check point inhibitor treatments induced tumor growth delay in selected models. FACS analysis of xenograft tumors revealed an increased percentage of tumor infiltrating T-cells. We identified a set of CDX and PDX models without interference with parallel injection of PBMC, T- or NK-cell preparations for the evaluation of immune cell engagers and other cell therapies.

**Conclusion**

We established human tumor-immune-cell models of different entities using CDX or PDX in combination with different donor derived immune cell subsets as effector cells.These models allow preclinical, translational studies on tumor immune biology as well as evaluation of new therapies, drug combinations and biomarker identification and validation.

### EACR23-0873

### Changes in gene expression and secretome of breast cancer upon cross-talk with tumour-associated macrophages.

A. Mazan[1], D. Mehlich[1], A. Marusiak[1]

*[1]The International Institute of Molecular Mechanisms and Machines- Polish Academy of Sciences, Laboratory of Molecular OncoSignalling, Warsaw, Poland*

**Introduction**

The cancer progression is driven not only by cancer cells themselves but also by interactions with tumour microenvironment (TME) associated cells. The largest group of immune cells in the TME are tumour-associated macrophages (TAMs), reaching up to 50% of a solid tumour mass. Additionally, we know that a high density of pro-tumourigenic TAMs is correlated with poor clinical outcomes in breast cancer. Interactions between TAMs and breast cancer cells result in enhanced cancer cell proliferation, the transition towards mesenchymal phenotype, promotion of angiogenesis, and elevated expression of the cytokines.

**Material and Methods**

We polarized monocytic THP1 cell line into M0, M1 or M2 macrophages and used them in a co-culture with breast cancer cell lines. We performed a set of experiments involving colony formation and migration assays to determine the phenotypic impact on breast cancer cells upon co-culture with macrophages. In addition, we wanted to explore the changes in secretome and gene expression under the condition of mixed culture with M2 macrophages. For this purpose, RNA sequencing and secretome mass spectrometry analysis were performed.

**Results and Discussions**

The co-culture of breast cancer cells with TAM-like M2 macrophages increased the proliferation of cancer cells. Moreover, our results indicated that breast cancer cell migration increased upon M2 co-culture. Conversely, the co-culture of breast cancer cells with M0 and M1 macrophages did not increase the proliferation or migration rate of cancer cells. Furthermore, we identified a few highly enriched candidates in the co-culture condition based on RNAseq and secretome analysis. From gene ontology analysis, we know that our targets are involved in migration, invasion, and metastasis processes.

**Conclusion**

Taken together, co-culture with M2 macrophages promotes the proliferation and migration of breast cancer cells. Based on our recent phenotypic findings and identified candidates, we will uncover and describe their role in the cross-talk between breast cancer cells and tumour-associated macrophages.

### EACR23-0891

### The effect of steatosis by ferroptosis on high fat and high oxidative stress in hepatocytes

C.S. Chen[1], S.C. Wang[2]

*[1]Kaohsiung Medical University, Medical Laboratory Science and Biotechnology, Kaohsiung, Taiwan*

*[2]Kaohsiung Medical University, Center for Liquid Biopsy, Kaohsiung, Taiwan*

**Introduction**

An iron-dependent mechanism of cell death, ferroptosis, distinct from apoptosis, occurs with intracellular iron accumulation and is associated with lipid peroxidation.Previous studies have shown that lipid peroxidation-induced ROS can lead to DNA damage and promote the progression of steatosis to advanced hepatocellular carcinoma (HCC). Therefore, we aim to study ferroptosis in a cell model with hepatocytes co-cultured with adipocytes and low dose $H_2O_2$ for a high-fat/high ROS environment *in vitro*; an animal model with high fat content and accompanying low dose carbon tetrachloride($CCl_4$) *in vivo* to observe the possibility of hepatocyte steatosis due to ferroptosis.

**Material and Methods**

Cell model: Pre-adipocytes, 3T3L1 were differentiated by adding IBMX, Dexamethasone, and Insulin (10 μg/ml). After differentiation, adipocyte culture medium(ACM) was collected. The XTT assay was used to examine the relationship between cell death and ferroptosis by a ferroptosis inducer (Erastin) in high-fat and high ROS co-culture environments.

Animal model: Mice were fed a Western diet and provided with high fructose/glucose containing sugar drinking water (WFSD) for 7 months, while receiving a monthly extra-low dose of $CCl_4$. Staining of liver sections was performed to observe histological abnormalities in each group.

qPCR: To detect the expression of iron transport-related genes.

**Results and Discussions**

© 2023 The Authors. Molecular Oncology is published by John Wiley & Sons Ltd on behalf of Federation of European Biochemical Societies

For the *in vitro* study, we obtained ACM for the high-fat environment and $H_2O_2$ for the ROS effect. They were used in the XTT cytotoxicity assay. ACM co-culture with the hepatocytic AML12 cell line increased the sensitivity of ROS-related cell cytotoxicity, which was related to ferroptosis by Erastin in the high-fat/high-ROS cell environment. However, these results can be suppressed by UAMC3203, a ferroptosis inhibitor. As for the animal model experiments, the mice had higher weight, Alanine Aminotransferase (ALT) levels, and developed tumors. Their liver section showed lipid accumulation, histological fibrosis, and iron accumulation in the WD and WD+$CCl_4$ groups, which corresponds to the IRP1 and IRP2 upregulated in both groups by qPCR result.

**Conclusion**

In conclusion, we have established the effect of ferroptosis on hepatic steatosis *in vivo* and *in vitro* in nonalcoholic steatosis hepatitis (NASH). These results may enable the development of precise health prevention strategies by inhibiting ferroptosis to prevent the occurrence of liver diseases including hepatic steatosis and HCC.

### EACR23-0908

### Semaphorin 4A-expressing bone marrow-derived myeloid cells promote tumor progression.

*S. Brundu*[1,2], *G. Sardo*[1,2], *Y. Qiu*[1,2], *G. Franzolin*[1,2], *C.F. Cojocaru*[1,2], *F. Maione*[3,4], *E. Giraudo*[1,2]
[1]*Candiolo Cancer Institute - IRCCS, Laboratory of Tumor Microenvironment, Candiolo, Italy*
[2]*University of Torino, Department of Science and Drug Technology, Torino, Italy*
[3]*Candiolo Cancer Institute – IRCCS, Laboratory of Cancer Epigenetics, Candiolo, Italy*
[4]*University of Torino, Department of Oncology, Torino, Italy*

**Introduction**

Semaphorins (Semas) and Plexins are a family of proteins first identified as axonal guidance molecules and recently discovered as regulator the tumor microenvironment and cancer growth. Accumulating evidences indicate that several Semas critically involved in the immune response and regulate tumor progression. We previously described that Sema4A, overexpressed in myeloid cells under inflammatory conditions, exerted a pro-angiogenic effect. Despite its established role in regulating immune cell functions, until now Sema4A role in tumor-associated inflammation and cancer progression is still controversial and poorly investigated.

**Material and Methods**

We used two mouse: i) an orthotopic mouse model of pancreatic ductal adenocarcinoma (PDAC); ii) a transgenic mouse model of spontaneous multistep tumorigenesis of HPV16-induced cervical cancer (HPV16/E2). Moreover, by means of lentiviral shRNA technology we efficiently silenced Sema4A in bone marrow (BM)-derived myeloid cells in tumor bearing PDAC or HPV16/E2 mice to study the role of Sema4A during tumor progression.

**Results and Discussions**

We observed increased levels of Sema4A and its receptors in PDAC and HPV16/E2 compared with normal tissues. Interestingly we noticed that Sema4A was mainly expressed by myeloid cells and particularly by pro-tumoral M2-like macrophages. In addition, Sema4A was expressed in wild type BM myeloid cells and up-regulated in PDAC or HPV16-derived BM. Stemming from these findings, we efficiently silenced Sema4A in BM cells in tumor bearing from the two models and we demonstrated that the lack of Sema4A expression significantly hampered tumor growth e metastasis spreading compared with controls.
Notably, we observed that Sema4A silencing induced a shift from M2 toward M1 anti-tumor phenotype, to decrease regulatory Tregs and to enhance the recruitment of $CD8^+$ T cells, along with induction of tumor vessel normalization in both PDAC and HPV16/E2 models.
Finally, we observed that Sema4A promotes proliferation and migration of PDAC and that the depletion of PlexinB1 in these cells, reverted these effects.

**Conclusion**

We demonstrated that Sema4A express by BM myeloid cells contributes to tumor progression by acting on tumor-associated macrophages and T cells in PDAC and cervical cancer, and that its receptor PlexinB1 may be mainly involved in Sema4A-induced tumor growth and invasiveness. In conclusion, Sema4A representing a novel predictive biomarker and a new potential therapeutic target to inhibit the progression of pancreatic and cervical cancers.

### EACR23-0923

### Role of the NADPH oxidase NOX4 in the liver tumor microenvironment

*R. Espinosa Sotelo*[1], *I. Peñuelas-Haro*[1], *M. Herranz-Itúrbide*[1], *D. Caballero-Díaz*[1], *E. Crosas-Molist*[1], *E. Bertran*[1], *I. Fabregat*[1]
[1]*Bellvitge Biomedical Research Institute IDIBELL, TGF-β and Cancer Group. Oncobell Program, L'Hospitalet de Llobregat- Barcelona, Spain*

**Introduction**

The progression of hepatocellular carcinoma (HCC) is known to be a complex process involving different mutations in hepatocytes, caused by a continuous inflammatory environment where Reactive Oxygen Species (ROS) likely contribute to an oxidative stress and its consequences. The NADPH oxidase NOX4, downstream from the Transforming Growth Factor (TGF)-β pathway, has been proposed as relevant regulator of liver tumor cell proliferation and invasion, playing a tumor suppressor function. However, whether NOX4 expression (either in the tumor cell or in the stroma cells) influences liver tumor microenvironment, regulating fibroblast activation or inflammation, remains unidentified.

**Material and Methods**

Wild Type (WT) or NOX4-/- mice (14 days-old) were treated either with PBS or Diethylnitrosamine (DEN), as a model of induced experimental hepatocarcinogenesis. Tissues (tumor and non-liver tumor areas) were gathered at 9 months and 11 months of treatment. Additionally, in order to know whether the expression of NOX4 is relevant for the observed effects, either in the tumor cells or in the immune cells, we have started doing in vitro experiments, 3D co-cultures of activated macrophages and HCC spheroids, where the expression of NOX4 will be modulated through silencing NOX4 with shRNA.

**Results and Discussions**

© 2023 The Authors. Molecular Oncology is published by John Wiley & Sons Ltd on behalf of Federation of European Biochemical Societies

Results in mice have shown significant differences in the inflammatory tumor microenvironment, pointing to a role for NOX4 in macrophage activation under liver tumorigenesis.

**Conclusion**

Loss of NOX4 in HCC might influence not only the tumor cell behaviour, but also the tumor microenvironment.

*Grants to IF from Ministry of Science, Innovation and Universities (cofounded by FEDER funds/Development Fund—a way to build Europe): RTI2018-094079-B-100, PID2021-1225510B-100. FPI fellowship to RE-S.: PRE2019-089144. Grants to Red REDOX: RED2018-102576-T. This study is funded by Spanish Association for Cancer Research (AECC): PRYGN211279FABR. The CIBEREHD, National Biomedical Research Institute on Liver and Gastrointestinal Diseases, is funded by the Instituto de Salud Carlos III, Spain. We thank CERCA Programme/Generalitat de Catalunya for institutional support.*

### EACR23-0954

### ER+ breast cancer drug-tolerant persister cells are metabolically reprogrammed and vulnerable to inhibition of oxidative phosphorylation

*S. Tau[1], H. Yang[1], A. Roberts[1], C. Dragnev[1], M. Carmichael[1], L. Cressey[2], E. Greiner[2], F. Kolling[3], A. Kettenbach[2], T. Miller[1]*

*[1]Dartmouth College, Molecular and Systems Biology, Lebanon, United States*

*[2]Dartmouth College, Biochemistry and Cell Biology, Lebanon, United States*

*[3]Dartmouth College, Biomedical Data Science, Lebanon, United States*

**Introduction**

Approximately 30% of patients with ER+ breast cancer experience recurrence. Minimal residual disease whereby cells persist despite endocrine therapy is associated with risk of recurrence. Mechanisms of resistance to endocrine therapy have been shown, but the biology underlying persistence during endocrine therapy and potential therapeutic vulnerabilities are poorly understood.

**Material and Methods**

Persister cells were generated through hormone deprivation and fulvestrant treatment *in vitro* and *in vivo*. Persister ontogeny in xenografts was evaluated by variant profiling of exomes and barcode composition. Persister vulnerabilities were ascertained by genome-wide CRISPR/Cas9 knockout screening. Metabolic states were profiled by proteomics and $^{13}$C metabolic flux analysis. Dependence upon oxidative phosphorylation (OXPHOS) was tested using IACS-010759 (IACS), an inhibitor of mitochondrial complex I.

**Results and Discussions**

Clonal composition by temporal SNP profiling revealed maintenance of clonal distribution in endocrine-persistent xenografts, while recurrent tumors showed marked shifts. Barcode profiling revealed that while rare clones were lost over time, clones abundant at baseline were maintained during persistence, suggesting that the ability to persist is an adaptive response. CRISPR/Cas9 knockout screening identified mitochondrial metabolism and OXPHOS as critical pathways in persister cells. Proteomics revealed decreased levels of glycolytic proteins and maintenance of mitochondrial proteins. Estrogen restoration reversed proteomic profiles toward glycolysis. Metabolomic flux analysis with $^{13}$C-glucose revealed decreased labeling of intermediates in glycolysis, the pentose phosphate pathway, and the tricarboxylic acid (TCA) cycle in persister cells compared to estrogen-driven control cells. In contrast, $^{13}$C-oleic acid labeling revealed increased labeling of TCA cycle intermediates in persisters, indicating alternative fuel source preferences to drive ATP synthesis in persister cells. Persisters were more sensitive to IACS treatment compared to parental cells due to an increased reliance on mitochondrial OXPHOS. Similarly, IACS was able to induce tumor regression in a PDX model persistent to fulvestrant treatment.

**Conclusion**

Metabolic reprogramming is an inducible phenotype in ER+ breast cancer endocrine-tolerant persister cells and OXPHOS is a metabolic vulnerability.

### EACR23-0959

### Fusion-derived M13SV1 hybrid cells displayed an abnormal high level of mitotic aberrations that may contribute to the reproduction of hybrid daughter cells with novel genotypic traits

*J. Weiler[1], T. Dittmar[1]*

*[1]Witten/Herdecke University, Immunology- Center for Biomedical Education and Resear ch ZBAF, Witten, Germany*

**Introduction**

Cancer cell formation is presumably one of the most serious consequences for the organism due to its common property to invade and destroy healthy tissue structures resulting in the physiological collapse of the individual. The knowledge about how a non-cancerous cell can convert into a cancer-forming cell may contribute to the development of new cancer therapies useful to prevent such a catastrophic outcome. In this study, we highlighted the consequences of aberrant cell fusion among breast epithelial cells leading to the evolution of polyploid hybrid cells whose further underwent abnormal mitoses to produce poly-aneuploid and genomic-instable daughter cells.

**Material and Methods**

Two M13SV1 breast epithelial cell populations, each expressing a distinct fluorescence-tagged H2B protein, were co-cultivated and spontaneously derived hybrid clones were picked based on their simultaneously expression of H2B-GFP and H2B-mCherry. qPCR of early and late passages hybrids was conducted to analyse changes in cell-cycle gene expression essential for G1/S-phase and M-phase progression. Long-term live imaging was used to analyse the individual cell fate of early and late passages hybrids across two generations. Confocal-laser scanning microscopy was performed to investigate DNA damage by immunostaining of γ-H2AX, as well as to acquire z-stacks images to visualize aberrant mitotic events in 3D. DNA damage in distinct cell-cycle stages was determined by co-staining with propidium iodide and immunolabelling of γ-H2AX via flow cytometry.

© 2023 The Authors. Molecular Oncology is published by John Wiley & Sons Ltd on behalf of Federation of European Biochemical Societies

**Results and Discussions**
Results indicated that cyclin D2 was abundantly overexpressed in all hybrids at low passage, while only 3 of 5 hybrids revealed a constant upregulation of cyclin D2 at late passage. Cell fate analysis of early and late passages hybrid cells showed a high frequency of mitotic aberrations, such as multipolar cell division, multi- and micronucleation or endomitosis. This unusually high number of aberrations remains at a similar level in the second cell generation, suggesting that genomic imbalance persists across generations. Data referred to DNA damage analysis of the same passages hybrids confirmed that high-number cell fate anomalies were associated with an elevated degree of DNA damage in those hybrids.
**Conclusion**
The study demonstrated that one single aberrant cell fusion event might give rise to the formation of hybrid cells with new genotypic variations by which each mitotic round could potentiate the risk to gain hybrid daughter cells with cancer-initiating properties.

### EACR23-0960
### Fecal miRNA profiles and metagenome composition in a mouse model for Lynch syndrome and in humans carrying the disease mutations

*G. Francescato[1], G. Ferrero[2], M. Beltrà Bach[2], S. Tarallo[3], C. Bruggeling[4], F. Penna[2], P. Costelli[2], A. Boleij[4], B. Pardini[3], A. Naccarati[3]*
*[1]Italian Institute for Genomic Medicine IIGM, Genetic and Molecular Epidemiology, Candiolo TO, Italy*
*[2]University of Turin, Department of Clinical and Biological Sciences, Turin, Italy*
*[3]Candiolo Cancer Institute- FPO-IRCCS, Genetic and Molecular Epidemiology, Candiolo, Italy*
*[4]Radboud Institute for Molecular Life Sciences RIMLS- Radboud University Medical Center, Department of Pathology, Nijmegen, The Netherlands*
**Introduction**
Lynch Syndrome (LS) is an inherited disorder defined by heterozygous germline mutations at DNA mismatch repair (MMR) genes leading to a dysfunctional post-replication repair system. Affected individuals present an increased lifetime risk of developing many types of cancers, mainly colorectal cancer (CRC), at earlier age compared to sporadic forms.
It is known that gut microbiome and epigenetic factors are involved in CRC development. In this sense, the concomitant analysis of stool host microRNA (miRNA) profiles and microbiome composition may allow the identification of specific fecal markers in LS that could reflect the gut alterations associated with the development of precancer/cancer lesions in these patients.
The aim of this study is the evaluation of the changes in fecal miRNAs released by the host and the microbial population residing in the gut during the carcinogenesis process induced in a mouse model for LS. Moreover, we will compare the results with those obtained from a cohort of human subjects affected by LS.
**Material and Methods**
We performed small RNA-sequencing (sRNA-seq) and shotgun metagenomics sequencing analyses in stool samples collected at five different time points (months of age) from a conditional knockout mouse with a tissue-specific inactivation of Msh2 in the intestinal mucosa (*$VCMsh^{loxP}$*).
In concomitance, we collected stool samples of 78 LS subjects. At the sampling, 36 out of 78 were negative for intestinal adenomas/lesions, 12 negative but with a previous history of lesions, 23 with a neoplasia and 6 developed a lesion during the follow-up. We used 22 stool samples from healthy subjects as controls.
**Results and Discussions**
In mice, preliminary results from sRNA-seq and metagenomics analyses showed several dysregulated miRNAs and differential microbial relative abundances, either between *$VCMsh2^{loxP}$* and controls, or at different time points.
In humans, we identified 38 fecal miRNAs whose levels significantly increased (n=17) or decreased (n=21) in LS patients when compared to healthy controls. In addition, levels of 23 miRNAs were observed as significantly altered in LS who presented a lesion at sampling compared to those with negative colonoscopy.
**Conclusion**
This is the first study characterizing the concomitant alterations in microbial composition and host fecal miRNome overtime in relation to the onset of cancerous/precancerous lesions in affected patients and an in vivo model that recapitulates human pathology.

### EACR23-0964
### Dual specificity phosphatase-13(DUSP13) is associated with disease progression in ER-positive breast cancers.

*X. Liu[1], Q. Dou[2], W.G. Jiang[1], L. Ye[1]*
*[1]Cardiff University School of Medicine, Cardiff China Medical Research Collaborative- Division of Cancer and Genetics, Cardiff, United Kingdom*
*[2]Barbara Ann Karmanos Cancer Institute, Departments of Oncology- Pharmacology and Pathology- School of Medicine- Wayne State University, Detroit, United States*
**Introduction**
Dual-specificity phosphatases (DUSPs) are a group of phosphatases that can dephosphorylate various substrates. Recent studies showed that DUSP13 prevented TGF-β1-promoted migration of lung cancer adenocarcinoma cells and a putative link with microvascular invasion in hepatocellular carcinomas. However, the exact biological function and implication in malignant tumours are yet to be investigated. The present study determined the expression of DUSP13 and ascertain the relationship between the expression and clinical outcomes.
**Material and Methods**
An established fresh frozen breast cancer tissue cohort with both breast cancer tissues (n=127) and normal background tissues (n=35) was used to quantitatively examine the transcript level of DUSP13. The RNA-seq data from TCGA breast clinical cohort was also used to detect the correlation between the expression of DUSP13 and clinical characteristics. The expression of DUSP13 was assessed with histological, prognostic, and clinical factors of patients using Mann-Whitney and ANOVA tests with Minitab. Kaplan-Meier test was conducted for the overall

© 2023 The Authors. Molecular Oncology is published by John Wiley & Sons Ltd on behalf of Federation of European Biochemical Societies survival (OS) analysis using SPSS and the Kaplan-Meier Plotter (www.Kmplot.com).

**Results and Discussions**

Increased DUSP13 transcript levels were observed in histological grade 2&3 tumours compared with grade 1 tumours (p=0.045) shown by Mann Whitney Test in breast tumour tissues. An association between the expression of DUSP13 and TP-53 mutation status was found in TCGA public cohort as DUSP13 was highly expressed in TP53-NonMutant compared with tumours with mutant TP53 (P=0.00068). With TCGA samples, increased DUSP13 expression was displayed in advanced diseases according to the TNM stages: Stage1-vs-Stage3 (P=0.046) and Stage3-vs-Stage4 (P=0.032). There is a link between the higher expression level of DUSP13 and lymph node metastasis in comparison with its expression in tumours without the involvement of regional lymph nodes (P=0.0003) in the TCGA cohort. High expression of DUSP13 was associated with shorter overall survival (OS) in patients with ER-positive breast cancer including both Luminal A (P=0.00063, HR=1.94) and Luminal B (P=0.0049, HR=2.43) subtypes and also poorer OS in patients who had endocrine therapies (P=0.0072, HR=1.48).

**Conclusion**

Taken together, increased DUSP13 expression is associated with poor histological grade and disease progression, especially in ER-positive breast cancers which show resistance to endocrine therapy, which provokes a full investigation.

### EACR23-0969
### IL-1β+ macrophages fuel pathogenic inflammation in pancreatic cancer

*N. Caronni[1], F. La Terza[1], F. Vittoria[1], L. Mezzanzanica[1], V. Cuzzola[1], P. Cappello[2], F. Novelli[2], M. Genua[1], G. Barbiera[1], R. Ostuni[1]*
*[1]IRCCS San Raffaele Scientific Institute, San Raffaele Telethon Institute for Gene Therapy SR-Tiget, Milano, Italy*
*[2]University of Turin, Department of Molecular Biotechnology and Health Sciences, Torino, Italy*

**Introduction**

Pancreatic ductal adenocarcinoma (PDAC) is a lethal disease with high resistance to therapies. Inflammatory and immunomodulatory signals co-exist in the tumor microenvironment (TME), leading to dysregulated reparative and cytotoxic responses. Tumor-associated macrophages (TAMs) control immune dynamics in the TME, but their heterogeneity and plasticity have hampered our understanding of the underlying mechanisms.

**Material and Methods**

We combined single-cell and spatial genomics with functional experiments to elucidate macrophage functions in human and murine models of PDAC.

**Results and Discussions**

We uncovered, both in human and murine PDAC, distinct tumor niches whereby a subpopulation of tumor cells establishes an inflammatory loop with interleukin (IL)-1b$^+$ TAMs, a subset of macrophages elicited by a local synergy between tumor-derived prostaglandin $E_2$ ($PGE_2$) and tumor necrosis factor (TNF)-a. The propagation of the inflammatory loop is facilitated by the spatial proximity between cancer cells and IL-1b$^+$ TAMs. These data link the spatial heterogeneity of tumor cells with the different immune populations that infiltrates PDAC. Interfering with the $PGE_2$-IL-1b axis elicited TAM reprogramming and antagonized tumor cell-intrinsic and -extrinsic inflammation, leading to PDAC control *in vivo*. IL-1b$^+$ TAMs are conserved across human cancers and correlate with patient survival in a context-dependent manner.

**Conclusion**

Targeting IL-1β$^+$ TAMs may represent a powerful therapeutic strategy to reprogram immune dynamics in cancer.

### EACR23-0973
### Hypoxic tumour cells survive and remain quiescent before giving rise to tumour relapse after radiotherapy

*A. Menegakis[1], C. Vennin[2], J. Ient[3], A. Groot[3], L. Krenning[1], M. Schubert[1], R. Klompmaker[1], A. Friskes[1], M. Ilic[1], R. Harkes[4], J.J. Sonke [5], M. De Jong[5], J. van Rheenen[2], M. Vooijs[3], R. Medema[1]*
*[1]Netherlands Cancer Institute NKI, Cell Biology, Amsterdam, The Netherlands*
*[2]Netherlands Cancer Institute NKI, Molecular Pathology, Amsterdam, The Netherlands*
*[3]Maastricht University Medical Centre, Department of Radiation Oncology Maastro-GROW School for Oncology and Developmental Biology, Maastricht, The Netherlands*
*[4]Netherlands Cancer Institute NKI, Bioimaging facility, Amsterdam, The Netherlands*
*[5]Netherlands Cancer Institute NKI, Department of Radiation Oncology, Amsterdam, The Netherlands*

**Introduction**

The presence of tumor hypoxia consists a negative prognostic factor leading to poor treatment outcome in solid tumors that are treated with radiotherapy. However, the way that hypoxia compromises the efficacy of therapy is not well understood, partly because efforts to tag and trace hypoxic cells have been unsuccessful. Here, we have validated a hypoxia reporter and utilize it to understand the dynamics of individual hypoxic cells prior, during and post-treatment.

**Material and Methods**

We used H1299 lung adenocarcinoma cells expressing a novel lineage-tracing reporter based on the HIF1a-CreERT2-UnaG reporter, which upon treatment with tamoxifen drives the sustained expression of UnaG in HIF1a-expressing cells. Using this, we can tag and trace hypoxic cells over time. We applied live-cell and multiphoton microscopy along with flow cytometry analysis and RNA-sequencing of sorted populations from multicellular spheroids to characterize and study the fate of the hypoxic cell population after irradiation. Next, we engrafted and treated H1299- HIF1a-CreERT2-UnaG tumors to visualize the contribution of hypoxic cells in tumor relapse.

**Results and Discussions**

We first confirmed that in 3D cultures, UnaG-positive (UnaG+) cells are located in the central part of the spheroids. Irradiation of H1299-HIFCre-ER-UnaG spheroids leads to a significant enrichment of UnaG+ cells,

© 2023 The Authors. Molecular Oncology is published by John Wiley & Sons Ltd on behalf of Federation of European Biochemical Societies indicating that the hypoxic cells are the main surviving population that drives spheroid regrowth. Upon IR, the hypoxic core survives while increased cell death is observed in the UnaG-negative (UnaG-) cells. The surviving UnaG+ cells remain arrested for a prolonged period before they start to proliferate and give rise to regrowing spheroids. We observed that UnaG+ cells lack active cell cycle markers in unperturbed spheroids and remain in an arrested state after IR up to 28 days before they acquire an active proliferative status driven by c-Myc upregulation.
In xenograft tumors, we found that UnaG-positive-cells coincide with pimonidazole-positive tumour areas and show absence of active cell cycle markers (Ki-67) in untreated tumours. Upon IR, regrowing tumours show significantly higher percentage of UnaG+ cells compared to untreated controls revealed with cell-by-cell analysis of whole tumour cross-sections.

**Conclusion**

Collectively, our data demonstrate the feasibility to track individual tumour cells that were hypoxic at the time of irradiation and provide proof that the hypoxic tumour cells survive and drive tumour relapse after irradiation.

### EACR23-0974

### B-cell-receptor inhibitors induce AID-mediated genome instability in Chronic Lymphocytic Leukaemia

*A. Gasparetto[1], C. Pighi[1,2], E. Genuardi[1], Q. Wang[3], R. Piazza[4], S. Ciavarella[5], M. Coscia[1], S. Ferrero[1], C. Voena[1], R. Chiarle[1,3]*

*[1]University of Turin, Department of Molecular Biotechnology and Health Sciences, Turin, Italy*

*[2]IRCCS Sacro Cuore Don Calabria Hospital, Department of Pathology, Negrar di Valpolicella - Verona, Italy*

*[3]Children's Hospital Boston - Harvard Medical School, Department of Pathology, Boston, United States*

*[4]University of Milano - Bicocca, Department of Medicine and Surgery, Milan, Italy*

*[5]IRCCS Istituto Tumori "Giovanni Paolo II", Hematology Unit - Oncology Department, Bari, Italy*

**Introduction**

Chronic Lymphocytic Leukemia (CLL) is a B-cell neoplasia, and it is the most frequent form of leukemia in Western countries. Activation-induced cytidine deaminase (AID) is a B-cell enzyme that physiologically targets immunoglobulin genes to induce B-cell maturation. Through off-target activity, AID has a much broader effect on genomic instability by initiating oncogenic chromosomal translocations and mutations often involved in the development and progression of neoplasia. AID expression is tightly regulated in B cells, and it is repressed by the transcription factors FOXO1 and BLIMP1 that are regulated by PI3Kδ activity. B-cell-receptor (BCR) inhibitors such as idelalisib and ibrutinib, currently used for leukemia or lymphoma therapy, inhibit PI3Kδ activity directly or indirectly through BTK inhibition, thereby increasing AID expression and, consequently, genomic stability in B cells, as we recently showed.

**Material and Methods**

Peripheral blood samples from 29 CLL patients treated for 12 months with BCR inhibitors were collected before and after treatment. A targeted DNA Sequencing of known AID off-target genes was performed for each sample to compare the AID-dependent mutations frequency before and after treatment. We generated four different clones from a primary human CLL line by knocking out (KO) the AID gene. Clones were treated with increasing concentrations of idelalisib and ibrutinib to induce drug resistance. Modification of cell signaling during resistance was evaluated by Western Blot. Whole Exome Sequencing (WES) and targeted DNA Sequencing of parental idelalisib or ibrutinib sensitive cells and the corresponding resistant cells were used to determine AID activity.

**Results and Discussions**

Patients treated with idelalisib and ibrutinib showed an increase of AID-dependent mutational rate in AID off-targets genes, such as BCL2, CD83, cMYC, IRF8, PAX5, PIM1, RhoH, TCL1A in case of idelalisib, and BIRC3, BTG2, LRMP in case of ibrutinib treatment. Those findings have been confirmed also in resistant CLL cell lines at genomic level. Moreover, CLL-resistant cells showed increased activation of pERK as compared to the sensitive cells.

**Conclusion**

We demonstrate that BCR inhibitors increase genomic instability in both CLL cell lines and patients by an AID-dependent mechanism. This effect could be involved in the development of drug resistance and should be carefully evaluated during long-term therapies. The identification of specific loci targeted by AID could be used to develop a biomarker for risk assessment.

### EACR23-0979

### Cancer-neuronal cross talk in Glioblastoma: studying how cortical neurons sustain tumor progression

*C. Saulle[1], F. Mirabella[1], E. Tagliatti[1], E. Fraviga[1], A. Sironi[1], L. Passoni[1], M. Matteoli[2], D. Pozzi[2], E. Stanzani[1]*

*[1]IRCCS Humanitas Research Center, Laboratory of Pharmacology and Brain Pathology, Milan, Italy*

*[2]Humanitas University, Department of Biomedical Sciences, Milan, Italy*

**Introduction**

Glioblastoma (GBM) is a highly aggressive and invasive brain tumor with rather unique features. It is known that GBM cells actively interact with surrounding tumor microenvironment, including brain resident cells. Recently, a direct functional communication between neurons and glioma cells has been proposed as novel mechanism exploited by tumor to sustain its proliferation and invasion into the brain parenchyma. In addition, GBM induces hyperexcitability of the surrounding neuronal network creating a vicious cycle. However, the mechanisms and the specific contribution of brain cells to the interplay between neurons and cancer cells are still unclear.

**Material and Methods**

To study the molecular mechanisms underlying GBM-neuronal crosstalk we set up an in vitro model of neuro-tumoral unit. Primary human GBM Stem-like Cell Lines (hGSCs) were established from patient post-surgical

© 2023 The Authors. Molecular Oncology is published by John Wiley & Sons Ltd on behalf of Federation of European Biochemical Societies specimens and were cocultured in presence of murine primary embryonic neurons and astrocytes (mNA).

**Results and Discussions**

As detected by flow-cytometry, 7 days of co-culture boosted hGSCs proliferation regardless to the molecular subtype. In addition, enhanced neuronal activity exacerbates hGSC proliferation while the blockade of neuronal firing was not associated to reduction of hGSC proliferation suggesting the possible contribute of both activity-dependent and independent mechanisms. We exploited genetically encoded intracellular Glutamate-Sensitive Fluorescent sensor (iGluSnFR) to investigate the ability of hGSCs to sense neuron-released glutamate. We found that hGSCs are capable to sense neuronal electrically evoked glutamate releases with kinetic properties resembling neuron-to-neuron synapses. The glutamate-induced intracellular signaling need to be further investigated. In parallel, neuronal network activity was measured in presence/absence of tumor cells using High Density-Multi Electro Array. After 24 hours of neuron-cancer co-culture, the mean firing rate of neurons was increased indicating that hGSCs promote network excitability.

**Conclusion**

In summary, here we described a GBM-neuron vicious cycle probably involving glutamate in which neurons boost hGSCs proliferation and cancer cells trigger neuron hyperexcitability. These results represent a solid starting point to investigate the molecular mechanisms underpinning cancer-neuronal crosstalk. We expect to unveil new potentially druggable pathways in GBM, which are central for the functional communications between cancer cells and neurons and for cancer progression.

### EACR23-0997
### EFFECT OF METFORMIN AND OXYGEN MICROBUBBLES THERAPY ON THE VASCULARITY, OXYGENATION AND AGGRESSIVENESS OF BREAST CANCER

*G. Dziurman[1], P. Hoła[1], A. Delalande[2], A. Drzał[1], C. Pichon[2], M. Elas[1]*
*[1]Jagiellonian University, Department of Biophysics and Cancer Biology, Kraków, Poland*
*[2]CNRS, Centre de Biophysique Moléculaire, Orleans, France*

**Introduction**

Low tissue oxygenation and hypoxic conditions, that develop during solid tumor growth, favor the formation of an aggressive tumor phenotype, which has a negative impact on almost all anticancer therapies used to date. Hypoxic cells change the metabolism of administered chemotherapeutic agents, and moreover they are three times more resistant to radiotherapy than cells with proper oxygenation. A new and innovative approach to this problem is the use of ultrasound-sensitive oxygen microbubbles, which in the cavitation process locally release oxygen to the neoplastic tissue, thus giving hope for increasing the level of oxygenation in this area. To normalize tumor vasculature, metformin therapy was introduced in the experiment. Metformin is a commonly used drug in type II diabetes with widely studied antitumor activity.

**Material and Methods**

To perform this experiment, electron paramagnetic spectroscopy (EPR) was used to determine the level of oxygen partial pressure in tissues and Doppler ultrasound imaging to visualize tumor vasculature. The experiments were carried out *in vivo* on 4T1 orthotopic murine breast cancer grown in Balb/c mice, and then *ex vivo*, from harvested tissues, to detect vessels, pericytes, perfusion and the markers of tumor aggressiveness.

**Results and Discussions**

Inhibition in tumor growth (400 $mm^3$ less than control group) and drastic reduction in VEGF levels (nearly two times smaller than other groups) were observed after the introduction of combination therapy. An increase and normalization of vascularization was observed in all treatment groups (up to 2.5 times more vascular volume was seen in the metformin-treated group), along with increased pericyte recruitment and decreased perfusion. The expected effect of increasing the oxygen partial pressure (mean values ranged from 10-15 mmHg for the OMB group, 7-12 mmHg for the metformin group, and 10-20 mmHg for the combination therapy group, compared to the control) in the tumor was obtained between the twelfth and eighteenth days of therapy. The unexpected effect, requiring further analysis, turned out to be a significant increase in the metastatic potential of the neoplasm, resulting in macroscopic metastases to the lungs along with the applied therapy.

**Conclusion**

The maximum values of oxygen partial pressure, on the fourteenth day after tumor inoculation, indicate then the emergence of a "therapeutic window", which may increase the effectiveness of planned radiotherapy.

### EACR23-1005
### Drug-tolerant persister cells arise and mediate resistance to targeted CHK1 inhibition

*L. Carmichael[1], A. Gao[1], K. Mitsopoulos[1], M. Bright[1], O. Rossanese[1]*
*[1]Institute of Cancer Research, Cancer Therapeutics, London, United Kingdom*

**Introduction**

Drug-tolerant persister cells (DTPs) are a subpopulation of quiescent cells that have been identified in several cancer cell lines following lethal exposure to targeted and cytotoxic therapeutics and shown to precede acquisition of diverse and clinically relevant drug resistance mechanisms. Much of the data comes from studies using tyrosine kinase inhibitors and it is unclear if this persister phenotype is broadly observed in response to drugs that act by varying anti-tumour mechanisms. Herein, we sought to investigate the emergence and progression of DTPs in response to treatment with the clinical candidate SRA737; a small molecule inhibitor of checkpoint kinase 1 (CHK1) that targets the DNA damage response (DDR) pathway.

**Material and Methods**

SK-N-AS cells were exposed to lethal concentrations of SRA737 for 7 and 50 days to generate DTP and drug-tolerant expanded persister (DTEP) populations, respectively. A dose-escalated model of SRA737 resistance

© 2023 The Authors. Molecular Oncology is published by John Wiley & Sons Ltd on behalf of Federation of European Biochemical Societies was established in parallel to investigate persister cell-specific mechanisms of drug resistance. Persister-derived and dose-escalated populations were investigated for changes in epigenetic histone modifications, CHK1 signalling, drug sensitivity and gene expression.

**Results and Discussions**

Slow-cycling DTPs at day 7 represent a large proportion of the starting population and have an increase in global histone H3 lysine 9 trimethylation ($H3K27_{me3}$). Day 50 DTEPs are marked by resumed proliferation, cross-resistance to other small molecule CHK1/CHK2 and DDR inhibitors and continued H3K27 hypermethylation, suggesting a role for epigenetic regulation in DTP emergence and progression. Inhibition of the H3K27 methyltransferase EZH2 using tazemetostat inhibits DTP-to-DTEP transition but fails to abrogate DTP prevalence or DTEP survival, confirming the requirement of epigenetic plasticity for persister cell progression. Gene expression analysis reveals distinct transcriptional profiles in DTP and DTEP populations compared to drug-naïve counterparts. Comparison to dose-escalated cells reveals a specific enrichment of genes associated with JAK-STAT signalling in persister-derived populations.

**Conclusion**

We have characterised the persister cell response within a novel and clinically relevant therapeutic context and identified EZH2 activity and JAK-STAT signalling as potential therapeutic targets to eradicate this persistent population. Studies are presently underway to evaluate the therapeutic efficacy of these strategies to abrogate DTP emergence, survival and progression.

### EACR23-1014
### Evaluating Blood-Brain Barrier Permeability Changes during Premetastatic Niche Formation for HER2+ Breast Cancer Brain Metastasis

*L. Santos[1], H. Ferreira[1], F. Tomatis[2], S. Almeida[1], A. Ribeiro[3], L. Ferreira[2], A.P. Silva[1], J.N. Moreira[4], A. Abrunhosa[5], C. Gomes[1]*

*[1]Faculty of Medicine- University of Coimbra, iCBR - Coimbra Institute for Clinical and Biomedical Rese arch, Coimbra, Portugal*

*[2]Centre for Neuroscience and Cell Biology - CNC, Institute of Interdisciplinary Research - IIIUC, Coimbra, Portugal*

*[3]Institute for Research and Innovation in Health - i3s, Institute of Molecular Pathology and Immunology of the U niversity of Porto - IPATIMUP, Porto, Portugal*

*[4]Centre for Neuroscience and Cell Biology - CNC, Faculty of Pharmacy of University of Coimbra, Coimbra, Portugal*

*[5]Institute for Nuclear Sciences Applied to Health - ICNAS, ICNAS Pharma- Unipessoal- Lda, Coimbra, Portugal*

**Introduction**

Up to 50% of HER2+ breast cancer patients eventually develop brain metastasis, with a median survival of less than 1 year after diagnosis. Brain metastasis formation occurs through a complex multistep process. Breast cancer cells modulate the brain microenvironment by secreting bioactive factors that will prepare the premetastatic niche and favor their colonization. Here, we aim to evaluate the structural changes occurring in the blood-brain barrier (BBB) during premetastatic niche formation, focusing on how these changes facilitate the transmigration of breast cancer cells.

**Material and Methods**

An in vitro BBB model was established to assess the effects of the secretome derived from HER2+ breast cancer cells and their brain-tropic variants. BBB integrity was evaluated by measuring the transendothelial flux of a 4kDa-fluorescent dye, the transendothelial electrical resistance (TEER), and the expression of tight and adherens junction proteins. The transmigration ability of breast cancer cells through the BBB was evaluated in vitro. Swiss $Fox^{1nu}$ nude mice were assigned into three groups. The first group was pretreated with the secretome derived from breast cancer cells, while the second group was injected orthotopically into the mammary gland to induce primary tumor formation. The third group was inoculated with brain-tropic breast cancer cells intracardially to induce brain metastasis. BBB integrity was assessed in vivo by near-infrared fluorescence imaging, and ex vivo by collagen IV and albumin immunostaining in the brain.

**Results and Discussions**

Brain-tropic cells secrete specific bioactive factors that disrupt the BBB in vitro and in vivo, facilitating their transmigration into the brain. In vivo, changes in BBB permeability were observed in animals harboring a localized primary tumor before the formation of brain metastasis, as evidenced by a decrease in collagen IV and an increase in albumin immunoreactivity. These changes were even more pronounced after brain metastasis formation, allowing the passage of at least 20kDa dextran into the brain, which is indicative of structural damages in BBB.

**Conclusion**

Our results emphasize the importance of BBB dysfunction as a crucial step in the development of brain metastases and highlight the contribution of tumor-secreted factors to this process.

### EACR23-1022
### New insight into orthotopic liver cancer with Kras G12D mutant

*Y. Jiang[1], F. Zhu[1], C. Ju[1], X. Zhang[1], H. Sun[1], J. Xu[1], D. Jia[2], H. Wang[1], C. Ju[2], J. Zhao[3], X. Gao[4]*

*[1]GemPharmatech Co.- Ltd., Department of Pharmacology, Nanjing, China*

*[2]GemPharmatech Co.- Ltd., R&D Department, Nanjing, China*

*[3]GemPharmatech Co.- Ltd., General Manager Office, Nanjing, China*

*[4]GemPharmatech Co.- Ltd., Management, Nanjing, China*

**Introduction**

Hepatocellular carcinoma (HCC) is a primary cancer of the liver with the highest morbidity and mortality in Eastern Asia. Previous reports suggested many pathways were involved, but the mechanism was unclear

**Material and Methods**

To obtain a comprehensive insight, the orthotopic HCC model of KPA mice was developed by crossbred B6-$Kras^{LSL\text{-}G12D}p53^{LSL\text{-}R172H}$ mice with B6-Alb cre mice. HCC occurs spontaneously in mice at 16 weeks of mice. The

© 2023 The Authors. Molecular Oncology is published by John Wiley & Sons Ltd on behalf of Federation of European Biochemical Societies incidence of spontaneous tumor would be up to 77% within 2 weeks.

**Results and Discussions**

This model successfully offered an *in vivo* mouse model for HCC study, but due to the prolonged and inconsistent onset time for tumorigenesis, and hard to be detected, it is difficult to apply in cancer therapy research. To further verify the quantification, liver cancer cells from the tumor of KPA model were separated and modified with a luciferase label. The growth of tumors could be monitored via biofluorescence reaction in subcutaneous and orthotopic models. The efficacy of Aezolizumab and bevacizumab, which were used as first-line systemic therapy of HCC in the clinic, were evaluated in C57BL/6J mice bearing KPA-luc cell line. Aezolizumab or bevacizumab alone, or their combination, significantly inhibited tumor growth in KPA-Luc bearing mice model.

**Conclusion**

Above all, the mKPA-Luciferase cell line is a new model for the development of hepatocellular carcinoma in preclinical therapies.

### EACR23-1029
### C57BL/6-KRASG12C mouse model for different cancer research

*H. Wang*[1], *X. Yang*[1], *Y. Jiang*[2], *X. Wu*[1], *M. Zhang*[1], *C. Ju*[1], *J. Zhao*[3], *X. Gao*[4]

[1]*GemPharmatech Co.- Ltd., R&D Department, Nanjing, China*
[2]*GemPharmatech Co.- Ltd., Department of Pharmacology, Nanjing, China*
[3]*GemPharmatech Co.- Ltd., General Manager Office, Nanjing, China*
[4]*GemPharmatech Co.- Ltd., Management, Nanjing, China*

**Introduction**

KRAS G12C is an oncogene driven mutation. The KRAS G12C mutation occurs in about 13% of NSCLC patients, and 1%-3% of colorectal and other solid tumors.

**Material and Methods**

To understand the relationship between the mutational activation of KRAS and tumorigenesis, and promote the development of KRAS-G12C inhibitors, we established B6-Loxp-Stop-Loxp Kras G12C(B6-*Kras*$^{LSL-G12C}$) strain. Floxed Stop sequences will be deleted in the mouse genome when Cre recombinase exists, oncogenic KRAS-G12C protein are expressed with endogenous levels following removal of the stop cassette, which allows to control of timing, location, and the multiplicity of tumor initiation.

**Results and Discussions**

B6-*Kras*$^{LSL-G12C}$ mice were crossed with the Lyz2-cre mice to develop spontaneous lung cancer model, which is different in previous studies that crossed with Sftpc-CreER treatment with Tamoxifen. Besides, the mice could develop lung cancer at 8-10 weeks age. In addition, when B6-*Kras*$^{LSL-G12C}$ mice were also crossed with Alb-cre mice, the offspring mice developed liver cancer around 10 months of age.

**Conclusion**

In general, B6-*Kras*$^{LSL-G12C}$ mice model can be used to study the occurrence and development of different cancer types.

### EACR23-1033
### Extracellular matrix remodelling and cancer cell signalling underpin tumour microenvironment heterogeneity and organisation in glioma

*M. Dinevska*[1], *S. Widodo*[2], *S. Mangolia*[3], *P. Neeson*[4], *D. Phillip*[4], *R. Ramsay*[4], *F. MacKay*[5], *M. Christie*[6], *S. Stylli*[2], *T. Mantamdiotis*[7]

[1]*The University of Melbourne, Department of Surgery, Preston, Australia*
[2]*The University of Melbourne, Department of Surgery, Melbourne, Australia*
[3]*WEHI, Bioinformatics division, Melbourne, Australia*
[4]*Peter MacCallum Cancer Centre, Sir Peter MacCallum Department of Oncology, Melbourne, Australia*
[5]*QIMR Berghofer, QIMR Berghofer Medical Research Institute, Brisbane, Australia*
[6]*The University of Melbourne, Department of Anatomical Pathology, Melbourne, Australia*
[7]*The University of Melbourne, Department of Microbiology and Immunology, Melbourne, Australia*

**Introduction**

Gliomas are the most prevalent type of primary brain cancer, with the most aggressive form glioblastoma (GBM) presenting with a median survival of only 15 months. Rapid tumour cell invasion and progression is a significant challenge for physicians, reducing treatment efficacy and inevitably leading to tumour recurrence. Cancer cells thrive by responding and adapting to cellular and non-cellular cues such as the extracellular matrix (ECM) within the tumour microenvironment. However, little is known about how the tumour microenvironment and ECM evolve during disease progression, and what impact they have on cancer cell signalling activation and the subsequent functional behaviour of cancer cells.

**Material and Methods**

Using multiplex immunohistochemistry, histopathological staining, and spatial analysis we investigated the composition and spatial relationship between cancer cells and their signalling activation patterns, immune cells and ECM deposition in low-grade, high-grade astrocytoma and GBM.

**Results and Discussions**

Our results demonstrate that low-grade astrocytoma tissue is largely devoid of infiltrating immune cells and ECM, while high-grade astrocytoma and GBM exhibits abundant immune cell infiltration, activation, and extensive ECM deposition and tissue remodelling. Spatial analysis indicates that most T-cells are restricted to perivascular regions dense in ECM, while macrophages penetrate deeper into tumour cell-rich regions. The tumour microenvironment is characterised by heterogeneous PI3K, MAPK and CREB signalling, with specific signalling profiles correlating with distinct pathological hallmarks, including angiogenesis, tumour cell density and tumour cell invasion. We identify that tissue remodelling is important in regulating the architecture of the tumour microenvironment and contributes to tumour progression.

© 2023 The Authors. Molecular Oncology is published by John Wiley & Sons Ltd on behalf of Federation of European Biochemical Societies

**Conclusion**
Overall, our results present compelling evidence that the accumulation of ECM plays an important role in glioma progression, which impacts both immune cell composition and distribution within the glioma tumour microenvironment, and cancer cell signalling activation and function. These findings support the view that targeting and modifying the ECM will be critical in improving existing therapies such as immunotherapy and current cytotoxic therapies.

### EACR23-1034
### Assessment of the effect of dimensionality, tumor matrix and air-liquid interface conditions on the growth, phenotype and behavior of lung tumor cells

*K. YANGIN*[1,2], *E. Öztürk*[1,2]
[1]*Koç University,*
*Engineered Cancer and Organ Models Laboratory- School of Medicine, İstanbul, Turkiye*
[2]*Koç University,*
*Koç University Research Center for Translational Medicin e KUTTAM, İstanbul, Turkiye*

**Introduction**
Non-small cell lung cancer(NSCLC) is the main cause of lung cancer related mortality. It is critical to implement translational research on reliable cancer models to understand complex molecular and pathophysiological responses in organ-level models *in vitro*. The role of tumor microenvironment has been emphasized in regulation of signaling events involved in tumorigenesis and metastasis which led to pursuit of approaches to engineer biomimetic models. Lung epithelia form sheet-like alveolar structures and reside under air-liquid interface(ALI) conditions in their microenvironment. We aimed to conduct a thorough study on the effect of culture dimensionality, tumor matrix and ALI on the phenotype and malignancy of lung tumor epithelium.
**Material and Methods**
Lung adenocarcinoma cells were used. Cells were grown as submerged and air-lifted in 2D, 2D combined with tumor ECM and embedded in 3D ECM conditions. After 4 weeks of ALI differentiation, samples were collected. Cells were monitored each week with microscopy for morphological assessment. Barrier integrity was assessed with dextran permeability and transepithelial electrical resistance analyses. qRT-PCR and immunostainings were performed for analyses of muco-ciliary and alveolar differentiation, EMT and inflammation.
**Results and Discussions**
Tumor cells grown in 2D and 2D combined with ECM conditions under ALI organized themselves in alveolar-mimicking structures whereas in 3D we observed that ALI caused formation of significantly larger clumps compared to non-ALI even though nutrient access was higher in the latter. 3D cancer cell growth led to weaker epithelial barrier function and integrity compared to monolayer models. We found that cells upregulated mucin family proteins under ALI which are marked to increase in adenocarcinomas of the lung and known to trigger EMT. Indeed, ALI culturing caused a significant increase in EMT markers independent of dimensionality. In 3D, cells upregulated alveolar markers whereas in 2D conditions, ALI triggered extensive bronchial differentiation in cells. ALI culturing led to a significant upregulation in inflammatory genes independent of dimensionality.
**Conclusion**
We assessed growth and phenotype of lung cancer cells in organotypic, lung-mimetic dimensional aspects in ALI conditions. Our results reveal that both dimensionality and ALI have crucial effects on tumor cell growth and expression of invasive signaling routes which reveal insights on how native tissue microenvironmental aspects translate to in vitro models for NSCLC.

### EACR23-1035
### Reconstitution of human T regulatory and NK cells in a Novel IL2-humanized NCG mouse model

*X. Zhang*[1], *M. Wu*[1], *H. Wang*[2], *W. Yu*[1], *F. Zhu*[1], *H. Sun*[1], *J. Xu*[1], *H. Wang*[1], *C. Ju*[2], *S.S. Chen*[3], *Z. Li*[3], *M.W. Moore*[3], *J. Zhao*[4], *X. Gao*[5]
[1]*GemPharmatech Co.- Ltd., Department of Pharmacology, Nanjing, China*
[2]*GemPharmatech Co.- Ltd., R&D Department, Nanjing, China*
[3]*GemPharmatech LLC, Marketing Department, Cambridge, United States*
[4]*GemPharmatech Co.- Ltd., General Manager Office, Nanjing, China*
[5]*GemPharmatech Co.- Ltd., Management, Nanjing, China*

**Introduction**
T regulatory cells (Tregs) are specialized subpopulation of T cells that suppress the immune response by inhibiting T cell proliferation and cytokine production. Natural killer (NK) cells are the predominant innate immune subset that mediates anti-tumor and anti-viral responses. Both Tregs and NK cells are two critical immune cells that play an important role in cancer immunotherapy. However, there is a lack of animal model that can be used to study the in vivo function of these two human immune subsets.
**Material and Methods**
Interleukin 2 is a key molecule that promotes the expansion and activation of lymphocytes, including Tregs and NK cells. We knocked in the human IL-2 gene on the NCG mouse and developed an excellent severe immunodeficient mouse model, NOD/ShiLtJGpt-Prkdc$^{em26Cd52}$Il2rg$^{em26Cd22}$/Gpt (NCG-hIL2). The ability of this model to support Treg and NK cells was then compared to NCG and tested with the reconstitution using human peripheral blood mononuclear cells (PBMC) and human hematopoietic stem cells (HSC).
**Results and Discussions**
In the PBMC engrafted mice, the presence of IL-2 supported the development of T cells, especially the Tregs, with 53.74% in Tregs in NCG-IL2 compared to 4.81% in NCG mice (test at 2 weeks of reconstruction). Similarly, the NK cells are also supported with those cohort engrafted with human HSC (91.63% in NK cells in NCG-IL2 compared to 0.53% in NCG mice, tested at 10 weeks after reconstruction). Notably, reconstituted NK cells expressed various NK receptors such as NKp30, NKp44, NKp46, NKG2D, and CD94 in NCG-IL2 mice. They produced comparable levels of granzyme when compared with human peripheral blood–derived NK cells, and a

© 2023 The Authors. Molecular Oncology is published by John Wiley & Sons Ltd on behalf of Federation of European Biochemical Societies considerable amount of perforin protein was detected in the plasma of huHSC-NCG-hIL2 mice.

**Conclusion**

In conclusion, humanized NCG-hIL2 mouse is an ideal model for the preclinical anti-tumor efficacy study of drugs targeting human Tregs and NK cells. The application scenarios and potential value of this model need to be further studied and explored.

### EACR23-1041
### The preclinical study of B7-H3 antibody in humanized mouse model

*S. Guo[1], L. Song[1], X. Qin[1], J. Xu[1], H. Sun[1], C. Ju[2], H. Wang[1], S.S. Chen[3], Z. Li[3], M.W. Moore[3], J. Zhao[4], X. Gao[5]*
*[1]GemPharmatech Co.- Ltd., Department of Pharmacology, Nanjing, China*
*[2]GemPharmatech Co.- Ltd., R&D Department, Nanjing, China*
*[3]GemPharmatech LLC, Marketing Department, Cambridge, United States*
*[4]GemPharmatech Co.- Ltd., General Manager Office, Nanjing, China*
*[5]GemPharmatech Co.- Ltd., Management, Nanjing, China*

**Introduction**

Immune checkpoint molecule B7-H3, also known as CD276, is a member of the B7-CD28 family of immunomodulatory proteins. It is a type I membrane protein with sequence similarities to the extracellular domain of programmed death-ligand 1 (PD-L1).

**Material and Methods**

B7-H3 is highly expressed in most human cancers, but has limited distribution in normal tissues, remaing elusive of its receptor. Due to its promising safety as a dominant tumor target, various strategies have been developed to modulate the effect of B7-H3 via monoclonal antibodies, bispecific antibodies, ADC, or CAR-T. To study the effect of these therapies in an immunocompetent mouse model, we established a double humanized B7-H3 and PD-L1 mouse model on BALB/c background (BALB/c-hB7-H3/hPD-L1).

**Results and Discussions**

In this model, the extracellular domain of murine fragments was replaced by the human counterparts while the trans-membrane and cytoplasmic domain were kept intact. When engrafted with CT26 colon cancer cells, which stably overexpress human B7-H3 and PD-L1 while endogenous murine counterparts were knocked out, the tumor growth was inhibited certain degree by anti-B7-H3 antibody (8H9 Biosimilar, 20mpk, TGI=18.56%) treatment while inhibited significantly after the monotherapy of anti-PD-L1 (Tecentriq, 3 mpk, TGI=55.89%, $p < 0.001$). The same dosage combination of anti-B7-H3 and anti-PD-L1 had a significant inhibition on tumor growth (TGI=76.85%, $p < 0.001$), and had a synergistic effect (CDI$<$0.7) compared with the monotherapy. Analysis of tumor infiltrating lymphocytes (TILs) at the end of the efficacy study showed that the proportion of CD45+ immune cells was significantly increased in all of the treated groups. The NK cells was significantly increased and Treg cells was significantly decreased especially in the combined treatment group.

**Conclusion**

Summary, the B7-H3 and PD-L1 double humanized mouse model can be used in the pre-clinical evaluation of mono or combined immune checkpoint blockade with anti-human B7-H3 and PD-L1 therapy.

### EACR23-1052
### Humanized mouse models for preclinical efficacy evaluation of ADCs

*F. Zhu[1], Q. Lu[1], Y. Fang[1], H. Sun[1], J. Xu[1], C. Ju[2], H. Wang[1], S.S. Chen[3], Z. Li[3], M.W. Moore[3], J. Zhao[4], X. Gao[5]*
*[1]GemPharmatech Co.- Ltd., Department of Pharmacology, Nanjing, China*
*[2]GemPharmatech Co.- Ltd., R&D Department, Nanjing, China*
*[3]GemPharmatech LLC, Marketing Department, Cambridge, United States*
*[4]GemPharmatech Co.- Ltd., General Manager Office, Nanjing, China*
*[5]GemPharmatech Co.- Ltd., Management, Nanjing, China*

**Introduction**

Antibody-drug conjugates (ADCs) are promising cancer treatments that include delivering toxic drugs to specific tumor cells. Xenograft tumor models based on immunodeficient mice are suitable for evaluating anti-tumor effect of ADCs. Meanwhile, tumor models of immunocompetent animals may be more suitable for evaluating antibody-mediated immune response of ADCs and the combined effect of ADCs and immunotherapy. Aiming on this issue, we developed humanized mouse models to evaluate the efficacy of ADCs in combination with immunotherapy.

**Material and Methods**

DS-8201a (Trastuzumab deruxtecan,T-DXd ) is a novel HER2-targeting ADC , which is composed of a humanized anti-HER2 antibody and a novel topoisomerase I inhibitor, exatecan derivative (DX-8951 derivative, DXd).

**Results and Discussions**

*In vitro* efficacy studies showed that DS-8201a effectively bound to the HER2-positive cell line SK-BR-3, resulting in strong endocytosis and induction of apoptosis. HER2-negative MDA-MB-468 cells were killed through a bystander effect both by DS-8201a *in vitro* and *in vivo*. *In vivo* efficacy studies demonstrated that ADCs significantly inhibit human tumor (NCI-N87, HT-29) growth in xenograft tumor models. In addition to human tumor cell lines, the efficacy of ADCs was also estimated in BALB/c-hPD1 engrafted with CT26-hHER2 and tumor volume was significant inhibited by the combination of DS-8201a and Keytruda (TGI= 91%), which showed more efficacy than mono-therapy (Keytruda:TGI=66%, DS-8201a:TGI=57%.). The evaluation of Fc segment-mediated effect (such as ADCC, CDC and ADCP) of ADCs was carried out in humanized immune system mouse models, such as huHSC-NCG-hIL15 and huHSC-NCG-hSGM3.

**Conclusion**

we established humanized mouse models to evalutate the synergistic anti-tumor effects of ADCs combining with immune checkpoint blockade antibodies.

© 2023 The Authors. Molecular Oncology is published by John Wiley & Sons Ltd on behalf of Federation of European Biochemical Societies

### EACR23-1055
### Unraveling vulnerabilities of drug-tolerant persister cells in Gastric Cancer

E. Puliga[1], C. Orrù[2], D. Conticelli[2], F. Maina[1], A. Morandi[3], S. Corso[2], S. Giordano[2]
[1]Candiolo Cancer Institute- IRCCS, Cancer Molecular Biology, Candiolo, Italy
[2]University of Turin, Department of Oncology, Turin, Italy
[3]University of Florence, Department of Experimental and Clinical Biomedical Sciences, Florence, Italy

**Introduction**
Cytotoxic drugs often fail to eradicate cancers due to the presence of treatment-persistent residual tumour cells that represent a reservoir for relapse. These "residual" cancer cells escape from chemotherapy-induced cell death by entering a reversible slow proliferation state, known as drug tolerant persister (DTP) state. Although improvement of treatment options achieved a survival benefit, Gastric Cancer (GC) is still endowed with a poor prognosis. Surgery and neo/adjuvant chemotherapy remain the keystone of GC treatment. However, most of patients with advanced cancer relapse even after complete surgical resection, suggesting the presence of DTP cells.

**Material and Methods**
GC DTP cells were obtained from primary cells upon chemotherapy regimens (FOLFIRI and FLOT). Cells were treated for 21 days (2 treatments /week for 3 weeks), with doses corresponding to the drug maximal plasmatic concentration. At the end of the treatment, cells were left for 10 days without drugs (washout). Three weeks later, when cells had regrown, they were re-challenged with chemotherapy.

**Results and Discussions**
DTP cells showed increased expression of LGR5, a putative gastric cancer stem cell marker. Additionally, they displayed decreased levels of PS6, used as readout of mTOR activation. However, scattered cells were positive for PS6, suggesting the presence of few cycling DTPs in the persister population. Interestingly, DTPs resulted positive to X-gal–based β-galactosidase staining, suggesting a chemotherapy-induced senescence status. We also investigated the ability of DTPs to transiently increase their genomic instability by altering DNA repair and replication mechanisms. Western blot analysis revealed downregulation of MSH6, MLH1 and MSH2 in DTPs vs the parental counterpart; interestingly, this downregulation was observed also in models in which the MMR system is already deficient (MSI cells). Metabolic analysis showed an important switch in the activation of the NRF2/KEAP1 anti oxidative pathway in DTPs vs parental cells. Other important metabolic differences were observed as well. These preliminary data strongly suggest that even in the chemotherapy setting, DTPs exploit a mechanism of adaptive mutability to survive the therapeutic pressure confirming their role of "reservoir of resistance".

**Conclusion**
Cancer persistence represents a major obstacle to cure cancer, thus, a deeper insight into DTP cells biology is crucial to target this mechanism and it is predicted to be endowed with important clinical implications.

### EACR23-1065
### NCG-MHC-dKO mice -- an excellent model for PBMC reconstitution and pharmacodynamic evaluation in the absence of GvHD

H. Wang[1], J. Xing[1], J. Fan[1], H. Hou[1], S.S. Chen[2], M.W. Moore[2], C. Ju[1], J. Zhao[3], X. Gao[4]
[1]GemPharmatech Co.- Ltd., R&D Department, Nanjing, China
[2]GemPharmatech LLC, Markting Department, Cambridge, United States
[3]GemPharmatech Co.- Ltd., General Manager Office, Nanjing, China
[4]GemPharmatech Co.- Ltd., Management, Nanjing, China

**Introduction**
Human immune cell reconstitution (peripheral blood mononuclear cells; PBMC or CD34+ hematopoietic stem cell; HSC) is commonly performed in immunodeficient mouse strains such as NCG and NCG derivatives. PBMC transplantation is preferred for faster engraftment, lower cost, and the presence of functional CD3+ T cell populations. Despite these advantages, PBMC reconstitution will lead to graft-versus-host disease (GvHD) occurrence around 4 weeks after PBMC engraftment, and specific donors need to be screened to delay the onset of GvHD as much as possible. GvHD significantly limits the study window required available to evaluate the efficacy of therapeutic agents, such as immune-oncology treatments.

**Material and Methods**
GvHD is an immune reaction triggered mainly by donor-derived memory and effector T cells attacking the host cells and tissues. Deletion of β2m gene (NCG-β2m-KO) reduces the occurrence of GvHD; however, β2m is not only in the MHC class I subunit but also in the FcRn subunit, of which deletion shortens the half-life of IgG, making it unsuitable for IgG antibody agent evaluation. In addition, the deletion of MHC class I or class II alone results in an imbalanced CD4/CD8 ratio. To solve these problems, we developed the NCG-MHC-dKO mouse model by knocking out the H2K1, H2D1, and H2Ab1 genes.

**Results and Discussions**
Compared with the β2m null mouse models, the NCG-MHC-dKO model significantly prolonged survival, and reduced GvHD occurrence. Furthermore, treatment with anti-PD-L1 antibodies significantly inhibited MDA-MB-231 tumor cell growth in PBMC-reconstituted NCG-MHC-dKO mice, similar to NCG mice.

**Conclusion**
Based on our preliminary data, the NCG-MHC-dKO mouse is a promising model for antibody and cell therapy agent evaluation, assessing treatment-related cytokine release syndrome, and evaluating long-term toxicities of cell therapies.

### EACR23-1068
### Mouse model of GvHD-an ideal model for evaluating the efficacy of preclinical anti-GVHD drugs

S. Zhang[1], M. Wu[1], F. Zhu[1], H. Sun[1], J. Xu[1], C. Ju[2],

© 2023 The Authors. Molecular Oncology is published by John Wiley & Sons Ltd on behalf of Federation of European Biochemical Societies

H. Wang[1], S.S. Chen[3], Z. Li[3], M.W. Moore[3], J. Zhao[4], X. Gao[5]
[1]GemPharmatech Co.- Ltd., Department of Pharmacology, Nanjing, China
[2]GemPharmatech Co.- Ltd., R&D Department, Nanjing, China
[3]GemPharmatech LLC, Marketing Department, Cambridge, United States
[4]GemPharmatech Co.- Ltd., General Manager Office, Nanjing, China
[5]GemPharmatech Co.- Ltd., Management, Nanjing, China

**Introduction**

The continuous optimization and development of CAR-T cell therapy has brought great hope to the treatment of hematological malignancies, and a great progress has been made so far. However, the acute Graft-versus-Host Disease (GVHD) has been found in the study and application of CAR-T cell therapy, which has hindered the development of CAR-T cell therapy. GVHD is a major complication of allogeneic transplantation, which is caused by T lymphocytes in the allogeneic donor graft attacking allotypic antigens in the recipient after transplantation, resulting in a series of adverse reactions and even death.

**Material and Methods**

Gempharmatech developed and validated two mouse models for studying and evaluating the efficacy of anti-GVHD drugs. The first model was established by transplanting bone marrow containing T cells and spleen cells from C57BL/6 mice into BALB/c mice irradiated with lethal doses. This model can simulate the clinical symptoms of patients after allogeneic hematopoietic stem cell transplantation, such as GVHd-specific damage, inflammation and T-cell infiltration. Surprisingly, the evaluation of anti-GVHD drugs based on this model successfully reflected the efficacy of anti-GVHD drugs in prolonging the survival time of patient mice and improving the adverse reactions.

**Results and Discussions**

In the second model, severely immunodeficient NCG mice were irradiated to accelerate GvHD production and then transplanted into human Peripheral Blood Mononuclear cells (PBMCs). Human T cells then reconstruct in mice, and recognizes and attacks mouse tissue, eventually producing GvHD. Pathological sections of the heart, liver and spleen of these mice showed infiltration of hCD45+ cells in these tissues. Remarkably, the severe weight loss and shortened life span of these mice were significantly alleviated after treatment with anti-GVHD drugs.

**Conclusion**

In conclusion, the above two models provide a good platform for the preclinical development and evaluation of anti-GvHD drugs and facilitate the further development of allogeneic hematopoietic stem cell transplantation for the treatment of hematologic malignancies.

### EACR23-1076
### NCG-M humanized mice -- an excellent model for human immune reconstitution of myeloid lineages

H. Wang[1], J. Fan[1], S. Guo[2], S. Li[2], S.S. Chen[3], J. Zhao[4], X. Gao[5], C. Ju[1]
[1]GemPharmatech Co.- Ltd., R&D Department, Nanjing, China
[2]GemPharmatech Co.- Ltd., Department of Pharmacology, Nanjing, China
[3]GemPharmatech LLC, Marketing Department, Cambridge, United States
[4]GemPharmatech Co.- Ltd., General Manager Office, Nanjing, China
[5]GemPharmatech Co.- Ltd., Management, Nanjing, China

**Introduction**

Mouse Models differ from humans in many aspects for specice differences, therefore, the results from the mouse models often do not translate well directly to the later. One approach to solve this problem is constructing human immune syetem reconstituted mice which could well mimic human immune system in mice.

Severe immunodeficient mice engrafted with human hematopoietic stem cells (HSC) have been extensively used with immune system related studies like oncolocy and autoimmune diseases to evaluate the efficacy of cancer therapies. However, because of murine cytokines could provide limited support to human immune cells, thus immunodeficient mice have limited development of human immune cells.

Increasing evidence has shown that myeloid cells, especially macrophages and dendritic cells, are critical for the induction of anti-tumor immunity.

**Material and Methods**

We developed a mouse model, NCG-M, that can support human T, B, NK and myeloid cells such that in vivo evaluation of agents that require the interplay between these immune cells can be examined. This model was genetically engineered on the severe immunodeficient strain NCG and can produce human granulocyte/macrophage colony stimulating factor 2 (GM-CSF, also known as CSF2), interleukin-3 (IL-3) and stem cell factor (SCF, also known as KITLG).

**Results and Discussions**

Upon human $CD34^{+}$ HSC cell engraftment, increased myeloid lineage cells, such as granulocytes, monocytes, neutrophils, macrophages, and dendritic cells, were observed in the NCG-M cohort compared to NCG mice.

**Conclusion**

The NCG-M mouse also supports the development of human T cells, and preliminary data showed increased B and NK cells. The NCG-M is an appropriate mouse model for studying the efficacy of therapeutic agents that require human T cells and myeloid cells.

### EACR23-1080
### Multiomic interrogation of endothelial and mural cells in brain metastasis reveals multiple immune-regulatory pathways

L. Bejarano Bosque[1,2], A. Kauzlaric[3], E. Lamprou[1,2], M. Ballabio[1,2], R. Colotti[4], M. Massara[1,2], K. Soukup[1,2], N. Fournier[3], J. Lourenco[3], J. Joyce[1,2,5]
[1]AGORA Cancer Research Center, Department of Oncology- University of Lausanne, Lausanne, Switzerland
[2]Ludwig Institute for Cancer Research, University of Lausanne, Lausanne, Switzerland
[3]Translational Data Science Group, Swiss Institute of Bioinformatics, Lausanne, Switzerland
[4]In Vivo Imaging Facility IVIF, Department of Research and Training- University of Lausa

© 2023 The Authors. Molecular Oncology is published by John Wiley & Sons Ltd on behalf of Federation of European Biochemical Societies nne, Lausanne, Switzerland
[5]L. Lundin and Family Brain Tumor Research Center, Departments of Oncology and Clinical Neurosciences- Centre Hospitalier Universitaire Vaudois, Lausanne, Switzerland

**Introduction**

Brain metastasis (BrM) represents the most common and aggressive brain malignancy, predominantly arising from non-small cell lung cancer, breast cancer and melanoma. Recent studies have revealed the importance of the brain tumor microenvironment (TME), notably diverse immune cells, which play important roles in regulating cancer progression in both primary and metastatic brain malignancies. The blood-brain barrier (BBB) is another critical TME component formed by endothelial cells, mural cells, astrocytic end-feet, and closely-associated microglial cells. Metastasizing cancer cells can utilise different strategies to traverse the BBB and once they have successfully seeded and colonized the brain, they can exploit the vasculature for their own benefit, forming the so-called blood-tumor barrier.

**Material and Methods**

To explore the mechanisms underlying tumor vascularization in BrM we performed an in-depth multiomic analysis of the principal components of the tumor vasculature combining single-cell (sc) and/or bulk RNA sequencing (RNA-seq) of sorted endothelial and mural cells isolated from human and mouse BrM and non-tumor samples; immunofluorescence imaging analysis of the spatial architecture of the TME; and functional studies using BrM mouse models to therapeutically target vascular regulators of tumor immunity.

**Results and Discussions**

Combining cell sorting and scRNA-seq we identified several endothelial and mural subtypes as particularly enriched in BrM patient samples. Further transcriptomic analyses in BrM vs. non-tumor samples reinforced the depiction of an altered vasculature characterized by increased extracellular matrix synthesis and modification, compromised transport systems, and increased interactions with immune cells, among others. These phenotypic changes were validated by immunofluorescence analysis of the spatial TME and mural cell depletion studies in BrM mouse models, which revealed mural cells as important regulators of BBB leakiness and immune cell infiltration. To further interrogate the role of the vascular components as immune-regulatory players, we examined the expression of multiple immune checkpoint molecules and took advantage of mouse BrM models to target vascular regulators of tumor immunity.

**Conclusion**

Our study provides a comprehensive understanding of the biology underlying vascularization in metastatic brain tumors, specifically highlighting the importance of vascular players as immune regulators and proposing novel therapeutic approaches for these devastating tumors.

### EACR23-1094
### Mapping the fate of post-hypoxic cancer cells and their role in vasculogenic mimicry

*S. Pearsall*[1], *K. Sawicka*[1], *I. Cannell*[1], *G. Hannon*[1]
[1]*Cancer Research UK Cambridge Institute, University of Cambridge, Cambridge, United Kingdom*

**Introduction**

Breast cancer is a heterogeneous, invasive disease and 90% of breast cancer deaths are a consequence of uncurable metastatic disease. We are investigating vasculogenic mimicry (VM) as a new therapeutic opportunity in breast cancer, which is linked to poor patient prognosis, metastasis and resistance to anti-angiogenic therapy (AAT). Tumour cells undergoing VM acquire endothelial-like characteristics to form pseudo blood vessels, endowing the tumour with an alternative circulatory system. We hypothesise that hypoxia promotes tumour-endothelial differentiation to establish a tumour-derived blood supply and re-oxygenate the tumour whilst providing a mechanism of escape into the blood stream.

**Material and Methods**

The fate of post-hypoxic VM proficient breast cancer cells was monitored by genetically engineering cell lines with a two-vector lentiviral Cre-Lox hypoxia reporter system, in which cells permanently switch fluorescence (DsRED to GFP or BFP to GFP) upon exposure to hypoxia. Hypoxia reporter cancer cells were mapped longitudinally and spatially *in vivo* using flow cytometry, histopathology and 3D imaging of hypoxia reporter tumours, in conjunction with fluorescently labelled perfused vasculature. The molecular mechanisms underpinning hypoxia-induced VM was explored using transcriptomic analyses of hypoxia naïve, hypoxic and post-hypoxic cell populations.

**Results and Discussions**

The utility of two fluorescent hypoxia reporter systems to sense hypoxia *in vitro* was validated in breast cancer cell lines demonstrating that cells switch fluorescence at $\leq$ 3% $O_2$. Following engraftment of hypoxia reporter cells into the mammary fat pad of mice, the percentage of tumour cells that experienced hypoxia increased from 26% to 75%, as tumours grew and outstripped their host vasculature. The spatial architecture of hypoxia naïve, hypoxic and post-hypoxic tumour cells and their contribution to VM *in vivo* can be elegantly mapped in 3D, using tissue clearing and light sheet imaging of the perfused vasculature following administration of intravenous fluorescent lectin into mice bearing hypoxia reporter tumours.

**Conclusion**

This work aims to better understand the molecular events that underpin hypoxia-driven tumour-endothelial differentiation and AAT resistance in breast cancer to identify new targetable options for VM. We propose that suppression of hypoxia adaptation mechanisms and/or VM signalling pathways will synergise with AAT to improve their efficacy and long-term survival benefit.

### EACR23-1104
### Communication between tumour cells and fibroblasts in the tumour microenvironment during melanoma progression

*S. Tauch*[1], *J. Hey*[2], *B. Kast*[1], *L. Weiß*[1], *N. Gengenbacher*[3], *H. Augustin*[3], *C. Plass*[2], *P. Angel*[1]
[1]*German Cancer Research Center, Signal Transduction and Growth Control, Heidelberg, Germany*
[2]*German Cancer Research Center,*

© 2023 The Authors. Molecular Oncology is published by John Wiley & Sons Ltd on behalf of Federation of European Biochemical Societies

Division of Cancer Epigenetics, Heidelberg, Germany
[3]European Center for Angioscience,
Department of Vascular Biology and Tumor Angiogenesis, Heidelberg, Germany

**Introduction**

Malignant melanoma (MM) relates to only about 5 % of all skin cancers, but accounts for about 65 % of skin cancer deaths, and, thus, represents the most aggressive and deadly form of skin cancer. Both, immunotherapy and targeted therapy, were developed to target melanoma cells directly; however, cancer progression, relapse and therapeutic resistance are also supported by cells of the tumour microenvironment (TME), such as cancer-associated fibroblasts (CAFs). Thus, researchers aiming to identify novel therapeutic targets for MM, have meanwhile also focus on CAFs and their interplay with MM cells. Clearly, relevant signalling cascades in the communication between tumour cells and melanoma- associated CAFs at the site of the primary tumour and especially in the TME of distant metastases remain to be explored. Therefore, in order to identify novel therapeutic targets in melanoma progression, we aim at uncovering and functionally analysing the genetic signatures and related signalling pathways of CAFs during MM progression and metastasis.

**Material and Methods**

We employed an optimised mouse model that faithfully mimics human MM development, progression and metastasis (Gengenbacher et al., Cancer Discovery 2021) to isolate cells from the tumour stroma of primary tumours and lung metastases applying an improved protocol for tissue dissociation at low temperatures and isolation of viable single cells. Subsequent scRNA-sequencing revealed unique marker gene expression patterns of CAF populations. Expression of specific genes was examined on murine tissue sections and in biopsies of human MM patients by IF staining.

**Results and Discussions**

The scRNA analysis enabled us to identify CAF sub-populations and their corresponding genetic signature, thereby revealing two key findings: 1) VEGFα CAFs in the TME at the site of the primary tumour with an angiogenesis-specific profile, and 2) a distinct signature for metastasis CAFs, which is related to SAA3 and its downstream signalling targets. Further analyses highlighting an additional SAA3- dependent interaction between CAFs and neutrophils will be discussed.

**Conclusion**

We performed scRNA analysis to define genetic programs characterising CAF sub-populations in the stroma of MM primary tumour and lung metastases. Thereby, we uncovered two, in MM previously undescribed, CAF populations, which both harbour potential TME-specific targets for MM therapy.

### EACR23-1110

### Expression of Olfactomedin gene is associated with disease progression and poor prognosis of Pancreatic cancer.

D. Shankla[1], C. Hao[2], Q. Dou[3], W.G. Jiang[1], L. Ye[1]
[1]Cardiff University- School of Medicine, Cardiff China Medical Research Collaborative- Division o f Cancer and Genetics, Cardiff, United Kingdom
[2]Peking University Cancer Hospital and Institute, Peking University Cancer Hospital and Institute and Key L aboratory of Carcinogenesis, Fucheng Street- Haidian District- Beijing, China
[3]Barbara Ann Karmanos Cancer Institute, Departments of Oncology- Pharmacology and Pathology- School of Medicine- Wayne State University, Detroit- MI 48201, United States

**Introduction**

Human Olfactomedin 1 (OLFM1) gene also known as noelin 1 and pancortin, is highly expressed in various organs, including brain, liver, small intestine, heart, lung and spleen. The abundance of OLFM1 in brain indicates an important role in the nerve system. Elevated expression of OLFM1 was evident in cervical neoplastic keratinocytes. However, biological function of OLFM1 and its role in malignant tumours remain largely unknown. The aim of the present study was to evaluate the involvement of OLFM1 in the disease progression of pancreatic cancer.

**Material and Methods**

OLFM1 transcript expression in a cohort of pancreatic cancer tumours (n=133) was determined in comparison with adjacent non-tumour pancreatic tissues (n=135) using real time PCR with a further validation in a gene expression array dataset (GSE71729). The pancreatic tissues were collected at Peking University Cancer Hospital with ethic approval from Peking University Cancer Hospital Research Ethic Committee and patients' informed consent. The implication of OLFM1 in disease progression and prognosis was analysed which was also further assisted by analysing the RNAseq data of the TCGA (The Cancer Genome Atlas) pancreatic cancer cohort, Kaplan-Meier survival analysis was also employed to evaluate the association with patients' survival.

**Results and Discussions**

Expression of OLFM1 was seen in the pancreatic tumours compared with adjacent normal pancreatic tissues ($p<0.05$). A further validation was conducted by analysing OLFM1 in the (GSE71729) dataset in which a down-regulation of OLFM1 was also seen in the primary tumours (n=145), $p<0.001$ in comparison with normal pancreatic tissues (n=31) with a significantly further reduction in the metastatic tumours from pancreatic cancer. Tumours located in either body or tail exhibited higher expression compared with tumours in the head of pancreas.  There was no significant change of OLFM1 transcript levels in the tumours of different T stages and grades in the Peking cohort. Kaplan-Meier survival analysis showed that lower expression of OLFM1 was associated with shorter overall survival with a median survival being 15.7 months, $p<0.001$ compared with that (30.4 months) of patients with a OLFM1 high expression tumour.

**Conclusion**

OLFM1 was down-regulated in pancreatic cancer. The reduced expression was associated with shorter OS and was also observed in the secondary tumours from pancreatic cancer. This provokes further investigation for its involvement in both tumorigenesis and distant metastasis of the disease.

### EACR23-1117

### Virtual microdissection of non-small cell lung cancer identifies transcriptome-based tumor-specific and immune-specific

© 2023 The Authors. Molecular Oncology is published by John Wiley & Sons Ltd on behalf of Federation of European Biochemical Societies

### molecular subtypes

S. Parab[1], F. Napoli[2], D. Corà[3], G. Doronzo[1], P. Bironzo[2], L. Manganaro[4], S. Bianco[4], L. Primo[1], L. Righi[2], G. Scagliotti[2], F. Bussolino[1]
[1]University of Turin, Oncology, Turin, Italy
[2]San Luigi, Oncology, Orbassano, Italy
[3]Piemonte Orientale University, Translational Medicine, Novara, Italy
[4]aizoOn Technology Consulting, Digital Engineering and Innovation division, Turin, Italy

**Introduction**

Non-small cell lung cancer (NSCLC) is a highly heterogenous disease with the largest number of cancer-related mortality worldwide, one of the reasons for this is the complex and diverse tumor microenvironment (TME) comprising of numerous cell types. Several studies have already highlighted the importance of TME in dictating progression steps and response to therapies; however, a transcriptome-based molecular subtyping of patients in lung adenocarcinomas (LUADs) and lung squamous cell carcinomas (LUSCs) can further determine the distinct tumor immune microenvironment (TiME), which can eventually provide a systematic overview to improve the diagnosis and prognosis of patients.

**Material and Methods**

To elucidate such nature of interactions between tumor cells and cells comprising the TME, we exploited the transcriptome of 300 early stages (Ib-IIIa) NSCLC recruited in the prospective observational clinical trial PROMOLE. With the help of a clustering approach, initially we performed a molecular-based virtual stratification/dissection on the NSCLC patients. Next, to elucidate the relative cell-type abundance, a deconvolution approach was applied to identify the possibility of tumor infiltrating immune cells within these subgroups. Immunohistochemistry (IHC) was then used to substantiate these predictions on tumor cells.

**Results and Discussions**

The resulting subgroups of LUADs and LUSCs are biologically well-characterized by mutational and gene expression profiles. Cell-type abundance approach identified samples which are enriched with tumor infiltrating immune cells like Neutrophils, Tcells, macrophages, etc. These findings were positively confirmed by IHC with multiple cell markers such as MPO, CD4, CD8, CD68, etc. Integrating these two results highlighted the proportion of TiME in the two different sub-populations along with shedding some light on the crosstalk happening between different cancer-/immune-cell lines.

**Conclusion**

The in-silico predictions on bulk RNA data by virtual micro-dissection, distinguished the two distinct NSCLC subtypes, each associated with clinical and molecular features. Furthermore, the immune cells infiltration suggests a possible role of infiltrating tumor immune cells with the prognosis of patients. Our analysis successfully performed an intra-sample and inter-sample comparison, which can unveil new prognostic markers that can provide relevant information for cancer immunotherapy.

### EACR23-1126
### POSTER IN THE SPOTLIGHT
### How a PI3K Interactor drives PDAC chemoresistance and metastasis while paving the way for PI3K-targeted therapy

A. Costamagna[1], P. Cappello[2], F. Novelli[2], E. Giovannetti[3], M. Martini[1]
[1]University of Torino, Molecular Biotechnology and Health Sciences, Torino, Italy
[2]University of Torino, Laboratory of Tumor Immunology-Center for Experimental Research and Medical Studies, Torino, Italy
[3]Amsterdam University Medical Centers, Department of Medical Oncology- Cancer Center Amsterd am-, Amsterdam, The Netherlands

**Introduction**

Pancreatic cancer is a major challenge in cancer treatment today. The initial steps towards invasive pancreatic cancer involve activating Kras mutations and the PI3K/AKT pathway. It's unclear which signaling pathways allow these early cancer cells to gain metastatic abilities after Kras activation, giving rise to the early metastatic spread observed in pancreatic cancer and leading to the high recurrence rates seen in operable PDAC patients. Recently, we found that the p130Cas protein plays a pivotal role in activating the PI3K-AKT pathway downstream of mutant Kras. We believe that p130Cas is key to activating the PI3K-AKT pathway and enabling metastatic properties of pancreatic cancer cells.

**Material and Methods**

To evaluate the impact of the p130Cas protein as a new regulator of PDAC metastatization, mouse models of PDAC (KrasG12D/Trp53R172H/CrePdx1 and KrasG12D/CrePdx1) were crossed with mouse strain carrying p130Cas floxed alleles. Functional 3D in vitro and in vivo experiments were performed with murine primary PDAC cells.

**Results and Discussions**

We found that p130Cas is required for the activation of the PI3K-AKT pathway downstream of mutant Kras, which promotes acinar metaplasia and tumor progression. We discovered that p130Cas binds to the PI3K-p85 regulatory subunit, thereby releasing its inhibitory effect on the PI3K-p110 catalytic subunit and allowing full activation of the PI3K-AKT pathway. Analysis of RNAseq datasets revealed that circulating tumor cells from patients with metastatic PDAC display a significant upregulation of the p130Cas gene and PI3K-AKT gene signature, suggesting a crucial role of this axis in promoting metastasis. We demonstrated that p130Cas levels control the ability of PDAC cells to grow as 3D organoids and their metastatic potential in an in vivo zebrafish model. We observed that organoids with higher p130Cas levels are more resistant to gemcitabine and that these cells are addicted to p130Cas-dependent PI3K-AKT activation, as the inhibition of the pathway has a significant impact on cells with high levels of p130Cas.

**Conclusion**

Our data suggests that the p130Cas-PI3K pathway contributes to both primary tumor chemoresistance and metastatic cell chemoresistance. Elimination of p130Cas-dependent cells with PI3K inhibitors may present a novel

© 2023 The Authors. Molecular Oncology is published by John Wiley & Sons Ltd on behalf of Federation of European Biochemical Societies therapeutic opportunity to specifically target aggressive metastatic cancer cells escaping from primary tumor or already in circulation and may potentially interrupt the way of tumor metastasis.

### EACR23-1131
### Unraveling the regulation of cancer-associated fibroblast heterogeneity in pancreatic ductal adenocarcinoma through functional genetic screening

I. Mora Martinez[1], R. Leite de Oliveira[2]
[1]Amsterdam UMC, Human Genetics- Cancer Center Amsterdam, Amsterdam, The Netherlands
[2]Amsterdam UMC, Center for Experimental and Molecular Medicine- Cancer Center Amsterdam, Amsterdam, The Netherlands

**Introduction**
Pancreatic ductal adenocarcinoma (PDAC) is a highly aggressive and devastating disease with limited treatment options and a poor prognosis, accounting for significant cancer-related mortality. PDAC microenvironment is characterized by a desmoplastic stroma rich in cancer-associated fibroblasts (CAFs), which play a crucial role in tumor progression. However, the heterogeneity of CAF subtypes and their precise regulation remains unclear. We hypothesized that identifying genes important for the establishment of tumor-promoting and tumor-restraining CAFs could allow for the manipulation of pro-tumor CAFs and overall achieve a more tumor-restraining stroma.
**Material and Methods**
To shed light on the regulation of CAF heterogeneity and its impact on PDAC biology, we conducted a functional genetic screening using spontaneously immortalized mouse pancreatic stellate cells as precursors of CAFs. Our cell model permits for the long-term culture and high throughput conditions necessary for genome-scale CRISPR screening, overcoming the shortcomings of primary cells frequently used as cell models in the field. Cell identity and phenotype was thoroughly characterized prior to the screen by RNAseq, qPCR and flow cytometry, showing expected response to known CAF activation stimuli.
**Results and Discussions**
Our CRISPR screen setting coupled with a FACS-based detection method allowed for the identification of tumor-promoting and tumor-restraining CAFs based on antibody staining of established markers of both phenotypes. Results from the screen will be presented, providing new insights into the regulation of CAF heterogeneity and its impact on PDAC biology.
**Conclusion**
Further investigation of the identified genetic factors may lead to the development of novel therapeutic strategies for PDAC patients. We are excited to share the experimental set-up, optimization steps, and initial findings of our work.

### EACR23-1139
### The RNA 5′ cap methyltransferase TGS1 regulates redox metabolism in Acute Myeloid Leukaemia

V. Miano[1], J.L. Miljkovic[2], S.L. Luan[1], L. Pandolfini[3], H.A. Prag[2], M.P. Murphy[2], I. Barbieri[4]
[1]University of Cambridge, Pathology - Division of Cellular and Molecular Pathology, Cambridge, United Kingdom
[2]MRC Mitochondrial Biology Unit, University of Cambridge, Cambridge, United Kingdom
[3]Istituto Italiano di Tecnologia, Center for Human Technologies - Central RNA Lab, Genova, Italy
[4]University of Turin, Molecular Biotechnology Center, Turin, Italy

**Introduction**
RNA modifications represent a new epigenetic mechanism controlling gene expression and are involved in the pathogenesis of several disease, including cancer. RNA modifications are specifically required for the growth of cancer cells and pharmacological inhibition of RNA modifying enzymes emerged as a promising approach for cancer therapy. Acute myeloid leukaemia (AML) remains a difficult disease to cure, and most patients relapse after chemotherapy-induced remission, indicating that second line treatments are desperately needed.
**Material and Methods**
To determine vulnerabilities amongst RNA-modifying enzymes, we performed a CRISPR-Cas9 dropout screen in mouse AML cells. Upon the identification of the Trimethylguanosine synthase (TGS1) as a the top target required for AML cell proliferation, we validated the screening in several human AML cell lines by shRNA interference. By $m^{2,2,7}G$ RNA-IP-Seq in human AML cell lines we identify TGS1 targets. The effects of TGS1-KD on AML cells metabolism were characterized by metabolites Mass Spectrometry (MS) analysis and by profiling the energetic and redox state of targeted cells.
**Results and Discussions**
TGS1 is responsible for the conversion of the m7G-cap to 2,2,7 trimethylguanosine ($m^{2,2,7}G$) on specific RNAs. Our RNA-IP analysis identified more than 500 modified mRNAs which are highly enriched for nuclear genes encoding for mitochondrial proteins, especially members of the cellular respiration and of the oxidative phosphorylation pathways. While mRNA levels of these targets are not affected by TGS1-KD, protein levels are significantly downregulated. Polysome profile confirmed that loss of cap hypermethylation reduced the enrichment of TGS1-targets in the actively transcribing polysome fraction, indicating a specific role of $m^{2,2,7}G$ in translation. We observed an increased oxidative stress in TGS1-depleted AML cells, characterized by high level of cellular Reactive Oxygen Species (ROS) and by the upregulation of ATF4 protein level. Despite TGS1-KD impairs the expression of the antioxidant enzyme GPX4, we couldn’t observe any consistent lipid oxidation upon TGS1-silencing, which could potentially trigger ferroptosis of targeted cells. Nevertheless, TGS1-targeted cells are sensitised to sublethal doses of a variety of drugs, including chemotherapeutical and ferroptosis-inducing agents.
**Conclusion**
We identified TGS1 as master regulator of cellular oxygen metabolism in AML cells and demonstrated that its targeting represents a potential new strategy to treat leukaemia patients.

© 2023 The Authors. Molecular Oncology is published by John Wiley & Sons Ltd on behalf of Federation of European Biochemical Societies

### EACR23-1159
### A single-cell atlas of the benign and metastatic omentum reveals tissue reprogramming in response to ovarian cancer colonization

*U. Lischetti*[1], *C.Y. Liang*[1], *R. Coelho*[1], *F. Singer*[2], *M. Carrara*[2], *F. Jacob*[1], *C. Beisel*[3], *V. Heinzelmann-Schwarz*[1]
[1]*University Hospital Basel and University of Basel, Department of Biomedicine, Basel, Switzerland*
[2]*ETH Zürich, NEXUS Personalized Health Technologies, Zürich, Switzerland*
[3]*ETH Zürich, Department of Biosystems Science and Engineering, Basel, Switzerland*

**Introduction**
High-grade serous ovarian cancer (HGSOC) has a high mortality rate due to its late diagnosis and high metastatic burden. The disease quickly spreads from the primary site through the abdomen and preferentially colonizes the omentum, which is a highly vascularized, immune-rich adipose tissue. Here, we study the single-cell atlas of the human omentum and aim to identify cellular and molecular mechanisms transforming the omental microenvironment upon tumor infiltration.
**Material and Methods**
The clinical cohort consists of 36 omental tissue samples from 15 patients. Biopsies were taken from different intra-omental locations derived from either benign patients or those containing omental metastases. Samples were freshly processed to isolate the non-adipose cell fraction for single-cell RNA sequencing that yielded over 113,000 cells. Immunofluorescence and cell type characterisation was applied to further validate findings.
**Results and Discussions**
Our cell atlas revealed that the non-malignant omentum is stable in cell type composition and cell states–consisting of mesothelial, T, and mesenchymal stem and progenitor cells. Upon metastatic colonization, the cellular landscape diversifies with increased immune cell infiltration concomitant with a loss of mesothelial and progenitor cells. Omentum samples distal to metastatic sites formed a premetastatic niche characterized by an increased presence of NET-forming neutrophils. The immune landscape in tumor-infiltrated omentum consisted of T cells with gained regulatory and exhausted phenotypes as well as macrophages that had acquired proangiogenic and tumor-associated features. Mesenchymal stem cells from the benign tissue forked into submesothelial fibroblasts and adipocyte progenitors. We also report on highly plastic mesothelial cells transitioning into cancer-associated cells upon metastasis supporting multiple protumorigenic functions. Trajectory and cell subtyping analysis gained insights into cancer-associated fibroblast heterogeneity and cellular origin. Finally, cell-cell communication analysis identified altered signaling pathways at distal and metastatic sites revealing midkine among others as important HGSOC-mediators for microenvironment reprogramming.
**Conclusion**
We characterized the benign omental tissue, revealed the distal omentum to function as a premetastatic niche and report on changes in cell-cell signaling that underlie tumor-initiated microenvironment reprogramming.

### EACR23-1169
### Fibroblast tissue of origin determines epithelial cancer progression

*T. Budden*[1], *S. Gurung*[1], *J. Marques*[2], *K. Harrington*[3], *M. Pederson*[3], *A. Viros*[1]
[1]*Cancer Research UK Manchester Institute, Skin Cancer and Ageing, Manchester, United Kingdom*
[2]*University of Edinburgh, Mass Spectrometry, Edinburgh, United Kingdom*
[3]*The Institute of Cancer Research, Targeted Therapy Team, London, United Kingdom*

**Introduction**
Squamous cell carcinomas (SCC) arise from epithelial cells in various tissues including the skin, head and neck, and lung. SCCs share many similar features including high mutation burden from environmental carcinogens such as UV, alcohol, and tobacco; as well as overlapping oncogenic driver mutations in *TP53*, *NOTCH1/2* and *CDKN2A*. Despite their molecular similarities, cutaneous SCC have a low rate of metastasis and favourable outcome compared to internal SCCs, which have a poor prognosis. Epithelial cells initiate transformation within the epithelium, before invading the connective tissue. The tumour microenvironment plays a role in tumour progression, so we tested whether fibroblasts from different tissues of origin contribute to the different outcomes of epithelial cancers.
**Material and Methods**
We conducted in vitro and in vivo assays investigating invasion, proliferation, transcriptomics, and metabolomics of human SCC and normal fibroblasts from the skin, oral mucosa, and lung. Bioinformatic analysis was also performed on SCC progression datasets.
**Results and Discussions**
Organotypic 3D invasion models show that fibroblasts from lung and oral mucosa drive more invasion in SCC cell lines, regardless of SCC tissue of origin, than skin fibroblasts. Similarly, lung and oral fibroblast secretome significantly increases spheroid invasion of SCC, while skin fibroblast secretome does not. RNA sequencing analysis of fibroblasts from different tissues shows that compared to dermal fibroblasts, oral and lung fibroblasts have higher expression of lipid metabolism and lipid synthesis pathways. We observed that stripping lipids from the fibroblast secretome, or targeting specific lipid pathways, inhibited the invasive phenotype conferred by oral and lung fibroblasts. We have found the lipid species secreted by lung and oral fibroblasts determine SCC invasion and compared the lipidomics of fibroblasts by tissue site. Finally, we show that lipid metabolism pathway gene expression increases progressively in lung and oral epithelia, preneoplastic precursor lesions and SCC; while skin SCC progression is associated with a loss of lipid metabolism.
**Conclusion**
Alterations to lipid metabolism contribute to cancer progression, and fatty acids are linked to metastasis of oral SCCs. Our research shows that lipids secreted by normal fibroblasts in the tumour microenvironment contribute to SCC early invasion and that differences in fibroblast lipid

© 2023 The Authors. Molecular Oncology is published by John Wiley & Sons Ltd on behalf of Federation of European Biochemical Societies metabolism by tissue site of origin contributes to different epithelial cancer outcomes.

### EACR23-1170
### Role of microenvironmental cues in the regulation of oncogene-dependent expansion of preneoplastic cells through cell competition.

*F. Napoletano*[1], *C. Dezi*[2], *L. Triboli*[2], *L. Braga*[3], *M. D'Agostino*[2], *G. Del Sal*[4]
[1]*University of Trieste, Department of Life Sciences, Trieste, Italy*
[2]*University of Trieste and ICGEB, Department of Life Sciences, Trieste, Italy*
[3]*ICGEB, Functional Cell Biology, Trieste, Italy*
[4]*University of Trieste- ICGEB Trieste and IFOM Milano, Department of Life Sciences, Trieste, Italy*

**Introduction**
Tumors develop through (epi)genetic changes that alter cell growth, and selection of cells that adapt to microenvironmental changes, including hypoxia, nutrient fluctuation and physical constraints. In different epithelia, including breast, esophagus and colon, clones of preneoplastic cells with cancer-driving mutations accumulate with age, and can evolve into cancer. Genes mutated in these clones include the tumor suppressor *TP53*, one of the most frequently mutated genes in cancer, with a high prevalence of oncogenic missense mutations (mutp53).
Preneoplastic cells with cancer-driving mutations, such as mutp53 cells, compete with normal neighbors for space and survival. Normal cells are able to outcompete preneoplastic cells, however certain conditions, such as inflammation or extracellular matrix alterations, promote preneoplastic cells expansion. How, in these conditions, competition between preneoplastic and normal cells is regulated is still poorly understood. Identifying the underlying mechanisms could provide opportunities to interfere with tumor initiation and evolution.
Work in our laboratory showed that microenvironmenal cues, including mechanical stress, play a key role in the stabilization and activation of mutp53 (Ingallina et al. Nat Cell Biol 2018). We posit that, in mutp53 preneoplastic cells, microenvironmental cues could promote mutp53 stabilization/activity and thus a mutp53-dependent program allowing preneoplastic cells expansion.
**Material and Methods**
To study the impact of microenvironmental cues on mutp53 human breast epithelial cells interacting with normal neighbors, we established *ad-hoc* co-culture models in which competing mutp53 and normal calls can be traced and isolated to identify molecular pathways that regulate cell competition.
**Results and Discussions**
We found that mutp53 human breast epithelial cells acquire competitive advantage over co-cultured normal neighbors, in a context-dependent manner, and isolated these cells to identify the underlying pathways by integrated mRNA/ncRNA/protein multi-omic analyses.
**Conclusion**
The identified pathways, by which mutp53 preneoplastic cells acquire competitive advantage over normal neighbors, will be functionally validated in mutp53 mosaic organoids, derived from $p53^{WM}$ mice (Zhang et al. Nat Comm 2018), exposed to microenvironmental challenges. Furthermore, we are taking advantage of *Drosophila melanogater*, a model of election to study cell competition, to also study how preneoplastic cells outcompete normal neighbors *in vivo.*

### EACR23-1175
### Overexpression of osteopontin or its mutants in murine shNF1/shp53 glioma cells alters cell behavior, transcription patterns and interactions with microglia

*I. Ciechomska*[1], *K. Jacek*[1], *A. Lenkiewicz*[1], *N. Ochocka*[1], *P. Magod*[2], *D. Friedmann-Morvinski*[2], *B. Kaminska*[1]
[1]*Nencki Institute of Experimental Biology of Polish Acade my of Sciences, Laboratory of Molecular Neurobiology- Neurobiology Cen ter, Warsaw, Poland*
[2]*Tel Aviv University, The School of Neurobiology- Biochemistry and Biophysics- The George S. Wise Faculty of Life Sciences, Tel Aviv, Israel*

**Introduction**
Osteopontin (OPN, encoded by the *SPP1* gene) acts via integrin receptors and the CD44 glycoprotein, and regulates adhesion, migration, invasion, chemotaxis and cell survival. We found that *SPP1* is overexpressed in malignant gliomas, human glioma cells, glioma stem cells (GSCs). While the CD44-binding domain of OPN was important in maintenance of self-renewal and pluripotency properties of GSCs, tumor-derived proteolytic fragments of OPN via the RGD motif participated in reprograming of glioma-associated microglia and macrophages. In this study, we explored if OPN is modulates properties of GSCs, tumor invasion and tumor-host interactions.
**Material and Methods**
We generated murine glioma spheres with constitutive Ras-Akt activation (lentiviral shNF1/shp53 expression) overexpressing a wild type (wt) or mutated *Spp1* (constructs RAE-OPN with the mutated integrin-binding RGD site or RAH-OPN with the mutated thrombin proteolysis site). We assessed proliferative and invasive potential of those glioma cells (estimated by BrdU assay and gelatin zymography), OPN production and processing (ELISA assay and Western blotting) and their transcriptomes. The interaction between glioma cells and microglia were studied in cell co-cultures.
**Results and Discussions**
Overexpression of wt or mutated *Spp1* changed morphology of glioma cells. Cells overexpressed OPN but processing of wt-OPN, RGD-OPN and RAH-OPN were different. Transcriptomic analyses showed various gene expression and distinct processes resulting from forced expression of *Spp1* or its mutants. Microglial cells in co-cultures with glioma spheres overexpressing OPN underwent amoeboid transformation (visualized by Phalloidin staining).
**Conclusion**
Altogether, we demonstrate that overexpression of OPN changes proliferation and invasive potential of NF1/p53

© 2023 The Authors. Molecular Oncology is published by John Wiley & Sons Ltd on behalf of Federation of European Biochemical Societies deficient-glioma cells and controls microglia activation in co-culture via the RGD motifs.
Supported by NCN grant N 2020/39/B/NZ4/02683.

### EACR23-1238
### Individual tumor cell-secreted factors differentially modulate the immune microenvironment in pancreatic cancer subtypes

*V. Gölling[1], S. Bärthel[2,3], C. Schmitt[2,3], S. Widholz[4], S. Jonathan[5], F. Meissner[5], R. Rad[4], M. Schmidt-Supprian[1], D. Saur[2,3]*
*[1]Institute of Experimental Hematology, Center for Translational Cancer Research TranslaTUM- School of Medicine- Technical University of Munich, Munich, Germany*
*[2]Institute of Translational Cancer Research and Experimental Cancer Therapy-, Center for Translational Cancer Research TranslaTUM- School of Medicine- Technical University of Munich, München, Germany*
*[3]Division of Translational Cancer Research, German Cancer Research Center DKFZ and German Cancer Consortium DKTK, Heidelberg, Germany*
*[4]Institute of Molecular Oncology and Functional Genomics, Center for Translational Cancer Research TranslaTUM- School of Medicine- Technical University of Munich, München, Germany*
*[5]Institute of Innate Immunity- Department of Systems Immunology and Proteomics, Medical Faculty- University of Bonn, Bonn, Germany*

**Introduction**
Pancreatic ductal adenocarcinoma (PDAC) is a devastating disease with poor prognosis, primarily attributed to its complex tumor heterogeneity with various molecular subtypes and immunosuppressive tumor microenvironment (TME) defined by T cell exclusion and attenuation.
To uncover new targetable inflammatory drivers, it is essential to gain a full understanding of the crosstalk between molecular PDAC subtypes and their respective immune cell subpopulations. In this study, we investigated the influence of tumor cell secreted factors, in particular cyto- and chemokines, on immune cell populations in mouse models for the classical and mesenchymal PDAC subtypes.
**Material and Methods**
We analyzed a cohort *of Kras*-driven mouse tumors by FACS and scRNA-sequencing to characterize the subtype-specific immune cell compartments. Our results revealed that both PDAC subtypes display a unique TME composition. We identified two major myeloid players, neutrophils with a myeloid-derived suppressor cell-like signature in classical and strongly alternatively activated macrophages in mesenchymal tumors, respectively. Furthermore, we discriminated subtype-specific secretion profiles of mouse PDAC cell cultures *in vitro* and found that they correlated with these main immune infiltrates. Candidate secreted factors were inactivated or overexpressed in PDAC cell lines, which were then orthotopically implanted in the pancreas to study successive tumor growth and immune composition.
**Results and Discussions**
Our studies revealed that mesenchymal PDAC-derived Csf1 did not affect macrophage accumulation in tumors *in vivo*, but impacted on macrophage activation/polarization. On the other hand, Cxcl5, mainly secreted by classical PDAC cells, was critical for neutrophil recruitment into the tumor. In both subtypes, changes in myeloid cell populations influenced T cell abundance and phenotype. In classical tumors, neutrophil reduction upon Cxcl5 depletion significantly increased T cell numbers and proliferation. However, none of the tested perturbations alone significantly affected tumor growth, underscoring the dynamic plasticity of PDAC.
**Conclusion**
Taken together, we identified PDAC subtype-specific signaling axes driven by secreted factors that shape their respective unique TME composition and emphasize T cell suppression facilitated by immunosuppressive myeloid subsets. Our analyses suggest that specific targeting and reprogramming of the immunosuppressive PDAC TME represents a promising option for future combinatorial immunotherapeutic approaches.

### EACR23-1250
### Evaluation of bufalin-induced macrophage plasticity in head and neck tumor microenvironment using 3D multicellular spheroid model

*N. MHAIDLY[1], S. saussez[2], G. Descamps[1], F. journe[1]*
*[1]university of Mons, Department of Human Anatomy and Experimental Oncology- Faculty of Medicine and Pharmacy- University of Mons, mons belgium, Belgium*
*[2]university of Mons, Department of Human Anatomy and Experimental Oncology- Faculty of Medicine and Pharmacy- University of Mons, mons, Belgium*

**Introduction**
Tumor-associated macrophages (TAMs) are key components of the tumor microenvironment (TME) and have been shown to play important roles in the progression of head and neck cancer (HNC). Depending on the tumor environment stimuli, macrophages have two different phenotypes: M1 macrophages have anti-tumor effects and M2 macrophages have pro-tumor actions to create a favorable environment for tumor progression.
**Material and Methods**
We choose the Liquid Overlay Technique (LOT) to generate a multicellular spheroid. This spheroid is created by combining cancer cells and monocytes isolated from peripheral blood mononuclear cells (PBMC) or differentiated in M1 or M2 macrophages in a 1:4 ratio Every spheroid volume was examined under microscope and quantified using Image J Software. Phenotypic characterization and apoptotic profile of monocytes in 3D co-culture was performed before and after bufalin treatment for 48 hours by analyzing the expression of cell surface markers by Real-Time PCR, immunofluorescence, and flow cytometry. Differences between experimental groups were analyzed using an independent t-test and one way ANOVA followed by Tukey’s post-hoc.

© 2023 The Authors. Molecular Oncology is published by John Wiley & Sons Ltd on behalf of Federation of European Biochemical Societies

**Results and Discussions**

Using this model, the functionality of the different subpopulations of macrophages to affect spheroid growth was proved. The comparison of their volumes at day 7 demonstrates a major difference in growth between the M1 and M2-containing spheroids, the latter having a volume three times larger than that of the M1-containing spheroids. Furthermore, the M1 spheroids presents a higher apoptotic profile than M2 ones. In addition, HNC cells in 3D culture recreates an invasive and immunosuppressive TME and induced polarization of macrophages into M2-like phenotype with high CD206 expression and low CD86 and HLA-DR levels. On the other hand, we showed that bufalin can function as an antitumor immune modulator that governs the polarization of TAMs from tumor-promoting M2 toward tumor-inhibitory M1.

**Conclusion**

The 3D co-culture constitutes a helpful tool to study tumor-immune cell interaction as well as macrophage plasticity, and to assess the effect of bufalin treatment. Because of its inhibitory impact on tumor cell growth, bufalin treatment could be combined with conventional therapies against cancer cells and could be beneficial in improving patient survival.

### EACR23-1270
### Transcriptional consequences of driver mutations in chronic liver disease

*N. Brzozowska*[1], *F. Rouhani*[1], *S. Brunner*[1], *S. Ng*[2], *M. Hoare*[3], *P. Campbell*[1]
[1]*Wellcome Sanger Institute, Cancer- Ageing and Somatic Mutation, Hinxton, United Kingdom*
[2]*University of California- Irvine, Biological Chemistry, Irvine, United States*
[3]*Early Cancer Institute, Department of Oncology, Cambridge, United Kingdom*

**Introduction**

Non-alcoholic fatty liver disease (NAFLD) is the most important risk factor for hepatocellular carcinoma. NAFLD is characterized by excessive hepatic steatosis, is often accompanied by obesity and metabolic syndrome and is estimated to affect one-quarter of the world's population. We previously sequenced 1590 whole genomes from healthy and diseased livers, focusing predominantly on NAFLD. We identified recurrent mutations in genes implicated in insulin signalling and fatty acid metabolism, notably FOXO1, CIDEB and GPAM. We hypothesized that mutations in these genes allow the mutant cells to evade lipotoxicity resulting from chronic caloric excess, thereby providing the cells with survival advantage. In this study we extend the sample cohort and identify new genes under positive selection in NAFLD. Furthermore, we integrate spatial transcriptomics and genomic profiling on adjacent tissue sections to characterize the gene expression profile of mutant clones.

**Material and Methods**

Liver tissue was collected from multiple Couinaud segments across 3 donors with NAFLD. To annotate driver mutations across tissue sections, 669 microbiopsies were collected using laser-capture microdissection and sent for whole exome or whole genome sequencing. To increase statistical power for identification of genes under positive selection, we combined somatic variants with those from 1560 previously sequenced genomes. To study functional consequences of driver mutations, we performed 10x Visium on adjacent tissue sections and applied differential gene expression analyses on wild-type vs mutant clones.

**Results and Discussions**

Through whole genome and exome sequencing, we identify seven additional genes under positive selection, three of which (INSR, FASN and A1CF) are implicated in insulin signalling and fatty acid metabolism pathways. Additionally, we identify somatic mutation hotspots in CHD4, a chromatin remodelling enzyme. Finally, we characterize the impact of driver mutations on global gene expression. FOXO1 and CHD4 mutant nodules show similar transcriptional profiles and are frequently mutated across the whole liver within the same patient, suggesting convergence on the same phenotypic properties. We describe transcriptional signatures associated with driver mutations.

**Conclusion**

Here we demonstrate that clonal expansions promoted in the context of chronic lipid overload can dominate the clonal architecture of the entire organ, without malignant transformation. We show how metabolism is reprogrammed in mutant clones.

### EACR23-1277
### PlexinB1 deficiency in the microenvironment inhibits tumor growth and metastatic dissemination in mouse models of triple negative breast cancer.

*G. Franzolin*[1,2], *S. Brundu*[1,2], *A. Curatolo*[1,2], *M. Ponzo*[2], *H. Suga*[3], *J. Takagi*[4], *L. Tamagnone*[5], *E. Giraudo*[1,2]
[1]*Candiolo Cancer Institute - IRCCS, Laboratory of Tumor Microenvironment, Candiolo, Italy*
[2]*University of Turin, Department of Drug Science and Technology, Turin, Italy*
[3]*University of Tokyo, Department of Chemistry- School of Science, Tokyo, Japan*
[4]*Osaka University, Laboratory for Protein Synthesis and Expression - Institute for Protein Research, Osaka, Japan*
[5]*Università Cattolica del Sacro Cuore, Department Life Sciences and Public Health, Rome, Italy*

**Introduction**

Semaphorins and Plexins, originally characterized as axon guidance cues, have been shown to play a critical role also in cancer development. A number of so-called "immune"-semaphorins play a major part in immunological diseases pathogenesis and in shaping the so-called tumor microenvironment (TME), that dynamically regulates cancer progression and impacts on the therapeutic outcome. SEMA4D for instance, was initially described for its role in the immune system, and its targeting in solid tumors has been attempted with controversial outcomes. Nevertheless, so far, the role of SEMA4D high-affinity receptor PlexinB1 (PLXNB1) in the TME has been poorly addressed.

**Material and Methods**

Our work focused on understanding the role of PLXNB1 in the TME and its contribution to tumor progression, in metastatic triple-negative murine breast carcinoma models.

© 2023 The Authors. Molecular Oncology is published by John Wiley & Sons Ltd on behalf of Federation of European Biochemical Societies

To this aim, we performed in vivo orthotopic tumor transplants of the syngeneic murine triple negative breast cancer (TNBC) cells (4T1 and Py230) in WT and PLXNB1 deficient animals (*Plxnb1*$^{-/-}$ mice).

**Results and Discussions**

Tumors growing in *Plxnb1*$^{-/-}$ mice displayed reduced primary tumor growth and metastatic dissemination. Interestingly, PLXNB1 deficiency in the TME induced tumor vessel "normalization", as indicated by increased pericyte coverage and reduced hypoxia. Remarkably, tumor-associated macrophages (TAMs) infiltrating *Plxnb1*$^{-/-}$ mice tumors, underwent a switch in their polarization status towards a pro-inflammatory phenotype, compared to the WT cohort. In addition, we observed a strong increase in the infiltration of cytotoxic T lymphocytes, together with a shift in the Th1/Th2 ratio, and an increase in the infiltrating CD11c$^{+}$ antigen presenting cells (APCs). Since PLXNB1-depletion changed the immune-suppressive phenotype of myeloid and T-cells, we tested its therapeutic impact in combination with immunotherapy. Remarkably, the efficacy of anti-PD-1 blocking antibodies was significantly improved in *Plxnb1*$^{-/-}$ mice, leading to a strong reduction in tumor growth and metastasis spreading. Finally, we demonstrated that pharmacologically inhibition of PLXNB1 significantly hindered tumor growth.

**Conclusion**

Taken together, our data suggest that PLXNB1 is a key regulator of metastatic breast cancer TME and represents a promising therapeutic and predictive marker and target for metastatic breast cancers alone and in combination with other immunomodulatory drugs, due to its ability of reverting the immune-suppressive TME milieu of TNBC.

### EACR23-1283
### A role for the glycerophosphodiesterase EDI3 in breast cancer metastasis

*A. Glotzbach*[1], *S. Lüke*[1], *Ö. Demirci*[1], *A. Gonscharow*[1], *K. Rohlf*[1], *J. Reinders*[1], *K. Edlund*[1], *J.G. Hengstler*[1], *R. Marchan*[1]

[1]*IfADo - Leibniz Research Centre for Working Environmen t and Human Factors, Toxicology, Dortmund, Germany*

**Introduction**

Metastasis remains a major problem for tumor therapy. In human tumors of endometrial and ovarian cancer patients elevated levels of the glycerophosphodiesterase EDI3 (endometrial carcinoma differential 3, *GPCPD1*) were found to be associated with metastasis and worse survival. EDI3 is a key enzyme in choline metabolism that hydrolyses glycerophosphocholine (GPC) to produce choline (Cho) and glycerol-3-phosphate (G3P). Altered choline metabolism has been reported in several malignancies, including breast cancer and is a metabolic hallmark of cancer. It was shown that EDI3 expression is particularly high in HER2+/ER- human breast tumors and cell lines and that silencing EDI3 led to reduced viability in these cells. In order to further investigate EDI3 in this specific breast cancer subtype, we established a doxycycline inducible EDI3 knockdown system in luciferase-expressing HCC1954 (HCC1954-luc) breast cancer cells, which allows us to investigate the effect of silencing EDI3 on processes important for metastasis both *in vitro* and *in vivo*.

**Material and Methods**

To create doxycycline inducible knockdown cell lines, HER2+/ER- HCC1954-luc cells, which express high levels of EDI3, were transduced with lentiviral particles containing EDI3-targeting shRNA oligos. The effect of silencing EDI3 on metabolite levels as well as on cellular processes that are relevant in the formation of metastasis was investigated *in vitro* using various cell assays. Subsequently, EDI3's role in tumor growth and metastasis was investigated in mice by *in vivo* bioluminescence imaging using both experimental and spontaneous metastasis models.

**Results and Discussions**

Mass spectrometry analysis showed that doxycycline-induced EDI3 knockdown led to an increase in the endogenous GPC/PCho ratio as well as to alterations in glycerophospholipid levels. *In vitro* studies revealed a significant reduction in colony formation, viability, and proliferation upon EDI3 knockdown induction. Using different mouse models we could show that silencing EDI3 led to a significant reduction in metastasis compared to the non-induced control. Furthermore, silencing EDI3 was associated with longer survival time.

**Conclusion**

The obtained results suggest that silencing EDI3 leads to reduced metastatic burden which makes it a potential therapeutic target to reduce the severity of metastatic disease and increase survival.

### EACR23-1286
### Drug-tolerant persister cell interactions with immune cells following oncogene-targeted therapy

*M. Honkanen*[1], *J. Rannikko*[2], *M. Hollmén*[2], *K. Kurppa*[1]

[1]*University of Turku, Institute of Biomedicine & MediCity Research Laboratory, Turku, Finland*

[2]*University of Turku, MediCity Research Laboratory, Turku, Finland*

**Introduction**

The long-term efficacy of oncogene-targeted therapies is hindered by acquired drug resistance. Resistance is frequently preceded by residual disease (RD) where tumour cells persist despite treatment and lay dormant for extended periods of time, followed by a gradual regrowth of a drug-resistant tumour.

The transcriptional co-regulator YES-associated protein (YAP) is an important regulator of cell plasticity and drug resistance in cancer. YAP activation induces non-genetic reprogramming of EGFR-mutated non-small cell lung cancer (NSCLC) cells *in vitro* and *in vivo*, enabling them to enter a reversible senescence-like state of dormancy and survive targeted treatment.

The drug-tolerant persister cells (DTPs) exhibit robust secretion of both immunostimulatory and immunosuppressive cytokines and chemokines *in vitro*, suggesting that these cells may directly modulate the immune response. The mechanisms underlying cancer cell survival in RD, particularly the interaction of DTPs with the immune system, remain poorly understood. We aim to investigate how DTPs interact with the tumour microenvironment, focusing on the hypothesis that they suppress the immune response via tumour-associated immunosuppressive macrophages.

© 2023 The Authors. Molecular Oncology is published by John Wiley & Sons Ltd on behalf of Federation of European Biochemical Societies

**Material and Methods**
By RNA sequencing, we have identified the YAP-driven secretome of EGRF-mutated NSCLC DTPs by comparing the expression of genes encoding secreted proteins in the presence and absence of an inhibitor of YAP/TEAD activity. Moreover, the effect of the DTP secretome on macrophage polarisation was studied by incubating THP-1 monocytic cells and primary monocyte-derived macrophages in DTP-conditioned media and assessing the effect on the expression of M1/M2 macrophage marker genes.
**Results and Discussions**
Our preliminary results demonstrate that the transcription of approximately 25 % of the secretome is driven by active YAP in two drug-tolerant EGFR-mutant NSCLC cell lines. Importantly, nearly half of these genes are also shared by both cell lines. Furthermore, these DTPs secrete soluble factors which induce the polarisation of macrophages to an immunosuppressive, protumorigenic M2-like phenotype.
**Conclusion**
Based on our preliminary data, DTPs show altered secretion of chemokines and cytokines that promote immune-suppressive signalling. DTPs also polarise macrophages toward an immunosuppressive M2-like phenotype. Thus, targeting this phenomenon could present a therapeutic opportunity to enhance the immune response against RD and improve the long-term efficacy of oncogene-targeted therapies.

### EACR23-1311
### The conundrum of 5-methylcytidine of RNA via NSUN2 in early colorectal cancer

*A. Bastem Akan*[1], *C.V. Billard*[1], *A.E. Hall*[1], *K.B. Myant*[1]
[1]*University of Edinburgh, Institute of Genetics and Cancer, Edinburgh, United Kingdom*

**Introduction**
Colorectal cancer (CRC), the second most mortal cancer, initiates with the loss of APC gene which is mutated in 80% of CRC cases. Despite all treatment strategies, relapse still occurs due to mainly poor knowledge of molecular mechanisms. To improve this, an extensive understanding of cancer biology is crucial. Epitranscriptomics (epiT) is a novel cancer mechanism. 5-methylcytidine ($m^5C$) is one of the well-characterized epiT in tRNA and rRNA but not in mRNA. $m^5C$ is regulated by NSUN2 (NOP2/Sun RNA Methyltransferase2). The role of NSUN2/$m^5C$ in CRC initiation has not been described in detail. Particularly, its function, transcriptomic and epiT footprints following Wnt hyper-activation in early CRC tumorigenesis are obscure. Herein, we primarily focus on NSUN2/$m^5C$ in early CRC.
**Material and Methods**
Nsun2 function was studied by knockdown in 3D mouse intestinal organoids and genetic deletions in VilCre/Lgr5Cre mouse models and functional molecular biology analysis. To characterize the mechanisms, we performed mRNA- and mRNA-Bisulphite-sequencing. We also implemented a meta-analysis in TCGA human datasets. All animal experiments were reviewed by the animal welfare and ethics board of the University of Edinburgh and UK Home Office.
**Results and Discussions**
We previously reported enriched RNA metabolism and upregulated NSUN2 in the $Apc^{KO}$ mouse intestine. Our results correlate with TCGA data regarding clinical relevance. To clarify the function of Nsun2, $Apc^{KO;shNsun2}$ 3D organoids diminished self-renewal capacity and stem cell signature.
$Apc^{KO}$;$Nsun2^{KO}$ mice intestine verified the in vitro data with a reduction in hyper-proliferation and CSC function. Transcriptomic analysis identified decreased expression of Wnt targets, RNA processing, and intestinal CSC signatures in $Apc^{KO}$;$Nsun2^{KO}$ crypts. Surprisingly, no changes were detected in global $m^5C$ levels in $Apc^{KO}$ or $Nsun2^{KO}$. However, our epiT data identified numerous changes in $m^5C$-mRNA sites both in vitro and in vivo $Apc^{KO}$/$Nsun2^{KO}$. We are currently analyzing their functions. Moreover, double-knockout mice exhibited better survival and less adenoma development. Our meta-analysis showing $NSUN2^{high}$ patients with poor survival is also in line with our in vivo tumor model.
**Conclusion**
Collectively, high NSUN2 expression might be an indicator of $Wnt^{high}$ early CRC. We also suggest NSUN2/$m^5C$ are critical mediators of CSC function in Wnt-dependent CRC initiation. Therefore, we reveal NSUN2/$m^5C$ as a potential target for further understanding CRC mechanisms to enhance treatment options.

### EACR23-1330
### Fatty Acid Metabolism as a Therapeutic Target in ER+ Breast Cancer Drug-Tolerant Persister Cells

*A. Goen*[1], *J.D. Marotti*[2], *J.A. Chen*[1], *T.W. Miller*[1]
[1]*Dartmouth College, Molecular and Systems Biology, Lebanon- NH, United States*
[2]*Dartmouth College, Pathology and Laboratory Medicine, Lebanon- NH, United States*

**Introduction**
The ER+ subtype constitutes ~65% of breast cancer (BC) cases. While endocrine therapies used in the adjuvant setting inhibit ER signaling, 1/3 of patients ultimately experience recurrence, implicating a drug-tolerant persister cancer cell (DTP) population as a source of disease recurrence. Mouse models of dormancy and human tumor transcriptional profiles suggested preferential use of fatty acids as a fuel source in DTPs, offering fatty acid metabolism as a potential therapeutic vulnerability.
**Material and Methods**
ER+ BC cells were treated ± 1 nM 17b-estradiol (E2) for 14 d to provide growing and DTP cell populations. Cells were then treated ± inhibitors of fatty acid transporter CPT1a or fatty acid synthase (FASN). Assay endpoints included levels of proteins and transcripts associated with fatty acid metabolism [CD36 receptor, FASN, CPT1a, fatty acid binding proteins 4 (FABP4), adipose triglyceride lipase (ATGL)], oxidative respiration, and glycolysis. BC cells were cultured with adipocytes or treated with adipocyte-condition medium; assay endpoints included fatty acid uptake, and levels of fat metabolism proteins and stored lipid. Orthotopic luciferase-labeled ER+ BC xenografts were induced in NSG mice with E2, which was removed for 90 d to induce tumor regression and yield DTP populations. Mice were randomized to treatment with vehicle or CD36 mAb, and assayed for DTP burden by imaging.
**Results and Discussions**

© 2023 The Authors. Molecular Oncology is published by John Wiley & Sons Ltd on behalf of Federation of European Biochemical Societies

DTP cells had increased lipid uptake compared to E2-treated controls. Estrogen deprivation increased levels of fatty acid metabolism-associated proteins, and FASN inhibition induced CD36 upregulation, indicating a potential compensatory mechanism between sources of fatty acids. Functional metabolic assays showed inhibition of FASN, CPT1a, or CD36 reduced the ability of DTPs to use fatty acid oxidation to drive respiration. Xenografts showed model-specific upregulation of FASN or CD36 after 90 d of estrogen deprivation compared to baseline. Treatment of mice bearing MCF-7 xenografts with the anti-CD36 mAb decreased DTP burden compared to vehicle control.

**Conclusion**

Preclinical modeling suggests that an abundant source of fatty acids (e.g., mammary fat pad or palmitate supplementation) drives DTPs to depend on lipids more than their proliferative counterparts, offering fatty acid metabolism as a therapeutic vulnerability in DTPs.

### EACR23-1336
### Dysregulation of Splicing Machinery as a novel source of diagnostic, prognostic, and therapeutic biomarkers in Craniopharyngiomas

*M.E. GARCÍA-GARCÍA[1,2,3], A.C. Fuentes-Fayos[1], D. Cano[4], I. Di Caro[1], J. Solivera[5], E. Cárdenas[6], J.P. Martínez-Barbera[7], A. Soto-Moreno[4], M.A. Galvez-Moreno[1], R.M. Luque[1]*
*[1]Maimonides Institute of Biomedical Research of Cordoba, Imibic, Cordoba, Spain*
*[2]Reina Sofia University Hospital, Hurs, Cordoba, Spain*
*[3]University of Cordoba, Department of Cell Biology- Physiology and Immunology, Cordoba, Spain*
*[4]Metabolism and Nutrition Unit- Hospital Universitario Virgen del Rocío, Instituto de Biomedicina de Sevilla IBIS, Seville, Spain*
*[5]Service of Neurosurgery, Hurs, Cordoba, Spain*
*[6]Service of Neurosurgery, Hospital Universitario Virgen del Rocío, Seville, Spain*
*[7]Institute of Child Health, Developmental Biology and Cancer Programme Birth Defects Research Centre, London, United Kingdom*

**Introduction**

Craniopharyngiomas (CP) are relatively benign epithelial tumors that typically arise in the sellar/suprasellar region and are classified in adamantinomatous (ACP) and papillary (PCP). Diagnosis is usually performed when tumor development is already advanced, and serious comorbidities are present. First-line therapy is usually surgery, but frequently resection is not complete, causing high rates of recurrence. Therefore, identification of alternative diagnostic, prognostic and therapeutic tools to improve CP management is necessary. Recently, growing evidence indicates that defects in the splicing process are frequent in cancer, leading to the appearance of altered spliceosome components (SCs), splicing factors (SFs) and/or aberrant splicing variants (SVs), which are associated to the development/progression/aggressiveness of various cancer types. Herein, we aimed to analyze the potential dysregulation of the splicing machinery and the associated functional consequences in CP.

**Material and Methods**

Expression profile of key splicing machinery components [i.e. 17 major/minor spliceosome components (SCs; n=13/4) and 28 splicing factors (SFs)] in ACP (n=36) and PCP (n=4) vs. control samples [normal pituitaries (NP, n=11)] was determined by microfluidic qPCR and IHC. Bioinformatics, RNAseq and functional approaches were performed to identify and explore the putative functional role of key SCs and SVs.

**Results and Discussions**

A substantial number of SCs and SFs were drastically altered in ACP vs. NP, and also when primary vs. recurrent ACP were compared. Specifically, 4 SFs were identified as the most discriminating diagnostic/prognostic factors, being corroborated in additional human cohorts. PRPF8 and RAVER1 expression was associated with key clinical parameters suggesting a potential oncogenic role in both ACP and PCP. Moreover, in vitro overexpression of these SFs in primary ACP-derived cells revealed a critical antitumor role through the modulation of the phosphorylation levels of key components of the MAPK, AKT, JAK/STAT, NF-kB and TGFB pathways. Finally, relevant SVs associated to CP development/progression (e.g., CLTA) were identified as potential oncogenic linkers of the observed effects in response to PRPF8/RAVER1 dysregulation.

**Conclusion**

A drastic splicing machinery-associated molecular dysregulation is observed in CP, which could potentially be considered as a source of novel diagnostic/prognostic biomarkers and therapeutic targets for CP, specially RAVER1 and PRPF8.

*Funding: PEER-0048-2020, FPU20/03954*

### EACR23-1349
### Targeting of astrocyte reactivity in brain tumors to prevent radioresistance

*P. Jeannot[1], T. Berg[1], J. Dawson[2], R. Elliott[2], N. Carragher[2], A. Pietras[1]*
*[1]Lund University, Division of Translational Cancer Research, Lund, Sweden*
*[2]University of Edinburgh - CRUK Scotland Centre and Edinburgh Cancer Research, Institute of Genetics and Cancer, Edinburgh, United Kingdom*

**Introduction**

Glioblastoma multiforme (GBM) is the most aggressive type of glioma and deadliest brain tumor. Despite aggressive treatments including surgery, chemo-, and radiotherapy, tumors invariably recur as incurable lesions, and the median survival is approximately 15 months after diagnosis. Recurrence is tightly linked to tumor cell resistance to ionizing radiation (IR), a feature that in turn is linked to stem cell characteristics of tumor cells. Previous results from our lab indicate that stromal astrocytes respond to IR with a reactive phenotype that in turn promotes therapeutic resistance of neighboring tumor cells. Our objectives were to i) identify pathways involved in IR-induced astrocyte reactivity; ii) identify compounds able to inhibit IR-induced astrocyte reactivity.

**Material and Methods**

© 2023 The Authors. Molecular Oncology is published by John Wiley & Sons Ltd on behalf of Federation of European Biochemical Societies

We used two complementary approaches to meet our objectives. For the first one, we used an antibody array to identify pathways that were activated following IR in primary human astrocytes. To validate their involvement in the induction of astrocyte reactive phenotype we used an image-based readout combining the changes in the expression of reactivity markers and in the morphology of astrocytes.
For the second approach, we used this readout to perform an image-based drug screen which aimed to identify compounds able to inhibit IR-induced astrocyte reactivity. The drug libraries used consisted of approved drugs to get candidates with potential for drug repurposing.

**Results and Discussions**

Using the first approach, we found two pathways that were involved in the induction of astrocyte reactivity and that their inhibition blocked IR-induced astrocyte reactivity *in vitro*.
The drug screens that we performed allowed us to identify 119 compounds inhibiting IR-induced astrocyte reactivity. Most of the compounds belong to 6 different drug classes. Interestingly, amongst them 4 classes are composed of brain-penetrant compounds that could make them good candidates for repurposing in GBM.
A confirmation screen is now ongoing for further validation of the compounds and the most efficient at inhibiting IR-induced astrocyte reactivity will be tested *in vivo* in a PDGFA/PDGFB-driven mouse model of GBM.

**Conclusion**

We developed a new approach to monitor astrocyte reactivity *in vitro* that allowed us to identify pathways involved in the induction of reactivity and compounds inhibiting it. Our future plans are to validate the ability of these compounds to inhibit the astrocyte reactivity *in vivo* and in this way to radiosensitize glioma tumor cells.

### EACR23-1369
### Development of Three- Dimensional Cell Culture Models of Adrenocortical Carcinoma

*S. Feely[1], P. Donlon[1], N. Mullen[1], A. Sorushanova[1], D. Finn[2], B. Hernan[2], O. Carroll[3], P. Owens[4], A. Pandit[3], C. Hantel[5], M. Dennedy[1]*
*[1]Lambe Insitute for Translational Research, Discipline of Pharmacology and Therapeutics, Galway, Ireland*
*[2]Galway Neuroscience Centre and Centre for Pain Researc h NCBES, Discipline of Pharmacology and Therapeutics, Galway, Ireland*
*[3]Science Foundation IrelandSFI Centre for Research in Me dical Devices CURAM, Biomedical Science Building, Galway, Ireland*
*[4]University of Galway, Centre for Microscopy and Imaging, Galway, Ireland*
*[5]University Hospital, Department of Medicine iv, Munich, Germany*

**Introduction**

Adrenocortical carcinoma (ACC) is associated with a poor prognosis (1). Current treatments are limited with surgical resection the only option for a complete cure. In addition, mitotane being the only pharmacological agent approved for the treatment of ACC. (2). There is a clear need for the development of translational therapies, however this is limited by pre-clinical disease models. Three-dimensional (3D) cell culture models can accurately reflect the tumour micro-environment but are lacking in ACC (3).

**Material and Methods**

In the current study, we developed and characterised novel 3D models of MUC-1, HAC15 and H295R within a type-1 Collagen matrix. Morphology of ACC cells in the 3D environment was imaged using confocal microscopy. Viability of each model was assessed by Sytox Blue staining and confocal microscopy. Metabolic activity was assessed via AlamarBlue staining with Ki67 detecting proliferation. Liquid chromatography tandem mass spectrometry and real time- polymerase chain reaction (RT-qPCR) was used to determine the steroidogenic capacity of ACC cells grown in the 3D models versus monolayer.

**Results and Discussions**

All cells were successfully cultured in a type 1 collagen matrix. H295R and MUC-1 models show optimum viability up until day 7 with a significant decrease occurring as far as day 21 to when the corresponding cell line was cultured in monolayer. HAC15 model maintained a constant level of viability over 21 days in culture. All three models increase their metabolic and proliferative activity over time. All were steroidogenic, with HAC15 and H295R cells showing an increased expression of aldosterone but a decrease in cortisol expression.
Each cell line in a 3D model showed behaviour reflective of the 3D tumour micro-environment. Each cell line in a 3D model showed: (i) lower overall viability compared to monolayer- reflective of cell turnover and necrosis in the 3D tumour microenvironment, and (ii) an increase in metabolic activity and proliferation reflective of cell turnover in the 3D microenvironment. Similar findings have been shown in the literature for other 3D cancer cell culture models. All were steroidogenic in nature.

**Conclusion**

In the current study, we successfully developed 3D cell culture models for ACC.This model will be used in the future studies to test novel and traditional therapeutics to test the translational relevance of this model to ACC, as a reliable, animal sparing model.

### EACR23-1375
### Developing tools to robustly visualise tumour-lined vasculogenic mimicry blood vessels.

*L. Deighton[1], K. Sawicka[1], G. Hannon[1], C. Caldas[1]*
*[1]University of Cambridge, Cancer Research UK Cambridge Institute, Cambridge, United Kingdom*

**Introduction**

Vasculogenic mimicry (VM) describes the ability of tumour cells to form blood vessels independent of angiogenic mechanisms. VM is associated with resistance to anti-angiogenic therapy (AAT), metastasis and poor patient prognosis. Despite this, the exact mechanisms of VM remain largely unknown and there is still speculation by critics about whether VM delivers a functional tumour-derived blood supply. Current available methods lack two features that are required to confidently and reliably visualise VM networks *in vivo:* 1) a platform to confirm that VM vessels are functional and can carry blood derived

© 2023 The Authors. Molecular Oncology is published by John Wiley & Sons Ltd on behalf of Federation of European Biochemical Societies from the host circulation; and 2) a positive and specific biomarker which unambiguously identifies VM vessels. We aim to develop methods for robust visualisation of VM networks in order to further study and characterise VM and its detrimental effect on tumour progression and resistance to therapeutics.

**Material and Methods**

An *in vivo* 3D vessel imaging modality to highlight functional vasculature and differentiate between host and tumour-lined vessels has been developed for use on breast cancer patient-derived tissue xenograft (PDTX) models. Functional vessels are labelled fluorescently with Lycopersicon esculentum (Tomato) Lectin, host vessels are stained with anti-CD31 and the tumour is optically cleared and imaged using light-sheet microscopy to generate the 3D vessel network.

VM models have been analysed by single-cell transcriptome sequencing to identify potential VM-participating cells and develop VM-specific biomarkers. Probe-based spatial transcriptomics and hyperplexed immunofluorescence was then utilized to spatially locate these cells and potential markers.

**Results and Discussions**

Using the 3D vessel imaging modality, PDTX models with VM networks have been identified. Within these VM models, single-cell transcriptomic data has identified a potential population of VM-participating tumour cells which express high levels of endothelial genes. Genes which are highly expressed within these cells have been spatially visualized using *in situ* probe-based spatial transcriptomics and hyperplexed immunofluorescence to validate them as VM-specific biomarkers.

**Conclusion**

The application of these techniques has allowed us to visualise and understand these fascinating pseudo-vessels on a deeper level to identify VM-specific markers. Enhanced characterisation of VM vessels means we may be able to develop drug targets against these deadly vessels to be used in combination with AAT to starve the tumour of a blood supply.

### EACR23-1403
### Recombinant monoclonal antibodies for cancer biology research

*A. Ball[1], Y.L. Hsieh[2], C.K. Huang[2], S. Huang[2], P.K. Hsieh[2], S.R. Wu[2], Y.Y. Chang[2], J. Liu[2], P.S. Peng[2], C.Y. Lin[2]*
*[1]GeneTex- Inc., Product Development, Irvine, United States*
*[2]GeneTex- Inc., Product Development, Hsinchu City, Taiwan*

**Introduction**

Recombinant monoclonal antibodies (rAbs) are rapidly supplanting standard polyclonal and hybridoma-generated monoclonal antibody reagents in both academic and clinical cancer research. These rAbs offer consistent performance and supply, in stark contrast to traditional nonrecombinant antibodies whose lack of dependability impacts data reproducibility. To increase the availability of thoroughly validated recombinant monoclonal reagents, GeneTex has established a recombinant antibody production platform combined with rigorous validation protocols. The goal is to develop reliable antibody products that function in various experimental applications routinely employed by cancer biology researchers.

**Material and Methods**

GeneTex's rAb production protocol involves a multi-parameter fluorescence-activated single cell sorting (FACS)-based process to identify and isolate antigen-specific IgG+ memory B cells obtained from an immunized rabbit (Starkie *et al.*, 2016). The heavy and light chain variable region genes from single cells are cloned into plasmids to produce full-length heavy and light chains coding for a specific IgG. These constructs are then co-transfected into mammalian cells for expression, thereby preserving natural pairing of the chains. This manufacturing approach allows application-specific testing of clones (e.g., for western blot, immunohistochemistry, and immunocytochemistry, etc.) during the antibody screening workflow. Validation is performed inhouse and, when feasible, with interested academic or industry/pharma cancer biologists who possess ideal samples for optimal assessment of the antibodies.

**Results and Discussions**

This production/validation system has created a series of well-validated recombinant antibodies for cancer biology research. These include reagents against PD-L1, hormone receptors such as ER alpha and androgen receptor variant ARV7, RAS mutants (e.g., RAS G12D), NRF2, SP1, 53BP1, B-Raf, E-cadherin, EpCAM, Claudin 3, Mucin 4, Vimentin, TSG101, SQSTM1/p62, and ATM pSer1981, among many others. Validation for multiple applications was performed, with knockdown or CRISPR-based knockout cell lysates being the primary mode of ensuring antibody specificity within GeneTex's 5+1 Pillar validation plan.

**Conclusion**

In summary, GeneTex is leveraging a FACS-based recombinant rabbit monoclonal antibody production platform to generate meticulously validated recombinant antibodies that will support reproducible cancer biology research.

### EACR23-1404
### Hijacking mechanosensing to block neuroendocrine progression in prostate cancer

*A. Brevi[1], M. Lorenzoni[1], Y. Yamada[2], M. Grioni[1], L.L. Cogrossi[1], M. Freschi[3], F. Demichelis[4], H. Beltran[2], M. Alfano[5], M. Bellone[1]*
*[1]Fondazione Centro San Raffaele, Division of Immunology Transplantation and Infectious Di seases, Milano, Italy*
*[2]Dana-Farber Cancer Institute, Department of Medical Oncology, Boston MA, United States*
*[3]IRCCS Ospedale San Raffaele, Unità Operativa Anatomia Patologica, Milano, Italy*
*[4]University of Trento, Cellular Computational and Integrative Biology CIBIO, Trento, Italy*
*[5]IRCCS Ospedale San Raffaele, Urological Research Institute Division of Experimental On cology, Milano, Italy*

**Introduction**

© 2023 The Authors. Molecular Oncology is published by John Wiley & Sons Ltd on behalf of Federation of European Biochemical Societies

Neuroendocrine (NE) differentiation in prostate cancer (PC) associates with metastasis and poor prognosis. This represents a relevant problem for PC patients since up to 20% of treated castration-resistant PC (CRPC) show the emergence of NEPC. Within tumor microenvironment, extracellular matrix (ECM) can influence tumor development and aggressivity especially through structural remodeling or altering its molecular composition. Here, we showed that NEPC can promote integrin α2 upregulation and YAP activation through interaction with surrounding exocrine cells or ECM enhancing cellular proliferation and metastasis.

**Material and Methods**

NEPC stem-like cells (PNE- SCs) are used *in vitro* to test the influence of prostate-derived extracellular matrix (ECM) from mice with PC or exocrine PC stem-like cells (PAC-SCs) on cell survival, proliferation and invasion. Proteomic and transcriptomic analysis are performed on ECM and stem-like cells respectively to highlight alterations in key pathways or variations in protein and gene expression. *In vivo* experiments with mouse models test the effect of blocking selected pathways in the perspective of potential novel therapeutic strategies.

**Results and Discussions**

PAC-SCs promote integrin α2 upregulation and YAP activation in PNE-SCs coculture, further increasing cell proliferation and invasion. PNE-SCs show improved survival and integrin α2 upregulation also when cultured with ECM from PC-bearing mice, a result that is not obtained when using ECM from wild-type littermates. Inhibition of RANK/RANKL or NF-kB signaling prevents integrin α2 upregulation while blocking YAP or integrin α2 itself impairs cellular invasion. Reduction of cell proliferation is also observed in human organoids derived from castration-resistant PC treated with YAP inhibitors. Similarly, *in vivo* experiments with YAP inhibition shows reduction of PNE-SCs growth and metastatic behavior in NEPC-bearing mice while the same strategy limited NEPC development in a castrated PC genetic model.

**Conclusion**

Our findings highlight the importance of mechano-perception in PC development and progression; in fact, both the composition and organization of the surrounding microenvironment may have profound influence on cellular behavior. Moreover, this work suggests the possibility to act on the integrin α2-YAP axis as a potential strategy for PC patients to improve standard-of-care treatment and prevent therapy-related NEPC development.

### EACR23-1406
### Hypoxia and Extracellular Vesicles involvement in drug resistance via oxidative stress modulation in a 3D Neuroblastoma model

N. Torriero[1], V. Zingales[2], A. Tognon[1], V. Consoli[3], L. Vanella[3], M.R. Esposito[1], E. Cimetta[1]

[1]*Fondazione Istituto di Ricerca Pediatrica Città della Speranza, Department of Industrial Engineering, Padova, Italy*

[2]*Fondazione Istituto di Ricerca Pediatrica Città della Speranza / Laboratory of Toxicology Faculty of Pharmacy University of Valencia /, Department of Industrial Engineering, Padova, Italy*

[3]*University of Catania, Department of Drug and Health Sciences Section of Biochemistry, Catania, Italy*

**Introduction**

Neuroblastoma (NB) is an embryonal malignancy of pre-scholar age originating from neural crest cells. High-Risk (HR) NB has very poor prognosis and requires multimodal treatment, with frequent use of Doxorubicin (Dox) as chemotherapic. The aim of the study is to understand the role of hypoxia and extracellular vesicles (EVs) in inducing NB resistance to treatment.

**Material and Methods**

We based our study on an optimized 3D model recapitulating *in vivo*-like tumor features such as the nutrient and oxygen gradients from the external periphery to the inner core of a mass. Spheroids were formed using SK-N-AS NB cells and cultured until they reached a diameter ~400-500μm. To better understand NB features, we isolated and characterized two sub-populations from each spheroid: the "core" and "periphery". Briefly, we transferred spheroids to gelatin coated 96-well plates and isolated the cells which had migrated on the gelatin after 48 hours ("periphery") from those still anchored to the main spheroid body ("core"). The separated sub-populations were further cultured in normoxic (normo) and hypoxic (hypo) conditions and the EVs they shed were purified and analyzed with Western Blotting (WB), Transmission microscopy (TEM) and Nanoparticle Tracking Analysis (NTA).

**Results and Discussions**

Oxygen gradients in the spheroids were confirmed via immunofluorescence analysis. Whole NB spheroids were treated with Dox for 48 hours to determine $IC_{50}$. WB and cell-cycle analyses on the separated core and periphery populations showed differences in terms of stemness and cell-cycle distribution. Correctly purified EVs shed from the sub-populations cultured both in normo and hypo conditions were used in combination with Dox on 2D SK-N-AS cells.

SK-N-AS treated with Dox and EVs derived from core cells cultured in hypoxia had a 10% higher resistance to the drug compared to Dox alone, suggesting the involvement of EVs and hypoxia in the modulation of drug resistance. Moreover, co-treatment with Dox and core-derived EVs decreased oxidative stress levels compared to treatment with Dox alone.

**Conclusion**

This study highlights the *in vivo* heterogeneities of NB mirrored by the two sub-populations isolated from *in vitro* 3D spheroids and offers new insights on the potential role of hypoxia and EVs in NB drug resistance and progression via modulation of oxidative stress.

### EACR23-1422
### Investigating the role of the cystine/glutamate antiporter xCT in the extracellular vesicle-mediated modulation of the pre-metastatic niche

A. Iacoviello[1], R. Ruiu[1], M. Arigoni[1], M. Tapparo[2], C. Landi[3], F. Cavallo[1]

[1]*University of Turin, Department of Molecular Biotechnology and Health Scienc*

© 2023 The Authors. Molecular Oncology is published by John Wiley & Sons Ltd on behalf of Federation of European Biochemical Societies es, Turin, Italy
[2]University of Turin, Department of Medical Sciences, Turin, Italy
[3]University of Siena, Functional Proteomics Lab- Department of Life Sciences, Siena, Italy

**Introduction**

Breast cancer is the most common malignancy among the women worldwide. Metastasis is mainly responsible for treatment failure and is the cause of most breast cancer deaths. The cystine/glutamate antiporter xCT is upregulated in breast cancer, with a role in redox homeostasis and cell metabolism. In addition to this, xCT has a role in the invasiveness of breast cancer cells. Indeed, we have recently highlighted that its lack in cancer cells strongly reduces the formation of lung metastases and affects the composition of the immune infiltrate within the lungs even before the formation of metastases. Interestingly, it was reported that xCT is involved in the release of extracellular vesicles (EV) by tumor cells through a glutamate-dependent pathway, and EV are recognized as important mediators of pre-metastatic niche formation.

**Material and Methods**

To study the role of xCT in the EV release we used 4T1 cells and $xCT^{KO}$ 4T1 cells, previously generated using CRISPR/Cas9 technology. EV were isolated from cell culture media of xCT-proficient and xCT-deficient cells through ultracentrifugation and analysed through Nanoparticle Tracking Analysis. EV were lysed to evaluate the presence of exosomal markers and xCT using Western Blot. 2D electrophoresis was performed to analyse differences in EV protein cargo. In addition, we also isolated EV from plasma of breast cancer patients through ExoQuick Plasma Precipitation kit and we evaluated the presence of xCT in EV using Western Blot.

**Results and Discussions**

Our first results demonstrate that the absence of xCT in 4T1 cells lead to a reduction in the release of small EV and to a strong alteration of the composition of their cargo.  Additionally, we observed that xCT is present both in EV derived from xCT WT 4T1 cells and EV isolated from plasma of breast cancer patients. On this base we will test the ability of xCT-proficient cells to propagate malignant and pro-tumoral properties through the EV-dependent transmission of xCT. The next step of this work will be the evaluation of the existence of a causal link between xCT-mediated EV release and the metastatic process, with a particular focus on the formation of pre-metastatic niche.

**Conclusion**

In conclusion, these results demonstrated the role of xCT in the release of EV and in the regulation of their cargo. These results provided the basis for a line of investigation to elucidate the role of xCT in inducing immunosuppression and favoring the formation of a pro-metastatic niche, through the modulation of EV formation.

### EACR23-1425
### Cell extrinsic D-2-hydroxyglutarate prevents the expression of inflammatory regulons in microglial cells of IDH-mutant gliomas

P. Pugliese[1], A. Laurenge[1], Q. Richard[1], K. Labreche[1], F. Bielle[1], A. Iavarone[2], M. Ceccarelli[3], M. Mallat[1], M. Sanson[1], L.J. Castro-Vega[1]
[1]Paris Brain Institute - ICM, Genetics and development of brain tumors, Paris, France
[2]University of Miami - Miller School of Medicine, Sylvester Comprehensive Cancer Center, Miami, United States
[3]University of Naples 'Federico II', Department of Electrical Engineering and Information Tec hnology, Naples, Italy

**Introduction**

Tumor-associated macrophages and microglia (TAMs) are highly abundant myeloid cells in gliomas and their phenotype and immune response are determined by ontogeny and microenvironment. TAMs display distinctive transcriptional programs and phenotypes according to the *IDH* mutation status of glioma cells but the underlying mechanisms remain largely unknown.

**Material and Methods**

To explore the mechanistic underpinnings of TAMs behavior linked to the *IDH* status, TAMs ($CD11B^+$) and glioma cells ($CD11B^-$) were purified by magnetic-activated cell sorting from 25 *IDH*-mutant and 11 *IDH*-wildtype gliomas. Bulk RNA and DNA were isolated from both cell populations and profiled for DNA methylome and transcriptome using MethylationEPIC array and RNA-seq, respectively. The contamination of the $CD11B^+$ fraction by tumor cells was determined by the detection of *IDH* and/or *TERT* mutations using droplet digital PCR. Only samples with a purity of at least 95% were used for analyses. Primary cultures of human microglial cells exposed to D-2-hydroxyglutarate (D-2-HG) were used for experimental validations.

**Results and Discussions**

Consistent with previous studies, *IDH*-mutant glioma cells were hypermethylated. TAMs from corresponding tumors exhibit decreased expression of pro-inflammatory pathways (mainly IFN-γ, IFN-α, and TNF-α signaling) and genes related to G2-M checkpoint, glycolysis and EMT. Strikingly, $CD11B^+$ myeloid cells in *IDH*-mutant gliomas exhibit promoter hypermethylation linked to decreased expression of genes involved in inflammatory responses and regulation of MHC class II together with hypermethylated promoters and enhancers enriched for binding motifs of core transcription factors driving myeloid cell differentiation such as SPI1, AP-1, CEBP-α/β and RUNX1. These findings were confirmed by comparing methylome data of $CD11B^+$ cells from *IDH*-mutant gliomas and from normal brains. Prolonged exposure of human microglial cells to D-2-HG partially recapitulates the observed methylation changes. Further transcriptome analyses show that this oncometabolite prevents the IFN-α/γ and LPS response pathways while inducing a metabolic shift toward oxidative phosphorylation.

**Conclusion**

Our findings provide a mechanistic rationale for the hyporesponsive state of microglial cells in *IDH*-mutant gliomas and support the concept that oncometabolites may disrupt the function of immune cells residing in the tumor microenvironment.

© 2023 The Authors. Molecular Oncology is published by John Wiley & Sons Ltd on behalf of Federation of European Biochemical Societies

### EACR23-1428
### Radiation Promotes Medulloblastoma Metastasis Through Inflammation

*C. Nor[1], K. Kharas[1,2], A. Rasnitsyn[1,3], M. Vladoiu[1,2], M. Taylor[1,2,4,5]*
*[1]The Hospital for Sick Children, Developmental and Stem Cell Biology, Toronto, Canada*
*[2]University of Toronto, Laboratory Medicine and Pathobiology, Toronto, Canada*
*[3]University of Toronto, Medical Biophysics, Toronto, Canada*
*[4]Texas Children's Hospital, Pediatric Neuro-Oncology Research Program, Houston, United States*
*[5]Baylor College of Medicine, Department of Pediatrics, Houston, United States*

**Introduction**
Medulloblastoma (MB) is the most common brain malignancy in children. Current treatment includes surgical resection, chemotherapy and radiation therapy (RT). While overall 5-year survival is 60-80%, recurrence is nearly universally fatal. MB metastasizes to the surface of the central nervous system (CNS), called the leptomeninges, and little is known about the biology of MB metastasis.

**Material and Methods**
In our first model, mice were injected subcutaneously with GFP-positive MB cells in the flank. Once a tumour was palpable, mice received either 1Gy of RT or sham treatment. There were two experimental arms: 1) peripheral blood was harvested 1h, 5h or 24h post-treatment or 2) flank tumours were resected, and the brain and spine were harvested at tumour recurrence. In our second model, genetically engineered mutant mice, who spontaneously develop MB, received 1Gy of RT or sham treatment once a tumour was detected by MRI. Brain tumours 5h or 24h post-treatment were harvested for omics analysis (bulk RNA-seq, scRNA-seq, proteo/phosphoproteomics and spatial transcriptomics).

**Results and Discussions**
In three non-overlapping infant medulloblastoma cohorts, patients recur locally without RT and metastatically after RT. In three xenograft mouse models, a single dose of radiation is sufficient to cause an increase in CNS metastasis incidence. Additionally, the number of circulating tumour cells and the expression of inflammatory cytokine and chemokine in the peripheral blood of flank tumour-bearing mice increases following RT. Multi-omics analyses of RT treated primary MB mouse tumours show that immune-related pathways are the most enriched compared to controls. Immunohistochemistry validated our omics observations showing increased macrophages, neutrophils and dendritic cells in the tumour following RT. Live intravital microscopy showed an increase in blood vessel permeability following RT. To interrogate the role of inflammation in metastasis, mice received one dose of lipopolysaccharide instead of RT and showed a significant increase in metastatic burden compared to controls. Administering the anti-inflammatory drug dexamethasone alongside radiation significantly abrogated RT's prometastatic effects.

**Conclusion**
RT is an integral part of standard-of-care treatment for MB; however, it may also have deleterious effects by promoting metastasis through inflammation. This work advances our knowledge of how the tumour niche responds to radiation and could inform approaches to prevent metastasis.

### EACR23-1437
### Enteric Neurons Impact the Colorectal Cancer Immune landscape

*S. Schonkeren[1], M. Thijssen[1], M. Idris[1,2], K. Wouters[1], L. Temmerman[1], N. Vaes[3], F. Verhaegen[4], L. Dubois[5], M. Gijbels[1,6], W. Boesmans[1], V. melotte[1,2]*
*[1]Maastricht University, Pathology, Maastricht, The Netherlands*
*[2]Erasmus MC, Dept. of clinical genetics, Rotterdam, The Netherlands*
*[3]Hasselt University, Dept. of neuroscience, Hasselt, Belgium*
*[4]Maastricht University, Maastro Clinic, Maastricht, The Netherlands*
*[5]Maastricht University, The M-Lab- Dpt of Precision Medicine, Maastricht University, The Netherlands*
*[6]UMC, Dept. of medical biochemistry- experimental vascular biolo gy, Amsterdam, The Netherlands*

**Introduction**
The impact of neuron-cancer crosstalk has become widely recognized over the last years and it is currently known that a higher abundance of nerves in, or surrounding, the tumor is associated with a worse patient prognosis. Recently we showed that the enteric neuron specific protein ndrg4 enhances cancer growth via the secretion of extracellular matrix proteins. Here, we aim to understand how enteric nerves influence the cellular and molecular colorectal cancer (CRC) environment.

**Material and Methods**
*Hand2$^{fl/+}$;Wnt1-Cre2* (hypo-innervated) and *Hand2$^{fl/+}$* (control) mice were subjected to a colitis-associated CRC induction protocol using azoxymethane and dextran sodium sulfate. To track tumor initiation, growth and burden, we acquired and analyzed computed tomography scans during cancer induction and histologically assessed tumor specimens post mortem. Isolated tumor tissues from hypo-innervated and control mice were subjected to RNA sequencing, followed by a gene set enrichment analysis for hallmarks and for *hallmark of cancer* genes. In addition, stool samples were analyzed for microbiota composition using 16S rRNA sequencing and flow cytometry is being performed to assess immune cell populations and tumor cell metabolism in the colon.

**Results and Discussions**
Despite the confirmed reduction in enteric neurons, *Hand2$^{fl/+}$;Wnt1-Cre2* mice presented with a similar tumor initiation, growth and burden as the *Hand2$^{fl/+}$* mice. Also, tumor stage was not changed in the hypo-innervated mice compared to control. RNA sequencing of tumors showed a high number of immunoglobulin-related differentially expressed genes and an enrichment of the gene ontology (defense) response to bacterium. Moreover, gene set enrichment analysis showed that the cancer hallmarks '*avoiding immune destruction*' and '*deregulating cellular energetics*' were enriched

© 2023 The Authors. Molecular Oncology is published by John Wiley & Sons Ltd on behalf of Federation of European Biochemical Societies when comparing both genotypes. Moreover, the population of B cells in the colon lamina propria of AOM/DSS-treated Hand2$^{fl/+}$;Wnt1-Cre2 mice was reduced compared to controls, and antigen-presentation in macrophages was upregulated.

**Conclusion**

A reduced number of enteric neurons prior to tumorigenesis does not directly impact murine tumor growth, but affects the colorectal cancer immune cell populations. Further research is needed to elucidate the enteric neuron – B cell – cancer crosstalk.

### EACR23-1440

### Sialyl-Tn in Triple Negative Breast Cancer Associates with Immunosuppression and Cancer Progression

*R. Adubeiro Lourenço[1,2,3], D. F. Barreira[1,2,3], C. Lopes[4], P. Granjo[1,2,3], A.S. Rodrigues[1,2,3], Z. Silva[1,2,3], M. Martins[4], A.R. Grosso[1,2], P.A. Videira[1,2,3]*

*[1]Associate Laboratory i4HB- Institute for Health and Bioeconomy, NOVA School of Science and Technology, Almada, Portugal*

*[2]UCIBIO - Applied Molecular Biosciences Unit, Department of Life Sciences- NOVA School of Science and Technology, Almada, Portugal*

*[3]CDG & Allies- Professionals and Patient Associations International Netw ork CDG & Allies-PPAIN, NOVA School of Science and Technology, Almada, Portugal*

*[4]Centro Hospitalar de Lisboa Central- EPE, Serviço de Anatomia Patológica, Lisboa, Portugal*

**Introduction**

The Sialyl-Tn (STn) antigen is a carbohydrate resulting from incomplete synthesis of branched O-glycan chains on proteins. It is absent in healthy tissues but aberrantly expressed in several types of cancer, such as breast cancer (BC) and usually associated with poor prognosis and immunosuppressive microenvironment. Triple-negative breast cancer (TNBC) is a particularly aggressive subtype with limited treatment options, highlighting the need for new biomarkers for targeted therapy. This study investigates the clinical and biological relevance of STn in TNBC and proposes promising candidates for targeted therapy.

**Material and Methods**

Immunohistochemistry was performed for STn and other cancer-related makers in 128 TNBC patient tissues. *In vitro* validation was performed on MDA-MB-231 cell line, overexpressing the *ST6GALNAC1* gene (MDA-STn+). Moreover, transcriptomics analysis based on *ST6GALNAC1* expression was performed on TNBC genetic data set from The Cancer Genome Atlas (TCGA).

**Results and Discussions**

STn expression in patients' tissues was associated with reduced overall survival and increased proliferation in MDA-STn+ cells, reinforcing its poor prognosis association. However, the c-myc expression, at both protein and gene levels, was reduced in the presence of STn and correlated negatively with the expression of STn/*ST6GALNAC1*. Functional enrichment analysis based on *ST6GALNAC1* expression identified potential mechanisms independent of c-myc that drive high proliferation, such as the PLC-activating GPCR and PKA signalling pathways. These pathways may lead to the phosphorylation of the transcription factor CREB, a competitor for the binding site of the coactivator CBP with c-myc, and increase expression of anti-apoptotic genes. Moreover, higher expression of *ST6GALNAC1* was associated with a relative pro-tumoral environment displaying a significant positive correlation with M2 macrophages, regulatory T cells, CD4 T cells and B cells. The significant enrichment of immune-related terms further supports this.

**Conclusion**

Targeting the tumour-specific antigen STn may hinder anti-apoptosis pathways, reduce tumour cell proliferation, and decrease pro-tumoral immune cell infiltration. In fact, the anti-STn mAb developed by us was able to revert the expression of immunosuppressive cytokines *in vitro*, suggesting that STn blockade could leverage the immune response. This study provides new insights into the potential of STn as a targetable biomarker for TNBC therapy.

### EACR23-1447

### Screening for microRNA dependencies of Enzalutamide-resistance in Prostate Cancer Cells

*J.T. Chow[1], B.Y. Lee[1], D.K.C. Lee[1], I. Grigore[1], M. Gabra[1], L. Salmena[1,2]*

*[1]University of Toronto, Department of Pharmacology and Toxicology- Faculty of Medicine, Toronto, Canada*

*[2]University Health Network, Princess Margaret Cancer Centre, Toronto, Canada*

**Introduction**

Prostate cancer (PCa) is the most common cancer in men worldwide. Localized PCa can be successfully treated with standard treatment consisting of surgery and radiotherapy, leading to a 5-year survival rate ≥90%. In recurrent cases, androgen deprivation therapy (ADT), such as the second generation androgen receptor antagonist Enzalutamide, is used to reduce tumour burden and provides excellent initial responses. Unfortunately, ADT invariably leads to castration-resistant PCa (CRPC), an incurable form of PCa that eventually leads to metastatic disease. Despite the introduction of new treatments, CRPC still persists and there is a need to identify drivers of castration-resistance in order to prevent the development of CRPC. microRNA (miRNA) are small non-coding RNA that can regulate diverse gene networks simultaneously. Recent studies have also shown that miRNA can play key roles in driving CRPC development. Despite numerous correlative studies, the functions of miRNA in PCa have not been studied in a comprehensive manner. As such, key miRNA that can suppress or drive CRPC development, and serve as putative therapeutic targets are largely uncharacterized. We hypothesize that perturbations of miRNA expression and the gene networks they control, are crucial for PCa progression and castration-resistance.

**Material and Methods**

We performed an Enzalutamide (Enz) CRISPR-Cas9 screen using our previously developed miRNA-focused CRISPR KO library, miRKOv2. Briefl LNCaP cells

© 2023 The Authors. Molecular Oncology is published by John Wiley & Sons Ltd on behalf of Federation of European Biochemical Societies transduced with miRKOv2 were split into three treatment arms, Vehicle, sub-lethal Enz (IC20) and lethal Enz (IC80), to identify Enz-sensitizing and Enz-resistant miRNA using the drugZ algorithm. Validation of candidate hits using knockout and overexpression will consist of in vitro growth assays in the presence of Enz and dose-response assays. Moreover, we are generating Enz-resistant LNCaP cells in order to profile miRNA alterations.

**Results and Discussions**

We have identified several hits from our Enz CRISPR-Cas9 screen and preliminary hit validation is underway. Resistance has been achieved in our in vitro model of Enz resistance and further tests for stability of resistance are ongoing.

**Conclusion**

A novel miRNA-focused CRISPR-Cas9 screen to identify miRNA dependencies of Enzalutamide resistance was recently completed. This screen, along with profiling of miRNA alterations in resistant cells will identify miRNA changes that sensitize or provide resistance to Enzalutamide and drive the development of CRPC.

### EACR23-1455
### Enteric glial cell phenotype in colorectal cancer

*M. Thijssen[1,2], M. Idris[1,3], M. Massen[1], J. van der Meer[1], A. Sacchetti[4], M. Weijenberg[5], P. van den Brandt[5], M. Alves[3], K. Smits[1], W. Boesmans[1,2], V. Melotte[1,3]*

*[1]Maastricht University Medical Center, Department of Pathology- GROW - School for Oncology and Reproduction, Maastricht, The Netherlands*
*[2]Hasselt University, Biomedical Research Institute BIOMED, Hasselt, Belgium*
*[3]Erasmus University Medical Center, Department of Clinical Genetics, Rotterdam, The Netherlands*
*[4]Erasmus University Medical Center, Department of Pathology, Rotterdam, The Netherlands*
*[5]Maastricht University Medical Center, Department of Epidemiology- GROW - School for Oncology and Reproduction, Maastricht, The Netherlands*

**Introduction**

Enteric glia are active players within the enteric nervous system as they regulate major gastrointestinal functions, influence the development of mucosal epithelial cells and are believed to bridge neuro-immune interactions. Recently a role for enteric glia in colorectal carcinogenesis has been proposed. Here, we aim to study the phenotype of enteric glial cells in the colorectal cancer (CRC) microenvironment.

**Material and Methods**

To investigate the phenotype of enteric glia in the CRC microenvironment, formalin-fixed, paraffin-embedded tumor tissues and corresponding healthy epithelium and adenoma tissues from the same CRC patients were immunohistochemically stained for the glial markers s100b and glial fibrillary acidic protein (GFAP). Moreover, two large CRC patient cohorts (n=447 and n=324 respectively) were evaluated for GFAP expression within the tumor area, and staining was associated with CRC patient characteristics. To profile enteric glial cells in CRC on a transcriptomic level, we used fluorescence-activated cell sorting (FACS) to enrich for enteric neural cells from normal and tumor tissue samples from CRC patients, and performed single-cell RNA sequencing (scRNA-seq).

**Results and Discussions**

GFAP positive enteric glial cells were found within the tumor stroma of carcinoma tissue, but not in the healthy epithelium or adenoma tissue from the same patient, indicating a change in glia cell phenotype in the CRC microenvironment. GFAP staining was not consistently associated with CRC patient characteristics, such as tumor stage, sublocation, differentiation grade and median survival. In the multivariate analysis, when compared to negative GFAP staining, high-density GFAP staining was associated with better survival in the study cohort (HR 0.56; p=0.030), but this association could not be validated in the in-cohort validation (HR=0.85; p=0.606). As expected, transcriptional profiling of enteric glial cells showed that distinct enteric glial cell clusters are present in the normal tissue of CRC patients. Moreover, cancer-associated enteric glia clustered differently, suggesting enrichment of specific enteric glial cell subtypes.

**Conclusion**

These findings indicate that the CRC environment induces changes in enteric glial cell phenotype. Future studies are necessary to identify the exact function of enteric glia in colorectal carcinogenesis.

### EACR23-1467
### Human organ-specific vasculature models to study cancer:endothelial cell interaction in metastasis formation.

*C. Cerutti[1], A. Luraschi[1], L. Bettinelli[1], V. Grazioli[1], I. Kasioulis[2], R. Nacho[3], G. Spinetti[4], A. Granata[2], M. Rasponi[5], P.G. Pelicci[1]*

*[1]IEO- European Institute of Oncology IRCCS, Department of Experimental Oncology, Milan, Italy*
*[2]University of Cambridge, Heart & Lung Research Institute- Cambridge Biomedical Campus, Cambridge, United Kingdom*
*[3]The Open University, Faculty of Science- Technology- Engineering & Mathematics, Milton Keynes, United Kingdom*
*[4]IRCCS MultiMedica, Laboratory of Cardiovascular Research, Milan, Italy*
*[5]Politecnico di Milano, Dept. of Electronics- Information- and Bioengineering, Milan, Italy*

**Introduction**

Introduction: Interaction of cancer cells with endothelial cells(ECs), which line blood vessels, is an early and critical event in metastasis formation. Breast cancer in the most common cancer in women worldwide, that after years of latency metastasise to the brain, lung and bone, causing 90% of cancer related death. Despite the contribution of animal models to cell-cell interaction and cancer studies, human organ-specific vasculature in vitro models to study breast cancer organ tropism are needed.

**Material and Methods**

Material and method: Here, we set-up microfluidic human vascular models of brain, lung and bone to study breast cancer cells interaction (firm adhesion and migration) with ECs under hemodynamic-like shear stress coupled to live-cell imaging.

© 2023 The Authors. Molecular Oncology is published by John Wiley & Sons Ltd on behalf of Federation of European Biochemical Societies

**Results and Discussions**
Results and discussion: We characterized the microfluidic human vascular models of brain, lung and bone by endothelial specific markers and cell junction expression, such as VE-cadherin and ZO1, by immunofluorescence. Permeability to 4KD dextran was also measured in the microfluidic brain model.
**Conclusion**
Conclusion: These models may provide an important tool to unravel the molecular mechanism of cancer cell interaction with organ-specific vasculatures and new targets to prevent and/or reduce breast cancer metastasis.

### EACR23-1470
### HGF-C-Met- β1 integrin crosstalk regulates tunneling nanotube formation in lung adenocarcinoma cells through the scaffolding protein paxillin, and downstream MAPK, PI3K and Arp2/3 signalling pathways.

*G. Awanis[1], S. Raveenthiraraj[1], R. Johnson[1], J. Gavrilovic[1], D. Warren[1], A. Sobolewski[1]*
*[1]University of East Anglia, School of Pharmacy, Norwich, United Kingdom*
**Introduction**
Tunneling nanotubes (TNTs) are long, thin intercellular extensions that transport intracellular organelles, signalling molecules & oncogenes between cells. TNTs are present in cancer & are implicated in aggressiveness & chemoresistance. Studies so far have focussed on how cellular stresses regulate downstream cell signalling &TNT formation. Not much is known about the upstream signalling pathways involved in TNT formation, especially in different cancers. Non small cell lung cancer (NSCLC) is one of the most common cancers & has a poor survival rate. Hepatocyte growth factor (HGF) & its receptor c-met are dysregulated in NSCLC & contribute to cancer cell growth, survival, & metastasis, but their role in TNT formation has not been investigated. The aim of this work was to determine whether HGF & its receptor c-met mediate adenocarcinoma cell communication & the transfer of intracellular organelles through TNTs.
**Material and Methods**
Lung adenocarcinoma A549 cells (ATCC) were seeded in 12-well plates at a density of 5000 cells/well & cultured for 72h in (DMEM,L-Glu, P/S, 10%FBS; 37ºC/5%$CO_2$) before 24h HGF treatment (≤700ng/ml). White light images were captured & the mean % of cells with TNTs, average number of TNTs/cell & the mean length of TNTs were analysed using image J software. Inhibitor studies (c-metR;PI3K;MAPK;ROCK;CDC42-GEF;Rac-GEF;Arp2/3 & β1 integrin Ab) included a 30min pre-incubation before HGF addition. Knockdown studies using non-targeting & paxillin siRNA were used to determine effects on HGF-induced TNTs. Immunofluorescent labelling with specific antibodies followed by confocal microscopy was used to determine expression of known TNT markers (eg f-actin,m-sec) & novel proteins (eg c-met).Significance was tested by one-way ANOVA & Tukey's multiple comparison test & experiments conducted at least 3 times.
**Results and Discussions**
HGF induced TNT formation in A549 cells in a concentration & time-dependent manner with maximal effects at 100ng/ml and 24h. TNTs expressed established markers α-tubulin, F-actin & m-sec & novel TNT proteins c-met, β1 integrin and paxillin. HGF-induced TNTs were able to transfer mitochondria & lipid vesicles. siRNA knockdown studies showed that paxillin was required to mediate HGF/c-met/β1 integrin crosstalk in HGF-induced TNTs along with the downstream signalling pathways MAPK and PI3K & Arp2/3.
**Conclusion**
Understanding HGF-induced TNT regulation provides a better understanding of cancer progression that will help the development of new, more effective therapies for NSCLC

### EACR23-1489
### Beta-galactosylceramidase modulates the pro-oncogenic features of melanoma cells in vitro

*D. Capoferri[1], M. Corli[1], D. Coltrini[1], M. Belleri[1], M. Presta[1]*
*[1]University of Brescia, Molecular and Translational Medicine, Brescia, Italy*
**Introduction**
β-galactosylceramidase (GALC) is a lysosomal hydrolase whose known physiologic function is to remove β-galactosyl- moieties from β-galactosyl-sphingolipids. Its expression in melanoma patients was demonstrated to positively correlate to the stage of the disease, suggesting a role of this enzyme in modulating the malignant features of this neoplasm.
**Material and Methods**
Melanoma cell lines A2058 and A375 were transduced by lentiviral vector to overexpress GALC (henceforth upGALC) and characterized *in vitro* for proliferation, motility, receptor activation, proteomic and lipidomic profile alteration, and conditioned media were collected from their culture supports to stimulate HUVEC cells. Inhibitors of GALC activity and internalization, as well as the use of a known GALC-inactive mutant, were employed.
**Results and Discussions**
upGALC cells exhibited a higher proliferation rate and a more motile phenotype when compared to controls. These features were accompanied by increased ground-state activation levels of receptor tyrosine kinases (RTKs) and of downstream intracellular signalling pathways, as well as from significant underrepresentation of mitochondria-related proteins in melanoma cell lysates and alteration of the lipidomic profile. Further, GALC-enriched conditioned media collected from upGALC cells induced pro-angiogenic responses in HUVEC cells. The use of specific inhibitors of its enzymatic activity and of GALC mutants devoid of any activity put the spotlight on the dependence of its pro-oncogenic features on its functionality.
**Conclusion**
Taken together, these data indicated for the first time that GALC expression and functionality in melanomas might not only be a passive byproduct of melanocyte transformation but could interact within a complex microenvironment to promote those malignant features that can be seen in advanced stage diseases.

© 2023 The Authors. Molecular Oncology is published by John Wiley & Sons Ltd on behalf of Federation of European Biochemical Societies

### EACR23-1501
### ALK-Rearranged Lung Adenocarcinoma Initially Presenting with Cutaneous Metastases: A Case Report

H. Dudipala[1], C. Townsend[1], N. Alavi[2]
[1]Boston University School of Medicine and Boston Medical Center, Department of Medicine, Boston, United States
[2]Boston University School of Medicine and Boston Medical Center,
Department of Hematology and Medical Oncology, Boston, United States

**Introduction**

Lung cancer is the leading cause of cancer incidence and mortality worldwide. The most frequent sites of metastasis of lung cancer are bone (34.3%), brain (28.4%), adrenals (16.7%), and liver (13.4%). Only 1-12% of patients develop cutaneous metastases. While adenocarcinoma is incredibly common, it is rare for a cutaneous manifestation of the disease to be the initial presenting complaint. Here we present a rare case of ALK-rearranged lung adenocarcinoma that initially presented as cutaneous skin metastases to the neck.

**Material and Methods**

A 60-year-old female presented to the emergency room with a large painful right neck ulcerating lesion that had been progressively growing for 8 months. The neck lesion was previously treated with steroids, antibiotics, and antifungal therapy. The patient presented again a few months later with shortness of breath secondary to a pericardial effusion, for which cytology was consistent with poorly differentiated adenocarcinoma with ALK rearrangement. An excisional lymph node biopsy was performed showing metastatic adenocarcinoma consistent with lung origin, with an EML4-ALK fusion, PD-L1 +, and negative for BRAF, EGFR, ERBB2, KRAS, MET, RET, and ROS1. The patient was diagnosed with Stage IV ALK-rearranged lung adenocarcinoma with lymphangitic carcinomatosis, and right neck dermal involvement. She was started on Alectinib, but later switched to Lorlatinib due to concern for treatment failure in the setting of increased CEA levels. Her course was further complicated by multiple pericardial and pleural effusions, resulting in respiratory decompensation. Given patient's disease progression and overall poor prognosis, the patient was transitioned to CMO and eventually succumbed to her disease.

**Results and Discussions**

Only 2.8% of non-small cell lung cancer patients show cutaneous metastases with initial presentation. Cutaneous metastases of lung cancer is a poor prognostic indicator and has a shorter survival time compared to other metastases, at less than 1 year. Our patient initially presented with a neck lesion that was treated as an infection, but a malignancy was only considered when the lesion progressed in size. Therefore, it is crucial to identify skin lesions early and consider them as a potential manifestation of an internal malignancy.

**Conclusion**

Skin manifestations can be difficult to identify and can appear deceptively benign. Timely biopsy of suspicious lesions and further imaging should be performed to prevent delays in diagnosis and treatment.

### EACR23-1516
### 3D in vitro models for advanced colorectal cancer

S. Vitale[1], F. Colonna[1], F. Calapà[2], F. Luongo[2], L. Villanova[1], L. Businaro[3], A. De Ninno[3], M. Biffoni[1], F. Giuliante[4], R. De Maria[2], M.E. Fiori[1]
[1]Istituto Superiore di Sanità, Oncology and Molecular Medicine OMM, Rome, Italy
[2]Università Cattolica del Sacro Cuore, Istituto di Patologia Generale, Rome, Italy
[3]Consiglio Nazionale delle Ricerche, Institute of Photonics and Nanotechnologies, Rome, Italy
[4]Fondazione Policlinico A. Gemelli, Hepatobiliary Surgical Unit, Rome, Italy

**Introduction**

Three-dimensional (3D) cell culture models have emerged as promising tools to bridge the gap between animals and cell culture systems. However, to generate reliable pre-clinical data and identify new effective therapeutics, in vitro models must be integrated with components of the tumor microenvironment (TME). TME has a determinant role in cancer development, it supports adaptation of disseminated cancer cells required for their survival and homing to distant sites and exerts a protective role reducing therapy efficacy.

**Material and Methods**

We established a living biobank of organoids and spheroids from liver metastases of 60 CRC patients. We also isolated hepatic Cancer Associated Fibroblasts (CAFs) and normal fibroblasts. Primary cultures were validated by STR identification, tumorigenicity assessment in mice and xenograft comparison with the tumor of origin. Isolated cells were analyzed for the expression of specific markers and by functional assays. Pre-clinical platforms were developed to perform drug screenings and detailed studies on tumor-stroma interactions. 3D co-culture methods were optimized, and custom chips were exploited to mimic interactive dynamics between CAFs and cancer cells.

**Results and Discussions**

A systematic comparison between spheroids and organoids defined specific features of each 3D model. Spheroid cultures are enriched in cancer stem cells, endowed with mesenchymal traits while organoids contain a higher percentage of differentiated cells, recapitulating tumor heterogeneity. Consistently, spheroids are more prone to migration/invasion than organoids, when co-cultured with CAFs in a 3D matrix.

The growth rate of spheroids and organoids were compared both in vivo and in vitro, and patient-specific drug sensitivities were screened recapitulating the therapeutic regimen of using the same chemotherapeutic agents (5FU, oxaliplatin, irinotecan) used for therapeutical treatment. We optimized high-throughput drug screening procedures with spheroids and different co-culture settings of organoids/spheroids and stromal cells to analyze reciprocal spatial organization and how cancer cells are molded by the niche in terms of secretome, specific protein expression and drug response.

**Conclusion**

We have developed a solid 3D co-culture system with primary cancer cells and CAFs, able to reproduce the

© 2023 The Authors. Molecular Oncology is published by John Wiley & Sons Ltd on behalf of Federation of European Biochemical Societies structure of the tumour of origin and the response to therapies of patients with metastatic CRC.

### Tumour Evolution and Heterogeneity

### EACR23-0046
### Mutational order and epistasis regulate the transcriptional consequences of FBXW7 mutations during early colorectal cancer

D.K.H. Chan[1], S.J.A. Buczacki[1]
[1]*University of Oxford, Nuffield Department of Surgical Sciences, Oxford, United Kingdom*

**Introduction**

Recent discoveries suggest that the transition from normal tissue to cancer is a continuum of cell states without any precipitous genetic events. Somatic mutations in cancer driver genes including *FBXW7* have been found in normal human colonic epithelium but their role in tumour initiation and evolution remain unknown. Here, using gene engineering and human colon organoids we study the molecular and phenotypic effects of *FBXW7* mutation during cancer evolution.

**Material and Methods**

Wildtype (W) adult human colon organoids were derived. To model pre-cancer and cancer, *APC*$^{-/-}$ (A) and *APC*$^{-/-}$/*TP53*$^{-/-}$ (AP) double mutant organoids were generated. *FBXW7*$^{-/-}$ (F) mutation was then introduced on W, A and AP backgrounds to generate F, AF and APF organoids respectively. F organoids were molecularly and phenotypically characterised. W-F organoid co-culture assays were performed to identify co-operative or competitive phenotypes. Bulk RNAseq and scRNAseq was used to detect transcriptomic differences amongst organoids across different genetic backgrounds and data validated.

**Results and Discussions**

Compared to W organoids, F organoids expectedly showed an upregulation of downstream degradation targets such as CCNE1, MYC, JUN and NOTCH. However, when W organoids were co-cultured with F organoids, no growth advantage of F organoids was observed. Bulk RNAseq showed the transcriptional impact of a *FBXW7*$^{-/-}$ mutation was surprisingly highly restricted when compared with W, with only 5 genes being differentially expressed.
However, *FBXW7*$^{-/-}$ mutation on different genetic backgrounds showed varying effects. Between AF and A, and APF and AP, 86 and 908 genes were differentially expressed respectively, proposing an epistatic effect of mutations in the *FBXW7* gene.
Most strikingly, when an *APC*$^{-/-}$ mutation was generated on *FBXW7*$^{-/-}$ (FA) organoids, RNAseq showed greater transcriptional similarity between FA, F and W organoids than with AF organoids, providing compelling evidence that the order in which mutations arise play a hitherto unappreciated role in determining tumour phenotype.

**Conclusion**

These data provide the first biological evidence of epistasis and mutational order as being deterministic of phenotype in a human model of cancer evolution. These observations may have profound implications for targetted therapies and cancer screening. Our findings further suggest that *FBXW7* mutations in normal epithelial tissue abrogate against the effect of a future *APC* mutation; commonly considered to be the first driver mutation acquired in CRC.

### EACR23-0095
### Heterogeneity of tumor-associated astrocytes in experimental gliomas defined by single-cell, spatial transcriptomics and immunohistochemistry

M. Ghosh[1], P. Pilanc[1], K. Jacek[1], P. Szadkowska[1], B. Kaminska[1]
[1]*Nencki Institute of Experimental Biology- Polish Academy of Science, Laboratory of Molecular Neurobiology, Warsaw, Poland*

**Introduction**

Compelling evidence shows that the composition of the tumor microenvironment (TME) modulates tumor progression and impacts therapy outcomes. The most aggressive and deadly primary brain tumors in adults are glioblastoma (GBM). Despite multimodal therapy including neurosurgery, radiation therapy and chemotherapy, the median survival of glioblastoma patients treated remains 15-16 months. GBM is one of the most immunosuppressed and heterogeneous tumors. Inter and intra-tumor heterogeneity is a major hurdle for precise diagnosis and treatment of brain tumor patients. TME heterogeneity due to heavy infiltration of immunosuppressive myeloid cells, activation of astrocytes and endothelial cells might contribute to tumor progression, treatment resistance and relapse. Therefore, we sought to understand spatial heterogeneity of astrocytes in a mouse model of GBM.

**Material and Methods**

We optimized the protocol for spatial transcriptomics (Visium, 10x Genomics) on fresh frozen sections of murine brains bearing intracranial GL261 gliomas and combined it with multiple immunohistochemistry (IHC) staining for detailed characteristics of mouse glioma TME. Deconvolution of TME was performed with public single-cell (sc) RNAseq datasets from GL261 gliomas.

**Results and Discussions**

We decoded several structural and functional clues allowing to dissect functionalities of tumor-associated astrocytes at distinct locations within TME. The analysis of morphology and location of astrocytes demonstrated their heterogeneity and various roles in shaping the local tumor microenvironment and revealed potential contributing factors. We demonstrated that the therapeutic intervention targeting glioma-myeloid cell interactions via integrins and aiming to convert “cold” into “hot” TME impacted the spatial distribution of astrocytes in murine gliomas.

**Conclusion**

Integration of single-cell and spatial transcriptomics combined with multi marker IHC revealed the tumor heterogeneity and substantial changes in astrocytes surrounding experimental gliomas. We show that the heterogeneous population of tumor-associated astrocytes could be an important therapeutic target. These findings

© 2023 The Authors. Molecular Oncology is published by John Wiley & Sons Ltd on behalf of Federation of European Biochemical Societies provide a rationale for development of novel combinatorial treatment to improve anti-glioma therapies.

### EACR23-0097

### Clinical implication and chemo-sensitivity of adjuvant chemotherapy in patients with poorly cohesive cell gastric cancer

*B.W. Kang[1], Y.S. Chae[1], J.H. Baek[1], J.G. Kim[1], M. Kim[2], A.N. Seo[2], O.K. Kwon[3], K.B. Park[3], J.Y. Park[3]*
*[1]Kyungpook National University Hospital, Oncology, Daegu, South Korea*
*[2]Kyungpook National University Hospital, Pathology, Daegu, South Korea*
*[3]Kyungpook National University Hospital, Surgery, Daegu, South Korea*

**Introduction**
Although poorly cohesive cells-gastric cancer (PCC-GC) represents a distinct pathological entity within the GC spectrum, there has been few data concerning the chemosensitivity of PCC-GC. The present study investigated the clinicopathologic characteristics and implication of chemotherapeutic agents for a relatively large cohort of PCC-GC patients in an adjuvant setting.
**Material and Methods**
This study retrospectively reviewed patients who were diagnosed with PCC-GC and underwent curative surgical resection at Kyungpook National University Chilgok Hospital between April 2011 and May 2022.
**Results and Discussions**
A total of 268 patients was analyzed. According to tumor staging, 115 (42.9%) cases were stage II and 153 (57.1%) were stage III. One hundred twenty-four (46.3%) patients were signet-ring cell carcinoma and most (94.4%) of the patients were diffuse-type. Two hundred-twenty three (83.2%) patients received adjuvant therapy. Among these patients, 139 (62.3%) received capecitabine/oxaliplatin and 84 (37.7%) received S-1 as their adjuvant chemotherapeutic agents, respectively. With a median follow-up duration of 38.9 (1.6 – 137.8) months, the estimated 5-year disease-free survival (DFS) and overall survival (OS) rates were 52.3% and 61.0% respectively. Survival was significantly higher in the adjuvant chemotherapy group than in the surgery only group. In the subgroup analysis, there was no significant difference in DFS or OS between types of adjuvant chemotherapy in both stage groups.
**Conclusion**
The current study found that adjuvant chemotherapy provided survival benefit for patients with resected stage II and III PCC-GC. However, the addition of oxaliplatin appeared to be less effective in these patients groups, supporting the fact that PCC-GC could have a different chemosensitivity profile.

### EACR23-0178

### Unravelling the clonal and transcriptional dynamics of metastatic breast cancer under therapeutic pressure via single-cell lineage tracing.

*A. Dalmasso[1], A. Cossa[1], N. Roda[2], P.G. Pelicci[1]*
*[1]IEO - Istituto Europeo di Oncologia, Department of Experimental Oncology, Milan, Italy*
*[2]VIB - KU Leuven Center for Cancer Biology, Dept. of Oncology, Leuven, Belgium*

**Introduction**
Triple negative breast cancer is characterized by poor differentiation, high proliferation and it is associated with high recurrence rates and poor overall survival. Chemotherapy remains the most used systemic treatment option. Chemotherapy was shown to induce a transcriptional reprogramming during treatment. Lineage tracing studies demonstrated that chemotherapy does not significantly alter the clonal composition of primary breast tumors. Rather, most cellular clones were shown to resist to neoadjuvant chemotherapy. Nevertheless, the specific transcriptional phenotype(s) conferring chemoresistance at primary tumor (PT) level, as well as at metastatic level, are still largely undetermined, specifically at single-clone resolution.
**Material and Methods**
A high-complexity genetic barcode library was employed to infect cells, then injected in immunocompromised mice. PT growth was monitored and resection was performed in the control groups, when palpable. In the other groups, PT was treated with three cycles of chemotherapy (adriamycin + cyclophosphamide A+C) and then removed. In all the experimental branches, metastasis formation was followed and in two branches metastatic cells were re-challenged with A+C, and infiltrated organs harvested. Collected tissues were then processed and undergone single-cell RNA sequencing, as well as bulk genomics analyses, to infer clonal dynamics and transcriptomic changes and trajectories.
**Results and Discussions**
A+C caused a drastic reduction of the clone number in the PT. However, pro-metastatic clones were detectable in different experimental branches. Metastatic seeding was different in the diverse experimental branches. Pro-metastatic clones were found to be endowed with specific transcriptional traits and trajectories, which were dramatically different with or without therapeutic pressure. Dominant clones, defined as clones that form the majority of the biomass, were characterised by different transcriptional features. From the single-cell data, gene lists were determined and the prognostic power was validated on publicly available patients cohorts.
**Conclusion**
The lineage tracing model set up so far represents a useful tool to unravel novel determinants of cancer cell escape under therapeutic pressure, as well as to study the transcriptional trajectories that distinguish expanding clones from the other parts of the tumor biomass.
The next steps will be the thorough validation of the clinical relevance of the candidate genes leading breast cancer dissemination under chemotherapy.

### EACR23-0205

### CTC-based workflow for dissecting pancreatic cancer heterogeneity at single-cell resolution

*C. Reina[1], B. Sabanovic[1], A. Awada[2], C. Moroni[2], A. Bartolini[3], P. Ananthanarayanan[1], S. Tondi[1], Y. Minamida[4], F. Okano[5], C. Heeschen[1]*
*[1]Candiolo Cancer Institute - FPO - IRCCS,*

© 2023 The Authors. Molecular Oncology is published by John Wiley & Sons Ltd on behalf of Federation of European Biochemical Societies

Pancreatic Heterogeneity Lab, Candiolo TO, Italy
[2]University of Turin, Molecular Biotechnology Center, Turin, Italy
[3]Candiolo Cancer Institute - FPO - IRCCS, Sequencing Facility, Candiolo TO, Italy
[4]Toray Industries Inc., New Frontiers Research Laboratories, Kamakura, Japan
[5]Toray Industries- Inc., Basic Research Center, Kamakura, Japan

**Introduction**

Pancreatic ductal adenocarcinoma (PDAC) is a rare and extremely lethal disease with unmet medical needs. It is usually diagnosed at an advanced stage, which further limits treatment options and aggravates prognosis. We have previously shown that targeting stemness using genetic tools enhances the response to standard therapy. Therefore, this precise targeting allows us to reprogram the underlying transcriptional circuitry that can prevent relapse and improve prognosis. For this purpose, we are studying PDAC at a single-cell resolution to build in-silico models of stemness and subsequently identify druggable therapeutic targets . The analysis of circulating tumor cells (CTCs) isolated from blood in contrast to tissue biopsies offers a snapshot of the tumor at any given time, including metastasis. As such, it reflects both spatial and temporal cancer heterogeneity at the genetic and epigenetic level. CTCs are rare cells among millions of blood cells and therefore are challenging to isolate and study. Here we established an innovative workflow for the detection and isolation of CTCs for single-cell RNA sequencing (sc-RNAseq) to explore heterogeneity of PDAC and identify novel therapeutic targets

**Material and Methods**

Blood collected from PDAC patients is processed by the Parsortix™ Cell Separation System (Angle, UK), a microfluidic device that separates, captures and harvests CTCs from blood based on size and deformability. Retrieved CTCs are fixed with dithiobis-succinimidyl propionate (DSP) and immunostained with a panel of fluorescence-labeled antibodies (anti-CD45-FITC, anti-CXCR4-AF555, anti-CAPRIN-1 bound to Donkey anti-rabbit secondary antibody AF-647) and nuclei are counterstained with Hoechst 33342.

**Results and Discussions**

We identified CTCs as CAPRIN-1+/CD45–/Hoechst+ cells. The subset of cancer stem cells among CTCs are identified by additional staining for CXCR4. Subsequently, CTCs were then enumerated and picked as single cells using the ALS CellCelector™ (Sartorius, Germany) into a lysis buffer prepared according to the SMART-Seq3 (SS3) protocol for scRNA-seq.

**Conclusion**

This workflow describes a successful isolation and detection of CTCs that opens new avenues of investigation of tumor heterogeneity. Subsequently, the customized SS3 protocol for scRNA-seq will provide full-length transcriptome coverage that introduces a 5'UMI sequence essential for in-silico isoform assignment and allelic origin determination of individual RNAs in single cells.

### EACR23-0262
### POSTER IN THE SPOTLIGHT

### Extremely rare human breast cancer cells identified by single cell clonal tracking rapidly expand, resist chemotherapy, and reconstitute the full tumour transcriptional landscape

L. Nguyen[1], Y. Eyal-Lubling[1], D. Guerrero Romero[1], S.F. Chin[1], R. Manzano Garcia[1], L. Allan[1], H. Bardwell[1], G. Lerda[1], W. Greenwood[1], E. Esmaeilishirazifard[1], O. Rueda[2], C. Caldas[3]
[1]University of Cambridge, CRUK Cambridge Institute, Cambridge, United Kingdom
[2]University of Cambridge, MRC Biostatistics Unit, Cambridge, United Kingdom
[3]University of Cambridge, CRUK Cambridge Institute- Department of Oncology- CR UK Cambridge Centre- Cambridge Experimental Cancer Medicine Centre ECMC and NIHR Cambridge Biomedical Research Centre, Cambridge, United Kingdom

**Introduction**

Late-stage breast cancer remains a challenge to eradicate due to the inevitability of treatment resistance. Intratumour heterogeneity contributes to this when a minor fraction of cells can resist treatment and drive progression.

**Material and Methods**

We developed an expressed lentiviral-based cellular barcoding strategy that allows us to track the clonal growth of 'barcoded' single cells as they expand to form clones *in vivo*, and simultaneously analyse their single cell transcriptomic profiles with single cell RNA sequencing. We applied this approach to analyse 113 tumours across 27 patient-derived tumour xenograft models grown in immunodeficient mice.

**Results and Discussions**

Overall, 1 in 267 cells formed clones *in vivo*. 19,943 barcode clones were identified and classified into 3 distinct Gaussian distributions based on their population doubling times. The fastest growing clones (doubling time <8.3 days) represented 19% of all clones detected (25% in ER+ models, and 16% in triple-negative models). MAPK signalling pathway, and genes involved in epithelial cell migration were among those significantly upregulated in these fast-growing clones. Only 1 in 19 clones further propagated upon serial transplantation into secondary mice. Of these, only 1 in 4 were able to regenerate cells spanning the full transcriptional landscape of the tumour. These extremely rare clones were derived from 1 in 90,000 (0.001%) originally barcoded cells. When assayed across multiple secondary mice, this activity was consistently observed across replicates indicating the ability to serially propagate and to regenerate the full transcriptional landscape of the tumour are an intrinsic program of these rare cell clones and not the result of other stochastic effects. Furthermore, these same clones were the only ones capable of resisting *in vivo* treatment with Carboplatin and Paclitaxel chemotherapy by altering their transcriptional cell states to adapt to this treatment pressure.

**Conclusion**

This is the first definitive evidence that a single cell can reconstitute the full transcriptional landscape of a tumour, and that such cell clones undergo rapid expansion and are responsible for resistance to chemotherapy. This supports the notion that extremely rare cells in human breast cancer

© 2023 The Authors. Molecular Oncology is published by John Wiley & Sons Ltd on behalf of Federation of European Biochemical Societies are ultimately responsible for disease progression and treatment resistance, and this should be considered in the future design of patient-tailored treatment strategies.

### EACR23-0394
### Platform toward identifying novel plasticity modulating treatment strategies in neuroblastoma.

*C. Roux*[1], *S. Hamer*[1], *A. Shea*[1], *C. English*[1], *E. Chen*[1], *A. Allo Anido*[1], *A. Bruna*[1]
[1]*The Institute of Cancer Research, Center of Evolution and Cancer, Belmont, United Kingdom*

**Introduction**
Non-genetic mechanisms are thought to play a key role in drug resistance and disease progression. Neuroblastoma represents an optimal model to study plastic versus Darwinian hereditable traits in evolutionary processes because of its high phenotypic heterogeneity and low genetic alterations. Neuroblastoma is classified into at least two phenotypic cell states: adrenergic (ADRN) and mesenchymal (MES). Evidence highlights the role of phenotypic transition in the absence of genetic changes between the two populations in therapy resistance and relapse. Though, the precise mechanisms underpinning these processes remain elusive. This project aims to unravel the role of phenotypic plasticity in directing adaptation to treatment and driving evolution by preceding selection of the fittest.

**Material and Methods**
We are using cell lines and patient-derived spheroids, which are stably imprinted in both ADRN and MES cell states, coupled with a flow cytometry approach and with a high complexity lentiviral barcoding system to track clonal evolution at single cell level.

**Results and Discussions**
We observed spontaneous and bidirectional plasticity between ADRN and MES cells. We characterized the impact of a selection of compounds, including epigenetic-modulating drugs, on phenotypic plasticity. Interestingly, we reported functional implications of each cell state that affect response to treatment, with front-line chemotherapy acting primarily on the more differentiated ADRN cells. Further, plasticity-modulating compounds can steer an enrichment of a transient intermediate cell state. This intermediate state is of high fitness yet highly unstable, with the potential to be a reservoir of drug tolerance. By applying a DNA-barcoding approach, we are tracking the evolution of individual clones to identify the trajectories of clonal evolution in response to treatment and match drug responses to cell identity by scRNA-seq. The phenotypic observations and the phylogenetic history given by the DNA barcodes indicate that there is a multimodal adaptive evolution in neuroblastoma in response to treatment.

**Conclusion**
We have generated empirical evidence that strongly supports the switching of cellular phenotypes towards fitter cell states, which may be an early cancer driver process in response to treatment. This new concept challenges natural selection as a sole cancer evolution driver and highlights the need to increase our understanding of molecular processed underpinning phenotypic plasticity to guide new therapeutic strategies.

### EACR23-0408
### Exploring cell states in high-grade serous ovarian cancer utilizing the cell of origin

*A. Pirttikoski*[1], *E. Pekcan Erkan*[2], *J. Hynninen*[3], *A. Vähärautio*[1]
[1]*University of Helsinki, Faculty of Medicine, Helsinki, Finland*
[2]*Tampere University, Faculty of Medicine and Health Technology, Tampere, Finland*
[3]*University of Turku, Department of Obstetrics and Gynecology, Turku, Finland*

**Introduction**
High transcriptional heterogeneity among cancer cells of a tumor is a characteristic feature in high-grade serous ovarian cancer (HGSOC) and it is increasingly recognized as a driver of tumor progression and drug resistance. This heterogeneity is organized into diverse cancer cell states which we hypothesize to originate and be co-opted from those present in normal epithelia. According to current scientific knowledge, the majority of HGSOC arise from fallopian tube epithelium (FTE).

**Material and Methods**
We used scRNA-seq samples from HGSOC primary tumors from 50 patients and from FTE from 7 healthy postmenopausal women to explore cell states related to HGSOC. Cell states were categorized into cancer and normal specific as well as shared epithelial cell states. They were characterized based on co-expressed gene modules that can be expressed in a combinatorial manner. Cell states were divided into early and late states based on their latent time in RNA velocity analysis. We utilized the L1000 data to identify potential drugs to target the cell states.

**Results and Discussions**
We identified cell states representing cellular processes such as oxidative phosphorylation (OXPHOS), inflammation and stress response which are relevant for both HGSOC and normal epithelia. We found that OXPHOS is represented mainly in initial states across normal and cancer cells which indicate that both epithelial stem cells and stem-like cancer cells use it for their metabolic needs. One of the cell states related to normal FTE was associated with significantly improved overall survival (OS) in HGSOC and it could be potentially induced with cyclin-dependent kinase inhibitors or inhibitors related to mitogen-activated protein kinase pathway. Inflammatory and stress signals were concentrated on terminal states, and those states predicted poor OS. Our results suggest that OXPHOS and inflammation are anticorrelated processes, even if their exact biological relationship remains unclear in the context of HGSOC.

**Conclusion**
Our study characterizes cell states in HGSOC taking into account their normal epithelial origin. We defined cell states through gene modules as this approach considers the complexity of cell states. We found OXPHOS-inflammatory response axis from initial to terminal cell states which plays potentially a relevant role in HGSOC development and treatment response.

© 2023 The Authors. Molecular Oncology is published by John Wiley & Sons Ltd on behalf of Federation of European Biochemical Societies

### EACR23-0588
### ecDNAs drive intra-tumoral copy-number heterogeneity and adaptation to environmental stress in PDAC organoids

A. Malinova[1], D. Schreyer[2], E. Fiorini[1], D. Pasini[1], M. Napoleone[1], S. D'Agosto[1], S. Andreani[1], S. Pedron[1], A. Scarpa[1], P. Bailey#[2], V. Corbo#[1]
[1]*University of Verona, Department of Diagnostics and Public Health, Verona, Italy*
[2]*Institute of Cancer Sciences- University of Glasgow, Institute of Cancer Sciences, Glasgow, United Kingdom*

**Introduction**

Genomic heterogeneity is a hallmark of pancreatic ductal adenocarcinoma (PDAC) and drives emergence of cells that can withstand selective pressures. Extrachromosomal DNA (ecDNA) amplifications can lead to a more effective increase in oncogene copy numbers than chromosomal amplifications. However, little is known about their role in PDAC, whether they are preserved in *in vitro* models, and how ecDNAs respond to environmental pressures.

**Material and Methods**

We used whole genome sequencing (WGS) and RNA sequencing on tissues and patient-derived organoids (PDOs) from the human cancer model initiative (HCMI). Findings were validated using fluorescent *in situ* hybridisation (FISH). *In vitro*, we depleted niche factors from the organoid medium and characterised organoids by FISH, pharmacological treatment, immunohistochemistry, and orthotopic transplantations into immunodeficient mice.

**Results and Discussions**

EcDNA amplifications containing known oncogenes were found in both tissues and PDOs. RNA sequencing showed that oncogenes amplified on ecDNA had higher expression than those on chromosomal DNA. The WGS-inferred structure of ecDNAs was conserved between PDOs and matched tissues. FISH on tissues and metaphases from PDOs confirmed the presence of ecDNA containing *MYC* and further evidenced extensive copy-number heterogeneity within individual cultures. The most common ecDNA amplification was *MYC*, a known WNT target. Depletion of WNT from the culture media severely affects long-term propagation of PDOs, and overexpression of *MYC* conferred WNT independency to PDOs. Therefore, we depleted WNT activators from PDO media to model the dynamics of ecDNA *MYC* amplification. Upon depletion, we observed a significant increase in ecDNA copy number in individual cells as well as an increase in the number of cells carrying the amplification. Conversely, the reintroduction of WNT activators led to a significant decrease of ecDNAs. Collectively, our data suggest that ecDNA amplifications are under strong selective pressure and drive adaptation to stress. Furthermore, PDOs displaying ecDNA *MYC* amplification showed higher *MYC* expression and *in vivo* produced larger tumours and metastases. Nonetheless, these PDOs were more sensitive to JQ1, a *MYC* transcription inhibitor.

**Conclusion**

ecDNAs are present in early staged PDAC and are preserved in PDOs. ecDNA *MYC* amplification can drive adaptation to suboptimal niche conditions in PDOs and PDOs with ecDNA amplifications display higher tumorigenicity, but also higher sensitivity to *MYC* inhibition.

### EACR23-0606
### Patient-derived co-cultures of TRACERx lung cancer organoids and autologous T-cells reveal heterogeneity in immune evasion between cancer subclones

K. Dijkstra[1], R. Vendramin[2], D. Karagianni[3], F. Gálvez-Cancino[3], K. Foster[3], M. Hill[4], R. Hynds[5], D. Pearce[4], V. Barbè[4], D. Moore[6], A. Rowan[4], O. Pich[4], M. Jamal-Hanjani[7], C. Hiley[4], K. Litchfield[2], J. Reading[3], B. Chain[8], E. Voest[1], S. Quezada[3], C. Swanton[4]
[1]*Antoni van Leeuwenhoek - Nederlands Kanker Instituut, Department of Molecular Oncology and Immunology, Amsterdam, The Netherlands*
[2]*University College London Cancer Institute- University College London, Tumour ImmunoGenomics and Immunosurveillance Laboratory, London, United Kingdom*
[3]*University College London Cancer Institute- University College London, Cancer Immunology Unit, London, United Kingdom*
[4]*The Francis Crick Institute, Cancer Evolution and Genome Instability Laboratory, London, United Kingdom*
[5]*Great Ormond Street UCL Institute of Child Health- University College London, Developmental Biology and Cancer Department, London, United Kingdom*
[6]*University College London Hospital, Department of Cellular Pathology, London, United Kingdom*
[7]*University College London Cancer Institute, Cancer Metastasis Laboratory, London, United Kingdom*
[8]*University College London, Division of Infection and Immunity, London, United Kingdom*

**Introduction**

Cancers display a remarkable heterogeneity in local immune context, with immunologically hot and cold regions coexisting in the same tumour. Branching tumour evolution results in genetically distinct cancer subclones, but the extent to which separate subclones differ in their capacity for immune evasion and the tumour-intrinsic mechanisms underlying any such heterogeneity remain largely unexplored.

**Material and Methods**

Here, we leverage the multi-region TRACERx lung cancer evolution study to generate a patient-derived study platform that allows the evaluation of T-cell responses to individual cancer subclones. We generated libraries of >20 separate non-small cell lung cancer (NSCLC) organoid lines per patient, based on isolating individual (clonal) organoids established from multiple spatially separated tumour regions. Each organoid subline was co-cultured with autologous tumour infiltrating lymphocytes (TIL) to evaluate how they differ in their capacity to elicit a T-cell response. We combined functional assays with DNA, RNA and TCR sequencing to perform an in-depth characterisation of 44 individual organoid lines derived from 6 separate tumour regions from two patients with NSCLC to identify the mechanistic basis driving subclonal immune evasion.

**Results and Discussions**

Our data reveal heterogeneity between organoid lines representing different cancer subclones in their

© 2023 The Authors. Molecular Oncology is published by John Wiley & Sons Ltd on behalf of Federation of European Biochemical Societies capacity to stimulate TIL. For patient 1, organoids derived from papillary tumour regions escaped T-cell detection in vitro, in contrast to organoids from the solid tumour compartment. Immune escape was antigen-dependent, suggesting that subclonal immune escape is driven by regional heterogeneity in antigen expression or presentation. For patient 2, T cell recognition strongly differed between separate clones established from one individual tumour region. Immune escape was antigen-independent and was associated with a transcriptional profile linked to quiescence.

**Conclusion**

These results show (i) that tumour evolution can give rise to distinct cancer clones with intrinsic differences in immune evasion capacity and (ii) provide an approach to prospectively identify and isolate immune evading subclones from cancer patients.

### EACR23-0619
### POSTER IN THE SPOTLIGHT
### Tracing the origin of pediatric cancer using single-cell whole genome sequencing

*S. Middelkamp[1], F. Manders[1], M. van Roosmalen[1], F. Peci[1], D. Montiel González[1], E. Bertrums[1], I. van der Werf[1], A. Brandsma[1], M. Belderbos[2], R. van Boxtel[1]*
*[1]Princess Máxima Center and Oncode Institute, Van Boxtel Lab, Utrecht, The Netherlands*
*[2]Princess Máxima Center, Belderbos Lab, Utrecht, The Netherlands*

**Introduction**

While single-cell RNA sequencing has revolutionized biomedical research, the development and application of single-cell whole genome sequencing (WGS) has lagged due to low accuracy and sensitivity caused by uneven and imprecise DNA amplification. Even the newest, most accurate whole genome amplification technologies, such as primary template-directed amplification (PTA), still generate hundreds of false positive artificial mutations, impeding reliable analyses of somatic mutations.

**Material and Methods**

We developed an innovative machine learning approach (PTATO) to accurately filter PTA artefacts from single-cell WGS data. This novel bioinformatic workflow enables analyses of single nucleotide variants, indels and structural variants in single-cell genomes at unprecedented resolution.

**Results and Discussions**

The exceptional performance of our method enables us to perform detailed retrospective genetic lineage tracing to study the origin and evolution of cancers. Uniquely, we can study most passenger mutations, and thereby characterize mutation patterns, in all types of single cells (including cancer cells and differentiated cells). In contrast to regular bulk WGS, which is limited to the study of highly clonal, early mutations, we can time the activity of mutational processes throughout the entire history of a cancer. We demonstrate that the origin of leukemic drivers occurred years before the diagnosis of pediatric acute myeloid leukemia (AML), and that some AML blasts are still able to differentiate into mature cell types. Additionally, we discovered that the processes causing mutational signature SBS18 (associated with oxidative stress) appear to be mostly active in (non-) leukemic differentiated hematopoietic cells and that they can be active during each stage of AML development in a subset of patients.

**Conclusion**

Our optimized single-cell WGS approach gives unprecedented views of the life histories of cancers, yielding novel insights in the mutagenic processes involved in carcinogenesis, cancer evolution and tumour heterogeneity.

### EACR23-0636
### The role of clonal interactions in the formation of intestinal tumours

*I. Sadien[1], S. Adler[1], F. Lourenco[1], R. Kemp[1], D. Winton[1]*
*[1]University of Cambridge, Cancer Research UK Institute, Cambridge, United Kingdom*

**Introduction**

Cancers are commonly held to originate from a single transformed cell. However, some reports have shown that some intestinal tumours in the colorectal cancer (CRC) predisposition syndrome Familial Adenomatous Polyposis (FAP) and mouse models of that condition may have a multiancestral structure. The aetiology of these polyclonal tumours is not understood.

**Material and Methods**

The clonal structure of intestinal tumours was investigated using a multicolour lineage reporter in a mouse model combining chemical mutagenesis in tissue primed with cancer driver events. Microdissected clones within polyclonal and monoclonal tumours were subjected to genomic and transcriptomic analysis. Tumour spheroids originating from monoclonal and polyclonal clones were used to functionally validate results from the transcriptomic analysis, with an aim to identify the mode of communication between clones.

**Results and Discussions**

We show that the clonal architecture of intestinal tumours depends on the underlying genetic background, with at least 40% of tumours in *Apc*$^{+/-}$ animals being polyclonal. Conversely, in *Kras*$^{G12D/+}$ animals, more than 85% of tumours were monoclonal. This suggests that activation of the RAS/MAPK pathway is sufficient to overcome a need for clonal interactions for tumour formation. Pharmacological inhibition of the RAS/MAPK pathway in Fbxw7$^{-/-}$ primed animals significantly reduced the number of tumours compared to vehicle-treated controls and strikingly led to an increase in the proportion of polyclonal tumours. Genomic analysis of 112 tumours in the *Apc*$^{+/-}$ model revealed that minor clones from polyclonal tumours and monoclonal tumours have different sets of mutations in *Apc,* hinting at a role for the level of Wnt activation in the development of polyclonal tumours. Transcriptomically, major clones had a secretory cell identity while minor clones were more stem cell-like, recapitulating the relationship between intestinal stem cells and their niche-sustaining secretory cells in homeostasis in the crypt.

**Conclusion**

Taken together, these data suggest that a significant proportion of intestinal tumours arise through clonal cooperation and that early RAS/MAPK pathway activation may obviate the need for this. The nature of

© 2023 The Authors. Molecular Oncology is published by John Wiley & Sons Ltd on behalf of Federation of European Biochemical Societies the *Apc* mutation influences the requirement for cooperation, although the exact mechanism for this remains to be elucidated. Uncovering this underlying mechanism may pave the way for new therapeutics aiming to reduce tumour burden and slow tumour growth in patients with FAP and a subset of sporadic CRCs.

### EACR23-0642
### Molecular characterisation of the evolution of premalignant lesions in the upper aerodigestive tract

*A. Lechner[1], J. Kumbrink[2], C. Walz[2], A. Jung[2], P. Baumeister[1], S. Flach[1]*
*[1]Hospital of the University of Munich- LMU Klinikum, Department of Otorhinolaryngology- Head and Neck Surgery, München, Germany*
*[2]LMU Munich, Institute of Pathology, München, Germany*

**Introduction**
Early relapse and development of metastatic disease are some of the primary reasons for the poor prognosis of patients with head and neck squamous cell carcinoma (HNSCC). HNSCC is a heterogeneous disease which may develop in large premalignant fields of genetically altered cells. Yet knowing which individuals will progress and develop clinically significant cancers within their lifetimes remains one of the most important challenges to reducing HNSCC morbidity and mortality. Here, we performed a focused analysis of the genome and immune microenvironment from multiple, matched normal squamous tissues, premalignant lesions and primary and recurrent tumours from seven patients with p16-negative HNSCC.
**Material and Methods**
We performed targeted panel Next Generation Sequencing (161 genes) to analyse somatic variants from sequentially collected, matched formalin-fixed paraffin-embedded tissue (normal, premalignant, HNSCC) from two patients. These samples in addition to samples from five further patients were analysed with the Nanostring PanCancer Immune Panel. Furthermore, we performed shallow whole genome sequencing (0.5x coverage) on samples from one patient. Patients were primarily treated with curative-intent surgery and received subsequent adjuvant treatment, if indicated.
**Results and Discussions**
The most frequently mutated gene was *TP53*. Other mutated genes included *NOTCH1*, *NOTCH3* and *CDKN2A* among others. A significant number of mutations were shared between dysplastic and malignant lesions. Pathways related to interferon alpha and gamma response were upregulated even in low-grade dysplastic lesions with increasing expression in higher grade dysplasia and carcinoma. SPINK5, a known tumour suppressor gene in HNSCC, was already downregulated even in low-grade dysplastic lesions, indicating an early deactivation in the evolution of the disease.
**Conclusion**
Genomic alterations as well as aberrant immune gene expression can be observed early on in the evolution of tumours of the upper aerodigestive tract, highlighting the potential for targeting early mechanisms of disease progression.

### EACR23-0696
### Tracing the trade-offs associated with the evolution of chemoresistance in epithelial ovarian cancer

*S. Goutham[1], R. Bhat[1]*
*[1]Indian Institute of Science, Department of Developmental Biology & Genetics, Bangalore, India*

**Introduction**
Resistance to chemotherapy a hallmark of epithelial ovarian cancer (EOC). A debilitating gynecological malignancy, EOC shows early resistance to the first-line drugs carboplatin and docetaxel, resulting in poor survival rates. While work by other groups focuses on the mechanisms underlying chemoresistance, we ask how cancer invasion is molded by the acquisition of drug resistance, distinguishing them phenotypically from their sensitive counterparts.
**Material and Methods**
Somatic selection experiments involving pulsed carboplatin treatment approach were performed to establish an isogenic chemoresistant variant of OVCAR3, a cell line typifying high-grade serous adenocarcinoma, the most aggressive type of EOC. Chemoresistance phenotype was ascertained using resazurin assay. Transwell migration and adhesion assays on Collagen I were used to study the invasion properties of Pt-res-OVCAR3. Fluorescence analysis was used to study population dynamics in coculture experiments.
**Results and Discussions**
Pt-res-OVCAR3 lines were found to have slower proliferation rates, lower adhesion to, and higher invasion potential on, Collagen I, compared to chemosensitive OVCAR3. We also observe that Pt-res-OVCAR3 cells form spheroids with distinct morphological differences compared to chemosensitive OVCAR3. Transcriptomic analyses performed on these cell lines revealed dysregulation of genes that are ontologically enriched for cell-extracellular matrix interactions. Combined with RNA sequencing observations, PCR-based screening approaches lead to the selection of genes of interest (FN1, CDH1, LGALS3BP – upregulated; CDH6 – downregulated in Pt-res-OVCAR3), whose roles in drug resistance has been hitherto unexplored. Preliminary results indicate involvement of LGALS3BP in the resistance, invasion and adhesion properties of Pt-res-OVCAR3. Coculture studies using fluorescently labelled chemo -sensitive and -resistant OVCAR3 variant lines reveal survival advantages of Pt-res-OVCAR3 in the absence of chemotherapeutic selection pressures, aided at least in part by ECM secreted by Pt-res-OVCAR3 cells.
**Conclusion**
Evolution of chemoresistance is a complex process accompanied by a myriad of genetic and phenotypic alterations resulting in a heterogenous tumor system. Understanding population dynamics in these bulk tumors, wherein drug-resistant and sensitive cancer cells coexist could provide valuable insights into disease progression, aiding in better models of translational relevance.

© 2023 The Authors. Molecular Oncology is published by John Wiley & Sons Ltd on behalf of Federation of European Biochemical Societies

### EACR23-0731
### Timing of breast cancer copy number gains reveals genomic subtype-specific temporal dynamics

K. Eason[1], C. Boursnell[1], R. Manzano[1], C. Young[1], P.A.W. Edwards[1], S.F. Chin[1], O. Rueda[2], C. Caldas[1]
[1]University of Cambridge, Cancer Research UK Cambridge Institute, Cambridge, United Kingdom
[2]University of Cambridge, Medical Research Council Biostatistics Unit, Cambridge, United Kingdom

**Introduction**

Breast cancer genomes are often marked by high levels of copy number aberrations (CNAs). We have previously defined CNA/transcriptome-based subtypes of breast cancer, referred to as "IntClust" subtypes. These subtypes have distinct prognoses and responses to treatment. However, it is unclear whether key driver CNAs occur at similar times between different subtypes. Recently published methods have allowed the calculation of "mutation timings" of CNAs to estimate their relative timings in the evolution of a tumour.

**Material and Methods**

WGS data from approximately 1000 breast cancer primary tumours and metastases from ICGC and Hartwig Medical Foundation were used to generate copy number profiles. The timing of CNAs was calculated using MutationTimeR.

**Results and Discussions**

We demonstrate that common copy number gains show substantial differences in timing between different subtypes; in particular, 1q and 8q gains show significant variation in timing despite being common events in multiple breast cancer subtypes, with IntClust 3 and 8 showing the earliest 1q gains and IntClust 9 showing the earliest 8q gains. Early copy-neutral loss-of-heterozygosity events are common in subtypes usually considered to have flat copy number profiles, such as IntClust 4. Amplifications that are predominant in one subtype tend to occur particularly early in that subtype compared to others, such as 8p11-12/*ZNF703* amplification in IntClust 6. Furthermore, in most subtypes, genes that are gained early in a tumour's evolution tend to be those that are characteristically highly expressed in that subtype as profiled at biopsy.

**Conclusion**

Our results establish that the timing of common CNAs is highly variable between different IntClust subtypes, potentially indicating their variable importance in driving the evolution of breast cancers of different subtypes. We suggest that the prevalence of early copy-neutral loss-of-heterozygosity events in subtypes with flat copy number profiles may signal their role in driving those subtypes. Further investigation into events that consistently occur early in particular subtypes may help identify drivers of subtypes with as-yet-unknown aetiology, potentially revealing novel therapeutic targets or identifying subtypes where patients could more likely benefit from existing therapeutics.

### EACR23-0799
### Tolerance to colibactin correlates with resistance to chemotherapy in colorectal cancer cells

A. Sogari[1], E. Rovera[1], N.M. Reilly[2], S. Lamba[2], E. Durinikova[2], A. Lorenzato[1], M. Avolio[3], E. Piumatti[4], M. Russo[1], S. Arena[1], L. Trusolino[1], F. Di Nicolantonio[1], M. Donalisio[5], D. Lembo[5], A. Bardelli[4]
[1]University of Torino - Candiolo Cancer Institute FPO-IRCCS, Department of Oncology - Satellite Candiolo, Candiolo TO, Italy
[2]Candiolo Cancer Institute FPO-IRCCS, Laboratory of Molecular Oncology, Candiolo TO, Italy
[3]Candiolo Cancer Institute FPO-IRCCS, Laboratory of Translational Cancer Medicine, Candiolo TO, Italy
[4]University of Torino - IFOM ETS- The AIRC Institute of Molecular Oncology, Department of Oncology, Torino TO - Milano MI, Italy
[5]University of Torino, Department of Clinical and Biological Sciences, Orbassano TO, Italy

**Introduction**

The bacterial genotoxin colibactin is enriched in colorectal cancer (CRC) and promotes the accumulation of mutations that drive tumorigenesis. However, systematic assessment of its impact on DNA damage response is lacking and the effect of colibactin exposure on response to other genotoxic agents (such as chemotherapy) is missing.

**Material and Methods**

We implemented an *in vitro* coculture system to assess the effect of colibactin on a representative panel of 40 CRC cell lines and in isogenic DDR KO cell lines we generated. We further validated our results in patient-derived organoids. Finally, we recapitulated prolonged exposure to colibactin occurring during tumorigenesis by chronically infecting sensitive cells until the emergence of a tolerant phenotype.

**Results and Discussions**

We found that different cell lines display specific sensitivity to colibactin's genotoxic stress, and that homologous recombination (HR) proficiency discriminates colibactin-tolerant cells, which display higher levels of RAD51 foci (as marker of activation of HR) compared to sensitive cells upon infection with colibactin. Screening of isogenic DDR KO cell lines revealed that inactivation of the intertwined pathways of HR (through KO of *ATM*) and replication stress (RS) response (KO of *ATRIP*) significantly sensitized cells to colibactin. In addition, restoration of HR activity was sufficient to induce a colibactin-tolerant phenotype in initially sensitive cell lines. Notably, thanks to a previous effort of pharmacological characterization of CRC cell lines in our lab, we unveiled a significant correlation between sensitivity to colibactin and irinotecan active metabolite SN38, but not oxaliplatin. We validated the same correlation in patient-derived organoids annotated for response to SN38. While colibactin, SN38 and oxaliplatin all induced RS in treated cells, we observed that colibactin and SN38 showed a similar DNA damage response which involved activation of ATM. Finally, chronic re-infection of sensitive CRC cells with colibactin selected a tolerant phenotype characterized by restoration of HR activity. Of translational relevance, colibactin-tolerant derivative cells acquired cross-resistance to SN38 and PARP inhibitor olaparib but not to oxaliplatin.

**Conclusion**

Our results support a model in which colibactin both promotes tumorigenesis and acts as an evolutionary

© 2023 The Authors. Molecular Oncology is published by John Wiley & Sons Ltd on behalf of Federation of European Biochemical Societies bottleneck which selects HR proficient CRC cells. Furthermore, our study provides preclinical evidence on colibactin's role in promoting chemoresistance in colorectal cancer.

### EACR23-0834
### Multi-region histogenomic analysis of prostate cancer delineates routes of intra-prostatic and metastatic spread, revealing genomic differences between distinct morphologies

*S. Rao[1], C. Verrill[1], L. Cerundolo[1], N. Khalid Alham[2], Z. Kaya[3], D. Wedge[4], I. Mills[1], D. Woodcock[1], I. Tomlinson[5], F. Hamdy[1]*
*[1]University of Oxford, Nuffield Department of Surgical Sciences, Oxford, United Kingdom*
*[2]University of Oxford, Department of Engineering Science, Oxford, United Kingdom*
*[3]University of Oxford, Nuffield Department of Orthopaedics- Rheumatology and Musculoskeletal Sciences, Oxford, United Kingdom*
*[4]University of Manchester, Manchester Cancer Research Centre, Manchester, United Kingdom*
*[5]University of Oxford, Nuffield Department of Medicine, Oxford, United Kingdom*

**Introduction**
Extension of prostate cancer beyond the primary site into the surrounding organs by local invasion or nodal metastasis is associated with poor prognosis. The emergence and evolution of cancer clones at this early stage of expansion and spread is poorly understood. Further, prostate cancers exhibit extensive histological heterogeneity but the relationship between genomic changes and histological appearance have not been studied in detail.

**Material and Methods**
We systematically sampled and performed whole genome sequencing on 42 prostate cancer samples from the prostate, seminal vesicles and regional lymph nodes of five treatment-naive patients with locally advanced disease who underwent radical prostatectomy. We computed cancer cell fractions from single nucleotide variants (SNVs; Mutect2) and copy number alterations (CNAs; Battenberg), using which we reconstructed the tumour phylogenies (dirichlet process clustering).

**Results and Discussions**
Phylogenetic analysis of the cancers enabled us to infer key molecular steps in cancer evolution in these individuals. We mapped the clonal composition of cancer sampled across the prostate in each individual and inferred the routes of cancer spread within the prostate and to seminal vesicles and lymph nodes. In four patients, we observed that regions of different morphologies (Gleason grade, cribriform transformation, amphicrine morphology, intraductal morphology) were composed of distinct clones harbouring CNAs as well as hundreds of SNVs exclusive to each morphology. These morphologies map to different branches of the phylogenetic trees. We identified specific genomic changes (e.g. 8p loss, 8q gain) in the transformation of adenocarcinoma to amphicrine prostate cancer during its spread from the apex of the prostate via the seminal vesicles to lymph nodes. We also found evidence of chromoplexy in a low grade region and whole genome doubling in a high grade region in another patient, with clones from the high grade region leading to lymph node metastases. In addition, we identified putative driver events in the spread to seminal vesicles and lymph node metastasis.

**Conclusion**
Taken together, these findings have implications for diagnosis and risk stratification, in addition to providing a rationale for further studies to characterise the genetic changes associated with morphological transformation. Our results demonstrate the value of integrating multi-region sequencing with histopathological data to study tumour evolution and identify mechanisms of prostate cancer spread.

### EACR23-0880
### Impact of RB1 alterations in the development of Glioblastoma with Primitive Neuronal Component

*E. Somenza[1], F. Pagani[1], M. Gryzik[1], R. Ronca[1], P.L. Poliani[1]*
*[1]University of Brescia, Department of Molecular and Translational Medicine, Brescia, Italy*

**Introduction**
Retinoblastoma-associated protein 1 (RB1) is a tumour suppressor protein downregulated in up to 60% of human cancers and recently identified as a hallmark of the rare glioblastoma variant, the Glioblastoma with Primitive Neuronal Component (GBM-PNC). GBM-PNC is a biphasic tumour composed of a glial component with classical GBM features along with nodules of cells displaying early neuronal differentiation. Our group has demonstrated that RB1 inactivation or pathway alterations are strictly correlated with the transcription factor Early B-cell Factor3 (EBF3) expression within the PNC component (paper in draft). Whereas not expressed in conventional GBM, MYC-driven EBF3 boosts early neuronal differentiation conferring a less differentiated phenotype and impairs the glial phenotype development.We investigated whether RB1 loss is sufficient to drive a conventional GBM toward the gain of a GBM-PNC phenotype in tumour organoids, a model of tumorigenesis in vitro.

**Material and Methods**
One *RB1* wildtype GBM stem cell population (GBM GSCs) was selected and transduced with a CRISPR/Cas9 lentiviral vector targeting human *RB1* exon 2.
Total proteins were extracted and quantified with the Bradford method; equal amounts separated on SDS-PAGE and transferred to PVDF for Western blot (WB).
Organoids were obtained seeding GSCs into Matrigel drops.
Immunostainings on FFPE organoids were performed using antibodies for EBF3, GFAP, and βIIITubulin.

**Results and Discussions**
In order to investigate RB1 role in the GBM-PNC development, we characterized *RB1* knockout GBM GSCs clones. Interestingly, *RB1* knockout clones showed lower expression of the glial marker GFAP and higher levels of

© 2023 The Authors. Molecular Oncology is published by John Wiley & Sons Ltd on behalf of Federation of European Biochemical Societies

EBF3 and βIIITubulin, as compared to the wildtype RB1+ control, an immunophenotypical profile related to an early neuronal phenotype, in both WB and organoids.
Indeed, we demonstrated that RB1 pathway alterations are shared between the two components of the GBM-PNC and may act as a predisposing factor for GBM-PNC determination.
In order to reinforce this hypothesis, we aim to assess if the ectopic expression of RB1 protein in a *RB1*-deleted GBM-PNC-derived GSCs can revert the phenotype into a conventional GBM.

**Conclusion**

Our results confirm that RB1 loss plays a role in the divergent phenotype of GBM-PNC components.
We will focus on the RB1-EBF3 axis (effects on downstream factors, cell viability, oxidative stress, apoptosis) that may play a key role as predisposing molecular feature for the development of the GBM-PNC with diagnostic and translational implications.

### EACR23-0966

### A multi-omics approach to investigate how the stool miRNome and microbiome reflect CMS subtyping for colorectal cancer classification.

*A. Gagliardi*[1,2], *G. Francescato*[1,3], *G. Ferrero*[3,4], *S. Tarallo*[1,2], *G. Piaggeschi*[1,2], *A. Francavilla*[1], *C. Di Battista*[1], *E. Berrino*[2,5], *C. Marchiò*[2,5], *F. Cordero*[4], *B. Pardini*[1,2], *A. Naccarati*[1,2]
[1]*Italian Institute for Genomic Medicine, Genetic and molecular epidemiology, Candiolo- Turin, Italy*
[2]*Candiolo Cancer Institute, Fpo-Irccs, Candiolo - Turin, Italy*
[3]*University of Turin, Department of Clinical and Biological Sciences, Turin, Italy*
[4]*University of Turin, Department of Computer Science, Turin, Italy*
[5]*University of Turin, Department of Medical Sciences, Turin, Italy*

**Introduction**

Colorectal cancer (CRC) is a complex disease with high inter and intra-tumoral heterogeneity, hindering its management. RNA-seq-based Consensus Molecular Subtypes (CMS) system has improved traditional tumor classification and clinical translation. However, a more in-depth description of each CMS (CMS1-4) integrating different omics could refine this classification. Moreover, the identification of CMS molecular markers in CRC surrogate tissues, such as stool miRNome and microbiome, deserves a deeper investigation. Besides a comprehensive description of CMS integrating RNA-seq, target genomic sequencing, small RNA-seq and immune infiltrate profile, we aim to investigate how each subtype is reflected in the stool miRNome and metagenome to identify CMS-specific signatures non-invasively.

**Material and Methods**

RNA-seq and small RNA-seq were performed on tumor tissues and adjacent mucosa collected in RNA later from 87 CRC patients recruited in a cross-sectional study. small RNA-seq and shotgun metagenomic sequencing were performed in stool samples of the same patients and healthy individuals (n=58).
Tumor samples were classified with CMScaller and characterized with the TruSight Oncology 500 cancer panel. Differential expression (DE) analyses in tissues and stool were performed with DESeq2. Relative abundances of microbial species were analyzed with MetaPhlAn and SIAMCAT.

**Results and Discussions**

Tumors were classified as CMS1(n=8), CMS2(n=27), CMS3(n=26) and CMS4(n=23), while 4 samples were not classified. CMS1 showed the highest number of SNV (n=290) and the highest fraction of frameshift variants, probably due to mutations in MMR genes. Paired DE analyses of mRNAs and miRNAs in tissues showed a major dysregulation of CMS1-2 compared to the other subtypes. In contrast, stool miRNome showed a similar dysregulation among CMS, with an overall miRNA up-regulation. CMS1 was the only subtype with miRNAs (n=7) altered in both stool and tissues. Preliminary metagenome analyses revealed that CRC patients (especially CMS1) have lower species richness than healthy individuals. In addition, association analyses showed different abundancies of several species between CMS, such as Fusobacterium nucleatum, which is more prevalent in CMS2 than in other subtypes.

**Conclusion**

Investigating CMS reflections in stool miRNome and metagenome could shed new light on the host gut-microbiota interactions and allow a non-invasive classification for personalized clinical management of CRC.

### EACR23-0999

### Analysis of clonal evoluton by single cell DNA sequencing monitors response/resistance trajectories and highlights novel therapeutic targets in acute myeloid leukemia

*S. Bruno*[1], *E. Borsi*[2], *L. Bandini*[2], *A. Patuelli*[2], *A. Poletti*[1], *G. Cristiano*[1], *A. Curti*[2], *E. Ottaviani*[2], *M. Cavo*[2], *S. Soverini*[1]
[1]*UNIBO-University of Bologna, DIMEC- Department of Medical and Surgical Science, Bologna, Italy*
[2]*IRCCS Azienda Ospedaliero-Universitaria di Bologna, DIMEC- Department of Medical and Surgical Science, Bologna, Italy*

**Introduction**

Acute myeloid leukemia (AML) is a highly heterogeneous hematologic neoplasia. The tyrosine kinase inhibitor midostaurin targeting mutant FLT3 provides a new therapeutic opportunity for a subset of AML patients (pts). However, drug resistance responsible for refractory and relapsed cases still represents a challenge. In this study we aimed to assess the potential of single cell analysis to dissect intratumor heterogeneity in FLT3-mutated AML, follow the evolution of clonal architecture under the selective pressure of midostaurin and highlight emerging resistant clones, ultimately enhancing personalized therapy.

**Material and Methods**

High throughput amplicon-based single-cell DNA sequencing (scDNA-seq) was performed on mononuclear cells isolated from 7 bone marrow samples of 2 pts before

© 2023 The Authors. Molecular Oncology is published by John Wiley & Sons Ltd on behalf of Federation of European Biochemical Societies and during midostaurin in combination with 3+7 chemotherapy using a targeted panel of 20 leukemia-associated genes on the MissionBio Tapestri platform.

**Results and Discussions**

Pt 1 was an 82-y-old man with AML secondary to myelodysplastic syndrome (MDS). At the time of MDS 2 independent clones, one carrying TET2 p.N191Kfs*4 alone and one carrying also the RUNX1 p.R201Q mutation were detectable. Parallel expansion of a RUNX1 homozygous clone and acquisition of the FLT3 p.D835H mutation marked the evolution to AML. Midostaurin therapy cleared the 2 FLT3 clones but the pt relapsed concomitantly with the branching evolution of new subclones that had additionally acquired KIT p.D816H, KIT p.D816V and RAS p.G12A mutations, respectively. Pt 2 was a 50-y-old woman with *de novo* AML. At diagnosis, she had evidence of 2 clones, one harboring KIT p.S821Y and one harboring NPM1 p.W288Cfs*12, TET2 p.N148Kfs*6, DNMT3A p.R822C and an FLT3-ITD mutation. Analysis of 2 serial follow-up samples showed the clearance of the quadruple-mutated clone and the reduction of the KIT mutant in parallel with achievement of response.

**Conclusion**

We here provide proof-of-principle evidence that high-throughput scDNA-seq may enable better molecular stratification and personalization of therapy in AML. In fact, scDNA-seq could successfully dissect the complex clonal dynamics of AML undergoing targeted therapy in correlation with clinical response. Longitudinal analysis with scDNA-seq better than analysis of individual MRD markers allows to follow disease evolution and highlight the emergence of clones harboring novel potential therapeutic targets in refractory or resistant pts.

### EACR23-1038
### Epithelial-to-mesenchymal transition is a major contributor to intratumor heterogeneity in lymph node breast cancer metastasis

*S.D. Sajib[1], Å. Florholmen-Kjær[2], V.H. Brun[3,4], E.S. Mortensen[5], A.M.K. Hovd[1], X. Tekpli[6], G.A. Calin[7,8], G.M. Mælandsmo[1,9], M. Kuijjer[10], E. Knutsen[1,11]*

*[1]UiT - The Arctic University of Norway, Department of Medical Biology, Tromsø, Norway*

*[2]University Hospital of North Norway, Department of Gastroenterological Surgery, Tromsø, Norway*

*[3]University Hospital of North Norway, Department of Breast and Endocrine Surgery, Tromsø, Norway*

*[4]UiT - The Arctic University of Norway, Institute of Clinical Medicine, Tromsø, Norway*

*[5]University Hospital of North Norway, Department of Pathology, Tromsø, Norway*

*[6]Oslo University Hospital, Department of Medical Genetics, Oslo, Norway*

*[7]The University of Texas MD Anderson Cancer Center, Department of Translational Molecular Pathology, Houston, United States*

*[8]The University of Texas MD Anderson Cancer Center, Center for RNA Interference and Non-Coding RNAs, Houston, United States*

*[9]Institute for Cancer Research- Oslo University Hospital, Department of Tumor Biology, Oslo, Norway*

*[10]Centre for Molecular Medicine Norway- University of Oslo, Computational Biology and Systems Medicine Group, Oslo, Norway*

*[11]University Hospital of North Norway, Centre for Clinical Research and Education, Tromsø, Norway*

**Introduction**

Breast cancer is a heterogenous disease, showcasing both large inter- and intratumor heterogeneity. Some of this heterogeneity can be explained by the plasticity of cancer cells. The differentiation program epithelial to mesenchymal transition (EMT) has been proposed as a mechanism for generating cancer cells with a plastic potential. For breast cancer to metastasize, the cancer cells will primarily travel via regional lymph nodes before entering into systemic circulation. However, the regulatory impact on cancer cells by lymph node microenvironment has not been studied in detail.

**Material and Methods**

To examine the transcriptional changes in breast cancer cells due to the microenvironment of regional lymph node, single cell RNA-sequencing (scRNA-seq) was performed on paired primary tumor and lymph nodes. An in-house pipeline was developed to conduct data analysis. Briefly, quality control, dimensional reduction, clustering, and annotation of cell types using both automated and canonical marker identification methods were performed. Malignant cells were identified by inferring copy number variation before generating a pseudotime trajectory model. Finally, gene set enrichment analysis was performed to analyze signaling pathway perturbations. An EMT gene signature was also established from scRNA-seq data analysis of the EMT model cell lines – HMLE, D492, and MCF10A. The signature was used to score the cancer cells based on their EMT states.

**Results and Discussions**

Primary tumor and lymph node samples from individual patients were combined for analysis, and unique cell clusters enriched for either primary tumor or lymph node metastasis were identified. Signaling pathway perturbation analysis between distinct cancer cell clusters revealed unique transcriptional programs distinct to the cell clusters. The EMT scoring signature classified cancer cells into pure epithelial and mesenchymal states, and the different states were associated with distinct cell clusters. Trajectory analysis showed that the clusters aligned according to a progression in EMT, suggesting that EMT can contribute to the intratumor heterogeneity in both primary tumor and lymph node metastasis. Further, the presence of cancer cell clusters located between the epithelial and mesenchymal cluster might point towards cells in an intermediated hybrid state.

**Conclusion**

ScRNA-seq can be utilized to visualize intratumor heterogeneity and to identify the underlying molecular mechanism affected by metastasis to the lymph nodes in breast cancer.

### EACR23-1114
### Exploiting stromal-epithelial crosstalk in intestinal homeostasis and colitis-association colorectal cancer

© 2023 The Authors. Molecular Oncology is published by John Wiley & Sons Ltd on behalf of Federation of European Biochemical Societies

Y. Zhou[1], A. Mandal[1], R. Owen[2], X. Lu[2], S. Buczacki[1]
[1]University of Oxford, Nuffield Department of Surgical Sciences, Oxford, United Kingdom
[2]University of Oxford, Oxford Ludwig Institute, Oxford, United Kingdom

**Introduction**

Paracrine communication between epithelial and neighbouring stromal cells is emerging as a key event required for the successful establishment of early colorectal cancer, but studies dissecting the precise interactions between these cellular components is lacking. Here, we have developed a novel *in vitro* primary human organoid co-culture system consisting of fluorescently labelled, gene-edited human colon organoids with primary human colonic fibroblasts that, for the first time, enables the precise dissection of this type of communication.

**Material and Methods**

Human colon organoids and paired primary human colonic fibroblasts derived from normal colon samples were lentivirally labelled. TP53 was knocked out in organoids using CRISPR-Cas9 system. Wild type or clonal *TP53$^{Null}$* organoids were then co-cultured with fibroblasts in basement membrane extract, phenotypes were observed, and cells subsequently extracted for downstream analyses.

**Results and Discussions**

Wild type adult human colon organoids when co-cultured with primary fibroblasts induced a profound change in organoid morphology generating larger organoids than observed in mono-culture. Bulk RNA sequencing following fluorescent activated cell sorting showed evidence of enrichment in extracellular remodelling processes in both epithelial and fibroblast components. Compared to mono-culture controls, single cell RNA sequencing (scRNAseq) of co-cultured wild type organoids showed an enrichment in a YAP-TAZ active population, which was compatible with a recently described regenerative stem cell (RSC) population. Next, we introduced a ***TP53$^{Null}$*** **mutation** into wild type colon organoids to model the earliest event in colitis associated cancer. Phenotypically, *TP53$^{Null}$* organoids retained the growth advantage seen when co-cultured with fibroblasts. scRNAseq showed that, whilst some changes appeared similar to that with WT co-culture, several additional *TP53$^{Null}$* specific changes also appear present. Strikingly, we see activation of the LGR5 stem cell population on fibroblast co-culture rather than the YAP/TAZ RSC population seen with WT co-culture.

**Conclusion**

Our data demonstrates that profound two-way interactions exist between epithelial and fibroblast components that affect cell identity and behaviour including modulation of the surrounding extra-cellular matrix. *TP53* mutations in epithelial cells alters this two-way interaction and further insights may provide putative therapeutic targets to prevent tumour initiation.

### EACR23-1121
### The origin and evolution of pediatric secondary neoplasms

M. Sanchez Guixe[1], F. Muiños[1], C. Lavarino[2], J. Mora[2], A. González-Pérez[1], N. López-Bigas[1]
[1]Institute for Research in Biomedicine, Cancer Science/Computational Biology, Barcelona, Spain
[2]Hospital Sant Joan de Déu, Developmental Tumor Biology Laboratory, Esplugues de Llobregat, Spain

**Introduction**

Pediatric cancer patients without known germline predisposing mutations that present with a second malignancy during their young age are rare. When this occurs it is often assumed that the second malignancy could be related to the treatment received for the first one. Other possible explanations for the second tumor include shared early driver mosaic mutations.

**Material and Methods**

In this study, we investigated the origin and evolution of second neoplasms from ten pediatric patients at Sant Joan de Déu Barcelona Children's Hospital, through whole-genome sequencing of the tumors and normal tissue samples. We investigated the timing of clonal expansion of the secondary neoplasms through treatment-related mutational signatures.

**Results and Discussions**

We have identified cases of secondary acute myeloid leukemia with clonal treatment-related mutations, pointing to an initiation of leukemogenesis posterior and likely related to the treatment. Solid secondary tumors showed no clonal treatment-related mutations, suggesting the possibility that the clonal expansion of the tumor occurred before the treatment. In a few cases we identified an early mosaic mutation shared by the two tumors that could explain the appearance of two tumors in a patient. Additionally, we conducted a lineage tracing study to an extremely rare case of two solid tumors without a shared driver event, to identify the relationship between the two tumors and its origin.

**Conclusion**

In conclusion, we show that mutational signatures can be used as barcodes to study the origin and evolution of secondary neoplasms. The study of driver mutations also sheds light onto this evolution.

### EACR23-1132
### Transcriptomic analysis reveals a metastatic site-specific differential expression profile in colon cancer patients

B. García Micó[1], F. Gimeno-Valiente[2], B. Martínez-Castedo[1], J. Martín-Arana[1], B. Palomar[3], C. Martínez-Ciarpaglini[3], V. Gambardella[1], D. Roda[1], A. Cervantes[1], N. Tarazona[1]
[1]Biomedical Research Institute INCLIVA, Medical Oncology, Valencia, Spain
[2]UCL Cancer Institute, Cancer Evolution and Genome Instability Laboratory, London, United Kingdom
[3]Biomedical Research Institute INCLIVA, Pathology, Valencia, Spain

**Introduction**

Although the prognosis of localized colorectal cancer (LCC) patients has improved with the addition of oxaliplatin to fluoropyrimidine-based chemotherapy, about 30% of them will develop metastases despite an optimal initial treatment. This phenomenon is probably related to intrinsic cellular mechanisms of resistance and to the

© 2023 The Authors. Molecular Oncology is published by John Wiley & Sons Ltd on behalf of Federation of European Biochemical Societies capability of resistant cells in developing metastases. The aim is to evaluate the organ-specific transcriptomic profile of metastatic lesions to improve personalised therapeutic approach.

**Material and Methods**

RNAseq of primary tumour tissue and paired metastasis from 15 patients with LCC at the Hospital Clínico Universitario in Valencia (Spain), was performed to identify dynamic changes in the expression profile. Differential expression of the transcriptomic data was carried out by DESeq2. Functional enrichment analysis was done by GSEA and hallmarks with FDR < 0.05 were selected.

**Results and Discussions**

RNAseq analysis exhibited 711 encoding genes differentially expressed among primary tumours and metastases, of them 554 were overexpressed in the metastatic site while 157 in primary tumours (FDR<0.05). Notably, a different expression pattern was observed among the different sites of metastases, obtaining three clearly separated clusters for peritoneal, lung and liver metastasis. Of the 554 genes overexpressed in the metastases, 503 of them were more expressed in liver, 48 in lung and 3 in peritoneal lesions; only 3 genes were found in both liver and lung metastases and just 1 in peritoneal and liver samples. Moreover, a GSEA analysis showed an enrichment of proliferative pathways in liver metastases, as well as INFα response and epithelial mesenchymal transition (EMT) pathways. Across lung metastases, an overexpression of immune response gene sets like INFα and γ and complement as well as metabolic pathways, *mTORC1* and *JAK-STAT* signalling, and EMT were found. Whereas, peritoneal metastases presented a downregulation of *P53* pathway, INFγ response or TNFα signalling. These results suggest that microenvironment organ-specific differences could contribute to growth restraint or progression of disseminated tumour cells.

**Conclusion**

Transcriptomic analysis showed significant differences between primary tumour and secondary lesions. Interestingly a specific transcriptomic pattern was observed according to the metastatic site suggesting the potential role of organ specific factors in developing metastasis.

### EACR23-1172

### The pan-cancer landscape of RNA variant signatures

*C. Martinez Ruiz[1], T.P. Jones[1], J.R.M. Black[1], M. Dietzen[1], N. McGranahan[1]*

*[1]University College of London- Cancer Institute, Oncology, London, United Kingdom*

**Introduction**

Somatic genomic alterations caused by different processes leave distinct signatures in the genome that can be deconvolved to shed light on the biology of tumours. To date, such signatures have focused on genomic modifications: single nucleotide variants, insertion-deletions and copy number variation. Here, we characterise the signatures observed in the RNA for 7,663 tumour samples from The Cancer Genome Atlas (TCGA) across 30 tumour types, using the nucleotide context of single nucleotide variants in the RNA sequence.

**Material and Methods**

We perform stringent variant calling on bulk RNA-seq data, resulting in a median of 500 RNA variants per sample. We then de-convolved different RNA variant signatures through Hierarchical Dirichlet Process on different nucleotide contexts.

**Results and Discussions**

We validate the presence of previously described RNA editing signatures caused by the activity of ADAR and APOBEC3A enzymes. We show that the activity of RNA editing by these enzymes varies across cancer types, with APOBEC3A RNA editing common in tumour types driven by viral activity and genomic instability. We show that aberrant ADAR activity is potentially linked to specific mutations within this gene. Additionally, using strandedness information, we recover signatures from expressed DNA mutations that were absent from the genomic data. These signatures correspond to mutations beyond the exon capture kits used in the whole-exome sequencing data from TCGA as well as from low frequency mutations not captured by DNA sequencing. Expressed DNA signatures therefore reveal mutational processes that had previously not been explored.

**Conclusion**

This work provides the first systematic analysis of RNA variant signatures across cancer types, providing a novel quantitative approach to explore cancer-specific processes beyond the genome.

### EACR23-1211

### Glioblastoma vessel co-option occurs as a resistance mechanism to chemoradiation via induction of a persister cell state

*C. Pichol-Thievend[1], O. Anezo[1], A. Pettiwala[1], G. Bourmeau[1], A. Ballestin Rodriguez[1], V. Madapusi Ravi[2], K. Joseph[2], F. Cavalli[3], G. Seano[1]*

*[1]Institut Curie Research Center, Tumor Microenvironment Lab U1021 - UMR3347, Orsay, France*

*[2]Medical Center University of Freiburg, Department of Neurosurgery, Freiburg, Germany*

*[3]Institut Curie Research Center, Computational Biology and Integrative Genomics of Cance r, Saint-Cloud, France*

**Introduction**

Glioblastoma (GB) is one of the deadliest types of human cancer. Despite a very aggressive treatment regime – including resection of the tumor, radiation and chemotherapy – its recurrence rate is more than 90%. Recurrence is mostly caused by the regrowth of highly invasive and resistant cells that spread from the tumor bulk and are not removed by resection. To develop an effective therapeutic approach, we need to better understand the underlying molecular and cellular mechanisms of chemoradiation resistance and tumor spreading in GB.

**Material and Methods**

To dynamically follow the changes occurring in GB post-therapy and track chemoradiation-resistant cells, we employed multiple bulk and single-cell RNA-Seq, phosphoproteome, in vitro and in vivo real-time imaging, organotypic cultures and functional assays, digital pathology, and spatial transcriptomics on patient material and preclinical models of GB.

© 2023 The Authors. Molecular Oncology is published by John Wiley & Sons Ltd on behalf of Federation of European Biochemical Societies

**Results and Discussions**
We demonstrated that chemoradiation and brain vasculature induce a transition to an invasive functional cell state, which we named VC-Resist. Better cell survival, G2M-arrest, senescence/stemness pathways' induction, make this GB cell state more resistant to therapy. Notably, these persister GB cells are highly vessel co-opting, allowing homing to the perivascular niche, which in turn increases their transition to this cell state. Molecularly, the transition to the VC-Resist cell state is driven by FGF-FGFR1 signaling that leads to activation of DNA damage repair, YAP, and Rho pathways.
**Conclusion**
These findings demonstrate that the perivascular niche and GB cell plasticity jointly generate a vicious loop that leads to resistance to therapy and brain infiltration during GB recurrence.

### EACR23-1212
### Deciphering mechanisms of therapeutic resistance in glioblastoma stem-like cells

*M. Prelli[1,2], F. De Bacco[1,2], F. Orzan[1], E. Casanova[1], G. Reato[1,2], G. Crisafulli[3], R. Albano[4], A. Bartolini[4], M. Macagno[5], V. Pessei[2,5], M. Panero[6], L. Casorzo[6], J. Erriquez[4], P. Cassoni[7], P. Zeppa[8], A. Melcarne[9], D. Garbossa[8], C. Boccaccio[1,2]*
*[1]Candiolo Cancer Institute- FPO-IRCCS, Laboratory of Cancer Stem Cell Research, Candiolo, Italy*
*[2]University of Turin Medical School, Department of Oncology, Candiolo, Italy*
*[3]IFOM ETS - The AIRC Institute of Molecular Oncology, BioInformatic Group BIG & Genomics of Cancer and Targeted Therapies Unit, Milan, Italy*
*[4]Candiolo Cancer Institute- FPO-IRCCS, Core Facilities, Candiolo, Italy*
*[5]Candiolo Cancer Institute- FPO-IRCCS, Laboratory of Cancer Epigenetics, Candiolo, Italy*
*[6]Candiolo Cancer Institute- FPO-IRCCS, Unit of Pathology, Candiolo, Italy*
*[7]University of Turin Medical School, Department of Medical Sciences, Turin, Italy*
*[8]University of Turin Medical School, Department of Neurosciences, Turin, Italy*
*[9]Città della Salute e della Scienza, Neurosurgery Unit, Turin, Italy*
**Introduction**
Glioblastoma (GBM) is believed to originate from cancer stem cells known as glioblastoma stem-like cells (GSCs). Due to genetic evolution, multiple GSC subclones can coexist in the same GBM and display varying degrees of resistance to conventional therapeutic treatments such as ionizing radiation (IR) and the alkylating agent temozolomide (TMZ). Selective pressure exerted by therapies can favor the growth of GSC subclones that drive GBM recurrence. Although Methyl Guanine Methyl Transferase (MGMT) expression is a known mechanism of TMZ resistance, the overall underlying resistance mechanisms are still elusive, and there are no therapeutic options for recurrent GBMs. To address these challenges, we have developed a protocol for selecting GSCs that are resistant to conventional therapies, which ideally represent the subclones driving tumor recurrence.
**Material and Methods**
Taking advantage of GBM surgery by ultrasonic aspiration, we virtually collected the whole tumor cell population from 28 GBMs. GBM cells were then cultured in a medium with high growth factor concentrations to preserve the majority of the original GSC subclones. Cells were then exposed to TMZ or IR conventional therapies, to simulate patient treatments. From single GBMs we obtained multiple long-term propagating cultures enriched in GSCs, named 'neurosphere (NS) families', which displayed resistance to the selective agent and whose features were characterized with genomic, transcriptomic and phenotypic analyses.
**Results and Discussions**
Starting from 28 GBM ultrasonic aspirates, we derived 9 NS families each including at least one member selected by therapeutic pressure with TMZ or IR (NS-TMZ and NS-IR respectively) in addition to an untreated control (NS-CTRL). Members of the same NS family displayed distinct genetic alterations and karyotypes, and different phenotypic features related to transcriptional profiles and biological properties such as clonogenicity and division symmetry. Interestingly, in a subset of cases, TMZ resistance correlated with increased EGFR expression and an anti-apoptotic protein expression profile. In this subset, cell death could be promoted by synthetic lethality between EGFR inhibitors and BH3 mimetics.
**Conclusion**
GSCs representative of GBM subclones resistant to conventional therapies have been isolated by ex-vivo selection. GSC genetic and molecular features correlating with therapeutic resistance are under characterization, revealing mechanisms that can be targeted to destroy resistant cells.

### EACR23-1213
### Spatial heterogeneity and temporal evolution of tumor acidosis provides evidence of cancer progression in a transgenic breast cancer murine model

*A. Anemone[1], E. Pirotta[2,3], A. Corrado[3], D. Ambrosecchia[1], D. Villano[1], W. Dastrù[1], A. Carella[3], F. Gammaraccio[1], D.L. Longo[3]*
*[1]University of Turin, Department of Molecular Biotechnology and Health Sciences, Torino, Italy*
*[2]University of Turin, Department of Pharmaceutical Science and Technology, Torino, Italy*
*[3]Institute of Biostructures and Bioimaging, National Research Council of Italy, Torino, Italy*
**Introduction**
Breast cancer remains the primary cause of cancer-related deaths in women. Typically, it progresses through several stages, culminating in the emergence of invasive carcinomas in advanced phases. While altered metabolism and vascularization are well known players during tumor progression, the role of tumor acidosis remains poorly understood, although the known correlation between acidity and invasiveness. In this study, we aim to evaluate the role of tumor acidosis and its spatial and temporal

© 2023 The Authors. Molecular Oncology is published by John Wiley & Sons Ltd on behalf of Federation of European Biochemical Societies changes throughout the stages of breast cancer using the transgenic PyMT mouse breast cancer model.

**Material and Methods**

FVB/N-Tg (MMTV-PyVT) (n=20) female transgenic mice (PyMT) were imaged every two weeks, from week 6 to 12 during the four distinct stages of tumor progression, from pre-malignant to malignant stages, with a Bruker Avance Neo 7T MRI scanner. A multislice CEST pH imaging acquisition to evaluate whole tumor pH and heterogeneity was acquired upon iopamidol i.v. injection (dose 4 g I/kg b.w.). After imaging session at each stage a group of mice was sacrificed and the mammary glands removed for histopathology and IHC staining for LDHA, CAIX, LAMP2 and GLUT1.

**Results and Discussions**

MRI T2w images of PyMT mice showed an increasing degeneration of the mammary gland tissue that was confirmed by H&E sections showing a transition from low-grade lesions in first stages (6 to 8 weeks) to an increased number and volume of tumors in late stages (10 to 12 weeks). An increasing mammary gland acidification during cancer progression was observed along stages (mean pH values of 7.1, 7.0, 6.9 and 6.8 respectively), reflecting underlying metabolic changes. The acidity score, index of spatial distribution and heterogeneity of acidosis, showed statistically significant differences at different stages, with an increased tumor pH heterogeneity associated to the late stage. Immunohistological staining showed increased expressions of LDHA and CAIX markers at different stages, confirming the changes of tumor altered metabolism and acidosis during progression.

**Conclusion**

Our results showed that MRI-CEST tumor pH imaging can successfully monitor *in vivo* both metabolic changes and spatial and temporal tumor acidosis evolution along breast cancer malignant progression in the PyMT murine model.

### EACR23-1231

### In vivo imaging of spatial tumor pH heterogeneity reveals a different invasive phenotype in two glioblastoma murine models

*A. Carella[1], E. Botto[2], A. Corrado[1], E. Pirotta[1], D. Villano[2], R. Gambino[2], F. Gammarraccio[2], E. Micotti[3], D.L. Longo[1]*

*[1]Italian National Research Council CNR, Institute of Biostructures and Bioimaging IBB, Torino, Italy*

*[2]University of Torino, Department of Molecular Biotechnology and Health Sciences, Torino, Italy*

*[3]Istituto di Ricerche Farmacologiche Mario Negri IRCCS-Department of Neuroscience, Laboratory of Biology of Neurodegenerative Disorders, Milano, Italy*

**Introduction**

Glioblastoma is the most aggressive brain tumor with a poor prognosis, despite recent advances in treatment options. A salient feature of solid tumor is tumor acidosis (caused by dysregulated metabolism and reduced perfusion), that is associated to cancer aggressiveness and resistance to therapy. However, few studies have investigated how tumor acidosis in glioblastoma models is associated to cancer invasiveness. In this study, we evaluated whether MRI-CEST tumor pH imaging, coupled to metabolite quantification and WB/IHC studies, can elucidate the invasiveness and metabolic alterations of two glioblastoma murine models.

**Material and Methods**

We investigated two glioblastoma models with different invasive phenotype upon stereotaxic injection (1.5mm ML to the bregma and 3.0 mm DV to the dura): U87 (1 x $10^6$ U87 cells into athymic nude mice), and GL261 (2x$10^5$ GL261 cells into C57BL/6 mice). Images were obtained with a 7T MRI Bruker scanner with the following scans: MRI-CEST tumor pH imaging of the whole tumors was obtained following Iopamidol injection; metabolites were assessed by single voxel spectroscopy (MRS) with a PRESS sequence with short (16ms) and long TE (135ms, for lactate quantification), VAPOR suppression, in a voxel size of 2x2x2mm$^3$. In addition, we acquired T1w contrast-enhanced images following Gd injection to delineate tumor borders. Western blot and IHC analysis for LDHA, LDHB, PDK1, LAMP2 and CAIX both *in cellulo* and in tumor sections were performed to assess the altered metabolism and acidosis.

**Results and Discussions**

Both the two glioblastoma models exhibited metabolic alterations, with the U87 model showing higher lactate levels and significant increased PDK1 expression, indicating a higher glycolytic-dependent phenotype compared to the less glycolytic phenotype of GL261. Moreover, the GL261 showed higher contrast enhancement suggesting increased vascularization in comparison to the U87 model. The U87 glioblastoma model showed a stronger extracellular acidification, correlated to the higher lactate levels, than the Gl261. Of note, the GL261 tumors showed increased tumor acidosis during tumor progression and higher spatial tumor pH heterogeneity, related to a more invasive phenotype.

**Conclusion**

In conclusion, our study confirms that the U87 glioblastoma model has a higher glycolytic-dependent phenotype than the GL261 model, associated to a more acidic microenvironment, but the increased spatial tumor pH heterogeneity can distinguish the two different invasive phenotypes.

### EACR23-1235

### Glioblastoma hybrid cell state conveys resistance to conventional therapies

*G. Bourmeau[1], O. Anezo[1], A. Ballestin[1], C. Pichol-Thievend[1], J. Reveilles[1], G. Seano[1]*

*[1]Institut Curie, Signalisation- Radiobiologie et Cancer, Orsay, France*

**Introduction**

Glioblastoma (GB) is a deadly disease and no therapeutic improvements have been made in the last 20 years. High phenotypic plasticity has been recognized as a major obstacle to efficiently treat GB. Single-cell sequencing revealed the coexistence of four different cell states within the same tumor. The transition from one subtype (or state) to another, such as from proneural (PN) to mesenchymal (MES), has been suggested as a mechanism for resistance to therapies. However, there is no prognostic value of the current GB states for patients, meaning that mechanisms of resistance may not lie in hardwire identities but in the transition from one subtype to another. Therefore, we

© 2023 The Authors. Molecular Oncology is published by John Wiley & Sons Ltd on behalf of Federation of European Biochemical Societies investigated the role of the hybrid cell states in GB resistance to therapy.

**Material and Methods**

To dynamically follow spatiotemporal changes in GB plasticity in real time, we transduced patient-derived GB cells (patient-derived cell lines with different mutational and phenotypic landscapes) using two specific GB-subtype fluorescent synthetic genetic tracing cassettes (Schmitt et al., Cancer Discovery, 2021). To follow the hybrid cell state dynamics and features, we functionally and transcriptionally investigated GB cells labeled with activated reporters for both the PN and MES subtypes. Using a combination of cytofluorimetry, live imaging, preclinical models, and bulk and single-cell RNA-sequencing, we visualized the single-cell GB plasticity and studied the changes in the hybrid state subpopulation proportion over time and under therapeutic stress.

**Results and Discussions**

We discovered that the PN/MES hybrid state (GB-hybrid) has a definite molecular and phenotypic identity and that it is strongly induced after conventional therapies. The GB-hybrid cell state is highly resistant to therapy and more proliferative. Importantly, when implanted in mice the GB-hybrid cells were more aggressive than the rest of the cells, which is consistent with the marked prognostic power for the GB-hybrid signature when tested in the TCGA database. Mechanistically, we showed that GB-hybrid cells were characterized by broad chromatin remodeling, an important increase in overall mRNA quantity per cell and intensification of the nuclear import/export machinery.

**Conclusion**

Here, we present a novel GB transition cell state between PN and MES subtypes/states. The GB-hybrid is at the same time highly proliferative and resistant to therapy, making it an interesting target to slow down GB recurrence after therapy.

### EACR23-1419

### Exploring the B(r)east: Investigating the Prevalence of Extrachromosomal DNA in Breast Cancer

*G. Stavrou*[1], *C. Bailey*[2], *M. Jamal-Hanjani*[1], *N. Kanu*[1], *C. Swanton*[2]

[1]*UCL Cancer Institute, Research Department of Oncology, London, United Kingdom*

[2]*The Francis Crick Institute, Cancer Evolution and Genome Instability Laboratory, London, United Kingdom*

**Introduction**

An important heterogeneous genomic feature prevalent in cancer is extrachromosomal DNA (ecDNA). Highly aggressive cancers are often driven by oncogene amplification and recent research suggests that ecDNA may play a pivotal role in driving tumour evolution. These circular structure have a very dynamic nature as they can be maintained outside of chromosomes in their circular form but can also reintegrate randomly back into the genome in the form of Homogeneously Stained Regions (HSR). It has been previously reported that approximately 25% of breast samples analysed contained ecDNA. Amplification of *ERBB2* in breast cancer samples on ecDNA has been observed albeit inconsistencies in the literature.

**Material and Methods**

2936 Whole Genome Sequencing data from the Genomics England breast cancer cohort were analysed using Amplicon Suite. Moreover, ten *ERBB2* amplified breast cancer datasets from the Cancer Cell Line Encyclopaedia were analysed using the same tools. In parallel to the in-silico analysis, a panel of nine *ERBB2* amplified breast cancer cell lines were used to detect *in-vitro* presence of ecDNA using metaphase Fluorescent In Situ Hybridisation. The *ERBB2* probe was used to determine the nature of *ERBB2* amplification in the selected cell lines.

**Results and Discussions**

Our analysis suggests an enrichment of ecDNA in metastatic patient samples when compared to primary breast cancer samples. Focally amplified genes amongst the patients were determined along with predictions of the cyclic nature of amplifications. Patients with focal amplifications in the form of ecDNA had poorer clinical outcomes compared to patients without any other forms of focal amplifications. The prevalence of ecDNA harbouring *ERBB2* in the patients with *ERBB2* amplification was 41%, with mean *ERBB2* copy number in patients with ecDNA at 12 copies. Similarly, we identified 9 breast cancer cell lines some of which contained up to 5 different species of ecDNA. In certain cell lines that were computationally predicted to harbour *ERBB2* on ecDNA, *ERBB2* amplification was detected *in-vitro* in the form of HSR

**Conclusion**

At present, we highlight the propensity of *ERBB2* to be amplified on ecDNA in both breast cancer patients and breast cancer cell lines. Amplification of *ERBB2 in-vitro* was in the form of HSR highlighting the propensity of certain cell lines to contain ecDNA. These cell lines can be used to further study the mechanisms of maintenance of ecDNA *in-vitro* which will shed light to some of the key questions in the field which remain unresolved.

### EACR23-1481

### Multiplexed in vitro and in vivo screening platform for clonal responses to targeted treatment strategies

*P. Dujardin*[1], *T. Mühlenberg*[2], *D. Hoffmann*[3], *S. Bauer*[2], *B.M. Grüner*[1]

[1]*University Hospital Essen- West German Cancer Center WTZ, Department of Medical Oncology-Cell Plasticity and Metastasis, Essen, Germany*

[2]*University Hospital Essen- West German Cancer Center WTZ, Department of Medical Oncology-Translational Sarcoma Research Group, Essen, Germany*

[3]*University Duisburg-Essen- Center for Medical Biotechnology ZMB, Bioinformtics and Computational Biophysics, Essen, Germany*

**Introduction**

Gastrointestinal stromal tumors (GIST) are the most common mesenchymal neoplasms of the gastrointestinal tract and are characterized by high resistance to conventional therapies. In recent years, the introduction of targeted therapies has revolutionized GIST treatment. However, virtually all patients eventually develop resistance to therapy. The development of new drugs and the adaptation strategies to overcome resistance is

© 2023 The Authors. Molecular Oncology is published by John Wiley & Sons Ltd on behalf of Federation of European Biochemical Societies complicated by the fact that the resistance mechanisms in GIST are highly diverse and are mediated by a large number of genetic mutations.

**Material and Methods**

We developed an *in vivo* preclinical study platform using an isogenic GIST cell line model and DNA barcoding technology previously developed by our group. In combination with mathematical modeling, this allows to capture the genetic heterogeneity observed in patients. Each sub-cell line harbors a different drug resistance mutation and is labeled with different six-nucleotide barcode sequences. Using this approach, the fate of individually labeled cell populations within a cell pool can be mapped both *in vitro* and *in vivo* to study the effect of subsequent treatment strategies on various resistance mutations.

**Results and Discussions**

By combining DNA cell barcoding, quantitative sequencing and a comprehensive model of tumor heterogeneity we performed high-throughput *in vivo* interrogation of multiple distinct genetic mutations under targeted therapies. As a result, we were able to determine how genetic heterogeneity in GIST influences therapeutic response in a time- and cost-efficient manner. We have applied this platform to study the clonal dynamics of the different sub-cell lines *in vitro* and *in vivo* under state of the art therapies and were able to track the clonal fitness advantages of different variants under selection conditions of the treatments.

**Conclusion**

DNA barcoding is a powerful tool for the identification of novel therapeutic compounds and drug targets and for the study of cancer biology and tumor heterogeneity during cancer progression and therapy. Technical advantages of DNA barcoding include, but are not limited to, use of smaller sample sizes, more comprehensive comparisons with higher sensitivity and lower variability, lower costs and significant reduction of animals needed for *in vivo* studies. Taken together, barcoding technology is a powerful tool that opens new possibilities for profound *in vivo* studies that have not been feasible so far.

### EACR23-1490
### Inhibition of exosome biogenesis reduces cell motility in heterogeneous sub-populations of paediatric-type diffuse high-grade gliomas

*G. pericoli[1], A. galardi[1], A. paolini[2], M. Helmer Citterich[3], L. pascucci[4], H. Peinado[5], E. de Billy[1], A. Masotti[2], A. Di Giannatale[1], M. vinci[1]*

*[1]Bambino Gesù Children's Hospital, Department of Onco-haematology- Gene and Cell Therapy, rome, Italy*
*[2]Bambino Gesù Children's Hospital, Multifactorial and Complex Phenotype Research Area, rome, Italy*
*[3]University of Rome "Tor Vergata", Department of Biology, Rome, Italy*
*[4]University of Perugia, Department of Veterinary Medicine, perugia, Italy*
*[5]Spanish National Cancer Research Centre, Microenvironment & Metastasis Group, Madrid, Spain*

**Introduction**

Paediatric-type diffuse High-Grade Gliomas (PDHGG) are highly heterogeneous tumours comprised of distinct cell sub-populations co-existing within the same tumour mass. We have shown that primary patient-derived sub-clones, as well as optical-barcoded single cell-derived clones (OBSCCs), function as an interconnected tumour network. Here, we investigated the role of the exosomes as a route of inter-clonal communication mediating PDHGG migration and invasion.

**Material and Methods**

Seven OBSCCs obtained from two patient-derived cell lines were characterised for their transcriptional profile by RNAseq and for their phenotypic behaviour assessed by proliferation, adhesion and 3D migration and invasion assays. Exosomes were isolated from OBSCCs conditioned media either by ultracentrifugation or by precipitation methods. Exosome primary characterisation was performed by western blot analysis, electron microscopy and nanosight particle-tracking analysis. The exosome cargo was analysed for miRNAs (exo-miRNA) by a miRNome PCR Panel. The phospholipase inhibitor GW4869 was used to block the exosome biogenesis.

**Results and Discussions**

OBSCCs displayed heterogeneous transcriptomic profiles and marked phenotypic differences including distinctive motility patterns, such as for example ameboid-like versus mesenchymal-like invasive phenotype. Live single-cell tracking analysis of 3D migration and invasion showed that OBSCCs in co-culture conditions display a higher speed and longer distance travelled compared mono-culture. Exosomes isolated from OBSCCs demonstrated to be actively internalized by recipient clones. The inhibition of the exosome biogenesis by GW4869 did not affect OBSCCs viability, but significantly reduced their motility in mono-culture and more prominently in co-culture conditions. Distinct exo-miRNAs were uniquely expressed by each sub-clone. Analysis of the exo-miRNAs highlighted a set of target genes regulating cell motility. These genes were differentially expressed in co-culture versus mono-culture conditions. Moreover, the expression levels of these genes (*e.g.* PTRZ, GLI3, NTRK2) were significantly modulated upon GW4869 treatment.

**Conclusion**

In conclusion, our study highlights the importance of exosomes in inter-clonal communication and it suggests that interfering with exosome biogenesis may be a valuable strategy to inhibit cell motility in PDHGG.

### Tumour Immunology

### EACR23-0015
### STING pathway activation by ATR inhibition potentiates the antitumor immune response to anti-PD-L1 antibody in small cell lung cancer.

*T. Sen[1]*

*[1]Tisch Cancer Institute- Mount Sinai, Oncological Sciences, New York, United States*

**Introduction**

© 2023 The Authors. Molecular Oncology is published by John Wiley & Sons Ltd on behalf of Federation of European Biochemical Societies

Small cell lung cancer (SCLC) is the most aggressive subtype of lung cancer, with a very poor prognosis and limited therapeutic options. Recent clinical trials of immune checkpoint blockade (ICB) combined with chemotherapy delivered only very modest benefit. Here, we identified that ataxia telangiectasia and rad3 related (ATR), the primary activator of the replication stress response, is highly enriched in SCLC and inhibition of ATR-induced DNA damage and apoptosis in multiple SCLC models.

**Material and Methods**

In this study, we performed genetic and pharmacological inhibition of ATR in a panel of human and murine SCLC models. Furthermore, we investigated the effect of ATR inhibition either alone or in combination with PD-L1 blockade either as a first-line or a second-line treatment in multiple immunocompetent mouse models of SCLC. The downstream effects of ATR inhibition was assessed by single-cell RNA sequencing, multicolor flow cytometry, western blot analysis, IHC, and genomic analysis.

**Results and Discussions**

In multiple immunocompetent SCLC mouse models, ATR inhibition (ATRi) remarkably enhanced the anti-tumor effect of PD-L1 blockade both as first- and second-line treatment regimen. Targeting ATR activated the cGAS/STING pathway, induced the expression of Type I and II interferon pathways, and caused significant infiltration of cytotoxic and memory/effector T-cells into tumors. Interestingly, ATRi also led to significant induction of MHC class I in SCLC *in vitro* and *in vivo* models.

Analysis of pre- and post-treatment clinical samples from a proof-of-concept study of a first-in-class ATR inhibitor, M6620 (VX970, berzosertib), and TOP1 inhibitor topotecan, in patients with relapsed SCLCs validated the induction of MHC class I and interferon pathway genes, for the first time in this disease.

**Conclusion**

Our findings highlight ATRi as a potentially transformative vulnerability of SCLC, paving the way for combination clinical trials with anti-PD-L1. Given the increasing importance of immunotherapy for the management of SCLC and that ATR inhibitors are already in clinical trials, combining an ATR inhibitor with PD-L1 blockade may offer a particularly attractive strategy for the treatment of SCLC and contribute to the rapid translation of this combination into the clinic.

### EACR23-0086

### Using gold nanoparticles and proton therapy to reprogram macrophages for improving cancer radiotherapy

S. Iyer[1,2,3], C. Michiels[2,3], A.C. Heuskin[1,3]
[1]*Laboratoire d'Analyse par Réaction Nucléaire (LARN), University of Namur, Belgium*
[2]*Unité de Recherche en Biologie Cellulaire (URBC), University of Namur, Belgium*
[3]*Namur Research Institute for Life Sciences (NARILIS), University of Namur, Belgium*

**Introduction**

Metallic nanoparticles have been observed to increase the radiosensitivity of cells, theoretically by synergistically releasing reactive oxygen species and secondary electrons when bombarded with radiation. Gold nanoparticles (AuNP) have also been observed to independently increase the reactive oxygen species production in cells, which could block pro-tumor (M2-like) polarization in macrophages and shift the macrophages towards the anti-tumor (M1-like) phenotype. This study aims to enhance the anti-tumor macrophage population by using gold nanoparticles alone or in conjunction with radiotherapy to reduce tumor load.

**Material and Methods**

Gold nanoparticles (AuNPs) were synthesized by reverse or direct Turkevich method to yield 15 or 50 nm diameter particles, respectively, and coated with either polyethylene glycol (AuNP-PEG) or polyvinylpyrrolidone (AuNP-PVP) via ligand replacement. M2 immortalized mouse bone marrow-derived macrophages (M2 iBMDM) were exposed to different concentrations of nanoparticles to determine the cytotoxicity. Internalization assay was performed to identify the particles having high uptake in M2 iBMDM.

**Results and Discussions**

All AuNPs were found to be stable and had a high polymer coating efficiency (60% and 67% for 15 nm PEG and PVP-coated particles, respectively). None of the particles induced cytotoxicity even at concentrations as high as 100 μg/mL. Internalization assay performed at low AuNP concentrations showed that the 15 nm AuNP-PVP have the highest exposed surface area after internalization in M2 iBMDM, followed by 50 nm AuNP-PVP while the AuNP-PEG had little to no internalization.

**Conclusion**

Gold nanoparticles are biocompatible at sizes 15 and 50 nm with no toxicity at high concentrations. They are non-toxic and readily endocytosed by M2-like macrophages. PVP is an optimal coating to increase the stability and uptake of AuNP in M2 iBMDM, while PEG is good for stability but prevents macrophage uptake. Further studies will be performed to check polarization status shift, ROS production, TrxR activity, and the effects of AuNP+proton irradiation on macrophages *in vitro* and *in vivo*.

### EACR23-0136

### Exploring the divergent oncogenic pathways in two transmissible cancers endangering the Tasmanian devil

*A. Schönbichler[1], A. Orlova[1], L. Kosack[2], C. Kreindl[1], J. Darby[3], C. Viczenczova[2], A. Koren[2], S. Kubicek[2], A. Flies[3], A. Bergthaler[4], R. Moriggl[1]*
[1]*University of Veterinary Medicine Vienna, Institute for Animal Breeding and Genetics, Vienna, Austria*
[2]*CeMM, Research Center for Molecular Medicine of the Austrian Academy of Sciences, Vienna, Austria*
[3]*University of Tasmania, Menzies Research Institute, Hobart, Australia*
[4]*Medical University of Vienna, Institute for Hygiene and Applied Immunology, Vienna, Austria*

**Introduction**

Two independent transmissible cancers, termed Devil Facial Tumour 1/2 (DFT1/2), threaten the existence of the Tasmanian devil. While individuals can reject skin transplants from each other, the transmitted cancer cells are able to escape a highly developed immune system. By investigating the active oncogenic pathways in DFT1/2

© 2023 The Authors. Molecular Oncology is published by John Wiley & Sons Ltd on behalf of Federation of European Biochemical Societies cells, we can explore a connection between cancer signalling and immune escape mechanisms. Therefore, this is a unique model system to study human cancer and tumour immunity.

**Material and Methods**

To identify active core cancer pathways and possible therapies, we performed several large-scale drug screens including DFT1/2 cell lines and healthy fibroblasts as a control. We validated the hits with both *in vitro* cell assays and xenograft models. We further investigated basal expression levels of involved target genes by RT-qPCR and Western blotting to validate the suggested signalling axes.

**Results and Discussions**

DFT1 and DFT2 are phenotypically similar in nature and our experimental approaches reveal that both tumours are largely kinase driven. Interestingly, however, they are controlled by disparate kinases and downstream pathways. In DFT1, members of the epidermal growth factor receptor (EGFR) family are highly upregulated and use aberrant levels of signal transducer and activator of transcription 3 (STAT3) as signalling partner to guarantee increased proliferation. Subsequently, this pathway hinders major histocompatibility complex I (MHCI) expression by capturing STAT1 and, thus, promotes immune evasion. DFT2, on the other hand, shows significantly enriched platelet-derived growth factor receptor alpha (PDGFRα) levels, suggesting this pathway is also able to deregulate immune activation. Supporting our data, we used clinically relevant drugs *in vitro* and in xenograft models to show that DFT1/2 are selectively killed compared to healthy control fibroblasts when targeting their specific vulnerabilities.

**Conclusion**

Our findings suggest that DFT1 and DFT2 use different driver pathways and employ connected mechanisms to evade the immune system. By targeting the divergent vulnerabilities pharmacologically, cancer cells can be killed selectively and immune recognition might be restored thereupon. Our gained knowledge is therefore not only relevant for protecting the Tasmanian devil as a species but can contribute to human cancer, metastasis and tumour immunity research.

### EACR23-0152

### Spatial Multiparametric Investigation of Mouse and Human Brain Tumor Microenvironment Identifies Critical Neuro-Oncological Processes in Brain Neoplasms

*R. Spada[1], E. Lopez[2], N. Zabinyakov[3], Q. Raza[3], C. Loh[3]*

*[1]Fluidigm, Commercial Operations EMEA, Les Ulis, France*

*[2]Standard BioTools, Commercial Operations EMEA, Les Ulis, France*

*[3]Standard BioTools, Research and Development, Markham, Canada*

**Introduction**

Brain cancer research presents challenges that require comprehensive assessment of the structural and cellular organization of the tumor microenvironment (TME). Lack of readily available tissue samples, insufficient multiparametric assessment, and tissue autofluorescence further limit identification of key neuro-oncological processes that dictate disease progression. Imaging Mass Cytometry™ (IMC™) offers unprecedented insight into the TME by uncovering the spatial distribution of 40-plus distinct molecular markers without data artifacts caused by autofluorescence. Here, we present a deep phenotypic spatial analysis of various mouse and human brain tumors and identify cellular composition and activation of immuno-oncological processes within the TME.

**Material and Methods**

Our high-plex brain cell phenotyping antibody panel is designed for imaging application on formalin-fixed, paraffin-embedded tissues. These neural research-specific panels consist of human and mouse cross-reactive clones and are compatible with Maxpar® Human and Maxpar OnDemand™ Mouse Immuno-Oncology IMC Panel Kits. They enable flexible panel design for brain-specific research goals, such as brain tumor classification, and assessment of neuronal inflammation, degeneration, and development. We applied the high-plex antibody panel on tissue microarrays containing a variety of human and mouse brain tumors. Normal brain tissues were used for comparative analysis as controls. The Hyperion™ Imaging System was utilized to digitize images from the tissues followed by quantitative analysis to assess the cellular composition of normal and cancerous brain TME.

**Results and Discussions**

We identified major cell populations that make up human and mouse brain matter, such as neurons, astrocytes, microglia, and oligodendrocytes. Various tumor cell phenotypes, resident and infiltrating cells, and resting and activated microglia were detected in multiple tumor subtypes. Additionally, we assessed vascular coverage, extracellular matrix composition, and activation of immune cells within the TME. Subsequent single-cell analysis provided a comprehensive and quantitative assessment of the brain TME in our samples.

**Conclusion**

Our phenotypic analysis resolved the brain TME to the single-cell level and provided insights into the spatial complexity of neuronal neoplasms. We quantified myeloid and lymphoid immune cell infiltration across normal, astrocytoma, and glioblastoma tissues.

### EACR23-0161

### Assessing the ability of cytokine-activated allogeneic NK cells to killing glioblastoma cultures reveals poor efficacy of killing.

*S. Graham[1], H.J. Kim[1], L. Robilliard[1], J. Yu[1], G. Finlay[1], K. Angel[2]*

*[1]University of Auckland, Molecular Medicine and Pathology, Auckland, New Zealand*

*[2]University of Auckland, Schol of Biological Sciences-, Auckland, New Zealand*

**Introduction**

The goal of this research was to develop a clear appreciation of the sensitivity of cultured glioblastoma cells to highly activated allogenic Natural Killer (NK) cells. The glioblastoma cells used in this study are originally from patient-derived tumours and the cells grow as a mixture of phenotypes expressing variable levels of

© 2023 The Authors. Molecular Oncology is published by John Wiley & Sons Ltd on behalf of Federation of European Biochemical Societies

CD133, A2B5, CD49F, and CD44. Most of the glioblastoma cells express nestin, vimentin, βIII-tubulin with heterogeneity for GFAP.

**Material and Methods**

The NK cells were isolated from PBMC from healthy donors and so were therefore allogeneic. Killing assays were then conducted after the NK cells were exogenously activated with IL-2 for 48 hours to gain enhanced killing activity.

**Results and Discussions**

Killing assays were initially conducted with physiologically relevant ET ratios (<1:5 ratio) and no killing was observed. Killing assays were repeated with higher ET up to 5:1 ratios. In some GBM cultures, some killing was observed over a 4-24 hour period of killing. With concern, the extent of killing was usually less than 20% meaning that the majority of the GBM cells were resistant to highly activated allogeneic NK cells. The inability of the activated allogeneic NK cells to killing the GBM at physiological relevant (low) ET ratios is particularly concerning. Moreover, resistance to killing of the majority of the GBM cells as very high ratios of 5:1 indicates the failure of the NK cells to recognise the GBM cells as tumorous.

**Conclusion**

We hypothesise that this occurs because the key activation signals are lacking to initiate killing and/or that the GBM cells express an overwhelming repertoire of suppressive ligands. Research is now underway to fully appreciate which ligands are expressed by the GBM cells to coordinate sensitivity to the NK cells.

### EACR23-0162

### Mapping expression of key NK-activation and suppressive ligands by cultured glioblastoma cells in order to identify targets for enhancing killing of GBM cells by NK cells.

*S. Graham[1], H.J. Kim[1], L. Robilliard[1], A. Anchan[1], J. Yu[1], G. Finlay[1], K. Angel[2]*

*[1]University of Auckland, Molecular Medicine and Pathology, Auckland, New Zealand*

*[2]University of Auckland, Biological Sciences, Auckland, New Zealand*

**Introduction**

We have found that the majority of glioblastoma cells are insensitive to recognition and killing by activated NK cells. This poses the question as to the molecular mechanisms of this insensitivity. In order for cells to be recognised by NK cells, they must express a repertoire of activation signals that indicate the cells being foreign, unhealthy, virally infected or cancerous. In addition, they must also down regulate expression of inhibitory ligands, which are typically highly expressed by healthy cells, and thus protect them from NK surveillance.

**Material and Methods**

We have used Nanostring technology (transcriptional analysis) to measure expression of known NK-regulatory ligands (activation and inhibition) expressed by the glioblastoma cells. Expression analysis has been conducted using a range of patient-derived glioblastoma cultures. In addition, expression of the respective regulatory receptors was conducted by flow cytometry.

**Results and Discussions**

Preliminary analysis reveals low mRNA levels of some activation ligands, which does provides some promise. However, at the mRNA level there is overwhelming expression of numerous suppressive ligands. Interestingly, the Nanostring analysis has also revealed multiple activation and inhibitory ligands that do not appear to be expressed by any of the glioblastoma cultures. Flow-cytometry experiments are currently underway to ascertain the actual cell-surface expression of the detected ligands. Flow-cytometry has been conducted on primary cytokine activated NK cells revealing the presence of their activation/inhibitory receptor repertiore.

**Conclusion**

The key next step is to ascertain which of the detected ligands represent targets that we can realistically manipulate to enhance the detection of the glioblastoma cells by the NK cells. The obvious approach is to "convert" the dominant inhibitory signals into activation signals. This will having the advantage of providing the activation signals that may well be completing lacking for some glioblastoma cells.

### EACR23-0164

### Oral ingestion of Lactobacillus-derived exopolysaccharide enhances immune-checkpoint blockade therapy mediated by tumor-infiltrating CCR6+ CD8+ T cells

*H. Kawanabe-Matsuda[1,2], K. Takeda[1], M. Nakamura[2], S. Makino[1,2], T. Karasaki[3], K. Kakimi[3], M. Nishimukai[4], T. Ohno[1,5], K. Okumura[1]*

*[1]Juntendo University, Graduate School of Medicine, Tokyo, Japan*

*[2]Meiji Holdings Co. Ltd, Co-Creation Center, Hachioji, Japan*

*[3]The University of Tokyo, Hospital, Tokyo, Japan*

*[4]Iwate University, Faculty of Agriculture, Morioka, Japan*

*[5]Tokyo Dental College, Oral Health Science Center, Tokyo, Japan*

**Introduction**

Intratumoral microbiota is now established as one of the critical parameters for the efficacy of immune-checkpoint blockade (ICB) therapies by direct effects on cancer cells and tumor-infiltrating immune cells. Besides, it is well known that gut microbiota and some probiotic supplementation improve the therapeutic efficacy against several types of cancer in distal organs, but their effecting mechanisms are still unclear. We have been studying the biological functions of exopolysaccharides (EPS) derived from non-commensal *Lactobacillus delbrueckii* subsp. *bulgarius* OL L1073R-1 (EPS-R1) and found that these EPS induced IFNγ production by mouse splenocytes. Here, we examined the effect of oral ingestion of EPS-R1 on the ICB therapies using mice.

**Material and Methods**

First, we examined the expression profile of chemokine receptors on gut T cells after the oral administration of EPS-R1 for two weeks. Then, we generated experimental tumor models by subcutaneous transplanting of several

© 2023 The Authors. Molecular Oncology is published by John Wiley & Sons Ltd on behalf of Federation of European Biochemical Societies syngeneic tumor cell lines and evaluated the combinational effect of EPS-R1 with ICB therapies. For the mechanistic study, tumor-infiltrating T cells and immune signatures were examined by flow cytometry and time-course bulk RNA-seq analysis, respectively. In addition, we analyzed the translational value based on the TCGA database.

**Results and Discussions**

Oral administration of EPS-R1 selectively induced $CCR6^+$ $CD8^+$ T cells in the small intestinal Peyer's patches, and these cells were distributed to the periphery. Moreover, the ingestion of EPS-R1 augmented the anti-tumor effects of anti-CTLA-4 or anti-PD-1 mAb against CCL20-expressing tumors, in which infiltrating $CCR6^+$ $CD8^+$ T cells were increased and produced IFNγ accompanied by a substantial immune response gene expression signature maintaining T-cell functions. These results suggest that IFNγ-rich tumor tissues conditioned by EPS-R1 improve ICB therapies. Of note, CCR6 gene expression correlated with favorable prognoses in some CCL20-producing cancers in humans.

**Conclusion**

We revealed that $CCR6^+$ $CD8^+$ T cell is a novel mediator connecting gut and tumor and a bacterial polysaccharide improves the efficacy of ICB therapies by the induction of these lymphocytes.

*(Reference: Kawanabe-Matsuda et al. Cancer Discov 2022;12:1336-55.)*

### EACR23-0234

### Investigating the role of demethylating agent decitabine in enhancing immune targeting of breast cancer cells.

M. Sultan[1], M. Hanes[2,3], P. Marcato[2,3], C. Giacomantonio[1]
[1]Dalhousie University, Department of surgery, Halifax- NS, Canada
[2]Dalhousie University, Department of microbiology and immunology, Halifax- NS, Canada
[3]Dalhousie University, Department of pathology, Halifax- NS, Canada

**Introduction**

Avoiding detection and destruction by immune cells is key for tumor initiation and progression. The important role of epigenetic modifications in tumorigenesis have been well established, yet their role in mediating immune evasion is only partly understood. Our previous work showed the ability of highly tumorigenic populations of breast cancer stem cells to epigenetically silence TAP1 which contribute to their ability to evade immune destruction. Furthermore, we showed that TAP1 expression is negatively correlated with hypermethylation in breast cancer in general. This suggests that targeting breast cancer cells with demethylating agents such as Decitabine could enhance their detection by immune cells.

**Material and Methods**

We are currently investigating the use of low dose intratumoral vs. Intraperitoneal decitabine treatment in targeting 4T1 tumors and characterizing changes in tumoral immune infiltration associated with either treatment modality. Additionally we are investigating TAP1 methylation status and its effect on patient survival when melanoma and urothelial tumors are treated with checkpoint inhibitors.

**Results and Discussions**

Our preliminary data suggests that intratumoral decitabine treatment can alter tumor growth and cause a shift in immune cell invasion. Interestingly, investigation of several TCGA datasets revealed that epigenetic silencing of TAP1 is evident in Melanoma and Urothelial tumors. Furthermore, high expression of TAP1 was associated with a significant increase in patient survival when Melanoma and Urothelial tumors were treated with checkpoint inhibitors.

**Conclusion**

Together, the findings from our ongoing breast cancer *in vivo* studies as well as the patient datasets will provide further insights on the role of decitabine in targeting tumor cells and its potential as combination therapy to enhance immunotherapy efficacy.

### EACR23-0239

### Altering Future Immune Challenges, Priming and Metastasis Through Long-Term Changes Driven By Acute Influenza Infection

R. Devlin[1], C. Pirillo[1], J. McCowan[1], A. Shergold[1], A. Bravo-Blas[1], S. Al-Khalidi[1], L. Carlin[2], E. Roberts[1]
[1]Beatson Institute for Cancer Research, Roberts' Lab, Glasgow, United Kingdom
[2]Beatson Institute for Cancer Research, Carlin/Leukocyte Dynamics Lab, Glasgow, United Kingdom

**Introduction**

Trained immunity has been shown to impact the course of subsequent infections. We therefore hypothesised that prior infections could alter immune responses to tumours.

**Material and Methods**

Specific pathogen free mice were given PR8 influenza A virus (IAV) and, at 28 days post-infection, their lungs and draining lymph nodes characterised with flow cytometry and imaging.

We then investigated the impact of this prior infection on subsequent tumours. At day 28 post-influenza, we gave mice carcinogen driven primary lung tumours through administration of urethane, or experimental metastasis through intravenous injection of MC38 or B16 tumour cells.

Finally, we experimentally increased cDC levels through administration of FLT3L daily for four days prior to tumour injection, thereby investigating cDC in isolation of other factors driven by infection.

**Results and Discussions**

The lung and lymph node of specific pathogen free mice 28 days post PR8 influenza A virus (IAV) infection showed significant changes in both the stromal and immune compartments, despite the virus being cleared from the lungs and bone marrow changes returning to baseline. Gross alterations in the organisation of immune cells within the lung were also evident at this late timepoint post-infection, with an increase in conventional dendritic cells (cDC) within the lung which were organised in clusters in the peribronchiolar space.

Following IAV infection resolution, mice developed more lung tumours either in response to a carcinogen, urethane, or intravenous injection of metastatic cells. However, despite this increased tumour burden, the raised levels of

© 2023 The Authors. Molecular Oncology is published by John Wiley & Sons Ltd on behalf of Federation of European Biochemical Societies cDC correlated with improved anti-metastasis CD8+ T cell priming even when tumour abundance was controlled for. This raised the possibility that increasing levels of cDC may improve anti-metastatic immune responses. Treatment with FLT3L prior to intravenous tumour injection not only increased cDC numbers but led to a highly significant decrease in metastatic seeding.

**Conclusion**

Influenza infection drastically reshapes the immune and stromal layout of the lung and lymph nodes in the long term, driving an increase in pulmonary cancer burden. As a part of this post-flu response, cDC and T-cell priming are increased, identifying a potential therapeutic target. We now aim to investigate how cDC numbers remain elevated, the role of flu in increasing tumour burden, and the potential of FLT3L as an anti-metastatic therapeutic.

### EACR23-0259
### Small extracellular vesicle-associated adenosine promotes pro-tumor neutrophil reprogramming in head and neck squamous cell carcinoma

*D. Rubenich[1,2], A. Głuszko[3], K. Franke[1], D. Schulz[1], H. Poeck[4], M. Federlin[5], R. Bauer[1], T. E. Reichert[1], E. Braganhol[2], N. Ludwig[1]*
*[1]University Hospital Regensburg, Department of Oral and Maxillofacial Surgery, Regensburg, Germany*
*[2]Universidade Federal de Ciencias da Saude de Porto Aleg re, Programa de Pós-Graduação em Biociências, Porto Alegre, Brazil*
*[3]Faculty of Medicine- Medical University of Warsaw, Chair and Department of Biochemistry, Warsaw, Poland*
*[4]University Hospital Regensburg and Leibniz Institute for I mmunotherapy LIT, Clinic and Polyclinic for Internal Medicine III, Regensburg, Germany*
*[5]University Hospital Regensburg, Department of Conservative Dentistry and Periodontology, Regensburg, Germany*

**Introduction**

Head and neck squamous cell carcinomas (HNSCCs) are clinically complex and molecularly heterogeneous. Current research considers tumor-derived small extracellular vesicles (TEX) as important regulators of HNSCC progression due to their accumulation and functions in the tumor microenvironment (TME) and at distant sites. The cargo of TEX includes components of the adenosinergic (ADO) pathway and TEX were shown to be a major source of ADO in the TME, ultimately mediating pro-tumor activities. This study tests the hypothesis that TEX interact with neutrophils (N0) and reprogram them via ADO receptors (P1R), ultimately inducing a tumor-associated phenotype.

**Material and Methods**

TEX produced by UMSCC47 HNSCC cells were isolated by size exclusion chromatography (SEC) and subsequently characterized. Primary human N0 were co-incubated with TEX for 48h and functional assays for migration, activation and lifespan of N0 were performed using transwell inserts, scanning electron microscopy (SEM), and FACS, respectively. The secretome of N0 in the presence and absence of TEX was evaluated by antibody arrays.

**Results and Discussions**

Isolated TEX ranged in size from 50-150 nm, carried CD63 and CD9 but not the negative marker Grp94. Also, TEX carried enzymatically-active CD39 and CD73. TEX closely interacted with N0, attaching to their cell surface, resulting in stimulation of N0 activation, transmigration, and elongation of their lifespan ($p < 0.05$). Treatment with TEX promoted the release of immunosuppressive markers by N0 including MCP-1 and CCL1/I-309 and decreased the release of inflammatory cytokines such as CXCL1, IFNγ and IL-6. The TEX-induced alterations of the secretome and the N0 activation were blocked by P1R antagonists ($p < 0.05$), especially selective $A_{2A}R$ antagonists, suggesting that TEX-associated ADO orchestrated these processes. Secretome analysis also implied the involvement of netosis since TEX increased levels of PAI-1. Indeed, NETs release was stimulated by TEX as observed by SEM.

**Conclusion**

Overall, data suggests that TEX are potent inducers of N0 activation and modulation via the ADO pathway, resulting in pro-tumor and immunosuppressive N0 phenotypes. Understanding the multifaceted role of neutrophils in HNSCC is of current interest and has the potential to unravel important biological aspects, which may become important for the development of future therapeutic targets.

### EACR23-0266
### Traces of Epstein Barr virus (EBV) are not involved in the pathogenesis of Diffuse Large B cell lymphoma (DLBCL)

*T. Mangiaterra[1], R. Alonso[2], M. De Dios Soler[3], E. De Matteo[1], S.M. Rodriguez Pinilla[2], P. Chabay[1]*
*[1]Instituto Multidisciplinario de Investigaciones en Patologí as Pediátricas IMIPP. CONICET- GCBA- Hospital de Niños Ricardo Gutierrez, Pathology Division, Ciudad Autonoma de Buenos Aires, Argentina*
*[2]IIS Hospital Universitario Fundación Jiménez Díaz, Pathology Department, Madrid, Spain*
*[3]Hospital Marie Curie, Division Patología, Ciudad de Buenos Aires, Argentina*

**Introduction**

Epstein-Barr virus-positive DLBCL (EBV+ DLBCL) was defined by the WHO, with＞80% EBERs+ cells, although 20% was used as cut off. The detection of traces of EBV was reported by more sensitive methods than EBERs in situ hybridization (ISH). The aim was to analyze immune response markers in EBV+ DLBCL, and to compare them in cases with or without traces of EBV infection.

**Material and Methods**

Fifty DLCBL biopsies cases were included. The expression of immune response genes was evaluated by Lymph2Cx. EBERs ISH was performed. LMP1 and EBNA2 transcripts were detected by double ISH with ViewRNA ISH Tissue 2-Plex Assay to define the presence of traces of EBV. LMP1+, EBNA2+, and double LMP1+/EBNA2+ cells were counted in tumor cells. Viral load was measured by RT- qPCR. Immunohistochemistry (IHC) for CD68 and LAG-3 at the TME, and PDL-1 in tumor cells was performed.

**Results and Discussions**

© 2023 The Authors. Molecular Oncology is published by John Wiley & Sons Ltd on behalf of Federation of European Biochemical Societies

EBV was detected by EBERs ISH in 12/50 cases. A cut-off of 20% of EBERs+ cells was used to define EBV+DLBCL. Viral transcripts were detected in 19/45 cases (5 EBV+ and 14 EBV-) with good quality for analysis. 19 and 5 cases expressed LMP1 and EBNA2 transcripts , respectively. Only EBV+DLBCL cases displayed a mean viral load above the limit of detection. When the expression of immune response genes was compared in EBV+ vs EBV- DLBCL, higher expression of PDL-1, LAG-3, CD68, CD274, among others, was observed. This increased expression was not observed when cases with traces of viral transcripts were compared with its negative counterpart. The expression of LAG-3 and CD68 in the TME and PDL-1 in tumor cells was assessed by IHC to validate gene expression results.PDL-1+ and CD68+cell count was higher in EBV+ DLBCL ($p<0.05$), whereas no difference was observed in LAG-3 expression. However, when cases with traces of EBV transcripts were compared with cases without them, no differences were proved in LAG-3, CD68 and PDL-1+cell count.

**Conclusion**

This study provides further evidence that traces of EBV could be detected by a sensitive method. However, since differences were only demonstrated in cases with more than 20% of EBERs+ cells as cut off, traces of EBV would not be involved in the pathogenesis of DLBCL, in contrast to the hit-and-run theory. In EBV+ DLBCL PDL-1 and CD68 expression is upregulated in tumor cells and in the TME, respectively.

### EACR23-0295
### CX3CR1 antagonism holds therapeutic utility to reverse the pro-tumourigenic effects of fractalkine in oesophageal adenocarcinoma

C. Marion[1,2], E. Mylod[1], F. O'Connell[1], J.V. Reynolds[3], M. Lowery[3], S. Maher[1], J. Lysaght[1], M.J. Conroy[2]
[1]*Trinity Translational Health Institute, Surgery, Dublin, Ireland*
[2]*Trinity College Dublin, Physiology, Dublin, Ireland*
[3]*St James's Hospital, Gastro-intestinal Medicine and Surgery, Dublin, Ireland*

**Introduction**

Oesophageal adenocarcinoma (OAC) is a poor prognosis cancer with a 5-year survival rate of ~20%. Current response rates to chemotherapy and/or radiotherapy are only ~30% and new therapeutics are urgently needed. Our group has reported that fractalkine, a pro-inflammatory chemokine, plays a role in the disruption of anti-tumour immunity in OAC via its recruitment of natural killer (NK) cells to the visceral adipose tissue (VAT). Furthermore, we have reported that antagonism of the fractalkine receptor CX3CR1 can prevent NK cell migration towards VAT and holds therapeutic potential to re-invigorate their infiltration and killing of OAC tumours. We propose that fractalkine may directly promote OAC tumour progression and here, we investigated its proliferative and pro-metastatic effects in this setting. In addition, we examined if CX3CR1 antagonism could block these effects in OAC.

**Material and Methods**

To determine the pro-tumourigenic effects of fractalkine and the anti-tumourigenic efficacy of CX3CR1 antagonism in OAC, the OE33 cell line and the metastatic OAC cell lines FLO-1 and FLO-$1^{LM}$ cell lines were pre-treated with CX3CR1 antagonist E6130 (0,1, 1, 4,9, 10, 100, 1000nM) and/or treated with recombinant fractalkine (0.1, 0.5, 1, 5, 10, 50, 100, 200ng/mL) for 24 hours. Cell viability and proliferation was assessed using a CCK8 and a BrdU assay.

**Results and Discussions**

Treatment with 100ng/mL and 200ng/mL fractalkine significantly increased OE33 cell viability and proliferation. Pre-treatment with E6130 attenuated these effects. Fractalkine significantly increased FLO-1 and FLO-$1^{LM}$ cell line viability at different concentrations, affecting FLO-1 cell line at lower concentrations. Interestingly, FLO-1 cells express higher basal levels of CX3CR1 on their surface, compared to their liver-metastatic counterparts FLO-$1^{LM}$. This may contribute to their higher sensitivity to fractalkine-mediated effects and warrants further investigation.

**Conclusion**

Our data demonstrate that high concentrations of fractalkine increases OAC tumour cell proliferation and viability. Furthermore, these novel findings indicate that CX3CR1 antagonism holds therapeutic potential to reverse these pro-tumourigenic effects. Future *in vitro* and *in vivo* studies will further elucidate the role of fractalkine in OAC tumour growth and metastasis and confirm the therapeutic utility of CX3CR1 antagonism in this hard-to-treat cancer.

### EACR23-0347
### Microglia-mediated Brain Tumor Regression is Regulated by T Cell Immunity

T.C. Sun[1], C.S. Chiang[1]
[1]*National Tsing Hua University, Department of Biomedical Engineering and Environment Sciences, Hsinchu city, Taiwan*

**Introduction**

Microglia are the resident immune cells in the central nervous system (CNS) and the most abundant immune cells in the brain tumor microenvironment (TME). To clarify microglia's role in brain tumor progression, we established an in vitro co-culture system with an astrocytoma cell line, ALTS1C1, and microglia cell line, BV2, to mimic the microglia-rich TME of brain tumors. We demonstrated that microglial cells would lead brain tumor cells to form colonies and protect them from the cytotoxicity of anti-tumor agents. However, how microglial cells affect brain tumor progression in vivo is still unclear. In this study, we aimed to examine further microglia's influence on brain tumor progression in an in vivo preclinical model.

**Material and Methods**

The astrocytoma cell line, ALTS1C1, and microglia cell line, BV2, were orthotopic intracranial co-implanted in fully immunocompetence mice, C57BL/6 as well as SCID mice, C.B17. The animal survival was recorded and analyzed by Kaplan-Meier analysis. The brain tumor microenvironment was analyzed by immunohistochemical (IHC) staining. To examine the systemic immunity, a re-challenge assay using the intramuscular implant was applied, and T cell function was evaluated by CTL assay.

© 2023 The Authors. Molecular Oncology is published by John Wiley & Sons Ltd on behalf of Federation of European Biochemical Societies

**Results and Discussions**

Our data show that the survival of ALTS1C1-bearing C57BL/6 mice was significantly improved by the presence of the microglial cell BV2. The mean surviving time was increased when one-tenth of BV2 was present during the tumor inoculation. Furthermore, the growth of the ALTS1C1 tumor was completely repressed when the composition of inoculated cells containing 50% of BV2 cells. When the same experiments were performed in immune-deficient C.B17 SCID mice, the mean survival time of ALTS1C1-bearing mice decreased. On the other hand, the mean surviving time of 50% BV2-containing ALTS1C1 was 28.8 days. These results indicate that host immunity plays an essential role in controlling the growth of ALTS1C1, especially in the presence of microglia. The IHC staining revealed four-fold more infiltrating CD8 T cells and two-fold more PD-1 expression in BV2-containing ALTS1C1 tumors. The re-challenge and CTLs assays demonstrated the development of systemic immunity in BV2-containing ALTS1C1 tumor-bearing mice. These results reveal that microglia-mediated ALTS1C1 regression is associated with T-cell immunity.

**Conclusion**

This study demonstrates that microglia would establish an anti-tumor microenvironment by recruiting T cells and polarizing them to become cytotoxic T cells.

### EACR23-0364
### Exercise-induced mobilization of immune cells in newly diagnosed breast cancer patients

*T. Koivula[1], S. Lempiäinen[1], J. Neuvonen[1], J. Norha[1], M. Hollmén[2], C.J. Sundberg[3,4], H. Rundqvist[5], H. Minn[6], P. Rinne[7], I. Heinonen[1]*

*[1]University of Turku and Turku University Hospital, Turku PET Centre, Turku, Finland*

*[2]University of Turku, MediCity Research Laboratory, Turku, Finland*

*[3]Karolinska Institutet, Department of Physiology and Pharmacology, Stockholm, Sweden*

*[4]Karolinska Institutet, Department of Learning-Informatics-Management and Ethics, Stockholm, Finland*

*[5]Karoliska Institutet, Department of Laboratory Medicine, Stockholm, Sweden*

*[6]Turku University Hospital, Department of Oncology and Radiotherapy, Turku, Finland*

*[7]University of Turku, Institute of Biomedicine, Turku, Finland*

**Introduction**

Acute exercise is a powerful stimulus for mobilizing the immune system in healthy individuals, but the phenomenon is less investigated in cancer patients. Exercise-induced mobilization of immune cells may have positive effects in cancer patients, improving the ability of the immune system to combat against tumor cells.

**Material and Methods**

19 women aged 36 to 68 with newly diagnosed grade I, II, and III breast cancer performed an acute 30-minute bicycle ergometer exercise at a self-selected power output. Blood samples were taken at rest, at 15-minute (E15) and at 30-minute (E30) time points during the exercise, and 30 minutes (P30) and 60 minutes (P60) post-exercise. Total leukocytes and their subsets were analysed using flow cytometry.

**Results and Discussions**

Mean heart rate during exercise was 127 bpm and mean heart rate percentage of age predicted maximal heart rate was 77 %. Acute exercise increased the number of total leukocytes, neutrophils, lymphocytes, monocytes, basophils, total T cells, $CD4^+$ T cells, T helper (Th) 1 cells, Th 2 cells, Th 17 cells, $CD8^+$ T cells, $CD4^-CD8^-$T cells, $CD56^+$ natural killer (NK) cells, and $CD14^-CD16^+$ monocytes (p<0.05 in all). These changes peaked at E15, except for NK and Th 1 cells, which peaked at E30 and at P30, respectively. Furthermore, the number of $CD19^+$ B cells decreased, while no change was observed in other subpopulations. Heart rate and heart rate percentage of age predicted maximal heart rate at E15 correlated positively with total monocyte, $CD14^-CD16^+$ monocyte, and basophil mobilization at E15. Moreover, lactate concentration at E30 correlated positively with lymphocyte and NK cell mobilization at E30. Further analysis showed that the grade of breast cancer did not correlate with any immune cell concentrations at baseline but correlated positively with basophil mobilization at E15 and with increase in neutrophils and decrease in myeloid derived suppressor cells between baseline and E30.

**Conclusion**

Our findings show that 30-minute acute exercise increased the number of total circulating leukocytes as well as several immune cell subsets in breast cancer patients. The mobilization of some immune cells appears to be related to the intensity of exercise and the disease state. It is possible that the positive effect of exercise on oncologic outcome might be partly due to immune cell mobilization as documented in this study.

### EACR23-0389
### Epithelial Ovarian Cancer is infiltrated by activated effector T cells co-expressing CD39, PD-1 and TIM-3 and by myeloid cells expressing inhibitory receptor ligands

*E. Tassi[1], A. Bergamini[2,3], J. Wignall[4], M. Sant'Angelo[5], E. Brunetto[5], C. Balestrieri[4], M. Redegalli[5], A. Potenza[4], D. Abbati[4], F. Manfredi[4], M.G. Cangi[5], E. Ruggiero[4], L. Bocciolone[2], G. Candotti[2], M. Candiani[2,3], G. Taccagni[5], C. Doglioni[3,5], G. Mangili[2], C. Bonini[1,3]*

*[1]IRCCS Ospedale San Raffaele, Experimental Hematology Unit and Cell Therapy Immuno monitoring Laboratory MITiCi- Division of Immunology- T ransplantation and Infectious Diseases, Milan, Italy*

*[2]IRCCS Ospedale San Raffaele, Department of Obstetrics and Gynecology, Milan, Italy*

*[3]Università Vita-Salute, San Raffaele, Milan, Italy*

*[4]IRCCS Ospedale San Raffaele, Experimental Hematology Unit - Division of Immunology-Transplantation and Infectious Diseases, Milan, Italy*

*[5]IRCCS Ospedale San Raffaele, Department of Surgical Pathology, Milan, Italy*

**Introduction**

Despite predicted efficacy, immunotherapy in epithelial ovarian cancer (EOC) has limited clinical benefit and the prognosis of patients remains poor. There is thus a strong need for better identifying local immune dynamics and

© 2023 The Authors. Molecular Oncology is published by John Wiley & Sons Ltd on behalf of Federation of European Biochemical Societies immune-suppressive pathways limiting T-cell mediated anti-tumor immunity.

**Material and Methods**

In this observational study we analyzed by immunohistochemistry, gene expression profiling and flow cytometry the antigenic landscape and immune composition of 48 EOC specimens, with a focus on tumor-infiltrating lymphocytes (TILs).

**Results and Discussions**

Activated T cells showing features of partial exhaustion with a $CD137^{+}CD39^{+}PD\text{-}1^{+}TIM\text{-}3^{+}CD45RA^{-}CD62L^{-}CD95^{+}$ surface profile were exclusively present in EOC specimens but not in corresponding peripheral blood or ascitic fluid, indicating that the tumor microenvironment might sustain this peculiar phenotype. Interestingly, while neoplastic cells expressed several tumor-associated antigens possibly activating tumor-specific TILs, myeloid cells provided both co-stimulatory and inhibitory signals and were more abundant in TILs-enriched specimens harboring the $CD137^{+}CD39^{+}PD\text{-}1^{+}TIM\text{-}3^{+}CD45RA^{-}CD62L^{-}CD95^{+}$ signature. WT-1 expression by cancer cells and stromal $CD137^{+}CD39^{+}$ TILs-infiltrate positively correlated with progression free survival.

**Conclusion**

Collectively, our data depict an EOC microenvironment where activated antigen-experienced T lymphocytes and myeloid cells co-exist and possibly cooperate to drive local immune response. These results suggest that the combined inhibition of multiple exhaustion-related pathways might be needed for effective immunotherapy in EOC, and identify CD39, PD-1 and TIM-3 as potential targets for the reinvigoration of anti-tumor immunity.

### EACR23-0405

### Metastatic colorectal carcinoma-associated fibroblasts display an IGFBP2-dependent immunosuppressive effect on the tumour microenvironment

*C. Ramos[1], N. Walterskirchen[1], C. Müller[1], M. Sachet[1], L. Unger[1], V. Gerakopoulos[1], T. Bachleitner-Hofmann[1], M. Bergmann[1], H. Dolznig[2], R. Oehler[1]*

*[1]Medical University of Vienna, Department of General Surgery- Division of Visceral Surgery, Vienna, Austria*

*[2]Medical University of Vienna, Institute of Medical Genetics, Vienna, Austria*

**Introduction**

The tumour microenvironment (TME) is a crucial factor in carcinogenesis, disease progression and resistance to therapy. Fibroblasts are the major source of extracellular matrix (ECM) proteins which are the major component of TME. As the most abundant stromal cell, fibroblasts play an essential role in primary and metastatic colorectal cancer (CRC). In addition to their supportive effect on tumour cells, there is growing evidence that fibroblasts also modulate the immune microenvironment in tumours. We investigated the differences in fibroblast-mediated immune modulation between primary CRC and peritoneal metastasis.

**Material and Methods**

Cancer associated fibroblasts were isolated from primary cancer (CAFs) and from peritoneal metastases (MAFs) from a total of 57 patients. Gene expression analysis was performed on CAFs and MAFs to identify differential gene expression. Additionally, differentially expressed proteins were validated by immunofluorescence, flow cytometry and ELISA. To test their possible immunomulatory effects, fibroblasts were co-cultured with monocyte-derived macrophages (Mφs) or triple co-cultured with Mφs and T cells. To reverse their immunosuppressive effects, Igfbp2 was silenced in MAFs via siRNA transfection.

**Results and Discussions**

Gene expression analysis of our CAFs and MAFs shows a significant change in CD38 and IGFBP2 expression. Additionally, the protein levels of both proteins were validated by flow cytometry and ELISA, respectively. Interestingly, we found that the ectoenzyme CD38 was consistently expressed on the surface of all MAFs, while it was absent from CAFs. Furthermore, the NAD+-hydrolase activity of CD38 was preserved in MAFs. Moreover, MAFs secreted higher levels of IGFBP2, CXCL2, CXCL6, CXCL12, PDGF-AA, FGFb, and IL-6. Triple co-culture assays revealed that this differential secretome induced a decreased activation of macrophages and a suppression of CD25 expression and proliferation of T-cells. Silencing of IGFBP2 abolished these immunosuppressive effects of MAFs. Interestingly, the knockdown of IGFBP2 impaired the CD38 expression, suggesting that CD38 is a downstream target of IGFBP2.

**Conclusion**

Taken together, these results show that MAFs contribute to an immunosuppressive TME in CRC metastases by modulating the phenotype of immune cells through an IGFBP2-dependent mechanism. Therefore, strategies targeting fibroblast co-expressing IGFBP2 and CD38 might be promising therapeutic target to improve the immunosuppressive state of carcinomatosis patients.

### EACR23-0505

### Effect of vinorelbine on the modulation of M2 macrophages in non-small cell lung cancer

*A. Al-Omar[1], M. Asadi[1], C. Muftuoglu[1], U. Mert[2], H. Karakus[3], A. Caner[1]*

*[1]Ege University, Basic Oncology- Translational Pulmonary Research Center, Izmir, Turkiye*

*[2]Ege University, Basic Oncology- Translational Pulmonary Research Center- Ataturk Health Care Vocational School, Izmir, Turkiye*

*[3]Ege University, Faculty of Medicine- Chest Diseases, Izmir, Turkiye*

**Introduction**

In the tumor microenvironments, M2 like TAMs play an important role in the treatment response and progression of cancer. They induce tumor proliferation and growth, angiogenesis, migration, invasion, and immunosuppression and cause drug resistance. Vinorelbine is a chemotherapy used to treat NSCLC patients. However, it remains unclear whether vinorelbine can modulate M2 macrophages to M1. In addition, we investigated the effect of the drug on the expression levels of pro-tumor genes in NSCLC cell line in the presence and absence of M2 macrophages.

**Material and Methods**

© 2023 The Authors. Molecular Oncology is published by John Wiley & Sons Ltd on behalf of Federation of European Biochemical Societies

Monocytes isolated from peripheral blood differentiated into macrophages. Macrophages were polarized to the M1 and M2 phenotype using LPS and IL4/IL10, respectively, and were identified by flow cytometry with specific biomarkers. MIF levels of M2 macrophages treated with vinorelbine were determined by flow cytometry. The NSCLC cell line was indirectly co-cultured with M2 macrophages. Then, wound healing assay was performed to examine cell invasion. Expression levels of genes and proteins involved in lung cancer progression were evaluated by RT-qPCR and western blot.

**Results and Discussions**

We detected that vinorelbine, at low concentrations, had the ability to significantly modify M2 macrophages towards M1. After indirect co-culture, M2 macrophages reduced the effect of vinorelbine in NSCLC cells. It also revealed that the M2 phenotype increased the invasive ability of cancer cells and upregulated the expression of some genes involved in cancer progression after vinorelbine treatment

**Conclusion**

Vinorelbine has a modulating effect on the reprogramming of M2 macrophages to M1. In addition, M2 macrophages reduce the effect of vinorelbine and cause cancer progression.

### EACR23-0513
### Hypoxia-inducible factor (HIF-1) orchestrates adenosine metabolism to promote liver cancer development

*J.W.S. Cheu*[1,2], *D.K.C. Chiu*[1], *K.K.L. Kwan*[1,2], *C. Yang*[1], *V.W.H. Yuen*[1,2], *C.C. Goh*[1], *N.N.Q. Chui*[1], *W. Shen*[1,2], *C.T. Law*[1], *Q. Li*[1], *M.S. Zhang*[1,2], *M.H.R. Bao*[1,2], *B.P.Y. Wong*[1,2], *C.Y.K. Chan*[1,2], *C.X. Liu*[1], *G.F.W. Sit*[1,2], *Z.Y. Ooi*[1], *H. Deng*[1], *I.O.L. Ng*[1,3], *C.C.L. Wong*[1,2,3,4,5]

[1]*The University of Hong Kong, Department of Pathology- Li Ka Shing Faculty of Medicine , Hong Kong, Hong Kong- China*

[2]*Centre for Oncology and Immunology, Centre for Oncology and Immunology, Hong Kong, Hong Kong- China*

[3]*The University of Hong Kong, State Key Laboratory of Liver Research, Hong Kong, Hong Kong- China*

[4]*The University of Hong Kong, ShenZhen Hospital, Hong Kong, Hong Kong- China*

[5]*Sun Yat-Sen University, Guangdong-Hong Kong Joint Laboratory for RNA Medicine, Guangzhou, China*

**Introduction**

Hypoxia is a major player in establishing an immunosuppressive tumor microenvironment (TME). Adenosine, an immunosuppressive metabolite in hypoxic TME, suppresses anti-tumorigenic immune cells and dampens the efficacy of immune checkpoint inhibitors (ICIs). Extracellular ATP is converted to adenosine by hypoxia-induced ectoenzymes CD39 and CD73. However, as an indispensable source of extracellular adenosine, the molecular mechanisms regulating intracellular adenosine metabolism and export remain largely elusive.

**Material and Methods**

Chromatin immunoprecipitation and luciferase reporter assays were performed to study the regulation of ADK and ENT4 by hypoxia inducible factor-1 (HIF-1). Extracellular adenosine level was determined by mass spectrometry. We demonstrated the immunosuppressive role of adenosine via multiple *in vitro* assays with immune cells. We also employed hydrodynamic tail vein injection to induce HCC tumor formation in immunocompetent mice and studied the role of ADK and the therapeutic outcomes of combination treatment of anti-PD-1 and adenosine receptor blockade.

**Results and Discussions**

We found that HIF-1 orchestrates adenosine efflux through two steps in hepatocellular carcinoma (HCC). First, HIF-1 activates transcriptional repressor MXI1, which inhibits adenosine kinase (ADK), resulting in the failure of adenosine phosphorylation to adenosine monophosphate (AMP). This leads to adenosine accumulation in hypoxic cancer cells. Second, HIF-1 transcriptionally activates equilibrative nucleoside transporter 4 (ENT4), pumping adenosine into the interstitial space of HCC, leading to elevated extracellular adenosine levels. Clinically, ENT4 was upregulated while ADK was downregulated in HCC patients. Multiple *in vitro* assays showed that adenosine promoted T cell apoptosis and regulatory T cell accumulation while suppressed T cell proliferation and cytotoxicity. Adenosine also favored MDSC accumulation and skewed macrophages to M2-like phenotypes while inhibited differentiation to dendritic cells (DC). Knockout of ADK *in vivo* skewed intratumoral immune cells to pro-tumorigenic and promoted tumor progression. Therapeutically, combination treatment of adenosine receptor antagonists and anti-PD-1 prolonged survival of HCC-bearing mice.

**Conclusion**

We illustrated the significance of the dual role of hypoxia in creating an adenosine-mediated immunosuppressive TME and offered a potential therapeutic approach that might synergize with ICIs in HCC.

### EACR23-0545
### Knockdown of MARCH7 suppresses progression and invasion of esophageal cancer cells via regulation of epithelial to mesenchymal transition and modulates immune response

*A. BANO*[1], *S. Singh*[2], *A. Saraya*[2], *P. Das*[3], *R. Sharma*[1]

[1]*Guru Gobind Singh Indraprastha University, University School of Biotechnology, New Delhi, India*

[2]*All India Institute of Medical Sciences- New Delhi, Gastroenterology, New Delhi, India*

[3]*All India Institute of Medical Sciences- New Delhi, Pathology, New Delhi, India*

**Introduction**

Ubiquitin E3 ligase MARCH7 has been shown to have a role in immune tolerance and neuronal development. However, the clinical and functional significance of MARCH7 in esophageal cancer remains unknown. Our study aimed to unravel the role of MARCH7 in esophageal cancer for the first time.

**Material and Methods**

The expression of MARCH7 in esophageal cancer (EC) was evaluated using an online tool, GEPIA. Quantitative Real-Time PCR (qRT-PCR) and immunohistochemistry (IHC) were employed to determine the expression levels of MARCH7 in esophageal squamous cell carcinoma (ESCC) tissues and distant matched non-malignant tissues. The

© 2023 The Authors. Molecular Oncology is published by John Wiley & Sons Ltd on behalf of Federation of European Biochemical Societies effect of knockdown of MARCH7 gene on proliferation and migration/invasion in EC cells was assessed using MTT assay, colony formation assay, and transwell assay. Furthermore, MARCH7-mediated regulation of epithelial to mesenchymal transition (EMT) was checked via western blot analysis. The correlation of MARCH7 with immune cell infiltration was first analysed using the online tool, TIMER. Next, MARCH7 protein expression and its correlation with the expression of tumor-infiltrating lymphocytes (TILs) such as CD8+ and PD-1+ cells was checked using IHC. Moreover, the expression of various genes involved in immune response signalling was checked post-MARCH7 silencing via qRT-PCR.

**Results and Discussions**

Analysis of MARCH7 mRNA expression using GEPIA revealed its significant upregulation in EC. IHC analysis showed significant upregulation of MARCH7 protein in 84% of ESCC tissues with nuclear and cytoplasmic expression as compared to distant matched non-malignant tissues ($p \leq 0.001$, AUC=0.983) . MARCH7 silencing led to the significant inhibition of proliferation, migration/invasion, and clonogenic potential of EC cells. Deregulation of various EMT markers was observed in EC cells post MARCH7 silencing. TIMER analysis showed a significant inverse correlation between TILs viz., CD8+ and PD-1+ cells. Interestingly, IHC analysis in clinical samples corroborated in-silico findings and established an inverse correlation between MARCH7 and PD-1 expression ($r = -0.560$, $p=0.005$) as well as MARCH7 and CD8 expression ($r = -0.633$, $p = 0.001$). Remarkably, MARCH7 silencing also led to the upregulation of genes involved in activating the immune response.

**Conclusion**

Our results show that MARCH7 silencing inhibits the proliferation, invasion, and clonogenic potential of EC cells and indicates its involvement in immunomodulation via regulation of TILs, and the expression of genes involved in immune response activation.

### EACR23-0570
### POSTER IN THE SPOTLIGHT
### Targeted CRISPR activation reconstitutes cGAS-STING pathway function in breast cancer

E. Lauridsen[1], S.H. Godsk[1], A.S. Hansen[1], T. Tramm[2,3], J. Ahrenfeldt[4], J.M. Gudbergsson[1], N.J. Birkbak[4], A. Etzerodt[1], M.R. Jakobsen[1]
[1]*Aarhus University, Biomedicine, Aarhus, Denmark*
[2]*Aarhus University, Clinical Medicine, Aarhus, Denmark*
[3]*Aarhus University Hospital, Pathology, Aarhus, Denmark*
[4]*Aarhus University Hospital, Molecular medicine, Aarhus, Denmark*

**Introduction**

Tumor escape is linked to epigenetic changes, steering the process from benign to malignant cancer. We and others have reported that expression of Stimulator of Interferon Genes (STING) can be found subdued in various cancers. STING is part of an innate immune pathway evolutionarily conserved to detect cytosolic DNA – which in cancer cells has been linked to micronuclei accumulation. Thus, silencing STING may indeed be favorable for cancer to prevent immune activation.

**Material and Methods**

Single-cell RNAseq (39 samples & 18 controls) and tissue microarrays (n=600) from breast cancer (BC) patients were used to study the STING pathway expression. The 4T1 breast cancer murine model was used for *in vitro* and *in vivo* studies. To restore STING expression, we developed and validated a CRISPR activation (CRISPRa) approach. For *in vivo* exploration, we simultaneously established CRISPRa mRNA delivery using lipid nanoparticles (LNP).

**Results and Discussions**

The single-cell RNAseq and tissue microarray data revealed that STING signals in BC primarily originated from stroma and immune cells, whereas cancer cells had low to no expression. We employed the 4T1 model to explore the effects of epigenetic reactivation of STING. General characteristics of 4T1 showed low/to none STING expression but high frequency of micronuclei and cGAS activity. To achieve gene specific reactivation, we refracted from using the unspecific and toxic demethylation agents and cMYC inhibitors, but instead developed a CRISPRa approach. Following STING reactivation, we saw that 4T1 cells had a time-dependent immune activation which correlated to STING protein levels. This immune response was confirmed to be dependent on cGAS sensing micronuclei DNA. To realize a therapeutical potential, we next developed a targeted LNP-delivery system that was evaluated *in vivo*. Using antibody-conjugation we were able to deliver CRISPRa components to 4T1 tumor cells in a targeted manner. The anti-tumoral effects of STING reactivation *in vivo* is currently ongoing and will be presented at EACR.

**Conclusion**

By reactivating STING in cancer cells with an otherwise intact DNA sensing machinery and micronuclei formation, we composed a toxic cocktail for the survival of the tumor. The immune profile induced by STING reactivation holds the potential for activating a broad immune response in the tumor microenvironment. With low toxicity and highly specificity, CRISPRa epigenetic regulation may become a cornerstone in the future of immunotherapies.

### EACR23-0577
### ANAEROCOCCUS SPECIES ASSOCIATED WITH INCREASING PROSTATE CANCER RISK

A. Bampalis[1], R. Hurst[1], D. Brewer[1], R. Mills[2], C. Cooper[1], J. Clark[1], M. Winterbone[1]
[1]*University of East Anglia, Norwich Medical School, Norwich, United Kingdom*
[2]*Norfolk and Norwich University Hospitals, Department of Urology, Norwich, United Kingdom*

**Introduction**

Interactions between the microbiome and the human body may cause or influence diseases. Up to 16% of cancers worldwide are caused by infectious agents. Inflammation as a result of infection can contribute to the development and progression of prostate cancer (PCa). There are unique microbial compositions that have been isolated from the urogenital system that differ between benign and PCa patients.

Five bacteria genera have been associated with prostate cancer risk and progression, called the Anaerobic Bacteria Biomarker Set (ABBS), including *Anaerococcus*.

© 2023 The Authors. Molecular Oncology is published by John Wiley & Sons Ltd on behalf of Federation of European Biochemical Societies

The research project aimed to investigate the presence of bacterial species belonging to ABBS and in particular the *Anaerococcus* genera in urine samples from participants with PCa compared with control groups.
**Material and Methods**
Urine samples were collected from study participants (n=290) after a digital rectal examination. Following collection, DNA was isolated from the samples. The nucleic acid isolation included a repeated bead beating method to ensure sufficient lysis of potential bacteria in the urine.
Following DNA extraction, the samples were tested for bacterial presence using qPCR assays. The bacterial species of interest belonged to the ABBS genera. The assays targeted ribosomal protein genes that offer high taxonomic distinction. PCR amplicons were checked and analysed using melt curve analysis, agarose gel electrophoresis and sequencing techniques as quality control measures.
**Results and Discussions**
Six bacteria species belonging to ABBS were detected across six clinical categories ranging from clinically benign to advanced cancer. *Anaerococcus lactolyticus* and a second *Anaerococcus* species were significantly associated with increased prostate cancer risk group ($\chi 2$ test for trend, $p<0.006$ and $p<0.003$ respectively).
The two species were also flagged as important features with the Boruta algorithm. Further research will investigate the potential for utilising bacterial presence as a prognostic tool for different risk groups.
**Conclusion**
Specific bacteria species were linked with PCa risk groups. Detection of specific bacteria in the urogenital tract may lead to the development of a prognostic test, that in combination with already established tests, can improve the accurate prognosis of the disease and better treatment strategies.

### EACR23-0582
### The spatiotemporal immune profile during mouse oral carcinogenesis

*K. Sellæg[1], R. Schwienbacher[1,2], A.M. Wirsing[1,2], A. Engan Aamodt[1], E. Hadler-Olsen[1,3], G. Berge[1], S. Norvoll Magnussen[1]*
*[1]University of Tromsø UiT - The Arctic University of Norw ay, Medical Biology, Tromsø, Norway*
*[2]University Hospital of North Norway, Clinical Pathology, Tromsø, Norway*
*[3]Troms and Finnmark County Muncipality, Public Dental Service Competence Centre of Northern Nor way, Tromsø, Norway*
**Introduction**
Only a minority of oral squamous cell carcinoma (OSCC) patients respond well to therapeutic targeting of the tumor immune microenvironment, possibly owing to an insufficient number of tumor-infiltrating lymphocytes (TILs). Tumor-associated (TA) high-endothelial venules (HEVs) are specialized lymphocyte-recruiting vessels that can be found in solid tumors. The presence of TA-HEVs and a strong immune infiltrate are both associated with a favorable prognosis in OSCC, and their presence have been shown to improve anti-tumor response in certain experimental cancer models. However, the regulation of HEVs in tumors is poorly understood. The aim of this study was to investigate how the lymphocytic infiltrate and TA-HEVs evolve during oral cancer progression.
**Material and Methods**
Histopathology and the immune infiltrate in the tongue mucosa was longitudinally observed using a carcinogen-induced (4-nitroqunioline 1-oxide; 4NQO) mouse model of oral carcinogenesis. Immunohistochemistry was used to characterize the immune infiltrates by T-cells (helper CD4+, cytotoxic CD8+, regulatory FoxP3+), B-cells (B220+), and PNAd-expressing HEVs, while histopathology was assessed in H&E-stained sections.
**Results and Discussions**
A time-dependent development of histological lesions was observed following 4NQO-exposure, and the incidence and severity of histological changes was more pronounced in the upper surface of the tongue. The lymphocytic infiltrate and number of TA-HEVs were significantly increased in 4NQO-treated mice compared to untreated controls and corresponded with the site and grade of histological lesions, indicating that these are regulated as an early event in tumor development.
**Conclusion**
Together, our results show that an adaptive immune response is initiated during transition of normal tongue mucosa through dysplasia and eventually oral cancer, accompanied by the induction of TA-HEVs, providing evidence that the 4NQO mouse model can be utilized for further studies of the tumor immune microenvironment and can permit the development of new treatment approaches.

### EACR23-0584
### The prognostic significance of IDO and ARG1 expression patterns in the colorectal cancer microenvironment

*H. Elomaa[1], M. Ahtiainen[2], S. Väyrynen[3], E.V. Wirta[4], T. Seppälä[5], J. Böhm[1], J.P. Mecklin[1], T. Kuopio[2], J. Väyrynen[6]*
*[1]Well Being Services County of Central Finland, Department of Education and Research, Jyväskylä, Finland*
*[2]Well Being Services County of Central Finland, Department of Pathology, Jyväskylä, Finland*
*[3]Oulu University Hospital, Department of Internal Medicine, Oulu, Finland*
*[4]Tampere University Hospital, Department of Gastroenterology and Alimentary Tract Sur gery, Tampere, Finland*
*[5]Helsinki University Hospital, Department of Surgery, Helsinki, Finland*
*[6]Oulu University Hospital, Cancer and Translational Medicine Research Unit, Oulu, Finland*
**Introduction**
The amino acid metabolism is altered in cancer, often including the upregulation of enzymes Indoleamine 2,3-dioxygenase (IDO) and Arginase-1 (ARG1). This can lead to T cell suppression by reducing the supply of indispensable amino acids. However, the expression patterns and prognostic significance of IDO and ARG1 in the colorectal cancer microenvironment are still incompletely understood.
**Material and Methods**

© 2023 The Authors. Molecular Oncology is published by John Wiley & Sons Ltd on behalf of Federation of European Biochemical Societies

Using a custom 10-plex immunohistochemistry assay combined with supervised machine learning-based digital image analysis, we identified monocytic cells, granulocytes, mast cells, and tumor cells and quantified IDO and ARG1 expression in the tumor microenvironment of 833 colorectal cancer patients. We assessed the prognostic value of densities and spatial patterns of $IDO^+$ and $ARG1^+$ cells using multivariable Cox regression models for cancer-specific survival.

**Results and Discussions**

IDO was mainly expressed on $CD14^+$ monocytic cells and tumor cells, whereas ARG1 was mostly expressed on $CEACAM8^+$ granulocytes. Higher density of $IDO^+$ monocytic cells associated with longer cancer-specific survival both in the tumor center ($P_{trend}$<0.001) and the invasive margin ($P_{trend}$<0.001) independent of disease stage, microsatellite instability status and other prognostic factors. Higher density of both $ARG1^+$ and $ARG1^-$ granulocytes associated with longer cancer-specific survival in univariable models. Granulocytes were, on average, located closer to tumor cells than monocytic cells. Furthermore, $IDO^+$ monocytic cells were located farther from tumor cells than $IDO^-$ monocytic cells, and $ARG1^+$ granulocytes were located farther than $ARG1^-$ granulocytes.

**Conclusion**

Our findings provided new insights into the infiltration patterns and prognostic value of ARG1 and IDO expressing cells in colorectal cancer. The results highlight the role of the immune microenvironment in colorectal cancer progression and have potential to inform future research, as well as biomarker and treatment development in this area.

### EACR23-0609
### POSTER IN THE SPOTLIGHT
### Androgen presence improves efficacy to cancer immunotherapy

*M.E. Vargas Delgado[1], L. Meier[1], J.S. Waizenegger[1], J. Oberbauer[1], N. Berenbrok[1], J. Engelmann[2], K. Riecken[3], H.W. Mittrücker[4], I. Ben-Batalla[1], S. Loges[1]*

*[1]German Cancer Research Center DKFZ- German Center for Lung Research DZL, DKFZ-Hector Cancer Institute at the University Medical Center Mannheim- Department of Personalized Oncology- University Hospital Mannheim- Medical Faculty Mannheim- University of Heidelberg- Mannheim- Division of Personalized Medical Oncology A420, Heidelberg, Germany*

*[2]University Medical Center Hamburg-Eppendorf, Department of Oncology- Hematology and Bone Marrow Transplantation with Section Pneumology- Hubertus Wald Comprehensive Cancer Center Hamburg, Hamburg, Germany*

*[3]University Medical Center Hamburg-Eppendorf, Research Department Cell and Gene Therapy- Department of Stem Cell Transplantation, Hamburg, Germany*

*[4]University Medical Center Hamburg-Eppendorf, Department of Immunology, Hamburg, Germany*

**Introduction**

Immune therapies have revolutionized the treatment of cancer, however, until now only a minority of patients derive long-term benefit. Notably, despite widely reported significant differences between the immune system of males and females, there continues to exist a knowledge gap regarding sex disparities in anti-cancer immune responses. Recent meta-analyses indicate that immune checkpoint blockade (ICB) treatment has higher efficacy in male patients compared to females, regardless of the cancer type.

**Material and Methods**

To investigate the mechanisms underlying sex-specific differences we used the syngeneic colorectal cancer model MC38. We inoculated tumor cells subcutaneously (s.c.) into male and female mice, and treated them with either IgG2a control or anti-PD1 antibody. We validated treatment effect of ICB by tumor growth kinetics. To asses a potential role of androgens in impacting sex differences to ICB response *in vivo*, we performed orchiectomy in male mice, as well as sham-surgery in male and female mice as controls, and also, we implanted s.c. placebo or testosterone pumps into male and female mice. We performed flow cytometry to characterize immune cell subsets in tumor, blood and spleen of MC38 bearing mice.

**Results and Discussions**

Our data showed significant advantage of males over females in tumor growth kinetics upon ICB. Furthermore, testosterone suppression in castrated males led to a lower response rate to ICB (71%) compared to sham males (92%), and more similar to sham females (58%). Moreover, testosterone supplementation in females led to enhanced response rate to ICB (85%) compared to placebo females (50%), resembling response rate of placebo males (90%). Overall, this demonstrated that regardless of biological sex, presence of testosterone positively impacts ICB therapy outcome. Additionally, immunophenotyping analysis showed that upon ICB females with testosterone had significantly increased intratumoral stem-like $CD8^+TCF1^+PD1^+$ T cells compared to placebo females, which in turn correlated with enhanced intratumoral terminal differentiated effector $CD8^+TCF1^-PD1^+$ T cells in both placebo males and females with testosterone, compared to placebo females.

**Conclusion**

These preliminary results suggest androgens can modulate anti-cancer immune responses by contributing to a more sustained anti-tumor $CD8^+$ T cell response and consequently better responses upon ICB. These findings are in concordance with male cancer patients responding better to ICB and warrant therapy escalation in female patients.

### EACR23-0610
### Combinations of tri-complex KRAS(ON) inhibitors with RAS companion inhibitors and immunotherapies improve anti-tumor activity and abrogate adaptive resistance in preclinical tumor models in vivo

*C. Blaj[1], M. Moreno Ayala[1], A. Salmon[1], M. Menard[1], N. Shifrin[1], P. Wig[1], H. Courtney[1], A. Kumamoto[1], J.A. Smith[1], E. Quintana[1]*

*[1]Revolution Medicines, Biology, Redwood City, United States*

**Introduction**

© 2023 The Authors. Molecular Oncology is published by John Wiley & Sons Ltd on behalf of Federation of European Biochemical Societies

First generation KRASG12C(OFF) inhibitors have been approved as targeted therapy for patients with non-small cell lung cancer harboring a KRASG12C mutation. However, efficacy is not durable, and tumors rapidly escape monotherapy. In addition to documented tumor cell intrinsic resistance mechanisms, remodeling of the TME and immunoediting have been reported in tumors treated with KRAS inhibitors and could play a role in acquired resistance.

**Material and Methods**

KRASG12C(ON) and KRASG12D(ON) tri-complex inhibitors drove anti-tumor immunity and achieved transient complete responses in traditionally hard to treat immune-evasive tumor models. We compared gene expression profiles and flow cytometric analyses of tumors during the early response to RAS(ON) inhibitors and later tumor re-growth to inform optimal treatment strategies, including combinations.

**Results and Discussions**

In a NSCLC model, and a GEM-derived PDAC model, the TME during initial response with RAS(ON) inhibitor monotherapy was remodeled in favor of anti-tumor immunity, with an increase in tumor infiltrating lymphocytes (TILs) and decrease in myeloid suppressor cells. However, multiple compromising adaptive mechanisms were observed in response to RAS inhibition, including upregulation of TGF-b and increased macrophage infiltration. In addition, an upregulation of RTKs was also observed in the resistant NSCLC (MET) and PDAC (PDGFB) preclinical tumors, consistent with clinical finding in patient tumors treated with KRASG12C(OFF) inhibitors.

The combination of mutant selective RAS(ON) inhibitors with a RASMULTI(ON) inhibitor favorably transformed the TME and improved anti-tumor activity in preclinical models. In addition, in an immune refractory model, the combination with a de novo engineered IL-2/IL-15 mimetic (NL-201®), that can attract and activate TILs, showed synergy with RAS(ON) inhibitors and translated into durable complete responses.

**Conclusion**

The observed changes in the TME after treatment with tri-complex RAS(ON) inhibitors have important implications for combination strategies involving mutant-selective RAS(ON) inhibitors, companion inhibitors and novel immunotherapies.

### EACR23-0612

### Cancer cell-elicited immune signals shape the immune landscapes of intestinal cancer subtypes and drive tumor progression

A.E. Zaurito[1], V. Brunner[1], D. Lucarelli[1], A. Sinha[2], R. Rad[3], F. Meissner[4], M. Schmidt-Supprian[5], M. Tschurtschenthaler[1], D. Saur[1]

*[1]Institute of Translational Cancer Research and Experimental Cancer Therapy, TranslaTUM-Center for Translational Cancer Research, Munich, Germany*

*[2]Research Department Proteomics and Signal Transduction, Max Plank Institute of Biochemistry, Munich, Germany*

*[3]Institute of Molecular Oncology and Functional Genomics, TranslaTUM - Center for Translational Cancer Research, Munich, Germany*

*[4]Institute of Innate Immunity, University Hospital Bonn, Bonn, Germany*

*[5]Institute of Experimental Hematology, TranslaTUM - Center for Translational Cancer Research, Munich, Germany*

**Introduction**

Colorectal cancer (CRC) is a highly heterogeneous disease with diverse molecular subtypes. Whereas the genetics of the disease and the main drivers have been studied intensively, it remains elusive how distinct genetic alterations affect the tumor microenvironment (TME) and how this, in turn, influences tumor development and progression. Therefore, we investigated the inflammatory TME and the tumor cell – immune cell crosstalk in CRC subtypes.

**Material and Methods**

For the systematic investigation of tumor subtype-specific immune mechanisms, we used genetic mouse models that recapitulate important molecular subtypes of CRC: classical (loss of *Apc*), serrated (activation of oncogenic *Kras*$^{G12D}$ or *Braf*$^{V637E}$) and mucinous routes (activation of *Pik3ca*$^{H1047R}$) of CRC pathogenesis. Along this unique collection of mice, we generated an extensive resource of tissue samples and corresponding 3D tumor-derived organoids (TDO) to study i) infiltrating immune cells at different stages of tumorigenesis (wild-type, hyperplasia, adenoma, and invasive cancer) by integrating single cell and tissue RNA-seq followed by deconvolution, flow-cytometry and multispectral imaging; ii) the transcriptome, proteome and phospho-proteome of tumor organoids to decipher communication networks of potential cell-to-cell interactions between tumor cells and cells of the TME; and iii) genetic alterations and the degree of microsatellite instability (MSI). To functionalize immune cell populations, we depleted distinct immune subsets in the different intestinal cancer models using *Rag2*$^{-/-}$;*Il2rg*$^{-/-}$ mice.

**Results and Discussions**

Our multiscale approach identified dynamic changes in the TME during cancer progression. We observed major context-dependent differences in the recruitment/exclusion of T and myeloid cells. Transcriptomics analysis from tumor tissue and TDO revealed genotype-specific chemokines expression patterns and immunomodulatory gene modules rewiring the TME. Finally, we uncovered the impact of adaptive immune system depletion on autochthonous tumor development and progression in different oncogenic backgrounds. While the absence of T, B and NK cells does not influence tumor initiation and progression in loss of *Apc*-driven tumors, we observed a pivotal role in tumor progression in *Pik3ca*$^{H1047R}$ background.

**Conclusion**

These findings indicate distinct context and tumor stage-specific mechanisms of immune cell exclusion and immunosuppression that drive tumor initiation and progression.

### EACR23-0719

### Type III IFN as a modulator of tumor-immune cell interactions

K. Raaby Gammelgaard[1], S. Godsk[1], A. Solhøj Hansen[1],

© 2023 The Authors. Molecular Oncology is published by John Wiley & Sons Ltd on behalf of Federation of European Biochemical Societies

A.G. Oliver[2], J.G. Pedersen[1], A. Etzerodt[1], E. Voest[3], M.R. Jakobsen[1]
[1]Aarhus University, Department of Biomedicine, Aarhus, Denmark
[2]Institut d'Investigacions Biomèdiques August Pi i Sunyer, Biomedical Research Institute, Barcelona, Spain
[3]Netherlands Cancer Institute, Molecular Oncology and Immunology, Amsterdam, The Netherlands

**Introduction**

Type III interferons (IFNl) elicit local antiviral effects around epithelial surfaces and has recently been shown to prevent development of severe COVID-19 and help control chronic hepatitis B. The IFNl receptor (IFNLR1) is expressed primarily on epithelial cells and a limited subset of immune cells, in contrast to type I IFN which is ubiquitously expressed. Albeit the well-established link between IFNLR1-IFNl signaling and epithelial cells, there is limited knowledge regarding type III IFN in relation to cancer. Here, we explore the IFNLR1- IFNl axis in the context of immune cell activation in cancer and how it modulates the immune function of cancer cells.

**Material and Methods**

Analysis of scRNA-seq data, qPCR and flow cytometry were used for investigating IFNLR1 expression in patient samples and *in vitro* models. T cells and monocytes were isolated from healthy donor PBMCs using EasySep separation kits. Patient-derived tumor organoids and matched normal organoids were used for studying tumor-macrophage interactions. The 4T1 syngeneic subcutaneous mouse tumor model was used for *in vivo* evaluation of IFNL therapy. Flow cytometry was used for evaluating macrophage polarization and immune cell composition and activation *in vivo*.

**Results and Discussions**

scRNA-seq data analysis from healthy donors and cancer patients showed IFNLR1 to be highest expressed in epithelial cells and monocytes/macrophages. IFNLR1 expression was downregulated in tumor organoids compared to healthy organoids from the same cancer patients. IFNl stimulation of macrophages significantly upregulated ISGs including CD80 and TNFa ($p<0.02$, n =4), and IFNl stimulation prevented tumor-induced upregulation of SIRPa on macrophages during co-culture with tumor organoids ($p=0.0041$, n=4).
Furthermore, IFNl stimulated macrophages showed increased capacity for activating CD8+ T cells by elevating their production of IFNg ($p=0.0215$, n=6) and Granzyme B ($p=0.0033$, n=6). This was confirmed *in vivo* where CD8a+ T cells from 4T1 tumors treated with IFNl had increased IFNg MFI ($p=0.0022$, n=3) compared to PBS treated mice. We hope to present data from an ongoing efficacy study at EACR 2023.

**Conclusion**

IFNλ1 has the ability to prime a wider immune response by targeting macrophages and hereby increasing the capacity for macrophages to activate CD8+ T cells. Given the well-tolerated profile during anti-viral treatments, IFNl shows potential clinical value and deserves further exploration in the clinic as an anti-cancer treatment to support existing immunotherapies.

### EACR23-0729
### Dissecting the role of lncRNAs in promoting Natural Killer cytotoxicity in Non-Small Cell Lung Cancer models

V. Fantini[1], F. Reggiani[1], F. Torricelli[1], E. Sauta[2], S. Strocchi[1], A. Grieco[1], A. Ciarrocchi[1], V. Sancisi[1]
[1]Azienda USL-IRCCS di Reggio Emilia, Laboratory of Translational Research, Reggio Emilia, Italy
[2]IRCCS Humanitas Clinical and Research Center, AI Center, Milan, Italy

**Introduction**

The use of immune therapy in the treatment of lung cancer during the past few years has been one of the most significant developments in the fight against this fatal illness. Natural killer (NK) cells are one of the main immune populations in the tumor microenvironment and can play a significant role in the development and spread of cancer. Long non-coding RNAs (lncRNAs) are pervasively transcribed in the human genome and have been shown to be implicated in many different biological processes. Although their role in cancer progression has been extensively studied, very few information is known about their involvement in tumor immune microenvironment.

**Material and Methods**

We treated tumor-derived primary NKs or NK92 cell line with inhibitors of bromodomain and extra-terminal domain proteins (BETi) JQ1 or OTX-015, followed by co-culture with NSCLC cell lines or primary patient-derived 3D cultures. NK cytotoxic activity was assessed by measuring cancer cell growth and NK cells activation markers (IFNg and CD107a) by flow cytometry. Gene expression reprogramming upon BETi treatment was explored by RNA-sequencing analysis. We selected the 13 lncRNAs involved in immune system pathways by a bioinformatic analysis of co-expression.

**Results and Discussions**

We and other have shown that BETi achieve their anti-cancer efficacy through a variety of mechanisms, including epigenetic reprogramming of the immune microenvironment. We demonstrated that BETi enhances the cytotoxic activity of NK cells in co-culture with NSCLC cell lines and in primary patient-derived 3D cultures. To dissect the molecular events underlying this effect, we performed a transcriptomic analysis. Strikingly, 15% of deregulated genes were non-coding transcripts, indicating that they may have a relevant, still underestimated role in regulation of NK cytotoxic activity. From the list of deregulated lncRNAs, we selected 13 transcripts showing a co-expression profile with coding genes of immunity system pathways. Most of these lncRNAs are poorly characterized within the immune system. Seven of these lncRNAs were discovered to be expressed in NK cells and other immune cell populations by single-cell analysis, suggesting that they may have a broader function in immune system regulation.

**Conclusion**

We showed that BETi increases the cytotoxicity of NK cells and changes their transcriptional profile. Among the deregulated genes, 15% are lncRNAs. We identified a set of 13 lncRNAs that may actively contribute to both the anti-tumor and cytotoxic activity of NK cells.

© 2023 The Authors. Molecular Oncology is published by John Wiley & Sons Ltd on behalf of Federation of European Biochemical Societies

### EACR23-0763
### Evaluation of oral health status in patients with Non-Hodgkin lymphoma

I. Besu Zizak[1], S. Matkovic[2], B. Mihaljevic[3], Z. Zizak[1]
[1]Institute of Oncology and Radiology of Serbia, Department of Experimental Oncology, Belgrade, Serbia
[2]Institute of Oncology and Radiology of Serbia, Department of Medical oncology, Belgrade, Serbia
[3]Clinic for Hematology- Clinical Center of Serbia, Lymphoma Center, Belgrade, Serbia

**Introduction**

The relation between oral health and general health is currently widely described. Systemic diseases may manifest in the oral cavity. Oral mucosal involvement may reflect activity or progression of the primary condition. Non-Hodgkin lymphoma (NHL) represents a heterogeneous group of malignant disease, characterized by a proliferation of lymphoid cells or their precursors. The purpose of this study was to evaluate the relationship between the status of the oral cavity in these patients, before therapy or after first relapse, again before applying a new therapy.

**Material and Methods**

The study included 60 patients with NHL. In 40 patients the disease was only diagnosed before therapy, while 20 patients were in the relapse stage, but before re-use therapy. Oral mucosal lesions were performed using a dental mirror and probe.

**Results and Discussions**

Changes in the oral cavity were present in 29 out of 60 (48.3%) patients. The changes were noted in 20 patients with newly diagnosed NHL and 9 patients who were in stage of relapse of disease, while 31 (51.7%) patients had no oral mucosal lesions. The main clinical oral manifestation was pale oral mucosa in 13 (44.8%) patients. In 8 (27.6%) patients was noticed atrophy of the tongue cover. In 4 (13.8%) cases were present coated tongue and purpura. Necrotizing periodontal diseases, dry mouth and white mouth changes were present in at 3 (10.3%) patients. Other mucosal lesions (hyperplasia of the oral mucosa, opalescent oral mucosa, hyperkeratosis of the tongue, ulcerations) were performed in one (3.4%) case.

**Conclusion**

It is supposed that oral mucosal lesions of these patients are due to the changes in their blood parameters and altered immune status as a consequence of the primary malignant disease.

### EACR23-0780
### Immunogenomic analysis of human brain metastases reveals diverse immune landscapes across genetically distinct tumors.

A. Alvarez-Prado[1,2], R. Maas[1,2], K. Soukup[1,2], F. Klemm[1,2], M. Kornete[1,2], F. Krebs[3], V. Zoete[3], S. Berezowska[4], M. Hegi[5], J. Joyce[1,2]
[1]University of Lausanne, Department of Oncology, Lausanne, Switzerland
[2]Ludwig Institute for Cancer Research, Lausanne Branch, Lausanne, Switzerland
[3]University of Lausanne and Swiss Institute of Bioinformati cs, Department of Oncology, Lausanne, Switzerland
[4]Centre Universitaire Vaudois CHUV, Department of Pathology, Lausanne, Switzerland
[5]Centre Universitaire Vaudois CHUV, Department of Neuroscience, Lausanne, Switzerland

**Introduction**

Brain metastases (BrMs) are the most common form of brain tumors in adults, and frequently originate from lung and breast primary cancers. BrMs are associated with a high mortality, emphasizing the need for more effective therapies. Genetic profiling of primary tumors is increasingly used as part of the effort to guide targeted therapies against BrMs, and immune-based strategies for the treatment of metastatic cancer are gaining momentum. However, the tumor immune microenvironment (TIME) of BrM is extremely heterogeneous and whether specific genetic profiles are associated with distinct immune states remains unknown.

**Material and Methods**

To address this critical question, we performed a comprehensive immunogenomic analysis of lung- and breast-BrMs by combining whole-exome and whole-genome sequencing of tumors; RNA-sequencing of cancer cells and purified immune populations (microglia, monocyte-derived macrophages, neutrophils, CD8+ and CD4+ T cells), encompassing > 170 transcriptomes; flow cytometry and immunofluorescence analyses.

**Results and Discussions**

Our data revealed that specific genetic drivers correlate with distinct immune landscapes in BrMs, with *TP53*-mutant lung-BrMs presenting with an increased $CD8^+$ T cell infiltration and activation, but a more immunosuppressive myeloid compartment; and hypermutated breast-BrMs showing a generally more pro-inflammatory microenvironment. These results support the incorporation of genetic profiling of BrMs as a means to potentially predict responses to current immunotherapies, and for the development of personalized immune-based interventions informed by the genetic makeup of the tumors.

**Conclusion**

This study showed that: (i) $TP53^{mut}$ lung-BrMs show higher immune cell infiltration than $TP53^{WT}$ lung-BrMs; (ii) $TP53^{mut}$ lung-BrMs show more activated CD8 T cells and more immunosuppressive TAMs; (iii) Focalized hypermutation (kataegis) is a relatively frequent event and occurs in 44% of the analyzed breast-BrMs; (iv) Kataegic breast-BrMs present a higher proportion of T cells and a more inflamed tumor immune microenvironment.

### EACR23-0800
### The CD39/CD73/adenosine pathway contributes to generating an immunosuppressive microenvironment in patients with Sézary Syndrome

Y. Yakymiv[1], S. Marchisio[1], E. Ortolan[1], V. Pullano[2], C. Leso[1], R. Senetta[3], M. Fia[4], P. Quaglino[4], A. Funaro[1]
[1]University of Torino, Laboratory of Immunogenetics- Department of Medical Sci ences, Torino, Italy
[2]University of Torino, Department of Medical Sciences, Torino, Italy
[3]University of Torino, Pathology Unit- Department

© 2023 The Authors. Molecular Oncology is published by John Wiley & Sons Ltd on behalf of Federation of European Biochemical Societies of Medical Sciences, Torino, Italy
[4]University of Torino, Dermatologic Clinic-Department of Medical Sciences, Torino, Italy

**Introduction**

Sézary Syndrome (SS) is an aggressive form of T-cell lymphoma characterized by clonal expansion of CD4+ T cells in the skin and peripheral blood and progressive impairment of the immune response. No curative treatments are available so far. Mogamulizumab (anti-CCR4) seems to be a promising avenue to achieve long-term disease control for patients with relapsed or refractory disease; however, relapses are frequent. Recently, we described the aberrant expression of CD39 and/or CD73 ectoenzymes in SS cells circulating in the blood and infiltrating the skin. CD39 and CD73 catalyze the breakdown of extracellular ATP into adenosine leading to immunosuppression. This observation prompted us to hypothesize the existence of an adenosinergic network orchestrated by malignant T cells and the tumor microenvironment contributing to tumor escape from the immune response.

**Material and Methods**

The expression of CD39 and CD73 was monitored in 20 SS patients by multiparametric flow cytometry at two-month intervals. The target region of the ENTPD1/CD39 gene was amplified by PCR using appropriate primers and sequenced by Sanger. For *ex vivo* analysis, PBMCs and primary CD4+ T-cells isolated from patients with SS were used.

**Results and Discussions**

In this study we showed that i) both proteins are biologically active, i.e., able to defosphorylate ATP by producing ADP and AMP, and to hydrolyze AMP by generating adenosine; ii) based on the expression of CD39 and/or CD73, three subgroups of patients can be identified: CD39-high, CD73-high, $CD39^{+}/CD73^{+}$; iii) the expression of CD39 in SS cells is genetically controlled. Indeed, genotypic analysis of SNP rs10748643 A/G in the ENTPD1/CD39 gene revealed that CD39high SS patients had GG or AG genotype; iv) CD39-high circulating SS T-cells interacting with vascular endothelium in an *ex vivo* system proved able to generate high concentrations of adenosine, which reduced the proliferation of circulating lymphocytes and suppressed the immune response, and iv) specific inhibitors of the CD39/CD73/ADO pathway restored the antitumor immune response and modulated the immunosuppression.

**Conclusion**

These results suggest that the CD39/CD73-mediated adenosinergic pathway contributes to generating an immunosuppressive environment in patients with SS, particularly in the CD39high subgroup, and could pave the way for the design of new therapeutic strategies combining immunotherapy with specific inhibitors of adenosinergic pathway.

### EACR23-0820

### Exploiting TLR2 and the cystine/glutamate antiporter xCT to develop a new combined therapy for breast cancer

A. Di Lorenzo[1], E. Bolli[1], C. Romiti[1], A. Amaolo[1], G. Ferrauto[1], E. Di Gregorio[1], F. Cavallo[1], L. Conti[1]
[1]University of Turin, Molecular Biotechnology and Health Sciences, Turin, Italy

**Introduction**

Breast cancer is still the leading cause of cancer death in women, due to relapses and metastases. Therefore, developing combination therapies targeting key cancer-inducing or cell-sustaining pathways is needed. We have previously demonstrated that mammary cancer stem cells (CSCs) overexpress the cystine-glutamate antiporterxCT and Toll-Like Receptor (TLR)2, which play a crucial role in their self-renewal and resistance to chemotherapy. Both xCT and TLR2 are promising targets for breast cancer therapy, and deeper characterization of their crosstalk may lead to the setup of effective combination therapies for breast cancer.

**Material and Methods**

The effects exerted by TLR2 activation on xCT expression and function were analyzed in vitro on mouse and human breast cancer cell lines in which TLR2 was either activated with endogenous or bacteria-derived ligands or silenced using specific siRNA. Moreover, xCT expression was analyzed in a mouse model of HER2-neu-induced mammary carcinogenesis on a TLR2 WT or KO background (TLR2WT-neuT and TLR2KO-neuT mice) and on cell lines derived from their tumors. The efficacy of a combined targeting of TLR2 and xCT, in association or not with chemotherapy, was tested in vitro on these cell lines.

**Results and Discussions**

TLR2 promoted CSC self-renewal and its deletion impaired mammary carcinogenesis in vivo. TLR2 induced the upregulation of xCT in breast cancer cells, and its silencing or deletion decreased xCT both in vitro and in mouse models of mammary cancer. Since xCT controls intracellular redox balance, TLR2 downregulation increased intracellular reactive oxygen species in breast cancer cells. TLR2 inhibitors synergized with xCT inhibitors in hindering breast cancer cell viability and inducing their apoptosis, and the association with doxorubicin further increased these results.

**Conclusion**

We demonstrated that TLR2 promotes breast CSC self-renewal, cancer progression and upregulates xCT expression in breast cancer cells, protecting them from oxidative stress. The use of TLR2 inhibitors in association with xCT inhibition (or immunotargeting) and chemotherapy may lead to the setup of more effective combination therapies for breast cancer. Moreover, we are currently investigating the possibility of using nanoparticles to deliver chemotherapy and TLR2 inhibitors inside the tumor in combination with xCT immunotargeting.

### EACR23-0877

### Influence of genetic background on immune cell composition and functionality in mouse glioblastoma microenvironment at single cell resolution

A.M. Lenkiewicz[1], K. Jacek[1], S. Cyranowski[1], M. Ghosh[1], P. Rosa[1], S. Baluszek[1], B. Kaza[1], P. Szadkowska[1], B. Gielniewski[2], M.G. Castro[3], B. Kaminska[1]
[1]Nencki IBD PAS, Laboratory of Molecular Neurobiology, Warsaw, Poland

© 2023 The Authors. Molecular Oncology is published by John Wiley & Sons Ltd on behalf of Federation of European Biochemical Societies

[2]Nencki IBD PAS, Laboratory of Sequencing, Warsaw, Poland
[3]University of Michigan Medical School, Departments of Neurosurgery and Cell and Developmental Biology, Michigan, United States

**Introduction**

Glioblastoma (GBM) is the most common and malignant primary brain tumor. GBM cells should be effectively recognized and destroyed by the immune system, but its antitumor activity is often inhibited by factors secreted by the tumor that contribute to the formation of the tumor immunosuppressive microenvironment (TME). Since TME plays a key role in cancer progression and immune evasion, and GBM patients with various genetic alterations differ in anticancer response and length of survival, we sought to explore potential links between specific genomic changes occurring in GBM and TME composition. The aim of this study is to investigate how the specific genetic background of gliomas affects tumor growth as well as the phenotype and heterogeneity of myeloid and lymphoid cells in mouse models of gliomas with genetic changes occurring in the GBM patients.

**Material and Methods**

We explored the combination of unique mouse models of gliomas reflecting human pathology (NRAS/shTP53/shATRX and PDGFB/shTP53/shATRX with wt or mutated IDH1) with high-dimensional technologies. To identify subtypes and functional diversity of immune cells in glioma TME we employed single-cell RNA and protein sequencing (CITEseq, Cellular Indexing of Transcriptomes and Epitopes by Sequencing) and Visium (10X Genomics) spatial transcriptomics. We characterized the populations of myeloid- and lymphoid cells and examined their unique transcription profiles, functional diversity and localization in TME.

**Results and Discussions**

Our results indicated that specific genetic background of gliomas affects tumor growth, appearance of symptoms and mice survival. By combining analysis of CITE-seq with spatial transcriptomics we characterized and described 33 phenotypes of immune cells, which then we localized spatially within TME in mouse gliomas. We have shown that the specific genetic background of gliomas changes the composition of the main populations within TME as well as their localization. Finally, Ligand-Receptor and CellChat analysis of our CITE-seq results revealed the interplay between GBM, myeloid cells and lymphocytes, indicated on potential factors responsible for accumulation and tumor-evoked reprograming of immune cells.

**Conclusion**

Understanding the interplay between GBM cells and myeloid and lymphoid populations is pivotal in creating new therapeutic strategies for GBM patients with various genetic alterations. Studies were supported by NSC grant 2020/39/B/NZ4/02683 (BK) and PACIFIC Call 1 PAS (MG).

### EACR23-0878
### Analysis of plasma CXCL13 levels in patients with triple-negative breast cancer

K. Horti-Oravecz[1], A. Bozsik[1,2,3], J. Papp[1,2,3], A. Patócs[1,2,3,4], V.K. Grolmusz[1,2,3,4]
[1]National Institute of Oncology, Molecular Genetics, Budapest, Hungary
[2]MTA, National Tumorbiology Laboratory, Budapest, Hungary
[3]Semmelweis University, Hereditary Cancers Research Group- Eötvös Loránd Rese arch Network, Budapest, Hungary
[4]Semmelweis University, Department of Laboratory Medicine, Budapest, Hungary

**Introduction**

Elevated immune infiltration is a frequent characteristic of triple-negative breast cancer (TNBC) and is essential for the clinical efficacy of immunotherapies. Tertiary lymphoid structures (TLSs) have been demonstrated to influence the prognosis of a variety of tumors. Former studies unveiled C-X-C Motif Chemokine Ligand 13 (CXCL13) as a major organizer of TLSs, while it is also a recognized plasma marker of germinal center activity. Moreover, CXCL13-producing T-cells were recently found to predict effective response to PD-L1 blockade in TNBC. Our aim was to investigate the peripheral plasma levels of CXCL13 in patients with TNBC and in healthy volunteers to assess if its concentration correlates with disease state or clinical characteristics.

**Material and Methods**

20 treatment-naïve women with TNBC and 20 age- and sex-matched healthy individuals were included in this pilot study at the Department of Molecular Genetics of the National Institute of Oncology following informed consent. Plasma was isolated from peripheral blood following centrifugation. Plasma CXCL13 levels were determined using the Quantikine Human CXCL13 ELISA kit (R&D Systems), according to the manufacturer's instructions with samples measured in duplicate. Statistical analysis was performed by the application of Student's T-test and Pearson's correlation coefficient.

**Results and Discussions**

Plasma CXCL13 levels did not differ significantly between patients with TNBC and healthy women ($55.6 \pm 54.4$ vs. $59.6 \pm 113.0$ pg/ml, $p = 0.89$). Plasma CXCL13 levels did not correlate with tumor burden, Ki-67 index or lymph node metastasis.

**Conclusion**

Plasma CXCL13 levels do not correlate with TNBC state or its basic clinical characteristics. Further studies are needed to investigate the role of CXCL13 in the tumor tissue.

Funding The authors would like to acknowledge the financial support of the National Research, Development and Innovation Office (NLP-17 and NKFIH-FK-21-138377). VKG is the recipient of the János Bolyai Postdoctoral Scholarship (BO/00141/21) and the New National Excellence Program Bolyai+ Scholarship (ÚNKP-22-5-SE-25).

### EACR23-0892
### Prostaglandin E2 (PGE2) controls inflammatory activation of macrophages

F.M. Vittoria[1,2], L. Mezzanzanica[1,2], F. Cilenti[1], M. Genua[1], V. Cuzzola[1,2], S. Barresi[1], F. La Terza[1], G. Barbiera[1], N. Caronni[1], R. Ostuni[1,2]
[1]Ospedale San Raffaele, SR-Tiget, Milan, Italy
[2]Università Vita-Salute San Raffaele, Università Vita-

© 2023 The Authors. Molecular Oncology is published by John Wiley & Sons Ltd on behalf of Federation of European Biochemical Societies

*Salute San Raffaele, Milano, Italy*

**Introduction**

$PGE_2$ is a lipid mediator with pleiotropic functions in tissue homeostasis and cancer. It drives acute inflammation but it also modulates tissue remodeling and suppresses the anti-tumor immune response by hijacking cytotoxic activities of NK and CD8 lymphocytes and by hampering macrophage activation. Previous studies in our laboratory demonstrated the ability of $PGE_2$ to antagonize the expression of type I IFN in macrophages exposed to inflammatory stimuli, by acting on chromatin remodeling. However, how $PGE_2$ controls additional inflammatory responses in macrophages remains completely uncharacterized.

**Material and Methods**

We performed transcriptomic analysis of mouse Bone Marrow-derived Macrophages (BMDMs) exposed to multiple inflammatory stimuli (LPS, TNFa and IL-1b), in the presence or absence of $PGE_2$. These experiments were combined with functional assays to dissect the mechanisms of action of $PGE_2$.

**Results and Discussions**

We found that co-exposure of BMDMs to $PGE_2$ and inflammatory stimuli resulted in increased induction of a set of genes encoding for key inflammatory molecules and tissue-reparative mediators, exemplified by *Il1b*. $PGE_2$-mediated synergistic effects with inflammatory stimuli resulted in increased IL-1b intracellular levels and higher IL-1b release, upon NLRP3-inflammasome activation, in co-stimulated BMDMs. Mechanistically, $PGE_2$ co-stimulation was not associated with increased activation of NF-kB signaling. Moreover, a permeable analog of cAMP, second messenger downstream $PGE_2$ receptors EP2 and EP4, is able to phenocopy the $PGE_2$-mediated synergistic effects with inflammatory stimuli on gene expression.

**Conclusion**

These analysis showed that $PGE_2$ exerts divergent activities on macrophages. While suppressing type I IFN and Interferon-stimulated genes, co-stimulation with $PGE_2$ increases the expression of a set of inflammatory and tissue reparative mediators, including *Il1b*. By identifying a set of genes differently regulated by $PGE_2$, our findings reconcile with the seemingly opposing functions of $PGE_2$ in macrophages. Overall, our data highlight the critical role of $PGE_2$ in driving immune-modulation and dysfunction in the tumor microenvironment, further supporting the therapeutic potential of $PGE_2$ blockade alone or in combination with immunotherapeutic approaches. Mechanistically, how $PGE_2$ controls inflammatory responses in macrophages is currently under investigation.

### EACR23-0926

### Single Cell RNA Sequencing of Head and Neck Cancer: Characterisation of HPV-Positive and HPV-Negative Subtypes

*B. Jenkins[1], I. Tracy[1], C. Hanley[1], G. Thomas[1]*
*[1]University of Southampton, Faculty of Medicine- Cancer Sciences, Southampton, United Kingdom*

**Introduction**

Head and neck squamous cell carcinoma (HNSCC) is the sixth most common cancer worldwide with an estimated annual incidence of ~900,000 (450,000 deaths). In the UK, HNSCC incidence has increased by 20% over the last decade (~12,500 cases/year; Cancer Research UK, 2016) with around 25% of cases relating to human papillomavirus (HPV) infection. Notably, HPV-driven tumours are associated with significantly better survival compared with HPV-negative tumours, and this correlates morphologically with high levels of tumour-infiltrating T-cells found in HPV-positive disease. The aim of this study was to characterise and compare HPV-positive and HPV-negative HNSCC by single cell RNA sequencing (scRNA-Seq).

**Material and Methods**

HNSCC and matched normal tissue samples were obtained and disaggregated from 10 treatment-naïve patients (7 HPV-positive, 3 HPV-negative), followed by scRNA-Seq (10X) and bioinformatics analysis. This new dataset comprised 54,801 cells from HNSCC and 33,196 matched-normal cells. The novel dataset was integrated with publicly available HNSCC scRNA-Seq data to increase sample numbers. The resulting integrated HNSCC dataset comprised 121,633 CD45-positive and 53,401 CD45-negative cells allowing comprehensive comparisons across 13 HPV-positive and 8 HPV-negative HNSCC patient tumours.

**Results and Discussions**

Fourteen broad cell populations were identified, including prominent populations of T cells, B cells and fibroblasts. Striking differences in immune populations were found in HPV-driven tumours, including enrichment for B cells and T cell subsets. Notably, we also uncovered diverse stromal cell phenotypes with distinct inflammatory and myofibroblastic cancer-associated fibroblast (CAF) subtypes, and a fibroblastic-reticular cell subset in HPV-positive cancers. Trajectory analysis suggested that in HNSCC, CAFs originate from both fibroblastic and mural cells.

**Conclusion**

Analysis of this novel dataset has improved understanding of the microenvironments in HPV-positive and HPV-negative HNSCC. Characterising the immune response in immune permissive, HPV-positive HNSCC may help develop future strategies for immunotherapy.

### EACR23-0932

### OSM cytokine promotes immune suppression in breast cancer by remodelling the myeloid compartment and reprogramming tumour metabolism

*M.M. Caffarel[1,2], P. Azcoaga[1,3], A. Abaurrea[1], P. Petit[1], F. Soler[1], S. Manzano[1]*
*[1]Biodonostia Health Research Institute, Oncology, San Sebastian, Spain*
*[2]Ikerbasque, Basque Foundation for Science, Bilbao, Spain*
*[3]CIC biomaGUNE, Molecular and Functional Biomarkers, San Sebastian, Spain*

**Introduction**

Understanding how cytokines orchestrate pro-malignant effects in the tumour microenvironment (TME) is key to design new therapeutic strategies to block tumour-promoting inflammation. We recently characterized the cytokine Oncostatin M (OSM), member of the IL-6 family, as a as central node for multicellular interactions within the

© 2023 The Authors. Molecular Oncology is published by John Wiley & Sons Ltd on behalf of Federation of European Biochemical Societies breast TME. We observed that myeloid-derived OSM activates an intriguing pro-tumoral signalling in cancer-associated fibroblasts (CAFs) and cancer cells, leading to increased myeloid recruitment (Araujo et al., *JCI*. 2022. doi:10.1172/JCI148667). In here, we investigated the role of OSM on immune suppression.

**Material and Methods**

We generated mouse breast tumours deficient in OSM signalling by crossing MMTV-PyMT mice with mice lacking the OSM receptor OSMR. The effect of OSM in the immune landscape and myeloid compartment of those tumours was analysed by flow cytometry and RNAseq. Our results were complemented with in vitro co-cultures of breast cancer (BC) cells CAFs and macrophages, in which we performed Seahorse and lactate secretion experiments. The clinical relevance of the results was assessed by staining of a TMA comprising 141 human BC samples and bioinformatic analysis of human breast tumors from TCGA.

**Results and Discussions**

Our data supports that OSM activates IL1 and IL8 secretion and promotes immune suppression by remodelling the myeloid compartment. In particular, OSM activates the expression of genes involved in neutrophil activation and myeloid derived suppressor cell (MDSC) signatures, promotes M2-like macrophage recruitment, and inhibits myeloid phagocytosis through up-regulation of the "do-not-eat me" signals (CD47-SIRPA) and down-regulation of the "do-eat-me" signal SLAMF7. Depletion of OSM signalling in a genetic BC model results in increased levels of cytotoxic CD8 and CD4 T cells and B lymphocytes. High levels of OSM and OSMR correlate with IL1, IL6 and IL8, and associate with increased myeloid recruitment and decreased T and B cell infiltration in human BC samples. In addition, OSM promotes, in cancer cells and CAFs, lactate secretion, increased glycolysis and hypoxic signalling, all features strongly associated with immune suppression.

**Conclusion**

Our work sheds light on the effects of OSM signalling in immune suppression, which could be mediated by reprogramming of the tumour metabolism, and supports that the OSM pathway could be a promising candidate for therapeutic targeting in breast cancer.

### EACR23-0993
### Multimodal Spatial Transcriptomics uncover distinct tumor microenvironment states and cell-cell communication networks in molecular pancreatic cancer subtypes

S. Bärthel[1,2], C. Falcomatà[1,2], R. Gindra[1,3], D. Lucarelli[1,3], C. Schmitt[1], V. Gölling[4], J. Swietlik[5], S.A. Widholz[6], M. Jesinghaus[7], F. Meissner[5], M. Schmidt-Supprian[4], D. Saur[1,2]

*[1]Institute of Translational Cancer Research and Experimental Cancer Therapy, Center for Translational Cancer Research TranslaTUM- School of Medicine- Technical University of Munich, Munich, Germany*

*[2]Division of Translational Cancer Research, German Cancer Research Center DKFZ and German Cancer Consortium DKTK, Heidelberg, Germany*

*[3]Institute of Computational Biology, Helmholtz Zentrum München- German Research Center for Environmental Health, Neuherberg, Germany*

*[4]Institute of Experimental Hematology, Center for Translational Cancer Research TranslaTUM- School of Medicine- Technical University of Munich, Munich, Germany*

*[5]Institute of Innate Immunity- Department of Systems Immunology and Proteomics, Medical Faculty- University of Bonn, Bonn, Germany*

*[6]Institute of Molecular Oncology and Functional Genomics, Center for Translational Cancer Research TranslaTUM- School of Medicine- Technical University of Munich, Munich, Germany*

*[7]Institute of Pathology, University Hospital Marburg, Marburg, Germany*

**Introduction**

The tumor microenvironment (TME) presents a complex heterocellular ecosystem comprising a diversity of immune and stromal cell populations that influence tumor progression, drug delivery and therapy outcome. High levels of inter- and intra-tumor heterogeneity in TME state are driven by molecular tumor phenotypes. Pancreatic ductal adenocarcinoma (PDAC), one of the most lethal cancer types, displays a high genetic heterogeneity along with a highly immunosuppressive TME. However, it remains largely elusive how distinct TME states are mechanistically influenced, and which molecular processes dictate the emergence of different modes of immunosuppression within the PDAC TME of molecular PDAC subtypes.

**Material and Methods**

Here, we performed a systematic analysis of functional associations between the major molecular PDAC subtypes, the classical and mesenchymal subtype, and their TME states. We describe the TME composition and cell-cell communication networks within subtypes using a multimodal integration of Spatial Transcriptomics (ST) with scRNA-seq, Secretomics and multiplexed histocytometry data. We generated ST data sets (10x Visium) of a *Kras*-driven mouse PDAC cohort and analyzed ST data sets from human PDAC samples. To delineate the TME composition, spatial niches and communities of divergent cell types, we computationally enhanced the spot-resolution of ST data sets using the BayesSpace toolkit, followed by cell type deconvolution and cell-cell communication analysis to identify subtype-specific TME communities and cell-cell communication networks.

**Results and Discussions**

To this end, we generated a large resource of PDAC mouse models which represent molecular subtypes of the disease and mimic the heterogeneity of TME states found in human PDAC cohorts. We use this resource to functionally investigate the diversity of the associated TME states. This analysis revealed that a set of subtype-specific secreted factors shape the immunosuppressive PDAC TME via direct and indirect cell-cell communication networks with immunosuppressive myeloid and T cells in molecular PDAC subtypes. Multimodal ST analysis delineated spatial subtype-specific TME communities as well as spatial communication patterns.

**Conclusion**

© 2023 The Authors. Molecular Oncology is published by John Wiley & Sons Ltd on behalf of Federation of European Biochemical Societies

We functionally investigated the spatial landscape of molecular PDAC subtypes and analyzed how subtype-specific secreted factors shape the TME composition and microenvironmental crosstalk, providing potential therapeutic vulnerabilities for more effective and rational combinatorial subtype-specific therapies.

### EACR23-1141
### Oncostatin M promotes a pro-tumorigenic inflammatory response in NASH-related HCC

*B. Foglia[1], S. Sutti[2], C. Rosso[3], C. Bocca[1], P. Carucci[4], S. Gaia[4], E. Bugianesi[3], E. Albano[2], M. Parola[1], S. Cannito[1]*
*[1]University of Torino, Dept. Clinical and Biological Sciences, Torino, Italy*
*[2]University Amedeo Avogadro of East Piedmont, Dept. Health Sciences and Interdisciplinary Research Cent er for Autoimmune Diseases, Novara, Italy*
*[3]University of Torino, Dept. Medical Sciences, Torino, Italy*
*[4]Città della Salute e della Scienza University-Hospital, Division of Gastroenterology, Torino, Italy*

**Introduction**
Oncostatin M (OSM) is a pleiotropic cytokine belonging to the IL-6 family that has been proposed to contribute to the progression of chronic liver diseases and hepatocellular carcinoma (HCC). High levels of OSM were found in cirrhotic patients with different etiology carrying HCC. In particular,OSM serum levels are significantly higher in patients carrying non-alcoholic steatohepatitis (NASH)-related HCC, as compared to those whit viral etiologies, and correlate with poor survival.
**Material and Methods**
This work discusses the role of OSM in relation to the development of NASH-related HCC taking advantage of: a) cohort of NASH patients with HCC; b) human THP1 macrophage cell lines exposed to human recombinant OSM (hrOSM); c) *Wild type* (wt) mice fed with a control diet (CSAA) or a lipogenic diet (CDAA) for 24 weeks to reproduce the NASH pathogenic phenotype (CSAA-CDAA protocol); d) *Wild type* (wt) and OSM Receptor β *knock out* (OSMRβ$^{-/-}$) mice treated with a protocol of NASH-related liver carcinogenesis (DEN/CDAA).
**Results and Discussions**
In patients with NASH-related HCC, OSM is expressed in relation to CD68$^{+}$ macrophages. In *in vitro* experiments (THP1 exposed to hrOSM) we found that OSM is able to promote an M2 pro-tumorigenic phenotype due to the activation of STAT3 and PI-3K/Akt signaling pathways. Accordingly, OSM expression (which was found increased in NASH-related liver tumors of wt mice) correlates with F4/80 gene expression. This data suggest an interplay between OSM and macrophages recruitment/functions in the tumor microenvironment. Wt mice treated with the DEN-CDAA protocol show a stronger promotion of the M2 phenotype compared with the M1 and in these mice OSM transcript levels correlate better with M2 macrophage polarization markers. As OSMRβ is fundamental for the activation of the OSM-related STAT3 and PI-3K/Akt signaling pathways, the OSMRβ$^{-/-}$ murine model was employed. The data obtained shows that the livers of these animals develop smaller tumors and this event may be due to: i) a decreased amount of M2 TAMs in the nodules (reduction of *CD163, PDL1, CD206*, *CCR2)* compared with wt mice; ii) an impairment of the angiogenic process (lower levels of *VE-cadherin, VEGFR2, CD105,* VEGF); iii) a reduction of tumor growth (decreased levels of PCNA, Ki67).
**Conclusion**
Experimental data highlight a pro-carcinogenic contribution for OSM in NASH, by promoting pro-tumorigenic inflammation, suggesting a possible role for the OSM-OSMRβ axes as therapeutic target for NASH-related HCC.

### EACR23-1148
### Single-cell Spatial Immune-profiling of Tumor Tissues by Highly Multiplexed Fluorescence Imaging

*T. Campbell[1], A. Christians[2], C. Jackson[3], K. Kwarta[1], K. Gamber[3]*
*[1]Canopy Biosciences, Product Management, Saint Louis, United States*
*[2]Canopy Biosciences, Research & Development, Hannover, Germany*
*[3]Canopy Biosciences, Research & Development, Saint Louis, United States*

**Introduction**
Highly multiplexed spatial biomarker analysis has demonstrated the potential to advance our understanding of the immune system and its role in cancer, from tumor initiation to metastatic progression. Previously, a trade-off between plex and spatial context meant that our understanding of immune cell involvement in cancer was limited to providing deep information on either cell phenotypes or their spatial context, but not both.
**Material and Methods**
ChipCytometry is a novel, highly multiplexed technology that preserves both plex and spatial context to deeply profile immune cell diversity at single-cell resolution. ChipCytometry uses commercially available antibodies and combines iterative immuno-fluorescent staining with high-dynamic range imaging to profile dozens of protein biomarkers in a single tissue specimen. Cellular phenotypes are identified with via flow cytometry-like hierarchical gating from standard multichannel OME-TIFF images, compatible with a variety of computational tools being developed for multiplexed analysis and visualization.
**Results and Discussions**
Here, we use ChipCytometry to identify and quantify key immune cell subtypes in FFPE tumor tissues. The results show precise expression levels for each biomarker in the assay in each individual cell in the sample. More than a dozen cellular phenotypes are quantified while maintaining spatial positioning of each cell. Spatial analysis shows quantifiable heterogeneity of immune cell infiltration within the tumor samples.
**Conclusion**
This study demonstrates the utility of the ChipCytometry platform for in-depth single-cell immune profiling in FFPE samples, revealing spatial relationships between cell types.

### EACR23-1225
### MOLECULAR PROFILING OF THE

© 2023 The Authors. Molecular Oncology is published by John Wiley & Sons Ltd on behalf of Federation of European Biochemical Societies

### PROTECTIVE ROLE OF EOSINOPHILS IN THE BLADDER CANCER MICROENVIRONMENT

M. Degoricija[1], I. Gabela[2], L. Frankovic[2], K. Vilovic[3], J. Korac Prlic[2]
[1]University of Split School of Medicine, Department of Medical Chemistry and Biochemistry, Split, Croatia
[2]University of Split School of Medicine, Department of Immunology and Medical Genetics, Split, Croatia
[3]University Hospital Split and University of Split School of Medicine, Department of Pathology, Split, Croatia

**Introduction**

Urothelial carcinoma is the most common histological type of bladder cancer and the ninth most common malignancy worldwide. Muscle-invasive urothelial carcinoma is considered immunogenic and responsive to immune checkpoint inhibitors. Tumor-infiltrating immune cells are key players in the tumor microenvironment thus having prognostic and predictive roles in cancer. Pharmacological modulation of the tumor microenvironment has great potential for cancer therapy. Eosinophils contribute to the formation of the tumor microenvironment due to their role in tissue repair and normalization of tumor vasculature, however, their role in bladder cancer remains elusive.

**Material and Methods**

To examine the role of eosinophils in the bladder cancer microenvironment, we used eosinophil-deficient male mice (ΔdblGATA1). Mice were administered N-butyl-N-(4-hydroxybutyl) nitrosamine (BBN) in drinking water to induce bladder cancerogenesis. Pathohistological analysis was performed to characterize carcinogenic changes in the bladder tissue and gene expression profiling of the BBN-treated bladder specimens was performed.

**Results and Discussions**

Administration of BBN to mice induces tumor growth which resembles the human basal-like subtype of urothelial carcinoma. This is the most common mouse model of bladder cancer because of histological similarity and high mutational burden, which are representative of human bladder cancer. Eosinophil-deficient mice develop more advanced bladder tumors with differential gene expression patterns associated with an altered tumor microenvironment.

**Conclusion**

Eosinophils are important constituents of the tumor microenvironment with a protective role in bladder cancer progression.

### EACR23-1227

### Cytotoxic chemotherapy dampens cancer-neoantigens specific immune responses in glioma patients

A. Picca[1], M. Baron[2], K. Labrèche[1], A. Bouzidi[3], A. Rousseau[4], V. Morin[4], F. Bielle[1], M. Touat[1], M. Sanson[1], B. Autran[4]
[1]Paris Brain Institute ICM, Team "Genetics and development of nervous system tumors", Paris, France
[2]AP-HP- Hôpital Pitié- Salpêtrière, Department of Clinical Haematology, Paris, France
[3]AP-HP- Hôpital Pitié- Salpêtrière, Department of Endocrine Biochemistry and Oncology, Paris, France
[4]AP-HP- Hôpital Pitié- Salpêtrière, Department of Immunology, Paris, France

**Introduction**

Despite major progress in molecular characterization, gliomas remain hard-to-treat cancers. Promising results in cancer neoantigen (NeoAg)-directed vaccines have been seen; however, final outcomes remain poor. A better comprehension of the factors associated with effectively immunogenic NeoAgs and detectable immune responses is needed.

**Material and Methods**

Patients were prospectively enrolled in the IDeATIon project (NCT03706625) devoted to tumors occurring in conditions of immunosuppression (including immune-privileged sites such as the CNS). Whole exome and RNA sequencing of tumor samples along with patients HLA typing were performed. Cancer NeoAg sequences and expression were predicted *in silico* using state of the art algorithms and personalized filters. For a subset of patients, peripheral blood mononuclear cells (PBMC) were collected and their reactivity to NeoAg-derived peptides was assessed *in vitro* using ELISpot assays to validate *in silico*-predicted immunogenicity.

**Results and Discussions**

Thirty-six prospective glioma patients (IDH mutant, n=10; IDH wildtype, n=26) were enrolled, including 21 mismatch-repair (MMR)-deficient (MMRd) gliomas (post-temozolomide, n=12; de novo, n=9). As expected, median tumor mutational burden (TMB) was significantly higher for MMRd compared to MMR-proficient (MMRp) gliomas (50 vs. 2 mutations/Mb, $p<0.001$).
The median number of predicted NeoAgs per tumor was also higher in MMRd vs. MMRp tumors (1540 vs. 58, $p<0.001$). The TMB and the number of predicted NeoAgs were highly correlated ($\rho=0.81$, $p<0.001$).
In vitro PBMC responses were assessed for best ranked peptides (median 48 peptides/patient, range 12-62) in 18 patients (10 MMRd, 8 MMRp). NeoAg-specific T-cell responses (>50 spot forming units/10e6 PBMC) were detected in 72% (13/18) of cases, including both MMRd (8/10) and MMRp (5/8) tumors. All non-responder patients (5/5, 100%) were under treatment with cytotoxic chemotherapies at the time of blood sampling, compared to 15% (2/13) of responders ($p=0.003$). NeoAg-specific responses were seen in 100% (11/11) of patients not under cytotoxic chemotherapy.

**Conclusion**

We developed and validated in vitro a robust and reliable pipeline for the identification of effectively immunogenic neoantigens using validated algorithms and personalized filters. Our analyses suggest that cytotoxic chemotherapies can profoundly dampen antitumor immune response.

### EACR23-1241

### Deciphering the role of Pin1 in the interplay between nuclear mechanotransduction and innate immunity in breast cancer

R. Bertolio[1], A. Rustighi[2], M. Guissouegou Doubi[2], L. Triboli[1], R. Zanatta[1], M. Albano[1], F. Napoletano[1], F. Benvenuti[3], V. Cancila[4], C. Tripodo[4], S. Piccolo[5], G. Del Sal[1]
[1]University of Trieste, Life Science, Trieste, Italy

© 2023 The Authors. Molecular Oncology is published by John Wiley & Sons Ltd on behalf of Federation of European Biochemical Societies

[2]International Centre for Genetic Engineering and Biotech nology - ICGEB, Cancer Cell Signaling, Trieste, Italy
[3]International Centre for Genetic Engineering and Biotech nology - ICGEB, Cellular Immunology, Trieste, Italy
[4]University of Palermo, Tumor Immunology Unit- Department of Health Science- Human Pathology Section- School of Medicine, Palermo, Italy
[5]University of Padua, Molecular Medicine, Padova, Italy

**Introduction**

Biomechanical alterations characterize most tumors and elicit cell responses contributing to tumor progression. Transduction of mechanical signals from the ECM to chromatin by cytoskeleton/Lamin nucleoskeleton connection allows adaptation of nuclear envelope (NE) structure, chromatin organization, and gene expression to mechanical cues. In mechanically challenged cells, lack of key mechanosensors causes NE/DNA damage, with cytosolic leakage of DNA, triggering cGAS/STING innate immunity pathway.
We recently found that in normal cells, the prolyl isomerase Pin1 plays a key role in maintaining NE and heterochromatin (HC), in response to mechanical stress (Napoletano et al., Cell Reports 2021). Pin1 loss/inhibition led to NE malformations, and HC relaxation, causing mobilization of transposable elements (TEs) and DNA damage, which led to IFN-I induction and cell death.
Cancer cells experience mechanical challenges during tumor progression and mount a nuclear mechano-protective response, whose failure causes NE/genome damage, leading to cGAS/STING/IFN-I pathway activation, which promotes immune surveillance.
In cancer, Pin1 is upregulated and amplifies tumorigenic pathways, while its depletion/inhibition curbs tumor growth, sensitizing to therapies.
We posit that Pin1 could maintain NE/genome integrity in cancer cells and that Pin1 loss/inhibition could cause NE/DNA damage and HC relaxation leading to TE hyperactivity, thus activating cGAS/STING/IFN-I.

**Material and Methods**

We assessed the role of Pin1 in mechanical response of cancer cells, using breast cancer (BC) cells 3D-cultured in matrices with defined composition and mechanical properties.
Also, we assessed the role of Pin1 in maintaining NE/genome integrity, generating Pin1 CRISPR KO BC cells and mouse models, in which Pin1 can be specifically knocked-out in cancer cells.

**Results and Discussions**

In mechanically challenged BC cells, Pin1 was recruited to the NE and required to maintain NE structure and HC condensation. Pin1 KO caused NE ruptures, HC relaxation, TE upregulation, DNA damage, and cGAS/STING/IFN-I activation.
In mouse models, Pin1 KO in cancer cells led to cGAS/STING activation and immune cells infiltration, with reduction of tumor mass. Importantly, similar effects were obtained with Pin1 inhibitors, which also synergized with ICB.

**Conclusion**

We have provided evidence that Pin1 could be a key regulator of mechanoresponse in cancer cells and treatment with Pin1 inhibitors could sensitize BC cells to ICB.

### EACR23-1312
### Accentuated autophagy shapes the immune responses in the tumor microenvironment

A. Chryplewicz[1,2], R. Elased[1,2], M. Tichet[1,2], D. Hanahan[1,2,3]
[1]EPFL, Swiss Institute for Experimental Cancer Research, Lausanne, Switzerland
[2]AGORA, Translational Cancer Research Center, Lausanne, Switzerland
[3]Ludwig Institute for Cancer Research, Lausanne Branch, Lausanne, Switzerland

**Introduction**

Several years ago, our lab reported the successful repurposing of two well-tolerated FDA-approved agents to target malignant progression in a genetically modified mouse model of GBM: a tricyclic antidepressant called imipramine (IM) and an anticoagulant agent targeting the purinergic receptor P2Y12 (Shchors et al.; Cancer Cell, 2015). This combination hyper-activated the cellular recycling system called autophagy to the level of inducing autophagy-associated cell death (AACD) in cultured cells and contributing to therapeutic benefit in vivo. In our most recent publication (Chryplewicz et al., Cancer Cell. 2022), we have extended upon the therapeutic benefit of IM by combining it with the murine analog of the anti-VEGF antibody bevacizumab (approved in the clinic to treat progressive GBM). Investigation of the therapeutic efficacy revealed increased autophagy in GBM tumors following the dual therapy and tumor microenvironment to be remarkably remodeled, orchestrating the infiltration of T lymphocytes. Motivated by our previous results, we are investigating the functional role and molecular mechanisms of immunostimulatory autophagy and assessing the importance of autophagic flux as an enhancer of immunity beyond GBM.

**Material and Methods**

In order to determine the functional role of autophagy in driving the immune responses, mouse cancer cells of different origins (glioma, pancreas, colon) were treated with a combination of autophagy-inducing drugs and co-cultured with mouse primary immune cells; T cells, bone-marrow-derived macrophages, and DCs. The underlying transcriptional and protein profiles were analyzed in vitro and in vivo. These findings were further confirmed in co-cultures with ATG3 and ATG7 knockdown or overexpressing cells.

**Results and Discussions**

We found that autophagic cancer cells were less immunosuppressive on T cell proliferation, which correlated with our findings in vivo where the survival benefit of mice treated with the autophagy inducers was abrogated in shATG3 tumors and associated with reduced CD8 T cell infiltration, in comparison with similarly treated ATG3-proficient tumors. In addition, macrophages co-cultured with autophagic cancer cells presented with a decreased M2-like profile, and DCs expressed higher levels of costimulatory molecules.

**Conclusion**

Our findings highlight the potential importance of autophagic flux in rendering the tumor microenvironment more immunostimulatory and could be potentially more broadly applicable across different tumor types.

© 2023 The Authors. Molecular Oncology is published by John Wiley & Sons Ltd on behalf of Federation of European Biochemical Societies

### EACR23-1316

### Reprogramming immunosuppressive tumor-associated macrophages potentiates standard-of-care therapy in melanoma

*M. Tichet*[1,2], *A. Chryplewicz*[1], *D. Hanahan*[1,2]
[1]*EPFL, Swiss Institute for Experimental Cancer Research ISREC, Lausanne, Switzerland*
[2]*Ludwig Institute for Cancer Research, Lausanne Branch, Lausanne, Switzerland*

**Introduction**

Cutaneous melanoma is a highly aggressive cancer capable of distant and lethal metastatic spread. Breakthroughs in treatment have come from understanding oncogenic signaling and cancer immunobiology. Targeted therapies successfully block MAPK signaling in BRAFV600e mutant melanoma with remarkably high clinical responses followed, by rapid relapse, whereas checkpoint inhibitors activating the immune response induce long-lasting responses, albeit only in a subset of patients. These limitations have driven interest in understanding innate and acquired resistance mechanisms.

**Material and Methods**

Using a refined, immunocompetent genetically-engineered mouse model of BRAF-driven melanoma (iBIP2), which phenocopies the human disease in its development, histopathology, and response to therapy, we focused on the tumor microenvironment (TME) seeking to elucidate resistance mechanisms by performing in vivo, in vitro and ex vivo analyses.

**Results and Discussions**

Our investigations revealed that tumor-associated macrophages (TAMs) are involved in tumor development and therapeutic resistance. We characterized this myeloid population and showed that TAMs are a major component of the TME, predominantly polarized toward a pro-tumoral “M2-like” phenotype while producing immunosuppressive factors and exhibiting extensive immunosuppressive capabilities. Combining conventional strategies with TAMs-reprogramming agents stimulated T cell-mediated anti-tumor immune responses, leading to improved survival and responsiveness to standard-of-care therapies. We then performed integrative transcriptomic analyses to understand, at the single-cell level, the complex interplay occurring in the TME and further validated our results in human melanoma.

**Conclusion**

Our work highlights the central role played by macrophages in melanoma resistance to therapy and demonstrates that pharmacologic reprogramming of macrophages represents a new therapeutic modality with the potential to elicit more effective anti-tumor immune responses against this devastating disease.

### EACR23-1323

### Quantification of immunoediting signal using HLA-I genotype and mutational signatures.

*N. Kherreh*[1], *C. Seoighe*[1]
[1]*University of Galway, School of Maths- Statistics and Applied Maths, Galway, Ireland*

**Introduction**

Neoantigens are mutated peptides that have the potential to initiate immune responses against tumors. These immunogenic mutations are reportedly removed by selection in a process referred to as immunoediting. Although it has been studied extensively, the influence of patient MHC-I genotype on the extent of immunoediting has not been quantified precisely and remains controversial.

**Material and Methods**

We developed a method to detect and quantify the immunoediting signal in tumors by comparing the observed and expected proportions of immunogenic mutations, given the patient-specific mutation signature activity profile and HLA-I genotype. We applied this method to data from TCGA.

**Results and Discussions**

In most tumor types, the signal of immunoediting was weak or absent. In a pan-cancer analysis we found slightly stronger evidence of immunoediting in the subclonal compared to clonal mutations, in line with previous reports. Overall, our results are consistent with at most 1% of mutations having been removed through immunoediting, suggesting that the effect of immunoediting on the cancer mutational landscape is, at most, marginal. Indeed, a weak signal of immunoediting persisted even when HLA alleles were shuffled randomly between patients, casting doubt on the existence of an HLA-I dependent immunoediting signal in the data.

**Conclusion**

Overall, there was no evidence that HLA-I dependent immunoediting makes a substantial contribution to the somatic mutations observed in cancer samples.

### EACR23-1333

### POSTER IN THE SPOTLIGHT

### Neutrophils mediate protection against colitis and carcinogenesis by driving IL-22 production by gd T cells.

*G. Grieco*[1], *S. Carnevale*[2], *A. Ponzetta*[3], *R. Carriero*[2], *C. Recordati*[4], *C. Tripodo*[5], *P. Kunderfranco*[2], *C. Garlanda*[6,7], *A. Mantovani*[2,8,9], *S. Jaillon*[2,7]
[1]*Humanitas University, Biomedical Sciences, via Manzoni 56- 20089 Rozzano- MI- Italy., Italy*
[2]*IRCCS Humanitas Research Hospital, IRCCS Humanitas Research Hospital, via Manzoni 56- 20089 Rozzano- MI- Italy., Italy*
[3]*Center for Infectious Medicine, Department of Medicine Huddinge, Karolinska Institutet- Stockholm- Sweden, Sweden*
[4]*Mouse and Animal Pathology Laboratory, Fondazione Filarete, Milano- MI- Italy., Italy*
[5]*Tumor Immunology Unit- University of Palermo, Department of Health Science- Human Pathology Section- School of Medicine, 90133- Palermo- Italy, Italy*
[6]*IRCCS Humanitas Research Hospital, IRCCS Humanitas Research Hospital, via Manzoni 56- 20089 Rozzano- MI- Italy, Italy*
[7]*Humanitas University, Department of Biomedical Science, 20072- Pieve Emanuele- MI- Italy, Italy*

© 2023 The Authors. Molecular Oncology is published by John Wiley & Sons Ltd on behalf of Federation of European Biochemical Societies

[8]Queen Mary University of London,
The William Harvey Research Institute,
London EC1M 6BQ UK., United Kingdom
[9]Humanitas University, Department of Biomedical Science, 20072- Pieve Emanuele- MI- Italy., Italy

**Introduction**

Neutrophils are the most abundant population of circulating leukocytes in humans and play important roles in defense against invading pathogens. An increasing body of evidence indicates that neutrophils are an important component of the tumor microenvironment but their role in cancer remains poorly understood and controversial.

**Material and Methods**

Neutrophils have been proposed to affect the pathogenesis of colitis and colitis-associated colorectal cancer (CAC). However, both detrimental and beneficial effects of neutrophils have been reported and their overall role is still debated.Therefore, we assessed the role of neutrophils in colitis and CAC by using a genetic mouse model of neutrophil deficiency (*Csf3r*$^{-/-}$ mice) challenged with classic models of colitis and colitis-associated colorectal cancer.

**Results and Discussions**

*Csf3r*$^{-/-}$ mice showed increased susceptibility to colitis and CAC development compared to *Csf3r*$^{+/+}$ control mice. Neutrophils were important in limiting bacterial infiltration and driving the induction of the IL-23/IL-22-dependent protective pathway. IL-23, which is produced mainly by macrophages, acts on lymphoid cells to induce IL-22 production, which in turn drive intestinal epithelial cells repair and mucosal healing. We found that *Csf3r*$^{-/-}$ mice displayed low or undetectable levels of IL-23 and IL22, which are rescued upon neutrophil adoptive cells transfer. We found that neutrophil deficiency was associated with altered polarization of gd T cells and impaired IL-22 production. We set out an *in vitro*model to assess the impact of neutrophils on the production of IL-23 by bone marrow-derived macrophages (BMDMs). Neutrophils amplified IL-23 expression by BMDMs stimulated with GM-CSF and TLR9 agonist. Mechanistically, the production of IL-23 was dramatically reduced by an inhibitor of reactive oxygen species. Finally, in patients with ulcerative colitis, signatures associated with epithelial cell development, proliferation and antimicrobial response were enriched in *CSF3R*$^{high}$ patients.

**Conclusion**

Collectively, our results support that neutrophils promote protection against colitis and CAC, driving the activation of the IL-23/IL-22 dependent tissue repair pathway

### EACR23-1385
### RANBP1 regulates the SGK1-dependent Th17 pathological differentiation: nuclear export hypothesis

C. Brescia[1], V. Dattilo[2], L. D'Antona[1,3], E. Chiarella[2], S. Audia[1], R. Iuliano[1,3], D. Scumaci[2], F. Trapasso[2,3], N. Perrotti[1,3], R. Amato[1,3]
[1]Magna Graecia University of Catanzaro Unicz- Germaneto CZ- Italy, Department of Health Science, Catanzaro, Italy
[2]Magna Graecia University of Catanzaro Unicz- Germaneto CZ- Italy,
Department of Experimental and Clinical Medicine- Medical School- University “Magna Graecia” of Catanzaro- Catanzaro- Italy, Catanzaro, Italy
[3]Magna Graecia University of Catanzaro Unicz- Germaneto CZ- Italy,
Medical Genetics Unit- University Hospital- Medical School- University “Magna Graecia” of Catanzaro- Catanzaro - Italy, Catanzaro, Italy

**Introduction**

Th17 lymphocytes, a special CD4$^{+}$ differentiation cluster, are involved in the development of cancer, autoimmune diseases and viral diseases (Tesmer LA et al., 2008). In cancer, increased Th17 and related cytokines levels have been observed (Tesmer LA et al., 2008). Th17 differentiation is dependent on several components, including the serine/threonine kinase SGK1(Wu C et al., 2013). SGK1 controls the RANBP1 expression, implicated in mitosis and nuclear transport (Amato R et al., 2013). The aim of my project is to define the RANBP1 role in the Th17 differentiation, also by establishing the potential role of RANBP1 as molecular target for a Th17 pharmacological modulation in cancer.

**Material and Methods**

Buffy coats from healthy donors were subjected to CD4$^{+}$ isolation. CD4$^{+}$ were undergone to Th17 differentiation protocol in the presence of adequate cytokine and co-stimulatory pool. Expression studies for RANBP1, SGK1, IL23R, IL17, RoRγ and FOXO1 were conducted by WB, IF on intact cells, Real-time PCR and FACS approaches. To determine the role of RANBP1 in Th17 maturation process, RANBP1 fluctuations have been regulated by lentiviral over-expression and stable gene silencing approaches. IF assays and differential nucleus-cytoplasmic extractions were performed to determine the role of RANBP1 modulation, on FOXO1 localization. Functional metabolomics studies were performed by intracellular Sea-Horse approach. Cytokine modulation studies were conducted by FluoroSpot approach.

**Results and Discussions**

We determined that RANBP1, similarly to SGK1, is modulated during early/late pathological Th17 differentiation. In addition, we demonstrated that RANBP1 play an essential and rate-limiting role in the differentiative process by modulating IL23R-SGK1-dependent signalling. We verified that the underlying mechanism consists in the SGK1/RANBP1-dependent regulation of the FOXO1 nuclear export. Recent evidence shows that RANBP1 impacts the secretory and metabolic framework of TH17 lymphocytes such that it alters Th17 plasticity and toxicity behaviours.

**Conclusion**

This new mechanism has completed the delineation of a crucial pathway for lymphocyte differentiation processes, which could lead to new outlets not only for understanding the basic mechanisms of Th17 plasticity, but also for future prospects of pharmacological modulation through targeted manipulation of this signalling pathway.

### EACR23-1401
### Dynamic single-cell RNA sequencing of 3D microphysiological systems reveals small cell lung cancer vulnerability to NK cell

© 2023 The Authors. Molecular Oncology is published by John Wiley & Sons Ltd on behalf of Federation of European Biochemical Societies

### attack

M. CAMPISI[1], S.R. Park[1], T. Michael[1], C. Paweletz[2], N. Mahadevan[1], D. Barbie[3]
[1]Dana-Farber Cancer Institute, Medical Oncology, Boston, United States
[2]Dana-Farber Cancer Institute, Belfer Center for Applied Cancer Science, Boston, United States
[3]Dana-Farber Cancer Institute, Medical Oncology, Boston, Italy

**Introduction**

Testing next-generation immune therapies remains challenging in animal models and requires sophisticated *ex vivo* systems to study human tumor biology and predict treatment response in real time. Here, we developed a novel 3-dimensional microphysiological model of the tumor microenvironment (TME) of vascularized tumor spheroids with endothelial cells and lung fibroblasts which revealed to be perfusable with NK cells and monocytes. We then developed a novel method of dynamic method to dissect at the single-cell transcriptomic level the effect of cGAS-STING agonism on each TME components to enhance immune cell infiltration and correlate our finding with spatial transcriptomics datasets. We have previously characterized the heterogeneity of Small Cell Lung Cancer demonstrating that neuroendocrine SCLC subpopulations (70% of SCLC) downregulate MHC I, thus we predicted that these cells might be vulnerable to adaptive NK cell therapies.

**Material and Methods**

Here we performed n=6 single-cell RNA seq samples in HUVEC-derived microvascular networks with human lung fibroblasts, H69, -/+ human monocytes perfused with NK cells, -/+ 2',3'-cGAMP (1 ug/mL) after 7 days in the microfluidic devices. Image-based viability assays were performed in the 3D spheroid system.

**Results and Discussions**

To dissect the influence of vascular barriers to immune cell homing, we analyzed by single cell analysis our microphysiological TME model of vascularized SCLC spheroids. We were able to confidently cluster all the cell input from the 3D device and their transcriptomes were profiled. Stromal cells (endothelial cells and fibroblasts) highly responded to STING agonism promoting NK cell extravasation and recruitment via chemoattractant CXCL10 and vascular activation by upregulation of adhesion molecules. Each cell type upregulated interferon signaling pathways, JAK/STAT3 signaling, cell migration, adhesion shown by differentially-expressed genes, hallmark and gene ontology analysis and unique upregulated genes. We validated in our systems that MHC-I low SCLC subpopulation are vulnerable to primary NK cells, and vascular priming is necessary for NK cell infiltration.

**Conclusion**

This microphysiological system can be used to recapitulate TIME biology and infer cross-talk interactions and identify therapeutic vulnerabilities while studying immune infiltration and killing in the TME, offering an accurate and well-controlled and adaptable platform for biomarker and therapeutic development.

### EACR23-1441
### Molecular profiling of the immunomodulatory role of mast cells in the bladder cancer microenvironment

M. Degoricija[1], I. Gabela[2], L. Frankovic[2], K. Vilovic[3], T. Feyerabend[4], H.R. Rodewald[4], J. Korac Prlic[2]
[1]University of Split School of Medicine, Department of Medical Chemistry And Biochemistry, Split, Croatia
[2]University of Split School of Medicine, Department of Immunology and Medical Genetics, Split, Croatia
[3]University of Split- School of Medicine- University Hospit al Split, Department of Pathology, Split, Croatia
[4]German Cancer Research Center, Division of Cellular Immunology, Heidelberg, Germany

**Introduction**

Bladder cancer is the fourth most common cancer in men in Europe. The tumor microenvironment plays an important role in the development and progression of bladder cancer. Immunotherapy is a promising treatment option for bladder cancer, as demonstrated by the successful use of immune checkpoint inhibitors for muscle-invasive bladder cancer. As an integral part of the tumor immune milieu, mast cells play a role in shaping the tumor microenvironment, displaying a dichotomous role in cancer that is both cancer-and stage-specific. The mechanisms by which mast cells influence bladder cancer formation and progression remain unclear.

**Material and Methods**

To address the role of mast cells in bladder cancer, we used the most common preclinical mouse model of bladder cancer induced by the carcinogen N-butyl-N-(4-hydroxybutyl) nitrosamine (BBN). BBN-induced bladder cancer resembles the human basal-like bladder cancer subtype with great similarities in pathohistology, immune tumor microenvironment, and a high mutational burden, specifically the Trp53 mutation. In this study, we investigated the role of mast cells in modulating the bladder cancer microenvironment by analyzing mast cell-deficient mice (Cpa3 Cre/+). Pathohistological analysis and gene expression profiling were performed on the BBN-treated bladder specimens.

**Results and Discussions**

Pathohistological analysis of the BBN-induced bladder cancer model revealed more advanced tumors in mast cell-deficient mice, whereas gene expression analysis revealed alterations in the tumor microenvironment. Mast cells, as part of innate immunity, are bladder tissue-resident immune cells with the potential to modulate the tumor microenvironment.

**Conclusion**

Our results demonstrated the protective role of mast cells in bladder cancer development by modulating the tumor microenvironment.

### EACR23-1444
### Subtype-specific secreted factors influence microenvironmental crosstalk in pancreatic cancer

C. Schmitt[1], S. Bärthel[1,2], S.A. Widholz[3], C. Falcomatà[1,2], V. Gölling[4], J.J. Swietlik[5], F. Meissner[5], R. Rad[3], M. Schmidt-Supprian[4], D. Saur[1,2]
[1]Institute of Translational Cancer Research and Experimen tal Cancer Therap, Center for Translational Cancer Research TranslaTUM- School of Medicine- Techn

© 2023 The Authors. Molecular Oncology is published by John Wiley & Sons Ltd on behalf of Federation of European Biochemical Societies ical University of Munich, Munich, Germany
[2]Division of Translational Cancer Research, German Cancer Research Center DKFZ and German Cancer Consortium DKTK, Heidelberg, Germany
[3]Institute of Molecular Oncology and Functional Genomics, Center for Translational Cancer Research TranslaTUM- School of Medicine- Technical University of Munich, Munich, Germany
[4]Institute of Experimental Hematology, Center for Translational Cancer Research TranslaTUM-School of Medicine- Technical University of Munich, Munich, Germany
[5]Institute of Innate Immunity, Department of Systems Immunology and Proteomics- Medical Faculty- University of Bonn, Bonn, Germany

**Introduction**

Pancreatic ductal adenocarcinoma (PDAC) displays a highly immunosuppressive and heterocellular tumor microenvironment (TME). It remains unknown how distinct genetic alterations influence the TME composition and thereby affect the crosstalk of tumor cells with immune cell subsets. Therefore, we aim to systematically determine the role of the tumor cell secretome, in particular chemokines, on the TME composition and tumor progression in molecular PDAC subtypes.

**Material and Methods**

We performed a widespread characterization of the TME composition within the two major transcriptional PDAC subtypes, namely the classical and mesenchymal subtype, by analyzing a cohort of *Kras*-driven mouse pancreatic tumors. The subtype-specific TME composition was analyzed by flow cytometry and scRNA-sequencing in vivo, as well as MS-based secretome analysis of *in vitro* mouse PDAC cell cultures. To functionally validate the thereby identified subtype-specific secreted factors, which might shape the distinct TME composition, we performed CRISPR-Cas9 mediated gene-editing as well as overexpression studies in PDAC mouse cell lines followed by orthotopic implantation into immunocompetent mice.

**Results and Discussions**

Our results reveal that both molecular PDAC subtypes display a unique TME composition and present distinct modes of immunosuppression. The classical subtype shows high infiltration of neutrophils, whereas the mesenchymal PDAC subtype displays more abundance in macrophage infiltration. In mesenchymal PDAC cells, we identified strong secretion of Csf1 and Il34, which are known to instruct macrophage recruitment and migration. In contrast, Cxcl5 and Cxcl1 emerged as candidate factors in classical PDAC cells and are associated to neutrophil recruitment and migration. Flow cytometry analysis of engrafted mouse PDAC samples showed that Cxcl5 knockout in classical cell lines causes a decrease of neutrophil infiltration. Accordingly, Cxcl5 overexpression in mesenchymal cell lines caused an increased neutrophil recruitment as well as a faster tumor progression *in vivo*. In contrast, modulation of tumor cell secreted Csf1 did not have a similarly strong effect on macrophage infiltration.

**Conclusion**

Our systematic analysis on subtype-specific secreted factors uncovers how tumor cell secreted factors shape the TME composition and instruct immune cell infiltration. These findings contribute to the understanding of PDAC subtype-specific biology and aid the identification of potential immunotherapeutic vulnerabilities.

### EACR23-1474
### IFNγ-induced tumor-CD8 equilibrium in melanoma latency

F. Zhao[1], B. Thier[1], S. Kwapik[1], M. Laudage[1], K. Griewank[1], P. Nalbant[2], T. Wölfel[3], F. Rambow[4], A. Sucker[1], D. Schadendorf[1], A. Paschen[1]
[1]University Hospital Essen, Dermatology, Essen, Germany
[2]University of Duisburg-Essen, Center for Medical Biotechnology, Essen, Germany
[3]Universitätsmedizin der Johannes Gutenberg-Universität, III. Medizinische Klinik und Poliklinik, Mainz, Germany
[4]University Hospital Essen, Tumor Bioinformatics, Essen, Germany

**Introduction**

Despite the presence of tumor-reactive CD8 T cells, many tumor cells persist.

**Material and Methods**

Here, we took advantage of autologous tumor-T cell patient models to gain more insights into the mechanisms mediating tumor persistence under CD8 T cell surveillance.

**Results and Discussions**

In multiple models, we observed that IFNγ released upon T cell activation contributed substantially to the anti-tumor effect of CD8 TILs by killing the majority of melanoma cells. However, minor tumor cell populations persisted upon IFNγ exposure and switched into a non-proliferative, dedifferentiated cell state that evaded T cell recognition. Strikingly, after IFNγ withdrawal, the persistent tumor cells resumed proliferation and regained T cell sensitivity, suggesting an IFNγ-driven tumor-immune equilibrium during melanoma latency. Moreover, by immunohistochemistry staining we detected tumor-immune equilibrium regions also in metastatic lesions.

**Conclusion**

Taken together, our results demonstrate the potential role of IFNγ in mediating tumor persistence in a CD8-rich microenvironment, highlighting the clinical importance to eliminate such persisters in order to achieve durable tumor control.

### EACR23-1487
### FMRP Upregulation in Cancer: Implications for Cancer-Associated Fibroblasts and Immune Modulation

S. Yucel[1], D. Hanahan[1]
[1]Agora Pôle De Recherche Sur Le Cancer, CMSO, Lausanne, Switzerland

**Introduction**

Our group shed light on the crucial role of FMRP in cancer biology beyond its traditional association with neuronal development. We reported that FMRP upregulation facilitates immune evasion in several murine and human tumors by modulating various immune cell subsets, particularly the suppression of effector T cells (Zeng et al., Science 2022). This finding suggests that targeting FMRP in cancer may have the potential to enhance immune responses against tumors and improve the efficacy of immunotherapy. Moreover, our study revealed that FMRP

© 2023 The Authors. Molecular Oncology is published by John Wiley & Sons Ltd on behalf of Federation of European Biochemical Societies also governs critical roles in modulating the stromal compartment of the tumor microenvironment. Specifically, we focused on cancer-associated fibroblasts (CAFs), a key component of the tumor microenvironment that plays a vital role in tumor progression and therapy resistance. We compared and contrasted the characteristics of CAFs from FMRP wild-type and knock-out tumors and found that FMRP upregulation in tumors modulates the abundance and secretory profile of immunosuppressive CAFs. Interestingly, we also identified that FMRP is expressed by CAFs in FMRP-null tumors, suggesting a compensatory mechanism conveying immune evasion in breast tumors.

**Material and Methods**

CAFs from murine pancreatic ductal adenocarcinoma (PDAC) and breast tumors were analyzed using flow cytometry and immunofluorescence. Co-culture experiments are conducted using CAFs from wild-type or FMRP-knock-out murine tumors with bone marrow-derived macrophages and CD8 T cells. The proteome-secretome and pro-tumorigenic phenotype were assessed following FMRP knockdown and/or overexpression. Inducible stable knockdown of FMRP was generated in PDAC and 4T1 cells to determine its immediate effect on fibroblasts and the expression of FMRP in fibroblasts.

**Results and Discussions**

We found that CAFs convey differential immunosuppressive profiles in FMRP WT versus knock-out tumors and govern differential secretory phenotype. Immunosuppressive CAF population increased in abundance and elevated the expression of FMRP in FMRP-null tumors suggesting a potential resistance mechanism.

**Conclusion**

In conclusion, our study demonstrates that FMRP plays a critical role in cancer biology by modulating both the immune and the stromal compartments of the tumor microenvironment. Targeting FMRP in cancer and CAFs, in particular, represents a promising therapeutic approach to improve cancer treatment outcomes.

### EACR23-1519

### High throughput immuno-oncology assay in matrix embedded, bioprinted 3D cell model

*R Bleach*[1], *R. Utama*[1], *D. Gerace*[1], *B. Aghaei*[1]

[1]*Inventia Life Science, Science, Sydney, Australia*

**Introduction**

Advanced 3D cell culture techniques have been adopted in many laboratories to better model in vivo tissue by recapitulating multi-cellular architecture and the presence of extracellular matrix features. Here, we demonstrated an immuno-oncology assay application using 3D cell cultures generated with the RASTRUM™ 3D Advanced Cell Model Platform.

**Material and Methods**

3D cell culture of both breast (MDA-MB-231 and MCF7) and lung (A549) cancer were prepared in RASTRUM Platform® using RASTRUM matrices® (Inventia Life Science). The printing design was prepared using RASTRUM Cloud® software then incubated in media to ensure tumour growth.

After incubation, both activated (CD3/CD28 T-cell activator) and non-activated HLA-mismatched peripheral blood mononuclear cells (PBMCs) in media were added into the 3D cell culture. Infiltration of immune cells into cell-laden hydrogels was analysed by immunofluorescent staining of immune cell markers such as CD3 and CD56. Immune cell cytotoxicity against tumour cells was quantified longitudinally by fluorescence imaging of TMRE-labelled target cells.

**Results and Discussions**

We used MDA-MB-231, MCF7, and A549 cancer cells in the most physiologically relevant RASTRUM Matrices® and tested their compatibility with an immunooncology application using PBMCs. We showed that all three cancer cells were growing (bright field) & remained viable (live/dead assay).

We demonstrated that relevant, activated immune cells invaded cell-laden hydrogels and destroyed target cells in a dose-dependent manner. Successful infiltration of the immune cells into the cell model was confirmed by immunofluorescence imaging, which showed the presence of both immune and cancer cells. After confirming the ability of the immune cells to infiltrate the 3D cell models, we also confirmed that the model is suitable for a quantifiable T-cell cytotoxicity assay. We showed that activated T-cells were more cytotoxic towards spheroids (associated with CD3+ T-cell infiltration) when compared to naive T-cells.

**Conclusion**

RASTRUM Platform is a suitable tool to generate relevant cell models for immuno-oncology assays. The models were confirmed to facilitate the infiltration of activated T cells to kill tumors in a 3D format. The ability to study this in a matrix-embedded 3D format provides a more physiologically relevant in vitro environment than 2D or 3D suspension cultures. Furthermore, the high-throughput nature of RASTRUM Platform enables such an assay to be scaled to allow researchers to conduct interventional drug screens.

© 2023 The Authors. Molecular Oncology is published by John Wiley & Sons Ltd on behalf of Federation of European Biochemical Societies
